# Comparative genomics reveals 104 candidate structured RNAs from bacteria, archaea, and their metagenomes

**DOI:** 10.1186/gb-2010-11-3-r31

**Published:** 2010-03-15

**Authors:** Zasha Weinberg, Joy X Wang, Jarrod Bogue, Jingying Yang, Keith Corbino, Ryan H Moy, Ronald R Breaker

**Affiliations:** 1 Howard Hughes Medical Institute, Yale University, P.O. Box 208103, New Haven, CT 06520-8103, USA; 2 Department of Molecular, Cellular and Developmental Biology, Yale University, P.O. Box 208103, New Haven, CT 06520-8103, USA; 3 Department of Molecular Biophysics and Biochemistry, Yale University, P.O. Box 208103, New Haven, CT 06520-8103, USA; 4 Current address: Department of Biology, University of Rochester, Rochester, NY 14627, USA; 5 Current address: School of Medicine, University of Pennsylvania, Philadelphia, PA 19104, USA

## Abstract

**Background:**

Structured noncoding RNAs perform many functions that are essential for protein synthesis, RNA processing, and gene regulation. Structured RNAs can be detected by comparative genomics, in which homologous sequences are identified and inspected for mutations that conserve RNA secondary structure.

**Results:**

By applying a comparative genomics-based approach to genome and metagenome sequences from bacteria and archaea, we identified 104 candidate structured RNAs and inferred putative functions for many of these. Twelve candidate metabolite-binding RNAs were identified, three of which were validated, including one reported herein that binds the coenzyme *S*-adenosylmethionine. Newly identified *cis*-regulatory RNAs are implicated in photosynthesis or nitrogen regulation in cyanobacteria, purine and one-carbon metabolism, stomach infection by *Helicobacter*, and many other physiological processes. A candidate riboswitch termed *crcB *is represented in both bacteria and archaea. Another RNA motif may control gene expression from 3'-untranslated regions of mRNAs, which is unusual for bacteria. Many noncoding RNAs that likely act in *trans *are also revealed, and several of the noncoding RNA candidates are found mostly or exclusively in metagenome DNA sequences.

**Conclusions:**

This work greatly expands the variety of highly structured noncoding RNAs known to exist in bacteria and archaea and provides a starting point for biochemical and genetic studies needed to validate their biologic functions. Given the sustained rate of RNA discovery over several similar projects, we expect that far more structured RNAs remain to be discovered from bacterial and archaeal organisms.

## Abstract 2

Novel motifs identified in a comparative genomic analysis of bacterial, archaeal and metagenomic data reveals over 100 candidate structured RNAs.

## Background

Ongoing efforts to identify and characterize various structured noncoding RNAs from bacteria are revealing the remarkable functions that structured RNAs can perform [1-3]. To detect novel RNA classes in bacteria and archaea, a variety of bioinformatics strategies have been used [4-12]. In our recent efforts to identify novel structured RNAs, we applied a scheme based on detecting RNA secondary structures upstream of homologous protein-coding genes [13,14]. However, this strategy is best suited to finding *cis*-regulatory RNAs, not noncoding RNAs. Also, some *cis*-regulatory RNAs such as c-di-GMP riboswitches [14,15] or *ydaO *motif RNAs [5] are not often found upstream of homologous genes [13].

We therefore implemented a search system that is independent of protein-coding genes. In brief, our system clusters intergenic regions (IGRs) [16] by using a BLAST-based method [17] and infers secondary structures by using CMfinder [18]. Then, as before [19,20], the identified structures are used in homology searches to find homologues that allow CMfinder to refine further its structural alignment. The resulting alignments are scored and then analyzed manually to identify the most promising candidates and to infer possible biologic roles.

This method was applied to all available bacterial and archaeal genome sequences, as well as metagenome (that is, environmental) sequences, and identified 104 candidate RNA motifs described in this report. Some additional RNAs will be reported later (unpublished data) that bind cyclic di-GMP or tetrahydrofolate, that represent diverse variants of hammerhead self-cleaving ribozymes, or that exhibit exceptional characteristics suggesting a novel or unusual biochemical function [21]. In this report, we provide biochemical evidence that members of one of the 104 RNA motifs bind *S*-adenosylhomocysteine (SAH) and *S*-adenosylmethionine (SAM) *in vitro*, and presumably regulate the downstream genes coding for SAM synthetase. The rest of this report provides predicted structures of selected motifs and hypotheses regarding their biologic roles. The remaining motifs, as well as additional information on the selected motifs, are presented in Additional File 1. Discussions about individual motifs are largely independent, but are grouped into common putative functional roles. A list of all 104 motifs is provided in Table 1 and Additional File 2. Multiple-sequence alignments of motifs, the organisms in which their representatives appear, and predicted flanking genes are available in printable format in Additional File 3, and alignments are provided in machine-readable format in Additional Files 4 and 5. Consensus diagrams for all motifs are depicted in Additional File 6. Selected motifs (Table 1) were submitted for inclusion in the Rfam Database version 10.1 [22].

**Table 1:** Motifs identified in this work

Motif	RNA?	*cis*-reg?	Switch?	Taxa	Rfam
6S-flavo	**Y**	N	N	Bacteroidetes	RF01685

*aceE*	?	y	?	γ-Proteobacteria	

Acido-1	**y**	n	n	Acidobacteria	RF01686

Acido-Lenti-1	**y**	n	n	Acidobacteria, Lentisphaerae	RF01687

Actino-pnp	**Y**	Y	N	Actinomycetales	RF01688

AdoCbl-variant	**Y**	Y	Y	Marine	RF01689

*asd*	**Y**	?	?	Lactobacillales	RF01732

*atoC*	**y**	y	?	δ-Proteobacteria	RF01733

Bacillaceae-1	**Y**	n	n	Bacillaceae	RF01690

*Bacillus*-plasmid	**y**	?	n	*Bacillus*	RF01691

Bacteroid-*trp*	**y**	y	n	Bacteroidetes	RF01692

Bacteroidales-1	**Y**	?	?	Bacteroidales	RF01693

*Bacteroides*-1	**y**	?	n	*Bacteroides*	RF01694

*Bacteroides*-2	?	n	n	*Bacteroides*	

Burkholderiales-1	?	?	n	Burkholderiales	

c4 antisense RNA	**Y**	N	N	Proteobacteria, phages	RF01695

c4-a1b1	**Y**	N	N	γ-Proteobacteria, phages	

Chlorobi-1	**Y**	n	n	Chlorobi	RF01696

Chlorobi-RRM	**y**	y	n	Chlorobi	RF01697

Chloroflexi-1	**y**	?	n	*Chloroflexus aggregans*	RF01698

Clostridiales-1	**y**	n	n	Clostridiales, human gut	RF01699

COG2252	?	y	n	Pseudomonadales	

*Collinsella*-1	**y**	n	n	Actinobacteria, human gut	RF01700

*crcB*	**Y**	Y	Y	Widespread, bacteria and archaea	RF01734

Cyano-1	**y**	n	n	Cyanobacteria, marine	RF01701

Cyano-2	**Y**	n	n	Cyanobacteria, marine	RF01702

Desulfotalea-1	?	n	n	Proteobacteria	

Dictyoglomi-1	**y**	?	?	Dictyoglomi	RF01703

Downstream-peptide	**Y**	y	y	Cyanobacteria, marine	RF01704

*epsC*	**Y**	y	y	Bacillales	RF01735

*fixA*	?	y	n	*Pseudomonas*	

Flavo-1	**y**	n	n	Bacteroidetes	RF01705

*flg*-Rhizobiales	**y**	y	n	Rhizobiales	RF01736

*flpD*	**y**	?	n	Euryarchaeota	RF01737

*gabT*	**Y**	y	?	*Pseudomonas*	RF01738

Gamma-*cis*-1	?	y	n	γ-Proteobacteria	

*glnA*	**Y**	Y	y	Cyanobacteria, marine	RF01739

GUCCY-hairpin	?	?	n	Bacteroidetes, Proteobacteria	

Gut-1	**Y**	n	n	Human gut only	RF01706

*gyrA*	**y**	y	n	*Pseudomonas*	RF01740

*hopC*	**y**	Y	?	*Helicobacter*	RF01741

*icd*	?	y	n	*Pseudomonas*	

JUMPstart	**y**	Y	?	γ-Proteobacteria	RF01707

L17 downstream element	**y**	y	n	Lactobacillales, *Listeria*	RF01708

*lactis*-plasmid	**y**	?	n	Lactobacillales	RF01742

Lacto-*int*	?	?	n	Lactobacillales, phages	

Lacto-*rpoB*	**Y**	y	n	Lactobacillales	RF01709

Lacto-*usp*	**Y**	?	?	Lactobacillales	RF01710

Leu/phe leader	**Y**	Y	N	*Lactococcus lactis*	RF01743

*livK*	**y**	y	?	Pseudomonadales	RF01744

Lnt	**y**	y	?	Chlorobi	RF01711

*manA*	**Y**	Y	y	Marine, γ-Proteobacteria, cyanophage	RF01745

*Methylobacterium*-1	**Y**	n	n	*Methylobacterium*, marine	RF01712

Moco-II	**y**	Y	?	Proteobacteria	RF01713

*mraW*	**y**	y	?	Actinomycetales	RF01746

*msiK*	**Y**	Y	?	Actinobacteria	RF01747

*Nitrosococcus*-1	?	n	n	*Nitrosococcus*, Clostridia	

*nuoG*	**y**	y	?	Enterobacteriales (incl. *E. coli *K12)	RF01748

Ocean-V	**y**	n	n	Marine only	RF01714

Ocean-VI	?	?	?	Marine only	

*pan*	**Y**	Y	?	Chloroflexi, Firmicutes, δ-Proteobacteria	RF01749

*Pedo*-repair	**y**	?	n	*Pedobacter*	RF01715

*pfl*	**Y**	Y	Y	Several phyla	RF01750

*pheA*	?	y	n	Actinobacteria	

PhotoRC-I	**y**	y	n	Cyanobacteria, marine	RF01716

PhotoRC-II	**Y**	y	n	Marine, cyanophage	RF01717

*Polynucleobacter*-1	**y**	y	?	Burkholderiales, fresh water/estuary	RF01718

*potC*	**y**	y	?	Marine only	RF01751

*psaA*	**Y**	y	?	Cyanobacteria	RF01752

*psbNH*	**y**	y	n	Cyanobacteria, marine	RF01753

*Pseudomon*-1	**y**	n	n	Pseudomonadales	RF01719

*Pseudomon*-2	?	n	n	*Pseudomonas*	

*Pseudomon*-GGDEF	?	y	?	*Pseudomonas*	

*Pseudomon*-*groES*	**y**	y	?	*Pseudomonas*	RF01721

*Pseudomon*-Rho	**y**	Y	n	*Pseudomonas*	RF01720

*Pyrobac*-1	**y**	n	n	*Pyrobaculum*	RF01722

*Pyrobac*-HINT	?	y	n	*Pyrobaculum*	

*radC*	**Y**	y	?	Proteobacteria	RF01754

Rhizobiales-1	?	n	N	Rhizobiales	

Rhizobiales-2	**y**	?	n	Rhizobiales	RF01723

Rhodopirellula-1	?	y	?	Proteobacteria, Planctomycetes	

*rmf*	**Y**	y	?	Pseudomonadales	RF01755

*rne*-II	**Y**	y	N	Pseudomonadales	RF01756

SAM-Chlorobi	**y**	Y	?	Chlorobi	RF01724

SAM-I-IV-variant	**Y**	Y	Y	Several phyla, marine	RF01725

SAM-II long loops	**Y**	Y	Y	Bacteroidetes, marine	RF01726

SAM/SAH riboswitch	**Y**	Y	Y	Rhodobacterales	RF01727

*sanguinis*-hairpin	?	n	n	*Streptococcus*	

*sbcD*	**y**	?	n	Burkholderiales	RF01757

ScRE	?	y	n	*Streptococcus*	

Soil-1	?	n	n	Soil only	

*Solibacter*-1	?	n	n	*Solibacter usitatus*	

STAXI	**y**	?	n	Enterobacteriales	RF01728

*sucA*-II	**y**	y	?	Pseudomonadales	RF01758

*sucC*	**Y**	Y	?	γ-Proteobacteria	RF01759

Termite-*flg*	**Y**	y	n	Termite hind gut only	RF01729

Termite-*leu*	**y**	?	?	Termite hind gut only	RF01730

*traJ*-II	**Y**	Y	n	Proteobacteria, *Enterococcus faecium*	RF01760

Transposase-resistance	?	y	n	Several phyla	

TwoAYGGAY	**y**	n	n	Human gut, γ-Proteobacteria, Clostridiales	

*wcaG*	**Y**	y	y	Marine, cyanophage	RF01761

Whalefall-1	**Y**	n	n	Whalefall only	RF01762

*yjdF*	**Y**	Y	Y	Firmicutes	RF01764

*ykkC*-III	**y**	Y	y	Actinobacteria, δ-Proteobacteria	RF01763

Columns are as follows. "RNA?" : is this motif likely to represent a biological RNA? "Y" = certainly, "y" = probably, "?" = ambiguous, "n" = probably not, "N" = no. "*cis*-reg" : is the motif *cis*-regulatory? "switch?" : is the motif a riboswitch? Additional annotation and justification is in Additional File 2. "Taxa" : common taxon/taxa carrying this motif. Many motifs are discussed only in Additional file 1. "Rfam" : accession numbers of motifs that were submitted to the Rfam database for version 10.1. Note: consensus diagrams of some motifs were presented as supplementary data of a previous report [21] under simplified names: Acido-1 (previously ac-1), Dictyoglomi-1 (dct-1), Gut-1 (gt-1), *manA *(manA), Termite-*flg *(tf-1) and Whalefall-1 (wf-1).

## Results and discussion

### Identification and analysis of RNA structures

Promising RNA motifs predicted by our automated bioinformatics procedure were subsequently evaluated manually (see Materials and Methods). As previously reported [14], we identified promising motifs by seeking RNAs that exhibit both regions of conserved nucleotide sequence and evidence of secondary structure. Evidence for the latter characteristic involved the identification of nucleotide variation between representatives of a motif that conserves a given structure. For example, one form of covariation involves mutations to two nucleotides that preserve a Watson-Crick base pair. Assessment of covariation can be complicated, because, for example, spurious evidence of covariation is sometimes a consequence of sequence misalignments. Therefore, final covariation assessments were performed manually.

*Cis*-regulatory RNAs in bacteria are typically located in 5' UTRs. However, transcription start sites for most genes have not been experimentally established. Therefore, when a motif commonly resides upstream of coding regions, we usually assume that it resides in 5' UTRs and is a *cis*-regulatory RNA. Additional analysis of our system and our scheme for naming motifs is described in Additional File 1.

### Riboswitch candidates

Riboswitches [1,2,23] are RNAs that sense metabolites and regulate gene expression in response to changes in metabolite concentrations. Typically, they form domains within 5' UTRs of mRNAs, and their ligand binding triggers a folding change that modulates expression of the downstream gene. Therefore, good riboswitch candidates are consistently located in potential 5' UTRs. Most known riboswitches require complex secondary and tertiary structures to form tight and highly selective binding pockets for metabolite ligands. Therefore, motifs that comprise the strongest riboswitch candidates have complex secondary structures and stretches of highly conserved nucleotide positions. Motifs were analyzed manually according to these criteria.

We identified a total of 12 RNA motifs that exhibited these characteristics. Here we report the validation of a new SAM/SAH-binding RNA class, and analysis of other riboswitch candidates. Experimental validation of cyclic di-GMP-II and tetrahydrofolate riboswitches will be reported elsewhere. Details describing additional experimental validation efforts and ligands tested with other riboswitch candidates are presented in Additional File 1.

#### SAM/SAH-binding RNA

The coenzyme SAM and its reaction by-product SAH are frequently targeted ligands for riboswitches. Three structurally unrelated superfamilies [24] of SAM-binding riboswitches [25] and one SAH-binding riboswitch class [26] have been validated previously. All discriminate against SAM or SAH by orders of magnitude, despite the fact that SAM differs from SAH only by a single methyl group and associated positive charge.

**Figure 1:**
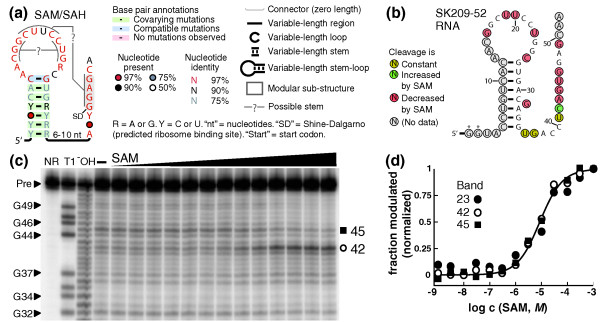
**SAM/SAH riboswitches**. **(a) **SAM/SAH motif consensus diagram. Possible additional base-pairing interactions are shown (Additional File 1). The legend applies to all other consensus diagrams in this report. **(b) **Sequence and proposed secondary structure of SK209-52 RNA. In-line probing annotations are derived from the data in **c**. Asterisks identify G residues added to improve *in vitro *transcription yield. **(c) **In-line probing gel with lanes loaded with 5' ^32^P-labeled RNAs subjected to no reaction (NR), partial digestion with RNase T1 (T1), partial digest under alkaline pH (^-^OH), in-line probing reaction without added compound (-), or in-line probing reactions with various concentrations of SAM. Selected bands in the RNase T1 partial digest lane (products of cleavage 3' of G residues) are numbered according to the nucleotide positions in **b**. Uncleaved precursor (Pre) and two internucleotide linkages whose cleavage rates are strongly affected by SAM (3' of nucleotides 42 and 45) are marked. The full gel image is provided in Additional File 1. **(d) **Plot of the normalized fraction of RNAs whose cleavage sites (linkage 23 not shown in **c **) have undergone modulation versus the concentration of SAM present during the in-line probing reaction. The curve represents an ideal one-to-one binding interaction with a *K*_D _of 8.6 μ*M*.

Our current search produced a motif, termed SAM/SAH (Figure 1a), that is found exclusively in the order Rhodobacterales of α-proteobacteria. The RNA motif is consistently found immediately upstream of *metK *genes, which encode SAM synthetase. Because known SAM-binding riboswitches are frequently upstream of *metK *genes [25], the element's gene association suggests that it may function as part of a novel SAM-sensing riboswitch class.

A SAM/SAH RNA from *Roseobacter *sp. SK209-2-6, called "SK209-52 RNA," was subjected to in-line probing [27] in the presence of various concentrations of SAM or SAH (Figure 1b,c). SK209-52 RNA appears to bind SAH with a dissociation constant (*K*_D_) of ~4.3 μ*M *and SAM with a *K*_D _of ~8.6 μ*M *(Figure 1d). Similar results were obtained with SAM/SAH RNA constructs from other species (data not shown). However, because SAM undergoes spontaneous demethylation, SAM samples contain at least some of the breakdown product SAH. Thus, apparent affinity for SAM could result from binding only of contaminating SAH [26]. However, binding assays based on equilibrium dialysis and molecular-recognition experiments indicate that SAM/SAH RNAs do bind SAM (Additional File 1).

It is interesting to note that SAM/SAH aptamers, which are the smallest of the SAM and SAH aptamer classes, presumably cannot discriminate strongly against SAH. This lack of discrimination may mean that genes associated with this RNA are purposefully regulated by either SAM or SAH. However, SAM is more abundant in cells than is SAH [28]. This fact, coupled with the frequent association of the RNA motif with *metK *gene contexts of SAM/SAH RNAs, suggests that their biologic role is to function as part of a SAM-responsive riboswitch.

#### *crcB *motif

**Figure 2:**
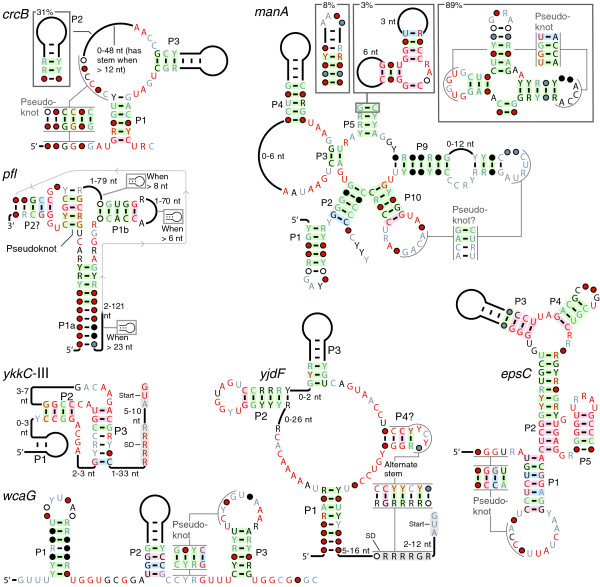
**Riboswitch candidates *crcB*, *yjdF*, *wcaG*, *manA*, *pfl*, *epsC*, and *ykkC*-III**. Annotations are as described in Figure 1a. The transcription terminators that often overlap *crcB *or *pfl *RNAs are not depicted because they are not consistent in all representatives. They are annotated in Additional File 3. Question marks signify base-paired regions ("P4?" in *yjdF*, "P2?" in *pfl*, and "pseudoknot?" in *manA*) with weaker covariation or structural conservation. The pseudoknot in the *epsC *motif was predicted by others (Wade Winkler, personal communication, 2009). A portion of this figure was adapted from the supplementary data of a previous publication [21].

The *crcB *motif (Figure 2) is detected in a wide variety of phyla in bacteria and archaea. Thus, *crcB *RNAs join only one known riboswitch class (TPP) [29], and few other classes of RNAs, that are present in more than one domain of life. The *crcB *motif consistently resides in the potential 5' UTRs of genes, including those involved in DNA repair (*mutS*), K^+^, or Cl^- ^transport, or genes encoding formate hydrogen lyase. In many cases, predicted transcription terminators overlap the conserved *crcB *motif. Therefore, if ligand binding of the putative riboswitch stabilizes the conserved structure predicted for these RNAs, higher ligand concentrations are expected to inhibit terminator stem formation and increase gene expression.

The *crcB *motif might regulate genes in response to stress conditions that can damage DNA and be mitigated by increased expression of other genes controlled by the RNAs (Additional File 1). If *crcB *RNAs are riboswitches, they presumably sense a metabolite present in organisms that is indicative of a common cellular condition in two domains of life.

#### *pfl *motif

The *pfl *motif (Figure 2) is found in four bacterial phyla. As with *crcB *RNAs, predicted transcription terminators overlap the 3' region of many *pfl *RNAs; thus, gene expression is likely increased in response to higher ligand concentrations. The genes most commonly associated with *pfl *RNAs are related to purine biosynthesis, or to synthesis of formyltetrahydrofolate (formyl-THF), which is used for purine biosynthesis. These genes include *purH*, *fhs*, *pfl*, *glyA*, and *folD*. PurH formylates AICAR by using formyl-THF as the donor. Formyl-THF can be synthesized by the product of *fhs *by using formate and THF as substrates. Formate, in turn, is produced in the reaction catalyzed by Pfl. The upregulation of Pfl to create formate for the synthesis of purines was observed previously [30]. Formyl-THF can also be produced from THF and serine by the combined action of GlyA and FolD. Thus, the five genes most commonly predicted to be regulated by *pfl *RNAs have a role in the synthesis of purines or formyl-THF. Most other genes apparently regulated by *pfl *RNAs (Additional File 3) encode enzymes that perform other steps in purine synthesis, or convert between THF or its 1-carbon adducts at least as a side effect (for example, *metH*) (Additional File 1).

#### *yjdF *motif

The *yjdF *motif (Figure 2) is found in many Firmicutes, including *Bacillus subtilis*. In most cases, it resides in potential 5' UTRs of homologues of the *yjdF *gene (Additional File 7), whose function is unknown. However, in *Streptococcus thermophilus*, a *yjdF *RNA motif is associated with an operon whose protein products synthesize nicotinamide adenine dinucleotide (NAD^+^) (Additional File 3). Also, the *S. thermophilus yjdF *RNA lacks typical *yjdF *motif consensus features downstream of and including the P4 stem. Thus, if *yjdF *RNAs are riboswitch aptamers, the *S. thermophilus *RNAs might sense a distinct compound that structurally resembles the ligand bound by other *yjdF *RNAs. Or perhaps these RNAs have an alternate solution to form a similar binding site, as is observed with some SAM riboswitches [24].

#### *manA *and *wcaG *motifs

The *manA *and *wcaG *motifs (Figure 2) are found almost exclusively in marine metagenome sequences, but are each detected in T4-like phages that infect cyanobacteria (Additional File 3). Also, two *manA *RNAs are found in γ-proteobacteria. Remarkably, many phages of cyanobacteria have incorporated genes involved in metabolism, including exopolysaccharide production and photosynthesis [31-33], and some of these cyanophages carry *manA *or *wcaG *RNAs. RNA domains corresponding to the *manA *motif are commonly located in potential 5' UTRs of genes (Additional File 3) involved in mannose or fructose metabolism, nucleotide synthesis, *ibpA *chaperones, and photosynthetic genes. Distinctively, *wcaG *RNAs typically appear to regulate genes related to production of exopolysaccharides or genes that are induced by high-light conditions. Perhaps *manA *and *wcaG *RNAs are used by phages to modify their hosts' metabolism [33], although they may also be exploited by uninfected bacteria.

#### *epsC *motif

RNA domains corresponding to the *epsC *motif (Figure 2) are found in potential 5' UTRs of genes related to exopolysaccharide (EPS) synthesis, such as *epsC *[34], in *B. subtilis *and related species. Different species use different chemical subunits in their EPS [35], which acts in processes such as biofilm formation, capsule synthesis, and sporulation [35-37]. If *epsC *RNAs are riboswitches, they might sense an intermediate in EPS synthesis that is common to all bacteria containing *epsC *RNAs. Signalling molecules also regulate EPS synthesis in some bacteria [36,38], and are therefore also candidate riboswitch ligands.

The *epsC *motif was discovered independently by another group and named EAR (W. Winkler, personal communication, 2009). This candidate has been shown to exhibit transcription antitermination activity, likely by directly interacting with protein components of the transcription elongation complex (W. Winkler, personal communication, 2009), and therefore, this RNA motif may not also function as a metabolite-binding RNA. Intriguingly, the JUMPstart sequence motif [39] is found in the 5' UTRs of genes related to polysaccharide synthesis and also is associated with modification of transcriptional elongation [40-43]. We detected a conserved stem-loop structure among JUMPstart elements (Additional File 1).

#### *ykkC*-III motif

The previously identified *ykkC *[5] and mini-*ykkC *[14] motifs are associated with genes related to those associated with *ykkC*-III, but these RNAs have distinct conserved sequence and structural features. The new-found *ykkC*-III motif (Figure 2) is in potential 5' UTRs of *emrE *and *speB *genes. *emrE *is the most common gene family associated with mini-*ykkC *and the second most common to be associated with *ykkC*, and *speB *is also associated with *ykkC *RNAs in many cases (Additional File 8). Although a perfectly conserved ACGA sequence in *ykkC*-III is similar to the less rigidly conserved ACGR terminal loops of mini-*ykkC *RNAs, the structural contexts are different (Additional File 1). All three RNA motifs have characteristics of gene-control elements that regulate similar genes, and perhaps respond to changing concentrations of the same metabolite. However, unlike mini-*ykkC*, whose small and repetitive hairpin architecture is suggestive of protein binding, both *ykkC *and *ykkC*-III exhibit more complex structural features that are suggestive of direct metabolite binding.

#### *glnA *and Downstream-peptide motifs

**Figure 3:**
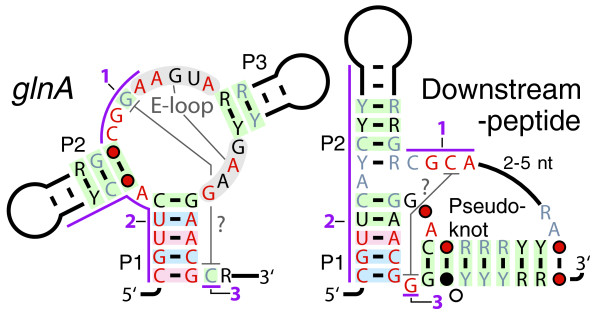
**Riboswitch candidates *glnA *and Downstream-peptide**. Annotations are as described in Figure 1a. Purple lines and numbers indicate conserved sequences or structures common to the two motifs.

The *glnA *and Downstream peptide motifs carry similar sequence and structural features (Figure 3), although the genes they are associated with are very different. Many genes presumably regulated by *glnA *RNAs are clearly involved in nitrogen metabolism, and include nitrogen regulatory protein P_II_, glutamine synthetase, glutamate synthase, and ammonium transporters. Another associated gene is PMT1479, which was the most repressed gene when *Prochlorococcus marinus *was starved for nitrogen [44]. Some *glnA *RNAs occur in tandem, which is an arrangement previously associated with more-digital gene regulation [45,46].

The Downstream-peptide motif is found in potential 5' UTRs of cyanobacterial ORFs whose products are typically 17 to 100 amino acids long and are predicted not to belong to a known protein family. We observe a pattern of synonymous mutations and insertions or deletions in multiples of three nucleotides (data not shown), supporting the prediction of a short conserved coding sequence. A previously predicted noncoding RNA called "yfr6" [47] is ~250 nucleotides in length and contains a short ORF. The 5' UTRs of these ORFs correspond to Downstream-peptide RNAs. Although only two full-length yfr6 RNAs were found, 634 Downstream-peptide RNAs were detected, suggesting that only the 5' UTR is conserved. Experiments on yfr6 showed that transcription starts ~20 nucleotides 5' to the proposed Downstream-peptide motif [47]. Also, a Downstream-peptide RNA resides in the potential 5' UTR of a gene that appears to be downregulated in response to nitrogen starvation [47]. A conserved amino acid sequence in predicted proteins associated with Downstream-peptide RNAs hints at a possible regulatory mechanism (Additional File 1). The proposed structural resemblance between *glnA *and Downstream-peptide RNAs suggests they may bind to chemically similar ligands, and previously conducted experiments suggest that both elements downregulate genes in response to nitrogen depletion.

### Cyanobacterial photosystem regulatory motifs

#### *psaA *motif

**Figure 4:**
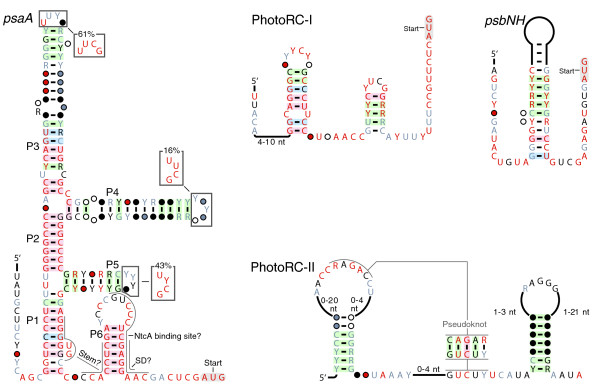
**Cyanobacterial motifs related to photosynthesis**. Annotations are as described in Figure 1a.

Representatives of the *psaA *motif (Figure 4) occur in the potential 5' UTRs of Photosystem-I *psaAB *operons in certain cyanobacteria. The motif includes three hairpins that often include UNCG tetraloops [48]. Although the regulation of *psaAB *genes in species with *psaA *RNAs has not been studied, multiple *psa *genes in *Synechocystis *sp. PCC 6803 are regulated in response to light through DNA elements that are presumably transcription factor-binding sites [49]. Photosynthetic organisms upregulate photosystem-I (*psa*) genes under low-light conditions to maximize energy output, but must reduce their expression under sustained high-light conditions, to avoid damage from free radicals [50]. *psaA *RNAs could be involved in this regulation, although we have not found this RNA element upstream of *psa *genes other than *psaAB*.

#### PhotoRC-I, PhotoRC-II, and *psbNH *motifs

Two distinct RNA structures (Figure 4) are associated with genes belonging to the photosynthetic reaction center family of proteins that are probably *psbA*. PhotoRC-I RNAs are present in known cyanobacteria and in marine environmental samples, whereas PhotoRC-II RNAs are detected only in marine samples and a cyanophage. These motifs and *psbNH *are further described in Additional File 1.

### Other motifs

#### L17 downstream element

The L17 downstream element (Additional File 6) is located downstream (within the potential 3' UTRs) of genes that encode ribosomal protein L17. In many cases, no annotated genes are located immediately downstream of the element. Although the motif might actually be transcribed in the opposite orientation, the structure as shown is more stable because it carries many G-U base pairs and GNRA tetraloops [48]. These structures would be far less stable in the corresponding RNA transcribed from the complementary DNA template. RNA molecules overlapping an L17 downstream element were recently detected by microarrays and designated SR79100 [51]. The expression of ribosomal proteins is frequently regulated by a feedback mechanism in which the protein binds an RNA structure in the 5' UTR of its mRNA, called a ribosomal leader [52]. We did not detect obvious similarity between the L17 downstream element and rRNA, although this situation is typical of ribosomal leaders [53]. Thus, the L17 downstream element could function in the 3' UTR and be part of a feedback-regulation system for L17 production. Regulation of a gene by a structured RNA domain located in the 3' UTR is highly unusual in bacteria. However, precedents include an element in a ribosomal protein operon that regulates both upstream and downstream genes [54], and regulation of upstream genes is observed in a phage [55] and proposed in *Listeria *[56].

#### *hopC *motif

The *hopC *motif (Additional File 6) is found in *Helicobacter *species in the potential 5' UTRs of *hopC/alpA *gene and co-transcribed *hopB/alpB *genes. Previous studies established that expression of the *hopCB *operon is increased in response to low pH [57]. The experimentally determined 5' UTRs of the *hopCB *operon mRNA in *H. pylori *60190 [57] contains a predicted *hopC *motif RNA. HopCB is needed for optimal binding to human epithelial cells [58] and is presumably involved in infection of the human stomach.

#### *msiK *motif

The *msiK *motif is always found in the potential 5' UTRs of *msiK *genes [59,60], which encode the ATPase subunit for ABC-type transporters of at least two complex sugars [61], and probably many more [62]. The motif comprises an 11-nucleotide bulge within a long hairpin. The 3' side of the basal pairing region includes a predicted ribosome binding site, which may be part of the regulatory mechanism. Existing data indicate that *msiK *genes are not regulated in response to changing levels of glucose [59,61], so perhaps the RNA participates in a feedback-inhibition loop by binding MsiK proteins (Additional File 1).

#### *pan *motif

The *pan *motif (Additional File 6) is found in three phyla and is present in the genetically tractable organism *B. subtilis*. Each *pan *RNA consists of a stem interrupted by two highly conserved bulged A residues. Most *pan *RNAs occur in tandem, and their simple structure and dimeric arrangement is suggestive of a dimeric protein-binding motif. The RNAs are located upstream of operons containing *panB*, *panC*, or aspartate decarboxylase genes, which are involved in synthesizing pantothenate (vitamin B_5_).

#### *rmf *motif

The *rmf *motif is found in the potential 5' UTRs of *rmf *genes in *Pseudomonas *species. These genes encode ribosome-modulation factor, which acts in the stringent response to depletion of nutrients and other stressors [63]. Because Rmf interacts with rRNA, the protein Rmf might bind to the 5' UTR of its mRNA. Alternately, because the RNA is relatively far from the *rmf *start codon, *rmf *RNAs might be noncoding RNAs that are expressed separate from the adjacent coding region.

#### SAM-Chlorobi motif

The SAM-Chlorobi motif is found in the potential 5' UTRs of operons containing all predicted *metK *and *ahcY *genes within the phylum Chlorobi. As noted earlier, *metK *encodes SAM synthetase, and in most other organisms, *metK *homologues are controlled by changing SAM concentrations that are detected by SAM-responsive riboswitches. In contrast, *ahcY *encodes *S*-adenosylhomocysteine (SAH) hydrolase, and this gene is known to be controlled by SAH-responsive riboswitches in some organisms [26]. Sequences conforming to a strong promoter sequences [64,65] imply that SAM-Chlorobi RNAs are transcribed (Additional File 1). However, preliminary analysis of several SAM-Chlorobi RNA constructs by using in-line probing did not reveal binding to SAM or SAH (Additional File 1).

#### STAXI motif

**Figure 5:**
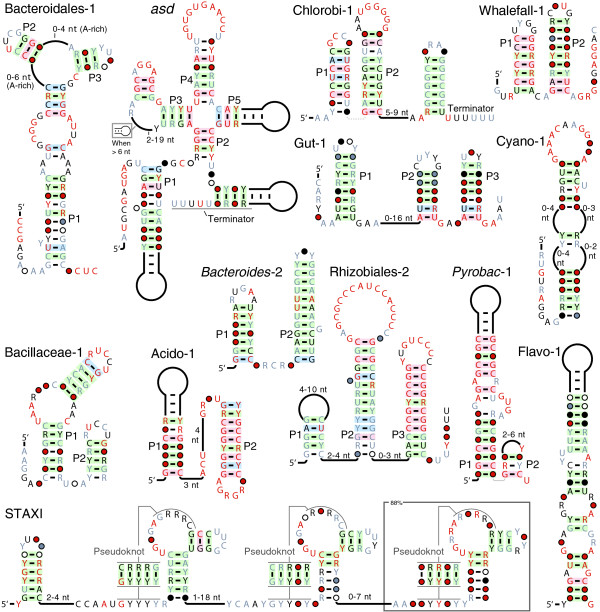
**Examples of other candidate RNAs**. Annotations are as described in Figure 1a. The Bacteroidales-1 motif has more conserved nucleotides than depicted (Additional File 6). A portion of this figure was adapted from the supplementary data of a previous publication [21].

The Ssbp, Topoisomerase, Antirestriction, XerDC Integrase (STAXI) motif is composed mainly of a pseudoknot structure repeated at least two and usually three times (Figure 5). Tandem STAXI motifs are frequently near to genes that encode proteins that bind or manipulate DNA, including single-stranded DNA-binding proteins (Ssbp), integrases and topoisomerases, or antirestriction proteins. Also, they are occasionally located near c4 antisense RNAs [66] (Additional File 1). Because genes proximal to STAXI representatives encode DNA-manipulation proteins, it is possible that the STAXI motif represents a single-stranded DNA that adopts a local structure when duplex DNA is separated, as occurs during DNA replication, repair, or when bound by some proteins. However, the UUCG tetraloops that frequently occur within the STAXI motif repeats are known to stabilize RNA, whereas the corresponding TTCG are not particularly stabilizing for DNA structures [67]. This suggests that the motif is more likely to serve its function as an RNA structure.

#### Noncoding RNAs

Several motifs that are most likely expressed as noncoding RNAs unaffiliated with mRNAs also were identified (Figure 5, Table 1). Gut-1 and whalefall-1 RNAs are found only in environmental sequences, and Bacteroides-2 is found in only one sequenced organism (Additional File 1). Thus, bacteria from multiple environmental samples express noncoding RNAs that are not represented in any cultivated organisms whose genomes have been sequenced [68,21]. Similarly, Acido-1 and Dictyoglomi-1 RNAs are found in phyla in which few genome sequences are available. Further observations regarding all noncoding RNA candidates can be found in Additional File 1.

#### Expansion of representatives of previously characterized structured RNAs

Existing homology search methods for RNAs frequently fail to detect representatives of known RNA classes whose sequences have diverged extensively. However, our computational pipeline occasionally reveals examples of such RNAs. Details regarding RNA representatives that expand the collection of 6S RNAs, AdoCbl riboswitches, SAM-II riboswitches, and SAM-I/SAM-IV riboswitches are provided in Additional File 1. The RNAs that expand the collection of the superfamily of SAM-I [69] and SAM-IV [24] riboswitches (Additional File 6) are typically found in metagenome sequences. These variant SAM-I/SAM-IV riboswitches share many of the structural features of both families (Additional File 6), but lack an internal loop in the P2 stem, which is present in SAM-I/SAM-IV riboswitches (Additional File 1).

## Conclusions

Numerous structured RNA candidates have been identified in the genomic and metagenomic DNA sequence data from bacteria and archaea. The predicted RNAs exhibit a great diversity of conserved sequences and structural features, and their genomic locations are indicative of a wide variety of mechanisms of action (for example, *cis *vs. *trans*) and putative biologic roles. Our findings suggest that the bacterial and archaeal domains of life will continue to be a rich source of novel structured RNAs.

Although some of the RNAs identified perform the same function as previously validated RNA classes (for example, 6S-Flavo RNA, SAM/SAH riboswitches), the vast majority of the predicted RNA motifs are likely to perform novel functions. Given that many of these RNAs are specific to certain lineages or uncultivated environmental samples, technologies that more rapidly make available DNA sequence information from additional lineages of bacteria and archaea are likely to accelerate the discovery of more classes of structured RNAs. This discovery rate might also be increased by improvements in computational analysis methods. These findings should yield a diverse collection of structured noncoding RNAs that will reveal a more complete understanding of the roles that RNAs perform in microbial cells.

## Materials and methods

### DNA sequence sources and gene annotations

The microbial subsets of RefSeq [70] version 25 or 32 (Additional file 9) were searched, along with metagenome sequences from acid mine drainage [71], soil and whale fall [72], human gut [73,74], mouse gut [75], gutless sea worms [76], sludge [77], Global Ocean Survey scaffolds [78,79], other marine sequences [80], and termite hindgut [81]. Locations and identities of protein-coding genes were derived from RefSeq or IMG/M [82] annotations, or from "predicted proteins" [83] in Global Ocean Survey sequences. However, genes in some sequences [74,80,81] were predicted by using MetaGene (dated Oct. 12, 2006) with default parameters [84]. Conserved protein domains were annotated by using the Conserved Domain Database version 2.08 [85].

Annotations for tRNAs and rRNAs were derived from the sources noted earlier, or were predicted by using tRNAscan-SE [86] run in bacterial mode. To detect additional rRNAs, annotated rRNAs whose descriptions read "ribosomal RNA" or "#S rRNA" (# represents any number) were used in WU-BLAST queries with command-line flags -hspsepQmax = 4000 -E 1e-20 -W 8 [13]. Other RNAs were detected with Rfam [22] and WU-BLAST, as described previously [13]. We also used published alignments of riboswitches [87] as queries with RAVENNA global-mode searches [19,20], selecting hits manually based primarily on E-values.

### Automated motif identification

To reduce false positives in sequence comparisons, the pipeline was run separately on related taxa or metagenome sources (Additional File 9). For each run, InterGenic Regions (IGRs) of at least 30 nucleotides were extracted between protein-coding, tRNA and rRNA genes.

To generate clusters, an early version of a recently described algorithm was used [16]. Specifically, IGRs were compared by using nucleotide NCBI BLAST [17] version 2.2.17 and parameters -W 7 -G 2 -E 2 -q -2 -m 8. Self-matches were ignored. BLAST scores below a parameter S (see later) were considered insignificant and were ignored. Each BLAST match defines two "nodes," corresponding to the matching sequences. Nodes that overlap by at least five nucleotides are merged, along with their BLAST homologies. A cluster consists of all nodes that have direct or indirect (transitive) BLAST matches. Closely related sequences that span multiple distinct elements in an entire IGR can lead to spurious node merges. Therefore, homologies with BLAST scores >100 are ignored.

If a node's length in nucleotides is L, and L < 500, then the node is extended on either side by (500-L)/2 nucleotides, but is constrained to remain within the original IGR. CMfinder can easily tolerate nodes of 500 nucleotides. When L > 1,000, nodes are shrunk by (L - 1,000)/2 nucleotides around the center. The L > 1,000 case is extremely rare. Only clusters with at least three members were reported.

For each pipeline run, we tried a range of values for the parameter S = 35, 40, ..., 85, and determined how many known RNAs were detected with each value. Based on these data, a set of S values was selected manually, and the union of clusters arising from each S was used as input to CMfinder [18]. CMfinder was used to predict motifs exactly as before [13]. Automated homology searches were then performed as described [13], except that covariance model scores used the null3 model [88]. Motifs were scored by using a previously established method [13], and by using tools comprising Pfold [89] to infer a phylogenetic tree, and then running pscore [90]. We also automatically eliminated motifs that had no covarying base-pair positions, that had an average G+C content <24%, that had representatives whose nucleotide coordinates overlapped the reverse-complements of other representatives on average by ≥30% of their nucleotides, or that had fewer than six positions that were ≥97% conserved (when sequences were weighted with the GSC algorithm). Source code is provided (Additional File 10).

### Manual analysis of motifs

The manual analysis of each candidate RNA motif proceeded essentially as described previously [14]. For motifs that were likely to be *cis*-regulatory, we routinely searched for articles referencing the locus tags of apparently regulated genes, by using Google Scholar [91]. We also used mutual information analysis [87] to predict additional base-pairing interactions. Motifs less likely to represent structured RNAs were rejected by using previously established criteria [14]. In motif consensus diagrams, covariation and levels of conservation were calculated using earlier protocols [14], but ≤10% noncanonic pairs were tolerated in alignment columns that correspond to conserved base pairs. RNAs were drawn with R2R (Z.W., R.R.B., unpublished software) and Adobe Illustrator.

### Assessing the novelty of motifs

To determine whether the predicted RNA structures were reported previously, we searched the Rfam database [22], and various articles not yet incorporated into Rfam that performed detailed analysis or experiments on new-found candidate RNAs [10,47,92-110]. Although some raw predictions of a previous report [9] overlap some of our RNA motifs (Additional File 11), these raw predictions have never been subjected to detailed evaluation. Additionally, extensive Google searches [111] for genes associated with *crcB *RNAs revealed that one of the 358 raw predictions of conserved elements on the RibEx web server [112] overlaps several of the *crcB *RNAs we found. This conserved element was called RLE0038 and was not previously subjected to detailed evaluation. We have not determined whether other coinciding predictions are present on this web server because its data are not available in a machine-readable format.

### In-line probing experiments

RNA constructs were prepared by *in vitro *RNA transcription by using T7 RNA polymerase and the appropriate DNA templates that were created by overlap extension of synthetic DNA oligonucleotides by using SuperScript II reverse transcriptase (Invitrogen), as instructed by the manufacturer. RNA transcripts were purified by using denaturing (8 *M *urea) polyacrylamide gel electrophoresis (PAGE). RNAs were eluted from the gel, dephosphorylated by using alkaline phosphatase, and 5' radiolabeled with [γ-^32^P] by using methods reported previously [26]. 5' ^32^P-labeled fragments resulting from in-line probing reactions were subjected to denaturing PAGE, and were imaged and analyzed as previously described [26].

### Equilibrium dialysis experiments

Equilibrium dialysis experiments were conducted in a Dispo-Equilibrium Biodialyzer (The Nest Group, Inc., Southboro, MA, USA), which comprises two chambers (A and B) separated by a 5,000-kDa MW cut-off membrane. Chamber A was loaded with 20 μl solution of 500 n*M *^3^H-SAM, and Chamber B was loaded with 20 μ*M *specified RNA in a buffer containing 50 m*M *MOPS (pH 7.2 at 20°C), 20 m*M *MgCl_2_, and 500 m*M *KCl. The chambers were equilibrated at 25°C for 10 h before a 3-μl aliquot was removed from each chamber. Radioactivity of the aliquots was measured with a liquid scintillation counter. Each experiment was repeated 3 times, and average B/A values and standard deviations were calculated.

## Authors' contributions

ZW and RRB conceived of the study, ZW prepared bioinformatics scripts, and RRB supervised the study. ZW, JY, KC, RM, and JXW analyzed motif predictions to infer conserved RNA structures. JXW, ZW, and JB tested riboswitch candidates by using in-line probing. JXW and JB conducted SAM/SAH experiments. ZW and RRB wrote the manuscript, with assistance from all authors.

## Supplementary Material

Additional file 1 **Supplementary results and discussion**. Additional analysis of motifs, including those not discussed in the manuscript, and in-line probing experiments on riboswitch candidates. Click here for file

Additional file 2 **Summary and evaluation of all motifs**. Table 1, with summary of supporting evidence, and numbers of representatives of each motif. Click here for file

Additional file 3 **Taxa of motif representatives, genes flanking representatives and annotated multiple-sequence alignments**. For each motif, this file shows the taxa of each motif representative, depicts genes flanking these representatives and describes conserved domains that the genes encode. Also, a multiple-sequence alignment is provided for each motif, and includes secondary structure and other annotations. Click here for file

Additional file 4 **Raw text alignment files, including annotation**. Raw alignments of RNAs, including annotations (for example, predicted transcription terminators, flanking sequences) in "Stockholm" text format. The alignment format and appropriate viewing programs are discussed on Wikipedia [113]. The Stockholm files can be retrieved from the .tar.gz archive file by using programs such as WinZip (Windows), StuffIt Expander (Mac), or tar/gzip (UNIX). Click here for file

Additional file 5 **Raw text alignment files, just the motifs**. Raw alignments of RNA motifs with minimal annotation and no flanking sequences, in "Stockholm" text format. The Stockholm files can be retrieved from the .tar.gz archive file by using programs such as WinZip (Windows), StuffIt Expander (Mac), or tar/gzip (UNIX). Click here for file

Additional file 6 **Consensus diagrams of all motifs**. Consensus diagrams depicting all motifs in high resolution. Click here for file

Additional file 7 **Alignment of YjdF proteins**. Multiple-sequence alignment of proteins predicted to be homologous to YjdF of *Bacillus subtilis*. Click here for file

Additional file 8 **Genes associated with *ykkC*, mini-*ykkC *and *ykkC*-III RNAs**. The frequencies with which various gene families are associated with *ykkC*, mini-*ykkC *or *ykkC*-III RNAs are listed. Click here for file

Additional file 9 **Partitioning of genomes and metagenomes**. Describes how genomes and metagenomes were divided into pipeline runs. Click here for file

Additional file 10 **Source code implemented as part of this project**. Source code files and a README.pdf file are provided to assist in detailed understanding of the methods. The files can be retrieved from the .tar.gz archive file, as described for Additional file 4. Click here for file

Additional file 11 **Overlap with previous raw predictions**. Overlaps of our RNA motifs with raw predictions of a prior study [9]. Tab-delimited text file. Click here for file
