# Supplementary material for: Comparative genomics reveals 104 candidate structured RNAs from bacteria, archaea, and their metagenomes
Source: Genome Biol. 2010 Mar 15;11(3):R31. doi: 10.1186/gb-2010-11-3-r31 (PMC2864571; doi:10.1186/gb-2010-11-3-r31)
Supplement: Additional file 1 — Supplementary results and discussion. Additional analysis of motifs, including those not discussed in the manuscript, and in-line probing experiments on riboswitch candidates. [file gb-2010-11-3-r31-S1.DOC]

Additional File 1 for:

Comparative genomics reveals 104 candidate structured RNAs from bacteria, archaea and their metagenomes

Zasha Weinberg^1§^, Joy X. Wang^1^, Jarrod Bogue^2,4^, Jingying Yang^2^, Keith Corbino^1^, Ryan H. Moy^2,5^, Ronald R. Breaker^1,2,3§^

^1^Howard Hughes Medical Institute, Yale University, P.O. Box 208103, New Haven, CT 06520-8103, USA.

^2^Department of Molecular, Cellular and Developmental Biology, Yale University, P.O. Box 208103, New Haven, CT 06520-8103, USA.

^3^Department of Molecular Biophysics and Biochemistry, Yale University, P.O. Box 208103, New Haven, CT 06520-8103, USA.

Present address: ^4^Department of Biology, University of Rochester, Rochester, NY 14627, ^5^School of Medicine, University of Pennsylvania, Philadelphia, PA 19104, USA.

^§^Corresponding authors

**Contents**

[General comments 5](#__RefHeading___Toc248925708)

[Applicability of the computational pipeline to find *cis*-regulatory RNAs 5](#__RefHeading___Toc248925709)

[Naming candidate RNA motifs 6](#__RefHeading___Toc248925710)

[Experimental analysis of SAM binding by SAM/SAH-binding RNAs 6](#__RefHeading___Toc248925711)

[Additional discussion of candidate RNA motifs 8](#__RefHeading___Toc248925712)

[*aceE* motif 8](#__RefHeading___Toc248925713)

[Acido-1 motif 8](#__RefHeading___Toc248925714)

[Acido-Lenti-1 motif 8](#__RefHeading___Toc248925715)

[Actino-*pnp* motif 8](#__RefHeading___Toc248925716)

[*asd* motif 9](#__RefHeading___Toc248925717)

[*atoC* motif 9](#__RefHeading___Toc248925718)

[Bacillaceae-1 motif 9](#__RefHeading___Toc248925719)

[*Bacillus*-plasmid motif 10](#__RefHeading___Toc248925720)

[Bacteroid-*trp* leader motif 10](#__RefHeading___Toc248925721)

[Bacteroidales-1 motif 10](#__RefHeading___Toc248925722)

[Bacteroides-1 motif 10](#__RefHeading___Toc248925723)

[Bacteroides-2 motif 10](#__RefHeading___Toc248925724)

[Burkholderiales-1 motif 10](#__RefHeading___Toc248925725)

[c4 antisense RNA motif 11](#__RefHeading___Toc248925726)

[c4 antisense RNA a1b1 motif 11](#__RefHeading___Toc248925727)

[Chlorobi-1 motif 11](#__RefHeading___Toc248925728)

[Chlorobi-RRM motif 11](#__RefHeading___Toc248925729)

[Chloroflexi-1 motif 12](#__RefHeading___Toc248925730)

[Clostridiales-1 motif 12](#__RefHeading___Toc248925731)

[*Collinsella*-1 motif 12](#__RefHeading___Toc248925732)

[*crcB* motif 12](#__RefHeading___Toc248925733)

[Cyano-1 motif 12](#__RefHeading___Toc248925734)

[Cyano-2 motif 12](#__RefHeading___Toc248925735)

[Desulfotalea-1 motif 13](#__RefHeading___Toc248925736)

[Dictyoglomi-1 motif 13](#__RefHeading___Toc248925737)

[Downstream-peptide motif 13](#__RefHeading___Toc248925738)

[Flavo-1 motif 13](#__RefHeading___Toc248925739)

[*fixA* motif 14](#__RefHeading___Toc248925740)

[*gabT* motif 14](#__RefHeading___Toc248925741)

[Gamma-*cis*-1 motif 15](#__RefHeading___Toc248925742)

[GUCCY hairpin motif 15](#__RefHeading___Toc248925743)

[Gut-1 motif 15](#__RefHeading___Toc248925744)

[*gyrA* motif 15](#__RefHeading___Toc248925745)

[*hopC* motif 15](#__RefHeading___Toc248925746)

[*icd* motif 15](#__RefHeading___Toc248925747)

[JUMPstart sequence motif 16](#__RefHeading___Toc248925748)

[Lacto-*int* motif 16](#__RefHeading___Toc248925749)

[Lacto-plasmid motif 16](#__RefHeading___Toc248925750)

[Lacto-*rpoB* motif 16](#__RefHeading___Toc248925751)

[*lactis*-plasmid motif 17](#__RefHeading___Toc248925752)

[Leu/phe-leader motif 17](#__RefHeading___Toc248925753)

[Lnt motif 17](#__RefHeading___Toc248925754)

[Methylobacterium-1 RNA motif 18](#__RefHeading___Toc248925755)

[Moco-II motif 18](#__RefHeading___Toc248925756)

[*mraW* motif 18](#__RefHeading___Toc248925757)

[*msiK* motif 18](#__RefHeading___Toc248925758)

[*nuoG* motif 19](#__RefHeading___Toc248925759)

[Ocean-V motif 19](#__RefHeading___Toc248925760)

[Ocean-VI motif 19](#__RefHeading___Toc248925761)

[*pan* motif 20](#__RefHeading___Toc248925762)

[*Pedo*-repair motif 20](#__RefHeading___Toc248925763)

[*pfl* motif 20](#__RefHeading___Toc248925764)

[*pheA* motif 21](#__RefHeading___Toc248925765)

[PhotoRC-I and PhotoRC-II motifs 21](#__RefHeading___Toc248925766)

[Polynucleobacter-1 motif 21](#__RefHeading___Toc248925767)

[*potC* motif 21](#__RefHeading___Toc248925768)

[*psaA* motif 21](#__RefHeading___Toc248925769)

[*psbNH* motif 21](#__RefHeading___Toc248925770)

[Pseudomon-1 motif 22](#__RefHeading___Toc248925771)

[Pseudomon-2 motif 22](#__RefHeading___Toc248925772)

[*Pseudomon*-GGDEF motif 22](#__RefHeading___Toc248925773)

[Pseudomon-*groES* motif 22](#__RefHeading___Toc248925774)

[*Pseudomon*-Rho motif 22](#__RefHeading___Toc248925775)

[*Pyrobac*-1 motif 22](#__RefHeading___Toc248925776)

[*Pyrobac*-HINT motif 23](#__RefHeading___Toc248925777)

[*radC* motif 23](#__RefHeading___Toc248925778)

[Rhizobiales-1 motif 23](#__RefHeading___Toc248925779)

[Rhodopirellula-1 motif 23](#__RefHeading___Toc248925780)

[*rmf* motif 23](#__RefHeading___Toc248925781)

[*rne*-II motif 24](#__RefHeading___Toc248925782)

[SAM-Chlorobi motif 24](#__RefHeading___Toc248925783)

[SAM-I/SAM-IV variant riboswitch motif 24](#__RefHeading___Toc248925784)

[SAM/SAH-binding RNAs 24](#__RefHeading___Toc248925785)

[*sanguinis*-hairpin motif 24](#__RefHeading___Toc248925786)

[*sbcD* motif 25](#__RefHeading___Toc248925787)

[ScRE (*Streptococcus* Regulatory Element) motif 25](#__RefHeading___Toc248925788)

[Soil-1 motif 25](#__RefHeading___Toc248925789)

[*sucA*-II motif 25](#__RefHeading___Toc248925790)

[*sucC* motif 25](#__RefHeading___Toc248925791)

[Solibacter-1 motif 25](#__RefHeading___Toc248925792)

[Termite-*flg* motif 26](#__RefHeading___Toc248925793)

[Termite-*leu* motif 26](#__RefHeading___Toc248925794)

[*traJ*-II motif 26](#__RefHeading___Toc248925795)

[Transposase-resistance motif 26](#__RefHeading___Toc248925796)

[TwoAYGGAY motif 27](#__RefHeading___Toc248925797)

[*wcaG* motif 27](#__RefHeading___Toc248925798)

[Whalefall-1 motif 27](#__RefHeading___Toc248925799)

[*yjdF* motif 27](#__RefHeading___Toc248925800)

[*ykkC*-III motif 28](#__RefHeading___Toc248925801)

[Additions to previously characterized RNA classes 28](#__RefHeading___Toc248925802)

[6S RNA 28](#__RefHeading___Toc248925803)

[AdoCbl and SAM-II riboswitches 29](#__RefHeading___Toc248925804)

[Ligand-binding experiments using in-line probing 29](#__RefHeading___Toc248925805)

[In-line probing experiments with a *pfl* RNA 29](#__RefHeading___Toc248925806)

[In-line probing experiments with *yjdF* RNA 30](#__RefHeading___Toc248925807)

[In-line probing experiments with SAM-Chlorobi RNA 30](#__RefHeading___Toc248925808)

[In-line probing experiments with *pan* RNA 31](#__RefHeading___Toc248925809)

[In-line probing experiments with *msiK* RNA 31](#__RefHeading___Toc248925810)

[In-line probing experiments with *gabT* RNA 32](#__RefHeading___Toc248925811)

[In-line probing experiments with *rmf* RNA 32](#__RefHeading___Toc248925812)

[In-line probing experiments with Downstream peptide RNA 33](#__RefHeading___Toc248925813)

# General comments

## Applicability of the computational pipeline to find *cis*-regulatory RNAs

The previous version of our pipeline aligned the potential 5′ UTRs of homologous protein-coding genes [13, 14]. This pipeline was thus designed to detect RNA motifs that are frequently in the potential 5′ UTRs of homologous genes. We call these “gene-associated” motifs. By contrast, the new pipeline compares (by nucleotide BLAST) the sequences of IGRs without regard for the type of protein-coding gene residing nearby. The new pipeline is thus directed at finding RNA motifs that are not gene-associated, i.e., are “gene-independent” motifs. Using this new pipeline, we did indeed find many gene-independent motifs, but we additionally found many gene-associated motifs, e.g., the *msiK* motif. It may seem surprising that gene-associated motifs like *msiK* were not detected by the previous pipeline, given that the previous pipeline was designed to find such motifs. The following factors probably contribute to the increase in motifs discovered by the new pipeline, including gene-associated motifs:

1. Newly released genome sequence data facilitates the discovery of motifs that are relatively uncommon. For example, the *msiK* motif is derived from a very compelling alignment produced by our newer pipeline, whereas the previous pipeline produced an unpromising prediction. This is most likely due to the fact that several additional genomes of Actinobacteria are now available, which provided more *msiK* motif representatives and resulted in a more convincing consensus sequence and secondary structure model. Similarly, the SAM-Chlorobi motif exhibits covariation only with the 11 Chlorobi genomes now available. We also observed that the older pipeline failed to detect SAM-III riboswitches [114], because these riboswitches often contain long and variable-length loops that make identification of the surrounding stem difficult for CMfinder. The pipeline now easily finds SAM-III riboswitches because many genome sequences are now available that carry SAM-III riboswitches containing short loops.
2. In some instances, too many UTRs of a given gene family are available and only a few of these carry the motif. For example, the previous pipeline originally identified SAM-IV riboswitches [24] based on 3 UTRs out of 54 UTRs of the COG0520 family sequenced from in Actinobacteria at the time of our analysis. Thus, most input data in this sequence cluster did not contain the motif. In contrast, the sequence clustering method in our current pipeline will likely partition the three SAM-IV RNAs into a different cluster from the other COG0520 UTRs, which reduces spurious sequences in the cluster. It should be noted, however, that one drawback to BLAST-based sequence clustering is that the accuracy of BLAST searches accuracy may be limited. Our frequent decision in the present work to group bacteria at the level of order, rather than the more-broad phylum or class, also might help to reduce spurious sequences in clusters.
3. The use of environmental sequences helped to find RNAs that are not well represented in organisms whose genomes have been fully sequenced. For example, representatives of SAM-I/IV riboswitches are present in RefSeq, but these few representatives are diluted among unrelated phyla, making their discovery using comparative sequence analysis unlikely. Fortunately, SAM-I/IV riboswitches are common in environmental sequences. A pipeline independent of protein-coding genes is helpful for the analysis of environmental sequences, since gene annotation is difficult when only fragmentary sequences are available.
4. Some protein coding regions are poorly annotated, and so clustering of IGRs based on gene homology is hindered. For example, the *yjdF* motif is almost always upstream of homologous *yjdF* genes, but these poorly annotated genes are not presented as a conserved domain in the Conserved Domain Database. Therefore, in the context of our previous pipeline, most *yjdF* motif representatives could not have been identified as residing upstream of homologous genes.

## Naming candidate RNA motifs

Relatively little is known about most of our new-found motifs, but we believe it is useful to give them a mnemonic name that reflects some current knowledge of the RNA, its source, or its associated genes. Thus, motifs present only in metagenome data are named after the environment from which they were identified, e.g., “whalefall-1 motif”. Similarly, some motifs are named after their exclusive or predominant taxon, e.g., “Bacteroidales-1 motif”. *Cis*-regulatory RNA candidates that appear to regulate a variety of heterologous gene families, are named after a single example gene, e.g., “*crcB* motif”. When the precise biological roles of these RNAs are better understood, we recommend that the class be renamed to more accurately reflect their functions. For example, the SAM/SAH-binding RNA identified in this work was originally named the *metK*-Rhodobacter motif, before its binding to ligands was confirmed and its riboswitch function was hypothesized based on its gene association and its proximity to possible expression platforms.

# Experimental analysis of SAM binding by SAM/SAH-binding RNAs

Note: the full gel image from in-line probing experiments described in the main text is available here as Fig. 1 (see end of this document).

As noted in the main text, it is difficult to draw definitive conclusions regarding SAM binding by aptamers that also tightly bind SAH. Since SAM can undergo spontaneous demethylation, all SAM samples will contain at least some SAH, and this contaminating by-product will increase with aging of the sample. Therefore, the *K*_D_ reported for SAM could largely reflect the binding of contaminating SAH.

To address this issue, we performed two experiments that indicate that SAM/SAH RNAs do bind SAM. First, we performed in-line probing assays using several close analogs of SAM (Figure 2a; end of this document), and all but one of these analogs are apparently bound by SK209-52 RNA with *K*_D_ values within 10 fold of that measured for SAH. This suggests that SAM/SAH RNAs cannot strongly discriminate against compounds like SAM that carry additional chemical groups on the thioether linkage of SAH. Therefore, these data imply that SAM/SAH RNAs likely bind SAM with an affinity that is biologically relevant.

Our second experiment used a strategy based on equilibrium dialysis that we previously applied to the analysis of SAH riboswitches [26]. For these experiments, SAM was obtained with radioactive ^3^H in its methyl group. When this ^3^H-SAM degrades spontaneously, it will lose the methyl group, resulting in non-radioactive SAH. In our experiments, two chambers called “A” and “B” are separated by a membrane with a 5,000 kDa molecular weight cutoff. Small molecules like SAM and SAH can pass through this membrane, but RNA molecules cannot. ^3^H-SAM is placed in chamber A, while SK209-52 RNA is placed in chamber B. If SK209-52 RNA binds SAM, more ^3^H-SAM will be found in chamber B than in chamber A, because of its association with the RNA. The relative amounts of radioactivity between chambers A and B will thus be indicative of SAM binding, but will not reflect SAH binding because the SAH in this experiment is not radioactive. As positive controls, we additionally performed this experiment with known SAM-binding RNAs called 156 *metA* [6] and 62 *metY* [115]. Finally, when a point mutation called “A48U” was applied to SK209-52 RNA, the mutated RNA exhibited a drastically reduced ability to bind SAM when compared to the wild-type RNA.

Our results show that significantly more radioactivity is present in chamber B when the known SAM-binding RNAs or when SK209-52 RNA is applied to chamber B (Fig 2b; end of this document). Therefore, SK209-52 RNA is binding ^3^H-SAM. As expected, when the A48U mutant is applied to chamber B, the amounts of radioactivity in the two chambers are roughly equal, showing that this mutant has a greatly reduced ability to bind SAM.

Additionally, we conducted equilibrium dialysis experiments with SK209-52 RNA and ^3^H-SAM in the presence of competing compounds that were not radiolabeled (Figure 3; end of this document). As expected, the B/A ratio was significantly above 1 when the experiment was performed without any competitor, or with methionine as a competitor. Methionine did not appear to bind in previous in-line probing experiments (data not shown), and no previously established SAM-binding riboswitch has detectable affinity for this amino acid, although methionine is a component of SAM. However, when either SAH or the sulfoxide derivative of SAM was used as competitors, the B/A ratio was much closer to a value of 1. Thus, SAH and the sulfoxide derivative of SAM—but not methionine—compete with SAM for the binding of SK209-52 RNA. These results are consistent with the conclusion that SK209-52 RNA binds SAM, SAH and the sulfoxide derivative of SAM.

# Additional discussion of candidate RNA motifs

In the text below, we comment on each motif identified in the current study. Notable characteristics derived by examining the sequence and structural features, or derived by literature analysis of the associated genes is presented. All motif consensus diagrams are available in Additional File 6.

## *aceE* motif

The *aceE* motif is found in the potential 5′ UTRs of *aceE* genes in *Pseudomonas* species. The *aceE* gene encodes pyruvate dehydrogenase, which can use pyruvate to synthesize coenzyme A that then participates in the citric acid cycle. Growth of *P. aeruginosa* in anaerobic conditions with nitrite as the sole electron acceptor leads to lower levels of *aceE* expression. However, this condition also leads to lower expression of other genes related to the citric acid cycle [116] that do not have predicted *aceE* RNAs. On the other hand, expression of *aceE* in a *P. aeruginosa* strain isolated from a cystic fibrosis patient differed from that of a strain isolated from a burn victim, yet other citric acid cycle genes were not differently regulated in this case [117].

## Acido-1 motif

The Acido-1 motif consists of two hairpins, with high sequence conservation in the linker between the hairpins, and in the terminal loop of the 3′ hairpin. Given its lack of association with genes, the motif appears to act in *trans*. Although only four sequences are predicted to have the Acido-1 motif, there is significant covariation. The motif appears to be restricted to Acidobacteria.

## Acido-Lenti-1 motif

The Acido-Lenti-1 motif is found in the phyla Acidobacteria and Lentisphaerae. In Lentisphaerae, it is sometimes located near group II introns.

## Actino-*pnp* motif

Actino-*pnp* motif representatives are predicted only in Actinobacteria. They are consistently in the potential 5′ UTRs of genes annotated as encoding a 3′-5′ exoribonuclease, such as polynucleotide phosphorylase or RNase PH. RNA leader structures have been reported upstream of polynucleotide phosphorylase genes in enterobacteria such as *E. coli* where they reduce gene expression when enzyme levels are high [118]. Since the enterobacterial *pnp* leader RNA does not appear to be structurally related to the Actino-*pnp* motif, we hypothesize that the Actino-*pnp* is a distinct structural solution to regulate expression of the enzyme.

## *asd* motif

The *asd* motif is often, but not always, in potential 5′ UTRs of genes, which suggests a possible *cis*-regulatory role. However, in two cases, non-homologous genes are downstream of an *asd* RNA, in the wrong orientation for the RNA to be in their 5′ UTRs (Additional File 3). Also, downstream of the motif in *Streptococus mutans* is a conserved transcription terminator, followed by a strong promoter that is, in turn, followed by the *asd* gene [119]. In *S. mutans*, no significant modulation in gene expression was observed in response to changing levels of amino acids for whose synthesis Asd participates (i.e., lysine, threonine, and methionine) [119]. In *Streptococcus pneumoniae* D39, a CodY binding site was predicted in between an *asd* RNA and the downstream *asd* gene [120]. CodY binds double-stranded DNA when there are high concentrations of branched-chain amino acids (BCAAs, i.e., leucine, isoleucine or valine). This binding event typically represses genes involved in synthesizing BCAAs, and repression was demonstrated using microarrays, protein expression and DNA binding. Thus, this *asd* gene is regulated in response to BCAAs, in a manner unrelated to the upstream *asd* RNA. If *asd* RNAs are *cis*-regulatory elements, they presumably sense a signal other than high BCAA concentrations.

Given these characteristics, the *asd* motif is more likely to correspond to a non-coding RNA at least in some instances. This possibility is consistent with the fact that there is a transcription terminator downstream of it, and we do not observe potential base pairing that might serve as an antiterminator that would respond to metabolite binding or other signals. Interestingly, genes upstream of *asd* RNAs are always transcribed in the same direction as the RNA, and the distance between these upstream genes and the *asd* RNA is always within about 200 base pairs, although it is not clear whether this observation is biologically relevant, or merely a coincidence.

RNA molecules overlapping an *asd* RNA were recently detected by microarrays and designated as SR914400 [51]. The RNAs were also detected by northern hybridization experiments as a roughly 170-nucleotide transcript. The abundance of this transcript was essentially constant in the conditions tested, which included four points in exponential or stationary phase. The transcription start site (TSS) was determined using 5′ RACE experiments. Interestingly, the TSS exactly corresponds to 5′ boundary that we determined by analysis of nucleotide conservation (Additional File 6).

## *atoC* motif

Motif representatives are in potential 5′ UTRs of genes encoding domains with oxidoreductase activity, response regulators containing DNA-binding domains, or FolK (folate synthesis).

## Bacillaceae-1 motif

This RNA likely functions in *trans* and is found in many gene contexts. In several cases is adjacent to a ribosomal RNA operon. The terminal loops of its two hairpins both have the consensus RUCCU, which is suggestive of binding to a homodimeric protein.

## *Bacillus*-plasmid motif

The *Bacillus*-plasmid motif occurs in species within the genera *Bacillus* and *Lactobacillus* species, and is usually found in plasmids. In a notable exception, the motif is found upstream of the *ydcS* gene in *B. subtilis.* The motif consists of a single hairpin where the 5′ and 3′ regions of the terminal loop are highly conserved. The interior part of the terminal loop is not highly conserved and can be as long as 38 nucleotides. *Bacillus*-plasmid candidate RNAs are typically upstream of genes annotated as *repA* or mobilization element genes, although the gene is typically 200-300 nucleotides 3′ of the RNA structure. Nonetheless, this arrangement is suggestive of a *cis*-antisense RNA that might regulate plasmid copy number [121], even though the motif does not resemble a known RNA of this type.

## Bacteroid-*trp* leader motif

This motif apparently controls *trpB* and *trpE* genes in Bacteroidetes, which are involved in tryptophan synthesis. The motif contains a region of two or more conserved tryptophan codons (UGG), and therefore is presumably a peptide leader that detects low levels of tryptophan by attenuation [122]. Although tryptophan attenuation leaders are known in Proteobacteria, none have been reported in Bacteroidetes. We did not create a consensus diagram of the bacteroid-trp leader, since it is a loosely conserved hairpin (Additional File 3).

## Bacteroidales-1 motif

Upstream sequences that conform to consensus promoters for *Bacteroides* [64] allow us to predict an approximate transcription start site for this RNA. These promoters are depicted in the Bacteroidales-1 alignment in Additional File 3.

## Bacteroides-1 motif

The *Bacteroides*-1 motif may act in *trans*. However, it is typically downstream of genes that are associated with synthesis of exopolysaccharides. Therefore the RNA candidate might regulate expression of the upstream genes by acting within their 3′ UTRs.

## Bacteroides-2 motif

This RNA is found almost exclusively in human gut bacterial sequences, except for the one species *Bacteroides capillosus* ATCC 29799. The genome sequence of this species was released in 2007, and metagenomics data was essential for identification by our bioinformatics pipeline.

## Burkholderiales-1 motif

The Burkholderiales-1 motif is present in some species in the order Burkholderiales. It is sometimes present in many copies in the same genome (e.g., 33 copies in *Polaromonas* sp. JS666). The genes immediately downstream of Burkholderiales-1 RNAs usually are oriented in the opposite direction. This arrangement would be expected if the Burkholderiales-1 motif were the reverse complement of a rho-independent transcription terminator. However, the motif’s reverse complement lacks the expected polyuridine stretch.

## c4 antisense RNA motif

The c4 antisense RNA was previously identified in P1 and P7 phages of *E. coli* [66]. We identified a motif within Pseudomonadales, and established many homologs in other Proteobacteria, as well as several phages, including phage P1. Our predicted structure is supported by covariation, and is consistent with the structure that was proposed based on the RNA present in P1 phage [66]. Our alignment indicates that c4 antisense RNA is found in the genomes of many bacteria, presumably from phage integration events. We also observe that the terminal loop of P2 is often the stable tetraloop GNRA, UNCG or CUUG: out of 492 unique C4 antisense RNA P2 sequences, 122 terminate in GNRA, 233 in UNCG and 19 in CUUG. The other sequences present in the terminal loop of P2 might also have high stability. In several cases, the 3′ half of the P1 stem overlaps the 5′ half of a predicted transcription terminator hairpin. It is possible that c4 antisense RNA sometimes functions as a ­*cis*-regulatory element, although these predicted transcription terminators may simply function constitutively.

## c4 antisense RNA a1b1 motif

The c4 antisense RNA (described above) is believed to regulate *ant* genes by binding to complementary RNA sites, one of which overlaps a potential ribosome-binding site [66]. c4 antisense RNA has two regions, called a′ and b′, that can base pair with sites designated a1, b1 and a2, b2. The a2, b2 sites are upstream of the *ant* gene and downstream of the c4 RNA. The a1, b1 sites are upstream of the c4 antisense RNA itself. It was proposed that the a1, b1 sites might compete with the a2, b2 sites for binding c4 RNA, and thereby free the a2, b2 sites, in turn allowing *ant* expression [66].

We found a motif that encompasses the a1 site, and is immediate 5′ to the b1 site. The motif consists of two hairpins whose structure is well supported by covariation. A third stem is sometimes found that connects a region several nucleotides 5′ to P1 with a sequence overlapping the 3′ part of P2 (not shown). Although this stem exhibits covariation when it is found, it is absent from many sequences, including those in DNA isolated from purified phage particles. No conserved secondary structure was previously proposed for the a1, b1 sites, but conserved structures are known for other targets of antisense RNAs, such as the *traJ*-I RNA mentioned below (see *traJ*-II motif).

## Chlorobi-1 motif

Chlorobi-1 RNAs are found only in the phylum Chlorobi and consist of two hairpins, with most nucleotide conservation found in their terminal loops. All known Chlorobi-1 RNAs have predicted transcription terminators downstream.

## Chlorobi-RRM motif

The Chlorobi-RRM motif is consistently in the potential 5′ UTRs of genes predicted to encode an RNA-binding protein, which suggests that it serves an auto-regulatory role for the gene.

## Chloroflexi-1 motif

The Chloroflexi-1 motif is present in three copies in *Chloroflexus aggregans*, a species in the phylum Chloroflexi. Although there is good covariation, the few sequences available make it difficult to assess the significance of the covariation. The fact that the three representatives are located near to one another on the chromosome suggests that the motif might be associated with a repetitive element.

## Clostridiales-1 motif

The Clostridiales-1 motif is a large four-stem structure that is very common in DNA sequences from microbes in the human gut, and is present in several bacteria in the order Clostridiales. The structure seems to be less conserved when predicted homologs are incorporated using sensitive “local” mode covariance model searches [123], but even within the sequences that are similar to one another, there is significant covariation.

## *Collinsella*-1 motif

There are only six representatives identified for the *Collinsella*-1 motif. Five are from environmental samples of the human gut, and the remainder is found in *Collinsella aerofaciens* ATCC 25986. The P3 stem is well supported by covariation, however the P1 and P2 stems might not be conserved.

## *crcB* motif

The structural characteristics and genetic distribution of this motif are strongly suggestive of riboswitch aptamer function. When considering ligand candidates, we considered two stress conditions under which cells up-regulate some of the genes presumably controlled by *crcB* RNAs. However, these conditions do not appear to account for all genes associated with the riboswitch candidate. Acidic pH stress typically induces K^+^ or Na^+^ transporters [124], though unfortunately also many other genes not associated with *crcB* RNAs. The response to oxidative stress involves upregulating two genes associated with *crcB* RNAs, *iscU* and GTP cyclohydrolase [125], but again others not relevant to *crcB* RNAs.

## Cyano-1 motif

Some Cyano-1 RNAs in *Prochlorococcus marinus* MED4 (RefSeq accession NC_005072) are near to noncoding RNAs detected previously, though no conserved secondary structure was proposed for these regions [96]. Specifically, Yfr10 is 200 nt distant in one case, Yfr12 is 80 nt distant in one case, Yfr18 is 170 nt distant in one case, and a Cyano-1 RNA overlaps ~60 nt of the 3′ end of the roughly 250-nt Yfr15.

## Cyano-2 motif

The Cyano-2 RNA consists of two structured regions separated by an internal region that has no apparent conserved structure. The sequence GCGA within terminal loops is common, and may form GNRA tetraloops in the subset of Cyano-2 RNAs in which the three immediate-flanking nucleotides both upstream and downstream of the tetraloop can form Watson-Crick base pairs. The second structured region has a highly conserved bulge. It is possible that the motif represents two distinct structures that are functionally associated. Cyano-2 RNAs usually occur in regions without any predicted genes or RNAs for the upstream 1 Kb, which is uncommon among known RNAs.

## Desulfotalea-1 motif

The Desulfotalea-1 motif has characteristics of a *trans*-acting RNA and, in many instances, is located near rRNA operons.

## Dictyoglomi-1 motif

The Dictyoglomi-1 motif is present in two copies in each of the two species sequenced from the phylum Dictyoglomi. The RNA is consistently in the potential 5′ UTRs of genes, but since it is far from the genes, it is unclear whether it represents a *cis*-regulatory element. The downstream genes are annotated as enzymes that hydrolyze glycosidic bonds. Dictyoglomi-1 RNAs conserve four E-loops, which are often associated with intermolecular interactions [126]. The structure prediction of the Dictyoglomi-1 motif is compromised by the lack of availability of diverged homologs. Detection of diverged homologs may reveal covariation within a longer structure.

## Downstream-peptide motif

The gene P9301_07111 in *Prochlorococcus marinus* str. MIT 9301 is apparently regulated by a Downstream-peptide RNA and is among the 20 most highly expressed genes in this organism, although nitrogen regulation was not tested in this case [127].The peptides encoded downstream of Downstream-peptide RNAs are difficult to align, and might not all be homologous. However, out of 429 ORFs that were not truncated by short sequencing reads, 360 encode a peptide with the amino acid motif YRG and 207 have the longer LTYRG. The YRG motif was suggested by previous analysis of ORFs associated with the yfr6 motif [47], which corresponds to a previously predicted noncoding RNA that overlaps Downstream-peptide RNAs (see main text). It is known that P_II_ proteins (also called GlnB) contain a conserved YRGxxY motif and are involved in regulating genes in nitrogen metabolism [128]. 355 out of 429 Downstream-peptides contain this YRGxxY arrangement. In most bacteria, the second Y is uridylated, though in Cyanobacteria, a serine residue after the G is phosphorylated. It is possible that the peptides associated with Downstream-peptide RNAs function in a way that is somehow related to the phosphorylation of P_II_ proteins, perhaps as a decoy.

A distinct ncRNA called yfr14 was detected that overlaps the reverse complement of yfr6 [96]. Therefore, these yfr14 RNAs in turn also overlap predicted Downstream-peptide RNAs.

## Flavo-1 motif

All but a few Flavo-1 RNAs are found in Flavobacteria, with others found in the same phylum, Bacteroidetes, or the related Spirochaetes.

## *fixA* motif

The *fixA* motif is consistently located in potential 5′ UTRs of *fixA* genes in certain *Pseudomonas* species. The *fixA* gene and the downstream *fixB* gene encode an enzyme required for carnitine reduction under anaerobic conditions [129].

## *gabT* motif

The *gabT* motif is found in the potential 5′ UTRs of *gabT* genes in the genus *Pseudomonas*. The motif is located downstream of *gabD* genes. Thus, the gene organization is always *gabD*, then the RNA, then *gabT*. In microarray experiments in various *Pseudomonas* species, *gabD* and *gabT* genes that are associated with *gabT* RNAs were shown to be induced by agmitine, putrescine, GABA [130], lysine, delta-aminovalerate [131] and iron depletion [132]. In all cases, both *gabD* and *gabT* genes were induced by approximately the same amount, suggesting that they form an operon. In the case of agmatine and putrescine, the region upstream of *gabD*—which does not contain the predicted RNA—was fused to a *lacZ* reporter, and yielded approximately the same induction as the genes. So, the regulation in response to the above metabolites is probably caused by an element in the region upstream of *gabD*.

GabT is annotated as a transaminase, and GabD as a dehydrogenase, but they appear to operate on multiple substrates in multiple pathways. In the catabolism of agmatine, putrescine and other metabolites, GabT catalyzes the transamination of gamma-aminobutanoate (GABA) to form succinate semialdehyde, which is then dehydrogenated to succinate by GabD, where it feeds into the citric acid cycle [130]. GabD was shown to catalyze the expected reaction *in vitro*, and both genes are induced by GABA, the substrate of GabT. However, the genes also play a role in lysine degradation. In this pathway, the gene product annotated as GabT transaminates delta-aminovalerate, which is dehydrogenated to glutarate by the annotated GabD. In support of this proposed activity, the combined proteins catalyze the expected two-step reaction *in vitro* [133], and both are induced by delta-aminovalerate [131], their starting metabolite.

If *gabT* RNAs are *cis*-regulatory elements, they are presumably regulating *gabT* in a manner independent of *gabD*. In most *gabT* RNAs, a second hairpin is located 3′ of the primary hairpin. This stem appears to overlap the Shine-Dalgarno sequence of the downstream *gabT* genes, although this part of the stem does have a few non-canonical pairs in some sequences. In two cases, this second hairpin is absent, and the apparent Shine-Dalgarno sequence is located six nucleotides 3′ of the primary hairpin. This arrangement suggests possible mechanisms by which the *gabT* gene could be regulated. Note that all *gabT* RNAs are upstream of *gabT* genes, so both *gabT* RNAs with and without the second hairpin should affect gene expression in the same direction (up or down) under similar changes in cellular conditions. Thus, for example, if the second hairpin sequesters the ribosome binding site given high concentrations of an effector molecule, the *gabT* RNAs lacking the second hairpin should also somehow sequester the ribosome binding site under these conditions.

## Gamma-*cis*-1 motif

The Gamma-*cis*-1 motif is found in a variety of γ-proteobacteria. The motif as depicted (Additional File 6) is a three-stem junction, but the pairing in the P2 stem is often weak. Overall, although there is some evidence of covariation among Gamma-*cis*-1 RNAs, it is not clear whether these sequences correspond to structured RNAs.

## GUCCY hairpin motif

The GUCCY hairpin is a short hairpin flanked by the consensus sequences GUC and CY. Due to its small size, there is a high risk of false positives in homology searches. Therefore, we were conservative in adding hits as homologs. Also, the difficulty in confidently assigning homologs makes it more difficult to conclude that the motif represents a conserved RNA. Assuming that this motif is genuine, we observe that there is a possible overrepresentation of genes that are classified as COG2827 nearby. COG2827 genes encode endonucleases containing a URI domain.

## Gut-1 motif

The Gut-1 motif is detected only in environmental sequences from the human gut, and not in any sequenced organism.

## *gyrA* motif

The *gyrA* motif consists of two hairpins that are generally supported by covariation, and is present in the order Pseudomonadales. The motif is always found in the potential 5′ UTRs of *gyrA* genes, and therefore we presume that it is a regulator of these genes. However, *gyrA* has been regarded as a housekeeping gene whose expression is constant in many conditions [134]. The *gyrA* gene encodes a subunit of DNA gyrase. Mutations in this gene are commonly associated with ciprofloxacin resistance in *Pseudomonas* [135]. The *gyrA* motif is also sometimes present upstream of *dnaJ* genes, which encode chaperones.

## *hopC* motif

The method by which *hopCB* transcript abundance is regulated is unknown, but it was speculated that a homopolymeric tract of 13 thymidines might be involved [57]. This tract is located upstream of the transcription start site, and does not overlap the *hopC* motif.

## *icd* motif

The *icd* motif is found in Pseudomondales, in the potential 5′ UTRs of *icd* genes, which encode isocitrate dehydrogenase. This arrangement suggests that it is a *cis*-regulatory element. However, the modest covariation makes it ambiguous as to whether the *icd* motif is a genuine RNA.

## JUMPstart sequence motif

The JUMPstart sequence is a conserved 39 bp element upstream of operons whose protein products are involved in the synthesis of polysaccharides [39]. Experiments on the promoter region of the Escherichia coli O7-specific lipopolysaccharide gene cluster confirmed that the conserved JUMPstart sequence is in the 5′ UTR of the mRNA [40]. We find a stem-loop structure that is conserved in many JUMPstart sequences (Additional File 6). No conserved RNA structures were previously reported in JUMPstart sequences.

A major feature of the JUMPstart sequence is the ops (operon polarity suppressor) element, which has the consensus GGCGGUAG [41]. The ops element enhances transcription of downstream genes—especially distal genes of the operon—when the protein factor RfaH is present [40-43]. Some JUMPstart representatives have an additional partial ops sequence. This partial ops sequences has the consensus GGUAG and overlaps the stem 5′-side and the loop. Deletion of either ops sequence reduced the RfaH-mediated transcription enhancement [40].

There are a diversity of JUMPstart sequences containing the stem loop structure, including most studied JUMPstart sequences. However, a sequence called “hly (pHly152)” contains a validated ops element [43], but lacks stems in the most typical location (Additional File 6). It is possible that there is more flexibility in the sequence and structural features that define the motif than we have allowed, for example by allowing more distance between the stem and the major ops element. Alternately, the stem-loop structure might function independently of RfaH-mediated transcription enhancement.

## Lacto-*int* motif

The Lacto-*int* motif is found upstream of phage integrase genes, though not always in their potential 5′ UTRs. It is present in purified phages and in bacterial genomes, where it is presumably associated with prophage sequences. The motif consists of two hairpins, of which the first is supported by covariation while the second is ambiguous. It is possible that the motif is actually an inverted repeat that forms in DNA to facilitate integration or excision.

## Lacto-plasmid motif

Lacto-plasmid RNAs are typically, but not always, located on plasmids, and are apparently restricted to Lactobacillales. In addition to their location on plasmids, they are sometimes present in apparent prophages.

## Lacto-*rpoB* motif

The Lacto­-*rpoB* motif is a hairpin with a highly conserved loop found in the order Lactobacillales. It is in the potential 5′ UTRs of *rpoB* genes, which encode the β subunit of RNA polymerase.

## *lactis*-plasmid motif

*Lactis*-plasmid representatives are located on plasmids in bacteria in the order Lactobacillales, mostly *Lactococcus lactis*. The candidate RNAs are typically located nearby to *repB* genes (though not necessarily in the potential 5′ UTR), although this might simply reflect the limited size of the plasmids. *repB* genes are involved in the replication of plasmids. Like the *Bacillus*-plasmid motif, *lactis*-plasmid RNAs might regulate plasmid copy number [121]. However, according to Rfam [22], many of the plasmids containing a *lactis*-plasmid RNA also contain predicted ctRNA-pND324 RNAs (Rfam accession RF00238) [136], so the *lactis*-plasmid RNAs likely perform another function.

## Leu/phe-leader motif

Detected only in the species *Lactococcus lactis*, the leu/phe-leader motif is supported by substantial covariation, and includes an ORF encoding a short peptide. Leu/phe-leader RNAs are consistently in the potential 5′ UTRs of genes. When the gene is *leuB* or *leuC*, the peptide includes are run of three leucine residues. A leucine peptide leader has already been identified in *L. lactis*, where low concentrations of leucine lead to stalling during translation and ultimately affect transcriptional attenuation [137]. Other leucine peptide leaders were previously identified upstream of a predicted amino acid transporter. We detected two additional homologous RNAs that had a run of three phenylalanine residues instead of the leucine residues. While one of these is upstream of a hypothetical protein, the other is upstream of *aroH*, which is predicted to encode 3-deoxy-7-phosphoheptulonate synthase, which catalyzes an early step in the synthesis of phenylalanine, tyrosine and tryptophan. We presume that this step is regulated via phenylalanine levels. All leu/phe-leader RNAs we identify have predicted transcription terminators where the 5′ part of the terminator stem overlaps the 3′ part of the P4 stem in the leader motif.

## Lnt motif

The Lnt motif is found in Chlorobi, where it is in the potential 5′ UTRs of genes predicted to encode apolipoprotein *N*-acyltransferases. The RNA structure consists of a single six-bp stem that is modestly supported by covariation. The terminal loop for this stem is not well conserved, but the region 3′ to it has significant sequence conservation. One representative of the Lnt motif has a G-U base pair, which on the reverse complement would be A-C. G-U wobble pairs are more energetically favorable, and therefore help identify the proper orientation for putative RNA motifs. This fact argues that the Lnt motif is more likely to be predicted on the correct strand. However, on the reverse strand, the motif is very close to the predicted start codon of bacteriochlorophyll A genes.

## Methylobacterium-1 RNA motif

This motif is largely found in marine metagenome sequences, although it is also present in *Methylobacterium* sp. 4-46, a kind of α-proteobacteria. The motif consists of three hairpins.

## Moco-II motif

The previously discovered Moco RNA element is a candidate riboswitch that is associated with genes involved in biosynthesis and utilization of molybdenum cofactor (Moco) and tungsten cofactor [14, 138]. The newly found Moco-II motif is also associated with Moco-related genes, including a molybdenum-binding domain (MoeA) and nitrate reductase. However, only 8 representatives of the Moco-II motif are known. Seven representatives are in δ-proteobacteria, with one possible diverged example in the β-proteobacteria division. The Moco-II motif consists of a hairpin with a conserved internal loop, and the hairpin is typically adjacent to a transcription terminator, suggesting a possible expression platform. The structure is supported by modest covariation, and by the motif’s presence upstream of genes that are not homologous, but are functionally related via Moco. The simple structure suggests that the motif might not be a riboswitch, but this cannot easily be tested because Moco is unstable.

## *mraW* motif

The *mraW* motif is found in a wide variety of Actinobacteria such as *Mycobacterium*. It is a hairpin with three moderately conserved stems, and poorly conserved internal loops. The terminal loop has a highly conserved CUUCCCC sequence. Motif representatives are always in front of a predicted *mraW* gene, and appear to control an operon with a highly conserved series of genes: *mraW*, a hypothetical membrane protein and *ftsI*, typically followed by one or more *mur* genes. *ftsI* and *mur* genes are known to be involved in peptidoglycan synthesis [139], so presumably the *mraW* motif is involved in the regulation of this process.

## *msiK* motif

An *msiK* null mutant was identified as *S. lividans* 10-164 [59]. Strain 10-164 grows poorly on cellobiose, maltose and other sugars, but its growth on glucose is similar to wild type [61]. It also imports glucose at wild-type levels, but has a reduced ability to import other sugars [59]. In wild-type *S. lividans* or *S. reticuli* cells, concentrations of the MsiK protein are highest when cells are grown on cellobiose, lower for other sugars, and very low when cells are grown on glucose. In contrast, *S. lividans* 10-164 expresses MsiK at very high levels for all sugars tested, including glucose [61].

One hypothesis for the role of *msiK* RNAs is that they directly or indirectly sense glucose levels. In this model, the presence of this fundamental sugar would imply that the import of other sugars is not necessary. However, the above experimental results imply that glucose is imported into strain 10-164 cells, but does not repress MsiK expression. One possibility is that *msiK* RNAs sense a small molecule that indicates sufficient levels of some sugars, but whose concentrations are not increased by glucose. Another hypothesis is that when MsiK protein levels are sufficient to supply an ATPase domain to the various ABC sugar importers, excess MsiK binds the *msiK* RNA in its 5′ UTR and thereby represses further MsiK expression. In this model, the mutated MsiK in strain 10-164 is unable to bind to the RNA, leading to constitutive expression. It has been hypothesized for a different ATPase that it might repress its expression only in the ATP-bound state, as this state is most likely when no substrate is being transported [140], so this might explain why the 10-164 mutation hinders both ATPase activity and the proposed RNA-binding function. A related model is that MsiK binds to another, unknown protein, which in turn binds to the *msiK* RNA. Other hypotheses are, of course, also possible (e.g., the RNA may be a binding site for a receptor protein that senses a change in cellular conditions).

## *nuoG* motif

This motif is found in enterobacteria upstream of *nuoG* genes, which encode a subunit of ubiquinone reductase. The downstream genes also encode subunits of this enzyme and presumably belong to the same operon. Since the motif is very small, there is a risk that homologs were not detected that would reveal that the structure is not conserved, as these homologs might have insignificant E-values in homology searches. The motif is present in most sequenced enterobacteria including the genus *Escherichia*, but not the closely related *Salmonella*. When we inspected the region upstream of a predicted *nuoG* gene in *Salmonella typhimurium* LT2 (an arbitrarily selected organism), we found sequences that loosely match the *nuoG* RNA motif, but could not fold into the consensus structure. However, again, since the motif is very small, it is unclear whether sequence-only matches in *Salmonella* are significant. It is possible that the *Salmonella* sequence is unrelated to the *nuoG* motif. Regardless, within the *nuoG* RNAs we do predict (Additional file 3), we see considerable covariation, despite sequence and length constraints that would reduce the possibility of spurious base pairing. Thus, it is ambiguous whether the *nuoG* motif represents an RNA structure.

## Ocean-V motif

The Ocean-V motif is found in only three sequences from marine environmental DNA samples. Although it is difficult to confidently assess its assignment as a structured RNA, even among these three sequences there is some covariation. The Ocean-V motif is not detected in any sequenced organism.

## Ocean-VI motif

The Ocean-VI motif is found frequently in marine environmental sequences, but is not detected in any known sequenced organism. The putative stems are highly conserved, and as a result there is only modest covariation. Ocean-VI RNAs are sometimes located downstream of non-homologous genes involved in methionine metabolism (metA, metK), but the upstream gene is often in the opposite orientation, so it is not clear that there is any functional association with methionine.

## *pan* motif

Although most *pan* RNAs occur in tandem pairs, those in δ-proteobacteria typically occur singly (Additional File 3). Note that there might be a technical bias in favor of pan RNAs containing two hairpins, since they are easier to find in homology searches.

## *Pedo*-repair motif

The *Pedo*-repair motif is found in five instances in *Pedobacter* sp. BAL39, and in no other available sequence. The *Pedo*-repair motif is a three-stem junction that is followed by an additional hairpin, which might be a rho-independent transcription terminator. There are additional stems that might be pseudoknots or stems involved in alternate structures. The motif is well supported by covariation, but the fact that it is present in only one species and only five sequences are available makes us reluctant to declare that it is certain to be a structured RNA. The motif is in the potential 5′ UTRs of operons that contain a *radC* gene, which is annotated as a DNA repair protein, or a *mcrC* gene, part of a predicted methyl-dependent restriction system.

## *pfl* motif

The main text noted that *pfl* RNAs are usually associated with genes involved in the synthesis of purines or that catalyze conversions between THF and its one-carbon adducts. On the basis of a previously published metabolism diagram [141], we found that most genes associated with *pfl* RNAs are involved in these metabolic processes (Fig. 4; see end of this document).

The *pfl* riboswitch candidate has been tested for ligand binding with a number of compounds (see later in this document), but the most promising candidate ligands seemed to be AICAR and PRPP. A build-up of AICAR could indicate insufficient levels of formyl-THF, without which formylation of AICAR cannot proceed. Many genes regulated by *pfl* RNAs could help to synthesize formyl-THF, as noted in the main text. High AICAR concentrations were a consequence of formyl-THF starvation in *Salmonella typhimurium* [142], although this phenomenon was not observed in *E. coli* [143]. Since *pfl* RNAs are often present in an organism that is closely related to an organism lacking *pfl* RNAs, this RNA distribution could be consistent with a scenario in which closely related organisms differ in whether they produce high levels of AICAR in response to folate stress.

Alternately, high levels of PRPP is apparently an indicator of purine starvation, since the *B. subtilis*-type PurR repressor detects purine levels by sensing PRPP [144]. Note that the *B. subtilis*-type PurR has a distinct mechanism and is not homologous to the PurR protein found in *E. coli*, although their biological roles are analogous. It was hypothesized that PRPP might be a good indicator because excess adenine is phosphorylated and PRPP synthetase is inhibited by ADP, so high levels of adenine should lead to low levels of PRPP [144]. However, as noted in the last section of this document, our experiments with these candidate ligands did not reveal evidence of binding.

## *pheA* motif

The *pheA* motif is usually located upstream of *pheA* genes, which encode chorismate mutase. In cases where no annotated *pheA* gene is present, it is possible that the small ORF corresponding to *pheA* genes was missed.

## PhotoRC-I and PhotoRC-II motifs

The genes associated with the PhotoRC-I motif in Synechococcus species are typically annotated as *psbA* genes. The *psbA* genes associated with these PhotoRC RNA motifs have not been studied, but *psbA* genes in related species have been studied. For example, multiple *psbA* paralogs are found *S. elongatus* PCC 7942 and are regulated transcriptionally and post-transcriptionally [27]. Just as *psbA* genes are observed in cyanophages [8], a PhotoRC-II RNA is found upstream of a *psbA* gene in a cyanophage. Presumably the phage gene is regulated in the same way as for host-encoded *psbA* genes in this case. Indeed, since PhotoRC-II RNAs were found only in metagenome or phage sequences, it is possible that all PhotoRC-II RNAs detected were derived from phages or prophages.

## Polynucleobacter-1 motif

The *Polynucleobacter*-1 motif is found in marine environmental samples, but is also detected in *Polynucleobacter* sp. QLW-P1DMWA-1. The 3′ half of the motif is not always detected, but the 5′ part is well conserved among the examples found. Most *Polynucleobacter*-1 candidate RNAs are downstream of genes classified into the family GOS11034 [83], and with possible homology to locus PSSM2_218 in cyanophage P-SSM2. However, we did not detect *Polynucleobacter*-1 representatives in any sequenced purified phage.

## *potC* motif

The *potC* motif is located in the potential 5′ UTRs of genes predicted to encode transporters or peroxiredoxins. The motif is detected in marine metagenome sequences only.

## *psaA* motif

Most highly conserved nucleotides in this structure are involved in base pairing. In contrast, most conserved positions in riboswitches do not reside in extended Watson-Crick base-paired structures. DNA corresponding to the motif might be bound by NtcA, a protein involved in nitrogen regulation that might also play a role in photosynthesis [145].

## *psbNH* motif

This motif is consistently found between *psbN* and *psbH* genes. Since the motif and its reverse complement are equally plausible, it is unclear which of these genes is regulated if the motif is a *cis*-acting regulatory RNA.

## Pseudomon-1 motif

The *Pseudomon*-1 motif is present in most species of *Pseudomonas*. It is consistently downstream of DNA polymerase I genes, and conceivably in their 3′ UTRs. It is usually, but not always, upstream of genes predicted to encode GTPases. However, these genes are in the opposite orientation to the *Pseudomon*-1 RNAs.

## Pseudomon-2 motif

The *Pseudomon*-2 motif has no apparent gene associations, so it probably corresponds to a *trans*-acting non-coding RNA. Although the alignment is supported by some covariation, the structure is not overall strongly conserved and therefore may not represent a structured RNA.

## *Pseudomon*-GGDEF motif

The *Pseudomon*-GGDEF motif is confined to *Pseudomonas syringae*, where it resides 5′ of genes predicted to encode cyclic di-GMP synthases. The previously identified cyclic di-GMP riboswitch is sometimes present upstream of cyclic di-GMP synthases. However, the sequences exhibiting the *Pseudomon*-GGDEF motif are closely related, and so it is difficult to evaluate the conservation of structure, or sequence identities, and therefore whether the motif is likely to encode a riboswitch. One stem is supported by covariation, but there are also a few instances of non-canonical base pairs. Therefore, it is not entirely clear that the motif corresponds to a structured RNA. Because of this, the consensus diagram is not given, but an alignment is available (Additional File 3).

## Pseudomon-*groES* motif

The *groES* and *groEL* operon is involved in the heat shock response in many bacteria. In *Pseudomonas aeruginosa*, experiments showed that transcription of this operon starts at one of two sites, termed P1 and P2 [146]. P1 is located at the 5′ end of the Pseudomon-*groES* RNA, while P2 is located inside the RNA motif (Additional File 6). Transcripts starting at P1 and P2 are both increased at roughly the same levels during heat shock [146]. Therefore, the RNA likely does not participate in this regulation. However, P1-initiated transcripts, which contain full-length RNA, might undergo additional regulation that is mediated by the RNA.

## *Pseudomon*-Rho motif

The *Pseudomon*-Rho motif consists of two hairpins with some sequence conservation that are consistently upstream of the gene encoding the Rho protein. Since the Rho protein interacts with RNA, it is possible that the RNA motif is part of an autoregulatory circuit to maintain appropriate levels of the Rho protein.

## *Pyrobac*-1 motif

The *Pyrobac*-1 motif is found in archaea in the genus *Pyrobaculum*. Given its lack of a gene association, we presume that it corresponds to a *trans*-acting RNA. Although many small nucleolar RNAs (snoRNAs) have been identified in archaea, *Pyrobac*-1 RNAs do not share typical features of either C/D box or H/ACA box snoRNAs.

## *Pyrobac*-HINT motif

The *Pyrobac*-HINT motif has only four known representatives, one for each of the four sequenced species in the genus *Pyrobaculum*. All four representatives are immediately upstream of a HINT protein (domain “cd01277”), which contains a histidine triad motif [147].

## *radC* motif

The *radC* motif is consistently in the potential 5′ UTRs of genes encoding proteins that operate on DNA, such as *radC* DNA repair proteins, integrases, methyltransferases that might operate on DNA and an anti-restriction protein. The most common gene is annotated as *radC*. The *radC* gene was initially thought to be involved in DNA repair, but the key mutation was later shown to be located in a different gene [148, 149]. However, while the function of *radC* is currently unknown, DNA repair is broadly related to the other functions associated with *radC* RNAs. Although the RNAs are associated with integrases, no *radC* RNA was detected in any sequenced purified phage.

## Rhizobiales-1 motif

The Rhizobiales-1 motif is present in many species of α-proteobacteria, especially those in the order Rhizobiales. It is commonly present in many copies per genome, as many as 92 in *Nitrobacter hamburgensis* X14, but 40 copies is a typical number. The motif consists of a hairpin with some conserved sequence features.

## Rhodopirellula-1 motif

The *Rhodopirellula*-1 motif is a hairpin with characteristic bulges, and sequence conservation surrounding its base. The terminal loop varies widely in size, and some long variants exist that do not appear to have a stable structure. The stem itself exhibits significant covariation, but has some non-canonical base pairs. Since many of these seem to be A-C pairs, it is possible that the true RNA may be the reverse complement of the motif, although that orientation also has several A-C pairs. *Rhodopirellula*-1 RNAs are generally in 5′ regulatory configurations to genes that are often short and hypothetical. In many cases where the motif appears not to be located 5′ of a coding region, it is possible that an undetected short hypothetical gene is actually present. The motif occurs in a few phyla, but is overwhelmingly the most dominant in Planctomycetes. Its name derives from the fact that it has 36 predicted instances in *Rhodopirellula baltica* SH 1. These occurrences tend to cluster together in the genome, although they are located at least ~500 nucleotides apart. *Rhodopirellula*-1 RNAs are also present in other species of Planctomycetes, some Proteobacteria and other assorted bacteria.

## *rmf* motif

NCBI GEO queries [150] revealed that the *rmf* gene in *Pseudomonas aeruginosa* (locus PA3049) is differentially regulated by azithromycin exposure [151] and by co-culturing with human airway epithelial cells [152]. The *rmf* RNA motif might play a role in this regulation.

## *rne*-II motif

The *rne*-II motif is consistently in the potential 5′ UTRs of RNase E genes. It is present in species of the family Pseudomonadaceae. A *cis*-regulatory RNA is known that is in the 5′ UTRs of RNase E genes in enterobacteria (e.g., *E. coli*) [153]. The enterobacterial motif is a complex structure that is a substrate for RNase E. Cleavage of the RNA by RNase E leads to reduced gene expression. We presume that the *rne*-II motif performs a similar function. We do not detect any similarity in sequence or structure to the previously identified element, other than the general observation that both structures have many stems.

## SAM-Chlorobi motif

As noted in the main text, sequences conforming to strong promoters are found upstream of all predicted SAM-Chlorobi RNAs, suggesting that the RNAs are transcribed. These promoter sequences were validated in Bacteroidetes [64, 65], which is a phylum that is related to the phylum Chlorobi [154], in which SAM-Chlorobi RNAs are found. Therefore, these conserved promoter sequences probably do, in fact, facilitate transcription of SAM-Chlorobi RNAs. These putative promoter sequences are marked in the SAM-Chlorobi motif sequence alignment in Additional File 3.

## SAM-I/SAM-IV variant riboswitch motif

SAM-I and SAM-IV riboswitches share features in their ligand-binding core, although they have several distinctions in their overall architecture [24]. One commonality is a pseudoknot formed by the tip of P2 binding to the junction 3′ to P3. SAM-I riboswitches have a kink turn that facilitates formation of this pseudoknot [155]. SAM-IV riboswitches have an internal loop with a distinct sequence that might also create a turn in P2. However, most of the new-found SAM-I/SAM-IV variant RNAs entirely lack an internal loop in their P2 stem. Moreover, a pseudoknot involving the tip of P2 may not exist, as we do not observe significant base-pairing potential.

## SAM/SAH-binding RNAs

A pseudoknot pairing is possible between the tip of the hairpin (CUUC) and the Shine-Dalgarno sequence. However, there is only one mutation observed in these sequences, and this mutation disrupts Watson-Crick pairing. Nucleotides on both sides of the putative pseudoknot do show modest reduction in cleavage in in-line probing experiments on SK209-52 RNA. When the 3′-most 5 nucleotides of this RNA are removed, ligand-mediated structure modulation is not observed (data not shown). This result is consistent with a pseudoknot interaction that stabilizes the nucleotides involved.

## *sanguinis*-hairpin motif

The *sanguinis*-hairpin motif is a hairpin that is found in *Streptococcus sanguinis* and *S. thermophilus*. In *S. sanguinis*, there are four repeats in one part of the genome, and three repeats in a nearby region. These repeat regions include short spacers whose sequences are not conserved.

## *sbcD* motif

The *sbcD* motif is in the potential 5′ UTRs of apparent operons that might include *sbcD* genes, and other DNA repair genes. Since the *sbcD* genes are not the immediately downstream gene, and since all *sbcD* RNA candidates are located in apparently syntenic regions, it is difficult to ascertain whether the *sbcD* motif is truly associated with *sbcD* genes. If it is, SbcD is thought to be involved in removal of palindromic DNA sequences, which can be problematic during replication [156]. Therefore, it is possible that *sbcD* RNAs might operate as DNA mimics, perhaps as a feedback system of regulation for SbcD, or they may conceivably operate as ssDNA structures. *sbcD* RNAs are usually, but not always, located in plasmids.

## ScRE (*Streptococcus* Regulatory Element) motif

The ScRE motif has only modest covariation, and some non-canonical nucleotides. Therefore its assignment as a structured RNA is tenuous. However, it is consistently located upstream of several non-homologous classes of protein-coding genes, and therefore is likely to be a functional *cis*-regulatory element.

## Soil-1 motif

The Soil-1 motif is found only in metagenomic DNA isolated from soil samples. The motif consists of two hairpins. Although the first often ends in a GNRA tetraloop, no covariation is evident. The second stem exhibits a moderate amount of covariation, but also carries non-canonical base pairs.

## *sucA*-II motif

The *sucA*-II motif is found in the potential 5′ UTRs of *sucA* genes in species of the genus *Pseudomonas*. SucA is part of an enzyme in the citric acid cycle that it responsible for creating succinate. A distinct RNA motif was previously identified upstream of *sucA* genes in certain β-proteobacteria [14].

## *sucC* motif

The *sucC* motif is a hairpin structure that is in the potential 5′ UTRs of an apparent *sucCD* operon in *Pseudomonas*. We also predict a potential *sucC* RNA in *Marinobacter* sp. ELB17, which is in a different order of γ-proteobacteria, but it is not clear if this sequence is a true homolog. In this species, *sucC* RNA is in the potential 5′ UTRs of a predicted polyphosphate kinase. However, while the *sucC* motif appears to correspond to a *cis*-regulatory RNA, it is not clear why polyphosphate kinases should be co-regulated with *sucCD* genes, suggesting that the predicted homology might be a false positive. The *sucC* motif is one of multiple motifs predicted that might be involved in regulating the citric acid cycle in *Pseudomonas*.

## Solibacter-1 motif

The *Solibacter*-1 motif is found in many copies in the species *Solibacter usitatus*. The motif includes a three-stem junction, but it is supported by only modest covariation, and has some nucleotide pairs that are not normally energetically favorable. In view of this observation, and the fact that it is present in many copies in one organism, the motif might correspond to a repetitive element.

## Termite-*flg* motif

The Termite-*flg* motif is found only in environmental sequences from a termite hindgut metagenome, and is not detected in any genome from a known species. It is in the potential 5′ UTRs of flagellar genes.

## Termite-*leu* motif

The Termite-*leu* motif is found only in metagenome samples from termite hindguts, and consists of two hairpins. It is sometimes in the potential 5′ UTRs of a variety of leucine-related genes: *leuA*, *leuB* and *ilvC*, but often the downstream gene is in the opposite orientation. While some of these genes are likely misannotations due to the challenges inherent in annotating metagenome fragments, some are homologous to known gene families, and are therefore probably correctly annotated. Therefore the motif might not be *cis*-regulatory.

Some *cis*-regulatory RNAs are peptide leaders [122], which contain a short ORF that encodes a peptide rich in some amino acid. When levels of this amino acid are low, ribosomal stalling leads to increased expression of the downstream gene. The product of this downstream gene is typically required to synthesize the given amino acid. Termite-*leu* RNAs upstream of *leuA* and *ilvC* genes contain a short ORF immediately 5′ to their first hairpin that is rich in codons for branched-chain amino acids (BCAAs) (i.e., leucine, isoleucine and valine). The *leuA* and *ilvC* gene products are involved in the synthesis of these related amino acids. Moreover, these short ORFs exhibit some mutations that change a codon for one BCAA to a codon for a different BCAA, a phenomenon that suggests that the BCAA-rich ORF might be functionally important. However, a Termite-*leu* RNA that is upstream of a *leuB* gene does not have a similar ORF.

## *traJ*-II motif

The *traJ*-II motif is typically found in the apparent 5′ UTRs of *traJ* genes. A previously identified motif, which we will call *traJ*-I (Rfam accession RF00243), was identified in *E. coli*, and the closely related genus *Salmonella* [157]. The *traJ*-II motif has no apparent similarities in sequence or structure to the earlier motif. It is present in α-, β- and γ-Proteobacteria, although in each bacterial class it is only present in a few species. This distribution might be the result of horizontal transfer via conjugation, the process in which *traJ* functions. Since *traJ*-I RNAs are targets for FinP antisense RNAs, it is natural to speculate that *traJ*-II RNAs are also targets of an antisense RNA. The *traJ*-II motif has no obvious expression platform, though annotated start codons are often 20-30 nucleotides 3′ to the *traJ*-II RNA. There is a possibility that the RNA may be expressed as the reverse complement, as there are fewer A-C mismatches in the reverse complementary sequences of *traJ*-II RNAs, and the P2 stem would have a structurally stable CUUG terminal loop.

## Transposase-resistance motif

The Transposase-resistance motif is typically found in the potential 5′ UTRs of genes, and these genes or surrounding genes often confer antibiotics resistance. Although there are few transposons and integrases, there are more than would be expected by chance. Resistance genes are: *emrE* (include drug exporters), nucleotidyltransferases (includes kanamycin resistance), pfam03595 transporters (include tellurite exporters), dihydropteroate synthase (target of sulfonamide drugs), the pfam 00903 domain (includes bleomycin resistance enzymes), beta-lactamase (penicillin resistance), aminoglycoside phosphotransferase and aminoglycoside acetyltransferase. In *Xanthomonas campestris*, a putative Transposase-resistance RNA is adjacent to a gene involved in synthesizing the pigment xanthomonadin. The motif is often present in plasmids. The motif’s association with a wide variety of resistance genes could be a result of these genes being carried by repetitive elements, plasmids or phages. The hairpin structure of the motif could represent the inverted repeats that are often associated with transposases. The Transposase-resistance motif is present in a wide variety of bacteria, but is predominantly in Enterobacteria such as *E. coli*.

## TwoAYGGAY motif

The TwoAYGGAY motif is named after its two terminal loops that have an AYGGAY subsequence. The motif is present in some Clostridia and γ-proteobacteria, but is more common in a human gut metagenome. The P1 stem that normally closes the structure is often very large (e.g., 24 base pairs with only 2 non-canonical/mismatching pairs). However, some representatives have very small P1 stems.

## *wcaG* motif

In two places *wcaG* RNAs carry a conserved UGGYG motif. Such duplicate short sequences are sometimes binding sites for a dimeric protein.

## Whalefall-1 motif

The Whalefall-1 motif is found only in metagenome sequences from whale fall (a whale carcass that has settled on the ocean floor). It consists of two hairpins, followed by a purine-rich sequence. Although this purine-rich sequence resembles a Shine-Dalgarno sequence, there is no strong evidence of a conserved gene immediately downstream of the motif. The terminal loop of the second loop often has a CUUG tetraloop.

## *yjdF* motif

Most predicted *yjdF* genes contain a *yjdF* RNA in their apparent 5′ UTR. In *Streptococcus thermophilus*, however, no *yjdF* gene is predicted. In *Bacillus anthracis*, RNA-seq experiments [158] suggest that transcription of *yjdF* RNA and the *yjdF* gene arises from readthrough of the upstream gene. Although expression levels of the *yjdF* gene appear to be modestly modulated in the conditions tested, this differential expression seems to correlate with the expression of the upstream gene. Similarly, when *B. subtilis* cells are grown in complex medium, tiling array experiments indicate that the upstream *manPA* genes are transcribed at similar levels to the *yjdF* RNA/gene, and form a single transcriptionally active region (TAR) [104]. However, during growth in minimal medium, the *manPA* genes are transcribed at much lower levels, while the *yjdF* gene mRNA is almost as abundant as during growth in complex medium [104]. Under minimal medium conditions, the TAR containing the *yjdF* gene [104] is predicted to begin five nucleotides upstream of the predicted start of the *yjdF* motif RNA.

## *ykkC*-III motif

The *ykkC*-III motif exhibits somewhat more A-C mismatches in the given orientation than does its reverse complement. However, the orientation that we have depicted seems more likely to be biological given the apparent *cis*-regulatory locations of the motif in the given orientation, and the fact that it is generally very close to predicted Shine-Dalgarno sequences.

As noted in the main text, *ykkC*-III RNAs carry ACGA sequences that resemble conserved sequences in the mini-*ykkC* motif (Fig. 5; end of this document). This could indicate a structural relationship between the motifs. In addition to a contiguous ACGA sequence, the *ykkC*-III motif has a possible split occurrence of ACGA (Fig. 5) that might fold into a similar conformation. However, some observations suggest that the common ACGA sequences might not be related. First, the structural contexts of the two ACGA occurrences within *ykkC*-III differ from the structure contexts within the mini-*ykkC* motif. Moreover the repetitive hairpin structure of mini-*ykkC* RNAs provides fewer opportunities for intricate binding sites than the pseudoknotted structure of *ykkC*-III. Thus, the ACGA sequences in mini-*ykkC* have a diminished ability to form complex tertiary interactions, as the *ykkC*-III ACGA sequences might. Second, we observe that representatives of both the *ykkC*-III and the mini-*ykkC* motifs are found in a similarly wide range of phyla. Given that their opportunity to diverge is presumably comparable, it is noteworthy that the ACGA sequences are perfectly conserved in *ykkC*-III representatives, whereas their conservation in mini-*ykkC* RNAs is much looser. If the ACGA sequences serve similar structural roles, it is unclear why so much more variability is permitted in mini-*ykkC* RNAs. However, at this point it is not possible to reach a definitive conclusion regarding the possible structural relationship between the two motifs.

# Additions to previously characterized RNA classes

## 6S RNA

6S RNA is known to be present in almost all bacteria and regulates genes by binding to RNA polymerase [159]. Two new motifs might represent diverged 6S RNAs. 6S-Flavo is found in Flavobacteria, which lack previously predicted 6S RNAs. Homology searches with 6S-Flavo detect a few known 6S RNAs, which is additional evidence that it represents 6S RNA. Our alignment might be partial, as we do observe some pairing potential that would extend the hairpin further, to make it more similar to 6S RNA lengths.

The Lacto-*usp* motif is found in five instances in the order Lactobacillales. It is consistently in the potential 5′ UTRs of operons containing a hypothetical gene and *usp* (Universal Stress Protein). Although these data suggest that Lacto-*usp* is a *cis*-regulatory RNA, three observations imply that it might correspond to 6S RNA. First, the four *Lactobacillus* species with Lacto-*usp* entirely lack a predicted 6S RNA. Second, the Lacto-*usp* motif conforms to the general structure of 6S RNA, a hairpin with large internal loops. Finally, 6S RNAs are already known that appear to be in the potential 5′ UTRs of operons containing *usp* genes in other Lactobacillales species. However, the Lacto-*usp* motif is noticeably shorter than most 6S RNAs, and we do not observe obvious potential to extend the alignment.

## AdoCbl and SAM-II riboswitches

We found a motif (Additional File 3) that resembles a previously identified class of riboswitches for adenosylcobalamin (AdoCbl) [160]. The main differences are a P6 hairpin that is even shorter than previously found [161], and a stem (which we call P13) that flanks a pseudoknot. Other variants of AdoCbl riboswitches have also been observed previously [162].

Variants of SAM-II riboswitches [6] reveal that long insertions are possible in this motif (Additional File 3), although longer insertions generally fold into a stable structure.

# Ligand-binding experiments using in-line probing

We tested some riboswitch candidates for metabolite binding using in-line probing experiments [163]. RNAs tested were transcribed *in vitro* from a DNA template using RNA polymerase T7. In the experiments described below, we did not observe any modulation in gel patterns that would suggest metabolite binding. However, we cannot rule out the possibility that the RNAs used were inactive due to problems with the constructs (e.g. constructs may be missing important nucleotides or constructs may carry additional nucleotides that cause misfolding). Also, it is possible that ligand-induced structural modulation did not result in noticeable changes in the spontaneous cleavage rates of internucleotide linkages that are visualized in in-line probing assays. Additionally, many of the motifs assayed were borderline riboswitch candidates (Table 1).

## In-line probing experiments with a *pfl* RNA

Experiments were performed with the following RNA encoded by *Clostridium acetobutylicum* ATCC 824:

5′-GGUAAAAUAAGAAAAUCAUGCAACUGGCGGAAAUGGAGUUCACCAUAGGGAGCAUGAUUAAUAUAAGAAUCGACCGCCUGGGUAAAUUAAUA-3′

The following metabolites were tested at 1 mM except where noted: AICAR riboside, AICAR ribotide, SAICAR, GAR, pyruvate, guanine (100 μM), hypoxanthine (40 μM), IMP, formate, THF, dihydrofolate, 5-formyl-THF, 10-formyl-THF, methylene-THF, methenyl-THF, methyl-THF, glutamate, glutamine, glycine, serine, aspartate, dUMP, homocysteine, SAM, AICA, CAIR, CoA, acetyl-CoA, alanine, NAD, NADH, NADP, NADPH, dCMP, histidine, d-ribose 5′-phosphate, adenine, d-ribose, PRPP, ppGpp, cAMP, HMP, ATP (600 μM), CTP (600 μM), GTP (600 μM), UTP (600 μM), AMP, ADP, GMP, GDP, UMP, UDP, uridine, and tryptophan.

Due to the instability of PRPP, it was also tested in an RNase protection assay with RNase T1 and V1. The use of an RNase permits a shorter incubation time than used for in-line probing. However, we did not observe any change in degradation patterns with either RNase.

## In-line probing experiments with *yjdF* RNA

Experiments were performed on the following three RNAs, encoded by *Bacillus subtilis*, which have different 3′ ends:

5′-GGUAAAGAAUGAAAAAACACGAUUCGGUUGGUAGUCCGGAUGCAUGAUUGAGAAUGUCAGUAACCUUCCCCUCCUCGGGAUGUCCAUCAUUCUUUAAUAUCUUUUAUGAGGAGGGAAUCGUU-3′

5′-GGUAAAGAAUGAAAAAACACGAUUCGGUUGGUAGUCCGGAUGCAUGAUUGAGAAUGUCAGUAACCUUCCCCUCCUCGGGAUGUCCAUCAUUCUUUAAUAUCU-3′

5′-GGUAAAGAAUGAAAAAACACGAUUCGGUUGGUAGUCCGGAUGCAUGAUUGAGAAUGUCAGUAACCUUCCCCUCCUCGG-3′

The following metabolites were tested with each RNA at 1 mM: NAD, NADH, NADP, NADPH, ADP-ribose, glutamine, nicotinamide, nicotinic acid, glutamate, beta-nicotinamide mononucleotide, and d-ribose 5′-phosphate.

## In-line probing experiments with SAM-Chlorobi RNA

Experiments were performed on the following four RNAs, encoded by *Chlorobium tepidum* TLS, which have different 3′ ends:

5′-ggAUUUUCCGGCAUCCCCAUUACCUAUGGACACGGUGCCAAAAGCUCUCUUGCGGGAGUUGUCCCCGGAGCUUGCCGAAAGGUUUCCCGUGUCCCGUUUGUCCCUCCGCGACAUUCACCUUCACGAGAAAACCGCAUCGGCAAACCGCCGGACACCUGCCGUUCUUGUCGUUCGAUUAACAAAAAACCGAAAGGGAAACUA-3′

5′-ggAUUUUCCGGCAUCCCCAUUACCUAUGGACACGGUGCCAAAAGCUCUCUUGCGGGAGUUGUCCCCGGAGCUUGCCGAAAGGUUUCCCGUGUCCCGUUUGUCCC-3′

5′-ggAUUUUCCGGCAUCCCCAUUACCUAUGGACACGGUGCCAAAAGCUCUCUUGCGGGAGUUGUCCCCGGAGCUUGCCGAAAGGUUUCC-3′

5′-ggAUUUUCCGGCAUCCCCAUUACCUAUGGACACGGUGCCAAAAGCUCUCUUGCGGGAGUU-3′

Lowercase letters represent G nucleotides that were added to improve transcription yield.

The following metabolites were tested at 1 mM: SAM, SAH, methionine, and homocysteine.

## In-line probing experiments with *pan* RNA

Experiments were performed on the following RNA encoded by *Geobacter metallireducens* GS-15:

5′-ggCAAAUUGAUACUGCCUGGAUUCGUACGAACCGGGACGGAUGGCAAUAGCCGCAACGACAAGGAAAUAGCUUUUUCUCUUGGUCUUGGUACAUGCGCCUCCGGAA-3′

Lowercase letters represent G nucleotides that were added to improve transcription yield.

The following metabolites were tested at 1 mM: pantothenate, CoA, and beta-alanine.

## In-line probing experiments with *msiK* RNA

Experiments were performed with the following RNA encoded by *Streptomyces coelicolor* A3(2):

5′- GGACUACACCACCACCUUCCUACAACGGAUCGUCCGGCACGUUCCUGCCGGUAGAAGGGGGCCCUUUCAC-3′

The following metabolites were tested at 1 mM: fructose, galactose, glucose, mannose, xylose, cellobiose, lactose, maltose, sucrose, trehalose, glucose-1-phosphate, glucose-6-phosphate, fructose-6-phosphate, and fructose-1,6-bisphophate.

## In-line probing experiments with *gabT* RNA

Experiments were performed with the following two RNAs, encoded by *Pseudomonas syringae* pv. tomato str. DC3000, which have different 3′ ends:

5′- ggUCUUGGCGGCCUGAAGGCUGCAGCAGUCGAUCAUCGUAUGCUGUUGCAGUUGAUCCAGCCCGCUUGAUCC-3′

5′-ggUCUUGGCGGCCUGAAGGCUGCAGCAGUCGAUCAUCGUAUGCUGUUGCAGUUGAUCCAGCCCGCUUGAUCCUUGAACCACGCCGACCGAUGAGCGGCGAAUGAGGAAUACA-3′

Lowercase letters represent G nucleotides that were added to improve transcription yield.

The following metabolites were tested at 1 mM except where noted: cAMP, cGMP, cyclic di-GMP, agmatine, putrescine, GABA, l-glutamine, l-glutamate, l-lysine, 2-oxoglutarate (200 μM), glutaric acid (200 μM), succinate (200 μM), and succinic semialdehyde (200 μM).

## In-line probing experiments with *rmf* RNA

Experiments were performed with the following four RNAs, encoded by *Pseudomonas syringae* pv. tomato str. DC3000, which have different 5′ and /or 3′ ends:

5′- gGCGCUUUGGUUAGAAAUCAACUCAGGUCAUUUCCGCAAUGGUUAUGGCAUCAAGGCCCGCCACGCCGGCAGCGGGCCCCAACGGCAGAAGACUCUGCCCGACCCCACCACGGGGUCUCAGGGAUAUUACAGUCAACAGA-3′

5′-ggAUCAUUCACAUCACCCUGCGCUUUGGUUAGAAAUCAACUCAGGUCAUUUCCGCAAUGGUUAUGGCAUCAAGGCCCGCCACGCCGGCAGCGGGCCCCAACGGCAGAAGACUCUGCCCGACCCCACCACGGGGUCUCAGGGAUAUUACAGUCAACAGA -3′

5′- gGCGCUUUGGUUAGAAAUCAACUCAGGUCAUUUCCGCAAUGGUUAUGGCAUCAAGGCCCGCCACGCCGGCAGCGGGCCCCAACGGCAGAAGACUCUGCCCGACCCCACCACGGGGUCUCAGGGAUAUUACAGUCAACAGACGAGGGCAUUACCCUAUGAGAAGA-3′

5′-ggAUCAUUCACAUCACCCUGCGCUUUGGUUAGAAAUCAACUCAGGUCAUUUCCGCAAUGGUUAUGGCAUCAAGGCCCGCCACGCCGGCAGCGGGCCCCAACGGCAGAAGACUCUGCCCGACCCCACCACGGGGUCUCAGGGAUAUUACAGUCAACAGACGAGGGCAUUACCCUAUGAGAAGA -3′

Lowercase letters represent G nucleotides that were added to improve transcription yield.

The metabolite ppGpp was tested at 1 mM.

## In-line probing experiments with Downstream peptide RNA

Experiments were performed with the following two RNAs, encoded by *Synechococcus* sp. CC9605, which have different 5′ and 3′ ends:

5′- gGCGACCACGUUCACCUCGUCUUCGGCGAGGCGCAGUUCGACUCAGGCCAUGGAACGGGGACCUGAGCUUG-3′

5′- gGCUACGCGACCACGUUCACCUCGUCUUCGGCGAGGCGCAGUUCGACUCAGGCCAUGGAACGGGGACCUGAGCUUGCUUCGAGGAACU -3′

Lowercase letters represent G nucleotides that were added to improve transcription yield.

The following metabolites were tested at 1 mM except where noted: cAMP, cGMP, cyclic di-GMP, agmatine, putrescine, GABA, l-glutamine, l-glutamate, l-lysine, 2-oxoglutarate (200 μM), glutaric acid (200 μM), succinate (200 μM), and succinic semialdehyde (200 μM).

**Figure 1**

**Complete gel image from in-line probing experiments.** Below is the full gel image from Figure 1 in the main text. Nucleotide positions 23, 42 and 45 are marked as in Figure 1 in the main text. Note that changes in band intensities with varying concentrations of SAM were quantitated by computer as described in Materials and Methods, and references therein.


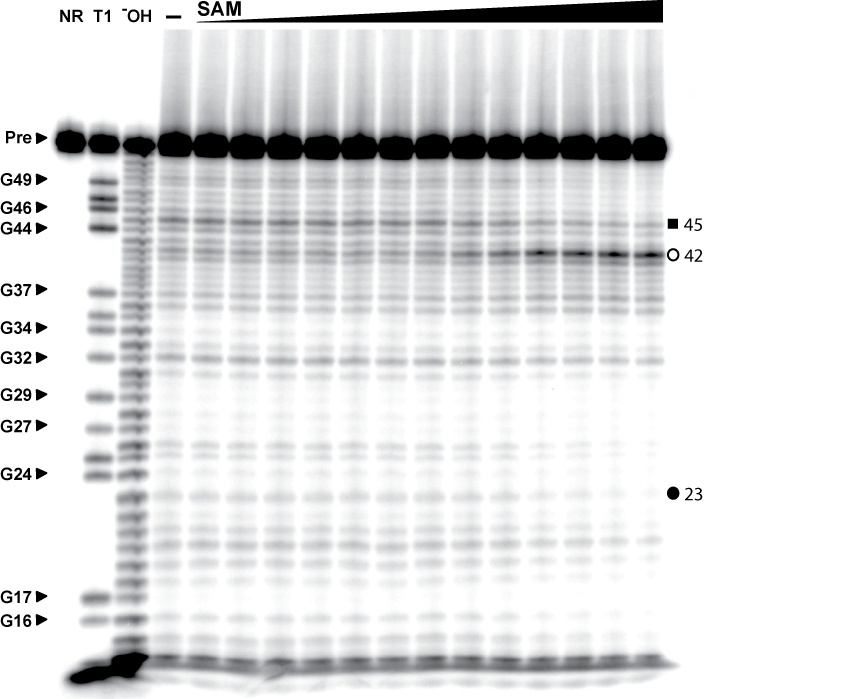


**Figure 2**

**Binding characteristics of SAM/SAH riboswitches. (a)** The dissociation constant (*K*_D_) was calculated for SK209-52 RNA when binding compounds related to SAM. The structures of SAM, SAH and the sulfoxide analog of SAM are marked. The horizontal dashed line indicates the *K*_D_ for SAM. This *K*_D_ was determined using a newer stock of SAM that presumably contained less SAH, resulting in a somewhat higher value. **(b)** Two previously established SAM-binding RNAs (156 *metA*, 62 *metY*) and SK209-52 RNA were subjected to equilibrium dialysis experiments. An SK209-52 RNA mutant, termed “A48U”, was also tested. The SAM209-52 structure is shown at left, with the A48U mutation. At right is shown the ratios of the counts/minute in chamber B (containing the RNA) divided by that in chamber A (containing SAM with a ^3^H-labeled methyl group). Error bars reflect three independent experiments. The horizontal dashed line indicates a ratio of 1, which is the expected value when no RNA is used, or when an RNA that does not bind SAM is used.


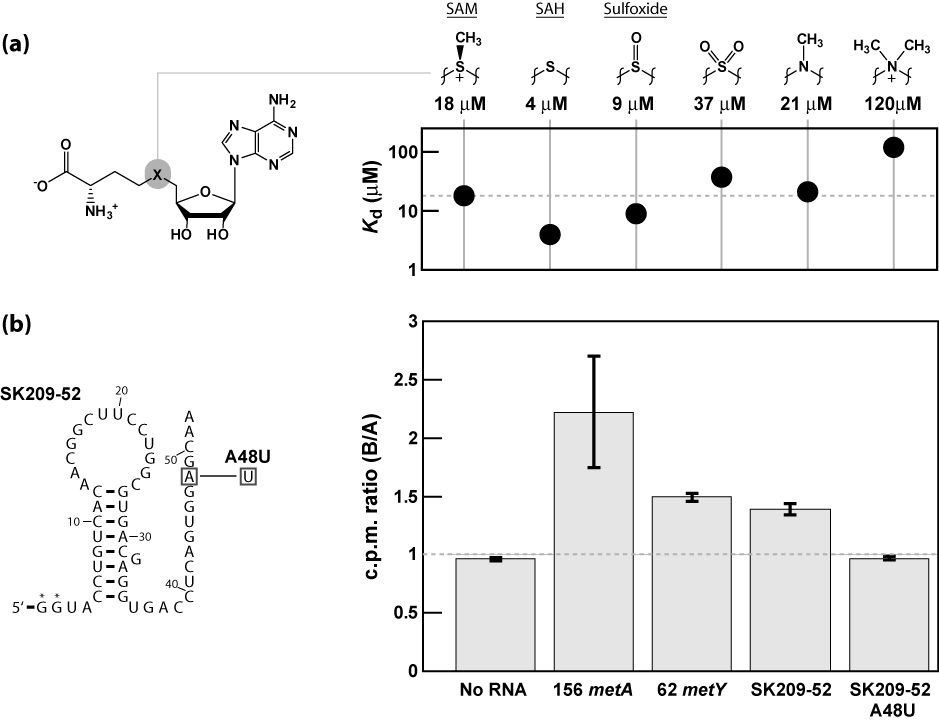


**Figure 3**

**Competitive equilibrium dialysis experiments with SAM/SAH-binding RNA.** SK209-52 RNA was further tested in equilibrium dialysis experiments in competition with other small molecules. In each experiment, 0.5 μM ^3^H-SAM was added. In addition, SK209-52 RNA was added to 40 μM, except for a negative control experiment (left bar). Some experiments (right three bars) included 200 μM of the indicated non-radiolabeled competitor. The structures of SAH and the sulfoxide analog of SAM are given in Figure 2. The amino acid l-methionine was also used. Error bars reflect three independent experiments.


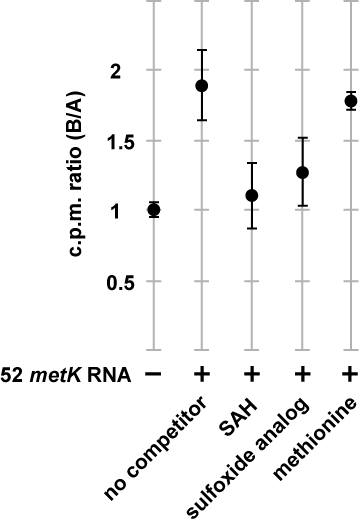


**Figure 4**

**Genes regulated by *pfl* RNAs in the context of purine and one-carbon metabolism.** This diagram is adapted from a previously published diagram regarding purine metabolism [141]. The apparent regulation by a *pfl* RNA of an *rpiB* gene was observed in more recent homology searches (unpublished data). Genes known to be regulated by *purR* in Gram-positive or Gram-negative bacteria were collected from previous reports [141, 144, 164]. The compound formyltetrahydrofolate is repeated three times in the diagram, as indicated by the label “same compound”.
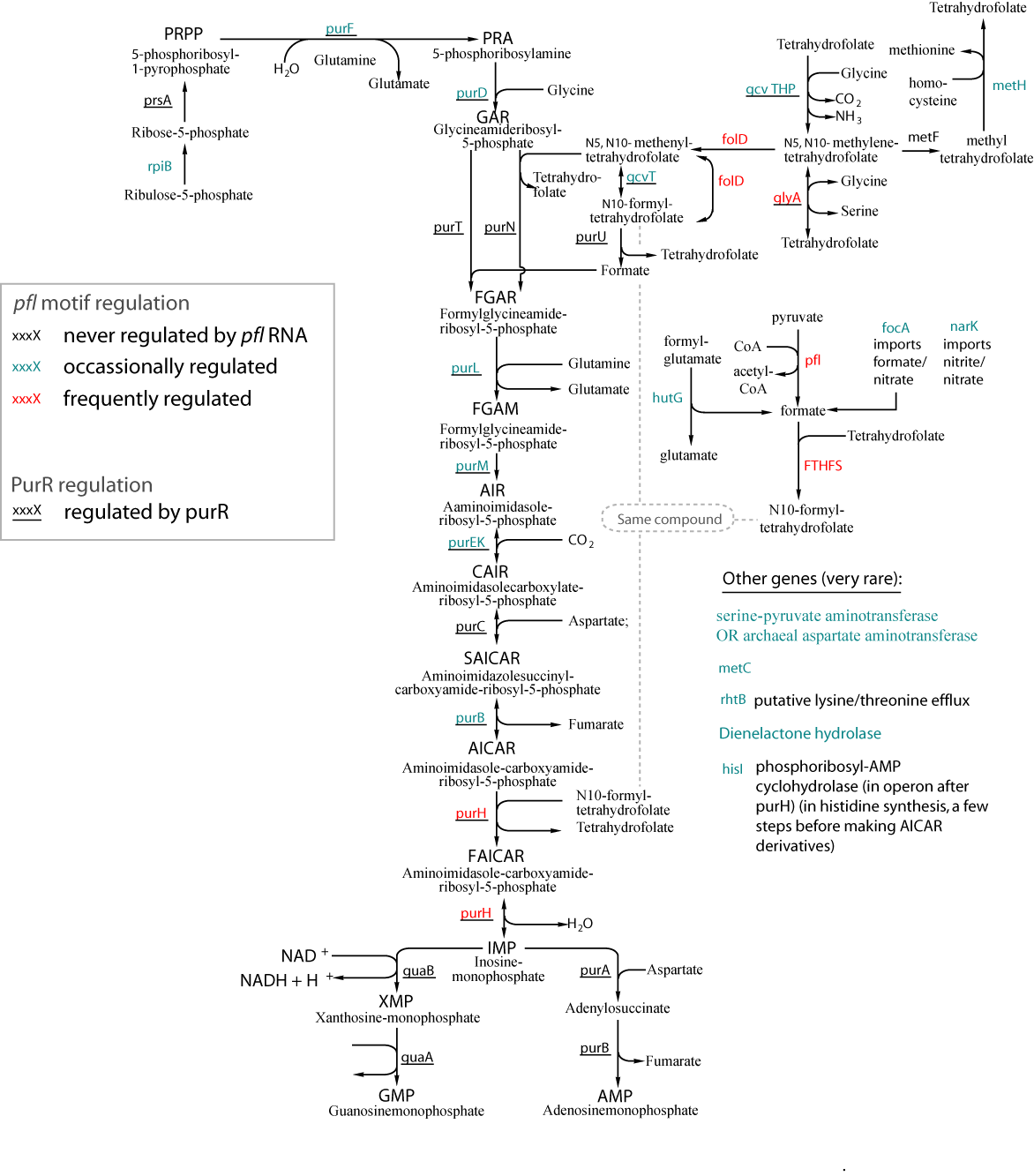


**Figure 5**

**Comparison of *ykkC*-III and mini-*ykkC* motifs.** Conserved sequences with the consensus ACGA present in the *ykkC*-III or mini-*ykkC* motifs are shaded blue. The *ykkC*-III motif contains AC and GA sequences that might together comprise an ACGA sequence. The mini-*ykkC* motif drawing is derived from a previous depiction [14].


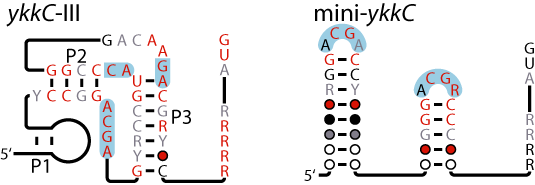


# References

References are listed in the manuscript.
