# Supplementary material for: Comparative genomics reveals 104 candidate structured RNAs from bacteria, archaea, and their metagenomes
Source: Genome Biol. 2010 Mar 15;11(3):R31. doi: 10.1186/gb-2010-11-3-r31 (PMC2864571; doi:10.1186/gb-2010-11-3-r31)
Supplement: Additional file 2 — Summary and evaluation of all motifs. Table 1, with summary of supporting evidence, and numbers of representatives of each motif. [file gb-2010-11-3-r31-S2.PDF]

Additional File 2 Summary of all RNA motifs supplementary for: Comparative genomics reveals 104 candidate structured RNAs from bacteria, archaea and their metagenomes

Zasha Weinberg, Joy X. Wang, Jarrod Bogue, Jingying Yang,
Keith Corbino, Ryan H. Moy, Ronald R. Breaker

March 22, 2010

The following table lists all RNA motifs predicted in this work, with annotation. For the "RNA?" column, the annotation codes are:

- Y/y/?/n/N: certainly, probably, ambiguous, probably not, definitely not. **Y** and **y** are capitalized to emphasize motifs that for which there is clear evidence of RNA structure.
- COV: strong covariation
- cov: modest covariation
- nc: modest non-canonicals
- seq: poor sequence conservation
- < : few seqs, so significance of covariation is unclear
- short: motif is short, so homology searches might not reveal sequences that break the model.
- V: Variable-length stems
- mod: modular stems
- tr : experimentally shown to be transcribed
- TFBS: predicted transcription-factor binding site implies that putative RNA is transcribed
- apt : experimental evidence of aptamer
- exp: other experimental evidence supporting RNA assignment

- hom(X): possible homology to RNA class X.
- fhom: presumable functional homolog to distinct RNA class in the same gene context
- tson: possibly transposon-associated, or associated with integrases
- loc: "local"-mode covariance model searches lead to reduced sequence conservation, perhaps simply because of unrelated hairpins, but this is cause for additional caution.
- rep: possibly repetitive (many representatives per genome)
- TT: predicted terminator associated with motif
- SD: overlap of Shine-Dalgarno implies a *cis*-regulatory role, which in turn implies RNA
- peptide: presence of short ORF with peptide corresponding to downstream amino acid synthesis genes implies RNA
- DNA?: some reason to suggest motif operates at the level of DNA.
- 4loop: common presence of terminal tetraloops (GNRA, UNCG, CUNG)
- Eloop: E-loop(s) is commonly present
- RBP: downstream gene encodes RNA-binding protein

For the "*cis*-reg" (does the motif represent a *cis*-regulatory RNA) column, annotation codes are:

- Y/y/?/n/N: (same as for "RNA?" column).
- one : always upstream of genes belonging to a single gene family. This arrangement is consistent with a *cis*-regulatory role for the motif, but if the organisms containing the motif representatives are closely related, the arrangement might simply reflect insufficient opportunities for the location of the motif to change via neutral rearrangements.
- > 1 : upstream of non-homologous genes. This phenomenon favors the hypothesis that it is functionally important for motif representatives to be upstream of protein-coding genes, because there were more events where motif representatives might have (under the null hypothesis) not been in a potential $5'$ UTR.
- alt : alternate hypothesis for function questions whether the motif is *cis*-regulatory
- over-TT : the motif commonly overlaps predicted transcription terminators, which would be consistent with a *cis*-regulatory function.
- over-SD : the motif commonly overlaps predicted Shine-Dalgarno sequences, which would be consistent with a *cis*-regulatory function.

The "switch?" column answers how likely it is that the motif represents a riboswitch. Evidence in favor of a *cis*-regulatory role (see above) also favors riboswitch function. Annotation codes are:

- Y/y/?/n/N: (same as for "RNA?" column)
- alt: alternate hypothesis that questions riboswitch function
- complex: motif structure is predicted to contain at least one multistem junction or pseudoknot.
- ss: secondary structure is not very complex
- phyla: motif displays high conservation of some nucleotide positions and is found in multiple phyla, suggesting a strong biochemical constraint consistent with riboswitch function.

- orders: motif present in multiple orders (of taxonomy) with some highly conserved nucleotide positions; see "phyla" annotation.

The "taxa/environments" column shows taxa or environmental samples in which motif representatives are commonly found.

The "#" columns is the total number of motif representatives predicted.

The "Rfam acc." column gives the Rfam Database accession for the motif, for motifs that are included in Rfam.

The motifs are as follows:

**motif**	**RNA?**	***cis*-reg?**	**switch?**	**Taxa / environments**	**#**	**Rfam acc.**
6S-flavo	**Y**<br>COV hom(6S)	N	N	Bacteroidetes	92	RF01685
aceE	? cov<br>nc	y	?	γ-Proteobacteria	20	
Acido-1	**y**<br>COV <	n	n	Acidobacteria	4	RF01686
Acido-Lenti-1	**y**<br>COV nc	n	n	Acidobacteria, Lentisphaerae	89	RF01687
Actino-pnp	**Y**<br>cov fhom	Y	N	Actinomycetales	100	RF01688
AdoCbl-variant	**Y**<br>COV V<br>mod hom(AdoCbl)	Y >1	Y complex	Marine	144	RF01689
asd	**Y**<br>COV V<br>mod TT	?	? complex	Lactobacillales	44	RF01732
atoC	**y**<br>cov TT	y	?	δ-Proteobacteria	8	RF01733
Bacillaceae-1	**Y**<br>COV nc	n	n	Bacillaceae	149	RF01690
Bacillus-plasmid	**y**<br>cov	?	n	*Bacillus*	32	RF01691
Bacteroid-*trp*	**y**<br>peptide	y	n alt	Bacteroidetes	47	RF01692
Bacteroidales-1	**Y**<br>COV 4loop<br>TFBS<br>TT	?	?	Bacteroidales	47	RF01693
Bacteroides-1	**y**<br>cov nc<br>4loop	?	n	*Bacteroides*	40	RF01694
Bacteroides-2	? COV<br>nc	n	n	*Bacteroides*	66	
Burkholderiales-1	? cov<br>nc loc<br>rep	?	n	Burkholderiales	88	
c4 antisense RNA	**Y**<br>COV V<br>4loop<br>exp hom(c4)	N	N	Proteobacteria, phages	593	RF01695
c4-a1b1	**Y**<br>COV tr	N	N	γ-Proteobacteria, phages	87	
Chlorobi-1	**Y**<br>COV TT	n	n	Chlorobi	15	RF01696

motif	RNA?	*cis*-reg?	switch?	Taxa / environments	#	Rfam acc.
Chlorobi-RRM	**y** cov RBP	y	n	Chlorobi	20	RF01697
Chloroflexi-1	**y** COV nc rep <	?	n	*Chloroflexus aggregans*	3	RF01698
Clostridiales-1	**y** COV nc 4loop V loc	n	n	Clostridiales, human gut	206	RF01699
COG2252	? cov nc V RBP	y	n	Pseudomonadales	11	
Collinsella-1	**y** COV nc loc	n	n	Actinobacteria, human gut	6	RF01700
crcB	**Y** COV V	Y >1 over-TT	Y phyla complex	Widespread, bacteria and archaea	449	RF01734
Cyano-1	**y** cov	n	n	Cyanobacteria, marine	276	RF01701
Cyano-2	**Y** COV nc V	n	n	Cyanobacteria, marine	57	RF01702
Desulfotalea-1	? cov nc	n	n	Proteobacteria	229	
Dictyoglomi-1	**y** cov Eloop	?	?	Dictyoglomi	4	RF01703
Downstream-peptide	**Y** COV V 4loop tr	y	y orders complex	Cyanobacteria, marine	634	RF01704
epsC	**Y** COV	y >1	y complex	Bacillales	35	RF01735
fixA	? cov nc	y	n	*Pseudomonas*	10	
Flavo-1	**y** COV V nc	n	n	Bacteroidetes	239	RF01705
flg-Rhizobiales	**y** cov 4loop	y	n	Rhizobiales	14	RF01736
flpD	**y** cov	?	n	Euryarchaeota	8	RF01737
gabT	**Y** COV nc mod SD	y SD	?	*Pseudomonas*	21	RF01738
Gamma-*cis*-1	? cov nc tson loc	y	n	γ-Proteobacteria	50	
glnA	**Y** COV V Eloop	Y >1	y orders complex	Cyanobacteria, marine	1039	RF01739
GUCCY-hairpin	? cov short	?	n	Bacteroidetes, Proteobacteria	651	
Gut-1	**Y**	n	n	Human	43	RF01706

motif	RNA?	*cis*-reg?	switch?	Taxa / environments	#	Rfam acc.
	COV nc V			gut only		
gyrA	**y** COV nc	y >1 SD	n	*Pseudomonas*	24	RF01740
hopC	**y** cov tr <	Y	?	*Helicobacter*	4	RF01741
icd	? cov nc	y	n	*Pseudomonas*	20	
JUMPstart	**y** COV nc tr hom(JUMPstart)	Y	?	γ-Proteobacteria	243	RF01707
L17 downstream element	**y** cov V	y (3′)	n alt	Lactobacillales, *Listeria*	82	RF01708
lactis-plasmid	**y** cov	?	n	Lactobacillales	14	RF01742
Lacto-*int*	? COV NC tson	?	n	Lactobacillales, phages	54	
Lacto-*rpoB*	**Y** COV	y	n	Lactobacillales	60	RF01709
Lacto-*usp*	**Y** COV hom(6S)?	? alt	?	Lactobacillales	6	RF01710
Leu/phe leader	**Y** COV V peptide hom(leu leader)	Y	N	*Lactococcus lactis*	9	RF01743
livK	**y** cov	y	?	Pseudomonadales	8	RF01744
Lnt	**y** cov	y	?	Chlorobi	10	RF01711
manA	**Y** COV V mod	Y >1	y phyla complex	Marine, γ-Proteobacteria, cyanophage	195	RF01745
Methylobacterium-1	**Y** COV nc	n	n	*Methylobacterium*, marine	36	RF01712
Moco-II	**y** cov TT <	Y TT	?	Proteobacteria	8	RF01713
mraW	**y** COV	y	?	Actinomycetales	60	RF01746
msiK	**Y** COV V SD	Y SD	? orders	Actinobacteria	136	RF01747
Nitrosococcus-1	? cov nc 4loop	n	n	*Nitrosococcus*, Clostridia	14	
nuoG	**y** COV V SHORT	y	?	Enterobacteriales (incl. *E. coli* K12)	86	RF01748
Ocean-V	**y** cov <	n	n	Marine only	3	RF01714
Ocean-VI	? cov 4loop	?	?	Marine only	308	

motif	RNA?	*cis*-reg?	switch?	Taxa / environments	#	Rfam acc.
pan	**Y**<br>COV nc<br>V TT	Y TT	?	Chloroflexi, Firmicutes, δ-Proteobacteria	121	RF01749
Pedo-repair	**y**<br>COV <	?	n	*Pedobacter*	6	RF01715
pfl	**Y**<br>COV V<br>mod TT	Y over-TT	Y phyla complex	Several phyla	234	RF01750
pheA	? cov<br>nc	y	n	Actinobacteria	29	
PhotoRC-I	**y**<br>cov 4loop	y	n	Cyanobacteria, marine	66	RF01716
PhotoRC-II	**Y**<br>COV	y	n	Marine, cyanophage	467	RF01717
Polynucleobacter-1	**y**<br>COV nc	y >1	?	Burkholderiales, fresh water/estuary	16	RF01718
potC	**y**<br>cov	y >1	? ss	Marine only	136	RF01751
psaA	**Y**<br>COV V<br>nc 4loop	y V	? orders? complex	Cyanobacteria	31	RF01752
psbNH	**y**<br>cov nc	y	n	Cyanobacteria, marine	39	RF01753
Pseudomon-1	**y**<br>COV TT	n	n	Pseudomonadales	23	RF01719
Pseudomon-2	? cov<br>NC	n	n	*Pseudomonas*	16	
Pseudomon-GGDEF	? cov<br>nc	y	?	*Pseudomonas*	7	
Pseudomon-*groES*	**y**<br>COV 4loop<br>tr	y	?	*Pseudomonas*	20	RF01721
Pseudomon-Rho	**y**<br>cov nc	Y	n	*Pseudomonas*	22	RF01720
Pyrobac-1	**y**<br>COV V	n	n	*Pyrobaculum*	8	RF01722
Pyrobac-HINT	? cov<br><	y	n	*Pyrobaculum*	4	
radC	**Y**<br>COV V<br>4loop	y	? complex	Proteobacteria	167	RF01754
Rhizobiales-1	? cov<br>nc loc<br>rep	n	N	Rhizobiales	795	
Rhizobiales-2	**y**<br>cov	?	n	Rhizobiales	16	RF01723
Rhodopirellula-1	? cov<br>nc rep	y	?	Proteobacteria, Planctomycetes	153	
rmf	**Y**<br>COV	y	?	Pseudomonadales	22	RF01755
rne-II	**Y**<br>COV V<br>mod fhom	y	N	Pseudomonadales	21	RF01756

motif	RNA?	*cis*-reg?	switch?	Taxa / environments	#	Rfam acc.
SAM-Chlorobi	**y**<br>cov TFBS	Y	?	Chlorobi	10	RF01724
SAM-I-IV-variant	**Y**<br>COV V<br>4loop<br>mod hom(SAM-I/IV)	Y	Y phlya complex	Several phyla, marine	473	RF01725
SAM-II long loops	**Y**<br>COV hom(SAM-II)	Y >1	Y phyla complex	Bacteroidetes, marine	162	RF01726
SAM/SAH riboswitch	**Y**<br>COV	Y over-SD?	Y apt	Rhodobacterales	64	RF01727
sanguinis-hairpin	? cov<br>nc rep	n	n	*Streptococcus*	12	
sbcD	**y**<br>cov V<br>TT <	?	n	Burkholderiales	8	RF01757
ScRE	? cov<br>nc	y	n	*Streptococcus*	59	
Soil-1	? cov<br>nc 4loop	n	n	Soil only	15	
Solibacter-1	? cov<br>nc rep	n	n	*Solibacter usitatus*	34	
STAXI	**y**<br>COV V<br>4loop<br>DNA?	?	n	Enterobacteriales	77	RF01728
sucA-II	**y**<br>cov V	y	?	Pseudomonadales	22	RF01758
sucC	**Y**<br>COV V<br>4loop	Y	?	γ-Proteobacteria	25	RF01759
Termite-*flg*	**Y**<br>COV nc	y	n	Termite hind gut only	13	RF01729
Termite-*leu*	**y**<br>COV V	?	?	Termite hind gut only	20	RF01730
traJ-II	**Y**<br>COV V<br>nc fhom	Y	n	Proteobacteria, *Enterococcus faecium*	71	RF01760
Transposase-resistance	? V COV<br>nc tson<br>loc	y	n	Several phyla	218	
TwoAYGGAY	**y**<br>COV nc<br>loc	n	n	Human gut, γ-Proteobacteria, Clostridiales	216	
wcaG	**Y**<br>COV	y >1	y complex	Marine, cyanophage	120	RF01761
Whalefall-1	**Y**<br>COV nc<br>4loop	n	n	Whalefall only	16	RF01762
yjdF	**Y**<br>COV V<br>SD 4loop	Y over-SD	Y orders complex	Firmicutes	110	RF01764
ykkC-III	**y**	Y >1	y phyla	Actinobacteria,	44	RF01763

**motif**	**RNA?**	***cis*-reg?**	**switch?**	**Taxa / environments**	**#**	**Rfam acc.**
	COV nc		complex	δ-Proteobacteria		
