# Supplementary material for: Comparative genomics reveals 104 candidate structured RNAs from bacteria, archaea, and their metagenomes
Source: Genome Biol. 2010 Mar 15;11(3):R31. doi: 10.1186/gb-2010-11-3-r31 (PMC2864571; doi:10.1186/gb-2010-11-3-r31)
Supplement: Additional file 6 — Consensus diagrams of all motifs. Consensus diagrams depicting all motifs in high resolution. [file gb-2010-11-3-r31-S6.PDF]

Additional File 6: Consensus diagrams of all RNA motifs supplementary for: Comparative genomics reveals 104 candidate structured RNAs from bacteria, archaea and their metagenomes

Zasha Weinberg, Joy X. Wang, Jarrod Bogue, Jingying Yang,
Keith Corbino, Ryan H. Moy, Ronald R. Breaker

February 27, 2010

Contents

6S-Flavo, *aceE*, **Acido-1, Acido-Lenti-1, Actino-pnp** **3**

AdoCbl-variant, *asd*, *atoC*, **Bacillaceae-1,** *Bacillus*-**plasmid** **4**

Bacteroidales-1, *Bacteroides*-**1,** *Bacteroides*-**2, c4 antisense RNA, c4 antisense RNA target a1b1** **5**

Chlorobi-1, Chlorobi-RRM, Chloroflexi-1, Clostridiales-1, *Collinsella*-**1** **6**

crcB, **Cyano-1, Cyano-2,** *Desulfotalea*-**1, Downstream-peptide** **7**

Dictyoglomi-1, *epsC*, *fixA*, **Flavo-1** **8**

flpD, *flg*-**Rhizobiales,** *gabT*, **Gamma-***cis*-**1,** *glnA*, **GUCCY-hairpin** **9**

Gut-1, *gyrA*, *hopC*, *icd*, **JUMPstart, L17 downstream element** **10**

lactis-**plasmid, Lacto-***int*, **Lacto-***rpoB*, **Lacto-***usp*, **leu/phe leader, Lnt** **11**

manA, *Methylobacterium*-**1,** *metK*-**Rhodobacter, Moco-II,** *msiK* **12**

Ocean-V, Ocean-VI, *pan*, *Pedo*-**repair** **13**

pfl, *psaA*, *pheA*, **PhotoRC-I, PhotoRC-II** **14**

Polynucleobacter-**1,** *potC*, **Pseudomon-1** **15**

psbNH, **Pseudomon-2, Pseudomon-***groES*, *Pseudomon*-**Rho,** *Pyrobac*-**1,** *Pyrobac*-**HINT** **16**

radC, **Rhizobiales-1, Rhizobiales-2, Rhodopirellula-1,** *rmf* **17**

rne-**II, SAM-Chlorobi, SAM-I-nil** **18**

SAM/SAH, *sanguinis*-**hairpin,** *sbcD*, **ScRE, Soil-1, Solibacter-1** **19**

STAXI, *sucA*-**II,** *sucC* **20**

Termite-*flg*, Termite-*leu*, *traJ*-II, TwoAYGGAY **21**

wcaG, **Whalefall-1,** *yjdF*, *ykkC*-**III** **22**

6S-Flavo, *aceE*, Acido-1, Acido-Lenti-1, Actino-pnp

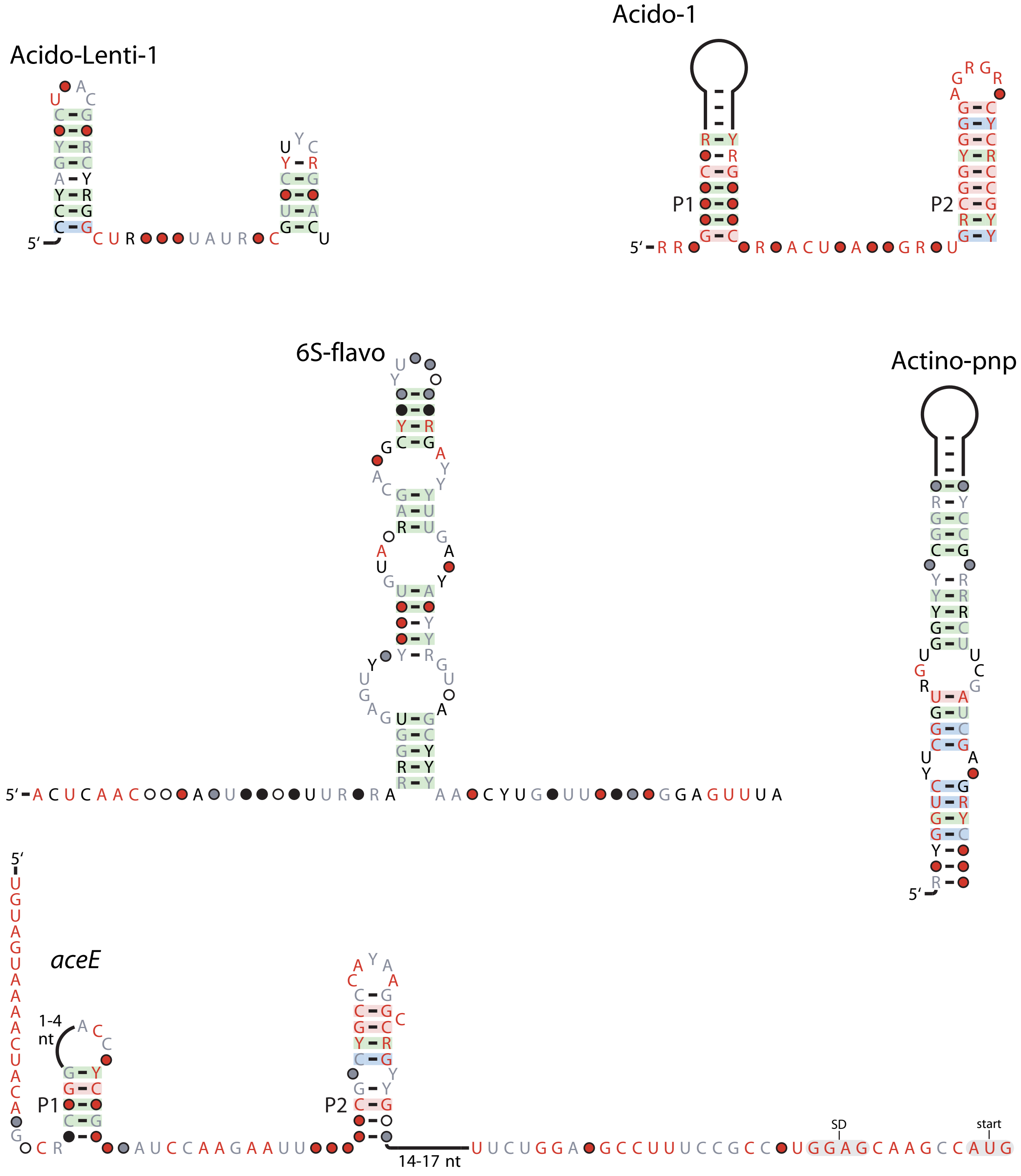

A portion of this figure was adapted from the supplementary data of a previous publication [21].

AdoCbl-variant, *asd*, *atoC*, Bacillaceae-1, *Bacillus*-plasmid

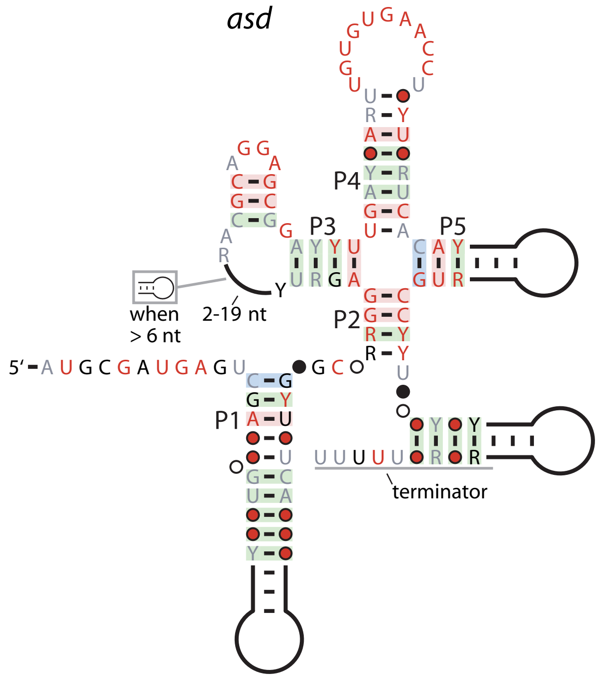

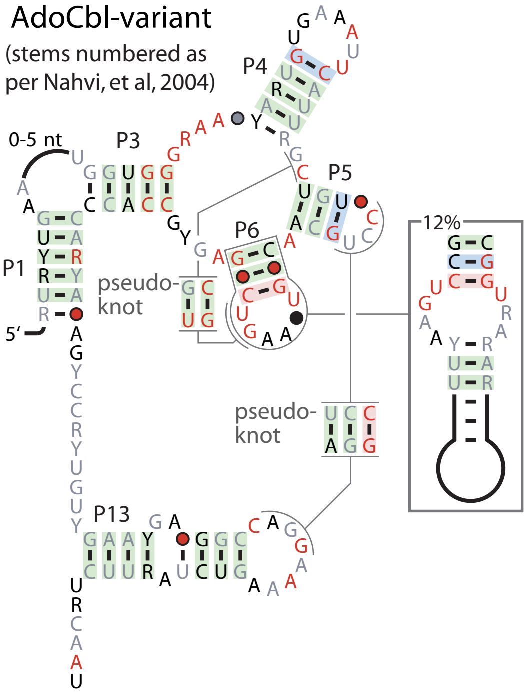

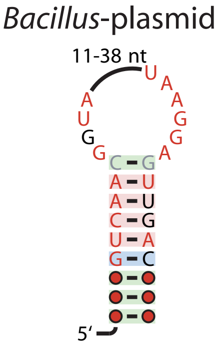

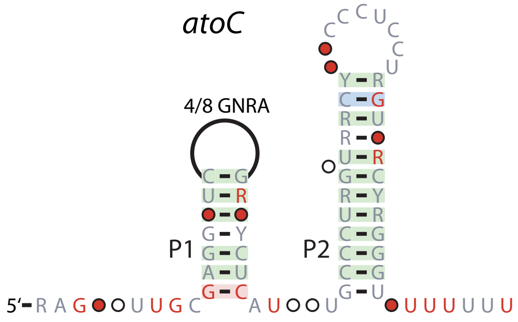

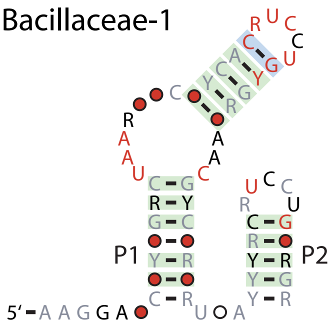

Bacteroidales-1, *Bacteroides*-1, *Bacteroides*-2, c4 antisense RNA, c4 antisense RNA target a1b1

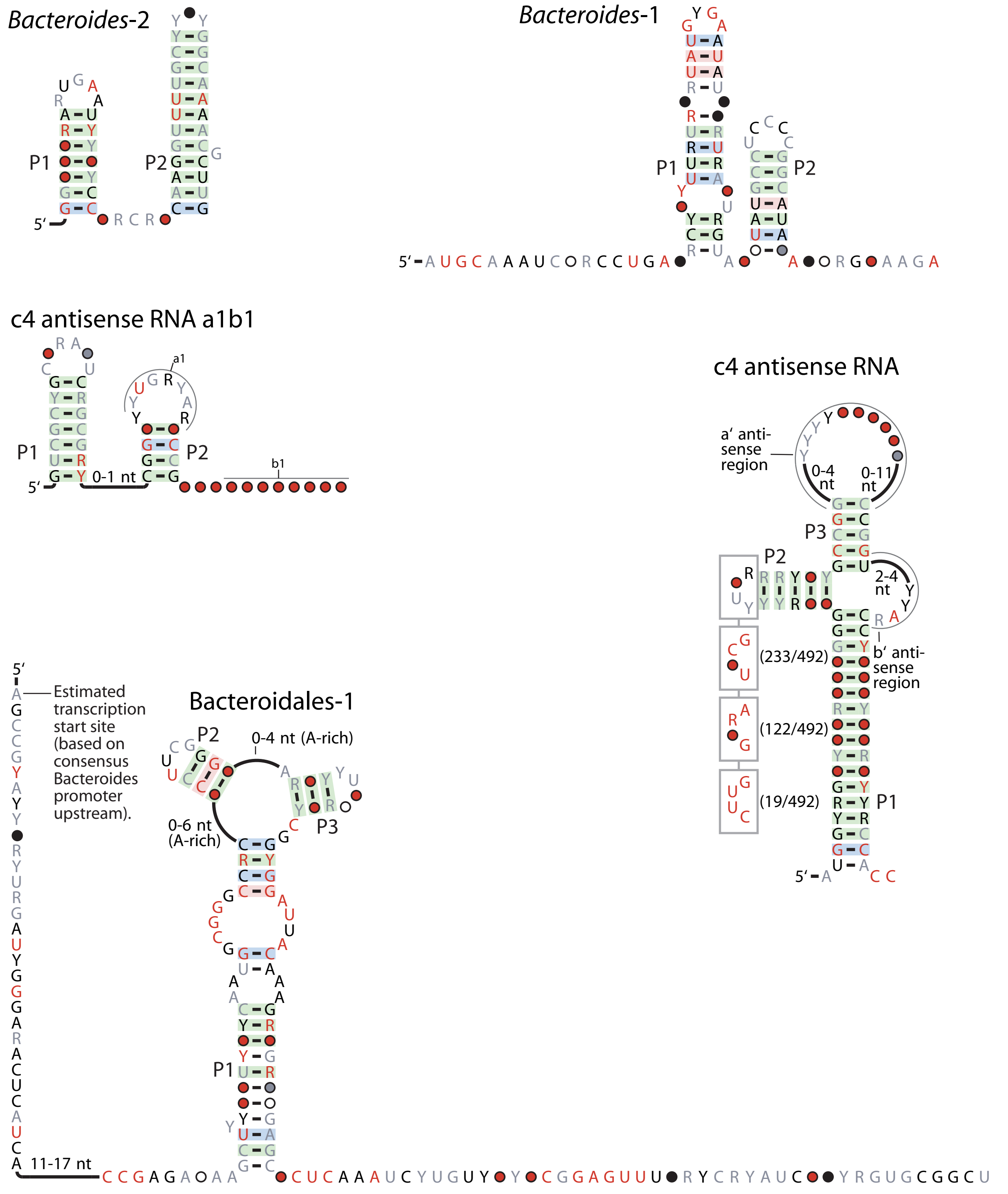

Chlorobi-1, Chlorobi-RRM, Chloroflexi-1, Clostridiales-1, *Collinsella*-1

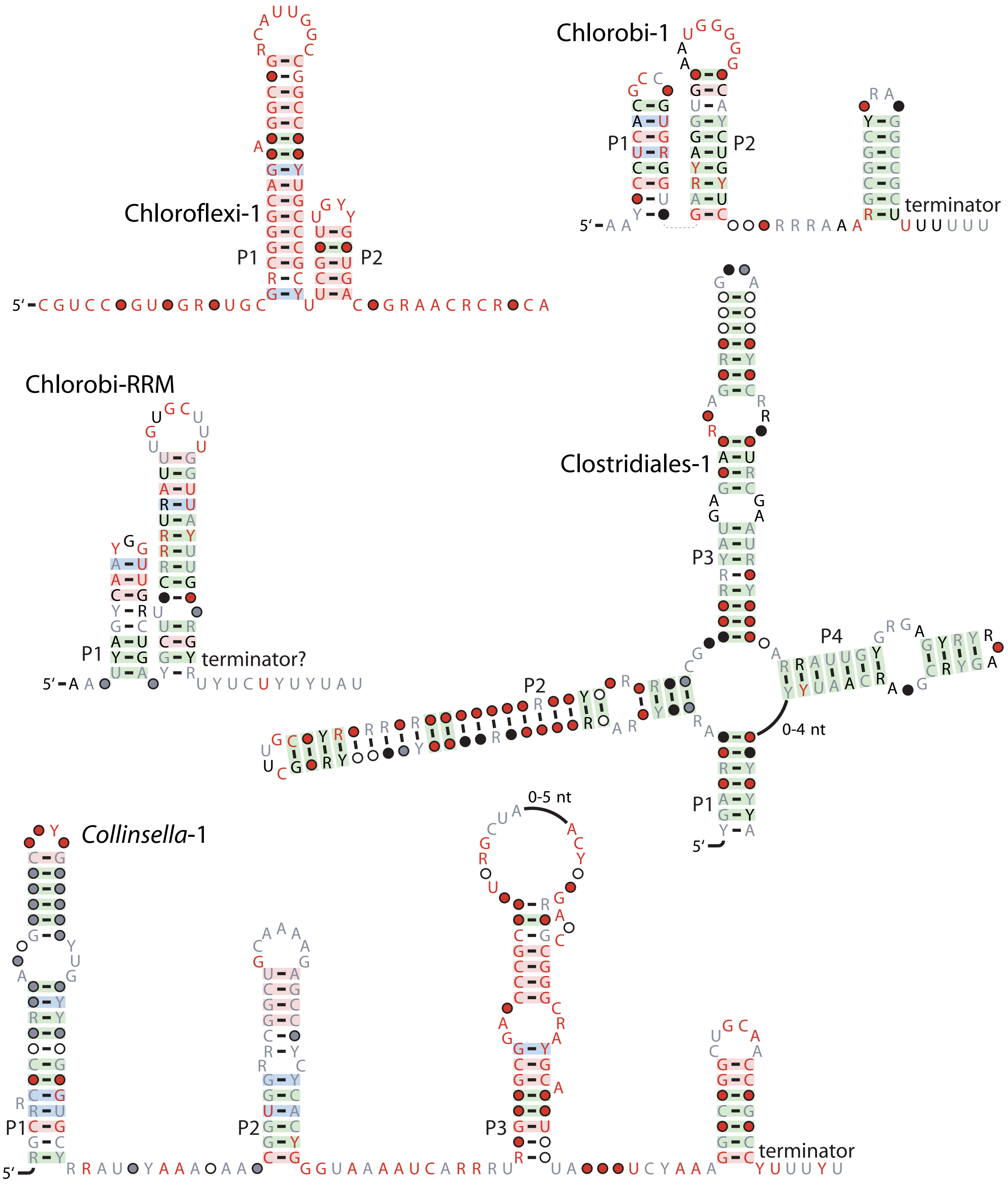

crcB, Cyano-1, Cyano-2, *Desulfotalea*-1, Downstream-peptide

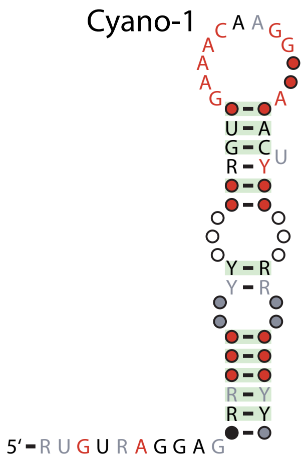

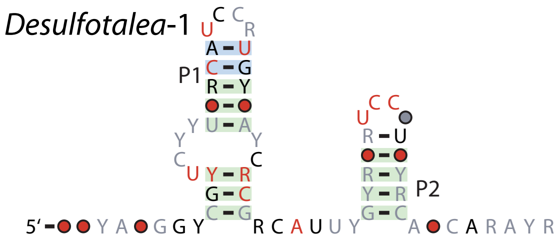

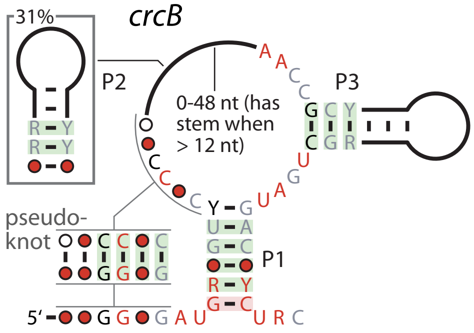

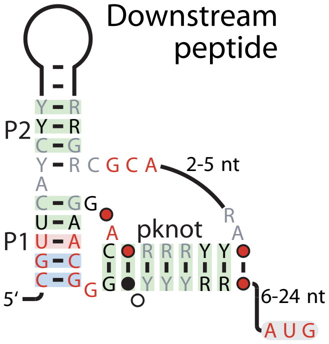

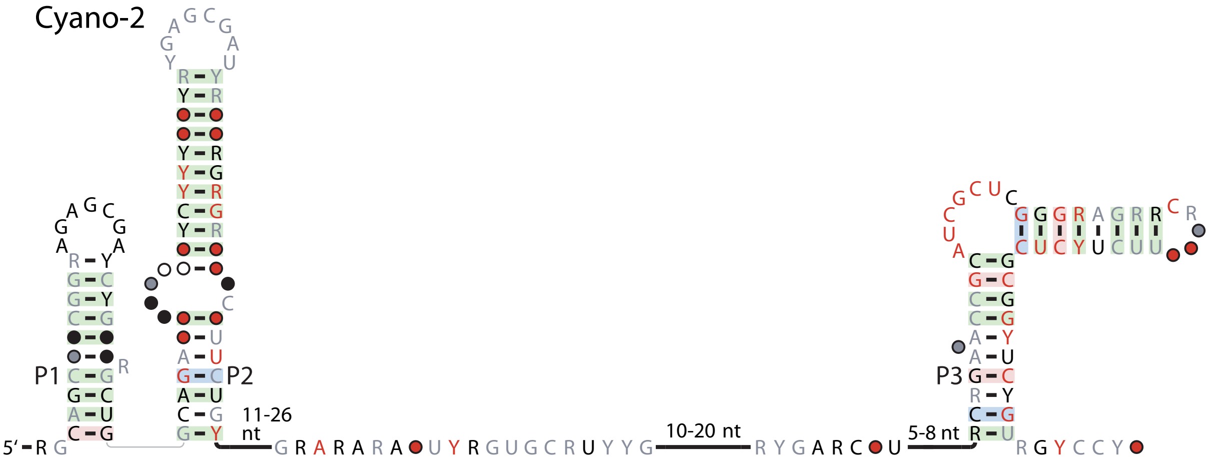

Dictyoglomi-1, *epsC*, *fixA*, **Flavo-1**

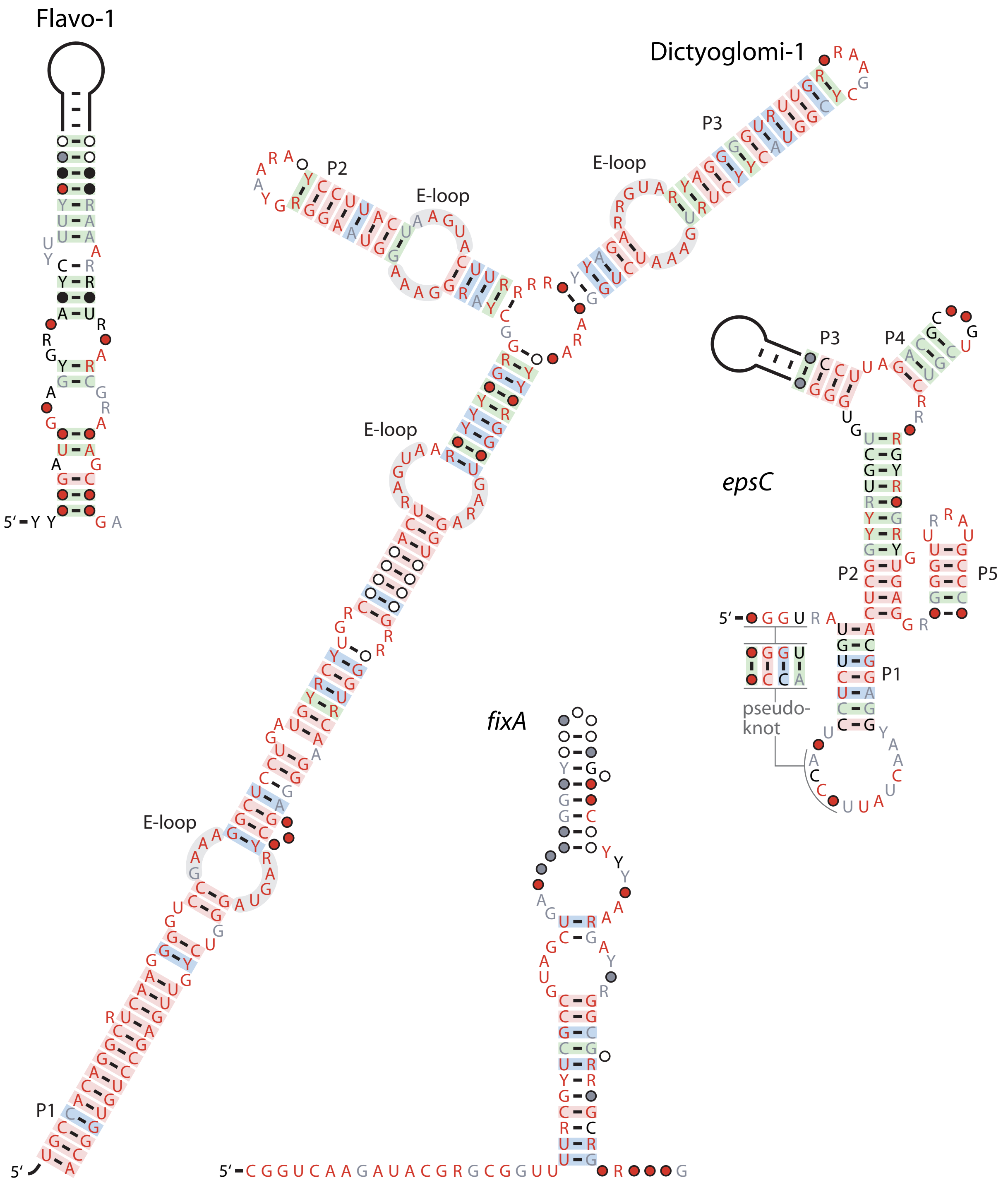

A portion of this figure was adapted from the supplementary data of a previous publication [21].

flpD, *flg*-Rhizobiales, *gabT*, Gamma-*cis*-1, *glnA*, GUCCY-hairpin

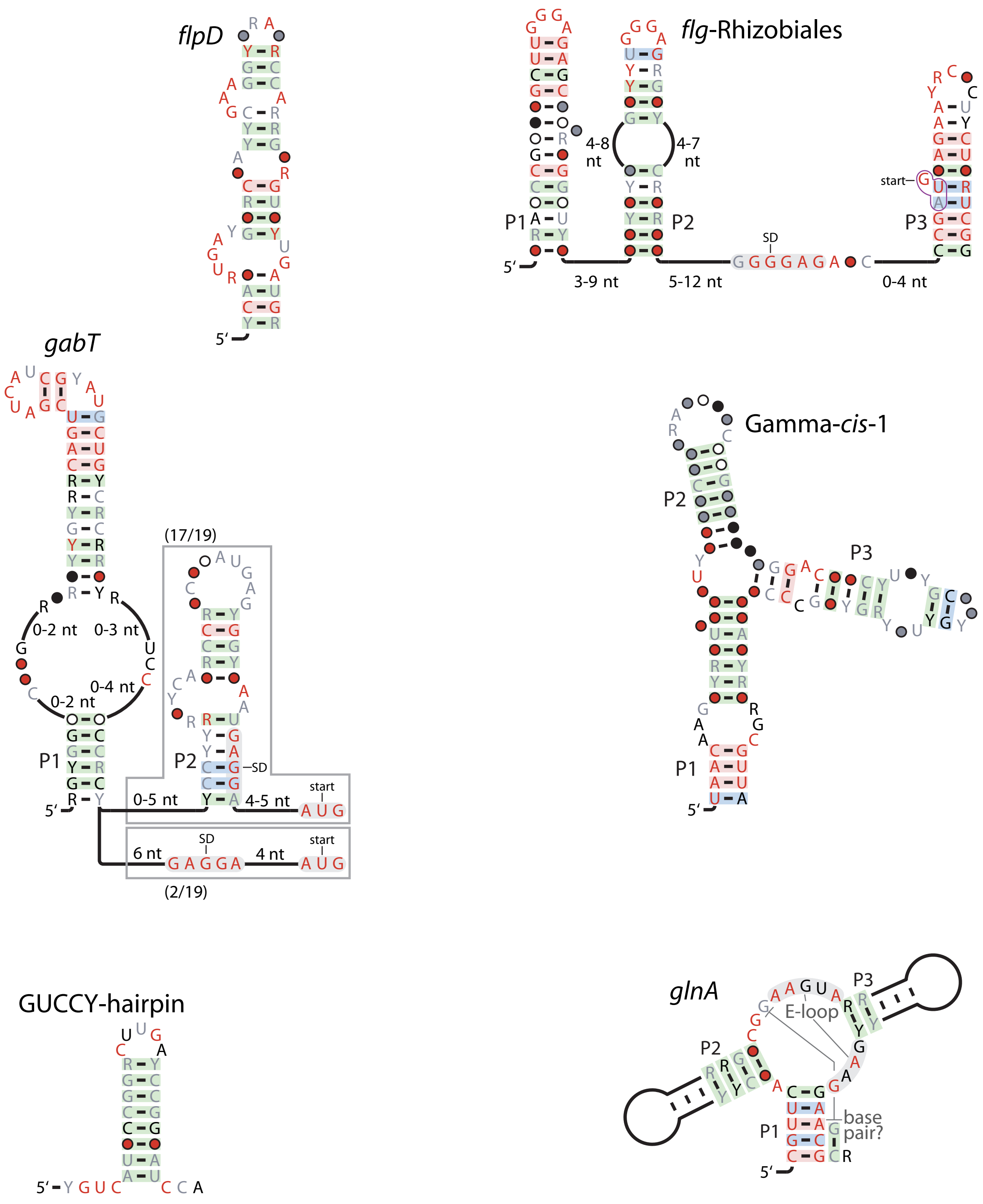

A portion of this figure was adapted from the supplementary data of a previous publication [21].

Gut-1, *gyrA*, *hopC*, *icd*, JUMPstart, L17 downstream element

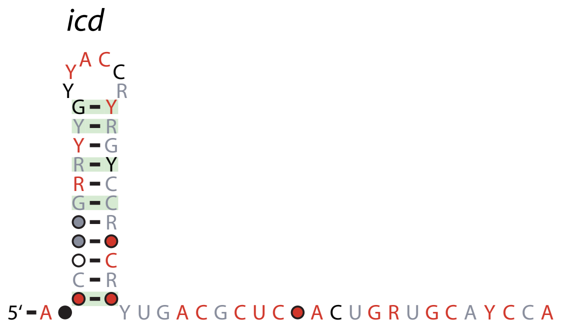

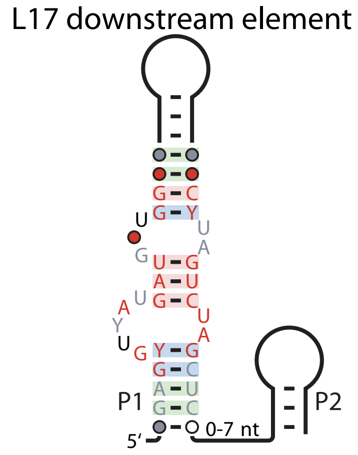

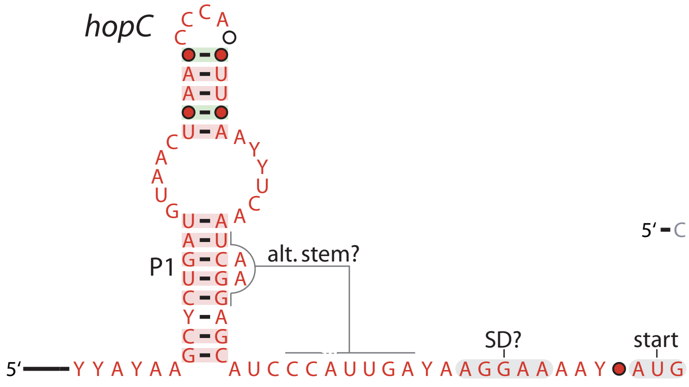

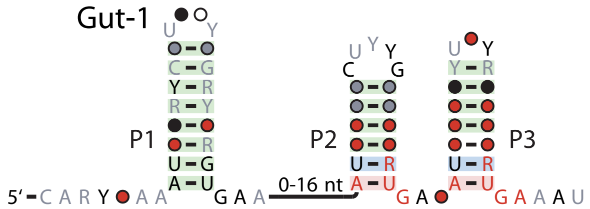

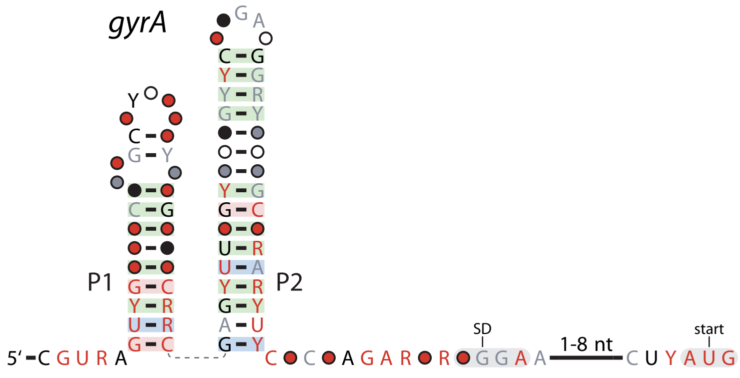

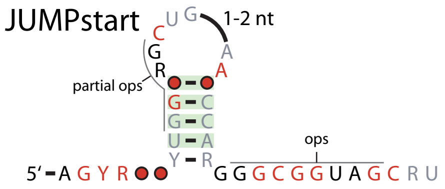

lactis-plasmid, Lacto-*int*, Lacto-*rpoB*, Lacto-*usp*, leu/phe leader, Lnt

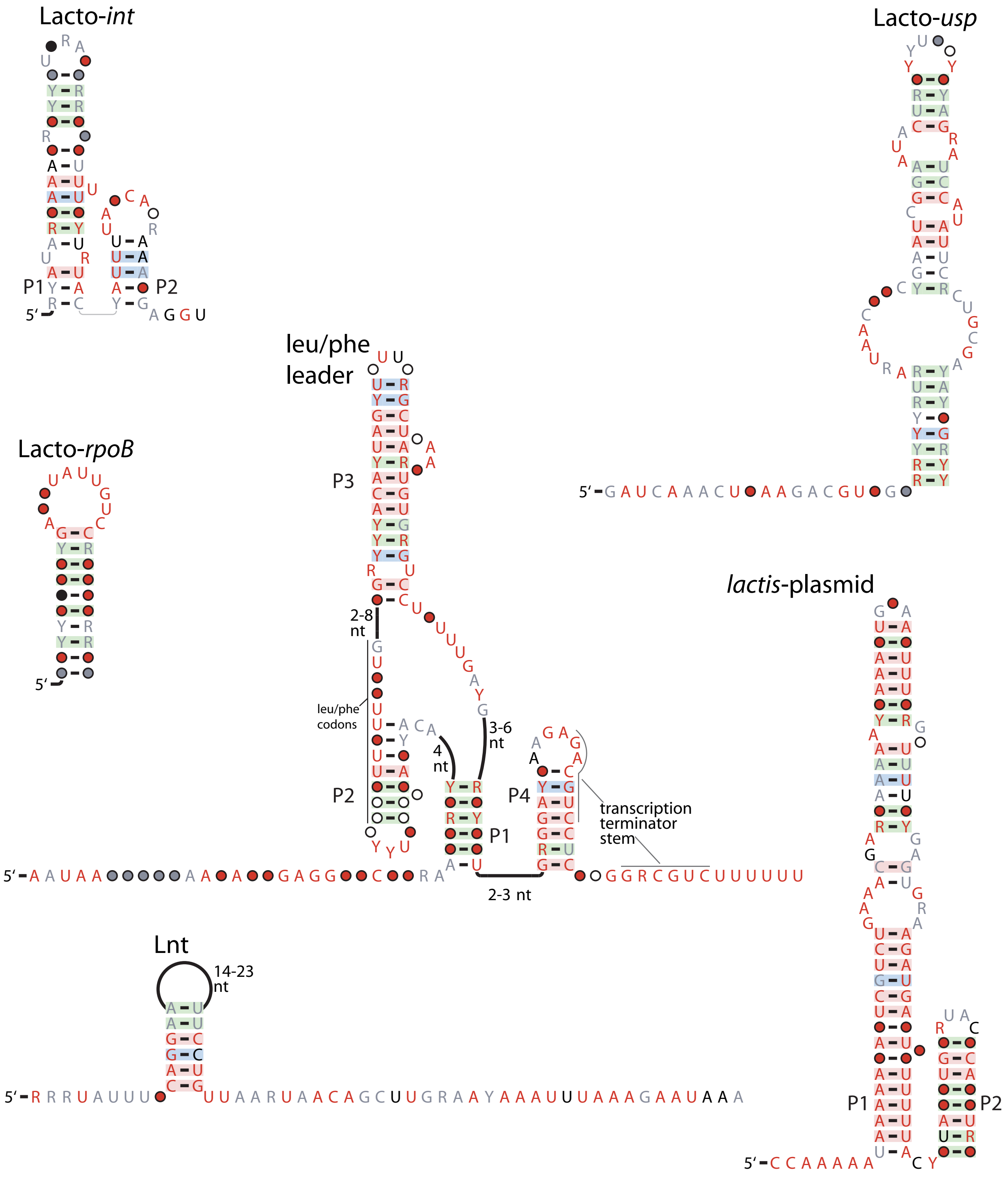

manA, *Methylobacterium*-1, *metK*-Rhodobacter, Moco-II, *msiK*

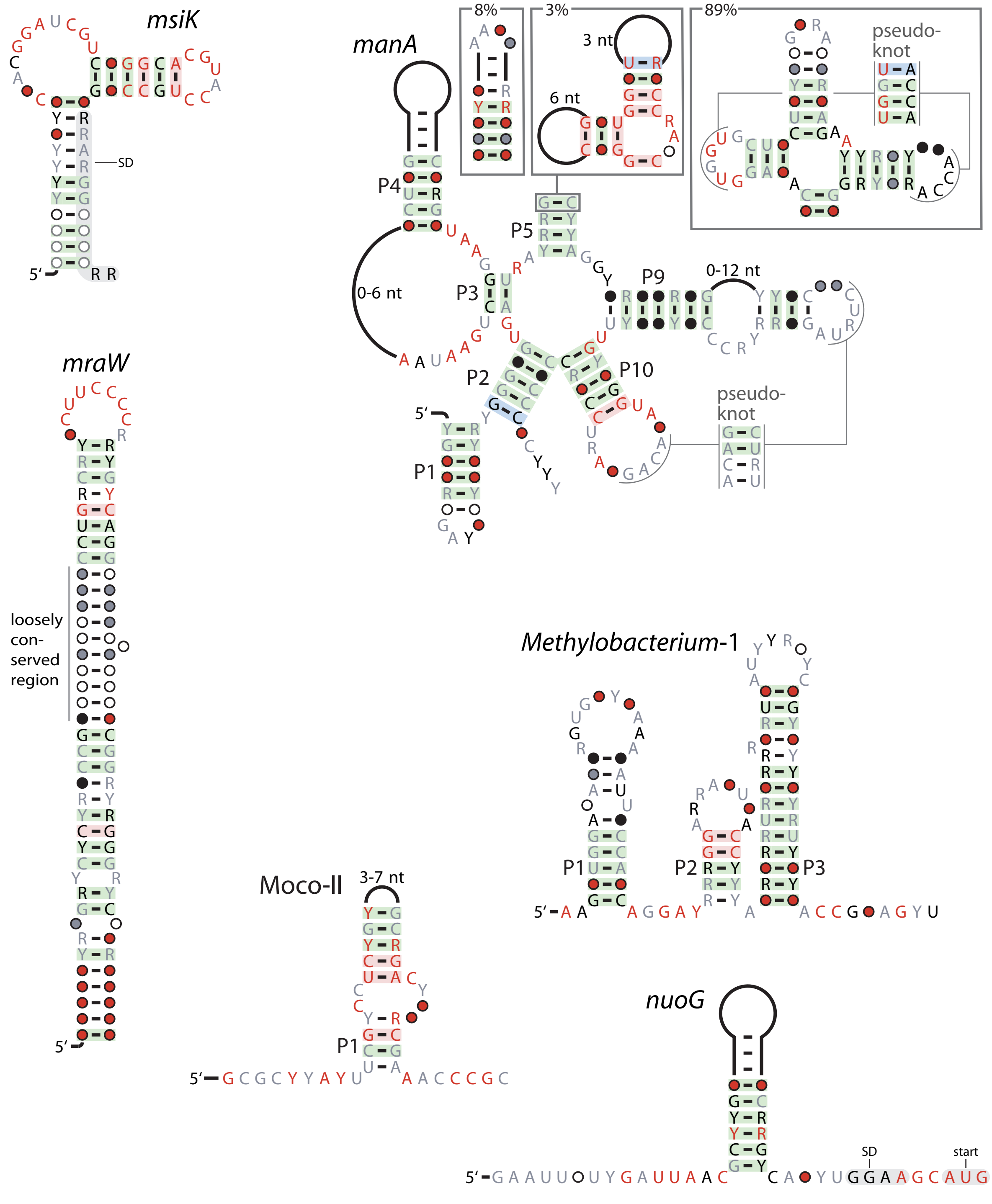

A portion of this figure was adapted from the supplementary data of a previous publication [21].

Ocean-V, Ocean-VI, *pan*, *Pedo*-repair

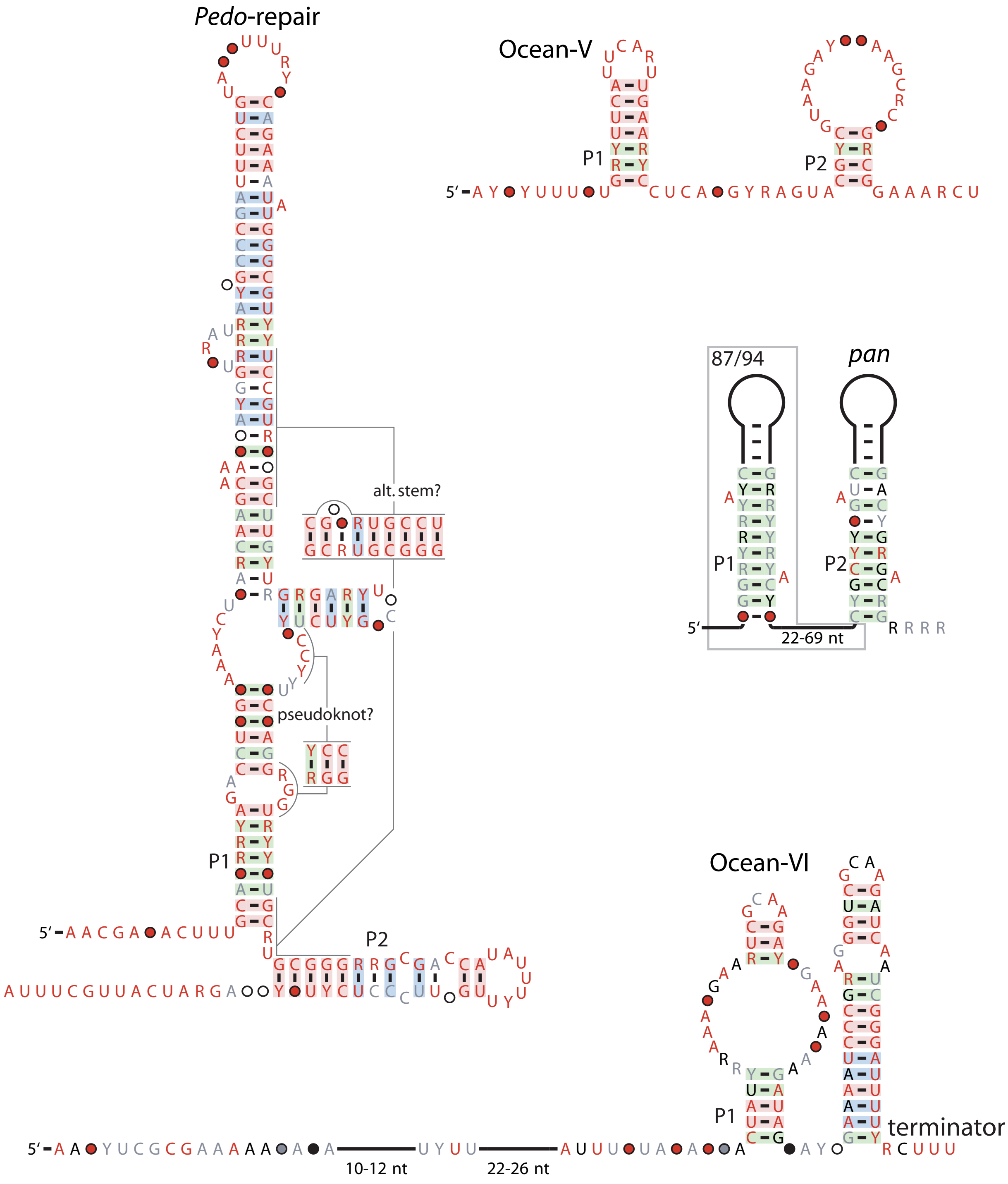

pfl, *psaA*, *pheA*, PhotoRC-I, PhotoRC-II

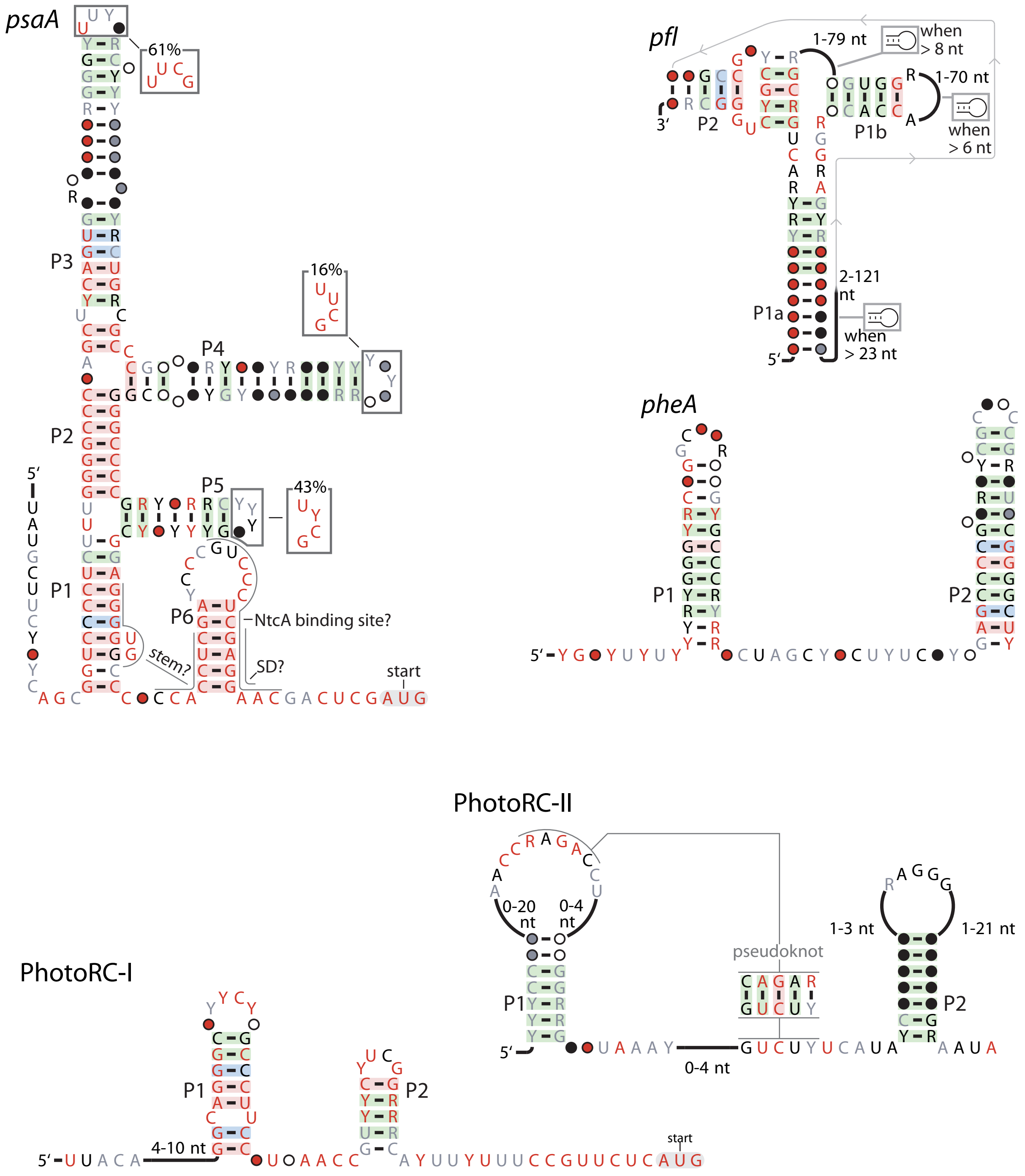

Polynucleobacter-**1**, *potC*, **Pseudomon-1**

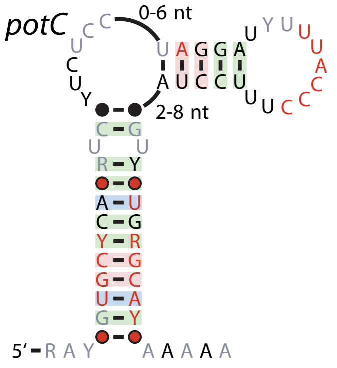

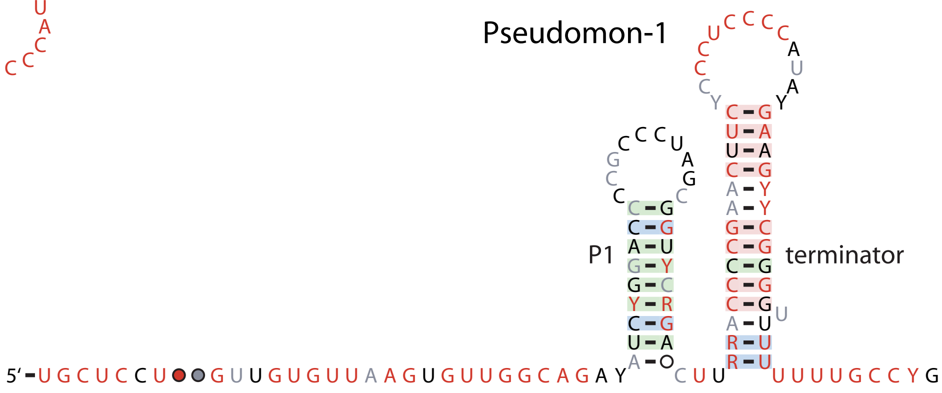

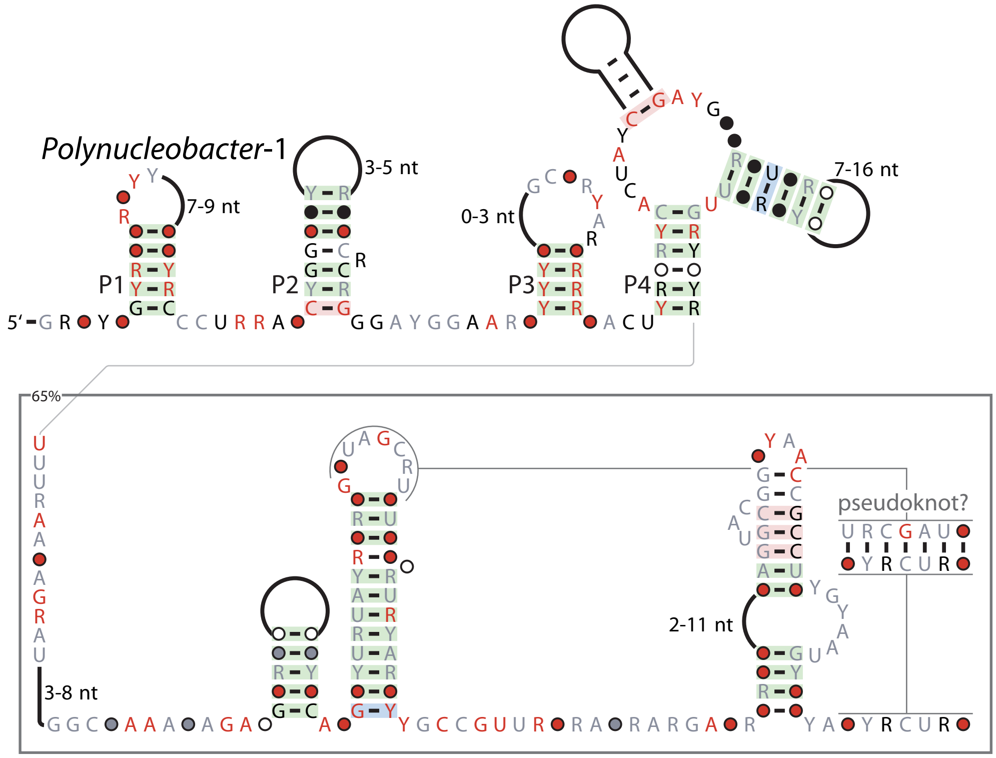

psbNH, Pseudomon-2, Pseudomon-*groES*, *Pseudomon*-Rho, *Pyrobac*-1, *Pyrobac*-HINT

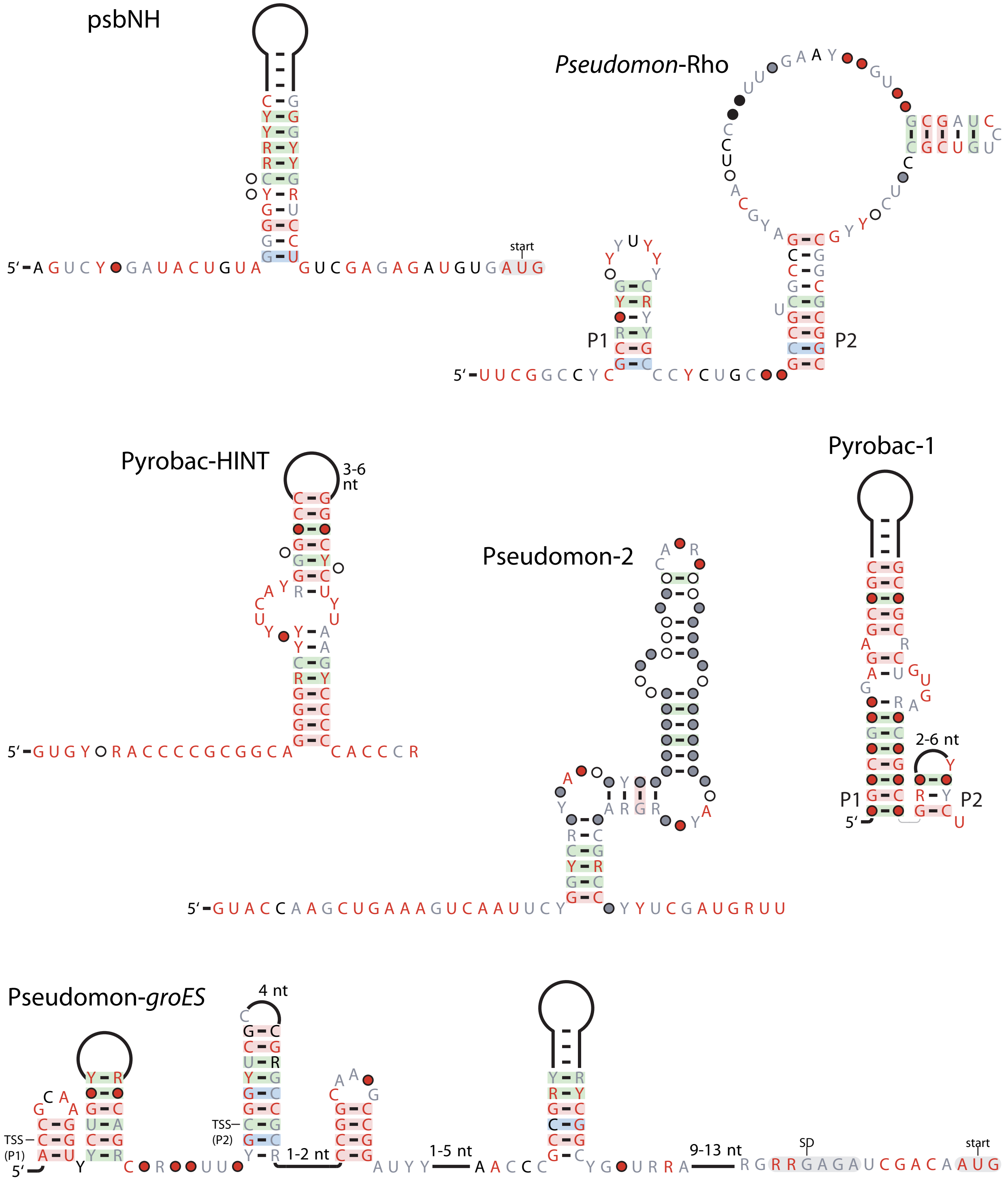

radC, Rhizobiales-1, Rhizobiales-2, Rhodopirellula-1, *rmf*

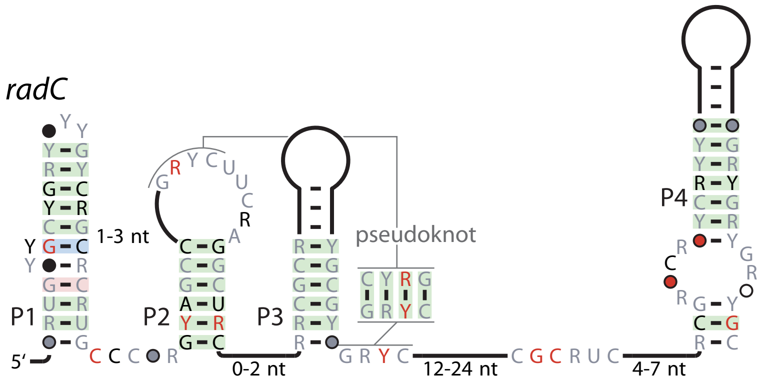

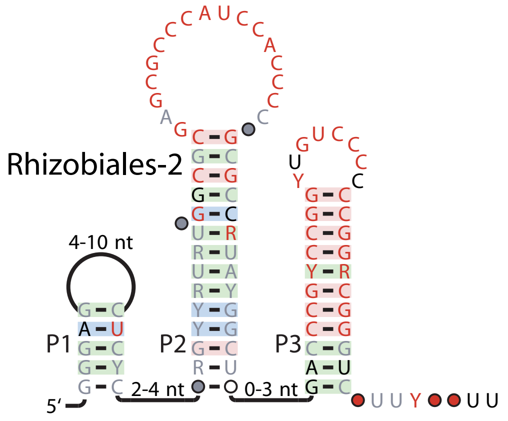

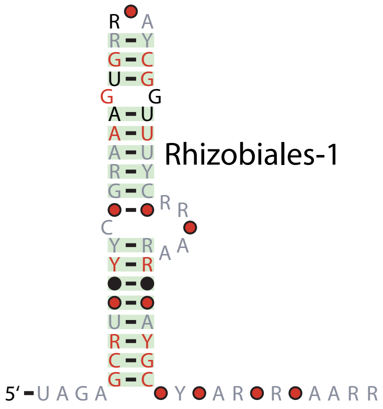

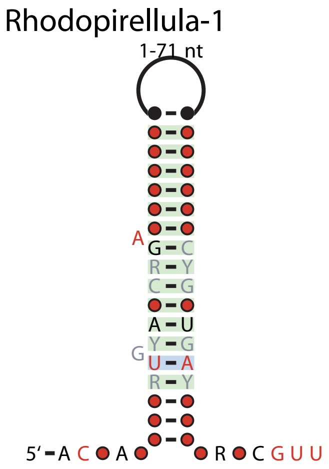

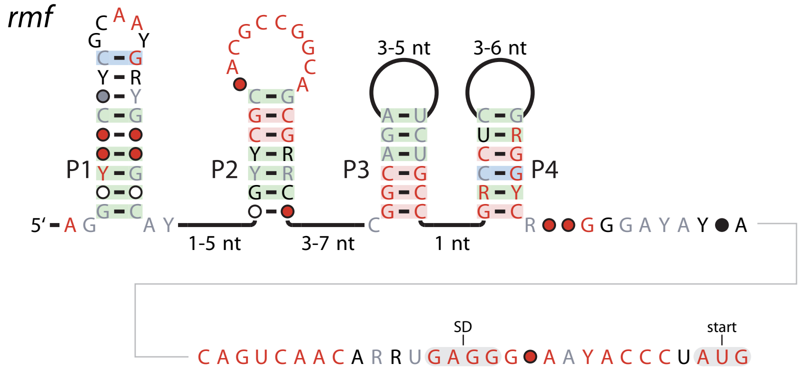

rne-II, SAM-Chlorobi, SAM-I-nil

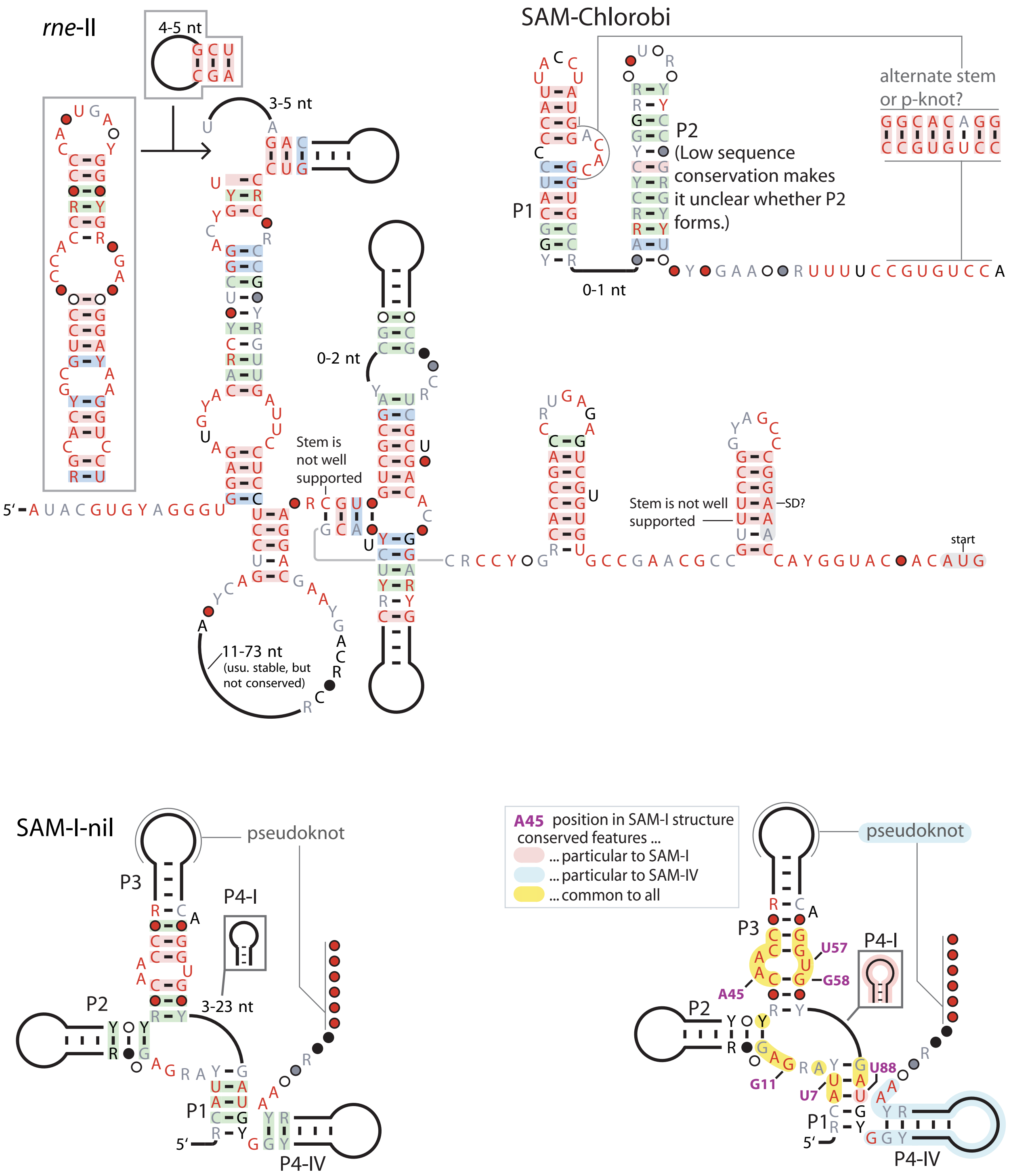

SAM/SAH, *sanguinis*-hairpin, *sbcD*, ScRE, Soil-1, Solibacter-1

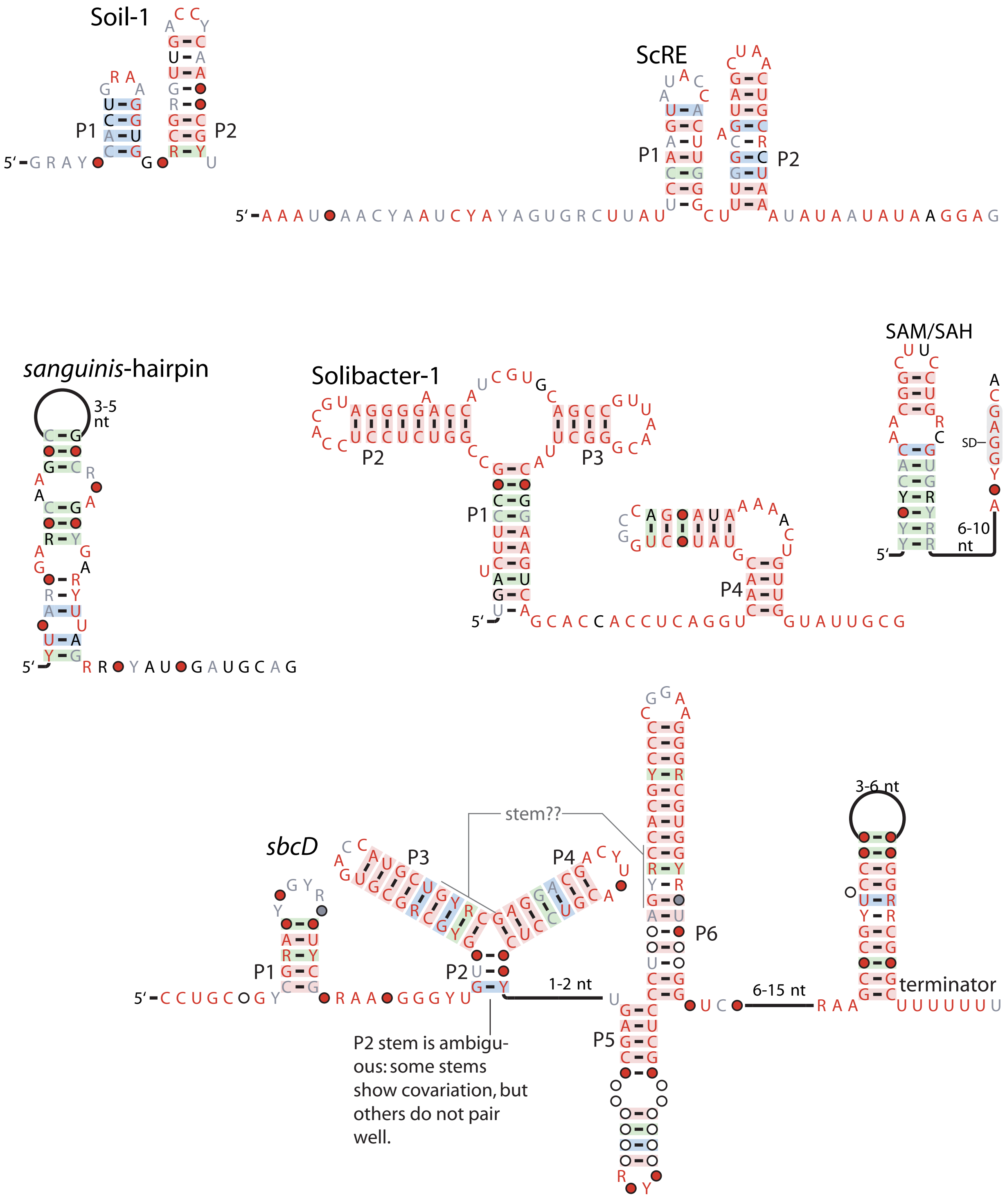

STAXI, *sucA*-II, *sucC*

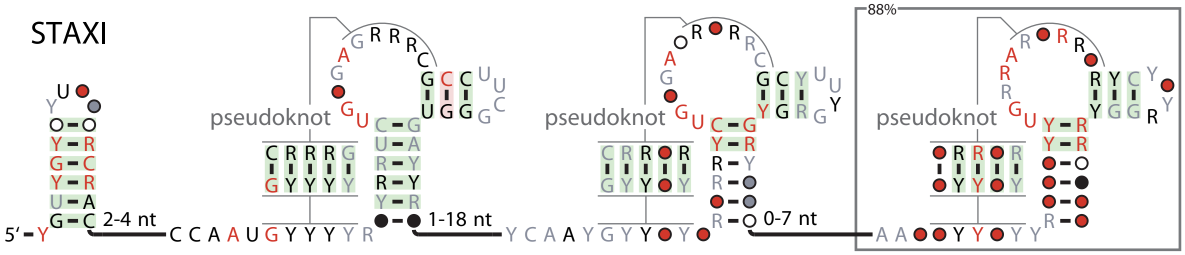

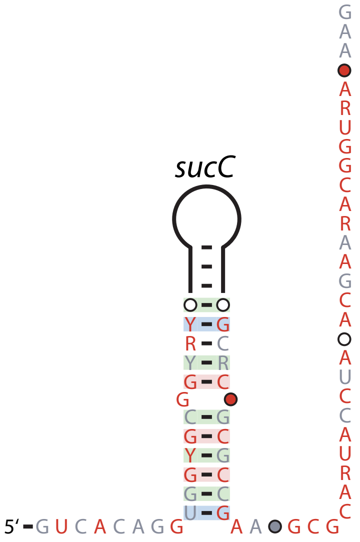

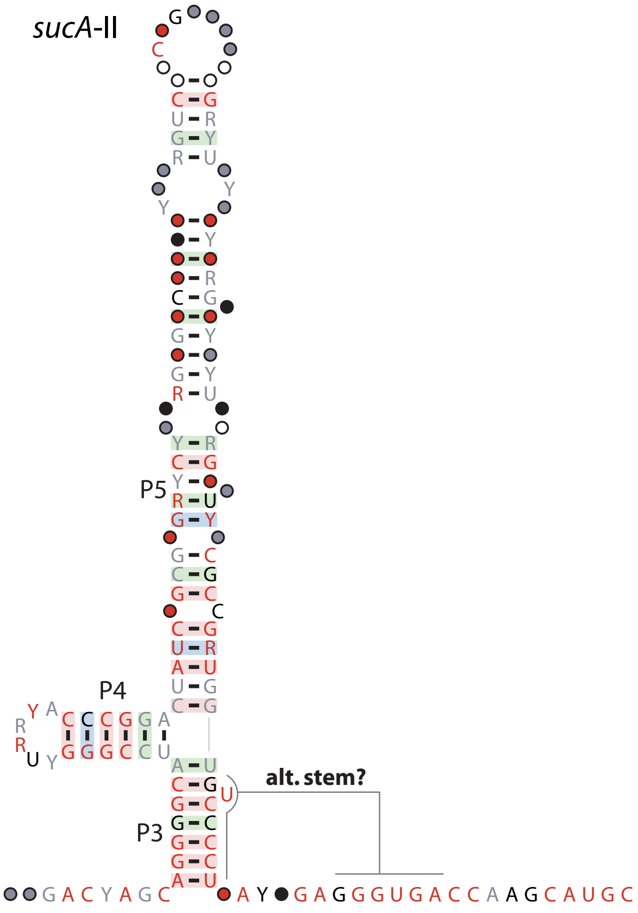

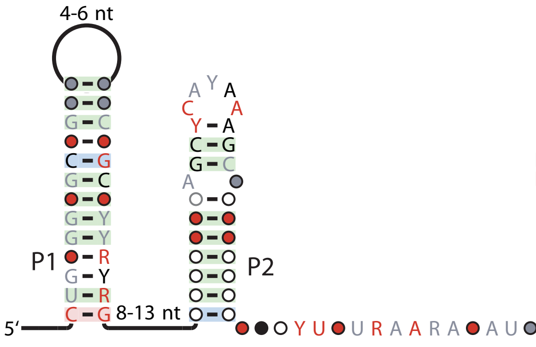

Termite-*flg*, Termite-*leu*, *traJ*-II, TwoAYGGAY

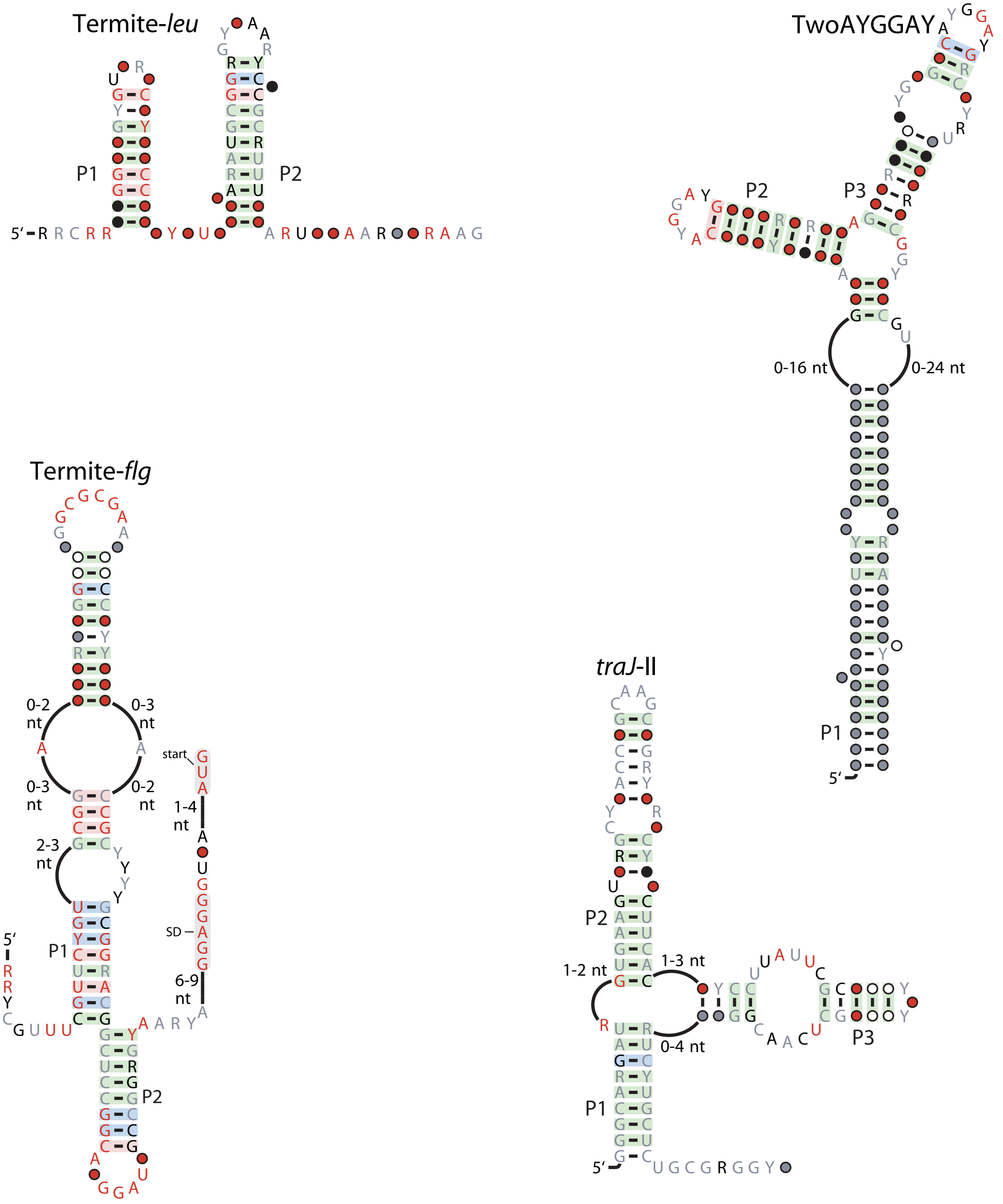

A portion of this figure was adapted from the supplementary data of a previous publication [21].

wcaG, Whalefall-1, *yjdF*, *ykkC*-III

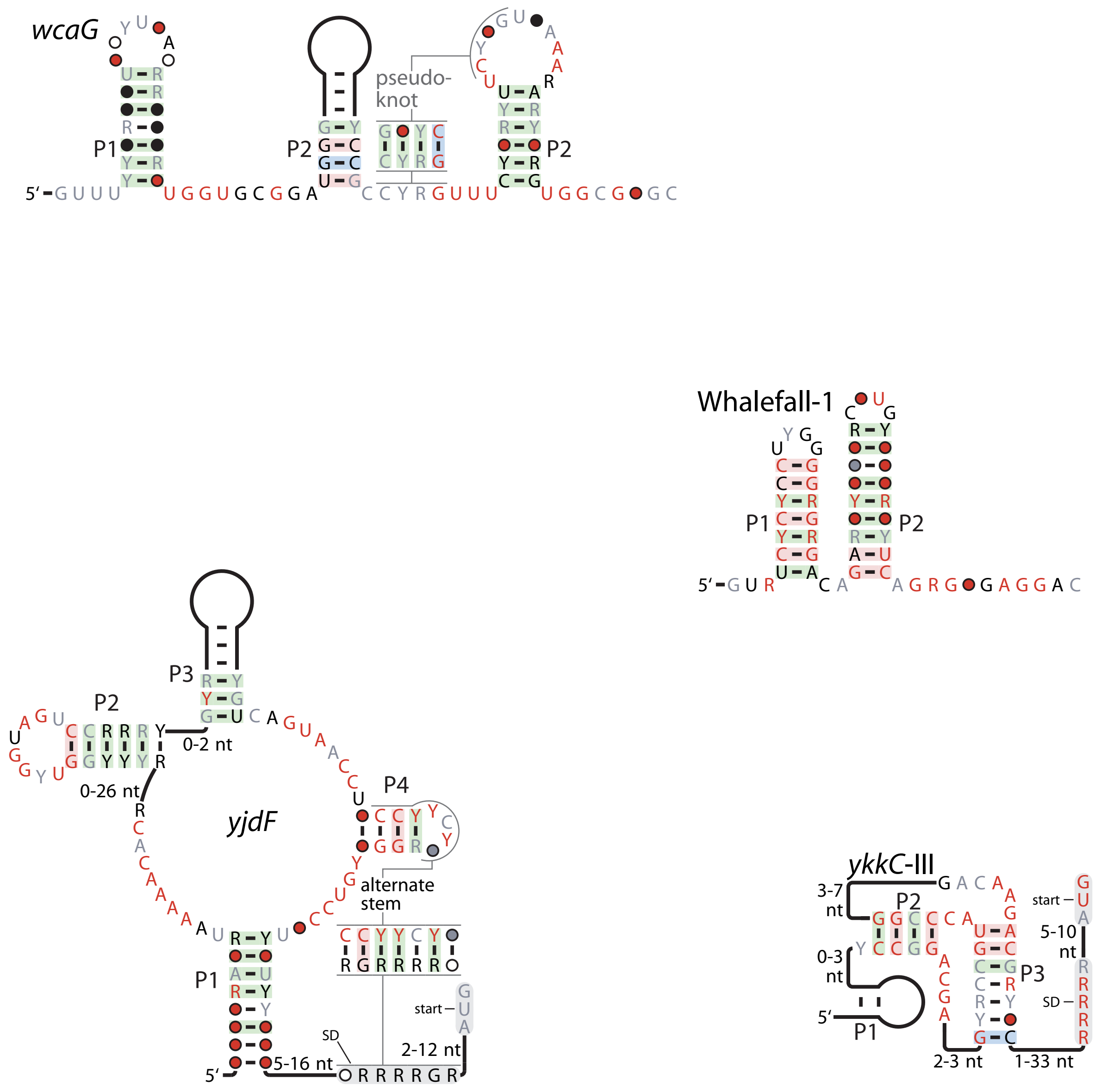

A portion of this figure was adapted from the supplementary data of a previous publication [21].

References

References are listed in the manuscript.
