# Supplementary material for: Comparative genomics reveals 104 candidate structured RNAs from bacteria, archaea, and their metagenomes
Source: Genome Biol. 2010 Mar 15;11(3):R31. doi: 10.1186/gb-2010-11-3-r31 (PMC2864571; doi:10.1186/gb-2010-11-3-r31)
Supplement: Additional file 8 — Genes associated with ykkC, mini-ykkC and ykkC-III RNAs. The frequencies with which various gene families are associated with ykkC, mini-ykkC or ykkC-III RNAs are listed. [file gb-2010-11-3-r31-S8.PDF]

Additional File 8: genes associated with *ykkC*, mini-*ykkC* and *ykkC*-III RNAs
supplementary for: Comparative genomics reveals 104 candidate structured RNAs from bacteria, archaea and their metagenomes

Zasha Weinberg, Joy X. Wang, Jarrod Bogue, Jingying Yang,
Keith Corbino, Ryan H. Moy, Ronald R. Breaker

November 17, 2009

Statistics were calculated on the first gene downstream of representatives of the *ykkC* (Barrick, *et al.*, 2004), mini-*ykkC* (Weinberg, *et al.*, 2007) or *ykkC*-III (this work) motifs, where such a gene was annotated. Only the first gene was considered, since operon prediction is often unreliable. By contrast, the first gene is almost certainly regulated by the RNA. The number of motif representatives associated with each conserved domain was calculated, and normalized by dividing it by the total number of domains counted. (Thus the ratios for a given motif will sum to 1.)

The following table lists conserved domains associated with at least one of the motifs with their CDD accession, and the first sentence of their description. Each motif is listed (for clarity "*ykkC*-I" refers to the motif of Barrick, *et al.*), along with the total number of first genes considered. The normalized ratios for each conserved domain and motif are given in the table. COG2076 is EmrE. Conserved domains are sorted in decreasing order of their average ratio.

(The table begins on the next page)

CDD	description	*ykkC*-I (#=203)	mini-*ykkC* (#=263)	*ykkC*-III (#=42)
COG2076	Membrane transporters of cations and cationic drugs [Inorganic ion transport and metabolism]	0.155	0.639	0.925
COG0715	ABC-type nitrate/sulfonate/bicarbonate transport systems, periplasmic components [Inorganic ion transport and metabolism]	0.260	0.010	0.000
COG3665	Uncharacterized conserved protein [Function unknown]	0.072	0.035	0.000
COG3382	Uncharacterized conserved protein [Function unknown]	0.105	0.000	0.000
COG0010	Arginase/agmatinase/formimionoglutamate hydrolase, arginase family [Amino acid transport and metabolism]	0.044	0.000	0.050
COG4770	Acetyl/propionyl-CoA carboxylase, alpha subunit [Lipid metabolism]	0.011	0.058	0.000
COG0511	Biotin carboxyl carrier protein [Lipid metabolism]	0.011	0.058	0.000
pfam02626	Allophanate hydrolase subunit 2.	0.011	0.055	0.000
pfam02682	Allophanate hydrolase subunit 1.	0.006	0.058	0.000
COG1051	ADP-ribose pyrophosphatase [Nucleotide transport and metabolism]	0.055	0.000	0.000
COG2252	Permeases [General function prediction only]	0.044	0.000	0.000
pfam00893	Small Multidrug Resistance protein.	0.011	0.032	0.000
pfam05232	Bacterial Transmembrane Pair family.	0.000	0.032	0.000
pfam05787	Bacterial protein of unknown function (DUF839).	0.000	0.000	0.025
COG1284	Uncharacterized conserved protein [Function unknown]	0.022	0.000	0.000
COG0387	Ca2+/H+ antiporter [Inorganic ion transport and metabolism]	0.000	0.019	0.000
COG0591	Na+/proline symporter [Amino acid transport and metabolism / General function prediction only]	0.017	0.000	0.000
cd01360	Adenylsuccinate lyase_1: Adenylsuccinate lyase (ASL)_subgroup 1.	0.017	0.000	0.000
COG0600	ABC-type nitrate/sulfonate/bicarbonate transport system, permease component [Inorganic ion transport and metabolism]	0.017	0.000	0.000
COG0531	Amino acid transporters [Amino acid transport and metabolism]	0.017	0.000	0.000
COG0041	Phosphoribosylcarboxyaminoimidazole (NCAIR) mutase [Nucleotide transport and metabolism]	0.011	0.000	0.000
COG0026	Phosphoribosylaminoimidazole carboxylase (NCAIR synthetase) [Nucleotide transport and metabolism]	0.011	0.000	0.000
COG2510	Predicted membrane protein [Function unknown]	0.011	0.000	0.000

CDD	description	*ykkC*-I (#=203)	mini-*ykkC* (#=263)	*ykkC*-III (#=42)
cd01558	D-Alanine aminotransferase (D-AAT_like): D-amino acid aminotransferase catalyzes transamination between D-amino acids and their respective alpha-keto acids.	0.006	0.000	0.000
COG1473	Metal-dependent amidase/aminoacylase/carboxypeptidase [General function prediction only]	0.006	0.000	0.000
COG1670	Acetyltransferases, including N-acetylases of ribosomal proteins [Translation, ribosomal structure and biogenesis]	0.006	0.000	0.000
COG0561	Predicted hydrolases of the HAD superfamily [General function prediction only]	0.006	0.000	0.000
COG0440	Acetolactate synthase, small (regulatory) subunit [Amino acid transport and metabolism]	0.006	0.000	0.000
cd01415	Prokaryotic and archaeal group of SAICAR synthetases represented by the Thermotoga maritima (Tm) SAICAR synthetase and E.	0.006	0.000	0.000
cd03267	Similar in sequence to NatA, the ATP-binding member of a bacterial ABC-type transporter called NatAB.	0.006	0.000	0.000
COG0683	ABC-type branched-chain amino acid transport systems, periplasmic component [Amino acid transport and metabolism]	0.006	0.000	0.000
cd01595	Adenylsuccinate lyase_like (ASL_like): This subgroup contains proteins similar to ASL and prokaryotic-type 3-carboxy-cis,cis-muconate cycloisomerase (pCMLE).	0.006	0.000	0.000
COG1001	Adenine deaminase [Nucleotide transport and metabolism]	0.006	0.000	0.000
COG1744	Uncharacterized ABC-type transport system, periplasmic component/surface lipoprotein [General function prediction only]	0.006	0.000	0.000
COG2188	Transcriptional regulators [Transcription]	0.006	0.000	0.000
COG0833	Amino acid transporters [Amino acid transport and metabolism]	0.006	0.000	0.000
COG0601	ABC-type dipeptide/oligopeptide/nickel transport systems, permease components [Amino acid transport and metabolism / Inorganic ion transport and metabolism]	0.006	0.000	0.000
COG3603	Uncharacterized conserved protein [Function unknown]	0.006	0.000	0.000
COG0731	Fe-S oxidoreductases [Energy production and conversion]	0.006	0.000	0.000
cd01907	Glutamine amidotransferases class-II (Gn-AT)_GlxB-type.	0.006	0.000	0.000
pfam06965	Na+/H+ antiporter 1.	0.000	0.003	0.000
