# Supplementary material for: Comparative genomics reveals 104 candidate structured RNAs from bacteria, archaea, and their metagenomes
Source: Genome Biol. 2010 Mar 15;11(3):R31. doi: 10.1186/gb-2010-11-3-r31 (PMC2864571; doi:10.1186/gb-2010-11-3-r31)
Supplement: Additional file 9 — Partitioning of genomes and metagenomes. Describes how genomes and metagenomes were divided into pipeline runs. [file gb-2010-11-3-r31-S9.PDF]

Additional File 9: Partitions of genomes and metagenomes supplementary for: Comparative genomics reveals 104 candidate structured RNAs from bacteria, archaea and their metagenomes

Zasha Weinberg, Joy X. Wang, Jarrod Bogue, Jingying Yang, Keith Corbino, Ryan H. Moy, Ronald R. Breaker

November 17, 2009

Partitions of genome sequences taken from RefSeq. The "incomplete genomes" columns refers to whether incomplete genomes from RefSeq were used ("Y"=yes, "N"=no). In RefSeq, the accessions of fully completed genomes start with the letters "NC_", while incomplete genomes typically start with "NZ_".

**name**	**RefSeq version**	**Incomplete genomes?**	**Taxon**
crenarch	25	Y	Archaea; Crenarchaeota
acido	25	Y	Bacteria; Acidobacteria
actino	25	N	Bacteria; Actinobacteria
bacteroid	25	Y	Bacteria; Bacteroidetes
cyano	25	N	Bacteria; Cyanobacteria
chlamyd	25	Y	Bacteria; Chlamydiae
chlorobi	25	Y	Bacteria; Chlorobi
chloroflexi	25	Y	Bacteria; Chloroflexi
clostridia	25	N	Bacteria; Firmicutes; Clostridia
deino	25	Y	Bacteria; Deinococcus-Thermus
delta	25	N	Bacteria; Proteobacteria; Deltaproteobacteria
epsilon	25	N	Bacteria; Proteobacteria; Epsilonproteobacteria
lacto	25	N	Bacteria; Firmicutes; Lactobacillales
mollicutes	25	N	Bacteria; Firmicutes; Mollicutes
plancto	25	Y	Bacteria; Planctomycetes
gamma-aeromon	25	Y	Bacteria; Proteobacteria; Gammaproteobacteria; Aeromonadales
gamma-alteromon	25	N	Bacteria; Proteobacteria; Gammaproteobacteria; Alteromonadales
gamma-chrom	25	Y	Bacteria; Proteobacteria; Gammaproteobacteria; Chromatiales
gamma-legion	25	Y	Bacteria; Proteobacteria; Gammaproteobacteria; Legionellales
gamma-oceano	25	Y	Bacteria; Proteobacteria; Gammaproteobacteria; Oceanospirillales
gamma-pasteur	25	N	Bacteria; Proteobacteria; Gammaproteobacteria; Pasteurellales
gamma-pseudomon	25	N	Bacteria; Proteobacteria; Gammaproteobacteria; Pseudomonadales
gamma-thio	25	Y	Bacteria; Proteobacteria; Gammaproteobacteria; Thiotrichales
gamma-vibrio	25	N	Bacteria; Proteobacteria; Gammaproteobacteria; Vibrionales
gamma-xanthomon	25	N	Bacteria; Proteobacteria; Gammaproteobacteria; Xanthomonadales
spiro	25	Y	Bacteria; Spirochaetes
thermatog	25	Y	Bacteria; Thermotogae
euryarch	32	N	Archaea; Euryarchaeota
aquifex	32	Y	Bacteria; Aquificae
dicty	32	Y	Bacteria; Dictyoglomi

name	RefSeq version	Incomplete genomes?	Taxon
bacillales	32	N	Bacteria; Firmicutes; Bacillales
erysipelo	32	Y	Bacteria; Firmicutes; Erysipelotrichi
fuso	32	Y	Bacteria; Fusobacteria
lenti	32	Y	Bacteria; Lentisphaerae
nitro	32	Y	Bacteria; Nitrospirae
caulobact	32	Y	Bacteria; Proteobacteria; Alphaproteobacteria; Caulobacterales
rhizo	32	N	Bacteria; Proteobacteria; Alphaproteobacteria; Rhizobiales
rhodobact	32	N	Bacteria; Proteobacteria; Alphaproteobacteria; Rhodobacterales
rhodospir	32	N	Bacteria; Proteobacteria; Alphaproteobacteria; Rhodospirillales
rickett	32	N	Bacteria; Proteobacteria; Alphaproteobacteria; Rickettsiales
sphingo	32	N	Bacteria; Proteobacteria; Alphaproteobacteria; Sphingomonadales
beta	32	N	Bacteria; Proteobacteria; Betaproteobacteria
gamma-acid	32	Y	Bacteria; Proteobacteria; Gammaproteobacteria; Acidithiobacillales
gamma-alteromon	32	N	Bacteria; Proteobacteria; Gammaproteobacteria; Alteromonadales
gamma-entero	32	N	Bacteria; Proteobacteria; Gammaproteobacteria; Enterobacteriales
tg1	32	Y	Bacteria; candidate division TG1
tm7	32	Y	Bacteria; candidate division TM7
verruco	32	Y	Bacteria; Verrucomicrobia

Partitions of metagenome sequences from environmental data not including GOS data:

name	description
env-sludge	All sludge sequences (Garcia-Martin, *et al.*)
env-acidmine	All acid mine drainage sequences (Tyson, *et al.*)
env-soil	All soil sequences (Tringe, *et al.*)
env-whalefall	All whalefall sequences (also *Tringe, et al.*)
env-wormgut	All gutless sea worm sequences (Woyke, *et al.*)
env-termite-gut	All termite gut sequences (Warnecke, *et al.*)
env-mammal-gut	All human and mouse gut sequences (Gill, *et al.*, Kurokawa, *et al.* and Turnbaugh, *et al.*)
env-ocean-HOTS	All marine sequences from DeLong, *et al.* only

Partitions of metagenome sequences from GOS data (Venter, *et al.*, and Rusch, *et al.*). Note the "predicate" column references parameters in GOS metadata downloaded from the CAMERA web site at http://camera.calit2.net.

name	predicate
env-gos-freshwater	salinity< 0.2
env-gos-hypersaline	salinity> 60
env-gos-mangrove	habitat='Mangrove'
env-gos-upwelling	habitat='Coastal upwelling'
env-gos-coral	habitat='Coral Atoll' or habitat='Coral Reef' or habitat='Coral Reef Atoll'
env-gos-fringing-reef	habitat='Fringing Reef'
env-gos-embayment	habitat='Embayment'
env-gos-warmseep	habitat='Warm Seep'
env-gos-openoceanbigcells	habitat='Open Ocean' and min_size_fraction>= 0.79
env-gos-eastpacificocean	habitat='Open Ocean' and geographic_location='Eastern Tropical Pacific'
