# Supplementary material for: Comparative genomics reveals 104 candidate structured RNAs from bacteria, archaea, and their metagenomes
Source: Genome Biol. 2010 Mar 15;11(3):R31. doi: 10.1186/gb-2010-11-3-r31 (PMC2864571; doi:10.1186/gb-2010-11-3-r31)
Supplement: Additional file 10 — Source code implemented as part of this project. Source code files and a README.pdf file are provided to assist in detailed understanding of the methods. The files can be retrieved from the .tar.gz archive file, as described for Additional file 4. [file gb-2010-11-3-r31-S10.GZ › 104-motifs-source-code/README.pdf]

Additional File 11: source code for pipeline system

from:

Comparative genomics reveals 104 candidate structured RNAs from bacteria, archaea and their metagenomes

Zasha Weinberg, Joy X. Wang, Jarrod Bogue, Jingying Yang, Keith Corbino, Ryan H. Moy, Ronald R. Breaker

Introduction

This archive file contains the source code specific to the system described in our paper. The source code is distributed under the terms of the GNU General Public License, without any warranty whatsoever. See the file "LICENSE" in this directory.

The purpose of the source code is to better document our methods. Due to the complexity of the overall system, it is not practical to provide a ready-to-install software suite without considerable additional software development. Therefore, the goal of this document is to give pointers to the most relevant parts of the source code. The source code within this archive file does not include source code of software integrated into the system, such as CMfinder or RAVENNA. These and other components can be downloaded separately.

Overall design of the system and source code directory structure

Most computation is done on a cluster administered by our university called "bulldogi" (this name is referenced in the code). This cluster has direct access to a MySQL database. Some computation is done on an independent server called "bliss" that is housed in the Breaker lab. Bliss has access to a separate MySQL database, and hosts a web server running Apache. However, most computation of likely research interest happens on the bulldogi cluster.

The directory "motifs_2007/pl" within this source code distribution houses the primary scripts used to run the pipeline. Ancillary scripts more focused on individual tasks are stored in the "exe" directory. The "motifs_2007/config" directory contains configuration files used to define data sets. Some configuration is also done in the "data/ravenna.config.tab", "bliss-scripts/Intergenic/Config.pm" and "bliss-scripts/Intergenic/AnalysisSettings.pm" files. IGR clustering is implemented in the Python and C code in "motifs_2007/Jeremy-code". This code was primarily written by Jeremy Gore. The source code in "bliss-scripts" is mostly directed at storing and drawing information on genome features (i.e., loci) stored in a MySQL database. It was written by Jeffrey E. Barrick, with some modifications by Zasha Weinberg. This source code is explained in more detail in a recent methods paper (Barrick, JE, Predicting Riboswitch Regulation on a Genomic Scale, *Methods in Molecular Biology* volume 540, 2009).

The main code to run the pipeline is in the file "motifs_2007/pl/ProcessPipeline.pl", and the perl module (".pm") files in that directory. The pipeline logic is implemented in a series of major and minor steps, and

the main loop in the pipeline starts with the code " for ($majorStepNum=0; $majorStepNum<scalar @pipelineSteps; $majorStepNum += 2) {" on line 411 of that file. Each step is implemented by a distinct Perl object within one of the ProcessPipeline_....pm files.

Some commonly referenced entities are as follows. A "super-db" is a database such as RefSeq that contains sequences that could be analyzed by the pipeline. A "group" defines a partition of these sequences, and a set of these groups is encapsulated by the "Groups" object. A pipeline "run" defines a set of parameters for a run of the pipeline, including a specific group. The set of runs current being processed is encapsulated by the “PipelineRuns” object. To be practical, the pipeline must run code in parallel on a cluster. This is accomplished using the $parallelWork object and functions defined in "ParallelBlock.pm". Note that this code is highly specific to the particular configuration of PBSPro software on the bulldogi cluster, and indeed all code is highly specific to the particular environment available in our infrastructure.

Annotated methods

The following indicates the parts of the code that implement each feature of the methods. The methods from the manuscript are repeated verbatim, but without references, and annotations are made in bolded text within square brackets [**like this**]. Some methods are taken as-is from a previously established protocol (Z. Yao, *et al.,* A computational pipeline for high-throughput discovery of *cis*-regulatory noncoding RNA in prokaryotes, *PLoS Comput Biol*, 3:e126, 2007.). Methods annotated as "Yao *et al* 2007." are documented in that work. Some text is replaced by elipses (“…”) or removed entirely where the text is not relevant to the source code.

DNA sequence sources and gene annotations

The microbial subsets of RefSeq version 25 or 32 were searched [**RefSeq sequences are downloaded in FASTA- and GenBank-format files. FASTA files are installed into searchable databases for RAVENNA (See RAVENNA documentation for how to edit the “data/ravenna.config.tab” file.) GenBank files are imported into a MySQL database. See the paper by JE Barrick, referenced above, for details.**], along with metagenome sequences [**To import environmental sequences, their FASTA-format sequences and any annotation files are processed by the script “motifs_2007/pl/EnvironmentalToGenBank.pl”. The output of this script is GenBank-format files that include gene and RNA annotation. The FASTA files and the resulting GenBank files are imported as per RefSeq (see above).**] from … Locations and identities of protein-coding genes were derived from RefSeq or IMG/M annotations, or from “predicted proteins” in Global Ocean Survey sequences. However, genes in some sequences were predicted using MetaGene (dated Oct. 12, 2006) with default parameters [**EnvironmentalToGenBank.pl can import GOS data, IMG/M data and can run MetaGene.**]. Conserved protein domains were annotated using the Conserved Domain Database version 2.08. [**Classifications are performed using the “exe/RpsblastParallel.pl” script, which uses the rpsblast command that is recommended by the Conserved Domain Database.**]

Annotations for tRNAs and rRNAs were derived from the sources noted above, or were predicted using tRNAscan-SE run in bacterial mode [**For RefSeq, tRNAscan-SE is run as part of the processing implemented by the “Step_FindRfamHitsByBlast” object in “motifs_2007/pl/ProcessPipeline_SuperDbProcessing.pm”. search for “tSE” to find the commands used to run tRNAscan-SE on the relevant IGRs. For environmental sequences, tRNAscan-SE is run by**

“EnvironmentalToGenbank.pl”]. To detect additional rRNAs, annotated rRNAs whose descriptions read “ribosomal RNA” or “#S rRNA” (# represents any number) were used [**This is implemented by the “Step_ExtractRibosomalRnas” object within ProcessPipeline_SuperDbProcessing.pm (see it’s “DoSubStep” method).**] in WU-BLAST queries with command-line flags -hspsepQmax=4000 -E 1e-20 -W 8 [**implemented by the “Step_FindRfamHitsByBlast” object in “motifs_2007/pl/ProcessPipeline_SuperDbProcessing.pm”. The blast command is set up on line 397.**]. Other RNAs were detected with Rfam, and WU-BLAST as described previously [**also implemented by the “Step_FindRfamHitsByBlast” object in “motifs_2007/pl/ProcessPipeline_SuperDbProcessing.pm”. The blast command for matching Rfam hits is set up on line 391.**]. We also used published alignments of riboswitches as queries with RAVENNA global-mode searches, selecting hits manually based primarily on E-values [**These hits are imported into the same data structures as for other RNAs.**].

Automated motif identification

To reduce false positives in sequence comparisons, the pipeline was run separately on related taxa or metagenome sources [**runs are defined within the file “motifs_2007/config/runs1.tab”, and groups are defined within the file “motifs_2007/config/pipeline_groups.tab”.**]. For each run, InterGenic Regions (IGRs) of at least 30 nucleotides were extracted between protein-coding, tRNA and rRNA genes. [**Extraction of IGRs from GenBank-format files is implemented by the script “motifs_2007/pl/IgrExtract.pl”, which is invoked from ProcessPipeline_SuperDbProcessing.pm (search for “IgrExtract”)**]

To generate clusters, an early version of a recently described algorithm was used. Specifically, IGRs were compared using nucleotide NCBI BLAST version 2.2.17 and parameters -W 7 -G 2 -E 2 -q -2 -m 8 [**parallel runs of blastn are implemented by the object “Step_JeremyRunBlast” within “ProcessPipeline_Jeremy.pm”. Actual blastall commands are formed in line 324. The object “Step_JeremyPrepareForBlast” formats sequences for use with NCBI blastall.**]. Self matches were ignored. BLAST scores below a parameter S (see below) were considered insignificant and ignored. Each BLAST match defines two “nodes”, corresponding to the matching sequences. Nodes that overlap by at least five nucleotides are merged, along with their BLAST homologies. A cluster consists of all nodes that have direct or indirect (transitive) BLAST matches. Closely related sequences that span multiple distinct elements in an entire IGR can lead to spurious node merges. Therefore, homologies with BLAST scores above 100 are ignored. [**All configurable parameters are defined in “motifs_2007/config/run1.tab” or “run2.tab”. Clustering is implemented by the “overcluster” command that is built by the “Makefile” in the directory “motifs_2007/Jeremy-code”. The .c and .h files within that directory comprise the program, which was written in C. The overall clustering process, including invocation of the Python scripts mentioned below, is implemented by the “Step_JeremyOverCluster” object within “ProcessPipeline_Jeremy.pm”, and in particular by the “DoSubStep” method of that object.**]

If a node’s length in nucleotides is L, and L<500, then the node is extended on either side by (500-L)/2 nucleotides, but is constrained to remain within the original IGR. CMfinder can easily tolerate nodes of 500 nucleotides. When L>1000, nodes are shrunk by (L-1000)/2 nucleotides around the center. The L>1000 case is extremely rare. Only clusters with at least three members were reported. [**Extraction of sequences corresponding to each pipeline is performed by the Python script “motifs_2007/Jeremy-code/procClusters.py”**]

For each pipeline run, we tried a range of values for the parameter S=35, 40, …, 85, and determined how many known RNAs were detected with each value [**Test results were collated by the script**

“motifs_2007/Jeremy-code/testClusters2.py”]. Based on these data, a set of S values was selected manually, and the union of clusters arising from each S was used as input to CMfinder. CMfinder was used to predict motifs exactly as before [**Invocation of CMfinder is done by the three steps in “ProcessPipeline_CMfinder.pm” The code is equivalent to the methods described by Yao *et al.* 2007**]. Automated homology searches were then performed as described, except that covariance model scores used the null3 model [**These automated searches were nicknamed “miniscans”, and the process is implemented by the object within “ProcessPipeline_Miniscan.pm”, which invokes the script “exe/MultiRavenna.pl”, which, in turn, uses the “exe/ravenna.pl” script that is part of RAVENNA. Readers wishing to replicate this functionality might prefer to use the Infernal software package (http://infernal.janelia.org) created by Sean Eddy’s group. Infernal implements the same heuristic profile HMM algorithm that were developed by RAVENNA, and is used by the automated miniscans, and Infernal also implements additional algorithms and optimizations that appear to result in even faster, and more accurate searches.**]. Motifs were scored using a previously established method [**The code in the “DoSubStep” method of “Step_CMfinderPostprocess” implements the scoring method that is identical to the method developed and documented by Yao *et al.* 2007**], and by using tools comprising Pfold to infer a phylogenetic tree, then running pscore [**This scoring method is implemented by the script NewMotifScore.pl, which is invoked by the “Step_ApplyAltScores” object within the file “ProcessPipeline_PrepareForWeb.pm”**]. We also automatically eliminated motifs that had no covarying base pair positions, that had an average G+C content less than 24%, that had representatives whose nucleotide coordinates overlapped the reverse-complements of other representatives on average by 30% or more of their nucleotides, or that had fewer than six positions that were at least 97% conserved (when sequences were weighted with the GSC algorithm). [**These tests are applied by the “MotifFailsCriteria” subroutine in newmotifs.pl. The GSC weighting is applied by code in RAVENNA that is invoked via the “cmzasha” command by the “Step_PrepareForWeb” object in ProcessPipeline_PrepareforWeb.pm. The GSC algorithm itself is implemented as part of the Infernal package (http://infernal.janelia.org).**]
