# Supplementary material for: Comparative genomics reveals 104 candidate structured RNAs from bacteria, archaea, and their metagenomes
Source: Genome Biol. 2010 Mar 15;11(3):R31. doi: 10.1186/gb-2010-11-3-r31 (PMC2864571; doi:10.1186/gb-2010-11-3-r31)
Supplement: Additional file 3 — Taxa of motif representatives, genes flanking representatives and annotated multiple-sequence alignments. For each motif, this file shows the taxa of each motif representative, depicts genes flanking these representatives and describes conserved domains that the genes encode. Also, a multiple-sequence alignment is provided for each motif, and includes secondary structure and other annotations. [file gb-2010-11-3-r31-S3.PDF]

Additional File 3: taxa, gene contexts and multiple-sequence alignments of all motifs supplementary for: Comparative genomics reveals 104 candidate structured RNAs from bacteria, archaea and their metagenomes

Zasha Weinberg, Joy X. Wang, Jarrod Bogue, Jingying Yang,
Keith Corbino, Ryan H. Moy, Ronald R. Breaker

Note: the presentation of this supplementary data on novel RNA motifs follows the pattern of our presentation of previously found conserved RNA motifs [14].

Contents

1 6S-flavo **16**
1.1 Taxa . . . 16
1.2 Notes . . . 17
1.3 Gene contexts . . . 17
1.4 Conserved domains . . . 19
1.5 Multiple-sequence alignment . . . 19

2 *aceE* **26**
2.1 Taxa . . . 26
2.2 Gene contexts . . . 27
2.3 Conserved domains . . . 27
2.4 Multiple-sequence alignment . . . 28

3 Acido-1 **30**
3.1 Taxa . . . 30
3.2 Gene contexts . . . 30
3.3 Conserved domains . . . 30
3.4 Multiple-sequence alignment . . . 31

4 Acido-Lenti-1 **32**
4.1 Taxa . . . 32
4.2 Gene contexts . . . 32
4.3 Conserved domains . . . 34
4.4 Multiple-sequence alignment . . . 35

5 Actino-pnp **39**
5.1 Taxa . . . 39
5.2 Gene contexts . . . 39
5.3 Conserved domains . . . 42
5.4 Multiple-sequence alignment . . . 42

6 AdoCbl-variant **49**
6.1 Taxa . . . 49
6.2 Notes . . . 49
6.3 Gene contexts . . . 49
6.4 Conserved domains . . . 53
6.5 Multiple-sequence alignment . . . 53

7 *asd* **64**
7.1 Taxa . . . 64
7.2 Gene contexts . . . 65
7.3 Conserved domains . . . 66
7.4 Multiple-sequence alignment . . . 67

8 *atoC* **71**
8.1 Taxa . . . 71
8.2 Gene contexts . . . 71
8.3 Conserved domains . . . 72
8.4 Multiple-sequence alignment . . . 72

9 Bacillaceae-1 **74**
9.1 Taxa . . . 74
9.2 Gene contexts . . . 74
9.3 Conserved domains . . . 77
9.4 Multiple-sequence alignment . . . 79

10 ***Bacillus*-plasmid** **86**
10.1 Taxa . . . 86
10.2 Gene contexts . . . 86
10.3 Conserved domains . . . 87
10.4 Multiple-sequence alignment . . . 88

11 Bacteroid-*trp* **91**
11.1 Taxa . . . 91
11.2 Gene contexts . . . 92
11.3 Conserved domains . . . 93
11.4 Multiple-sequence alignment . . . 94

12 Bacteroidales-1 **98**
12.1 Taxa . . . 98
12.2 Gene contexts . . . 98
12.3 Conserved domains . . . 99
12.4 Multiple-sequence alignment . . . 100

13 *Bacteroides*-1 **104**
13.1 Taxa . . . 104
13.2 Gene contexts . . . 105
13.3 Conserved domains . . . 106
13.4 Multiple-sequence alignment . . . 107

14 *Bacteroides*-2 **111**
14.1 Taxa . . . 111
14.2 Gene contexts . . . 112
14.3 Conserved domains . . . 113
14.4 Multiple-sequence alignment . . . 114

15 Burkholderiales-1 **119**
15.1 Taxa . . . 119
15.2 Gene contexts . . . 119
15.3 Conserved domains . . . 121
15.4 Multiple-sequence alignment . . . 124

16 c4 antisense RNA **130**
16.1 Taxa . . . 130
16.2 Gene contexts . . . 134
16.3 Conserved domains . . . 146
16.4 Multiple-sequence alignment . . . 149

17 c4-a1b1 **182**
17.1 Taxa . . . 182
17.2 Gene contexts . . . 183
17.3 Conserved domains . . . 185
17.4 Multiple-sequence alignment . . . 186

18 Chlorobi-1 **190**
18.1 Taxa . . . 190
18.2 Notes . . . 191
18.3 Gene contexts . . . 191
18.4 Conserved domains . . . 191
18.5 Multiple-sequence alignment . . . 192

19 Chlorobi-RRM **194**
19.1 Taxa . . . 194
19.2 Gene contexts . . . 194
19.3 Conserved domains . . . 195
19.4 Multiple-sequence alignment . . . 195

20 Chloroflexi-1 **197**
20.1 Taxa . . . 197
20.2 Gene contexts . . . 197
20.3 Conserved domains . . . 198
20.4 Multiple-sequence alignment . . . 198

21 Clostridiales-1 **199**
21.1 Taxa . . . 199
21.2 Gene contexts . . . 199
21.3 Conserved domains . . . 203
21.4 Multiple-sequence alignment . . . 206

22 COG2252 **224**
22.1 Taxa . . . 224
22.2 Gene contexts . . . 224
22.3 Conserved domains . . . 224
22.4 Multiple-sequence alignment . . . 225

23 ***Collinsella*-1** **226**
23.1 Taxa . . . 226
23.2 Gene contexts . . . 227
23.3 Conserved domains . . . 227
23.4 Multiple-sequence alignment . . . 227

24 *crcB* **228**
24.1 Taxa . . . 228
24.2 Notes . . . 234
24.3 Gene contexts . . . 234
24.4 Conserved domains . . . 244
24.5 Multiple-sequence alignment . . . 245

25 Cyano-1 **272**
25.1 Taxa . . . 272
25.2 Gene contexts . . . 273
25.3 Conserved domains . . . 278
25.4 Multiple-sequence alignment . . . 280

26 Cyano-2 **299**
26.1 Taxa . . . 299
26.2 Gene contexts . . . 299
26.3 Conserved domains . . . 301
26.4 Multiple-sequence alignment . . . 301

27 Desulfotalea-1 **307**
27.1 Taxa . . . 307
27.2 Gene contexts . . . 307
27.3 Conserved domains . . . 312
27.4 Multiple-sequence alignment . . . 315

28 Dictyoglomi-1 **330**
28.1 Taxa . . . 330
28.2 Gene contexts . . . 331
28.3 Conserved domains . . . 331
28.4 Multiple-sequence alignment . . . 331

29 Downstream-peptide **332**
29.1 Taxa . . . 332
29.2 Notes . . . 333
29.3 Gene contexts . . . 333
29.4 Conserved domains . . . 346
29.5 Multiple-sequence alignment . . . 347

30 ***epsC*** **391**
30.1 Taxa . . . 391
30.2 Gene contexts . . . 392
30.3 Conserved domains . . . 393
30.4 Multiple-sequence alignment . . . 394

31 ***fixA*** **397**
31.1 Taxa . . . 397
31.2 Gene contexts . . . 397
31.3 Conserved domains . . . 398
31.4 Multiple-sequence alignment . . . 398

32 Flavo-1 **400**
32.1 Taxa . . . 400
32.2 Gene contexts . . . 400
32.3 Conserved domains . . . 405
32.4 Multiple-sequence alignment . . . 409

33 *flg*-Rhizobiales **429**
33.1 Taxa . . . 429
33.2 Gene contexts . . . 430
33.3 Conserved domains . . . 430
33.4 Multiple-sequence alignment . . . 431

34 *flpD* **433**
34.1 Taxa . . . 433
34.2 Gene contexts . . . 433
34.3 Conserved domains . . . 433
34.4 Multiple-sequence alignment . . . 434

35 *gabT* **435**
35.1 Taxa . . . 435
35.2 Gene contexts . . . 436
35.3 Conserved domains . . . 436
35.4 Multiple-sequence alignment . . . 437

36 Gamma-*cis*-1 **439**
36.1 Taxa . . . 439
36.2 Notes . . . 440
36.3 Gene contexts . . . 440
36.4 Conserved domains . . . 441
36.5 Multiple-sequence alignment . . . 442

37 *glnA* **446**
37.1 Taxa . . . 446
37.2 Gene contexts . . . 446
37.3 Conserved domains . . . 467
37.4 Multiple-sequence alignment . . . 469

38 GUCCY-hairpin **557**
38.1 Taxa . . . 557
38.2 Gene contexts . . . 557
38.3 Conserved domains . . . 570
38.4 Multiple-sequence alignment . . . 579

39 Gut-1 **611**
39.1 Taxa 611
39.2 Gene contexts 611
39.3 Conserved domains 612
39.4 Multiple-sequence alignment 613

40 ***gyrA*** **617**
40.1 Taxa 617
40.2 Gene contexts 618
40.3 Conserved domains 619
40.4 Multiple-sequence alignment 619

41 ***hopC*** **621**
41.1 Taxa 621
41.2 Gene contexts 622
41.3 Conserved domains 622
41.4 Multiple-sequence alignment 622

42 ***icd*** **623**
42.1 Taxa 623
42.2 Gene contexts 624
42.3 Conserved domains 625
42.4 Multiple-sequence alignment 625

43 JUMPstart **627**
43.1 Taxa 627
43.2 Gene contexts 629
43.3 Conserved domains 637
43.4 Multiple-sequence alignment 639

44 L17 downstream element **646**
44.1 Taxa 646
44.2 Gene contexts 648
44.3 Conserved domains 650
44.4 Multiple-sequence alignment 650

45 ***lactis*-plasmid** **655**
45.1 Taxa 655
45.2 Gene contexts 655
45.3 Conserved domains 656
45.4 Multiple-sequence alignment 656

46 Lacto-*int* **658**
46.1 Taxa . . . 658
46.2 Gene contexts . . . 659
46.3 Conserved domains . . . 660
46.4 Multiple-sequence alignment . . . 660

47 Lacto-*rpoB* **665**
47.1 Taxa . . . 665
47.2 Gene contexts . . . 666
47.3 Conserved domains . . . 667
47.4 Multiple-sequence alignment . . . 668

48 Lacto-*usp* **671**
48.1 Taxa . . . 671
48.2 Gene contexts . . . 671
48.3 Conserved domains . . . 672
48.4 Multiple-sequence alignment . . . 672

49 Leu/phe leader **673**
49.1 Taxa . . . 673
49.2 Notes . . . 673
49.3 Gene contexts . . . 674
49.4 Conserved domains . . . 674
49.5 Multiple-sequence alignment . . . 674

50 ***livK*** **676**
50.1 Taxa . . . 676
50.2 Gene contexts . . . 676
50.3 Conserved domains . . . 677
50.4 Multiple-sequence alignment . . . 677

51 Lnt **679**
51.1 Taxa . . . 679
51.2 Gene contexts . . . 679
51.3 Conserved domains . . . 680
51.4 Multiple-sequence alignment . . . 680

52 ***manA*** **681**
52.1 Taxa . . . 681
52.2 Gene contexts . . . 682
52.3 Conserved domains . . . 686
52.4 Multiple-sequence alignment . . . 687

53 ***Methylobacterium*-1** **705**
53.1 Taxa . . . 705
53.2 Gene contexts . . . 705
53.3 Conserved domains . . . 706
53.4 Multiple-sequence alignment . . . 707

54 Moco-II **711**
54.1 Taxa . . . 711
54.2 Gene contexts . . . 712
54.3 Conserved domains . . . 712
54.4 Multiple-sequence alignment . . . 713

55 ***mraW*** **714**
55.1 Taxa . . . 714
55.2 Gene contexts . . . 715
55.3 Conserved domains . . . 716
55.4 Multiple-sequence alignment . . . 717

56 ***msiK*** **721**
56.1 Taxa . . . 721
56.2 Gene contexts . . . 722
56.3 Conserved domains . . . 724
56.4 Multiple-sequence alignment . . . 725

57 ***Nitrosococcus*-1** **732**
57.1 Taxa . . . 732
57.2 Gene contexts . . . 732
57.3 Conserved domains . . . 733
57.4 Multiple-sequence alignment . . . 733

58 ***nuoG*** **734**
58.1 Taxa . . . 734
58.2 Gene contexts . . . 736
58.3 Conserved domains . . . 739
58.4 Multiple-sequence alignment . . . 740

59 Ocean-V **743**
59.1 Taxa . . . 743
59.2 Gene contexts . . . 743
59.3 Conserved domains . . . 743
59.4 Multiple-sequence alignment . . . 744

60 Ocean-VI **745**
60.1 Taxa . . . 745
60.2 Gene contexts . . . 745
60.3 Conserved domains . . . 751
60.4 Multiple-sequence alignment . . . 752

61 *pan* **761**
61.1 Taxa . . . 761
61.2 Gene contexts . . . 763
61.3 Conserved domains . . . 766
61.4 Multiple-sequence alignment . . . 767

62 ***Pedo*-repair** **774**
62.1 Taxa . . . 774
62.2 Gene contexts . . . 774
62.3 Conserved domains . . . 774
62.4 Multiple-sequence alignment . . . 774

63 *pfl* **776**
63.1 Taxa . . . 776
63.2 Notes . . . 779
63.3 Gene contexts . . . 779
63.4 Conserved domains . . . 784
63.5 Multiple-sequence alignment . . . 785

64 *pheA* **806**
64.1 Taxa . . . 806
64.2 Gene contexts . . . 807
64.3 Conserved domains . . . 808
64.4 Multiple-sequence alignment . . . 808

65 PhotoRC-I **810**
65.1 Taxa . . . 810
65.2 Gene contexts . . . 811
65.3 Conserved domains . . . 812
65.4 Multiple-sequence alignment . . . 813

66 PhotoRC-II **816**
66.1 Taxa . . . 816
66.2 Gene contexts . . . 817
66.3 Conserved domains . . . 826
66.4 Multiple-sequence alignment . . . 827

67 ***Polynucleobacter*-1** **859**
67.1 Taxa . . . 859
67.2 Gene contexts . . . 859
67.3 Conserved domains . . . 860
67.4 Multiple-sequence alignment . . . 860

68 ***potC*** **862**
68.1 Taxa . . . 862
68.2 Gene contexts . . . 863
68.3 Conserved domains . . . 865
68.4 Multiple-sequence alignment . . . 866

69 ***psaA*** **876**
69.1 Taxa . . . 876
69.2 Gene contexts . . . 876
69.3 Conserved domains . . . 877
69.4 Multiple-sequence alignment . . . 877

70 ***psbNH*** **882**
70.1 Taxa . . . 882
70.2 Gene contexts . . . 882
70.3 Conserved domains . . . 883
70.4 Multiple-sequence alignment . . . 883

71 ***Pseudomon*-1** **887**
71.1 Taxa . . . 887
71.2 Gene contexts . . . 887
71.3 Conserved domains . . . 888
71.4 Multiple-sequence alignment . . . 888

72 ***Pseudomon*-2** **890**
72.1 Taxa . . . 890
72.2 Gene contexts . . . 891
72.3 Conserved domains . . . 891
72.4 Multiple-sequence alignment . . . 892

73 ***Pseudomon*-GGDEF** **894**
73.1 Taxa . . . 894
73.2 Gene contexts . . . 894
73.3 Conserved domains . . . 894
73.4 Multiple-sequence alignment . . . 895

74 *Pseudomon*-*groES* **896**
74.1 Taxa . . . 896
74.2 Gene contexts . . . 897
74.3 Conserved domains . . . 897
74.4 Multiple-sequence alignment . . . 897

75 *Pseudomon*-**Rho** **900**
75.1 Taxa . . . 900
75.2 Gene contexts . . . 900
75.3 Conserved domains . . . 901
75.4 Multiple-sequence alignment . . . 901

76 *Pyrobac*-**1** **903**
76.1 Taxa . . . 903
76.2 Gene contexts . . . 904
76.3 Conserved domains . . . 904
76.4 Multiple-sequence alignment . . . 904

77 *Pyrobac*-**HINT** **906**
77.1 Taxa . . . 906
77.2 Gene contexts . . . 906
77.3 Conserved domains . . . 906
77.4 Multiple-sequence alignment . . . 906

78 *radC* **907**
78.1 Taxa . . . 907
78.2 Gene contexts . . . 908
78.3 Conserved domains . . . 912
78.4 Multiple-sequence alignment . . . 912

79 Rhizobiales-1 **926**
79.1 Taxa . . . 926
79.2 Gene contexts . . . 927
79.3 Conserved domains . . . 942
79.4 Multiple-sequence alignment . . . 954

80 Rhizobiales-2 **1001**
80.1 Taxa . . . 1001
80.2 Gene contexts . . . 1001
80.3 Conserved domains . . . 1002
80.4 Multiple-sequence alignment . . . 1002

81 Rhodopirellula-1 **1004**
81.1 Taxa . . . 1004
81.2 Gene contexts . . . 1004
81.3 Conserved domains . . . 1008
81.4 Multiple-sequence alignment . . . 1009

82 ***rmf*** **1020**
82.1 Taxa . . . 1020
82.2 Gene contexts . . . 1020
82.3 Conserved domains . . . 1021
82.4 Multiple-sequence alignment . . . 1021

83 ***rne*****-II** **1023**
83.1 Taxa . . . 1023
83.2 Gene contexts . . . 1023
83.3 Conserved domains . . . 1024
83.4 Multiple-sequence alignment . . . 1024

84 SAM-Chlorobi **1028**
84.1 Taxa . . . 1028
84.2 Gene contexts . . . 1028
84.3 Conserved domains . . . 1029
84.4 Multiple-sequence alignment . . . 1029

85 SAM-I-IV-variant **1030**
85.1 Taxa . . . 1030
85.2 Notes . . . 1031
85.3 Gene contexts . . . 1031
85.4 Conserved domains . . . 1041
85.5 Multiple-sequence alignment . . . 1043

86 SAM-II long loops **1083**
86.1 Taxa . . . 1083
86.2 Gene contexts . . . 1084
86.3 Conserved domains . . . 1088
86.4 Multiple-sequence alignment . . . 1088

87 SAM/SAH riboswitch **1100**
87.1 Taxa . . . 1100
87.2 Gene contexts . . . 1101
87.3 Conserved domains . . . 1103
87.4 Multiple-sequence alignment . . . 1103

88 *sanguinis*-hairpin **1107**
88.1 Taxa . . . 1107
88.2 Gene contexts . . . 1107
88.3 Conserved domains . . . 1108
88.4 Multiple-sequence alignment . . . 1108

89 *sbcD* **1109**
89.1 Taxa . . . 1109
89.2 Gene contexts . . . 1110
89.3 Conserved domains . . . 1110
89.4 Multiple-sequence alignment . . . 1110

90 ScRE **1112**
90.1 Taxa . . . 1112
90.2 Gene contexts . . . 1112
90.3 Conserved domains . . . 1114
90.4 Multiple-sequence alignment . . . 1114

91 Soil-1 **1117**
91.1 Taxa . . . 1117
91.2 Gene contexts . . . 1118
91.3 Conserved domains . . . 1118
91.4 Multiple-sequence alignment . . . 1118

92 *Solibacter*-1 **1120**
92.1 Taxa . . . 1120
92.2 Gene contexts . . . 1120
92.3 Conserved domains . . . 1121
92.4 Multiple-sequence alignment . . . 1122

93 STAXI **1126**
93.1 Taxa . . . 1126
93.2 Gene contexts . . . 1127
93.3 Conserved domains . . . 1129
93.4 Multiple-sequence alignment . . . 1129

94 *sucA*-II **1134**
94.1 Taxa . . . 1134
94.2 Gene contexts . . . 1134
94.3 Conserved domains . . . 1135
94.4 Multiple-sequence alignment . . . 1136

95 ***sucC*** **1138**
95.1 Taxa . . . 1138
95.2 Notes . . . 1139
95.3 Gene contexts . . . 1139
95.4 Conserved domains . . . 1140
95.5 Multiple-sequence alignment . . . 1140

96 Termite-*flg* **1143**
96.1 Taxa . . . 1143
96.2 Gene contexts . . . 1143
96.3 Conserved domains . . . 1144
96.4 Multiple-sequence alignment . . . 1144

97 Termite-*leu* **1146**
97.1 Taxa . . . 1146
97.2 Notes . . . 1146
97.3 Gene contexts . . . 1146
97.4 Conserved domains . . . 1147
97.5 Multiple-sequence alignment . . . 1147

98 ***traJ*-II** **1149**
98.1 Taxa . . . 1149
98.2 Gene contexts . . . 1150
98.3 Conserved domains . . . 1152
98.4 Multiple-sequence alignment . . . 1153

99 Transposase-resistance **1157**
99.1 Taxa . . . 1157
99.2 Gene contexts . . . 1158
99.3 Conserved domains . . . 1162
99.4 Multiple-sequence alignment . . . 1163

100 TwoAYGGAY **1175**
100.1 Taxa . . . 1175
100.2 Gene contexts . . . 1176
100.3 Conserved domains . . . 1180
100.4 Multiple-sequence alignment . . . 1182

101 ***wcaG*** **1196**
101.1 Taxa . . . 1196
101.2 Gene contexts . . . 1197
101.3 Conserved domains . . . 1199
101.4 Multiple-sequence alignment . . . 1200

102 Whalefall-1 **1209**
102.1 Taxa . . . 1209
102.2 Gene contexts . . . 1209
102.3 Conserved domains . . . 1210
102.4 Multiple-sequence alignment . . . 1210

103 *yjdF* **1212**
103.1 Taxa . . . 1212
103.2 Notes . . . 1214
103.3 Gene contexts . . . 1214
103.4 Conserved domains . . . 1216
103.5 Multiple-sequence alignment . . . 1217

104 *ykkC*-III **1225**
104.1 Taxa . . . 1225
104.2 Notes . . . 1226
104.3 Gene contexts . . . 1226
104.4 Conserved domains . . . 1228
104.5 Multiple-sequence alignment . . . 1228

1 6S-flavo

1.1 Taxa

The taxonomy of each organism containing a putative 6S-flavo RNA is listed, with abbreviations identifying each hit (e.g., "Eco-1-1" and "Eco-1-2" might hypothetically represent two distinct RNAs in *E. coli*). The abbreviations will be used to identify each individual 6S-flavo RNA in Sections 1.3 and 1.5.

abbrev. of hits	taxonomy of species
ueu-1-1	Bacteria Bacteroidetes environmental samples unidentified eubacterium SCB49
Fba-1-1	Bacteria Bacteroidetes *Flavobacteria bacterium* BBFL7
Fba-2-1	Bacteria Bacteroidetes Flavobacteria Flavobacteriales *Flavobacteria bacterium* BAL38
Cat-1-1	Bacteria Bacteroidetes Flavobacteria Flavobacteriales Flavobacteriaceae *Croceibacter atlanticus* HTCC2559
Ddo-1-1	Bacteria Bacteroidetes Flavobacteria Flavobacteriales Flavobacteriaceae *Dokdonia donghaensis* MED134
Fjo-1-1	Bacteria Bacteroidetes Flavobacteria Flavobacteriales Flavobacteriaceae *Flavobacterium johnsoniae* UW101
Fps-1-1	Bacteria Bacteroidetes Flavobacteria Flavobacteriales Flavobacteriaceae *Flavobacterium psychrophilum* JIP02/86
Lbl-1-1	Bacteria Bacteroidetes Flavobacteria Flavobacteriales Flavobacteriaceae *Leeuwenhoekiella blandensis* MED217
Pir-1-1	Bacteria Bacteroidetes Flavobacteria Flavobacteriales Flavobacteriaceae *Polaribacter irgensii* 23-P
Psp-1-1	Bacteria Bacteroidetes Flavobacteria Flavobacteriales Flavobacteriaceae *Polaribacter sp.* MED152
Pto-1-1	Bacteria Bacteroidetes Flavobacteria Flavobacteriales Flavobacteriaceae *Psychroflexus torquis* ATCC 700755
Rbi-1-1	Bacteria Bacteroidetes Flavobacteria Flavobacteriales Flavobacteriaceae *Robiginitalea biformata* HTCC2501
Fba-3-1	Bacteria Bacteroidetes Flavobacteria *Flavobacteriales bacterium* HTCC2170
Psp-2-1	Bacteria Bacteroidetes Sphingobacteria Sphingobacteriales Sphingobacteriaceae *Pedobacter sp.* BAL39
Gka-1-1	Bacteria Firmicutes Bacillales Bacillaceae *Geobacillus kaustophilus* HTA426
Dps-1-1	Bacteria Proteobacteria Deltaproteobacteria Desulfobacterales Desulfobulbaceae *Desulfotalea psychrophila* LSv54
env-1 to env-76	environmental samples

1.2 Notes

Superscript numbers are used to annotate any 6S-flavo RNA that has special characteristics (described below). These numeric annotations will be used in Sections 1.3 and 1.5. The meaning of the numbers are as follows.

[1] overlaps known 6S

1.3 Gene contexts

Each 6S-flavo RNA (indicated by "RNA→") is listed with its surrounding genes. Environmental sequences and some RefSeq entries lack gene annotations, and no genes are listed for such sequences. The direction of each gene is indicated with an arrow (→), and each conserved domain in the gene is colored. Conserved domains associated with more than one 6S-flavo RNA are assigned a color; other domains are gray. Information about these conserved domains is given in Section 1.4. The accession number of the sequence containing each 6S-flavo RNA is given. Accessions beginning with "NC_" or "NZ_" are RefSeq, while other accession refer to environmental samples. Nucleotide coordinates are given for the 5′ and 3′ boundaries of each. If the 5′ coordinate is greater than the 3′ coordinate, the RNA is present on the reverse-complement strand of the containing genomic DNA sequence. Each hit is denoted by an abbreviation (like "Eco-1-1") that refers to a taxonomy given in Section 1.1. Superscript numbers refer to annotations listed in Section 1.2

abbrev.	Seq. accession		5′ at	3′ at	genes
Gka-1-1	NC_006510.1	+	1316460	1316575	HNHc (cd00085)RVT (pfam00078)→ **RNA**→ COG1418 (COG1418)→
[1]Dps-1-1	NC_006138.1	-	3243257	3243154	hypo→ **RNA**→ COG1418 (COG1418)→ TyrS (COG0162)→
Psp-2-1	NZ_ABCM01000031.1	-	12076	11976	DUF710 (pfam05164)→ **RNA**→ COG1094 (COG1094)COG1418 (COG1418)→ hypo→ DUF962 (pfam06127)→
env-1	JCVI_SCAF_1101668538900	+	204	329	**RNA**→ ? (GOS6223)→ COG1418 (COG1418)? (GOS3546)→
env-2	JCVI_SCAF_1096627580171	-	788	663	←? (GOS819) **RNA**→ ? (GOS6223)→
Fba-2-1	NZ_AAXX01000003.1	-	68580	68477	DUF710 (pfam05164)→ **RNA**→ COG1418 (COG1418)→
Fjo-1-1	NC_009441.1	+	128358	128466	DUF710 (pfam05164)→ **RNA**→ COG1418 (COG1418)→
Fps-1-1	NC_009613.1	+	1319608	1319714	hypo→ **RNA**→ COG1418 (COG1418)→
Ddo-1-1	NZ_AAMZ01000005.1	+	144676	144798	hypo→ **RNA**→ COG1418 (COG1418)→
Cat-1-1	NZ_AAMP01000001.1	+	1492344	1492454	hypo→ **RNA**→ COG1418 (COG1418)→ COG3956 (COG3956)→ hypo→
env-3	JCVI_SCAF_1101667205009	+	99	201	**RNA**→
env-4	JCVI_SCAF_1096626999289	+	468	571	? (GOS14297403)→ **RNA**→ ←GalK (COG0153)? (GOS427)
env-5	JCVI_SCAF_1101668678955	+	1063	1165	**RNA**→
env-6	JCVI_SCAF_1096628195137	+	483	586	? (GOS14297403)→ **RNA**→ ? (GOS399)→
env-7	JCVI_SCAF_1096628404094	-	1203	1100	? (GOS14297403)→ **RNA**→ ←? (GOS427)
env-8	JCVI_SCAF_1096627264411	+	1413	1515	? (GOS14297403)→ **RNA**→ ←? (GOS427)
env-9	JCVI_SCAF_1096627821315	+	9	112	**RNA**→
env-10	JCVI_SCAF_1101668383300	-	699	596	? (GOS14297403)→ **RNA**→ ←GalK (COG0153)? (GOS427)
env-11	JCVI_SCAF_1096627338603	+	565	668	? (GOS14297403)→ **RNA**→ ←GalK (COG0153)? (GOS427)
env-12	JCVI_SCAF_1101668386025	+	733	835	**RNA**→
env-13	JCVI_SCAF_1096627171768	+	137	240	**RNA**→ ←GalK (COG0153)? (GOS427)
env-14	JCVI_SCAF_1096627054710	-	1014	911	? (GOS14297403)→ **RNA**→ ←GalK (COG0153)? (GOS427)
env-15	JCVI_SCAF_1101668193208	+	744	847	? (GOS14297403)→ **RNA**→ ←GalK (COG0153)? (GOS427)
env-16	JCVI_SCAF_1096627335608	+	1071	1178	**RNA**→
env-17	JCVI_SCAF_1101668380480	-	494	389	? (GOS14229324)→ **RNA**→ ? (GOS6223)→
env-18	JCVI_SCAF_1096627117432	-	1990	1886	? (GOS14229324)→ **RNA**→ COG1418 (COG1418)? (GOS3546)→
env-19	JCVI_SCAF_1096627377246	-	1148	1045	**RNA**→ ? (GOS6223)→
env-20	JCVI_SCAF_1101668293410	-	1017	911	? (GOS11001734)→ **RNA**→ ←MIP (cd00333)? (GOS38)
env-21	JCVI_SCAF_1096627074548	+	918	1023	? (GOS13021950)→ **RNA**→ ←MIP (cd00333)? (GOS38)

env-22	JCVI_SCAF_1096627999429	+	1628	1734	**RNA**→
Psp-1-1	NZ_AANA01000001.1	-	543738	543632	hypo→ **RNA**→ COG1418 (COG1418)→
env-23	JCVI_SCAF_1101668090991	-	586	483	? (GOS13021950)→ **RNA**→ ? (GOS6223)→
env-24	JCVI_SCAF_1101668418675	-	371	268	**RNA**→
env-25	JCVI_SCAF_1101668709204	+	19	122	**RNA**→
env-26	JCVI_SCAF_1096627858119	-	1234	1130	DUF710 (pfam05164)? (GOS13021950)→ **RNA**→ ←? (GOS427)
env-27	JCVI_SCAF_1101668394845	+	499	603	DUF710 (pfam05164)? (GOS13021950)→ **RNA**→ ←? (GOS427)
env-28	JCVI_SCAF_1101668404018	+	803	909	**RNA**→
env-29	JCVI_SCAF_1096627168612	-	1095	989	DUF710 (pfam05164)? (GOS13021950)→ **RNA**→ ? (GOS3546)→
env-30	JCVI_SCAF_1101667286162	-	859	756	**RNA**→
Pir-1-1	NZ_AAOG01000003.1	-	188107	187981	hypo→ **RNA**→ COG1418 (COG1418)→
env-31	JCVI_SCAF_1096626676035	-	150	43	**RNA**→
env-32	JCVI_SCAF_1101668679737	+	757	863	? (GOS11001734)→ **RNA**→ ? (GOS6223)→
env-33	JCVI_SCAF_1101668337420	+	157	263	**RNA**→ COG1418 (COG1418)? (GOS3546)→
env-34	JCVI_SCAF_1096627384620	+	3535	3641	? (GOS11001734)→ **RNA**→ COG1418 (COG1418)? (GOS3546)→
Lbl-1-1	NZ_AANC01000006.1	-	17405	17297	hypo→ **RNA**→ COG1418 (COG1418)→
env-35	JCVI_SCAF_1096627496052	+	600	708	? (GOS13021950)→ **RNA**→ ? (GOS3546)→
env-36	JCVI_SCAF_1101668019466	+	1316	1423	**RNA**→
env-37	JCVI_SCAF_1096627276620	-	2625	2518	DUF710 (pfam05164)? (GOS11001734)→ **RNA**→ ←? (GOS8166)
Fba-3-1	NZ_AAOC01000004.1	+	42714	42822	hypo→ **RNA**→ COG1418 (COG1418)→
Rbi-1-1	NZ_AAOI01000003.1	+	345742	345849	hypo→ **RNA**→ COG1418 (COG1418)→
ueu-1-1	NZ_ABCO01000011.1	+	67885	68005	hypo→ **RNA**→ COG1418 (COG1418)→
Pto-1-1	NZ_AAPR01000008.1	+	9568	9681	DUF710 (pfam05164)→ **RNA**→ COG1418 (COG1418)→
env-38	JCVI_SCAF_1096627866490	-	199	92	**RNA**→
env-39	JCVI_SCAF_1096627081943	-	715	607	? (GOS11226237)→ **RNA**→ ? (GOS6223)→
env-40	JCVI_SCAF_1101668406192	-	337	229	**RNA**→
env-41	JCVI_SCAF_1096627372169	+	1137	1244	? (GOS13021950)→ **RNA**→ ? (GOS3546)→
env-42	JCVI_SCAF_1101668084529	-	593	486	? (GOS13021950)→ **RNA**→ ? (GOS3546)→
env-43	JCVI_SCAF_1101668684262	-	708	600	? (GOS11001734)→ **RNA**→ ←MIP (cd00333)? (GOS38)
env-44	JCVI_SCAF_1096627447502	-	1390	1281	**RNA**→ ? (GOS6223)→
env-45	JCVI_SCAF_1096626860696	+	794	902	? (GOS11001734)→ **RNA**→ ←MIP (cd00333)? (GOS38)
env-46	JCVI_SCAF_1096627318269	-	2667	2558	? (GOS11001734)→ **RNA**→ ←MIP (cd00333)? (GOS38)
env-47	JCVI_SCAF_1101668259956	-	1026	918	? (GOS11001734)→ **RNA**→ ←MIP (cd00333)? (GOS38)
env-48	JCVI_SCAF_1101668072492	+	15	123	**RNA**→
env-49	JCVI_SCAF_1096626909345	+	172	280	**RNA**→ COG1418 (COG1418)? (GOS3546)→
env-50	JCVI_SCAF_1101667353886	+	33	141	**RNA**→
env-51	JCVI_SCAF_1096627999061	+	1137	1245	? (GOS13021950)→ **RNA**→ ←MIP (cd00333)? (GOS38)
env-52	JCVI_SCAF_1096626292370	-	517	409	? (GOS13021950)→ **RNA**→ ←MIP (cd00333)? (GOS38)
env-53	JCVI_SCAF_1096628393098	+	669	776	? (GOS11001734)→ **RNA**→ COG1418 (COG1418)? (GOS6906)→
env-54	JCVI_SCAF_1096628290523	-	503	396	? (GOS11001734)→ **RNA**→ ? (GOS3546)→
env-55	JCVI_SCAF_1101668445732	-	630	524	**RNA**→ ←? (GOS8279)
env-56	JCVI_SCAF_1096626973177	-	2237	2131	? (GOS11440210)→ **RNA**→ ←? (GOS8279)
env-57	JCVI_SCAF_1096627033120	-	201	95	**RNA**→
env-58	JCVI_SCAF_1096627273815	+	981	1087	? (GOS11440210)→ **RNA**→ ←? (GOS8279)
env-59	JCVI_SCAF_1101668174794	+	977	1083	? (GOS11440210)→ **RNA**→ ←? (GOS8279)
env-60	JCVI_SCAF_1096627172476	-	756	650	? (GOS11440210)→ **RNA**→ ←? (GOS8279)
env-61	JCVI_SCAF_1101668074176	-	372	266	**RNA**→
env-62	JCVI_SCAF_1096626853002	+	1470	1576	? (GOS11001734)→ **RNA**→ ←? (GOS8279)
env-63	JCVI_SCAF_1096627219240	+	1647	1751	? (GOS14483285)→ **RNA**→ ←? (GOS8279)
env-64	JCVI_SCAF_1096628003170	+	183	287	**RNA**→ ←? (GOS8279)
env-65	JCVI_SCAF_1096627276151	-	547	443	? (GOS14483285)→ **RNA**→ ←? (GOS8279)
env-66	JCVI_SCAF_1101667344488	-	491	387	? (GOS14483285)→ **RNA**→ ←? (GOS8279)

env-67	JCVI_SCAF_1096627107045	-	1479	1375	? (GOS14483285)→ **RNA**→ ←? (GOS8279)
env-68	JCVI_SCAF_1096627107851	+	1008	1112	? (GOS14483285)→ **RNA**→ ←? (GOS8279)
env-69	JCVI_SCAF_1101668544692	-	1224	1118	**RNA**→ ←? (GOS8279)
env-70	JCVI_SCAF_1101667566873	-	455	349	? (GOS14578054)→ **RNA**→ ←? (GOS8279)
env-71	JCVI_SCAF_1096627260888	+	18194	18300	? (GOS14578054)→ **RNA**→ ←? (GOS8279)
env-72	JCVI_SCAF_1096627328300	+	3516	3625	**RNA**→
env-73	JCVI_SCAF_1096628305661	+	1761	1870	**RNA**→
Fba-1-1	NZ_AAPD01000002.1	-	349281	349171	hypo→ **RNA**→ COG1418 (COG1418)→
env-74	JCVI_SCAF_1101668047041	-	1267	1140	**RNA**→ COG1094 (COG1094)? (GOS3546)→
[1]env-75	JCVI_SCAF_1096627350108	-	3515	3405	? (GOS13021950)→ **RNA**→ COG1418 (COG1418)? (GOS3546)→
env-76	JCVI_SCAF_1096627431273	-	1609	1478	? (GOS13021950)→ **RNA**→ ? (GOS6223)→ COG1418 (COG1418)? (GOS3546)→

1.4 Conserved domains

Conserved domains found in protein-coding genes listed in Section 1.3 are listed, with the first sentence in their description from the Conserved Domain Database. Conserved domains associated with more than one 6S-flavo RNA are assigned a color, while others are shown in gray. The number in parentheses is the number of occurrences of the given gene.

u cd00085 (1) HNH nucleases; HNH endonuclease signature which is found in viral, prokaryotic, and eukaryotic proteins.
d cd00333 (8) Major intrinsic protein (MIP) superfamily.
d COG0153 (6) Galactokinase [Carbohydrate transport and metabolism]
D COG0162 (1) Tyrosyl-tRNA synthetase [Translation, ribosomal structure and biogenesis]
D COG1094 (2) Predicted RNA-binding protein (contains KH domains) [General function prediction only]
D COG1418 (24) Predicted HD superfamily hydrolase [General function prediction only]
D COG3956 (1) Protein containing tetrapyrrole methyltransferase domain and MazG-like (predicted pyrophosphatase) domain [General function prediction only]
u GOS11001734 (11) ?
u GOS11226237 (1) ?
u GOS11440210 (4) ?
u GOS13021950 (12) ?
u GOS14229324 (2) ?
u GOS14297403 (8) ?
u GOS14483285 (5) ?
u GOS14578054 (2) ?
D GOS3546 (13) ?
d GOS38 (8) ?
D GOS399 (1) ?
d GOS427 (10) ?
D GOS6223 (9) ?
D GOS6906 (1) ?
d GOS8166 (1) ?
u GOS819 (1) ?
d GOS8279 (15) ?
u pfam00078 (1) Reverse transcriptase (RNA-dependent DNA polymerase).
u pfam05164 (8) Family of unknown function (DUF710).
D pfam06127 (1) Protein of unknown function (DUF962).

1.5 Multiple-sequence alignment

Each 6S-flavo RNA is denoted by an abbreviation (like "Eco-1-1") that refers to a taxonomy given in Section 1.1. Superscript numbers refer to annotations listed in Section 1.2. The alignment includes sequences containing the RNA motif, as well as flanking sequence. The 6S-flavo RNA itself is denoted by the line underneath marked 5′ and 3′ on either end. Nucleotides in the flanking sequences (i.e., not part of the motif) are written in gray letters. Stems of predicted rho-independent transcription terminators are shaded yellow. (Note: terminator predictions have not been analyzed manually, and many are likely to be false positives.) Start codons are shaded green. (Note: start codons are frequently misannotated, especially in environmental samples.) Nucleotides proposed to basepair as part of the consensus structure are shaded in color when they comprise Watson-Crick or G-U pairs. Otherwise they are shaded gray. Conserved stems are also indicated at the bottom of the alignment by angle brackets, where matching $<$ and $>$ denote base-paired columns. Below these angle brackets, the symbol "2" denotes base pairs exhibiting covariation, "1" denotes base pairs exhibiting compatible mutations, "0" denotes base pairs that are not observed to mutate and "?" denotes base pairs that have a significant frequency of non-canonical nucleotides for Watson-Crick or G-U pairs ($>$ 5%). Below these base pair annotation is the consensus sequence: "R" =

"A" or "G", "Y" = "C" or "U", red nucleotides: nucleotide identity conserved more than 97% of the time, black nucleotides: 90%, gray nucleotides: 75%, red circle (•): nucleotide is present 97% of the time, black circle (•): 90%, gray circle (•): 75%, white circle (◦): 50%. All percentages of sequences just described (e.g. 97% conserved) assume that sequences have been weighted by the GSC algorithm implemented by the Infernal software.

```
          alignment positions 1···99

 Gka-1-1  AUAAGCCUAAAAAAGGACGGCGUGCGGAACGGUCUUGUUCCGUUGUCAUCCUCUAGGCGCGUCGGUGUAAUAUCUUGACAAUUCCUAUUGCCGCCUUUA
1Dps-1-1  UAGGUUUUCGUUUAAGCAAAAAUUUGCUAUCCUAGUGAGUGUAAGUUUAGAAGAAAAGAUGAUAUUCCCCUGUGCUGUUGGUGAUUGCUAAAGUGUGUU
 Psp-2-1  UAAAUGAUUUUUUUUCCAGAUAGGAUCGUUCUUUAUAUACAGUUAAGAUUAGCACAAUGAUAUAGCCGCAGCUAAAUUUGUGGGUUUCACUAUUUAAAA
 env-1    GUAAUAUUGAUGCUAUUGAAGCUAAAAUAUCAGCUAUACUAGAACGUUCUUUUACAUAAAACUAUAAAUAGUUUCCCGUAUUAAUUCAAUAAGUUGUUA
 env-2    NNNNNNNNNNNNNNNNNNNNNNNNNNNNNNNNNNNNNNNNNCGUUCUUUUACAUAAAAUUAUAAUAUAGUUUCCCGUAUUAAUUCAAUAAGUUGUUA
 Fba-2-1  AGAUUACAAAAUUUGGAUAAAAAAAUUGAGCGAGUUACUUUCUAAAUAAAUACGUUCUUACAAUAACAAAGAUACUGCCUACAUUAGUAUAUAUUUGAUA
 Fjo-1-1  AAGAAUUAAAAGAUUAAAUUCGCUUUUAGAUCAAUAUCUCGAAAAUUAAACGUUCUUUACAGAAACUAAGAUACUGCCUACAUUAGUUCACAAUUGGUA
 Fps-1-1  AAAGCUUAGCAAACUAAAUGAAUUAUUAGGCAAAUAUUUAGAUAAAUAACACGUUCUUUACAUACACUAAAUACUACCUACAUUAGUACCUAUUUGAUA
 Ddo-1-1  UAGAUUACAAAAGCUGGCAGACCUCCUUGAUGAUAGUUUGUCAUAACGUUCUUUACAUACAACAGCAUAUUACUGCCUGCAUUGAUUAUUAUUUGAUA
 Cat-1-1  UUGAAAAACUUGAACCUCUUUCUGGAACAACAAUUAACCUAACAUUACGUUCUUUAAAAUAAACAAGCAUUACUGCCUGUGUUAGCAACCUUUUUGAUA
 env-3    ..AGAGAAGUUAAAUAAAAUUAAAGAAUUGAUAGAUUCAAGACUUUAGUUCAACGUUCAUUAAAAUAAAUAUUUUACCUACAGCAGAUUAAAAUUGGUA
 env-4    AGGAGAGAAGCUAAAUAAAAUUAAAGAAUUGAUAGAUUCAAGACUUUAGUUAAACGUUCAUUAAAAUAUAAUUUUACCUACAGCAGAUUUAAAUUGGUA
 env-5    AAUAAAAUAGAAGAAUUAAAAAAUUUAAUUGACUCAAGAUUAGAAAAUUAAAACGUUCAUUAAUAUAUAUAAUUUACCUACAGCAGAUUAAAUUUGGUA
 env-6    AUAAAAUAGAAGAGUUAAAAAAAUUUAAUUGACUCAAGAUUAGAAAAUUAAAACGUUCAUUAAAAUAUAUAAUUUUACCUACAGCAGAUUAAAUUUGGUA
 env-7    AUAAAAUAGAAGAGUUAAAAAAAUUUAAUUGACUCAAGAUUAGAAAAUUAAAACGUUCAUUAAAAUAUAUAAUUUUACCUACAGCAGAUUAAAUUUGGUA
 env-8    AUAAAAUAGAAGAAUUAAAAAAAUUUAAUUGACUCAAGAUUAGAAAAUUAAAACGUUCAUUAAAAUAUAUAAUUUUACGUACAACAGAUUAAAUUUGGUA
 env-9    ............................................................................................UUUGGUA
 env-10   AUAAAAUAGAAGAAUUAAAAAAAUUUAAUUGACUCAAGAUUAGAAAAUUAAAACGUUCAUUAAAAUAUAUUAUUUUACCUACAGCAGAUUAAAUUUGGUA
 env-11   AUAAAAUAGAAGAAUUAAAAAAAUUUAAUUGACUCAAGAUUAGAAAAUUAAAACGUUCAUUAAAAUAUAUAAUUUUACCUACAGCAGAUUAAAUUUGGUA
 env-12   AAUAAAAUAGAAGAAUUAAAAAAAUUUAAUUGACUCAAGAUUAGAAAAUUAAAACGUUCAUUAAAAUAUAUAAUUUACCUACAGCAGAUUAAAUUUGGUA
 env-13   CGUGGAAAAAAUUGACAAUAUUAAAAAAAUUAAUUGAGUCAAGAUUAAAAUAUUAACGUUCAUUAAAAUAUAUUUUACCUACAGCAGAUUAAAUUUGGUA
 env-14   AUACAGAAGAAAAACUUAAAGAACUUAUUGAGUUAAUUGAUAAAAAUAUCAAUUAACUACGAUCUUAUUAUAUUGACCUUAGGCAGAUAAUCAUUGGUA
 env-15   AAUAACGAAGAAAAACUUAAAGAACUCAUUGAAUUAAUUGAUAAAAAUAUCAAUUAACUACGAUCUUAACAUAUUGACCUAGGCAGAUAACCAUUGGUA
 env-16   UAUUGAGUUAAAUGAAAUUAAUAAUAUGAUUUCUGAUUGUUUAAAAUCUUAACGUUCAUUAAAAUAAAUUUUACUACCUACAUCAGAUUUAAUUUGGUA
 env-17   AUAAGUUAAAUUCAAUUAAUGAUUUAGUUAUUCAGAGUUUAGAUUCUUAACGUUCAUUAAAAUAAAAUUAUAUUACCUACAUCAGAUUUUAAUUUGGUA
 env-18   AUAAGUUAAAUUCAAUUAAUGAUUUAGUUAUUCAGAGUUUAGAUUCUUAACGUUCAUUAAAAUAAAAUUAUAUUACCUACAUCAGAUUUUAAUUUGGUA
 env-19   CAUGAGUUAAUUAAUAUUAAUAAUUUAUUAUCCCAAUGUUUAGAUUCUUAACGUUCAUUUAAAUAAAUUAAUACUACCUACAUCAGAUUUAAUUUGGUA
 env-20   CUUAAAAAAACUUUCAAAUAUUAAUGAAUUGAUUCAAGAUCAUAUUACUAAUUUUUUGUAAAUUUCAUUAAUUAUCUGCGUUAGUUUAUUUUUUUGAUA
 env-21   UUUAAAAAAUAUAUCAAAUUUAAACGAACUGAUUAAAGAACAUAUUGCUAAUUUUUUGUAAAUUUCAAUAAUUAUCUGCGUUAGCUUAUUUUUUUGAUA
 env-22   UAAUUUAAAAAAAAUAUCAAAUAUAAAUGAAUUGAUUAAAGAGCAUAUUGCUAAUUUUUUGUAAAUUACAAAUGUCUGCGUGAGCUUAUUUUUUUGAUA
 Psp-1-1  AAAAGUUAAAUGAGCUUACCAAUUUGGUAAAAACUCAUUUAGAAUAAGUUCAUUAAAAUACAUUAACAACACACUGCCUACGUUAGUAAUUGUUUAUUA
 env-23   AAGCGAUUGGAGACUUUAAAUAAGUCUGUUCAGGAGAUUAUUGAUUUAUAACACGUUCUUUUAAAUAAUUAAUUACUGCCUACAUCAAAUUUUUUGAUA
 env-24   ACAAAGAAUAGAAAAAUUAAAUCAUUCUAUUUCUGAAAUUUUUAACACAUAAACGUUCAUUUAAAUAUUAAUUUAAAGCCUAUAUCAAUCUUUUUGAUA
 env-25   ....................................................................................CACCAAUUUUUUUGAUA
 env-26   AGCUUAGUAAAAAACUGGAUGAUUUAAAUAAAUCAAUUCAAGAGGUUAUUAAUCCAUAAACGUUCAUUUAAAUUAUUGCCUACACCAAUUUUUUUGAUA
 env-27   AGCUUAGUAAAAAACUGGAUGAUUUAAAUAAAUCAAUUCAAGAGGUUAUUAAUCCUUAAACGUUCAUUUAAAUUAUUGCCUACACCAAUUUUUUUGAUA
 env-28   AAAAGACUUAAAAAGAUUAAUCAGUCCAUUAAAGAAAUUAUUGACCUUUAGUACGUUCAUUUAAAUAAUACAUUAUUGCCUACAUCAAUUUUUUUGAUA
 env-29   AAAAGACUUAAAAAGAUUAAUCAGUCCAUUAAAGAAAUUAUUGACCUUUAGUACGUUCAUUUAAAUAAUACAUUAUUGCCUACAUCAAUUUUUUUGAUA
 env-30   .....................................................................................AUCAAUUUUUUUGAUA
 Pir-1-1  UAAACUAGAAGAGUUAACUAAUUUACUCUCAACGCAUUUAAAAUAAGUUCUUUAAAAUACAUUAACAACACACUGCCUACGUUAGUAAUUGUUUGAUA
 env-31   CGUGGAGGAUAGAUUAUCUAACCUGAUAAAUAUUCUUGAUGAAAAGAGUUAAGUUCUUUAAAUAAUAUCAUAAAACUACCUACAUUAGUUUUUUUUGUA
 env-32   AUGUGGAAGUUAAAAUUUCUGAAUUAAUUAAAUCUUUUGAUCAAACUGACUAGUUUUAUAUUGUAGUAUAAUAAACUGCCUACAUUAGUUUUUUUUGUA
 env-33   AUGUAGAAAUUAAAAUUUCUGAAUUAAUUAAAUCUUUUGAUCAAACUGACUAGUUUUAUAUUGUAGUAUAAUAAACUGCCUACAUUAGUUUUUUUUGUA
 env-34   AUGUGGAAGUUAAAAUUUCUGAAUUAAUUAAAUCUUUUGAUCAAACUGACUAGUUUUAUAUUGUAGUAUAAUAAACUGCCUACAUUAGUUUUUUUUGUA
 Lbl-1-1  AGCAACUGAAAAACUUAGAGUUGCUGGUUAAAAGUCAGUUAUCCUAACGUUCUUUAAAAUAGAAUUACUUACUGCCUACAUUGGUUACAUUUUUUGGUA
 env-35   AUCAAAGAUCUCGUUGAUCUUAUCAACGUUCAUAUGGUGACUUACUAAGUUCUUUAAAAUUAUAUACAACACUGCCUGUAUUAGUUGACAUUUUUGAUA
 env-36   GUUCAUAAAAAAAUUGUACUUUUAUGCAAGUAUUUAACGGAGCAAUUAGCUUCCUAAAUACCAAAAAUACCUACCCUACACAAGGUACAUUUUUUGAUA
 env-37   UUCACAAAAAAAUUGUACUUUUAUCCAAGUAUUUAACGGAGCAAUUAGCUUCCUAAAUACCAAAAAAAUCUUACCCUACAUCAGGUAUAUUUUUUGAUA
 Fba-3-1  UGCGAGCAUUGGACGAGUUGGUAAAUUCAAAGCUUGGUAUUAAAUAAGUUCUUUUAAAUAACAAUAAGUUACUGCCCACAUUGGUAUUAUCUUUUGACG
 Rbi-1-1  UCCGGUCGCUGGACAGCCUGAUCGCUUCCACGCUGAGCGCGCAAUAAGUUCUUUUACAUAGCAAUAGAUACUGCCCACAUUGGUAUUAUUUUUUUGACA
 ueu-1-1  GAACGCCUUAAAGCGUUAAACCAAUUGCUUCACGAGAAAAUAUCGUAACGUUCAUUACAUAACAUUAGGUAACUGCCUAUAUUAAUAUACAUUUUGGUA
 Pto-1-1  AACAAAGCUUGUAGAACUGAGUGAGAUGAUAAAAGAGCAGUUAAACUAACGUUCAUUACAUAAAUCGACACUGCCUGUAUUAGUCGUACCUUUUUGAUA
 env-38   UCAAGAAUUAAUUGAUCUCAUUGACGUUCAUAUUGCUACUUACUAACGUUCUUUAACAUAACAAUACAAUACUGCCUGUAUUAGUAAUAUUUUUUGAUA
 env-39   UAAAACCAAUGAAUUGAUUGAAUUAAUUGAUUUACAUCUCUCCAAUUACUAAGUUCUUUAAAUAAAAUACAAUCUGCCUGUAUUGGUGAUUUUUUGAUA

          ...................................................................................................
          ...................................................................................................
          •••••••••••••••••••••••••••••••••••••••••••••••••••••••••••••••••••••••••••••••Y••••••••••YYYUUGRUA
```

```
env-40    UAAAACCAAUGAAUUGAUCGAAUUGAUUGAUUUACAUCUCUCCAAUUACUAAGUUCUUUAAAUAAAAUACAAUCUGCCUGUAUUGGUGAUUUUUUGAUA
env-41    CAAGACCAACGAAUUGAUUGAAUUGAUCGAUUUACAUCUUUCCAAUUACUAAGUUCUUUAAAUAAAAUACAUACUGCCUGUAUUGGUGAUUUUUUGAUA
env-42    UAAAAUCUUAGAAUUAAUAGAUUUAAUUGAUGUCCACAUGGCUGCAUAUUAAGUUUAAUUGACAGCUAUACAAACUACCUGUAUUAGUAUUUUUUGAUA
env-43    UCUGAGGACAAAAUAGAGGAGUUGAUUGAUAUGAUAAAUAUUCACAUGUCAACAUAUUAAAUUUUUUAAAUUAAUGCCUGUAUUAGUAUUUUUUGAUA
env-44    AAAUUCAAGAACUUAUAGGCUUGAUAGAUCUCCAUUUAGCUGCUUACUAAGUUCUGUAAACAGCAAUACAACACUGCCUGUAUUAGUAUUUUUUGAUA
env-45    AAAUAGAAGAGUUAAUUGAUAUGAUUGAUAUUCAUUUGACUUCAUAUUAAGGUUUAAAGUAAUUAAUACAGUAAUGCCUGUAUUAGUAUUUUUUGAUA
env-46    AUGAUAAAGUCCAGGAGCUAAUUGAUAUUAUAGAUGUUCAUUUAUCUGCAUAUUGAAUCAAAAUCUAAUUAUUUAUGCCUGUAUUAGUAUUUUUUGAUA
env-47    AUGAUAAAGUCCAGGAGCUAAUUGAUAUUAUAGAUGUUCAUUUAUCUGCAUAUUGAAUCAAAAUCUAAUUAUUUAUGCCUGUAUUAGUAUUUUUUGAUA
env-48    .....................................................................................UAUUUUUUUGAUA
env-49    AAAUCAAAGAACUGAUAGAUAUGAUUGAUGUUCAUUUGGACGCUUAUUAAGUUCUGAAGACAAUAGUACAACACUGCCUGUAUUAGUAUUUUUUGAUA
env-50    ..................................................................UUAUUUAUGCCUGUAUUAGUAUUUUUUGAUA
env-51    CGAUGAAGAAAAAAUUAAGAAUUUAAUAGAAAUGAUUGAUAUACACUUAGGCACUUAUAAAUAAAAUUAAAGUUAAGCCUGUGCUAGUUUUUUUGAUA
env-52    AAGUUCAAGAGCUAAUUGAUUUAAUUGAUAUUCAUAUGACAACAUAUUAACUUAUUUUUGAAACUAAAUAGAAACUACCUGUAUUAGUAUUUUUUGAUA
env-53    AAGAUAGCCUCAAUGAUAGCAUUGAUAGACGUUCAUAUGGCUGCCUAUUAAAGUUCUUUUUUAUAAUAAUACAUACUGCCUGUAUUAGUUUUUUUGAUA
env-54    AAGAUAUCAUCAAUGAUAGCGUUGAUAAACGUUCAUAUGGCUGCCUAUUAAAGUUCUUUCUUAUAAUAAUACAUACUGCCUGUAUUAGUUUUUUUGAUA
env-55    AUUGAAUUAGAAAAAAUGGUAUUAAAAAUGAUAAAUAAUAUUGAUAAUUCAAUUUAAACUUCAUACAUUUAACUUAACCUGUAUGAGUGAUUUUUGGUA
env-56    AUUGAAUUAGAAAAAAUGAUAUUAAAAAUGAUAAAUAAUAUUGAUAAUUCAAUUUAAACUUCAUACAUUUAACUUAACCUGUAUGAGUGAUUUUUGGUA
env-57    AUUGAAUUAGAAAAAAUGGUAUUAAAAAUGAUAAAUAAUAUUGAUAAUUCAAUUUAAACUUCAUACAUUUAACUUAACCUGUAUGAGUGAUUUUUGGUA
env-58    AUUGAAUUAGAAAAAAUGGUAUUAAAAAUGAUAAAUAAUAUUGAUAAUUCAAUUUAAACUUCAUACAUUUAACUUAACCUGUAUGAGUGAUUUUUGGUA
env-59    AUUAAAUUGGAAGAAAUGGUAUUAAAAAUGAUAAAUAAUAUUGAUAAUUCAAUUUAAACUUCAUACAUUUAACUUAACCUGUAUGAGUGAUUUUUGGUA
env-60    AUUAAAUUAGAAGAAGUGGUAUUAAAAAUGAUAAAUAAUAUUGAUAAUUCAAUUUAAACUUCAUACAUUUAACUUAACCUGUAUGAGUGAUUUUUGGUA
env-61    UAGAAGUUGAGAAAGUUAUUUUAAAAAUGAUAGAUGAUAUUGACAAAUCCAUUUUAACUUCUUAAAUUUACUUAGACCUGUAUUAGUGAUUUUUUGGUA
env-62    UAGAAGUUGAGAAAGUUAUUUUAAAAAUGAUAGAUGAUAUUGACAAAUCCAUUUUAACUUCUUAAAUUUACUUGAACCUGUAUUAGUGAUUUUUUGGUA
env-63    GAGUGAAGUUGAAAAAAUGAUCUUAAAAAUCAUUGAUGAUAUUGAUAAAUCCAUUUAAAUUCUUAAAUUUAUGAUAACCUGUAUUAGUGAUUCUUGGUA
env-64    AGUGAAGUUGAAAAAAUUAUCUUAAAAAUUAUUGAUGAUAUUGAUAAAUCCAUUUUAACUUCUUAAAUUUACUUCAACCUGUAUUAGUGAUUCUUGGUA
env-65    AGUGAAAUUGAAAAAAUUAUACUAAAAAUUAUUGAUGAUAUUGAUAAAUCCAUUUUAACUUCUUAAAUUUACUUCAACCUGUAUUAGUGAUUCUUGGUA
env-66    AGUGAAGUUGAAAAAAUUAUCUUAAAAAUUAUUGAUGAUAUUGAUAAAUCCAUUUUAACUUCUUAAAUUUACUUCAACCUGUAUUAGUGAUUCUUGGUA
env-67    AGUGAAGUUGAAAAAAUUAUCUUAAAAAUUAUUGAUGAUAUUGAUAAAUCCAUUUUAACUUCUUAAAUUUACUUCAACCUGUAUUAGUGAUUCUUGGUA
env-68    AGUGAAGUUGAAAAAAUUAUCUUAAAAAUUAUUGAUGAUAUUGAUAAAUCCAUUUUAACUUCUUAAAUUUACUUCAACCUGUAUUAGUGAUUCUUGGUA
env-69    AUAAAAUUGAUGAGAUGAUAACAAAUAUGAUUGAUUCUAUUGAUAAUAAAAUAUAAGUUCUUAAAUUUAUAAUCAGCCUGUUUUAGUGAUACUUUGGUA
env-70    AUGAAAUUGAUGAAAAGAUCACAAAAAUGAUUGAUUCUAUUGAUAAUAUAAUAUAAGUUCUUAAAUUUAUUGUAAGCCUGUAUUAGUGAUACUUUGGUA
env-71    AUGAAAUUGAUGAAAAGAUCACAAAAAUGAUUGAUUCUAUUGAUAAUAUAAUAUAAGUUCUUAAAUUUAUUGUAAGCCUGUAUUAGUGAUACUUUGGUA
env-72    AAAAAAUUGCGUACAAUUGAUGAAAUGUUAUCUGCGAUAACAUAGCUACAUACGAUCAUUGAUUACAAUGCAUAGCCUGCAUUAGUUUUAUUUUUGAUA
env-73    AAAAAAUUGCGUACUAUUGAUGAGAUGUUAUCCGAGAUAACAUAGCCACAUACGAUCAUUGAUUACAAUAUACAGCCUGCAUUGGUUUUAUUUUUGAUA
Fba-1-1   AACAAUUAGAAGUCUUGAGUCAGUUAUUGUCACAACAAUUAUCUUAGACGUUCUUUAAACAUAAACAGUUACUACCUGCACUUGUUUCAUUUUUUGGUG
env-74    GUCCCAAGCCGGCAAAGCGCUGGCAGAUGUGGAAGCUGUACUAGAUGCAGCCCUACAAAACGGAUAAACACUUUCCCGUACUUGCUAUUUUUUGUGCUA
1 env-75  AUGCCCUGCAAAAGCUGGAAGACAAGAUUCAGGCCGCGCUGAACUAGCAACGUUCUUUAACACAUUUUUUCCCGCAUCCUGAGCAAUUGAAUUUUGCUA
env-76    UUCAGGAAUUGGAUAAACUCAUUGGAAAGCUGGGUGCUUUCAAGUGAGUUGAAGCGUUCUUUUAUAGCGAUUUCCCGCAUAGGCCUACCUGCUUUGGUA
          ..................................................................................................
          ..................................................................................................
          ••••••••••••••••••••••••••••••••••••••••••••••••••••••••••••••••••••••••••••••Y••••••••••YYYUUUGRUA
```

alignment positions 100 · · · 259

```
Gka-1-1   CAAUGAACAUGUAUAUUUGU....CCAUACGC.GCGGCAGG.GCCA.UAC.GCCCGC..CC.....G....C...UGCGGCGGACCG...CCUUC.GU......GCGGAA.GGC.UGCGC.GGG.UC.G..AUUGUGAUGCC....AAGCGACUGAGCUA
1 Dps-1-1 GAGCCAACUCUCA.AUAAAA.....GGGCGUU.GUCUCUUG.UCAG.UAAAGGUGAA..UC.....G...............GUGA.................CGAAUUGGC.UGAGC.UGA.UA.AUAUGAUGCCCACC.UAUUAAUUUAGGUUUAA
Psp-2-1   AACUUAACAC....UUCAAUAUUUAUAGAAUU.AAGAGG....GUG.UAUUUCAUCU..GC.....A...............UUUAU.................UGCACCUGA.AAAUG.A......CUUAAAUUCUAAUUAUGUAACAGUUGAGGUUU
env-1     AACUCAACAUAUAUAAAAUU.......GGAUUAGAUUCGUC.UUUU.UAA.AAACAA.GCC.....U....C.AACCCUAUGGGU.....CCUUC.GGGUGUCAGUGGAAGUUU..GAUG.AUU.GGUGGUCGGUCCAUCCG.UGAU.UUACCGGAGUUU
env-2     AACUCAACAUAUAUAAAAUU.......GGAUUAGAUUCGUC.UUUU.UAA.AAACAA.GCC.....U....C....AACCCAAUGGGUCCUUCGG.GUGUC..AGUGGAAGUUU..GAUG.AUU.GAUGGUCGGUCCAUCCG.UGUU.UUACCGGAGUUU
Fba-2-1   AACUCAACAC.UA.ACAA.A.UUA.AACGAGU.GAAUCUUU.GCAACUAU.AAC.AG.GCC.....C...............UUUAGU...............GGGAA.GUU.UGAAC.UGC.AG.GUUAGCUUAAAUCU.UG.UAUA.ACAGAGUUU
Fjo-1-1   AACUCAACAC.UA.ACAA.U.UUAGAAUGAGC.AAAUCGUC.GCUACUAU.AGU.AU.GCC.....C....AG..........CUUUGA.............CCGGGAA.ACU.UGAAC.AGU.GA.GUUAGCUCAAAACU.UGUC.UUUACG.AGUUU
Fps-1-1   AACUCAACAC.UA.ACAA.A.UUAAAAUGAGC.AAAUCAUC.GUUACUAU.AGC.AA.GCC.....A....A..........UUUAGU..............UUGGAA.GCU.UGAAC.AAC.GA.GUUAACUCAAAACU.UGUCAAUC.CG.AGUUU
Ddo-1-1   AACUCAACAG.UU.AUUU.U.UUAUAAAGAGU.GACUCUAA.GUUG.UAA.AAGCAC.GCC.....G....U.UACU.AGA..CUUGU....UCU.AGGUA..GACGGAACCUU..GAUC.AGC.UUUAU.AGCUCUAAUCCUUACUAUU..AGGAGUUU
Cat-1-1   AACUUAACAAUCA.AUUA.U.UUGAAAAGGGU.AAGUUUUA.AUUG.UAAAAAC.AA.GCC.....G....U..........CUUAGA..............ACGGAA.GUU.UGAUC.AGU.UAUGU.AGCCCUAAACUUUAUUAUUUAUGAAGUUU
env-3     AACUCAACAC.UA..UUUUU.UUAAAAAAGGU.GAGUC.UG.UAUGCUA..GAGCGC.GCC.....CUAAC...............................GGGAUUUUC..GAAU.AUA.CA.GUUAGCCUUAAACU.UGUA.UAACAGGAGUUU
env-4     AACUCAACAC.UA.UUUUUU.UUAAAAAAGGU.GAGUC.UG.UAUGCUA..GAGCGC.GCC.....CUAAG...............................GGGAUUUUC..GAAU.AUA.UA.GUUAGCCUUAAACU.UGUA.UAACAGGAGUUU
env-5     AACUCAACAC.UA..UUUUU.UUAAAAAAGGU.GAGUC.UG.UGUGCUA..GAGCGC.GCC.....CUAAG...............................GGGAUUUUC..GAAU.AUU.CA.GUUAGCCUUAAACU.UGUA.UAACAGGAGUUU
env-6     AACUCAACAC.UA.UUUUUU.UUAAAAAAGGU.GAGUC.UG.UAUGCUA..GAGCGC.GCC.....CUCAG...............................GGGAUUUUC..GAAU.AUU.CA.GUUAGCCUUAAACU.UGUA.UAACAGGAGUUU
env-7     AACUCAACAC.UA.UUUUUU.UUAAAAAAGGU.GAGUC.UG.UAUGCUA..GAGCGC.GCC.....CUCAG...............................GGGAUUUUC..GAAU.AUU.CA.GUUAGCCUUAAACU.UGUA.UAACAGGAGUUU
env-8     AACUCAACAC.UA..UUUUU.UUAAAAAAGGU.GAGUC.UG.UAUGCUA..GAGCGC.GCC.....CUAAG...............................GGGAUUUUC..GAAU.AUU.CA.GUUAGCCUUAAACU.UGUA.UAACAGGAGUUU

          ...........................<<<<<.......<<.<<<......<<<.....<<.....<....<<<<<<<<<<<<.......>>>>>.>>>>>..>>>>>...>>>.......>>>.>>.....>>>>>.......................
          ...........................22222.......?2.?22......222.....22.....2....2??????????22.......22???.?????..?2222...222.......22?.2?.....22222.......................
          AACUCAAC○○-•A-○U•••○•-UUR•RARGGU-GAGUY○Y•-••UG-UA○-RAGCA•-GCY-----•-----○-----------YU○○○---------------○•RGAYYYUU--GA•Y-A•Y-YR-GU○AGCYYYAA•CY-UG•U-U••○•GGAGUUU
          5′
```

```
env-9     AACUCAACAC.UA.UUUUUU.UUAAAAAAGGU.GAGUC.UG.UAUGCUA..GAGCGC.GCC.....CUAAG..................................GGGAUUUUC..GAAU.AUU.CA.GUUAGCCUUAAACU.UGUA.UAACAGGAGUUU
env-10    AACUCAACAC.UA.UUUUUU.UUAAAAAAGGU.GAGUC.UG.UAUGCUA..GAGCGC.GCC.....CUAAG..................................GGGAUUUUC..GAAU.AUU.CA.GUUAGCCUUAAACU.UGUA.UAACAGGAGUUU
env-11    AACUCAACAC.UA.UUUUUU.UUAAAAAAGGU.GAGUC.UG.UAUGCUA..GAGCGC.GCC.....CUAAG..................................GGGAUUUUC..GAAU.AUU.CA.GUUAGCCUUAAACU.UGUA.UAACAGGAGUUU
env-12    AACUCAACAC.UA..UUUUU.UUAAAAAAGGU.GAGUC.UG.UAUGCUA..GAGCGC.GCC.....CUAAG..................................GGGAUUUUC..GAAU.AUU.CA.GUUAGCCUUAAACU.UGUA.UAACAGGAGUUU
env-13    AACUCAACAC.UA.UUAAAU.UUAAAAAAGGU.GAGUC.UG.UGUGCUA..GAGCGC.GCC.....CUCAG..................................GGGAUUUUC..GAAU.AUU.CA.GUUAGCCUUAAACU.UGUA.UAACAGGAGUUU
env-14    AACUCAACAC.UA.AUUU.UAUUAAAAAAGGU.GAGUC.UG.UUUGCUA..GAGCGU.GCC.....UGAAG..................................AGGAUUAUC..GAAU.AUU.CA.GUUAGCCUUAAACC.UGUA.UAAACAGAGUUU
env-15    AACUCAACAC.UA.UUUUAA.UUAAAAAAGGU.AAGUC.UG.UUUGCUA..GAGCGU.GCC.....UGAAA..................................AGGAUUGUC..GAAU.AUU.CA.GUUAGCCUUAAACC.UGUA.UAAACAGAGUUU
env-16    AACUCAACGC.GA.AUUA.A.UUAAAAAGAGU.GAGUC.CA.AGCCCUA..GAGCGU.GCC.....C....UA..........AUCUU...............UUGGGAUUUUC..GAAU.GCU.UG.GUUAGCUUUAAUCU.UGUA.UAAAUGGAGUUU
env-17    AACUCAACGC.UA.AUAA.A.UUAAAAAGAGU.GAGUC.UA.GAUCCUA..GAGCGU.GCC.....U....U...........UUUUU................AAGGAUUAUC..GAAU.AUU.UA.GUUAGCUCUAAACU.UGUA.UAAAUGGAGUUU
env-18    AACUCAACGC.UA.AUAA.A.UUAAAAAGAGU.GAGUC.UA.GAUCCUA..GAGCGU.GCC.....U....U...........UUUU.................AAGGAUUAUC..GAAU.AUU.UA.GUUAGCUCUAAACU.UG.UAUAAAUGGAGUUU
env-19    AACUCAACAC.UA.AUAA.A.UUAAAAAGAGU.GAGUC.UA.AAUGCUA..GAGCGU.GCC.....U................UUUUU.................AGGAUUUUC..GAAU.AUU.UA.GUUAGCUCUAAACU.UG.UAUAAAUAGAGUUU
env-20    AACUCAAC...GAAAUUU.U.UAAUAAAGGGU.GAGUCCUU.UUGG.UAA.AAGCAU.GCU.....U....U...........CUUGA................AAAGACCCUU..GAUC.CAA.AG.GUUAGCCUAAAUCC.UGUUUAUAUGGGAGUUU
env-21    AACUCAAC...GAAAUUU...UAAUAAAGGGU.AAGUCCGU.UUGG.UAA.AAGCAA.GCU.....U....U...........UCUGA................AAAGAUCCUU..GAUC.CAA.AG.GUUAGCCCAAAUCC.UGUUUACAUAGGAGUUU
env-22    AACUCAAC...GAAAUUU.U.UAAUAAUGGGU.AAGUCCGU.UUGG.UAA.AAGCAA.GCU.....U....U...........CAUGA................AAAGAUCCUU..GAUC.CAA.AG.GUUAGCCCAAAUCC.UGUUUAUAUAGGAGUUU
Psp-1-1   AACUCAACAC.UA..UUC.UAUUAUGAAGGAU.GAGUCUUA.ACUACUA..AAGCAA.GCC.....A....C...........CUUGA................GUGGAUCCUU..GAAC.AGU.UA.GUUAGUCCUAUAUA.UGUCAUU.AUGGAGUUU
env-23    AACUCAACAC.GA.AUUU.U.UUAUAAUGGGU.GAGUCAUU.ACUGCUA..AAGCGC.GCC.....A....U.................U..............AUGGAUACUU..GAUC.AGU.AU.GUUAGCCUAAAUCU.UGUUUUAAUAAGAGUUU
env-24    AACUCAACGC.GA.AUUUUU.UUAUAAAGGGU.GAGUCUUU.UCUGCUA..AAGCAU.GCC.....AGUA...................................UGGAUCCUU..GAUC.AGU.UA.GUUAGCCUUAAUCU.UGUU.UUAUGAGAGUUU
env-25    AACUCAACGC.GA.AUUU.U.UUAUAAAGGGU.GAGUCAUU.UCUGCUA..AAGCAC.GCCUAAUU........................................GGAUCCUU..GAUC.AGU.AU.GUUAGCUCUAAUCU.UGUUUUAAUGAGAGUUU
env-26    AACUCAACGC.GA.AUUUUU.UUAUAAAGGGU.GAGUCAUU.UCUGCUA..AAGCAC.GCCUAAUU........................................GGAUCCUU..GAUC.AGU.AU.GUUAGCUCUAAUCU.UGUUUUAAUGAGAGUUU
env-27    AACUCAACGC.GA.AUUUUU.UUAUAAAGGGU.GAGUCAUU.UCUGCUA..AAGCAC.GCCUAAUU........................................GGAUCCUU..GAUC.AGU.AU.GUUAGCUCUAAUCU.UGUUUUAAUGAGAGUUU
env-28    AACUCAACGC.GA.AUUUUU.UUAUAAAGGGU.GAGUCUAU.UCUGCUA..AAGCAA.GCC......................UUAAUAG................GGAUCCUU..GAUC.AGU.UA.GUUAGCUCUAAACU.UGUUAAUACAAGAGUUU
env-29    AACUCAACGC.GA.AUUUUU.UUAUAAAGGGU.GAGUCUAU.UCUGCUA..AAGCAA.GCC......................UUAAUAG................GGAUCCUU..GAUC.AGU.UA.GUUAGCUCUAAACU.UGUUAAUACAAGAGUUU
env-30    AACUCAACGC.AA.AUCU.U.UUAUAAAAGGU.GAGUCAAU.AUUGUUA..AAGCGU.GCC......................UUUAC..................GGAUACUU..GAUC.AGU.AU.GUUAGCCUUAAUCU.UGUAUUAAUAGGGGUUU
Pir-1-1   AACUCAAC...GA.GUAUCUAUUAUAAAGGAU.AAGUCUCA.GUUGCUA..AAGCAA.GCC.....A....U.AGAAUUCGAAUUUA...UUCUU.UUUCA..GGUGGAUCCUU..GAUC.AGC.UA.GUUAGUCCUAAACC.UG.UAAUAAUGGAGUUU
env-31    AACUCAACGC.UA.AUUA.A.UUAUAAAGGGU.GAGUUUAG.GUUGCUA..AAUCGU.GCU.....G....AA..........AUUU................UUCAGAUUUUU..GAUC.AGU.CUUGU.AGCCCUAAUCC.UGUU.AAACUGGAGUUU
env-32    AACUCAACGC.UA.AUCA.A.UUAAGAAGGGU.GAGUUUAG.AUUGUUA..AAUCGU.GCU.....G....A...........UUUAU................UCAGAUUUUU..GAUC.AGU.CUUGU.AGCCCUAAACC.UGUU.AAAAUGGAGUUU
env-33    AACUCAACGC.UA.AUCA.A.UUAAGAAGGGU.GAGUUUAG.AUUGUUA..AAUCGU.GCU.....G....A...........UUUAC................UCAGAUUUUU..GAUC.AGU.CUUGU.AGCCCUAAACC.UGUU.AAAAUGGAGUUU
env-34    AACUCAACGC.UA.AUCA.A.UUAAGAAGGGU.GAGUUUAG.AUUGUUA..AAUCGU.GCU.....G....A...........UUUAC................UCAGAUUUUU..GAUC.AGU.CUUGU.AGCCCUAAACC.UGUU.AAAAUGGAGUUU
Lbl-1-1   AACUCAACAC.UAUAUUC.U.UUAAGAGGGAU.GAGUCUGA.GUUG.UAA.AAGCGC.GCC.....G....U...........CUUUGA...............ACGGAUUCUU..GAUC.AGC.UC.GUUAGUCCUAUGAU.UUAU.UUUAAGGAGUUU
env-35    AACUCAAC...GAAGUUUACAUUAAAAGAGGU.GAUUUGCC.GUUG.UAA.GAGCAA.GCU.....G................UUUUGUA...............CAGAUCCUC..GAUC.AGU.GG.GGUAGCCUCAAACCUGUCUAAUUAAG.GGUUU
env-36    AACUCAACGC.UA.AUUUAA.UUCGAAGAGGU.GAGUUUUG.GGUG.CAC.AAGCAA.GCC.....G....U...........UUUAU................ACGGACCCUU..AAAC.ACA.CA.GGUAGCCUCAAACA.UGUU.UAACUCGAGUUU
env-37    AACUCAACGC.UA.AUUUAA.UUCGAAGAGGU.GAGUUUUG.GGUG.CAC.AAGCAA.GCC.....G................UUCUAAA...............CGGAUCCUU..AAAC.ACG.CA.GGUAGCCUCAAACA.UGUU.UAAUUCGAGUUU
Fba-3-1   AACUCAACAU.UA.AUUUGU.UUAAAAAGGGU.GAGUUUAG.AUUG.UAA.AAGC.AGGCC.....U....U...........CUCGAUA..............AAGGAUCCUU..GAUC.AGU.CU.GGUAACCCUAAACC.UGUU.UUC.UGGAGUUU
Rbi-1-1   AACUCAACAU.UA.CUUUAA.UUAGAAAGGGU.GAGUUUAG.GUUG.UAA.AAGCAA.GCC.....U....U...........CUUUGA...............AAGGAUCCUU..GAGC.AAU.CU.GUUAGCCCUAAACC.UGUU.UAUA.GGAGUUU
ueu-1-1   AACUCAACAC.UA.AUUCUU.UUAAAAAGGGU.GAGUUAAA.GUUG.UAA.AAG.AAUGCC.....G....CUAGA..AC...CUUG......GU.UCUCG..GGCGGACCCUU..GAUC.AGC.UU.GGGAGCCCUAAACU.UUUU.UAAAUGGAGUUU
Pto-1-1   AACUCAACAU.UU.ACUA.U.UUAAAAAGGGU.GAGUUUCA.GUUGCUA..AAGCAA.GCC.....G....C..CAC......UUCG..........GUGC..AGCGGAUCCUU..GAUC.AGC.UG.GGUAUCCCUAAACC.UGUU.UACACGGAGUUU
env-38    AACUCAACGAAGA..UUAAA.UUCAAAGAGGU.GAGUCACU.AUUG.UAA.AAGCAU.GCU.....G....C...........CUAGA................GCAGAUCCUU..GAUC.AAU.GG.GGUAGCCUCAAACC.UGUU.UAUAUGGAGUUU
env-39    AACUCAACGA.GC.UUUAAU.UUAAGAGAGGU.GAGUUAUG.ACUG.UAA.AAGCAU.GCU.....G....C...........CUUGA................GCAGAUCCUU..GAUC.AGU.UGUGU.AGCCUCAUACC.UGUUUAUUUAGGAGUUU
env-40    AACUCAACGA.GC.UUUAAU.UUAAGAGAGGU.GAGUUAUG.ACUG.UAA.AAGCAU.GCU.....G....C...........CUUGA................GCAGAUCCUU..GAUC.AGU.UGUGU.AGCCUCAUACC.UGUUUAUUUAGGAGUUU
env-41    AACUCAAC...GA.GCUUUAAUUAGAAGAGGU.GAGUUAUA.AUUG.UAA.AAGCAA.GCU.....G....C...........CUUGA................GCAGAUCCUU..GAUC.AGU.UAUGU.AGCCUCAAACC.UGUUUAUUUAGGAGUUU
env-42    AACUCAAC...GAGCUUUUAAUUCAGAGAGGU.GAGUUUCU.UUUG.CAA.AAGCAA.GCU.....G....C...........CUUGA................GCAGAUCCUU..GACC.AUA.GGUGU.AGCCUCAAACU.UGAU.UUUUAGGAGUUU
env-43    AACUCAAC...GAGCUUUUAAUUAUGAGAGGU.GAGUUUCA.UUUG.UAA.AAGCAA.GCU.....G....U...........CUUAGA...............ACAGAUCCUU..GAUC.AAU.UGUGU.AGCCUCAAACU.UGAU.UUUUAGGAGUUU
env-44    AACUCAAC...GAGCUUUUAAUUCAGAGAGGU.GAGUUUCA.GUUG.UAA.AAGCAA.GCU.....G....U...........CUUUUGA..............ACAGAUCCUU..GAUC.AAU.UGUGU.AGCCUCAAACU.UGAU.UUUUAGGAGUUU
env-45    AACUCAAC...GAGCUUUUAAUUCAGAGAGGU.GAGUUUCA.UUUG.UAA.AAGCAA.GCU.....G....U...........CAUUGA...............ACAGAUCCUU..GAUC.AAU.UGUGU.AGCCUCAAACU.UGAU.UUUUAGGAGUUU
env-46    AACUCAAC...GAGCUUUUAAUUCGGAGAGGU.GAGUUUCA.UUUG.UAA.AAGCAA.GCU.....G....U...........CUUGUA...............ACAGAUCCUU..GAUC.AAU.UGUGU.AGCCUCAAACU.UGAUUUUUUAGGAGUUU
env-47    AACUCAAC...GAGCUUUUAAUUCAGAGAGGU.GAGUUUCA.UUUG.UAA.AAGCAA.GCU.....G....U...........CUUGUA...............ACAGAUCCUU..GAUC.AAU.UGUGU.AGCCUCAAACU.UGAU.UUUUAGGAGUUU
env-48    AACUCAAC...GAGCUUUUAAUUCAGAGAGGU.GAGUUUCA.GUUG.UAA.AAGCAA.GCU.....G....C...........CUUGUA...............GCAGAUCCUU..GACC.AAU.UGUGU.AGCUUCAAACU.UGAU.UUUUAGGAGUUU
env-49    AACUCAAC...GAGCUUUUAAUUCAGAGAGGU.GAGUUUCA.GUUG.UAA.AAGCAA.GCU.....G....C...........CUUGUA...............GCAGAUCCUU..GACC.AAU.UGUGU.AGCCUCAAACU.UGAU.UUUUAGGAGUUU
env-50    AACUCAAC...GAGCUUUUAAUUCAGAGAGGU.GAGUUUCA.GUUG.UAA.AAGCAA.GCU.....G....U...........CUUGUA...............ACAGAUCCUU..GACC.AAU.UGUGU.AGCCUCAAACU.UGAU.UUUUAGGAGUUU
env-51    AACUCAAC...GAGCUUUUAAUUAGAAGAGGU.GAGUUUCA.UUUG.UAA.AAGCAA.GCU.....G....U...........CUUUGA...............GCAGAUCCUU..GAUC.AAU.UGUGU.AGCCUCAAACU.UGAU.UUUUUGGAGUUU
env-52    AACUCAAC...GAGCUUUUAAUUCAGAGAGGU.GAGUUUCC.UUUG.UAA.AAGCAU.GCU.....A....C...........CCUUGA...............GUAGACCCUU..GAGC.AUU.GGUGU.AGCCUCAAACU.UGAU.UUUUAGGAGUUU
env-53    AACUCAACGAGUU.AUUA.A.UUAAGAGUGGU.GAGUUUCA.AUUG.UAA.AAGCAA.GCU.....G....U...........CUUGA................ACAGAUCCUU..GAUC.AGU.UGUGA.AGCCACAAACC.UGAU.UUUUAGGAGUUU
env-54    AACUCAAC...GAGUUAUUAAUUAAGAGUGGU.GAGUUUCA.AUUG.UAA.AAGCAA.GCU.....G....U...........CUUGA................ACAGAUCCUU..GAUC.AGU.UGUGU.AGCUACAAACC.UGGU.UUUAUGGAGUUU
env-55    AACUCAACGAGUA..UUAAA.UUAAGAAAGGU.GAGUU.UA.AAUGCUA..AAGCAA.GCU.....A....U...........CUUGA................AUAGAUCCUU..GAAU.AUU.UA.AUUAGCCUUAAUCC.UGUU.UUUUAGAAGUUU
env-56    AACUCAACGAGUA..UUAAA.UUAAGAAAGGU.GAGUU.UA.AAUGCUA..AAGCAA.GCU.....A....U...........CUUGA................AUAGAUCCUU..GAAU.AUU.UA.AUUAGCCUUAAUCC.UGUU.UUUUAGGAGUUU
env-57    AACUCAACGAGUA..UUAAA.UUAAGAAAGGU.GAGUU.UA.AAUGCUA..AAGCAA.GCU.....A....U...........CUUGA................AUAGAUCCUU..GAAU.AUU.UA.AUUAGCCUUAAUCC.UGUU.UUUUAGGAGUUU
env-58    AACUCAACGAGUA..UUAAA.UUAAGAAAGGU.GAGUU.UA.AAUGCUA..AAGCAA.GCU.....A....U...........CUUGA................AUAGAUCCUU..GAAU.AUU.UG.AUUAGCCUUAAUCC.UGUU.UUUUAGGAGUUU
env-59    AACUCAACGAGUA..UUAAA.UUAAGAAAGGU.GAGUU.UA.AAUGCUA..AAGCAA.GCU.....A....U...........CUAGA................AUAGAUCCUU..GAAU.AUU.UG.AUUAGCCUUAAUCC.UGUU.UUUUAGGAGUUU
env-60    AACUCAACGAGUA..UUAAA.UUAAGAAAGGU.GAGUU.UA.AAUGCUA..AAGCAA.GCU.....A....U...........CUAGA................AUAGAUCCUU..GAAU.AUU.UG.AUUAGCCUUAAUCC.UGUU.UUUUAGGAGUUU
env-61    AACUCAAC...GA.GUAUUAAUUAAGAAAGGU.GAGUU.UA.AAUGCUA..AAGCAA.GCU.....A....U...........CUUCGA...............AUAGAUCCUU..GAAU.GUU.UA.AUUAGCCUUAAUCC.UGUU.UAUUAGGAGUUU
env-62    AACUCAAC...GA.GUAUUAAUUAAGAAAGGU.GAGUU.UA.AAUGCUA..AAGCAA.GCU.....A....U...........CUUCGA...............AUAGAUCCUU..GAAU.GUU.UA.AUUAGCCUUAAUCC.UGUU.UAUUAGGAGUUU

          ...........................<<<<<.......<<.<<<......<<<.....<<.....<....<<<<<<<<<<<<.......>>>>>.>>>>>..>>>>>...>>>.......>>>.>>.....>>>>>.......................
          ...........................22222.......?2.?22......222.....22.....2....2?????????22.......22???.?????..?2222...222.......22?.2?.....22222.......................
          AACUCAAC○○-●A-○U●●○●-UUR●RARRGGU-GAGUY○Y●-●●UG-UA○-RAGCA●-GCY-----●----○----------YU○○○---------------○●RGAYYYUU--GA●Y-A●Y-YR-GU○AGCYYYAA●CY-UG●U-U●●○●GGAGUUU
          5′
```

```
env-63    AACUCAAC...GA.GUAUUAAUUAAGAAAGGU.GAGUU.UA.AAUGCUA..AAGCAA.GCU.....A....U..........CUAGA...............AUAGAUCCUU..GAAU.AUU.UA.AUUAGCCUUAAUCC.UGUU.UUU.UGGAGUUU
env-64    AACUCAAC...GA.GUAUUAAUUAAGAAAGGU.GAGUU.UA.AAUGCUA..AAGCAA.GCU.....A....U..........CUUGA...............AUAGAUCCUU..GAAU.AUU.UA.AUUAGCCUUAAUCC.UGUU.UUU.AGGAGUUU
env-65    AACUCAAC...GA.GUAUUAAUUAAGAAAGGU.GAGUU.UA.AAUGCUA..AAGCAA.GCU.....A....U..........CUUGA...............AUAGAUCCUU..GAAU.GUU.UA.AUUAGCCUUAAUCC.UGUU.UUU.GGGAGUUU
env-66    AACUCAAC...GA.GUAUUAAUUAAGAAAGGU.GAGUU.UA.AAUGCUA..AAGCAA.GCU.....A....U..........CUUGA...............AUAGAUCCUU..GAAU.GUU.UA.AUUAGCCUUAAUCC.UGUU.UUU.AGGAGUUU
env-67    AACUCAAC...GA.GUAUUAAUUAAGAAAGGU.GAGUU.UA.AAUGCUA..AAGCAA.GCU.....A....U..........CUUGA...............AUAGAUCCUU..GAAU.GUU.UA.AUUAGCCUUAAUCC.UGUU.UUU.AGGAGUUU
env-68    AACUCAAC...GA.GUAUUAAUUAAGAAAGGU.GAGUU.UA.AAUGCUA..AAGCAA.GCU.....A....U..........CCUGA...............AUAGAUCCUU..GAAU.GUU.UA.AUUAGCCUUAAUCC.UGUU.UUU.AGGAGUUU
env-69    AACUCAACGAGUAUACAA...UUAAAAAAGGU.AAGUU.CA.AGUGUUA..AAGCAA.GCU.....A....U..........CUAGA...............AUAGAUCCUU..GAUC.AUU.UG.AUUAGCCUUAAUCC.UGUU.UAAAAGGAGUUU
env-70    AACUCAACGAGUAUACAA...UUAAAAAAGGU.GAGUA.CA.AGUGUUA..AAGCAA.GCU.....A....U..........CUAGA...............AUAGAUCCUU..GAUC.AUU.UG.AUUAGCCUUAAUCC.UGUU.UAAAAGGAGUUU
env-71    AACUCAACGAGUAUACAA...UUAAAAAAGGU.GAGUA.CA.AGUGUUA..AAGCAU.GCU.....A....U..........CUAGA...............AUAGAUCCUU..GAUC.AUU.UG.AUUAGCCUUAAUCC.UGUU.UAAAAGGAGUUU
env-72    AACUCAACAC.UA.AUUCAA.UUAAAAGAGGU.GAGUUUAA.GUUG.CAA.AAGCAA.GCC.....G....C..........CACCGA..............GUGGAUCCUU..GAAC.AAU.UU.GGUAGCCUCAAACC.UGUUUAUUUCAGAGUUU
env-73    AACUCAACAC.UA.AUUCAA.UUAAAAGAGGU.GAGUUUAA.GUUG.CAA.AAGCAA.GCC.....G....C..........CUUCGA..............GCGGAUCCUU..GAAC.AAU.UU.GGUAGCCUCAAACC.UGUUUAUUUCAGGGUUU
Fba-1-1   AACUCAACACAUA.AUUC.U.UUAAAAAAGGU.GAGUCGUG.GUUG.UAA.AAGCAA.GCC.....G....GAC........CUAGGA..........G...CCGGAUCCUU..GAUC.AAU.CA.GUUAACCUUAAACUU...UAUUUAAGGAGUUC
env-74    AACUCAACGAGCA......CGUUGUUAUGGAC.UUCGCUCGAGACGUUAC.GAGCAG.GCC.....A....C......ACCUCUUUGAGGGAGUUUACUCC..AGUGGAAGGUC.AAGGC.GUCACG.GCUAGUACAGAUCC.UGAUAUAU.AGGAGUUU
env-75    AACUCAACAC.GA.....UUACCAAUAGGUGU.UUAGCUCG.GGCUUUCUAGGUCAG.GCC.....C....C..........CACGACA.............GGGGAAGACCGAAAUU.GUC.UG.GUAAAUGCCGAUCC.UGUC.UAAACGGAGUUU
env-76    AACUCAACAGUUA.....ACCUAAAAAGGAGU.CCAGCUCG.UUUUGGAGAUGUCAU.GCC.....C....UUUUGG.AAAAGCUCGGC.UUUUU.ACUGA..GAGGGAAGACU.GAAACUCAG.CG.GUGGAUUCCGAUCC.UGUC.UAAUUGGAGUUU
          ..........................<<<<<......<<.<<<......<<<.....<<.....<....<<<<<<<<<<<<.......>>>>>.>>>>>..>>>>>...>>>.......>>>.>>.....>>>>>.......................
          ..........................22222......?2.?22......222.....22.....2....2??????????22.......22???.?????..?2222...222.......22?.2?.....22222.......................
          AACUCAACoo-•A-oU••o•-UUR•RARRGGU-GAGUYoY•-••UG-UA•o-RAGCA•-GCY-----•----o----------YUooo---------------o•RGAYYYUU--GA•Y-A•Y-YR-GUoAGCYYYAA•CY-UG•U-U•••o•GGAGUUU
          5′
```

alignment positions 260 · · · 419

```
Gka-1-1   AUGGAUUGAAAACGGGAAUGUCCAUCUGCGCCGGUCAUGGCGGUGAGAAGAGAGAGCGAAAGGAAACCGAACAGCAAGAGGAGGUGAAAUGAUGGGUUCGAUCAUCAUCUCCGCUUUGCUUGCCUUAGUCAUUGGUGCCGUUGUUGGCUUUUUUGUUCGC
Dps-1-1   CUAUCUUAGCGACACGGCAGUGUGGGGGAUUUUUAUGAUUUUUCAGAGAUCUUGUUUGGAUCCUGUGGGAUAUAGUUUUAUCUCAAAAUAUAAAUGCAAAUAAAUGUGGAAGCAAGAGUAGAUCGUUGUGAUAAGACUCUUGCUGGUGCAUUAAAAUAAU
Psp-2-1   UGAAUACAUGAGACUUAUUGAAUUAGUUGCGGUUUUUUUUUUAACAAAAUUUGGGAUAUCCGUGGGCUUUUGAGCAUGAGAUGCAUCAGGGGGCACGUAAAGAGCAUAUAUAAAUACAGAUGAAUUAACACAAUGGAAAUAGUAGGAAUAAUUGGAUAUGU
env-1     AAUUAACAGAAUUAAUACGGGUUUCUAUUUUUACAACAACACUAAGUAUGACUACAGAAAUGAUAAUAACAAUAGCAAUUACGAACAUUAUAGCCUUAAUAAUAGGGUAUAUAAGUGCGUCUUCAGUUCUACGUAAAUCAAUAGAACGUAAAUCUGAAAA
env-2     AAUUAACAGGAUUAAUGCGGGUUUCUAUUUUUACAACAACAUUAAGUAUGACUUCAGAAAUAAUGAUAACAUUAGCAAUUACAAGUAUUAUAGCCUUGGUAAUAGGUUAUAUAUUUGCGUCUACAGUACUUCGUAAAUCAAUAGAACGUAAAUCUGAAAA
Fba-2-1   AUACAAACACCUAAUGUAGGCUUUUUUUAUAUAAUUUUUUAACACAUAUAAUGGAUAUACUAAGCAUAAUAAUUGGAAUUGUUGUCGGGAUUGCCGGAGGUUUUGGAAUUGCUAAAUUUUUAGAAAAAAGCAAUGUUUCCAACCUUAUUAAAAACGCAAA
Fjo-1-1   AUUCAAGCAAUUAAUGUAGGCUUUUUUUAUAUAUAAACCCUAACAAACAUGGACAUCAUAACGAUCAUUAUUGGUAUUGUAGGUAUUGCGGCAGGUUUUGCAAUAGCUAAAAUUAUCGAGAAAAGUAAUAUUUCAAACCUAAUCAAAAACGCUAAAAAAG
Fps-1-1   AUACAAUACCCGCUGAUGUAGGUUUUUUUUAUAUAUAACUUAAAAACAAAUAUGGACAUUAUAACAAUAAUAAUAGCAGUAAUUGCUGGAAUAGGCGGCGGAUUUGGAAUUUCAAAAUUUUUAGAAAAAAGUGGCGUUUCUAAUUUAAUAAAAAACGCAA
Ddo-1-1   AUACGAAAUGCACUAUCAAUGUGGGCUUUUUUUAUAUCUCAUCGCGAGAUAGUGAGGCUUAAAAGCCUUAACAAUCAACCAUCAGUAGCGCCUGUAAAGGCAUUAUAAUAUUAAGUCAAAAUUAUGGAACAUAACGCACUCUUGUUUAUAGUAGGUGUGA
Cat-1-1   AUACAAAACCAAUGCUAAUACAGGCUUUUUUUAUACCGUAAACUGAAAUGAUUUUUCAUAAACGGGUUUUUAUUUAAACAUACAUAUAUGAGCACUACAAUACUUUACUUUACCAUUAUUGGCGUUAUAGGCGUUGCAAUUGGGUAUAUCAUUGCUAAAA
env-3     AUACAAUCAAUCUGUUUGUAGGUUUUUUAUUUUCACACCAUCACAUAUUGUAAUUUCUAUUGGUGUCAAGUCAAUUGUAAAUUUGUCUUUGUAGCAUUUGUAUGCUCUUUCUAUAAACUCAUCUUUAAAAGAUUUUCAACAAGAUUUAUUGUACAGCCA
env-4     AUACAAUCAAUCUGUUUGUAGGUUUUUUAUUUUUACACCAUCGCAUAUUGUAAUUUCGAUUGGAGUCAAGUCAAUUCUAAAUUUGUCUUUAUAGCUUUUGCAUGCUUUUUCUAUAAACUCAUUUUUAAAAGAUUUUCAACAAGAUUUAUUGUACAUCCA
env-5     AUACAACUAAUCUGUUUGUAGGUUUUUUAUUUUAACUCCAUCACAAACAUUUAUUUCAAUUGGGGUUAAAUGAAUAGAAAACUUUUCAAAGUAGCUUUUGCUAGCUUCUUCAAUAAACUCAUCUGUAAAUCUUUUAUGAAUUAGGUUAAUUGUACAA...
env-6     AUACAACUAAUCUGUUUGUAGGUUUUUUAUUUUAACUCCAUCACAAAUAUUGAUCUCAAUGGGGGUUAAAUUAAUAGAAAACUUUUCAAAGUAGCUUUUGCUAGCUUCUUCAAUAAACUCAUCUAUAAAUCUUUUAUGAAUUAGGUUUAUUNNNNNNNN
env-7     AUACAACUAAUCUGUUUGUAGGUUUUUUAUUUUAACUCCAUCACAAAUAUUGAUCUCAAUGGGGGUUAAAUUAAUAGAAAACUUUUCAAAGUAGCUUUUGCUAGCUUCUUCAAUAAACUCAUCUAUAAAUCUUUUAUGAAUUAGGUUUAUUGUACAACCA
env-8     AUACAACUAAUCUGUUUGUAGGUUUUUUAUUUUAACUCCAUCACAAACAUUUAUUUCAAUUGGGGUUAAAUUAAUAGAAAACUUUUCAUAGUAGCUUUUGCUAGCUUCUUCUAUAAACUCAUCUAUAAAUCUUUUAUGAAUUAGGUUUAUUGUACAACCA
env-9     AUACAACUAAUCUGUUUGUAGGUUUUUUAUUUUAACUCCAUCACAAACAUUUAUUUCAAUUGGGGUUAAAUUAAUAGAAAACUUUUCAUAGUAGCUUUUGCUAGCUUCUUCAAUAAACUCAUCUAUAAAUCUUUUAUGAAUUAGGUUUAUUGUACAACCA
env-10    AUACAACUAAUCUGUUUGUAGGUUUUUUAUUUUAACUCCAUCACAAACAUUUAUUUCAAUUGGGGUUAAAUUAAUAGAAAACUUUUCAUAGUAGCUUUUGCUAGCUUCUUCAAUAAACUCAUCUGUAAAUCUUUUAUGAAUUAGGUUUAUUGUACAACCA
env-11    AUACAACUAAUCUGUUUGUAGGUUUUUUAUUUUAACUCCAUCACAAACAUUUAUUUCAAUUGGGGUUAAAUUAAUAGAAAACUUUUCAUAGUAGCUUUUGCUAGCUUCUUCAAUAAACUCAUCUAUAAAUCUUUUAUGAAUUAGGUUUAUUGUACAACCA
env-12    AUACAACUAAUCUGUUUGUAGGUUUUUUAUUUUAACUCCAUCACAAAUAUUGAUUUCAAUUGGGGUUAAAUUAAUAGAAAACUUUUCAAAGUAGCUUUUGCUAGCUUCUUCAAUAAACUCAUCUAUAAAUAUUUUAUGAAUCAGGUUUAUUGUACAACCA
env-13    AUACAACUAAUCUGUUUGUAGGUUUUUUAUUUUAACUCCAUCACAAAUAUUUAUUUCAAUUGGGGUCAAAUUAAUAGAAAACUUUUCAAAGUAGCUCCUGAUAGCUUCAUCAAUAAACUCAUCCGUAAAUUUUUUAUGAAUUAGGUUUAUCGUACAACCA
env-14    AUGCAAUCUUUCUGCCUUUAGGUCUUUUUUAUAGUUAUGCCAUUGUCAAGUUCUGCAAGGAUUGGUGUAGCCUCUUUUCCAAAUUUUCUGUAGUAAGCUUUACAAGAUUCAUCUACAAAUUGGCCUAUGAAGUCAUCUUGUAUCAAGUUUAUUGUACAGC
env-15    AUGCAAUCUUUCUGCCUUUAGGUCUUUUUUAUCGAGAUGCCAUUGUCAAGUCCUGCAAGGAUUGGUGUCGCCUCUUUUCCAAAUCUUCUGCGAUAAGCUUCACAAGAUUCAUUCACAAAUUGGUCUAUAAAGUCAUCCUGGAUCAAGUUUAUUGUACAAC
env-16    AUACAAUCAAUCUGAUUGUAGGUUUUUAAAUUAAACUUAUGAAUGAAAUUAUAUUUAUUGUAAUAGGGGUUGUAAUUGGAUUUACAGUAACCUAUUUAUUAAAAAAAGCAUCAAACAAAAAAUUACUUAGUGAAAGCCAAAUUGAGUCUCAAAAAAUUAU
env-17    AUGCAAUAAAUCUGUAUGUAGGUUUUUUUUUAAAAAAAAAAUCAUGAAUGAAUUAAUUUAUAUAGUAAUUGGAUCUUUAAUCGGAGGAGUUGCUCUUUUUAUAAUCAAUAAAAUUUCAAAUAAAAAAUCCACUGAUAAUGCUAAGUCUAGUGCUGAUAAA
env-18    AUGCAAUCAAUCUGUAUGUAGGUUUUUUUUUAAAAUAAAAUCAUGAAUGAAUUAAUUUAUAUAGUAAUUGGAUCUUUAAUUGGAGGAGUUACUCUUUUUAUAAUCAAUAAAAUUUCAAAUAAAAAAUCCAUUGAUAAUGCUAAGACUAGUGCUGAUAAAA
env-19    AUGCAAUCAAUCUGUAUGUAGGUUUUUUUUAAAAUAAUUAUGGAAUAUUUAGUUUAUAUAUCAAUAGGCUUUUUUCUUGGUGGAAUUACCGGUUUUUUUAUUACCAAAAUUUUUAAUAAAAGAUACCUUCAGGAUAGGAUUUCUUCUGCUGAUAAAAUAU
env-20    AUACAAAACCUAAGUUAGCGCAGAUUUUUAUAUCAAAAAGCUGUAGGAUAAUCCAGCAAAUACAGCUCCGCAGAUAGGACCUGCAAUUGGAACCCAGCUGUAUGCCCAGUCUGCAUCUUUUUCUUUUCUUGGUAAUAAUUGAUAAACAAUCCUUGGACCC
env-21    AUACAAAACCUAGGUUGGCGCAGAUUUUUAUAUUAACACGCUGUAGCAUAAUCCUGAAAUAACAGCCCCUAAUAUAGGACCUAUCACAGGAACCCAGCUGUACGCCCAAUCUGCGUUUUUUUCUUUUCUAGGCAAAAAUUGAUAAACAAUUCUUGGACC
env-22    AUACAAAACCUAAGUUUACGCAGAUUUUUAUACUAAUAAGGUAUAGCAUAACCCCGAAAAUACAGCUCCAAAUAAAGGACCUAAAACAGGAAUCCAGCUGUAAGACCAAUCUGCGUUUUUUUGUUUUCUUGGCAAUAAUUGGUAAACAAUUCUUGGACCU
Psp-1-1   AAAAAAAACCAACUAAUGUAGGCUUUUUUUAUAUACAAAUUUUAAUUAUGGAAGGAAUAAUACUUCCCCUUAUUUUGGGAGUUUUAGUAGGAAUCGCUUUAGGUUUUAUAAUAUUUAAAUCAAUUGAGAAAUCUAAAGGAAAGAAGAUUUUAAAUGGCAC
env-23    AUACAAACAUUCACGAUGUAGGCAUUUUUUUUACAAAAAACUAUGGAUACAGAAAUUUUUACAUUAUUGAACAUAAUUAUAGUACUGAUUGGAAUUACCGUUGGAUUUUUAAUUUCUAAAAUAUUAGAAAAAUCUUCGGUUUCAAAAACACUUAUCAAUG
env-24    AUACAAAACAUUCAUGAUGUAGGCUUUUUUAUUUUCAUAAUUAAGCUUUAUACUAUUAACACUCUCUUAAUCUUUUGGCUGCAAAUUCUGGUGUAAUAUCCCUUUGCGGACCAGCAAACAUUUCGUAUCCAACCAUAAAUUUUUGAUUUUUGAGGACCU
env-25    AUACAAAACAUUCAUGGUGUAGGCUUUUUUUAUAAUAUUUAGUAAUAAAUUUAAAAUUUGUUUCAAAUUUAUUUUUUAACAACUAUUCCAUCUCCAAUUUUAACAAUAUCAUAAGUUAAAAUUAAGCCAAAUCUAUUUUUGUAUGAAUUAGUAACUAAUU
env-26    AUACAAAACAUUCAUGGUGUAGGCUUUUUUUAUAAUAUUUAUUAAUAAAUUAUAAAUUUAAAAUUUGUUUCAAAUUUAUUUUUUAACAACUAUUCCAUCUCCAAUUUUAACAAUAUCAUAAUUUAAUAUCAAGCCAAAUCUAUUUUUGUAUGAAUUAGUA
env-27    AUACAAAACAUUCAUGGUGUAGGCUUUUUUUAUAAUAUUUAGUAAUAAAUUAUAAAUUUAAAAUUUGUUUCAAAUUUAUUUUUUAACAACUAUUCCAUCUCCAAUUUUAACAAUAUCAUAAGUUAAAAUCAAGCCAAAUCUAUUUUUGUAUGAAUUAGUA
env-28    AUACAAAACAUUCAUGAUGUAGGCUUUUUUUUUAACCAAAAAAUAAAAAUUAUGGAUUUUUUUACAAUACAAAACUUAGUUCUUUUAAUAUUAGGACUUAUAGCUGGUUUUAUUAUUUCAAAGCUUCUAGAGAAGUCUGCAAUAAAUAAAUCAAUACAAA
env-29    AUACAAAACAUUCAUGAUGUAGGCUUUUUUUUUAACCAAAAAAUAAAAAUUAUGGAUUUUUUUACAAUACAAAACUUAGUUCUUUUAAUAUUAGGACUUAUAGCUGGUUUUAUUAUUUCAAAGCUUCUAGAGAAGUCUGCAAUAAAUAAAUCAAUACAAA

          ................................................................................................................................................................
          ................................................................................................................................................................
          AYRCAA•R••Y••••••••••R••YUUUUU•••••••••••••••••••••••••••••••••••••••••••••••••••••••••••••••••••••••••••••••••••••••••••••••••••••••••••••••••••••••••••••••
        3′
```

```
env-30    AUACAAAACACAUGUGAUGUAGGCUUUUUUUUAAAAGAAAAAACAUGGGAACAGAAAUUUUUACAAUUACAAAUAUAAUUAUAGCAUUAACCGGAGUUUUAUCUGGCUUUUUCAUUUCUAAAAUUUUAGAAAAGUCAUCUGCUUCAAGUAUUAUCUAA
Pir-1-1   AUAAAAACCACACUUUCGUAGGCUUUUUUUUAUAUAAAAUUUAAAUUAUGGACGGAAUUUUACUUCCCAUUAUUGUGGGAAUUAUAGGGAUUGCAAUUGGUUUUAUUAUUGCAAAAGGAAUUGAGAAAUCUAAAGGGAAAAAAUUAUUAAACACAACCCG
env-31    AAAUAAAAAAGCUUUUGUAGGUUUUUUUUUAAAAAUUAAAUU..........................................................................................................
env-32    AAAUAAAAUGACUUUUGUGGGUUUUUUAUUUAAUAAAUUUUAAUAUGGAUAUAACAACAAAUGUGAUUAUUUCAUUACUAAUAGGUUUUGUGCUGGGUUUGGUAUUUUUAAAAUUACUUAACAAAAACAAAUCUUUAGGGCUAAUUAACAAAGCAAAAAA
env-33    AAAUAAAAUGACUUUUGUGGGUUUUUUAUUUAAUAAAUUUUAAUAUGGAUAUAACAACAAAUGUGAUUAUUUCAUUACUUAUAGGUUUUGUGCUGGGUUUGGCAUUUUUAAAAUUACUUAACAAAAACAAAUCUUUAGGGCUAAUUAAUAAAGCAAAAAA
env-34    AAAUAAAAUGACUUUUGUAGGUUUUUUAUUUAAUAAAUUUUAAUAUGGAUAUAACAACAAAUGUGAUUAUUUCAUUACUUAUAGGUUUUGUGCUGGGUUUGGCAUUUUUAAAAUUAAUUAACAAAAACAAAUCUUUAGGGCUAAUUAACAAAGCAAAAAA
Lbl-1-1   ACUCAAAACACUUCCAAUGUAGGCUUUUUUUAUAUAUACACUAACUCAAUGGGAAAUACUGUACUUAUAAUCGUAGGUGUCAUCUUAAUUGCCGGCACACUAGGAUUUUUACUAGCAAAAUUGCUAGAGAAAAAUAAUGCCUCUCAAAUUGUAAGCAAUG
env-35    AUACAAAACUUCAUAACUAAUACGGGCUUUUUUAUUUAGUAAGACAUGGAAGCAGACAUCCUAAUCAUUAUUAUCGCGGGCAUCNNNNNNNNNNNNNNNNNNNNNNNNNNNNNNNNNNNNNNNNNNNNGCUCAACAAAACCAAGUCGUUAGCU
env-36    AUGCAAAACUUGCCUUGGGUAGGUUUUUUAAUAAUUGGCAGGUAAUUUAUUUUUCUCGAAUAGUUUGUGAAUCCAUAGGAUAACAACCCCAAAGGCAACUGCUGAAAUAAUAAGCAAUAUAUUUUGAAGCCCUGAAACACUAAAGGAUAACUUGACCAAA
env-37    AUGCAAAACUUGCCAGGUGUAGGUUUUUUUAAUAAUUGACCGGUAAUUUAUUCUUUUCGAAUAGUUUGUGAAUCCACAGAAUAACAACUCCAAAGCCAACAGCUGAUAUAAUAAGCAAUAUAUUUUGUAGUCCAGUAACACUAAAGGAUAAUUUGACCAA
Fba-3-1   GUACAAAACUUCUCCAAUGUGGGCUUUUUUUAUACUAAAUUUUGAAAUUAUGGACAGUACCACACUUAUAUUAGUCGGAAUCGCGGGCCUUGUUAUAGGAUUUGUGAUUGCCAAGUUUAUGGAAAAAGGCAAAGCAUCUAAGACCAUUACAAAUGCGAAA
Rbi-1-1   GUACAAAACUUCCAUCAAUGUGGGCUUUUUUUAUGCUUUCGGGGGUUGCCCGGAGGCGUAUUCUGAUUCAGGUGACACUGCCGGAACCGCAAACUAAAUUGACAGAUCAUGGACAUUACAACGAUAGCCAUCGCCCUGGUGGUUGGGUUGGGGAUCGGUU
ueu-1-1   ACACAAAUCACAUUAUUAAUAUAGGCUUUUUUAUGUUUUAUAGUGAACACAUUAAUUAAUAUUAAAGUGACAUUAUACCUACUGUUGUAGGUGUUUUAAAUAAAUAAUACCCAUAAAUAAUUAAAUUAUAACCAUCAAAUGAUCGAAUAUAUAAUACCAG
Pto-1-1   UAUACAAAACUAGGCUAGUACAGGCUUUUUUUAUAUAUAUAAUUAUGGAUAUAACAUUUAUUAUAAUAGCAGCUAUAGCUGGCCUUAUCAUAGGAUUUUAAUAUCAAAAGCCAUAGAUAGCAAAAGUGCAACAGGGACUGUAAAAACAGCUGAAAAAG
env-38    AUACAAAACUCAUCGCUAAUGCAGGCUUUUUAUUUUAAACAAUAUGGAAGCAUACAUCCCUUAUUUACUUGCCCUUUUCGUAGGCAUCGUU.....................................................
env-39    AUACGAAAACUCUUUGCUGAUACAGGCUUUUUUAAUGUUAUAAAAAACGAAUUAAAUGGAAGUAAGUACCAAUAUGAUAAUAUUUAUAGCUAUCGCCAUACUGAUCGGAGUGGGCAUGGGAAUCUUUAUCCAGCGCAGAAAAAAAUAUCAUACAAUUGGAC
env-40    AUACGAAAACUCUUUGCUAAUACAGGCUUUUUUAAUGAUAUAAAAAACGAAUUAAAUGGAAGUAAGUACCAAUAUGAUAAUAUUUCUAGCUAUCGCCAUACUGAUUGGAGUGGGCAUGGGAAUCUUUAUCCAGCGCAGAAAAAAUACCAUACAAUUGGAC
env-41    AUACAAAAAUUCUUUGCUGAUACAGGCUUUUUAAUGAUUUAAAACCAACCAAAUGGAAGUAAACACAAGCAUAAUAAUACUCACCACCAUCGCUAUUGUGAUCGGAAUAGGCUUGGGGAUUAUUAUACAACGUAGAAAAAACAACAAACUGCUCGUCGAU
env-42    AUACAAAGAACCCGCUAAUACAGGUUUUUUUUAAAAAAGAAAAUAAAGAUGGAAUCAUAUUUAAUAGUUAUAAGUAUUGGAAUAGGAUCACUUUUAAUAGGAUUUAUUAUUGGUGUAAAUUCUAGAAAAGGAAAAAAUAAAUCUCUUUUAGAGGAUUUGAAA
env-43    AUACAAAAGGCACGCUAAUACAGGUUUUUUAUGUCAAAAUAAAUUUUUAAAAACUAAUUAGUAUUAUAUGUAAUAUUCCUGCAGUAGCUCCUCCAAACAUUGGGCCUAUUAUUGGAAUCCAGCUAUAACUCCAAUCAGCAUUUUUAUUUUUCCUAGGUAA
env-44    AUACAAAAAGCACGCUAAUACAGGUUUUUUAAACGGAAAAAAAUGGAAACUGAUUUCAAUUUUAUACUUGUACUAAUACCCUUAGCCAUAGGGGUUUUACUUGGUACAAUAUUAAAAAAAGGGAAGCAUAAACGCUUGUUGGAGUCUGCAAAAAAAGAAG
env-45    AUACGAAAGGCACGCUAAUACAGGUUUUUUUAAAAAUCAAAUUUCACAUCUAGCAUAUCUAAAUAUUUACUAGUAUCCCAUAUAUAAUCUGCAUGCUCCCCCAACCAAUGGCCUAUAACUGGAAUCCAACUAUAUUCCAAUCAGCAUCUUUAUU
env-46    AUACAAAGAUACGCUAAUACAGGUUUUUUUAAAUCUUAGAUAAAAUAGAAAAUAUUAAACCAGCUGUUGCGCCUCCAAAAAAGGGACCUAAAAUAGGAACCAAACUGUAACUCCAGUCUGCAUUUUUGUUUUUUCUUGGUAAUAGUUGAUAAACUAAAC
env-47    AUACAAAGAUACGCUAAUACAGGUUUUUUUUAAAUCUUAGAUAAAAUAGAAAAUAUUAAACCAGCUGUUGCGCCUCCAAAGAAGGGACCUAAAAUAGGUACCAAACUGUAACUCCAGUCUGCAUUUUUGUUUUUUCUUGGUAAUAGUUGAUAAACUAAAC
env-48    AUUCAAGAAGCACGCUAAUACAGGUUUUUUUUAACAAAUGAUGAGAAGAUGGAAUCCUAUAUAUACUUUAUACCGGCACUGAUCACACUUCUAGUAGGAUUAUUACUAGGAGGACUUUUUAAAAAAGGAACUCAAAAAAAAAUAGUAGAACAGGCUAAGA
env-49    AUACAAAAAGCCCGCUAAUACAGGUUUUUUUAACAAAUGAUUAGAAAAAAUGGAAUCCUACAUAUCUUAUAUACCCACGGUUAUCGCACUCUUUGUGGGCUUGUUUUUGGGGGUGCUCUUAAAAAAAGGGAAAACAUAAAAAUUUACUAGAAGCUGCAACC
env-50    AUACAAAAAUACGCUAAUACAGGUUUUUUUAAAAAUUAGAUAAAAAGAGAAUAUAUUAAACCCGCUGCUGCACCCCCAAAAAAGGGACCAAAAAUUGGUACCCAACUGUAAUCCCAAUCCGCAUUUUUAUUUUUUCUUGGUAAUAAUUGAUAAACUAAACG
env-51    AUACAAAAAAUCUUUCUGGCGCAGGUUUUUUCAUUUGACUAUUUCCAUAGACUUAUUUUCAGAAUAAAACAAAAAGAAAGGACUACAUUAAAAAAUUAUAAAUUAUCCCUGCUAUUGCUCCUCCUAAAAAAGGUCCUAGAAUUGGAACCCAGCUAUAAUU
env-52    AUACAAAAAACGCGCUAAUGCAGGUUUUUUAAAAAAAUAGUAAAUAGAAAUUUAUUUAAAGGAUAGUGUAAAUCAAACCAGCUAUAGCUCCUCCUGUAAAUGGUCCUAAAAUUGGAACCCAACUAUAAGCCCAGUCUGCGUCUUUGUUUUUUCUCGGAAG
env-53    AUACAAAAACGUGCUAGUACAGGCAUUUUUAAAUUAAAAAAAUGGUAAAUACACUAUCAAUAAUAUUUACACUUAUAGGACUGGUUCUUGGAGUUGGUCUUGGCUUUAUCUUAAGAAAGAAAAAANNNNNNNNNNNNNNNNNNNNNNNNNNNNNNNNNNNN
env-54    AUACAAAAAGCGCGCUAAUACAGGCAUUUUUAAAUAAAAAAAUGGUAAAUCCACUAUCAAUAAUAUUUACACUUAUAGGAUUGGUUCUUGGAGUUAGUCUUGACUUUAUCUUAAGAAAAAAAAAAAUUAAAAGUAUUGAUUGAAAAAGCAAUGCAAGAGGC
env-55    ACGCAAUCGCACUCUCAUACAGGUUUAUUUAUGUUAAGAGGUUUCCUUCCUCAUCCCAUUUUGCAGUUUCAUUUCUUUUUGAUUGAUCAACACACUUGGAUAUCCAUUCUUCAUCAUAUGAUACAGAAUGUUUUUUUAAUACUUCAUCGGGUACUCCAUC
env-56    ACGCAAUCGCACUCUCAUACAGGUUUAUUUAUGUUAAGAGGUUUCCUUCCUCAUCCCAUUUUGCAGUUUCAUUUCUUUUUGAUUGAUCAACACACUUGGAUAUCCAUUCUUCAUCAUAUGAUACUGAAUGUUUUUUUAAUACUUCAUCAGGUACUCCAUC
env-57    ACACAAUCGCACUCUCAUACAGGUUUAUUUAUGUUAAGAGGUUUCCUUCCUCAUCCCAUUUUGCAGUUUCAUUUCUUUUUGAUUGAUCAACACA.................................................
env-58    ACACAAUCGCACUCUCAUACAGGUUUAUUUAUGUUAGGAGGUUUCCUUCCUCAUCCCAUUUUGCAGUUUCGUUUCUUUUUGAUUGGUCAACACACUUCGAUAUCCAUUCAUCAUCAUAUGAUACAGAAUGUUUUUUUAAUACUUCAUCUGGUACUCCAUC
env-59    ACGCAAUCGCACUCUCAUACAGGUUUAUUUAUGUUAAGAGGUUUCCAUCCUCAUCCCAUUUUGCAGUUUCGUUUCUUUUUGAUUGAUCAACACACUUGGAUAUCCAUUCUUCAUCAUAUGAUACUGAAUGUUUUUUUAAUACUUCAUCAGGUACUCCGUC
env-60    ACGCAAUCGCACUCUCAUACAGGUUUAUUUAUGUUAAGAGGUUUCCAUCCUCAUCCCAUUUUGCAGUUUCAUUUCUCUUUGAUUGAUCAACACACUUGGAUAUCCAUUCUUCAUCAUAUGAUACUGAAUGUUUUUUUAAUACUUCAUCAGGUACUCCAUC
env-61    ACGCAAUACUCACUUUAAUACAGGUUUUUUUUAAACCUUAAGUUAAUAGAUUCCCUUCUUCAUCCCAUUCUGCAAGAUCAUUUCUUUUACUUUGAUCUACACAUCUGGAAAUCCAGUCUUCAUCAUAGGAUACUGAAUAUUCUUUAAGGACAUCUUCUGG
env-62    ACACAAUACUUAUUUAAUACAGGUUUUUUUUAAUUCCUACGUUAGUAGAUUCCCAUCUUCAUCCCAUUCUGCAACAUCAUUUCUUUUACUUUGAUCUACACAUCUGGAAAUCCAGUCUUCAUCAUAGGAUACUGAAUAUUCUUUAAGGACAUCUUCUGGA
env-63    ACGCAAUACUCACUCUAAUACAGGUUUGUUAUUUUAUCAGGUAAGAAGAUUGCCGUCUUCAUCCCAAACAGCAGUUUCAUUUCUUUUAGAUUGAUCAACACACUUUGCGAUCCACUCUUCAUCAUAUGAUACAUCAUGAUCUUUUAGGACAUCUUCAGGA
env-64    ACGCAAUACUCACUCUAAUACAGGUUUGUUAUUUAAUCAAGUUAGAAGAUUGCCAUCUUCAUCCCAAACAGCAGUUUCAUUUCUUUUAGAUUGGUCAACACACUUUGCAAUCCAUUCUUCAUCAUAUGAUACACUAUGAUCCUUAAGGACAUCUUCAGGA
env-65    ACGCAAUACUCACUCUAAUACAGGUUUGUUAUUUAAUUAAGUUAGAAGAUUACCAUCUUCAUCCCAAAUAGCAGUUUCAUUUCUUUUGGAUUGGUCAACACACUUUGCAAUCCAUUCCUCAUCAUAUGAUACAUUAUGAUCUUUAAGUACAUCUUCAGGA
env-66    ACGCAAUACUCACUCUAAUACAGGUUUGUUAUUUUAUUAGGUAAGAAGAUUUCCAUCCUCGUCCCACUCUGCUAAGUCAUUUCUUUUGGAUUGAUCAACACACCUGGAAAUCCAUUCUUCAUCAUAUGAAACUGAGUAAUCUUUUAGAAUUUCUUCAGGG
env-67    ACGCAAUACUCACUCUAAUACAGGUUUGUUAUUUUAUUACGUAAGAAGAUUUCCAUCUUCGUCCCACUCGGCUAAGUCAUUUCUUUUGGAUUGAUCAACACACCUAGAAAUCCACUCUUCAUCAUAUGAAACUGAGUAAUCUUUUAGAAUUUCUUCAGGA
env-68    ACGCAAUACUCACUCUAAUACAGGUUUGUUAUUUAUUAAGUUAGAAGAUUACCAUCUUCAUCCCAUACAGCAGUUUCAUUUCUUUUGGAUUGGUCAACACAUCUUGAAAUCCAUUCUUUAUCAUAUGAUACGUCAUGAUCUUUAAGGACAUCUUCUGGAA
env-69    ACGCAAAUUCGCUAAAUGCAGGCUUUUUAUGUUAAAAGAUUCCCGUCCUCAUCCCAUUCGGCAAUCUCUCCUCUUUUACUUUGAUCAAUACACUUAUCAAUCCAUUCCUUAUCAUAGGAGACUAUUCCUUUAUAUUGCUCCAAUACUUCAUCAGGAACUC
env-70    ACGCAAAUUCACUAAAUACGGGCUUAUUUUAUGUUAAGAGGUUCCCUUCUCCAUCCCACUCGGCAAUUUCUCCUCUCUUACUUUGAUCAACACAUUUAUCAAUCCAUUCUUUAUCAUAGGAGACAAUACCUUUAUACUGUUCUAAAACAUCAUUAGGCAC
env-71    ACGCAAAUUCACUAAAUACGGGCUUAUUUUAUGUUAAGAGAUUCCCUUCUUCAUCCCACUCGGCAAUUUCUCCUCUCUUACUUUGAUCAACACAUUUAUCAAUCCAUUCUUUAUCAUAGGAGACAAUACCUUUAUAUUGAUCUAAAACAUCAUUAGGCAC
env-72    AUACAAAACUCAACUAGUGUAGGCUUUUAUUAAUAGUUGGUCGGAAUUUUAUUUUUUUCAAACAUCAUGUGAAUCCAUAAGAUCAACACCCCAAAGCCAACUGCUGUAACGAUGAGAACGGAAUUCAAUAAUCCCUCAGUUCCAAAAGAUAGCUUUACUA
env-73    AUACAAAACUCUACUAGUGUGGGCUUUUUUAAUAGUUGGUCGGAAUUUUAUUUUUUCUCAAA.................................................................................
Fba-1-1   AUUCAAAACCCAAACUUGUGCAGGUUUUUUUAUAUAUAUUAACCAAUGGAAACAUCAUUAAUGACACCAAUCAUAUAUGCUAUUGUAGCAUUAGUAGUAGGCCUAGCGAUUGGCUAUUUUAUAGCUAAAUCGAUAGUUGAAAAAGGCAAAGCCUCACAAC
env-74    AGACAACAUGCAUAGCAGGUGCGGGAUUUUUUAAAAACAGUAAACCCAAUAGUGGUAUGGUAAUGACAAUAAUUGCUGCUGUAGUAGGUCUUUUAAUCGGUUUAGCUGGAGGCUAUGUUCUCUGGCAAAACGCAAUGAAAAAACGCGGUGCCCUCAUGAU
1 env-75  AGACAAAAACUUGCCAGGGUGCGGGUUUUUUAUUUUUAACAAUACUGAGAAAUGGAGCUAAGCGCAAUAUUGAUAUCCGCUCUUCCCGCCCUCCUGCUGGGCUUUGGAUUGAGCUGGUGGUGGAACCGCCAGAGGGAAGCCAAAAAGGCCAAUGGCCUUU
env-76    ACGCAAACUCUGUGGUCUGUGCGGGUUUUUUUUAAUUCAGAAACAGAUAUGUCAUACACCAUCAUCAUUGCUGCCAUUGGGUCGUUGCUUGCAGGAUUCAUAACUGCCACGCUCAUCGCGCGUCAAAGGGAAAAGAAACAAGUUGGCGGAUUGAUGGCCGA
          ..........................................................................................................................................
          ..........................................................................................................................................
3'        AYRCAA•R••Y••••••••••R••YUUUUU•••••••••••••••••••••••••••••••••••••••••••••••••••••••••••••••••••••••••••••••••••••••••••••••••••••••••••••
```

alignment positions 420 · · · 559

```
..........................................................................................................................
..........................................................................................................................
••••••••••••••••••••••••••••••••••••••••••••••••••••••••••••••••••••••••••••••••••••••••••∘∘∘∘∘∘∘∘∘∘∘∘∘∘∘∘∘∘∘∘∘∘∘∘∘∘∘∘∘∘∘∘
```

```
Gka-1-1   AAAUCGAUUGCCGAAGCGAAAAUCGGCGGGGCGAAAGCCGCUGCCGAACAGCUGAUUGAGGAGGCGAAACGCGAGGCGGACGCCUUGAAAAAAGAGGCGCUUCUCGAAGCGAAAGACGAGAUUCAUAAACUGCGUACGGA
1 Dps-1-1 UAAGAAGAUUGAUUCCAACAGAAAUUCUGUUGAGUAAUAUACGGAUGUUGAAAGGUUGGAUAUGGGUUUUCUCUUAUCUCCUCUUUUCAGUGAGGAUAACGGCAUAUUGGGGAGAAGGUUUUUUUGUUUGUAGGGUGACU
Psp-2-1   ACUUGCCGGCCUUGCAGUUGGUAUUGUCGUAGGCAGGUAUCUGCUGCGCGACUUGCUGAAAAAGCAGGAGAUUGCUGCACAGACGAAAGUGAAGAAGAUGCUUAAAGAUGCGGAAAGUAAAGCAGAGAUCCUGAAGAAGG
env-1     CAUAUUAAAAGACGCUAAAAAAGCCUCAGAACAGAUAAAAAAAGAUAAAAUACUUCAAGCAAAAGAAAAGUUUAUAGAACUUAAAAGUGAACAUGAAAAAUUCAUCAACAGUCGUAAUCAGAAAAUUUCAGAUCUGGAAU
env-2     CAUUUUAAAAGACGCAAAAAAAGCCUCAGAGCAGAUAAAAAAGGAUAAAAUACUUCAAGCAAAAGAAAAGUUUAUAGAACUUAAAAGUGAACACGAGAAAUUCAUAAACAGUCGAAACCAAAAAAUUUCAGAUUUAGAAU
Fba-2-1   AAAAGAAGCAGGUUCUAUUUUAAAAGACGCUAAAACAGAAGCCGAAGCCACUAAAAAAGAAAAAAUUCUUCAAGCAAAAGAGAAGUUUUUAGAACUUAAAGCGGAACACGAACAAGUUAUUUUAGCUCGAGAUAAAAAAA
Fjo-1-1   AAGCAGCUUCCAUUUUAAAAGAUGCUAAUUUAGAAGCCGAAAACAUAAAAAAAGACAAAAUACUUCAGGCAAAAGAGCGUUUUAUCGAGUUAAAAUCUGAACACGAACAAGUUAUUUUAGCACGCGAUAAAAAAGUGGCC
Fps-1-1   AAAAAGAAGCUUCUUCUAUCUUAAAAGAUGCAAAUUUAGAAGGAGAAAACAUAAAAAAAGAUAAAAUACUACAAGCAAAAGAGAAAUUUAUCGAACUGAAAUCUGAACACGAACAAGUAAUUCUAACUCGCGAUCGCAAG
Ddo-1-1   UUGCUCUUGCAUUAGGAUUCCUUAUUGCAAAAAUGUUAGAACGUAACAAUGCAUCACAAAUCAUUAAACGCGCAAAGAAAAGUGCUCAGGGCAUCUUAAAAGAAGCUAAGUCUGAGGGCGAGUCUAUCAAAAAAGAUAAA
Cat-1-1   UGAUAGAAAGAUCUGCAUCAUCUAAGAUCAUUACAGAUGCAGAAAAAUCUGCAGCUACGAUCAUAAAAGAUGCUAAAACAGAAGGCGAAAGCAUAAAGAAAGAUAAAAUACUUCAAGCUAAAGAAAAGUUUAUCGAGCUA
env-3     CCAAAUCCUCCCCCCAUCAUUCUAGCACCUAAAAUUUUAUCAUAUGACUUAGUUAAAUCCACUAAAAAAUCUAAUUCAUCACAACUAACUUCAUAAAGAUCUUUUAAGCCUGAAUGUGAUUUAUACAUCAAUUCACCCAG
env-4     CCAAAUCCUCCCCCCAUCAUUCUAGCACCUAAAAUUUUUUCAAAUGGCUUUGUCAAAUCCACCAAAAAAUCUAAUUCAUCACAACUAACUUCAUAAAGGUCUUUUAACCCUGAAUGUGAUUUAUACAUUAAGCUACCUAG
env-5     ..............................................................................................................................................
env-6     NNNNNNNNNNNNNNNNNNNNNNNNNNNNNNNNNNNNNNNNNNNNUCUAUGAUAAUACUAUAGUGUUCUUUCUUUCUGAUAAUGGCGCACCUAAAAAAAAAAUGGACGGAUAAAGAUGGAGCCAAUUCACCUUUUAGUGGUUAC
env-7     CCAAAUCCUCCACCCAUCAUUCUGGAUCCUAAUAUCUCGUCAUGCGCUUUAGUUAGUCAACUAAAUAAUCUAAUUCAUUACAACUAACUUCAUAUAAAUCUUUUAAACCUGCAUGGGAUUGAUACAUUAAAUUUCCUAU
env-8     CCAAAUCCUCCACCCAUCAUUCUGGAUCCUAUUAUCUGGUCAUACGCUUUAGUUAGGUCAACUAAAUAAUCUAAUUCAUUACAACUAACCUCAUACAAAUCUUUUAAACCUGCAUGGGAUUGAUACAUUAAAUUUCCUAU
env-9     CCAAAUCCUCCACCCAUCAUUCUGGAUCCUAUUAUCUGGUCAUACGCUUUAGUUAGGUCAACUAAAUAAUCUAAUUCAUUACAACUAACCUCAUACAAAUCUUUUAAACCUGCAUGGGAUUGAUACAUUAAAUUUCCUAU
env-10    CCAAAUCCUCCACCCAUCAUUCUGGAUCCUAUUAUCUGGUCAUGCGCUUUAGUUAGGUCAACUAAAUAAUCUAAUUCAUUACAACUAACCUCAUACAAAUCUUUUAAACCUGCAUGGGAUUGAUACAUUAAAUUUCCUAU
env-11    CCAAAUCCUCCACCCAUCAUUCUGGAUCCUAUUAUCUGGUCAUAAGCUUUAGUUAGGUCAACUAAAUAAUCUAAUUCAUUACAACUAACCUCAUACAAAUCUUUUAAACCUGCAUGGGAUUGAUACAUUAAAUUUCCUAU
env-12    CC............................................................................................................................................
env-13    CCAAAUCCUCCACCCAUCAUUCUAGAUCCUAUUAUCUGGUCAUGCGCUUUAGUUAAGUCAACUAAAUAAUCUAAUUCAUUACAACUAACCUCAUACAAAUCUUUUAAACCUGCAUGAGAUUGAUACAUUAAAUUCCCUAU
env-14    CGCCAAAUCCACCUCCCAUCAUCCUUGAACCAAUAAUUUUUUGGUGAUUUCUAGUUAAGUCAACUAAAAAAUCUAAUUCUUCGCAACUAACUUUAUAAAGUUUUGAUAACCCAUAAUGUGACUCAUACAUCAGGUUCCCG
env-15    CGCCAAAACCACCUCCCAUCAUUCGUGAACCAAUAAUUUUGUGGUGAUUCCUAGUUAAGUCAACCAGAAAAUCUAAUUCUUCGCAGCUAACUUUAUAAAGUUUUGAUAACCCAAAAUGUGACUCAUACAUCAGUUUUCCC
env-16    UAGCAAUGCUAAAAAUGAAGCUGAAAAUUUAAAAGCCAAUAAAAUUUUUCAAGCUAAAGAGAAAUUUUUAGAGCUAAAAUCAGAACACGAGAAGUAUAUAAUUUCAAAAGAAUCUGAGCAUAAUGAAAAAAAGCAAG...
env-17    AUUUUAAACGAAGCACGUGCAGAAGCUGAUAAUAUAAAGAACAAUAAAAUCCUUCAGGCAAAAGAAAAAUUUUUAGAAUUAAAGUCUGAACAUGAGAAAUAUAUACUUUCUAAAGAUGCAGAACAUAAUGAUAAAAAGCA
env-18    UAUUAAAUGAAGCACGUGCAGAAGCUGAUAAUAUAAAGAAUAAUAAAAUCCUUCAGGCAAAAGAAAAAUUUUUAGAAUUAAAGUCUGAACAUGAGAAAUAUAUACUUUCUAAAGAUGCAGAACAUAAUGAUAAAAAGCAA
env-19    UAAUUGAUGCGCAGAUUGAAGCUGAAAAUAUAAAAAAUAAUAAAAUUUUACAAGCGAAAGAAAAAUUCUUAGAUUUAAAAUCUCAGCAUGAAAAGUAUAUUUUAUCAAAAGAAGCAGAAAACAAAGAAAAAAACAAAAU
env-20    AAAUCUCUAGCUGGGUUUAUAGCAUAGCCUGUAGUGCCGCCGAGUCCCAUUCCUAUAACCCAAACUAAAAUUCCAACUGGUAAUGACUCCAAUGAACCUAUUCCAAAAUUUAUAGUCUCAAUGUUUUCAAAAGCAAUAUU
env-21    UAAAUCUCUUGCUGGAUUUAUAGCAUAACCUGUAGUUCCUCCGAGUCCCAUGCCUAUAACCCAAACUAAAAUUCCAACUGGUAAUGAUUCCAGUGAUCCAAUUCCAAAAUUUACAAUUUCAAUGUUUUCGAAAGUAAUAU
env-22    AAAUCUCUUGCUGGAUUUAUAGCGUACCCCGUGGUUCCACCCAGUCCCAUA...........................................................................................
Psp-1-1   AAGAAAAGAAGCUAGCUCUAUUCUAAAAGAAGCAAAAAUUGAUGCAGAAUCAAUUAAAAAAGAUAAAAUUUUACAAGCAAAAGAAAAGUUUAUAGAAUUAAAAUCUGAACACGAGAAAGUAAUUUUAUCUAGAGAAAAGA
env-23    CUAAAACAGAAGCCCAAAGAAUUAUUAAUUCCUCAGAAAUUGAGGCUGAAAAUUUAAGGAAAGACAAAAUAUUUCAAGCCAAAGAAAGAUUUUUGGAAUUAAAGUCUGAACAUGAAAAACAUAUUUUAAAUCGUGAAAAA
env-24    UAAUAAUGGAGGGUAGAAAGACAUGUGCAUAUGCCAAUGUUUAUUUGCCUUUUUGUCUGUUGGUGAUUGGUGAAUUCCCAUUGAGUAUGGAAACGCACAUCCAAAUA..................................
env-25    CAGUAAAUUCUUCAAUAAAAAACUCAUCAAUAAGAUUAAUUGUACACCCUCCGAAUCCUCCCCCCAUCAUCCUAGACCCAAUAACUUGUUCAAAAUCUUGAGUGUAGUCAACUAAAAAAUCUAAUUCCUCACAGCUUACA
env-26    ACUAAUUCAGUAAAUUCUUCAAUAAAAAAAUUCAUCAAUAAGAUUAAUUGUACACCCUCCGAAUCCUCCCCCCAUCAUCCUUGACCCAAUAACUUGUUCAAAAUCUUGAGUGUAGUCAACUAAAAAAUCUAAUUCCUCACA
env-27    ACUAAUUCAGUAAAUUCUUCAAUAAAAAAUUCAUCAAUAAGAUUAAUUGUACAUCCUCCGAAUCCUCCCCCCAUCAUCCUAGACCCAAUAACUUGUUCAAAAUCUUGAGUGUAGUCAACUAAAAAAUCUAAUUCCUCACA
env-28    AUGCUAAAAAUGAAGCGAAUAAUAUUCUUAAAUACUGCUAAAAUCGAGGGUGAGAAUAUAAAAAAA............................................................................
env-29    AUGCUAAAAAUGAAGCGAAUAAUAUUCUUAAAUCUGCUAAAAUCGAGGGUGAGAAUAUAAAAAAAGAUAAAAUAUUUCAAGCUAAAGAAAGGUUUUUGGAACUAAAAUCUGAACAUGAAAAGCACAUAUUAAAUAAAGAA
env-30    UGCUAAAAAUGAAGCAGAGAGAAUAAUUAAAUCUUCAGAAAUUGAAGGAGAAAAUUUAAAAAAAGAUAAAAUUUUUCAAGCGAAAGAAAGUUUUUAGAAUUAAAAUCUCAACACGAAAAACAUAUAUUAGAUCGCGAAA
Pir-1-1   GAAAGAAGCUUCUGCGAUUUUGAAAGAGGCAAAGAUUGAUGCAGAUGCAAUUAAAAAAGGAUAAGAUUCUACAAGCAAAAGAAAAAUUUAUAGAAUUAAAGUCUGAGCAUGAAAAAGUAAUCUUAAGCAGGGAAAAGAAAA
env-31    ..............................................................................................................................................
env-32    AGAUGCUCAAAGAAUCAUUGAUGAUGCUAAUGAAAAAGGAGAGAGUAUCAAAAAGAAUAAGAUUAUACAAGCAAAGGAGAAAUUUUUAGAAUUAAAAUCUGAACAUGAAAAAAUUAUCUUUUCAAGAGAAGAUAAAAUUA
env-33    AGAUGCUCAACGAAUUAUUGAUGAUGCUAAUGAAAAAGGAGAGAGUGUCAAAAAGAACAAGAUUAUUCAAGCAAAGGAAAAAUUUUUAGAAUUAAAAUCUGAACAUGAAAAAAUUAUCUUUUCAAGAGAAGACAAAAUUA
env-34    AGAUGCUCAACGAAUUAUUGAUGAUGCUAAUGAAAAAGGAGAGAGUGUCAAAAAGAACAAGAUUAUUCAAGCAAAGGAAAAAUUUUUAGAAUUAAAAUCUGAACAUGAAAAAAUUAUCUUUUCAAGAGAAGAUAAAAUUA
Lbl-1-1   CAAAAAAAGAAGCUGCAAACAUUGUAAAAGAAGCAAAGAGCGAGGGAGAAUCUAUUAAAAAGGAUAAGAUCCUUCAGGCAAAAGAAAAGUUUAUCGAGCUUAAAUCUGAGCACGAAAAAGUGAUUCUUUCACGUGAUAAA
env-35    AGACAAUUAGAGCAAAAAGAAAACGCACUCACCAAGAAGACCGAAAAAGCCGAUCAAAAGCAAGAAGAGCUCGACGCACUUCACAAGGUACAGGUAGAAAAACUCGAACAACUUGCUCAGUAUUCUGCCGAGGAAGCCAA
env-36    AUGGUCGAGAUUACAAAUCCAGAGUUGCGAAUGACCUUAUUGAACUUGUCCGUAUGGGCAAAAGAAAACAACACCAAUAGC.............................................................
env-37    AAUGGUGGAGAUUACAAAACCUGAAUUGCGAAUCACUUUGUUAAACUUAUCGGUAUGUGCAAACGAAAACAACACCAAAAGCACGUCUACCAAGAUGAGCACAGUAAAGAAAUCAUCAAAAAACACAGCAUUCACAUCAA
Fba-3-1   AAAGAUGCAAAUUCUAUUUUAAAAGAAGCUAAGACUGAAGGCGAGAAUAUCAAGAAAGAUAAAAUCUUUCAAGCCAAGGAAAAAUUUUUGGAGCUCAAGGCCGAACAUGAGAAAGUAAUAAUAAAUAAGAACAAGAAAAU
Rbi-1-1   UUGCCAUUGCCAAGCUCCUUGAAAAGGGACAGGCUUCCAAGACCAUUGCCGCGGCCAAAAAGGAAUCCGCGGCCAUCCUGAAGGAUGCCCGGGCCGAAGGGGAGAAUAUCAAAAAGAAAAGAUUUACCAGGCCAAGGAG
ueu-1-1   UAAUAGUUGCAACUGUUGUAGGUCUUCUUAUAGGCUUUUUUAUAGCAAAACAAAUGGCAAAAAGCAGUGCCUCUAGUACAGUUGUCGCCGCAGAGAAAGAGGCAGAGGCUAUCAAAAAAGAGGCAAAUGCUGUAAAAAAG
Pto-1-1   AAGCCAAUAAACUCUUAAAAGAAGCUGAGAAAGAAGGGGUGAAUAUCAAAAAAGAUAAAAUCCUUCAGGCUAAAGAAAAAUUUAUAGAGCUUAAAUCUGAACAUGAAAAAGUAAUUCUUUCUAGGGAUAAGAAAAUUAAU
env-38    ..............................................................................................................................................
env-39    GAUGCUAAUACAGAGUUCAAGGAAAUAAUCAAUCAAGCUAAAAGACAGGCAGAUCAAAUUAAAAAGUGAAAAAAUACUUCAGGCCAAGGAACGCUUUAUCGAGUUGAAGUCCGAGCAUGAGAAGGUUAUCUUUCAAAGAGA
env-40    GAUGCUAAUACAGAGUCCAAGGAAAUAAUCGAUCAAGCUAAAAGACAGGCAGAUCAGAUAAAAGGAAA..........................................................................
env-41    GCCAAUGCAGAGGCAAAGGAAAUUCUGAAUCAAUCGAGAAGGGAAGCAGAUCGUAUCAAAAGCGAAAAAAUACUUCAGGCCAAAGAGCGCUUUAUCGAAUUAAAAUCUGAACACGAAAACGUAAUUUUUCAGAGGGAAAA
env-42    CGCGAAGGGCAGGAGUUGUUGCAAAAAGCAAGAAUUGAAGCUGAAGCAAUUAAAAAGGACAAAGUCCUUGAAGCAAAAGAACAUUUUAUUGAACUCAAAUCAAAAUACGAUAUUGAGAUUAAUCAUAGAGAAAAAAAAAU
env-43    AAAAGAAUAAACCAAACGAGGGCCAAAGUCUCUAGCAGGGUUUAUAGCAUAUCCAGUAGUACCUCCAAGAGCAAUUCCAAUAAGCCAAACUAAAAUUCCAACCGGUAAAGCAUCUAAGGAACCUAAGCCAAAGUUUAGUA
env-44    UAGACGAAAUGAAGCACAAGCACAACUCGAUGCUGAAGCGAUUAAAAAAGAAAAAAUACUUCAAGCCAAAGAGCAUUUUAUUGAACUCAAAUCAAAACACGAUAGCGAAAUACACCAAAGAGAAAAAAGAAUUACAGAA
env-45    UUUUCGAGGUAAAAAAGAAUAGACUAAACGUGGACCAAAAUCACGAGCUGGAUUAAUGGCGUAUCCUGUAGUACCACCAAGUGCAAUUCCAAUAAGCCAAACCAAAAUUCCAACAGGUAGUGCAUCUAGGGAACCUAAAC
env-46    GCGGGCCAAAAUCUCUUGCUGGAUUAAUUGCAUAACCUGUUGUACCUCCAAGUGAGAUUCCAAUAAGCCAAACUAAUAUUCCUAUAGGUAAUGCAUCAAUUGAACCCAGACCAAAAUUCUGAAUUUCACUACCACUUAUU
env-47    GCGGGCCAAAAUCUCUUGCUGGAUUAAUUGCAUAACCUGUUGUACCUCCAAGUGAGAUUCCAAUAAGCCAAACUAAUAUUCCUAUAGGUAAUGCAUCAAUUGAGCCCAAGCCAAAAUUCUGAAUUUCACUACCACUUAUU

          ..............................................................................................................................................
          ..............................................................................................................................................
          ●●●●●●●●●●●●●●●●●●●●●●●●●●●●●●●●●●●●●●●●●●●●●●●●●●●●●●●●●●●●●●●●●●●●●●●●●●●●●●●●●●●●●●●●●●●●●●●●●●●●●●○○○○○○○○○○○○○○○○○○○○○○○○○○○○○○○○○○○○
```

```
env-48   GUCAGGCCGAUGAACUUCUGAGAAAAGCCCAGCUAGAAGCGGAAGCCACAAAAAAAGAAAAAAUGCUUCAAGCCAAGGAACACUUUAUUGAAUUGAAGUCCAAACACGAUAGUGCGAUCCAUCAACGUGAAAAGAGAAUA
env-49   AAAGAAGCUGAUGAACUCCUGAGAAAUGCAAAGUUAGAAGCAGAAUCCACCAAAAAAGACAAAAUGCUUCAAGCUAAGGAACACUUUAUUGAAUUGAAGUCUAAACACGAUAGUGAGAUCCACCAGCGUGAAAAAAGAAU
env-50   UGGGCCAAAGUCUCUUGCUGGAUUAAUUGCAUAACCUGUUGUACCUCCAAGAGAUAUUCCAAUGAGCCAUACUAAUAUGCCUAUAGGAAGGGCAUCAAGCGAGCCCAAACCAAAAUUCUGAAUUUCACUGCCACUAAUUU
env-51   CCAUUCAGCAUUUUUGUUUUUUCGGGGAAGCAACUGGUAGAUCACUCUUGGCCCAAAGUCCCUAGCCGGAUUAAUUGCAUAACCUGUAGUCCCACCAAGGGCCAUUCCAAUGAGCCAAACCAAAAUCCCAAUUGGUAAUG
env-52   UAAUUGAUAUACUAAACGAGGUCCAAAAUCACGUGCAGGAUUUAUUGCAUAACCAGUAGUCCCCCCUAAAGCAAUACCAAUUACCCAGACUAAUAUUCCUACAGGAAGUGCAUCCAAGGCACCUAGGCCGAAGUUUUCAA
env-53   NNNNNNNNNNNNNNNNAGAAACACCUCAUGCUCUUUUAGGUAUGGAAUGGGCUGAAAAAUAUGGAGAAAAAGCUGAGGUAUGUAAUGCCAUUGGAGCCCAUCAUGAUGAAAUUGAAAUGACCUCUUUGUUGUCACCUAUU
env-54   AAAUCAAAUUAAGAAUAAAGCCAAAUCUGAAGCUGAAAGCAUGAAAAAAGAAAAGAUCCUUCAAGCUAAAGAACGUUUUAUUGAAUUAAAAUCUGAAUACGAAAAGGUGAUUUUAGAUAGAGAAAAGAAAAUUAUUUCUA
env-55   CCAAACAUUAGGAAUGUUUCCUUUAUAACCUAGGUCUUUCAUUCCUACCUCUGAUGUAAACCAACCUGUCACAACAAAGUAUCUAUAUGUCGCAAAAAAGCUAGCAGCCUCUGAAAGUUCAUCGCUAAUAUUAUCUUGAU
env-56   CCAAACAUUAGGAAUGUUUCCUUUAUAACCAAGGUCUUUCAUUCCAACCUCUGAUGUAAACCACCCUGUCACAACAAAGUGUCUGUAUGUUGCAAAAAAGCUAGCAGCUUCUGAAAGUUCAUCGCUAAUAUUAUCUUGAU
env-57   ............................................................................................................................................
env-58   CCAAACAUUUGGAAUGUUUCCUUUAUAACCUAGGUCUUUCAUUCCUACCUCUGAUGUAAACCACCCAGUAACAACAAAGUAUCUGUAUGUUGCAAAAAAGCUAGCAGCCUCUGAAAGUUCAUCGCUAAUAUUAUCUUGAA
env-59   CCAAACAUUAGGAAUGUUUCCUUUAUAACCUAGGUCUUUCAUUCCCACUUCUGAUGUAAACCACCCUGUCACAAUAAAGUGUCUGUAUGUUGCAAAAAAGCUAGCAGCCUCUGAUAGUUCAUCGCUAAUAUUAUCUUGAU
env-60   CCAAACAUUAGGAAUGUUUCCUUUAUAACCAAGGUCUUUCAUUCCCACCUCUGAUGUAAACCACCCUGUCACAACAAAGUGUCUGUAUGUUGCAAAAAAGCUAGCAGCUUCUGAAAGUUCAUCGCUAAUAUUAUCUUGAU
env-61   GACACCGUCCCAAACAUUUGGAGUAUUACCUUUAUAACCUAAAUCAUUAAUUCCUAUCUCUGAGGUAAAAUAUCCAGUAAGAACCAUACCUCUGUAAACUGCAAA...................................
env-62   ACACCGUCCCAAACAUUUGGAGUAUUACCUUUGUAACCUAAAUCAUUAAUUCCUAUCUCUGAGGUAAAAUAUCCAGUAAGAACCAUACCUCUGUAAACUGCAAAUAAUCUAAUAGCUUCUUGUAAGUCUUCAUUUUUAUU
env-63   ACUCCGUCCCAUACAUUAGGUAUGUUUCCCUUAUAUCCAAGAUCUCGCAUUCCAACCUCUGAUGUAAACCAUCCAGUUACAACCAUAUAUCUGUAGGUAGCAAAGAAACUUGCAGCUUCCUUAAAGUCUUCCUCAAUGCC
env-64   ACUCCAUCCCAUACAUUAGGUAUGUUACCCUUGUAUCCUAAAUCACGCAUUCCAACCUCUGAUGUAAACCACCCUGUCACAACCAUGUAUCUGUAUGUAGCAAAGAAACUUGCAGCUUCUUUAAGGUUUCUUCAAUACC
env-65   ACUCCAUCCCAUACAUUAGGUAUGUUGCCUUUAUAUCCUAAAUCCUGCAUUCCAACCUCUGAUGUAAACCAUCCGGUCACAACCAUAUAUCUAUAUGUAGCAAAAAAACUUGCAGCUUCCUUCAUGUCUUCUUCAAUAUC
env-66   ACACCAUCCCAAACAUUUGGAGUAUUCCCCUUAUAUCCAAGAUCUUUAAUGCCAAUCUCAGAUGUAAAAUAUCCUGUUAAAACCAUUCCCCUAUAUACAGCAAAUAGUCUUAUUGCCUCUUGAAGGUCCUCAUCUUUAUU
env-67   ACACCAUCCCAAACAUUAGGGGUAUUCCCUUUAUACCCAAGAUCUUUAAUGCCAAUCUCAGAUGUAAAAUAUCCUGUUAAAACCAUUCCCCUAUAUACAGCAAAUAGUCUUAUUGCCUCUUGAAGGUCCUGAUCUUUAUU
env-68   CUCCAUCCCAUACAUUUGGAAUGUUUCCCUUGUAUCCAAGAUCUCGCAUACCAACCUCUGAUGUAAACCAUCCAGUUACUACCAUAUAUCUAUAUGUAGCAAAGAAACUUGCAGCUUCUUUUAGGUUUUCUUCAAUACCA
env-69   CAUCCCAAACAUUAGGUGUAUUACCUUUAUAUCCUAGAUCUUUAAUUCCUACUUCAGAGGUGAAAUAUGCAGUUACAAUCAUACCUCUGUAUGUAGCAAAUAGUUUUACACUUUCUUUUUGAUCUUUUAGUGAUUUAUCU
env-70   ACCAUCCCAUACAUUAGGUGUAUUUCCUUUAUAGUCUAGAUCUUUAAUUCCAAUUUCAGAAGUAAAAUAUCCUGUAACAACCAUACCUCUAAAUGUUGCAAAAAGUUUUACACUCUCUUUUUGAUCUUUUAAAGACUCAU
env-71   ACCAUCCCAUACAUUUGGUGUAUUUCCUUUAUAGUCUAGAUCUUUAAUACCAAUUUCAGAAGUAAAAUAUCCUGUAACAACCAUACCUCUAAAUGUUGCAAAAAGUUUUACACUUUCUUUUUGAUCUUUUAAAGACUUAU
env-72   AUAUUGUAGAAAUCACAAAUCCUGAGUUU...............................................................................................................
env-73   ............................................................................................................................................
Fba-1-1  UUAUAUCCGACGCUCAAAAAGAGGCAAAGUCUCUAUUAAAAGAAGCUCAAGUAGAAGGAGAAAAAAUUAAAAACGAAAAAACUUUUAAAGCAAAAGAAAAGUUUAUCGAACUUAAAUCGCAACAUGAAAAGGAAAUCAUG
env-74   UACUGAGGCGAAAAAAGCGGCCGAGCAGCUUAAAAAAAGAUAGGGCACUAGAAGCGAAAGAACGUUUCUUGAACCUUAAAGAAGAGCACGAACGUGCAGUACAGAAGCGCGAUCAAAAAUUAGCCGAACGUCAGAAUUCAU
1 env-75 UGGAAGAAGCCCGGAAGGAGGCAGAAACCAUAAAAAAAAGACAAAAUCCUUCAGGCCAAGGAAAAGUUCCUCGAACUAAAGGCCGAGCACGAAAAGGUCAUUUACAAGCGGGAACAAAAGCUGAACGACCGCCUCAACAGC
env-76   CGCCAAGAAAGAAGCAGAGGCUCUCAAGAAAGAAAAGAUCCUACAGGCCAAGGAAAAGUUCAUUGAGCUCAAGUCCGAGCACGAGAAGACCAUUCAGUCCCGGGAGCGAAAAAUGGGCGAUGCGGAAAAGCGCAUCAAGG
         ............................................................................................................................................
         ............................................................................................................................................
         ●●●●●●●●●●●●●●●●●●●●●●●●●●●●●●●●●●●●●●●●●●●●●●●●●●●●●●●●●●●●●●●●●●●●●●●●●●●●●●●●●●●●●●●●●●●●●●●●●●●●●●●○○○○○○○○○○○○○○○○○○○○○○○○○○○○○○○○○○○
```

2 *aceE*

2.1 Taxa

The taxonomy of each organism containing a putative *aceE* RNA is listed, with abbreviations identifying each hit (e.g., "Eco-1-1" and "Eco-1-2" might hypothetically represent two distinct RNAs in *E. coli*). The abbreviations will be used to identify each individual *aceE* RNA in Sections 2.2 and 2.4.

**abbrev. of hits**	**taxonomy of species**
Avi-1-1	Bacteria Proteobacteria Gammaproteobacteria Pseudomonadales Pseudomonadaceae *Azotobacter vinelandii*
Pae-1-1	Bacteria Proteobacteria Gammaproteobacteria Pseudomonadales Pseudomonadaceae *Pseudomonas aeruginosa* 2192
Pae-2-1	Bacteria Proteobacteria Gammaproteobacteria Pseudomonadales Pseudomonadaceae *Pseudomonas aeruginosa* C3719
Pae-3-1	Bacteria Proteobacteria Gammaproteobacteria Pseudomonadales Pseudomonadaceae *Pseudomonas aeruginosa* PA7
Pae-4-1	Bacteria Proteobacteria Gammaproteobacteria Pseudomonadales Pseudomonadaceae *Pseudomonas aeruginosa* PACS2
Pae-5-1	Bacteria Proteobacteria Gammaproteobacteria Pseudomonadales Pseudomonadaceae *Pseudomonas aeruginosa* PAO1
Pae-6-1	Bacteria Proteobacteria Gammaproteobacteria Pseudomonadales Pseudomonadaceae *Pseudomonas aeruginosa* UCBPP-PA14
Pen-1-1	Bacteria Proteobacteria Gammaproteobacteria Pseudomonadales Pseudomonadaceae *Pseudomonas entomophila* L48
Pfl-1-1	Bacteria Proteobacteria Gammaproteobacteria Pseudomonadales Pseudomonadaceae *Pseudomonas fluorescens* Pf-5
Pfl-2-1	Bacteria Proteobacteria Gammaproteobacteria Pseudomonadales Pseudomonadaceae *Pseudomonas fluorescens* Pf0-1
Pme-1-1	Bacteria Proteobacteria Gammaproteobacteria Pseudomonadales Pseudomonadaceae *Pseudomonas mendocina ymp*
Ppu-1-1	Bacteria Proteobacteria Gammaproteobacteria Pseudomonadales Pseudomonadaceae *Pseudomonas putida* F1
Ppu-2-1	Bacteria Proteobacteria Gammaproteobacteria Pseudomonadales Pseudomonadaceae *Pseudomonas putida* GB-1
Ppu-3-1	Bacteria Proteobacteria Gammaproteobacteria Pseudomonadales Pseudomonadaceae *Pseudomonas putida* KT2440
Ppu-4-1	Bacteria Proteobacteria Gammaproteobacteria Pseudomonadales Pseudomonadaceae *Pseudomonas putida* W619
Pst-1-1	Bacteria Proteobacteria Gammaproteobacteria Pseudomonadales Pseudomonadaceae *Pseudomonas stutzeri* A1501

Psy-1-1	Bacteria Proteobacteria Gammaproteobacteria Pseudomonadales Pseudomonadaceae *Pseudomonas syringae pv. phaseolicola* 1448A
Psy-2-1	Bacteria Proteobacteria Gammaproteobacteria Pseudomonadales Pseudomonadaceae *Pseudomonas syringae pv. syringae* B728a
Psy-3-1	Bacteria Proteobacteria Gammaproteobacteria Pseudomonadales Pseudomonadaceae *Pseudomonas syringae pv. tomato str.* DC3000
env-1	environmental sample

2.2 Gene contexts

Each *aceE* RNA (indicated by "RNA→") is listed with its surrounding genes. Environmental sequences and some RefSeq entries lack gene annotations, and no genes are listed for such sequences. The direction of each gene is indicated with an arrow (→), and each conserved domain in the gene is colored. Conserved domains associated with more than one *aceE* RNA are assigned a color; other domains are gray. Information about these conserved domains is given in Section 2.3. The accession number of the sequence containing each *aceE* RNA is given. Accessions beginning with "NC_" or "NZ_" are RefSeq, while other accession refer to environmental samples. Nucleotide coordinates are given for the 5′ and 3′ boundaries of each. If the 5′ coordinate is greater than the 3′ coordinate, the RNA is present on the reverse-complement strand of the containing genomic DNA sequence. Each hit is denoted by an abbreviation (like "Eco-1-1") that refers to a taxonomy given in Section 2.1.

**abbrev.**	**Seq. accession**		**5′ at**	**3′ at**	**genes**
Avi-1-1	NZ_AAAU03000012.1	-	97225	97146	**RNA**→ TPP_E1_EcPDC_like (cd02017)→ AceF (COG0508)Biotin_lipoyl (pfam00364)→
Pae-3-1	NC_009656.1	+	5928905	5928986	**RNA**→ COG3958 (COG3958)TPP_E1_EcPDC_like (cd02017)→
Pae-5-1	NC_002516.2	+	5636028	5636109	**RNA**→ COG3958 (COG3958)TPP_E1_EcPDC_like (cd02017)→
Pae-2-1	NZ_AAKV01000094.1	-	140786	140705	**RNA**→ COG3958 (COG3958)TPP_E1_EcPDC_like (cd02017)→
Pae-4-1	NZ_AAQW01000001.1	+	5844740	5844821	**RNA**→ COG3958 (COG3958)TPP_E1_EcPDC_like (cd02017)→
Pae-1-1	NZ_AAKW01000064.1	-	169964	169883	**RNA**→ COG3958 (COG3958)TPP_E1_EcPDC_like (cd02017)→
Pae-6-1	NC_008463.1	+	5907425	5907506	**RNA**→ COG3958 (COG3958)TPP_E1_EcPDC_like (cd02017)→
Pme-1-1	NC_009439.1	-	633058	632979	**RNA**→ COG3958 (COG3958)TPP_E1_EcPDC_like (cd02017)→ AceF (COG0508)Biotin_lipoyl (pfam00364)→
Pst-1-1	NC_009434.1	+	4168863	4168945	**RNA**→ TPP_E1_EcPDC_like (cd02017)→ AceF (COG0508)Biotin_lipoyl (pfam00364)→
env-1	soil_Contig41332	+	1133	1211	**RNA**→
Pfl-2-1	NC_007492.1	-	536189	536111	**RNA**→ AceE (COG2609)→ AceF (COG0508)Biotin_lipoyl (pfam00364)→
Pfl-1-1	NC_004129.6	-	593917	593840	**RNA**→ AceE (COG2609)→ AceF (COG0508)Biotin_lipoyl (pfam00364)→
Psy-2-1	NC_007005.1	-	579052	578973	**RNA**→ AceE (COG2609)→
Psy-3-1	NC_004578.1	+	5686568	5686647	**RNA**→ AceE (COG2609)→
Psy-1-1	NC_005773.3	-	593005	592926	**RNA**→ AceE (COG2609)→
Ppu-4-1	NZ_AAVY01000020.1	+	111124	111201	**RNA**→ ←hypo (cons)
Pen-1-1	NC_008027.1	+	5464828	5464905	**RNA**→ AceE (COG2609)→ AceF (COG0508)Biotin_lipoyl (pfam00364)→
Ppu-1-1	NC_009512.1	-	420581	420504	**RNA**→ AceE (COG2609)→ AceF (COG0508)Biotin_lipoyl (pfam00364)→
Ppu-3-1	NC_002947.3	-	410786	410709	**RNA**→ AceE (COG2609)→ AceF (COG0508)Biotin_lipoyl (pfam00364)→
Ppu-2-1	NZ_AAXR01000011.1	+	22482	22559	**RNA**→ ←hypo (cons)

2.3 Conserved domains

Conserved domains found in protein-coding genes listed in Section 2.2 are listed, with the first sentence in their description from the Conserved Domain Database. Conserved domains associated with more than one *aceE* RNA are assigned a color, while others are shown in gray. The number in parentheses is the number of occurrences of the given gene.

cd02017 (9) Thiamine pyrophosphate (TPP) family, E1 of E.
COG0508 (8) Pyruvate/2-oxoglutarate dehydrogenase complex, dihydrolipoamide acyltransferase (E2) component, and related enzymes [Energy production and conversion]
COG2609 (8) Pyruvate dehydrogenase complex, dehydrogenase (E1) component [Energy production and conversion]
COG3958 (7) Transketolase, C-terminal subunit [Carbohydrate transport and metabolism]
pfam00364 (8) Biotin-requiring enzyme.

Duplicate sequences: the following putative homologs are not shown in the alignment because their sequences are identical to a homolog already shown: Pae-2-1

2.4 Multiple-sequence alignment

Each *aceE* RNA is denoted by an abbreviation (like "Eco-1-1") that refers to a taxonomy given in Section 2.1. The alignment includes sequences containing the RNA motif, as well as flanking sequence. The *aceE* RNA itself is denoted by the line underneath marked 5′ and 3′ on either end. Nucleotides in the flanking sequences (i.e., not part of the motif) are written in gray letters. Stems of predicted rho-independent transcription terminators are shaded yellow. (Note: terminator predictions have not been analyzed manually, and many are likely to be false positives.) Start codons are shaded green. (Note: start codons are frequently misannotated, especially in environmental samples.) Nucleotides proposed to basepair as part of the consensus structure are shaded in color when they comprise Watson-Crick or G-U pairs. Otherwise they are shaded gray. Conserved stems are also indicated at the bottom of the alignment by angle brackets, where matching < and > denote base-paired columns. Below these angle brackets, the symbol "2" denotes base pairs exhibiting covariation, "1" denotes base pairs exhibiting compatible mutations, "0" denotes base pairs that are not observed to mutate and "?" denotes base pairs that have a significant frequency of non-canonical nucleotides for Watson-Crick or G-U pairs (> 5%). Below these base pair annotation is the consensus sequence: "R" = "A" or "G", "Y" = "C" or "U", red nucleotides: nucleotide identity conserved more than 97% of the time, black nucleotides: 90%, gray nucleotides: 75%, red circle (•): nucleotide is present 97% of the time, black circle (•): 90%, gray circle (•): 75%, white circle (•): 50%. All percentages of sequences just described (e.g. 97% conserved) assume that sequences have been weighted by the GSC algorithm implemented by the Infernal software.

alignment positions 1 · · · 95

```
Avi-1-1    CCUGAUAUCGGCCAGACUUUCCAAAGCGGUUGAGCCCGACCCGUGGGGUAAUUUUACAAAAAUCCUCGGAGGGGGGCUGAAAGCGCCAUUGUUUU
Pae-3-1    GGCUCUACAGCGGGCCUUAUAGCCUGACAGCGACAAUCGAAAUGUAGUUUUACUACUAAAAAUUGUAUCCAGAGGGCUGUUUCUCAGACUGAUCU
Pae-5-1    GGCUCUACAUCGGGCCUUAUAGCCUGACAGCGACAAUCGAAAUGUAGUUUUACUACUAAAAAUUGUAUCCAGAGGGCUGUUUCUCAGACUGAUCU
Pae-4-1    GGCUCUACAUCGGGCCUUAUAGCCUGACAGCGACAAUCGAAAUGUAGUUUUACUACUAAAAAUUGUAUCCAGAGGGCUGUUUCUCAGACUGAUCU
Pae-1-1    GGCUCUACAUCGGGCCUUAUAGCCUGACAGCGACAAUCGAAAUGUAGUUUUACUACUAAAAAUUGUAUCCAGAGGGCUGUUUCUCAGACCGAUCU
Pae-6-1    GGCUCUACAUCGGGCCUUAUAGCCUGACAGCAACAAUCGAAAUGUAGUUUUACUACUAAAAAUUGUAUCCAGAGGGCUGUUUCUCAGACUGAUCU
Pme-1-1    CCCGCUGGCGCAGGGCGCGCGCGGCCGGCAGAACCGCGGAUUGUAGUUUUACUACUAAAGAAUUGUAUCCAGAGGGCUGAAUCCGGCGUCGGAAU
Pst-1-1    GCCCCGCCCGUGCCGUUUGAAACGCACGACGACGCCGCUGACCUGUAGUUUUACUACUAAAGAGUAUUUCGGGGGGCUGAUCCCGACGUCGUUUU
env-1      GAUUAUUCUCACAACGCAGCUGGAGGCCACAAGCCGCUCAUCUGUAGUUUUACUACUCGUAUAUACAUUCGAAAGGCUGAAAUGCCCGAUGAUUU
Pfl-2-1    GAUUAUUCUCACAACGCAGUCGGAGGCCACAAGCCUUUCAUCUGUAGUUUUACUACUCGUCUAUACAUUCGAAAGGCUGAAAUGCCGGAUGAUUU
Pfl-1-1    GAUUAUUCUCACAACGCAGCAGGAGGCCACAAGCCGCUCUUCUGUAGUUUUACUACUCGUAUAUACAUUCGAAAGGCUGAAAUGGCCGACGAUUU
Psy-2-1    GUUUUAAGCUACACGACAACAGGAGCGAUUGAGUCGCUUCUCUGUAGUUUUACUACUGGGUCCGAUACUCGGAGGGCUGAAAUCGUCGUCGAUUU
Psy-3-1    GUUUUAAGUUACAAGACAACAGGAGCGAUUGAGUCGCUUCUCUGUAGUUUUACUACUGGGUCCGAUACUCGGAGGGCUGAAAUCGUCGUCGAUUU
Psy-1-1    GUUUUAAGUUACAAGACAACAGGAGCGAUUGAGUCGCUUCUCUGUAGUUUUACUACUGGGUCCGAUACUCGGAGGGCUGAAAUCGUCGUCGAUUU
Ppu-4-1    GUUUUUCCUCACAAGCCAAGGUGCGACCAAACGUCGCAUUUGUGUAGUUUUACUACUAUCCCGACCGUGCAAAAGCAUGAAAUGCGAAAGCAAAU
Pen-1-1    GUUUAUCCUCACAACCCGGGAUGCGACAAACCGUCGCAUUUGUGUAGUUUUACUACUACCCCGUAGGUGCAAAAGCAUGAAAUGCGAAAGCAUUU
Ppu-1-1    GUUUUUCCUCACAAGCCAAGGUGCGACCAAACGUCGCACAGAUGUAGUUUUACUACUACUCGUGUCGUGUAAAAGCAUGAAAUGCGAAAGCAAAU
Ppu-3-1    GUUUUUCCUCACAAGCCAAGGUGCGACCAAACGUCGCACAAAUGUAGUUUUACUACUACUCGUGUCGUGUAAAAGCAUGAAAUGCGAAAGCAAAU
Ppu-2-1    GUUUUUCCUCACAAGCCAAGGUGCGACCAAACGUCGCACAAAUGUAGUUUUACUACUACUCGCGUCGUGCAAAAGCAUGAAAUGCGAACGCAAAU
           ...............................................................................................
           ...............................................................................................
           ••Y••U•YY••••R••Y•R•••R••R••R••RR•C•Y••••••••RG•UU••YU••Y•R••••••Y•Y•YRRRRGGCUGAA•Y••••R•YG•UYU
                                                                                                         5′
```

alignment positions 96 · · · 255

```
Avi-1-1   GUAGUAAAACUACAAGACAGUCGGAUCACC.CGCCGACCA.....CUAACAA..CAAUCCCGCAGCCGCCCAUAAGGCCGGCUGUGUAUCCGG.GCUCCCAACUCAGGGAUGCCUUCCG.CUAUGGAGCAAGACAUGCAAGACAUGCAAGACCUUGACCC
Pae-3-1   GUAGUAAAACUACAAGUCGCCGGGU...ACACCCCCGGAGAACAUCCAAGAA..UUAGC.UGCGGUCGCCCACGAGGCCGGCCGCUCACCUAU.GGCAUCCGAUUCUGGAAGCCUUUCCGCCCUGGAGCAAGCCAUGCAAGACCUCGAUCCCGUCGAAAC
Pae-5-1   GUAGUAAAACUACAAGUCGCCGGGU...ACACCCCCGGAGAACAUCCAAGAA..UUAGC.UGCGGUCGCCCACGAGGCCGGCCGCUCACCUAU.GGCAUCCGAUUCUGGAAGCCUUUCCGCCCUGGAGCAAGCCAUGCAAGACCUCGAUCCCGUCGAAAC
Pae-4-1   GUAGUAAAACUACAAGUCGCCGGGU...ACACCCCCGGAGAACAUCCAAGAA..UUAGC.UGCGGUCGCCCACGAGGCCGGCCGCUCACCUAU.GGCAUCCGAUUCUGGAAGCCUUUCCGCCCUGGAGCAAGCCAUGCAAGACCUCGAUCCCGUCGAAAC
Pae-1-1   GUAGUAAAACUACAAGUCGCCGGGU...ACACCCCCGGAGAACAUCCAAGAA..UUAGC.UGCGGUCGCCCACGAGGCCGGCCGCUCACCUAU.GGCAUCCGAUUCUGGAAGCCUUUCCGCCCUGGAGCAAGCCAUGCAAGACCUCGAUCCCGUCGAAAC
Pae-6-1   GUAGUAAAACUACAAGUCGCCGGGU...ACACCCCCGGAGAACAUCCAAGAA..UUAGC.UGCGGUCGCCCACGAGGCCGGCCGCUCACCUAU.GGCAUCCGAUUCUGGAAGCCUUUCCGCCCUGGAGCAAGCCAUGCAAGACCUCGAUCCCGUCGAAAC
Pme-1-1   GUAGUAAAACUACAAG.CGGCCGGUCCUAC.CCCCGGUAA...AUCCAAGAA..UUUCAACUCGCCCGCUCAAAAAGCCGG.CGAGAUCCAACUGGCCUCCGAUUCUGGAAGCCUUUCCGCCCUGGAGCAAGCCAUGCAAGACCUCGAUCCGAUCGAAAC
Pst-1-1   GUAGUAAAACUACA.GACGUCCGGUCCUAC.CUCCGGCACUC.AUCCAAGAACAUUUCA.CGCG.CCGCCCACAAAGCCGGCAGCGAAG.CA..GGCGACCGAUUCUGGAAGCCUUUGCGCCAUGGAGCAAGCCAUGCAAGAUCUCGAUCCCGUCGAAAC
env-1     GUAGUAAAACUACACG.CCGCCGGAACAACCUCUCGGCA....AUCCAAGAA..UUUAU.AUCGUCUGCCCACAAGGCCAGUCGC.AAA.CUCAGGCAACCGAUUCUGGAAGCCUUUCCGCCCUGGAGCAAGCCAUGCAAGACCUCGAUCCCGUCGAAAC
Pfl-2-1   GUAGUAAAACUACACG.CCGCCGGAACAACCUGUCGGCA....AUCCAAGAA..UUUUAAAUCGUCUGCCCACAAGGCCAGUCG..CAAACUCAGGCAACCGAUUCUGGAAGCCUUUCCGCCCUGGAGCAAGCCAUGCAAGACCUCGAUCCCGUCGAAAC
Pfl-1-1   GUAGUAAAACUACACG.CCGCUGGAUCAAC.CUCCGGCA....AUCCAAGAA..UUUAUAAUCGUCUGCCCAAAAGGCCAGUCG..CAAACUCAGGCAACCGAUUCUGGAAGCCUUUCCGCCCUGGAGCAAGCCAUGCAAGACCUCGAUCCCGUCGAAAC
Psy-2-1   GUAGUAAAACUACACGCCGCCGGAUCCCAC.CCUCCGGAA...AUCCAAGAA..UUUAU.GUCGUCUGCCCACAAGGCCAGUCG.CAAA.CUCAGGCAACCGAUUCUGGAAGCCUUUCCGCCCUGGAGCAAGCCAUGCAAGACCUCGAUCCCGUCGAAAC
Psy-3-1   GUAGUAAAACUACACGCCGCCGGAUCCCAC.CCUCCGGUA...AUCCAAGAA..UUUAU.GUCGUCUGCCCACAAGGCCAGUCG.CAAA.CUCAGGCAACCGAUUCUGGAAGCCUUUCCGCCCUGGAGCAAGCCAUGCAAGACCUCGAUCCCGUCGAAAC
Psy-1-1   GUAGUAAAACUACACGCCGCCGGAUCCCAC.CCUCCGGUA...AUCCAAGAA..UUUAU.GUCGUCUGCCCACAAGGCCAGUCG.CAAA.CUCAGGCAACCGAUUCUGGAAGCCUUUCCGCCCUGGAGCAAGCCAUGCAAGACCUCGAUCCCGUCGAAAC
Ppu-4-1   GUAGUAAAACUACACGCCGCCGGGUC..AC.ACUCCGGUA...AUCCAAGAA..UUCAC.GACGUCUGCCCAUAAGGCCAGUCG.CAAA.CUCAGGCCCCCGAUUCUGGUGGCCUUUCCGCCCUGGAGCAAGCCAUGCAAGACCUCGAUCCAAUCGAAAC
Pen-1-1   GUAGUAAAACUACACCGCGCCGGAU..GAC.UGUCCGGUA...AUCCAAGAA..UUCAC.GACGUCUGCCCAUAAGGCCAGUCG.CAAA.CUCAGGCCAUCGAUUCUGGUGGCCUUUCCGCCCUGGAGCAAGCCAUGCAAGACCUCGAUCCAAUCGAAAC
Ppu-1-1   GUAGUAAAACUACAACGCG.CCGGA.AGACACCCCGGUA....AUCCAAGAA..UUCAC.GACGUCUGCCCAUAAGGCCAGUCG.CAAU.CUCAGGCAAUCGAUUCUGGUUGCCUUUCCGCCCUGGAGCAAGCCAUGCAAGACCUCGAUCCAAUCGAAAC
Ppu-3-1   GUAGUAAAACUACAACGCG.CCGGA.AGACACCCCGGUA....AUCCAAGAA..UUCAC.GACGUCUGCCCAUAAGGCCAGUCG.CAAU.CUCAGGCAAUCGAUUCUGGUUGCCUUUCCGCCCUGGAGCAAGCCAUGCAAGACCUCGAUCCAAUCGAAAC
Ppu-2-1   GUAGUAAAACUACAACGCG.CCGGA.AGACACCCCGGUA....AUCCAAGAA..UUCAC.GACGUCUGCCCAUAAGGCCAGUCG.CAAU.CUCAGGCAAUCGAUUCUGGUUGCCUUUCCGCCCUGGAGCAAGCCAUGCAAGACCUCGAUCCAAUCGAAAC
          ...................<<<<<.........>>>>>......................<<<<.<<<<<.....>>.>>>.>>>>..........................................................................
          ...................?2202.........2022?......................??0?.1200?.....?0.021.?0??..........................................................................
          GUAGUAAAACUACA∘G∘CR•C•GG•∘∘∘AC-C•YC•G••∘---AUCCAAGAA--UU•••-••CG∘CYGCCCAYAAGGCCRGYYG∘∘•A•∘C•∘∘GGC••CCGAUUCUGGA•GCCUUUCCGCC•UGGAGCAAGCCAUGCAAGACCUCGAUCC•RUCGAAAC
                                                                                        3′
```

alignment positions 256 · · · 415

```
Avi-1-1   CAUCGAGACCCAGGAGUGGCUGGACUCGCUCGAAUCAGUCCUCGACCACGAAGGCGAAGAGCGCGCCCAUUAUCUGCUGACCCGGAUGGGCGAACUGGCCACUCGCACGGGCACCCAACUGCCCUAUGCGAUCACCACCCCCUAUCGCAACACCAUUCCG
Pae-3-1   CCAGGAAUGGCUGGACGCCCUGGAGUCCGUACUCGACCGCGAAGGUGAAGACCGCGCUCAUUACCUGAUGACCCGCAUGGGCGAGCUGGCCAGCCGUAGCGGCACCCAGCUGCCCUACGCGAUCACCACGCCCUACCGCAACACCAUUCCGGUAACCCAC
Pae-5-1   CCAGGAAUGGCUGGACGCCCUGGAGUCCGUACUCGACCGCGAAGGUGAAGACCGCGCUCAUUACCUGAUGACCCGCAUGGGCGAGCUGGCCAGCCGUAGCGGCACCCAACUGCCCUACGCGAUCACCACGCCCUAUCGCAACACCAUUCCGGUAACCCAC
Pae-4-1   CCAGGAAUGGCUGGACGCCCUGGAGUCCGUACUCGACCGCGAAGGUGAAGACCGCGCUCAUUACCUGAUGACCCGCAUGGGCGAGCUGGCCAGCCGUAGCGGCACCCAACUGCCCUACGCGAUCACCACGCCCUAUCGCAACACCAUUCCGGUAACCCAC
Pae-1-1   CCAGGAAUGGCUGGACGCCCUGGAGUCCGUACUCGACCGCGAAGGUGAAGACCGCGCUCAUUACCUGAUGACCCGCAUGGGCGAGCUGGCCAGCCGUAGCGGCACCCAACUGCCCUACGCGAUCACCACGCCCUACCGCAACACCAUUCCGGUAACCCAC
Pae-6-1   CCAGGAAUGGCUGGACGCCCUGGAGUCCGUACUCGACCGCGAAGGUGAAGACCGCGCUCAUUACCUGAUGACCCGCAUGGGCGAGCUGGCCAGCCGUAGCGGCACCCAACUGCCCUACGCGAUCACCACGCCCUACCGCAACACCAUUCCGGUAACCCAC
Pme-1-1   CCAGGAAUGGCUGGACGCCCUUGAAUCCGUCCUCGACCGUGAGGGUGAAGACCGUGCCCAUUACCUGAUGACCCGUCUGGGCGAGCUGGCCACCCGUAGCGGUGCGCAACUGCCCUACGCGAUCACCACGCCGUACCGCAACACCAUCCCGGUCACCCAC
Pst-1-1   CCAGGAAUGGCUGGACGCCCUCGAGUCCGUCCUCGACCGCGAGGGUGAAGACCGCGCUCACUACCUGAUGACCCGGAUGGGCGAACUGGCCACACGUAGCGGUACUCCGCUGCCCUACGGCAUCACCACGCCGUACCGCAACACCAUCCCCGUCACCCGU
env-1     CCAGGAAUGGCUGGACGCCCUGGAAUCGGUUCUCGACAAAGAAGGCGAAGACCGCGCUCACUAUCUGAUGACCCGUAUGGGCGAACUCGCAACCCGAGCGUCCUCAAC......................................................
Pfl-2-1   CCAGGAAUGGCUGGACGCCCUGGAAUCGGUUCUCGACAAAGAAGGCGAAGACCGUGCUCACUAUCUGAUGACCCGUAUGGGUGAACUGGCAACCCGCAGCGGUUCGCAGCUCCCUUACGCCAUCACCACGCCUUACCGCAACACGAUCCCCGUUACCCAC
Pfl-1-1   CCAGGAAUGGCUGGACGCCCUGGAAUCGGUUCUCGACAAAGAAGGCGAAGACCGUGCUCACUAUCUGAUGACCCGUAUGGGCGAACUCGCGACCCGCAGCGGCUCGCAACUGCCCUACGCCAUCACCACGCCAUACCGCAACACGAUUCCGUCACCCAC
Psy-2-1   CCAGGAAUGGCUGGACGCCCUGGAAUCGGUUCUCGACAAAGAAGGCGAAGACCGCGCUCACUACCUGAUGACCCGUUUGGGCGAGCUGGCCACCCGUAGUGGUUCGCAGCUGCCGUACGCCAUCACCACGCCAUAUCGCAACACCAUUCCUGUCACCCAC
Psy-3-1   CCAGGAAUGGCUGGACGCCCUGGAAUCGGUUCUCGACAAAGAAGGCGAAGACCGCGCUCACUACCUGAUGACCCGUCUGGGCGAGCUGGCCACCCGUAGUGGUUCGCAGCUGCCGUACGCCAUCACCACGCCAUACCGCAACACGAUUCCUGUCACCCAC
Psy-1-1   CCAGGAAUGGCUGGACGCCCUGGAAUCGGUUCUCGACAAAGAAGGCGAAGACCGCGCUCACUACCUGAUGACCCGUUUGGGCGAGCUGGCCACCCGUAGUGGUUCGCAGCUGCCGUACGCCAUCACUACGCCAUACCGCAACACGAUUCCUGUCACCCAC
Ppu-4-1   CCAGGAAUGGCUGGAUGCCCUGGAGUCGGUCCUCGACAAAGAAGGCGAAGACCGCGCUCAUUACCUGAUGACCCGCAUGGGCGAGCUGGCCACCCGCAGUGGCUCCCAGCUGCCAUACGCGAUCACCACGCCAUACCGCAACACCAUCCCUGUCACCCAC
Pen-1-1   CCAGGAAUGGCUGGAUGCCCUGGAGUCGGUCCUCGACAAAGAAGGCGAAGACCGCGCUCAUUACCUGAUGACCCGUAUGGGCGAGCUGGCCACCCGUAGUGGCUCCCAGCUGCCGUAUGCCAUCACCACGCCGUACCGCAACACCAUCCCUGUCACCCAC
Ppu-1-1   CCAGGAAUGGCUGGAUGCCCUGGAGUCGGUCCUCGACAAAGAAGGCGAAGACCGCGCUCAUUACCUGAUGACCCGUAUGGGCGAGCUGGCCACCCGUAGUGGCUCCCAGCUGCCGUAUGCGAUCACCACGCCAUACCGCAACACCAUCCCUGUCACCCAC
Ppu-3-1   CCAGGAAUGGCUGGAUGCCCUGGAGUCGGUCCUCGACAAAGAAGGCGAAGACCGCGCUCAUUACCUGAUGACCCGUAUGGGCGAGCUGGCCACCCGUAGUGGCUCCCAGCUGCCAUAUGCGAUCACCACGCCAUACCGCAACACCAUCCCUGUCACCCAC
Ppu-2-1   CCAGGAAUGGCUGGAUGCCCUGGAGUCGGUCCUCGACAAAGAAGGCGAAGACCGCGCUCAUUACCUGAUGACCCGUAUGGGCGAGCUGGCCACCCGCAGUGGCUCCCAGCUGCCGUAUGCGAUCACCACGCCAUACCGCAACACCAUCCCUGUCACCCAC
          ................................................................................................................................................................
          ................................................................................................................................................................
          CCAGGAAUGGCUGGAYGCCCUGGARUC•GUYCUCGAC•R•GARGGYGAAGACCGCGCYCAYUACCUGAUGACCCG••UGGCGARCUGGCCA••CG•R•YR•Y•C•••R••••C•YA••Y••YC••YRCG•Y•••C••C••C••Y••Y•••RYCAYYC••
```

alignment positions 416 · · · 482

```
Avi-1-1   GUGACCCACGAAGCGCACAUGCCCGGCGACCUGUUCAUGGAACGCCGCAUCCGCUCCCUGGUGCGCU
Pae-3-1   GAAGCACGCAUGCCCGGCGACCUGUUCAUGGAACGCCGGAUCCGCUCCCUGGUACGCUGGAACGCCC
Pae-5-1   GAAGCACGCAUGCCCGGCGACCUGUUCAUGGAACGCCGGAUCCGCUCCCUGGUGCGCUGGAACGCCC
Pae-4-1   GAAGCACGCAUGCCCGGCGACCUGUUCAUGGAGCGCCGGAUCCGCUCCCUGGUGCGCUGGAACGCCC
Pae-1-1   GAAGCACGCAUGCCCGGCGACCUGUUCAUGGAACGCCGGAUUCGCUCCCUGGUGCGCUGGAACGCCC
Pae-6-1   GAAGCACGCAUGCCCGGCGACCUGUUCAUGGAGCGCCGGAUCCGCUCCCUGGUGCGCUGGAACGCCC
Pme-1-1   GAAGCGCGCAUGCCCGGCGACUUGUUCAUGGAGCGCCGCAUCCGCUCGCUGGUGCGCUGGAACGCC.

          ...................................................................
          ...................................................................
          G•RRC•CRCR•R•C••RCR••CY•••CR••••RY•C•••R••CGCY•••U••••Y•YY•GR•••••∘
```

```
Pst-1-1   GAAGCCAAGAUGCCCGGCGACCUGUUCAUGGAGCGUCGAAUCCGCUCCCUGGUCCGCUGGAAUGCUC
env-1     ...................................................................
Pfl-2-1   GAAGCACGCAUGCCUGGCGACCUGUUCAUGGAACGCCGCAUUCGCUCGCUGGUACGCUGGAACGCG.
Pfl-1-1   GAAGCACGCAUGCCUGGCGACCUGUUCAUGGAACGCCGCAUUCGCUCGCUGGUACGUUGGAACGCG.
Psy-2-1   GAAGCACGCAUGCCUGGCGACCUGUUCAUGGAACGCCGCAUUCGCUCGCUGGUGCGCUGGAACGCAU
Psy-3-1   GAAGCACGCAUGCCUGGCGACCUGUUCAUGGAACGCCGCAUUCGCUCGCUGGUGCGCUGGAACGCAU
Psy-1-1   GAAGCACGCAUGCCUGGCGACCUGUUCAUGGAACGCCGCAUUCGCUCGCUGGUGCGCUGGAACGCAU
Ppu-4-1   GAAGCACGCAUGCCUGGCGACCUGUUCAUGGAACGCCGCAUUCGCUCGAUGGUGCGUUGGAACGCGC
Pen-1-1   GAAGCACGCAUGCCUGGCGACCUGUUCAUGGAACGCCGCAUCCGCUCGAUGGUGCGUUGGAACGCCC
Ppu-1-1   GAAGCACGCAUGCCUGGCGACCUGUUCAUGGAACGCCGCAUUCGCUCGAUGGUGCGUUGGAACGCGC
Ppu-3-1   GAAGCACGCAUGCCUGGCGACCUGUUCAUGGAACGCCGCAUUCGCUCGAUGGUGCGUUGGAACGCGC
Ppu-2-1   GAAGCACGCAUGCCUGGCGACCUGUUCAUGGAACGCCGCAUUCGCUCGAUGGUGCGUUGGAACGCCU
          ...................................................................
          ...................................................................
          G•RRC•CRCR•R•C••RCR••CY•••CR••••RY•C•••R••CGCY•••U••••Y•YY•GR•••••○
```

3 Acido-1

3.1 Taxa

The taxonomy of each organism containing a putative Acido-1 RNA is listed, with abbreviations identifying each hit (e.g., "Eco-1-1" and "Eco-1-2" might hypothetically represent two distinct RNAs in *E. coli*). The abbreviations will be used to identify each individual Acido-1 RNA in Sections 3.2 and 3.4.

**abbrev. of hits**	**taxonomy of species**
Aba-1-1	Bacteria Acidobacteria Acidobacteriales Acidobacteriaceae *Acidobacteria bacterium* Ellin345
Sus-1-1 to Sus-1-3	Bacteria Acidobacteria Solibacteres Solibacterales Solibacteraceae *Solibacter usitatus* Ellin6076

3.2 Gene contexts

Each Acido-1 RNA (indicated by "RNA→") is listed with its surrounding genes. Environmental sequences and some RefSeq entries lack gene annotations, and no genes are listed for such sequences. The direction of each gene is indicated with an arrow (→), and each conserved domain in the gene is colored. Conserved domains associated with more than one Acido-1 RNA are assigned a color; other domains are gray. Information about these conserved domains is given in Section 3.3. The accession number of the sequence containing each Acido-1 RNA is given. Accessions beginning with "NC_" or "NZ_" are RefSeq, while other accession refer to environmental samples. Nucleotide coordinates are given for the 5′ and 3′ boundaries of each. If the 5′ coordinate is greater than the 3′ coordinate, the RNA is present on the reverse-complement strand of the containing genomic DNA sequence. Each hit is denoted by an abbreviation (like "Eco-1-1") that refers to a taxonomy given in Section 3.1.

**abbrev.**	**Seq. accession**		**5′ at**	**3′ at**	**genes**
Aba-1-1	NC_008009.1	-	235844	235785	AcnA_IRP_Swivel (cd01580)AcnA_IRP (cd01586)→ **RNA**→ SUL1 (COG0659)→
Sus-1-1	NC_008536.1	+	3413964	3414030	hypo→ **RNA**→ ←MecR1 (COG4219)
Sus-1-2	NC_008536.1	+	3480719	3480784	hypo→ **RNA**→ TPR (cd00189)→
Sus-1-3	NC_008536.1	+	3481695	3481760	TPR (cd00189)→ **RNA**→ type IV pilus assembly PilZ→

3.3 Conserved domains

Conserved domains found in protein-coding genes listed in Section 3.2 are listed, with the first sentence in their description from the Conserved Domain Database. Conserved domains associated with more than one Acido-1 RNA are assigned a color, while others are shown in gray. The number in parentheses is the number of occurrences of the given gene.

`uD` cd00189 (2) Tetratricopeptide repeat domain; typically contains 34 amino acids [WLF]-X(2)-[LIM]-[GAS]-X(2)-[YLF]-X(8)-[ASE]-X(3)-[FYL]-X(2)-[ASL]-X(4)-[PKE]
`u` cd01580 (1) Aconitase A swivel domain.
`u` cd01586 (1) Aconitase A catalytic domain.
`D` COG0659 (1) Sulfate permease and related transporters (MFS superfamily) [Inorganic ion transport and metabolism]
`d` COG4219 (1) Antirepressor regulating drug resistance, predicted signal transduction N-terminal membrane component [Transcription / Signal transduction mechanisms]

3.4 Multiple-sequence alignment

Each Acido-1 RNA is denoted by an abbreviation (like "Eco-1-1") that refers to a taxonomy given in Section 3.1. The alignment includes sequences containing the RNA motif, as well as flanking sequence. The Acido-1 RNA itself is denoted by the line underneath marked 5′ and 3′ on either end. Nucleotides in the flanking sequences (i.e., not part of the motif) are written in gray letters. Stems of predicted rho-independent transcription terminators are shaded yellow. (Note: terminator predictions have not been analyzed manually, and many are likely to be false positives.) Start codons are shaded green. (Note: start codons are frequently misannotated, especially in environmental samples.) Nucleotides proposed to basepair as part of the consensus structure are shaded in color when they comprise Watson-Crick or G-U pairs. Otherwise they are shaded gray. Conserved stems are also indicated at the bottom of the alignment by angle brackets, where matching < and > denote base-paired columns. Below these angle brackets, the symbol "2" denotes base pairs exhibiting covariation, "1" denotes base pairs exhibiting compatible mutations, "0" denotes base pairs that are not observed to mutate and "?" denotes base pairs that have a significant frequency of non-canonical nucleotides for Watson-Crick or G-U pairs (> 5%). Below these base pair annotation is the consensus sequence: "R" = "A" or "G", "Y" = "C" or "U", red nucleotides: nucleotide identity conserved more than 97% of the time, black nucleotides: 90%, gray nucleotides: 75%, red circle (•): nucleotide is present 97% of the time, black circle (•): 90%, gray circle (•): 75%, white circle (○): 50%. All percentages of sequences just described (e.g. 97% conserved) assume that sequences have been weighted by the GSC algorithm implemented by the Infernal software.

alignment positions 1 · · · 99

```
Aba-1-1  GACGGAAACAUUCCGAGCUACGCUGCGCAUUGAUACACCCCAGGAAGUGCAGUAUUUCCGUCACGGCGGAAUUCUGCAUUACGUGGUGCGGCAGUUGGC
Sus-1-1  AGCCCACCGCCACAUGCUCAAUCUCAAGGAGACCGCCUCGCACACGGGCAGUUUUCCAAAAUCGGAAGUAGGGGGUGGUUUUAAGGGGUAGGGCAGGUU
Sus-1-2  GCGGGAACUGGGGCUGAAGUUGGUGAAGCAGACGCGAAUGUACCCCGUGAUCGUGGUGGAUCACAUCGCGGAGAAGCCGGUGGAGUGACCCGUAAAAAG
Sus-1-3  CGGUGAGACGAUCUCCGCACUCAAACAGGCGCCUUCGCCGGAAAACCUGGCGAAAGUGACUUCGCUGCUCCAAGGAUUGAAAUAGGAGCCCUAAAAAGA
         ...................................................................................................
         ...................................................................................................
         RR•••A••••••C••R•Y•••••U••R•R••R•Y•C•YC•YA•R••G•••••••••Y••R•Y••R••G•RR•••••••••••R•GG•GY•••R•••R••
```

alignment positions 100 · · · 259

```
Aba-1-1  GGGAGCAGCGAA...GAA.......UUAGUUGCGAAACUGAUUGACU.GACGGCGGGAGAGAUCCCGCCGUUUUGCUUUGUCCCAAUGUGAGUACCACCUUCCCGUGAUUCCCUUCAGCUACUCUGCUUGCUAGCUUGAUAAAAAGGCCCGUAUUUUCCC
Sus-1-1  UAAAGGGUCCG..CCUGGGUAGUA..CGGACCCUGCACUCAGAGGGUUGGCGGCGGGAGGGGUCUCGCCGCCAGGGAUGCUGUUAAAUUCAAUAUACCGAUUUCAGAGCCGUUUGUCCACAAUUAUUUUUAGCGACUCGAGUCUCCCGUUUGUUCCUGGC
Sus-1-2  UAAUGGCCCCGAGC.UGGGUA...CUCGGGGCCUGCACUCAGAGGGU.GGCGGUGGGAGGGGACUCACCGCCAGGAUGCUGUCUGUGUUGAUCUAAGAUUAAUAUUUUAAGACCUCCUUGUCAACUAUUAUUUUGAUGAUUUCGCAAAAAAGUUUUCCGC
Sus-1-3  UAAUGGCCCCGAGC.UGGGUA...CUCGGGGCCUGCACUCAGAGGGU.GGCGGUGGGAGGGGACUCACCGCCUGGGAUGCUGUCUGUUUGAUCUAAUAUUAGCAAUUUAAGGCCUUCUUGUCUAGUAUUAUUUUAUAAAAAACGCAGGACCGAAAAAGUC
         ....<<<<<<<<<...........>>>>>>>>>...............<<<<<<<<<......>>>>>>>>>........................................................................................
         ....02220?200...........002?02220...............120002010......010200021........................................................................................
         •RR•G•••C•R○-○-•RR○○○----○YRG•••C•R•ACU•A••GR•U-GRCGGYGGGAGRGR•CYCRCCGYY••G••U••U•YYRR••U•A•Y•••••••••Y•R•••••••YY•Y••••••U••••UU••••••YU•R•R•••••••••Y•••UYYY••C
          5′——————————————————————————————————————————————————————————————3′
```

alignment positions 260 · · · 419

```
Aba-1-1  AAUGCACGAGUGGCUACCGAAGUCCGUUCUUGCGCUGCGUGAUUACUCCCGCCAGCGCUUUGUUGCCGACCUGCUUGCCGGCAUUACUGUGGGGCUGGUUGCGCUUCCGCUGGCGAUGGCGUUUGCCAUUGCUUCUGGCGUGCCGCCGCAGAGCGGACUG
Sus-1-1  UGUAGAUUACGUUUCACGGAACGAUGAUUUCGUCGCCCGGUUUCAGCCAAAUAUUCUGUGCAGGAUUCUUGCCGGUCUUGACCUCUUCGUAGCGAAACCGGAACUCCGUGACCUUGCCGCCUUCCCUCCGGACAAUCCGAAUCUCCUUCGUAUUCGCGGA
Sus-1-2  CUCAGGGGGGCUUUUCCCCGUCAUCGUGAUUGUGUAUGCUAUAUCAGCUCUGAGGGAGGGCGGGAAAUGCGCAUCACUCUGUUGAUGGUGCUGGGUUUAACCGUGGGCUGGGCGCAGUCGGAUCCGGGAGAGGUUCUUCGGCAAUGGCUGGAGGAAGGCC
Sus-1-3  CCGGUUAGUACUGGUUAAUGUUAUCUUCCGGGGAUUCCCUAACUCUCUGUUUUUAAAUAAGAUAUCCGGAUGACAGGUACUCGCGCCGAGGGGGUGAUAGCGGCCUCGAAAUAACUAAAGACAGCUUGAGCGUUCUCCGUACCCCUGCAAUUCUGUCUUU
         ................................................................................................................................................................
         ................................................................................................................................................................
         ••YR•R••R••••YY•C••R••••YG•YYUYGY••Y•C••••YY••YC•••Y••••••••YR••RYY••Y•Y•••••YY•RC•Y••Y•R•RG•G••R•••••RCYYC••••••••R••R••YUY•YY•••R•Y••U•••R••CY•Y••YR•A•Y•R••••
```

alignment positions 420 · · · 471

```
Aba-1-1   UAUUGCGCGAUCGUCGCCGGGUUCCUCGUCUCGGCGUGCGGUGGUUCGCUGA
Sus-1-1   CUCCAGAAAUCCCCCGGCCUCGACCAGCGCCCGCAGGACUGUCGUCGGGUAC
Sus-1-2   GGGCAGCCGUACAGGCCGGCCAGUUCGACGCCGCGAUCGCCAGGUUUCAGAA
Sus-1-3   GGAAGGUGGGCGCAUGCAAAUGUUUGACAGCCACGAUGACCGGUCGAUGAAC
          ....................................................
          ....................................................
          ●●●YR●●●R●YC●●YG●●●●●●●YY●●●●●YCG●●R●R●●●●●GUY●●●●R●
```

4 Acido-Lenti-1

4.1 Taxa

The taxonomy of each organism containing a putative Acido-Lenti-1 RNA is listed, with abbreviations identifying each hit (e.g., “Eco-1-1” and “Eco-1-2” might hypothetically represent two distinct RNAs in *E. coli*). The abbreviations will be used to identify each individual Acido-Lenti-1 RNA in Sections 4.2 and 4.4.

**abbrev. of hits**	**taxonomy of species**
Aba-1-1 to Aba-1-11	Bacteria Acidobacteria Acidobacteriales Acidobacteriaceae *Acidobacteria bacterium* Ellin345
Lar-1-1 to Lar-1-56	Bacteria Lentisphaerae Lentisphaerales Lentisphaeraceae *Lentisphaera araneosa* HTCC2155
Rba-1-1 to Rba-1-2	Bacteria Planctomycetes Planctomycetacia Planctomycetales Planctomycetaceae *Rhodopirellula baltica* SH 1
Gur-1-1	Bacteria Proteobacteria Deltaproteobacteria Desulfuromonadales Geobacteraceae *Geobacter uraniireducens* Rf4
env-1 to env-19	environmental samples

4.2 Gene contexts

Each Acido-Lenti-1 RNA (indicated by “RNA→”) is listed with its surrounding genes. Environmental sequences and some RefSeq entries lack gene annotations, and no genes are listed for such sequences. The direction of each gene is indicated with an arrow (→), and each conserved domain in the gene is colored. Conserved domains associated with more than one Acido-Lenti-1 RNA are assigned a color; other domains are gray. Information about these conserved domains is given in Section 4.3. The accession number of the sequence containing each Acido-Lenti-1 RNA is given. Accessions beginning with “NC_” or “NZ_” are RefSeq, while other accession refer to environmental samples. Nucleotide coordinates are given for the 5′ and 3′ boundaries of each. If the 5′ coordinate is greater than the 3′ coordinate, the RNA is present on the reverse-complement strand of the containing genomic DNA sequence. Each hit is denoted by an abbreviation (like “Eco-1-1”) that refers to a taxonomy given in Section 4.1.

abbrev.	Seq. accession		5′ at	3′ at	genes
Lar-1-1	NZ_ABCK01000026.1	+	37913	37958	hypo→ **RNA**→ **RNA**→ ←PDZ_serine_protease (cd00987)
Lar-1-2	NZ_ABCK01000026.1	+	37713	37758	(shown above)
Lar-1-3	NZ_ABCK01000029.1	-	8929	8884	←AslA (COG3119) **RNA**→ ←hypo
Aba-1-1	NC_008009.1	-	1267162	1267117	←hypo **RNA**→ ←SpoU (COG0566)
Lar-1-4	NZ_ABCK01000007.1	-	4868	4823	COG1073 (COG1073)COG1647 (COG1647)→ **RNA**→ ←rve (pfam00665)
Lar-1-5	NZ_ABCK01000002.1	-	270653	270608	←AslA (COG3119) **RNA**→ **RNA**→ **RNA**→ ←COG1413 (COG1413)COG2133 (COG2133)
Lar-1-6	NZ_ABCK01000002.1	-	270461	270416	(shown above)
Lar-1-7	NZ_ABCK01000002.1	-	270845	270800	(shown above)
Lar-1-8	NZ_ABCK01000002.1	+	237476	237521	hypo→ **RNA**→ **RNA**→ AslA (COG3119)→
Lar-1-9	NZ_ABCK01000002.1	+	237916	237961	(shown above)
Lar-1-10	NZ_ABCK01000002.1	+	217312	217357	hypo→ **RNA**→ COG2133 (COG2133)DUF1080 (pfam06439)→
Lar-1-11	NZ_ABCK01000002.1	+	210449	210494	PulG (COG2165)→ **RNA**→ ←RVT (pfam00078)
Lar-1-12	NZ_ABCK01000002.1	+	212552	212597	←hypo **RNA**→ hypo→
Lar-1-13	NZ_ABCK01000005.1	-	275464	275419	RVT (pfam00078)→ **RNA**→ ←AslA (COG3119)
Lar-1-14	NZ_ABCK01000001.1	-	419681	419636	hypo→ **RNA**→ hypo→
Lar-1-15	NZ_ABCK01000012.1	+	53466	53511	EFh (cd00051)→ **RNA**→ hypo→
Lar-1-16	NZ_ABCK01000012.1	-	104879	104834	←hypo **RNA**→ ←AslA (COG3119)
Lar-1-17	NZ_ABCK01000012.1	-	106309	106264	←hypo **RNA**→ ←hypo
Lar-1-18	NZ_ABCK01000010.1	+	121151	121196	hypo→ **RNA**→ **RNA**→ **RNA**→ hypo→
Lar-1-19	NZ_ABCK01000010.1	+	120473	120518	(shown above)
Lar-1-20	NZ_ABCK01000010.1	+	120955	121000	(shown above)
Lar-1-21	NZ_ABCK01000006.1	+	14314	14359	←AslA (COG3119) **RNA**→ **RNA**→ **RNA**→ ←RpoE (COG1595)
Lar-1-22	NZ_ABCK01000006.1	+	14204	14249	(shown above)
Lar-1-23	NZ_ABCK01000006.1	+	14425	14470	(shown above)
Lar-1-24	NZ_ABCK01000001.1	-	416780	416735	COG1943 (COG1943)→ **RNA**→ **RNA**→ **RNA**→ **RNA**→ ←COG3618 (COG3618)
Lar-1-25	NZ_ABCK01000001.1	-	416706	416661	(shown above)
Lar-1-29	NZ_ABCK01000001.1	-	416632	416587	(shown above)
Lar-1-31	NZ_ABCK01000001.1	-	416558	416513	(shown above)
Lar-1-26	NZ_ABCK01000001.1	-	431760	431715	←hypo **RNA**→ **RNA**→ ←vWFA (cd00198)
Lar-1-27	NZ_ABCK01000001.1	-	431565	431520	(shown above)
Lar-1-28	NZ_ABCK01000080.1	+	409	454	hypo→ **RNA**→ hypo→
Lar-1-30	NZ_ABCK01000001.1	-	417523	417478	RVT (pfam00078)→ **RNA**→ COG1943 (COG1943)→
Lar-1-32	NZ_ABCK01000006.1	+	103015	103060	←hypo **RNA**→ hypo→
Lar-1-33	NZ_ABCK01000006.1	+	85512	85557	COG0400 (COG0400)→ **RNA**→ **RNA**→ ←AslA (COG3119)
Lar-1-34	NZ_ABCK01000006.1	+	85251	85296	(shown above)
Lar-1-35	NZ_ABCK01000006.1	+	107180	107225	RVT (pfam00078)→ **RNA**→ ←AslA (COG3119)
Lar-1-36	NZ_ABCK01000016.1	+	16051	16096	←probable cytochrome c precursor **RNA**→ **RNA**→ ←COG3828 (COG3828)
Lar-1-37	NZ_ABCK01000016.1	+	16291	16336	(shown above)
Lar-1-38	NZ_ABCK01000007.1	-	84866	84821	←COG1413 (COG1413)COG2133 (COG2133) **RNA**→ **RNA**→ ←tRNA_Me_trans (pfam03054)
Lar-1-40	NZ_ABCK01000007.1	-	85099	85054	(shown above)
Lar-1-39	NZ_ABCK01000006.1	+	115823	115868	←COG3533 (COG3533) **RNA**→ hypo→
Lar-1-41	NZ_ABCK01000004.1	+	95064	95109	AslA (COG3119)→ **RNA**→ ←Metallophos (pfam00149)
Lar-1-42	NZ_ABCK01000001.1	-	246374	246329	←AslA (COG3119) **RNA**→ **RNA**→ ←RVT (pfam00078)
Lar-1-47	NZ_ABCK01000001.1	-	246515	246470	(shown above)
Lar-1-43	NZ_ABCK01000020.1	+	22700	22745	VanZ (pfam04892)→ **RNA**→ **RNA**→ **RNA**→ **RNA**→ WcaJ (COG2148)→
Lar-1-44	NZ_ABCK01000020.1	+	21790	21835	(shown above)
Lar-1-45	NZ_ABCK01000020.1	+	22585	22630	(shown above)
Lar-1-46	NZ_ABCK01000020.1	+	22470	22515	(shown above)
Lar-1-48	NZ_ABCK01000006.1	+	178011	178056	ThiF_MoeB_HesA_family (cd00757)→ **RNA**→ ←hypo
Lar-1-49	NZ_ABCK01000029.1	+	14774	14819	RVT (pfam00078)→ **RNA**→ ←hypo
Lar-1-50	NZ_ABCK01000001.1	+	226211	226256	LamGL (smart00560)→ **RNA**→ **RNA**→ **RNA**→ **RNA**→ hypo→
Lar-1-51	NZ_ABCK01000001.1	+	226073	226118	(shown above)
Lar-1-52	NZ_ABCK01000001.1	+	226142	226187	(shown above)
Lar-1-53	NZ_ABCK01000001.1	+	225878	225923	(shown above)
env-1	JCVI_SCAF_1096628177727	+	654	698	**RNA**→
[illegible]	[illegible]	[illegible]	[illegible]	[illegible]	[illegible]

4.3 Conserved domains

Conserved domains found in protein-coding genes listed in Section 4.2 are listed, with the first sentence in their description from the Conserved Domain Database. Conserved domains associated with more than one Acido-Lenti-1 RNA are assigned a color, while others are shown in gray. The number in parentheses is the number of occurrences of the given gene.

u cd00051 (1) EF-hand, calcium binding motif; A diverse superfamily of calcium sensors and calcium signal modulators; most examples in this alignment model have 2 active canonical EF hands.
d cd00189 (1) Tetratricopeptide repeat domain; typically contains 34 amino acids [WLF]-X(2)-[LIM]-[GAS]-X(2)-[YLF]-X(8)-[ASE]-X(3)-[FYL]-X(2)-[ASL]-X(4)-[PKE]
d cd00198 (1) Von Willebrand factor type A (vWA) domain was originally found in the blood coagulation protein von Willebrand factor (vWF).
u cd00757 (1) ThiF_MoeB_HesA.
d cd00987 (1) PDZ domain of tryspin-like serine proteases, such as DegP/HtrA, which are oligomeric proteins involved in heat-shock response, chaperone function, and apoptosis.
u d cd02953 (2) DsbD gamma family; DsbD gamma is the C-terminal periplasmic domain of the bacterial protein DsbD.
D COG0021 (1) Transketolase [Carbohydrate transport and metabolism]
u COG0034 (1) Glutamine phosphoribosylpyrophosphate amidotransferase [Nucleotide transport and metabolism]
d COG0046 (1) Phosphoribosylformylglycinamidine (FGAM) synthase, synthetase domain [Nucleotide transport and metabolism]
d COG0150 (1) Phosphoribosylaminoimidazole (AIR) synthetase [Nucleotide transport and metabolism]
d COG0235 (1) Ribulose-5-phosphate 4-epimerase and related epimerases and aldolases [Carbohydrate transport and metabolism]
u COG0312 (1) Predicted Zn-dependent proteases and their inactivated homologs [General function prediction only]
u COG0352 (1) Thiamine monophosphate synthase [Coenzyme metabolism]
u COG0400 (1) Predicted esterase [General function prediction only]
u COG0529 (1) Adenylylsulfate kinase and related kinases [Inorganic ion transport and metabolism]
d COG0566 (1) rRNA methylases [Translation, ribosomal structure and biogenesis]
u COG0677 (1) UDP-N-acetyl-D-mannosaminuronate dehydrogenase [Cell envelope biogenesis, outer membrane]
d COG1004 (1) Predicted UDP-glucose 6-dehydrogenase [Cell envelope biogenesis, outer membrane]
u COG1073 (1) Hydrolases of the alpha/beta superfamily [General function prediction only]
u d COG1413 (2) FOG: HEAT repeat [Energy production and conversion]
u COG1506 (1) Dipeptidyl aminopeptidases/acylaminoacyl-peptidases [Amino acid transport and metabolism]
d COG1595 (1) DNA-directed RNA polymerase specialized sigma subunit, sigma24 homolog [Transcription]
d COG1620 (1) L-lactate permease [Energy production and conversion]
u COG1647 (1) Esterase/lipase [General function prediction only]
uD COG1943 (2) Transposase and inactivated derivatives [DNA replication, recombination, and repair]
u COG2085 (1) Predicted dinucleotide-binding enzymes [General function prediction only]
uDd COG2133 (3) Glucose/sorbosone dehydrogenases [Carbohydrate transport and metabolism]
D COG2148 (1) Sugar transferases involved in lipopolysaccharide synthesis [Cell envelope biogenesis, outer membrane]
u COG2165 (1) Type II secretory pathway, pseudopilin PulG [Cell motility and secretion / Intracellular trafficking and secretion]
u COG2814 (1) Arabinose efflux permease [Carbohydrate transport and metabolism]
d COG2877 (1) 3-deoxy-D-manno-octulosonic acid (KDO) 8-phosphate synthase [Cell envelope biogenesis, outer membrane]
uDd COG3119 (10) Arylsulfatase A and related enzymes [Inorganic ion transport and metabolism]
d COG3205 (1) Predicted membrane protein [Function unknown]
u COG3533 (1) Uncharacterized protein conserved in bacteria [Function unknown]
d COG3550 (1) Uncharacterized protein related to capsule biosynthesis enzymes [General function prediction only]
d COG3577 (1) Predicted aspartyl protease [General function prediction only]
d COG3618 (1) Predicted metal-dependent hydrolase of the TIM-barrel fold [General function prediction only]
u COG3666 (1) Transposase and inactivated derivatives [DNA replication, recombination, and repair]
u d COG3828 (2) Uncharacterized protein conserved in bacteria [Function unknown]
u COG4972 (1) Tfp pilus assembly protein, ATPase PilM [Cell motility and secretion / Intracellular trafficking and secretion]
u GOS1134 (1) ?
d GOS11523 (1) ?
u GOS11714918 (2) ?
u d GOS1212 (4) ?
d GOS1254 (1) ?
D GOS13384922 (1) ?
d GOS2400 (1) ?
u GOS4150 (1) ?
d NOG30274 (1)
u d pfam00078 (6) Reverse transcriptase (RNA-dependent DNA polymerase).
d pfam00149 (1) Calcineurin-like phosphoesterase.
u pfam00551 (1) Formyl transferase.
d pfam00665 (1) Integrase core domain.

d pfam01507 (1) Phosphoadenosine phosphosulfate reductase family.
d pfam03054 (1) tRNA methyl transferase.
d pfam03720 (2) UDP-glucose/GDP-mannose dehydrogenase family, UDP binding domain.
u pfam04892 (1) VanZ like family.
D pfam06439 (1) Domain of Unknown Function (DUF1080).
u smart00560 (1) LamG-like jellyroll fold domain;

Duplicate sequences: the following putative homologs are not shown in the alignment because their sequences are identical to a homolog already shown: env-8, env-9

4.4 Multiple-sequence alignment

Each Acido-Lenti-1 RNA is denoted by an abbreviation (like "Eco-1-1") that refers to a taxonomy given in Section 4.1. The alignment includes sequences containing the RNA motif, as well as flanking sequence. The Acido-Lenti-1 RNA itself is denoted by the line underneath marked 5′ and 3′ on either end. Nucleotides in the flanking sequences (i.e., not part of the motif) are written in gray letters. Stems of predicted rho-independent transcription terminators are shaded yellow. (Note: terminator predictions have not been analyzed manually, and many are likely to be false positives.) Start codons are shaded green. (Note: start codons are frequently misannotated, especially in environmental samples.) Nucleotides proposed to basepair as part of the consensus structure are shaded in color when they comprise Watson-Crick or G-U pairs. Otherwise they are shaded gray. Conserved stems are also indicated at the bottom of the alignment by angle brackets, where matching < and > denote base-paired columns. Below these angle brackets, the symbol "2" denotes base pairs exhibiting covariation, "1" denotes base pairs exhibiting compatible mutations, "0" denotes base pairs that are not observed to mutate and "?" denotes base pairs that have a significant frequency of non-canonical nucleotides for Watson-Crick or G-U pairs (> 5%). Below these base pair annotation is the consensus sequence: "R" = "A" or "G", "Y" = "C" or "U", red nucleotides: nucleotide identity conserved more than 97% of the time, black nucleotides: 90%, gray nucleotides: 75%, red circle (•): nucleotide is present 97% of the time, black circle (•): 90%, gray circle (•): 75%, white circle (•): 50%. All percentages of sequences just described (e.g. 97% conserved) assume that sequences have been weighted by the GSC algorithm implemented by the Infernal software.

alignment positions 1 ⋯ 160

Lar-1-1	AAAGGUCUCUUGGGAUCGCCAAGAACCAGCUUGGAAAUCGCAGACUACGUGCCCCGUAGGUGUCGGACUUUCAGCCCUCCUUCGUUUAAUCACGUCAGUUCGUGUGGCUCACGC.CACACGCUACAUUAUGUCGGGCUUCCAGCCCUGAAUCAGCUUAUA
Lar-1-2	GCAUGGAAACGGUUUUUUCAUUAAUUUAGGGCCCAGGCUUGGGUCCGGAAUAAUAUGCCGGACUCACAGGUGAGCCCUCCUUCGUUUAAUCACGUCAGAUCGUGUGGCUCACGC.CACACGCUACAUUAUGUCGGGCUUCUAGCCCUGAAUCAGCUUAUG
Lar-1-3	UGGGAAUUAAUCUAGGGCCCAAGCUUGGGUCCGACACUCUAUGCCGGACUCACAUCCGCGAGGUAUUGCCAUACGGGCAGAUAAAAACUAUUUCCCCAUUCGUGUGGCUAACGC.CACACGCUAAUAUCUAUCGGUGCUCUGCACCUUUAAAGUAUUUUC
Aba-1-1	UCCGGAUAAACUUCAAUCUAGGUCGAAUCACUUUCGGGUCUUCGCGUCGAUCAAGCGCGUGACGCCCCUGACGGGGCUGUUAAAGAUUUGUGCGGCUUUUCCCAGCGCUCACGC.GCUGGGCUACCAACUGGCGCCACUACGUGGCUUGCCUCGCGACUA
Lar-1-4	CUGUGUUCUCGGAGUCCUCUGUGGUGAAACUUUAUCAUUAAAAAUGAGCCGGACUCUAUGCCGUACCUUCGGCACUCAGAUAUUUGCUUCAAUUAAAUUUCCUAGCUCUCACAA.GCUAGGCUGUGUUAUGUCGUACCUUUGGUACUUAAAGCAUAACUG
Lar-1-5	GCCUUGGGGGUAAAAUGUAAGAUUAAAGGAGAAUGCCAAAGGCAUGGCAUAGAGCCCUAUACCGUACCUUCGGCACUCGAAUUUAUAUUCAUUAUUAUUUACUAGCACUGACGU.GCUAGGCUGAGUUAUGCCGUACCUUCGGUACUGAUGAUGAAUGCC
Lar-1-6	GCCUUGGGGGUAAAAUGUAAGAUUAAAGGAGAAUGCCAAAGGUACGGUAUAGGGCUCUAUACCGUACCUUCGGCACUCGAAUUUAUAUUCAUUAUUAUUUACUAGCACUGACGU.GCUAGGCUGAGUUAUGCCGUACCUUCGGUACUGAUGAUGAAUGCC
Lar-1-7	CUUAAGUUAUUUAUAUGUGUUAAUCUGAGUUUAUCGGUGGAUGAAUUUUCUUUUCUUUAUGCCGUACCUUCGGCACUCGAAUUUAUAUUCAUUAUUAUUUACUAGCACUGACGU.GCUAGGCUGAGUUAUGCCGUACCUUCGGUACUGAUGAUGAAUGCC
Lar-1-8	GUUGGCAGUUUUAUCUGUGUUAAUCGGAGUCAUCUGUGGAUGAAUUGUAUUUCUCUAUGUCGUACCUUCGGCACUCACAUUUUUUUGAUCGAUCUUAUCUCCUAGCUCUCACGA.GCCAGGCUGAACUAUACCGUACUCUCGGUACUCAUAAGGAAUGCU
Lar-1-9	GUUGGCAGUUUCAUCUGUGUUAAUCGGAGUCAUCUGUGGAUGAAUUGUAUUUCUCUAUGUCGUACCUUCGGCACUCACAUUUUUUUGAUCGAUCUUAUCUCCUAGCUCUCACGA.GCCAGGCUGAACUAUACCGUACUCUCGGUACUCAUAAGGAAUGCU
Lar-1-10	AUUCGUAGUUUUAUCUGUGUUAAUCUGCGUUCAUCUGUGGAUGAAUUUUGUUUCUCUGUGUCGUACCUUCGGCACUCACUUUUUUUGAUCGACCUUAUCUCCUAGCUCUCACGA.GCCAGGCUGAACUAUAACGUACCUUCGGUACUCACAAGGAAUGCC
Lar-1-11	AUCAAAACUCUUUACUAAAUUCAUUUUUAUAGGUAAAUCGGCUGCGUACCAUUUUUCUUAGUCGCAAUGGUAGGAGCUUUUUUUAUUUUAAACUUAUCUCCUAGCUCUCACGA.GCCAGGCUGAACUAUACCGUACCUUCGGUACUCACGUGGAAUGCC
Lar-1-12	CGGUCAACCCUGACGAGGUCUUUCACCUCGCUGAUGUGUGUACGCAUAGGCGUACUAUAUCGUACCUUUGGUACUCAGGUUUUUUGAUCGACCUUAUCUCCUAGCUCUCACGA.GCCAGGCUGAACUAUACCGUACCUUCGGUACUCACAUGGAAUGCG
Lar-1-13	CCUUGGGUGACGGAAUGCACGAUUCAAAUUGAAUGCCAAGGGCAUGACAUAGAGCUCUAUACCGUACCUUCGGCACUCACUUUUUUUAUCGACCUUAUCUCCUAGCUCUCACGA.GCCAGGCUAGCGUAUGCCGUACCUUAGGUACUCAAAACUGCUCAU
Lar-1-14	CAAAACCCCAAAGACCAAUCCCUCUUCGCCGUCUACCAAUACCAAGGCCCGGUAGCUUUUGACGAUAUUCAGGGGAGGGCGCUAUGCCAUUCCGUUACACCUAGCACUUCCGU.GCCAGGCUGAGUUAUGCCGUCCCAUUGGGACUCAAAUUGAAUGCC
Lar-1-15	GCCUUGGGGCAUAUUUCUGGGGAAAGAGAGAAUGCCGAAGGUAUGAAAUAGUUACUUAUACCGUCCCGUUGGGACUCAUUUUUUAUCUAUUCUGCCCACACCUAGCACUUGCGU.GCUAGGCUGAGUUAUGUCGUCCCCUUGGGACUCAAACUGAGUGCC
Lar-1-16	GACGGAUAUUAAAAUAUCCUUAUCAUGUUGUUAACAUUGAUUGUUUAUUUUUAACAAUAUGUUGUCCUCUUGGAACUCAGUUUUUGUUUUAUCUACGUACCUAGCACUUUCGU.GCCAGGCUGUACUAUAUCGUCCCUUUGGGACUCUAAUUGAAUGCC
Lar-1-17	GACGGAUAUUAAAAUAUCCUUAUCAUGUUGUUAACAUUGAUUGUUUAUUUUUAACAAUAUGUUGUCCUCUUGGAACUCAAACUUUUGUUUUACCUGCAUACCUAGCACUGUCGU.GCCAGGCUGUACUAUACCGUCCCUUUGGGACUCUGAUUAAAUGCC
Lar-1-18	UGGGAUAAUGACAGAAGUUCAUGCAUCAGAAUGCCGAAGGCAUGACAUAGAGCUCUAUGCCGUUCCUUCAGAACUCACAAUAGUGUUUUUGUUUAGUAGUCCUAGCCCUUACGG.GCCAGGCUAAAUUAUGCCGUGCUUUUAGCACUUCUUUUGAAUGCC
Lar-1-19	GCAGCAGUUCACUUCAAAUCAAUGGUGACACGGGGAUAGCUUAAACAUAUCACUCUAUGCCGUUCCUUCAGAACUCACAAUAGUGUUUUUGUUUAGUAGUCCUAGCCCUUACGG.GCCAGGCUAAAUUAUGCCGUGCUUUCAGCACUCAAUUUACCUAAU
Lar-1-20	UGGGAUAAUGACAAAAGUUCAUUCAUCAGAAUGCCGAAGGUAUGAUAUAGAGCUCUAUGCCGUCCCUUCAGAACUCACAAUAGUGUUUUUGUUUAGUAGUCCUAGCCCUUACGG.GCCAGGCUAAAUUAUGCCGUGCUUUCAGCACUUAUUUUGAAUGCC
Lar-1-21	GCGAGCCAGGCUAAGUUAUGUCGUGCUUUCAGCACUCAAAAGCAAUUUGAUUGAUUUCCUGAUUGUUUUAAAUGACUCACUUUAUUGUUUGUUUUGUUCACCUAGCUCUGGCGA.GCCAGGCUAAGUUAUGUCGUGCUUUCAGCACUCAAAAGCAAUCUG
Lar-1-22	UUUUUUAUGAAGUAUUUGCUUCCGUAGUGGAGAAGUCAUGGGGGGGGGAUAGAGCUCUAUGCCGUUCUUUCAGAACUCUCUUUAUCGUUUGUUUUGUUCACCUAGCUCUGGCGA.GCCAGGCUAAGUUAUGUCGUGCCUUCAGCACUCAAAAGCAUUUG
Lar-1-23	CGAGCCAGGCUAAGUUAUGUCGUGCUUUCAGCACUCAAAAGCAAUCUGAUUGAUUUCCUGCUGGUUUCUAAAUAACUCCCUUUAUUGUUUGUUUUGUUCACCUAGCUCUGGCGA.GCCAGGCUAAGUUAUGUCGUGCCUUCAGCACUCAAAAAUGAUUUA
Lar-1-24	UAUGAUGUUGAAUAUAACGAAAAAUAUUUAUGGGAGUGAGUUUUGAUUUGGAAGUUAUACCGUCCCGUUGGGACUCACAUUAACAACCGUCACUUUAUUUCCUAGCACUUGCGU.GCCAGGCUGAGUUAUGCCGUGCUUUCAGCACUCAAUGAUUAAUCG
Lar-1-25	UCACAUUAACAACCGUCACUUUAUUUCCUAGCACUUGCGUGCCAGGCUGAGUUAUGCCGUGCUUUCAGCACUCAAUGAUUAAUCGCAAUUCAAAUUUAGACCUAGCACUUGCGU.GCCAGGCUGAGUUAUGCCGUGCUUUCAGCACUCAAUGAUUAAUCA
Lar-1-26	UUAUAUUUAGUUUUUUCAAAAUUAUUAAAUGCAUAAAUCAUUCGAUGUUGAAGCAUUAUUUACACUUUUGGUAUUCACAUUAACAACAGUAUUUAUUUCCUAGCACUUUCGU.GCCAGGCUGAGUUAUGCCGUGCUUUCAGCACUAAAUUGAAUGCG
Lar-1-27	UUGGGCAUAAAUCGGAUGAUAAAACAAAUGAAUGCCGAAGGUAUGGUAUAGGAUUUAUACCGUCCCGUUGGGACUCACAUUAACUACGGUCACUUUAUUUCCUAGCACUUUCGU.GCCAGGCUGAGUUAUGCCGUGCUUUCAGCACUCACAAUUAUUCGG
Lar-1-28	UUGGGGAAAUAUGUAAAUUCAAAACGAGAAUGCCGAAGGUAUGACAUAGGGAUUUAUGCCGUCCCCGUUGGGACUCAUCAUUAAUAAUCAUCAUUUAAUUCCUAGCACUUACGU.GCCAGGCUGAGCUAUGCCGUGCUUUCAGCACUCAAUGAUUAAUCG
Lar-1-29	AUGAUUAAUCGCAAUUCAAAUUUAGACCUAGCACUUGCGUGCCAGGCUGAGUUAUGCCGUGCUUUCAGCACUCAAUGAUUAAUCACAAUUCAAAUUUAGACCUGGCACUGCCGU.GCCAGGCUGAGUUAUGCCGUGCUUUCAGCACUCAAUGAUUAAUCG
Lar-1-30	UGGGAAAAAUAUGUAACUCGCACAAAGAGAAUACCGAAGGUAUGACAUAGAGAUCUAUGCUGUCCCGUUAGGACUCAUCAUUAACAGCCAUCAUUUAAUUCCUAGCACUGCCGU.GCCAGGCUGAGUUAUGGCGUGCUUUCAGCACUCAAUGAUUAAUCA
Lar-1-31	AUGAUUAAUCACAAUUCAAAUUUAGACCUGGCACUGCCGUGCCAGGCUGAGUUAUGCCGUGCUUUCAGCACUCAAUGAUUAAUCGCAAUUCGUAUUUAGACCUAGCACUGCCGU.GCCAGGCUGAGUUAUGCCGUGCUUUCAGCACUUAAUUGAAUAC
Lar-1-32	AGAACUCCAGGAACAUUAAAGAUGGUAGUUUUGAAUUAAAUAUUGAGAGUGAGUUAUGCCGUCCCUUCGGGACUCCUUAUUCUUUCAAUGACGCAUAGACCUAGCACUUCCGU.GCCAGGUUAAUGUAUGGCGUGCCUUCGGCACUCAACAAUUAACCA
Lar-1-33	AGCCUUGGGUGAUUUAUGAUAAACAAAUUGUGAAUGCCGAAGGUAUGAUAUACGGCGCUAUGCCGUCCCUUCGGGACUCAUUAUUUUGUUACUUAUAGACUGGCACUUCCGU.GCCAGGCUAAUGUAUGUCGUGCUUUCAGCACUCAAUAAUUAACCG
Lar-1-34	GUAGAUUUUUGAGGUUUCUCAAGGAAGAGGUGUUUUUAUGAGGGGUGAGGAGUGGGCUAUGCCGUCCCUUCGGGACUCUAUUUUUUGUUGCGUAUAGACCUAGCACUUCCGU.GCUAGGCUAAUGUAUGUCGUGCUUUCAGCACUCAAACUAAUGCCA
Lar-1-35	GCCUUGGGAAUCUGUGAUAAACAAAUUGUGAAUGCGGCCGGUAUGAUAUAGGCCCUAUGUCGUCCCUUUGGGACUCGUUAUUUUUCUUUGUGGCGUGUAGACCUAGCACUCCCGU.GCCAGGCUAAUGUAUGUCGUGCCUUCAGCACUUAAAUGAAAGUG
Lar-1-36	GCUUGGGUUAGGGUUGUUGGCCAUGCAUUGAAUGCCGAAGGUAUGGUAUAGGAAUUUAUGGCGUUCCUUCAAAACUCGCAAUGGAGUUAGAUUUCGCAAACCUAGUUCUGACGA.ACCAGGCUCAGUUAUGCCGUGCUUUCAGCACGCGAUUUUGCUAAU
Lar-1-37	GUUGGGGUCUCGUGUGGUAAGACAUAGGAAUGCCGAAGGUAUGGCAUAAGUAUUUAUGCCGUUCCUUCAGAACUCGCAAUGGAGUUAGAUUUCCCAAAACCUAGUUCUGACGA.AUUAGGCUCAGCUAUGCCGUGCUUUCAGCACUCGGUUUGUCUAAU
Lar-1-38	AAACCCCGCGCCACAGCGGCUCUGCCUGAGUAUGUUGAUUUAAUUUAUCGAACUCUAUGCCGUUCCUUCAGAACUCACUAAUAAUCAGUUAUUUACAUUCUCUAGCUCUCACGA.GCUAGGCUAAAUCAUGGCGUGCUUUCAGCACUCUAUUUUUCUAUA
Lar-1-39	CUUGGGGAAGAGAGGUGAUUAGUGUUUUGAAUGCCGUAGGUAUGGCAUAGGCCCUAUGCCGUUCCUUCAGAACUCCUGAAUCUUUGAUCAUAUGUUAUGCGUAGUUCUCACGA.ACUAGGCUAAGUUAUGGCGUGCUUUCAGCACUCAAAAAAUUCAAC
Lar-1-40	AAACUCUGCGCCACAGCGGCUCUGCCUGAGUGUUUUGAUUUAAUAUAUCGAACUCUAUGCCGUUCCUUCGGAACUCGUUAUUAUAGGCCAUAUACAGACUCUAGUUCUCACGA.ACUAGGCUAAGUUAUGGCGUGCUUUCAGCACUCUAUUUAGUCGGC
Lar-1-41	GCCUUGGUGGAAAUGGAUAAUGAUAUUUUGUGAAUGCCGAAGGUAUGAUAUAGAGCCCUAUGUCGUUCCUUCAGAACUCAUGUUUUCAUUGAUUUUUUACCUAGUUCUCACGA.ACUAGGCUUAGUUAUGGCGUGCCUUCAGCACUCAAAACAAGACUA
Lar-1-42	GAACUCGUGAUUUUCCUUGUUUCACAUAUACCUAGCUCCACGAGAUAGGCUAAUUUAUACCGUGCUUUCAGAACUCGUGAUUUUCCUUGUUUCACAUAUACCUAGCUCUCACGA.GCUAGGCUAAUUUAUGCCGUUCCUUCAGAACUCGUGAUUUUCCUU
Lar-1-43	GUAGGCUAAUUUAUACCGUGCUUUCAGCACUCAAAUUAAGCAUGGAUUAGAUCACUAUAGCGUUCCUUCAGAACUCGUGAUUUUCCUUGUUUCACCUAUACCUAGCUCUCACGA.GCUAGGCUAAUUUAUACCGUGCUUUCAGCACUCAAAAGUAAAUCU
Lar-1-44	UUAAAGACUUUAGCCGAUCUAUUAUUUAACUAAAAUUCAGCAUGGAUUAGAUCACUAUAGCGUUCCUUCAGAACUCGUGAUUUUCCUUGUUUCACCUAUACCUAGCUCUCACGA.GCUAGGCUAAUUUAUACCGUGCUUUCAGCACUCAAAAGUAAAUCU
Lar-1-45	GUAGGCUAAUUUAUACCGUGCUUUCAGCACUCAAAUUAAGCAUGGAUUAGAUCACUAUAGCGUUCCUUCAGAACUCGUGAUUUUCCUUGUUUCACCUAUACCUAGCUCUCACGA.GCUAGGCUAAUUUAUACCGUGCUUUCAGCACUCAAAUUAAGCAUG
Lar-1-46	GUGAUAAUAAUGUACAUAGUCAUAUUAUGAACACUUUAAGCAUGGAUUAGAUCACUAUAGCGUUCCUUCAGAACUCAUGAUUUUCCUUGUUUCACCUAUACCUAGCUCUCACGA.GCUAGGCUAAUUUAUACCGUGCUUUCAGCACUCAAAUUAAGCAUG
Lar-1-47	CCUGCAGUUACCUGCCACCUGUCAUUUUAGUGCCAAUUAGUGUGGAUUAGAGCACUAUGCCGUUCCUUCAGAACUCGUGAUUUUCCUUGUUUCACAUAUACCUAGCUCUCACGA.GCUAGGCUAAUUUAUACCGUGCUUUCAGAACUCGUGAUUUUCCUU
Lar-1-48	UGGAAUAAUAAUGUACAUAGUCAUAUUAUGAAUGCUGUAAGCAUGGAUUAGAGCACUAUGCCGUUCCUUCAGAACUCGUAAUUUUUUUGUUUCACAUAUACCUAGCUCUCACGA.GCUCGGCUAAUUUAUACCGUGCUUUCAGCACUCAAAAGUAAAUCU
Lar-1-49	GGGGAUAAUGACAAAAGUUCAUUCAUGCAGAAUGCUGUAAGUAUGAAAUAGAGAUCUAUACCGUUCCUUCAGAACUCAAUUACUUUUUCAAAAUCACAGACCUAGUUCUGACGA.ACUAGGCUGAUCUAUACCGUUCCUUCAGAACUCGAAGUCCAUCAA
Lar-1-50	UCGGGACUCUCAUUUUACUCUAUCUUCUAAUCCUAGCUCUCACGAGCCAGGCUAUGUUAAGCCGUCCCUUCGGGACUCUCAUUUUACUCUACCUUCUAAUCCUAGCUCUGACGA.GCUAGGCUAAGUUAAUACGUACUUACAGCACUCCAAUCAAUCUG
Lar-1-51	CUUGGGGAGAUCACAAACCCAAACAGAAGUGAAUGCCGAAGGCAUGGAUUAGGGCCUUAACUCGUUCCUUCAGAACUCCUAUAUUUAUCACAUACGCUAAUCCUAGCUCUCACGA.GCCAGGCUAUGUUAAGCCGUCCCUUCGGGACUCUCAUUUUACUCU
Lar-1-52	CAGAACUCCUAUAUUUAUCACAUACGCUAAUCCUAGCUCUCACGAGCCAGGCUAUGUUAAGCCGUCCCUUCGGGACUCUCAUUUUACUCUAUCUUCUAAUCCUAGCUCUCACGA.GCCAGGCUAUGUUAAGCCGUCCCUUCGGGACUCUCAUUUUACUCU
Lar-1-53	GUUCUCUGCAGAGUAAAAACUCUCUGCACUAAUGCCGAAGGCAUGGAUUAGGACCUUAACUCGUUCCUUCAGAACUCCUAUAUUUAUCACAUACGCUAAUCCUAGCUCUCACGA.GCCAGGCUAUGUUAAGCCGUCCCUUCGGGACUCAAAUAUGAAUGC
env-1	AAAAUCCAAGUAGAAAAAUACCUUGUAUCGUGAAUUUAUUGUAAUUGGCUAAAGAGGUGUUGCGUCCUUUCAGGACUUUUUCGAGGCUUGUGAUUGAUCAGGGAUUUC.AU.CCCUGGCUAAUAUAUUUGUGCUUUCAGCACUUCUUUGAUCGAGA
Aba-1-2	CCACGCCCGCAUGGGCGGCUUCGGAGAUCUUGGCAAUCAAGUCGCCCUUCAGCGCCCGCCGGUCCGUUAUGUAAUAGAGGAUCAACCCAACAUUCUAGCUCUCGCUUCGGAAGU.GGUGGGCUAUUGCAUCGCGUGCCUUCGGCACUGCCUUACUGCUUC
env-2	UUGGCGUUCUCCUUUGGUGUAGUCCAACUCUAGGAUAGGAGGCUUUGCAAAAAUUCGAUUCUGUCGCCCCUGCGGGGCUCGUCACCUUAACUACUGUCCCACGGCUGACGC.UGUGGGCUACUCUGUGACGCCGCUACGCGGCUGAUUUAGCUCGCG
Aba-1-3	CGCGAUUGAGAGCGAGUUGGUUUGAUGUGGCCGGGAAUUCCGUCCCCUGAUGGGGGCUCCAAUGCGGCUAAGAUACCGGUUCUUUCGUGGAUUCGACGUGCCACCACUCACGU.GGUGGGCUAUCUCGUUCCGAGCCUGUGGCACUGUGAGGCAAUCCG
Aba-1-4	CGUUGCAGGUUGGAUCGUGAACCAUUUCUUACAAGGGGAACUGAGCUGAAAGCGCAUACUGUCCGCAGAGGCGGGCUCGUGUAGGCCACACACACGAAACCCACCUCUUACGA.GGUGGGCUAUUUUCUUGCGUGCCUUCGGGACUCAUUACGAGUAG
Aba-1-5	AAGGGGAGGCUUCCGCGGACGCGGCCGAUCUUGCCGGGAGAGGUGAUCGCGGAGUGAUGUCGUCCGCUAAAGCGGACUCUCGACGUGGAGCUUGGCUUGUCCCACCACUCUCGU.GGUGGGCUUAUUUAUUGCGUCGCUGCGCGACUCUCUGAUUUCUUA
Aba-1-6	UCGUGCAGCUUGUCGAAUAGGUCGUCGGUCUCUUCGAUCAUCCGUUUGCCCCGCUGUGUGGCGUCCGCUGACGCGGACUCUGUGAUUCGCUGACCCGUUCCCACCUCUUACGA.GGUGGGCUAUUGCAUUUCGUCGCUUCGCGACUGGUUGUCGGCGC
Aba-1-7	UCGGGCGUGAUGGUCGUCACGCGAUGUUGUGAGAUGCGCCGAAGUGUCGUCCCCUGAAGGGUUCUCGGGAGACCGAGAGAAGCUAGACCGUGCCCGUGUUCCCUCCACUCACGU.GGCGGGCUAUUUCAUUGCGUCGCUACGCGACUGGGGUUGCGGGAG
Aba-1-8	CAGGUGCGACGAACGAUUCAACAAAGGCAGCGGCCCCGAAUGCCGUCCCAUGAAUGGGACUCCGACCUUUUGUUUAGCUUCUUUCCUUGCUUCGUCUCCGUUUUCCCACCACUCACGU.GGUGGGCUAUUUCAUUCCGUCGCUGCGCAACUGAGACUUUGGUGC
Aba-1-9	UGGAUGGGGGCAAGGAAUCGGUGAUUUCGGACACAGGUUGGUGCGAUGGAUCACGAGGAGGUGUGUAAACAACGGGUGCCGUGGGGUGAAGCUUUCCCACCGCUUACGC.GGUGGGCUAUUACGUUGCGUCGCUUCGCGACUGUUGAGAUGCGGAU
Aba-1-10	GAUGGCGACGAAGAACAGCGCCAGCAGAUGUGUUUUGCUAAUCACCAUGCAGUUGGAUGCCGUCGCGCUGCAGGGGACGUCUGACUAUCUCGCAUUUCUUCCCACCGCUUACGC.GGUGGGCCAUUGCAUGGCGUCGCUGCGCGACUGGAAAUGUGGAC
Aba-1-11	AAAUGGCGUUGAGCAUCCUCACGACGAAAACCGGCGGGAUGUUGUAGGUUCCCGGGGCGUCCCCUGAAGGGGACGUCGGUUUCUUUUCCUCUUCCAUUUCCCACCGCUUACGC.GGUGGGCUAUUUCAUUCCGUCGCUGCGCGACUGGGAUUUCGGCGC
env-3	UUGCGCGUGGGCCCUCCCCUUUAGGGGCUGGGGGCGCGGGUGGGCUCGUAGCGUUCACAAAUGGCGCACUUACAGCGCUUGAAUCCUGGUUCCUUCCCUCCUAGGGCUUACGC.CCUAGGCUAAUAAGUAGCGCGCCUACAGCGCUUUCUAUUUAUGU
env-4	CCACUGACCACUAAAAAUCCUUUAGGGGUUGGGGGCGCGGGUGGGGCGCAACUUUCACAAAUGGCGCACCUACAGCGCUUGAAUUUCGUUUAUUCCCAUCCUAGGGCUUACGC.CCUAGGCUAAUAAAGUACGGGCCUUCAGCCCUUCCUUAUUUUUG
env-5	CCACUGACCACUAAAAAUCCUUUAGGGGUUGGGGGCGCGGGUGGGGCGCAACUUUCACAAAUGGCGCACUUACAGCGCUUGAAUUUCUGUUUAUUCCCAUCCUAGGGCUUACGC.CCUAGGCUAAUAAGGUACGGGCCUUCAGCCCUUCUUUAUUUUCUG

alignment positions 161 ··· 320

Lar-1-1	UUUAUUUUUUCAUAGCCUCUUCAGUGUGCAUUAAAAAUGUGGACAAAAAGGUCUUUUGGGAUCGCCAAGCACUUGCUUGGCAAACAGGGGGCUAAAAAAGGCACGUAGUUCAAGCUUCAGCUUGCUUAAAAAAACAUAUAAAACCUUCUCCUCAUAACAG
Lar-1-2	UUUUUUCCACAGUCUCUUUGUUUUUCCUAAAAUGCGGGCAAAAAGGUCUCUUGGGAUCGCCAAGAACCAGCUUGGAAAUCGCAGACUACGUGCCCCGUAGGUGUCGGACUUUCAGCCCUCCUUCGUUUAAUCACGUCAGUUCGUGUGGCUCACGCCACAC
Lar-1-3	CACACUGCCUCGACAGCCUUGAGCUAUAUUAUUCCUUUGACACUAAAAUUGACAUCUUUUUAUCUAUCUUGUUGCCAGAUCAAUAGCUUAUUUCUUACCACGCUUAUUGGCAUCUGGAUUACCCUCGUCACCACCGCGUUUCACCAUAACAGGAAACUGC
Aba-1-1	CUUACGGUUUUUCUUUUUAGACGUUGUACCCGGCGAUCCGGCACCCGCCACCUUCGCGCGCCGCUGCCGCGAAACUUCGUACAUCACGAUGCCGCCGGCCACGGAAACAUUAAGCGACGAGACCUCGCCCGCCAUCGGGAUUUUCACCAGCAGAUCGCAU
Lar-1-4	UCAAGUCCUGAAAAAAUAGUUGUCACUUUUGUUUAUGUUAAUUGACUAAUAUUUAUGCACAUUGUGUUUCAUGAACAACACUUGGAAAUGAAUAUUCCAAAGCUGUAUGAGGGCGACAAUUAUUGUAUAAGUAAAUAGCCUGCUUAAUUGCUUUUUUAGCA
Lar-1-5	GCAGGCAUGCUAUAAAAUAGCCCAAUGGCGUGAGCCUUGGGGGUAAAAUGUAAGAUUAAAGGAGAAUGCCAAAGGUACGGUAUAGGGCUCUAUACCGUACCUUCGGCACUCGAAUUUAUAUUCAUUAUUAUUUACUAGCACUGACGUGCUAGGCUGAGUU
Lar-1-6	GCAGGCAUGCUAUAAAAUAGCCCAAUGGCGUGAGCCUUGGGGGUAAAAUGUAAGAUUAAAGGAGAAUGCCAAAGGCAUGGCAUAGAGCCCUAUACCGUACCUUCGCACUAGAUUUAUUAAUCUGAUAACUAAAAGCGAAGCGAUCUGACAACUGAUGACU
Lar-1-7	GCAGGCAUGCUAUAAAAUAGCCCAAUGGCGUGAGCCUUGGGGGUAAAAUGUAAGAUUAAAGGAGAAUGCCAAAGGCAUGGCAUAGAGCCCUAUACCGUACCUUCGGCACUCGAAUUUAUAUUCAUUAUUAUUUACUAGCACUGACGUGCUAGGCUGAGUU
Lar-1-8	GAAGGUACGGCAUAGGUUCUUAUACCCUAGCUACCACUCAACGACUCAAGGAAUGCUGAAGGCAUGGCAUAACUCAGCCCAAUGGCGUCAGCCUUGGGAGAUGGAACACACGAUUCAAGUUGAAUGCCAAGGGCAUGGCAUAGGGCCUUAUACCCUAGCC
Lar-1-9	GAAGGUACGGCAUAAAGUACCCACAUAUGACUAAGGUUAACACAAAAGGUCGGACUAAAGAACUAUAGAGAAUUAAUUGUCUAACCUAUCAAUUAAAGACAAUGCGCAGCAGGAGCUACGCGGUCCAAUAAAAAACUUUGUAUCUUGGCGCCUUCGCGAG
Lar-1-10	GAAGGCAUGGUAUAACUUAGCCCAAUGGCGUAAGCCUUGGGGGGUGGAAUAACACAAUUAAAAUUGAAUGCCGAAGGCAUGGCAUAAAGCUUUAUACCUCUCCUUCAGAACCCGAAGUCCAUCUAGGACUUUGUCCUUGAUGAAUUUUUACUGUUAAAUA
Lar-1-11	GAAGGCAUGGUAUACCUUAGCCCAAUGGCGUCAGCCUUGGGGGAUGAAAUACACGAUGAAAAUUGAAUACCAAGGGCAUGACAUAGAUCUCAUAAGGGUAGGGAAGACUCCUUGCGGAGUCUCCCCUCCUUCCGAACCGUACGUGCGGUUCUCCCGCAUA
Lar-1-12	GAAGGCAUGGUAUACCUUAGCCCAAUGGCGUCAGCCUUGGGGGAUGGAAUACACGAUGAAAAUUGAAUGCCAAGGGCAUGGCAUAAAGCCUUAUACCGUACCCUGGGUACUCCGGCACUCUAUUUUUUCGAUCAAAAAAUCUCAUUUUUGAAUCGAGUUU
Lar-1-13	UUGGAAUGCCAAGGGCAUGACAUAGAGCUCUAUGCCGUACCUUCGGCACUGAAUGUGCUUAUUUCUUACGUACUUUUUGAGGGAUUUCUACGACUUGGUAUUUUUCUCGAAGAUUGGCUAAUUCGAUUUUCAGCUUUGCGAUUUCCGCAGCGUAUUCAGG
Lar-1-14	GAAGGCAUGAUAUAUUGAUCUAUGCCGUUCCGUUGGGACUUGGUUUGAAUGCCGAAGGCAUGAUAUAGUACGCCUAUGCGUACACAACAUCAGCGAGGUGAAAGACCUCGUCAGGGUUGACCGUCAGCCCUGUAGUCGAAGUCAACUGCGUCGUUGUGAA
Lar-1-15	GAAGGCAUGACAUAAUAUAGCCCAAUGGCGUGAGCCUUGGGAUAAGUAUAUGAUUCCCACAAAGAGAAUGCCGAAGGUAUGAUAUAGGGAUCUAUGCCGUCCCCUUGGGACUCAAACUGAGUGCCGAAGGCAUGACAUAAUAUAGCCCAAUGGCGUGAGC
Lar-1-16	GAAGGCAUGGCAUAUUUCAGCCCAAUGGCGUCAGCCUUGGCUAUGAUUGGAUAAUAAUAUAAUUGAAUGCCGAAGGCAUGGCAUAGAAAGCCUACACUUUCAACUUACUGAAAUAAUCAUUUGAGUCGAUCGCCUUUUAGGGAGUAGGCUUGGCUUUCUU
Lar-1-17	GAAGGCAUGGCAUAUUUCAGACCAAUGGCGUCAGCCUUGGGUAUGAUUGGAUAAUAAUAUAAUUGAAUGCCGAAGGCAUGGCAUAGAAAGACUGCACUUUCAACUUACUGAAAUAAUCAUUUGAGUCGAUCGCCUUUUAGGGAGUAGGCUUGGCUUUCUU
Lar-1-18	GAAGGCAUGACAUAGACCUCCUACCCUUUCUUCACAACUCGAUGCCCAUCUAAUUCAUCGCCAAAUAGCUAGCUUCACAACCUAAAAAAAAUAAUAUUUGUUUCAAAUACUCGUUACGCUGACUGCAUCCACUCCUCUAGUUUAGUCAGAAGAACACAACA
Lar-1-19	AAAUCAAGCCUUGUUGUACCGUACUUUCUGCACUUAUUUUGAAUGCCGAAGGCAUGAUAUAACACAGCCCAAUGGCGUAAGCCUUGGGAUAAUGACAGAAGUUCAUUCAUCAGAAUGCCAAAGGCAUGACAUAGAGCUCUAUGCCGUUCCUUCAGAACUC
Lar-1-20	GAAGGCAUGAUAUAACACAGCCCAAUGGCGUAAGCCUUGGGAUAAUGACAGAAGUUCAUGCAUCAGAAUGCCGAAGGCAUGACAUAGAGCUCUAUGCCGUUCCUUCAGAACUCACAAUAGUGUUUUGUUUAGUAGUCCUAGCCCUUACGGGCCAGGCUA
Lar-1-21	AUUGAUUUCCUGCUGGUUUCUAAAUAACUCCCUUUAUUGUUUGUUUUGUUCACCUAGCUCUGGCGAGCCAGGCUAAGUUAUGUCGUGCCUUCAGCACUCAAAAAUGAUUUACUUUAUUGAGGGCUAUUCUAUCAAACUGAUUUAAUACUGAUUUUCAGGA
Lar-1-22	AUUGAUUUCCUGAUUGUUUUAAAUGACUCACUUUAUUGUUUGUUUUGUUCACCUAGCUCUGGCGAGCCAGGCUAAGUUAUGUCGUGCUUUCAGCACUCAAAAGCAAUCUGAUUGAUUUCCUGCUGGUUUCUAAAUAACUCCCUUUAUUGUUUGUUUUGUU
Lar-1-23	CUUUAUUGAGGGCUAUUCUAUCAAACUGAUUUAAUACUGAUUUUCAGGACCACUUGUCGUUGAGGAUGCGGAUUUCGUGACAGAGGGCAUCCUUGACGCGUUGUUUAUAAACGUAGAUGGUGGCUUCGGCAAUGCCGGUGAUUUCUGCGAUUUCCGCAGU
Lar-1-24	CAAUUCAAAUUUAGACCUAGCACUUGCGUGCCAGGCUGAGUUAUGCCGUGCUUUCAGCACUCAAUGAUUAAUCACAAUUCAAAUUUAGACCUGGCACUGCCGUGCCAGGCUGAGUUAUGCCGUGCUUUCAGCACUCAAUGAUUAAUCGCAAUUCGUAUUU
Lar-1-25	CAAUUCAAAUUUAGACCUGGCACUGCCGUGCCAGGCUGAGUUAUGCCGUGCUUUCAGCACUCAAUGAUUAAUCGCAAUUCGUAUUUAGACCUAGCACUGCCGUGCCAGGCUGAGUUAUGCCGUGCUUUCAGCACUCAUAAUUGAAUACCGAAGGUAUGCC
Lar-1-26	UAAGGCAUGUUAUAAUGUAGCCCAAUGGCGUGAGCCUUGGGCAUAAAUCGGAUGAUAAAACAAAUGAAUGCCGAAGGUAUGGUAUAGGAUUUAUACCGUCCCGUUGGGACUCACAUUAACUACGGUCACUUUAUUUCCUAGCACUUUCGUGCCAGGCUGA
Lar-1-27	AGGAUUAUAUAUUAACGAAAUUUACGCCAGUACCAUUCGAGGCAGAAGAAGAAAAGAAUAAGGCUUAGGAUAUAAAAGUUAUCCCAGAGAGGGGUUCUUUUGACUUGGAGUGAGACUGAAUUAACACGGCCAGUUUGAUUCAUUUUAGUGAGCCAAUCAG
Lar-1-28	CAAUUCGUAUUUAGACCUAGCACUGCCGUGCCUGGCUGAGUUAUGCCGUCCCGUUGGGGCUUGGUUUGAAUGCCGAAGGCAUGAUAUAGUACGCCUAUGCGUACACAACAUCAGCGAGGUGAAAGACCUCGUCAGGGUUGACCGUCAGCCCUGUAGUCGA
Lar-1-29	CAAUUCGUAUUUAGACCUAGCACUGCCGUGCCAGGCUGAGUUAUGCCGUGCUUUCAGCACUCAUAAUUGAAUACCGAAGGUAUGCCAUAGGGUUCUAUGUCAUAUUCUUGGCGUUCAAAGUAGUAUUUUUUAAAUCUGGUAGAAUUUGGCGGCGUUGGCG
Lar-1-30	CAAUUCGUAUUUAGACCUAGCACUGCCGUGCCAGGCUGAUCUAUGCCGUCCCGUUGAGGCAGAUACGAAUUAUACCAAAAUUAAUUGAGUAUCACCUGAGAAAAUUUGCUUUUGAUUUCAUGUCUACAUAAUUUAAAGUCAUAAGGAAAGGAGAAACCAU
Lar-1-31	CGAAGGUAUGCCAUAGGGUUCUAUGUCAUAUUCUUGGCGUUCAAAGUAGUAUUUUUUAAAUCUGGUAGAAUUUGGCGGCGUUGGCGCCGAAGACUUUGGCUUCUGCAGUGUCUGAGAACUGAGUUGUGAAGUCGUGGGCGAUUUUGUAGGUUUCUGAAUA
Lar-1-32	CAAUGAGUUCUAUUAGCACUUCCGUGCUAGGCUGAGUUAUAUCGUCACGCUAGGACUCUCAAAUUAUUGUUGUAUUUGAAUGCCGAAGGCAUGAUAUAGUACGCCUAUGCGUACACAACAUCAGCGAGGUGAAAGACCUCGUCAGGGUUGACCGUCAGCC
Lar-1-33	CAGUGAGUACUUCUUACAAUUCCGUGCCAGGCUAAUGUAUGUCGUGCUUUCAGCACUUAAACUGAAUGCGGUCAGUAUGAUUAUUCAGCUUCAGAUUUUUUCCGGUAAUCACCUUUGGGAUUGCCGUCAAAGUUCGUACAGAUACGGGCUGGAUCUAUUU
Lar-1-34	AAGGCAUGGUAUAAUAUAGCCCAAUGGCGUGAGCCUUGGGAAAAAUGUGGGCCUCCCAAAAAGAGGUGCCGAAGGUAUGAUAUAAUACAGGCCAAUGGCGUGAGCCUUGGGUGAUUUAUGAUAAACAAAUUGUGAAUGCCGAAGGUAUGAUAUACGGCGC
Lar-1-35	GUCGGUAUGAUUAUUCAGCCGCAGAUGUUUUUCGGUAAUCACCUUUGGGGUUGCCGUCAAAGUUCGUGCAGAUACGAGCUGGAUCUAUUUUUUGGAGCUUAAAGUCCUGCAGGCGUUUUAAGUCCUUAUCCCGUUUCUUGGUUAGCGCAGAAAUUUUAGU
Lar-1-36	GAGUUAGACUAUGUAGUGGCGUUUGAACAUCACUUGUCUUGAAUGCUGAAGGCAUGAAAUAAUAAAGCCCAAGGCGUGAGCCUUGGGGGUCUCGUGUGGUAAGACAUAGGAAUGCCGAAGGUAUGGCAUAAGUAUUUAUGCCGUUCCUUCAGAACUCGCA
Lar-1-37	AAACUAGGCUCAGUUAUGGGGUGUAUUCAGCACUCGAAUUCUAUAUAUUAUUCAUGAACCAGGCUGAGCUAUGCCGUGCUUUUAGCACUUCUGGCAUCGGUAUUUAUAUGAUAGGGUAACUAUUUAACUGGCUUGGGUAGGAGGCCGAGUUUGGCGCUUG
Lar-1-38	ACUCCGCGGCCGAGGCAGAUGAGGUCGUCGUAAAAGAUGCAGGAUUGACCUGGGGCAAUGCCCUUAUCUUUUUCAGCGAGCUUAAUGAAAACGCGACUUUCGCCGAGAUCCAUGAUGCGGGCUUCAACGAGUUGAGGGCCAUGACGGAGUUUUACUUUGA
Lar-1-39	UGAGCGCUAGAUAUUUCAUCCAGUGACUUAAAGAUAGCCAGAAACUUCCUUAAUUACCCUAGUUUUGAUGGCUCUAAGAAGAUUUUUCUAAGUUCUUGUUUGCACUGGUUUAUUUCAAAAAUGUGUUAUUUUACGGGCAUUAAAUUUUAAAGGAAUCACA
Lar-1-40	AUCUCAACCAGCUCUGCUCGUGACUAUUUUACUUUUAAACAUGUAGUGAGAAGUGCCGUUUUGUAUACAUAUCAAAACCCCGCGCCACAGCGGCUCUGCCUGAGUAUGUUGAUUUAAUUUAUCGAACUCUAUGCCGUUCCUUCAGAACUCACUAAUAAUC
Lar-1-41	AAAGUUUUAAUCCUCUGCGCCCCAGCGACUCUGUGUGAUAUGAAAUAUACCAUAGGCAAGAUUCCUACGAGCCCUAAAACAGCAAGCCCUUAAGCCUGUAACUUCUCGGUGUUCUCUGUGCAAUCUGUGGUGAAAUUUUUCUUGAAACAUAAGUACUAAU
Lar-1-42	GUUUCACAUAUACCUAGCUCUCACGAGCUAGGCUAAUUAUAAGGGUAGGGAAGACUCCUUGCGGAGUCUCCCCUCCUUCCGAACCGUACGUGCGGUUCUCCCGCAUACGGCUCUCCAGUCAGUGCUUACUCCGAAGAGACUGAAAUUCCAGUUUCCAGGC
Lar-1-43	AAUUCAUCGCCAGAUUAAUAGAAACUCAAAACUCAAAACUCAAAACUCAAAACUCAAAACUCAAAACUCAAAACUAACCAUGUCAACAACACAAACUCAACAACCUGAUUUUAUUCAGCGGCACUUAGCCACAAUUGAUUUAUUCACAAUAAAUUUUGUGGCAGUUU
Lar-1-44	AAUUCAUCGCCAGAUUAAUAAAAACUAAAAACUCAAAACUCAAAACUCAAAACUCAAAACUCAAAACUAGUUAUGUCGACAACACAAACUCAACAACCUGAUUUUAUUCAGCGGCACUUAGCCACAAUUGAUUUCCCAUUUUAGUGAUGAUUAGCGUAG
Lar-1-45	GAUUAGAUCACUAUAGCGUUCCUUCAGAACUCGUGAUUUUCCUUGUUUCACCUAUACCUAGCUCUCACGAGCUAGGCUAAUUUAUACCGUGCUUUCAGCACUCAAAAGUAAAUCUAAUUCAUCGCCAGAUUAAUAGAAACUCAAAACUCAAAACUCAAAA
Lar-1-46	GAUUAGAUCACUAUAGCGUUCCUUCAGAACUCGUGAUUUUCCUUGUUUCACCUAUACCUAGCUCUCACGAGCUAGGCUAAUUUAUACCGUGCUUUCAGCACUCAAAUUAAGCAUGGAUUAGAUCACUAUAGCGUUCCUUCAGAACUCGUGAUUUUCCUUG
Lar-1-47	GUUUCACAUAUACCUAGCUCCACGAGAUAGGCUAAUUUAUACCGUGCUUUCAGAACUCGUGAUUUUCCUUGUUUCACAUAUACCUAGCUCUCACGAGCUAGGCUAAUUUAUGCCGUUCCUUCAGAACUCGUGAUUUUCCUUGUUUCACAUAUACCUAGCU
Lar-1-48	AGUUCAUCGCCAGAUUAAUAGCUCUGACGAGGCUGUGUGAAAAAUAAAAAAACGAAAUAUCUAUAUUGUUAGGGCUCACGUGUGAGUCCGCUAAAAUGUAGCAUGCUCCGUGAGGGGCAUGAAAAUGGGUUCGGAAUUAAGCCAGGGCCCAAACUUGGGU
Lar-1-49	UUACUCGCCGAAUUAAUAGAGCUUGGUGUUCCCAGGAAUACUGUCACUCUUUAGUCUUGUUCUUAGUUUGUCCCGCUUCAUGAGUGAUUAUGUCUUUAAAAGCUUUAUUUGUAAAUGAACCAGCUCGUUACUUUGGCAUUUUGAGUACUUGUGGCUCUAA
Lar-1-50	AUUCCUUAUCGAAGUAAUAACUAAUAGUUAUUUAUUUUGCCUGAGUGAUUUUCAGGGUGAAGAUAUUUUUAGAGAGUGUUUUAGUUGGGUUCAGUUGAGGAAUAAUGACUUCAAUUUCCUUAGCGGUCUUGGCAAAGUUGAAUUAAAAUGGAAAUGAAAA
Lar-1-51	AUCUUCUAAUCCUAGCUCUCACGAGCCAGGCUAUGUUAAGCCGUCCCUUCGGGACUCUCAUUUUACUCUACCUUCUAAUCCUAGCUCUGACGAGCUAGGCUAAGUUAAUACGUACUUACAGCACUCCAAUUCAAUCUGAUUCCUUAUCGAAGUAAUAACU
Lar-1-52	ACCUUCUAAUCCUAGCUCUGACGAGCUAGGCUAAGUUAAUACGUACUUACAGCACUCCAAUUCAAUCUGAUUCCUUAUCGAAGUAAUAACUAAUAGUUAUUUAUUUUGCCUGAGUGAUUUUCAGGGUGAAGAUAUUUUUAGAGAGUGUUUUAGUUGGGUU
Lar-1-53	CGAAGGCAUGGCUUAACUUAGCCCAAUGGCGUCAGCCUUGGGAGAUCACAAACCCAAACAGAAGUGAAUGCCGAAGGCAUGGAUUAGGGCCUUAACUCGUUCCUUCAGAACUCCUAUAUUUAUCACAUACGCUAAUCCUAGCUCUCACGAGCCAGGCUAU
env-1	AGAUUUUUCUUGGAUUAAAUAUACAAACACGUAGGGUACGAGCUCUGCUUCGUACCGAAAAAUGUAUAAAGGCCGAUGCGGAGAUCGGCCACUACGAGUGAAUNNNNNNNNNNNNNNNNNNNNNNNNNNNNNNNNNNNNNNNNNNNNNNNNNUCGAAU
Aba-1-2	UCCCGCCCAUACUCCGUCAAAAACGCGCGGACUUUUUCCGGUACGUAACUGCUCUUGCCGUCCUCGAGGUCCGACGUAUCUUGCGACUUCAGCAGCUUCCCAUCUGCGGUAAGCACAUACAAAUGCGGGCAGCCCUUCAGCUUGGGAAACUGCGCCAGGA
env-2	AAGCGAGCGAUACAAACUAGCCCACGGCGACAGGGGUUGUUGCCCAACGCGAAAUCGCAAAGAUGCUGCGAUUCCCCUCCUCCUAGAGGAGGGAUGGCUGCGACCAGUAAGAAAAAGGUCCCGUUCCGAAGCUCGAACAGGCGGGGUGGUCAAAACAUUU
Aba-1-3	CCGUUGUGACCUUCGCACAAUCGCUUGUGAUAACGGUCACUGCCCCUAACCUUGUCUCGUCCUACGCUUUACACGCCAUGUCCAUCAUUGACUCCGCCACCAAGACCGUUGUCCGCGAUUACACCGUUGAGGAACUGAAAGACCAGGCCGCAUUAAUGCG
Aba-1-4	CUGACUUUUCGGUCGCCCUUCACAAUGCGCCCGAUCGUAUAGCCCUUCUCCCCAGCGCGCUCCAGCACUGUCUGUACCUUCUUGAACUUCUUCGCCGGAAUCACCAGCACCAUCCCGAUACCCAUGUUGAAGGUGCGGAGCAUCUCGUCCUGGCUGAUAU
Aba-1-5	GCGAAUCACCAACUUCGUAAACGGAUACACAUAUGCCUCCAAGGUCACGACGAUUCCAACUAGCGCGGCCAGAGCAAUCGAGUGGAAGAAGACGUAGCGGAGGAUUUUGCCUUCGUGGCCGUACCACUCGGUGGCGGUGCUAGCGACAACGAUGCUUUGG
Aba-1-6	UUGGAGCGCUCUGGUGGUUCAAAUACUUCGGCGCGAAUUGUAUCGCGCUCUUUGAGAUUCGCAGGGGCUAAAGCCCUCUUUCUUACUUCGCGCUGCGGCAGCGCUGAAGCGCUGCCCUGAUACAAAGCAAAUUAGCUCCGCUGCGUUCGCGGUUCGGCAG
Aba-1-7	GCGCUUUGGUUUGAUGGCCGAGAAGAUCAAUGGGGAGAUAACGUAUUGCCAGGUUAGAGCGGCCACGAGCACGGCGGCGCUGAUAUAGCGGACACGAGUUUCACGGGCUAGCGGGAGUAGGACGAGCGAGGCGAUGAUCGCGACGGCAGCGACCGAUGAC
Aba-1-8	GGUUGGCGAAGUGGGAUUGGCGCCGACGGCGCGCUUGGAUUCGGCAGCGGCGGUUUCGGCGCGCGCGGCGGGCUUUUUCCCGGGGAGAGUCGCGGUGUUCGCCGCGGUUUUUGCGGUUUCGCGUUCGGUUUUUUUGGCGGCGACUACUUCGAGUUUCUUU
Aba-1-9	UCAGCGUUUAUUCGAGUUGUGGUAGUCGAAGUUGACGAGGCCGUCGCGGUAGUCGAUGGUCAGGCGGAACUGGCUUAGGAGUGGGAAGCCCAGGAUGCCGCCGAGGGCGAUGCCUUCGCCUUCGCUGAGUUUAUCCAUGCUGAUGGCGACCAUGCGGAAG
Aba-1-10	GAAUCGCAUCUUCGCGCCACUCUGCCGAUAAAUCGGCGGGCUACCUCGAAGCGGAUGGAAUCAAGACGCUGCGGGUGGCGUAACGGCUUCGCGGAUGCGGAGCAGCGAAGUCAGGACUCCGCGCAGAAGUUUGAGGUCGAGAGCGUUGGCGCCGUCUGAC
Aba-1-11	GGUCGGCGGAGUUGGAUUGGCGCCGACGGCGACUUUGCGCUCGGAUGCGGUGGUUUCGGCGCGCGCGGCGGGCUUUUUCCCGGGGAGAGUCGCGGUGUUCGCCGCGGUUUUUGCGGUUUCGCGUUCGGUUUUUUUGGCGGCGACUACUUCGAGUUUCUUU
env-3	GAUUAACCCUAAAAGUGUUUGAUAGCCCUAUCGUAUUUUCCCCCGAAUUCGCCAAAAGCGAACACAGUAAUCAAAUCACCCUUCUCACUCCUCUCACCUUCUCACCCUUCUCAUCUGAUCACUGAAAACUUAAACCCUUUAAACCUUUCACGGAAGCUUC
env-4	AUCAACCCUAUAAGCGUUUGCAAGACCUAUUGCCGUUGCCAUCAAAUACCCGCAUCCCCAAAUACCCAAAUACCGCUCCUUCCGUAGUCUCCGUCCACUCACCCUUUUCAUCUCCUCAUCCACUAA..............................
env-5	AUUAACCCUAUAAGCGUUUGCAAGACCUAUUGCCGUUGCCAUCAAAUACCCGCAUCCCCAAAUACCCAAAUACCGCUCCUUCCGUAGUCUCCGUCCACUCACCCUUUUAUCUCCUCAUCCACUAAAGUCGCAGAUCCCGACAAAGCGAAGCUUUCGUCA

alignment positions 321 · · · 447

Lar-1-1	AUAAGUUUACUUAUAUUCCACAUUGAAUUCUUGGAAAGCGCUAUGCUCACCACCACCCUUGCUGCUAAUUGGAGAUACUUUCCACAUGAGCCAACGGAAUUCUCCGAGUGAUUCACCAUCAGUGGCU
Lar-1-2	GCUACAUUAUGUCGGGCUUCCAGCCCUGAAUCAGCUUAUAUUUAUUUUUUCAUAGCCUCUUCAGUGUGCAUUAAAAAUGUGGACAAAAAGGUCUUUUGGGAUCGCCAAGCACUUGCUUGGCAAACAG
Lar-1-3	UUAUCCCACCAUUUACUGUAAGCUUGUUUAAGUUCAUCCACGAGUUCAGGGUGAGCAUUCGCAAGAUCAUAUAGAUUGCUUGGAUCCUUUUGACAUCAAAGAGCUGCCAAGCAUCUGAAACUCCCC
Aba-1-1	UUCUUGCGCACGAGAUCGUGCAGGCCCUUGCCUUCCGCGCCGAGCACCAGCGCGAGGCUCAUGUUGUAGUCGAUCUCGUCGUAAUUCUGCGUGCCGCGCUCGUCCAGUCCUACGGUCCACACGUUGU
Lar-1-4	UCUUUAAAGUUCUUAAAUGUUUCGCGUAAAUGAUAUUCAUACUUCAGUAUUCCGUUGACUCUUUCAGCUUUUGAAUUUUCAUAGCAAUGGUUUUCUUCAGUCAUACUGACAGGAAGAUUUCUAUUCG
Lar-1-5	AUGCCGUACCUUCGGUACUGAUGAUGAAUGCCGCAGGCAUGCUAUAAAAUAGCCCAAUGGCGUGAGCCUUGGGGGUAAAAUGUAAGAUUAAAGGAGAAUGCCAAAGGCAUGGCAUAGAGCCCUAUAC
Lar-1-6	UUCUUCAAUUCUUUCAGCGAAGCGGUCUUACAGCGAAGCGGUCUUACAAUGCGUAGCAUGGUCUUUCAGCGAAGCGGUCUUAAUGCGCAGCAUGGUCUUGUAGCGCAGCGUUCUAAAAGCGCGAAGC
Lar-1-7	AUGCCGUACCUUCGGUACUGAUGAUGAAUGCCGCAGGCAUGCUAUAAAAUAGCCCAAUGGCGUGAGCCUUGGGGGUAAAAUGUAAGAUUAAAGGAGAAUGCCAAAGGUACGGUAUAGGGCUCUAUAC
Lar-1-8	UAUCACUCAACGACUCAUUGAUUUUUGCUAUUUAUACUUGAUCACGUAGAGGCGCUAAGACGCAGAGUUUUUUCCCGGGAGCGCAGGAAUCUUGCCUGCUUUAAGAAGUCAUCAAUUAUCCGUUGGC
Lar-1-9	AGAAAUCUAAAGUCUUUGUUACUCUGACAACUGCAGGCGAAGCCGAUCUGCAGUGAGCUUGCGAACGAUCUUUCAGCGUAGCGAUCUUACAGUGAGCCUGCGAACGGUCUUCCAGCGCGAAGCACGG
Lar-1-10	UCCUCUCAACCCACACUAUUCUUGUAUCGACCUAGCCAAGCCCCUAGCUUAGAAAAUGAAUGUAAUCUGAUCAUUAGACGAUGGCUAGAUUUAAAGAAAAAUAGAAAAAAUAUUAUAAAUAAUUUCA
Lar-1-11	CGGCUCUCCAGUCAGUGCUUACUCCGAAGAGACUGAAAUUCCAGUUUCCAGGCUUCCUCCAGUGAAAAGAAGCCUAGAUCUGUAAAGUAGGCUUUAUUGUAUUUACGAGUAUCAAUCAUGCGAUGAG
Lar-1-12	UGGAAAUCACACUUGUCAUCGAUAAAUCUAUGGAUAAUUUGAUGGCCGGUGGAGGGAUUGUUCGUAACUUUUAAUACUCAUACUUAAGAUUAUGAGUUCAGAUUUCUUAUAAGCGAUCACGAAAAAC
Lar-1-13	GUUGCCGUAUUGACUCGUCAUUUCUUUGGGAUCAUUUUUCAGAUCAAAAAACUCCCAUUCUUCGCCAUUUGGCACAUCAAUACCAUAAAAACGAAUGAGUUUGAAGCGCUUGCCAGAGACGCCUUCA
Lar-1-14	ACGGGGUGGGUAGUAGGGCCUAGACGAAAUAUCAGUCCGUAGGAUGACGAACGCGUUUCGGCCGGUCAGGGUGUCGAGCCUGCUAUAUCAUGGCGAAGACCAGAGAGCGGGAGGAAGAGCUGCGUAG
Lar-1-15	CUUGGGAUAAGUAUAUGAUUCCCACAAAGAGAAUGCCGAAGCUAUGGCAUAGGUGUUGAUACCUUGGUAAUUCUCAGUUUAUAUAAUGGCUUCCUGGCUUAAUAGCUUUGAACUCAAAAAAGUAAUU
Lar-1-16	UGGUUUCUUUUUCUUAGACUUUUUUCUUGGUUGUACUAUUGAAUAAAUCGCAUCCUCAGACUCAAGUUUCCGUUUAAGACGAUCGAGUUUUUCGGGAUAUUGCUUAGCUAAAUUCUUGGUUUCUCCC
Lar-1-17	UGGUUUCUUUUUCUUAGACUUUUUUCUUGGUUUUACUAAUGAAUAAAUCUCAUCCUCAGACUCAAGUUUACGUUUAAGACGAUCUAACUUUUCAGGAUACUGCUUUGCCAUCCCGAAAAGGACCUAA
Lar-1-18	GCACCUUCAGAACACUCUGCAGUGAUGCUAAAAAAAAAAUAAGCUAAGGAUAUUAAAUGAAAAAAAUAACAAUGUUGCUGAUCAUACUUUUUACAAGUAUCGCAUGUGCAAGUACUCAAGUUAAACCC
Lar-1-19	ACAAUAGUGUUUUUGUUUAGUAGUCCUAGCCCGGGCCAGGCUAAAUUAUGCCGUGCUUUCAGCACUCAAUUUACCUAAUAAAUCAAGCCUUGUUGUACCGUACUUUCUGCACUUAUUUUGAAUGCCG
Lar-1-20	AAUUAUGCCGUGCUUUUAGCACUUCUUUUGAAUGCCGAAGGCAUGACAUAGACCUCCUACCCUUUCUUCACAACUCGAUGCCCAUCUAAUUCAUCGCCAAAUAGCUAGCUUCACAACCUAAAAAAAU
Lar-1-21	CCACUUGUCGUUGAGGAUGCGGAUUUCGUGACAGAGGGCAUCCUUGACGCGUUGUUUAUAAACGUAGAUGGUGGCUUCGGCAAUGCCGGUGAUUUCUGCGAUUUCCGCAGUGGAUUUUCCCUUGAGA
Lar-1-22	CACCUAGCUCUGGCGAGCCAGGCUAAGUUAUGUCGUGCCUUCAGCACUCAAAAAUGAUUUACUUUAUUGAGGGCUAUUCUAUCAAACUGAUUUAAUACUGAUUUUCAGGACCACUUGUCGUUGAGGA
Lar-1-23	GGAUUUUCCCUUGAGAGUUAAGUCAAAGCACUGCAUGACUUUCUCGGAAAAGUUCCCUUGAAUAUUGCUGUAGGCUCGAUCGGAGAUAUGCGCCCGCCAUUCUUUUUCGGCAAUAUCAUAGAUCUCU
Lar-1-24	AGACCUAGCACUGCCGUGCCAGGCUGAGUUAUGCCGUGCUUUCAGCACUCAUAAUUGAAUACCGAAGGUAUGCCAUAGGGUUCUAUGUCAUAUUCUUGGCGUUCAAAGUAGUAUUUUUUAAAUCUGG
Lar-1-25	AUAGGGUUCUAUGUCAUAUUCUUGGCGUUCAAAGUAGUAUUUUUUAAAUCUGGUAGAAUUUGGCGGCGUUGGCGCCGAAGACUUUGGCUUCUGCAGUGUCUGAGAACUGAGUUGUGAAGUCGUGGGC
Lar-1-26	GUUAUGCCGUGCUUUCAGCACUCACAAUUAUUCGGAGGAUUAUAUAUUAACGAAAUUUACGCCAGUACCAUUCGAGGCAGAAGAAGAAAAGAAUAAGGCUUAGGAUAUAAAAGUUAUCCCAGAGAGG
Lar-1-27	AGAAUUUCUGACGUUGAAUUUCACUACCAUUGGUUUUUUCACUGAGGGCUCGGAGCAAUUUAAUGUUAGUGGUGAUAUCAUUGAGUUCAUUAUGCUCCGAAGAGACGUAGAUCUUGGCAGAGAGGGA
Lar-1-28	AGUCAACUGCGUCGUUGUGAAACGGGGUGGGUAGUAGGGCCUAGACGAAAUAUCAGUCCGUAGGAUGACGAACGCGUUUCGGCCGGUCAGGGUGUCGAGCCUGCUAUAUCAUGGCGAAGACCAGAGA
Lar-1-29	CCGAAGACUUUGGCUUCUGCAGUGUCUGAGAACUGAGUUGUGAAGUCGUGGGCGAUUUUGUAGGUUUCUGAAUAGCUAGCGUUCAAGAGACAAACGGGCCAGUCGGAGCCGUACAUAAUGCGAUCUU
Lar-1-30	GAGUCAAUCAUUAUGUCAAAAUUAUGUGCAUAUAAUCUUUCACAUUAAUUUUGUGAAUCCAGUAAAAAUACCUUCAGAAAUAGAAAGAGAUUUAUAUGCUUAUCUUGCAGAAAUAUGUAAAAGGCAU
Lar-1-31	GCUAGCGUUCAAGAGACAAACGGGCCAGUCGGAGCCGUACAUAAUGCGAUCUUCGCCAAAGGCGUUAAAGAUGACUUCCAUGUAUGGCGUAAAAUCUUCUUGAUCCCAGCCUGGCUUUGUUUCCGUA
Lar-1-32	CUGUAGUCGAAGUCAACUGCGUCGUUGUGAAACGGGGUGGGUAGUAGGGCCUAGACGAAAUAUCAGUCCGUAGGAUGACGAACGCGUUUCGGCCGGUCAGGGUGUCGAGCCUGCUAUAUCAUGGCGA
Lar-1-33	UUUGGAGCUUAAAGUCCUGGAGGCGUUUUAAGUCCUUAUCUCGUUUCUUUGUUAGCGUAGAAAUUUUAGUCUGAUCAUCGCCACUUUGCUGAAUUAACUUUUCAUGUUUGGCCUUUAUAAGUUCCAU
Lar-1-34	UAUGCCGUCCCUUCGGGACUCAUUAUUUUUGUUACUUAUAGACCUGGCACUUCCGUGCCAGGCUAAUGUAUGUCGUGCUUUCAGCACUCAAUAAUUAACCGCAGUGAGUACUUCUUACAAUUCCGUG
Lar-1-35	CUGAUCAUCGCCACUUUGCUGAAUUAACUUUUCAUGUUUGGCCUUUAUAUCUUCCAUUUGUGAAGCGAUAUUCUUGUAUUGGGCAUCAAAGACAUCGUCAAUUUUUUCACCCCCGACUUUCUCUAAA
Lar-1-36	AUGGAGUUAGAUUUCCCAAAACCUAGUUCUGACGAAUUAGGCUCAGCUAUGCCGUGCUUUCAGCACUCGGUUUGUCUAAUAAACUAGGCUCAGUUAUGGGGUGUAUUCAGCACUCGAAUUCUAUAUA
Lar-1-37	GGGUGGCGUCGGCUUCUAGGUCACCGCAGGCAUACUGAAUGCCAGAUAGCAUGUGCUGGAGAGCGACUUUGUCGAAGUAGGUCGCUUCGUUGUGACCAAGGUUCGUAUAGAAUACGCGUGAACCUGU
Lar-1-38	GAUUGGUAUUUUUAGGAAAUUUUCCUGAAGUCCAGUGCACUGUAUUGACAGUGACUUCGUCGCGAAGCUGAUCUUCCAGACCAUUUUUGUGGGAGAUAUAUACAAUGUUGGUCGCCAAAUCUUUUUU
Lar-1-39	UGAACCUCGAGCAAUACAUUUCCUGGCUGAAUCAUGCAGUGAGUCAAGCUGAUCGUCCCUAUUGGGUUGAUUUGGCUUAUCAAUCAUUUCAAAAAGGCUGGUAUAGAGAAAUCUUAGGUCGUCACGA
Lar-1-40	AGUUAUUUACAUUCUCUAGCUCUCACGAGCUAGGCUAAAUCAUGGCGUGCUUUCAGCACUCUAUUUUUCUAUAACUCCGCGGCCGAGGCAGAUGAGGUCGUCGUAAAAGAUGCAGGAUUGACCUGGG
Lar-1-41	CAAAAACUUAGUGUCUUGGCGCCUUCGCGAGGGAAACCUAAGAAUAACUCCCAUGUGCUAGUUCACAAAAAACAUUAUUAUCACGCCAAGACGCUAAGUCGCCAAGAAGGACAAUUUGAAUUAUUUC
Lar-1-42	UUCCUCCAGUGAAAAGAAGCCUAGAUCUGUAAAGUAGGCUUUAUUGUAUUUACGAGUAUCAAUCAUGCGAUGAGAACCUUUCUUGCGAUUAUAUUUGGCUAUAAUACUGCGCAGUCUUCUGCGUGUA
Lar-1-43	UUCUUGACUACCACAUGGAUUACAUCAAUGUCUUUCAGCAAUUAGCUUUGUUUGUAGGUAUUAAUAUUUCUUGGUUCUUAACUGCCUACUUCCACAAGGCAUAUAGGAUUCGUAAUGUGAUGGGUUU
Lar-1-44	AUUAGUGGUUUAUAUCUUAAAGGCUUUAGCCGAUCUUACCGCGCAGCGUUCUUGCAGGGAACGAAGUGACCGAUCUUGCAGGCUUUAGCCGGUCUUUCAGUUCGCUUGCGAACGGUCUUGUAGCGCA
Lar-1-45	CUCAAAACUCAAAACUCAAAACUAACCAUGUCAACAACACAAACUCAACAACCUGAUUUUAUUCAGCGGCACUUAGCCACAAUUGAUUUAUUCACAAUAAAUUUUGUGGCAGUUUUCUUGACUACC
Lar-1-46	UUUCACCUAUACCUAGCUCUCACGAGCUAGGCUAAUUUAUACCGUGCUUUCAGCACUCAAAAGUAAAUCUAAUUCAUCGCCAGAUUAAUAGAAACUCAAAACUCAAAACUCAAAACUCAAAACUCAA
Lar-1-47	CUCACGAGCUAGGCUAAUUAUAAGGGUAGGGAAGACUCCUUGCGGAGUCUCCCCUCCUUCCGAACCGUACGUGCGGUUCUCCCGCAUACGGCUCUCCAGUCAGUGCUUACUCCGAAGAGACUGAAAU
Lar-1-48	UCGACACUCUAUGCCGGACUCACAUCCGUGAGGUAUUGCCAUACGGGCGGAUAAAAACUAUUUCCCCAUUUGUGCGGCUGCACCACACGCUAUUGUCUAUCGGUACUCUGCACCUAAAAGAGUGUUU
Lar-1-49	CCCAAUAAGCUGAGAAACCCUCGGGGAAAGUAUGGUUGAGAGCCUGGCCUGCUUUGAGCUCAAAUUUCUUGUACUCAACCCAUAGUCCAGUGCCGUCGAUAUCAACUUCAAGUUUGAUCGUCGUUUC
Lar-1-50	GCUGAUUUUACUCAGCUUCUAUUAUUCUACAGGGCUAAUUAUGUAGUGAUUAAUAAGUUUUUUUAAAUAAAGUUUGUGUUCCCCUUUUAAGUAUAAUAAUUUAAUUUGUGUAGCGGUGUUAUUAUAU
Lar-1-51	AAUAGUUAUUUAUUUUGCCUGAGUGAUUUUCAGGGUGAAGAUAUUUUUAGAGAGUGUUUUAGUUGGGUUCAGUUGAGGAAUAAUGACUUCAAUUUCCUUAGCGGUCUUGGCAAAGUUGAAUUAAAAU
Lar-1-52	CAGUUGAGGAAUAAUGACUUCAAUUUCCUUAGCGGUCUUGGCAAAGUUGAAUUAAAAUGGAAAUGAAAAGCUGAUUUUACUCAGCUUCUAUUAUUCUACAGGGCUAAUUAUGUAGUGAUUAAUAAGU
Lar-1-53	GUUAAGCCGUCCCUUCGGGACUCUCAUUUUACUCUAUCUUCUAAUCCUAGCUCUCACGAGCCAGGCUAUGUUAAGCCGUCCCUUCGGGACUCUCAUUUUACUCUACCUUCUAAUCCUAGCUCUGACG
env-1	UGGAGCUAGUUUCGCUUGUGCUAGGUCUCUUGGGUAAUGAUUUCUCAGUUGCACCCGAACGACACUGUUUGCUCCUUCCACGUGGUUUAACCAUUCGUAGUUGCUGGCACUCUCCAUUUGGACUUCU
Aba-1-2	ACUUGUCGUUCUUGUUAUCGGCGCCGAAAUUCACGUACAUCACGACAAAAUUCUUGUCGCGAAGCUCGCGCAGGUCGGCGUGAUCGGUAAAGAACUUAUCCAAGUAGCCGCACCACUUGCACCAGGU
env-2	CCGACCACCCCGUCCGCGCCUUUCUAACGUUUGGUGGGCGCGUCCACCCCUCCUCUAGAGGAGGGGAAUCGCAGCGGCCUUGCGAUUUCAUCUCGUUGGGCAGCAACCCCGUAGCCCGUGGGUUGGG
Aba-1-3	CGGCUACAACCUCGUCGCGCUUUGCGCCGCCGGUUCCGGCCACGCCGGGGGCACGCUGUCCAUCAUGGACAUUACUGCCGCCCUUUAUCUCAGCGUUGCCAAUCAUGAUCCCAAGAAUCCGAACUGG
Aba-1-4	UCCCGAGCUGCUGCAUGUGGGUAAAGAUCGGCAGCACCGGCCACGAUCCCAGUUCCACCACCGCGCCCGUGCCCUUGGGCAGCACGCGCGGCAGGUUCUCGGUAAUGCCGCCGCCUGUAAUGUGUGC
Aba-1-5	GCGUCGAUCAUUUUGCCCAUGACCCCGCCGGCGCUGUUGGCGGCGCACAUGAGCGUGGGUGAGAGAUUCAGUUGCUGCGCGCUGAUUUUCUGAAGGCUCCCGAAGAGGACGUUGGAUGCGGUAUCCG
Aba-1-6	CGCUGAAGCGCUGCCCUGAUACAAAGCAAAUUAGCUCCGCUGCGUUCGCGGUGCGGCAGCGCUCAAGCGCUGCCCUGAUACAAAGCAAAUCAGCUCCGCUGCAGAAGUUCGGGCACGAGGUGCUCGG
Aba-1-7	GUCGCCACCAGGAAAGCUGGGCGCUUCGGUUUCGAGAUCGCAACGACCGGGCUAAUCUUGUCGAGUUCGGCGCGGAGGUCGGCGUAGCGAUCGGUGUCGCCAGGGAUUUGAAUAUUGCGUCCGCCGC
Aba-1-8	UCUUCGGCGAUGUCGGCGGCGAUUUCUUCUUCGAGGAGGUCGGCUUCGUCGUCGUCGUAUUCGGCGGCUUGGCGGUCGAAGCCGGAGACAUCGAGGUCGAUGUGGCGCACGUUGAUGGCGGCGGUUU
Aba-1-9	UUGUCCUGGGUGAAGCGGCCGAACUGGAUCUGAACGUUACUGGCGACGUAAACGUCGCGAACGUUACCGGAGAGGCCGCGCACAGAUAAGCCGGGGGCGGGCUGAAGUUUGGAUUUGGAGACGUUAU
Aba-1-10	UUGGCGUGCGGCGGGUCGUCAUGAACCUCCAUGAAGACGCCAUCCACGCCGGCGGCAACGGCGGCGCGGGAGAGGACCGGAAUGAACUCGGGCUGUCCACCGGAGACGGCAUGGCCGUCUUUGCCGG
Aba-1-11	UCUUCGGCGAUGUCGGCGGCGAUUUCUUCUUCGAGGAGGUCGGCUUCGUCGUCGUCGUAUUCGGCGGCUUGGCGGUCGAACCCGGAGACAUCGAGGUCGACGUGGCGCACGUUGAUGGCGGCGGUUU
env-3	CAAAGCAUUCGCUAGUAGUCUAUGGUUUUAUUGCUCAUCAGCUAAUCGCUAAAGGCCAAUAGCCAAAGGCUAAUCGCUAAUCGCUAACAGCCAAUAGCCAAAGGCCUUCAUGGUCCUGAUUCAGACA
env-4	....
env-5	GGAGCGGACACGGUCUAUCUCACCGACGCUGACCACUGAUCUUUUUCUCCGUAUUUUUCCCCAAUACCCACAUCAUCAGAUCAUCAUUCUCACCUUCUUAUCUGACCACUGAAAACUGAACACUGAU

5 Actino-pnp

5.1 Taxa

The taxonomy of each organism containing a putative Actino-pnp RNA is listed, with abbreviations identifying each hit (e.g., "Eco-1-1" and "Eco-1-2" might hypothetically represent two distinct RNAs in *E. coli*). The abbreviations will be used to identify each individual Actino-pnp RNA in Sections 5.2 and 5.4.

**abbrev. of hits**	**taxonomy of species**
Aod-1-1	Bacteria Actinobacteria Actinobacteridae Actinomycetales Actinomycineae Actinomycetaceae *Actinomyces odontolyticus* ATCC 17982
Mgi-1-1	Bacteria Actinobacteria Actinobacteridae Actinomycetales Corynebacterineae Mycobacteriaceae *Mycobacterium gilvum* PYR-GCK
Mle-1-1	Bacteria Actinobacteria Actinobacteridae Actinomycetales Corynebacterineae Mycobacteriaceae *Mycobacterium leprae* TN
Msm-1-1	Bacteria Actinobacteria Actinobacteridae Actinomycetales Corynebacterineae Mycobacteriaceae *Mycobacterium smegmatis str.* MC2 155
Msp-1-1	Bacteria Actinobacteria Actinobacteridae Actinomycetales Corynebacterineae Mycobacteriaceae *Mycobacterium sp.* JLS
Msp-2-1	Bacteria Actinobacteria Actinobacteridae Actinomycetales Corynebacterineae Mycobacteriaceae *Mycobacterium sp.* KMS
Msp-3-1	Bacteria Actinobacteria Actinobacteridae Actinomycetales Corynebacterineae Mycobacteriaceae *Mycobacterium sp.* MCS
Mul-1-1	Bacteria Actinobacteria Actinobacteridae Actinomycetales Corynebacterineae Mycobacteriaceae *Mycobacterium ulcerans* Agy99
Mva-1-1	Bacteria Actinobacteria Actinobacteridae Actinomycetales Corynebacterineae Mycobacteriaceae *Mycobacterium vanbaalenii* PYR-1
Mav-1-1	Bacteria Actinobacteria Actinobacteridae Actinomycetales Corynebacterineae Mycobacteriaceae Mycobacterium Mycobacteriumavium complex (MAC) *Mycobacterium avium* 104
Mav-2-1	Bacteria Actinobacteria Actinobacteridae Actinomycetales Corynebacterineae Mycobacteriaceae Mycobacterium Mycobacteriumavium complex (MAC) *Mycobacterium avium subs*[illegible]
Mbo-1-1	Bacteria Actinobacteria Actinobacteridae Actinomycetales Corynebacterineae Mycobacteriaceae Mycobacterium Mycobacteriumtuberculosis complex *Mycobacterium bovis* AF2122[illegible]
Mbo-2-1	Bacteria Actinobacteria Actinobacteridae Actinomycetales Corynebacterineae Mycobacteriaceae Mycobacterium Mycobacteriumtuberculosis complex *Mycobacterium bovis* BCG st[illegible]
Mtu-1-1	Bacteria Actinobacteria Actinobacteridae Actinomycetales Corynebacterineae Mycobacteriaceae Mycobacterium Mycobacteriumtuberculosis complex *Mycobacterium tuberculosis* [illegible]
Mtu-2-1	Bacteria Actinobacteria Actinobacteridae Actinomycetales Corynebacterineae Mycobacteriaceae Mycobacterium Mycobacteriumtuberculosis complex *Mycobacterium tuberculosis* [illegible]
Mtu-3-1	Bacteria Actinobacteria Actinobacteridae Actinomycetales Corynebacterineae Mycobacteriaceae Mycobacterium Mycobacteriumtuberculosis complex *Mycobacterium tuberculosis* [illegible]
Mtu-4-1 to Mtu-4-2	Bacteria Actinobacteria Actinobacteridae Actinomycetales Corynebacterineae Mycobacteriaceae Mycobacterium Mycobacteriumtuberculosis complex *Mycobacterium tuberculosis* [illegible]
Mtu-5-1	Bacteria Actinobacteria Actinobacteridae Actinomycetales Corynebacterineae Mycobacteriaceae Mycobacterium Mycobacteriumtuberculosis complex *Mycobacterium tuberculosis* [illegible]
Mtu-6-1	Bacteria Actinobacteria Actinobacteridae Actinomycetales Corynebacterineae Mycobacteriaceae Mycobacterium Mycobacteriumtuberculosis complex *Mycobacterium tuberculosis* [illegible]
Nfa-1-1	Bacteria Actinobacteria Actinobacteridae Actinomycetales Corynebacterineae Nocardiaceae *Nocardia farcinica* IFM 10152
Rsp-1-1	Bacteria Actinobacteria Actinobacteridae Actinomycetales Corynebacterineae Nocardiaceae *Rhodococcus sp.* RHA1
Ace-1-1	Bacteria Actinobacteria Actinobacteridae Actinomycetales Frankineae Acidothermaceae *Acidothermus cellulolyticus* 11B
Fal-1-1	Bacteria Actinobacteria Actinobacteridae Actinomycetales Frankineae Frankiaceae *Frankia alni* ACN14a
Fsp-1-1	Bacteria Actinobacteria Actinobacteridae Actinomycetales Frankineae Frankiaceae *Frankia sp.* CcI3
Fsp-2-1	Bacteria Actinobacteria Actinobacteridae Actinomycetales Frankineae Frankiaceae *Frankia sp.* EAN1pec
Kra-1-1	Bacteria Actinobacteria Actinobacteridae Actinomycetales Frankineae Kineosporiaceae *Kineococcus radiotolerans* SRS30216
Bli-1-1	Bacteria Actinobacteria Actinobacteridae Actinomycetales Micrococcineae Brevibacteriaceae *Brevibacterium linens* BL2
Cmi-1-1	Bacteria Actinobacteria Actinobacteridae Actinomycetales Micrococcineae Microbacteriaceae *Clavibacter michiganensis subsp. michiganensis* NCPPB 382
Lxy-1-1	Bacteria Actinobacteria Actinobacteridae Actinomycetales Micrococcineae Microbacteriaceae *Leifsonia xyli subsp. xyli str.* CTCB07
Aau-1-1	Bacteria Actinobacteria Actinobacteridae Actinomycetales Micrococcineae Micrococcaceae *Arthrobacter aurescens* TC1
Asp-1-1	Bacteria Actinobacteria Actinobacteridae Actinomycetales Micrococcineae Micrococcaceae *Arthrobacter sp.* FB24
Sar-1-1	Bacteria Actinobacteria Actinobacteridae Actinomycetales Micromonosporineae Micromonosporaceae *Salinispora arenicola* CNS205
Str-1-1	Bacteria Actinobacteria Actinobacteridae Actinomycetales Micromonosporineae Micromonosporaceae *Salinispora tropica* CNB-440
Nsp-1-1	Bacteria Actinobacteria Actinobacteridae Actinomycetales Propionibacterineae Nocardioidaceae *Nocardioides sp.* JS614
Pac-1-1	Bacteria Actinobacteria Actinobacteridae Actinomycetales Propionibacterineae Propionibacteriaceae *Propionibacterium acnes* KPA171202
Ser-1-1	Bacteria Actinobacteria Actinobacteridae Actinomycetales Pseudonocardineae Pseudonocardiaceae *Saccharopolyspora erythraea* NRRL 2338
Sav-1-1	Bacteria Actinobacteria Actinobacteridae Actinomycetales Streptomycineae Streptomycetaceae *Streptomyces avermitilis* MA-4680
Sco-1-1	Bacteria Actinobacteria Actinobacteridae Actinomycetales Streptomycineae Streptomycetaceae *Streptomyces coelicolor* A3(2)
Tfu-1-1	Bacteria Actinobacteria Actinobacteridae Actinomycetales Streptosporangineae Nocardiopsaceae *Thermobifida fusca* YX
env-1 to env-60	environmental samples

5.2 Gene contexts

Each Actino-pnp RNA (indicated by "RNA→") is listed with its surrounding genes. Environmental sequences and some RefSeq entries lack gene annotations, and no genes are listed for such sequences. The direction of each gene is indicated with an arrow (→), and each conserved domain in the gene is colored. Conserved domains associated with more than one Actino-pnp RNA are assigned a color; other domains are gray. Information about these conserved domains is given in Section 5.3. The accession number of the sequence containing each Actino-pnp RNA is given. Accessions beginning with "NC_" or "NZ_" are RefSeq, while other accession refer to environmental samples. Nucleotide coordinates are given for the 5′ and 3′ boundaries of each. If the 5′ coordinate is greater than the 3′ coordinate, the RNA is present on the reverse-complement strand of the containing genomic DNA sequence. Each hit is denoted by an abbreviation (like "Eco-1-1") that refers to a taxonomy given in Section 5.1.

abbrev.	Seq. accession		5′ at	3′ at	genes
env-1	JCVI_SCAF_1096628304755	-	1856	1796	RNA→
env-2	JCVI_SCAF_1096627653623	+	419	480	RNA→ ? (GOS2596)→ RNase_PH (pfam01138)PNPase (pfam03726)? (GOS2596)→
env-3	JCVI_SCAF_1101668161176	+	1157	1215	RNA→
env-4	JCVI_SCAF_1101668134089	-	962	904	RNA→ RNase_PH (pfam01138)? (GOS2596)→
Fsp-1-1	NC_007777.1	-	4254111	4254042	RNA→ Pnp (COG1185)→ PqqL (COG0612)→ DapB (COG0289)→
Fsp-2-1	NZ_AAII01000067.1	-	33426	33356	RNA→ Pnp (COG1185)→ PqqL (COG0612)→ DapB (COG0289)→
Fal-1-1	NC_008278.1	-	6238131	6238064	RNA→ Pnp (COG1185)→ PqqL (COG0612)→ DapB (COG0289)→ DnaK (COG0443)→ hypo→ hypo→
Ace-1-1	NC_008578.1	-	1705830	1705770	RNA→ Pnp (COG1185)→ PqqL (COG0612)→ DapB (COG0289)→ COG1233 (COG1233)→
Nsp-1-1	NC_008699.1	-	3377420	3377345	RNA→ Pnp (COG1185)→ PqqL (COG0612)→
env-5	JCVI_SCAF_1096627678551	-	471	413	RNA→ RNase_PH (pfam01138)? (GOS2596)→
Aau-1-1	NC_008711.1	+	1715342	1715414	RNA→ Pnp (COG1185)→ PqqL (COG0612)→ hypo→
Kra-1-1	NC_009664.1	+	1567631	1567701	RNA→ Pnp (COG1185)→ PqqL (COG0612)→ DapB (COG0289)→ conserved hypothetical secreted protein→
Aod-1-1	NZ_AAYI02000004.1	-	1711891	1711833	RNA→ Pnp (COG1185)→
Tfu-1-1	NC_007333.1	+	925836	925906	RNA→ Pnp (COG1185)→ PqqL (COG0612)→
Rsp-1-1	NC_008268.1	+	7150693	7150751	RNA→ Pnp (COG1185)→
Pac-1-1	NC_006085.1	-	1592080	1592022	RNA→ Pnp (COG1185)→
Nfa-1-1	NC_006361.1	-	4102358	4102295	RNA→ Pnp (COG1185)→ PqqL (COG0612)→
env-6	JCVI_SCAF_1101668152286	-	1046	983	RNA→ RNase_PH (pfam01138)? (GOS2596)→
env-7	JCVI_SCAF_1101668623901	-	761	687	RNA→ RNase_PH (pfam01138)RNase_PH_C (pfam03725)? (GOS2596)→
env-8	JCVI_SCAF_1096627457080	-	848	776	RNA→ RNase_PH (pfam01138)? (GOS2596)→ ? (GOS2596)→
env-9	JCVI_SCAF_1101668138734	-	842	771	RNA→ RNase_PH (pfam01138)? (GOS2596)→
env-10	JCVI_SCAF_1101668248408	-	1068	995	RNA→ Pnp (COG1185)? (GOS2596)→
env-11	JCVI_SCAF_1101668243999	-	995	922	RNA→ RNase_PH (pfam01138)? (GOS2596)→
env-12	JCVI_SCAF_1096626897378	-	1600	1521	RNA→ Pnp (COG1185)? (GOS2596)→
env-13	JCVI_SCAF_1101668100121	-	648	584	RNA→ RNase_PH (pfam01138)? (GOS2596)→
env-14	JCVI_SCAF_1101668160289	-	798	727	RNA→ RNase_PH (pfam01138)RNase_PH_C (pfam03725)? (GOS2596)→
env-15	JCVI_SCAF_1101668116854	-	1286	1224	RNA→ Pnp (COG1185)? (GOS2596)→
env-16	JCVI_SCAF_1101668148428	-	765	703	RNA→ RNase_PH (pfam01138)? (GOS2596)→
env-17	JCVI_SCAF_1101668122106	-	1127	1062	RNA→ Pnp (COG1185)? (GOS2596)→
env-18	JCVI_SCAF_1101668118458	-	673	600	RNA→ RNase_PH (pfam01138)? (GOS2596)→
env-19	JCVI_SCAF_1096626901761	+	1019	1083	RNA→ Pnp (COG1185)? (GOS2596)→ PqqL (COG0612)? (GOS289)→
env-20	JCVI_SCAF_1101668114308	-	1382	1311	RNA→ Pnp (COG1185)? (GOS2596)→
env-21	JCVI_SCAF_1096627654472	-	1030	960	RNA→ RNase_PH (pfam01138)? (GOS2596)→ RNase_PH (pfam01138)? (GOS2596)→
env-22	JCVI_SCAF_1101667162011	-	110	50	RNA→
env-23	JCVI_SCAF_1101668104815	+	53	112	RNA→
env-24	JCVI_SCAF_1101668107913	-	827	768	RNA→ RNase_PH (pfam01138)? (GOS2596)→
env-25	JCVI_SCAF_1096627446429	+	920	978	RNA→
env-26	JCVI_SCAF_1096626895935	+	2004	2064	RNA→ ←? (GOS11987980)
env-27	JCVI_SCAF_1096627298438	-	941	881	RNA→ ←? (GOS11987980)
env-28	JCVI_SCAF_1101668121219	+	759	819	RNA→ ←? (GOS11987980)
env-29	JCVI_SCAF_1096626198158	+	767	823	RNA→

env-30	JCVI_SCAF_1096627650139	-	515	457	**RNA**→ RNase_PH (pfam01138)? (GOS2596)→
env-31	JCVI_SCAF_1101668247038	+	1346	1402	**RNA**→
env-32	JCVI_SCAF_1096627006102	-	1734	1676	**RNA**→ Pnp (COG1185)? (GOS2596)→
env-33	JCVI_SCAF_1101667164550	-	365	309	**RNA**→ ? (GOS2596)→
env-34	JCVI_SCAF_1101668245159	-	532	468	**RNA**→ RNase_PH (pfam01138)? (GOS2596)→
env-35	JCVI_SCAF_1096627382872	-	2493	2429	**RNA**→ Pnp (COG1185)? (GOS2596)→
env-36	JCVI_SCAF_1096627672664	-	306	242	**RNA**→
env-37	JCVI_SCAF_1101668233903	-	759	700	**RNA**→ RNase_PH (pfam01138)? (GOS2596)→
env-38	JCVI_SCAF_1096627005160	-	593	531	**RNA**→ RNase_PH (pfam01138)? (GOS2596)→
env-39	JCVI_SCAF_1096627015732	-	327	265	**RNA**→
env-40	JCVI_SCAF_1096627667722	+	80	142	**RNA**→
env-41	JCVI_SCAF_1101668120237	-	602	542	**RNA**→ RNase_PH (pfam01138)? (GOS2596)→
env-42	JCVI_SCAF_1101668151658	+	624	697	**RNA**→ RNase_PH (pfam01138)? (GOS2596)→
env-43	JCVI_SCAF_1101668143468	+	1190	1249	**RNA**→
env-44	JCVI_SCAF_1096627298000	+	870	946	**RNA**→ Pnp (COG1185)? (GOS2596)→
env-45	JCVI_SCAF_1101668115969	-	1250	1184	**RNA**→ Pnp (COG1185)? (GOS2596)→
env-46	JCVI_SCAF_1101668253034	+	836	893	**RNA**→ RNase_PH (pfam01138)? (GOS2596)→
env-47	JCVI_SCAF_1101668115647	+	1399	1470	**RNA**→
env-48	JCVI_SCAF_1101668141007	-	840	769	**RNA**→ RNase_PH (pfam01138)? (GOS2596)→
Bli-1-1	NZ_AAGP01000012.1	-	38273	38199	**RNA**→ Pnp (COG1185)→ PqqL (COG0612)→
Asp-1-1	NC_008541.1	+	1593772	1593848	**RNA**→ Pnp (COG1185)→
Sar-1-1	NZ_AAWA01000012.1	+	89375	89448	**RNA**→ Pnp (COG1185)→ PqqL (COG0612)→
Str-1-1	NC_009380.1	+	1548720	1548793	**RNA**→ Pnp (COG1185)→ PqqL (COG0612)→
env-49	JCVI_SCAF_1096627357583	-	353	293	**RNA**→
Sco-1-1	NC_003888.3	+	6259624	6259683	**RNA**→ Pnp (COG1185)→ PqqL (COG0612)→ DapB (COG0289)→ hypo→
Sav-1-1	NC_003155.3	-	3089217	3089156	**RNA**→ Pnp (COG1185)→ PqqL (COG0612)→ DapB (COG0289)→ hypo→
Ser-1-1	NC_009142.1	-	6641639	6641570	**RNA**→ Pnp (COG1185)→
Mav-2-1	NC_002944.2	-	3223271	3223201	**RNA**→ Pnp (COG1185)→ PqqL (COG0612)→ NPD (pfam03060)→
Mav-1-1	NC_008595.1	-	3804947	3804877	**RNA**→ Pnp (COG1185)→ PqqL (COG0612)→
Mle-1-1	NC_002677.1	+	1013687	1013757	**RNA**→ Pnp (COG1185)→ PqqL (COG0612)→
Mtu-3-1	NC_009565.1	-	3104733	3104663	**RNA**→ Pnp (COG1185)→ PqqL (COG0612)→ NPD (pfam03060)→
Mbo-2-1	NC_008769.1	-	3046226	3046156	**RNA**→ Pnp (COG1185)→ PqqL (COG0612)→ NPD (pfam03060)→
Mtu-4-1	NC_009525.1	-	3104746	3104676	**RNA**→ Pnp (COG1185)→ PqqL (COG0612)→ NPD (pfam03060)→
Mtu-2-1	NC_002755.2	-	3087501	3087431	**RNA**→ Pnp (COG1185)→ PqqL (COG0612)→ NPD (pfam03060)→
Mtu-5-1	NC_000962.2	-	3092778	3092708	**RNA**→ Pnp (COG1185)→ PqqL (COG0612)→ NPD (pfam03060)→
Mtu-1-1	NZ_AAKR01000096.1	-	9117	9047	**RNA**→ Pnp (COG1185)→ PqqL (COG0612)→ NPD (pfam03060)→
Mtu-6-1	NZ_AASN01000042.1	-	38563	38493	**RNA**→
Mtu-4-2	NZ_AAYK01000241.1	-	32936	32866	**RNA**→
Mbo-1-1	NC_002945.3	-	3049327	3049257	**RNA**→ Pnp (COG1185)→ PqqL (COG0612)→ NPD (pfam03060)→
Msp-3-1	NC_008146.1	+	2248687	2248748	**RNA**→ Pnp (COG1185)→ PqqL (COG0612)→ PenP (COG2367)→ COG3012 (COG3012)→
Msp-1-1	NC_009077.1	+	2189966	2190027	**RNA**→ Pnp (COG1185)→ PqqL (COG0612)→ PenP (COG2367)→ COG3012 (COG3012)→
Msp-2-1	NC_008705.1	+	2267671	2267732	**RNA**→ Pnp (COG1185)→ PqqL (COG0612)→ PenP (COG2367)→ COG3012 (COG3012)→
Mul-1-1	NC_008611.1	+	2397606	2397677	**RNA**→ Pnp (COG1185)→
Msm-1-1	NC_008596.1	+	2735853	2735921	**RNA**→ Pnp (COG1185)→ PqqL (COG0612)→ PenP (COG2367)→
Mva-1-1	NC_008726.1	+	2507941	2507999	**RNA**→ Pnp (COG1185)→ PqqL (COG0612)→ PenP (COG2367)→
Mgi-1-1	NC_009338.1	-	4277016	4276958	**RNA**→ Pnp (COG1185)→ PqqL (COG0612)→ PenP (COG2367)→
Lxy-1-1	NC_006087.1	+	918955	919031	**RNA**→ Pnp (COG1185)→
env-50	JCVI_SCAF_1096626904161	-	355	298	**RNA**→
env-51	JCVI_SCAF_1096626894801	-	1400	1343	**RNA**→ Pnp (COG1185)? (GOS2596)→
env-52	JCVI_SCAF_1101667060140	-	382	325	**RNA**→ ? (GOS2596)→
env-53	JCVI_SCAF_1101668772299	+	1225	1288	**RNA**→
env-54	JCVI_SCAF_1096627358323	-	174	111	**RNA**→

env-55	JCVI_SCAF_1096627364885	+	1283	1346	**RNA**→ Pnp (COG1185)? (GOS2596)→
Cmi-1-1	NC_009480.1	-	2312598	2312542	**RNA**→ Pnp (COG1185)→
env-56	JCVI_SCAF_1096627387932	-	1167	1111	**RNA**→ Pnp (COG1185)? (GOS2596)→
env-57	JCVI_SCAF_1096627357648	+	1000	1056	**RNA**→ Pnp (COG1185)? (GOS2596)→ PNPase_KH (cd02393)RNase_PH_C (pfam03725)? (GOS2596)→
env-58	JCVI_SCAF_1101668632187	+	995	1051	**RNA**→ RNase_PH (pfam01138)? (GOS2596)→
env-59	JCVI_SCAF_1096626905106	+	1093	1151	**RNA**→ Pnp (COG1185)? (GOS2596)→
env-60	JCVI_SCAF_1096627298538	-	918	863	**RNA**→ RNase_PH (pfam01138)? (GOS2596)→

5.3 Conserved domains

Conserved domains found in protein-coding genes listed in Section 5.2 are listed, with the first sentence in their description from the Conserved Domain Database. Conserved domains associated with more than one Actino-pnp RNA are assigned a color, while others are shown in gray. The number in parentheses is the number of occurrences of the given gene.

cd02393 (1) Polynucleotide phosphorylase (PNPase) K homology RNA-binding domain (KH).
COG0289 (7) Dihydrodipicolinate reductase [Amino acid transport and metabolism]
COG0443 (1) Molecular chaperone [Posttranslational modification, protein turnover, chaperones]
COG0612 (31) Predicted Zn-dependent peptidases [General function prediction only]
COG1185 (53) Polyribonucleotide nucleotidyltransferase (polynucleotide phosphorylase) [Translation, ribosomal structure and biogenesis]
COG1233 (1) Phytoene dehydrogenase and related proteins [Secondary metabolites biosynthesis, transport, and catabolism]
COG2367 (6) Beta-lactamase class A [Defense mechanisms]
COG3012 (3) Uncharacterized protein conserved in bacteria [Function unknown]
GOS11987980 (3) ?
GOS2596 (45) ?
GOS289 (1) ?
pfam01138 (25) 3' exoribonuclease family, domain 1.
pfam03060 (8) 2-nitropropane dioxygenase.
pfam03725 (3) 3' exoribonuclease family, domain 2.
pfam03726 (1) Polyribonucleotide nucleotidyltransferase, RNA binding domain.

Duplicate sequences: the following putative homologs are not shown in the alignment because their sequences are identical to a homolog already shown: Mbo-2-1, Msp-1-1, Msp-2-1, Mtu-1-1, Mtu-2-1, Mtu-4-1, Mtu-4-2, Mtu-5-1, Mtu-6-1

5.4 Multiple-sequence alignment

Each Actino-pnp RNA is denoted by an abbreviation (like "Eco-1-1") that refers to a taxonomy given in Section 5.1. The alignment includes sequences containing the RNA motif, as well as flanking sequence. The Actino-pnp RNA itself is denoted by the line underneath marked 5′ and 3′ on either end. Nucleotides in the flanking sequences (i.e., not part of the motif) are written in gray letters. Stems of predicted rho-independent transcription terminators are shaded yellow. (Note: terminator predictions have not been analyzed manually, and many are likely to be false positives.) Start codons are shaded green. (Note: start codons are frequently misannotated, especially in environmental samples.) Nucleotides proposed to basepair as part of the consensus structure are shaded in color when they comprise Watson-Crick or G-U pairs. Otherwise they are shaded gray. Conserved stems are also indicated at the bottom of the alignment by angle brackets, where matching < and > denote base-paired columns. Below these angle brackets, the symbol "2" denotes base pairs exhibiting covariation, "1" denotes base pairs exhibiting compatible mutations, "0" denotes base pairs that are not observed to mutate and "?" denotes base pairs that have a significant frequency of non-canonical nucleotides for Watson-Crick or G-U pairs (> 5%). Below these base pair annotation is the consensus sequence: "R" = "A" or "G", "Y" = "C" or "U", red nucleotides: nucleotide identity conserved more than 97% of the time, black nucleotides: 90%, gray nucleotides: 75%, red circle (•): nucleotide is present 97% of the time, black circle (•): 90%, gray circle (•): 75%, white circle (◦): 50%. All percentages of sequences just described (e.g. 97% conserved) assume that sequences have been weighted by the GSC algorithm implemented by the Infernal software.

alignment positions 1 · · · 99

env-1	....GAUUUAUCGAU
env-2	UCCGUCGCUAAUCGACGGUUUCGAGACAACUACAUACCAAUCUUCGAGGUCGGCAACGGCCAAGAGGGAUUAUUGGAACACUCUCGCUAGUGCGAAGUA
env-3	GGAAGUGAGCUGUGUCGGUUUCGUGCCGACACAGCAAGCACAAAAUCAAGACUCGCAGUUUUGCGAUAACCGCGGAUAGCACGCACAUUCGAGCAGUUU
env-4	UAUUUAAUUGAUUUAAUUCAGCUAAAUAGAUUCGGCAAGCACAAAGUAAGACUCGCUAAUUAGCGAAAACCGCGGAUAGCACUCACCUUCAAACUGUUU
Fsp-1-1	UCUGACGCAUCGGGGGUAGGUACCGAAUGCGUCGAGCCGUCGGCGGAGUCGUCCGCUCGGCGGUGUGGAGGACCUGGCCGGGUCCGUGGUGGUGCGAGC
Fsp-2-1	CCUCCGGCACAUCGGGGGUAGAUACCGGUGUGCCGGGCCGCCGGCGGCGAAUGGCCGCCGGCGGCCGGCAGGACCCAGCCGGGUCCGCGCGGCAUGAGC
Fal-1-1	CCUCUGACGCAUCGGGGGUAGAUACCAGAUGCGUCGCCCGUCGGCGGAGGUCUCCGUCGGCGGAUCGGAGGACCUGACCGGGUCCGUGGCGGCGAGAGC
Ace-1-1	UCUCGGCUGCCGGCACCGUCAUGACGGCGGCGCGCGGCCGGCGUCCGUGGCGCACAAUCACACAAGGAAAGACCAGCUGGAGCCCAGCCGACCUCUGGC
Nsp-1-1	CAGGGCGGCGGCCGGCGCGUUUCCUCUUCUCGGAGGAACUUGGCUAGAUUCACCGUGAACAACAUCUGAAAACACCCAGGAGCGACCCGCCCCGUUCUG
env-5	AUUGAUAUCGAGCGCUACCGCUCGAUUGUCGAUCGCCUUAGCCUGCGCCGAUAACAAACACACAAAUACAAAACAAGCGCGAGAACUCCGCUCAAUAGG
Aau-1-1	CUGCGCCGCUAGUCUGCCUUUAGGGGCGGCCCGCUCCGUUUCGGAACGGGCCGCCUUCUUCACCAACAACUAAAAACAGGAAUCAACCGCAUCGCGCAU
Kra-1-1	UCGGGUCCACCCCUGUUGCGCAUUCCCGCCGCACUCGCGGCAGACUCCUGAGUGCACGACCCAGGAGCACACGCACACCCAGCGGCACCGCGCAGCGUC
Aod-1-1	CCUGCGCCGCUGAUUUUGGGCGGCCCGCCUCCCGUAGCGGAGACGGGCCGGUUUUCACACGCCCGCCUCGGGCAAACCGUUAAUUCUCGCGCGAAAGC
Tfu-1-1	AACAGUCAGGCAUUCCCGCGCUCCCACCCUCGGCCGUCUCUAGCCACGGUGGCAUCUUCCGGUAGGCGAGGAGAACACCCUCGCCACGGUGUCGAGAAA
Rsp-1-1	UCACUCCGGUAGCCGGCGGGGCCUUGCUUAGACUGGGCCUCGCUGGCUAUCGGCGUUAGCUGCCGAGGGCAGCACUACCGUAUGCACCCGAGAAUGUGG
Pac-1-1	ACCGCGUGCAUCGCGGCAAUUCGUGGGAUUGGUCGCUGAUGCCGUAGACUCAUAACCACACUAAGAAUCACUGUAACCGUCGACUGCCUCCCCAUGACA
Nfa-1-1	AGACGUAGAAUCGGCCCCGUUGGCUAUUCGUGUAUGAGGGGUGGUUACCUCCCCCGGGCACGAACAACAUGUGCACAGCCGUAUGCACCCGCCGCGUGG
env-6	GUGCUUUGAUUGAACGUUUAGGUAUUCGUCGCUAACUCAUUUAAUUUAUUAUUCGAAAAACCAAUCGGGUAAUUAGUUAGAAGAAAUAAAGAAGAUGAC
env-7	CACUGAGCGAUUGCACUGAGCGAUUGCACUGAGCGAAUGCACUGACAACCGAAUAGCGAAAACCAAACCGCAAGGAUCCGGGGCGCUACGGUGCGCCGU
env-8	GGAAUUCGCCGUUAAUUUUUAAAAGUUAAUAAGUAGUACUUGGUAGGAAAGUUGGGCCUCAUACGGAGGCCUGACUGGAGCAUCAGCGCCCACGAGGAU
env-9	AUUUUGCAAUAUCUUGCAAAGACCGAUGUAACACGUUACAGAGCGAUUAUCGAAAAACUCGGUAUUCGUCGCUAAUUUAGCAAAACUCACUCAUCAGUG
env-10	CUUGCAAUACCUUGCAAAGACCGACAUCAAUCGCUACCGCGCAAUUAUUGAAAAACUCGGCAUCCGCCGAUAAACAAAUUAAACCUACGGGGGCGUAAG
env-11	CUUGCAAUACCUUGCAAAGACCGACAUCAAUCGCUACCGCGCAAUUAUUGAAAAACUCGGCAUCCGCCGAUAAACAAAUUAAACCUACGGGGGCGUAAG
env-12	CCUUCAGUACCUUGCGAAGACAGAUAUCAACCGUUACCGCGCAAUUAUCGAGAAGCUCGGUAUUCGCCGCUAAUUAAGUAAAACCAAUCUGCCGUAGAG
env-13	CCUUCAAUACCUAGCGAAGACAGAUAUCAACCGUUACCGCGCAAUUAUCGAAAAACUCGGUAUUCGCCGCUAAUUAAGUACAACCAAUCUGCCGUAGAG
env-14	CCUUCAAUACCUAGCGAAGACAGAUAUCAACCGUUACCGCGCAAUUAUCGAGAAGCUCGGUAUUCGCCGCUAAUUAAGUAAAACCAAUCUGCCGUAGAG
env-15	UUCAAUAUCUUGCUAAGACAGAUAUCAACCGUUACCGCGUGAUUAUCGAGAAGCUCGGUAUUCGCCGCUAAUUAAUUAAUACCUACACCACUUCAAGGC
env-16	UUCAAUACCUUGCUAAGACAGAUAUCAACCGUUACCGCGUGAUUAUCGAGAAGCUCGGUAUUCGCCGCUAAUUAAGUAAUACCUACACCACUUCAAGGC
env-17	AUCAAUCGUUAUCGUGCGAUUAUCGAAAAGCUCGGUAUUCGCCGCUAAUUAAAUAACUACCCACCAGGGGAGUUUGAAGUUUCCAGUUUUAUUUGGAAG
env-18	AGACAGAUAUCAAUCGUUAUCGCGCAAUUAUCGAGAAGCUCGGUAUUCGCCGCUAAUUAAUCCAGUACAAAGUUAGAUAAGAACUACACGCCGGGGAC
env-19	AAAGACAGAUAUUGCGCGAUACCGCGCGAUCAUCGACAAACUCGGCAUCCGCCGUUAGUUUGCUGAACAAUUAAAUAAUCACCCAGCGCAACGCUAGAG
env-20	AUUCUUCAGUACCUCGCAAAGACAGAUAUUAAUCGCUACAGAGCGAUUAUUGAAAAACUCGGCAUUCGCCGUUAAUUAGACACACCUACCCGCCGACGG
env-21	UUGCAGUACCUCGCUAAAACUGACAUUGAUCGCUAUAGAGCGAUUAUCGAAAAGCUCGGUAUUCGUCGUUAAUUAAACGCAAAACUUUAUUUGCAAGAG
env-22	GUACCUCGCCAAAACAGAUAUUGAACGCUAUAGAGCGAUAAUCGAAAAGCUCGAUCUUCGCAGAUAAGUUAAUUCAACUAAUAACUUUAUUGUUCAGAG
env-23	....CUCGAUCUUCGCCGCUAAGAAAAAAAUCAGCAAAAACUUUAUUUAAAAGAG
env-24	UACCUCGCCAAGACAGAUAUUGAGCGCUAUAGAGCGAUUAUCGAAAAGCUCGAUCUUCGCCGCUAAGAAAAAAAUCAGCAAAAACUUUAUUCAAAAGAG
env-25	AUCGCGCAAUUAUCGAGAAACUCGGUAUCCGUCGCUAAUAGCAAACCCAGUAGUUCUUAUCUAAGAACUCUGUAAUUAACACGACGCCUCGCAUCAAGU
env-26	UACCUCGCCAAGACGGACAUUGAGCGCUAUAGAGCGAUAAUCGAAAAGCUCGAUCUUCGCCGUUAAAUAAAAAUCAGCAAAAAACUUUAUCGCUAAGAG
env-27	AUACCUCGCCAAGACAGACAUUGAGCGCUAUAGAGCGAUUAUCGAAAAGCUCGAUCUUCGCAGAUAAAUAAAAUCAGCAAAAAACUUUAUCGUUAAGAG
env-28	UACCUCGCCAAGACGGAUAUUGAGCGCUAUAGAGCGAUUAUCGAAAAGCUCGAUCUUCGCCGUUAAAUAACAAUCAGCAAAAAACUUUAUCGCUAAGAG
env-29	UGCAAAGAAAGAUAUCGCUCGUUAUCGCGCGAUAAUCGACAAACUCGGCAUCCGCCGUUAGUUUGAUAAAAAGUUAAUAGAAAACUUCGCGGGCUAGUU
env-30	GACCGAUAUCGAGCGUUAUCGCGCGAUCAUCGACAAGCUCGGCAUCCGCCGUUAGUUUGAAUUCAACAAUUUCAUAACCCAACUCUGCGCGGGCCGGAA
env-31	UUGCAAAGACCGAUAUCGCUCGUUAUCGCGCGAUCAUCGACAAGCUCGGCAUCCGCCGCUAGUUUGAAAUAAUUUAAUAAAUCUCGCCACGGGCCGGAU
env-32	CUAAGACAGAUAUCGCUCGCUAUCGUGCGAUUAUCGACAAGCUCGGCAUCCGCCGUUAGUUUGCUUUUAAAAAUUUAAUAUCUCCUACGCGGGCUAGAA
env-33	UUGCAAAGACCGAUAUCGCUCGUUACCGCGCGAUCAUCGACAAGCUCGGCAUCCGCCGAUAGUUUGAAAUAACUUAAUAAAUCUCGCCACAGGCCGGAA
env-34	AAAGACAGAUAUCGAGCGUUACCGCGCGAUUAUCGACAAACUCGGCAUCCGCCGUUAGUUUGUUUUAAAACUACAUAUUUACCCAGCGCAAGACCGGAU
env-35	AAAGACAGAUAUCGAGCGUUACCGCGCGAUUAUCGACAAACUCGGCAUCCGCCGUUAGUUUGUUUUAAAACUACAUAUUUACCCAGCGCAAGACCGGAU
env-36	AAAGACAGAUAUCGAGCGUUACCGCGCGAUUAUCGACAAACUCGGCAUCCGCCGUUAGUUUGUUUUAAAACUACAUAUUUACCCAGCGCAAGACCGGAU
env-37	UAUUCGCCGCUAAAAAAUAACGAAAACUGAAUAACAGAAAAACUAACUAACAACAACCAAGGUUUUUAGUCGGUGCAAUUAAUUUGUAUCGAUUGCAGA
env-38	CGGUAUUCGUCGCUAACUUAAAAAAAUACAAUUUCAUAUAGAUAAAAAUAAAUAACAACUAGCUACAACCUAGAUUUCAAUUUUUUAAAUUUUAAAUAAA
env-39	AAAAACACAAUUUCAUAUAGAUAAAAAUAAAUAAACUAGCUACAACCUAGAUUUCAAUUUUUUAAAUUUUAAAAUAAAAAAAUUGCAAAAAUUGCAAAA
env-40	.....................AAAAUACAAUUUCAUACAGAGAAAAAUAAAUAACAACUAGCUACAACCUAGAUUUCAAUUUUUUAAAUUUUAAAUAAA
env-41	UCUUCAGUACCUCGCAAAGACAGAUAUCAAUCGUUAUCGCGCAAUUAUCGAGAAGCUCGGUAUUCGCCGCUAAUUAAGAAUUAACUACCCGCCGGGGGA
env-42	UUGCAGUACCUUGCAAAGACAGAUAUCAACCGUUAUCGCGCAAUUAUCGAGAAGCUCGGUAUUCGCCGCUAAUUAAUUAUUAACUACACGCCGGGGAC
env-43	UCUUCAGUACCUCGCAAAGACAGAUAUCAAUCGUUAUCGCGCGAUUAUCGAAAAGCUCGGUAUUCGUCGCUAAUUAAGUAAGCACUGCGUCCCGAGGAG
env-44	UCAAAGACAGAUAUCAAUCGUUAUCGCGUAAUUAUCGAAAAGCUCGGUAUUCGCCGUUAAUUAAUUUACCUACCUGACCGCACUCUUGAAGUAACGGAG
env-45	AUAUCAAUCGUUAUCGUGCGAUUAUCGAAAAGCUCGGUAUUCGCCGCUAAUUAAAUAACUACCCGCCAGGGGAGUUUGGUUUCCAGUUUUAUGUGGAAG
env-46	UUUUGCAGUACCUGGCAAAGACCGACAUCAAUCGCUACCGCGCAAUUAUUGAAAAACUCGGCAUCCGCCGAUAAACAAUUAAACCUACGGGGACGGCAU
env-47	AUUUUGCAAUAUCUUGCAAAGACCGAUGUAACACGUUACAGAGCGAUUAUCGAAAAACUCGGUAUUCGUCGCUAAAUUAGCAAAACUCACUCAUCAGUG
env-48	AUUUUGCAAUAUCUUGCAAAGACCGAUGUAACACGUUACAGAGCGAUUAUCGAAAAACUCGGUAUUCGUCGCUAAAUUAGCAAAACUCACUCAUCAGUG
	....
	....
	●●R●●

```
Bli-1-1    GCCCGGAGACUGCCCAUGGCAUGCCCGGGUGGAAUGCGAGGCAAUGGCUCACAGGGGCAACUGCGGUAGACUUCAACGUGGACAACCAUGCCAUGGACG
Asp-1-1    CUGCGCCGCUAGUCAGACCUGAGGGGCGGCCCCUUCCGUGACGGAAAGGGCCGCGUUCUUCAACCAAAACUAAACAGGAGGAUCAACCGCAUCACGCAU
Sar-1-1    GGGAGUGGCCGACCGGGCCGCUCCCCCGAUCCGUACACCGCCAGGAACUACGACAACCAGGGCCGCACGACCACCCGACCCACCGGGAGCCGGUCAGCG
Str-1-1    GGAGGGGCCGACCGGCCGCUCCCCCGUUCCGGUUCACCGCCAGAACCCUCCGACAACCAGGGCCGCACGACCCCCCGACACGCCGGGAGCCGGUCAGCG
env-49     GGUGCAGGCCGAUAAGGAGGGGGACUGCGCCGCUAGGUGCAGGCCGGUUAAUUGAAAAGGAUGACGAUCACACGGGUGUCGUGUGCGGAUGUGGUGAUG
Sco-1-1    GGGCCGCUCCCUUUGCUGUACGUGCGCGCUGUCGCCGCCCCUUUGUAGUGUGGUAACAGAAGAACAAGACGUACGAGGAGAAGCGGACCGCACCGCCGC
Sav-1-1    GUAGCACAACGCAAUACGUACGACACACAACGUGGUACACCGACAGCGCACAGCGAUGACGGCACGCCACACCCAACGAGGAGAAGCGCACCUCGCCGC
Ser-1-1    GGGAGUGGCCGGUCGGCCGCUCCCCUCGGUGCAUGAAGAGGGGGCGGCGCCGCCCGCAGAACUGCAGAGAGCUCACGGUGACAGCGGGCAAGCGUC
Mav-2-1    AGCAACCGGUGCGGUCACGCAGAUCCGCGUGUGCCGUUCCAGCGUGUCACUACGCACCUUUCGAGGGGGCACCUUCGAGGGAGCAGCCCGGUCUGCAUC
Mav-1-1    AGCAACCGGUGCGGUCACGCAGAUCCGCGUGUGCCGUUCCAGCGUGUCACUACGCACCUUUCGAGGGGGCACCUUCGAGGGAGCAGCCCGGUCUGCAUC
Mle-1-1    GCCGGUUCGGUCCAAGCAAGUGGUGUGGUUCUCGCAAAUCCGCGUGUUCCGCUCCAGUGUGUCACUGUGUAAGUCCGAGGGCGCUGCUCUGUCUGUAUC
Mtu-3-1    GCCGGUUCGGCCCAGCAAAUGGUGCGGUACGCGCAGAACCGCGUGUGCCGUUCCAGCGAGUCACUAUGCAUAUCCAAGGGAGCACCCCCGGCCUGUAUC
Mbo-1-1    GCCGGUUCGGCCCAGCAAAUGGUGCGGUACGCGCAGAACCGCGUGUGCCGUUCCAGCGAGUCACUAUGCAUAUCCAAGGGAGCACCCCCGGCCUGUAUC
Msp-3-1    CAUUGGUGUGUUCGACGCGAUGCGCGAACACAAUAUUGGGUGCGAAACACGCGGUCCGCGGGCUUCGUUCCCGUGUGUCAUGCCAGGAGCCGCUGUAUC
Mul-1-1    CCCAUUCGGCCCAGGCACUGUGGUACGGUUCACGCAGAUCCGCGUGCGCCGUUCCAGCGAGUCGCUACGUAUGUCCGAGGGAGCAGCCCUGCCUGUAUC
Msm-1-1    GUCUGCUCAGUGGGCGCUCGUUGGUCGUACGAACGUCAGGUGCGACACCCGCGAUCCGUGGGUGACGUUCCUGCGAGUCACGUCAGGGCCAGCCUGAUC
Mva-1-1    CAUGGGUGCGUUCCGAUGCCAACGGCACGAACAACUGGGUGCGGUCCUCGCAGAUCCGCGUGCACCGUUCCCGCGUGACAUGACACAAACCGCCCGAUC
Mgi-1-1    AGGUGCGUUCGACUGCCAUCGGCACGAACACACAUGGGUGCGGUCCUCGCAGACCCGCGUGCACCGUUCCCGCGUGACACGACAAGGAACCGCCUGAUC
Lxy-1-1    UCUGUCGUCAUACGCAGCCGUCCUCGCGGCGGAAUGCAGCGUCCCCUGGUAGGAUUCUGGUGGUCGGCCGCGUGCCGGCCGCCGGAGCAGGCUGGCAGC
env-50     GUAUCGCUUUUGGGGGACAAUCGCGCUACCCUAAAGCAGUAGGUGAAUCUCACCUGCACACAACUGAAUAUGGCUCUCUACGGGAGUAGGUGGGUCAGG
env-51     GUAUCGCUUUUGGGGGACAAUCGCGAUACCCUAAACCAGUAGGUGAAUCUCGCCCGCACACAACUGAAUAUGGCUCUCUACGGGAGUAGGUGGGUCAGG
env-52     GUAUCGCUUUUGGCGGACAAUCGCGAUACCCUAAACCAGUAGGUGAAUCUCACCUGCACACAACUGAAUAUGGCUCUCUACGGGAGUAGGUGGGUCAGG
env-53     GCUAACGCGCUCCUGCUUCCUGCCUGCUACCCUUAAGCAGGCGCACAGUCGUGCGCGAGAAGAAAAAACUGCAUAAAGACGCAUUCGAAGAAGACAAUU
env-54     GCUAACGCGCUCCUGCUUCCUGCCUGCUACCCUUAAGCAGGCGCACAGUCGUGCGCGAGAAGAAAAAACUGCAUAAAGACGCAUUCGAAGAAGACAAUU
env-55     GCUAACGCGCUCCUGCUUCCUGCCUGCUACCCUUAAGCAGGCGCACAGUCGUGCGCGAGAAGAAAAAACUGCAUAAAGACGUAUUCGAAGAAGACAAUU
Cmi-1-1    UCCCGGGCCGGCGAGGGGCCGGGUGCUACCAUGUCCGAAGUGAUCCAGCGGACGCCCAGCGUCACGAUGCGAUCACGACAGAUGGAGCUGGCCGGCAGG
env-56     AAUUCAGCUUUGAUAAAAAAGCGUCCAUCUUUUGGUGGAGGGUUUUUUAUGUCUUAUUUCUGGCUUGACUGCUAGGCUGAACGUGGAGUUAGGCCGAGG
env-57     UAACUCAGUUUUGAUAAAAAAGCCUCCAUCAUUUGGUGGAGGGUUUUUAUGUUUUUAUUUGCGCUUGCCUGCUAGGCUUACAAAGGAGUUAGGCCGAGG
env-58     AAUUCAGUCUUAACAAAAAGCCUCCAUCUCUUGGUGGAGGGUUUUUUAUGUUUUAUUUCUGGCUUGACUGCUAGGCUGAACGCGGAGUUAGGCCGAGG
env-59     UAGAUGAUUUUCAAUUACCCCCGGGAAACCGCCGGGGGUUUUUGCUUCUAGGCAUUUUUGACCCUAAGUUGUUACGCUUGUAAUACGGAGGAGCCCGAA
env-60     GGUAUCCGCCGCUAACUAGCAAAACACUGUUAUUGACCUAGAUUCCUAGGCAAUUUUGCCCUGCCCUGUUAGGCUCGUAAGGAGCGAUGAGGACAGGUU
           ....................................................................................................
           ....................................................................................................
           ••••••••••••••••••••••••••••••••••••••••••••••••••••••••••••••••••••••••••••••••••••••••••••••••R••
```

alignment positions 100 · · · 259

```
env-1      GAUCGAUCAUCGAUGAUCGAGCAUCGA.....................UCACGUCGA.....................UCGAUGAUCGAUGAUCGAUGAU.CG.AUCAUCGAUCAUCGAAGAUCGUUAAGCGACCAUCGAUCAUCGAUGACCGAUCCUG
env-2      ACGUGGUCCUCGGUAGUGGUUCA.CAC.A....A.C............UUUUCU.................G...U.UGUG.UGUGCUUCGAUCGAUGAU.CA.UCGACUCAACUAGUCAAAAGUCUUUGCCUUCAAUCCCUUAUCGCUUCGCGUGCCU
env-3      GGUCGGUCCUCGGUAGUGAA.GUUCGG.C....U.C............AAU....................G...A.GCCGCGC.UUCUCGAUCGAAGAU.CG.ACAUGUGAUUUGAAUGCAUUUCACUCGUUUGAAGUGUGGUGUUAUCCCUUUAUGA
env-4      GGUCGGUCCUCGGUAGUGAA.GUUCGG.C....U.C............GAU....................G...A.GCCGCGC.UUCUCGAUCGAAGAU.CG.ACAUGUGACUUGAAUGCAAUUCACUCGUGCGAAGUGCAGUGUUAUCCCUUUAUGA
Fsp-1-1    CGCCGGUCCUCGGUCGUGGU.CGCCGG.GA...U.G..G..GU......ACGG..............GCACCGG.A.CCCGGGG.ACUUCGACUGAGCAC.CG.GCAUCCUCGUUGGCGUGGACCCGGCCGUAGAAGAGGCUCGGCCGCGUGGAUCCGG
Fsp-2-1    CGCCGGUCCUCGGUCGUGGC.CGCCGG.GA...C.G..A.GCGCC....GUC.............GGUGC.C...G.CCCGGAG.ACUUCGACUGAGCAC.CG.GCAUCCUCCAUGGCGCGGGCACGGCCCACGAUGAGGAUGGAGUAUCGAGUGGACG
Fal-1-1    CGCCGGUCCUCGGUCGUGGU.CGCCGG.G....A.GCGG.GC.......UUC.................GUCC...UCCCGGGG.GCUUCGACUGAGCAC.CG.GCAUCCUCGGGACGCGGACCCGGCCGAAGAACAAGAUCGGCUGCGUGGACCCGGC
Ace-1-1    CGCCGGUCCUCGGUCGUGGU.CU.CCG.GG...A.G..C.........GCAA..................GC...U.GCGG.AG.GCUUCCAUCGAAGAC.CG.GCCACCACCUCCGGCGCGGCGGGCUCCCCAAUCCCCAAGGAGGAGUUCGUGGCCG
Nsp-1-1    GCUCGGUCCUCGGUAGUGGC.UCUCAGCA....U.C.....CGGCCGGCUCU.........GCCG..CGCAG...A.UCUGCGG.GCCUCGAUCGAAGAC.CG.GCUCGCAGCAGCGGGACGCCGCGGAUCGCUCCGCGACAGAAGAGGGACACACCCA
env-5      UUGGGGUCCUCGGUAGUGGC.UCUCGGAU....A..............UUUA...................U.GCCGACG.GCUUCGAUCGAUGAU.CU.CCAUCGCACAUAGCUAAUACUCGCGAGAUAUGGAGAAGACCCAUGCUCGGGUCACG
Aau-1-1    UCGCGGUCCUCGGUAGUGAU.UCCCGG.GAA..A.C..C.AUC.....UGUGAC.............GAUGA...UGCCCGUGG.AUCUCGAUCGAUGAC.CG.GGUGUCGUACAGGCCAGGAAAAAAGAAGAGGCCUGGGUUGAUGCGGCGGACUUCC
Kra-1-1    GACCGGUCCUCGGUAGUGGC.CUCCGG.A....A.G..C.........AGCACGACCACCA.........GC...U.UCCGUGG.GCCUCGAUCGAAGGC.CG.GGGCGCGGGUGCCGCGCACACCGGAGGAGGAACCCCUUGGAGGGUCCUGAGAU
Aod-1-1    CGCCGGUUUUCGACAGUGGC.CUACGG.A....G...............CGUU.....................UAUCCGUGG.GCUUCGAUCGAAGGC.CG.AAGUGGAGCGCGACACCAACAAACCAAGGAGAAACGCCCGAUGAUGGAAGGCUCC
Tfu-1-1    GGCCGGUCCUCGGUAGUGGU.UCUCCG.AC...A.CG.C.CC.......GCACCA.............GGGG...UCUCGGGGA.ACUUCGAUCGAUGGC.CG.GCCGUCGGGGCCAUCGCGGUACGGCUGCGGCGGUCCGCCCGGCCUACCGCGGAGU
Rsp-1-1    UGUCGGUCUUCGGUAGUGGU.CUUCCG.G....A.C............ACG....................G...U.CCGGGGG.ACUUCGAUCGACGGC.CG.CCUCCGCACAGCGACAUCCCGAGAACUCGAGAGGGGGAUGUCCCUCAGGUGCGCA
Pac-1-1    CAUGGGUCCUCGGUAGUGGC.CACCGG.G....G..............CGCGA.....................U.CCCGGCG.GCUUCGAUCGAAGAC.CG.GUGUGGUCUCUAAAGGGGCGCCUUGUGGAGCGCCUGCGGGCGGUGGUCGGCCCUG
Nfa-1-1    CGUCGGUCUUCGGUAGUGGCCUCUCGA.G....U.G..G.........ACAU..................CC...G.CCCGUCGGGCUUCGAUCGAUGAC.CG.CCUCCGCGUCGCCGUUUCCAAGGGGGAUCGGCCGGGUGUGCGCGUCGUGGCCAGG
env-6      CUUCGGUCUUCGGUAGUGAU.GUCCGG.A....A.A..A.........AAGUUC...............UU...U.UCCGUGC.GUCUCGAUCGAUGAC.CG.GGUCUAAAAUUAGUUUUAAAUUAUUUCUUUUAAUAAAAUUACCCCUGUAAAGAAA
env-7      CGUCGGUCCUCGGUAGUGGC.CCUCGAAGGAUU.C....GCGC.....CCA..............GCGC.GAG.AACCCGGGG.GCCUCGAUCGAAGAC.CG.CCACUGGACGACUGCCGUGGUUGCCCCUGCUUGCAAACCAAGGAAACAGGAGGCA
env-8      AAAUGGUCCUCGGUAGUGGC.UCACGA.A....A.UGCUGAUU.....CAGAA.............AAUAA...U.UUCGUGG.ACUUCGAUCGAAGAU.UCAUUUGCCUUAUGUGGUGCGCUGACUUAAUUAAAGGAGCGCCCUAUGGAGGGUCAAG
env-9      AAUCGGUCCUCGGUUGUGGC.UCCCGG.GCCAAA.U..U.UA.......UUAAUU.............UAAA...U.CCCGUGG.GCUUCCAUCGAAGAU.CC.GUUACUGAUGCGUGUUCAUUAGAAAUGGAGUCGACCUAUGGAAGGUCAAGAUGUU
env-10     UCAUGGUCUCCGGUAGUGGU.UUUCAG.A....U.AAUUAGAUUU....AAU.............AAAUC.U...A.UCUGAGA.ACUUCGAUCGUAGAUUCA.UUUCUCUCGUCGCCCGUGGGUUCGAAGAGAAUGGAGCGACCCAUGGAGGGUCAAG
env-11     UCAUGGUCUCCGGUAGUGGU.UUUCAG.A....U.AAUUAGAUCU....AAU.............AAAUC.U...A.UCUGAGA.ACUUCGAUCGUAGAUUCA.UUUCUCUCGUCGCCCGUGGGUUCGAAGAGAAUGGAGCGACCCAUGGAGGGUCAAG

           .<<<<<<..<<<<...<<<.<<.<<<.<....<.<..<.<<<<<<<<<............>>>>>>>>>>>...>.>>>>.>>.>>>...>>>>..>>>.>>.>>.......................................................
           .???1211..1120...222.2?.222.?....2.2..?.2222??02?............?20??2222?2...2.?222.?2.222...0211..112.1?.??.......................................................
           •R•YGGUCYUCGGURGUGGY-YY∘C GG-R----∘-∘-------------•••∘-----------------∘∘---∘-YCCG∘RR-RCUUCGAUCGA•GRY-C•-••••Y•••••••••••••••••••••••••••••••••••••••••••••••••••
            5′————————————————————————————————————————————————————————————————————————————————————3′
```

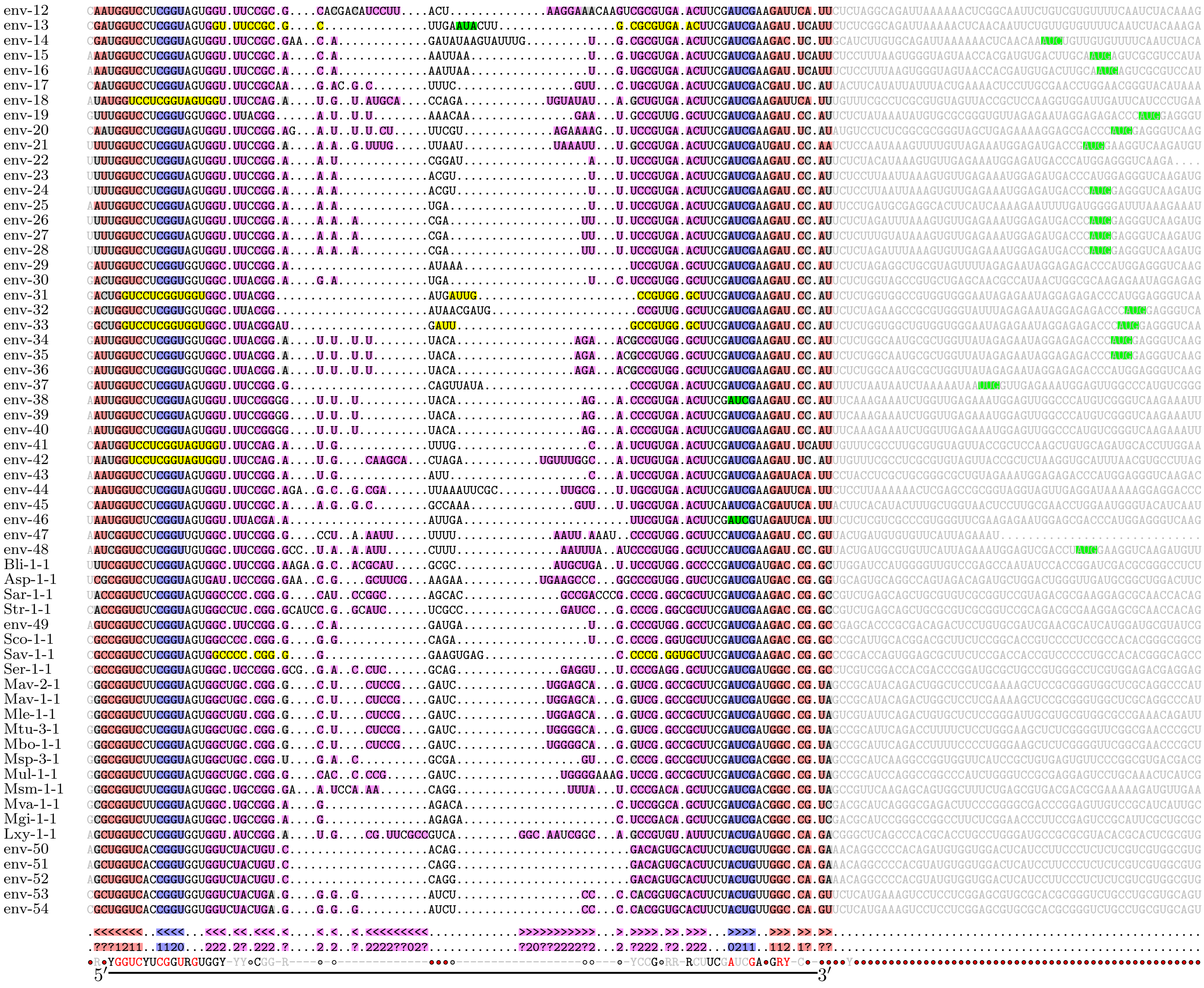
env-12
env-13
env-14
env-15
env-16
env-17
env-18
env-19
env-20
env-21
env-22
env-23
env-24
env-25
env-26
env-27
env-28
env-29
env-30
env-31
env-32
env-33
env-34
env-35
env-36
env-37
env-38
env-39
env-40
env-41
env-42
env-43
env-44
env-45
env-46
env-47
env-48
Bli-1-1
Asp-1-1
Sar-1-1
Str-1-1
env-49
Sco-1-1
Sav-1-1
Ser-1-1
Mav-2-1
Mav-1-1
Mle-1-1
Mtu-3-1
Mbo-1-1
Msp-3-1
Mul-1-1
Msm-1-1
Mva-1-1
Mgi-1-1
Lxy-1-1
env-50
env-51
env-52
env-53
env-54
5′
3′

```
env-55   CGCUGGUCACCGGUGGUGGUCUACUGA.G....G.G..G.........AUCU.................CC...C.CACGGUGCACUUCUACUGUUGGC.CA.GUUCUCACGAAAGUCCUCCUCGGAGCGUGCGCACGCGGGUCUGCCUGCGUGCAGU
Cmi-1-1  AGCUGGUCGUUGGUGGUGGUAUUCCGA.G...................UCG........................CUCGGAGCAUUCCCACUGAUGGC.CA.GACAUGCGCCCGCCGCCGUCGGCCACCGGCCGACGAGCGGAACCAGCCGCGCACG
env-56   GUUGGGUCAUCGGUGGAAAA.AUCUGG.A...................GUUCU.......................UCCAGGU.CUUUUGACUGAGGAC.CA.AACCUUCUAGGUGGGCUACGGAUGGUUCACCGCUCCCACUUGCUAGGUGAUGCCU
env-57   GUUGGGUCAUCGGUGGAAAA.AUCUGG.A...................AACGU.......................UCCAGGU.CUUUUGACUGAGGAC.CA.AACCUUCUAGGUGAGCUACGGAUGGUUCACUGCUCCCAUUUGCUAGGCGAUGCUU
env-58   GUUGGGUCAUCGGUGGAAAA.AUCUGG.A...................ACUUU.......................UCCAGGU.CUUUUGACUGAGGAC.CA.AACCUUCUAGGUGAGCUACGGAUGGUUCACCGCUCCCAUUUGCUAGGUGAUGCCU
env-59   GGCGGGUCCUCGGUGGAAGA.UUUUGG.G...................UCACGGC.....................CCCAGAA.UUUUUUACUGACGAC.CA.GCACUUCCUCCUCCUAGCACGCAUGCGAAAAGCCGAUUUGGUUUUUUGCGUUUGU
env-60   UGCUGGUCUUCGGUGGUGGAUCUUGGG.....................AAAU.........................CCCAAGUUUUUCUACUGACGAU.CA.GGCACGCUUUUCAAGUCUUGAGAAGCAAAAAAUCUCUCGCCUCAUUCUCGCUAUG
         .<<<<<<<..<<<<...<<<.<<.<<<.<....<.<..<.<<<<<<<<<............>>>>>>>>>>>...>.>>>>.>>.>>>...>>>>..>>>.>>.>>..................................................
         .???1211..1120...222.2?.222.?....2.2..?.22222??02?............?20??2222?2...2.?222.?2.222...0211..112.1?.??..................................................
         •R•YGGUCYUCGGURGUGGY-YY∘CGG-R----∘-∘------------•••∘-----------------∘∘---∘-YCCG∘RR-RCUUCGAUCGA•GRY-C•-••••Y••••••••••••••••••••••••••••••••••••••••••••••••••••
          5′———————————————————————————————————————————————————————————————————————————————————————————3′
```

alignment positions 260 · · · 419

```
env-1    GAUGAUAUAUACAGUACAUACAUAAUCAUGUCUAGAUGAUGUUUUUAAAAAUAUGCUUAUUUCUUGUAUCUUGUUAUUAAAUUAAAAGUAUAUAUAUAUAUCCACAGCGCAGCAAAGCUGCGACCCCGCCACCGCGCCAAGAACAAAGUAACAAAGUAGC
env-2    CGAUCUCAUCUGAGAGCACCGUGCUCGCUAUGAGUUUUAAAACCUGAUCGCGAGCGAUGAGAACGCACGAAAAGGAGACCCAAGAAGUGGGUAAUCAAAUAGUAGGAAAAGAAGUACAGAGCGCUGUUGCGACAAUCGACAAUGGCAAAUUCGGCAAGCG
env-3    AGAGGGAGGUUACCCAUGGAGGGUAACGAAGUACAGACCAGCGUUGCUGUGAUUGACAAUGGGUCAUUCGGAAAGCGCACAGUUAAGUUCGAAACCGGACGUCUGGCACGUCAGGCAAACGGAACCGCAGUUGUUUACAUGGAUGACGACACAAUGUUGC
env-4    AGAGGGAGGUUACCCAUGGAGGGAACCGAAGUACAGUCAAGCGUUGCUGUGAUUGACAAUGGGUCAUUCGGAAAGCGCACAAUAAAGUUCGAAACCGGACGUCUGGCACGCCAGGCAAAUGGAACUGCAUCAGUCUAUUUAGAUGAUGAAACCAUGUUGC
Fsp-1-1  CCGAGGAAGAGGAUGGAGUGGUUCGAGUGGACGACUCGACACAUGCGGCCGAAGCGGUCAUCGCGACCCCGGCCGGAGACCGGACGGUUCGGUUCGAGACCGGACGUCUCGCCAAGCAGGCCGCCGGCUCCGCCGUCGCCUACCUGGGCGACACGAUGGU
Fsp-2-1  ACUCGACACAGGCGGCAGAGGCCGUCAUCACGACCCCGACCGGGGCCCGCACGGUCCGUUUCGAGACCGGCCGGCUGGCCAGGCAGGCCGCUGGCGCGGUCGUCGCCCACCUCGGCGACACGAUGGUCCUCUCGGCGACGACGGUCAGCCGCUCGCCGAA
Fal-1-1  CGAAGAAGAGGAUGGAGUGGUUCGAGUGGACGACUCGACACAUGCGGCCGAGGCGGUCAUUGCGACCCCGGCCGGAAACCGUACGGUGCGCUUCGAGACCGGUCGGCUGGCCAGGCAGGCCGCCGGUUCCGCCGUCGCCUACCUGGGCGACACGAUGGUG
Ace-1-1  ACCACGACGUCCACACUGCCGAGGCCGUCAUCGACAACGGCUCAUUCGGCGUCCGCACCGUGCGGUUUGAGACCGGGCGGCUCGCCCGGCAAGCCGGCGGUUCCGCCGUCGCCUAUCUCGGCGACACGAUGGUGCUCUCGGCAACCACGGCAAGCAAGGA
Nsp-1-1  UGACCGACCCCGAGAUCUCUGCCGUCGAGACCGUUCUGGACAACGGCAAGUUCGGCACCCGCACCGUCAAGUUCGAGACCGGCCUACUGGCCCGGCAGGCUGCCGGCGCCGUGACCGCCUACCUCGACGAGGACACCAUGCUCCUGUCGGCCACCACGGC
env-5    ACACACAAGUUCAUUCAUCAGAAGCAGUUAUCGAUAACGGGAAGUUUGGAAAGCGAAUCAUUCGUUUCGAGACUGGUCGAUUAGCUCGUCAGGCAGCAGGCUCAGCAGUAGUUACCCUCGAUGAGGACACCACACUGCUCUCUGCGACAACCGCAUCAAA
Aau-1-1  GCUAACAGAAACGGAGGUGACUCUCUAUGGAGGGUCCCGAAAUCCAGUUCUCAGAAGCCAUUAUCGACAACGGACGUUUCGGCAAGCGAGUCAUUCGCUUCGAAACCGGCCGCCUUGCCAAGCAGGCAGCCGGCGCGUCGAUGGUCUACAUCGACGAAGA
Kra-1-1  CACCGCCGCCGAAGCCGUCAUCGACAACGGCUCGUUCGGCACCCGCACCAUCCGCUUCGAGACCGGGCGCCUCGCCAAGCAGGCCGCCGGGUCCGCCGCGGUCUACCUCGACGGCGAGUCCUUCCUGCUGAGCGCGACCACCGCCGGCAAGUCCCCGAAG
Aod-1-1  GACAUCAUCGCCGCAGAGGCGAUUAUCGAUAACGGCCGUUUCGGUAAGCGCACCGUGCGCUUCGAAACCGGCCGUCUCGCCAAGCAGGCCGCCGGCUCCGCCCUGGCCUACCUGGACGAUUCCACCACCGUUCUGUCCGCCACCACGGUGGGCAAGAACC
Tfu-1-1  CCUCACGACUCUCGAGACGGACACCUACGGGGCACGGACGCGGGAUCUUCGUUAUCCGUGUAGGAGGUCGCCCAUGGAGGGCGUGUAUUCUGCCGAGGCCGUCAUCGACAACGGCCGUUUCGGCACCCGUACGAUCCGCUUCGAGACCGGUCGCCUGGCC
Rsp-1-1  GUUCUCGCCAGGAGGGUGAGAACGCCCCCGCGGGUACGGCGAAGACGAGAGGGAAGUGAAAAGAAGAGAUGACAGAGAAUUCCGCAGUAGAGGUCGAUGAGGGCGUGUUCGAAUCCACCGCCGUGAUCGACAACGGGUCCUUCGGAACCCGCACCAUUCG
Pac-1-1  CGAAAGGAUAUCCCUGUGGAGGGACCUGGACUCGAGUUCACCGAGGCCAUUAUCGAUAACGGCAAGCUUGGUAAGCAUGCUGUCCGUUUUGAGGCGGGACUGCUGGCUCAGCAGGCGGAUGGAUCCGCCGCCGUCUACCUUGAUGGUGACACUAUGCUGC
Nfa-1-1  AGGGCUACCGCGCGACCGUGCCGGGUACGGCUGACGACAGCCGGAUCGCGUCCGACGGACGCUCGAUCCGGCGAGACGAGAGGUUGAGAGAAGACAAUGACGCAGACAAGUGAACGCAAGUCCUCGGCCGUGGAGGUCGAGCCGGGUGUGUUCGAAUCGG
env-6    ACAGAGGAAAGCCCAUGACGGGUUCAGCAAUACAUAAAUCAACGGCUGUUAUUGAUAACGGUCCGUUUGGAAAAGUAAAUAUCCGCUUUGAAACAGGACGCUUAGCUCAACAAGCAGGCGGUGCUGUUCAAGCAUUCUUAGAUGACGACACGAUGGUUUU
env-7    CCCGUGGAGGGUUCCCAGAUCGCGACGGCCGAGGCCGUCAUCGACAACGGCGCGUUCGGAACGCGCACCAUCACCUUCGAGACCGGGCGAUUGGCUCGGCAGGCCGCCGGAUCGGUAGCCGUGUACCUCGACGGUGGCACCAUGCUGCUGAGCGCCACGA
env-8    ACGUAAAAUCAGCAGUAGCCGUUAUCGAUAACGGAAAGUUCGGAAAGCGCGAGAUCAAGUUCGAAACAGGACGCCUCGCUAAGCAAGCAGCAGGAGCUGCAGCUGUUUAUCUCGAUGAUCAGACAAUGAUCUUCUCGGCAACAACAGCAUCAAAGACACC
env-9    AAGACCGCCGUUGCAGUAAUUGAUAACGGCAAAUAUGGAAAGCGCGAAAUCCGGUUUGAAACCGGCCGCCUAGCACGUCAAGCGUCAGGAACUGCGCUUGUUUAUUUAGAUGAUGAUUCAAUGUUGCUCUCUGCAACAACUGCUGGAAAGCAGCCAAAGG
env-10   ACGUUAAGCAUGCCGUUGCAGUUAUAGAUAACGGAAAAUUCGGAAAGCGCGAGAUUCGUUUCGAGACUGGCCGCUUGGCAAGACAAGCAGCUGGUUGCGCAACCGUUUAUUUAGAUGAUGAGACCAUGUUGCUUUCUGCAACUACUGUCUCAAAGUCACC
env-11   ACGUUAAGCAUGCCGUUGCAGUUAUAGAUAACGGAAAAUUCGGAAAGCGCGAGAUUCGUUUCGAGACUGGCCGCUUGGCAAGACAAGCAGCUGGUUGCGCAACCGUUUAUUUAGAUGAUGAGACCAUGUUGCUUUCUGCAACUACUGUCUCAAAGUCACC
env-12   AGGAGGACCCAUGGAGGGUCAAGAGGUUCAAUCAGCCGUUGCAGUUAUUGAUAACGGAAAAUUCGGAAAACGAGAGAUUCGUUUUGAAACAGGUCGCCUAGCGCGCCAAGCAGCAGGAGCAGCAGCUGUUUACUUAGAUGAUCAAACAAUGAUCUUCUCA
env-13   GGAGGACCCAUGGAGGGUCAAGAGGUUCAGUCAGCCGUUGCAGUUAUUGAUAACGGAAAAUUCGGAAAACGAGAGAUUCGUUUUGAAACAGGUCGCCUAGCGCGCCAAGCAGCUGGAGCUGCAGCUGUUUAUCUAGAUGAUCAAACAAUGAUCUUCUCUG
env-14   AAGAGGAGGACCCAUGGAGGGUCAAGAGGUUCAGUCAGCCGUUGCAGUUAUUGAUAACGGAAAAUUCGGAAAACGAGAGAUUCGUUUUGAAACAGGACGCCUAGCGCGCCAAGCAGCAGGAGCUGCAGCGGUUUACUUAGAUGAUCAAACAAUGAUCUUC
env-15   GUAAUAAAGGAGGACCCAUGGAGGGUCAAGAGGUUCAGUCAGCCGUUGCGGUUAUUGAUAACGGAAAAUUUGGAAAACGAGAGAUUCGUUUUGAAACAGGUCGCCUAGCGCGCCAAGCAGCAGGAGCAGCAGCAGUUUACUUAGAUGAUCAAACAAUGAU
env-16   AGUAAUAAAGGAGGACCCAUGGAGGGUCAAGAGGUUCAGUCAGCCGUUGCAGUUAUUGAUAACGGAAAAUUUGGAAAACGAGAGAUUCGUUUUGAAACAGGUCGCCUAGCGCGCCAAGCAGCAGGAGCAGCAGCAGUUUAUUUAGAUGAUCAAACAAUGA
env-17   UUAAGGAGGACCCAUGGAGGGUCAAGAAGUACAGUCAUCAGUUGCCGUUAUCGAUAAUGGCAAAUUCGGAAAGCGCGAAAUCCGCUUUGAAACAGGACGCCUAGCGCGUCAAGCAGCAGGAGCCGCAGCAGUUUAUCUAGAUGAUCAAACAAUGAUCUUC
env-18   UUAGCCCCUUGGAAUAGAAACACGAAUCAAAGGAGGACCCAUGGAGGGUCAAGACGUUAAGUCAGCAGUAGCCGUUAUCGAUAACGGAAAGUUCGGAAAGCGCGAGAUCAAGUUCGAAACAGGACGCCUCGCAAAGCAAGCAGCAGGAGCUGCAGCUGUU
env-19   CAAGACGUUAAGUCUGCCGCUGUCACAAUUGACAACGGAAAAUUUGGAAAACGAGAAAUUCGUUUCGAAACUGGUCGCCUAGCGCGCCAAGCUGCAGGAACUGCAGUUGUUUAUCUAGAUGAUGAAACUAUGUUGCUAUCAGCAACUACUGCUUCAAAGU
env-20   AGGUUCAGACCGCAGUAGCCGUUAUUGAUAACGGAAAGUUUGGAAAACGAGAGAUUCGUUUUGAAACAGGACGUCUAGCACGUCAAGCAGCAGGAGCAGCAGCAGUUUAUUUGGAUGAUCAAACAAUGAUCUUCUCAGCAACAACUGCAUCAAAGACUCC
env-21   AAAAAGCGCCGUUGCCAAAAUUGAUAACGGUAAGUUUGGAAAGCGGGAGAUCCGCUUUGAAACUGGACGUCUAGCACGUCAAGCAGCGGGUACCGCUGUUGUUUAUUUAGAUGAUGACAGCAUGUUGCUAUCUGCAACUACUGCAUCAAAAAAUCCGAAG
env-22   ................................................................................................................................................................
env-23   UGCAAUCAGCCGUUGCAAAAAUAGACAACGGUAAAUUUGGAAAGCGGGAGAUCCGCUUUGAAACUGGCCGACUAGCUCGGCAAGCAGCGGGAACCGCUGUUGUGUACUUAGAUGAUGACAGCAUGUUGCUAUCUGCAACUACUGCAUCAAAAAAUCCGAA
env-24   UGCAAUCAGCCGUUGCCAAAAUAGACAACGGUAAAUUUGGAAAGCGGGAGAUCCGCUUUGAAACUGGCCGACUAGCUCGGCAAGCAGCGGGAACCGCUGUUGUGUACUUAGAUGAUGACAGCAUGUUGCUAUCUGCAACUACUGCAUCAAAAAAUCCGAA
env-25   GGAGCGACCUAUGGAAGGUCAAGAUGUGAAGUUCGCCGUUGCCAAGAUUGAUAACGGCAAGUUUGGAAAAAGAGAGAUCCGCUUUGAAACCGGGCGCCUAGCUCGUCAAGCAGCAGGUACCGCAGUCGUUUAUCUCGACGAUGAUUCAAUGUUGCUUUCA
env-26   UGCAAUCAGCCGUUGCAAAAAUAGACAACGGUAAAUUUGGAAAGCGGGAGAUCCGCUUUGAAACUGGCCGACUAGCUCGGCAAGCAUCGGGAACAGCCGUUGUUUAUUUGGAUGAUGACAGCAUGUUGCUAUCUGCAACUACUGCAUCAAAGAAACCAAA
env-27   UUAAUUCAGCCGUUGCAAAAAUAGAUAACGGUAAAUUUGGAAAGCGGGAGAUCCGCUUUGAAACUGGCCGACUUGCUCGGCAAGCAGCAGGAACUGCUGUUGUGUACUUAGAUGAUGACAGCAUGUUGCUAUCUGCAACUACUGCUUCAAAAAAUCCGAA
env-28   UGCAAUCAGCCGUUGCAAAAAUAGACAACGGUAAAUUUGGAAAGCGGGAGAUCCGCUUUGAAACUGGCCGACUAGCUCGGCAAGCAGCGGGAACAGCCGUUGUUUAUUUAGAUGAUGACAGCAUGUUGCUUUCUGCAACUACUGCAUCAAAAAAUCCGAA
env-29   ACGUUAAGUAUGCCGUUGCAAAAAUAGACAACGGCAAAUUUGGGAAACGUGAAAUACGUUUCGAAACUGGUCGACUGGCGCGUCAGCUGCUGGAACCGCAGUUGUGUAUCUAGAUGA.....................................
env-30   ACCCAUGGAGGGUCAAGACGUUAAGUCUGCCGUUGCCACAAUCGACAACGGAAAAUUUGGGAAACGAGAGAUUCGUUUCGAAACUGGUCGCCUCGCGCGACAGGCUGCGGGAACUGCAGUCGUGUAUCUUGAUGAUGAGACGAUGCUGCUCUCUGCCACA
env-31   GACGUUAGUUAUGCCGUUGCAAAGAUAGAUAACGGCAAAUUUGGGAAACGAGAGAUUCGUUUCGAAACCGGGCGCCUAGCGCGCCAAGCUGCAGGAACUGCAGUCGUUUAUCUCGAUGAUGAAACUAUGU...............................
env-32   AGACGUUAAGUCUGCCGUUGCCACAAUUGACAACGGAAAGUUUGGAAAACGAGAGAUUCGUUUUGAAACCGGACGCCUUGCGCGCCAAGCUGCAGGAACUGCAGUCGUAUUUCUUGAUGAUGAAUCAAUGUUGCUAUCGGCAACAACUGCUUCAAAAUCA
env-33   GACGUUAGUUAUGCCGUUGCAAAAAUUGAUAACGGCAAAUUUGGAAAACGAGAGAUUCGUUUUGAAACUGGGCGUUUAGCGCGCCAAGCUGCGGGAACCGCAGUCGUUUAUUUGGAUGAUGAGACGAUGUUGCUAUCAGCAACAACUGCUUCAAAGAAUC
env-34   ACGUUAAGUCUGCCGUUGCAACAAUUGACAACGGAAAGUUUGGAAAACGAGAGAUUCGUUUUGAAACCGGCCGCCUAGCGCGCCAAGCUGCAGGAACUGCAGUUGUUUACCUCGAUGAUGAUUCGAUGUUGCUCUCUGCAACAACCGCAUCAAAAUCACC
env-35   ACGUUAAGUCUGCCGUUGCAACAAUUGACAACGGAAAGUUUGGAAAACGAGAGAUUCGUUUUGAAACCGGCCGCCUAGCGCGCCAAGCUGCAGGAACUGCAGUUGUUUACCUCGACGAUGAUUCGAUGUUGCUCUCUGCAACAACCGCAUCAAAAUCACC

         ................................................................................................................................................................
         ................................................................................................................................................................
         •••••••••••••••••••••••••••••••••R••••••••••••••••••••••••••••••••••••••••••••••••••••••••••••••••••••••••••••••••••••••••••••••••••••••••••••••••••••••••••••
```

```
env-36   ACGUUAAGUCUGCCGUUGCAACAAUUGACAACGGAAAGUUUGGAAAACGAGAGAUUCGUUUUGAAACCGGCCGCUUAGCGCGCCAAGCUGCAGGAACUGCAGUUGUUUACCUCGAUGAUGAUUCGAUGUUGCUCUCUGCAACAACCGCAUCAAAAUCACC
env-37   UCAAGAAAUUCACUCUGCCGUUGCAAAAAUAGAUAACGGCAAAUUUGGAAAACGACAGAUUCGAUUUGAAACUGGACGUUUAGCAAGACAGGCUGCCGGUUGCGCAGUGGUUUAUUUAGAUGAUGACAGCAUGUUGCUCUCGGCUACAACUGUCUCUAAA
env-38   CACUCUGCCGUUGCGAAAAUCGACAAUGGCAAAUUCGGAAAACGUCAGAUUCGUUUUGAAACUGGACGUUUAGCAAGACAGGCUGCCGGUUGCGCAGUUGUCUAUCUCGACGAUGACAGCAUGUUGCUUUCGGCAACAACUGUUUCCAAAAAACCAAAAG
env-39   CACUCUGCCGUUGCGAAAAUCGACAAUGGCAAAUUCGGAAAACGUCAGAUUCGUUUUGAAACUGGACGUUUAGCAAGACAAGCUGCCGGUUGCGCAGUUGUCUAUCUCGAUGAUGACAGCAUGUUGCUUUCAGCAACAACUGUUUCCAAAAAACCAAAAG
env-40   CACUCUGCCGUUGCGAAAAUCGACAAUGGCAAAUUUGGAAAACGUCAGAUUCGUUUUGAAACUGGACGUUUAGCAAGACAGGCUGCCGGUUGCGCAGUUGUCUAUCUCGAUGAUGACAGCAUGUUGCUUUCGGCAACAACUGUUUCCAAAAAACCAAAAG
env-41   UUUGAAACAAAGGAGGACCCAUGGAGGGUCAAGACGUUAAGACAGCAGUAGCCGUUAUCGAUAACGGAAAGUUCGGAAAGCGCGAGAUUAAGUUCGAAACAGGACGCCUCGCAAAGCAAGCAGCAGGAGCUGCAGCUGUGUAUCUCGAUGAUCAGACAAU
env-42   AAAAAGAAGGAAACGAAUCAAAGGAGGACCCAUGGAGGGUCAAGACGUUAAGUCAGCAGUAGCCGUUAUCGAUAACGGAAAGUUCGGAAAGCGCGAGAUCAAGUUUGAAACUGGUCGCCUCGCAAAGCAAGCAGCAGGAGCUGCAGCUGUAUAUCUAGAU
env-43   GUUAAGACAGCAGUAGCCGUUAUUGAUAACGGAAAGUUUGGUAAGCGCGAGAUUCGCUUUGAAACAGGGCGACUUGCAAAGCAGGCAGCAGGAGCUGCCGCUGUAUAUCUAGAUGAUCAGACAAUGAUCUUCUCAGCAACAACAGCUUCGAAGACACCAA
env-44   AUGGAGGGUCAAGAAGUUCAGUCAGCCGUUGCAGUUAUUGAUAACGGAAAAUUUGGUAAGCGUGAGAUUCGCUUCGAAACAGGACGACUAGCUCGUCAAGCAGCAGGAGCCGCAGCUGUUUAUCUAGAUGAUCAAACAAUGAUCUUUUCAGCUACAACAG
env-45   UAAAGGAGGACCCAUGGAGGGUCAAGAAGUACAGUCAUCAGUUGCCGUUAUCGAUAACGGCAAAUUCGGAAAGCGCGAAAUCCGCUUUGAAACAGGACGCCUUGCGCGUCAAGCAGCCGGAGCUGCCGCAGUUUACUUAGAUGAUCAGACGAUGAUCUUC
env-46   ACGUUAAGCAUGCCGUUGCAGUUAUAGACAACGGAAAGUUCGGCAAGCGAGAGAUUCGCUUUGAGACUGGUCGACUAGCAAAGCAAGCAGCUGGUUGUGCCACAGUUUAUUUAGAUGAUGAAACAAUGUUGCUAUCGGCGACUACAGUUUCAAAGUCACC
env-47   ........................................................................................................................................................................
env-48   AAGACCGCCGUUGCAGUAAUUGAUAACGGCAAAUUUGGAAAGCGCGAAAUCCGGUUUGAAACCGGCCGCCUAGCACGUCAAGCGUCAGGAACUGCGCUUGUUUAUUUAGAUGAUGAUUCAAUGUUGCUCUCUGCAACAACUGCUGGAAAGCAGCCAAAGG
Bli-1-1  UUCGCCUUCAUUCUUCGAAGGUGUCCGCGUGCCGGUUACAAACAAGGAGAAUACGUGGGACUCAACCCUGAAUCCGCCGUAGGCGACAUUGACAAUGGCAGCUAGGGUUCACACACCAUCCGCUUCGAAACAGGACGGCUUGCACAGCAGGCCGCCGGUU
Asp-1-1  CGUUAACCAAGAAACGGAGGUGACUCUCUUGGAGGGUCCCGAAAUCCAGUUCUCAGAAGCCGUCAUUGACAAUGGCCGCUUCGGCAAGCGUGUAAUCCGCUUCGAAACCGGCCGCCUUGCCAAGCAGGCAGCCGGCGCAGCCAUGGUGUACAUCGACGAA
Sar-1-1  AUGACCGAGAGUAACCUCGGCGCCCAGUCUCGUACCGCCACGAUCGACAACGGUGCCUUCGGCACCCGGGUGAUCACCUUUUCCACCGGCCGGCUGGCCCGCCAGGCCGCCGGCUCCGUCGUCGCCCAGCUCGGCGAGACGGUCGUCCUCUCCGCCACCA
Str-1-1  AUGACCGAGAGCAACCUCGGUACCGAGUCUCGUACCGCCACGAUCGACAACGGGGCCUUCGGCACCCGUGUGAUCACCUUCUCCACCGGUCGGCUGGCCCGCCAGGCCGCCGGCUCCGUCGUCGCCCAGCUCGGCGAGACGGUCGUCCUCUCCGCCACCA
env-49   ACGAUGAAGGAGUAACCCGUGGACGGGUCCACUAUCACCGCCACCGAGGCGGUUCUUGACAACGGGCCGUUCGGCUCGCGAACAGUUCGUUUCGAAACCGGCCGCCUGGCUCGCCAGGCGGGUGGUGCGGUGUCGGCGUUCUUGGACGACGACACCAUGC
Sco-1-1  UGGGACAAAAGACGACAAGCAUCGGAGAAACCACUAGUGGAGAACGAGACCCACUACGCCGAAGCCGUGAUCGACAACGGCGCGUUCGGCACCCGCACCAUCCGCUUCGAGACGGGCCGCCUGGCCCGUCAGGCCGCCGGUUCCGCCGUGGCGUACCUGG
Sav-1-1  CGGGACAAAAGACGAAAAGUAUCGGAGAAAACGCUAGUGGAGAACGAGACCCACUACGCCGAGGCCGUCAUUGACAACGGUUCCUUCGGCACCCGCACCAUCCGCUUCGAGACGGGCCGCCUGGCCAAGCAGGCCGCCGGCUCCGCCGUCGCGUACCUGG
Ser-1-1  AAACCAACGUGACUGACGUGCAGUCACUGGACGAGGGCGUGUACGAAGCGACCGCGGUGCUGGACAACGGCGCCUUCGGCACCCGUACCGUCCGAUUCGAGACCGGCCGGCUGGCCAAGCAGGCCGCCGGCAGCGUCGUCGCCUACCUCGACGACGACAC
Mav-2-1  UCGGGUCGGUGAGUGAUCCUGCAAACCCUCUCCAGUUGCGCGUGAUGACGCGAAACAGCCGAAUAAGAGAGGCCGUACGGACGUCUAUGUCUGUCGCUGAAAUUGAAGAAGGCGUGUUCGAGGCGACCGCCACCAUCGACAACGGGAGCUUCGGCACCCG
Mav-1-1  UCGGGUCGGUGAGUGAUCCUGCAAACCCUCUCCAGUUGCGCGUGAUGACGCGAAACAGCCGAAUAAGAGAGGCCGUACGGACGUCUAUGUCUGUCGCUGAAAUUGAAGAAGGCGUGUUCGAGGCGACCGCCACCAUCGACAACGGGAGCUUCGGCACCCG
Mle-1-1  AAUAACGAGAGAGGCUGUGCGGACGGUUAUGUCUGUAGCUGAAAUCGAAGAGGGCGUGUUCGAGGCAACCGUUACUAUCGAUAACGGGAGUUUCGGCACCCGCGCCAUCCGUUUUGAGACCGGGCGCUUAGCCUUGCAAGCCGCUGGCGCAGUGGUGGCC
Mtu-3-1  GGAUUGCGCGUGACGACGCGAAACAGCUCAAUAAUCCAGAGAGGCCGCACGGACGUCAUGUCUGCCGCUGAAAUUGACGAAGGCGUGUUCGAGACGACCGCCACCAUCGACAACGGGAGCUUUGGCACCCGGACCAUCCGCUUCGAGACCGGCCGAUUGG
Mbo-1-1  GGAUUGCGCGUGACGACGCGAAACAGCUCAAUAAUCCAGAGAGGCCGCACGGACGUCAUGUCUGCCGCUGAAAUUGACGAAGGCGUGUUCGAGACGACCGCCACCAUCGACAACGGGAGCUUUGGCACCCGGACCAUCCGCUUCGAGACCGGCCGAUUGG
Msp-3-1  CGAAACAGCUGAAUGAAUUCAGAGAGGCUGUACGGACGUCAAUGUCUGUAGUUGAAAUUGAAGACGGUGUGUACGAAUCGACCGCCGUCAUCGACAACGGGAGCUUCGGCACCCGCACCAUCCGUUUCGAGACCGGCCGGCUGGCCCAGCAGGCCGCCGG
Mul-1-1  GGUUGCGUGUGACGCCGCGAAACAGCUGAAUAACCCAGAGAGGGCCGCACGGACGUUAUGUCUGUAGCUGAAACUGAAGAAGGCGUGUUCGAAGCAACCGCCACCAUCGACAACGGGAGCUUCGGCACCCGCACCAUCCGUUUCGAGACUGGCCGAUUGG
Msm-1-1  UUGAAAGAGGCCACUGGACUAAAUGUCUGUAGUCGAACUUGAAGACGGUGUGUACGAAUCGACCGCUGUCAUCGACAACGGGAGCUUCGGCACCCGCACCAUCCGUUUCGAGACCGGGCGCCUCGCCCAGCAGGCCGCCGGCUCCGCCGUCGCCUACCUC
Mva-1-1  GCGUGAUCACGCGAAACCGCUGAUGUGAAUCAGAGAGGCCAUACGGACGUCCAUGUCUGUAGUUGAAAUUGAAGACGGCGUGUUCGAAUCCACCGCCACCAUCGACAACGGGAGCUUCGGCACCCGCACCAUUCGCUUCGAGGCCGGCCGGCUGGCCCAG
Mgi-1-1  GUGUCCACGCGAAACAGUUGAAGGACGAUCUAGAGAGGCCGUACGGACGUCUAUGUCUGUAGUUGAAAUCGAAGAGGGCGUGUUCGAAUCCACCGCCACCAUCGACAACGGGAGCUUCGGCACCCGCACCAUCCGUUUCGAGACCGGCCGGCUGGCCCGC
Lxy-1-1  UCGAACUGCCUGCUCGCUCCACCGCUCCAUACGAAGAGCAAAGGAGAGUCGCGCCCAAACGGGGCCGCGACGUUAAACAUAAGGAGAAAGACCUCUUGGAAGGUCCCGAAAUCACAUUCGCCGAAGCCAUGCUCGACAACGGCCGCUUCGGCGCCCGCAC
env-50   CCGCCAUGCCCGGCCGAUCGGUUUGGCAUGUUCGAAGAGAUAUAAGGAGAUGUGACCUUAAUGGAAGGUCCAGAAAUCAAGUUCGCUGAAGCUGUUUUGGAUAACGGCAAGUUCGGAAAGCGCACCGUGCGCUUUGAAACGGGCCGUUUGGCCCAGCAAG
env-51   CCGCCAUGCCCGACCGGUCGGUUGGGCAUGUUCGAAGAGAAAUAAGGAGAUGUGACCUUAAUGGAAGGUCCAGAAAUCAAGUUCGCUGAAGCCGUAUUGGAUAACGGCAAGUUCGGAAAGCGCACCGUGCGCUUUGAAACGGGUCGUUUGGCCCAGCAAG
env-52   CCGCCAUGCCCGACCGGUCGGUUGGGCAUGUUCGAAGAGAAAUAAGGAGAUGUGACCUUAAUGGAAGGUCCAGAAAUCAAGUUCGCUGAAGCCGUAUUGGAUAACGGCAAGUUCGGAAAGCGCACCGUGCGCUUUGAAACGGGUCGUUUGGCCCAACAAG
env-53   GCAACGAUGAAUAAGGAGAAAACA................................................................................................................................................
env-54   GCAACGAUGAAUAAGGAGAAAACACCUCAUGGAAGGUGACGACAUCACCUGGUCCGA...............................................................................................................
env-55   GCAACGAUGAAUAAGGAGAAAACACCUCAUGGAAGGUGACGACAUCACCUGGUCCGAAGCUGUUUUAGAUAACGGAAAAUUCGGAACCAGAACCGUUCGUUUUGAAACCGGGCGUCUCGCCCAGCAAGCACAGGGGGCGGUUGCCGCCUACCUCGACGAG
Cmi-1-1  UGCACCCGGAUCCCGCAUCAGGCGGGCGCCGGGGUGUCGCCUCGCCCGCUCCUCCUCUGUCGCAGGAGGUCCGCAUCCACACGGGAUGCAGGACGAGCGCCCGACGGCGUCGCCGAGAGGCGCAACACGAAGGAGAGAGACCUCAAUGGAAGGUCCAGA
env-56   GGCUAUGUCUUAAAGAAGGAGUGACCUUGGAGGGUCCAGAAAUUAAAUCAGCCGAAUGCGUUAUCGACAACGGAAAAUUCGGCAAGCGAACAGUUCGAUUUGAAACCGGCAGACUUGCCCAACAGGCGCAGGGUUCGGCGGUUGCAUACAUCGAUGAAGA
env-57   AGCCGUGUCUUAAAGAAGGAGUGACCUUGGAGGGUCCAGAAAUUAAAUCAGCCGAAUGCGUAAUUGAUAAUGGAAAGUUCGGCACCAGAACAGUUCGAUUUGAAACCGGAAGACUUGCCCAGCAGGCACAGGGUUCUGCAGUUGCAUACAUCGAUGAAGA
env-58   GGCUAUGUCUUAUAGAAGGAGUGACCUUGGAGGGUCCAGAAAUUAAAUCAGCCGAAUGUGUGAUUGACAACGGAAAAUUCGGCAAGCGAACAGUUCGAUUUGAAACCGGCAGACUUGCCCAGCAGGCACAGGGUUCGGCGGUUGCAUACAUCGAUGAAGA
env-59   UGCGUGCGCGUAAGGAUAAGGAGGGGCCAUGGAAGGCCCAGAGAUUAAAUUCGCAGAAGCCGUGAUUGAUAACGGCAAAUUCGGAAAGCGCACCAUUCGCUUCGAGGCAGGGCGCCUUGCCCAGCAGGCAGCAGGUGCUGCGGCCGUCUACAUCGACGAG
env-60   CGCAUUUACCCAACAGUUGGGUGAAAUUAGUGCGCAUUACAAGAACAAAGAGGAGGACCAUGGAAAGUCCAGAAAUUAAAUCAGCCGAAACAGUAAUUGAUAACGGCAAGUACGGAAAGCGAACCAUUCGCUUUGAAGCUGGAAGACUGGCGCAACAGG
```

alignment positions 420 ··· 506

```
env-1    AAGUCCAUAUGAAAAAUUGAUCAAAACACUCGAAACGAUUAAAAACGAAGCGAAAACUAUCGACCAAAAUCGAUUAAAAAUCGAUCA
env-2    CGAGAUCAGAUUCGAAACUGGUCGACUCGCCCAGCAGGCGUCAGGGAGCGCAACCGUCUUUCUUGAUGAUGAGACGAUGUUGCUCGC
env-3    .......................................................................................
env-4    UUUCUGCAACAACUGCAGGAAAAUCACCAAAGGAUCAAUUUGAUUUCUUUCCACUAACUGUAGAUGUUGAAGAACGCAUGUACGCAG
Fsp-1-1  GCUCUCGGCGACGACGGUCAGCAAGCAGCCCAAGGAACAGCUCGACUUCUUCCCGCUGACGGUGGAUGUCGAGGAGCGGAUGUACGC
Fsp-2-1  GGAGCAGCUGGACUUCUUCCCCCUGACGGUGGACGUCGAGGAGCGGAUGUACGCGGCCGGGCGCAUCCCCGGCUCGUUCUUCCGCCG
Fal-1-1  CUCUCGGCGACGACGGUCAGCAAGCAGCCCAAGGAACAGCUCGACUUCUUCCCCCUGACGGUGGACGUCGAGGAGCGGAUGUACGCC
Ace-1-1  ACCUCGAGAGCAGUUCGACUUCUUCCCGCUCACGGUCGAUGUUGAGGAGCGGCUCUAUGCGGCGGGCCGGAUUCCCGGCUCGUUCUU
Nsp-1-1  CGGCAAGCACCCGAAGGACCACUUCGACUUCUUCCCGCUCACCAUCGACGUCGAGGAGCGGAUGUACGCCGCGGGCAAGAUCCCCGG
env-5    GAAUCCUAAAGAUCAAUUCGAUUUCUUCCCGCUUACCGUUGAUGUGGAAGAACGUAUGUAUGCAGUGGGCCGAAUUCCCGGUUCAUU
Aau-1-1  CACCGCACUGCUCUCGGCAACCACCGCUGGCAAGCAGCCGCGCGAAGGUUUCGACUUCUUCCCGCUGACGGUUGAUGUUGAAGAGCG
```

Kra-1-1	GACCAGUUCGACUUCUUCCCCCUGACGGUGGACGUCGAGGAGCGCAGCUACGCCGCCGGCAAGAUCCCCGGCUCGUUCUUCCGCCGC
Aod-1-1	CCAAGGACCAGUUCGACUUCUUCCCGCUGACCGUCGACGUUGAAGAGCGUCAGUACGCCGCGGGUCGCAUCCCCGGUUCCUUCUUCC
Tfu-1-1	CGCCAGGCCACAGGGUCGGCUACGGCCUACCUUGACGACGAGACGAUGGUCCUGUCGGCGACGACCGUGUCGAAGCGCCCCAAGGAG
Rsp-1-1	UUUCGAGACCGGUCGCCUCGCGCAGCAGGCAGCCGGCGCCGUCGUCGCCUACCUGGACGACGAGACGAUGCUGCUGUCCGCGACGAG
Pac-1-1	UGUCGGCCACUACGGCCGCUAAAACUCCUCGCGAUUCCAUCGACUUUUUCCCGCUGACGGUCGACGUCGAGGAGCGCAUGUAUGCCG
Nfa-1-1	UCGCGCUGAUCGACAACGGCAGCUACGGCACCCGCACGGUGCGGUUCGAGACCGGCAGGCUGGCGCGCCAGGCCGCCGGGUCGGUCG
env-6	GUCUGCAACAACUGCAGGUAAAGAACCAAAAGACUCAUUUGAUUUUUUCCCAUUAACAAUCGAUGUUGAAGAAAAAAUGUAUGCACU
env-7	CGGCGUCCAAGCAGCCCAAGGAUCAAUUCGACUUCUUCCCGCUCACGGUCGACGUCGAGGAGCGGAUGUACUCCGUGGGGCGCAUCC
env-8	UAAGGAUCAAUUCGACUUCUUCCCACUCACAGUGGAUGUUGAAGAGAAGAUGUAUGCCAUCGGCAAGAUUCCUGGUUCAUUCUUCCG
env-9	AUCAAUUCGAUUUCUUCCCACUAACCGUGGAUGUUGAAGAACGUAUGUAUGCCGCAGGAAAGAUUCCAGGAUCUUUCUUCCGUCGCG
env-10	AAAAGACCAAUUCGAUUUCUUCCCACUUACUGUGGAUGUCGAAGAGCGUAUGUAUUCACUCGGAAAGAUCCCAGGAUCAUUUUUUCG
env-11	AAAAGACCAAUUCGAUUUCUUCCCACUUACUGUGGAUGUCGAAGAGCGUAUGUAUUCACUCGGAAAGAUCCCAGGAUCAUUUUUCCG
env-12	GCAACAACAGCAUCAAAGACUCCAAAGGAUCAAUUUGAUUUCUUCCCAUUAACAGUUGAUGUUGAAGAGAAGAUGUAUGCAGUUGGU
env-13	CGACAACAGCAUCAAAAACUCCAAAGGAUCAAUUUGAUUUCUUCCCUUUAACAGUUGAUGUUGAAGAGAAGAUGUAUGCAGUUGGUC
env-14	UCAGCAACAACCGCAUCAAAGACACCUAAGGAUCAAUUUGAUUUCUUCCCUUUGACAGUUGAUGUUGAAGAGAAGAUGUACGCAGUU
env-15	CUUCUCAGCAACAACUGCAUCAAAGACUCCUAAAGAUCAAUUUGAUUUCUUUCCUUUAACAGUUGAUGUUGAAGAAAAGAUGUAUGC
env-16	UCUUCUCAGCAACAACUGCAUCAAAGACUCCUAAAGAUCAAUUUGAUUUCUUUCCUUUAACAGUUGAUGUUGAAGAAACGAUGUAUG
env-17	UCAGCAACAACUGCCUCAAAGACUCCAAAAGAUCAAUUCGACUUCUUUCCUUUAACAGUAGAUGUUGAAGAGAAGAUGUAUGCAGUC
env-18	UAUCUCGACGAUCAGACAAUGAUCUUCUCGGCAACAACAGCAUCAAAGACACCUAAGGAUCAAUUCGACUUCUUUCCACUCACAGUG
env-19	CACCUAAAGAUCAAUUCGACUUCUUCCCAUUAACAGUUGAUGUGGAAGAGCGCAUGUAUGCAAUUGGAAAGAUCCCAGGAUCAUUUU
env-20	AAAAGAUCAAUUCGAUUUCUUUCCUUUGACAGUAGAUGUUGAAGAGAAGAUGUAUGCAGUUGGUCGUAUUCCAGGAUCAUUCUUCCG
env-21	GAUCAAUUUGAUUUCUUCCCAUUAACUGUAGAUGUGGAAGAGCGCAUGUCUGCAGCUGCAAAAAUUCCUGGCUCAUUCUUNNNNNNN
env-22	....
env-23	GGAUCAAUUUGAUUUCUUCCCACUUACAGUUGAUGUGGAAGAGCGAAUGUAUGCCGCUGGCAAAAUUCCUGGAUCAUUCUUUCGUAG
env-24	GGAUCAAUUUGAUUUCUUCCCACUUACAGUUGAUGUGGAAGAGCGAAUGUAUGCCGCUGGCAAAAUUCCUGGAUCAUUUUUUCGUAG
env-25	GCAACAACCGCUUCAAAGCAACCGAAAGACCAA..
env-26	GGAUCAAUUUGAUUUCUUCCCUCUAACUGUUGAUGUAGAAGAGCGAAUGUAUGCCGCUGGAAAAAUUCCAGGGUCAUUUUUCCGUCG
env-27	AGAUCAAUUUGAUUUCUUUCCACUAACCGUAGAUGUAGAAGAGAGAAUGUAUGCUGCUGGGAAAAUACCUGGUUCUUUUUUCCGUCG
env-28	GGAUCAAUUUGAUUUCUUCCCACUAACAGUUGAUGUGGAAGAGCGAAUGUAUGCUGCUGGAAAAAUUCCAGGAUCAUUUUUCCGUCG
env-29	....
env-30	ACUGCUUCAAAAUCACCACGCGAAUCAUUCGAUUUCUUCCCGCUCACGGUUGAUGUCGAAGAGCGCAUGUAUUCAGUAGGAAAGAUC
env-31	....
env-32	CCUAAAGAUCAAUUCGAUUUCUUUCCUCUGACAGUAGAUGUGGAAGAGAGAAUGUAUUCGGUUGGUCGAAUUCCGGGAUCGUUCUUC
env-33	CAAAAGAUCAAUUCGAUUUCUUCCCACUCACUGUUGAUGUGGAAGAGCGAAUGUAUUCGGUGGGAAGAAUCCCUGGUUCUUCUCCGU
env-34	ACGCGAUCAAUUCGACUUCUUCCCACUCACUGUUGAUGUGGAAGAGAGAAUGUACGCCGUCGGAAAGAUUCCAGGAUCAUUUUUCCG
env-35	ACGCGAUCAAUUCGACUUCUUCCCGCUCACUGUUGAUGUGGAAGAGAGAAUGUACGCCGUAGGAAAGAUCCCAGGAUCAUUUUUCCG
env-36	ACGCGAUCAAUUCGACUUCUUCCCACUC...
env-37	AAUCCGAAAGAUCAAUUUGAUUUCUUCCCAUUAACAGUUGAUAUCGAAGAGAGAAUGUAUGCCGCAGGCAGAAUUCCCGGUUCAUUC
env-38	AUCAAUUUGAUUUCUUUCCACUAACAGUUGAUGUGGAAGAACGUAUGUAUGCAGCCGGAAAAAUUCCUGGCUCAUUCUUUAGACGUG
env-39	AUCAAUUUGAUUUCUUUCCACUCACAGUUGAUGUGGAAGAACGUAUGUAUG....................................
env-40	AUCAAUUUGAUUUCUUUCCACUCACAGUUGAUGUGGAAGAACGUAUGUAUGCAGCUGGAAGAAUUCCUGGCUCAUUCUUUAGACGCG
env-41	GAUCUUCUCAGCUACAACAGCAUCAAAGACACCUAAGGAUCAAUUCGACUUCUUCCCACUAACAGUGGAUGUUGAAGAGAAGAUGUA
env-42	GAUCAAACAAUGAUCUUCUCAGCAACAACAGCAUCAAAGUCACCUAAGGACCAAUUCGACUUCUUCCCACUUACAGUGGAUGUCGAA
env-43	AAGAUCAAUUCGACUUCUUUCCUUUGACAGUAGAUGUUGAAGAGAAGAUGUACGCAGUCGGAAAGAUCCCAGGUUC...........
env-44	CAUCAAAGACUCCAAAAGAUCAAUUCGAUUUCUUUCCUUUAACAGUAGAUGUUGAAGAGAAGAUGUAUGCAGUUGGUCGUAUUCCGG
env-45	UCUGCUACAACCGCAUCUAAGACUCCAAAAGAUCAAUUCGACUUCUUCCCAUUAACAGUAGAUGUUGAAGAGAAGAUGUAUGCAGUU
env-46	AAAAGAUCAAUUCGACUUCUUCCCAUUAACCGUGGAUGUCGAAGAGCGUAUGUAUUCAAUCGGAAAGAUCCCUGGAUCAUUCUUCCG
env-47	....
env-48	AUCAAUUCGAUUUCUUCCCACUAACUGUGGAUGUUGAAGAACGUAUGUAUGCCGCAGGAAAGAUUCCAGGAUCUUUCUUCCGUCGCG
Bli-1-1	CUGCCGUCGCCUACCUCGACGACGAAACCAUGCUGUUCUCGGCCACCUCGGCGGGCAAGAACCCCCGUGAGGGCUUCGACUUCUUCC
Asp-1-1	GACACCGCGCUGCUGUCCGCAACCACCGCCGGCAAGCACCCGCGUGAAGGCUUUGACUUCUUCCCGCUGACCGUUGACGUCGAAGAG
Sar-1-1	CCGUCGGCCGGCAGCCGAAGGAGCAGUUCGACUUCUUCCCGCUGACCGUCGACGUCGAGGAGCGGAUGUACGCCGCGGGCCGGAUCC
Str-1-1	CCGUUGGCCGGCAGCCGAAGGAGCAUUUCGACUUCUUCCCGCUGACCGUCGACGUCGAGGAGCGGAUGUACGCGGCGGGCCGGAUCC
env-49	UGUUGAGCACGACGACGGCGUCUAAGUCGCCGAAGGAGCAUUUCGAUUUCUUUCCGCUGACCGUCGAUGUGGAGGAGCG........
Sco-1-1	ACGACGACACCAUGGUGCUGUCGGCCACCAGCGCCUCCAAGAACCCGAAGGACCAGCUCGACUUCUUCCCCCUGACGGUGGACGUCG
Sav-1-1	ACGACGACACCAUGGUGCUGUCGGCCACCACCGCCUCCAAGAAGCCCAAGGAAAAUCUCGACUUCUUCCCCCUCACGGUGGACGUCG
Ser-1-1	CAUGCUGCUCUCGGCCACCACGGCCUCCAAGCAGCCGAAGGAGCACUUCGACUUCUUCCCGCUGACCGUCGACGUCGAGGAGCGGAU
Mav-2-1	CACCAUCCGCUUCGAGACCGGCCGGUUGGCCCAGCAGGCCGCCGGCGCCGUCGUCGCCUACCUCGACGACGAGAACAUGCUGUUGUC
Mav-1-1	CACCAUCCGCUUCGAGACCGGCCGGUUGGCCCAGCAGGCUGCCGGCGCCGUCGUCGCCUACCUCGACGACGAGAACAUGCUGUUGUC
Mle-1-1	UACCUGGACGCCGACAAUAUGCUCUUGUCGGCGACCACCGCCAGCAAGAACCCCAAGGAGCAAUUCGACUUCUUCCCGCUUACGGUC
Mtu-3-1	CCUUGCAGGCCGCCGGCGCGGUGGUCGCCUACCUCGACGACGACAACAUGCUGCUGUCGGCGACCACCGCCAGCAAGAACCCCAAAG
Mbo-1-1	CCUUGCAGGCCGCCGGCGCGGUGGUCGCCUACCUCGACGACGACAACAUGCUGCUGUCGGCGACCACCGCCAGCAAGAACCCCAAAG
	....
	....
	●●●○○○○○○○○

```
Msp-3-1   CGCCGUCGUCGCCUACCUCGACGACGAGACCAUGCUGCUGUCGGCCACCUCGGCCAGCAAGAGCCCGAAGGACCACUUCGACUUCUU
Mul-1-1   CCCAGCAGGCCGCCGGCUCGGUGGUCGGAUACCUCGACGACGAGAACAUGUUGCUGUCGGCGACCACCGCCAGCAAGAACCCCAAAG
Msm-1-1   GAUGACGAGACCAUGCUGCUGAGCGCGACGACCGCCAGCAAGAACCCCAAGGACCACUUCGACUUCUUCCCGCUCACGGUGGACGUC
Mva-1-1   CAGGCUGCCGGAGCCGUCGUCGCCUACCUCGACGACGAGACCAUGCUGCUGAGCGCGACGACCGCCAGCAAGAGCCCCAAAGAGCAC
Mgi-1-1   CAAGCCGCCGGCGCCGUCGUCGCCUACCUCGACGACGAGACCAUGCUGCUGAGCGCCACGACCGCCAGCAAGCAGCCCAAGGAGCAU
Lxy-1-1   CGUCCGGUUCGAGACCGGGCGCAUCGCUCAGCAGGCCCAGGGCGCUGUCGCCGCCUACCUCGACGAGGAAACCAUGCUGCUGAGCGC
env-50    CCCAGGGUGCGGUUGCCGCUUACCUGGAUGAAGAAACCAUGUUGCUCUCGGCAACGAGUGCCUCGAAGAAUGCCCGUGAGGGUU...
env-51    CCCAGGGUGCGGUUGCCGCAUACCUCGAUGAAGAAACCAUGUUGCUCUCGGCAACGAGUGCCUCGAAGAAUGCCCGCGAGGGUUUUG
env-52    CCCAGGGUGCGGUUGCCGCAUAUCUGGAUGAAGAAACCAUGUUGCUCUCGGCAACGAGUGCCUCGAAGAAUGCCCGCGAGGGUUUUG
env-53    .......................................................................................
env-54    .......................................................................................
env-55    GAGACAAUGCUCCUCUCGGCGACCAGUGCUGGCAAAAACCCCAGGGAAGGCUUUGAUUUCUUCCCGCUCACUGUUGAUGUGGAAGAG
Cmi-1-1   GAUCAAGUUCGCCGAGGCAGUCCUCGACAACGGCAAGUACGGAACGCGCACGGUCCGGUUCGAGGCCGGCCGCCUCGCGCAGCAGGC
env-56    AACUAUGCUUUUCAGCGCAACAACCGCUUCAAAGCAACCAAAAGAUCAAUUCGAUUUUUUCCCGCUAACAAUUGACGUCGAGGAGCG
env-57    AACUAUGCUUUUCAGCGCAACAACCGCUUCAAAGCAACCAAAAGAUCAAUUUGACUUUUUCCCGCUAACAAUUGACGUCGAGGAGCG
env-58    AACUAUGCUUUUCAGCGCAACAACCGCUUCCAAGCAACCAAAAGAUCAAUUUGAUUUUUUCCCACUAACAAUUGAUGUCGAGGAGCG
env-59    GAGACAAUGCUCUUCUCGGCAACCAGCGCUUCAAAGCAGCCCAAGGAGCAGUUCGACUUCUUCCCGCUGACCAUCGACGUUGAAGAG
env-60    CGCAAGGUUCAGCAGCCAUCUACCUAGACGGCGAAACAAUGCUCUUUUCGGCAACUAGCGCAAGCAAGCAGCCAAAAGAUCAGUUCG
          .......................................................................................
          .......................................................................................
          ●●●●●●●●●●●●●●●●●●●●●●●●●●●●●●●●●●●●●●●●●●●●●●●●●●●●●●●●●●●●●●●●●●●●●●●●●●●●●●○○○○○○○○
```

6 AdoCbl-variant

6.1 Taxa

The taxonomy of each organism containing a putative AdoCbl-variant RNA is listed, with abbreviations identifying each hit (e.g., "Eco-1-1" and "Eco-1-2" might hypothetically represent two distinct RNAs in *E. coli*). The abbreviations will be used to identify each individual AdoCbl-variant RNA in Sections 6.3 and 6.5.

**abbrev. of hits**	**taxonomy of species**
env-1 to env-144	environmental samples

6.2 Notes

Superscript numbers are used to annotate any AdoCbl-variant RNA that has special characteristics (described below). These numeric annotations will be used in Sections 6.3 and 6.5. The meaning of the numbers are as follows.

[1] Overlaps AdoCbl riboswitch sequence that was already detected as such, although the original sequence is not aligned well, and therefore probably fits the variant motif better.

[2] Near predicted rRNA.

6.3 Gene contexts

Each AdoCbl-variant RNA (indicated by "RNA→") is listed with its surrounding genes. Environmental sequences and some RefSeq entries lack gene annotations, and no genes are listed for such sequences. The direction of each gene is indicated with an arrow (→), and each conserved domain in the gene is colored. Conserved domains associated with more than one AdoCbl-variant RNA are assigned a color; other domains are gray. Information about these conserved domains is given in Section 6.4. The accession number of the sequence containing each AdoCbl-variant RNA is given. Accessions beginning with "NC_" or "NZ_" are RefSeq, while other accession refer to environmental samples. Nucleotide coordinates are given for the 5′ and 3′ boundaries of each. If the 5′ coordinate is greater than the 3′ coordinate, the RNA is present on the reverse-complement strand of the containing genomic DNA sequence. Each hit is denoted by an abbreviation (like "Eco-1-1") that refers to a taxonomy given in Section 6.1. Superscript numbers refer to annotations listed in Section 6.2

abbrev.	Seq. accession		5′ at	3′ at	genes
env-1	JCVI_SCAF_1101668560197	+	868	993	**RNA**→
env-2	JCVI_SCAF_1096628287862	-	1414	1296	**RNA**→ ? (GOS4369)→ ? (GOS4665)→ Thy1 (pfam02511)? (GOS5954)→
env-3	JCVI_SCAF_1101668518363	-	1418	1300	**RNA**→ BtuB (COG4206)? (GOS1720)→
env-4	JCVI_SCAF_1101668460391	-	384	266	**RNA**→ ? (GOS1720)→
env-5	JCVI_SCAF_1101668181603	+	993	1106	**RNA**→ ? (GOS1720)→
env-6	JCVI_SCAF_1096628002478	+	1267	1385	**RNA**→ ? (GOS496)→
env-7	JCVI_SCAF_1096626931125	+	391	507	**RNA**→ BtuB (COG4206)? (GOS1720)→
env-8	JCVI_SCAF_1096627830159	+	1185	1300	**RNA**→ ? (GOS4369)→
env-9	JCVI_SCAF_1096627408381	+	141	256	**RNA**→ ? (GOS4369)→ ? (GOS4665)→
env-10	JCVI_SCAF_1096628254439	+	357	495	**RNA**→ ? (GOS1720)→
env-11	ABEF01043191.1	-	931	798	**RNA**→ hypo→
env-12	JCVI_SCAF_1101668133785	-	328	197	**RNA**→
env-13	ABEF01008614.1	+	1	139	**RNA**→
env-14	JCVI_SCAF_1101668593526	+	801	929	**RNA**→ ? (GOS1720)→
env-15	JCVI_SCAF_1101668492791	+	611	749	**RNA**→ ? (GOS920)→
env-16	JCVI_SCAF_1096626110926	+	473	604	**RNA**→ ? (GOS11983570)→
env-17	JCVI_SCAF_1101668495477	+	564	702	**RNA**→ ? (GOS1720)→
env-18	JCVI_SCAF_1096627476376	+	351	479	**RNA**→ ? (GOS9454)→
env-19	JCVI_SCAF_1101668174084	-	1891	1775	**RNA**→ ? (GOS4369)→ ? (GOS4665)→
env-20	JCVI_SCAF_1096627290449	+	2935	3064	**RNA**→
env-21	JCVI_SCAF_1096626307088	-	364	236	**RNA**→ ←? (GOS11790770)
env-22	ABEF01048062.1	+	1373	1515	**RNA**→ hypo→
env-23	JCVI_SCAF_1101668472248	+	19	147	**RNA**→ BtuB (COG4206)? (GOS1720)→
env-24	JCVI_SCAF_1096628149541	-	147	19	**RNA**→
env-25	JCVI_SCAF_1096627100734	-	553	425	**RNA**→ ? (GOS1720)→
env-26	JCVI_SCAF_1096627885039	+	1487	1630	**RNA**→
env-27	JCVI_SCAF_1101668176557	+	771	916	**RNA**→ ? (GOS4369)→
env-28	JCVI_SCAF_1096627126031	-	1664	1524	**RNA**→ BtuB (COG4206)? (GOS1720)→
env-29	JCVI_SCAF_1096628223806	+	1694	1835	**RNA**→
env-30	JCVI_SCAF_1101668002322	+	1360	1487	**RNA**→
env-31	JCVI_SCAF_1096627253662	+	2472	2603	**RNA**→ BtuB (COG4206)? (GOS1720)→
env-32	JCVI_SCAF_1096626931276	-	1019	889	**RNA**→ ? (GOS1720)→
env-33	JCVI_SCAF_1096627339561	+	642	772	**RNA**→ ? (GOS1720)→
env-34	JCVI_SCAF_1101668048187	-	1134	1004	**RNA**→ BtuB (COG4206)? (GOS1720)→
env-35	JCVI_SCAF_1096627699890	-	422	290	**RNA**→ ←? (GOS11714930)
env-36	JCVI_SCAF_1096626643666	+	1300	1432	**RNA**→ ←? (GOS11714930)
env-37	JCVI_SCAF_1096627719709	-	1443	1311	**RNA**→ ? (GOS4369)→
env-38	JCVI_SCAF_1096628259790	+	1208	1350	**RNA**→ ? (GOS4369)→
env-39	JCVI_SCAF_1096626962232	-	1959	1818	**RNA**→ ? (GOS920)→ TonB_dep_Rec (pfam00593)? (GOS4665)→
env-40	JCVI_SCAF_1096627991926	-	141	13	**RNA**→
env-41	JCVI_SCAF_1096627270303	-	1972	1838	**RNA**→ ? (GOS920)→ BtuB (COG4206)? (GOS4665)→

env-42	JCVI_SCAF_1096627854245	-	1394	1264	**RNA**→ ←? (GOS11790770)
env-43	JCVI_SCAF_1101668501944	+	36	167	**RNA**→ ligand_gated_channel (cd01347)? (GOS1720)→
env-44	JCVI_SCAF_1096627301998	+	1198	1329	**RNA**→ ? (GOS1720)→
[2]env-45	JCVI_SCAF_1096628403513	-	1599	1469	**RNA**→ ? (GOS4369)→
env-46	JCVI_SCAF_1096627684153	+	893	1024	**RNA**→ ? (GOS920)→
env-47	JCVI_SCAF_1096627808730	-	815	683	**RNA**→ ? (GOS4369)→
env-48	JCVI_SCAF_1101668713690	-	379	251	**RNA**→ ←? (GOS11790770)
env-49	JCVI_SCAF_1096627256224	+	234	364	**RNA**→ BtuB (COG4206)? (GOS1720)→
env-50	JCVI_SCAF_1096627698506	+	1315	1453	**RNA**→
env-51	JCVI_SCAF_1101668212159	-	162	24	**RNA**→
env-52	JCVI_SCAF_1096627993344	-	1348	1220	**RNA**→ ? (GOS4369)→ BtuB (COG4206)? (GOS1720)→
env-53	JCVI_SCAF_1096627699886	-	468	339	**RNA**→ ? (GOS1720)→
env-54	JCVI_SCAF_1101668502439	-	1211	1082	**RNA**→ BtuB (COG4206)? (GOS1720)→
env-55	JCVI_SCAF_1096628156220	-	1539	1410	**RNA**→ ? (GOS4369)→ ? (GOS9454)→ ? (GOS11245591)→
env-56	JCVI_SCAF_1096627830576	-	1617	1488	**RNA**→ ? (GOS4369)→ BtuB (COG4206)? (GOS1720)→
env-57	JCVI_SCAF_1101667038075	+	579	710	**RNA**→
env-58	JCVI_SCAF_1096627982330	-	566	436	**RNA**→ ? (GOS4369)→
env-59	JCVI_SCAF_1096626313314	+	459	590	**RNA**→ ? (GOS11094971)→
env-60	JCVI_SCAF_1096627886130	+	747	877	**RNA**→ ? (GOS11622442)→
env-61	JCVI_SCAF_1096628272437	+	1440	1571	**RNA**→
env-62	JCVI_SCAF_1096628236348	-	418	295	**RNA**→ ? (GOS1720)→
env-63	JCVI_SCAF_1096628165183	+	63	187	**RNA**→ ? (GOS4369)→
env-64	JCVI_SCAF_1101667474120	+	18	144	**RNA**→ ? (GOS4369)→
env-65	JCVI_SCAF_1096627032404	-	590	462	**RNA**→ ? (GOS920)→
env-66	JCVI_SCAF_1096628065383	+	343	471	**RNA**→ ? (GOS4369)→
env-67	JCVI_SCAF_1096627581275	-	1700	1572	**RNA**→ ? (GOS920)→ TonB_dep_Rec (pfam00593)? (GOS4665)→
env-68	JCVI_SCAF_1096626922281	-	1134	1007	**RNA**→ ? (GOS1720)→
env-69	JCVI_SCAF_1096628084481	+	445	586	**RNA**→ ? (GOS4369)→
env-70	JCVI_SCAF_1101668356142	-	614	484	**RNA**→ ? (GOS4369)→
env-71	JCVI_SCAF_1096626133132	-	131	1	**RNA**→
env-72	JCVI_SCAF_1096626933891	-	1319	1189	**RNA**→ BtuB (COG4206)? (GOS496)→
env-73	JCVI_SCAF_1096627001005	+	640	770	**RNA**→ ? (GOS1720)→
env-74	JCVI_SCAF_1096628223711	+	1507	1637	**RNA**→ ←? (GOS11081972)
env-75	JCVI_SCAF_1096627431207	-	1457	1326	**RNA**→ ? (GOS1720)→ TonB_dep_Rec (pfam00593)? (GOS1720)→
env-76	JCVI_SCAF_1096628197778	-	1231	1100	**RNA**→ ? (GOS1720)→
env-77	JCVI_SCAF_1096627578146	+	73	202	**RNA**→ ? (GOS4369)→ ? (GOS4665)→
env-78	JCVI_SCAF_1101668011934	+	125	250	**RNA**→ ? (GOS920)→ ? (GOS496)→
env-79	JCVI_SCAF_1096627867925	+	240	367	**RNA**→ ? (GOS4369)→ ? (GOS1720)→
env-80	JCVI_SCAF_1101668659621	+	480	607	**RNA**→ ? (GOS4369)→
env-81	JCVI_SCAF_1101668182195	+	1359	1486	**RNA**→
env-82	JCVI_SCAF_1101668012817	-	471	342	**RNA**→ ? (GOS1720)→
env-83	JCVI_SCAF_1096627570490	+	1297	1426	**RNA**→ ? (GOS920)→
env-84	JCVI_SCAF_1101668471885	-	643	515	**RNA**→ ←? (GOS11790770)
env-85	JCVI_SCAF_1101668459580	+	711	851	**RNA**→ ? (GOS920)→
env-86	JCVI_SCAF_1096626582089	+	673	814	**RNA**→
env-87	JCVI_SCAF_1096627925949	+	429	560	**RNA**→ ? (GOS1720)→ BtuB (COG4206)? (GOS496)→
env-88	JCVI_SCAF_1096626980542	-	1015	884	**RNA**→ ? (GOS1720)→
env-89	JCVI_SCAF_1096626944243	+	159	290	**RNA**→ BtuB (COG4206)? (GOS1720)→
env-90	JCVI_SCAF_1096627815144	-	269	138	**RNA**→
env-91	JCVI_SCAF_1096628098054	+	83	213	**RNA**→ ←? (GOS11081972)
env-92	JCVI_SCAF_1101667521383	-	602	472	**RNA**→ ? (GOS4369)→
env-93	JCVI_SCAF_1096628238228	-	371	246	**RNA**→ ? (GOS4369)→

env-94	JCVI_SCAF_1096627993984	-	1344	1214	**RNA**→ ? (GOS1720)→ ? (GOS1720)→
env-95	JCVI_SCAF_1096627324500	-	188	58	**RNA**→
env-96	JCVI_SCAF_1101668283861	+	141	271	**RNA**→ BtuB (COG4206)? (GOS1720)→
env-97	JCVI_SCAF_1096627835374	-	1102	977	**RNA**→ BtuB (COG4206)? (GOS1720)→
env-98	JCVI_SCAF_1096627226916	-	899	771	**RNA**→ ? (GOS1720)→
env-99	JCVI_SCAF_1096628254797	-	1323	1194	**RNA**→ ? (GOS920)→ ? (GOS4665)→
env-100	JCVI_SCAF_1101667554535	-	534	405	**RNA**→ ←? (GOS11790770)
env-101	JCVI_SCAF_1101667253413	+	477	606	**RNA**→ ←? (GOS11790770)
env-102	JCVI_SCAF_1101667515587	+	475	603	**RNA**→ ? (GOS4369)→
env-103	JCVI_SCAF_1096627126564	+	969	1097	**RNA**→ ? (GOS920)→
env-104	JCVI_SCAF_1096627441792	+	1339	1467	**RNA**→ ? (GOS4369)→
env-105	JCVI_SCAF_1096627799570	+	634	764	**RNA**→ ? (GOS1720)→
env-106	JCVI_SCAF_1096628243783	+	51	179	**RNA**→ ? (GOS1720)→ ? (GOS4665)→
env-107	JCVI_SCAF_1096627898401	+	1404	1546	**RNA**→
env-108	JCVI_SCAF_1101668677996	-	164	34	**RNA**→
env-109	JCVI_SCAF_1101667429719	+	358	489	**RNA**→ ? (GOS1720)→
env-110	JCVI_SCAF_1101668547925	-	1002	875	**RNA**→ ? (GOS1720)→
env-111	JCVI_SCAF_1101667533745	+	172	299	**RNA**→ ? (GOS4369)→
[1]env-112	JCVI_SCAF_1101667573135	+	469	599	**RNA**→ ? (GOS1720)→
env-113	JCVI_SCAF_1096628056260	+	1623	1753	**RNA**→
env-114	JCVI_SCAF_1096627507316	-	520	390	**RNA**→ ? (GOS11094971)→
env-115	JCVI_SCAF_1096626884209	+	524	655	**RNA**→ ? (GOS1720)→
env-116	JCVI_SCAF_1101668488469	-	924	793	**RNA**→ ? (GOS496)→
env-117	JCVI_SCAF_1096628393525	+	1318	1448	**RNA**→ ←? (GOS11081972)
env-118	JCVI_SCAF_1096628194870	-	1569	1438	**RNA**→ ? (GOS4369)→ AsnB (cd00712)? (GOS729)→
env-119	JCVI_SCAF_1101668691878	+	589	719	**RNA**→ ? (GOS4369)→
env-120	JCVI_SCAF_1096628389621	-	777	647	**RNA**→ ? (GOS1720)→
env-121	JCVI_SCAF_1101668181310	+	918	1047	**RNA**→ ? (GOS920)→
env-122	JCVI_SCAF_1096626342623	+	28	158	**RNA**→ ? (GOS1720)→
env-123	JCVI_SCAF_1096628133600	-	1261	1131	**RNA**→ ? (GOS920)→
env-124	JCVI_SCAF_1096627559529	-	1746	1616	**RNA**→ ? (GOS4369)→ ? (GOS4665)→
env-125	JCVI_SCAF_1096626626183	+	425	555	**RNA**→ ? (GOS1720)→
env-126	JCVI_SCAF_1096628274095	+	1673	1803	**RNA**→
env-127	JCVI_SCAF_1096627184957	+	1942	2072	**RNA**→
env-128	JCVI_SCAF_1096627599646	+	1443	1573	**RNA**→
env-129	JCVI_SCAF_1101668433054	-	312	182	**RNA**→
env-130	JCVI_SCAF_1096628072499	+	592	722	**RNA**→ ? (GOS1720)→
env-131	JCVI_SCAF_1096627825342	-	570	441	**RNA**→ ? (GOS4369)→
env-132	JCVI_SCAF_1096627794834	-	1956	1826	**RNA**→ ? (GOS1720)→ FepA (COG4771)? (GOS4665)→
env-133	JCVI_SCAF_1096628049855	-	1223	1093	**RNA**→ ? (GOS9454)→ 5S_rRNA→
env-134	JCVI_SCAF_1096628121198	-	484	354	**RNA**→ ←? (GOS11081972)
env-135	JCVI_SCAF_1101667419775	+	622	753	**RNA**→ ? (GOS11983570)→
env-136	JCVI_SCAF_1096628119255	+	580	711	**RNA**→ ? (GOS4665)→
env-137	JCVI_SCAF_1096628284445	-	760	634	**RNA**→ ? (GOS4369)→
env-138	JCVI_SCAF_1096627115721	+	242	372	**RNA**→ BtuB (COG4206)? (GOS1720)→
env-139	JCVI_SCAF_1101668527442	-	1012	862	**RNA**→ ? (GOS1720)→
env-140	JCVI_SCAF_1101668519723	+	854	994	**RNA**→
env-141	JCVI_SCAF_1096626860966	+	57	194	**RNA**→ BtuB (COG4206)? (GOS496)→
env-142	JCVI_SCAF_1096628185343	-	860	742	**RNA**→ ? (GOS1720)→
env-143	JCVI_SCAF_1096627388192	-	1248	1134	**RNA**→ BtuB (COG4206)? (GOS1720)→
env-144	JCVI_SCAF_1101668310910	+	603	724	**RNA**→ ? (GOS1720)→

6.4 Conserved domains

Conserved domains found in protein-coding genes listed in Section 6.3 are listed, with the first sentence in their description from the Conserved Domain Database. Conserved domains associated with more than one AdoCbl-variant RNA are assigned a color, while others are shown in gray. The number in parentheses is the number of occurrences of the given gene.

cd00712 (1) Glutamine amidotransferases class-II (GATase) asparagine synthase_B type.
cd01347 (1) TonB dependent/Ligand-Gated channels are created by a monomeric 22 strand (22,24) anti-parallel beta-barrel.
COG4206 (19) Outer membrane cobalamin receptor protein [Coenzyme metabolism]
COG4771 (1) Outer membrane receptor for ferrienterochelin and colicins [Inorganic ion transport and metabolism]
GOS11081972 (4) ?
GOS11094971 (2) ?
GOS11245591 (1) ?
GOS11622442 (1) ?
GOS11714930 (2) ?
GOS11790770 (6) ?
GOS11983570 (2) ?
GOS1720 (53) ?
GOS4369 (31) ?
GOS4665 (12) ?
GOS496 (6) ?
GOS5954 (1) ?
GOS729 (1) ?
GOS920 (13) ?
GOS9454 (3) ?
pfam00593 (3) TonB dependent receptor.
pfam02511 (1) Thymidylate synthase complementing protein.

6.5 Multiple-sequence alignment

Each AdoCbl-variant RNA is denoted by an abbreviation (like "Eco-1-1") that refers to a taxonomy given in Section 6.1. Superscript numbers refer to annotations listed in Section 6.2. The alignment includes sequences containing the RNA motif, as well as flanking sequence. The AdoCbl-variant RNA itself is denoted by the line underneath marked 5′ and 3′ on either end. Nucleotides in the flanking sequences (i.e., not part of the motif) are written in gray letters. Stems of predicted rho-independent transcription terminators are shaded yellow. (Note: terminator predictions have not been analyzed manually, and many are likely to be false positives.) Start codons are shaded green. (Note: start codons are frequently misannotated, especially in environmental samples.) Nucleotides proposed to basepair as part of the consensus structure are shaded in color when they comprise Watson-Crick or G-U pairs. Otherwise they are shaded gray. Conserved stems are also indicated at the bottom of the alignment by angle brackets, where matching < and > denote base-paired columns. Below these angle brackets, the symbol "2" denotes base pairs exhibiting covariation, "1" denotes base pairs exhibiting compatible mutations, "0" denotes base pairs that are not observed to mutate and "?" denotes base pairs that have a significant frequency of non-canonical nucleotides for Watson-Crick or G-U pairs (> 5%). Below these base pair annotation is the consensus sequence: "R" = "A" or "G", "Y" = "C" or "U", red nucleotides: nucleotide identity conserved more than 97% of the time, black nucleotides: 90%, gray nucleotides: 75%, red circle (•): nucleotide is present 97% of the time, black circle (•): 90%, gray circle (•): 75%, white circle (∘): 50%. All percentages of sequences just described (e.g. 97% conserved) assume that sequences have been weighted by the GSC algorithm implemented by the Infernal software.

alignment positions 1 ··· 160

```
env-1  AUGCAAGAGUAAAUAUAAACGACUGCAUAGAUCGCUUUUGUAAGUACUAAAUUGAGGGUUGCACAUAACGGUAAAAGUUGUUACUUUGAAAAAUGAAAAGUAAAUAAGUUUAUAUG.UGAUGAAGUAGAGUGAA.....AUUCUCUCACUGU.CGCGCAA
env-2  CCAUAUUUAUUGGUGUGGUAAUUUGCAAAAAAUAAUUACAACUGUGUUAUAGAUCACAUUGACAGAAGUGGCUUUGUGCGAUAUAAUAUUACAUAAGACUGUGCUAAAAG.CAUAG.UGGGAAAGUGACGUGAA.....AUUCGUCCACAUUACUUGAUA
env-3  CAAAUCAUUAUAAAGCAAUGAAUCCAAAUUUACCAACAUUUAAUGGAAUACCUAAUCCAUUUCACUUGUAUAAAAAAGAAAAAUAACAUAUAAUAAUAAUGUGCUAAAAG.CAUGG.UGGGAAAGUGACGUGUA.....AUUCGUCCACAUUACUUGAUA
env-4  UUUUUGUUAUCAGGCAUCGGCUAAUAUUUAUUGUACUACAAAUACCACAGAAAUCAAAUCUUCAUUUGACAGAACAGUGUUUCUACCAUAUAAUAGUAAUGUGUUAAAAG.CAUAG.UGGGAAAGUGACGUGUA.....AUUCGUCCACAUUACUUGAUG

       ....................................................................................................<<<<<<........<<.<<<.....<<<<<............>>>>>..<<<....>>>.
       ....................................................................................................?22222........?2.222.....?2221............1222?..221....122.
       .....................................................................................................................................................<<<....
       .....................................................................................................................................................022....
       ...............................................................................................................................................<<...........
       ...............................................................................................................................................22...........
       ••••••••••••••••••••••••••••••••••••••••••••••••••••••••••••••••••••••••••••••••••••••••••••••••••••RURYUGAA∘∘∘Y•UGG-UGGGRAA∘YARUGUGAA-----AUUCAUURGCUGU•CCUGCAA
                                                                                                           5′
```

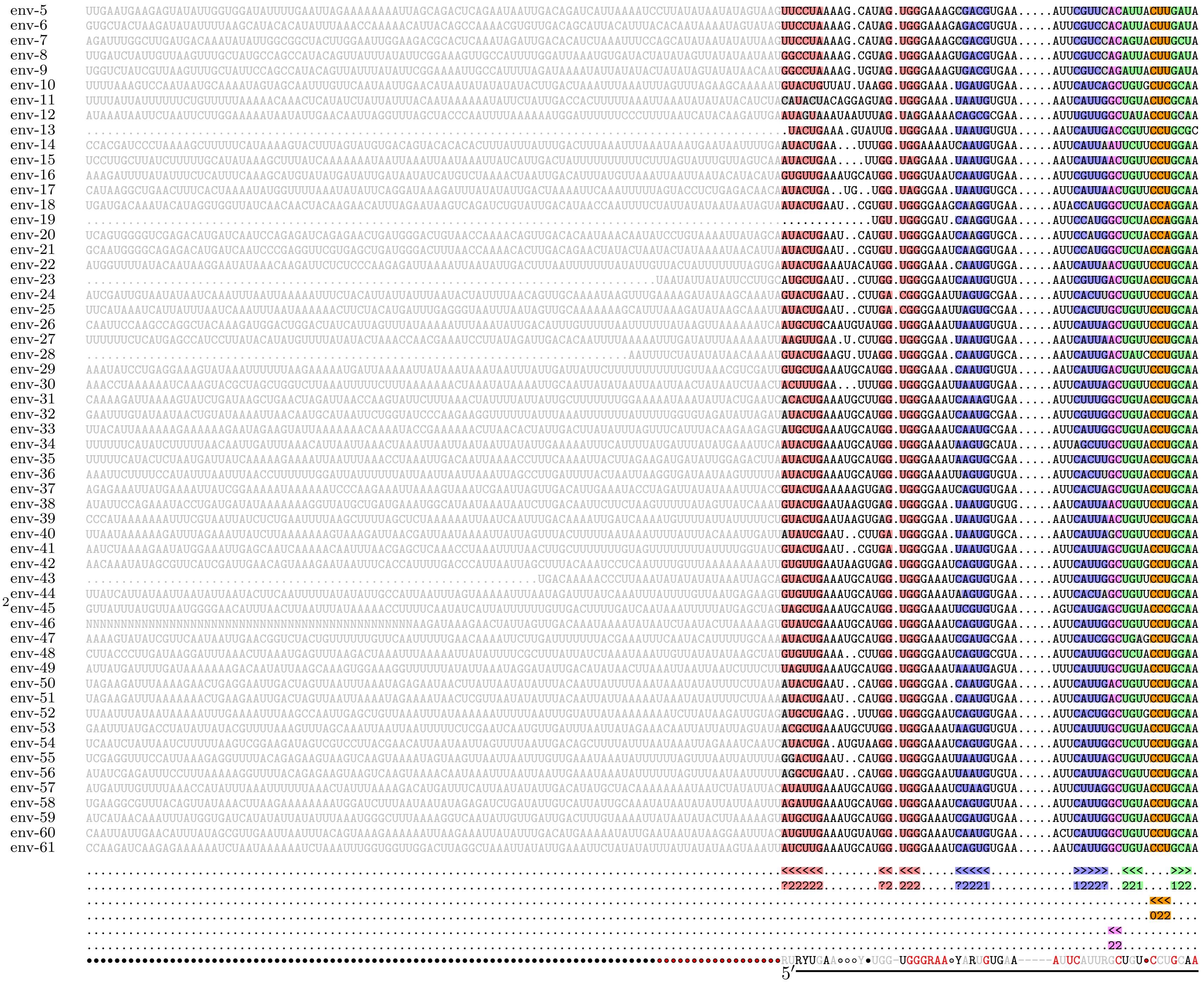
env-5
env-6
env-7
env-8
env-9
env-10
env-11
env-12
env-13
env-14
env-15
env-16
env-17
env-18
env-19
env-20
env-21
env-22
env-23
env-24
env-25
env-26
env-27
env-28
env-29
env-30
env-31
env-32
env-33
env-34
env-35
env-36
env-37
env-38
env-39
env-40
env-41
env-42
env-43
env-44
2 env-45
env-46
env-47
env-48
env-49
env-50
env-51
env-52
env-53
env-54
env-55
env-56
env-57
env-58
env-59
env-60
env-61
5′

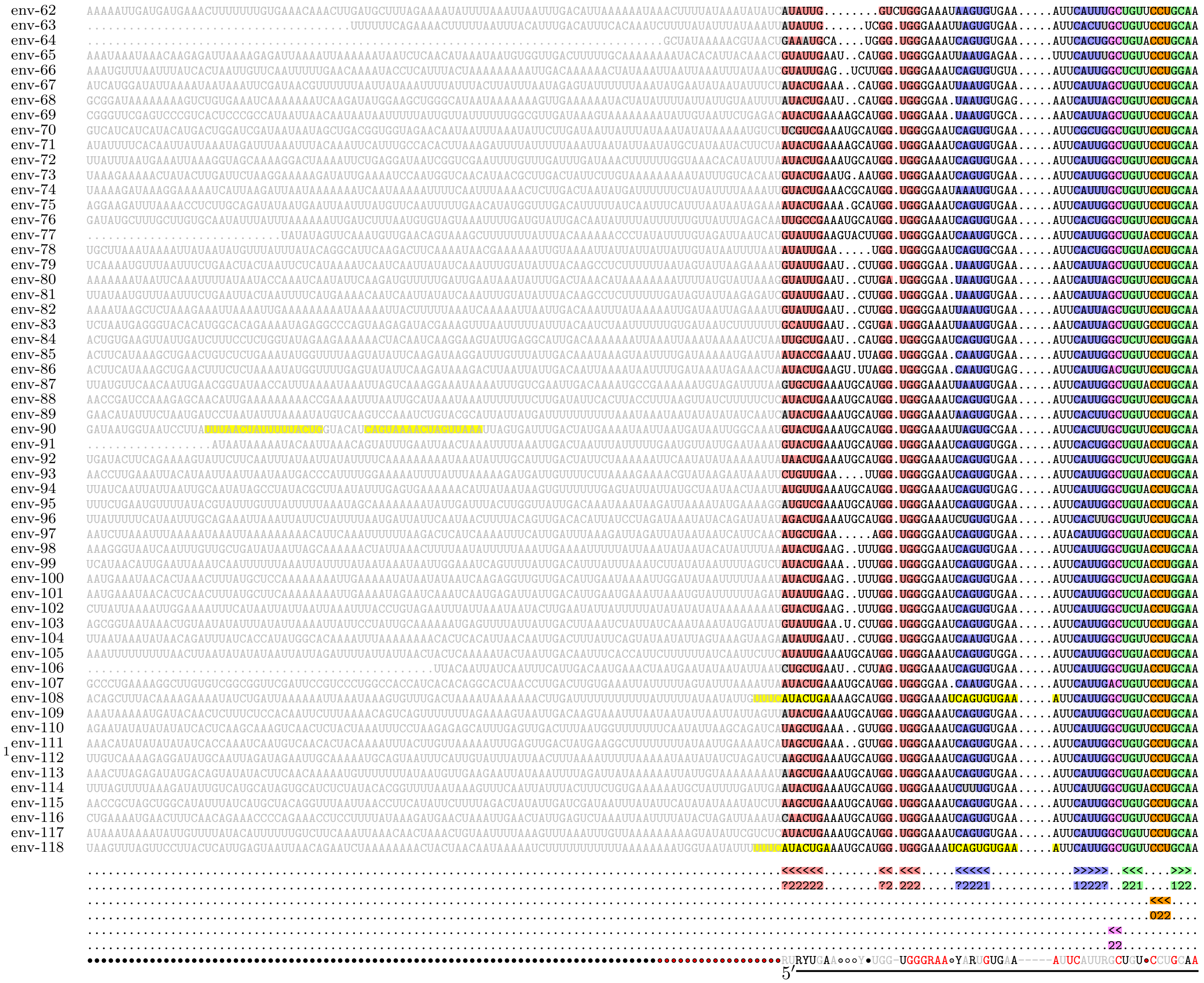
env-62
env-63
env-64
env-65
env-66
env-67
env-68
env-69
env-70
env-71
env-72
env-73
env-74
env-75
env-76
env-77
env-78
env-79
env-80
env-81
env-82
env-83
env-84
env-85
env-86
env-87
env-88
env-89
env-90
env-91
env-92
env-93
env-94
env-95
env-96
env-97
env-98
env-99
env-100
env-101
env-102
env-103
env-104
env-105
env-106
env-107
env-108
env-109
env-110
env-111
env-112
env-113
env-114
env-115
env-116
env-117
env-118
5′

```
env-119  AGAAUUAAGUGUAGGAGUAACAAUUGACAAUUUCAUAUAUUAAUAAAGUUUUCUAUACCUUUUGCUUUUAAAUUUCAUUAAUUUAGUGUAAAUAAGAACCAUACUGAAAUGCAUGG.UGGGAAAUCAGUGUGAA.....AUUCAUUGGCUGUACCUGCAA
env-120  AAUCUACACUUGUCUUAAUUAUUNNNNNNNNNNNNNNNNNNNNNNNNNNNNNNNNNNNNNNNNNNNNNNNNNNNNNNNUUUUUUAUAGGAUGAUAUAAUUAUCACAUACUGAAAUGCAUGG.UGGGAAAUCAGUGUGAA.....AUUCAUUGGCUGUUCCUGCAA
env-121  UUCAUAAGGCUGAGUUAUCUUUAAGGUAUGGUUUUAAAUAUAUUCAGGACAAAGAUUUGUUUAUUGACAAAUAAUUUUUGUAUAGUAGUUAGACGGAAUAAUACCGAAAUGCAUGG.UGGGAAAUCAGUGUGGA.....AUUCAUUGGCUGUCCCUGCA.
env-122  .........................................................................AAUUUUAUUUAUAGUAUUCAAAAAAUAAUACUGAAAUGCAUGG.UGGGAAAUCAGUGUGAA.....AUUCAUUGGCUGUCCCUGCAA
env-123  UUCAUAAAGCAGAAUUGUCAUUAAAAUAUGGUUUUAAAUAUAUUCAGGACAAAGAUUUAUUUAUUGACAAGUAAUUUUACUUUUAGUAUUAAACGAAUUAAUACCGAAAUGCAUGG.UGGGAAAUCAGUGUGAA.....AUUCAUUGGCUGUCCCUGCAA
env-124  AUACCUAGUAUUUCUAUACAACCAGUUAAAUUGUGCAACAAGGAUUUUUGCCGUAAGCUAAAUAGAUUGCAUUAAAGUUAUCGUUAUUAUAUCAUAAGAUAUACUGAAAUGCAUGG.UGGGAAAUCAGUGUGAA.....AUUCAUUGGCUGUACCUGCAA
env-125  AUUUAUUUUUGAAAAUAAGAUAGACUCUUUAAACUCAAAGAAAUAAAAUUUAACCUCUUUUUUAAUAUUGACAAAAUAUAUUUGUAAAAUAAUAUUAAAAUGACUGAAAUGCAUGG.UGGGAAAUCUGUGUGAA.....ACUCAUAGGCUGUACCUGCAA
env-126  CCAAGAAAACGUUUUAGAAUGGAGAAAAAGAAGAAAAAAAAGAUAUGACUCUGUCAAAUAAAAUAUUUGACUUUUUUUCAUUUUAUAAUAUUUAAAACUUAGGCUGAAAUGCAUGG.UGGGAAAUCAGUGUGAA.....AUUCAUUGGCUGUUCCUGCAA
env-127  AAAACUACGUAAAAAUUAAUUAAAAAUUGAUUAAUUAAAUACUCAAUCAAUUUUAAUUUAUAUUAUUGCAAAUUUAUAUUUUACCCAAUAUAACUCAGCUGUACUGAAAUGCAUGG.UGGGAAAUCAAUGUGUA.....AUUCAUUGGCUGUACCUGCAA
env-128  UUAAAAUACAUUUGCUCAAAAAUUUUAGAUUUUUAUAAGAUCAAAGCUUAUUUUAAGAAAAUCACUUGAUUUUUGAUUAACAUUAAUAUAAAUAGAUUUUGUACUGAAAUGCAUGG.UGGGAAAUCAGUGUGUA.....AUUCAUUGGCUGUGCCUGCAA
env-129  CAAAAACCCAUCAAAAUGAAAAAUUUUUUUACAAACAAUCCUUUAUUUUAGUAAAUUUUUUAAGGUUUACAGAGUUAAUAUUUUGUAUAAAAAAAAGAUAAUACUGAAAUGCAUGG.UGGGAAAUCAGUGUGAA.....AUUCAUUGGCUGUUCCUGCAA
env-130  UUCUUCUCAAAUAUCUUUUUUUCAUUUAUUUUAUCUAAAAAGUUGUAUUUUGCUAAAAAAAUUUAUUUGACAUUUAUAUUAUAAAUUAUAAAUGUAACUGAUACUGAAAUGCAUGG.UGGGAAAUCAGUGUGAA.....AUUCAUUGGCUGUACCUGCAA
env-131  GAGAAAGGUAAACUAAUUAAAAUCGGAAAAACCAAUUCAGGUUUUAAGAAAUUAGGAAUAAAUAUUUAUUGACUAAAUUGAUUAUUAUUGUAACUUAUAAUUGCUGAAAUGCAUGG.UGGGAAAUCAGUGUGAA.....AUUCAUUGGCUGUACCUGCAA
env-132  ....................................................................................................AUACCGAAAUGCUUGG.UGGGAAAUCAGUGUGAA.....AUUCAUUGGCUGUACCUGCAA
env-133  AUACGCUAUUUCUAUACUUUUAGACGUAAUCUUCAAUAAGGAUAAUUUGACAUAUUAUCUAGUUAUUGCAAUAUUUUUCUACGUGUUGUAUUUAAAUAAUGUGCUGAAAUGCAUGG.UGGGAAAUCAGUGUGAA.....AUUCAUUGGCUGUUCCUGCAA
env-134  AUCUUAAAAUCAAAAAUCUUGCUUAUUAGCCUAUAAUUGUUGAUUUUAUGUUAACUUGAUCACCUAUUGACAUUUUUGAGGAAAUUUUUAUUGUACAAGUGUAUUGAAAUGCAUGG.UGGGAAAUCAGUGUGAA.....AUUCGCUGGCUGUUCCUGCAA
env-135  GAGACAAGGCGAAAUUAAUUUUGUUAUCAAAACAAUGAGAUCUUUUUUUAGAAAGAAUUAAUUACUUGCUAUUUAUUUAAUAAUCAUUAUAAAAAUAAAUGUACUGAAAUGCAUGG.UGGGAAAUCAGUGUGAA.....AUUCAUUGGCUGUUCCUGCAA
env-136  GAGACAAAGCGAAAUUAAUUUUGUUAUCAACACGAUAAGAUCUUUUUUUAGAAAAAAUUAAUUACUUGCAAAUUGUUUUACAAUCAUUAUAAAAACCAAUGUACUGAAAUGCAUGG.UGGGAAAUCAGUGUGAA.....AUUCAUUGGCUGUUCCUGCAA
env-137  GCAAGAAGGGACAAUGCGAUAAUGAAUUUAAAAAUAUUAGUAGAAAAAAAAAGUGACAUUUUUAUUUGAUUUCUUUAUUUAUUUAAUCUAAAUACUAAGCAUAUUGAU....AUGG.UGGGGAAUCAGUGUGAA.....AUACAUUGGCUGUACCUGCAA
env-138  UCAAUUAAUCUUUUACAAUUAUUUGUUUAUAUUUAAAUAGUUUAUAUACGUUGAUUUAUAUGAUGUUUGACAAAUUAAUUUAAUUUAAUUAAUAUAAUUAAAGUCGAAAUGCUUGG.UGGGAAAUCAGUGUGAA.....AUUCACUGGCUGUACCUGCAA
env-139  AUGCAAAAACAGGUGAAAAUUUUGUAAAAUAUGCUUUAUCUAAUUAAUAAAUUUUACUUAAUAAACUUGAAAAUUAUAAUUUUUCAAUAUAAACGAAAUACACUGAAGUACAUGG.UGGGAAA.UAAUGUGAG.....AAUCAUUAACUGUUCGUGCAA
env-140  UAAUAACAAAUUUCCUUCAACAGAACAACUAGCCACCAUAGAUGGAGUGGUUUUUAGAAAAUAAUUUGACUUUAUAAAAAAAAAAAUAGUACCAAGUUUUUAUACUGAAAG.CAUGG.UGGAAAA.CAAUGUGUG.....AUUCAUUGGCUGUACUCGCAA
env-141  ............................................UUAUACUGUAUUCUUUAGCUAUAUAUUUACUCAAUAGAGGUAUUGGUGUAAAAAAUUAACUCUCAC.GAGGU.UGGGAAA.GUCAGUGAAUACAUAAUCUGACACUGCCCCGCAA
env-142  ACAACGGUUAAUCGCUUUUGUCUCUANNNNNNNNNNNNNNNNNNNNNNNNNNNNNNNNNNNNNNNNNNNNNNNNGGGGUUGGUAUAAAAAAAUUAGAUGUGCAUUU...GAAGU.AGGGAAAAUUCUGU.AA.....AUUCAGAUACUACACCCGUAA
env-143  UAUUCUUAGCUCUUGUCAUAGUUCUUGUUUUUGCUGUUUUUUUAUUUAUAAUUGGUAUUCAAAUACUAAACAAAGGGGUUGGUAUAAAAAAAUUAGAUGUGCAUUUGAA......GU.AGGGAAAAUUCUGUAAA......UUCAGAUACUACACCCGUAA
env-144  ACAUAUUCUUAGCUCUUGCGAUAGUUCUUGUCUUUGCUGUCAUGUUAUUUAUAAUUGGAGUACAAAUACUAAACAAAGGGGUUGGUAUAAAAAAAUUAGAUGUGCAUUU...GAAGU.AGGGAAAAUUCUGU.AA.....AUUCAGAUACUACACCCGUAA
         ....................................................................................................<<<<<<........<<.<<<.....<<<<<.............>>>>>..<<<....>>>.
         ....................................................................................................?22222........?2.222.....?2221.............1222?..221....122.
         ......................................................................................................................................................<<<....
         ......................................................................................................................................................022....
         ..................................................................................................................................................<<..........
         ..................................................................................................................................................22..........
         ••••••••••••••••••••••••••••••••••••••••••••••••••••••••••••••••••••••••••••••••••••••••••••••••••••RURYUGAA∘∘∘Y•UGG-UGGGRAA∘YARUGUGAA-----AUUCAUURGCUGU•CCUGCAA
                                                                                                             5′
```

alignment positions 161 · · · 320

```
env-1    CGGU.........UAAA.................GUCCGAAC.UCACGUAUAAG.CUAAAGACGUAGCU...AGGCU.CGCGGAA.AGC..U.A....AGCAGU.UACUCAAAAAGUAAUUGGUCCCGCUAGAGCGGGAUUUUUUUAUCCCCUCCUA
env-2    AGGU.........CAUA.................GUCCGAAUGCCACCUAGCAC.AGAA..........C..AGAG.CAAGGAGACUC..GAAUGG....UAA.GAUUGAUUAUAUUAAUCUUGGUAUGGACAUCUAUAGCCCAUGCCAAAACAGA
env-3    CGGU.........UAUA.................GUCCGAAUGCCACCUAGCACAA.............AGUAGAG.CAAGGAGACUCAAUGA..U....UAG.AAUAAUUCUAAUAUUAUUCUUUAUAUGGAUUACUACGGUUCAAGCAGAAAUU
env-4    CGGU.........UAUA.................GUCCGAAUGCCACCUAACACAA.............AGUAGAG.CAAGGAGACUCAAUGA..U....UAG.AAUAACUCUAAUAUUAUUCUUUGUAUGGAUUACUACGGUUCAAGCAGAGAUU
env-5    CGGU.........UAUA.................GUCCGAAUGCCACCUAGGAA.CGU...........AUA.GAG.CAAGGAGACUC.......G....UGA.AAAGAAUAUUAAUGGCAAUAUUCUUAGCAUGGUUUAGUACGGCCAAUGCUGA
env-6    CGGU.........GAUA.................GUCCGAAUGCCACCUAGGAAUAGA...........U..AGAG.CAAGGAGACUCAAUGA..A....UAA.AGUAACUACAGCCAUGGCAACUGUGCUUUGGAUUUUGUGGACGUCUGCACAU
env-7    CGGU.........CAUA.................GUCCGAAUGCCACCUAGGAA........UGU.....AACAGAG.CAAGGAGACUCGAUGA..G........AAUAUUAUUAAUAUUCUUAGUGUGGAUUACCACGGCCCAUGCUGAGAUUGUA
env-8    CGGU.........UAUA.................CUCCGAAUGCCACCUAGGCC...........AUA.CAACGAG.CAAGGAGACUC..A.A..CAU.....GAUAAGAUUAAUUCUUAUAUUAAUCUUGGUAUGGGCUAAUGUUGCCUAUUCU
env-9    CGGU.........UAUA.................CUCCGAAUGCCACCUAGGCC...........AUA.CAACGAG.CAAGGAGACUC..A.A..CAU.....GAUAAGAUUAAUUCUUAUAUUAAUCUUGGUAUGGGCUAAUGUUGCCUAUUCC
env-10   CGGU.AAA......UUAG.............UUAAAGUCCGAAGGCCACUCAGUAU.AGUCCGGUGUUAGA.AAGUAAU.CGAGAAGAAUU..ACA..A.UCUUAC.AAUGAAAAAUUAGCAACGGCAUAAAAUUAUAAUUUGUUUUUUAUUAAAAUAU
env-11   CGGU.........AAAAAAA...............GCCCGGAAACCACAUAAAGU.AUACCACUGUUGGA.UGAAGAC.CGAGAAAAGUC..A.C..G.UUCCAC.AAUUUUCAUAUUAGCAGUGUAUUAACAAAAAGAUUACGAAAACCUUAAAUUG
env-12   CCGU.........UAAA.................GUCGGAGAGCCACCCAGUAU.AGCCCAGUGUUGAA.UGAAGGC.CAGGAAAAGUC..U.A..G.CUCUGC.AACUAAUUUUGGCAUCGGCAAAUUUAUUAAAAUAGUUUAAGAAUUUGUUUGC
env-13   UGGUA.AGAA....UAAAN..........UUUUA..GUCCAGACACCAC.CAGUAU.AGCCCUCUGUCGAA.UGACGAC.CAGGAAAAGUC..U.A..G.UUCUAC.AAUGAAAAAUUAACAGCGGCAUUAAAAAAGGAAGAGCAAUUAAAAGUUUAAC
env-14   CCGU.........UAAA.................GUCGGAGCACCACCCUGUAG.AGUCCGGUGUUGAA.UGAUGAA.CAGGAAAAUUC..U.A..G.UUCUAC.AAUUUUAAUUAACAACGGCACUAAAUAACGAGCAAUAAGUAUUUAUUGUUCA
env-15   CGGUGA.......AAAAGUAAGAUA........AA.GUCCGAGCACUACCCAGUAU.AGUCCCCUGUCGAA.CAAUGGC.CAGGAAAAGUC..U.A..G.UUCUAC.AAUGAAAAAUUAGCAGUGGCAUUAACAGUAAAAAAUUAAAGCUAUGGCUUUA
env-16   CGGU.........UAUA.................GUCCGAGCGCCACCCAACAU.AGUCCGAC.UAGAA.UGAAGGC.CAGGAAAAGUC..U.A..A.UUCUGUUAACGGCAAAACGUAAAAAAAAAGAGAGUUUGCCGUGAAAUCAAAAAUUUAUC
env-17   CGGUAAAAAC....UAAA...........GUUUAA.GUCCGAGCGCUACCCAGUAU.AGUCCCCUGUCGAA.UGAUGGC.CAGGAAAAGUC..U.A..G.UUCUGC.AAUGCAAAAUUAGCAGUGGCAUUAACUUAAAUUUUAAGGUGAAAACCUUAAU
env-18   CCGU..........AUA.................GUCGGAGUGCCAACCAGUAU.AGUCCGCUGUCGAA.UGAAGGC.CUGGGAAAGUC..U.A..A.UUCUAC.AAUGUAAAGUUAACAGUGGCGAUAAACGUUAACACGUUUAAUAGGAUACUGU
env-19   CCGU.........AA.A.................GUCGGAGUGCCAACCAGUAU.AGUCCGCUGUUGAA.UGAGGGC.CUGGGAAAGUC..U.A..G.UUCUAC.AAUGUAUAUAAUUGCAGUGGCAUAUAGAAAGGCUACUGUUAUGAAAAAAUAU
         <<<...<<<<<<<<...........>>>>>>>>.....>>>....>>>>>>>>>>>............<<<.<..<<<<.........>>>..>....>.>>>...............................................
         220...?222?020...........020?222?.....022....2222?22222?............222.2..?222.........222..?....2.222...............................................
         ........................................................................................>>>...............................................................
         ........................................................................................220...............................................................
         ..................................>>..............................................................................................................................
         ..................................22..............................................................................................................................
         C•GU----------•A-A-----------------GUC•GAGYGCCACCCARYA•-AGYCCRYUGUYGAA-YGA•GGC-CAGGAAAAGUC--U-A--R-UUCURC-AAU•••AA•••R•••••R••••••••••••••••••••••••••••••••••••
                                                                                                                                    3′
```

```
env-20   CCGU..........AA.A...................GUCGGAGUGCCAACCAGUAUAAGUCCGCUGUCGAG.CGAGGGC.CUGGGAAAGUC..U.A..G.UUCUAC.AAUGUAAAGUUAACAGUGGCAUGAAACGUUAACUCGUUUAAUGAGAACUGUU
env-21   CCGU..........AA.A...................GUCGGAGUGCCAACCAGUAU.AGUCCGCUGUCGAA.CGAGGGC.CUGGGAAAGUC..U.A..G.UUCUGC.AAUUUAAAAUUAACAGUGGCGUAAAUGAAAGGCUACUGUUAUGAAAAAAUAU
env-22   CGGUAAGAAC....AAGA............GUUUAA.GUCCGGGCACCGCCUAGUAU.AGACCCCUGUUGAA.UGAAGAC.CAGGAAAUGUC..U.A..G.UUCUAC.AAUGAAAAAUUAACAGCGGCAUUAUUUUAUUAAUAUAUUUUUACAGUUUUAG
env-23   CCGU..........AA.A...................GUCGGAGUGCCACCCAGCA..AGUCCGUUGAUGAA.UGAAGGC.CAGGAAAAGUC..U.A..G.UUCUUCUAAUAUUUUAAUUAGCAUCGGCAUAGAUGGAAAGGACAUUAUGUCGAGGUUAAU
env-24   CCGU...........AUA...................GUCGGAGUGCCGUCCAGUAU.AGUCCGCUGUUGAG.CGAUGGC.CAGGAAAAGCC..U.A..G.UUCUGC.AAUUUAAUUGACAGUGGCUUUAAAGGU.........................
env-25   CCGU...........AUA...................GUCGGAGUGCCGUCCAGUAU.AGUCCGCUGUUGAG.CGAUGGC.CAGGAAAAGCC..U.A..G.UUCUGC.AAUUUAAUUGGCAGUGGCUUUAAAGGUAAAAGUUAGAUGAAAAAAUCUAACA
env-26   CGGUAAUAAC....AU.UU...........GUUUAA.GUCCGAGAGCCACCCAGCAU.AGACCCCUGUUGAA.UGAAGGC.CAGGAAAAGUC..U.A..G.UUCUAC.AAUGAAAAAUUAGCAGAGGCAUUAUCAAAGGAAAUUAAAGCAAAAGCUUUAA
env-27   CGGUAAUAAC.UU.AAUA.........AAUGUUAAA.GUCCGAGUGCCGCCCAACUU.AGUCCUUUGUUGAA.UGAAGGC.CAGGAAAAGUC..U.A..G.UUCUAC.GAUGAAAAAUUAGCAGAGGCUUAACAAAAUAUAAGGAGAAAUUAUGCCUAGG
env-28   CGGUAAGAAU....UA.C............AUUCAA.GUCCGAGCGCCACCCAGUAU.AGUCCGCUGUUGAA.UGAUGGC.CAGGAAAAGUC..U.A..G.UUCUAC.AAUUUUAAUUAGCAACGGCAAAUAUUAACCCGCAAAAAGGUUUUUUGCGAUA
env-29   CGGUAAAAAC....GAAA............GUUUA..GUCCGAGCGCCACCCAGCAU.AGCCCGCUGUUGAA.CGAUGAU.CAGGAAAAAUC..U.A..G.UUCUAC.AAUGAAAAGUUAGCAGUGGCAUAGAAAAAUUA....................
env-30   CGGU..........AA.A...................GUCCGAGCGCCACCCAGAGU.AGCCCGCUGUUGAA.UGAAGGC.CAGGAAAAGUC..U.A..G.UUCUAC.AAUUUAAAUUAACAUCGGCACUAAAUUAAUUAGUUUUAUAUUUUUUAUAAAA
env-31   UCGU..........GAAA...................GUCGGAGCGCCACCCAGUGC.AGUCCUUUGUCGAA.UGAUGGC.CAGGAAAAGUC..U.A..A.UUCUGC.AAUGAAAAAUUAACAGAGGCAUAAAUUAAACUAAUUAGGAAUUUUAUUUCCU
env-32   CCGU..........AA.A...................GUCGGAGCGCCACCCAGUAG.AGACCGCUGUCAAA.CGAAGGC.CAGGGAAAGUC..U.A..G.UUUAAC.AAUUUAAAUUAACAGUGGCAAAUAUUGAAGUUUAAAAACAAAUAGGAGCCAC
env-33   CCGU..........AA.A...................GUCGGAGCGCCACCCAGCAA.AGUCCCCUGUUGAA.CUAAGAC.CAGGAAAAGUU..A.G..G.UUCUAC.AAUGCAAAGUUAUCAGAGGCAUCGUAUUUAACUAAGAGUUUAUACUCUUAUA
env-34   CCGU..........GA.A...................GUCGGAGCGCCACCCAGUAU.AGACCCCUGUUGAA.UUAAGGC.CAGGAAAAGUC..U.A..C.UUCUAC.AAACGUGCAGUGGCAUACCGAAAGGAUGCUACAUGCAAAUAAAAUAUUUUUU
env-35   CCGU..........AA.A...................GUCGGAGCGCCACCCAGUAU.AGACCCCUGUUGAA.UUAAGGC.CAGGAAAAGUCCCU.A..C.UUCUAC.AAAUUUGCAGUGGCAUAACUGAAAGGAUGCUACAUGCACANNNNNNNNNNN
env-36   CCGU...........AGA...................GUCGGAGCGCCACCCAGUAU.AGACCCCUGUGGAA.UUAAGGC.CAGGAAAAGUU..UUA..A.UUUUUACAAAUUUGCAGUGGCACAAACGAAAGGAUGCUACAUGCAAAAAAAAUGCUAUA
env-37   CGGU..........UAAA...................GUCCGAGCGCCACCCAGUAU.AGUCCGCUGUCGAA.UUAAGGC.CAGGAAUAGUC..U.U..ACUUCUAC.AAUUAAAAAUUACAACGGCAUAAUUUAAUACUAAGGCACUACGUGCAGGGGU
env-38   CGGUAAAAC.....CUAAAA...........GUUAA.GUCCGAGCGCCACCCAGUAU.AGCCCGCUGUUGAA.UGAAGGC.CAGGAAAAGUC..U.A..G.UUCUGC.AAUUAAAAAUUAGCAUCGGCAUACAAAUAAAACUAUUUGCUUAUACAAAGCA
env-39   CGGUAAGGA.....AAAAA............UCUAA.GUCCGAGCGCCACCCAGUAU.AGUCCGCUGUCGAA.UGAAGGC.CAGGAAAAGUC..U.A..A.UUCUAC.AAUUUAAAAUUAGCAGUGGCAAAAAAAAAUGAGGCCAUGUGCUAAGGAUAGU
env-40   CGGU..........AAAA...................GACCGAGUGCCAUCCGAUAC.AGACCGUUGUCGAA.UGAAGGC.CAGGAAAAGUC..U.A..G.UUCUAC.AAUUUAAAAAAUUAGCAGCGG...............................
env-41   CGGU..........GAAAAAAAA..............GUCCGAGUGCCAUCCAGUAU.AGUCCGCUGUUGAG.UGAAGGC.CAGGAAAAGUC..U.A..G.CUCUACGAUUAUUAAAUAGACAUCGGCAUUAAAAACUUAAACACUAUUAAUCUUGAUUA
env-42   CCGU..........AA.A...................GUCGGAGCGCCACCCAACAU.AGUCCGCUGUUGAA.UGAUGGC.CAGGAAAAGCC..U.A..G.UUCUGC.AAUUUUUAAAUUGACAUCGGCGUUUUGUUAAUUGUGGAAUUAUUUUCUACGC
env-43   CUGU..........UAUA...................GUCAAAGCGCCACCCAGUAC.AGCCCGUUGUUGAA.CGAAGGU.CAGGAAAAAUC..U.A..G.UUCUGC.AAUUUUUAAAUUAACAGCGGCAUAAAUUAAAAUCCUAGUUUCUAGGAUUGUA
2 env-44 CGGU..........AA.AA..................GUCCGAGCGCCACCCAACAU.AGUCCGAUGUCGAA.UGAAGGC.CAGGAAAAGUC..U.A..A.UUCUAC.AAUUUAAAUAUUAGCAGCGGCAUAAAUUAAUCAAUUUCUAAAUUAAUUUAGA
env-45   CCGU..........AUA....................GUCGGAGCGCCACCCAGUUA.AGUCCGCUGUUGAA.UGAUGGC.CGGGAACAGUC..U.A..G.UUCUAC.AAUGAAAAAUUAGCAUCGGCACUUAAAUUAAUCAGGUUGUCUAAUUAGGCGA
env-46   AGGU..........UAAA...................GUCCUAGCGCCACCCGAUAU.AGUCCGCUGUUGAA.UGAAGGC.CAGGAAAAGUC..U.A..G.UUCUGC.AAUUUAAAUUAGCAGUGGCGAAUGUUGAAGUGUUUACAAACAAAAGGAGAGC
env-47   CCGU..........UAAA...................GUCGGAGCGCCACCCAGUAU.AGUCCGUUGUUGAA.CGAGGGC.CAGGGAAAGUC..U.A..G.UUCUACUAAUGUUUAUUAGCAUCGGCAUAUUUAUUAACUUGUGAAUUUAAUUGUUCACA
env-48   CCGU..........AA.A...................GUCGGAGUACCACCCAACAU.AGCCCGCUGUUGAA.UGAAGGC.CAGGAAAAGUC..U.A..G.UUCUAC.UAUUAAAAAUUGACAUCGGCAAAAUAUUAACGGUUUAAGUUAAAACUUAACU
env-49   CCGU..........AU.A...................GUCGGAGCGCCACCCAACUA.AGUCCGCUGUUGAA.UGAAGGC.CAGGAAAAGUC..U.A..G.UUCUAC.AAUGAAAAAUUGACAUCGGCAUUGUAUAAAAAAUAAAUAUCUUGAAAGGAAG
env-50   CGGUAAAAGU....AAA.............AUUGA..GUCCGAGUGCCACCCAGUAA.AGUCCGCUGUCGAG.UGAAGGC.CAGGAAAAGUC..U.A..A.CUCUGC.AAUAUUAAAUUGACAUCGGCAAUAGUAAAAAAAUUAAUCUUAAUAAGAUUAA
env-51   CGGUAAAAGU....AAA.............AUUGA..GUCCGAGUGCCACCCAGUAA.AGUCCGCUGUCGAG.UGAAGGC.CAGGAAAAGUC..U.A..A.CUCUGC.AAUAUUAAAUUGACAUCGGCACUAGUAGAAAA....................
env-52   CCGU..........AA.A...................GUCGGAGUGCCACCCAGUAA.AGUCCUUUGUCGAA.UGAUGGC.CAGGAAAAGUC..U.A..G.UUCUGC.AAUUUAAAAUUAACAGAGGCAAAAAAAAUUUUAAGGAGAAAUUUAUGUCUC
env-53   CCGU..........AA.A...................GUCGGAGCGCCGCCCAGUGA.AGUCCUUUGU.GAA.CGAAGGC.CAGGAAAAGUC..U.A..G.UUCUAC.AAUUAAAAAUUAGCAGAGGCAUUAAAUAUUUAAGGGAAAUAAUUUCCUGGAA
env-54   CCGU..........AA.A...................GCCGGAGCUCCACCCAGUAG.AGACCGAUGUUGAA.UGAAGGC.CAGGAAAAGUC..U.A..G.UUCUGC.AAUUAAAAAUUAACAGCGGCAUUAAUUAAUGUAACUCCUAAAAUUUAGGAGU
env-55   CGGU..........UAAA...................GUCCGAGUGCCACCCAGUAA.AGUCCGGUGUUGAA.CGAAGGC.CAGGAAAAGUC..U.A..G.UUCUGC.AAUUAAAGUUGGCAGCGGCAUUUAUUUGAAAUUCUUAAAUAUUUAAGAAUUU
env-56   CGGU..........UAAA...................GUCCGAGUGCCACCCAGUAA.AGUCCGGUGUUGAA.CGAAGGC.CAGGAAAAGUC..U.A..G.UUCUGC.AAUUAAAGUUGGCAGCGGCAUUUAUUUAAAAUUCUUAAAUAUUUAAGAAUUU
env-57   CCGU..........AA.A...................GUCGGAGCGCCACCCAGUAU.AGUCCGCUGUUGAA.CGAGGGC.CAGGGAAAGUC..U.A..G.UUCUACUAAUAAUAAUUAGCAUCGGCAUAUAAUUAACAAAUUGGAGAAAAAUUUUCUCC
env-58   CCGU..........AA.A...................GUCGGAGCGCCACCCAAUCU.AGCCCGGUGUUGAA.UGAUGGC.CAGGAAAAGUC..U.A..G.UUCUAC.AAUUUUAGUUAGCAUCGGCAUUAACUAAUUAGUUUCAUAUUUUAUAUGAGAC
env-59   CCGU..........AA.A...................GUCGGAGCGCCACCCAGCAU.AGUCCGUUGUCGAA.UGAAGGC.CAGGGAAAGUC..U.A..A.UUCAACGAAAUAAAUAAUUAGCAGCGGCAUAAAAAUAUAAAUUCUUGUUAAAUCAAGAA
env-60   CGGU..........AA.AA..................GUCCGAGCACCACCCAACAU.AGUCCGUUGUCGAA.UGAA.GC.CAGGAGAAGUC..U.A..A.UUCUGC.AAUGAAAAAUUAACAACGGCANNNNNNNNNNNNNNNNNNNNNNNNNNNNNNN
env-61   CCGU..........AA.A...................GUCGGAGCGCCACCCAAGAU.AGUCCGUUGUUGAA.UGAUGGC.CAGGGAAAGUC..U.A..G.UUCUACUAAUACUUAAUUAGCAUCGGCAUUAACUAAGGUGAUGUAUAAAACAUUGCUUG
env-62   CGGU..........UAAA...................GUCCGAAUGCCAACCAAUAA.AGUCCGCUGU.GAA.UGAUGGC.CAGGAAAAGUC..U.A..G.UUCUAC.AAUGAAAAAUUAACAUCGGCAUAAUUUAAAAAUCUCUCUGAUUAAUGUCAGA
env-63   CGGU..........UAAA...................GUCCGAAUGCCACCCAAUAA.AGUCCGCUGU.GAA.UGAAGGC.CAGGAAAAGUC..U.A..A.UUCUAC.AAUGAAAAAUUGACAUCGGCACAAAUAAAAAUCUCUAACCCAAUUUAGAGAG
env-64   CCGU..........AA.A...................GUCGGAGCGCCACCCAGUUA.AGUCCGCUGUCGAA.UGAGGGC.CAGGAAAAGUC..U.A..A.UUCUAC.AAUGAAAAAUUAGCAUCGGCAUAAUUAAAACUCUUUAAAAAAAGAGAAAUU
env-65   CCGU..........AA.A...................GUCGGAGUGCCACCCAAUAU.AGUCCGCUGUUGAA.UGAAGGC.CAGGAAAAGUC..U.A..G.UUCUAC.AAUUUUUAAAUUAGCAUCGGCGUAAAUAGAAAUUUAUCUCAUGGAGGAGAUG
env-66   CCGU...........AUA...................GUCGGAGUGCCACCCAAUAU.AGUCCGCUGUUGAA.UGAUGGC.CAGGAAAAGUC..U.A..G.UUCUGC.AAUGAAAAAUUAGCAUCGGCAUUAAAAUUAACUAGUUUCAUAUUUAUAUA
env-67   CGGU..........AA.A...................GUCCGAGUGCCACCCAGUAG.AGUCCACUGUUGAA.UGAAGGC.CAGGAAAAGUC..U.A..A.UUCUGC.AAUUUUAAAUUAACAGCGGCAUUAAGUAAACCGGGUUAGAGUUAAAACUCUU
env-68   CGGU..........AA.A...................GUCCGAGUGCCACCCAGUAG.AGACCGCUGUUGAA.UGAAGGC.CAGGAAAAGUC..U.A..G.UUCUGC.AAUGAAAAAUUGACAUCGGCAUACAAUUAAUUGCUCUAAUUUUGAGCAAUU
env-69   CGGUAAAGAU....AA.A............AUCUAA.GUCCGAGCGCCACCCAGUAU.AGUCCGCUGUUGAA.UGAAGGC.CAGGAAAAGUC..U.A..G.UUCUAA.GAUGAAAAAUUAACAACGGCAUAUUUUGUAAAAAAAAGUUUGUAUAAAACUU
env-70   CCGU..........AU.A...................GUCGGAGCGCCACCCGAUAA.AGUCCGCUGUUGAA.UGAAGGC.CAGGAAAAGUC..U.A..G.UUCUAC.AAUUUAAAAUUAGCAGAGGCAAAACAUAAAAGGAAACAAUUCUAUGAAAGGA
env-71   CCGU..........AA.A...................GUCGGAGCGCCACCCAGUAU.AGACCCCUGUCGAA.UGAUGGC.CAGGAAAAGCC..U.A..A.UUCUAC.AAUACUUAA...........................................
env-72   CCGU..........AA.A...................GUCGGAGCGCCACCCGGUAU.AGACCCCUGUCGAA.UGAUGGC.CAGGAAAAGCC..U.A..G.UUCUGC.AAAAUAGCAGUGGCGAAAAGUGAAUUAAAGGAGGCCACAUGCUAAAAAGAUU
env-73   CCGU..........AAAA...................GUCGGAGCGCCACCCAGUAC.AGUCCGCUGUUGAA.CGAAGGC.CAGGAAAAGUC..U.A..G.UUCUAC.AAUUUAAUUAGCAGUGGCAAUAUUAUUAGGAGCUUUUGCUCAUAGAAAGGCC
env-74   CCGU..........AA.A...................GUCGGAGCGCCACCCAGUAU.AGUCCGCUGUUGAA.CGAAGGC.CAGGAAAAGUC..U.A..G.UUCUGC.AAUUAAAUUUAGCAGUGGCUUUAAAAAAUAAAUUGGAACUUACAUUCCGAUU
env-75   CCGU..........AA.A...................GUCGGAGCGCCACCCAGUAU.AGUCCGCUGUCGAA.UACGAGC.CAGGAAGAGCU..U.AUUA.UUCUAC.AAUGAAAAAUUAACAUCGGCAUUAAAAAUUUUAUUAUUAAUUUAAAUAAUA
env-76   CGGU..........UAAA...................GUCCGAGCGCCACCCGGUAA.AGCCCGCUGUUGAA.UGAAGGC.CAGGAAAAGUC..U.A..A.UUCUAC.AAUGUAAAAUUAACAACGGCAUUUAUAAAUAAUUAGGUUCAUAAAUUAUGGA

         <<<...<<<<<<<<............>>>>>>>>.....>>>....>>>>>>>>>>>............<<<.<..<<<<.........>>>..>....>.>>>.......................................................
         220...?222?020............020?222?.....022....2222?22222?............222.2..?222.........222..?....2.222.......................................................
         ..................................................................................>>>..........................................................................
         ..................................................................................220..........................................................................
         .....................................>>........................................................................................................................
         .....................................22........................................................................................................................
         C•GU----------•A-A------------------GUC•GAGYGCCACCARYA•-AGYCCRYUGUYGAA-YGA•GGC-CAGGAAAGUC--U-A--R-UUCURC-AAU•••AA•••R•••••R••••••••••••••••••••••••••••••••••
                                                                                                                          3′
```

```
env-77   CCGU..........AA.A..................GUCGGAGUGCCACCCAAUAU.AGCCCGCUGA.GAA.UGAUGGC.CAGGAAAAGCC..U.A..G.UUCUAC.AAUGAAAAAUUGACAUCGGCAUAUUUUAUUAACGUAAGGUUAAAACCUAACA
env-78   CCGU..........AA.A..................GUCGGAGCGCCGCCCAGUAU.AGUCCGCUGUUGAA.CAAUGGC.CAGGAAAAGUC..U.A..G.UUCUAC.AAUUAAAAUUGCAUCGGCAUAUUAUCAAAAUGUAAAGUAUUUAUACUUUAUA
env-79   CGGU..........AA.A..................GUCCGAGUACCACCCAAUAU.AGUCCGCUGUUGAA.CGAUGGC.CAGGAAAAGUC..U.A..G.UUCUGC.AAUUUAAAUUAGCAUCGGCAUUAAAUUAACUAGUUUCAUAAAUUUUUAUGAA
env-80   CGGU..........AA.A..................GUCCGAGUGCCAUCCAAUAU.AGUCCGCUGUUGAA.CGAGGGC.CAGGAAAAGUC..U.A..G.UUCUAC.AAUUUUAAUUAACAACGGCAUAAAUUAAAACAGCCUUAAGAUUUUAAGGUGA
env-81   CGGU..........AA.A..................GUCCGAGUGCCGCCCAAUAU.AGUCCGCUGUUGAA.UGAUGGC.CAGGAAAAGUC..U.A..G.UUCUAC.AAUUUUAAUUAACAUCGGCAUUAAAUAAAUAAGUUUCUAUUUAGAAACUAAU
env-82   CGGA..........UAAA..................GUCCGAGUGCCACCCAAUAU.AGUCCGCUGUUGAA.CAAUGGC.CAGGAAAAGUC..U.A..G.UUCUAC.AAUUUUAAUUAGCAUCGGCAUUAAUUAACAAGCUUCAUAUUUUUUAUGAGGC
env-83   CGGU..........UAAA..................GUCCGAGUGCCAUCCAAUGU.AGUCCGCUGUUGAA.UGAGGGC.CAGGGAAAGUC..U.A..A.UUCUAC.AAUUUUAAUUAACAACGGCAAAUUUUUAAUUGUGAGACUCAAAAAGUCUCAA
env-84   CCGU..........AA.A..................GUCGGAGUACCACCCAGUAA.AGUCCGCUGUUGAA.UGAUGGC.CAGGAAAAGUC..U.A..G.UUCUAC.AAUUAAAAAUUAGCAACGGCAUAAUUAAUAACUCAAAUUUAUAUUUGAGAAG
env-85   CGGUGUGAACG...AA.A...........GUUUAA.GUCCGAGCGCCACCCGGUAU.AGUCCGCUGUUGAA.CGAA.GC.CAGGAAAAGUC..U.A..G.UUCUGC.AAUGCAAAAUUAGCAGUGGCAUUAAAUUUUGUGCUUAUGCUAAAAAAAAUAU
env-86   CGGUGUGAAC....GAAA...........GUUUAA.GUCCGAGCGCCACCCAGUAU.AGCCCGCUGUUGAA.CGAAGGC.CAGGAAAAGUC..U.A..G.UUCUAC.AAUGCAAAAUUAGCAGUGGCAUAAAAUUUUAUGCUUAUGCUAAAAAGAUUAG
env-87   CCGU..........AA.A..................GUCGGAGCGCCACCCAGUAA.AGUCCGCUGUUGAA.UGAAGGC.CAGGAAAAGUC..U.A..A.UUCUUCUAAUUAUUAUUAGCAUCGGCAUUAUAUUAAUUAGUUUCAUAAUUUUAUGGAAC
env-88   CGGU..........UAUA..................GUCCGAGCGCCACCCAGUAU.AGUCCGCUGUUGAA.CAAUGGC.CAGGAAAAGUC..U.A..G.UUCUAC.AAUUAAAUUUAGCAGUGGCUUUUAUUAAUUAGAACUUAAUGUUCUACAAAAG
env-89   CGGU..........UAAA..................GUCCGAGCGCCACCCAGUAA.AGUCCGCUGUUGAA.UGAAGGC.CAGGAAAAGUC..U.A..G.UUCUAC.AAUUGAAAGAUUAGCAUCGGCAUAAAUAAACAAAAACUUUUUUUAAGAAAAG
env-90   CGGU..........UAUA..................GUCCGAGCGCCACCCAGUAU.AGUCCGCUGUUGAA.UGAAGGC.CAGGGAAAGUC..U.A..G.UUCUAC.AAUUAAGUUAGCAGUGGCUUUUUAAGUAAAGGUUAGAUUAAAAAAAUCUAAC
env-91   CCGU..........AA.A..................GUCGGAGCGCCACCCAGUAU.AGUCCGCUGUUGAA.CGAGGGC.CAGGAAUAGUC..U.A..G.UUCUGC.AAUAUUUAAUUAGCAGUGGCAAAAUUAAAUUAUAUUAAGCUUUUGCUUAAUA
env-92   CCGU..........AA.A..................GUCGGAGCGCCACCCAGUUA.AGCCCUUUGCUGAA.CGAAGGC.CAGGAAAAGUC..U.A..G.UUCUAU.AAUGAAAAAUUAGCAACGGCAUUAAAUAAAUACCUCAUUAAGUAGUGGGGUC
env-93   CCGU..........AA.A..................GUCGGAGUGCCGCCCAACAG.AGUCCGCUGU.GAA.UGAUGGC.CAGGAAAAGUC..U.A..G.UUCUAC.AAUGAAAAAUUAGCAGUGGCUAAAAAAAGGAGAUAGCUACAUGCUAAAAAAU
env-94   CCGU..........AA.A..................GUCGGAGCGCCGCCCAACAU.AGUCCGCUGUUGAA.UGAUGAC.CAGGGAAAGUC..U.A..A.UUCUAC.AAUGAAAAAUUGACAUCGGCAUUGAGUAAGGGAACUAAAUUUAAAUUUUAGU
env-95   CCGU..........AA.A..................GUCGGAGCGCCACCCGAUGA.AGCCCUUUGUUGGA.UGAAGGC.CAGGGAAAGUC..U.A..G.UUCUAC.AAUGAAAAAUUAACAGAGGCAUUAAAUUAACAAAUGUGAGUUUACUCACUAA
env-96   CCGU..........AU.A..................GUCGGAGCGCCACCCAGUCU.AGUCCGCUGUUGAA.UGAAGGC.CAGGAAAAGUC..U.A..A.UUCUGC.AAUGUAAAAUUAGCAUCGGCAUAAUAUUAAUUUUAAUCUUAAGGAGAAGAUU
env-97   CCGU..........AA.A..................GUCGGAGCGCCACCCAGCAU.AGUCCGCUGUUGAA.UGAAGGC.CAGGAAAAGUC..U.A..G.UUCUAC.UAUUAAAAUAAGCAUCGGCAUAAAUUAAACUGGUUAAAGUAAAAACUUUAAU
env-98   CCGU..........AA.A..................GUCGGAGCGCCACCCAGUAU.AGUCCGCUGUUGAA.UGAAGGC.CAGGAAAAGUC..U.A..G.UUCUAC.AAUUUUUAAAUUGACAUCGGCAUAAAAUUAAAAACUUCUUUACACGAUUAAA
env-99   CCGU..........AA.A..................GUCGGAGCGCCACCCAGUAU.AGUCCGUUGUUGAA.UGAAGGC.CAGGGAAAGUC..U.A..G.UUCUACUAAUAAUAAUUAGCAUCGGCAUAUGAUUAACAAAAUUGGAGAAAAAUUUUCUC
env-100  CCGU..........AA.A..................GUCGGAGCGCCACCCAGUAU.AGUCCGUUGUUGAA.UGAAGGC.CAGGGAAAGUC..U.A..G.UUCUACUAAUAAUAAUUAGCAUCGGCAUAUAACUAACAAAAUUGGAGAAAAAUUUUCUC
env-101  CCGU..........AA.A..................GUCGGAGCGCCACCCAAUAU.AGUCCGUUGUUGAA.UGAGGGC.CAGGGAAAGUC..U.A..G.UUCUACUAAUAAUAAUUAGCAUCGGCAUAUAAUUAAUAAAAUUGGAGAAAAAUUUUCUC
env-102  CCGU..........AA.A..................GUCGGAGCGCCACCCAGUAU.AGUCCGCUGUUGAA.UGAUGGC.CAGGGAAAGUC..U.A..A.UUCUAC.AAUUUUAAUUAGCAACGGCAUUAAAUAAAUACGCAAUUAAUUUGCGACAAA
env-103  CCGU..........AA.A..................GUCGGAGUGCCACCCAAUAU.AGUCCGCUGUUGAA.UGAUGGC.CAGGAAAAGUC..U.A..G.UUCUAC.GAUUUUAAUUAACAUCGGCACUAAAUUAACUAAUUCCAUUUUUUUUUGGAA
env-104  CCGU..........AA.A..................GUCGGAGUGCCACCCAAUAG.AGACCGCUGUUGAA.UGAUGGC.CAGGAAAAGUC..U.A..A.UUCUAC.AAUUUUAAUUAACAUCGGCAUAAAUUAAUUUGUUUCAUGUAAAUUUAAACAU
env-105  CCGU..........AA.A..................GUCGGAGCGCCACCCAAUAU.AGUCCGCUGUCGAA.UGAAGGC.CAGGAAAAGUC..U.A..A.UUCUAU.AAUGCAAAAUUAGCAGAGGCAUAAAGGUAAAAAAAAUAUUUAGUCGUCUUAAA
env-106  CCGU..........AA.A..................GUCGGAGUGCCACUCAGUAA.AGUCCGCUGUUGAA.UGAAGGC.CAGGAAAAGUC..U.A..G.UUCUAC.AAUUAAAAAUUAACAUCGGCAAUAUUUUAAACGUUAUUAAUUAUUAUUACGU
env-107  CGGUGUGAAC....AU.AA..........GUUUGA.GUCCGAGCGCCACCCAGUAU.AGCCCGCUGUUGAA.CGAAGGC.CAGGAAAAGUC..U.A..G.UUCUGC.AAUGUAAAAUUAACAGUGGCAUAAAGUUUUAUGCUUAUGUUAAAAAAAAUAU
env-108  CCGU..........AA.A..................GUCGGAGCGCCACCCAGUAU.AGUCCGCUGUUGAA.UGAUGGC.CAGGAGGUGUC..U.A..A.UUCUAC.AAUUUAAAAUUAGCAGUGGCUUUAAAAUGAACGUUAAGAUUA.........
env-109  CCGU..........AA.A..................GUCGGAGCGCCACCCAGUAA.AGUCCGCUGUUGAA.UGAGGGC.CAGGGAGAGUC..U.A..G.UUCUACUAAUAUCUAUUAGCAUCGGCACUAACUAAACUUGUUUCAUUAAUUAAUGAAAC
env-110  CCGU..........AA.A..................GUCGGAGCGCCACCCAGCUA.AGUCCGCUGU.GAA.UGAAGGC.CAGGAAAAGUC..U.A..G.UUCUAC.AAUGAAAAAUUAACAUCGGCAUUAAAUUAACUUAAUUUCAUAUAUUUUUAUG
env-111  CCGU..........AA.A..................GUCGGAGCGCCACCCAGCUA.AGUCCGCUGU.GAA.UGAGGGC.CAGGGAAAGUC..U.A..G.UUCUAC.AAUGAAAAAUUAACAUCGGCAGAAAUGAGGAGCAAUCUUAACAAGAAAGGAU
1 env-112  CCGU..........AA.A..................GUCGGAGCGCCACCCAGCUA.AGUCCUCUGUUGAG.CGAAGGC.CAGGAAAAGUC..U.A..G.UUCUAC.AAUGAAAAAUUAGCAGCGGCAUUGAAAUAAAACUAUAAAAAGUUUUAAAGGA
env-113  CCGU..........AA.A..................GUCGGAGCGCCACCCAGCUA.AGUCCUUUGUUGAA.UGAUGGC.CAGGAAAAGUC..U.A..G.UUCUAC.AAUGCAAAAUUAGCAGAGGCAUAUUUGUGAGGUAUAAAAUUCCAAAGCUAAG
env-114  CCGU..........AA.A..................GUCGGAGCGCCACCCAGUAG.AGUCCGCUGUUGAA.UGAAGGC.CAGGAAAAGUC..U.A..G.UUCUGC.UAUUUAAAUUGACAUCGGCAUUAAAUAAAUAAACUUCUUAAUUAUAAGAAGU
env-115  CCGU...........AGA..................GUCGGAGCGCCACCCAGCUU.AGUCCGCUGUUGAA.UGAUGGC.CAGGAAAAGUC..U.A..A.UUCUACUAAUGUUAAUUAGCAUCGGCAAAUUAUUAACUGUGAGAUUAAAUUCUUACAUA
env-116  CGGU..........AA.AA.................GUCCGAGCGCCACCCAGUUA.AGUCCGCUGUUGAA.UGAAGGC.CAGGAAAAGUC..U.A..A.UUCUAC.AAUGAAAAAUUAGCAUCGGCAUAAAAAUAUAAUGCCUUUUUAAGGUAGUGGA
env-117  CGGU..........AA.A..................GUCCGAGCGCCACCCAGUAU.AGUCCGGUGUUGAA.UGAAGGC.CAGGAAAAGUC..U.A..A.UUCUGC.AAUGAAAAAUUGACAACGGCACUAUUACUAAUGGACGCUUAAUGUCCAAAUG
env-118  CGGU..........AAUA..................GUCCGAGCGCCACCCAGUAG.AGUCCGAUGUUGAA.UGAAGGC.CAGGAAAAGUC..U.A..A.UUCUAC.AAUGAAUAAUUAGCAGCGGCAUAUAUUUUUAAAAUUCUUUCUUGAUAUGUUU
env-119  CCGU..........AA.A..................GUCGGAGCGCCACCCAGUAA.AGACCGUUGUCGAA.UGAAGGC.CAGGAAAAGUC..U.A..G.UUCUAC.UAUUUUAAAUAGCAUCGGCGUUAAAUUAAAAUCAUCUUGUUAUUUCAAGAUG
env-120  CCGU..........AA.A..................GUCGGAGCGCCACCCAGUAU.AGCCCGUUGUUGAA.UGAAGGC.CAGGAAAAGUC..U.A..G.UUCUAU.AAUUUAAAAUUAGCAUCGGCAUAUUAUUAACUUUAAUUGGAGAGAAGUUAUC
env-121  CCGU..........AA.A..................GUCGGAGCGCCACCCGGUAU.AGCCCGCUGUUGAA.CGAAGGC.CAGGAAAAGUC..U.A..G.UUCUGC.AAUGCAAAAUUAGCAGUGGCAUAAAAAAUUGUGCUUAUGCUAAAAAAGUUAA
env-122  CCGU..........AA.A..................GUCGGAGCGCCACCCAGUAU.AGCCCGCUGUUGAA.CGAAGGC.CAGGAAAAGUC..U.A..G.UUCUGC.AAUGCAAAAUUAGCAGUGGUAUAAAAAAUUAUGCUUAUGCUAAAAAAAAUUAA
env-123  CCGU..........AA.A..................GUCGGAGCGCCACCCGGUAU.AGCCCGCUGUUGGA.CGAUGAC.CAGGAAAAGUC..U.A..G.UUCUGC.AAUGCAAAAUUAGCAGUGGCAUAGAAAAUUAUGCUUAUGCUAAAAAGAUUAA
env-124  CCGU..........AG.A..................GUCGGAGCGCCACCCAGUAU.AGUCCGCUGUUGAA.UAAUGGC.CAGGGAAAGCC..U.A..G.UUCUAC.AAUGAAAAAUUAGCAUCGGCAUAAUUAAUAACAAAAGGCCUUAGGUUUUUAA
env-125  CCGU..........AA.A..................GUCGGAGCGCCACCCAGUUA.AGUCCGCUGUUGAA.UGAUGGC.CAGGAAAAGUC..U.A..G.UUCUGC.AAUGAAAAAUUAGCAUCGGCAUCAAUGAAUUGAGCCUCUAUGUUUUUAGAGG
env-126  CCGU..........AA.A..................GUCGGAGCGCCACCCAGCUU.AGUCCGCUGAUGAA.UGAUGGC.CAGGAAAAGUC..U.A..A.UUCUAU.AAUGAAAAAUUAACAGAGGCAUAAAUAAACCAAAAUUAUUAUCUCAAAUUGA
env-127  CCGU..........AA.A..................GUCGGAGCGCCACCCAGUAU.AGUCCGCUGUUGAA.CAAUGGC.CAGGAAAAGUC..U.A..G.UUCUAC.AAUGAAAAAUAAACAUCGGCGUAUUAUUAAUUUAUAUUGGAGAAAAUUUAUC
env-128  CCGU..........AA.A..................GUCGGAGCGCCACCCAGUAU.AGUCCGCUGUUGAA.UGAUGGC.CAGGAAAAGUC..U.A..G.UUCUAC.AAUUAAAAAUUGACAUCGGCAAUUAUUAACGUGGAACACCAAGUGUUUUACA
env-129  CCGU..........AA.A..................GUCGGAGCGCCACCCGGUAU.AGUCCGCUGUUGAA.UGAUGGC.CAGGAAAAGUC..U.A..G.UUCUAC.AAUGAGAAAUUAACAACGGCAUAAAUAAAUGUGUUUCUAAAAGAAACAAAGG
env-130  CCGU..........AA.A..................GUCGGAGCGCCACCCAGUAA.AGUCCGCUGUUGAA.UGAUGGC.CAGGAAAAGUC..U.A..G.UUCUGC.GAUGAAAAAUUAGCAUCGGCAUAAAAAUAAAUAGAUUUGGACACUAUGUGUC
env-131  CCGU..........AA.A..................GUCGGAGCGCCACCCAGUA..AGUCCGCUGUUGAA.UGAGGGC.CAGGAAAAGUC..U.A..G.UUCUAC.AAUGAAAAAUUAGCAACGGCAUAUAUUAAUAAUUGCUAGAAAUAGCAUAACC
env-132  CCGU..........AA.A..................GUCGGAGCGCCACCCGGUAA.AGUCCGCUGUUGAA.UGAAGGC.CAGGAAAAGUC..U.A..A.UUCUGC.AAUGAAAAAUUGACAACGGCACAUGAUAAAAACAAAGUUAUAACUUUGUAAA
env-133  CCGU..........AA.A..................GUCGGAGCGCCACCCAGCAU.AGUCCGCUGUUGAA.UGAAGGC.CAGGAAAAGUC..U.A..A.UUCUAC.GAUGAAAAAUUAACAACGGCAUUUAAUAAACGUGAAAACGUUAAACAAAUAU

         <<<...<<<<<<<<............>>>>>>>>.....>>>....>>>>>>>>>>>............<<<.<..<<<<.........>>>..>....>.>>>..........................................................
         220...?222?020............020?222?.....022....2222?22222?............222.2..?222.........222..?....2.222..........................................................
         ................................................................................>>>...............................................................................
         ................................................................................220...............................................................................
         ....................................>>...........................................................................................................................
         ....................................22............................................................................................................................
         C•GU----------•A-A------------------GUC•GAGYGCCACCCARYA•-AGYCCRYUGUYGAA-YGA•GGC-CAGGAAAAGUC--U-A--R-UUCURC-AAU•••AA•••R•••••R••••••••••••••••••••••••••••••••••••••••
                                                                                                                3′
```

```
env-134  CCGU.........AA.A..................GUCGGAGCGCCACCCAGUAU.AGUCCGCUGUUGAA.UGAAGGC.CAGGAAAAGUC..U.A..G.UUCUAC.AAUUAGAAAUUAACAUCGGCAAAAGUUAAGAAAAAGAAGUUAAAACUUAAUA
env-135  CCGU.........AA.A..................GUCGGAGCGCCACCCAGUAU.AGUCCGCUGUUGAA.UGAUGGC.CAGGAAAAGUC..U.A..G.UUCUACUAAUGUUUCUUAGCAGUGGCUUUUAUAAACUUAAUUAGAAUUAAUAUUCUAAU
env-136  CCGU.........AA.A..................GUCGGAGCGCCACCCAGUAU.AGUCCGCUGUUGAA.UGAUGGC.CAGGAAAAGUC..U.A..G.UUCUACUAAUGUUUCUUAGCAGUGGCUUUUAAAAACUAAAUUAGAAUUAACAUUCUAAU
env-137  CCGU.........AA.A..................GUCGGAGCGCCACCCAAUAU.AGUCCGCUGUUGAA.UGAAGGC.CAGGAAAAGUC..U.A..G.UUCUGC.AAUGAAAAAUUAGCAUCGGCAUAAAUUAAUAUAACUUCUAAAAUUUAGAAGU
env-138  CCGU.........AA.A..................GUCGGAGCGCCACCCGAUAA.AGUCCACUGUUGAA.UGAAGGC.CAGGAAAAGUC..U.A..G.UUCUAC.AAUGAAAAGUUAGCAGAGGCAAAAAUUAAUCUAAAGAGAAGGAUUUCUCUCU
env-139  CGGU.AUUUUUUAUUUAA.......AUAAAAAAAA.GUCUGAGCGCCACCCAGUGU.AGUCCGCUGUUGAA.UGAAGGC.CAGGAAAUGUC..U.A..G.UUCUACUAAUAAUAUUAGCAACGGCAUCGAAACUAAUUUAACUCCUAAAAUUUAGGGGU
env-140  CGGUAAGAAC....GU.A...........GUUUAA.GUCCGAAUGCCAUCCAGUAU.AGUCCGCUGUUGAA.UGAAGGC.CGAGGAAAGUC..U.A..G.UUCUAC.AAUUUAAAUAUUAGCAGUGGCAUGAAAAAUUAAGCUUAUGCUAAGAAAAUUA
env-141  CGGUGA........ACAACA................GUCCGAAUGCCAACGAGGUG.AGACC........A.ACAUUAG.CGGUGGUGCUAUGAAAAUUAUCUUAAUCAUAUAAAAAUAUAAACUAUUACUUUCUGAAGAUUGCUGUUAUGCGUCAUGU
env-142  CGGUUA........AA.A..................GUCCGAACGCCUACAUGAAA...............UGAUGCC.CAUGAAGCGGG..G.AGGCUUCUUAAGGAUGGGAGAAAAACUUUUAUGGAUCUCAGCAAUCCUAUUAUUUAGCAAUUCU
env-143  CGGU.........UAAAA..................GUCCGAACGCCUA.CAAGAAAUGAUGCCCAU........GAAG.CGGGGAGGCUU..C.A.......UAA.GAUGGGAAAAAAACUUUUAUUGAUCUCAGCAGUCCUAUUAUUUAGCAAUACU
env-144  CGGUUA........AA.A..................GUCCGAACGCCUACAUGAAA.UGAUGCCCAU........GAAG.CGGGGAGGCUU..CUUAA.....UGAUGGGGGAAAAACUUUUAUGGAUCUCAGCAGUCCUAUUAUUUAGCAAUUCUUUU
         <<<...<<<<<<<<...........>>>>>>>>.....>>>....>>>>>>>>>>>...........<<<.<..<<<<.........>>>..>....>.>>>..................................................
         220...?222?020...........020?222?.....022....2222?22222?...........222.2..?222.........222..?....2.222..................................................
         ...............................................................................>>>..............................................................................
         ...............................................................................220..............................................................................
         ....................................>>...........................................................................................................................
         ....................................22...........................................................................................................................
         C•GU----------•A-A------------------GUC•GAGYGCCACCCARYA•-AGYCCRYUGUYGAA-YGA•GGC-CAGGAAAAGUC--U-A--R-UUCURC-AAU•••AA•••R•••••R••••••••••••••••••••••••••••••••••••
                                                                                                                              3′
```

alignment positions 321 · · · 480

```
env-1    AGUCUGCGUUUUAAUCAAUAAACAAAAAUGCAGACACAUUUGUUAAAACGACUAUCAGCAUCUUUCAUUAUUUUAAUUGGGCUAUUUUUUGCAGCUGGUGUUACUUAUGGCUUGUAUCAUUUCAUGCAGAUCUCAUCUCAUGAAGUGAGUAGUGUAAGCG
env-2    AGAUUGCAGUUGGAACAAUGAUGUUCCUUGUAUUACAAUUAUUAAGCCGAACAGUAAUAUUAUAAAUGACAAAAUUUCUCCUUCUCAGAUAAUAACAAAAGAACAAAUUAAAAAACAUAAUCUUGUAGAUCUUAAAAGUAUUUUUAAGUUUACAAACAGC
env-3    GUAAACAUAGAAAAACGUGGUAUUGCAGACGACAUUAUAAAAUGUGAAUGGGAAAACCAAAAAGGUGUUCCUUGUGUCACAAUUAAAAAAACAAUACCAAAUUCAAAUGCAAUAUCUGAUAAAGUUUCUCCCACAAUUAUAAUCACUAAAAAACAAAUUG
env-4    GUAAACAUAAAAGAACGUGGUGUUGCAGACGACAUUAUAAAAUGUGAAUGGGAAAACCAAGAGGGUGUUCCUUGUGUCACAAUUAGAAAAACAUUACCAAAUUCAAAUGCAAUAUCAGAUAAAGUUUCUCCCACAACUAUAAUCACUAAAAAACAAAUUG
env-5    GAUAGAAAUUGUUAAAGUUGAAAAACGUGGUGUAGCAGAUGAUCUUAUCACUUGCGAAUGGAACAAUAAAGAAGGUGUGCCAUGUGUCGCAAUUACUUUACCUAUUUCAAAUUCAAAUAAAAUAUCAGAAAAAAUUUCUGCAAAAACAAUUAUUACAAAA
env-6    GCGGAUCCACUUAAAAUUACCAUAACUAUCCCUUAUGUUCCAAGCCAUGAUAGUUUAAAAACAACAAACGUGGAAAGCUUUGAAGCGUCAUCAUACAAAAAUAAAAUUACAAUUGGAGAUGUACUUGACACCAAUUCAUCAGUUCAAUCAACUAGAUCCG
env-7    AAAGUUGAAAAACGUGGUGUAGCUGAUGAUCAAAUCAAGUGCGAAUGGAACAACAGUGAAGGGGUGCCCUGUGUUACAAUCAGUAAAAGACCCAACUCAAACAUAAUCAGUAAUAAAAUAAAUCCCACCCAGAUUAUAACACCAAGUGAUAUCGAAAAGU
env-8    AAAGAAAAUGAUUGUAAAUGGGAUGAUGAUAUUCCUUGUCUUACAAUUUAUCCAAACAUAAACAAUUCAAAUGCAUUAGGUGAUAAAAUAACACCUACAUACAAAAUUAAAAAAUCUGAAAUUGAAAAAUAUAAUCUAAUUGACUUACCUAAAGUAUUAA
env-9    AAGGAAGAUGAUUGUAAAUGGGAUGACGAUAUUCCUUGUCUUACAAUAUAUCCAAACAUAAACAACUCAAACGCAUUAGGUGAUAAGAUAACACCUACACUUACAAUUAAAAAAUCUGAAAUUCAAAAGUAUAAUCUAAUUGAUUUACCUAAAGUAUUAA
env-10   AAAAAACGGUGGAGAUUGCUAAAUGUUAAUUAGGUAUUUUGUUUUAUUUUUUUUAUUAAUUUCUAGUUUUAAUAUUGUAAAUGCUAGAGAUUGUAAUUGGGAUAAUGAAAAUAAUAUUCCGUGUUUAGAAAUUAAAUCUAAUGUUUCGAAUACAUCACGA
env-11   AAUCAUUAAGGUAAUAAUGGGCUGUUAUGAAAGAUAAAAAAAUUUUUAUAUUUAUAGUUUUAAUUAACCUAUUUUUUUUAAAGCAAUCUUUUUCACAAGACAUUCCAGUAAUAGUUAUUGCGCCAGGAAAAACUCCUCAGUCUCUAAAUACUGUAGGUAG
env-12   UGAAUGAAAAAAUUAAAAUUUAUUAUAUGUUUAUUAUAUUUAAGUUUUUUUUUCUUAAAUAACUUAUCAUCUGAAGAAAUACCAGUAAUUGUAAUUUCAGCAGGAAAAACUGCUCAAACAUUAAACUCAGUUGGAAGCAGUAUUGAAAUCAUA.......
env-13   UUUAAGAAUUAGUUAAUCAGAUGUCAUUGUUAGCAAAAUUUAUAAUCUUUAU............................................................................................................
env-14   AUGAAUAGGUUUGUUUAUGUUGUUGAGAAUAAUUGUAUUAACUUCUUUUUUUAUAUUUCUAAAUGUUAAAGCAGAAGAUUUAAAAAAUUGUAAAUGGGAUAAUAGAAAUGGUAUUCCAUGUAUUGCUAUAAGCAAGACUUCUAAUACAUCAUAUUUAUCA
env-15   ACGAAUUUAGAAAAGUUAUAUGUACAUAAAGCUAAAAUUAUUAUUUUUAUUUUUAUUAUUAAUUUCCCGUUUAAUAAAGUUUUGUCUGCAGACAUUCCUAUUAUUGUUAUUGCGCCUAGUAAAAAAGCUCAGUCUGUUUCGACUGUAGGAACUUCUGUA
env-16   UUUUAUCAUUUCUUAUAGUUUUUAAUAGUUUAGUGUUUACAAAUUUUUUAAAUGCAUCAGAAAUUCCAGUAAUUGUUAUUGCGCCAAGUAAAAAAGCUCAAUCAAUAUCUACCGUCGGUUCUUCUGUAAUUGUCUUAGACGAAGAAUUUAUAGAAAAUUC
env-17   AAAAUGGAGUGUCGCGAUGUUAAAAAAAAUAAAAGUAUCUUUAUUAAGUAUAAUUUUAAUUUUUUCUUUUAAUCAAGUAUUCGCCGCAGAUAUACCUGUAAUUGUUAUUUCAUCAAGUAAAAAAGCACAAUCAACUUCGAUAGUAGGAACUAGCGUGACU
env-18   UAUGAAAAAAACAAAACAAAAAUUUGUAUUGUUCCUGUACUUUAUAGGCUUGAUAGCCUUGAGCAGAUUAAUACCUCAUCCACCAAACUUUACGCCUGUUAUUGCUAUGGCUGUCUUUAUGCCAUACUUGACUCGUAAUGUUUAUUCAGCGAUGCUAGUA
env-19   CUACUGUUUUUCGUAUUGCUUAUGUUCACGGGUGUGACAUAUGCUGACGAUUGUAAGUGGGACAACGAUGUUCCCUGCACAACAAUCUAUCCAAAGUAUACGAACAAUUCUAAUGCUCUUGGAGACAAGAUAACACCCACAAACACAAUCACUAAAUCAG
env-20   AUGAAAAAAACAAA..................................................................................................................................................
env-21   CUAAUAUUUUUCGUGUUGCUUAUGUUCACGGGUGUGGCAUAUGCUGACGAUUGUAAGUGGGACAACGACGUUCCCUGUACAACAAUCUAUCCAAAGUAUACGAACAAUUCAAAUGCUCUUGGCGACAAGAUUACACCCACAAGCACAAUCACUAAAUCAG
env-22   CUUUAUGAAUUAAAAAAAGGAAUACACGUGCUAAAAGUUGUUGUUUCACUAUUAAUUAUUAUAUUUUCGUUUCAAAAUUUAUUUGCUGCCGAUAUACCUAUUAUAGUUAUUGCACCAAGCAAAAAAGUACAAUCGAUUUCAACUGUUGGUUCAUCCGUUU
env-23   UUUAAUUUUGGUUAACAUAUUAUUUUUUAAUAAUUUUGUUUUAUCAGCUGAUAUAGAAAAUUGUUCUUGGGAUAACAAAGAUCAAAUCCCAUGUUUAGAAGUCACAAGUUAUAUCACAAAUAGCUCUGAGCACUCUAAAUCUAGUGUAAAUAAAAUAGUA
env-24   ................................................................................................................................................................
env-25   AAAAAAAGGACUGUUAAUGAAAAAUUUUUUAAUAGUAAUAUUAUCUUUACUUAUUUUUUUUCCUCUAGGAGGCGAGGAAUUGAAAAAUUGUAAAUGGAAAAACGAAAGUGGUACUCCGUGUUUAACUAUUUUUUCUGCACCUAAUACUUCUAAAAUAACA
env-26   UAGAUGAUUUAGAGUAAGGAAAAUGUGCAAAAGCUAAUAAUAACAAUAUUAAUAUUUAUUUUUUCUUUCAGCAAAGUAUUUGCUGCAGAUAUUCCUGUAAUAGUUAUAGCGCCAAGCAAAAAAGCACAAUCUACUUCUA.....................
env-27   CUAAUUAUAUAUAUACUUUUUAAUUUAAUAUUUUCAAAUCUAAUAUUUGCUGCGGAUAAAAAAUUCUAAUUACACAUGUAGAUGGGAUAACAAAAAUAAAAUUCCAUGUAUAGAGGUUAUUAGUUUAACUCCAAAUUCAUCAAACUUUUCAAAGUCUGGAA
env-28   AUGGAGGUAGAAAUAUGCUGUUGAGAAUUUUUUUAAUAAUCUUUUUUUUACAUUUUACAAAUGUUAAUGCUGAAAAUCUUAAAAAAAUGUGAAUGGAAAAAUAAUGGAGGCAAACCAUGCUUAACUAUAAGCAAAACUCCAAAUAGUUCUAUUUACUCUGA
env-29   ................................................................................................................................................................
env-30   CUUAACCUAAGUGCGUAUAAUGAUGUUAAGAUUGCUUAUUUCUAUUUAUAUUAUUUUAGUUUCUAGUUCAGUUUUUUCUGAUGAAAUUCCUGUAAUAGUUAUUUCUGCUGGU...............................................
env-31   AAAGGAGAAAAAAAAUGCUGAUGUUAAAGGGACUUAUCUUUUUUAUAUUAUUUUUUAUAAAUAUUUCAUUUGCUGAAGAAGUUAGUAAAAAUAGUGAAACUUGUUUUUGGACAACCGAACUAAAAUUUCCAUGUUUGGAAAUUGUUAGCAAUAUACCAA
env-32   AUGCUAAAUAUUAUAAAAUUAUAUUCUUUUUCACUAAUUAUAAUUCUUUUUUUUAAUAAAGCUUUUUCGGAAGAUGUACCUGUUAUCGUAAUCGCUCCAGGUAAAACACCCCAAUCUUUGAGUACAGUAGGAAGUACAAUAACCGUUAUUGAUGGUGAAA
env-33   AAAGAAAGAUGGCAUGAUGAAAAUAUUAAUUUUAUUAUUAAUUAAUUUUAUUAUAUUUAAUAAUUCUUUUGCAAAUAAACAAGAUGCUUCAUGUGUUUGGGACAAUAAAAAUAAAAUUAGCUGUAUAGAAAUAACCAGUCAUAUAUCAAAUAACUCCACA
env-34   GUUACAACAUUAAUACUCUGUCUAAGCUUUCCAAGCUUUGUCGUCGGUCAAGACAUUCCCGUGAUAGUAAUCGCACCAAGUAAAAAACCACAAUCAAUUUCAACUGUUGGAUCUUCAGUAGUUGUUUUAGAUGAAAAAUUUUUGAAAAAACAAAUGAUU
env-35   NNNNNNNNNNNNNNNNNNNNNNNNNNNNNNNNNNNNNNNNNNNCAAAAUCUGGAGAAAAAUUAAAUACAAAUUAUUUAGUAUCAUCUGAAAAAUUUCAGAAUGAUGGAAUUUCAGCAAUGAGUCAUAAUGAAGAAAAAGACGCUUAUAGAAACAAUUCUUUUUU

         ................................................................................................................................................................
         ................................................................................................................................................................
         ................................................................................................................................................................
         ................................................................................................................................................................
         ................................................................................................................................................................
         ................................................................................................................................................................
         ••••••••••••••••••••••••••••••••••••••••••••••••••••••••••••••••••••••••••••••••••••••••••••••◦◦◦◦◦◦◦◦◦◦◦◦◦◦◦◦◦◦◦◦◦◦◦◦◦◦◦◦◦◦◦◦◦◦◦◦◦◦◦◦◦◦◦◦◦◦◦◦◦◦◦◦◦◦◦◦◦◦◦◦◦◦◦◦◦◦
```

```
env-36   UUUUAACAAUCUUUAUAUAUCUUUUAAGUAUAAAUAAUUUUUUAUUUAGCGAGGAUAUUCCAAUAAUUGUAAUUGCGCCAAGUAAGAAAACA.........................................................
env-37   UUAAUUUUUAAUUGUGAGAAAUAAAAUCUUUAAAAAUAAUUUUAUUGUAUUCAAUUUUUUUAUUGCCUAUUAGCGCAGAAGUAAUUGAGAAGUGUACAUGGGAUAAUCGUGUUGGAACACCUUGCUUAAUUAUAAGUAAAACACCUAAUACAUCUGAAUAU
env-38   AAACGAAAGGCUAAUAAUGAAAAAGUUAUUAAUAUUAAAUAUUUUCUUAUUUUUUUUUUACAAUAAUAGAGGCUAAUGAAUUACCAAAUUGUGAGUGGAAUAAUUCUGAUGGUAAUCCUUGUUUAACGAUUUUUUCAGCUCCAAAUACUUCUGAUAUCUCC
env-39   AUUAAUAUUUAUUUUUAUAUUUCAGUUAAAUUCCUUUGCUUUUUCAAAGGAUAUUCCAGUUAUAGUUAUUUCUCCGGGUAAAACACCUCAAUCAAUUAAUACUGUUGGAAGUUCUGUUGAAGUAAUAGAUGGAGAAAAAAAUUGAAAGUUCCUCUUAUUUU
env-40   ..........................................................................................................................................
env-41   AUAGUAAAAAAUGGAGGAGUGCCGAAUGAUUAUUAGAUUUUAUCUAAUUUUAAUCAUUAUAAUAUUUUCACACAAUACGAGCUUUGCCAAAGAUAUACCAAUAAUAGUCAUAGCGCCAAGUAAAACAAAUCAAUCAUAUAGUUCUGUUGGAACUUCUGUG
env-42   AACAUAGAGGCAAAGAUGAUCAAGAUUUUAAGUUUUUUUUUAAUAUUUAAUUCUAUAGUAUUUAACAACAUUCUUAAUGCAGAAGAUAUACCAAUUUUAGUUAUAUCUCCUGGAAAAACUGUUCAAUCGAAAAGUAUUGUGGGAAGUGAUGUGCAAGUCG
env-43   AGGACAUGUUGUAUGUUAAGAAUAUUUAUAUUUUUAUAUUUAAUUUUUUUUUACUUUGAAUGCUAGUGCAUCACACAAGUGUAAAUUUAAUAAUAAAAAAUGGAACAUCUUGUUUAAUUAUAAGCAAAACUAAUAAUGCAUCAAAAAUCUCAGAAGAAGGAG
env-44   AAUAAAGAGGUAUAAAAUGUCAAAGUUAAUAUGUAUCGCAACAUUUGCUUUUAUUUAUUUUUCAAAUUUUUUUUAUGCUUUUGCCUUAGAUAGUUGUGAAUGGGAUAAUAGAGAGGGAGUUCCAUGUAUAGCAAUAUCAAAAACUCCCAAUACUUCAGAU
2 env-45 UCUUAAAAAAAGGGCCGAUUAUGAAAAAGGUAAUUACAUUAGGUAUAAUUUAUCUUUUGAUGCUUCCUUUGAAAGCUGAAGAACUUCCAAGCUGUAAAUGGAACAAUUCAGACGGAAUACCUUGUGUUACAAUAUUUUCAGCUCCUAAUACAUCUAAAAUU
env-46   UGCAUGCUAAAGACAAUAAAAUUAUAUUCUUUUUUAAUUAUUAUAACUCUUUUUUUUAAUAAAGCAUUUUCUAAAGACUUACCUGUGAUUGUAAUUUCACCUGGUAAAACCCCGCAGUCUUUAAGCACAGUUGGAAGUACUGUUACUGUUAUAGACGGAG
env-47   AAAAAAGAUAGGUCGAUAUGAUAAAACUAAUAUUUUAGUAUUCACAGUUCUAAUUUAUUUUCCUUUAGAGGCAAAAGAAUUAAAAAAAUUGUGAGUGGAAAAAUGAUAACGGAACACCUUGUUUAACAAUUUUUUCUGCUCCAAAUACCUCAGAAUUAAA
env-48   UAAGGAGAUAUUAUGCUUAAAAAUUUUUUUUAUUAUCUUACUAUUUUUUUUAUUACUAUUAAUUUUAAAGUUUACUCUGAAAUUCCAAUUAUAGUAAUCACACCUAGCAAAAAGUCACAAUCUUUGUCUUCAGUUGGAACUUCUAUACAAGUAAUAAGUG
env-49   AUAUGCCGAAGUUAAAAUAUUUUAUAACAUUUAUAAUCUUAGUUUCAUACUGUUUUAAAUUUGAUGCAAAAGCCCUUGAUAUUCCUAUAAUAGUCAUUACACCGAAUAAAAACCCACAAUCUUAUAGUACUGUAGGUAGUUCAGUUUCAAUAAUUAACCA
env-50   CUUAGGAGAUGUCAUAUGUUUUUAAGAGCCAAAAUAAUUUUUUCAUACUAAUAUCAAAUUUAUAUGCUGAAAAAUUUGAAAACUGCAAAUGGGACAAUAUGAAUGAAAUAAGUUGUGUAGAAAUAAUUGGUCAUAUAUCAAAUUAUUCUAAAUAUUCAC
env-51   ..........................................................................................................................................
env-52   GGUUAAUAGUUUGCUUAUUUUUAAGCUUAAUUUAUUCCAAUGCUUCUUUUGGUGUUGAAAAUUCUAAUAAUUAUUCUUGUAAGUGGGAUAAUAAGUCUUCAUUGCCAUGCAUCGAAAUAUCAAGUUUAACUUCUAAUUCCUCAAAUUUUUCUAAAUUAGG
env-53   AGGAUUAAGUGUCUAGACUAAUUUUUGUAUUAAUAUUACAAAUUUUUUAUUUUCAAAUUUCUUUUACUAAAGAAGAAGUUUAUAAUUGCAAAUGGGACAACAAGAACAAUACCCCAUGUAUAGAAAUCAUAAAUGAUAUAUCUAACUCUUCUGAAUUUGC
env-54   UUUUAAAAAAAGAGUAAUUUUAUGCUGUUAAGAGUAAUUUUUAUUUUAAUAUUAAUUAGUAUUACUUCUGCCGCUUUAUACGCAAAGGAAAUACCUAUAAUUGUUAUUUCUGCUGGAAAAUCUACUCAAUCAUAUAGCACCGUAGGUAGCGAUGUAGAAGU
env-55   GGGAGAUAACAUGCUGAAGAGGCUGAUUUUGAUAUUUAUUUUUUUUAUAACUUCAUCAAAUGCUGAAUAUAUAAAAAAUUGUAAUUUUAAAAAUGAAAAUGGAACUCCAUGUAUUACUAUAAGCAAAACACCCAAUAGUUCAUCUUUUUCCGACAAGGGA
env-56   AGGAGAAAAAAAUGCUGAUAAGACUGAUUUUGAUAUUUAUUUUUUUUAUAACUUUAUCAAAUGCUGAAGAUAUAAAAAAUUGUACUUUUAAAAAUGAAAAUGGAACUCGAUGUAUUACUAUAAGCAAAACUCCCAAUAGCUCAUCUUUUUCCGACAAGGGA
env-57   AAUAAACUUAUAGGUCGAUUAUGAAAAAAUUUAUCUACUUUUUUCUUUUAAUAUCGUUCUUAAAUUUUACAAAUUUAUUAGCAAAAGAUAUUCCCGUUAUAGUAAUAUCGCCAGGCAAGACGAUUCAAUCAAAAGGUAUCGUAGGGUAGCGAUGUUGAGG
env-58   CUAACAUUGGAUGUGUCGUAUGCUGAAUGAAACGCUACAUAUAAGACUAUUAUUUAUAUUAAUAUUAUUAACCUUUUCUUUAGACGCCCUUUCAUUAGAAAAUUGCAAAUGGACCAAUAAUAAUGGUGUUCCAUGCACAACUGUUAGCAAAACACCUAAC
env-59   UUUUUGGAGGUUUAUGUACAAAAAGUUGUUAAUUGUUUUCUUUCUAAUAAAUAUUUUAUUUUCGGCUGAAGCAUUAGAAAAAUGUGAUUUUUAUAAUAAUGAUGGUGUACCAUGUAUUAUUAUUUCUAAAACACCAAAUACAUCAAGUUUAAAUAAAGAU
env-60   NNNNNNNNNNNNNNNNNNNUUUUUUAAUACACAGUUCUUUAGGAAAAGAAAUAGAUAAGAAAAAAAUCUUGCUCAUGGGAUAAUAGGUCUAAAAUACCAUGUGUAGAAAAUAAAAGGUUAUAUACCAAAUUCCUCAAAAUUUUCGAGAUCAGGAAUUAAU
env-61   AAAGAACUGGUGUAUGAUUUGUAGAAAUUUAAAAAUAUUAUUCUGUUUUGUAUUACUUUUAUUAUCUAAAACUAGUAAAGCUGAAGAUAUACCUA....................................................
env-62   GAGAAAUAAUAUAGCUGAAUGUUUUUAAGAAUUUUAAUUACUUUUACAAUUUUAAUAUCAAAUUUAUAUGCUGAAGACUUAGAAAAUUGUAAAUGGGAUAAUAGAAAAGGUGUGCCAUGUUUAACAAUAAGCAAAACUCCUAAUACUUCCUCUUUUAAUG
env-63   AGAGGUUAUUGCUGAAUGUUAUUAAGAAUUCUAAUUAUAUUACUAAUUUUUAUAUCAAGUUUAUAUGCUGAAGACUUAGAAAACUGCAAAUGGGACAAUAGAGAAGGUGUGCCAUGUUUAACAAUAAGCAAAACCCCUAAUACCUCAUCUUAUAACGACA
env-64   AAAUGAAAAGGAUAUUUUAUGCUUAUCAGAGUGAUGUUAAUUUUUUUAUUAUUUUAUACAAAUUUAAAUGCUGAAAUUUUAGAAAAUUGCAAGUGGGUAAAUAAAGACGGUAAGCCAUGCUUAACUAUAACUAAAACACCAAAUACCUCAGAGUUUAACC
env-65   UGUCGAGGCUAAAAUAUUUAUUAACUAUUAUUUUUUUUAUCUUAUUUUGUUUUCAAUUUACUGCUAGGGCUCUUGAUAUUCCAGUUAUUGUAAUAGCUCCUAAUAAAAGUCCUAAAUCUUAUAGUGCAGUAGGUAGUUCAGUUUCAAUAAUUGAUCAAGA
env-66   UGGAGCUUAACAAUGGAUAUGUUGAAUGAAUUAUUUAAAAGCACUUUUUAUUUUAUUUAUGUUAAUCUCUGCUUCUAAUGCAGUUCCAUUAGAGAAUUGUGAAUGGAACAAUAGUAAAGGAACUCCAUGCAUUACAAUAACUAAAACUCCGAACACAUCC
env-67   AACCUUAUUAAUAUUAUGAGGUUGUUAGUGAGAAUAUUAUUUUUAAGUCUAAUAUUAUUUUUAAUUUCUUUUGAGUCGGUAAGAGUUUAUGCAAAAGAUAUUCCUAUAAUAGUUAUAGCGCCAAGUAAAAUAGACCAAUCAUAUAGUACAGUAGGUACAU
env-68   UAAAAUGGGUGUGAUGUUUAAAAUUAUAACAAAAAUUGCGGUAUAUUUUAUUUUAAUUUUUAAUAUUUUAAGUGUAAACGUUUCGGCACUUGAGGAAUGUAAGUGGAAUAACAAAGAAGGUAUUCCUUGUUUAACUAUAAGUAAAACUCCUAACACCUCA
env-69   UUAAAAAAAAAAGGCCGUUAUGAAAAUUUUUUUUAUAUUUAUAAUUUUAGUUUUAAGUUUCUUGCCUCUAAAAAGUAAGGAAUUAAAUAAUUGUGAGUGGAAGGAUCAAUCUGGUACUCCAUGUUUAACAAUUUUUUCUGCCCCCAAUACUUCAGAAAUUA
env-70   UGUUUAUGUCUCUUUAUAAAAAAAUAUUUUUAAUAUUAGCGUAUUUUUUAUUUUUAAGUAAUCACUCUUUAUCAGCCCAAAAAAAAAGAAAAUAAUUGUAGUUGGGAUAAUGAAAAUGAUAUUCCAUGUAUAUCUAUAGUUAAAAAAACUUCUAACUCCUC
env-71   ..........................................................................................................................................
env-72   AGUAUCAUUUUUAAGUUUAUUACUUUUAAACACACUAUUUACAAUCCCAGUUUUAGGAAAAGACACGCCAAUUAUUGUAAUUGCACCUAGUAAAAAACCACAAUCAGUCUCAACAGUUGGUUCGUCUGUAAUUAUUUUAGAUGAAAAAUUUUUUGAAGAA
env-73   AUAUGUUAAAUAAAAAUAUAAUCACUUUAAGUAUUAUAUCUAUUUUGUUUUUUAAUAAAGUUUUAUGCGAGGAGAUCCCAACAAUUGUAAUUGCUCCUAGUAAACAGCCACAAUCUAUUUCUACUGUUGGAACAUCCGUAGUAGUUUUGGAUGAAAAAUA
env-74   AACCAAAGGAGAGCUAAUGCUAAAAAAAAAUUUUAGUAAAUUUUUUGUUUUUAACAUUUUUAUUCAAUAGCGUAUCAUUUUCUCAAGACAUUCCGAUUAUUGUAAUAGCACCAAGCAAAAAACCUCAGUCAGCAU.........................
env-75   AUAACGAGGGAGAUUAUGUUGUAUGGUAAUUACAAGAAUAUUAAUUAUCUUAGGUUUAUUUCUUUUAAGCAGUAAAGCAUUUUCUUUAGAAAACUGUGAAUGGAAUAAUCAUAAAGGCACGCCUUGUCUAACAAUAACUAAAACACCAAACACAUCUUAU
env-76   CUUAGAAAAUUAGAAAAGGAGUCGUUGUGCUACUAAGGGCUUUUAUAUUAAUCUUCAUAUUAAUUUCAAAUCUGUACGCAGAAAAUUUAAAAAAAUUGUGAAUGGGAUAAUAGAGAAGGCACUCCCUGUAUUGUUAUAAGCAAAACGCCAAAUACAUCAUC
env-77   AAAAAAAUAAGUCGAUAUGAAAAAGAUAAUUUUAAUCUUAGUAGUUUUAUCAAUAUAUUUUCCUAUACAUAGUAAAGAGCUUGAAAAUUGUGAAUGGAAUAAUAAAAAAGGCAUGCCUUGUCUUACAUUAUUUUCUGCACCCAACACUUCGAAUUUAACU
env-78   AGAACAGAUAGGCCGAUAUGAAAAUAAUUCACCUAAUUUUACUUACUUCUUUAACUUUAACUUCUUUUAAUAAAAUUUUUGCCGAAGAUAUUCCAAUUUUAGUAAUCUCACCAAGUAAAACCCCACAAUCAGUCUCUACAGUUGGAACUUCUGUAACUGU
env-79   AUUUUUAUAUAGGUGCGAUUGAAUGAAAAUUAUAAAAUUACUUUUAGUUCUUGUUUUAUCAAUUUUUACUUUAAAUGCCUCUGCAUUAGAAAAUUGUAAGUGGAAUAAUAAAAAAGGAGUACCUUGUUUAACUAUAAGCAAAACUCCAAAUACCUCAGAA
env-80   AUAAUAGGAUGCUGUAUGAAAAUUAAAAUAUUUUUCUUAACAAUUUUUUUAACUCUAAAUUUAAUAACUUUAAAUUUAAAGGCUAAUGAUCAAUUAGAUUGUAGUUUUACAAAAAGUUCUGUUCCAUGUAUCGAAAUAAGCUCUAAUAUUUCUAAUAUUU
env-81   AAAAGGAUAUGUUUAUGUUUGUUAAAAUAUUUAUAACAAUUUUACUAUUU.....................................................................................
env-82   UGAACAAUGGAUGUGUCAAAUGAUGUUAAGAGUAUUUAUAUUUUUUUAUUUUAUUUUAAUUACAAGCUCAGUUUUUUCAAAAGAAAUUCCUAUAAUUGUAAUCUCAGCAAGCAAAAAACCACAGUCUUUAUCAACUAUAGGUACGUCAACAACUAUUCUU
env-83   UGUAAAAGUGGGUCGUUAUGAAAAAAUUUUUUUUAUAUUUAUUUUUAUUUUUUAAUUUUGUCUUUUUUAAAUCAUUCGCAAAAGAUAUUCCAAUUAUAGUAAUAUCUCCCGGAAAAACAUUACAAUCUAAAAGCAUUGUGGGUAGUGAUAUUAGUGUCAU
env-84   AAAGGAUUAAGUCGUUGAAUGAGUAAAAUAAUUUUAUAUAUAUAUAUAUUUCUCUUUAUUUCUAAAAAUCUUGUCUAUGCAAUCGAUAUACCCGUGAUAGUAAUUGCUCCAAGUAAAUCGUCGCAAUCAUAUAGUUCAGUCGGGAGUUCAGUUUCAUCUA
env-85   UUAGUUUAUUAUUAUUUUUAAUUUUUCCCGGCAAAAAUACUAUUUGCUGGGGAUAUACCAAUAAUAGUUAUUGCGCCCGGUAAAAGUGUUCAGUCCUAUAGCACAGUAGGAAGUUCAGUUUCUGUAAUGAUAGUAAAGAUAUAAAAAAUUCAAACGAUUA
env-86   UAAAAUUAUUAAUAAUUU.......................................................................................................................
env-87   UUAACAAUGGAUGAUGAACGAUGAUGAUAAAGUUAUUUUUAAUAUUAAUAUUUUCAAUUUUAGUUUCAAAUGUAUCUGCUUUAGAAAAUUGUAAAUGGAACAACAGUAAGGGUAUACCUUGUACAACAAUUACAAAACACCUAAUACUUCAAAAUAUAA
env-88   AAAGGUAAGUCAAUGCUAAAAAAAACUAUAACAAGUUUAAUUUUUUUAAUAUUCAUUUCCAAAAGCAGUCUAUUAUUUUCUGAGGACAUUCCCAUAAUUGUCAUUGCACCAAGUAAAAAACCUCAAUCAGCAUCGACUGUAGGAACCUCAGUAGUAGUUC
env-89   GUUUUAAAUUAAUAGGAUGUCAAAUGCUUUAUAGAAUUUUAAUAAUUUUAAUAUUUCUAAUUUCAAAUUUAAAUGCUGAUAAUUUGGAAAAAUGCGAAUGGGAUAAUAGAGAGGGUAUUGCAUGUUUGACUAUAAGCAAAACACCAAACACAUCUGCUUA
env-90   ACAACAAAAGGACUGUUAAUGAAAAAGUUAUAUAUAAUAAUACUAUCUGUACUAAUUUACUUACCGUUAGAAAGCAAAGAAUUAGAAAAUUGUA....................................................
env-91   AACGAAGGGGAGUCACAUGCUAAGUAAAAUUAAAAACCUAAUAGUAUUUCUAGUAAUAGUUCUAUUUGCUACCCAACUUUAUUCUAAGGAUUUUCCUGUUAUUGUUAUAGCUCCAAGUAAAAAACCGCAAUCUAUAUCUACAGUUGGAACAUCUGUGGUA
env-92   UAAUUUUAGGAGAUGUUUAUGCUAACAAGAGUUAUAAUAUUUUUUAUAUUAACAAUAACAAGCCUGCACGCAGAAAAUUUAAAAAAAUUGUGCUUGGGAUAAUAGAAAAGGAAUUCCUUGUAUUGUUGUUUCAAAAACUCCAAAUUCAUCUGAUUAUUCUG
env-93   UUUAAAAAAAAUAACAUUUGUAUUAAUAAUAUUAACAGCUUUUUCUUUUGUGAGUGUUAAUGCAAAAGAAAUCCCUGUUAUUGUAAUUGCACCAAGUAAAACACCUCAAUCUAUUUCUACGGUAGGAACGUCUGUAAAAGUAUUAGAUGAACAAGACUUGA

         ..........................................................................................................................................
         ..........................................................................................................................................
         ..........................................................................................................................................
         ..........................................................................................................................................
         ..........................................................................................................................................
         ..........................................................................................................................................
         ••••••••••••••••••••••••••••••••••••••••••••••••••••••••••••••••••••••••••••••••◦◦◦◦◦◦◦◦◦◦◦◦◦◦◦◦◦◦◦◦◦◦◦◦◦◦◦◦◦◦◦◦◦◦◦◦◦◦◦◦◦◦◦◦◦◦◦◦◦◦◦◦◦◦
```

```
env-94  UUAAUGUUGGGGUUAAUAUGAAAAAAAUUUUUAAGUUAUUAAUAAUAAAAACGUUUUUGAUUAAUUUUAAUUUAGCUUUUGCAAAAGAUAUACCUGUUUUAGUUAUAUCGCCUGGUAAAACAAUACAAUCAAAAAGCACUGUGGGAAGUGACGUUGAAGU
env-95  GGGGUUUUUUGUGA..................................................................................................................................................
env-96  UGUCGGUACUAAAAUUCUUAAUAACUCUAAUACUAACAUUUUUAAAUUUAAUUAGUUUUUCUGCGAAAGCUCUUGAUAUUCCAACAAUUGUAAUUGCUCCUAACAAAAGUCCACAAUCUUAUAGCACUGUGGGUAGCUCAAUAACAUCUCUUGAUAGUGA
env-97  CAUAACAUUAUGAGGUACUUANNNNNNNNNNNNNNNNNNNNNNNNNNNNNNNNNNNNNNNNNNNNNNNNNNNAAUUUUAAAUGUAUUAACUGCAAAAGCUGAAAAUUUAUCUAAUUGUAAAUGGGAUAAUAGAGAUGGAGUACCAUGUGUAAUAGUAACC
env-98  GAAGAAAAGAGAGUGAAUGCUUAUGUUUUUAAAGUUUAAUUUAUAUUUUAUUAUUUUUAUUUUUUGUUUUCGCUAAAAAUUCAAGCAAAAGAAAUACCAAUUAUUGUAAUUUCAGCAGGUAAAUCUAAUCAAUCAAUAAGUGCCGUAGGAAGCGAUAUA
env-99  CAAUGAAACUAUAGGUCGAUUAUGAAAAAAUUUAUCUACUUUUUCUUUUAAUAAUGUUCUUUAAUUUUUCACAUUUAUUAGCAAAAGAUAUUCCCGUUAUAGUAAUAUCACCAGGAAAGACACUUCAAUCAAAAGGUAUCGUAGGUAGCGAUGUUGAGG
env-100 CAAUGAAACUAUAGGUCGAUUAUGAAGAAAUUUAUCUACUUUUUCUUUUAAUAAUAAUCUUAAAUUUUACACAUUUAUUAGCAAAAGAUAUUCCCGUUAUAGUAAUAUCGCCAGGUAAGACACUUCAAUCAAAAGGUAUCGUAGGUAGCGAUGUUGAGG
env-101 CAAUGAAUCUAUAGGUCGAGUAUGAAAAAAUUCAUCUACUUUUUCUUUUAAUAAUGUUUUUAAAUUUUACAAAUUUAUUAGCAAAAGAUAUUCCCGUUAUAGUAAUAUCGCCAGGUAAAACACUUCAAUCAAAAGGUAUCGUAGGUAGCGAUGUCGAGG
env-102 AAGAGGUUAUAUGCUGUUGAGAUAUUUUUUAAUAUUUUUUACUUUUUACUUUACUAAUGUUAGUGCUCAAAACUUAAAAGAAUGUGACUGGAAAAAUGAUAAAGGUAUACCGUGCAUAACGGUAAUGAAAACUCCAAAUAGUUCUGUUUAUUCAGAAAUU
env-103 UUAUUAAAAAAAUUGGAUGUAUAUUAUGAUGUUAAAAAGUUUUUUUUCUUAUAUUUUGUACUAUUUACAACUUCUAUUUUUUCAAAAGAGAUUCCUGUAAUUGUAAUUUCACCUUCAAAAUCUCCUCAAUCAUAUAGUUCUGUUGGCAGUCAAGUUACA
env-104 GAAACUUUAAAUGUUUGGAAUGCCGAAUGAGCAUUAGAAUAUUAUUAUUUUUUCUGUUUUUUUUAUCAAAUGUUCAAGCUAUGGAAAACUGCAAAUGGAAUAAUGAAAAAGGGGUUCCGUGUGUUACCAUUAGCAGAACACCAAACACUUCCUUGUAUAA
env-105 GUUACUUAGGAUGAUAUUAAAAAUGGAUAUUUAUGCUUAAAAAAAAAUUNNNNNNNNNNNNNNNNNNNNNNNNNNNNNNNNNNNNNNNNNNNNNNNNNNNCAAGAAGAACAAAAUCAAAUUAUGACACAAGCACUAGUGAUGAAAAUGGUUUUGCUCAUG
env-106 UGAUGAAAGGGUGCUGAUGAUGGUUAGAUUUUUAAUAUUUUUAAUUUGAAUUUUUUACAUUUAAAUUAUUUAUUUUCAAAAGAUAUACCAGUAAUAGUAAUUUCACCCGGAAAAACUAUUCAAUCAAAAAGUAUAGUAGGAAGCGAUAUCGAAGUCAUA
env-107 UUAGAUUAUUAUUAUUAUUAAUUUUUCCGGCAAAGAUGCUGUUUGCUGCAGAUAUACCAAUAAUAGUUA...........................................................................................
env-108 ................................................................................................................................................................
env-109 UUAAAAGGAUGUACAGAAUGCUGGUAAGAUUGUUAUUUGUAUUGUUUUUUAUUUUUUUUUCAAAUUUUGUAUUUUCUAAAGAUAUUCCAAUAGUUGUUAUUAAAGCAAGUAAAAAACCUCAGUCGCUUUCUACUGUAGGUACAUCUGUGACUGUCCUUGA
env-110 AAAUUUGGAAAGGUGUUAUAUUUAUGAAAAUCACAAAAAAUAUUUUUAAUAUUUAUUAUAUCAAUUUUUACUUUUAAUGCCUCAGCAUUAGAAAAUUGUAAAUGGGAUAACAAAAAAGGAGUGCCUUGCUUAACUAUAAGCAAAACUCCAAAUACCUCAG
env-111 GUUAAAUGUAUUUAAAAUUAAUUGUAAUUUUUUUAAUACUUACAAUCAAUUCUUACGCUGAUGAAUUGAAGAAAUGUAAGUGGGAUAAUAGAGAAGGAGUUCCCUGUAUUGUAAUUAGCAAGACACCAAAUACUUCAGAUAUAUCUGAAGAAACAAUAAA
1 env-112 GAUUAAUUGAAAAAAAUAUUUUUUUUUAUUUUAAGUUUGUUGAUUUUAAAAAGCUCUAAUGCUCUAGCGCUAGAAAAAUGUAAAUGGAACAAUAAAGAUGGAGUGCCUUGUAUAACCGUUAGCAAAACAACGAAUAGUUCUUAUUACAACCAAAACAAUA
env-113 ACUAUUU.........................................................................................................................................................
env-114 UAAAAAAAUGAGGUGGCGAAUGUUAGAAAGGCUUUUACUUAUUUUAUUUUCAAUAAGUUUUUGUUCAAGUGCUAUUGCUUUAGAAAAAUGUAAAUGGAAUAAUCAAAAAGGAGUUCCUUGUAUUACAAUAAGUAAAACACCAAAUACUUCUUUAUAUAAU
env-115 ACAAUAAAAGGCCGGCAAAUGAAAAAAAUUUUUUUAGUUUAUUUAUUUUAACUUUUUCCUCAAUAGAAGCUUUAAGUUUAGAAAAAUGUGAAUGGAAAAAUACGCAAGGGAAGCCUUGUCUUAUUAUCACUAAAACCCCCAAUACAUCCUUUUUUGAUG
env-116 GAAUUGAUUAUGUUAAUGCUAUUAAAAAUUAUAUUUUUCUUUAUUUCUUUUAUAUCUUUUUCACACGCUAAUGAUCUUAAAAAUUGUGAGUGGAAUAAUAGCAAAGGAAGUCCAUGUAUAACAAUCAGCAAAACUCCAAAUACUUCUAAGUAUUCAGAGG
env-117 AAAGGGGCCGUUAUGUUAUUUAGAAACUUUAUUUUUUUUAUGAUUUUGUUUUAUUCAAGCACUAUUUUCGCUAAAGAUAUUCCCAUAAUUGUAAUUUCACCUGGUAAAACUUUACAAUCCAAAAGUGUUGUAGGAAGUGAUGUAGAGGUCAUAGAUAAAA
env-118 CAAGAAAGAAUAACUGAUAGGUUAAAAAGGAUUUGGUUUAUAUGCUGAAAAAGUUUUUUCUUUUUUUAUUAUUAAUUACAAUUACCACCAAUAUAUAUUCCCAAGAGUUGAAAAAAUGUGAAUGGGAAAAUAAAAAAGGUAAACCUUGUAUAACUAUAAA
env-119 UUUUAAAAGAGGAGAAAUGCUGCAUGUAUUUAAAAUUUAUAUGUCUUUUAUUGCUAUUAAUUAUUUUUAAGUCUGGAAAUAUUCACGCCAAAAGUAUUCCUGUAGUAGUAAUUGCGCCGAGUAAAAAACCACAAUCAAUAUCGACUGUUGGUACAUCUAU
env-120 UCCAAUAGACAAAUAGGUCGAUUAUGAAAAAACUUAAUUAUCUCAUACUUCUAUUAAUGCUUUUUAAUUUUACGAAAUUAUUAGCAAAAGAUAUCCCCAUUAUAGUCAUAUCGCCAGGCAAGACAAUUCAAUCAAAAGGUAUUGUGGGUAGCGAUGUUGA
env-121 UAAAAAUAUUAAUAAUUUUAAGUUUUCCGUUUAAUGCAUUAUAUGCAGCGGACAUUCCGAUAAUUGUUAUAGCUCCAAGUAAAAAAGCGCAAUCUGUUUCAACCGUAGGAACAUCUGUAACAGUGCUUGAUGAAACAUUUUUUAAAAACUCAAAUGAACU
env-122 UAAAAAUACUUAUAAUUUUAAGCUUUCCUUUUAAUGCUGUGUCAGUUGCUGACAUACCAAUAAUUGUUAUAGCCCCAAGUAAAAAAGCGCAAUCUGUUUCAACUGUAGGAACAUCCGUAACAGUGCUUGAUGAAACAUUUUUUAAAAAUUCAAAUGAGUU
env-123 UAAGUUUAUUAAUAAUUUUUACUUUUCCGUUUAAUGUAGUCUUUGCAACGGACAUACCGAUAAUUGUUAUUGCGCCUAGCAAGAAAGCGCAAUCAAUUUCAACAGUAGGAACAUCUGUAACUGUACUUGAUGAAGUAUUUUUUAAAAAUUCAAACGAAUU
env-124 AAAUAUGCAGAGCUAAAAUGAAAAAAAUAUUUCUUAGUUUUAUUCUAUUAAUCUUAACCUCAAUAGAAGCUUUAAGUUUAGAAAAGUGUGAAUGGAAAAAUACCAAAGGUAUACCCUGUGUAAUUAUUACUAAAACUCCUAAUACAUCUUUUUUUGAUGA
env-125 UAUGAGAAGGAGUGACUUAUGCUUAUAAGAAUUUUUACAAUAUUAUUUACAUUUAUUUGUAGUGCUAAUUCGAUAGAAAAUUGUAAGUGGAACAAUAAAAAAGGCACUCCAUGUAUCACAAUUAAUAAGACAUCAAAUUCUUCUUUAUAUAAUGCUCAAG
env-126 GAUGAUAAAAAAAGGAAAACAUGUGCUUAGUGUUAGAUUACUUUUAUUUUUAUUAAUAUCUUUAUUAUUUAGUU......................................................................................
env-127 UCCAUGAACUAGAUAGGCUGAUUAUGAAAAUAUUUAAAUAUAUUUUAAUUUUUACAAUUUUUAUUAAUUAUACAAAUUUAUUAGCAAAAGAUAUACCUAUUAUAGUUAUAUCACCAGGUAAGACACUCCAAUCUAAAGGUA..................
env-128 AAAAAAAGGAUUGGCCGAUAUGAAAAACUUAAUAUUAGUAUUGAUAGGUGUAUUCGUAUUUUUACCUUUAAAUGGUAAAGAAUUGCAGGAUUGUAAAUGGAAAAAUGAACAAGGUACACCUUGUUUGACAAUUUUUUCUGCUCCUAAUACGUCAAAAUUU
env-129 AGAUAUGCUUAUGUUAAUUAAGUUUAUAAUUUUAAUUUUUUUAUUUUUAUGUUCUUUUAAAGCAUUUGCUUUAGAAAAUUGUAAAUGGGACAAUAGAAAAGGUGUUCCGUGUUUAACAAUUAGUAAAACUUCUAAUAC.....................
env-130 UAGAUNNNNNNNNNNNNNNNNNNNNNNNNNNNNNNNNNNNNNNNNNNNNNNNNNNNAAAAAUAUUUAAAAAAUAGUAAUGAGUAUUUUCUUGGAGAUAUAUUGUCAUCUAACUCAACAAGCACAAAUUUUUUUCAAAGUGGUGGUCAUGGAACAGUUUCA
env-131 GCGAGGCUAAUUAUGAAAAAAUUUUUAAAGUUCUUAUCAAUAUUUUUUUUACUUACGAGUGUUGAGGCGAAAGAUUUGCCUAAUUGUGAAUGGAGCAAUCCCAGUGGAACUCCGUGUUUGACCAUCUUUUCAGCUCCUAAUACCUCAUCUAUCAACGAAA
env-132 UACGGAGGUACGUUGUGAAAAUUAUAUUAUUUAUUACUACUUUUUUUUUAACAUAUGUUUUCCAAAUUCAGGCUAAAGAGCUUGAAAAUUGUCAGUGGAAAAACGAGAUUGGAAUUCCUUGUUUAACAAUAUUUUCUGCGCCGAAUACUUCAAAUUUAAG
env-133 AGGUAGCCGUUAUGAAAUUUUUAAUAAUUUUAUUAACUUUUUCCAUAAGUUUUAUUAAUGCUUACGGAGAAGAAUUAAAUAAAUGUGAGUGGAAAAAUGUUGCUGGAAAACCAUGUNNNNNNNNNNNNNNNNNNNNNNNNNNNNNNNNNNNNNNNNNNNN
env-134 AGAGAGCCGAUAUGCGGAAAAAUUUUUUUAUACUAGCAGUCUUAAUCCUAAGUUUCCUACCUGUAGAAAGUAAAGAAUUACAUAAUUGUAAAUGGAAAAAUAAAUCUGGAACACCCUGUUUAACAAUUUUUUCUGCGCCAAAUACUUCAAAGAUCACAGA
env-135 UACAAUUAAAGGUUAACCAAUGCUAAAAAAAAACUUUAGUCAAAUUGAUUUUUUUGUUAUUGUUUUUAAUAAUACUUUAUUAUCACAAGAUAUUCCUAUUAUCGUAAUUGCUCCUAGUAAGAAACCUCAAUCAGCUUCAACAGUUGGAACUUCGGUUGUA
env-136 UACUAUGAAAGGUUAACCAAUGCUAAAAAAAAAUUUUAAUUAAAUUGAUUUUUUUUGUUUUUGUUUUUAAUAACAUUUUACUAUCACAAGAUAUUCCUAUUAUCGUAAUUGCUCCAAGUAAGAAACCCNNNNNNNNNNNNNNNNNNNNNNNNNNNNNNN
env-137 UUGUAAAAGGAGUUUAUUUUAUGUUGUUGAGAGUAAGUUUUAUUUUAUUACUACUUAUAUCAAAUUUGUAUGCUGAAAAUUUAAAAAACUGUGAAUGGAAUAAUUCUGAUGGUAUUCCAUGUACGGUGAUCAGCAAAACACCAAAUACUUCAUCGUAUAA
env-138 UAGAAAGUUAGAAAAUGUCUUUGGUAUUGAACUUUUUUUUUAUUUUUUUACUUCUAAUUUUUACAAAUAUUUCAUCAGCAACAAAUAAUGUAAUCACAGAUAAAUGUAAUUGGGAUAACAAGCUAAUCACACCGUGUAUUGAAAUUAUUAAAAAAAUUCC
env-139 UUAAAAAAAAGUUUAUUUUAUGUUGUUAAGAGUAAGUUUUUUUCUAAUAUUACUUAUAUCAAAUUUAUAUGCUGAAAAUUUAAAGAAUUGUGAAUGGGAUAAUAAAGAGGGUGCUCCAUGUGUGGUAGUCAAUAAGACGCAAAAUACCUCAGAAUUUUCU
env-140 AUAAAGAUAAUAAUAAUUUUAACUUUUCCCUUUUAAUGCCAGUU....................................................................................................................
env-141 AUAACUGUGACACCUGUUUGUAAUAAUUAUUAACUUUCAUAAAUGAUGUUUCAUAAUAUCUAUAGAUAUUGUUGUAUCUAAAUGUAGUAAAUAAUUUUUGCUCAUAACUAUAAACAAGUAAUAGUCUCAAAAAAAAUUUAAAUUAAUUAACUUAUAAAGA
env-142 UUUGCAUAUGCUGAUUCAACAUUAUACGAAAUAACUGUAACACCUGAAAAAAAACCAACAAGACUCAUAGACACUCUUAAUUCUGUAGAGAUUAUUACAUCCGAUGAUAUAAAAACAUCUGGUUUUAUAACUAUGGAAGAAGUUUUGAAUAGCUCUAGCU
env-143 UUUGCAUAUGCUGAUUCAACAUUAUACGAAAUAACUGUAACACCAGAAAAAAAACCAACAAGACUCAUUGAUACUCUUAAUUCUGUAGAGAUUAUUACAUCUGAUGAUAUAAAAACAUCUGGUUUUAUAACUAUGGAAGAAGUUUUGAAUAGCUCUAGCU
env-144 ACAUAUGCUGAUUCAACAUUAUACGAAAUAACUGUAACACCUGAAAAAAAACCAACAAGACUCAUAGACACUCUUAAUUCUGUAGAGAUUAUUACAUCCGACGAUAUAAAAACAUCUGGUUUUAUAACUAUGGAAGAAGUUUUGAAUAGCUCUAGCUCUA
        ................................................................................................................................................................
        ................................................................................................................................................................
        ................................................................................................................................................................
        ................................................................................................................................................................
        ................................................................................................................................................................
        ................................................................................................................................................................
        ●●●●●●●●●●●●●●●●●●●●●●●●●●●●●●●●●●●●●●●●●●●●●●●●●●●●●●●●●●●●●●●●●●●●●●●●●●●●●●●●●●●●●●●●●●●○○○○○○○○○○○○○○○○○○○○○○○○○○○○○○○○○○○○○○○○○○○○○○○○○○○○○○○○○○○○○○○○○○
```

alignment positions 481 $\cdots$ 577

```
env-1  CAUCAGUUCAAGAUGAAGACACAGAUGAGCUAGUCCAGGGCACAGCAUCUACAUUAUUUCCCCAACGCCAAUAGCAACCACUUCUUCACUUGGAUA
env-2  ACGAUUGCUGUGCAAUCUGGACCUACAGGACAACAGACUUCUGUGUUCAUGAGAGGAACCAACAGCAAUCAUACUCUGGUAUUGUUAAAUGGCAUAC
env-3  AAGAAAAUAACUUAAUUGAUUUACCUAAAGUUUUAAAAUUUAUAAAUGGCGUUAACAUAACACAGUCAGGCUCGACUGGACAGCAAACAUCUGUGUU
env-4  AAAAGAAUAAUUUAAUUGACUUACCCAAAGUUUUAAAUUUUGUUAAUGGCAUUAAUAUAACA...................................
env-5  CAAGAAAUAGAAGCAAAUAACAUCAAGGAUCUAAAAUCUGUAAUGGCCCACGUCAACAAUGUGAUGGCUGUUCAGUCAGGUCCCACAGGACAGCAAA
env-6  GGCCUCGAGGACAAACUGUUUCGACGUUCACUAGGGGCACAAAUUCUAAUCACACUCUUUUCAUGAUCAACGGAUCACCGAUCACAGACCAUUCAAC
env-7  AUAAUCUCACCGAUUUAACAAAAGUUCUAAAAUUUGUAAACAAUGUGAAUGUUGUUCAGUCGGGACCAACCGGACAGCAGUCGUCGGUCUUUAUGCG
env-8  ACUAUGUUCAAGGCCUAGACAUAACUCAAUCAGGUCCGACAGGUCAACAAUCAUCUUUAUUUUUAAGAGGCACAAACUCUAAUCACACAUUAGUAUU
env-9  ACUAUGUUCAAGGUUUAGACAUAACUCAAUCAGGUCCUACAGGUCAACAAUCAUCGGUGUUUUUAAGAGGCACUAAUUCUAAUCAUACAUUAGUAUU
env-10 UACAGUUUUGAAAGUAUUAAAAAAAUAACGAUUACGAGAAAACAAAUUGAAC.............................................
env-11 UACUGUUACAGUUAUUGAUGUGGAUAAAAUUAACGAGUCCUCACAUGUUUCUUUAGCACAAAUUAUUGACGACAGCUCGACAAGCACAAACAUGUUU
env-12 .................................................................................................
env-13 .................................................................................................
env-14 GAAGGAUCAGUUAAUAAAAUUGUUAUUAA....................................................................
env-15 ACUGUUUUUGAUGAAAAAUUUUUUAAAAAUUCAAAUAAAUUAUUUUUAGGAGAUGUUUUAAAAAACAAUUCAACCAGUAUUAAUUUUUUUCAAAAUG
env-16 AAACGAAUUUUUUUUAGGAGAUUUG........................................................................
env-17 GUACUGGAUGAAAAUUUUUUUAAAAAUACUAAUCACUUAUUUUUAGGGGACGUUUUAAAAGGAAAUUCAAACAGUGUAAAUUUCUUUCAAAGUGGCG
env-18 CCAUUGUUGGCUAUGUUUGUGUCGGAUCUGUAUUUGGGGUUCCACUCCAGUAUGUUUUGGGUUUACAUUAGUAUCUUGUUAGGCACAACAUUGAGUC
env-19 AAAUACAAAAGUACAAUCUCAUAGACUUACCUAAGGUAUUGAACUAUGUGCAAGGAUUAGACAUCACACAGUCUGGACCAACAGGACAACAGAGUUC
env-20 .................................................................................................
env-21 AAAUACAAAAGUACAAUCUCAUAGACUUACCU.................................................................
env-22 CAGUUUAUGAUAAAAGUUACAUCGAAGGUACAAAUGGCUUUUUCUUAGGAGAUGUUUUGGAAACGGGACUACUAGUUUUAACUAUUUUCAAACAGG
env-23 AUAAAUAAAAAUCAAAUAAUUGAAUCUGGAGCUAUUGAUUUAAUAGACGUACUUAAUACAGUUCCUAAUAUAAACAUUACCCAAAGCGGUCCUAGGG
env-24 .................................................................................................
env-25 GAAGGUGUUCUUGGAAAAACAGUUAUUACAAAAAAACAAAUUAUCGAGUCUGGCUAUGAAGACGUGAGAAGUGUUUUAGAAUAUAUAGCUGGCGUAG
env-26 .................................................................................................
env-27 UUAACAAAACUAUAAUUACAAAAAAACAAAUCGAAGAAAGUGGUGCAGUAGAUUUAAUUGACGUCUUAGAAUCUGUGCCCGAUAUUAAUAUCACUCA
env-28 GAUUGGUAUUAAUAAACAAAUUAUUACAAAACAAGAUAUAAUUGAUUCAGGUGCUAUAGACUCAAAUGAUAUUCUAAACUUAGUAUCUGGACUUGAU
env-29 .................................................................................................
env-30 .................................................................................................
env-31 AUUCAUCAGAAUUGUCUAAAAGUGGUAUUUAUAAAACUACGAUUUCAAAAAAAGAAAUUGACGAAAGUGGAGCAAUUGAUUUAAUCGAUAUUUUACA
env-32 CUAUAAGAAAUUCAAAAGAAAUUUUUCUUGGCGCAAUAAUUGAUCAAGAGUCUGGAAGCACAAAUACUUUCCAAGAUGGUGGACACGGCACUAACAU
env-33 UAUUCCAAAUCUGGUAUUAACAAAAUCAUAAUAAACAAAAAGCAAAUUGAAGAAAGUGGUGCAGUAGAUCUAAUUGACGUGUUAAAAACUAUUCCAG
env-34 AUUUUUUAGGAGAUAUCCUCUCUAAUAAUUCAACUUCAAUAAAUUUUUUUCAAUCUGGAGGACAUGGUACAGCUUCAGCAAUUCAAUUAAGGGGAUU
env-35 GGGAAAGUUUGAUAAUCAAUUUAAUGAUAGUUUGGAAAUUGCCGGAACGGUCAGAUUACGUGAUACUUAUCUUCAGUAUGAUAAAG...........
env-36 .................................................................................................
env-37 UCAUCAGAUGGGGUUAAUAAAAUAGUUAUAACCAAGCAAGAUAUGAUAAAUGCAGGCGCCUCAGAUAUCAAUGAUGUUCUUAAGCUAAUACCCGGAU
env-38 CAGGAAAGUUUAGGUAAAAUUGUAAUUACCAAACAACAAAUGAUUAAUUCAGGCUAUCAAGAUGUAAGGAGUGUUCUAGAGAAUGUACCUGGUGUAG
env-39 ACGUUGGCUGAUGUUAUUAAUGAUAACUCAACUAGCACAAAUUUUUUUCAAGCUGGAGGUAUUGGGAGUAAUACGGGCAUUCAACUUAGGGGCUUAG
env-40 .................................................................................................
env-41 ACCACUCUAAAUAAUAAAGAAAUUCAGGAGGCUGGGGAAUUUUUUUUAGCAGAUGUUUUAAAUGAUAGUUUACCAGGAAUGAAUUAUUUUAGAUCGG
env-42 UUGAUAAAAAAAUUUUAGAAAAUUCAAACCAAUUCUUUAUCGGAGAUAUUUUAGAUAAUAAUUUAAAUGGAAUAAAUUAUUUUCAAUCAGGUGGAUA
env-43 UAAUUAAAACAGUAAUAACCAAGCAAGAUAUAGAAAAUUCUGGAUAUACUAAUUUAACUGAUAUUCUAAAAACAAUUAGUGGGCUCAAUGCUUAUCA
env-44 GUUUCUGAAAAAUCUAUAAAUAAAACAGUAAUAAAUAGAGAAGAUAUUGAAAAGAGUGGAGCAAUAGAUGUCGUAGAUUUAUUAAAAUAUUUAGAUG
2 env-45 UCGGAGGGAAGUCUAGGAAAAACAAUCAUUACCAAAAAACAAUUAAUUGAGUCUGGUUAUGAUGACGUUAGAAGUGUUCUUGAAAAUGAAAUAGGGG
env-46 AAACAAUCAGAAAUUCUAAUGAAAAUUUUCUUGGUGCAAUCAUCGACCAAAAAUCAGGAAGCACUAAUACUUUUCAGGAUGGAGGCCAUGGUACGAA
env-47 UGAAGGUUCACUUAACAAAGUGAUUAUUACAAAAAAACAGAUGGUGGACUCAGGGUAUGAGGAUGUCAGAGGUGUUUUAGAGUAUGUAGCUGGUUUA
env-48 AAAAGGAAAUUGAAAAAUUCUGGCGAGUUUUUUCUUGGUGAUAUACU..................................................
env-49 AGAAACUAUUUCCAAUUCUAAUAAUUUUUUUUUAGGUGAAACUCUUGAUAAAAAAUUUACCAGGAAUGAAUUAUUUUAGAUCAGGCGGUUACGGAACC
env-50 AAUUUGGAAUUAACAAAGUUAU...........................................................................
env-51 .................................................................................................
env-52 AAUAAACAAAACAGUAAUAACUAAAANNNNNNNNNNNNNNNNNNNNNNNNNNNNNNNNNNNNNNNNNNNNNNNNNNNAUCAACGAUCAAUCAACACU
env-53 AAAAAGGGGGAUUAAUAAAGUUGUUAUUUCAAAAAAACAAAUUGAUGAAAUGGGAGCUAUUGAUUUAAUUGAUGUACUGGCACAAGUGCCAGACAUU
env-54 UAUAGAUUCUGAAGCUUUAGAAAAAUCAGAACAUAAUUUUUUAGGGGAUAUACUUGAUAACGCUAUGCCUGGUGCGAAUUAUUUUCAAAGUGGGGGG
env-55 AUAAAUAAAAUAGUUAUAAAUAAAGAGGAUAUUAACAAUUCUGGCGCAUUAGAUGCAAAUGAUGUGCUUAAAUAUAUUUCAGGUUUAGAUGUUUUUC
env-56 AUAAACAAAAUAGUUAUAAAUAAAGAAGAUAUUAACAAUUCUGGCGCAUUAGAUACAAAUGAUGUACUUAAGUAUAUUUCGGUUUGGAUGUCUUUC

       .................................................................................................
       .................................................................................................
       .................................................................................................
       .................................................................................................
       .................................................................................................
       .................................................................................................
       ∘∘∘∘∘∘∘∘∘∘∘∘∘∘∘∘∘∘∘∘∘∘∘∘∘∘∘∘∘∘∘∘∘∘∘∘∘∘∘∘∘∘∘∘∘∘∘∘∘∘∘∘∘∘∘∘∘∘∘∘∘∘∘∘∘∘∘∘∘∘∘∘∘∘∘∘∘∘∘∘∘∘∘∘∘∘∘∘∘∘∘∘∘∘∘∘∘
```

```
env-57   UUGUUGAUAGAAAUACUAUUUCAAAUUCAAGUGAGCUUUUCAUUGGUGAUAUCUAGACAGUA......................................
env-58   ACCUCUUCUUAUAAUGCCCAGAAUGUAGCUAAAAAAGUUUUUACCAAGCAACAAAUUAUUCAGUCGGGGGCUGUAAGUACUUUGGAUUUAUUAAAAA
env-59   GUUGUAAACAAAAUAAUUAUAACCAAAGAAGUGAUAGAUAAAAUGAAUGCUAUUGAUGCUAAUGAUGUUUUAAGAAAGAUUCCAGGGCUGGAUGUUU
env-60   AAAAAAGUAAUAACAAAAAAACAAAUAAUUGAAAGUGGUGCUGUUGAUUUAAUAGAUGUAUUAAAAUCAUUACCUGAUAUCAAUAUCACUCAAUCAG
env-61   ....................................................................................................
env-62   GUGACAAAGUAGUUAAAAAAGUUUUUACAAGGAAAGAUAUAGAAAUAACAGGUGCCAAAGAUACAUUUGAUCUUCUUAAAUUAAUUCCUGG......
env-63   ACAAUGUAAUUAAAAAAGUUUUUACCAGGAAAGAUAUAGAAUUAAUUGGUGCUAAAGAUACUUUUGAUCUUCUUAAAUUAAUUCCUGGACUUGACUA
env-64   AAAUUGGUGUUAAUAAAAUAAUCAUUAAUAAGCAAGAGAUUGUCAAAUCUGGAGCAUUAGAUGUUAAUGACAUAUUAAAAUUGGUUUCAGGUCUUGA
env-65   AACAAUUGAAAAUUCAAAUAACUUUUUUUUAGGUGAAACUCUUAAUAAUAAUUUACCAGGGAUGAAUUAUUUUAGAUCAGGAGGUUAUGGAACUAUU
env-66   UCUAUAAAUUCUCAAGGCAUAACUAAGAAGGUUUUUACUAGACAACAGAUAGAAGAUUCUGGUGCAACAACUGCCUUAGAAUUGAUAAAUAAAAUUU
env-67   CUGUUACAUCUUUGAAUAAUAAAGAGAUACAAGGAAAUGGAAAAUUUUUUUUGGGUGAAGUUUUAAAUGAUAAUUUACCCGGCAUGAAUUAUUUUAG
env-68   AAUUUUAAUCAGCAAGGCGUCAGAAAAAUUAUAAUCAAUAAAGAAGAUAUGAUUGCUUAUGGUGCACAAGAUGUAAAUGAUGCGCUUAAAAUGAUUU
env-69   CAGAGGGCACACUUGGUAAAACUGUUAUCACAAAACAACAAAUAAUAGAAUCCGGAUAUGAUGACGUAAGAAGUCUAUUAGAGCAAGUUCCCGGGAU
env-70   AAAGUAUUCAUCUGGUGGAAUUAAUAAAGUUAUAAUAACCAAAAAAGAGAUUGAUGAUAUGGGGGCUAUGGAUUUAAUAGAUGUCCUUAAAAGAAUA
env-71   ....................................................................................................
env-72   ACUAACGAUUAUUUUCUAGGAGAUGUGAUUUCUAAUAAUUCAACUUCAAUAAACUUUUUCCAAUCUGGUGGACACGGAACUGCUUCAGCUAUUCAAU
env-73   UUUUAAAAAUACAAAAGAAUUUUUUCUAGGUGAUGUUUUAGCAGGAAGCUCAACUAGUUCAAACUUUUUCCAAAGUGGGGGGUAUGGCGCAACGUCA
env-74   ....................................................................................................
env-75   UUAAACAGCGAGUCUAUCAAUAAAAAAAAUUUAUACAAAAAAAAAUGAUUUUGGAUUCUGGAGCAAAAUCAACAAUAGAUUUAAUUAAAUAUGUAUCUG
env-76   UUUUUCUGAAGGAUCAGUAAGUAAAAUUAUCAUUAAUCGNNNNNNNNNNNNNNNNNNNNNNNNNNNNNNNNNNNNNNNNNNNNNNNNNNNNNAGACUGA
env-77   GAGAAAACUUUAGGCAAAACAGUUAUCACAAAAAAACAAAUGGUAGAGUCUGGUUACAAUGAUGUAAGGGGGAUUCUAGAACACAUAGAUGGUAUUG
env-78   GCUCAAUGAAAAAGAUCUUGAGAAUUCUAAUAGAUCUUUUUUAGGGGAAUCUUUGAACAAGUCAGUUACGGGUUUAAAUUUGUUUCAAACAGGUGGA
env-79   UUUAAUCAAGGGAGUGUAAAUAAAAUAAUUAUAACCAAACAAGAUAUUGUUAAUUCUGGAGCGGUAGAUACAAACGAUAUAUUUAAACUUAUUCCUG
env-80   CAGAAUUUAGUAAAAAAAGUUUAAAUAAAACAACUAUAAAUAGACAAGAAAUUGAGGAAAGUGGCUCAGUUGAUUUGAUCGAUGUAUUAAACAAACA
env-81   ....................................................................................................
env-82   GAUGAAAAAUUUUUUAAUAAUUCUACUGAAUAUUUUUUAGGAGACGCAUUAAGCACGUCCACAACAGGUGCUAAUUUCUUUCAAAGUGGUGGACACG
env-83   UGAUGGAAAUACUAUUUCAAAUUCUAAUGAACUUUUUAUUGGUGAUAUUUUGGAUGGUAAUCUUAAUGGAAUGAAUUAUUUUCAAUCAGGUGGUCAC
env-84   UAGAUAGUAAAAGAAUUGAGAAAUCUAAUGAAUUUUUUAUUAGUGAUAUUCUUGAUAAUAAUUUGCCCGGUAUGAAUAAUUUUAGAUCAGGAGGUUA
env-85   UUUUUUAGGAAAUGUUCUUGAUAAUAAUUUGCCAGGUAUGAACUAUUUUCGUCAAGGAGGUCACGGAGCUGUAAUGGGCAUCCAAUUAAGGGGACUU
env-86   ....................................................................................................
env-87   UUCUAAUGGAAUAAAUAAGACUAUUUUUACCAAGCAACNNNNNNNNNNNNNNNNNNNNNNNNNNNNNNNNNNNNNNNNNNNNNNNNNNAUAGUUGA
env-88   UUGAUGAGGAAUUUUUAAAGAAUUCUAAUGAGUAUUUUUUAGGAGAUAUUUUAUCUGGAGGCACUACUAGCGCAAAUUUUUUUCAAAAUGGAGGACA
env-89   UAAUAAUAAUAAUGUUAUUAAAAAAGUUUUUAACAAACAAGAAAUUGAGGCUGCUGGAGCAAAAGAUACUUUAGAUCUUCUAAAAUUAAUUCCAGGA
env-90   ....................................................................................................
env-91   GUUCUGGAUGAAAAGUUUUUAGAAAAUACUAAUGAAUUUUUUUUAGGAGAUGUUUUAGCAGGAAAUUCGACUAGUAUAAAUUUUUUUCAAAACGGAG
env-92   GAGCCGGUAUUAAUAAAACAAUAAUUACUAAAGAAGAAAUAAUAAGAUCGGGCGCUGUAGACACAAACGAUAUAUUAAAAUCAAUUCAAGGUUUAGA
env-93   UUGGGUCUAAAGAUUUUUUUUUAGGUGAUUUGUUGAAUAAUU..........................................................
env-94   UAUUGAUGUCAAGACUAUUAAACAAUCAAAUGAGCAAUUUAUUGGAGAUAUAUUGGAUAAUAAUUUAAAUGGUAUAAAUUAUUUUCAAUCAGGUGGU
env-95   ....................................................................................................
env-96   AACUAUAGAAAGUUCAAAUAAUUAUUUCUUAGGAGAAGUUUUAAAUAAAAAUUUACCAGGCAUGAACUAUUUUAGAUCAGGAGGUCAUGGCACAGUU
env-97   AAAACCAACAAUUCUUCAAAAAUUUCUGAAACUGGAAUUAAUAAAAAAAAUAAUAACUAAAGAAGAUAUAGAAAACUCUGGAGCAACUAGUGUAAGUG
env-98   ACAGUUUUGGACUCAAAAACUAUUUAUGAAUCAGAACAUAAUUUUUUAGGAGAUAUUAUUAAUGAAAAAAUACCUGGUAUGAAUUUUUUUCAGAGUG
env-99   UUGUUGAUAGAAAUACUAUUUCAAAUUCAAGUGAGCUUUUCAUUGGUGAUAUCUUAGACAGUAAUCUUAAUGGUCUAAAUUAUUUUCAACAAGGCGG
env-100  UUGUUGAUAGAAAUACUAUUUCAAAUUCAAGUGAACUUUUCAUUGGUGAUAUCUUAGACAGUAAUCUUAAUGGUCUAAAUUAUUUUCAACAAGGCGG
env-101  UUGUUGAUAGAAAUACUAUUUCAAAUUCAAGUGAACUUUUCAUUGGUGAUAUCUUAGACAGUAAUCUUAAUGGUCUAAAUUAU..............
env-102  GGAAUAAAUAAAACUGUCAUUUCUAAGCAAGAUAUAAUUGAUUCAGGUGCUGUAGAUACUAACGACGUACUCAAAUUAAUUUCUGGUCCUGACGUUU
env-103  GUCAUUGACUCAGAAACAAUUAAAAACUCUUCAGACUCUUUUUUAACUGAUCUUCUAAAUAAUGAAGUUCAAGGAAUGAAUAUUUUUUCAUUAGGUG
env-104  UUCUCUUGGGGUAAAUAAACAAAUUUUUACUAAAGAACAAAUUACUGAGUCAGGCGCAACUACUGCAUUAGAUUUAUUAAAGAAAGUCUCAGGAUUA
env-105  ACAAAAUGUACAUAUUUCAAACUAGUCUUAGUAAUCUAACUAAAAAUUCUGAAAAUUUAUUGACUUUUCAUUUGCAUCAUUAUGACAGAGAAUAUGA
env-106  GAUAGUGAAACUAUCUCAUCUUCUAAUCAACACUUUAUCGGAGAUGUUUUGGAUAAUAAUUUAAAUGGAAUUAAUUAUUUUCAACAAGGAGGCAGUG
env-107  ....................................................................................................
env-108  ....................................................................................................
env-109  UGAAAAUUUUUUUAAUGGCACAAGUGAGUACUUUUUAG..............................................................
env-110  AAUUUAAUCAAGGGAGUGUAAAUAAAAUAAUUAUAACUAAACAAGAUAUUAUUAAUUCUGGAGCGGUAGAUACAAACGAUGUAUUAAAACUUAUUCC
env-111  CAAAAUUAUAAUAAACAAAAACCAAAUAGAACAAAGUGGAGCAGUAGAUGUAAUAGAUGUUCUUAAGUAUGUCGAUGGAUUAGAAAUUAAACAGAAU
1 env-112  UUAAUAAAAUAAUUAUAACAAAAAAGGAUAUAUUAAAUUCAGGAGCAAUUGACACCAUUGAUGUUUUAAAACUUGUA....................
env-113  ....................................................................................................
env-114  UCUCAAGGAAUAAAUAAAAAAAUUUUCACUAGACAACAAAUUAUUGAAUCCGGAGCAACUACAGCAGUAGAUGUUCUUAAAAAAGUAAAUGGUUUGG

         ....................................................................................................
         ....................................................................................................
         ....................................................................................................
         ....................................................................................................
         ....................................................................................................
         ....................................................................................................
         °°°°°°°°°°°°°°°°°°°°°°°°°°°°°°°°°°°°°°°°°°°°°°°°°°°°°°°°°°°°°°°°°°°°°°°°°°°°°°°°°°°°°°°°°°°°°°°°
```

```
env-115 AAAAUAAUAUUAAUAAAAUAAGUAUUAAUAAACAAGAUAUAAUUAAUUCAGGUGCAUUGGAUGUAAAUGAUGUUUUAAAAAUUGUAAACGGUCUUGA
env-116 AUUUUGUUAGCAAAACAAUAAUUUCAAAAGAAAAAAUUGAUAUUAUAAAUGCGACUGACGUAAACGAUUUACUUAAACAAAUUCCUGGAUUAGAUAU
env-117 AAACUUUACAAGACUCAAAUGAGUAUUUCAUAGGUGAUAUUUUGGAUAAUAAUCUAAAUGGAAUGAAUUAUUUUCAACAAGGUGGUAUAGGAACAGU
env-118 UAAAACCUCAAAUACCUCAACUUACUCAGAAAAAGGUAUUAGUAAAAUUGUAAUAACAAAAGAAGAAAUUGUUAAAAGUGGAGCAAUAGAUGUCAUU
env-119 CUCGAUUCUAGAUGAAAAUUUUUUUAAAAACUCAAAAGAAAUAUUUUUAGGGGACGUUUUAUCAAAUAACAGUAAUAGUAUUAAUUUUUUUCAAAGU
env-120 GGUUGUUGAUAAAAAUACUAUCUCAAAUUCAAACGAACUUUUCAUUGGCGAUAUUUUAGACAAUAAUCUAAAUGGUCUAAAUUAUUUUCAGCAGGGC
env-121 UUUUUUAGGAGAUGCUUUAGCAACUAACUCAACCAGCAUUAAUUUCUUUCAAAAUGGUGGUCAUGGACAAACUUCGGCUAUUCAAUUAAGAGGUAUG
env-122 UUUUUUAGGAGAUGCUAUAGGAACGAACUCAACUAGUAUAAAUUUUUUUCAAAGUGGUGGUCACGGACAAACUUCAGCUAUUCAAUUGAGAGGUUUG
env-123 UUUUUUAGGAGAUGUUUUAGCAAAUACCUCAACUUCUGUUAAUUUUUUUCAAANNNNNNNNNNNNNNNNNNNNNNNNNNNNNNNNNNNNNNNNNNNN
env-124 AAAAAAUAUAAAUAAGAUAACUAUAACAAAACAAGAUAUAAUUAACUCAGGUGCAUUGGAUGUGAACGAUGUACUAAAAAUGGUUAAUGGGCUUGAC
env-125 GAAUAAAUAAAAAAGUUUUUACAAAAAAACAGAUUGCUGAGUCUGGUGCAACCUCAGCCUUAGAUUUGUUAAAAAAGGUUUCAGGCCUAGACUAUUA
env-126 .................................................................................................
env-127 .................................................................................................
env-128 UCAGAGGAAUCACUUAACAAAAUUGUA......................................................................
env-129 .................................................................................................
env-130 GCUAUACAACUUAGAGGUCUACCAAAAAGAUAUUCUACAGUUUAUAUUGAUGGUGUUAAAAUGUCUGAUCCAUCAAAUGUUUCCAAUGAUUUUGAUU
env-131 AAAAUUUAGGUAAAACAGUAAUUACAAAAAAACAAAUGAUAAAUUUUGGUUAUGAAGACGUAAGAAGUUUACUUGAAAAUGUUGUUGGCUUGGAUAU
env-132 UGAGAAAACUUUGGGAAAAACAGUUAUUACAAAACAACAAAUGAUAGACUCUGGUUAUGAAGAUGUAAGAGGAAUUCUAGAGUAUGUAGAUGGUAUC
env-133 NNNNNNNGGGCAAAAGUAGAUAGCUCAGAUAUAAUGACCCUGGUGCUAUCAAAGGAAAUAUUAGCUAAUACAUGGUCAAUAAAUAUACUGAAUCUGA
env-134 AGGAACACUUGGCAAAACAGUGAUAACAAAAAAACAAAUGAUCGAGUCCGGAUAUGAUGAUCUUAGGAGUCUAUUAGAACAAGUUGCUGGAAUUGAU
env-135 GUUCUAGAUGAAGAAUUUUUAAAAAAGUCUAAUGAAUUUUUUUUUAGGAGAUGUUUUGUCAAGCGGUACAACUAGUGCAAACUUUUUU.........
env-136 NNNNNNNNNNNNNNNNNNUUAUUUAGAUUAUCAAAAGCGUUUCACAGAAAAUAUUUUUGCUACAUUUGGCUCUAGGUUUGAUGACAAUUCAAUUGCA
env-137 UGGCCAGACAAUAAAUAAAGUAGUUUUUACCAAACAGCAAAUUAAUGAGUCAGGUUCCACUACUGCUUUAGAUUUGAUAAAAAAAGUUUCGGGUUUG
env-138 AAAUUCAUCUGAAUUUACUAAGAAUGGCAUUAAAAAAAUAAUUAUAUCAAAAAAACAAAUUGAUGAAGUUGGUGCAGUUGAUUUAAUAGAUGUUUUG
env-139 GAACUUGGGGUUAAUAAAAUAGUUAUCACCAAAGAAGAGAUUAAUAAUUCAGGAGCUGUUGAUGUUAAUGACGUGCUUAAACUUGUUUCUGGACUUG
env-140 .................................................................................................
env-141 GAAUAUUAUGAACUUUAAAAAAAUUUUAUUACUAAAUAUUUUACUUAUAUCUUUUACUUUUUCGCACGCAAGUGAAACUUUAAAUCCAAUUGUAGUA
env-142 CUAUAAGUAUUGGAAGUAAUGGUGGAUUUGGUCAAACAAAAUCAAUAUUUAUGAGAGGUACGGAGAGUAAUCACACAAAAGUACUUGUUAAUGGUGU
env-143 CUAUAAGUAUUGGAAGUAAUGGUGGAUUUGGUCAAACAAAAUCAAUAUUUAUGAGAGGCACAGAGAGUAAUCAUACAAAAGUUCUUGUCAAUGGCGU
env-144 UAAGUAUUGGAAGUAAUGGUGGAUUUGGUCAAACAAAAUCAAUAUUUAUGAGAGGUACAGAGAGUAAUCACACAAAAAUUCUUGUUAAUGGUGUGGA
        .................................................................................................
        .................................................................................................
        .................................................................................................
        .................................................................................................
        .................................................................................................
        .................................................................................................
        °°°°°°°°°°°°°°°°°°°°°°°°°°°°°°°°°°°°°°°°°°°°°°°°°°°°°°°°°°°°°°°°°°°°°°°°°°°°°°°°°°°°°°°°°°°°°°°°°
```

7 *asd*

7.1 Taxa

The taxonomy of each organism containing a putative *asd* RNA is listed, with abbreviations identifying each hit (e.g., "Eco-1-1" and "Eco-1-2" might hypothetically represent two distinct RNAs in *E. coli*). The abbreviations will be used to identify each individual *asd* RNA in Sections 7.2 and 7.4.

**abbrev. of hits**	**taxonomy of species**
Lla-1-1	Bacteria Firmicutes Lactobacillales Streptococcaceae *Lactococcus lactis subsp. cremoris* MG1363
Lla-2-1	Bacteria Firmicutes Lactobacillales Streptococcaceae *Lactococcus lactis subsp. cremoris* SK11
Lla-3-1	Bacteria Firmicutes Lactobacillales Streptococcaceae *Lactococcus lactis subsp. lactis* Il1403
Sag-1-1	Bacteria Firmicutes Lactobacillales Streptococcaceae *Streptococcus agalactiae* 18RS21
Sag-2-1	Bacteria Firmicutes Lactobacillales Streptococcaceae *Streptococcus agalactiae* 2603V/R
Sag-3-1	Bacteria Firmicutes Lactobacillales Streptococcaceae *Streptococcus agalactiae* 515
Sag-4-1	Bacteria Firmicutes Lactobacillales Streptococcaceae *Streptococcus agalactiae* A909
Sag-5-1	Bacteria Firmicutes Lactobacillales Streptococcaceae *Streptococcus agalactiae* CJB111
Sag-6-1	Bacteria Firmicutes Lactobacillales Streptococcaceae *Streptococcus agalactiae* COH1
Sag-7-1	Bacteria Firmicutes Lactobacillales Streptococcaceae *Streptococcus agalactiae* H36B
Sag-8-1	Bacteria Firmicutes Lactobacillales Streptococcaceae *Streptococcus agalactiae* NEM316
Smu-1-1	Bacteria Firmicutes Lactobacillales Streptococcaceae *Streptococcus mutans* UA159
Spn-1-1	Bacteria Firmicutes Lactobacillales Streptococcaceae *Streptococcus pneumoniae* D39
Spn-2-1	Bacteria Firmicutes Lactobacillales Streptococcaceae *Streptococcus pneumoniae* R6
Spn-3-1	Bacteria Firmicutes Lactobacillales Streptococcaceae *Streptococcus pneumoniae* SP11-BS70
Spn-4-1	Bacteria Firmicutes Lactobacillales Streptococcaceae *Streptococcus pneumoniae* SP14-BS69
Spn-5-1	Bacteria Firmicutes Lactobacillales Streptococcaceae *Streptococcus pneumoniae* SP18-BS74
Spn-6-1	Bacteria Firmicutes Lactobacillales Streptococcaceae *Streptococcus pneumoniae* SP19-BS75
Spn-7-1	Bacteria Firmicutes Lactobacillales Streptococcaceae *Streptococcus pneumoniae* SP23-BS72
Spn-8-1	Bacteria Firmicutes Lactobacillales Streptococcaceae *Streptococcus pneumoniae* SP3-BS71
Spn-9-1	Bacteria Firmicutes Lactobacillales Streptococcaceae *Streptococcus pneumoniae* SP6-BS73
Spn-10-1	Bacteria Firmicutes Lactobacillales Streptococcaceae *Streptococcus pneumoniae* SP9-BS68
Spn-11-1 to Spn-11-2	Bacteria Firmicutes Lactobacillales Streptococcaceae *Streptococcus pneumoniae* TIGR4
Spy-1-1	Bacteria Firmicutes Lactobacillales Streptococcaceae *Streptococcus pyogenes* M1 GAS
Spy-2-1	Bacteria Firmicutes Lactobacillales Streptococcaceae *Streptococcus pyogenes* M49 591
Spy-3-1	Bacteria Firmicutes Lactobacillales Streptococcaceae *Streptococcus pyogenes* MGAS10270
Spy-4-1	Bacteria Firmicutes Lactobacillales Streptococcaceae *Streptococcus pyogenes* MGAS10394
Spy-5-1	Bacteria Firmicutes Lactobacillales Streptococcaceae *Streptococcus pyogenes* MGAS10750
Spy-6-1	Bacteria Firmicutes Lactobacillales Streptococcaceae *Streptococcus pyogenes* MGAS2096
Spy-7-1	Bacteria Firmicutes Lactobacillales Streptococcaceae *Streptococcus pyogenes* MGAS315
Spy-8-1	Bacteria Firmicutes Lactobacillales Streptococcaceae *Streptococcus pyogenes* MGAS5005
Spy-9-1	Bacteria Firmicutes Lactobacillales Streptococcaceae *Streptococcus pyogenes* MGAS6180
Spy-10-1	Bacteria Firmicutes Lactobacillales Streptococcaceae *Streptococcus pyogenes* MGAS8232
Spy-11-1	Bacteria Firmicutes Lactobacillales Streptococcaceae *Streptococcus pyogenes* MGAS9429
Spy-12-1	Bacteria Firmicutes Lactobacillales Streptococcaceae *Streptococcus pyogenes* SSI-1
Spy-13-1	Bacteria Firmicutes Lactobacillales Streptococcaceae *Streptococcus pyogenes str.* Manfredo
Ssa-1-1	Bacteria Firmicutes Lactobacillales Streptococcaceae *Streptococcus sanguinis* SK36
Ssu-1-1	Bacteria Firmicutes Lactobacillales Streptococcaceae *Streptococcus suis* 05ZYH33
Ssu-2-1	Bacteria Firmicutes Lactobacillales Streptococcaceae *Streptococcus suis* 89/1591
Ssu-3-1	Bacteria Firmicutes Lactobacillales Streptococcaceae *Streptococcus suis* 98HAH33
Sth-1-1	Bacteria Firmicutes Lactobacillales Streptococcaceae *Streptococcus thermophilus* CNRZ1066
Sth-2-1	Bacteria Firmicutes Lactobacillales Streptococcaceae *Streptococcus thermophilus* LMD-9
Sth-3-1	Bacteria Firmicutes Lactobacillales Streptococcaceae *Streptococcus thermophilus* LMG 18311

7.2 Gene contexts

Each *asd* RNA (indicated by "RNA→") is listed with its surrounding genes. Environmental sequences and some RefSeq entries lack gene annotations, and no genes are listed for such sequences. The direction of each gene is indicated with an arrow (→), and each conserved domain in the gene is colored. Conserved domains associated with more than one *asd* RNA are assigned a color; other domains are gray. Information about these conserved domains is given in Section 7.3. The accession number of the sequence containing each *asd* RNA is given. Accessions beginning with "NC_" or "NZ_" are RefSeq, while other accession refer to environmental samples. Nucleotide coordinates are given for the 5′ and 3′ boundaries of each. If the 5′ coordinate is greater than the 3′ coordinate, the RNA is present on the reverse-complement strand of the containing genomic DNA sequence. Each hit is denoted by an abbreviation (like "Eco-1-1") that refers to a taxonomy given in Section 7.1.

abbrev.	Seq. accession		5′ at	3′ at	genes
Lla-3-1	NC_002662.1	-	1668839	1668682	hypo→ **RNA**→ MIP (cd00333)→ Asd (COG0136)→ COG1051 (COG1051)→
Lla-1-1	NC_009004.1	+	839782	839941	hypo→ **RNA**→ MIP (cd00333)→ Asd (COG0136)→ COG1051 (COG1051)→ COG1051 (COG1051)→
Lla-2-1	NC_008527.1	-	1641549	1641391	hypo→ **RNA**→ MIP (cd00333)→ Asd (COG0136)→ COG1051 (COG1051)→
Spy-10-1	NC_003485.1	-	972072	971922	Cls (COG1502)→ **RNA**→ ATP-cone (pfam03477)→
Spy-1-1	NC_002737.1	-	996147	995997	Cls (COG1502)→ **RNA**→ ATP-cone (pfam03477)→
Spy-4-1	NC_006086.1	-	919140	918990	Cls (COG1502)→ **RNA**→ ATP-cone (pfam03477)→
Spy-9-1	NC_007296.1	-	916998	916848	Cls (COG1502)→ **RNA**→ ATP-cone (pfam03477)→
Spy-5-1	NC_008024.1	-	1020618	1020468	Cls (COG1502)→ **RNA**→ ATP-cone (pfam03477)→
Spy-2-1	NZ_AAFV01000017.1	-	782	632	Cls (COG1502)→ **RNA**→ ATP-cone (pfam03477)→
Spy-8-1	NC_007297.1	-	914543	914393	Cls (COG1502)→ **RNA**→ ATP-cone (pfam03477)→
Spy-11-1	NC_008021.1	-	986220	986070	Cls (COG1502)→ **RNA**→ ATP-cone (pfam03477)→
Spy-6-1	NC_008023.1	-	946984	946834	Cls (COG1502)→ **RNA**→ ATP-cone (pfam03477)→
Spy-13-1	NC_009332.1	+	878900	879050	Cls (COG1502)→ **RNA**→ ATP-cone (pfam03477)→
Spy-12-1	NC_004606.1	-	1038560	1038410	Cls (COG1502)→ **RNA**→ ATP-cone (pfam03477)→
Spy-7-1	NC_004070.1	-	908040	907890	Cls (COG1502)→ **RNA**→ ATP-cone (pfam03477)→
Spy-3-1	NC_008022.1	-	989456	989306	Cls (COG1502)→ **RNA**→ ATP-cone (pfam03477)→
Smu-1-1	NC_004350.1	+	935481	935617	Cls (COG1502)→ **RNA**→ Asd (COG0136)→ DHDPS (cd00950)→
Ssu-2-1	NZ_AAFA02000005.1	+	26347	26496	hypo (cons)→ **RNA**→ ←PflA (COG1180)
Ssu-1-1	NC_009442.1	+	684117	684266	hypo→ **RNA**→ ←PflA (COG1180)
Ssu-3-1	NC_009443.1	+	683851	684000	hypo→ **RNA**→ ←PflA (COG1180)
Sth-3-1	NC_006448.1	-	1153119	1152969	COG4912 (COG4912)→ **RNA**→ Asd (COG0136)→
Sth-2-1	NC_008532.1	-	1185932	1185782	COG4912 (COG4912)→ **RNA**→ Asd (COG0136)→
Sth-1-1	NC_006449.1	-	1150990	1150840	COG4912 (COG4912)→ **RNA**→ Asd (COG0136)→
Ssa-1-1	NC_009009.1	-	1221575	1221437	RimL (COG1670)→ **RNA**→ Asd (COG0136)→ DHDPS (cd00950)→ hypo→
Sag-4-1	NC_007432.1	-	1119613	1119477	Cls (COG1502)→ **RNA**→ fibrinogen-binding protein, putative→
Sag-7-1	NZ_AAJS01000010.1	+	44065	44201	Cls (COG1502)→ **RNA**→
Sag-6-1	NZ_AAJR01000023.1	+	31271	31407	Cls (COG1502)→ **RNA**→ fibrinogen-binding protein→
Sag-5-1	NZ_AAJQ01000001.1	+	73469	73605	Cls (COG1502)→ **RNA**→ Unknown→
Sag-2-1	NC_004116.1	-	1063292	1063156	Cls (COG1502)→ **RNA**→ ←hypo
Sag-8-1	NC_004368.1	-	1133913	1133777	Cls (COG1502)→ **RNA**→ fbsA→
Sag-1-1	NZ_AAJO01000112.1	+	4936	5072	Cls (COG1502)→ **RNA**→ fibrinogen receptor FbsA→
Sag-3-1	NZ_AAJP01000010.1	+	35943	36079	Cls (COG1502)→ **RNA**→ fibrinogen-binding protein→
Spn-9-1	NZ_ABAA01000002.1	+	167881	168023	tRNA-Thr→ **RNA**→ Asd (COG0136)→ DHDPS (cd00950)→
Spn-5-1	NZ_ABAE01000003.1	-	127675	127533	tRNA-Thr→ **RNA**→ Asd (COG0136)→ DHDPS (cd00950)→
Spn-11-1	NC_003028.2	+	956780	956922	tRNA-Thr→ **RNA**→ Asd (COG0136)→ DHDPS (cd00950)→
Spn-11-2	NZ_AAGY02000049.1	+	10416	10558	COG4708 (COG4708)→ **RNA**→ Asd (COG0136)→ DHDPS (cd00950)→
Spn-1-1	NC_008533.1	+	912569	912711	tRNA-Thr→ **RNA**→ Asd (COG0136)→ DHDPS (cd00950)→
Spn-2-1	NC_003098.1	+	905038	905180	tRNA-Thr→ **RNA**→ Asd (COG0136)→ DHDPS (cd00950)→
Spn-8-1	NZ_AAZZ01000006.1	+	11563	11705	tRNA-Thr→ **RNA**→ Asd (COG0136)→ DHDPS (cd00950)→
Spn-7-1	NZ_ABAG01000003.1	+	110887	111029	tRNA-Thr→ **RNA**→ Asd (COG0136)→ DHDPS (cd00950)→
Spn-6-1	NZ_ABAF01000013.1	+	125	268	hypo→ **RNA**→ Asd (COG0136)→ DHDPS (cd00950)→
Spn-10-1	NZ_ABAB01000021.1	-	32130	31987	tRNA-Thr→ **RNA**→ Asd (COG0136)→ DHDPS (cd00950)→
Spn-3-1	NZ_ABAC01000013.1	+	112	255	hypo→ **RNA**→ Asd (COG0136)→ DHDPS (cd00950)→
Spn-4-1	NZ_ABAD01000016.1	+	11693	11836	tRNA-Thr→ **RNA**→ Asd (COG0136)→ DHDPS (cd00950)→

7.3 Conserved domains

Conserved domains found in protein-coding genes listed in Section 7.2 are listed, with the first sentence in their description from the Conserved Domain Database. Conserved domains associated with more than one *asd* RNA are assigned a color, while others are shown in gray. The number in parentheses is the number of occurrences of the given gene.

cd00333 (3) Major intrinsic protein (MIP) superfamily.
cd00950 (14) Dihydrodipicolinate synthase (DHDPS) is a key enzyme in lysine biosynthesis.
COG0136 (20) Aspartate-semialdehyde dehydrogenase [Amino acid transport and metabolism]
COG1051 (4) ADP-ribose pyrophosphatase [Nucleotide transport and metabolism]
COG1180 (3) Pyruvate-formate lyase-activating enzyme [Posttranslational modification, protein turnover, chaperones]
COG1502 (22) Phosphatidylserine/phosphatidylglycerophosphate/cardiolipin synthases and related enzymes [Lipid metabolism]
COG1670 (1) Acetyltransferases, including N-acetylases of ribosomal proteins [Translation, ribosomal structure and biogenesis]
COG4708 (1) Predicted membrane protein [Function unknown]
COG4912 (3) Predicted DNA alkylation repair enzyme [DNA replication, recombination, and repair]
pfam03477 (13) ATP cone domain.

Duplicate sequences: the following putative homologs are not shown in the alignment because their sequences are identical to a homolog already shown: Sag-1-1, Sag-7-1, Sag-8-1, Spn-11-2, Spn-2-1, Spn-4-1, Spy-13-1, Spy-2-1, Spy-5-1, Spy-6-1, Spy-7-1, Spy-9-1, Ssu-3-1

7.4 Multiple-sequence alignment

Each *asd* RNA is denoted by an abbreviation (like "Eco-1-1") that refers to a taxonomy given in Section 7.1. The alignment includes sequences containing the RNA motif, as well as flanking sequence. The *asd* RNA itself is denoted by the line underneath marked 5′ and 3′ on either end. Nucleotides in the flanking sequences (i.e., not part of the motif) are written in gray letters. Stems of predicted rho-independent transcription terminators are shaded yellow. (Note: terminator predictions have not been analyzed manually, and many are likely to be false positives.) Start codons are shaded green. (Note: start codons are frequently misannotated, especially in environmental samples.) Nucleotides proposed to basepair as part of the consensus structure are shaded in color when they comprise Watson-Crick or G-U pairs. Otherwise they are shaded gray. Conserved stems are also indicated at the bottom of the alignment by angle brackets, where matching < and > denote base-paired columns. Below these angle brackets, the symbol "2" denotes base pairs exhibiting covariation, "1" denotes base pairs exhibiting compatible mutations, "0" denotes base pairs that are not observed to mutate and "?" denotes base pairs that have a significant frequency of non-canonical nucleotides for Watson-Crick or G-U pairs (> 5%). Below these base pair annotation is the consensus sequence: "R" = "A" or "G", "Y" = "C" or "U", red nucleotides: nucleotide identity conserved more than 97% of the time, black nucleotides: 90%, gray nucleotides: 75%, red circle (•): nucleotide is present 97% of the time, black circle (•): 90%, gray circle (•): 75%, white circle (◦): 50%. All percentages of sequences just described (e.g. 97% conserved) assume that sequences have been weighted by the GSC algorithm implemented by the Infernal software.

alignment positions 1 · · · 157

```
Lla-3-1    UACGAUAGGAUUUACUAUUUGUAUCCUUAGUUUUGUGGGAACGGUAGGAAUUUUGAUUGGCUUCUUGUUUCAUUUAGCAUAAUAUGUUAAAAUUUAACUAUGCGAUGAGUCGAAUAAUUCUACUUU.GUAGAAUUUCGAA.GC.UAAGGAAUAC...
Lla-1-1    UACAAUAAGAUUUGCUAUUUGUGUUAUAAGUUUUGUGGGUUCAGGCUCAAUAUUGCUUGCCUUCUUGUUUCAUUUGAAUUAAUAUGAUAGAAUAGGUCUAUGCGAUGAGUCGAAUAAUUCUACUUUUGUAGAAUUUCGAA.GC.UAAGGAGUGC...
Lla-2-1    UACAAUAAGAUUUGCUAUUUGUGUUAUAAGUUUUGUGGGUUCGGGCUCAAUAUUGCUUGCCUUCUUGUUUCAUUUGAAUUAAUAUGAUAGAAUAGGUCUAUGCGAUGAGUCGAAUAAUUCUACUUUUGUAGAAUUUCGAA.GC.UAAGGAGUGC...
Spy-10-1   AUUUGACGCAACGUCCUUGGUAUCAAAAAUUGAUAGGAUUAUUAGUACGAACAAUUGCCCCUCUCUUGUAAUUUUCGUAAAAUAUGUUAUAAUAUCUGUAUGCGAUGAGUCGAUU..GUGACUGCAAAGUCACUUUUGC..GCA.GGGGAGGUCAU.
Spy-1-1    AUUUGACGCAACGUCCUUGGUAUCAAAAAUUGAUAGGAUUAUUAGUACGAAUAAUUGCCCCUCUCUUGUAAUUUUCGUAAAAUAUGUUAUAAUAUCUGUAUGCGAUGAGUCGAUU..GUGACUGCAAAGUCACUUUUGC..GCA.GGGGAGGUCAU.
Spy-4-1    AUUUGACGCAACGUCCUUGGUAUCAAAAAUUGAUAGGAUUAUUAGUACGAACAAUUGCCCCUCUCUUGUAAUUUUCGUAAAAUAUGUUAUAAUAUCUGUAUGCGAUGAGUCGAUU..GUGACUGCAAAGUCACUUUUGC..GCA.GGGGAGGUCAU.
Spy-8-1    AUUUGACGCAACGUCCUUGGUAUCAAAAAUUGAUAGGAUUAUUAGUACGAACAAUUGCCCCUCUCUUGUAAUUUUCGUAAAAUAUGUUAUAAUAUCUGUAUGCGAUGAGUCGAUU..GUGACUGCAAAGUCACUUUUGC..GCA.GGGGAGGUCAU.
Spy-11-1   AUUUGACGCAACGUCCUUGGUAUCAAAAAUUGAUAGGAUUAUUAGUACGAACAAUUGCCCCUCUCUUGUAAUUUUCGUAAAAUAUGUUAUAAUAUCUGUAUGCGAUGAGUCGAUU..GUGACUGCAAAGUCACUUUUGC..GCA.GGGGAGGUCAU.
Spy-12-1   AUUUGAAGCAACGUCCUUGGUAUCAAAAAUUGAUAGGAUUAUUAGUACGAACAAUUGCCCCUCUCUUGUAAUUUUCGUAAAAUAUGUUAUAAUAUCUGUAUGCGAUGAGUCGAUU..GUGACUGCAAAGUCACUUUUGC..GCA.GGGGAGGUCAU.
Spy-3-1    AUUUGACGCAACGUCCUUGGUAUCAAAAAUUGAUAGGAUUAUUAGUACGAACAAUUGCCCCUCUCUUGUAAUUUUCGUAAAAUAUGUUAUAAUAUCUGUAUGCGAUGAGUCGAUU..GUGACUGCAAAGUCACUUUUGC..GCA.GGGGAGGUCAU.
Smu-1-1    AAUUAAUUGGUUUGGUUGUUAAGUUGGUAGCCCCCUCUUGUGAUGUUCAAUUUUUUGAUGGCUUGGCAAUUCAGACGAUUUGUGUUAUACUAAAACUAUGCGAUGAGUCGAUU..GUGGU.UUAU.ACCACAUACG....C.UAAGGAGAUCA..
Ssu-2-1    CUAUUGGAAAACAGGCUGGUGAUAGAGUACAAGUAACCAUUCAAGAACGAAUGGAGUAAUCAGGCUUGCAAAUCCUUGAGAUUAUGGUAGAAUAGAUUCAUGCGAUGAGCCGAGUA.GUGAC..UUU.GUCACUUUCGAC.GCU.AGGGAGGUCU..
Ssu-1-1    CGUGUCAACAUCUCAGCGCAGUGGUUGAUUGGCAGAUUUGUUCGUAUUUUAUACUCCAAAUCUGACCUAAUCAACUGUGCGGGGGUGGAAGACGAACUCUUGGUUGAC..AAGUU.GAGUUCUUUCCCACUCCCUUCGACGC.UAGGGAGGUCU..
Sth-3-1    CAUGCUAGUCAAAUGGCAUCUCUUAGUAUUAGGGAAGCAAGAAAGUAUUUGUAGAAUUUAACCGCUUGACUCUGUUACCUAUUAUGUUAGAAUAGGAGUAUGCGAUGAGUCGAUU..GGCUC.UUUA.GAGCCUUUUGC..GC.UGAGGAGGU....
Sth-2-1    CAUGCUAGUCAAAUGACAUCUCUUAGUAUUAGGGAAGCAAGAAAGUAUUUGUAGAACUCAACCGCUUGACUCUGUUACCUAUUAUGUUAGAAUAGGAGUAUGCGAUGAGUCGAUU..GGCUC.UUUA.GAGCCUUUUGC..GC.UGAGGAGGU....
Sth-1-1    CAUGCUAGUCAAAUGGCAUCUCUUAGUAUUAGGGAAGCAAGAAAGUAUUUGUAGAAUUUAACCGCUUGACUCUGUUACCUAUUAUGUUAGAAUAGGAGUAUGCGAUGAGUCGAUU..GGCUC.UUUA.GAGCCUUUUGC..GC.UGAGGAGGU....
Ssa-1-1    CAAAAAGAAGGUCAGGAGUGGAAGGAUAGACAAGUUUUUGUUUAUAUUUUAGAUUAAACACCUUGACAAAACGGAAAAAGAUAAGGUAAACUAUAUUUCGUGCGAUGAGUCGAUA..UGCAGAUUUU.CUGCAAAUUGAA.GCA.UGGGAGGUCA..
Sag-4-1    UAAUUUAUUUUUAAUUUAAUAUUAAAAGUAAACUGAAGAAUCUAGUUAUAUUUAAAAAGUAAAGGUUGCAUUUUAACUAAAUUAUGUUAAACUACUGUUAUGCGAUGAGUUGAUAU.GUGGU..UUU.ACCACUAUUGC..GC..AGGGAGAUUA..
Sag-6-1    UAAUUUAUUUUUAAUUUAAUAUUAAAAGUAAACUGAAGAAUCUAGUUAUAUUUAAAAAGUAAAGGUUGCAUUUUAACUAAAUUAUGUUAAACUACUGUUAUGCGAUGAGUCGAUAU.GUGGU..UUU.ACCACUAUUGC..GC..AGGGAGAUUA..
Sag-5-1    UAAUUUAUUUUUAAUUUAAUAUUAAAAGUAAACUGAAGAAUCUAGUUAUAUUUAAAAAGUAAAGGUUGCAUUUUAACUAAAUUAUGUUAAACUACUGUUAUGCGAUGAGUCGAUAU.GUGGU..UUU.ACCACUAUUGC..GC..AGGGAGAUUA..
Sag-2-1    UAAUUUAUUUUUAAUUUAAUAUUAAAAGUAAACUGAAGAAUCUAGUUAUAUUUAAAAAGUAAAGGUUGCAUUUUAACUAAAUUAUGUUAAACUACUGUUAUGCGAUGAGUCGAUAU.GUGGU..UUU.ACCACUAUUGC..GC..AGGGAGAUUA..
Sag-3-1    UAAUUUAUUUUUAAUUUAAUAUUAAAAGUAAACUGAAGAAUCUAGUUAUAUUUAAAAAGUAAAGGUUGCAUUUUAACUAAAUUAUGUUAAACUACUGUUAUGCGAUGAGUCGAUAU.GUGGU..UUU.ACCACUAUUGC..GC..AGGGAGAUUA..
Spn-9-1    GAUAUGUAUAGGGAAACUCUUGAAUACGAGAGUUUUUUGUUAUACUCGAUCGAAAAAAUUUCUUAUUGACAAAAUCAAGUAUCUCUAGUAAUAUAGAAUAUGCGAUGAGUCGAUAG.GUAGUCUUCGGACUACUAUUGA..GCAUAAGGAGGUCA..
Spn-5-1    CAUACGGCAAGGCGACGCUGACGAAGUUUGAAGAGAUUUUCGAAGAGUAUAAAGAGUUUCGUCUGUUGACAAAAGUCAUUCUCUUUAGUAGAAUAGUAGAUGCGAUGAGUCGAUAG.GUAGUCUUCGGGCUACUAUUGA..GCAUAAGGAGGUCA..
Spn-11-1   GAUAUGUAUAGGGAAACUCUUGAAUACGAGAGUUUUUUGUUAUACUCGAUCUGAAAAAUUUCUUAUUGACAAAAUCAAGUAUCUCUAGUAAUAUAGAAUAUGCGAUGAGUCGAUAG.GUAGUCUUCGGACUACUAUUGA..GCAUGAGGAGGUCA..
Spn-1-1    AUAUGUAUAGGGAAACUCUUGAAUACGGGAGUUUUUUGUUAUACUCGAUCGAAAAAAUUUCUUAUUGACAAAAUCAAGUAUCUCUAGUAAUAUAGAAUAUGCGAUGAGUCGAUAG.GUAGUCUUCGGACUACUAUUGA..GCAUAAGGAGGUCA..
Spn-8-1    GAUAUGUAUAGGGAAACUCUUGAAUAUGAGAGUUUUUUGUUAUACUCGAUCGAAAAAAUUUCUUAUUGACAAAAUCAAGUAUCUCUAGUAAUAUAGAAUAUGCGAUGAGUCGAUAG.GUAGUCUUCGGACUACUAUUGA..GCAUAAGGAGGUCA..
Spn-7-1    GAUAUGUAUAGGGAAACUCUUGAAUACAAGAGUUUUUUGUUAUACUCGAUCGAAAAAAUUUCUUAUUGACAAAAUCAAGUAUCUCUAGUAAUAUAGAAUAUGCGAUGAGUCGAUAG.GUAGUCUUCGGACUACUAUUGA..GCAUAAGGAGGUCA..
Spn-6-1    CAUACGGCAAGGCGACGCUGACGAAGUUUGAAGAGAUUUUCGAAGAGUAUAAAGAGUUUUGGCUGUUGACAAAAAUGACUCUAUCUAGUAGAAUAGUAGAUGCGAUGAGUCGAUAG.GUAGUCUUCGGGCUACUAUUGA..GCAUAAGGAGGUCA..
Spn-10-1   GAUAUGUAUAGGGAAACUCUUGAAUACGAGAGUUUUUUGUUAUACUCGAUCGAAAAAAUUUCUUAUUGACAAAAUCAACUAUCUCUAGUAAUAUAGAAUAUGCGAUGAGUCGAUAG.GUAGUCUUCGGGCUACUAUUGA..GCAUAAGGAGGUCA..
Spn-3-1    CAUACGGCAAGGCGACGCUGACGAAGUUUGAAGAGAUUUUCGAAGAGUAUAAAGAGUUUCGUCUGUUGACAAAAGUCAUUCUCUUUAGUAGAAUAGUAGAUGCGAUGAGUCGAUAG.GUAGUCUUCGGGCUACUAUUGA..GCAUAAGGAGGUCA..
           ..............................................................................................................<<<<<..<<<<<<....>>>>>>>>>>>.......<<<<<<<<....
           ..............................................................................................................120??..22222?....?22222??021.......?2000222....
           ●R●●●●R●●●●●●●●●●●●●●●●●●R●●●●●●●●●●●●●●●●●A●●●●●●●●●●●●●●●●●●●●●UYR●●●●●●●●●●●●A●YRY●●●RRA●●●●●●●UAUGCGAUGAGUCGA●●○-GU●●Y○○UY●○●●●ACU●UYG●--GC-○RRGGAGRUY○--
                                                                                                     5′
```

alignment positions 158 · · · 317

```
Lla-3-1   UGGGAAAUUGG....GUUUUCCG....CGCAGGAAGCGUGAGUAUUUGUCAAAUUGUGUGAACCUUUUUGACA.....CACU.U.UU...AGUGCCUUUAAAAGCCCUC......GAAA.......GAGGGCUUUUUUU..ACGUAACGAUUAAAAUAA
Lla-1-1   UGGGAAAUUGA....GUUUUCCG....CGCAGGAAGCGUGAGCAUUUGUCAAAAUGUGUGAACCUUUUUGACA.....CACU.U.UU...AGUGCCUUAAAA.AGCCCUU.....GUAA......AAGGGCUUUUUUUGUGAUUUUAUUUAUUGAAAG
Lla-2-1   UGGGAAAUUGA....GUUUUCCG....CGCAGGAAGCGUGAGUAUUUGUCAAAAUGUGUGAACCUUUUUGACA.....CACU.U.UU...AGUGCCUUAAAAAGCCCUU......GUAA.......AAGGGCUUUUUUUU.GUGAUUUUAUUUAUUGAA
Spy-10-1  ......................UAA.UGCGGGAAGCC...GACUUUGAUAAUCUGUGUGAACCAUUUUAUCACAAUCUUAUUUA.UUAAGAAUGCCCCUU...GGAUUGAGA....UAU....UCUCAAUCCUUUUUUU..AAUUUUAUUAUCUUAGUU
Spy-1-1   ......................UAA.GGCGGGAAGCC...GACUUUGAUAAUCUGUGUGAACCAUUUUAUCACAAUCUUAUUUA.UUAAGAAUGCCCCUU...GGAUUGAGA....UAU....UCUCAAUCCUUUUUUU..AAUUUUAUUAUCUUAGUU
Spy-4-1   ......................UAA.GGCGGGAAGCC...GACUUUGAUAAUCUGUGUGAACCAUUUUAUCACAAUCUUAUUUA.UUAAGAAUGCCCCUU...GGAUUGAGA....UAU....UCUCAAUCCUUUUUUU..AAUUUUAUUAUCUUAGUU
Spy-8-1   ......................UAA.GGCGGGAAGCC...GACUUUGAUAAUCUGUGUGAACCAUUUUAUCACAAUCUUAUUUA.UUAAGAAUGCCCCUU...GGAUUGAGA....UAU....UCUCAAUCCUUUUUUU..AAUUUUAUUAUCUUAGUU
Spy-11-1  ......................UAA.GGCGGGAAGCC...GACUUUGAUAAUCUGUGUGAACCAUUUUAUCACAAUCUUAUUUA.UUAAGAAUGCCCCUU...GGAUUGAGA....UAU....UCUCAAUCCUUUUUUU..AAUUUUAUUAUCUUAGUU
Spy-12-1  ......................UAA.GGCGGGAAGCC...GACUUUGAUAAUCUGUGUGAACCAUUUUAUCACAAUCUUAUUUA.UUAAGAAUGCCCCUU...GGAUUGAGA....UAU....UCUCAAUCCUUUUUUA..AUUUUAUUAUCUUAGUUA
Spy-3-1   ......................UAA.GGCGGGAAGCC...GACUUUGAUAAUCUGUGUGAACCAUUUUAUCACAAUCUUAUUUA.UUAAGAAUGCCCCUU...GGAUUGAGA....UAU....UCUCAAUCCUUUUUUU..AAUUUUAUUAACUUAGUU
Smu-1-1   ......................UAA.UGCAGGA.GCA...GAUCUUGGCAAGUUGUGUGAACCUGCUUGCCA.....CACUUAAUCAAAGUGCCUUUU...CCCUUGU.....AAUA......ACAAGGGCUUUUUUAUUGAAUUCAAUAGUUUAUG
Ssu-2-1   UCA.....UUUAAU......UGA.AA.CGCAGGA.GCG...GACCUUGAAUAGUUGUGUGAACCUUCUAUUCU.....CAUCG..GAAACGAUGCCCU.....GGCCCACC....UUUAU....GGUGGGCCUUUUUU...AUAUACUAAGUAUGCUUC
Ssu-1-1   UCA.....UUUAAU......UGA.AA.CGCAGGA.GCG...GACCUUGAAUAGUUGUGUGAACCUUCUAUUCU.....CAUCG..GAAACGAUGCCCUUG...CCCACC......UUUAU......GGUGGGCUUUUUGUAUACUAAGUGAGGCUGGGC
Sth-3-1   CAA.....CAUUAAA.....UUGUAA.CGCAGGA.GCG...GACCUUGAGUAGUUGUGUGAACCUGCUGCUCU.....CAUC...GAAA.GAUGCCUCUUA..UCCCUUGCC....GUUU...GGUAGGGGAUUUUU....CUGUUAUUCUUGCUCUUU
Sth-2-1   CAA.....CAUUAAA.....UUGUAA.CGCAGGA.GCG...GACCUUGAGUAGUUGUGUGAACCUGCUACUCU.....CAUC...GAAA.GAUGCCUCUUA..UCCCUUGCC...GUUU....GGUAGGGGAUUUUU....CUGUUAUUCUUGCUCUUU
Sth-1-1   CAA.....CAUUAAA.....UUGUAA.CGCAGGA.GCG...GACCUUGAGUAGUUGUGUGAACCUGCUACUCU.....CAUC...GAAA.GAUGCCUCUUA..UCCCUUGCC...GUUU....GGUAGGGGAUUUUU....CUGUUAUUCUUGCUCUUU
Ssa-1-1   ......................UAA.CGCAGGA.GCG...GACCUUGAUCAGUUGUGUGAACCUGCUGAUCA.....CAC....AAAA..GUGCCCUUA...UCCCCAGUC...UACG....GAUUGGGGAUUUUUCU..GUUGCUCUUGAUGAAAUG
Sag-4-1   ......................UGAACGCAGGA.GCG...GAUCUUGAUAAGUUGUGUGAACCUUCUUAUCA.....CACUU..GAAAAGGUGCCCUUAGC.UUACUA......CUAC.......UUGUAAUUCCUUA..CAAACUGUGGUAAGUAGC
Sag-6-1   ......................UAAACGCAGGA.GCG...GAUCUUGAUAAGUUGUGUGAACCUUCUUAUCA.....CACUU..GAAAAGGUGCCCUUAGC.UUACUA......CUAC.......UUGUAAUUCAUUA..CAAACUGUGGUAAGUAGC
Sag-5-1   ......................UAAACGCAGGA.GCG...GAUCUUGAUAAGUUGUGUGAACCUUCUUGUCA.....CACUU..GAAAAGGUGCCCUUAGC.UUACUA......CUAC.......UUGUAAUUUCUU..ACAAAUUGUGGUAAGUAG
Sag-2-1   ......................UAAACGCAGGA.GCG...GAUCUUGAUAAGUUGUGUGAACCUUCUUGUCA.....CACUU..GAAAAGGUGCCCUUAGC.UUACUA......CUAC.......UUGUAAUUUCUU..ACAAAUUGUGGUAAGUAG
Sag-3-1   ......................UAAACGCAGGA.GCG...GAUCUUGAUAAGUUGUGUGAACCUUCUUAUCA.....CACUU..GAAAAGGUGCCCUUAGC.UUACUA......CUAC.......UUGUAAUUUCUU..ACAAACUGUGGUAAGUAG
Spn-9-1   ......................UAA.CGCAGGA.GCG...GAUCUUGAUGAGUUGUGUGAACCUGCUCAUCA.....CAU....GAAG..AUGCCUCUUA..GUCCCUAGUC..UAGC...GACU.GGGAUUUUUAUU..UUCCAAAAAAUGAUGAAA
Spn-5-1   ......................UAA.CGCAGGA.GCG...GACCUUGAUGAGUUGUGUGAACCUGCUCAUCA.....CAU....GAAG..AUGCCUCUUA..GUCCCUAGUC..UAGC...GACU.GGGAUUUUUAUU..UUCCAAAAAAUGAUGAAA
Spn-11-1  ......................UAA.CGCAGGA.GCG...GACCUUGAUGAGUUGUGUGAACCUGCUCAUCA.....CAU....GAAG..AUGCCUCUUA..GUCCCUAGUC..UAGC...GACU.GGGAUUUUUAUUUUCCAAAAAAUGAUGAAAAA
Spn-1-1   ......................UAA.CGCAGGA.GCG...GACCUUGAUGAGUUGUGUGAACCUGCUCAUCA.....CAU....GAAG..AUGCCUCUUA..GUCCCUAGUC..UAGC...GACU.GGGAUUUUUAUUUUCCAAAAAAUGAUGAAAAA
Spn-8-1   ......................UAA.CGCAGGA.GCG...GACCUUGAUGAGUUGUGUGAACCUGCUCAUCA.....CAU....GAAG..AUGCCUCUUA..GUCCCUAGUC..UAGC...GACU.GGGAUUUUUAUUUUCCAAAAAAUGAUGAAAAA
Spn-7-1   ......................UAA.CGCAGGA.GCG...GACCUUGAUGAGUUGUGUGAACCUGCUCAUCA.....CAU....GAAG..AUGCCUCUUA..GUCCCUAGUC..UAGC...GACU.GGGAUUUUUAUUUUCCAAAAAAUAAUGAAAAA
Spn-6-1   ......................UAA.CGCAGGA.GCG...GACCUUGAUGAGUUGUGUGAACCUGCUCAUCA.....CAU....GAAG..AUGCCUCUUA..AUCCCUAGUC..UAGC...GAUUGGGGAUUUUUAU...UUUCCAAAAAAUGAUGAA
Spn-10-1  ......................UAA.CGCAGGA.GCG...GACCUUGAUGAGUUGUGUGAACCUGCUCAUCA.....CAU....GAAG..AUGCCUCUUA..AUCCCUAGUC..UAGC...GAUUGGGGAUUUUUAU...UUUCCAAAAAAUGAUGAA
Spn-3-1   ......................UAA.CGCAGGA.GCG...GACCUUGAUGAGUUGUGUGAACCUGCUCAUCA.....CAU....GAAG..AUGCCUCUUA..AUCCCUAGUC..UAGC...GAUUGGGGAUUUUUAU...UUUCCAAAAAAUGAUGAA
          <<<<<<<<.......>>>>>>>>....<<<.....>>>....>>>><<<<<<<<..........>>>>>>>>.....<<<<<......>>>>>>>>>.....<<<<<<<<<<........>>>>>>>>>>...........................
          22200000.......00000222....200.....002....2220?02220??..........??02220?.....10222......22201002?.....2222??2220........0222??2222...........................
          ---------------------∘AA-CGCAGGA-GCG---GAYYUUGAY•ARUUGUGUGAACCU•YU•RUCA------CAY∘∘--••AR∘∘RUGCCYYU•∘--•Y•YY•∘∘----∘•••-----∘∘∘RR•R•UUUUUU∘--•••••••••••••••••
                                                                                                                                                            3′
```

alignment positions 318 · · · 477

```
Lla-3-1    UUAAUAAAAAAAUCUUGUUAUAAUCAGAUGAAUACAAUGCUGAAAAAAUAUAUAAACAAUCUAUUGUAGGAGUUAAUCAUAUUUUCGAUGUUAUUAUUAGCAAGAGUUAAGAAAAAUAUGAUAAAAUUGAAGUGUCAACAUCUUUAUAUAUUUUUCAAAA
Lla-1-1    UUAACUAGACUAAAAAUGUUUCUGCUAAAGAAUUUUUUAACACUAUAAUUGUAUUUUGCAAUGUUAUUAUUAGCAAGAGUUAAGAAAAAUAUGAUAAACUUAUAGUGUCACAUCUUUUAUGUAUUUUUAAAAAUAGAAAGGAAACUAUCAUGGAUGUUUC
Lla-2-1    AGUUAACUAGACUAAAAAAGUUUCUGCUAACGAAUUUUUUAACACUAUAAUUGUAUUUUGCAAUGUUAUUAUUAGCAAGAGUUAAGAAAAAUAUGAUAAAAUUAUAGUGUCACAUCUUUUAUGUAUUUUUAAAAAUAGAAAGGAAAAUAUCAUGGAUGUU
Spy-10-1   AAAAUUCAGAAUGUAUCAGUGAUUACUGUGUUUCAAAAUAAGAAAGUAGUUUUUCGGGUAUGAGUGCUAUGACUUGACAAAUGCUAAUAACAUUUGCAUAAUGUAAGUAGAGAUGAAAACUUAUGAAAAGAGAUGAUCUUAUGCAAAUUAUUAAGCGUGA
Spy-1-1    AAAAUUCAGAAUGUAUCAGUGAUUACUGUGUUUCAAAAUAAGAAAGUAGUUUUUCGGGUAUGAGUGCUAUGACUUGACAAAUGCUAAUAACAUUUGCAUAAUGUAGGUAGAGAUGAAAACUUAUGAAAAGAGAUGAUCUUAUGCAAAUUAUUAAGCGUGA
Spy-4-1    AAAAUUCAGAAUGUAUCAGUGAUUACUGUGUUUCAAAAUAAGAAAGUAGUUUUUCGGGUAUGAGUGCUAUGACUUGACAAAUGCUAAUAACAUUUGCAUAAUGUAAGUAGAGAUGAAAACUUAUGAAAAGAGAUGAUCUUAUGCAAAUUAUUAAGCGUGA
Spy-8-1    AAAAUUCAGAAUGUAUCAGUGAUUACUGUGUUUCAAAAUAAGAAAGUAGUUUUUCGGGUAUGAGUGCUAUGACUUGACAAAUGCUAAUAACAUUUGCAUAAUGUAGGUAGAGAUGAAAACUUAUGAAAAGAGAUGAUCUUAUGCAAAUUAUUAAGCGUGA
Spy-11-1   AAAAUUUAGAAUGUAUCAGUGAUUACUGUGUUUCAAAAUAAGAAAGUAGUUUUUCGGGUAUGAGUGCUAUGACUUGACAAAUGCUAAUAACAUUUGCAUAAUGUAAGUAGAGAUGAAAACUUAUGAAAAGAGAUGAUCUUAUGCAAAUUAUUAAGCGUGA
Spy-12-1   AAAUUCAGAAUGUAUCAGUGAUUAUUGUGUUUCAAAGUAAGAAAGUAGUUUUUUGGGUAUGAGUGCUAUGACUUGACAAAUGCUAAUAACAUUUGCAUAAUGUAAUUAGAGAUGAAAACUUAUGAAAAGAGAUGAUCUUAUGCAAAUUAUUAAGCGUGAU
Spy-3-1    AAAAUUCAGAAUGUAUCAGUGAUUACUGUGUUUCAAAAUAAGAAAGUAGUUUUUCGGGUAUGAGUGCUAUGACUUGACAAAUGCUAAUAACAUUUGCAUAAUGUAAGUAGAGAUGAAAACUUAUGAAAAGAGAUGAUCUUAUGCAAAUUAUUAAGCGUGA
Smu-1-1    GACUUUUUUUUUUGUAUAAAAAGUAGUAUAAUGUCUAUUAUUAGAAUGAAUUAGAAUAAAGAGGUAAACUAUGGCUACACAGUUGCUAUCGUUGGUGCUACAGGCGCCGUUGGAACUCGUAUGAUUCAACAAUUGGAACAAUCGACACUUCCAGUUGAC
Ssu-2-1    AGAUACUCUAUCAGAAACAUAUUUCAGUAUGUGAUUUCUAUAGGAAUGCUAUUGGCUCAGCCGAGGAAGUUUUUGUAUUUCUCAAAGUUAGAAAUAACAGUUGAUAUUAUGUUUGAUGAAGAUUGCUUGAUAGUCUUUUAAAUCUUCUGGAUGGAGAGCC
Ssu-1-1    AAAAAGCCCUGGCCCACUUCUCAGUGUUCGUGUCAACAUCUCAGCGCAGUGGUUGAUUGGGUUUAACAGUCCAGUGGACUGUUAAAGGUAGGAGAUAAGAUUUGCGAAGCAAAUCUCAGCAAUUCGUGUUUUACACUCGAAAUCUAACCUAAUCAACUGU
Sth-3-1    UAACCCUUUUCAACUGGGCUUAAACAUAGUAUAAUAGAUUCAUUAAACUAAAUUGAUAGAAAAUUCCGAAAGAGGUUUAACUAUGGGAUACACAGUUGCUGUUGUCGGUGCUACAGGUGCCGUUGGUGCUCAAAUGAUCAAAAUGUUGGAAGAAUCGACG
Sth-2-1    UAACCCUUUUCAACUGGGCUUAAACAUAGUAUAAUAGAUUUAUUAAACUAAAUUGAUAGAAAAUUCCAAAAGAGGUUUUACUAUGGGAUACACAGUUGCUGUUGUCGGUGCUACAGGUGCCGUUGGUGCUCAAAUGAUCAAAAUGUUGGAAGAAUCACCA
Sth-1-1    UAACCCUUUUCAACUGGGCUUAAACAUAGUAUAAUAGAUUCAUUAAACUAAAUUGAUAGAAAAUUCCGAAAGAGGUUUAACUAUGGGAUACACAGUUGCUGUUGUCGGUGCUACAGGUGCCGUUGGUGCUCAAAUGAUCAAAAUGUUGGAAGAAUCAACA
Ssa-1-1    AGUCUGCAGGAGAAAGUUGAGUUAGUGUAGUUUAUCAAAAUAUCGCACUUAUAUUCUUCAGAAAUAUUUGCAUUUUUCCCUAAAAAUAGUAUAAUACUAACAAUAAAAAUUUUUUGAAAAUUCAGAGAGGUUAUCGCAUGGGAUAUACAGUUGCUGUAGU
Sag-4-1    UGAAAAGCAAAAAAAAGAAAGAACCAGUUUGGUUCUUUCUUUUUUUUGCAUAAAUAAGUCACAAUUUCCUUCUAAAAAUUAUGUCUUUACUUAACUUUAAUUGAAUAUGCUACCAUCACAUUCUUUGUAAAAUUUUUAAAUAAUCUAGUUUCUGAUGGUU
Sag-6-1    UGAAAAGCAAAAAAGAAAGAACCAGUUUGGUUCUUUCUUUUUUUUGCAUAAAUAAGUCACAAUUUCUUUCUAAAAAUUAUGUUUUUACUUAACUUUAAUUGAAUAUGCUACCAUCACAUUCUUUGUAAAAUUUUUAAAUAAUCUAGUUUCUGAUGGUUUA
Sag-5-1    CUGAAAAGCAAAAAAGAAAGAACCAAACUGGUUCUUUCUUUUUUGCAUAAAUAAGUCACAAUUUCCUUCUUAAAAUUAUGUCUUUACUUAACUUUAAUUGAAUAUGCUACCAUCACAUUCUUUGUAAAAUUUUUAAAUAAUCUAGUUUCUGAUGGUUUAG
Sag-2-1    CUGAAAAGCAAAAAAGAAAGAACCAGUUUGGUUCUUUCUUUUUUGCAUAAAUAAGUCACAAUUUCCUUCUUAAAAUUAUGUCUUUACUUAACUUUAAUUGAAUAUGCUACCAUCACAUUCUUUGUAAAAUUUUUAAAUAAUCUAGUUUCUGAUGGUUUAG
Sag-3-1    CUGAAAAGCAAAAAAGAAAGAACCAAACUGGUUCUUUCUUUUUUUUGCAUAAAUAAGUCACAAUUUCCUUCUAAAAAUUAUGUCUUUACUUAACUUUAAUUGAAUAUGCUACCAUCACAUUCUUUGUAAAAUUUUUAAAUAAUCUAGUUUCUGAUGGUUU
Spn-9-1    AAGCUUUGCAAUUCAUGGAAAAAGUAGUAUAAUACUUCUAUUAUAGAAAUUUUUAGAAAAUUCCGAAAGAGGUUAUUUAUGGGAUAUACAGUUGCUGUAGUCGGCGCGACAGGUGCUGUCGGUGCUCAGAUGAUAAAAAUGUUGGAAGAAUCAACACUUC
Spn-5-1    AAGCUUUGCAAUUCAUGGAAAAAGUAGUAUAAUACUUCUAUUAUAGAAAUUUUUAGAAAAUUCCGAAAGAGGUUAUUUAUGGGAUAUACAGUUGCUGUAGUCGGCGCGACAGGUGCUGUCGGUGCUCAGAUGAUAAAAAUGUUGGAAGAAUCAACACUUU
Spn-11-1   GCUUUGCAAUUCAUGGAAAAAGUAGUAUAAUACUUCUAUUAUAGAAAUUUUUAGAAAAUUCCGAAAGAGGUUAUUUAUGGGAUAUACAGUUGCUGUAGUCGGCGCGACAGGUGCUGUCGGUGCUCAGAUGAUAAAAAUGUUGGAAGAAUCAACACUUCCA
Spn-1-1    GCUUUGCAAUUCAUGGAAAAAGUAGUAUAAUACUUCUAUUAUAGAAAUUUUUAGAAAAUUCCGAAAGAGGUUAUUUAUGGGAUAUACAGUUGCUGUAGUCGGCGCGACAGGUGCUGUCGGUGCUCAGAUGAUAAAAAUGUUGGAAGAAUCAACACUUCCA
Spn-8-1    GCUUUGCAAUUCAUGGAAAAAGUAGUAUAAUACUUCUAUUAUAGAAAUUUUUAGAAAAUUCCGAAAGAGGUUAUUUAUGGGAUAUACAGUUGCUGUAGUCGGCGCGACAGGUGCUGUCGGUGCUCAGAUGAUAAAAAUGUUGGAAGAAUCAACACUUCCA
Spn-7-1    GCUUUGCAAUUCAUGGAAAAAGUAGUAUAAUACUUCUAUUAUAGAAAUUUUUAGAAAAUUCCGAAAGAGGUUAUUUAUGGGAUAUACAGUUGCUGUAGUCGGCGCGACAGGUGCUGUCGGUGCUCAGAUGAUAAAAAUGUUGGAAGAAUCAACACUUCCA
Spn-6-1    AAAGCUUUGCAAUUCAUGGAAAAAGUAGUAUAAUACUUCUAUUAUAGAAAUUUUUAGAAAAUUCCGAAAGAGGUUAUUUAUGGGAUAUACAGUUGCUGUAGUCGGCGCGACAGGUGCUGUCGGUGCUCAGAUGAUAAAAAUGUUGGAAGAAUCAACACUU
Spn-10-1   AAAGCUUUGCAAUUCAUGGAAAAAGUAGUAUAAUACUUCUAUUAUAGAAAUUUUUAGAAAAUUCCGAAAGAGGUUAUUUAUGGGAUAUACAGUUGCUGUAGUCGGCGCGACAGGUGCUGUCGGUGCUCAGAUGAUAAAAAUGUUGGAAAAAUCAACACUU
Spn-3-1    AAAGCUUUGCAAUUCAUGGAAAAAGUAGUAUAAUACUUCUAUUAUAGAAAUUUUUAGAAAAUUCCGAAAGAGGUUAUUUAUGGGAUAUACAGUUGCUGUAGUCGGCGCGACAGGUGCUGUCGGUGCUCAGAUGAUAAAAAUGUUGGAAGAAUCAACACUU
           ................................................................................................................................................................
           ................................................................................................................................................................
           ••R••••••••••••R••••••••••••••••••••••Y••••••••••••••••••••••••••••••••••••••••••••••••••••••••••••••••R••••••••••••••••••••••••••R••••••••••••••••••••••R••••••
```

alignment positions 478 · · · 599

```
Lla-3-1    UAGAAAGGAAAUUAUCAUGGAUGUUUCAUGGAUGACAAAAUAUAUUGCUGAGGGAUUGGGAACUAUGCUUCUCGUCUUGCUUGGUAAUGGUACAAUUGCUGGGGUAACCCUCAAAGGCUCUA
Lla-1-1    AUGGAUGACAAAAUAUAUUGCUGAGGGAUUGGGAACUAUGCUUCUCGUUUUGCUUGGUAAUGGUACAAUUGCGGGAGUAACACUCAAAGGCUCUAAAAAUGAGGGCAUGGGUGGCCUUGCAA
Lla-2-1    UCAUGGAUGACAAAAUAUAUUGCUGAGGGAUUGGGAACUAUGCUUCUCGUUUUGCUUGGUAAUGGUACAAUUGCGGGAGUAACACUCAAAGGCUCUAAAAAUGAGGGCAUGGGUGGCCUUGC
Spy-10-1   UGGCCACGUUGCUGAUUUUGAUCCAGAUAAAAUUUAUCAAGCAAUUAUUAAAGCAGCAAGGACGGUCUAUGUUAUUGAUGACACGUGGCGACAAAACUUAACACAUGUCACUAAAAAAGUAG
Spy-1-1    UGGCCACGUUGCUGAUUUUGAUCCAGAUAAAAUUUAUCAAGCAAUUAUUAAAGCAGCAAGGACGGUCUAUGUUAUUGAUGACACGUGGCGACAAAACUUAACACAUGUCACUAAAAAAGUAG
Spy-4-1    UGGCCACGUUGCUGAUUUUGAUCCAGAUAAAAUUUAUCAAGCAAUUAUUAAAGCAGCAAGGACGGUCUAUGUUAUUGAUGACACGUGGCGACAAAACUUAACACAUGUCACUAAAAAAGUAG
Spy-8-1    UGGCCACGUUGCUGAUUUUGAUCCAGAUAAAAUUUAUCAAGCAAUUAUUAAAGCAGCAAGGACGGUCUAUGUUAUUGAUGACACGUGGCGACAAAACUUAACACAUGUCACUAAAAAAGUAG
Spy-11-1   UGGCCACGUUGCUGAUUUUGAUCCAGAUAAAAUUUAUCAAGCAAUUAUUAAAGCAGCAAGGACGGUCUAUGUUAUUGAUGACACGUGGCGACAAAACUUAACACAUGUCACUAAAAAAGUAG
Spy-12-1   GGCCACGUUGCUGAUUUUGAUCCAGAUAAAAUUUAUCAAGCAAUUAUUAAAGCAGCAAGGACGGUCUAUGUUAUUGAUGACACGUGGCGACAAAACUUAACACAUGUCACUAAAAAAGUAGU
Spy-3-1    UGGCCACGUUGCUGAUUUUGAUCCAGAUAAAAUUUAUCAAGCAAUUAUUAAAGCAGCAAGGACGGUCUAUGUUAUUGAUGACACGUGGCGACAAAACUUAACACAUGUCACUAAAAAAGUAG
Smu-1-1    AAGGUACGGCUUUUGUCAUCUUCACGUUCUGCAGGUAAAGUUUUGCAAUAUAAAGAUCAAGAUGUCACGGUUGAAUUAACUACGAAAGAUUCCUUUGAAGGUGUUGAUAUUGCGCUUUUUUC
Ssu-2-1    UUGACAUCGGUCAUAGCAUAGGUUCGAUUGAGAAGCUUAUACUUAUUUUCUCCAAAUUGAUGGAAGGGGAGUAGUUGAACUUUGUCAAUCUUGAGUUGAUUAAAGAGACAGGCAAAUUGUUC
Ssu-1-1    GCGGGGGUGGGAGUGAAACAGUCUGGGGAUAGACUGUUUCAGCUCAACAACUUAAAACGAAAAAUUGUUGACGAACCUUUUUUGACUUAGUCGAUGUCUUUCCCACUCCCAUUUCAGUAUGU
Sth-3-1    CUUCCAAUCGAUAAAAUACGCGACCUUGCGUCUGCUCGAUACGCAGGAAAAGUCUUGCAGUUUAAAGGUCAAGACGUGACCAUCGAAGAAACGACGGAAGACGCAUUUGAAGGUGUAGACAU
Sth-2-1    CUUCCAAUCGAUAAAAUUCGCUACCUUGCGUCUGCUCGUUCUGCAGGAAAAGUCUUGCAGUUUAAAGGUCAAGACGUGACCAUCGAAGAAACGACGGAAGACGCAUUUGAAGGUGUAGACAU
Sth-1-1    CUUCCAAUCGAUAAAAUUCGCUACCUUGCGUCUGCUCGUUCUGCAGGAAAAGUCUUGCAGUUUAAAGGUCAAGACGUGACCAUCGAAGAAACGACGGAAGACGCAUUUGAAGGUGUAGACAU
Ssa-1-1    AGGUGCCACUGGUGCCGUUGGAUCUCAAAUGAUUAAAAUGCUGGAAGAAUCUAGCCUUCCAAUUGAUAAAAUACGCUAUCUGGCGUCUGCUCGUUCAGCUGGAAAAGUUCUCCAGUUCAAAG
Sag-4-1    UAGAUGAAGUAUUAAAAAUAUACUAUUAUCUCAUUGUAAAUCCUAAUGUUAGUAUGACUAUCUAUCAUGUUUUAUAAUAUUGAAGGAAAAUUUAAAAAUAUCAUGUUUUAGAUAUCAACUAU
Sag-6-1    GAUGAAGUAUUAAAAAUAUACUAUUAUCUCAUUGUAAAUCCUAAUGUUAGUAUGACUAUCUAUCAUGUUUUAUAAUAUUAAAGGAAAAUUUAAAAAUAUCAUGUUUUAGAUAUCAACUAUUU
Sag-5-1    AUGAAGUAUUAAAAAUAUACUAUUACCUCAUUGUAAAUCUUAAUGUUAGUAUGACUAUCUAUCAUGCUUUAUAAUAUUAAAGGAAAAUUUAAAAAUAUCAUGUUUUAGAUAUCAACUAUUUA
Sag-2-1    AUGAAGUAUUAAAAAUAUACUAUUACCUCAUUGUAAAUCUUAAUGUUAGUAUGACUAUCUAUCAUGCUUUAUAAUAUUAAAGGAAAAUUUAAAAAUAUCAUGUUUUAGAUAUCAACUAUUUA
Sag-3-1    AGAUGAAGUAUUAAAAAUAUACUAUUAUCUCAUUGUAAAUCCUAAUGUUAGUAUGACUAUCUAUCAUGUUUUAUAAUAUKGAAGGAAAAUUUAAAAAUAUCAUGUUUUAGAUAUCAACUAUU
Spn-9-1    CAAUCGAUAAAAUCCGUUACCUUGCUUCUGCACGUUCAGCAGGUAAGUCAUUGAAAUUUAAAGAUCAAGAUAUUACAAUUGAAGAAACGACUGAAACAGCUUUUGAAGGAGUUGAUAUUGCU
Spn-5-1    CAAUCGAUAAAAUCCGUUACCUUGCUUCUGCACGUUCAGCAGGUAAGUCAUUGAAAUUUAAAGAUCAAGAUAUUACAAUUGAAGAAACGACUGAAACAGCUUUUGAAGGAGUUGAUAUUGCU
Spn-11-1   AUCGAUAAAAUUCGUUACCUUGCUUCUGCACGUUCAGCAGGUAAGUCAUUGAAAUUUAAAGAUCAAGAUAUUACAAUUGAAGAAACGACUGAAACAGCUUUUGAAGGAGUUGAUAUUGCUCU
Spn-1-1    AUCGAUAAAAUCCGUUACCUUGCUUCUGCACGUUCAGCAGGUAAGUCAUUGAAAUUUAAAGAUCAAGAUAUUACAAUUGAAGAAACGACUGAAACAGCUUUUGAAGGAGUUGAUAUUGCUCU
Spn-8-1    AUCGAUAAAAUUCGUUACCUUGCUUCUGCACGUUCAGCAGGUAAGUCAUUGAAAUUUAAAGAUCAAGAUAUUACAAUUGAAGAAACGACUGAAACAGCUUUUGAAGGAGUUGAUAUUGCUCU
Spn-7-1    AUCGAUAAAAUCCGUUACCUUGCUUCUGCACGUUCAGCAGGUAAGUCAUUGAAAUUUAAAGAUCAAGAUAUUACAAUUGAAGAAACGACUGAAACAGCUUUUGAAGGAGUUGAUAUUGCUCU
Spn-6-1    CCAAUCGAUAAAAUUCGUUACCUUGCUUCUGCACGUUCAGCAGGUAAGUCAUUGAAAUUUAAAGAUCAAGAUAUUACGAUUGAAGAAACGACUGAAACAGCUUUUGAAGGAGUUGAUAUUGC
Spn-10-1   CCAAUCGAUAAAAUCCGUUACCUUGCUUCUGCACGUUCAGCAGGUAAGUCAUUGAAAUUUAAAGAUCAAGAUAUUACGAUUGAAGAAACGACUGAAACAGCUUUUGAAGGAGUUGAUAUUGC
Spn-3-1    CCAAUCGAUAAAAUCCGUUACCUUGCUUCUGCACGUUCAGCAGGUAAGUCAUUGAAAUUUAAAGAUCAAGAUAUUACGAUUGAAGAAACGACUGAAACAGCUUUUGAAGGAGUUGAUAUUGC
           ..........................................................................................................................
           ..........................................................................................................................
           ••R•••••••••••••••••••Y••••••••••••••••••••••••••••••R•••••••••••••••••Y••••••••••••••R••••••R•••••••••••••••••••••••••Y••
```

8 *atoC*

8.1 Taxa

The taxonomy of each organism containing a putative *atoC* RNA is listed, with abbreviations identifying each hit (e.g., "Eco-1-1" and "Eco-1-2" might hypothetically represent two distinct RNAs in *E. coli*). The abbreviations will be used to identify each individual *atoC* RNA in Sections 8.2 and 8.4.

abbrev. of hits	taxonomy of species
Gbe-1-1	Bacteria Proteobacteria Deltaproteobacteria Desulfuromonadales Geobacteraceae *Geobacter bemidjiensis* Bem
Glo-1-1	Bacteria Proteobacteria Deltaproteobacteria Desulfuromonadales Geobacteraceae *Geobacter lovleyi* SZ
Gme-1-1	Bacteria Proteobacteria Deltaproteobacteria Desulfuromonadales Geobacteraceae *Geobacter metallireducens* GS-15
Gsp-1-1	Bacteria Proteobacteria Deltaproteobacteria Desulfuromonadales Geobacteraceae *Geobacter sp.* FRC-32
Gsu-1-1 to Gsu-1-2	Bacteria Proteobacteria Deltaproteobacteria Desulfuromonadales Geobacteraceae *Geobacter sulfurreducens* PCA
Gur-1-1	Bacteria Proteobacteria Deltaproteobacteria Desulfuromonadales Geobacteraceae *Geobacter uraniireducens* Rf4
Ppr-1-1	Bacteria Proteobacteria Deltaproteobacteria Desulfuromonadales Pelobacteraceae *Pelobacter propionicus* DSM 2379

8.2 Gene contexts

Each *atoC* RNA (indicated by "RNA→") is listed with its surrounding genes. Environmental sequences and some RefSeq entries lack gene annotations, and no genes are listed for such sequences. The direction of each gene is indicated with an arrow (→), and each conserved domain in the gene is colored. Conserved domains associated with more than one *atoC* RNA are assigned a color; other domains are gray. Information about these conserved domains is given in Section 8.3. The accession number of the sequence containing each *atoC* RNA is given. Accessions beginning with "NC_" or "NZ_" are RefSeq, while other accession refer to environmental samples. Nucleotide coordinates are given for the 5′ and 3′ boundaries of each. If the 5′ coordinate is greater than the 3′ coordinate, the RNA is present on the reverse-complement strand of the containing genomic DNA sequence. Each hit is denoted by an abbreviation (like "Eco-1-1") that refers to a taxonomy given in Section 8.1.

abbrev.	Seq. accession		5′ at	3′ at	genes
Gsu-1-1	NC_002939.4	+	1810322	1810409	**RNA**→ AtoC (COG2204)→
Glo-1-1	NZ_AAVG01000035.1	+	27997	28086	**RNA**→ HPPK (cd00483)→ COG3350 (COG3350)→ Transaldolase_FSA (cd00956)→ REC (cd00156)GSPII_E_N (pfam05157)→
Ppr-1-1	NC_008609.1	-	292999	292905	**RNA**→ FolK (COG0801)→ COG3350 (COG3350)→ Transaldolase_FSA (cd00956)→
Gbe-1-1	NZ_AAZA01000001.1	+	121278	121368	**RNA**→ COG1146 (COG1146)RocR (COG3829)Fe_hyd_lg_C (pfam02906)→
Gsu-1-2	NC_002939.4	+	3694623	3694713	**RNA**→ COG1144 (COG1144)RocR (COG3829)Fe_hyd_lg_C (pfam02906)FeS (pfam04060)→ hypo→
Gme-1-1	NC_007517.1	+	67665	67752	**RNA**→ COG1146 (COG1146)RocR (COG3829)Fe_hyd_lg_C (pfam02906)FeS (pfam04060)→
Gsp-1-1	NZ_AASH01000003.1	-	37660	37569	**RNA**→ COG1146 (COG1146)FhlA (COG3604)Fe_hyd_lg_C (pfam02906)FeS (pfam04060)→
Gur-1-1	NC_009483.1	-	4839082	4838991	**RNA**→ COG1144 (COG1144)AtoC (COG2204)Fe_hyd_lg_C (pfam02906)FeS (pfam04060)→

8.3 Conserved domains

Conserved domains found in protein-coding genes listed in Section 8.2 are listed, with the first sentence in their description from the Conserved Domain Database. Conserved domains associated with more than one *atoC* RNA are assigned a color, while others are shown in gray. The number in parentheses is the number of occurrences of the given gene.

cd00156 (1) Signal receiver domain; originally thought to be unique to bacteria (CheY, OmpR, NtrC, and PhoB), now recently identified in eukaroytes ETR1 Arabidopsis thaliana; this domain receives the signal from the sensor partner in a two-component systems; contains a phosphoacceptor site that is phosphorylated by histidine kinase homologs; usually found N-terminal to a DNA binding effector domain; forms homodimers
cd00483 (1) 7,8-dihydro-6-hydroxymethylpterin-pyrophosphokinase (HPPK).
cd00956 (2) Transaldolase-like fructose-6-phosphate aldolases (FSA) found in bacteria and archaea, which are member of the MipB/TalC subfamily of class I aldolases.
COG0801 (1) 7,8-dihydro-6-hydroxymethylpterin-pyrophosphokinase [Coenzyme metabolism]
COG1144 (2) Pyruvate:ferredoxin oxidoreductase and related 2-oxoacid:ferredoxin oxidoreductases, delta subunit [Energy production and conversion]
COG1146 (3) Ferredoxin [Energy production and conversion]
COG2204 (2) Response regulator containing CheY-like receiver, AAA-type ATPase, and DNA-binding domains [Signal transduction mechanisms]
COG3350 (2) Uncharacterized conserved protein [Function unknown]
COG3604 (1) Transcriptional regulator containing GAF, AAA-type ATPase, and DNA binding domains [Transcription / Signal transduction mechanisms]
COG3829 (3) Transcriptional regulator containing PAS, AAA-type ATPase, and DNA-binding domains [Transcription / Signal transduction mechanisms]
pfam02906 (5) Iron only hydrogenase large subunit, C-terminal domain.
pfam04060 (4) Putative Fe-S cluster.
pfam05157 (1) GSPII_E N-terminal domain.

8.4 Multiple-sequence alignment

Each *atoC* RNA is denoted by an abbreviation (like "Eco-1-1") that refers to a taxonomy given in Section 8.1. The alignment includes sequences containing the RNA motif, as well as flanking sequence. The *atoC* RNA itself is denoted by the line underneath marked 5′ and 3′ on either end. Nucleotides in the flanking sequences (i.e., not part of the motif) are written in gray letters. Stems of predicted rho-independent transcription terminators are shaded yellow. (Note: terminator predictions have not been analyzed manually, and many are likely to be false positives.) Start codons are shaded green. (Note: start codons are frequently mis-

annotated, especially in environmental samples.) Nucleotides proposed to basepair as part of the consensus structure are shaded in color when they comprise Watson-Crick or G-U pairs. Otherwise they are shaded gray. Conserved stems are also indicated at the bottom of the alignment by angle brackets, where matching < and > denote base-paired columns. Below these angle brackets, the symbol "2" denotes base pairs exhibiting covariation, "1" denotes base pairs exhibiting compatible mutations, "0" denotes base pairs that are not observed to mutate and "?" denotes base pairs that have a significant frequency of non-canonical nucleotides for Watson-Crick or G-U pairs (> 5%). Below these base pair annotation is the consensus sequence: "R" = "A" or "G", "Y" = "C" or "U", red nucleotides: nucleotide identity conserved more than 97% of the time, black nucleotides: 90%, gray nucleotides: 75%, red circle (•): nucleotide is present 97% of the time, black circle (•): 90%, gray circle (•): 75%, white circle (∘): 50%. All percentages of sequences just described (e.g. 97% conserved) assume that sequences have been weighted by the GSC algorithm implemented by the Infernal software.

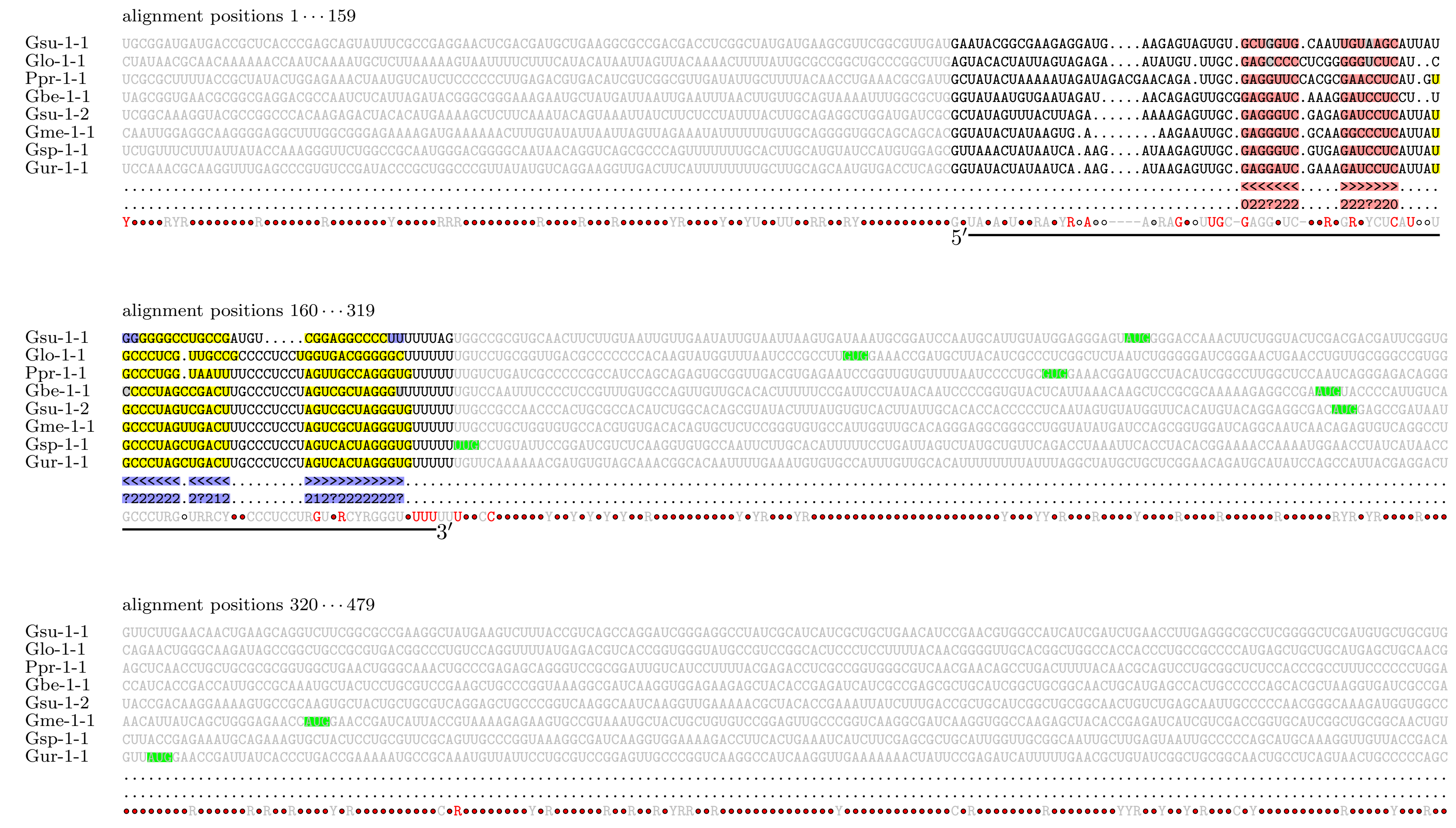

	alignment positions 480 · · · 499
Gsu-1-1	AGGCGAAACGGCUCGACCAU
Glo-1-1	GAUAGAACGGGAGAUCUUUG
Ppr-1-1	AUUGCUGCGUCGACUCCUUA
Gbe-1-1	UAACGUUGCGGUAACCAACG
Gsu-1-2	GACAAAGUGGGUGUUACCGA
Gme-1-1	CUCAGCAACUGCCCCCAGCA
Gsp-1-1	AGGUGGGGGUCGUGGAGAAC
Gur-1-1	ACGCCAAAGUCGUCACCGAC
	....
	....
	R•••R•R•••••••••••••

9 Bacillaceae-1

9.1 Taxa

The taxonomy of each organism containing a putative Bacillaceae-1 RNA is listed, with abbreviations identifying each hit (e.g., “Eco-1-1” and “Eco-1-2” might hypothetically represent two distinct RNAs in *E. coli*). The abbreviations will be used to identify each individual Bacillaceae-1 RNA in Sections 9.2 and 9.4.

**abbrev. of hits**	**taxonomy of species**
Bam-1-1 to Bam-1-2	Bacteria Firmicutes Bacillales Bacillaceae *Bacillus amyloliquefaciens* FZB42
Bcl-1-1	Bacteria Firmicutes Bacillales Bacillaceae *Bacillus clausii* KSM-K16
Bco-1-1 to Bco-1-6	Bacteria Firmicutes Bacillales Bacillaceae *Bacillus coahuilensis m4-4*
Bha-1-1 to Bha-1-4	Bacteria Firmicutes Bacillales Bacillaceae *Bacillus halodurans* C-125
Bli-1-1 to Bli-1-46	Bacteria Firmicutes Bacillales Bacillaceae *Bacillus licheniformis* ATCC 14580 (DSM 13)
Bpu-1-1 to Bpu-1-2	Bacteria Firmicutes Bacillales Bacillaceae *Bacillus pumilus* ATCC 7061
Bpu-2-1 to Bpu-2-6	Bacteria Firmicutes Bacillales Bacillaceae *Bacillus pumilus* SAFR-032
Bsp-1-1 to Bsp-1-35	Bacteria Firmicutes Bacillales Bacillaceae *Bacillus sp.* NRRL B-14911
Bsu-1-1 to Bsu-1-16	Bacteria Firmicutes Bacillales Bacillaceae *Bacillus subtilis subsp. subtilis str.* 168
Bsu-2-1 to Bsu-2-8	Bacteria Firmicutes Bacillales Bacillaceae *Bacillus subtilis subsp. subtilis str.* JH642
Bsu-3-1 to Bsu-3-8	Bacteria Firmicutes Bacillales Bacillaceae *Bacillus subtilis subsp. subtilis str.* NCIB 3610
Bsu-4-1 to Bsu-4-8	Bacteria Firmicutes Bacillales Bacillaceae *Bacillus subtilis subsp. subtilis str.* SMY
Gka-1-1 to Gka-1-2	Bacteria Firmicutes Bacillales Bacillaceae *Geobacillus kaustophilus* HTA426
Oih-1-1 to Oih-1-5	Bacteria Firmicutes Bacillales Bacillaceae *Oceanobacillus iheyensis* HTE831

9.2 Gene contexts

Each Bacillaceae-1 RNA (indicated by “RNA→”) is listed with its surrounding genes. Environmental sequences and some RefSeq entries lack gene annotations, and no genes are listed for such sequences. The direction of each gene is indicated with an arrow (→), and each conserved domain in the gene is colored. Conserved domains associated with more than one Bacillaceae-1 RNA are assigned a color; other domains are gray. Information about these conserved domains is given in Section 9.3. The accession number of the sequence containing each Bacillaceae-1 RNA is given. Accessions beginning with “NC_” or “NZ_” are RefSeq, while other accession refer to environmental samples. Nucleotide coordinates are given for the 5′ and 3′ boundaries of each. If the 5′ coordinate is greater than the 3′ coordinate, the RNA is present on the reverse-complement strand of the containing genomic DNA sequence. Each hit is denoted by an abbreviation (like “Eco-1-1”) that refers to a taxonomy given in Section 9.1.

**abbrev.**	**Seq. accession**		**5′ at**	**3′ at**	**genes**
Bli-1-1	NC_006270.3	+	1210076	1210143	hypo→ **RNA**→ ←DegV (COG1307)

Bli-1-2	NC_006322.1	+	1209927	1209994	hypo→ **RNA**→ ←DegV (COG1307)
Bli-1-3	NC_006270.3	+	3243766	3243832	←hypo **RNA**→ ←Ndh (COG1252)
Bli-1-4	NC_006322.1	+	3243618	3243684	←YuiA **RNA**→ ←Ndh (COG1252)
Bli-1-5	NC_006270.3	+	800841	800907	MOSC (pfam03473)3-alpha (pfam03475)→ **RNA**→ ←hypo
Bli-1-6	NC_006322.1	+	800647	800713	MOSC (pfam03473)3-alpha (pfam03475)→ **RNA**→ ←YflJ
Bli-1-7	NC_006270.3	+	712173	712240	CypX (COG2124)→ **RNA**→ hypo→
Bli-1-8	NC_006322.1	+	711979	712046	CypX (COG2124)→ **RNA**→ ANTAR (pfam03861)→
Bsu-1-1	NC_000964.2	-	3773345	3773279	hypo→ **RNA**→ FdhD-NarQ (pfam02634)→
Bsu-1-2	NZ_ABQK01000005.1	-	2754870	2754804	**RNA**→
Bsu-3-1	NZ_ABQL01000005.1	-	2754915	2754849	**RNA**→
Bsu-2-1	NZ_ABQM01000009.1	-	598692	598626	**RNA**→
Bsu-4-1	NZ_ABQN01000009.1	-	598702	598636	**RNA**→
Bsu-1-3	NC_000964.2	+	318564	318631	Sugar_tr (pfam00083)→ **RNA**→ ←AraJ (COG2814)
Bsu-1-4	NZ_ABQK01000002.1	+	148540	148607	**RNA**→
Bsu-3-2	NZ_ABQL01000002.1	+	124330	124397	**RNA**→
Bsu-2-2	NZ_ABQM01000002.1	+	148540	148607	**RNA**→
Bsu-4-2	NZ_ABQN01000004.1	+	92219	92286	**RNA**→
Bsu-1-5	NC_000964.2	+	514393	514460	COG3428 (COG3428)→ **RNA**→ ←Rhomboid (pfam01694)
Bsu-1-6	NZ_ABQK01000004.1	+	53417	53484	**RNA**→
Bsu-3-3	NZ_ABQL01000004.1	+	53417	53484	**RNA**→
Bsu-2-3	NZ_ABQM01000006.1	+	22575	22642	**RNA**→
Bsu-4-3	NZ_ABQN01000006.1	+	53419	53486	**RNA**→
Gka-1-1	NC_006510.1	+	32949	33016	tRNA-Ala→ **RNA**→ 23S ribosomal RNA→
Gka-1-2	NC_006510.1	-	2946074	2946006	16S ribosomal RNA→ **RNA**→ 23S ribosomal RNA→
Bpu-2-1	NC_009848.1	+	138237	138304	←Polysacc_deac_1 (pfam01522) **RNA**→ 16S ribosomal RNA→
Bpu-2-2	NC_009848.1	+	905312	905382	hypo→ **RNA**→ 16S ribosomal RNA→
Bpu-2-3	NC_009848.1	-	2738313	2738246	←COG3859 (COG3859) **RNA**→ 16S ribosomal RNA→
Bpu-2-4	NC_009848.1	+	9503	9570	GyrA (COG0188)→ **RNA**→ 16S ribosomal RNA→
Bpu-2-5	NC_009848.1	+	531946	532013	tRNA-Gly→ **RNA**→ 16S ribosomal RNA→
Bpu-1-1	NZ_ABRX01000002.1	-	133	66	←COG3859 (COG3859) **RNA**→
Bpu-1-2	NZ_ABRX01000010.1	-	918	851	conserved protein YgxA→ **RNA**→
Bpu-2-6	NC_009848.1	+	555169	555236	BofA (pfam07441)→ **RNA**→ 16S ribosomal RNA→
Bha-1-1	NC_002570.2	+	561399	561462	16S ribosomal RNA→ **RNA**→ 23S ribosomal RNA→
Bha-1-2	NC_002570.2	+	52868	52931	16S ribosomal RNA→ **RNA**→ 23S ribosomal RNA→
Bha-1-3	NC_002570.2	+	119801	119864	tRNA-Ala→ **RNA**→ 23S ribosomal RNA→
Bha-1-4	NC_002570.2	+	1020202	1020265	16S ribosomal RNA→ **RNA**→ 23S ribosomal RNA→
Bli-1-9	NC_006270.3	+	3080604	3080669	←MetK (COG0192) **RNA**→ PEPCK_ATP (cd00484)PckA (COG1866)→
Bli-1-10	NC_006322.1	+	3080457	3080522	←MetK (COG0192) **RNA**→ PEPCK_ATP (cd00484)PckA (COG1866)→
Bsu-1-7	NC_000964.2	+	35162	35228	ribosomal RNA-23S→ **RNA**→ ribosomal RNA-5S→
Bsu-1-8	NZ_ABQK01000001.1	+	35162	35228	**RNA**→
Bsu-3-4	NZ_ABQL01000001.1	+	35162	35228	**RNA**→
Bsu-2-4	NZ_ABQM01000001.1	+	35162	35228	**RNA**→
Bsu-4-4	NZ_ABQN01000001.1	+	35162	35228	**RNA**→
Bli-1-11	NC_006270.3	+	163014	163080	23S ribosomal RNA→ **RNA**→ 5S ribosomal RNA→
Bli-1-12	NC_006322.1	+	162818	162884	23S ribosomal RNA→ **RNA**→ 5S ribosomal RNA→
Bli-1-13	NC_006270.3	-	3118594	3118528	23S ribosomal RNA→ **RNA**→ 5S ribosomal RNA→
Bli-1-14	NC_006322.1	-	3118447	3118381	23S ribosomal RNA→ **RNA**→ 5S ribosomal RNA→
Bli-1-15	NC_006270.3	+	616707	616773	23S ribosomal RNA→ **RNA**→ 5S ribosomal RNA→
Bli-1-16	NC_006322.1	+	616513	616579	23S ribosomal RNA→ **RNA**→ 5S ribosomal RNA→
Bli-1-17	NC_006270.3	+	14778	14844	23S ribosomal RNA→ **RNA**→ 5S ribosomal RNA→
Bli-1-18	NC_006322.1	+	14582	14648	23S ribosomal RNA→ **RNA**→ 5S ribosomal RNA→
Bli-1-19	NC_006270.3	+	100058	100124	23S ribosomal RNA→ **RNA**→ 5S ribosomal RNA→

Bli-1-20	NC_006322.1	+	99862	99928	23S ribosomal RNA→ **RNA**→ 5S ribosomal RNA→
Bli-1-21	NC_006270.3	+	39477	39543	23S ribosomal RNA→ **RNA**→ 5S ribosomal RNA→
Bli-1-22	NC_006322.1	+	39281	39347	23S ribosomal RNA→ **RNA**→ 5S ribosomal RNA→
Bli-1-23	NC_006270.3	-	2767403	2767336	←MGS1 (COG2256) **RNA**→ **RNA**→ **RNA**→ COG1959 (COG1959)→
Bli-1-25	NC_006270.3	-	2767338	2767272	(shown above)
Bli-1-27	NC_006270.3	-	2767468	2767401	(shown above)
Bli-1-24	NC_006322.1	-	2767256	2767189	←MGS1 (COG2256) **RNA**→ **RNA**→ **RNA**→ COG1959 (COG1959)→
Bli-1-26	NC_006322.1	-	2767191	2767125	(shown above)
Bli-1-28	NC_006322.1	-	2767321	2767254	(shown above)
Bcl-1-1	NC_006582.1	+	1440503	1440568	←hypo **RNA**→ ribosomal RNA-16S→
Oih-1-1	NC_004193.1	-	423489	423422	hypo→ **RNA**→ ←DUF72 (pfam01904)
Oih-1-2	NC_004193.1	-	3404930	3404863	COG3333 (COG3333)→ **RNA**→ G_glu_transpept (pfam01019)→
Oih-1-3	NC_004193.1	+	2452262	2452329	←hypo **RNA**→ ←TlyC (COG1253)
Oih-1-4	NC_004193.1	-	2350186	2350119	23S ribosomal RNA→ **RNA**→ 5S ribosomal RNA→
Oih-1-5	NC_004193.1	+	675233	675300	23S ribosomal RNA→ **RNA**→ 5S ribosomal RNA→
Bam-1-1	NC_009725.1	+	92818	92883	16S ribosomal RNA→ **RNA**→ 23S ribosomal RNA→
Bam-1-2	NC_009725.1	+	98703	98768	16S ribosomal RNA→ **RNA**→ 23S ribosomal RNA→
Bsu-1-9	NC_000964.2	-	634985	634919	←ribosomal RNA-16S **RNA**→ ←tRNA-Gly
Bsu-1-10	NZ_ABQK01000004.1	-	174009	173943	**RNA**→
Bsu-3-5	NZ_ABQL01000004.1	-	174009	173943	**RNA**→
Bsu-2-5	NZ_ABQM01000006.1	-	143167	143101	**RNA**→
Bsu-4-5	NZ_ABQN01000006.1	-	174011	173945	**RNA**→
Bsu-2-6	NZ_ABQM01000009.1	+	3026	3092	**RNA**→
Bsu-1-11	NC_000964.2	+	3177677	3177743	←ribosomal RNA-16S **RNA**→ COG3859 (COG3859)→
Bsu-3-6	NZ_ABQL01000005.1	+	2159247	2159313	**RNA**→
Bsu-1-12	NC_000964.2	-	9813	9747	←ribosomal RNA-16S **RNA**→ ←GyrA (COG0188)DNA_topoisoIV (pfam00521)DNA_gyraseA_C (pfam03989)
Bsu-1-13	NZ_ABQK01000001.1	-	9813	9747	**RNA**→
Bsu-3-7	NZ_ABQL01000001.1	-	9813	9747	**RNA**→
Bsu-2-7	NZ_ABQM01000001.1	-	9813	9747	**RNA**→
Bsu-4-6	NZ_ABQN01000001.1	-	9813	9747	**RNA**→
Bsu-1-14	NZ_ABQK01000005.1	+	2159201	2159267	**RNA**→
Bsu-1-15	NC_000964.2	-	946035	945969	←ribosomal RNA-16S **RNA**→ ←hypo
Bsu-1-16	NZ_ABQK01000004.1	-	485060	484994	**RNA**→
Bsu-3-8	NZ_ABQL01000004.1	-	485060	484994	**RNA**→
Bsu-2-8	NZ_ABQM01000006.1	-	454224	454158	**RNA**→
Bsu-4-7	NZ_ABQN01000006.1	-	485065	484999	**RNA**→
Bsu-4-8	NZ_ABQN01000009.1	+	3033	3099	**RNA**→
Bli-1-29	NC_006270.3	-	920927	920861	←16S ribosomal RNA **RNA**→ ←hypo
Bli-1-30	NC_006322.1	-	920778	920712	←16S ribosomal RNA **RNA**→ ←YgxA
Bli-1-31	NC_006270.3	+	3123307	3123373	←16S ribosomal RNA **RNA**→ ←PurR (COG1609)
Bli-1-32	NC_006322.1	+	3123159	3123225	←16S ribosomal RNA **RNA**→ ←PurR (COG1609)
Bli-1-33	NC_006270.3	-	9902	9836	←16S ribosomal RNA **RNA**→ ←GyrA (COG0188)DNA_topoisoIV (pfam00521)DNA_gyraseA_C (pfam03989)
Bli-1-34	NC_006322.1	-	9706	9640	←16S ribosomal RNA **RNA**→ ←GyrA (COG0188)DNA_topoisoIV (pfam00521)DNA_gyraseA_C (pfam03989)
Bli-1-35	NC_006270.3	-	611992	611926	←16S ribosomal RNA **RNA**→ ←tRNA-Gly
Bli-1-36	NC_006322.1	-	611798	611732	←16S ribosomal RNA **RNA**→ ←tRNA-Gly
Bli-1-37	NC_006270.3	-	34600	34534	←16S ribosomal RNA **RNA**→ ←BofA (pfam07441)
Bli-1-38	NC_006322.1	-	34404	34338	←16S ribosomal RNA **RNA**→ ←BofA (pfam07441)NOG18015 (NOG18015)
Bli-1-39	NC_006270.3	+	925587	925653	23S ribosomal RNA→ **RNA**→ 5S ribosomal RNA→
Bli-1-40	NC_006322.1	+	925438	925504	23S ribosomal RNA→ **RNA**→ 5S ribosomal RNA→
Bsp-1-1	NZ_AAOX01000001.1	-	32371	32305	←COG1971 (COG1971) **RNA**→ ←SUA5 (COG0009)SUA5 (pfam03481)
Bsp-1-2	NZ_AAOX01000004.1	+	108232	108299	←L-lactate dehydrogenase **RNA**→ ←hypo
Bsp-1-3	NZ_AAOX01000018.1	-	15801	15737	hypo→ **RNA**→ ←TrkG (COG0168)

Bsp-1-4	NZ_AAOX01000026.1	+	47929	47995	AmpS (COG2309)→ **RNA**→ ←MurC (COG0773)
Bsp-1-5	NZ_AAOX01000016.1	-	93298	93232	group-specific protein→ **RNA**→ **RNA**→ ←IDI-2_FMN (cd02811)
Bsp-1-35	NZ_AAOX01000016.1	-	93112	93046	(shown above)
Bsp-1-6	NZ_AAOX01000033.1	-	19237	19171	←GlyRS_alpha_core (cd00733) **RNA**→ ←RecO (COG1381)
Bsp-1-7	NZ_AAOX01000015.1	-	33444	33377	←COG5401 (COG5401) **RNA**→ ←RecQ (COG0514)
Bsp-1-8	NZ_AAOX01000019.1	-	82190	82124	←Ala_racemase (cd00430) **RNA**→ ←LolA (COG2834)
Bsp-1-9	NZ_AAOX01000026.1	+	29664	29729	←TyrS (COG0162) **RNA**→ MrcB (COG0744)→
Bsp-1-10	NZ_AAOX01000011.1	-	44591	44525	BetT (COG1292)→ **RNA**→ ←PaaX (COG3327)
Bsp-1-11	NZ_AAOX01000014.1	-	110839	110773	←hypo **RNA**→ ←hypo
Bsp-1-12	NZ_AAOX01000014.1	-	110652	110586	←hypo **RNA**→ ←hypo
Bsp-1-13	NZ_AAOX01000041.1	-	673	607	←hypo **RNA**→ ←hypo
Bsp-1-14	NZ_AAOX01000017.1	+	99694	99760	AcoB (COG0022)→ **RNA**→ ←dihydrolipoamide acetyltransferase
Bsp-1-15	NZ_AAOX01000006.1	-	139439	139373	SplA→ **RNA**→ ←hypo
Bsp-1-16	NZ_AAOX01000027.1	-	25525	25460	hypo→ **RNA**→ ←MutY (COG1194)
Bsp-1-17	NZ_AAOX01000017.1	+	72416	72482	stage III sporulation protein AH→ **RNA**→ ←MA (smart00283)
Bsp-1-18	NZ_AAOX01000009.1	-	76759	76693	DUF520 (pfam04461)→ **RNA**→ ←RimL (COG1670)
Bsp-1-19	NZ_AAOX01000001.1	-	33520	33454	←Wzb (COG0394) **RNA**→ ←hypo
Bsp-1-20	NZ_AAOX01000070.1	+	3409	3475	←BglG (COG3711) **RNA**→ ←COG2252 (COG2252)
Bsp-1-21	NZ_AAOX01000004.1	+	227007	227073	hypo→ **RNA**→ ←VanW (pfam04294)
Bsp-1-22	NZ_AAOX01000016.1	-	19511	19445	←hypo **RNA**→ ←COG4918 (COG4918)
Bsp-1-23	NZ_AAOX01000050.1	-	9503	9437	←COG3881 (COG3881) **RNA**→ ←hypo
Bsp-1-24	NZ_AAOX01000022.1	-	85956	85890	COG1988 (COG1988)→ **RNA**→ ←hypo
Bsp-1-25	NZ_AAOX01000020.1	-	52596	52530	←COG0742 (COG0742) **RNA**→ ←hypo
Bsp-1-26	NZ_AAOX01000014.1	+	92801	92867	RHOD_Lact_B (cd01523)GloB (COG0491)→ **RNA**→ DUF81 (pfam01925)→
Bsp-1-27	NZ_AAOX01000032.1	-	33782	33716	hypo→ **RNA**→ ←COG1511 (COG1511)
Bsp-1-28	NZ_AAOX01000004.1	+	240854	240920	←Lig (COG0272) **RNA**→ ←UvrD (COG0210)
Bsp-1-29	NZ_AAOX01000020.1	-	14455	14389	LdcC (COG1982)→ **RNA**→ hypo→
Bsp-1-30	NZ_AAOX01000016.1	-	10629	10563	OYE_YqiM_FMN (cd02932)→ **RNA**→ ←hypo
Bsp-1-31	NZ_AAOX01000009.1	-	145067	145001	←hypo **RNA**→ ←hypo
Bsp-1-32	NZ_AAOX01000009.1	-	75104	75038	FabG (COG1028)→ **RNA**→ ←PurR (COG1609)
Bsp-1-33	NZ_AAOX01000004.1	+	234474	234540	←PutP (COG0591) **RNA**→ ←Aldedh (pfam00171)
Bsp-1-34	NZ_AAOX01000048.1	+	12	78	**RNA**→ ←Ribosomal_S18 (pfam01084)
Bli-1-41	NC_006270.3	+	58035	58101	SASP (pfam00269)→ **RNA**→ IspE (COG1947)→
Bli-1-42	NC_006322.1	+	57839	57905	SASP (pfam00269)NOG16872 (NOG16872)→ **RNA**→ IspE (COG1947)→
Bli-1-43	NC_006270.3	+	176604	176667	tRNA-Glu→ **RNA**→ tRNA-Val→
Bli-1-44	NC_006322.1	+	176409	176472	tRNA-Glu→ **RNA**→ tRNA-Val→
Bli-1-45	NC_006270.3	+	926189	926254	tRNA-Glu→ **RNA**→ tRNA-Val→
Bli-1-46	NC_006322.1	+	926040	926105	tRNA-Glu→ **RNA**→ tRNA-Val→
Bco-1-1	NZ_ABFU01000013.2	+	842	907	16S ribosomal RNA→ **RNA**→ 23S ribosomal RNA→
Bco-1-2	NZ_ABFU01000131.1	+	643	708	**RNA**→ tRNA-Ile→
Bco-1-3	NZ_ABFU01000134.1	-	1560	1495	16S ribosomal RNA→ **RNA**→ tRNA-Ile→
Bco-1-4	NZ_ABFU01000133.1	+	2038	2104	tRNA-Gly→ **RNA**→ 16S ribosomal RNA→
Bco-1-5	NZ_ABFU01000135.1	+	2040	2106	tRNA-Ala→ **RNA**→ 16S ribosomal RNA→
Bco-1-6	NZ_ABFU01000002.2	-	740	674	hypo→ **RNA**→ 16S ribosomal RNA→

9.3 Conserved domains

Conserved domains found in protein-coding genes listed in Section 9.2 are listed, with the first sentence in their description from the Conserved Domain Database. Conserved domains associated with more than one Bacillaceae-1 RNA are assigned a color, while others are shown in gray. The number in parentheses is the number of occurrences of the given gene.

u cd00430 (1) Alanine racemase.
D cd00484 (2) Phosphoenolpyruvate carboxykinase (PEPCK), a critical gluconeogenic enzyme, catalyzes the first committed step in the diversion of tricarboxylic acid cycle intermediates toward gluconeogenesis.
u cd00733 (1) Class II Glycyl-tRNA synthetase (GlyRS) alpha subunit core catalytic domain.
u cd01523 (1) Member of the Rhodanese Homology Domain superfamily.
d cd02811 (1) Isopentenyl Diphosphate:dimethylallyl diphosphate isomerase type 2 (IDI-2) FMN-binding domain.
u cd02932 (1) Old yellow enzyme (OYE) YqjM-like FMN binding domain.
d COG0009 (1) Putative translation factor (SUA5) [Translation, ribosomal structure and biogenesis]
u COG0022 (1) Pyruvate/2-oxoglutarate dehydrogenase complex, dehydrogenase (E1) component, eukaryotic type, beta subunit [Energy production and conversion]
u COG0162 (1) Tyrosyl-tRNA synthetase [Translation, ribosomal structure and biogenesis]
d COG0168 (1) Trk-type K+ transport systems, membrane components [Inorganic ion transport and metabolism]
u d COG0188 (4) Type IIA topoisomerase (DNA gyrase/topo II, topoisomerase IV), A subunit [DNA replication, recombination, and repair]
u COG0192 (2) S-adenosylmethionine synthetase [Coenzyme metabolism]
d COG0210 (1) Superfamily I DNA and RNA helicases [DNA replication, recombination, and repair]
u COG0272 (1) NAD-dependent DNA ligase (contains BRCT domain type II) [DNA replication, recombination, and repair]
u COG0394 (1) Protein-tyrosine-phosphatase [Signal transduction mechanisms]
u COG0491 (1) Zn-dependent hydrolases, including glyoxylases [General function prediction only]
d COG0514 (1) Superfamily II DNA helicase [DNA replication, recombination, and repair]
u COG0591 (1) Na+/proline symporter [Amino acid transport and metabolism / General function prediction only]
u COG0742 (1) N6-adenine-specific methylase [DNA replication, recombination, and repair]
D COG0744 (1) Membrane carboxypeptidase (penicillin-binding protein) [Cell envelope biogenesis, outer membrane]
d COG0773 (1) UDP-N-acetylmuramate-alanine ligase [Cell envelope biogenesis, outer membrane]
u COG1028 (1) Dehydrogenases with different specificities (related to short-chain alcohol dehydrogenases) [Secondary metabolites biosynthesis, transport, and catabolism / General function prediction only]
d COG1194 (1) A/G-specific DNA glycosylase [DNA replication, recombination, and repair]
d COG1252 (2) NADH dehydrogenase, FAD-containing subunit [Energy production and conversion]
d COG1253 (1) Hemolysins and related proteins containing CBS domains [General function prediction only]
u COG1292 (1) Choline-glycine betaine transporter [Cell envelope biogenesis, outer membrane]
d COG1307 (2) Uncharacterized protein conserved in bacteria [Function unknown]
d COG1381 (1) Recombinational DNA repair protein (RecF pathway) [DNA replication, recombination, and repair]
d COG1511 (1) Predicted membrane protein [Function unknown]
d COG1609 (3) Transcriptional regulators [Transcription]
d COG1670 (1) Acetyltransferases, including N-acetylases of ribosomal proteins [Translation, ribosomal structure and biogenesis]
D COG1866 (2) Phosphoenolpyruvate carboxykinase (ATP) [Energy production and conversion]
D COG1947 (2) 4-diphosphocytidyl-2C-methyl-D-erythritol 2-phosphate synthase [Lipid metabolism]
D COG1959 (2) Predicted transcriptional regulator [Transcription]
u COG1971 (1) Predicted membrane protein [Function unknown]
u COG1982 (1) Arginine/lysine/ornithine decarboxylases [Amino acid transport and metabolism]
u COG1988 (1) Predicted membrane-bound metal-dependent hydrolases [General function prediction only]
u COG2124 (2) Cytochrome P450 [Secondary metabolites biosynthesis, transport, and catabolism]
d COG2252 (1) Permeases [General function prediction only]
u COG2256 (2) ATPase related to the helicase subunit of the Holliday junction resolvase [DNA replication, recombination, and repair]
u COG2309 (1) Leucyl aminopeptidase (aminopeptidase T) [Amino acid transport and metabolism]
d COG2814 (1) Arabinose efflux permease [Carbohydrate transport and metabolism]
d COG2834 (1) Outer membrane lipoprotein-sorting protein [Cell envelope biogenesis, outer membrane]
d COG3327 (1) Phenylacetic acid-responsive transcriptional repressor [Transcription]
u COG3333 (1) Uncharacterized protein conserved in bacteria [Function unknown]
u COG3428 (1) Predicted membrane protein [Function unknown]
u COG3711 (1) Transcriptional antiterminator [Transcription]
uD COG3859 (3) Predicted membrane protein [Function unknown]
u COG3881 (1) Uncharacterized protein conserved in bacteria [Function unknown]
d COG4918 (1) Uncharacterized protein conserved in bacteria [Function unknown]
u COG5401 (1) Spore germination protein [General function prediction only]
u NOG16872 (1)
d NOG18015 (1)
u pfam00083 (1) Sugar (and other) transporter.
d pfam00171 (1) Aldehyde dehydrogenase family.
u pfam00269 (2) Small, acid-soluble spore proteins, alpha/beta type.
d pfam00521 (3) DNA gyrase/topoisomerase IV, subunit A.
D pfam01019 (1) Gamma-glutamyltranspeptidase.
d pfam01084 (1) Ribosomal protein S18.
u pfam01522 (1) Polysaccharide deacetylase.
d pfam01694 (1) Rhomboid family.
d pfam01904 (1) Protein of unknown function DUF72.
D pfam01925 (1) Domain of unknown function DUF81.
D pfam02634 (1) FdhD/NarQ family.
u pfam03473 (2) MOSC domain.
u pfam03475 (2) 3-alpha domain.
d pfam03481 (1) SUA5 domain.
D pfam03861 (1) ANTAR domain.
d pfam03989 (3) DNA gyrase C-terminal domain, beta-propeller.
d pfam04294 (1) VanW like protein.
u pfam04461 (1) Protein of unknown function (DUF520).
u d pfam07441 (3) SigmaK-factor processing regulatory protein BofA.
d smart00283 (1) Methyl-accepting chemotaxis-like domains (chemotaxis sensory transducer).

Duplicate sequences: the following putative homologs are not shown in the alignment because their sequences are identical to a homolog already shown: Bam-1-2, Bha-1-3, Bha-1-4, Bli-1-10, Bli-1-12, Bli-1-14, Bli-1-16, Bli-1-18, Bli-1-2, Bli-1-20, Bli-1-22, Bli-1-24, Bli-1-26, Bli-1-28, Bli-1-30, Bli-1-32, Bli-1-34, Bli-1-36, Bli-1-38, Bli-1-4, Bli-1-40, Bli-1-42, Bli-1-44, Bli-1-46, Bli-1-6, Bli-1-8, Bsu-1-10, Bsu-1-13, Bsu-1-16, Bsu-1-2, Bsu-1-4, Bsu-1-6, Bsu-1-8, Bsu-2-1, Bsu-2-2, Bsu-2-3, Bsu-2-4, Bsu-2-5, Bsu-2-7, Bsu-2-8, Bsu-3-1, Bsu-3-2, Bsu-3-3, Bsu-3-4, Bsu-3-5, Bsu-3-7, Bsu-3-8, Bsu-4-1, Bsu-4-2, Bsu-4-4, Bsu-4-5, Bsu-4-6, Bsu-4-7

9.4 Multiple-sequence alignment

Each Bacillaceae-1 RNA is denoted by an abbreviation (like "Eco-1-1") that refers to a taxonomy given in Section 9.1. The alignment includes sequences containing the RNA motif, as well as flanking sequence. The Bacillaceae-1 RNA itself is denoted by the line underneath marked 5′ and 3′ on either end. Nucleotides in the flanking sequences (i.e., not part of the motif) are written in gray letters. Stems of predicted rho-independent transcription terminators are shaded yellow. (Note: terminator predictions have not been analyzed manually, and many are likely to be false positives.) Start codons are shaded green. (Note: start codons are frequently misannotated, especially in environmental samples.) Nucleotides proposed to basepair as part of the consensus structure are shaded in color when they comprise Watson-Crick or G-U pairs. Otherwise they are shaded gray. Conserved stems are also indicated at the bottom of the alignment by angle brackets, where matching < and > denote base-paired columns. Below these angle brackets, the symbol "2" denotes base pairs exhibiting covariation, "1" denotes base pairs exhibiting compatible mutations, "0" denotes base pairs that are not observed to mutate and "?" denotes base pairs that have a significant frequency of non-canonical nucleotides for Watson-Crick or G-U pairs (> 5%). Below these base pair annotation is the consensus sequence: "R" = "A" or "G", "Y" = "C" or "U", red nucleotides: nucleotide identity conserved more than 97% of the time, black nucleotides: 90%, gray nucleotides: 75%, red circle (•): nucleotide is present 97% of the time, black circle (•): 90%, gray circle (•): 75%, white circle (•): 50%. All percentages of sequences just described (e.g. 97% conserved) assume that sequences have been weighted by the GSC algorithm implemented by the Infernal software.

alignment positions 1 · · · 99

```
Bli-1-1    AGACAGCUGACGCUGAGGCCUGCUUUUAAAAAGCAGGCCUCAGCGUGUCGACAAACCCUCGCAUUCGUUGUCAGGCCUGCUAAGUCGGUGCUCACGAAU
Bli-1-3    AGUUUUUAGCCUGAAAUAAAAAGCCUGCAGAGGAAGCUGCAGGCUUCAGCGUGUCGACAAACCCUCGUUGUCAGACCUGCUAAGUCGGUGCUCAUGAAU
Bli-1-5    CGCAUAAAAACAAGCCGUGUCUGAAGAGGAACACGGCUUUCAGCGUGUCGACAAACCCUCGCAUUCGUUGUCAGGCCUGCUAAGUCGGUGCUCACGAAU
Bli-1-7    CGGCGCCUAACUAAUUAGAAAAGCACCGAAAAGGUGCUUUCAGCGUGUCGACAAACCCUCGCAUUCGUUGUCAGGCCUGCUAAGUCGGUGCUCACGAAU
Bsu-1-1    AGAAUCAGCAUAAUCAAACAUAGAGUCCUUUAUACAGAGGUGUCUCAGCGUGUCGACAAACCCCGCGUUCGUUGUCAAUGCACAUUGGUGCUCACGUGU
Bsu-1-3    AACUGAGCUAGAAUAGGAAAAGCACCUCUUAAAAGAGGUGCUUUCAGCGUGUCGACAAACCCUCGCAUUCGUUGUCAGGACUGCGCUUCGGUGCUCAAU
Bsu-1-5    AAGUGGUACUCAUAUGAAAAAGCGGACGGUUAAUACCGCCGCUCCAGCGUGUCGGCAAACCCUCGCAUUCGUUGUCAGGACUGCGCUUCGGUGCUCAAU
Bsu-4-3    AAGUGGUACUCAUAUGAAAAAGCGGACGGUUAAUACCGCCGCUCCAGCGUGUCGGCAAACCCUCGCAUUCGUUGUCAGGACUGCGCUUCGGUGCUCAAU
Gka-1-1    UAGCAGAUUCGAGGAAGUGGGAUUCCUCGAUGCAAGACUGCAGAGAAGCAAAUUCAAGAAGCAGUCUCGUUCCUUGAAAACUAGAUAACCGGAAAAGCG
Gka-1-2    AGCCGCCGAAGGUGGGGCAAGUGAUUGGGGUGAAGUCGUAACAAGGUAGCCGUACCGGAAGGUGCGGCUGGAUCACCUCCUUUCUAAGGAGAAAAGCGG
Bpu-2-1    AUCUUUGAAAACUAAACAAGACAAAACGUACCUGUUAAUUCGAGUUUUUAUAAAAAAAUCCUAUGAUGUUUCAUAGGUAGUCAGUCAAACGCUGACGAGG
Bpu-2-2    AUCUUUGAAAACUAAACAAGACAAAACGUACCUGUUAAUUCGAGUUUUUAUAAAAAAAUCCUAUGAUGUUUCAUAGGUAGUCAGUCAAACGCUGACGAGG
Bpu-2-3    UCUUUGAAAACUAAACAAGACAAAACGUACCUGUUAAUUCGAGUUUUUAUAAAAAAAAUCCUAUGAUGUUUCAUAGGUAGUCAGUCAAACACUGACGAGG
Bpu-2-4    UUCUUUGAAAACUAAACAAGACAAUACGUACCUGUUAAUUCGAGUUUUUAUAAUAAAUCCUAUGAUGUUUCAUAGGUAGUCAGUCAAACGCUGACGAGG
Bpu-2-5    UCUUUGAAAACUAAACAAGACAAAACGUACCUGUUAAUUCGAGUUUUUAUAAAAAAAAUCCUAUGAUGUUUCAUAGGUAGUCAGUCAAACGCUGACGAGG
Bpu-1-1    UCUUUGAAAACUAAACAAGACAAAACGUACCUGUUAAUUCGAGUUUUUAUAAAAAAAAUCCUAUGAAACAUCAUAGGUAGUCAGUCAAACGCUGACGAGG
Bpu-1-2    UCUUUGAAAACUAAACAAGACAAAACGUACCUGUUAAUUCGAGUUUUUAUAAAAAAAAUCCUAUGAAACAUCAUAGGUAGUCAGUCAAACGCUGACGAGG
Bpu-2-6    UUCUUUGAAAACUAAACAAGACAAAACGUACCUGUUAAUUCGAGUUUUUAUAAAAAAAUCCUAUGAUGUUUCAUAGGUAGUCAGUCAAACGCUGACGAGG
Bha-1-1    ACUCACGAACGAGACGUCAAGAAUUCCAUGACGCUUUUGAUGAAUAACAAAGAGUCUCAAGUAGAUCGAGGAAGCAAGGGUUUGAGGAAUGGAGCGUAU
Bha-1-2    ACUCACGAACGAGACGUCAAGAAUUCCAUGACGCUUUUGAUGAAUAACAAAGAGUCUCAAGUAGAUCGAGGAAGCAAGGGUUUGAGGAAUGGAGCGUAU
Bli-1-9    UGACGAUACUCGGGCUUCAUACGCUCUCGUUUUGAAACAAUUUCCAUUAUGCUAUUCUUGACGAGAUUCUGAGUCGAAAUUCCCCUUAGCAGAAUUUUU
Bsu-1-7    CAUGGAGCUGACAGAUACUAAUCGAUCGAGGACUUAACCACAUUUUGAAUGAUGUCACACCUGUUAUCUAGUUUUGAGAGAACACCUCUCUAAAGGCGG
Bli-1-11   AUGGAGCUGACAGAUACUAAUCGAUCGAGGACUUAACCUAAAUAUUUGUGAAUCGUCUGUCCGUUAUCUAGUUUUGAGAGAACACCUCUCAGAAAGCCA
Bli-1-13   CAUGGAGCUGACAGAUACUAAUCGAUCGAGGACUUAACCUAAAUAUUUGUGAAUCGUCUGUCCGUUAUCUAGUUUUGAGAGAAUGCUCUCAGAAAGCCA
Bli-1-15   AUGGAGCUGACAGAUACUAAUCGAUCGAGGACUUAACCUAAAUAUUUGUGAAUCGUCUGUCCGUUAUCUAGUUUUGAGAGAACACCUCUCAGAAAGCCA
Bli-1-17   CAUGGAGCUGACAGAUACUAAUCGAUCGAGGACUUAACCUAAAUAUUUGUGAAUCGUCUGUCCGUUAUCUAGUUUUGAGAGAAUGCUCUCAGAAAGCCA
Bli-1-19   AUGGAGCUGACAGAUACUAAUCGAUCGAGGACUUAACCUAAAUAUUUGUGAAUCGUCUGUCCGUUAUCUAGUUUUGAGAGAACACCUCUCAGAAAGCCA
Bli-1-21   AUGGAGCUGACAGAUACUAAUCGAUCGAGGACUUAACCUAAAUAUUUGUGAAUCGUCUGUCCGUUAUCUAGUUUUGAGAGAACACCUCUCAGAAAGCCA
Bli-1-23   UUUGCUGUGAGGAACGCCGAGCACCUUUGUUCUGGUGUGAAAAGGACUCGGCUAAGGGCUUUCACAUCCUGUGAAAAAAGCCGAGUACCUUCGUCCUGG

           ...................................................................................................
           ...................................................................................................
           ●●●●●●●●●●●●●●●●●●●●●●●●●●●●●●●●●●●●●●●●●●●●●●●●●●●●●●●●●●●●●●●●●●●●●●●●●●●●●●●●●●●●●●●●●●●●●●●●
```

```
Bli-1-25   AUCCUGUGAAAAAAGCCGAGUACCUUCGUCCUGGUGUGAAAGGAGACUCGGCUAAGGUCUUUCGCGUCCUGCGAAAAACGCCGGUCACCUUCGUCCUGG
Bli-1-27   AUGCCUUAUGAGGAUGCACCGCGGCGCUUUCUGCGCCUGAACGGGACUCGGCUUAAGGGCUUUUGUUUGCUGUGAGGAACGCCGAGCACCUUUGUUCUG
Bcl-1-1    UCGCAACCGAGUCCUUUGAAAACUGAACAAAAGCCAAGCGUAAAAGAGAUACAAGGUAUCUCGUCAAUCAUAGUGAGCCACAACUUCGGUUGUGCAAUG
Oih-1-1    CGGAAGCAAUCAAAAUAUAUCGUAAAAGUGUUCAAGGUGGCGCUGCUCCCCGUACCGAAACGUAUGUAGUUAAUACGUGAGGAACUAAACAACAAAGCC
Oih-1-2    AUCGAUGGGAUAUGGGAUCUAUAGUAAUAUGAAGAUAAGUAAGAAAGUGAAAGAAUAAUAGAAAAGGUUGUUCAAAAAGUGUGGUGAAAAUGACACAUA
Oih-1-3    UACAUAUUUUUAAUCACUGCAUCAAAGGUAAAACAAAAAAGCUUCGGAGUAGAAAAAUUCUACACACGAAGCUUUAAAUUCUUUUAGUACUAGCUGUUC
Oih-1-4    CGCAAGGAAGAGAAGGAAGUCUGAUAGUUGAUAGAGACUGGAGCUAGAAUAUAUUGAUGUCACGUUACUCUUAUUUAGUUUUUAGGGUACAAUCCUAAA
Oih-1-5    CACGUGCAGCUGACGGAUACUAAUUGAUCGAGGACUUAUCUAAGUUCUUAAUAUUGAUGUCACGUUACUCUUAUUUAGUUUUUAGGGUACAAUCCUAAA
Bam-1-1    GAUCACCUCCUUUCUAAGGAUUUUAACGGAAUAUAAGACCUUGGGUCUUAUAAACAGAACGUUCCCUGUCUUGUUUAGUUUUGAAGGAUCAUUCCUUCA
Bsu-1-9    CCGCCGCUAACAUCAGGGAGCAAGCUCCCAUCUGUUCGCUCGACUUGCAUGUAUUAGGCACGCCGCCAGCGUUCGUCCUGAGCCAGGAUCAAACUCUCC
Bsu-2-6    GUCCGCCGCUAACAUGGGAGCAAGCUCCCACCUGUCCGCUCGACUUGCAUGUAUUAGGCACGCCGCCAGCGUUCGUCCUGAGCCAGGAUCAAACUCUCC
Bsu-1-11   GUCCGCCGCUAACAUCGGAGCAAGCUCCCACCUGUCCGCUCGACUUGCAUGUAUUAGGCACGCCGCCAGCGUUCGUCCUGAGCCAGGAUCAAACUCUCC
Bsu-3-6    GUCCGCCGCUAACAAUGGAGCAAGCUCCCACCUGUCCGCUCGACUUGCAUGUAUUAGGCACGCCGCCAGCGUUCGUCCUGAGCCAGGAUCAAACUCUCC
Bsu-1-12   CCGCCGCUAACAUCAGGGAGCAAGCUCCCAUCUGUCCGCUCGACUUGCAUGUAUUAGGCACGCCGCCAGCGUUCGUCCUGAGCCAGGAUCAAACUCUCC
Bsu-1-14   GUCCGCCGCUAACAUCGGAGCAAGCUCCCACCUGUCCGCUCGACUUGCAUGUAUUAGGCACGCCGCCAGCGUUCGUCCUGAGCCAGGAUCAAACUCUCC
Bsu-1-15   CCGCCGCUAACAUCAGGGAGCAAGCUCCCAUCUGUCCGCUCGACUUGCAUGUAUUAGGCACGCCGCCAGCGUUCGUCCUGAGCCAGGAUCAAACUCUCC
Bsu-4-8    GUCCGCCGCUAACAUCGGAGCAAGCUCCCACCUGUCCGCUCGACUUGCAUGUAUUAGGCACGCCGCCAGCGUUCGUCCUGAGCCAGGAUCAAACUCUCC
Bli-1-29   CCGCUGACCUAAGGGAGCAAGCUCCCGUCGGUCCGCUCGACUUGCAUGUAUUAGGCACGCCGCCAGCGUUCGUCCUGAGCCAGGAUCAAACUCUCCAUG
Bli-1-31   CCGCUGACCUAAGGGAGCAAGCUCCCGUCGGUCCGCUCGACUUGCAUGUAUUAGGCACGCCGCCAGCGUUCGUCCUGAGCCAGGAUCAAACUCUCCAUG
Bli-1-33   CCGCUGACCUAAGGGAGCAAGCUCCCGUCGGUCCGCUCGACUUGCAUGUAUUAGGCACGCCGCCAGCGUUCGUCCUGAGCCAGGAUCAAACUCUCCAUG
Bli-1-35   CCGCUGACCUAAGGGAGCAAGCUCCCGUCGGUCCGCUCGACUUGCAUGUAUUAGGCACGCCGCCAGCGUUCGUCCUGAGCCAGGAUCAAACUCUCCAUG
Bli-1-37   CCGCUGACCUAAGGGAGCAAGCUCCCAUCGGUCCGCUCGACUUGCAUGUAUUAGGCACGCCGCCAGCGUUCGUCCUGAGCCAGGAUCAAACUCUCCAUG
Bli-1-39   UAAGAUCCCUGAAAGAUGAUCAGGUUGAUAGGUCUGAGGUGGAAGUGUGGCGACACAUGGAGCUGACAGAUACUAAUCGAUCGAGGACUUAACCUAAUA
Bsp-1-1    CCCCAAAAAACGAAGUCAUCCAUUCUCCUCCUUGGACUUGCUAAUUCAGCAUAUGCACGUCCAAGGGGAAAUAGAAUUAGAAAAGCGGAGGCGACUGUU
Bsp-1-2    UCAUUUCCCUCCUUAAGUUUCUAUUUUACUUAGUGUACAUUUCUAUAGCAGCGGCUGAUCUCCUUCCUGCAGACGGCCUUUUUACAAAGAAAAGCGCAA
Bsp-1-3    UCCUCGAAAAAAAACAAAAACCCCAAAGCUCCCCCUUCAUGCACCCAGAAAAAGACGUGAGCAGGGGAACGGAUUAAACGAAGUGCGGAAGCGGCUUGC
Bsp-1-4    CCUGAUCCGGAAGGAUGGAAGAUUCGUCAUCCCUGAACUCGAUGCGCUGAACCCUGAAAAUCUUAAAUAAACAGGAAUAAAAGCGCAAGCUCCUUGAUC
Bsp-1-5    CUCCUGGAUAAAAGGCGUUCUUUGCCUUUAAUCCAGGAGUGGCUUAUGACUCGGGCUAGCGCCCGGAACUGGACAAGUAGAAAAGCGGAGGCGACUGUU
Bsp-1-6    AGAGCGGAAGGUUGUUCUUUAACCUUCUGAUCUGAUUGGCUUAAGACUCGAGGGGCUAACGCCUGCAGCUGGACAAUAUCGAAAAGCGGAAGGCGUUUG
Bsp-1-7    AAUAAAAGGCGUUCUUUGCCUUUAAUUUCUAAGUGGCUUAUGACUCGAGGGGCUAGCGCCCGGAACUGGACAAAACUGAAAUGUGGAGGCGGCUGUUCA
Bsp-1-8    AGAAUGAAAGGCGUUCUUUGCCUUUCAUUCUUGGGUGGCUUAUGACUCGAGGCUCUAGCCGCCGGAGCUGGACAAUAAGAAAUGCGGAGGGCGCUUGCU
Bsp-1-9    GACGGAAGGUUGUUCUUUAACCUUCUGGCCUGAUUGGCUUAUGACUCGAGGGGCUAGGCUUUGGAGCUGGACAAUUUGAAAUGCGGAGGCGACUGUUUA
Bsp-1-10   CCAUAUCCCUUAUCAAAGCAUUGUAUAAGGAAUCCAAAGAACUGGGGAUCGGUCAGAUAAAAAGACGGAAGCAGUAGAAAGGCGGAAGGCGCUUGCCCA
Bsp-1-11   GAUAGAAGGCGUUCUUUGCCUUCAAUCCUGAAGUGGCUUAUGACUCGAGGGGCUAGCGCCCGGAGCUGGACAAAUCGAAAUGCGGAGGCGGCUUGUUCA
Bsp-1-12   ACGGAAGGUUGUCCUUUAACCUUCUGGCCUGAUUGGCUUAUGACUCGAGGCUCUAGCCGCCGGAGCUGGACAAAUCGAAAUGCGGAGGCGGCUUGCUCA
Bsp-1-13   GAAAGUAUCCGUAUAAAACCAAACUCACUAUUUCAUAAAUAUGUGGGAAAUGCUGUAUUAUGCCUGAUGGGGGAAAGAAAAGCGGAGGGCGCUUGUUCA
Bsp-1-14   GACGGAAGGUUGUUCUUUAACCUUCUGGCCUGAAUGGCUUAUGACUCGAGCACCUAGGAGCCGGAGCUGGACAGUAAGAAAAGCGGAGGCGACUGUUCA
Bsp-1-15   AGGAUAAAAGGCGUUCUUUGCCUUUAAUCCUUGCGUGGCUUAUGACUCGAGGCUCUAGCCGCCGGAGCUGGACAAAUAGAAAAGCGGAAGCGGCUGUUC
Bsp-1-16   AGAAGAAAUCGAAAACCGCGGCGGCCUUAAAAGGCAGGAAGCUGACUAUACACAUGGGACACGGGUCAGUUACGAGUAGACAUGCGGAAAAGCCUGGUU
Bsp-1-17   CAACGAAAUCAUCCAAAUGGUAAAAGGCGAAAUCCCGCAGCUGCAGGUAGCAUCAGUAGAAUUUGAACCUGGGAAAUAGAAAAGCGGAGGGCGCUUGCU
Bsp-1-18   GGACGACCUUCAGCAGGUAAUAGCUGCUGUAAGAGAAGCCGACCUGACAGUCGAUGUGCAGUUUAUAAAUUACAGAUAGAAAAGCGGAAAAGCCUUGCU
Bsp-1-19   GGAUAAAAGGCGUUCUUUGCCUUCAAUCCUAAAGUGGCUUAUGACUCGAAGGGCUAGCACCCGGAGCUGGACAACACGAAGUGCGGAAGGGGCUUGCUC
Bsp-1-20   GUUUUCAUAUCCUCUUCCUCUUUUCAGUCUCCAUCAAGUAGUUUCAUGUUAUAAAAGACAAAAUCGGAAGUCAAUAAAAAGAAAAGCGGAGGCGGCUGU
Bsp-1-21   GCUCUUGGCAUGACCGCCGUUAUUAUUGAAGGCCCCGGACUUGAGCCUUCUUCGCAGCUUCAGCCUCUCUGAAGAACAAAAGCGCAAGCGCCUUGAUCA
Bsp-1-22   CUAUGGUUCUCUAGCCUUAUUCGGCUUUUUUGAAACCUGAAUUGAUAAAAAAUUCCAUUUUGAGUUUUGAGCCGGAACAAAAGCGCAAGCGCCUUGAUC
Bsp-1-23   CCACCUUCCCGAACAUUAUUAUUGUCUGCCCCCGCCCUUGAAUUAUGAGGCUAAAAAUUUACCUUAACUAACAGGAACAAAAGUGUAAGCGCCUUGAUC
Bsp-1-24   UUACGAUCCCCGAUUCUACAUCAAUGGCCAAUCCUUCUUAUUCGAAGCGAUAAAAAAAGAAGCAGGGCAAUAAAGAACAAAAGCGCAAGCGCCUUGAUC
Bsp-1-25   CUUGGCUUAUGACUCGAGGGGGUAGGCGCUGGAGCUGGACAGUGACCGAAGUAGAGAAAUACCACAUUCUUCUUUGCAGAAAAGCGGAAGCGCCUUGCU
Bsp-1-26   UAUGGGCAGAAUCACUCCUGAUGAAGAAGAGCAGCGUGAAAUGGAAAUAGGUCCAAACCGCUGUGCUGUCAGGUAAUUAGAAAAGCGCAAGCGCCUUGC
Bsp-1-27   GAGCAGCACGAAUGCUAUGGGACUAAAUGUGAUAAGUGUCUUGACUUGAACUGACUGGAAUUCUAUAAAAUUAUAAGAAAAGCGCAAGCGCCUUGCUCA
Bsp-1-28   AUUGAUUCAGCAGAUUAUGCAGUUCUUUUACUCUUGCUUCGGCAUUUUGAAGGUCCAUAUUUUCCAGCCCUUUCAUUAGAAAAGCGCAAGCGCCUUGCU
Bsp-1-29   GGGACCUGAGGACUACGAUUUCAAAUUCCUGCGUGUGAUUAAAGAGCAUAAAGCGAUCAGGUAAGAAUAUGGAACAAAAGCGCAAGCGCCUUGAUCCAC
Bsp-1-30   AGGGCGGAAGGUUGUUCUUUAACCUUCUGGCCUGAUUGGCUUAUGACUCGAGGGGCUAGCGCCCGGAGCUGGACACUAAGAAAAGCGGAGGCGACUGUU
Bsp-1-31   ACCUUCUUUGCAUAAGUCAGGCAAGUCAAACACUUGGCGUUCGCCGGCUUUGACCGGCGGACCGCUUUGUUUUUGUAUUGAAAUGCGGAGGCGGCUUGC
Bsp-1-32   UAUACCUCGCAAGCGACGACUCCUCCUACGUAUCCGGCCAAAUGCUUCAUAUUAAUGGCGGGAAGGUUGUUAAUGGUUAGAAAAGCGGAAGGCGCUUGC
Bsp-1-33   GAGGGUAAAACUAAUAAAAUUAUACACAAUUAAUUUAAUAGUAUAAAUAUUAAGAUAAAAACAUGACUAUCCUACUAGAAGAGCGCAAGCGCCUUGAUC
Bsp-1-34   ...................................................................................GGAGGAUCCU
Bsp-1-35   AGGACGGAAGGUUGUGCUUUAACCUUCUGGCCUGCUUGGCUUAUGACUCGAGCACCUAGGAGCCGGAGCUGGACAAAUAGAAGAGCGGUGGCGACUGUU
Bli-1-41   GCGACCUGGGAAUCAUGGCGAGGGGCGCAUUCGCAAUCUGAAGGAAUGACCGCAUGCCAAAGGCGAGGCAUCCUGCGCAGGCGCAUGCAUUUGUACUUG
Bli-1-43   AUCCCGUACGGGUCACCAUUCUUUUUUGGACGGCACGCUGGGAAAAGGAAGGCAUGCUAAAGGGAGUAACAUUCUGUUGCAACGCGCACUUGUACUUCA
Bli-1-45   CACCGCCCUUUCACGGCGGUAACACGGGUUCGAAUCCCGUACGGGUCACCAUUCUUUUUUGGACAGCACGCUGAAAAGUAUCAGGUUUACACCAAAGCU
Bco-1-1    GACAGAUGAUUGGGGUGAAGUCGUAACAAGGUAGCCGUAUCGGAAGGUGCGGCUGGAUCACCUCCUUUCUAAGGAAUAUUCAUACAUCUACGAUGUAUG

           .............................................................................................
           .............................................................................................
           .............................................................................................
```

```
Bco-1-2    GACAGAUGAUUGGGGUGAAGUCGUAACAAGGUAGCCGUAUCGGAAGGUGCGGCUGGAUCACCUCCUUUCUAAGGAAUAUUCAUACAUCUACGAUGUAUG
Bco-1-3    GACAGAUGAUUGGGGUGAAGUCGUAACAAGGUAGCCGUAUCGGAAGGUGCGGCUGGAUCACCUCCUUUCUAAGGAAUAUUCAUACAUCUACGAUGUAUG
Bco-1-4    GCUCCAAUUAAUGCUUGAUUGAACUUUGAAAACUGAAAAGACAACACGUCAACGUUAAAAUUAGUACCCAUGAGGUACAAAACUUAUUUAUACUUCGGU
Bco-1-5    UCGCAUUUUUGAGGCGAUGAUUGAACUUUGAAAACUGAAAAGAUAACACGUCAACGUUAAAACUAGUACCCAUGAGGUACAAAACAAUUUACACUUCGG
Bco-1-6    GCUCAAGGGCGACGGACAUUGAACUUUGAAAACUGAAAAGACAACACGUCAACGUUAAAAUUAGUACCCUUGAGGUACAAAAACAAUUUACACUUCGGU
           ...................................................................................................
           ...................................................................................................
           •••••••••••••••••••••••••••••••••••••••••••••••••••••••••••••••••••••••••••••••••••••••••••••••••••
```

alignment positions 100 · · · 259

```
Bli-1-1    AGGAGAAGG.AUCGGCG.GCUAAUCGCC.CAGGCGUCCUGCCUG...AACGCCGCCGU.CGUCACUUCCUGUGAAAGUCCGCUCCGAUGCUCGGCCUUCCUAGACUGAAAGGGUUUUGGGUCACGCUGAAAGAAGGAUGACAAAAUCCCAAAACUAAGAG
Bli-1-3    AGAGGAAGG.AUCGGCG.GCUAACCGCC.CAGGCAUCCUGCCUG...AACGCCGCCGUCA.UCACCUUCUGUGA.AAGUCUGAUCAGAAAAAUUUAAACUUGCCUUUUUCAGAACAAGUCCCGGCCCGCCGAUCAUGAAAAGCGAGCGGUUGUCGAUGA
Bli-1-5    AGAGGAAGG.AUCGGCG.GCUAA.CCGC.CAGGCGUCCUGCCUG...AACGCCGCCGU.AAUCACCUCCUGUGAAAGUCUGCUUCGAUGCUCGGCCUUCCUAGACUGCAAGGGUUGCGGGUCACGCUGAGCUUAAAGGAUGUUUAUUUUUGACGCUUUC
Bli-1-7    AGAGGAAGG.AUCGGCG.GCUAACCGCC.CAGGCGUCCUGCCUG...AACGCCGCCGU.CAUCACCUCCUGUGAAAGUCCGCUCCGAUGCUCGGCCUUCCUAGACUGCAAGGUUUUCAAUCACGCUGAAAAGGUGACGAAAUCCUAAAACUUUAAACCGU
Bsu-1-1    UAUAAAAGG.AACGGCG.GCUAA.AUGCUUAGGCGUCCUGCCUAA...ACACCGCCGUCA.UCACAUCCUGUAA.AAGCCCUUUAUAUAGAGGGCUCUUUUUGUGUCUCAGAAUUCGACAAAAUCCGAGGAGUGUGAAAAAAUAUAAAGGCAGGAACAUU
Bsu-1-3    GCGAAGUGG.AUCAACG.GCUAA.AUUCUCAGGCGUCCUGCCUAA...ACGCCGUCGU.CAUCACAUCCUGUGAAUGUCCGCUCCUAUGCUCGUCCUUCCUAGACUUCAAGGGUUUUCAAUCACGCUGAAAAGAUUUUGUCAACAAUCUGAGGCACCUCU
Bsu-1-5    GCGAAGUGG.AUCAACG.GCUAA.AUUCUCAGGCGUCCUGCCUAA...ACGCCGUCGU.CAUCACAUCCUGUGAAAGUCCGCUCCUAUGCUCGUCCUUCCUAGACUUCAUGGGUUUUCAAUCACGCUGAAAAGAUGACAAAAUCCUAAAAUGAAAACCAU
Bsu-4-3    GCGAAGUGG.AUCAACG.GCUAA.AUUCUCAGGCGUCCUGCCUAA...ACGCCGUCGU.CAUCACAUCCUGUGAAAGUCCGCUCCUAUGCUCGUCCUUCCUAGACUUCAUGGGUUUUCAAUCACGCUGAAAAGAUGACAAAAUCCUAAAAUGAAAGCCAU
Gka-1-1    GAGGCUCGU.AAUUUCG.CCUAAAGGCAGCCCGCGUCCUGCGGGC..AACGGCGAAAU.AUCGCCAUCGUGGCG..AUCGCGAUGGGCAAAUGUUCUUCGCCUGGAGGGAUGUUCACAUCCCGAAGGCGAAGGUUAUUUGACCCCGAGCGGCGGCGAGCC
Gka-1-2    AAGCGCCGU.AAUUUCG.CCUAAAGGCAGCCCGCGUCCUGCGGGC..AACGGCGAAAU.AUCGCCAUCCUGGCG.AUCUCGAAGCCAAAUGUUCUUCACCCGUUGGGGUGCUUGCACCCCGAGGGGAAGGUUAUUUGGCAGAAGAGAGCCAGGCGCUGG
Bpu-2-1    CAGGAUGUG.AUCACAU.GCUAA.GUUC.GCUACAUCGUGUAGC...AACGCAUGUGUGAUAUGCAUCCUGCAUACCUCGGAGAGUUUGAUCCUGGCUCAGGACGAACGCUGGCGGCGUGCCUAAUACAUGCAAGUCGAGCGAACAGAAGGGAGCUUGCU
Bpu-2-2    CAGGAAAUG.AUCACAU.GCUAA.GU.UCGCUACAUCGUGUAGCGACAACGCAUGUGUGAUAUGCAUCCUGCAUACCUCGGAGAGUUUGAUCCUGGCUCAGGACGAACGCUGGCGGCGUGCCUAAUACAUGCAAGUCGAGCGAACAGAAGGGAGCUUGCU
Bpu-2-3    CAGGAGAUG.AUCACAU.GCUAA.GUUC.GCUACAUCGUGUAGC...AACGCAUGUGUGAUAUGCAUCCUGCAUACCUCGGAGAGUUUGAUCCUGGCUCAGGACGAACGCUGGCGGCGUGCCUAAUACAUGCAAGUCGAGCGAACAGAAGGGAGCUUGCU
Bpu-2-4    CAGGAAAUG.AUCACAU.GCUAA.GUUC.GCUACAUCGUGUAGC...AACGCAUGUGUGAUAUGCAUCCUGCAUACCUCGGAGAGUUUGAUCCUGGCUCAGGACGAACGCUGGCGGCGUGCCUAAUACAUGCAAGUCGAGCGAACAGAAGGGAGCUUGCU
Bpu-2-5    CAGGAAAUG.AUCACAU.GCUAA.GUUC.GCUACAUCGUGUAGC...AACGCAUGUGUGAUAUGCAUCCUGCAUACCUCGGAGAGUUUGAUCCUGGCUCAGGACGAACGCUGGCGGCGUGCCUAAUACAUGCAAGUCGAGCGAACAGAAGGGAGCUUGCU
Bpu-1-1    CAGGAAAUG.AUCACAU.GCUAA.GUUC.GCUACAUCGUGUAGC...AACGCAUGUGUGAUAUGCAUCCUGCAUACCUCGGAGAGUUUGAUCCUGGCUCAGGACGAACGCUGGCGGCGUGCCUAAUACAUGCAAGUCGAGC...................
Bpu-1-2    CAGGAAAUG.AUCACAU.GCUAA.GUUC.GCUACAUCGUGUAGC...AACGCAUGUGUGAUAUGCAUCCUGCAUACCUCGGAGAGUUUGAUCCUGGCUCAGGACGAACGCUGGCGGCGUGCCUAAUACAUGCAAGUCGAGCGGACAGAAGGGAGCUUGCU
Bpu-2-6    CAGGAAAUG.AUCACAU.GCUAA.GUUC.GCUACAUCGUGUAGC...AACGCAUGUGUGAUAUGCAUCCUGCAUACCUCGGAGAGUUUGAUCCUGGCUCAGGACGAACGCUGGCGGCGUGCCUAAUACAUGCAAGUCGAGCGAACAGAAGGGAGCUUGCU
Bha-1-1    AGGAAAAGG.A.ACCAC.GCUAA.GGGC.GUGACAUCGUGUCAC...AACGCCUGGU..ACCUACAUCUUGUAG.GCAACAUGACCGAAAAUCCCGAAGCUGACAAAGAGAUACGAAGAGAAUCUGACGUUAUUUGGUUAAGUUAGAAAGGGCGCACGGU
Bha-1-2    AGGAAAAGG.A.ACCAC.GCUAA.GGGC.GUGACAUCGUGUCAC...AACGCCUGGU..ACCUACAUCCUGUAG.GCAACAUGACCGAAAAUCCCGAAGCUGACAAAGAGAUACGAAGAGAAUCUGACGUUAUUUGGUUAAGUUAGAAAGGGCGCACGGU
Bli-1-9    CCUGACUGG.UUGCCCG.GCUA..GACCUUUCACGUCCUGUGAAA..AACGCCCAGU..ACCUUCCUCCUGAAG.GCAGUUUUUUUAGCAAUUUUUGGAUCUCGAUUUAUGCAAUAAAAAAACCUUUUCCUAUUCGGUGAGGAAAGGGUCUGGUAUUGCG
Bsu-1-7    AAGGUAAGG.AAACUCC.GCUAA.GGGCUCUCACAUCCUGUGAGA..AACGCCCAGU..ACCUUCAUCCUGAAG.GCAUUUGUUUGGUGGCGAUAGCGAAGAGGUCACACCCGUUCCCAUACCGAACACGGAAGUUAAGCUCUUCAGCGCCGAUGGUAGU
Bli-1-11   AAGGCAAGG.AAACUCG.GCUAA.AGGCCCUCACAUCCUGUGAGG..AACGCCCAGU..ACCUUCGUCCUGAAG.GCAUAGUUUGGUGGCGAUAGCGAAGAGGUCACACCCGUUCCCAUGCCGAACACGGAAGUUAAGCUCUUCAGCGCCGAUGGUAGUU
Bli-1-13   AAGGCAAGG.AAACUCG.GCUAA.AGGCCCUCACAUCCUGUGAGG..AACGCCCAGU..ACCUUCGUCCUGAAG.GCAUAGUUUGGUGGCGAUAGCGAAGAGGUCACACCCGUUCCCAUGCCGAACACGGAAGUUAAGCUCUUCAGCGCCGAUGGUAGUU
Bli-1-15   AAGGCAAGG.AAACUCG.GCUAA.AGGCCCUCACAUCCUGUGAGG..AACGCCCAGU..ACCUUCGUCCUGAAG.GCAUAGUUUGGUGGCGAUAGCGAAGAGGUCACACCCGUUCCCAUGCCGAACACGGAAGUUAAGCUCUUCAGCGCCGAUGGUAGUU
Bli-1-17   AAGGCAAGG.AAACUCG.GCUAA.AGGCCCUCACAUCCUGUGAGG..AACGCCCAGU..ACCUUCGUCCUGAAG.GCAUAGUUUGGUGGCGAUAGCGAAGAGGUCACACCCGUUCCCAUGCCGAACACGGAAGUUAAGCUCUUCAGCGCCGAUGGUAGUU
Bli-1-19   AAGGCAAGG.AAACUCG.GCUAA.AGGCCCUCACAUCCUGUGAGG..AACGCCCAGU..ACCUUCGUCCUGAAG.GCAUAGUUUGGUGGCGAUAGCGAAGAGGUCACACCCGUUCCCAUGCCGAACACGGAAGUUAAGCUCUUCAGCGCCGAUGGUAGUU
Bli-1-21   AAGGCAAGG.AAACUCG.GCUAA.AGGCCCUCACAUCCUGUGAGG..AACGCCCAGU..ACCUUCGUCCUGAAG.GCAUAGUUUGGUGGCGAUAGCGAAGAGGUCACACCCGUUCCCAUGCCGAACACGGAAGUUAAGCUCUUCAGCGCCGAUGGUAGUU
Bli-1-23   UGUGAAAGG.AGACUCG.GCUAA.GGUCUUUCGCGUCCUGCGAAA..AACGCCG.GUC.ACCUUCGUCCUGGUGUGAAAGGAGACUCGGCUAAGGUCUUUCGCGUCCUGCGAAAAACGCCGAGUACCUUCGUCCUGAAGGCAUGAUUUAUUAUAGGUUAG
Bli-1-25   UGUGAAAGG.AGACUCG.GCUAA.GGUCUUUCGCGUCCUGCGAAA..AACGCCGAGU..ACCUUCGUCCUGAAG.GCAUGAUUUAUUAUAGGUUAGAGGUGUUUAUUUUGAAAAUAUCAACUAAAGGCAGAUACGGGCUGACGAUUAUGAUUGAGCUUGC
Bli-1-27   GUGUGAAAA.GGACUCG.GCUAA.GGGCUUUCACAUCCUGUGAAA..AAAGCCGAGU..ACCUUCGUCCUGGUGUGAAAGGAGACUCGGCUAAGGUCUUUCGCGUCCUGCGAAAAACGCCGGUCACCUUCGUCCUGGUGUGAAAGGAGACUCGGCUAAGG
Bcl-1-1    AAGCUAAGG.AUCUUCG.GUUAA.GGUC.GCGACGUCCUGUCGC...AACACCGAAGC.CAACACAUCC.GUGUGUAAGUAAACUUUUUUAACGGAGAGUUUGAUCCUGGCUCAGGACGAACGCUGGCGGCGUGCCUAAUACAUGCAAGUCGAGCGGACA
Oih-1-1    AACAGAAGG.ACCUUCU.GCUAA.AAUCGCCCACGUCCUGUGGGC..AACGCAGAAAU.CACCACAUCCUGUGG.GUGCUCUUUUUGAACAAACCUAAAAUAAAUCUAAUUGUUUAGGUGCCAAACCUUGAUAAUCUAUCCCCAACAUCUCCUGUAAUUG
Oih-1-2    UCAGGGGGG.ACCUUCU.GCUAA.AAUCGCCCACGUCCUGUGGGC..AACGCAGAAAU.CACCACAUCCUGUGG.AAGCUCGUUAGCUCAGCUUUAAGUUCCUCACGUAUUCAUUACAUACGUUCUGGUACGGGGAGCUACGCCGCCUCGAACUUCUCGG
Oih-1-3    AAAAAGGAG.ACUUUCU.GCUAA.AAUCGCCCAUGUCCUGUGGCU..AAAGCAAAAAUCA.CCACAUCCUGUGG.AAGCUCAUUUUUUACCCUACUUUUCGAACAAUCUUUAAGCUAUUUUAGUUAUUUUAACUCGUUUAAUUUGGAAGCCAUCUUUUUC
Oih-1-4    AAAAUAAGG.AAGUUCG.UUUAA.AACCGUUCACGUCCUGUGAAC..AACACCGAACG.ACCUACAUCCUGUAG.GUCUGGUAGCGAUAGCGAAGAGGUCACACCUGUUCCCAUGCCGAACACAGAAGUUAAGCUCUUCAGCGCCGAUGGUAGUUGGGGC
Oih-1-5    UAAAUAAGG.AAGUUCG.UUUAA.AACCGUUCACGUCCUGUGAAC..AACACCGAACG.ACCUACAUCCUGUAG.GUCUGGUAGCGAUAGCGAAGAGGCCACACCUGUUCCCAUGCCGAACACAGAAGUUAAGCUCUUCAGCGCCGAUGGUAGUUGGGGC
Bam-1-1    AAACAUAGG.AACACCU.GCUAA.AUGC.GCCACUUCCUGUGGC...AACGCAUGUGU.UAGCAUGUCCUAUGC.GUUGUUCUUUGAAAACUAGAUAACAGAAGUAAUUCACAUUCAAUUAGUAAUGCAAGAUAUCACGUAGUGAUUCUUUUUAACGGUU
Bsu-1-9    GAUAAAUGG.AUCACAG.GUUAA.GUC.CACCGCAUCCCGCGGUG...ACACCUGUGUGACCUGCAUCGUGCAG.GCCCUAGUUUGACUGACUACGCACAUCGCUGUGCGAUUUUCAAAAACUGAAUUAACAGGUACGUUUUGUCUUGUUUAGUUUUCAA
Bsu-2-6    GAUAAAUGG.AUCACAG.GUUAA.GUU.CACCGCAUCCUGCGGCG...ACACCUGUGUGACCUGCGUCGUGCAG.GCCCUCUUNNNNAUACGCACAUCGCAGUGCGAUUUUUAAAAACUGAAUUAACAGGUACGUUUUGUCUUGUUUAGUUUUCAAAGA
Bsu-1-11   GAUAAAUGG.AUCACAG.GUUAA.GUU.CACCGCAUCCUGCGGCG...ACACCUGUGUGACCUGCGUCGUGCAG.GCCCUGUUUGAUGAUACGCACAUCGCAGUGCGAUUUUUAAAAACUGAAUUAACAGGUACGUUUUGUCUUGUUUAGUUUUCAAAGA
Bsu-3-6    GAUAAAUGG.AUCACAG.GUUAA.GUU.CACCGCAUCCUGCGGCG...ACACCUGUGUGACCUGCGUCGUGCAG.GCCCUAUUUNNUAAUACGCACAUCGCAGUGCGAUUUUUAAAAACUGAAUUAACAGGUACGUUUUGUCUUGUUUAGUUUUCAAAGA
Bsu-1-12   GAUAAAUGG.AUCACAG.GUUAA.GUU.CACCGCAUCCUGCGGCG...ACACCUGUGUGACCUGCGUCGUGCAG.GCCCUAGUUUGACUGACUACGCACAUCGCUGUGCGAUUUAUAAAAAUGAAUUAACAGGUACGUUUUGUCUUGUUUAGUUUUCAAA
Bsu-1-14   GAUAAAUGG.AUCACAG.GUUAA.GUU.CACCGCAUCCUGCGGCG...ACACCUGUGUGACCUGCGUCGUGCAG.GCCCUCUUUNNNNNUACGCACAUCGCAGUGCGAUUUUUAAAAACUGAAUUAACAGGUACGUUUUGUCUUGUUUAGUUUUCAAAGA
Bsu-1-15   GAUAAAUGG.AUCACAG.GUUAA.GUU.CACCGCAUCCUGCGGCG...ACACCUGUGUGACCUGCGUCGUGCAG.GCCCUAGUUUGACUGACUACGCACAUCGCUGUGCGUUUUUUAAAAACUGAAUUAACAGGUACGUUUUGUCUUGUUUAGUUUUCAA
Bsu-4-8    GAUAAAUGG.AUCACAG.GUUAA.GUU.CACCGCAUCCUGCGGCG...ACACCUGUGUGACCUGCGUCGUGCAG.GCCCUNNNNNNNNNUACGCACAUCGCAGUGCGAUUUUUAAAAACUGAAUUAACAGGUACGUUUUGUCUUGUUUAGUUUUCAAAGA
Bli-1-29   AAGUAAUGG.AGCGCAG.AUUAA.GUUC.GUCACAUCCUGUGAC...AACAUCUGCAUGACCUGCAUCGUGCAG.GCCCCUGACUACGCACAUUCGCAUGUACGAUUUAUUUAAUGAAUUAACAGGUACGUUUUGUCUUGUUUAGUUUCAAAGAUCAUU
Bli-1-31   AAGUAAUGG.AGCGCAG.AUUAA.GUUC.GUCACAUCCUGUGAC...AACAUCUGCAUGACCUGCAUCGUGCAG.GCCCCUGACUACGCACAUUCGCAUGUACGAUUUAUUUAAUGAAUUAACAGGUACGUUUUGUCUUGUUUAGUUUCAAAGAUCAUU
Bli-1-33   AAAUAAUGG.AGCGCAG.AUUAA.GUUC.GUCACAUCCUGUGAC...AACAUCUGCAUGACCUGCAUCGUGCAG.GCCCCUGACUACGCACACUUACAUGUGCGAUUUAUAAUGAAUUAACAGGUACGUUUUGUCUUGUUUAGUUUCAAAGAACUUCGC

           ............<<<<<.<<........<<<<<<.....>>>>>>.....>>>>>>>...<<<<<.....>>>>.>....................................................................................
           ............?22?2.22........222221.....122222.....222?22?...?2222.....2222.?....................................................................................
           •••••AAGG-A•C•Y•G-RCUAA-R••C-•YCACRUCCUGYGR•---AACGYC•R•RU∘AYYYRCRUCCUG•RG-RY•Y•R•••••••••••••••••••••••••••••••••••••••••••••••••••••••••••••••••••••••••••••••
            5′————————————————————————————————————————————————————————————————————3′
```

```
Bli-1-35  AAAUAAUGG.AGCGCAG.AUUAA.GUUC.GUCACAUCCUGUGAC...AACAUCUGCAUGACCUGCAUCGUGCAG.GCCCCUGACUACGCACAUUCGCAUGUACGAUUUAUUUAAUGAAUUAACAGGUACGUUUUGUCUUGUUUAGUUUUCAAAGAUCAUU
Bli-1-37  AAAUAAUGG.AGCGCAG.AUUAA.GUUC.GUCACAUCCUGUGAC...AACAUCUGCAUGACCUGCAUCGUGCAG.GCCCCUGACUACGCACACUCAAAUGUGCGAUUUAUAAAAAUGAAUUAACAGGUACGUUUUGUCUUGUUUAGUUUUCAAAGAACUU
Bli-1-39  AAAUAAGUG.AACGCUG.GCUAA.AUAC.GCCACAUCGUGUGGC...AACGCCUGCAUGACCCACAUCCUGUGG.GCCUCGUCUGCCCGUUAUCUAGUUUUGAGAGAAUAAUUUCUCAAAAAAUGCACUUGCAUUUUUCAAAUACAUAGUAUAAUGAUC
Bsp-1-1   CUAUCGAGG.AACGCGG.ACUAA.GUAC.GCCACGUCGUAUGGC...AACGUCCUCAUGACCCGCUUCCUGCGG.GCCUAAAAAAAUGCCAGGCACCUCCCGCGGACAAAUCACAUUGAAAUGUCCACAGACGGUGCCUGGCACUAAAAUUAUUUAAUGAU
Bsp-1-2   GCGCCUUGGUCACGCAG.ACUAA.GAAC.GCCACGUCCUGUGGC...AACGUCUGCAUGACCCCCAUCCGGGGG.AGCCUCACUCCAGGAAAGGCGUUCUUUGCCUUUUUGGGAGGAUUGGCUUGUGACCUCGAGGGGGUAGGCGCAGCUGCUAGACAGAA
Bsp-1-3   CCACUAAGG.AACGCAG.ACUAA...AA.ACCACGUACUGCGGC...AACGUCUGCAUAACCCACAUCCCGUGG.CUUAUGAUGACUCGAGGCCCGGGUUUUGGGGCUUUUGCCCCGAUAAAAAAGGAUAUAAGCAUGGUUAUAUGGUUUUACCCAAUGA
Bsp-1-4   ACCUUAAGA.AACGCAG.ACUAA.GAAC.GCCACGUCCUGUGGC...AACGUCUGCAUGGCUCCCAUGCUGGAG.CCCUCAAGACGAACCUCCCGGAAACCUUUUUGGGUUGUUUGGCUAGUGACCUGGGCGCAGCUGCUGGACGUGGAUCAAGAAUUGA
Bsp-1-5   CCAACGAGG.AACGCAG.GCUAA.GGGC.GCCACGUCCUGUGGC...AACGCCUGCAUGACCCGCAUACUGCGG.GCCUCAAGCAUAAGACGAGCAGGACGGAAGGUUGUGCUUUAACCUUCUGGCCUGCUUGGCUUAUGACUCGAGCACCUAGGAGCCG
Bsp-1-6   CUGUCAAGG.AACGCAG.ACUAA.AAAC.GCCACGUCCUGUGGC...AACGUCUGCAUGACCCGCGUCCUGCGG.GCCUCAAGCAUAAGACGAGCCGGACGGGAGGUUGUUCUUUAACCUCCCGGCCGGAUUGGCUUAUGACUCGAGCCAAUGGCCCCUG
Bsp-1-7   GAUUAGAGG.AACGCAGGGCUAA.AACC.GCCACGUCCUGUGGC...AACGCCUGCAUGAACCCGCGUCCUGCGG.GCCCAGCAUAAGCCGCUCCAGGAUAAAAGGCGUUCUUUGCCUUUAAUCCUGGAGCGGCUUAUGACUCGAGCACCUAGGAGCCGGA
Bsp-1-8   CAAUCAAGG.AACACAG.GCUAA.GAGC.GCCACGUCCUGUGGC...AACGCCUGCAUGACCCGCGUCCUGCGG.GCCUCAAGCAUAAGACGAGCAGGACGGAAGGUUGUCUUUAACCUUCUGGCCUAAUUGGCUUAUGACUCGAGGGCUAGCGCCCG
Bsp-1-9   GAAUUAAGG.AACGCAG.GCUAAAAACG..CCACGUCCUGUGG....AACGCCUGCAUGACCCGCGUCCUGCGG.GCCCAGCAUAAGGCGCUUUGGAAUUGAAGGCGUUCUUUGCCUUCGGUUCCAAAGUGACUUAUGACUCGAGCACCUAGGAGCCGGA
Bsp-1-10  GAAUUAAGG.AACGCAG.GCUAA.AGGC.GCCACGUCCUGUGGC...AGCGCCUGCAUGACCCGCGUCCUGCGG.GCCCAGCAUAAGACGCGCAAGAAUAAAAGGCGUUCUUUGCCUUUAAUUCUUGCGUGGCUUAUGACUCGAGGCUCUAGCCGCUGGA
Bsp-1-11  GAAUCAAGG.AACGCAG.GCUAA.AACC.GCCACGUCCUGUGGC...AACGCCUGCAUGACCCGCAUCCUGCGG.GCCCAGCAUAAGACGAGCAGGACGGAAGGUUGUCCUUUAACCUUCUGGCCUGAUUGGCUUAUGACUCGAGGCUCUAGCCGCCGGA
Bsp-1-12  GAAUCAAGG.AACGCAG.GCUAA.AACC.GCCACGUCCUGUGGC...AACGCCUGCAUGACCCGCAUCCUGCGG.GCCCAGCAUAAGACGAGCAGGACGGAAGGUUGUCCUUUAACCUUCUGGCCUGAUUGGCUUAUGACUCGAGGGGCUAGCGCCCGGA
Bsp-1-13  GAAUAAAGG.AACGCAG.GCUAA.AGAC.GCCACGUCCUGUGGC...AACGCCUGCAUGACCCGCAUCCUGCGG.GCCCAGCAUAAGGCGCUUUGGAAUGGAAGGCGUUCUUUGCCUUCGGUUCCAAAGUGACUUAUGACUCGAGCACCUAGGAGCCGGA
Bsp-1-14  GAAUUAAGG.AACGCAG.GCUAA.AAAC.GCCACGUCCUGUGGC...AACGCCUGCAUGACCCGCGUCCUGCGG.GCCCAGCAUAAGACGAGCAGGACGGAAGGUUGUCUUUAACCUUCUGGCCUGAUUGGGUUAUGACUCGAGUACCUAGGAGCCGGA
Bsp-1-15  GGAUUAAGG.AACGCAG.GCUAA.AGAC.GCCACGUCCUGUGGC...AACGCCUGCAUGACCCGCGUCCUGCGG.GCCCAGCAUAAGACGCUUUGGAAUUGGAGGCGUUCUUUGCCUUCGGUUCCAAAGUAGCUUAUGACUCGAGGCUCUAGCCGCCGGA
Bsp-1-16  CAAUAGAGG.AACGCGG.ACUAA.GAGC.GCCACGUCCUGUGGC...AACGUCCGCAUGACCCGCAU.CUGUGG.GCCUCAUGCAUAAGACGAGCAGGUCGGAAGGUUGUUCUUUACCUUCUGGCCUGUUUGGCUUAUGACUCGAGGUUCCAGCGCCCGG
Bsp-1-17  CAAUAAAGG.AACGCGG.ACUAA.GAAC.GCCACGUCCUGUGGC...AACGUCCGCAUGACCCGCAUCCUGCGG.GCCUCAAGCUUAAGACGAGCAGAGCGGAAGGUUGUUCUUUAACCUUCUGAUCUGAUUGGCUUAAGACUCGAGGGGCUAGCUCCCG
Bsp-1-18  CGAUAAAGG.AACGCGU.ACUAA.GAAC.GCCACGUCCUGUGGC...AACGUCCGCAUGACCCACAUCCUGUGG.GCAUCAAGCUUAAGACGAGCAGAGCGGAAGGCGCUUGUUUAGUUAAAAUCCCCGGAUGCCAGAGGCAUGCGGGGAUUUUAAUUU
Bsp-1-19  AGAUUAAGG.AUCGCGG.ACUAA.AAAC.GCCACGUCCUGUGGC...AACGUCCGCAUGACCCGCAUCCUGCGG.GCCCAGCAUAAGGCGCUUAGGAAUAAAAGGCGUUCUUUGCCUUUAAUUCCUAAGCGCUUAUGACUCGAGGCUCUAGCCCCUGGA
Bsp-1-20  UCACUAAGG.AACGCAG.GCUAA.GUAC.GCCACGUCCUGUGGC...AGCGCCUGCAUGGCCCACUUCCUGUGG.GCCUCAAGGAUAAGACAAUCCUAUACACAAAAACAAAAAGAGCCCCCAGUAUAAGGAGGCUCCCCAAAUAAUUAUUUCAAGAAAA
Bsp-1-21  CCCUUAAGG.AACGCAG.ACUAG.GAAU.GCCGCAUCCUGUGGC...AACGUCUGCAUGGCCAGCUUCCUGGGG.GCCUUAAGACGAACAUCCCGGAAAGGCGUUCUUUGCCUUUUUGGGAUGUUUAGCUUGUGAACUUGGUGCAGCUGCUGGACGUGGA
Bsp-1-22  ACCUUAAGG.AACGCAG.ACUAA.GAAC.GCCACGUCCUGUGGC...AACAUCUGCAUGACCAGCUUCCUGGGG.GCCUCAAGACGAACCUCCCGGAAAGGCGUUCUUUGCCUUUUUGGGAGGUUCGGCUUAUGACUUCGGCGCUGCUGCUGGACGUGGA
Bsp-1-23  ACCUUAAGG.AACGCAG.ACUAA.GAAC.GCCACGUCCUGUGGC...AACGUCUGCAUGACCAGCUUCCUGGGG.GCCACAAGGCAAACUCCCGGAAAGGCGUUCUUUGCCUUUUUGGGAGGUUUGCCUUGUGACCUCGGCGCAGCUGCUGGACGUGGA
Bsp-1-24  ACUUUAAGG.AACGAAG.ACUAA.GAAC.GCCGCGUCCUGUGGC...AACAUCUGCAUGACCAGCUUCCUGGGG.GCCUCAAGACGAACCUCCCGGAAAGGCGUUCUUUGCCUUUUUGGGAGGUUUGGCUUGUGACCUCGGCGCAGCUGCUGGACGUGGA
Bsp-1-25  CACUUAAGG.AACGCAG.ACUAA.GAAC.GGCACGUCCUGUGGC...AACGUCUGCAUGACCCCCAUCCUGGGG.GCCUUAAGACAAGACACCCGAAAAGGCGUUCUUUGCCUUUUGGGGGGGCUUGGCUUAUGACUCGAGGGGCACGCGCUGGAUUAG
Bsp-1-26  UACUAAAGG.AACGCAG.ACUAA.GAAC.GCCACGUCCUGUGGC...AACGUCUGCAUGACCCCCAUCUUGGGG.GCCUCAAGGCGAUCUCCCAGAAAGGCGUUCUUUGCCUUUUUGGGAGGAUUGACUUGUGACCUCGAGGGGGCGCAGCUGCUAGACA
Bsp-1-27  CCCUUAAGG.AACGCAG.ACUAA.GAAC.GCCACGUCCUGUGGC...AACGUCUGCAUAACCCCCAUCCUGGGG.GCCUCAAGACGAUCAUCCCGGAAAGGCGUUCUUUGCCUUUUUGGGAUGAUUGGCUUGUGACCUCGAGGGGGUAGGCGCUGGAGCU
Bsp-1-28  CCCUAAAGG.AACGCAG.ACUAA.GAAC.GCCACGUCCUGUGGC...AACGUCUGCAUGACCCCCAUCCUGGGG.GCCUCAAGACGAUCUCCCUAAAAGGCGUUCUUUGCCUUUUUGGGAGGAUUGGCUUGUGACCUCGAGGGGGUAGGCGCUGGAGCU
Bsp-1-29  CCCUUAAGG.AACGCAG.ACUAA.AAAC.GCCACGUCCUGCUGC...AACGUCUGCAUGACCCCCAUCCUGGGG.GCCUCAAGACGAACCUCCCGGAAAGGCGUUCUUUGCCUUUUUGGGAGGUUUGGCUUGUGACCUCGGCGCAGCUGCUGGACGUGG
Bsp-1-30  CAACUAAGG.AACGCAG.ACUAA.AAAC.GCCACGUCCUGUGGC...AACGUCUGCAUGACCCACAUCCUGUGG.GCCUCAAGCAUAAGACGCUUUGGAAUUGAAGGUGUUCUUUACCUUCGGUUCCAAAGUGGCUUAUGACUCGAGGCUCUAGCCGAUG
Bsp-1-31  CCAUUAAGG.AGCGCAG.ACUAA.GAAC.GCCACGUCCUGUGGC...AACGUCUGCAUGACCCACAUCCUGUGG.GCCUCAAGCAUAUGACGCGGUAGAAUAAAAGGCGUUCUUUGUCUUUAAUUCUACCGUGGCUUAUGACUAGAGGCUCCAGCCGCUG
Bsp-1-32  UCACUAAGG.AACGCAG.ACUAA.GAAC.GCCACGUCCUGUGGC...AACGUCUGCAUGACCCACAUCCUGUGG.GCCUCAAGCAUAAGACGCUUUAGGAUAGAAGGCGUUCUUUGCGUUCUUUGCCUUCAAUCCUAAAGUGGCUUAUGACUCGAGGGGC
Bsp-1-33  ACCCUAAGG.AACGCAG.ACUAA.GAAC.GCCACGUCCUGUUGC...AACGUCUGCAUGACCCGCAUCCUGCGG.CCCUCAAGAGGAACUCCCCGGAAAGGGGUUCUUUGCCUUCUUGGGAGGUUCGUCUUGUGACCUCGAGGGUGUAGUCGCUGGAGCUG
Bsp-1-34  UUAUAAAGG.AACGCAG.ACUAA.AAAC.GCCACAUCCUGCGGC...AGCGUCUGCAUGACCCGAAUCCUGUGG.GCCUCAAAGAUCCAAAACAAUCAUCUAUGUAUGAAAAAAGAAGGACACCCAUAAGGCGCCCUUCUUUUAUUAUAUUAUUCACC
Bsp-1-35  CUAUCGCCG.AGCGCAG.GCUAA.GGGC.GCCAAGUCCUGUGGC...AACGCCUGCAUGACCUGCAUCCUGUGG.GCCUCAAGCACAAGACUCCUGGAUAAAAGGUGCUCUUUGCCUUUAAUUCAGGAGUGUCUUUUGACACGAGGGGCUGGGCUUUCGG
Bli-1-41  CGAGGAAUG.ACCGCAU.GCUAA.GAGC.GAGGCGUCCUGCCUC..AGGCGCAUGCAU.UUGUACGUCCUGUAC.UUCAAAAUAUGUUACAAUGUGGAUACUUAUGUUUAGAUGGAGAAGUAGGUGAAUGGAUUGCGUAUUUUAGAGAAGGCGCCGGCUA
Bli-1-43  AAUGCAAGG.AAGGCAU.GCUAA.AAGC.GCAACGUCCUGUUGC...AACGCAUGCAC.UUGUACGUCCUGUA...CUUCGGAGGAUUAGCUCAGCUGGGAGAGCAUCUGCCUUACAAGCAGAGGGUCGGCGGUUCGAUCCCGUCAUCCUCCACCAUUUU
Bli-1-45  UUUUAAAGG.AAGGCAU.GCUAA.GAUC.GCAACGUCCUGCUGC...AACGCAUGCAC.UUGUACGGUCCUGUAC.UUCGCAGGAUUAGCUCAGCUGGGAGAGCAUCUGCCUUACAAGCAGAGGGUCGGCGGUUCGAUCCCGUCAUCCUCCACCAUUUCUA
Bco-1-1   ACAAAAAUG.AACUUCU.ACUAA.GUUC.GCCACGUCAUGUGGC...AACGUAGAAGUCA.GCGCGUCGUGCGC.AAGUACGUUACGUUGUUGUCUUUUCAGUUUUGAAGGUUUAAUCCUUCAAAAGUUUGUACUUUGAAAACUAGAUAAAGCAACACAU
Bco-1-2   ACAAAAAUG.AACUUCU.ACUAA.GUUC.GCCACGUCCUGUGGU...AACGUAGAAGUCA.GCGCGUCGUGCGC.AAGUACGUUGCGUUGUUGUCUUUUCAGUUUUGAAGGUUCAAUCCUUCAACUUUAUAGCGAAGUAAGAAUGGGCCUAUAGCUCAGC
Bco-1-3   ACAAAAAUG.AACUUCU.ACUAA.GUUC.GCCACGUCCUGUGGU...AACGUAGAAGUCA.GCGCGUCGUGCGC.AAGUACGUUGCGUUGUUGUCUUUUCAGUUUUGAAGGUUCAAUCCUUCAACUUUAUAGCGAAGUAAGAAUGGGCCUAUAGCUCAGC
Bco-1-4   AUAAAAAUG.AACGUCG.AUUAA.GUUU.GCUACGUCCUGUAGC...AACAUCGACAUCAUGUGCAAAGUGAGC.UUGCUCAAACUUUUUAUGGAGAGUUUGAUCCUGGCUCAGGACGAACGCUGGCGGCGUGCCUAAUACAUGCAAGUCGAGCGAGGCC
Bco-1-5   UGUAAAAUG.AACGUCG.AUUAA.GUUC.GCUACGUCCUGUAGC...AACAUCGACAUCAUGCACAUCAUGUGC.AAAGUGAGCUUGCUCAAACUUUUUAUGGAGAGUUUGAUCCUGGCUCAGGACGAACGCUGGCGGCGUGCCUAAUACAUGCAAGUCG
Bco-1-6   GUAAAAAUG.AACGUCG.AUUAA.GUUC.GCUACGUCCUGUAGU...AACAUCGACAUCAUGCACAUCAUGUGC.AAAGUGAGCUUGCUCAAACUUUUUAUGGAGAGUUUGAUCCUGGCUCAGGACGAACGCUGGCGGCGUGCCUAAUACAUGCAAGUCG
          ...........<<<<<.<<........<<<<<<.....>>>>>>.....>>>>>>>...<<<<<.....>>>>.>...........................................................................
          ...........?22?2.22........222221.....122222.....222?22?...?2222.....2222.?...........................................................................
          ●●●●●AAGG-A●C●Y●G-RCUAA-R●●C-●YCACRUCCUGYGR●---AACGYC●R●RU○AYYYRCRUCCUG●RG-RY●Y●R●●●●●●●●●●●●●●●●●●●●●●●●●●●●●●●●●●●●●●●●●●●●●●●●●●●●●●●●●●●●●●●●●●●●●●●●●●●●●
           5′——————————————————————————————————————————————————————————————————3′
```

alignment positions 260 · · · 419

```
Bli-1-1   CCGUUUUGGGAUUUUGUCAACAAUCUAAGGCCUGCUUUUAUAAAGCAGGCCUCUGUUUUAUCUUAUCUUUUGUCUUUCCCCGGAAAAAAGACCGCCAAUGUUCCGGGUCCGGCGUGGGCGCCGACCGCUGAACCGAUCAUGUUGACGACGAUCUCCUCCG
Bli-1-3   UUUUUUUCAUCAUGGACGCCGUCGUUCCCGUCAUCUUUCUGCCGAAUGCGACGCCGAUUGCAUCGUGUUCGCCGAGAGAAGCGACAGAGCCUUUGUUAUCAAAUGAGAACGUUUCCGUUUCUUUGCCGUGAAUCAGUGCGGCCAGAUUGUUUGCGCACGU
Bli-1-5   AAGCAGGUUGGCCAAAAAGUGUUUUUUAUAGCUUUGAAGCUUUCUUCCUUCAUACUUCGUCAUUCCCAGCUUUUUCAGUUCUUCUACAUACCAGCCUUUGCUUCCGUAAUGCAUGGCAGAAAACUCCCUUCUAUAUUUCGAUGCAAUUGGGAUUCUAACA
Bli-1-7   UUUAGGAUUUCGUCAACAAUCUGCCACGUUGUUUCCCAAGAAGGUGUUUUUUUACGGACAGGGCAUUUCUUUCAUUGCAUAAGCCGGUCAGCAGGAAUUUGACUGGAUAAGCCUCGUGCUUUAAAAUGAAAGUAAUAAAAAUGAAUACUCCAAAGGGGAG
Bsu-1-1   GCGGAGGGGAAGAGACAUUGAACGAUAAAGUAACUGCGGUCAGGGAUGUAUGGCGCUAUGAACAAGGGGAAAUAAGCAAAGUAGAAGACCGGAUGGUGACGGAAUUCCCUUUGACAGUGAUACUGAAUGGCAGUGAAUUUGUGACGCUCGUUUGUACGCC
Bsu-1-3   UGAAUGAGGUGCUUUUUUACAAUUGCGCCCGGGCUGUGGAUUGGCGUUUCCCGGUUUUACUGAUUGUCUGGUAGAGCGUCAUAGAAAGUAUGAACAGAAUGGCAGUAAAUCCUGUGACAGCUCCGAAAUGCUGUGUGCUUUGAUAUAAAAAACUGCAAU
Bsu-1-5   UUUAGGAUUUUGUCAACAAUCUGAAGCGGCGGGUUAAUACGGCCGCUCUUUUUUUGCACGCAUAAAAACGAAAGAAGAAACCCGCCGCAUAGUCCAAACAAAUGGGCCAUCAUGUUAAUAUUGGAGUUGAUAAACGACAUGAGAACGGCAAAUGCGAGCA
          ..................................................................................................................................................................
          ..................................................................................................................................................................
          ●●●●●●●●●●●●●●●●●●●●●●●●●●●●●●●●●●●●●●●●●●●●●●●●●●●●●●●●●●●●●●●●●●●●●●●●●●●●●●●●●●●●●●●●●●●●●●●●●●●●●●●●●●●●●●●●●●●●●●●●●●●●●●●●●●●●●●●●●●●●●●●●●●●●●●●●●●
```

Bsu-4-3	UUUAGGAUUUUGUCAACAAUCUGAAGCGGCGGUUAAUACCGCCGCUCUUUUUUUUGCACGCAUAAAAACGAAAGAAGAAACCCGCCGCAUAGUCCAAACAAAUGGGCCAUCAUGUUAAUAUUGGAGUUGAUAAACGACAUGAGAACGGCAAAUGCGAGCA
Gka-1-1	GAAGCUAGACAGUAAGGAAGAAGCCGAGAGCGCAAUAGGUUAAGCUGGAAAGGGCGCACGGUGGAUGCCUUGGCACUAGGAGCCGAUGAAGGACGGGGCAAACGCCGAAACGCUUCGGGGAGCUGUAAGCAAGCGUUGAUCCGGAGAUGUCCGAAUGGGG
Gka-1-2	AGCUAGACAUCAGUCGUUAUUCAAAGCAAAACGCGCUUCUGUUUCGUUCAGUUUGAAGGAAUGAAAAAUUCCUUCAGUUGCUUUGCGUCUGCGUCUACAAGUGAAUUCAGAGAAGCUGGAUUCCUCGACGCAAAGCUGCAAGGAAGCUGUUUCAACGUA
Bpu-2-1	CCCGGAUGUUAGCGGCGGACGGGUGAGUAACACGUGGGUAACCUGCCUGUAAGACUGGGAUAACUCCGGGAAACCGGAGCUAAUACCGGAUAGUUCCUUGAACCGCAUGGUUCAAGGAUGAAAGACGGUUUCGGCUGUCACUUACAGAUGGACCCGCGGC
Bpu-2-2	CCCGGAUGUUAGCGGCGGACGGGUGAGUAACACGUGGGUAACCUGCCUGUAAGACUGGGAUAACUCCGGGAAACCGGAGCUAAUACCGGAUAGUUCCUUGAACCGCAUGGUUCAAGGAUGAAAGACGGUUUCGGCUGUCACUUACAGAUGGACCCGCGGC
Bpu-2-3	CCCGGAUGUUAGCGGCGGACGGGUGAGUAACACGUGGGUAACCUGCCUGUAAGACUGGGAUAACUCCGGGAAACCGGAGCUAAUACCGGAUAGUUCCUUGAACCGCAUGGUUCAAGGAUGAAAGACGGUUUCGGCUGUCACUUACAGAUGGACCCGCGGC
Bpu-2-4	CCCGGAUGUUAGCGGCGGACGGGUGAGUAACACGUGGGUAACCUGCCUGUAAGACUGGGAUAACUCCGGGAAACCGGAGCUAAUACCGGAUAGUUCCUUGAACCGCAUGGUUCAAGGAUGAAAGACGGUUUCGGCUGUCACUUACAGAUGGACCCGCGGC
Bpu-2-5	CCCGGAUGUUAGCGGCGGACGGGUGAGUAACACGUGGGUAACCUGCCUGUAAGACUGGGAUAACUCCGGGAAACCGGAGCUAAUACCGGAUAGUUCCUUGAACCGCAUGGUUCAAGGAUGAAAGACGGUUUCGGCUGUCACUUACAGAUGGACCCGCGGC
Bpu-1-1	....
Bpu-1-2	CCCGGAUGUUAGCGGCGGACGGGUGAGUAACACGUGGGUAACCUGCCUGUAAGACUGGGAUAACUCCGGGAAACCGGAGCUAAUACCGGAUAGUUCCUUGAACCGCAUGGUUCAAGGAUGAAAGACGGUUUCGGCUGUCACUUACAGAUGGACCCGCGGC
Bpu-2-6	CCCGGAUGUUAGCGGCGGACGGGUGAGUAACACGUGGGUAACCUGCCUGUAAGACUGGGAUAACUCCGGGAAACCGGAGCUAAUACCGGAUAGUUCCUUGAACCGCAUGGUUCAAGGAUGAAAGACGGUUUCGGCUGUCACUUACAGAUGGACCCGCGGC
Bha-1-1	GGAUGCCUUGGCACUAGGAGCCGAAGAAGGACGCGACGAACGGCGAAACGCCUCGGGGAGCUGUAAGUAAGCUUUGAUCCGGGGAUAUCCGAAUGGGGGAACCCACCAUCCGUAAUGGGAUGGUACCCAUACCUGAAUCUAUAGGGUAUGAGGAGGCAGA
Bha-1-2	GGAUGCCUUGGCACUAGGAGCCGAAGAAGGACGCGACGAACGGCGAAACGCCUCGGGGAGCUGUAAGUAAGCUUUGAUCCGGGGAUAUCCGAAUGGGGGAACCCACCAUCCGUAAUGGGAUGGUACCCAUACCUGAAUCUAUAGGGUAUGAGGAGGCAGA
Bli-1-9	UGCCUUUCACUCUUAUCGCUCAAGGUAUUGAACACCUUGCAUCAGGUUAGCACCGUAGUUGUCUCAGAAACUGUUUUUUUCCAUUAAUAAGAGAAACCGGUUGCUGGGUUUCAUUGGGCCUGUCCCUCCACCAGCUCGGGAUAAGAGUAUCCGCUCAAUG
Bsu-1-7	CGGGGGUUUCCCCCUGUGAGAGUAGGACGCCGCCAAGCAAGCUUAAACCCAGCUUAAUGAGCUGGGUUUUUUGUUUACUCAAGAUCGAGAAAAUCAAACAAAUACAAACACCGAGGGCUUAUAUAGCCCAUAUACUGAUUUAAAGUUUCAAAAAGGUCUA
Bli-1-11	GGGGGCUUCCCCCUGUGAGAGUAGGACGCCGCCAAGCUGUUAUAUAUCUUUACCCUGCAUGAAUUGAAGCUUUUCUUAUUCCGCAGUAGCUCAGUGGUAGAGCUAUCGGCUGUUAACCGAUCGGUCGCAGGUUCGAAUCCUGCCUGCGGAGCCAAUGCUU
Bli-1-13	GGGGGCUUCCCCCUGUGAGAGUAGGACGCCGCCAAGCGCUUAAGAAGACCAGCUUAUAUAAGCUGGUGUUUUUUGCGGGUGUUCGAUUGUAUAUACCGGAAGGGCAGGAGUGCAUAGACGAUUUCAACCCUUCAGGCCCCGCGAAACGGUGUGAAGAAUA
Bli-1-15	GGGGGCUUCCCCCUGUGAGAGUAGGACGCCGCCAAGCUGUUUAUAUUAUCCCGGGGUGGAGCAGUUCGGUAGCUCGUCGGGCUCAUAACCCGAAGGUCGCAGGUUCAAAUCCUGCCCCCGCAACCAAAUAUAAUUUGCAUACAAUCAAACAAAAUAAAUA
Bli-1-17	GGGGGCUUCCCCCUGUGAGAGUAGGACGCCGCCAAGCGCUAAGAGGACCAGCUUAUAUAAGCUGGUCUUUUUUGUUGUUUUCUUUUUUUCGAAUAAGCAAUUCAGCGAUAUAAUAAGGGAACUUUGACUCGACUAUAUGUAAAAACUGUUGGAUGAUGAC
Bli-1-19	GGGGGCUUCCCCCUGUGAGAGUAGGACGCCGCCAAGCGCUUAGAAAGACCAGCUUAUAUAAAAGCUGGUCUUUUUCUUUUCCCUAAGGUUUUAUGACAGCUAAAAGUGGAAAUAAUAAAGGACGAAGAGCAAUUAGUUGAAAAAAAAACGAAAACCACU
Bli-1-21	GGGGGCUUCCCCCUGUGAGAGUAGGACGCCGCCAAGCGCUAAGAGGACCAGCUUAUAUAAGCUGGUCUUUUUAUUUGGAACCUCAUUGAUACAAACACCUAACACUUCCCGGCUACAUAUACUAAUCUAACCUUCAAAAACUUUCUUAAUUUUGUAUAC
Bli-1-23	AGGUGUUUAUUUUGAAAAUAUCAACUAAAGGCAGAUACGGGCUGACGAUUAUGAUUGAGCUUGCGAAAAAGCAUGGGGAAGGCCCGACUUCUUUGAAAAGUAUUGCUCAGACAAACGAUUUAUCGGAACAUUAUUUGGAACAGCUUGUUUCACCUUUGCG
Bli-1-25	GAAAAAGCAUGGGGAAGGCCCGACUUCUUUGAAAAGUAUUGCUCAGACAAACGAUUUAUCGGAACAUUAUUUGGAACAGCUUGUUUCACCUUUGCGGAACGCCGGGCUUGUGAAAAGCAUUCGCGGGGCCUACGGCGGCUACAUUCUUGGUGAGGAGCCU
Bli-1-27	UCUUUCGCGUCCUGCGAAAAACGCCGAGUACCUUCGUCCUGAAGGCAUGAUUUAUUAUAGGUUAGAGGUGUUUAUUUUGAAAAUAUCAACUAAAGGCAGAUACGGGCUGACGAUUAUGAUUGAGCUUGCGAAAAAGCAUGGGGAAGGCCCGACUUCUUUG
Bcl-1-1	GAAGGGAGCUUGCUCCCGGACGUUAGCGGCGGACGGGUGAGUAACACGUGGGCAACCUGCCCCUUAGACUGGGAUAACUCCGGGAAACCGGAGCUAAUACCGGAUAAUCCUUUCUCCACCUGGAGAGAGGGUGAAAGAUGGCUUCGGCUAUCACUAGGG
Oih-1-1	CUUUGCAUUAUUUGCAGCAUGCCCUCCAGAGUUAUUAUUGAACAGUACUAUUACAUCGUGAUUGGGCUUAUUUAAUUGAGUCACUUCAUUCGCUAGUGUUAAUAGUUCUUCUUUGUUAUAGUCAUAGAGAUACCGGACCUCUCGCCAGUUUUCAUUCCCA
Oih-1-2	UCCUUUUCAUCCUCCUUUUUGAACACGUAAUAGAAAGGGAGAUUUGAUGAAUUUUUUUCAUACCGAAACAUAUCUUAUACAAAUCCAAGGCAUGCAACCUAUGCAAAAAAUGGAAUGGUGGCAACAUCGCAGCCACUUGCAUCAGAAGCCGGACUGUCG
Oih-1-3	AAUAAGUGCAAAUUCAUGUCCUUCAUAUUCCAUAACAGUUCCAACAUCUGCUUCUAAAUUAUGAGUAAAGAACCAUCCACCAAUUGUAUCUACUUCAUCGUCUUCUAAAUAGAUACUUAACCUUUGAUUUACUUCAUCUAAACUUAGUCUACCUGAAACA
Oih-1-4	UUUGCCCCUGCAAGAGUAGGACGCCGCCAGGCAACGAAAGAAAAGACGGAUUAGAUUUAAAUAUCUAAUCCGUCUUUUCUUUUGGCUCUAUAAUAUUUGUUGGGGUAAAAGAAAAAUAAAAAACACGCAAUCUCCUAUUUUUGUUAUCAUUGUUUAAACC
Oih-1-5	UUUGCCCCUGCAAGAGUAGGACGCCGCCAGGCAACGAAAGAAAAGACGGAUUAGAUUUAAAUAUCUAAUCCGUCUUUUUCUUUUUUUGUUAGGGUUGAAAAAAAGGGCUCUACAAUAGUGGCUCUAUAAUAUUUGUUUGAGUAAAAGAAAACUAAAAAA
Bam-1-1	AAGUUAGAAAGGGCGCACGGUGGAUGCCUUGGCACUAGGAGCCGAUGAAGGACGGGACGAACACCGAUAUGCUUCGGGGAGCUGUAAGCAAGCUUUGAUCCGGAGAUUUCCGAAUGGGGAAACCCACCACUCGUAAUGGAGUGGUAUCCAUAUCUGAAUA
Bsu-1-9	AGAUCAUUCAAUAAUGGAGCGGGUGAUGAGAAUCGAACUCACGACAUCAGCUUGGAAGGCUGAGGUUUUACCACUAAACUACACCCGCGUGGUAUCUUAUAUGGCGCGCCCGAGAGGAGUCGAACCCCUAACCUUUUGAUCCGUAGUCAAACGCUCUAUC
Bsu-2-6	UCAUUUCGUUACUUCUCAAUGAAGCAGCUUUACUAAUAUAACAUUCAGCUUCUUUUUUGUCAACAACUUUUUUCAAGUUGUUUUGAAGCAUCUAGUUUGUGUCGCGUCUGCAUCAGCGACGUUUAAUAAUAUAACACCUGUAUUUAUUUAUUGCAAUAGU
Bsu-1-11	UCAUUUCGUUACUUCUCAAUGAAGCAGCUUUACUAAUAUAACAUUCAGCUUCUUUUUUGUCAACAACUUUUUUCAAGUUGUUUUGAAGCAUCUAGUUUGUGUCCGCGUGCAUCAGCGACGUUUAAUAAUAUAACACCUGUAUUUAUUUAUUGCAAUAGU
Bsu-3-6	UCAUUUCGUUACUUCUCAAUGAAGCAGCUUUACUAAUAUAACAUUCAGCUUCUUUUUUGUCAACAACUUUUUUCAAGUUGUUUUGAAGCAUCUAGUUUGUGUCCGCUGUGCAUCAGCGACGUUUAAUAAUAUAACACCUGUAUUUAUUUAUUGCAAUAGU
Bsu-1-12	GAACUUGUGUGCUUCUCUCAAAGCGACUACUUAAUAGUAACAUUUUUAGUUAACUAGGUCAAUACUUUUUUGAAAAAGUUUUUACUAGUCAUAAUGGUCAUGCUUGUGUCUUUAAAAAGACAACAGAAAUGAUUAUACCAUAAUUUUUUAUGACUGUAAAG
Bsu-1-14	UCAUUUCGUUACUUCUCAAUGAAGCAGCUUUACUAAUAUAACAUUCAGCUUCUUUUUUGUCAACAACUUUUUUCAAGUUGUUUUGAAGCAUCUAGUUUGUGUCCGCGUGUGCAUCAGCGACGUUUAAUAAUAUAACACCUGUAUUUAUUUAUUGCAAUAGU
Bsu-1-15	AGAUCAUUUUCACUUCUUGUUGAAGCGACUUUAUUAAUAUAACAUUUUGACUUUCUUUUGUCAAAUGUUUUUUUGAUUUAUUUUCCCGCCGCUGUGAGCUUGUUUUCUCAGAAGCGCAUCAGCGACGUUUAAUAAUAUAACACGGUCAAGAAUCAGAGUC
Bsu-4-8	UCAUUUCGUUACUUCUCAAUGAAGCAGCUUUACUAAUAUAACAUUCAGCUUCUUUUUUGUCAACAACUUUUUUCAAGUUGUUUUGAAGCAUCUAGUUUGUGUCCGCUGUGCAUCAGCGACGUUUAAUAAUAUAACACCUGUAUUUAUUUAUUGCAAUAGU
Bli-1-29	UCCGCUUCUGUCCGAAGCGACUUUAUUAGUAUAACAUUUUCAACGAGAAGUGUCAAAUGUUUUUUUGAUUUUUCGUCGCUGCUGCUUUCUGUUCAAAAACCGUUCCGCUAACAGCGACGCUUAAUAAUAUAUCACCGUUAUAAAUUAGUUGCAACACUUU
Bli-1-31	UCCGCUCCUGUCCGAAGCGACUUUAUUAAUAUAACAUUCUAUCAAUGUGAAUGUCAACAACUUUUUUUAAAAGUUUUUUUGAAGUUGUAUUUAUAAAGCCUUCUUUUUAGGGCGGAUACUAAUAUAACAUCUAUUUUCGUUUUGCACAAUAGCUUUUUUA
Bli-1-33	UGCCGCUCUCAAAGCGACCACUUAAAUAUAACAUUUAUAAGACAAGCGGUCAAUACUUUUUUAAAAAAAGUUUUUGUUGUUGCUUUUAUAAGCGACCAAGAGAAAUUAUAGCACAUUAUGUUCCUGAUGAAAAGAUUAUAUUUGAAAGAUUGAAUAGAAA
Bli-1-35	CUAAAAAUGGAGCGGGUGAUGAGAAUCGAACUCACGACAUCAGCUUGGAAGGCUGAGGUUUUACCACUAAACUACACCCGCAUCGCGAAUAUAUGGCGCGCCCGAGAGGAGUCGAACCCCUAACCUUUUGAUCCGUAGUCAAACGCUCUAUCCAAUUGAG
Bli-1-37	CGUUGCCGCUCUCAAAGCAACUAUAAUAUCUUAACAUUUAAUCGUGAUGUCGUCAACAACUUUUUUUCAAAAAAAUUAUUCGUCAUUAGCGACAAAUAUUAAUUUAACACCUUUAAAUAUAUUUGUCAACAAAAAAUUAUAUUGCGAUAAAUUGUUUAAU
Bli-1-39	UUUGUCACUAAAUUGUCUGGUGAUGAUGCCCAAGAGGUCACACCCGUUCCCAUGCCGAACACGGAAGUUAAGCUCUUCAGCGCCGAGGUAGUUGGGGGCUUCCCCCUGCGAGAGUAGGACAUCGCCAGGCAGGCAUUAUUCCCAGUAGCUCAGUGGUA
Bsp-1-1	UCGAUGGCCUGCUGCUUUGAGAAGCCGGUUCAUAAUGGCUUCACCCGACACCUUCUGCGGGGAAGGAUUCGCUGAAGAUCACAUCCACUCCAGAGCUGUUGAAGCUCCGUAAUGCUUCGUAAAGGGAGGCUGCCACUGUUUCCAGAUUCGCUCUCUUGCCG
Bsp-1-2	GACAAAGUAGAAAAGAUAUACUUUCUUAUCUUAUAAGAUGCAAAAAGCCCGCCGUCUUGCGGCAGGGCUUCCUUUCCAUCAUUCACUUUUUUCGCUGGAAUCGGAGUCUUGUUUCUUUUCAGCUUUAUAGGCUUUCUUGAAAUCACUGCUGUAGUUCAC
Bsp-1-3	UGACUCUUUCCUUUGGAUAAUGAUAGAUUGACUUCUUUUCUUUUCCUCCAAUGAUGAAUAAGAAGGAAGUCAGCCCAAUUCUUCCAAUGAACAUCAGUAUGAUGAUCACGCACUUCCCGACCGCUGACAGAUCGGGGGUUAUCCCCAUCGACAAUCCUGU
Bsp-1-4	AAAUUAUACACAAGUCUUACAAUUUAUAAAUUCCUUAACAGCAAAAAAGGAUGCAGCAUGAGCUGCAUCCUUUUUCUUAUGUAGCCAAAAGGCCUUUCCGGAGAGCGCCAGGAGACAGCCUGCCCAACGCUUCUGCACGGAACUUAUAAAGUGCCCUCA
Bsp-1-5	GAGCUGGACAAAGAAGAGCGGUGGCGACUGUUCUAUCGCCGAGCGCAGGCUAAGGGCGCCAAGUCUGUGGCAACGCCUGCAUGACCUGCAUCCUGUGGGCCUCAAGCACAAGACUCCUGGAUAAAAGGUGCUCUUUGCCUUUAAUUCAGGAGUGUCU
Bsp-1-6	CAGCUGGACAAUCGAAAUGCGGAAAAGCCUUGUUCAGGCCCGACAAGCUUAAGACGCCUAAGGAUGGAAGGCGUUCUUUGCCUUCUGUCCUUAGGUGGCUUAAGACUCGAGGCCUAAGCUUUCCAACUGACAAUCGAAACGCGGAGGGGCCUGUAUCG
Bsp-1-7	GCUGGACAACGAAAUGUGGAGGGCGCUUGUUCUAUUAAGGAACGCGACUAGGAACUCCAAAUCCAGCGCCAUGCACAGGCGCCGCUUUCUGCCCAAUCCAAAAAAGCAGACCUGUAAAGGCCUGUUGUUUUACCAUAUUCAUGAUGUGACGGCUGCUUC
Bsp-1-8	GAGCUGGACAAAUGAAAUGCGGAGGCGACUGUUCAGGUGCGACAAGCAUAAGACGAUUUGGGAUUGAAGGCGUUCUUUGCCUUCGGUUCCAAAGUGGCUUAUGACUCGAGCACCUAGGAGCCGGAGCUGGACAAAUCGAAAUGCGGAGGCGGCUUUCUCC
Bsp-1-9	GCUGGACAAUCGAAAUGCGGAAAAGCCUUGUUCAGCCCCGACAAGCAUAAGACGAUCAGGACGGAAGGUGUUCUUUAACCUUCUGGCCUGAUUGGCUUAUGACUCGAGGGGCUAACCCCCUAACCAGGGCACCCUGAACUGCAGAUAUUUGCGAGUUUU
Bsp-1-10	GCUGGACAAUGAAAAGCGGAGGGCGCUUGCUCAGCCCCGACAAGCUUAAGACAAGCAGAUUGGAAGGUUGUUCUUUAAUUUGCUUGGCUUAAGACUCGAGGGGCUAGCGCCCGGAGCUGGACAAUGAAAAGCGGAAGCGGCUUGCUCAGAAUUAAGGAAC
Bsp-1-11	GCUGGACAAAUCGAAAUGCGGAGGCGGCUUGCUCAGAAUCAAGGAACGCAGGCUAAAACCGCCACGUCCUGUGGCAACGCCUGCAUGACCCGCAUCCUGCGGGCCCAGCAUAAGACGAGCAGGACGGAAGGUUGUCCUUUAACCUUCUGGCCUGAUUGGC
Bsp-1-12	GCUGGACAAAUCGAAAUGCGGAGGGCGCUUGUUCAGCCCCGACAAGCAUAAGACGCUUCAGGAUAGAAGGCGUUCUUUGCCUUCAAUCCUGAAGUGGCUUAUGACUCGAGGCUCUAGCCGCCGGAGCUGGACAAAUCGAAAUGCGGAGGGCGCUUGUUCA
Bsp-1-13	GCUGGACAAAAAGAAAAGCGGAGGGCGCUUGUUCUAUAAGCAGGAAUCUAUCUGCGUAACUGAAUAAGUAGAAAAAAUUGGAAGCCCAAAUCAAAUGCAUGCAUAUGUUGAAUUAUUUUUUCAAUAAAAAACCCCGGCAAAAGCCGAGGCCUGAUUCAUAU
Bsp-1-14	GCUGGACAGUAAGAAAAGCGGAGGGCGCUGCUCUUAGCAGCGCCGCCCAUAAAAUAUCUAUAGCAAUGGAGGGUAUGCCAAUUCGCUAUUGAACAAAUAAAAAUGCCGCAGCUUGGUGAGAGUGUGACAGAGGCACCAUCUCCAAGUGGCUUGUUUCUGU
Bsp-1-15	GCUGGACAAUAGAAAAGCGGAAAUGCCUUGUUCCAGGAACACGGCAUAAAUACUCUGCGUUAUGCCAACAAAAAGAAGCCUGACAAAGGUUCAGGCUUCUUCCCAUUAAUCGUGCAGCAUCAAAUCUUUGGAAGCUCUCUUAUUUCAGCAGUCAUACCUG
Bsp-1-16	AGCUGGACAAGUAGAAAAGCGAAAAAUCCUUGUUUCUAAUUCACAAAAAAACAGCAGGGCUGCCCCCUGCUGUUCUAUUUAUGCUCCUGCACGUGCUUGAGCAUUUUCUGAUGGGAAACCGGAAAGGCAAAUUCCUUCAGCUCUUCUUCGGUUACAAGC
Bsp-1-17	GAGCUGGACAAAUAGAAAAGCGGAGGGCGCUCUCUCAAAACACCACUCUUGCAGAUAUGCUGAAAAGAGGCAGUGCAUCACUGAAUGCACUGCCUCUUUAAUGUUUGAUGCACACUAGGAUAAUAAAUCUGCCGGGUUUCACUUACUUUUUCUGAAACUC
	....
	....
	....

```
Bsp-1-18   UAUUUUACAAACACAUACAUCAAAAGCUCAUCAGAAUAAGAGCCAUCAAAGUUCUUCACAUGCCUGAUCCUUCUGCCUUCCUCCUGGAAUCCCAUUUUUUUAUAUAAAUGAAUGCCCCGUUCAUUAUGUGAGAACACUUCCAGGCAUACUUUUUCAAUGC
Bsp-1-19   GCCGGACAAACCGAAAUGCGGAGACGGCUUGCCCAGGGCCGACAAGCCUAAGACGCGCUAGAAUAAAAGGCGUUCUUUGCCUUUAAUUCUAGCGUGGCUUAUGACUCGAGGCCCUAGCCGUCGCAGCUGGACAAUACGAAAUGCGGAGGGGGCUUGCUCA
Bsp-1-20   UAAAGUAUAGGACAAACACUGCAAACAGGAAGUACAUGAUCGGGUGGAUCUCUUUAGCUUUGCCUUUUACGAUCAUGGUGAUUGGUAGAAGAUAAAGCCGAUUGCGAUUCCGGUUGCAAUGCUGUAUGUGAGCGGCAUGGCUAUGAUCGUCAGGAAGGC
Bsp-1-21   UCAAGGAUUGAUAAUAUUCCACAAGACUUACAAUUUUUAAAUUUCCUGCACAAUAAGGAAGCCCGCUCUGUUAAUCCGGGCGGGCUGUUCCUUCCAGAAGAGGCUCGUACAUCAUAAUGGCAUGGUUCUCUGUUACAUAUUCGUCUUCAUAAAGCUUUCC
Bsp-1-22   UCAAGAAUUGAAAAUUAUCCACAAGUCUUCCAAUUUAUAAAUUCUUCAACUACAAAAAAACAGACCGCAUUGCGGCCUGCCUCAGGAAAUCUUAUUAAUGAAGCUCAUUCUCGGAUUCAAGUAUUGAUUCGGGCUUUUCAGCUGAAAACAAUUGGCUUGA
Bsp-1-23   UCAAGAGUUGGAAAUUUUCCACAAGUCUUUCAAAUUGUAAAAUCUUUAACAGAAAAAACCUGCCAGGCGACUGCGCCGGACAGGUUCAUUCUCAUUCUUUUUCAGCUCUUUCAAUGAUUUCAUUUCUUUCUCCGCCAUCUGAAAGCUCUUCAGAAAAUUG
Bsp-1-24   UCAAGAAUUGAUAGUUAUCCACAAGACUUACCAUUUAUAAUUUCGUUAACAACGAAAAAGUAUGGGCAGAGAAAAUAGAGCCCAUACUUUUUUUAAUGUCGGGCAAAAUAGCACUAAUUGGAUUCGUCUAUUAACAUAGAAUGGAGUGCUAUUAUGAAUU
Bsp-1-25   UAAAUUUCCUUAACUGCAAAAAAUGUUCCCAAAAGUGUAUAACCGUGUUCUUUAUGGAAAUAAUGUAAGUAACAACAUCUAUUCGAGGGUGUUGUUGCCAACCGCGGAGAAGACUUACGUUUCCCCAUAAGUUCUUCCUCCGGUUUCUCCUCUCCCUUUG
Bsp-1-26   GUAAUCUAAGUGAAAAUUGCCGUUUUUUUAUGAGGGAAAAGCCUGCCGUUUGAAUUUUCACAGCCGGCAGGCUUUUUCCUGAAAAGGGAGGAUGAGAACCAUGAGCAUUGAAUUAAUCAUCGUCAUUUUCCUGCUUGGCUUUGCUGGAUCGUUUAUUUCC
Bsp-1-27   AGACAGUAAUCGAAGUUAAAAAAAUAUACUUUCUUAUUUUACCAAAAAGACCCGGCCGGCGGCCGGGUCUUUCUCUGUCUCUAUUAUUCUGCGAUUGCUGCGUACUUUCUUUUAUGCAUGAUAGUGAAAUAAGUGAUCGUGCCAGCAAUGAAUGCGAGCA
Bsp-1-28   AGACAGUAAUCGAAGAUAAAAAAUAAACUUUCUUACCAUUAGAAAAACUCGGCCGCUGCCCGCCUCCAAUUAGGCCUGGCAGCUCCGCAGCAAUUAUGCUUUUUUAUCCUAUAUUUCUGUACUGUAUCAUCCUUUUGUAAUCGGAGCGAAUUUUGCCAGC
Bsp-1-29   AUCAAGAAUUGAUAAUAAUCCACAAGGAUUACUAAUUAUAAAUUCCUAAACUUUAAAAAAGAAAGCAUGCAAUUUUUGCAUGCUUUCUUCUAUAAAAUCAUUUUUAAUGCUGAUUCUAAUUGCUUUUCUCUUUUUGUCCGCCUUUUCUGCAAUUGCAUUU
Bsp-1-30   GAGCGGGACAAUAAGAAAAGCGGAGGGCGCUUGCCCUCCUCGAGAUACAAAAAGAGCGUGCAGCCACAUAAGGCUGCACGCUCUUCUUUCAUUCUUCCUUCCAAAUCCGCUCCAGAACAUGUCCCUCACCACUGAUGGCAAUCCUGAAAACAUCAGGAUU
Bsp-1-31   CAGCUGGACAAAUUGAAAUGCGGAAGGCGCUUGCUCAGCCCCGAAAAUCAUGAUUCCUAUUCAUUAAAAAGCCAGCCCGAAGGCUGGCUUUUUUGCGUGCUUAUUACUUUGUGAAGCCAAUCAUCUUGAUUUCUUUUAUAGACUUUACAGAAAUCGUUUC
Bsp-1-32   UAGCGCCUGGAGCUGGACAAGUAGAAAAGCGGAGGGCGCUGCACCUUUAUAAAAACAAUUGCAGAUCAAUCAUGCCAGCCGGAAGCACAACUUCCGGCUGGCCUUUUAUUAUCGGAAAAAAUGAUUAUAUUAUCGAAAUCAAAAUUUUAUUAUCGAAAUAU
Bsp-1-33   GACGUUGAUCAAGAAAUACUAAUCAUCCACAAGAAUUACAAUUUAUAAAUUCCUUAACAAGCACAAAAAGCCCGCAUCCCAUAUUGGGAUGCGGGCAUGUCCGGCGGAUUAGUACAUUUCAGAAGUUGUCUUCGCCUGCAUAUGCAGAAGGAUAUAAUCA
Bsp-1-34   UGAAACGUAAGGCAGUAAUGCCAUGAUACGCGCACGCUUGAUAGCAACAGUCAAUUUACGUUGGUAUUUAGCGUUUGUACCAGUUACACGGCGAGGUAAAAUUUUACCGCGCUCAGAGAUGAAUUUUUUCAGAAGAUCUACAUCUUUGUAGUCGAUGUGA
Bsp-1-35   GCUGGACAAUAGAAAAGCGGAGGGCGCCUUGCUCAGCCCCGACAAGCAUAAGACAUACAAAAAUAAAAGGCGUUCUUUGCCUUUAAUUCUUGGGUGGCUUAUGACUCGAGGGGCUAGCUCCUGGAGAUGGACAAUAGAAAAGCGGAGCGACUGUUCCAAC
Bli-1-41   AAAUUAAUUUGUCGCUCGACGUUCGCCACAAACGUCCAGAUGGGUAUCAUGAAGUAGAGAUGGUCAUGACGACGAUUGAUUUGGCGGAUCGGGUCGAAUUAACAGAGCUGGCAGAAGAUAAAGUAACGGUUUCUUCGCAUAACCGCUUUGUUCCCGACGA
Bli-1-43   UCGAAGCGGUUUUAGACACAACUGCGCCGGUAACUGCAUAAGAUACCGUAUCAUGCCGGUCUAGCUCAAUUGGUAGAGCAACUGACUUGUAAUCAGUAGGUUGGGGGUUCAAGUCCUCUGGCCGGCACUGUUUUUGGAGGGGUAGCGAAGUGGCUAAACG
Bli-1-45   UGAAAAUCAUAUUAUCGCGGGGUGGAGCAGUUCGGUAGCUCGUCGGGCUCAUAACCCGAAGGUCGCAGGUUCAAAUCCUGCCCCCGCAACCAAAUUACUCAUUAAUGGUCCGGUAGUUCAGUUGGUUAGAAUGCCUGCCUGUCACGCAGGAGGUCGCGGG
Bco-1-1    GAAGAUAUAAUAUUUGCCAUUUGGCAAACCGAGUAUCGUCACUGAUGGAUUUUAACCGAUUUUUAAAGAAGUGAUCGCUCACUUCGAAUUGAGUUUUUAGGUUAAGUUAAUAAGGGCGCACGGUGGAUGCCUUGGCACUAGGAGCCGACGAAGGACGGGA
Bco-1-2    UGGUUAGAGCGCACGCCUGAUAAGCGUGAGGUCGGUGGUUCGAGUCCACUUAGGCCCACCAUUUUCAUUUUUACAACUUAAAAAAAUGGGGCCUUAGCUCAGCUGGGAGAGCGCCUGCUUUGCACGCAGGAGGUCAGCGGUUCGAUCCCGCUAGGCUCCA
Bco-1-3    UGGUUAGAGCGCACGCCUGAUAAGCGUGAGGUCGGUGGUUCGAGUCCACUUAGGCCCACCAUUUUCAUUUUUACAACUUAAAAAAUGGGGCCUUAGCUCAGCUGGGAGAGCGCCUGCUUUGCACGCAGGAGGUCAGCGGUUCGAUCCCGCUAGGCUCCAC
Bco-1-4    UUCGGGCCUAGCGGCGGACGGGUGAGUAACACGUGGGUAACCUGCCUGUAAGACUGGGAUAACUUCGGGAAACCGAAGCUAAUACCGGAUAAUAUUUUUGGUUGCAUGACCGAAAAUUGAAAGGUGGCUUUUAGCUAUCACUUACAGAUGGACCCGCGGC
Bco-1-5    AGCGAGGCCUUCGGGCCUAGCGGCGGACGGGUGAGUAACACGUGGGUAACCUGCCUGUAAGACUGGGAUAACUUCGGGAAACCGAAGCUAAUACCGGAUAACAUUUUUGGUUGCAUGACCGAAAAUUGAAAGGUGGCUUUUAGCUAUCACUUACAGAUGG
Bco-1-6    AGCGAGGCCUUCGGGCCUAGCGGCGGACGGGUGAGUAACACGUGGGUAACCUGCCUGUAAGACUGGGAUAACUUCGGGAAACCGAAGCUAAUACCGGAUAACAUUUUUGGUUGCAUGACCGAAAAUUGAAAGGUGGCUUUUAGCUAUCACUUACAGAUGG
           ................................................................................................................................................................
           ................................................................................................................................................................
           ••••••••••••••••••••••••••••••••••••••••••••••••••••••••••••••••••••••••••••••••••••••••••••••••••••••••••••••••••••••••••••••••••••••••••••••••••••••••••••••••
```

alignment positions 420 · · · 475

```
Bli-1-1    GCUGGAAUUCGGCUUCGAUCAUCCGUUUGACUUCUUCGGCCGUCUCUUCGUCAUCU
Bli-1-3    UUCGCCUUGCUGCAUGGAAAUCUGCGCAGUCGGCGGAUAAGGCCUUUCGGUUUCUU
Bli-1-5    UCUAUAGAAAAACGCUGAAAAGGGAUAGGCGGCAAUUUUUAAAAACUGAGUCUUUC
Bli-1-7    UAGCUGUCACAGUAAAGUCGUCAUUACGGGAUGUCCUAGAUCCUCGGCUUUACUGG
Bsu-1-1    UGAGCAUAUCGAAGAGCUUGUCAUCGGAUUUCUCGCAUCAGAGGGUGUUAUACGAU
Bsu-1-3    ACUUGUACCGACAGUGGUUCCGGCAUACAUAGCGGCAUUGGAAAGGGAAGCGAUGG
Bsu-1-5    GUGUGAUGAUCAUUUUGGAAUGCUCCUGCCCAAUUAACUCAUUGCGAAAAAGAACC
Bsu-4-3    GUGUGAUGAUCAUUUUGGAAUGCUCCUGCCCAAUUAACUCAUUGCGAAAAAGAACC
Gka-1-1    GAACCCACUGUCCGUAAUGGGGCAGUAUCCAUGCCUGAAUCCAUAGGGCAUGGAGG
Gka-1-2    GUUGCUUUGCAUCUGUGCCUGCGUCUGCUAGCAGGUUCAGGGAAGCAAGAUUCCUC
Bpu-2-1    GCAUUAGCUAGUUGGUGGGGUAAUGGCUCACCAAGGCGACGAUGCGUAGCCGACCU
Bpu-2-2    GCAUUAGCUAGUUGGUGGGGUAAUGGCUCACCAAGGCGACGAUGCGUAGCCGACCU
Bpu-2-3    GCAUUAGCUAGUUGGUGGGGUAAUGGCUCACCAAGGCGACGAUGCGUAGCCGACCU
Bpu-2-4    GCAUUAGCUAGUUGGUGGGGUAAUGGCUCACCAAGGCGACGAUGCGUAGCCGACCU
Bpu-2-5    GCAUUAGCUAGUUGGUGGGGUAAUGGCUCACCAAGGCGACGAUGCGUAGCCGACCU
Bpu-1-1    ........................................................
Bpu-1-2    GCAUUAGCUAGUUGGUGGGGUAAUGGCUCACCAAGGCGACGAUGCGUAGCCGACCU
Bpu-2-6    GCAUUAGCUAGUUGGUGGGGUAAUGGCUCACCAAGGCGACGAUGCGUAGCCGACCU
Bha-1-1    CCUGGGGAACUGAAACAUCUAAGUACCCAGAGGAAGAGAAAGCAAAUGCGAUUUCC
Bha-1-2    CCUGGGGAACUGAAACAUCUAAGUACCCAGAGGAAGAGAAAGCAAAUGCGAUUUCC
Bli-1-9    AAAUAUCUUAUCUUAAAAUGGCUUGUUAUGUCAAUAUCGGCCGAAAUAAAUUUGAA
Bsu-1-7    AAUAGUGUAUACAAUAUCCGUUUUAAGGGGAGGCUAACUGUACGGAGGUGGAGAAG
Bli-1-11   CCAUAGCUCAGCAGGUAGAGCACUUCCAUGGUAAGGAAGAGGUCAGCGGUUCGAGC
Bli-1-13   GGCAAGCUGUCUUCAUCUUCUCUGGCCCGUGUUCUGGGUGUUCAAAUCCGAAUUUU
Bli-1-15   AAAAUAUGGUCCGGUAGUUCAGUUGGUUAGAAUGCCUGCCUGUCACGCAGGAGGUC
Bli-1-17   AUAAUCAUCGCUGGCACUUUGAAGAAGAAGGUCAUACCACCAUUCUGUUUCAUACC
Bli-1-19   UAUAGUAAAGUUAAAGUCAAAUAUAGUCAAAGUCAAGUGAAGGAGGGGGCUGAGUG
Bli-1-21   AAAUAUUGUUCUUUGGGAAUACUGAAACCCAUGGAGGUGGAAUGGAUGAGUAAGUC
           ........................................................
           ........................................................
           ••••••••••••••••••••••••••••••••••••••••••••••••••••••••
```

```
Bli-1-23   GAACGCCGGGCUUGUGAAAAGCAUUCGCGGGGCCUACGGCGGCUACAUUCUUGGUG
Bli-1-25   GCGGCGAUUACGGCGGGCGAUAUCAUUCGCGUCCUGGAAGGGCCGAUCAGCCCGGU
Bli-1-27   AAAAGUAUUGCUCAGACAAACGAUUUAUCGGAACAUUAUUUGGAACAGCUUGUUUC
Bcl-1-1    GAUGGGCCCGCGGCGCAUUAGCUAGUUGGUAAGGUAACGGCUUACCAAGGCAACGA
Oih-1-1    GAUCUAGUCCAACCAUGAAAAUUCCGUCCAUGCAAUCGAAUAAUUGUUGUAUCAGG
Oih-1-2    AUUCUAAAAAAAAGGUGGUAAUGCGAUUGACGCUGCAAUUGCUACAGCGGCAUGUUU
Oih-1-3    AGAAAUGUAUUUUCGUCUACCGUUUCAAAAUCCGGUGUUUCAUCUUCAUCAAACUC
Oih-1-4    ACUAAACCAAACAAAAAGGAGAUGCGUGCAAUUGAAUUCUAACAUAACAUUACCUG
Oih-1-5    CACACAAUCUCCUAUUUUUAUUAUCAUUGUUUAAGCUACUAAACAAACCAAAAAGG
Bam-1-1    CAUAGGAUAUGAGAAGGCAGACCCGGGGAACUGAAACAUCUAAGUACCCGGAGGAA
Bsu-1-9    CAAUUGAGCUACGGGCGCUUAUUGAGCGACUUAACUAUCAUACUAUCAUUUGAUGU
Bsu-2-6    UUUUUAAAAAAAAAUCUAUAAAACAAAUAAACUAUAAUCCUCGCCAUGAUUUCUAAA
Bsu-1-11   UUUUUAAAAAAAAAUCUAUAAAACAAAUAAACUAUAAUCCUCGCCAUGAUUUCUAAA
Bsu-3-6    UUUUUAAAAAAAAAUCUAUAAAACAAAUAAACUAUAAUCCUCGCCAUGAUUUCUAAA
Bsu-1-12   GGUUAUGACACAAAAAAGCGCAGCUAUUUCAGCUGCGCUUUUUUUCACACUUCUUC
Bsu-1-14   UUUUUAAAAAAAAAUCUAUAAAACAAAUAAACUAUAAUCCUCGCCAUGAUUUCUAAA
Bsu-1-15   AACACCUUUUUUAAAAGAAUAUCCUCGGUGAUAGACAGCCUGUCCUUUCGUUUCUA
Bsu-4-8    UUUUUAAAAAAAAAUCUAUAAAACAAAUAAACUAUAAUCCUCGCCAUGAUUUCUAAA
Bli-1-29   UUUUAAUAAAAUAUCCUCGGUGAAAAAUCUUUUGGCCCUUUGUCUCUAUCGGCUUU
Bli-1-31   AAAAAAUAAUAAUCAAAUGAAAUUCCCGAUCAUCCCGGACCAGCCGACAAUUGACG
Bli-1-33   AACAUGCAGCCAUUUGGCUGCAUGUUUUUAUGAUUCAUCCGCUUCCUGUUCUUCUG
Bli-1-35   CUACGGGCGCUUCAUCAGGCGACUUAACUAUAAUACUAUGAAUAUCAGUUAAAGUC
Bli-1-37   GACAAUAAGAGCUGCUACACCGGGAACCCCCAAUAAUCCGCUCACUCCGGUUGUUA
Bli-1-39   GAGCUAUCGGCUGUUAACCGAUCGGUCGCAGGUUCGAAUCCUGCCUGCGGAGCCAU
Bsp-1-1    CAUAUAAAUGUAAAAUCGCUGUCGUAAAAUUUUUCAUUUUCGGCCGUUGUAAGCAC
Bsp-1-2    UUCCUGGGUAUCGGAGAGCCCGUUGUUCUGCUCUGUGUUGAACCCUUUGUAUGGUG
Bsp-1-3    CGUUCCGAAGGCCGAACACACUUCAAAAAUAAUAGCUGUCACGCUGUGGGCCUCAG
Bsp-1-4    UAUGCUUCCUGGAAUUUCUGGAUGUCUCCCGCUCCCAUAAAGAGGAUGACACUGUU
Bsp-1-5    UUUGACACGAGGGGCUGGGCUUUCGGGCUGGACAAUAGAAAAGCGGAGGGCGCCUU
Bsp-1-6    GAGAAUGACUGUGAGCUCCCCUCAGGCAUUAUCCUUUUUCAAAGAAACCGCCCAUC
Bsp-1-7    AUUUUUAAUCAGUACUGCUUUGAUUGUAAAAUAGUCGAUUUCAUCCCCGAGGAGCU
Bsp-1-8    GUAAAUCGGCCUCCGCAAAAAGAAGGCAGAUGCCCGUUCCAGAGCAUCUGCCUUUC
Bsp-1-9    CAACUAUAUUUGCGGGUUUUUCAAUAUAUUUGCGACAUUUCUGAUAUAUUUGCGAG
Bsp-1-10   GCAGGCAAAAGGCGCUACGUUCUGUGGCAGCGCCUGCAUGAGGGGGUUUAGUGACU
Bsp-1-11   UUAUGACUCGAGGGGCUAGCGCCCGGAGCUGGACAAAUCGAAAUGCGGAGGGCGCU
Bsp-1-12   GCGCCGACAAGCAUAAGACGCUUCAGGAUAGAAGGCGUUCUUUGCCUUCAAUCCUG
Bsp-1-13   AUUAAACAUUCAGGAAGCUGCCUACCUGGUCUUCCAGGAGCAGGUUUCCGACAAAG
Bsp-1-14   CGGCGAUACAGUAAAUAAAUACGACCCUUUAGCUGAGGUCAUGACAGAUAAAGUGA
Bsp-1-15   UUCCGCAAAGGGGGCAUAAUAAGUCAUCGGAUGCGAAAUCUUUCCUCAUCCACCCA
Bsp-1-16   UUCAGAUCGCUCCGUUCUUCUUCCAGGGAAAGCAGUGUCCCUUUAUAAACAUGGAU
Bsp-1-17   CACCAGAUCCUCCAGUGAAUGAUGAGCCUUUUUUAUCGACGAGAAGAUGUCUUCGU
Bsp-1-18   CCGGCUCCUCUUUAGCCCAUUCCAGGCAGGUGCCGAUCAGCUGCUUUCCGAUGCCU
Bsp-1-19   GCCCUGACAAGCAUAAGACGCUUUAGGAUAGAAGGCGUUCUUUGCCUUCAAUCCUA
Bsp-1-20   UGGAACAGCAAUUUCGAAUCUCUUCCAGUCAAUCUGCCCAAGCGACGAGACCAUCA
Bsp-1-21   CUGCCGGUUAUAGGACCUGCGGAAGAUUUCAUUAUGGCGGACAUAUCCGCCCCAGU
Bsp-1-22   UCGGAAUAAUCGAUUGUCAUGCUUUCAUCGAAAAAGACUUCUUUUGAUUUUUCUAU
Bsp-1-23   GAGUUCUUCUCCGUUUCCGAUCAGCUCUGAUGAUUUAAUUUCUGCUGCGGGGAGAU
Bsp-1-24   UCAUCUGGGGAAUUGCCGGAAUACUGACCGUACUGGUCAUCGCUUUGCUGCUUUCC
Bsp-1-25   CCUUCUGUCCCUAUGGGAUAUAGAAGGGCCCUGCAGGAAUGCAGGGUUUUUUUCUG
Bsp-1-26   GGGCUGCUCGGCAUAGGCGGUGCGAUCAUCAAUUUUCCUAUGCUGCUGUAUAUACC
Bsp-1-27   UAAAGCCAAGGAGGAUGGCUGCAUUAUGCCACAUGAAGGCAAAGUCUCCGCUCGAU
Bsp-1-28   AGGCGUUUGAUGCCGGUCGGGCUCGGGAAGGCGAUAUCGAGCUCUGUUCCUUCUCC
Bsp-1-29   CGCAUACAGAACCGUUACCUUUUCAUCUUCAAAGUGAUCGAUUGUUGAAUUGCAAU
Bsp-1-30   GGACAUUUUCAUCAGCACAUCAAAGCCGCUCUGUUCGUCGAAAUAUCUGAGCAGGA
Bsp-1-31   UAAGGAUUGAUCCAUAAGUUUUAUUUCUACCCUAUCUUCUGAAGGCAUGCUCUGCA
Bsp-1-32   GCAUUUUUUUAUCGAAAUCCUAACACCAAUCACCAAUAGCAUCACCGUCUUCUGAA
Bsp-1-33   GGUCCUCCAGCUUUUGAAUCUGUACCAGACAUGUUGAAACCGCCGAAUGGCUGGUA
Bsp-1-34   GUGAUUCCGUUUGCUGUGAAGUAGCAAACUUUACGGCGCUUUGCACGUCCGCCUCU
Bsp-1-35   GAGGAACGCAGGCUAAGGGUGCCGCGUUCUGUGGCAGCUUCUUAAAAGGGCCUUUG
Bli-1-41   UCAGCGAAACCUUGCUUAUCAGGCUGCAAUGCUCAUGAAAGAACGCUACGGCAUUA
Bli-1-43   CGGCGGACUGUAAAUCCGCUCCCUCAGGGUUCGGCGGUUCGAAUCCGUCCCCCUCC
Bli-1-45   UUCGAGUCCCGUCCGGACCGCCAUUUAAAUAGAGGCUCGGUAGCUCAGUUGGUAGA

           ........................................................
           ........................................................
```

```
Bco-1-1   CUAACACCGAUAUGCUUCGGGGAGCUGUAAGUAAGCUUUGAUCCGGAGAUUUCCGA
Bco-1-2   CCACUUGUACUUUGAAAACUAGAUAAAGCAACACAUGAAGAUAUAAUAUUUGCCAU
Bco-1-3   CACUUGUACUUUGAAAACUAGAUAAAGCAACACAUGAAGAUAUAAUAUUUGCCAUU
Bco-1-4   GCAUUAGCUAGUUGGUGAGGUAACGGCUCACCAAGGCAACGAUGCGUAGCCGACCU
Bco-1-5   ACCCGUGGCGCAUUAGCUAGUUGGUGAGGUAACGGCUCACCAAGGCAACGAUGCGU
Bco-1-6   ACCCGCGGCGCAUUAGCUAGUUGGUGAGGUAACGGCUCACCAAGGCAACGAUGCGU
          ........................................................
          ........................................................
```

10 *Bacillus*-plasmid

10.1 Taxa

The taxonomy of each organism containing a putative *Bacillus*-plasmid RNA is listed, with abbreviations identifying each hit (e.g., "Eco-1-1" and "Eco-1-2" might hypothetically represent two distinct RNAs in *E. coli*). The abbreviations will be used to identify each individual *Bacillus*-plasmid RNA in Sections 10.2 and 10.4.

**abbrev. of hits**	**taxonomy of species**
Bpu-1-1 to Bpu-1-2	Bacteria Firmicutes Bacillales Bacillaceae *Bacillus pumilus*
Bsu-1-1 to Bsu-1-6	Bacteria Firmicutes Bacillales Bacillaceae *Bacillus subtilis*
Bsu-2-1	Bacteria Firmicutes Bacillales Bacillaceae *Bacillus subtilis subsp. subtilis str.* 168
Bce-1-1 to Bce-1-2	Bacteria Firmicutes Bacillales Bacillaceae Bacillus Bacilluscereus group *Bacillus cereus* 03BB108
Bce-2-1	Bacteria Firmicutes Bacillales Bacillaceae Bacillus Bacilluscereus group *Bacillus cereus* E33L
Bce-4-1	Bacteria Firmicutes Bacillales Bacillaceae Bacillus Bacilluscereus group *Bacillus cereus subsp. cytotoxis* NVH 391-98
Bce-3-1	Bacteria Firmicutes Bacillales Bacillaceae Bacillus Bacilluscereus group *Bacillus cereus* W
Bmy-1-1 to Bmy-1-4	Bacteria Firmicutes Bacillales Bacillaceae Bacillus Bacilluscereus group *Bacillus mycoides*
Bth-1-1	Bacteria Firmicutes Bacillales Bacillaceae Bacillus Bacilluscereus group *Bacillus thuringiensis*
Bth-2-1	Bacteria Firmicutes Bacillales Bacillaceae Bacillus Bacilluscereus group *Bacillus thuringiensis serovar israelensis*
Bth-3-1	Bacteria Firmicutes Bacillales Bacillaceae Bacillus Bacilluscereus group *Bacillus thuringiensis serovar israelensis* ATCC 35646
Lbr-1-1	Bacteria Firmicutes Lactobacillales Lactobacillaceae *Lactobacillus brevis* ATCC 367
Lca-1-1	Bacteria Firmicutes Lactobacillales Lactobacillaceae *Lactobacillus casei* ATCC 334
Lcu-1-1	Bacteria Firmicutes Lactobacillales Lactobacillaceae *Lactobacillus curvatus*
Lhe-1-1	Bacteria Firmicutes Lactobacillales Lactobacillaceae *Lactobacillus helveticus subsp. jugurti*
Lpl-1-1 to Lpl-1-3	Bacteria Firmicutes Lactobacillales Lactobacillaceae *Lactobacillus plantarum*
Lsa-1-1	Bacteria Firmicutes Lactobacillales Lactobacillaceae *Lactobacillus sakei*
Pac-1-1	Bacteria Firmicutes Lactobacillales Lactobacillaceae *Pediococcus acidilactici*
env-1 to env-2	environmental samples

10.2 Gene contexts

Each *Bacillus*-plasmid RNA (indicated by "RNA→") is listed with its surrounding genes. Environmental sequences and some RefSeq entries lack gene annotations, and no genes are listed for such sequences. The direction of each gene is indicated with an arrow (→), and each conserved domain in the gene is colored. Conserved domains associated with more than one *Bacillus*-plasmid RNA are assigned a color; other domains are gray. Information about these conserved domains is given in Section 10.3. The accession number of the sequence containing each *Bacillus*-plasmid RNA is given. Accessions beginning with "NC_" or "NZ_" are RefSeq, while other accession refer to environmental samples. Nucleotide coordinates are given for the 5′ and 3′ boundaries of each. If the 5′ coordinate is greater than the 3′ coordinate, the RNA is present on the reverse-complement strand of the containing genomic DNA sequence. Each hit is denoted by an abbreviation (like "Eco-1-1") that refers to a taxonomy given in Section 10.1.

abbrev.	Seq. accession		5′ at	3′ at	genes
Lpl-1-1	NC_003894.1	-	3083	3036	hypo→ **RNA**→ ←hypo
Lpl-1-2	NC_004944.1	-	2644	2584	hypo→ **RNA**→ Rep_3 (pfam01051)→
Lbr-1-1	NC_008499.1	-	332	272	hypo→ **RNA**→
Pac-1-1	NC_004832.1	+	264	324	**RNA**→ Rep_3 (pfam01051)→
Lcu-1-1	NC_003320.1	+	3074	3134	hypo→ **RNA**→ Rep_3 (pfam01051)→
Lsa-1-1	NC_004942.1	+	149	209	**RNA**→ Rep_3 (pfam01051)→
Lca-1-1	NC_008526.1	-	360213	360153	hypo→ **RNA**→ UPF0236 (pfam06782)→
env-1	BABE01000260.1	+	3320	3379	Mob_Pre (pfam01076)→ **RNA**→ Rep_trans (pfam02486)→
env-2	BABE01002635.1	+	459	518	hypo→ **RNA**→
Lpl-1-3	NC_002123.1	+	1928	1989	Rep_1 (pfam01446)→ **RNA**→
Bce-1-1	NZ_ABDM01000007.1	+	167738	167800	**RNA**→
Bmy-1-1	NC_006870.1	+	3291	3366	Peptidase_S26 (pfam00461)→ **RNA**→ ←hypo
Bce-1-2	NZ_ABDM01000060.1	+	278	352	**RNA**→
Bmy-1-2	NC_006869.1	+	120	192	**RNA**→ Mob_Pre (pfam01076)→
Bth-1-1	NC_001446.1	+	6441	6496	hypo→ **RNA**→ Mob_Pre (pfam01076)→
Bth-3-1	NZ_AAJM01000209.1	-	3338	3283	←hypo **RNA**→ Mob_Pre (pfam01076)→
Bsu-1-1	NC_001764.1	+	5114	5169	←HTH_YyaN (cd01109) **RNA**→ Rap40→
Bpu-1-1	NC_004932.1	+	376	432	**RNA**→ TPR (cd00189)→
Bpu-1-2	NC_001858.1	+	104	159	**RNA**→ TPR (cd00189)→
Bsu-1-2	NC_002075.1	+	6989	7044	hypo→ **RNA**→ hypo→
Bsu-1-3	NC_001765.1	+	5173	5228	←SoxR (COG0789) **RNA**→
Bsu-1-4	NC_006825.1	+	5969	6024	←SoxR (COG0789) **RNA**→
Bsu-1-5	NC_007956.1	+	5968	6023	←SoxR (COG0789) **RNA**→
Bsu-1-6	NC_001766.1	+	6354	6409	hypo→ **RNA**→ Rap60→
Bth-2-1	NC_002091.1	+	5075	5141	collagen-like protein→ **RNA**→
Bsu-2-1	NC_000964.2	+	535441	535494	COG2946 (COG2946)→ **RNA**→ hypo→
Bce-2-1	NC_007104.1	+	1986	2052	hypo→ **RNA**→ hypo→
Bce-4-1	NC_009674.1	-	2388712	2388646	←Tra8 (COG2826) **RNA**→ Mob_Pre (pfam01076)→
Bmy-1-3	NC_005703.1	+	594	660	**RNA**→ Mob_Pre (pfam01076)→
Bmy-1-4	NC_005704.1	+	3044	3110	←putative Rep protein **RNA**→
Bce-3-1	NZ_ABCZ01000046.1	-	1174	1108	**RNA**→
Lhe-1-1	NC_001379.1	+	65	124	**RNA**→ RepA_N (pfam06970)→

10.3 Conserved domains

Conserved domains found in protein-coding genes listed in Section 10.2 are listed, with the first sentence in their description from the Conserved Domain Database. Conserved domains associated with more than one *Bacillus*-plasmid RNA are assigned a color, while others are shown in gray. The number in parentheses is the number of occurrences of the given gene.

D cd00189 (2) Tetratricopeptide repeat domain; typically contains 34 amino acids [WLF]-X(2)-[LIM]-[GAS]-X(2)-[YLF]-X(8)-[ASE]-X(3)-[FYL]-X(2)-[ASL]-X(4)-[PKE]
u cd01109 (1) Helix-turn-helix transcription regulator YyaN.
u COG0789 (3) Predicted transcriptional regulators [Transcription]
u COG2826 (1) Transposase and inactivated derivatives, IS30 family [DNA replication, recombination, and repair]
u COG2946 (1) Putative phage replication protein RstA [DNA replication, recombination, and repair]
u pfam00461 (1) Signal peptidase I.
D pfam01051 (4) Initiator Replication protein.
uD pfam01076 (6) Plasmid recombination enzyme.
u pfam01446 (1) Replication protein.
D pfam02486 (1) Replication initiation factor.
D pfam06782 (1) Uncharacterised protein family (UPF0236).
D pfam06970 (1) Replication initiator protein A (RepA) N-terminus.

Duplicate sequences: the following putative homologs are not shown in the alignment because their sequences are identical to a homolog already shown: Bsu-1-5

10.4 Multiple-sequence alignment

Each *Bacillus*-plasmid RNA is denoted by an abbreviation (like "Eco-1-1") that refers to a taxonomy given in Section 10.1. The alignment includes sequences containing the RNA motif, as well as flanking sequence. The *Bacillus*-plasmid RNA itself is denoted by the line underneath marked 5′ and 3′ on either end. Nucleotides in the flanking sequences (i.e., not part of the motif) are written in gray letters. Stems of predicted rho-independent transcription terminators are shaded yellow. (Note: terminator predictions have not been analyzed manually, and many are likely to be false positives.) Start codons are shaded green. (Note: start codons are frequently misannotated, especially in environmental samples.) Nucleotides proposed to basepair as part of the consensus structure are shaded in color when they comprise Watson-Crick or G-U pairs. Otherwise they are shaded gray. Conserved stems are also indicated at the bottom of the alignment by angle brackets, where matching < and > denote base-paired columns. Below these angle brackets, the symbol "2" denotes base pairs exhibiting covariation, "1" denotes base pairs exhibiting compatible mutations, "0" denotes base pairs that are not observed to mutate and "?" denotes base pairs that have a significant frequency of non-canonical nucleotides for Watson-Crick or G-U pairs (> 5%). Below these base pair annotation is the consensus sequence: "R" = "A" or "G", "Y" = "C" or "U", red nucleotides: nucleotide identity conserved more than 97% of the time, black nucleotides: 90%, gray nucleotides: 75%, red circle (•): nucleotide is present 97% of the time, black circle (•): 90%, gray circle (•): 75%, white circle (◦): 50%. All percentages of sequences just described (e.g. 97% conserved) assume that sequences have been weighted by the GSC algorithm implemented by the Infernal software.

alignment positions 1 · · · 99

```
Lpl-1-1   UAUAUAAACAAGUUUGAAACCGCUGAGAAUAAAAAAUAAAGGCCUUAAAAAGGAGCAUGUUAGUCAAAUUCUUAUUAAGUCUUAGAAAAACUAAUUGUU
Lpl-1-2   AUAUGUAAAUAUGCUUAAAACUGCUGAGAAUUGAAAAUAAAGGCCUUAAAAGGGACAUAGCAGUUAAAUUCUUAUUAUGUUUUAGAAAAGCUAACUGUU
Lbr-1-1   GUAUAUGAAUAGUCUUAAAAUCGCUAGAAACGAAAAAUAAGGCCCUUAAAAACGAACAUAGCAGCUAAAAUCUUGUUGAGAUUCAAAAAACUAACUGUU
Pac-1-1   GUAUAUGAAUGGUCUUAAAAUCGCUAGAAACGAAAAAUAAGACCCUUAAAAACGAACAUAGCAGCUAAAAUCUUUUUUAGAUUCAAAAAACUAACUGUU
Lcu-1-1   UAUAUGAAUAGUCUUAAAAUUGCUAGAAACGAAAAAUAAGGCCCUAAAAAAACGAACAUAGCAGUUAAAUCUUUAUCAAGUUUUAGAAAAGCUAACUAUU
Lsa-1-1   UAUAUGAAUAGUCUUAAAAUUGCUAGAAACGAAAAAUAAGGCCCUAAAAAAACGAACAUAGCAGUUAAAUCUUUAUCAAGUUUUAGAAAAGCUAACUAUU
Lca-1-1   UAUAUAAACAGUCUCAAAAUCGCUAGAAACGAGAAAUAAGGCCCUUAAAAACGAAUAUAGCAGUUAGAUUCUUGUUAAGAUUCAAAAAAACUAACUGUU
env-1     AAAAAUAUUAGAUCAAGAGAAAACGUUGGCUAGUCAGCAGAAUGCCCCAGAACGUAAUAUAGGGCGUUCUGAGAAUGAGAUGGAGUGGUAGUUAUACGG
env-2     AGAAGUGUUAGAGCAGGAGAAAACCUUAUUUUAUCGUCAAAACGUCUCACAGCGUAAAUUAGAGCGUUCUGAAGAUGAGAUCGAGUGGUAGUUACGAUG
Lpl-1-3   CUAGCAUGCUGGAACAUGUUACAAAAAUUAAGUUCAGUCGUUUACACUCCUUCGAACUUAAUCCUUGUACCAUGUUGCAAUUAAAACCGAGUACUAGCU
Bce-1-1   GGUGAACAUCAAGAAGAAUUCAAAGAAAUUUAUGAUUCAAUUCAAAGUGAAGUAUUAAACGUAAUGAAUAGUUAUAAGUUGCAUUCGUAAAAUAAUCUG
Bmy-1-1   AUAAUGAGGACAGCGAAGUAUAAUAUUCCAUAAGUUGGGACAAAAAAGGAGUCUGUAUUCAGGCCCUUUUUUAUUUACCAUAAGAGGUAGAUAUACAGU
Bce-1-2   GAUAUUGAAGCGAAACCAAUUUCACGUACAGAUAUUGUACAAAAAUUAAUACAAGCAAAAUACGAUAGUCUUACAAACGAUUAAAGGUAGUUAUAUCGU
Bmy-1-2   UAAAAAAGAGAAGGAUUAUAAAAAUCUUUCUCUUUUUUAUUUAUAAACUAUUAUCUAGUGUAAUAAAUUUUAAAAAAAGAAACCGGAUUUAAUAUGGCU
Bth-1-1   GAAUUGUUUCAAAAACACCACUCCUAUUGGUUUUUAUCUAUAUAUUUCUAAAAAAUAUAAGGAUUCCUGCAAGUAAAAUAUAAAAAAGAAAAACUAGGU
Bth-3-1   GAAUUGUUUCAAAAACACCACUCCUAUUGGUUUUUAUCUAUAUAUUUCUAAAAAAUAUAAGGAUUCCUGCAAGUAAAAUAUAAAAAAGAAAAACUAGGU
Bsu-1-1   AUGAUGAAUUAAGUUUCGUGUUUGUUUUGUGUUGUUUCGGAAAAGUGUGCACUCAAAAGGAGCGUUACUGUUUAAAAAAUGGAGUGAAAAACAUAUGGC
Bpu-1-1   AUGGAUGAAUUAAGUUCGUGUUUGUUUUGUGUUGUUUCGGAAAGGUGUGCACUCAAAAGGAGCGUUACUGUUUAAAAAAUGGAGUGAAAAACAUUCGGC
Bpu-1-2   CUUUGAAUUAAGUUUCGUGUUUGUUUUGUGUUGUUUCGGAAAAGUGUGCACUCAAAAGGAGCGUUACUGUUUAAAAAAAUGGAGUGAAAAACAUAUGGC
Bsu-1-2   CAGGGGCUUAUAAUAUUCAUAGUUGUUGUUAGAACCUGAACCCUUUUACCGUUUUUGGCGGUGAGGGUUCUUUUUUUUAGGCAGUGAUGCGAGUUGGGG
Bsu-1-3   CAGGGCUUAUAAUAUUCAUAGCUGUUGUUAGAACCUAAACCCUUUUACCGUUUUUGGCGGUGAGGGUUCUUUUUUUUUAGGCAGUGAUGCGAUUUUGGC
Bsu-1-4   AGGGGCUUAUAAUAUUCAUAGCUGUUGUUAGAACCUAAACCCUUUUACCGUUUUUGGCGGUGAGGGUUCUUUUUUUUUAGGCAGUGAUGCGAUUUUGGC
Bsu-1-6   AGAAAUCCUUCUUAUUAGUUUAUUCAACUUUCUAUAUUUAUUAUACAUGUGAAAAUUUUAUGUUUCAAGGGUGGUUUUAUUUAAACGAGUUUUUAUGGC
Bth-2-1   GAUACAAGAAAAAAGAUUUUAUGACGCUAGAUAGAAAGUUUAAUAUGCAAUUCAUUGCGGUCGCAAAACGAUUACUGGAGUAGGGAAAGAUUUAGAGAU
Bsu-2-1   UAAAAAAAUGUUAGAUGUGUAUAUGGCUGAUGUUGCUGACAUGGUCGUUUAAAACCAAGCUGCUAAACCUUAAUAAACACAGGUGAUGCGAUUAAACGU
Bce-2-1   CGUUUUAAGGGUAUAUAGCAAGUUUUAAUCGUAUUCGUUUUAUAGAAAAAAAGAAUUAAAAAGGCUAGUGCUGAAAUAGAUAAGAGGUAGUUGCAUGGC
Bce-4-1   UGACUAUUCACAAUAUUAAUUUACCAUAAUUCAAGAUUUUUAAAUUAGGAUAAUUAUGUAUAUUUUGUGUAAUGUUCUAUUUUUCCGACUCUACAUGGC
Bmy-1-3   CUUUAUAUUCCAAUUAGUAUUUUUGUGUUAGCUAUUGGUUUGCUUUCCUUUUUUGAAUAUAGAAAAAGGAAAGUAAAUGAAAAGGGGUAAUUACAUGGU
Bmy-1-4   AUAGAAUGUUGAUUUUAUAAAUCAAAAGAGAGGGGAACCUAUGUAGUGAUACUUGAGUAGAAAAUUAGGUUUUUAAUUAGGUAGAGGUAGCUGCAUAGC
Bce-3-1   UUUAAACGUAAACAAAUUCCCUAUAUAUGUAAACGGAACCCGAUUUUAGAAAUAUAAGACUGAAUAUUCUGAUUUUUGGGUAGUGUUCUAAAAUAAAAA
Lhe-1-1   ....................................CAGCUGUAUUAUAGCACGAAAAAAUUUUUAGCAAUGUAAAAUUCUAAAAAAUCUAAAAAAGAC
          ...................................................................................................
          ...................................................................................................
          •••••••••••R••••••••••••••••••••••••••••••••••••••••••••R•••••••••••••••••••••R•••RRRRR••R••••••R•Y
```

alignment positions 100 · · · 259

```
Lpl-1-1  UGCUGUCAAUGG.UAGCGU.............................GAGAGCAU.AAGGAAUUAACAG.CUUUUAACUAGUUUUUGCACCUUUUAGCGUAUAAUGCUGCUUAGGUUGCUUGUAAGCAUACAAAAAGGCCUAUCAAAUUAAUGACGGCC
Lpl-1-2  UGCUGUCAAUGG.UAGCGG..........AC.GAGC.....AAAGCGUGGGAGCAU.AAGGAAUUGACAG.CUCUAAACCAGUCUUAACACUGAAUUGGCGAAAGCCAAAGUUUCUAUAAAACUUUGCUUUCCUGCCUAACGGCGAGUGAAAAAACGGUC
Lbr-1-1  UGCUGUCAAUGG.UAGCGG..........AC.GAGC.....AAAGCGUGGGAGCAU.AAGGAAUUGACAG.CUCUAAACCAGUCUUAACACUGAAUUGGCGAAAGCCAAAGUUUCUAUAAAACUUUGCUUUCCUGCCUAACGGCGAGUGAAAAAGCGGUU
Pac-1-1  UGCUGUCAAUGG.UAGCGG..........AC.GAGC.....AAAGCGUGGGAGCAU.AAGGAAUUGACAG.CUCUAAACCAGUCUUAACACUGAAUUGGCGAAAGCCAAAGUUUCUAUAAAACUUUGCUUUCCUGCCUAACGGCGAGUGAAAAAGCGGUC
Lcu-1-1  UGCUGUCAAUGG.UAGCGG..........AC.GAGC.....GUAGCGUGGGAGCAU.AAGGAAUUGACAG.CUCUAAACCAGUCUUAACACUGAAUUGGCGAAAGCCAAAGUUUCUAUAAAACUUUGCUUUCCUGCCUAACGGCGAGUGAAAAAGCGGUU
Lsa-1-1  UGCUGUCAAUGG.UAGCGG..........AC.GAGC.....GUAGCGUGGGAGCAU.AAGGAAUUGACAG.CUCUAAACCAGUCUUAACACUGAAUUGGCGAAAGCCAAAGUUUCUAUAAAACUUUGCUUUCCUGCCUAACGGCGAGUGAAAAAGCGGUU
Lca-1-1  UGCUGUCAAUGG.UAGCGG..........AC.GAGC.....GUAGCGUGGGAGCAU.AAGGAAUUGACAG.CUCUAAACCAGUCUUAACACUGAAAUGGCGAAAGCAAAGUUUUAUAGAAACUUUGCUUUCCUGCCUAACGGCGAGUGAAAAAGCGGUCA
env-1    UGCAGUCAACGG.UACCGG..........A..GGCG.....GUCUGUGCCGAGGAU.AAGGAGUUGACGG.CUAAACCACUAAUCCCUGAAGCUGACAUUUGCUAAAAAAUGGCAAGUCCAUUUUUUGGCCAACUUAGCGUUAGCGAAGUUUGGGGGCGC
env-2    UGCAGUCAACGG.UACCGG..........AG.GCGG.....UUUGUGC.CGAGGAU.AAGGAGUUGACGG.CUAAACCACUAGCCUAUUAAGCUGACAUUUGCUAAAAAAUGGCCAGUCCAUUUUUUGGCCAACCUAGCGUUAGCGAAGUUUGGGGCGC
Lpl-1-3  GGUAGUCAACGG.UACCGG..........AACGACC.....AACGGGAGCGAGGAU.AAGGAGUUGACUAGCUA.ACUAAACCCAGACACUGAAUUGGCUGAAAGCCAAAGUUUCCUAAACUUUGCUUUCCUGCCUUCACGACGAGUGAUGGGGUGAGUU
Bce-1-1  AUUAGUCAACGGCUAGCGG..........ACAACCG.....UAAGGGAGGGGCAUU.AAGGAGUUGACUA.AAUAACACCUCCAACCCCUCAAAAAAUGAGGGAUUGGGGGUGUUAUUUUUUCUUUUAAUACUUUAAUAUUGUUGUAAUUUAUAUUUGCC
Bmy-1-1  GUCUGUCAACGG.UAAGCGGACAGAAUGAA..GCUGUAAGCGAAAUGAAGGAGCAUUAAGGAGUUGACAG.ACUUACUAUGCAACCCUCUUGUGGUUGUCAAAAGGUACCCUUUUGACCCUGCCUACUGGCGAGGGAGCCCCUCAAGAAGUGAGGGGUUG
Bce-1-2  GUGUGUCAACGG.UAAGCGGACAGAUCGAAGCGAUAGC...GAAGAGAAGGAGCAUUAAGGAGUUGAUAC.ACUCACAAUGCAACCCUUUUAUGGUUGUCAAACGGUACCCGUUUGACCCUGCCUACCGGCGAGGGAGCCCCUCAAGGAAUUGAGGGGUU
Bmy-1-2  AUAUGUCAACGG.UAAGCGGACGAACGAAGCAAAGC.....GAAGUGAAGGAGCAUUAAGGAGUUGACAU.AUUUACCACACCCAAGACCCUCUUGUGUGGGUCAAACGGUACCCGUUUGACCCUGCCAACCGGCGAGGGAGCCCCUCAAGGAUUGAGGG
Bth-1-1  GUUUGUCAACGG.UAAGCG.............GACC.....GAAGGGAGC...AUU.AAGGAGUUGACAA.ACUCAUGACACUAAGACCCUCUUGGAUUGUCAAAACGGAAACGUUUUGGCCUGCCAACCGGCGAGGGAGCCCCUCAAGGAUUGAGGGGU
Bth-3-1  GUUUGUCAACGG.UAAGCG.............GACC.....GAAGGGAGC...AUU.AAGGAGUUGACAA.ACUCAUGACACUAAGACCCUCUUGGAUUGUCAAAACGGAAACGUUUUGGCCUGCCAACCGGCGAGGGAGCCCCUCAAGGAUUGAGGGGU
Bsu-1-1  AUGAGUCAACGG.UAAGCG.............GAAC.....GAAGUGAGC...AUU.AAGGAGUUGACUC.AUUCACGACACCCAAAAGCCUGUUAAUCAAACAAACCCCGUUUGUUUGCGCCUGCCGAAGGGGCAUCCCCCGAAGAUCGGGGGUAGGGG
Bpu-1-1  AUGAGUCAACGG.UAAGCG.............GAACG....AAAGUGAGC...AUU.AAGGAGUUGACUC.AUUCACUACAUCCAAAGCCUAUUGAUUCAAACAAACCCCGUUUGUUUGCGCCAGCCGAAGGGGCAUCCCCCGAAGAUCGGGGGUAGGGG
Bpu-1-2  AUGAGUCAACGG.UAAGCG.............GAAC.....GAAGUGAGC...AUU.AAGGAGUUGACUC.AUUCACUACACCCAAAGCCUAUUGAUUCAAACAAACCCCGUUUGUUUGCGCCAGCCGAAGGGGCAGCCCCCGAAGAUCGGGGGUAGGGG
Bsu-1-2  GUGAGUCAACGG.UAACCG.............GACC.....GUAGGGAGG...AUU.AAGGAGUUGACUC.GCUCAGCGCCACCCGAACCCUUUCAGCACUCAAACAAACCCGUUUGUUUGACGCCAACCGGCGAGGGAGCCCCCGAAGAUGCGGGGG
Bsu-1-3  GUGAGUCAACGG.UAACCG.............GACC.....GUAGGGAGG...AUU.AAGGAGUUGACUC.GCUCAGCGCCACCCGAACCCUUUCAGCACUCAAACAAACCCGUUUGUUUGACGCCAACCGGCAGGGAGCCCCCGAAGAAGCGGGGGUU
Bsu-1-4  GUGAGUCAACGG.UAACCG.............GACC.....GUAGGGAGG...AUU.AAGGAGUUGACUC.GCUCAGCGCCACCCGAACCCUUUCAGCACUCAAACAAACCCGUUUGUUUGACGCCAACCGGCGAGGGAGCCCCCGAAGAUGCGGGGG
Bsu-1-6  GUGAGUCAACGG.UAACCG.............GACC.....GUAGGGAGG...AUU.AAGGAGUUGACUC.GCUCAGUGCUACCCGAACCCUUUCAGCACUCAAACAAACUCGUUUGUUUGACGCCAACCGGCGAGGGAGCCCCCGGAGAUGCGGGGG
Bth-2-1  AAGUGUCAACGG.UACCGG..........ACCGAAC.CCGAAAGGGGCGGGAGGAU.AAGGAGUGGACACGUGCAUCGGAUAGUUCAUCCUCUUGGCAGUUUCUCCAAACAUGUUGUUUGGGGAACGCCAACUGGCGAGGGAGCCUCCCAGGAUUGGGAG
Bsu-2-1  AUGUGUCAACGG.UACCGG..........AA.............CGAAGUGAGGAU.AAGGAGUUGACAU.AUUCAGCGCUACCCAAACCCUCUUGGCAUUGAACAAACAACCGUUUGUUCCUGCCUACCGGCGAGGGAGUCCCCGAUGAUUCGGGGA
Bce-2-1  GUGUGUCAACGG.UACCGG..........ACCGAACCCGAAAGGGUGCGGGAGGAU.AAGGAGUUGACAC.ACUUACUAUGCGACCCUCUGGGCAUUGAACAAACUACGGUUUGUUCCUGCCAACCGGCGAGGGAGCCCCUCAAGGAUUGAGGGGUUGGG
Bce-4-1  GUGUGUCAACGG.UACCGG..........ACUGAACCCGUAAGGGUGAGGGAGGAU.AAGGAGUUGACAC.ACUCACUAUGCGACCCUCUUGGUUUGUCAAAACGUUUACGUUUUGGCCUGCCAACUGGCGAGGGAGUCCCUCAAGGAUUGAGGGGUUGG
Bmy-1-3  GUGUGUCAACGG.UACCGG..........ACCGAACCCGUAAGGGGGUGGGAGGAU.AAGGAGUUGACAC.ACUCACUAUGCAACCCUCUUGGCUUGUCAAAACGGUUACCGUUUUGGCCUGCCAACCGGCGAGGGAGUCCCUCAAGGAUUGAGGGGUUG
Bmy-1-4  GUGUGUCAACGG.UACCGG..........ACCGAACCCGUAAGGGGGCGGGAGGAU.AAGGAGUUGACAC.ACUCACUAUGCAACCCUCUUGUGGUUGUCAAAAGGUACCCUUUUGACCCUGCCAACUGGCGAGGGAGCCCUCAAGGAUUGAGGGGUUG
Bce-3-1  UCGUGUCAACGA.UACCGG..........ACCGAGCCCGAAAGGGAUAGGGAGGAU.AAGGAGUUGAUAC.GUCCACUACAACCAAACCCUCUUGCAGAACCAAAACGUUCCUCGUUUUGGCCUGCCAACCGGCGAGGGAGCCCCUCAAAGAUUGAGGGG
Lhe-1-1  AAAUGUCAACGG.UACCGG..........ACUGAGC.....UU.GCGAGGGAGGAU.AAGGAGUUGACAU.UUU.GCUGUCAAAACACUUGAAAACCAGGAGAAAGAAUUUAUUCUUUCUUCCUGCCUAACGGCGAGUGAUAAAAAAUGGGCUAUUCUAA
         .<<<<<<<<<....................................................>>>>>>>>.>........................................................................................
         .222100002....................................................20000122.2........................................................................................
         ●●●●GUCAACGG-UA●●●G----------○○-GA●●-----R●RG●G●G●GR○●●U-AAGGAGUUGAC●●-●YYYA●●RY●●●●●●●●CYY●●●●●●●●●●R●●R●●●●●●●●YY●●Y●●●●Y●●Y●●●●●●●●●●●●●●Y●●●●●R●●●●●●R●●R●●●
          5′—————————————————————————————————————————————————————————————————————3′
```

alignment positions 260 · · · 419

```
Lpl-1-1  UCUUUAUUGAUUGGAAUUUUCACACUAUUAUGAAGUAGAUGUGUCAUUAUGUCGGAGACACAAGCUUGAUUAUAGCACUAUGCAAACAUGUUGCAACCACUAAAAAAGUGAGCACCCGGGUAAGUGCUCACUAAGAAGUUGUCACUUCAAAACGAAAUAU
Lpl-1-2  AAGCUGGUUCAGCUUGACGGGGUGCGGGGCGUCAGCGCCCGUAUAAAUGUGGCUUGCCACACCUUUUUAGGCAACGAACGAAGUGAGGCGCAAAGAGCGUAGCGAAUGGAGUUUAAUGUGAGCCGGUUUUUGGCUCACUCCUUUGUGUUUUUGAUUUUAG
Lbr-1-1  AAGCUGGCUCAGCUUGGACGGGGUGCGGGGCGUCAGCGCCCGUAUUAUAUGUGGCUUGCCACACCUUUUUAGGCAACGAACAGAGUGAGGCGCAAGGAGCAUAGCGACUGAAGUUUAAUGUGAGCUGGUUUUAGCUCACUCCUUUGUGUUUUUUGCUUCU
Pac-1-1  AAGCUGGCUCAGCUUGGACGGGGUUCGGGGCGUCAGCGCCCGAAUUAAUGUGGCUUGCCACACCUUUUAGGCAACGAACAGAGUGAGGCGCAAGGAGCAUAGCGACUGGAGUUUAAUGUGAGCCUGUUUUUUGGCUCACUCCUUUGUGUUUUUGUUUUAG
Lcu-1-1  AAGCUGGCUCAGCUUGGACGGGGUUCGGGGCGUUAGCGCCCGAUUAAAUGUGGCUUGCCACACCUUUUAAGCAACGAACAGCGUGAGGCGCAAGGAGCUGUGCGACUGGAGUUUAAUGUGAGCCGUUUUUUGGCUCACUCCUUUGUGUUUUUUGAUUCUA
Lsa-1-1  AAGCUGGCUCAGCUUGGACGGGGUUCGGGGCGUUAGCGCCCGUAAUAAAUGUGGCUUGCCACACCUUUUUAGGAAACGAACAAAGUGAGGCGCAAAGAGCGUAGCGAAUGGAGUUUAAUGUGAGCCGGUUUUUGGCUCACUCCUUUGUGUUUUUAUUUUA
Lca-1-1  AGCUGGUUCAGCUUGGACGGGGUUGGGGGCGUCAGCGCCCGAAUAAACGUGGCUUGCCACACCUUAUCUGGCAACGAACAGAGUGAGGCGCAAGGAGCGUAGCGACUGGAGUUGAAUGUGAGCCAGUUUUUAGCUCACUCCUUUGUGUUUUUUUGUUUCU
env-1    CCUUUUCAAAGGGUGCGGUUUUAUGUUUUUGGAUAUUUGCAUAGCUAAAGAGAAGUGCUAUUAUUGAGUUAUCAAAUAAAUAAACUGAAAAAAGAGCAAAAAAAUAAAGUCGAGCAACGCUGGACACGUUCUCGACCUUUACGGAAACUACUUCACCUAG
env-2    GCUUUUCAAAGGGUGCGGUUUUAUUUUUUGGAUAUUUGCAUAGCUAAAGAGAAGUGCUAUUAUUGAGUUAGCAAACAAAUGAACUGAAAAAAGAGUAAAAAAAUAGUCGAGCAACGCUACCAACGUUCUCGACCUUUACGGAAACUAAUCGCACUAGCUC
Lpl-1-3  UGAGCGGGAGCACCCGCUCAAGUUUUGCGGUCAAGCUGGUUCAGCUUGGUCGGGGUUCGGGGCGACAGCGCCCGAGGCUUGAAACUGGCGAUUUUUGCCAGUUCCCUCUUAUCUUGAUACAUACUAGACACAAUUAUUUUUUCAAAACUGCCUUUAAG
Bce-1-1  AAAAAGCGUAUUGUUUUCUACUUUGCUUAAUAGAAUUGUUCCGUUAAAACUAACAUAUUUUUUGUUAUUAUGUAAGCAUUAGCUAUACCUACUUUUGCAACAUAAAUAAAAUUAUGUUCUUCAAGAUAUUUAAUAGUUUUAUAUAUUGUUGUUCUACCUU
Bmy-1-1  GGGAGUUCGAUUAAAUGGGGUCUGGGGAAGAGUUCCCCAGUGGUUUGGGCAAAGCCCAAGGUUUGUUUUUGCCAUGCUAAGCAUGGCUCGGCAAAGCCGAAAAGCGUCGCAGACCCCUAUCUAUUUUGAUGCGUUAGCGUCAAAAUACCUAUAGGGUUA
Bce-1-2  GGGGAGUUUGAUAAAUGGGGUCUGGGGAAGGGUUCCCCAGUGGUUUGGGCAAGGCCCACGGUUUUGGGGGUGUUUGUAGGGGCUUGCCCCCUCAAGGUUUUUAUGGGGCGGAAUCGCCACAUCAAGCAGUAUUUGGGUACCAAAUACGAAGCUUGCUC
Bmy-1-2  GGUGGGGAGUUGGAUAUAGGGGUCUGGGGAAGAGUUCCCCAGUGGGUUUGGGCGAUGCCCAAGGUUUGAUUUUGUCAUGCUGAGCAUGGCUCGGCGAAGCCGACAAGCGUCGCAGACCCCUAUCAAUUUUGAUGCGUUAGCGUCAAAAUACGUAUAGGGU
Bth-1-1  UGGGGAGUUCGAUUGAUGGGGUCAAGGGGAAAAUUUCCCCUUGUAGGUUCGGACAAAGUCCGAGGUCUUUUAGCCAUGCUAAGCAUGGUUCGGCGAAGCCGACAAGCGUCGCAGACCCCUAUCGAUUUUGAUGCGUUACGUCAAAAUACGUAUAGGGUUA
Bth-3-1  UGGGGAGUUCGAUUGAUGGGGUCAAGGGGAAAAUUUCCCCUUGUAGGUUCGGACAAAGUCCGAGGUCUUUUAGCCAUGCUAAGCAUGGUUCGGCGAAGCCGACAAGCGUCGCAGACCCCUAUCGAUUUUGAUGCGUUAGCGUCAAAAUACGUAUAGGGUU
Bsu-1-1  GUUUGAAUAUUUGCCAUCCAACGUGCUAGCGUUGGCGGGUUUGGGCAGAGCCCAAGAAAGGCGGUCGGAUGAUGAAAAUGACAAGAGAGCGUUUUGAAAAAGGCAUGAGAUAUGCGAAAGCGACACAUGCUAUUGAAGGAAUUUAUCUUACAGCUGACGA
Bpu-1-1  GUUUGAAUAUUUGCGAUCCAACGGGCUGACCGUUGGCGGGUUUGGGCAGAGCCCAAAAAGAGUCAUUGGAGAACCAGUGCCCACUUAUGCACCAACAGAAUGCCAGGGCAUUGUACCGUUGGGAGAUUGAUGAAAAAAUUUGGUGGAGAUGAUAGAAUAA
Bpu-1-2  GUUUGAAUAUUUGCGAUCCAACGGGCUGACCGUUGGCGGGUUUGGGCAGCGCCCGAAAAGAGUCACUAGGGAACCAGUGCCCACUUAUGCACCUAAAUAGGUGCUGUGUGUUUUCUCUCCGUUCAAAGCAGAAUGCCAGGGCAUUGUACCGUUGAGAAUU
Bsu-1-2  UUGGGGGGAUUGAAUGCUGGCAUCCAACGGCCGUCCGUUGGUGGGUUUGGGCAAAGCCCAAGAACUGUUGAAAGGCUCGUUGAGAAUAAAGAAUGCUUUUCAGGAUGCUUAGAAUCGUUUCUGAGAGCUUCAAAUAAAAAAGAUGACCUUUUAUAGGGGG
Bsu-1-3  GGGGGGAUUGAAUGCUGGCAUCCAACGGCCGUCCGUUGGUGGGUUUGGGCAAAGCCAAGAACUGUUGCAAGGCUCGUUGAGAAUAAAGAAUGCUUUUCAGGAUGCUUAGAAUCGUUUCUGAGAGCUUCAAAUAAAAAAGAUGACCUUUUAUAGGGGGAAG
Bsu-1-4  UUGGGGGGAUUGAAUGCUGGCAUCCAACGGCCGUCCGUUGGUGGGUUUGGGCAAAGCCCAAGAACUGUUGCAAGGCUCGUUGAGAAUAAAGAAUGCUUUUCAGGAUGCUUAGAAUCGUUUCUGAGAGCUUCAAAUAAAAAAGAUGACCUUUUAUAGGGGG
Bsu-1-6  UUGGGGGGAUUGAAUGUUGGAAUCCAACGGCCAUCCGUUGGUGGGUUUGGGCAAAGCCCAAGGUUCUACUUUAACUCUUGAUGCAAUUUGUUGUUUUUGUCGAGCAAGACAAUUCCUUCAGGGCUUCGUUCUGGGAAGUGUUGUGUGAGAUUUAGCUUUU
Bth-2-1  GUUGGUGGGUGGAUGUUUGUAUUGCCAUCCAAGGGGCUUCCCUUGGUCGGCGUUUGGGGCGAAACCCCAAGGUCUUUGUUGCGCUGUGGCGUAACUUGGCGAAGCCAAAGAGCCUCGCAGAGCCUUUUCAUUGUGUACGAGUAACGGAGUGAAGUGUCA
Bsu-2-1  AUAGGGGGAUAGAUUAACAGCAAUCCAACGAGCUACCGUUGGUGGGUUUGGGUGAAACCCAAGAACUAACUUGGAGGGAUAAGAUGCAUAAUGUCAAUCUGUUGAAUCAGGCUGGACUGGAAAAUGCUUUGGAAUCAGUUGGCUGUUUAGAUAUUGUAGA
Bce-2-1  GAGUUCGAUUAAGGGGGUCUGGGGAAGAGUUCCCCAGUGGGUUUGGGCAACGCCCAAGGUUUUUAUUCGGCAAGCCGAAUCCUCCGUAGGAGCACACUCUUUACGAUAGUAAAGUAUAGUGUGCUAUACUUUACAUGGAAGUCCACCGAAAAACAGUAGA
Bce-4-1  GGAGUUCGAUUGAAGGGGUCAAGGGGGAUUCUCCCCCUUGUAGGUUCGGACAAAGUCCGAGGUUUUUUGCCAUGUUAAACAUGGGUCGGCGGAGCCGACAAGCGUCGCAGACCCCUAUGUAUUUUGAUGCGUUAGCAUCAAAAUACAUAUAGAUUUUACA
Bmy-1-3  GGGAGUUCGAUUGAGGGGGUCAAGGGGAAGAGUUCCCCUUGUAGGUUCGGACAAAGUCCGAGGUCUUUUUUUGCCAUGCAGGGCAUGGAUCGGCGAAUGCCGAAGAGCCUCGCAGAGCACGUCCAAAUGAAAUUUGGUAUAACGUGCUACACCAUUUAUG
Bmy-1-4  GGGAGUUGGAUUAAGGGGGUCUGGGGAAGAGUUCCCCAGCAGGUUUGGACAGAGUCCAAGGUUUUGUUUUUUGUCAUGCGAAGCAUGGCUUGGCGAUAGCCAAAACAGCCUCGUAGAGCCUUUUCAUUGUGUACAUGAAGCGUAGCGUAAUGUCACAAUA
Bce-3-1  UUGGGGAGUUUGAUUCAGGGGGUCUGGGGAAGAGUUCCCCAGUAGGUUUGGACAACGUCCAAGGUCUGAUUUGGUGAAGCCAAAUUCCCCUUUAGGGGCACACUCUUUACGAUAGUAAAGUAUAGUGUGCUAUACUUUACAUGGAAGUCCACCGAAAAAC
Lhe-1-1  UAGCCCGUUACCCGUUCAAAAAUUGAACCUUCAGUUGCAAAAGUAGAAAAAUUCGUUCUUUGUGUGAGUAUGAAGUUACUCAUAGAAAGAUGAAUAACUGGAGCAUUUUUAGAAUGUUACCUAUGACUACUUUUGCAUGUAUUAAAUAUUGGAAUGGUAU
         ................................................................................................................................................................
         ................................................................................................................................................................
         ●●●●●●●●●●●●●●●●●●●●●●●●●●R●●●●●●●●Y●●●●●●●●●●●●R●●●●●●●●●●●●●●●●Y●●●●●●●●●●●●●●●RR●●●●●●●●●●●●●●●●●●R●●●●●●●●●●●●●●●●●●●●●●●●●●●●●●●●●●●●●●●●●●●●●●●●●●●●●●●●●
```

alignment positions 420 · · · 480

```
Lpl-1-1   CAUGAGUCCCGACUGAUAGGGACAACUUUAUUUUAGAUCAUUCUAAUUAAAUUGCAAUAUU
Lpl-1-2   GUCUUGAUCUCGUACAGUGGUGCCUCUUAUAUACCUCUUUUAUAAACCUCUUUUAAACCUC
Lbr-1-1   AGGUUUUGAUCUCGUACAGUGGUGCCUCUUUU.............................
Pac-1-1   AUUUUGCCCUCGUACAGUAGUGCAUCUUUUAAACCUCUUUUAUAAACCUCUUUUAAACCUC
Lcu-1-1   GGUUUUGAUCUCGUACAGUGGUGCCUCUUAUAUACCUCUUUUAUAAACCUCUUUUAAACCU
Lsa-1-1   GGUUUUGAUCUCGUACAGUGGUGCCUCUUAUAUACCUCUUUUAUAAACCUCUUUUAAACCU
Lca-1-1   AGGUUUUGGUCUCGUACAGUAGUGCCUCUUUUAUACCUCUUUUAUACCUCUUUUAUAAACC
env-1     CUCCGUAACUCAGGUUCAUUAUACGAAUAUUUUUAGGUUUCUUCAAGCUAGAGAUUUUUGG
env-2     CGUAACUUCUUAAUCAUUAUACGAAUAUUUUUAGAUUUCUUCAAGACUAGAGAUUUUUUGG
Lpl-1-3   CCUUGACACGUCUGGCCUAAAGCGUAUUAUGAUGGUAUAAAACGAAACAAUAAAAAAAACCC
Bce-1-1   UUUUGAAUUCUUCACUUAAAAGUUACUGAGAAGCCAUUACUAGGGGGUGUUAUAAAAUGCC
Bmy-1-1   CGUCAAUUGACACAAUUUCGUCGCCUUGCUAGACUUAUUGUAUCAAUUAAUCUAGGGGAAG
Bce-1-2   AUCCGACUGAUUUUUACUAUAAUGGGAGUACAAAUGUACGACAAUUAUAGUAAAAAUCUUA
Bmy-1-2   UACGUCAAUUGACACAAUUUCGUCGCCUUGCUAGACUUAUUGUAUCAAUUAAUCUAGGGGA
Bth-1-1   CGUUAAUUGCUUUGCUAGACUGAUGGUAUCAAUUAAUCUAGGGAAAGUGGUGGCACUUUAU
Bth-3-1   ACGUUAAUUGACAUCAUUUCGUCGCUUUGCUAGACUGAUGGUAUCAAUUAAUCUAGGGAAA
Bsu-1-1   GGAAGAAUUAUUAUGGCAGCAUGCAUCCGGUCAGAUCACAGAUGAGGAAUUUGAGAGAAAG
Bpu-1-1   GAGAGGAUUUAUUCAUCUGAUUGUUUGGCGAAGGUGCGAAGCGAAAAGGCGGCGGUAGUCG
Bpu-1-2   UUGGAAGAUGAAUGGUAUAAUAGAUUCCUAAUAUCCUGUUCCUAGUCAGUUUGGCGAAGGU
Bsu-1-2   AAGCUCUUAAAAAUGAAUGUAGGGGCAUUUAAACACGUUUAAAAAUAAAAAAAGCAGACUC
Bsu-1-3   CUCUUAAAAUUGAAUGUAGGGGCAUUUAAACACGUUUAAAAAUAAAAAAAGCAGACUCUUU
Bsu-1-4   AAGCUCUUAAAAUUGAAUGUAGGGGCAUUUAAACACGUUUAAAAAUAAAAAAAGCAGACUC
Bsu-1-6   GAUCAGAAAGCAACUCCUGCAUUUUCUUUUGUGCUUCAGCGAGCGUUUCGAUUUUCAAUUU
Bth-2-1   UAAUACAGAAAAGGUUACUUUGCGAAAGUAACAAA..........................
Bsu-2-1   AGAUUUAAUAGAAAAGAUGCAGGAAUAUGUUUUGUAUCAUACCGAAACUGCCGAAAGAUUU
Bce-2-1   GAAGUAUAUAUACUUUAUAUGGUUCUAGGGACUGAUAAGGACGUAGAACCAUAUGGAAAAG
Bce-4-1   UAAAUUGACUCCAUUUCGUCGCUUUGCUAGACUUAUGGUAUCAAUUCAUCUAGGGGAAGUG
Bmy-1-3   AGUGUAGCUAUCACCCAUGUGUUAUGAUAGUACCAAAAAGUGGAGGGGUCCUCAUGGCAAA
Bmy-1-4   CAGAAAAGGUUACUUUGGCAAAGUAACA.................................
Bce-3-1   AGUAGAGAAGUAUAUAUACUUUAUAAAGUGGUCAGGCGCAAAAUAAAGAGAAAAAUUUAGG
Lhe-1-1   GAAUGCUCGAUAUGCUAGAGGAUCAUUGUCAUUAACGUUUUACAGGAAAAGUAUUGCUGAU
          .............................................................
          .............................................................
          .............................................................
```

11 Bacteroid-*trp*

11.1 Taxa

The taxonomy of each organism containing a putative Bacteroid-*trp* RNA is listed, with abbreviations identifying each hit (e.g., "Eco-1-1" and "Eco-1-2" might hypothetically represent two distinct RNAs in *E. coli*). The abbreviations will be used to identify each individual Bacteroid-*trp* RNA in Sections 11.2 and 11.4.

**abbrev. of hits**	**taxonomy of species**
Bca-1-1	Bacteria Bacteroidetes Bacteroidia Bacteroidales Bacteroidaceae *Bacteroides caccae* ATCC 43185
Bfr-1-1	Bacteria Bacteroidetes Bacteroidia Bacteroidales Bacteroidaceae *Bacteroides fragilis* NCTC 9343
Bfr-2-1	Bacteria Bacteroidetes Bacteroidia Bacteroidales Bacteroidaceae *Bacteroides fragilis* YCH46
Bov-1-1	Bacteria Bacteroidetes Bacteroidia Bacteroidales Bacteroidaceae *Bacteroides ovatus* ATCC 8483
Bth-1-1	Bacteria Bacteroidetes Bacteroidia Bacteroidales Bacteroidaceae *Bacteroides thetaiotaomicron* VPI-5482
Bun-1-1	Bacteria Bacteroidetes Bacteroidia Bacteroidales Bacteroidaceae *Bacteroides uniformis* ATCC 8492
Bvu-1-1	Bacteria Bacteroidetes Bacteroidia Bacteroidales Bacteroidaceae *Bacteroides vulgatus* ATCC 8482
Pdi-1-1	Bacteria Bacteroidetes Bacteroidia Bacteroidales Porphyromonadaceae *Parabacteroides distasonis* ATCC 8503
Pme-1-1	Bacteria Bacteroidetes Bacteroidia Bacteroidales Porphyromonadaceae *Parabacteroides merdae* ATCC 43184
ueu-1-1	Bacteria Bacteroidetes environmental samples unidentified eubacterium SCB49
Fba-1-1	Bacteria Bacteroidetes *Flavobacteria bacterium* BBFL7
Fba-2-1	Bacteria Bacteroidetes Flavobacteria Flavobacteriales *Flavobacteria bacterium* BAL38
Cat-1-1	Bacteria Bacteroidetes Flavobacteria Flavobacteriales Flavobacteriaceae *Croceibacter atlanticus* HTCC2559
Fjo-1-1	Bacteria Bacteroidetes Flavobacteria Flavobacteriales Flavobacteriaceae *Flavobacterium johnsoniae* UW101

Fps-1-1	Bacteria Bacteroidetes Flavobacteria Flavobacteriales Flavobacteriaceae *Flavobacterium psychrophilum* JIP02/86
Gfo-1-1	Bacteria Bacteroidetes Flavobacteria Flavobacteriales Flavobacteriaceae *Gramella forsetii* KT0803
Lbl-1-1	Bacteria Bacteroidetes Flavobacteria Flavobacteriales Flavobacteriaceae *Leeuwenhoekiella blandensis* MED217
Pir-1-1	Bacteria Bacteroidetes Flavobacteria Flavobacteriales Flavobacteriaceae *Polaribacter irgensii* 23-P
Psp-1-1	Bacteria Bacteroidetes Flavobacteria Flavobacteriales Flavobacteriaceae *Polaribacter sp.* MED152
Pto-1-1	Bacteria Bacteroidetes Flavobacteria Flavobacteriales Flavobacteriaceae *Psychroflexus torquis* ATCC 700755
Rbi-1-1	Bacteria Bacteroidetes Flavobacteria Flavobacteriales Flavobacteriaceae *Robiginitalea biformata* HTCC2501
Fba-3-1	Bacteria Bacteroidetes Flavobacteria *Flavobacteriales bacterium* HTCC2170
Asp-1-1	Bacteria Bacteroidetes Sphingobacteria Sphingobacteriales Cyclobacteriaceae *Algoriphagus sp.* PR1
Chu-1-1	Bacteria Bacteroidetes Sphingobacteria Sphingobacteriales Flexibacteraceae *Cytophaga hutchinsonii* ATCC 33406
Mma-1-1	Bacteria Bacteroidetes Sphingobacteria Sphingobacteriales Flexibacteraceae *Microscilla marina* ATCC 23134
Psp-2-1	Bacteria Bacteroidetes Sphingobacteria Sphingobacteriales Sphingobacteriaceae *Pedobacter sp.* BAL39
env-1 to env-21	environmental samples

11.2 Gene contexts

Each Bacteroid-*trp* RNA (indicated by "RNA→") is listed with its surrounding genes. Environmental sequences and some RefSeq entries lack gene annotations, and no genes are listed for such sequences. The direction of each gene is indicated with an arrow (→), and each conserved domain in the gene is colored. Conserved domains associated with more than one Bacteroid-*trp* RNA are assigned a color; other domains are gray. Information about these conserved domains is given in Section 11.3. The accession number of the sequence containing each Bacteroid-*trp* RNA is given. Accessions beginning with "NC_" or "NZ_" are RefSeq, while other accession refer to environmental samples. Nucleotide coordinates are given for the 5′ and 3′ boundaries of each. If the 5′ coordinate is greater than the 3′ coordinate, the RNA is present on the reverse-complement strand of the containing genomic DNA sequence. Each hit is denoted by an abbreviation (like "Eco-1-1") that refers to a taxonomy given in Section 11.1.

**abbrev.**	**Seq. accession**		**5′ at**	**3′ at**	**genes**
Psp-2-1	NZ_ABCM01000008.1	+	235022	235164	**RNA**→ TrpE (COG0147)→ PabA (COG0512)→ indole-3-glycerolphosphate synthase→
Asp-1-1	NZ_AAXU01000004.1	+	467000	467130	**RNA**→ TrpE (COG0147)→ Ribosomal_S23p (pfam05635)→ PabA (COG0512)→ TrpD (COG0547)→ IGPS (cd00331)→ TrpF (COG0135)→ TrpB (COG0133)→
Rbi-1-1	NZ_AAOI01000005.1	+	7798	7916	**RNA**→ TrpE (COG0147)→ PabA (COG0512)→ TrpD (COG0547)→ TrpC (COG0134)→ TrpF (COG0135)→ TrpB (COG0133)→ trp_synt_A (cd00346)→
Mma-1-1	NZ_AAWS01000005.1	-	77275	77138	**RNA**→ TrpE (COG0147)→
Cat-1-1	NZ_AAMP01000001.1	-	1429786	1429665	**RNA**→ TrpE (COG0147)→ PabA (COG0512)→ TrpD (COG0547)→ IGPS (cd00331)→ TrpF (COG0135)→ TrpB (COG0133)→
env-1	JCVI_SCAF_1101668064648	+	1028	1145	**RNA**→ ? (GOS681)→
env-2	JCVI_SCAF_1096627072666	+	1417	1534	**RNA**→ TrpE (COG0147)? (GOS681)→
env-3	JCVI_SCAF_1101668438486	-	379	262	**RNA**→
Fba-1-1	NZ_AAPD01000009.1	+	45681	45788	**RNA**→ TrpE (COG0147)→ hypo→ PabA (COG0512)→ TrpD (COG0547)→ IGPS (cd00331)→ TrpF (COG0135)→ TrpB (COG0133)→ TrpA (COG0159)→
Chu-1-1	NC_008255.1	+	3762321	3762447	**RNA**→ TrpE (COG0147)→ PabA (COG0512)→
Pir-1-1	NZ_AAOG01000004.1	-	65950	65830	**RNA**→ TrpE (COG0147)→
Psp-1-1	NZ_AANA01000002.1	+	232794	232908	**RNA**→ TrpE (COG0147)→ PabA (COG0512)→ TrpD (COG0547)→ hypo→ IGPS (cd00331)→ Mug (COG3663)→ TrpF (COG0135)→ TrpB (COG0133)→ TrpA (COG0159)→
Lbl-1-1	NZ_AANC01000011.1	+	62850	62973	**RNA**→ TrpE (COG0147)→ PabA (COG0512)→
env-4	JCVI_SCAF_1096628041415	-	689	564	**RNA**→ Anth_synt_I_N (pfam04715)? (GOS681)→
env-5	BABB01000545.1	-	1746	1604	**RNA**→ TrpB (COG0133)→ hypo→
env-6	BAAU01027801.1	-	741	599	**RNA**→ TrpB (COG0133)→
env-7	BAAY01005429.1	-	1262	1120	**RNA**→ TrpB (COG0133)→ hypo→
Bvu-1-1	NC_009614.1	+	5029321	5029462	**RNA**→ TrpB (COG0133)→ TrpE (COG0147)→ PabA (COG0512)→ TrpD (COG0547)→ TrpC (COG0134)→ TrpF (COG0135)→ trp_synt_A (cd00346)→
env-8	BAAU01000768.1	+	936	1077	**RNA**→ TrpB (COG0133)→ hypo→
env-9	BAAV01013420.1	+	190	331	**RNA**→ TrpB (COG0133)→

env-10	BAAX01007295.1	-	1158	1017	**RNA**→ TrpB (COG0133)→
env-11	BABG01003022.1	+	1510	1651	**RNA**→
env-12	BABB01001584.1	-	2460	2322	**RNA**→ TrpB (COG0133)→ TrpE (COG0147)→
Bun-1-1	NZ_AAYH02000042.1	-	71718	71577	**RNA**→
env-13	BABA01003390.1	+	487	628	**RNA**→ TrpB (COG0133)→ hypo→
env-14	BABG01015768.1	+	769	915	**RNA**→
Bfr-2-1	NC_006347.1	+	3050356	3050502	**RNA**→ TrpB (COG0133)→
env-15	BAAU01006342.1	-	1825	1679	**RNA**→ TrpB (COG0133)→ hypo→
Bfr-1-1	NC_003228.3	+	3113186	3113332	**RNA**→ TrpB (COG0133)→
env-16	BAAZ01029283.1	-	197	51	**RNA**→
Bca-1-1	NZ_AAVM02000001.1	-	509313	509169	**RNA**→ TrpB (COG0133)→ TrpE (COG0147)→ PabA (COG0512)→ TrpD (COG0547)→ IGPS (cd00331)→ TrpF (COG0135)→ TrpA (COG0159)→
Bov-1-1	NZ_AAXF02000052.1	+	120432	120576	**RNA**→
Bth-1-1	NC_004663.1	-	657806	657662	**RNA**→ TrpB (COG0133)→ TrpE (COG0147)→ PabA (COG0512)→ TrpD (COG0547)→ IGPS (cd00331)→ TrpF (COG0135)→ TrpA (COG0159)→
Pme-1-1	NZ_AAXE02000021.1	+	155182	155313	**RNA**→ TrpB (COG0133)→ TrpE (COG0147)→ PabA (COG0512)→ TrpD (COG0547)→ IGPS (cd00331)→ TrpF (COG0135)→ TrpA (COG0159)→
env-17	BABD01002850.1	-	1611	1480	**RNA**→ TrpB (COG0133)→
env-18	BABB01001973.1	-	2743	2604	**RNA**→ TrpB (COG0133)→ TrpE (COG0147)→
Pdi-1-1	NC_009615.1	-	693568	693428	**RNA**→ TrpB (COG0133)→ TrpE (COG0147)→ PabA (COG0512)→
ueu-1-1	NZ_ABCO01000006.1	-	190508	190390	**RNA**→ TrpE (COG0147)→ PabA (COG0512)→ TrpD (COG0547)→ TrpC (COG0134)→ TrpF (COG0135)→ TrpB (COG0133)→ TrpA (COG0159)→
env-19	JCVI_SCAF_1096627859334	+	530	635	**RNA**→ ? (GOS681)→ TrpE (COG0147)? (GOS681)→
Pto-1-1	NZ_AAPR01000002.1	+	281378	281492	**RNA**→ TrpE (COG0147)→ PabA (COG0512)→ TrpD (COG0547)→ IGPS (cd00331)→ TrpF (COG0135)→ TrpB (COG0133)→ TrpA (COG0159)→
Fba-2-1	NZ_AAXX01000005.1	-	34839	34711	**RNA**→ TrpE (COG0147)→
Fjo-1-1	NC_009441.1	+	5912046	5912172	**RNA**→ TrpE (COG0147)→ hypo→ PabA (COG0512)→
Fps-1-1	NC_009613.1	-	621441	621313	**RNA**→ TrpE (COG0147)→
env-20	JCVI_SCAF_1101668030442	+	227	342	**RNA**→ TrpE (COG0147)? (GOS681)→ TrpE (COG0147)? (GOS681)→
Fba-3-1	NZ_AAOC01000005.1	+	43902	44018	**RNA**→ TrpE (COG0147)→ PabA (COG0512)→ TrpD (COG0547)→ TrpC (COG0134)→ TrpF (COG0135)→ TrpB (COG0133)→ TrpA (COG0159)→ hypo→ ArgE (COG0624)→
env-21	wf3_AHAI20157_g2	-	837	716	**RNA**→ TrpE (COG0147)→
Gfo-1-1	NC_008571.1	+	2785164	2785282	**RNA**→ TrpE (COG0147)→ PabA (COG0512)→

11.3 Conserved domains

Conserved domains found in protein-coding genes listed in Section 11.2 are listed, with the first sentence in their description from the Conserved Domain Database. Conserved domains associated with more than one Bacteroid-*trp* RNA are assigned a color, while others are shown in gray. The number in parentheses is the number of occurrences of the given gene.

cd00331 (8) Indole-3-glycerol phosphate synthase (IGPS); an enzyme in the tryptophan biosynthetic pathway, catalyzing the ring closure reaction of 1-(o-carboxyphenylamino)-1-deoxyribulose-5-phosphate (CdRP) to indole-3-glycerol phosphate (IGP), accompanied by the release of carbon dioxide and water.
cd00346 (2) Tryptophan synthase alpha chain; Tryptophan synthase is a bifunctional pyridoxal-5'-phosphate (PLP)-dependent tetrameric (2 alpha and 2 beta subunits) enzyme that catalyzes the last 2 steps of the L-tryptophan synthesis, the condensation of indole-3-glycerol phosphate (IPG) and L-serine to L-tryptophan and water.
COG0133 (26) Tryptophan synthase beta chain [Amino acid transport and metabolism]
COG0134 (4) Indole-3-glycerol phosphate synthase [Amino acid transport and metabolism]
COG0135 (12) Phosphoribosylanthranilate isomerase [Amino acid transport and metabolism]
COG0147 (29) Anthranilate/para-aminobenzoate synthases component I [Amino acid transport and metabolism / Coenzyme metabolism]
COG0159 (8) Tryptophan synthase alpha chain [Amino acid transport and metabolism]
COG0512 (18) Anthranilate/para-aminobenzoate synthases component II [Amino acid transport and metabolism / Coenzyme metabolism]
COG0547 (12) Anthranilate phosphoribosyltransferase [Amino acid transport and metabolism]
COG0624 (1) Acetylornithine deacetylase/Succinyl-diaminopimelate desuccinylase and related deacylases [Amino acid transport and metabolism]
COG3663 (1) G:T/U mismatch-specific DNA glycosylase [DNA replication, recombination, and

repair]
GOS681 (7) ?

pfam04715 (1) Anthranilate synthase component I, N terminal region.
pfam05635 (1) S23 ribosomal protein.

Duplicate sequences: the following putative homologs are not shown in the alignment because their sequences are identical to a homolog already shown: env-9

11.4 Multiple-sequence alignment

Each Bacteroid-*trp* RNA is denoted by an abbreviation (like "Eco-1-1") that refers to a taxonomy given in Section 11.1. The alignment includes sequences containing the RNA motif, as well as flanking sequence. The Bacteroid-*trp* RNA itself is denoted by the line underneath marked 5′ and 3′ on either end. Nucleotides in the flanking sequences (i.e., not part of the motif) are written in gray letters. Stems of predicted rho-independent transcription terminators are shaded yellow. (Note: terminator predictions have not been analyzed manually, and many are likely to be false positives.) Start codons are shaded green. (Note: start codons are frequently misannotated, especially in environmental samples.) Nucleotides proposed to basepair as part of the consensus structure are shaded in color when they comprise Watson-Crick or G-U pairs. Otherwise they are shaded gray. Conserved stems are also indicated at the bottom of the alignment by angle brackets, where matching < and > denote base-paired columns. Below these angle brackets, the symbol "2" denotes base pairs exhibiting covariation, "1" denotes base pairs exhibiting compatible mutations, "0" denotes base pairs that are not observed to mutate and "?" denotes base pairs that have a significant frequency of non-canonical nucleotides for Watson-Crick or G-U pairs (> 5%). Below these base pair annotation is the consensus sequence: "R" = "A" or "G", "Y" = "C" or "U", red nucleotides: nucleotide identity conserved more than 97% of the time, black nucleotides: 90%, gray nucleotides: 75%, red circle (●): nucleotide is present 97% of the time, black circle (●): 90%, gray circle (●): 75%, white circle (○): 50%. All percentages of sequences just described (e.g. 97% conserved) assume that sequences have been weighted by the GSC algorithm implemented by the Infernal software.

alignment positions 1 · · · 144

```
Psp-2-1   CCCUGAUCCUGAUUGUAGCUGCAAUCAUAGCAAUGGUGAAAUUUGACCCUACACGUACCUUUUGGGGGUAUCAUAAAAUUUCACAAUUUAAUUUGGUCUUGUAAGAUUUAUUUAUAUUUUUGUUGCUCAUGA.UUAAC.A...
Asp-1-1   UUACAAGGAUUGCAUCUUUUGGAGCAGCGAAAUUUCUGAAGUGAAGAAUUUUUUUUGAAAAAAAAAUGACUUGAUGCUUUGUACUUCAUAAUCCCUUUCUAAAUUUGCUGCCAUUCUUAUGAAAACAUUCGCA.AACAA.A...
Rbi-1-1   AAUAUGCAGGACGAGCACAUGCAGUUUAAAAUAACUGACUGAUAAACGCCGGGGUCCGCUCCGGCGUUUUUUUUGAAACGGGGUUUGACAAUACCGAAUUCCUUUCUAUAUUUGACUCCGAUGAAAACAUUGA.UUACA.A...
Mma-1-1   GAAUGAUCAGGGGUGAUAAGUAGUGUUAAAAUUUAACUAGAAAAAUGUUUGCUUUUAUGGAAGUGCUUUUUUAGGUUUGCGACCAUAAAGUAAAACAAAAUUGCUUUGAAUAUGAAUGAAAAAAGUUUACAUA.UUACA.A...
Cat-1-1   CUUUGCAUUUAGAAAUGUGCCGUUUUUAAUAGAUGUACGACGUAAUUUAAGUUUACAUAGGAAUCUUAUCUUAAAAUUAUCUAAAAUAGUUUUAAAACAUUAUAAUCCUGAGUAUAUUUGGCGCAUAUUAUGA.AAGCA.AACA
env-1     UAAGUUUUGAUUUUGAAUUAUUAGACCAAUCUGUUUGAGUCAAAUCCAUUUUGAAUAUUUAAUUUUAAAAUUAUCAAUAAUUUUUGUAAUCUAUAAUUUUCGUUGCUAUAUUUGAAAUGAUUAUGAAUUUAA.AAUUA.A...
env-2     UGGGUUUUGAUUUUGAAUUAUUAGACCAAUCUGUUUGAGUCAAAUCCAUUUUGAAUAUUUAAUUUUAAAAUUAUCAAUAAUUUUUGUAAUCUAUAAUUUUCGUUGCUAUAUUUGAAGUGAUUAUGAAUUUAA.AAUUA.A...
env-3     UGGGUUUUGAUUUUGAAUUAUUAGACCAAUCUGUUUGAGUCAAGUCCAUUUUGAAUAUUUAAUUUUAAAAUUAUCAAUAAUUUUUGUAAUCUAUAAUUUUCGUUGCUAUAUUUGAAGUGAUUAUGAAUUUAA.AAUUA.A...
Fba-1-1   CUUUUAAAAAUAUGAACGAUCCUUCAGGUCGUGCGGUAAGUGGUGUACAUGAUGAAGGAUAUUAAAAAAAUGUUAGAUACUUGUAGGUUUAACUAUCCAUACUUACUUUUACACUCUAUAUGAAAAACACAAA.UCAUA.....
Chu-1-1   AGCCGGGAAUGAACGUAUACAUGCAGAUCUUUAAAAGAAAUACAGAAGCGUAUCUAUGUAUAAGCUAAUUUUUGGUGGUGCCAUUUGGAAAUUGAAAAUUCCCCCAUAUCUUUGUAACCAUGAAAACAAUUA.CAGGA.A...
Pir-1-1   AACGCUAGAAUUUAGAUAAGAAAUUUUUGGAUGUUUGUCAAUUUGUAAAAUUUUGUUAAAACGGAUAAAAUAAUUUAGAAAGGUUGUUUAAUUUAAAAAUCUACCCUACUUUUGUACUCGACAUGAAAAAUA.AUACA.A...
Psp-1-1   AGUUAUAAUAUGUAAUUAAAUGUUCUAAAAUCUUUAGUUCUGUUUUAAAAUUCUUAAAUUCUUAAAAUAUAUCCUAUAUAAUUUGCGCAAAUAAAAAACCAUUUUUACCUUUGUAACAUAAUGAAAAACAGUA.UA....A...
Lbl-1-1   CACGGGAUAAAACCAUCUACGACGACUUUACUUUAGACAUCAACUUGUCUAUCUAAUAUUUUUAAAAAGUAAUUUUAAAUUUUGUUUGUCGCUUAAGCAUCAAUUUUUAUCUUUGAUGCUCAAAUUAUGAAAAACAAAACA...
env-4     AAUUUCGUCUAAAAUUAAUAAUCGAUUAUGGGUUAGUUUAAACUUUUAACAAAUAAUUAAAUGCUAUUUGUGUUUGCGAAAUAAAAUUUGAUUUCUAUAUUUACACUUCAAAACAAACAAAUGAACACAAUUU.UAAAU.C...
env-5     GAAAGUGGAAGAAGUGAAAGGGAAAAUACACGAGGCUGCAAAUAAAUAAAAAAAAAGUAAGUUUUUCUUUGGAGUUUAAAACAAAACGCUUACAUUUGCCCCAAAAUAAAAACAGAUUUUAUGAAUAGAAUCA.UGGUA.A...
env-6     AAAGUUGGAGAAGUGAAAGGGGAAAAUACACGAGGCUGCAAAUAAAUAAAAAAAAAGUAAGUUUUUCUUUGGAGUUAAAAACAAAACGCUUACAUUUGCCCCAAAAUAAAAACAGAUUUUAUGAAUAGAAUCA.UGGUA.A...
env-7     GAAAGUGGAAGAAGUGAAAGGGAAAAUACACGAGGCUGCAAAUAAAUAAAAAAAAGGUAAGUUUUUCUUUGGAGUUUAAAACAAAACGCUUACAUUUGCCCCAAAAUAAAAACAGAUUUUAUGAAUAGAAUCA.UGGUA.A...
Bvu-1-1   GAAAGUGGAAGAAGUGAAAGGGAAAAUACACGAAGCUGCAAAUAAAUAAAAAAAAAGUAAGUUUUUCUUUGGAGUUUAAAACAAAACGCUUACAUUUGUCCCAAAAUAAAAACAGAUUUUAUGAAUAGAAUCA.UGGUA.A...
env-8     GAAAGUGGAAGAAGUGAAAGGGAAAAUACACGAAGCUGCAAAUAAAUAAAAAAAAAGUAAGUUUUUCUUUGGAGUUUAAAACAAAACGCUUACAUUUGCCCCAAAAUAAAAACAGAUUUUAUGAAUAGAAUCA.UGGUA.A...
env-10    GAAAGUGGAAGAAGUGAAAGGGAAAAUACACGAAGCUGCAAAUAAAUAAAAAAAAAGUAAGUUUUUCUUUGGAGUUUAAAACAAAACGCUUACAUUUGCCCCAAAAUAAAAACAGAUUUUAUGAAUAGAAUCA.UGGUA.A...
env-11    GAAAGUGGAAGAAGUGAAAGGGAAAAUACACGAAGCUGCAAAUAAAUAAAAAAAAAGUAAGUUUUUCUUUGGAGUUUAAAACAAUACGCUUACAUUUGUCCCAAAAUAAAAACAGAUUUUAUGAAUAGAAUCA.UGGUA.A...
env-12    AGUAUCCGCCAUAAACAACAAAACUUUAAGAAAAAAGUUCUCAUAAGAAAAAAAGAAAGAUUUGCUUUGAUUAUUAAAACAAUGUGCUUAUAUUUGCCUCGGUCAAAAAUAAAAAAGAUAAUGAAAACAAUGU.UCGUA.A...
Bun-1-1   GUGCUUAUAUUUGUCGCGAAAACGAAGACAGGCUGCACCUCAGCAUAAAAAAUGAACAAGUUCAUUUUAUUAUACAUUCGGUUUGCACUGUCUUUGCCCCCGAUAAAACAAAAAAAGUCAAUGAAAACAAUGU.UAGUA.A...
env-13    GUGCUUAUAUUUGUCGCGAAAACGAAGACAGGCUGCACCUCAGCAUAAAAAAUGAACAAGUUCAUUUUAUUAUACAUUCGGUUUGCACUGUCUUUGCCCUCGAUAAAACAAAAAAAGUCAAUGAAAACAAUGU.UAGUA.A...
env-14    ACAAUUCGUUUUUGGGGACAAAACAAAAAAUAAAAAGGAGGGCAAUAAAAAAAAGAAAGAUUUUAUUUGUUAUUCCAAACAAAGUAUCUAUAUUUGCCCCCGUUAAAUAACAAUUAGCAAUGAUGACAAUGC.AAGUA.A...
Bfr-2-1   ACAAUUCGUUUUUGGGGACAAAACAAAAAAUAAAAAGGAGGGCAAUAAAAAAAAGAAAGAUUUUAUUUGUUAUUCCAAACAAAGUAUCUAUAUUUGCCCCCGUUAAAUAACAAUUAGCAAUGAUGACAAUGC.AAGUA.A...
          ........................................................................................................................................<<<.....
          ........................................................................................................................................2??.....
          ●●●●●●●●●●●●●●●●●●●●●●●●●●●R●●●●●●●●●●●●●R●●●●●●●●R●●●●●●●●●●●●●●●●●●●●●●●●●●●●●●●●●●●●●●●●●●●●●●●●●●Y●●●●●●●●●●●●●R●●●●●R●RR●●A●●●●●●-●●R●A-A---
                                                                                                     5′———————————————————————————————————————————
```

```
env-15   CAAUUCGUUUUUGGGGACAAAACAAAAAAUAAAAAGGAGGGCAAUAAAAAAAAAGAAAGAUUUUAUUUGUUAUUCCAAACAAAGUAUCUAUAUUUGCCCCCCGUUAAAUAACAAUUAGCAAUGAUGACAAUGC.AAGUA.A...
Bfr-1-1  ACAAUUCGUUUUUGGGGACAAAACAAAAAAUAAAAAGGAGGGCAAUAAAAAAAAAGAAAGAUUUUAUUUGUUAUUCCAAACAAAGUAUCUAUAUUUGCCCCCGUUAAAUAACAAUUAGCAAUGAUGACAAUGC.AAGUA.A...
env-16   CAAUUCGUUUUUGGGGACAAAACAAAAAAUAAAAAGGAGGGCAAUAAAAAAAAAGAAAGAUUUUAUUUGUUAUUCCAAACAAAGUAUCUAUAUUGGCCCCCCGUUAAAUAACAAUUAGCAAUGAUGACAAUGC.AAGUA.A...
Bca-1-1  AGAUCAGGGGGAAAAUUGUUCUAUAUCAGAAAAAUGAAGGGGAAAAUGAAAAAAAGUCAUUUUUUGUUUGUUGUUUCAAACAAAGUGUCUAUAUUUGCUCCCAUUAAAUAACAAAUGACAAUGAGAACUAUAU.UAGUA.A...
Bov-1-1  AGUUCAGGUUGAAAAUUGUUCUAUAUCAGAAAAAUGAAGGCAAAAGAGAAAAAAAGUCAUUUUUUGUUUGCUGUUUCAAACAAAGUGUCUAUAUUUGCCCCCAUAAAAUUACAAAAGACAAUGAGAACAAUGU.UAGUA.A...
Bth-1-1  AGAUCAGGCAGAAAAUUGUUCUAUAUCAGAAAAAUGAAGAGGAAAAAGAAAAAAAGUAAGAUUUUGUUUGUUGUUUCAAACAAAGUGUCUAUAUUUGUCCCCGAUAAAUAACAAAAGACAAUGAGAACAAUGU.UAGUA.A...
Pme-1-1  AGAGGGACAACUGAGACUGCCAUACAAAAGUUCUAUGAUUAUAUAACAAAAAAAACAGCCGAAUUAUUUGCAGUUUGUCAGAGAAUGCAUAUAUUUGCUGCCGUAAUAACAACAAAAGUCAUGAGAACAAUUA.UAGUA.A...
env-17   GAGAGGGACAACUGAGACUGCCAUACAAAAGUUCUAUGAUUAUAUAACAAAAAAAACAGCCGAAUUAUUUGCAGUUUGUCAGAGAAUGCAUAUAUUUGCUGCCGUAAUAACAACAAAAGUCAUGAGAACAAUUA.UAGUA.A...
env-18   AAUAGAGCAGUUUUAAUAGACCUUCUGAUCUUUUUUAUCUCAUUCAUAAAAAAAAGUACGAUUUUAUUUGCUGUUUGUUAAAGAUUCCAUAUAUUUGCUGCCGUAAUAACAAAACAGAGUCAUGAGAACAUUCG.UAGUA.A...
Pdi-1-1  CAAUAGAGCAGUUUUAAUAGACCUUCUGAUCUUUUUUAUCUCAUUCAUAAAAAAAAACACGAUUUUAUUUGCUGUUUGUCAAAGAUUCCAUAUAUUUGCUGCCGUAAUAACAAAACAGAGUCAUGAGAACAUUCGUAGUA.A...
ueu-1-1  UUUCUUUGAUAAUUUAGGAGACAAAACAAUAUCAGAUAAUUUCACACUUGACGUAGUUUUAAAAUUUUAAGUAAAAUUUAAGUUGUAUGUUUUCAUAUAGUUAUUAUAUUUGAAACAUAAUUAUGCAAACAAC.AAACA.A.UC
env-19   AGCUUUUUUGAUGGACUAAAAGACCGUGCUAUUUAUGACGAAUUCGAUUUAAACGUAACUUUAUCAUUCUAAAAAUAUUGGCAGAUAAAAAAAAAGCUGCUUAUAUUUGAUCUGAAAUGAAAAACACAAAACAA.UA...UA...
Pto-1-1  UGUGUCAGGAAGGCUCGAACAUAAACAAGGAAUUAUAAAGGCUUGUGAUUAAAAUAAGCAACAAAAUUUUAACACCUGUCCAAAUCAGGGGAAAUGAAUAUUAUUCUUAUGUUUGCAAGAUGCUUAAAACAAA.UAACAAA...
Fba-2-1  UUUUUGAGAAAGUAUAUGCGAUUAUGAAUGAGGCAAAUCCAACAAAAUAUCCAAGUUUGUAUUAAAAAAUUUGUUUAUUCAAAAUUUUAUUCGAAAUAUUUGCAACAUACAAUUAAGAAAAUGAUUUCAAUUU.UAAAC.A...
Fjo-1-1  UUUUAGUCAAAAGUUAGAAUUUAAAAUUUAACAAAUUUCAAAGAUUAGAAAAGUUUUUUUUGAAAAUUUAUGUAUUCUAAUUCAAAUUACAUUUAUAUCUUUGUCACAUCAAAAUAAAGAAAUGAGAACAAUA.AUGAACA...
Fps-1-1  UACCCUCACAAACCCAAUACAAACCUCGUAGAGCAAUCUUCGAGGUUUUUUUUCAUAAAAAAAUAAAAAAAAAAUUUGCAAGUAAAAAAAUUAAUUGCUACAUUUGAACUUUAAUUAAGAACAUGAACACAUUUU.UAAAC.A...
env-20   UCUUUGAUGAUUUGGGCGACAAGGCUAUCUCAAACAAUUUUGACCUAAAUGUUAUGAUGAAAUUUUAAUUGUCUUUAUAGUGUCAAUUUAAAUUUGAUUUAUAUAUUUGACAAUAAGAAUAUGAAAACAAUAA.AUACA.A...
Fba-3-1  UUGGUAUUUUAAUAAACUUGACCUCACAAGUUAAGCCCCAUAUGUUCAAAAACAUAUUGGGCUUAUUAUUUUAUAAAAAACGUAUUUGUUGUUUUAAAAAUCAUUUCUAUAUUUGAGGCAAUGAAAAACAGAA.CAAAU.A...
env-21   CGGGAAGCUUCUUUGAUAACUUGGGAGACACCAUGAUCUAUGACGAUUUUAUNUUAGAUGUAAAUCUUAAAUUUUAAUUAAAUUAUUGUAGAGAAACAAAACGUUUCUAUAUUUGCAACAAUGUUUAAAAUAA.CUGUA.C...
Gfo-1-1  GACAAUUUUGAGCUUGAUAUAAAUCUAAAAUUCUAAGAAUUAAAAUCCUGAAAUUAAUCUUUCAGGAUUUUAUUUUAAAAAAUAUUUGUUUAGAAAAAUUUCAUCUCUAUCUUUGAAGUCAUGUUUAAAAUGA.AAAAA.A...
         ...............................................................................................................................<<<.....
         ...............................................................................................................................2??.....
         ●●●●●●●●●●●●●●●●●●●●●●●●●●●R●●●●●●●●●●●●●●R●●●●●●●●●R●●●●●●●●●●●●●●●●●●●●●●●●●●●●●●●●●●●●●●●●●●●●●●●●●●Y●●●●●●●●●●●●●●R●●●●●R●RR●●A●●●●●●-●●R●A-A---
                                                                                                                      5′
```

alignment positions 145 · · · 304

```
Psp-2-1  AUAUACAU.ACUUGGCAAUGGCGCAGUAAUUCUGAAA.AGAGUUCCGUCGU.AGUCU..U..GU.GUACCU.UAU.AGUUU...........AUCAACCUAAACCAAACCACAAAUAAGCCCGGCGUAACACACGCCGGGCUUUUGUUUUUACGCGAUU
Asp-1-1  .....UAU.UGGUGGUGGCAUUCUUAUUCUUUCCAAAAGGAAGUUUAGAA...AGCCCCU..GC.AAU....AAAAUUUGCGUUACGAUAU....AGUAU.............AAAAGGCUUACUGGAUUUCCGGUAAGCCUUUUUUAUUGAAAAUAACC
Rbi-1-1  ...CAAAC.UGGUGGUGGGUAUCUGAUCGCGGAACGUCGUGACCUG.......AGCCCCC..CU.GUGU.......UUGUCCACCAUACCU........................AAGGCUUGUCUGUCACGACAGGCCUUUUUUAAUUUUGUCAACAA
Mma-1-1  G...AUGU.UGGUGGUGG...CAC...AAAAGAGAAC.UCUCUUCUGUGAA.UAGCCCCG..UU.GUA....UAC.GUGUAAAACU.UUAUUACAUAAAGAUA.....UAAGCAAAAAGGCUUCCUGUUCACAGGAAGCCUUUUGCGUUUUGGGGUAGC
Cat-1-1  AUACAUCU.UGGUGGU..GGAACAACUCUAGACAAUA.GUCGUGAG....C.CA.CGCUU..AGUAUG....UAA.AUACAAUACC..........................AAAAGCCUGUCGUCACGACAGGCUUUUAUUUUUUAUACCUAUGUA
env-1    AUACUAAU.UGGUGGCAG.U.CAC....UUAAUUUUC.AUUAA...GUCGA.UAACUCU...UU.UAGUU..UAUAGUAAA......UAAU.....................AAAAGGCUUGUUAAUCGACAAGCCUUUUUUAAUUUAAAAAUGACAGU
env-2    UUACUAAU.UGGUGGCA.AU.CGC....UUAAUUUUC.AUUAG...GUCGA.UAACUCU...UU.UAGUU..UACAGUAAA......UAAU.....................AAAAGGCUUGUUAAUCGACAAGCCUUUUUUAAUUUAAAAAAGACAGU
env-3    AUACUAAU.UGGUGGCA.AU.CGC....UUAAUUUUC.AUUAA...GUCGA.UAACUCU...UU.UAGUU..UAUAGUAAA......UAAU.....................AAAAGGCUUGUUAAUCGACAAGCCUUUUUUAAUUUAAAAAAGACAGU
Fba-1-1  ...CAUGG.UGGUGGAA.AUAUAC.....AGCUCAAC.AGCC....GUAAA.GU.CACCC..GU.AUGU.......UUAUA......UAAC......................AAAAGGUCUGCUUAACGGCAGACCUUUUUUAUUUUAUCAAUUUUAG
Chu-1-1  CUAAACAA.UUGUGGUG.GUGCGU....GAACUAAUC.AGUUC...GUGAA.CA.UGCUG..CU.GU.........GUCCUG.....UUUGAAAAUAUAUCAA........AAAAGAGGCUUGCUGUUCACGGCAAGCCUCUUUAUUUUAUUAGGAUUU
Pir-1-1  ACAAAACU.UGGUGGUGG.AACUC....UCUUAAUUCAUAAGU...GAGAU.GAAUCCUAUAUG.UUU....AUA.ACAUA...........AUA................AAAAAAGGCUUAUCUCACGAUAAGCCUUUUUUAUUAUUAAAGUUCCGG
Psp-1-1  ACAUAUCU.UGGUGGUG.GAACUC....UCUUAACUAAUAAGU...GAGUU.UA.UACCU.....GUA....UGU.A...A...........AAAUAUAU...........UAAAAAGGCUUAUCUCACGAUAAGCCUUUUUCAAUUCAAUACAAUGAAG
Lbl-1-1  GUAAAUAC.U..UGGUG.GUGGAUCUCGCAACAGUAA.UGUCGUGAAACAA.C..CU..U..GU.AUU....UAAAAUUUA...........AAUAACUG.............AAAGGCCUGUCUCACGACAGGCCUUUUUGUUUCGCCUAACAACCA
env-4    AUA...CU.UGGUGGUGGAACUUC....AAGAUUUCA.AUCUUCGUGAAGU.AAACCCUU..AG........UAU.AUUUA......AUUUAAAAUAUUU............AAAAAAGGCUUGUCAUUCACGACAAGCCUUUUUUGUUUAUAAAAACUUA
env-5    AUACAUAU.UGGUGGC..GU.UUC.UUACAACUAAACAAGUCGUAAGGGAACCA.GACC...AU.GUA....UAU.AUACCUGACA.UUCUAAUAAGGAUAUUCAAACU....GAAGGCCUGUCGUACUUACGACAGGCCUUUUUUCGUUAGUGCAAUU
env-6    AUACAUAU.UGGUGGC..GU.UUC.UUACAACUAAACAAGUCGUAAGGGAACCA.GACC...AU.GUA....UAU.AUACCUGACA.UUCUAAUAAGGAUAUUCAAACU....GAAGGCCUGUCGUACUUACGACAGGCCUUUUUUCGUUAGUGCAAUU
env-7    AUACAUAU.UGGUGGC..GU.UUC.UUACAACUAAACAAGUCGUAAGGGAACCA.GACC...AU.GUA....UAU.AUACCUGACA.UUCUAAUAAGGAUAUUCAAACU....GAAGGCCUGUCGUACUUACGACAGGCCUUUUUUCGUUAGUGCAAUU
Bvu-1-1  AUACAUAU.UGGUGGC..GU.UUC.UUACAACUAAACAAGUCGUAAGGGAA.CCAGACC...AU.GUA....UAU.AUACCUGACA.UUCU.AAAAGGAUAUUCAAACU....GAAGGCCUGUCGUACUUACGACAGGCCUUUUUUCGUUAGUGCAAUU
env-8    AUACAUAU.UGGUGGC..GU.UUC.UUACAACUAAACAAGUCGUAAGGGAA.CCAGACC...AU.GUA....UAU.AUACCUGACA.UUCU.AAAAGGAUAUUCAAACU....GAAGGCCUGUCGUACUUACGACAGGCCUUUUUUCGUUAGUGCAAUU
env-10   AUACAUAU.UGGUGGC..GU.UUC.UUACAACUAAACAAGUCGUAAGGGAA.CCAGACC...AU.GUA....UAU.AUACCUGACA.UUCU.AAAAGGAUAUUCAAACU....GAAGGCCUGUCGUACUUACGACAGGCCUUUUUUCGUUAGUGCAAUU
env-11   AUACAUAU.UGGUGGC..GU.UUC.UUACAACUAAACAAGUCGUAAGGGAA.CCAGACC...AU.GUA....UAU.AUACCUGACA.UUCU.AAAAGGAUAUUCAAACU....GAAGGCCUGUCGUACUUACGACAGGCCUUUUUUCGUUAGGCCAAUA
env-12   AUACAUAU.UGGUGGUG.GCAUCCGUUACAACUCCGGCAGUCGUAAGGGAG.CAGUGCUC..GU.AUGUAUAUAU.GUACA......AUAU.AGAUAUAU...........AAAAGAGCCCUGUCGCACCAAGCGACGGGGCUCUUUUAUUUAAACGAUG
Bun-1-1  AUACAUAU.UGGUGGUG.GCACCCGUUACAACUCCGACAGUCGUAAGGGAG.CAGCGCUC..GU.AUG....UAU.AUAUG..........UAAUAUAUAGUAAAAAACAAAAAGAGCCCUGUCGCACCAAGCGACGGGGCUUUUUUCGUUUAAGCAAU
env-13   AUACAUAU.UGGUGGUG.GCACCCGUUACAACUCCGACAGUCGUAAGGGAG.CAGCGCUC..GU.AUG....UAU.AUAUG..........UAAUAUAUAGUAAAAAACAAAAAGAGCCCUGUCGCACCAAGCGACGGGGCUUUUUUCGUUUAAGCAAU
env-14   AUACAUAU.UGGUGGUG.GCGCCCGUUACAACUCCGACAGUCGUAAGGGAG.CAGCGCUC..GU.AUG....UAU.AUACC.....UCAUUAAAGAUAUAACAGAAUUUAGAAAGAGCCCUGUCGCAACCUGCGACGGGGCUCUUUUUUUUAACCCCAA
Bfr-2-1  AUACAUAU.UGGUGGUG.GCGCCCGUUACAACUCCGACAGUCGUAAGGGAG.CAGCGCUC..GU.AUG....UAU.AUACC.....UCAUUAAAGAUAUAACAGAAUUUAGAAAGAGCCCUGUCGCAACCUGCGACGGGGCUCUUUUUUUUAACCCCAA
env-15   AUACAUAU.UGGUGGUG.GCGCCCGUUACAACUCCGACAGUCGUAAGGGAG.CAGCGCUC..GU.AUG....UAU.AUACC.....UCAUUAAAGAUAUAACAGAAUUUAGAAAGAGCCCUGUCGCAACCUGCGACGGGGCUCUUUUUUUUAACCCCAA
Bfr-1-1  AUACAUAU.UGGUGGUG.GCGCCCGUUACAACUCCGACAGUCGUAAGGGAG.CAGCGCUC..GU.AUG....UAU.AUACC.....UCAUUAAAGAUAUAACAGAAUUUAGAAAGAGCCCUGUCGCAACCUGCGACGGGGCUCUUUUUUUUAACCCCAA
env-16   AUACAUAU.UGGUGGUG.GCGCCCGUUACAACUCCGACAGUCGUAAGGGAG.CAGCGCUC..GU.AUG....UAU.AUACC.....UCAUUAAAGAUAUAACAGAAUUUAGAAAGAGCCCUGUCGCAACCUGCGACGGGGCUCUUUUUUUUAACCCCAA
Bca-1-1  AUACAUAU.UGGUGGUG.GCGUCCGUUACAACUCCGACAGUCGUAAGGGAG.CAGCGCUC..GU.AUG....UAU.AUACC.....UCAUUAAAGAUAUAACAGAUUU..GAAAGAGCCCUGUCGCAACUUGCGGCAGGGCUUUUUUAUUUGCCCGUUU
Bov-1-1  AUACAUAU.UGGUGGUG.GCACCCGUUACAACUCCGACAGUCGUAAGGGAG.CAGUGCUC..GU.AUG....UAU.AUACC.....UCAUUAAAGAUAUAACAGAUUU..GAAAGAGCCCUGUCGCAACUUGCGGCAGGGCUCUUUUUAUUUGCCCAUU

         <<<<<<<<..<<<<....<<.<<<.<<<<.<<<.....>>>.>>>>>>>.>.>..>>>>...>>.>>>....>>>..>>>...............................................................................
         ????????..11??....??.???.????.222.....222.???????.?.?..??11...??.???....???..??2...............................................................................
         ○YAY●UR●-UGGUGG●R-○●○YYY----○○RY●●●●○●●Y●●Y○○R○○●○-○R○Y●CY●--●●-●UR----UA○-RU●Y●------○○○○-○○○○○○○○------------AAAGGCYUGUC●●●Y●●●●●R●●●●YYUUUUYY●●Y●●R●●●●●●
                  codons
                                                                                                                                  3′
```

```
Bth-1-1   AUACAUAU.UGGUGGUG.GCGCCCGUUACAACUCCGACAGUCGUAAGGGAG.CAGUGCUC..GU.AUG....UAU.AUACC......UCAUUAAAGAUAUAACAAAUUU..GAAAGAGCCCUGUCGCAACCUGCGACAGGGCUCUUUUAUUUGCCCGUU
Pme-1-1   AUACAUAU.UGGUGGUGGCGCUCU.UUACAACUCAUAAGGUCGUAAGGAAC.CAGUCCUC....AUG....UAU.AUACUA.....UCAU....GAUAUAAU.........AAAAGGUCUGUCGCACUUGCGGCAGGCCUUUUUUCGUUUAGGGCAAU
env-17    AUACAUAU.UGGUGGUGGCGCUCU.UUACAACUCAUAAGGUCGUAAGGAAC.CAGUCCUC....AUG....UAU.AUACUA.....UCAU....GAUAUAAU.........AAAAGGUCUGUCGCACUUGCGGCAGGCCUUUUUUCGUUUAGGGCAAU
env-18    AUACAUAU.UGGUGGUGGCGCUCCG.UACAACUCAAAAGGUCGUAAGGAAC.GAAGACUC..GU.AUG....UAU.AUAUAAACACUACAU.AAAGAUAU...........AAAGAGGUCUGUCGCACUUGCGGCAGGCCUCUUUCUUUUUAUGGCAA
Pdi-1-1   AUACAUAU.UGGUGGUGGCGCUUCG.UACAACUCAAAAGGUCGUAAGGAAC.GAAGACUC..GU.AUG....UAU.AUAUAAACACUACAU.AAAGAUAU...........AAAGAGGUCUGUCGCACUUGCGGCAGGCCUCUUUCUUUUUAUGGCAA
ueu-1-1   UUACUUGG.UGGUGGAA.UAACUC....UCGACAAAA.AUCGU...GAGCU.AAGCACUA..UU.UGG....UAU.AUAUC......AUAC....................AAAAGGCUUGUCAUUCACGACAAGCCUUUUUUUUUAUUUAAAAAUGA
env-19    ...CUUGG.UGGUGGAA.CUAUCG......AUAUCCAAUGUCGUGA...AA.C..CCCUA..GU.GUG....UAAAA...A......AUAC....................AAAGGCUUGUCUUCACGACAAGCCUUUUUUUUAUGAUGAAUAAGAC
Pto-1-1   U..CUUGG.UGGUGGUC.UUUAUU......ACGUCAAACGUCGUG.ACUUA.GA.UCCUG..CU.AUG....UAA.AUAUA......UCAU....................AAAGGCUUGUCUAUCACGACAAGCCUUUUUCAUUUAUAACAUUUG
Fba-2-1   AUACUUGG.UGGUGGUC.UAACUU......ACGUCGAACUUCGUGAGCGA...A.UCCUG..CUAUAG....UAUCAUUUAAAACC......AAAUACAU...........AGAAAAGGCUUGUCAAUCACGACAAGCCUUUUUUAUUGCCAAAAAUUA
Fjo-1-1   AUACUUGG.UGGUGGAA.CAAUUU......ACGUCAAACGUCGUGAACGA...AGCUUCC..UA.UGG....UAU.UUUCA......AAACUAUAAAUAU.............AAAAGGCUUGUCAUCACGACAAGCCUUUUUUUUGGCCGGAACUUUA
Fps-1-1   AUACUUGG.UGGUGGA..AUAAUUU.....ACGUCAAACGUCGUGAACUAA.UC.CUCUA..UA.UUG....UAU.UUUUA......AAACUCAAAAUAU...........AAAAAAGGCUUGUCAAUCACGACAAGCCUUUUUUUUGUGCAAAAAACCA
env-20    AUACUUGG.UGGUGGAAA.ACCUU......ACGUCAAACUUCGU..GAGCUAAAGCACUA..UUGGUA....UAA.AUAUAC............................AAAAGGCUUGUCAAUCACGACAAGCCUUUUUUUUGUUUUCAAUAGUA
Fba-3-1   AUACAUGG.UGGUGGAG.UAACUU......ACGAAAAACGUCGU..GAGCA.AAGCAUCA....UUGUAG.UAA.AACUA................UAU.............AAAAGGCUUGUCUAUCACGACAAGCCUUUUUUAAUUAUAAAAACACA
env-21    AUACUUGG.UGGUGGAA.UAACUU......ACGUCAAACGUCGU..GAGCU.AGCUACUA..GU.UUG....UAUAAUCUA...........AUAUAUAA...........AAAAGGCUUGUCAUUCACGACAAGCCUUUUUUCAUGCUUUAAAUUUA
Gfo-1-1   AUAACUGG.UGGUGGAA.UAACUU......ACGUCAAACUUCGU..GAGCU.A..AACUG..CU.AUU....UUCAAUAUA..............GAAAU...........UAAAAAGGCUUGUUUCACGACAAGCCUUUUUUUAUUGCAUAAAAUCGAC
          <<<<<<<<..<<<<....<<.<<<.<<<<.<<<.....>>>.>>>>>>>.>.>..>>>>...>>.>>>....>>>..>>>..........................................................................
          ????????..11??....??.???.????.222.....222.???????.?.?..??11...??.???....???..??2..........................................................................
          ∘YAY•UR•-UGGUGG•R-∘•∘YYY----∘∘RY••••••∘••Y••Y∘∘R∘∘•∘-∘R∘Y•CY•--••-•UR----UA∘-RU•Y•------∘∘∘∘-∘∘∘∘∘∘∘∘------------AAAAGGCYUGUC•••Y•••••R••••YYUUUUUYY••Y••R••••••
                   codons
```

alignment positions 305 · · · 464

```
Psp-2-1   UUGAAAAAAAGCCCGUAGGAUACGACGUAGAGAAAAGAGAGAGCGGGAUAUUGAUGAUGACAAGAAAAACGGUGACACUGCGCUGAGUGCCAAUGAGUCGCCAAUAAAACGAAAUAAAAUUAUGCUGAUGAACAAUUAUAAGAUCAAUACCAUUCAUAAA
Asp-1-1   ACGAAAUGACUCAAGUAAAACUCCCGAUUUUAACCACUUAUAGAAAGCGAUUAGCGGAUACAAUUACUCCUGUGAGUAUUUAUCUGCAAAUCCGAGAUCGAUUUGCCAAUCCGAUUCUUUUGGAGAGUUCUGAUUACCAUGGACACGAAAAUAGCUAUUC
Rbi-1-1   GCUCAAUAUGGCAUACGAACUCCAUACACACCACAAGAAAAUCCUCGCGGACACCCUGACGCCGGUGAGCGUCUACCUGAAGGUACGCGACCGGUUCCCGGGCAGCCUCCUGCUGGAGAGCAGCGACUACCACGCCAACGACAAUUCGUUUUCCUAUAUC
Mma-1-1   CCUAAUGAGAAACAUUAAGCCAUUAACGACAAGUAGUUGUAAGUUACGAAUAGUCAAUAUAAAAACUUGCCGCUACUGACAACACACACACAAGAAAUAUUUGACAAAACACAAUGAAAAAGUACGCAUUAAAAACCCGUUACCGUAAACUACUUGCCGACA
Cat-1-1   CAAACUUAAAACUUACAGCACUAAAAUACUUGCAGACACUAUUACACCAGUUUCUGUAUACCUAAAAGUUAGAGAUCGUUUUCCUAACAGUAUUUUAUUAGAAAGUAGUGAUUACCGAGCAAAUGAUAAUACAUUUAGUUAUAUAUGCUGCAACCCGAUU
env-1     GUUUAAAAUAAAAACAUAUAGAAAAAAAAUACUUGCUGAUACUUUAACCCCAGUCAGCAUCUAUCAAAAGAUUAGAGAUAAAUAUUCAAACUCUGUCUUGUUAGAAAGUAGUGAUUAUGGAGCCAAUGAUAACAGUUAUGCUUAUGUAUGUUUUAACCCA
env-2     GUAUAAAAUCAAAACAUAUAGAAAAAAAAUACUUGCUGAUACUUUAACUCCUGUCAGUAUCUAUCAAAAGAUUAGGGAUAAAUACUCAAACUCUGUCUUGUUAGAAAGUAGUGAUUAUGGAGCCAAUGAUAACAGUUAUGCUUACGUGUGUUUUAACCCA
env-3     GUAUAAAAUCAAAACAUAUAGAAAAAAAAUACUUGCGGACACUUUAACUCCUGUCAGUAUCUAUCAAAAGAUUAGGGAUAAAUACUCCAACUCUGUCUUGUUAGAAAGUAGUGAUUAUGGAGCCAAUGAUAACAGUUAUGCUUAUGUAUGUUUCAACCCA
Fba-1-1   AAACCUCAUAUGCAUCAUUAUAAAGCAAAAUCCACGCAACAAACGAUAUUAGCAGACAUGCUAACUCCUGUUGCUAUAUACAUGCGUUUACGUGAUAAGUAUCCCGGUAGCAUCUUAUUAGAAAGCAACGAAUAUGGAGAACGCAGUAAUAGCUAUUCCU
Chu-1-1   AGGAAAUUUCAAUUAUGAAAACAUAUACAUUAAAGCUUACCUAUAAAAAAAUACUUGCCGAUACUACUACACCGGUAAGCAUGUAUUUAAAAUUGCGCGACAAGUUUCCGCAAACCAUGCUGCUGGAAAGUUCCGAUUACCAUGGCAACCAGGAUAUUUA
Pir-1-1   AACGAAAUGAAAAAAAUACAGUUUAAAACAAUUUAUAAGACAAAAAUAGCAGAUACCAUAACUCCUGUAGGAUUGUAUUUGCGUUUUAGAGACGCUUUUGCAAACACGCUUUUAUUAGAGAGUUCUGACUAUCACAGUAAAGAAGAAAGUUUUCUUAUA
Psp-1-1   AAUAUAAAAUUUAAAACAAUUCAUAAAACUAAAAUUGCAGAUACUAUAACUCCUGUGGGUUUGUAUUUACGAUUUAGAGAUAAGCAUGCGAAUACGCUUUUACUAGAGAGUUCAGACUAUCAUAGUAAAGACGAAAGUUUCACAUUUAUUGCUAUUGAAC
Lbl-1-1   AAAAAUGAAAUAUACCUUAAAAACGCACAGCAAGAAGAUUCUUGCCGAUACCAUCACUCCGGUUUCGGUAUACCUUCGCCUGCGAGAUCGCUUCCCCAACAGUCUGCUUCUCGAGAGUAGCGACUACCGGGCUAACGACAAUACCUUCAGCUACAUCUGU
env-4     UGAAAACAUUUAGUCUUUACACACAUUACAAAAAAAUAUUGGCAGAUACCAUUACACCUGUAAGUAUCUACUUAAAAAUUCGUGAUAAAUACCCAAAUAGUAUCUUGCUAGAAAGUAGCGAUUAUCAUGCUAACGACAACAGUUUUCUUACAUCUGUUU
env-5     AACAAUCAAAAUUUAAUAUAGAACGACAAUGAAAACUUAUCAGGUAAACAAAGAAGGUUAUUAUGGCGAAUUUGGCGGGGCCUAUAUUCCCGAAAUCCUUCAUCGCUGUGUGGAAGAGUUAAAAAAUGCCUAUUCCAAAGUAUUGGAAAGUGAAGGAUUC
env-6     AACAAUCAAAAUUUAAUAUAGAACGACAAUGAAAACUUAUCAGGUAAACAAAGAAGGUUAUUAUGGCGAAUUUGGCGGGGCCUAUAUUCCCGAAAUCCUUCAUCGCUGUGUGGAAGAGUUAAAAAAUGCCUAUUCCAAAGUAUUGGAAAGUGAAGGAUUC
env-7     AACAAUCAAAAUUUAAUAUAGAACGACAAUGAAAACUUAUCAGGUAAACAAAGAAGGUUAUUAUGGCGAAUUUGGCGGGGCCUAUAUUCCCGAAAUCCUUCAUCGCUGUGUGGAAGAGUUAAAAAAUGCCUAUUCCAAAGUAUUGGAAAGUGAAGGAUUC
Bvu-1-1   AACAUUAAAAUAUAGAACGACAAUGAAAACUUAUCAGGUAAACAAAGAAGGUUAUUAUGGCGAAUUUGGAGGGGCGUAUAUUCCCGAAAUCCUUCACCGCUGUGUGGAAGAAUUGCAGAAUGCCUAUUCCAAAGUAUUGGAAAGUGAAGGAUUCAAACAA
env-8     AACAUUAAAAUAUAGAACGACAAUGAAAACUUAUCAGGUAAACAAAGAAGGUUAUUAUGGCGAAUUUGGAGGGGCGUAUAUUCCCGAAAUCCUUCACCGCUGUGUGGAAGAAUUGCAGAAUGCCUAUUCCAAAGUAUUGGAAAGUGAAGGAUUCAAACAA
env-10    AACAUUAAAAUAUAGAACGACAAUGAAAACUUAUCAUGUAAACAAAGAAGGUUAUUAUGGCGAAUUUGGAGGGGCGUAUAUUCCCGAAAUCCUUCACCGCUGUGUGGAAGAAUUGCAGAAUGCCUAUUCCAAAGUAUUGGAAAGUGAAGGAUUCAAACAA
env-11    AACAUUAAAAUAUAGAACGACAAUGAAAACUUAUCAGGUAAACAAAGAAGGUUAUUAUGGCGAAUUUGGAGGGGCGUAUAUUCCCGAAAUCCUUCACCGCUGUGUGGAAGAAUUGCAGAAUGCCUAUUCCAAAGUAUUGGAAAGUGAAGGAUUCAAACAA
env-12    UAUAUAGCUAACAGUUCGCAACCGGUAACUGGCAGCCAACAAUUAAAUAAUAAAACAAUAUAUGGUUAGUUUCUUAGUUGACAAAGAAGGUUAUUACGGAGAAUUCGGCGGAGCAUACGUUCCCGAAAUACUCCACAGGUGUGUAGAGGAAUUGCAGAAA
Bun-1-1   AAUGCGUUUUGAUUUCUGAAAUAGUAAAUAGUAAAUCGUCAAAUAGUAAAUAACAUGAUGAAUUUCUUAGUUGACAAAGAAGGUUAUUACGGAGAGUUCGGCGGGGCAUACGUUCCCGAAAUCCUCCACAAAUGUGUAGAAGAGUUGCAGAACACCUACC
env-13    AAUGCGUUUUGAUUUCUGAAAUAGUAAAUAGUAAAUCGUCAAAUAGUAAAUAACAUGAUGAAUUUCUUAGUUGACAAAGAAGGUUAUUACGGAGAGUUCGGCGGGGCAUACGUUCCCGAAAUCCUCCACAAAUGUGUAGAAGAGUUGCAGAACACCUACC
env-14    AUACAAAUUACAAAUAAAUAUAGGAUUAUGAAAAGUUUUUUAGUUGAUCAGGAUGGCUAUUACGGAGAAUUUGGAGGUGCUUAUGUACCUGAAAUCCUCCACAAGUGUGUUGAAGAGUUGCCGAAUACUUAUCUCGACGUAAU.................
Bfr-2-1   AUACAAAUAACAAAUAAAUAUAGGAUUAUGAAAAGUUUUUUAGUUGAUCAGGAUGGCUAUUACGGAGAAUUUGGAGGUGCUUAUGUACCUGAAAUCCUCCACAAGUGUGUAGAAGAGUUGCAGAAUACUUAUCUCGACGUAAUAGAGAGCGAGGACUUCA
env-15    AUACAAAUAACAAAUAAAUAUAGGAUUAUGAAAAGUUUUUUAGUUGAUCAGGAUGGCUAUUACGGAGAAUUUGGAGGUGCUUAUGUACCUGAAAUCCUCCACAAGUGUGUAGAAGAGUUGCAGAAUACUUAUCUCGACGUAAUAGAGAGCGAGGACUUCA
Bfr-1-1   AUACAAAUAACAAAUAAAUAUAGGAUUAUGAAAAGUUUUUUAGUUGAUCAGGAUGGCUAUUACGGAGAAUUUGGAGGUGCUUAUGUACCUGAAAUCCUCCACAAGUGUGUAGAAGAGUUGCAGAAUACUUAUCUCGACGUAAUAGAGAGCGAGGACUUCA
env-16    AUACAAAUAACAAAU.................................................................................................................................................
Bca-1-1   CUAAACAUCGAAAAAAAUAACAUAUAAAAAUAGAUAGGAUUAUGAAAAGUUUUUUAGUUGACCAGGAUGGCUAUUACGGAGAGUUCGGCGGUGCUUAUGUACCGGAAAUUCUCCACAAAUGUGUAGAGGAACUGAAGAAUAAGUACCUCGAAGUACUUGA
Bov-1-1   UUCGAACAGAUUUAGAUAAUAACAUAAAAAAAUAGAUAGGAUUAUGAAAAGUUUUUUAGUUGACCAGGAUGGCUAUUACGGAGAAUUCGGUGGUGCUUAUGUACCGGAAAUCCUCCACAAAUGUGUUGAGGAACUGACAAACAAGUACCUCGAAGUGAUU
Bth-1-1   UUUGAAAAUCGAAUAAAUAAUAAUAAAUAAAAAUAGGAUUAUGAAAAGUUUUUUAGUUGAUCAGGAUGGUUACUACGGAGAGUUCGGCGGCGCUUAUGUUCCGGAAAUUCUCCACAAAUGUGUAGAGGAACUGAAGAAUACGUACCUCGGAGUGCUCGAA
Pme-1-1   UACCAAGAUUCGAAAAGACAAAAAAUAAAUAAUAACAGAUAGAACGAUGAAAACGUAUCAAGUUGACGAAAAUGGGUAUUAUGGUGAGUUUGGAGGAGCAUAUGUGCCCGAAAUACUUCACCAAUGUGUGGAAAACUUGCAGAAGAAGUAUCUGGAAGUA
env-17    UACCAAGAUUCGAAAAGACAAAAAAUAAAUAAUAACAGAUAGAACGAUGAAAACGUAUCAAGUUGACGAAAAUGGGUAUUAUGGUGAGUUUGGAGGAGCAUAUGUGCCCGAAAUACUUCACCAAUGUGUGGAAAACUUGCAGAAGAAGUAUCUGGAAGUA
env-18    AUUCAAAAGGAAAAUAACUAAAAUAGAUAAAAGAUGAAAACGUAUCAAGUUGACAAGAAUGGUUACUAUGGUGAGUUCGGCGGGGCUUAUGUUCCCGAGAUCCUUCAUCAAUGCGUGGAAAAUUUACAGAAUACCUAUCUGCAAGUGUUGGAGAGCGAUA
Pdi-1-1   AUUCAAAAGGAAAAUAACUAAAAUAGAUAAAAGAUGAAAACGUAUCAAGUUGACAAGAAUGGUUACUAUGGUGAGUUCGGCGGGGCCUAUGUUCCCGAGAUCCUUCAUCAAUGCGUGGAAAAUUUACAGAAUACCUAUCUGCAAGUGUUGGAGAGCGAUA
ueu-1-1   AAUACAAUCUUAAAACAUACAGUAAAAACCUACUAGCAGAUACACUUACACCUGUAUCCGUUUAUCUGCGUUUACGCGAUAAAUUUCCUAAUAGUUUAUUAUUAGAGAGUAGUGAUUAUCACGCAAAUGAUAAUAGUUUUAGUUAUAUCUGUUGCAAUCC

          ................................................................................................................................................................
          ................................................................................................................................................................
          •••••••••••••••••••••••R••••••R•••••••••••••••••••••••••••••••••••••••••••••••••••••••••••••••••••••••••••••••••••••••••••••••••••••••••••••••••••••••••••••••
```

```
env-19    AGUAUAUAGCCUAAAAACCAACUACAAAAAAAUACUUGCAGACACCCUAUCGCCAGUCAGUAUUUAUUUGAAAAUCAGGGAUAAAUUUCCAAAUAGUAUGUUGCUAGAAAGUAGUGACUAUCAUGGUAAUGACAAUAGUUUUUCAUACAUAUGCUGCAAC
Pto-1-1   AACAAACACUAUGAGUUAUUCACUUAAAACCACUCACCAAAAGCUUCUGGCCGAUACCUUUACGCCAGUAAGUGCCUACCUAAAGAUAAGGGAUAGAUACCCGAAUUCUCUUCUUUUGGAAAGUAGCGAUUACCAUACCAACGAUAACAGCUUCAGUUAU
Fba-2-1   AACAAAUAUGAAAAAAUACAAGUUACAUACUAAUUACAAACAAAUUCUAGCUGACACUAUUACGCCAGUAAGUGUUAACUUAAAAAUUCGCGAUAAGUUUCCUAAUAGUAUUUGUUAGAAAGUUCUGAUUAUCAUGCGAAUGACAACAGUUUUCUUAC
Fjo-1-1   ACAGAAAAAUAUAUUAAAACUAAAAAACAACAUACAUUGAAACCGUUUAUACUCAACACACAUUACAAACAAAUUCUGGCAGAUACCAUUACGCCGGUUAGUAUUUAUUUUAAAAUAAGAGAUAAAUUUCCAAAUAGUUUAUUGUUGGAAAGCAGUGAUU
Fps-1-1   AAACAAUCUAUAUGAAAACUUAUAAUCUAAAAACAAAAUUUAAACAAAUUCUUGCAGACACCAUCACACCUGUAAGCAUUUACCUAAAAAUUCGAGACAAAUUUCCUAACAGUUUAUUGUUAGAAAGUAGCGAUUAUCAUGCAAAUGACAAUAGUUUUUC
env-20    GGCAUUUCAAAAAAAGAAUAAAAUUUAAGAUGAAAUAUCAAUUACAUACUCAUUCAAAAAAACUAUUGGCAGACACCUUAACACCGGUGUCGGUUUACCUGCGUAUACGUGAUAAAUUUCCAAAUAGCCUGCUGUUGGAAAGCAGUGACUAUCAUGCCAA
Fba-3-1   UGUCGAAAUAUCAAUUUAAAUCACAUCACAAGAAAAUACUUGCAGAUACUAUUACACCUGUAAGUGUCUAUUUGAAAAUACGGGAUCGAUUUCCAAAUAGUAUUCUUUUGGAAAGUAGUGACUAUCAUGCCAAUGAUAACAGUUUUUCUUAUAUCUGCUG
env-21    AAAUUUACUAUGUAUACGUUAACCACUUCCUCCAAAAAAAUUAUUGCCGAUACCAUCACUCCGGUGAGUAUUUAUUUAAAAUUAAGAGACAAAUACCCCCACAGUCUUCUUUUAGAGAGUAGUGAUUACCACGCCAAUAAUAAUAGUUUUUCAUACAUCU
Gfo-1-1   UUUACAUGUUUAAAUUAAAUACAAAAUACAAGAAGAUCCUUGCCGACACUAUCACUCCGGUUAGCAUCUACCUGAAAUUGCGCGAUAAAUACCCUAAUAGCCUGUUGCUUGAGAGUAGUGAUUAUCACGCCAGUGACAAUAGUUUCUCUUAUAUCUGUUG
          ................................................................................................................................................................
          ................................................................................................................................................................
          ●●●●●●●●●●●●●●●●●●●●●●●●R●●●●●●R●●●●●●●●●●●●●●●●●●●●●●●●●●●●●●●●●●●●●●●●●●●●●●●●●●●●●●●●●●●●●●●●●●●●●●●●●●●●●●●●●●●●●●●●●●●●●●●●●●●●●●●●●●●●●●●●●●●●●●●●
```

alignment positions $465 \cdots 569$

```
Psp-2-1   AAGCUGCUUGCAGACACUACAACGCCGGUGAGCAUCUAUUUAAGGUUAAGGGAUGUAUUUCCCAAUACGAUCCUGCUGGAGAGUUCCGAUUACCAUAGCCGCGAG
Asp-1-1   CUAUAUCUGUUUUAAUCCAUUGGCUACUUUUACUUUCAAUGCAGGUAAAGUAGAAGAAACUCUUCCUCUUGGACAUUCGGUUUCGUAUGAGGUUAACUCAGAUCA
Rbi-1-1   UGCUGCAACCCGAUUGCCUCCAUCCGGGUACAGCACGAGACGAUCUUCCUGGAAUACCCGGACGGGAAACGCGAACAGCGCCCCGUGGGCCCGGAUACGGAUAUC
Mma-1-1   CCCUUACACCGGUGAGCAUUUACCUAAAGUUGCGCGAUAAAUUUGCCAACGCUUUGUUGCUCGAGAGCUCAGACUACCACGGGGCAGACAAUAGUUUUUCCUACA
Cat-1-1   GCUUCAAUAAAUAUACAAAAUGAAGAAAUUAUCAAACAGUUUCCAGACGGCACAAAAGAACAGUCUUCAAUAAAUGAGACUACUAAUGUCCCACUAGAGAUUGAA
env-1     AUAUCAUCAUUUAAAGUAUAUAAAAAUUUAAUUGAAAAAAAAAUAUCCAGACAAUUCAACUAAACAAACUAAGUUGACAAAAUCUAAUUUAGUUGUUGAUGAAUUA
env-2     AUAUCAUCAUUUAAAGUUUAUAAUAAUUUAAUUGAAAAAAAAAUAUCCAGACAAUUCAACUCAACAAACUAAAUUGACAAAAACUAAUUUAGUUGUUAAUGAAUUA
env-3     AUAUCAUCAUUUAAAGUUUAUAAUAAUUUAAUUGAAAAAAAAAUAUCCAGACAAUUCAACUCAACAA......................................
Fba-1-1   UCAUUUGUUGUAAUCCUGUAGCUCAAUUCUCUGUUACUAAUACAAAAAUUGAAAGCCAAUUUCCUGAUGGAACAAUAGAGCAAUCAGACUUCAGUUCGAGUUAUG
Chu-1-1   UUCCUAUAUCUGCUGCAAUCCCAUUGCAGGGAUAAAAAUUGACAAAGGAUAUUUUACAGAAUAUUAUCCGGAUGGCACAUUGAAUAAUCUGGAUAUUGAAGAAAC
Pir-1-1   UCGCAAUAGAGCCUGUGCUUACCAUGAAGGUUGAUGAUGCACAAUAUACAGUUUUUCACAAAGGAACAGAGAUUGAUGCGCAACCGGUAGAUAAGAACUUUUAUC
Psp-1-1   CUAUUGUCUCUAUUAAAGCUGAAAAUCAUCAUUUGAGUUUUUCUCAUAAAGGUUUAGAGAUUGCAAAUGAAGAAAUUGGUAGAAAUUUCAAUGAAAUAUUCGAAA
Lbl-1-1   UGUAAACCUAUCGCUUCGAUCACCGUAAAGGAUGAAAUUAUUACGGAAAGUUUCCCUGACGGAAAAACCACAACCACAAGCAUCGACUCCACUACCGAUGUAGUU
env-4     UAACCCAAUUGCUUCAAUUAAAGUAGACGAAAAAGCAAUUACACAAACGUUUCCUGACGGAAGUACGAUGAUUAAUAAGGUUAAAAAUGUAACGCAGAUGAUUCA
env-5     AAAAAAGAAUUCCACCAGUUACUACACGACUAUGUGGGACGCCCUUCACCUCUAUACCUGGCAAACCGGCUAAGCGAACAAUACGGCUGUAAAAUCUACCUGAAA
env-6     AAAAAAGAAUUCCACCAGUUACUACACGACUAUGUGGGACGCCCUUCACCUCUAUACCUGGCAAACCGGCUAAGCGAACAAUACGGCUGUAAAAUCUACCUGAAA
env-7     AAAAAAGAAUUCCACCAGUUACUACACGACUAUGUGGGACGCCCUUCACCUCUAUACCUGGCAAACCGGCUAAGCGAACAAUACGGCUGUAAAAUCUACCUGAAA
Bvu-1-1   GAAUUCCACCAGUUACUACGCGACUAUGUAGGACGCCCUUCACCUCUUUACCUGGCAAACCGGCUAAGCAAGAAAUAUGGCUGUAAGAUCUACCUGAAACGUGAA
env-8     GAAUUCCACCAGUUACUACGCGACUAUGUAGGACGCCCUUCACCUCUUUACCUGGCAAACCGGCUAAGCAAGAAAUAUGGCUGUAAGAUCUACCUGAAACGUGAA
env-10    GAAUUCCACCAGUUACUACGCGACUAUGUAGGACGCCCUUCACCUCUUUACCUGGCAAACCGGCUAAGCAAGAAAUAUGGCUGUAAGAUCUACCUGAAACGUGAA
env-11    GUAUUCCACCAGUUACUACGCGACUAUGUAGGACGCCCUUCACCUCUUUACCUGGCAAACCGGCUGACAGCUUCAGC............................
env-12    ACUUAUCUCACAGUCCUGCAAAAUAAAGAUUUCAAGCAGGAAUACGACCGGUUGCUGCGCGACUACGUAGGACGUCCCUCACCGCUCUACCAGGCACGGCGUCUG
Bun-1-1   UCAAGGUGCUGCAAAGUGAGGACUUCAAGCGUGAAUACGACCAGUUGCUGCGCGACUACGUGGGACGCCCUUCCCCACUCUACUUGGCACACCGCUUGUCCGAGA
env-13    UCAAGGUGCUGCAAAGUGAGGACUUCAAGCGUGAAUACGACCAGUUGCUGCGCGACUACGUGGGACGCCCUUCCCCACUCUACUUGGCACACCGCUUGUCCGAGA
env-14    .........................................................................................................
Bfr-2-1   AGAAAGAGUUCGACCAAUUGUUGCGUGACUACGUAGGACGCCCUUCCCCGCUCUAUCCGGCCCGCCGCCUGUCGGAGAAGUACGGUUGCAAGAUGUAUCUGAAAC
env-15    AGAAAGAGUUCGACCAAUUGUUGCGUGACUACGUAGGACGCCCUUCCCCACUCUAUCCGGCCCGCCGCCUGUCAGAGAAGUACGGUUGCAAGAUGUAUCUGAAAC
Bfr-1-1   AGAAAGAGUUCGACCAAUUGUUGCGUGACUACGUAGGACGCCCUUCCCCACUCUAUCCGGCCCGCCGCCUGUCGGAGAAGUACGGUUGCAAGAUGUAUCUGAAAC
env-16    .........................................................................................................
Bca-1-1   GAGUGAGGAAUUCAAGAAAGAAUUCGAACAGUUGUUACGUGAUUACGUAGGACGUCCUUCCCCACUCUAUCCGGCAAGGCGCCUUUCGAAAAAAUAUGGUUGCAA
Bov-1-1   GAAAGCGAAGAGUUUAAAAAGGAAUUUGAGCAGUUAUUACGCGACUACGUAGGACGCCCUUCCCCACUCUAUCUGGCAAAACGCCUUUCCGAAAAGUAUGGUUGC
Bth-1-1   AGCGAAGAUUUCAAAAAGGAGUUUGACCAGUUGCUGCGUGACUACGUAGGCCGCCCUUCCCCACUCUAUCUCGCCCGCAGACUUUCAGAAAAGUAUGGUUGCAAA
Pme-1-1   UUGGAAAGCGACAGUUUCAAGAAUGAAUUCAACCAGUUACUUCGGGACUAUGUCGGCCGCCCUUCUCCUCUCUAUCAGGCAAAAAGGCUGAGUGAGAUGUAUGGC
env-17    UUGGAAAGCGACAGUUUCAAGAAAGAAUUCAACCAGUUACUUCGGGACUAUGUCGGCCGCCCUUCUCCUCUCUAUCAAGCAAAAAGGCUGAGUGAGAUGUAUGGC
env-18    GCUUCAAGGAAGAGUUCGAUCAGUUAUUGCGUGAUUAUGUAGGCCGCCCCUCCCCUCUUUACUUGGCCAAACGGUUGAGCGAGAAAUACGGAUGUAAGAUCUAUU
Pdi-1-1   GCUUCAAGGAAAAGUUCGAUCAGUUAUUGCGUGAUUAUGUAGGCCGCCCCUCCCCUCUUUACUUGGCCAAACGGUUGAGCGAGAAAUACGGAUGUAAGAUCUAUU
ueu-1-1   AAUUGCCUCAAUAAAAAUUGCCAACGGUCAAAUUACACAGCAGUAUCCUGAUGGUUCUUCGGAAAUUUUAAACAUCAAAAAAUCUACAGAUGUUCCAAAAGUAAU
env-19    CCUAUUGCAACAAUCAAAAUUCAAGCUAAUACCUUAACCAAAACAUNNNNNNNNNNNNNNNNNNNNNNNNNNNNNNNNNNNNNNNNNNNNNNNNNNNNAAAAAAGA
Pto-1-1   AUCUGUUGUAAUCCUAUUGCCCAUUUCAAGGUUGAAAAUGAACGGAUAGAACUCAAGUAUCCUGAUGGCAGCUAUAAACAUAUCGAUAUUGAUGCUGAGGUAAAU
Fba-2-1   AUCUGUUUCAAUCCAAUUGCUUCUAUUAAAGUGGAAAAUCAAAAAAUUGAAACAAAUUUUCCAGAUGGUUUUUCAGAAAUUAUACCAAUUGAUGCAGCUGUAAAC
Fjo-1-1   AUCACGGAAACGACAACAGUUUCUCGUAUAUCUGCUGUAAUCCUAUUGCAACAAUAAAAAUUGAAAACGAAACCAUUUCAAAAACUUUUCCAGACGGAACUUCAG
Fps-1-1   CUAUAUCUGUUGCAAUCCAAUUGCUUCAAUAAAAAUAGAAAACGAAACUAUUUUCAAAUCAUUUCCUGAUGGAUCUUCAGAGAAAAUUGAAAUUAAUAACCAAAC
env-20    CGACAAUAGCUUUAGUUAUAUAUGCUGCAAUCCAAUUGCCUCAAUAAACAUUCAAAAUGAAGUAAUUACACAACAGUUUCCCGACGGAAAAGAACAAAUAUCGAA
Fba-3-1   UAACCCGAUUGCAUCGAUUAAGAUUGAGGACGAAACGAUAAUCGAGCAAUAUCCAGAUGGAAAAAGCGCUUCAAAGGAAAUUGGGCCUGACACCGAUGUGGUUAA

          .........................................................................................................
          .........................................................................................................
          ●●●●●●●●●●●●●●●●●●●●●●●●●●●●●●●●●●●●●●●●●●●●●●●●●●●●●●●●●●●●●●●●●●●●●●●●●●●●●●●●●●●●●●●●●●●●●●●●●●●●●
```

```
env-21    GUUGUAAUCCUAUUGCAUCUAUAAAUGUUAAAAAUGAAAUGAUUAAUAUUCAAUUUCCAGAUGGAAAGUUAACAGAGAAAGCUAUUACAGAAGAAGUUAAUGUAA
Gfo-1-1   UAACCCCAUCGCUUCAUUUAAAAUUGAAAAUGAUGUAAUUACUACUACUUUUCCAGAUGGAAACAGAGAAAUUUUAAAUAUUGACAAGCAAGUUUCUGUUCCUCA
```

12 Bacteroidales-1

12.1 Taxa

The taxonomy of each organism containing a putative Bacteroidales-1 RNA is listed, with abbreviations identifying each hit (e.g., “Eco-1-1” and “Eco-1-2” might hypothetically represent two distinct RNAs in *E. coli*). The abbreviations will be used to identify each individual Bacteroidales-1 RNA in Sections 12.2 and 12.4.

**abbrev. of hits**	**taxonomy of species**
Bca-1-1	Bacteria Bacteroidetes Bacteroidia Bacteroidales Bacteroidaceae *Bacteroides caccae* ATCC 43185
Bco-1-1 to Bco-1-2	Bacteria Bacteroidetes Bacteroidia Bacteroidales Bacteroidaceae *Bacteroides coprocola* DSM 17136
Bdo-1-1	Bacteria Bacteroidetes Bacteroidia Bacteroidales Bacteroidaceae *Bacteroides dorei* DSM 17855
Beg-1-1	Bacteria Bacteroidetes Bacteroidia Bacteroidales Bacteroidaceae *Bacteroides eggerthii* DSM 20697
Bfi-1-1	Bacteria Bacteroidetes Bacteroidia Bacteroidales Bacteroidaceae *Bacteroides finegoldii* DSM 17565
Bfr-1-1	Bacteria Bacteroidetes Bacteroidia Bacteroidales Bacteroidaceae *Bacteroides fragilis* NCTC 9343
Bfr-2-1	Bacteria Bacteroidetes Bacteroidia Bacteroidales Bacteroidaceae *Bacteroides fragilis* YCH46
Bin-1-1	Bacteria Bacteroidetes Bacteroidia Bacteroidales Bacteroidaceae *Bacteroides intestinalis* DSM 17393
Bov-1-1	Bacteria Bacteroidetes Bacteroidia Bacteroidales Bacteroidaceae *Bacteroides ovatus* ATCC 8483
Bpl-1-1	Bacteria Bacteroidetes Bacteroidia Bacteroidales Bacteroidaceae *Bacteroides plebeius* DSM 17135
Bst-1-1	Bacteria Bacteroidetes Bacteroidia Bacteroidales Bacteroidaceae *Bacteroides stercoris* ATCC 43183
Bth-1-1	Bacteria Bacteroidetes Bacteroidia Bacteroidales Bacteroidaceae *Bacteroides thetaiotaomicron* VPI-5482
Bun-1-1	Bacteria Bacteroidetes Bacteroidia Bacteroidales Bacteroidaceae *Bacteroides uniformis* ATCC 8492
Bvu-1-1	Bacteria Bacteroidetes Bacteroidia Bacteroidales Bacteroidaceae *Bacteroides vulgatus* ATCC 8482
Pdi-1-1	Bacteria Bacteroidetes Bacteroidia Bacteroidales Porphyromonadaceae *Parabacteroides distasonis* ATCC 8503
Pjo-1-1	Bacteria Bacteroidetes Bacteroidia Bacteroidales Porphyromonadaceae *Parabacteroides johnsonii* DSM 18315
Pme-1-1	Bacteria Bacteroidetes Bacteroidia Bacteroidales Porphyromonadaceae *Parabacteroides merdae* ATCC 43184
Pgi-1-1	Bacteria Bacteroidetes Bacteroidia Bacteroidales Porphyromonadaceae *Porphyromonas gingivalis* ATCC 33277
Pgi-2-1	Bacteria Bacteroidetes Bacteroidia Bacteroidales Porphyromonadaceae *Porphyromonas gingivalis* W83
env-1 to env-27	environmental samples

12.2 Gene contexts

Each Bacteroidales-1 RNA (indicated by “RNA→”) is listed with its surrounding genes. Environmental sequences and some RefSeq entries lack gene annotations, and no genes are listed for such sequences. The direction of each gene is indicated with an arrow (→), and each conserved domain in the gene is colored. Conserved domains associated with more than one Bacteroidales-1 RNA are assigned a color; other domains are gray. Information about these conserved domains is given in Section 12.3. The accession number of the sequence containing each Bacteroidales-1 RNA is given. Accessions beginning with “NC_” or “NZ_” are RefSeq, while other accession refer to environmental samples. Nucleotide coordinates are given for the 5′ and 3′ boundaries of each. If the 5′ coordinate is greater than the 3′ coordinate, the RNA is present on the reverse-complement strand of the containing genomic DNA sequence. Each hit is denoted by an abbreviation (like “Eco-1-1”) that refers to a taxonomy given in Section 12.1.

abbrev.	Seq. accession		5′ at	3′ at	genes
env-1	BABD01027605.1	-	926	751	←hypo **RNA**→ RRM (smart00360)→
env-2	BAAY01005444.1	-	242	57	**RNA**→
Pgi-1-1	NC_010729.1	-	146751	146582	←MMT1 (COG0053) **RNA**→ ←hypo
Pgi-2-1	NC_002950.2	-	2190292	2190123	←MMT1 (COG0053) **RNA**→ ←hypo
env-3	BAAX01000158.1	-	508	314	HisB (COG0241)IGPD (pfam00475)→ **RNA**→ Lum_binding (pfam00677)→
env-4	BAAX01010365.1	+	733	913	ThrA (COG0460)→ **RNA**→
env-5	BAAX01004754.1	+	420	598	**RNA**→ RibC (COG0307)→
Pdi-1-1	NC_009615.1	+	788079	788256	Ribosomal_L20 (pfam00453)→ **RNA**→ RRM (smart00360)→
env-6	BABA01004120.1	+	1238	1415	Ribosomal_L20 (pfam00453)→ **RNA**→ RRM (smart00360)→
env-7	BABB01008998.1	+	97	274	**RNA**→ RRM (smart00360)→
Pjo-1-1	NZ_ABYH01000068.1	+	291	468	**RNA**→
Pme-1-1	NZ_AAXE02000021.1	-	125990	125813	hypo→ **RNA**→ hypo→
env-8	BABD01027604.1	+	632	810	Ribosomal_L20 (pfam00453)→ **RNA**→
env-9	BABD01016942.1	+	749	930	Ribosomal_L20 (pfam00453)→ **RNA**→
Bpl-1-1	NZ_ABQC02000016.1	+	77157	77332	Ribosomal_L20 (pfam00453)→ **RNA**→ COG2706 (COG2706)→
Bco-1-1	NZ_ABIY02000096.1	-	615	440	RVT (pfam00078)→ **RNA**→ hypo→
Bco-1-2	NZ_ABIY02000127.1	-	14645	14470	COG3344 (COG3344)→ **RNA**→ hypo→
env-10	BABD01000999.1	+	665	840	Ribosomal_L20 (pfam00453)→ **RNA**→ COG1144 (COG1144)→
env-11	BABG01004849.1	+	451	627	**RNA**→ FldA (COG0716)→
Bvu-1-1	NC_009614.1	-	4991729	4991553	Ribosomal_L20 (pfam00453)→ **RNA**→ FldA (COG0716)NuoI (COG1143)→
env-12	BAAU01004407.1	-	862	686	Ribosomal_L20 (pfam00453)→ **RNA**→ FldA (COG0716)→
Bdo-1-1	NZ_ABWZ01000013.1	+	484277	484453	**RNA**→
env-13	BAAX01034787.1	-	345	169	Ribosomal_L20 (pfam00453)→ **RNA**→
env-14	BAAY01001699.1	+	365	541	hypo→ **RNA**→ FldA (COG0716)→
env-15	BABB01000835.1	-	8418	8242	hypo→ **RNA**→ FldA (COG0716)→
env-16	BABA01025825.1	-	358	177	**RNA**→ hypo→
Bca-1-1	NZ_AAVM02000004.1	+	418226	418403	←hypo **RNA**→ FldA (COG0716)NuoI (COG1143)→
Bfi-1-1	NZ_ABXI01000084.1	+	5207	5384	**RNA**→
Bth-1-1	NC_004663.1	+	512928	513105	Ribosomal_L20 (pfam00453)→ **RNA**→ NuoI (COG1143)→
Bov-1-1	NZ_AAXF02000045.1	+	146907	147084	Ribosomal_L20 (pfam00453)→ **RNA**→ NapF (COG1145)→
Bfr-1-1	NC_003228.3	+	1976767	1976943	Ribosomal_L20 (pfam00453)→ **RNA**→ FldA (COG0716)NuoI (COG1143)→
Bfr-2-1	NC_006347.1	+	1960011	1960187	Ribosomal_L20 (pfam00453)→ **RNA**→ FldA (COG0716)NuoI (COG1143)→
env-17	BABB01013913.1	+	442	618	Ribosomal_L20 (pfam00453)→ **RNA**→
Bin-1-1	NZ_ABJL02000008.1	-	781990	781813	Ribosomal_L20 (pfam00453)→ **RNA**→ ←PaaK (COG1541)
env-18	BAAZ01000710.1	+	2540	2717	Ribosomal_L20 (pfam00453)→ **RNA**→
env-19	BABA01003411.1	+	651	828	Ribosomal_L20 (pfam00453)→ **RNA**→ ←hypo
env-20	BAAY01021114.1	+	550	727	Ribosomal_L20 (pfam00453)→ **RNA**→
Bun-1-1	NZ_AAYH02000043.1	+	16479	16656	Ribosomal_L20 (pfam00453)→ **RNA**→ ←PaaK (COG1541)
env-21	BAAX01004326.1	-	2389	2212	**RNA**→ ←PaaK (COG1541)
env-22	BABA01001035.1	-	1522	1345	Ribosomal_L20 (pfam00453)→ **RNA**→ ←PaaK (COG1541)
env-23	BAAZ01019253.1	-	445	268	Ribosomal_L20 (pfam00453)→ **RNA**→
env-24	BABG01009248.1	+	549	726	Ribosomal_L20 (pfam00453)→ **RNA**→
env-25	BABD01001370.1	-	2435	2258	**RNA**→ ←PaaK (COG1541)
env-26	BAAU01008698.1	-	740	563	Ribosomal_L20 (pfam00453)→ **RNA**→ ←hypo
env-27	BABB01001469.1	+	28	205	**RNA**→ ←hypo
Bst-1-1	NZ_ABFZ02000019.1	+	401830	402007	Ribosomal_L20 (pfam00453)→ **RNA**→ hypo→
Beg-1-1	NZ_ABVO01000025.1	+	18262	18439	**RNA**→

12.3 Conserved domains

Conserved domains found in protein-coding genes listed in Section 12.2 are listed, with the first sentence in their description from the Conserved Domain Database. Conserved domains associated with more than one Bacteroidales-1 RNA are assigned a color, while others are shown in gray. The number in parentheses is the number of occurrences of the given gene.

u COG0053 (2) Predicted Co/Zn/Cd cation transporters [Inorganic ion transport and metabolism]
u COG0241 (1) Histidinol phosphatase and related phosphatases [Amino acid transport and metabolism]
D COG0307 (1) Riboflavin synthase alpha chain [Coenzyme metabolism]
u COG0460 (1) Homoserine dehydrogenase [Amino acid transport and metabolism]
D COG0716 (8) Flavodoxins [Energy production and conversion]
D COG1143 (5) Formate hydrogenlyase subunit 6/NADH:ubiquinone oxidoreductase 23 kD subunit (chain I) [Energy production and conversion]
D COG1144 (1) Pyruvate:ferredoxin oxidoreductase and related 2-oxoacid:ferredoxin oxidoreductases, delta subunit [Energy production and conversion]
D COG1145 (1) Ferredoxin [Energy production and conversion]
d COG1541 (5) Coenzyme F390 synthetase [Coenzyme metabolism]
D COG2706 (1) 3-carboxymuconate cyclase [Carbohydrate transport and metabolism]
u COG3344 (1) Retron-type reverse transcriptase [DNA replication, recombination, and repair]
u pfam00078 (1) Reverse transcriptase (RNA-dependent DNA polymerase).
u pfam00453 (24) Ribosomal protein L20.
u pfam00475 (1) Imidazoleglycerol-phosphate dehydratase.
D pfam00677 (1) Lumazine binding domain.
D smart00360 (4) RNA recognition motif;

Duplicate sequences: the following putative homologs are not shown in the alignment because their sequences are identical to a homolog already shown: Bco-1-2, Bfr-2-1, env-15, env-21, env-22, env-7

12.4 Multiple-sequence alignment

Each Bacteroidales-1 RNA is denoted by an abbreviation (like "Eco-1-1") that refers to a taxonomy given in Section 12.1. The alignment includes sequences containing the RNA motif, as well as flanking sequence. The Bacteroidales-1 RNA itself is denoted by the line underneath marked 5′ and 3′ on either end. Nucleotides in the flanking sequences (i.e., not part of the motif) are written in gray letters. Stems of predicted rho-independent transcription terminators are shaded yellow. (Note: terminator predictions have not been analyzed manually, and many are likely to be false positives.) Start codons are shaded green. (Note: start codons are frequently misannotated, especially in environmental samples.) Nucleotides proposed to basepair as part of the consensus structure are shaded in color when they comprise Watson-Crick or G-U pairs. Otherwise they are shaded gray. Conserved stems are also indicated at the bottom of the alignment by angle brackets, where matching $<$ and $>$ denote base-paired columns. Below these angle brackets, the symbol "2" denotes base pairs exhibiting covariation, "1" denotes base pairs exhibiting compatible mutations, "0" denotes base pairs that are not observed to mutate and "?" denotes base pairs that have a significant frequency of non-canonical nucleotides for Watson-Crick or G-U pairs ($> 5\%$). Below these base pair annotation is the consensus sequence: "R" = "A" or "G", "Y" = "C" or "U", red nucleotides: nucleotide identity conserved more than 97% of the time, black nucleotides: 90%, gray nucleotides: 75%, red circle (•): nucleotide is present 97% of the time, black circle (•): 90%, gray circle (•): 75%, white circle (◦): 50%. All percentages of sequences just described (e.g. 97% conserved) assume that sequences have been weighted by the GSC algorithm implemented by the Infernal software.

(Features specific to this RNA motif) Canonical *Bacteroidetes* promoters are detected, and we have labeled their -33 motif ("TTTG"), -7 motif ("TAnnTTTG") and rough transcription start site ("TSS").

alignment positions 1 · · · 159

```
env-1    ........................................................................GGGCAGGGUUACCCCACGUGUG.CGAGGGGAA.CUGACGAGUAC..AUCAAACCCCGA.ACA.AGCUC.GGUUCGC.AAGGGCGG..
env-2    UUUG..ACAUAUGCAAUAGUUGACGUAUCUUUGCA.GUA..GAAUAUGG..........................GGGUUGUUCCGGUUUGAUC.GGGAUACUC.AUCAAAAUAAA..ACGAA..ACCGAGG.A.AGUUUCUCUUGUU.AAUGGCGGG.
Pgi-1-1  AUUG..CAAAUCUAAACAAUGUUGCUAACUUUGCA.AUACAGAGAAGACGAGGAAU...................AAGCCGCACUGU.UAGAUCGGGGAAACUC.AUCAUAACAA.....CUAUUCCGAGG.ACAAUU.UCAUCGCU.GAUGGCGGG.
Pgi-2-1  AUUG..CAAAUCUAAACAAUGUUGCUAACUUUGCA.AUACAGAGAAGACGAGGAAU...................AAGCCGCACUGU.UAGAUCGGGGAAACUC.AUCAUAACAA.....CUAUUCCGAGG.ACAAUU.UCAUCGCU.GAUGGCGGG.
env-3    UUUG.GCUGCAUCAGAAGAAAGCAGUAUCUUUGCAAUA..CAUAA..............................GGGUCGUUUCGGUUAGAUU.GGGAUACUC.AUCACAUUACAAAUUAUGAAACCGAGA.A.GACUUCUCUUGCC.UAUGGCGGG.
env-4    CUUG.UCAGAAUAAAAUGAAAGUAUUAACUUUGCA.UUA..GAAAACAGAAACAG.ACACUCAACCCAUAGGGGCGCGCCAC....GUUCGAUC.GGGAUACUC.AUCAAUAAAAA..AAUCGAAACCGAGAAG..ACUUCUUUUGCC.AAUGGCGGG.
env-5    UUUG..UAGAAACAGAAAAUAGCAGUAUCUUUGCA.ACA..AAGAAAUAAAGGACAGUAAAGAUU..........GGACCGUAUCGGUUAGAUC.GGGAUACUC.AUCAAUAAAAA..AACGAAAACCGAGAUG..AUUUCUAUUGCU.AAUGGCGGG.
Pdi-1-1  UUGG..UCAUGUCGAUACGAUUACAUAUCUUUGUA.ACAA.GAAAAAGAUAAACAGGGAAUGAGUU.........AAGCCGCACUUGUUCGAUU.GGGAAACUC.AUCAGUUUAU....UUUAAUUCCGAGACAUAGCUCUUGUCGCC.AAUGGCGGG.
env-6    UUGG..UUAUGUCGAUACGAUUACAUAUCUUUGUA.ACAA.GAAAAGAUAAACAGGGAAUGAGUU..........AAGCCGCACUUGUUCGAUU.GGGAAACUC.AUCAGUUUAU....UUUAAUUCCGAGACAUAGCUCUUGUCGCC.AAUGGCGGG.
Pjo-1-1  UUUG.GUGGUUUCAUUCCUAUUACAUAUCUUUGUG.AUA..AGAAAAAUGAGU......................UAGCCGCACAUGUUCGAUU.GGGAAACUC.AUCAGUUUA....UUUUAAUUCCGAGACACAGCUCUUGUCGUC.AAUGGCGGG.
Pme-1-1  UUUG.GUGGUUUCAUUCCUAUUGCAUAUCUUUGUG.AUA..AGAAAAAUGAGU......................UAGCCGCACAUGUUCGAUU.GGGAAACUC.AUCAGUUUA....UUUUAAUUCCGAGACACAGCUCUUGUCGUC.AAUGGCGGG.
env-8    UUUG.GUUAUGUCGAUACGAUUACAUAUCUUUGUA.ACAA.GAAAAAGAUNAACAGGGAAUGAGUU.........AAGCCGCACUUGUUCGAUU.GGGAAACUC.AUCAGUUUAUU..UUAAU..UCCGAGACAUAGCUCUUGUCGCC.AAUGGCGGG.
env-9    UUUG.GUGGUUUCAUUCCUAUUGCAUAUCUUUGUG.AUA..CGAACAAUGAGU......................UAGCCGCACAUGUGCGAUU.GGGAAACUC.AUUCAGUUUAU..UUUAUU.UCCGAGACAAAGCUCUUGUCGUC.AAUGGCGAG.
Bpl-1-1  UUUG..UAUAUUCAAAUAUUUGUCGUAUAUUUGUC.AUA..GAAAAAUUG.........................AAGCCGUACUUGCAGAAUC.GGGAAACUC.AUCAAAUCUA...AAGAAAUACCGAGACA.AGCUUCUACUUCC.AAUGGCGGG.
Bco-1-1  UUUG..UUUUUUCAGAUUAAAGUAUUAUUUUUGUA.AUA..CAAGAAUG..........................AAGCCGCACUUGCAGAAUC.GGGAAACUC.AUCAAAUUAU...AAGAAAUACCGAGA.ACAGCUUCAUCUUCC.AAUGGCGGG.
env-10   UUUG..UUUUUUCAGAUUAAAGUAUUAUUUUUGUA.AUA..CAAAAAUG..........................AAGCCGCACUUGCAGAAUC.GGGAAACUC.AUCAAAUUAU...AAGAAAUACCGAGA.ACAGCUUCAUCUUCC.AAUGGCGGG.
env-11   CUUG.UUUAUAUCAUAAAAAGGACGUAUAUUUGUC.AUA..CGGAAAGAGAUGAAAAGC................GAGCCGCAUUAGUUGGAUU.GGGAAACUC.AUCAAACUAA...UCUGAAAACCGAGACAUAGCUUCAUCUUCC.AAUGGCGGG.
Bvu-1-1  CUUG.UUUAUAUCAUAAAAAGGACGUAUAUUUGUC.AUA..CGGAAAGAGAUGAAAAGC................GAGCCGCAUUAGUUGGAUU.GGGAAACUC.AUCAAACUAA...UCUGAAAACCGAGACAUAGCUUCAUCUUCC.AAUGGCGGG.
env-12   CUUG.UUUAUAUCAUAAAAAGGACGUAUAUUUGUC.AUA..CGGAAAGAGAUGAAAAGC................GAGCCGCAUUAGUUGGAUU.GGGAAACUC.AUCAAACUAA...UCUGAAAACCGAGACAUAGCUUCAUCUUCC.AAUGGCGGG.
Bdo-1-1  CUUG.UUUAUAUCAUAAAAAGGGCGUAUAUUUGUC.AUA..CGGAAAGAGAUGAAAAGC................GAGCCGCAUUAGUUGGAUU.GGGAAACUC.AUCAAACUAA...UCUGAAAACCGAGACAUAGCUUCAUCUUCC.AAUGGCGGG.
env-13   CUGG.UUUAUAUCAUAAAAAGGACGUAUAUUUGUC.AUA..CGGAAAGAGAUGAAAAGC................GAGCCGCAUUAGUUGGAUU.GGGAAACUC.AUCAAACUAA...UCUGAAAACCGAGACAUAGCUUCAUCUUCC.AAUGGCGGG.
env-14   CUUG.UUUAUAUCAUAAAAAGGACGUAUAUUUGUC.AUA..CGGAAAGAGAUGAAAAGC................GAGCCGCAUUAGUUGGAUU.GGGAAACUC.AUCAAACUAA...UCUGAAAACCGAGACAUAGCUUCAUCUUCC.AAUGGCGGG.
env-16   CUUGUUUCUUUCUAUAGAUACUCAUUACCUUUGUU.UAU..AGUAUAUA..........................UAGCCGCAUUGAUUGGAUC.GGGAUACUCUAUCUAUUUUU...AUGAUAUACCGCGACU.AGCUUCGCUUCUCUAUGG.CGGGC
Bca-1-1  CUUG.CUAUUCUCCGGGAUUCUUAUUAUAUUUGUG.AUA..CAAAAAUAU.........................UAGCCGUAUCGACUGGAUC.GGGAAACUC.UUCAAAUUAAU..CUGAUAUACCGAGA.A.AGCUUCGAUCUUC.AAUGGCGGG.
Bfi-1-1  CUUG.CUAAUAUCAAGGAUUCUUAUUACAUUUGUG.AUA..CAAAAAUAU.........................UAGCCGUAUCGACUGGAUC.GGGAAACUC.UUCAAAUUAAU..CUGAUGCACCGAGA.A.AGCUUCGUUCUUC.AAUGGCGGG.
Bth-1-1  CUUG.CUAUUCUCAGAGAAUCUUAUUACAUUUGUG.AUA..GAAAAAUAU.........................UAGCCGUAUCGACUGGAUC.GGGAAACUC.UUCAAAUUAAU..CUGAUGCACCGAGA.A.AGCUUCGUUCUUC.AAUGGCGGG.
Bov-1-1  CUUG.UUAGUCUCAUAGAUUCUUAUUACAUUUGUG.AUA..CAAAAAUAU.........................UAGCCGUAUCGACUGGAUC.GGGAAACUC.UUCAAAUUAAU..CUGAUGCACCGAGA.A.AGCUUCGUUCUUC.AAUGGCGGG.
Bfr-1-1  CUUG.UUUAUCUCACAAAAACUAUCUAUAUUUGUU.GUA..GUAAAAUAU.........................UAGCCGCAUUGAUUGGAUC.GGGAAACUC.AUCAAUUAA....ACUGAAAACCGAGACA.AGCUUCGCUUCUC.AAUGGCGGG.
env-17   CUUG.UUUAUCUCACAAAAACUAUCUAUAUUUGUU.GUA..GUAAAAUAU.........................UAGCCGCAUUGAUUGGAUC.GGGAAACUC.AUCAAUUAA....ACUGAAAACCGAGACA.AGCUUCGCUUCUC.AAUGGCGGG.
Bin-1-1  CUUG.UUUCUUUGACAGAAACUUACUACAUUUGUU.GUA..GAAAAUA...........................UAGCCGCAUUGAUUGGAUC.GGGAAACUC.AUCAAACUAUU..AUGAAAUACCGAGACA.AGCUUCGCUUCUC.GAUGGCGGG.
env-18   CUUG.UUUCUUUGACAGAAACUUACUACAUUUGUU.GUA..GAAAAUA...........................UAGCCGCAUUGAUUGGAUC.GGGAAACUC.AUCAAACUAUU..AUGAAAUACCGAGACA.AGCUUCGCUUCUC.GAUGGCGGG.
env-19   CUUG.UUUCUUUGACAGAAACUUACUACAUUUGUU.GUA..GAAAAUA...........................UAGCCGCAUUGAUUGGAUC.GGGAAACUC.AUCAAACUAUU..AUGAAAUACCGAGACA.AGCUUCGCUUCUC.GAUGGCGGG.
env-20   CUUG.UUUCUUUCCUAGAAACUCAUUACCUUUGAA.GUA..GAAAAAA...........................UAGCCGCAUUGAUUGGAUC.GGGAAACUC.AUCAAUUUUU...AUGAAAUACCGAGACA.AGCUUCAGUUCUU.AAUGGCGGG.
Bun-1-1  CUUG.UUUCUUUCAUAGAAACUCAUUACCUUUGUU.AUA..GAAAAAA...........................UAGCCGCAUUGAUUGGAUC.GGGAAACUC.AUCAAUUUUU...AUGAAAUACCGAGACA.AGCU.UCGCUUCUCAAUGGCGGG.
env-23   CUUG.UUUCUUUCAUAGAAACUCAUUACCUUUGUU.AUA..GAAAAAA...........................UAGCCGCAUUGAUUGGAUC.GGGAAACUC.AUCAAUUUUU...AUGAAAUACCGAGACA.AGCU.UCGCUUCUCAAUGGCGGG.
env-24   CUUG.UUUCUUUCAUAGAAACUCAUUACCUUUGUU.AUA..GAAAAAA...........................UAGCCGCAUUGAUUGGAUC.GGGAAACUC.AUCAAUUUUU...AUGAAAUACCGAGACA.AGCU.UCGCUUCUCAAUGGCGGG.
env-25   CUUG.UUUCUUUCGUAGAAACUCACUACCUUUGAA.GUA..GAAAAAA...........................UAGCCGCAUAGAUUGGAUC.GGGAAACUC.AUCAAUUCUA...AUGAAAUACCGAGACA.AGCUUCAGUUCUC.AAUGGCGGG.
env-26   CUUG.UUUCUUUCGUAGAAACUCACUACCUUUGAA.GUA..GAAAAAA...........................UAGCCGCAUUGAUUGGAUC.GGGAAACUC.AUCAAUUCUA...AUGAAAUACCGAGACA.AGCUUCAGUUCUC.AAUGGCGGG.
env-27   ..................AACUCACUAC.CUUGAAAGUA..GAAAAA............................UAGCCGCAUUGAUUGGAUC.GGGAAACUC.AUCAAUUCUA...AUGAAAUACCGAGACA.AGCUUCAGUUCUC.AAUGGCGGG.
Bst-1-1  CUUG.UUUCUUUCGUAGAAACUCACUACCUUUGAA.GUA..GAAAAAA...........................UAGCCGCAUUGAUUGGAUC.GGGAAACUC.AUCAAUUCUA...AUGAAAUACCGAGACA.AGCUUCAGUUCUC.AAUGGCGGG.
Beg-1-1  CUUG.UUUCUUUCCUAGAAACUCAUUACCUUUGAA.GUA..GAAAAAA...........................UAGCCGCAUUGAUUGGAUC.GGGAAACUC.AUCAAUUUUU...AUGAAAUACCGAGACA.AGCUUCAGUUCUU.AAUGGCGGG.
         ........................................................................................................................................<<<.<<<<<<<<<..<<......
         ........................................................................................................................................?21.???2?2221..20......
         .......................................................................................................................................................<<<<<<<.
         .......................................................................................................................................................?10???1.
         YUUG-○Y●●●●YYR●R●●●●●●●●●UAY●UUUGY●-RUA--●RRAAR○○○-------------------------●AGCCGYAYY●RYURGAUY-GGGARACUC-AUCAR●●YA●---○●●●●A○●CCGAGA○A-AGCUYY●●UY●YC-AAUGGCGGG-
         TTTG                     TAnnTTTG        TSS                                <<<<<<<<<<<<  <<<  <<    >>  >>>
                                                                                    5′
```

alignment positions 160 · · · 319

```
env-1    CCGC......CCC...UUUC...GGAU...AAGCUAA.GCCACGUCGG.AUCA.CAAA.GGUGACAAAGCACUCAAAUCCGG...UCA.CACGGAGUUGGU.ACAUAUCAACAGUGCGGCUUUUACAAAUAGGGAAGAGGAUCUGAAAUGAGAUUCUCUU
env-2    CCACAUAAAACCC...UUAU...GGGACUAAGCUGUAUAGC.CGGUGG.AUUA.CAAA.GAC.GGAGAGCUCUCAAAUCCUA...UUAGCUCGGAGUUUCAUCACAUGAGUGGGGCAAGCCCUUUUCGUUUAAUUCCCUUUACAUUUAUAUAUGCAAU
Pgi-1-1  CCGC......ACC...UUCG...GGU.....UGCUUU.GCA.CAGCGG.AUUA.CAAA.GGUGGUGGAAUCCUCAAAUCUUG...UCU.UUCGGAGUUU.A.CGCUAUCCGCAGUGCGGCUUUUCAUAUAAGCAUAAGAAAUGAGGGUGUGUCGAAAAUC
Pgi-2-1  CCGC......ACC...UUCG...GGU.....UGCUUU.GCA.CAGCGG.AUUA.CAAA.GGUGGUGGAAUCCUCAAAUCUUG...UCU.UUCGGAGUUU.A.CGCUAUCCGCAGUGCGGCUUUUCAUAUAAGCAUAAGGAAUGAGGGUGUGUCGAAAAUC
env-3    CCACAAUAAGCCGGACUUCG.UUCGGU.GAAGCUGCAAAGC.CGGUGG.AUUA.CAAA.AGCGGG.GGGCUCUCAAACUUAUUGCUAAGUUCGGAGUUUCA.UCACAUCAUCGG.GCGACCCUUUUCUUUUAUUCUUAGAACUUUGUCAUUGAAUACUCU
env-4    CCAUAUAAAACC....UCAGU...GGACUAAGCUGCAAAGC.CGGUGG.AUUA.CAAA.GGCGGA.GAGCUCUCAAAUCUUG...UAUACUCGGAGUUUCA.UCACAUCAC.UGGGCGUCCCUUUUUAAACUAUACGAGGAGAUUACGCAGAAAUGCUGU
env-5    CCACAU....UCC...UUCG...GGAU...AGCUGUAGAGC.CGGUGG.AUUA.CAAA.GGC.GUAGAACUCUCAAAUCUUA...UUAGCUCGGAGUUUCA.UCACAUCA.CGAUGCGGUCUUUUAGUUAGAAGCGGUGGAUUCCUAUUGACAAGUAUGG
Pdi-1-1  CCGCG.....CCC...UUCG...GGGU...AUGCUUA.GCA.CGGCGG.AUUA.CAAA.GGUGGCAAAGCACUCAAAUCCUG...UCA.UACGGAGUUUGU.UCAUAUCUACAGUGCGGCUUUUUCAAAUAGGGAAGAGGAUCUUUAAUUAGAUUCUCUU
env-6    CCGCG.....CCC...UUCG...GGGU...AUGCUUA.GCA.CGGCGG.AUUA.CAAA.GGUGGCAAAGCACUCAAAUCCUG...UCA.UACGGAGUUUGU.UCACAUCUACAGUGCGGCUUUUUCAAAUAGGGAAGAGGAUCUUUAAUUAGAUUCUCUU
Pjo-1-1  CCGCA.....CCC...UUCG...GGGC...AUGCUUA.GCA.CGGCGG.AUUA.CAAA.GAUGGCAAAGCACUCAAAUCCUG...UCA.UACGGAGUUUGU.UCAUAUCUACGGUGCGGCUUUCUUUACAUAGGAGGAUAUGUCAGAACAGGCAUAUCCU
Pme-1-1  CCGCA.....CCC...UUCG...GGGC...AUGCUUA.GCA.CGGCGG.AUUA.CAAA.GAUGGCAAAGCACUCAAAUCCUG...UCA.UACGGAGUUUGU.UCAUAUCUACGGUGCGGCUUUCUUUACAUAGGAGGAUAUGUCAGAACAGGCAUAUCCU
env-8    CCGCA.....CCC...UUCG...GGGU...AUGCUUA.GCA.CGGCGG.AUUA.CAAC.GGUGGCAAAGCACUCAAAUCCUG...UCC.UACGGAGUUUGUUCCUAUUCUACCGUGCGGCUUUUUCCAAUAGGGAGAGGAUCUUUAAUUGGAUUCUCUU
env-9    CCGAA.....ACC...UUCGA..GGGC...AUGCUUC.ACAACGGCGGUAUUAACAAG.GAUGGCAAAGAACUCAAUUCCUG...UCA.UAACGAGUUUGU.CCUAUUCUAC.GUGCGGUUUCUUUACUAGG............................
Bpl-1-1  CCAC......GCC...UUCG...GGU....AGCAUUUAUGC.CGGUGG.AUUA.CAAA.GGAGG.GGAGCACUCAAAACCUG...UCA.UUCGGAGUUUCG.UCACGCCUCGGGUACGGCUUCUCCUUUUUAUAAAAGCGAAGAAUCUUGGGUUUAGCGA
Bco-1-1  CCAC......GCC...UUCG...GGU....AGCAUUUAUGC.CGGUGG.AUUA.CAAA.GGGGA.GAGCACUCAAAUCAUG...UUU.AUCGGAGUUUCG.UCACGCCUCUGGUGCGGCUUUUCUUUUAUAGUAAGCGAAAGAUUUUAUAUCGAUUUGA
env-10   CCAC......GCC...UUCG...GGU....AGCAUUUAUGC.CGGUGG.AUUA.CAAA.GGGGA.GAGCACUCAAAUCAUG...UUU.AUCGGAGUUUCG.UCACGCCUCUGGUGCGGCUUUCCUUUUAUAGUAAGCGAAGGAUUUUAUAUCGAUUUGA
env-11   CCAC......GCC...UUCG...GGU....AGCAUUUAUGC.CGGUGG.AUUA.CAAA.GGAGGA.GAGCACUCAAAACCUG...UCA.UUCGGAGUUUCA.UCGCAUCACUGGUGCGGCUUCAUUAUAAGGCGAUGCUAAUUCAAAAGAUUAGCAUCGC
Bvu-1-1  CCAC......GCC...UUCG...GGU....AGCAUUUAUGC.CGGUGG.AUUA.CAAA.GGAGGA.GAGCACUCAAAACCUG...UCA.UUCGGAGUUUCA.UCGCAUCACUGGUGCGGCUUCAUUAUAAGGCGAUGCUAAUUCAAAAGAUUAGCAUCGC
env-12   CCAC......GCC...UUCG...GGU....AGCAUUUAUGC.CGGUGG.AUUA.CAAA.GGAGGA.GAGCACUCAAAACCUG...UCA.UUCGGAGUUUCA.UCGCAUCACUGGUGCGGCUUCAUUAUAAGGCGAUGCUAAUUCAAAAGAUUAGCAUCGC
Bdo-1-1  CCAC......GCC...UUCG...GGU....AGCAUUUAUGC.CGGUGG.AUUA.CAAA.GGAGGA.GAGCACUCAAAACCUG...UCA.UUCGGAGUUUCA.UCGCAUCACUGGUGCGGCUUCAUUAUAAGGCGAUGUUAAUUCAAGAGAUUAGCAUCGC
env-13   CCAC......GCC...UUCG...GGU....AGCAUUUAUGC.CGGUGG.AUUA.CAAA.GGAGGA.GAGCACUCAAAACCUG...UCA.UUCGGAGUUUCA.UCGCAUCACUGGUGCGGCUUCAUUAUAAGGCGAUGCUAAUUCAAAAGAUUAGCAUCGC
env-14   CCAC......GCC...UUCG...GGU....AGCAUUUAUGC.CGGUGG.AUUA.CAAA.GGAGGA.GAGCACUCAAAACCUG...UCA.UUCGGAGUUUCA.UCGCAUCACUGGUGCGGCUUCAUUAUAAGGCGAUGUUAAUUCAAGAGAUUAGCAUCGC
env-16   CUACG.....CCC...UUCG...GGGU...UACUCUUGAGU.CGGUGG.AUUA.CUUAGUAGUAGGUAGUACUCNUAUCAGU...CUA.UACGGAGUUUCA.UCACAUUAACCGUGCGGCGUUCUUAUAAAGAGGAACGCUUCUCGCUUGCCUANCCGGU
Bca-1-1  CCACA.....UCC...UUCG...GGAC...AGCUUUA.AGC.CGGUGG.AUUA.CAAA.GGGGA.CGGGCACUCAAAUCUUG...UUCUAUCAGAGUUUCA.UCACAUCGUCGGUACGGCUUUUUUAUAUACUCUCCUAUGAUAUUUUAUUUCUCCGGUA
Bfi-1-1  CCACA.....UCC...UUCG...GGAC...AGCUUUA.AGC.CGGUGG.AUUA.CAAA.GAGGA.CGAGCGCUCAAAUCUUG...UUCUAUCAGAGUUUCA.UCACAUCGUCGGUACGGCUUUCUUAUAUACUUUUCUAUGAUAUUUUAUUUCUCAGGUA
Bth-1-1  CCACA.....UCC...UUCG...GGAC...AGCUUUA.AGC.CGGUGG.AUUA.CAAA.GAGGG.CGAGCGCUCAAAUCUUG...UUCUAUCAGAGUUUCA.UCACAUCGUCGAUGCGGCUUUUUUAUAUACUCUUCUAUGAUAUUUUAUUUCUCAGGUA
Bov-1-1  CCACA.....UCC...UUCG...GGAC...AGCUUUA.AGC.CGGUGG.AUUA.CAAA.GAGGG.CGAGCGCUCAAAUCUUG...UUCUAUCAGAGUUUCA.UCACAUCGUCGAUGCGGCUUUUUUUAUAUACUUUUCUAUGAUAUUUUAUUUCUCCGGU
Bfr-1-1  CCACA.....UCC...UUCG...GGAU...AACUUUAUAGU.CGGUGG.AUUA.CAAA.GAGGA.CGGGCACUCAAAUCAUG...UCA.UUCGGAGUUUCA.UCACAUCAUCGGUGCGGCUUUUUAAAUAAAAGGAAGAGGGCGGUAAUCAUCUAAAGAC
env-17   CCACA.....UCC...UUCG...GGAU...AACUUUAUAGU.CGGUGG.AUUA.CAAA.GAGGA.CGGGCACUCAAAUCAUG...UCA.UUCGGAGUUUCA.UCACAUCAUCGGUGCGGCUUUUUAAAUAAAAGGAAGAGGGCGGUAAUCAUCUAAAGAC
Bin-1-1  CCACG.....CCC...UUCG...GGGU...AACUUUG.AGU.CGGUGG.AUUA.CAAA.GAGGA.CGAGCGCUCAAAUCAUG...UCA.UUCGGAGUUUCA.UCACAUCAUCGGUGCGGCUUUUUUAUAAAGGGGAACGGUAAUCUUGGUUGGUUACCGU
env-18   CCACG.....CCC...UUCG...GGGU...AACUUUG.AGU.CGGUGG.AUUA.CAAA.GAGGA.CGAGCGCUCAAAUCAUG...UCA.UUCGGAGUUUCA.UCACAUCAUCGGUGCGGCUUUUUUAUAAAGGGGAACGGUAAUCUUGGUUGGUUACCGU
env-19   CCACG.....CCC...UUCG...GGGU...AACUUUG.AGU.CGGUGG.AUUA.CAAA.GAGGA.CGAGCGCUCAAAUCAUG...UCA.UUCGGAGUUUCA.UCACAUCAUCGGUGCGGCUUUUUUAUAAAGGGGAACGGUAAUCUUGGUUGGUUACCGU
env-20   GCACG.....CCU...UUCG...GGGU...AACUCUUGAGU.CGGUGG.AUUA.CAAA.GAGGAC.GAGCACUCCCAUCAUG...UCG.UACCGAGUUUCA.UCACCUCAUCGGUGCGGAGUUUCUAUUANACAGGAGCCGCGACCUUGGUGACUACAGN
Bun-1-1  CCACG.....CCC...UUCG...GGGU...AACUCUUGAGU.CGGUGG.AUUA.CAAAGAGGAG..GAGCACUCAAAUCAUG...UCA.UACGGAGUUUCA.UCACAUCAUCGGUGCGGCUUUCUAUAAAAGAGGAACGGUAAUCUGUUUGGAUAACCGU
env-23   CCACG.....CCC...UUCG...GGGU...AACUCUUGAGU.CGGUGG.AUUA.CAAAGAGGAG..GAGCACUCAAAUCAUG...UCA.UACGGAGUUUCA.UCACAUCAUCGGUGCGGCUUUCUAUAAAAGAGGAACGGUAAUCUGUUUGGAUAACCGU
env-24   CCACG.....CCC...UUCG...GGGU...AACUCUUGAGU.CGGUGG.AUUA.CAAAGAGGAG..GAGCACUCAAAUCAUG...UCA.UACGGAGUUUCA.UCACAUCAUCGGUGCGGCUUUCUAUAAAAGAGGAACGGUAAUCUGUUUGGAUAACCGU
env-25   CCACG.....CCC...UUCG...GGGU...AACUCUUGAGU.CGGUGG.AUUA.CAAA.GAGGAC.GAGCACUCAAAUCAUG...UCA.UACGGAGUUUCA.UCACAUCAUCGGUGCGGCUUUCUAUAAAAGAGGAACGGUAAUCUUGGUUGACUACCGU
env-26   CCACG.....CCC...UUCG...GGGU...AACUCUUGAGU.CGGUGG.AUUA.CAAA.GAGGAC.GAGCACUCAAAUCAUG...UCA.UACGGAGUUUCA.UCACAUCAUCGGUGCGGCUUUCUAUAAAAGAGGAACGGUAAUCUUGGUUGACUACCGU
env-27   CCACG.....CCC...UUCG...GGGU...AACUCUUGAGU.CGGUGG.AUUA.CAAA.GAGGAC.GAGCACUCAAAUCAUG...UCA.UACGGAGUUUCA.UCACAUCAUCGGUGCGGCUUUCUAUAAAAGAGGAACGGUAAUCUUGGUUGACUACCGU
Bst-1-1  CCACG.....CCU...UUCG...GGGU...AACUCUUGAGU.CGGUGG.AUUA.CAAA.GAGGAC.GAGCACUCAAAUCAUG...UCA.UACGGAGUUUCA.UCACAUCAUCGGUGCGGCUUUCUAUAAAAGAGGAACGGUAAUCUUGGUUGACUACCGU
Beg-1-1  CCACG.....CCC...UUCG...GGGU...AACUCUUGAGU.CGGUGG.AUUA.CAAA.GAGGAC.GAGCACUCAAAUCAUG...UCA.UACGGAGUUUCA.UCACAUCAUCGGUGCGGCUUUCUAUAAAAA............................
         <<<<......<<<<<......>>>>>.....<<<....>>>...>>>>......>>..>>>>>>>>>>>>..........................................................................................
         0121......20200......00202.....222....222...1210......02..1222?2???12?..........................................................................................
         ..............................................................................>>>>...>>>........................................................................
         ..............................................................................1???...01?........................................................................
         CCRC○-----•CC---UUCG---GG•○---AR•YYU•○R•Y-CGGYGG-AUUA-CAAA-GR•GR○○GAGC•CUCAAAUCYUG---UY•-Y•CGGAGUUUU•R-YCRYAUC••YRGUGCGGCUUUYYY•••••R••••R••R•••••••••••••••••••
                                                                                                                    >>>>>>>>>>>>
                                                                                                                                 3′
```

alignment positions 320 · · · 479

```
env-1     CUUUAAAGCCGGUUCACGCAGAUAGUCUCGUUAGCUAAUAUAAGUAGUCUUCUGAAGAUAAAGUGAAUUAUUUGGAAAAUAAAUUAGGUUUAAUAGUGGGUAAUCCAAAAAGAAGGCUUUCCUUGGUAGCAACAUCCUAAAGAUAGUUUGUCUGGCUCUU
env-2     GGUUCCCCGCUUCAUAGCGUACA.........................................................................................................................................
Pgi-1-1   GCUUCAGCAGCGUUUUCGGCACACUUUUUUAUGCCAGAAAGGUUGUACUGAGAGCCUGCAUAGCCGUAAGCCACAUAGCAAUCCGGCGUAUUCUUAUUUCAAAAGGACGAUGCGGCAAAAGCGAUGCUACUUGCCGAUGGAUUGGAUUCGCCUUCGCACG
Pgi-2-1   GCUUCAGCAGCGUUUUCGGCACACUUUUUUAUGUCAGAAAGGUGUACUGAGAGCCUGCAUAGCCGUAAGCCACAUAGCAAUCCGGCGUAUUCUUAUUUCAAAAGGACGAUGCGGCAAAAGCGAUGCUACUUGCCGAUGGAUUGGAUUCGCCUUCGCACGU
env-3     CAUCAUUCUUUGAACAAUAUUCAACAUCAUAGAAAACAUUCAUCAACAUUUUAAAUUCAUACGAAUAUCACAUCAUGUUUAGCGGAAUCAUAGAAAACAUGGCUACUGUCGUGGCUGUGAAAAGGGACAAGGAGAAUAUCGACCUUACACUGGAAUGCCC
env-4     AAUCUCCUUUUUUUGUUACUAUUCUAUAUUUCUACCGAGUAUACUCUUAAUUUAGUAUAAUAAAUUUUGGCACGUACGAAAUAUUUCGUUACUUGUCGAAAGACGAAUCACCC............................................
env-5     AACACACCGCUUCUUUUUGUAUGUAGUAAAUAAACUGAAUAAUAAAUUUUAUUUGAAUUAUGUUCUCAGGAAUUGUUGAAGAGUUUGCCAUCGUGACAAAAAUCAGGAAAUAUGGCGAAAACAUUGAUUUCACAUUUAAAUGCUCUUUCACCGACGAGCU
Pdi-1-1   CUUUUUUAUGCGGUUCACGCAGAUAGUCUCGUUAGCUCAUAUAAGUAGUCUUCUGAAGAUAAAGUGAAUUAUUUUGAAAAUAAAUUAGGUUUUAUAGUUGGUAAUCCAAAAAGAAUGCUUUCCUUUGUAGCAACAAACAAAAGAUAGUUUGUCUUGCUCU
env-6     CUUUUUUAUGCGGUUCACGCAGGUAGUCUCGUUAGCUCAUAUAAGUAGUCUUCUGAGGAUAAAGUGAAUUAUUUUGAAAAUAAAUUAGGUUUUAUAGUUGGUAAUCCAAAAAGAAUGCUUUCCUUUGUACCAACAAACAAAAGAUAGUUUGUCUUGCUCU
Pjo-1-1   CUUUUUGUGCCUAAUAAAGCAUAGCUUUUUGUUUGAAAUAGGAGCUUGUCUGUUUUUUCUUCGUAUAUUUACCCUCCAAGAAUUAAACACACUAAACUAAAUAUUUAUGUUAUUGCCAAAACAAUCUCACAAUUGUGUAACCGGGCUGGCUGUCGCCUGC
Pme-1-1   CUUUUUUUGUGCCCCGUCAAGCAUAGUUUUAGACGGUGUAACGCUAUGGGUUAGGGGAGCUAAAGCAUCGCUUUUUGUUUGAAAUAGGAGCUUGUCCGUUUUUUCUUCGUAUAUUUACCCUCCAAGAAUUAAACACACUAAACUAAAUAUUUAUGUUACU
env-8     CUUUUUAUGCGGUUCACGCCAAAUAGUCUCUUUGGCUCAUAUACGUUGUCUUCUGAAGAUCACGUGAAUUUUUUUGACCCCUCACUAGCGUUAUAUAGUUGGUUAUCCGGAAGAAUGCUUCCUUUGUAGCACCA........................
env-9     ................................................................................................................................................................
Bpl-1-1   GUCAAGGUUCUUCGUUUUUUUUAUGAGUUUACUUUUGGAACGUUGUUUACUUAGAUAAUAUAAGAACUAUGAGGAAGCACAUUUUAAUGUCGUUGUGUGCCGGCUCUCUUUUGUUGGGGGCUUGUGCUGGAAAAGGUGAGCAGAAAAUGAGUGUGUCACAA
Bco-1-1   UAUAUAAUCUUCGCUUUAUUUUUUUUAUCAUGCUUUUUAGGGAUUCCUUUGAUUUUUAAUUGCAGUUUAUAUGAUAUUUUAUUUUUCUGGAACAGGUAAUUCGCGAUGGGUGGCUGAAUCUCUUGGAAAAAGAUUGGGGAUGCGGUAGAGGAUAUAGCA
env-10    UAUAUAAUCUUCGCUUUAUUUUUUAUCAUGCUUUUUAGUGAUUCCUUGAUUUUUAAUUGCAGUUUAUAUGAUAUUUUAUUUUUCUGGAACAGGUAAUUCGCGAUGGGUGGCUGAAUUUCUUGGGAAAAGAUUGGAGGAUGCUGUAGAGGAUAUAGCAGAU
env-11    UUCUUUUUAUAAAAAUAGUACGGAUAUGAUAUUUUAUUUUUCAGGUACAGGUAAUUCGGCUUGGGUGGCAAGGCAGCUGGCGGAAGGACAGAAUGAGGAAUUGUUGUCCAUAGCUAUGGAAAUAGACAGGAACAAAGCGUAUAAGCUGAAAGAAGGGGAA
Bvu-1-1   UUCUUUUUAUAAAAAUAGUACGGAUAUGAUAUUUUACUUUUCAGGUACAGGUAAUUCGGCUUGGGUGGCAAGGCAGCUGGCGGAAGGACAGAAUGAGGAAUUGUUGUCCAUAGCUAUGGAAAUAGACAGGAACAAAGCGUAUAAGCUGAAAGAAGGGGAA
env-12    UUCUUUUUAUAAAAAUAGUACGGAUAUGAUAUUUUACUUUUCAGGUACAGGUAAUUCGGCUUGGGUGGCAAGGCAGCUGGCGGAAGGACAGAAUGAGGAAUUGUUGUCCAUAGCUAUGGAAAUAGACAGGAACAAAGCGUAUAAGCUGAAAGAAGGGGAA
Bdo-1-1   UUCUUUUUUAUAAAAAUAAUACGGAUAUGAUAUUUUAUUUUUCAGGUACAGGUAAUUCAGCUUGGGUGGCAAGGCAGCUGGCGGAAGGACAGAAUGAGGAAUUGCUCUCCAUAGCUGUGGAAAUAGAUAAGAAUAAGGAGUAUAAACUAAGAGAAGGAGA
env-13    UUCUUNUUUAUAAAAUAGUUCACAUCUGAUAUGUUAUUUUUCAGGUACAGUAUUCGUUACACGUGCGAUGGAUCACCCGGAAGUCUGCUACUACGCUGCACCUGGAAAAAAUUCUANNNUAUAUAUAACUAAUA........................
env-14    UUCUUUUUUAUAAAAAUAAUACGGAUAUGAUAUUUUAUUUUUCAGGUACAGGUAAUUCAGCUUGGGUGGCAAGGCAGCUGGCGGAAGGACAGAAUGAGGAAUUGUUCUCCAUCGCUGUGGAAAUAGAUAAGAAUAAGGAGUAUAAACUAAGAGAAGGAGA
env-16    AGCUUCGCCCUNGNUCCUGCGCGAUGCCACAACGUGUAAAAAUAAAAAAAAAAAAAAAAGAGAAAAAAAAAAAAAAAAAUAAUAAAANNNNAAACAAAAAAUAUAUUAGUUGUUUUUCUUUUUUUUUUGUUUUGGUUUUUUUUUU................
Bca-1-1   CAGGUAACUCUGAAUGGAUUGCAAAUCAGCUCUCUAAAGGACAAAAUGAAGAUUUGGUCUUUAUCCCUGAGGCGUUGAAAAAUGAGGCGCUGGAGUUCGAUUUGCAGAAAGAUGAAAAGAUUGGUUUUGUGUUUCCGAUUUACUCAUGGGCUCCUCCUGA
Bfi-1-1   CAGGUAAUUCAAAGUGGAUCGCUAAUCAGCUGUCUAAGGAACAAAAAGAGGAUUUAGUUUUCAUUCCCGACGCAUUGAGGAGUGGAGUGUUGGAGUUUUGUCUUAAAGAGGAAGAAACGAUUGGAUUCGUGUUUCCUAUUUACUCGUGGGCGCCUCCUGA
Bth-1-1   CAGGUAACUCUAAAUGGAUUGCUAAUCAGCUUUCUAAAGAACAACAGGAAGAUUUGUUUUUUAUCCCGGACGCAUGGAAGAAUGACACAUGGGAGUUCUCUUUGCGGGAGGAUGAGAAGAUCGGUUUUGUCUUUCCGGUCUAUUCGUGGGCGCCUCCCGU
Bov-1-1   ACAGGUAAUUCUAAGUGGAUUGCAAAUCAGCUUUCCAAAGAGCAAAAAGAAGAGUUGGUUUUUAUUCCUGAUGCGCUAAAGAAUGGAGCAUUGGAGUUUAGUUUGCAGGCAGAUGAAAAGAUCGGCUUUGUAUUUCCUAUCUAUUCGUGGGGACCUCCUG
Bfr-1-1   UACCGUCCUUUUUUUAUGCUGUUUUCUUUAAUACAUAUACAUUAUAGUUAUGAUAUUCUAUUUUUCAGGAACUGGAAAUUCUAAAUGGAUUGCGGAGCAGAUCGCUAAGGCACAAAACGAAGUGCUUGUUUUUAUGCCGAAUGCCAUCAGAGACGGAAUA
env-17    UACCGUCCUUUUUUUAUGCUGUUUUCUUUAAUACAUAUACAUUAUAGUUAUGAUAUUCUAUUUUUCAGGAACUGGAAAUUCUAAAUGGAUUGCNGAGCAGAUCGCUAAGGGCACAAAACGAGUGCUUGUUUUUAUGCCGAAAUGCAUCAGAGACNGAAUA
Bin-1-1   UCCUUUUUCUUUGUACUUUCUACAAUCCAAAGAUUUCCGCUUAACCUCUAUUAAGCGAUGUCGUAACUCAAAUAUUGUCAAUAGCAGAUUUAUAUAAUAUUAACGGAAGUAGGUUAGGAGGCUGCUUUGAGGUAGUGAUAUGCAAUAAAAGGACUUUCGU
env-18    UCCUUUUUCUUUGUACUUUCUACAAUCCAAAGAUUUCAGCUUAACCUCUAUUAAGCGAUGUCGUAACUCANAUAUUGUCAAUAGUAGAUUUAUAUAAUAUUAACGGAAGUAGNNGUAGAGGCUGCUUUGAGGUAGUGAUAUGCAAUAAAAGGACUUUCGU
env-19    UCCUUUUUCUUUGUACUUUCUACAAUCCAAAGAUUUCAGCUUAACCUCUAUUAAGCGAUGUCGUAACUCAAAUAUUGUCAAUAGUAGAUUUAUAUAAUAUUAACGGAAGUAGGUUAGGAGGCUGCUUUGAGGUAGUGAUAUGCAAUAAAAGGACUUUCGU
env-20    GCCUUUUCUCUGUUGUCCCCUUUCCUCUUAUUUGGUAUCCCCUUAGUCUUUCACACUUACAGGCGUUUCCUCGCGUUUGGGGGAGAACCCUUUUUCCAGCGCUUCUUUGGGCGGACGGGCUCCUCUUUAAUGGGGGGAAGGUUCGUUU...........
Bun-1-1   UCCUCUUUCGUUUUAUGCGGAUGUGGGUGAUUGUGAAACGAUGAUACGUGAUGCUGAAACAGAUGAUGUGUCAAGGGAAGAUAGAUGAUGUGUUUAUACAAGACAGACCAUCUGUAUCGGGUGAACAGAUGGUCUGUUUUGAAAAGAGUUUUUUUAAGUU
env-23    UCCUCUUUCGUUUUAUGCGGAUGUGGGUGAUUGUGAAACGAUGAUACGUGAUGCUGAAACAGAUGAUGUGUCAAGGGAAGAUAGAUGAUGUGUUUAUACAAGACAGACCAUCUGUAUCGGGUGAACAGAUGGUCUGUUUUGAAAAGAGUUUUUUUAAGUU
env-24    UCCUCUUUCGUUUUAUGCGGAUGUGGGUGAUUGUGAAACGAUGAUACGUGAUGCUGAAACAGAUGAUGUGUCAAGGGAAGAUAGAUGAUGUGUUUAUACAAGACAGACCAUCUGUAUCGGGUGAACAGAUGGUCUGUUUUGAAAAGAGUUUUUUUAAGUU
env-25    UCCUUUUUCUUUUGUCGUUAUUUUACGGUUUUGCUUCUUGAAGAUCAGAGAGUUCAGUAAAAAUUGGAUUGGUUGCAGUGAGGGGGAAAAGUCCAGGCUUUUGCUCGUUUAACUCCAGGUGUUUACUCCCGAAAUGCCUGAAGUUUUAGGUUGGAAUGCC
env-26    UCCUUUUUCUUUUGUCGUUAUUUUACGGUUUUGCUUCUUGGGAUCAACGUAACAAUCUGAGGGAUUUUAUAUUUAAGCAUAUAAUUUAGCAAGUCUAUAUAAGAAAAAAGCUCUAUCUCUUACUCCUCUGAACUGUGCUCUAAAUGCUUUGAUUUUAGCA
env-27    UCCUUUUUCUUUUGCCGUUAUUUUAUGGUUUUGCUUCUUGAAGAUCAGAGAGUUCAGUAAAAAUCGGAUUGGUUGCAGUGAGGGGGAAAAGUCGGGUCAACGUAAAAACCUGAGGGAUUCAUAGUUUAUGCAUAAAUUUUCGCCAGUCUGAACAGGAAGA
Bst-1-1   UCCUUUUUCUUUUGUCGUUAUUUUACGGUUUUGCUUCUUGAAGAUCAGAGAGUUCAGUAAAAUCGGAUUGGUUGCAGUGAGGGGGAAAAGUCCAGGCUUUUGCUCGUUUAACUCCAGGUGUUUACUCCCGAAAUGCCUGAAGUUUUAGGUUGGAAUGCCC
Beg-1-1   ................................................................................................................................................................
          ................................................................................................................................................................
          ................................................................................................................................................................
          ................................................................................................................................................................
          ................................................................................................................................................................
          ●●Y●●●●Y●YY●●●●●●●●Y●●●●●●Y●●●●●●●●●●●●●●●●●●●●●●●●●●●●●●●●●●●●●●●●●●●●●●●●●●●●●●●●●●●●●●●●●●●●●●●●●●●●●●●○○○○○○○○○○○○○○○○○○○○○○○○○○○○○○○○○○○○○○○○○○○○○○○
```

alignment positions 480 · · · 586

```
env-1     GAUCUUCGAGUUUCUAUUCUCAGUAAUGAUAAUAUAUUCUUCUAGAAAUCGAUCUUCACGAACAUCACUUGUUUUAUUAUUAAUUUAUUUUUAAUUUUUUUCGUAUG
env-2     ...........................................................................................................
Pgi-1-1   UCUUUGGAUAUUUCCAAGAACAUAUAACCCGACUUGCCGGAGUGAAUGUCAGCUUCGUUUUCCUGAUAUUUCUUGUUGGCAUAUUCUUUCGACUUCUCGAAAGUCAG
Pgi-2-1   CUUUGGAUAUUUCCAAGAACAUAUAGCCCGACUUGCCGGAGUGAAUGUCAGCUUCGUUUUCCUGAUAUUUCUUGUUGGCAUAUUCUUUCGACUUCUCGAAAGUCAGC
env-3     GUUUGCUGGCGAGUUGAGUAUCGACCAGAGUGUGGCGCACAAUGGAGUAUGCCUCACGGUCGUGGCUUUAGAAGGCAACACCUAUACAGUUACUGCCAUGAAGGAAC
env-4     ...........................................................................................................
env-5     GAAGAUUGACCAGAGCGUGGCGCACAACGGUGUAUGCCUCACCGUGGUGGAGGUUAAGAACGGCACGUAUACCGUUACGGCAAUGAAGGAGACUCUCGACCGCUCCA
Pdi-1-1   UUAUCUUCGAGUUUCUAUUCUCAGUAAUGAUAAUAUAUUCUUCUAGAAAUCGAUCUUCACUAACAUCACUUGUUUUAUUAUUAAUUUAUUUUUAAUUUUUUUCGUAU
env-6     UUAUCUUCGAGUUUCUAUUCUCAGUAAUGAUAAUAUAUUCUUCUAGAAAUCGAUCUUCACUAACAUCACUUGUUUUAUUAUUAAUUUAUUUUUAAUUUUUUUCGUAU
Pjo-1-1   UCGUUGCUAUUGGUUGCCUGCUCUCCGAAGGAAGCCCAGCAGGUCAAUCUUAUUCCCAAACCGGAACGGAUGACAAUGACCGGAGGAACCUUCAGGGUCGAUAGUCU
Pme-1-1   GCCAAAACAGUCUCGCAAUUAUGUAACCGGGCUGGCUGCCGCUUGCUCGUUGCUAUUGAUUGCCUGCUCUCCGGAGGAAGUCCGGCAGGUCAAUCUUAUUCCCAAAC
env-8     ...........................................................................................................
env-9     ...........................................................................................................
Bpl-1-1   GAACCGGAUACUUUAUUGAUGUUGGUGGGCAGUUAUGCAAGUGCCGAUCAGGAAGGGAUAAAGACGUAUCGUUUCAACCAGGAGACGGGAGAGGCUGUGCUGGCUAG
Bco-1-1   GAUGUGAAAAUUGAGAAAGAAGAAUAUACCCUGUCAGAAGGAGAAAUAUUAGGGUUUGUUUUUCCUGUUUAUGCAUGGGCACCUCCUACUAUUGUGAUGGAUUUUAU
env-10    GUGAAAAUUGAGAAAGAAGAAUAUACCCUACCAGAAGGAGAAAUAUUAGGGUUUGUUUUUCCUGUUUAUGCAUGGGCACCUCCUACUAUUGUGAUGGAUUUUAUUUC
env-11    AAAGUAGGUUUUGUUUUUCCUGUAUAUGCAUGGGGCCCUCCCAAGAUAGUACUUCGUUUUAUUCAUCAGCUAAAGUUGGAUAAGCCGGGAUACCUUUUCUUUGUCUG
Bvu-1-1   AAAGUAGGUUUUGUUUUUCCUGUAUAUGCAUGGGGACCUCCCAAGAUAGUACUUCGUUUUAUUCAUCAGCUAAAGUUGGAUAAGCCGGGAUACCUUUUCUUUGUCUG
env-12    AAAGUAGGUUUUGUUUUUCCUGUAUAUGCAUGGGGCCCUCCCAAGAUAGUACUUCGUUUUAUUCAUCAGCUAAAGUUGGAUAAGCCGGGAUACCUUUUCUUUGUCUG
Bdo-1-1   AAAAGUAGGUUUUGUCUUCCCUGUGUAUGCAUGGGGACCUCCGGAGAUUGUACUUCGUUUUAUCCGUCGGUUGAAGAUGACUAAGCCGGAAUAUCUUUUCUUUGUCU
env-13    ...........................................................................................................
env-14    AAAAGUAGGUUUUGUCUUCCCUGUGUAUGCAUGGGGACCUCCGGAGAUUGUACUUCGUUUUAUCCGUCGGUUGAAGAUGACUAAGCCGGAAUAUCUUUUCUUUGUCU
env-16    ...........................................................................................................
Bca-1-1   AAUUGUACUGCGCUUUAUCAAUCGAAUUUCUUUGAAAGAAUACCAGAAUCAGUAUUUGUUUUUUGUUUGUUCUUGUGGGGAUGAUACGGGACUUACUCAACAAGUGU
Bfi-1-1   GAUUGUAUUGAACUUUAUCCAAAAACUCUCGCUGAAGGGGUACAGAGGGCAGUAUUUGUUCUUUGUAUGUUCUUGUGGAGAUGAUAUCGGCUUGACUCAACAGGUUC
Bth-1-1   GAUUGUACAGGAGUUUAUUCGGAAGCUGGUUCUGAAGGGGUAUCAGCAGCAAUAUCUGUUCUUUGUGUGUUCGUGUGGAGACGAUACGGGGCUGACCCGGCAGGUGA
Bov-1-1   AAAUUGUACUUAAUUUUAUUCGGCAACUCUCUUUAAAGGGGUAUAAAAGACAAUAUUUGUUUUUUGUUUGUUCCUGUGGGGAUGAUACCGGGCUUACGCAGCAGGUG
Bfr-1-1   GAAGAGUUUGUGUUGGCGGAUGAUGAAAAAGUAGGUUUUGUUUUCCCUGUUUAUUCAUGGGGACCUCCGUUGAGCGUAUUGCGGUUCUUGGAUUGGAUUACUUUAUC
env-17    NAAAAAGUUGUGUUGNNCGAUGAUGAAAAGUNNAGUUUGUUUCCCCCUGUUAUCAUGGGGACCUCGUUGAGCGUAUUGCGGGUCUUGGAUGGNAUACUUUAUCUAAU
Bin-1-1   UUCUUUUGCCAGUGUUUGUUAGGUCAAAUGCUACAUCUCAGUGCUCUCAAUGCCACACCUUAACAGGACAAAUGCCACAGGUUGCAGACAGUGAAGUGUGGAGUUAA
env-18    UUCUUUUGCCAGUGUUUGUUAGGGUCAAUGCUACAUCUCAGUGCUCUCAAUGCCACACCUUAACAGGACAAAUGCCACAGGNUGCAGACAGUGAAGUGUGGAGUUAA
env-19    UUCUUUUGCCAGUGUUUGUUAGGUCAAAUGCUACAUCUCAGUGCUCUCAAUGCCACACCUUAACAGGACAAAUGCCACAGGUUGCAGACAGUGAAGUGUGGAGUUAA
env-20    ...........................................................................................................
Bun-1-1   UAUUUGUUGUCCCGUAAGUCUUUUACCCUGACGGCUUUUCCUUCGCUCUGCGGCAAGGAACCUUUCUUUACCAACCGUAACUUCGGAGUUACCAGAAUCUCGUCUUU
env-23    UAUUUGUUGUCCCGUAAGUCUUUUACCCUGACGGCUUUUCCUUCGCUCUGCGGCAAGGACCUUUCUCACCAACC.................................
env-24    UAUUUGUUGUCCCGUAAGUCUUUUACCCUGACGGCUUUUCCUUCGCUCUGCGGCAAGGACCUUUCUUUACCAACCGUAACUUCGGAGUUACCAGAAUCUCGUCUUUC
env-25    CGGUGUUUGGGAAGUAAAUUCCGGGAGUUUCGGGAGUAUAAUGCAGGGAUAUUUACCGGUAUGGCACGGCUUCAUUAACGGUUGAAACUGGACAUACCGGUAGAAUA
env-26    UUGAAUGACUCUGCUGCGGCAUUGGUUGCCCUCCUUUCAAAGAAAUUGAUUAUAUCCAGAUAAUGUGUUUGUAUAGAACGUGCGACCCUUCCAAAGGUGAGGAACCC
env-27    AAGCUCUGUCCUUUACUCCUCUGAAUUGUGUGCGGAACUCCUUUAUUUUUGCAUUGAACGAUUCGGAAGCAGCGUUUGUUGAACGUCUGUCAAAGAAGUUGAUUAUU
Bst-1-1   GGUGUUUGGGAAGUAAAUUCCGGAAGUUUCGGGAGUAUAAUGCAGGGAUAUUUACCGGUAUGGCACGGCUUCACUAACGGUUGGAACUGGACAUACCGGUAGAAUAU
Beg-1-1   ...........................................................................................................
          ...........................................................................................................
          ...........................................................................................................
          ...........................................................................................................
          ...........................................................................................................
          ∘∘∘∘∘∘∘∘∘∘∘∘∘∘∘∘∘∘∘∘∘∘∘∘∘∘∘∘∘∘∘∘∘∘∘∘∘∘∘∘∘∘∘∘∘∘∘∘∘∘∘∘∘∘∘∘∘∘∘∘∘∘∘∘∘∘∘∘∘∘∘∘∘∘∘∘∘∘∘∘∘∘∘∘∘∘∘∘∘∘∘∘∘∘∘∘∘∘∘∘∘∘∘∘∘∘∘
```

13 *Bacteroides*-1

13.1 Taxa

The taxonomy of each organism containing a putative *Bacteroides*-1 RNA is listed, with abbreviations identifying each hit (e.g., "Eco-1-1" and "Eco-1-2" might hypothetically represent two distinct RNAs in *E. coli*). The abbreviations will be used to identify each individual *Bacteroides*-1 RNA in Sections 13.2 and 13.4.

abbrev. of hits	taxonomy of species
Bca-1-1 to Bca-1-3	Bacteria Bacteroidetes Bacteroidia Bacteroidales Bacteroidaceae *Bacteroides caccae* ATCC 43185
Bco-1-1	Bacteria Bacteroidetes Bacteroidia Bacteroidales Bacteroidaceae *Bacteroides coprocola* DSM 17136
Bdo-1-1	Bacteria Bacteroidetes Bacteroidia Bacteroidales Bacteroidaceae *Bacteroides dorei* DSM 17855
Beg-1-1	Bacteria Bacteroidetes Bacteroidia Bacteroidales Bacteroidaceae *Bacteroides eggerthii* DSM 20697
Bfr-1-1 to Bfr-1-2	Bacteria Bacteroidetes Bacteroidia Bacteroidales Bacteroidaceae *Bacteroides fragilis* NCTC 9343
Bfr-2-1 to Bfr-2-2	Bacteria Bacteroidetes Bacteroidia Bacteroidales Bacteroidaceae *Bacteroides fragilis* YCH46
Bin-1-1	Bacteria Bacteroidetes Bacteroidia Bacteroidales Bacteroidaceae *Bacteroides intestinalis* DSM 17393
Bov-1-1 to Bov-1-3	Bacteria Bacteroidetes Bacteroidia Bacteroidales Bacteroidaceae *Bacteroides ovatus* ATCC 8483
Bst-1-1	Bacteria Bacteroidetes Bacteroidia Bacteroidales Bacteroidaceae *Bacteroides stercoris* ATCC 43183
Bth-1-1 to Bth-1-3	Bacteria Bacteroidetes Bacteroidia Bacteroidales Bacteroidaceae *Bacteroides thetaiotaomicron* VPI-5482
Bun-1-1	Bacteria Bacteroidetes Bacteroidia Bacteroidales Bacteroidaceae *Bacteroides uniformis* ATCC 8492
Bvu-1-1	Bacteria Bacteroidetes Bacteroidia Bacteroidales Bacteroidaceae *Bacteroides vulgatus* ATCC 8482
env-1 to env-20	environmental samples

13.2 Gene contexts

Each *Bacteroides*-1 RNA (indicated by "RNA→") is listed with its surrounding genes. Environmental sequences and some RefSeq entries lack gene annotations, and no genes are listed for such sequences. The direction of each gene is indicated with an arrow (→), and each conserved domain in the gene is colored. Conserved domains associated with more than one *Bacteroides*-1 RNA are assigned a color; other domains are gray. Information about these conserved domains is given in Section 13.3. The accession number of the sequence containing each *Bacteroides*-1 RNA is given. Accessions beginning with "NC_" or "NZ_" are RefSeq, while other accession refer to environmental samples. Nucleotide coordinates are given for the 5′ and 3′ boundaries of each. If the 5′ coordinate is greater than the 3′ coordinate, the RNA is present on the reverse-complement strand of the containing genomic DNA sequence. Each hit is denoted by an abbreviation (like "Eco-1-1") that refers to a taxonomy given in Section 13.1.

abbrev.	Seq. accession		5′ at	3′ at	genes
Bco-1-1	NZ_ABIY02000058.1	+	8754	8822	GalE (COG1087)→ **RNA**→ EriC (COG0038)→
env-1	BABD01000872.1	-	1354	1286	GalE (COG1087)→ **RNA**→ EriC (COG0038)→
Bth-1-1	NC_004663.1	-	2410752	2410667	←hypo **RNA**→ **RNA**→ ←hypo
Bth-1-2	NC_004663.1	-	2410987	2410907	(shown above)
env-2	BAAV01015564.1	+	659	744	Amidase_3 (pfam01520)→ **RNA**→ **RNA**→ hypo→
env-3	BAAV01015564.1	+	424	504	(shown above)
Bca-1-1	NZ_AAVM02000011.1	-	62850	62766	COG3292 (COG3292)HTH_ARAC (smart00342)→ **RNA**→ Cons_hypoth698 (pfam03601)→
Bov-1-1	NZ_AAXF02000043.1	+	119625	119709	AmiC (COG0860)→ **RNA**→ **RNA**→ ←COG4799 (COG4799)
Bov-1-2	NZ_AAXF02000043.1	+	119356	119436	(shown above)
Bca-1-2	NZ_AAVM02000011.1	-	71894	71814	AmiC (COG0860)→ **RNA**→ ←hypo
Bth-1-3	NC_004663.1	+	768695	768775	GalE (COG1087)→ **RNA**→ ←IspE (COG1947)
Bca-1-3	NZ_AAVM02000001.1	-	1013664	1013584	GalE (COG1087)→ **RNA**→ ←IspE (COG1947)
env-4	BABG01016634.1	-	466	385	hypo→ **RNA**→ ←hypo
Bov-1-3	NZ_AAXF02000048.1	+	20925	21006	GalE (COG1087)→ **RNA**→ COG4680 (COG4680)→
env-5	BABB01010445.1	-	346	262	←hypo **RNA**→
Bfr-1-1	NC_003228.3	+	3358325	3358409	hypo→ **RNA**→ ←COG3871 (COG3871)
env-6	BABA01000991.1	+	1142	1226	hypo→ **RNA**→ ←IclR (COG1414)
Bfr-2-1	NC_006347.1	+	3456921	3457005	←hypo **RNA**→ ←COG3871 (COG3871)
env-7	BAAZ01000465.1	+	2353	2434	GalE (COG1087)→ **RNA**→ ←IclR (COG1414)
Bin-1-1	NZ_ABJL02000008.1	-	538328	538247	GalE (COG1087)→ **RNA**→ ←IclR (COG1414)
env-8	BABD01020940.1	+	232	313	hypo→ **RNA**→
env-9	BABG01021432.1	+	589	670	GalE (COG1087)→ **RNA**→
Bun-1-1	NZ_AAYH02000045.1	-	87516	87435	GalE (COG1087)→ **RNA**→ hypo→
env-10	BAAU01022191.1	-	354	273	GalE (COG1087)→ **RNA**→
env-11	BABD01004454.1	+	837	918	GalE (COG1087)→ **RNA**→
env-12	BABB01007489.1	-	586	505	hypo→ **RNA**→ hypo→
Bst-1-1	NZ_ABFZ02000018.1	+	247284	247365	GalE (COG1087)→ **RNA**→ AtpD (COG0055)→
env-13	BAAU01022945.1	+	559	640	GalE (COG1087)→ **RNA**→
Bfr-1-2	NC_003228.3	-	3042077	3041996	GalE (COG1087)→ **RNA**→ ←putative lipoprotein
Bfr-2-2	NC_006347.1	-	2980257	2980176	GalE (COG1087)→ **RNA**→ ←hypo
env-14	BAAY01000403.1	+	207	288	**RNA**→ AtpD (COG0055)→
Beg-1-1	NZ_ABVO01000039.1	+	26199	26280	**RNA**→
env-15	BAAX01008592.1	-	313	232	hypo→ **RNA**→
env-16	BABB01000399.1	-	1462	1380	**RNA**→ hypo→
env-17	BAAU01002748.1	-	674	588	GalE (COG1087)→ **RNA**→ hypo→
Bdo-1-1	NZ_ABWZ01000015.1	+	81732	81818	**RNA**→
env-18	BAAV01002879.1	-	654	568	GalE (COG1087)→ **RNA**→ hypo→
Bvu-1-1	NC_009614.1	-	4788592	4788506	GalE (COG1087)→ **RNA**→ hypo→
env-19	BABA01008930.1	-	1160	1074	hypo→ **RNA**→ hypo→
env-20	BABG01024910.1	+	369	455	hypo→ **RNA**→ hypo→

13.3 Conserved domains

Conserved domains found in protein-coding genes listed in Section 13.2 are listed, with the first sentence in their description from the Conserved Domain Database. Conserved domains associated with more than one *Bacteroides*-1 RNA are assigned a color, while others are shown in gray. The number in parentheses is the number of occurrences of the given gene.

D COG0038 (2) Chloride channel protein EriC [Inorganic ion transport and metabolism]
D COG0055 (2) F0F1-type ATP synthase, beta subunit [Energy production and conversion]
u COG0860 (2) N-acetylmuramoyl-L-alanine amidase [Cell envelope biogenesis, outer membrane]
u COG1087 (18) UDP-glucose 4-epimerase [Cell envelope biogenesis, outer membrane]
d COG1414 (3) Transcriptional regulator [Transcription]
d COG1947 (2) 4-diphosphocytidyl-2C-methyl-D-erythritol 2-phosphate synthase [Lipid metabolism]
u COG3292 (1) Predicted periplasmic ligand-binding sensor domain [Signal transduction mechanisms]
d COG3871 (2) Uncharacterized stress protein (general stress protein 26) [General function prediction only]
D COG4680 (1) Uncharacterized protein conserved in bacteria [Function unknown]
d COG4799 (1) Acetyl-CoA carboxylase, carboxyltransferase component (subunits alpha and beta) [Lipid metabolism]
u pfam01520 (1) N-acetylmuramoyl-L-alanine amidase.
D pfam03601 (1) Conserved hypothetical protein 698.
u smart00342 (1) helix_turn_helix, arabinose operon control protein;

13.4 Multiple-sequence alignment

Each *Bacteroides*-1 RNA is denoted by an abbreviation (like "Eco-1-1") that refers to a taxonomy given in Section 13.1. The alignment includes sequences containing the RNA motif, as well as flanking sequence. The *Bacteroides*-1 RNA itself is denoted by the line underneath marked 5′ and 3′ on either end. Nucleotides in the flanking sequences (i.e., not part of the motif) are written in gray letters. Stems of predicted rho-independent transcription terminators are shaded yellow. (Note: terminator predictions have not been analyzed manually, and many are likely to be false positives.) Start codons are shaded green. (Note: start codons are frequently misannotated, especially in environmental samples.) Nucleotides proposed to basepair as part of the consensus structure are shaded in color when they comprise Watson-Crick or G-U pairs. Otherwise they are shaded gray. Conserved stems are also indicated at the bottom of the alignment by angle brackets, where matching < and > denote base-paired columns. Below these angle brackets, the symbol "2" denotes base pairs exhibiting covariation, "1" denotes base pairs exhibiting compatible mutations, "0" denotes base pairs that are not observed to mutate and "?" denotes base pairs that have a significant frequency of non-canonical nucleotides for Watson-Crick or G-U pairs ($> 5\%$). Below these base pair annotation is the consensus sequence: "R" = "A" or "G", "Y" = "C" or "U", red nucleotides: nucleotide identity conserved more than 97% of the time, black nucleotides: 90%, gray nucleotides: 75%, red circle (•): nucleotide is present 97% of the time, black circle (•): 90%, gray circle (•): 75%, white circle (•): 50%. All percentages of sequences just described (e.g. 97% conserved) assume that sequences have been weighted by the GSC algorithm implemented by the Infernal software.

alignment positions 1 · · · 99

```
Bco-1-1  GCUGAAACUUCGUGAAAAAGGAGUUCAGUAAUCGGUUGAAUGAAAAAUGAUAAAAAAACGUUAAUAUAUAAAACCAAAACGUUUGAAGAAAUGUUUUAUU
env-1    GCUGAAACUUCGUGAAAAAGGAGUUCAGUAAUCGAUUGAAUGAAAAAUGAUAAAAAAACGUUAAUACAUAAAACCAAAACGUUUGAAGAAAUGUUUUAUU
Bth-1-1  GAUGUGAAUCGGGGUACCGUUUUUUUUGUUAAAAAGUCGUCUUAACUUCAUUUUUUUCAGAACUCUUGUGAACAAAAUCCGAAUUGGUGCGUUAUUUAU
env-2    GAUGUGAAUCGGGGUACCGUUUUUUUUGUUAAAAAGUCGUCUUAACUUCAUUUAUUUCAGAACUCUUGUGAACAAAAUCCGAAUGGGUGCGUUAUUUAU
Bca-1-1  AUAAAGAGGUGUGGUUGCUGUUUUCCUUGUUAAAAUCCCGUUCCUGCCUCAUUUUCUUUAAACUUCUGUGAACAAAAUCCGGAUUAGUCUGUUUUUUAU
Bov-1-1  UGAAUUCCUUAAAGGGCGUGCUAUUUCAUUUUCUUAUCAUAUUAUUGAAUAAAAUCUUAUCAAUCCUGUGAACAAAAUCCGGAAAUGUAUGUUAUUUAU
Bov-1-2  CAAAUGGCUGAUGGAAGGCUUCCUGAAAGACUAUAAGGAGAAGAAAUAACAUUUUUACUUCUCCUUUCGUGAACAAAAUAAAAAUCUGAAUGUUUUAUA
Bca-1-2  GCAAAAUGGCUAAUGGAGGGCUUUCUGAAAGAUUAUAAAGAGAAAAAUAAAUAUAAAACAUCUUUUUCGUGAACAAAACCGAAAUAGGAAUGUUUUAUA
env-3    CAAAUGGUUGAUGGAAGGCUUUCUGAAAGAUUUUAAAGGGAGAAAAUAAACUUCAAUCUCUUUUUUUCGUGAACAAAACGAAAAUCGGGAUGUUAUAUA
Bth-1-2  CAAAUGGUUGAUGGAAGGCUUUCUGAAAGAUUUUAAAGGGAGAAAAUAAACUUCAAUCUCUUUUUUUCGUGAACAAAACGAAAAUCGGGAUGUUAUAUA
Bth-1-3  UUGGCAGCUGAAACUCCGUGAAAGAGGCAUUCAGUAAAAAGAUAUUUGUUUUAAUUAACAUAAAAGUUUAAACAAAUCGUUCAUUCGAUUUGUUUAUUA
Bca-1-3  GUGAAAGAGGUAUUCAAUAAGAUAUAUUUAAUUAUUUGUUGUAAAAACAUGAAAAUAUAAACAAAAUAGAAACAAAUCGUGCAUGCGAUUUGUUUAAUU
env-4    UGGCAGCUGAAACUCCGUGAAAGAGGGAUUCAAUAAAAGAGAUUUGUUUUAAAAUAAACAUAAAAGAUUAAACAAAUCGUGCAUGCGAUUUGUUUAUCU
Bov-1-3  UGGCAGCUGAAACUCCGUGAAAGAGGGAUUCAAUAAAAGAGAAACUUGUUUUAAAUAACAUAAAAGUUUAAACAAAUCGUGCAUGCGAUUUGUUUAAUU
env-5    UGAGAGCCGGUCAUAGACUUGGGGAAAUACAUAUAACUCGAAAAAAAAUACAUUUUUUUUGCUCACUCCUGAACAAAAUGAUUUCUUCUGCGUUUAUAAU
Bfr-1-1  GAGAGCCGGUCAUAGACUUGGGGAAAUACAUAUAACUCGAAAAAAAAUACAUUUUUUUUUGCUCACUCCUGAACAAAAUGAUUUCUUCUGCGUUUAUAAU
env-6    CGAGAGCCGGUCAUAGACUUGGGGAAAUACAUAUAACUCGAAAAAAAAUACAUUUUUUUUGCUCACUCCUGAACAAAAUGAUUUCUUCUGCGUUUAUAAU
Bfr-2-1  GAGAGCCGGCCAUAGACUUGGGGAAAUACAUAUAACUCGAAAAAAAAUACAUUUUUUUUUGCUCACUCCUGAACAAAAUGAUUUCUUCUGCGUUUAUAAU
env-7    GAAGCUUCGUGAGAGGGGUAUUCAAUAAGCGAUAAAAGAGAUUUUUGUUUUUAUUAACAAAUAGGGAAUGAACAAAUGCUUCCCCGCAUUUGUUUAUAU
Bin-1-1  GAAGCUUCGUGAGAGGGGUAUUCAAUAAGCGGUAAAAGAGAUUUUUGUUUUUAUUAACAAAUAGGGAAUGAACAAAUGCUUCCCCGCAUUUGUUUAUAU
env-8    AGUUGAAGCUUCGUGAACGAGGUAUUCAAUGACAAACUUUGAAUUUGUGUUUUUUAACAAAUAAGCAAUAAACAAAUGCCUCCCCGCAUUUGUUUAUAA
env-9    AGUUGAAGCUUCGUGAAAGAGGUAUUCAAUGACAAACUUUGAAUUUGUGUUUUUUAACAAAUAGGCAAUAAACAAAUGCCUCCCCGCAUUUGUUUAUAA
Bun-1-1  AGUUGAAGCUUCGUGAAAGAGGUAUUCAAUGACAAACUUUGAAUUUGUGUUUUUUAACAAAUAGGCAAUAAACAAAUGCCUCCCCGCAUUUGUUUAUAA
env-10   AGUUGAAGCUUCGUGAAAGAGGUAUUCAAUGACAAACUUUGAAUUUGUGUUUUUUAACAAAUAGGCAAUAAACAAAUGCCUCCCCGCAUUUGUUUAUAA
env-11   CAGUUGAAGCUUCGUGAGAGAGGCAUUCAAUAAGAAUAAAGAUUUUGAACUUUUUAACAUACAGAAAAUAAACAAAUGCUUCCCCGCAUUUGUUUAUUA
env-12   CAGUUGAAGCUUCGUGAGAGAGGCAUUCAAUAAGAAUAAUGAUUUUAAACUUUUUAACAUACAGAAAAUAAACAAAUGCUUCCCCGCAUUUGUUUAUUA
Bst-1-1  AGUUGAAGCUUCGUGAGAGAGGUAUUCAAUAAGAAUAAUGAUUUUAAACUUUAUUAACAUACAGAAAAUAAACAAAUGCUUCCCCGCAUUUGUUUAUUA
env-13   CAGCUGAAGCUUCGUGAGAGAGGCAUUCAAUAAGAAUAAAGAUUUUGAACUUUUUAACAUACAGAAAAUGAACAAAUGCUUCCCCGCAUUUGUUUAUUA
Bfr-1-2  UGGCAGUUGAAACUUCGUGAAAGAGGUAUCCAAUAAAUAAGAUAUUGUUUUAAUUAACAUAUAAGACAUGAACAAAUGUUUUUAUACAUUUGUUUAUAG
Bfr-2-2  GGCAGUUGAAACUUCGUGAAAGAGGUAUCCAAUAAAUAAGAAUAUUGUUUUAAUUAACAUAUAAGACAUGAACAAAUGUUUUUAUACAUUUGUUUAUAG
env-14   UUGAAACUUCGUGAAAGAGGUAUUCAAUGACGAAUAAAUGAGUUUGUCUUUUUUUAACAGAUAGGAUAUAAACAAAUGCGUUCCUGCAUUUGUUUAUUA
Beg-1-1  UUGAAACUUCGUGAAAGAGGUAUUCAAUGACGAAUAAAUGAGUUUGUCUUUUUUUAACAGAUAGGAUAUAAACAAAUGCGUUCCUGCAUUUGUUUAUUA
env-15   CUGGCAGCUGAAACUCCGUGAGAGAGGCAUACAAUAAGAAGAGUUUAUGUUACAUUAACAUACAAGAAAUAAACAAAUACAUAAGUUAUUUGUUUAUAU
env-16   ........................................................................................GGCGUUUAAUA
env-17   GGAACUGGCAGCUGAAACUAAGAGAAAGAGGAAUUCAAUAAGAAUUAAGAUAUUUUUAGAAAGACCGAUGAACAAAGGACGCUAGUGUGGCGUUUUAUA
Bdo-1-1  GGAACUGGCAGUUGAAGCUAAGAGAAAGAGGAAUUCAAUAAGAAUUAAGAUAUUUUUAGAAAGACCGAUGAACAAAGGACGCUAGUGUGGCGUUUUAUA
env-18   GGAACUGGCAGUUGAAGCUGAGAGAAAGAGGAAUUCAAUAAGAAUUAAGAUAUUUUUAGAAAGACCGAUGAACAAAGGACGCUAGUGUGGCGUUUUAUA
Bvu-1-1  GGAACUGGCAGCUGAAACUAAGAGAAAGAGGAAUUCAAUAAGAAUUAAGAUAUUUUUAGAAAGACCGAUGAACAAAGGACGCUAGUGUGGCGUUUUAUA
env-19   GGAACUGGCAGCUGAAACUAAGAGAAAGAGGAAUUCAAUAAGAAUUAAGAUAUUUUUAGAAAGACCGAUGAACAAAGGACGCUAGUGUGGCGUUUUAUA
env-20   GGAACUGGCAGCUGAAACUAAGAGAAAGAGGAAUUCAAUAAGAAUUAAGAUAUUUUUAGAAAGACCGAUGAACAAAGGACGCUAGUGUGGCGUUUUAUA
         ...................................................................................................
         ...................................................................................................
         ●●●●●●●●●●●●●●●●●●●●●●●●●●●●●●●●●●●●●●●●R●●●●●●●●●●●●●●●●●●●●●●●●●●●●RAAC●AA●●●●●●●●●●●●●●YGUUU●●●●
```

alignment positions 100 · · · 259

```
Bco-1-1   GAUGCAGACG...CUUAA...AACACUUGGAUGUAUGUGAAUAUGUGUAAGU...GUUAC..CUG....UUC........CAGAAG....UGGAAAAGGAGCGUUAAUGCAUAGUAGUUUUGUCGUGUUUUAUUUUGUGUUUGUGUUGUGACUGUUUCUC
env-1     GAUGCAGACG...CUUAA...AACACUUGGAUGUAUGUGAAUAUGUGUAAGU...GUUAC..CUG....UUC........CAGAAG....UGGAAAAGGAGCGUUAAUGCAUAGUAGUUUUGUCGUGUUUUAUUUUGUGUUUGUGUUGUGACUGUUUCUC
Bth-1-1   UAUGCAAAUC.GCCUGAUAAAGCUUCUUAUGGGUAUGCGAAUAUAGAUAAGUCA.AGCAAG.GUAUGCCUCCCC.GGCAUUACA......GGUAAGAUUGCAUAGUAGUUUUGUCGUGUUUUAUUUUGUGUUUGUGUUGUGACGGUGCUGCGAUGUGAAU
env-2     UAUGCAAAUCA.CCUGAUAAAGCUUCUUAUGGGUAUGCGAAUAUAGAUCAGUCA.AGCAAG.AUAUGCCUCCCC.GGCAUUACA......GGUAAGAUUGCCUAGUAGUACGGUCGUGUACUAUAACGAGUAGGUGUAGUGACAGUCUUCGAUAUGAUUC
Bca-1-1   UAUGCAAAUC.GCCUGAUAAAGCUUCUUAUGGGUAUGUGAAUAUAGAUAAGUCA.AGCAAGA.UAUGCCUCCCC.GGCA.UA.AC.....AGGAAGAUUGCAUAGUAGUUUUGUCGUGUUUUAUUUUGUGUUUGUGUUGUGACGGUGCUGCGAUGUGAAU
Bov-1-1   UGUGCAAAUC.GCCUGAUAAAGCUUCUUGUGUGUAUGCGAAUAUAGAUAAGUCA.AGCAAGG.UAUGCCUCCCC.GGCA.UG.AC.....AGGAAGAUUGCAUAGUAGUUUUGUCGUGUUUUAUUUUGUGUUUGUGUUGUGACGGUGCUGCGAUGUGAAU
Bov-1-2   UAUGCAAAUC.GCCUGAUG..GCUACUUGUAGGUAUGCGAAUAUAUAUAGGCU..AGUGAA..UAUGCCUCCCC.GGCA.UA.AC.....AGGAAGAUUGCAUAGUAGUUUUGUCGUGUUUUAUUUUGUGUUUGUGUUGUGACGGUGCUGCGAUGUGAAU
Bca-1-2   UAUGCAAAUC.GCCUGAUG..ACUACUUGUAGGUAUGCGAGUAUAUAUGAGUC..GGUGAA..UAUGCCUCCCC.GGCA.UA.AC.....AGGUAGAUUGCAUAGUAGUUUUGUCGUGUUUUAUUUUGUGUUUGUGUUGUGACGGUGCUGCGAUGUGAAU
env-3     UGUGCAAAUC.GCCUGAUG..GCUGCUUGUAGGUAUGCGAAUAUAUAUGAGUC..AGUGAA..UAUGCCUCCCC.GGCA.UA.AC.....AGGAAGAUUGCAUAGUAGUUUUGUCGUGUUUUAUUUUGUGUUUGUGUUGUGACGGUGCUGCGAUGUGAAU
Bth-1-2   UGUGCAAAUC.GCCUGAUG..ACUGCUUGUAGGUAUGCGAAUAUAUAUGAGUC..AGUGAA..UAUGCCUCCCC.GGCA.UA.AC.....AGGAAGAUUGCAUAGUAGUUUUGUCGUGUUUUAUUUUGUGUUUGUGUUGUGACGGUGCUGCGAUGUGAAU
Bth-1-3   GAUGCUAAUCAGCUUGA....UCUGUUUGUGAGUAUGUGAAUAUGAGUAAAU...GGUAC..UUAUGCCUCUCC.GGCA.UAGACA....GGUAUGAUUGCAUAGUAGUUUUGUCGUGUUUUAUUUUGUGUUUGUGUUGUGACGGUACUACGACGGGAGU
Bca-1-3   GAUGCAAGUCGACCUGAA...GCUGUUUGUGAGUAUGUGAAUAUACAUGGAU...AGUAC..UUAUACCUCCCC.GGUA.UAGAC.....AGGGAGAUUGCAUAGUAGUUUUGUCGUGUUUUAUUUUGUGUUUGUGUUGUGACGGUACUACGACGUGAGU
env-4     GAUGCUAAUCGACCUGAA...GCUGUUUAUGAGUAUGCGAAUAUACGUAAAU...GGUAC..UUAUGCCUCCCC.GGCA.UAGACA....GGAAAGAUUGCAUAGUAGUUUUGUCGUGUUUUAUUUUGUGUUUGUGUUGUGACGGUACUACGACGUGAGU
Bov-1-3   GAUGCUAAUCGACCUGAA...GCUGUUUAUGAGUAUGCGAAUAUACGUAGAU...AGUAC..UUAUGCCUCCCC.GGCA.UAGACA....GGAAAGAUUGCAUAGUAGUUUUGUCGUGUUUUAUUUUGUGUUUGUGUUGUGACGGUACUACGACGUGAGU
env-5     UAUGCAAAUCGGCCUGAA...GCGCUUUAUGCCUAUGGGAAUAUCCAUAAUAGU.CGCACCG.UAUGCCUCCCC.GGCA.UAGACA....GGUAAGAUUGCAUAGUAGUUUUGUCGUGUUUUAUUUUGUGUUUGUGUUGUGACGGUGCUGCGAUGUGAAU
Bfr-1-1   UAUGCAAAUCGGCCUGAA...GCGCUUUAUGCCUAUGGGAAUAUCCAUAAUAGU.CGCACCG.UAUGCCUCCCC.GGCA.UAGACA....GGUAAGAUUGCAUAGUAGUUUUGUCGUGUUUUAUUUUGUGUUUGUGUUGUGACGGUGCUGCGAUGUGAAU
env-6     UAUGCAAAUCGGCCUGAA...GCGCUUUAUGCCUAUGGGAAUAUCCAUAAUAGU.CGCACCG.UAUGCCUCCCC.GGCA.UAGACA....GGUAAGAUUGCAUAGUAGUUUUGUCGUGUUUUAUUUUGUGUUUGUGUUGUGAAUAAGCCUUGUCGGGAGA
Bfr-2-1   UAUGCAAAUCGGCCUGAA...GCGCUUUAUGCCUAUGGGAAUAUCCAUAAUAGU.CGCACCG.UAUGCCUCCCC.GGCA.UAGACA....GGUAAGAUUGCAUAGUAGUUUUGUCGUGUUUUAUUUUGUGUUUGUGUUGUGACGGUGCUGCGAUGUGAAU
env-7     GUUGCAAAUCAGCCUGAU...GCUGUUUAUGUAUAUGUGAAUAUCCGUAACU...AGUAC..UUAUGCCUCCCC.GGCA.UAGACA....GGUAAGAUUGCAUAGUAGUUUUGUCGUGUUUUAUUUUGUGUUUGUGUUGUGAAUAAGCCUUGUCGGGAGA
Bin-1-1   GUUGCAAAUCAGCCUGAU...GCUGUUUAUGUAUAUGUGAAUAUCCGUAACU...AGUAC..UUAUGCCUCCCC.GGCA.UAGACA....GGUAAGAUUGCAUAGUAGUUUUGUCGUGUUUUAUUUUGUGUUUGUGUUGUGAAUAAGCCUUGUCGGGAGA
env-8     UGUGCAAAUCAGCCUGAA...GCUGUUUAUGUAUAUGUGAAUAUCCGUAACU...AGUAC..GUAUGCCUCCCC.GGCA.UAGACA....GGUAAGAUUGCAUAGUAGUUUUGUCGUGUUUUAUUUUGUGUUUGUGUUGUGAAUAAGCCUUGUCGAGAGA
env-9     UGUGCAAAUCAGCCUGAA...GCUGUUUAUGUAUAUGUGAAUAUCCGUAACU...AGUAC..GUAUGCCUCCCC.GGCA.UAGACA....GGUAAGAUUGCAUAGUAGUUUUGUCGUGUUUUAUUUUGUGUUUGUGUUGUGAAUAAGCCUUGUCGAGAGA
Bun-1-1   UGUGCAAAUCAGCCUGAA...GCUGUUUAUGUAUAUGUGAAUAUCCGUAACU...AGUAC..GUAUGCCUCCCC.GGCA.UAGACA....GGUAAGAUUGCAUAGUAGUUUUGUCGUGUUUUAUUUUGUGUUUGUGUUGUGAAUAAGCCUUGUCUCUCGA
env-10    UGUGCAAAUCAGCCUGAA...GCUGUUUAUGUAUAUGUGAAUAUCCGUAACU...AGUAC..GUAUGCCUCCCC.GGCA.UAGACA....GGUAAGAUUGCAUAGUAGUUUUGUCGUGUUUUAUUUUGUGUUUGUGUUGUGAAUAAGCCUUGUCGAGAGA
env-11    CAUGCAAAUCGGCCUGAC...GCUGCUUAUGUAUAUGUGAAUAUCCGUAAUU...AGUAC..AUAUGCCUCCCC.GGCA.UAGACA....GGUAAGAUUGCAUAGUAGUUUUGUCGUGUUUUAUUUUGUGUUUGUGUUGUGAAUAAGCCUUGUCGCGAGA
env-12    CAUGCAAAUCGGCCUGAU...GCUGCUUAUGUAUAUGUGAAUAUCCGUAAUU...AGUAC..AUAUGCCUCCCC.GGCA.UAGACA....GGUAAGAUUGCAUAGUAGUUUUGUCGUGUUUUAUUUUGUGUUUGUGUUGUGAAUAAGCCUUGUCGCGAGA
Bst-1-1   CAUGCAAAUCGGCCUGAU...GCUGCUUAUGUAUAUGUGAAUAUCCGUAAUU...AGUAC..AUAUGCCUCCCC.GGCA.UAGACA....GGUAAGAUUGCAUAGUAGUUUUGUCGUGUUUUAUUUUGUGUUUGUGUUGUGAAUAAGCCUUGUCGCGAGA
env-13    CAUGCAAAUCGGCCUGAC...GCUGCUUAUGUAUAUGUGAAUAUCCGUAAUU...AGUAC..AUAUGCCUCCCC.GGCA.UAGACU....GGUNAGAUUGCAUAGUAGUUUUGUCGUGUAUAAUUUUGUGUAUAGUGUUGUGAAUAAGCCUUGUCGCGAG
Bfr-1-2   GAUGCAAAUCGGCCUGAA...GCUGUUUAUGUGUAUGUGAAUAUCCAUAAUU...AGUAC..UUAUGCCUCCCC.GGCA.UAGACA....GGUAAGAUUGCAUAGUAGUUUUGUCGUGUUUUAUUUUGUGUUUGUGUUGUGACAGUACUACGACGUGAGU
Bfr-2-2   GAUGCAAAUCGGCCUGAA...GCUGUUUAUGUGUAUGUGAAUAUCCAUAAUU...AGUAC..UUAUGCCUCCCC.GGCA.UAGACA....GGUAAGAUUGCAUAGUAGUUUUGUCGUGUUUUAUUUUGUGUUUGUGUUGUGACAGUACUACGACGUGAGU
env-14    CAUGCAAAUCAGCCUGAA...GCUGCUUAUGUGUAUGUGAAUAUCCAUAAUU...AGUAC..GUAUGCCUCCCC.GGCA.UAGACA....GGUACGAUUGCAUAGUAGUUUUGUCGUGUUUUAUUUUGUGUUUGUGUUGUGAUAAGCCUUGUCGAGAGAC
Beg-1-1   CAUGCAAAUCAGCCUGAA...GCUGCUUAUGUGUAUGUGAAUAUCCAUAAUU...AGUAC..GUAUGCCUCCCC.GGCA.UAGACA....GGUACGAUUGCAUAGUAGUUUUGUCGUGUUUUAUUUUGUGUUUGUGUUGUGAUAAGCCUUGUCGAGAGAC
env-15    AGUGCAAAUCGGCCUGAA...GCCAUUUAUGUAUAUGUGAAUAUCCAUACUC...GGUAC..AUAUGCCUCCCC.GGCA.UAGACA....GGUAAGAUUGCAUAGUAGUUUUGUCGUGUUUUAUUUUGUGUUUGUGUUGUGACGGUACUACGACGUGAGU
env-16    GAUGCAAAUAA.CCUGAA...CCCCUUCUGGGAUAUGUGAAU..ACCUGAUUGNCGGUAG..UUAUGUUACUCAAAACA.UAGAGC....AGGCUAAAAAAUUGCAUAGUAGUUUUGUCGUGUUUUAUUUUGUGUUUGUGUUGUGGCUGUUCUCCGACGU
env-17    GAUGCAAAUA.ACCUGAAACC.CCUCUUGGA..UAUGUGAAUAC..CUGAUUGGCGGUAG..UUAUGUUACUCAAAACA.UAGAGCAGGCUAAAAAAUUGCAUAGUAGUUUUGUCGUGUUUUAUUUUGUGUUUGUGUUGUGGCUGUUCUCCGACGUGAGU
Bdo-1-1   GAUGCAAAUA.ACCUGAAACC.CCUCUUGGA..UAUGUGAAUAC..CUGAUUGGCGGUAG..UUAUGUUACUCAAAACA.UAGAGCAGGCUAAAAAAUUGCAUAGUAGUUUUGUCGUGUUUUAUUUUGUGUUUGUGUUGUGGCUGUUCUCCGACGUGAGU
env-18    GAUGCAAAUA.ACCUGAAACC.CCUCUUGGA..UAUGUGAAUAC..CUGAUUGGCGGUAG..UUAUGUUACUCAAAACA.UAGAGCAGGCUAAAAAAUUGCAUAGUAGUUUUGUCGUGUUUUAUUUUGUGUUUGUGUUGUGGCUGUUCUCCGACGUGAGU
Bvu-1-1   GAUGCAAAUA.ACCUGAAACC.CCUCUUGGA..UAUGUGAAUAC..CUGAUUGGCGGUAG..UUAUGUUACUCAAAACA.UAGAGCAGGCUAAAAAAUUGCAUAGUAGUUUUGUCGUGUUUUAUUUUGUGUUUGUGUUGUGGCUGUUCUCCGACGUGAGU
env-19    GAUGCAAAUA.ACCUGAAACC.CCUCUUGGA..UAUGUGAAUAC..CUGAUUGGCGGUAG..UUAUGUUACUCAAAACA.UAGAGCAGGCUAAAAAAUUGCAUAGUAGUUUUGUCGUGUUUUAUUUUGUGUUUGUGUUGUGGCUGUUCUCCGACGUGAGU
env-20    GAUGCAAAUA.ACCUGAAACC.CCUCUUGGA..UAUGUGAAUAC..CUGAUUGGCGGUAG..UUAUGUUACUCAAAACA.UAGAGCAGGCUAAAAAAUUGCAUAGUAGUUUUGUCGUGUUUUAUUUUGUGUUUGUGUUGUGGCUGUUCUCCGACGUGAGU
          ....................<<<..<<<<<.<<<<....>>>>.>>>>>.....>>>....<<<<<<<......>>>>.>>>...............................................................................
          ....................?22..1212?.?001....100?.?2121.....22?....?120222......2220.21?...............................................................................
          •AUGCAAAUC∘RCCUGA•---RCY•YUURUR•RUAUGYGAAUAU••RURA•U---RGUA•--∘UAUGCCUCCCC-GGCA-UA∘A•∘----RG•AAGAUUGCAUAGUAGUUUUGUCGUGUUUUAUUUUGUGUUUGUGUUGUGA••G••CU•YG•YG•GARY
           5′————————————————————————————————————————————————————————————————————————————3′
```

alignment positions 260 · · · 419

Bco-1-1	GACGUGAGUCGGGAAACAGUUUUUUGUUUGCAGAAUUUUGUAUAACUUUGCAGCCGAUUUUGAAUUCUGUCCGAUUUUACCGAUUAUUCAGGCUGAUUCAAAGUCAGGGGAAUGGGACUUCCCUGUUAUAAACCGCUAAUAAAGUAGCUGAUAGUUCCUG
env-1	GACGUGAGUCGGGAAACAGUUUUUUGUUUGCAGAAUUUUGUAUAACUUUGCAGCCGAUUUUGAAUUCUGUCCGAUUUUACUGACUGUUCAGGCUGAUUCAAAGUCAGGGGAAUGGGACUUCCCUGUUAUAAACCGCUAAUAAAGUAGCUGAUAGUUCCUG
Bth-1-1	CGAGGUGCCGUUUUUUUGUUAUAACCGCUUUGGGGGUAUAACCAGGGAUUAUCCGACAUAAUAAAAUUCUGUGAUUCAGUCUGCCGGAAAAUGUCUUCCUUUGCAAAAAAGAAAUAAUUUAGUAUCACUUAAAUCAAAUCAUUUAUGUUCAAUGAAAAGA
env-2	GAGGUGCCAAUAUUGGCUAUACCGCUAUGGGGUUAUACCAGGGAUUAUCCGACAUCUUAAAUUCUG...
Bca-1-1	CGAGGUACCGUUUUUUAUUAUGGAUAGGUAUAACCGGAGAUUAUCCGGUAUAAAAAAAUACUGUGAACCGGUUUGAUGAAAAAUGGCUUCCUUUGCAGAGUAAUAAACAGAGUAAUAAAUUCAGUAUCACUAAAAUCUAGUAAUUUAUGUUCAACGAAAA
Bov-1-1	CGAGGUGCCGUUUUUUUUGUCACAUAUAAAGAAACGGCAGGCUUACAAAUAAGAAACGAGCUUACAAAUCUCCUAAAGUAUAUCAUAGAAUCUUUGCAAGCUCGUUGUAUGAGUGGUGCUUUUGCGCACCUUAUCUAGUUGUUAUAAAGGCAUAUUACCA
Bov-1-2	CGGGGUACCGUUUUUUCGUUUCUCUUGAAUUCCUUAAAGGGCGUGCUAUUUCAUUUUCUUAUCAUAUUAUUGAAUAAAAUCUUAUCAAUCCUGUGAACAAAAUCCGGAAAUGUAUGUUAUUUAUUGUGCAAAUCGCCUGAUAAAGCUUCUUGUGUGUAUG
Bca-1-2	CGGGGUACCGUUUUUAUGUAUGUGACAGAAAGAAAUUGUAAAUGGCUGCAUUAAAAAAAGAAUAAGCACGAAACCAUAAUUUAUUCCGGCUUCGUGCUCACUCUUUACUUAUAUACUGCAAUCAGGCAGUACUAUUUCCAUUCGCUGUGCUGUUCCAACA
env-3	CGGGGUACCGUUUUUUUUGUUAAAAAGUCGUCUUAACUUCAUUUAUUUCAGAACUCUUGUGAACAAAAUCCGAAUGGGUGCGUUAUUUAUUAUGCAAAUCACCUGAUAAAGCUUCUUAUGGGUAUGCGAAUAUAGAUCAGUCAAGCAAGAUAUGCCUCCC
Bth-1-2	CGGGGUACCGUUUUUUUUGUUAAAAAGUCGUCUUAACUUCAUUUUUUUCAGAACUCUUGUGAACAAAAUCCGAAUUGGUGCGUUAUUUAUUAUGCAAAUCGCCUGAUAAAGCUUCUUAUGGGUAUGCGAAUAUAGAUAAGUCAAGCAAGGUAUGCCUCCC
Bth-1-3	CGUGGUACCGUUUUUUGUUACUCUUUUACAGCCUGCCUUUAAAACAGAAUACGUUAGUUCCCCAAUCAGUUUCAGGUAAAGUCCCUUCCGGCUCGAACAAUCCAUAUACGGAAGAACCGGAACCGCUCAUAGAGGCAUAAACAGCCCCUUGCCUGUAUAG
Bca-1-3	CGUGGUACCGUUUUUUGUUUAUUCGUUUGGAGUAGGUUCUAUUUUGUAAAAUAUUUAUGCCAUAUUGAUUUAUAAUCCAUAACUGUUUAGGGUUCUUCUUCAGAUUUGGUGGUAAUUUAAUCAGUCGGUGGUAUCUCCAUUCCCCUCCUUCGGGGAAGGA
env-4	CGUGGUACCGUUUUUUGUUCGCCCAUACUGGGCGAACAAAACUAUGUUUAUAGCUUUCCUUUGAAAUAGAAAACGUCGCUUUCUCCCAUUGUUUCCGGUAAAGAAGUUCCGGGGGCGAACAGUCCGAACACUGAAGAGCCGGAGCCGCUCAUGGAGGCGU
Bov-1-3	CGUGGUACCGUUUUUUUGGUACGCCCAGCAUGGAUGUUCUGUUUUAUAAAAUAUUUAGCACUAAUUCUUUUGUUAUCCAAAAUUAUUUUGUAAUUUUGUAUCAAAUGAAUAAGAUAUGAGAGUAGUAUCACAUAAGAAAUUGAAAGAUUUCUACGAGACG
env-5	CGAAGUACCGUUUUUUUGUACCUACCUCUCAGAAGCUAAAAGAUUUACCAUAAAAGAAGCAGAAUACAGGGUAACGGGUUAAAUGUUCCGAUGGACGAAUUGUCCGUCGAUCCAGAACGUGGCAUGCUCAGAACGGAACUUCAGUAAAACGUAUUCCGGA
Bfr-1-1	CGAAGUACCGUUUUUUUGUACCUACCUCUGAGAAGCUAAAAGAUUUACCAUAAAAGAAGCAGAAUACAGGGUAACGGGUUAAAUGUUCCGAUGGACGAAUUGUCCGUCGAUCCAGAACGUGGCAUGCUCAGAACGGAACUUCAGUAAAACGUAUUCCGGA
env-6	CAGGGCUUAUUUUUUUUCUUGUUCUUCUAUUGCCUUAUUCAGUAAAAAAGUCGUUUCUUUAAGUUUUUCGACAAUCUUUAUUUUAGUAGUUUCGUCAAACCGAAUAUCUGGCAAAUAAAUCCCCAGACUUGCGAUGAUUGUCUUGUUUUUAUAAAUAGGA
Bfr-2-1	CGAAGUACCGUUUUUUUGUACCUACCUCUCAGAAGCUAAAAGAUUUACCAUAAAAGAAGCAGAAUACAGGGUAACGGGUUAAAUGUUCCGAUGGACGAAUUGUCCGUCGAUCCAGAACGUGGCAUGCUCAGAACGGAACUUCAGUAAAACGUAUUCCGGA
env-7	CAGGGCUUAUUUUUUUUCUUGUUCUUCUAUUGCCUUAUUCAGUAAAAAAGUCGUUUCUUUAAGUUUUUCGACAAUCUUUAUUUUAGUAGUUUCGUCAAACCGAAUAUCUGGCAAAUAAAUCCCCAGACUUGCGAUGAUUGUCUUGUUUUUAUAAAUAGGA
Bin-1-1	CAGGGCUUAUUUUUUAUUCUUGUUCUUCUAUUGCCUUAUUCAAUAGAAAUGUUGUUUCUUUAAGUUUUUCGACAAUAUUUAUUUUAGUAAUUUCUUCAAACCGAAUAUCUGGCAAGUAAAUCCCCAGACUUGCGAUGAUCGUCUUGUUUUUAUAAAUAGG
env-8	CAAGGCUUAUUUUUUGUAUAUUAUACCUUAAAUCCGCAACUCCAUUCCCGAAGUGCUACCUACAUAAAGAGAAAUGGACUUUUGAUUCGUAAGAUAACCAUUAAGUCCUCGCGUCCGUUUCAAUGCGCAUACCAUCCCCUUACCCCAUAAAGCGACUUG
env-9	CAAGGCUUAUUUUUUGUAUAUUAUAUACAGAAUCUCUGUUGUCAAUUCAGUGUUUUUUCAUAAGAUGGGUAGGCAAUUCCUUUUUCUUACUAAUUGUUAACUGACGUUAUCAUGUCUGUAACAAUUUGUUCCAAUUGUAGUAUUAAUCGUUCAAAUAAGU
Bun-1-1	CAAGGCUUAUUUUUUGUAUAUUAUAUACAGAAUCUCUGUUGUCAAUUCAGUGUUUUUUCAUAAGAUGGGUAGGCAAUUCCUUUUUCUUACUAAUUGUUAACUGACGUUAUCAUGUCUGUAACAAUUUGUUCCAAUUGUAGUAUUAAUCGUUCAAAUAGU
env-10	CAAGGCUUAUUUUUUGUAUAUUAUAUACAGAACCUCUGUUGUCAAUUCAGUGUUUUUUCAUAAGAUGGGUGGGCAAUUCCUUUUUCUUGCUAAUUGUUAACUGACGUUAUCAUGUCUGUAACAAUUUGUUACACUUGUAGUGUUAAUCGUUCAAAAUAU
env-11	CAGGGCUUAUUUUUU..
env-12	CAGGGCUUAUUUUUUAGGAGAAUACGGAUGGCUUUUUAUGAUUAGGUGCAUUACCGAUUGUUAACCAACGUUAUUAUGUGUGGAACAAAUUGUUCCCAUUGUAGUAUUAAUCGUUCAAAAUAGUGUAAUUUAUAGAAAAAAUGCUUUUAAGGAGAACAAU
Bst-1-1	CAGGGCUUAUUUUUUAGGAGAAUACGGAUGGCUUUUUAUGAUUAGGUGCAUUACCGAUUGUUAACCAACGUUAUUAUGUGUGGAACAAAUUGUUCCCAUUGUAGUAUUAAUCGUUCAAAAUAGUGUAAUUUAUAGAAAAAAUGCUUUUAAGGAGAACAAU
env-13	ACAGGGCUUAUUUUUAAGAGAAUACGGAUGGCUUUAUAUGAUUAGGUGCAUUACCGAUUGUUAACCAAGCGUUUUUAUGUGUGGAAUAAAUUGUUACCAUUGUAGUAUUAAUCGUUUCAAAUAGUGUAAUUUAUAGCAAAAAAUGCUUUUGAGGAGAAC
Bfr-1-2	CGUGGUACUGUUUUUUUUAUUAUAAACUUUAUUUUAUCUAUGAGAUUUUAUUUUGUCUGACUAUUGGAUAGAAAAAACUCUCCUCUUUGAAGGAGUUCAAAGAGGAGAGUCGUUAUUAUUUUACCAAUACUACUACCGGGUUGGAUUCUUCAUUCAAAUU
Bfr-2-2	CGUGGUACUGUUUUUUUUAUUAUAAACUUUAUUUUAUCUAUGAGAUUUUAUUUUGUCUGACUAUUGGAUAGAAAAAACUCUCCUCUUUGAAGGAGUUCAAAGAGGAGAGUCGUUAUUAUUUUACCAAUACUACUACCGGAUUUGAUUCUUCAUUCAAAUUC
env-14	AAGGCUUAUUUUUUUAUGCUAAGAUGAUUCCUUCCCUAUUUCCAUCAUAUUUUGGUUCAUUGUGAUUUUAUCAGUCGAGGCAAGAAUCCGGAUGCAGAUAUGGAUUGGGAUGAAUAAUCUCUUCAAUUAGGUGCAUUACUAAUUGUUAACCAACGUUAUU
Beg-1-1	AAGGCUUAUUUUUUUAUGCUAAGAUGAUUCUUUCCCUAUUUCCAUCAUAUUUUGGUUCAUUGUGAUUUUAUCAGUCGAGGCAAGAAUCCGGAUGCAGAUAUGGAUUGGGAUGAAUAAUCUCUUCAAUUAGGUGCAUUACUAAUUGUUAACCAACGUUAUU
env-15	CGUGGUACCGUUUUUAUGUUAUAUCCUAUCUUAUUUAAUUUUGAUAUUUCUUUUUUUCUUACCUUUGCGAGCAUUACUUUUAUGAAUACGACAUGUAUCUGACACUUUUUCUCAAUUUUGUAAAAUAGGUAAUCUACCAGGCGAUUCACCCGGGAGAUCUG
env-16	GAGUCGGGGGACAGUUUUUUUNGACUAUUUAGUUGUUUUUUAGUUAUUUAUGGCUAUAUUUGCCGAAAAUAAUCACGCACUACUUUAUGAAAACAAAAUUUUGGAUCAUCUGCUUAUCAGUUUUCUUUGUGUCUUGCUUGCGUAAUAUGCAUGAUAUAUA
env-17	CGGGGGACAGUUUUUUUUCUGACUAUUUAGUUGUUUUUUAGUUAUUUAUGGCUAUAUUUGUCGAAAAUAAUCACGCACUACUUUAUGAAAACAAAAUUUUGGAUCAUCUGCUUAUCAGUUUUCUUUGUGUCUUGCUUGCGUAAUAUGCAUGAUAUAUAU
Bdo-1-1	CGGGGGACAGUUUUUUUUGACUAUUUAGUUGUUUUUUAGUUA..
env-18	CGGGGGACAGUUUUUUUUGACUAUUUAGUUGUUUUUUAGUUAUUUAUGGCUAUAUUUGCCGAAAAUAAUCACGCACUACUUUAUGAAAACAAAAUUUUGGAUCAUCUGCUUAUCAGUUUUCUUUGUGUCUUGCUUGCGUAAUAUGCAUGAUAUAUACACG
Bvu-1-1	CGGGGGACAGUUUUUUUUCUGACUAUUUAGUUGUUUUUUAGUUAUUUAUGGCUAUAUUUGUCGAAAAUAAUCACGCACUACUUUAUGAAAACAAAAUUUUGGAUCAUCUGCUUAUCAGUUUUCUUUGUGUCUUGCUUGCGUAAUAUGCAUGAUAUAUAUA
env-19	CGGGGGACAGUUUUUUUUCUGACUAUUUAGUUGUUUUUUAGUUAUUUAUGGCUAUAUUUGUCGAAAAUAAUCACGCACUACUUUAUGAAAACAAAAUUUUGGAUCAUCUGCUUAUCAGUUUUCUUUGUGUCUUGCUUGCGUAAUAUGCAUGAUAUAUAUA
env-20	CGGGGGACAGUUUUUUUUCUGACUAUUUAGUUGUUUUUUAGUUAUUUAUGGCUAUAUUUGUCGAAAAUAAUCACGCACUACUUUAUGAAAACAAAAUUUUGGAUCAUCUGCUUAUCAGUUUUCUUUGUGUCUUGCUUGCGUAAUAUGCAUGAUAUAUAUA
	....
	....
	CR•GGY•Y••UUUUU••Y•••

alignment positions 420 · · · 496

```
Bco-1-1   CCGGAGUUGGUCUUUGGAGUAAGACUGGCAGGAAAAUCUAUAUCUGCUUGUCUGAAAAAAGAAUCCGGCUUCCGUUU
env-1     CCGGAGUUGGUCUUUGGAGUAAGACUGGCAGGAAAAUCUAUAUCUGCUUGUCUGAAAAAAAAGAAUCCGGCUUCCGUU
Bth-1-1   GAAGCAGCAUGCUGCACGGUGUCCUGUUGAUAGCAUUAUUUUCUUGUGCUGCGUUUUAUAUUGGCGAGAUGUCUUUU
env-2     .............................................................................
Bca-1-1   AAGAAGCAGCAUGUUGCACGGUGUCUUGUUGAUAGCACUGUUCUCUUGUGCGGCGUUUUACAUUGGUGAGAUGUCUU
Bov-1-1   UGCUUCUUGGGCGGGUUUGUCUGCAUCUUGUUUUCCGUCAUUUCCAGUGCCUGAAUCAGACGUUUGCGAGUCUGUCU
Bov-1-2   CGAAUAUAGAUAAGUCAAGCAAGGUAUGCCUCCCCGGCAUGACAGGAAGAUUGCAUAGUAGUUUUGUCGUGUUUUAU
Bca-1-2   UCGGGUUUGUAUCAAUCUCACUUUGCGGAAUAGGCCAUACCAAAUCUUGUGCUCCACGUAGAUUAUUGCGUUCGACU
env-3     CGGCAUUACAGGUAAGAUUGCCUAGUAGUACGGUCGUGUACUAUAACGAGUAGGUGUAGUGACAGUCUUCGAUAUGA
Bth-1-2   CGGCAUUACAGGUAAGAUUGCAUAGUAGUUUUGUCGUGUUUUAUUUUGUGUUUGUGUUGUGACGGUGCUGCGAUGUG
Bth-1-3   CUCGGCUUUAAUUUCUCCGAUCACCGGAUGCUGGGGGAAUACGCUGUCUUCAAAAUCAUUGAUUAAUAUCUCUUUCC
Bca-1-3   GGGGUGGCUGAAAGCCGGGGUGGUAGGUGGAUACACAAUGCUGAUAAUAACCGACUUACGCAUUCACCUACCACCUC
env-4     AGAUCGCUCCUUGAUGAUAGAGUUCUCUUUUAAUUUCCCCAAUAACGGGAUGUUGCGGGAAUACACUUGCUUCAAAA
Bov-1-3   AAAGGGUGUGAAGAUUCACGUGUUGCUUUAGAACGUUGGUAUGAUAUAGCGGAAAAAGCAGAAUGGCGGAAUUUGUC
env-5     UCAGUCGGUCCCUUUGGGAAAUGGGCGAUAAACCAGUC.......................................
Bfr-1-1   UCAGUCGGUCCCUUUGGGAAAUGGGCGAUAAACCAGUCUUGCCAAUACUUCUGUUUGAGUCCGGCAUCUGUCACUAC
env-6     GUUGCUAGGCUAACAACAUGGUUGGCAUUGGAAGUGAUUUCCACUUCUUUAGCUCUAAUUUCACUUAAGGCAGCAAC
Bfr-2-1   UCAGUCGGUCCCUUUGGGAAAUGGGCGAUAAACCAGUCUUGCCAAUACUUCUGUUUGAGUCCGGCAUCUGUCACUAC
env-7     GUUGCUAGGCUAACAACAUGGUUGGCAUUGGAAGUGAUUUCCACUUCUUUAGCUCUAAUUUCACUUAAGGCAGCAAC
Bin-1-1   AGUUGCUAGGCUGACAACAUGGUUAGCAUUGGAAGUGAUUUCUACUUCUUUGGCUCUAAUUUCAGUUAAGGCAGCAA
env-8     AGGCAAUACGCAAUCAGUGGCCAUCAAGUGAUGUAAAUCAUCCGGAAGAAGUUUUGAAGGCUUCUGCAUAUGCAUGG
env-9     CUUUUUUGUUCAAAAAAUGCUAUACCCGGUAACAACUACCUGCAAAUUGUUUAAACUUUUGCGUUGUUGUUUAAGGA
Bun-1-1   CUUUUUUGUUCAAAAAAUGCUAUACACGGUAACAACUACAUGCAAAUUGUUUAUACAUUUGCGUUGUUGUUUAUGUA
env-10    CUUUUUGUUAACAANAGCUGGCGAUCACCCGGGAGAUCNUCNACGACGC............................
env-11    .............................................................................
env-12    UAGACGUGAUUUGUUUAUACAUUUGCGAAGUUGUUUAUGUAACUAUUUUGAAUGAACAAACAGUCUGGCGCGAAUGU
Bst-1-1   UAGACGUGAUUUGUUUAUACAUUUGCGAAGUUGUUUAUGUAACUAUUUUGAAUGAACAAACAGUCUGGCGCGAAUGU
env-13    AAUUUGAGUUGAUUGUGAAAAAUUUGACGAGUUUGUUAUGUACUUAUUUGAAUGAACAAACGUCUGGAGCAAAUAUA
Bfr-1-2   CUUCAGGACGCCUAGUUCUUUUUCUAUUUUGACUUCCGGAUGUUCCUCUAGCCAAGUAAGAAUGUCAGCAGCUUGAA
Bfr-2-2   UUCAGGACGCCUAGUUCUUUUUCUAUUUUGACUUCCGGAUGUUCCUCUAGCCAAGUAAGAAUGUCAGCAGCUUGAAC
env-14    AUAUAUGGAAUAGAUUGUUCUUAUUGUAGUAUAAAACGUUCAAAAUCGUAUAAUUUAUAGAAAAAAAGCUUCUAAGG
Beg-1-1   AUAUAUGGAAUAGAUUGUUCUUAUUGUAGUAUAAAACGUUCAAAAUCGUAUAAUUUAUAGAAAAAAAGCUUCUAAGG
env-15    CUACGACG.....................................................................
env-16    CACGGGGGGAGGGGAAGAAGGGGAGGAAAAAAUCAAUAAAGACGAUUUUUUUGAUUUUACUACUGUCCGUAAUAGUC
env-17    ACGGGGGAAGAGGAAGAAGAGGAGGAAAAAAUCAAUAAAGGCGAUUUUUUUGAUUUUACUACUGUCCGUAAUAGUCA
Bdo-1-1   .............................................................................
env-18    GGGGGAGGGGAAGAAGGGGAGGAAAAAAUCAAUAAAGACGAUUUUUUUGAUUUUACUACUGUCCGUAAUAGUCAACU
Bvu-1-1   CGGGGGGAGAGGAAGAAGAGGAGGAAAAAAUCAAUAAAGACGAUUUUUUUGAUUUUACUACUGUCCGUAAUAGUCAA
env-19    CGGGGGAAGAGGAAGAAGAGGAGGAAAAAAUCAAUAAAGGCGAUUUUUUUGAUUUUACUACUGUCCGUAAUAGUCAA
env-20    CGGGGGAAGAGGAAGAAGAGGAGGGAAAAAUCAAUAAAGACGAUUUUUUUGAUUUUACUACUGUCCGUAAUAGUCGA
          .............................................................................
          .............................................................................
          ●●●●●●●●●●●●●●●●●●●●●●●●●●●●●●●●●●●●●●○○○○○○○○○○○○○○○○○○○○○○○○○○○○○○○○○○○○○○○
```

14 *Bacteroides*-2

14.1 Taxa

The taxonomy of each organism containing a putative *Bacteroides*-2 RNA is listed, with abbreviations identifying each hit (e.g., “Eco-1-1” and “Eco-1-2” might hypothetically represent two distinct RNAs in *E. coli*). The abbreviations will be used to identify each individual *Bacteroides*-2 RNA in Sections 14.2 and 14.4.

abbrev. of hits	taxonomy of species
Bca-1-1 to Bca-1-9	Bacteria Bacteroidetes Bacteroidia Bacteroidales Bacteroidaceae *Bacteroides capillosus* ATCC 29799
env-1 to env-57	environmental samples

14.2 Gene contexts

Each *Bacteroides*-2 RNA (indicated by "RNA→") is listed with its surrounding genes. Environmental sequences and some RefSeq entries lack gene annotations, and no genes are listed for such sequences. The direction of each gene is indicated with an arrow (→), and each conserved domain in the gene is colored. Conserved domains associated with more than one *Bacteroides*-2 RNA are assigned a color; other domains are gray. Information about these conserved domains is given in Section 14.3. The accession number of the sequence containing each *Bacteroides*-2 RNA is given. Accessions beginning with "NC_" or "NZ_" are RefSeq, while other accession refer to environmental samples. Nucleotide coordinates are given for the 5′ and 3′ boundaries of each. If the 5′ coordinate is greater than the 3′ coordinate, the RNA is present on the reverse-complement strand of the containing genomic DNA sequence. Each hit is denoted by an abbreviation (like "Eco-1-1") that refers to a taxonomy given in Section 14.1.

abbrev.	Seq. accession		5′ at	3′ at	genes
env-1	BABD01006177.1	+	420	476	←hypo **RNA→** ←OmpR (COG0745)
env-2	BABB01012787.1	-	923	862	**RNA→** ←Metalloenzyme (pfam01676)
env-3	BABD01026837.1	+	161	220	**RNA→** **RNA→** ←Mur_ligase_C (pfam02875)
env-4	BABD01026837.1	+	346	405	(shown above)
env-5	BABA01023012.1	-	391	329	←hypo **RNA→** **RNA→** ←hypo
env-8	BABA01023012.1	-	188	126	(shown above)
env-6	BABD01023490.1	+	297	359	←hypo **RNA→** **RNA→** ←S1 (smart00316)
env-9	BABD01023490.1	+	500	562	(shown above)
env-7	BABB01003135.1	-	807	746	←hypo **RNA→** ←S1 (smart00316)
env-10	BABB01003677.1	+	906	956	NtpB (COG1156)→ **RNA→** ATP-synt_D (pfam01813)→
env-11	BABB01017599.1	+	202	262	**RNA→** ←hypo
env-12	BABD01023181.1	+	345	406	hypo→ **RNA→** GTP_EFTU (pfam00009)→
env-13	BAAY01006515.1	-	627	567	ModA (COG0725)→ **RNA→** ModC (COG4149)→
env-14	BABG01020158.1	-	574	517	←zf-CHC2 (pfam01807) **RNA→** ←hypo
env-15	BABB01010706.1	+	437	494	←hypo **RNA→**
env-16	BABD01029485.1	+	372	431	**RNA→** GIYc (smart00465)→
env-17	BAAX01015758.1	+	773	834	←hypo **RNA→** ←OmpR (COG0745)
env-18	BAAV01027752.1	+	834	892	←HAMP (smart00304) **RNA→**
env-19	BAAZ01007067.1	+	359	417	←hypo **RNA→** ←OmpR (COG0745)
env-20	BAAY01006259.1	-	378	317	hypo→ **RNA→** **RNA→** ←hypo
env-24	BAAY01006259.1	-	593	532	(shown above)
env-21	BABD01002881.1	-	1028	971	←Acetyltransf_1 (pfam00583) **RNA→** ←IlvH (COG0440)
env-22	BABA01027753.1	+	861	922	hypo→ **RNA→**
env-23	BABB01009884.1	-	396	339	**RNA→** ←YqgFc (smart00732)
Bca-1-1	NZ_AAXG02000041.1	-	20454	20396	hypo→ **RNA→** ←Upp (COG0035)
env-25	BABA01007724.1	-	485	426	←hypo **RNA→** ←hypo
env-26	BABB01011836.1	-	318	260	Leu tRNA→ **RNA→**
Bca-1-2	NZ_AAXG02000019.1	-	42448	42391	HIS2 (COG1387)→ **RNA→** hypo→
env-27	BAAX01027037.1	+	205	262	←hypo **RNA→** ←hypo
env-28	BAAX01027037.1	+	435	492	←hypo **RNA→** ←hypo
env-29	BABA01007680.1	-	711	650	←Enolase_N (pfam03952) **RNA→** ←Metalloenzyme (pfam01676)
env-30	BABD01023182.1	+	19	79	**RNA→** ←EFG_C (pfam00679)LepA_C (pfam06421)
env-31	BAAZ01005461.1	-	884	827	←Rnc (COG0571) **RNA→** ←Rnc (COG0571)
env-32	BABD01023870.1	+	89	150	←hypo **RNA→** ←DHHA1 (pfam02272)
env-33	BABD01024680.1	-	99	41	hypo→ **RNA→**
env-34	BAAZ01009952.1	+	406	522	COG1937 (COG1937)→ **RNA→** HMA (cd00371)→
Bca-1-3	NZ_AAXG02000006.1	+	58384	58443	hypo→ **RNA→** ←rubredoxin_SM (cd00729)Rubrerythrin_like (cd01046)
env-35	BAAZ01029945.1	-	420	361	←hypo **RNA→** ←hypo

env-36	BAAZ01015734.1	-	284	229	←hypo **RNA**→
Bca-1-4	NZ_AAXG02000006.1	+	93579	93636	hypo→ **RNA**→ ZntA (COG2217)CopZ (COG2608)→
env-37	BAAZ01029758.1	+	489	548	Ile tRNA→ **RNA**→
env-38	BAAV01030571.1	+	354	413	←RbfA (COG0858) **RNA**→
env-39	BAAX01027502.1	+	395	456	hypo→ **RNA**→ ←SIS (pfam01380)
env-40	BAAY01013091.1	+	1353	1413	barnase (cd00933)→ **RNA**→ barnase (cd00933)→
env-42	BAAY01022414.1	-	154	97	hypo→ **RNA**→
env-41	BAAY01022414.1	-	366	309	(shown above)
Bca-1-5	NZ_AAXG02000011.1	+	55260	55319	←hypo **RNA**→ ←AlaS (COG0013)tRNA-synt_2c (pfam01411)DHHA1 (pfam02272)
Bca-1-6	NZ_AAXG02000010.1	-	39737	39678	hypo→ **RNA**→ hypo→
env-43	BAAZ01022445.1	-	602	542	**RNA**→ hypo→
env-44	BAAY01009092.1	-	436	376	DRTGG (pfam07085)→ **RNA**→ **RNA**→ POLIIIAc (smart00481)→
env-49	BAAY01009092.1	-	642	582	(shown above)
env-45	BABA01009539.1	+	34	94	**RNA**→ POLIIIAc (smart00481)→
env-46	BAAZ01022446.1	-	519	459	**RNA**→ **RNA**→ ←Dtyr_deacylase (cd00563)
env-47	BAAZ01022446.1	-	308	248	(shown above)
env-48	BABB01011814.1	+	103	163	**RNA**→ ←Dtyr_deacylase (cd00563)
env-50	BAAX01006424.1	+	687	744	←MazF (COG2337) **RNA**→ ←Ala_racemase (cd00430)
env-51	BABA01022941.1	+	154	213	**RNA**→ ←EF_TS (pfam00889)
env-52	BABE01019904.1	+	317	376	hypo→ **RNA**→ hypo→
env-53	BABA01013052.1	-	751	690	←hypo **RNA**→ **RNA**→ ←hypo
env-54	BABA01013052.1	-	535	474	(shown above)
Bca-1-7	NZ_AAXG02000047.1	-	151807	151748	←hypo **RNA**→ ←EFP (pfam01132)
Bca-1-8	NZ_AAXG02000045.1	-	57489	57431	hypo→ **RNA**→ ←PheT (COG0072)tRNA_bind_bactPheRS (cd02796)
Bca-1-9	NZ_AAXG02000047.1	-	23814	23754	YtcJ_like (cd01300)→ **RNA**→ SdaA (COG1760)→
env-55	BABD01004318.1	+	1157	1216	←hypo **RNA**→ hypo→
env-56	BABG01015003.1	-	206	147	**RNA**→
env-57	BABG01015003.1	-	458	399	**RNA**→

14.3 Conserved domains

Conserved domains found in protein-coding genes listed in Section 14.2 are listed, with the first sentence in their description from the Conserved Domain Database. Conserved domains associated with more than one *Bacteroides*-2 RNA are assigned a color, while others are shown in gray. The number in parentheses is the number of occurrences of the given gene.

D cd00371 (1) Heavy-metal-associated domain (HMA) is a conserved domain of approximately 30 amino acid residues found in a number of proteins that transport or detoxify heavy metals, for example, the CPx-type heavy metal ATPases and copper chaperones.
d cd00430 (1) Alanine racemase.
d cd00563 (2) D-Tyrosyl-tRNAtyr deacylases; a class of tRNA-dependent hydrolases which are capable of hydrolyzing the ester bond of D-Tyrosyl-tRNA reducing the level of cellular D-Tyrosine while recycling the peptidyl-tRNA; found in bacteria and in eukaryotes but not in archea; beta barrel-like fold structure; forms homodimers in which two surface cavities serve as the active site for tRNA binding
d cd00729 (1) Rubredoxin, Small Modular nonheme iron binding domain containing a [Fe(SCys)4]
uD cd00933 (2) Barnase, a member of the family of homologous microbial ribonucleases, catalyses the cleavage of single-stranded RNA via a two-step mechanism thought to be similar to that of pancreatic ribonuclease.
d cd01046 (1) Rubrerythrin-like domain,similar to rubrerythrin, a nonheme iron binding domain found in many air-sensitive bacteria and archaea, and member of a broad superfamily of ferritin-like diiron-carboxylate proteins.
u cd01300 (1) YtcJ_like metal dependent amidohydrolases.
d cd02796 (1) tRNA-binding-domain-containing prokaryotic phenylalanly tRNA synthetase (PheRS) beta chain.
d COG0013 (1) Alanyl-tRNA synthetase [Translation, ribosomal structure and biogenesis]
d COG0035 (1) Uracil phosphoribosyltransferase [Nucleotide transport and metabolism]
d COG0072 (1) Phenylalanyl-tRNA synthetase beta subunit [Translation, ribosomal structure and biogenesis]
d COG0440 (1) Acetolactate synthase, small (regulatory) subunit [Amino acid transport and metabolism]

u d COG0571 (2) dsRNA-specific ribonuclease [Transcription]
u COG0725 (1) ABC-type molybdate transport system, periplasmic component [Inorganic ion transport and metabolism]
d COG0745 (3) Response regulators consisting of a CheY-like receiver domain and a winged-helix DNA-binding domain [Signal transduction mechanisms / Transcription]
u COG0858 (1) Ribosome-binding factor A [Translation, ribosomal structure and biogenesis]
u COG1156 (1) Archaeal/vacuolar-type H+-ATPase subunit B [Energy production and conversion]
u COG1387 (1) Histidinol phosphatase and related hydrolases of the PHP family [Amino acid transport and metabolism / General function prediction only]
D COG1760 (1) L-serine deaminase [Amino acid transport and metabolism]
u COG1937 (1) Uncharacterized protein conserved in bacteria [Function unknown]
D COG2217 (1) Cation transport ATPase [Inorganic ion transport and metabolism]
u COG2337 (1) Growth inhibitor [Signal transduction mechanisms]
D COG2608 (1) Copper chaperone [Inorganic ion transport and metabolism]
D COG4149 (1) ABC-type molybdate transport system, permease component [Inorganic ion transport and metabolism]
D pfam00009 (1) Elongation factor Tu GTP binding domain.
u pfam00583 (1) Acetyltransferase (GNAT) family.
d pfam00679 (1) Elongation factor G C-terminus.
d pfam00889 (1) Elongation factor TS.
d pfam01132 (1) Elongation factor P (EF-P).
d pfam01380 (1) SIS domain.
d pfam01411 (1) tRNA synthetases class II (A).
d pfam01676 (2) Metalloenzyme superfamily.
u pfam01807 (1) CHC2 zinc finger.
D pfam01813 (1) ATP synthase subunit D.
d pfam02272 (2) DHHA1 domain.
d pfam02875 (1) Mur ligase family, glutamate ligase domain.
u pfam03952 (1) Enolase, N-terminal domain.
d pfam06421 (1) GTP-binding protein LepA C-terminus.
u pfam07085 (1) DRTGG domain.
u smart00304 (1) HAMP (Histidine kinases, Adenylyl cyclases, Methyl binding proteins, Phosphatases) domain;
d smart00316 (2) Ribosomal protein S1-like RNA-binding domain;
D smart00465 (1) GIY-YIG type nucleases (URI domain);
D smart00481 (2) DNA polymerase alpha chain like domain; DNA polymerase alpha chain like domain, incl.
d smart00732 (1) Likely ribonuclease with RNase H fold.

14.4 Multiple-sequence alignment

Each *Bacteroides*-2 RNA is denoted by an abbreviation (like "Eco-1-1") that refers to a taxonomy given in Section 14.1. The alignment includes sequences containing the RNA motif, as well as flanking sequence. The *Bacteroides*-2 RNA itself is denoted by the line underneath marked 5′ and 3′ on either end. Nucleotides in the flanking sequences (i.e., not part of the motif) are written in gray letters. Stems of predicted rho-independent transcription terminators are shaded yellow. (Note: terminator predictions have not been analyzed manually, and many are likely to be false positives.) Start codons are shaded green. (Note: start codons are frequently misannotated, especially in environmental samples.) Nucleotides proposed to basepair as part of the consensus structure are shaded in color when they comprise Watson-Crick or G-U pairs. Otherwise they are shaded gray. Conserved stems are also indicated at the bottom of the alignment by angle brackets, where matching $<$ and $>$ denote base-paired columns. Below these angle brackets, the symbol "2" denotes base pairs exhibiting covariation, "1" denotes base pairs exhibiting compatible mutations, "0" denotes base pairs that are not observed to mutate and "?" denotes base pairs that have a significant frequency of non-canonical nucleotides for Watson-Crick or G-U pairs ($>$ 5%). Below these base pair annotation is the consensus sequence: "R" = "A" or "G", "Y" = "C" or "U", red nucleotides: nucleotide identity conserved more than 97% of the time, black nucleotides: 90%, gray nucleotides: 75%, red circle (•): nucleotide is present 97% of the time, black circle (•): 90%, gray circle (•): 75%, white circle (•): 50%. All percentages of sequences just described (e.g. 97% conserved) assume that sequences have been weighted by the GSC algorithm implemented by the Infernal software.

alignment positions 1 · · · 160

```
env-1  CUUGUACCCAUAUCCCCACACGGUGGUGAUAUAAACCGGGUUCGUGGCGUCCCUCCCCAAAGAGCUCGCUCUUUGGGGACCCCUCUUUUUUCCGAUGCUCGGGGCAA.....................................................
env-2  GGUCAGUUGACAUUCAGGGUUUCCCCAUUCAUCUCGGCGGGUUUUCCAAGAUACCCACCGCGGUCGCCGGGCGGGGGCCCCCGGUUCUUUUAUCUGUUGGGGGCCAA.....................................................
env-3  CCAGCGGUGGCCUGAGCGGCCGAUCCUCCGCCGGAUGUCCGCCGUAUCCCAUUGUCCAUGCGGCGCCGCCCACGGCUGACCGCCGCAUGUCACGCGGACCGCCCGAA.....................................................
env-4  GUGCUCCGCCACGAUCUCCCGUUCGUCCAUGUGGUGCUUCUCGUGGUUCACGCCCCACAAAAUCUCCGAUUUUGUCGGGACCCCGCAUUUGACUCAAAUCGCCCGAA.....................................................
env-5  CUCCCCCUUUUGGCUUCUUCACGGUGAUGGAAAUCCGCUUUUUCUUCUCGCCGCCCCUCGCGGCCAUGGCCGCGCGGGGAUCCCUCAUUUCCCUGAACUCGCCCGAA.....................................................
env-6  CUCCCCCUUUUGGCUUCUUCAUGGUGAUGGAAAUCCGCUUUUUCUUCUCGCCGCCCCCCGCGGCCAUGGCCGCGCGGGGAUCCCUCAUUUCCCUGAACUCGCCCGAA.....................................................
env-7  CUCCCCCUUUUGGCUUCUUCAUGGUGAUGGAAAUCCGCUUUUUCUUCUCGCCGCCCCCCGCGGCCAUGGCCGCGCGGGGGCCCCUCAUUUCACUGAACUCGCCCGAA.....................................................

       ...................................................................................................<<<<<<<.....................................................
       ...................................................................................................1222222.....................................................
       ••••••••••••••••••••••••••••••••••••••••••••••••••••••••••••••••••••••••••••••••••••••YUY•RY•U•CYCGG•••RA-----------------------------------------------------
                                                                                                   5′
```

```
env-8     UUCAGCCCCUUGGCUUUUUCAUGGUGAGGGAAAUCCGCUUCUUCUUCUCGUCGCCCCGCGUGGCCCCGGCCGCACGGGGGCCCCUCAUUUCACACAACCCGCCCGAA.....................................................
env-9     UUCAGCCCCUUGGCUUUUUCAUGGUGAGGGAAAUCCGCUUCUUCUUCUCGUCGCCCCGCGUGGCCCCGGCCGCACGGGGGCCCCUCAUUUCACUCAGCCCGCCCGAA.....................................................
env-10    UUGAGAAAUACCUCCCGAAAAAGGAGGGGUAAUCCAUGCCCGCGAUUAACGUGAACCCCAUACGGCCGGCCGCAUGGGGGCCCCGGGAUUUCCCAUCCUCGGGCGGA.....................................................
env-11    CGGUUAAAACAGUCCGUCGAACAGGGUAAUCUGACUCGUAUCCGGCAUGGCCCAGGGGCGGCCGCGCCGUCCCUGGGGUCCCGUUUCCCUCCAUCUGGCGGGACGAA.....................................................
env-12    AAAUCCGUAAUUUUUCGAUAAUUGCCCACAUUGACCACGGCAAAUCAAAUCCCGCGGAGUCGGCCGACUCCGCGGGUACCCCGUGUUAUUUUGCAUCCUCGGCGGAA.....................................................
env-13    GGUCACUUAUGGAUUGGUACCCCUUAUGGAACUCGCUGCGCAUCGCGGCGCUCGUCCCAUUGGGCUGCCGCCCAACGGGAGCCCUGAUUUGAAAAGCACGGUCGGAA.....................................................
env-14    CGGAAUGGCCAAAAGACUUCCUCCUUUCAAAUCGGGAUCACGAGGGCAGGCCGUUCCCACGGGUCAGGCCCCGUGGGAGCCCUGAGGAUUUUAAAUGCUCGGGCGAA.....................................................
env-15    CGAUCUGCUCGAUCUGCUGCAUCACUGCUUCCUUGGACGGGCCGCCGCAGCUUCCCACACAGGCGGCCUGCGUGGGGGCCCCGUUUUGAUAAAAAUGCUCGGCGGAA.....................................................
env-16    UACGAGUCAUGACCUUUGACGAAUUAAAAGAAAAAGCCCAUGCCCAUUCCCCAAACGAACGGGUUCGUUUGGGGCCCCCGGAGGAUGAUUUGACAUCCUCGGCGGGA.....................................................
env-17    ACUUGUAUCCGUAUCCCCACACCGUGGUGAUAUACGUGGGAUUCUGCACGUUGCUUCCCAUCCCGCGGCGCGGGAUGGACCCCUUUCCUCAGCUUGCGCGGCGGAA.....................................................
env-18    CGUGGCUUUUCGUUUUUGACUUCCCUCCGGCACGAAGCAUCUCACCCUUUCUUUUCCCAUUUUGCGGGGCAAAAUGGGAGCCCCUUCAUCUUACUUGCGCGGCGGAA.....................................................
env-19    CCGCUGAUAGCCGUGCCCCACAGGGGCGUAAGAUAGACCGGAUUCAUGGGGGCGUCUCAUCCCCUAUUGUACGGGGACCCCUUGUGCUUUCGACCUGCUGGAGCGAA.....................................................
env-20    AGAACGGAACCUUUUUUAAUAGCUGACGCCCUGCCAGAUCAUCGCGUCGGCGCUCCCAAAACACAGGGUGUUUUGGGAACCCUGUGGCCUUCACAUGCUCGGGGCAA.....................................................
env-21    GGGAGCUUUACAGGAAGUUCUUGCCCAGAUAGAACUCGGUCUGCAUGUCCCAAACGCGCUGGCGCGUUUGGGAGCCCUCCGGGCGGAAUUCACAUCCUCGGGGCAA.....................................................
env-22    GCGUGGAUUCAGCGGUACAUCGAACCGAACCGGGAAUAUGAAUAAAAAGCAGGUGUCCCNCCGGGACACCUGCUUUUUUCUGCGUCUAUUUCACAUGCUCGGGGCAA.....................................................
env-23    GCCUGAAGGUCAGAUAUCCCUCCAGAAUCAGCGUCGCCGCCACCGCGUCCCUCCCAUCCGGCCAUGCCGGAUGGGAACCCUGAUGCCUUUUGGCAUGCUCGGGGCAA.....................................................
env-24    GCGUGGAUUCAGCGGUACAUCGAACCGAACCGGGAAUAUGAAUAAAAAGCAGGUGUCCGCCGGGACACCUGCUUUUUUCUGCGUCUAUUUCACAUGCUCGGGGCAA.....................................................
Bca-1-1   UUGAAGCUGUAUGGGUGUCUUACUUGGUACCGAAGAUGCGGUCGCCGGCGUCAGCCCCAGCCGGCUGAUGCCAGCCGGGGACCCCGAAUCUUACAUCCUCGGGGCAA.....................................................
env-25    UCGAUGGCUUCGAUCUGCCGCUCCACCGCCUCUUUGGCCGGGCCGCCGCAGCGCCCCACGGGGCCGGCCCCGUGGGGACCCCCGUUGAUUUCACCUGCUCGGGACAA.....................................................
env-26    CCGCCCUUUGCAAAAAAGAAAGACAUGACCACAGGUCAUGUCUUUCUUUUUUGGUUCCGGCCGCCGGAGGCGGCCUCCAGUCCUCGGUAUUUGAAUGCUCGGGGUAA.....................................................
Bca-1-2   AACGGAGGUACUGUUAUGACAAUUGCACUUGGUUCCGAUCACGGCGGCUUUGCUCCCCAUGGAGCAGGCUCCAUGGGGACCCCUCCGAUUUUAUUUGCUCGGGGCAA.....................................................
env-27    UGGUCUGUUCAUCCCAGACACUGGCCUGCACCUCGCCGAUGUGGGCCUUGAGCCUCGCCCGGCCGUGGCCGGGCGGGGACCCCACAGAUUUUAUCUGCGCGGGGCGA.....................................................
env-28    UGGUCUGUUCAUCCCAGACACUGGCCUGCACCUCGCCGAUGUGGGCCUUGAGCCUCGCCCGGCCGUGGCCGGGCGGGGACCCCACAGAUUUUAUCUGCGCGGGGCGA.....................................................
env-29    GGUCAGUUGACAAUCAGCGUCUCCCCAUCCAUCUCGGCAGGCUUCUCCAGAUACCCCACGCGGCUGGCCGCGCGGGGGCCCCGGGUCUUUUUAUCUGCCCGGGGCAA.....................................................
env-30    ..................................................................................UCUCCAUUUCACAUGCUCGGGCGGA.....................................................
env-31    CCCCAGCCCUCAGCUUCUCUAUCGCGGCCUUGGCCGCGUUCUGCUCCGCCGCCCAAACCGCCGCGGGCAGUUUGGGGCCCCCGUUUCUCUCCAUCUGUCCGGGCGGA.....................................................
env-32    ............UACCGCCGCCCAUGGCGCUGUCGGGCUUGCCGCCCGCCCCAAACAGCGGGAACCGCUGUUUGGGGAACCCCGCCAUUUUUAAAUGCUCGGGCGGA.....................................................
env-33    GUACCUUACCUCCAAAACGAAAGGACAUCCGAAAGGAUGUCCUUUCGUUUUGGGCUCCGUCGCCCAAAGGCGGCUACACCCUUCGGUAUUUUAAUGCUCAGGGCGGC.....................................................
env-34    UGAAGCAGAAAUUCAAUGUCACAGGCAUGACCUGUUCCGCCUGCUCCGUUCCCCGCGGCAUGUCCCAUGCCCCGGGGGGCCCAAAGCUUUUUAUCUGCUCGGGCGAAACACCCGCAAGGGGUACGCCGCAUCCGUACAGCGGCAGAGCCUCUAACGGCUG
Bca-1-3   UAGCCGAAGUAGCGUUUCAGCAGGCCCUCAAAAGCCUUGCCGUGACGGCCCAUUCCCUAUGGGACUCGUCCCAUGGGGUCCCCGCUGAUUUUAUCUGCUCGGGCGGA.....................................................
env-35    UUUCUCCGCCAGAUAGCUCUUCAGCUGGUCGACGGGCAGCCGCACCUGCCCUCCCGCAAAAGCACUGCUUUUGCGGGACCCCUGUCCCAUCUCAUUCCUCGGGCGGA.....................................................
env-36    UACUCGUCCCGGUAGCCGAAGUUCUUGAGCAAAUUCACCUGGUCUCUCCCGUUUGCCCACGGGACGUCCCGUCCCGCGGGGCCCCGAUUCUCUCUUCCUCGGGCGGA.....................................................
Bca-1-4   UUGAAACAGAAAUUUGAUGUUACCGGCAUGACCUGUUCGGCCUGCUCCCUUCCCCACAGGACGUGCCGUCCUGCGGGGACCCCGUUUUCUCUAUCUGCUCGGCGGAA.....................................................
env-37    ACGACUGAUAAUCGUGAGGUCGGUGGUUCGAGUCCACUUGGGCCCACCAUCCCCAUUUGAUUUCAUCAAAUGGGGACCCCGAAGGGGCUUUGACAUCCUCGGCGGAA.....................................................
env-38    GAAGUGGUUUACUGGGGGAUCUCCUCCAUCGUGAAGGCCUCGAUCAUGUCGCCUGCCCCAUGAAACGGCGUUUCAUGGGGACCCCGGACAGAAUGUCCUUGGCGGAA.....................................................
env-39    UCCACCGUCACGCUCUUAGCAAGGUUCCGGGGCUUAUCAAUAUCGCAGCCCUCCCACAAAAGCUGCGCUUUUGCGGGAACCCUAUGAUUUAACUCAAAUCGUCCGGA.....................................................
env-40    CCGACUACCGGCACCAAAAAAGAAGCACUGGCAAAGCCAGUGCUUCUUUUUUGGGUUCCGCCGCCUUUGGGCGGCUCCACCCGGUUCAUUUUAUCUGCUCGGGCGGA.....................................................
env-41    CAAGGCCGGCAGGUCAAUGNCCUGCCGGCCUUGGCUUGUCAAAAAUCCUUAAUGGGACAUUCUCCGCUGGGGAGCGGCUCCAUCAAAGCGCUGCGGACACGGGCGGA.....................................................
env-42    CGUUCCUCGCGGAAGUAGCUCAGACCCAGGUUUGCAGGCGGCUGCUUUGAUCCCCUGUCCGGCCAAGGCCGGACAGGGGACCCGGAUUUCAUUUUCCUCCGGGCGGA.....................................................
Bca-1-5   ACAGCCCCAGCUUCUCCGCCACCAGAUCGUCCACCUUGGCAAGCGCAUCGUCCCCCACGCGGCCAUGCCGCGCGGGGACCCCAUCCCUUUUUAUCUGCUCGGGCGGA.....................................................
Bca-1-6   GCCGAGAAUGUCCACGUUGUAGGGCGUGAUGAUGUAGGUGGAGAUGGCCGCCCCAGCCGGUGCUCCGCACCGUCCGGGGACCCCGUCCCUGUAUCUGCUCGGGCGGA.....................................................
env-43    UAACGGGCCGUUUUUUUUAGUGAUUACUCUUGUCAUUUUGUUCCCGAUCGUCCCAGGGGGUUUUCCCUUCUUUGGGACUCCGGUUCCUUUUUUAUUUGCCGGGGCGUA.....................................................
env-44    GAAAAGGAGUUUUACAUGAAGUAUUACUACGAUCUCCACCUUCACUCCUGCCUGUCACCCCACCCGGCUCAGCCGGGCGGGGGCCCCUUUUUUCUCCCUCGGGCGGA.....................................................
env-45    ...................................................................AGGCCUGAGCUUGUCGCCCCUUUUUUCUCCCUCGGGCGGA.....................................................
env-46    GUCACCAACCCGUUAUUUGGCGGGUACACGUGCAUAUCCGAGCAGAACGUCCCAGGGGGGUCCCGCCUCGGGGGGACCCCGGUUGCCGUUUUAUAUGCCGGGGCGGA.....................................................
env-47    UCGUCACCGGCCCGUCGUUCUGCGAAUACACCUGCAUAUCCGCCCCGAACGCCCCACGGGGCUCCCACCCCGCGGGGACCCCGGUUCCCUUUAUCUGCCGGGGCGGA.....................................................
env-48    UUUUUUUUUUAAGAAUAAAUAAAAAAAAUUAUGUAGAAAUAAAGCNNNNGCAGCUGAGCUACUGCGCAGGAUCUAGGCUGAAGCUUGUCCCUUUAUCUGCCGGGGCGGA.....................................................
env-49    AAAAGCAAAUUCUACACAAAGUAUUUCCACGAUUUUCACCUACACUCCUGCCUGUCGCCCCACCCGGCAACGCCGGGCGGGGACCCCCUUUUGCUUCCUCGGGCGGA.....................................................
env-50    GUUUUCCAUAUACAAUCUGGGGACCCGUUUGCUCACCGCACAGGUCAGUAUCCCACCGGGAUCUCGAUCCCGGCGGGAGCCCUUUCAUUGGACUCAAAUGGUCCGAA.....................................................
env-51    CCAAUCUGCGGUUUACUUGCCCAGCUGCUUAGCGAUCUCUUCGGCGAAGUUCCGCCCCACCCGGCAAGCCGGGCGGGGAGCCCCGAUUUUCAUUCGAUUGGGGCGAA.....................................................
env-52    CUCCUUUGUGGACGCCUGCACGGAAGAGCACUUCGCGGCCAUGAGCCGCAGUUCCCCACCCGGCUUCCGCCGGGCGGGGGCCCCGAUUUUUUAUCUGCGCGGGCGAA.....................................................
env-53    UUUUCUCCAGCAUAGUCUUGAUCAGCUUCGCAAUGUGAGCACCCGCCGUCCCCCGUGCGGCUGGCCGCACGGGGCCCCCAAGCGUCUCUUUUAUCUGCUCGGGCAAA.....................................................
env-54    UCUUCUCCAGCAUGGUCUUGAUCAGCUCCGCGAUAUGCGCGCCCGCCGUUCCCCGUGCGGCUGGCCGCACGGGGCCCCCAAGCGUCUCUUUCAUCUGCUCGGGCGAA.....................................................
Bca-1-7   UGCUGUGUCUGAACUCAGGCCUUGCAGCGCUCCAGAUACUCGCCGGUGCGGGUGGUCCGUCCAAAUUAAAAUUUGUCCGGGAACCCUUUUCCACUUCCGCGGGCGGA.....................................................
Bca-1-8   CAGCUCGCUCUCCAGGAGGGCAAGCACACUCUUGACGUCCUCAUCCGCCGCCCCGUGAAAGGCUCCGCCUUUCACGGGGACCCCAGGAUUCUAUCUCAUUGGGCGGA.....................................................
Bca-1-9   CCUUGAACAUUUUCGAUAUCAUGGGCCCCGUCAUGGUGGGGCCCAUCCCCAGAGCACGCUGACGCGUACUCCGGGGAACCCCGCUCUUUUUCAUCUGCUGGGGCGGA.....................................................
env-55    CCCGCCUCCACCAAAACGAAAGGACAUCCGAAAGGAUGUCCUUUCGUUUUGGGCUCCGACCGCCGAAGGCGGUCUCCGCCCUUCGGUGAUUCAAAUGCUCGGGAUGG.....................................................
env-56    GAGAUCCGGCCCUUACUCCGCCAGGGCGGCAGUGAUGAUGGCCAUGGAAUAGACCCGCAAAAUACAUAUUUUGCGGGAACCCAGUUUUUUUUAGAUUGCUCAGGGCGG.....................................................
env-57    GAGAUCCGGCCCUUACUCCGCCAGGGCGGCGGUGAUGAUGGCCAUGGAAUAGACCCGCAAAAUACAUAUUUUGCGGGAACCCAGUUUUUUUUAGAUUGCUCAGGGUGG.....................................................
          ....................................................................................................<<<<<<<.....................................................
          ....................................................................................................1222222.....................................................
          ••••••••••••••••••••••••••••••••••••••••••••••••••••••••••••••••••••••••••••••••••••••••YUY•RY•U•CYCGG•••RA-----------------------------------------------------
                                                                                                              5′
```

alignment positions 161 · · · 320

```
env-1    ....AUGAAUUGCCCCU.CCGC.CAAUGCUUC..GCA....C....UGC..GAAGC.AUUGACGCGCUGCCGCGCGCCCCGCUCUGCGGGGCCCCGGACGACGCCCCUGGGAGCCUGCUUAUCCGCCCCACUUGUACCCAUAUCCCCACACGGUGGUGAU
env-2    ....GGGA.UUUGCCCGGACAU.UAAGGGUUU..GCCGUC.G....GGCC.AAACCGCUUGGACGGCGCACAGCGCCGCCCGCCNUGCGCGGGAUAGCNNUGGAAAGCCCCUUAAUCAAAAUCAGAGUCUCCCCCAUCCAUCUCGGCGGCUUUUCCAG
env-3    ....GUGAAUUCAGCCG.AUUU.CAAGGGUUU..GCC.UG.C....GGC..AAACCGCUUGACGCCGGACGCCCGGCGGUCACUUCCCCGCCAAGUGCUCCGCCACGAUCUCCCGUUCGUCCAUGUGGUGCUUCUCGUGGUUCACGCCCCACAAAAUCUC
env-4    ....GUGAAUUCAGCCG.AUUU.CAAGGGUUU..GCC.UG.C....GGC..AAACCGCUUGACGCCGGACGUCCGGCGGUUACUUCCCCGUCAAGUGCUCCGCCACGAUCUCCCGUUCGUCCAUGUGGUGCUUCUCGUGGUUCACUUCCUGGUAGGUCUC
env-5    ....GUGAAUUCGGGCG.GGGU.CAGGGUUCC.GGCC.UGCC....GGCC.GGAACGCUUGGCGCGGCAUGCGCCGUGUCCCGCCUGCGGCGGGGCCUGUCAAGGACGGUUCAGCCCCUUGGCUUUUUCAUGGUGAGGGAAAUCCGCUUCUUCUUCUCGU
env-6    ....GUGAAUUCGGGCG.GGGU.CAGGGUUCC.GGCC.UGCC....GGCC.GGAACGCUUGGCGCGGCAUGCGCCGUGUCCCGCCUGCGGCGGGGCCUGUCAAGGCCGGUUCAGCCCCUUGGCUUUUUCAUGGUGAGGGAAAUCCGCUUCUUCUUCUCGU
env-7    ....GUGA.UUCGGGCG.AGUU.CAGGGUUCC.GGCC.UGCC....GGCC.GGAACGCUUGGCGCAGCAUGCGCCGCGUCCCGCCUGCGGCGGGGCCCGUCAAGGCCGGUUCAGCCCUUUGGCUUCUUCAUGGUGAGGGAGAUCCGCUUCUUCUUUUCGU
env-8    ....GUGAAUUCGGGCG.AGUU.CAAGGUUCC.GGCC.UGCC....GGCC.GGAACGCUUGGCGCGGCAUGCGCCGCGUCCCGCCUGCGGCGGGGCCCGUCAAGGCCGGUUCAGCCCUUUGGCUUCUUCAUGGUGAGGGAGAUCCGCUUCUUCUUUUCGU
env-9    ....GUGAAUUCGGGCG.GGUU.CAAGGUUCC.GGCC.UGUC....GGCC.GGAACGCUUGGCGCGGCAUGCGCCGCGUCCCGCCUGCGGCGGGGCCUGUCAAGGCCGGUUCAGCCCUUUGGCUUCUUCAUAGUGAGGGAAAUCCGCUUUUUCUUCUCGU
env-10   ....AUGAAUCCGCCCU.GCGG.....AAAUC..GCC.UG.C....GGC..GAUUU.....UGCGGCGCAAAGGCGCCGUCCCGCGCGGCGGGGCCCCUCUGAAGUCUGAUGGUGGCGCAAGGAGAGGAGGGGUAAGCCAUGCCUGCGAUUAACGUAAA
env-11   ....AUCAAUUCGCCCC.GCCAUCGAGGUUUU..GCC.UG.C....GGC..AAAACGCUCGGACGCUACAGGCAGCGGACGGCAUGCCGGUUAAAACAGUCCGUCGAACAGGGUAAUCUGACUCGUAUCCGGCAUGCCGUCCAGAGCACCAGCGGCCUUG
env-12   ....GUAAAUUCCGCCU.UCGG.CAAGGUUUU..GUC.CG.GGG..GAC..AAAACGCUUGGACGCGGCAGGCCGCCGCGCCCCGCUGCGCGGGGUUCCGGGUGGAUUUGCCGAAGGGAGGAGUAUGCCAUGACUGACCAAUCCAAAAUCCGCAACUUUU
env-13   ....GUGAAUUCCGUCC.GCGC.CAAGGUUUU..GCC.UGUC....GGC..AAAACGUUUGAACGCCGCAUUUGCGGCGGCGUGGAGAUCGCCUGAAUCUCCCGAACUCCAGAAAAUCGGAGGUCACUUAUGGAUUGGUAUCCCUUAUGGAACUCGCUGC
env-14   ....GUCAAUUCGCCCU.GCGC.AAAGGUUUU..GCC.UU.U....GGC..AAAAC.CUU.GUACGCGCUGCGCGCGCCCCAUCCAUACGGAUGGGUCCCAUCGCCGGCCCCUAUUUGACGGACCAGGCGGUUGGAAUAAACAGCCUGCUGAACUGCUC
env-15   ....AUGAAUUCCGCCU.GCGC.CAAGGUUU......UGCUCCCGC......AAACGCUUGUACGCGCCUGCAGGCGCGCCCCGCCGUUUGCGGGGGGCAAAAGUCUCGGCGGNCUUACCCCUUCGGCUCCGNNCACAAUGGCUCCAUCUGCUCGAUCUG
env-16   ....GUGAAUCCCGCCU.GCGG.CGAGGUUUG..GCC.UG.C....GGC..CAAACGCUCGGACGGCGCGAAGCGCCGGGACGCUGCCGCGUCCAGCGGCAGGGCAUGAGAGACCCAGGCAGAGAGGAGGGUAUACUGCCCCGAUUAUGACUUUCGAUGA
env-17   ....GUAAAUUCCACCUUGCGC.CAAGGUUUU..GGC.UUCC....ACC..AAAACGCUUGUACGGGCCAUCGGCCCGCCCCGCUCCGCGGGGCCCCGGACAACGCCCGGAGCCGCUCAGAACCCCCACUUGUAUCCGUAUCCCCACACCGUGGUGAUGU
env-18   ....AUGAAUUCCGCUU.GCGC.CAAGGUUUU..GGC.U..C....GCC..AAAAACUUGUACGCGCCUGACGGGCCACCCCGCCGUGCGGGGCCCGGAACAAUGUGCGUCAGUCUUAUCGUUUCACGCGGCCCAUUUAUAGACAUAACCCCAAAACGG
env-19   ....AUAAAUCCGCUCU.GGGC.CAAGGGUUU..GCC.UG.C....GGC..AAGCC.CUUGUGCGGCGCAGACGCCGCCCCGCUGCGCGGGGCCCACCCAAGCCGCCCCCGGGCCGCCGGCUCAAAAGCCCCACUUAUAGCCGUAUCCCCACACGGUCGU
env-20   ....AUGAAUUGCCCCU.GCGC.CAAGGUUUU..CGC.CU.CUG..GCG..AAAACGCUUGUACGGCGCUUGCCACGCCGCCCCAUCUUGCGAUGGGGUUCCGGUGGCCGACGGGUCUCAGAAGGCAGAAGCCUUUUUAGUAACUCACGCCCUGCCAGAU
env-21   ....GUGAAUUGCCCCU.GCGG.CAAGGUUUC..GC..UG.C.....GC..GAAACGCUUGGACGCCGCAGAGCGGCGGCUCGCUGCGCUCGCAGGAACAGACCGGCCGGAGCUACAGGAAGUUCUUGCCCAGAUUAAACUCGGUCUGUUCCUUGGUCUC
env-22   ....AUGAAUUGCCCCU.GCGC.CAAAGGUUU.CGCC.UC.C....GGCG.AAAACCCCUGGACGGGGCUUGCCCCUCCGCCCCAACUUGCAAUGGGGUUCCGGGGGCCAACCGGCCCAAGGAAAAGUAAACUUUUUUAAUAACGAAACCCUAGCAAAUU
env-23   ....GUGAAUUGCCCCU.GCGC.CAAGAUUUU..GC..UC.C.....GC..AAAAUACUUGUAUGGCGCAAGCGCCGCCCCGCUGCGCGGGAAACCGUGGACGCCUCUUUUACUCACAGCCGCUCCGCCGCCUGAAGGUCAGAUAUCCCUCCAGAAUCAG
env-24   ....AUGAAUUGCCCCU.GCGC.CAAGGUUUU.CGCC.UC.C....GGCG.AAAACGCUUGUACGGCGCUUGCCGCGCCGCCCCAUCUUGCGAUGGGGUUCCGGUGGCCGACGGGCCGCAGGAGAACGGAACCUUUUUAAUAGCUGACGCCCUGCCAGA
Bca-1-1  ....AUGAAUUGCCCCU.GCGG.CAAGGUUUU..GCC.UG.A....GGC..AAAAC.CUUGGACGCGCCAAAAGGCGCGGUGCCGGGCGACGCCGAAAGAAAACUUACUUCGUGCCGAAGAUGCGGUCGCCGGCGUCGCCCAGGCCGGGGACAAUGUAGC
env-25   ....UUCAAUUGCCCCU.GCGC.CAAGGUUUU..GCC.UG.C....GGC..AAAACGCUUGUGCGCGCCGCUCGGCGCGAUCGUCUCCGGCGGCCCGUUCCCCCAUCAUGUCCGCUCCCUCACAAACUGCUCGAUGGCUUCGAUCUGCCGCUCCACUGCC
env-26   ....AUAAAUUGCCUCU.GCG..CAAGGUUUU..ACU.UG.C....GGC..AAAACACUUGUGCGGCGCAUAAGCACCGCCGCCCGGAAGGCCAAUGGAAUAUCUCGAACUUAGAAUAUCAAGCGCAAAAACACCAGAGCGGCAGCAUCCCCUCGACCAG
Bca-1-2  ....AUGAAUUGCCCCU.GCGC.CAAGGUUUU..GCG....C....GGC..AAAACGCUUGACGGCGCAAAGCGCCGCCCGGCUCCGCCGGGGAGAGCGCAAGCCGCUCACUCCGUUCACACAAACAACAUUACAAAUAUGGAGGUAACAACAUGAUU
env-27   ....AUGAAUCGCCCCG.GCAU.CAAGGUUUU..GC.AGA.......GC..AAAACGCUUGGACGCGCCCAAAGGCGCGGGCCUGGGCAAGGCCCAAUCGGCGUCAGAGCAGAAUUACCCCGGCGUCGCGGCAGGCCUGGAUGGUCUGUUCAUCCCAGAC
env-28   ....AUGAAUCGCCUCG.GCAU.CAAGGUUUU..GC.AGA.......GC..AAAACGCUUGGACGCGCCCAAAGGCGCGGGCCUGGGCAAGGCCCAAUCGGCGUCAGAGCAGAAUUACCCCGGCGUCGCGGCAGGCCUGGAUGGUCUGUUCAUCCCAGAC
env-29   ....GUGAAUUGCCCCG.GCAU.CAAGGUUUU.GGCC.GA.C....GGCC.AAAACACUUGGACGGCGCACAGCGCCGCCCGCCUGCGGCGGGGAUGGGCCUGGAAAAGCCCUUAAUUCAGAAUCAGCGUCUCCCCGUCCAUCUCGGCGGGCUUUUCCAG
env-30   ....GUGAAUCCGCCCU.GCGC.CAAGGGUUU.CGCU.CU.C....GGCG.AAACC.CUUGUACGGCGCAAGCGCCGCCCCACCUUGUGGGGCCCCAGAGGCCAAAAAGAAGAUGCCGUCAUUCCUCGUCCAACUUGAGAACCGCCACCGCAGCGUUAAG
env-31   ....GUGAAUCCGCCCG.GCAU.CCAGAUUUU..GC..UC.C.....GC..AAAAUGCUUGACGCCGCUGACGCGGCGGCUCGCUUCCGCUCGCAAGAGAGGCGGAGCGGCCGGUAUUCCCCAGCCUUUAACUUCUCUAUCGCAGCCUUGGCCGCGUUCU
env-32   ....GUGGAUCCGCCCU.GCGC.CAAGGUUUU.CGCC.UA.C....GGCG.AAAACGCUUGUUCAGGCGGCGGACUGCGCCCGCCAGCUUAGGCCGGCUUACAGGCCCAGCUUCUGAGACACGAAAUCGUCCACGCAGGCCAGGGCGUUAUCCAGCUUGG
env-33   ....A..AAGCCGCCCCUUCGC.CGAGGUUUU..GCC.UG.C....GGC..AAAACGCUCGAACGCCGCAAAAGCGGCGCGGCCCAGAAGGGCCGUAGUAAACGAUUUA................................................
env-34   CGCAGUGAAUUCGCCCG.GCAU.CGAGGUUUU..GCC.UC.C....GGC..AAAACGCUCGGGGCGCGCCAUCCGGCGCGGAUGGGCGGAGCAUUCUCGCCUUAGAAAGUCUGGUGCAUCCCUAUGAAACAGAAAUUCAAUGUUACAGGCAUGACCUGCU
Bca-1-3  ....GUGAAUCCGCCCG.GCAU.CGAGGUUUU..GCC.AC.G....GGC..AAAACGCUCGGUGACGAGGCCCGCCACGACAAAAAAUCCCCGGACCUUGCGGUCCGGGGACUUUUCACUCUAUUGGCUUAGCCGAAGUAACGCUUCAGCAGGCCCUCGA
env-35   ....AUGAAUCCGCCCU.CCGG.CGAGAUUUU..GCC.UC.C....GGC..AAAAUGCUCGGACGCCGCGCCGCGGCGUUCUGGAGCAGGAUCGACACAGGCAUCAAUAUGCCAGUUUCUCCGCCAGAUAGCUCUUCAGCUGGUCGAUAGGCAGCCGCAC
env-36   ....AUGAAUCCGCCCU.GCGG.CAAGGUUUG.....CUCUG.........CAAACGCUUGGCGCGCCGGGGCGCGGCCCGCUCCCGCGGGCAAGAAAACCGGCUUGGGUCCGCCGGAGCGCGUCACUCGUCCCGUAGCCGAAGUUCUUGAGCAAUUACC
Bca-1-4  ....AUGAAUUCCGCCG.GCAU.CAAGAUUUU..GC..UC.C.....GC..AAAAUGCUUGGGACGCGCCACUAGGCGCGGUGUGGGCGGAGCCUUUUCUGAAAGUGUGGUGUUCCUUUUGAAACAGAAAUUUGAUGUUACCGGCAUGACCUGUUCGGCU
env-37   ....AUGAAUUCCGCCU.GCGG.CAAGGUUUU..GGC.UG.C....GCC..AAAACGCUUGGAGCGCGGGGCUUCCCGCGUCCCAUCUUCGAUGGGUCCCAAUGAGAGGCCUGCAGAUGGGAAGUUCGAGUCCAGAUUCAAGCAUUCAAGCGAUUGAGUG
env-38   ....AUGAAUUCCGCCU.ACGG.CAAGGUUUU..GCU.UG.C....AGC..AAAACGCUUGGGACGCGUGGGGCGCGAGCCGCUUGCGCGGCAGGAAAGCGACAAAAGGCCUGCGGUAAGGCGAUGCCGACUGCUUACUGAGGGAUCUCCUCCAACGUGA
env-39   ....AUGAAUCCGUCCG.AUUU.CAAGAUUUU..GCGCUAUG....CGC..AAAAUGCUUGUGCGCCGCACUUGCGGGCGAGCCAGCGCGGCUGUAUCCGGUUCGGAAACGGGUUUAUUCCACCGUCACGCUCUUAGCAAGGUUCCGGGGCUUAUCAAUG
env-40   ....GUGA.UCCGCCCG.GCAU.CAAGAUUUU.UGCC.GA.A....GGCA.AAAAUGCUUGGCACGCCGCACUCGCGGCGAAUUUUUUUCAGACUUCGGGUUUGACUUUCCUGUCCCUCUUUUUGGGUUCCGCCGCCUUUGGCGGCU............
env-41   ....GUGAAUCCGCCCU.UCGG.CAAGGUUUU..GCU....G....GGC..AAAACGCUUGGGACGCGGCUGCGCCGCGGGGGAAAAGCAGCAGGGGAGCCGACUCAGGAACCGGCAGCUUACCGUUCCUCGCGGAAGUAGCUCAGACCCAGGUUUGCAG
env-42   ....AUGAAUCCGCCCU.GCGG.CAAGGUUUU..GC..UG.C.....GC..AAAACGCUUGGGACGCAGCUGCGCUGCGGGGGAAAAGCAGCAGACAGACGCCGGAUGACGAAAGCCUUACCGUUCCUCUCGGAAAUAGGUCAAGCCCAGACUUGCAGGC
Bca-1-5  ....GUGAAUCCGCCCG.GCAU.CAAGGUUUU..GGC.UU.C....GCC..AAAACGCUUGGGACGCCGCACUCGCGGCGGAGCUGGACGACACGUAUUCUGGUGCCACUCUUACAGCCCCAGCUUCUCCGCCACCAGGUCGUCCACCUUGGCCAGCGCA
Bca-1-6  ....GUAAAUCCGCCCG.GCAU.CAAGGUUUU..GGC.UC.C....GCC..AAAACGCUUGGUACGGCGUAACCGCCGCCCCACCUGCGGCGGGGCCCGGUGGCCAUUUCUCAGAAAUACAGCCCGUUGUUCUCCAGCUCGUCGAUUAAAUCGCCGAGAA
env-43   ....GUGAUUCCGUCCCGGCAU.CGAGGUUUU..GGU.GG.A....GCC..AAAACGCUUGGACCUUCUGGGGGCGCGGGGGCGAUUUCGUCCCUACAUCUGGUCGCUGUCCAGCUGAAUCGUCACCGGCCCGUCGUUUUGUGUAUACACCUGCAUAUUU
env-44   ....GUGAAUCCGCCCU.GCGG.CGAGGUUUU..CGC.UCCG....GCG..AAAACGCUCGGGCGCGCCUUCCGGCGCGUUUCGGGCGACAGGCACGACGCCUUCCCGUUAUCUCGAAAAGGAGUUCUACAUGAAGUAUUACUACGAUCUCCACCUCCAU
env-45   ....GCGAAUCCGCCCU.GCGG.CGAGGUUUU..CGC.UCCG....GCG..AAAACGCUCGGGCGCGCCUUCCGGCGCGUUUCGGGCGACAGGCACGACGCCUUCCCGUUAUCUCGAAAAGGAGUUCUACAUGAAGUAUUACUACGAUCUCCACCUCCAU
env-46   ....GUGAAUCCGCCCCGGCAU.CGAGGUUUU..GGC.GG.A....GCC..AAAACGCUCGGACGCGCCGGGGGCGCGGGGGCGAGUUCGGCCCUACAUCUGGUCGGUGUCCAGCAGAAUCGUCACCGGCCCGUCGUUCUGCGAAUACACCUGCAUAUCC
env-47   ....GUGAAUCCGCCCCGGCAU.CGAGGUUUU..GGC.UC.C....GCC..AAAACGCUCGGACGCGCCGGGGGCGCGGGGGCGAGUUCGGCCCUACAUCUGGUCGGUGUCCAGCAGAAUGGUCACCGGUCCGUCGUUCUGCGAGUACACCUGCAUGUCU
env-48   ....GUGAAUCCGCCCCGGCAU.CGAGGUUUU..GGC.UC.C....GCC..AAAACGCUCGGACGCGCCGGGGGCGCGGGGGCGAGUUCGGCCCUACAUCUGGUCGGUGUCCAGCAGAAUGGUCACCGGUCCGUCGUUCUGCGAGUACACCUGCAUGUCU
env-49   ....GUGAAUCCGCCCU.GCGG.CGAGGUUUU..CGCCUC.C....GCG..AAAACCCUCGGCGCGCCUUGUGGCGCGCUUCGGGCGAUAAGCACGAUGCCGUUUUGUUAUCCUGAAAAGGAGUUUUACAUGAAGUAUUACUACGAUCUCCACCUUCACU
env-50   ....AUGAAUUCGGCCC.AUUU.CAAGGUUUU..GC..UC.C.....GC..AAAACGCUUGUACGCCGCAUUUGCGGCGGCGAACUGACCUGCUUACAGCAGCCGCAGGCUUCGCUCGAUCUCCUUGCCGUUUUCCAUAUACAAUCUGGGGACCCGCUUG
env-51   ....AUGAAUCCGCCCC.AAUC.CAAGGUUUU..GCC.UC.C....GGC..AAAACGCUUGUACGCGCCAUGCGGCGCGGCCCGCCGGAGGCGGGCGCAGGAGAACGCGCAGGCAUGGAAAAGCGCCGGACACUCAGUGUCCGGCGCUCGAAGCCAAUCU
env-52   ....CUGAAUUCGCCCG.GCAU.CAAGGUUUU..GCC.UC.C....GGC..AAAACGCUUGUACGCGCCACCCGGCGCGGGCGGCUCAUCCACUCGCUAAACGAGGUCUGCUUAUGAAUUCACUGCGCAUCAUAGACGCCUGCACGGAGGAACACUUUGC
env-53   ....CUAAAUUCGCCCG.GCAU.CAAGGUUUU..GCC.CG.CAG..GGC..AAAACGCUUGUACGCGCGAUGCGCGCCCCGUGGAGGCGUCCGUAUUUCACCGCAGAUCAAUGCCGCUGGCCUUCUUCUCCAGCAUGGUCUUGAUCAGCUCCGCGAUAUG

         .........>>>>>>>.......<<<<<<<<<..<<<.........>>>..>>>>>.>>>>...................................................................................................
         .........2222221.......122222222..222.........222..22222.2221...................................................................................................
         ----RUGAAUYY•YCC•-RCR•-CAAGGUUUU--GCY-Y•-Y----GGC--AAAACGCUUG•R•••••••••••••••••••••••••••••••••••••••••••••••••••••••••••••••••••••••••••••••••••••••••••••••••
         ------------------------------------------------------------------3′
```

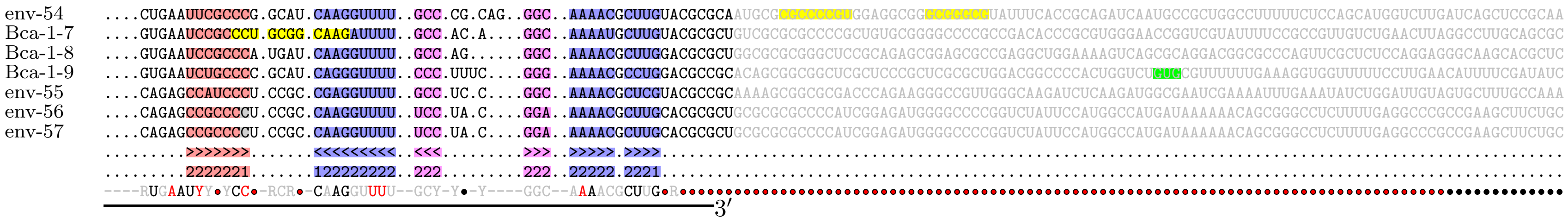

alignment positions 321 · · · 480

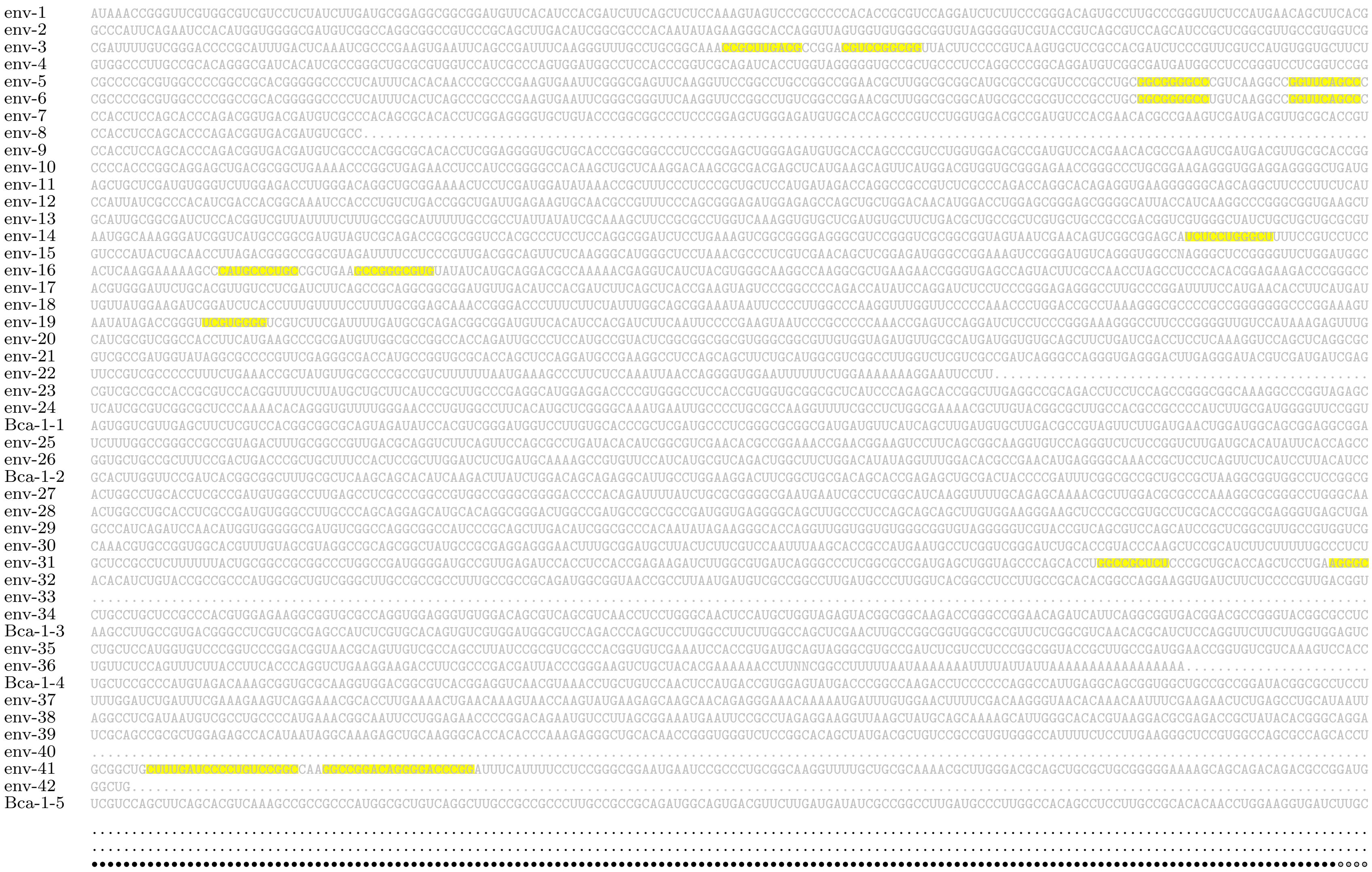

```
Bca-1-6   UAUCCACGUUGUAGGGCGUGAUGAUGUAGGUGGAGAUGGCCACCUUCUUGAUUUUGCCCUCGUUGGCGUAGGCCACGCCGGACAGGAAGUCCAGCAGGCGGCGGGCGAUAUCCUUGUUGGUCUGCUCCAGAUUGAGCACCACGGUGCGCUUCUCACGCAG
env-43    CCCCCGAAGGCCCCACGGGGUUCCCAUCUUGCGGGGACCCCGGUUCCCUUUAUCUUCCGUGGCGGCCUGAAUCUGCCCCGGCAUCGGGGUUUCGGCUGGGCCGGAGCCCUUUGGCGUGUUGGGGGCGGGUGGGCGAGUUUGUUCACAGAUCUACUGGCCA
env-44    UCCUGCCUGUCGCCCUGCGGCAGUGAGGACAUGACCCCCGUCAACCUGGCGGCCAUGUGCGCGCUGGCGGGGCUGGAUAUUGUGGCCCUUACCGACCACAACACCUGCGGCAACUGCGCCGCCUUCUGCCGGGCGGCGGAGGAGCGGGGCCUGCUGGCUC
env-45    UCCUGCCUGUCGCCCUGCGGCAGUGAGGACAUGACCCCCGCCAACCUGGCGGCCAUGUGCGCGCUGGCGGGGCUGGAUAUUGUGGCCCUUACCGACCACAACACCUGCGGCAACUGCGCCGCCUUCUGCCGGGCGGCGGAGGAGCGGGACCUGCUGGCUC
env-46    GCCCCGAACGCCCCACGGGGCUCCCACCCCGCGGGGACCCCGGUUCCCUUUAUCUGCCGGGGCGGAGUGAAUCCGCCCCGGCAUCGAGGUUUUGGCUCCGCCAAAACGCUCGGACGCGCCGGGGGCGCGGGGGCGAGUUCGGCCCUACAUCUGGUCGGUG
env-47    GCGCCGAACUCGCCGUGCUCCACCCGGAAACCCCGCCCGGCGCACUCGGCCAUAAAUUUCUCAUAGAGGGGGAUGGCCACCGGCGGCUUGGCGGCCCCGGUGAAGCCGGGGCGGCGGGACUUGGUAUCGGCAUAGAGGGUGAACUGGCUCACCACC....
env-48    GCGCCGAACUCGCCGUGCUCCACCCGGAAACCCCGCCCGGCGCACUCGGCCAUAAAUUUCUCAUAGAGGGGGAUGGCCACCGGCGGCUUGGCGGCCCCGGUGAAGCCGGGGCGGCGGGACUUGGUAUCGGCAUAGAGGGUGAACUGGCUCACCACCAGCA
env-49    CCUGCCUGUCACCCCACCCGGCUCAGCCGGGCGGGGGCCCCUUUUUUCUCCCUCGGGCGGAGUGAAUCCGCCCUGCGGCCGAGGUUUUCGCUCCGGCGAAAACGCUCGGGCGCGCCUUCCGGCGCGUUUCGGGCGACAGGCACGACGCCUUCCCGUUAUCU
env-50    CUCACCGCGCAGGUCAAUUCGUAGGGGAUCGUGUUCAGAAUUGCGGCCAGAGAAUCUACGCUCUGGGUCUGGCCGAAGAUCUCCACCGUAUCCCCCACCUUUACCUCCGGCAGGUCCGUCAGGUCCACCAUGCACAUAUCCAUGCAGAUGCGGCCCCGCU
env-51    GCGGUUUACUUGCCCAGCUGCUUAGCGAUCUCUUCGGCGAAGUUCUCCUGCUUCUUCUCGAUGCCCUCGCCCUUCUCAAAGCGGACGGCGUUCUUGAAGGUAAUGGUUCCGCCCAGAGUCUUGGCAACGGAGGCGAUAUACUGCUCCACGGUCAUGGCGU
env-52    GGCCAUGAGCCGCAUUCACGCCCGGGGCUGGCACGCCGCUUACCCCGGCCACGUGCCCGACGACUAUCUGAGGGACGUGAUCACCGAAGAUCACUGGAUUCCCUUUUUCCGGGAGGACUACGCCACCGGGCGGUGCCGGGGGCUGCUGCUCUAUGACGGG
env-53    CGCGCCCGCCGUUCCCCGUGCGGCUGGCCGCACGGGGCCCCCAAGCGUCUCUUUCAUCUGCUCGGGCGAACUGAAUUCGCCCGGCAUCAAGGUUUUGCCCGCAGGGCAAAACGCUUGUACGCGCAAUGCGCGCCCCGUGGAGGCGGGCGGGCGUAUUUCA
env-54    UGUGCGCGCCCGCCUCCACCGCGGUGGUACCCUGGCGGUGGAUGUUGGAGAUCACCGUGCGCUUGGACUCAGGUAUGCCGAUGUGGGGCUUGUAGGUCAGGUAGGCUGACAUGGACUCCGCCGUAACCAGGCCAGGCCGCUCUCCCACCAGCAGGCACAC
Bca-1-7   UCCAGAUACUCGCCGGUGCGGGUGUCGAUCUUGAUGCGGUCGCCGGGGUUGAUGAACAGGGGCACCUUGAUCUCCGUGCCGGUCUCCAGGGUGGCGGGCUUGGUCACGUUGGUGGCGGUGUCGCCGCGGAAGCCGGGAUCGGUCUCGGUGACCUCCAGCU
Bca-1-8   UUGACAUCCUCAUCAGCCUCGGCGGCGGUGAGGGAGCGGUCGUCGGCCCGGAGGACCAGGUUGAAGGCCACGCUCUUCUUGCCGGCGGGGAUGGGAGCGCCGGUGUAGAUGUCGAAGAGCUCGACCUCCUUCAGCAGGCCCUUGGCGCCCUUGAAGAUGC
Bca-1-9   AUGGGCCCCGUCAUGGUGGGGCCGUCCAGCUCCCACACGGCGGGAGCGGUGCGCAUCGGCCUCAUCACCCGCAAGCUGCUGGGCCGUCCCCCCGUACGGGCCGAACUGCUGCUCCACGGCUCCUUUGCCGCCACUGGCAAGGGCCACGGCACCGACCGUG
env-55    AAGAAAGACACGCUAAUAGCGCGUCUUUCUUUUUGGGCUUCGCGGGCCGAGGGCCCGCUUCGCCCUUCGGGAUCAAAAUGCUCAGGGCGGCAAAGCCGCCCCUCCGCCGAGGUUUUGCCUCCGGCAAAACGCUCGUACGGCGCAUUAGCGCCGCCGACGA
env-56    GGAGAUCCGGCCCUACUCCGCCAGGCGACGAUUCACCCGGAGUCUGCUACUACGC.........................................................................................................
env-57    GGAGAUCCGGCCCUUACUCCGCCAGGGCGGCAGUGAUGAUGGCCAUGGAAUAGACCCGCAAAAUACAUAUUUUGCGGGAACCCAGUUUUUUUAGAUUGCUCAGGGCGGCAGAGCCGCCCCUCCGCCAAGGUUUUUCCUACGGAAAAACGCUUGCACGCGC
          ................................................................................................................................................................
          ................................................................................................................................................................
          ●●●●●●●●●●●●●●●●●●●●●●●●●●●●●●●●●●●●●●●●●●●●●●●●●●●●●●●●●●●●●●●●●●●●●●●●●●●●●●●●●●●●●●●●●●●●●●●●●●●●●●●●●●●●●●●●●●●●●●●●●●●●●●●●●●●●●●●●●●●●●●●●●●●●●○○○○
```

alignment positions 481 · · · 529

```
env-1     AUGCCGUACUCCACCUGAGUCAGCUUCACCCGCUGUCCGUUUUUCUCCA
env-2     GCGGUAAUGAGCGACACGCCGCCCAUCCGGGAGGUGGCCUCCACCCACC
env-3     CGUGGUUCACUUCCUGGUAGGUCUCGUGGCCCUUGCCGCACAGGGCGAU
env-4     GGGUUGUCGGAGGUCACCACCACAAAGUCCGCCAGCUCCGCCGCCACGC
env-5     UUUGGCUUCUUCAUGGUGAGGGAGAUCCGCUUCUUCUUUUCGUCCACCU
env-6     UUUGGCUUCUUCAUAGUGAGGGAAAUCCGCUUUUUCUUCUCGUCCACCU
env-7     GCCGGUGAGCUCCAUGCCGGGCUUCAAAUCCUUCAUCUCCAGCACGUCG
env-8     .................................................
env-9     GCCGGUGAGCUCCCUGCCGGGCUUCAAAUCCUUCAUCUCCAGCACGUCG
env-10    CGCGCCCACGGCUCCUUCACCGUGGCCUCCGCCCUCAUGUCCACCGAGA
env-11    UGUCUGGCAGGAAGUUGACCGCGUGAGAGCGGUAGAGAUCCAUGUGCUC
env-12    CAACUAUCAGGCUGCCGACGGGGAGACCUAUGAGCUGAACCUGAUCGAU
env-13    GUUCGGCCCCAAGCGCGUCAUCGGCGCGUGGGUGCUCGAAACGUUCGGC
env-14    CCCUUGGCCAGGGGGUUGGUAUAGACGCUUUCAAACAUGAUUCCCGCAG
env-15    AAAAAUGAACAACCGGCCCAAGAUCAAUAAAGCUUCCCGAAAGGCAUCC
env-16    AUGGUGAGCCAGAUUGACCACUUCGACGUGAUUGUGGCCGACUCGGAGU
env-17    GGAAUACUCCACCUGGGUCAGCUUGAUUCGCUGGCCGUUCUUCUCCAGC
env-18    GGCCCCCC.........................................
env-19    AUGAUCGAGUAUUCCACCUGGGUAAGCUUGACCCGCUGACCGCUUUUCU
env-20    AUGGAGUUCUGGCUCAUCUCCAGGCCGGAGGUGGCCACGCCGCCGGCGU
env-21    GCCCGGAAGAUGUUGGCGAUCUGGAUGAUCUCGUUGCGGAUGUCGGGGG
env-22    .................................................
env-23    UCGGCCCGGGGGCCUUCAGUGCCGUCCAUAUUCCGGGGGAAGCCCAUUA
env-24    GGCCGACGGGUCUCAGAAGGCAGAAGCCUUUUUAGUAACUCACGCCCUG
Bca-1-1   GCCGCCGGUGGCCAGCAUGGGAUCCAGAAUGAUGACGUCACGCUCGGAG
env-25    GCCCCACGAUCAUAUACGCCUCCCGGAAGGGCAUACCCUUUUUGACCAG
env-26    GAGGGACG.........................................
Bca-1-2   AGUGCGAAAAGGGCAUUGUGGUGUGCACCACCGGCAUCGGCAUCUCCAU
env-27    GGCCCAAUCGGCGUCAGAGCAGAAUUACCCCGGCGUCGCGGCAGGCCUG
env-28    UGGUCCAGGGCGGCGGCGUCCACCCGGAUGCCCAUGGAGGACAGCUCAG
env-29    GCGGUAAUGAGCGACACGCCGCCCAUCCGGGAGGUGGCCUCCACCACCU
env-30    UUCUGCUUCUCCAGCAGCUUCUUUUUCCGGGUGAUGUCGCCGCCGUAGC
env-31    GGUCUUGUAAUCGCGGCUGGCGCUCUUCUCCGCCUCCCGGUCCAGGAUG
env-32    GGCCAGAACGCCCACGCCGUUGGGGGCCUUGUCCCGGAGCAGGUCGCCC
env-33    .................................................
env-34    CCUCCCCGGCCCCGCCGGGAAGGCCGCCCCGGCCGCCCGGCCCGCCGAC

          .................................................
          .................................................
          ○○○○○○○○○○○○○○○○○○○○○○○○○○○○○○○○○○○○○○○○○○○○○○○○○
```

```
Bca-1-3   GGUCACGACCUCGCCCAGCAGCUCGGCAAACUUGGCAGCGUGCUCGGCC
env-35    AUCCAGUCCUUGCUGAGCUCCGCCUGGAUCUCACGGGCCUUUUCGGAGA
env-36    .................................................
Bca-1-4   GUCCCCUGCCCGCCGGGAAACAGUCCCCCGCUGUGCAGCAGGGGCCCUC
env-37    CAAACGCUUGAGAACCAGAGCGAGACCCUUCUUUGUAGAGCGGAGAGUA
env-38    AGGCGACAAAAAGAGCCUUGAGGUAAAGCAAUACCGGACUGCUUACUUG
env-39    CGGCUCCACGGGCCUUUACCUCCACCACGUUGCUCAUGGCCUUGUCAAA
env-40    .................................................
env-41    ACGAAAGCCUUACCGUUCCUCUCGGAAAUAGGUCAAGCCCAGACUUGCA
env-42    .................................................
Bca-1-5   CGUCGUUGACGGCGGACAGCACCGCCACCACGUUGGGCUGCUUGUCCCG
Bca-1-6   GUGGUCGGCAAUCUCGGCGGCGUUCUCGAAGCGGUCGGGCUUCACCAGC
env-43    UUGAGCAUUAUGGUGACCGCUGAGUAGUUGUAGGACUGCACGUGCCCCU
env-44    UGGCGGGGAUGGAGCUGUGCACCCGGGAGGAGAUCCACGUAGUCUGUCU
env-45    UGGCGGGGAUGGAGCUGUGCACCCGGGAGGAGAUCCACGUAGUCUGUCU
env-46    UCCAGCAGAAUGGUCACCGGUCCGUCGUUCUGCGAGUACACCUGCAUGU
env-47    .................................................
env-48    GCGCGCCCCCCACCGCCUCCAGGUUCAGGUUCAUCUUCUCGUUCUCGUC
env-49    CGAAAAGGAGUUCUACAUGAAGUAUUACUACGAUCUCCACCUCCAUUCC
env-50    GCGGUGCCGGGCCAUAGGCCGUCUGCACCGCCAUGCGGUUGGAAAGCCC
env-51    UAUCCUUCACGAACUCCUGCUGCAGCAGGCAGUUCUCCUUGUAGAACUU
env-52    GAGAACCCCGUCUGCUGCUGCACCUGCGGGCCGGUGCGGCUGGGGCCCA
env-53    CCGCAGAUCAAUGCCGCUGGCCUUUUUCUCCAGCAUGGUCUUGAUCAGC
env-54    CGCCUCCGCGCCGGUCAGCUCCCCGAUGUGGUCGGAGGCGCCCACCCGG
Bca-1-7   CGACGAACGUGGGGGGCUCCACGGAGAAGAUCUUGCCCUUGUAGGAGUU
Bca-1-8   ACUCCUCAAGCUGGGCCACGGUGACGGCGGCGUCGCACACCACAGCGAU
Bca-1-9   CCCUGGUGGCCGGUCUGCUGGGCAUGGCCCCCGAUGACCCGGACAUUCC
env-55    AUCACCCGGGAAGAUCUGCUACGACGC......................
env-56    .................................................
env-57    UGCGCGCGCCCCAUCGGAGAUGGGGCCCCGGUCUAUUCCAUGGCCAUGA
          .................................................
          .................................................
          ooooooooooooooooooooooooooooooooooooooooooooooooo
```

15 Burkholderiales-1

15.1 Taxa

The taxonomy of each organism containing a putative Burkholderiales-1 RNA is listed, with abbreviations identifying each hit (e.g., "Eco-1-1" and "Eco-1-2" might hypothetically represent two distinct RNAs in *E. coli*). The abbreviations will be used to identify each individual Burkholderiales-1 RNA in Sections 15.2 and 15.4.

**abbrev. of hits**	**taxonomy of species**
Rme-1-1	Bacteria Proteobacteria Betaproteobacteria Burkholderiales Burkholderiaceae Cupriavidus *Ralstonia metallidurans* CH34
Aav-1-1 to Aav-1-24	Bacteria Proteobacteria Betaproteobacteria Burkholderiales Comamonadaceae *Acidovorax avenae subsp. citrulli* AAC00-1
Asp-1-1 to Asp-1-2	Bacteria Proteobacteria Betaproteobacteria Burkholderiales Comamonadaceae *Acidovorax sp.* JS42
Cte-1-1 to Cte-1-2	Bacteria Proteobacteria Betaproteobacteria Burkholderiales Comamonadaceae *Comamonas testosteroni* KF-1
Psp-1-1 to Psp-1-33	Bacteria Proteobacteria Betaproteobacteria Burkholderiales Comamonadaceae *Polaromonas sp.* JS666
Rfe-1-1	Bacteria Proteobacteria Betaproteobacteria Burkholderiales Comamonadaceae *Rhodoferax ferrireducens* T118
Mpe-1-1 to Mpe-1-7	Bacteria Proteobacteria Betaproteobacteria Burkholderiales *Methylibium petroleiphilum* PM1
env-1 to env-18	environmental samples

15.2 Gene contexts

Each Burkholderiales-1 RNA (indicated by "RNA→") is listed with its surrounding genes. Environmental sequences and some RefSeq entries lack gene annotations, and no genes are listed for such sequences. The direction of each gene is indicated with an arrow (→), and each conserved domain in the gene is colored. Conserved domains associated with more than one Burkholderiales-1 RNA are assigned a color; other domains are gray. Information about these conserved domains is given in Section 15.3. The accession number of the sequence containing each Burkholderiales-1 RNA is given. Accessions beginning with "NC_" or "NZ_" are RefSeq, while other accession refer to environmental samples. Nucleotide coordinates are given for the 5′ and 3′ boundaries of each. If the 5′ coordinate is greater than the 3′ coordinate, the RNA is present on the reverse-complement strand of the containing genomic DNA sequence. Each hit is denoted by an abbreviation (like "Eco-1-1") that refers to a taxonomy given in Section 15.1.

abbrev.	Seq. accession		5′ at	3′ at	genes
env-1	JCVI_SCAF_1096628042959	+	1204	1263	←GD_AH_N (pfam04292)? (GOS3923) **RNA→** ←? (GOS381)
Rme-1-1	NC_007973.1	+	1410864	1410915	←VapI (COG3093) **RNA→** ←HyaC (COG1969)
Aav-1-1	NC_008752.1	+	2544313	2544364	←COG2908 (COG2908) **RNA→** ←cyclophilin_EcCYP_like (cd01920)
Aav-1-2	NC_008752.1	-	3804316	3804265	MA (smart00283)→ **RNA→** ←TPR (cd00189)COG4976 (COG4976)
Psp-1-1	NC_007948.1	+	2957718	2957769	DppC (COG1173)→ **RNA→** DppD (COG0444)→
env-2	JCVI_SCAF_1096627195847	+	73	124	**RNA→** ←Bug (pfam03401)? (GOS6036)
Mpe-1-1	NC_008825.1	-	604396	604345	←FliM (COG1868) **RNA→** ←FliL (COG1580)
Aav-1-3	NC_008752.1	-	368521	368470	←TufB (COG0050) **RNA→** ←FusA (COG0480)
env-3	JCVI_SCAF_1096627691218	+	96	147	**RNA→** ←PaaB (pfam06243)? (GOS13617)
Psp-1-2	NC_007948.1	-	2342985	2342933	←PhoU (COG0704) **RNA→** ←PstB (COG1117)
Psp-1-3	NC_007948.1	+	609731	609783	←LivH (COG0559) **RNA→** ←LivG (COG0411)
Psp-1-4	NC_007948.1	+	4969255	4969308	←SucD (COG0074) **RNA→** ←SucC (COG0045)
Aav-1-4	NC_008752.1	-	1318518	1318468	←RecO (COG1381) **RNA→** ←Era (COG1159)
env-4	JCVI_SCAF_1096626880474	-	686	635	←CcmA (COG4133)? (GOS1277) **RNA→**
env-5	sludgePhrap_Contig9288	+	699	750	←FcbC (COG0824) **RNA→** ←Trunc_globin (cd00454)
Aav-1-5	NC_008752.1	-	716470	716399	←NarG (COG5013) **RNA→** ←NarK (COG2223)
Psp-1-5	NC_007948.1	-	4983586	4983535	←PotA (COG3842) **RNA→** ←AfuA (COG1840)
Psp-1-6	NC_007948.1	+	260351	260403	←BaeS (COG0642) **RNA→** ←OmpR (COG0745)
Asp-1-1	NC_008782.1	-	137440	137389	←AcetylCoA_hydro (pfam02550) **RNA→** ←CaiD (COG1024)
Rfe-1-1	NC_007908.1	-	2308753	2308702	←COG4239 (COG4239) **RNA→** ←COG4239 (COG4239)
Psp-1-7	NC_007948.1	-	4082160	4082108	←PotC (COG1177) **RNA→** ←PotB (COG1176)
Psp-1-8	NC_007948.1	+	4172154	4172206	rpsA (cd03322)→ **RNA→** ←TetR_N (pfam00440)
Psp-1-9	NC_007948.1	-	3565843	3565791	←PDC_hydrolase (cd01311) **RNA→** ←Bug (pfam03401)
Aav-1-6	NC_008752.1	+	5064250	5064302	←ProA (COG0014) **RNA→** ←HolA (COG1466)
Aav-1-7	NC_008752.1	+	3868943	3869014	←LivH (COG0559) **RNA→** ←LivK (COG0683)
Aav-1-8	NC_008752.1	+	1006441	1006492	←PulJ (COG4795) **RNA→** ←GSPII_IJ (pfam02501)
Aav-1-9	NC_008752.1	-	293325	293273	←hypo **RNA→** ←Sms (COG1066)
env-6	JCVI_SCAF_1096627900356	+	1222	1274	←? (GOS4375) **RNA→** ←? (GOS482)
Psp-1-10	NC_007948.1	-	1868152	1868101	←putative transmembrane protein **RNA→** ←ERG3 (COG3000)
Psp-1-11	NC_007948.1	+	4685970	4686021	←Sun (COG0144) **RNA→** ←HcaD (COG0446)
Psp-1-12	NC_007948.1	-	2218449	2218398	Bug (pfam03401)→ **RNA→** fumarate reductase/succinate dehydrogenase flavoprotein-like→
Psp-1-13	NC_007948.1	-	3918470	3918418	P4Hc (smart00702)NOG10085 (NOG10085)→ **RNA→** MhpC (COG0596)→
Psp-1-14	NC_007948.1	-	3119380	3119329	←CaiD (COG1024) **RNA→** ←FadB (COG1250)3HCDH (pfam00725)
Psp-1-15	NC_007948.1	-	1086185	1086134	←DUF484 (pfam04340) **RNA→** **RNA→** ←DapF (COG0253)
Psp-1-16	NC_007948.1	-	1086353	1086302	(shown above)
Psp-1-17	NC_007948.1	+	4740918	4740970	←RpoN (COG1508) **RNA→** ←YhbG (COG1137)
Psp-1-18	NC_007948.1	+	4909353	4909403	COG3118 (COG3118)→ **RNA→** ←SAICAR_synt_Sc (cd01414)
Aav-1-10	NC_008752.1	+	305651	305728	←BaeS (COG0642) **RNA→** ←OmpR (COG0745)
Psp-1-19	NC_007948.1	-	2444860	2444811	OYE_like_FMN (cd02933)→ **RNA→** ←Sbp (COG1613)
Psp-1-20	NC_007948.1	-	3818209	3818158	←TrpF (COG0135) **RNA→** ←PseudoU_synth_EcTruA (cd02570)
Psp-1-21	NC_007948.1	-	4510367	4510315	←SpeE (COG0421)NOG08837 (NOG08837) **RNA→** ←Pcm (COG2518)

Psp-1-22	NC_007948.1	-	299862	299811	←Bug (pfam03401) **RNA**→ ←AraD (COG0235)
Cte-1-1	NZ_AAUJ01000010.1	-	158321	158270	←TauB (COG1116) **RNA**→ ←TauC (COG0600)
env-7	soil_XZS57751_b1	+	47	98	**RNA**→ ←DUF299 (pfam03618)
Psp-1-23	NC_007948.1	+	2754283	2754334	←Lpd (COG1249) **RNA**→ ←AceF (COG0508)
env-8	JCVI_SCAF_1096627888312	+	254	305	←? (GOS310) **RNA**→ ? (GOS11414712)→
env-9	soil_XZS84935_y1	-	408	358	←hypo **RNA**→ ←ABC-type amino acid transport/signal transduction systems, periplasmic component/domain
Psp-1-24	NC_007948.1	+	2502627	2502678	←COG0436 (COG0436) **RNA**→ ←HTH_1 (pfam00126)LysR_substrate (pfam03466)
Psp-1-25	NC_007948.1	+	3896820	3896871	←Glyoxalase (pfam00903) **RNA**→ ←PutA (COG1012)
env-10	JCVI_SCAF_1101667310215	+	123	174	**RNA**→ ←OmpA (COG2885)? (GOS5249)
Psp-1-26	NC_007948.1	-	4175473	4175422	ArgB (COG0548)→ **RNA**→ OmpR (COG0745)→
env-11	soil_XZS39501_b1	-	355	304	←CoxS (COG2080) **RNA**→ ←Coenzyme F390 synthetase(EC:6.2.1.30)
Psp-1-27	NC_007948.1	-	715115	715064	←hypo **RNA**→ ←hypo
env-12	soil_Contig12405	+	553	604	←CPSase_L_chain (pfam00289) **RNA**→ ←hypo
env-13	soil_Contig7686	+	65	116	**RNA**→ ←8 Dehydrogenases with different specificities (related to short-chain alcohol dehydrogenases)(EC:1.1.1.100)
Aav-1-11	NC_008752.1	+	445572	445649	COG3556 (COG3556)→ **RNA**→ ←PsiF repeat protein
Asp-1-2	NC_008782.1	+	4080009	4080061	RhtB (COG1280)→ **RNA**→ protein of unknown function DUF6, transmembrane→
Aav-1-12	NC_008752.1	-	1020613	1020537	←DppD (COG0444) **RNA**→ ←DppC (COG1173)
Psp-1-28	NC_007948.1	+	754991	755043	←5-nucleotidase (pfam06189)NOG04570 (NOG04570) **RNA**→ ←GpmB (COG0406)
Aav-1-13	NC_008752.1	+	200376	200453	←COG3415 (COG3415)rve (pfam00665) **RNA**→ ←COG3181 (COG3181)
Aav-1-14	NC_008752.1	+	4536891	4536963	←TesB (COG1946) **RNA**→ ←thiolase (cd00751)
Aav-1-15	NC_008752.1	+	3771060	3771132	COG0354 (COG0354)→ **RNA**→ ←FcbC (COG0824)ElaA (COG2153)
Aav-1-16	NC_008752.1	+	839913	839990	←PaaK (COG1541) **RNA**→ ←LivF (COG0410)
Aav-1-17	NC_008752.1	+	3303992	3304069	←Chorismate_synt (pfam01264) **RNA**→ ←COG0517 (COG0517)
Psp-1-29	NC_007948.1	-	1380999	1380948	←HemN (COG0635) **RNA**→ ←COG0127 (COG0127)
env-14	JCVI_SCAF_1101667585797	+	31	79	? (GOS6292)→ **RNA**→ ? (GOS6292)→
Aav-1-18	NC_008752.1	-	136019	135968	←MinC_C (pfam03775)MinC_N (pfam05209) **RNA**→ ←PssA (COG1183)
Aav-1-19	NC_008752.1	-	4739072	4739021	DUF11 (pfam01345)→ **RNA**→ ←OmpR (COG0745)
Aav-1-20	NC_008752.1	+	289331	289407	IclR (COG1414)→ **RNA**→ ←Gst (COG0625)
Aav-1-21	NC_008752.1	+	3596677	3596754	←COG4174 (COG4174) **RNA**→ ←OppA (COG4166)
Aav-1-22	NC_008752.1	-	2551526	2551449	←PP-ATPase (cd01992) **RNA**→ ←AccA (COG0825)
Aav-1-23	NC_008752.1	+	1025874	1025950	←RpiR (COG1737) **RNA**→ ←G6PD_N (pfam00479)G6PD_C (pfam02781)
Aav-1-24	NC_008752.1	-	4952147	4952098	←COG2872 (COG2872) **RNA**→ ←Qor (COG0604)
Psp-1-30	NC_007948.1	-	3676473	3676421	←HisM (COG0765) **RNA**→ **RNA**→ **RNA**→ ←PBPb (smart00062)
Psp-1-31	NC_007948.1	-	3676570	3676518	(shown above)
Psp-1-32	NC_007948.1	-	3676667	3676615	(shown above)
Mpe-1-2	NC_008825.1	+	3690150	3690202	tRNA-Leu→ **RNA**→ tRNA-Leu→
Mpe-1-3	NC_008825.1	-	3496095	3496043	←COG1189 (COG1189) **RNA**→ ←AdoHcyase (cd00401)
Mpe-1-4	NC_008825.1	-	2782748	2782697	←COG2232 (COG2232) **RNA**→ ←methylene tetrahydromethanopterin dehydrogenase/methylenetetrahydrofolate dehydrogenas [illegible]
Mpe-1-5	NC_008825.1	-	876440	876389	←PRA-CH (pfam01502) **RNA**→ ←HisF (COG0107)
Mpe-1-6	NC_008825.1	-	509303	509252	←LpxC (pfam03331) **RNA**→ ←FtsZ_type1 (cd02201)
env-15	JCVI_SCAF_1101668241578	-	314	263	←Hmp (COG1018)NapF (COG1145)? (GOS259) **RNA**→ ←? (GOS398)
Psp-1-33	NC_007948.1	-	2521468	2521417	←COG4239 (COG4239) **RNA**→ ←COG4239 (COG4239)
Mpe-1-7	NC_008825.1	-	1032969	1032918	←D-glucarate_dehydratase (cd03323) **RNA**→ ←Bug (pfam03401)
Cte-1-2	NZ_AAUJ01000017.1	-	1110	1059	←CaiC (COG0318) **RNA**→ ←Bug (pfam03401)
env-16	soil_XZS33563_y1	+	529	580	←hypo **RNA**→ ←ABC-type sugar transport system, periplasmic component
env-17	JCVI_SCAF_1096627140474	+	981	1032	←HisKA (cd00082)? (GOS336) **RNA**→ ←OmpR (COG0745)? (GOS4556)
env-18	JCVI_SCAF_1096628011002	+	1578	1631	←WcaG (COG0451)? (GOS4294) **RNA**→

15.3 Conserved domains

Conserved domains found in protein-coding genes listed in Section 15.2 are listed, with the first sentence in their description from the Conserved Domain Database. Conserved domains associated with more than one Burkholderiales-1 RNA are assigned a color, while others are shown in gray. The number in parentheses is the number of occurrences of the given gene.

u cd00082 (1) His Kinase A (dimerization/phosphoacceptor) domain; Histidine Kinase A dimers are formed through parallel association of 2 domains creating 4-helix bundles; usually these domains contain a conserved His residue and are activated via trans-autophosphorylation by the catalytic domain of the histidine kinase, they subsequently transfer the phosphoryl group to the Asp acceptor residue of a response regulator protein.
d cd00189 (1) Tetratricopeptide repeat domain; typically contains 34 amino acids [WLF]-X(2)-[LIM]-[GAS]-X(2)-[YLF]-X(8)-[ASE]-X(3)-[FYL]-X(2)-[ASL]-X(4)-[PKE]
d cd00401 (1) S-adenosyl-L-homocysteine hydrolase (AdoHycase) catalyzes the hydrolysis of S-adenosyl-L-homocysteine (AdoHyc) to form adenosine (Ado) and homocysteine (Hcy).
d cd00454 (1) Truncated hemoglobins (trHbs) are a family of oxygen-binding heme proteins found in cyanobacteria, eubacteria, unicellular eukaryotes, and plants.
d cd00751 (1) Thiolase are ubiquitous enzymes that catalyze the reversible thiolytic cleavage of 3-ketoacyl-CoA into acyl-CoA and acetyl-CoA, a 2-step reaction involving a covalent intermediate formed with a catalytic cysteine.
u cd01311 (1) 2-pyrone-4,6-dicarboxylic acid (PDC) hydrolase hydrolyzes PDC to yield 4-oxalomesaconic acid (OMA) or its tautomer, 4-carboxy-2-hydroxymuconic acid (CHM).
d cd01414 (1) Eukaryotic, prokaryotic and archaeal group of SAICAR synthetases represented by the Saccharomyces cerevisiae (Sc) SAICAR synthetase.
d cd01920 (1) cyclophilin_EcCYP_like: cyclophilin-type A-like peptidylprolyl cis- trans isomerase (PPIase) domain similar to the cytosolic E.
u cd01992 (1) N-terminal domain of predicted ATPase of the PP-loop faimly implicated in cell cycle control [Cell division and chromosome partitioning]
d cd02201 (1) FtsZ is a GTPase that is similar to the eukaryotic tubulins and is essential for cell division in prokaryotes.
d cd02570 (1) PseudoU_synth_EcTruA: Pseudouridine synthase, Escherichia coli TruA like.
u cd02933 (1) Old yellow enzyme (OYE)-like FMN binding domain.
u cd03322 (1) The starvation sensing protein RpsA from E.
u cd03323 (1) D-Glucarate dehydratase (GlucD) catalyzes the dehydration of both D-glucarate and L-idarate to form 5-keto-4-deoxy-D-glucarate (5-KDG) , the initial reaction of the catabolic pathway for (D)-glucarate.
u COG0014 (1) Gamma-glutamyl phosphate reductase [Amino acid transport and metabolism]
d COG0045 (1) Succinyl-CoA synthetase, beta subunit [Energy production and conversion]
u COG0050 (1) GTPases - translation elongation factors [Translation, ribosomal structure and biogenesis]
u COG0074 (1) Succinyl-CoA synthetase, alpha subunit [Energy production and conversion]
d COG0107 (1) Imidazoleglycerol-phosphate synthase [Amino acid transport and metabolism]
d COG0127 (1) Xanthosine triphosphate pyrophosphatase [Nucleotide transport and metabolism]
u COG0135 (1) Phosphoribosylanthranilate isomerase [Amino acid transport and metabolism]
u COG0144 (1) tRNA and rRNA cytosine-C5-methylases [Translation, ribosomal structure and biogenesis]
d COG0235 (1) Ribulose-5-phosphate 4-epimerase and related epimerases and aldolases [Carbohydrate transport and metabolism]
d COG0253 (1) Diaminopimelate epimerase [Amino acid transport and metabolism]
u COG0318 (1) Acyl-CoA synthetases (AMP-forming)/AMP-acid ligases II [Lipid metabolism / Secondary metabolites biosynthesis, transport, and catabolism]
u COG0354 (1) Predicted aminomethyltransferase related to GcvT [General function prediction only]
d COG0406 (1) Fructose-2,6-bisphosphatase [Carbohydrate transport and metabolism]
d COG0410 (1) ABC-type branched-chain amino acid transport systems, ATPase component [Amino acid transport and metabolism]
d COG0411 (1) ABC-type branched-chain amino acid transport systems, ATPase component [Amino acid transport and metabolism]
u COG0421 (1) Spermidine synthase [Amino acid transport and metabolism]
u COG0436 (1) Aspartate/tyrosine/aromatic aminotransferase [Amino acid transport and metabolism]
uD COG0444 (2) ABC-type dipeptide/oligopeptide/nickel transport system, ATPase component [Amino acid transport and metabolism / Inorganic ion transport and metabolism]
d COG0446 (1) Uncharacterized NAD(FAD)-dependent dehydrogenases [General function prediction only]
u COG0451 (1) Nucleoside-diphosphate-sugar epimerases [Cell envelope biogenesis, outer membrane / Carbohydrate transport and metabolism]
d COG0480 (1) Translation elongation factors (GTPases) [Translation, ribosomal structure and biogenesis]
d COG0508 (1) Pyruvate/2-oxoglutarate dehydrogenase complex, dihydrolipoamide acyltransferase (E2) component, and related enzymes [Energy production and conversion]
d COG0517 (1) FOG: CBS domain [General function prediction only]
u COG0548 (1) Acetylglutamate kinase [Amino acid transport and metabolism]
u COG0559 (2) Branched-chain amino acid ABC-type transport system, permease components [Amino acid transport and metabolism]
D COG0596 (1) Predicted hydrolases or acyltransferases (alpha/beta hydrolase superfamily) [General function prediction only]
d COG0600 (1) ABC-type nitrate/sulfonate/bicarbonate transport system, permease component [Inorganic ion transport and metabolism]
d COG0604 (1) NADPH:quinone reductase and related Zn-dependent oxidoreductases [Energy production and conversion / General function prediction only]
d COG0625 (1) Glutathione S-transferase [Posttranslational modification, protein turnover, chaperones]
u COG0635 (1) Coproporphyrinogen III oxidase and related Fe-S oxidoreductases [Coenzyme metabolism]
u COG0642 (2) Signal transduction histidine kinase [Signal transduction mechanisms]
d COG0683 (1) ABC-type branched-chain amino acid transport systems, periplasmic component [Amino acid transport and metabolism]
u COG0704 (1) Phosphate uptake regulator [Inorganic ion transport and metabolism]
Dd COG0745 (5) Response regulators consisting of a CheY-like receiver domain and a winged-helix DNA-binding domain [Signal transduction mechanisms / Transcription]
u COG0765 (1) ABC-type amino acid transport system, permease component [Amino acid

transport and metabolism]
u d COG0824 (2) Predicted thioesterase [General function prediction only]
d COG0825 (1) Acetyl-CoA carboxylase alpha subunit [Lipid metabolism]
d COG1012 (1) NAD-dependent aldehyde dehydrogenases [Energy production and conversion]
u COG1018 (1) Flavodoxin reductases (ferredoxin-NADPH reductases) family 1 [Energy production and conversion]
u d COG1024 (2) Enoyl-CoA hydratase/carnithine racemase [Lipid metabolism]
d COG1066 (1) Predicted ATP-dependent serine protease [Posttranslational modification, protein turnover, chaperones]
u COG1116 (1) ABC-type nitrate/sulfonate/bicarbonate transport system, ATPase component [Inorganic ion transport and metabolism]
d COG1117 (1) ABC-type phosphate transport system, ATPase component [Inorganic ion transport and metabolism]
d COG1137 (1) ABC-type (unclassified) transport system, ATPase component [General function prediction only]
u COG1145 (1) Ferredoxin [Energy production and conversion]
d COG1159 (1) GTPase [General function prediction only]
u d COG1173 (2) ABC-type dipeptide/oligopeptide/nickel transport systems, permease components [Amino acid transport and metabolism / Inorganic ion transport and metabolism]
d COG1176 (1) ABC-type spermidine/putrescine transport system, permease component I [Amino acid transport and metabolism]
u COG1177 (1) ABC-type spermidine/putrescine transport system, permease component II [Amino acid transport and metabolism]
d COG1183 (1) Phosphatidylserine synthase [Lipid metabolism]
u COG1189 (1) Predicted rRNA methylase [Translation, ribosomal structure and biogenesis]
u COG1249 (1) Pyruvate/2-oxoglutarate dehydrogenase complex, dihydrolipoamide dehydrogenase (E3) component, and related enzymes [Energy production and conversion]
d COG1250 (1) 3-hydroxyacyl-CoA dehydrogenase [Lipid metabolism]
u COG1280 (1) Putative threonine efflux protein [Amino acid transport and metabolism]
u COG1381 (1) Recombinational DNA repair protein (RecF pathway) [DNA replication, recombination, and repair]
u COG1414 (1) Transcriptional regulator [Transcription]
d COG1466 (1) DNA polymerase III, delta subunit [DNA replication, recombination, and repair]
u COG1508 (1) DNA-directed RNA polymerase specialized sigma subunit, sigma54 homolog [Transcription]
u COG1541 (1) Coenzyme F390 synthetase [Coenzyme metabolism]
d COG1580 (1) Flagellar basal body-associated protein [Cell motility and secretion]
d COG1613 (1) ABC-type sulfate transport system, periplasmic component [Inorganic ion transport and metabolism]
u COG1737 (1) Transcriptional regulators [Transcription]
d COG1840 (1) ABC-type Fe3+ transport system, periplasmic component [Inorganic ion transport and metabolism]
u COG1868 (1) Flagellar motor switch protein [Cell motility and secretion]
u COG1946 (1) Acyl-CoA thioesterase [Lipid metabolism]
d COG1969 (1) Ni,Fe-hydrogenase I cytochrome b subunit [Energy production and conversion]
u COG2080 (1) Aerobic-type carbon monoxide dehydrogenase, small subunit CoxS/CutS homologs [Energy production and conversion]
d COG2153 (1) Predicted acyltransferase [General function prediction only]
d COG2223 (1) Nitrate/nitrite transporter [Inorganic ion transport and metabolism]
u COG2232 (1) Predicted ATP-dependent carboligase related to biotin carboxylase [General function prediction only]
d COG2518 (1) Protein-L-isoaspartate carboxylmethyltransferase [Posttranslational modification, protein turnover, chaperones]
u COG2872 (1) Predicted metal-dependent hydrolases related to alanyl-tRNA synthetase HxxxH domain [General function prediction only]
d COG2885 (1) Outer membrane protein and related peptidoglycan-associated (lipo)proteins [Cell envelope biogenesis, outer membrane]
u COG2908 (1) Uncharacterized protein conserved in bacteria [Function unknown]
d COG3000 (1) Sterol desaturase [Lipid metabolism]
u COG3093 (1) Plasmid maintenance system antidote protein [General function prediction only]
u COG3118 (1) Thioredoxin domain-containing protein [Posttranslational modification, protein turnover, chaperones]
d COG3181 (1) Uncharacterized protein conserved in bacteria [Function unknown]
u COG3415 (1) Transposase and inactivated derivatives [DNA replication, recombination, and repair]
u COG3556 (1) Predicted membrane protein [Function unknown]
u COG3842 (1) ABC-type spermidine/putrescine transport systems, ATPase components [Amino acid transport and metabolism]
u COG4133 (1) ABC-type transport system involved in cytochrome c biogenesis, ATPase component [Posttranslational modification, protein turnover, chaperones]
d COG4166 (1) ABC-type oligopeptide transport system, periplasmic component [Amino acid transport and metabolism]
u COG4174 (1) ABC-type uncharacterized transport system, permease component [General function prediction only]
u d COG4239 (4) ABC-type uncharacterized transport system, permease component [General function prediction only]
u COG4795 (1) Type II secretory pathway, component PulJ [Intracellular trafficking and secretion]
d COG4976 (1) Predicted methyltransferase (contains TPR repeat) [General function prediction only]
u COG5013 (1) Nitrate reductase alpha subunit [Energy production and conversion]
D GOS11414712 (1) ?
u GOS1277 (1) ?
d GOS13617 (1) ?
u GOS259 (1) ?
u GOS310 (1) ?
u GOS336 (1) ?
d GOS381 (1) ?
u GOS3923 (1) ?
d GOS398 (1) ?
u GOS4294 (1) ?
u GOS4375 (1) ?
d GOS4556 (1) ?
d GOS482 (1) ?
d GOS5249 (1) ?
d GOS6036 (1) ?

uD GOS6292 (2) ?
u NOG04570 (1)
u NOG08837 (1)
u NOG10085 (1)
d pfam00126 (1) Bacterial regulatory helix-turn-helix protein, lysR family.
u pfam00289 (1) Carbamoyl-phosphate synthase L chain, N-terminal domain.
d pfam00440 (1) Bacterial regulatory proteins, tetR family.
d pfam00479 (1) Glucose-6-phosphate dehydrogenase, NAD binding domain.
u pfam00665 (1) Integrase core domain.
d pfam00725 (1) 3-hydroxyacyl-CoA dehydrogenase, C-terminal domain.
u pfam00903 (1) Glyoxalase/Bleomycin resistance protein/Dioxygenase superfamily.
u pfam01264 (1) Chorismate synthase.
u pfam01345 (1) Domain of unknown function DUF11.
u pfam01502 (1) Phosphoribosyl-AMP cyclohydrolase.
d pfam02501 (1) Bacterial type II secretion system protein I/J.
u pfam02550 (1) Acetyl-CoA hydrolase/transferase.
d pfam02781 (1) Glucose-6-phosphate dehydrogenase, C-terminal domain.
u pfam03331 (1) UDP-3-O-acyl N-acetylglycosamine deacetylase.
u d pfam03401 (6) Bordetella uptake gene (bug) product.
d pfam03466 (1) LysR substrate binding domain.
d pfam03618 (1) Domain of unknown function (DUF299).
u pfam03775 (1) Septum formation inhibitor MinC, C-terminal domain.
u pfam04292 (1) D-galactarate dehydratase / Altronate hydrolase, N terminus.
u pfam04340 (1) Protein of unknown function, DUF484.
u pfam05209 (1) Septum formation inhibitor MinC, N-terminal domain.
u pfam06189 (1) 5'-nucleotidase.
d pfam06243 (1) Phenylacetic acid degradation B.
d smart00062 (1) Bacterial periplasmic substrate-binding proteins; bacterial proteins, eukaryotic ones are in PBPe
u smart00283 (1) Methyl-accepting chemotaxis-like domains (chemotaxis sensory transducer).
u smart00702 (1) Prolyl 4-hydroxylase alpha subunit homologues.

15.4 Multiple-sequence alignment

Each Burkholderiales-1 RNA is denoted by an abbreviation (like "Eco-1-1") that refers to a taxonomy given in Section 15.1. The alignment includes sequences containing the RNA motif, as well as flanking sequence. The Burkholderiales-1 RNA itself is denoted by the line underneath marked 5′ and 3′ on either end. Nucleotides in the flanking sequences (i.e., not part of the motif) are written in gray letters. Stems of predicted rho-independent transcription terminators are shaded yellow. (Note: terminator predictions have not been analyzed manually, and many are likely to be false positives.) Start codons are shaded green. (Note: start codons are frequently misannotated, especially in environmental samples.) Nucleotides proposed to basepair as part of the consensus structure are shaded in color when they comprise Watson-Crick or G-U pairs. Otherwise they are shaded gray. Conserved stems are also indicated at the bottom of the alignment by angle brackets, where matching < and > denote base-paired columns. Below these angle brackets, the symbol "2" denotes base pairs exhibiting covariation, "1" denotes base pairs exhibiting compatible mutations, "0" denotes base pairs that are not observed to mutate and "?" denotes base pairs that have a significant frequency of non-canonical nucleotides for Watson-Crick or G-U pairs (> 5%). Below these base pair annotation is the consensus sequence: "R" = "A" or "G", "Y" = "C" or "U", red nucleotides: nucleotide identity conserved more than 97% of the time, black nucleotides: 90%, gray nucleotides: 75%, red circle (●): nucleotide is present 97% of the time, black circle (●): 90%, gray circle (●): 75%, white circle (○): 50%. All percentages of sequences just described (e.g. 97% conserved) assume that sequences have been weighted by the GSC algorithm implemented by the Infernal software.

alignment positions 1 · · · 99

```
env-1     CUUCCGCAUGCAUGCGGAUGGUCAAAGGACUGCUCAUCUCAGUGUUCCAUGGCGCAAGGCGCCGUCAGCGGCCGGCAGAAUCGGCGCAGCGCAAGCCAG
Rme-1-1   ACACCCACCGGCGGAAGAACCGGUGCGGCAGGGUGCGAUGAACGUGGACCAAUGCGCGUCAUGAUGAAGAAACAGAACGAGAGAGAUGCCUUGAUCAAG
Aav-1-1   AUGACCAUACGGGAGCAGGAGUGCGCUUGCGCCAGGCUCAUGGCCCUCUCACCCGCGCGGCGCCCGCGGUCGGAAGGCCGCGGAGCAGGCCACGCCAGC
Aav-1-2   CCCGCCGAGAAAAAAGCGCGCCCGAGGCAGGCUCGGGGCGCCAGCGUUGGACAGGCAGUGCGCCACCCAUCCGAAGGCCGCGGAGCAGGCCAUGUCAGC
Psp-1-1   UGGCCUUCAACCUGCUCGGAGACGGACUGCGCGAUGCGUUCGAUCCAAAACUUAAACGAUGAACAGGAUCCACCCACCCUUCAUGAGCCUCUUCGCAAG
env-2     ...........................CCCGACAUCCUGGUAUCCAUUUCCUUCACAUACCGCCCACGACCUGCGCACCCGCGAUGCACCACUGGCCG
Mpe-1-1   CCCCUCGUCCGACGAGGACGUCAGCCGGUCCCCCGAGGGGACGCGGGCCGGCUUGGGAGCGGCCCAGCGCUCGGCCCGCAGGCACGGCACGACGCGAGC
Aav-1-3   CCACGGGCAACGAAGGGCAUGCGAUCGCAGACAGAAAUUGAUUUCCUAGCGCCACCCCGACCUUCCUGUUCGAAAGGCCGCGGAGCAGGCCACGCCAGC
env-3     .....GGCAGGUGUCCGCCAGUUGCAGGACGUAGAUACAUCGGGGAUCAAGCAUCUUGGUUCUCGCAGUUCGAUUCGCGGCCCGUGGGUUCAGCCCAGC
Psp-1-2   CUGGGUCGAGAGGUGUUUAUCAGGCAUGUUGUUCCCUUUGGGUUUAUUUGCCGCUGCAAUCGGAGCCGCAACGCAUGAGACGCUCAGUGCCGAGCCAGC
Psp-1-3   GCUGCCACGUGGUUUUAUCGCUUCCCGCCUUUGGGGGGAGGUCGACAUGGGGGCUCGCAAGCUUUGAGCGAAGGCGUGCAGCUUGGCGCCCACAUCAGC
Psp-1-4   CUGCCCGGUCUUGCCGGUGAUGCCUUGCGUGAUGACUUUGGUGUGUUGGUUGAUGUAAAUGCUCAUGCUGUCCUCACAAGGUGAAUGCUAUUGUCAAGU
Aav-1-4   UCCCGAAAUCGGAUAACCGAAGAUGCAUGCCGGGACGACGGCACAUGGGCUACGUGCGGCCUCGACCCGUCAGAAGGCCGCGGAGCAGGCCAUGCCAGC
env-4     CCGCGAAACAGCAGCCGCGUGCCCCGCCGGCAGGCGAGGGCCUGUAGCCGAAGGAGAGGUGUGGCAUUCACGUCAGCAGCCUCCAUCGAAGGUGCCAGC

          ...................................................................................................
          ...................................................................................................
          ●●●●●●●●●●●●●●●●●●●●●●●●●●●●●●●●●●●●●●●●●●●●●●●●●●●●●●●●●●●●●●●●●●●●●●●●●●●●●●●●●●●R●YY●●●YCRR●
```

```
env-5      AAUGGCAGCGUCAUCUCGAACGUCAGCUGCUUGUCCUUGGGGAUCUCUAUGCGCAUAUCAACUCUCCGUCGCACCGCAGGCCACAACCAAAACCCAGC
Aav-1-5    AAAGUCGUCAUGUCAUCCGUCGUGUUGACGCUGCGCCCGCUUCGCCGGGAGCUGGCACGCCAUCACGCAUCCGGUGGCCGCGGAGCAGGCCGCGCCAGC
Psp-1-5    CUUUCCAGUAACACCGCGGAACCGGCUUUGCCGGGCCGCAGGUGUUGCCCCUUGAGGGGGAUCCGGCUACACGCAGUGAGCCGAGACGGGGGAAACUCA
Psp-1-6    CAGCACAAAAAAUGCCGCCAGCCCGGUAACCAGGAAAAAAACCGAACAGCAGGCGAAUACCUAAACGCAUACCUGUUCCCGCACACAGGCUUUUGCCAGC
Asp-1-1    GCGCUCAUAUCAGUCAUCCAUGGCGCAGCGCGCCACCCGCAACCCAUGAAACUGGCGCGACGGCACCGGUCCGAUGGCCGCGGAGCAGGCCAUGCCAGC
Rfe-1-1    UCACAAAAACCAGCAGGCUCCAAUAGCCCAAACGGUUACGCUUGAACCGCAACCAGGCCCGACGGCCCGGCGAUGGAGACUUCGGGGGCGUGCUCGCGG
Psp-1-7    CGGCGCGCAGCCAGGCCGGGCGGGGCAGGGAGGCGAUCAUUCGAUGAUUCCCCAGCCUGAUGCGCACCCGCAGUGUUGGCGCAGGCGGUGAAAUGCCAG
Psp-1-8    CUUCCAGGGGACAGGCGCCUUGGGGCCGGCAAAGCCGGACCCCGGCCCCCACUGGAAAAGAAUAGCCCUUAGCUGGCCUCCCCCGGGGGGCUUGCCAGC
Psp-1-9    CAGGGCGGAGCCGAAGGGGGCGGGGGUCAAAGCCAUCUCAGUUGUCCACCGCGCUGCGCGCCACCCGUAGCCCACGAAACUGGCGCGACUCAGCCCAGC
Aav-1-6    ACAGCCUGCAGGCCUGGGUGGACAAGGCACGGGCCCAAAUCGGGGGCAUGCACUGACGUGCUUCACCCGUCCAAUGGCCGCGGAGCAGGCCACGCCAGC
Aav-1-7    AGGGAUGAGAACAUGGUGAAGGAAAAAACAUCGCGAACGGAGGAUCUGCAGCCAUGUCCGUCAGCGUCAUGGCGCAAGCACUGCGCGGCCCAGGCCAGC
Aav-1-8    GUACGUGUCCUGAAGAUCCAGAGAUCAAAGCCAUGUCGACUGGGUACGCUAUCUGCACGGCCUUCCUGGUCCGGAGGCCGCGGAGCAGGCCAUGCCGGC
Aav-1-9    UGCUUACAGGAGAUGCCGCAACCGCGGCCAGGCAUUGCAAUGCCUCACGCACCAGCACGGCUCCACCGCUCGGAAGGCCGCGGAGCAGGCCAUGCCAGC
env-6      CAUCAGACGAAGAAGCCGUUCCAUCAUGUUCAUCGGUCUCUCUCCUCGGGUGCUUUUUGAAUCAUUCGUGAGCCGCGUGCAAACCGUGUGCUUCGCGGG
Psp-1-10   GUAGCUCCCUGCCCCCCGAGGGGGCCGCCCGCCUGUGGCCCGGCAAAGCCGGUUCCACGGCUCAUGCUUGGUUGAAGAGUUCAGGGAUCACAACCCGGU
Psp-1-11   UGUAGCGCCCUGCCCCCCGAGGGGGCCGCCCGCCUGUGGCCCGGCAAAGCCGGUUCCACGGCUCAUGCUGGGUUUAAGAGUUCAGGGGACACUACCAGC
Psp-1-12   CUGCGUGUAGCUCUCGAGGCCUUGCAGGCCGCGGCUCGCGAGACGCUCCGCCUCUGUCGCCUGUCCAUGCUGGGUUAAAGACUUCAGGGAGCACAACCA
Psp-1-13   AGGGGACGGCGCCUGUGGGCCGGCGUAGCCGGACCCACGGCCCCCGCUGGUGGAGUCGGGCUACACGCAGUGAACAGGGGUGACGAAUCCCCCGCCAGC
Psp-1-14   CACUGGUACUUUUGAUUGCGCCUGCCAUGUCAGUUUUUCCAAUGCAUCGAAAUGAGAAAUAGGCAGGGACCGAGUCCCCGUACAAGUCCACUUACCAAU
Psp-1-15   GUGAGCCUGGACGGGGGUGGUUUUAUAGCUCCGCCUGCAGUCCGGCAAAGCCGGUCCUGCGGCUCAUGCUGGGUUGAAGAGUUCAGUGGGCACACGAGU
Psp-1-16   AGCCGCAGGCUCCAGCCCCUCGGGGGCCGCCCGCCUGCAGUCCGGCAAAGCCGGUCCUGCGGCUCAUGCUGGGUUGAAGAGUUCAGUGGGCACACGAGU
Psp-1-17   CGGCGUGAGCGCGAGAUGCUGCGAGACGCGGAGAGAAAGCCCUUGCUUCACAACCCCUCCAGGUAUCCACAAAAGCUGGGCGGGGUGGAUUUUUCAAGC
Psp-1-18   CCCGUGGAGGCCUUCGGCCUCCUCCCCCUCGCAGGGGGCGGCGCCUAGGGCCGGCAAAGCCGGACCCUGGCCCCCACUGGGUUUGAUGUCUUGUUAAGC
Aav-1-10   GCCAUGGCGGCUGAUGCUGCGGCCUGCGGCAUUGCAGGCGGCCCGUGAACCGCGCCCCGCCGCCACCCGUGCGGUGGCCGCGGAGCAGGCCAAGCCAGC
Psp-1-19   CAAUGAGCAGCACGGAUUGUUGUACGGACAUUUUUUAUUUCCUUGAUGUGCCCUGGGCCUUGGAUAACCAAGCGCCGUGUGACUGUUCUCCCCUACCAG
Psp-1-20   GGUACGGGUGGGAGGCGGGGUAUUUUGCGUUUUCAUGCCGAGGCCUCCAUGGCGCGAAGCGACACCCGCUGCUUAUGACAGUGGCGCUGCACAGUCCAG
Psp-1-21   CCGCCACGUGCAAGCUCGCGUGUCCAGGUCAUGGGCAUCUCAGUUGCUCCACCGCGCUUCGCACGAGCCGCGACGCAUGAAACGCGAGGCCUGUGCGGG
Psp-1-22   CCGUCGCGGCCGCAAGCGCAAGCAGGGCCGCGCAAAUUCGAUGGGUUGUAAGGGGUUUCAUUUUCGUUCCUUGGUUGGAGUCAAACGAGCGCUUCAAGC
Cte-1-1    ACAGUCCCUUGGCAGUCUUGAAGGUCUGCUCCACCCCGUGGAUCUCGAUGUACUUGGUGCUGAGAGAUGUAUGCAUGAGGUACUCCUUGUUUUUUCCGA
env-7      .....................................................UUGUGUAUUGAAUGCUAUGACGUUCCGCGACGGCGCCUGUUCGCG
Psp-1-23   ACGUCGAAUUGUUUGGACAUGUUGUUCUUUCAAUAAAGCUGCCGGUGGCAAUACCUCGAUUUGCUCUCGCACCGGCGAGCGAAGCAAAGCCUGUUCGCG
env-8      CCGGCCGCGACGGUGCCGACAAGAAUGGCCAACAAGGUCAUAUCAGUAUUCAGUUACUCGGUAGAACAAGACCUGCGGAGCACGGGCGGUCGAACCGAG
env-9      CCUGGCUGCGCCAGGUCCAGCGGCGGGUCUUGGGCGGCGCACGCAUCGUCGUCAUGGGAGUCGCCUUCUAGCGGCCCGCAGGGCUCGCGCCCUGCAAGC
Psp-1-24   CGUGAAGAUCGUGGUGCCGACGGCGGGCAGCUUGGUUUCCAGUUUGGGGGUGCGUUCGAGGAUAGUGGUCAUGUCUGCAAAGUGAGGGUUUUUUCAAGC
Psp-1-25   GUAAGUGAUUUCGUCGAUGCCCAGUACGCUCAUGGUGUCAGUUCUCCAUGUGAUGUCAGUUGUUCCACGGGCUGCUCGCAACGUGCACGCCAUUCCAGC
env-10     UUCCCGGCCAUGACCUGCGUGGAGGUCAGCGCGCUGGUGGAGGAACGGGCAAAGGUGGAGAUCGAAGUCACCGCAGUCGUCCCAGACUGACAGUUCCAG
Psp-1-26   CGGAGAAAAGAGAGUGAACUUUUCCCUGUGUAGAGUGCUUCCGAUUCAAUCGUUACAGUCCACUAGACCCAUGAUGGCGCGUCCGGACCCUUCUCAAGC
env-11     CUCCAGCAGCAGCGCGCUUCCGGGCGCGGCGAUCUCGUGCGCACGCCCGUUCACGUUGAGAAAAAGAAUUUGUUUGGCCAUUGAAUUACUCUUUUCGUG
Psp-1-27   CGCGCACUGCAGUGAAGUCGGCAAAGCGCUUCUGCAGAUUCCGGAUUCUGAUUUCACUCAUGGUCAAACUCCGGGCGCAGGACGGCGGACUCGUUCGCG
env-12     UUCGCCGCGGUUCGCAAUCAGGAUCUUCUUGAACAUCUCACUCCUCCGACCGCGCAGCGCGCGGUUCCACUUGGCAUAAAAGCGCGGUGCCUGCUCGAG
env-13     ...................................GAUUCGUUGCAGUUUUCAAGCCGGUCUUCCCAUCUACACCGCCGGAAGUCCGCCAGUCUCGGG
Aav-1-11   GCACAGGCAUAAGGGGGCAAAACCUAACCUCUCCAGCAUGCUUCGCCAGUGGCCUUCACCCCCAAAGAUUCAGAAGGCCGCGGAGCAGGCCAUGCCAGC
Asp-1-2    CAUGGCGGUGGUGCUGCUGGCCACGGCGCUGUGGAUGGUGGCCGCAUGAGACCGGCGCAUCCUCAUUGGUCGAAAGGCCGCGGAGCAGGCCAAGCCAGC
Aav-1-12   GGGACAUGGCAAACCAUCCAACCGUUAUGCAUUGCGCUUCGGCACGGCGCUCGAAACUGGCGCGGCCCGACCUGUGGCCGCGGAGCAGGCCACGCCAGC
Psp-1-28   CCAGAUUUGCUGCCAUGUUGUGAUGCCUUGGUGUUGUUAGGUCGGUUUUUGGUAUGGCUUCAUUUGGCGUUGAUCGCCCGGGUGUGGCGCGAUACCAGC
Aav-1-13   CCUGUCCUGAGUUGCUGGGGCCUGGUAACCACAGCUUCCCAGGUCGGGCCUGGAUGAACAACCUAUUGAGACAUCACAACUAGCGCGGCCCAGGCGAGC
Aav-1-14   AGAGAAUCGGUCAUGGUGAGCGAGUGUGCCGUGGUUGCAAACGGUUGGCCGUCGUACCGGUGACUGGACAAAGGCAGAGCGCCCACGGUAUUCGCCAGC
Aav-1-15   GCCCUGAUCUUCCGGCUGCCUGAGCGGGCGCCUUGCGAAAGAUGUCCGAUACCGCAACGGCCUUUCCCGUCAGAGGGCCGCGGAGCGGGCCACGCCAGC
Aav-1-16   UAUGUACAUGUCCCCGCCACCCAUAUUUGCCUGUAGCUCAUUGCCCAUGAACAGGCGCAGCGCCACCUAUCGAGAGGCCGCGGAGCAGGCCAUGCCAGC
Aav-1-17   CAGAAAGGGGGGCCGCAGCCGAGGCAACAGCCAUCAGGCGCAGCCCUUGAACAGCCCCGCCCUCACCCGUCAAAAGGCCGCGGAGCAGGCCAAGCCAGC
Psp-1-29   GCCAGCUGCAGCGUACCGCUGCGCAUGUAGUGCUGGAUGUCUUUCACGCGAUCAAUCCCCACGGUUCGCGAUGUCGGCAACCAGCGUGGCUUGGCCAGC
env-14     .....................................................................UUCGUUGAUUUGGCGCUACCGAGGCCAGC
Aav-1-18   GGGCUACCGACAGUCAGGAGGCAUUCUCAAGCCCGGCGGGCCCCUCACGCACGCCCGGAGCCUGCCCCGUCAGAGGGCCGCGGAGCAGGCCACGCCAGC
Aav-1-19   GGCGCCAGCCCCCCGGUUCAAACAUUUCAAAAACGAUGGACAGCAUGCGUUAAGGCACGGCCUUUCCCGUCAGAAGGCCGCGGAGCAGGCCACGCCAGC
Aav-1-20   GACGCGGCGCAUCCCCCCGUAUGCACCUCGUGCCACCACGUUCCAUGGAAGCCCGCACGGCGCCACCCCUCCGAUGGCCGCGGAGCAGGCCAUGCCAGC
Aav-1-21   UCCAAAGCUUCAGUGAUCGACGGCGCUACGCGCCAUCCACGCACCAUGAAACCGCCGCGGCACGCCCCUUCCGCUGGCCGCGGAGCAGGCCAUGCCAGC
Aav-1-22   ACCCGAAUGCAGCAGUCGGCGGUAAUCCAAAACCCCGGGGCCAGUGGAGGCCCCCAGCGGCCUUACCUGUCUGAAGGCCGCGGAGCAGGCCAAGCCAGC
Aav-1-23   UGCGAAGGCUGGAGGGCACUGGAUGAGUGCCACUGCCCAUAGCCCGUGAAACUGGCGCGGCCUCACCCGUCGAAUGGCCGCGGAGCAGGCCACGCCAGC
Aav-1-24   UCGUGGGUCAUCCGGCGAUGGUAGCGCCGUCCUCCUGCACGCCGCUGGGACGUCCUGCGGCCAGCCCUGUCCGAAGGCCGCGGAGCAGGCCAUGCCAGC
Psp-1-30   CCUGCCGCCGUGGAACCGGCUUCGCCGGGCCACCAGCGGCGCCCCCUGCAAGGGGGAGGGGGCUACGCAAAGCGAGCCCCAACAGGGGUGUGCAACUCC
Psp-1-31   CAGGCCGCCGUGGAACCGGCUUUGCCGGGCCACCAGCGGCGCCCCCUGCAAGGGGGAGGGGGCUACGCGAAGCGAGCCCCAACAGGGGUGUGCAACUCC
Psp-1-32   AAGGGCCACUGACUCAAGACCGAGAUGAAGUCCAACUCAACCCGCAUCUGGCUUCACUCCUUCUAAAAAGUAACGACAGUCUCUUCUGCAUCUCUACCA

           ..................................................................................................
           ..................................................................................................
           ••••••••••••••••••••••••••••••••••••••••••••••••••••••••••••••••••••••••••••••••••••••R•YY•••YCRR•
```

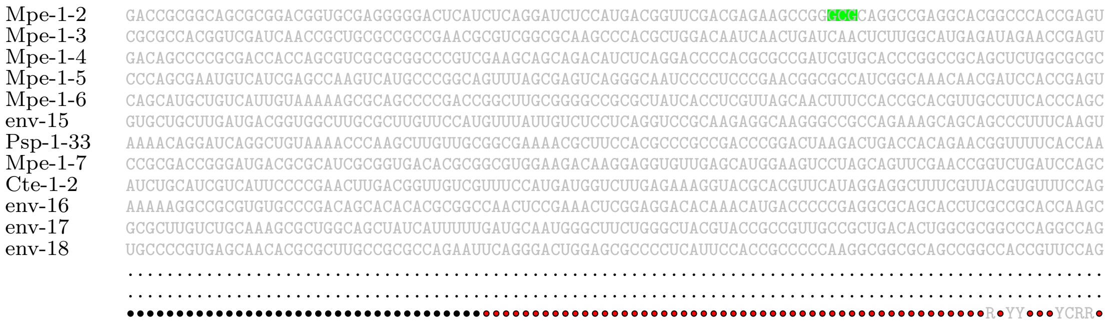

alignment positions 100 · · · 259

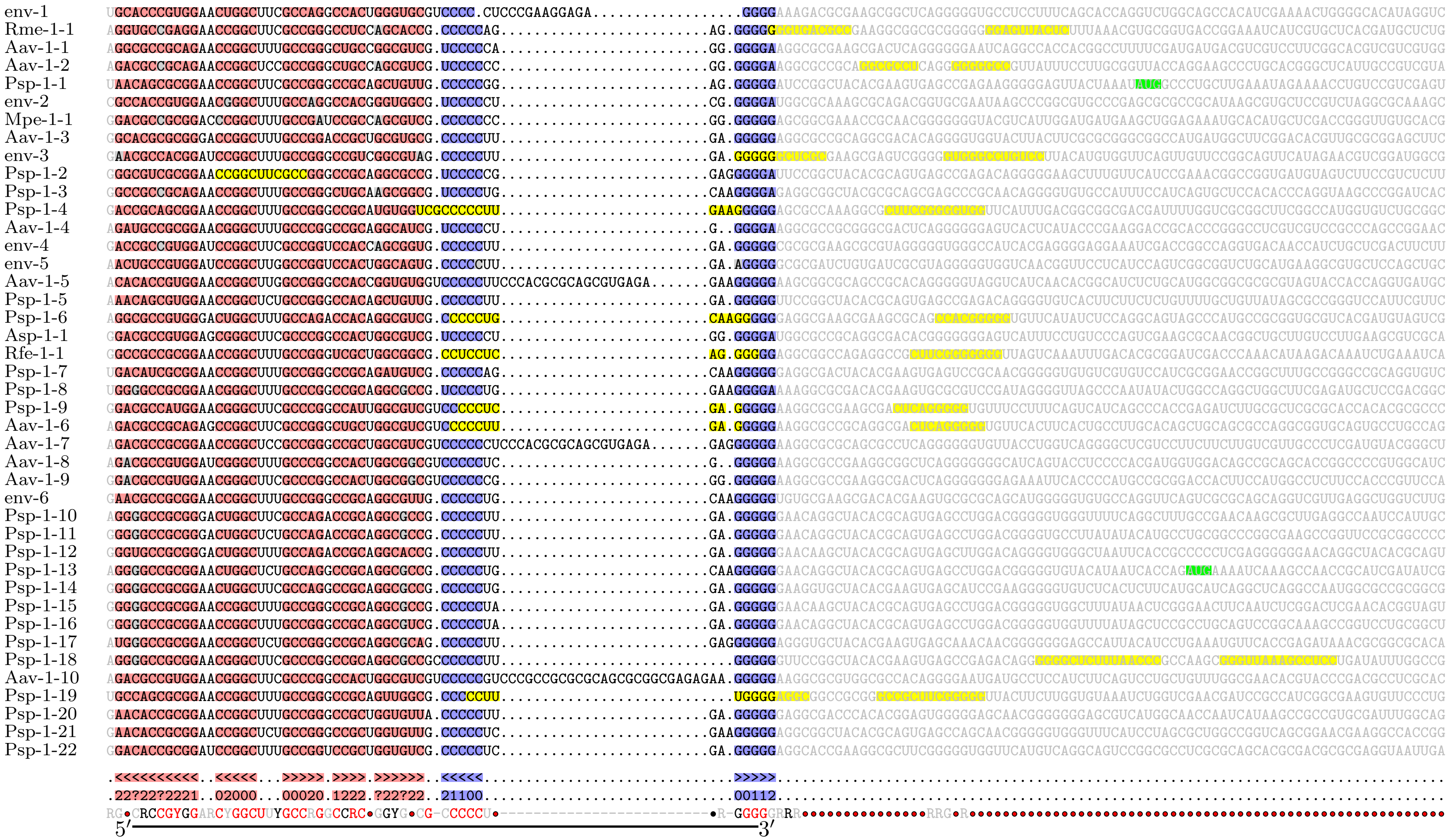

```
Cte-1-1   GGCCACCGUGGCGCCGGCUUUGCCGGGCCACCGGUGGUG.CCCCCUU.........................GA.GGGGGUGACGCGCCCGAAGGCGCGGCUCAGGGGUGGGUUAAGUCUUGACCUCUUCAAACGUAAAGGCCGAGGCCAGCUUGACCAG
env-7     GGCCACCGCGGAUCCGGCUCUGCCGGUCCGCCGGUGGCG.CCCCCUU.........................GA.GGGGGAGGCGCCGGAGGCGCUUCGGGGGUGUUUCUAAUACUCCAGCCGUUCCGGCCGGAUUUCCUGCAGGAUGGUGGUGGCGAUU
Psp-1-23  AGACACCGCGGAACCGGCUUUGCCGGGCCGCAGGUGUCG.CCCCCUU.........................GA.GGGGGGAGGCCGCUACACGCAGUGAGCGGCAACAGGGGCGUCAAAUAUCAAACAGCAAGCGGGCCGGAUCUUCCAGCGCUUCCUU
env-8     UGACACCGCGGAACCGGCUGUGCCGGGCCGCCGGUGUCG.CCCCCUU.........................GA.GGGGGUCGCGCAAAGCGCGGCGGGGGGUCUCGUUUCAGGCCACGCCGUGCUGCUUGAACCAGGCCAGGGCACGCUUCCAGCCGUC
env-9     GACCGCCGCGGAACCGGCUUCGCCGGGCCGCUGGCGGUG.CCCCCU..........................GA.GGGGGAGGCGGCCGAAGGCCGCUUCGGGGGGGAUCAACGUACCGGCGGGGCGUACAUGAAGCCGCCCUUGCUCCACAGGUUGUUG
Psp-1-24  GGCCGCCGCGGAACCGGCUUCGCCGGGCCGCUAGCGGCG.CCCCCUU.........................GA.GGGGGAAACGGCCGCAGGUCGGUUCGGGGGUGGAUCAAAUCUCAUAGUCGUUGAUGCGGCCUGCCAUGGCCUUGGUGAUAAGGGC
Psp-1-25  GGCCGCCGCGGAACCGGCUUCGCCGGGCCGCUGGCGGCG.CCCCCUC.........................GA.GGGGGGAGGCGGCUACACGCAGUGAGCAGGCAACGGGGGUGGUUUUCAUUCGGCCCACGCCAGCGGUUGUUCGUUGAGGCCCCAG
env-10    UGCCGCCGCGGAACCGGCUUUGCCGGGCCUCUGGCGGCG.CCCCCUU.........................GA.GGGGGAAGCGCCGAAGGCGCUUCGGGGGUGGGCUUAACUCCCGACGACCUCGAUCAUCACGCGACGAUCGGCCUGCAUGCACUGG
Psp-1-26  AGCCACCGUGGAACCGGCUUUGCCGGGCCACAGGUGGCG.CCCCCUU.........................GA.GGGGGAGGCGCCGAAGGCGCUUCGGGGGGGAGCCUAGAUGAGACUCCUGCUUGUUGAAGACGAUGUGAUGCUGGCCAGCGGCAUC
env-11    GGCAAUCGCGGAACCGGCUUUGCCGGGCCGCUGGUGGCG.CCCCCUU.........................GA.GGGGGAGGCGGCCGCAGGCCGCUUCGGGGGUGGGCUUCAUACUUCCUUGGGCCGAAGGUCGCGCACCCGCACGGCCUUGCCCUGC
Psp-1-27  GGCCACUGCGGAACCGGCUUUGCCGGGCCGCUGGUGGCG.CCCCCUU.........................GA.GGGGCUGAGGGCCGCGGCUCCAAGCCUGCCUGCGCAGGCUUGGACGGCUCCGAAGAACCGCCGCCUCUGGCGACGGUGCUGGGCG
env-12    UGCCACCGCGGAGCCGGCUUCGCCGGGCCGCUGGUGGCG.CCCCCUU.........................GA.GGGGGAGGCGCCGCAGGCGCUUCGGGCGUGGGCAGGCUUCACCCCAACUCCCGCGGCAUGGGCCGCCCCCUGAAGAAUUCACCGA
env-13    AGCCACCGCGGAACCGGCUUUGCCGGGCCGCUGGUGGCG.CCCCCUU.........................GA.GGGGGAGAGGGCGAAGCCCGAGGGGGCAUCGGCCGCAGGCCGCUUCGGGGGUGGGCUUAUUCACCCGGCCAUUGUCAGCCCGCCG
Aav-1-11  AGAUGCCGUGGAACCGGCUUUGCCGGGCCACCGGCAUCGUCCCCCUUCCCGCCGCGCGCAGCGCGGCGAGA.GAAGGGGGAAAGGCGGCGCAGCCGCCUCAGGGGGAUGUCACUUAUUACUCAGGCAUUCCUUCAUGAACGCCUUGCGCUCGUCACCCUU
Asp-1-2   GGACGCCGUGGAGCUGGCUUGGCCAGGCCACCGGCGGCGUCCCCCUG.........................GA.GGGGGAAGGCGCGAAGCGACUCAGGGGGUGAUUCGGAAUCACGCGGCCUGCUGCUGGGCUUGCUGGGCGUGGCGAUCUUCGCGCU
Aav-1-12  AGACGCCGCGGAACUGGCUUCGCCAGGCCGCUGGCGUCGUCCCCCUGCCCGCCGCGCGCAGCGCGGCGAGAGC..GGGGGGAAGCGGCGCAGCCGCUCAGGGGGGUGUGCUCCAUUUCUAGCGGAGGCGGGGGUUCAGCGCAUCGCGCAGCCAGUCCCCC
Psp-1-28  AGACGCCGCGGAACUGGCUUUGCCAGGCCGCUGGCGUCG.CCCCCUG.........................CAAGGGGGAGGCGGUUACACGAAGUGAGCAAGCAACGGGGGUGUUUCAUUUGAGAUCCAGUGUGCACCAUUGUCCAUAAGCUGGCGCG
Aav-1-13  AGACGCCGUGGAACUGGCUCCGCCAGGCCACCGGCGUCGUCCCCCUUCCCGCCGCGCGCAGCGCGGCGAGA.GAAGGGGGAAGCGGCGCAGCCGCUCAGGGGGUUGGUUCAAUACCGGCUUGCCGGAUCAAAGUGCGCCAGUGCGCCAGCUGCUGCGCCA
Aav-1-14  AGACGCCGUGGAUCUGGCUUCGCCAGGCCACCGGCGUCGUCCCCCUUCCCGCGCGCCAGCGCGAGA......GAAGGGGGAAGCGGCGAAGCCGCUCAGGGGGAUGUACUCACACCCUUUCGAAGAUGCCCGCAGCGCCCUGCCCCAUGCCCACGCACAU
Aav-1-15  GGACGCCGUGGAACGGGCUCCGCCCGGCCACCGGCGUCGUCCCCCUUCCCGCGCGCCAGCGCGAGA......GAAGGGGGAAGCGGCGUCAGCCGCUCAGGGGGAUGUCACCAGCGAGAUGCUCAUGUUCACAUGGGGAAGGCCCGCCUCUUCAUAGCGC
Aav-1-16  AGACGCCGUGGAACCGGCUUCGCCGGGCCACUGGCGUCGUCCCCCUUCCCGGCGUGCGAAGCACGGCGAGA.GAAGGGGGAAGGCGCGCAGCGCCACAGGGGGUAGUUCAUCAGAUCAGGCAAGCCACCGCUUGCGCCGCUUGUAGCUCUUCGCAUCCUU
Aav-1-17  AGACGCCGUGGAACCGGCUUUGCCGGGCCACUGGCGUCGUCCCCCUUCCCGAUGCGCGCAGCGCAGCGAGA.GAAGGGGGAAGCCGCGCAGCGGCUCAGGGAGUAGCCUCUUUUUCCUCUGGCCAGUCCCGGAUAUAGGCUUUCAGCAUCUGGUUCUCGA
Psp-1-29  AGACGCCGUGGAACUGGCUUUGCCAGGCCACUGGCGUCG.CCCCCUU.........................GA.GGGGGGAGGCGACCCACACGCAGUGGGGAGCAACGGGGGUGGUUCAUAUCCACCACCGCUCGCGCAUCAAAUCCAUCAUCUGGC
env-14    AGACGCCGUGGAACUGGCUUCGCCAGGCCACUGGCGUCG.UCCCCU.............................GGGGGAAGCGGCGAAGCCGCUCAGGGGGUGGUCGGUUUCAGCCGUUCAGUUCGUCGGCGCGGGCUUGUGCUGCCGCGAAGUCCAG
Aav-1-18  AGACGCCGUGGAACCGGCUUUGCCGGGCCACUGGCGUCG.UCCCCCU.........................UG.GGGGGGGAAGGCGCCGAAGGCGACUCAGGGGGGGUCAAAACUUCGGUAUGCGGAUCCGGCUGAUCAUGAGCGAGCCGGACACCGC
Aav-1-19  AGACGCCGUGGAACCGGCUCCGCCGGGCCACUGGCGUCG.UCCCCCU.........................CG.GGGGAAGGCGCCGAAGGCGACACAGGGGGGCAACAUGUUCCGGGCCACUGGCGUCGUCCCCCUUCAGGGGGAAGGCGCCGAAGGC
Aav-1-20  AGACGCCGUGGAACCGGCUUUGCCGGGCCACUGGCGUCGUCUCCCUGCCCGCCGUACGCAGUACGGCGAGAGC..GGGGGAAGCGGCGCAGCCGCUCAGGGGGGUGUCCUUUCUUUCAGGUCAGUGCUCGCUGUACCGCCGGGCGCGCCGCCACGCGCUG
Aav-1-21  AGGCGCCGUGGAACCGGCUUCGCCGGGCCACCGGCGUCGUCCCCCUGCCCGACGUGCGCAGCACGGCGAGAGCG.GGGGGAAGGCGCGCAGCGCCUCAGGGGGGUGGACUCUCACUUCAGGCUCCACCACGAAAACAUCGCCCACUCCUCGGCUUUCGCA
Aav-1-22  AGACGCCGUGGAACCGGCUCCGCCGGGCCACCGGCGUCGUCCUCCUGCCCGCCGUGCGCAGCACGGCGAGAGCG.GGGGGAAGGCCCGCAGGGCCUCGGGGGGGUGCUUCACCUUUCUGCGCGGGUAUCGUUGAACCGGCCGUAGCUCUGAAGCCGCUCG
Aav-1-23  AGACGCCGUGGAACCGGCUUUGCCGGGCCACCGGCGUCGUCCCCCUGCCCGCCGUGCGCAGCACGGCGAGAGC..GGGGGGAUGCGGCGCAGCCGCUCAGGGGGGUGUUUCAAGACUCCUCCAUCCAGCUAUGCCCCUCGCGCGCCACCAUGGCGCUCGC
Aav-1-24  GGACGCCGUGGAACCGGCUUUGCCGGGCCACUGGCGGCG.UCCCCUG............................GGGGAUGGCGCGAAGCGCCUCAGGGGGCUACCAGCACCAGCUUGCCGAUGGUCCGGCCGCUCUCGAUGCGGGCGUGCGCCUCGCG
Psp-1-30  UGCCGCCGUGGAACCGGCUUUGCCGGACCACCAGCGGCG.CCCCCUG.........................CAAGGGGGGAGGGGGCUACGCAAAGCGAGCCCCAACGGGGGUGUGCAACAUUUACUGUGGCAAAUCACCGGCAGGGCGACCCAGCCAU
Psp-1-31  UGCCGCCGUGGAACCGGCUUCGCCGGGCCACCAGCGGCG.CCCCCUG.........................CAAGGGGGAGGGGGCUACGCAAAGCGAGCCCCAACAGGGGUGUGCAACUCCUGCCGCCGUGGAACCGGCUUUGCCGGACCACCAGCGG
Psp-1-32  GGCCGCCGUGGAACCGGCUUUGCCGGGCCACCAGCGGCG.CCCCCUG.........................CAAGGGGGAGGGGGCUACGCGAAGCGAGCCCCAACAGGGGUGUGCAACUCCUGCCGCCGUGGAACCGGCUUCGCCGGGCCACCAGCGG
Mpe-1-2   GGACGCCGCGGGUCCGGCUCCGCCGGCCCGCCAGCGUCG.CCCCCUU.........................CGAGGGGGAGCGGCGCAGCCGCAACGGGGUGUUCAAAGUGUCCAGGUGUCGAGGAUGAAGGCGUCGGCCUUGCGGUGCAGCAGGAUGA
Mpe-1-3   GGACGCCGCGGGUCCGGCUCCGCCGGCCCGCCAGCGUCG.CCCCCUU.........................CGAGGGGGAGCGGCGCAGCCGCAACGGGGGUGGGUGUCAAUACCGAUAGGUGUCGGCCUUGUACGGACCGGACUUGCUGACACCGAUG
Mpe-1-4   AGACGCCGUGGAUCCGGCUCUGCCGGUCCACCAGCGUCG.CCCCCUC.........................GA.GCGGGAGGCGCCGCAGGCGCUUCGGGGAUGGGUCAUUCCUGCGCUUCGGAGGUGGGUCACGUCUCGGCGAGCACUUCGCGCGCCA
Mpe-1-5   GGACGCCGUGGAUCCGGCUCCGCCGGUCCACUAGCGUCG.CCCCCUC.........................GA.GGGGGAGCGGCGCAGCCGCAACGGGGAUGGGCCACACACGCAACGGGGGUGGAUUACAUCCUGACGGGGAUGCCGUGCUCGGCCA
Mpe-1-6   AGGCGCCGCGGGUCCGGCUCCGCCGGCCCGCCAGCGCCG.CCCCCUC.........................GA.GGGGGAGCGCCGAAGGCGCAACGGGGGUGGUCGUCAUCAAUCGGCUUGCUUGCGCAGGAACGCCGGGAUCUCGAUCUCGUCCAUG
env-15    GGCCGCCGCGGAUCCGGCUUUGCCGGUCCGCCAGCGGCG.UCCCCUC.........................GA.GGGGGGAGGCGGCUUCGCCGCCUCGGGGGUGGUCAAUUGAACCGCACGUAUUCGAAAUCGACCGGCUGGCGGUUGAUGCCGACGA
Psp-1-33  GGCCGCCGCGGAUCGGGCUCCGCCCGUCCGCCAGCGGCG.CCCCCUC.........................GG.GGGGGAGGCGGCCAACGGCCGCUUCGGGGGGGUCAGUCAAAUUUAACACGCGGGUCUACCCAGACAUAGCAGAGAUCGCUGACCA
Mpe-1-7   AGCCGCCGCGGAUCCGGCUCCGCCGGUCCGCCAGCGGCG.CCCCCUC.........................GA.GGGGGAGCGCCGAAGGCGCAACGGGGGUGGGCCAAUCAAUCUGCAGUGAUCUUGCGCGUCUCGAUCACGUUCUUCCAGCGCGCGU
Cte-1-2   UGCCGCCGCGGAUCCGGCUUUGCCGGUCCGCUGGGGGCG.ACCCCUU.........................GA.GGGGGUAGCGCCGCAGGCGCUUCGGGGGUGGGUCGACCUCACUCGAACUUGAUCGGCACGGUUUCCAUGAUCUUCUUGAUGUUGU
env-16    GGACGCCGCGGCACCGGCUUGGCCGGGCCGCCGGCGGCG.CCCGCUU.........................GA.GGGGGUGAACGGGACAGGGACCGUCCAGUGGUCUGUCCCUCGCCAGCUAACGCGCAGCAGCUUCUGGGGUGGGCUCGUUUACCGG
env-17    CGCACCCGUGGAGCUGGCUUUGCCAGGCCACCGGGUGCG.UCCCCUU.........................GG.GGGGAAGGCGCAAAGCGACUCAGGGGGGCCUCCUAUCCAGGGAUCUUUUCCAGGCAGUAGCCCAGCCCGCGCACUGUGGCAAUGC
env-18    GGCACCCGUGGAGCCGGCUAUGCCGGUCCACUGGGUGCGCCCCCCUU.........................GAGGGGGGAGACGCGCAGCGUCUCGGGGGGUGCUUCU..................................................
          .<<<<<<<<<<..<<<<<...>>>>>.>>>>.>>>>>>..<<<<<..............................>>>>>................................................................................
          .22?22?2221..02000...00020.1222.?22?22..21100..............................00112................................................................................
          RG•CRCCGYGGARCYGGCUUYGCCRGGCCRC•GGYG•CG-CCCCCU•------------------------•R-GGGGGRRR••••••••••••••RRG•R••••••••••••••••••••••••••••••••••••••••••••••••••••••••••
           5′―――――――――――――――――――――――――――――――――――――――――――――――――――――――――――――――――――――――――3′
```

alignment positions 260 · · · 419

```
env-1     ACCAGCAUCAGCGCCACGAUGAGCGCACUGUAGAACGGCCAGAUGGUCUUCAUCACCUCUCCCACCGACACGCCCCCGAUGGUGCAGCCGAUGAACUGGGUGGUGCCCACGGGUGGCGUGUUCAGGCCCAGGGCGCAGUUGAUGAGCAUGACGAUGCCGA
Rme-1-1   GCGGCCCAUGAUGUCUUCCCGGAUCGCCGCGUAGAUGUGCAGCAUCACGAACACCACGAUGCCCCACAUGCCCAGGCGGUGCCAGGUGUGCACGUCCUGGCUCUGGCCCAUGAGCGGGAUCACCCAGCCGAACAGGUGGUCUUGCCAGGAGCCGGCCUGC
Aav-1-1   AAGCCCUUGCGGCCCGUUUUCACGCCCUUGAUGGCGUCCACGAUCUCGGUGCCCUUGACCACCUUGCCGAACACGGCGUAGCCCCAGCCCUGGGCGGACGGCGCGGUGUGGUUCAGGAAGCCGUUGUCGGCCACGUUGAUGAAGAACUGCGCGGUGGCCG
Aav-1-2   GCGCAGCAGGAGAUCCUCGAUCUCCACCGACGUGAAGCCCGCCGCCCGGCACACGGCCAGGGCGUGGCUGCGCUUGUGGGCGUAGCGGCCACUGGCCUGCAGCACCAGGUCCGGGCCCUCUUCGGGAGCGGCUUCGCACGAGAAGAGGAAGUCGCCGUUC
Psp-1-1   UUCCCUCGCAAAACGGGAUCAUGUAUGCCGUUGACGGCGUCAGCCUGUCGCUCGAAGAGGGCGAAGUGCUGGGUAUCGUCGGUGAAUCCGGCUCCGGUAAAAGCGUCACGAUGAUGGCGCUGAUGGGGCUCGUGGCCUAUCCGGGACGGUGCGCGCCGA
env-2     GCAGCCAUAGCACCGGCUAUGGCCAGCAUUUGCAACAACGACGGUGUGCGCUUUUGCGGAGCGAGCGGGCAUGGCGGGUUUGUUUGCAACAUCUUGGGGGUUAGUCCAAUUUGGCGCCAGACGCCUUCACCACGGCCGCCCAGCGCUUGACCUCGCUGCU
Mpe-1-1   GCCGGGGCCUUCUUGCGCUUCUUCUUGACCGGCUUUUCUUCGGCAUCGGUGUCGGCCGCCGCGUCAUGCGCCGGCGCGUCGUCGACCUCGAUCUCGAUGCCCAGGGGCCGCACCGACUCGCGCAUGAUCUCGGCCGCCAGCUUUUCCUUGCCAGCACGCU
Aav-1-3   GCUGUAGUGCUUGAACUCCAUCGUGUACGUGGCACGGCCUUGCGUCGCGGAGCGCAGCGCGGUCGAGUAGCCGAACAUUUCCGACAGGGGCACUUCGGCCUUGAUGGCCUUGCCGCCGCCGACCAUGUCGUCCAUGCCCUGCACCAUGCCGCGGCGGGAC

          ................................................................................................................................................................
          ................................................................................................................................................................
          ••••••••••••••••••••••••••••••••••••••••••••••••••••••••••••••••••••••••••••••••••••••••••••••••••••••••••••••••••••••••••••••••••••••••••••••••••••••••••••••••
```

env-3	AUAGACCUUGUCGUCGGCCGGAUCGAAGAGGCUGCCCUUCUCCACCGGGUCACUGGCGACGAUCUGCUCCGAGCGCACGACCCAGAUGCUCGUGCCUUCCUGGCGGCGGGUGUAGACGUCGCGCGCCAUCUGGAUGGCCAUCGCCGCAUCGGCGGCAUGC
Psp-1-2	UGCGCUUGGGUUUGAAGAAGAGGUCUUCCGUGGCACCAAAUUCAACAAGAUCGCCCAGGUACAUAUAGGCCGUGUAAUCGCUGCAGCGGGCGGCCUGCUGCAUGUUGUGCGUGACGAUCACCACCGUGUAGUCGUUUUUCAGCUCGGCAAUCAGUUCCUC
Psp-1-3	GUCGGGGUUGUUGCGGACUUCAUCGGGGGUGCCGUCGCCAAUCUUCUUGCCGUAGUCCAGCACCACCACGCGGUCGGAAAUGUCCAUCACCACGCCCAUGUCGUGCUCGAUCAAUACCACGGUGGUGCCGAACUCGUCGUUCACGUCGAGGACAAAGCGG
Psp-1-4	AAUGAUGGGCAGACCGGAGUCGGCCAGCAUUUUCUUGCCCAGGUCUUCGUUGGUGCCCUUCAUGCGCACGACCAGCGGAACCGACAGGUUCACGGCCUUGCAGGCGGUGAUGACGCCGUCGGCGAUGGUGUCGCACUUCAUGAUGCCGCCGAAGAUAUUG
Aav-1-4	GCACCUUGACCCAGACCUCCAGGAACACCUUGGCGCCCAGCAGCUUUUCGAGCUCCAUGCGGGCGUCGGUGCUGAUGCGCUUGAGGCGCUCGCCCUUUUCGCCGAUCACCAUGGCCUUGUGGCCAUCGCGCUCCACGACGAUCGUCGCCGCGACGCGGAC
env-4	UCGCACCAAAGUCGAACUGCCGCACGCGCGCGAGCAGGCGGUUCUCCAGGUCGGGCGCGUUGAGUUCGGUGGAGACGAUGCGCACAUCCACGACAUCGCCUGAGGGCGCGAUGGUCAGGCGCAGCACCACCUUGCCCUGCAGCGACGGGUCUUCGCGCAG
env-5	UCGCGCAGGGGCUCAGGCACCCCGCACUCGCGCAUGGCCUGGCUCAUGCAGGCCACCCAUUGGUUGCGCUCCUGCAGGCCGAUGGCAAACGGCAGGUGGCGUGCCCGCAGUUGCGGGUGGCCAAAGCGCUGCACAUAGAGGUCGGGGCCGCCCAGCCAAC
Aav-1-5	CGAUGCAGAGCGCGUAGAACGCGGCGAACGCCCACAGCGCCAGUUCCGGCCCCCCCGUCGCGGCGAUGGAGCUGCCGUAGCUCUUGGGGAUGAAGAAGCCGCCGUAGGCGCCCAGCGCGCCCGCGAAGCCCAGGGCCGCGGCGCCUUCGGUGUUGCCCUC
Psp-1-5	AGGAUGCGUUCGCGGUUGUCGGCGGCCCAGGUGAAAUCCAUCUUCACGAGGCGCGCUUCAUAGUCCUUGGGCACGUUGGCCAGUGGCGGCGCCAGGCCGGGCUGCGCCGUGAUGGCGAAGUUCUUGCCGUACAGCAUCAUGGCGUCCUUGCUGGAAGCCC
Psp-1-6	CGCUCCGGCGCGGCCUCCUGCAGCUUGGCGCGCAGGGUCUUGAUGUGGGUGUCCACCGUGCGGUCGGUGCUGUCCGAGUCGGCACCCCAGACUGCGCCCAGCAAUUCGUCGCGUGAGCGUAUGCGGCCGCGAUGGUGCAGCAGGUCGGCGAGCAGGCCGA
Asp-1-1	CCGCGUCCUGGUGGUAGUCGGAGCCACGGCACACAGAUUGCGCAUAGGACUCCAGUUCGGCGAUGGUGCGCGCGUCGCUCUCGAAGGACUGGUUCAUGAUGGUCUUGGCCAUGCCGAUGGCAGCGGUGGAGGCCUCGCCAAACCGCUGCGCCAGCGCCAA
Rfe-1-1	GCUUGGUCACCAGACCAAUCAGCGUAAAGAAGUAAAGGGUGCCCAGCACCACCGGGUAAUCGCGCCGGAUCACACUCUCGUAACUCAGCAGCCCCAAGCCAUCGAGCGAAAACAGCGUCUCGAUCAGCAAGGAGCCGGUAAAAAAAGCGCCAAUAAACGC
Psp-1-7	GCCCCCUGCCAGGGGGAGGCCGCUACACGAAGUGAGUCGCAACGGGGGUGUGUCAUGUCUUCCUCAUCACUUCACCGAAUUUCUGGCCCGUGAGCUUCAGGCCCAGCCAGACGAUGGCGGUGGACAGCCCCAGCAGCAAAAAGCCGAAGGCAGCGCCC
Psp-1-8	AGGCGCUUGAAGCCCGAGCGGGCAAAGCGCUGCAAACGGCCGACCAUGAAGGCCACGAUCACCGAGGCACGCACCUGAGCAUCGACCGUGGGCGUGGCGGCGCCGCUGGCGGCCGCUGCGUCGCAGGCUCUGCUUGAGGCUGGACUCGAGCUUGUCGA
Psp-1-9	AUUGUUCCUGCUGGGCCGCGAUGAACUUGCCGGCGCUUUGCUGCGAGGUAUCGGCAAACACGUCACCGCCCAAUUGGCGCACGCGGCCCAGCACCUCGGGGUCGGCCAGUGCCUUGCGAAUGGCGGCCACCAGCGUGGCCUUUGCGUCCGCCGGCAGGCC
Aav-1-6	CCAUCGGCGGGCCAGUCGGGCACCUUGAGCCCCUUCACGAUGCCGUCCACGAUGUGGGCGGACUGCAGCAGGCGCGAUGCGGCGGCAUCGGAAAAACGGGGCACGAUGCGCUCGAAGAGCCGCUCUUUCGUCCCCCAGACGCGGUUUUCGCGCAGGGCCA
Aav-1-7	CCACGGCUUGGCCUUGACCGGGCCGGGCGUCUUCCACACCACGUUGAACUGGCCGUCGGCCUUGAUCUCGCCGAUGAACACGCUCUUGUGCAGGUGGUGGUUCUUCUCGUCCAUCUUGCUGACGAUGCCCGAGGGCGCCUUGAAGGUCUGGCCGGCCAUG
Aav-1-8	GAAAACCUGGGCAUCGACCCGGCGGAACUGCGGAUUGGGCGUCGGCCGCACGGCCACCGCCACGUCAUAGCGCAGGCCGGCCUGCUCGCAGGGCACGGUGCUGUCCCCGAUGGAAGGCAUCUGGCGUGCGAGCCGCACCUUCACGAGUUCGUUCUCCGCG
Aav-1-9	CCGCAUGGAUCGUCAGGCCCUCGAUGGGCUUCCUGGGCGCGUUGGCCUUGGGCACCACGGCCAUCGUGAAACCGAGCUUGGCGGCCUCCUUCAGGCGCUCCUGGCCGCGCGGCGCAGGGCGCACCUCGCCCGCCAGCCCGACUUCGCCGAAAGCGAUGAA
env-6	GAGCGCGUCUGCGCGUCGACCGUCUUCACGAUGAUCGCGGCGUAGGUGCUGUAGUCUUGGCCAGACUUGGUCUUCUUGGGCAGGUUGCCGCUGAUCACCACGCUGCCGCUCGGCACGCGGCCGUACGAGAUCUCGCCGGUGUCGCGAUUGAAGAUCGGGG
Psp-1-10	UGCGACCAGAAGCCUUUGCCGUAAUCACGGUUGGCUUCGACCUGGUCACGGAUACCGGUGGGGUCAUAACGCUGCUCGAAGUUAGCUGUGCCAAACAGCACAUCCCACCAGGGCAGCAGCACGCCAAAGUUAUGUCCACCCAAGACAGUUUUUUCCGCCU
Psp-1-11	UGCUUGCACUUCGGGAGAGCCCUCGGGAGCCGGUCUCAGGCCAAGGUAUCGCCAGAUAUUGCUGGCCCAGCUCAGCGCGUAAACGCCUUCAAGCUCGUAGGCCCGGCGCUGACACCGCCGGAGCCGGCCACGAUUUUGAAGGUUUUUUCAGCGGCC
Psp-1-12	GAGCCUGGACGGGGUCCAACUUCAACUUCAUCACCGAACCCGCUCGCACCCUGCCCGUCUAUGGCGAAUUUGACGUGGUCGUGGUGGGCGGCGGCCCUGCCGGCCUGGCGGCCAGCGUGAGUGCCGCAAAGCAUGGCGCACGCACCCUGCUGGUGGAGC
Psp-1-13	AAAUCGAGGACACUUACCCGGGCGAUGCCACGGGCCGGCCUGCGGUGCUGCUCAUCAUGGGCCUGGGCAUGCAGCUGAUCGCCUGGCCGCCGGAGCUGGUGCAGGGCCUGCUGGACGCCGGCUUUCGCGUGGUGCGCUUUGACAAUCGCGAUACCGGGUU
Psp-1-14	GCGCAGCCACGGGGAAGGGCGGUAGCGCUGGUCGCCAUAGACGGUUUGCAUGUUGAACAGCACCUCCAGCACGUUGGUGGGCCCGUAGCGGUCGCCCAUGGCGAGCGGCCCGAGCGGGUAGCCCAGCCCCAGCGUCACGGCGGUUUCAAGAUCUUUCGGG
Psp-1-15	GGCCGGGCCGGUCAUCAUGACCGGGGCAUCGAAGCCGCCCGCCCAUUCAAUCGUCAGCUGGCCGCCGCGGGUCUGCACGUCCACCCGCGCAUCGAGCAGGCCCCAGCGUAUGCCGGCCACCACCGCCGCACAGGCGCCGGUGCCGCAGGCCAGUGUUUCG
Psp-1-16	CAUGCUGGGUUGAAGAGUUCAGUGGGCACACGAGUGGGGGCCGCGGAACCGGCUUUGCCGGGCCGCAGGCGCCGCCCCCUAGAGGGGGGAACAAGCUACACGCAGUGAGCUGGACGGGGGUGGCUUUAUAACUCGGGAACUUCAAUCUCGGACUCGAAC
Psp-1-17	UCGGCGUUGUUCACAAUCUCGGAGGGGGCGCCGGCAGCCAGCACAUGGCCGUCACUGAUGAUGUAGGCAUGGUCGCAAAUGCCCAGCGUUUCGCGCACAUUGUGGUCGGUGAUCAGCACGCCAAUGCCGCGACUUCAGGAAACCGAUGAUGCGCUGGA
Psp-1-18	CGGUUUUUGCCACCACAUCGGGCGGUAGCUGCGGCGAGGGCGGGGUCUUGUCCCAGGGCUUGCCGUUGAUGCGCACGGCCUCCAGCCAGUCGCGCACGAACUGCUUGUCAUAGCUGGGCGGGUUCAGCCCGGCUGCAUAGGCGGCUUCGUAGCCUUCGAU
Aav-1-10	GGUCAGGAUGCGGCGGGGGUUCUUGGGGUCGGACUCGAUCGCGGCGCGGAUGCGCCCCAUGUGCACGUCGAUGGAGCGGUCGAAGGCUUCCAGUUCGCGGCCGCGCACGGCCUCCAUGAUCUGGUCGCGCGUGAGCACGCGGCCGGCCCGCUCCGCGAGC
Psp-1-19	UCUGCCUUGGUCCAGCCACCAAAGGCCUGGUCGAUGGUGAACAGGUUCAGCUUGGCGAACUGCUUGGCGUACUUGGCCUUGGCCUUCCCGGAAACCGCCGGGCGGUAGAAGUUCUUGCCCGCGAUGUCCUGGCCUUCUUCCGAAUAGAGGUACUUCAGGU
Psp-1-20	GCCAUAGUGGUCUUCAUAGACUGGCCCCAGGAAAUAGAGGCCGUUGGGUGAAAAGGUGGGGGCGGCGGCAUCCCGGCUGCGUGCGGCCAUCACGUCCAGCAGCCAGCCAGGCUCAUGGCUGCCUUGACCUAUGGCGACCAGGCAGCCCAUGAUGUUGCGG
Psp-1-21	CAGGAUCUGCCGGGUCGACACCGUCCCAUCGCGGUUCUUCUCGACCAGCAUCAGUUGCUGGACCAUGAAUGCUGCGCCCACCGGAAUCACCAUCCUGCCGCCGGCCUUGAGCUGGCGGAUGAGUGGCGGCGGAAUGUGGCUGGCCGCCGCGGUGACGAGA
Psp-1-22	AGGUCGCAGCGAUGUACUUGGGACUGCCGCGAUAGCUACGGGCCUCGCGCGCGAGUUCCGGCUUCACCCGCCUGAUCGGCCCCACCGCGCGCGCCAUGAGUUGCAGCUUGGCCGCGCGCUCCACGUAGAACGCGAGCACCGCCGCCUCCUCGAUCGUCUU
Cte-1-1	GGCCCACUCCAGCACCAGGCCCACGAUGCCGAUCACGAAGAUGGCGAUGAUGAUGUUCUUGACAUUGAGGUUGUUCCACUCGUCCCAGACCCAGAAGCCUAUGCCCACGCCGCCAGUCAGCAUCUCGGCGGCCACGAUCACCAGCCAGGCCGUGCCCACG
env-7	UCCUCGAUCGACUUGGUCGUCGUGCUCAGCCAGCGGAUGCCCGCCCGGCGCAUCAUCGCCUCCGCCUCGGCCACCUCCAUCCGGCAAUUCUCCAGCGAUGCGUACCGGGAGUUGGGCCGGCGCUCGUUGCGGAUCUCCGCCAGGCGUUCGGGCUGGAUGC
Psp-1-23	CAUGGCCACCAGGCCCAGCACGGCUUCGCGGCCAUCAAUGAUGCGGUGGUCAUAGCUCAUGGCCAGGUAGUUCAUCGGGCGAACCACAAUCUGGCCGUUCUCGACGACUGCCCGGUCUUUCGUGGCGUGCACGCCCAGAAUGGCGGACUGCGGUGGGGUUG
env-8	CUCGGCCGGCUCCUUGCGGAAGCUCGGCCGGUAGUCGGCGUGGAAGGCGUGCGGUGCGUCCGGGUACACCACGAAGGUCGACGCCUUGGCCGCCGCGCUGCCGCCCGCCAAGGCCGAUUUCAUCUUAUCAACCGUGUCAAGCGGGAUGCCGGUGUCGGCC
env-9	GCGCCGCGUGGCAGCUUCAGGGCUGACUUCGGACCCACGUUGCGCUCGAAGAUCUCGCCGUAGUUGCCCACCGCGUUGAUGGCGCGGUAGGCCCAGUCCUUGUCCAGGCCCAGCAGCUUGCCGGUGUCUUCCGACGUGCCCAGGAUGCGCUGCACCACCG
Psp-1-24	GCGGUCCAGCUUGUCGCUGAGCAGCUCGGCAAAUUUAUAAACGAAAUUGCGCAGGUAGGCGCUGCGUUGAAAGCGACGCGGGCCACGUUCAUGCCAAACAACAUGCCGGCCGGCUUGGCCACGAGGUCGCUGUUGGUGCCGUCAUCCAUGAUGCCAUC
Psp-1-25	UACAGGCUUUUUUGCUCCAUGUACUGCAGGAUCCCGAGGCGGCCCUUCUCGCUCCAGGCCGCUGUCGCGCCAGCCGCCGAACGGCGUCGAGAUGGAGAACUGCUUGUAGGUGUUGAUCCACACGGUGCCGGCCUGCACCGCGCGGCCGAUGCGCCAGG
env-10	ACCAGCUCUUCACGGCCCAGCUUGUCACUGCAGGCAUUGGCCGUGAGGGGCUCGCGCUUGCCGGCGCUCUUGAUCUGCACGUUCACGGCCUUGACGCCCAGCGACUCCAGGUAGGCCUUGACCGUCUGGGCGCGGGCCAUGGCCAGCUUUUCGUUGUAGU
Psp-1-26	AAGCUGGGGCUCAGCGACGCCGGAUAUGCGGUCGACUGGGUCGGCAGCGCCGAGCGCGCCGAAGAGGUGUUGCAGGGCGAGACCUUCGAUGCCGCCAUCAUUGACAUCGGCCUGCCCCGGCUCGACGGGCUGGAACUCACGCGGCGCCUGAGGCGCGGCG
env-11	GAACGCGGAAUGGUGCCGGGCGCCUGCACCACGACCCUGGUCGUGAUGCCGACAAUCGACUUGAUGUGGUGGGUGACGUCGCGCGCGAUGGCGUCGAACUGGUCGGCGCCUGUGCCGGCCAUCGCCUCGACCUCGAUGUGCACAUGGUCCAGGAUCCCGC
Psp-1-27	CCGAAGGCGCUUCGGGGUGGGCCUUCCUUGCGGCAGUUUGCCGGCGAUGGAAUAGCCGAUCACGCGGCCAGCAGCACUGUGAAGGCAUAGCUGUAGACCUCGAUGGACCAGGGCUGCAUCAACAUCACCAGGCCCAGGCCGAUCAGGGUGGCGGUGCC
env-12	GUCCGCGCACCACCUUGCGCAGGAACCGGACCAGCACCACCAGCAGGAUCACCGCCAGCACGACGCUGACGACCAGCACGACGCCGAACGCCACCGGGUGCGUGGCCGACAGCCACAGCAUGAAGACCACCAGCCCGUCCUCCGCCAGCGACACCGCCAC
env-13	CUGACGCUGAGCACCUGGCCGGUGAUGUAGCGCGCACGGUCGCUGCCGAAGAACAGGAUGGCGUCGGCGAUUUCCUCGGGCUGGCCCAGGCGCCGGAACGGGAUGGCCUUGGUCAGCGCUUCCUUCACCUUGUCGGGCACGACGGCCAUCAGCGGCGUGU
Aav-1-11	GAGCGCCUUGGUCUUCGCAUCGGCGUUGCAGGUCUUCAUCUUGUCCUGCUGGGUGGCGGGCUUCUUGGCCGAGAGGCACUCCUUCAUGAAGGCCUUGCGCUCGUCGCCCUUCUUGUCCUUGGCCUCGGCGUUGCAGGUGGUCAUCUUGCCCUGCUGCGCG
Asp-1-2	CACCCUGCCCAUGACGCGCCUGGCCGUCGGCACGCCCGAGGCACCGCUGAUGUCCGGCCUGUUCCUGGCCAUGGGCCGCGCGGCGGUGGCCGGGCUGCUGUCGGCCGCGCUGCUGCUGUGGCAGCGCGCGCCAUUGCCGCGGCGCGGCGACUGGUGGCCG
Aav-1-12	AGCAGGUUCACGGAGAGCGCGAUCAGCACCAGCAUCACGCCCGGGAACACCGUGAUCCACCAUUCGCCCGAGAACAGAUAGUCGUUGCCGAUGCGGAUCAGCGUGCCGAGCGACGGCGAAGUGGGCGGCGCGCCCACUCCCAGGAAGGACAGCGUGGCCU
Psp-1-28	UCCGCCGGCCACUGGUCAGCGUGCGCGACCUGGCGCACGCCGCGCAUGAGCAGAUGCACGGCCGCAUGACGCCCGCAUGCGUGAUCCACAGCGUGUCUGCCGGGCCCUGCAAUGCGUCGAAGGCAGAGGCGACGCGCGCCAUCACCUGGCUGACGCUUU
Aav-1-13	CGUACUGCGCGAAUUCCUCCGGCGUCUUGCUGGGCCAGACCUGGAAGCCGAUCUGCGCGAGCUUGUCCUGCGUGUCCCUGUCGGCCAGCACGGCCUGCAGCUCGGUGUUCAGGCGCUGCACCACGGCGGGCGGCAUGCCGGCCGGGCCGAAGAUGCCGUU
Aav-1-14	CGUGACCAUGCCGUAUUUCAGGUUCUUUCUGCGCAGCGCAUGCACGACCGUGGCCGAGCGGAUGGCGCCCGUGGCGCCCAGCGGAUGGCCCAGCGCGAUGGCGCCGCCCAUCGGAUUCACCUUGGCCGGGUCCAGGCCCAGCUGGCGCAGCACGGCGAGC
Aav-1-15	UCGCCUUCCACCUCGAAGCCUGCACGGCGGUAGAAGUUCUUCGGCGGUGCAUUGCGCGUGCAGCGUGACCCGCCUGUCGCCCGCGCCGCGCGCCUCGACCAGCGCAUCCAGCACCGCCUGCCCCACCGCUGCCCGCGCACCGCGCGGUCCACGGCCA
Aav-1-16	GAAACUCUUGCGCUCGUCCCCGCCCAUGCCCAGGUAGAACUCCUUCACGUCCUCGUUGUUGGCAAGGUCGGAAGCGGCGCCGUCCAUCACCACGCGGCCGCUUUCGAGGAUGUAGCCGUAGUCCGCGUACUUCAGCGCCAUGUGCGUGUUCUGCUCGGCC
Aav-1-17	AGUUCUGGCUGUCCACCACGGCCUUGGCCACGUCGUAGAAGCUGACCACGCCCAUGAGCAUCUUCUUGUCCAUGACCGGCAUGUAGCGCGCGUGGCGGUUGAGCAUCAUGCGGCGCACCUCGUCCAUUUCGGUUUCGAGCGUGCAGGUGAGGGGCGCGUC
Psp-1-29	GCGUGGCCCGGCCGCGGUGACUGUUGGCGUUCUUGACUUCCACCGGCAACUGCGCAAAGGUCUGGCCGAAUUCCGGAAUGAACAUGACCGGGUCAAAGCCAAAGCCGUUGCUGCCCACCGGCUCACGGGCAAUGAGGCCCACCACCCGCCCGCAGGCGAU
env-14	GUCGCCGCUGUAGAUGGCGCGGCCGCAGAUCACACCUUCCACGCCCUCGUCUUCCACGGCGCACAACUGCUCGAUGUCGGCCAUGUUCGACAGGCCGCCCGAGGCGAUCACGGGAAUGGUCAGCGCCUGGGCCAGUUUGACGGUGGCGUCGAUGUUGAUG
Aav-1-18	GAACAGCAGCACGAAGGGUGCAGCGUGAAGCCCCCCACCAGCAGCUGCCGAACCACAGGUUCUCGCGCACGGCGCCGAGCGAUGCGGCCAGUGCCAUGAUCCCACGAGCACGAUGGACGUGGGGAUCGGCGGCCCUCGAAGUACUUCACCUUGCCC
Aav-1-19	GACUCAGGGGUGCCGCUAUUUCCAGCCGAUAACCCACCCCGUACACCGCCGUGAUCACGUAGCCAUGGUCCGCGCCAGCGCCAGCUUGGCGCGCAGGCGGGAUAUGUGCGUGCCAGAGAACGCGAGGUGCGUCGUGGAUGCCGCCCCAUAUCGCCU
Aav-1-20	AGAGUGAGCGUGGACCUUGGGAAAGCGCGCGAUGUCCACCCCGUCGCUGGCCAGCCAGCCGGCCACGGUGAACAGGUAGCCGUCGGCGACCGUGUACUGGUCGCCCAUCACCCAGGGGCCGUCGCCGAGGCUGUGCUGCUCGAUCAGCUCGAAGCACUCG
Aav-1-21	UAGGGCGGCAUGGGCGCGUGGUAGGCCAGACGCUGCUGGUUGUACACGAUGCGGUGCGCGGACAGUUGCCACUGCGGGAUGAGGUAGUGGCUGUGCAUGAUGACGCGGUCCAGCGCCCGGCACGCGGGCAGCAGUUCGGCCUUGGAUUUCGCCCGCGUCA

```
Aav-1-22  UAGCGGCGGUCCAGCAGUUCGCGCGGCUUGAGGUCCGCCAGCUGGCGGUGGGCGUCGCCCAGCGCGCGCUUGAGGAAGGCGGCCAUCUGCUUGUGGUCGCGGUGGGCGCCGCCCACGGGCUCGCUCACGAUCUUGUCCACCAGGCCCAGGGCCUUGAGGC
Aav-1-23  GGCACUGGGCCCCCAGCUGCCGGCGGCGUAGGGCCGUGGGCCGUCUUCCGAGUCGCGCCAGGAUUGCAUGAUGGGCUCGACCCAGCGCCACGCCGCUUCCUGCUCGUCGGCGCGCACGAAGAGGUUCAGCCGCCCGGCGAUCACGUCGAGCAGCAGGCGU
Aav-1-24  CAGCGUGGCGGCCGAGAGCGGGCCCAGGGUGUCGGUGAGCGUGCUGCGCAGGCGGCCCGCGCGGGCGAGUUCCGCCACUUCGGCCAGCAGUCGGCCCUGCGCGGCCAUGUCGGGCGUGCCGAACAUCGGGCGCGUGAACAUCAUCUCCCAGUGCAGCGAG
Psp-1-30  UUCACGGCCAUCUUGUCAAUGUCGCCUGCCUUUUUGCCCGUUGCGAUGAUUUCAUUGACCUUCAGGCGCAGCUUGUCUUCGCCCUUGCCCACGCCAAUGAAGUUGGGCGAGUCCUUGAGCAGCAGUUUGUACUCGGCAGCGAGUUUCGGGUUUUUGGCCA
Psp-1-31  CGCCCCCUGCAAGGGGGGAGGGGGCUACGCAAAGCGAGCCCCAACGGGGGUGUGCAACAUUUACUGUGGCAAAUCACCGGCAGGGCGACCCAGCCAUUUCACGGCCAUCUUGUCAAUGUCGCCUGCCUUUUUGCCCGUUGCGAUGAUUUCAUUGACCUUC
Psp-1-32  CGCCCCCUGCAAGGGGGAGGGGGCUACGCAAAGCGAGCCCCAACAGGGGUGUGCAACUCCUGCCGCCGUGGAACCGGCUUUGCCGGACCACCAGCGGCGCCCCCUGCAAGGGGGGAGGGGGCUACGCAAAGCGAGCCCCAACGGGGGUGUGCAACAUUUA
Mpe-1-2   UGUCCAGCACGUCCAGCGGACUGAUCGGGCGGAUGCCCUCGAUCAGCGCGCCCUCGCCGUGGCCGUAGAGCGCCUGCAGCGCGAAGCGGCAGGCGUAGACCUUCCCGCCCUCGGCGAUGAACUUGGCGAUCUGGUUGUUGAAGUUCUGGUGACCGGGGAA
Mpe-1-3   UACGCAGCCUGCUCGUCGGUGAGCUCGGUCAGCAUCGCGCCGACCUUCUUCAGGUGCAGGCGCGCCACCUUCUCGUCCAGGUGCUUGGGCAGCACAUAGACCUUGCCGACCUGGUAGUCGGCGCUCUUCGUGAAGAGCUCGAUCUGCGCGAUGGUCUGGU
Mpe-1-4   CCGCCAGCGCCUCGGGGAAGCUCAGGUAGACCGGCUUCUCCGCCCCGCGCAUGCGCACCAGCAGGCGGUGCUGCACCUGGUACUUCACGUUGCCGAUCGCCAGCGCGCCGAUGCCCACCGCGCGGCUGCCGGGCAGCAGCUUGCCGCUGUCCAUCACGCC
Mpe-1-5   UCAAGGCCUUGGCCUGGCCGACGGUGAACGUGCCGUAGUGGAAGAUGCUGGCGGCCAGCACCGCAUCGGCGCCACCGACCUGGAUGCCCUCGGCGAGGUGCUCCAGCGCGCCGACGCCGCCCGAGGCGAUCACCGGCACCGGCACCGCGUCGCUGACCGC
Mpe-1-6   CCGUUGCUCGCCAGCGCAUCCACCUUCGCCGCGGCCUGGGUGCGCCCGCUGCGCCACACGCUCGGCACCUGCAUGCCGGCGUAGUCGUGCUGGGUCGGCGCGGCGGACAGCCCGCCCUGCGUGUGCAGCGGCUGGUUCAGCACCGGGAUGUUGUCGGUGC
env-15    CGGGAGGCGCGAUCCAGUUGGCGAAUUUGCCCGGCUCGACGACGCGGCCCAUGAGGCUGGCGACGUAGGCACGGUCCUGCGCGCUGGGCAGCCACUCGUCGGCCUUGGCGUUCCACUCGGCCUCGGAGACCACGCGGCCCUCGGGCGAGACCUUGACGCC
Psp-1-33  GCUUGGUCACCAGGCCGAUCAGCGUGAAGAGAUAGAGCGUGCCCAGCACCACGGGGUAGUCCCGCCGGAUCACGCUCUCAUAGCUGAGCAGGCCCAGGCCGUCCAGCGAAAACAGGGUUUCAAUCAGCAGCGAACCGGUGAAAAAAGCUCCGAUAAACGC
Mpe-1-7   ACUCGGCCUGCUGGAAAGCGGCGAACUGCUCGGGCGUGUUGGCCACCACCUCGAUGCCGAUCUCGGCCAGCUUGUGCUUCACCUGCGCGUCGUUGAGCGCGGCGACGAGCGCGGCGUGCAGCUUGCCCUUCACGUCGGCCGGCAGGCCCUUCGGCGCGGC
Cte-1-2   UCGACUCGCGCGUGGUGAUGCGGGUCACUUCAUCGGGCCCGGCCACUGCAAGCGGCACGGAGAACGCGACGAACUUCUCGGCCAUGCCCGGUGCCGACAGGCCCUUGCGCACGGUCUCGGUCAGCUUGGCGGCCACGUCGGCCGGCGUGCCGGGCGGCGC
env-16    ACCUUGCCGGCCUUCCAGGCGUCGAGCAGCGUCUGGUAGGCGAUCGUCUGGCCCUUGGGCUUUUCAUUCGCCAGCUUGGCCCACGGCGCGGCCUUGUCGCUCAGAUGCUUCUUCGGGUCUUCCUUGGGGUUGAGCUUGGGCGGGCAGCGGGCCAUGCCGG
env-17    GAAUCGGGCCCUUCUCGAUCUUCUUGCGCAGGCGGUGGAUGUAAACCUCAAUCGCGUUGUUGCUCACCUCUUCGCCCCACUCGCAGAGGCGCUCCACCAGCUGCUCCUUGCUCACCAGGCGGCCGGCGCGCUGCAGCAGCACCUCCAGCAGGCCCAGCUC
env-18    ......................................................................................................................................................
          ......................................................................................................................................................
          ......................................................................................................................................................
          ......................................................................................................................................................
```

alignment positions 420 · · · 479

```
env-1     ACUGCUCGGUGCUCAUGCCGAACUGCUGCA..............................
Rme-1-1   GCGCCUUCGCUGUAGAGCGCAAAGCCGGUCACGACCAUGAACACCGACAGCAUCAGGUAC
Aav-1-1   AGUGCGGAUCGCUGGUGCGGGCCAUGGCCACGGUGUACUUGUCGUUCUUCAGGCCGUUCU
Aav-1-2   GGCACGAUCAGGCGGAAGGCGUCGGGCACGGUGUCCGACAGGUCGCCCGCGUAGAUGAAG
Psp-1-1   CAAACUCCGCUUUGCCGGCCAUGACCUGCUGGCCCUGUCGGCCAGCGAGCGCAGCAAGCU
env-2     CACAAACGCGCCAAACUGUUGCUGCGUCAUGCCGCCGUAGUCCGCGCCGUUGCUGGCCCA
Mpe-1-1   CGAGCAGCUCGCCCGAGGUCUUGUGCGACAGGAUCAUCAGCACGCCGUUGCGGAUGGCCG
Aav-1-3   GAGAGGUCGCCCAUCACGGUACCGGCGUAGUCUUCGGGCGUCUCCACUUCCACGGCCAUC
env-3     AGGCUGCCGCAGUGCUUGUGGUCCAGACCCGCCUUGCUGCGGACGAAGACUUCCCACAGC
Psp-1-2   CACCUUGGCCGUGGAAAUGGGGUCCAGCGCCGAGCAAGGCUCAUCGAGCAGCAACACCUC
Psp-1-3   CACAUGUCCUGCUUUUCUUCGACGUUCAUGCCGGCCAUGGGCUCGUCAAGCAGCAGGACC
Psp-1-4   ACCAGGAUGCCCUUGACCUCGGGGUUCUUCAGCAUGAUCUUGAAGGCUUCGGUGACUUUC
Aav-1-4   AAAGCGCUUGUGCGUCUUGCUGGGCUCUUCCUCGAACUUGUCGAUCACCACGGUGGAGGU
env-4     CGCGCGGUUGUAGAUGGCGUAGAUGGCGCCCUUGUGGCGUUCGAAGACGAGCUUGAUCUC
env-5     CACACAAGAACCAGAAGAGUUUGUCGCGGGCGGCAUCCAGCGUGGGGCCAUGCGUGGCGC
Aav-1-5   GCGGGUGGCCUGGGCGCGGGCCCCGGCGCCGCCCGUGCGUACUUCGGCCAGGCGCAGGUU
Psp-1-5   AGUCGGCCAGUUUUUUGGCGGCAUCCAGUUUUUUGGUGCCCUUGUGGAUGGCAAACGCCU
Psp-1-6   ACUCCCGGCGUGUCAGGUUGAGCGGACGGCCAUGCAGGCGCAUGCGCUGGCCCGCUGCGU
Asp-1-1   CGCGGCCUCGGUCAGCGCGGUGGCGUCGUCCACGAUGUCGAACACCAUGCCCAGUUGCUU
Rfe-1-1   CGCCGGGAAGCCGGUGAUGAUAGGAAUCAGCGCAUUGCGAAAAACGUGCUUCCACAGCAC
Psp-1-7   UGUUCCCAGUUGAAACGGGUAAUGAACUGGGUGUAAAUCUGCUCGGUAAACCAUAGCGAG
Psp-1-8   AGAACUGGUUCAUGCGCUCUUGCAACCGGUCAUUUUCAAACACCAGUGCAUCGCCCACCA
Psp-1-9   UGCAGGGGCCAGCAGCGGGUUCCACUCCAGCACCUCGAAGCCGGCCAAUCCGGCCUCCGC
Aav-1-6   UGGGCAGGGGCUUGCCGGCAUUCACCGCAUCCUUCACGCGCUUCAGGGACCGGAUGUCUU
Aav-1-7   GCGGCGAUCACCUUGUCCACGUCGGUGCUCUUGGCCUUCUCCACGGCCUGCUUCCACAUG
Aav-1-8   CAGAUGUGCGCGAGCAUCACGUCGGACUGGCGCGCGGCAUUGCGGGCCAGCGCCGAGGUG
Aav-1-9   GCCCUUGGGCAGGGGCUUGCCGCGCAGGCUGCUGGUGAUGGCCAGCAUCACCGCCAGGUC
env-6     UGCUCUGGCCGAUGUACACGCCCAUGCCGAGCACGGAGUUCUCUUCGACGAUCACGCCUU
Psp-1-10  UCACGGUUUCAUGCCCGAUACCGAUGCUGUGAUGCAAGCGGUGAAAACGCGGGCUGACCC
Psp-1-11  ACAUACUCGUGCACGCUGGCCACGUGGAUCACAUUCUUGUUGUCGACGAAGCUGUAGCAG
Psp-1-12  GCUACGGCUUCCUGGGCGGCAUGGGCACGGCGGGCGGCGUCACCAAUUUUGCGGGCCUGU
Psp-1-13  AAGCCAGCACUUUGACCACCUGGGCACACCCAACCUGCUGUGGGAGGGACUCAAGUACCG
Psp-1-14  CUGCAGAUGCCUUGCUGGCAGAUAUCACUGGCAAUGUUGACGAUGGUGGCCACGACGCGC
Psp-1-15  CCAGCGCCGCGCUCGUACACCCGCAGGCGGAUGCGGCUGCGCGAGACGACCUGCAUGAAG
Psp-1-16  ACGGUAGUGGCCGGGCCGGUCAUCAUGACCGGGGCAUCGAAGCCGCCCGCCCAUUCAAUC
Psp-1-17  UUUCGAUCACGGCGAUCGGGUCGAUGCCGGCAAAGGGCUCGUCGAGCAGGAUGAAGCGCG
Psp-1-18  GGGCCAGUAGCGUGACGAAUCGGGUGUCAGCACCUCGUCCAUCAGCGUCAGCGUGCCGUU

          ............................................................
          ............................................................
          ............................................................
```

```
Aav-1-10   GCCACGAGCAGGUCGAACUGGUAGGAGGUGAGGUCGGCCGGUUGCCCGCCCACCGUGACG
Psp-1-19   AUUCCUCGGCGACGGCACGCGUGCCUUUUUUGUCGACGUUCUUGUCGACGACGGCCACAG
Psp-1-20   AUCAUGUGGUGCAAAAAGGCGUUGGCUUCGAACUCGAAGUGCCAGUACUUUGAACCGGUA
Psp-1-21   AUGGCAUCGUACGGCGCGUGUUCCUCCCAGCCGUGGUAGCCGUCGCCCAGCCGCACCUGG
Psp-1-22   CGCCGCGGUCAGCAGGCCGUGGUGCGCGAGCAGCACCGCCUGCUUGUCGCCCAGCGCGGC
Cte-1-1    GCCAGGCGCACGCCGGUCAGCAUAUAGGGCAGCACCGCAGGAAACAGAAUGGUGGUGGCG
env-7      UCAGUCCGAAGAUCUUCUUGUGGUGGGCGCCCAGCGCGGAGGGCAGCUGCUGGCGUUCGA
Psp-1-23   AUGAUGGGCGUGGACAGCAUGGAGCCGAACACACCGCCGUUGGAGAUCGAGAAGGUACCA
env-8      GCGCCGUAGAGCCCCAGCACCGGGCCGUGCAGCUGGCCGGCCACGUCCACCGGGUUCUUC
env-9      GGUCAUUGCUGUCGGCUUGAGCUGUCGACGUUGGCCUGGGUGAUGCCGUACUCCUCGCUU
Psp-1-24   UCGGCCACAAUCCCCACGCCCAGCCCCAGGCGCACAUAGGUCUUGAUCACAUCAGAGUCA
Psp-1-25   CGCGCUUGUAGUCGCGCGUCCAGAUGCCGGCGGCCAGCGCGUAGAGGCUGUCGUUGGCCU
env-10     CGUCACCGCCGAUGCGGUCGGUGUAGCCGGCAAUCGUCAGCGACUGGAUGUUGCGGUAGC
Psp-1-26   GCUUGGCCAUGCCGGUGCUCAUUCUGACGGCGCGCGAUGCGCUGCAGGAUCGCGUGCAGG
env-11     GGCGCUCGACUACCAGCUGGUAGUGCGGCGCGAGGUUCGGCCGGCCGACCAGCACGGACU
Psp-1-27   ACGUUCCCAGGGCGCGCGGCGCAGCCAGCGGGGGGAUUUGCGGGACUUCAUUUUCGAAUG
env-12     GUUGGAGAACGGCUCCGGCGAGGUAUUGACUGCCGCCCGCGUGGUCAUCUUGGUGGCGUG
env-13     CGGUCGGGCCGGGGCAAACGC........................................
Aav-1-11   GUGUGGGCCUUGGCGGAUGCCGCGCCGGGUGUGGACGCCGGUGCGGACGCCAUGGCCGCG
Asp-1-2    CUGGCCGUGACGGCGCUGGGCGUGGUGUUCGGCUUUCCGCUGCUCACCUCCAUCGCCAUG
Aav-1-12   CGGUGAUGAUGGCCGUCGCCACCUGGAUCGUGGCCAGCACCAUCACCGGCCCGAGCACAU
Psp-1-28   CCCCGUCGCCACCGACGGCGUAGUUCGCAAAGUCGUCGGUCCAUGCCUGCAACUCGGCCG
Aav-1-13   CCACGACGUCAGGUCGAAACCCUGCACCGUCUGGCCGAUGGGUGGCACGCCCGGCAGCAG
Aav-1-14   GACUGCGCGGCGAAGGCCUCGUUCAGCUCGAACCAGUCGAUGUCCUGCUGAUUCAGGCCG
Aav-1-15   UGCGGCCGAUGCGUGCCUCGCCCGGCGCCGGCUGCAGCAGCCGGCCGGUGGCCACGGGCA
Aav-1-16   AGCAGGAAGGUGACCUUCUCGCGCGUAUUGAGGUCGCGCACGAUGUGGAAGACCUCGUCC
Aav-1-17   GUCCAUGGCCGUGCGCACGAGCAUGGUGCCCACGGUGCCGCCGUUCUUCACGACGGCCUG
Psp-1-29   CAGGGGCUCGGGGUCCAUCUCGGAUCGCACCGCCACCAGGGUGCUCACCAGCGCCGCGCG
env-14     CCCGAGAGCAUGCCGUCACGGCCGAUGUCGGUGUAGAUGAUGGAUUCGACGCCCCAGUCC
Aav-1-18   GAGCCUUCGGAGAGCGUUUCCGCCGUGACGUUGUAGCGCGCGAGGCGCGAGACGCCGCAG
Aav-1-19   CGCGCAGGUGCUCGCGCGACAGCAGCCGUCCCGCGUUCUGGAACAUGAACAGCGCCAGCU
Aav-1-20   GUCAUGUUCUGCGGCACCUUGCGCUGCAUCGCGGCGAUGGCCUCGGGUUCGUCCGCCCAG
Aav-1-21   ACGCGAUCGCGAGGGCAUCGACCGCCGGGCUCUUCACCCCCGAGUAAUUGCUGCUGCCCU
Aav-1-22   GGUGCGCGGUGAUGCCCAUGGCAUCGGCCGCGUCCUGGGCCUUGUCGCCGGUCUUCCAGA
Aav-1-23   UCGUAGGCGCCCACCCGCUCCGUGCCGAAGCGCUGCUCGAAGUCCAGGUCCAGGUGGACG
Aav-1-24   AUGCUCUUGCGCUUGAGCGGCAUCACGUCGAGCGAGGGCAUGUCGUCGAUCACCGCGAUG
Psp-1-30   UCAUGUUGCCGGCGACUGAGGCACCCGUGGCAAUCAGUUGCACCUGGCCUGCGACGAACG
Psp-1-31   AGGCGCAGCUUGUCUUCGCCCUUGCCCACGCCAAUGAAGUUGGGCGAGUCCUUGAGCAGC
Psp-1-32   CUGUGGCAAAUCACCGGCAGGGCGACCCAGCCAUUUCACGGCCAUCUUGUCAAUGUCGCC
Mpe-1-2    GGCCUCGUCGCCGAGCGUCGGGAAGCCGCGCUUGACACCCAGCGUCACGCCGGGGCCGUA
Mpe-1-3    UGGCGAACGACGAGCUCAUCACGAAGCUCGGGUGGCCGGUGGCGCAGCCCAGGUUGACCA
Mpe-1-4    CACGCCGGCGAUGCCCUCCGGCGGCACCGCGUUCACGUCGGCCGCCACCAGCAGCCGCGU
Mpe-1-5    GCGCGUCAGCGGCAGGUUGAAGCCGAUCUGCGUGCCGUCGCGGUCCAUGCUGGUCAGCAG
Mpe-1-6    CGGUCCGCUGCAGCGUGGCGUUGUGCACCACCGUCAUCGGCGCCUGCACACGGCGGGCCG
env-15     GGCCAGUGCGCCGAUGUUGCGG.......................................
Psp-1-33   GGCGGGAAACCCUGUGAUGAUGGGAAUCAGCGCAUUGCGAAACACAUGCUUCCAGAGCAC
Mpe-1-7    GACUGCCUGCCACGAGUAGACGUCGACGCCCUUCACGCCGCUCUCGACCAGCGUCGGCAC
Cte-1-2    CCACAGCGCGAAGUUGAUGUCCUGCACAUAGCCCGGCACGAAUUUGUUGAGCGGCACCGC
env-16     CACGUUCGAGCCGGGACAGCACGUCGUCCAUCUCCUCGGCCAGCGUGUCCAUCGCCUUCU
env-17     GCGGGCCGACAGCUCCACCAUCUUGCCGUCAAUGGUGGCUACGCGACCGGCCUGGUCGUA
env-18     .............................................................
           .............................................................
           .............................................................
           •••••••••••••••••••••••••••••••••••••••••••••••••••••••••••••
```

16 c4 antisense RNA

16.1 Taxa

The taxonomy of each organism containing a putative c4 antisense RNA RNA is listed, with abbreviations identifying each hit (e.g., "Eco-1-1" and "Eco-1-2" might hypothetically represent two distinct RNAs in *E. coli*). The abbreviations will be used to identify each individual c4 antisense RNA RNA in Sections 16.2 and 16.4.

**abbrev. of hits**	**taxonomy of species**
Bce-1-1 to Bce-1-2	Bacteria Proteobacteria Betaproteobacteria Burkholderiales Burkholderiaceae Burkholderia Burkholderia cepacia complex *Burkholderia cenocepacia* MC0-3
Bce-2-1	Bacteria Proteobacteria Betaproteobacteria Burkholderiales Burkholderiaceae Burkholderia Burkholderia cepacia complex *Burkholderia cenocepacia* PC184
Bdo-1-1 to Bdo-1-2	Bacteria Proteobacteria Betaproteobacteria Burkholderiales Burkholderiaceae Burkholderia Burkholderia cepacia complex *Burkholderia dolosa* AUO158
Bmu-1-1	Bacteria Proteobacteria Betaproteobacteria Burkholderiales Burkholderiaceae Burkholderia Burkholderia cepacia complex *Burkholderia multivorans* ATCC 17616
Bsp-1-1	Bacteria Proteobacteria Betaproteobacteria Burkholderiales Burkholderiaceae Burkholderia Burkholderia cepacia complex *Burkholderia sp.* 383
Bvi-1-1 to Bvi-1-3	Bacteria Proteobacteria Betaproteobacteria Burkholderiales Burkholderiaceae Burkholderia Burkholderia cepacia complex *Burkholderia vietnamiensis* G4
Bma-1-1	Bacteria Proteobacteria Betaproteobacteria Burkholderiales Burkholderiaceae *Burkholderia mallei* 2002721280
Bma-2-1	Bacteria Proteobacteria Betaproteobacteria Burkholderiales Burkholderiaceae *Burkholderia mallei* ATCC 10399
Bma-3-1	Bacteria Proteobacteria Betaproteobacteria Burkholderiales Burkholderiaceae *Burkholderia mallei* ATCC 23344
Bma-4-1	Bacteria Proteobacteria Betaproteobacteria Burkholderiales Burkholderiaceae *Burkholderia mallei* FMH
Bma-5-1	Bacteria Proteobacteria Betaproteobacteria Burkholderiales Burkholderiaceae *Burkholderia mallei* GB8 horse 4
Bma-6-1	Bacteria Proteobacteria Betaproteobacteria Burkholderiales Burkholderiaceae *Burkholderia mallei* JHU
Bma-7-1	Bacteria Proteobacteria Betaproteobacteria Burkholderiales Burkholderiaceae *Burkholderia mallei* NCTC 10229
Bma-8-1	Bacteria Proteobacteria Betaproteobacteria Burkholderiales Burkholderiaceae *Burkholderia mallei* NCTC 10247
Bma-9-1	Bacteria Proteobacteria Betaproteobacteria Burkholderiales Burkholderiaceae *Burkholderia mallei* PRL-20
Bma-10-1	Bacteria Proteobacteria Betaproteobacteria Burkholderiales Burkholderiaceae *Burkholderia mallei* SAVP1
Bph-1-1 to Bph-1-2	Bacteria Proteobacteria Betaproteobacteria Burkholderiales Burkholderiaceae *Burkholderia phytofirmans* PsJN
Bxe-1-1 to Bxe-1-2	Bacteria Proteobacteria Betaproteobacteria Burkholderiales Burkholderiaceae *Burkholderia xenovorans* LB400
Bok-1-1	Bacteria Proteobacteria Betaproteobacteria Burkholderiales Burkholderiaceae Burkholderia pseudomallei group *Burkholderia oklahomensis* C6786
Bok-2-1	Bacteria Proteobacteria Betaproteobacteria Burkholderiales Burkholderiaceae Burkholderia pseudomallei group *Burkholderia oklahomensis* EO147
Bps-1-1	Bacteria Proteobacteria Betaproteobacteria Burkholderiales Burkholderiaceae Burkholderia pseudomallei group *Burkholderia pseudomallei* 1106a
Bps-2-1	Bacteria Proteobacteria Betaproteobacteria Burkholderiales Burkholderiaceae Burkholderia pseudomallei group *Burkholderia pseudomallei* 1106b
Bps-3-1	Bacteria Proteobacteria Betaproteobacteria Burkholderiales Burkholderiaceae Burkholderia pseudomallei group *Burkholderia pseudomallei* 112
Bps-4-1	Bacteria Proteobacteria Betaproteobacteria Burkholderiales Burkholderiaceae Burkholderia pseudomallei group *Burkholderia pseudomallei* 14
Bps-5-1 to Bps-5-3	Bacteria Proteobacteria Betaproteobacteria Burkholderiales Burkholderiaceae Burkholderia pseudomallei group *Burkholderia pseudomallei* 1655
Bps-6-1 to Bps-6-2	Bacteria Proteobacteria Betaproteobacteria Burkholderiales Burkholderiaceae Burkholderia pseudomallei group *Burkholderia pseudomallei* 1710a
Bps-7-1 to Bps-7-2	Bacteria Proteobacteria Betaproteobacteria Burkholderiales Burkholderiaceae Burkholderia pseudomallei group *Burkholderia pseudomallei* 1710b
Bps-8-1 to Bps-8-2	Bacteria Proteobacteria Betaproteobacteria Burkholderiales Burkholderiaceae Burkholderia pseudomallei group *Burkholderia pseudomallei* 305
Bps-9-1	Bacteria Proteobacteria Betaproteobacteria Burkholderiales Burkholderiaceae Burkholderia pseudomallei group *Burkholderia pseudomallei* 406e
Bps-10-1	Bacteria Proteobacteria Betaproteobacteria Burkholderiales Burkholderiaceae Burkholderia pseudomallei group *Burkholderia pseudomallei* 668
Bps-11-1 to Bps-11-3	Bacteria Proteobacteria Betaproteobacteria Burkholderiales Burkholderiaceae Burkholderia pseudomallei group *Burkholderia pseudomallei* 7894
Bps-12-1	Bacteria Proteobacteria Betaproteobacteria Burkholderiales Burkholderiaceae Burkholderia pseudomallei group *Burkholderia pseudomallei* 9
Bps-13-1 to Bps-13-2	Bacteria Proteobacteria Betaproteobacteria Burkholderiales Burkholderiaceae Burkholderia pseudomallei group *Burkholderia pseudomallei* 91
Bps-14-1	Bacteria Proteobacteria Betaproteobacteria Burkholderiales Burkholderiaceae Burkholderia pseudomallei group *Burkholderia pseudomallei* B7210
Bps-15-1 to Bps-15-2	Bacteria Proteobacteria Betaproteobacteria Burkholderiales Burkholderiaceae Burkholderia pseudomallei group *Burkholderia pseudomallei* BCC215
Bps-16-1 to Bps-16-2	Bacteria Proteobacteria Betaproteobacteria Burkholderiales Burkholderiaceae Burkholderia pseudomallei group *Burkholderia pseudomallei* K96243
Bps-17-1 to Bps-17-2	Bacteria Proteobacteria Betaproteobacteria Burkholderiales Burkholderiaceae Burkholderia pseudomallei group *Burkholderia pseudomallei* Pasteur 52237
Bps-18-1	Bacteria Proteobacteria Betaproteobacteria Burkholderiales Burkholderiaceae Burkholderia pseudomallei group *Burkholderia pseudomallei* S13
Bth-1-1 to Bth-1-2	Bacteria Proteobacteria Betaproteobacteria Burkholderiales Burkholderiaceae Burkholderia pseudomallei group *Burkholderia thailandensis* Bt4
Bth-2-1 to Bth-2-2	Bacteria Proteobacteria Betaproteobacteria Burkholderiales Burkholderiaceae Burkholderia pseudomallei group *Burkholderia thailandensis* MSMB43
Bth-3-1 to Bth-3-3	Bacteria Proteobacteria Betaproteobacteria Burkholderiales Burkholderiaceae Burkholderia pseudomallei group *Burkholderia thailandensis* TXDOH
Reu-1-1 to Reu-1-2	Bacteria Proteobacteria Betaproteobacteria Burkholderiales Burkholderiaceae Cupriavidus *Ralstonia eutropha* H16
Reu-2-1	Bacteria Proteobacteria Betaproteobacteria Burkholderiales Burkholderiaceae Cupriavidus *Ralstonia eutropha* JMP134
Rpi-1-1 to Rpi-1-6	Bacteria Proteobacteria Betaproteobacteria Burkholderiales Burkholderiaceae *Ralstonia pickettii* 12D
Rpi-2-1 to Rpi-2-5	Bacteria Proteobacteria Betaproteobacteria Burkholderiales Burkholderiaceae *Ralstonia pickettii* 12J
Rso-1-1 to Rso-1-5	Bacteria Proteobacteria Betaproteobacteria Burkholderiales Burkholderiaceae *Ralstonia solanacearum* GMI1000
Rso-2-1 to Rso-2-3	Bacteria Proteobacteria Betaproteobacteria Burkholderiales Burkholderiaceae *Ralstonia solanacearum* UW551
Cte-1-1 to Cte-1-2	Bacteria Proteobacteria Betaproteobacteria Burkholderiales Comamonadaceae *Comamonas testosteroni* KF-1
Dac-1-1	Bacteria Proteobacteria Betaproteobacteria Burkholderiales Comamonadaceae *Delftia acidovorans* SPH-1
Pna-1-1 to Pna-1-2	Bacteria Proteobacteria Betaproteobacteria Burkholderiales Comamonadaceae *Polaromonas naphthalenivorans* CJ2
Cvi-1-1 to Cvi-1-2	Bacteria Proteobacteria Betaproteobacteria Neisseriales Neisseriaceae *Chromobacterium violaceum* ATCC 12472
Nmu-1-1	Bacteria Proteobacteria Betaproteobacteria Nitrosomonadales Nitrosomonadaceae *Nitrosospira multiformis* ATCC 25196
Dps-1-1 to Dps-1-2	Bacteria Proteobacteria Deltaproteobacteria Desulfobacterales Desulfobulbaceae *Desulfotalea psychrophila* LSv54
Asa-1-1	Bacteria Proteobacteria Gammaproteobacteria Aeromonadales Aeromonadaceae *Aeromonas salmonicida subsp. salmonicida* A449
Sde-1-1	Bacteria Proteobacteria Gammaproteobacteria Alteromonadales Alteromonadaceae *Saccharophagus degradans* 2-40
Sba-1-1	Bacteria Proteobacteria Gammaproteobacteria Alteromonadales Shewanellaceae *Shewanella baltica* OS185
Sba-2-1	Bacteria Proteobacteria Gammaproteobacteria Alteromonadales Shewanellaceae *Shewanella baltica* OS195
Sfr-1-1	Bacteria Proteobacteria Gammaproteobacteria Alteromonadales Shewanellaceae *Shewanella frigidimarina* NCIMB 400

Dno-1-1 to Dno-1-4	Bacteria Proteobacteria Gammaproteobacteria Cardiobacteriales Cardiobacteriaceae *Dichelobacter nodosus* VCS1703A
Esa-1-1 to Esa-1-2	Bacteria Proteobacteria Gammaproteobacteria Enterobacteriales Enterobacteriaceae Cronobacter *Enterobacter sakazakii* ATCC BAA-894
Esp-1-1 to Esp-1-3	Bacteria Proteobacteria Gammaproteobacteria Enterobacteriales Enterobacteriaceae *Enterobacter sp.* 638
Eco-1-1	Bacteria Proteobacteria Gammaproteobacteria Enterobacteriales Enterobacteriaceae *Escherichia coli*
Eco-2-1 to Eco-2-3	Bacteria Proteobacteria Gammaproteobacteria Enterobacteriales Enterobacteriaceae *Escherichia coli* 101-1
Eco-3-1 to Eco-3-14	Bacteria Proteobacteria Gammaproteobacteria Enterobacteriales Enterobacteriaceae *Escherichia coli* 53638
Eco-4-1 to Eco-4-2	Bacteria Proteobacteria Gammaproteobacteria Enterobacteriales Enterobacteriaceae *Escherichia coli* APEC O1
Eco-5-1 to Eco-5-3	Bacteria Proteobacteria Gammaproteobacteria Enterobacteriales Enterobacteriaceae *Escherichia coli* B
Eco-6-1 to Eco-6-4	Bacteria Proteobacteria Gammaproteobacteria Enterobacteriales Enterobacteriaceae *Escherichia coli* B171
Eco-7-1	Bacteria Proteobacteria Gammaproteobacteria Enterobacteriales Enterobacteriaceae *Escherichia coli* B7A
Eco-8-1 to Eco-8-5	Bacteria Proteobacteria Gammaproteobacteria Enterobacteriales Enterobacteriaceae *Escherichia coli* E110019
Eco-9-1 to Eco-9-3	Bacteria Proteobacteria Gammaproteobacteria Enterobacteriales Enterobacteriaceae *Escherichia coli* E22
Eco-10-1 to Eco-10-4	Bacteria Proteobacteria Gammaproteobacteria Enterobacteriales Enterobacteriaceae *Escherichia coli* E24377A
Eco-11-1 to Eco-11-2	Bacteria Proteobacteria Gammaproteobacteria Enterobacteriales Enterobacteriaceae *Escherichia coli* F11
Eco-12-1 to Eco-12-2	Bacteria Proteobacteria Gammaproteobacteria Enterobacteriales Enterobacteriaceae *Escherichia coli* O157:H7 EDL933
Eco-13-1 to Eco-13-3	Bacteria Proteobacteria Gammaproteobacteria Enterobacteriales Enterobacteriaceae *Escherichia coli* O157:H7 str. Sakai
Eco-14-1	Bacteria Proteobacteria Gammaproteobacteria Enterobacteriales Enterobacteriaceae *Escherichia coli* SECEC SMS-3-5
Eco-15-1 to Eco-15-2	Bacteria Proteobacteria Gammaproteobacteria Enterobacteriales Enterobacteriaceae *Escherichia coli* UTI89
Plu-1-1 to Plu-1-2	Bacteria Proteobacteria Gammaproteobacteria Enterobacteriales Enterobacteriaceae *Photorhabdus luminescens subsp. laumondii* TTO1
Sen-1-1	Bacteria Proteobacteria Gammaproteobacteria Enterobacteriales Enterobacteriaceae *Salmonella enterica subsp. enterica serovar* Paratyphi A str. ATCC
Sen-2-1	Bacteria Proteobacteria Gammaproteobacteria Enterobacteriales Enterobacteriaceae Salmonella Salmonella enterica subsp. entericaserovar Dublin *Salmonella enterica subsp. en*
Sen-3-1	Bacteria Proteobacteria Gammaproteobacteria Enterobacteriales Enterobacteriaceae Salmonella Salmonella enterica subsp. entericaserovar Hadar *Salmonella enterica subsp. ent*
Sen-4-1 to Sen-4-5	Bacteria Proteobacteria Gammaproteobacteria Enterobacteriales Enterobacteriaceae Salmonella Salmonella enterica subsp. entericaserovar Heidelberg *Salmonella enterica subsp.*
Sen-5-1	Bacteria Proteobacteria Gammaproteobacteria Enterobacteriales Enterobacteriaceae Salmonella Salmonella enterica subsp. entericaserovar Heidelberg *Salmonella enterica subsp.*
Sen-6-1	Bacteria Proteobacteria Gammaproteobacteria Enterobacteriales Enterobacteriaceae Salmonella Salmonella enterica subsp. entericaserovar Kentucky *Salmonella enterica subsp.*
Sen-7-1 to Sen-7-3	Bacteria Proteobacteria Gammaproteobacteria Enterobacteriales Enterobacteriaceae Salmonella Salmonella enterica subsp. entericaserovar Newport *Salmonella enterica subsp. e*
Sen-8-1 to Sen-8-4	Bacteria Proteobacteria Gammaproteobacteria Enterobacteriales Enterobacteriaceae Salmonella Salmonella enterica subsp. entericaserovar Newport *Salmonella enterica subsp. e*
Sen-9-1 to Sen-9-4	Bacteria Proteobacteria Gammaproteobacteria Enterobacteriales Enterobacteriaceae Salmonella Salmonella enterica subsp. entericaserovar Saintpaul *Salmonella enterica subsp.*
Sen-10-1 to Sen-10-3	Bacteria Proteobacteria Gammaproteobacteria Enterobacteriales Enterobacteriaceae Salmonella Salmonella enterica subsp. entericaserovar Typhi *Salmonella enterica subsp. ent*
Sen-11-1 to Sen-11-3	Bacteria Proteobacteria Gammaproteobacteria Enterobacteriales Enterobacteriaceae Salmonella Salmonella enterica subsp. entericaserovar Typhi *Salmonella enterica subsp. ent*
Sen-12-1	Bacteria Proteobacteria Gammaproteobacteria Enterobacteriales Enterobacteriaceae Salmonella Salmonella enterica subsp. entericaserovar Virchow *Salmonella enterica subsp. e*
Sty-1-1	Bacteria Proteobacteria Gammaproteobacteria Enterobacteriales Enterobacteriaceae *Salmonella typhimurium*
Sty-2-1	Bacteria Proteobacteria Gammaproteobacteria Enterobacteriales Enterobacteriaceae *Salmonella typhimurium* LT2
Spr-1-1 to Spr-1-2	Bacteria Proteobacteria Gammaproteobacteria Enterobacteriales Enterobacteriaceae *Serratia proteamaculans* 568
Sbo-1-1 to Sbo-1-4	Bacteria Proteobacteria Gammaproteobacteria Enterobacteriales Enterobacteriaceae *Shigella boydii* BS512
Sbo-2-1 to Sbo-2-2	Bacteria Proteobacteria Gammaproteobacteria Enterobacteriales Enterobacteriaceae *Shigella boydii* Sb227
Sdy-1-1 to Sdy-1-4	Bacteria Proteobacteria Gammaproteobacteria Enterobacteriales Enterobacteriaceae *Shigella dysenteriae* 1012
Sdy-2-1 to Sdy-2-3	Bacteria Proteobacteria Gammaproteobacteria Enterobacteriales Enterobacteriaceae *Shigella dysenteriae* Sd197
Sfl-1-1 to Sfl-1-2	Bacteria Proteobacteria Gammaproteobacteria Enterobacteriales Enterobacteriaceae *Shigella flexneri* 2a str. 2457T
Sfl-2-1 to Sfl-2-3	Bacteria Proteobacteria Gammaproteobacteria Enterobacteriales Enterobacteriaceae *Shigella flexneri* 2a str. 301
Sfl-3-1 to Sfl-3-2	Bacteria Proteobacteria Gammaproteobacteria Enterobacteriales Enterobacteriaceae *Shigella flexneri* 5 str. 8401
Sso-1-1 to Sso-1-2	Bacteria Proteobacteria Gammaproteobacteria Enterobacteriales Enterobacteriaceae *Shigella sonnei* Ss046
Sgl-1-1 to Sgl-1-4	Bacteria Proteobacteria Gammaproteobacteria Enterobacteriales Enterobacteriaceae *Sodalis glossinidius*
Sgl-2-1 to Sgl-2-18	Bacteria Proteobacteria Gammaproteobacteria Enterobacteriales Enterobacteriaceae *Sodalis glossinidius str. 'morsitans'*
Ybe-1-1 to Ybe-1-2	Bacteria Proteobacteria Gammaproteobacteria Enterobacteriales Enterobacteriaceae *Yersinia bercovieri* ATCC 43970
Yen-1-1 to Yen-1-4	Bacteria Proteobacteria Gammaproteobacteria Enterobacteriales Enterobacteriaceae *Yersinia enterocolitica subsp. enterocolitica* 8081
Yfr-1-1	Bacteria Proteobacteria Gammaproteobacteria Enterobacteriales Enterobacteriaceae *Yersinia frederiksenii* ATCC 33641
Yin-1-1 to Yin-1-2	Bacteria Proteobacteria Gammaproteobacteria Enterobacteriales Enterobacteriaceae *Yersinia intermedia* ATCC 29909
Ymo-1-1	Bacteria Proteobacteria Gammaproteobacteria Enterobacteriales Enterobacteriaceae *Yersinia mollaretii* ATCC 43969
Ype-1-1 to Ype-1-3	Bacteria Proteobacteria Gammaproteobacteria Enterobacteriales Enterobacteriaceae *Yersinia pestis* Angola
Ype-2-1 to Ype-2-2	Bacteria Proteobacteria Gammaproteobacteria Enterobacteriales Enterobacteriaceae *Yersinia pestis* Antiqua
Ype-9-1 to Ype-9-3	Bacteria Proteobacteria Gammaproteobacteria Enterobacteriales Enterobacteriaceae *Yersinia pestis biovar* Antiqua str. B42003004
Ype-10-1 to Ype-10-3	Bacteria Proteobacteria Gammaproteobacteria Enterobacteriales Enterobacteriaceae *Yersinia pestis biovar* Antiqua str. E1979001
Ype-11-1 to Ype-11-2	Bacteria Proteobacteria Gammaproteobacteria Enterobacteriales Enterobacteriaceae *Yersinia pestis biovar* Antiqua str. UG05-0454
Ype-12-1 to Ype-12-3	Bacteria Proteobacteria Gammaproteobacteria Enterobacteriales Enterobacteriaceae *Yersinia pestis biovar* Mediaevalis str. K1973002
Ype-13-1 to Ype-13-3	Bacteria Proteobacteria Gammaproteobacteria Enterobacteriales Enterobacteriaceae *Yersinia pestis biovar* Microtus str. 91001
Ype-14-1 to Ype-14-3	Bacteria Proteobacteria Gammaproteobacteria Enterobacteriales Enterobacteriaceae *Yersinia pestis biovar* Orientalis str. F1991016
Ype-15-1 to Ype-15-3	Bacteria Proteobacteria Gammaproteobacteria Enterobacteriales Enterobacteriaceae *Yersinia pestis biovar* Orientalis str. IP275
Ype-16-1 to Ype-16-3	Bacteria Proteobacteria Gammaproteobacteria Enterobacteriales Enterobacteriaceae *Yersinia pestis biovar* Orientalis str. MG05-1020
Ype-3-1 to Ype-3-3	Bacteria Proteobacteria Gammaproteobacteria Enterobacteriales Enterobacteriaceae *Yersinia pestis* CA88-4125
Ype-4-1 to Ype-4-3	Bacteria Proteobacteria Gammaproteobacteria Enterobacteriales Enterobacteriaceae *Yersinia pestis* CO92
Ype-5-1 to Ype-5-3	Bacteria Proteobacteria Gammaproteobacteria Enterobacteriales Enterobacteriaceae *Yersinia pestis* FV-1
Ype-6-1 to Ype-6-3	Bacteria Proteobacteria Gammaproteobacteria Enterobacteriales Enterobacteriaceae *Yersinia pestis* KIM
Ype-7-1 to Ype-7-3	Bacteria Proteobacteria Gammaproteobacteria Enterobacteriales Enterobacteriaceae *Yersinia pestis* Nepal516
Ype-8-1 to Ype-8-3	Bacteria Proteobacteria Gammaproteobacteria Enterobacteriales Enterobacteriaceae *Yersinia pestis* Pestoides F

Yps-1-1	Bacteria Proteobacteria Gammaproteobacteria Enterobacteriales Enterobacteriaceae *Yersinia pseudotuberculosis* IP 31758
Yps-2-1	Bacteria Proteobacteria Gammaproteobacteria Enterobacteriales Enterobacteriaceae *Yersinia pseudotuberculosis* IP 32953
Msp-1-1 to Msp-1-2	Bacteria Proteobacteria Gammaproteobacteria Oceanospirillales *Marinomonas sp.* MWYL1
Hin-1-1	Bacteria Proteobacteria Gammaproteobacteria Pasteurellales Pasteurellaceae *Haemophilus influenzae* 22.1-21
Hin-2-1	Bacteria Proteobacteria Gammaproteobacteria Pasteurellales Pasteurellaceae *Haemophilus influenzae* 22.4-21
Hin-3-1 to Hin-3-3	Bacteria Proteobacteria Gammaproteobacteria Pasteurellales Pasteurellaceae *Haemophilus influenzae* 3655
Hin-4-1	Bacteria Proteobacteria Gammaproteobacteria Pasteurellales Pasteurellaceae *Haemophilus influenzae* 86-028NP
Hin-5-1 to Hin-5-2	Bacteria Proteobacteria Gammaproteobacteria Pasteurellales Pasteurellaceae *Haemophilus influenzae* PittAA
Hin-6-1	Bacteria Proteobacteria Gammaproteobacteria Pasteurellales Pasteurellaceae *Haemophilus influenzae* PittEE
Hin-7-1 to Hin-7-2	Bacteria Proteobacteria Gammaproteobacteria Pasteurellales Pasteurellaceae *Haemophilus influenzae* PittGG
Hin-8-1	Bacteria Proteobacteria Gammaproteobacteria Pasteurellales Pasteurellaceae *Haemophilus influenzae* PittHH
Hin-9-1	Bacteria Proteobacteria Gammaproteobacteria Pasteurellales Pasteurellaceae *Haemophilus influenzae* PittII
Hin-10-1	Bacteria Proteobacteria Gammaproteobacteria Pasteurellales Pasteurellaceae *Haemophilus influenzae* R2846
Hin-11-1	Bacteria Proteobacteria Gammaproteobacteria Pasteurellales Pasteurellaceae *Haemophilus influenzae* R2866
Hin-12-1	Bacteria Proteobacteria Gammaproteobacteria Pasteurellales Pasteurellaceae *Haemophilus influenzae* R3021
Hso-1-1 to Hso-1-2	Bacteria Proteobacteria Gammaproteobacteria Pasteurellales Pasteurellaceae Histophilus *Haemophilus somnus* 129PT
Hso-2-1	Bacteria Proteobacteria Gammaproteobacteria Pasteurellales Pasteurellaceae Histophilus *Haemophilus somnus* 2336
Mha-1-1 to Mha-1-2	Bacteria Proteobacteria Gammaproteobacteria Pasteurellales Pasteurellaceae *Mannheimia haemolytica* PHL213
Pmu-1-1	Bacteria Proteobacteria Gammaproteobacteria Pasteurellales Pasteurellaceae *Pasteurella multocida subsp. multocida str.* Pm70
Aba-1-1 to Aba-1-24	Bacteria Proteobacteria Gammaproteobacteria Pseudomonadales Moraxellaceae *Acinetobacter baumannii* ATCC 17978
Asp-1-1 to Asp-1-4	Bacteria Proteobacteria Gammaproteobacteria Pseudomonadales Moraxellaceae *Acinetobacter sp.* ADP1
Avi-1-1 to Avi-1-3	Bacteria Proteobacteria Gammaproteobacteria Pseudomonadales Pseudomonadaceae *Azotobacter vinelandii*
Pae-1-1	Bacteria Proteobacteria Gammaproteobacteria Pseudomonadales Pseudomonadaceae *Pseudomonas aeruginosa* 2192
Pae-2-1	Bacteria Proteobacteria Gammaproteobacteria Pseudomonadales Pseudomonadaceae *Pseudomonas aeruginosa* C3719
Pae-3-1	Bacteria Proteobacteria Gammaproteobacteria Pseudomonadales Pseudomonadaceae *Pseudomonas aeruginosa* PA7
Pae-4-1 to Pae-4-2	Bacteria Proteobacteria Gammaproteobacteria Pseudomonadales Pseudomonadaceae *Pseudomonas aeruginosa* PACS2
Pae-5-1	Bacteria Proteobacteria Gammaproteobacteria Pseudomonadales Pseudomonadaceae *Pseudomonas aeruginosa* PAO1
Pae-6-1	Bacteria Proteobacteria Gammaproteobacteria Pseudomonadales Pseudomonadaceae *Pseudomonas aeruginosa* UCBPP-PA14
Pen-1-1 to Pen-1-30	Bacteria Proteobacteria Gammaproteobacteria Pseudomonadales Pseudomonadaceae *Pseudomonas entomophila* L48
Pfl-1-1 to Pfl-1-9	Bacteria Proteobacteria Gammaproteobacteria Pseudomonadales Pseudomonadaceae *Pseudomonas fluorescens* Pf-5
Pfl-2-1 to Pfl-2-29	Bacteria Proteobacteria Gammaproteobacteria Pseudomonadales Pseudomonadaceae *Pseudomonas fluorescens* Pf0-1
Pme-1-1 to Pme-1-2	Bacteria Proteobacteria Gammaproteobacteria Pseudomonadales Pseudomonadaceae *Pseudomonas mendocina ymp*
Ppu-1-1 to Ppu-1-10	Bacteria Proteobacteria Gammaproteobacteria Pseudomonadales Pseudomonadaceae *Pseudomonas putida* F1
Ppu-2-1 to Ppu-2-5	Bacteria Proteobacteria Gammaproteobacteria Pseudomonadales Pseudomonadaceae *Pseudomonas putida* GB-1
Ppu-3-1 to Ppu-3-13	Bacteria Proteobacteria Gammaproteobacteria Pseudomonadales Pseudomonadaceae *Pseudomonas putida* KT2440
Ppu-4-1 to Ppu-4-27	Bacteria Proteobacteria Gammaproteobacteria Pseudomonadales Pseudomonadaceae *Pseudomonas putida* W619
Pst-1-1	Bacteria Proteobacteria Gammaproteobacteria Pseudomonadales Pseudomonadaceae *Pseudomonas stutzeri* A1501
Psy-1-1 to Psy-1-6	Bacteria Proteobacteria Gammaproteobacteria Pseudomonadales Pseudomonadaceae *Pseudomonas syringae pv. phaseolicola* 1448A
Psy-2-1 to Psy-2-5	Bacteria Proteobacteria Gammaproteobacteria Pseudomonadales Pseudomonadaceae *Pseudomonas syringae pv. syringae* B728a
Psy-3-1 to Psy-3-3	Bacteria Proteobacteria Gammaproteobacteria Pseudomonadales Pseudomonadaceae *Pseudomonas syringae pv. tomato str.* DC3000
Vba-1-1	Bacteria Proteobacteria Gammaproteobacteria Vibrionales unclassified Vibrionales *Vibrionales bacterium* SWAT-3
Vch-1-1	Bacteria Proteobacteria Gammaproteobacteria Vibrionales Vibrionaceae *Vibrio cholerae* MZO-3
Vch-2-1	Bacteria Proteobacteria Gammaproteobacteria Vibrionales Vibrionaceae *Vibrio cholerae* NCTC 8457
Vch-3-1	Bacteria Proteobacteria Gammaproteobacteria Vibrionales Vibrionaceae *Vibrio cholerae* O395
Vfi-1-1 to Vfi-1-2	Bacteria Proteobacteria Gammaproteobacteria Vibrionales Vibrionaceae *Vibrio fischeri* ES114
Vsh-1-1	Bacteria Proteobacteria Gammaproteobacteria Vibrionales Vibrionaceae *Vibrio shilonii* AK1
Vsp-1-1	Bacteria Proteobacteria Gammaproteobacteria Vibrionales Vibrionaceae *Vibrio splendidus* 12B01
Sma-1-1 to Sma-1-5	Bacteria Proteobacteria Gammaproteobacteria Xanthomonadales Xanthomonadaceae *Stenotrophomonas maltophilia* R551-3
Xax-1-1	Bacteria Proteobacteria Gammaproteobacteria Xanthomonadales Xanthomonadaceae *Xanthomonas axonopodis pv. citri str.* 306
Xca-1-1	Bacteria Proteobacteria Gammaproteobacteria Xanthomonadales Xanthomonadaceae *Xanthomonas campestris pv. campestris str.* ATCC 33913
Xor-1-1	Bacteria Proteobacteria Gammaproteobacteria Xanthomonadales Xanthomonadaceae *Xanthomonas oryzae pv. oryzae* KACC10331
Xor-2-1	Bacteria Proteobacteria Gammaproteobacteria Xanthomonadales Xanthomonadaceae *Xanthomonas oryzae pv. oryzae* MAFF 311018
Xfa-1-1 to Xfa-1-2	Bacteria Proteobacteria Gammaproteobacteria Xanthomonadales Xanthomonadaceae *Xylella fastidiosa* 9a5c
Xfa-2-1	Bacteria Proteobacteria Gammaproteobacteria Xanthomonadales Xanthomonadaceae *Xylella fastidiosa* Ann-1
Xfa-3-1	Bacteria Proteobacteria Gammaproteobacteria Xanthomonadales Xanthomonadaceae *Xylella fastidiosa* Dixon
Sen-13-1 to Sen-13-2	CVM23701Bacteria Proteobacteria Gammaproteobacteria Enterobacteriales Enterobacteriaceae Salmonella Salmonella enterica subsp. entericaserovar 4,[5],12:i:- *Salmonella ente*
Sen-14-1 to Sen-14-2	GA_MM04042433Bacteria Proteobacteria Gammaproteobacteria Enterobacteriales Enterobacteriaceae *Salmonella enterica subsp. enterica serovar* Javiana str
Sen-15-1 to Sen-15-3	HI_N05-537Bacteria Proteobacteria Gammaproteobacteria Enterobacteriales Enterobacteriaceae Salmonella Salmonella enterica subsp. entericaserovar Weltevreden *Salmonella e*
Sen-16-1 to Sen-16-2	SC-B67Bacteria Proteobacteria Gammaproteobacteria Enterobacteriales Enterobacteriaceae Salmonella Salmonella enterica subsp. entericaserovar Choleraesuis *Salmonella enter*
Eph-2-1	Viruses dsDNA viruses, no RNA stage Caudovirales Myoviridae *Enterobacteria phage phi*P27
Eph-1-1	Viruses dsDNA viruses, no RNA stage Caudovirales Myoviridae *Enterobacteria phage* SfV
Hph-1-1	Viruses dsDNA viruses, no RNA stage Caudovirales Myoviridae *Haemophilus phage* Aaphi23
Eph-3-1	Viruses dsDNA viruses, no RNA stage Caudovirales Myoviridae P1-like viruses *Enterobacteria phage* P1
Eph-4-1	Viruses dsDNA viruses, no RNA stage Caudovirales Myoviridae P2-like viruses *Enterobacteria phage* P4
Api-1-1	Viruses dsDNA viruses, no RNA stage Caudovirales Podoviridae *Acyrthosiphon pisum secondary endosymbiont phage* 1

Eph-5-1	Viruses dsDNA viruses, no RNA stage Caudovirales Podoviridae *Enterobacteria phage* VT2-Sakai
Eph-6-1	Viruses dsDNA viruses, no RNA stage Caudovirales Podoviridae P22-like viruses *Enterobacteria phage phi*V10
Sph-1-1	Viruses dsDNA viruses, no RNA stage Caudovirales *Salmonella phage* ST64B
Kph-1-1	Viruses dsDNA viruses, no RNA stage Caudovirales Siphoviridae *Klebsiella phage phi*KO2
Pcd-1-1	Viruses dsDNA viruses, no RNA stage Caudovirales Siphoviridae Lambda-like viruses unclassified Lambda-like viruses *Phage cdt*I (Bacteriophage cdtI)
Sco-1-1	Viruses dsDNA viruses, no RNA stage Caudovirales Siphoviridae Lambda-like viruses unclassified Lambda-like viruses *Stx*2 converting phage II (Stx2 converting bacteriophage I
Eph-7-1	Viruses dsDNA viruses, no RNA stage Caudovirales Siphoviridae N15-like viruses *Enterobacteria phage* N15
Yph-1-1	Viruses dsDNA viruses, no RNA stage Caudovirales Siphoviridae *Yersinia phage* PY54
PGi-1-1	Viruses *Phage* Gifsy-1
env-1 to env-52	environmental samples

16.2 Gene contexts

Each c4 antisense RNA RNA (indicated by "RNA→") is listed with its surrounding genes. Environmental sequences and some RefSeq entries lack gene annotations, and no genes are listed for such sequences. The direction of each gene is indicated with an arrow (→), and each conserved domain in the gene is colored. Conserved domains associated with more than one c4 antisense RNA RNA are assigned a color; other domains are gray. Information about these conserved domains is given in Section 16.3. The accession number of the sequence containing each c4 antisense RNA RNA is given. Accessions beginning with "NC_" or "NZ_" are RefSeq, while other accession refer to environmental samples. Nucleotide coordinates are given for the 5′ and 3′ boundaries of each. If the 5′ coordinate is greater than the 3′ coordinate, the RNA is present on the reverse-complement strand of the containing genomic DNA sequence. Each hit is denoted by an abbreviation (like "Eco-1-1") that refers to a taxonomy given in Section 16.1.

**abbrev.**	**Seq. accession**		**5′ at**	**3′ at**	**genes**
Aba-1-1	NC_009085.1	+	1492888	1492976	EriC (COG0038)→ **RNA**→ putative transcriptional regulator→
Aba-1-2	NC_009085.1	-	2365091	2365008	hypo→ **RNA**→ hypo→
Hin-3-1	NZ_AAZF01000005.1	+	72866	72949	hypo→ **RNA**→ hypo→ hypo→ hypo→ hypo→ hypo→
Hin-5-1	NZ_AAZG01000002.1	+	114094	114177	hypo→ **RNA**→ hypo→ hypo→ hypo→ hypo→ hypo→
Hin-7-1	NC_009567.1	-	760122	760036	hypo→ **RNA**→ hypo→ hypo→ hypo→ COG5519 (COG5519)→
Hso-1-1	NC_008309.1	+	565968	566054	hypo→ **RNA**→ hypo→ hypo→ hypo→
Hso-2-1	NZ_AACJ01000013.1	-	2187	2101	Transposase_2 (pfam01385)Transposase_35 (pfam07282)→ **RNA**→ hypo→
Pmu-1-1	NC_002663.1	+	2002795	2002883	hypo→ **RNA**→ hypo→ hypo→ hypo→ hypo→ COG5519 (COG5519)→
Hin-3-2	NZ_AAZF01000002.1	-	232050	231964	hypo→ **RNA**→ hypo→ hypo→ hypo→ hypo→ hypo→ hypo→ Pox_D5 (pfam03288)→
Hin-2-1	NZ_AAZJ01000012.1	+	9811	9897	hypo→ **RNA**→ hypo→ hypo→ hypo→ hypo→ hypo→ COG5519 (COG5519)→
Mha-1-1	NZ_AASA01000084.1	+	6161	6256	**RNA**→
Mha-1-2	NZ_AASA01000084.1	+	6623	6711	**RNA**→
Pfl-2-1	NC_007492.1	+	3001178	3001267	←hypo **RNA**→ hypo→
env-1	JCVI_SCAF_1096626333345	-	508	421	**RNA**→
Dps-1-1	NC_006139.1	+	46573	46654	←related to TraJ protein **RNA**→ hypo→
Dac-1-1	NZ_AAVD01000003.1	-	489428	489345	Twin-arginine translocation pathway signal→ **RNA**→ hypo→
Sgl-2-1	NC_007712.1	-	4059279	4059192	INT_P4 (cd00801)→ **RNA**→ hypo→
Pfl-2-2	NC_007492.1	-	317345	317260	hypo→ **RNA**→ hypo→
Sbo-1-1	NZ_AAKA01000001.1	-	39540	39453	hypo→ **RNA**→ hypo→
Pna-1-1	NC_008781.1	-	380216	380131	RVT (pfam00078)→ **RNA**→ hypo→
Pna-1-2	NC_008781.1	-	1018953	1018867	hypo→ **RNA**→ hypo→ hypo→ hypo→ COG4643 (COG4643)→ hypo→
Xfa-1-1	NC_002488.3	-	2387378	2387297	←hypo **RNA**→ COG3617 (COG3617)→
Sma-1-1	NZ_AAVZ01000004.1	-	59163	59071	HTH_1 (pfam00126)LysR_substrate (pfam03466)→ **RNA**→ COG4487 (COG4487)→ SIR2-like (cd01406)→ protein of unknown function DUF87→
Dno-1-1	NC_009446.1	-	1080812	1080727	INT_P4 (cd00801)→ **RNA**→ virulence-associated protein VapG3→ VirE (pfam05272)→
Dno-1-2	NC_009446.1	+	824732	824817	DnaC (COG1484)→ **RNA**→ RusA (pfam05866)→ hypo→ hypo→ hypo→
Dno-1-3	NC_009446.1	-	299743	299661	virulence-associated protein VapH→ **RNA**→ virulence-associated protein VapH→ virulence-associated protein VapG1→ VirE (pfam05272)→

Dno-1-4	NC_009446.1	-	292912	292832	←VapI (COG3093) **RNA**→ virulence-associated protein VapG2→
Esp-1-1	NC_009436.1	-	1205701	1205615	FepA (COG4771)→ **RNA**→ hypo→
Plu-1-1	NC_005126.1	+	4085959	4086042	hypo→ **RNA**→ hypo→ Nlp (COG3423)→
Xax-1-1	NC_003921.3	-	1645	1553	hypo→ **RNA**→ hypo→
Aba-1-3	NC_009085.1	-	2570672	2570588	←RimL (COG1670) **RNA**→ putative transcriptional regulator→
Xfa-3-1	NZ_AAAL02000013.1	+	8678	8770	COG3617 (COG3617)→ **RNA**→ COG3617 (COG3617)→ Ssb (COG0629)→
Xfa-2-1	NZ_AAAM03000068.1	+	11383	11475	COG3617 (COG3617)→ **RNA**→ COG3617 (COG3617)→ Ssb (COG0629)→ hypo (cons)→ INT_Lambda_C (cd00800)→
Xfa-1-2	NC_002488.3	-	1588569	1588477	LpxD (COG1044)→ **RNA**→ COG3617 (COG3617)→ Ssb (COG0629)→ hypo→ INT_Lambda_C (cd00800)→
Sma-1-2	NZ_AAVZ01000007.1	+	44241	44329	←hypo **RNA**→ hypo→
env-2	JCVI_SCAF_1096628066130	+	205	288	**RNA**→
env-3	JCVI_SCAF_1096627028318	+	1583	1668	←MoaA (COG2896)? (GOS255) **RNA**→
Sma-1-3	NZ_AAVZ01000011.1	+	25481	25567	←hypo **RNA**→ hypo→
Sma-1-4	NZ_AAVZ01000001.1	+	108170	108255	COG4700 (COG4700)→ **RNA**→ hypo→ hypo→
Plu-1-2	NC_005126.1	+	989054	989139	←hypo **RNA**→ Nlp (COG3423)→
env-4	sludgeOz_Contig11344	+	5032	5114	(,,,)→ **RNA**→ ←(,,,)
Aba-1-4	NC_009085.1	+	871749	871832	Flavin_Reduct (pfam01613)→ **RNA**→ TetR_N (pfam00440)→
Bph-1-1	NZ_AAUH01000032.1	+	20757	20848	COG1981 (COG1981)→ **RNA**→ hypo (cons)→
Bxe-1-1	NC_007951.1	-	4245368	4245279	COG1981 (COG1981)→ **RNA**→ hypo→
env-5	JCVI_SCAF_1096627192337	-	13368	13279	←COG0702 (COG0702)? (GOS4294) **RNA**→ ? (GOS11386653)→
env-6	JCVI_SCAF_1101668744231	-	1049	962	**RNA**→ Nlp (COG3423)? (GOS11363276)→ RimL (COG1670)→
Spr-1-1	NZ_AAUN01000013.1	+	38258	38343	Glyco_hydro_1 (pfam00232)→ **RNA**→ DNA_methylase (pfam00145)→ Vsr (cd00221)→
Pfl-2-3	NC_007492.1	+	523624	523710	hypo→ **RNA**→ hypo→
env-7	JCVI_SCAF_1096628137543	+	1077	1163	? (GOS1771)→ **RNA**→ COG3617 (COG3617)? (GOS11900477)→
Pfl-2-4	NC_007492.1	+	965543	965629	←SBP_bac_5 (pfam00496) **RNA**→ hypo→
Msp-1-1	NC_009654.1	-	652730	652647	←hypo **RNA**→ hypo→ hypo→
Msp-1-2	NC_009654.1	-	619642	619558	←hypo **RNA**→ hypo→
env-8	sludgeOz_Contig11395	-	8077	7988	COG3645 (COG3645)COG3646 (COG3646)→ **RNA**→ (,,,)→ (,,,)→
Aba-1-5	NC_009085.1	+	871633	871716	Flavin_Reduct (pfam01613)→ **RNA**→ TetR_N (pfam00440)→
Pfl-1-1	NC_004129.6	+	5946544	5946629	Acetyltransf_1 (pfam00583)→ **RNA**→ hypo→
Psy-1-1	NC_005773.3	-	1124857	1124771	hypo→ **RNA**→ putative inner membrane protein→
Pae-5-1	NC_002516.2	-	1348513	1348430	←hypo **RNA**→ hypo→
Pae-2-1	NZ_AAKV01000003.1	-	6640	6557	←hypo **RNA**→ hypo→
Pae-6-1	NC_008463.1	+	4286083	4286166	←hypo **RNA**→ hypo→
Pae-1-1	NZ_AAKW01000002.1	-	898	815	←hypo **RNA**→ hypo→
Pae-4-1	NZ_AAQW01000001.1	-	1918648	1918565	←hypo **RNA**→ hypo→
Ppu-4-1	NZ_AAVY01000017.1	+	134946	135034	←Glycos_transf_2 (pfam00535) **RNA**→ hypo→ hypo→ hypo→
Dps-1-2	NC_006138.1	+	446698	446777	←hypo **RNA**→ ←hypo
Asa-1-1	NC_009348.1	+	3150898	3150985	putative phage regulator→ **RNA**→ putative phage regulator→ hypo→
env-9	JCVI_SCAF_1101668263049	-	1534	1447	**RNA**→
Bsp-1-1	NC_007510.1	-	2729355	2729271	←hypo **RNA**→ hypo→ Phage_AlpA (pfam05930)→
Pen-1-1	NC_008027.1	+	2652833	2652926	←RpoE (COG1595) **RNA**→ hypo→
Cvi-1-1	NC_005085.1	+	4158872	4158962	AlpA (COG3311)→ **RNA**→ hypo→ hypo→ hypo→
Sdy-2-1	NC_007606.1	+	1757441	1757528	BsuBI_PstI_RE (pfam06616)NOG08008 (NOG08008)→ **RNA**→ COG3646 (COG3646)→ hypo→
Sbo-1-2	NZ_AAKA01000020.1	-	58210	58123	Transposase_27 (pfam03400)→ **RNA**→ hypo→
env-10	BABG01026403.1	+	226	313	**RNA**→ hypo→ hypo→ hypo→
Cte-1-1	NZ_AAUJ01000008.1	-	30413	30331	COG4748 (COG4748)→ **RNA**→ COG3646 (COG3646)→
Cte-1-2	NZ_AAUJ01000008.1	+	21804	21888	hypo→ **RNA**→ COG3646 (COG3646)→
Sgl-2-2	NC_007712.1	+	2663915	2664000	←hypo **RNA**→ hypo→
Sen-8-1	NZ_ABEW01000010.1	+	115026	115110	**RNA**→
Vfi-1-1	NC_006840.1	-	2244353	2244268	hypo→ **RNA**→ COG3617 (COG3617)→
Vfi-1-2	NC_006840.1	-	2243662	2243579	COG3617 (COG3617)→ **RNA**→ ←SmpB (COG0691)

Sde-1-1	NC_007912.1	+	342124	342208	←Secretion protein HlyD **RNA**→ hypo→
Sba-1-1	NC_009665.1	-	896014	895930	←COG3941 (COG3941) **RNA**→ COG3646 (COG3646)→
Sba-2-1	NZ_AATK01000033.1	-	24005	23923	←COG5283 (COG5283) **RNA**→ COG3646 (COG3646)→
Eco-3-1	NZ_AAKB01000016.1	+	10920	11003	POLAc (smart00482)→ **RNA**→ COG3561 (COG3561)→
Vch-2-1	NZ_AAWD01000002.1	+	57743	57826	←hypo (cons) **RNA**→ COG3617 (COG3617)→
Vch-3-1	NC_009456.1	-	1033991	1033907	←hypo **RNA**→ COG3617 (COG3617)→
Vsp-1-1	NZ_AAMR01000038.1	-	33213	33129	←hypo **RNA**→ COG3646 (COG3646)→
Vch-1-1	NZ_AAUU01000060.1	-	5711	5630	hypo→ **RNA**→ transposase ORF-A, IS-type→
Vba-1-1	NZ_AAZW01000012.1	-	43784	43701	COG3335 (COG3335)→ **RNA**→ KilA-N (pfam04383)→
Sfr-1-1	NC_008345.1	-	1972071	1971987	←hypo **RNA**→ COG3646 (COG3646)→
Vsh-1-1	NZ_ABCH01000029.1	+	41299	41379	←hypo **RNA**→ KilA-N (pfam04383)→
Sgl-1-1	NC_007185.1	+	1478	1562	←RepA protein **RNA**→ HTH_LUXR (smart00421)→
Sgl-2-3	NC_007714.1	-	16189	16105	←replication associated protein RepA1 **RNA**→ HTH_LUXR (smart00421)→
Sgl-1-2	NC_007184.1	+	1478	1562	←putative replication-associated protein **RNA**→ HTH_LUXR (smart00421)→
env-11	BAAW01000646.1	+	4186	4274	MarC (pfam01914)→ **RNA**→ hypo→
Eco-5-1	NZ_AAWW01000074.1	-	5013	4925	←YD repeat protein **RNA**→ hypo→
Sen-11-1	NC_004631.1	+	4519523	4519609	←hypo **RNA**→ hypo→ Nlp (COG3423)→
Sen-10-1	NC_003198.1	+	4536714	4536800	←hypo **RNA**→ hypo→ Nlp (COG3423)→
Sen-12-1	NZ_ABFH01000014.1	+	112132	112219	**RNA**→
Esp-1-2	NC_009436.1	+	4152361	4152449	MarC (pfam01914)→ **RNA**→ hypo→
Sgl-2-4	NC_007712.1	+	3395263	3395347	←hypo **RNA**→ COG3646 (COG3646)→
Sgl-2-5	NC_007712.1	-	1233127	1233043	HTH_LUXR (smart00421)→ **RNA**→ COG3646 (COG3646)→
Sgl-2-6	NC_007712.1	-	2063246	2063161	hypo→ **RNA**→ hypo→ hypo→
Sgl-2-7	NC_007712.1	+	3106295	3106377	hypo→ **RNA**→ hypo→
Sgl-2-8	NC_007712.1	-	1814121	1814038	ArtM (COG4160)→ **RNA**→ COG3617 (COG3617)→
Sdy-1-1	NZ_AAMJ01000005.1	+	122662	122749	←HicB (pfam05534) **RNA**→ COG3646 (COG3646)→
Sbo-1-3	NZ_AAKA01000020.1	+	568	655	**RNA**→ COG3646 (COG3646)→
Eco-10-1	NZ_AAJZ01000001.1	-	4185666	4185579	COG3646 (COG3646)→ **RNA**→ COG3646 (COG3646)→
Sfl-1-1	NC_004741.1	+	1891549	1891636	hypothetical bacteriophage protein→ **RNA**→ hypothetical bacteriophage protein→
Sfl-2-1	NC_004337.1	-	1419385	1419298	COG3646 (COG3646)→ **RNA**→ COG3646 (COG3646)→
Eco-3-2	NZ_AAKB01000057.1	+	7419	7506	COG3646 (COG3646)→ **RNA**→ COG3646 (COG3646)→
Eco-3-3	NZ_AAKB01000097.1	-	235	148	**RNA**→
Eco-3-4	NZ_AAKB01000020.1	+	9022	9109	COG3646 (COG3646)→ **RNA**→ COG3646 (COG3646)→
Sfl-3-1	NC_008258.1	+	771649	771736	COG3646 (COG3646)→ **RNA**→ COG3646 (COG3646)→
Sfl-2-2	NC_004337.1	+	913450	913537	COG3646 (COG3646)→ **RNA**→ COG3646 (COG3646)→
Sfl-1-2	NC_004741.1	+	908250	908337	putative bacteriophage protein→ **RNA**→ putative bacteriophage protein→ hypothetical bacteriophage protein→
Eco-6-1	NZ_AAJX01000004.1	+	60275	60362	COG3646 (COG3646)→ **RNA**→ COG3646 (COG3646)→ hypo→ COG0640: Predicted transcriptional regulators→
					hypo→ Dam (pfam05869)→ LexA_DNA_bind (pfam01726)→ RusA (pfam05866)→ KilA-N (pfam04383)→ hypo→
Pcd-1-1	NC_009514.1	+	36578	36665	**RNA**→
Sso-1-1	NC_007384.1	+	1984424	1984511	Transposase_27 (pfam03400)→ **RNA**→ COG3646 (COG3646)→
Sbo-1-4	NZ_AAKA01000012.1	-	22926	22839	COG3646 (COG3646)→ **RNA**→ COG3646 (COG3646)→ hypo→ hypo→
Sfl-3-2	NC_008258.1	+	2768141	2768228	putative bacteriophage protein→ **RNA**→ putative bacteriophage protein→
Eco-3-5	NZ_AAKB01000061.1	+	3762	3849	COG3646 (COG3646)→ **RNA**→ COG3646 (COG3646)→ hypo→ hypo→
					hypothetical proteinCould not find sequence in database.→
Eco-13-1	NC_002695.1	+	2598583	2598670	hypo→ **RNA**→ hypo→ putative derepression protein→ hypo→
Eco-12-1	NC_002655.2	+	2673794	2673881	unknown protein encoded by prophage CP-933T→ **RNA**→ unknown protein encoded by prophage CP-933T→
					unknown protein encoded by prophage CP-933T→ putative replication protein for prophage CP-933T→
					StbA (pfam06406)→ putative stability/partitioning protein encoded within CP-933T→
Eco-3-6	NZ_AAKB01000056.1	+	37	124	**RNA**→
Sen-13-1	NZ_ABAO01000085.1	-	1827	1740	**RNA**→
Sph-1-1	NC_004313.1	+	30105	30192	**RNA**→
Sen-16-1	NC_006905.1	+	1315152	1315239	←HTH_XRE (smart00530)NOG17530 (NOG17530) **RNA**→ COG3646 (COG3646)→ hypo→
Eco-2-1	NZ_AAMK01000006.1	+	59258	59345	COG3646 (COG3646)→ **RNA**→ COG3646 (COG3646)→

env-12	BABF01000198.1	-	474	387	COG3646 (COG3646)→ **RNA**→ COG3646 (COG3646)→
Eco-15-1	NC_007946.1	+	4997122	4997209	COG3646 (COG3646)→ **RNA**→ COG3646 (COG3646)→ hypo→ putative replication protein→ hypo→
					Dam (pfam05869)NOG18143 (NOG18143)→ LexA_DNA_bind (pfam01726)→ RusA (pfam05866)→
					KilA-N (pfam04383)NOG17190 (NOG17190)→ hypothetical protein YdfU→ putative Q protein→
Eco-4-1	NC_008563.1	+	5012458	5012545	COG3646 (COG3646)→ **RNA**→ COG3646 (COG3646)→
Eco-6-2	NZ_AAJX01000031.1	+	41435	41522	COG3646 (COG3646)→ **RNA**→ COG3646 (COG3646)→ hypo→ hypo→
Eco-6-3	NZ_AAJX01000103.1	-	3179	3092	hypo→ **RNA**→ hypo→ hypo→ hypo→
Eco-2-2	NZ_AAMK01000026.1	+	15471	15558	MarR (COG1846)→ **RNA**→ hypo→ hypo→ hypo→ StbA (pfam06406)→
Eco-8-1	NZ_AAJW01000006.1	+	162043	162130	hypo→ **RNA**→ hypo→ hypo→ hypo→
Sdy-1-2	NZ_AAMJ01000008.1	+	123989	124076	hypo→ **RNA**→ hypo→ hypo→ hypo→
Eco-7-1	NZ_AAJT01000067.1	-	18116	18029	hypo→ **RNA**→ hypo→ hypo→ hypo→ StbA (pfam06406)→ hypo→
Eco-6-4	NZ_AAJX01000034.1	+	25727	25814	hypo→ **RNA**→ hypo→ hypo→ hypo→ StbA (pfam06406)→ hypo→
Eco-9-1	NZ_AAJV01000026.1	+	43700	43787	←COG0043: 3-polyprenyl-4-hydroxybenzoate decarboxylase and related decarboxylases **RNA**→
					←COG0043: 3-polyprenyl-4-hydroxybenzoate decarboxylase and related decarboxylases
Eco-3-7	NZ_AAKB01000017.1	+	49998	50085	COG0583: Transcriptional regulator→ **RNA**→ hypo→ hypo→ hypo→ hypo→
Eco-15-2	NC_007946.1	-	942027	941938	HflC (COG0330)→ **RNA**→ ←hypo
Eco-2-3	NZ_AAMK01000002.1	+	372673	372760	←hypo **RNA**→ ←hypo
Eco-10-2	NZ_AAJZ01000001.1	-	4973644	4973559	hypo→ **RNA**→ hypo→
Spr-1-2	NZ_AAUN01000029.1	+	36100	36187	hypo→ **RNA**→ COG3617 (COG3617)→
Sty-2-1	NC_003197.1	-	2762765	2762682	antiterminator-like protein→ **RNA**→ antirepressor-like protein→
PGi-1-1	NC_010392.1	-	34214	34131	**RNA**→
Sen-9-1	NZ_ABAN01000002.1	+	164	247	**RNA**→
Sen-9-2	NZ_ABAN01000025.1	-	4031	3948	**RNA**→
Sen-14-1	NZ_ABEH01000004.1	-	43689	43606	**RNA**→
Sen-13-2	NZ_ABAO01000067.1	-	2472	2389	**RNA**→
Sen-7-1	NZ_ABEN01000001.1	-	2762799	2762716	**RNA**→
Sen-8-2	NZ_ABEW01000016.1	+	12612	12695	**RNA**→
Sen-15-1	NZ_ABFF01000012.1	+	15475	15558	**RNA**→
Eco-10-3	NZ_AAJZ01000001.1	-	4209920	4209837	hypo→ **RNA**→ ←Caudo_TAP (pfam02413)
Sso-1-2	NC_007384.1	-	2936419	2936336	antitermination protein Q-like protein→ **RNA**→ putative bacteriophage protein→
Ype-7-1	NC_008149.1	+	1777837	1777924	phage antitermination protein→ **RNA**→ COG3617 (COG3617)→
Ype-8-1	NC_009381.1	-	1164990	1164903	phage antitermination protein→ **RNA**→ COG3617 (COG3617)→
Ype-6-1	NC_004088.1	-	2450258	2450171	hypo→ **RNA**→ COG3617 (COG3617)→
Ype-4-1	NC_003143.1	+	2369225	2369312	hypo→ **RNA**→ hypo→ COG3617 (COG3617)→
Ype-13-1	NC_005810.1	+	2151105	2151192	hypo→ **RNA**→ hypo→ COG3617 (COG3617)→
Ype-15-1	NZ_AAOS01000087.1	-	11026	10939	hypo→ **RNA**→ COG3617 (COG3617)→
Ype-5-1	NZ_AAUB01000062.1	+	10162	10249	**RNA**→
Ype-16-1	NZ_AAYS01000003.1	-	187708	187621	**RNA**→
Ype-12-1	NZ_AAYT01000003.1	-	330911	330824	**RNA**→
Ype-9-1	NZ_AAYU01000001.1	+	387329	387416	**RNA**→
Ype-10-1	NZ_AAYV01000003.1	+	195033	195120	**RNA**→
Ype-14-1	NZ_ABAT01000021.1	-	71591	71504	**RNA**→
Ype-3-1	NZ_ABCD01000001.1	-	1281217	1281130	hypothetical phage protein→ **RNA**→ hypothetical phage protein→ COG3617 (COG3617)→
Ype-1-1	NZ_AAKS01000038.1	+	24399	24486	hypo→ **RNA**→ COG3617 (COG3617)→
Eco-3-8	NZ_AAKB01000062.1	+	3801	3885	COG1522: Transcriptional regulators→ **RNA**→ hypo→
Eco-3-9	NZ_AAKB01000008.1	-	102865	102778	COG1763: Molybdopterin-guanine dinucleotide biosynthesis protein→ **RNA**→
					COG1763: Molybdopterin-guanine dinucleotide biosynthesis protein→ hypo→
Yen-1-1	NC_008800.1	-	2104229	2104144	hypo→ **RNA**→ Transposase_31 (pfam04754)→
Yen-1-2	NC_008800.1	-	4498927	4498841	←COG3177 (COG3177) **RNA**→ hypo→
Yps-1-1	NC_009708.1	-	1069976	1069890	←HTH_XRE (cd00093) **RNA**→ hypo→
Ype-13-2	NC_005810.1	+	4074558	4074644	←RelE (COG2026) **RNA**→ ←INT_P4 (cd00801)
Yps-2-1	NC_006155.1	+	3687840	3687926	←RelE (COG2026) **RNA**→ ←HP1_INT_C (cd00797)
Ype-2-1	NC_008150.1	-	451213	451127	←RelE (COG2026) **RNA**→ hypo→

Ype-7-2	NC_008149.1	+	3467455	3467541	←RelE (COG2026) **RNA**→ hypo→
Ype-8-2	NC_009381.1	-	711648	711562	←HTH_XRE (cd00093) **RNA**→ hypo→
Ype-6-2	NC_004088.1	+	3599632	3599718	hypo→ **RNA**→ hypo→ hypo→
Ype-4-2	NC_003143.1	+	969600	969686	←RelE (COG2026) **RNA**→ ←INT_P4 (cd00801)
Ype-1-2	NZ_AAKS01000008.1	+	55766	55852	←HTH_XRE (cd00093) **RNA**→ hypo→
Ype-15-2	NZ_AAOS01000023.1	-	12408	12322	←HTH_XRE (cd00093) **RNA**→ hypo→
Ype-5-2	NZ_AAUB01000028.1	-	1859	1773	**RNA**→
Ype-11-1	NZ_AAYR01000002.1	+	390330	390416	**RNA**→
Ype-16-2	NZ_AAYS01000001.1	+	283755	283841	**RNA**→
Ype-12-2	NZ_AAYT01000006.1	+	182777	182863	**RNA**→
Ype-9-2	NZ_AAYU01000005.1	-	204312	204226	**RNA**→
Ype-10-2	NZ_AAYV01000007.1	+	132679	132765	**RNA**→
Ype-14-2	NZ_ABAT01000002.1	-	94342	94256	**RNA**→
Ype-3-2	NZ_ABCD01000004.1	-	31787	31701	←RelE (COG2026) **RNA**→ ←INT_P4 (cd00801)
Yen-1-3	NC_008800.1	-	1858079	1857993	←hypo **RNA**→ ←hypo
Ybe-1-1	NZ_AALC01000034.1	+	29766	29853	Transposase_31 (pfam04754)→ **RNA**→ hypo→
Yin-1-1	NZ_AALF01000002.1	+	162645	162731	←hypo **RNA**→ hypo→
Yen-1-4	NC_008800.1	-	1859900	1859814	hypo→ **RNA**→ Transposase_31 (pfam04754)→
Yin-1-2	NZ_AALF01000002.1	+	164423	164510	hypo→ **RNA**→ hypo→
Sbo-2-1	NC_007613.1	+	1140537	1140623	hypo→ **RNA**→ hypo→ Nlp (COG3423)→
env-13	BAAW01012961.1	-	630	545	**RNA**→ hypo→
Sen-16-2	NC_006905.1	+	1315804	1315890	←HTH_XRE (smart00530)NOG17530 (NOG17530) **RNA**→ COG3646 (COG3646)→ hypo→
Api-1-1	NC_000935.1	-	28158	28072	**RNA**→
Sgl-2-9	NC_007712.1	-	1935487	1935401	←hypo **RNA**→ INT_Lambda_C (cd00800)→
Sdy-1-3	NZ_AAMJ01000022.1	+	3716	3799	hypo→ **RNA**→ hypo→
Eco-3-10	NZ_AAKB01000065.1	+	5191	5278	**RNA**→
Eph-2-1	NC_003356.1	+	9612	9699	**RNA**→
Eco-3-11	NZ_AAKB01000061.1	+	2450	2537	hypo→ **RNA**→ hypo→ hypo→ COG3646 (COG3646)→ hypo→ hypo→<br>hypothetical proteinCould not find sequence in database.→
Eco-3-12	NZ_AAKB01000061.1	+	3106	3193	hypo→ **RNA**→ hypo→ COG3646 (COG3646)→ hypo→ hypo→<br>hypothetical proteinCould not find sequence in database.→
Sdy-1-4	NZ_AAMJ01000006.1	-	84023	83936	hypo→ **RNA**→ hypo→ hypo→ hypo→ hypo→<br>COG0543: 2-polyprenylphenol hydroxylase and related flavodoxin oxidoreductases→ hypo→ hypo→<br>COG4643 (COG4643)COG5519 (COG5519)→
Eco-8-2	NZ_AAJW01000030.1	-	27742	27658	Dam (pfam05869)→ **RNA**→ COG3561 (COG3561)→ COG3646 (COG3646)ANT (pfam03374)→
Sdy-2-2	NC_007606.1	-	2215306	2215222	COG3561 (COG3561)→ **RNA**→ COG3561 (COG3561)→ COG3646 (COG3646)ANT (pfam03374)→
Eco-10-4	NZ_AAJZ01000001.1	-	4210661	4210579	DUF892 (pfam05974)→ **RNA**→ hypo→
Sfl-2-3	NC_004337.1	+	1400922	1401006	putative bacteriophage protein→ **RNA**→ Transposase_8 (pfam01527)→ rve (pfam00665)→<br>hypothetical bacteriophage protein→
Sen-10-2	NC_003198.1	-	1915250	1915166	hypo→ **RNA**→ putative bacteriophage protein→
Sdy-2-3	NC_007606.1	-	553748	553664	hypo→ **RNA**→ putative bacteriophage protein→
Eco-9-2	NZ_AAJV01000004.1	-	34572	34487	DUF892 (pfam05974)→ **RNA**→ hypo→
Eph-1-1	NC_003444.1	+	27704	27791	**RNA**→
Eco-13-2	NC_002695.1	+	1263291	1263373	NinE→ **RNA**→ COG3561 (COG3561)→ COG3646 (COG3646)ANT (pfam03374)→ NinG→ hypo→<br>antitermination protein Q→
Eco-8-3	NZ_AAJW01000017.1	+	44676	44758	Dam (pfam05869)→ **RNA**→ COG3561 (COG3561)→ ANT (pfam03374)→
Eph-5-1	NC_000902.1	+	17279	17361	**RNA**→
Sco-1-1	NC_004914.1	+	49167	49249	**RNA**→
Sen-15-2	NZ_ABFF01000008.1	-	134074	133992	**RNA**→
env-14	BABF01001575.1	+	2753	2840	hypo→ **RNA**→ COG3646 (COG3646)→
Eco-9-3	NZ_AAJV01000109.1	-	1078	991	COG3646 (COG3646)→ **RNA**→ COG3646 (COG3646)→
Sen-2-1	NZ_ABAP01000068.1	-	6237	6150	**RNA**→
Sen-15-3	NZ_ABFF01000019.1	+	53810	53897	**RNA**→
Eco-11-1	NZ_AAJU01000058.1	-	222	135	**RNA**→

env-15	BABF01001366.1	+	2134	2221	**RNA**→
Sen-9-3	NZ_ABAN01000056.1	-	12998	12911	**RNA**→
Eco-4-2	NC_008563.1	+	2745296	2745380	hypo→ **RNA**→ COG3617 (COG3617)→
Eph-6-1	NC_007804.2	-	20363	20278	**RNA**→
env-16	BAAX01012263.1	-	1243	1160	**RNA**→ COG3617 (COG3617)→
Sgl-1-3	NC_007182.1	+	21311	21396	Tpn1 protein→ **RNA**→ hypo→
Sgl-1-4	NC_007183.1	+	21311	21396	Tpn1 protein→ **RNA**→ hypo→
Sgl-2-10	NC_007713.1	+	50694	50779	←Antirestrict (pfam03230) **RNA**→ ←Transposase_31 (pfam04754)
Pfl-2-5	NC_007492.1	+	1502980	1503070	←UbiE (COG2226) **RNA**→ hypo→
Ppu-1-1	NC_009512.1	+	339637	339726	←hypo **RNA**→ hypo→
Pfl-2-6	NC_007492.1	-	5257618	5257533	←AcrA (COG0845)NOG08737 (NOG08737) **RNA**→ hypo→
env-17	sludgeOz_Contig11395	+	4620	4705	FOG(,,,)→ **RNA**→ COG3646 (COG3646)→
env-18	sludgeOz_Contig11626	-	4444	4358	TPR (cd00189)→ **RNA**→ COG3646 (COG3646)→
env-19	sludgeOz_Contig11626	+	29442	29528	←(,,,) **RNA**→ COG3646 (COG3646)→
env-20	sludgeOz_Contig9633	-	3083	2999	**RNA**→
Bth-1-1	NZ_ABBH01000502.1	+	7145	7238	**RNA**→
Bps-11-1	NZ_ABBO01000018.1	-	1738	1645	**RNA**→
Rso-1-1	NC_003295.1	+	1663851	1663938	hypo→ **RNA**→ hypo→ LT_GEWL (cd00254)→
Sgl-2-11	NC_007712.1	-	1527580	1527499	←hypo **RNA**→ COG3646 (COG3646)→ COG3646 (COG3646)NOG18702 (NOG18702)→ hypo→
Bxe-1-2	NC_007951.1	+	915998	916090	COG3415 (COG3415)rve (pfam00665)→ **RNA**→ hypo→
Bph-1-2	NZ_AAUH01000005.1	-	66833	66741	←COG3781 (COG3781) **RNA**→ hypo (cons)→
Ppu-4-2	NZ_AAVY01000026.1	-	50658	50567	GlpC (COG0247)GlcD (COG0277)→ **RNA**→ hypo (cons)→ hypo→
Pen-1-2	NC_008027.1	-	5667972	5667886	←hypo **RNA**→ hypo→ hypo→
Pst-1-1	NC_009434.1	-	813410	813328	hypo→ **RNA**→ hypo→
Sgl-2-12	NC_007712.1	-	2086052	2085968	←hypo **RNA**→ COG3617 (COG3617)→
Eph-7-1	NC_001901.1	-	28388	28301	**RNA**→
env-21	BAAW01005016.1	+	464	551	**RNA**→ hypo→ hypo→
Esa-1-1	NC_009778.1	-	2971361	2971274	Phage_cap_P2 (pfam05125)→ **RNA**→ hypo→
Sen-11-2	NC_004631.1	+	4674209	4674296	phage immunity repressor protein→ **RNA**→ phage immunity repressor protein→ hypo→ hypothetical P4 phage protein→
Sen-10-3	NC_003198.1	+	4691320	4691407	phage immunity repressor protein→ **RNA**→ phage immunity repressor protein→ hypo→ hypothetical P4 phage protein→ COG3378 (COG3378)COG4643 (COG4643)→
Yph-1-1	NC_005069.1	+	23719	23806	**RNA**→
Sgl-2-13	NC_007712.1	+	2901789	2901873	hypo→ **RNA**→ hypo→
Sgl-2-14	NC_007712.1	-	2897417	2897333	hypo→ **RNA**→ RusA (pfam05866)→
Eco-3-13	NZ_AAKB01000013.1	-	22127	22040	Phage_AlpA (pfam05930)→ **RNA**→ hypo→
Sen-7-2	NZ_ABEN01000001.1	+	4718071	4718158	**RNA**→
Esp-1-3	NC_009436.1	+	3381272	3381359	putative CI repressor→ **RNA**→ putative CI repressor→ hypo→ conserved hypothetical P4 phage protein→ COG3378 (COG3378)COG4643 (COG4643)→
Ybe-1-2	NZ_AALC01000015.1	+	69245	69332	hypo→ **RNA**→ hypo→ hypo→ hypo→ COG3378 (COG3378)COG4643 (COG4643)→
Ymo-1-1	NZ_AALD01000007.1	+	16582	16669	hypo→ **RNA**→ hypo→ hypo→ hypo→ COG3378 (COG3378)COG4643 (COG4643)→
Esa-1-2	NC_009778.1	-	596095	596008	AlpA (COG3311)→ **RNA**→ hypo→
Yfr-1-1	NZ_AALE01000013.1	-	3159	3075	hypo→ **RNA**→ hypo→ hypo→ hypo→ COG3378 (COG3378)COG4643 (COG4643)→
Sen-9-4	NZ_ABAN01000037.1	-	23094	23007	**RNA**→
Sen-5-1	NZ_ABEL01000004.1	-	418202	418115	**RNA**→
Sen-4-1	NZ_ABEM01000001.1	-	708764	708677	**RNA**→
Sen-7-3	NZ_ABEN01000001.1	+	2885196	2885283	**RNA**→
Sen-8-3	NZ_ABEW01000002.1	-	653315	653228	**RNA**→
Eco-13-3	NC_002695.1	+	318667	318754	putative CI repressor→ **RNA**→ putative CI repressor→ hypo→ Cnr-like protein→ COG3378 (COG3378)COG4643 (COG4643)→
Eco-12-2	NC_002655.2	+	318668	318755	putative regulator encoded in prophage CP-933I→ **RNA**→ putative regulator encoded in prophage CP-933I→
env-22	BAAX01008057.1	-	541	455	hypo→ **RNA**→ hypo→ hypo→
env-23	BAAW01000581.1	-	4187	4100	AlpA (COG3311)→ **RNA**→ hypo→ hypo→ hypo→ COG3378 (COG3378)COG4643 (COG4643)→

Sen-11-3	NC_004631.1	+	2741095	2741182	hypo→ **RNA**→ hypo→ hypo→ hypo→
Eco-5-2	NZ_AAWW01000009.1	+	31532	31619	putative bacteriophage protein→ **RNA**→ putative bacteriophage protein→ hypo (cons)→ hypo (cons)→
Eco-5-3	NZ_AAWW01000018.1	+	30662	30749	putative bacteriophage protein→ **RNA**→ putative bacteriophage protein→ hypo (cons)→ hypo (cons)→
Eco-14-1	NZ_ABAQ01000005.1	-	134828	134741	**RNA**→
Eco-11-2	NZ_AAJU01000002.1	-	106093	106006	hypo→ **RNA**→ hypo→
Eph-4-1	NC_001609.1	-	8507	8420	**RNA**→
Sbo-2-2	NC_007613.1	-	4221428	4221341	putative bacteriophage protein→ **RNA**→ putative bacteriophage protein→
Eco-8-4	NZ_AAJW01000039.1	+	10939	11026	hypo→ **RNA**→ hypo→
Sgl-2-15	NC_007713.1	+	13096	13181	hypo→ **RNA**→ CitB (COG2197)→
Sty-1-1	NC_005014.1	-	120268	120181	**RNA**→ hypo→
Eco-1-1	NC_007635.1	-	97837	97750	hypo→ **RNA**→ hypo→ HTH_LUXR (smart00421)→
Sen-6-1	NZ_ABAK01000021.1	+	44344	44431	**RNA**→
Sen-4-2	NZ_ABEM01000011.1	-	16405	16318	**RNA**→
Sen-4-3	NZ_ABEM01000012.1	+	9911	9998	**RNA**→
Sen-4-4	NZ_ABEM01000008.1	+	9908	9995	**RNA**→
Sen-4-5	NZ_ABEM01000008.1	+	101276	101363	**RNA**→
Kph-1-1	NC_005857.1	-	32623	32536	**RNA**→
Ype-2-2	NC_008150.1	-	654903	654816	putative prophage protein→ **RNA**→ putative prophage protein→ COG4643 (COG4643)→ repA (cd01125)→
Ype-7-3	NC_008149.1	+	3265707	3265794	prophage protein→ **RNA**→ prophage protein→ COG4643 (COG4643)→ repA (cd01125)→
Ype-8-3	NC_009381.1	+	2954292	2954379	prophage protein→ **RNA**→ prophage protein→ COG4643 (COG4643)→ repA (cd01125)→
Ype-6-3	NC_004088.1	+	3396658	3396745	hypo→ **RNA**→ hypo→ COG4643 (COG4643)→ repA (cd01125)NOG13495 (NOG13495)→
Ype-4-3	NC_003143.1	-	1238167	1238080	putative prophage protein→ **RNA**→ putative prophage protein→ COG4643 (COG4643)→ repA (cd01125)NOG13495 (NOG13495)→
Ype-13-3	NC_005810.1	+	1144762	1144849	putative prophage protein→ **RNA**→ putative prophage protein→ COG4643 (COG4643)→ repA (cd01125)→
Ype-1-3	NZ_AAKS01000002.1	+	71350	71437	hypo→ **RNA**→ hypo→
Ype-15-3	NZ_AAOS01000089.1	+	4167	4254	hypo→ **RNA**→ hypo→ COG4643 (COG4643)→ repA (cd01125)→
Ype-5-3	NZ_AAUB01000092.1	+	7617	7704	**RNA**→
Ype-11-2	NZ_AAYR01000002.1	+	184692	184779	**RNA**→
Ype-16-3	NZ_AAYS01000002.1	+	420406	420493	**RNA**→
Ype-12-3	NZ_AAYT01000002.1	+	577828	577915	**RNA**→
Ype-9-3	NZ_AAYU01000002.1	-	73126	73039	**RNA**→
Ype-10-3	NZ_AAYV01000015.1	-	22777	22690	**RNA**→
Ype-14-3	NZ_ABAT01000001.1	+	556501	556588	**RNA**→
Ype-3-3	NZ_ABCD01000002.1	+	736077	736164	putative prophage protein→ **RNA**→ putative prophage protein→ COG4643 (COG4643)→ repA (cd01125)→
env-24	BABF01000165.1	+	10514	10601	COG3561 (COG3561)→ **RNA**→ hypo→ hypo→ hypo→ hypo→ hypo→ hypo→ hypo→
Sen-1-1	NC_006511.1	+	4467249	4467336	phage immunity repressor protein→ **RNA**→ phage immunity repressor protein→ hypo→ hypothetical P4 phage protein→ COG3378 (COG3378)COG4643 (COG4643)→
Sen-14-2	NZ_ABEH01000010.1	+	117001	117088	**RNA**→
env-25	JCVI_SCAF_1096627954892	+	938	1021	**RNA**→
Ppu-4-3	NZ_AAVY01000013.1	+	86112	86198	←OYE_YqiM_FMN (cd02932) **RNA**→ hypo→
Ppu-2-1	NZ_AAXR01000007.1	+	203630	203715	←OYE_YqiM_FMN (cd02932) **RNA**→ hypo (cons)→
Pen-1-3	NC_008027.1	-	5194676	5194589	←LivK (COG0683) **RNA**→ hypo→
Nmu-1-1	NC_007614.1	-	68166	68083	AlpA (COG3311)NOG19993 (NOG19993)→ **RNA**→ hypo→ hypo→ hypo→
env-26	sludgePhrap_Contig2337	+	52	135	**RNA**→
Avi-1-1	NZ_AAAU03000001.1	-	771062	770978	COG5659 (COG5659)→ **RNA**→ COG3617 (COG3617)→
Pae-4-2	NZ_AAQW01000001.1	+	6009993	6010074	MviM (COG0673)→ **RNA**→ hypo→
Ppu-2-2	NZ_AAXR01000002.1	-	340097	340011	KefB (COG0475)Kch (COG1226)→ **RNA**→ hypo (cons)→
Pen-1-4	NC_008027.1	+	427349	427440	←COG2944 (COG2944) **RNA**→ hypo→
Pen-1-5	NC_008027.1	-	4670418	4670332	←PhnF (COG2188) **RNA**→ hypo→ hypo→
Ppu-4-4	NZ_AAVY01000014.1	-	30948	30861	←AcrA (COG0845) **RNA**→ hypo→ hypo→
Pen-1-6	NC_008027.1	+	1026431	1026518	COG4625 (COG4625)→ **RNA**→ hypo→ hypo→
Pen-1-7	NC_008027.1	+	619442	619529	SGNH_hydrolase_peri1 (cd01825)→ **RNA**→ hypo→ hypo→
Ppu-4-5	NZ_AAVY01000023.1	+	201	288	**RNA**→ hypo→ hypo→

Ppu-3-1	NC_002947.3	+	335774	335862	←hypo **RNA**→ hypo→
Ppu-3-2	NC_002947.3	-	2302915	2302823	hypo→ **RNA**→ hypo→
Ppu-4-6	NZ_AAVY01000003.1	-	8342	8252	OprD (pfam03573)→ **RNA**→ hypo (cons)→ hypo→
Ppu-1-2	NC_009512.1	+	4379908	4379996	←ComEA (COG1555) **RNA**→ hypo→
Ppu-4-7	NZ_AAVY01000059.1	-	821	732	←hypo (cons) **RNA**→ hypo→ hypo→
Ppu-3-3	NC_002947.3	-	2038016	2037928	WecB (COG0381)→ **RNA**→ ←hypo
Ppu-4-8	NZ_AAVY01000023.1	-	18560	18473	hypo→ **RNA**→ repA (cd01125)→
Ppu-4-9	NZ_AAVY01000025.1	+	47012	47100	plasmid pRiA4b ORF-3-like→ **RNA**→ ←CynX (COG2807)
env-27	JCVI_SCAF_1101668291644	-	1191	1104	**RNA**→
Pen-1-8	NC_008027.1	-	3518484	3518397	←hypo **RNA**→ hypo→
Ppu-1-3	NC_009512.1	+	770187	770275	←tRNA-OTHER **RNA**→ hypo→
Ppu-4-10	NZ_AAVY01000026.1	-	49745	49657	hypo→ **RNA**→ hypo (cons)→ hypo→
Ppu-4-11	NZ_AAVY01000021.1	-	79306	79219	←COG1943 (COG1943) **RNA**→ hypo→ hypo→
Pen-1-9	NC_008027.1	-	793129	793043	←hypo **RNA**→ ←hypo
Pen-1-10	NC_008027.1	+	980078	980164	←GGDEF (cd01949) **RNA**→ hypo→ hypo→
Pfl-2-7	NC_007492.1	+	5006731	5006819	COG1721 (COG1721)→ **RNA**→ hypo→
Xor-1-1	NC_006834.1	-	1745312	1745231	TOPRIM_primases (cd01029)NOG06080 (NOG06080)→ **RNA**→ hypo→
Xor-2-1	NC_007705.1	-	1727613	1727532	TOPRIM_primases (cd01029)→ **RNA**→ hypo→ hypo→
env-28	JCVI_SCAF_1096628042224	+	713	793	? (GOS12118414)→ **RNA**→
Xca-1-1	NC_003902.1	+	3556259	3556339	TOPRIM_primases (cd01029)→ **RNA**→ hypo→ hypo→ hypo→ hypo→ hypo→ hypo→
Sma-1-5	NZ_AAVZ01000020.1	-	14481	14397	TonB family protein→ **RNA**→ ←SSL2 (COG1061)
Bdo-1-1	NZ_AAKY01000047.1	+	15904	15993	←hypo **RNA**→ hypo→
Pfl-2-8	NC_007492.1	-	753624	753536	hypo→ **RNA**→ hypo→
Pfl-1-2	NC_004129.6	-	3142100	3142011	←COG3666 (COG3666) **RNA**→ hypo→
Ppu-4-12	NZ_AAVY01000025.1	+	46883	46967	plasmid pRiA4b ORF-3-like→ **RNA**→ ←CynX (COG2807)
Pen-1-11	NC_008027.1	+	28932	29019	SUA5 (COG0009)→ **RNA**→ hypo→
Ppu-3-4	NC_002947.3	+	81574	81663	hypo→ **RNA**→ hypo→
Ppu-2-3	NZ_AAXR01000002.1	-	88895	88806	SUA5 (COG0009)→ **RNA**→ hypo (cons)→
Ppu-4-13	NZ_AAVY01000019.1	+	172	260	**RNA**→ hypo (cons)→
Ppu-1-4	NC_009512.1	+	93169	93257	SUA5 (COG0009)→ **RNA**→ hypo→
env-29	JCVI_SCAF_1096627487547	-	493	405	? (GOS11335753)→ **RNA**→ ? (GOS11335753)→
Psy-3-1	NC_004578.1	-	447442	447360	←ComJ (COG2961) **RNA**→ hypo→
Psy-1-2	NC_005773.3	+	5451361	5451443	←ComJ (COG2961) **RNA**→ ←hypo
Psy-1-3	NC_005773.3	+	4631607	4631691	hypo→ **RNA**→ hypo→
Pfl-2-9	NC_007492.1	-	3357442	3357358	hypo→ **RNA**→ hypo→
Pfl-2-10	NC_007492.1	+	2049523	2049609	ABC_Class2 (cd03227)NOG22115 (NOG22115)→ **RNA**→ hypo→ hypo→
Pfl-2-11	NC_007492.1	+	3662342	3662427	←hypo **RNA**→ hypo→
Pfl-2-12	NC_007492.1	-	2779539	2779454	hypo→ **RNA**→ hypo→
env-30	JCVI_SCAF_1101668291562	+	916	1003	R_hydratase (cd03449)→ **RNA**→ ? (GOS11008091)→
Psy-2-1	NC_007005.1	+	4827916	4828000	hypo→ **RNA**→ hypo→
Pfl-2-13	NC_007492.1	-	2641456	2641372	←hypo **RNA**→ hypo→
Psy-2-2	NC_007005.1	+	5651663	5651744	←ComJ (COG2961) **RNA**→ hypo→
Pfl-2-14	NC_007492.1	-	4317777	4317693	←COG4104 (COG4104) **RNA**→ COG3547 (COG3547)→
Psy-3-2	NC_004578.1	-	6094972	6094888	DnaJ (pfam00226)→ **RNA**→ hypo→
Pfl-2-15	NC_007492.1	-	2739890	2739805	←DUF72 (pfam01904) **RNA**→ hypo→
Avi-1-2	NZ_AAAU03000001.1	+	1076949	1077035	←DUF891 (pfam05973) **RNA**→ hypo→ hypo→ hypo→ TOPRIM_primases (cd01029)→ hypo→
Ppu-2-4	NZ_AAXR01000001.1	-	951433	951347	hypo→ **RNA**→ hypo (cons)→
Pen-1-12	NC_008027.1	-	2115228	2115143	←RhtB (COG1280) **RNA**→ ←ACR_tran (pfam00873)
Pfl-2-16	NC_007492.1	+	3960594	3960682	←COG5606 (COG5606) **RNA**→ hypo→ hypo→
Pfl-2-17	NC_007492.1	+	2273321	2273407	CsgG (COG1462)→ **RNA**→ hypo→ hypo→
Pfl-2-18	NC_007492.1	+	5173901	5173991	Gcd (COG4993)→ **RNA**→ hypo→
Pfl-1-3	NC_004129.6	-	6137446	6137360	←Plasmid_killer (pfam05015) **RNA**→ hypo→

Pen-1-13	NC_008027.1	+	1922676	1922764	COG4178 (COG4178)→ **RNA**→ hypo→
Pfl-2-19	NC_007492.1	-	2414243	2414159	←hypo **RNA**→ hypo→ hypo→
Pfl-2-20	NC_007492.1	-	854931	854844	Tar (COG0840)→ **RNA**→ hypo→
Pfl-2-21	NC_007492.1	-	351669	351583	←COG1724 (COG1724) **RNA**→ hypo→
Pfl-1-4	NC_004129.6	+	1157583	1157668	hypo→ **RNA**→ hypo→
env-31	JCVI_SCAF_1096626143183	+	172	261	**RNA**→
env-32	JCVI_SCAF_1096627151993	-	460	372	DszC (cd01163)? (GOS26)→ **RNA**→ ? (GOS11008091)→
env-33	JCVI_SCAF_1096627846063	+	1373	1459	←PelB (COG3866)? (GOS3774) **RNA**→
env-34	JCVI_SCAF_1096627605558	+	194	280	**RNA**→ ←? (GOS3431)
Pfl-2-22	NC_007492.1	+	5919175	5919260	lyase, putative→ **RNA**→ hypo→ COG1476 (COG1476)→
env-35	JCVI_SCAF_1096627781829	+	501	587	**RNA**→ COG4680 (COG4680)? (GOS13828)→
Pfl-1-5	NC_004129.6	-	1753283	1753198	←filamentous hemagglutinin, intein-containing, putative **RNA**→ hypo→
Psy-1-4	NC_005773.3	+	369048	369143	TauA (COG0715)→ **RNA**→ hypo→
Psy-2-3	NC_007005.1	+	363735	363830	TauA (COG0715)→ **RNA**→ hypo→ hypo→
Pen-1-14	NC_008027.1	-	4962315	4962229	←LipA (COG1075) **RNA**→ hypo→
env-36	JCVI_SCAF_1096627876825	-	932	845	**RNA**→
Pfl-2-23	NC_007492.1	+	2239520	2239607	LysR (COG0583)→ **RNA**→ hypo→
Pfl-2-24	NC_007492.1	-	1747486	1747402	←hypo **RNA**→ hypo→
Pfl-2-25	NC_007492.1	-	1740090	1740006	hypo→ **RNA**→ hypo→
Psy-2-4	NC_007005.1	+	4545144	4545229	←DUF1349 (pfam07081) **RNA**→ COG3617 (COG3617)→ adh_short (pfam00106)→
env-37	JCVI_SCAF_1096627936442	+	472	555	**RNA**→ ? (GOS13412611)→
env-38	JCVI_SCAF_1101667131354	+	39	129	**RNA**→
env-39	JCVI_SCAF_1096628172702	+	1077	1159	**RNA**→
Pfl-1-6	NC_004129.6	+	5731958	5732043	hypo→ **RNA**→ COG5519 (COG5519)TOPRIM_primases (cd01029)→ chromosome segregation SMC protein, putative→
Ppu-3-5	NC_002947.3	+	759603	759688	Acetyltransf_1 (pfam00583)→ **RNA**→ ←GntP_permease (pfam02447)
env-40	JCVI_SCAF_1096628052827	+	1532	1617	? (GOS11069324)→ **RNA**→ ? (GOS11725977)→
Pen-1-15	NC_008027.1	-	791092	791007	tmRNA involved in trans-translation→ **RNA**→ hypo→
Ppu-4-14	NZ_AAVY01000025.1	+	47399	47484	plasmid pRiA4b ORF-3-like→ **RNA**→ ←CynX (COG2807)
Pen-1-16	NC_008027.1	-	4960291	4960206	hypo→ **RNA**→ hypo→
Ppu-4-15	NZ_AAVY01000004.1	+	95972	96057	DTW (pfam03942)→ **RNA**→ ←HlyD (pfam00529)
env-41	JCVI_SCAF_1101668395415	-	172	86	**RNA**→
Psy-3-3	NC_004578.1	+	3480015	3480104	lipoprotein, putative→ **RNA**→ COG3617 (COG3617)→
Pae-3-1	NC_009656.1	-	373488	373402	INT_P4 (cd00801)→ **RNA**→ hypo→ hypo→ transcriptional regulator, AlpA family→ Bbp36→ zf-CHC2 (pfam01807)→ hypo→ hypo→ COG5519 (COG5519)→
Ppu-4-16	NZ_AAVY01000008.1	-	10570	10486	Aes (COG0657)→ **RNA**→ hypo (cons)→
Pfl-1-7	NC_004129.6	-	2447607	2447522	←hypo **RNA**→ hypo→
Pfl-2-26	NC_007492.1	+	2338723	2338805	ArgE (COG0624)→ **RNA**→ hypo→
Pfl-2-27	NC_007492.1	-	3665249	3665164	←Acetyltransf_1 (pfam00583)NOG15906 (NOG15906) **RNA**→ hypo→
env-42	JCVI_SCAF_1101667357642	+	288	376	**RNA**→ ? (GOS14070030)→
env-43	JCVI_SCAF_1101667148170	-	172	86	**RNA**→
Pfl-1-8	NC_004129.6	+	6960256	6960341	AspA (COG1027)→ **RNA**→ hypo→
Avi-1-3	NZ_AAAU03000001.1	-	851854	851768	hypo→ **RNA**→ hypo→ hypo→
Psy-1-5	NC_005773.3	-	5617115	5617032	DnaJ (pfam00226)→ **RNA**→ hypo→
Psy-2-5	NC_007005.1	-	5828644	5828559	DnaJ (pfam00226)→ **RNA**→ hypo→
Pfl-2-28	NC_007492.1	+	6230633	6230715	←hypo **RNA**→ hypo→
env-44	JCVI_SCAF_1096627418547	+	617	700	**RNA**→
Pfl-1-9	NC_004129.6	-	4859973	4859886	hypo→ **RNA**→ hypo→
Ppu-4-17	NZ_AAVY01000010.1	+	155069	155158	hypo→ **RNA**→ hypo (cons)→
Ppu-4-18	NZ_AAVY01000002.1	+	296110	296200	OprD (pfam03573)→ **RNA**→ hypo (cons)→
Pen-1-17	NC_008027.1	+	3966788	3966882	hypo→ **RNA**→ hypo→
Pen-1-18	NC_008027.1	+	3960618	3960708	←NUDIX (pfam00293) **RNA**→ hypo→
Ppu-4-19	NZ_AAVY01000010.1	+	153965	154055	Oligopeptide transporter OPT superfamily→ **RNA**→ hypo (cons)→

Ppu-4-20	NZ_AAVY01000002.1	+	273462	273557	←conserved secreted protein with internal repeats **RNA**→ hypo (cons)→
Pen-1-19	NC_008027.1	+	5582506	5582595	←DUF1234 (pfam06821) **RNA**→ hypo→
Pen-1-20	NC_008027.1	-	910398	910306	←HemA (COG0373) **RNA**→ hypo→
Pen-1-21	NC_008027.1	-	1493171	1493083	←LysR (COG0583) **RNA**→ hypo→
Ppu-3-6	NC_002947.3	+	5390633	5390719	hypo→ **RNA**→ hypo→
Pen-1-22	NC_008027.1	+	3854037	3854123	←hypo **RNA**→ hypo→
Ppu-3-7	NC_002947.3	-	2858046	2857965	hypo→ **RNA**→ yedG domain protein→
Ppu-1-5	NC_009512.1	+	3625062	3625148	hypo→ **RNA**→ hypo→
Ppu-1-6	NC_009512.1	+	4999895	4999981	←PsiE (pfam06146) **RNA**→ hypo→ hypo→
Pen-1-23	NC_008027.1	-	3291941	3291852	hypo→ **RNA**→ hypo→
Pen-1-24	NC_008027.1	+	1180765	1180854	DUF479 (pfam04336)→ **RNA**→ hypo→
Pen-1-25	NC_008027.1	+	4896084	4896172	FecA (COG4772)→ **RNA**→ hypo→
Ppu-4-21	NZ_AAVY01000003.1	+	347373	347461	←ACAD_fadE5 (cd01153) **RNA**→ hypo (cons)→
Ppu-4-22	NZ_AAVY01000026.1	-	47910	47825	hypo (cons)→ **RNA**→ hypo (cons)→
Ppu-4-23	NZ_AAVY01000018.1	+	10366	10453	←lipoprotein, putative **RNA**→ hypo (cons)→
Ppu-1-7	NC_009512.1	-	1439621	1439537	←Rick_17kDa_Anti (pfam05433) **RNA**→ hypo→ hypo→
Ppu-1-8	NC_009512.1	+	1545409	1545496	←Transposase_mut (pfam00872) **RNA**→ hypo→ hypo→
Ppu-3-8	NC_002947.3	-	5149014	5148927	hypo→ **RNA**→ hypo→
Pen-1-26	NC_008027.1	-	2996471	2996385	hypo→ **RNA**→ hypo→ hypo→
Ppu-2-5	NZ_AAXR01000004.1	+	377787	377878	←COG3714 (COG3714) **RNA**→ hypo (cons)→
Ppu-4-24	NZ_AAVY01000008.1	-	10944	10858	Aes (COG0657)→ **RNA**→ hypo (cons)→
Ppu-4-25	NZ_AAVY01000001.1	+	243198	243282	←AbgB (COG1473) **RNA**→ hypo (cons)→
Ppu-3-9	NC_002947.3	-	1995754	1995667	dTDP_sugar_isom (pfam00908)→ **RNA**→ COG1835 (COG1835)→
Pen-1-27	NC_008027.1	+	2592733	2592818	FhuE (COG4773)→ **RNA**→ hypo→ hypo→
Ppu-3-10	NC_002947.3	-	2855914	2855828	←hypo **RNA**→ ←DltE (COG0300)
Ppu-3-11	NC_002947.3	-	1420040	1419951	←Rick_17kDa_Anti (pfam05433) **RNA**→ hypo→
Pen-1-28	NC_008027.1	+	4355591	4355676	←hypo **RNA**→ hypo→ hypo→
Psy-1-6	NC_005773.3	-	2874551	2874467	GAF (pfam01590)→ **RNA**→ COG3617 (COG3617)→
Ppu-4-26	NZ_AAVY01000050.1	-	3054	2970	←hypo **RNA**→ hypo (cons)→ hypo→ hypo (cons)→
Ppu-3-12	NC_002947.3	-	4856630	4856545	←hypo **RNA**→ ←PBPb (smart00062)
Ppu-1-9	NC_009512.1	+	1788565	1788652	hypo→ **RNA**→ hypo→
Ppu-3-13	NC_002947.3	+	5989252	5989335	KefB (COG0475)Kch (COG1226)→ **RNA**→ hypo→
Ppu-1-10	NC_009512.1	+	5795249	5795332	KefB (COG0475)Kch (COG1226)→ **RNA**→ hypo→
Pen-1-29	NC_008027.1	+	5708208	5708293	←VapI (COG3093) **RNA**→ hypo→
env-45	JCVI_SCAF_1096627055672	-	3101	3015	**RNA**→ ? (GOS11222950)→
env-46	JCVI_SCAF_1096628071788	-	497	410	HisM (COG0765)? (GOS4027)→ **RNA**→
Pme-1-1	NC_009439.1	+	3776559	3776646	←hypo **RNA**→ hypo→ hypo→
Pme-1-2	NC_009439.1	-	1667390	1667307	COG2085 (COG2085)→ **RNA**→ hypo→
Rso-2-1	NZ_AAKL01000002.1	-	62829	62742	hypo→ **RNA**→ hypo→ hypo→ DnaG (COG0358)→
Rpi-1-1	NZ_ABDZ01000005.1	-	76964	76878	Ogr_Delta (pfam04606)→ **RNA**→ protein of unknown function DUF1484→ zf-CHC2 (pfam01807)TOPRIM_primases (cd01029)→
env-47	JCVI_SCAF_1096628063291	-	1161	1075	Ogr_Delta (pfam04606)? (GOS14311108)→ **RNA**→
Reu-1-1	NC_008314.1	-	532510	532418	hypo→ **RNA**→ ←hypo
Rso-2-2	NZ_AAKL01000071.1	-	17467	17379	Hypothetical protein→ **RNA**→ Hypothetical Protein→
Reu-2-1	NC_007347.1	+	2423671	2423759	Ogr_Delta (pfam04606)NOG22117 (NOG22117)→ **RNA**→ hypo→ hypo→ DnaG (COG0358)NOG06313 (NOG06313)→
Rso-1-2	NC_003295.1	+	1013264	1013352	Ogr_Delta (pfam04606)NOG22117 (NOG22117)→ **RNA**→ hypo→ hypo→ DnaG (COG0358)NOG06313 (NOG06313)→
Rso-1-3	NC_003295.1	-	3475955	3475871	hypo→ **RNA**→ hypo→
Bth-3-1	NZ_ABBD01000499.1	-	2644	2554	**RNA**→
Bps-8-1	NZ_AAYX01000007.1	+	10966	11055	hypo (cons)→ **RNA**→ hypo (cons)→ hypo (cons)→
Bps-16-1	NC_006350.1	-	1334602	1334513	hypo→ **RNA**→ hypo→
Bps-13-1	NZ_ABBK01000286.1	-	12447	12358	**RNA**→

Bps-11-2	NZ_ABBO01000209.1	+	19106	19197	**RNA**→
Bth-3-2	NZ_ABBD01000806.1	+	5334	5425	**RNA**→
Bth-2-1	NZ_ABBM01000179.1	+	20561	20652	**RNA**→
Bvi-1-1	NC_009256.1	-	463132	463042	hypo→ **RNA**→ hypo→ hypo→ hypo→ hypo→ hypo→
Bth-3-3	NZ_ABBD01000762.1	-	4854	4764	**RNA**→
Bps-16-2	NC_006350.1	-	98311	98220	hypo→ **RNA**→ hypo→ hypo→ hypo→
Bps-10-1	NC_009074.1	-	98244	98153	hypo→ **RNA**→ hypo→ hypo→ hypo→
Bps-4-1	NZ_ABBJ01000023.1	-	9760	9669	**RNA**→
Bps-13-2	NZ_ABBK01000021.1	-	11685	11594	**RNA**→
Bps-7-1	NC_007434.1	-	323597	323506	hypo→ **RNA**→ hypo→
Bps-1-1	NC_009076.1	-	114154	114063	hypo→ **RNA**→ hypo→ hypo→ hypo→
Bps-6-1	NZ_AAHS02000063.1	-	18204	18113	hypo→ **RNA**→ hypo→ hypo→
Bps-17-1	NZ_AAHV02000110.1	-	2933	2842	hypo→ **RNA**→ hypo→ hypo→
Bps-2-1	NZ_AAMB01000054.1	+	10782	10873	hypo→ **RNA**→ hypo→ hypo→
Bps-12-1	NZ_ABBL01000019.1	-	9742	9651	**RNA**→
Bps-14-1	NZ_ABBN01000016.1	-	9766	9675	**RNA**→
Bps-11-3	NZ_ABBO01001513.1	+	7645	7736	**RNA**→
Bps-15-1	NZ_ABBR01000147.1	+	5365	5456	**RNA**→
Bps-8-2	NZ_AAYX01000008.1	+	9285	9376	hypo→ **RNA**→ hypo (cons)→ hypo (cons)→ hypo (cons)→
Bps-5-1	NZ_AAHR02000044.1	-	42873	42782	hypo→ **RNA**→ hypo→ hypo→
Bps-18-1	NZ_AAHW02000026.1	-	10700	10609	hypo→ **RNA**→ hypo→ hypo→
Bok-2-1	NZ_ABBF01000782.1	+	19995	20086	**RNA**→
Bok-1-1	NZ_ABBG01000072.1	+	36101	36192	**RNA**→
Bce-1-1	NZ_AAVA01000001.1	-	268317	268226	←hypo **RNA**→ hypo (cons)→
Bps-5-2	NZ_AAHR02000018.1	-	20965	20874	←PinR (COG1961) **RNA**→ hypo→ hypo→
Bth-1-2	NZ_ABBH01000753.1	+	1638	1728	**RNA**→
Bps-7-2	NC_007434.1	+	1344790	1344879	hypo→ **RNA**→ hypo→ COG4643 (COG4643)COG5519 (COG5519)→
Bps-5-3	NZ_AAHR02000028.1	-	56606	56517	←hypo **RNA**→ hypo→ hypo→ hypo→ COG4643 (COG4643)DUF927 (pfam06048)→ COG5519 (COG5519)→
Bps-6-2	NZ_AAHS02000008.1	+	100667	100756	←hypo **RNA**→ hypo→ hypo→ hypo→ hypo→ COG4643 (COG4643)COG5519 (COG5519)→
Bps-17-2	NZ_AAHV02000008.1	-	73985	73896	←hypo **RNA**→ hypo→ hypo→ hypo→ hypo→
Bdo-1-2	NZ_AAKY01000125.1	+	82257	82346	hypo→ **RNA**→ hypo→ hypo→ hypo→ hypo→
Bps-9-1	NZ_AAMM02000028.1	+	49678	49767	←Transposase_8 (pfam01527) **RNA**→ hypo→ hypo→ hypo→ hypo→
Reu-1-2	NC_008314.1	+	273926	274013	CheZ (COG3143)→ **RNA**→ hypo→
Cvi-1-2	NC_005085.1	-	675562	675477	←hypo **RNA**→ hypo→ hypo→ zf-CHC2 (pfam01807)→
Bps-15-2	NZ_ABBR01001020.1	-	7221	7131	**RNA**→
Rpi-2-1	NZ_AAWK01000002.1	+	203495	203583	←hypo (cons) **RNA**→ hypo→
Rpi-1-2	NZ_ABDZ01000003.1	+	406374	406462	←putative cytoplasmic protein **RNA**→ hypo→
env-48	JCVI_SCAF_1096628075889	-	1758	1670	**RNA**→
env-49	sludgePhrap_Contig14725	+	1993	2074	**RNA**→
Bce-1-2	NZ_AAVA01000004.1	+	244809	244900	hypo→ **RNA**→ repA (cd01125)→
Bma-10-1	NC_008785.1	-	443372	443281	←CorB (COG4536) **RNA**→ Transposase_8 (pfam01527)→ rve (pfam00665)→
Bma-8-1	NC_009080.1	+	3220377	3220468	←CorB (COG4536) **RNA**→ Transposase_8 (pfam01527)→ rve (pfam00665)→
Bma-7-1	NC_008836.1	-	1333874	1333783	←CorB (COG4536) **RNA**→ Transposase_8 (pfam01527)→ rve (pfam00665)→
Bma-3-1	NC_006348.1	-	2633521	2633430	←CorB (COG4536) **RNA**→ Transposase_8 (pfam01527)→
Bma-2-1	NZ_AAHN02000004.1	+	44523	44614	←CorB (COG4536) **RNA**→ Transposase_8 (pfam01527)→ rve (pfam00665)→
Bma-5-1	NZ_AAHO01000003.1	-	120218	120127	←CorB (COG4536) **RNA**→ Transposase_8 (pfam01527)→ rve (pfam00665)→
Bma-4-1	NZ_AAIQ02000005.1	-	72568	72477	←CorB (COG4536) **RNA**→ Transposase_8 (pfam01527)→ rve (pfam00665)→
Bma-6-1	NZ_AAIR02000007.1	-	72522	72431	←CorB (COG4536) **RNA**→ Transposase_8 (pfam01527)→ rve (pfam00665)→
Bma-1-1	NZ_AANX02000033.1	+	54491	54582	←CorB (COG4536) **RNA**→ ←rve (pfam00665)
Bma-9-1	NZ_AAZP01000002.1	-	127546	127455	**RNA**→
Bth-2-2	NZ_ABBM01000536.1	-	13915	13824	**RNA**→

Bvi-1-2	NC_009254.1	-	633610	633518	←hypo **RNA**→ hypo→ CysI (COG0155)→ DUF934 (pfam06073)→
env-50	JCVI_SCAF_1096627390482	+	71935	72027	←COG1848 (COG1848)? (GOS13071826) **RNA**→ ←INT_SG1_C (cd01183)? (GOS13019564)
Bce-2-1	NZ_AAKX01000021.1	-	71214	71126	hypo→ **RNA**→ COG0840: Methyl-accepting chemotaxis protein→
Bps-3-1	NZ_ABBP01000530.1	-	16827	16739	**RNA**→
Bmu-1-1	NZ_AAVB01000029.1	+	2859	2950	TrfA (pfam07042)→ **RNA**→ hypo (cons)→
Bvi-1-3	NC_009227.1	-	216892	216802	hypo→ **RNA**→ hypo→ hypo→ hypo→
env-51	JCVI_SCAF_1096627389595	-	53519	53428	? (GOS14593241)→ **RNA**→ COG5642 (COG5642)? (GOS11073445)→ COG5654 (COG5654)? (GOS10365)→
Rso-1-4	NC_003295.1	-	2052344	2052257	hypo→ **RNA**→ hypo→
Rpi-2-2	NZ_AAWK01000002.1	-	1178	1090	←hypo (cons) **RNA**→ hypo→
Rpi-1-3	NZ_ABDZ01000003.1	-	186473	186388	hypo→ **RNA**→ hypo→
Rso-2-3	NZ_AAKL01000003.1	-	1035	948	←Hypothetical Protein **RNA**→ Hypothetical Protein→
Rso-1-5	NC_003295.1	-	3705084	3704998	hypo→ **RNA**→ hypo→ hypo→ hypo→
Rpi-1-4	NZ_ABDZ01000005.1	-	62708	62621	hypo→ **RNA**→ hypo→
Rpi-2-3	NZ_AAWK01000008.1	-	223652	223566	COG2105 (COG2105)→ **RNA**→ hypo→
Rpi-1-5	NZ_ABDZ01000027.1	-	11058	10972	COG2105 (COG2105)→ **RNA**→ hypo→
Rpi-2-4	NZ_AAWK01000002.1	-	155352	155263	←COG2944 (COG2944) **RNA**→ hypo (cons)→
Rpi-1-6	NZ_ABDZ01000003.1	-	358829	358740	←hypo (cons) **RNA**→ hypo (cons)→ hypo (cons)→
Rpi-2-5	NZ_AAWK01000002.1	+	340004	340091	←type I restriction system endonuclease **RNA**→ hypo→
env-52	JCVI_SCAF_1101667386172	+	658	747	**RNA**→
Pen-1-30	NC_008027.1	+	2692820	2692908	COG2863 (COG2863)→ **RNA**→ hypo→
Ppu-4-27	NZ_AAVY01000024.1	+	72189	72273	FhuE (COG4773)→ **RNA**→ hypo (cons)→ hypo→
Pfl-2-29	NC_007492.1	-	4210726	4210638	←COG3950 (COG3950) **RNA**→ hypo→
Sgl-2-16	NC_007712.1	-	1219801	1219714	KilA-N (pfam04383)NOG17190 (NOG17190)→ **RNA**→ DnaC (COG1484)→
Sgl-2-17	NC_007712.1	+	444749	444836	←Transposase_9 (pfam01548)Transposase_20 (pfam02371) **RNA**→ ←Phage_portal (pfam04860)
Eco-8-5	NZ_AAJW01000085.1	-	4756	4672	hypo→ **RNA**→ hypo→ hypo→
Eco-3-14	NZ_AAKB01000008.1	-	102388	102303	hypo→ **RNA**→ hypo→
Sen-3-1	NZ_ABFG01000007.1	+	330034	330119	**RNA**→
Sgl-2-18	NC_007712.1	+	1663464	1663548	←Porin_1 (pfam00267) **RNA**→ putative phage replication protein→
Eph-3-1	NC_005856.1	+	48337	48422	**RNA**→
Sen-8-4	NZ_ABEW01000015.1	-	43415	43331	**RNA**→
Aba-1-6	NC_009085.1	-	3754463	3754380	←HTH_ARAC (smart00342) **RNA**→ putative transcriptional regulator→
Aba-1-7	NC_009085.1	-	1620947	1620860	surface antigen→ **RNA**→ ←LysR (COG0583)
Aba-1-8	NC_009085.1	+	1471058	1471146	HtrB (COG1560)→ **RNA**→ TetR_N (pfam00440)→
Aba-1-9	NC_009085.1	-	3063911	3063825	←hypo **RNA**→ TetR_N (pfam00440)→
Asp-1-1	NC_005966.1	+	2750060	2750143	←hypo **RNA**→ hypo→ COG3152 (COG3152)→
Aba-1-10	NC_009085.1	+	1261816	1261900	NlpA (COG1464)→ **RNA**→ AcrR (COG1309)→
Aba-1-11	NC_009085.1	-	2432140	2432056	←Lrp (COG1522) **RNA**→ putative transcriptional regulator→
Aba-1-12	NC_009085.1	+	1493304	1493388	EriC (COG0038)→ **RNA**→ putative transcriptional regulator→
Aba-1-13	NC_009085.1	-	3419053	3418968	←hypo **RNA**→ hypo→
Aba-1-14	NC_009085.1	+	872109	872195	Flavin_Reduct (pfam01613)→ **RNA**→ TetR_N (pfam00440)→
Aba-1-15	NC_009085.1	+	590336	590422	COG5001 (COG5001)→ **RNA**→ putative transcriptional regulator→
Aba-1-16	NC_009085.1	-	2114540	2114456	COG0385 (COG0385)→ **RNA**→ putative transcriptional regulator→
Aba-1-17	NC_009085.1	-	2055349	2055265	HTH_XRE (cd00093)→ **RNA**→ putative transcriptional regulator→
Aba-1-18	NC_009085.1	+	1581044	1581128	PaaI_thioesterase (cd03443)→ **RNA**→ TetR_N (pfam00440)→
Aba-1-19	NC_009085.1	-	2778777	2778689	MoeA_N (pfam03453)→ **RNA**→ AcrR (COG1309)→
Aba-1-20	NC_009085.1	-	3904932	3904848	←RhtB (COG1280) **RNA**→ ←AgaS (COG2222)
Asp-1-2	NC_005966.1	+	962260	962346	←Acetyltransf_1 (pfam00583) **RNA**→ hypo→
Aba-1-21	NC_009085.1	-	1060224	1060139	Sugar_tr (pfam00083)→ **RNA**→ hypo→
Asp-1-3	NC_005966.1	+	3357993	3358079	←hypo **RNA**→ COG3152 (COG3152)→
Asp-1-4	NC_005966.1	-	920344	920258	←FhaC (COG2831) **RNA**→ ←hypo
Aba-1-22	NC_009085.1	-	98547	98462	←KAS_I_II (cd00834) **RNA**→ ←COG3981 (COG3981)
Aba-1-23	NC_009085.1	+	1469454	1469538	HtrB (COG1560)→ **RNA**→ TetR_N (pfam00440)→

Hin-11-1	NZ_AADP01000001.1	+	917452	917538	COG0419: ATPase involved in DNA repair→ **RNA**→ COG3561 (COG3561)→
Hin-1-1	NZ_AAZD01000004.1	-	76634	76548	hypo→ **RNA**→ COG3561 (COG3561)→
Hin-9-1	NZ_AAZI01000003.1	-	134972	134886	hypo→ **RNA**→ COG3561 (COG3561)→
Hin-7-2	NC_009567.1	+	169172	169257	hypo→ **RNA**→ COG3561 (COG3561)→
Hin-10-1	NZ_AADO01000003.1	+	1933	2018	COG1201: Lhr-like helicases→ **RNA**→ COG3561 (COG3561)→
Hin-12-1	NZ_AAZE01000005.1	+	1255	1340	hypo→ **RNA**→ COG3561 (COG3561)→
Hin-3-3	NZ_AAZF01000003.1	-	38900	38815	hypo→ **RNA**→ COG3561 (COG3561)→
Hin-5-2	NZ_AAZG01000005.1	-	122796	122711	fumarate/nitrate reduction transcriptional regulator→ **RNA**→ COG3561 (COG3561)→
Hin-8-1	NZ_AAZH01000001.1	+	84396	84481	hypo→ **RNA**→ ←hypo
Hin-4-1	NC_007146.1	-	1576614	1576529	hypo→ **RNA**→ COG3561 (COG3561)→
Hin-6-1	NC_009566.1	+	965902	965987	hypo→ **RNA**→ COG3561 (COG3561)→
Hph-1-1	NC_004827.1	-	6364	6280	**RNA**→
Hso-1-2	NC_008309.1	+	1602795	1602876	hypo→ **RNA**→ COG3617 (COG3617)→ Haemophilus-specific protein, uncharacterized→ Haemophilus-specific protein, uncharacterized→ Haemophilus-specific protein, uncharacterized→ RecT (pfam03837)→ hypo→ hypo→ DNA_methylase (pfam00145)→ Haemophilus-specific protein, uncharacterized→ AlpA (COG3311)→
Aba-1-24	NC_009085.1	+	871520	871603	Flavin_Reduct (pfam01613)→ **RNA**→ TetR_N (pfam00440)→

16.3 Conserved domains

Conserved domains found in protein-coding genes listed in Section 16.2 are listed, with the first sentence in their description from the Conserved Domain Database. Conserved domains associated with more than one c4 antisense RNA are assigned a color, while others are shown in gray. The number in parentheses is the number of occurrences of the given gene.

cd00093 (5) Helix-turn-helix XRE-family like proteins.
cd00189 (1) Tetratricopeptide repeat domain; typically contains 34 amino acids [WLF]-X(2)-[LIM]-[GAS]-X(2)-[YLF]-X(8)-[ASE]-X(3)-[FYL]-X(2)-[ASL]-X(4)-[PKE]
cd00221 (1) Very Short Patch Repair (Vsr) Endonuclease.
cd00254 (1) Lytic Transglycosylase (LT) and Goose Egg White Lysozyme (GEWL) domain.
cd00797 (1) Phage HP1 integrase, C-terminal catalytic domain.
cd00800 (3) Lambda integrase, C-terminal catalytic domain.
cd00801 (6) Bacteriophage P4 integrase.
cd00834 (1) Beta-ketoacyl-acyl carrier protein (ACP) synthase (KAS), type I and II.
cd01029 (6) TOPRIM_primases: The topoisomerase-primase (TORPIM) nucleotidyl transferase/hydrolase domain found in the active site regions of bacterial DnaG-type primases and their homologs.
cd01125 (10) Hexameric Replicative Helicase RepA.
cd01153 (1) Putative acyl-CoA dehydrogenase (ACAD).
cd01163 (1) Dibenzothiophene (DBT) desulfurization enzyme C (DszC).
cd01183 (1) INT_SG1, DNA breaking-rejoining enzymes, integrase/recombinases subgroup 1, C-terminal catalytic domain.
cd01406 (1) Sir2-like: Prokaryotic group of uncharacterized Sir2-like proteins which lack certain key catalytic residues and conserved zinc binding cysteines; and are members of the SIR2 superfamily of proteins, silent information regulator 2 (Sir2) enzymes which catalyze NAD+-dependent protein/histone deacetylation.
cd01825 (1) SGNH_peri1; putative periplasmic member of the SGNH-family of hydrolases, a diverse family of lipases and esterases.
cd01949 (1) Diguanylate-cyclase (DGC) or GGDEF domain: Originally named after a conserved residue pattern, and initially described as domain of unknown function 1 (DUF1).
cd02932 (2) Old yellow enzyme (OYE) YqjM-like FMN binding domain.
cd03227 (1) ABC-type Class 2 contains systems involved in cellular processes other than transport.
cd03443 (1) PaaI_thioesterase is a tetrameric acyl-CoA thioesterase with a hot dog fold and one of several proteins responsible for phenylacetic acid (PA) degradation in bacteria.
cd03449 (1) (R)-hydratase [(R)-specific enoyl-CoA hydratase]
COG0009 (3) Putative translation factor (SUA5) [Translation, ribosomal structure and biogenesis]
COG0038 (2) Chloride channel protein EriC [Inorganic ion transport and metabolism]
COG0155 (1) Sulfite reductase, beta subunit (hemoprotein) [Inorganic ion transport and metabolism]
COG0247 (1) Fe-S oxidoreductase [Energy production and conversion]
COG0277 (1) FAD/FMN-containing dehydrogenases [Energy production and conversion]
COG0300 (1) Short-chain dehydrogenases of various substrate specificities [General function prediction only]
COG0330 (1) Membrane protease subunits, stomatin/prohibitin homologs [Posttranslational modification, protein turnover, chaperones]
COG0358 (3) DNA primase (bacterial type) [DNA replication, recombination, and repair]
COG0373 (1) Glutamyl-tRNA reductase [Coenzyme metabolism]
COG0381 (1) UDP-N-acetylglucosamine 2-epimerase [Cell envelope biogenesis, outer membrane]
COG0385 (1) Predicted Na+-dependent transporter [General function prediction only]
COG0475 (3) Kef-type K+ transport systems, membrane components [Inorganic ion transport and metabolism]

COG0583 (3) Transcriptional regulator [Transcription]
COG0624 (1) Acetylornithine deacetylase/Succinyl-diaminopimelate desuccinylase and related deacylases [Amino acid transport and metabolism]
COG0629 (3) Single-stranded DNA-binding protein [DNA replication, recombination, and repair]
COG0657 (2) Esterase/lipase [Lipid metabolism]
COG0673 (1) Predicted dehydrogenases and related proteins [General function prediction only]
COG0683 (1) ABC-type branched-chain amino acid transport systems, periplasmic component [Amino acid transport and metabolism]
COG0691 (1) tmRNA-binding protein [Posttranslational modification, protein turnover, chaperones]
COG0702 (1) Predicted nucleoside-diphosphate-sugar epimerases [Cell envelope biogenesis, outer membrane / Carbohydrate transport and metabolism]
COG0715 (2) ABC-type nitrate/sulfonate/bicarbonate transport systems, periplasmic components [Inorganic ion transport and metabolism]
COG0765 (1) ABC-type amino acid transport system, permease component [Amino acid transport and metabolism]
COG0840 (1) Methyl-accepting chemotaxis protein [Cell motility and secretion / Signal transduction mechanisms]
COG0845 (2) Membrane-fusion protein [Cell envelope biogenesis, outer membrane]
COG1027 (1) Aspartate ammonia-lyase [Amino acid transport and metabolism]
COG1044 (1) UDP-3-O-[3-hydroxymyristoyl] [3-hydroxymyristoyl]
COG1061 (1) DNA or RNA helicases of superfamily II [Transcription / DNA replication, recombination, and repair]
COG1075 (1) Predicted acetyltransferases and hydrolases with the alpha/beta hydrolase fold [General function prediction only]
COG1226 (3) Kef-type K+ transport systems, predicted NAD-binding component [Inorganic ion transport and metabolism]
COG1280 (2) Putative threonine efflux protein [Amino acid transport and metabolism]
COG1309 (2) Transcriptional regulator [Transcription]
COG1462 (1) Uncharacterized protein involved in formation of curli polymers [Cell envelope biogenesis, outer membrane]
COG1464 (1) ABC-type metal ion transport system, periplasmic component/surface antigen [Inorganic ion transport and metabolism]
COG1473 (1) Metal-dependent amidase/aminoacylase/carboxypeptidase [General function prediction only]
COG1476 (1) Predicted transcriptional regulators [Transcription]
COG1484 (2) DNA replication protein [DNA replication, recombination, and repair]
COG1522 (1) Transcriptional regulators [Transcription]
COG1555 (1) DNA uptake protein and related DNA-binding proteins [DNA replication, recombination, and repair]
COG1560 (2) Lauroyl/myristoyl acyltransferase [Cell envelope biogenesis, outer membrane]
COG1595 (1) DNA-directed RNA polymerase specialized sigma subunit, sigma24 homolog [Transcription]
COG1670 (2) Acetyltransferases, including N-acetylases of ribosomal proteins [Translation, ribosomal structure and biogenesis]
COG1721 (1) Uncharacterized conserved protein (some members contain a von Willebrand factor type A (vWA) domain) [General function prediction only]
COG1724 (1) Predicted periplasmic or secreted lipoprotein [Cell motility and secretion]
COG1835 (1) Predicted acyltransferases [Lipid metabolism]
COG1846 (1) Transcriptional regulators [Transcription]
COG1848 (1) Predicted nucleic acid-binding protein, contains PIN domain [General function prediction only]
COG1943 (1) Transposase and inactivated derivatives [DNA replication, recombination, and repair]
COG1961 (1) Site-specific recombinases, DNA invertase Pin homologs [DNA replication, recombination, and repair]
COG1981 (2) Predicted membrane protein [Function unknown]
COG2026 (6) Cytotoxic translational repressor of toxin-antitoxin stability system [Translation, ribosomal structure and biogenesis / Cell division and chromosome partitioning]
COG2085 (1) Predicted dinucleotide-binding enzymes [General function prediction only]
COG2105 (2) Uncharacterized conserved protein [Function unknown]
COG2188 (1) Transcriptional regulators [Transcription]
COG2197 (1) Response regulator containing a CheY-like receiver domain and an HTH DNA-binding domain [Signal transduction mechanisms / Transcription]
COG2222 (1) Predicted phosphosugar isomerases [Cell envelope biogenesis, outer membrane]
COG2226 (1) Methylase involved in ubiquinone/menaquinone biosynthesis [Coenzyme metabolism]
COG2807 (3) Cyanate permease [Inorganic ion transport and metabolism]
COG2831 (1) Hemolysin activation/secretion protein [Intracellular trafficking and secretion]
COG2863 (1) Cytochrome c553 [Energy production and conversion]
COG2896 (1) Molybdenum cofactor biosynthesis enzyme [Coenzyme metabolism]
COG2944 (2) Predicted transcriptional regulator [Transcription]
COG2961 (3) Protein involved in catabolism of external DNA [General function prediction only]
COG3093 (2) Plasmid maintenance system antidote protein [General function prediction only]
COG3143 (1) Chemotaxis protein [Cell motility and secretion / Signal transduction mechanisms]
COG3152 (2) Predicted membrane protein [Function unknown]
COG3177 (1) Uncharacterized conserved protein [Function unknown]
COG3311 (5) Predicted transcriptional regulator [Transcription]
COG3335 (1) Transposase and inactivated derivatives [DNA replication, recombination, and repair]
COG3378 (8) Predicted ATPase [General function prediction only]
COG3415 (1) Transposase and inactivated derivatives [DNA replication, recombination, and repair]
COG3423 (6) Predicted transcriptional regulator [Transcription]
COG3547 (1) Transposase and inactivated derivatives [DNA replication, recombination, and repair]
COG3561 (17) Phage anti-repressor protein [Transcription]
COG3617 (29) Prophage antirepressor [Transcription]
COG3645 (1) Uncharacterized phage-encoded protein [Function unknown]
COG3646 (56) Uncharacterized phage-encoded protein [Function unknown]
COG3666 (1) Transposase and inactivated derivatives [DNA replication, recombination, and repair]
COG3714 (1) Predicted membrane protein [Function unknown]
COG3781 (1) Predicted membrane protein [Function unknown]
COG3866 (1) Pectate lyase [Carbohydrate transport and metabolism]
COG3941 (1) Mu-like prophage protein [General function prediction only]
COG3950 (1) Predicted ATP-binding protein involved in virulence [General function prediction only]

COG3981 (1) Predicted acetyltransferase [General function prediction only]
COG4104 (1) Uncharacterized conserved protein [Function unknown]
COG4160 (1) ABC-type arginine/histidine transport system, permease component [Amino acid transport and metabolism]
COG4178 (1) ABC-type uncharacterized transport system, permease and ATPase components [General function prediction only]
COG4487 (1) Uncharacterized protein conserved in bacteria [Function unknown]
COG4536 (9) Putative Mg2+ and Co2+ transporter CorB [Inorganic ion transport and metabolism]
COG4625 (1) Uncharacterized protein with a C-terminal OMP (outer membrane protein) domain [Function unknown]
COG4643 (21) Uncharacterized protein conserved in bacteria [Function unknown]
COG4680 (1) Uncharacterized protein conserved in bacteria [Function unknown]
COG4700 (1) Uncharacterized protein conserved in bacteria containing a divergent form of TPR repeats [Function unknown]
COG4748 (1) Uncharacterized conserved protein [Function unknown]
COG4771 (1) Outer membrane receptor for ferrienterochelin and colicins [Inorganic ion transport and metabolism]
COG4772 (1) Outer membrane receptor for Fe3+-dicitrate [Inorganic ion transport and metabolism]
COG4773 (2) Outer membrane receptor for ferric coprogen and ferric-rhodotorulic acid [Inorganic ion transport and metabolism]
COG4993 (1) Glucose dehydrogenase [Carbohydrate transport and metabolism]
COG5001 (1) Predicted signal transduction protein containing a membrane domain, an EAL and a GGDEF domain [Signal transduction mechanisms]
COG5283 (1) Phage-related tail protein [Function unknown]
COG5519 (9) Superfamily II helicase and inactivated derivatives [DNA replication, recombination, and repair]
COG5606 (1) Uncharacterized conserved small protein [Function unknown]
COG5642 (1) Uncharacterized conserved protein [Function unknown]
COG5654 (1) Uncharacterized conserved protein [Function unknown]
COG5659 (1) FOG: Transposase [DNA replication, recombination, and repair]
GOS10365 (1) ?
GOS11008091 (2) ?
GOS11069324 (1) ?
GOS11073445 (1) ?
GOS11222950 (1) ?
GOS11335753 (2) ?
GOS11363276 (1) ?
GOS11386653 (1) ?
GOS11725977 (1) ?
GOS11900477 (1) ?
GOS12118414 (1) ?
GOS13019564 (1) ?
GOS13071826 (1) ?
GOS13412611 (1) ?
GOS13828 (1) ?
GOS14070030 (1) ?
GOS14311108 (1) ?
GOS14593241 (1) ?
GOS1771 (1) ?
GOS255 (1) ?
GOS26 (1) ?
GOS3431 (1) ?
GOS3774 (1) ?
GOS4027 (1) ?
GOS4294 (1) ?
NOG06080 (1)
NOG06313 (2)
NOG08008 (1)
NOG08737 (1)
NOG13495 (2)
NOG15906 (1)
NOG17190 (2)
NOG17530 (2)
NOG18143 (1)
NOG18702 (1)
NOG19993 (1)
NOG22115 (1)
NOG22117 (2)
pfam00078 (1) Reverse transcriptase (RNA-dependent DNA polymerase).
pfam00083 (1) Sugar (and other) transporter.
pfam00106 (1) short chain dehydrogenase.
pfam00126 (1) Bacterial regulatory helix-turn-helix protein, lysR family.
pfam00145 (2) C-5 cytosine-specific DNA methylase.
pfam00226 (3) DnaJ domain.
pfam00232 (1) Glycosyl hydrolase family 1.
pfam00267 (1) Gram-negative porin.
pfam00293 (1) NUDIX domain.
pfam00440 (8) Bacterial regulatory proteins, tetR family.
pfam00496 (1) Bacterial extracellular solute-binding proteins, family 5.
pfam00529 (1) HlyD family secretion protein.
pfam00535 (1) Glycosyl transferase.
pfam00583 (4) Acetyltransferase (GNAT) family.
pfam00665 (10) Integrase core domain.
pfam00872 (1) Transposase, Mutator family.
pfam00873 (1) AcrB/AcrD/AcrF family.
pfam00908 (1) dTDP-4-dehydrorhamnose 3,5-epimerase.
pfam01385 (1) Probable transposase.
pfam01527 (10) Transposase.
pfam01548 (1) Transposase.
pfam01590 (1) GAF domain.
pfam01613 (4) Flavin reductase like domain.
pfam01726 (2) LexA DNA binding domain.
pfam01807 (3) CHC2 zinc finger.
pfam01904 (1) Protein of unknown function DUF72.
pfam01914 (2) MarC family integral membrane protein.
pfam02371 (1) Transposase IS116/IS110/IS902 family.
pfam02413 (1) Caudovirales tail fibre assembly protein.
pfam02447 (1) GntP family permease.
pfam03230 (1) Antirestriction protein.
pfam03288 (1) Poxvirus D5 protein-like.

pfam03374 (4) Phage antirepressor protein.
pfam03400 (2) IS1 transposase.
pfam03453 (1) MoeA N-terminal region (domain I and II).
pfam03466 (1) LysR substrate binding domain.
pfam03573 (2) outer membrane porin, OprD family.
pfam03837 (1) RecT family.
pfam03942 (1) DTW domain.
pfam04336 (1) Protein of unknown function, DUF479.
pfam04383 (5) KilA-N domain.
pfam04606 (4) Phage transcriptional activator, Ogr/Delta.
pfam04754 (4) Putative transposase, YhgA-like.
pfam04860 (1) Phage portal protein.
pfam05015 (1) Plasmid maintenance system killer protein.
pfam05125 (1) Phage major capsid protein, P2 family.
pfam05272 (2) Virulence-associated protein E.
pfam05433 (2) Rickettsia 17 kDa surface antigen.
pfam05534 (1) HicB family.
pfam05866 (4) Endodeoxyribonuclease RusA.
pfam05869 (4) DNA N-6-adenine-methyltransferase (Dam).
pfam05930 (2) Prophage CP4-57 regulatory protein (AlpA).
pfam05973 (1) Bacterial protein of unknown function (DUF891).
pfam05974 (2) Protein of unknown function (DUF892).
pfam06048 (1) Domain of unknown function (DUF927).
pfam06073 (1) Bacterial protein of unknown function (DUF934).
pfam06146 (1) Phosphate-starvation-inducible E.
pfam06406 (4) StbA protein.
pfam06616 (1) BsuBI/PstI restriction endonuclease C-terminus.
pfam06821 (1) Protein of unknown function (DUF1234).
pfam07042 (1) TrfA protein.
pfam07081 (1) Protein of unknown function (DUF1349).
pfam07282 (1) Putative transposase DNA-binding domain.
smart00062 (1) Bacterial periplasmic substrate-binding proteins; bacterial proteins, eukaryotic ones are in PBPe
smart00342 (1) helix_turn_helix, arabinose operon control protein;
smart00421 (5) helix_turn_helix, Lux Regulon; lux regulon (activates the bioluminescence operon
smart00482 (1) DNA polymerase A domain;
smart00530 (2) Helix-turn-helix XRE-family like proteins ;

Duplicate sequences: the following putative homologs are not shown in the alignment because their sequences are identical to a homolog already shown: Bma-1-1, Bma-2-1, Bma-3-1, Bma-4-1, Bma-5-1, Bma-6-1, Bma-7-1, Bma-8-1, Bma-9-1, Bps-1-1, Bps-10-1, Bps-11-3, Bps-12-1, Bps-13-1, Bps-13-2, Bps-14-1, Bps-15-1, Bps-17-1, Bps-17-2, Bps-2-1, Bps-4-1, Bps-5-3, Bps-6-1, Bps-6-2, Eco-12-1, Eco-12-2, Eco-4-1, Eco-5-3, Eco-6-4, Eco-8-3, Eph-5-1, Hin-1-1, Hin-5-2, Hin-9-1, PGi-1-1, Pae-1-1, Pae-4-1, Sco-1-1, Sen-10-1, Sen-10-3, Sen-14-1, Sen-15-1, Sen-4-1, Sen-4-2, Sen-4-3, Sen-4-5, Sen-6-1, Sen-7-1, Sen-8-2, Sen-9-2, Sgl-1-4, Sph-1-1, Xfa-2-1, Xor-2-1, Ype-1-2, Ype-1-3, Ype-10-1, Ype-10-2, Ype-10-3, Ype-11-1, Ype-11-2, Ype-12-1, Ype-12-2, Ype-12-3, Ype-13-1, Ype-13-3, Ype-14-1, Ype-14-2, Ype-14-3, Ype-15-1, Ype-15-2, Ype-15-3, Ype-16-1, Ype-16-2, Ype-16-3, Ype-2-1, Ype-3-1, Ype-3-2, Ype-3-3, Ype-4-1, Ype-4-2, Ype-4-3, Ype-5-1, Ype-5-2, Ype-5-3, Ype-6-1, Ype-6-2, Ype-6-3, Ype-7-2, Ype-7-3, Ype-8-1, Ype-8-2, Ype-8-3, Ype-9-1, Ype-9-2, Ype-9-3, env-12

16.4 Multiple-sequence alignment

Each c4 antisense RNA RNA is denoted by an abbreviation (like "Eco-1-1") that refers to a taxonomy given in Section 16.1. The alignment includes sequences containing the RNA motif, as well as flanking sequence. The c4 antisense RNA RNA itself is denoted by the line underneath marked 5′ and 3′ on either end. Nucleotides in the flanking sequences (i.e., not part of the motif) are written in gray letters. Stems of predicted rho-independent transcription terminators are shaded yellow. (Note: terminator predictions have not been analyzed manually, and many are likely to be false positives.) Start codons are shaded green. (Note: start codons are frequently misannotated, especially in environmental samples.) Nucleotides proposed to basepair as part of the consensus structure are shaded in color when they comprise Watson-Crick or G-U pairs. Otherwise they are shaded gray. Conserved stems are also indicated at the bottom of the alignment by angle brackets, where matching < and > denote base-paired columns. Below these angle brackets, the symbol "2" denotes base pairs exhibiting covariation, "1" denotes base pairs exhibiting compatible mutations, "0" denotes base pairs that are not observed to mutate and "?" denotes base pairs that have a significant frequency of non-canonical nucleotides for Watson-Crick or G-U pairs (> 5%). Below these base pair annotation is the consensus sequence: "R" = "A" or "G", "Y" = "C" or "U", red nucleotides: nucleotide identity conserved more than 97% of the time, black nucleotides: 90%, gray nucleotides: 75%, red circle (•): nucleotide is present 97% of the time, black circle (•): 90%, gray circle (•): 75%, white circle (◦): 50%. All percentages of sequences just described (e.g. 97% conserved) assume that sequences have been weighted by the GSC algorithm implemented by the Infernal software.

alignment positions 1 ··· 99

```
Aba-1-1   AUUGUUAAUGUGAAAAGAUAAUAUUUAAAAACAAUUGUUCUAAUUUGAUACGAAUAGGAUUUUAUAUUCUUGACGUCAAGAAAAGUGAAUAAUAUCCUU
Aba-1-2   CGGGUGUUUGGAUUGGUCUCCAAAAGUUUCUCAAGGUCGAAAGACCGUAUUUAGCGGUUUUAUUUUGCCUAUAAUUUUCUACACCCUGUGGAAAAUGCC
Hin-3-1   CGAACGACAGCACGCCAUUAGACCGUGCUUUUUUUGUUCGUAACAUUCGCACACCAAAAGAAUAUGCGGAUUUUGUUUUCAAUCUAAAUCCGAUCAUUC
Hin-5-1   CGAACGACAGCACGCCAUUAGACCGUGCUUUUUUUGUUCGUAACAUUCGCACACCAAAAGAAUAUGCGGAUUUUGUUUUCAAUCUAAAUCCGAUCAUUC
Hin-7-1   ACAUUUUACAGGCGAACGAUAGCACGCCAUUUAACCGUGCUUUUUUUGUUCGUAGCGUUUACGCAUCUAUCCAAAAUAAUCUAUCUUUUGAUAGACUAU
Hso-1-1   GUAGGAAGCUGAAAAAUACAAAGGCGAAUAGCACGCCUCAAGCGUGUUUUUUUAUUCGUAAUUUACGCACACCUAAAAUAAAUCUAGCAAUAGAUCUAU
Hso-2-1   GUGAGAAGCUGAAAAAUACAAAGGCGAAUAGCACGCCUCAAGCGUGUUUUUUUAUUCGUAAUUUACGCACACCUAAAAUAAAUCUAGCAAUAGAUCUAU
Pmu-1-1   CGAAUAAUAAACAUACGGCGAACAGUAGCACGCCAUUUAACCGUGCUUUUUUUGUUCGUGAAGUUCGCACACCUAAAAUAAAUCCAGCAAUGGAUCUAU
Hin-3-2   UGGCGCAGAGCACGCCCAAAGCGUGUUUUUUUAUGCGUAACAUUCGCACACCCAAAGAAUUUGCGGAUUUUGUUUUAUUGAUCAAAUCUACAAAUCUC
Hin-2-1   AAUUGGCGCAGAGCACGCCCAAAGCGUGUUUUUUUAUGCGUAACAUUCGCACACCUAAAGAAUAUGCGGAUUUUGUUUUACAUCUAAAUCCGAUCAUUC
Mha-1-1   CUGAACUACAUAAUACGGCGAACAGUAGCACGCCUUUAACCCGUGCUAUUUUUAUUCGUAGCUUACACACACCUAAAGAACGCCGAAAUAAGGCGUGUU
Mha-1-2   ACUCGAAAGAACAUCAGGCGAACAAUAGCACGCCUUUAACUCGUGCUUUUUUGUUCGUAGCUCACGCACACCCAAAGAAUGCCAAAAUAAGGCGUGUU
Pfl-2-1   CGUCGCUGCAAAAUCAGCGGCUUAGGUUUGGUCACCUAAAAUAACAGCCCUCAAGUGUGCCAUUAUUCGCUCCGUGGACAUCUAUGUCCUCGAAGUGCA
env-1     AAUUAUCCAUAGUAAAGGAGUACGUCGCAAAAGGGCGUGCCGGGUUUAGCCACUCGAAUAUAUCUGCGGCGCUCAGCCGUGUCGUCUUUAGAUGCGGCA
Dps-1-1   ACGUCACCAGUCUCUAUUUUAUAGGUGGUUUUUUUUAUGCCUUUUGUUUAUGGCUGUAGUAUUGUGUAGACAUUUAUCACAGCAUUGAACCGCACAGAA
Dac-1-1   CCCUGAAGGAACGCUUUGCGCAGGCGCCCUGGGGGCUGUACCACAUGGGGCUGCACGCGCAGCAGCCCCAGGGCUGACCUCAAGACUGCGCAUGGUUCC
Sgl-2-1   GUAAAACGGGUGCCGGGUUUAGUAGCCCGAGUGUCAGAGCGGACAGAAUAAGCCGCUCAAAAGUGCGGUUUUUUACGUCUGUUUCCUGCGUUGUGCCC
Pfl-2-2   GACUGUCGUUGCCAAAUCAGCGACUCGGGUUUGGUCACCUGAAAAUGAGAGAGAAACUUCGCAUGCCCUUGUAUGCUUGCCGCCUGAUUCAUGCGGCAC
Sbo-1-1   GUGGAAACCCGAACACACUCAGGGCGACACAGACGCCGAAAGCGUCUUUUUUUGUGUCCGAUCACUACGCACAUCAAAUCAGCGGCCUAGUCCGUACUG
Pna-1-1   ACAUCAAAAAAAGGUGUGGCCGAGAUUGGUCUCUCGAAUCACUCUGCGACAAAAGCCGCAGUUUUCCCGCAGAACCUGCGGCUUCAUCAUUUUUGCCUC
Pna-1-2   CGGUGCUGAAAAACACCACGGGUUUGACGACCUGUUGACAUCCUGCGACGUAAGCCGCAGCCACCGCAUAUGGUCUGCGGCUUUUGAAUUUGUGCCUCC
Xfa-1-1   AGAAGCCUGAAUCCCCAGCGCGAAAAGCGCCCAUUUCCGAUGUUGGCGCUUUUUCAUUGCUCGCUGCCGUCGGCAGUGGGCCUUGCGUACAGCGCAACU
Sma-1-1   GUGGCAGCGGGUCUGCAAACCCGUGAUGAGAGAUGCACCUGUAGUUUGCGCUUGCCCGUCGGCACCGUCCUUAACCGAUGCCGGCGGGCAAUCCGUCGG
Dno-1-1   UAUAGUCCACUCGCUACAGUAAGCUGUAGUCGGGAUUGGCAUCUCGUUGAUACGAAAGCGUGUACAGCGCUUAUUAAGCGCAUUUUUUAUGCCUCAAAU
Dno-1-2   UAAUCGAGUUGCCGCAGCAAUAUCUGCGGUCGAGAUUAGCCUCUCGUUGAUACGAAAGCGUGUGCAGCGCUUCUACCGCGCUAUUUUUAUGCCUAAAAU
Dno-1-3   CUCGCUACAGUAAGCUGUAGUCGGGAUUGGAACCCCGUUGAUACGAAAGCGUGUACAGCGCUUAUUAAGCGCAUUUUUUAUGCCCAAAAUUUGUCUAUG
Dno-1-4   CAAUACAUUACAUUCUGCGAUCGGGGUUGACCUCCCGUUGAUACACAAGCGUGUGCAGCGCUUAUUAAGCGCAUUUUUUAUGCCCAAAAUUUGUCUAUG
Esp-1-1   GGUCGUAAAGUGCGUGCAUUGCGGCACAUCCGCAAUCGGGUGUAGGAACCCAGACUUCACGAUACGACAAUGUUUGUCGCAACAGUGCGUGUCCCAUUA
Plu-1-1   AAGUAGUGUUGUCAUUGGCAAAAUCCAGUGACCAAGGUUUUGCAGCCUUGCGUUCAUGGACGCUGGUAAGAAGUUUUUACCAAUGUGCCUGCUAUCGCC
Xax-1-1   GCUGCACAAUCAGCGAUUCGGCCUAUCAAACCGACAAAAGGUGGAAAAGAGCGCGCAAGCGCCCAUCGUUCGAUGCUGGCGCUUUUAUUUGGCUCACCA
Aba-1-3   UUUGCCUGCUGGUCACAUUACCAGUCAGCUUUUGACAGAGCUGGUACAAAAGGCGCACAACAACGUAUUAUUUCGUUUUGUGCGUACUCCUAAGUCACG
Xfa-3-1   UCCUGCGCUGGCAAAUUCUAGCGCCGGGUUUGGCAACCCGAUGAACGGCGCUUCAAAAGCGCCCCGCAUCCCGAGUGGCGCUUUUUUGUUGCCUCCCAC
Xfa-1-2   UACUGCGCUGGCAAAUUCUAGCGCCGGGUUUAGCGGCCCGAUGAAAGGCGCUUCAACAGCGCCCCACAUCCCGAGUGGCGCUUUUUUGUUGCCUCCUAC
Sma-1-2   AAACGGGUGGCCGGGCCUCGAAAACCCGAGCUAUUAGGAUGUUGUUUGCACUUGCCAGCCGGCAUCGCCUUGCGUGGUGUCGGCUGGCAUUCCGUCUGC
env-2     GCAUCGGUGGUCGGGACUCGAAAGCCCGUUUUGUUGGGAAGUAGUUUGCGCUUGCCAGCCGGCACCACCGCGCGUGGUGCCGGCUGGCAAUCCGUCUGC
env-3     GGAAAAGGGUGGCCGGGCUUCGAAUCCCGUGUUGGCCUUGAGUAAGUGCGCUUGCCAGCCGGCGUCACCAUGCGUGGCGCCGGCUGGCAAUCCGUCUGC
Sma-1-3   GAAAAGGGUGGCCGGGCUUCGAACACCCGAGUUGAUGUUGAUGUAUGCGCUUGCCAGCCGGCGUCACCUCAGCGUGGCGCCGGCUGGCAAUCCGUCUGC
Sma-1-4   UGCAUCGGUGGUCGGGAUUCGAAACCCCGGUUGUGUGGAUGUUGCUCGCACUUGCCAGCCGGCACCGCUACGCGCGGUGUCGGCUGGCAAUCCGUCGGC
Plu-1-2   UUGCGGACUUGACGCCGCAGCAAAAGGGCAGUACGACGAUUACAGGCUCAUGCCUGCUAGCAGCGCAGUGAAUCUGACACGGCAUUUUAUAGAUUGAAA
env-4     CCUGUCGCUGCACCUCAGCGAUACGGGUUUGGAAGCCCGGAGAAUAGGCGGACAAGCCGCCUGCAUCGCGGCUUUUUCAUGUCCGUCGGCAUGGCUCC
Aba-1-4   AAGAAGGACGCCUCGUGUGUGCUGGGUACUCAAAGCCAGUCUGUUAACCUUCUUUCGUUUCGCCGCCAUUCUUUAAGAAUAACUGGGUGGGCUUCAUUA
Bph-1-1   CGCACCAUCGGCACCAACCGGUGGUCGGGUUUGACAGCCCGCGCUUGAAACCGCACACCCGCCCAGGCGGGUCGUGCGUCUAUCUCUGCACGUCUGUAU
Bxe-1-1   UUCCGAACCACCGGCACCAACCGGUGGUCUGGUUUAGCAGCCAGUGCCAAAAACCGCACACCCGCCCAGGCGGGCGUGCGUCUGCCUCUGCACGUCUAA
env-5     AAGUCGUCGGGAUACACCCGGUGACCAGGUUUAGCAGCCUGUAGUCGAAAACCGCACACCCGCUCACGCGGGGCGUGCGUCUACCUCUGCACGUCUAUA
env-6     UUGUAGCCCUUACUAAAUCGUAAGAGUGGGGAUUUGCAGCCCCGACUAAUGACACGCCGUUCAUGUACUUGAACGGUUCGCACUCGCUCGCCUGUUUUU
Spr-1-1   GUCUGUUGGCUGCGGCACAAUCCGCAGCCGGAGUUAGCGUUCCGAAUCCCCCAACCCUGUCCGAAUAUGAUAUAAAUUCGUGCAUGCCGAUGCGCACCC
Pfl-2-3   UGUCUCCGCGAGUGGAGACAGGGCGUAGGAGCCCUGAUUUCAGAGCAGUGCAUAAGCGUCGUUGCCCGAUUUGCGGCAGUCUUCGCUCGACCGUAAGAU
env-7     CGCUGCAAAUUCAGUGACAGGGCGUAGGAACCCUUUUAUGUACCCUUGGCGUCAUAGCGCCACCGUCUGAUUGCGGCGUUUUUUUUGUGCCCGUACUAU
Pfl-2-4   CAGAAGACUCUGCGAUCGGGAGGUGAGAUUCCCGGUUACUGAAUUUGGAAACCAGAGCAAGCCGCCCCAUAAUCGCCGCCAGUUCAUGCUGUCGGCAGU
Msp-1-1   UUAAGGGUGUUGGCAAAAUCCAACAUGAGGUUUUAGCAGACCUGAACUACAAGGACGCACAUCACCGCCGCGUCGCGGUUUUUUUGUGCCAUAAUGCUU
Msp-1-2   UUAAGAGUGUUGGCAAAAUCCAACAUCAGGUUUUAGCAGACUUGAAUCCAAAACGACGCACAAUCCGCCGCGUUGCGGUUUUUUUGUGCCAUAAUGUUG
env-8     AGCAUUGCCUGCGGUCGGGUUUCGCAGCCUGAAUACAAUACAAGGCGCACAAGCGCCAAUUUCUUCGGUGCUUUUUUUAUUUGUAGCACCCGAAGUUCU
Aba-1-5   CGAGAGGGAGACGCCUUAGUGCGUACUGAAUCCUUGGGGCAGUCUGUCAACUUCCUUUCGUUCUACUGCCAUUAUUUGAAAUGAUUGGUGGAAACUUCU
Pfl-1-1   UCGCUGCAAAAUCAGCGAACAGGCUUGGCGGCCUGAUAGAAUCGAGGCGCAACAGCGCCCCACCCACAUGCGGGGCGCUUUUUUUUGCGCCCGCAUACCU
Psy-1-1   CGCUGCGAACUCAGCGACCGGGCUUGGCGGCUCGAACAGUACAAGGCGCAACAGCGCCCCCAUUACGAUUGCAGGCGCUUUUUUUGUGCCCGCAUUCUC
Pae-5-1   UGACAGCCCGAAGAGACGGCGCAAUGCGCCGAGCGGUGCAUGCGAAAGCAGCGAAUGCUUUUGCAGGCAUCGGCUCGACCGCAGUUUUCCAACUGCGGG
Pae-2-1   UGACAGCCCGAAGAGACGGCGCGAUGCGCCGAGCGGUGCAUGCGAAAGCAGCGAAUGCUUUUGCAGGCAUCGGCUCGACCGCAGUUUUCCAACUGCGGG
Pae-6-1   UGACAGCCCGAAGAGACGGCGCGAUGCGCCGAGCGGUGCAUGCGAAAGCAGCGAAUGCUUUUGCAGGCAUCGGCUCGACCGCAGUUUUCCAACUGCGGG
Ppu-4-1   GUCUGAUUGUUCCUGAAACCGCUGCCCAUUAGCCUCUUUGGGUCGCUGUCGGUUCAGCGAUCGGGUGUGGAAACCCGCCAAAAGCAUUUCGGGUUUGUC
Dps-1-2   GCCGAGUUUGGACGCUCGGACGCAAACGGUGGAUACAUGCCACCACUAUUGGUGGUUUUUUUAUGCCCUUUACGGGUAUACUGUAUCCGUCGCAUAGUC
Asa-1-1   GAAUCUUGGAGCCGACCCACGCUCUUGCGUGGUUUUUUGUCGGUGCUCGUUCCUCAUUUCUUGGGGCGUUGCUCGCCUACCCGGUCAGCAGCCUCUACA
env-9     .......................CUUGCGUGGUUUUUUGUCGGUACUCGUUCCUCAUUUCUUGGGGCGCUGCUGGCCUACCAGGUCGGUGGCCUCUACA

          ..................................................................................................
          ..................................................................................................
          ●●●●●●●●●●●●●●●●●●●●●●●●●●●●●●●●●●●●●●●●●●●●●●●●●●●●●●●●●●●●●●●●●●●●●●●●●●●●●●●●●●●●●●●●●●●●●●
```

```
Bsp-1-1    GCCCGUCGACGUACACAACGUUGACCGGGUUUGACAGCCCGAAUGCAUGCGGCAGACAACCGCCGCGAUGGCGGUUUUUCUUCGUUUGAGAACGCGCAU
Pen-1-1    GUCUCGCCGGGUCAUUGCCAAAUCAACGACCGGGUCUGCAAGCCUGCGCGGUAUUACGAAGGUGCUCACCAUGGGCCCCGGCGCUUUGCCGUGGGUUUC
Cvi-1-1    GUCAAUCCCAGCGCCAGGCUUGGCGGCCUGUUAGACUCAAGGCGGAUUGCCGCCAUAUGCGGCAUUUUUUAUGUCCGUCAAUUGCCAUUGGUGCGCCCU
Sdy-2-1    ACACCUCAUAAAACGGGUGUCGGGAUUGCUACCCCGCUGACAAUCGAGGCGAUACAGACGCGCCAGCGUCUUUUUUUGUAUCGACAUACACGCACAUCU
Sbo-1-2    UCCCUUAUAAAACGGGGAUCGGGAUUACCACCCCGCUGAAGCAAAAGGCGACACAUGACGCGCCAGCGUCUUUUUUGUUGUCUGUACACACGCACACCU
env-10     CCUUAUAAAACGGGGAUCGGGAUUGGCCUCCCGCUGGAGUUAGAGGCGAUACACGACGCGCCAGCGUCUUUUUUUUUGUAUCGGCGCACACGCACACCU
Cte-1-1    UGGUCGCCCGGUAUCAGUAGGCGCAGCAGCCGCGCCCAUACAAACAAUGUUGGUCUGCGGCUUUUUGCUUUCAUGAGCAUGCUGUGCACGCAUGGCUCC
Cte-1-2    CUUGGCGGCCCGGAACGUAGGCGCAGCAGCCGCGCCCAUACAAACAAUGUUGGUCUGCGGCUUUUUGCUUUCUUGAGCAUGCUGUGCACGCAUGGCUCC
Sgl-2-2    AGUCAAUCUGCAAAUUCAGGUGCCGGGUUUCGUAGCCCGCCGAGAUUGUCGCGACAAAAUGACACGCAGCGUGUUUUUUUACGUCACAAUCCAGCUAAA
Sen-8-1    CGCCAGCAAAAUCUGGCGUCGGGAUUGGUCUCCCGGAUUACUACUCAACGCAUACCGCGUUAAGCGGUUUUUUUAUGCGCUAAGCACGGCUACAUUCGC
Vfi-1-1    UGGCAAAAUCCAAUGACAGAAUUGAUACUCUGAAAUGAUAACAAGGCGUAUAUCGCCAGCAUCUUGCUGGUUUUUUUAUGCGUAUGCUUUAGCACAUCU
Vfi-1-2    UCCCAAGCAUAGUAAGAGAACCUGGUUUCUUUAAAGUGAAGGAUUUGCUUGAAAUAGCGAAUGCAGUAAAUGAACAACUUCGGUUAAUCUGUAAAUAAU
Sde-1-1    AGCACUAAAGUAGCCCCGCUUGGUAACAAACCAAGUCGGGAUUGAGACCCCGAUAACACAAGGACCACAAGACGUUUAAAGCGUUUAUCUGUGGGCAGC
Sba-1-1    UGGCAAAAUCCAGUGCCGGGAUUGAGACCCCGCAAUAUCACAGGCGCAAUCAUGUCGCCAGCCUAGUGCUGGUUUUUUAUUGCGUGACUCGGCGCACCA
Sba-2-1    UGGCAAAAUCCAAUGCCGGGUUUCGCACCCCGCAGUAUCACAGGCGCAAUCAUGUCGCCAGCCUAGUGCUGGUUUUUUAUUGCGUGACUCGGCGCACCU
Eco-3-1    AAUCUCCCAAAUGGAGAUCGGGAUUUGCAACCCGGAAUUGUUAGGGGCGACACCAGACGCGCCAGCGUCUUUUUUAUUGUCGCAAGCUUUGCCACGUUC
Vch-2-1    UUUUACUCAAGGUACACAAGUACCAGCGUGAGCUGGUUUUUUUGUGUAUGGCUUCAGCACAUUUAAACAUAGUCGUUUCGCAUAGAAACCGUACUAUCU
Vch-3-1    UUUUCUAUCGGUACACAAGUACCAGCUUUUUGCUGGUUUUUUUGUGUAUGGCUUCAGCACAUUUAAACAUAGUCGUUUCGUAUAGAAACCGUACUAUCU
Vsp-1-1    GUUAACCAAAGGCGCACAGACGCCAGCUUUUGCUGGUUUUUUUAUGUGUGGCUUCAGCACAUCUAUACAUAGACGUUUCGCAUAAGAAUCGUACUAUCU
Vch-1-1    CAAAAUCCAAUGACGGGAUUGGUCCCCCGAUUUAGUAAACUUUGGCGCACGAUCGCCAGCUCUUCGCUGGUUUUUUUGUACGUGUAAUUCGGCACAUCU
Vba-1-1    AUACACCCAAGGCGCAUAGACGCCAGCUUUUGCUGGUUUUUUUAUGUGUGGCUUCAGCGCAUCCAAAUAUAGGCAAUUCGUUCAGGGAUUGUACUAGCA
Sfr-1-1    UGGCAAAAUCCAAUGCCGGGAUUCGAACCCCGCUAUAAUCAAGGCGCAGUUGUCGCCAGCCUUUAGAGCUGGUUUUUUAUUGCGUGACUCGGCGCACCA
Vsh-1-1    CAAGGCGCAUAGACGCCAGCUUCCUGCUGGUUUUUUUAUGUGCGGCUUCGGCACACCUGCACAUGGUGAAUUUGCUAGCAAUAUGAGCAAAUAGCACCU
Sgl-1-1    CCUCUUAUUUUCUUUUCAGGGGUCGGGAUUCUAACCCCGCGAUAUAAGUCUGGUGAAUUUGUGGUUGUACUACAUUUUACCGGCAUCCUGAGCACGCCA
Sgl-2-3    GCACCUCUUUUCUUUUCAGGGGUCGGGAUUCUAACCCCGCGAUAUAAGUCUGGUGAAUUUGUGGUUGUACUACAUUUUACCGGCAUCCUGAGCACGCCA
Sgl-1-2    CCUCUUAUUUUCUUUUCAGGGGUCGGGGUUCUAACCCCGCGAUAUAAGUCUGGUGAAUUUGUGGUUGUACUACAUUUUACCGGCAUCCUGAGCACGCCA
env-11     GCCUUAUCUUCUCGCUGCACCUGCAAAAUCAGGUGCCAGAAUUAGCCUUCUGAAAAUCAUACUGGCGACAUAUACUUCUAUGUCGUGCGACCGAUUUCG
Eco-5-1    CAUUGUAUUGUUGCGCCGCGGCUGCAAAAUCAGGCGUCGGGAUUGGCAUCCUGUAAUACUUCACUGACAACACAAUAUUUUGUGUUGCGUAUGGUCACA
Sen-11-1   GCACCUGCAAAUUCAGGUGCCGGGAUUAGCCUCCUGAUUGACUUCAUCGACGACACAAAGGCGCGUCUGCGCUUUUUUUGUGUCGUGCGUUCUGCUACA
Sen-12-1   GCACCUGCAAAUUCAGGUGCCGGGCUUGACCUCCUGACUAACUUCAUCGACGACACAAAGGCGCGUCUGCGCUUUUUUUGUGCCGUGCGUUUGGCUACA
Esp-1-2    GUAUCUUCUCGCCACGCCUGCAAAAUCAGGCGUCGGGAUUAGCCUCCUGAACUACUUCUUCGGUGACACAAGUACUUUUGUGUCGCGCGCUUAGCUACA
Sgl-2-4    CUGCAAAUUCAGGUGCCGGGUUUCGCAGCCCGCCGAGAUUGUCGCGACAAAAUGACACGCCGCAAGCGUGUUUUUUAUGUCGCACAUCUAGCCGCAUCA
Sgl-2-5    AUCUGCAAAUUCAGGUGCCGGGAUUGAUACCCCGCCGAGAUUCACCGCGAUAGACUGACACGCCGCGAGCGUGUUUUUUUAUGUCGCAAUCUAGCCACA
Sgl-2-6    CAUCUGCAAAUUCAGGUGCCGGGAUUAGGAGCCUCGAAGGAAUCACCGCGACAGAGUAACACGCCGCGAGCGUGUUUUUUAUGUCGCAAUCUAGCCUCA
Sgl-2-7    AUCUGGAAAUUCAGGUGCCGGGAUUGGUCUCCCGCCGAGAUUCACCGCGACAGAAUGACACGCCGCGAGCGUGUUUUUUAUGUCGCACAUUUAGCCACA
Sgl-2-8    AUCUGCAAAUUCAGGUGCCGGGGUUCGCGUCCUGCCGGAAGUAACUGCGACAGAAUGACACGCCGCGAGCGUGUCUUUUUAUGUCGCAAUCUAGAUACA
Sdy-1-1    CACCUCAUAAAACGGGUGCCGGGAUUAGCACCCCGAAAGUUACUCGAGCGCAUAACCGCGCUGAAGCGGUUUUUUUAUGCGUAAUGCACAGCCACAUUC
Sbo-1-3    CACCUCAUAAAACGGGUGCCGGGAUUAGCACCCCGAAAGUUACUCGAGCGCAUAACCGCGCUGAAGCGGUUUUUUUAUGCGUAAUGCACAGCCACAUUC
Eco-10-1   CACCUCAUAAAACGGGUGCCGGGCGUGGAAACCCGAUGUUCACUAACGCGCAUAACCGCGCUCAGGCGGUUUUUUUAUGCGUAAUGCACAGCCACAUUC
Sfl-1-1    CACCUCAUAAAACGGGGGCCGGGCGUGGAAACCCGAACGUUACUGGAGCGCAUAGCCGCGCUGAAGCGGUUUUUUUAUGCGUAAUGCACAGCUCCAUUC
Sfl-2-1    CACCUCAUAAAACGGGGGCCGGGCGUGGAAACCCGAACGUUACUCGAGCGCAUAGCCGCGCUGAAGCGGUUUUUUUAUGCGUAAUGCACAGCUCCAUUC
Eco-3-2    CACCUUAUAAAACGGGUGCCGGGCGUGGAAACCCGAACGUUACUCGAGCGCAUAGCCGCGCUGAAGCGGUUUUUUUAUGCGUAAUGCACAGCUCCAUUC
Eco-3-3    CACCUUAUAAAACGGGUGCCGGGCGUGGAAACCCGAACGUUACUCGAGCGCAUAGCCCCGCUGAAGCGGUUUUUUUAUGCGUAAUGCACAGCUCCAUUC
Eco-3-4    CACCUCAUAAAACGGGUGCCGGGAUUGCUACCCCGAAGUCAUUCACGGCGCAUAACCGCGCUCAGGCGGUUUUUUUAUGCGUAAUGCACAGCCACAUUC
Sfl-3-1    CACCUCAUAAAACGGGUGCCGGGAUUGCUACCCCGACGAUGACUACUGCGCAUAACCGCGCUCAGGCGGUUUUUUUAUGCGUCAAGCACAGCCACAUUC
Sfl-2-2    CACCUCAUAAAACGGGUGCCGGGAUUGCUACCCCGACGAUGACUACUGCGCAUAACCGCGCUCAGGCGGUUUUUUUAUGCGUCAAGCACAGCCACAUUC
Sfl-1-2    CACCUCAUAAAACGGGUGCCGGGAUUGCUACCCCGACGAUGACUACUGCGCAUAACCGCGCUCAGGCGGUUUUUUUAUGCGUCAAGCACAGCCACAUUC
Eco-6-1    CACCUUAUAAAGCGGGUGCCGGGAUUGGCGUCCUGGAGAAGGCUACCGCGUACAACCGCGUAGAUGCGGUUUUUUUGUACGCAUUAUUUUGUCACGCCC
Pcd-1-1    CACCUUAUAAAGCGGGUGCCGGGAUUGGCGUCCUGGAGAAGGCUACCGCGUACAACCGCGUAGAUGCGGUUUUUUUGUACGCAUUAUUUUGUCACGCCC
Sso-1-1    ACACAACCGCAAAAUCGGUUGUCGGGAUUGGCGUCUCGGAUAACAUCACAACGCAUAGCCGCGUUUGCGGUUUUUUUUGCGUUAAGCAUUGCCACAUUC
Sbo-1-4    CCGCACCUUAUAAAGCGGGUGCCGGGAUUGGCGUCCUGAAAUUCGCACAUGCGCAUAACCGCGCUUCAGCGGUUUUUUUGCGCACGUUUCCUCACAUCC
Sfl-3-2    CACCUUAUAAAGCGGGGGCCGGGAUUGGCGUCCUGAAAUUCAAUAUAGAGCAUAACCGCGCUCAUGCGGUUUUUUCGUGUCAUGAGCAUUGCUACGCCC
Eco-3-5    CACCUUAUAAAGCGGGUGCCGGGAUUGGCGUCCUGAAAUUCAAUAUAGAGCAUAACCGCGCUCAUGCGGUUUUUUCGUGUCAUGAGCAUUGCUACGCCC
Eco-13-1   AAACGGGUGCCGGGAUUCUCAACCCGAUACAGAGCAAAGCGCAUAACCGCGCUAGCGGUUUUUUUGUGCGUACUGUAUUGCCACGUCUUUUUCGCGUCA
Eco-3-6    .................................................................UGCGGUUUUUUUGUGUCAUGAGCAGCAUUACGCCC
Sen-13-1   CACCUCAUAAAGCGGGUGCCGGGCGUGGAAACCCGGAAUUCAAUAUAGAGCACAACCGCGCUCAUGCGGUUUUUUCUUGUCAUGAGCAUUGCUACGCCC
Sen-16-1   CACCUUAUAAAGCGGGUGCCGGGCGUGGAAACCCGGAAUUCAAUAUAGAGCACAACCGCGCUCAUGCGGUUUUUUCUUGUCAUGAGCAUUGCUACGCCC
Eco-2-1    CACCUUAUAAAGCGGGUGCCGGGCGUGGAAACCCGGAAUUCACCAAAGCGCACAACCGCGCUCUUGCGGUUUUUUUGUGUAAUGAGCAGCAUUACGCCC
Eco-15-1   CACCUUAUAAAGCGGGUGCCGGGCGUGGAAACCCGGAAUUCACCAAAGCGCACAACCGCGCUCUUGCGGUUUUUUUGUGUCAUGAGCAGCAUUACGCCC
Eco-6-2    CACCUUAUAAAGCGGGUGCCGGGCGUGGAAACCCGAAAUUCAAUAUAGAGCACAACCGCGCUCAUGCGGUUUUUUCGUGUCAUGAGCAUCGUUACGCCC
Eco-6-3    AAACGGGUGCCGGGUUUCGCAGCCCGCUGACAAACGUAGCGCACAACCGCGCCAGCGGUUUUUUUGUGCGUACUGUAUCGCCACGUCUUUUUCGCAUCA
Eco-2-2    AAACGGGUGCCGGGUUUCGCAGCCUGCUGACAAACAUAGCGCACAACCGCGCCAGCGGUUUUUUUGUGCGUACUGUAUCGCCACGUUUUUUUCGCACAC
```

Eco-8-1	AAACGGGUGCCGGGUUUCGCAGCCUGCUGACUACACAAGCGCACAACCGCGCCAGCGGUUUUUUUGUGCGUACUGUAUUGCCACGUUUUUUUCGCGUCA
Sdy-1-2	AAACGGGUGCCGGGUUUCGCAGCCUGCUGACUACACAAGCGCACAACCGCGCCAGCGGUUUUUUUGUGCGUACUGUAUUGCUACGUUUUUUUCGCGUCA
Eco-7-1	AAACGGGUGCCGGGUUUCGCAGCCUGCUGACUACACAAGCGCACAACCGCGCCAGCGGUUUUUUUGUGCGUACUGUAUUGCCACGUUUUUUUCGCGUCA
Eco-9-1	AAACGGGUGCCGGGAUUUCCACCCCGCUGACUAGCAAAGCGCACAACCGCGCCAGCGGUUUUUUUGUGCGUACCGUAUCGCCACGUCUUUUUCGCGUCA
Eco-3-7	AAACGGGUGCCGGGAUUGAGACCCCGCUGACAAACAUAGCGCACAACCGCGCCAGCGGUUUUUUUGUGCGUACUGUAUCGCCACAUCUUUUUCGCACAC
Eco-15-2	AACGGGUGCCGGGAUUGGAACCCCGCUGAUCAACAAAGCGCACAACCGCGCCAGCGGUUUUUUUGUGCGUACCGUAUCGCCGCAUAUUUUUUCGCACAC
Eco-2-3	AAACGGGUGCCGGGAUUGGAACCCCGCUGACAACCAAAGCGCACAACCGCGCCAGCGGUUUUUUUGUGCGUACCGUAUCGCCACGUCUUUUUCGCACAC
Eco-10-2	GCCGCAAAAUCGGUUGCCGGGAUUUGCACCCCGGAUAUCUAAAAGGCGACAACGACGCGCCAGCGUCUUUUUUAUUGUCGUGCGCACAGCCACAUCCGC
Spr-1-2	AGCCGCAAAAUCGGUUGCCGGGUUUCGCAGCUCGAACAUCACGGAAGCGCACGACCGCGCCAGCGGUUUUUUUAUGUGUGAAGCAUGGCCACAUUUACG
Sty-2-1	GCCAGCAAAAUCUGGCGUCGGGAUUGGCGUCCCGGAAUUUCACCGCGACAGAGACACGCCGCGAGCGUGUUUUUUAUUGUCGUUUGUAUACGCGCAUUU
Sen-9-1	GCCAGCAAAAUCUGGCGUCGGGAUUGGCGUCCCGGAAUUUCACCGCGACAGAGACACGCCGCGAGCGUGUUUUUUAUUGUCGUUUGUAUACGCGCAUCU
Sen-13-2	GCCAGCAAAAUCUGGCGUCGGGAUUGGCGUCCCGGAAUUUCACCGCGACAGAGACACGCCGCGAGCGUGUUUUUUAUUGUCGUUUGUAUACGCGCAUUU
Eco-10-3	CACGGCUGGCUCGUUGCCCGUAAAGAACAACUGGUGGAAAGGCUGAUGCAGCUUUAGCAGAUAUAACAAGUGUUUUUUCUCGUAUGCACGCGCACAUAU
Sso-1-2	GCCAGCAAAAUCUGGCGUCGGGAUUGGAACCCCGGAUAGAGACCGCGACAGACACACGCCGCGAGCGUGUUUUUUAUUGUCGUAUGCACGCGCACAUCU
Ype-7-1	UACACCUGCAAAAUCAGGUGUCGGGAUUUGCACCCUGGAUAUCACAACAACGCAUAGCCGCGUUUGCGGUUUUUUUAUGCGUAACGUAUGACUACAUCA
Ype-1-1	UACACCUGCAAAAUCAGGUGUCGGGAUUUGCACCCUGGAUAUCACAACAACGCAUAGCCGCGUUUGCGGUUUUUUUAUGCGUAAAGUAUGACUACAUCA
Eco-3-8	UCAGCAAAAUCUGGCGUCGGGAUUGGAACCCCGGAUAUUCAACCGCGACAGACACACGCCGCGAGCGUGUUUUUUAUUGUCGUAAGCACACGCACAUCU
Eco-3-9	GUGCACCUUAUAAAACGGGUGCCGGGAUUAGCCUCCUGAAAACAACUGAAGCGCACAGCACGCGCCCCGCGUGUUUUUUUGUGCCGCAUAGCCACACCU
Yen-1-1	GGUUAUAGUCACACCGCAGCGGUAAAAUCCUUUACCGGAUUUGGCAGUCCGGCAUACACCAAAGCGCAUGGCGUAAAUACGGUUACAUUGUUAAUAAGC
Yen-1-2	AGCGGCAAAAUCCGUUGCUGGGUUUAGCAGCCUGAGACAAAACAGAGCGCACAACCGCGCAGGCGGUUACUAUGUGCGGGUACAGUUACACCUGUGAAA
Yps-1-1	CAGCGGCAAAAUCCGUUGCUGGGUUUAGCAGCCUGAGACAAAACAGAGCGCACAACCGCGCAGGCGGUUACUAUGUGCGGGUACAGUUACACCUGUGAA
Ype-13-2	CAGCGGCAAAAUCCGUUGCUGGGUUUAGCAGCCUGAGACAAAACAGAGCGCACAACCGCGCAGGCGGUUACUAUGUGCGGGUACAGUUACACCUGUGAA
Yps-2-1	CAGCGGCAAAAUCCGUUGCUGGGUUUAGCAGCCUGAGACAAAACAGAGCGCACAACCGCGCAGGCGGUUACUAUGUGCGGGUACAGUUACACCUGUGAA
Yen-1-3	GGCAAAAUCCGCUGCCGGAUUUGACAGUCCGGAAUUCAUUGAAACGCACAACCUUAGUUUCGGCUAUUAUGUGCGGGCACAGUGACACCUGUAAUAAGU
Ybe-1-1	CGCAGCGGAAAAAUCUGCUGCCGGGUUUGGAACCCCGUAUUUCAACAGGGCGCAUAACUGCGUAAGCGGUUAUUAUGUGCGGAGCACGGCUACAUCUGU
Yin-1-1	GGGUUAUAGUCACACCGCAGCGGUAAAAUCCUUUACCGGAUUUGGCAGUCCGGCAUAGACCAAAGCGCAUGAUAUAGAUACGGUUACAUGGUAAUAAGC
Yen-1-4	GUUAUAGUCAGCACCGCUGCGGUAAAUUCCUUUACCGGAUUUGGCGGUCCGGCAUAAGCCAAAGCGCAUGACGUAAAUACGGUUACAUUGGUAAUAAGC
Yin-1-2	GGCAAAAUCCGCGGUCGAGUUUGGAACCCCGGAAAUUACCAAAGCGCACGACCGUAGACACGGCUAUUAUGUGCGGGCACAGUGACACCUGUAAUAUGC
Sbo-2-1	GCAAAAUCAGGUGCCGGGAUUGGAACCCCGAAAUAUAACCAUACUGGCGACACGUGACGCGUCUGCGUCUUUUUUUAUGUCGCGCGCUUAGCUACACCA
env-13	GCACCUGCAAAUUCAGGUGCCGGGAUUGGAACCCCGAGAAAUGUUACAGGCGACAUACGACGCGCCCGCGUCUUUUUUUAUGUCGUGCGCUCAGCUAUA
Sen-16-2	CGUUGCUGUAAAUCCAGCAACCGGGUUUAGCAGCCUGGAACACAUCACAGCGCACAACCGCGCCUGCGGUUUUUUUGUGUGCAAUGCCCCGUCACAUUU
Api-1-1	UGCAAAAUCAGGUGUCGGGUUUGAGACCCCGUAUUAGCUAACAAGGCGCAUAGCACGCAGUAAGCGUGUUUUUUUUAUGCGUCAGGCUGCCAUAUCAAA
Sgl-2-9	CACCUGCAAAUUCAGGUGUCGGGUUUGAGACCCCGGUUAUCUAAGAAGGCGCAUAACACGCCGAGAACGUGUUUUUUUAUGCGUCAGUCCGCCAUAUCA
Sdy-1-3	UCUAUUUCAGGAAGGAGUGUCCGUUAAACUCAGGCUACCUCAAAACGGUGCAUAACCGCGCUGGCGGUUUUUUUAUGCGCUAAGCACAGUCACAUUCGC
Eco-3-10	CACCUUAUAAAACGGGUGCCGGGAUUUGCACCCCGGAAUUGAUCAAGGCGAUAUAUGACGCGCCAGCGUCUUUUUUAUCGUCCACGCUCACGCACGCCA
Eph-2-1	CACCUUAUAAAGCGGGUGCCGGGAUUGGAACCCCGGAAUUGCUCGAGGCGAUAUACGACGCGCCAGCGUCUUUUUUAUCGUCCAUGCUCGCGCACGCCA
Eco-3-11	CCCCUUAUAAAGCGGGGGCCGGGAUUGGAACCCCGGAAUUGAUCGAGGCGAUAUACGACGCGCCAGCGUCUUUUUUAUCGUCCGCGCUCACGCACGCCA
Eco-3-12	CACCUUAUAAAGCGGGUGCCGGGAUUUGCACCCCGGAAUUGUCAACGGCGAUAUACGACGCGCCAGCGUCUUUUUUAUCGUCCGCGCUCACGCACGCCA
Sdy-1-4	UGUACGAGGCGACACAUGACGCGCCGAGCGUCUUUUUUUGUGCCGUUAGCCAGACUCACACUUUUUUCAGUGAUGCGGUUAUAAUCCGCGUCGCUUGCA
Eco-8-2	CGCCAGCAAAAUCUGGCGUCGGGAUUAGCACCCCGGAUAUCGAAACGGUGCAUAACCGCGCUGGCGGUUUUUUUAUGCGCUAAGCACAGUCACAUUCGC
Sdy-2-2	CCAGCAAAAUCUGGCGUCGGGAUUGAGACCCCGGAUGUUUACGGAGCGAUAUGAGACGCGCCCGCGUCUUUUUUAUAUCGUUUGCACAGUCACAUUCGC
Eco-10-4	AGCAAAAUCUGGCGUCGGGAUUGGAACCCCGGAUGUUUACGGAGCGAUAUGAGACGCGCCCGCGUCUUUUUUCAUAUCGUUUGCACAGUCACAUUCGC
Sfl-2-3	CAGCAAAAUCUGGCGUCGGGAUUGGCGUCCCGGAUGUUUACGGAGCGAUAUGAGACGCGCCCGCGUCUUUUUUCAUAUCGUUUGCACAGUCACAUUCGC
Sen-10-2	CAGCAAAAUCUGGCGUCGGGAUUGGCGUCCCGGAUGUUUACGGAGCGAUAUGAGACGCGCCCGCGUCUUUUUUCAUAUCGUUUGCACAGUCACAUUCGC
Sdy-2-3	CAGCAAAAUCUGGCGUCGGGAUUGGCGUCCCGGAUGUUUACGGAGCGAUAUGAGACGCGCCCGCGUCUUUUUUCAUAUCGUUUGCACAGUCUCAUUCGC
Eco-9-2	CACGCGCCAGCAAAAUCUGGCGUCGGGAUUGGCGUCCCGGAUGAAAAAGGCGACAACAGACGCGCCAGCGUCUUUUUUAUUGUCGUUUGCACUGUCACA
Eph-1-1	CACCUUAUAAAGCGGGUGCCGGGAUUGGCGUCCUGGAAUUGAUCAAGGCGAUAUAUGACGCGCCAGCGUCUUUUUUAUCGUCCGCAUUUGCUCACAUCA
Eco-13-2	GCCAGCAAAAUCUGGCGUCGGGAUUGGCGUCCUGGAUAGAGACCGCGACAGAUACACGCCGCGAGCGUGUUUUUUAUUGUCGUAUGCACGCGCACAUCU
Sen-15-2	GCCAGCAAAAUCUGGCGUCGGGAUUGGCGUCCCGGAUAGAAACCGCGACAGACACACGCCGCGAGCGUGUUUUUUAUUGUCGUAUGCACGCGCACAUCU
env-14	CACCUUAUAAAGCGGGUGCCGGGAUUGGCGUCCUGAAAUUGUCAACGGCGAUGUAUGACGCGCCAGCGUCUUUUUUAUCGUCCGCAUUUGCUCACAUCC
Eco-9-3	CACCUUAUAAAGCGGGUGCCGGGAUUGGCGUCCUGAAAUUGUCAACGGCGAUGUAUGACGCGCCAGCGUCUUUUUUAUCGUCCGCAUUUGCUCACAUCC
Sen-2-1	CACCUCAUAAAGCGGGUGCCGGGAUUGGCGUCCUGGAAUUGCAUACGGCGACAAUGGGCGCGUUAGCGUCUUUUUUGUUGCUACAGCUCAGCUAUACCC
Sen-15-3	CACCUCAUAAAGCGGGUGCCGGGAUUGGCGUCCUGGAAUUGCAUACGGCGACAAUGGGCGCGUUAGCGUCUUUUUUGUUGCUACACCUCAGCUAUACCC
Eco-11-1	CACCUUAUAAAGCGGGUGCCGGGAUUGGCGUCCUGGAAAUGUUAUCGGCGAUAUAUGACGCGCCAGCGUCUUUUUUAUCGUCUGCGUCUGCGCACACCC
env-15	CACCUUAUAAAGCGGGUGACGGGAUUGGCGUCCUGGAAAUGUUAUCGGCGAUAUAUGACGCGCCAGCGUCUUUUUUAUCGUCUGCGUCUGUGCACACCC
Sen-9-3	CACCUUAUAAAGCGGGUGCCGGGAUUGGCGUCCUGGAAUUGUUAUCGGCGACAAAUGACGCGCCUGCGUCUUUUUUAUUGUCGCAGCUCGGCUAUAUCC
Eco-4-2	GCACUGGCAAAAUCCAGUGCCGGGAUUGGCGUCCCGGAUUACUAAAAGGCGCAUUCACCGCGCAAGCGGUUUUUUUAUGCGUAUAGCACGGCCACAUUC
Eph-6-1	GCACUGGCAAAAUCCAGUGCCGGGAUUGGCGUCCCGGGUUACUAAAAGGCGCAUUCACCGCGCAAGCGGUUUUUUUAUGCGUAUAGCACGGUCACAUUC
env-16	CACUGGCAAAAUCCAGUGCCGGGAUUGGCGUCCCGGGUUACUAAGUGGCGCAUACCACGCCAGACGUGGUUUUUUUAUGCGUUAAGCACAGCUAUAUCC
Sgl-1-3	UCAUACCUUUGAUAAAUCAGAGGUCGGGAUUGAUACCUCGAAGCCAAAUACCAGUAUGAUAUACUUAUGGUAUAUUAUACCGGCAUCAGAAGCUCAUCA
Sgl-2-10	UCAUACCUUUGAUAAAUCAGAGGUCGGGAUUGAUACCUCGAAGCCAAAUACCAGUAUGAUAUACUUAUGGUAUAUUGUACCGGCAUCAGAAGCUCAUCA
Pfl-2-5	GCUGAUGAAUCGGCGAUCGGGAUUGGAACCCCGGCGACUUCCCAAAAAAGCAAAAGCAGCUUUCAUGACGUAUGCUUGCCCGCCUUCAUGGUGUGCACA

```
Ppu-1-1    CGCCGAAGUAACGCUGUUGUCGGGGAAGUGGCUCACGGAUACCGUUUUCGGGUCGCUCUGGCGACUGGGUGUGAGAACCCGACUUUGAACGUAUCGGCU
Pfl-2-6    UCGCUGCCAAUUCAGCGAACAGGCGUGGAAACCUGAUUUCAAGUAGUUGCAAAGCAUCUCCCCGUAAUCGUUGCCGAUGUCUUCUUUCGUCUGCAAACU
env-17     AAAAAACCUUAUCGGGAUUGCAACCCCGUCUAAUUACCAAGCGUACAGCCGCGCUAAGCGGUUUUUUUGUGCCAGCAUAAAACUAUUUUCACAAAAAAU
env-18     AAAAACCUUAUCGGGAGUAGAAACCCGCCGAUUUGCAGAGCGUACAGACCGCGCUAAGCGGUUUUUUUGUGCCAACGCUAAACUAUUGCCACAAAAAUU
env-19     AAAAACCUUAUCAGGAUUGGAACCCUGCCGGAAUAAAGAGCGUACAGACCACGCUAAGUGGUUUUUUUGUGCCUGCAAUAAACUAUUGCCACAAAAGAU
env-20     ...................................GGGGCGCUGGAUGACAGACGCGCAGCGGUUUUUUUGUGCCACUGAAAACUAUUGUCACAAAAAAU
Bth-1-1    GCUGAAACAACAGCGGCUCGGGCGUCGCGGCUCGAUAUGCAAAAGGCGGACGACCGCCUCCCGGCGGUAUUUUUUCGUCCGUGUGCCUUCGCACGUCUC
Bps-11-1   GCUGAAACAACAGCGGCUCGGGCGUCGCGGCUCGAUACGCAAAAGGCGGACGACCGCCUCCCGGCGGUAUUUUUUCGUCCGUGUGCCUUCGCACGUCUC
Rso-1-1    UCGCGUAGACAAACGCGAUCGGGCUUGGCCGCUCGACUACCAGAGGCGCGAUCCCGUCGUGAGCGGCGGUAUCAUCUGCGCCGUUGCCUUCGCGCGCCC
Sgl-2-11   GCACUUGCAAAUUCAGGUGCCGGGAUUCGCAACCCGCCGUGUGUACCGGCCAAUAACACGCCGAGAGCGUGUUUUUUUAUUGGUCAAAACCUAGUCACA
Bxe-1-2    AGUUCGCCGUCGUUCAAGGACGGUCAGGCUUGACAGCCUGGAAACCAAACCCGCACGCUUGCCCGGGCAAGCAUGUGCGUCUGUUUCUGCACGUCCCUU
Bph-1-2    UCAAUUCGCCGUCGUUCAAGGACGGUCAGGUUUGACAGCCUGCAACCAAACCCGCACGCUUGCCCGGGCAAGUCUGUGCGUCUGUUUCUGCGCGUCCAU
Ppu-4-2    CUGCCGUUUCGCUGUUGUUGCUCACAACCAUCAGGGCUAACUUCCCUGGGUCGUCAUCUUUGACGAUUUGGGUGUGAGAGCCCGCAAACUUCGGCAGUA
Pen-1-2    UUGCGGAAGCUUCGAUGUUGUCGGGGAAAUGCCCCAGGCAUAACGUCUUCGGGUCGCUUUCGGGCGACCGGGAUUGAGACCCCUGGAUCAAUAGAUGCC
Pst-1-1    CAGCACUCCAAUAGGUUUGUGCUCCCCUUUAAAGGGGACGGGAUUGGCGUUCCGGUCUGAGACAGGGCUAAGCGAGGCUUAGCUCUGAGCAUGGCUGUG
Sgl-2-12   AUAUGCAAAUUCAGGUGCUGGGAUUGUCACCCCGAUGAAUAAACCGCGCCAAUAGACACGCCGCGAGCGUGUUUUUUUAUUGGCCCCAGCCAAGUCACA
Eph-7-1    CUGCAGCAAAAUCUGCAGCCGGGCCUCUCAACCCCGAAUGACAAGAAGCGCACAACACGCGCCAGCGUGUUUUUUUGUGUGUUAAAUCUGCGCAUACCU
env-21     AUUCACAGGCGACACAACACGCGCCUUGCGUGUUUUUUUACGUCGUAGCUCAGGCACACCUAUAUUUCGGGCUGUGGUGUUUACACCGUGGUUCCAGUC
Esa-1-1    AUCUAACGGCGACACCAGACGCGCCUUGCGUCUUUUUUUGUGUCAUUGCCUUUGCGCAUCUGUUACUUGCACUGCGGUUUCUUUGCCGUUGUGGCUAUC
Sen-11-2   AUCUAACGGCGACACCUGACGCGCCAUGCGUCUUUUUUUGUGUCUAUGCCCUCGUGCACCUGUUACUUGCGCAAUGGUUUCAUGACCUUUGCAGCUAUC
Yph-1-1    CGUUGCAGCAAAAUCUGCGACCGGGCGUAGGAACCUGAACCCCAUCGGCGGCACAUACACGCUAGGCGUGUUUUUUUGUGUCUAUGCCUGAGCACAGCC
Sgl-2-13   CAGCGGCAAAUUCCGUUGCCGGGUUUGAGACCCCGUAUAUCGAUCUGGUGCCAAGACACGCACCCAAAGCGUUUUUUUUGUUGCCUGUAGCCCAUCAUU
Sgl-2-14   CAGCGGCAAAUUCCGUUGCCGGGUUUGAGACCCCGUAUAUCGUAUCGGUGCCAGAACACGCACCCAAAGCGUGUUUUUUGUUGCCUGUAGUUACCCAUU
Eco-3-13   ACUUCAAGGCGACAUAUGACGCGCCAUGCGUCUUUUUUUACGUCGUUGCUUCGGCACACCUGUUUUUCUGGCUGUGGUUUUUAAACCGUAGCCUCUGUC
Sen-7-2    ACUUAUUGGCGACACCAGACGCGCCGUGCGUCUUUUUUUACGUCGUUGCUCAGGCACACCCAUUUUUCGGGCUGCGGUGCUUAUACCUUAGUCCCUGUC
Esp-1-3    UAACUAUGGCGACAUCAGACGCGCCUUGCGUCUUUUUUUAUGUCUGUGCCUUGAUGCAUCCAUUCAUUACACAAUGGUUAAUUUACCACUGUGUAUAUC
Ybe-1-2    UAAUCUUGGCGACACAACACGCGCCUGGCGUGUUUUUUUAUGUCGUGGCCUUGGCCUACCAGUUUUUUGCGCAGUGGUUUCUAUACCGCUGUCGCUAUC
Ymo-1-1    ACUUAUUGGCGACUCAACACGCGCCAUGCGUGUUUUUUUAUGUCGUGGCCUCGGCCUACCAGUUUUUUACGCAGUGGUUUCUAUACCGCUGUCGUUAUC
Esa-1-2    ACUUAUUGGCGACACAACACGCGCCAUGCGUGUUUUUUUGUGUCGUUGCCUCAGUGCGCCAUUUUUUAGCGCAGCGGUUCUUAUGCCGUUGUGCCAUCA
Yfr-1-1    ACUCAAUGGCGACACAACACGCGCCAGGCGUGUUUUUUUAUGUCGUGGCCUCGGCCUACCAGUUUUUUGCACAGUGGUUUCUACACCGCUGUCGCUAUC
Sen-9-4    ACGCGCCAGGCGUGUUUUUUUGUGUCGCAGCUUUGACUCACCUAAAAUUUAUGGUGUGGUGGCUGAUUGUAUUUUUUUAUUUUUUACGCCACAUCAUCA
Sen-5-1    ACGCGCCAGGCGUGUUUUUUUGUGUCGCAGCUUUGACUCACCUAAAAUUUAUGGUGUGGUGGCUGAUUGUAUUUUUUUAUUUUUUACGCCACAUCAUCA
Sen-7-3    AGGCGACACGACACGCGCCAUGCGUGUUUUUUUAUGUCGUUGCUCAGGCACACCCAUUUUCAGGGCUGUGGUGUUUAUUCGUGCACCAGAGUCCUUUUC
Sen-8-3    AGGCGACACGACACGCGCCAUGCGUGUUUUUUUAUGUCGUUGCUCAGGCACACCCAUUUUCAGGGCUGUGGUGUUUAUUCGUGCACCAGAGUCCUUUUC
Eco-13-3   UGGCGACAAUUGACGCCCCUUGCGUCUUUUUUUACGUCGUAGCUCAGGCACAACCAUUUUCAGGGCUGUGGUGUUUAUGCUUACACCAUGGUUCUAUCG
env-22     UGGCGACAACUGACGCGCCUUGCGUCUUUUUUUACGUCGUAGCUCAGGCACACCCAUUUUCCGGGCUGUGGUGUUGUGUGUACACCGUGGUUCUUUCA
env-23     UGGCGACAACUGACGCGCCUUGCGUCUUUUUUUACGUCGUAGCUCAGGCACACCCAUUUUCCGGGCUGUGGUGUUGUGUGUACACCGUGGUUCUUUCA
Sen-11-3   UUUGUGUCGCAAUCAACGCCACAGAGCGCCAGAUUAUGGUGUGGCGUGUGGUUUGCCGUGCAGGUAUGAUCCUGUUCGCAAUCGCAUGUUAUGCCACUG
Eco-5-2    UUUGUGUCGCAAUCAACGCCACAGAGCACCAGAUUAUGGUGUGGCGUGUGGUUUGCCGUGCAGGUAUGAUCCUGUUCGCAAUCGCAUGUUAUGCCUCUG
Eco-14-1   UUUGUGUCGCAAUCAACGCCACAGAGCACCAGAUUAUGGUGUGGCGUGUGGUUUGCCGUGCAGGUAUGAUCCUGUUCGCAAUCGCAUGUUAUGCCACUG
Eco-11-2   UUUGUGUCGCAAUCAACGCCACAGAGCGCCAGAUUAUGGUGUGGCGUGUGGUUUGCCGUGCAGGUAUGAUCCUGUUUGCAAUCGCAUGUUAUGCCACUG
Eph-4-1    CAAUCAAUGCCACAGAGCGCCAGAUUAUGGUGUGGUGUGUGGUCAGUCGUGCAGAUGGCAUUCCGUGCAUCCUGCCCGCGUCCGCUCAUUAUGCCGCAG
Sbo-2-2    CAAUCAAUGCCACAGAGCGCCAGAUUAUGGUGUGGUGUGUGGUCAGUCGUGCAGAUGGCAUUCCGUGCAUCCUGCCCGCGUCCGCUCAUUAUGCCGCAG
Eco-8-4    CAAUCAACGCCACAGAGCGCCAGAUUAUGGUGUGGUGUGUGGUCAGUCGUGCAGAUGGCAUUCCGUGCAUCCUGCCCGCGUCCGCUCAUUAUGCCGCAG
Sgl-2-15   AUACACCAACCUCUAUUAAGAUAUCCUUGCUGCUUUCCUUGGCGACAUAGACACCAUUUGCGUCUUUUUUAUGUCGUAAAAUGCUGAGUGAGACCUGUA
Sty-1-1    UAGGAACCCGCGUAGUCAUCGUCGGCGCAGAAGUUAAUUUCUUCAUUUGGCGUCGUUUUUUGCCAUACCAUAAAGACAGGAACUUGUAUUUCUUGUCGU
Eco-1-1    UAGGAACCCGCGUAGUCAUCGUCGGCGCAGAAGUUAAUUUCUUCAUUUGGCGUCGUUUUUUGCCAUACCAUAAAGACAGGAACUUGUAUUUCUUGUCGU
Sen-4-4    UAGGAACCCGCGUAGUCAUCGUCGGCGCAGAAGUUAAUUUCUUCAUUUGGCGUCGUUUUUUGCCAUACCAUAAAGACAGGAACUUGUAUUUCUUGUCGU
Kph-1-1    CGCUGCAGCAAAUUCUGCAGCCGGGCGUAGGAACCCGUAUCCAGUCAGCGCACAACACGCGCCUGCGUGUUUUUUUGUGUGUAAUACCUGUGCACGCCA
Ype-2-2    CGGCAAAAUCCGUAGCCGGAAUUAGCGUUCCGAAAAUCUUCUUGAUACCUAGCCUCGUCAUUCAGACAUGGUCUUUUAUAGGUAUGGCUUGCGCACACC
env-24     UUCUGAAGGCGACAUAUGACGCGCCGAGCGUCUUUUUUUAUGUCGUAGGUCUGACUCACCCAUUUUUUGGGCGUUGGUGUAUAAUCCAGCGCCUUUGUC
Sen-1-1    CAACAUUGGCGACACCGGACGCGCCUUGCGUCUUUUUUUGUGUCUAUGCCUCAGCGCAUCCAUUUUUGAUCCAGAGAUUUAUCUGUCUAUGGAUCCUUC
Sen-14-2   CAACAUUGGCGACACCGGACGCGCCUUGCGUCUUUUUUUGUGUCUAUGCCUCAGCGCAUCCAUUUUUGAUCCAGAGAUUUAUGUGUCUAUGGAUCCUUC
env-25     GUUUUUCCGCUGCGGCAAAAUCCGCAGCCGGAGUUAGCGUUCCGAAUCUGACAUACUACCGCUGUAUUAUGUAAUAAUGCAGCAGCUUCAAUUCACGCC
Ppu-4-3    CUCGCGGCUGAUUGUCCCUGGAAACCCCACCCUUUAGGCUUUUCGUGUCGCUGAUGUUUGGCGUCCGGGAGUGGAAUCCUGUCAAUUACUGAGUUGCUG
Ppu-2-1    UCGCGUCUGAUUGUUGCUGGAAUACUGAUUCUCUAGCCUUGGCUGGUCGCUGAUCGUCAGCGUCCGGGGGUGGAAUCCCGACCCAUUACCGAGUAGCUG
Pen-1-3    CCGUGCCGCACCGCAGUAGUUUCUGCGGUUCGGGAUUGGCGUCCCGCUGUCAGAACCGGGCACAUCGCACCUGCGAUAGCGUGUCCUGCAUGGUUGCAC
Nmu-1-1    ACUGCAAAUUCAGUGGUCGAGAUUGGCGUCUCGGUACAAAGGCGGACACAACCGCCUCUUGGCGGUUUUUUUAUUUACGUCCGUUGUACGGCAAGCCC
env-26     ..................................................AGCGGGUUGGCUGCNAUGAUCGGUUUUUUUCGUCCGCAGCAUGGCACUCC
Avi-1-1    CGCCAAAUCGGCAGCCUGGGCUUGGCGGCCCGGAUAAAACGGGCGGUCACAAAACCGCUGUAAAAAAGCGGUUUUUUUGUGCCCGCGGCAUGGCUGCAC
Pae-4-2    UUGCUGCCGCAAAUUCGGCAGCCGGGAUUGGCGUCCCGUGGAUUAGGCGGACACACCGCCUUAAGAGCGGUUUUUUUGUGUCCGCAGCAUGGCUGGCCU
Ppu-2-2    CCGUCUGAUUGUCCGGGAAAGGCCUGCUCUCUAGGCUGACCGGGUCGCUGUCGGUUCAGCGAUCGGGUGUGGAAACCCGGCAGGUAAUCGGAACAGCUG

           ....................................................................................................
           ....................................................................................................
           ●●●●●●●●●●●●●●●●●●●●●●●●●●●●●●●●●●●●●●●●●●●●●●●●●●●●●●●●●●●●●●●●●●●●●●●●●●●●●●●●●●●●●●●●●●●●●●●●
```

Pen-1-4	CGGUCCGGCUUCCAAGGUAUUUAUUCGCAGUUAACUUUUUCCGGUCGCCGCACAUUCGGUGACCGGGCGUGAGAACCCGGCGAUUUACUUGGAGGCAUG
Pen-1-5	AAGCCGCUGCGUUGUCGGGGAAAUGCCCCGGGGAUAACUUCACCGGGUCGCCGUAUUUGGUGACCGGGUGUGAGAACCCGUUCCACGAAGAUCGCCGCU
Ppu-4-4	AUGGCGUCUGAUAGGCGGGGAACUGUUUCAGCGAUAACUUCACGGUUCGCCACAACGGCGAGCGGCCGUGAGAAGCCGCAGCUGUAUCCAGGAUGCCAU
Pen-1-6	GAAUUGGCUGGAAAUUACUCCGAGGGUAACGUCGUCGGGUCGUCAAAUUUGGCGACCGGGUGUGAGAAGCCCGCAACGUGGUACACAGUCAUGGCUUUG
Pen-1-7	GCUCUGAAUUGGCUGGGAAAUGCCCCGGGGGUAACGUCGCUGGGUCGUCAAAUUUGGCGACCGGGUGUGAGAAGCCCGUUGUGUUUCGAACAGGCUGGC
Ppu-4-5	UCGGAUGCCGCCUGAUAGGCGGGGAAGGACUACAGCGAUAACUUCACGGUUCGCCCAUAUGGCGAGCGAGUGUGAGAACUCGUUGUUGGUAGUCGGCCG
Ppu-3-1	CCGCCGAAGUAACGCUGUUGUCGGGGAAGUGGCUCACGGAUACCGUUUUCGGGUCGCUCUGGCGACUGGGUGUGAGAACCCGACUUGAAUAAGCGUUAC
Ppu-3-2	CCGAACUAUCGCUGUUGUCGGGGAAACACCUCACGGAUAACGUUUCCGGGUCGCUUCGGUGACCGGGUGUGAGAACCCGACAGAGAUUUUACGCAGCAC
Ppu-4-6	CUGCUGAAGUAUCGCUGUUGUCGGGGAAAUGCCUCAGGGAUAACGUUUUCGGGUCACUUCGGUGACCGGGUGUGAGAACCCGACUGAUACAAGGCAUCG
Ppu-1-2	AAGCAGCUGAAUUGUCGGGGAAAUGCCCCAGGGAUAACGUCACCGGGUCGCCAUGUUGGUGACCGGGUGUGAGAACCUGAUGCGAGUUACCUGGCUUGC
Ppu-4-7	UCACUGUUGGCGGGGAAGUGCCCCAGGGAUACCGUUCCUGGGUCGCCAAUACCCUGGUGACCGGGUGUGAGAAACCCGUUGUGGAGCUACUUGGCUAGC
Ppu-3-3	AAGCAGCUGAAUUGUCGGGGAAAUGCCUCAGGGAUAACGUCACAGGGUCGCCAUGUUGGUGACCGGGUGUGAGAACCUGAUGCGAGCUACCUGACUUGC
Ppu-4-8	UCGCUGCAAAUUCAGCGACCGGGCGUAGGAACCCGAAUUCAAGAAGGCGCAUAGGCGCCCCAUUUCAAUCGCAGGCGCUUUUUUGUGCCCGCGAUAUU
Ppu-4-9	AUGGUGUGCUGGAUUCCUUUCGCUCCAGUCGGCCAACCCGCGUACCGCCGCCACCCGAACUCACCCACUGCAUUUGCUUUUUGGGGAGGCGCCGUUUC
env-27	UGGCAAAAUCCAUGAUCGGGUGUAGGAACCCAACAGAAAACUUGGCGCACAGGCGCCCCCAUUACGAUUGCUGGCGUCUUUUUUUACGCCCGCAGUUAA
Pen-1-8	AGCAAAACCUGCGGCCAGGUGUAGGAACCUGCUGGUAAACUUGGCGCACGCACUAGGCGCCCCAUCACCACGGCUGGCGCCUUUUUGCGCUUGCUGCG
Ppu-1-3	UAAAUCCCGUGACCGGGCGUAGGAACCCGUUGGUAAACUUGGCGCACGCACUAGGCGCCCCUGCACCACAGCUGGCGCUUUUUUUGCGUCUGCCGUGCU
Ppu-4-10	CUGCCGUUUCGCUGUUGUUGCUCACAACCAUCAGGGCUAACUUCCCUGGGUCGUUUUCGAACGACAGGGUGUGAGAACCCCGUAAACAUGACAGCAGCC
Ppu-4-11	AGGUGCCGUCUUGGCUGGCAAGCGCUCCGGGAAUAAUGUCCUCGGGUCGCUUCACAAGGCGAUCGGGCGUGAGACACCCGGUUCACAUUCGGUAGCGCC
Pen-1-9	GUCACGUAGCCGGGGAAAUACCCCAGGGAUAACGUCACUCCGUCGCCACAUGUGGUGACCGGGGUGUGAGAACCCCGAAGCUCAAGAAAUUCGGCCUUC
Pen-1-10	GAAGGGUCCGGAUAUGCCGGGGAAUUCAUCAGGGCUAACGUCGUCGGGUCGCCAAAUGGGUGACCGGGUGUGAGAACCCGACGAGAUGACUGUUAGCCA
Pfl-2-7	ACUUGAUCCCCGUCGCUGCCAAUCAGCGGCCGGGUUUAGUCGCCCGAGGCAAGAUAGGUUUGCAAACGCAUCGUCCAGUCGGGUAUUCCCUAUCCCUGC
Xor-1-1	CCGGGAUUGGUCUCCCGAUUACUCAGGCGCACCAGCGCCCAUCGAUCGAUGCAAGGCGCUUUUUUGUUGCCCUGCAUCGCGCCGGGCACGUGCUUGCCA
env-28	CCGGGAUUGGUCUCCCGGAUUCCAAGGCGCACCAGCGCCCAUCUCCCGAUGCAAGGCGCUUUUUUGUUGCCCUGCAUCGCAGCGGGCACGUGCCGGCCA
Xca-1-1	CGGGCUUGGCAGCCUGAAUACAAGCGGCGCAAUAGCGCCCAUCGACCGAUGUCUGGCGCUUUUUAUUGCCCGACGUCGAGUUGGGCGCAUGCCUGCCA
Sma-1-5	AAGGGUGGCGCGGGACUCGAAAACCCGUCUAUGUACCAGAUGUAUGCGCUUGCCGGCCGGCGUCACUUUUUAUGUGGCGCCGGCUGGCAAUCCGUUCGC
Bdo-1-1	CUGAGACAACAGCGGUCGGGUUUGGCGACCUGAUUUCUAGAGGCGGACGACGACCGCCAUAUGGCGGUAUUUUUUCGUCUGUAUACUGCGCACGCGUCC
Pfl-2-8	AUAGGCUUUGGGGGUCGCUGACGCAUCAGCGGCCGGGUCUGGAAAACCCGAUUUCGACCAACCAUGCUCGUGGCAUACUGCGGCCCUUUUCAGCUCGCA
Pfl-1-2	CACUCGCCCCAAUGGCGACCGGGUUUGGCGACCUGGCAACUCACCCACAGGCACUUCCGCUUGCAAGCUUUGCCGAGGACCCUUUAUGUUCACGGCGAU
Ppu-4-12	AGAUUGUUCCAGUCGCCGAAUCAUUGGCGACGCGAUCCUGAUUCGUAGAAGCCGAAAAGCUCAUCGGCAUCCAGGCGGCAUUUUGCCAGGCUUGCUUUU
Pen-1-11	UUACCUUCUCAGUGGGAACUCCUGCCUCUAGGCUCCAGACCGUCGCCGAAUAACUUGGCGAUCGGGUGUGGUAACCCGGUAACAGGAAGGUAUCAGAGU
Ppu-3-4	CCAUCACCUUCUCAGUGGGAACCUCUGCCCCUAGGCUCAAACCGUCGCCGAACAACUUGGCGAUCGGGUGUGGUAACCCGGGUUCUGGGUGAUCUUGGC
Ppu-2-3	CCGUCACCUUCUCAGCGGGAACCUCUGCCCUUAGGCUUAAACCGUCGCCGAAUAACUUGGCGAUCGGGUGUGGUAACCCGGGUUCAGAGAGAUCUUGGC
Ppu-4-13	CAUCACCUUCUCAGUGGGAAGUCCUGUCUCUAGGCUUCAGACCGUCGCCGAACAACUUGGCGAUCGGGUGUGGUAGCCCGGAUGCAUCAUUUCCAUGAC
Ppu-1-4	CCAUCACCUUCUAAGUGGGAACCUCUGCCUCUAGGCUCUAUCCGUCGCCGAAUAACUUGGCGAUCGGGUGUGGUAGCCCGGACGCAUUUUUUCCAUGAC
env-29	CCAUCACCUUCUAAGUGGGAACCUCUGCCUCUAGGCUCUAUCCGUCGCCGAAUAACUUGGCGAUCGGGUGUGGUAGCCCGGAUGCAUUUUUUCCAUGAC
Psy-3-1	CUCUGCGCCCGUCGCUGCAUAUUCAGCGAUGAGGAUUAGUAGCCCUCGUUAUCCAGGCGCAUCGCGCCACUUCGUGCAAGGUGCUUUCAACGGCCUGCA
Psy-1-2	UGCGCCCGUCGCUGCAUAUUCAGCGAUGAGGAUUAGUAGCCCUCGUUAAAUCCAGGCGCAUUGCGCCACCUUGUGCAGGUGCCCUUUCACGGCCUGCAU
Psy-1-3	UAGCCUUCAACGGUCGCUGCCCAAUCAGCGAUCAGGUUUGGUAGACCUGCUUUUGCUGGCGCAAUGUGCCACCUCUGCGAGGCGAUCCUUUUCGUCUCG
Pfl-2-9	GUUUUAUCGUUGUCCCGUCGCUGAAAAGAACAGCGAUCGGGUUUGGUAGCCCGGGUCUUUAAGUGCAGAGUGCUCCAUUUUGUAGAGUCUUUUGUCUCU
Pfl-2-10	AUAUCGUUUCCCUGUCACUGAAAAACAGUGAUCGGGUUUGGUAGCCCGUCUGUAAUAGCUGUGUGGCAACACCGUAUGCAGUCCCUUUCUUGGGGCUCG
Pfl-2-11	GUCUUAUUGUUUCCGGGUCACUGAAAAACAGUGAUCGGGUUUGGUAGCCCGCACAGGAGAGUUGCACAGCAUCACCAUUCGCGGCGUCGUUUCGUCUCG
Pfl-2-12	UUUUAUCGUUUCCGGGUCACUGAAAAACAGUGAUCGGGUUUGGUAGCCCGAGACAAACACAAGAUGCAGAGCACACCAUCGCGGGCGGUCAAUCGCCCCG
env-30	UCGUGGGUCGGUCGCUGAUAAAGCAGCGAUCGGGUUUGGUAGCCCCGGUUUCACACAUGGCGCAAAACGUCACCUUACGCAGAUGCCUUCUGGCGCCUGU
Psy-2-1	AGCCUUCAUCGGUCGCUGCCCAAUCAGCGAUCAGGUUUGGUAGACCUGAGUGACCUGUGCGCAAUGCGCCACCUUCUCAAGGCGCAUUUUUUCGGCCUG
Pfl-2-13	UUUUAUCGUUUCCCGGUCACUGAAAAACAGUGAUCGGGUUUGGUAGCCCGGAUUACCUGUCGCAUCGCAACACCAGAUUGCAGGGACUCUUUCGUCCUC
Psy-2-2	UGCGCCCGCCGCUGCAUAUUCAGCGGUGAGGAUUAGUAGCCCUCGAUGUGAUUAGGCGCAAUGCGCCACCUUGUGCAAGGCGCUUGUUUUCGGCCUGCA
Pfl-2-14	GCUUUUAUCGUUCUCGGGUCACUGAAAACCAGUGAUCGGGUUUGGUAGCCCGUGUGUUAGAUGUACAGCGUCAUCGUCACCGGUCGUGCUUUCGAUCGG
Psy-3-2	CUUCUACUCUCCGCCCGUCGCUGCCCAUCAGCGAUCGGGUUUAGUAGCCCGCAGUGAGUUAAGGCGCAACACAGCCGUGUUUCAUCAGGUUUUGUCCCG
Pfl-2-15	CGGUAACUUGGAUCGGGUCACUGAUUUCCAGUGAUCGGGUUUAGCAGCCCGGUUCAUCUACCACGGUUGCACAUCUCACCUUGUCAGGUUUUUACCUGC
Avi-1-2	CACGGUCAAUCCCGUGGCCGGCCGUCGCGGGCCGAUUCAGCAAACGGCGCACCAGCGCCCCAAGACCAUUGCAGGCGCUUUUUUUGUGUCCGCAAUGUC
Ppu-2-4	UGUCAAUCCAGCAGCCGGGUUUGGUAGCCCGCGAUCAAUCAAGGCGCACAAGCGCCCGCUCUGCGAUACAGUUGGCGCUUUUUUGUGCCCGGCUGUUUC
Pen-1-12	CACAAUCCUUGUGGGGGUGGGAGGCACGAAGUGUAUCCGCACAUGACCCCGCCUUGAAACGUGUUCCCGAACGGCGCAUUUUUUGUGCCCGCUCGUUUC
Pfl-2-16	AACUUGAUUCGCAUCACUGAAUGGUCAGUGAACGGGUUUAGUCGCUCGGUGUUCUCCAACUUCGCAUGCAGUCAUUCAGUCAGGUGCUUUUGCGCCUGC
Pfl-2-17	GUCGCUGCCAAUUCAGCGAUCGGGUCUGGAAACCCGGAUGUGUGAGAAAGCGAAAUCGCUUUUCAUAACGUAUGCUUGCGCACCUUCAUUGUGUGCACG
Pfl-2-18	AUAGGCUUUGGGGGUCGCUGCAAAUUCAGCGAACGGGCGUCGCGGCCCGAUAACUGAUGCAUAAUCGCCAACCUCACGGCACCUCGGUGCCUGUUGUUU
Pfl-1-3	UUCGCGCCGUCGCUGCCAAUUCAGCGGCCGGGUUUGGUCACCCGAAAUUAGAAGGCGCACGCGCCACCCUGGUUUGCAGGCACUUUUUCGUGUCCGCAC
Pen-1-13	CUCGCCCAGUCGUUGCCAAUUCAACGACCGGGUGUGCAAGCCCGUACAGGUACAGCGCGAAAGCGCCAUGUGAACCUGUCGCUAUCUGCGACAAGGGUU
Pfl-2-19	GGGUCUGUCGCUGAACGAAUUUGGCGAUAGGGUUUCGCAGCCCUAGCGAGCUAAGUCCACACCCGGCACAAUGCGGCCCGGCUACUUGCCUGUGCUUCA
Pfl-2-20	GCGUAAUUUGAUUGCGUCACUGAUUUCCAGUGAUCGAACUUAGCGGUUCGGCAUGCGUUCACGGUUGUACACGUCCGAUCCAGUCAGGCAUUCGCCUGC
Pfl-2-21	CUGCCAACUCAGCGACCGGGCCUGAGAAUCCGCGUAAGAUGCAGGCGCACCAGCGCCCCAACCCACGUUUGCCGGCGUUUUUUUUGUGCCUGCAUUUCC
Pfl-1-4	UCGCUGCCAACUCAGCGGCCGGGCUUGGUAACCCGUGUUGAUACAGGCGCAACAGCGCCCCUUGACGAUUGCAGGCGUUUUUUUUGCGCCCGCGUUUCC

....

....

•••

```
env-31    CUUGUGCGCUUGGGGGGUGGUCUCUAAGGUGGGUCGGUCACUGAAUUUCUCGGUGAUCGGGUUUAGUAGCCCGAACUUCCCAACACAAUAUGCACAAGC
env-32    CUAAGGUUGCUCGGUCGCUGACAACUCAGUGAUCGGGUUUAGUAGCCCGAGUGACAACAUAAAGUGCAUGAGCCUCAUCUCUCAGAUGUUUUGUCUGU
env-33    CUUGCGCCUGUUGGAAUCGAUGGCUAGUCUGCGCUCCGUCACUGCUCAUAGUGAUCGGGUUUAGUCGCUCGGAUGGCACAAGGCGCAUGGUCACAUGAA
env-34    CUCUAAGGUUCUUCGGUCGCUGAAUGUUCAGCGGUCGGGUUUAGUAGCCCGAAGAACGGCGUAAAGUGCACGAGCUUCAUCUAGCAGACUUUUGUCUGU
Pfl-2-22  ACUUGAUUCGGGUCACUGAUUCGCAGUGAUCGGGUUUAGUAGCCCGAGGGUUCGCGAGGCGUAUUUGCACUCCAGAAAGUGAGAUAUCUCGGUAUCUGC
env-35    UAAGGUUCGUCUGCCGCUGCCCAUCAGCGGUCGGGUUUAGUAGCCCGAUUUGAUCAACGGCGUAUGGCUGUCCUAUCAGUCAGGCAUCUCGAUGUCUGU
Pfl-1-5   UAGUCGAGGGGUCGUUGAAAGGACGACCGGGUUUGGCGACCUGACACCAGAAUUGAUGCAAAAGCCUCUGCUUGCAGGUACUUUUGCUCCCGCACAAUC
Psy-1-4   CUUGUGCCUGCCAACUCUGCGCACUAGGCUGCGUCGGUCGCUGAUUUUUCAGCGGUCGGGUUUAGCAGCCUGUUUGUGGAUUACAAGUGGCGCAAGUGC
Psy-2-3   UUGUGCCUGCCAACUCUGCACACUAGGCUGCGUCGGUCGCUGAUUUUUCCAGCGAUCGGGUUUAGCAGCCUGUUUGUGGAUGACAAGUGGCGCAAGUGC
Pen-1-14  UCGCCGAAGCAUCGGCGACGCGACCUUGGCCGGUCGGAAUCGAAAGGCGCAGCAGCGCCCAGUCUUCAUCGCAGGCGCUUUUUUUGUGCCCGCGAUGUC
env-36    .................................................................................CGCUUUUUCUUGUCUGU
Pfl-2-23  UUCUAUUGUUUGGCGGUCGCUGAAAAGCAGCGAUCGGGUUUAGCGAUCCGACUCCAUACAGACGCAACAACACCCAAUCCCAAUUGCAGUCUGCACUUG
Pfl-2-24  GGGUCGGUCGCUGACGCAUCAGCGAUCGGGUUUAGCGACUCGAACUUUCUCAGCGCAUCAGCGUUCAUGGCUUCGUUGUGGGACUCUUCGUCCGCAAUU
Pfl-2-25  GGGUCGGUCGCUGACGCAUCAGCGAUCGGGUUUAGCGACUCGAACUUUCUCAGCGCAUCAGCGUUCAUGGCUUCGUUGUGGGACUCUUCGUCCGCAAUU
Psy-2-4   CCCCGCCGUUCGCUGCCAAAUCAGCGGCCGGAUUUGACGAUCCGUUCUAUCCAGGCGUGUCAGCGCCCCACACACUGCAUCAAGCUAAACUUGAAGUAC
env-37    CCGGCCCGUCGCCCAACAAGGGCGAUCGGAUUUGGCGAUCCGUUGACCGUAUGCGAAAGCUUUCUGCUUGCUUGCAGGGGCUUCCGUACCCGCAUCGCU
env-38    .............................................................CCUGCUCCUUUGCUGGUGCUUUUGCACGCGUAAGGCU
env-39    UUCGGCGGUCGUCCAUAUGGCGACACGGGUUUGGCGACUCGGGUGAAUGGUGCAAAAGCUUCUGCUUUUUUGCAGGUUCUUUUGCGUCCGCAAUAGCUG
Pfl-1-6   UCGCUGCAAAUUCAGCGAACGGGUUUGGCGACCCGGAUAGAAAAGGCGCACAGGCGCCCCCAUUACGAUUGCAGGCGCUUUUUUGUGCCCGCAUGUCC
Ppu-3-5   UCGCGGCCAACUCCGCGACCGGGUUUGGCGACCCGAACGAAAAGGCGCAUCGGCGCCCCCGACCACGAUUGCAGGCGCUUUUUUGCGUCCGCAGUUUC
env-40    UCGCGGCCAACUCCGCGACCGGGUUUGGCGACCCGAACGAAAAGGCGCAUCGGCGCCCCCGAUCACGAUUGCAGGCGCUUUUUUGCGUCCGCAGUUUC
Pen-1-15  UCGCGGCCAUUUCCGCAACCGGGUUUGGCGACCCGACCGAAAAAGGCGCAACGGCGCCCCGACCACGAUUGCAGGCGCUUUUUUGUGUCCGCAGUUUC
Ppu-4-14  GUCGCAACCAAUUGUGCGACCGGGUUUGGCGACCCGUAUGAAAAGGCGCAACGGCGCCCCGAUCACGAUUGCAGGCGCUUUUUUGCGUCCGCUGUCUC
Pen-1-16  AGUCGCGGCAAAUUCCGCGACCGGGUUUGGCGACCCGGAUAAACUUGGCGCAGCAGCGCCCCACACGAUUGCAGGCGCUUUUUUGCGUCCGCAGCCUC
Ppu-4-15  UGAGACGUUCCAAAGAAAUCACGGUGAAGGCUGACACCUGCACUGUCAGUGCCGUUCUCUCCACACGAUUGCAGGCGCUUCGUGUGCGCCCGCAGCCUC
env-41    UCCGCCGGUCGCCCAUAUGGCGAUUCAGGUUUGGCGACCUGACGGUAGAGAAUGCGCAAGCUGACGGCUUUUUUGCAGACACUUGUGCGCCCGCAAAGU
Psy-3-3   CGCCGUUCGCUGCAAAACCAGCGGCUGGGUUUGGCGACCCAAAGAAAAGACAAAAAUACGCACCAUCGCAAAGCAGCAGAUUCUAUCGGUCUGACUUUC
Pae-3-1   CGCCGCAAAAUCGGCGACCGGGUUUAGCAGCCCGUUUCGACAAAGGCGCACCAGCGCCCAAUUCCCCAUUGCAGGCGUUUUUUUUGUGUCUGCAAUGCC
Ppu-4-16  CUUUAGUCUCGGCACGUCGUUGUCACUCAACGACUGGGUUAGCCGCCCAAAAAUCGUCGGACACUACAGCCGUGCAAUACCGAGAAAUCGGCAUUAGCG
Pfl-1-7   UCGCGGCAAAAUCCGCGAACGACCUUGGCCGGUCGAAUUACAACGACGCAUCAGCGUCCAAAACACCAUUGCAGACGCUUUUUUUGUGCCUGCGGUUCU
Pfl-2-26  AUAGUUCGGGCUCGCGGAAAACUCCGCGAACGACCUUGGCCGGUCGAUACAGGAACGUACAGUUGUCCAACAUAUUCAGCGAUGUUCGCCUGAGUUGUU
Pfl-2-27  UGUGGUGCGGUCGCUGACGAAUCAGUGAUCGGAUUUGACCGUCCGCCCAACAUCAAGGCGCACCAGCGUUCUGCACGUGUUCAGGCAUUGUCUGAAAUC
env-42    CGCCUCGUCAUCGAAAUCACGAUGACCGGACGUGCAAGUCCGGCUCGCAAAGGUGCACAAGCACCCCACAUCUCAACGCCCGUUUUAGUGAUGGGCGUU
env-43    CUGCGUACACCGCUGCACACGCAGCGGUCGGGCGUCGCGGCCCGGAUUGAUUAAGCAUUGCCUCCUUUCAGUUGACGCUUUUUUAUCGUCUACUGUGCG
Pfl-1-8   GGCCGCUGCCAAAUCAGCGGCCCGGAUGUGGAAGUCUGGGAUAAAGGACGUGCAGUCGUCUCAUUCGAACACCCUUGCCGAUGCGGCUUGGGUGAACUC
Avi-1-3   CACGGUCAAUCCCGUGGCCGGGUUUGGCGACCCGAAGACUACACGGCGCACCAGCGCCACCAAGACCAUUGCCGGCGCUUUUUUGUGCCCGCAAUGUC
Psy-1-5   UAAUCUCCGCCCGUCGCUGCAUAUCAGCGAUCGGGUUUAGUCGCCCGGAUUUGAGUUAAGGCGUAUCGCGUCGCUGUUUGGUCAGGUCUUCUUCCUGC
Psy-2-5   UAAUCUCCGUCCGUCGCUGCCUAUCAGCGAUCGGGUUUAGUCGCCCGGAUUUGAGUUAAGGCGUAUCGCGUCGCUGUUUGGUCAGGUCUUCUUCCUGC
Pfl-2-28  UCUGUGUGUCGCCGCAAAAUCGGUGACUCGGACGUGCAAGUCCGGUUCAGGUGCACAAUCGCAAUGGCAUUUCGCCGAGCCUUUUCCUAUGCUCGCGGA
env-44    CUCGCUCUGUCAUCGAAUGUUCGGUGACUGGACGUGCAAGUCCGACGACGGUAAUACGCAUCAGCCUUAACUCUCAACGUCUGUAUUUCAGGCAUUGAU
Pfl-1-9   GUUCCGGAGCCAUCGCCCAAUCGAUGGCUCGGAUGUGCAAGUCCGACUGUUACCAGACGCAUAAGCGCUCAGUCAUGCCUGAGCGAUUUCAUGUUCGCC
Ppu-4-17  UCCGUUGCGGUCGCCCUGCUGGCGCUCUAGUCUCGGCCUGUUGCUGCACGUCAGCGACAUGGCUUUGACAGGCCGUAACGGUAUGUAGGUAGGCAGCGC
Ppu-4-18  UCCGUUGCGGUCGCUUGGGCUGCAUUCUAGUCUCGGCGGGUCGCUGUUGUUCAGCGAUCGGCUUUGACAGGCCGCGACGGUAUUAUGCAUACGGCUUGC
Pen-1-17  UUGCGGUCGCCCUGGCGGGGCUCUAGUCUCGGCCUGUCGUUGCACAUCAACGACACAGCUUUGACAGGCUGUGACGGUACAAUCACGCUGGCAGUCUCC
Pen-1-18  CCCGUUGCGGCAGCCCAGGCAGGGCUCUAGUCUCGGCGAGUCGCUGGUGUUCAGCGACCGGCUUUGACAGGCCGCGACGGUAUUAAGCAUGCGGCUAGC
Ppu-4-19  UCCGUUGCGGUCGCCCUGCUGGCGCUCUAGUCUCGGCCUGUUGCUGCACAUCAGCGACACGGCUUUGACAGGCCGUAACGGUUUAAGCACAUGGCUUUC
Ppu-4-20  GUUGCGGUCGCUUGGGCUGCGUUCUAGUCUCGGCAGGUCGUUGCUCAUCAACGAUACAGCUUUGACAGGCUGUGACGGUAAAUCACGCAGACGGUCUCC
Pen-1-19  CCGUUGCGGCUGCUCAGGGAGCGCUCUAGUCUCGGCGAGUCGUUGCAUAUCAACGACACAGCUUUGACAGGCUGCGACGAAAAAUCGUGUACGGCUCGC
Pen-1-20  CCAUUGCGGUGGUCUGGGUUGGCCUCUAGUCUCGGGCGGUCGCUGCACAAUCAGCGAUCGGCUUUGACAGGCCGCUCAGAAAAAAACCUCAUGGCUUGC
Pen-1-21  GUUGCGGUCGUUCUGGCACGGCUCUAGUCUCGGCGGGUCGCUUGAAUCAGCGACCGGCUUUGACAGGCCGACUUCCAAGAGCAACUUCAACGGCUUUGC
Ppu-3-6   UUUCCGUUGCGGUCAUUUUUUCAGGGUUUUAGUCUCGGUGGGUCGUUGCUCAUCAACGACCGGCUUUGACAGGCCGCAACGAUACACAGCGCGCAUAAC
Pen-1-22  UUCCGUUGCGAUCGCCCUGUCAUCGCUCUAGUCUCGGCAGGUCGCUGCAAAUCAGCGAUCGGCUUUGACAGGCCGCGACGCGACACCUGCACUGCUUGC
Ppu-3-7   CUUUGCACAUGGAAAUAGUCAGCCCUAGUCUCGGCCUGUCGCCACCCAUUGGCGGCACGGUUUGACAGACCGUGGUUCACUGAGAGCAUGUGCGAUGC
Ppu-1-5   CGUUGCACAUGGAAAUAGUCAGCCCUAGUCUCGGCCUGUCGCCACCCAUUGGCGGCACGGUUUGACAGACCGUGGUUCACUGAGAGCAUGUGCAAUGC
Ppu-1-6   UGCCAUUGUGGCGUUCGGGCAUGCGCACUAGUCUGGGGUCGUCGCUGCCAAUCAGCGACCGGCUUUGACAGGCCGCCCUGAACUAACCACAUGGCUUUG
Pen-1-23  UUUCCGUUGCGGUCGUUCUGCCACGGCUCUAGUCUCGGCGGGUCGCUUGAAUCAGCGACCGGCUUUGACAGGCCGCUCUGAACAGACCGCAUGGCCUUG
Pen-1-24  UUUCCGUUGCGGUCGUUUUGGCAUGGCUCUAGUCUCGGCGGGUCGCUUGAAUCAGCGACCGGCUUUGACAGGCCGCUCUGAACAGACCGUAUGGCCUUG
Pen-1-25  GCCAUUGCGGUGUUCCCGGUUGGCCUCUAGUCUCGGCCGGUCGCUGCGAAAUCAGUGACCGGCUUUGACAGGCCGACCUAGGUUCAUUCCCAUGGCUCG
Ppu-4-21  GCCAUUGCGGUGCUCUGGGUAGGGCCCUAGUCUCGGGUGGUCGCUGCCGAAUCAGUGACCGGCUUUGACAGGCCGCUUAGGUCAAACCGAAUCGCUUUG
Ppu-4-22  AGCCAUUGUGGUGUUUCAGGCGAGGCACUAGUGUCGGCUUGUCGUUGCCAGGCAACGAUCGGCUUUGACAGGCCGUCCUGAACUAACCACAUGGCUUUG
Ppu-4-23  CCCAUUGCGAGCGCUCGGAUUGCGGCCUAGUCUCGGUUCGUCGUUGACACCUCAGCGACCGGCUUUGACAGGCUGACCGAUUCAAGGGUCCUUUGCAGC
Ppu-1-7   AACGGUUGCGACGGAUUCGGUCGCGCUCUAGUCUCGGCUCGUCGUUGUGAAAUCAGCGACCGGCUUUGACAGGCCGACUACUCAAGUUUCCGUUGCAGC

          ..................................................................................................
          ..................................................................................................
```

```
Ppu-1-8     CCAUUGUGGUGUUAGGAGUUGGCCACUAGUCUCAGCCGGUCGUUGCCAUUCAACGACCGGCUUUGACAGGCCGACUCAAAUUGCAAUCCAUAGCUUUGC
Ppu-3-8     CCAUUGUGCAGUUCCGAGUUGGCCACUAGUCUCGCUUGGUCGUUGCCAUUCAACGACCGGCUUUGACAGGCCGACUCAAAUUGCAAUCCAUAGCUGUAC
Pen-1-26    CCGUUGCGGUGGCCAUUUUCGCCCUCUAGUCUCGCCUGGUCGCUGCAAUCAGCGACGCGACUUUGACAGGUCGCGAUGAACUGUAUGCGUACGGUCUGC
Ppu-2-5     AUUGCGGCCUCUCUGGCAGCGCUCUAGUCUCGGCCUGUCGUUGCACAUCAACGACACGGCUUUGACAGGCUGGAAUUGAAAGAAGGUGCACUCGCUUUG
Ppu-4-24    UUUCUUUGCACAUGGCCAACGUGGAUUCUAGUCUCGGCCGGUCGCUGCCAAUUCAGCGACCGGUUUUGACAGACCGUCUUCGUUGUCCACAUGUGCACA
Ppu-4-25    UCGUUGCACGCGCUAAACGUGGGCCAUAGUCUCGGCCGGUCGCUGCCAAUUCAGCGACCGGCUUUGACAGGCCGUCGAUUGUUGUCCGUGUGCAUACGC
Ppu-3-9     UUUCCAUUGCACACGUUAAACGUGAGACUUAGUCUCGACCCGUCGCUGCCAAAUCAGUGACCGGUUUUGGCAGGCCGUCAAUUGUUGUCCGUGUGCUUA
Pen-1-27    AGCCCUUGCCCCAGGCCAACCCGGUCACUAGUCUCGGCCAGCCGCUGCCAAUUCAGCGGCCGGUUUUGACAGACCGUAGACGUUGCCCUCAUAGGCUCG
Ppu-3-10    UUUCGUUGCACAUGGAAAUAGUCAGCCCUAGUCUCGGCCUGUCGCUGCCAAUUCAGCGACCGGUUUUGACAGACCGCUGACGUUGUCCUCAUGUGCUCG
Ppu-3-11    AAACGGUUGCGACGGAUUCGGUCGCGCUCUAGUCUCGGCUCGUCGUUGCAAAAUCAACGGCCGGCUUUGACAGGCCGACGAUUUCCCGCACACGUGCAC
Pen-1-28    CAAGCCAUUGCAGCCACACGAUGCGCGGCCUAGUCUCGACCCGUCGUUGCCAACUCAACGACCGGCUUUGACAGGCCGAAUCUCUCCGAGCACGUGCGC
Psy-1-6     AAGGUGCGCCGUCGCUGCAAAAUCAGCGGCCGGAUUUAGCGAUCCGAAUACCUCACAGCUAGCUUAUGUAUGCUUCCAGCCUCAGUGCAUUUGAAGCAA
Ppu-4-26    UCUGAUUGCCCCUGGAAUCCUCACCCUCUAGCCUGAUUGGGUCGCUGAAUGGCUCAGCGAUCGGGCGUGGAAACCCGGCAAGAUGCAUGGCUGUUGUCG
Ppu-3-12    ACUCCCGCCUGAUUGUCCCGGUCAACCUCGCCCACUAGGCUUUUUCGGUCGCUGUCAGUUCAGCGAUCGGGUGUGGAAGCCUGGGUGGUUUGGCGGCUG
Ppu-1-9     CGUCUGAUUGUCCCGGUAAACCUCGCCCACUAGGCUUUCGCGGUCGCUGUCAGUUCAGCGAUCGGGUGUGGAAGCCUGGGUGGCACAUGGGACAGUUGU
Ppu-3-13    CGCCCGUCUGAUUGUCCGGGGAAAGCCAGCUCUCUAGGCUGACCAGGUCGCUGUCGGUUCAGCGAUCGGGUGUGGAAACCCGGAUGUGAUGAAGGGCUG
Ppu-1-10    CGCCCGUCUGAUUGUCCAGGGAAGGCCUGCUCUCUAGGCUAACCGGGUCGCUGUCGGUUCAGCGAUCGGGUGUGGAAACCCGGAUGUGAUGAAGGGCUG
Pen-1-29    UUCCCGUCUGAUUGUCCGGGGAAAUCACGCCCUCUAGGAUCUCCCAGUCGCUGUCGGUUCAGCGAUCGGGUGUGGAAACCCAGUAGUCCUUAGAAGGUG
env-45      CGUGGUCAAUCCCACGACCGGGUGUGACAACCCGAAUCGUUCGAGGCGCGGUAGCGCCAUAGCCUAAGUAGCCAGCACUCCAAGUGCAUGGUUACUUAA
env-46      UCAAUCCCGUGACCGGGUGUGGUAGCCCGAUUCACACGAAGGCGCGGUAGCGCCAUAGUCGAGAGCAGGCGCUUUUUUGUGCCUGCUGUUUUCUCGUG
Pme-1-1     GUCAAUCCCACGGCCGGGUGUGGUAGCCCGAUUCACACGAAGGCGCGGUUGCGUCAUAAACGAGAGUAGGCCCUUCUCAGGAUCUGCUGUUUUCUCGUG
Pme-1-2     CAAUCCCGUGACCGGGUGUGGUAACCCGUGGUAAUCAAGGCGCGAAGCGCCAUAGCUCUGAAAUAGCCGGCGUUUUUUAUGCCUGCUGUUUUCGUGCU
Rso-2-1     GCCAUCGAAACAGCGAUGGUCGGGUGUGGAAACCCGACAUCUACAGGCGGACGGCCGCUUUCGCGGCUUUUUUUCGUCCGUAUCGCCUUUGCGCGUCC
Rpi-1-1     UCACCGAAACAGCGGUGGCCGGGCGUGAGAACCCGAUUCCUACAGGCGGACAGGCCGCGUUCGCGGCAUUUUUUUCGUCCGUAUCGCCUUCGCACGCCC
env-47      UCACCGAAACAGCGGUGGCCGGGCGUGAGAACCCGAUUCCUACAGGCGGACAGGCCGCGUUCGCGGCAUUUUUUUCGUCCGUAUCGCCUUCGCACGCCC
Reu-1-1     ACUGGAGCCAUCACCGAGACAACGGUGAUCGGGCGUACCAACCCGGAUCUUUACAGACGGCCGUCUCAUACGGCCAAGUUCGCGCGUCCACGUCUCCUC
Rso-2-2     CUGUCGCCGUCAUCGAACAACGAUGACCGGGAGUGGUAACCCGAGCAACGUGCGGCGACCGGCCAGCGUCCUAUCCGUAGCAAGCAUGCACGCUUACAA
Reu-2-1     CCGAGACAACGGUGGCCGGGCGUCGAAACCCGAAUUGCACAAGGCGGACGGCCGCCAAUAGCGGCUAUUUUUACGUCCGUCACGUCUUCGCACGCCUUU
Rso-1-2     GUCGAAACAGCGAUGGCCGGGUGUGGAAACCCGAACAAAGUGAGGCGGAUGCCCGCCGUUAGGCGGUUUUUUUACGUCCGUGUGCCGUCGCGCGUCUUC
Rso-1-3     GUCACCGUCAUCGAACAACGGUGACCGGGAGUGGUAACCCGAGCAACGCACGGCGACCGGUGGCCGCAGCCUAUCCGCAGCACGUCUGCACGCUUACAA
Bth-3-1     CUGAGACAACAGCCGCCGGGUUUGGCGACCUGAGAUCUACAGGCGGACGACCGCCAAAGCGCGGUUUUUUUACGUCCGCACGCCUCUGCGCGCACCUAC
Bps-8-1     UGUGGCUGAGACAACAGCCGCCCGGGGUGACAGCCGGCAAAUGUUGGCGGACGACCGCCGUAGUGCGGUUUUUUUACGUCCGUAUGCCUUCGCACACCU
Bps-16-1    UGUGGCUGAGACAACAGCCGCCCGGGGUGACAGCCGGCAAAUGUUGGCGGACGACCGCCGUAGUGCGGUUUUUUUACGUCCGUAUGCCUUCGCACACCU
Bps-11-2    CGCGUCGCUGAGACAACAGCGGCCCGGUUUGGCGACUGGAGUUUGCAAAAGGCGGACAACCGCCGCAGCGCGGUUUUUUUACGUCCGCACGCUAAGCGC
Bth-3-2     GUCGCUGAGACAGCAGCGACCCGGAUUGGCGUCCGGAAUCUGAAAACGGCGGACGACCGCCAUGUGGCGGUAUUUUUUCGUCCGCGCGUCAACGCACGC
Bth-2-1     GUCGCUGAGACAGCAGCGACCCGGAUUGGCGUCCGGAAUCUGAAAACGGCGGACGACCGCCAUUUGGCGGUAUUUUUUCGUCCGCGCGUCAACGCACGC
Bvi-1-1     GGCGUCGCUGAAACAACAGUGACCCGGUUUGGCGACCGGAAUUUGUCAAAGGCGGACAACCGCCUGAGCGCGGUUUUUUUGCGUCCGCACAUCAUGUGC
Bth-3-3     CGCGUCGCUGAGACAACAGCGACCCGGUUUGGCGACCGGAAUUUGUCAAAGGCGGACAACCGCCUGAGCGCGGUUUUUUUGCGUCCGCACAUCAUGUGC
Bps-16-2    GGUGUCGCUGAAACAGCAGCGAUCCGGUUUGGCGACCGGAAUUGUUCCAAGGCGGACAGCCGCCGACGUGCGGUUUUUUUACGUCCGUACACCUUGCGC
Bps-7-1     GGUGUCGCUGAAACAGCAGCGAUCCGGUUUGGCGACCGGAAUUGUUCCAAGGCGGACAGCCGCCGACGUGCGGUUUUUUUACGUCCGUACACCUUGUGC
Bps-8-2     AAAGCCUUACUUUCGGACAUAUUCCGACACCCUGUCCGUAAGGGUUCCAAGGCGGACAGCCGCCGACGUGCGGUUUUUUUACGUCCGUACACCUUGCGC
Bps-5-1     GGUGUCGCUGAAACAGCAGCGAUCCGGUUUGGCGACCGGAAUUGUUCCAAGGCGGACAGCCGCCGACGUGCGGUUUUUUUACGUCCGUACACCUUGUGC
Bps-18-1    GGUGUCGCUGAAACAGCAGCGAUCCGGUUUGGCGACCGGAAUUGUUCCAAGGCGGACAGCCGCCGACGUGCGGUUUUUUUACGUCCGUACACCUUGCGC
Bok-2-1     CGCGUCGCUGAGACAACAGUGACCCGGUUUGGCGACCGGAGAUGCUCCAAGGCGGACAACCGCCUUCGCGCGGUUUUUUUACGUCCGUACACCUUGCGC
Bok-1-1     CGCGUCGCUGAGACAACAGUGACCCGGUUUGGCGACCGGAGAUGCUCCAAGGCGGACAACCGCCUUCGCGCGGUUUUUUUACGUCCGUACACCUUGCGC
Bce-1-1     CGCGUCGCUGAGACAACAGUGACCCGGUUUGGCGACCGGAGAUGCUCCAAGGCGGACAACCGCCUUCGCGCGGUUUUUUUACGUCCGUACACCUUGCGC
Bps-5-2     ACGUCGUUGAGACAACAGCGACCCGGGGUGACAGCCGGAAUAGUUCCAAGGCGGACAACCGCCUUCGCCGCGGUUUUUUUUACGUCCGUACACCUUGCGC
Bth-1-2     AGCGUCGCUGAGACAACAGCGAUCCGGGUUGGAAGCCGGCAAAUUCCCGAGGCGGACAGCCGCCUCAGCGCGGUUUUUUUGCGUCCGCACACUAAGCGC
Bps-7-2     UGACACAGAAGCGACCGGGUUUGGAAGCCCGAUUGUUCCAAGGCGGACGACCGCCGCAGUGCGGUUUUUUUACGUCCGUGUGCCUUCGCGCGCCUCUAC
Bdo-1-2     UGAGACAACAGCGACCGGGAUUUGCAGCCCGACAGUUCCAAGGCGGACGACCGCCGCAGUGCGGUUUUUUUACGUCCGUGUGCCUUCGCGCGCCUCUAC
Bps-9-1     UGAGACAACAGCGACCGGGAUUUGCAGCCCGACAGUUCCAAGGCGGACGACCGCCGCAGUGCGGUUUUUUUACGUCCGUGUGCCUUCGCGCGCCUCUAC
Reu-1-2     UCGUCGCUGCAUAGUGGCGACCGGGUUUAGCAGCCUGAGUGAGAGGGAAGCCGACCGCCUUCUGGCGGUAUCCUUACAUCCGCCAUACUGCGCACGCCU
Cvi-1-2     UGUUCAAUAGCGCGACCGGGUUUGGCGACCUGAACAGUAAGGCGGACAGCCGCCUAUUGCGGCUAUUUUUACGUCCGUCACACACCGUUGGUGCGUCCU
Bps-15-2    CUGAAACAACAGCGAUCGGGUUUGGCGACCUGGAUUCCAUAGGCGGACAACCGCCGCAGUGCGGUUUUUUUACGUCCGUAUGCCUUUGCACGCCUGUAC
Rpi-2-1     AUUCCCCCGCUGCCGCACCAACGGCAGCCUGGUUUAGCGACCUGAAACACCAAGGCGGACGAUUGCUGCAUGCAGUCGCACUGCUGCUUCCGUUCGCGC
Rpi-1-2     AUUCUCUCGCUGCCGCACCAACGGCAGCCUGGUUUAGCGACCUGAAACACCAAGGCGGACGAUUGCUGCAUGCAGUCGCACUGCUGCUUCCGUUCGCGC
env-48      .....................................................................................CUGCUUCGUUCGCGC
env-49      CUCGUGUCGCUGCACAUCAGCGAUGCGGGUUUGGCGACCCGGAUUCAGAGGCGGACGGCCGCCUAGAUGCGGUUUUUUUACGUCCGUCGCAUGGCAUCG
Bce-1-2     UGAAACAGCAGCGGCCGGGUUUGGCGACCUGUUCAAUGUGAGGCGGACAACCGCCGCAGUGCGGUUUUUUUACGUCCGUAUGCCUACGCGCGCCCAUAC
Bma-10-1    CGGAAACACCGUGGCCGGGUUUGGCGACCCGAUUAUUAGGAGGCGGACGAGCCGCCGCAUGUGCGGCUUUUUCGUCUGCAUGCCUUUGCGCGCCUAUGC
Bth-2-2     CGGAGACACCGUGGCCGGGUUUGGCGACCCGAUUAUUAGGAGGCGGACGAGCCGCCGCAUGUGCGGCUUUUUCGUCUGCAUGCCUUUGCGCGCCUAUGC
Bvi-1-2     CGCACCUGAAACAACAGGUGCCGGGUUUGGCGACCCGAACGGUUAAUGCGCACAGCCGCGUUCUGCGGCUUUUUUGUGCGGUGUGCCUUUGCACGUCGG

            ....................................................................................................
            ....................................................................................................
            ....................................................................................................
```

```
env-50      CGUACCUGAAACAACAGGUGCCGGGUUUGGCGACCCGAACGGUUAAUGCGCACAGCCGCGCUCAGCGGCUUUUUUGUGCGAAGUGCCUGUGCACGUCGG
Bce-2-1     ACGGGUGCCGGGUUUGGCGACCUGCUAUGCUCUCUGGCGCAACGAAGCCGCGCCGUCACUCGACACGCGGCUUUUGUGUUUGCGUUCGCCUUCGCGCGC
Bps-3-1     CAGCGGCCGGGUUUGGCGACCUGCUAUGCUCUCUGGCGCAACGAAGCCGCGCCGUCACUCGACACGCGGCUUUUGUGUUUGCGUUCGCCUUCGCGCGCC
Bmu-1-1     CGCCUGAGACAGCAGGCGUCGGGUUUGGCGACCCGUCUAAUCAAAGCGCACAAGACCGCGCCCGCGGUCUUUUUUGUGCCGAAGGCCUUUGCGCGCCGG
Bvi-1-3     ACGUCUGAAACAGCAGACGUCGGGUUUGGCGACCUGAACAAACCAAAGCGCACACGCCGCGCCAAGCGGCUUUUUUGUGCCUGUGCCUCUGCACGCCGG
env-51      GCACUUCAGACAACGAGUGCCGGGUUUGGCGACCCGCUUCAUACAAGCGCACGGCCGCGCUAUGCGGCUUUUUUUGUGCGUGUUGCCUCUGCGCGCCGU
Rso-1-4     CCCUGAAACAGCAGGGACCGAGUUUGGCGACUCGAUUAACAACGGCGGACAGCCGCCACACGCGGCAUUUUUUUCGUCCGUCACGCCCUCGCGCGCCUA
Rpi-2-2     UGUCGCACCAACGGCAGCCGGGUCUGGCGAUCCGACCGCGCAACACUCAAAGAAGACGGACAGCUGCUGAUGCGGUUGCGUCCGUACACGCUCGCGCUA
Rpi-1-3     CCCCCGCUGCUGCUCCAACGGCAGCCGGGUCUGGCGAUCCGCGGUUUUUCCAAGAGGGGCUGAUGUCAUGCACGAUCAGCCCUGAGCACUCGCUCGCGC
Rso-2-3     CCGCACCAACGGCAGCCGGGUCUGGCGAUCCGCGCUGUUCUCUCCAAGAGGGGCUGACGCUUCAUGCGGUCAGCCUCAACGCACUCGCUCGCGCGUUUG
Rso-1-5     CCGCACCAACGGCAGCCGGGUCUGGCGAUCCGCGCUGUUCUCUCCAAGAGGGGCCGACGCUUCAUGCGAUCAGCCUCAAUGCACUCGCUCGCGCGUUUU
Rpi-1-4     CCAUCCCGCUGCCGCACCAACGGCAGUCGGGUUUGGCGACUCGACAUACUCACAACGAGCAUCCGCUUGGCGGAUACGCUCGGGCACACUCGCGCAUGC
Rpi-2-3     GCGAUAUAAUUCGCGCGCUGUUCAUUCGAGCAGCCGGGUUUAGCAGCCCACCAGAUGUGCCAUCUAGACGGACAAUGUCCGGUGCUUGCACACGCCUAU
Rpi-1-5     GCGAUAUAAUUCGCGCGCUGUUCAUUCGAGCAGCCGGGUUUAGCAGCCCACCAGAUGUGCCAUCUAGACGGACAAUGUCCGGUGCUUGCGCACGCCUAU
Rpi-2-4     ACCAACGGUGACUGGGUUUAGCAGCCCAUCCCUUUUUCCCAGACGGACAGCCGCCUUUCGCGCGGCAUUUUUGUGUCCGGUGCUUGUGCACGCCUAUGU
Rpi-1-6     CACCAACGGUGACUGGGUUUAGCAGCCCACCCCUUUUUCCCAGACGGACAGCCGCCUCCGCGCGGCAUUUUUGUGUCCGGUGCUUGUGCACGCCUAUGU
Rpi-2-5     UCCGCUGCCGCACCAACGGUAGCCGGGUGUAGCGACCUGAUCUAUGCAGUACCUCUGGACGGCACUCGCACAGCGAUUCGCCGGCAGGCACUCGCGCGC
env-52      UUGGCGAGACGCCCCGCAAAUCAGACCUGCGCCGCCGGCCCCAUCGCUAUAAUCCCGCCGCUGCCGCGCCAAGGGCAGUGUGUCGGCAGGCACGCGCGG
Pen-1-30    UAGUCUCGCCGGGUCGUUGCCAAAUCAACGACCGGGCCUGCAAGCCCGUCCGCAAAAGAUUCACUGCCAAUACAGCAACCUGUAAUCGUGUUGCUUGUU
Ppu-4-27    CGGCCUAGUCUGGGCCCGUCGUUGCCAAAUCAGCGACCGGGCCUGCAAGCCCGCAGACGAAUCCCAGCGCUAUCCGAGGUGCCUAGUGUCGGCAGCUUC
Pfl-2-29    CCGCUGCAAAAUCAGCGGUCGGAUGUGGAAGUCCGGUGAAUCCCGAAAGCGCAUGUGCGCCUGUAAACACUUGUUGGCGCCUUUUAUGCCAACAUUGUU
Sgl-2-16    UUAACAAAACGGGUGCCGGGUUUGACAGCCCAAGUCAAAGAGCGGAUAUAACACGCCGCUUUAGUGCGGUUUUUUACGUCCGAAAUCAGCGUUACGCCC
Sgl-2-17    CCCCUCAAUGGGCAACCGGGUUUAGCAUCCCGAGUGAAGAAGCGAACAUAUGCCGCUUCAAAGUGCGGUUUUUUUACGUCCGAAACCUGCGUUGCGCCC
Eco-8-5     CCCACAUGGCGACCGGGUUUAGCAGCCUGAAUACAUCUGGCGGACAGCCGCCCACAUCCGAUAAGCGGUUUUUUUGUGUCCGUAAACCUACCCAUACCC
Eco-3-14    GCCCACAUGGCGACCGGGUUUAGCAGCCUGAAUAAAUUGGCGGACAGCCGCCCAUAUCCGAUAAGCGGUUUUUUUGUGUCCGUAAACCUACCCAUACCC
Sen-3-1     GCACGUGCAAAUUCAGGUGCCGGGGUUUAGCAGCCUGACUAUCAACGCGGACAGCCGCCGUUUUUCGUAGUGCGGUUUUUUUACGUCCGUAACUGCCCC
Sgl-2-18    CAAAAGUCGGUGUCGGGUUUGACAGCCCGAACCAUACAGCGGACGGCCGCUAUUUCUCCUUGCGGCUUUUUUACGUCCUUCAAUACCACAGUUGCGUCC
Eph-3-1     CAAAUUCUGCGACCGGUUUUGACAGCCUGAAUGUUAGUGCGGACAACCGCAGAUUUCCGAUAUUGCGGUAUUUUUGUGUCCGUAAACCGCGUUACGCCC
Sen-8-4     AAAUCCUGCGACCAGGUUUGACAGCCUGAAUGUACAUGCGGACAACCGCUAACUCCGUAAUGCGGUAUUUUUAUGUCCGUAGUAAACCACGUUACGCCC
Aba-1-6     UGCCUGCUGGCCUACAUUGCCAGUCGAUUUUGACAGAUCGAUAUAACACACAAGACAUCAAAAAGUUCUCUUUUUGGUGGCGAACUCGUUCGUCCCCCU
Aba-1-7     CUUAUGAUGCUCGCGCUGACCAAUUUUGUCAGUCGGUUUUAGCAGACCGUCUACCUAAGUGCACUAAAAACACGUUUUUUGGUGUGACGCCCCUCGUGC
Aba-1-8     CUUUGCCUGCUGGCCACAUUGCCAGUCGGUUUUAGCAGGCCGUAUUUACCCAAAGCACACGAAAAAGUCUCUCUUUUUUGUGGGCAUCCCUACGUGCCC
Aba-1-9     UAUCCUUUGCCUGCUGGCCACAUUGCCAGCCGGCUUUGACAGGCUGAUUCAAACGAGCAUUAAAGGUUUUAUACUUUUAGUGUGUACACCCUCGUGCCC
Asp-1-1     UAUCAUUUCGCUGCUGGCCUACAUUGCCAGUCGGUUUUAGCAGACCGUUUCCUAAGAGCACUAAAAAAUCUCAAUUUUUUGGUGUGUAUCCUCGUGCUC
Aba-1-10    UACUAUUCUUCGCAUGCUGGCCACAUUGCCAGUCGGUUUUAGCAGACCGUUCCGAAAAGCAUUAAAAGAGCAAUCUUUUAGUGACGAACCCUCGUGCCC
Aba-1-11    CCUUUGCCUGCUGGCCUACAUUGCCAGUUGGUUUUAGCAGACCAUUAGCCAAAGAACACGAAAAAGUAAUCACUUUUUUGUGGGAAUCCCUGCGUGCCC
Aba-1-12    CCUUUGCCUGCUGGCCUACAUUGCCAGUCGACUUUGACAGGUCGGAUAGCCAACGCACACGAAAAAGUCUCUCUUUUUUGUGGGCAUCCCUGCGUGCCC
Aba-1-13    AUCCUUUGCGUGCUGGCCUACAUUGCCAGUUGGUUUUGACAGACCAUUUCUCAGAGAUAUUAAAAGAUCUUCUUUUAAUGUCUAGCUUCUCGUUCCCUU
Aba-1-14    UGCUGGCCACAUUGCCAGCCGGUUUUAGCAGACCGCAUAGUAAGAGCAUCAGAUUAUUCCUUCUGAGGAGUAGUCUUAUGUGUAUCAAUCGUCCCUCGC
Aba-1-15    UGCUGGCCUACAUUGCCAGCCGGUUGUCGCAGACCGAUUAGUAAAGGCAUCAGAUUAUUCCCUCUGAGGAGUAGUCUUGUGUGUACAAUCGUCCCUCGC
Aba-1-16    GGCCUACAUUGCCAGUCGGUUUUGACGGACCGAGUUUGUAAGACCACAUCAGAGCUAUUCCUUCUGAGGGAUACUCAUAUGUGCACACCCCGUUCCCUC
Aba-1-17    UGGCCUACAUUGCCAGACGGUUGUCGCAGACCGUAUUAAGACCGCAUCAGGAUUAUUUCUUCUGAGGAAUAGUUUUGUGCGUAAACUCCCAGUUCCCUC
Aba-1-18    UGGCCACAUUGCCAGCCGGUCGUCGCGGACCGUUGUAAGAUCACAUCAGAUUUAUUCCUUUCUGAGGGAUAGUCUCAUGUGUAAACUCCUAGUCUCCUC
Aba-1-19    GCCACAUUGCCAGUCGGUUUUAGCAGACCGUGAUGAAAGAUCGCAUCAGAGUUAUUCCUAUCUGAGGGAUACUCUUAUGUGUAAACUCCUAGUCUCCUC
Aba-1-20    GCUGGCCUACAUUGCCAGUCGGUUUUAGCAGACCGUACCCUAGCGUGUCGGGUUAUACUUCCUCAGUAUUACCCGUGUGGAACCUUUGUGCCCCCUUUU
Asp-1-2     CCGGCUGGCCCAAAUCGCCAGUGGGUUUUGACAGGCCCGUUCAAACAAAGAUGGUUCAACAAUUUAGUUUGUGAGCCAAAAACCCGUUCGUCCCCCUUU
Aba-1-21    GCCUACAUUGCCAGUCGGUUUUGACAGACCGAGUAUACAGCGUAUCAGGUUCAUACUUCCUGAGUAUGACACUUGUGCGCAACCUGCACCUCCCCCUUA
Asp-1-3     GCCUACAUUGCCAGUCGGUUUUGACAGACCGUUCAUACAGCGCAUCAGGGUAUAUAGUCUUCUGAGUAUUACCCUUAUGUGUAAAAACCUUCUCGUCCC
Asp-1-4     UGGCCUACAUUGCCAGCCGGUUUUGACAGACCGUUCAUCUAGCGCAUCAGAGUUUGCUCUUCUGAGUAUUACCCUUAUGUGUAAAAACCUUUUCGUCCC
Aba-1-22    UAUUCCGUUGCUGGCCUACAUUGCCAGACGGUUUUGACAGACCGUAACUCGUAGCGAAUUGUAGUUCUACUAUAAUUCGUUAGACCCUUGCGUCCCCUU
Aba-1-23    AACAAUAAUUUUUAAUUAUUUAUCCUUAGUUUUUAAUGAUCUAAUUUAAAACAAAAGUCAUUUUGUGUGCUUGACUGUAUGGGGCUUGGCUACUAACCU
Hin-11-1    UUUCUGAAUAACUUGGAGCGGUGAAAAGAUAGACGCUCAAGCGUCUUUUUUUAUAGCCGUAAAACAGCAAAUCUACCUUUUUCGGAAAUUUCCGAAAAA
Hin-7-2     UUCUGAAUAACUUGGAGCGGUGGAAAGACAGACGCUCAAUGCGUCUUUUUUUAUCGCCACAAAACAGCUAUUCUACCUUUUUCAUAAAUUUAUGAAAAA
Hin-10-1    CCUGAAUGAUUAGAGCAGUGAAAAAGACAGUCGCUCAAAAGCGACUUUUUUUAUAGCCGCAAAACAGCAAAUCUACCUUUUCCCCAAAUUUGGUGAAAA
Hin-12-1    UUCUGAAUAACUUGGAGCGGUGGAAAGACAGACGCUCAAUGCGUCUUUUUUUAUCGCCACAAAACAGCUAUUCUACCUUUUUCAUAAAUUUAUGAAAAA
Hin-3-3     UUCUGAAUAACUUGGAGCGGUGGAAAGACAGACGCUCAAUGCGUCUUUUUUUAUCGCCACAAAACAGCUAUUCUACCUUUUUCAUAAAUUUAUGAAAAA
Hin-8-1     UUCUGAAUAACUUGGAGCGGUGGAAAGACAGACGCUCAAUGCGUCUUUUUUUAUCGCCACAAAACAGCUAUUCUACCUUUUUCAUAAAUUUAUGAAAAA
Hin-4-1     UUCUGAAUAACUUGGAGCGGUGGAAAGACAGACGCUCAAUGCGUCUUUUUUUAUCGCCACAAAACAGCUAUUCUACCUUUUUCAUAAAUUUAUGAAAAA
Hin-6-1     UUUCUGAAUAACUUGGAGCGGUGGAAAGACAGACGCUCAAUGCGUCUUUUUUAUUGCCACAAAACAGCAAUUCUACCUUUUUCAUAAAUUUAUGAAAAA
Hph-1-1     AAAUUAUAGUCGCCUAAAUGGCGAUUUUUUUAUAGCCGAAAAUCAGCAAAUCAAACCCUUUAAAGGGGUACAUCAAUUUGAUACCCCCCUUUCAAAAGU
Hso-1-2     AAAUGAUAGUCGCCUAAAUGGUGGCUUUUUUUAUAGCCGAAAAUCAGAAAAUCAAACCUUUAAAAAGGGUAACUCAAUUUGAGACCCCCUUUAAAAAGU
Aba-1-24    GAUAAUUUAAUAAAUCUAAAAAAUAAUAAUAAAUUUAAUAACUUACGUUAUUUUUCAAUAUUGACACAAGCAGUCUUCCAAAUUAUUCUCGUCUGCUUA
```

alignment positions 100 · · · 259

```
Aba-1-1  UCUUUCUG.GUA.G.A.GU..GAAAA.GG.GG.AUAUC...UUUUG....GAUGU.GCCAG....UUUCUAGAUG..........UCUGGUC.UGCU.A.ACCCU.UCUU...C....AC..UC.U.AU.CAUUGUCAUUAUU...UUUAGAUAAUAAAC
Aba-1-2  CUGUUAUG.GUG.G.G.UU..AGGC..GG.AA.GUGC....UUCG......GCAC.GCUAG....ACCCUUGAG...........ACUAGUAAGACC.A.AUUCC..GUU...U....AA..CC.U.GC.CACCCUAAUUGAUUGGUCUCAAUUUUGGUG
Hin-3-1  UCUCAAUG.GUA.G.A.GC..GUAA..UG.AG.CCGUC...UAU......GACGG.GCUGUC...UUCCUGUGAU..........GGCAGUU.UUCC.A.CCUUG..CUA...C....GC..UC.U.AC.CGCCC..GACCGUGGAAAGUCUAGCGGUAG
Hin-5-1  UCUCAAUG.GUA.G.A.GC..GUAA..UG.AG.CCGUC...UAU......GACGG.GCUGUC...UUCCUGUGAU..........GGCAGUU.UUCC.A.CCUUG..CUA...C....GC..UC.U.AC.CGCCC..GACCGUGGAAAGUCUAGCGGUAG
Hin-7-1  UCUCAAUG.GUA.G.C.GC..GUAA..UG.GG.CAACC...CUUAGC...GGUUG.GCUGCU...UUCCUGUAAA..........GGCAGUU.UGUC.A.CCCCG..UUA...C....GU..GC.U.AC.CGCCC..GACCGUGACAAGUCUAGCGGUAG
Hso-1-1  UCUCAAUG.GUA.G.A.GC..GUAA..AG.GG.CAACCG..CUUG....CGGUUG.GCUAUG...UUCCUUUGUA..........CGUAGUU.UCCU.A.CCCCU..UUA...C....GC..UU.U.AC.CGCCC..GACCGUAGGAAGUCUAGCGGAAA
Hso-2-1  UCUCAAUG.GUA.G.A.GC..GUAA..AG.GG.CAACCG..CUUG....CGGUUG.GCUAUG...UUCCUUUGUA..........CGUAGUU.UCUC.A.CCCCU..UUA...C....GC..UU.U.AC.CGCCC..GACCGUGAGAAGUCUAGCGGAAA
Pmu-1-1  UCUCAAUG.GUA.G.C.GU..GCGA..UU.GG.CAACG...UUUAAU...CGUUG.GCUGUG...UUCCGUAUGU..........CGCAGUU.UUCC.A.CCCAG..UCG...C....AC..GU.U.AC.CGCCCUAAAGCGUGGAAACUUUUAGCGGUA
Hin-3-2  UCUCAAUG.GUA.G.C.GU..GUAG..CG.GU.AAAGG...UUCGC....CCUUU.GCUGUG...UUCCAAUUGU..........CGCAGUU.UUCG.A.GACCG..UUA...C....AC..GU.U.AC.CGCCAA.AGCCUCGAAACUUUACGCGGUAA
Hin-2-1  UCUCAAUG.GUA.G.C.GU..GUAG..CG.GU.AAAGG...UUCGC....CCUUU.GCUGUG...UUCCAAUUGU..........CGCGGUU.UUCG.A.GACCG..UUA...C....AC..GU.U.AC.CGCCAA.AGCCUCGAAACUUUACGCGGUAA
Mha-1-1  CGUCUAUG.GUA.G.C.GU..GUAG..CG.GG.CAAGG...UUCGC....CCUUG.GCUGUU...UUUGGUUCGCCGUUUGU...AGCAGUUUUCUC.A.CCCCG..UUA...C....AC..GC.U.AU.CGCCCUGCACCGUGAGAAGUGUAGCCGAUA
Mha-1-2  CGUCUAUG.GUA.G.C.GU..GUAG..CG.GG.CAAGG..UUUCGC....CCUUG.ACUGCU...UACCUGAUGU..........UGCAGUU.UUCC.A.CCCCG..UUA...C....AC..GC.U.AU.CGCCCUACAUUGUGGAAAAUGUUAGCGAUA
Pfl-2-1  UUGCAGUG.GCG.G...CUGUGUGC..AG.GG.UCGCC...UUCG.....GGCGU.GCUGAG...UUGGGUUGUU..........CUCGGUU.GACC.A.AGCCU..GCG...C..ACAG..UC.C.GC.CUCCCCAUCGUUUGGUCACGGUGUCGGCGG
env-1    UUUUUAUG.CCU.G.G.GC..GUAG..UG.GG.AGUCC...GCAA.....GGACU.GCCGGG...UUCCGUGGAU..........ACCGGUU.GGCU.A.ACCUG..CUA...C...UGC..CC.A.GG.CACCCGUUUAGCC...ACGGGUCUUUGGGU
Dps-1-1  UUUCUAGG.GCG.G.G.CU..AUA...UGUGG.GAGC....UUCG......GCUC.GCUGG....UUCCGGUGA............CCAGCC.UACC.A.ACCCA...UA...U....AG.UCC.C.GC.CCUUUUUUGUUUGG..UAGCAAGAAAAGG
Dac-1-1  GUUU..CG.GCG.G.G.GC..UGUG..CA.GG.ACCUCC..GCAA....GGGGGU.GCCGG....CUCCUUUGC............CCGGCC.CGCC.A.ACCUG..CAC...A....GC..CC.U.GC.CACCCGUUGGCGGCGGCAAGCUCAGCAUAG
Sgl-2-1  CUAUAAUGGUGA.G.G.CG..UAAC..GA.GG.GAGC....CUCUGC....GCUC.GCCGGG...UUCUCUGAC...........ACCGGUC.UACU.A.ACCUC..GAU...A....CG..UC.U.CUCCACCCCGUUUAGUA...GCGGGGAGCGAGG
Pfl-2-2  GCUUUAUG.GCG.G...CUG.UGUU..UG.GG.ACGC....UUCG......GCGU.GCCUGGAG.UUCUCUCUCU...........CUGGUU.GACC.A.ACCCG..AAC...A...CAG..UC.C.GC.CUCCUA.UCGUUUGGUCAUGAUGUUGGCGG
Sbo-1-1  UAUCAAUG.GUG.G.C.GU..UAGU..GG.GG.CUACG...UUCG.....CGUAG.GCCGGU...UUCCUUUGUG..........UCCGGUAUUUCC.A.CCCCU..GCU...A....AC..GU.C.AC.CGCCAUUUCAGAGCGUGGAAACUCAGGCGG
Pna-1-1  CAGUUUUG.GCG.G.C.UC..GGA...UG.GG.GGAGC...CUCG.....GCUCC.GCCGGU...UUUCGCAAGAGUG.......CCCGGUA.GACC.A.ACCCG...UU...C....GA..GC.U.GC.CGCCC..CCGAUUGGUCUCGGGUGCGGCGG
Pna-1-2  AGUUUUUG.GCG.G.C.UC..GAA...UG.GG.AGAGC...GCAA.....GCUCU.GCCGGU...UUCCGCAAGGAUGU......CCCGGUU.CGUC.A.ACCCG...UU...C....GA..GC.U.GC.CGCCC..UCAUUUGACGAUGGGUGCGUCGG
Xfa-1-1  CGUCUAUG.GCG.G.G.UG..GCGU..GG.AG.AGACU...UUCG.....AGUCU.ACCGG......UCCUGGGG...........UCGGUC.UUCU.A.ACUCC..GCG...U....CG..UC.C.GC.CUCCAU.GUUUAGAAGCGUGGCGGCGGACU
Sma-1-1  CCUUCCUG.GCG.G.G.CG..GUGC..GU.GG.GGGCC...CUCG.....GGCCC.ACCGG......UCUAUGGUGCUAUUUCAUCCCGGU..UUGC.A.GCCAC..GCA...U....CG..UC.C.GC.CACCUCUUCGCGGUGGCGCUGUUAAGCACA
Dno-1-1  UUGUUAUG.GCA.G.U.GU..GCGU..GG.GA.GAACC...GCAA.....GGUUC.GCCGU....UUCUUUCGU...........AGCGGUAGUCUC.A.AUCCU..GCG...U....AC..AC.U.GU.CGCCCUCUUGCUUGAGACCAAUGGCGGCAG
Dno-1-2  UUGUUAUG.GCG.G.C.AU..ACGU..AG.GA.GAACC...GCAA.....GGUUC.GCCGU....UUCUUUCGU...........AGCGGCAAGGCU.A.AUCCU..GCG...U....AU..GC.U.GU.CGCCCUCUUAUUUAGCCUUAACGGCGGCAG
Dno-1-3  U...UAUG.GCA.G.C.AU..ACGU..GG.GA.GAACC...GAAA.....GGUUC.GCCGU....UUCUUUCGU...........AGCGGUAGUUCC.A.AUCUC..GCG...U....AU..GC.U.GU.CGCCCUCUUAUUUGGAACUAAUGGCGGCAG
Dno-1-4  U...UAUG.GCA.G.C.AU..ACGU..GG.GA.GAACC...GAAA.....GGUUC.GCCGU....UCCUUGUGU...........AGCGGUAAGGUC.A.AUCCU..GCG...U....AU..GC.U.GU.CGCCC..UUAUUUGACCUUAAUGGCGGCAG
Esp-1-1  AGAUCAUG.GCG.G.C.GC..UGGU..GG.AG.CAGC...CGAAAA.....GCUG.GCCGU....UUUUAUCGUG..........UACGGUAUUCCU.A.CCUCC..GUC...A....GC..GC.U.AC.CAUCCCUGAGCGUCGUAACUCGUGUGGUGG
Plu-1-1  CCUCUAUG.GCG.G.U.UC..AGGC..AG.GG.GAGGU...UAAC.....ACCUC.GCCGG......AAAUGAAC..........GCCGGUC.UGCA.A.ACCCU..GUU...U....GA..AU.C.GC.CACCACCAAAGAGGGAAGCAGGAAUAUGAA
Xax-1-1  GUUCUAUG.GUG.G.G.CG..GUGC..GC.GG.AGGCU...GCAA.....GGCCU.GCCGGU...UAUCGCUCUUUUCCUA....UCCGGUUUUGAU.A.GCCGC..GUA...C....CG..UC.C.AC.CACCUU.UCCUGCGAGUACACUCGCUCCUG
Aba-1-3  CCUUUAUG.GUG.A.G.GC..UAGG..AG.GG.GCACG...CUUG.....CGUGC.GCUGGU...UUCCUUUUGU..........ACCAGUC.UGUC.A.ACCCU..UCU...A....GU..CU.U.GC.CACCA..UUAUUUGACAGUAAUUGGUAAGA
Xfa-3-1  GAUUUACG.GUG.G.G.GC..AGUC..AG.GA.AGACC...GCAA.....GGUCU.GCCGG....UUCAUGCGCAAGCUCCGUU.CCCGGUU.UGCC.A.AUCCU..GCC...U....GU..CC.C.AU.CACCA..UGGGGUUUGGCAACGUCUGGUGA
Xfa-1-2  AGUUUAUG.GUG.G.G.GC..AGUC..AG.GA.AGACC...GCAA.....GGUCU.GCCGGU...UUAUGCGCAAGCUCCUUU..CCCGGUU.CGCU.A.AUCCU..GCC...U....GU..CC.C.AU.CACCA..UGGGGUUUAGCGACGUCUGGUGA
Sma-1-2  CGGCUAUG.GCG.G.G.CG..GGGC..GU.GG.GGGC....CUUGU.....GCCC.GCCGGUUUCUUCCUAAUAG..........CCCGGUU.UUCG.A.GCCAC..GCU...C....UG..CC.C.GC.CACCUUUAUCGGUGGCGGCUUCCGCCUGCU
env-2    CGGCUAUG.GCG.G.G.CG..GGGC..GU.GG.GGGU....CCUGU.....ACCC.GCCGGU...UCCCAACAU............CCGGUU.UUCG.A.GCCAC..GCU...C....UG..CC.C.GC.CACCUUUAUCGGUGGCGGCGUCCGCCUGCA
env-3    CGGCUAUG.GCG.G.G.CG..GUGC..GUUGG.GGGCG...UUCG.....CGCCC.GCCGG......CUUGAGGCCAA.......CCCGGA..UUCG.A.ACCAC..GUA...C....CG..CC.C.GC.CACCUU.UCGGGUGGCGGCUACCGAUUGCC
Sma-1-3  CGGCUAUG.GCG.G.G.CG..GUGC..GU.GG.GGGCU...UCCG.....GGCCC.GCCGGAC..UGACAUCAAC...........CCGGUG.UUCG.A.ACCAC..GUA...C....CG..CC.C.GC.CACUUUUAUGGGUGGCGGCUACCGAUUGCC
Sma-1-4  CAGCUAUG.GCG.G.G.CG..GUGC..GU.GG.AGGC....CUUGC.....GCCU.GCCCGG...UCAUCCACAC..........ACGGGGU.UUCG.A.ACCAU..GCA...C....CG..UC.C.GC.CACCUUUAUCGGUGGCGGCUUCUGCCUGCA
Plu-1-2  GAGUAAUG.GUG.G.U.UC..AGGC..GG.GG.GAGGC...UUCG.....GCCUU.GCCGGA...AGGUAGCUA............CCGGUACUGUG.A.ACCCU..GUC...U....GA..AU.C.AC.CAUCAUCAAUUUUCUCAAUUAAUGGAAGGG
env-4    CAGUUAUG.GGC.G.G.UC..GUG...UG.GG.GCAUC...UUCG.....GGUGC.GCCGG....UUCCUAUUC............CCGGUC.UUCC.A.ACCCG...CA...C....GG.CCU.G.CC.CUCCCCGUUUGGAAGCGGGGAAGGCGGAAA
Aba-1-4  CCAUUAUG.GUA.G.GGUG..AAAG..UGGGG.ACGC...CUCUGU.....GCGU.ACUGG....UUCUUGAG.............UCAGUC.UGCU.A.CUCCA..CUU...U....UG.UCC.U.GU.CACCA..UUACUUAAUUUACAUCUGUUCCA
Bph-1-1  UUUCUAUG.GGC.G.G.GCUGUGGU..GG.AG.GAGCC...GCAA.....GGCUC.GCCGGA...UUGUUUCAAG..........CCCGGUC.UGUC.A.ACUUC..GCU...A.CAUGC..CC.G.CU.CACCCUCUUUGCAAGCGAGUGUCGGGCGAU
Bxe-1-1  UUUCAAUG.GGC.G.G.GCC.GUGG..UG.GG.GAGCC...GAAA.....GGCUC.GCCGGU...AUGUUUUUGG..........CCCGGUC.UGCU.A.ACCCG..CUA...C..GUGC..CC.G.CU.CACCCUCCUUGCAAGCGAGGGUCGGGCGAU
env-5    UUUCAAUG.GGC.G.G.GCC.GUGG..UG.GG.GAGCC...GCAA.....GGCUC.GCCGGU...UUGUUUUCGA..........CCCGGUC.UGCU.A.ACCCG..CUU...C..GUGC..CC.G.CU.CACCCUUUUCCAAGCGAGUGUCGGGCGAUC
env-6    UGUCAAUG.GUG.G.GUCG..GGGC..GU.GG.GAGCC...UUAC.....GGCUC.GCCAGU...CGUCAUUAG............CUGGUACUGCA.A.ACCAU..GUU...C....CG.AUU.C.AC.CACCAAAGGAAAUGGAGAUGGAAAAACAGG
Spr-1-1  AAACAAUG.GUG.G.C.UC..GGGU..GG.UG.CUUCC...UUCG.....GGAAG.GCCGGG...AUCCGUUGGG..........GCCGGUA.CGCU.A.ACACU..GCU...C....GA..GC.U.AC.CACCCC.GAGUUUAGCGUCUCUUCAGGUAG
Pfl-2-3  CCUUUGCG.GCG.G.U.UA..UGCG..CG.GG.CACGC...UUCG.....GCGUG.GCUGAG...UGCUCUGGUC...........UCAGUCCUCCU.A.CCCCG..CGC...A....UG..GC.U.GC.CACCCAAUCCUGUAGGAGGGAAACGGUGUG
env-7    CAGUCGCG.GCG.G.C.UA..UGCG..CG.GG.CACGU...UUCG.....ACGUG.GCUGAG...AUCCCUGGGUA.........CUCGGU..UCCU.A.CCCCG..CGU...A....UG..GC.U.GC.CACCCAUGCCCGUAGGAAGGCUGUUGGCAG
Pfl-2-4  UCUUUAUG.GCG.G.C.UA..UGUA..CG.GG.CGGAC...UUCG.....GUCCG.GCCGGG...UUGGUUUCCUU.........ACCGGUAAUCUC.A.CUCCG..UAC...A....UA..GU.U.GC.CACCU..CAUUUCUCGAGUGAGAUCGGAAA
Msp-1-1  GCGUUAUG.GUG.G.U.UGU.GCGU..GG.GG.AGAC....UUCG......GUCU.GCCAGG...UACCUUGUG...........ACUGGUC.UGCU.A.ACCCU..GCG...U...GCA..GC.C.AC.CACCC..AUGUUUAGCAGUGUGGGUUGUGG
Msp-1-2  CCUUUAUG.GUG.G.C.UGU.GUGU..GG.GG.AGAC....UUCG......GUCU.GCCAGC...UUUCGUUUUG..........ACUGGUC.UGCU.A.ACCCU..GUA...C...GCA..GC.C.AC.CACCU..AUAUUUAGCAGUGUAGGUAGUGG
env-8    CCUCUUUG.GUA.G.U.GU..GCGU..GG.GG.AGACC...UUCG.....GGUCU.GCCGGUUU.UGCCUUGUGUU.........GCCGGUC.UGCG.A.ACCCC..GCG...C....AU..GC.U.GC.CACCAAUUUAUUUCGCAGUGAAUUGUGGCA
Aba-1-5  CUAUUAUG.GCG.A.AAUG..AAAG..AA.GG.ACGC....CUCGU.....GUGU.GCUGGG...UACUCAAAG............CCAGUC.UGUU.A.ACCUU..CUU...U....CG.UUU.C.GC.CGCCA..UUCUUUAAGAAUAACUGGGUGGG
Pfl-1-1  GCGUUAUG.GCG.G.G.UU..GCGU..GG.GA.GAACC...UUCG.....GGUUC.GCCGG....UUCCUUGAUU..........CCCGGUC.CGCC.A.AUCCU..GCG...C....AA..UC.C.GC.CACCCUGAUCUGCUUGGCGGCAGACGGUGA
Psy-1-1  GCGUUAUG.GCG.G.G.UU..GCGC..AG.GA.GCACC...CUCG.....GGUGC.GCCGGU...CUCCUUGUAC..........GCCGGUC.CGCC.A.AUCCU..GUG...C....AA..UC.C.GC.CACCCUAAUCUGCUUGGCGGCAGACCGUGG
Pae-5-1  CCUUCAUG.GCG.G.A.UC..GCGU..GG.GG.AGACC...CUCGU....GGUCU.GCCGG....UUCCGUUUC............CCGGUC.UGUC.A.ACCCU..GCG...C....GA..UC.CUGC.CUCCA..UCGAUUGACAGCGAAGGCGGCAG
Pae-2-1  CCUUCAUG.GCG.G.A.UC..GCGU..GG.GG.AGACC...CUCGU....GGUCU.GCCGG....UUCCGUUUC............CCGGUC.UGUC.A.ACCCU..GCG...C....GA..UC.CUGC.CUCCA..UCGAUUGACAGCGAAGGCGGCAG
Pae-6-1  CCUUCAUG.GCG.G.A.UC..GCGU..GG.GG.AGACC...CUCGU....GGUCU.GCCGG....UUCCGUUUC............CCGGUC.UGUC.A.ACCCU..GCG...C....GA..UC.CUGC.CUCCA..UCGAUUGACAGCGAAGGCGGCAG
Ppu-4-1  GUGUAAUG.GCG.G.C.UG..UACC..CG.GA.AGGC....CUCGU.....GCCU.ACCGAG...UUUUCCUGAAUGCA......CUCGGGU.UUCC.A.CUCCG..UGU...A....CA..GC.U.GC.CACCCU.UCCUGUGGAAACGGAAGAGGCAG

         ......<<.<<<.<.<.<<..<<<<..<<.<<.<<<<<<<........>>>>>>>.<<<<<.........................>>>>>..........>>>>..>>>...>....>>..>>.>.>>.>>............................
         ......?1.222.2.2.22..2222..22.22.2222220........0222222.22222.........................22222..........2222..222...2....22..22.2.22.1?............................
         ●●●Y●AUG-GYR-G-●-Y●--●R●●--●G-GG-●●RYY---YU●R-----RRY●Y-GCCGG○---YYYY●●●●●○---------○CCGGU●-Y●YY-A-RCCY●--●●Y---●----●R--●Y-Y-R-C-CACC●○○●●●●●Y●●●R●●●●●●●●R●●●●
         5′                                                                                                                                       3′
```

```
Dps-1-2   GCUUUUGG.GUG.A.C.UG..UGU....G.GG.AGACC...UUCG.....GGUCU.GCCGA....UUCCGUUUG..........AUCGGUC.GUCC.A.ACCC....GC...A....UA..GU.C.GC.CCUUUU.UUUUGUUUGGACGCAAAAAAUGG
Asa-1-1   GCGUUAUG.GCG.G.U.UC..GGGU..GA.GG.GCCCC...GCAA.....GGGGC.GCUGGA...CUCCAAGGUC.........UCCAGUUACGCC.A.ACCUC..GUC...C....GG..GC.U.GC.CACCAGAAGAUUGGCGUCUCUGUGGCAG
env-9     GUGUUAUG.GCG.G.U.UC..GGGC..GA.GG.GCCUC...GAAA.....GGGGC.GCUGGG...UUCCGUAGUG.........ACCAGUUACGCC.A.ACCUU..GUC...C....GG..GC.U.GC.CACCAGUUGGAGAUUGGCGUCUUCCGGUGG
Bsp-1-1   CUUCUAUG.GGU.G.G.CGC.GAGU..GG.AG.AGGC....UUCG......GCCU.GCCGGG...UUCCGUAUGC.........CCCGGUC.UGUC.A.ACUUC..GCU...C..GUUG..CC.G.CC.CACC...CCGUUUGACAGCGGGGCGGUGCG
Pen-1-1   GCGCAAUG.GUG.G.C.UA..CGUG..CG.GG.UGACC...UCUU.....GGUCA.GCCGGG...UUUUCGCCUCGUAAUGC...CCCGGUC.UUGC.AGGCCCG..UAC...G....UA..GC.U.GC.CACCCU.UCACCUGCAAGUGGAGCGGCGGC
Cvi-1-1   UUUCUAUG.GGC.U.G.GCCAUGGC..AG.GA.GCGUG...CUUG.....CACGC.GCCGGU...CACCUUGAGU.........CCCGGUC.CGCC.A.AUCCU..GCC...AUUGUGC..CA.G.CC.CACCC..CGCUUGGCGGCGGGAGGCUGGUG
Sdy-2-1   UAUCAAUG.GUG.G.G.CU..GUAU..GG.GG.CCGAC...UUCA.....GUCGG.GCUGGU...UCCCUUGAUU.........GCCAGUUGUAGC.A.ACCCU..GUA...C....AG..UC.C.AC.CGCCAGCGAGCUUGCUACCUCCUGCGGUGG
Sbo-1-2   UAUCAAUG.GUG.G.G.CU..GUAU..GG.GG.CUGAC...UUCG.....GUCAG.GCUGGU...CUCCUUUUGC.........GCCAGUUGUGGU.A.ACCCU..GUA...C....AG..UC.C.AC.CGCCAGCGAGCUUACCACCUCCUGCGGUGG
env-10    CAACAAUG.GUG.G.G.CU..GUAU..GG.AA.CUUCC...UUCG.....GGAAG.GCUGGU...UUCCUCUAAC.........ACCAGUAAGGCC.A.AUUCC..GUA...C....AG..UC.C.AC.CGCCAGCAAGAUUGGCCUCUUGGGCGGUGG
Cte-1-1   GCUUUACG.GUG.G.G.CC..GUGU..GG.GG.ACACCC..GCGA....GGGUGU.GCCGG.....UCCUACUG...........CCGGUC.GACC.A.ACCCU..GCA...C....GG..UC.C.AC.CACCU..UCUCUUGGUCGGGAAGGUGAUGG
Cte-1-2   GCUUUACG.GUG.G.G.CC..GUGU..GG.GG.ACACCC..GCGA....GGGUGU.GCCGG....UUCCUACGG...........CCGGUC.CGCC.A.ACCCU..GCA...C....GG..UC.C.AU.CACCUC.UGUUUGGCGGCAGUGGGAUGG
Sgl-2-2   UCUCUAUG.GUG.G.A.CU..GGAU..GG.GG.CAGCC...GAAA.....GACUG.GCCGGU...UGGUUGUAU...........CCGGUAACGCU.A.ACUCC..AUU...C....AU..UU.C.AC.CACCAAUCAGAGGUUAGCGUCUCUUGGUGG
Sen-8-1   GAUUUAUG.GUG.G.G.CU..GUGU..GA.GG.GCUUC...UUAG.....GAAGC.GCCGGG...UUUGAGUAG...........CCGGUAAGACC.A.ACCUU..GCA...C....AG..UU.C.AC.CACCCG.UCGAUUGGUCUCGGCAGUGGUGA
Vfi-1-1   AAAUUAUG.GUG.G.G.CC..GAGU..GA.GG.CCGA....UUCGU.....UCGG.GCCGUU...UUCCUUGUGU.........CGCGGUAGUAUC.A.ACCUU..GCU...C....GG..UU.C.GC.CACCAA.AUGAUUGAUACCUUUGAGUGGUG
Vfi-1-2   AAAUUAUG.GUG.G.G.CU..GAGU..GA.GA.GCAC....UUCG......GUGC.GCCGU....AUCCUUGGA..........AGCGGUAUGACU.A.AUCUU..GCU...C....AG..UU.C.GC.CACCCAAUAAUUAGUAUCAUUUAUUAAUAC
Sde-1-1   CUGCUAUG.GUG.G.G.CU..GUAU..GG.GG.CACCC...UUAG.....GGGUG.GCCGC....UUCCUUGUGU..........GCGGUAGUCUC.A.ACCCU..GUG...C....AG..CC.C.AU.CACCAU.AAGAUUGAGACCUUGUGAUGAUG
Sba-1-1   CUUCUAUG.GUG.A.G.CU..GGGC..GG.GG.CAACC...UUCG.....GGUUG.GCCGC....UUCCUGUGAU..........GCGGUAGUCUC.A.ACCCC..GUU...C....AG..CU.C.AU.CACCAC.UAGAUUGAGACCGACUGGUGAUG
Sba-2-1   CUACUAUG.GUG.A.G.CU..GGGU..GG.GG.CAACC...UUCG.....GGUUG.GCCGU......UCCUGUG...........ACGGUAGUGCG.A.ACCCU..GCU...C....AG..CU.C.AU.CACCAAUUGAUUCGCACUAGUUGGUGAUGA
Eco-3-1   GCAUUAUG.GCG.G.G.CU..GGC...GG.GG.GCGC....UUCG......GCGC.GCCGGU...UUCCUCUAAC.........GCCGGUAUUGCA.A.ACCUC...GU...C....AG..UU.C.GC.CACCCAUAUGAGAUUUGCAACUCAGUGGUG
Vch-2-1   GCAUUAUG.GUG.G.G.CU..GGGC..AG.GG.GCGC....UUCG......GCGC.GCCGC....UUCCUUGAGU..........GCGGUAGUACU.A.ACCUU..GUU...C....AG..UU.C.AC.CACCCAUUGAUUAGUACCUCUGUGUGGUGA
Vch-3-1   GCAUUAUG.GUG.G.G.CU..GGGC..AG.GG.GCGC....UUCG......GCGC.GCCGUG...UUCCGAUAG..........AGCGGUAGUACU.A.ACCUU..GUC...C....AG..UU.C.AC.CACCCAUAGAUUAGUACCUUUGAGUGGUGA
Vsp-1-1   GAAUUAUG.GUG.A.G.CU..GGGC..GA.GG.CAGC....UUUAU.....GCUG.GUCGC....UUCCUUUGGU..........GCGAUAGGUCC.A.ACCUU..GCU...C....AG..UU.C.AC.CACCCAAAGAUUGGACCUUUGCGUGGUGA
Vch-1-1   AAAUUAUG.GUG.G.G.CU..GGGC..GA.GG.CAGC....UUCA......GCUG.GCCGU....UUCCAAGGUU..........GUGGUAGGACU.A.ACCUC..GUU...U....AG..UU.C.AC.CACCA..GAGAUUGGUCCUGUAAACCUCCC
Vba-1-1   AUAUUAUG.GUG.G.G.CU..GGGC..GA.GG.CAGC....UUCG......GCUG.GCCGU....AACCUUGGG..........AGCGGUAGGACU.A.ACCUU..GUU...C....AG..CU.C.AC.CACCCGAAGAUUAGUCCCUUUGUGUGGUGA
Sfr-1-1   CUACUAUG.GUG.G.G.CU..GGGU..GA.GG.CAGCC...AUUU.....GGCUG.GCCGC....UUCCUUGAU...........GCGGUAGUUCG.A.ACCUU..GCU...C....AG..CU.C.AU.CACCCAACGAUUCGAACCGUUUGGUGAUGA
Vsh-1-1   CGCCAAUG.GUG.G.G.CU..GGAC..GA.GG.CAGC....UUCG......GCUG.GCCGU....UUCCUUGAGU..........ACGGUACUGCG.A.ACCUC..GUU...C....AG..UU.C.AU.CACC...AUUGAUGUCGCAGUUCUUGGUGA
Sgl-1-1   UAUCUAUG.GUG.G.G.CU..GGAU..GG.GG.GAGCC...GAGA.....GGCUC.GCCGUU...UACUUGUAU..........CCGGUUGUUAG.A.ACCCU..GUU...C....GG..CU.C.AC.CACCAA.UGGAGUGAAUGCUCAAAGGAAUU
Sgl-2-3   UAUCUAUG.GUG.G.G.CU..GGAU..GG.GG.GAGCC...GAGA.....GGCUC.GCCGUU...UACUUGUAU..........CCGGUUGUUAG.A.ACCCU..GUU...C....GG..CU.C.AC.CACCAA.UGGAGUGAAUGCUCAAAGGAAUU
Sgl-1-2   UAUCUAUG.GUG.G.G.CU..GGAU..GG.GG.GAGCC...GAGA.....GGCUC.GCCGUU...UACUUGUAU..........CCGGUUGUUAG.A.ACCCU..GUU...C....GG..CU.C.AC.CACCAA.UGGAGUGAAUGCUCAAAGGAAUU
env-11    UCUCAAUG.GCG.G.G.UC..GAGC..GG.GG.GCGUC...GCAA.....GACGC.GCCGGU...AUCCAGUAUG.........ACCGGUAAGGCU.A.ACUCC..GCU...C....GA.UUC.U.GC.CACCCGCGAGAUUAGCCUCUCCGGUGGUGG
Eco-5-1   UCUCAAUG.GUG.G.GAUC..GGGC..GG.GG.GCGUC...GUAA.....GACGC.GCCGG....UGUCAGUGAG.........GCCGGUAAUGCC.A.ACUCC..GUC...C....GG.UUU.C.AC.CACCCGUGAAAUUGGCAUUUCCGGUGGUGG
Sen-11-1  UCUCAAUG.GUG.G.G.CC..GGGC..GG.GG.GCGCU...GCAA.....AGCGC.GCCGG....UGUCGAUGAG.........GCCGGUAAGGCU.A.ACCCC..GUC...C....GG.UUC.C.AC.CACCAU.GAGAUUAGCCUCUCCGGUGGUGG
Sen-12-1  UCUCAAUG.GUG.G.G.CC..GGGC..GG.GG.GCGCU...GCAA.....AGCGC.GCCGG....UGUCGAUGAG.........GCCGGUAAGGUC.A.ACCCC..GUC...C....GG.UUC.C.AC.CACCCGCGAGAUUGACCUCUCCGGUGGUGG
Esp-1-2   CUCCAAUG.GUG.G.G.CC..GAGC..GG.GG.GCGUC...GCAA.....GACGC.GCCGGU...AUCCGAAGAG.........GCCGGUAAGGCU.A.ACCCU..GUU...C....GG.UUC.C.AC.CGCCAGCGAGAUUAGCCUCUCCAGUGGUGG
Sgl-2-4   AAUCAAUG.GUG.G.G.CU..GGAU..GG.GG.CAGCC...GUCA.....GGCUG.GCCGG......AUGACAAU.........CCCGGUACUGCG.A.ACCCU..GUU...C....AG..UU.C.AC.CACCAGUAAGUUUCGCAGCUUGUGUGGUGA
Sgl-2-5   UCUCAAUG.GUG.G.A.CU..GGAU..AG.GG.CUACC...GAAA.....GGUAG.GCCGG......ACGGUGAA.........CCCGGUAGUAUC.A.ACCCU..GUU...C....AG..UU.C.AC.CACCAAUAUAGAGAUUGAUACCUCUUGGUG
Sgl-2-6   UCUCAAUG.GUG.G.G.CU..GGAU..AG.GG.CAGCC...GCAA.....GGCUG.GCCGG......AUGGUGAU.........CCCGGUAGUUCCUA.ACCCU..GUU...C....AG..CU.C.GC.CACCAAUGAGAUUAGGAACCUCAUGGUGGU
Sgl-2-7   UCUCAAUG.GUG.G.G.CU..GGAU..GG.GG.GAGCC...GAAA.....GGCUC.GCCGG......AAGGUGAA.........CCCGGUAAGACC.A.ACUCC..GUU...C....AG..UU.C.GC.CACCC..UAGAUUGGUCUCGACGGUGGUGA
Sgl-2-8   UCUCAAUG.GUG.G.G.CU..GGAU..GG.UG.GAGCC...GAAA.....GGCUC.ACCGG......UUGGUUAC.........UCCGGUA.CGCG.A.ACUCC..GUC...C....AG..CU.C.GC.CACCCUCUAGAUUCGCGUCUUACGGUGGUG
Sdy-1-1   AAAUUAUG.GUG.A.G.GC..GUGC..GG.GG.CAGCC...GCAA.....GGCUG.GCCGGG...UUCUCGAGUG.........ACCGGUAGUGCU.A.ACCCU..GUG...C....GU..CU.C.AC.CACCCAUGAGAUUAGCACCUCUGGUGGUGA
Sbo-1-3   AAAUUAUG.GUG.A.G.GC..GUGC..GG.GG.CAGCC...GCAA.....GGCUG.GCCGGG...UUCUCGAGUG.........ACCGGUAGUGCU.A.ACCCU..GUG...C....GU..CU.C.AC.CACCCAUGAGAUUAGCACCUCUGCUGGUGA
Eco-10-1  AGAUUAUG.GUG.G.G.AC..GUGC..AG.GG.CAGCC...GCAA.....GGCUG.GCCGGG...UUCGUUAGUG.........ACCGGUAUUUCC.A.CCCCU..GUA...C....GU..CU.C.AC.CACCCUUAUGGUCGUGGAAAACCUUGGUGG
Sfl-1-1   AAAUUAUG.GUG.G.G.AU..GUGC..AG.GG.CAGUC...GCAA.....GACUG.GCCGGG...UUCUGGAGUG.........ACCGGUAUUUCC.A.CCCCU..GUA...C....GU..CU.C.AC.CACCCUUAUGGUCGUGGAAAACCUCGGUGG
Sfl-2-1   AAAUUAUG.GUG.G.G.AU..GUGC..AG.GG.CAGUC...GCAA.....GACUG.GCCGGG...UUCUCGAGUG.........ACCGGUAUUUCC.A.CCCCU..GUA...C....GU..CU.C.AC.CACCCUUAUGGUCGUGGAAAACCUCGGUGG
Eco-3-2   AAAUUAUG.GUG.G.G.AU..GUGC..AG.GG.CAGUC...GCAA.....GACUG.GCCGGG...UUCUCGAGUG.........ACCGGUAUUUCC.A.CCCCU..GUA...C....GU..CU.C.AC.CACCCUUAUGGUCGUGGAAAACCUCGGUGG
Eco-3-3   AAAUUAUG.GUG.G.G.AU..GUGC..AG.GG.CAGUC...GCAA.....GACUG.GCCGGG...UUCUCGAGUG.........ACCGGUAUUUCC.A.CCCCU..GUA...C....GU..CU.C.AC.CACCCUUAUGGUCGUGGAAAACCUCGGUGG
Eco-3-4   AAAUUAUG.GUG.G.G.GC..GUGC..GG.GG.CAGUC...GCAA.....GACUG.GCCGGG...UUCCGUGAUG.........ACCGGUUGUAGC.A.ACCCU..GUA...C....GU..CU.C.AC.CACCCAUGAGAUUGCUACCUCCGGUGGUGA
Sfl-3-1   AGAUUAUG.GUG.G.G.GC..GUGC..AG.GG.CAGCC...GCAA.....GGCUG.GCCGGU...UUCGGUAGUC.........ACCGGUUGUAGC.A.ACCCU..GUG...C....GU..CU.C.AC.CACCCAUGAGAUUGCUACCUCUGGUGGUGA
Sfl-2-2   AGAUUAUG.GUG.G.G.GC..GUGC..AG.GG.CAGCC...GCAA.....GGCUG.GCCGGU...UUCGGUAGUC.........ACCGGUUGUAGC.A.ACCCU..GUG...C....GU..CU.C.AC.CACCCAUGAGAUUGCUACCUCUGGUGGUGA
Sfl-1-2   AGAUUAUG.GUG.G.G.GC..GUGC..AG.GG.CAGCC...GCAA.....GGCUG.GCCGGU...UUCGGUAGUC.........ACCGGUUGUAGC.A.ACCCU..GUG...C....GU..CU.C.AC.CACCCAUGAGAUUGCUACCUCUGGUGGUGA
Eco-6-1   AAAUUAUG.GUG.G.G.GC..GUAC..AG.GG.GCAUC...GCAA.....GAUGC.GCCGGU...AAGGGUAGCC.........GCCGGUAACGCC.A.ACUCU..GUA...C....GU..CU.C.AC.CACCUCUAUGAUUGGCGUCUUAUGUGGUGA
Pcd-1-1   AAAUUAUG.GUG.G.G.GC..GUAC..AG.GG.GCAUC...GCAA.....GAUGC.GCCGGU...AAGGGUAGCC.........GCCGGUAACGCC.A.ACUCU..GUA...C....GU..CU.C.AC.CACCUCUAUGAUUGGCGUCUUAUGUGGUGA
Sso-1-1   GCAUUAUG.GUG.G.G.GC..GUGC..AG.GG.GCGGA...GAAA.....UCCGC.GCCGGG...UUUUGUGAUG.........ACCGGUUACGCC.A.ACCCU..GUA...C....GU..CU.C.AC.CACCUCCCUGAUUGGCGUCAGAGGUGGUGA
Sbo-1-4   AAAUUAUG.GUG.G.G.GC..GUGC..AG.GG.GCAUC...GAAA.....GAUGC.GCCGGG...GUCAUGUGCG.........ACCGGUUACGCC.A.ACCCU..GUA...C....GU..CU.C.AC.CACCUCUGUGAUUGGCGUCCCAUGUGGUGA
Sfl-3-2   AAAUUAUG.GUG.G.G.GC..GUGC..AG.GG.GCAUC...GCAA.....GAUGC.GCCGGG...UUCUAUGUUG.........ACCGGUUACGCC.A.ACCCU..GUA...C....GU..CU.C.AC.CACCUCUGUGAUUGGCGUCCCAUGUGGUGA
Eco-3-5   AAAUUAUG.GUG.G.G.GC..GUGC..AG.GG.GCAUC...GCAA.....GAUGC.GCCGGG...UUCUAUGUUG.........ACCGGUUACGCC.A.ACCCU..GUA...C....GU..CU.C.AC.CACCUCUGUGAUUGGCGUCCCAUGUGGUGA
Eco-13-1  GAAUUAUG.GCG.G.G.GC..GUAC..GG.GG.CCGAC...UUCG.....GUCGG.GCCGGA...UUCUUUGCUC.........UCCGGUGUUGAG.A.ACCCU..GUA...C....GU..CU.C.GC.CAGCCCGAGAUUCUCAACUCUGGAUGGUGA
Eco-3-6   AAAUUAUG.GUG.G.G.GC..GUGC..AG.GG.CCAAC...UUCG.....GUUGG.GCCGGG...UUCUUUGGUG.........ACCGGUU.UCUC.ACCCCCU..GUA...C....GU..CU.C.AC.CACCUAUUGCCGUGAGAAGCUUUGGUGGUG
Sen-13-1  AAAUUAUG.GUG.G.G.GC..GUGC..AG.GG.CCAGU...UUCG.....GCUGG.GCCGGG...UUCUAUGUUG.........ACCGGUAUUUCC.A.CCCCU..GUA...C....GU..CU.C.AC.CACCUAUAAGGUCGUGGAAAGCCUUGGUGG
Sen-16-1  AAAUUAUG.GUG.G.G.GC..GUGC..AG.GG.CCAGU...UUCG.....GCUGG.GCCGGG...UUCUAUGUUG.........ACCGGUAUUUCC.A.CCCCU..GUA...C....GU..CU.C.AC.CACCUAUAAGGUCGUGGAAAGCCUUGGUGG
Eco-2-1   AAAUUAUG.GUG.G.G.GC..GUGC..AG.GG.CCAAC...UUCG.....GUUGG.GCCGGG...UUCUUUGGUG.........ACCGGUAUUUCC.A.CCCCU..GUA...C....GU..CU.C.AC.CACCAAUAAGGUCGUGGAAAGCCUUGGUGG

          ......<<.<<<.<.<.<<..<<<<..<<.<<.<<<<<<<........>>>>>>>.<<<<<........................>>>>>..........>>>>..>>>...>....>>..>>.>.>>.>>...............................
          ......?1.222.2.2.22..2222..22.22.2222220........0222222.22222........................22222..........2222..222...2....22..22.2.22.1?...............................
          •••Y•AUG-GYR-G-•-Y•--•R••--•G-GG-••RYY---YU•R-----RRY•Y-GCCGG∘---YYYY•••••∘----------∘CCGGU•-Y•YY-A-RCCY•--••Y---•-----•R--•Y-Y-RC-CACC•∘∘•••••Y•••R••••••••R••••
          5′                                                                                                                                         3′
```

```
Eco-15-1   AAAUUAUG.GUG.G.G.GC..GUGC..AG.GG.CCAAC...UUCG.....GUUGG.GCCGGG...UUCUUUGGUG.........ACCGGUAUUUCC.A.CCCCU..GUA...C....GU..CU.C.AC.CACCAAUAAGGUCGUGGAAAGCCUUGGUGG
Eco-6-2    AAAUUAUG.GUG.G.G.GC..GUGC..AG.GG.CCAAC...UUCG.....GUUGG.GCCGGG...UUCUAUGUUG.........ACCGGUAUUUCC.A.CCCCU..GUA...C....GU..CU.C.AC.CACCUAUAUGGUCGUGGAAAGCCUUGGUGG
Eco-6-3    GAAUUAUG.GUG.G.G.GC..GUAC..GG.GG.CCGAC...UUCG.....GUCGG.GCCGGG...UUCUGCGUUU.........GCCGGUACUGCG.A.ACCUC..GUA...C....GU..CU.C.GC.CACCCACAGUUUCGCAGCUCUGGAUGGUGA
Eco-2-2    GAAUUAUG.GCG.G.G.GC..GUAC..GG.GG.CCGAC...UUCG.....GUCGG.GCCGGG...UUCUAUGUUU.........GCCGGUACUGCG.A.ACCUC..GUA...C....GU..CU.C.GC.CACCCACAGUUUCGCAGCUCUGGAUGGUGA
Eco-8-1    GAAUUAUG.GCG.G.G.GC..GUAC..GG.GG.CCGAC...UUCG.....GUCGG.GCCGGG...UUCUUGUGUA.........GCCGGUACUGCG.A.ACCUC..GUA...C....GU..CU.C.GC.CACCCACAGUUUCGCAGCUCUGGAUGGUGA
Sdy-1-2    GAAUUAUG.GCG.G.G.GC..GUAC..GG.GG.CCGAC...UUCG.....GUCGG.GCCGGG...UUCUUGUGUA.........GCCGGUACUGCG.A.ACCUC..GUA...C....GU..CU.C.GC.CACCCACAGUUUCGCAGCUCUGGAUGGUGA
Eco-7-1    GAAUUAUG.GCG.G.G.GC..GUAC..GG.GG.CCGAC...UUCG.....GUCGG.GCCGGG...UUCUUGUGUA.........GCCGGUACUGCG.A.ACCUC..GUA...C....GU..CU.C.GC.CACCCACAGUUUCGCAGCUCUGGAUGGUGA
Eco-9-1    GAAUUAUG.GUG.G.G.GC..GUAU..GG.GG.CCGAC...UUCG.....GUCGG.GCCGGG...UUCUUUGCUA.........GCCGGUUGUGGA.A.ACCCU..GUA...C....GU..CU.C.AC.CACCCCGAGUUUUCCACCUCUGGAUGGUGA
Eco-3-7    GAAUUAUG.GUG.G.G.GC..GUAC..GG.GG.CCGAC...UUCG.....GUCGG.GCCGGG...UUCUAUGUUU.........GCCGGUAGUCUC.A.ACCCU..GUA...C....GU..CU.C.GC.CACCCCGAGUUUGAGACCUCUGGAUGGUGA
Eco-15-2   GAAUUAUG.GUG.G.G.GC..GUAC..GG.GG.CCGAC...UUCG.....GUCGG.GCCGGGUU.UUCUUUGUUG.........ACCGGUAGUUCC.A.ACCCU..GUA...C....GU..CU.C.AC.CACCCCGAGCUUGGAACCUCUGGAUGGUGA
Eco-2-3    GAAUUAUG.GUG.A.G.GC..GUAU..GG.GG.CCGAC...UUCG.....GUCGG.GCCGGG...UUCUUUGGUU.........GCCGGUAGUUCC.A.ACCCU..GUA...C....GU..CU.C.AC.CACCCCGAGCUUGGAACCUCUGGAUGGUGA
Eco-10-2   GAUUUAUG.GUG.G.G.GC..GUGU..AG.GG.GAGUC...GAAA.....GGCUC.GCCGGG...UCCUUUUAG...........CCGGUAGUGCA.A.ACCCU..GCA...U....GU..CU.C.AC.CACCCAAAGAUUUGCACCUGAGGGGGGUGA
Spr-1-2    AAUCAAUG.GUG.G.G.GC..GUGC..AG.GG.GAGCC...GAAA.....GGCUC.GCCGGG...UUCUUUCGUG.........ACCGGUACUGCG.A.ACCUU..GUA...C....GU..CU.C.AC.CACCCCGAGUUUCGCAGCAAAAGGUGGUGA
Sty-2-1    GAAUUAUG.GUG.G.G.GC..GUAU..GG.GG.GAGCC...GAAA.....GGCUC.GCCGG......UCGGUGAU..........CUGGUUACGCC.A.ACCCU..GUA...C....GU..CU.C.AC.CACCCAAUCCGAUUGGCGUCGGCGGUGGUG
Sen-9-1    GAAUUAUG.GUG.G.G.GC..GUAU..GG.GG.GAGCC...GAAA.....GGCUC.GCCGG......UCGGUGAU..........CCGGUUACGCC.A.ACCCU..GUA...C....GU..CU.C.AU.CACCCAAUCCGAUUGGCGUCGGCGGUGGUG
Sen-13-2   GAAUUAUG.GUG.G.G.GC..GUAU..GG.GG.GAGCC...GAAA.....GGCUC.GCCGG......UCGGUGAU..........CCGGUUACGCC.A.ACCCU..GUA...C....GU..CU.C.AC.CACCCAAUCCGAUUGGCGUCGGCGGUGGUG
Eco-10-3   GAAUUAUU.GUG.G.G.GC..GCAU..GG.GG.GAGCU...GAAA.....AGCUC.GUCGG......UCGGUUUC..........CCGGUAGUUCC.A.ACCCU..GUG...C....GU..CU.C.AC.CACCCGAUGAUUGGAACCUGACGGUGGUGA
Sso-1-2    GAAUUAUG.GUG.G.G.GC..GCAU..GG.GG.GAGCU...GAAA.....AGCUC.GCCGG......UCGGUUUC..........CCGGUAGUUCC.A.ACCCU..GUG...C....GU..CU.C.AC.CACCCGAUGAUUGGAACCUGACGGUGUUGA
Ype-7-1    GAACAAUG.GUG.G.G.GC..GUAC..GG.GG.GAGCU...GAAA.....GGCUC.GCCGGG...UGUUGUUGUG.........ACCGGUAGUGCA.A.ACCUC..GUA...C....GU..CU.C.AC.CACCCAAUGAUUUGCACCAGACGGUGGUGA
Ype-1-1    GAACAAUG.GUG.G.G.GC..GUAC..GG.GG.GAGCU...GAAA.....GGCUC.GCCGGG...UGUUGUUGUG.........ACCGGUAGUGCA.A.ACCUC..GUA...C....GU..CU.C.AC.CACCCAAUGAUUUGCACCAGACGGUGGUGA
Eco-3-8    GAAUAAUG.GUG.G.G.GC..GUAU..GG.GG.GAGCU...GAAA.....GGCUC.GCCGG....UUGGUUGAU...........CCGGUAGUUCC.A.ACCCU..GUA...C....GU..CU.C.AC.CACCCAAUGAUUGGAACCUGACGGUGGUGA
Eco-3-9    UAUCAAUG.GUG.G.G.CU..GUAC..GG.GG.GCGGA...GCAA.....UCCGC.GCCGGU...UUCUUCAGUG.........UCCGGUAAGGCU.A.ACCCU..GUA...C....AG..UC.C.GC.CACCAGCGAAAUUAGCCUUUUGCGUGGCGG
Yen-1-1    AAGAAAUG.GUG.A.G.CU..GGGC..GG.GG.GCAUC...GUAA.....GGUGC.GCCAG....UUUUUGGGUG.........ACUGGUC.UGUC.A.ACCCC..AUC...C....AG..CU.C.AG.CACCUACAGAGAUGAAAAUCUCAAGAUGGC
Yen-1-2    AAGCAAUG.GUG.G.G.CU..GGAC..GG.GG.GCAUC...GAAA.....GAUGC.GCCGGG...UUCUCUGUUG.........ACCGGUC.UGCU.A.ACCCC..GUU...C....AG..UC.C.AC.CACCCAAAGGGAUUAGCAGCUCUUGGUGGU
Yps-1-1    AAACAAUG.GUG.G.G.CU..GGAC..GG.GG.GCAUC...GAAA.....GAUGC.GCCGGG...UUCUCUGUUG.........ACCGGUC.UGCU.A.ACCCC..GUU...C....AG..UC.C.AC.CACCCAAAGGGAUUAGCAGCUCUUGGUGGU
Ype-13-2   AAACAAUG.GUG.G.G.CU..GGAC..GG.GG.GCAUC...GAAA.....GAUGC.GCCGGG...UUCUCUGUUG.........ACCGGUC.UGCU.A.ACCCC..GUU...C....AG..UC.C.AC.CACCCAAAGGGAUUAGCAGCUCUUGGUGGU
Yps-2-1    AAACAAUG.GUG.G.G.CU..GGAC..GG.GG.GCAUC...GAAA.....GAUGC.GCCGGG...UUCUCUGUUG.........ACCGGUC.UGCU.A.ACCCC..GUU...C....AG..UC.C.AC.CACCCAAAGGGAUUAGCAGCUCUUGGUGGU
Yen-1-3    AAGCAAUG.GUG.A.G.CU..GGGC..GG.GG.GCAUC...GCAA.....GAUGC.GCCGGG...UUCUUCGAUG.........ACCGGUC.UGUC.A.ACCCC..GCC...C....AG..UU.C.AC.CACCCAUAAGAAAUUGACAGUUCUUGGUGG
Ybe-1-1    AAUCAAUG.GUG.A.G.CU..GGGC..AG.GG.GCAUC...GCAA.....GAUGC.GCCGGA...UUCUCUGUUG.........ACCGGUAGUUCC.A.ACCUU..GCU...C....AG..UU.C.AC.CACCCAUUAAGAGAUUGGAACCUCGGGCGG
Yin-1-1    AAGAAAUG.GUG.A.G.CU..GGGU..GG.GG.GCAUU...GCAA.....GAUGC.ACCGG....UUUCUUGGUG.........GCCGGUAGUUCC.A.ACCUC..GCU...C....AG..UU.C.AC.CACCCAUAGAGAUGAAAAUCUCAAGAUGGU
Yen-1-4    AAGAAAUG.GUG.A.G.CU..GGGU..GG.GG.GCAUU...GCAA.....GAUGC.ACCGG....UUUCUUGGUG.........GCCGGUAGUUCC.A.ACCUC..GCU...C....AG..UU.C.AC.CACCCAUAAAGAGAUUGGAACCUCAAGCUA
Yin-1-2    AAGAAACG.GUG.A.A.CU..GGGU..GG.GG.GCAUC...GCAA.....GAUGC.GCCGGG...UUCUUUGGUA.........ACCGGUAGUUCC.A.ACCUC..GCU...C....AG..UU.C.AC.CACCCAUAAAGAGAUUGGAACCUCAAGGUG
Sbo-2-1    AAAUUAUG.GUG.G.G.CU..GGGC..GG.GG.GCACC...GUAA.....GGUGC.GCCGG....UUCCAGUAUG.........GCCGGUAGUUCC.A.ACCUC..GUU...C....AG..UC.C.AC.CACCCAUGAGAUUGGAACCUCUGGUGGUGG
env-13     CCUCAAUG.GUG.G...CU..GGGC..GG.GG.GCACC...GUAA.....GGUGC.GCCGGU...UGCCUGUAAC.........GCCGGUAGUUCC.A.ACCCC..GUU...C....AG..UC.C.AC.CACCAU.GAGAUUGGAACCUCUGGUGGUGG
Sen-16-2   GAAUUAUG.GUG.G.C.UU..GGGU..AG.GG.GAGCC...GAAA.....GGCUC.GCCGGG...UUCUGUGAUG.........ACCGGUC.UGCU.A.ACCCU..GCU...C....AG..GU.C.AC.CACCAGUUGUUUAGCAGCAGCUUGUGGUGA
Api-1-1    AAUCAAUG.GUG.G.G.CU..GGAU..GG.GG.GAGCC...GAAA.....GGCUC.GCUGG....GUCCUUGUUA.........GCCGGUAGUCUC.A.ACCCU..GUU...C....AG..UU.C.AC.CGCCAAUAUGAGUUUGAGACCUCAAAGGUG
Sgl-2-9    GAAUAAUG.GUG.G.G.CU..GGAU..GG.GG.GAGCC...GCAA.....GGCUC.GCCGG....GUCCUUCUUA.........GCCGGUAGUCUC.A.ACCCU..GUU...C....AG..CU.C.GC.CACCAAUAUGAGUUUGAGACCUCUAGGUGG
Sdy-1-3    GAUUUAUG.GUG.G.G.CU..GUGU..GG.GG.GAGUU...GAGA.....GACUC.GCCGG....AUGUUUCGA...........CCGGUUACGCC.A.ACCCU..GCA...C....AG..UU.C.AC.CACCCA.GCGAUUGGCGUCGCAGGUGGUGA
Eco-3-10   GAAUUAUG.GUG.G.G.CU..GGGC..AG.GG.GAGCC...GAAA.....GGCUC.GCCGGU...UUCCUUGAUC.........GCCGGUAGUGCA.A.ACCCU..GUU...C....AG..UC.C.GC.CACCAGCGAGAUUUGCA............
Eph-2-1    GAAUUAUG.GUG.G.G.CU..GGGC..AG.GG.GAGCC...GAAA.....GGCUC.GCCGGU...UUCCUUGAGC.........GCCGGUAGUUCC.A.ACCCU..GUU...C....AG..UC.C.GC.CACCAGCGAGCUUGGAACCUCCGGUGGUGG
Eco-3-11   GAAUUAUG.GUG.G.G.CU..GGGC..AG.GG.GAGCC...GAAA.....GGCUC.GCCGGU...UUCCUUGAUC.........GCCGGUAGUUCC.A.ACCCU..GUU...C....AG..UC.C.GC.CACCAGCGAGCUUGGAACCUCCGGUGGUGG
Eco-3-12   GAAUUAUG.GUG.G.G.CU..GGGC..AG.GG.GAGCC...GAAA.....GGCUC.GCCGGU...CUCCGUUGAC.........GCCGGUAGUGCA.A.ACCCU..GUU...C....AG..UC.C.GC.CACCAGCGAGAUUUGCACCUCCGGUGGUGG
Sdy-1-4    AAAUUAUG.GUG.G.G.CU..GGAC..AG.GG.CAGCC...GAAA.....GGCUG.GCCGGU...UUCCUUGUAC.........GCCGGUACUGCG.A.ACUCU..GUU...C....AG..UC.C.AC.CACCAGCGAGUUUCGCAGCUCCGGUGGUGG
Eco-8-2    GAUUUAUG.GCG.G.G.CU..GUGU..GG.GG.GAGCC...GAAA.....GGCUC.GCCGG....AUGUUUCGA...........CCGGUAGUGCU.A.ACCCC..GCA...C....AG..UU.C.GC.CACCACGAUGAUUAGCACCUGACGGUGGCG
Sdy-2-2    GAUUUAUG.GUG.G.G.CU..GUGU..GG.GG.GAGCC...GAAA.....GGCUC.GCCGGU...UUCCGUAC............CCGGUAGUCUC.A.ACCCU..GCA...C....AG..CU.C.GC.CACCAGAUUAUUGAGACCAGAAGGUGGCGA
Eco-10-4   GAUUUAUG.GCG.G.G.CU..GUGU..GG.GG.GAGCC...GAAA.....GGCUC.GCCGGU...UUCCGUAC............CCGGUAGUUCC.A.ACCCC..GCA...C....AG..UU.C.GC.CACCA..UGAUGAUUGGAACCUGAAGGUGG
Sfl-2-3    GAUUUAUG.GCG.G.G.CU..GUGU..GG.GG.GAGCC...GAAA.....GGCUC.GCCGGU...UUCCGUAC............CCGGUUACGCC.A.ACCCU..GCA...C....AG..UU.C.GC.CACCAGUUCGAUUGGCGUCGUCGGUGGCGA
Sen-10-2   GAUUUAUG.GCG.G.G.CU..GUGU..GG.GG.GAGCC...GAAA.....GGCUC.GCCGGU...UUCCGUAC............CCGGUUACGCC.A.ACCCU..GCA...C....AG..UU.C.GC.CACCAGUUCGAUUGGCGUCGUCGGUGGCGA
Sdy-2-3    GAUUUAUG.GCG.G.G.CU..GUGU..GG.GG.GAGCC...GAAA.....GGCUC.GCCGGU...UUCCGUAC............CCGGUUACGCC.A.ACCCU..GCA...C....AG..UU.C.GC.CACCAGUUCGAUUGGCGUCGUCGGUGGCGA
Eco-9-2    UCUCAAUG.GUG.G.G.CU..GUGU..GG.GG.GCGGA...GCGA.....UCCGC.GCCGG....UUCCUUUUUC..........CCGGUUACGCC.A.ACCCU..GCA...C....AG..UU.C.AC.CACCAUACUGAUUGGCGUCAGCAGUGGUGA
Eph-1-1    AAGUUAUG.GUG.G.G.CU..GGGC..GG.GG.GCAUC...GAAA.....GAUGC.GCCGGU...UUCCUUGAUC.........ACCGGUUACGCC.A.ACCCC..GUU...C....AG..UU.C.AC.CACCAGCGAAAUUGCCGUUUCCGGUGGUGG
Eco-13-2   GAAUUAUG.GUG.G.G.CU..GUGU..GG.GG.GCGGA...GAGA.....UCCGC.GCCGG......UCGGUUUC..........CCGGUUACGCC.A.ACCCU..GCA...C....AG..UU.C.AC.CACCAG.ACGAUUGGCGUCGUCGGUGGUGA
Sen-15-2   GAAUUAUG.GUG.G.G.CU..GUGU..GG.GG.GCACC...GAAA.....GGUGC.GCCGG......UUGGUUUC..........CCGGUUACGCC.A.ACCCU..GCA...C....AG..UU.C.AC.CACCAG.ACGAUUGGCGUCGUCGGUGGUGA
env-14     AGAUUAUG.GUG.G.G.CU..GGGC..GG.GG.GCACC...GAAA.....GGUGC.GCCGGU...CUCCGUUGAC.........GCCGGUUACGCC.A.ACCCC..GUC...C....AG..UU.C.AC.CACCAGUGAAAUUGGCGUUUCCGGUGGUGG
Eco-9-3    AGAUUAUG.GUG.G.G.CU..GGGC..GG.GG.GCACC...GAAA.....GGUGC.GCCGGU...CUCCGUUGAC.........GCCGGUUACGCC.A.ACCCC..GUC...C....AG..UU.C.AC.CACCAGUGAAAUUGGCGUUUCCGGUGGUGG
Sen-2-1    AAAUUAUG.GUG.G.G.CU..GGGU..GG.GG.GCACC...GAAA.....GGUGC.GCCGGU...UUCCGUAUGC.........GCCGGUUACGCC.A.ACCCU..GCU...C....AG..UU.C.AC.CACCAGCGAAAUUGGCGUUUCCGGUGGUGG
Sen-15-3   AAAUUAUG.GUG.G.G.CU..GGGU..GG.GG.GCACC...GAAA.....GGUGC.GCCGGU...UUCCGUAUGC.........GCCGGUUACGCC.A.ACCCU..GCU...C....AG..UU.C.AC.CACCAGCGAAAUUGGCGUUUCCGGUGGUGG
Eco-11-1   AAAUUAUG.GUG.G.G.CU..GGAC..GG.GG.GCACU...GAAA.....GGUGC.GCCGGU...UUCCGAUAAC.........GCCGGUUACGCC.A.ACCCC..GUU...C....AG..UU.C.AC.CACCAGCGAAAUUGGCGUUUCCGGUGGUGA
env-15     AAAUUAUG.GUG.G.G.CU..GGAC..GG.GG.GCACC...GAAA.....GGUGC.GCCGGU...UUCCGAUAAC.........GCCGGUUACGCC.A.ACCCC..GUU...C....AG..UU.C.AC.CACCAGCGAAAUUGGCGUUUCCGGUGGUGA
Sen-9-3    AAAUUAUG.GUG.G.G.CU..GGGU..AG.GG.GCACC...GAAA.....GGUGC.GCCGGU...UUCCGAUAAC.........GCCGGUUACGCC.A.ACCCU..GCU...C....AG..UU.C.AC.CACCAGUGAAAUUGGCGUUUCCGGUGGUGG
Eco-4-2    GUAUUAUG.GUG.G.G.CU..GUGU..GG.GG.GCACC...GAAA.....GGUGC.GCCGGG...UCCUUUUAG...........CCGGUUACGCC.A.ACCCU..GCA...C....AG..UU.C.AC.CACCAA.CCGAUUGGCGUCGGUAGUGGUGA

           ......<<.<<<.<.<.<<..<<<<..<<.<<.<<<<<<<........>>>>>>>.<<<<<........................>>>>>..........>>>>..>>>...>....>>..>>.>.>>.>>............................
           ......?1.222.2.2.22..2222..22.22.2222220........0222222.22222........................22222..........2222..222...2....22..22.2.22.1?............................
           •••Y•AUG-GYR-G-•-Y•--•R••--•G-GG-••RYY---YU•R-----RRY•Y-GCCGG○---YYYY•••••○----------○CCGGU•-Y•YY-A-RCCY•--••Y---•----•R--•Y-Y-RC-CACC•○○•••••Y•••R••••••••R••••
            5′                                                                                                                                                    3′
```

```
Eph-6-1    GUAUUAUG.GUG.G.G.CU..GUGU..GG.GG.GCACC...GAAA.....GGUGC.GCCGG....GUCCUUUUAG..........CCGGUUACGCC.A.ACCCU..GCA...C....AG..UU.C.AC.CACCAACGCAUUGGCGUAGGUAGUGGUGAU
env-16     GAAUUAUG.GUG.G.G.CU..GGGC..AG.GG.GUCC....GAAA......GGAC.GCCGG....UACCACUUAG.........GCCGGUA.CGCC.A.ACCUU..GUC...C....AG..UU.C.AC.CACCAGUAAUUGGCGUUGCGGUGGUGAUUA
Sgl-1-3    AAUCAAUG.GUG.G.G.CU..GGAC..AG.GG.GAACU...GAAA.....GGUUC.GCCGU....AGCUUUGGC...........ACGGUAGUAUC.A.CCCCU..GUU...C....AG..UU.C.AC.CACCAGCGCAGCUUGGAUGAAGCAUGGUGG
Sgl-2-10   AAUCAAUG.GUG.G.G.CU..GGAC..AG.GG.GAACU...GAAA.....GGUUC.GCCGU....AGCUUUGGC...........ACGGUAGUAUC.A.CCCCU..GUU...C....AG..UU.C.AC.CACCAGCGCAGCUUGGAUGAAGCAUGGUGG
Pfl-2-5    CUGUUAUG.GCG.G.C.UG..UGCG..CG.AG.AGAC....UUCGA.....GUCU.GUCGGG...CUUUUGGCUUUUUUCG....CCCGGG..UUCC.A.ACUCG..UGU...A....CA..GC.U.GC.CACCUCUUCGAUUGGAACCGAGUAGGUCAG
Ppu-1-1    GCGUAAUG.GCG.A.C.UG..UGCG..CG.GG.CAGAC...CUUG.....GUCUG.GCCGAGC..UUCCUACGUUCA........CUCGGUU.UCUC.A.CCUCG..UGC...A....CG..GU.U.GC.CGCCUUUGUUCCGUGAGAAAGAAUGAACAG
Pfl-2-6    GCGUCGCG.GCG.G.C.UG..UGCG..CA.GG.CAGAC...UUCG.....GUCUG.GCCGGU...UGCCUACUU...........AUCGGUU.UUCC.A.CCCUG..CGU...A....CA..GC.U.GC.CACCUUUUGUCGUGUGGAAACGACAAGGGA
env-17     CGUCUAUG.UGG.G.A.CU..GUAC..GA.GG.CAACC...UUCG.....GGUUG.GCCGG....UGCUUGGUAG..........CCGGUUGUUGC.A.ACCUC..GUA...C....AG.CUC.C.CG.CACCUC.UGCUGCAACCAGACUGCGGGUAG
env-18     CCUCUAUG.CGG.G.A.CU..GUGC..GG.GG.CAGCC...UUCG.....GGCUG.GCCGG....UACUCUGUAG..........CCGGUUUUUCU.A.CCCCC..GUA...C....AG.CUC.C.CG.CACCUAAUUUGUAGAAAGAUUAAUGCGGGU
env-19     CGUCUAUG.UGG.G.A.CU..GUGC..GG.GG.CAGCC...UUCG.....GGCUG.GCCGGU...UACUCUUUA...........CCGGUAGUUCC.A.ACCUC..GCA...C....AG.CUC.C.CG.CUCCUCUCGUUGGAACCAGAAUGCGGGUAG
env-20     CGUCUAUG.UGG.G.A.CU..GUGC..GG.GG.CAGCC...UUCG.....GGCUG.GCCGG....UUCGUAUGCU..........CCGGUAUUUCC.A.CCCCC..GUA...C....AG...C.U.CC.CGCCCUUAAUCUGUGGAAAGAUUAAAGCGG
Bth-1-1    CUUCAAUG.GCG.G.G.CCUUGGCUGAGG.GG.ACACC...UUCG.....GGUGU.GCCGGU...UUCCUUUUGC.........GCCGGUC.CGCG.A.ACCCCAAAGC...C.UUGCG..CC.U.GC.CACCA..UCGCUUCGCGGCGGUGAUGGCGG
Bps-11-1   CUUCAAUG.GCG.G.G.CCUUGGCUGAGG.GG.ACACC...UUCG.....GGUGU.GCCGGU...UUCCUUUUGC.........GCCGGUC.CGCG.A.ACCCCAAAGC...C.UUGCG..CC.U.GC.CACCA..UCGCUUCGCGGCGGUGAUGGCGG
Rso-1-1    UUUCAAUG.GCG.G.G.UCG.AGGU..GG.GG.ACACC...UUCG.....GGUGU.GCCGG....UUCUGGUAG...........CCGGUC.GGCC.A.ACCCC..GCU...U..UCGG..UC.G.GC.CGCCCGUAUCUUGGCCGAUGCAUGGCGGAC
Sgl-2-11   GAACAAUG.GUG.G.C.UC..AGGU..GG.GG.GAGCC...GUAUAA...GGCUC.GCCGG.........GGUAC..........ACCGGUAUUGCG.A.ACCCC..GCU...U....GG..GC.U.AC.CACCA..GUGAUUCGCAACUCUGAUGGUAG
Bxe-1-2    UUGCAAUG.GGC.G.G.GCCGUGAU..AG.GG.AGACC...UUCG.....GGUCU.GCUGGU...UUGGUUUGGU..........GCCAGUC.UGUC.A.ACCUU..GUC...ACUGUGC..CC.G.CU.CACCACCGCGUAAGCGCGUGUGUGGGCGAU
Bph-1-2    CUGCAAUG.GGC.G.G.GCCGUGAU..AG.GG.AGACC...UUUUG....GGUCU.GCUGG....UUUGGUUUGG..........GCCAGUC.UGUC.A.ACCUU..GUC...ACUGUGC..CC.G.CU.CACCACCGCGUAAGCGCGUGUGUGGGCGAU
Ppu-4-2    CUGCUAUG.GCA.G.C.CGC.AACG..CG.GG.CAGGC...UUCG.....GCCUG.GCUGGC...UUAUGCCGAUCUUU......GCCGGUC.UCUC.A.CCCCG..CGU...U....CG..GC.U.GC.CACCUUAUUCUGUGAGAGGAAGACGAAGGG
Pen-1-2    ACUCCAUG.GCA.G.C.CG..CACG..CG.GG.CAGGC...UUCG.....GCCUG.GUCGGGU..UUCCUAUUGG..........CCCGGG..UCUC.ACCCCCCG..CGU...G....UU..GC.U.GC.CACCAU.UUCCGUGAGACGAAUAGGACGGC
Pst-1-1    UCUUUAUG.GGC.G.G.GC..CGUG..CG.GG.GCAUC...UUCG.....GAUGC.GCCGG......CCCUCAG...........ACCGGUA.CGCC.A.ACCCG..UGC...G....GU..CU.G.CU.CACCCAAUUGGCGUUGGGUGGCAGAACUAU
Sgl-2-12   UAUCAAUG.GUG.G.C.UC..AGGU..GG.GG.GAACC...GAAA.....GGUUC.GCCGG....UUGGUUUAU...........CCGGUUGUGAC.A.ACCCU..GUC...U....GG..GC.U.AC.CACCAAUGAGAUUGUCACCUCGGGUUUAUA
Eph-7-1    GAAUUAUG.GUG.G.C.UC..AGAU..GG.GG.CCAAC...UUCG.....GUUGG.GCCGGU...UUCUUCUUGU..........ACCGGUGUUGAG.A.ACCCU..GUC...U....GG..GC.U.AC.CACCCCUAUAGAGAUUCUCAACUCUGGUGG
env-21     AAUAAAUG.GUG.G.C.UC..GAGU..GG.GG.CAGUC...UUCG.....GACUG.GCCGGU...AUUCUGUGAA..........GCCGGUACUGCA.A.ACCCU..GUU...C....GG..GU.C.AC.CACCAAUGAGAUUUGCAGCUCCGGUGGUGG
Esa-1-1    GUGUAAUG.GUG.G.C.UC..AGGC..GG.GG.CUGAC...UUCG.....GUCAG.GCCGGU...AUCCGUUAGA..........GCCGGUAUUUCC.A.CCCCC..GUC...U....GG..GC.U.AC.CACCAGUGAGCGUGGAAACUCCGGUGGUAG
Sen-11-2   GAGUAAUG.GUG.G.C.UC..AGGC..GG.GG.CCGAC...UUCG.....GUCGG.GCCGGU...AUCCGUUAGA..........GCCGGUAUUUCC.A.CCCCC..GUC...U....GG..GC.U.AC.CACCACUGAGCGUGGAAACUCCGGUGGUAG
Yph-1-1    AAAUUAUG.GCA.G.U.CC..AGGC..GG.AG.CAGCU...UUCG.....AGCUG.GCCGGU...AUCCGUUGGG..........GCCGGUAUUCCU.A.CCUCC..GUU...U....GG..GC.U.GC.CACCCCAUAGAGUGUAGGAACUCUGGUGGU
Sgl-2-13   C...AAUG.GUG.G.C.UC..AGGC..GG.GG.CAGCC...UUCG.....GGCUG.GCCGGA...UUCCAGAUUG..........ACCGGUAGUCUC.A.ACCCC..GUC...U....GG..GC.U.GC.CACCAAUGAGCUUGAGACCUCUGGUGGUAG
Sgl-2-14   C...AAUG.GUG.G.C.UC..AGGC..GG.GG.CAGCC...UAAG.....GGCUG.GCCGGG...UUCCGAUACG..........ACCGGUAGUCUC.A.ACCCC..GUC...U....GG..GC.U.GC.CACCAAUGAGCUUGAGACCUCUGGUGGUGG
Eco-3-13   AGAUAAUG.GUG.G.U.CC..GGGC..GG.GG.CAGCC...UUCG.....GGCUG.GCCGGU...UUCCUUGAAG..........GCCGGUAUUCCU.A.CCCCC..GUC...C....GG..GC.U.AC.CACCCAUGAGCGUAGGAACUCCAGUGGUAG
Sen-7-2    AGAUAAUG.GUG.G.U.CC..GGGC..GG.GG.CAGCC...UUCG.....GGCUG.GCCGGU...UUCCAAUAAG..........GCCGGUAUUCCU.A.CCCCC..GUU...C....GG..GU.C.AC.CACCCAUGAGCGUAGGAACUCCGGUGGUGG
Esp-1-3    UGUGAAUG.GUG.G.C.UC..AGGC..GG.GG.CAGCC...UUCG.....GGCUG.GCCGGU...UCUCAUAGUU..........ACCGGUAUUCCU.A.CCCCC..GUC...U....GG..GC.U.AC.CACCCAUGAGUGUAGGAACUCCGGUGGUAG
Ybe-1-2    AAGCAAUG.GUG.G.C.UC..AGGC..GG.GG.CAGCU...UUCG.....GGCUG.GCCGGU...UUCCAAGGUU..........GCCGGUAUUCCU.A.CCCCC..GUC...U....GG..GC.U.AC.CACCCAUGAGUGUAGGAACUCCGGUGGUAG
Ymo-1-1    AAGCAAUG.GUG.G.C.CC..AGGC..GG.GG.CAGCC...UUCG.....GGCUG.GCCGGU...UUCCAAUAAG..........GCCGGUAUUCCU.A.CCCCC..GUC...U....GG..GC.U.AC.CACCCAUAAGUGUAGGAACUUCGGUGGUAG
Esa-1-2    UAAUUAUG.GUG.G.C.UC..AGGC..GG.GG.CAGCC...UUCG.....GGCUG.GCCGGU...UUCCAAUAAG..........GCCGGUAUUCCU.A.CCCCC..GUC...U....GG..GC.U.AC.CACCCUUGAGCGUAGGAACUCUGGUGGUAG
Yfr-1-1    AAGCAAUG.GUG.G.C.UC..AGGC..GG.GG.CAGCU...UUCG.....GGCUG.GCCGGU...AUCCAUUGAG..........GCCGGUU.UCUC.A.CCCCC..GUC...U....GG..GC.U.AC.CACCC..AAGUGUGAGAACUCAAGUGGUAG
Sen-9-4    GUAUUAUG.GUA.G.U.CC..AGGC..GG.GG.CAGCU...UUCG.....GGCUG.GCCGGU...AUCCUUCAGA..........GCCGGUAUUCCU.A.CCCCC..GUC...U....GG..GC.U.AC.CACCCAUGAGCGUAGGAACUCCGGUGGUAG
Sen-5-1    GUAUUAUG.GUA.G.U.CC..AGGC..GG.GG.CAGCU...UUCG.....GGCUG.GCCGGU...AUCCUUCAGA..........GCCGGUAUUCCU.A.CCCCC..GUC...U....GG..GC.U.AC.CACCCAUGAGCGUAGGAACUCCGGUGGUAG
Sen-7-3    UGAUAAUG.GUA.G.U.CC..GGGC..GG.GG.CAGCC...UUCG.....GGCUG.GCCGGU...UUCCUUGAAG..........GCCGGUUACGCC.A.ACCCC..GUU...C....GG..GC.U.GC.CACCAGUGAAAUUGGCGUUUCCGGUGGUAG
Sen-8-3    UGAUAAUG.GUA.G.U.CC..GGGC..GG.GG.CAGCC...UUCG.....GGCUG.GCCGGU...AUCCUUGAAG..........GCCGGUUACGCC.A.ACCCC..GUU...C....GG..GC.U.GC.CACCAGUGAAAUUGGCGUUUCCGGUGGUAG
Eco-13-3   AGAUAAUG.GUA.G.U.CC..GGGC..GG.GG.CAGCC...UUCG.....GGCUG.GCCGGU...UUCCAAUAAG..........GCCGGUUACGCC.A.ACCCC..GUU...C....GG..GC.U.GC.CACCAGUGAAAUUGGCGUUUCGGUGGUAG
env-22     AGAUAAUG.GCA.G.U.CC..GGGC..GG.GG.CAGCC...UUCG.....GGCUG.GC.GGU...UUCCAAUAAG..........GCCGGUUACGCC.A.ACCCC..GUU...C....GG..GC.U.GC.CACCAGUGAAAUUGGCGUUUCGGUGGUAG
env-23     AGAUAAUG.GCA.G.U.CC..GGGC..GG.GG.CAGCC...UUCG.....GGCUG.GCCGGU...UUCCAAUAAG..........GCCGGUUACGCC.A.ACCCC..GUU...C....GG..GC.U.GC.CACCAGUGAAAUUGGCGUUUCGGUGGUAG
Sen-11-3   AGUCAAUG.GUA.G.C.UC..AGGC..GG.GG.CAGCC...UCCG.....GGCUG.GCCGGU...UUCCGAUAAC..........GCCGGUAUUCUC.A.CCCCC..GUC...U....GG..GC.U.AU.CGCCAUCGAGCGUGAGAACUCCGGCGAUAG
Eco-5-2    AGUCAAUG.GUA.G.C.UC..AGGC..GG.GG.CAGCC...UCCG.....GGCUG.GCCGGU...UUCCGAUAAC..........GCCGGUAUUCUC.A.CCCCC..GUC...U....GG..GC.U.AU.CGCCAUCGAGCGUGAGAACUCCGGCGAUAG
Eco-14-1   AGUCAAUG.GUA.G.C.UC..AGGC..GG.GG.CAGCC...UCCG.....GGCUG.GCCGGU...UUCCGAUAAC..........GCCGGUAUUCUC.A.CCCCC..GUC...U....GG..GC.U.AU.CGCCAUCGAGCGUGAGAACUCCGGCGAUAG
Eco-11-2   AGUCAAUG.GUA.G.C.UC..AGGC..GG.GG.CAGCC...UCCG.....GGCUG.GCCGGU...AUCCGUUAGA..........GCCGGUAUUCUC.A.CCCCC..GUC...U....GG..GC.U.AU.CGCCAUCGAGCGUGAGAACUCCGGCGAUAG
Eph-4-1    AGUCAAUG.GUA.G.C.UC..AGGC..GG.GG.CAGCC...UCCG.....GGCUG.GCCGGU...AUCCUGUGAA..........GCCGGUAUUCUC.A.CCCCC..GUC...U....GG..GC.U.AU.CGCCAUCGAGCGUGAGAAGUCCGGCGAUAG
Sbo-2-2    AGUCAAUG.GUA.G.C.UC..AGGC..GG.GG.CAGCC...UCCG.....GGCUG.GCCGGU...AUCCUGUGAA..........GCCGGUAUUCUC.A.CCCCC..GUC...U....GG..GC.U.AU.CGCCAUCGAGCGUGAGAAGUCCGGCGAUAG
Eco-8-4    AGUCAAUG.GUA.G.C.UC..AGGC..GG.GG.CAGCC...UCCG.....GGCUG.GCCGGU...AUCCUGUGAA..........GCCGGUAUUUCC.A.CCCCC..GUC...U....GG..GC.U.AU.CGCCAUCGAGCGUGGAAAGUCCGGCGAUAG
Sgl-2-15   AAACAAUG.GUG.G.C.UC..AGGC..GG.GG.CACCC...GUAA.....GGGUG.GCCGGU...AUCCAAGGAA..........GCCGGUU.UCUC.A.CUCCU..GUC...U....GG..GC.U.AC.CACCUC.UAGUGUGAGAACUUUGGUGGUAG
Sty-1-1    CAUCUAUG.GUA.G.A.UC..AGGC..GG.GG.CAGGC...AUUA.....GCCUG.GCCGGU...UUCCGAUGAC..........GCCGGUAUUCCU.A.CCCCC..GUC...U....GG..GC.U.AU.CACCAAUGAGCGUAGGAACUAGGUGAUAGG
Eco-1-1    CAUCUAUG.GUA.G.C.UC..AGGC..GG.GG.CAGGC...AUUA.....GCCUG.GCCGGU...UUCCGAUGAC..........GCCGGUAUUCCU.A.CCCCC..GUC...U....GG..GC.U.AU.CACCAAUGAGCGUAGGAAGUCGGUGAUAGG
Sen-4-4    CAUCUAUG.GUA.G.C.UC..AGGC..GG.GG.CAGGC...AUUA.....GCCUG.GCCGGU...UUCCGAUGAC..........GCCGGUAUUCCU.A.CCCCC..GUC...U....GG..GC.U.AU.CACCAAUGAGCGUAGGAAGUCGGUGAUAGG
Kph-1-1    GAAUUAUG.GUG.G.C.UC..AGGC..AG.GG.CAGCU...UUCG.....GGCUG.GCCGGU...AGCUGAUUGG..........GCCGGUAUUCCU.A.CCCUU..GUC...U....GG..GU.C.AC.CACCAACGAGUGUAGGAACUCCGGUGGUGA
Ype-2-2    CGUCAAUG.GUG.G.C.CC..AGGC..GG.GG.GCUCC...GAAA.....GGAGC.GCCGGU...UUCCAAGAAG..........UCCGGUAACGCU.A.ACUCC..GUC...U....GG..GC.U.AC.CACCCAAGGGAUUAGCGUCUCAGGUGGUAG
env-24     AAUCAAUG.GUG.G.C.UC..AGGC..GG.GG.GCUUC...UUCG.....GAAGC.GCCGGU...AUCCUUCAGA..........GCCGGUUACGCC.A.ACCCU..GUC...U....GG..GC.U.AC.CACCAGCGAAAUUGGCGUUUCCGGUGGUAG
Sen-1-1    GUGUAAUG.GUG.G.C.UC..AGGC..GG.GG.GCUUC...UCAC.....GAAGC.GCCGGU...UUCCAAUGUU..........GCCGGUUACGCC.A.ACCCC..GUC...U....GG..GC.U.AC.CACCAGUGAAAUUGGCGUUUCCAGUGGUAG
Sen-14-2   GUGUAAUG.GUG.G.C.UC..AGGC..GG.GG.GCUUC...UCAC.....GAAGC.GCCGGU...UUCCAAUGUU..........GCCGGUUACGCC.A.ACCCC..GUC...U....GG..GC.U.AC.CACCAGUGAAAUUGGCGUUUCCAGUGGUAG
env-25     AAUAAAUG.GUG.G.C.UC..AGGC..GG.GG.GCUUC...GUAA.....GAAGC.GCCGG....UUCGUAUGUC..........CCGGUGACGCU.A.ACCCC..GUC...U....GG..GC.U.AC.CACCC..GAGUUUAGCGUCUCUGGUGGUAG
Ppu-4-3    UCGUCAUG.GCA.G.U.UG..UGCG..CG.GG.AGGC...CGCAAA.....GCCU.ACCGGG...UUUACUCGUA..........GCCUGGA.UUCC.A.CACCG..CGU...A....CA..AC.U.GC.CACCCAUCCGUGUGGAAUCGGACAGGUCGG
Ppu-2-1    UCGUCAUG.GCA.G.U.UG..UGCG..CG.GA.AGGC...CGCAAA.....GCCU.ACCGGG...UUUGCUCGUA..........GCCUGGA.UUCC.A.CACCG..CGU...A....CA..AU.U.GC.CACCCU.UCGUGUGGAAUCGGAGAAGGCGG

           .....<<.<<<.<.<.<<..<<<<..<<.<<.<<<<<<<........>>>>>>>.<<<<<........................>>>>>..........>>>>..>>>...>....>>..>>.>.>>.>>...........................
           .....?1.222.2.2.22..2222..22.22.2222220........0222222.22222........................22222..........2222..222...2....22..22.2.22.1?...........................
           •••Y•AUG-GYR-G-•-Y-•--•R•••--•G-GG-••RYY---YU•R-----RRY•Y-GCCGG∘---YYYY•••••∘----------∘CCGGU•-Y•YY-A-RCCY•--••Y---•-----•R--•Y-Y-R-C-CACC•∘∘••••Y•••R••••••••R••••
           5′                                                                                                                                                           3′
```

```
Pen-1-3     CCGUUAUGGGCG.G.A.GC..CGUG..UGUGG.GAGCC...UUCG.....GGCUC.ACCGG......ACCUUCUG..........ACCGGCAACGCC.A.ACCCG..CAC...G....GC..UC.C.GUCCACCCAUAUUGGCGUAUGGGCACGGAGAGA
Nmu-1-1     UUUUUACG.GGC.G.G.GU..CGUG..UG.GG.GAGCC...UUCG.....GGCUC.GCCGG....UUCCUUUGU...........CCGGUA.CGCC.A.ACUCG..CAC...G....AU.UCC.G.CC.CACCCG.AUUGGCGUCGGGGAAGCGGAGAA
env-26      CAGUUUCG.GGC.G.G.GC..CGUG..CG.GG.AGACC...UUCG.....GGUCU.GCCGG....UUCCUGUAU...........CCGGUU.CGCC.A.ACCCG..CAC...G....GU.UCC.G.CC.CACCCG.UUUGGCGACGGGGAAGCGGACUU
Avi-1-1     CCGUUAUG.GGC.G.G.GC..CGUG..UGUGG.GGGCC...UUAC.....GGCCC.GCCGG....UUCCCGUUU...........CCGGUC.CGCC.A.ACCCG..CAC...G....GU.UCC.G.CU.CACCCA.UCUUGGCGGAUGGGCGCGGCAGA
Pae-4-2     A...UAUG.GGC.G.G.GC..CGUG..UG.GG.GCUGC...GCAA.....GCAGC.GCCGG....UUCCUAAUC...........CCGGUA.CGCC.A.ACCUG..CAC...G....GU.UCC.G.CC.CACCCUGAUUGGCGUCAGGGCAGCGGGGUC
Ppu-2-2     UCGUCAUG.GCA.G.U.UG..CACG..CG.GG.AGGCC...GUAU.....GGCCU.GCCGGG...GUUCCGAUAG.........CCCGGCU.UUCC.A.CCCCG..CGU...G....CA..GC.C.GC.CACCCUUCGCGUGGAAACGGAUGGUGCCGG
Pen-1-4     CUGUUAUG.GCA.G.C.UG..UGCG..CG.GG.CAGGC...UUU......GCCUG.GCCGGA...AUGUAGCCUCCAACUUC...GCCGGU..UCUC.A.CCCCG..CGC...A....UG..GC.U.GC.CACCCAUUCCCGUGAGAAGGUUGUGUGGUG
Pen-1-5     CUGCUAUG.GCA.G.C.CG..UGCG..CG.GG.CAGG....UUCGU.....CCUG.ACCGGUU..UUCCUUCGUG.........GCCGGGU.UCUC.A.CUCCG..CGC...A....UG..GC.U.GC.CACCCAAAUCAUGUGAGAACGAUCAAGUGG
Ppu-4-4     UUUUCAUG.GCA.G.C.CG..UGCG..UG.UG.CGGGC...UUCG.....GUCCGGGCCGCG...GUUCCUGGAC..........GCGGUCUUCUC.A.CCUCG..CGU...A....CG..GC.U.GC.CACCCAAUCCGUGAGAAGAACAAGGUGGUG
Pen-1-6     CUUUCAUG.GCA.G.C.CG..UGCG..UG.GG.CGGAC...UUUG.....GUCCG.ACCGCG...UUUCUGUGUC.........CGCGGACUUCUC.A.CCUCG..CGC...A....CG..GC.U.GC.CACCCACAUCUGUGAGAAGGAUUCGGGCGG
Pen-1-7     CAGUCAUG.GCA.G.C.UG..UGCG..CG.UG.CAGGC...UUCG.....GCCUG.GCCGUG...GUGUUCGGGA.........CACGGUCUUCUC.A.CCUCG..CGC...A....CG..GC.U.GC.CACCCACAUCUGUGAGAAGGAUUCGGGCGG
Ppu-4-5     UUUUUAUG.GCA.G.C.UG..UGCG..CG.UG.CAGGC...UUAG.....GCCUG.ACCGUGG..UGACUCCAAC..........ACGGGCUUCUC.A.CCUCG..CGC...A....CG..GC.U.GC.CACCCCAAUCUGUGAGAAGGACCAGGGUGG
Ppu-3-1     CCGCCAUG.GUG.G.U.CG..UACG..UG.GG.CAGAC...CUCG.....GUCUG.GCCGGGC..UUCCUUAUUCA........CUCGGUU.UCUC.A.CCCUG..CGU...A....CG..GC.U.GC.CGCCCCAAAUCCGUGAGAAAGAUUUUUGGC
Ppu-3-2     CUCUAAUG.GCG.G.C.CG..UACG..UG.GG.CAGGC...UUCG.....GCCUG.GCCGAGUCAUUGCGUAUUUCUC.......CUCGGUU.UCUC.A.CCCCG..CGU...A....CG..GC.U.GC.CACCCUCGAUCCGUGAGAAAGAUUCAGGUG
Ppu-4-6     UCGUUAUG.GCG.G.C.CG..UGCG..CG.GG.CAGGC...UUAC.....GUCUG.GCCGAG...UUUUGCCUUUGUUC......CUCGGUU.UCUC.A.CCCUG..CGU...A....CG..GC.U.GC.CACCCACAAUCCGUGAGAAAGAUUCAGGUG
Ppu-1-2     CUGUCAUG.GCA.G.U.CG..UACG..CG.GG.CAGAC...UUCG.....GUCUG.GCCGAGCC.UUGUAGCUCC..........CUCGGUU.UCUC.A.CCCCG..UGU...A....CG..AC.U.GC.CACCCUGGUCCGUGAGAAAGAUCUGCGUGG
Ppu-4-7     CUGUUAUG.GCG.G.U.CG..UACG..CG.GG.CAGAC...UUCG.....GUCUG.GCCGAG...CUUUGUAGCUCC........CUCGGUUUUCUC.A.CCCCG..CGU...A....UG..AC.U.GC.CACCUAGAUCUGUGAGAAAGAUCCGCGUGG
Ppu-3-3     CUGUCAUG.GCA.G.U.CG..UACG..CG.GG.CAGAC...UUCG.....GUCUG.GCCGAG...CUUUGUAGCUCC........CUCGGUU.UCUC.A.CCCCG..UGU...A....CG..AC.U.GC.CGCCCUGGUCCGUGAGAAAGAUCUGCGUGG
Ppu-4-8     UAGUUAUG.GCG.G.C.CG..UGCG..UG.GG.CAGAC...UUCG.....GUCUG.GCCGGG...GCCCUUCUUG.........CCCGGUAUUCCU.A.CCCCA..CGC...A....UA..GC.U.GC.CACCCAAUCCCGUAGGAAGGAUUCUGGCAG
Ppu-4-9     AUGUCAUG.GCG.G.C.UG..UGUG..UG.GG.CAGGC...UUCG.....GCCUG.GCCGGGU..UUCCAAGUUC.........CCCGGUAUUCCU.A.CCCCA..UAC...A....AA..GC.U.GC.CACCCCCCAAUCCUGCCGGAAGUAGCGAUU
env-27      GUGUUAUG.GCG.G.C.CG..UGUG..UG.GG.CAGGC...UUCG.....GCCUG.GCCGGG...UUCCAAGUUU.........CCCGGUAUUCCU.A.CCCCA..CGC...A....CG..GC.U.GC.CACCCAAUCCCGUAGGAAGGACCGUGGCAG
Pen-1-8     AUGUCAUG.GCG.G.C.UG..UGUG..UG.GG.CAGGC...UUCG.....GCCUG.GCCGGG...UUCCAAGUUU.........CCCGGUAUUCCU.A.CCCCA..CGC...A....CA..GC.U.GC.CACCCAAUCCCGUAGGAAGGAUCGUGGUAG
Ppu-1-3     GUGUCAUG.GCG.G.C.UG..UGUG..UG.GG.CAGGC...UUCG.....GCCUG.GCCGGGU..UUCCAAGUUU.........CCCGGUAUUCCU.A.CCCCG..CAC...A....CA..GC.U.GC.CACCCAAUCCCGUAGGAAGGAUCGUGGCAG
Ppu-4-10    UCGUAAUG.GCG.G.C.CC..UGCG..UG.GG.CAGAC...CUCG.....GUCUG.GCCGGAU..UUCCCAUGUUU.........ACCGGUU.UCUC.A.CCCCA..CGC...A....UG..GC.U.GC.CACCCAAUCCCGUGAGAAAGGAUCGCGUAG
Ppu-4-11    GCUCCAUG.GCG.G.U.CG..UGCG..CG.GG.CAGGC...UUCG.....GUCUG.GCCGGGGU.UACCGUAUGU.........CCCGGG..UCUC.A.CCCCG..UGC...A....CG..AC.U.GC.CACCCAAAUCCGUGAGACGAGGAGGAAGGC
Pen-1-9     CUUCCAUG.GCA.G.C.CG..UGUG..CG.GG.CAGGC...UUCG.....GUCUG.ACCGGG...UUUCUUGUGC.........CCCGGAU.UCUC.A.CCCUG..UUC...A....CG..GC.U.GC.CACCCUUUGUCUUGUGAGAAUUGCCUUGGG
Pen-1-10    UCUUCAUG.GCA.G.C.CG..UGUU..CG.GG.CAGAC...CUCUG....GUCUG.GCCGGG...UUUCAUGUCC..........UCGGUU.UCUC.A.CCCCG..UAC...A....CG..GU.U.GC.CACCCAAUCCCGUGAGAAAGGUUCAAGUGG
Pfl-2-7     ACUCGAUG.GUG.G.C.UG..UGAG..CA.GG.GUGCC...CCCG.....GGCAC.GCCGGAAA.AGCCUACUUC.........CCCGGUC.GACU.A.ACCUG..CUU...A....CA..GC.U.GC.CACCCCCUCGUUUAGUCGCGAAGCGGUAAC
Xor-1-1     UCUUUAUG.GCG.G.G.CG..GUA...CGUGG.GGGGC...UUCG.....GCCCC.GCCGG....UCCCUGAGU...........CCGGUA.GACC.A.ACCCG...UG...C....CG..UC.C.GC.CACCCC.GAUUGGUCUCGGGUUGACGGACU
env-28      GUUUUAUG.GCG.G.G.CG..GUA...CGCGG.GGGGC...UUCG.....GCCCC.GCCGG......CCCUUGG..........ACCGGUA.GACC.A.ACCCG...UG...C....CG..UC.C.GC.CACCCC.GAUUGGUCUCGGGGUGGCGGGAUU
Xca-1-1     GUUCUAUG.GCG.G.G.CG..GUG...CGUGG.AGGGC...UUCG.....GCCCU.GCCGG....UUCCGUUUGU..........CCGGUC.UGCC.A.ACCCG...CA...C....CG..UC.C.GC.CACC...UUGCUUGGCAGCAAGUGGGCGGA
Sma-1-5     GUUCUAUG.GCG.G.G.CG..GUG...CGUGG.GGAUC...UUCG.....GAUCC.GCCGG......UCUGGUACAU.......ACCGGUU.UUCG.A.GCCCG...CA...C....CG..UC.C.GC.CACCUUUAUUGGUGGCGCUGCUUUCUGUCC
Bdo-1-1     AUUUUAUG.GGC.G.G.GCGGUGGU..AG.GG.AGACC...CUCG.....GGUCU.GCCGGA...AUCCUCUAGA.........UCCGGUU.CGCC.A.ACCUU..GUC...A.UUCGC..CU.G.CC.CACCC..CCGUUUGGCGACGGGAUGGCAGG
Pfl-2-8     GUGUUAUG.GCG.G.C.UG..UGCG..CG.GG.AGGCU...CUUG.....GGCCU.GCCGGG...UUGGAUGGUUG........ACCGGUUUUUCC.A.GUCCG..UGC...A....CA..GC.U.GC.CACCCUUUCGCCUGGAAAACGAAAGAUGGG
Pfl-1-2     CUUUCAUG.GCG.G.C.UG..UACG..CG.GG.AGAUC...CUCG.....GAUCU.GCCGAU...UAUUGUGGGAUGA......GUCGGCU.CGCC.A.ACCCG..CGU...A....UA..GC.U.GC.CACCUAUUCGACUGGCGACGACAGGACAGC
Ppu-4-12    CGUCUAUG.GCG.G.U.GC..UGCG..CG.GG.ACACC...UGAU.....GGUGU.GCUGGA...UUCCUUUCGC.........UCCAGUC.GGCC.A.ACCCG..CGU...A....CC..GC.C.GC.CACCC..GAACUCACCCACUGCAUUUGCUU
Pen-1-11    CGUCUAUG.GCG.G.C.UG..UGCG..CG.GG.AGGCU...UUCG.....AGCCU.GCCGG....GUCUGGUACCUC........CCCGGUU.UACC.A.CCCCG..UGU...A....CA..GC.U.GC.CACCCAUUCGUGUGGUAACGGACGAGGUGU
Ppu-3-4     AGUCCAUG.GCG.G.C.UG..UUCG..CA.GG.AGGC....UUCGU.....GCCU.GCUGGG...UUUUAGAUCUCCCA......CCCGGUU.UACC.A.CCCUG..CGU...A....CA..GC.U.GC.CACCCAUUCGUGUGGUAACGGACUGGGUGG
Ppu-2-3     AGUCCAUG.GCG.G.C.UG..UUCG..CA.GG.AGGC....UUCGU.....GCCU.GCUGGG...UUUUAGAUCUCUCA......CCCGGUU.UACC.A.CCCUG..CGU...A....CA..GC.U.GC.CACCCAUUCGUGUGGUACCGACUGGGUGG
Ppu-4-13    AGUCUAUG.GCG.G.C.UG..UGCG..CG.GG.AGGCU...UUCG.....AGCCU.GCCUGG...AUUGGAAAAUGC........CCGGGUC.UACC.A.CCUCG..CGG...A....CA..GU.C.GC.CACCCAAUUCACGUGGUAGUGAUUGGUAGC
Ppu-1-4     AGUCUAUG.GUG.G.C.UG..UGCG..UG.GG.AGGCU...UUCG.....AGCCU.GCCUGG...AUUGGAAAAUGC........CCGGGUC.UACC.A.CCUCG..CGU...A....CA..GU.C.GC.CACCCAUUCACGUGGUAGUGAGGUUGGCG
env-29      AGUCUAUG.GUG.G.C.UG..UGCG..UG.GG.AGGCU...UUCG.....AGCCU.GCCUGG...AUUGGAAAAUGC........CCGGGUC.UACC.A.CCUCG..CGU...A....CA..GU.C.GC.CACCCAUUCACGUGGUAGUGAAGGUUGGCG
Psy-3-1     GUUUUAUG.GUG.G.U.CG..UGCG..CG.GG.GCAUC...UUCG.....GAUGC.ACCGGG...GUUCCUGGA............CCGGGC.UACU.A..CCUG..CGC...A....UG..AC.C.AC.CACCCU.CGCCUGGUAGCCGACGGUAAUGG
Psy-1-2     UUUUUAUG.GUG.G.U.CG..UGCG..UG.GG.GCAUC...UUCG.....GAUGC.ACCGGG...GUUCCUGGA............CCGGGC.UACU.A..CCUG..CGC...A....UG..AU.C.AC.CACCCU.CGCUUAGUAGCCGACGGUGAUGG
Psy-1-3     UCUUUAUG.GUG.G.C.CG..UGCG..CA.UG.GCGUC...UUCG.....GAUGC.ACCGGG...UGCCAGCAG............CCGGUC.UACC.A.ACUUG..CAC...A....CG..GC.C.GC.CACCUUAUCGUUUGGUAGCGAUUAGUGACG
Pfl-2-9     GUUUUAUG.GUG.G.U.CA..UUCG..CA.GG.GCACC...UUCG.....GGUGC.GCCGUU...UCUCCUAAGG.........CCCGGUC.UACC.A.ACCUG..CGU...C....UG..GC.C.AC.CACCA..UCGUUUGGUAGCGAGAGUGGUGG
Pfl-2-10    GUUUUAUG.GUG.G.U.CA..UGCG..UG.GG.GCUCA...UUCG.....UGGGC.GCCGGG...UUCCUAUUGCA........GCCGGUC.UACC.A.ACCCG..CGU...A....UG..GC.C.GC.CACCCA.UCGUUUGGUAGCGUGGGUGAUGG
Pfl-2-11    GCUUUAUG.GUG.G.U.CA..UGCA..CA.GG.GCACC...UUCG.....GGUGC.GCCGGG...UUCCUCAUGU.........GCCGGUC.UACC.A.ACCUG..AGC...A....UG..GC.C.GC.CACCUU.CGUUUGGUAGCGAAUUUGGAUGG
Pfl-2-12    GUUUUAUG.GUG.G.C.CA..UGCA..CA.GG.GCGUC...UUCG.....GGCGC.GCCGGU...CUCCUUGUGU.........CCCGGUC.UACC.A.ACCUG..CGU...A....UG..GC.C.AC.CACCCA.ACGUUUGGUAGCGUGUGGGUGAU
env-30      GUUUUAUG.GUG.G.C.CA..UGCG..CA.GG.GCGCU...UUCG.....AGCGC.GCCGGUUU.UUCCAUGUGU.........GCCGGUC.UACC.A.ACCUU..CGU..AA....UG..GC.C.AC.CACCC..UCGUUUGGUAGCGAAUGUGAUGG
Psy-2-1     CGUUUAUG.GUG.G.C.CG..UGCG..CU.UG.GCGUC...UUCG.....GACGC.ACCGGC...UUGCCUGGUC.........ACCGGUC.UACC.A.ACCUG..CGU...A....CG..GC.C.GC.CACCC..UGAUCGUUUGGUAGCGAAAGGGG
Pfl-2-13    GUUUUAUG.GUG.G.C.CA..UGCG..UA.GG.GCGUC...UUCG.....GACGC.GCCGGG...UAUCCAGGUG.........ACCGGUC.UACC.A.ACCUC..CGU...A....UG..GC.C.AC.CACCC..UCGUUUGGUAGCGAGGCUGAUGG
Psy-2-2     UCUUUAUG.GUG.G.C.CG..UGCG..UG.GG.GCGUC...UUCG.....GAUGC.ACCGG....UUCCUAAUC..........ACCGGGC.UACU.A..CCUG..CGU...G....CG..GC.C.GC.CACCU..UCGAUUAGUAGCCGAAGUGACGA
Pfl-2-14    GGUCUAUG.GUG.G.U.CA..UGUA..CA.GG.GCGUC...UCCG.....GGCGC.GCCGG....CUCCUAACAC...........CCGGUC.UACC.A.ACCUG..UUC...A....UG..GC.C.GC.CACCCUAAUGAGAUGGUCAGCCCAGUACGG
Psy-3-2     AUGCUAUG.GCG.A.C.UG..UGCG..CU.UG.GCACC...UUCG.....GGUGC.GCCGG....UUCUUAGCUC.........ACCGGUC.UACU.A.ACUUG..CGU...A....CA..GU.U.GC.CGCCUU.UCGUUUAGUAGCGAGCAGGCAGC
Pfl-2-15    AUUUUGCG.GUG.G.C.UG..UGCG..CA.UG.GCACC...UUCG.....GGUGC.GCCGGG...UUUGGUGAUG.........ACCGGUC.UGCU.A.ACUUG..CGC...A....CA..GC.U.GC.CACCUU.CCGUUUAGCAGCGAGACGGUUGC
Avi-1-2     GUGUCAUG.GCG.G.C.UG..UGCG..UG.GG.AGGGC...UUCG.....GCCCU.GCCGGG...UCCCGUUUGC.........CCCGGUC.CGCG.A.ACCCG..CGC...A....CA..GU.C.GC.CACCAGUUACCGCGUCGCGGCGGUCGGUGG
Ppu-2-4     GUUUUAUG.GCG.G.C.UG..UGCG..UA.GG.GCGC...CUCCGU.....GCGC.GCCGGU...UUCCUUGAUU.........CCCGGUC.UACC.A.ACCUG..CGU...A....CA..GC.C.GU.CACCACUUCGUUUGGUAGCGAUCUGUGGCG
Pen-1-12    GUUCAAUG.GUG.G.C.UG..UGCG..UG.GG.GCGCA...CUCG.....UGCGA.GCCGGU...UUCCUUGAUU.........CCCGGUU.GACC.A.ACCCG..CGU...A....CA..GC.C.GU.CGCCAU.UCGUGUGGUGUCCAGAUGCAGCA
Pfl-2-16    GCUCAAUG.GUA.G.C.UG..UGCG..CA.GG.GCGCC...UUCG.....GGUGC.GCCGGUAA.AGCUUGGAGU.........CCCGGUC.GACU.A.ACCUG..CGC...A....UA..GC.U.GC.CACCCAAUCGUUUAGUCGCGAAAUGGGAGC
Pfl-2-17    CCGUUAUG.GCG.G.C.UG..UGCG..CG.GG.AGACA...UUUA.....UGUCU.GCCGGG...UCGCGUUCUC.........CCCGGAU.UUCC.A.GCCCG..CGU...A....CA..GC.U.GC.CACCCAUUCUUCCUGGAAGUCGAUUGGGCU

            ......<<.<<<.<.<.<<..<<<<..<<.<<.<<<<<<<........>>>>>>>.<<<<<.........................>>>>>..........>>>>..>>>..>....>>..>>.>.>>.>>...........................
            ......?1.222.2.2.22..2222..22.22.2222220........0222222.22222.........................22222..........2222..222..2....22..22.2.22.1?...........................
            •••Y•AUG-GYR-G-•-Y•--•R••--•G-GG-••RYY---YU•R-----RRY•Y-GCCGG○---YYYY•••••○----------○CCGGU•-Y•YY-A-RCCY•--••Y---•----•R--•Y-Y-RC-CACC•○○•••••Y•••R••••••••R••••
            5′———————————————————————————————————————————————————————————————————————————————————————————————————————————————————————————————3′
```

```
Pfl-2-18   GUUUCAUG.GCG.G.C.UG..UGCG..CG.GG.AUACCUUCCUUG...GAGGUAU.GCCGA....UUCCUCAGUU..........AUCGGUC.CGCG.A.ACCCG..CGU...A....CA..GC.U.GC.CACCUCAGAUUACGUCGCGGCAAUCGAAGG
Pfl-1-3    GCUUAAUG.GUG.G.GCUG..UGCG..CG.GG.ACACC...UUCG.....GGUGU.GCCGGG...UGCCUUCUUC...........CCGGUU.GACC.A.ACCUG..UGC...A....CA.GUC.C.GC.CACCCA.UCGUUUGGUCACGAUUGGGGCGG
Pen-1-13   CUUCAAUG.GCG.G.C.UA..UGCGU.UG.GG.AGACC...UUCG.....GGUCU.GCCGGG...UUCCUGUAUC..........CUCGGUC.UUGC.A.CCCCU.GCGC...A....UA..GC.U.GC.CACCUAUUCGAGUGCAAGCGGAUUUCGGCA
Pfl-2-19   UUUUAAUG.GCG.G.U.UG..UGCG..UG.GG.AGACC...UUCG.....GGUCU.GCCGG....GUCUUAGCUC..........CUCGGUC.UGCG.A.ACCCG..CGC...A....UA..AC.U.GC.CACCCA.ACUCGUUUCGCAGCGAGCAGUGA
Pfl-2-20   ACUUGAUG.GUG.G.C.UG..UGCG..CA.UG.GCAUC...UUCG.....GAUGC.GCCGGG...UUGUGAUGCUU.........ACCGGGC.CGCU.A.ACUUG..CGC...A....CA..GC.U.GC.CUCCCUUUGCUCAGCGGCGAAAUGGGCGC
Pfl-2-21   GUGCAAUG.GCG.G.C.UG..UGCG..UG.GG.AGGCU...UUCG.....AGCCU.GCCGGG...UUCCUGUAUC..........UCCGGUU.UCUC.A.GCCUG..CGC...A....UA..GC.U.GC.CACCCUUUCGCCUGAGAACGAAUUGGCAGC
Pfl-1-4    GUGCAAUG.GCG.G.C.UG..UGCG..UG.GG.AGGG....CUCGU.....CCCU.GCCGGG...UUCCUGUAUC..........UCCGGUU.UACC.A.ACCUG..CGC...A....CG..GC.U.GC.CACCCAUUCGCUUGGUAACGAACGUGGCAG
env-31     CAAUUAUG.GCG.G.C.UG..UGCG..UA.GG.GCGCC...UUCG.....GGCGC.GCCGGG...UUUGUGUGGGAUU.......ACCGGUC.UACU.A.ACCUG..CGC...A....CA..GC.U.GC.CACCCAACUUGUUUAGUAGCGAUGCGGGUG
env-32     GCUUGAUG.GUG.G.C.UG..UGCG..CA.GG.GCGUC...CUCG.....GGCGC.GCCGGC...UUUGUGUGUUUC........CCCGGUC.UACU.A.ACCUG..UGC...A....CA..GC.U.GC.CACCCUUUCGUUUAGUAGCGAAAAGGUGUC
env-33     GUUUUAUG.GUG.G.C.UG..UGCG..CA.GG.GCACC...UUCG.....GGUGC.GCCGGAU..UUACCUUGUG..........GCCGGUC.GACU.A.ACCUG..CGU...A....CA..GC.C.GC.CACCCA.UCGUUUAGUCGCGACAGGUGUUG
env-34     GUUUGAUG.GUG.G.C.UG..UGCG..UA.GG.GCAUC...UUCG.....GGUGC.GCCGGCU..UUGCGUUGUUC.........CCCGGUC.UACU.A.ACCUG..CGC...A....CA..GC.U.GC.CACCC..UCGUUUAGUAGCGAGGUAAGGGC
Pfl-2-22   GCUUGAUG.GUA.G.C.UG..UACG..CA.GG.GCGCU...UUCG.....GGCGC.GCCGG....UUCGCGUCUC..........CCCGGUC.UACU.A.ACCUG..CGU...U....CA..GC.U.GC.CACCCCAUCGUUUAGUAGCGAUCCGGUGUC
env-35     CCUCAAUG.GUG.G.C.UG..UACG..CA.GG.GCACU...UUCG.....GGUGC.GCCGGU...UUCGUUGAUCC.........GCCGGUC.UACU.A.ACCUG..CAU...A....CA..GC.C.GC.CACCCA.UUGUUUAGUAGCUCUGGAAGGUG
Pfl-1-5    GCUUCAUG.GCG.G.C.UG..UGCG..UG.GG.AGAUCC..UUUU....GGGUCU.GCCGGC...UUAUCUGGUG...........CCGGUU.CGCC.A.ACCUG..CGU...A....CA..GC.U.GC.CACCU..UUCGUUUGGCGACGGUAGGACA
Psy-1-4    CGUUUAUG.GCG.G.C.UU..UGCG..UG.GG.GCACC...UUCG.....GGUGC.GCCGGU...UGCUUGCGCUUGUUCCAC..ACCGGUC.UGCU.A.ACCCG..CGU..CA....AA..GU.C.GC.CACCCUUGUUUGUUAGCCGCAGGCAUCUG
Psy-2-3    CGUUUAUG.GCG.G.C.UU..UGCG..UG.GG.GCACC...UUCG.....GGUGC.GCCGGU...UGCUUGCGCUUGUUCCAC..ACCGGUC.UGCU.A.ACCCG..CGU..CA....AA..GU.C.GC.CACCCUUGUUUGUUUAGCAGCAGGCAUCUG
Pen-1-14   GCGUCAUG.GCG.G.U.GG..UGCG..CG.GG.ACACC...UUCG.....GGUGU.GCCGGG...CCCCUUUCGU..........CCCGGUC.GGCC.A.ACCCG..CGU...A....CU..GC.U.GC.CACCUAAACUUGCUUGGCCGCGAGCGGUGG
env-36     GUUUUAUG.GUG.G.C.UG..UGCG..CG.GG.GCGCU...UUCG.....AGUGC.GCCGGG...UGCCUAACGU..........CCCGGUC.UUCC.ACACCUG..CGU...A....CA..GC.C.GC.CACCCAUUACGUGGAAGUAGUGCUUGGCGG
Pfl-2-23   GCGUAAUG.GUG.G.C.UG..UGCG..UG.GG.AGACC...UUCG.....GGUCUACCCGGGG..UUUCUGUGAC...........CCGGAU.CGCU.A.ACCUG..CGU...A....CA..GC.C.GC.CACCUUUACUUGUUUAGCGAUGAGUCGUGG
Pfl-2-24   CCGUUAUG.GCG.G.C.UG..UAUG..CG.GG.AGACC...UUCG.....GGUCU.ACCGGGG..UGCCUGAGA............CCGGUU.CGCU.A.ACCUG..CGU...A....CA..GC.U.GC.CACCCU.UACUUGUUUAGCGACAGGAAAG
Pfl-2-25   CCGUUAUG.GCG.G.C.UG..UAUG..CG.GG.AGACC...UUCG.....GGUCU.ACCGGGG..UGCCUGAGA............CCGGUU.CGCU.A.ACCUG..CGU...A....CA..GC.U.GC.CACCCU.UACUUGUUUAGCGACAGGAAAG
Psy-2-4    GUGCUAUG.GCG.G.C.UG..UGCG..UC.GG.AGACC...UUCG.....GGUCU.GCCGGU...UUCCUGGAU...........ACCGGUU.CGUC.A.ACCUU..CGC...A....CA..GC.U.GC.CACCCAUUCGUUUGACGACGUCUUGUGGCA
env-37     GCUUUAUG.GCA.G.C.UG..UGCG..CG.GG.AGACC...UUCG.....GGUCU.GCCGG....AACUCGGUC............CCGGUU.CGCC.A.ACCUG..CGU...A....CA..GC.U.GU.CACCCAUUCGUUUGGCGACGAUAGGUGACA
env-38     GCUUUAUG.GCG.G.C.UG..UGCG..CG.GG.AGACC...UUCG.....GGUCU.GCCGG......CCUUUGCUUUAUUUC...ACCGGUU.CGCC.A.ACCUG..CGU...A....CA..GC.U.GC.CACCCAUUCGUUUGGCGACGUGUGAGUGGU
env-39     CUUUCAUG.GUG.G.C.UG..UGCG..CG.GG.AGACC...UUCG.....GGUCU.GCCGG....UUCCAUUC.............CCGGUU.CGCC.A.ACCUG..CGU...A....CA..GC.U.GC.CACCCAAUUCGUUUGGCGACGAAUAAGUGU
Pfl-1-6    GUUUUAUG.GUG.G.C.UG..UGCG..UG.GG.AGACC...UUCG.....GGUCU.GCCGGG...UUCCUUUUCC...........CCGGUU.CGCC.A.ACCUG..CGC...A....CA..GC.U.GC.CACCCUAAUUCGUUUGGCGACGAUCAGUGG
Ppu-3-5    GUGUAAUG.GCG.G.C.UG..UGCG..UG.GG.AGACC...UUCG.....GGUCU.GCCGAG...UUCCUUUUCC...........UCGGUU.CGCC.A.ACCUG..CGU...A....CA..GC.U.GC.CACCCUAAUCGUUUGGCGACGGUCAGCGUG
env-40     GUGUAAUG.GCG.G.C.UG..UGCG..UG.GG.AGACC...UUCG.....GGUCU.GCCGAG...UUCCUUUUCC...........UCGGUU.CGCC.A.ACCUG..CGU...A....CA..GC.U.GC.CACCCUAAUCGUUUGGCGACGGUCAGCGUG
Pen-1-15   GUGUAAUG.GCG.G.C.UG..UGCG..UG.GG.AGACC...UUCG.....GGUCU.GCCGGG...UUCCUUUUCC...........UCGGUU.CGCC.A.ACCUG..CGU...A....CA..GC.U.GC.CACCCUAUUCGUUUGGCGACGAUGGUGUGG
Ppu-4-14   GUGUAAUG.GCG.G.C.UG..UGCG..UG.GG.AGACC...CUCG.....GGUCU.GCCGGG...UUCCUUUUCC...........UCGGUU.CGCC.A.ACCUG..CGU...A....CA..GC.U.GC.CACCCUAAUCGUUUGGCGACGGUCAGCGUG
Pen-1-16   GUGCCAUG.GCG.G.C.UG..UGCG..CG.GG.AGACC...UUCG.....GGUCU.GCCGGG...UUCCAAGUUC...........CCGGUU.CGCC.A.ACCUG..CGU...A....CA..GC.U.GC.CACCCAUUGUUUGGCGACAGUGCGUGGUGG
Ppu-4-15   GUGCCAUG.GCG.G.C.UG..UGCG..UG.GG.AGGCC...UUCG.....GGUCU.GCCGGG...UUCCAAGUUC...........CCGGUU.CGCC.A.ACCUG..CGU...A....CA..GC.U.GC.CACCCUCGCGAAUAACCCCGCGAACGGGCC
env-41     CGUUUAUG.ACG.G.C.UG..UGCG..CG.GG.AGACC...UUAG.....GGUCU.GCCGGU...UUUCUCUACU..........GCCGGUU.CGCC.A.ACCUG..CGU...A....CA..GC.U.GU.CACCCAUACGUUUGGCGACGUUUGAGUGAU
Psy-3-3    GCGCUAUG.GCG.G.C.UG..UGUG..CG.GG.AGACC...UUCG.....GGUCU.GCCGGGGUUUGUCUUUUUG..........CCCGGUU.CGCC.A.ACCCU..CGC...A....CA..GC.U.GC.CACCCAUUCGUUUGGCGACGAUUCGUGGCG
Pae-3-1    GAGUUAUG.GCG.G.C.UG..UGCG..CG.GG.GCACC...UUCG.....GGUGC.GCCGGG...UUCCUUUGUC..........CCCGGUC.UGCU.A.ACCCG..CGU...A....CA..GC.U.GC.CUCCCAUCGUUUAGCAGCGAUUCGUGGCAG
Ppu-4-16   CCGUUAUG.GCG.G.C.UG..UGCG..UG.GG.GCACC...UUCG.....GGUGC.GCCGGG...UGCCUGACGU..........CCCGGUC.GGCU.A.ACCCG..CGU...A....CA..GC.U.GC.CACCC..AUUCGUUUAGCCGCGAUUGGUGG
Pfl-1-7    GUGUUAUG.GCG.G.C.UG..UGUG..CG.GG.ACACC...UUCG.....GGUGU.GCCGG....UGUCGUUGUA..........GCCGGUC.GGCC.A.ACCCU..CGC...A....CA..GC.U.GC.CACCCUCAAUUUGCUUGGCCGCAAACGGUG
Pfl-2-26   GUGUUUUG.GCG.G.C.UG..UGCG..UG.GG.ACACC...UUCG.....GGUGU.GCCGG....UUCCUGUAU............CCGGUC.GGCC.A.ACCCG..CGU...A....CG..GC.U.GC.CACCCU.CUUCGUUUGGCCGCGAAUGGUGA
Pfl-2-27   AUGUCGCG.GUG.G.C.UG..UGCG..UG.GG.ACACC...UUCG.....GGUGU.GCCGGA...UUCCUUGAUG..........UCCGGUC.GGUC.A.ACCCG..CGU...A....CA..GC.U.GC.CACCCU.GUUUGUUUGACCGCGAACGGUGG
env-42     UAGUUAUG.GCG.G.C.UG..UGCG..CU.GG.AGACC...UUCGG....GGUCU.GCCGGG...UUUCCUUUGC..........CCCGGUC.UUGC.ACACCUG..CGU...A....CA..GC.U.GC.CACCCUCAUUCGCGUGCAAGCGAACGGUGA
env-43     GCGCUAUG.GUG.G.C.UG..UGCG..CG.GG.AUACC...UUCG.....GGUAU.GCCGGU...UUCCUUAAUC..........UCCGGUC.CGCG.A.ACCUG..CGC...A....CA..GC.U.GC.CACCCUCUAUCGCAUCGCGGCAUCAGUGG
Pfl-1-8    GAUCUAUG.GCG.G.C.UG..UGCG..UA.GG.GCACC...UUCG.....GGUGC.GCCGG....UUCCUUUAGC...........CCGGUC.UUCC.ACACCUG..CGC...A....CG..GC.U.GC.CACCCUCUCAUGUGGAAGUGAGAUCCGGCG
Avi-1-3    CUGUCAUG.GCG.G.C.UG..UGCG..CG.GG.AGACC...GAAA.....GGUCU.GCCGGG...UUCCGUGUGU..........CCCGGUU.CGCC.A.ACCUG..CGC...A....CA..GU.C.GC.CUCCAAUGACCGUUUGGCGACGGUCGGGGG
Psy-1-5    ACUUUAUG.GCG.G.C.UG..UGCG..CGAGG.GCACC...UUUG.....GGUGC.GCCGG......UCUUAGCU..........CCCGGUC.GACU.A.ACCUU..CGC...A....CA..GC.U.GC.CACCUC.ACUCGUUUAGUCGCGACCCCAGU
Psy-2-5    ACUCUAUG.GCG.G.C.UG..UGCGU.GA.GG.GCACU...UUCG.....GGUGC.GCCGG......UCUUAGCU..........CCCGGUC.GACU.A.ACCUU.UCGC...A....CA..GC.U.GC.CACCUCAAUCGUUUAGUCGCGACCCAGUGG
Pfl-2-28   UCGUUAUG.GCG.G.C.UG..UGCG..UC.GG.AGACC...UUUG.....GGUCU.GCCGGG...CACCUGGA.............CCGGUC.UUGC.ACACCAA..CGC...A....CA..GC.U.GC.CACCU..AUUUCGAAGUGCAAAGCGAAACG
env-44     UCGUUAUG.GCA.G.U.UG..UACG..CG.GG.AGAUC...UUCG.....GAUCU.ACCGG......ACUCCGUC...........ACGGUC.UUGC.ACACCCG..CGU...A....CU..GC.U.GC.CACCCUAAUUCGUGUGCGAGCGAACGGUUG
Pfl-1-9    GUGUUAUG.GCG.G.C.CG..UGCG..CG.GG.AGAUC...UUCG.....GAUCU.GCCGGG...GUUCUGGUAA..........CUCGGUC.UUGC.ACACCCG..CGU...A....CG..GC.U.GC.CACCUUAUUCGUGUGCAAGCGAACGGUGUC
Ppu-4-17   CUUCAAUG.GCG.G.C.UG..UGUG..UG.GG.GCACG...CUUG.....CGUGC.GCCGGAAC.UUCCUAUGUCC.........GCCGGUC.UGUC.A.ACCUG..CAC...A....CA..GC.U.GC.CACCCAAACUGUUUGACAGCAGGAUAGGUG
Ppu-4-18   CUUCAAUG.GCG.G.C.UG..UACG..UGCGG.GCACG...CUUG.....CGUGC.GCCGGAAC.UUGCAUAGUCC.........GCCGGUC.UGUC.A.ACCCA..UGU...A....CA..GC.U.GC.CACCCUUCUGUUUGACAGCGGAUAGGUGAC
Pen-1-17   GUUUAAUG.GUG.G.C.UG..UACG..UGUGG.GCACG...CUUG.....CGUGC.GCCGG....AACUUUUGUGUGAUUUACC.GCCGGUC.UGUC.A.ACCCA..CGU...A....CA..GC.U.GC.CACCCU.UCUGUUUGACAGCAGGCUGGUGG
Pen-1-18   CCUCGAUG.GCG.G.C.UG..UACG..UGCGG.GCACG...CUCG.....CGUGC.GCCGG....AACUUUGCUUAUUCC....GCCGGUC.UGUC.A.ACCCG..CGU...A....CA..GC.U.GC.CACCCU.UCUGUUUGACAGCAGACAGGUGG
Ppu-4-19   CUUCUAUG.GUG.G.C.UG..UGCG..UG.GG.GCACG...CUUG.....CGUGC.GCCGG....AACUUUGCUUAUUCC....GCCGGUC.UGUC.A.ACCCA..CGU...A....CA..GC.U.GC.CACCCUUCUGUUUGACAGCGGCGAGGUGAC
Ppu-4-20   UUUUUAUG.GUG.G.C.UG..UGCG..UGUGG.GCACG...CUUG.....CGUGC.GCCGGAGCUUUUCGUGAUUUUACC....GCCGGUC.UGUC.A.ACCCA..CGU...A....CA..GC.U.GC.CACCCCACUGUUUGACAGCAGGUGGGUGAC
Pen-1-19   CCUCUAUG.GCG.G.C.UG..UGCG..UGCGG.GCACG...CUUG.....CGUGC.GCCGGA...AUUUUACGAUUUUC.....GCCGGUC.UGUC.A.ACCCA..CGC...A....CA..GC.U.GC.CACCC..UCUGUUUGACAGCAGACAGGUGG
Pen-1-20   GCUCUAUG.GCG.G.C.UG..UGUG..UG.GG.GCAC....CUCGU.....GUGC.GCCGGG...UUUUUUGGCUUUUUCCU...GCCGGUC.UGUC.A.ACCCA..UGC...A....CA..GC.U.GC.CACCCUCUUGUUUGACAGCAGAUGGUGUCG
Pen-1-21   UUUAUAUG.GCG.G.C.UG..UAUG..UG.GG.GCAC....UUCGU.....GUGC.GCCGGG...GUUUUGCUCUUGCUG....ACCGGUC.UGUC.A.ACCCA..UGU...A....CA..GC.U.GC.CACCC..UCUUGUUUGACAGCAGACGGUGG
Ppu-3-6    CGUUCAUG.GCG.G.U.UG..UGCA..UG.GG.GCACG...CUUG.....CGUGC.GCCGGU...UUCUGUGUUC..........GCCGGUC.UGUC.A.ACCCA..UGC...A....CA..GC.U.GC.CACCAUUCCGUUUGACAGCGGGUUGGCGGU
Pen-1-22   CCUCUAUG.GCG.G.C.UG..UGCA..UG.GG.GCACG...CUUG.....CGUGC.GCCGGU...UUUUGUGUCC..........GCCGGUC.UGUC.A.ACCUA..UGC...A....UA..GC.U.GC.CACCUACCUGUUUGACAGCAGUCCGGUAGC
Ppu-3-7    CAGUUAUG.GCA.G.C.UG..UGCG..UG.GG.UUAC...CUUCGU.....GUGU.GCAGGG...AUUCUCAGUGCC........CCCGGUC.UGUC.A.ACCCA..CAA...A....CA..GC.U.GC........ACUGAGAGGGUUUAUGCUGAACA
Ppu-1-5    CAGUUAUG.GCA.G.C.UG..UGCG..UG.GG.GCAC...CUUCGU.....GUGC.GCCGGG...AUUCUCAGUGCC........CCCGGUC.UGUC.A.ACCCA..CAA...A....CA..GC.U.GC.CACCC..UUCCGUUUGACAGCGGUACUGCG

           ......<<.<<<.<.<.<<..<<<<..<<.<<.<<<<<<<........>>>>>>>.<<<<<.........................>>>>>..........>>>>..>>>...>....>>..>>.>.>>.>>...........................
           ......?1.222.2.2.22..2222..22.22.2222220........0222222.22222.........................22222..........2222..222...2....22..22.2.22.1?...........................
           •••Y•AUG-GYR-G-•-Y•--•R••--•G-GG-••RYY---YU•R-----RRY•Y-GCCGG∘---YYYY•••••∘----------∘CCGGU•-Y•YY-A-RCCY•--••Y---•-----•R--•Y-Y-RC-CACC•∘∘•••••Y•••R•••••••••R••••
           5′                                                                                                                                                  3′
```

```
Ppu-1-6    CCUUUAUG.GCA.G.C.UG..U.CA..UG.GG.GCAC....CUCGU.....GUGC.GCCGG......CUUUUGGUUCUGUUC...ACCGGUC.UGUC.A.ACCCA..UGU...A....CA..GC.U.GC.CGCCU..CAUUGUUUGACAGCAGGAGGUGG
Pen-1-23   CCGUUAUG.GCA.G.C.UG..UGCG..UG.GG.GCAC....CUCGU.....GUGC.GCCGGU...UUCUGGUCUUGUUC......ACCGGUC.UGUC.A.ACCCA..UGU...A....CA..GC.U.GC.CACCUUCUUGUUUGACAGCAGGCUGUGGUA
Pen-1-24   CCGUUAUG.GCA.G.C.UG..UGCG..UG.GG.GCAC....CUCGU.....GUGC.GCCGGU...UUCUGGUCUUGUUC......ACCGGUC.UGUC.A.ACCCA..UGU...G....CA..GC.U.GC.CACCUUCUUGUUUGACAGCAGGCUGUGGUA
Pen-1-25   CUCUUAUG.GCG.G.C.UG..UGCG..UG.GG.GCAC....UUCGU.....GUGC.GCCGGG...UUUUGGGAUGCCU.......ACCGGUC.UGUC.A.ACCCA..UGU...A....CA..GC.U.GC.CACCUUCUUGUUUGACAGCAGGCGUGUUGC
Ppu-4-21   CCUUUAUG.GCA.G.C.UG..UGCA..UG.GG.GCAC....UUCGU.....GUGC.GCCGGG...UUUUGGUUUGCCU.......ACCGGUC.UGUC.A.ACCCA..UGU...A....CA..GC.U.GC.CACCCUCUUGUUUGACAGCAGGUGUGUAGU
Ppu-4-22   CCGUUUUG.GCA.G.C.UG..UGCA..UG.GG.GCAC....CUCGC.....GUGC.GCCGGU...UUUGGUCUGUUC........ACCGGUC.UGUC.A.ACCCA..UGU...A....CA..GC.U.GC.CGCCU..UUCUGUUUGACAGCAGAGGGUGG
Ppu-4-23   GCUUCAUG.GCA.G.C.UG..UACA..UG.GG.GCAC...CUUCGU.....GUGC.GCCGGG...UUCCUUGGGUC.........ACCGGUC.UGUC.A.ACCCG..UGU...A....UA..GC.U.GC.CUCCAUUCGUUUGACAGCGUUUAAAGGUAG
Ppu-1-7    GCUUAAUG.GCA.G.C.UG..UGCA..UG.GG.GCACA....UUCG.....UGUGC.GCCGG....GUCCUUGGGU..........ACCGGUC.UGUC.A.ACCCA..UGU...A....UA..GC.U.GC.CUCCAU.UCGUUUGACAGCGUUGCCAGGCG
Ppu-1-8    CUUCAAUG.GUG.G.C.UG..UGCG..UG.GG.GCGCC...UUCG.....GGUGC.GCCGGG...UUUCUCAAUUUG........ACCGGUC.UGUC.A.ACCCG..UGC...A....CA..GC.U.GC.CACCCU.UUGUUUGACAGCGAAUGGCGGUA
Ppu-3-8    CUUCAAUG.GUG.G.C.UG..UGCG..UG.GG.GCGCC...UUCG.....GGUGC.GCCGGG...UUUCUCAAUUUG........ACCGGUC.UGUC.A.ACCCG..UGC...A....CA..GC.U.GC.CACCCU.UUGUUUGACAGCGAAUGGCGGUA
Pen-1-26   CCUCAAUG.GUG.G.C.UG..UGCG..UA.GG.GCACG...UUCG.....CGUGC.GCCGGU...CUUCUCAGUUCC........UCCGGUC.UGUC.A.ACCUA..CGC...A....CA..GC.U.GC.CACCC..ACCGUCUGACAGCAGGUGGAUG
Ppu-2-5    CCUCAAUG.GCG.G.C.UG..UGCG..UGUGG.GCACG...UUCG.....CGUGC.GCCGGUC..UUUCUUUCUUUCC......ACCGGUC.UGUC.A.ACCCG..CGC...A....UA..GC.U.GC.CACCCAACCGUUUGACAGCGGGCGGAUGGC
Ppu-4-24   CUGUUAUG.GUG.G.U.UG..UACG..UGCGG.GCACC...CUCU.....GGUGC.GCCGAG...UGCCAACGUC..........UCGGUC.UGUC.A.ACCCG..CGU...A....CA..GC.U.GC.CACCUCAUUGUUUGACAGCAAGGCCGUGGU
Ppu-4-25   U...UAUG.GCG.G.C.UG..UACG..UGCGG.GCAC...CUUCGU.....GUGC.GCCGAG...UGCCAACAAU.........CUCGGUC.UGUC.A.ACCCG..CGU...A....CA..GC.U.GC.CACCCUCUUGUUUGACAGCAAGGCGGCUUG
Ppu-3-9    CGCUUAUG.GCG.G.C.UG..UACG..UGCGG.GCCC...CCUCAU.....GUGU.GCCGAG...UGCCAACAAU.........CUCGGUC.UGUC.A.ACCCA..AGU...A....CA..AC.U.GC.CACCCUCCCGUUUGACAGCGAGGUGGAUAG
Pen-1-27   CUUUCAUG.GCG.G.C.UG..UACG..UGUGG.GCAC....CUCGU.....GUGC.GCCGAG...UGCCAACGUC..........UCGGUC.UGUC.A.ACCCG..CGU...A....CA..GC.U.GC.CACCCUCUUGUUUUGACAGCAAAGUGUGGC
Ppu-3-10   CUUUCAUG.GCG.G.C.UG..UACG..UGUGG.GCACC...CUGU.....GGUGC.GCCGAG...UGCCAACGUC..........UCGGUC.UGUC.A.ACCCG..CGU...A....CA..GC.U.GC.CACCAUCUUGUUUGACAGCAAGGUUGAGGU
Ppu-3-11   UGUUUAUG.GCG.G.C.UG..UACG..UG.GG.GCAC...CUUCGU.....GUGC.GCCGAG...UUACUCCGGGAUC......CUCGGUC.UGUC.A.ACCCA..UGU...A....CA..GC.U.GC.CACCCUCUUGUUUGACAGCAAGCUUGCGGC
Pen-1-28   CCCUCAUG.GCG.G.C.AG..UACG..UG.GG.GCAC....CUCGU.....GUGC.GCCGGG...UCUCGGAGUC.........CUCGGUC.UGUC.A.ACCCA..CGU...A....CC..GC.U.GC.CACCCUCUUGUUUGACAGCAAGCCUGUUGC
Psy-1-6    CUGUUAUG.GCG.G.C.UG..UGCG..UC.GG.AGACU...CCAG.....GGUCU.GCCGGC...GUUGUGAGGU.........GCCGGUU.CGCU.A.ACCUU..CGC...A....CA..GC.C.GC.CACCC..AUUUCGUCCAGUGAUGUGCAGCC
Ppu-4-26   A...UAUG.ACG.G.C.UG..UACG..CG.GG.AGGUC...UUCG.....GGCCU.GCCGGG...UUAACCAUGC.........ACCGGUCUUUCC.A.CCCCG..UGU...A....CA..GC.U.GU.CACCCUAAUGUGUGGAAACGGACGGGUGGC
Ppu-3-12   UCGAUAUG.ACG.G.U.UG..UGCG..UG.GG.AGGCC...UUCG.....GGCCU.GCCGGG...UUUGCCAUACC.........CUGGUC.UUCC.A.CCCCG..CGU...A....CA..AC.U.GU.CACCC..UAAGUGUGGAAGCGAAAGGGUGC
Ppu-1-9    CAUUUUUG.GCG.G.C.UG..UGCG..CG.GG.AGGUA...UUCG.....UACCU.GCCGGGUC.UACCGUGUGC.........CUGGUC.UUCC.A.CCCCG..CGU...A....CA..GU.U.GC.CACCCUAAUGUGUGGAAGCAGAAGGGGACA
Ppu-3-13   CCGUUAUG.GCA.G.U.UG..UGCG..CG.UG.AGGCC...GUUU.....GGCCU.ACCGGC...UUGCCUUCUC.........ACCGGUU.UUCC.A.CCCCG..CGC...A....CA..AC.U.GC.CGCC...UUCUGCGUGGAAACGGGUGGUUG
Ppu-1-10   CCGUUAUG.GCA.G.U.UG..UGCG..CG.UG.AGGCC...GUUU.....GGCCU.ACCGGC...UUGCCUUCUC.........CCCGGUU.UUCC.A.CCCCG..CGC...A....CA..AC.U.GC.CGCC...UUCUGCGUGGAAACGGUGGUUG
Pen-1-29   CCGUAAUG.GCA.G.U.UG..UAUG..CG.GG.AGGCC...UCU.....GGCCU.ACCGGG...UGACCUUCUU.........UCCGGAU.UUCC.A.CCCCG..UGU...A....CA..AC.U.GC.CGCCCUCUGCGUGGAAACGGACGGUUGCAG
env-45     UUUCUAUG.GUG.G.A.CC..GUGU..GG.GG.CAGGC...UUUU.....GCCUG.GCCGGU...UCCCUCGGAC.........GCCGGUU.UUCC.A.CCCCC..GCA...C....GG..UC.G.GC.CACCCAUAGCCGAGUGACAACGGCGGGUGA
env-46     CAUUUAUG.GCG.G.G.CC..GUGU..GA.GG.CAGGC...UUCG.....GCCUG.GCCGGC...GUCCUUCGUGG.........GUCGGUC.UACC.A.CCCUU..GCA...C....GG..UC.G.GC.CUCCAUAUCGUGUGGUAGCGAAUGGAACGC
Pme-1-1    CAUUUAUG.GCG.G.G.CC..GUGU..GA.GG.CAGGC...UUUU.....GCCUG.GCCGGCC..UUCCUUCGUGG........GUCGGUC.UACC.A.CCCUU..GCA...C....GG..UC.G.GC.CUCCAU.UCGUGUGGUAGCGAAUGGCUGCG
Pme-1-2    U..CUAUG.GCG.G.G.CC..GUGU..GA.GG.CGGGC...UUCG.....GCCCG.GCCGGU...UCCCUUGGUU.........CCCGGUU.UACC.A.CCCUU..GCA...C....GG..UC.G.GC.CUCCAU.UCGUGUGGUAACGAGUGGGCGCG
Rso-2-1    UUUUUAUG.GCG.G.G.CC..GGGC..GG.GG.CCAGC...UUCG.....GCUGG.GCCGGU...UGCCUGUAGG..........CCGGUU.UUCC.A.CCCCC..GCCGUCU....GG..CC.C.GC.CACCC..UCAUGUGGAAAUGAGCGGCGGGC
Rpi-1-1    UUUUUAUG.GCG.G.G.CC..GGGC..GG.GG.CCAGC...UUCG.....GCUGG.GCCGGU...UUCCUGUAGG..........CCGGUU.UCUC.A.CCCCC..GCUGUCU....GG..CC.C.GC.CACCC..UCACGUGAGAAUGAGCGGCGGGU
env-47     UUUUUAUG.GCG.G.G.CC..GGGC..GG.GG.CCAGC...UUCG.....GCUGG.GCCGGU...UUCCUGUAGG..........CCGGUU.UCUC.A.CCCCC..GCUGUCU....GG..CC.C.GC.CACCC..UCACGUGAGAAUGAGCGGCGGGU
Reu-1-1    AGUUUUUG.GCG.G.G.CC..GGGC..GG.GG.CAGCC...GAAA.....GGCUG.GCCGGA...UUACUCUGUGAAG.......ACCGGUU.UGGU.A.CCCCC..GCUGUCU....GG..CC.C.GC.CACCCUUCAUUUUUAUCCAUCAGGGGGGUG
Rso-2-2    UGUGCAUC.GCG.G.G.CC..GGGC..GG.GG.CAGCC...UUCG.....GGCUG.GCCGG....UUUCUACGUU.........GCCGGUU.UUCG.A.CCCCC..GCUGUCU....GG..CC.C.GC.CACCCCUCACGUCGAAAUGAGCGGCGGGCC
Reu-2-1    UUUCUAUG.GCG.G.G.CC..GGGC..GG.GG.CAGCC...UUCG.....GGCUG.GCCGG....UACCUUGUGC.........GCCGGUU.UUCG.A.CCCCC..GCU.GUC....GG.GCC.C.GC.CACCCCUCACGUCGAAAUGAGCGGCGGGCC
Rso-1-2    UUUUUAUG.GCG.G.G.CC..GGGC..GG.GG.CAGCC...UUCG.....GGCUG.GCCGGU...GGCCUCCUUU.........GCCGGUU.UUCC.A.CCCCC..GCU.GUC...AGG..CC.C.GC.CACCC..UCAUGUGGAAAUGAGCGGCGGGC
Rso-1-3    UAAGCGCC.GCG.G.G.CC..GGGC..GG.GG.CAGCC...UUCG.....GGCUG.GCCGGU...UUCUACGUUU..........CCGGUU.UGUC.A.CCCCC..GCCGUCU....GG..CC.C.GC...CCC..UUAUGUGACAAUGAGCGGCGGGC
Bth-3-1    GAUCAAUG.GGU.G.G.GCCGUGGC..GG.GG.AGACG...CUUG.....CGUCU.GCCGGU...UUCCUGUAGG..........CCGGUU.CGCC.A.ACCCU..GUC...A.CGUGC..CC.G.CC.CACCCCAUUUGGCGAUGGAAGCGGGUUUC
Bps-8-1    UUUCUAUG.GGU.G.G.GCCAUGGU..GG.GG.GAGCG...CUUG.....CGCUC.GCCGG....UGCCAACGUU..........CCGGUC.UGUC.A.ACCCU..GUC...A.UGUGC..CC.G.CC.CACCCCAUUUGACAGUGGGGCGCGGGUUU
Bps-16-1   UUUCUAUG.GGU.G.G.GCCAUGGC..GG.GG.GAGCG...CUCG.....CGCUC.GCCGG....UGCCAACGUU..........CCGGUC.UGUC.A.ACCCU..GUC...A.UGUGC..CC.G.CC.CACCCCAUUUGACAGUGGGGCGCGGGUUU
Bps-11-2   CUUCAAUG.GUC.G.G.GCCUUGGC..GG.GG.GUGCC...UUCG.....GGCAC.GCCGGU...UUCCUUUUGC.........GCCGGUU.CGCC.A.ACCCU..GUC...A.UGUGC..CC.G.GC.CACCCCAUUUGGCGAUGGGGCCGGGUCCCG
Bth-3-2    CUUCAACG.GUC.G.G.GCCUUGGC..GG.GG.GUGCC...CUCG.....GGCAC.GCCGGU...UUCCGUUUUC.........GCCGGUA.CGCC.A.ACCCU..GUC...A.UGUGC..CC.G.GC.CACCCCAAUUGGCGUUGGGAGCCGGGUUCG
Bth-2-1    CUUCAACG.GUC.G.G.GCCUUGGC..GG.GG.GUGCC...CUCG.....GGCAC.GCCGGU...UUCCGUUUUC.........GCCGGUA.CGCC.A.ACCCU..GUC...A.UGUGC..CC.G.GC.CACCCCAAUUGGCGUUGGGAGCCGGGUUCG
Bvi-1-1    CUUCUAUG.GUC.G.G.GCC.UGAU..GG.GG.GUGCG...UUCG.....CGCAC.GCCGGU...UUCCUUUGAC.........GCCGGUU.CGCC.A.ACCCU..GUC...A.UGUGC..CC.G.GC.CACCCCAUUUGGCGAUGGGAGCCGGGUUCG
Bth-3-3    CUUCUAUG.GUC.G.G.GCC.UGAU..GG.GG.GUGCG...UUCG.....CGCAC.GCCGGU...UUCCUUUGAC.........GCCGGUU.CGCC.A.ACCCU..GUC...A.UGUGC..CC.G.GC.CACCCCAUUUGGCGAUGGGAGCCGGGUUCG
Bps-16-2   CUUCAAUG.GUC.G.G.GCCUUGGU..GG.GG.GUGCG...UUCG.....CGCAC.GCCGGU...UUCCUUGGAC.........GCCGGUU.CGCC.A.ACCCU..GUC...A.UGUGC..CC.G.GC.CACCCCAUUUGGCGAUGGGGGCCGGGUUCG
Bps-7-1    CUUCAAUG.GUC.G.G.GCCUUGGU..GG.GG.GUGCG...UUCG.....CGCAC.GCCGGU...UUCCUUGGAC.........GCCGGUU.CGCC.A.ACCCU..GUC...A.UGUGC..CC.G.GC.CACCCCAUUUGGCGAUGGGGGCCGGGUUCG
Bps-8-2    CUUCAAUG.GUC.G.G.GCCUUGGU..GG.GG.GUGCG...UUCG.....CGCAC.GCCGGU...UUCCUUGGAC.........GCCGGUU.CGCC.A.ACCCU..GUC...A.UGUGC..CC.G.GC.CACCCCAUUUGGCGAUGGGGGCCGGGUUCG
Bps-5-1    CUUCAAUG.GUC.G.G.GCCUUGGU..GG.GG.GUGCG...UUCG.....CGCAC.GCCGGU...UUCCUUGGAC.........GCCGGUU.CGCC.A.ACCCU..GUC...A.UGUGC..CC.G.GC.CACCCCAUUUGGCGAUGGGGGCCGGGUUCG
Bps-18-1   CUUCAAUG.GUC.G.G.GCCUUGGU..GG.GG.GUGCG...UUCG.....CGCAC.GCCGGU...UUCCUUGGAC.........GCCGGUU.CGCC.A.ACCCU..GUC...A.UGUGC..CC.G.GC.CACCCCAUUUGGCGAUGGGGGCCGGGUUCG
Bok-2-1    CUUCUAUG.GUC.G.G.GCCUUGGU..GG.GG.GUGCG...UUCG.....CGCAC.GCCGGU...UCCCUUGGAC.........GCCGGUU.CGCC.A.ACCCU..GCC...U.UGUGC..CC.G.GC.CACCCCAUUUGGCGAUGGGGCCGGGUUCGU
Bok-1-1    CUUCUAUG.GUC.G.G.GCCUUGGU..GG.GG.GUGCG...UUCG.....CGCAC.GCCGGU...UCCCUUGGAC.........GCCGGUU.CGCC.A.ACCCU..GCC...U.UGUGC..CC.G.GC.CACCCCAUUUGGCGAUGGGGCCGGGUUCGU
Bce-1-1    CUUCUAUG.GUC.G.G.GCCUUGGU..GG.GG.GUGCG...UUCG.....CGCAC.GCCGGU...UCCCUUGGAC.........GCCGGUU.CGCC.A.ACCCU..GCC...U.UGUGC..CC.G.GC.CACCCCAUUUGGCGAUGGGGCCGGGUUCGU
Bps-5-2    CUUCUAUG.GUC.G.G.GCCUUGGU..GG.GG.GUGCG...UUCG.....CGCAC.GCCGGU...UCCCUUGGAC.........GCCGGUC.UGUC.A.ACCCU..GCC...A.UGUGC..CC.G.GC.CACCCCGUUUGACAGCGGGAACCGGGUUCG
Bth-1-2    CUUCAAUG.GUC.G.G.GCCUUGGU..GG.GG.GUGCG...UUCG.....CGCAC.GCCGGU...GUCCUCGGGU.........UCCGGUC.UUCC.A.ACCCU..GCC...A.UGUGC..CC.G.GC.CACCC..GUUUGGAAGCGGGAGCCGGGUAG
Bps-7-2    GAUCAAUG.GGU.G.G.GCGACGGU..GG.GG.AGGC....UUCG......GCCU.GCCGGU...UCCCUUGGAC.........GCCGGUC.UUCC.A.ACCCU..GCU...G.UCUGC..CC.G.CC.CACCCCAUUUGGAAGUGGGGAGCGGGUUCA
Bdo-1-2    GAUCAAUG.GGU.G.G.GCGACGGU..GG.GG.AGGC....UUCG......GCCU.GCCGGU...UCCCUUGGAC.........GCCGGUC.UGCA.A.ACCCU..GCC...G.UCUGC..CC.G.CC.CACCCCAAUUUGCAGUGGGGAGCGGGUUCA
Bps-9-1    GAUCAAUG.GGU.G.G.GCGACGGU..GG.GG.AGGC....UUCG......GCCU.GCCGGU...UCCCUUGGAC.........GCCGGUC.UGCA.A.ACCCU..GCC...G.UCUGC..CC.G.CC.CACCCCAAUUUGCAGUGGGGAGCGGGUUCA
Reu-1-2    CUUCCAUG.GGC.G.G.GCCAUGGC..GG.GG.GAGCC...CUCG.....GGCUC.GCCGG....CUCCUUCUC..........ACCGGUC.UGCU.A.ACCCU..GCC...U.UGCGC..CC.G.CC.CGCCC..CGAUUAGCAGCGGGGUAGCGGGA
Cvi-1-2    UUUCUAUG.GCG.G.GCCG..UGGU..GG.GG.AUACC...UACG.....GGUAU.GCCGG....UUCCUUACUG..........CCGGUU.CGCC.A.ACCCU..GCC...A....UGCGCC.C.GC.CACCC..UCGUUUGGCGACGAGGACCGGGC

           ......<<.<<<.<.<.<<..<<<<..<<.<<.<<<<<<<.......>>>>>>>.<<<<<.......................>>>>>..........>>>>..>>>...>....>>..>>.>.>>.>>..........................
           ......?1.222.2.2.22..2222..22.22.2222220.......0222222.22222.......................22222..........2222..222...2....22..22.2.22.1?..........................
           •••Y•AUG-GYR-G-•-Y-•-•R•••-•G-GG-••RYY---YU•R-----RRY•Y-GCCGG∘---YYYY•••••∘----------∘CCGGU•-Y•YY-A-RCCY•--••Y---•-----•R--•Y-Y-RC-CACC•∘∘•••••Y•••R••••••••••R••••
           5′                                                                                                                                                 3′
```

```
Bps-15-2   AAUCUAUGGGCG.G.G.UGA.UGGC..AG.GG.AGACC...UUCG.....GGUCU.GCCGGU...UUCCUAUGGG...........CCGGUU.CGCC.A.ACCUU..GUC...A..UUCG..CC.C.GCUCACCCCAUUUGGCGAUGGGGCGCGGGUCUC
Rpi-2-1    CUACUUUG.GCG.G.G.CC..GGAU..GG.UG.AGACC...UUCG.....GGUCU.ACCGGU...UUCCUUGGG...........ACCGGGU.CGCU.A.ACACC..AUC.GUC...AGG..CC.C.GC.CACCCUCGUUUAGCGCCGAGAUGGUGGGGC
Rpi-1-2    CUACUUUG.GCG.G.G.CC..GGAU..GG.UG.AGACC...UCCG.....GGUCU.ACCGGU...UUCCUUGGG...........ACCGGGU.CGCU.A.ACACC..AUC.GUC...AGG..CC.C.GC.CACCCUCGUUUAGCGCCGAGAUGGUGGGGC
env-48     CUACUUUG.GCG.G.G.CC..GGAU..GG.UG.AGACC...UCCG.....GGUCU.ACCGGU...UUCCUUGGG...........ACCGGGU.CGCU.A.ACACC..AUC.GUC...AGG..CC.C.GC.CACCCUCGUUUAGCGCCGAGAUGGUGGGGC
env-49     C..CUAUG.GGC.G.GGCC..GUGU..GG.GG.AGACC...UUCG.....GGUCU.GCCGG......CCCUCUG...........ACCGGUU.CGCC.A.ACCCC..GCA...C....GGUUCC.G.CC.CACCC..GUUUGGCGACGGGAAAGCGGAAA
Bce-1-2    GAUCAACG.GGU.G.G.GCCUGGGU..GG.GG.AGACC...UUCG.....GGUCU.GCCGGU...UGCCUCCAUU..........GCCGGUU.CGCC.A.ACCCC..GCU...C.AGUGC..CC.G.CC.CACCCCAUUUGGCGAUGGAAGGCGGGCCUU
Bma-10-1   GAUCAAUG.GGU.G.G.GCCUUGGC..AG.GG.AGACC...UUCG.....GGUCU.GCCGGU...UGCCUCCUAU..........GCCGGUU.CGCC.A.ACCCU..GCC...U.UGUGC..CC.G.CC.CGCCCAUUUGGCGAUGGGAGAGCGGGUCUU
Bth-2-2    GAUCAAUG.GGU.G.G.GCCUUGGC..AG.GG.AGACC...UUCG.....GGUCU.GCCGGU...UGCCUCCUAU..........GCCGGUU.CGCC.A.ACCCU..GCC...U.UGUGC..CC.G.CC.CGCCCAUUUGGCGAUGGGAGAGCGGGGCUU
Bvi-1-2    UUUCUAUG.GGC.G.G.GCCUGGGU..GG.GG.AGACC...UUCG.....GGUCU.GCCGGU...GUUCAUUAGCC.........GCCGGUU.CGCC.A.ACCCC..GCU...C.UGCGC..CC.G.CU.CACCCCGUUUGGCGACGGGAGCGGGCUGAA
env-50     UUUCUAUG.GGC.G.G.GCCUGGGU..GG.GG.AGACC...UUCG.....GGUCU.GCCGGU...GUUCAUUAGCC.........GCCGGUU.CGCC.A.ACCCC..GCU...C.UGCGC..CC.G.CU.CACCCCGUUUGGCGACGGGGAUCGGGUUGA
Bce-2-1    CGUUUAUG.GGU.G.G.GCGUGGGU..GG.GG.AGACC...UUCG.....GGUCU.GCCGGU...AUCCAGAGAC..........GCCGGUU.CGCC.A.ACCCC..GCU...C.ACUGC..CC.G.CC.CACC...CCGUUUGGCGACGGGACGCGGGU
Bps-3-1    GUUUUAUG.GGU.G.G.GCGUGGGU..GG.GG.AGACC...UUCG.....GGUCU.GCCGGU...AUCCAGAGAC..........GCCGGUU.CGCC.A.ACCCC..GCU...C.ACUGC..CC.G.CC.CACC...CCGUUUGGCGACGGGACGCGGGU
Bmu-1-1    UUUCUAUG.GGC.G.G.GCCAAGGC..GG.GG.AGAC...CUUCGA.....GUCU.GCCGGU...UUCUUUGAUU..........UCCGGUU.CGCC.A.ACCCU..GCC...U.UGUGC..CC.G.CU.CACCCCAUUUGGCGAUGGGGCGCGGGAAA
Bvi-1-3    UUUCAAUG.GGC.G.G.GCCUGGGU..GG.GG.CGACC...UUCG.....GGUCG.GCCGGU...UUCUUUGGUU...........CCGGUU.CGCC.A.ACCCU..GCC...U.UGCGC..CC.G.CU.CACCCCAUUUGGCGAUGGAAGCGGGAUCA
env-51     CUUCUAUG.GGC.G.G.GCCUUGGU..GG.GG.AGACC...UUAG.....GGUCU.GCCGGC...UGCUUGUGUG..........ACCGGUU.CGCC.A.ACCCU..GCC...G.UGCGC..CC.G.CU.CACCUCAUUUGGCGAUGAGAAGCCGGGUAA
Rso-1-4    UUUCUUUG.GCG.G.G.CC..GGGC..GG.GG.AGACC...UUCG.....GGUCU.GCCGG....UUCCGUUGUU...........CCGGUU.CGCC.A.ACCCC..GCCGUCU....GG..CC.C.GC.CACCCCUCAUUUGGCGAUGAGCGGCGGGCC
Rpi-2-2    GCUUUAUG.GCA.G.G.CC..GGGU..GG.GG.AGACC...UUCG.....GGUCU.GCCGGU...UUUCUUUGGGG.........ACCGGUU.CGCC.A.GCCCC..GCC.GUC....GG.GCC.U.GC.CACCC..UCGUCUGGCGACGGAGUGGUGAG
Rpi-1-3    GUUUUAUG.GCG.G.G.CC..GGGU..GG.GG.AGACC...UUCG.....GGUCU.GCCGGU...UUUCUUGGAG...........CCGGUU.CGCC.A.GCCUC..GCC.GUC....GG.GCC.C.GC.CACC...UCGUCUGGCGACGAAUGACGGGC
Rso-2-3    UUUCCAUG.GCG.G.G.CC..GGGU..GA.GG.AGACC...UUCG.....GGUCU.GCCGG....UUUCUUGGAG..........GCCGGUU.CGCC.A.GCCUC..ACC.GUC....GG.GCC.C.GC.CACCUC.GUCUGGCGACGAAUGGCUGGCCU
Rso-1-5    UUUCUAUG.GCG.G.G.CC..GGGU..GA.GG.AGACC...UUCG.....GGUCU.GCCGG....UUUCUUGGAG..........GCCGGUU.CGCC.A.GCCUC..GCC.GUC....GG.GCC.C.GC.CACCU..CGUCUGGCGACGAAUGGCGGGCC
Rpi-1-4    CUCCUAUG.GCG.G.G.CC..GGGU..GG.GG.AGACC...GCAA.....GGUCU.GCCGGU...UGCCGUGAGU..........ACCGGUU.CGCC.A.GCCCC..GCC.GUC...AGG..CC.C.GC.CACCU..UCGUCUGGCGACGAGAAGGCGGA
Rpi-2-3    GCUUCAUG.GCG.G.G.CC..GGGC..GG.UG.AGACC...UUCG.....GGUCU.GCCGG....UUUCUAGAUG..........GCCGGUC.UGCU.A.ACGCC..GUC.GUC...AGG..CC.C.GC.CACCC..UCGUUUAGCAGCGAGCGGCUGGC
Rpi-1-5    GCUUCAUG.GCG.G.G.CC..GGGC..GG.UG.AGACC...UUCG.....GGUCU.GCCGG....UUUCUAGAUG..........GCCGGUC.UGCU.A.ACGCC..GUC.GUC...AGG..CC.C.GC.CACCC..UCGUUUAGCAGCGAGCGGCUGGC
Rpi-2-4    GUUUCAUG.GCG.G.G.CC..GGGC..GG.UG.AGACC...UUCG.....GGUCU.GCCGGU...AGCUGGGAUAGG........GCCGGUC.UGCU.A.ACGCC..GUC.GUC...AGG..CC.C.GC.CACCC..UCGUUUAGCAGCGAGCGGCGAGC
Rpi-1-6    GUUUUAUG.GCG.G.G.CC..GGGC..GG.UG.AGACC...UUCG.....GGUCU.GCCGGU...AGCUGGGAUAGG........GCCGGUC.UGCU.A.ACGCC..GUC.GUC...AGG..CC.C.GC.CACCC..UUGUUUAGCAGCGAGCGGCGAGC
Rpi-2-5    UUUCAAGG.GCG.G.G.CC..GGGU..GG.GG.AGACC...UUCG.....GGUCU.GCCGGU...GCUCCAGAGA..........ACCGGUU.CGCU.A.CCCCC..GCCGUCU....GG..CC.C.GC.CACCC..CCGUGUAGCGACGAGCUGGCGGG
env-52     CAUCAAGG.GCG.G.G.CC..GGGC..GG.GG.AGACC...UUCG.....GGUCU.GCCGGU...GCUCCAGAGA..........GCCGGUU.UGCU.A.CCCCC..GCC.GUC...AGG..CC.C.GC.CACCCGUGUGGUAACGAGUUGGCAGGCCUC
Pen-1-30   GUGUCAUG.GCG.G.C.UG..CGUA..UG.GG.AGGCC...UUUG.....GGCCU.GCCGGU...UUUCUCUUUUGC........GCCGGUC.UUGC.AGACCUG..UAC...G....UA..GC.U.GC.CACCCU.AUCCAUUGCAAGGGUGGCGUGGU
Ppu-4-27   GGCCAAUG.GUG.G.C.UG..CGUG..CU.GG.AGAUC...UUCG.....GAUCU.GCCGGU...UUUCGUCUGU...........CCGGUC.UUGC.AGACCUG..CAC...G....UA..GC.C.GC.CACCC..AUCAAAUGCAAGUGAUGUCGUGG
Pfl-2-29   UGCUUAUG.GCA.G.C.UA..UGCG..CA.GGAGCAC...GCUCGA.....GUGC.ACCGGG...UUUUUUCGGGGU........CCCGGAC.UUCC.ACACCUG..CGC...A....AA..GC.U.GC.CACCC..UCUCGUGGAAGGGAGCUCGGCAG
Sgl-2-16   CAAUAAUG.GCGUG.A.UG..UAAC..GG.GG.GAGCC...GUAA.....GGCUC.GCCGGA...UUUCUCUUUG..........ACCGGUC.UGUC.A.ACCCU..GUU...A....CG..UCUC.GC.CACCCU.GUUUGACAGCAGGUAGCGAGAUU
Sgl-2-17   CUAUAAUG.GUG.G.GACG..UAAC..GG.GG.GAGCC...UUCG.....GGCUC.GCCGG....CUUCAUCUUCUUC.......ACCGGUA.UGCU.A.ACCCC..GAU...A....CG.UCU.C.GC.CACC...UCGUUUAGCAUCGAGGAGCGAGG
Eco-8-5    GCAUUAUG.GCG.G.GGCG..UAAC..GG.GG.GAGC...CUUUGU.....GCUC.GCUGG....UUUCCAGAUG..........ACCAGUC.UGCU.A.ACCCU..GUU...A....CG.UCU.C.GC.CACC...AUGUUUAGCAGCGUAGUAGCGAG
Eco-3-14   GCAUUAUG.GCG.G.GGCG..UAAC..GG.GG.GAGC...CUUUGC.....GCUC.GCUGGU...UUCCCAAUUU..........GCCAGUC.UGCU.A.ACCCU..GUU...A....CG.UCU.C.GC.CACC...CCGUUUAGCAGCGUAGUAGCGAG
Sen-3-1    GUAUUAUG.GUG.G.GGCG..UAAC..GG.GG.GAGCG...UUCG.....CGCUC.GCCGG....UUCCGUUGAU..........GCCGGUC.UGCU.A.ACCCU..GUU...A....CG.UCU.C.AC.CACCC..CGUUUAGCAGCGGUCUGGUGAGG
Sgl-2-18   AAAUUAUG.GUG.G.GGCG..UAAC..GG.GG.GAGCG...CUUG.....CGCUC.GCCGGG...UUCUGUGUGG...........CCGGUC.UGUC.A.ACCCU..GUU...A....CG.UCU.C.GC.CUCC...CUGUUUGACAGCGGGGUGGUGAG
Eph-3-1    GAAUUAUG.GUG.G.GGCG..UGAU..GG.GG.AGGC....UUCG......GCCU.GCUGG....UUUCACUAAC..........GCCAGUC.UGUC.A.ACCCU..GUC...A....CG.UCC.U.GC.CACCUGUUUGACAGCGGGUAGCAGGUUGUU
Sen-8-4    AAAUUAUG.GUG.G.GGCG..UGAU..GG.GG.AGGC....CUCG......GCCU.GCUGG....UUUCAUGUAC..........GCCAGUC.UGUC.A.ACCCU..GUC...A....CG.UCC.U.GC.CACCCU.GUUUGACAGCGGGGAGCAGGUUG
Aba-1-6    UCGUAGCG.GUA.G.GGCG..AGGG..AG.GG.ACGC....CUUGU.....GCGU.GCUGA....UUCUUGUGUG...........UCAGUC.UGUC.A.ACCUU..CUC...U....CG.UUC.U.AC.CACCA..UCAUUUGACAGUGAUCGGGUAGA
Aba-1-7    CUUUGAUG.GAG.G.AACG..GGAG..UG.GG.ACACC...CUUG.....GGUGU.GCUGG....UUUCUUAGGU..........AUCAGUC.UGCU.A.ACCCU..CUU...U....CG.UUU.C.GC.CACCAUAAUAAUGAGCAAUGAGUGGGCAUA
Aba-1-8    CCUCUGCG.GCG.G.AACG..GGAG..GG.GG.ACAUC...UUCG.....GAUGU.GCUGAU...UUUUGGGUAA..........AUCAGUC.UGCU.A.ACCCC..UUU...U....CG.UUU.C.GC.CACCAUAAUCAUUUAGCAGUGAUGUGGCGA
Aba-1-9    CCUCUGCG.GCG.G.AACG..GGAG..GG.GG.ACACC...UUUG.....GGUGU.GCUGG......CCUCGUUUG.........ACCAGUC.UGUC.A.ACCCU..CUU...U....CG.UUC.U.GC.CACCAUAAUUUAAUGCUUUGGCAGAACUC
Asp-1-1    CCUCUGCG.GUA.G.AACG..GGAG..GG.GG.ACACC...UUCG.....GGUGU.GCUGA....UUCUUAGGU............UCAGUC.UGCU.A.ACCCC..CUU...U....CG.UUC.U.GC.CACCA..UCAUUUAGCAGUGAUCUGGUAGA
Aba-1-10   CCUCUACG.GCG.G.AACG..GGAG..GG.GG.ACACC...UUCG.....GGUGU.GCUGGG...UUUUUUCGG............CCAGUC.UGCU.A.ACCCC..CUU...U....CG.UUC.U.GC.CACCU..UAAUUUAAAUGUUUUGGCAGAAC
Aba-1-11   CCUCUGCG.GCG.G.AACG..GGAG..GG.GG.ACACC...UUCG.....GGUGU.GCUGGA...UUCUUUGGCU...........CCAGUC.UGCU.A.ACCCU..CUU...U....CG.UUU.C.GC.CACC...AUUAUUUAGCAGUAAUGUGGUGA
Aba-1-12   CCUCUGCG.GUG.G.AACG..GGAG..GG.GG.ACACC...UUCG.....GGUGU.GCUGA....UCUUUUGGCU..........AUCAGUC.UGUC.A.ACCUU..CUC...U....CG.UUU.C.AC.CACC...AUUAUUUGACAGUAAUGUGGUGA
Aba-1-13   CCGUAGCG.GUA.G.GACG..GAAG..UG.GG.ACACU...UUCG.....AGUGU.GCUGG......CCUCUGAG..........ACCAGUC.UGUC.A.ACCCA..CUU...U....CG.UUC.U.GC.CACCAUAAUUCUAAAUUAAUGCUUUGGCAG
Aba-1-14   UCGUUGCG.GUA.G.GACG..AGUG..AG.GG.ACACC...UUCG.....GGUGU.GCUGAU...UUCUUACUA............UCAGUC.UGCU.A.ACCCU..UGC...U....CG.UUC.U.GC.CACCAUAAUUCCAAAUGAAUGCUUUGGCAG
Aba-1-15   UCGUUGCG.GUA.G.GACG..AGUG..AG.GG.ACACC...UUUG.....GGUGU.GCUGA....UUCUUUACU...........AUCAGUC.UGCG.A.ACCCU..UGC...U....CG.UUC.U.GC.CACCAUAAUCUUUUAUUAAUGCUUUGGCAG
Aba-1-16   CCGUAGCG.GUA.G.GACG..GGAG..UG.GG.ACACC...UUCG.....GGUGU.GCUGG....AGUCUUAC.............CCAGUC.CGUC.A.ACCUG..CUU...C....CG.UUC.U.GC.CACCAUAAUUUAUAAUGCUUUGGCAGAACU
Aba-1-17   CCGUAGCG.GUA.G.GACG..GGAG..AG.GG.ACACC...UUUG.....GGUGU.GCUGG....AGUCUUAA.............CCAGUC.UGCG.A.ACCCU..CUU...U....CG.UUC.U.GC.CACCAUAAAUUUUAUGCUCUGGCAGAACUC
Aba-1-18   CCGUAGCG.GUA.G.GACG..GGAG..AG.GG.ACACC...CUCG.....GGUGU.GCUGGU...UUAUCUUAC............CCAGUC.CGCG.A.ACCCU..UUC...U....CG.UUC.U.GC.CACCA..UAAUUUUAAUGUCUGGCAGAACU
Aba-1-19   CCGUAGCG.GUA.G.GACG..GGAG..AG.GG.ACACC...UACG.....GGUGU.GCUGGU...UAUAUCUUUC..........ACCAGUC.UGCU.A.ACCCU..UUC...U....CG.UUC.U.GC.CACCAUAAUUUUAAUGCUUUGGCAGAACUC
Aba-1-20   UCUCUAUG.GUA.G.GGCG..GAAG..GG.GG.ACGCG...UUCG.....CGCGU.GCUGG......UCGCUAGG...........UCAGUC.UGCU.A.ACCCU..CUU...U....CG.UUC.U.AC.CACCCUAAAUUAGUUCGCGGUAAUCUGGUG
Asp-1-2    UCUCUAUG.GUA.G.GGCG..GAAG..GG.GG.ACGCG...CUUG.....CGCGU.GCUGGU...UUCUUUGUUU..........GCCAGUC.UGUC.A.ACCCU..CUU...U....CG.UUC.U.AC.CACCC..UCAUUUGACAGUGAGAUGCUAGA
Aba-1-21   CCGUUGCG.GUA.G.GACG..GGAG..GG.GG.ACGCG...CUCG.....CGCGU.GCUGG......UCUGUAUA..........CCCAGUC.UGUC.A.ACCCC..UUC...U....CG.UUC.U.AC.CACCCUAAUCAAUUGACGGUGAUUUUUGGU
Asp-1-3    CCUUUGCG.GUA.G.AACG..GGAG..GG.GG.ACGCG...UCCG.....CGCGU.GCUGG....UUCUGUAUGU...........CCAGUC.UGUC.A.ACCCU..CUU...U....CG.UUC.U.GC.CACCAUAAUCAAUUGACAGUGAUUCGGUAG
Asp-1-4    CCUUUGCG.GUA.G.AACG..GAAG..GG.GG.ACGCG...UCCG.....CGCGU.GCUGG....UUCUAGAUGU...........CCAGUC.UGUC.A.ACCCU..CUU...U....CG.UUC.U.GC.CACCAUAAUCAAUUGACAGUGAUUCGGUAG
Aba-1-22   UCUUUGCG.GUA.G.GACG..GGAG..GG.GG.ACGC....CCCGC.....GCGU.GCUGG....UUCUACGAGU...........CCAGUC.UGUC.A.ACCCC..CUU...U....CG.UUC.U.AC.CACCCUAAUUAAUUCGCGGUAAUCUGGUGG
Aba-1-23   UCGUAGCG.GUA.G.GACG..GAGG..UG.GG.ACGC....CUUGU.....GCGU.GCCGG....UUUCUAGAUG..........ACUGGUC.UGUC.A.ACCCG..CCU...U....CG.UUC.U.AC.CACCA..UCAUUUGAUAGUGAUUUGGUAGA
Hin-11-1   GUCCAAUG.AUG.A.G.CU..GAAU..GA.GG.GGAUC...GAAA.....GAUCC.GCCGUU...UUGCUCUUAAG.........UACGGUACUGCG.A.ACCUC..GUU...C....AG..UU.C.AU.CACCA..ACUAUUCGCAGUGGUUCGUGAUG

           ......<<.<<<.<.<.<<..<<<<..<<.<<.<<<<<<<........>>>>>>>.<<<<<.........................>>>>>..........>>>>..>>>...>....>>..>>.>.>>.>>............................
           ......?1.222.2.2.22..2222..22.22.2222220........0222222.22222.........................22222..........2222..222...2....22..22.2.22.1?............................
           •••Y•AUG-GYR-G-•-Y•--•R••--•G-GG-••RYY---YU•R-----RRY•Y-GCCGG∘---YYYY•••••∘----------∘CCGGU•-Y•YY-A-RCCY•--••Y---•----•R--•Y-Y-RC-CACC•∘∘•••••Y•••R••••••••R••••
           5′                                                                                                                                 3′
```

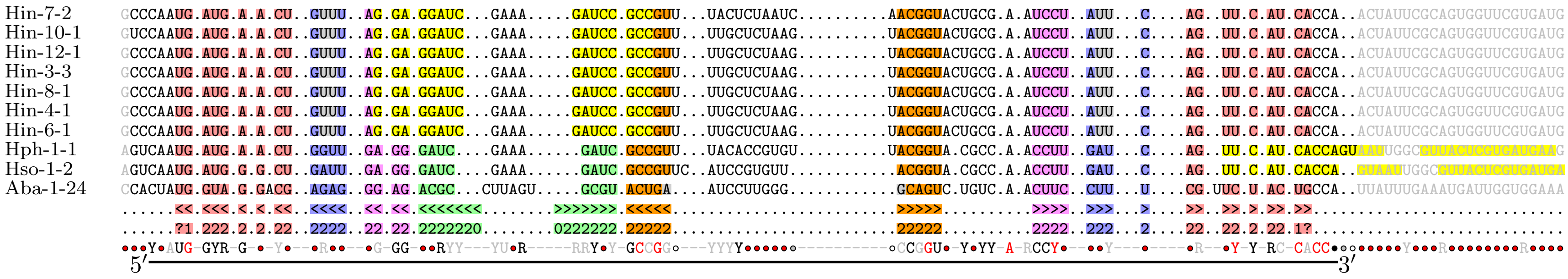

alignment positions 260 · · · 419

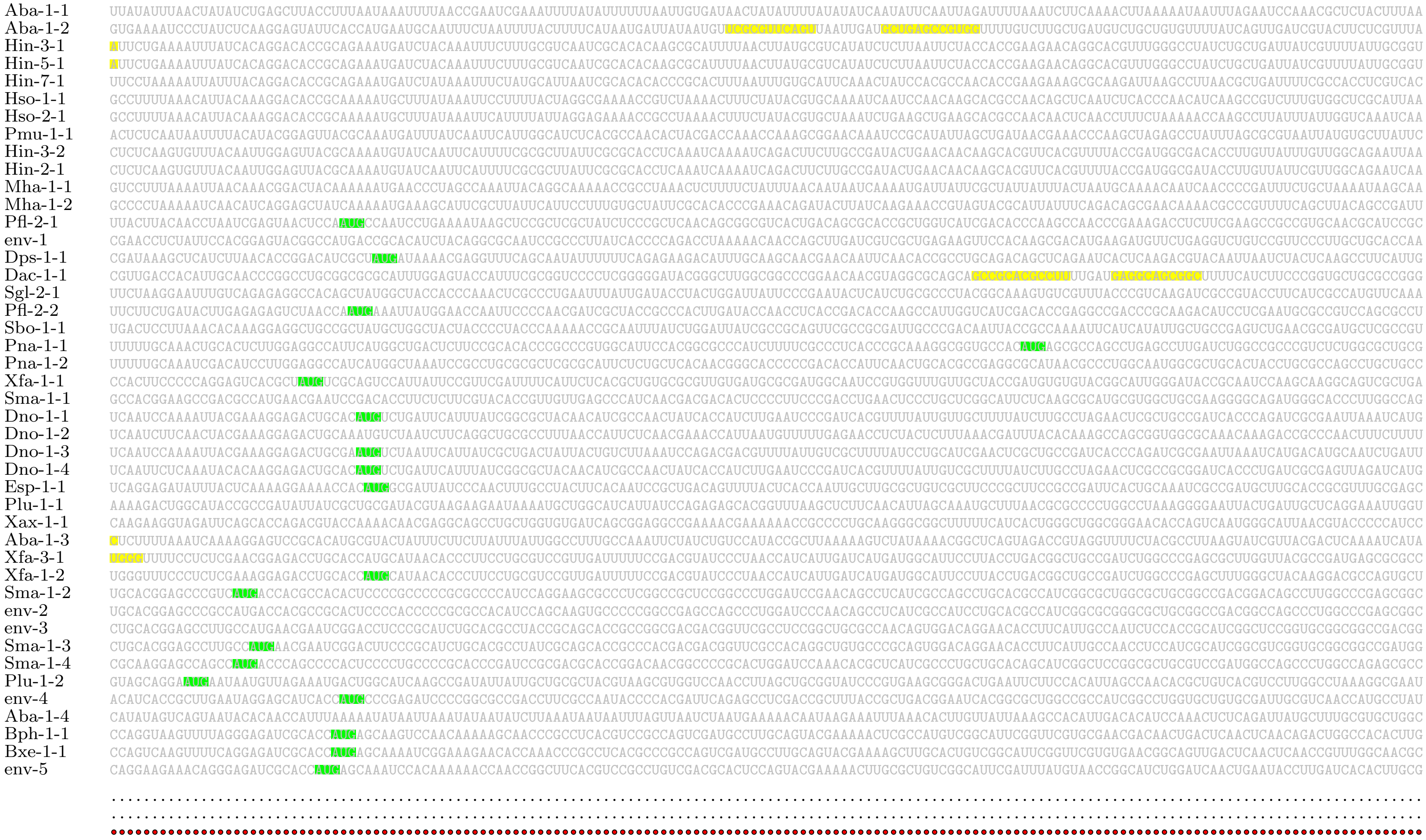

```
env-6      ACUGGCAUAAGGCAGACAUCAUAGCUGCGCUACAUAAGAAAGGUUUGUCACUUGCCUCACUGAGUAGGGCUAACGGUCUUUCAAGCAGCACUCUGGCAAAUGCACUGACACGUGACUGGCCUAGAGGCGAGAUCAUCAUCGCUAAUGCAAUUGGUGAAGA
Spr-1-1    CGGUAUUUCUCUCCCCAAUGGAAAUGACCGAUAUGGAAAACAUUAGCCAAAACAUAGCGUCAGAAACAGCUACAAGCACCCUUGCUCUCUAUCGAGAACUGUUCAAUCAAAUUGAUUUGCAUGACUUAACACCAUCACAGCUUCUCGAUCUGGCGGCAUU
Pfl-2-3    CGCCUCUUUCACUACCAGAGGAUUAAACAAUGAGCACGCUUCAUCCAGAUCCACCCUACGAGCCCUUCAUCCCCCACCCCGACAACCGCCUCAUGGCGCUGACCAGCAACUGCAACGACAUGCCCACCCUGUUCAUCGACACCCACGCCCCGCUCGACAU
env-7      CUCCUGUUCAUACCCGGAGUUGUCCCAAUGCAAUCACUUACCCCUGAUCCACCGAUCACACCCAAAGUCUUCACCCGCCACCAUCACCAGCUGCACGCUGUGUUGAUAGACAACCAGCCAUGGUUCAGCGCCUGCGACAUCGGCCACCUGAUGGGCCUGC
Pfl-2-4    UGAGUGGCGCAAAUUCAUAAGGAAAUGCUCCAUGAACAAGCCCGUACCCGACCCACCCCUCGAAUCACCAACGCCCCUCGAAGAAGCCAUGCGCGCCGAAGACCUCGCCCGCAACCGCGAAGCCAUCAAACGCGCCCUCGACUAUUACCUGUGCCCCAAU
Msp-1-1    UUUAAAGUCUUAAUCACAAGGAUUCAAACCAUGAAUAACGCUAUUGUUCGUUCACAGCACCACCACGCCCUUUUUCCGUCUAUCGGCACCCUUUCCGCUCUACCUACUCAAAGAACAGGCAUUGGCCGUAUUGUCGCGGUUUGCGUCGAAAGCGACGAAC
Msp-1-2    UUUAAAUGUAUCAAAACGAGAUUUAAACCUAUGACUAAUUCAGAUAAUUCCCCAUCCGAUACCAAUACACUAGAUUUUGAUCUAUCCCCUUCCGCUCGCUCCACCAUCACAAAGCAAAUGCAUUGCAUGAGUUAUGGUCUAGACGCGCUAGAUGGACUUA
env-8      GUGGUGGUUUAACCAACACAAGGUCACAAUCAUGAGCACUCCCUUAUUCGUGCCGCGCGGGGAUAUUACGCCCGCCGAAUGCCGCGAUUAUGUCCGUAUUUACGCUUUUCAAGCGCAAGCCUUAACCCGAUUGAUUGAUGAUGCUCUGAUCGAGAGCGUU
Aba-1-5    CUUCAUUACCAUUAUGGUAGGGUGAAAGUGGGGACGCCUCUGUGCGUACUGGUUCUUGAGUCAGUCUGCUACUCCACUUUUGUCCUGUCACCAUUACUUAAUUUACAUCUGUUCCACAUAUAGUCAGUAAUACACAACCAUUUAAAAAUAUAAUUAAAUA
Pfl-1-1    CGGUCCUUCGAAUCAAGGAGUCGUGACCAUGAACUGCUUCUGCAAUCUGUUUCCCCAACACCUCCCGCAACACAAAACCGGAGGUGCCCGAUGACCACUCAAUCGCCCCUGACCACCCUCGGUGAAAUCCGCUUUUCCCACUGCGGCGAACACUCGCAUC
Psy-1-1    CGGUUCCUCAGUACAAGGAGCCUCACCUACCUGCGUCGGCCAGCGGACAAGCUCACCGUGCCGCCGCUCGCUUUGGCCUCGCCGCCGCAGCGGGUGAGCUGGCCACUGCGAUGGGGAUAACAGGCUGGCCUGACGGUACUGCCACCACUGCUGCUCAGGU
Pae-5-1    GUCAUGUGACUCUCAGAAACGGAGUACCCAGCAUGACGAAGGAUGAAAAGGAAAAGACCCACGUCGACGCAAUCAUCGAACGCUACAAGGACCUGAUGGUCGAGAUUCCCCCGCCGACCGGCAACCCGGUCUCUCCCUGCUCUGGCCGGUUCCCGCCCA
Pae-2-1    GUCAUGUGACUCUCAGAAACGGAGUACCCAGCAUGACGAAGGAUGAAAAGGAAAAGACCCACGUCGACGCAAUCAUCGAACGCUACAAGGACCUGAUGGUCGAGAUUCCCCCGCCGACCGGCAACCCGGUCUCUCCCUGCUCUGGCCGGUUCCCGCCCA
Pae-6-1    GUCAUGUGACUCUCAGAAACGGAGUACCCAGCAUGACGAAGGAUGAAAAGGAAAAGACCCACGUCGACGCAAUCAUCGAACGCUACAAGGACCUGAUGGUCGAGAUUCCCCCGCCGACCGGCAACCCGGUCUCUCCCUGCUCUGGCCGGUUCCCGCCCA
Ppu-4-1    ACUCAAGCGUUCAGGAGUUGCUCAUGAAAAAAAUCGUCCCCGAUCCACCCCAUAAUUUCGACCUUCCCUCCGACAAGACCCUCUCCAGCGCCAUCAGCGAGCGCAUCGUGCCAAUCGAUCACGUGGUCAUGAACGUCACCCAUUACCUGAUGCUGGCCUA
Dps-1-2    GCUUAGACCACUUCCAAACGGAGCUAGUACCAUGCGUAAAGACCAUAUUGCUGUAGACCACCUCUCUCAACUCCCUGAAGAUUUUCUCCUAGCUAUCCAAAAACCAGAAGGACUCCCCAUCAAUCCUAUCGGGAUUGCCCUGGAGCGUUGCAAAUCCAUU
Asa-1-1    UAACAAGCGACUGACCUUGGAGGCUGCCAUCAUGGCUACUGUCCCCACCCCUGCUACAUCCAAAAUCUUCACUUUCCUGAUUGCCUCAAACUCCUGCCGGCUGGCAGACCUGAGCCGUAUCCGCACCAUUUCCGCCAUGGCUGACACCGAAGUCCAAGCC
env-9      CAGAGAAAAUCUGACCACUACGGAGGCUGCCAUCAUGGCUACUGUCCCCACCCCUGCUUUACCCAAAAUCUUCACCUUCCUGAUCCAGCGUCCUCGUACUUGCCGCCUGGCCGAGCUUCGGCGUAUCCGCACUAUCACCAUCAUCGCCAAUACCGAGGCU
Bsp-1-1    AUCACAUACGGAGCCACCAUGAACGCCAACGUUCAAUCUCUUGCUUUCCCUAUCGGUAGUAUUCCUGAUCAAGUGGCGGUGUCGUCACGCGAUAUCGCGACCAUCUAUGCCCUGCAGGGCGAACUCCGUACGUGCAUCUUUGCCCUUCGUGGUGCUCUCG
Pen-1-1    GGUCCGACUUCCGCAUUGCGAGGUGUUUAUGCUCAAGAUCGUUCCCGAUCCACCCCAUUCCACCGAGUCUCCUUACUUUCUCGAAGACACCCUGGUUCACGCCACUGAAUACGUCAUGUGUGCCUUGGCGGUGACGCAUCAGUCCUUGACCCACCUGCCC
Cvi-1-1    AAACCAGACUCAAGGAGACAUGCACCAUGUCUAGCAUCUCCCUGCGCGCUCCCCGCGCCCAUCUCUCAGUUCACCCUGUCCCAGAAAAAGCUACCACUGCUCUGAUAAAGCAGAGCAAGUCUUCCAAUGCCAACGGUAUCGGUAUCCCGUUUGCCUUGCG
Sdy-2-1    UUACCAAUCACCAAUCACCAAUCACCAAUCACCAAUCACCAAUCACCAAUCAAGGAGGCUGCCAUUAUGGCUACUAUCCCUGCCCUUUCUCAACCUGAAAUCACCAUUUCCAAUGGCCGCGCUGUUACUACGUCCUUUGCCGUUGCCGAAUAUUUUUGCA
Sbo-1-2    GUAUAUCCCAACGCAAAAGGAGGCUGCCAAAUGGCUACUAUCCCUGCCCUCGCACAUACUCAGAACGCCUUUAUCUGGCGCUUUAUCAUCGUUAAUCCGAGUGAUUACCGCGUCGUUCACGUCGUGGCGUCAACCGAACGAGAAGCGCGUAACUAUUGCC
env-10     GUAUAAUCCCAAAGUCAGAGGAGCCUUUCACAUGGCUGUAUCCGCACGCCCUUACUUUGUCUGGCGCUUUAUGCAGUGCCUGACAGACAGCAUUGCAAUAUUCACCGUUACCGCUGCCACUGAGCGCGAAGCACGCGCCCGGCUGCCGCACGCACAUCUG
Cte-1-1    GUUCAUACCUUGUUCAGUAGGAGCCUCAUCAUGGCUGACAGCAGCAUUGGUUCGUCCGUAACCGCCCUUUCCGCUCGUCCCGAGCUUUCCGUCGAUCACGGAACCAUCACCACCACCAGUCUUCAAAUCGCAGAGCACUUUGGCAAGCGCCACGCUGAUG
Cte-1-2    UUGAUAACUUGUUCCGUAGGAGCCUGUCAUGGCUAACAUCACCACUGCUUCGUCUGUAGAUGCCGUUUCUGCUCGCCCAGAGCUUUCCAUCCAUAACGGCAUCGUCACCACCACAAGUCUUCAAGUCGCACAGUUCUUUGGCAAAUGCCAUGACAACGUG
Sgl-2-2    UGAGUAGUUCAAAUACAACCAUCGGAGGCUAUCAUGGCCCUUCCAGCAUUAGCAGGAAACUUGCACAUUGAAGAACCUCGCGUAUUUAUCAACAAUGGUAAAGCUCUAACCACGUCUAGAGACGUUGCUGAUUACUUUAGCAAGCGUCACGAAAGGGUUU
Sen-8-1    UGGUUAACCUCAUGAGGUGAUACUAUGACUACUCAAAUUAAUGCAGAUAACCUUUCCCCUAUUACUCAUAACCAGAUCCCUGUCAUAACCACUGAACUGUUGGCGCAGCUUUACGGCACUGAACCUGUGCGUAUUCGCCAGAAUCAUCAUGAGAACAAAG
Vfi-1-1    AUUUAUAAAAAUUAGACACUUGGAGGUCGUUAUGACCAAUCAACUUACUUUUCAAAAUACUCAUUUCAAUCUUAUUGAACAAAAUAAUAAAAUUUGGCUAUCUGCAUCUCAAAUUGGAGCGGCUUUAGGUUAUAAAGAUACAAGUGCUUUAAAUCGAAUU
Vfi-1-2    CUAUCGAAAAACAGCCGCCAUUUAACCGCCAUUAAAACGAAAAACCCCUGUAACCUAUUGAUUACAGGGGUUUAAAUUGGUGGAGCUGGGGGGAUUUGAACCCCCGUCCGAAAAUCGUUCACCGUUGGCGCUACAUGUUUAGUCGAUCUUUGAUUUCACC
Sde-1-1    GGAUAUCAUUUUUCUCACAAGGAUUAUUAUGCAAAAUUAUCAAAACCCUAUCAUCCAAGAUAGCACUACAGAUACCUUAUUCUACGCACAAUCUGUGCUUGCAGUCUUGCAAGAAUAUUACGCAGCCGUGAACCUUCCCCAGCGCGAUCAAGAAGAGAAU
Sba-1-1    AUUUAUCUCGAUCACAGGAGUGUCACCCAUGAACCUACAACAGUCUAAAAACACCCCCGCACAACUGGUAUUUAUUAACGGCCUGCAAACCAUUACUAGCUCGCUGAUCGUUGCCGAUUAUUUUGGCAAACGGCACAGCCAUGUUUUGCGCAAAAUUGAC
Sba-2-1    UUACUCUCGAUCACAGGAGUGUCACCCAUGAACCUACAACAGCCUAAAAACACCGCCGCGCAACUGGUAUUUAUUAACGGCCUGCAAACCAUUACUAGCUCGCUGAUCGUUGCCGAUUAUUUUGCUAAACGUCACUGUGAUGUUUUACGUAAACUAGACC
Eco-3-1    GCGGUGAAUUUCACCGUUAGAGGUAACUGUGAUGGCAGCUCAACUCAUCCCCGUUUUCAACGGCGCUAUCGCCAACGAAAUCACCCUGCUCUGCAAUGCUCGCGAUCUGCACGCAUUCCUUGGGGUAGGGAAAGUGUUUGCAGCAUGGAUUACAUCACGA
Vch-2-1    UUUUAAAACCAUACUCAAGGAGGUCACUAUGACUUCAGCACUGACUUUUCAAAACACCCAUUUUGAUGUGAUCGAACAGCACAACCAGCUUUGGCUAUCUGCUAGUGAUAUCGCCAAUGCUCUUGGCUAUAAAUCCCCGAAAUCAAUUUCUAACAUUUAU
Vch-3-1    GUAACUAAAAUUUACUCUAUCGGAGGUCACUAUGACUUCAGUACUGACUUUUCAGGACACCCAUUUUGAUGUAGUUGAACGUAACAAUCAGUUGUAUCUCGAUGCGUACCAAAUUGGUACGGCACUAGGUUAUAGCGAUCCAAGAACUGCUGUACGUAAA
Vsp-1-1    UUUAGAAAUCAUUACCAAAGGAGGUCGCUAUGACCAAUCAAAUCACAAUUACUUUAGACUCUUCUGAUCUUGUCUUUGAACACUCCGGUGAUCUUGUCACCACAUCUAAACUAGUUUCUCAGGCUUUUGGUAAGCAACAUAAAGAUGUUUUGCGUAAGAU
Vch-1-1    CUAAACUAGCGGCAUACCAUUUUAGAGUUCUUCGGCUUACACUGAGCAAAUAAGGAGAACUCGCGAUGAAAAAAUCACGUUAUACCGAAACACAGAUCGUUAAGAAGUAGAAGGUGGCCGUCUCAUCAAAGAAGUCUGUAGGGAAUAUGGGAUAUCCUAU
Vba-1-1    UUUAAAGAGCAUCCCCAAGGAGGCCAUCAUGCCAAAUCUAACAAUCCUUUCUAAAGACAUUCGCACACUCGAUAAUUUGUAUUCGCUAAACGAUCUGCAUAAAGCGAGUGGUGGACACAAUCGACAUCGUCCAAGUAUUUUUAUCAAGAAUCAGCAAACU
Sfr-1-1    UUACUCACGAUAUCAAGGAGUGUCACCCAUGAACUUACAACAGUCUAAAAACUACCCUGCACAACUGGUCUUUAUUAACGGCCAACAAACCAUUACCAAUUCACUGAUCGUUGCCGACUACUUUGGCAAAAAACACUGUGACGUUUUGCGUAAAAUUGAC
Vsh-1-1    UGAUAUAAAUUAUUACUCAAGGGCUUUUCACAUGACGACUCUAACUAUUCUUUCAAAAGAUAUUCGCACACUCGAUGGACUUUAUUCGCUUAAUGAUCUUCACAAAGCCAGUGGUGCCAAUAAAAAUCAUCAACCUUCCAAUUUCGUUCGCCAAGCAAAC
Sgl-1-1    UUCAUGGAAGGAAAAAUAAAAAAAGAGACUAUCGGACCACUUAUAUCAUUCAUGGAAAAAAGCAAGACCCCAUGGGGAAUUAAGGAUCGCGAGUCUCGAUUUAUAUAUAUGAAUAGUGCUGCCUUGCAUUUUGGAAAUCUGCCAAAAAAUUUUAACCCUG
Sgl-2-3    UUCAUGGAAGGAAAAAUAAAAAAAGAGACUAUCGGACCACUUAUAUCAUUCAUGGAAAAAAGCAAGACCCCAUGGGGAAUUAAGGAUCGCGAAUCUCGAUUUAUAUAUAUGAAUAGUGCUGCCUUGCAUUUUGGAAAUCUGCCAAAAAAUUUUAACCCUG
Sgl-1-2    UUCAUGGAAGGAAAAAUAAAAAAAGAGACUAUCGGACCACUUAUAUCAUUCAUGGAAAAAAGCAAGACCCCAUGGGGAAUUAAGGAUCGCGAGUCUCGAUUUAUAUAUAUGAAUAGUGCUGCCUUGCAUUUUGGAAAUCUGCCAAAAAAUUUUAACCCUG
env-11     UUUAAAUAAAACAUACUGGAGGCUGCCCUAUGGCGACUACCCCUCUCUUUUCACACUCUUUAUUUACCCUGCCCUUUAGCCAUGCCACGGACUUCACCGAACUGGCGGAUAACUGCGAGCGUUUUGUCGAAGGGCUGGUUGAAUGCCAUGACCCGGUUGA
Eco-5-1    AGAUCAUCAUCACUCACUGGAGGCUGCCCCUAUGGCAACUACCCUCACUUCUGAAUGCAAUUUACUCGUCAUUCCCUUCAAUUCCCUUCAGCGCCGCUACCAACUUCACCACUCUGGCGGAUUACUGCGAGCAUUUCGCUGAAACGAUCAUCGAGUGCCA
Sen-11-1   UGAUAAACACUGCUCAUCGGAGGAUGCCUUAUGGCUACGACCCUCACCCAGACACACCCGCUACUCUCCGUUCCCUUCAAUGCCGCCACGGAUUUCACCGUACUGGCAGAACACUGCAAACAGUUCGCCGAAAUCCUGAUCGAACACGACGAUCCCACGC
Sen-12-1   UGAUAAACACUGCUCAUCGGAGGAUGCCUUAUGGCUACGACCCUCACCCAGACACACCCGCUACUCUCCAUUUCCUUCAAUGCCGCCAUGGAUUUCACCGUACUGGCAGAACACUGCAAACAGUUCGCCGAAACCCUGAUUGAAAGCGACGAUCCGGCGC
Esp-1-2    AUAAAAACAACUCUUUGGAGGCUGUCAUAUGACAACGACCCUUAGCUAUUCUCAUUCUUUAUUUACCGUUCCAUUCGAUAGCAACACCGAUUUCACCGAGCUGGCCGACAACUGCGAGCGGUUUGUCGACGUGCUGGUUGAAUGCGAAGACCCGACACAA
Sgl-2-4    UUAAAUCCAGAUUGUCACCGGAGGUCGCUAUGACCCUUCACACACCCCUUGCUGCACCCAAAGUUACUAUCUACAACGGUAAAGCUGUAACCACUUCUCAGGACGUUGCUGAUUACUUCGGUAAGCUGCAUAAAGACGUGCUCAAGAAGAUCGAUUCAUU
Sgl-2-5    GUGAUUAUAAGUUCACUAUCUGGAGGUCAGCAUGACGCUUCAAACAUCUGUUACCACACCAAAAGUUACCAUUCAUAACGGUAAAGCUGUUACUACGUCGCUUGACGUUGCUGAAUAUUUUCAAAAACGUCAUGACGAUGUUUUGAAGAAGCUGCGCGCA
Sgl-2-6    GAUUACAUCAAGAUCACUAUCGGAGGCUAUUAUGGCCCUUCAACGAUCUCUUACUGCACCUAAAGUUAGCGUUCACAACGGCAGAGCCGUAACUACAUCAGUAGACGUUGCUGAUUACUUCGGCAAGCUACAUAAAAACGCCAUCCAGAAGAUAGAAUCC
Sgl-2-7    AUAAGUUUAGUUCACUAUUCGGAGGCUAUCAUGGCUCUUCACACAUCUCUUGCCAUACCUAAAGUUACCAUUCAUAACGGUAAAGCCGUUACAACGUCUAUUGCUGUCGCUGAAUAUUUUGAAAAGCAGCACAAGGACGUACUAAAAAAAGAUCAGAUCAC
Sgl-2-8    AUAACUUCAAGUAACCAUUUGGAGGUCAUCAUGACCACACUAUCUAUUGUUCCAUUCACUUUUGAAAAGCAUGAAGUACGCACAACUAUUUUAAACGGCGAACCGUGGUUUGUUGGCAUUGACGUGUGUUCCGCGCUCGGAAUAAGUAACAAUAGAGAUG
Sdy-1-1    GUUAAUUAACUUAUCACUUGAGGAUGUCAUUAUGGCUACUACCCUUUCUCACCCUGACGUAACCAUCGAAAAUGGUCGCGCCGUCACUACUUCUAUUUCGAUUGCUGAGUUUUUUGGCAAGCAGCAUCACCAUGUUGUUCAGAAAAUAGAAUCCCUCGAA
Sbo-1-3    GUUAAUUAACUUAUCACUUGAGGAUGUCAUUAUGGCUACUACCCUUUCUCACCCUGACGUAACCAUCGAAAAUGGUCGCGCCGUCACUACUUCUAUUUCGAUUGCUGAGUUUUUUGGCAAGCAGCAUCACCAUGUUGUUCAAAAAAUAGAAUCCCUCGAA
Eco-10-1   UGAGUUAAUCAAAUUCACUAACGAGGCUGCUAUCAUGGCUACUAUCCCUACCCUUUCUCAACCUGACGUAACCAUCGAAAAUGGUCGCGCUGUCACUACGUCUGUUGCGAUCGCCGAGUUCUUUGGCAAACGCCACGACAAUGUUAUUCAAAAAAUAAAA
Sfl-1-1    UGAGUUAAUUAACUUAUCACUGGAGGAUGCCAUCAUGGCUACUAUCCCUGCCCUUUCUCACCCUGACGUAACCAUCGAAAACGGUCGCGCUGUCACUACGUCUGUUGCGGUUGCAGAGUUCUUCCACAAACGACACGACAAUGUGUUACGUGCCAUCGCA
Sfl-2-1    UGAGUUAAUUAACUUAUCACUCGAGGAUGCCAUCAUGGCUACUAUCCCUGCCCUUUCUCACCCUGACGUAACCAUCGAAAACGGUCGCGCUGUCACUACGUCUGUUGCGGUUGCAGAGUUCUUCCACAAACGACACGACAAUGUGUUACGUGCCAUCGCA
Eco-3-2    UGAGUUAAUUAACUUAUCACUCGAGGAUGCCAUCAUGGCUACUAUCCCUGCCCUUUCUCACCCUGACGUAACCAUCGAAAAUGGUCGCGCUGUCACUACGUCUAUUGCGAUCGCAGAGUUCUUUGGCAAGCGUCACGACAAUGUGUUACGUGCCAUCGCA
Eco-3-3    UGAGUUAAUUAACUUAUCACUCGAGGAUGCCAUCAUGGCUACUAUCCCUGCCCUUUCUCACCCUGACGUAACCAUCGAAAAUGGUCGCGCUGUCACUACGUCUAUUGCGAUCGCAGAGUUCUUG....................................
Eco-3-4    GUUAAUCAAAUUCAUCGCGGAGGCUGCCAUCAUGGCUACUACCCUUUCUCACCCUUGUGUAACCGUAGAAAACGGUCGCGCUGUCACUACGUCUGUUGCGGUUGCAGAGUUCUUCCACAAACGACACGACAAUGUGUUACGUGCCAUCGCAAAUAUUGAA

           ................................................................................
           ................................................................................
           ................................................................................
```

Sfl-3-1	GUCAUUUAAACUGACUAUCGAGGCUGCCAUCAUGGCUACUAUCCUUACCCUUUCUCACCCUGACGCAACCAUCGAAAAUGGUCGCGCUGUCACUACGUCUGUUGCGGUUGCGAAGUUCUUCCGGAAAAUGCACAAAAACGUUAUUCAGAAGAUCGAAACU
Sfl-2-2	GUCAUUUAAACUGACUAUCGAGGCUGCCAUCAUGGCUACUAUCCUUACCCUUUCUCACCCUGACGCAACCAUCGAAAAUGGUCGCGCUGUCACUACGUCUGUUGCGGUUGCGGAGUUCUUCCGGAAAAUGCACAAAAACGUUAUUCAGAAGAUCGAAACU
Sfl-1-2	GUCAUUUAAACUGACUAUCGAGGCUGCCAUCAUGGCUACUAUCCUUACCCUUUCUCACCCUGACGCAACCAUCGAAAAUGGUCGCGCUGUCACUACGUCUGUUGCGGUUGCGGAGUUCUUCCGGAAAAUGCACAAAACGUUAUUCAGAAGAUCGAAACUC
Eco-6-1	GUUUUUUAAGCUUGCUACCGAGGCUGCCAUUAUGGCUACGAUCCCAACCUUUGUUCACCCUGAAAUCACGAUCAUCAAUGGUCGUGCUGUCACUACAUCUCUUGCAGUUGCUAACUACUUUACUAAACGGCAUGAGCGGGUUUUAGAUAGAAUUCGAAAC
Pcd-1-1	GUUUUUUAAGCUUGCUACCGAGGCUGCCAUUAUGGCUACGAUCCCAACCUUUGUUCACCCUGAAAUCACGAUCAUCAAUGGUCGUGCUGUCACUACAUCUCUUGCAGUUGCUAACUACUUUACUAAACGGCAUGAGCGGGUUUUAGAUAGAAUUCGAAAC
Sso-1-1	UCAUCCAAUUAAUUGGAGUCAUCACAUAUGAAUACAGCUAUAACUUUUUCUAACGAGUCACCAAAAGUCUUUUUCAAAGAUGGUCGCCUGGUUACCACCUCUCAAGCUGUUGCCGACUACUUCGAUAAACAACAUAAACACGUUUUAGCUAAGAUUGACA
Sbo-1-4	GUUUUCAAAAUUCGCACAUGAGGAUGUCACUAUGGCAACCACCCCUACCCAAACUCACCCUAAAAUUGAUGUUAUCCAUGGGAAGGCUGUUACCUCUUCUUUGGCCGUUGCCGAAUAUUUCUGCAAGCAGCACAAAAACGUUAUUCAAAAAAUCCAGACG
Sfl-3-2	GUUCUUUGAAUUCAACAUAGGGGCUGUCACCAUGACUACUCUCCCAACCCUCUCUCAACCUGAAAUUGCCAUCGUUGAUGGUUAGGCUGUUACUUCAUCCCUGGCUGUUGCCAACUUCUUCUCUAAACGUCAUGACGAUGUUCUGAAAAAGAUCCGCAUU
Eco-3-5	GUUCUUUGAAUUCAACAUAGGGGCUGUCACCAUGACUACUCUCCCAACCCUCUCUCAACCUGAAAUUGCCAUCGUUGAUGGUCAGGCUGUUACUUCAUCCCUGGCUGUUGCCAACUUCUUCUCUAAACGUCAUGACGAUGUUCUGAAAAAGAUCCGCAUU
Eco-13-1	GUUAUUUCUAUCACCGAGCAAAGAGGCCACACCAUGGCAGACCGCAAACAGCACCGCGCUAUCGCGGAGCGUCGUCACAUCCAGACUGAAAUCAACCGCAGACUUUCCCGCGCAUCACGCGUCGCGCAAAUCAUGCACAUCAAUAUGCUGCAUGAGCGCA
Eco-3-6	AGUUAUUAAAAAUCACCAAAGAGGCUGCCAUCAUGGCUACGAUCCCAACCCUCACUCAACCUGAAAUUGCCAUCGUUGAUGGUCAGGCUGUUACUUCAUCCCUGGCUGUUGCCAACUUCUUCUCUAAACGUCAUGACGAUGUUCUGAAAAAGAUCCGCAU
Sen-13-1	UGAGUUCAUUGAAUUCAACAUAGAGGCUGCCACUAUGGCUACUGUCCCAACCCUCGCUCAACCUGAAAUUAGAAUUAUUAACGGCCAAGCCGUUACUUCCUCCCUGGCUGUUGCCGACUACUUCAUCAAGCGUCACGCUGAUGUUAUCCGUAAAAUAGAA
Sen-16-1	UGAGUUCAUUGAAUUCAACAUAGAGGCUGCCACUAUGGCUACUGUCACAACCCUCGCUCAACCUGAAAUUAGAAUUAUUAACGGCCAAGCCGUUACUUCCUCCCUGGCUGUUGCCGACUACUUCAUCAAGCGUCACGCUGAUGUUAUCCGUAAAAUAGAA
Eco-2-1	UGAGUUAUUAAAAAUCACCAAAGAGGCUGCCAUCAUGGCUACGAUCCCAACCCUCACUCAACCUGAAAUUGCCAUCGUUGAUGGUCAGGCUGUUACUUCAUCCCUGGCUGUUGCCAACUUCUUCUCCAAACGUCAUGACGAUGUACUGAAAAAGAUCCGC
Eco-15-1	UGAGUUAUUAAAAAUCACCAAAGAGGCUGCCAUCAUGGCUACGAUCCCAACCCUCACUCAACCUGAAAUUGCCAUCGUUGAUGGUCAGGCUGUUACUUCAUCCCUGGCUGUUGCCAACUUCUUCUCCAAACGUCAUGACGAUGUACUGAAAAAGAUCCGC
Eco-6-2	UGAGUUCAUUGAAUUCAACAUAGGGGCUGUCACCAUGACUACUCUCCCAACCCAAUCUCACCCUGAAAUCACGAUUAUCAAUGGUCGCGUUGUCACCACAUCUCUUGCAGUAGCUAAUUACUUUACUAAACGGCAUGAGCGGGUUUUAGAUAGAAUUAGA
Eco-6-3	GUUUUCAAAACUUACACACGUAGAGGCCACACCAUGGCAAACCGCAAACAGCACCGCGCUAUCGCGGAGCGUCGUCACAUCCAGACUGAAAUCAACCGCAGACUUUCCCGCGCAUCACGCGUCGCGCAAAUCAUGCACAUCAAUAUGCUGCAUGAGCGUA
Eco-2-2	GUUUUCAAAACUUACACACAUAGAGGCCACACCAUGGCAAACCGCAAACAGCACCGCGCUAUCGCGGAGCGUCGUCACAUCCAGACUGAAAUCAACCGCAGACUUUCCCGCGCAUUCCGCGUCGCUAAAAUCAUGCACAUCAAUAUGCUGCAUGAGCGUA
Eco-8-1	GUUUUCACAACUUACUACACAAGGGGCCACACCAUGGCAAACCGCAAACCACACCGCGCUAUCGCGGAGCGUCGUCACAUCCAGACUGAAAUCAACCGCAGACUUUCCCGCGCAUCACGCGUCGCGCAAAUCAUGCACAUCAAUAUGCUGCAUGAGCGCA
Sdy-1-2	GUUUUCACAACUUACUACACAAGGGGCCACACCAUGGCAAACCGCAAACCACACCGCGCUAUCGCGGAGCGUCGUCACAUCCAGACUGAAAUCAACCGCAGACUUUCCCGCGCAUCACGCGUCGCGCAAAUCAUGCACAUCAAUAUGUUGCAUGAGCGCA
Eco-7-1	GUUUUCACAACUUACUACACAAGGGGCCACACCAUGGCAAACCGCAAACCACACCGCGCUAUCGCGGAGCGUCGUCACAUCCAGACUGAAAUCAACCGCAGACUUUCCCGCGCAUCACGCGUCGCGCAAAUCAUGCACAUCAAUAUGUUGCAUGAGCGCA
Eco-9-1	GUUUUUAAAACUUACUAGCAAAGAGGCCACACCAUGGCAAACCGCAAACAGCAUCGCGCUAUCGCGGAGCGUCGUCACAUCCAGACUGAAAUCAACCGCAGACUUUUCCGCGCAUUCCGCGUCGCGCAAAUCAUGCACAUCAAUAUGCUGCAUGAACGCA
Eco-3-7	GUUUUCAAAACUUACACACAUAGAGGCCACAUCAUGGCAAACCGCAAACAACAGCGCGCAUACGCUGCGCGUCGUCACAUCCAGACUGAAAUCAACCGCAGACUUUUCCGCGCAUCACGCGUCGCGCAAAUCAUGCACAUCAAUAUGCUGCAUGAACGCA
Eco-15-2	GUAUUCAAAACUUACCAACAAAGAGGCCACACCAUGGCAAACCGCAAACAGCACCGCGCUAUCGCGGAGCGUCGUCACAUCCAGACUGAAAUCAACCGCAGACUUUUCCGCGCAUUCCGCGUCGCGCAAAUCAUGCACAUCAAUAUGCUGCAUGAACGCA
Eco-2-3	GUUUUCAAAACUUACAACCAAAGAGGCCACACCAUGGCAAACCGCAAACAGCACCGCGCUAUCGCGGAGCGUCGUCACAUCCAGACUGAAAUCGAUCGCAGACUCACCCGCGCCGCACACAUCGCCUUUAUCAUGCAAUCCAACACAUUGCACAGACUCA
Eco-10-2	UUAUCCGACUAAAAGGAAAUCGCUAUGACAACUCAAGUAUCCGCAGCAGCCAUCUCCCCGAUUAUCCACAACCAACUUCCCGUCCUCACCACCGAAUUACUCGCGAACCUCUAUGGCACAGAGCCGGAUUACAUUCGUAAAAACCAUAACCGGAACUCAG
Spr-1-2	CUCCAAAAUCACGAAAGGAGUUAAACCAUGACUAAUCAACUCGCAUUCCGCGAUACUCAAUUCAACGUUGUAAACCACGUCAAUCAAAUCUGGUUAACAAGUAAAGAGCUAGCCGCCGCCUUAAAGUAUUCCAGCGCAAAAUCUGUAACUGACAUAUAUA
Sty-2-1	AUAAACAAAACAAUCACCGGAGGGCGUCAUUAUGACCACUCAAAUCUCUGUCGAAACUCUUUCCCCGAUUACCCAUAACCAAAUUCCCGUUAUUACUACCGAACUUUUGGCGCACUUAUACGGCACAAAAAUCAAAAACAUUUCUGAUAACUUUCUGAAC
Sen-9-1	AUAAACAAAACAAUCACCGGAGGGCGUCACCAUGACCACUCAAAUCUCUGUCGAAACUCUUUCCCCGAUUACCCAUAACCAAAUUCCCGUUAUUACUACCGAACUUUUGGCGUACUUAUACGGCACAAAAAUCAAAAACAUUUCUGAUAACUUUCUGAAC
Sen-13-2	AUAAACAAAACAAUCACCGGAGGGCGUCAUUAUGACCACUCAAAUCUCUGUCGAAACUCUUUCCCCGAUUACCCAUAACCAAAUUCCCGUUAUUACUACCGAACUUUUGGCGCACUUAUACGGCACAAAAAUCAAAAACAUUUCUGAUAACUUUCUGAAC
Eco-10-3	UAGUUUAAGUUUAGAAACCACUAGAGGACGUCAUUAUGACAACUCAAAUCUCUGUUGAAACCAUAUCCCCGCUCAUCCAUAACCAGAUUCCUGUUAUCAUCACCGAACUUUUGGAUCAGCUUUAGGUGCCAAGAUCAACAACAUCAGAGUCAAUUACACC
Sso-1-2	CAGUUUCAGGUUUGCAGUUUACAUUUCCCCGCGGUGCUCAAAACACCUCGAAAGCGGUAUCCACACCCGAUAGCCAUGUGGUUUUUUUGUGUCCAGAAUUCUUGGUUUAUGACCGGGUGUGCGGCUAAUACAAUACCAGCAAUGGAAAUACGCCCGCCGA
Ype-7-1	UCAUCCUAUAUGUAGGAGAAUCACAACUAUGAAUACUCAAUUAUCCUUUCGCAACACUCAAUUCGAUAUUGCCAAUCACUCUGGACAAAUCUGGUUACGUGGGACUGAAAUUGCCAAAGCGCUUGGCAUGGAAAAAUCAGAUGCAGUAAGCCAGAUAUAC
Ype-1-1	UCAUCCUAUAUGUAGGAGAAUCACAACUAUGAAUACUCAAUUAUCCUUUCGCAACACUCAAUUCGAUAUUGCCAAUCACUCUGGACAAAUCUGGUUACGUGGGACUGAAAUUGCCAAAGCGCUUGGCAUGGAAAAAUCAGAUGCAGUAAGCCAGAUAUAC
Eco-3-8	UAGUUAAAUCCAUCAACCAGAGGGCGUUAUCAUGACGACUCAAGUUUCUGUCGAAACUCUUUCCCCGAUCACACACAACCAGCUCCCAGUUGUCACUACAGAGUUACUGGCCAAACUAUAUGGCUGUAGCGUCGAGUGUAUUCAUAGAAAUCACCACCGU
Eco-3-9	UUAUCUUAACCACACUGAGGAGGCUGCCAACAUGGCUACUACCCCUACCCUCGUACAUUCUCAAACUGCCUUUAUCUGGCGCUUUAUUGCCUUUGGCGCGUCAGAACAUCAAAUCAUCCACGUAACCGCCUGGACGGAACGCGAAGCGCGUAGCCGUUGC
Yen-1-1	GAUCUAAAUCCCGCCUACCAGGAAACGAACAUUAUUUAUUCAACGGCCAGAUGGGUGACCCAAUAACUCGUCGUCGAACAAGGAAGCACUGGUGAAAAUUGCAGAAGCACUUGAGCUAGAAGGCUAUCUGAAAGGAUACAUUGAAAGUUAUCAGGAAAGU
Yen-1-2	GGGAUAAAGCCAUCAACUGAGAGGUUGUCAUCAUGGAUUCGACGACUAAAGCCCCUUGCCGUACCGCUUUUUUUCACCUGCCAGCAUCACUGGCCGCCACUCUCCGGCCGGAGGUGCGCCAUGUACGAUAACUCGCCCCGCGAAGUGGAAGACCUCAUUG
Yps-1-1	GGGAUAAAGCCAUCAACUGAGAGGUUGUCAUCAUGGAUUCGACGACUCAAGCCCCUUGCCGUAAAGCUUUUCUUCACCUGCCAACAUCACUGGCCGCCACUCUCCCGCCGGAGGUGCGCCAUGUACGAUAAUACGCCCCGCGAAGUGGAAGAAGUCAUUG
Ype-13-2	GGGAUAAAGCCAUCAACUGAGAGGUUGUCAUCAUGGAUUCGACGACUCAAGCCCCUUGCCGUAAAGCUUUUCUUCACCUGCCAACAUCACUGGCCGCCACUCUCCCGCCGGAGGUGCGCCAUGUACGAUAAUACGCCCCGCGAAGUGGAAGAAGUCAUUG
Yps-2-1	GGGAUAAAGCCAUCAACUGAGAGGUUGUCAUCAUGGAUUCGACGACUCAAGCCCCUUGCCGUAAAGCUUUUCUUCACCUGCCAACAUCACUGGCCGCCACUCUCCCGCCGGAGGUGCGCCAUGUACGAUAAUACGCCCCGCGAAGUGGAAGAAGUCAUUG
Yen-1-3	UGAUAAUUGUCCCCAUCGAAGAGGUAGUCAUCAUGGAUCUGACGACUAAACACCUGUCAUUAUUUACCAGCAAUAUCAUUGUUAUUCCCACACCUGUUGCUUCAUCUGCCCCUCUGCUGCCGGAGGUGCGCUAUGUACGAUGACACCCCCUGCGACGUAG
Ybe-1-1	UGAGUUUAACCACCUUCAACUGAGAGGUUGUCAUCAUGGAUUCGACGACUAAGCCCCCUUGCAAUACCAUUACUUACCCACCUGUAUCGCUCCCUAUCACCCUCCUGUUGGAGGUGCGCCAUGUACGAUGAUUCGCCCCGCGAAUUAGAAGAACUAAUCG
Yin-1-1	GAGUAUCAACACCGUCAACUGUGAAAGACUAAUAGCAACUCGGCGUAGUACAAGGAAGCACUGAUGGCAAUUGCAGAAUCACUUGAACUAGAAGGCUAUCUGAAAGGAUACACUGAAAGCUAUCAGGAAAGUCAGCGGGAGACGAUACUGAAAAUCGCCC
Yen-1-4	GCGAUCUAAAUCCCGUCGACCAAGAAACGACCAUUAUUAAUUCAACGGCCAGAUGGGUGAGCCAAUAACUCGUCCGUAGUACAAGGAAGCACUGAUGACAAUUGCAGAAUCACUUGAACUAGAAGGCUAUCUGAAAGGAUACACUGAAAGCUAUCAGGAA
Yin-1-2	GUGAUUAGUCCCCCUACCAAAGAGGUAGUCAUCAUGGAUCUGACGACUAAACACCUGUCAUUAUUUACCAGCAAUAUCAUUGUUAUUCCCACACCUGUUUCUUCAUCUGCCCCUCUGCUACCGGAGGUGCGCUAUGUACGAUGACACCCCUGCGAAGUAG
Sbo-2-1	UUUUCAAACCACAUACUGGAGGCUGCCUUUAUGGCUACGAUCCCCACUCAGACACACCCGUUACUUGCUGUUCCCUUCAGUGCUGCCACAGAUUUUACCGUACUGGCAGACCACUGCGAAAACGUCGCCGAAACCCUGAUCGAAAGCGAAGAUCCGGUGC
env-13	GUUUUUAACCCGUUACAGGAGGCUGCCACUAUGGCUACUACCCUCAUCCAGACACACCCGUUACUUUCCGUUCACUUCAAUGCCGCUACGGACUUCACCGUCCUUGCCGAUCACUGCGAAAAUUUCGCCGAAACCCUGAUAGAAAACGACGAUCCCACGC
Sen-16-2	UAAGUCAAAACUCAUCACAGGGGCUGUCGUUAUGACUACCAUCCCAACCCUCGAUCAACCUGAACUUUGCGUUAUUGAUGGCAAAGUCGUUACUUCCUCACUGGCUGUUGCCGAUUAUUUCCAUAAGCCACAUAAAGACGUACUGGCUAAGAUUUCCCGC
Api-1-1	GUGAUUAUCCUUAACUAACAAGGAAAUCAUUACUAUGACUACUUUAGUUUUCCGCAAUACUGUUCUUGAAACCAUAUCUCAUAAUGGGCAGAUUUGGUUUACGUCUAGCGUGCUUGCUAAGGCAUUACAAUACUCCAGCAGUAAAUCCGUUACCGAUCUU
Sgl-2-9	UGAUGUUCCUUAACUAAGAAGGAAAUCAUUACUAUGACAACCUUAAUGUUCCGUAACACCAUUCUUGAAACCAUUCCCCACAACGGGCAGAUCUGGUUCACCUCUACCGAACUUGCUAAAGCACUCGAGUAUGCAGAUCCUAAAGCAGUUUCCGGGCUUU
Sdy-1-3	GUUAAAAACCAUCGAAACGCGAGGUCGUUAUGACUGUUCAGAUUUCUGUCAAAAACCUUUCCCCUGUUACCUAUAACCAGAUUCCUGUUAUUACCACCGAACUUUUGGCGCAGCUUUACGGCACUGAGCCGGUGCGUAUUCGCCAGAAUCAUCAUGAGAA
Eco-3-10	......
Eph-2-1	AAGUUUUUCACUGCUCAAGGAGGCUGCCAUCAUGGCUACAGUCCCAACUUCCCCAUACCUGAAAAUCGAAGUCGUCAACGGCAAGGCCGUUAUUUUCUCCCUGCAUGUUGCCUGCCACUUUAAGCGCAUGCACCAGAACAUCGUCGACAAAAUCGAGUAU
Eco-3-11	AAGUUUUUCACCGAUCAAGGAGGCUGCCAUCAUGGCUACGAUCCCAGUUUCCCCAUUCCUCAAAAUCGAAGUCGUCAACGGCAAGGCCGUUAUUUUCUCUUUGCAUGUAGCCUGCCAUUUCAAGCGCAUGCACCAGAACAUCGUCGACAAAAUCGAGUAU
Eco-3-12	AAGUUUUCCACAGUCAACGGAGGCUGCCAUCAUGGCUACAGUCCCAACCCACUCAUUCCUCAAAAUUGAGACCGUCAACGGCAAGGCCGUUAUUUUCUCUUUGCAUGUGGCCUGCCACUUUAAGCGCAUGCACCAGAACAUCGUCGACAAAAUCGAGUAU
Sdy-1-4	GAAGAACUCCCAGUACAAGGAGGCUGCCACAUGCUGGCUACUACCACUACCCAAAAACCGCAAUUUAUCUGGAUUAUCGCCGCAGUUCGCCGCGAUUGCCCGACAAUUACCGCCAAAAUUCAUCAUAUUGCUGCCGAGUCUGAACGCGAUGCUCGCCGUU
Eco-8-2	AGGUAAAAAUUAUCGAAACGCGAGGUCAUUAUGGCUGUUCAAAUUUCUGUCGAAAACCUUUCCCCUGUUACCUAUAACCAGAUCCCCGUAAUUACUACUGAACUGUUGGCUCACCUUUACGGAACAAAAAUCAAAAACAUUUCUGAUAACUUUCUGAACA
Sdy-2-2	UAAUGACCAAAUGUACGGAGUUAUCGUUAUGCUCACUCAAAUUUCUGUCGACACUCUUCCUGCAAUCACCCACAACCAGAUCCGCGUUAUUACAACUGAACUUCUGGCGCAGUUAUACGGAACAAAAAUCAAAAACAUUUCUGAUAAUUUUCUGAACAAC
Eco-10-4	CGAUAAUUUUCAAAUGUACGGAGUUAUCGUUAUGCCCACUCAAAUUUCUGUCGACACUCUUCCUGCAAUCACCCACAACCAGGUCCCCGUUAUUACAACUGAACUUCUGGCGCAGUUAUACGGAACAAAAAUCAAAAACAUUUCUGAUAACUUUCGGAAC
Sfl-2-3	UAAUUUCCAAAUGUACGGAGUUAUCGUUAUGACCACUCAAAUCUCUGUUGAAACUCUCUCCCCGAUCACCCAUAACCAGUGAGGUAGCCUGAGUUUAACGGACACUCCUUCCUGAAAUAGAAUGGCAUCAGAAGGAGCUAAUAAUGAGCAGAAAAACCCA
Sen-10-2	UAAUUUCCAAAUGUACGGAGUUAUCGUUAUGACCACUCAAAUCUCUGUUGAAACUCUCUCCCCGAUCACCCAUAACCAGAUUCCUGUUAUUACCACCGAACUUUUGGCGCAGCUUUACGGCACUGAGCCGGUGCGUAUUCGCCAGAAUCAUCAUGAGAAC

......

......

......

Sdy-2-3	UAAUUUCCAAAUGUACGGAGUUAUCGUUAUGACCACUCAAAUCUCUGUUGAAACUCUCUCCCCGAUCACCCAUAACCAGAUUCCUGUUAUUACCACCGAACUUUUGGCGCAGCUUUACGGCACUGAGCCGGUGCGUAUUCGCCAGAAUCAUCAUGAGAAC
Eco-9-2	UGAUUCACAUAGAAAAAGGAUCAUCCUGUGGCUAUUCAAAUCUCAGUUGAAACUCUUUCCCCGCUCCCCCAUAACCAAAUCCCUGUCAUUACUACCGAGCUUUUGGCACAACUUUAUGGCACCGAAACUAAUAACAUCAAAGUGAACUAUACACGCAAUG
Eph-1-1	AAGUAUUUCACCGAUCAAGGAGGCUGCCAUCAUGGCUACUGUCCCAGCCCUCACUCGUCUGAAUGAUGAAGACUUACAUAAACUCAGUUAUGUAACAACUGCACUACGUGCUCUGCGCAAGGUAACUCUUUCGGAUCCCCAGGCGCAUCAGGUUUUGGUA
Eco-13-2	GUUAUUAAGAAACCACCAGAGGGCGUCAUUAUGACAACUCAAAUUUCUGUUGAAACUCUCUCCCCGAUCACCCAUAACCAGAUUCCUGUUAUUACCACCGAACUUUUGGCGCAGCUUUACGGCACUGAGCCGGUGCGUAUUCGCCAGAAUCAUCAUGAGA
Sen-15-2	GUUAUUUAGAAACCACUCUAGGGCGUCAUUAUGACAACUCAAAUCUCUGUCGAAACUCUUUCCCCGAUUACCCAUAACCAAAUUCCCGUUAUAACUACCGAACUUUUGGCGCAGUUAUACGGCACAAAAAUCAAAAACAUUUCUGAUAACUUUCUGAACA
env-14	AAGUUUUUCACUGUCAACGGAGGCUGCCAUCAUGGCUACGAUCCCAGCCCUCACUCAACCUGAAAUCACCAUUGACAACGGCCAGGCCGUUACCACUUCUUUGGCUGUUGCCAACUUCUUCUCCAAGCGUCACGACGAUGUGCUGAAAAAGAUCCGCACU
Eco-9-3	AAGUUUUUCACUGUCAACGGAGGCUGCCAUCAUGGCUACGAUCCAAGCCCUCACUCAACCUGAAAUCACCAUUGACAACGGCCAGGCCGUUACCACUUCUUUGGCUGUUGCCAACUUCUUCUCCAAGCGUCACGACGAUGUGCUGAAAAAGAUCCGCACU
Sen-2-1	AAGUUAUCCAUUGCAUACGGAGGCUGCCAUCAUGGCUACGAUCCCUGCCUUAGUACAACCUGAACUUUGCAUUAUUGCAGGCAAAGUUGUUACUUCUUCUCUGGCUGUUGCUAGUUAUUUCGGCAAACAACACAAAAAUGUCAUUCAAAAAAUUGCGUCU
Sen-15-3	AAGUUAUCCAUUGCAUACGGAGGCGGCCAUCAUGGCUACAGUCCCAACUCUCGCUCAACCUGAAAUCAGUGUUAUUAACGGGCAAGCCGUUACUUCUUCCCUUGCUGUUGCCGACUACUUCACCAAACGUCAUGACGAUGUUCUGAAAAAGAUCCGCGCA
Eco-11-1	AGGUAAUUCACUGUUAUCGGAGGCUGCCAUCAUGGCUACUGUCCCAGCCCUCACUCGUCUGAAUGAUGAAGACUUACAUAAACUCAGUUAUGUAACAACUGCACUACGUGC..
env-15	AGGUAAUUCACUGUUAUCGGAGGCUGCCAUCAUGGCUACUGUCCCAGCCCUCACUCGUCUGAAUGAUGAA..
Sen-9-3	AGUCACAUCCAAGUUAUCGGAGGCGGCCAUCAUAGCUACAGUCCCAACUCUCGCUCAACCUGAAAUCAGUGUUAUUAACGGGCAAGCCGUUACUUCUUCCCUUGCUGUUGCCGACUACUUCACCAAACGUCAUGACGAUGUUCUGAAAAAGAUCCGCUCA
Eco-4-2	UUAACCAGACUAAAAGGUAACCACUAUGACAGCUACAAAAAGCACGUCCAUUUUUUCUUUCGAAUCCCAAGCCGAUAUACGGGUAAUCGUCAUUAAUGGUGAGCCAUGGUUUAUCGCUUCAGAUGUUUGUCGGGCUAUAGGCAUAGCAAACCAUCGAGAU
Eph-6-1	UAACCAGACUAAAAGGUAACCACUAUGACAGCUACAAAAAGCACGUCCAUUUUUUCUUUCGAAUCCCAAGCCGAUAUACGGGUAAUCGUCAUUAAUGGUGAGCCAUGGUUUAUCGCUUCAGAUGUUUGUCGGGCUAUAGGCAUAGCAAACCAUCGAGAUG
env-16	AAAUCACUAAGUGGAGAUAACCACCAUGGCUAAUGCUCAAACUUCCAUCUUCAAAUUUGAAUCUGUUAACCCUAUCCGUUCCAUCAUUAUCGAUGGCCAACCAUGGUUUGUAGCCCAAGACGUUUGUAGUGCGCUGCGUAUCCNAAACGUCACCCAAGCA
Sgl-1-3	UGGUCUUUUUUCCCUGCUUAGGAAAAGGAUUAUCUUAUGAACAUGAAUGGAAAAUAACAGGAAACAAUGGUCUAUAAAAGACCAUGAAUCACGGUAUUUAUUUAUUAAUAAAUCAGCAUUACAAUACUAUGGCAUCCCUUUAAACUUCGAUUAUCAUGGA
Sgl-2-10	UGGUCUUUUUUCCCUGCUUAGGAAAAGGAUUAUCUUAUGAACAUUAAUGGAAAAUAACAGAAAACAAUGGUCUAUAAAAGACCAUGAAUCACGGUAUUUAUUUAUUAAUAAAUCAGCAUUACAAUACUAUGGCAUCCCUUUAAACUUCGAUUAUCAUGGA
Pfl-2-5	CUCCACCUAUCGACUAGAAAGAAGCCAUUAUGAAGAAGCCAACCCCGAAUCCCCCCGAAGUCGAAACCGACCCAACAUCCCCGUACGUAUCGAUCGACUGCAGAAAACUCCACGACGCCGCCAACCGGGCCCUCGAUCACUACCUCUGCCCCGGCGCCCA
Ppu-1-1	GGCAACUCCUUUUGAACUAGGGAGUUUGAAAAGUGAAAACGAUCGAUUCUGAUCCCAACAGCCCCAAGCCAAUCACAACCGCAGGCGUGGAAACCUUCCUCGCAGCCGGUAACCCAUCCCUCAAUCUGCUACGCGUAGAGCCCGGCGUCCCCGUCGACGC
Pfl-2-6	UGGCUCCAUAGCCAAGUAGGAGCUUUUCAUGGACAAACUGAUACCCGACCCACCCCUCGAAUCACCCACCCCACUCGAAGAGGCCAUGCGCGCCGAAGACCUCGCCCGUAACCGCGAAGCAAUCAAACGCGCCCUCGACUACUACCUCUGCCCCAACCCC
env-17	UUCAAACCUUUACCAAGGACACUACCAUGUCAAACCUAGUCACGUCCCCCGAACUUACCAUUUUGAAUGGUGUUGCUUUCUGUACCAGCCUGCAAGUUGCUGAUCAUUUCGAUAAACGUCAUGACAAUAUAGUGCGCGAUAUUGAAGCAACUAUUACGCA
env-18	AGUCCAAACUUUACAGAGGCUACUAAGAUGACUACUUCACAUCUCCCCCUAAACACGUCCAGCGAAAACACCGAAAUCGAAGCCCCCGAACUAAUGCUGAUUGCAGGCAAAGCUUUUUGCACGAGCCUGCAAGUCGCGGAACAUUUCGAGAAACGUCAUG
env-19	UCCAACUUAAAAAGAGACUACUCAUCAUGACUCAUACGUCACUUAUCACUCAGCCUGAACUCACUAUUCUGAAUGGCGUUGCUUUCUGCACUAGCCUGCAAGUGGCUGAACACUUCGAUAAAACACACGCGAAUGUGUUGAAAGAUAUUGAACGAACUAU
env-20	GUAGUCCAAUUAGCAUACGGAACUACUACGAUGACUACUUCACAUCUCCCCCUCAGCACGUCCAGCGAAAACAUCGAAAUCGAAGCCCCCGAACUGAUGCUGAUUGCAGGCAAAGCUUUUUGCACGAGCCUGCAAGUGGCGGAACAUUUCGAGAAACAGC
Bth-1-1	GUUUCGAAACCGCUGCAAAAGGAGGCCGCACCCAAUGCGCCAGUCCACGCACGCCCGUACCGGGCAGCAAUCCCACCUCAUUCGAUCCAUCGUCCGUGCAGCCUUGCGCGACGCGGCAACCGCCAGCACGUAUCAAGACGCGCUCGACGCAACCGGCGCG
Bps-11-1	GUUUCGAAACCGCUGCAAAAGGAGGCCGCACCCAAUGCGCCAGUCCACGCACGCCCGUACCGGGCAGCAAUCCCACCUCAUUCAAUCCAUCGUCCGCGCAGCACUGCGCGACGCGGCAACCGCCAGCACGUAUCAAGACGCCCUCGAUGCAACCGGCGCG
Rso-1-1	CUCCACCCAUUACCAGGAGUCCGCAUCAUGCACUGCACCGCCGCUCAUUCUGAGCAAACCCGUCAUUCUUCCCAGUCCCCUCUCUGCUCUACCGAGUACGCGGCAAGGCGUGCGCGUAUCAGCGGCAAGCUAUUCGCGGUGCGCUGGCCCGAGGUCCGUG
Sgl-2-11	CGUUACCCAAAUGUACCAUUGGAGGUCAUUAUGACCAUUCAUACCAGCCUUGCUGCACCUAAAGUUACUAUCCACGAUGGUAAAGCUAUUACCACCACCGAUGACGUUGCCGCUUACUUCGGCAAACAACCACAUCAUGUUGUACAAAAAGUAGAAUCCC
Bxe-1-2	CGAAUACGUUUCAGGGAGAUCGCGCCAUGACUAAAUCCGUCAAGCAUUCACGCACCACGUCGCGCCCACCCGUUGAUGCAUUUCAAUACGAAAAACUAGCCUUAUCCGCAUUCGGACUGGUUGAACGGCAGCUAGGCCAACUGGACAAACUGGUCACGCU
Bph-1-2	CGAAUACGUUUCAGGGAGAUCGCGCCAUGAGUAAAUCCGCCAAGCAUUCACGCAUCACGUCGCGCCCACCGGUUGACGCACUUCAAUACGAAAAGCUCGCCUUAUCGGCCUUUGGACUGCUUGAACGGCAGCUGAGUCAACUGGACAAGUUGGUCACACU
Ppu-4-2	CAGCUCAAAUCCAGCCACGAUCGGAUCUGCACCAUGAAGAAAAUCGUACCCGACCCACCCCGCUCCAAACUCCUCACCAACCCGUACUUCUCCAUCCACACCGACAUGCCCCCUCCAGACGCCCUGGCCCACGCCAGCGAACUGCUGCGCGGCGUCGCCG
Pen-1-2	AGUUCCUGGCAUUUCAAUAGGAGCUUCCAGAAUGAAAAAGAUCGUCCCCGAUCCACCUUCCACCCCAUUGAUCCCCUUCGAACCCCCACCCCUCGCGCUGCAAUCCCCGCUCGGCAUCAACGAAUGCCACAGCAUGCUCCACACCCUGAGCCUGAUCAUG
Pst-1-1	UCCGUUCUGAGGAUCACACCAUGAAUGAAACCAUCUCUUCGCAAAGCGAAGCCAGCCGAGCCGUUUCCAAUAGCUGCGAUGCCAAUAGCGGCACAGGAAAAUGCUUCACCCCACUCCGUCACGAUGAACCUCACAGCUUGAUCGGCUAUCUCAGCAGCGC
Sgl-2-12	GCGAGUACCGCUAUAAACCACUGGAGGUCAUUAUGACCGCAUUAUCUAUCACACCAUUCACUUUUGAAGAUCACCUGGUGCGCACCGUCGUCAUUAACGAUGAGCCGUGGUUCUUUGCUGUAGAUGUAUACAGCGCCCUUGAUAUUCAAAACCCCUCUAA
Eph-7-1	UAGCACCCUAUACAAGAUAGGAAUGCAUACCAUGUUCAAAUUCAAAUUUGCAGCUGUCGUCCGCACGGACAAAAAAUCCCAUAUCCACCGCCUUUCCACCAUUGCAUCAUCCGAGCGAGAAGCUCGUCGCCAGUUCGCCAGCCGUUUUGUCCUCGUUCUG
env-21	CGUUAACCGCUAUUCACAGGAGGUUGCCCUAAUGGCUACGACCCUCACCCAGUCUCACCCCAAAUUUAUCUUAAGUGUUUGCCGCCGUUCGCCGCGCAGACCGUAAGCCGCGUAUCUGUAUGCUCCGCACCGUUUGCCCGGUGACGAGCAGGCCGCCCGU
Esa-1-1	CGUUACCCGCUAUCUAACGGAGGUUGCCACCAUGGCUACAGUCCUCGCUUCAUCACAUCCUGAAUUUACCUUUCUCUUUCUUGCCGUUCGCCGCGCUGACUGCGGUGCGCAGCCAUUAGCUGUUCGUGUUAAUGCCGGUACUGAACGUGCUGCACGUGCG
Sen-11-2	CAUAACCUGCUAUCUAACGGAGGUUGCCCUUAUGGCUACGAUCCUCACCCCGUCAUACCCGCAGUUUGUCUUUGUGUUUGCCGCUGUUCGUCGCGCAGACCGUAAACCCCGUAUCUGUAUGCUUCGCACCGUUGCCGGUGAUGAACAGGCCGCACGUCUU
Yph-1-1	AGCAUCACUACCCAUCGGAGUUCAUACAAUGUACACAUUCAAAUUCGCGGCAAUUUGCCGCACCGACCGAAAAAAUCACAUCCAUCAUUUUUCCACUAUUGCCGAUACAGAGCAGGCCGCUCGUCGCCAGCUAACUGGAAAAUUCGUGCUAUUUUUCUGU
Sgl-2-13	CAAUAGAUUUCUCAAUCUGGAGGCUGCCAUAUGGCUACUACUACCCCUAUCCAAACCUCGCCCGUUGUUACGAUCGCUGAUGGCCACGUUGUCACGACUUCAAUCGCUGUUGCGGAGUAUUUUCAAAAGCCCCACAAAGACGUUUGGCAAAGAUCGCAC
Sgl-2-14	UUGUUUUACCAUCGUAUCGGAGGCUGUCAUAAUGACUACUACCCUCACCCAAGCUAAGACUAUUGAAAUCGUAGAGCCGAAAGUUUUGAACAGAACGGUAAAAUUUUAACUACUUCCUUUGAUGUUGCAGCAUAUUUCCGUAAACGCCAUGACGAUGUA
Eco-3-13	CUGUAACUGUUACUUCAAGGAGGCUGCCAUCAUGGCUACAACCCUUACCCCCUCACACCCUGAAUUUGUCUUUGUGUUUGCCGCUGUCCGUCGCGCAGACCGCCAUCCCCGUAUCUGUAUGCUUCGCACCGUCGCCGGUGAUGAACGCAGCGCCCGCCGU
Sen-7-2	CAUUUACCGCGACUUAUUGGAGGUUGCCCUUAUGGCUACGAUCCUCGCCCCUUCACAUCCUCAAUUUAUAUUUGUUUUUGCCGCCGUUCGUCGUGCAGAACGUAAACCACGUAUCAGUAUGCUGCGUAUCGUCGCCAGCAAUGAGCAUCAUGCACGUCUU
Esp-1-3	CUGUUACCAGCUAACUAUGAAGGUUGCCCUUAUGGCUACGAUCCUCACCCCGUUACACCCGCAAUUUGUCUUCGUGUUUGCCGCUAUUUGUCGCGCAGAUCGUAAACCCCGUAUCUGUAUGCUUCGCACCGUGGCCGGUGACGAGCACGCCGCACGCCGU
Ybe-1-2	CGUUAAUCCGCUAACCUUGGAGGCUGCCACUAUGGCUACAUCCCUCACCCCGUUACACCCGCAAUUUAUCUAUCUGUUUGCUGCUGUUCGCCGUACUGAAUUAACAGCGCGUCCCUGCAUGUUGCGUACCACCGCCAGCAGUGAGAAGAAUGCCCGCCUG
Ymo-1-1	CAAUAACCAAUACUUAUUGGAGGCUGCCACUAUGGCUACAUCCCUCACCCCGUUACACCCGCAAUUUAUCUAUCUGUUUGCUGUUGUUCGCCGUACUGAAUUAACGGCGCGUCCCUGCAUGUUGCGUACCACUGCCAGCAGUGAGAAGAAUGCUCGCCUG
Esa-1-2	CAGUUACAGCUACUUAUUGGAGAUUGUCCCUAUGACAACGGCCCCUGCUGCCACUCAUCCCGAAUUCACCUUUAUCUUUGCCGCCGUGCGCCGCACCGAUAUCUUCGCGCGCCCCUGCAUGCUGCGUACCGUUGCCGGAGAUGAGCGCACCGCCCGUGCC
Yfr-1-1	CAAUAACCAUGACUCAAUGGAGGCUGCCACUAUGGCUACAUCCCUCACCCCGUUACACCCGCAAUUUAUCUAUCUGUUUGCUGCUGUUCGCCGUACUGAAUUAACAGCGCGUCCCUGCAUGUUGCGUACCAUCGCCAGCAGUGAGAAAAAUGCUCGCCUG
Sen-9-4	CGUUACCCGCUAUCUGAAGGAGGUUGCCAUUAUGGUUACAGUCCCUGCUUUAUCUCACCCUGAAUUUACCUUUGUCUUUCUGGCUGUCCGCCGUACAGACCGUGAUGCCCGUCCACGUCCUGUGCGUGUAAUCGCAGACUGUGAGCAUGCUGCACGCCUU
Sen-5-1	CGUUACCCGCUAUCUGAAGGAGGUUGCCAUUAUGGCUACAGUCCCUGCUUUAUCUCACCCUGAAUUUACCUUUGUCUUUCUGGCUGUUCGCCGUACAGACCGUGAUGCCCGUCCACGUCCUGUGCGUGUAAUCGCAGACUGCGAGCAUGCUGCACGCCUU
Sen-7-3	UUUUUCAAACUACUUCAAGGAGGCUGCCAUCAUGGCUACUACCCUCACCCAGUCACACCCGCAGUUUGUCUUUGUGUUUGCCGCCGUUCGUCGCGCAGACCGUAAACCCCGUAUCUGUAUGCUUCGCACCGUUGCCGGUGAUGAACACGCCGCACGUCUU
Sen-8-3	UUUUUCAAACUACUUCAAGGAGGCUGCCAUCAUGGCUACUACCCUCACCCAGUCACACCCGCAGUUUGUCUUUGUGUUUGCCGCUGUUCGUCGCGCAGACCGUAAACCCCGUAUCUGUAUGCUUCGCACCGUUGCCGGUGAUGAACACGCCGCACGUCUU
Eco-13-3	CAAUAACCGCUACUUAUUGGAGGCUGCCAUCAUGGCUACAAUCCUCACCCCGUCACACCCGCAAUAUGUCUUUGUGUUUGCCGCAAUCCGCCGUGCUGACACUCACCCCCGCAUCUGUAUGCUUCGCACUGUCUCCUGCGAUGAGCGUUCCGCGCGCCGU
env-22	CAAUAACCGCUACUUAUUGGAGGCUGCCAUCAUGGCUACAAUCCUCACCCCGUCAUACCCGCAAUAUGUCUUUGUGUUUGCCGCAAUCCGCCGUGCUGACACUCAACCCCGCAUUUUUAUGCUUCGCACUGUAGCCUGCGAUGAGCGUUCCGCGCGUCUU
env-23	CAAUAACCGCUACUUAUUGGAGGCUGCCAUCAUGGCUACAAUCCUCACCCCGUCAUACCCGCAAUAUGUCUUUGUGUUUGCCGCAAUCCGCCGUGCUGACACUCAACCCCGCAUUUUUAUGCUUCGCACUGUAGCCUGCGAUGAGCGUUCCGCGCGUCUU
Sen-11-3	CGUCAUUUACGCUGUUAUCGGAGGUUGCCUUAUGGCUACGACCCUUACCCCCUCACAUCCUGAAUUUGUCUUUGUGUUUGCGGCUGUCCGUCGCGCAGACCGUCAUCCCCGUAUCUGCAUGCUUCGCACCGUCGCCGGUGAUGAACGCAGCGCCCGCCGU
Eco-5-2	CGUCAUUUACGCUGUUAUCGGAGGUUGCCUUAUGGCUACGACCCUUACCCCUUCACACCCUGAAUUUGUCUUUGUGUUUGCGGCUGUCCGUCGCGCAGACCGUCAUCCCCGUAUCUGCAUGCUUCGCACCGUCGCCGGUGAUGAACGCAGUGCCCGCCGU
Eco-14-1	CGUCAUUUACGCUGUUAUCGGAGGUUGCCUUAUGGCUACGACCCUUACCCCCUCACACCCUGAAUUUGUCUUUGUGUUUGCGGCUGUCCGUCGCGCAGACCGUCAUCCCCGUAUCUGUAUGCUUCGCACCGUCGCCGGUGAUGAACGCAGCGCCCGCCGU
Eco-11-2	CGUAACCUGCUAUCUAACGGAGGCUGCCAUCAUGGCUACAACCCUUACCCCCUCACACCCUGAAUUUGUCUUUGUGUUUGCGGCUGUCCGUCGCGCAGACCGUCAUCCCCGUAUCUGCAUGCUUCGCACCGUCGCCGGUGAUGAACGCAGCGCCCGCCGU
Eph-4-1	CGUUAUUUGCUAUUCACAGGAGGCUGCCAUCAUGGCUACAACCCUUACCCCCUCACACCCUGAAUUUGUCUUUGUGUUUGCGGCUGUCCGUCGCGCAGACCGUCAUCCCCGUAUCUGUAUGCUUCGCACCGUCGCCGGUGAUGAACGCAGUGCCCGCCGU
Sbo-2-2	CGUUAUUUGCUAUUCACAGGAGGCUGCCAUCAUGGCUACAACCCUUACCCCCUCACACCCUGAAUUUGUCUUUGUGUUUGCGGCUGUCCGUCGCGCAGACCGUCAUCCCCGUAUCUGUAUGCUUCGCACCGUGGCCGGUGAUGAACGCAGUGCCCGCCGU
Eco-8-4	CGUUAUUUGCUAUUCACAGGAGGCUGCCAUCAUGGCUACAACCCUUACCCCCUCACACCCUGAAUUUGUCUUUGUGUUUGCGGCUGUCCGUCGCGCAGACCGUCAUCCCCGUAUCUGCAUGCUUCGCACCGUCGCCGGUGAUGAACGCAGCGCCCGCCGU
	....
	....
	●●●

```
Sgl-2-15   CAAGUCCAGUUAUUCCUUGGAGGUCGCUAUCAUGGCUACUACCCCCCUUAAAACUCGCCCUAAAAAUUUGACGCCGCGCCAACGUGCAUCCCUUCUGAAAAUAGGGGGGAUCGCAUGAACAUCCCCAUCAAGCAAUUUUGCCAAAGUCUCACGAUCCCCU
Sty-1-1    GUUUUUAGUUGUCAUCGGAGACCUUUGCUAUGUCACCUCAACUGAUACAGAAACUCCCGGCCAUCACUCUGUUGGAAGGCAUGUUUCCCGAGCUAUCAACAAACCAGCUUAAAGUCUGCGUUUUUUACGCAAUGGGUGUUCCUUACGACGCCAUUGCUCA
Eco-1-1    GUUUUUAGUUGUCAUCGGAGACCUUUGCUAUGUCACCUCAACUGAUACAGAAACUCCCGGCCAUCACUCUGUUGGAAGGCAUGUUUCCCGAGCUAUCAACAAACCAGCUUAAAGUCUGCGUUUUUUACGCAAUGGGUGUUCCUUACGACGCCAUUGCUCA
Sen-4-4    GUUUUUAGUUGUCAUCGGAGACCUUUGCUAUGUCACCUCAACUGAUACAGAAACUCCCGGCCAUCACUCUGUUGGAGGCAUGUUUCCCGAGCUAUCACAAACCAGCUUAAAGUCUGCGUUUUUUACGCAAUGGGUGUUCCUUACGACGCCAUUGCUCAGA
Kph-1-1    UCUUAACCAUCAGCUAGAGGGGUUACCCUAUGUUCACAUUCAAAUUUGCCGCGAUCUGCCGUACCGAUAAAAAAUCCCAUAUUCAUCAUCUGUCCACUAUCGCCUCAUCCGAGCGAGAAGCCCGUCUCCAGUUCGCCAGCCGUUUUGUUCUCGUUCUGUC
Ype-2-2    UCAUUACAACUACUUCUUGGAGGCGGCCCAAUGGCUACUACCCUUCACUGUCUGUACCCGCAAUACGUUCGUACCCAUCCGGGGGUGUGCUAUGCAUAACUCUUCCCCGCUCACGAUUGAAGAAAUUGUUGAUCACUGCCACGCACUGGUAUUGGCAAUG
env-24     CGUAACCUGCUAUCUGAAGGAGGCUGCCACAUGCUGGCUACUACCCCUACCCAAAAAACGCAAUUUAUCUGGAUUAUCGCCGCAGUUCGCCGCGAUUGCCCGACAAUUACCGCCAAAAUUCAUCAUAUUGCUGCCGAGUCUGAACGCGAUGCUCGCCGUU
Sen-1-1    CAGUCACAUGCAAACAUUGGAGGUUGCCACUAUGGCUACGACCCUCACCACGUCACACUCGCAGUUUGUCUUUGUAUUUGCCGCCGUUCGUCGCGCAGACCGUAAACCCCGUAUUUGUAUGCUUCGCACCGUUGCCGGUGAUGAGCACGCCGCACGCCUU
Sen-14-2   CAGUCACAUGCAAACAUUGGAGGUUGCCCUUAUGGCUACGAUCCUCACCCCGUCACACCCGCAGUUUGUCUUUGUGUUUGCCGCCGUUCGUCGCGCAGAUCGUAAACCCCGUAUCUGUAUGCUCCGCACCGUUGCCGGUGAUGAACACGCUGCACGUCGU
env-25     UCAUUAAUCAAUGACAUACGGAGAUGCACCCAUGACAAAGGUUCCCACUACAGAAUCCGUUGCAAGCACCCUUACCCUCUAUCGCCAACUGUUUAACCACCUCGAUCUCAGCGCUUUCAAUCCCUCAGAGCUCUUAGACCUCGCCGCACUCUCUGCCGAA
Ppu-4-3    UCAAUCAGUAACCUUACGAGUCGUUCACUAUGAAAAAACUAGUCCCCGAUCCGCCCCACCAUUUCGACCUUCCCUCCGACAAGACCCUGACCAACGCCGUGAGCGAUGGCAUCGUGCCAAUCGAUCACGUGUUGAUGAACGUCACCCACUACCUGAUGCU
Ppu-2-1    UCAAUCAGCAAAUCUACGAGUGAUUGAUAUGAAACAGACAGUUCCUGAUCCACCCCUCAUUUCUCCUCCUCUGACAACACACUGCAAGCAUUGAGGCUUUAUUCACCGGCCUUCGCGGGUGAGCCCGCUCCCGCAAGUGCUGUGCAUGACUUAAUGUAUG
Pen-1-3    CACUUCAGAAGGCUGCAAGGAGUCGCACCAUGCUCAAGAAGAUCGUUCCAGAUCCACCGUUCGCAUUUGUCGAGAUCGCCACCUUUCAUCGAGAGGUGCAGCCAUGAACCAGGAAUCGCUGGUCAACCACAGUCCCCGCAAAUCCCGUCCCGUCACCGCU
Nmu-1-1    CAUCAACCGCUUACAAAGGAGUCUUGCUAUGCCUAAGGCAUUCAAGGGUGCGUCCGCGCCACAUCUCAAAGCUGUUCAACCUCUCCCUGCCAAAUCAAUCCGGUCCCGCAGCGACCUUUCUUUCGCGGCCCCGGAUGAACACGAUCUUCUGCGCAUUUGG
env-26     CAUACCGCUAUACAGGAGUCUUGCCAUGCAACCCACCCCUACGCCCCUUGCGGGCGCUCCCCUGUUCCAUCGUGCCUACGACCUUGCCGCCUUGAUCGGCGAUUGCCGGUCGGUCGCACUCGCGCUACCGGCAAUCAUUCCCGCCGAAAGCGAGGAGAUG
Avi-1-1    CAAGCUAAAACGGGAAACUCACCAUGUCGGACACAAACCUCCAGCUCGGAACAGUCGAUUUUCACGGCCAAACUCUGGCCGUAAUUACCGGACCGGCCGGCGAACGCCUUGUCGCCAUGAAGCCCAUCUGCGAAGCCAUCGGCCUCCAGUGGGAGGCCCA
Pae-4-2    UCAAACCUUGAUUAGGAACUCAGUCAUGACCCAUCGUCGCAUCCUUACCCUCAAUCCGUCUAAAGCUCGCGCACGCUUCCAUCGCGCCUGCGCAAUUGCCGCCCUCCAUAGCAAUUCAUCUCUCGCCGUUCGCCUACGUCGUUACAACGCAGCCAUGAAG
Ppu-2-2    CCAACCUCUAUCGGAGUUUGGCUAUGAAAAAGAUUGUUCCCGAUCCACCCCUCCCCAACACCACACAACGCCCUUUCAGCCGCUGCGAUGCCGGCCAUCCCCCAUUGUUCACCGUAAACCCCGGCGUCUCAGCCCACGACGCCCUGGUACAUGUCGCCCA
Pen-1-4    GCCUCCUUGAGCCGGUAAUGGAGGCAAUACCCAUGAAAAAGAUCGUCCCUGACCCACCCCUGAAGCUGAACCCCACCACCGAGCAGUCCUUCUGCACCUGCCAGUCCAGCCACCCGCCGAUCUUCACCGUGCGCCCCGGCGUCGACGCCGCCGACGCUUU
Pen-1-5    CGGUUCCUGUCAUCCACGAAAGGAACUUCAUGAUGAAAAAAAUCGACCCCGACCCACCCCGCAUCGCCCCCUUCAUCACCAUCCGCCCCACCCUCACCCGCGAGGAGGCGAUGGCCGCUGCCGUCGAGGUAGCCACCGCCAUUUCCGACGUACUCGAUGU
Ppu-4-4    GCUUUCCAAUAGCAUCUUCAGGAAAGUCAUCAUGAAAAAAAUCGUUCCCGAUCCACCGGACUCAUUCCCGAUCCCUUACAUCACAAUCAUCGCCGACCUCUCCCCGGAAGACGCCAGGUCUCACGCUGCCGCGCUGAUGGACUGCCUCUGUAAAACCAGC
Pen-1-6    UGUUUUCUUGGUUUUGACACAGAGAACACAGAUGAAAAAGCAUGUACCUGACCCACCUUACCCAAUCCCCUUUGUCACCAUCAUUAGCGACCUCCCUCCCGAGGAAGCCGUGGCCCACGCCAACAAGCUGAUGCACACCCUCAGCGACACCAUCCACGCC
Pen-1-7    UGUUUUCUUGGUUUUGACACAGAGAACACAGAUGAAAAAGCAUGUACCUGACCCACCUUACCCAAUCCCCUUUGUCACCAUCAUUAGCGACCUCCCUCCCGAGGAAGCCGUGGCCCACGCCAACAAGCUGAUGCACACCCUCAGCGACACCAUCCACGCC
Ppu-4-5    UUGCUCCUAGAAAUGUCAUUGGAGUUCACCAUGAAAAAAAUCGUACCCGAUCCACCCGACAGCUUCCCCAUUCCCUACAUCUCGAUAAUCGCCGACCUUUCCCUUGAAGAUGCCAAGGCCCAUGCCGCCGCGCUUAUGGACAGCCUUUCGGGCACCAUCG
Ppu-3-1    AGUCUCCUUCCGUGAAUAAGGAUUUGCCAUGAAAAAAGAUGUUUCUGAGCCAAUCACAACCGCAGGCGUGGAAACCUUCCUCGCAGCCGUUAACCCAUCCCUCAAUCUCCUACGCGUAGAGCCCGGCGUCCCCGUCGACGCGGCCUACGAGCAUGUCUCG
Ppu-3-2    GCAGUGCCCUUCUGUUUCAGAGGAAUACGCCAUGAAGAAGAUCGUCCCCGAUCCUCCUGCAAUCCUGACAGCCCGCCCCUUCUACACCAUCAACCAGGACACGCCUUCGGUUGACGCCCUGACCCACACCCUCCAGUUGAUGGCCGGCAUCGAAGACUCG
Ppu-4-6    GCGGUUUCCAUAAGCAGAACAAAGGUUACAAGCCAUGAAGAAGAUCGUACCCGACCCACCCAGCCGUAAACCCGUCACUACCCCAUUCUUCACCGUCCAGUCCGACAUGUACCCGCCCGACGCCCUGGCCCAUGCCAGCGAGCUGUUGCGCGGCGUCAUC
Ppu-1-2    CAGUUCCACUUACCAGGAGCAACUACCAUGAAGAACAACAACCCAUCCCCCCUCCUCACCGCAGGCAUCGAAACCUUCCUCGAAGCCGGCAACCCCACCCUGGACCUGCUCCGCGUCCAACCCGGCAUCCCCGUCGAUGACGCCUAUGAACACGUCUCCG
Ppu-4-7    CGGUUUCCAACUCAACGGAGCUGCUGCCAUGAAGAAAAUCGUUCCAGACCCACCUCGUCCUCUCAUCUCCACCCCUUACUUCACCAUCCACAGCGACAUCUCCCCUCCCGACGCCAUCGCCCACGCCGGCCAGCUUUUGCGUGUCGUAGUCGAAACCCUG
Ppu-3-3    CAGUUCCACCUAUCAGGAGCAACUACCAUGAAAAACAACAACCAAUCCCCCCUCCUCACCGCAGGCAUCGAAACCUUCCUCGAAGCCGGCAACCCCACCCUGGACCUGCUCCGCGUCCAACCCGGCAUCCCCGUCGAUGACGCCUAUGAACACGUCUCCG
Ppu-4-8    CUCCUCAAAUCAAGAAGGAGCUACCUCCAUGUUUGCUCGACUAUUCAGCCUCACUACGCCUGAAAUCCAGUCAAAAAUUGAGCUUCACCGCGCUCGCGCAGUCGCCCAGCUCAAAAGCAAAUCCAGCCUCAAAGUACGCCAUGAGCGCUACAACUCCGAA
Ppu-4-9    AGGGUGCUGAGUAACAUCCCAAGCCUGAUCCUUGCGGGCGACCGUGACAGUUCAGCAUGAUUGUCGCGCCCUAGCGCGCUAUGGCGUGCUGUCGUGACAACUUCUACGGCUCAUGAUGCACUUUCGGACCACACUGAACCUUGCGGAAGCUGGCCUGGAG
env-27     UUCCAAAAUUCAACUUGGAGCGCCACCUAUGUCCGCCCUCAUUCCGUCCGCAAUUCGCGCAUUUGCUCACCGCCGCAUGGCCCUGAGCGCCCUCCGCGCAAACUCCAGCCUCGCAACCCGCCUCAAGCGUUACUCGCACCACAUGAGCAUUGCCCGCUCC
Pen-1-8    CUCCAAUCCAAAACUUGGAGUCUCAUGCAUGCUUAUGCGCAUUCUGAGCAGGAUCCUCGCAUCCGCUCUCCGCCGCCCAACCCCUGCGGCUACCCCAUCCCCAACCUUCUACCCGAUCGAUAGCCAUUCCCUUGCCACCCGCGCCAAUCGGGAGGUGUCC
Ppu-1-3    CUCCAACUAAAACUUGGAGCCUCAUGCAUGCUUAUCUCAAGUCCAAGCAGGACCCUCGCAUCCGUUCUCCUCCGCCGUUCCUCAACGGCCCUCAGCUGCUUCACCCUCAACCAACCCCGCUUCGUCUCCACCCAACCGGAGGCCCGCCAUGUCUGACCAA
Ppu-4-10   GCAGCUCCUGUUGAGUAAUCAAACUGGGAUCUGCACCAUGAAGAAAAUCGUACCCGACCCACCCCGCUCCAAACUUCUCACCAACCCGUACUUCUCCAUCCACACCGACAUGCCCCCUCCAGAUGCCCUGGCCCACGCCAGCGCACUGCUGCGCGGCGUC
Ppu-4-11   AGGUCAGCCCAACAUACGGAGAUCCGCCAUGAAAAAGCUCGUCCCAGACCCACCUCCAUCCGCCCUGCUUCUACUUGAUCCCCCGCCAUCUCGCUCCCGGAACCGCCCAACACCCAAGAGUGCAACGCACUCAUCUGCGCUUUGACCCUGACCAUAAAA
Pen-1-9    GUGGUGCUUCCUUGAGACAGCACAAGAGUUCCAACAAUGAAAAAGCUCGUCCCAGACCCACCCCACAUAGCGUCCUACCUCACCAUCUCCCCCACCCUCACCCGCGAAGAAGCCAUGGCCUCUGCCGUCGAGGUAGCCACCGCCAUUUCCGAUGUACUCG
Pen-1-10   CGAUUUCUUUGAAUCGACAUGAGCGAUUCAGAAUGAAAAAGCGCGUACCUGACCCACCCAUCCCCUACAUCUCCAUCAUCAGCCACCUCACCGUCGACGAAGUCCGCGAUCACGCCGCGGUCCUGAUGUCGACGAUCCACAAGACUGCCGGUGUCUACCU
Pfl-2-7    AGCCCAACUUAUUAGGAAGUUAGGUAUGUUCAAAGCUACACCAAAUCCGCCUUCGGAUAUCGAUCCAACUGACAAGACUGAUCAAACCGAUCCGGUCUCCCCCUACGCCUCCGUCGAUUCAAAAAAACUCCACGACGCGGCCCAUCGGGCCCUCGACUUU
Xor-1-1    CCAAUCUACUCAGGAGUUCAGCAUGUCCUACCACACCCAAGAAGCGCCCGCGUCUGCGGCGCGCCAAGUCACCCAUUAUUUCAGCCUGAUCGCCAACACCCUCGAAUGGAACCAUGCCGCCUGGCAGAGCCUGAUGGCUCGCCUGGAAGGCACUGGUAAG
env-28     CCAUCAUCCAAGGAAUCCGAACGAUGUCACAUGACACCCAAGUCACGCCCGGCGUGGCGCCUGCGCGGCAGCUCGCCANNNNNNNNNNNNNNNNNNNNNNNNNNNNNNNNNNNNNNNNNNNNNNNNNNNGCGCGACGCCGAAGCGUUCCGAACUGACUGG
Xca-1-1    UUCCAGACCACAAACGGAAUCCAAAAUGUCCUACGACACCCAAGAAGCGCCCGCGUCUGCGGCGCGCCAGGUCGCACACUAUUUCGGCCUGAUCGCCAACACCCUUGAAUGGAACCAUGCCGCGUGGCUGAGCCUGAUGGCUCGCCUCGAAGGCACUGGC
Sma-1-5    UGUACGGAGCAUUGCCAUGAACACAUCGGACUUCCGUUCCCUGCACGCGCAGUACGAUCCGGACAACGCCGAACCCGAGCGCGAACCGUCCGUCGAUCCCAACGCCUUCGUCGCCACGCUGCACCGCAUUGGCAGCGGUGUGGCUGCGGACGGCCAGCCU
Bdo-1-1    UAUCCCACUCUAGAGGAUGCAUCAUGCCGACCACCAAUUCCGCAAUUUCGUCCCCCAAGCCGACUACAACGCUCCUUCUUUUCGCGGCGCUUUGACGUGCGAUCUUUUCGCCUCCGACGUUGGUGGCGACCAUGCCACGGUACCGUCGAAUCUGAUCGG
Pfl-2-8    AGCUUCAACUCAACCAGCCAUCGAGUAAUGUUUUCAUGAAGAAACCGACGCCCAAUCCACCCGAAACUGACGAAACCUCUCCCUACGAAUCCGCCGAUUCCAGGAAACUCCACGAAGCCGCCGAACGCGCCCUCGAUCACCACUUCAAUCCCCUCCCGAA
Pfl-1-2    UCCCUUACCCACAAGCGGAGGUUCCCAUGACUCAGCAAUACAUCCCCAUCACCGGCAUCGACUGCCUGAUCCCCUGCCUCUUUAUCGACAAGACCGCCCCCUUGGACGUGCUGCACGCCAACGGCACCGCACGUUUACUGGCCGUGACUCAACUGCUGGA
Ppu-4-12   UUUGGGGGAGGCGCCGUUUCAUGUCAUGGCGGCUGUGUGUGGGCAGGCUUCGGCCUGGCCGGGUUUCCAAGUUCCCCGGUAUUCCUACCCAUACAAAGCUGCCACCCCCCAAUCCUGCCGGAAGUAGCGAUUAGGGUGCUGAGUAACAUCCCAAGCCUG
Pen-1-11   CAGCCCUACUCUGGUAGAGGUUGCCAUGACCAAAAAGAUUGUCCCCGAUCCACCACUUCCUAACACCACAACUCACCCCUUCGGCCGCUGCGACGCCGGCCACCCUCCACUCUUCACCGUAAACCCCAACAUCGAAGCCCACGACGCCCUGGUACACGUA
Ppu-3-4    GGCCAUGAAGCGAGAUCUAGUGAUGAAGAAGAUUGUUCCUGAUCCACCCCUACCCUGCACCUCCACCCGCCCCUUCGGCCGCUGCGAUGCCGGCCACGAUCCACUCUUCACCGUCAACCCAAACAUUUCUGCCGAGGACGCUCUGGUCCACGUAGCCCUA
Ppu-2-3    GCCAUGAAGCGAGAUCUAAUGAUGAAGAAAAUUGUUCCAGAUCCACCCCCUACCCUGCACCUCCACCCGCUCCUUCGGCCGCUGCGACGCCGGCCAUGACCCGCUCUUCACCGUCAACCCGAACAUUUCCGCCGAGGACGCUCUGGUCCACGUAGCCCUA
Ppu-4-13   GGCAUUGAGGAGCAUUUUCCAUGAUCAAGAAACUCGUCCCAGAUCCACCACUUCCCUGCACUUCCACCCGCCCCUUCGGCCGCUGCGACGCCGGCCAUUCAGCGCUGUUCACCGUAAACCCCGACAUUUCCGCCGCAGACGCCUUGGUCCACGUAGCCCU
Ppu-1-4    GCCUUGAGGAGCAUUUUCCAAUGAUGAAGAAAAUAGUCCCCGAUCCACCUCUCCCAUGCACCUCCACCCGCCCCUUCGGCCGCUGCGAUGCCGGCCACGAUCCACUCUUCACCGUCAACCCAAACAUUUCUGCCGAGGACGCUCUGGUCCACGUAGCCCU
env-29     GCCUUGAGGAGCAUUUUCCAAUGAUGAAGAAAAUAGUCCCCGAUCCACCCCUACCCUGCACCUCCACCCGCCCCUUCGGCCGCUGCGAUGCCGGCCACGAUCCACUCUUCACCGUCAACCCAAACAUUUCUGCCGAGGACGCUCUGGUCCACGUAGCCCU
Psy-3-1    UCUUUUAAAACUCCAGGAGAUACAUCACAUGCUCAAACUCGUCCCCGAUCCACCCCCUUACCGCGCCAACCCCACCAUCAGCCAUGAAGACGCACUGAUGUAUGCCAGUGACCUGCUGCGCUGCGCCGCCACCAGUGCCUACGAAUUCAGCGACGGCAUG
Psy-1-2    UCUUCUAAAACUCCAGGAGAUACAUCACAUGCUAAAAAUCGUCCCCGACCCACCCCUCUUCAACGCCCGCCCCAAAGUCAGCCAUGAAGAUGCGCUGAUGUAUGCCAGCGACCUGCUGCGCUGUGCAGCAACCAGCGCUUACGAAUUCAGCGACAGCAUG
Psy-1-3    GCUCCCCCCAAGUUCUGCUGGAGUUGCAAACAUGCCCCAUAAAAUCGUCCCCGACCCGCCCACCGCCCUGUUCACCGUCAACGCAUCCGUCAGCCAGGAAGAAGCGCUCAACUACGCUUCGGAUCUGCUGCGCUGCAUCAUCACGACGGCCUAUGAGUCC
Pfl-2-9    CUCCAUAUAUGUGCCUUAGGAGUUCCACCAUGAUCAAACCAACACCCAACCCACCCGAAGCCGAUUCAACGUCUCCCUACGAAACCCUCGAUUCAAAGAAAUUCCACGAAGCCGCCGAACGCGCCCUCGAUCACUACCUCAAUCCGCUGCUCCCCAGAAA
Pfl-2-10   CUCCUUGAAGUCUGCGAUAGGAGUUACAUCUAUGUUCAAAGUCACACCCAACCCACCAGAAACCGAUUCAAUCCCCUACGACGCCGCACUCGAAGCCGAAAACAUAAAAACGGCAACCGAACGGGCGAUCAAUCAUUACCUCGAUCCUGGGGCGCAGAAG
Pfl-2-11   CCUCCCUUUUUGAAGCAUGAGGAGUUACAUCUAUGUUCAAACCUACGCCCAAUCCCCCCGAAACCGAUCCGGUCUCCCCCUACAAAUUCCCCGACUCCCGAACCCUGAACGAAGCCGCCGAACGCGCCCUCGACCACUACCUCACCCCGCAACAACGCAU
Pfl-2-12   GGCUCCUGAUGACGACACAAGGAGUUUCACCAUGAUCAAACCAACACCCAACCCACCCGAAACCGACCCCACUUCCCCCUACGAAUCCCCCAACUCCAAAAAACUCCACGACGCCGCCGAGCGUGCGCUGGAUCACUACCUCUUCCCCGCCGCAGGAUC
env-30     UUCCCUUAGCCACAUAUGGAGCGUCACUAUGUUCAAGGUCACACCAAACCCACCCGUAACCGAAACGGUCUCACCCUACGAAUCCUCCGAUUCCCGAAAACUCAGCGAAGCCGCCGAACGCGCCCUCGACCACUACCUGAACCCCGCCAGCCAAAUCAUG

           ................................................................................................................................
           ................................................................................................................................
           ................................................................................................................................
```

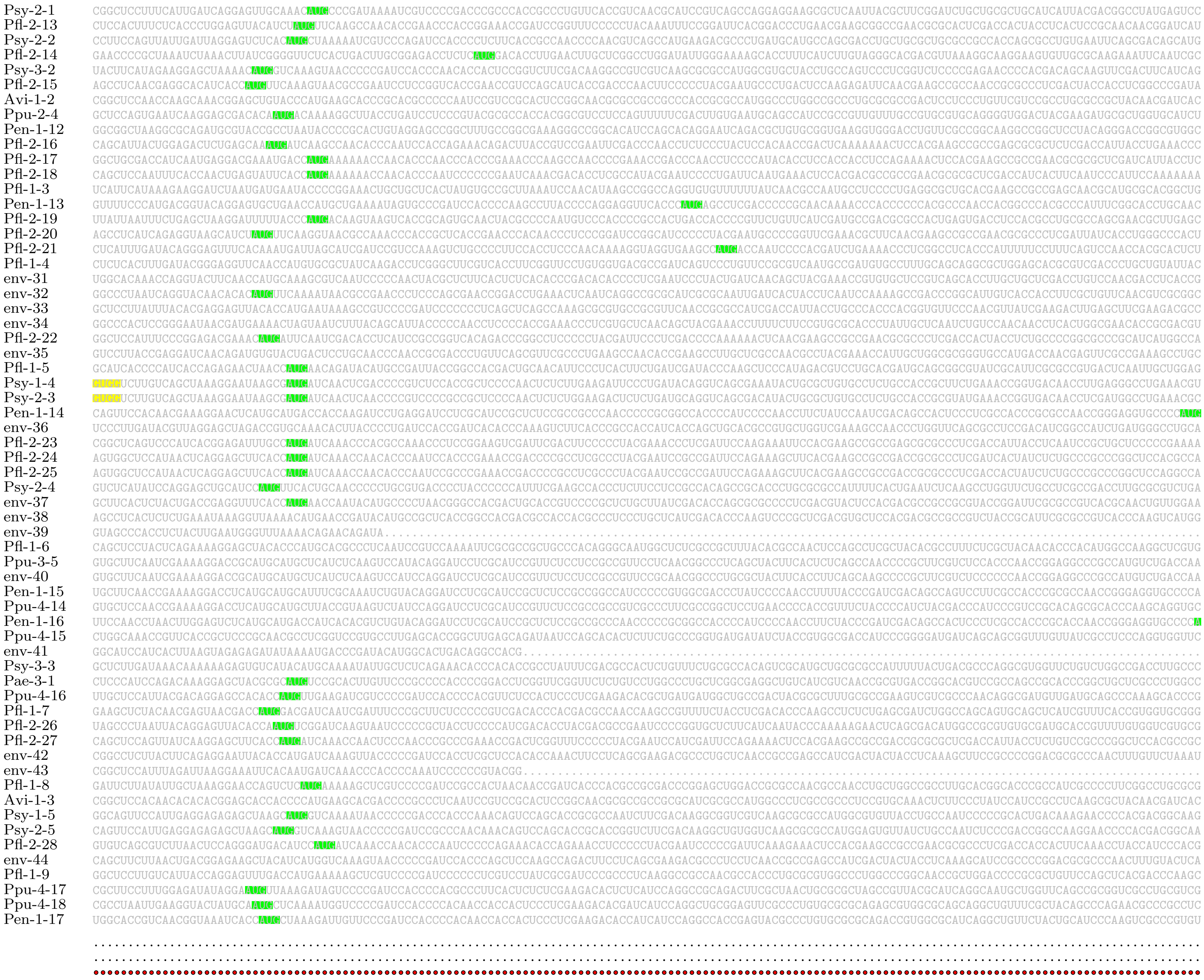
Psy-2-1
Pfl-2-13
Psy-2-2
Pfl-2-14
Psy-3-2
Pfl-2-15
Avi-1-2
Ppu-2-4
Pen-1-12
Pfl-2-16
Pfl-2-17
Pfl-2-18
Pfl-1-3
Pen-1-13
Pfl-2-19
Pfl-2-20
Pfl-2-21
Pfl-1-4
env-31
env-32
env-33
env-34
Pfl-2-22
env-35
Pfl-1-5
Psy-1-4
Psy-2-3
Pen-1-14
env-36
Pfl-2-23
Pfl-2-24
Pfl-2-25
Psy-2-4
env-37
env-38
env-39
Pfl-1-6
Ppu-3-5
env-40
Pen-1-15
Ppu-4-14
Pen-1-16
Ppu-4-15
env-41
Psy-3-3
Pae-3-1
Ppu-4-16
Pfl-1-7
Pfl-2-26
Pfl-2-27
env-42
env-43
Pfl-1-8
Avi-1-3
Psy-1-5
Psy-2-5
Pfl-2-28
env-44
Pfl-1-9
Ppu-4-17
Ppu-4-18
Pen-1-17

Pen-1-18	CUGCCCUCAAAGGAAUAAGAAGAUGCUAAAGAUCGUCCCCGAUCCACCCCACAACCACCACUCCCUCGAAGACACCAUCAUCCAGGCCACCGAGUACGCCCUGUGCGCGCAGACCGUGGCGCAUCAGGCUGUUCUACUGCAUCCCAAGUCGCCCGUGUCG
Ppu-4-19	GGCAUCCAUAGAGGUAAUAAGCGAUGUUGAAGAUGGUUCCUGAUCCACCCCACGCCCUUCACUCUCUCGAAGACACUCUCAUCCAGGCCGCAGACUUCGCUAACUGCGCGCUAGCCGUUACGCAUCAGGCAAUGCUGGUUCAGCCGCGGUCGCCUGCGUC
Ppu-4-20	GGCCCCGCUAACGGUAAAAUCAUUAUGUUAAAGGUCGUCCCCGAUCCACCCCACAACCACCACUCCCUCGAAGACACGAUCAUCCAGGCUGCGGAGUUCGCCCUGUGCGCGCAGAGCGUGGCGCAGCAGGCUGUUUCGCUACAGCCCAGAACGCCCGCCU
Pen-1-19	CCGCACCGGAAAGGAAAAUCGUUAUGCUGAAGAUCGUCCCCGACCCACCCCACAACCCCCACUCCCUCGAAGACACCCUCAUCCAGGCCACCGAGUACGCCCUGUGUGCCUUGACCGUGGCCCAUCAGGCGGUACUGGCCCAGCCCAGGUCCCCUGCCAC
Pen-1-20	GCCCUUUCUUCAGGAAAAAUACCGAUGCUUAAGAUUGUCCCCGAUCCACCCCACAACCCUCACUCCCUUGAAGACACCCUCAUCCAGGCCACCGAGUACGCCCUGUGCGCGCUGACCGUGGCCCAUCAGGCCGUGCACGCCCAGCCCAGAUCACCGGCCA
Pen-1-21	UGGUCCCACUCAUCAGCAAGAGGUCGUCAUGUUAAAGAUCGUCCCCGACCCACCCCAUCCUCACUCCCUUGAAGACACCCUCCUCCAGGCCAGCGAAUACGCCCUGUGCGCAUUGACCGUGGCCCAUCAGGCCGUGCAUGCCCAGCCCAGGUCACCGGCC
Ppu-3-6	GGCCCCUAUCGAGAACACAGUAAAAUGCUAAAGAUCGUUCCUGACCCGCCUCACCACCUCCACUCCCUGGAAGACACGAUCAUGAUGGCCUCGGACUACGCGCUCUGUGCAGAAGCCGUAGCCCAGCAGGCAAUACUGAUGCAGCCCAAGUCACCCGCAU
Pen-1-22	GGCCCCAUCAAAGGGCACAAAACCAUGCUGAAGAUAGUUCCCGAUCCACCCCACAACCACCACUCCCUCGAAGACACCAUCAUCCAGGCCACCGAGUACGCCCUGUGUGCGCAGACCGUGGCGCAUCAGGCUGUUCUGCUGCAUCCCAAGUCGCCCGUGU
Ppu-3-7	UGUAUUCGAUUCAUGUACCCCACCUACGGGCAGUUUCUGCCUGAGUGCUUCAGUGAGAACUCGAAAUCCACGGGUUCUCGUACUGGAGACCUUAUGGUAUGGCGGGCUUGUAGAGGGCAGUUGAACGCAGGGCUAUUUCAGCACAGGUCAGAUCGCCUGU
Ppu-1-5	GCAGCCCUGUAAGGCACUGAGAGGGUUUAUGCUGAAAAUAGUACCUGAUCCACCCCACAACGUUCUUUCCUUGGAAGACACCCUGGUUCAGGCUACGGACUACGCGUUAUGCGCGGCGACCGUCGUGCAUCAGGCAAUGCUGCUGCAGCCCAAGUCCCCC
Ppu-1-6	UGGCCCCAACUUUGAACAGGCCUUCACCAUGCUGAAAGUAGUUCCCGAUCCACCCCAUAACCCCCAUUCCCUCGAAGACACCCUGAUCCAGGCCACCGACUACGCGUUGUGCGCAGCCACCGUGGUGCAUCAGGCGUUGCUGCUUCAGCCCAAGUCACCG
Pen-1-23	GUCCUCAUUCACGAACAGGGCUAUGACCAUGCUGAAGAUUGUUCCCGAUCCACCCCAUCACCCUCACUCCCUCGAAGACACCCUUAUCCAGGCCACCGAGUACGCCCUGUGCGCACUGACCGUGGCCCAUCAGGCCGUGCACGCCCAGCCCAGGUCACCA
Pen-1-24	GUCCUCAUUCACGAACAGGACUAUGACCAUGCUAAAGAUCGUUCCUGAUCCACCCCAUCACCCUCACUCCCUCGAAGACACCCUCAUCCAGGCCACAGAGUACGCCCUGUGCGCGCUGACCGUGGCCCAUCAGGCCGUGCACGCCCAGCCCAGAUCACCG
Pen-1-25	GGCCCUUUUCAGGUACCCCAAAAUGCUGAAGAUUGUCCCCGACCCACCCCAUCACCACCACUCCCUCGAAGACACCCUCAUCCAGGCCACCGAGUACGCCCUGUGCGCCUUGACCGUGGCCCAUCAGGCGGUGCUGGCCCAGCCCAGGUCCCCGGCCACG
Ppu-4-21	GGCCUUUAUCACUAGGUGAACCAUGAUGCUGAAGAUAGUCCCCGAUCCACCCUAACGCCCAUUCCCUUGAAGACACCCUGAUCCAGGCCACGGAUCACGCGCUGUGUGCCGCAACCGUGGUGCACCACGCCAUGCAGCUGCAGCCCAGGUCGCCGGCUUC
Ppu-4-22	UGGCCUCAACUUGAACAGGCCUAAAACAUGCUGAAAAUAGUCCCCGAUCCACCCCACCUACUCCACUCCCUGGAAGACACGCUGAUGAUGGCCGCUGACUACGCCCUGUGCGCCGAAGUCGUCGCCCAGCAGGCAAUGCUGAUGCAGCCCAAGUCACCUG
Ppu-4-23	CUCCACCUGACUCAAGGAGCAUUCAUGCGCAAGAUUGUCCCCGAUCCACCGUCCCCCCUCGAUCCCUCGUACAUGCUACAGGACACCCUGGUACAGACCUCGGAAUACGUGAUCUGCGCCUUGUCCGUAGCCCGCCAGUCCGUGCGACUGACACCGACCU
Ppu-1-7	GCUCCACUACCCAAGGAGCUUACAUGCGCAAGACAGUCCCCGAUCCACCCUACUCCCCCCAUUCCACCCAGAUCCUGCAAGACACCCUGGUCCAGUCCUCAGAAUACGUACUCUGCGCCCUGUCCGUGGCCCGCCAAUCCGUGCAACUCAAACCCACCGC
Ppu-1-8	GUCCCAACCACAAAUUGAGGGUUACACCAUGCUUAAGAUAGUCCCCGAUCCACCCUGCAACCUCCACUCCCUGGAAGACACGCUGCUGAUGGCCGUGGAUUACGCGCUAUGCGCCGAAGCCGUUGCCCAGCAAGCGGUUUUGAUGCAACCGAGAUCACCC
Ppu-3-8	GUCCCAACCACAAAUUGAGGGUUACACCAUGCUGAAAAUAGUCCCCGAUCCACCUUACAAUCUUCACUCCCUUGAAGACACACUGCUGAUGGCAGCGGACUACGCACUGUGCGCGGAAGCCGUUGCUCAGCAAGCCGUUUUGAUGCAGCCGAGAUCACCC
Pen-1-26	GUCGCUCGACUGAGGAACUGAGAAUGCUGAAGAUAGUCCCCGAUCCCCCCACCGUCCUUGAAGACACCCUCGUCCAGACCUCCGAACACGUCCGCUGCGCCCUGGCCGUCGCCCAGCAAUCCAUCCCGCUCAUUUCCAGAUCACCCGGCUCGAUGCUCGU
Ppu-2-5	AGCCAAACAGGAAAGAAAGGAGCGAGUCAUGCUGAAAAUUGUCCCCGACCCACCCCACAACCCCCAUUCCCUCGAAGACACCCUGAUCCAGGCUACCGACUACGCACUAUGCGCGGCCACCGUGGUGCAUCAGGCGUUGCUGCUUCAGCCCAGAUCACCG
Ppu-4-24	AGCUCCACCGCAACGCUCAGUUUCUGCGAGCUAAAUGUUGACUGCCAACAGGUUACAAGCGGUUACAUCGAACAGCUGAAAAAUCAGAUUUAACCGCUUUCUUGUAGGGCAUUUCCAAAGAUACUGCCGGCGCCUGUUUUCCGUUGCGCACGCAAAAAG
Ppu-4-25	CAGCUCAACCAUUGUUGGAGCGUAAGCAUGCUGAAGAUAGUCCCCGAUCCACCCUACACUUCCCACUCCCUGGAAGACACACUCAUGAUGGCUGCUGACUACGCACUUUGCGCAGAAGCCGUUGCCCAGCAGGCGAUGAUGAUGCAACCCAAAUCGCCCU
Ppu-3-9	CAGCUCAACCAUUGCUGGAGUGUAACCAUGUUGAAAAUAGCUCCUGAUCCACCUCUCAGCACCAUUUGUCUUGAAGAUAUUGGUCCAGCCACAAUUUACGCGCUAUGCGCCGCUAUCCCCGUGCAUCAAGCGAUGUUGCUGCAGCCCAAGGCCCCAGCGU
Pen-1-27	GGCUCCAUCUCAUACGUUGGAGUCUAAUCAUGCUGAAGAUUGUCCCCGAUCCACCUUCCCCCCAAGACCACGCCCACUUCCUCGAAGACACCCUCGUGCGCGCCCUCGAGUACGCCUUCUGCGCACUCACGGUCGCCAACCAGGCCGUGCAGCUGAAACC
Ppu-3-10	AGCUCCACUUUCCGACGUUGGAGUUACGCCAUGCUCAAGAUCGUUCCAGACCCACCCCACCGGUCCCUCUGCCUUGAAGACACCCUGAUCCAGGCCACGGAUUACGCGCUUUGCGCUGCAACUGUCGUGCAUCAGGCAAUGCUGCUGCAGCCCAAGUCAC
Ppu-3-11	AGCUCCAAUGAUCCUGGAGAUGUUUAUGCGCAAGAUGGUCCCUGAUCCACCCCUCGAUACCACCCAGUUCCUGCAAGACACUCUGGUCCAGUCCUCAGAGUACGUACUCUGCGCCCUGUCCGUGGCCCGCCAAUCCGUGCAACUCAAACCCACCGCCCAC
Pen-1-28	AGCACCACCGACUCCGGAGAUGCAUAUGCGCAAGAUGGUUCCAGAUCCGCCGACACACCUUGGCAACUCCCCGCAUCAGAUCGAAGACUUGCUGGUUCAAAUUGCUGAAUACCUUGUCUGUGCCCUAACCGCCGCCCAGCACGCCGCGAUGAUCCAUACC
Psy-1-6	GGCUCAUUUUCAUGACAGGAGCUUUGCCGCGAUGGAUCUCCCAUUCCCCCUUCUUCCCUGCACCGAAAACCACGAACCCACCCUCUUCCUGCGCCACCGUCGCAUGUUGCGUGCUGUUGUCAUGCACGCAGAAGCCUGGUUCCCGCUGGAAGAUAUCGCC
Ppu-4-26	ACUUCACACUGCAUGGUGUUUUGAUGAAAAAAAUAGUCCCCGAUCCACCCCGCCUCAAACUGUUCAACACCCUCUAUUCCUCCAUCCACCCCGAACUGAUCCCACCCGAGGCCUUGGCGGUCGCCAGCGAGAUGCUGCUGGGUAUCUCUGAAGUCGUCGG
Ppu-3-12	UAUCAGCCCGGUAUGGUGAACUCAUGAAAAAACUGGUCCCCGAUCCGCCCCACGUCUUCGAUCUUCCCCAAGGCAAAUCCCUUUCCCGCGCGAUCAGCGAAGGCAUCGUCCCGAUGGAGUUCGCGUUGAUGAAUGUUUCCCACUACCUGAUGUUCGCCUA
Ppu-1-9	ACACAGCCAACACGGAGCUGUCAUGAAAAAACUGGUCCCCGAUCCACCCCACGUCUUCGAUCUUCCCCAAGGCAAAUCCCUUUCCCGCGCGAUCAGCGAAGGCAUCGUCCCGAUGGAGUUCGCGUUGAUGAAUGUUUCCCACUACCUGAUGUUCGCCUAC
Ppu-3-13	CAGUUCAAUCCUGGAGAAGGAAUUGCCAUGAAAAGAAUCGUCCCCGAUCCACCCCUCCCCAACACCACACAACGCUCUUUCAGCCGCUGCGAUGCCGGCCAUCCCCCAUUAUUCACCGUGAACCCAGGCGUCUCGACCCACGAUGCCCUGGUGCAUGUCG
Ppu-1-10	CAGUUCAAUCCUGGAGAAGGAAUUGCCAUGAAAAGAAUCGUCCCCGAUCCACCCCUCCCCAACACCACACAACGCUCUUUCAGCCGCUGCGAUGCCGGCCAUCCCCCAUUGUUCACCGUAAACCCCGGCGUUUCCGCCCACGAUGCCCUGGUGCAUGUCG
Pen-1-29	UCAAACCUCAAGAAGGAAUCUGCAAUGAAAAAGAUCGUCCCCGACCCACCCCUCCCCGACACCCGCCACCACCCCUUCGGCAAAUGCGACGCCGGCCACCCACCGCUGUUCGCGGUCAACCCCGACAUCAACGCCCACGACGCCCUGGUGCAUGUCGCCC
env-45	CGGCUCCUUAAUCGUCUGAGGAGCAUAAGGAAAAUGCGCUAUCCCCUUUUUACCCAAGAGCUCCGCCUUGGGCUUCAGGUCUUACUGUCCACCUCCGAUGUUGAGGUGAACCAUGGCUGAGUUCGAAUCCGGUGUUGUACCUUUUCCGAUGCGGCGAAGC
env-46	GGCUCCUUAAAUCCACGAAGGAGCAUAAGGAAAAUGCACUAUCCCCCUUUUACCCAAGGGCUCCGCCCUGGGCUGCUGGCUGUCGUGUCGACUUCCGUUCUGGAGGUGCCGCAUGGCCAGGUCUGAAUCCGUCGUUAUCCCCUUCCCCACACCACGCAAC
Pme-1-1	GCUCCUUAAUCUCACGAAGGAGCAUAAGGAAAAUGCGCUACCCCCUUUUUACCCGAGAGCUCCGCCUUGGGCUUCUAUUACUGCUGCACACCUCUGACCUGGAGGUGUGCCAUGGCUGAGUUCGAAUCCUGCAUUGUACCUUUUCCGCUGCAGCGAAGCC
Pme-1-2	GCUCCUUAAGUGAACCAAGGAGCAUAAGGAAAAUGCACUAUCCCCCUUUUACCCAAGGGCUCCGCCCUGGGCUGCUGGCUGUUGUGCCAACUUCCGUUUUGGAGGUGCUGCAUGACCAAGUCUGAAUCCGUCGUUAUCCCCUUCCCCACACUACACAAUC
Rso-2-1	UUUCCCACUACAGGAAGCCUCAAUGCUUAAUGCUCCCGCUCGUCCUGAGCAACCCGCUUUCCCUCAAAUCCUCGCUAUCGUCCGCACCGCCUUGCGCGAUGCCGUCGCCGCGCCGACUGACCGCGCCUCGCUCGAUGUGGCAGGCGCUGCGCUGUUGGCC
Rpi-1-1	CUCCACUUCUACAGGAGGCCGCAAUGCCUAACGCUUUCGCUCGUCCUGAGCAAACCGCUUUCCCGCAAAUCCUUGCCAUCGUCCGCGCGGCACUGCGCGACGCCGUUGCUGCGCCUGAUGACCGCGUAUCGCUCGAUGUUGCCGGCGCCGCACUCGUCGC
env-47	CUCCACUUCUACAGGAGGCCGCAAUGCCUAACGCUUUCGCUCGUCCUGAGCAAACCGCUUUUCCGCAAAUCCUUGCCAUCGUCCGCGCGGCACUGCGCGACGCCCGUGCUGCGCCUGAUGACCGCGUAUCGCUCGAUGUUGCCGGCGCCGCACUCGUCGC
Reu-1-1	UUCCAACUUUGAUAGGAGUUCGCAAUGGAAGAGCAAACCGCGUCGAAACAACAAACCGUCUUGAUCGCCAAUCCGGAAGAUUGUCGCGAAUCGCUGAAUUGCAUCAGUGCUGGAUUGGACAGGGUGCUAGUGCUGUUGGAAGUGGAAAGCGAAUGUUCGG
Rso-2-2	UCCACCAAACGUAGAGGUUCCUAUGCCUGAUGCCCUUGCUCGUCCUGAGCAGCCAGCUUUCCCCAUCAGUUUCGCCAUCAUCGGCCAUACGGCGUUGCCAAGCGUUGUGCUUUGCCGUGCCAGCCGAGCCUUUCCUGCGUCCACUGUCCGGACGGAGGCA
Reu-2-1	UCCUACCAUGCACAAGGAGGCCGCAAUGCCUAACGUCUUCGCUCGUCCUGAGCAAUCCAAUCCCCAUUACAUCGCCACAGUGCUAAGGGAACUCAGCGCCCUUGCCAACUUCAUGCAUGCCGAUGGAAGGGAAGCCUUCGCCGGCCUGUCGCUGCAAUGC
Rso-1-2	CUUCCCCCAAAAAAGGAGGCCGCAAUGCUUGAUGCUCCCGCUCGUCCAGAGCAACCCGCUUUCCCCCAAAUUCUCGCUAUCGUCCGCACCGCCUUGCGCGAUGCCGUCGCCGCACCGACAGACCGCGCCUCGCUCGAUGUGGCAGGCGCCGCGCUGCUGG
Rso-1-3	CUUCACCAAACGUAGAGGUCCCUAUGCCUGAUGCCCUUGCUCGUCCUGAGCAACCAGCUUUCCCCAUCAGCUUCUCCACCACCAUUCGCCUGCGCGUCCCGAGCCUUGUAUGUUGUGGCGCCGGCCGAGCUCUUCCUGCCCCCGUUGAGCAAACGGAGGC
Bth-3-1	CUCACUACAGGAGGCCGCUAUGCGUGCUAACGCCCCUGCUCGUCCUGAGCAAUCGCAACUUCCCCCGUCGUAUUCCGAAAUCAUCAAUCGCGCACUGCGCGAAGCCGCACUCGCGCCCACCGUCUACGAUGCGCUCGACAUUUGCGGCGCCGUGAUGGCG
Bps-8-1	CAACAUCAACGUUGGAGGCCGCUAUGCGUGCUAACGCCCCCGCUCGUCCUGAGCAAUCGCAACUCCCCUCUUCGUAUCGCGAAAUCAUCAAUCACGCACUGCGCGAAGCCGCGCUUGCGCCCACCGUUUUCGAUGCGCUCGACAUUUGCGGCGCCGUGAU
Bps-16-1	CAACAUCAACGUUGGAGGCCGCUAUGCGUGCUAACGCCCCCGCUCGUUCUGAGCAAUCGCAACUCCCCUCUUCGUAUCGAGAAAUCAUCAAUCACGCACUGCGCGAAGCCGCGCUUGCGCCCACCGUUUUCGAUGCGCUCGACAUUUGCGGCGCCGUGAU
Bps-11-2	AACCCAGCAAAAGGAGGUCGCACCCAAUGCGCCAGCCCUCGCACGCCCGUACCGGGCAGCAAUCCCACCUCAUUCAAUCGAUUGUCCGCGCAGCACUGCGCGACGCGGCAACCGCCAGCACGUAUCAAGACGCGCUCGACGCGACCGGCGCGGCACUGGC
Bth-3-2	UUACCACGAAAACGGAGGUCGCACCCAAUGCACAAGUGCACACACGCCCCUAUCAGGCGAAACGUUCCCCUUAUCCAUUCGAUCAUUCGCGCCGCACUGCGCGAAGCGGCCACUGCCGACACGUACCAAACCGCGCUCGAUGCGACCGGCGCGGCACUCG
Bth-2-1	UUACCACGAAAACGGAGGUCGCACCCAAUGCGCAAGUGCACACACGCCCAUACCAGGCGAAACGUUCCCCUUAUCCAUUCGAUCAUCCGUGCCGCACUGCGCGAAGCAGCCACUGCCGACACGUACCAAACCGCGCUCGAUGCGACCGGCGCGGCACUCG
Bvi-1-1	UCAACCCGUCAAAGGAGGUCGCACCCAAUGCGCCAGCCGUCGCACGCCCGUACCGGGCACCAAACCCACCUCAUUCAAUCCAUCGUCCGCGCAGCACUGCGCGACGCAGCAACCGCCAACACGUAUCAAGACGCGCUCGAUGCAACCGGCGCGGCACUGG
Bth-3-3	UCAACCCGUCAAAGGAGGUCGCACCCAAUGCGCCAGCCCUCGCACGCCCGUACCGGGCAGCAAUCCCACCUCAUUCAAUCCAUCGUCCGCGCAGCACUGCGCGACGCGGCAAUCGCCAGCACGUAUCAGGACGCGCUCGAUGCGACCGGCGCGGCACUGG
Bps-16-2	CCAACCCGUCCAAGGAGGUCGCACCCAAUGCGCCAGCCGACGCACGCCCAUUCCGGGCGAAACGUUCCCCUUAUCCAUUCGAUCAUCCGUGCCGCACUGCGCGAAGCGGCCACCGCCGACACGUACCAAACCGCGCUCGAUGCGACCGGCGCGGCACUCG
Bps-7-1	CCAACCCGUCCAAGGAGGUCGCACCCAAUGCGCCAGCCGACGCACGCCCAUUCCGGGCGAAACGUUCCCCUUAUCCAUUCGAUCAUCCGUGCCGCACUGCGCGAAGCGGCCACCGCCGACACGUACCAAACCGCGCUCGAUGCGACCGGCGCGGCACUCG
Bps-8-2	CCAACCCGUCCAAGGAGGUCGCACCCAAUGCGCCAGCCGACGCACGCCCAUUCCGGGCGAAACGUUCCCCUUAUCCAUUCGAUCAUCCGUGCCGCACUGCGCGAAGCGGCCACCGCCGACACGUACCAAACCGCGCUCGAUGCGACCGGCGCGGCACUCG
Bps-5-1	CCAACCCGUCCAAGGAGGUCGCACCCAAUGCGCCAGCCGACGCACGCCCAUUCCGGGCGAAACGUUCCCCUUAUCCAUUCGAUCAUCCGUGCCGCACUGCGCGAAGCGGCCACCGCCGACACGUACCAAACCGCGCUCGAUGCGACCGGCGCGGCACUCG
Bps-18-1	CCAACCCGUCCAAGGAGGUCGCACCCAAUGCGCCAGCCGACGCACGCCCAUUCCGGGCGAAACGUUCCCCUUAUCCAUUCGAUCAUUCGCGCCGCGCUGCGCGAAGCAGCUACCGCCGACACGUACCAAACCGCGCUCGAUGCGACCGGCGCGGCACUCG
Bok-2-1	CAACCCGUCCAAGGAGGUCGCAUUCAAUGCGCCAGCCCUCGCACGCCCGUACCGGGCAGCAAUCCCACCUCAUUCGAUCCAUCGUCCGCGCAGCACUGCGCGACGCGGCAACGGCCAGCACGUAUCAAGACGCGCUCGACGCAACCGGUGCGGCAUUGGC

```
Bok-1-1    CAACCCGUCCAAGGAGGUCGCAUUCAAUGCGCCAGCCCUCGCACGCCCGUACCGGGCAGCAAUCCCACCUCAUUCGAUCCAUCGUCCGCGCAGCACUGCGCGACGCGGCAACGGCCAGCACGUAUCAAGACGCGCUCGACGCAACCGGUGCGGCAUCGGC
Bce-1-1    CAACCCGUCCAAGGAGGUCGCAUUCAAUGCGCCAGUCCACGCACGCCCAUACCGGGCAGCAAUCCCACCUCAUUCAAUCCAUCGUCCGCGCAGCACUGCGCGACGCGGCAACCGCCAGCACGUAUCAGGACGCGCUCGACGCAACCGGCGCGGCACUGGC
Bps-5-2    UCAACCCGUCCAAGGAGGUCGCAUUCAAUGCGCCAGUCCACGCAUGCCCGUACCGGGCAGCAAUCCCACCUCAUUCAAUCCAUCGUCCGCACAGCACUGCGCGACGCGGCAACCGCCAACACGUAUCAGGACGCGCUCGAUGCGACCGGCGCGGCACUGG
Bth-1-2    CUCAACCCACCCGAGGAGGUCGCACCCAAUGCGCCAGCCGACGCACGCCCAUUCCGGGCGAAACGUUCCCCUUAUCCAUUCGAUCAUUCGCGCCGCACUGCGCGAAGCGGCCACCGCCGACACGUACCAAACCGCGCUCGAUGCGACCGGCGCGGCACUC
Bps-7-2    ACCCGUCCAAGGAGGUCGCAUUCAAUGCGCCGGUCCACGCACGCCCAUACCGGGCAGCAAUCCCACCUCAUUCGAUCCAUCGUCCGCGCAGCACUGCGCGACGCGGCAACGGCCAGCACGUAUCAAGACGCGCUCGACGCAACCGGCGCGGCAUUGGCCG
Bdo-1-2    ACCCGUCCAAGGAGGUCGCAUUCGAUGCGCCAGUCCACGCACGCCCAUACCGGGCAGCAAUCCCACCUCAUUCAAUCCAUCGUCCGCGCUGCACUGCGCGACGCGGCAACCGCCAGCACGUAUCAGGACGCGCUCGACGCAACCGGCGCGGCACUGGCCG
Bps-9-1    ACCCGUCCAAGGAGGUCGCAUUCAAUGCGCCAGUCCACGCACGCCCGUACCGGGCAGCAAUCCCACCUCAUUCAAUCCAUCGUCCGCGCAGCACUGCGCGACGCGGCAACCGCCAGCACGUAUCAAGACGCGCUCGAUACGACCGGCGCGGCACUGGCUG
Reu-1-2    CUGAACCUGUAUGAGAGGGAGUAGUGCCAUGUAUCAGUCUUUCCUUGCCCAUUCCGGGCGCUUGAUUAUCCCCCGCGCUUUCUCGCUGCAUCAGCGCCCGCAGACGUCGCAUGCGGAGGUGCGCCAUGUAUAGCCAUAGUCAUCACGGUAUUACUGCGGA
Cvi-1-2    UUAACGCUCAGUAGGGAGGCUCAACAUGCCCCAUAUCGCCCUGCGCGCCAUCCGCGCCCUAUUUCCGCAAGUUACCCGUCCCGUUUCCACGCUGCCCAACGAGGCCCAAGCUCGCGCGCUCGCCGCUUUGCUGGUCGCCCAGGGGCAACGCGCCUUGAUU
Bps-15-2   CACAUCCAUAGGAGUCCGCAAUGCUGCGCCAUAUGCCCGCUCGCCCCAGAGCACCCAAGCCGUAAUUGAACAAAUCACCAACGACGCGCUGCGUGCUGCAGCGCUUGCGCCGACCUACAUGCAAGCGCUCGACAUCACUGGCGCCGCCCUACUCGCCGU
Rpi-2-1    UCCUUCAAUCCCAAGGAGGCUCCAUGCCUAUUACAGACGAGACGCGCCCAGCGUCGUACCCAUCCCCGCCAGCACCGUCGCCUAGUCCACCGCCCACGUCCUCCGACACAGGCAGGCUCACCAAGAACCGCACCUACCUGAACGGCGCCAUCGCGGCACA
Rpi-1-2    UCCUUCAAUCCCAAGGAGGCUCCAUGCCUAUUGCAGACGAGACGCGCCCAGCGUCGUACCCGUCCCCGCCAGCACCGUCGCCUAGUCCACCGCACACGUCAUCGGACACAGGCAGGCUGACCAAGAACCGCACCUACCUGAACGGCGCCAUCGCGGCACA
env-48     UCCUUCAAUCCCAAGGAGGCUCCAUGCCUAUUGCAGACGAGACGCGCCCAGCGUCGUACCCGUCCCCGCCAGCACCGUCGCCUAGUCCACCGCACACGUCAUCGGACACAGGCAGGCUGACCAAGAACCGCACCUACCUGAACGGCGCCAUCGCGGCACA
env-49     AACAAACCGCUCUCAGAGGAUCAUUGCCAUGACCACCACCGAUUUGGUGCCGGUAUUCGUCGGCGCUCUCGCAGGACAAGCCACUCCACUCUGUAACGCCCGCGACCUGCACGCCGCCCUCGGCGUCGGCCGCGACUUCGCCACCUGGAUCAAGCAGCGC
Bce-1-2    CACUUGAAAAUGGAGGCCGCAAUGUCUAAUGCCGUCGCUCGUCCUGAGCUCCCCCUUGUCGACCUUAUCUCCCGUGCGUGGACGAUUCACAACAUCGUCAAGACGCUGCACGCCGCUCUUCCCGAAGACGAGAACGAUUGCGACAUUCCUACGCGCUGUC
Bma-10-1   ACGUUUUCAUAGGAGGCCCGACAUGCAUCAAACCCCCGCUUGUUCAGAACCCUCGGAACCUACCCUAGUCGAUAUCGCGCUCGUCGGUGUCGAUAACCUGUCUGCUUUAUCGAGGUUGCUGUCAGCCAAGGACGGCGCAGCGUUAUUUUCCGGUUUGGAU
Bth-2-2    ACGUUUUCAUAGGAGGCCCGACAUGCAUCAACCCCCCGCUUGUUCAGAACUCUCGGAACCUACCCUAGUCGAUAUCGCGCUCGUCGGUGUCGAUAACCUGUCCGCUUUAUCGAAGUUGCUCUCAGCCAAGGAUGGCGUAGCGUUAUUUUCCGGUUCGGAU
Bvi-1-2    ACCCUGCUAAUGAGGCCGCCCAUGCACAGCCCCGCGCCUUACCCUCCCCCGCCUAAUCGCAUUGGUCCUCGCCCUCGGUGCCACGAGCACGUCCGCGUGGAUCUUGAUCGUCGCGGGUAUUGAACGCGGCGGCACAUCGGCCGAACGGAUCGCGUGGGCC
env-50     CUCCCAGCUAAUGAGGCCGCACAUGCACAGCCACCCUUCCGACCGGCGUAUCGCGUCGCGCUCUACCCUUCCCCGUCUCAUCGCGUUGAUCCUCGCACUCGGCGCCACGGGCACGUCCGCGUGGAUCUCGAUCGUCGCCGGCAUUGAACGCGGCGGCACC
Bce-2-1    UUCGAUCCUCGUCUCUGGAGGCCGCACAAUGCGCCCUAACGCCCCCGCUCGUCCUGAGCAGCUGCAAUCUUCGCUCGAAAUCAUCCGCACCGCCCUGCGCGCGGCAGCGCUCGCGCCGACUGAUCAGGUCGCGCUCGACGUCACCGGCGACGCGCUGCGC
Bps-3-1    UUCGAUCCUCGUCUCUGGAGGCCGCACCAUGCGCCCUAACGUCCUCGCUCGUCCCGAGCAACCGCAAUCCCCACUCGAAAUCAUCCGCGCCGCCCUGCGCGCCGCCGCGCUCGCGCCGACCUAUCAGGAUGCGCUCGACGCCACCGGCGACGCGCUGCAC
Bmu-1-1    AUCGCAAAUCAAAGAGGCCAAUCCGUGAAGAACAUCUCGCCUGAAUCCGGGCAGGCAUUGCCCCGUCGUCUCCCGUACCCUUCAUCAAGACUCUUCGGAUUGCUGCUCGCGGUGCUAUCGACCGGCACAUCCCUCUGGAUAUCCGUCGUCGCCGGAGAGG
Bvi-1-3    CCCGCAAACCAGAGAGGCCAUAUCAUGCGCAAUUUUGCACCCGCUCGCGAGCCGUCGCCACUCCAUACUCCUGCCGUCUCGGCCCGCGUCUUCGCACUGCUUCUCGCCUCACUGGCGACGGGCACUUCCGCAUGGAUCUCUAUCGUGGCUGGGACCGAAC
env-51     CGCUCACACAAGAGGCCGCACCGUGCAUCCACAUUCAAUCCUGCCGCUCAAGUCGCUGCUUUCCCCCACGCUCAUGCAAUCGGCGCCGGCAGUGGCCGACUGCGCAAUUAACCCCGUCGACGUCGCCCGUGUCGCGACUGUGCACCGCGCACCUGCUCGC
Rso-1-4    UCCAUCAACAACGGAGGCCACCAUGCCUAAAGUCCACGCUCGUCCUGAGCAAACCCAAACCCCGCACGCGGAAGACACGCAGGAUCUGCGCUACACCAUCAACACCAUUGAUGCGAUGUCGCAGAGCGGCUUUUCGGAAAUCGCUGCGAUUGCAGGGCUC
Rpi-2-2    CCUCCUGCAACCCCAAAGAGGCCAUCAUGCCAAGCAACCAAAGCGGGCACCGCCCCGCGUCUUACCCAUCUCCACCCGAACCACCCACCCACGCGCCCACGUCAUCCGACACAGGUACGCUCACCAAAAACCGCACCUACCUGAACGGCGCCAUCGCAGC
Rpi-1-3    CUCUUAACCAUCCAAGAGGCUGUCAUGACUGCAAAUCACAGCGGGCACCGCCCCGCGUCUUACCCAUCUCCACCCGAACCACCCACGCACGCGUCCACGUCAUCCGACACAGGUAGGCUCACCAAAAAUCGCACCUACCUGAACGGCGCAAUCGCAGCGC
Rso-2-3    CUCACCAACUCCAAGAGGCUGUCAUGACUGCAAACCACAGCGGGCAUCGCCCCGCAUCAUACCCAUCCCCGCCACACGCACACCCCCGCACUUCCCACCGCACCGGAGGCUCCCAAUGACCUCCAAACGCCGCAUCUACCUAACCGGUGCACUCGCCGGC
Rso-1-5    UCUCACUCACUCCAAGAGGCUGUCAUGACUGCAAACCACAGCGGGCACCGCCCCGCGUCAUACCCAUCCCCACCGCACCAACACCCCCGCACUUCCCACCGCCCCGGAGGCCCCCAAUGACCUCCAAACGCCACAUCUACCUAACCGGCGCACUCGCCGC
Rpi-1-4    CCUCCAAUCAAUACUCACGGAGGCGCCUAUGCCUAUUGCAGACGAGACGCGACCAGCGUCCUACCCAUCUCCACCCGCAUCACCCAGCCCACCGCCCUCGUCAUCCGACACAGGUAGGCUCACCAAGAACCGUACCUACCUGAACGGCGCAAUCGCAGCG
Rpi-2-3    CUCUUCAUCUAGGAGACGUCAUGUCUAUCGCAUCAAGCGGGCAUCGCCCCGCGUCUUAUCCUUCCCCACCGUCUUGCAUCCCCUGGCAUCCGUUUUACGGAGGUGCACGUUGAACGCGCUACGGCACUACCAAUCGGGCGCCCUCGCAGCCAAGGAUUUU
Rpi-1-5    CUCUUCAUCUAGGAGACGUCAUGUCUAUCGCAUCAAGCGGGCAUCGCCCCGCGUCUUACCCCUCGCCACCGCCCUGCAUCUCCUGGCAUCCGCUUUACGGAGGUGCACGUUGAACGCGCUACGCCACUACCAAUCGGGCGCCCUCGCUGCCAAGGAUUUU
Rpi-2-4    CUCCCUCAACCUAUCCCCGGAGGCCACAUGGCUACCGCACUCAGCGGGUAUCGCCCCGCGUCUUAUCCUUCCCCUCCACCUUGGCGCUGCAGGCUUCCCACGCAACCGGGAGGUGCCGCAUGUCGCUGACCAAAAACCGCCUCUACCUUGACGGUGCCGU
Rpi-1-6    CUCCAUCAACCUAUCCCCGGAGGCCACAUGGCUACCGCACUCAGCGGGCAUCGCCCCGCGUCUUACCCUUCCCCACCGCCUUGGCGCUGCAGGCUUCCCACACAACCGGGAGGUGCCGCAUGUCGCUGACCAAAAACCGCCUCUACCUGGAUGGCGCGGU
Rpi-2-5    CCUCCAACCACCUCUGGAGGCUCUAUGCCAAAUGCUGAAAGCGGGCACCGCCCCGCGUCGUAUCCAUCUCCACCCGCACCACCCAGCACUGCGCCCACGUCCUCCGACACAGGCAGGCUCACCAAAAACCGCACCUACCUGAACGGCGCAAUCGCAGCGC
env-52     CCUCGGCGCGC.........................................................................................................................................
Pen-1-30   GGCUUUGAUCCCAUGCAAAAGAGGAUUCACCAUGCUCAAGAUCGUGCCGGAUCCACCGGCUCCAACCGAAUCUCCCCAUUUCCUCGAAGACACGCUGGUGAGUGCCACUGAGUACGUCAUGUGCGCGCUGGCGGUCACUCAUCAGUCCUUGACCCACCUG
Ppu-4-27   UGGUCCAUCAGUACACAGACGAGAUUCCAUCAUGUUAAAAAUUGUCCCCGAUCCGCCCCACGAUUCCCAUUCCCUUGAAGACACCCUGAUCCAGGCCACGGACUAUGCGCUGUGCGCCACGACUGUGGUGCACCAGGCGCUGCUAUUGCACCCCAAGUCC
Pfl-2-29   UUUUUUCUCUAACUGCACGAGAUCUUCACGCAUGAAAAAAAUUGAUUCGAACGCAGCCGAAAACCCCGGGAACCCGGAUUCCCCCGAACACAUCGCCAACCCCAAACCCCGCUACAUCCCCAUCACCGGCAUCGACUGCCCGAUUCCCUGUCUCCUGAUC
Sgl-2-16   UGGAAAACUUCAAAGAGAGGGCAACACUAUGGUUGCUACGACCCAAACUCGUUCUGAAUUUAUUGAUACCUACUGGAUUAUUCCAGAGUACUCCACCACGCCUUACGGCAAAGUGUCGUUUACACGUCAAGAUCGCCGCACCUUCAAUUCCAUGUUCAAA
Sgl-2-17   UCCUUAUGGAUCCGAAGAAGAGGCCACAACACUAUGGCUACCACCCAAGCUCGCCCUGAGUUUAUUAUACCUACUGGAUUAUCCCCGAAUACGAGACCACGCCUUACGGCAAAGUGUCGCUCACACGCCAAGACCGCCGUACUUUCAUCGCCAUGUUUAA
Eco-8-5    ACUCCUUAAAAUUCAUCUGGGAGCCUUUCACAUGGCUGUAUCCGCACGCCCUUAUUUUGUCUGGCGCUUUAUGCAGUGCCUGACAGACAGCAUUGCAAUAUUCACCGUUACCGCAGCCACUGAACGCGAAGCACGCGCCCAGCUGCCGCACGCACAUCUG
Eco-3-14   ACUCCUUAACAUUAAAUUGGGAGCCUUUCACAUGGCUGUAUUCACACGCCCUUACUUUGUCUGGCGCUUUAUGCAGUGCCACGGUAAAAACAUCCGCCUUCACACCGUUACCGCCGCCAGUGAGCACGAAGCCCGUGCGCAGCUGCCGCACGCACAUCUG
Sen-3-1    UUUGCAAAAUCUAAUCAACGAGGCUGCCACUAUGGCUACUACCCCCAUCCAGACACACCCGUUACUUGCCGUUUCCUUCAACGCCGCCACGGAUUUCACCGUACUGGCCGAUCACUGCGAAAACUUUGCCGAAACUCUGAUCGAAAGCGACGAUCCGGCG
Sgl-2-18   GUUGAAAAACUUUACCACACAGGAGGCCAUUACUAUGGCUACUAUCCCCACCUGCACUUUGCCUGAAAACCUGUCCCUCGACUGUUACCGCAAACUACAACGCGCGCGUGAAACCGCCCGUUAUCUGUUCCUCGAUCUUCGCACCCGCACCCCUGACGAA
Eph-3-1    AAACCUGUUAGUGAGGCCGUAACUAUGGUUAAUGCCAAUCCUUGCACACGCCCAGAAUUCAUCUGGGGUUUCUAUUCCUGUAAAAAACACCACUAUCACUUCGUUAUCGCAGCAACUGAAGACGAAGCACGCUCUCAAUUGCCUGAUGGCCCCUGCAUUU
Sen-8-4    UUAAAUCUGUACAUGAGGCCGUAACUAUGGUUAAUGCCAAUCCCUACUCACGCCCGGAGUUUAUCUGGCGCUUCUAUUCCUGCCAAAAACAUAACUAUCACCUCGUCAUAGCUUCGACUGAAGCAGAAGCUCGUUCACAGCUUCCGGACGCCCCUUGUAU
Aba-1-6    ACUCCUUUACACACAAGGAGUCCGCACAUGCGUACUAUUUCAUCUCUCUCAUUGGCAACCUUCGCCAAAAUCUUUGACUCUAUACCCAAUUGGUCUUUAAGACGGUUUAACCGACUCUAGGUUUUAAGCCAUAGGUCAUUACGACUAAAAAAUCAUAGCA
Aba-1-7    ACUUCUUUUAACCAAGGAGUCACUCAUGACUAUUAUUCUAUAUUUUUUAAAUUCUUUUAUUAAACCUACUUACAUUCAACCUCUUUUACUCAAGCAGUCAUUUCUUAGAGUUCUUUCUCAUCCUCUGUUUAAAAGAACUAGCUACUAUAGAGUUCGAAAA
Aba-1-8    AACUUCUUUCUUACCCAAAGGAGUUCGCACAUGCGUACUAUUUCUUAUUUAUUGGCCUUUGCCAAAUUCUCUUUAUUUAUACCACCGGAACAGUCUUUAAAACGGCUCUAGGUUUUCUACACCUUAAGAAUCGUCACGACUAAAAAUCAUAUCAACAACA
Aba-1-9    CCAAUAAAAAGGAGUUGGCUUUGCACAUCCAUUAUUUCGUGGCUCUCAAUGCCAAAAGCUAUAACGACACCACAUAAACUUUAAAGCAGUUUGAGGGCCAUUAGGCUUUUCAGGCUUUAAAUCGUCACAACCAAAAACUUAGCAACAAUAAAACUUGAGA
Asp-1-1    ACUUCUUAAUACCUAAGGAGUCAUACACAUGGCUGAUAUUUAUCGGUUGUCCAUUCCAUUCUCUGAAUUAUCUUAUCCAGCCUUACUGACGUUAUUGCCUGAUUAGUUCAUGACCUCUGUUCAUCAAGAGGGUCUCAACAUGAAACGAUUCAUCAGUAUG
Aba-1-10   UCCCAAUAAAAAGGAGUUGGCUUUGCACAUCCAUUAUUUCUUGGCACGCAUUGCCAUAAGCUAUAACGACACAACUUAAAAAUUUAAAGCAGCUUGAUAGCCUGUAGGGUUUUCAUGCUUUAAGUCAUUACGGCUCAAAAAACUUGCAUUAACUAUAAAA
Aba-1-11   AACUUCUUUAUAGCCAAAGGAGUUCGCUAUGCGUACUAAUUCUUCUCAUUUAUUGGUUUUUGCCAAAGUCUUUCUGUCUAUACCACUUAAACAGUCUAUAAAGCGGUUGAAUCGACUCUAGGUUUUUAAAUCUAAACAUCGUUACGACUAAAAAAAUCAUA
Aba-1-12   AACUUCUUUAUAGCCAAAGGAGUCCGCUAUGCGUACUAAUUCUUCUCAAUUUUUGGUCUUUGUCAAAAUCUCUCUAUUUAUACCGUUUAAAUACUCUAUAAAUUGGUUUAAUCGACUCUAGGUUUUUACACCAAAACAUCGUUACGACUAAAAAAAUCAUA
Aba-1-13   AACUCCCAAUAUAAGGAGUUGGCUAUGCAUAUUCUUUUUACACCUUCCGAUUAGUUUUAUAGGGUUUGAUAAACUUCUGGUUUCUCAAGCUUUAGUUUUUUUCGUCUAAAAAUUUAACUUAAUAAAUAUGUCAUGUGAGCUACACCGUGGCAGGGCUUUG
Aba-1-14   AACUCCCAAAAUCAGGAGUUGGCUAUGCACAUCCAUCAUUUCUUGGCCUUGAGCGCCAAAAGCUAUAACGACACAGCUUAAAAAAUUACAGCAGCUUGAACGCCAUAAGGUUUUCAGGCUUUAAGUCAUUUUUACUUAAAACUUAGCAAAACAAAACUUG
Aba-1-15   AACUCCCCAAAUAAGGAGUUGGCUAUGCAUACUAUUUUUACACCUCCCAGUUAGUCUUUAUGCAGUUUGAUCACCUUUAGGUUUUCAGGCGUCAGUUCUUCACGACUAAAAAUCAUAUCAACAAUAAAACUUGAGAUGCGUUAGGGCAGUCUUUGGGGCC
Aba-1-16   CCCAAAAGAAGGAGUUGGCUAUGCACAUUCAUAAAUUAUUGGCACUCACUGUCAAAAUCUAUCUCGACACAACUUAAAAUUUUAAAGCAGUUUGAUGAGCCAUAGGCUUUUCAGGCUUUAAAUCAUUACGUCUAAAACUUAGCAACAAUAAAACUUGAGA
Aba-1-17   CAUAAAUAAGGAGUUGGCUUUGCGCAUCCAUCAUUUCUUGGCACCCAUUGCCAAAAGCUAUAACAACACAGCUUAAAACAUUUAACGCAGCUUGAUCGCCGUAAGGCUUUUUCAGGCUUUAGCUCGUUAUUACUUAAAACUUCGAACAACUAAAACUUGA
Aba-1-18   CCCAAUAAAAAGGAGUUGGCUUUGCACAUCCAUUAUUUCUUGGCUCUCAAUGCCAAAAGCUAUAACGACACAGCUUAAAAAAUUAAAGUAGCUUGAACGCCUUAAGGUUGUCAGGCUUAAAGUCAUUUUUACUUAAAACUUAGCAACAACAAAUUUGAGAU
Aba-1-19   CCAAUAAAAAGGAGUUGGCUUUGCACAUCCAUUAUUUCUUGGCAACCUUUGCCAAAAGCUAUAACGACACCACAUAAACUUUAAAGCAGUUUGAGGGCCAUUAGGCUUUUCAGGCUUUAAAUCGUCACAACCAAAAACUUAGCAACAAUAAAACUUGAGA

           ........................................................................................................................................................
           ........................................................................................................................................................
           ••••••••••••••••••••••••••••••••••••••••••••••••••••••••••••••••••••••••••••••••••••••••••••••••••••••••••••••••••••••••••••••••••••••••••••••••••••••••
```

Aba-1-20	GUAGAACUCCAUUUUUAAGGAGUUGGCCAUGCCAUUAUUAAAACUCAUUUUCUCUUUUAGUUAACUAUUUUAUUUAUUUAUUUAAAAAUUUGGGUUUUUUGCGACCUGUUUUUUUAAAUUUAUAAAUUAUUUCUAUUUUAAAAUUUUAAUUAUUUUAUUUUCCUA
Asp-1-2	ACUUCUUUUUCAAACAAAGGAGUACGCUAUGUGCUUUUCAAAUCUUUUAUCGUAUUUUUUUAUACGGAAUCCCUUAAAAAAGAGUUACCCACAUUUCUAUCAACUCAUCGUUACAAAUACUCAAAGCGCAUUAUCGAUAUUAGAAAAAAUGCAUGUCAAA
Aba-1-21	GGUAGAGCUCCAUACAUUUUAAGGAGUUGACCAUGCCACGAUAUAAACUUAUCCCCUCUCUCUUAUAAACUUUUUAUUUAUUUAUUUAAAAUUUGGAUUUUCUAUUAUCUAAAUUUUCAAAUCUAUAAAUUAUUUUAUUUUAAAAUUUAAGUCAUUUUAUUUU
Asp-1-3	AACUUCUAAUACAUACAGGAGUAGUCACCAUGACUCACAUCGCCCGUUUAUUUCUUUGCUUACCGCAUCCAUCCAUUUUGUCUUUGUGCUUAUCCAAACGCAGAUUGCCCACACCUUCAUGAUCUUUUUUUAAAUUCAGAUCACGGCAUGUUUACGUUUC
Asp-1-4	AACUUCUAAUACAUCUAGGAGUAGUCACCAUGACUCACAUCGCUCGUUUAUUUCUUUGCUUAUCGCAUCCAUUCAUUUUGUCUUUGUGGUUAUUCAAACGCAGAUUGCCCACACCUUCAUGAUCUUUUUUUAACUUCAGAUCACGGCAUGUUUACGUUUC
Aba-1-22	UAGAACUCCAUUUUUUAAGGAGUUGACUAUGCCAUUUUUCAAACUCAUUUUCUCUCUUAUUUAACUAUUUUAUUGAUUUAAAAAUUUGGUUUUUUACGCCCUAUUUUUUAAGUUUAUAGAUUGUUUUUUAUUUUAAAAUUUUAAUUAUUUUAUUUUCCUGU
Aba-1-23	ACCGCAGAUUUUAGUAUCUAAACUAGCUUGUGACGUUGUGGAAGUUAAGAAAUACGCGUCAUUUGAGUAUGAACAAGUUAGAUAUCUUCAGAAUGCUGUCACGAAUAUUAUGAAUAUUAAACAAAACCAAAUUGUUAAGGUCUUUACUAUUAUUACUGCU
Hin-11-1	AGUUUUAAAACUUACUUAGAGAAUCACAAAAUGACACAUUUAAAUCUUAUCCCUGUUUUUAACGGAUUAAUCCAAAAUCAACCUGUUCAACUUUGCAACGCUCGUGAACUUCACGCAUUCCUAGAAAUACAAACUCGUUACAAUGACUGGAUCAAAAAC
Hin-7-2	AUUUCUAAAACUUUGAUUAGAGAAUCACAAAAUGACAAAUUCAAACUUAAUCCCUGUUUUUAACGGCUUAAUCCAAAAUCAACCUGUUCAGCUUUGCAACGCACGCGAACUUCACGCAUUCGUAAAAAGCAAACAAGAAUACGCAACUUGGAUCAAAAAC
Hin-10-1	AGUUUUAAAACUUAACUUAGAGUACAUCACAAUGACACACUUAAACUUAAUUCCUGUUUUUAAUGGCUUAAUCCAAAAUCAACCCGUUCAACUUUGCAACGCUCGUGAACUUCACGCAUUCGUAGAAAGCAAACAGCAAUACACUGAUUGGAUCAAAAAC
Hin-12-1	AGUUUUAAAACUUAACUUAGAGUACAUCACAAUGACACACUUAAACUUAAUUCCUGUUUUUAAUGGCUUAAUCCAAAAUCAACCCGUUCAACUUUGCAACGCUCGUGAACUUCACGCAUUCGUAGAAAGCAAACAGCAAUACACUGAUUGGAUCAAAAAC
Hin-3-3	AGUUUUAAAACUUAACUUAGAGUAUAUCACAAUGACAAACUCAAACUUAAUUCCUGUUUUUAAUGGCUUAAUCCAAAAUCAACCCGUUCAAAUUUGCAACGCUCGUGAACUUCACGCAUUCCUAGAAAUACAAACUCGUUACAAUGACUGGAUCAAAAAC
Hin-8-1	AGUUUUAAAACUUACUUAGAGAAUCACAAAAUGACAAACUCAAACUUAAUUCCUGUUUUUAACGGCUUAAUCAACCUGUUCAACUUUGCAACGCUCGUAAACUUCACGCAUUCCUAGAAAUACAAACUCGUUACAAUGACUGGAUCAAAAACCGCAUCA
Hin-4-1	AGUUUUAAAACUUAACUUAGAGUACAUCACAAUGACACACUUAAACUUAAUUCCUGUUUUUAAUGGCUUAAUCCAAAAUCAACCCGUUCAACUUUGCAACGCUCGUGAACUUCACGCAUUCGUAGAAAGCAAACAGCAAUACACUGAUUGGAUCAAAAAC
Hin-6-1	AGUUUUAAAACUUAACUUAGAGUACAUCACAAUGACACACUUAAACUUAAUUCCUGUUUUUAAUGGCUUAAUCCAAAAUCAACCUGUUCGACUUUGCAACGCCCGUGAACUUCACGCAUUCGUAGAAAGCAAACAGCAAUACACUGAUUGGAUCAAAAAC
Hph-1-1	UUUUAAAACUUAAACACAGAGACAGUCAGAAUGACUACAUUAACUUUUCAAAGCACAACUCUUUCGGCUAUUCAUCAAAACAAUCAAAUUUGGCUAACGGUUACAGAGAUUGGAAAAGCAUUAGGCUAUUCAGAUCCUUUUAAGUCGGUCAAAAAUAUCU
Hso-1-2	GUUUAAAACUUAAACACGGAGACAGUCAAAUGACUACAUUAACAUUUCAAAACACUUCUCUUUCGGUUAUCAAUCAGAACAAUCAAAUUUGGUUCUCUGCCUUAGACAUUGGCAAAGCACUUGGUUAUUCAAAUGGCGAUAUUGGUGUCAAAAAUAUCU
Aba-1-24	CUUCUCUAUUAUGGCGAAAUGAAAGAAGGACGCCUCGUGUGUGCUGGGUACUCAAAGCCAGUCUGUUAACCUUCUUUCGUUUCGCCGCCAUUCUUUAAGAAUAACUGGGUGGGCUUCAUUACCAUUAUGGUAGGGUGAAAGUGGGGACGCCUCUGUGCGU
	....
	....
	•••

alignment positions 420 ··· 546

Aba-1-1	UACGAUUGUCACUUCCUACGCUUGACGAUAACAAAAGUGAAUACUAUCCUUUGCCUGCUGGCCUACAUUGCCAGUCGACUUUGACAGGUCGGAUAGCCAACGCACACGAAAAAGUCUCUCUUUUUUG
Aba-1-2	UUACGUGAUUUGGAUGAAAAGGGGUUGGCAGAUUGCCACACCCCUACAAAUGGUGGAAAUCAAAAGAUUAAAUUUGUUAAUGAGCCAAAUCUUUAUCGGAUCAUCUUUCGUUCAAAUAAACCAGAAG
Hin-3-1	GGUAGCGAGAAUCAAUCCUCAAAAUAACCGCACUUUAGCGGCACUUCCUACCCUUUCUGUACCUGUAACACAAGGGGGCGCAAAUGUUUAAUCAAUCAAUUUUAUCUGCACUUCAAGAGAUGGCGGA
Hin-5-1	GGUAGCGAGAAUCAAUCCUCAAAAUAACCGCACUUUAGCGGCACUUCCUACCCUUUCUGUACCUGUAACACAAGGGGGCGAAAAUGUUUAAUCAAUCAAUUUUAUCUGCACUUCAAGAGAUGGCGGA
Hin-7-1	CGUUGCAAAAAUCAACCCGAAAUUUAACCGCACUUUAGCGAUGAAAGGGGGAUUUAUGCGUAAUCUUUCCCUUCAUUUAAUUGAUGAAAUUCAAAGCGAAAUAACCCGAUUUGAUACGUUGAACACC
Hso-1-1	UACAAGGGGGCGUUAUGUCUAAUCGCAACCUAUCAAACGAAAUCACCACGCUUAAAGCUGCAUUAAAAAGUUUGCUUGAUGAUUUAUCUGCAGCCGAAAUCUAUCUCAAUAAUGACCGCACUCAAUG
Hso-2-1	UACAAGGGGGCGUUAUGUCUAAUCGCAACCUAUCAAACGAAAUCACCACGCUUAAAGCUACAUUAAAAAGUUUACUUUCAACUUUUCAAACUAGCCGAGCAAAGCGACAAGUUAAAAUCCAAAUAUU
Pmu-1-1	CUUUAGGUCGCUUACCAAACACCGCAAAAAAUGACCGUACUUUAGAGGUUAAAGGGGGUAUUUAUGCGUAAUGUGAAAAAAGCAUUGUCAAGAUCUGUUAUUGCUAGUAGUAUUCAAGGUACUACUA
Hin-3-2	CCAAAACCCACUGAAAAAUAACCGCACUUUUUCAACUAAUGGGAAAAAUCACAGCUUGCCAGCAGGGCAGAAUGUGGGUAGUAUUGAAAGCACUACUACUAUCGAGGGCAACCGCAACCCUUAUCAA
Hin-2-1	CCAAAACCCACGGAAAAAUAACCGCACUUUUUCAACUAAUGGGAAAAAUCACAGCUUGCCAGCAGGGCAGAAUGUGGGUAGUAUUGAAAGCACUGAAACCAAAGACGGCAACCGCAACCGUCAUUUA
Mha-1-1	AAAUCAAGAUUUAUGCUUGACAACCCUCAAUCGUUCGCCUUAUGCUAGCCGUGCUUUUGCAAAAUCAAAAGCCGAGUGUGGAAACUCGAAAGAACAUCAGGCGAACAAUAGCACGCCUUUAACUCGU
Mha-1-2	AUCGCUUACUACUCCCCCGCCCUGUUGCAAAAAUCCGCCUAAAUUCUACCGCUUACACGGUGAAAGGGGGGAUUUAUGCUUAAUUAUUCCGAUAUUGAACAAAUCCGCUAUUUUAUGGAUCAAGCCG
Pfl-2-1	GCCGCAGCCGACCUGCUCGAAACCCUGCACUGCCUGGUGCUUCAAACACGCCGACGUCCAGGACAUUCCCCACAUCACCCAUGCGCUGUACCUGCUGACGCAGGAUGGCAGUGAUCUGCUGCAGGUUG
env-1	GGGAUUUCACGGAAAGCAAUUUUGCUUUCCGUGAGGAAAUUCGACGACACAGGACCGGAGCGACAAGAAAAAUAUGGGUUGAGAUGACCCGUGACGGCUUCUCACUUUUAGUGAUGGGCUUCACGGG
Dps-1-1	GCUUUCAGUCAAGACGGGCUAUGCAUGGCCUUCUCAGGGCUUUCUCGCCAACGAGAUAGGACGCUCACUUCGUACCGUUAAAUCACAUAUAGCCAAGCUCAAGAGCCUUGGACUCAUAACCGUCAAG
Dac-1-1	CACGGUUCCGUUUUUGGCGGGGCUGUGCGGGACCUCCGCUAGGGGGUGCCAGUUCAUGCCAAAGGAGCCGCCAUGCCUGACCUCGAUGCGCCCAGCGGAGCACACGCCUCCCCGCCUUUCUUCUCUU
Sgl-2-1	GAUAGCCGCCUAAUCUGGGCAGGGCAUCAACGUGUGCAGGACUUCACUGAGAACGUUAAACGUACCGAAUCGCCAAAGCUGCCCGUCGAAGGGACAGAGUCACGCUCACAAUUCAACCAAUCUCCCG
Pfl-2-2	GCGCGCCUCAAGCGACCUGCUGCAAACCCUCUACUGCCUCUGCUUCAAACACGCCGACGUCGAAGACAUCCCGCACAUCACCCACGCCCUUUAUCUGCUGACCAAAGACGGCUUUGACCUGUUACAG
Sbo-1-1	UCUCUGGUGCGCGAUCACGUCUGCUUUUUUGCUGGUCGUAUCCGCAUGGAGGUGGUGAAGUGGCACACUGAAUUUGGCCACCUGAACAGAGGUGAUAUGCUCACCUCAGAACAACACAGGUGCUCCA
Pna-1-1	CCCUGCCCCGUGGCAUUUCCAAGCUGGUGCUGCAGUGCUUUUGCCACCACGUCAACAACGAAUCCGGCCUGAGCUGGCCGUCCAUCGCCCGCGUGGCGCUGAUGUGCGGCCUGAGCGCGCGCGCCGU
Pna-1-2	AAUCUGCCCGCAGCAGCCCGCAAGGCCAUGCAAGCGCUCGCCGCCCUGAACCAGCUUCAAGGGAAGGCUUGAGUCAUGGAAAACACACCUGUCACGCCCGUUAUGGGCUACCUGGCACAAAACUGGA
Xfa-1-1	UCAUUUAGAUGAUGAUGAGAAAUCUAACCAAUCGUUAGGUUUAGCUGGGAAGCCAGUCAUCAUCAUUUCCGAGUCUGGCCUAUACGCCUUGGUACUCCGCUCCAGAAAGCCAGAAGCACGUAAGUUC
Sma-1-1	AAACCGAUGUGUCGCCCGGGCGUCGCGUGAGCCUGGCGAGGAGUGAGCGCGCGAUGGUGGGGGCGCUGACGGUGCUGGAGCUGCUGCAUGCAGCAGACCGCUGCCGGGUGUCAGCGGACCCGGAACG
Dno-1-1	ACAUGCAAUCUGAUUUAUUGCGUCUUGCCUAUCAUUUAACGCAAGCGUUAAUUGCGCAAAAUGCCGCGGCAAUCGCAGCAUUGGAGGAUUAAGUCAUGGAAAGAUUGGCUCAUAUUACUGCCAUUGA
Dno-1-2	UACAAAAUAAAGAAACACAAGCGUUAAUCGCUGAAAUUGAAAGAGAAAAUUAUAAUGCUUGUAAUCCAACAUUUAAAAAUGAAAGCGGAAAUCAUAAUGUUGGUGUUCCAACAUUUAAAAUCGAAGC
Dno-1-3	UAUUGCGUCUUGCCUAUCAUUUAACGCAAGCGUUAAAUGCGCAAAAUGCCGCGGCAAUCGCAGCAUUGGAGGAUUAAGUCAUGGAAAGAUUGGCUCAUAUUACUGCCACUGAGGAAGAGCAAAAAAA
Dno-1-4	AGACACAAGCUGAUUUAUUGCGUCUUGCCUAUCAUUUAACGCAAGCAUGCUUUGAAAAAAAUGCCGCGGAAACCGCAGCAUUGGAGGAUUAAGUCAUGAAUAAAUUUAUUGCAAGUUAUGUGCUGUU
Esp-1-1	GUACUGGUUGAAACGGACGAUACCGAAACACGCCGUGCGCUGUGCGAGCGUUUGCUGGAUGCGCUUGGUCAGUUGCAUUCACUCUGCAGCGAUCUCUCGCCCUAUCUGGUAAAGCAUUUGCUCGAAG
Plu-1-1	GUCACCCCUGCUGAAAUAUGGCCCAGUCGGUAUUUUGACGAAAAUAAGCAGCCUAUCAAACGAACUGUCCGAAAUGAUUUACCAAACAAUUCUGAUAUCACAUAAGUUAUUUCUAUAUCCGAAAAAG
Xax-1-1	UAAUCCCGCCGGCGAAGCCGUGCAAGAGGAAAAGGAUGCAAAGGCCUAAACGCCGAACACCUUGCCCCGCUGCCGUUCAUCGGCGCGGGACAUGCCAGCAGUAGAUCCAGGCUCCCACACGCCAGGC
Aba-1-3	GCAACAAUAAACCCGAGAUAGGCGAGCACACCAUUUUGUGCGGUUUUCGCAUGCCUGUAUUUAGCACUCAUUUAAGAGUGACGGCAUUUUUAUCUCUUUUAUAUACAACAAAAAAUUAUUUUAAGC
Xfa-3-1	GUUUCCCGUAUCUAUAACCGUCAUUCCGAAGAGUUCACCGUCGAGAUGAGCUUGGUGGUCAAUUUGACCACCAAGGGGUUUGGAAGUGGCAACUCUGAAAAACCUACCCGACUCUUCAGCCCCCGCG
Xfa-1-2	GUCUUGCGUAUCUACUCGCGCCAUACCGACGAGUUCACCAGUGAAAUGAGCUUGACGGUCAAUUUGACCGUCAAGGGUUUCGGUUGCGGCAACUCUGAAAAACCUGUCCGACUCUUCAGCCCUCGCG
Sma-1-2	AUCACCUGCGCAGCCGGCAGAUGCAGUUGUCCGACGCCGACUGCGCGCUCACCGGCCAACGCAUCGUGCAGGAGAUCCUGCUGGCGGCCGAGCGCACGCGCCAGAACGGCGAGCCGGAGCAGUACGU
env-2	ACCAUCUACGCAGCCGGCAGAUGCAGUUGUCCGACGCCGACUGCGCACUCACCGGCCAACGCAUCGUGCAGGAGAUCCUGCUGGCGGCCGAGCGCACGCGCCAGAACGGCGAGCCGGAGCAGUACGU
env-3	ACAACCCUGGCCGGAGCGUCACCAGCUGCCCGGCCGCUGCCUCGCCCUCGCCGAUGCCGAUUGCGCUCUGAACGGGUUGCGGGUGGUGCUGGAAAUGCUGCUGGCAGCCGAACGCACCCGGCAGAAC
Sma-1-3	CCAACCCUGGCCGGAGCGGCAUCAGCUGCCCGGGCGCUGCCUGGCCCUGGCCGAUGCCGAUUGCGCGUUGAGCGGGUUGCGGGUAGUGCUGGAAAUGCUGCUGGCGGAGCGCACCCGGCAGAAC
	....
	....
	•••

Sma-1-4	ACCAGAUGCCCGGCCGACGGCUCGCCCUCGCCGACACCGACUGUUCGCUGGCCGGCCUUCGCGUGGUAUUGGAGAUCCUGCUGGCCGCACAGCGGGCCCGAGAGAACGGCGAGCUGGAGCAAUACGU
Plu-1-2	GGAUUAUCGCUAAUUAUCUCAACAUUCAUUUAUCUGAAAUCUGGCCUAGUCGUUAUUUUGAUAUGGACAGUAAUCUUAUUGAGAGCAAAGUUCGCGAGAAAUCAGCAGAAUAAUUAUUCUAAUACCG
env-4	CUCAUCCUCAAUGACGCGCCGACGAUCGAGCCGGAUGAUUCGACAGACGGCCUGAUCGAGGCACUCAAUCGCCUGCAGGAUGCCAUCCACGGCUCAUCCGACGAGAUUGCGGGCGCCUUGGCUGCCU
Aba-1-4	CACAUUGCCAGCCGGUUUUAGCAGACCGCAUAGUAAGAGCAUCAGAUUAUUCCUUCUGAGGAGUAGUCUUAUGUGUAUCAAUCGUCCCUCGCUCGUUGCGGUAGGACGAGUGAGGGACACCUUCGGG
Bph-1-1	CGACCUCGAUAUAUAGGAAUCCUGCACUGACGAACCACGAACGACGCAGCCGUCAAGUGCUGCUCGAGUUGCUGGUUGAAACGUGCGAGGAGUAUGAGCGGGAAGUCGAAUGCGACCGCGAGUUGUA
Bxe-1-1	UUGCGACCUCGAUAUAUAGGAAUCCUGCACUGACGACCCACGAACGACGCAGCCGGCAAGUGCUGCUUGAGUUGCUCGUGGAAACGUGCGAGGAAUACGAGCGUGAAGUGGAAUGCGACCGCGAGCU
env-5	UCCUCAAUAUGUAGGAAUCCUCCCAUUACACUUGAAGAAAGACGACGUCGUCAGACGCUUCUUGAAUUGCUGGUGGACACGUCUGAGCGAUAUCAGCAGGAACUCGAAUGCGACCGCGAGUUGUACG
env-6	ACCUCAAGAAAUAUGGCCAACCCGAUAUUUUGAUAAGGCAGGAAUGCCGAUCAAGAGAAUCAUAAGAAAACCGGUUUAAAUGAAAAAGGCUGGCAUUUUUGCUGGCCUUAUAUACCUCCUGGCUCUG
Spr-1-1	GUCGGCAGAACAAACGGAAGGUUUGUGCAACGGGUUGAUGCUAUUCAAUGAGCUUUUGCAGACAAGUUCCCAGCCUAAUGCCGUCGACUGGGGGCAGCUAAAUAGUUAUCUGAAUGCAACCGCGCAU
Pfl-2-3	CCUGAUGGACGCCGCCAACUACCGCAUCCGCGCCGUGAUCCAGGUCCUGGAAAACAUGUGCAUGCGCGGCUCGGUCGAGUGCGACUCGGUCAUCCUCAGCGACUUCGCCCAGCUCUGCGUGAUUCCG
env-7	ACCUGAACCCUGCGUUACUGCGCAAGCUCGAUCCCGACCAACAACUCACCCUGCCGCUGAUCACUCAUGGCCACUGUGCGCCGACGUUGAUGGUCAGUGAGUCUGGCGUUUAUGCGCUGCUUAUCUA
Pfl-2-4	CCUUCCAAACCGCGCCCGAGCACGAUGUAUAUGGUCACGCCCAACGUCGACACCGAAAGCCUGCUGGCCAACGCCUGCGAAUCCCUGGCCUCGGCCAAUACCAUCGCCAGCAAUUUCGCCAACGAAC
Msp-1-1	CACUCUUUGUGCAAACGCCGAAUGGCGCCAUCUUUUCUAUCGGCGGUUUAUCCGACCGACAAAAGCAACAACUCCUUACCCUGUUACCCGACCUUAUUCAUACCCGUGUCCGCUUUCGGUACCGCCA
Msp-1-2	GCUUAAUACUGGCCGAUUCAAUAGAUAACGAUACUGGCACCACAGGUGACCAAGUAACAGGCCUACUAAAGUGCAUUGAAUACAGCCUUUUAAACAGCCAAAGAAAAAUCAUCAAAGCAUUACAAGA
env-8	GCGGGUGAAAUUUCCAAAGACACUCAAGACCACCUAGGCUGGGCCUUAUGGGCACUGAGCGAUGUGUUGCGCGCGCAGAUUGCGUUGUGAGCGGUUGGAGGGGGUGGGGUUCACCACCGUCGACA
Aba-1-5	UAAUAUCUUAAAUAAUAAUUUAGUUAAUCUAAUGAAAAACAAUAAGAAAUUUAAACACUUGUUAUUAAAGCCACAUUGACACAUCCAAACUCUCAGAUUAUGCUUUGCGUGCUGGCCACAUUGCCAG
Pfl-1-1	CGCUGUUUCGAGUGAAUGCCGACGUGCCGAUCAGCGACGCCCUGGAACACGCCUCGACCCUGUUGCAUCUGGCAAAGCAAUUCACCCUGGAGGCGGCCGUGGAGCCCCGGUACGACCGCUAUGCUUG
Psy-1-1	UUGCCUCAACGCAUGGAUGAACGAGCGUGGCGGCGUCGGUAACUUCGAGGGUGACGCAAUCGUGUCGCGACUGCGCCAGGUCAUCGAGCGCUUCGGCGAGAGCCGAUUUACCCGCUGGGAAUCGGCA
Pae-5-1	GCCCGCCAUCGACAAGGGCGUCCGCCAGGCCGAGAACUGGCUUGCCGACCAGAUCGAAGGCCAGCUCUGGACCGCCUUCGCCUUCGGCCGCGACAGCCUGCCCACACCGAUGCAGAAGACCGCCUUC
Pae-2-1	GCCCGCCAUCGACAAGGGCGUCCGCCAGGCCGAGAAUUGGCUUGCCGACCAGAUCGAAGGCCAGCUCUGGACCGCCUUCGCCUUCGGCCGCGACAGCCUGCCCACACCGAUGCAGAAGACCGCCUUC
Pae-6-1	GCCCGCCAUCGACAAGGGCGUCCGCCAGGCCGAGAACUGGCUUGCCGACCAGAUCGAAGGCCAGCUCUGGACCGCCUUCGCCUUCGGCCGCGACAGCCUGCCCACACCGAUGCAGAAGACCGCCUUC
Ppu-4-1	CAACCACUGCCACAUGGCCCUCUACGCAAUCGAGGAUGAGGCCACCCGUGAGUUGAUGGUAAACGGGCUGCGGGCCAUGCAGAUUGCCUGGGGGCAGUCGGAUGCGCUGUCGCUGGCCUUGGAGCGU
Dps-1-2	CUCAACAGUUUGCAAUCAUGCGGAGAAGAACCCCACUUCACGAUUCAACAUAGCGAUGUCUUAGGUUGCCUUAAUGCUAUCGAGGCCAAUAUCCAUAUGGUUGGCAAGAUGAUCAACCAUGCAGUGG
Asa-1-1	CGCGCCGCCCUGGCAGGUUUGCCGUUGGUGUUUGUCAGCCGCCGUCCGUCUGGGGAGGUGGCCGCAUGAACCCGACCCAUUCCCCCCGCCUAGCUGAACUGCUGGCGCAACUGGCCGUACUGCUGGA
env-9	CAAGCCCGCGCCAACUUGCCCGGCUUGUCGUUGGUGUUUAUGUCCCGCCCCCCUAGCAAGGGGGUGGCCGCAUGACCCUGUCCACUCCAUCCCGCCUAGAGGCACUGCUCGAGCAGUUUGCUGACGC
Bsp-1-1	AGCGGCAGAUCCAGCUCGAUACGAUCCCCGGUGCCGAGUGGUUUUGGCAACGAGCGGAGGCUGCGACUGACGAACUCGACGCCGCGAUCAACAAGGCUAUCGGGUGGGAUUGAAUGCGGCGCGUCUC
Pen-1-1	AAGUCACCCGCCACGCUGGUGAUGCUGACGGUGAUGCACGAACUCGAUGCUACCCGCACGUUGUUGGAGUCGGCGUUGGCUCAGCUACAGAUGAGCGCCCGGCCACCGUCCUACACGCUGCACUGAC
Cvi-1-1	CCAAAUGUCGAUGGUCGACAUCACGCGCUAUCAGCCCAUUGGGAGGCAAAUCAUGAUCACCAGUAAAUCCAUCCUGGCCCAGAUCGAACAAGCCUUGUUCUCCGUAGGCCAUACCACCCAACAACAA
Sdy-2-1	AGCGACACGAUAACGUAUUACGUGCCAUUGCAAAUAUUGAAUGCUCAGCGAAAUUUAACACCCUCAAUUUUGAGGACGUUACCUACACCGACGCCAAAGGCGAACAACGCCCAAUGUACCAAAUCAC
Sbo-1-2	CGUCCGGUUGUGUUGCCGUAUUCGCUGCCCGUAUUCGUCAGGGGGUAAGUCAUGCUUAAGCGAUUCCAGAUAACCGCACGCGCUGUAAAUCAUCAAGGCCACGUUAUUGAUAUUCGGCAAAACGUGA
env-10	AUUUUUGUCGCCCGUAUCCGUCAGGAGGUGACUCAUGCUUAAAACCUUCCGCGUCUUUUCCCGUGCUGUUNACAAGCGCGGCCAUACCAUCGGCAUCACACAGAACGUGAAGGCCGAAAACGUCCAG
Cte-1-1	UGAUUCGCGCGAUCAAAAGACUCGAUGCUCCUGCGGAAUUCACUGAACGCAAUUUUGCGUUGAGCGAUUUCACGGACUCAACAGGACGCACGCUUCCGGCUUAUCGCAUCACCCGCGACGGCUUUAC
Cte-1-2	UUGCGCGCUAUCCGUGCUUUGAUUCGUGAAAUGGCUUCGGAAGGCCGUCCCCUCAAUUUUGAGGAGACGGUCGAUGUCCGAACCAAUCCCAGCGGUGGCGCACCAAUUUCUACACCUGCUUACCGCA
Sgl-2-2	UAGAUAAAAUCAAAAACCUUGAUUGUUAAGCGAUUUUUAACCAGCACAAUUUUGUGCUAGUUGAUUACGUCGAUGCAAAAGGCGAAAAACGCCCGAUGUUUGAAAUGACCAAAGACGGCUUCGUCUU
Sen-8-1	UACGCUUUGUUGAAGGGAAACACUUUUUCAAAGUUGUUGGUAAUGACCUUAAAGAAUUGCGGGUAGCUUUAAACUACUCACAAAAUCCAGUUUCCCCCAAAGCCCGCUCCCUCAUCCUAUGGACAGA
Vfi-1-1	UAUGCCAGAAAUAUCGAUGAGUUUAGUUCUUCUAUGAGUGGUUCGGUCAAAUUGACCGACCCUAGAGGCGUCGAACAAAACGCAAGAAUAUUUUCUCUUCGUGGCGCUCAUUUGCUUGCUAUGUUCU
Vfi-1-2	GUAAAACUGCGAACCGACACGCUAUCUUAUGGCUAUCCUGAAUUAUAAUUCGAAGUUCAUCUCUCAGGCGGGAGAAUCCGUUCUAGCUCGUUUGGGUUUGAUCCCCUGUUAUUCCCCGUCUUACAAG
Sde-1-1	GUAAGUUGCGGCCUACAUUGGAUUCUACGCUGUGUGGACAACGCACUAGAGUUCGAAAUCAAGCGGCUUAGCCUCGGUGAAUAAGGACCAAUAAACAUCCUUCCACCAUCGAGUAUUGAGUUGUUAC
Sba-1-1	GAAAUACUUGUCGAAGCUCCCAGUGAAUUUACGUCAGCCCAUUUUUGGGCUAACGUGCAAAAUCAACAGGUUGGCACCAGUCAGCGCGAUUUAAAGUGUUACCACCUGACCAAAGACGGGUUUAUGU
Sba-2-1	AAAUACUUAUUGAAGCACCCAGCGAAUUUACGUCUACGCAUUUUUGCGUAAACCUGCAAAAUCAACAGGUUGGAAAUAAUCAGCGCGAUUUACGUAAUUACCAAAUGACCAAAGAUGGUUUUAUGUU
Eco-3-1	AUUGCAGACUAUAAAUUCGUUGAAAAUCAUGAUUAUAUCGUAACGUUUUCCAAAACUGGAAAGCGUAAAAAUGUUCUUUGUAAGGACUACCACCUCACCCUCGAUACAGCCAAAGAACUGGCGAUGG
Vch-2-1	GCUCGGUAUUCAGAUGAGUUUUCACAAGGAAUGACAUUGGUCAUCAAUUUGAUGACCAAUGGUAUAAAUGGAAGUAAACGCAGGAAUGAAACAAGAAUUUUCUCACUGCGCGGCGCUCACCUCAUCG
Vch-3-1	AUUUUUAAUCGUAACAAAGAUGAGUUUUCCAGUGGAAUGUCUGAGGUCAUCAAUUUGAUGACCUCAGGAAACUACCAAAAAUCCGUACGUAUUUUCUCACUGCGCGGCGCUCACCUCAUCGCCAUGU
Vsp-1-1	AGAAUCUUUAGAUUGCUCUAAAGAAUUCACUGAGCGCAAUUUUACGCUCAGUGAUUAUCGAGACGGUUCUGGUCGUAAACUUCCUCUUUGGGAAAUGACCAAAGACGGUUUUAUGUUCUUGGUUAUG
Vch-1-1	GAGACGUAUUACAACUGGAAAGCAAAAUACGAUGGUAUGGAAGCAUCCGAUAUUAAACGACUCAAAGAACUGGAAGAUGAAAAUCGUCGUCUGAAAGCGAUGUUUGCUGAGUUAAGUUUAGAGCACA
Vba-1-1	CAAGAACUGGUUAAUGAAAUUGAGCAAAGCAGGAAUUCCUGCUUUGCUGUAAAUACCAAACGUGGCGGUUGCAAUUCUGGGACUUGGGUAUGCAAAGAACUUGUUUACUCUUAUGCGAUGUGGAUUA
Sfr-1-1	CGUAUUCUAGUCGAUGCCCCCAAUGAAUUUACGUCAACGCAUUUUUGCGUCAACGUGCGAAAUCAACAGGUUGGCACCAGUCAGCGUGAUUUAAAGUGCUACCACCUGACCAAAGACGGUUUUAUGU
Vsh-1-1	ACCCAAGAACUAAUACAAGAAGUUGCCAGCUCCUCAGAACUGAGGAGCACAAUAAAGACUCAGAAUGGUAAGGGAACUUGGGUAUGCAAAGAACUCGUCUACGCCUACGCGAUGUGGAUUAGCCCUA
Sgl-1-1	AAGGUCUUUUCGACUCAGACUUCCCUGCUGAAUGGUCUGAACUUUCAACUCAAUUUAUCGAGCAUGAUAGGCUAGCAGAAAAAACGGGAGAUCAUAUAGAAAUCAUUCAAACCCAUUAUUUUUAUGG
Sgl-2-3	AAGGUCUUUUCGACUCAGACUUCCCUGCUGAAUGGUCUGAACUUUCAACUCAAUUUAUCGAGCAUGAUAGGCUAGCAGAAAAAACGGGAGAUCAUAUAGAAAUCAUUCAAACCCAUUAUUUUUAUGG
Sgl-1-2	AAGGUCUUUUCGACUCAGACUUCCCUGCUGAAUGGUCUGAACUUUCAACUCAAUUUAUCGAGCAUGAUAGGCUAGCAGAAAAAACGGGAGAUCAUAUAGAAAUCAUUCAAACCCAUUAUUUUUAUGG
env-11	AAAACUGGCGAUUUGUGCACGUCUGUCGGCCUGUCUCGCGUUAUUGCAACCGACCUUAACGGAGCCUGUACCGCAGCAUCUGCAGGCCAGUCUGACGGUAGAAACAUUCCCGACCCGUUUUCCCCUU
Eco-5-1	CGAUCUGGCGCUGAAAAUGGCGCCCUGCGGCAGGCUUAGUACCUGUCUUGCACUGCUGCGGCCCACGCUGGGCGAACCGAUCCCGCCGCACCUGAUCGACCGCUUUACUGUGGAUGUUCUUCCUGAU
Sen-11-1	UGAAAAUGGCACUUUGCGGCAGGCUUAAUGCCUGUCUGCAACUUCUUCAGCCCUCACUGCUUGAUCCGAUCCCGGAGCACAUGAUGGACAGCCUGAUCGUUGAUACGCUCCCCACCCGCUCACCGCG
Sen-12-1	UACGUCUGGCGCUUUGCGGACGGCUUGUCGCCUGCCUUUCGCUGCCGCGCCCCACGCUGAUCGAUCCCAUCCCGCCGCACCUGGUUGAAAGCCUGACCACCGAUACCCUUCCCGCCAGUUCAACCCA
Esp-1-2	AAAAUGGCACUGUGCGGACGGCUCUCUGCCUGCCUCGACCUGCUUCAGCCAACGCUGCUGGAACCGGUGCCUGAACAUUUAAAAGCGAGCCUGACGGUCGAUGAUCUGCCGCUGGACGUGCCAGUCU
Sgl-2-4	AGAUUGUUCGCCUACAUUUACAUCAGCGAACUUUUGCGCUGAUGUUCAAAAAGUAGACAUUGGCAACGAUGCAACCCGCGAUUCCAAAAUCUUCACCAUGACCAAAGACGGCUUCGUCUUCCUCGUC
Sgl-2-5	CUGGAUUGUUCUGCUGAUUUUAGCGCCCGCAAUUUUGCGGGGGCAGAAUACACCGACGAACAGAACAAACCUCGCCCGAUGUUCGAGAUGACCAAAGAUGGCUUCGUCUUCCUCGUCAUGGGCUUUA
Sgl-2-6	AUUGAUUGUUCUGAAAAAUUUAACCGGCUGAAUUUUCAGCCCGUUGAUUACAUCGAUGCAAAAGGCGAAAAACACCCGAUGUUCAAGAUGACCAAAGACGGCUUCGUCUUCCUCGUCAUGGGCUUUA
Sgl-2-7	UCGAAUGCUCAGCAGCUUUCACGACGGCGAACUUUUGCGCCGUCGGAGUGGAAGUACAGGCUGGCUUCGACAAACGUGAAAUUGAAACCUAUGAAAUGACAAAAGACGGCUUCGUCUUCCUCGUUAU
Sgl-2-8	CCUUAAGCAAGUUGGACGACGACGAAAAGACGACCGUCGCUUUAACCGACAGUCAGCCAGGGACUGGGGCUCAACGUAUUUCUUUGAUAUCCGAACCCAGCAUGUUUACCUUGGUAUUACGCUGCCG
Sdy-1-1	UGCUCUGAGCAAUUCUUAACCCGCAACUUUUCGCGGGUUAAAUUCGAACACCGAGGCAAUACCUAUAACGCCUACCAAAUCACCAAAAACGGCUUCGUUUUUCUGGUGAUGGGCUUCACCGGCAAAA

Sbo-1-3	UGUUCUGAACUAUUUUUAACCAGCAACUUUUCGCGGGUUAAAUUCGAACACCGCGGCAAUUCUUAUAACGCCUACCAAAUCACCAAAAACGGCUUCGUUUUCCUGGUGAUGGGCUUCACGGGCAAAA
Eco-10-1	UUACUGGAUUGCUCCCCUGAAUUCACUGCCCUUAAUUUUAAGGUGAGCGAAUACACCGACUCAACCGGGCGCAAACUCCCUAUGUACCAAAUCACCAAAAACGGCUUCGUUUUCCUGGUGAUGGGCU
Sfl-1-1	AAUACUGAAUGCUCACCUGAAUUUAACGCCCUCAAUUUUGAGGACGUCACCUACACCGACACCAAAGGCGAAAAACGCCCAAUGACCAAAUCACCAAAAACGGCUUCGUUUUCCUGGUGAUGGGCUU
Sfl-2-1	AAUACUGAAUGCUCACCUGAAUUUAACGCCCUCAAUUUUGAGGACGUCACCUACACCGACACCAAAGGCGAAAAACGCCCAAUGUACCAAAUCACCAAAAACGGCUUCGUUUUCCUGGUGAUGGGCU
Eco-3-2	AAUACUGAAUGCUCACCUGAAUUUAACGCCCUCAAUUUUGAGGACGUCACCUACACUGACGCCAAAGGCGAAAAACGCCCGAUGUACCAAAUCACCAAAAACGGCUUCGUUUUCCUGGUGAUGGGAU
Eco-3-3	....
Eco-3-4	UGCUCAGAUAAAUUUACUGCCCUCAAUUUUGAGGCGAGCGAAUACACCGACUCAACCGGGCGCAAACUCCCAAUGUACCAAAUCACCAAAAACGGCUUCGUUUUCCUGGUGAUGGGCUUCACUGGCA
Sfl-3-1	CUCGAAUGCUCGCGGGAGUUUAACCGGCUUAAUUUUAAGCCCGUCACCUAUACCGACGCCAAAGGCGAAAAACGCCCAAUGUACCAAAUCACCAAAAACGGCUUCGUUUUCCUGGUGAUGGGCUUCA
Sfl-2-2	CUCGAAUGCUCGCCGGAGUUUAACCGGCUUAAUUUUAAGCCCGUCACCUAUACCGACGCCAAAGGCGAAAAACGCCCAAUGUACCAAAUCACCAAAAACGGCUUCGUUUUCCUGGUGAUGGGCUUCA
Sfl-1-2	UCGAAUGCUCGCCGGAGUUUAACCGGCUUAAUUUUAAGCCCGUCACCUAUACCGACGCCAAAGGCGAAAAACGCCCAAUGUACCAAAUCACCAAAAACGGCUUCGUUUUCCUGGUGAUGGGCUUCAC
Eco-6-1	CUUGAAUGCUCCGCUGAAUUUGCUGAACACAAUUUUGUGUUAAGUGGUUAUAUCGACGCUUCAGGCCGCAAACUACCUUGCUAUCAAAUAACCCGCGAUGGCUUUGCGUUUCUUGCUAUGGGUUUCA
Pcd-1-1	CUUGAAUGCUCCGCUGAAUUUGCUGAACACAAUUUUGUGUUAAGUGGUUAUACCGACGCUUCAGGCCGCAAACUACCUUGCUAUCAAAUAACCCGCGAUGGCUUUGCGUUUCUUGCUAUGGGUUUCA
Sso-1-1	CACUCGACUGCUCUCCCGAAUUUACAUCAGCCAACUUUUCGGCCGAUGUUCAGACUGUCGAAAUUGGCAACGGCGCAGAACGUGAAUCACGAUGCUACCUGAUCACAAAAGACGGCUUCAUGUUCCU
Sbo-1-4	CUUGAGUGCUCUGUUGAAUUCACUGAGCUGAAUUUUCAGCCCAGUGAUUACACCGAUUGCACAGGCCGCAAACUACCUUGCUAUCAAAUCACCCGCGAUGGCUUCGCGUUCCUUGCUAUGGGCUUUA
Sfl-3-2	UUGGAUUGUUCUCCAGAGUUUUGUGCCCGCAAUUUUGCGGAGACAUCAAUUUUGGUACGCCAGCCCAACGGCGGUACUCGCAAACUUCCCUGCUACCAAAUUACCCGCGACGGCUUCGCCUUCCUUG
Eco-3-5	UUGGAUUGUUCUCCAGAGUUUUGUGCCCGCAAUUUUGCGGAGACAUCAAUUUUGGUACGCCAGCCCAACGGCGGUACUCGCAAACUUCCCUGCUACCAAAUCACCCGCGACGGCUUCGCCUUCCUUG
Eco-13-1	GCCACGCACUAUCAAACAUUUAUUCCGCCUCUGUUUUCAGCUAUCUGGCGGAUGAUCUGCACGAGUUUCAACAGCUCAUCCAGCAGCAAAACAAACUCCAUUAAUUCCUGUUCCGGGCCUUUCCUGC
Eco-3-6	UUUGGAUUGUUCUCCAGAGUUUUGUGCCCGCAAUUUUGCGGAGACAUCAAUUUUGGUACGCCAGCCCAACGGUGGUACUCGCAAACUUCCCUGCUACCAAAUCACCCGCGACGGCUUCGCCUUCCUU
Sen-13-1	UCUCUCGAAUGUUCCACUCUAUUUCGUCAACGCAAUUUUGCGUUUACAUCGAUUUCAAUAAAUCAGCCCAACGGCGGUACCCGCAAACUCCCAUGCUAUCAAAUCACCCGCGAUGGUUUUGCGUUCC
Sen-16-1	UCUCUCGAAUGUUCCACUCUAUUUCGUAAACGCAAUUUUGCGUUUACAUCGAUUUCAAUAAAUCAGCCCAACGGCGGUACUCGCAAACUCCCAUGCUAUCAAAUCACACGCGAUGGUUUUGCUUUCC
Eco-2-1	ACGCUUGAAUGCUCUGCAUCAUUCACUGCCCGCAAUUUUUCGGUGAGUGAUUACACCGAUUGCACAGGCCGCAAACUACCUUGCUAUCAAAUAACCCGCGACGGCUUUGCGUUUCUUGCUAUGGGGU
Eco-15-1	ACGCUUGAAUGUUCCGCAUCAUUCACUGCCCGCAAUUUUUCGGUGAGUGAUUACACCGAUUGCACAGGCCGCAAACUACCUUGCUAUCAAAUAACCCGCGACGGCUUUGCGUUUCUUGCUAUGGGUU
Eco-6-2	AACCUCGAAUGUUCCGCUGAAUUUACUGAACACAAUUUUGUGUUAAGUGAAUACACCGACGCAUCAGGCCGCAAACUACCUUGCUAUCAAAUCACCCGCGAUGGCUUCGCGUUCCUUGCCAUGGGCU
Eco-6-3	GCUGCGAACUUUCAAACCUCUAUUCAUCCGCUGUUUUCAGUUAUCUGGCGGAUGAUCUGCGCGAGCUUCAGCAGCUCAUCCAGCAGCAAAACAAACUCCAUUAAUUCCUGUUCCGGGCCUUUCCUGC
Eco-2-2	GCUGCGAACUUUCAAACCUCUACUCAUCCGCUGUUUUCAGUUAUCUGGCGGAUGAUCUGCGCGAGCUUCAGCAGCUCAUCCAGCAGCAAAACAAACUCCAUUAAUUCCUGUUCCGGGCCUUUCCUGC
Eco-8-1	GCCACGCACUAUCAAACAUUUAUUCCGCCUCUGUUUUCAGCUAUCUGGCGGAUGAUCUGCACGAGCUUCAACAGCUCAUCCAGCAGCAAAACAAACUCCAUUAACUCCUGUUCCGGGCCUUUCCUGC
Sdy-1-2	GCCACGCACUAUCAAACAUUUAUUCCGCCUCUGUUUUCAGCUAUCUGGCGGAUGAUCUGCACGAGCUUCAACAGCUCAUCCAGCAGCAAAACAAACUCCAUUAAUUCCUGUUCCGGGCCUUUCCUGC
Eco-7-1	GCCACGCACUAUCAAACAUUUAUUCCGCCUCUGUUUUCAGCUAUCUGGCGGAUGAUCUGCACGAGCUUCAACAGCUCAUCCAGCAGCAAAACAAACUCCAUUAAUUCCUGUUCCGGGCCUUUCCUGC
Eco-9-1	GUCACGCACUAUCGAACAGCUAUUCCGCCUCUGUUUUCAGCUAUCUGGCGGAUGAUCUGCGCGAGCUUCAGCAGCUCAUCCAGCAGCAAAACAAACUCCAUUAAUUCCUGUUCCGGGCCUUUCCUGC
Eco-3-7	GCCACGCGCUGUCAGACAGCUACUGCGCCGCUGUAUUCAGCUAUCUGGCGGAUGAUCUGCACGAGCUUCAACAGCUCAUCCAGCAGCAAAACAAACUCCAUUAAUUCCUGUUCCGGGCCUUUCCUGC
Eco-15-2	GUCACGCACUAUCGAACAGCUAUUCCGCCUCUGUUUUCAGCUAUCUGGCGGAUGAUCUGCGCGAGCUUCAGCAGCUCAUCCAGCAGCAAAACAAACUCCAUUAAUUCCUGUUCCGGGCCUUUCCUGC
Eco-2-3	ACAGCACUAUUUCAGCCGACUACUGCGCCGCUGUAUUCAGCUAUCUGGCGGAAGACCUCCUGUCUCUUCAGGAUCUCAUCCAGCAGCAAAACAAACUCCAUUAAUUCCUGUUCCGGGCCUUUCUUGC
Eco-10-2	AGCGAUUCGUGGUCGGGAAGCAUUACUUUUUGCUCGAAGGUGAGGAGUUGCGCGAAUUUAAGCACAGCAUGUCUUUAAGACCUCCUGUGAAAAUCGCCAGAAAUGUCCGCUCUCUCAUCCUCUGGAC
Spr-1-2	ACAAAAAUGAAGAUGAAUUUACGGAUGCUAUGUCAUUGGUCGUCGAAUCAAUGACCAAUGGGAUAAACGGCAGCAAGCGCCGCAUGAAGGUUAGAGUUUUCUCCCUGCGCGGUGCCCACCUGAUCGC
Sty-2-1	AACACCACGCGAUUCGUUGUAGGAAAGCAUUUUUUUAAAAUUGAAAAAAACGAAUUACGCGAGUUCAAGAACAGACCCGAAACAAUCGGGUUAGUUGGUAAAAAUGCCCGUUCCCUAAUCCUCUGGA
Sen-9-1	AACACCACGCGAUUCGUUGUAGGAAAGCAUUUUUUUAAAAUUGAAAAAAGCGAAUUACGCGAGUUCAAGAACAGACCCGAAACAAUCGGGUUAGUUGGUAAAAAUGCCCGUUCCCUAAUCCUCUGGA
Sen-13-2	AACACCACGCGAUUCGUUGUAGGAAAGCAUUUUUUUAAAAUUGAAAAAAACGAAUUACGCGAGUUCAAGAACAGACCCGAAACAAUCGGGUUAGUUGGUAAAAAUGCCCGUUCCCUAAUCCUCUGGA
Eco-10-3	CGUAACUCUGAGCGGUUUGUUUGUGGGAGGCACUACUUCAAAUUAGAGGGAAAUGAUUUGCGAGAAUUUAAGAACAAACUGCGACAAAACACAACACGAAUGAGGCGUGUUGUCGCACGUAAAAGUC
Sso-1-2	CUUUCGACGGUUUUGAGCGCCCGGUCACCCUCUCAAAAGGGGUAAAUCAAAAUAUUCGAAAGGACAUGUCUAUGAAUCGCACGUCUAUUGAAAAACUCCCGUCACUUACGCAUAACCAUCUUCCUGU
Ype-7-1	AAUCGAAAUUCCGACGAAUUUACAGAAGCCAUGACCUUGACCCUCAAAUUGAGUGUAAAGGGGUUUGGUAACGGAAAGUCAGCAAAAGACGUUAGAGUUUUCUCCCUGCGCGGCGCUCAUUUGAUCG
Ype-1-1	AAUCGAAAUUCCGACGAAUUUACAGAAGCCAUGACCUUGACCCUCAAAUUGAGUGUAAAGGGGUUUGGUAACGGAAAGUCAGCAAAAGACGUUAGAGUUUUCUCCCUGCGCGGCGCUCAUUUGAUCG
Eco-3-8	AAUAAAGAGCGAUUUGCAGAAGGGAAGCACUACAUAAUUGCGAAAGGCGCAGACCUCCAAAAUUUGAAAAUCUCUUUAAGGGAUUUUCAAACGAUCGCCCCAAAUGUCCGAAAGCUCAUACUCUGGA
Eco-3-9	CCGUCUGGUUGUGUUGCUGUAUUCGCUGCCCGUAUUCGUCAGGGGGUGGCCCAUGUGUAAAAACCGCUUGCAAAAAAUUAUCACAGGGCUGUAUUCUUCCUGUGUCGCCCACAUGGCGACCGGGUUU
Yen-1-1	CAGCGGGAAACGAUACUGAAAAUUGCCCGGUCUUUACUGGCCGAAGGUGUUGAUCGCGCUCUGGUAAAGGAAGUCACUGGCCUACGUGAUGAAGACCUGACGCAAUAACGCGUUAACCCCCAUUCUA
Yen-1-2	AUCAUUGCCGUGCGCUCAUUUACGCCGUUGUCGUGCUUGAUCAACCCGCUGCAAAAGAAAUACUCAAUCUCAUCCUAUGGCAGCAAAUAGACCUGCUAUAUCAAACUUAUCAUCACGCCACCAGCGA
Yps-1-1	AUCACUGCCGUGCACUGAUCUACGCCAUCAUCACACUCGAAUCCCCGGAAGUGAAAGAAAUACUCAACGUUGUCCUACAACAGCAAAUAGAUCUGCUACAUCAAACUUAUCAUCAAGCCACCAGCGA
Ype-13-2	AUCACUGCCGUGCACUGAUCUACGCCAUCAUCACACUCGAAUCCCCGGAAGUGAAAGAAAUACUCAACUUUGUCCUACAACAGCAAAUAGAUCUGCUAAGCCACCAGCGAGCCUCUCGAGGCCGAAU
Yps-2-1	AUCACUGCCGUGCACUGAUCUACGCCAUCAUCACACUCGAAUCCCCGGAAGUGAAAGAAAUACUCAACUUUGUCCUACAACAGCAAAUAGAUCUGCUACAUCAAACUUAUCAUCAAGCCACCAGCGA
Yen-1-3	AAGAACUUAUCGAUCACUGCCGUGCGUUGAUUUACGCCAUCAUUUCCGUUGAUCGGGCCGAUGCUAAAGAGAUUCUUUCGCUUAUUUUGUGGCAACAAAUUGAUGCGUUACACAGUACUUAUCUGCG
Ybe-1-1	ACCACUGCCGCGCCCUGAUUUACGCCAUCGUCACCCUUGAUCGAGCAGAUACAAAAGAAGUUCUCUCGCUUAUUUUAUGGCAACAGAUUGACGUGUUACGCAGAACUUAUGAGCGAGAUACUCAUGA
Yin-1-1	GGUCUUUACUGGCCGAAGGUGUUGAUCGCACUCUGGUAAAGGAAGUCACUGGCCUACGUGAUGAAGACCUGACGCAAUAACGCGUUAACCCCCAUUCUAACGGGCGAUGUUGUGUUAGACGUUGCCU
Yen-1-4	AGUCAGCGGGAGACGAUACUGAAAAUCGCCCGGUCUUUACUGGCCGAAGGUGUUGAUCGCACUCUGGUAAAGGAAGUCACUGGCCUACGUGAUGAAGACCUGACGCAAUAACGCGUUAACCCCCAUU
Yin-1-2	AAGAGCUUAUCGACCACUGCCGUGCGCUGAUUUACGCCAUCGUUACCCUUGAUCGUGCCGAUGCUAAAGAGGUUCUUUCUCUUAUUUUGUGGCAGCAAAUUGAUGCGUUGCACAGUACUUAUCUACG
Sbo-2-1	UGCGGCUGGCGCUUUGCGGGCGACUUGCCGCCUGUCUCACUUUGCUGCAACCCACGCUGAACGACCCCAUCCCGUCUCAUCUGACUGACAGCCUGACCGUUGAUGCUCUACCUGCCGAUAUGCCCCG
env-13	UCAAAAUGGCGCUUUGCGGCAGGCUCAACGCCGCUCUGACAUUGCUCCAGCCUACGCUGUUAGAACCCGUCCCGCCCCAUCUUGUUGAAAGCCUGACCGUCGAUACUCUCCCGGCGAAUUCCCCCGA
Sen-16-2	CUGGAUUGUUCUGUCGAAUUUACCGAGCGAAAUUUCUCGCUCAGCAAAUACACCGAUGUAACAGGGCGUAAGUUACAGUGUUAUCACAUCACCAGAAACGGUUUUGCGUUCCUGGCCAUGAGUUUUA
Api-1-1	UAUCACAAAAAUUCUGAUGAAUUCGCGGAUCAUAUGUCCAAGGUCGUUGAUUCGACGACCUUAGGAAAAUCUCGUAACAAAACAAGAAUUUUUUCUCUACGUGGGGCACACUUAAUCGCCAUAUUUU
Sgl-2-9	AUAACAGCAACGAAGAUAAAUUCGCAACAGGCAUGUCAUUGGUGGUCGAAUCAACCACCAAUGGAAUAAACAAUAGUUUACGUAAAAUUAAAACCAGCAUUUUCUCCCUGCGGUGCACAUCUCGUCG
Sdy-1-3	CAAAGUACGCUUCGUUGAAGGGAAACACUUUUUCAAAGUUGUUGGUAAUGACCUUAAAGAAUUGCGGGUAGCUUUAAACUACUCACAAAAUCCCAUCUCUCCCAAAAACCGCUCCCUCAUUCUCUGG
Eco-3-10	....
Eph-2-1	CUGAACUGCUCACGUGAAUUCUUCUCCCGUAAUUUCAUUCCGGGUACUUACCACGUGUAUGGCGAUUCCCUGCGUGGUUAUUACAUCACCCUUGAUGGGCUGAUGAUGCUUCAGCUUGGGUUAAGUC
Eco-3-11	CUGAACUGCUCGCGCGAAUUUUUUACCCGCAAUUUCAUACCGGGUACUUACCACAUCUAUGACGACUCUCUGCGUGGUUAUUACAUCACCCUUGAUGGUCUGAUGCUGCUUCAGCUUGGGUUAAGUC
	....
	....
	••

Eco-3-12	CUGAACUGCUCGCGCGAAUUUUUUACCCGCAAUUUCAUACCGGGUACUUACCACAUCUAUGACGACUCUCUGCGUGGUUAUUACAUCACCCUUGAUGGUCUGAUGCUGCUUCAGCUUGGGUUAAGUC
Sdy-1-4	CUCUGGUGCGCGAUCACAUCUGCUUUUUUGCUGGUCGUAUCCGCAUGGAGGUGGCACAUGAUUAAAACCUACGAUGUGCAUAUGGAUCCCCUCGAACGCACAAGCCAGAUCAUCACACUGACAGAAG
Eco-8-2	ACACGACGCGAUUCAUGCCCGGAAAGCAUUACUUUAAAAUUGAAAAAAACGAAUUACGCGAGUUUAAGAACAGACCCGAAACAAUCGGGUUAGUUGGUAAAAAUGCCCGCUCCCUCAUCCUCUGGAC
Sdy-2-2	ACGAUGCGAUUCGUGGUAGGAAAGCAUUACUUUAAAAUUGAAAAAAACGAAUUACGCGAGUUUAAGAACAGACCCGAAACAAUCGGGUUAGUUGGUAAAAAUGCCCGCUCCCUCAUCCUCUGGACAG
Eco-10-4	AACACGACGCGAUUCGUGGUAGGAAAGCAUUACUUUAAAAUUGAAAAAAACGAAUUACGCGAGUUUAAGAACAGACCUGAAACAAUCGGGUUAGUUGGUAAAAAUGCCCGUUCCCUUAUCCUCUGGA
Sfl-2-3	ACGUUACUCUAAAGAGUUCAAAGCCGAAGCUGUCAGAACGGUUCUUGAAAAUCAACUUUCGAUCAGUGAAGGCGCUUCCCGAUUAUCCCUUCCUGAAGGCACUUUAGGACAAUGGGUUACCGCCGCC
Sen-10-2	AAAGUACGCUUCGUUGAAGGGAAACACUUUUUCAAAGUUGUUGGUAAUGACCUUAAAGAAUUGCGGGUCGCUUUAAACUACUCACAAAAUCCAGUUUCACCCAAAGCCCGCUCCCUCAUCCUCUGGA
Sdy-2-3	AAAGUACGCUUCGUUGAAGGGAAACACUUUUUCAAAGUUGUUGGUAAUGACCUUAAAGAAUUGCGGGUAGCUUUAAACUACUCACAAAAUCCCAUCUCUCCCAAAGCCCGCUCCCUCAUCCUCUGGA
Eco-9-2	CCGAGCGUUUUGUUUGUGGGAAACACUACUUCAAAUUGGAAGGAGCUGAGUUGCGGGAAUUUAAGAACAAGGUUACUCAAAGUAACUUAGUUGCACCGCGUACAAAGCACCUCAUCCUCUGGACAGA
Eph-1-1	GAAACCCUUCUUAACUUGCAGGCUGAACGUAUUCGUCUGGCGGAUAAGGCUAAUUUUCAUAUUCACCGUCUCCUGAAUAUCAGCGGAGGGCAUCGUCAUGCUUAAUCCGUUGAGCCUCAAUAUUCGC
Eco-13-2	ACAAAGUACGCUUCGUUGAAGGGAAACACUUUUUCAAAGUUGUUGGUAAUGACCUUAAAGAAUUGCGGGUAGCUUUAAACUACUCACAAAAUUUGCGGGUUACUUUAAGUAACUCACAAAAUUUGCA
Sen-15-2	ACACCACGCGAUUCGUAGUAGGAAAGCAUUACUUUAAAAUUGAAAAAAACGAAUUACGCGAGUUCAAAAACAGACCCGAAACAAUCGGGUUAGUUGGUAAAAAUGCCCGUUCCCUAAUCCUCUGGAC
env-14	CUGGAUUGUUCCCCAGAGUUUUGUGCCCGCAAUUUUGCGGAGACAUCGAUUUCGGUAAAUCAACCGAACGGUGGUACACGCAAGCUCCCUUGCUAUCAAAUCACACGAGACGGUUUUGCGUUUCUUG
Eco-9-3	CUGGAUUGUUCCCCUGAGUUUUGUGCCCGCAAUUUUGCGGAGACAUCGAUUUCGGUAAAUCAACCGAACGGUGGUACACGCAAGCUCCCUUGCUAUCAAAUCACACGAGACGGUUUUGCGUUUCUUG
Sen-2-1	CUUGAAUGCUCUGCCGAAUUUACUGAGCUGAAUUUUCAGCUCAGUGAGUACAUCGACGCAUCAGGCCGCAAACUACCUUGCUAUCAAAUAACCCGCGACGGCUUUGCUUUCCUUGCUAUGGGCUUUA
Sen-15-3	CUUGAUUGCUCUCCUGAGUUUACUGCCCGCAAUUUUGCGGUGAGUGAUUACACCGACGCAUCUGGCCGCAAACUUCCCUGCUACAACAUCACCCGCGACGGCUUUGCUUUCCUUGCUAUGGGCUUUA
Eco-11-1	....
env-15	....
Sen-9-3	CUUGAUUGCUCUCCUGAGUUUACUGCCCGCAAUUUUGCGGUGAGUGAUUACACCGACGCAUCUGGCCGCAAACUUCCCUGCUACAACAUCACCCGCGACGGCUUUGCUUUCCUUGCUAUGGGCUUUA
Eco-4-2	GCUGUUCGAAAACUUGAUGAUGAUGAGAAGGGCGUCGCUUCAACCGACACCCCUGGCGGUGAGCAAGAAUCGAUCAUCAUCUCCGAGUCAGGCCUCUACACACUGAUCCUCCGCUGCCGCGACGCAG
Eph-6-1	CUGUUCGAAAACUUGAUGAUGAUGAGAAGGGUGUCGCUUCAACCGACACCCCUGGCGGUGAGCAAGAAUCGAUCAUCAUCUCCGAGUCAGGACUCUACACACUGAUCCUCCGCUGCCGCGAUGCGGU
env-16	CUUGAAAAACUGGAUGAUGAUGAAAGGUCUAUGUUCAACAUAGGGCAUGAACAUCGUGCAAUUUUUGACAGCCGAGUAAAAGAGAUCAACAUCAUCUCCGAGUCAGGCCUCUACACACUGAUCCUCC
Sgl-1-3	UGCCGAGAUGAGGAACUUCCUCAUCCUGGUGCAGAGUUAGCUUCAGAAUUUCAAAAAAAUGACAAAAUAAUUCUUAAUAAAGAAAUACCAAUCACUGAAAUUGAAACUCAUAUUUACGGGAAAAUGA
Sgl-2-10	UGCCGAGAUGAGGAACUUCCUCAUCCUGGUGCAGAGUUAGCUUCAGAAUUUCAAAAAAAUGACAAAAUAAUUCUUAAUAAAGAAAUACCAAUAACUGAAAUUGAAACUCAUAUUUACGGGAAAAUGA
Pfl-2-5	UGUCAUGGGCACGACCAACCAGCCCGAUCCGAUGUACUUCGCCAACCCGGCCUACGACACCGAAUCCCUGCUGGCCAACGCCAGCGAAUCACUGGGCUCGGCAUCCGAGAUGCUCAACAACUUCGCC
Ppu-1-1	GGCCUACGAGCAUGUCUCGAUCCUGAUGGGCUACAUCAAGCACCUGGUGCGCGAGGGGGAUAUGGAGGACGACCAUAAAUUCCUGGGGGCUGCCGAUUACCUGUGCGACAUGGCCAAGGCGCUGAUG
Pfl-2-6	ACCAAGCCGCGCCCGAGCACGAUGUACAUGGUCACUCCUAACGUCGACACCGAAAGCCUGCUGGCCAAUGCCUGCGAGUCCCUGGCGUCGGCCAGUACCAUCGCCAGCAAUUUCGCCAAUGAGCUUU
env-17	AGUCAUUGAUUCUUCCGAAGACCUCAAAUCUGAGGGCACCGAAAAGUUCUUUCAAAAAGCCACCUACACCCUCACCAAUAACCUCGGCCACGAAGUCAAAAAACCCAUGUACCUCCUCACUCGCGAC
env-18	CUGAUGUUUUGCGCGAUAUUGAAGCAAUUAGCACGCAAGUUAUUGAAAAUCGCUAUAAACGCAAAUCUGCGUUCAUAGAAGAUUUGUUUGAAAAGGCGGAAUACGAGGUUCAUACCGGCCUGAAUAC
env-19	UGAACAAGUUAUUGAAACUUCCGAAGAAGUAAAAUCUUACUCCACCGAAAACCUCUUCCAAAAAGCUACCUACACCCUCACCAAUAACCUCGGCCACGAAGUCAAAAAACCCAUGUACCUCCUCACC
env-20	AUAAGAAUGUUUUGCGCGAUAUUGAGGAAAUUAUCACGCAAGUCAUUGAUAAUCGCUAUAAGCUCAAAUCUGAGCGCAUAGAUGAACUGUUCGAGAAGGCGGAAUACGAGGUUCAUACCGGCCUGAA
Bth-1-1	GCACUGGCCGCGAUCGCGGCGCUCGUCCGUGCGGAGGUGCGUCAUGGCUGAGCUGCGCUGCAAGAUCGGCGAUCUGGCGAUCGUCACGAAGUGCGAUGCUCGCUCGCGUAUCGGCAUGCUGGUCGAG
Bps-11-1	GCACUGUCCGCAAUUGCAGCGCUCGUUCGUGCGGAGGUGCGUCAUGGCUGACCUGCGCUGCAAGAUUGGCGAUCUGGCGAUCGUCACGCAAUGCGAUGCGCGCUCACGCAUUGGCAUGCUGGUUGAG
Rso-1-1	AGCAGAUGCCCGAAGAGGCCACCAUUGGCGACUACCGCAUCAAGUGCGAGGCCGACUGGAGGGAGGCCCAUUUGUUUCCAGAGCGAACCCUGGGUGCGUUCCUUGACGGGUUCAAUGCGGAAGUGGC
Sgl-2-11	UUGAAUGUUCAGAAGAUUUUAUCAUCCGCAACUUUUCGCGAAUGAUAAAAAAUGUACAACUUGCCAAAGGAGCAACGCGUGAGGUCGUCUACUAUGAAAUGACCAAGGACGGCUUCGUCUUCCUCGU
Bxe-1-2	UGCGGCUUCGAUAGUUAGGAAUCCGGCUAUAACGCGAGAAGAAAGACACCGACAGCGGAGAUUGCUGGAAGUGCUGGUGGACACCGUCGAGGACUACCAACGCGAAGUCGAGAAUGACCGUGAGUUA
Bph-1-2	CGCGGCUUCAAUAGUUAGGAGUCCAGCUGUGACGCGAGAAGAAAGACACCGGCAACGGAAAUUGCUGGAACUGCUGUUGGACACGGGCGAGGACUACCAACGCGAAGUCGAGAUGGACCGUGACUUA
Ppu-4-2	AAACCAUCGACGAACACUGCCGCACCCACGCCGGCGAACCGGGCCUGAGCAUGCUCAGCAACGCCGAGCACGCCUGCGAAACAUCCAGAGCCCUGAUCGAGCAUGCCUUGACUGGCCUCUACAACGC
Pen-1-2	CACGAAACCGUGAACGUCUUCCUCGACAGCCAGCCCGGCCGUGCGCGCGAUGCGAUGGGCAUGAACAUCCGCCUGCUGUGCCGCUUCAUGACGGUAUUGCACGACCAGACGAUGACGGAGGGAGCAA
Pst-1-1	GUCAGCGCCCGAUCUUUACGAUGCAGCAACGCUUCGUCACACCGCUGCCCUCGGGGUUCUUUGUGUACUCAGCAGCGCGCCAGACCUGAAUGGGCUAAGCACAUCAAACCUGCAGAGUUGCAUCCAG
Sgl-2-12	AGCCUUGAAAAUACUGGAUAGCAAUGAACGGUCUAACUUUAAGUUAGGGCGUCAGGGAGAUGCGAACAUCAUAUCCGAGUCCGGCAUGUUCACCUUGGUAUUACGCUGCCGUUACGCUGUGAAGCAA
Eph-7-1	UCAGCCCGUAUCCCGGUCAGCGAGGUGAUCGCAUGAACCAGAUUCAGUUAAACGCCCAGGGCCUGCUGGAGACGAUUGAGGAACGCCUGCUGCAGGUUGAAGCGCUUGUGGGCUCUGCUCACCGCAC
env-21	CGCUCUCUCGUUCGCGAUUACGUGCUCUCCCUUGCUGCCCGUCUGCCGGUUGCGGAGGUAUGCCAUGCAUAACACUCAGAGCGUCUCAGCGCGUCGUGAUGUUCAGGAACAUCUCAGCCCUGAGUCA
Esa-1-1	CAACUCAUUUCGGAUUAUGUGUUGUGUUUUGCCGGACGGCUUCCUGUCUGCCAGCGCCGGGAGGUACGGGCAUGAAAACGUAUCAGCAACCUGUCUGUACUCUCAGUGAACAGGGCGAAAACCGGCU
Sen-11-2	UCCCUCGUUCGCGAUUACGUCCUCUCGUUCGCUGGUCGUCUGCCGGUUGCGGAGGUGCGCGAUGAGACACACCACCAUUACCGCCCGUGACCUCGAAUGUCUGGAGCAUAUGCGCAACGUCGGCCA
Yph-1-1	GCUCGCCUACCAGUUAUUGGGGGUGCAGCAUGAUCCACGAUGUUCGGCAGCAAAUUUAAUCUCUAACUGUCAUAAAGGGAAAGUCAGCAACUUAUCUACUUAAUAGCCGCAUAUUAAGGCGGCUAUC
Sgl-2-13	GUCUGGAAUGUUCGGCUGAAUUCACUGAGCGAAAUUUUUCGCCCAGUGAAUACACCGACUCAACAGGAAGGCAACUCUCCCCUGCUACCAGAUCACCCGAGACGGCUUCGCAUUCCUGGCAAUGGGU
Sgl-2-14	CUAAAAAAAAUUCGAGCCGUUAUAGCCGAUUGUGAGCGUGACUAUCAUGUCAGCAAUUUUGCGGAGAUGUUCCAAGAGGUCGAAGUUGCCAAUGGAGCAGUGCGUAAAAGCCCGUACUUUGAACUCAC
Eco-3-13	UCCCUUGUCCGUGACUAUGUGCUCUCCCUUGCUGCCCGUCUGCCGGUGAUGGGGGUGUCCCGUGCGUAAUAAAAAAGCCCCUCAGACCGUCUCAGCGCGUCAUGACGCCCGUGAACACCUCAGCAUU
Sen-7-2	UCACUGGUCCAUGAGUAUGUGCUUUGCCUUGCUGCUCGCCUGCCUGUCCGGGAGGUGAUGUGAUGAAUCACGCCGCAAUCUCUUAUGACGAUAUUGUCCGCCUGAAGCAUCUUCGUAACGUGGGGGA
Esp-1-3	UCCCUUGUUCGUGAUUACAUCCUCUCGUUUGCUGGCCGUCUGCCGGUGGCGGAGGUGCACGCAUGAAACAUCCCGUGAUCACCCUGAAAGACCUUGAAUGCCUUGAGCACCUGCGCAACGUCGGCCA
Ybe-1-2	CGUUUAACCCGUGACUACAUCCUGUCGUUUGCUGGCCGCCUGCCACUGGGGGAAGUCGCAUGAAUACCCUUGCCGCCCGUUAUCUCAGCGAUACGCAGUACACCAUUCCUCACGCUGAUUUCUUACG
Ymo-1-1	CGUUUAACCCGUGACUACAUCCUGUCGUUUGCUGGCCGCCUGCCACUUGGGGAGGUCGCAUGAAUACCCUUGCCGCCCGUUAUGACCGUGAUACGCAGUACAGCAUCCCUCACGCUGAUUUUUUACG
Esa-1-2	CGCCUCGUUCGCAACUACGUUCUCUACUUUGCCGGUCGCCUGCCGGUUAAGGCGGUGGCGGCAUGAACACCCUAGCUGAACGCCAUAACGGCCAGUCGGCCUGCUCCAUUUCGCAUGCUGAUUACCU
Yfr-1-1	CGUUUAACCCGUGACUACAUCCUGUCGUUUGCUGGCCGUCUGCCACUGGGGGAAGUCGCAUGAAUACCCUUGCCGCCCGUUAUGACUGUGAUACGCAGUACAGCAUCCCUCACGCCGAUUUCUUACG
Sen-9-4	AAACUUGCCACUGAAUUUAUCCUGAGCUUUGCUGCCCGUAUCCCUGUGAAAAACGCCGGGGAGGUGGUUGCAUGAAAUCCAUGAAUUUUAAGCAAGGCUGCAUAUUGAGUGAACUGGGGGAAAGUCG
Sen-5-1	AAACUUGCCACUGAAUUUAUCCUGAGCUUUGCUGCCCGUAUCCCUGUGAAAAGUGCCGGGGAGGUGGUUGCAUGAAAUCCAUAAAUUUUAAGCAAGGCUGCAUAUUGAGCGAACUGGGUGAGAGUCG
Sen-7-3	UCCCUCGUUCGCGAUUACGUCCUCUCGUUUGCUGGCCGUCUGCCGGUUGCGGAGGCGCACGCAUGAGACACACCACCAUUACCGCCCGAGACCUCGAAUGUCUGGAGCAUAUGCGCAACGUCGGCCA
Sen-8-3	UCCCUCGUUCGCGAUUACGUCCUCUCGUUUGCUGGCCGUCUGCCGGUUGCGGAGGUGUGCGCAUGAGACACACCACCAUUACCGCCCGUGACCUCGAAUGUCUGGAGCAUAUGCGCAAUGUCGGCCA
Eco-13-3	UUGCUAGUCCGAGAUUAUGUACUCUCCCUUUCUGCACGGCUGCCUGCCGGGGAGGUGACGCUAUGAACCAGUUUGAGAUUUCCUACGACGAUGUUGUAAGGCUGAAACAUUUACGCAAUGUGGGUGA
env-22	UCGCUGGUCCGUGAUUAUGUGCUCUCCCUUUCUGCACGCCUGCCUGCCGGGGAGGUGAUGCUAUGAACCAGUUUGAGAUCUCCUACGACGAUGUUGUAAGGCUGAAACAUUUACGCAAUGUGGGUGA
env-23	UCGCUGGUCCGUGAUUAUGUGCUCUCCCUUUCUGCACGCCUGCCUGCCGGGGAGGUGAUGCUAUGAACCAGUUUGAGAUCUCCUACGACGAUGUUGUAAGGCUGAAACAUUUACGCAAUGUGGGUGA
	....
	....
	••

```
Sen-11-3   UCCCUUGUCCGUGACUAUGUGCUCUCCCUUGCUGCCCGUCUGCCGGUGGUGGAGGUGUCCCGUGCGUAAUAAAAAAGCCCCUCAGACCGUCUCAGCGCGUCAUGACGCCCGUGAACACCUCAGCAUU
Eco-5-2    UCCCUUGUCCGUGACUAUGUGCUCUCCCUUGCUGCCCGUCUGCCGGUGGUGGAGGUGUCCCGUGCGUAAUAAACAAGCUCCUCAGACCGUCUCAGCACGUCAUGACGCCCGUGAACACCUCAGCAUU
Eco-14-1   UCCCUUGUCCGUGACUAUGUGCUCUCCCUUGCUGCCCGUCUGCCGGUGGUGGAGGUGUCCCGUGCGUAAUAAAAAAGCCCCUCAGACCGUCUCAGCGCGUCAUGACGCCCGUGAACACCUCAGCAUU
Eco-11-2   UCCCUUGUCCGUGACUAUGUGCUCUCCCUUGCUGCCCGUCUGCCGGUGGUGGAGGUGUCCCGUGCGUAAUAAAAAAGCCCCUCAGACCGUCUCAGCGCGUCAUGACGCCCGUGAACACCUCAGCAUU
Eph-4-1    UCCCUUGUCCGUGACUAUGUGCUCUCCCUUGCUGCCCGUCUGCCGGUGGUGGAGGUGUCCCGUGCGUAAUAAAAAAGCCCCUCAGACCGUCUCAGCGCGUCAUGACGCCCGUGAACACCUCAGCAUU
Sbo-2-2    UCCCUUGUCCGUGACUAUGUGCUCUCCCUUGCUGCCCGUCUGCCGGUGGUGGAGGUGUCCCGUGCGUAAUAAAAAAGCCCCUCAGACCGUCUCAGCACGUCAUGACGCCCGUGAACACCUCAGCAUU
Eco-8-4    UCCCUUGUCCGUGACUAUGUGCUCUCCCUUGCUGCCCGUCUGCCGGUGGUGGAGGUGUCCCGUGCGUAAUAAAAAAGCCCCUCAGACCGUCUCAGCGCGUCAUGACGCCCGUGAACACCUCAGCAUU
Sgl-2-15   GUGGUUUUACUGAUGCGCAAUCUCAGGAUUAUACUAAUGCCGCGAUGACCGAUAUUCUGGGGAUGACGGUAGAAUCUUCUCAGGUGCUGGAUGUUUUCAUACGCACGAGAAUCAUAAGAAAUUGCC
Sty-1-1    GAACUGUCGGCUUUCUCCCGAAACGGUACGAACCUACCUGAAGUGAAGCCUGAAGAAUUUAAAUCUGGAAGGAUAUGAUGCACUUCGCUCUGCUGUGCUGAUGCGAACUUUCGUUUUCAUGAUUAGC
Eco-1-1    GAACUGUCGGCUUUCUCCCGAAACGGUACGAACCUACCUGAAGCGAAGCCUGAAGAAUUUAAAUCUGGAAGGAUAUGAUGCACUUCGCUCUGCUGUGCUGAUGCGAACUUUCGUUUUCAUGAUUAGC
Sen-4-4    ACUGUCGGCUUUCUCCCGAAACGGUACGAACCUACCUGAAGCGAAGCCUGAAGAAUUUAAAUCUGGAAGGAUAUGAUGCACUUCGCUCUGCUGUGCUGAUGCGAACUUUCGUUUUCAUGAUUAGCAA
Kph-1-1    AGCCCGUAUCCGGGUUAGCGGGGUGGCCGCAUGAAUCAGGUGCAGCUAAACACCCAGGGCCUGCUUGAAUCGAUUGAGGAGCGCCUGGCGCAGAUAGAAGCGUUGGUUUCCUCCGCCCAUCGGACGA
Ype-2-2    CUCGAAAUUACCGAUCCUACAGCGAAAGAACUGUUGCUAUUUAUUCUGGCUGAACGGCUGGAUUUGCUGCAACUGAUGCUGGAUGAAGUGCCGGAUGCAGAGGAGGCUAACCAUGAGUAGGUUUGUU
env-24     CUCUGGUGCGCGAUCACAUCUGCUUUUUUGCUGGUCGUAUCCGCAUGGAGGUGGCACAUGAUUAAAAACUACGAUGUGCAUAUGGAUCCCCUCGAACGCACAAGCCAGAUCAUCACACUGACAGAAG
Sen-1-1    UCUCUUGUUCGCGAUUACGUCCUCUCGUUUGCUGGCCGUCUGCCGGUUGCGGAGGAGCACGCAUGAGACACACCACCAUUACCGCCCGUGACCUCGAAUGUCUGGAGCAUAUGCGCAAUGUCGGCCA
Sen-14-2   UCCCUCAUUCGCGAUUACGUCCUCUCGUUUGCUGGCCGCCUGCCGGUUGCGGAGGUGCGCGCAUGAGACACACCACCAUUACCGCCCGUGACCUCGAAUGUCUGGAGCAUAUGCGCAAUGUCGGCCA
env-25     CAGGCGGAAGGUCUGUGCCACGGCCUGAUGCUAUUCGGCCAUUUACUGCAAAAGCACCCACAGCUGGAUACUGAAGGCUGGGAACAGCUGAAUCGCUACCUCAACGCCACGGCGCAUCUAGUUCCUG
Ppu-4-3    CGCUUACAACCAUUGCCAUCGAGUCCUCGAUGCCGUCGAAGAUGACAGCUCCCGGGAAUCGCUUGUGAAUGGGCUGCGUGCCCUGCAGAUUGCCUGGGGCCAAGCAGAUGCGCUGUCAUUGGCCUUG
Ppu-2-1    UGGUCCCCCGUGGGAUCGGGUUCACCCGCAAAGACGCCAGGACAGACUCUCGAAAAAUAUCAGGAGAGGAUCAAUGAGCACCGAAGACACCCAAUUCACCGUCGGCAAGACCACUUUCCUUCAAAGU
Pen-1-3    UGUUCCCACUUUGGCACCUGCAAUCACGCCCAUGAUCCGAUUCUCUCGGUACGUGCUGGCGUUGAUAGCGAGGAUGCGCUGGUGUGCGCGGUGGCGGCGUUGAAGGCGGCGUAUGAGACCAAUGCUG
Nmu-1-1    CGUGUCCCCCACGAUCCAGAAGGGUACUGGCACGAGGGUGUUGAUAUCGGGUUGCAAUAUUUCGCAGAAAUUGUCGCGCUCGCCGAAGUUGACGAAUACGAGGCCUGCUUAGCCCUGGAGUACGUUU
env-26     ACGCUCCCCCGCCUGCAGCACCUCACCAACCUGGCCAGCGCCGUCAGCCGCCUGCUCGGCCCCGCCGAGGAUCUCGCCUACGCGCUCGGUUCCGACCUCCACGACCUGCACCGCCAACUCACGAAAG
Avi-1-1    GCUCAAACGCAUCAAGCGCCACCCCGUCCUUUCACAAGGUAUGUCCAUCAUGACCACCCCUUCCGCCGGCGGCGAUCAGGAAACCACCUGCCUCCCCCUCGACUACCUCAACGGCUGGCUGUUCGGC
Pae-4-2    CAGGCUCGCGCACUGGAAACGCAGGAGGCGAGCCAUGCGUAAUCUCACUUUUGUUGCCGAUAAGGUCGGAAAUAACCUGAGUACCGCUAGCAUUGCACUGGAGACAAUCGCAAACCUGCUGGGCGCC
Ppu-2-2    GUACCUGCGCGGUGCCUACGACUGUGGCUACAAAGCCCUGGAGCAUCUGGAUGAGACAGGCAAAAGCCUGUUCUGGAGCAACCUAAACUCACUCGAAAUGGCAGAAGGGCUGGUUGAGGCACUGCUG
Pen-1-4    GGUCCAUGCCAGCAUGCUGGCCCGCGCCAUCCAGGAGAUCGCCGACAACUACGCCCUGCAUCACGCCCCCGAAGCUAACCGGGCGAUGAUCUGGUCGAUCCUGCAUUCGGCGGAAACGCUCAGGGCA
Pen-1-5    CUAUUUCAAGACCGAACCCAGCGACUAUCGCGACCGCCUGUUCACUGCCAGCGAUUACCUCGGGCAACUCGCCUGCGCUUUGCUCGAGCACAGGCCGGAGGUACAGCCAUGACCAGGGGGCAUACCG
Ppu-4-4    CAGCUCUACCUCGACACCGAAGCCGAAGACCAUCGCUACGCGCUGCUGGAAAACCUCGGCAUUCUCACUGAACUGCUGCGUGUCCUUGUCGCCCAUAUGGCGAUGCAAGGAGAUCAUCCAUGAGUGA
Pen-1-6    UACACCCACUGCCCACGCGAUGAACGCCUGGACGUGCUGAUGGACAGCGCCGAAAUCCUCGCUCAACUGGUCAUCGCGUUGGUCCGCCACGCCCGGGCCAAGGGGGCGCCGGUAUGAGCGACGACUU
Pen-1-7    UACACCCACUGCCCACGCGAUGAACGCCUGGACGUGCUGAUGGACAGCGCCGAAAUCCUCGCUCAACUGGUCAUCGCGUUGGUCCGCCACGCCCGGGCCAAGGGGGCGCCGGUAUGAGCGACGACUU
Ppu-4-5    AGCUCUACCUCAGCACCGGGGCCGAAGACCAACGCAAUGUGUUGCUGGAUAACAUGGGCAUCCUCACCGAGCUCCUGCGGGCCCUUUUCGGCCAUAUGAGCACGUUGGAGAGGGUGCAUGAGUGAUG
Ppu-3-1    AUCCUGAUGGGCUACAUCAAGCACCUGGUGCGCGAGGGCGAUAUGGAGGACGACCAUAAAUUCCUGGGGGCAGCCGAUACCUGUGCGACAUGGCCAAGGCGCUGAUGAACGAUAUCGAGAUCGCCAA
Ppu-3-2    CUGGACGAAUACAUCUGCGCCAAUGCCGGUGAACCCGGUAUCAACAUGCUGGUCAACGCAGUGCAUCACGUGCAGAUGACCAGAGCGCUGGCCGAGUUGCUGCUACACCGGCAGACCGGGGAAAUCA
Ppu-4-6    GAAACCAUCGACGAACACUGCCGCGCCCGCGCAGGCGAACCUGGCCUGAACAUGCUGAGCAAUGCCAUGCAUGCCAGCGAGAUCGCCCAUUCGCUGGUCGAGCACGUGCACGCUCGGCUUUUUUGCC
Ppu-1-2    UCCUGCUGGGCUACUGCAAGCACCUGGUACGCGAAGGUGACAUGGAAGACGACCACAAGAUGCUGGGCGCAGCGGACUACCUGCUGGCCACCGCAAAAGCCCUGAUGAACGACAUCGAGAUUGCGAA
Ppu-4-7    GACGACCACUGCCGUGACCACGUAGGCGAGCCCGGCCUGAACCUGCUGGCAAACGCCAACCAUGCGGCGUAUUCGGCCUAUGUGCUGAUCCAGCAUGCGAAGGGGCGUUUGGACGAUGCGCAGGAUG
Ppu-3-3    UCCUGCUGGGCUACUGCAAGCACCUGGUACGCGAAGGUGACAUGGAAGACGACCACAAGAUGCUGGGCGCAGCGGAUUACCUGCUGGCCACCGCAAAAGCCCUGAUGAACGACAUCGAGAUUGCGAA
Ppu-4-8    AUGGCGCGUGCUCGCUUCUUUGAGUCUUUGCUGGUUGGGGUGCCCAAUGAAGCUCCUUACUACUGACCACACGUCUUUCUUCGCUCCACCACACGCAUUCCGGAAAAAUGACCUCUUUUCGGUGAA
Ppu-4-9    GGAUAAAUUCAUCGGGUCGCAACCAAUUGUGCGACCGGGUUUGGCGACCCGUAUGAAAAGGCGCAACGGCGCCCCGAUCACGAUUGCAGGCGCUUUUUUGCGUCCGCUGUCUCGUGUAAUGGCGGC
env-27     CUAGAGUCUGCCGGGGGUGUGCAAUGAAUACUCCCGCCCGCACCAUCCAAGAGAUUGCCCACGACACGCUUGAGGAUCUCGAAAGCGCCCAGAUCGCCCUGCGCAAGGUUGAGGCCGUUCUCUAUGC
Pen-1-8    CAUGACUGACCACGAACGGCUGUCUUCCAUCCAGAGCUACGUCUGGACCCUCGAAUUGCUCGGCGAGGCGCUGGUCCAGCAUGACGAGAUGCUGGAAUGCGAGCACAACCCGCAGCUGAGCUUCCGC
Ppu-1-3    GAACGCCUCUCCACCAUCCAAAGCUACGCCUGGACCCUCGAACUGCUAGGCGAGGCCCUGGUCCAGCACGACGAAAUGCUCGAGUGCGAACACAACCCGCGGCUGAGUUUUCGCAACACGGCCGGUA
Ppu-4-10   GCCGAAACCAUCGACGAACACUGCCGCACCCACGCCGGCGAGCCGGGCCUGAGCAUGCUCAGCAACGCCGAGCAUGCCUGCGAGACGUCCAGAGCCCUGAUCGAGCAUGCCUUGACUGGCCUCUACA
Ppu-4-11   CAGACCUCCAGCGUGCUGCUCGACAGCCCGCAAGGCCCUGUGCGUGACGCCAUGGGCAUGAACAUCCGCCUGCUGUGCCGGAUGAUCAACGCGCUGAACGAGCACGCCGGUGCGCAAGGAGCAUCCC
Pen-1-9    AUGUCUACUUCAAGACCGAACCCGGCGCCUACCGCGACCGCCUGUUCACCGCCAGCGACUACCUCGGCCAGCUCGCCUGCGCGCUGCUCGAACACAAGCCAGGAGCGCAGCCAUGACCGUUACCCCA
Pen-1-10   GGCGUUCAAACCCGAAGACCGCCCCGAAGUGCUGGUGGAUAACCUCUGCAUCCUCGCCCAACUGCUCCAAUGCCUGUUCGAACACGCCAGGUCGCAGGAGGUGGCGCCAUGAGCGAAAAGCCCGAAC
Pfl-2-7    UACCUCAACCCCAAAGCUGAAAAAUCACGCAACUCCGUGGAUCGUGGCAUGCAGAUGUUCAAAGUCGAUCCGGACAUCAACCCGGAAGCCAUCGCCAUCCAGACCUACGAAACCUUUUCCUCGGUCA
Xor-1-1    GCAAUCCACGCCCUCACACUUGCCGACGUAGCUGCAGCCAUCGCUGAUGUCGACGCGUUGCUGAGCGAGGCGCAACGAUGAACGCCAACAAGCAGUUUCGCGUCUGCGCUGGCGUCGUCCUCAGCUU
env-28     AUGGAUUGGCUCUGGCUGUGCUUUGGAACGCACGGCAUGAUCGCCCUCACCUUCUGGUUCGGCUCGUUGUUCGCGAACCAGAUCCGCAGCGCCCACAAGUCCUUCCCUUUCCUCGAAGCCACGGGCG
Xca-1-1    AAGGCUAUCCAAGCCCUCACGCUUGCCGACGUUGAAGCCGCGAUUGCUGCCGUUGAUACCGCGUUUGCGGAGGCGCAGCGAUGAGUGCCAACAAGCAAUUUCGCGUCUGCCCAGGCGUCAUCCUCAG
Sma-1-5    UGGCCGGAGCGGCAUCAACUGCCUGGGCGUUGCCUUCAGCUGGCGGAUGCGGAUUGCGCGCUGGCGGGGCUGCGUGUGGUGGCGGAGCUGAUGUUGGCUGCGGAGCGGACUCGGCAAAAUGGUGCGC
Bdo-1-1    CGACAUUCAACGUGCGUCCGACGCGAUUCGAACGAUUUCCCGUCUUGUGCACAACAGCCUCUGCGAGCCGGCCAUGUCAGGUGCCGAGCCGCUCGGUUUGUCGGCACAUCUCGGGCUGAUGAACGCG
Pfl-2-8    AAAAUGCCCCGGCCGUAAACCGAGCCAGAUGUUUCAGAUCUCACCGGACAUGGACGACGAAAGCCUGCUGGCCCACGCCUGCGAAUCCCUGGCCUCGGCCAGCGUCAUGGCCAGUGAUGUGGCGGCC
Pfl-1-2    AAGCCUCUCCCGCGCAGAGUUCAAAAGUGCUGACGACACCGAUCUGCAACAUAUCUCCAACGUAGCGGCCAUUCUGCUGCGUGACAGUUGCGACCUGUUGGGAGUGAUGGGCUGGCGGCUUCAAGCC
Ppu-4-12   AUCCUUGCGGGCGACCGUGACAGUUCAGCAUGAUUGUCGCGCCCUAGCGCGCUAUGGCGUGCUGUCGUGACAACUUCUACGGCUCAUGAUGCACUUUCGGACCACACUGAACCUUGCGGAAGCUGGC
Pen-1-11   GCGCUCUACCUGCGCUGUGCCUACGACACCGGCAUCAAGGCACUCGACCACCUGCGCGACGAGGGGCGUGGCAUGUACUGGUCGAGCCUGCAUUCGCUGGAAAUGGCGGAGGGGUUGGUGGAGGCGA
Ppu-3-4    UACCUGCGCAGCGCUUACGAAACCGGCUACAAAGCUUUGGACUACAUGCGUGAAGAAGGCCGUGGCAUGUUCUGGUCGAACCUGCAUGCGAUUGAGAUGGCAGAGGGUGUGAUCGAGGCGAUACUCG
Ppu-2-3    UACCUGCGCAGCGCCUACGAAACCGGGUACAAAGCCCUGGACUACAUGCGUGAAGAAGGCCGUGGCAUGUUCUGGUCGAACCUGCAUGCGAUUGAAAUGGCGGAGGGUGUGAUUGAGGCGAUACUCG
Ppu-4-13   CUACCUGCGCUGUGCCUACGAGACUGGCUACAAGACCCUCGAUUACCUGCGCGAAGAAGGCCGCGGCAUGUACUGGUCGAACCUGCAUGCGAUCGAGAUGGCGGAGGGGUUGGUAGAGGCGAUGCUU
Ppu-1-4    GUACCUGCGCAGCGCUUACGAAACCGGCUACAAAGCUUUGGACUACAUGCGUGAAGAAGGCCGUGGCAUGUUCUGGUCGAACCUGCAUGCGAUUGAGAUGGCAGAGGGUGUGAUCGAGGCGAUACUC
env-29     GUACCUGCGCAGCGCUUACGAAACCGGCUACAAAGCUUUGGACUACAUGCGUGAAGAAGGCCGUGGCAUGUUCUGGUCGAACCUGCAUGCGAUUGAAAUGGCAGAGGGUGUGAUCGAGGCGAUACUC
Psy-3-1    ACCGGCGCGCAGCGAGACAUGACGCUGACGAUCAUGCACCUGGUGGAAAUGGCAAAAGUCAUGGUCGACAACACGAUAGAAAACCUGCAAACCCAAUGACCACGACCUGAGCUGAACUGAAAACGAC

           ...............................................................................................................................
           ...............................................................................................................................
           •••••••••••••••••••••••••••••••••••••••••••••••••••••••••••••••••••••••••••••••••••••••••••••••••••••••••••••••••••••••••••••••
```

```
Psy-1-2    ACCGGCGCGCAAAGAGAUAUGACGCUCACUAUCAUGCACCUGGUGGAAAUGGCAAAGGUCAUGGUCGACAACACAAUAGAAAACCUGCAAAUCGAAUAAGCGCGACCUGAACUGAAAACGACGUACC
Psy-1-3    GGCGACAGCACUCAGGGCACCAGCCGUGAUCUGGCGUUUUCGGUAUUGCACAUGGCCGAGAUGGCCAAAGCCAUGGUUGACCGCUCACUGGAAUGCAUCGUUUGACCAGAUGACAACCCGGGGCCAC
Pfl-2-9    ACCGCUGCUCAAACCCAACGCCCGCUACCUCAUCGCCCCCGGCAUCGACAGCGAAGAACUGCUGGCCGAUGCCUGCGAGACGCUGACCUCUGCCAAAGUCAUGGCGACCGACUUCGCCGGACUGGUG
Pfl-2-10   ACAUCCAAGCCAUCGCGCAAGCCCGGCAGGAUUUAUCUGGUCAAUCCAACGGUGGAUGACGAGACGCUGCUGGUCGAAGCGUGCGAAACGCUGUCGUCGGCCAGUGACAUGGCCCGGGUUAUUGGCG
Pfl-2-11   CAUGGGCAGCCACCACAAACACGACCCCAUGUACAUGGCCAACCCGGCGUACAACACCGAAUCCCUGCUGGCCAACGCCAGCGAAUCCCUGGGAUCGGCCAGCGAAAUGCUCAACAACUUUGCGGCC
Pfl-2-12   AUGUCGUCCUUGAACGAGCCCGAGCGGAUGUACCUGGCCAAUCCGAAAUACAACACCGAAUGCCUGCUGGCCAACGCCAGCGAAAACCUCGGUGCAGCCAGUGAGAUGCUGAGCAAUUUCGCGGCCA
env-30     GCCACGCCCUACACACCCAACGCCAUGUUCCUGGCCAACCCCAAAAUCGCCACCGAGGAAUUGCUGGCGAACGCCAGUGAAUCACUGGGUUCGGCAACGGUGAUGUUGGGGGAUGUGGCCGCGCUGG
Psy-2-1    GGCGACAGCACCCAAGGCACCAGCCGUGACCUGGCGUUUUCGGUGUUGCACAUGGCCGAGAUGGCCAAAGCCAUGGUUGAUCGUUCACUGGAAUGCAUCGUUUGAGCAGAUGACAACCGGGGCCGCG
Pfl-2-13   GGGCAGCCAUCACAAACACGACCCCAUGUACCUGGCCAACCCGGCCUACAACACCGAAUCCCUCCUGGCCAACGCCAGCGAAUCACUGGGGUCGGCCAGCGAAAUGCUCAACAACUUUGCGGCGACU
Psy-2-2    ACCGGCACCCAGCGCGACAUGACGCUGUCGAUCAUGCACCUGGUGGAAAUGGCCAAGGUCAUGGUCGACAGAACCAUAGACAACCUGCAAGCCGAAUGACCGCAUGAACUGAAAACGACGCACUUGU
Pfl-2-14   AACCGCUUGCUCGAGUUCGCAGCAAGGCUGAAGCCCUGCACCAUAGCGAUGGAGUCUUGUGGCGGUUCACACUGGCUUGCCAGAAAGCUCUUGUCUUUCGGUCAUCAAGUGAAAUUGAUUGCGCCGC
Psy-3-2    CCUGGAAGCGACGUUGGUGCAUGCGCUGGAUUUCCUGCGUUGCGCAUCGGCCACCGCUUACGAACUCGGCGAUGAAUUGACCGGCUCACAACGCGAUCUGGCCUUCGCCUCCAUGCACAUGGUGGAG
Pfl-2-15   UCAGCCCACAUGAUGGCCGCGCCCUACUACCCCAACCGCCUCUACCAGGCCAACCCGGCCAGCAACAACGAAUCCCUGCUCGCCGACGCCGUCGAAACCCUGGGCUCGGCCAACGUCAUGCUCAACA
Avi-1-2    AUGACCAAGGCCCGCGCCCUGGAAGCGCAGGAGGUGCUGGCAUGAGCACCAUCGUCCGCACUACCGCCGCCAUCGCCGACAAGGCCCUGGAAACCAUCCGCUACGGCAAGGAUAACGUCCGCUGGUU
Ppu-2-4    GUCCACGCUGCUCAAGGGGGCGUUUGCGACCAACCUCAAGGCGUUGGAGUUGGCCAAGGGGACUUGUCGUGAUCUGUUGUUGAGCAAUGACCAUGGGCUGGAUUCGGCAAAGGCGGUGGUGGAGGCU
Pen-1-12   GUUGCUCGAUCCCAUGCAGAACCUCAGUGGCCCUGCGGCUCGCCCGGUUCUUCGCGCCGAUGCGCCCACUGGUACAGCGCCGGCAACACCAGCAAGGUCAACGCCGUCGACGACAGGAUGCCGCCGA
Pfl-2-16   GCCGCCCCGAAACAACACUGCCCCGGCCGCAUGUUCCUCGUCGCCCCCGACCUGGAUCAGCAUGCCCUGCUAGCCCACGCCUGCGAAUCGAUGACUUCGGCCAGCGUGAUGCUCAGCGACUUCGCCG
Pfl-2-17   UGCCCCGGCGCCCACAUCAUGGGCAGCGUCAACGAACCCGCCCCGAUGUACCUCGCCAACCCGGCCUACAACACCGAAUCCCUGCUCGCCAACGCCAGCGAAUCCCUGGGCUCGGCCAGCGAAAUGC
Pfl-2-18   UGCCCCGGUCGCCGACCGAGCCAGAUGUUCCAGAUCUCGCCAGACAUGGACGAUGAAAGCCUGUUGGCCCAUGCCUGUGAGUCCCUGGCUACCGCAAGUGUCAUGGCCAGUGAUGUAGCGGCUUUUG
Pfl-1-3    CCAACCUGCUGGAAACCCUCUGCUGCCUGAAUUUCAGCCACGGCGAUGUGAAGGACAUCCCGCACAUCAUCGACGUGCUCUAUGUACUGACCCAGGAUGGUUGCGACCUGCUGGAGGCGGCGAAAUG
Pen-1-13   GACGCCCACCCACCCAUGUUCUCGGUACACGCCGGUAUCGACGGCGAGGAUGCGCUGGUAUGUGCCGUGGCCGCGCUGCGGGCGGCCUAUGAAACCAAUGCGAUGGCCUUGGAAAGGGCGGGCGAGC
Pfl-2-19   GCAGUGCUCAAAUACCUCGACCUCAUGGCCUGCAUCCACCUCCCAGACCACGCCGAACAAGACAUCAACAUAGUUACCACUACAGCCAGAAUCCUGGUUCAGGACGUGCGCGAUGUAUUUAGAGUGA
Pfl-2-20   CAGCGCCCACAUCAUGGCCGCGCCCUACAAACCCAACACCCUCUACCAGGCUCACCCCGAAGCCGACACCGAAUCCCUGCUGGUCGACGCCAGCGAAUCCCUCGGCUCCGCCACCGUCAUGCUCAAC
Pfl-2-21   UUCGCACCACCCCCGGCGUCCCGGUCGCCACCGCCCUCUCCCACGCAUCCCAUCUGCUGCACAUUUCCAAACUGCUCACCUCAGAUACCUCUGCCGCCAACGAUCCAGAACUCCACGCCUGCGCCUC
Pfl-1-4    GCCAAGAAACUCACCCUGGAUGCGGCCAUGGAAGACCAGGGCGAGCGCUACGCCUGGGCCGCGCAUUUUCUGGCGGAAAUGGGCAAGGCGGUGAUCGAUGACGUGGGAUUGGGAAUGAAGGAAGAGG
env-31     GCAAACACCGCGACGUGGCUCUCGCCAUUCAUCAACUCAGCGAGUUAUCAGUCUUGAUGGUCGGCAAAGCCAUGGACCAGCAAACACCACGCACCUAAAGCCUAACUCGGUUAACCCUGUGGG....
env-32     CGAUGCCAGCAACGAAACCCUGAUCACCAACAGCUACGAAACCUUUUCCUCGGUCACCGCCUUACUGCUCGACCUGUCAGAAGACUUGACCGGAAAACAGCGCGACGUGGCGCUGGCGAUUCACCAG
env-33     CUGCUCUACACCGCCAGCUUGCUCGACAGCGCUUCGGCCACCACGCUCAAUUGCAGUGAGAUGCUGGCCAGCCCCGAACGGGCGAAGAUUCUGGCGGUGUGGCAUUUGCUGGAGAUGGCCAAGACCA
env-34     GGCCCUGGCCAUCCAUCAAUUGAGUGAGCUCGGCGUAAUGCUGGUCGGCCAGAUGAUGGACCGCGAAUCCCCGUAGCCUCAAGGUAAACGCGGACAANNNNNNNNNNNNNNNNNNNNNNNNNNNN
Pfl-2-22   CGGUCAACGAACCCGACCGCAUGUACUUCGCCAACCCCGCCUACAACACCGAAUCCCUGCUGGCCAACGCCAGCGAAACCCUCGGCUCCGCCGGCGAAAUACUCAACAACUUCGCCGCCACCCUCAC
env-35     CUUCUCAACAUCGCUACCUGGCCUUGGCCAUUCAGCAAAUGAUCGAACUGGGGCUGAUGCUGGUAGAAGCGGCGCAAGACCGCGUGGACCGCGCCGCGUAGGUUUNNNNNNNNNNNNNNNNNNNNN
Pfl-1-5    AACUUCGCGAUUUCUGAUGACGCGCCCAAAGGUGCAGUGGUGCUGCAGGAACUGUCGCUGGUGUGCUGCAUUUUGCUGCGCGAUGGAUGUGAUUUGAUAGAUGUGACGACAAGGCGAAUGCAGGAGC
Psy-1-4    GAUCUUGCGUUCUCGGUGGUGCAUCUGAUCAACAUGGCCAAGGCCGAGCUGGAGCGCUCGCUGGAAUGUGUUCAGAGCCACUGACAGCCCCUGACGAACCUAGCAGUUCUACGAGUAGCCGCUGCGG
Psy-2-3    GAUCUGGCGUUCUCGGUGGUGCAUCUGAUCAAUAUGGCCAAGGCCGAGCUGGAGCGCUCGCUGGAAUGUGUUCAGAACCCUGACAGCCCUGAUGAAACCUAGCAGUUCUACGAGUAGCCGCUAAGGU
Pen-1-14   CCUGACCAUGAACGCCUGUCCACCCUCCAGAGCUACCUCUGGACCCUCGAACUGCUCGGCGAGGCGCUGGUGCAGCAUGACGAGGUGCUGGAGUGCGAGCACAACCCGCAGCUGAGCUUUCGCAACA
env-36     CCUGAACCCUGCGCUACUGCGCAAGCUCGAUCCCGACCAACAACACACCCUGGCGCUGAUCACUCAUGGCCACUGUGCGCCGACGUUGAUGGUCAGUGAGUCUGGCGUCUACGCGCUGCUUAUCUAC
Pfl-2-23   CCGCUGCUCAAACCCAACACCCGCUACCUCAUCGCACCCGGCAUCGACAGCGAAGAACUGCUGGCCGACGCCUGCGAAACCCUGACUUCGGCCAAAAUCAUGGCCACCGACUUCGCCGGGCUGGUGG
Pfl-2-24   CCACCGCGCAAGACCCGCAAAAUGUACGCCGUCACCGCGGACUUCAAAAACGAAGAGCUGCUGGUCGAUGCCUGCGAAACCCUGGCUUCAGCCAGAACCAUCGUCAAUGACUUCACCCACCUCAUGC
Pfl-2-25   CCACCGCGCAAAACCCGCAGAAUGUACGCCGUCACCGCAGACUUCAAAAACGAAGAACUGCUGGCCGAUGCCUGCGAAACCCUGGCCUCAGCCAGGACCAUCGUCAAUGACUUCACCCACCUCAUGC
Psy-2-4    UGGGCAGGGCGCUGGAUGAGCGCGCCACGCUCAAGCUCGAUGCGGAUCAGCGUCGCGAGGUCUGGCUGGAAGCUCAUGGCGAGUGCCAGCGGCAGUUGAUGAUCAGCGAGUCUGGCGUGCUGGCGCU
env-37     AACCUGGCCUUCCGCGAACAGCUCAGCAGCGAUGCUGUGGUGCUUCAAGACUUCGCCCAACUACUGGCUAUACCGCUGCGGGAUGGGUGUGAUUUGCUGGAUGUCAUUAUUCGCAGGCUCAAUGCCG
env-38     AAAACCUGGCGUUUCGCGAAGGCCUGAACACCGACAACCUCGUGCUCCAGGACUUCGCCCUGCUCUGCGCCAUUCCGUUGCGUGAUGGCUGUGAUUUGCUGGAUGUGAUCAGCCGACGCCUACAGGC
env-39     ..................................................................................................................................
Pfl-1-6    CCCUGGAAAGCGCAGGGGGUGCCCAAUGAGCACUCAGCGCAGCCUUGCCCUGCCCGAAGACCUGCUGUGCGUCAGCAAUGACGUUGAAGCCGGUAUCCCUAUCGACGCCAUCACCUGCGCGAUUGAG
Ppu-3-5    GAACGCCUCUCCACCAUCCAAAGCUACGCCUGGACCCUCGAACUGCUAGGCGAGGCCCUGGUCCAGCACGACGAAAUGCUCGAGUGCGAACACAACCCGCGGCUGAGUUUCGCAACACGGCCGGUA
env-40     GAACGCCUCUCCACCAUCCAAAGCUACGCCUGGACCCUGGAACUGCUAGGCGAGGCCCUGGUCCAGCACGACGAAAUGCUCGAGUGCGAACACAACCCGCGGCUGAGCUUCCGCAACACGGCCGGUA
Pen-1-15   UGACUGACCAUGAACGCCUGUCUACCCUCCAGAGCUACGUCUAGACUCUCGAAUUGCUCGGUGAGGCGCUGGUCCAGCAUGACGAGGUGCUGGAGUGCGAGCACAACCCGCAGUUGAGCUUUCGUAA
Ppu-4-14   CCCAUGACUGACCAGGAACGCUUGUCCACAAUCCAGAGCUACGCCUGGACUCUCGAACUGCUCGGCGAAGCCUUGGUGCAGCAUGACGAGAUGCUCGAGUGUGAACACAACCCUCACUUGAGCUUCC
Pen-1-16   UGCCUGACCAUGAACGCCUGUCUACCCUCCAGAGCUACGUCUGGACCCUCGAACUGCUCGGUGAGGCGCUGGUGCAGCAUGACGAGGUGCUGGAGUGCGAGCACAACCCGCAGCUGAGCUUUCGCAA
Ppu-4-15   UUCUCGGUGCGCACCUGAAUCAGGUAAAACGCAUUGCCCUUGUCAUCGGUCACGGUAUCGGCACUGAUCAACUCAAGCUUGGCCUUCAUGCCGCCAUAAAUGGUGUAGUCAUACGCGCUGAACUUCA
env-41     ..................................................................................................................................
Psy-3-3    GGCUGAUGGGCAAGGCGCUGGACCAGCGCGCCACGCUCAAGCUUGAUGCGGAUCAGCGUCGCGAGGUCUGGUUGCAGGCCAAUGGCGAGUGCCAGCGGCAAUUGAUGAUCAGCGAGUCCGGCACGCU
Pae-3-1    GAUAUGGCCUAUGCCGUGUCGAUGGUCUCCGAACUGGAACGGCAAGCCGUCAUCGGGGGUGCCAAAUGACCGGCCUGACCUCAACUCAAGUGAUCGAUAACGCCCGCCAGCGGAUCGAAGCCGCGCA
Ppu-4-16   UGUCCGUGAUGAUCAUGACCUCGAUGCACGAGCUUGAUGCCCUGCGCAAAUUGCUCGAGUCAGCACUGGCCCAAAUACAAAAGCCAGCCGACCCGCAGACGCUGCAUUAAUCCUCAUCAUGAUCUGC
Pfl-1-7    AUCUGAUGGACACGCUGUCCACCCUUAACCUGAAAGACAUUGCCGACUGCGAUUUGACGCGGGUCACCCGAGGGGUGCAUUUGCUCACCCGGGAGGGUUGCGCGGUGCUGGAGGUGAUUCAGUGGCG
Pfl-2-26   CGAUCUGGCAGACACGUUUUCCAGUCUCGACCUCAAGGGCAUUUCCGAUUGUGAUCUGACGCGCAUUACCAGCGCGGUGCAUCUGCUGAUGCGAGAGGGCUGCGCCAUUCUGAACGUGAUUCAGACG
Pfl-2-27   CCCCACGCAGAACCCGCGGGAUGUAUGCCGUGACGGCGGACAACAAAAACGAAGAACUGCUGAUUGAUGCCAGUGAAACACUCGCUUCGGCCCAAACCAUUGCCCAGAACAUCGCCAGUCUGGUGCC
env-42     GACAACCUUAGCUUCGAAGACGCCCUCGCCCAUGCGGCCGACUUGCUGCAUUGCGCGGUGGAGACGGCGCACGGGUCGGCUGAAGCGAUGAAUGCGCAACAGCGAGCGGUGAUGCAUUUGGUGGGGA
env-43     ..................................................................................................................................
Pfl-1-8    ACGCCCAGGGCCACCCGCUGUUCGCCGUACAGCCCCAGGUGAAUGCCGAAGACGCCCUGAUGCACGUUUCCUUGCUGCUCAAAUGCGCCGAAGAGGUGUCCGAUGAAAUCACCGAACGGGCCAGCGG
Avi-1-3    AUGGCCAAGGCCCGCGCCCUGGAAGCCAAGGCAAACGCCACGCCGACCCGCAACCCUCGCACCGCGCUGUCCUGGAUCAAGUCCGGCCAGUCCGUCCGCAUCGAUGCCCUGAACCUGCCCGACCAUC
Psy-1-5    UUCGACUUCAUCAGCCUGGAAGCAACCCUGGUGCAUGCGCUGGAUUUCCUGCGUUGUGCAUCGGCUACUGCCCACGAACUGGGCGAUGAAUUGGCCGGUGCGCAACGCGACCUGGCGUUUGCCUCCA

           ..................................................................................................................................
           ..................................................................................................................................
```

Psy-2-5	GUUCGACUUCAUCAGCCUGGAAGCCACCCUGGUGCACGCGCUGGAUUUCCUGCGCUGCGCAUCGGCCACCGCUCACGAACUCGGCGAUGAACUGACCGGUUCGCAGCGCGAUCUGGCGUUCGCUUCC
Pfl-2-28	CCCAAAACGCCUGGGUGGCCUGUUCGCCCUCUGCCCCAACGCCGACGCCGAAGCGCUCAUGGCCAACGCCUCCGAAGACCUCCUGUCCAUCAGCGCCUUCGCCGCCAACCUGGCCGACGACCUCGAC
env-44	AUGACAA...
Pfl-1-9	CAAACCCGUUUCCCCCGACUCGCUGUUCAGCGUGCGCGAAGGCGUCAGCGCCGAAGAGGCCUUGGUGCAUGUGGCGCUGCUGCUCAAAUGCGCCGAAGAGGUCUGCGAUGAAAUCACCCAACAGGGC
Ppu-4-17	GCCCUGAUGAUGGCGUCAACACAUGAACUCGAGUCGCUGCGUGUGCUGCUCGAGGCGGCAUUGAUUCAGGUCCAGAUGCCGGCUGAGCCGCGAACGCUGCACUAAAUCGAUAACCCUGUGUUGCCUG
Ppu-4-18	GGUUCUGAUGAUGGCCUCGAUGCAUGAGCUCGAGUCGCUACGCGUGCUGCUCGAAUCAGCCCUGAUCCAGCUGCAGAUGCCGCCCGAGCCGCGCACUCUGCACUAGGCCGUUAAGCCUGUUUUGCCU
Pen-1-17	CGAUUCUGGUCAUGGCCGCGAUGCAUGAAAUGGAGGCGCUGCGGGUGCUGCUCGAGUCGGCGUUGAUUCAGGUGCAGAUGCCGCAGGUGGAGCCUCGGCCGUUGCAUUGAGCCGGUAUGUCGUGAGU
Pen-1-18	AUUCUGGUCAUGGCUGCGAUGCAUGAAAUGGAGGCGCUGCGGGUGCUGCUUGAGUCGGCGUUGAUUCAGGUGCAGAUGCCGCAGGUGGAGCCUCGGACGUUGCAUUAAGUCGGUAUGUAUGGAAAAG
Ppu-4-19	GGCCCUGAUGAUGGCGUCAACACAUGAACUCGAGUCGCUGCGUGUGCUGCUCGAGGCGGCAUUGAUUCAGGUCCAGAUGCCGGCUGAGCCGCGAACGCUGCACUAAAUCGAUAACCCUGUGUUGCCU
Ppu-4-20	CGGUUCUGAUGAUGGCCUCGAUGCAUGAGCUCGAGUCGCUACGCGUGCUGCUCGAAUCAGCCCUGAUCCAGCUGCAGAUGCCGCCCGAGCCGCGCACUCUGCACUAGGCCGUUAAGCCUGUUUUGCC
Pen-1-19	GAUCCUGAUGAUGGCGUCGAUGCACGAAGUCGAAUCGCUGCGCGUGCUGCUCGAAUCGGCGUUGAUCCAGGUGCAGAUGCCCAAGGCCCCACAGACGCUGCACUAGCCCGCGCCCGGCCCUGUUCGC
Pen-1-20	CGAUCCUGAUGAUGGCGUCGAUCCACGAAGUCGAAUCGCUGCGCGUGCUGCUUGAGUCGGCGUUGAUCCAGGUGCAGAUGCCCAAGGCUCCGCAGACGCUGCAUUAGGCCGCGCCCGGCCCUGUUCG
Pen-1-21	ACAAUCCUGAUGAUGGCGUCGAUCCACGAAGUCGAAUCGCUGCGCGUGCUGCUCGAAUCGGCGUUGAUCCAGGUGCAGAUGCCCAAGGCUCCGCAGACGCUGCAUUAGGUGGCGCCCGGCCCUGUUC
Ppu-3-6	CGGUUUUGAUCAUGGCCUCGAUGCACGAGCUUGAAACCCUGCGGCGGCUACUCGAAUCCGCGCUGACGCAAAUACAGCAAACAACCAACCCGCAACCGAUGCAUUGAUUCGCAUACCGAGCGGAAAA
Pen-1-22	CGAUUCUGGUCAUGGCCGCGAUGCAUGAAAUGGAGGCGCUUCGGGUGCUGCUCGAAUCGGCGUUGAUUCAGGUGCAGAUGCCGCAGGUGGAGCCUCGGACGUUGCAUUAAGCCGGUAUGUCGUGAGU
Ppu-3-7	GCUGACUUGAUUGGAGAAUCCUUGGUUCUUUGCACGCAAUGAUGGGAUUGGGGACUUGAACGCAUUAGAGCAACAAAUCAAUUAGUUACAAGACUUUAUAUGGGCAGCAAGUUCAGAUAGUAUCUU
Ppu-1-5	GCGUCGAUUUUGAUGAUGACCUCGAUGCAUGAACUGGAGACCUUGAGAGCUUUGCUCGAGUCCGCAAUAGCCCAAGUGCAGAUGCCCAGUGAGCCUCGGACUAUGCAUUGACGUGUCGCUGAUGUAC
Ppu-1-6	GUUUCGAUCCUGAUGAUGACCUCGAUGCAUGAGCUGGAGGCGCUGCGGGCUCUGCUUGAGUCGGCACUGGUUCAGGUGCAGAUGCACGCUGAGCCCAGGACUUCGCACUAAGGCUGGUUAAUGUGCC
Pen-1-23	GCCACGAUUCUGAUGAUGGCGUCGAUCCACGAAGUCGAAUCGCUGCGUGUGCUGCUCGAAUCGGCGUUGAUCCAGGUGCAGAUGUCGGCACAGCCGCAGGCGCUGCACUAGGCCCUUGCCGGCCCUG
Pen-1-24	GCCACGAUCCUGAUGAUGGCGUCGAUCCACGAAGUCGAAUCGCUGCGGUUGCUGCUCGAAUCGGCGUUGAUCCAGGUGCAGAUGUCGGCGCAGCCGCAGACGCUGCACUAAGCCCUUGCCGGCCCUG
Pen-1-25	AUCCUGAUGAUGGCGUCGAUGCACGAAGUCGAAUCGCUGCGCGUGCUGCUCGAAUCAGCGUUGAUCCAGGUCCAGAUGCCAGCGCAGCCGAGGACGCUGCACUAAGCCCUUGCCGGCCCUGUUCGCC
Ppu-4-21	GAUCCUGAUGAUGACCACGAUGCAUGAACUGGAGGCGCUGCGGGCUUUGCUUGAGUCUGCAUUGGUUCAGGUGCAGAUGCCCGCUGAGGCCAGACCUGUGCAUUGAGGCUGCGUAAUGUGUUGCCUG
Ppu-4-22	UGUCGUUGUUGAUCAUGACCUCAAUGCACGAGCUCGAGACGCUACGCAAAUUGCUCGAAGCGGCGCUGAUCCAGAUCCAGAAACCCGCCGAUCCGCAAACGCUGCACUGACCCGCAUCAUGAUCUGC
Ppu-4-23	CGCACAACUCGAUCGUGAUGCGGGCGGUCAUCCAUGAAAUGGAAGUUGCGCAUGCGCUGCUGGAAUCGGCGCUGAUGCAACUGCACAGGCAAGCGGAUUCGCCCACGGAGCCACAGACAUUGCACUA
Ppu-1-7	CCACAGCUCGAUCGUGAUGCAGGCCGUGAUCCAUGAAAUGGAAGCGGUACAGGGGCUGGUCGAGUCGGCGUUGAUGCAGUUGCAGAUGCGGCCGCAUUUACCCACAGAGCCUCACACCUUGCAUUAG
Ppu-1-8	GUCUCGGUAUUGAUGAUGGCGUCAAUGCACGAGCUGGAAACCCUUCGCAAGUUGCUUGAAUCAGCGCUGGCUCAAGUCCAAAAACCAGCAGAUCCGCAGACGCUGCAUUAAUCCGCAUCAUGAUUGG
Ppu-3-8	GUCUCGGUGUUGAUGAUGGCCUCAAUGCACGAGCUGGAGACCCUCCGCAAGUUGCUCGAAUCGGCGCUGGCUCAAGUCCAAAAGCCAGCGGACUCUCAGCCGUUGCAUUAACCGCAUCAUGACUCGC
Pen-1-26	GCUGGCUGCUUUGCACGAGAUGGAAACGGUGAAGGCCUUGCUGGAUUCGGCACUGGCCCAAGUGCAGGCGACCCUGCACUGAUGGUUGAAAUACCCACUUCAAGGAGAUCCCCAUGACACCGGAAGA
Ppu-2-5	GCUUCGAUCCUGAUGAUGACCUCCAUGCAUGAACUGGAGGCGCUGCGGGCUUUGCUGGAGUCGGCCUUGGUUCAGGUGCAAAUGCCCGUAGAGCCCAGGACUUCGCACUAAGGCUGCUUAAUGUGUU
Ppu-4-24	UGGACUUUAGUCUCGGCACGUCGUUGUCACUCAACGACUGGGUUAGCCGCCCAAAAAUCGUCGGACACUACAGCCGUGCAAUACCGAGAAAUCGGCAUUAGCGCCGUUAUGGCGGCUGUGCGUGGGG
Ppu-4-25	CCUCCUUGUUGAUCAUGACCUCCAUGCACGAGCUCGAAACCUUGCGCAAAUUGCUUGAGGUGGCCUUGGCGCAAGUGCAAAUGCCGACAGAGCCGAGAACGCUGCACUAAGUCGCAUCAUGUCAGGC
Ppu-3-9	CGAUUGUGAUGAUGACGUCCAUGUAUGAACAGGAGACGCUGCUGCCCUUGCUUUGGGCCGCAGCUGCUGCAGUUACAUAUGCCCUCUUAGCCAAAAACGUCUCAUUGAGGCUUAACUGAUUGUGUCG
Pen-1-27	CAGGUCGCCCGGUGCGAUCCUGAUGCUGGCCGCUGCCCACGAAAUGGAGGCGGUGCGCACGUUGCUGGAGUCCGCCCUGGCCCAGGUGCAGCACUCGGCCCAAACGCCGAGCGAGCCACCCACGCUG
Ppu-3-10	CCGCGUCGAUUCUGAUGAUGACCUCGAUGCAUGAACUGGAGACCUUGAGAGCUUUGCUCGAGUCCGCAAUAGCCCAAGUGCAGAUGCCCAGUGAACCUCGGACUAUGCAUUGACGUGUCGCUGAUGU
Ppu-3-11	AGCUCGAUCGUGAUGCAGGCCGUGAUCCAUGAAAUGGAAGCGGUACAGGGGCUGGUCGAGUCGGCGUUGAUGCAGUUGCAGAUGCGGUCGCAUUUACCCACAGAGCCUCACACCUUGCAUUAGCAGG
Pen-1-28	AAACCACCUGGCCAGGUCAUGGCAUUGACCGCCGUCCACGAAAUCGACAGCGCACGCACUUUGGUCGAGAUGGCCUUGAGCCGUGUACAGGCAAGGCACUGAUCGCGCCCCCCCGCGAACAAGGAG
Psy-1-6	CGCCUGAUGGGCAAGCGUCUGGACGAGCGCAACACCCGCAAGCUCGACGCCGAUCAGCGCCGCACCGCAUGGUUGUUGACCCACGGCGAAUGGCAAAAAUGCCUGCUGAUCAGCGAGUCUGCGGUAU
Ppu-4-26	CGAGUAUUGCCGCGCGCAUACCGGCGAGCCGGGCGUGUACAUGCUCACAAAUGCGGUGCAUAGCGCCGAUACCGCACAUGCAUUGAUCGAGCAUGCGCUUGAGCGCAUGUGAAGGUUUGGGGGCAAU
Ppu-3-12	CAGCGAUAGCCGGCGGGCGCUUGAGCGUAUUCAGGACGAAGAAACCAGGCAGUUAUUGGAGCAUGGGUUGCGGGCCAUGCAGAUUGCCUGGGGGCAGGCGGAUGCUGUCAGCGUGGCGUUUGAACGU
Ppu-1-9	AGCGAUAGCCGGCGGGCGCUUGAGCGCAUUCAGGACGAAGAAACCAGGCAGUUGUUGGAGCAUGGUUUGCGGGCCAUGCAGAUUGCCUGGGGGCAGGCGGAUGCUGUCAGCUUGGCGUUUGAACGUA
Ppu-3-13	CCCAAUACCUGCGCGGUGCCUACGACUGUGGCUACAAAGCCUUGGAGCAUCUGGAUGACACAGGCAAGAGCCUGUUCUGGAGCAACCUGAACGCACUCGAAAUGGCAGAAGGGUUGGUUGAGGCAUU
Ppu-1-10	CCCAAUACCUGCGCGGUGCCUACGACUGUGGCUACAAAGCCUUGGAACAUCUGGAUGACACAGGCAAGAGCCUGUUCUGGAGCAACCUGAACGCACUCGAAAUGGCAGAAGGGUUGGUUGAGGCAUU
Pen-1-29	AGUACCUGCGCAGCGCCUACAACGUUGGCUACAAAGCCCUCGACCAUAUGGACGACACGGGCAAGAGCCUGUUGUGGGCCAACCUGCAUGCGGUCGAGAUGGCCGAGGGGCUGACGGAGGCGAUGCU
env-45	CCGCUGCGUGAAUCAGAACGUUCCCCCUUAGCCUCUGAAGCAGCAGCUGAACGACGCGGUGAAAUGCUCAGCAAUUUAUUGGAUCAGUUGGCCGAGGCAGACGGUUUGCAGCCAACAGACCUGCGCG
env-46	CCCUUGCAUAGCCAUGUGGCCGACGAGUGUCCGCACCAGGCUGCGGCUGAGUAUCGGGCGGCGUUGCUGGCCAAGCUGUUGCGGCGGGUGCCGGAGCCGGAGUUGGCAAGCACCACCAAUGCGUUGC
Pme-1-1	CGCUGCAUGAAUCCGAAGGCUCUCAUCUACCCGCUGAAGCUGCAGCUGAACUGCGUGGCGUCAUGCUCGGCAAUCUGCUGGAUCAACUGGCUGAACCUGAUGGGCUGCAGCCGGCAGACCUGCGCAU
Pme-1-2	CCUUGCAUAGCCAUGUGGCCGACGACUGCCCCUUGCAGGCAGCAGCCGAGUAUCGGGCAGCGUUGCUGGCCAAGCUAUUGCGGCGAAUGCCGGAGCCGGAGUUGGCGAGCACUACCCACGCGCUAUU
Rso-2-1	GUCGCCGCUAUCGUUCAAGCGAGGCGGCACUAUGGGUAAGCGCAAUCGCCCGCCGCAUUGGAUGGCCCUGGAAGCGCAACGCUCGCUCACGGCACAGCUUGCGCAGCAGACCCCCGCCAAAACGCAA
Rpi-1-1	CGUAGCCGCAAUCGCAAAGGCAGAGGUGGCACAUGGAUAAGCACAUGCAUGCCGCGCCGAGCCUGGCGAUGAUGCAGCAGCGCAAGCUCGUUUCCCAGCUCGUGCACCAGGUGCAGCAGACAAGCAA
env-47	CGNNNNNNNNNNNNNNNNNNNNNNNNNNNNNNNNNNNNNNNNNNNNNNNNNNNNNGUAGCACGCGCCACUUCCAAGAAAAGUAGCUCGCAUUUUGCGAGCUCCUUUCUUCCGGAGUUGUCGCCAUCUCC
Reu-1-1	AUGCUUGCUUUGGUAUCCGGUGUCUGGUGGCGAUGAUUAAGGCGAAGUUUGACCGGACGGCGGGGGAGAUUUGUCCGGUGGAGUGAGUGGUGGUGCUGAUGUGGCCGGGGGUGUUGCUCCGGCGUGA
Rso-2-2	AGGCAUGCGUGAUCAGAAACACUCCCCGCAGACGCUCGCGCUCGCACAACAGCGACAACUGAUCGCUCAACUUGCGACACAUGCGGGACGAAUCGGCAAGCGCGCCAAGACGCCAGUCAUCGCGACG
Reu-2-1	CAGCGCGAAUUGCAGGAUGCCUUUCAACGACUGACCGUGGAACUGCAGCGGAUGAAUGGCGCGGAGGUGCAACAUGGCUAAUUGUGAACAGCACCUGCAGAACGGCUACCUCGCCUUCUCCCGCCAC
Rso-1-2	CCGUCGCCGCCAUCGCUCAAGCGAGGCGGCGCCAUGGGUAAGCGCAACCACCCGCCGCAUUGGAUGGCCCUGGAAGCGCAACGCUCGCUCACUGCACAGCUUGCACAGCAAGCCCCCGCCCAAACGC
Rso-1-3	GAUGCAUGCGUGAACAGACACAGUCCCCGCAGAUGCUUGCGUUUGCCCGGCAACACCAACUGAUCGCCCAACUUGCGGCACAAGCAGGACGGAUCGGUAAGCGUGCCAAACCGCCAGUCGCUGCGAC
Bth-3-1	CAGCUCGCCGAACUCUGCCGUCAGGAGGAAGCGAGCCAUGCGUGACGCCAACAUGAUUGAAGCCCUUCGCUACGCGCUGGCGAAGCAAGUACCCGACAUGGAGCGCGGGUUCGUCAUCCAAACGAAC
Bps-8-1	GGCGCAGCUCGCGGAACUCUGCCGCCAAGCGGAGGUGUCAGCAUGAGCGACAUUUCCAUCGAGGGCAAGGCCGCACAAUUGAGCGCGCUGCUGACGAGCAUGUACGGCGAGGGAUUCGUCACAUUCA
Bps-16-1	GGCGCAGCUCGCCGAACUCUGCCGUCAGGAGGAUGCGAGCCAUGCGUGACGCCAACAUGAUUGACGCCCUUCGCUACGCUCUGGCGAAGCAAGUACCCGACAUGGAGCACGGGUUCGUCAUCCAAAC
Bps-11-2	CGCAAUCGCGGCGCUCGUGCGUGCGGAGGUGCGUCAUGGCUGAGCUGCGCUGCAAGAUCGGCGAUCUGGCGAUCGUCACGAAGUGCGAUGCUCGCUCGCGUAUCGGCAUGCUGGUUGAGGUGGCAUC
Bth-3-2	UCGCCAUCGCGGCGCUCGUGCGUGCGGAGGUGCGGCAUGGCUGAUGCGUUGUCGAUCCACAUGAACGACGGCCGCCGCAUCGAAUUCGCCGGCGCGCUCGCGUUGUCUCACUUCGUCGCGAGCCGCG
Bth-2-1	UCGCCAUCGCGGCGCUCGUGCGCGCGGAGGUGCGGCAUGGCUGACGCGUUGUCGAUCCACAUGAACGACGGCCGGCGCAUCGAAUUCGCCGGCACGCUCGCGUUGUCUCACUUCGUCGCGAGCCGCG
Bvi-1-1	CCGCGAUCGCAGCACUCGUUCGCGCGGAGGUGCGCCAUGGCUGACCUGCGCUGCAAGAUUGGCGAUCUGGCGAUCGUCACGAAAUGCGAUGCUCGCUCGCGUAUCGGCAUGCUGGUCGAGGUGGCAU
	....
	....
	•••

Bth-3-3	CCGCGAUCGCGGUGCUUGUCCGUGCGGAGGUGCGCCAUGGCUGAGCUGCGCUGCAAGAUUGGCGAUCUCGCGAUCGUCACGAAAUGCGACGCACGCGCUCGCAUCGGCAUGCUGGUCGAGGUGGCAU
Bps-16-2	UCGCCAUCGCGGCGCUCGUGCGCGCGGAGGUGCGGCAUGGCUGACGCGUUGUCGAUCCACAUGAACGACGGCCGCCGCAUCGAAUUCGCCGGCGCACUCGCGUUGUCUCACUUCGUCGCGAGCCGUG
Bps-7-1	UCGCCAUCGCGGCGCUCGUGCGCGCGGAGGUGCGGCAUGGCUGACGCGUUGUCGAUCCACAUGAACGACGGCCGCCGCAUCGAAUUCGCCGGCGCACUCGCGUUGUCUCACUUCGUCGCGAGCCGUG
Bps-8-2	UCGCCAUCGCGGCGCUCGUGCGCGCGGAGGUGCGGCAUGGCUGACGCGUUGUCGAUCCACAUGAACGACGGCCGCCGCAUCGAAUUCGCCGGCGCACUCGCGUUGUCUCACUUCGUCGCGAGCCGUG
Bps-5-1	UCGCCAUCGCGGCACUCGUGCGCGCGGAGGUGCGGCAUGGCUGACGCGUUGUCGAUCCACAUGAACGACGGCCGCCGCAUCGAAUUCGCCGGCGCACUCGCGUUGUCUCACUUCGUCGCGAGCCGUG
Bps-18-1	UCGCCAUCGCGGCGCUCGUGUGCGCGGAGGUGCGGCAUGGCUGACGCGUUGUCGAUCCACAUGAACGACGGCCGCCGCAUCGAAUUCGCCGGCGCACUCGCGUUGUCUCACUUCGUCGCGAGCCGUG
Bok-2-1	CGCAAUCGCAGCGCUCGUCCGUGCAGAGGUGCGCCAUGGCUGAGCUGCGCUGCAAGAUUGGCGAUCUGGCGAUCGUCACGAAGUGCGAUGCACGCGCUCGCAUCGGCAUGCUGGUUGAGGUGGCAUC
Bok-1-1	CGCAAUCGCAGCGCUCGUCCGUGCAGAGGUGCGCCAUGGCUGAGCUGCGCUGCAAGAUUGGCGAUCUGGCGAUCGUCACGAAGUGCGAUGCACGCGCUCGCAUCGGCAUGCUGGUUGAGGUGGCAUC
Bce-1-1	CGCGAUCGCCGCGCUCGUCCGUGCGGAGGUGCGUCAUGGCUGAGCUGCGCUGCAAGAUCGGCGAUCUGGCGAUCGUCACGAAAUGCGAUGCUCGCUCGCGUAUCGGCAUGCUGGUCGAGGUGGCAUC
Bps-5-2	CCGCAAUCGCGGCGCUCGUCCGUGCGGAGGUGCGUCAUGGCUGACCUGCGCUGCAAGAUUGGCGAUCUGGCGAUCGUCACGAAGUGCGAUGCUCGCUCGCGUAUCGGCAUGCUGGUCGAGGUGGCAU
Bth-1-2	GUCGCCAUCGCGGCGCUCGUGCGCGCGGAGGUGCGGCUUGGCUGACGCGUUGUCGAUCCACAUGAACGACGGCCGCCGCAUCGAAUUCGCCGGCACGCUCGCGUUGUCCCACUUCGUCGCGAGCCGU
Bps-7-2	CGAUUGCCGCGCUCGUCCGUGCGGAGGUGCGCCAUGGCUGAGCUGCGCUGCCAGAUCGGAGAUCUGGCGAUCGUCACGCAAUGCGAUGCUCGCUCGCGUAUCGGCAUGCUGGUCGAGGUGGCAUCGG
Bdo-1-2	CAAUCGCGGCGCUCGUCCGUGCGGAGGUGCGUCAUGGCUGAGCUGCGCUGCAAGAUUGGCGAUCUGGCGAUCGUCACGAAGUGCGAUGCUCGCUCGCGUAUCGGCAUGCUGGUCGAGGUGGCAUCGG
Bps-9-1	CGAUCGCGGCGCUCGUUCGUGCGGAGGUGUGCCAUGGCUGAGCUGCGCUGCAAGAUCGGCGAUUUGGCGAUCGUCACCCAAUGCGAUGCUCGCGCACGCAUCGGCAUGCUGGUUGAGGUGGCAUCGG
Reu-1-2	ACACAAUGGUGUCGACAUGCUCGUGACCGCCCAUAGCCCGGGGAAAAUCCGUUGAGUCUUGCGGUGCAACGUGCGGCGCAGCUUCAUGGGCUUCUGCUCAUGGCUAGCGAUCAUGGUGCCUCGAGU
Cvi-1-2	CAUCGGCACCGCCGCCGGUUUCGCCGUGGAGGUGAUCGUAUGAGCGCCCUGCCCCUUCUCGCCCCGCUGGCCCUCGCCGCCAUCCAACGCGAACUGAUCCUCCUGCAGCAACUGGCCACCCAGCCGGA
Bps-15-2	CGCAACGCUCGCCCGGGUGGAGGUGCUCCAUGGCUAAGCUGACCCCCCAAGAGGCCGUUGUCGACGCACAAGAGGAUCUCGCAUCAAGCAUCCUUAUCGUCGGUCAGUUGGCAGAGCUUUUUUCCAC
Rpi-2-1	GGCGUACCUCAAGCGGACUCGCACCGCCAUGUGCACGCACGGCCAAUGCCGGCCGGGCAUGCUGCGUGAGCAGCUUCAACACCUCUCUGCACACGCAUUCCCGCCGGCGUUCAUCAAAGGCUUUGUG
Rpi-1-2	GGCGUAUCUCAAGCGAACUCGCACCGCCAUGUGCACGCACGGCCAAUGCCGGCCGGGCAUGCUGCGCGAACAGCUUCAAGUGUUCUCUACGCAGUCGUUCCCGCCGGCGUUCAUCAAAGGCUUUGUG
env-48	GGCGUAUCUCAAGCGAACUCGCACCGCCAUGUGCACGCACGGCCAAUGCCGGCCGGGCAUGCUGCGCGAACAGCUUCAAGUGUUCUCUACGCAGUCGUUCCCGCCGGCGUUCAUCAAAGGCUUUGUG
env-49	AUCGCUGAGUAUGGAUUUGCGGAAGGCGAGGACUUUUCCCCGAUUCGGGGAAAAGUCGCGGAGCCGACCUGCUCCGACUACCACCGGUACCGGCUCGAUUGAUCC......................
Bce-1-2	UCAUUCGUCACGUUCUGGAACUGACCGAGCCGCUCGCGCUCGAUUUGAUGGGCGCCGAAUGGAGGACGCGUAAUGCAUAACUCGAAUAUUCAGGGGUUUGACGCUCAAUCGGCGCUGCUGGAACUGG
Bma-10-1	UUAGACGAGCAAAAAGCGAUCUUCGACCUUUACGGCGUUACGCUCACGGCAGUUCGCAGCACGCUUCAUGAACUCAGAGCGGAGAGUCGCCCGGAGGUGCUGCAUGGCUGAGAAAUUUAGGCGAACG
Bth-2-2	UUGGACGAGCAAAAAGCGAUCUGCGACCUUUACGGCGUUACGCUCACCGCAGUUCGCAGCGCGCUUCAUGAACUCAGAGCGAAGAAUUGCCCGGAGGUGCUGCAUGGCUGAAAAAUUUAGACGCACA
Bvi-1-2	GCUGUUGCCCUCGUCGUCCUACUUGCCGCCCACCUUCUACCCGCCUUGACGCGGGGCGAGCCGCUGCAGAUCAAAUUGCCUGCGAUGGCGCUCUGGUUGGUGUGCCUCGUUGGCGCUGGCUACGGCC
env-50	UCGGCCGAACGCGCCGCAUGGGCCGCUGUCGCGCUCGUGGUGCUACUGGCCGCUCACCUGCUUCCCGCGCUGACGCGCGGCGAGCCGUUGCUGAUCAAGCUUCCGGCCUUGGCGCUUUGGGGGGUGU
Bce-2-1	CGCCUGGCUGAAAUCGCCCGCGCGGAGGUGGCUCAUGGCUAAGCCCGACCUCGAACUGCCGUUCAACCAGUUCUCGCAGCGCGCCGUCGAUCAACUGCGCGCCGGCCGUAUCGAUCCGGAGCCGCUG
Bps-3-1	CGCCUGGCCGAACUUGCUCGCGCGGAGGUGUCGUGAUGAGCGGCGAAAACUUCCUCGACUCCCGAUUUCCGGUCUUCAGCGAACGUGUGUCCGAUGCUGCCGCAAACGAUUCGCUCGUCAGGCCGC
Bmu-1-1	AACGCGGGGGAUCUUCGAUUGAGCGAGGAGUGUGGGCAGCAAUCGGGCUAGUUCUGCUCGUCGCAGCGCACCUUAUCCCUGCGGUGACGCGCGGUACGGCCGCUCGUAUUCGGUUGCCGGCGUUGCU
Bvi-1-3	GCGGGGGCUUGCUGUCUGAGCGUCUGGCGUGGACUGCGGUCGGCCUUGUCAUGCUGCUCGGCGCGCACCUGAUCCCGGCGCUCACACGCGGUACCGCCGCGCGCGCCCGCAUCCCGGCAUUGCUGCU
env-51	GCUGCGUCGUCGCUGAGAUGGUGUGCUGCAGCGCUCGCGAUGGCUGCGACUGGUACAUCUGCGUGCGUAUCAUUCCUUGCCUCAUGGGAGCGCGGUGGCCAGUUUUCCGAACGGCUGGUUUGGGUUG
Rso-1-4	GCCCUGUCCAGGCUGGAGACGCCGGAAGGGUACCUGCACCUGGAAGACAUCGCCUACGCGCUGCAAGCCAUCCGCAACAAAGCACGAGACAUCGAGAACUGCAUCAACGCGGAAGCUGAGAGCGUUG
Rpi-2-2	GCAGGCGUAUCUCAAGCGCACGCGCUCCGCCAUGCGCUCACACGGCCAAUGCCGCCCGGGCAUGCUGCGCGAGCAGCUUCAACAUCUGUCCGCGCACGCAUUCCCCCCGGCAUUUAUCAAAGGCUUU
Rpi-1-3	AGGCGUAUCUCAAACGCACGCGCACCGCCAUGCGCAGCCACGGCCAAUGCCGCCCGGGCAUGCUGCGCGAGCAGCUUCAGCAUCUGUCCGCACACGCAUUCCCGCCGGCGUUUAUCAAAGGCUUUGU
Rso-2-3	CGCGAGUUCCUGCGCCGCACCCAAUCCGACCUACACGUCCAUCAGCAGUACCUGCCGGAGUCAUUGCGCUGGGAGAUGGUCUCCGCGACUGCGUCGCAGCCACCGGAGUUUCUGGCGGGCUUUGUCG
Rso-1-5	CCGCGACUUCCUGCGCCGCACGCAAUCCGACCUGCACACCCAUCAGCAGUACCAGCCGGAGUCAUUGCGCUGGGAGAUGGUCUUUGCAACCGCAUCGCAGCCGCCUGAGUUUCUGGCGGGCUUUGUC
Rpi-1-4	CAGGCGUAUCUCCGGCGCACACGCGCCGCCAUGCGCAGCCACGGCCAAUGCCGCCCGGGCAUGCUGCGCGAGCAGCUUCAACAUCUGUCCGCGCACGCGUUUCCGCCGGCUUUUAUCAAAGGCUUUG
Rpi-2-3	CUUUGCCGUACGCACGUUGAUGCGCGUGCCGGCCGGCCCUUUGUGGCUAUGCGGCUACGCUCGAAAAUCGACGGCAUCACGCAUGCCCUGCCGCGCGAGUUUCGGGCCGGUUUCAUCGAUGCGAUCU
Rpi-1-5	CUUUGCCGUACGCACGUUGAUGCGCGUGCCGGCCGGCCCUUUGUGGCCAUGCGCCUACGCUCGAAAAUCGACGGCGUCACGCAUGCACUGCCGCGCGAGUUUCGGGCCGGUUUCAUCGAUGCGAUUU
Rpi-2-4	CUCGGCGCGCGCGUUUCUGUGCCGCACGCACAGCGUCAUGCGUGACCCUGGGCAUUGCCGGCCCGGCAGGCUGCGGGAGCAGCUCACGUACUUUUCGGAACACGCGUAUCCGCCGGCAUUUGUGAAA
Rpi-1-6	GUCAGCGCGCGCGUUUCUGUGCCGCACGCACAGCGUCAUGCGCGACCCUGGGCAUUGCCGGCCCGGCAGGCUGCGCGAGCAGCUCACGUACUUUUCGGAACACGCGUAUCCGCCGGCAUUCGUGAAA
Rpi-2-5	AGGCGUAUCUCAAGCGCACACGCACCGCCAUGCGCACACACGGCCAGUGCCGCCCCGGCAUGCUGCGCGAGCAGCUUCAACAUCUGUCCGCGCACGCAUUUCCGCCGGCUUUUAUCAAAGGCUUUGU
env-52	....
Pen-1-30	CCCAAGUCACCGGCCACGCUGGUGAUGCUGACAGUCAUGCACGAGCUCGAUGCCACCCACACGCUGCUGGAGUCGGCAUUGGCUCAGCUACAGAUGAGCGCCCGGCCGCCGUCCUACACGCUGCACU
Ppu-4-27	CCGGCAUCGAUCUUGAUGAUGACCUCGCUGCAUGAAAUGGAGGCGCUACGGGUUUUGCUCGAAUCGGCGCUGAUCCAGGUGCAGAUGCCCACAGGCCCUCGGACCUUGCAUUAAGCCUUAAAUGAUC
Pfl-2-29	GACCGCGAAGCCCCGGUAGACGUACUCCACGGCACUGCCGCCUACCGCUUGCGCAGCGUGACGCAGUUGUUGGAAACACUCUCUUUGAGCGACGGCAUCGGCCACGACGCCUUGCUGCUGCAGGACU
Sgl-2-16	GAUAGCCGCCUAAUAUGGGCUGGUCGUCAACCUGUGCAAGCUGGUAUGGGGGGAAAUUAACAUGAGAAUUGUUGAUACCUCGACUGCACAGCAGGAAAAAAUAAGACCAGAAAUUAGUCAUGAAAGC
Sgl-2-17	AGAUAGCCACCUUAUCUGGGCUGGCCGCAAGCUGGUUCGGGAGGCGGCCCAUGGCAAAGCAUUAAAAAGCCCCUCUUUGACCUGUCUCAAAUACAGGAAUUCAGUGAUGUCUUCCCGGAAAUGACGC
Eco-8-5	AUUUUUGUCGCCCGUAUUCGUCAGGGGGUAGGCUUAUGAGUCAGGAAAUCACACUACAACAGGCAGCAGAACGCGCCCACCAAAUCGAAGUUAUUUGCGCACUGGCAGAGGAUUGCCCUGGCAUGAU
Eco-3-14	AUUUUUGUCGCCCGUAUUCGCCAGGGAGAAAAUUUGUGAUGCAUAAAAUCCCUUUUGAAGUAUUAAUCCACUCUGAAAACGCAUUAAUUAAAGCGCGUGAAAUGAAUGCGCUGUUAGGAAAAUUAAU
Sen-3-1	UUACGUCUGGCGCUUUGCGGACGGCUUGCCGCCGGUCUCGCGCUGCUGCGCCCCACGCUGAACGAUCCCAUCCCGCCCCACUUGGUUGAAAGCCUGACCACCGACACCCUUCCCGCCAGUUACACCU
Sgl-2-18	CUGCUACAAUAUUACUUGCCGUUUAUUUUUUCCUGGCUGGCGGAUGAUCUUAACGCCAUUCAUCACGAGUUAACGGCGCGCGGCUUAUUUGCUGCUUUCCAGACGCAACCGGGAGGCGGCGAUGGUA
Eph-3-1	UUACUGCCCGUUUUUCUACUAACUCGCGCAAUUCACUUAGUUACUGGAACCUCCCCUUCUCUGCCGACGUUCAGGGGGGUUUAUGAAAAAACCUCUCGUCACCCGUAAUGAAAUAGCCGAAGCGAUC
Sen-8-4	UUUCUCUGCCCGAUUUUCAACUGAAGCACAUCAAUACUUUAACUGCUGGAAUGCCCAGAUUAUUGGCUGGCAAGGGGGGCAGUGAUGAAAACCUCCCUCAUUACCCGUGAAGAAAUGAUCGAAGCUA
Aba-1-6	ACAAUAAAACCCGAUAUAGGCGAGCACACCAUUUUUGUGCAGUUUUUGCAUGCCUGUAUUUAACGCUCAAUUUAAGAGCGACGGCAUUUCUAUCUCUUUUAUUUACAACAAAAAAUUAUUUUAAACG
Aba-1-7	AUUUUUACUUAAUCUUUUUGUGUACCCAUCUUGAUAUACAAGAUUGGGGCAGAAGUUUUAUGUAUUCAAAAUCACAUAAAGAUUAAAACUUCUAUCAACAUCACUUAACGAUAACAACAACCCAUGA
Aba-1-8	AAACUCGAGAUAGGCGAGCACACCACUUGUGCGGCUUUUGCAUGCCUGUAUUUAGCACUCAUUUUAAGAGUGACGGCAUUUCUAUCUCUUUACAUACAACAAGAAUUUAUAACGGUAAAACUAUGCC
Aba-1-9	UGUGCCAAGCACAGUCUUUAUAGGCUUUGCUGUGCCUUUUUUUCUCCCAAAAAAGUAUCUCAUUUACAAACAACAAGAAUUUAUAACGGUAAAACUAUGCCAAAUUUAGUUCUCCCAACACGUGCCU
Asp-1-1	GUGAUGAUGUGCGUAUGGUUGUGUGGCUGUGAUUCUCAGCCACCUGUAUUUGUCGACUUUGAAGAACUACAGCCCAAUAACUUCUUCUCGUCAUCCACACAUAAAACACAAAUCAGCAUUCAAGCAU
Aba-1-10	CUUGAGAUGUGCCAAGCACAGUCUUUAUGGCUUUGCUGUGCCUUUUUUUCUCCCAAAAAAGUAUCUCAUUUAAAACAACAAGAAUUUAUAACGGUAAAACUAUGCCAAAUUUAGAAACCUCUUCCAA
Aba-1-11	GCAACAAUAAACCCGAGAUAGGCGAGCACAUCUUUAUUGUGCGGCUUUUGCAUGUCUGUUUUUAGCGCUCAAUUUAAGAGUGACGGCAUGUCUAUCUCUUUUAUAUACAACAAAAAAUUAUUUUAAG
Aba-1-12	GCAACAAUAAACCCGAGAUAGGCGAGCACAUCUUUAUUGUGCAGCUUUUACAUGCCUGUAUUUAGCACUCAAUUUAAGAGUGACGGCAUGUCUAUCUCUUUUAUAUACAACAAAAAAUUAUUUUAAA
	....
	....
	....

Aba-1-13	UUCUGCCUAAAAAAGCCAAUCCUCCAUGACUAUGUCACUGGAUAUAACGAUGAAGAAUAAAAUUUUGAUGGGUUUGGUUAGUGUCAGUGUUUUGUUGAUUGCUGGCUGUUCUUCCGAUUUCGAAAAG
Aba-1-14	AGAUGUGCCAAGCACCGUCUUACGGCUUUGCUGUGCCUUUUUUUCUCCCAGAAAGGGCACAGGCUUUUUAUCUCAUUUAGAAACAACAAGAAUUUAUAACGGUAAAACUAUGCCAACUUUAGAAAUA
Aba-1-15	UUUGCAUGCUUUUUUUGCUUAGAGCAAAAGUUGCAGUCGUUUUAUCUCUUUUAAACACAACAAGAAAUUAUUUUAUAACGGUAACUAUAUGCCAUAUUCAGAUCUCUCAUUUCGCGCGUUAAGCGUG
Aba-1-16	UGUGCCAAGCACAGUCUUUACGGCUUUGCUGUGCCUUUUUUUCUCCCAGAAAAGGCACAGGCUUUUUAAUCUCUUUUAGAAACAACAAGAAUUUAUAACGGUAAAACUAUGCAAAAUUUAAAAACUU
Aba-1-17	GAUGUGCUGGGCACAGUCUUUAGGGCUUUGCUAUGCCUUUUUUUCUCCCAAAAAAGUCAGCAGUUUUUUUAUAUCUCAUUUAUAUACAACAAAAAUUUAAUUUAUAACGGUAAAAAUAUGCCAAAUU
Aba-1-18	GUGCCAAGCACAGUCUUUGUGGCUUUGCUGUGCCUUUUUUUCUCCCAAAAAGGGCACAGGCUUUUUUAUCUCUACAUACAACAAAAAUUUUUAUAACGGUAAAACUAUGCCAAAUUUAGUUCUCCCA
Aba-1-19	UGUGCCAAGCACAGUCUUUAUAGGCUUUGCUGUGCCUUUUUUUCUCCCAAAAAAGUAUCUCAUUUACAAACAACAAGAAUUUAUAACGGUAAAACUAUGCCAAAUUUAGUUCUCCCAACACGUGCCU
Aba-1-20	UGUUUGUGGGAGGAUUAAUUAUUAUUUAAAAAAUUAUUUUAAUAUUUAAUUGAACCAUAUUUUUAUUUUAUUUAAAUUUAUAAAUAAUCAUGAGAAUAAUAUGAAACUUCAUAAAUUUAUAUUGAUU
Asp-1-2	UAUAGAUUUAUCCAAGACUAUAAAUAAUCGACAUGAUCAAUUUUAAAAGACUAAAUCUUAAGAAUGCUUGGGAAGUAAGAUGUUAUGAAUACAUGGGAAUGGGAUAAAACCAAGUUUUUUAUCCAGC
Aba-1-21	AUUGCUUUUAUAGGCGGGUUAACUAUUGCCUAAAAAAAAUGAUUUUAAAACUUAAAAUAUAGCGGUUUAUUUUUUCUCAUUGAGAUAUCCCCAAAUCAUCACGAGAAUAAUAUGAAAUUCUAUAAAAC
Asp-1-3	AUUUUUAAUAUCUGGAAUGCUUUUCCAUAGGUUCGCUCGUCCUCAUUAUUUCUGGCGCCAAAAAAAAUCAUUCCAUUUCAAAAAAUAAGGAUAACAAUAGAUGAAAGGCAAAAUACUCGAUUAUUCCG
Asp-1-4	AUUUUUAAUAUCUGGAAUGCUUUUCCAUAGGUUCGCUCGUCCUAAAAAUUUCUGGCGCCAAAAAAAAUCAUUCCAUUUCAAAAAAUAAGGAUAACAAUACAGAUGAAAGGCAAAAUACUCGAUUAUUC
Aba-1-22	AUGUGUAGGAGGAUUAAUUAUUACCUGAAAAAAUUAUUUUAAUUUUUAAACUGAUUAUAUUUUAUUCUACUCAUAGAUAUAAAUCAUUAUGAGAAUAACAUAACAAUUUAUACACCUUUUUUGGCUUU
Aba-1-23	GUGUUUUUAUCACUAACAUUGGUAGGUACAUUUUAUGGGAUGGACUUUGAUGUAAUGCCUGAAUUAUCUUGGGAGCACGGUUUUUUAUAUUGUAUGAUACUUACUUUAGCAGUAGCGAUAAUACCUU
Hin-11-1	CGCAUCAACGAAUAUGGAUUCAUCCAAGAUGAAGACUACCUCGUCAUUACCGAACGCACCAAUGGACGCCCACGCAAGGAAUAUCACAUCACCCUCGAUAUGGGCAAAGAACUCAGAAAUUAAUUGC
Hin-7-2	CGCAUCAACGAAUAUGGCUUCAUCCAAGAUGAAGACUACUUCGUCAUCACCGAACGCACCAACGGACGCCCACGCAAGGAAUAUCACAUCACCCUCGAUAUGGGCAAAGAACUCAGAAAUUAAUUGC
Hin-10-1	CGCAUCAACGAAUAUGGCUUCAUCCAAGAUGAAGACUACCUCGUCAUUACCGAACGCACCAACGGACGCCCACGCAAGGAAUAUCACAUCACCCUCGAUAUGGGCAAAGAACUCAGAAAUUAAUUGC
Hin-12-1	CGCAUCAACGAAUAUGGCUUCAUCCAAGAUGAAGACUACCUCGUCAUUACCGAACGCACCAACGGACGCCCACGCAAGGAAUAUCACAUCACCCUCGAUAUGGGCAAAGAACUCAGAAAUUAAUUGC
Hin-3-3	CGCAUCAACGAAUAUGGCUUCAUCCAAGAUGAAGACUACCUCGUCAUUACCGAACGCACCAAUGGACGCCCACGCAAGGAAUAUCACAUCACCCUCGAUAUGGGCAAAGAACUCAGAAAUUAAUUGC
Hin-8-1	ACGAAUAUGGCUUCAUCCAAAAUGAAGACUACCUCGUCAUCACCGAACGCACCAACGGACGCCCACGCAAGGAAUAUCACAUCACCCUCGAUAUGGGCAAAGAACUCAGAAAUUAAUUGCACCAUUU
Hin-4-1	CGCAUCAACGAAUAUGGCUUCAUCCAAAAUGAAGACUACCUCGUCAUCACCGAACGCACCAACGGACGCCCACGCAAGGAAUAUCACAUUACCCUCGAUAUGGGCAAAGAACUCAGAAAUUAAUUGC
Hin-6-1	CGCAUCAACGAAUAUGGCUUCAUCCAAGAUGAAGACUACCUCGUCAUUACCGAACGCACCAAUGGACGCCCACGCAAGGAAUAUCACAUCACCCUCGAUAUGGGCAAAGAACUCAGAAAUUAAUUGC
Hph-1-1	ACGACCGCCACGCCGACGAAUUCACCCCACAAAUGACCGCACUUAUUGAUAUGCGGACAAAUGGCGGAAUCCAAAAAGUGCGCAUCUUCUCCUUGCGCGGCGCACACCUCAUCGGCAUGUUAAGCCA
Hso-1-2	AUAAUCGACAUCAAGAUGAAUUCACUCCUUGUAUGACGACUCUUAUUGACACUCAAACAAAUGGCGGAAUUCAGAAAGUCCGUAUUUUCUCAUUACGUGGCACUCAUCUAAUUGGAAUGUUAAGCCA
Aba-1-24	ACUGGUUCUUGAGUCAGUCUGCUACUCCACUUUUGUCCUGUCACCAUUACUUAAUUUACAUCUGUUCCACAUAUAGUCAGUAAUACACAACCAUUUAAAAAUAUAAUUAAAUAUAAUAUCUUAAAUA

17 c4-a1b1

17.1 Taxa

The taxonomy of each organism containing a putative c4-a1b1 RNA is listed, with abbreviations identifying each hit (e.g., "Eco-1-1" and "Eco-1-2" might hypothetically represent two distinct RNAs in *E. coli*). The abbreviations will be used to identify each individual c4-a1b1 RNA in Sections 17.2 and 17.4.

**abbrev. of hits**	**taxonomy of species**
Eco-1-1	Bacteria Proteobacteria Gammaproteobacteria Enterobacteriales Enterobacteriaceae *Escherichia coli* E110019
Eco-2-1	Bacteria Proteobacteria Gammaproteobacteria Enterobacteriales Enterobacteriaceae *Escherichia coli* O157:H7 str. Sakai
Sen-1-1	Bacteria Proteobacteria Gammaproteobacteria Enterobacteriales Enterobacteriaceae Salmonella Salmonella enterica subsp. entericaserovar Newport *Salmonella enterica subsp. er*
Sen-2-1	Bacteria Proteobacteria Gammaproteobacteria Enterobacteriales Enterobacteriaceae Salmonella Salmonella enterica subsp. entericaserovar Newport *Salmonella enterica subsp. er*
Sen-3-1 to Sen-3-2	Bacteria Proteobacteria Gammaproteobacteria Enterobacteriales Enterobacteriaceae Salmonella Salmonella enterica subsp. entericaserovar Saintpaul *Salmonella enterica subsp. e*
Sty-1-1	Bacteria Proteobacteria Gammaproteobacteria Enterobacteriales Enterobacteriaceae *Salmonella typhimurium* LT2
Sbo-1-1	Bacteria Proteobacteria Gammaproteobacteria Enterobacteriales Enterobacteriaceae *Shigella boydii* BS512
Pae-1-1	Bacteria Proteobacteria Gammaproteobacteria Pseudomonadales Pseudomonadaceae *Pseudomonas aeruginosa* 2192
Pae-2-1	Bacteria Proteobacteria Gammaproteobacteria Pseudomonadales Pseudomonadaceae *Pseudomonas aeruginosa* C3719
Pae-3-1	Bacteria Proteobacteria Gammaproteobacteria Pseudomonadales Pseudomonadaceae *Pseudomonas aeruginosa* PA7
Pae-4-1	Bacteria Proteobacteria Gammaproteobacteria Pseudomonadales Pseudomonadaceae *Pseudomonas aeruginosa* PACS2
Pae-5-1	Bacteria Proteobacteria Gammaproteobacteria Pseudomonadales Pseudomonadaceae *Pseudomonas aeruginosa* PAO1
Pae-6-1	Bacteria Proteobacteria Gammaproteobacteria Pseudomonadales Pseudomonadaceae *Pseudomonas aeruginosa* UCBPP-PA14
Pen-1-1 to Pen-1-12	Bacteria Proteobacteria Gammaproteobacteria Pseudomonadales Pseudomonadaceae *Pseudomonas entomophila* L48
Pfl-1-1 to Pfl-1-2	Bacteria Proteobacteria Gammaproteobacteria Pseudomonadales Pseudomonadaceae *Pseudomonas fluorescens* Pf-5
Pfl-2-1 to Pfl-2-2	Bacteria Proteobacteria Gammaproteobacteria Pseudomonadales Pseudomonadaceae *Pseudomonas fluorescens* Pf0-1
Pme-1-1 to Pme-1-2	Bacteria Proteobacteria Gammaproteobacteria Pseudomonadales Pseudomonadaceae *Pseudomonas mendocina ymp*
Ppu-1-1 to Ppu-1-4	Bacteria Proteobacteria Gammaproteobacteria Pseudomonadales Pseudomonadaceae *Pseudomonas putida* F1
Ppu-2-1	Bacteria Proteobacteria Gammaproteobacteria Pseudomonadales Pseudomonadaceae *Pseudomonas putida* GB-1
Ppu-3-1 to Ppu-3-10	Bacteria Proteobacteria Gammaproteobacteria Pseudomonadales Pseudomonadaceae *Pseudomonas putida* KT2440
Ppu-4-1 to Ppu-4-7	Bacteria Proteobacteria Gammaproteobacteria Pseudomonadales Pseudomonadaceae *Pseudomonas putida* W619

Psy-1-1 to Psy-1-6	Bacteria Proteobacteria Gammaproteobacteria Pseudomonadales Pseudomonadaceae *Pseudomonas syringae pv. phaseolicola* 1448A
Psy-2-1 to Psy-2-5	Bacteria Proteobacteria Gammaproteobacteria Pseudomonadales Pseudomonadaceae *Pseudomonas syringae pv. syringae* B728a
Psy-3-1 to Psy-3-3	Bacteria Proteobacteria Gammaproteobacteria Pseudomonadales Pseudomonadaceae *Pseudomonas syringae pv. tomato str.* DC3000
Sen-4-1	CVM23701Bacteria Proteobacteria Gammaproteobacteria Enterobacteriales Enterobacteriaceae Salmonella Salmonella enterica subsp. entericaserovar 4,[5],12:i:- *Salmonella enter*
Sen-5-1	GA_MM04042433Bacteria Proteobacteria Gammaproteobacteria Enterobacteriales Enterobacteriaceae *Salmonella enterica subsp. enterica serovar* Javiana str
Sen-6-1	HI_N05-537Bacteria Proteobacteria Gammaproteobacteria Enterobacteriales Enterobacteriaceae Salmonella Salmonella enterica subsp. entericaserovar Weltevreden *Salmonella en*
Eph-1-1	Viruses dsDNA viruses, no RNA stage Caudovirales Myoviridae P1-like viruses *Enterobacteria phage* P1
Eph-2-1	Viruses dsDNA viruses, no RNA stage Caudovirales Myoviridae P2-like viruses *Enterobacteria phage* P4
Api-1-1	Viruses dsDNA viruses, no RNA stage Caudovirales Podoviridae *Acyrthosiphon pisum secondary endosymbiont phage* 1
Eph-3-1	Viruses dsDNA viruses, no RNA stage Caudovirales Podoviridae *Enterobacteria phage* VT2-Sakai
Eph-4-1	Viruses dsDNA viruses, no RNA stage Caudovirales Podoviridae P22-like viruses *Enterobacteria phage phi*V10
Kph-1-1	Viruses dsDNA viruses, no RNA stage Caudovirales Siphoviridae *Klebsiella phage phi*KO2
Sco-1-1	Viruses dsDNA viruses, no RNA stage Caudovirales Siphoviridae Lambda-like viruses unclassified Lambda-like viruses *Stx*2 converting phage II (Stx2 converting bacteriophage II
Eph-5-1	Viruses dsDNA viruses, no RNA stage Caudovirales Siphoviridae N15-like viruses *Enterobacteria phage* N15
Yph-1-1	Viruses dsDNA viruses, no RNA stage Caudovirales Siphoviridae *Yersinia phage* PY54
PGi-1-1	Viruses *Phage* Gifsy-1
env-1 to env-6	environmental samples

17.2 Gene contexts

Each c4-a1b1 RNA (indicated by "RNA→") is listed with its surrounding genes. Environmental sequences and some RefSeq entries lack gene annotations, and no genes are listed for such sequences. The direction of each gene is indicated with an arrow (→), and each conserved domain in the gene is colored. Conserved domains associated with more than one c4-a1b1 RNA are assigned a color; other domains are gray. Information about these conserved domains is given in Section 17.3. The accession number of the sequence containing each c4-a1b1 RNA is given. Accessions beginning with "NC_" or "NZ_" are RefSeq, while other accession refer to environmental samples. Nucleotide coordinates are given for the 5′ and 3′ boundaries of each. If the 5′ coordinate is greater than the 3′ coordinate, the RNA is present on the reverse-complement strand of the containing genomic DNA sequence. Each hit is denoted by an abbreviation (like "Eco-1-1") that refers to a taxonomy given in Section 17.1.

abbrev.	Seq. accession		5′ at	3′ at	genes
Pme-1-1	NC_009439.1	-	1667511	1667459	COG2085 (COG2085)→ **RNA**→ hypo→
Psy-1-1	NC_005773.3	-	2874654	2874602	GAF (pfam01590)→ **RNA**→ COG3617 (COG3617)→
Pfl-2-1	NC_007492.1	-	4210840	4210788	←COG3950 (COG3950) **RNA**→ hypo→
Ppu-3-1	NC_002947.3	+	81504	81557	SUA5 (COG0009)→ **RNA**→ hypo→
Eph-1-1	NC_005856.1	+	48217	48269	**RNA**→
Pme-1-2	NC_009439.1	+	3776440	3776492	←hypo **RNA**→ hypo→ hypo→
env-1	JCVI_SCAF_1096627055672	-	3216	3164	**RNA**→
env-2	JCVI_SCAF_1096628137543	+	962	1014	? (GOS1771)→ **RNA**→ COG3617 (COG3617)? (GOS11900477)→
Eph-2-1	NC_001609.1	-	8705	8653	**RNA**→
Ppu-4-1	NZ_AAVY01000023.1	-	18674	18622	hypo→ **RNA**→ repA (cd01125)→
Yph-1-1	NC_005069.1	+	23607	23659	**RNA**→
Sbo-1-1	NZ_AAKA01000001.1	-	39678	39626	hypo→ **RNA**→ hypo→
Kph-1-1	NC_005857.1	-	32735	32683	**RNA**→
Eph-5-1	NC_001901.1	-	28502	28449	**RNA**→
Psy-3-1	NC_004578.1	+	3479907	3479959	lipoprotein, putative→ **RNA**→ COG3617 (COG3617)→
Psy-2-1	NC_007005.1	+	4545039	4545091	←DUF1349 (pfam07081) **RNA**→ COG3617 (COG3617)→ adh_short (pfam00106)→
Psy-1-2	NC_005773.3	-	1124972	1124920	hypo→ **RNA**→ putative inner membrane protein→
Pae-6-1	NC_008463.1	+	4285944	4285996	←hypo **RNA**→ hypo→
Pae-1-1	NZ_AAKW01000002.1	-	1037	985	←hypo **RNA**→ hypo→
Pae-5-1	NC_002516.2	-	1348652	1348600	←hypo **RNA**→ hypo→
Pae-2-1	NZ_AAKV01000003.1	-	6779	6727	←hypo **RNA**→ hypo→

Pae-4-1	NZ_AAQW01000001.1	-	1918787	1918735	←hypo **RNA**→ hypo→
Ppu-3-2	NC_002947.3	+	759489	759541	Acetyltransf_1 (pfam00583)→ **RNA**→ ←GntP_permease (pfam02447)
env-3	JCVI_SCAF_1096628052827	+	1418	1470	? (GOS11069324)→ **RNA**→ ? (GOS11725977)→
Pae-3-1	NC_009656.1	-	373603	373551	INT_P4 (cd00801)→ **RNA**→ hypo→ hypo→ transcriptional regulator, AlpA family→ Bbp36→ zf-CHC2 (pfam01807)→ hypo→ hypo→ COG5519 (COG5519)→
Pfl-1-1	NC_004129.6	+	5946430	5946482	Acetyltransf_1 (pfam00583)→ **RNA**→ hypo→
env-4	JCVI_SCAF_1096627616436	+	788	840	←BRCT (smart00292)? (GOS11076434) **RNA**→ COG3617 (COG3617)→
Pfl-1-2	NC_004129.6	+	5731844	5731896	hypo→ **RNA**→ COG5519 (COG5519)TOPRIM_primases (cd01029)→ chromosome segregation SMC protein, putative→
Psy-1-3	NC_005773.3	+	4631506	4631559	hypo→ **RNA**→ hypo→
Psy-2-2	NC_007005.1	+	4827814	4827867	hypo→ **RNA**→ hypo→
Pfl-2-2	NC_007492.1	+	2273208	2273260	CsgG (COG1462)→ **RNA**→ hypo→ hypo→
Ppu-1-1	NC_009512.1	+	1788494	1788546	hypo→ **RNA**→ hypo→
Ppu-3-3	NC_002947.3	-	4856696	4856644	←hypo **RNA**→ ←PBPb (smart00062)
Ppu-3-4	NC_002947.3	+	5989185	5989237	KefB (COG0475)Kch (COG1226)→ **RNA**→ hypo→
Ppu-4-2	NZ_AAVY01000017.1	+	134874	134926	←Glycos_transf_2 (pfam00535) **RNA**→ hypo→ hypo→ hypo→
Psy-2-3	NC_007005.1	+	5651557	5651610	←ComJ (COG2961) **RNA**→ hypo→
Psy-3-2	NC_004578.1	-	447545	447492	←ComJ (COG2961) **RNA**→ hypo→
Psy-1-4	NC_005773.3	+	5451255	5451308	←ComJ (COG2961) **RNA**→ ←hypo
Psy-3-3	NC_004578.1	-	6095069	6095018	DnaJ (pfam00226)→ **RNA**→ hypo→
Psy-2-4	NC_007005.1	-	5828745	5828694	DnaJ (pfam00226)→ **RNA**→ hypo→
Psy-1-5	NC_005773.3	-	5617216	5617165	DnaJ (pfam00226)→ **RNA**→ hypo→
Ppu-4-3	NZ_AAVY01000018.1	+	10292	10344	←lipoprotein, putative **RNA**→ hypo (cons)→
Ppu-4-4	NZ_AAVY01000002.1	+	296036	296087	OprD (pfam03573)→ **RNA**→ hypo (cons)→
Pen-1-1	NC_008027.1	+	3960544	3960595	←NUDIX (pfam00293) **RNA**→ hypo→
Pen-1-2	NC_008027.1	+	3853964	3854015	←hypo **RNA**→ hypo→
Pen-1-3	NC_008027.1	-	910473	910421	←HemA (COG0373) **RNA**→ hypo→
Ppu-3-5	NC_002947.3	-	1995826	1995774	dTDP_sugar_isom (pfam00908)→ **RNA**→ COG1835 (COG1835)→
Ppu-1-2	NC_009512.1	+	4999822	4999873	←PsiE (pfam06146) **RNA**→ hypo→ hypo→
Ppu-3-6	NC_002947.3	-	1420112	1420060	←Rick_17kDa_Anti (pfam05433) **RNA**→ hypo→
Ppu-1-3	NC_009512.1	-	1439694	1439642	←Rick_17kDa_Anti (pfam05433) **RNA**→ hypo→ hypo→
Pen-1-4	NC_008027.1	+	4896010	4896062	FecA (COG4772)→ **RNA**→ hypo→
Pen-1-5	NC_008027.1	+	1180693	1180743	DUF479 (pfam04336)→ **RNA**→ hypo→
Pen-1-6	NC_008027.1	-	3292013	3291963	hypo→ **RNA**→ hypo→
Pen-1-7	NC_008027.1	-	1493248	1493198	←LysR (COG0583) **RNA**→ hypo→
Pen-1-8	NC_008027.1	+	2592660	2592712	FhuE (COG4773)→ **RNA**→ hypo→ hypo→
Ppu-3-7	NC_002947.3	-	2855987	2855935	←hypo **RNA**→ ←DltE (COG0300)
Ppu-4-5	NZ_AAVY01000008.1	-	11017	10965	Aes (COG0657)→ **RNA**→ hypo (cons)→
Ppu-4-6	NZ_AAVY01000001.1	+	243123	243175	←AbgB (COG1473) **RNA**→ hypo (cons)→
Ppu-3-8	NC_002947.3	-	5149089	5149038	hypo→ **RNA**→ hypo→
Pen-1-9	NC_008027.1	+	4355520	4355572	←hypo **RNA**→ hypo→ hypo→
Ppu-3-9	NC_002947.3	+	5390561	5390612	hypo→ **RNA**→ hypo→
Ppu-2-1	NZ_AAXR01000004.1	+	377710	377762	←COG3714 (COG3714) **RNA**→ hypo (cons)→
Pen-1-10	NC_008027.1	+	5582431	5582483	←DUF1234 (pfam06821) **RNA**→ hypo→
Ppu-1-4	NC_009512.1	+	3624986	3625038	hypo→ **RNA**→ hypo→
Ppu-3-10	NC_002947.3	-	2858122	2858070	hypo→ **RNA**→ yedG domain protein→
Ppu-4-7	NZ_AAVY01000026.1	-	47983	47932	hypo (cons)→ **RNA**→ hypo (cons)→
Pen-1-11	NC_008027.1	+	2692719	2692771	COG2863 (COG2863)→ **RNA**→ hypo→
Pen-1-12	NC_008027.1	+	2652730	2652782	←RpoE (COG1595) **RNA**→ hypo→
env-5	JCVI_SCAF_1101668291562	+	813	865	R_hydratase (cd03449)→ **RNA**→ ? (GOS11008091)→
Psy-1-6	NC_005773.3	+	368971	369023	TauA (COG0715)→ **RNA**→ hypo→
Psy-2-5	NC_007005.1	+	363657	363710	TauA (COG0715)→ **RNA**→ hypo→ hypo→
Eph-4-1	NC_007804.2	-	20476	20424	**RNA**→

env-6	BAAX01012263.1	-	1357	1305	**RNA**→ COG3617 (COG3617)→
Eco-1-1	NZ_AAJW01000017.1	+	44561	44613	Dam (pfam05869)→ **RNA**→ COG3561 (COG3561)→ ANT (pfam03374)→
Eco-2-1	NC_002695.1	+	1263176	1263228	NinE→ **RNA**→ COG3561 (COG3561)→ COG3646 (COG3646)ANT (pfam03374)→ NinG→ hypo→ antitermination protein Q→
Eph-3-1	NC_000902.1	+	17164	17216	**RNA**→
Sco-1-1	NC_004914.1	+	49052	49104	**RNA**→
Sen-3-1	NZ_ABAN01000025.1	-	4146	4094	**RNA**→
Sen-5-1	NZ_ABEH01000004.1	-	43804	43752	**RNA**→
Sen-4-1	NZ_ABAO01000067.1	-	2587	2535	**RNA**→
Sen-1-1	NZ_ABEN01000001.1	-	2762914	2762862	**RNA**→
Sen-2-1	NZ_ABEW01000016.1	+	12497	12549	**RNA**→
Sen-6-1	NZ_ABFF01000012.1	+	15360	15412	**RNA**→
Sen-3-2	NZ_ABAN01000002.1	+	49	101	**RNA**→
Sty-1-1	NC_003197.1	-	2762880	2762828	antiterminator-like protein→ **RNA**→ antirepressor-like protein→
PGi-1-1	NC_010392.1	-	34329	34277	**RNA**→
Api-1-1	NC_000935.1	-	28276	28224	**RNA**→

17.3 Conserved domains

Conserved domains found in protein-coding genes listed in Section 17.2 are listed, with the first sentence in their description from the Conserved Domain Database. Conserved domains associated with more than one c4-a1b1 RNA are assigned a color, while others are shown in gray. The number in parentheses is the number of occurrences of the given gene.

cd00801 (1) Bacteriophage P4 integrase.
cd01029 (1) TOPRIM_primases: The topoisomerase-primase (TORPIM) nucleotidyl transferase/hydrolase domain found in the active site regions of bacterial DnaG-type primases and their homologs.
cd01125 (1) Hexameric Replicative Helicase RepA.
cd03449 (1) (R)-hydratase [(R)-specific enoyl-CoA hydratase]
COG0009 (1) Putative translation factor (SUA5) [Translation, ribosomal structure and biogenesis]
COG0300 (1) Short-chain dehydrogenases of various substrate specificities [General function prediction only]
COG0373 (1) Glutamyl-tRNA reductase [Coenzyme metabolism]
COG0475 (1) Kef-type K+ transport systems, membrane components [Inorganic ion transport and metabolism]
COG0583 (1) Transcriptional regulator [Transcription]
COG0657 (1) Esterase/lipase [Lipid metabolism]
COG0715 (2) ABC-type nitrate/sulfonate/bicarbonate transport systems, periplasmic components [Inorganic ion transport and metabolism]
COG1226 (1) Kef-type K+ transport systems, predicted NAD-binding component [Inorganic ion transport and metabolism]
COG1462 (1) Uncharacterized protein involved in formation of curli polymers [Cell envelope biogenesis, outer membrane]
COG1473 (1) Metal-dependent amidase/aminoacylase/carboxypeptidase [General function prediction only]
COG1595 (1) DNA-directed RNA polymerase specialized sigma subunit, sigma24 homolog [Transcription]
COG1835 (1) Predicted acyltransferases [Lipid metabolism]
COG2085 (1) Predicted dinucleotide-binding enzymes [General function prediction only]
COG2863 (1) Cytochrome c553 [Energy production and conversion]
COG2961 (3) Protein involved in catabolism of external DNA [General function prediction only]
COG3561 (2) Phage anti-repressor protein [Transcription]
COG3617 (6) Prophage antirepressor [Transcription]
COG3646 (1) Uncharacterized phage-encoded protein [Function unknown]
COG3714 (1) Predicted membrane protein [Function unknown]
COG3950 (1) Predicted ATP-binding protein involved in virulence [General function prediction only]
COG4772 (1) Outer membrane receptor for Fe3+-dicitrate [Inorganic ion transport and metabolism]
COG4773 (1) Outer membrane receptor for ferric coprogen and ferric-rhodotorulic acid [Inorganic ion transport and metabolism]
COG5519 (2) Superfamily II helicase and inactivated derivatives [DNA replication, recombination, and repair]
GOS11008091 (1) ?
GOS11069324 (1) ?
GOS11076434 (1) ?
GOS11725977 (1) ?
GOS11900477 (1) ?
GOS1771 (1) ?
pfam00106 (1) short chain dehydrogenase.
pfam00226 (3) DnaJ domain.
pfam00293 (1) NUDIX domain.
pfam00535 (1) Glycosyl transferase.

pfam00583 (2) Acetyltransferase (GNAT) family.
pfam00908 (1) dTDP-4-dehydrorhamnose 3,5-epimerase.
pfam01590 (1) GAF domain.
pfam01807 (1) CHC2 zinc finger.
pfam02447 (1) GntP family permease.
pfam03374 (2) Phage antirepressor protein.
pfam03573 (1) outer membrane porin, OprD family.
pfam04336 (1) Protein of unknown function, DUF479.
pfam05433 (2) Rickettsia 17 kDa surface antigen.
pfam05869 (1) DNA N-6-adenine-methyltransferase (Dam).
pfam06146 (1) Phosphate-starvation-inducible E.
pfam06821 (1) Protein of unknown function (DUF1234).
pfam07081 (1) Protein of unknown function (DUF1349).
smart00062 (1) Bacterial periplasmic substrate-binding proteins; bacterial proteins, eukaryotic ones are in PBPe
smart00292 (1) breast cancer carboxy-terminal domain;

Duplicate sequences: the following putative homologs are not shown in the alignment because their sequences are identical to a homolog already shown: Eph-3-1, PGi-1-1, Sco-1-1, Sen-1-1, Sen-2-1, Sen-5-1, Sen-6-1

17.4 Multiple-sequence alignment

Each c4-a1b1 RNA is denoted by an abbreviation (like "Eco-1-1") that refers to a taxonomy given in Section 17.1. The alignment includes sequences containing the RNA motif, as well as flanking sequence. The c4-a1b1 RNA itself is denoted by the line underneath marked 5′ and 3′ on either end. Nucleotides in the flanking sequences (i.e., not part of the motif) are written in gray letters. Stems of predicted rho-independent transcription terminators are shaded yellow. (Note: terminator predictions have not been analyzed manually, and many are likely to be false positives.) Start codons are shaded green. (Note: start codons are frequently misannotated, especially in environmental samples.) Nucleotides proposed to basepair as part of the consensus structure are shaded in color when they comprise Watson-Crick or G-U pairs. Otherwise they are shaded gray. Conserved stems are also indicated at the bottom of the alignment by angle brackets, where matching < and > denote base-paired columns. Below these angle brackets, the symbol "2" denotes base pairs exhibiting covariation, "1" denotes base pairs exhibiting compatible mutations, "0" denotes base pairs that are not observed to mutate and "?" denotes base pairs that have a significant frequency of non-canonical nucleotides for Watson-Crick or G-U pairs (> 5%). Below these base pair annotation is the consensus sequence: "R" = "A" or "G", "Y" = "C" or "U", red nucleotides: nucleotide identity conserved more than 97% of the time, black nucleotides: 90%, gray nucleotides: 75%, red circle (●): nucleotide is present 97% of the time, black circle (●): 90%, gray circle (●): 75%, white circle (∘): 50%. All percentages of sequences just described (e.g. 97% conserved) assume that sequences have been weighted by the GSC algorithm implemented by the Infernal software.

alignment positions 1 · · · 160

```
Pme-1-1  UUGGUGAAGCACGGAUGACCCACAAUCGGCGGACGCUCCAGGCGUCCGCUUUUGGCCGAUAGCAGCCACUCCACGCCUUGCCCUCUCCCUUACCCCGCGCUACAGUCCACCCC.GUCACGGUCAAU.CCCGUGAC.CGGGUGUGGUAA.CCCGUGGUAAU
Psy-1-1  AUUUUGCAGGAAAGGAUGAAGUCGCUCGACUGAAAAACCCCUUCGCCCUGUGUGAAGUUUCCGAGAAGACUUCAGUUGCUUGCGCGGGCUGUAUCGAGGAUUUAAGGUGCGCC.GUCGCUGCAAAA.UCAGCGGC.CGGAUUUAGCGA.UCCGAAUACCU
Pfl-2-1  AUUCCCACCGGAAUGAGCUGCGCAACACCCUUCAAAUCCAUCGGAUUUUUCCUGCAACGCGCGGCGGCACUGAGGAACUGGUGCUCCGAGAGCCGUGCCGAUAACCUUUCCGA.GCCGCUGCAAAA.UCAGCGGU.CGGAUGUGGAAG.UCCGGUGAAUC
Ppu-3-1  UUUUCUGAUUAUUGCCAAAAUGUCUGUCCAGCUGGGCACUUCGGAAAUAUCCUACGACCCGCGUCCUCUGCCAUCACCUUCUCAGUGGGAACCUCUGCCCCUAGGCUCAAACC.GUCGCCGAACAACUUGGCGAU.CGGGUGUGGUAA.CCCGGGUUCUG
Eph-1-1  AACUGUGUAUUUAUUAAACAAAUAAUGCUCUAAUAAAUUUGUAUUUUUAAGUCGCGAAUGCUAUCUUUUCGCAUCAUAUUGACCUUUUAAUCGUUCAGGCUUAUAGUUCCACC.GUCGUAGCAAAU.UCUGCGAC.CGGUUUUGACAG.CCUGAAUGUUA
Pme-1-2  AGACGAGUUCGAACAUGCCGCCGCCGAAUCAGAGGCCGAGGAGUUACCCGUCUCCAAUCUCAGCUACGCCAGAGUAGGUUGCCAUCCUGAAACCCUCGGGCUAGAGUGCGCCC.GUCGUGGUCA.AUCCCACGGC.CGGGUGUGGUAG.CCCGAUUCACA
env-1    ..................................................ACCACAAUCCAAACCAGACCAUGCCUGGUUGCCAUCCUGAAACCUGCAGGCUAGAGUUCGGCC.GUCGUGGUCAAU.CCCACGAC.CGGGUGUGACAA.CCCGAAUCGUU
env-2    UCCCACAGUGGUUUUGUGCAAGGUUUGAACAGGGUGUCGUCGGAGAUUUCCCACGUGUUGUCGCGCUGUGCGUGAACUUGUUGGACGGUCUUGGGGUGGCUAGUCUUUGGCCU.GUCGCUGCAAAU.UCAGUGAC.AGGGCGUAGGAA.CCCUUUUAUGU
Eph-2-1  GCCGGACGCAUUGCCGGACGCAAACGGGGGUACGACGCAUGAUGAUGCCCGCUCUGCAAAAACUCCCUUUUUCUGGCUUGCCUUUUUCCGGCAUUUGCGGAUAUAGUUUUUCG.CCUGCCGCAAAA.UCGGCAGC.CGGGCGUGAGAA.CCCGAGCAAUU
Ppu-4-1  CCCUUCAACGGCAGUCAUGCCCAUGCCAUAGGACGGGCACCACUGAGGGCACGACUCUAGAAAAGCCGCGUACCCCUGUUGCUAUACACGGACAAACCGGAUAUCUUUUCGUC.GUCGCUGCAAAU.UCAGCGAC.CGGGCGUAGGAA.CCCGAAUUCAA
Yph-1-1  AUUUAAGUAAAACACCUUAAAACACGCAAAGCAACACAAUAAACGCAAAAGAUUAAAAUUUAAGCACAAUGAUUUUUAUUGACGAGAUGUGUUGAUGUCGCUAUGGUUAUGAC.GUUGCAGCAAAA.UCUGCGAC.CGGGCGUAGGAA.CCUGAACCCCA
Sbo-1-1  AGGCGCUUACCCUCAACAAAAAGAUGGUGAGCCGUGCACAGGAAAAACUCUUAAAGGGUUACGGUUUUAUCUGGUAUGACCACCUAGCAGAGAAAAGCUGUUAUAGUUCAAGC.GUUGCUGCAAAA.UCAGCAAC.CGGGCGUGGAAA.CCCGAACACAC
Kph-1-1  CUGAUCAGUUAGUAAAGCAAACUAUAAAAACCCCUGUUGCCGUGACUGGUCACAGGGUAAAAUCUCCAUGUAAUUUAUUGACGUGCGUUGCGCUUUGGCGGUAAAGUUAGCCC.GCUGCAGCAAAU.UCUGCAGC.CGGGCGUAGGAA.CCCGUAUCCAG
Eph-5-1  CUGAUCAGUCAGUAAAGCAAACUAUAAAAACACCUGUGCACGUGACUGGUCACGGGGUAAAAUAUCCACCUAAAUUAUUGACGUGCGUUCUGCUUUGGCGGUAGAGUUACCCC.GCUGCAGCAAAA.UCUGCAGC.CGGGCCUCUCAACCCCGAAUGACA
Psy-3-1  CAUUGCGCCGCAGCCCCCCCAACAACCCGGCAAACCCUCCAGACUUUCGUUCUGCUCGACGAGCUACUAUCCCCCCUUGCCACCCCACCCCCACCACGCUAACCUCCCCGCCG.UUCGCUGCAAAA.CCAGCGGC.UGGGUUUGGCGA.CCCAAAGAAAA
Psy-2-1  ACAACGCAAAACCUCACGCACCUUACCCACCCAAGAAAAACGACAUUUCCUACGCCCCAAACCCACUUCCACACCCUUGCCACCCCACCCCUGCCACGCUAACCUCCCCGCCG.UUCGCUGCCAAA.UCAGCGGC.CGGAUUUGACGA.UCCGUUCUAUC
Psy-1-2  AACCGGAGGCGUUGAGGUAAAUCUACAUGCACGUCAAAUGUAUUUCGCAUCGAGCGCGAGUGCAAGUCGCUCAAACAGUUGCACAUCCCAAAACUGGACGUUAUUCUCUGGCC.GUCGCUGCGAAC.UCAGCGAC.CGGGCUUGGCGG.CUCGAACAGUA
Pae-6-1  AACGCCGGGUUAUUUCCAUGAAUCGCUUCAGCCAACGCCUUGCCUGCCGAUAGCCUCUGCCGUCACCCGUUCCCGCCCUUGCUCCCCGCCUUGCCUGCCCCUAUCCUGCGCCC.GUCACGGCCAAA.UCCGUGAC.CGGGUUUGACAG.CCCGAAGAGAC

         ..................................................................................................................<<<<<<<.......>>>>>>>.<<<<.........>>>>.......
         ..................................................................................................................2222222.......2222222.2212.........2122.......
         ●●●●●●●●●●●●●●●●●●●●●●●●●●●●●●●●●●●●●●Y●●●●●●●●●●●●●●●●●●●YY●●●●●●●●●Y●Y●●●Y●●UURY●●●●●●●●●●●Y●●●●●●YUA●●●UY●●Y●●-GUCGCYGC●RA∘-UCRGCGRY-CGG●YYUGRYAR-●CCG●●●●●●●
                                                                                                             5′————————————————————————————————————————————————————————3′
```

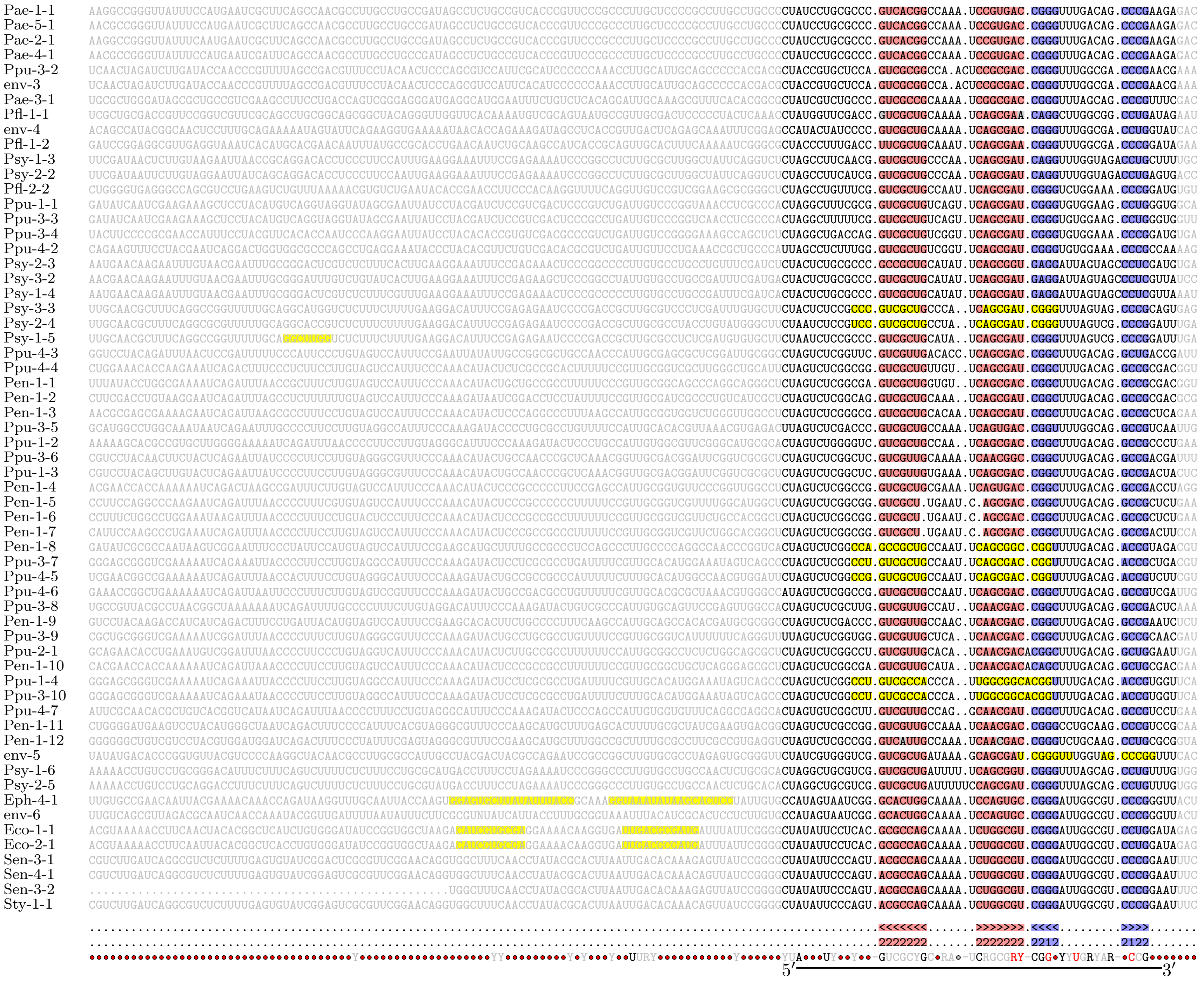
Pae-1-1
Pae-5-1
Pae-2-1
Pae-4-1
Ppu-3-2
env-3
Pae-3-1
Pfl-1-1
env-4
Pfl-1-2
Psy-1-3
Psy-2-2
Pfl-2-2
Ppu-1-1
Ppu-3-3
Ppu-3-4
Ppu-4-2
Psy-2-3
Psy-3-2
Psy-1-4
Psy-3-3
Psy-2-4
Psy-1-5
Ppu-4-3
Ppu-4-4
Pen-1-1
Pen-1-2
Pen-1-3
Ppu-3-5
Ppu-1-2
Ppu-3-6
Ppu-1-3
Pen-1-4
Pen-1-5
Pen-1-6
Pen-1-7
Pen-1-8
Ppu-3-7
Ppu-4-5
Ppu-4-6
Ppu-3-8
Pen-1-9
Ppu-3-9
Ppu-2-1
Pen-1-10
Ppu-1-4
Ppu-3-10
Ppu-4-7
Pen-1-11
Pen-1-12
env-5
Psy-1-6
Psy-2-5
Eph-4-1
env-6
Eco-1-1
Eco-2-1
Sen-3-1
Sen-4-1
Sen-3-2
Sty-1-1
5′
3′

```
Api-1-1   CUGGGCCAUCAGGUCAUCGUACUGGACAGUCAGGAUCUAAGCAGCAUCUUACCCGCUGUGAACUGAAAAUUAACUUAUUGACGAAAGAAGAAAUCAUGCGCUAUAGUUUAUUU.GCAUCUGCAAAA.UCAGGUGU.CGGGUUUGAGAC.CCCGUAUUAGC
          ..................................................................................................................<<<<<<<.......>>>>>>>.<<<<.........>>>>.......
          ..................................................................................................................2222222.......2222222.2212.........2122.......
          ••••••••••••••••••••••••••••••••••••••Y•••••••••••••••••••YY•••••••••Y•Y•••Y••UURY•••••••••••Y••••••YUA•••UY••Y••-GUCGCYGC•RA∘-UCRGCGRY-CGG•YYUGRYAR-•CCG•••••••
                                                                                                    5′————————————————————————————————————————————————————3′
```

alignment positions 161 · · · 320

```
Pme-1-1   CAAGGCGCGAAGCGCCAUAGCUCUGAAAUAGCCGGCGUUUUUUAUGCCUGCUGUUUUCGUGCUUCUAUGGCGGGCCGUGUGAGGCGGGCUUCGGCCCGGCCGGUUCCCUUGGUUCCCGGUUUACCACCCUUGCACGGUCCGCCUCCAUUCGUGUGGUAA
Psy-1-1   CACAGCUAGCUUAUGUAUGCUUCCAGCCUCAGUGCAUUUGAAGCAACUGUUAUGGCGGCUGUGCGUCGGAGACUCCAGGGUCUGCCGGCGUUGUGAGGUGCCGGUUCGCUAACCUUCGCACAGCCGCCACCCAUUUCGUCCAGUGAUGUGCAGGCGGCUC
Pfl-2-1   CCGAAAGCGCAUGUGCGCCUGUAAACACUUGUUGGCGCCUUUUAUGCCAACAUUGUUUGCUUAUGGCAGCUAUGCGCAGGAGCACGCUCGAGUGCACCGGGUUUUUUCGGGGUCCCGGACUUCCACACCUGCGCAAAGCUGCCACCCUCUCGUGGAAGGG
Ppu-3-1   GCUGAUCUUGGCAGUCCAUGGCGGCUGUUCGCAGGAGGCUUCGUGCCUGCUGGGUUUUAGAUCUCCCACCCGGUUUACCACCCUGCGUACAGCUGCCACCCAUUCGUGUGGUAACGGACUGGGUGGGGCAUGAAGCGAGAUCUAGUGAUGAAGAAGAUU
Eph-1-1   GUGCGGACAACCGCAGAUUUCCGAUAUUGCGGUAUUUUUGUGUCCGUAAACCGCGUUACGCCCGAAUUAUGGUGGGGCGUGAUGGGGAGGCUUCGGCCUGCUGGUUUCACUAACGCCAGUCUGUCAACCCUGUCACGUCCUGCCACCUGUUUGACAGCGG
Pme-1-2   CGAAGGCGCGGUUGCGUCAUAAACGAGAGUAGGCCCUUCUCAGGAUCUGCUGUUUUCUCGUGCAUUUAUGGCGGGCCGUGUGAGGCAGGCUUUUGCCUGGCCGGCCUUCCUUCGUGGGUCGGUCUACCACCCUUGCACGGUCCGCCUCCAUUCGUGUGGU
env-1     CGAGGCGCGGUAGCGCCAUAGCCUAAGUAGCCAGCACUCCAAGUGCAUGGUUACUUAAUUUCUAUGGUGGACCGUGUGGGGCAGGCUUUUGCCUGGCCGGUUCCCUCGGACGCCGGUUUGUCACCCCCGCACGGUCCGCCACCCAUAGCCGAGUGACAAC
env-2     ACCCUUGGCGUCAUAGCGCCACCGUCUGAUUGCGGCGUUUUUUUUGUGCCCGUACUAUCAGUCGCGGCGGCUAUGCGCGGGCACGUUUCGACGUGGCUGAGAUCCCUGGGUACUCGGUUCCUACCCCGCGUAUGGCUGCCACCCAUGCCCGUAGGAAGGC
Eph-2-1   CACAGGCGACAUAUGACGCGCCACGCGUCUUUUUUUGUGUCGCAAUCAAUGCCACAGAGCGCCAGAUUAUGGUGUGGUGUGUGGUCAGUCGUGCAGAUGGCAUUCCGUGCAUCCUGCCCGCGUCCGCUCAUUAUGCCGCAGAGUCAAUGGUAGCUCAGGC
Ppu-4-1   GAAGGCGCAUAGGCGCCCCAUUUCAAUCGCAGGCGCUUUUUUGUGCCCGCGAUAUUUAGUUAUGGCGGCCGUGCGUGGGCAGACUUCGGUCUGGCCGGGGCCCUUCUUGCCCGGUAUUCCUACCCCACGCAUAGCUGCCACCCAAUCCCGUAGGAAGGA
Yph-1-1   UCGGCGGCACAUACACGCUAGGCGUGUUUUUUUGUGUCUAUGCCUGAGCACAGCCAAAUUAUGGCAGUCCAGGCGGAGCAGCUUUCGAGCUGGCCGGUAUCCGUUGGGGCCGGUAUUCCUACCUCCGUUUGGGCUGCCACCCCAUAGAGUGUAGGAACUC
Sbo-1-1   UCAGGGCGACACAGACGCCGAAAGCGUCUUUUUUUGUGUCCGAUCACUACGCACAUCAAAUCAGCGGCCUAGUCCGUACUGUAUCAAUGGUGGCGUUAGUGGGGCUACGUUCGCGUAGGCCGGUUUCCUUUGUGUCCGGUAUUUCCACCCCUGCUAACGU
Kph-1-1   UCAGCGCACAACACGCGCCUGCGUGUUUUUUUGUGUGUAAUACCUGUGCACGCCAGAAUUAUGGUGGCUCAGGCAGGGCAGCUUUCGGGCUGGCCGGUAGCUGAUUGGGGCCGUAUUCCUACCUUGUCUGGGUCACCACCAACGAGUGUAGGAACUCCG
Eph-5-1   AGAAGCGCACAACACGCGCCAGCGUGUUUUUUUGUGUGUUAAAUCUGCGCAUACCUGAAUUAUGGUGGCUCAGAUGGGGCCAACUUCGGUUGGGCCGGUUUCUUCUUGUACCGGUGUUGAGAACCCUGUCUGGGCUACCACCCUAUAGAGAUUCUCAAC
Psy-3-1   GACAAAAAUACGCACCAUCGCAAAGCAGCAGAUUCUAUCGGUCUGACUUUCGCGCUAUGGCGGCUGUGUGCGGGAGACCUUCGGGUCUGCCGGGGUUUGCUUUUUGCCCGGUUCGCCAACCCUCGCACAGCUGCCACCCAUUCGUUUGGCGACGAUUCG
Psy-2-1   CAGGCGUGUCAGCGCCCCACACACUGCAUCAAGCUAAACUUGAAGUACGUGCUAUGGCGGCUGUGCGUCGGAGACCUUCGGGUCUGCCGGUUUCCUGGAUACCGGUUCGUCAACCUUCGCACAGCUGCCACCCAUUCGUUUGACGACGUCUUGUGGCAGU
Psy-1-2   CAAGGCGCAACAGCGCCCCCAUUACGAUUGCAGGCGCUUUUUUGUGCCCGCAUUCUCGCGUUAUGGCGGGUUGCGCAGGAGCACCCUCGGGUGCGCCGGUCUCCUUGUACGCCGGUCCGCCAAUCCUGUGCAAUCCGCCACCCUAAUCUGCUUGGCGGC
Pae-6-1   GGCGCGAUGCGCCGAGCGGUGCAUGCGAAAGCAGCGAAUGCUUUUGCAGGCAUCGGCUCGACCGCAGUUUUCCAACUGCGGGCCUUCAUGGCGGAUCGCGUGGGGAGACCCUCGUGGUCUGCCGGUUCCGUUUCCCGGUCUGUCAACCCUGCGCGAUCCU
Pae-1-1   GGCGCGAUGCGCCGAGCGGUGCAUGCGAAAGCAGCGAAUGCUUUUGCAGGCAUCGGCUCGACCGCAGUUUUCCAACUGCGGGCCUUCAUGGCGGAUCGCGUGGGGAGACCCUCGUGGUCUGCCGGUUCCGUUUCCCGGUCUGUCAACCCUGCGCGAUCCU
Pae-5-1   GGCGCAAUGCGCCGAGCGGUGCAUGCGAAAGCAGCGAAUGCUUUUGCAGGCAUCGGCUCGACCGCAGUUUUCCAACUGCGGGCCUUCAUGGCGGAUCGCGUGGGGAGACCCUCGUGGUCUGCCGGUUCCGUUUCCCGGUCUGUCAACCCUGCGCGAUCCU
Pae-2-1   GGCGCGAUGCGCCGAGCGGUGCAUGCGAAAGCAGCGAAUGCUUUUGCAGGCAUCGGCUCGACCGCAGUUUUCCAACUGCGGGCCUUCAUGGCGGAUCGCGUGGGGAGACCCUCGUGGUCUGCCGGUUCCGUUUCCCGGUCUGUCAACCCUGCGCGAUCCU
Pae-4-1   GGCGCGAUGCGCCGAGCGGUGCAUGCGAAAGCAGCGAAUGCUUUUGCAGGCAUCGGCUCGACCGCAGUUUUCCAACUGCGGGCCUUCAUGGCGGAUCGCGUGGGGAGACCCUCGUGGUCUGCCGGUUCCGUUUCCCGGUCUGUCAACCCUGCGCGAUCCU
Ppu-3-2   AGGCGCAUCGGCGCCCCCGACCACGAUUGCAGGCGCUUUUUUGCGUCCGCAGUUUCGUGUAAUGGCGGCUGUGCGUGGGAGACCUUCGGGUCUGCCGAGUUCCUUUUCCUCGGUUCGCCAACCUGCGUACAGCUGCCACCCUAAUCGUUUGGCGACGGU
env-3     AGGCGCAUCGGCGCCCCCGAUCACGAUUGCAGGCGCUUUUUUGCGUCCGCAGUUUCGUGUAAUGGCGGCUGUGCGUGGGAGACCUUCGGGUCUGCCGAGUUCCUUUUCCUCGGUUCGCCAACCUGCGUACAGCUGCCACCCUAAUCGUUUGGCGACGGU
Pae-3-1   AAAGGCGCACCAGCGCCCAAUUCCCCAUUGCAGGCGUUUUUUUUGUGUCUGCAAUGCCGAGUUAUGGCGGCUGUGCGCGGGGCACCUUCGGGUGCGCCGGGUUCCUUUGUCCCCGGUCUGCUAACCCGCGUACAGCUGCCUCCCAUCGUUUAGCAGCGAU
Pfl-1-1   CGAGGCGCAACAGCGCCCCACCCACAUGCGGGCGCUUUUUUUGCGCCCGCAUACCUGCGUUAUGGCGGGUUGCGUGGGAGAACCUUCGGGUUCGCCGGUUCCUUGAUUCCCGGUCCGCCAAUCCUGCGCAAUCCGCCACCCUGAUCUGCUUGGCGGCAG
env-4     GGCGGGACACACCGCACUUCCUACCAGAUUUGGCGGUUUUUUUGUGUCCNNNNNNNNNNNNNNNNNNNNNNNNNNNNNNNNNNNNNNNNNNNNNNNNNNUAGGGCGACGGUCUUAAACGACCAUCACAGCAGGAGUUAUGCCAUGCAUAACGACACCCA
Pfl-1-2   AAGGCGCACAGGCGCCCCCAUUACGAUUGCAGGCGCUUUUUUGUGCCCGCAUGUCCGUUUUAUGGUGGCUGUGCGUGGGAGACCUUCGGGUCUGCCGGGUUCCUUUUCCCCGGUUCGCCAACCUGCGCACAGCUGCCACCCUAAUUCGUUUGGCGACGA
Psy-1-3   UGGCGCAAUGUGCCACCUCUGCGAGGCGAUCCUUUUCGUCUCGUCUUUAUGGUGGCCGUGCGCAUGGCGUCUUCGGAUGCACCGGGUGCCAGCAGCCGGUCUACCAACUUGCACACGGCCGCCACCUUAUCGUUUGGUAGCGAUUAGUGACGGCUCCCCC
Psy-2-2   UGUGCGCAAUGCGCCACCUUCUCAAGGCGCAUUUUUUCGGCCUGCGUUUAUGGUGGCCGUGCGCUUGGCGUCUUCGGACGCACCGGCUUGCCUGGUCACCGGUCUACCAACCUGCGUACGGCCGCCACCCUGAUCGUUUGGUAGCGAAAGGGGCGGCUCC
Pfl-2-2   GAGAAAGCGAAAUCGCUUUUCAUAACGUAUGCUUGCGCACCUUCAUUGUGUGCACGCCGUUAUGGCGGCUGUGCGCGGGAGACAUUUAUGUCUGCCGGGUCGCGUUCUCCCCGGAUUUCCAGCCCGCGUACAGCUGCCACCCAUUCUUCCUGGAAGUCGA
Ppu-1-1   CAUGGGACAGUUGUCAUUUUUGGCGGCUGUGCGCGGGAGGUAUUCGUACCUGCCGGGUCUACCGUGUGCCUGGUCUUCCACCCCGCGUACAGUUGCCACCCUAAUGUGUGGAAGCAGAAGGGGACAACACAGCCAACACGGAGCUGUCAUGAAAAAACUG
Ppu-3-3   UGGCGGCUGUCGAUAUGACGGUUGUGCGUGGGAGGCCUUCGGGCCUGCCGGGUUUGCCAUACCCCUGGUCUUCCACCCCGCGUACAACUGUCACCCUAAGUGUGGAAGCGAAAGGGUGCUAUCAGCCCGGUAUGGUGAACUCAUGAAAAAACUGGUCCCC
Ppu-3-4   UGAAGGGCUGCCGUUAUGGCAGUUGUGCGCGUGAGGCCGUUUGGCCUACCGGCUUGCCUUCUCACCGGUUUUCCACCCCGCGCACAACUGCCGCCUUCUGCGUGGAAACGGGUGGUUGCAGUUCAAUCCUGGAGAAGGAAUUGCCAUGAAAAGAAUCGUC
Ppu-4-2   CAUUUCGGGUUUGUCGUGUAAUGGCGGCUGUACCCGGAAGGCCUCGUGCCUACCGAGUUUUCCUGAAUGCACUCGGGUUUCCACUCCGUGUACAGCUGCCACCCUUCCUGUGGAAACGGAAGAGGCAGACUCAAGCGUUCAGGAGUUGCUCAUGAAAAAA
Psy-2-3   UUAGGCGCAAUGCGCCACCUUGUGCAAGGCGCUUGUUUUCGGCCUGCAUCUUUAUGGUGGCCGUGCGUGGGGCGUCUUCGGAUGCACCGGUUCCUAAUCACCGGGCUACUACCUGCGUGCGGCCGCCACCUUCGAUUAGUAGCCGAAGUGACGACCUUCC
Psy-3-2   AGGCGCAUCGCGCCACUUCGUGCAAGGUGCUUUCAACGGCCUGCACUUUUAUGGUGGUCGUGCGCGGGGCAUCUUCGGAUGCACCGGGGUUCCUGGACCGGGCUACUACCUGCGCAUGACCACCACCCUCGCCUGGUAGCCGACGGUAAUGGUCUUUUAA
Psy-1-4   CCAGGCGCAUUGCGCCACCUUGUGCAGGUGCCCUUUCACGGCCUGCAUUUUUUAUGGUGGUCGUGCGUGGGGCAUCUUCGGAUGCACCGGGGUUCCUGGACCGGGCUACUACCUGCGCAUGAUCACCACCCUCGCUUAGUAGCCGACGGUGAUGGUCUUC
Psy-3-3   UUAAGGCGCAACACAGCCGUGUUUCAUCAGGUUUUGUCCCGAUGCUAUGGCGACUGUGCGCUUGGCACCUUCGGGUGCGCCGGUUCUUAGCUCACCGGUCUACUAACUUGCGUACAGUUGCCGCCUUUCGUUUAGUAGCGAGCAGGCAGCUACUUCAUAG
Psy-2-4   GUUAAGGCGUAUCGCGUCGCUGUUUUGGUCAGGUCUUCUUCCUGCACUCUAUGGCGGCUGUGCGUGAGGGCACUUUCGGGUGCGCCGGUCUUAGCUCCCGGUCGACUAACCUUUCGCACAGCUGCCACCUCAAUCGUUUAGUCGCGACCCAGUGGCAGUU
Psy-1-5   GUUAAGGCGUAUCGCGUCGCUGUUUUGGUCAGGUCUUCUUCCUGCACUUUAUGGCGGCUGUGCGCGAGGGCACCUUUGGGUGCGCCGGUCUUAGCUCCCGGUCGACUAACCUUCGCACAGCUGCCACCUCACUCGUUUAGUCGCGACCCCAGUGGCAGUU
Ppu-4-3   CAAGGGUCCUUUGCAGCGCUUCAUGGCAGCUGUACAUGGGGCACCUUCGUGUGCGCCGGGUUCCUUGGGUCACCGGUCUGUCAACCCGUGUAUAGCUGCCUCCAUUCGUUUGACAGCGUUUAAAGGUAGCUCCACCUGACUCAAGGAGCAUUCAUGCGCA
Ppu-4-4   AUUAUGCAUACGGCUUGCCUUCAAUGGCGGCUGUACGUGCGGGCACGCUUGCGUGCGCCGGAACUUGCAUAGUCCGCCGGUCUGUCAACCCAUGUACAGCUGCCACCCUUCUGUUUGACAGCGGAUAGGUGACCGCCUAAUUGAAGGUACUAUGCAAUGC
Pen-1-1   AUUAAGCAUGCGGCUAGCCCUCGAUGGCGGCUGUACGUGCGGGCACGCUCGCGUGCGCCGGAACUUUGCUUAUUCCGCCGGUCUGUCAACCCGCGUACAGCUGCCACCCUUCUGUUUGACAGCAGACAGGUGGCUGCCCUCAAAGGAAUAAGAAGAUGCU
Pen-1-2   ACACCUGCACUGCUUGCCCUCUAUGGCGGCUGUGCAUGGGGCACGCUUGCGUGCGCCGGUUUUUGUGUCCGCCGGUCUGUCAACCUAUGCAUAGCUGCCACCUACCUGUUUGACAGCAGUCCGGUAGCGGCCCCAUCAAAGGGCACAAAACCAUGCUGAA
Pen-1-3   AAAAACCUCAUGGCUUGCGCUCUAUGGCGGCUGUGUGUGGGGCACCUCGUGUGCGCCGGUUUUUUGGCUUUUUCCUGCCGGUCUGUCAACCCAUGCACAGCUGCCACCCUCUUGUUUGACAGCAGAUGGUGUCGGCCCUUUCUUCAGGAAAAAUACCGA
Ppu-3-5   UUGUCCGUGUGCUUACGCUUAUGGCGGCUGUACGUGCGGGCCCCCUCAUGUGUGCCGAGUGCCAACAAUCUCGGUCUGUCAACCCAAGUACAACUGCCACCCUCCCGUUUGACAGCGAGGUGGAUAGCAGCUCAACCAUUGCUGGAGUGUAACCAUGUUG
Ppu-1-2   CUAACCACAUGGCUUUGCCUUUAUGGCAGCUGUCAUGGGGCACCUCGUGUGCGCCGGCUUUUGGUUCUGUUCACCGGUCUGUCAACCCAUGUACAGCUGCCGCCUCAUUGUUUGACAGCAGGAGGUGGUGGCCCCAACUUUGAACAGGCCUUCACCAUGC
Ppu-3-6   CCCGCACACGUGCACUGUUUAUGGCGGCUGUACGUGGGGCACCUUCGUGUGCGCCGAGUUACUCCGGGAUCCUCGGUCUGUCAACCCAUGUACAGCUGCCACCCUCUUGUUUGACAGCAAGCUUGCGGCAGCUCCAAUGAUCCUGGAGAUGUUUAUGCGC
Ppu-1-3   AAGUUUCCGUUGCAGCGCUUAAUGGCAGCUGUGCAUGGGGCACAUUCGUGUGCGCCGGGUCCUUGGGUACCGGUCUGUCAACCCAUGUAUAGCUGCCUCCAUUCGUUUGACAGCGUUGCCAGGCGGCUCCACUACCCAAGGAGCUUACAUGCGCAAGACA
Pen-1-4   UUCAUUCCCAUGGCUCGCUCUUAUGGCGGCUGUGCGUGGGGCACUUCGUGUGCGCCGGGUUUUGGGAUGCCUACCGGUCUGUCAACCCAUGUACAGCUGCCACCUUCUUGUUUGACAGCAGGCGUGUUGCGGCCCUUUUCAGGUACCCCAAAAUGCUGAA
Pen-1-5   CAGACCGUAUGGCCUUGCCGUUAUGGCAGCUGUGCGUGGGGCACCUCGUGUGCGCCGGUUUCUGGUCUUGUUCACCGGUCUGUCAACCCAUGUGCAGCUGCCACCUUCUUGUUUGACAGCAGGCUGUGGUAGUCCUCAUUCACGAACAGGACUAUGACCA
Pen-1-6   CAGACCGCAUGGCCUUGCCGUUAUGGCAGCUGUGCGUGGGGCACCUCGUGUGCGCCGGUUUCUGGUCUUGUUCACCGGUCUGUCAACCCAUGUACAGCUGCCACCUUCUUGUUUGACAGCAGGCUGUGGUAGUCCUCAUUCACGAACAGGGCUAUGACCA
          ................................................................................................................................................................
          ................................................................................................................................................................
          •••••••••••••••••Y••••••••••••••••••••••••••••••••••••••••••••••••••••••••••••••••••••••Y•••••••••••••••••••••••••••••••••••••••••••••••••••••••
```

```
Pen-1-7   AGAGCAACUUCAACGGCUUUGCUUUAUAUGGCGGCUGUAUGUGGGGCACUUCGUGUGCGCCGGGGUUUUGCUCUUGCUGACCGGUCUGUCAACCCAUGUACAGCUGCCACCCUCUUGUUUGACAGCAGACGGUGGUGGUCCCACUCAUCAGCAAGAGGUC
Pen-1-8   UGCCCUCAUAGGCUCGCUUUCAUGGCGGCUGUACGUGUGGGCACCUCGUGUGCGCCGAGUGCCAACGUCUCGGUCUGUCAACCCGCGUACAGCUGCCACCCUCUUGUUUUGACAGCAAAGUGUGGCGGCUCCAUCUCAUACGUUGGAGUCUAAUCAUGCU
Ppu-3-7   UGUCCUCAUGUGCUCGCUUUCAUGGCGGCUGUACGUGUGGGCACCCUGUGGUGCGCCGAGUGCCAACGUCUCGGUCUGUCAACCCGCGUACAGCUGCCACCAUCUUGUUUGACAGCAAGGUUGAGGUAGCUCCACUUUCCGACGUUGGAGUUACGCCAUG
Ppu-4-5   UGUCCACAUGUGCACACUGUUAUGGUGGUUGUACGUGCGGGCACCCUCUGGUGCGCCGAGUGCCAACGUCUCGGUCUGUCAACCCGCGUACAGCUGCCACCUCAUUGUUUGACAGCAAGGCCGUGGUAGCUCCACCGCAACGCUCAGUUUCUGCGAGCUA
Ppu-4-6   UUGUCCGUGUGCAUACGCUUAUGGCGGCUGUACGUGCGGGCACCUUCGUGUGCGCCGAGUGCCAACAAUCUCGGUCUGUCAACCCGCGUACAGCUGCCACCCUCUUGUUUGACAGCAAGGCGGCUUGCAGCUCAACCAUUGUUGGAGCGUAAGCAUGCUG
Ppu-3-8   UUGCAAUCCAUAGCUGUACCUUCAAUGGUGGCUGUGCGUGGGGCGCCUUCGGGUGCGCCGGGUUUCUCAAUUUGACCGGUCUGUCAACCCGUGCACAGCUGCCACCCUUUGUUUGACAGCGAAUGGCGGUAGUCCCAACCACAAAUUGAGGGUUACACCA
Pen-1-9   CCGAGCACGUGCGCCCCUCAUGGCGGCAGUACGUGGGGCACCUCGUGUGCGCCGGGUCUCGGAGUCCUCGGUCUGUCAACCCACGUACCGCUGCCACCCUCUUGUUUGACAGCAAGCCUGUUGCAGCACCACCGACUCCGGAGAUGCAUAUGCGCAAGAU
Ppu-3-9   ACACAGCGCGCAUAACCGUUCAUGGCGGUUGUGCAUGGGGCACGCUUGCGUGCGCCGGUUUCUGUGUUCGCCGGUCUGUCAACCCAUGCACAGCUGCCACCAUUCCGUUUGACAGCGGGUUGGCGGUGGCCCCUAUCGAGAACACAGUAAAAUGCUAAAG
Ppu-2-1   AAGAAGGUGCACUCGCUUUGCCUCAAUGGCGGCUGUGCGUGUGGGCACGUUCGCGUGCGCCGGUCUUUCUUUCUUUCCACCGGUCUGUCAACCCGCGCAUAGCUGCCACCCAACCGUUUGACAGCGGGCGGAUGGCAGCCAAACAGGAAAGAAAGGAGCG
Pen-1-10  AAAUCGUGUACGGCUCGCCCUCUAUGGCGGCUGUGCGUGCGGGCACGCUUGCGUGCGCCGGAAUUUACGAUUUUCGCCGGUCUGUCAACCCACGCACAGCUGCCACCCUCUGUUUGACAGCAGACAGGUGGCCGCACCGGAAAGGAAAAUCGUUAUGCU
Ppu-1-4   CUGAGAGCAUGUGCAAUGCCAGUUAUGGCAGCUGUGCGUGGGGCACCUUCGUGUGCGCCGGGAUUCUCAGUGCCCCCGGUCUGUCAACCCACAAACAGCUGCCACCCUUCCGUUUGACAGCGGUACUGCGGCAGCCCUGUAAGGCACUGAGAGGGUUUAU
Ppu-3-10  CUGAGAGCAUGUGCGAUGCCAGUUAUGGCAGCUGUGCGUGGGUUACCUUCGUGUGUGCAGGGAUUCUCAGUGCCCCCGGUCUGUCAACCCACAAACAGCUGCACUGAGAGGGUUUAUGCUGAACAUGUAUUCGAUUCAUGUACCCCACCUACGGGCAGUU
Ppu-4-7   CUAACCACAUGGCUUUGCCGUUUUGGCAGCUGUGCAUGGGGCACCUCGCGUGCGCCGGUUUUGGUCUGUUCACCGGUCUGUCAACCCAUGUACAGCUGCCGCCUUUCUGUUUGACAGCAGAGGGUGGUGGCCUCAACUUGAACAGGCCUAAAACAUGCUG
Pen-1-11  AAGAUUCACUGCCAAUACAGCAACCUGUAAUCGUGUUGCUUGUUGUGUCAUGGCGGCUGCGUAUGGGAGGCCUUUGGGCCUGCCGGUUUUCUCUUUUGCGCCGGUCUUGCAGACCUGUACGUAGCUGCCACCCUAUCCAUUGCAAGGGUGGCGUGGUGGC
Pen-1-12  UUACGAAGGUGCUCACCAUGGGCCCCGGCGCUUUGCCGUGGGUUUCGCGCAAUGGUGGCUACGUGCGGGUGACCUCUUGGUCAGCCGGGUUUUCGCCUCGUAAUGCCCCGGUCUUGCAGGCCCGUACGUAGCUGCCACCCUUCACCUGCAAGUGGAGCGG
env-5     ACAUGGCGCAAAACGUCACCUUACGCAGAUGCCUUCUGGCGCCUGUGUUUUAUGGUGGCCAUGCGCAGGGCGCUUUCGAGCGCGCCGGUUUUUCCAUGUGUGCCGGUCUACCAACCUUCGUAAUGGCCACCACCCUCGUUUGGUAGCGAAUGUGAUGGUU
Psy-1-6   AUUACAAGUGGCGCAAGUGCCGUUUAUGGCGGCUUUGCGUGGGGCACCUUCGGGUGCGCCGGUUGCUUGCGCUUGUUCCACACCGGUCUGCUAACCCGCGUCAAAGUCGCCACCCUUGUUUGUUUAGCCGCAGGCAUCUGGUGGUCUUGUCAGCUAAAGG
Psy-2-5   AUGACAAGUGGCGCAAGUGCCGUUUAUGGCGGCUUUGCGUGGGGCACCUUCGGGUGCGCCGGUUGCUUGCGCUUGUUCCACACCGGUCUGCUAACCCGCGUCAAAGUCGCCACCCUUGUUUGUUUAGCAGCAGGCAUCUGGUGGUCUUGUCAGCUAAAGG
Eph-4-1   AAAAGGCGCAUUCACCGCGCAAGCGGUUUUUUUAUGCGUAUAGCACGGUCACAUUCGUAUUAUGGUGGGCUGUGUGGGGGCACCGAAAGGUGCGCCGGGUCCUUUUAGCCGGUUACGCCAACCCUGCACAGUUCACCACCAACCGAUUGGCGUAGGUAGU
env-6     AAGUGGCGCAUACCACGCCAGACGUGGUUUUUUUAUGCGUUAAGCACAGCUAUAUCCGAAUUAUGGUGGGCUGGGCAGGGGUCCGAAAGGACGCCGGUACCACUUAGGCCGGUACGCCAACCUUGUCCAGUUCACCACCAGUAAUUGGCGUUGCGGUGGU
Eco-1-1   ACCGCGACAGAUACACGCCGCGAGCGUGUUUUUUAUUGUCGUAUGCACGCGCACAUCUGAAUUAUGGUGGGCUGUGUGGGGGCGGAGAGAUCCGCGCCGGUCGGUUUCCCGGUUACGCCAACCCUGCACAGUUCACCACCAGACGAUUGGCGUCGUCGGU
Eco-2-1   ACCGCGACAGAUACACGCCGCGAGCGUGUUUUUUAUUGUCGUAUGCACGCGCACAUCUGAAUUAUGGUGGGCUGUGUGGGGGCGGAGAGAUCCGCGCCGGUCGGUUUCCCGGUUACGCCAACCCUGCACAGUUCACCACCAGACGAUUGGCGUCGUCGGU
Sen-3-1   ACCGCGACAGAGACACGCCGCGAGCGUGUUUUUUAUUGUCGUUUGUAUACGCGCAUCUGAAUUAUGGUGGGGCGUAUGGGGGAGCCGAAAGGCUCGCCGGUCGGUGAUCCGGUUACGCCAACCCUGUACGUCUCAUCACCCAAUCCGAUUGGCGUCGGCG
Sen-4-1   ACCGCGACAGAGACACGCCGCGAGCGUGUUUUUUAUUGUCGUUUGUAUACGCGCAUUUGAAUUAUGGUGGGGCGUAUGGGGGAGCCGAAAGGCUCGCCGGUCGGUGAUCCGGUUACGCCAACCCUGUACGUCUCACCACCCAAUCCGAUUGGCGUCGGCG
Sen-3-2   ACCGCGACAGAGACACGCCGCGAGCGUGUUUUUUAUUGUCGUUUGUAUACGCGCAUCUGAAUUAUGGUGGGGCGUAUGGGGGAGCCGAAAGGCUCGCCGGUCGGUGAUCCGGUUACGCCAACCCUGUACGUCUCAUCACCCAAUCCGAUUGGCGUCGGCG
Sty-1-1   ACCGCGACAGAGACACGCCGCGAGCGUGUUUUUUAUUGUCGUUUGUAUACGCGCAUUUGAAUUAUGGUGGGGCGUAUGGGGGAGCCGAAAGGCUCGCCGGUCGGUGAUCUGGUUACGCCAACCCUGUACGUCUCACCACCCAAUCCGAUUGGCGUCGGCG
Api-1-1   UAACAAGGCGCAUAGCACGCAGUAAGCGUGUUUUUUUUAUGCGUCAGGCUGCCAUAUCAAAAAUCAAUGGUGGGCUGGAUGGGGGAGCCGAAAGGCUCGCUGGGUCCUUGUUAGCCGGUAGUCUCAACCCUGUUCAGUUCACCGCCAAUAUGAGUUUGAG
          ....................................................................................................................................................................
          ....................................................................................................................................................................
          ●●●●●●●●●●●●●●●●●Y●●●●●●●●●●●●●●●●●●●●●●●●●●●●●●●●●●●●●●●●●●●●●●●●●●●●●●●●●●●●●●●●●●●●●●●●●●Y●●●●●●●●●●●●●●●●●●●●●●●●●●●●●●●●●●●●●●●●●●●●●●●●●●●●●●●●
```

alignment positions 321 · · · 457

```
Pme-1-1   CGAGUGGGCGCGGCUCCUUAAGUGAACCAAGGAGCAUAAGGAAAAUGCACUAUCCCCCUUUUACCCAAGGGCUCCGCCCUGGGCUGCUGGCUGUUGUGCCAACUUCCGUUUUGGAGGUGCUGCAUGACCAAGUCUGA
Psy-1-1   AUUUUCAUGACAGGAGCUUUGCCGCGAUGGAUCUCCCAUUCCCCCUUCUUCCCUGCACCGAAAACCACGAACCCACCCUCUUCCUGCGCCACCGUCGCAUGUUGCGUGCUGUUGUCAUGCACGCAGAAGCCUGGUUC
Pfl-2-1   AGCUCGGCAGUUUUUUCUCUAACUGCACGAGAUCUUCACGCAUGAAAAAAAUUGAUUCGAACGCAGCCGAAAACCCCGGGAACCCGGAUUCCCCCGAACACAUCGCCAACCCCAAACCCCGCUACAUCCCCAUCACC
Ppu-3-1   GUUCCUGAUCCACCCCUACCCUGCACCUCCACCCGCCCCUUCGGCCGCUGCGAUGCCGGCCACGAUCCACUCUUCACCGUCAACCCAAACAUUUCUGCCGAGGACGCUCUGGUCCACGUAGCCCUAUACCUGCGCAG
Eph-1-1   GUAGCAGGUUGUUAAACCUGUUAGUGAGGCCGUAACUAUGGUUAAUGCCAAUCCUUGCACACGCCCAGAAUUCAUCUGGCGUUUCUAUUCCUGUAAAAAACACCACUAUCACUUCGUUAUCGCAGCAACUGAAGACG
Pme-1-2   AGCGAAUGGCUGCGGCUCCUUAAUCUCACGAAGGAGCAUAAGGAAAAUGCGCUACCCCCUUUUUACCCGAGAGCUCCGCCUUGGGCUUCUAUUACUGCUGCACACCUCUGACCUGGAGGUGUGCCAUGGCUGAGUUC
env-1     GGCGGGUGACGGCUCCUUAAUCGUCUGAGGAGCAUAAGGAAAAUGCGCUAUCCCCUUUUUACCCAAGAGCUCCGCCUUGGGCUUCAGGUCUUACUGUCCACCUCCGAUGUUGAGGUGAACCAUGGCUGAGUUCGAAU
env-2     UGUUGGCAGCUCCUGUUCAUACCCGGAGUUGUCCCAAUGCAAUCACUUACCCCUGAUCCACCGAUCACACCCAAAGUCUUCACCCGCCACCAUCACCAGCUGCACGCUGUGUUGAUAGACAACCAGCCAUGGUUCAG
Eph-2-1   GGGGCAGCCUCCGGGCUGGCCGGUAUCCUGUGAAGCCGGUAUUCUCACCCCCGUCUGGGCUAUCGCCAUCGAGCGUGAGAACUCCGGCGAUAGCGUUAUUUGCUAUUCACAGGAGGCUGCCAUCAUGGCUACAACCC
Ppu-4-1   UUCUGGCAGCUCCUCAAAUCAAGAAGGAGCUACCUCCAUGUUUGCUCGACUAUUCAGCCUCACUACGCCUGAAAUCCAGUCAAAAAUUGAGCUUCACCGCGCUCGCGCAGUCGCCCAGCUCAAAAGCAAAUCCAGCC
Yph-1-1   UGGUGGUAGCAUCACUACCCAUCGGAGUUCAUACAAUGUACACAUUCAAAUUCGCGGCAAUUUGCCGCACCGACCGAAAAAAUCACAUCCAUCAUUUUUCCACUAUUGCCGAUACAGAGCAGGCCGCUCGUCGCCAG
Sbo-1-1   CACCGCCAUUUCAGAGCGUGGAAACUCAGGCGGUGACUCCUUAAACACAAAGGAGGCUGCCGCUAUGCUGGCUACUACCCCUACCCAAAAACCGCAAUUUAUCUGGAUUAUCGCCGCAGUUCGCCGCGAUUGCCCGA
Kph-1-1   GUGGUGAUCUUAACCAUCAGCUAGAGGGGUUACCCUAUGUUCACAUUCAAAUUUGCCGCGAUCUGCCGUACCGAUAAAAAAUCCCAUAUUCAUCAUCUGUCCACUAUCGCCUCAUCCGAGCGAGAAGCCCGUCUCCA
Eph-5-1   UCUGGUGGUAGCACCCUAUACAAGAUAGGAAUGCAUACCAUGUUCAAAUUCAAAUUUGCAGCUGUCGUCCGCACGGACAAAAAAUCCCAUAUCCACCGCCUUUCCACCAUUGCAUCAUCCGAGCGAGAAGCUCGUCG
Psy-3-1   UGGCGGCUCUUGAUAAACAAAAAAGAGUGUCAUACAUGCAAAAUAUUGCUCUCAGAAACACCCACACCGCCUAUUUCGACGCCACUCUGUUUCUGCGCCACAGUCGCAUGCUGCGCGCCAUUUUUACUGACGCCCAG
Psy-2-1   CUCAUAUCCAGGAGCUGCAUCCAUGUUCACUGCAACCCCCUGCGUGACCCCUACCCCCCAUUUCGAAGCCACCCUCUUCCUCCGCCACAGCCGCACCCUGCGCGCCAUUUUCACUGAAUCUCAAGCCUGGUUCUGCC
Psy-1-2   AGACCGUGGCGGUUCCUCAGUACAAGGAGCCUCACCUACCUGCGUCGGCCAGCGGACAAGCUCACCGUGCCGCCGCUCGCUUUGGCCUCGCCGCCGCAGCGGGUGAGCUGGCCACUGCGAUGGGGAUAACAGGCUGG
Pae-6-1   GCCUCCAUCGAUUGACAGCGAAGGCGGCAGGUCAUGUGACUCUCAGAAACGGAGUACCCAGCAUGACGAAGGAUGAAAAGGAAAAGACCCACGUCGACGCAAUCAUCGAACGCUACAAGGACCUGAUGGUCGAGAUU
Pae-1-1   GCCUCCAUCGAUUGACAGCGAAGGCGGCAGGUCAUGUGACUCUCAGAAACGGAGUACCCAGCAUGACGAAGGAUGAAAAGGAAAAGACCCACGUCGACGCAAUCAUCGAACGCUACAAGGACCUGAUGGUCGAGAUU
Pae-5-1   GCCUCCAUCGAUUGACAGCGAAGGCGGCAGGUCAUGUGACUCUCAGAAACGGAGUACCCAGCAUGACGAAGGAUGAAAAGGAAAAGACCCACGUCGACGCAAUCAUCGAACGCUACAAGGACCUGAUGGUCGAGAUU
Pae-2-1   GCCUCCAUCGAUUGACAGCGAAGGCGGCAGGUCAUGUGACUCUCAGAAACGGAGUACCCAGCAUGACGAAGGAUGAAAAGGAAAAGACCCACGUCGACGCAAUCAUCGAACGCUACAAGGACCUGAUGGUCGAGAUU
Pae-4-1   GCCUCCAUCGAUUGACAGCGAAGGCGGCAGGUCAUGUGACUCUCAGAAACGGAGUACCCAGCAUGACGAAGGAUGAAAAGGAAAAGACCCACGUCGACGCAAUCAUCGAACGCUACAAGGACCUGAUGGUCGAGAUU
Ppu-3-2   CAGCGUGGUGCUUCAAUCGAAAAGGACCGCAUGCAUGCUCAUCUCAAGUCCAUACAGGAUCCUCGCAUCCGUUCUCCUCCGCCGUUCCUCAACGGCCCUCAGCUACUUCACUCUCAGCCAACCCCGCUUCGUCUCCA
env-3     CAGCGUGGUGCUUCAAUCGAAAAGGACCGCAUGCAUGCUCAUCUCAAGUCCAUCCAGGAUCCUCGCAUCCGUUCUCCUCCGCCGUUCCGCAACGGCCCUGCGCUACUUCACCUUCAGCAAGCCCCGCUUCGUCUCCC
Pae-3-1   UCGUGGCAGCUCCCAUCCAGACAAAGGAGCUACGCGCAUGUCCGCACUUGUUCCCGCCCCACCCCGGACCUCGGUUCGGUUCUCUGUCCUGGCCCUGCUCGGCGAGGCUGUCAUCGUCAACCGCGUGACCGGCACGU
Pfl-1-1   ACGGUGACGGUCCUUCGAAUCAAGGAGUCGUGACCAUGAACUGCUUCUGCAAUCUGUUUCCCCAACACCUCCCGCAACACAAAACCGGAGGUGCCCGAUGACCACUCAAUCGCCCCUGACCACCCUCGGUGAAAUCC
env-4     AGGUGCGCCCGCACCAACCGUAUACGCCUUCUCAACAAACGAACUCCGAGUUGUCAUGAUCAAAGACGAACCAUGGUUCGUCGCAGUUGAUGUGUGUAACGCACUGAGCAUCGGAAAUCCGUCUGACAGCAUUCGAU
Pfl-1-2   UCAGUGGCAGCUCCUACUCAGAAAAGGAGCUACACCCAUGCACGCCCUCAAUCCGUCCAAAAUUCGCGCCGCUGCCCACAGGGCAAUGGCUCUCGCCGCUUUACACGCCAACUCCAGCCUCGCUACACGCCUUUCUC
          ............................................................................................................................................
          ............................................................................................................................................
          ●●●●●●●●●●●●●●●●●●●●●●●●●●●●●●●●●●●●●●●●●●●●●●●●●●●●●●●●●●●●●●●●●●●●●●●●●●●●●●●●●●●●●●●●●●●●●●●●●●●●●●●●●●●●●●●●●●●●●●●●●●●●●●●●●●●●●●●●
```

Psy-1-3	CAAGUUCUGCUGGAGUUGCAAACAUGCCCCAUAAAAUCGUCCCCGACCCGCCCACCGCCCUGUUCACCGUCAACGCAUCCGUCAGCCAGGAAGAAGCGCUCAACUACGCUUCGGAUCUGCUGCGCUGCAUCAUCACG
Psy-2-2	UUUCAUUGAUCAGGAGUUGCAAACAUGCCCGAUAAAAUCGUCCCCGACCCGCCCACCGCCCUGUUCACCGUCAACGCAUCCGUCAGCCAGGAGGAAGCGCUCAAUUACGCUUCGGAUCUGCUGCGCUGCAUCAUUAC
Pfl-2-2	UUGGGCUGGCUGCGACCAUCAAUGAGGACGAAAUGACCAUGAAAAAACCAACACCCAACCCACCCGAAACCCAAGCCAACCCCGAAACCGACCCAACCUCCCCAUACACCUCCACCACCUCCAGAAAACUCCACGAA
Ppu-1-1	GUCCCCGAUCCACCCCACGUCUUCGAUCUUCCCCAAGGCAAAUCCCUUUCCCGCGCGAUCAGCGAAGGCAUCGUCCCGAUGGAGUUCGCGUUGAUGAAUGUUUCCCACUACCUGAUGUUCGCCUACAGCGAUAGCCG
Ppu-3-3	GAUCCGCCCCACGUCUUCGAUCUUCCCCAAGGCAAAUCCCUUUCCCGCGCGAUCAGCGAAGGCAUCGUCCCGAUGGAGUUCGCGUUGAUGAAUGUUUCCCACUACCUGAUGUUCGCCUACAGCGAUAGCCGGCGGGC
Ppu-3-4	CCCGAUCCACCCCUCCCCAACACCACACAACGCUCUUUCAGCCGCUGCGAUGCCGGCCAUCCCCCAUUAUUCACCGUGAACCCAGGCGUCUCGACCCACGAUGCCCUGGUGCAUGUCGCCCAAUACCUGCGCGGUGC
Ppu-4-2	AUCGUCCCCGAUCCACCCCAUAAUUUCGACCUUCCCUCCGACAAGACCCUCUCCAGCGCCAUCAGCGAGCGCAUCGUGCCAAUCGAUCACGUGGUCAUGAACGUCACCCAUUACCUGAUGCUGGCCUACAACCACUG
Psy-2-3	AGUUAUUGAUUAGGAGUCUCACAUGCUAAAAAUCGUCCCAGAUCCACCCCUCUUCACCGCCAACCCCAACGUCAGCCAUGAAGACGCCCUGAUGCAUGCCAGCGACCUGCUGCGCUGCGCCGCCACCAGCGCCUGUG
Psy-3-2	AACUCCAGGAGAUACAUCACAUGCUCAAACUCGUCCCCGAUCCACCCCCUUACCGCGCCAACCCCACCAUCAGCCAUGAAGACGCACUGAUGUAUGCCAGUGACCUGCUGCGCUGCGCCGCCACCAGUGCCUACGAA
Psy-1-4	UAAAACUCCAGGAGAUACAUCACAUGCUAAAAAUCGUCCCCGACCCACCCCUCUUCAACGCCCGCCCCAAAGUCAGCCAUGAAGAUGCGCUGAUGUAUGCCAGCGACCUGCUGCGCUGUGCAGCAACCAGCGCUUAC
Psy-3-3	AAGGAGCUAAAACAUGGUCAAAGUAACCCCCGAUCCACCCAACACCACUCCGGUCUUCGACAAGGCCGUCGUCAAGCGCGCCAUGGCGUGCUACCUGCCAGUCCCUCGGUCUCCCCGAGAACCCCACGACAGCAAGU
Psy-2-4	CCAUUGAGGAGAGAGCUAAGCAUGGUCAAAGUAACCCCCGAUCCGCCCAACAAACAGUCCAGCACCGCACCGGUCUUCGACAAGGCUGUGGUCAAGCGCGCCAUGGAGUGUUAUCUGCCAAUCUCCCGACCGGCCAA
Psy-1-5	CCAUUGAGGAGAGAGCUAAGCAUGGUCAAAAUAACCCCCGACCCACCCAAACAGUCCAGCACCGCGCCAAUCUUCGACAAGGCGGUCGUCAAGCGCGCCAUGGCGUGUUACCUGCCAAUCCCCCGCACUGACAAAGA
Ppu-4-3	AGAUUGUCCCCGAUCCACCGUCCCCCCUCGAUCCCUCGUACAUGCUACAGGACACCCUGGUACAGACCUCGGAAUACGUGAUCUGCGCCUUGUCCGUAGCCCGCCAGUCCGUGCGACUGACACCGACCUCGCACAAC
Ppu-4-4	UCAAAAUGGUCCCCGAUCCACCCCACAACCACCACUCCCUCGAAGACACGAUCAUCCAGGCUGCGGAGUUCGCCCUGUGCGCGCAGAGCGUGGCGCAGCAGGCUGUUUCGCUACAGCCCAGAACGCCCGCCUCGGUU
Pen-1-1	AAAGAUCGUCCCCGAUCCACCCCACAACCACCACUCCCUCGAAGACACCAUCAUCCAGGCCACCGAGUACGCCCUGUGCGCGCAGACCGUGGCGCAUCAGGCUGUUCUACUGCAUCCCAAGUCGCCCGUGUCGAUUC
Pen-1-2	GAUAGUUCCCGAUCCACCCCACAACCACCACUCCCUCGAAGACACCAUCAUCCAGGCCACCGAGUACGCCCUGUGUGCGCAGACCGUGGCGCAUCAGGCUGUUCUGCUGCAUCCCAAGUCGCCCGUGUCGAUUCUGG
Pen-1-3	UGCUUAAGAUUGUCCCCGAUCCACCCCACAACCCUCACUCCCUUGAAGACACCCUCAUCCAGGCCACCGAGUACGCCCUGUGCGCGCUGACCGUGGCCCAUCAGGCCGUGCACGCCCAGCCCAGAUCACCGGCCACG
Ppu-3-5	AAAAUAGCUCCUGAUCCACCUCUCAGCACCAUUUGUCUUGAAGAUAUUGGUCCAGCCACAAUUUACGCGCUAUGCCGCGCUAUCCCCGUGCAUCAAGCGAUGUUGCUGCAGCCCAAGGCCCCAGCGUCGAUUGUGAU
Ppu-1-2	UGAAAGUAGUUCCCGAUCCACCCCAUAACCCCCAUUCCCUCGAAGACACCCUGAUCCAGGCCACCGACUACGCGUUGUGCGCAGCCACCGUGGUGCAUCAGGCGUUGCUGCUUCAGCCCAAGUCACCGGUUUCGAUC
Ppu-3-6	AAGAUGGUCCCUGAUCCACCCCUCGAUACCACCCAGUUCCUGCAAGACACUCUGGUCCAGUCCUCAGAGUACGUACUCUGCGCCCUGUCCGUGGCCCGCCAAUCCGUGCAACUCAAACCCACCGCCCACAGCUCGAU
Ppu-1-3	GUCCCCGAUCCACCCUACUCCCCCCAUUCCACCCAGAUCCUGCAAGACACCCUGGUCCAGUCCUCAGAAUACGUACUCUGCGCCCUGUCCGUGGCCCGCCAAUCCGUGCAACUCAAACCCACCGCCCACAGCUCGAU
Pen-1-4	GAUUGUCCCCGACCCACCCCAUCACCACCACUCCCUCGAAGACACCCUCAUCCAGGCCACCGAGUACGCCCUGUGCGCCUUGACCGUGGCCCAUCAGGCGGUGCUGGCCCAGCCCAGGUCCCCGGCCACGAUCCUGA
Pen-1-5	UGCUAAAGAUCGUUCCUGAUCCACCCCAUCACCCUCACUCCCUCGAAGACACCCUCAUCCAGGCCACAGAGUACGCCCUGUGCGCGCUGACCGUGGCCCAUCAGGCCGUGCACGCCCAGCCCAGAUCACCGGCCACG
Pen-1-6	UGCUGAAGAUUGUUCCCGAUCCACCCCAUCACCCUCACUCCCUCGAAGACACCCUUAUCCAGGCCACCGAGUACGCCCUGUGCGCACUGACCGUGGCCCAUCAGGCCGUGCACGCCCAGCCCAGGUCACCAGCCACG
Pen-1-7	GUCAUGUUAAAGAUCGUCCCCGACCCACCCCAUCCUCACUCCCUUGAAGACACCCUCCUCCAGGCCAGCGAAUACGCCCUGUGCGCAUUGACCGUGGCCCAUCAGGCCGUGCAUGCCCAGCCCAGGUCACCGGCCAC
Pen-1-8	GAAGAUUGUCCCCGAUCCACCUUCCCCCCAAGACCACGCCCACUUCCUCGAAGACACCCUCGUGCGCGCCCUCGAGUACGCCUUCUGCGCACUCACGGUCGCCAACCAGGCCGUGCAGCUGAAACCCAGGUCGCCCG
Ppu-3-7	CUCAAGAUCGUUCCAGACCCACCCCACCGGUCCCUCUGCCUUGAAGACACCCUGAUCCAGGCCACGGAUUACGCGCUUUGCGCUGCAACUGUCGUGCAUCAGGCAAUGCUGCUGCAGCCCAAGUCACCCGCGUCGAU
Ppu-4-5	AAUGUUGACUGCCAACAGGUUACAAGCGGUUACAUCGAACAGCUGAAAAAUCAGAUUUAACCGCUUUCUUGUAGGGCAUUUCCCAAAGAUACUGCCGGCGCCUGUUUUCCGUUGCGCACGCAAAAAGUGGACUUUAG
Ppu-4-6	AAGAUAGUCCCCGAUCCACCCUACACUUCCCACUCCCUGGAAGACACACUCAUGAUGGCUGCUGACUACGCACUUUGCGCAGAAGCCGUUGCCCAGCAGGCGAUGAUGAUGCAACCCAAAUCGCCCUCCUCCUUGUU
Ppu-3-8	UGCUGAAAAUAGUCCCCGAUCCACCUUACAAUCUUCACUCCCUUGAAGACACACUGCUGAUGGCAGCGGACUACGCACUGUGCGCGGAAGCCGUUGCUCAGCAAGCCGUUUUGAUGCAGCCGAGAUCACCCGUCUCG
Pen-1-9	GGUUCCAGAUCCGCCGACACACCUUGGCAACUCCCCGCAUCAGAUCGAAGACUUGCUGGUUCAAAUUGCUGAAUACCUUGUCUGUGCCCUAACCGCCGCCCAGCACGCCGCGAUGAUCCAUACCAAACCACCUGGCC
Ppu-3-9	AUCGUUCCUGACCCGCCUCACCACCUCCACUCCCUGGAAGACACGAUCAUGAUGGCCUCGGACUACGCGCUCUGUGCAGAAGCCGUAGCCCAGCAGGCAAUACUGAUGCAGCCCAAGUCACCCGCAUCGGUUUUGAU
Ppu-2-1	AGUCAUGCUGAAAAUUGUCCCCGACCCACCCCACAACCCCCAUUCCCUCGAAGACACCCUGAUCCAGGCUACCGACUACGCACUAUGCGCGGCCACCGUGGUGCAUCAGGCGUUGCUGCUUCAGCCCAGAUCACCGG
Pen-1-10	GAAGAUCGUCCCCGACCCACCCCACAACCCCCACUCCCUCGAAGACACCCUCAUCCAGGCCACCGAGUACGCCCUGUGUGCCUUGACCGUGGCCCAUCAGGCGGUACUGGCCCAGCCCAGGUCCCCUGCCACGAUCC
Ppu-1-4	GCUGAAAAUAGUACCUGAUCCACCCCACAACGUUCUUUCCUUGGAAGACACCCUGGUUCAGGCUACGGACUACGCGUUAUGCGCGGCGACCGUCGUGCAUCAGGCAAUGCUGCUGCAGCCCAAGUCCCCCGCGUCGA
Ppu-3-10	UCUGCCUGAGUGCUUCAGUGAGAACUCGAAAUCCACGGGUUCUCGUACUGGAGACCUUAUGGUAUGGCGGGCUUGUAGAGGGCAGUUGAACGCAGGGCUAUUUCAGCACAGGUCAGAUCGCCUGUGCUGACUUGAUU
Ppu-4-7	AAAAUAGUCCCCGAUCCACCCCACCUACUCCACUCCCUGGAAGACACGCUGAUGAUGGCCGCUGACUACGCCCUGUGCGCCGAAGUCGUCGCCCAGCAGGCAAUGCUGAUGCAGCCCAAGUCACCUGUGUCGUUGUU
Pen-1-11	UUUGAUCCCAUGCAAAAGAGGAUUCACCAUGCUCAAGAUCGUGCCGGAUCCACCGGCUCCAACCGAAUCUCCCCAUUUCCUCGAAGACACGCUGGUGAGUGCCACUGAGUACGUCAUGUGCGCGCUGGCGGUCACUC
Pen-1-12	CGGCGGUCCGACUUCCGCAUUGCGAGGUGUUUAUGCUCAAGAUCGUUCCCGAUCCACCCCAUUCCACCGAGUCUCCUUACUUUCUCGAAGACACCCUGGUUCACGCCACUGAAUACGUCAUGUGUGCCUUGGCGGUG
env-5	CCCUUAGCCACAUAUGGAGCGUCACUAUGUUCAAGGUCACACCAAACCCACCCGUAACCGAAACGGUCUCACCCUACGAAUCCUCCGAUUCCCGAAAACUCAGCGAAGCCGCCGAACGCGCCCUCGACCACUACCUG
Psy-1-6	AAUAAGCGAUGAUCAACUCGACCCCGUCUCCACCGCUCCCCAACUCUCUUGAAGAUUCCCUGAUACAGGUCAGCGAAAUACUGCGCUGUGCCUCUGCCACCGCUUCUGAAACCGGUGACAACCUUGAGGGCCUGAAA
Psy-2-5	AAUAAGCGAUGAUCAACUCAACCCCGUCCCCGCCGCUCCCCAACUCUCUGGAAGACUCUCUGAUGCAGGUCAGCGACAUACUGCGCUGUGCCUCUGCCACCGCGUAUGAAACCGGUGACAACCUCGAUGGCCUGAAA
Eph-4-1	GGUGAUUAACCAGACUAAAAGGUAACCACUAUGACAGCUACAAAAAGCACGUCCAUUUUUUCUUUCGAAUCCCAAGCCGAUAUACGGGUAAUCGUCAUUAAUGGUGAGCCAUGGUUUAUCGCUUCAGAUGUUUGUCG
env-6	GAUUAAAAUCACUAAGUGGAGAUAACCACCAUGGCUAAUGCUCAAACUUCCAUCUUCAAAUUUGAAUCUGUUAACCCUAUCCGUUCCAUCAUUAUCGAUGGCCAACCAUGGUUUGUAGCCCAAGACGUUUGUAGUGC
Eco-1-1	GGUGAGUUAUUAAGAAACCACCAGAGGGCGUCAUUAUGACAACUCAAAUUUCUGUUGAAACUCUCUCCCCGAUCACCCAUAACCAGAUUCCUGUUAUUACCACCGAACUUUUGGCGCAGCUUUACGGCACUGAGCCG
Eco-2-1	GGUGAGUUAUUAAGAAACCACCAGAGGGCGUCAUUAUGACAACUCAAAUUUCUGUUGAAACUCUCUCCCCGAUCACCCAUAACCAGAUUCCUGUUAUUACCACCGAACUUUUGGCGCAGCUUUACGGCACUGAGCCG
Sen-3-1	GUGGUGAUAAACAAAACAAUCACCGGAGGGCGUCACCAUGACCACUCAAAUCUCUGUCGAAACUCUUUCCCCGAUUACCCAUAACCAAAUUCCCGUUAUUACUACCGAACUUUUGGCGUACUUAUACGGCACAAAAA
Sen-4-1	GUGGUGAUAAACAAAACAAUCACCGGAGGGCGUCAUUAUGACCACUCAAAUCUCUGUCGAAACUCUUUCCCCGAUUACCCAUAACCAAAUUCCCGUUAUUACUACCGAACUUUUGGCGCACUUAUACGGCACAAAAA
Sen-3-2	GUGGUGAUAAACAAAACAAUCACCGGAGGGCGUCACCAUGACCACUCAAAUCUCUGUCGAAACUCUUUCCCCGAUUACCCAUAACCAAAUUCCCGUUAUUACUACCGAACUUUUGGCGUACUUAUACGGCACAAAAA
Sty-1-1	GUGGUGAUAAACAAAACAAUCACCGGAGGGCGUCAUUAUGACCACUCAAAUCUCUGUCGAAACUCUUUCCCCGAUUACCCAUAACCAAAUUCCCGUUAUUACUACCGAACUUUUGGCGCACUUAUACGGCACAAAAA
Api-1-1	ACCUCAAAGGUGGUGAUUAUCCUUAACUAACAAGGAAAUCAUUACUAUGACUACUUUAGUUUUCCGCAAUACUGUUCUUGAAACCAUAUCUCAUAAUGGGCAGAUUUGGUUUACGUCUAGCGUGCUUGCUAAGGCAU

18 Chlorobi-1

18.1 Taxa

The taxonomy of each organism containing a putative Chlorobi-1 RNA is listed, with abbreviations identifying each hit (e.g., "Eco-1-1" and "Eco-1-2" might hypothetically represent two distinct RNAs in *E. coli*). The abbreviations will be used to identify each individual Chlorobi-1 RNA in Sections 18.3 and 18.5.

**abbrev. of hits**	**taxonomy of species**
Cli-1-1 to Cli-1-3	Bacteria Chlorobi Chlorobia Chlorobiales Chlorobiaceae Chlorobium/Pelodictyon group *Chlorobium limicola* DSM 245
Cph-1-1 to Cph-1-2	Bacteria Chlorobi Chlorobia Chlorobiales Chlorobiaceae Chlorobium/Pelodictyon group *Chlorobium phaeobacteroides* BS1
Cph-2-1 to Cph-2-3	Bacteria Chlorobi Chlorobia Chlorobiales Chlorobiaceae Chlorobium/Pelodictyon group *Chlorobium phaeovibrioides* DSM 265 (Prosthecochloris vibrioformis
Plu-1-1 to Plu-1-3	Bacteria Chlorobi Chlorobia Chlorobiales Chlorobiaceae Chlorobium/Pelodictyon group *Pelodictyon luteolum* DSM 273
Pae-1-1 to Pae-1-4	Bacteria Chlorobi Chlorobia Chlorobiales Chlorobiaceae *Prosthecochloris aestuarii* DSM 271

18.2 Notes

Superscript numbers are used to annotate any Chlorobi-1 RNA that has special characteristics (described below). These numeric annotations will be used in Sections 18.3 and 18.5. The meaning of the numbers are as follows.

[1] Accepted with E-value 1.9 in global miniscan.

18.3 Gene contexts

Each Chlorobi-1 RNA (indicated by "RNA→") is listed with its surrounding genes. Environmental sequences and some RefSeq entries lack gene annotations, and no genes are listed for such sequences. The direction of each gene is indicated with an arrow (→), and each conserved domain in the gene is colored. Conserved domains associated with more than one Chlorobi-1 RNA are assigned a color; other domains are gray. Information about these conserved domains is given in Section 18.4. The accession number of the sequence containing each Chlorobi-1 RNA is given. Accessions beginning with "NC_" or "NZ_" are RefSeq, while other accession refer to environmental samples. Nucleotide coordinates are given for the 5′ and 3′ boundaries of each. If the 5′ coordinate is greater than the 3′ coordinate, the RNA is present on the reverse-complement strand of the containing genomic DNA sequence. Each hit is denoted by an abbreviation (like "Eco-1-1") that refers to a taxonomy given in Section 18.1. Superscript numbers refer to annotations listed in Section 18.2

**abbrev.**	**Seq. accession**		**5′ at**	**3′ at**	**genes**
[1]Pae-1-1	NZ_AAIJ01000001.1	-	66513	66450	←hypo (cons) **RNA**→ LMWPc (cd00115)→
Cph-2-1	NC_009337.1	-	146066	146001	hypo→ **RNA**→ ←Pol_V (cd01700)
Cph-2-2	NC_009337.1	+	970580	970645	hypo→ **RNA**→ chlorosome envelope protein B→
Pae-1-2	NZ_AAIJ01000007.1	-	10387	10320	←AtpD (COG0055) **RNA**→ ←TPR (cd00189)COG2956 (COG2956)COG4783 (COG4783)
Cph-1-1	NZ_AAIC01000009.1	+	56947	57014	←RadC (COG2003) **RNA**→ ←LDH-like_MDH (cd01339)
Cph-1-2	NZ_AAIC01000002.1	-	18795	18730	PAD_porph (pfam04371)→ **RNA**→ ←EXOIII (smart00479)
Pae-1-3	NZ_AAIJ01000012.1	-	17426	17357	PAD_porph (pfam04371)→ **RNA**→ **RNA**→ ←LDH-like_MDH (cd01339)
Pae-1-4	NZ_AAIJ01000012.1	-	17100	17033	(shown above)
Plu-1-1	NC_007512.1	-	1700572	1700507	hypo→ **RNA**→ **RNA**→ hypo→
Plu-1-2	NC_007512.1	-	1700346	1700281	(shown above)
Plu-1-3	NC_007512.1	-	1700120	1700054	hypo→ **RNA**→ ←LDH-like_MDH (cd01339)
Cph-2-3	NC_009337.1	-	1489337	1489270	PAD_porph (pfam04371)→ **RNA**→ ←LDH-like_MDH (cd01339)
Cli-1-1	NZ_AAHJ01000012.1	-	10407	10340	hypo→ **RNA**→ **RNA**→ **RNA**→ hypo→
Cli-1-2	NZ_AAHJ01000012.1	-	10694	10627	(shown above)
Cli-1-3	NZ_AAHJ01000012.1	-	10125	10058	(shown above)

18.4 Conserved domains

Conserved domains found in protein-coding genes listed in Section 18.3 are listed, with the first sentence in their description from the Conserved Domain Database. Conserved domains associated with more than one Chlorobi-1 RNA are assigned a color, while others are shown in gray. The number in parentheses is the number of occurrences of the given gene.

D cd00115 (1) Substituted updates: Aug 22, 2001
d cd00189 (1) Tetratricopeptide repeat domain; typically contains 34 amino acids [WLF]-X(2)-[LIM]-[GAS]-X(2)-[YLF]-X(8)-[ASE]-X(3)-[FYL]-X(2)-[ASL]-X(4)-[PKE]
d cd01339 (4) LDH-like structure and DMH enzymatic activity; member of the family of NAD-dependent 2-hydroxycarboxylate dehydrogenases.
d cd01700 (1) Pol V was discovered in Escherichia coli as Umuc and UmuD proteins induced by UV.
u COG0055 (1) F0F1-type ATP synthase, beta subunit [Energy production and conversion]
u COG2003 (1) DNA repair proteins [DNA replication, recombination, and repair]
d COG2956 (1) Predicted N-acetylglucosaminyl transferase [Carbohydrate transport and metabolism]
d COG4783 (1) Putative Zn-dependent protease, contains TPR repeats [General function prediction only]
u pfam04371 (3) Porphyromonas-type peptidyl-arginine deiminase.
d smart00479 (1) exonuclease domain in DNA-polymerase alpha and epsilon chain, ribonuclease T and other exonucleases

18.5 Multiple-sequence alignment

Each Chlorobi-1 RNA is denoted by an abbreviation (like "Eco-1-1") that refers to a taxonomy given in Section 18.1. Superscript numbers refer to annotations listed in Section 18.2. The alignment includes sequences containing the RNA motif, as well as flanking sequence. The Chlorobi-1 RNA itself is denoted by the line underneath marked 5′ and 3′ on either end. Nucleotides in the flanking sequences (i.e., not part of the motif) are written in gray letters. Stems of predicted rho-independent transcription terminators are shaded yellow. (Note: terminator predictions have not been analyzed manually, and many are likely to be false positives.) Start codons are shaded green. (Note: start codons are frequently misannotated, especially in environmental samples.) Nucleotides proposed to basepair as part of the consensus structure are shaded in color when they comprise Watson-Crick or G-U pairs. Otherwise they are shaded gray. Conserved stems are also indicated at the bottom of the alignment by angle brackets, where matching < and > denote base-paired columns. Below these angle brackets, the symbol "2" denotes base pairs exhibiting covariation, "1" denotes base pairs exhibiting compatible mutations, "0" denotes base pairs that are not observed to mutate and "?" denotes base pairs that have a significant frequency of non-canonical nucleotides for Watson-Crick or G-U pairs (> 5%). Below these base pair annotation is the consensus sequence: "R" = "A" or "G", "Y" = "C" or "U", red nucleotides: nucleotide identity conserved more than 97% of the time, black nucleotides: 90%, gray nucleotides: 75%, red circle (●): nucleotide is present 97% of the time, black circle (●): 90%, gray circle (●): 75%, white circle (○): 50%. All percentages of sequences just described (e.g. 97% conserved) assume that sequences have been weighted by the GSC algorithm implemented by the Infernal software.

alignment positions 1 · · · 99

```
1Pae-1-1  GUUGUCAGAGGUUGAGAUGUGUUAGCGUGUUGCCUGAUGUAAUAUUGAUAUAUAAUAUAUUUUUCAUCGUUUUUAAUAGGGUUUUAUUGUAUUAUACGU
Cph-2-1   UGAACUGCUCGACCAAGCUCAGGAGUACCGUUGCUGCCCUGUUGAACUCAUAGAAGACGAGGAUGCAGAUAAUGGAUAAUCGCCGCUAAAGCGGAAAAG
Cph-2-2   AUGGGAUGCUGUAAACCGUUUUUCUACUUUCAGUUAUCACUUCUUCAGAGCCAUCAUUUCAACUUUGGCAUACCAUUUGCAUUAUCAACAGUGAACAUA
Pae-1-2   UCAACACGCCACAUCGCAUAUUCUGUCACUUAUAAGCACGUCAUUUCGCACAUCAUGCCGCACAUGCAGCCAUUGGCACGGAUCUUGCAAAAUUAAUAG
Cph-1-1   AAAAGGCUGGGGUUUUCAUGGCGAAUUUAUUUAAGUUAUUAUUUAAUAGUGUUUUAUCUUGUUGAUUGUUUUUUUUGGCAUGUUGUUUGCUUAUUUCUC
Cph-1-2   GUGCGGGAGUGGCUUAUUUCACUAUUACUACGCUAUCCACUCUUUGCUUAUACUUCGUGUUGUUUUGGCACAUUCAUUGCUAUUAUUAUUGUAGUUAAG
Pae-1-3   UAAGCUGUUUGACUAAUUUGUUACAAUUAAGUCAUUUAUGUCUUUGAUGCUUUUGUUGCUUGUUUUUGGCACAGAGCUUGCACUGUAUUAUGUGAAAAU
Pae-1-4   CUCUAUUCUUCUAAGUCAUUUUAUUGUUAUGGUUUAUGUGUUUCUUUGUUAUUUUUUUUGGUUGUUUGGCACAGACUUUGAUCAAUAGUAGACAGAAAC
Plu-1-1   UUGUGGCCAGCAACUCCCUGCAUACAGCGUGUUGCUGGCUUUUGGUGCUAUUUUUUUUAUGUAUCACGAUCUUUGGCACGGCACUUGCAUCUAUAAUCA
Plu-1-2   AUCGUCUCACAAUGGAUCCGGCAGUGUACGAUGAGGGACGGUUCUGUAUGGGAAAAUGGGGAUUUUCUGGCGUUGGCACGGCAAUUGCAAGUAUAAUCA
Plu-1-3   AUCGUCUCACAAUGGAUCCGGCAGUGUACGAUGAGGGACGGUUCUGUAUGGGAAAAUGGGGAUUUUCUGGCGUUGGCACGGCAAUUGCAAGUAUAAUCA
Cph-2-3   GGGCGAUUUUUAUUUCCCCCUAACUUCUUGUUUAAAAAUGAGUUAAAGAAAAUAUUUUCUUUUUCUCAUGGUUUGGCACGGUGGUUGCUAUUUCUUUUA
Cli-1-1   GUGCUUCCGUUCGGAAAGGGUUUUUUGUCAAAGGAGUGUUGUGUUAUCUGGCCGGUUGCUUUUUUUUGUUGUUUGGCACGGUGAUUGCAUGAUAUGUAA
Cli-1-2   UUCCUGGGUUUUACAUUAUGUAAGUGUUUUUGUUAUAAUAGUAUAAACAUUUAGUGGCCGUUUUGGUGUCUUUUGGCACGGUGAUUGCUUGAUAUAUAA
Cli-1-3   GCGCUUCCUUUCGGAAAGGUUUUUUUGUCGAAGGAGUGUUGUUUUAUCUUACCGGUCGCUUUUUAUGAUUUUUUGGCACGAUGCUUGCAUAACACUAUA
          ...................................................................................................
          ...................................................................................................
          ...................................................................................................
          ...................................................................................................
          ●●●●●●●●●Y●●●●●●●●Y●●●●●●●●Y●●●●●●●●●●Y●●YYY●●●●●●●●●●●●●●●●●●YU●●●R●●●●UYRRY●●R●●●●●U●●●●●●●●●●●●●
```

alignment positions 100 · · · 259

```
1Pae-1-1  GAUAAUACUUCGCGCAAGUGAAGU.GUGUAGGUCU.GUGGGGGAUUUCUGCAC....GGGUAAAAGCAGCACAGAA..GUGCUGCUUUUGCUGUUGUAAGCCGGUUUAUUCACUUCUUCAGAGCAUUUGACAUAAUCCCUGCGGAUUGUUUUCUUUGCUU
 Cph-2-1  GAAAAAGCCUCAUGCAAAUGAGGUGGAGCAUGCGUAAUGGGGGACGCAUGCUC....ACCAGAAAACGGCC.GAAA...GGCCGUUUUCUGGUGAGCAUGCUUCAACACCGCACAAGGCAGAAACUCACCGCACCGUCAGAAUUUCAUCCCACUGCGUGC
 Cph-2-2  UGAAAUUCCUCACGCCAGUGAGGUUGAGUUGGUGUAAUGGGGACACCAACUC....UCAGAGAGACGGCU.UCAC...GGCCGUUUCUCUGUUUCACCACUUUCACGACCGAUAAUCUUCAAACACAAGCCGUUAUGAAAACCAGAAACAUCAGGCGCG
 Pae-1-2  CAUCAUGCCUCACGCCCGUGAGGUAGAGUAGGAGGAAUGGGGGCCUCCUGCUCAC..UGUUUAAAGCGGCCACAC...UGGCCGCUUUUUUUGUUUGUGGGUAGGGAAUUGUUCGGGUUGGGGGCUGGAUAACGUCACACAUCACCGGCAGCAAAAAGCA
 Cph-1-1  UGUAAUGCCUCACGCCCGUGGGGUAGGACAGGUGUAAUGGGGGACACCUGUUCAC..ACUAAAAGCGUCCCGUAUACUCGGGGGCGCUUUUUUUUAUGGUUCUUUUUUUGUAGAGUCAGGCAAGCAGCGAGGCGAGAUUAUUGCAGUUCUCUUCCACGAUC
 Cph-1-2  UUUUCAGCCUCACGCCCGUGGGGUUGGGCAGGCGUAAUGGGGGACACCUGCUC....GAAGAAAAGCGGUCUGCAA...GGCCGCUUUUUUUUUGCCCGCUCGGUUGUUUCCGUUCAGGAGUCUUCGUUUUUGGCGCAAUGUGUUUAGAGCAGUUUUGUC
 Pae-1-3  GAAAUUACCUCACGCCAGUGGGGUAGAGUAGCUGGAAUGGGGGCCAGCUGCUCACUUUAAAAAAAGCGACCGCAAC..CGGUCGCUUUUUUUAUUUCCAGAAUAGGCUUCUUUCGAUAUCCUGUCGUGUAUCUCACUAUGAGAGUGAUGCGCGUUUGCAA
 Pae-1-4  AGAAUUGCCUCACGCCAGUGGGGUAGAGCAGCUGGAAUGGGGGCCAGCUGCUCAA..CAAAAAAAGCGACCGUAAC..CGGUCGCUUUUUUUAUGCUUUUUUACAAGAAGAGGUCAAGGGAAAGGAAAGAAUAGAAAAAAGCGGUCUGGAUGACCGCUUU
 Plu-1-1  UGAAACUCCUCACGCCCGUGGGGUGGAGCAGGUGUAAUGGGGGACACCUGUUC....GAAAAAAAGCGGCU.GAAA...GGCCGCUUUUUUUUUAUCCCCCUGAAGUGCCUGCUUUCAUUCGGGUAUGGAACAGGAUCGUCUCACAAUGGAUCCGGCAGU
 Plu-1-2  UGAAACUCCUCACGCCCGUGGGGUGGAGCAGGUGUAAUGGGGGACACCUGUUC....GAAAAAAAGCGGCU.GAAA...GGCCGCUUUUUUUUUAUCCCCCUGAAGUGCCUGCUUUCAUUCGGGUAUGGAACAGGAUCGUCUCACAAUGGAUCCGGCAGU
 Plu-1-3  UGAAACUCCUCACGCCCGUGAGGUGGAACAGGUGUAAUGGGGGACACCUGUUCA...UAAAAAAAGCGGCC.GAAU...GGCCGCUUUUUUUAUGUAUUCAAGCAAUGGAUUGAUCCGGUAUGCUCAGCCAAGCAGUGCAUCAAGGUUCCUGCAGUUCUG
 Cph-2-3  UGAAAUACCUCACGCCCGUGGGGUUGAGCAGAUGUAAUGGGGGACAUCUGCUCAC..AAAAAAAAGCGGCU.GAAA...AGCCGCUUUUUUUUUAGCUCUUUUUCUUCUCCUCAAAUCGCAGAAGCAAGGCUUUUGCAGUUCUUCUCCACGAUUGCCGC
 Cli-1-1  UGAAAUUCCUCACGCCCGUGGGGAAGAACAGGUGGAAUGGGGGCCAUCUGUUCAA..GAAAAAAGGAGGUC.GAAA...GACCUCUUUUUUUUCGUUUUUGAAGCAUCGGUUCUGUCGCAGUCAUGUUGAACGGCAGUUGUAUCGAGCAGAUACAGCUUU
 Cli-1-2  UGAAACUCCUCACGCCCGUGGGGAAGAGCAGGUGGAAUGGGGGCCAUCUGUUCAA..GAAAAAAGGAGGUC.GAAA...GACCUCUUUUUUUUUCUUCAUUUUUGAAGCAUCGAUUCUGUCGCAGUUAUGCUGUACGGACAUCUGUAUCAGGCAGAUACA
 Cli-1-3  UGAAAUUCCUCACGCCAGUGAGGAAGAACAGGUGGAAUGGGGGCCAUCUGUUCAC..AAAAAAAAGCGGCU.UUCG...GGCCGCUUUUUUUGUUUGUGUCAUUCCAUGGUUUUUUCAGGAGAGAACAGCCUGAAGGCUAUUGCAGUUUUCCUCAACUAA
          .....<<<<<<<<....>>>>>>>><<<<<<<<<<.......>>>>>>>>>>.........................................................................................................
          .....??021012....210120??0222222?02.......20?2222220.........................................................................................................
          ..............................................................<<<<<<<<......>>>>>>>>.......................................................................
          ..............................................................2222222?......?2222222.......................................................................
          •R•AAY•CCUCACGCC•GUGRGGU•GARYAGGUG•AAUGGGGG•CAYCUGYUC∘∘--•RRRAAARGCGGCY-•RA•---GGCCGCUUUUUUU•U•Y•Y••••••••••••••Y••••••••R••••R•••Y••••••••••••••••••••••••••••
          5'―――――――――――――――――――――――――――――――――――――――――――――――――――――――――3'
```

alignment positions 260 · · · 419

```
1Pae-1-1  ACCUUAUACGUAUUCUGAUCAAACAGGUCACACGCACAGUAUGCUUCUCCGUGUAUUAUUUGUCUGUUAUGAAAAUAUCUGCCGUUCUCCUAUGGCGGAAGGUAUUUUUUCGGCUCUCGUCAGGCGCGAUGCACGCGCAGAGGGUAUUAUUGUCGAAUCG
 Cph-2-1  UGCACCGCGGUGAAAGACGCUGCUGCACCGGCCUCCACGCAUUCAUAUCCUCCGCCGCCAACCGUAUCGACGACUUUCCAUAACGCCUGUUGACCUCAUCCAUCACCGCCAUCAGCCUCGUGCCGCGAUCCUCGUCCGCCCUCUCUUCAAAGAGCGACAU
 Cph-2-2  AUAAAGGCUACAGAAUCUUCCGGGAAGCAGUUCUGUCGAGGGCAAUAACCUCUCUGCUCAGGAAGGCAAAAAGGAAAGAGUUUUGUGAACGGGAAUGCUACUGAGCUUUGUUUUUUAAAGCGGAAGGGGUAUACUGAUUAUAGAAGCAGGGAUGCAUACA
 Pae-1-2  UAGCGAGGAACGAGUGGCGCUUAUUGAUGUCCGAGUGUAUGUGGUUGUUAUGUAACAAUAUUUUCUAUCUCGUUCUUUGUUUCCCAUAAGCCGUCCAAACACAAACAACAACUACCGGCUCUAUUUCAAUUUAUAUAAAUCCUCAAUGUCUUCGAAAUAG
 Cph-1-1  GCCGCGGAUUUUCUCAGAGCGUCAAGUUCAGGGGCAUCGAGGUCGAUUUCAAGGAUCUGUUCAAUGCCGUUCUUUCCAAGCUUGACCGGUGCGCCUAUGUAGACAUUGUUGAUACCGUACUGGCCUUCAAGCAGUGCUGAACAGGGGAGUAUGCGUUUGC
 Cph-1-2  AGCAGUUCUCUUGUCGCUCGAAUAUCGGAGAACGCGUCAUGGGCCUCAAGCCUGAUGCCAUAGUGACGACAGACUGUUUCGAGCUUGUAGUCAGGCAGAUGCAGGGCUCCGGUGAAAUCUUUCAUAAACAGGACGGGAAGGGGGUCAAGCCUUCUCCAGU
 Pae-1-3  GACGAUAAUUGAGAAAAUAUCUCAGCUUGAUACCUGUCAGAUUUAUGGCUGGAAAUCUGAUUAUUCUUGCUCUAUUCUUCUAAGUCAUUUUAUUGUUAUGGUUUAUGUGUUUCUUUGUUAUUUUUUUGGUUGUUUGGCACAGACUUUGAUCAAUAGUAG
 Pae-1-4  GCAUGUGUCAGGCUCUGAUGCCUCAGUUUGAUCUGCUUACGAAAGCAGAUCGGCGAGAUUGCUGCAGUUUGAUUCAACAAUGGAUGCCGAUUGACGCAGGGCUUCAAGUUCGGCGGUGUCCAGAUUGAUCUCAAGAACUUCCUCAAUACCGUUUUUGCCU
 Plu-1-1  GUACGAUGAGGGACGGUUCUGUAUGGGAAAAUGGGGAUUUUCUGGCGUUGGCACGGCAAUUGCAAGUAUAAUCAUGAAACUCCUCACGCCCGUGGGGUGGAGCAGGUGUAAUGGGGGACACCUGUUCGAAAAAAAGCGGCUGAAAGGCCGCUUUUUUUUU
 Plu-1-2  GUACGAUGAGGGACGGUUCUGUAUGGGAAAAUGGGGAUUUUCUGGCGUUGGCACGGCAAUUGCAAGUAUAAUCAUGAAACUCCUCACGCCCGUGAGGUGGAACAGGUGUAAUGGGGGACACCUGUUCAUAAAAAAAGCGGCCGAAUGGCCGCUUUUUUUA
 Plu-1-3  CUCUACAAUGGAAGCCGACUGCUGCAAUGCCUUGAGUUCGUCCGCUGAGAGAUUGAUUUCCAGAAUCUGCUCCACGCCCUUUCUGCCGAGUUUGACCGGAGCGCCGCAGAAGACGUUCUGGAUGCCGAACUGGCCUUUCAGGCAUGUGGUGCAGGGGAGG
 Cph-2-3  UGAGGCCUGAAGUGCCUUGAGUUCAUUGUCGUCAAGGUUGAUUUCAAGAAUCUGCUCGACACCCUCUUUUCCCAGCUUGACAGGAACACCGCAGAAUACAUUAUUGAUGCCGUAUUGUCCCCCGAGGCAGGUGGUGCAGGGGAGAAUGCGCUUGCGGUCU
 Cli-1-1  GAGGCGAAGAGAGACGUUAUGACUCUGCUGCGCUUCCUUUCGGAAAGGUUUUUUUGUCGAAGGAGUGUUGUUUUAUCUUACCGGUCGCUUUUUAUGAUUUUUUGGCACGAUGCUUGCAUAACACUAUAUGAAAUUCCUCACGCCAGUGAGGAAGAACAGG
 Cli-1-2  AUCUUGAGGCAAAGAGAGACGUUGUGUCUCUGCUGUGCUUCCGUUCGGAAAGGGUUUUUUGUCAAAGGAGUGUUGUGUUAUCUGGCCGGUUGCUUUUUUUUGUUGUUUGGCACGGUGAUUGCAUGAUAUGUAAUGAAAUUCCUCACGCCCGUGGGGAAGA
 Cli-1-3  AGCUGCGGAUUUCUGAAGUGCUUCAAGUUCAUAUUCUGAAAGAUUGAUUUCAAGAAUCUGUUCAAUACCGUUUUUGCCAAGUUUGACCGGAACGCCGCAGAACACUCCGUCGAUACCGAACUGGCCGUUCAGCAUAGUUGUACAGGGGAGAAUGCGUUUG
          ................................................................................................................................................................
          ................................................................................................................................................................
          ................................................................................................................................................................
          ................................................................................................................................................................
          ••••••••••••R••••••••••••••••Y••••••••••Y••••••••R•••••••••••••••••••••••••••••••••••Y••Y•••••••••••••••••••••••••••••••••••••••••Y••••••••••R••••••••••••••••••••••••••
```

alignment positions 420 · · · 474

```
1Pae-1-1  GCAGGGACGGUAUGCUAUCAGAAAGGAUCUCUUCCGGAUAGCCGUGCUGUAAU..
 Cph-2-1  AGUCGUUUCCGGCUGGUUCCGCGAUACCAUUCCGCUCAGCAUCACCCCGGCUUUG
 Cph-2-2  UGAGACUUUAUUAUGUUGUUUUUUUUAAACCAUUAAACCAAUAAUACCAUGGACA
 Pae-1-2  GGCUCAAUGAAAUCUGCCAGCAUCUCCUCGUUGUUACCAUAAAGUGCGAAAUAG.
 Cph-1-1  GAUCGUUGACGAUGGCAUCGAUCAUUUCUACCGCGGAUGAUGCCGGAGCAU....
 Cph-1-2  UCACGUACGAUCCUAUUCCGUAUUUGUCGCCGUGCAUUUUGAACCACUGCUGUA.
 Pae-1-3  ACAGAAACAGAAUUGCCUCACGCCAGUGGGGUAGAGCAGCUGGAAUGGGGGCC..
 Pae-1-4  AUUUUUACCGGGACACCGAUAAAGACAUUAUCCAUGCCAUAUUGGCCGGUAAC..
 Plu-1-1  AUCCCCCUGAAGUGCCUGCUUUCAUUCGGGUAUGGAACAGGAUCGUCUCACAAUG
 Plu-1-2  UGUAUUCAAGCAAUGGAUUGAUCCGGUAUGCUCAGCCAAGCAGUGCAUCAAGGUU
 Plu-1-3  AUGCGCUUGCGGUCCUUUACGAUCGACUCGAUCAUUUCCACGGCUGAGGCUGCCG
 Cph-2-3  UUGACUAUCGACUCAAUCAUUUCAACUGCUGAUGCUGCCGGUGCAUAGAAUGCCG
 Cli-1-1  UGGAAUGGGGGCCAUCUGUUCACAAAAAAAAGCGGCUUUCGGGCCGCUUUUUUUG
 Cli-1-2  ACAGGUGGAAUGGGGGCCAUCUGUUCAAGAAAAAAGGAGGUCGAAAGACCUCUUU
 Cli-1-3  CGAUCUUUAACGAUGGCUUCAAUCAUUUCAACUGCUGAAGCUGCAGGCGCGUAAU
          .......................................................
          .......................................................
          .......................................................
          .......................................................
          •••••Y•Y•R••••••Y•••••••••••••••••••••••••••••••••••∘∘
```

19 Chlorobi-RRM

19.1 Taxa

The taxonomy of each organism containing a putative Chlorobi-RRM RNA is listed, with abbreviations identifying each hit (e.g., "Eco-1-1" and "Eco-1-2" might hypothetically represent two distinct RNAs in *E. coli*). The abbreviations will be used to identify each individual Chlorobi-RRM RNA in Sections 19.2 and 19.4.

abbrev. of hits	taxonomy of species
Cte-1-1 to Cte-1-2	Bacteria Chlorobi Chlorobia Chlorobiales Chlorobiaceae Chlorobaculum *Chlorobium tepidum* TLS
Cch-1-1	Bacteria Chlorobi Chlorobia Chlorobiales Chlorobiaceae Chlorobium/Pelodictyon group *Chlorobium chlorochromatii* CaD3
Cfe-1-1 to Cfe-1-2	Bacteria Chlorobi Chlorobia Chlorobiales Chlorobiaceae Chlorobium/Pelodictyon group *Chlorobium ferrooxidans* DSM 13031
Cli-1-1 to Cli-1-2	Bacteria Chlorobi Chlorobia Chlorobiales Chlorobiaceae Chlorobium/Pelodictyon group *Chlorobium limicola* DSM 245
Cph-1-1 to Cph-1-2	Bacteria Chlorobi Chlorobia Chlorobiales Chlorobiaceae Chlorobium/Pelodictyon group *Chlorobium phaeobacteroides* BS1
Cph-2-1 to Cph-2-2	Bacteria Chlorobi Chlorobia Chlorobiales Chlorobiaceae Chlorobium/Pelodictyon group *Chlorobium phaeobacteroides* DSM 266
Cph-3-1 to Cph-3-2	Bacteria Chlorobi Chlorobia Chlorobiales Chlorobiaceae Chlorobium/Pelodictyon group *Chlorobium phaeovibrioides* DSM 265 (Prosthecochloris vibrioformis
Plu-1-1 to Plu-1-2	Bacteria Chlorobi Chlorobia Chlorobiales Chlorobiaceae Chlorobium/Pelodictyon group *Pelodictyon luteolum* DSM 273
Pph-1-1 to Pph-1-2	Bacteria Chlorobi Chlorobia Chlorobiales Chlorobiaceae Chlorobium/Pelodictyon group *Pelodictyon phaeoclathratiforme* BU-1
Pae-1-1 to Pae-1-2	Bacteria Chlorobi Chlorobia Chlorobiales Chlorobiaceae *Prosthecochloris aestuarii* DSM 271
Hch-1-1	Bacteria Proteobacteria Gammaproteobacteria Oceanospirillales Hahellaceae *Hahella chejuensis* KCTC 2396

19.2 Gene contexts

Each Chlorobi-RRM RNA (indicated by "RNA→") is listed with its surrounding genes. Environmental sequences and some RefSeq entries lack gene annotations, and no genes are listed for such sequences. The direction of each gene is indicated with an arrow (→), and each conserved domain in the gene is colored. Conserved domains associated with more than one Chlorobi-RRM RNA are assigned a color; other domains are gray. Information about these conserved domains is given in Section 19.3. The accession number of the sequence containing each Chlorobi-RRM RNA is given. Accessions beginning with "NC_" or "NZ_" are RefSeq, while other accession refer to environmental samples. Nucleotide coordinates are given for the 5′ and 3′ boundaries of each. If the 5′ coordinate is greater than the 3′ coordinate, the RNA is present on the reverse-complement strand of the containing genomic DNA sequence. Each hit is denoted by an abbreviation (like "Eco-1-1") that refers to a taxonomy given in Section 19.1.

abbrev.	Seq. accession		5′ at	3′ at	genes
Hch-1-1	NC_007645.1	+	455464	455534	**RNA**→ RRM (smart00360)→
Cph-3-1	NC_009337.1	-	1000654	1000582	**RNA**→ RRM (smart00360)→ BaeS (COG0642)→
Cte-1-1	NC_002932.3	-	127933	127857	**RNA**→ RRM (smart00360)→
Pae-1-1	NC_011059.1	-	1344490	1344426	**RNA**→ RRM (smart00360)→
Cli-1-1	NC_010803.1	+	2339792	2339872	**RNA**→ RRM (smart00360)→
Cfe-1-1	NZ_AASE01000008.1	+	61479	61554	**RNA**→ RRM (smart00360)→
Cph-1-1	NC_010831.1	-	1507528	1507452	**RNA**→ RRM (smart00360)→
Cte-1-2	NC_002932.3	+	1802605	1802684	**RNA**→ ←hypo
Cph-3-2	NC_009337.1	-	178328	178253	**RNA**→ RRM (smart00360)→
Cph-1-2	NC_010831.1	-	100324	100249	**RNA**→ RRM (smart00360)→
Plu-1-1	NC_007512.1	-	94199	94127	**RNA**→ RRM (smart00360)→
Pae-1-2	NC_011059.1	-	134270	134199	**RNA**→ RRM (smart00360)→
Cli-1-2	NC_010803.1	-	100693	100622	**RNA**→ RRM (smart00360)→
Cph-2-1	NC_008639.1	+	3066282	3066355	**RNA**→ RRM (smart00360)→
Cch-1-1	NC_007514.1	+	2033621	2033693	**RNA**→ RRM (smart00360)→
Plu-1-2	NC_007512.1	+	1162497	1162569	**RNA**→ RRM (smart00360)→ BaeS (COG0642)→ OmpR (COG0745)→

Cfe-1-2	NZ_AASE01000039.1	+	9214	9286	**RNA**→
Pph-1-1	NC_011060.1	+	1458931	1459003	**RNA**→ RRM (smart00360)→
Pph-1-2	NC_011060.1	+	2187184	2187256	**RNA**→ hypo→ RRM (smart00360)→ hypo→
Cph-2-2	NC_008639.1	+	1452008	1452080	**RNA**→ RRM (smart00360)→

19.3 Conserved domains

Conserved domains found in protein-coding genes listed in Section 19.2 are listed, with the first sentence in their description from the Conserved Domain Database. Conserved domains associated with more than one Chlorobi-RRM RNA are assigned a color, while others are shown in gray. The number in parentheses is the number of occurrences of the given gene.

COG0642 (2) Signal transduction histidine kinase [Signal transduction mechanisms]

COG0745 (1) Response regulators consisting of a CheY-like receiver domain and a winged-helix DNA-binding domain [Signal transduction mechanisms / Transcription]

smart00360 (18) RNA recognition motif;

19.4 Multiple-sequence alignment

Each Chlorobi-RRM RNA is denoted by an abbreviation (like "Eco-1-1") that refers to a taxonomy given in Section 19.1. The alignment includes sequences containing the RNA motif, as well as flanking sequence. The Chlorobi-RRM RNA itself is denoted by the line underneath marked 5′ and 3′ on either end. Nucleotides in the flanking sequences (i.e., not part of the motif) are written in gray letters. Stems of predicted rho-independent transcription terminators are shaded yellow. (Note: terminator predictions have not been analyzed manually, and many are likely to be false positives.) Start codons are shaded green. (Note: start codons are frequently misannotated, especially in environmental samples.) Nucleotides proposed to basepair as part of the consensus structure are shaded in color when they comprise Watson-Crick or G-U pairs. Otherwise they are shaded gray. Conserved stems are also indicated at the bottom of the alignment by angle brackets, where matching < and > denote base-paired columns. Below these angle brackets, the symbol "2" denotes base pairs exhibiting covariation, "1" denotes base pairs exhibiting compatible mutations, "0" denotes base pairs that are not observed to mutate and "?" denotes base pairs that have a significant frequency of non-canonical nucleotides for Watson-Crick or G-U pairs (> 5%). Below these base pair annotation is the consensus sequence: "R" = "A" or "G", "Y" = "C" or "U", red nucleotides: nucleotide identity conserved more than 97% of the time, black nucleotides: 90%, gray nucleotides: 75%, red circle (●): nucleotide is present 97% of the time, black circle (●): 90%, gray circle (●): 75%, white circle (○): 50%. All percentages of sequences just described (e.g. 97% conserved) assume that sequences have been weighted by the GSC algorithm implemented by the Infernal software.

alignment positions 1 · · · 99

```
Hch-1-1  UGGAAAUGGGAAUGUUCCAUAGUUAAAUCAUUUGACGUUUUGCCACUUUUGUUCCAGAUUUGUAUGCAUUGUGCGCAGCCGCGAAAGCGGGCCUGCAAC
Cph-3-1  AAACCCGUGGGCUCAGAGGUCAUUGUCAAUAAACCGACCCAAAUCAAUUGAAAACAUUUUCUUGUUGUUUAUCAACUGCAAUUAAACUAAACUCUGUCU
Cte-1-1  AGGCGAAAAAGAAUCGUUUCUGAACGAAUUUUCACAGUGGAAUCGCCCGGUGAAAACGUUUUUCUUUUUGAAAACGAUUUUUUAAUGAGGUAAAUUAUA
Pae-1-1  AUUCUUCGUUAUGCUGAUAGGCGCACAUUGGUUUGUGAGAACGGAGGGCUGUUGUUUUUUGUUGUUGGUUUAUUUUUAUAAAAACGUAUAUUGGUACGU
Cli-1-1  GCAAGGUCUGCCGUUUGCACCUGAAAGAUGCCCGACAGCCGACGGAAUAAUAGUUUUGGAUUAUUUUCCUUUUGUUUUAUACAAAAAUAAAAGGUAUAA
Cfe-1-1  AGAACCCCAGGAUAAGCGUUCAUGCACGGCAUUGUAUAGAGUGCAUCUAUAUAAUAUUUUAAUUUCCCUUGCUUCUUGAAGAAAUAAAGCGUACUCUUG
Cph-1-1  ACAGGAAGGAAUUCUCUGCUUGCAAUGCUCCAAAGGGAUUGUCCUGUGUUGCGUGUAUAUUAUUUUCUUCUUGUUAUAUGGCAAGUAAAAGAGUAUUAU
Cte-1-2  CCGGAAGGUCUGCUCCCUGCAUCUGAAAGAACGGCAACAUGGAAGUAUAUGAACUUCAGAUCAUCACGGAUUUGUAUGUUCACAUAAUAAAGAUUAUUA
Cph-3-2  AGCGCCUGCUCAUUCCUGCGCCAUGAUAAAGCCCGGCGAGAGGGGGGGGGUGAAUUUUCCUCUUGGUAAAAAUUACGGAAAACGGUAAAUUAACGAUUA
Cph-1-2  ACCCAUGGGUUUACCGGUCAGGAGGAACAGACAAGAGCACGGUUUAUUCUGCCUGUGAAAUUUUUUCUUUUGAAAUACGCAGAUCGGUAGAGUAAAUUA
Plu-1-1  UUACGGCAGAGGAAGUGACGCGGACGAGGCAAAAACAACCCGUCCAGGGCGAUUUUAUUUUUUUGAAAACAAAUUCGCAUUACCAUAAAUUACCGCUUA
Pae-1-2  GACUGAAAGCAAAAAAUCGAGCGUAAAUGUUAAGGAUUUUUUUCAUUCCAGCGCAUUUUCUUUUUGAAAACUGCUUGACAGAAGAGUAAAUUGCGGUUG
Cli-1-2  AAAAUAAAUUACGAUUUUGUAUAUUUUUAUCCGUAUUUCAGUUUAUUCCGGAUGUUUUUAUUUUUGAAAACGACUGCUAAUUGCGGUAAAUUAGGUUAU

         ...................................................................................................
         ...................................................................................................
         ●●●●●●●R●●●●●●●●●●●●●●●●●●●●●●●●●●●●●●●Y●●●●●●●●●●●●Y●UUU●●●●●●●●●●●●●●●●●●●●●●●RR●●●●●●●●●
```

```
Cph-2-1 AACUGCCAUGCAGCCCCUGUGGCGGUUAUCGGGUAAAAUUAUUUGCCUUUCGUGAAUUUUUAUUUUUGAAAUCCUGUAGUAAUUAGGGUAAAUUGAAGC
Cch-1-1 UAGAAUAGCUGUAAAACGAGACAAAUAUCACUGUCUCUACCUUCAUCUUAAUAAUUAUAUAUUUGAAAAUGAACAUCUUUUGGGUUAAACUUAGCAUAG
Plu-1-2 GCGGCUGUGGUUCCGGCAGGAGUUCUUUUACUGAAGAAAUAAUCCGCUGAGGAAAUUUUCUUCUUGUAAACCACCGUUAAUAAAGGUAAACUCCAUACA
Cfe-1-2 AACAUUCACGAUAGUCCUCCCUGUAAAGCAAGGGCGUAUUACGGCAGUGGUUGUAAUUAUUUUUUGAAAUACAUUAUUAAUUACGGUAAAUUGUGCUCA
Pph-1-1 UAAAAAUACAAGAGCCUCAUUCGGUGCUUAUUUCAACAAAUAUGCUGUCUAUGCAUUUUAUGUUUGAAAACGAUCAGUAAUUACGUUAAACUUCGUAUA
Pph-1-2 AAACAGACAAAUGGAGCAGAGGAGAUCCCGAUGAUGAAAAUUUGACACCGGAUGAAUUAUCUGUUUGAGAUGAAGAAUAAUUACGUUAAACUUGGUACA
Cph-2-2 AUACGGGAACAGUAUCGAAGCAAUGCCUGAUGAUAACUAUUAUUGUUCCAUCGCAUUAUAAGUUUGAUAAUCAUCGGUAAUUACGUUAUACUUCGCUUA
        ....................................................................................................
        ....................................................................................................
        ••••••R••••••••••••••••••••••••••••••••••••••Y••••••••••••Y•UUU•••••••••••••••••••••••RR•••••••••
```

alignment positions 100 ⋯ 259

```
Hch-1-1 CGAA...UUAG.CCAGCGGUUGG..CUAGUC..ACU..GACGAU..AAU.U.G.GAGCGUUUG.GUU.UUCGUC..AGUG..UGAUUGCUACA.GGCGCUCGUGACAAAUAAUGAGAUGGUGAGAUGAAUAUUUAUGUUGGAAACCUGUCCUACCAGGUA
Cph-3-1 GAAU...UCAG.CCAAUGGUUGG..UUGAGA..AGUGUGCGGGU..AAU.U.G.GUGCUAU.G.GUU.AUCUGCA.AGA..CUAUCUUU.UUAUCACUGCACGAGUUUACUGAGAUUAGACAAUGAAUAUUUAUGUCGGCAAUCUGCCUUACACCGUUUC
Cte-1-1 AAAUCAUUUAG.CCAGUGGUUGG..CUGAUC..GCU.UGCGAGA.AAAC..AG.GUGCUUU..GGUUAUCUCGU..GGCG..UUUCUUUUUUAUCGAAGCACAUGUUUCUACCGUCCCACAAUGAACAUUUACAUUGGCAAUCUGCCAUACAGCGUUACC
Pae-1-1 UAA....CCAC.AGAAUGGUU.....UGG.C..GCU.UUCAGGU..GAU.U.U.GUGCUCUUG.AUU.AUUUGUA.AGCG......UAUAGUAUGAUCAAAGCACAAAGUUUCACAUAGAAAAUAUGAACAUUUACAUUGGCAAUCUGGAUUACGGUGUU
Cli-1-1 UAGUUUGUUAGGCCAACAGUUGG.UCUGAAA..GCU.UUCAAGU..GAU.U.U.GUGCUUUUG.GUU.AUUUGAC.AGCCUUUCUCUUUUUGAUCAAAGCACGAUUACUAUGAAAAAAGCAUGAACAUUUACAUUGGCAACCUUGAUUACAACGUAACAG
Cfe-1-1 GAAG...CAAGACCAACGGUUGG.UCUGAAA..ACU.UUCAAGU..GAU.U.U.GUGCUUUUG.GUU.AUUUGAC.AGUG..UCUCUUUUUGAUCAAGGCACGAACUACUAUAUGAAAUUAUGAAUAUUUACAUUGGCAAUCUUGAUUACAGCGUAACUG
Cph-1-1 CAAUCG.UUAG.CCAAUGGUUGG..UUGAUC..GCUG.UCCGAU..GAU.U.U.GUGCUUUUG.AUU.GUUUGAU.GGCGUUUCUCUCU.UUAUUAAAGCACACAUUACCAUGAGGUAAACAUGAACAUUUAUAUUGGCAAUUUGGAUUACGGCGUAAGU
Cte-1-2 UAAACUUUCUGGACAACGGUUGGGCCAGA.G..GCU.UUCAAGU..GAU.U.U.GUGCUUUUG.AUU.AUUUGGC.AGCUUUUCUCUUU.CUAUCAAAGCACCAAGACCUUUAAAAAGCAUGAACAUCUACAUCGGCAAUCUCGACUAUAACGUCACUGA
Cph-3-2 GAAA...UCAU.CCAAUGGUUGG..CUGAUC..GCUG.ACAGGU..UAU..GU.GUGCUUU..GGUU.ACCUGUUGAGCGGUUUCUUUUUUCUACAAAUGCACGAGUAUACCGAGACCAAACAAUGAAUAUUUAUGUAGGUAACCUGCCUUAUUCCAUCAC
Cph-1-2 CAACA..UCAG.CCAAUGGUUGG..CUGAUC..GCUAU.CGGAU..AAC..GU.GUGCUUUU.GGUU.AUUUGAC.AGCGGUUCUCUUU.CUAUCAAAGCACACGUCAUCUUAUAUCAAAAAUGAACAUUUACAUUGGCAAUCUACCCUACAGCUUAUCU
Plu-1-1 GAAA...CCAG.CCAAUGGUUGG..CUGGUC..GCU.UACAGGU..AAU.U.U.GUGCCUU.G.GUU.AUCUGCAGAGCG..CCUCUUU.CUAUCAAUGCACCAUGUAUACCAAGACCUAAAAAUGAAUAUUUAUAUUGGCAAUCUGCCGUACUCCGUGU
Pae-1-2 UA.....UUAG.UCAAUGGUUGA..CUGACC..GCA.UUCAAAU..AAU.U.UUGUGCUUUUG.GUU.GUUUGUC.UGCG..CUUCUUU.CUAUCAAAGCACAUGUUUCUACGAGAUCAACAUGAACAUUUACAUUGGUAACCUGCCCUACAACAUCACU
Cli-1-2 UAA....UUAG.CCAAUGGUUGG..CUGAUC..GCG.UUCAAAU..AAU.U.U.GUGCCUU.G.GUU.GUUUGCA.UGCG..UUUCUUUUCUAUCAAUGCACAUGUUUACUGAGACUAACAAUGAAUAUCUACAUUGGCAAUUUACCCUACUCCGUUAGG
Cph-2-1 UAAU...UUAG.CCAGUGGUUGG..CUGAUC..GCGAUUCAAAU..AAU.U.U.GUGCUUU.G.GUU.AUUUGCU.UGCG..UCUCUAUUCUAUCAAUGCACAUGUUUACUGAGACUAACAAUGAAUAUUUACAUUGGUAAUUUGCCUUAUCAAGUCACU
Cch-1-1 UAA....UUAG.UCAAUGGUUGA..CUGAUUC.GCUACUCAAGU..AAU.U.U.GUGCGUU.G.GUU.ACUUGAC.GGUA..UCUCUUU.UUCUACCAAUGCAUAGAUACACUGAGACGAACAAUGAAUAUUUACAUUGGCAAUCUGGCUUACAGCGUUA
Plu-1-2 UAAA...UCAG.CCAAUGGUUGG..CUGAUCGCGCUAU.CAAGU..AAU.U.G.GUGCUUU.G.GUU.ACUUGCC...UG.CUCUCUUU.UUAUCAAUGCACGCGUAUACUGAGACUGAUAAUGAACAUUUAUGUUGGCAAUCUGCCUUACACCAUUUCC
Cfe-1-2 GAAA...UCAG.CCAAUGGUUGG..CUGAUC..GCUAUGCAAGU..AAU.U.U.GUGCUUU.G.GUU.ACUUGCC.GGCG..UCUCUUU.CUAUCAAUGCACAUGCUUACUCAGACUAACAAUGAAUAUUUACAUUGGCAAUUUGCCUUACACUGUUUCU
Pph-1-1 GAAU...UCAG.UCAAUGGUUGG..CUGAUC..GCUAUUCAAGU..AAU.U.U.GUGCUUU.G.GUU.ACUUGCC.GGUA..UCUCUCC.CUAUCAAUGCACAUGUACACAGAGAUUAAAAAUGAAUAUUUACAUCGGUAAUUUGGCUUAUACUGUUUC
Pph-1-2 GAAA...UCAG.UCAAUGGUUGA..CUGAUC..GCUAUUCAAGU..AAU.U.U.GUGCUUU.G.GUU.ACUUGAC.GGUA..UCUCUUU.CUAUCAAUGCACAUGCAUACUGAAACUAACAAUGAAUAUUUACAUUGGCAAUUUGGCUUACACUGUUACU
Cph-2-2 GAUG...UCAG.UCAAUGGUUGA..CUGAUC..GCUAUUCAGGU..AAU.U.U.GUGCUUU.G.GUU.ACUUGUC.GGUA..UCUCUUU.UUUUCAAUGCACAUGUACUAUGAGAAUUAAUAAUGAAUAUUUACAUUGGCAAUUUGGCUUACACGGUUUC
        ......<<<<<<<<<...>>>>.>>>>>.<..<<<..<<<<<<..<<<.<..........>.>>>.>>>>>>..>>>>.........................................................................
        ......222?2?001...100?.2?222.?..202..?22222..102.0..........0.201.22222?..202?.........................................................................
        •AA•---UYAG-YCAAYGGUUGR--CUGA•Y--GCU-U•CRRRU--RAU-U-U-GUGCUUU-G-GUU-AYUUG••-RGYR--UYUCUYU-YUAU•A••GCAC••R••••Y•••RR•••R••R••R••••Y••••••••••••Y••••Y••••Y••••••
         5′                                                                                 3′
```

alignment positions 260 ⋯ 419

```
Hch-1-1 ACAGAAGACGAUUUGCGGGAAGCGUUUGCGGCCUAUGGCGAUAUCAGCAACGUCAAUAUCAUCCGCGAUAGAGAUACCGGUCAAUCCAAAGGGUUCGGUUUCGUGGAAAUGUCCGACAACGGGCAGGCGGAAGAGGCGAUUCAGAAGCUUAACGAAAGCA
Cph-3-1 UGAAGAUGACCUUCGUGACGCCUUUUCCGAAUUCGGACAGGUAGAGAGCGCAAACAUCAUCAUGGACAAAUUUUCGGCCGCUCAAAAGGCUUCGGUUUUGUUGAAAUGUCUGAUGACGAUGCUGCACGCGAAGCCAUUGAAGCCAUGCACAACAAGGAC
Cte-1-1 GAUGAAGACCUGCGCGACAAAUUCUCCGAGUUCGGGCAGGUGCAUAGCGCCAACAUCAUCACUGACAAAUUUUCUGGUCGCUCCAAAGGCUUCGGGUUCGUUGACAUGCCGAACGAAAGUGAAGCUCGCGAAGCGAUCGAUGCCAUGAACGACAAGGACU
Pae-1-1 ACAGAAGAAGAUCUUCGUGAGGCCUUUGGCGAAUUCGGUGAUGUUUCAAGCGCAAACAUCAUAACUGACAAAUUUACCGGCCGUUCAAAAGGCUUCGGUUUUGUUGAAAUGUCGAGCAGUGAUGACGCCAACGAAGCUAUCGAAUCUCUGAAUGACACGG
Cli-1-1 AAUCUGACCUUCGCGAAACAUUUGGCGAAUUCGGUGAAGUUUCUAACGCGAGUGUGAUCACCGAUAAAUUCACCGGACGUUCAAAAGGCUUUGGCUUUGUUGAAAUGCCUAACAACGCAGAAGCUUCUGAAGCCAUCUCUUCUCUGAACGACAGUGACUU
Cfe-1-1 AAGCUGAUCUUCGUGAGACUUUCGGCGAAUUCGGCGAAGUUUCAAGCGCAAGUGUCAUCAACGACAAAUUCACCGGCCGUUCAAAGGGUUUUGGUUUUGUUGAGAUGGCAAAUGAUGCAGAAGCUGCUUCUGCAAUCGCUGACCUCAACGACAGUGACCU
Cph-1-1 GAAGAAGAUUUGCGCGAAGCUUUUGGCGAGUUUGGAGAAGUCUCCAGCGCAAACAUCAUUUCAGACAAAUUCACAGGCCGUUCCAAAGGUUUUGGUUUUGUUGAAAUGCCGAAUGACGAAGCAAACCAGGCAAUCGAUGCAUUGAACGAUACCGACC
Cte-1-2 AGCAGACCUGAGCGGCGCAUUUGGCGAAUUCGGCACGGUUUCCAAAGCAAACGUCAUCAUCGACAAGUUCACUGGCCGUUCAAAAGGAUUCGGAUUCGUUGAAAUGCCUGACGACGCAGAGGCAAACGAAGCGAUUUCCAGCUGAACGAAAGCAGCCUC
Cph-3-2 CGAAGAUGAUCUUCGCGACACCUUUUCCGCUUUCGGUCAGGUCGACAGCGCAAACAUCAUCACCGACAAAUUCUCAGGCCGUUCAAAAGGCUUCGGUUUUGUUGACAUGCCUAACGACAGUGAAGCACGCGAAGCAAUAGAGUCAAUGCACGAGAAAGAG
Cph-1-2 GAAGAGGAACUCAGCGAUGCAUUCGCUCAAUUCGGUCAGGUAGACAGAGCGAACAUUAUCAUGGAUAAAGAAACCGGCCGCUCGAAAGGCUUCGGAUUUGUUGAAAUGCCAAACGACGAUGAAGCACAAAAUGCUAUCGAUUCUUUGCAUGACAGUGAUC
Plu-1-1 CAGAAGAUGAUCUUCGCGAUGCAUUCUCCCAGUUCGGGCAGGUCGACAGCGCAAACAUCAUCAUGGACAAAUUUUCGGACGCUCAAAGGGGUUCGGCUUUGUUGACAUGCUGAGCGACGAGGCAGGCCGUGCCGCCAUCGAGGCGAUGAACGAGCAGGA
Pae-1-2 GAAGACGACCUUCGUGAAGCCUUCAAUGAAUUCGGACAGGUAGACAGCGCAAACAUCAUUAUCGACAAAUUCCGGCCGCUCAAAAGGCUUCGGUUUUGUUGAAAUGCCAAAUGACGAAGAUGCUGAAAAAGCAAUCGACUCAUUGAACGACACCGAUU
Cli-1-2 GAAGACGAUCUUCGUGAUGUAUUCUCCCAGUUCGGACAGGUCGACAGCGCUAACAUCAUCACCGACAAGUUUUCAGGCCGCUCGAAAGGGUUUGGGUUUGUUGACAUGCCUAUCGACGGUGAAGCCCGCGAAGCUAUCGAAUCCAUGAACGACAAGGAUC
Cph-2-1 GAAGAUGAUCUUCGCGAUGCAUUCUCGCAGUUCGGACAGGUUGACAGCGCAAACAUCAUCACCGACAAGUUUUCAGGCCGUUCGAAAGGGUUCGGGUUUGUUGACAUGCCUCAUGAUGACGAAGCCCGUGAAGCUAUCGAAUCAAUGAAUGACAAGGAUU
Cch-1-1 CUGAAAAUGACCUGCGUGACGCAUUCGGUCAAUUUGGACAGGUAGAAAGCGCAAGUAUUAUUACCGACAAAUUUUCAGGGCGCUCUAAGGGCUUUGGUUUUGUGGACAUGCCAAACGAUAGUGAAGCUCGCGAAGCAAUCGGAGCAAUGAAUGAAAAAGA
Plu-1-2 GAAGAUGACCUUCGUGACGCUUUCUCCGAGUUUGGAGAAGUAGCAAGCGCAAACAUCAUCACUGACAAAUUCUCCGGCCGCUCCAAGGCUUCGGUUUUGUCGAGAUGCCUGAUGACGAUGCAGCUCGCGAAGCCAUUGAGUCCAUGCACAACAAGGACU
Cfe-1-2 GAAGAUGAUCUUCGCGAUGCGUUCUCCCAGUUCGGACAGGUCGAUAGCGCAAACAUCAUUACCGACAAGUUUUCAGGCCGUUCCAAAGGGUUUGGAUUUGUUGACAUGCCUAAUGACAGUGAAGCUCGUGAAGCCAUCGAAUCAAUGAAUGACAAAGAUU
Pph-1-1 UGAAGAUGAUCUUCGUGAUGCUUUCUCCGAAUUCGGAGAGGUUGCAAAUGCGAGCAUCAUCAAUGACAAAUUUUCAGGUCGCUCAAAGGAUUUGGAUUUGUUGAGAUGCCUAAGGACAGUGAAGCACGUGAGGCUAUCGAAUCAAUGAAUGGAAAGGAU
Pph-1-2 GAAGAUGAUCUGCGUGAUGCCUUCUCCGAAUUCGGACAGGUCGAGAGCGCAAGUAUCAUAAACGACAAAUUUUCAGGCCGUUCCAAGGGGUUCGGAUUUGUCGACAUGCCUAACGAAAGUGAAGCUCGUGAAGCCAUCGAAUCAAUGAAUGAGAAAGAUU
Cph-2-2 CGAAGAUGAUCUCCGUGACGCCUUCUCCCAGUUCGGGCAGGUCGAUAGCGCUAACAUCAUCAACGACAAAUUUUCUGGCCGUUCCAAAGGAUUCGGUUUCAUUGAAAUGUCUAAUGACAGCGAAGCUCGCGAAGCUAUCGAAUCAAUGAAUGAUCAGGAU
        ..............................................................................................................................................................
        ..............................................................................................................................................................
        •RRRRR••••YYY•••R••••YY•YY•••••••••••R•R•••••RR••••R••••Y•••••••R•RA••••YY••••••YY•••RRR••••••••YU••••R•R•••••••••RR••R•RRR•••••••RRR•••••••••••••••••RR•RR•R•••
```

alignment positions 420 · · · 493

```
Hch-1-1  ACCUGAAAGGCCGUAACAUGAAAGUUAACGAGGCCCGUCCCCGGGAAGAAAAGCCAAAGCGUAAUUUCAGACCU
Cph-3-1  UUCAUGGGCCGUUCCAUCAUGGUCAACGAGGCAAAGCCCCGCGAAGAAAGAGCUCCCCGCCGCGAUCAUUACUG
Cte-1-1  UUAAAGGCCGCACCAUAAAAGUGAACGAGGCAAGGCCUCGCGAGCAGCGUCCUCCCAGAAGAGAGCAAUACUGA
Pae-1-1  AUCUCAAUGGUCGCACCAUCAAAGUUAACGAGGCAAAACCUCGUGCUGAGCGCCCUGCAAGACGUUACCAGUAU
Cli-1-1  GAACGGCAGAACCAUCAAGGUAAACGAGGCAAAGCCACGCGAGCAGCGCCCGAUUUCCCGCCCGAGGUACUAAG
Cfe-1-1  GAACGGCAGAACCAUCAAGGUUAACGAGGCAAAGCCCCGUGAAGAGCGUCCAGCAUCCCGCUCAAGGUAUUAAG
Cph-1-1  UUAAUGGUCGUUCUAUCAAGGUCAACCAGGCAAGACCUCGCGAAGAACGUCCGUCAAGACCUCGUUACUGAGCA
Cte-1-2  AACGGAAGAAAAAUCAGGGUGAACGAAGCAAAGCCUCGUGAAGAGCGCCCGGCUGCCCGCCCCAGGUACUGAGC
Cph-3-2  CUCAGCGGACGCACCAUCACGGUCAACGAAGCAAGACCCCGUGAAGAACGCCCUGCAAGAAGGAUUACUGAUC
Cph-1-2  UGAACGGCCGCUCUAUCAAAGUCAAUCAGGCAAGGCCCCGUGAAGAGCGACCCCCUCGGAGAGACAACUACUGA
Plu-1-1  GUUCAAAGGCCGAACCAUCAAAGUGAACGAGGCAAGGCCCCGGGAAGAAAGGGCUCCCAGAAGGAGCAGCUACU
Pae-1-2  UUAAUGGCCGCACCAUCAAAGUCAACCAGGCAAGACCCCGCGAAGAACGUCCUGUACGCAGAGAUCGUUAUUGA
Cli-1-2  UUAAAGGCCGUACCAUAAAGGUUAACGAAGCCAGGCCACGUGAAGAACGCCCCGCAAGAAGAGACCGCUAUUGA
Cph-2-1  UGAAAGGUCGUACUAUAAAAGUCAAUGAAGCAAGGCCGCGCGAAGAAAGGCCGGCAAGAAGAGAUCGCUAUUAA
Cch-1-1  ACUCAAUGGUCGCCCAAUAAAGGUAAACGAGGCAAAACCUCGUGAGGAGAGACCAGCAAGAAGAGAUCGUUACU
Plu-1-2  UCAAGGGCCGUUCCAUCAUGGUCAACGAGGCUAAGCCCCGCGAAGAACGAGCUCCCCGCCGCGAUCGUUACUGA
Cfe-1-2  UGAAAGGCCGCACUAUAAAGGUAAACGAAGCGAGGCCUCGCGAAGAAAGGCC......................
Pph-1-1  UUGAACGGUCGCACGGUAACGGUAAACGAAGCGAAACCUCGCGAAGAGAGACCUCGUGAAGAAAGAUCGCCAAG
Pph-1-2  UGAACGGCCGCACCAUAAAGGUGAACGAAGCAAAGCCCCGUGAAGAACGGCCAGCAAGAAGAGAUCGCUACUAA
Cph-2-2  UUGAAAGGUCGCACCAUCAAGGUGAACGAAGCCAAACCUCGCGAAGAAAGAUCAGAGAGACGGGACCGCUAUUA
         ..........................................................................
         ..........................................................................
         •••••RR••••••Y••••R•R••••••RRR••RR•••YY•••RRRRR•R••••Y••••R••••R••••••••••
```

20 Chloroflexi-1

20.1 Taxa

The taxonomy of each organism containing a putative Chloroflexi-1 RNA is listed, with abbreviations identifying each hit (e.g., "Eco-1-1" and "Eco-1-2" might hypothetically represent two distinct RNAs in *E. coli*). The abbreviations will be used to identify each individual Chloroflexi-1 RNA in Sections 20.2 and 20.4.

**abbrev. of hits**	**taxonomy of species**
Cag-1-1 to Cag-1-3	Bacteria Chloroflexi Chloroflexales Chloroflexaceae *Chloroflexus aggregans* DSM 9485

20.2 Gene contexts

Each Chloroflexi-1 RNA (indicated by "RNA→") is listed with its surrounding genes. Environmental sequences and some RefSeq entries lack gene annotations, and no genes are listed for such sequences. The direction of each gene is indicated with an arrow (→), and each conserved domain in the gene is colored. Conserved domains associated with more than one Chloroflexi-1 RNA are assigned a color; other domains are gray. Information about these conserved domains is given in Section 20.3. The accession number of the sequence containing each Chloroflexi-1 RNA is given. Accessions beginning with "NC_" or "NZ_" are RefSeq, while other accession refer to environmental samples. Nucleotide coordinates are given for the 5′ and 3′ boundaries of each. If the 5′ coordinate is greater than the 3′ coordinate, the RNA is present on the reverse-complement strand of the containing genomic DNA sequence. Each hit is denoted by an abbreviation (like "Eco-1-1") that refers to a taxonomy given in Section 20.1.

**abbrev.**	**Seq. accession**		**5′ at**	**3′ at**	**genes**
Cag-1-1	NZ_AAUI01000002.1	+	27961	28045	hypo→ **RNA**→ hypo→
Cag-1-2	NZ_AAUI01000026.1	+	1295	1378	←RarD (COG2962) **RNA**→ ATS1 (COG5184)→
Cag-1-3	NZ_AAUI01000026.1	+	3992	4075	ATS1 (COG5184)→ **RNA**→ hypo→

20.3 Conserved domains

Conserved domains found in protein-coding genes listed in Section 20.2 are listed, with the first sentence in their description from the Conserved Domain Database. Conserved domains associated with more than one Chloroflexi-1 RNA are assigned a color, while others are shown in gray. The number in parentheses is the number of occurrences of the given gene.

u COG2962 (1) Predicted permeases [General function prediction only]
uD COG5184 (2) Alpha-tubulin suppressor and related RCC1 domain-containing proteins [Cell division and chromosome partitioning / Cytoskeleton]

20.4 Multiple-sequence alignment

Each Chloroflexi-1 RNA is denoted by an abbreviation (like "Eco-1-1") that refers to a taxonomy given in Section 20.1. The alignment includes sequences containing the RNA motif, as well as flanking sequence. The Chloroflexi-1 RNA itself is denoted by the line underneath marked 5′ and 3′ on either end. Nucleotides in the flanking sequences (i.e., not part of the motif) are written in gray letters. Stems of predicted rho-independent transcription terminators are shaded yellow. (Note: terminator predictions have not been analyzed manually, and many are likely to be false positives.) Start codons are shaded green. (Note: start codons are frequently misannotated, especially in environmental samples.) Nucleotides proposed to basepair as part of the consensus structure are shaded in color when they comprise Watson-Crick or G-U pairs. Otherwise they are shaded gray. Conserved stems are also indicated at the bottom of the alignment by angle brackets, where matching < and > denote base-paired columns. Below these angle brackets, the symbol "2" denotes base pairs exhibiting covariation, "1" denotes base pairs exhibiting compatible mutations, "0" denotes base pairs that are not observed to mutate and "?" denotes base pairs that have a significant frequency of non-canonical nucleotides for Watson-Crick or G-U pairs (> 5%). Below these base pair annotation is the consensus sequence: "R" = "A" or "G", "Y" = "C" or "U", red nucleotides: nucleotide identity conserved more than 97% of the time, black nucleotides: 90%, gray nucleotides: 75%, red circle (•): nucleotide is present 97% of the time, black circle (•): 90%, gray circle (•): 75%, white circle (•): 50%. All percentages of sequences just described (e.g. 97% conserved) assume that sequences have been weighted by the GSC algorithm implemented by the Infernal software.

alignment positions 1 · · · 99

```
Cag-1-1  AAUUGGGCGACGGCAGAGCGUUGUAUCGCACCACGCCGGUGGACGUGGUGAUGUCAGCGCAGGGUCAGUACACCUACCGAUGAUUAUCAGAUGAGAACA
Cag-1-2  ACUGGGCGACGGUAGACCGCUGUAUCGCACCACGCCGGUGGAAGUGGUGACCUCAGCGUCAUGGGGAGUAUACCUACCGAUGAUUGUUAGAUGAGUUCA
Cag-1-3  ACUGGGCGACGGCAGACCGCUGUAUCGCACCACGCCGGUGGAUGUGGUGACUUCAGCGUCAUGGGGAGUAUACCUACCGAUGAUUAUCAGAUGAGUUCA
         ...................................................................................................
         ...................................................................................................
         A•U•GG••R••G••RR••••U••••Y••••C••••C•G••GR••••G••R•Y•Y•R•••CA•GG••AGUAYACCUACCGAUGAUURUYAGAUGAG••CA
```

alignment positions 100 · · · 259

```
Cag-1-1  ACGUCCGGUGGAUUGCGACGGGCAGGAAGGCAGGCAUUGGCCGGCCUUCCUGCCCGCUUUCGGUUGCCGCUGACGGGAACACGUCAGGACGACACAGAACGGGUAUCGGCACGAGGAAGCAGCAGCGCGUAUUGAAAUACCAUUGUUCAUGAGCAGUUGU
Cag-1-2  CCGUCCCGUCGGCUGCGGCGGGCAGCACGGCCGACAUUGGCCGGCCG.GUUGCCCGCCUUCGUUUGUUGAUGACCGAAACGCAACACCGCCACGACGCCGAGGGCUUGGCCCGUCGAAAGCGAGGAUCGCUAUGCCGCCAUCGCAUCACCGGCGGAACGU
Cag-1-3  CCGUCCCGUGGGAUGCGGCGGGCAGCACGGCCGACAUUGGCCGGCCG.GUUGCCCGCCUUCGUUUGUUGAUGACCGAAACGCAACACCGCCACGACGCCAAGACACAAAGAGUUGUAUUCCCAAAGUAGGGUUGUCUCAGGUAUCGGAUUCGGUGUGUUG
         ................<<<<<<<<<<.<<<<<<........>>>>>>.>>>>>>>>>>.<<<<<....>>>>>.......................................................................................
         ................1?00000012.2000?0........0?0002.21000000?1.00020....02000.......................................................................................
         •CGUCC•GU•GR•UGCGRCGGGCAG•A•GGC•GRCAUUGGCCGGCC•-•YUGCCCGCYUUCG•UUGYYG•UGAC•GRAACRCR•CA••RC•ACR••G•••RGR••Y•RR•••••••A••••••RR•••G•••UG•••Y•••••Y•••••Y•R••R••Y••
          5′————————————————————————————————————————————————————————————————————————3′
```

alignment positions 260 · · · 419

```
Cag-1-1  UGAGUUCGAGUCAACGGUAUACGACUGAACAGUUGGCUCAGUUUCUCGAAGUAGUUUAUCGAGAUGAACAGACAAGCCUCUGUCGUCGUUCGUUACCUCCACAGCCCAACUUAACCUUUUCGCUAGGCAAAGGCUGGUACAAUCUAGAACGCACUGACAA
Cag-1-2  GACCACGCGCGGCCCCCCGCUCCCGGCGAAUGGACGCCGGGGCGCCGCGCAAAGAACCAAGUUUUCCCGCUUGACCCUAGGCCGGCAAACUGUUAUCCUACAGCCGUAGGAACCGACCGCAUCCGAGCUGCCGCCGCUGCCGGCGUUGCAACGUAUCGUU
Cag-1-3  UUUCUUGUCACGGCCUCCAGCAGCGCAUACGCUGGCAUCGGUGCGCAGGUGAUCGUUCCCGAUGCUUCGUCAUUGCAACCUCCGUGCGCCACCUAACACAUGUUCGAUUUGCAUAUGGGAUGGAUGGGAGACACCACGAGUACAACCGGGCGGUGUGUUC
         ................................................................................................................................................................
         ................................................................................................................................................................
         •••••Y••••••••C••YR•••••••••A••••••••Y•RG••••Y••R•R•••••Y•••G•••Y•••••••••••••••••Y•••••••Y••••R•Y••YUA•C•Y•••••C••••••••••••••••••••RG••R••RCYR••R••R•••••R••••RY••••••
```

alignment positions 420 · · · 485

```
Cag-1-1  UGGCAUACGCUGGCAGUGACAGCGGAUCAAGCGGAUGUUCACGUCUUUAAUUCGUCUGCAACGUCG
Cag-1-2  UUGUAAAGCAGUAGAUGAAAUCCGUCGGAGGCUGCAAUGAACCACUACACCAUGCUGCGCUGGCUA
Cag-1-3  AAUCACGAACUCGUCUACAAAGCGCACUGCGCGACCAGAUUUGUACUUUUUGCACAAAUUAAAUCU
         ..................................................................
         ..................................................................
         •••YA•R•••••R•••••A•••CG••••R•GC•R••R••••Y•••Y•Y••Y•YRY•••••••RYY•
```

21 Clostridiales-1

21.1 Taxa

The taxonomy of each organism containing a putative Clostridiales-1 RNA is listed, with abbreviations identifying each hit (e.g., "Eco-1-1" and "Eco-1-2" might hypothetically represent two distinct RNAs in *E. coli*). The abbreviations will be used to identify each individual Clostridiales-1 RNA in Sections 21.2 and 21.4.

abbrev. of hits	taxonomy of species
Csp-1-1	Bacteria Firmicutes Clostridia Clostridiales Clostridiaceae *Clostridium sp.* L2-50
Csp-2-1	Bacteria Firmicutes Clostridia Clostridiales Clostridiaceae *Clostridium sp.* SS2/1
Eve-1-1 to Eve-1-8	Bacteria Firmicutes Clostridia Clostridiales Eubacteriaceae *Eubacterium ventriosum* ATCC 27560
Aca-1-1	Bacteria Firmicutes Clostridia Clostridiales Lachnospiraceae *Anaerostipes caccae* DSM 14662
Ceu-1-1 to Ceu-1-4	Bacteria Firmicutes Clostridia Clostridiales Lachnospiraceae *Coprococcus eutactus* ATCC 27759
Dfo-1-1 to Dfo-1-3	Bacteria Firmicutes Clostridia Clostridiales Lachnospiraceae *Dorea formicigenerans* ATCC 27755
Rla-1-1 to Rla-1-58	Bacteria Firmicutes Clostridia Clostridiales Ruminococcaceae *Ruminococcus lactaris* ATCC 29176
Rob-1-1 to Rob-1-2	Bacteria Firmicutes Clostridia Clostridiales Ruminococcaceae *Ruminococcus obeum* ATCC 29174
env-1 to env-128	environmental samples

21.2 Gene contexts

Each Clostridiales-1 RNA (indicated by "RNA→") is listed with its surrounding genes. Environmental sequences and some RefSeq entries lack gene annotations, and no genes are listed for such sequences. The direction of each gene is indicated with an arrow (→), and each conserved domain in the gene is colored. Conserved domains associated with more than one Clostridiales-1 RNA are assigned a color; other domains are gray. Information about these conserved domains is given in Section 21.3. The accession number of the sequence containing each Clostridiales-1 RNA is given. Accessions beginning with "NC_" or "NZ_" are RefSeq, while other accession refer to environmental samples. Nucleotide coordinates are given for the 5′ and 3′ boundaries of each. If the 5′ coordinate is greater than the 3′ coordinate, the RNA is present on the reverse-complement strand of the containing genomic DNA sequence. Each hit is denoted by an abbreviation (like "Eco-1-1") that refers to a taxonomy given in Section 21.1.

abbrev.	Seq. accession		5′ at	3′ at	genes
env-1	JCVI_SCAF_1096628330702	+	195	362	**RNA**→
env-2	JCVI_SCAF_1101668573117	+	473	642	**RNA**→
env-3	JCVI_SCAF_1096627924491	+	482	642	**RNA**→
env-4	JCVI_SCAF_1096628322047	-	625	462	**RNA**→
env-5	JCVI_SCAF_1096628334677	-	1111	952	**RNA**→
env-6	JCVI_SCAF_1096628293756	-	644	486	**RNA**→
env-7	BABB01000778.1	+	1764	1911	GroEL (cd03344)→ **RNA**→ DegV (COG1307)→
env-8	BAAV01034327.1	+	142	292	**RNA**→ ThiS (cd00565)→
Ceu-1-1	NZ_ABEY02000022.1	-	37900	37738	COG3968 (COG3968)→ **RNA**→ TopA (COG0550)→

Eve-1-1	NZ_AAVL02000037.1	+	174692	174858	←hypo **RNA**→ MarR (pfam01047)→
env-9	BAAV01001515.1	-	1917	1759	3HCDH (pfam00725)→ **RNA**→ SCAD/SBCAD (cd01158)→
env-10	BAAV01002929.1	-	1019	863	hypo→ **RNA**→ ←ABC_MsbA_C (cd03251)
env-11	BAAU01003785.1	+	137	308	hypo→ **RNA**→ ←hypo
env-12	BAAY01014504.1	+	21	201	**RNA**→ ←EAL (cd01948)
env-13	BAAU01003080.1	+	9	185	**RNA**→ hypo→
env-14	BAAU01005865.1	-	1412	1225	PurL (COG0047)→ **RNA**→ hypo→
env-15	BABG01004091.1	+	375	562	←hypo **RNA**→ ←GlgA (COG0297)
env-16	BAAV01002036.1	-	1046	870	←hypo **RNA**→ hypo→
env-17	hgutS8_s8_169515	-	1029	842	←HisC (COG0079) **RNA**→ hypo→
env-18	hgutS8_s8_169849	+	467	654	←UPF0029 (pfam01205) **RNA**→ Transgly (pfam00912)→
env-19	BABB01002899.1	-	2303	2116	**RNA**→ ←GlgA (COG0297)
Eve-1-2	NZ_AAVL02000036.1	-	99484	99318	←hypo **RNA**→ ←hypo
env-20	BAAX01023897.1	-	957	790	**RNA**→ ArgC (COG0002)→
Eve-1-3	NZ_AAVL02000035.1	+	139817	139954	←HTH_XRE (cd00093)COG1917 (COG1917) **RNA**→ HIS2 (COG1387)→
Eve-1-4	NZ_AAVL02000031.1	+	48980	49147	hypo→ **RNA**→ hypo→
env-21	BABD01004363.1	-	554	380	hypo→ **RNA**→
Eve-1-5	NZ_AAVL02000027.1	+	21462	21630	GlgB (COG0296)→ **RNA**→ hypo→
env-22	BAAV01016969.1	-	686	523	hypo→ **RNA**→ hypo→
env-23	BAAX01018853.1	+	543	708	←DppD (COG0444) **RNA**→
env-24	BAAU01028694.1	+	160	335	hypo→ **RNA**→ hypo→
env-25	BAAZ01005512.1	-	486	314	hypo→ **RNA**→
Rob-1-1	NZ_AAVO02000012.1	+	105629	105797	hypo→ **RNA**→ MET17 (COG2873)→
Rob-1-2	NZ_AAVO02000013.1	-	48190	48022	IlvA (COG1171)→ **RNA**→ ArgE (COG0624)→
Eve-1-6	NZ_AAVL02000023.1	-	56132	55966	←HrcA (COG1420) **RNA**→ ←DegV (COG1307)
env-26	BABE01004765.1	-	1080	911	**RNA**→ DnaD (pfam04271)→
env-27	BABA01009489.1	+	308	486	GuaB (COG0516)→ **RNA**→ AdcA (cd01017)→
env-28	BAAZ01027624.1	-	869	703	**RNA**→ ←COG2070 (COG2070)
env-29	BAAV01006167.1	+	863	1032	PRA-CH (pfam01502)→ **RNA**→
env-30	BABD01002878.1	-	1413	1244	←hypo **RNA**→ ←nuc_hydro_3 (cd02653)
env-31	BAAY01020707.1	-	871	700	hypo→ **RNA**→ ←MurI (COG0796)
env-32	BABG01005618.1	+	356	523	←hypo **RNA**→ ←RimI (COG0456)
Eve-1-7	NZ_AAVL02000037.1	+	286875	287041	enolase (cd03313)→ **RNA**→ EriC (COG0038)→
env-33	BAAV01006120.1	+	617	787	←Nth (COG0177) **RNA**→ hypo→
env-34	BAAY01008255.1	-	580	413	←DMRL_synthase (pfam00885) **RNA**→ ←RibA (COG0807)
Eve-1-8	NZ_AAVL02000035.1	-	75876	75709	GreA (COG0782)→ **RNA**→ LysU (COG1190)→
Rla-1-1	NZ_ABOU02000027.1	-	54252	54086	←LeuS (COG0495) **RNA**→ ←COG0385 (COG0385)
env-35	BABG01000170.1	-	596	450	←hypo **RNA**→ hypo→
Dfo-1-1	NZ_AAXA02000013.1	+	250273	250440	←hypo **RNA**→ ←hypo
env-36	BABG01013486.1	+	1030	1197	PheS (COG0016)→ **RNA**→
Rla-1-2	NZ_ABOU02000020.1	-	5236	5069	←GGDEF (cd01949) **RNA**→ ←hypo
Rla-1-3	NZ_ABOU02000026.1	-	3951	3784	←HTH_XRE (cd00093) **RNA**→ hypo→
env-37	BABG01029695.1	-	208	75	GRS1 (COG0423)→ **RNA**→
Rla-1-4	NZ_ABOU02000048.1	+	174081	174247	LeuA (COG0119)HTH_ARAC (smart00342)→ **RNA**→ ←hypo
Rla-1-5	NZ_ABOU02000036.1	-	12225	12058	←hypo **RNA**→ ←hypo
Rla-1-6	NZ_ABOU02000034.1	-	13978	13811	←CLECT (cd00037) **RNA**→ ←hypo
Rla-1-7	NZ_ABOU02000050.1	-	2046	1878	←COG1725 (COG1725) **RNA**→ ←CstA (COG1966)
Rla-1-8	NZ_ABOU02000006.1	-	26217	26050	CSD (pfam00313)→ **RNA**→ ←hypo
Rla-1-9	NZ_ABOU02000050.1	-	140438	140271	←FtsX (pfam02687)ABC_MJ0796_Lo1CDE_FtsE (cd03255) **RNA**→ ←hypo
Rla-1-10	NZ_ABOU02000010.1	+	11853	12020	←PP_kinase (pfam02503) **RNA**→ ←COG4667 (COG4667)
Rla-1-11	NZ_ABOU02000050.1	+	30313	30480	←NptA (COG1283) **RNA**→ Relaxase (pfam03432)→
Rla-1-12	NZ_ABOU02000034.1	-	1573	1406	←LGT (pfam01790) **RNA**→ ←COG0012 (COG0012)

Rla-1-13	NZ_ABOU02000032.1	-	102267	102100	←hypo **RNA**→ ←ComFC (COG1040)
Rla-1-14	NZ_ABOU02000050.1	-	152044	151877	hypo→ **RNA**→ ←LysR (COG0583)
Rla-1-15	NZ_ABOU02000036.1	-	26948	26781	←GatC (COG0721) **RNA**→ ←hypo
Rla-1-16	NZ_ABOU02000033.1	+	1730	1897	←COG1032 (COG1032) **RNA**→ hypo→
Rla-1-17	NZ_ABOU02000034.1	+	55546	55713	hypo→ **RNA**→ ←IbpA (COG0071)
Rla-1-18	NZ_ABOU02000028.1	-	138359	138192	←hypo **RNA**→ COG1418 (COG1418)→
Rla-1-19	NZ_ABOU02000049.1	+	14048	14215	←hypo **RNA**→ ←Uup (COG0488)
Rla-1-20	NZ_ABOU02000055.1	+	90388	90555	←hypo **RNA**→ ←hypo
Rla-1-21	NZ_ABOU02000049.1	+	54819	54986	←GalM (COG2017) **RNA**→
Rla-1-22	NZ_ABOU02000035.1	-	71253	71086	COG3481 (COG3481)→ **RNA**→ TrmA (COG2265)→
Rla-1-23	NZ_ABOU02000053.1	+	15696	15863	PulA (COG1523)→ **RNA**→ ←DnaJ (COG0484)
Rla-1-24	NZ_ABOU02000046.1	+	32106	32273	CYK3 (COG5279)→ **RNA**→ CarA (COG0505)→
Rla-1-25	NZ_ABOU02000031.1	+	28256	28423	←ClpA (COG0542) **RNA**→ ←hypo
Rla-1-26	NZ_ABOU02000028.1	-	127880	127713	hypo→ **RNA**→ hypo→
Rla-1-27	NZ_ABOU02000049.1	+	30234	30401	←COG1873 (COG1873) **RNA**→ hypo→
Rla-1-28	NZ_ABOU02000017.1	+	92862	93029	←hypo **RNA**→ HTH_XRE (cd00093)COG0436 (COG0436)→
Rla-1-29	NZ_ABOU02000015.1	+	694	861	←GlpR (COG1349) **RNA**→ ←MscL (pfam01741)
Rla-1-30	NZ_ABOU02000046.1	-	647	480	←PTS_IIA_fru (cd00211)BglG (COG3711) **RNA**→ ←MscL (pfam01741)
Rla-1-31	NZ_ABOU02000037.1	-	8075	7919	COG1434 (COG1434)→ **RNA**→ ←ThiH (COG1060)
env-38	BAAU01002076.1	-	1922	1755	←Beta-lactamase (pfam00144) **RNA**→ ←hypo
env-39	BABG01002268.1	-	1271	1104	GatC (COG0721)→ **RNA**→ Amidase (pfam01425)→
env-40	BABG01000453.1	-	2218	2049	hypo→ **RNA**→ GATase1_GMP_Synthase (cd01742)GuaA (COG0519)→
env-41	BAAZ01002221.1	+	1348	1515	←Sialidase (cd00260) **RNA**→
Rla-1-32	NZ_ABOU02000047.1	-	15912	15745	←FN3 (cd00063)Sialidase (cd00260) **RNA**→ ←hypo
env-42	BAAU01024958.1	+	207	374	hypo→ **RNA**→ hypo→
env-43	BABG01002811.1	-	1092	925	UvrD (COG0210)→ **RNA**→ hypo→
env-44	BABG01000343.1	-	2063	1896	GGDEF (cd01949)→ **RNA**→ Prc (COG0793)→
env-45	BABG01000353.1	+	3907	4074	Iso_dh (pfam00180)→ **RNA**→ hypo→
env-46	BAAU01001385.1	+	1948	2124	PldB (COG2267)→ **RNA**→ PlsC (COG0204)→
Dfo-1-2	NZ_AAXA02000010.1	-	9926	9759	hypo→ **RNA**→ CysE (COG1045)→
env-47	BABG01021235.1	-	944	778	**RNA**→ ←hypo
Rla-1-33	NZ_ABOU02000007.1	+	34416	34583	←AdeC (COG1001) **RNA**→ ←hypo
Rla-1-34	NZ_ABOU02000026.1	+	37629	37796	SDH_alpha (pfam03313)→ **RNA**→ ←hypo
Rla-1-35	NZ_ABOU02000035.1	+	2399	2566	←AcnA (COG1048) **RNA**→ ←hypo
Rla-1-36	NZ_ABOU02000028.1	+	3639	3806	←hypo **RNA**→ ←hypo
Rla-1-37	NZ_ABOU02000023.1	+	2814	2981	HTH_XRE (cd00093)→ **RNA**→ ←hypo
Rla-1-38	NZ_ABOU02000039.1	-	74302	74135	←DUF177 (pfam02620) **RNA**→ ←hypo
Rla-1-39	NZ_ABOU02000036.1	-	901	734	←ARA1 (COG0656) **RNA**→ ←hypo
Rla-1-40	NZ_ABOU02000032.1	-	95004	94837	←UvrA (COG0178) **RNA**→ ←hypo
Rla-1-41	NZ_ABOU02000048.1	-	41861	41694	←HGD-D (pfam06050) **RNA**→ ←hypo
Rla-1-42	NZ_ABOU02000029.1	-	8103	7936	COG3335 (COG3335)COG3415 (COG3415)→ **RNA**→ ←hypo
Rla-1-43	NZ_ABOU02000029.1	-	251	83	←FeoA (pfam04023) **RNA**→ ←hypo
Rla-1-44	NZ_ABOU02000032.1	-	41539	41372	←hypo **RNA**→ ←hypo
Rla-1-45	NZ_ABOU02000042.1	+	30676	30843	hypo→ **RNA**→ ←hypo
Rla-1-46	NZ_ABOU02000052.1	-	12141	11974	Sms (COG1066)→ **RNA**→ hypo→
Rla-1-47	NZ_ABOU02000051.1	-	49001	48834	←Cof (COG0561) **RNA**→ ←hypo
Rla-1-48	NZ_ABOU02000020.1	-	7403	7236	←EutG (COG1454) **RNA**→ ←hypo
Rla-1-49	NZ_ABOU02000034.1	-	8847	8680	←hypo **RNA**→ ←hypo
Rla-1-50	NZ_ABOU02000045.1	-	33335	33167	←GreA (COG0782) **RNA**→ ←hypo
Rla-1-51	NZ_ABOU02000035.1	+	23673	23840	←Cyt_C5_DNA_methylase (cd00315) **RNA**→ ←hypo
Rla-1-52	NZ_ABOU02000028.1	+	88980	89147	←hypo **RNA**→ ←hypo
Rla-1-53	NZ_ABOU02000053.1	+	313	480	**RNA**→ ←hypo

Rla-1-54	NZ_ABOU02000026.1	-	49611	49444	←GlgP (COG0058) **RNA**→ ←hypo
env-48	BABG01006456.1	+	435	583	hypo→ **RNA**→ ←hypo
Rla-1-55	NZ_ABOU02000037.1	-	173	6	←hypo **RNA**→
Rla-1-56	NZ_ABOU02000047.1	-	5367	5200	←PAP2_Aur1_like (cd03386) **RNA**→ ←hypo
Rla-1-57	NZ_ABOU02000056.1	+	160	327	**RNA**→ ←hypo
Rla-1-58	NZ_ABOU02000047.1	-	13649	13478	←hypo **RNA**→ ←hypo
env-49	BABG01003277.1	+	999	1150	LysR_substrate (pfam03466)→ **RNA**→ COG1561 (COG1561)→
env-50	BABA01019112.1	-	596	425	**RNA**→ hypo→
env-51	BAAY01010317.1	+	1179	1351	DctQ (COG1593)→ **RNA**→
env-52	BAAZ01003651.1	-	1125	947	NorM (COG0534)→ **RNA**→ ←Pro tRNA
env-53	hgutS8_s8_162567	-	687	514	**RNA**→ ←hypo
env-54	BAAU01002871.1	-	989	818	←hypo **RNA**→ MarR (pfam01047)→
env-55	BABG01026309.1	-	776	604	hypo→ **RNA**→ **RNA**→ hypo→
env-95	BABG01026309.1	-	567	389	(shown above)
env-56	BAAU01018938.1	-	559	388	LysR_substrate (pfam03466)→ **RNA**→ PseudoU_synth_RluCD_like (cd02869)→
env-57	BABG01019443.1	-	442	271	**RNA**→ hypo→
env-58	BABG01006738.1	-	1128	957	ManC (COG0662)→ **RNA**→ OYE_like_FMN_family (cd02803)→
env-59	BABB01006594.1	-	1215	1043	←hypo **RNA**→ ArcC (COG0549)→
env-60	BAAU01010790.1	-	206	35	←AraC_binding (pfam02311) **RNA**→
env-61	BAAX01004650.1	+	1582	1753	hypo→ **RNA**→ ManX (COG2893)→
env-62	BABB01010898.1	-	183	12	hypo→ **RNA**→
env-63	BAAX01029733.1	-	904	738	hypo→ **RNA**→ hypo→
env-64	BAAX01029732.1	+	509	679	FAD-oxidase_C (pfam02913)→ **RNA**→
env-65	BABG01034003.1	+	219	392	**RNA**→
env-66	BABA01033985.1	-	198	26	**RNA**→ hypo→
env-67	BAAU01012045.1	+	8	179	←hypo **RNA**→ ←COG5263 (COG5263)
env-68	BAAX01011900.1	-	516	344	←pyridoxal_pyridoxamine_kinase (cd01173) **RNA**→ hypo→
env-69	BABA01008401.1	-	371	199	hypo→ **RNA**→ ←DbpA (pfam03880)
env-70	BAAV01014729.1	+	399	571	hypo→ **RNA**→ hypo→
env-71	BABG01028078.1	+	59	230	←hypo **RNA**→ ←FepC (COG1120)
env-72	BAAU01021894.1	-	499	327	**RNA**→ ←hypo
env-73	BAAU01019454.1	+	338	511	ErfK_YbiS_YhnG (pfam03734)→ **RNA**→ hypo→
env-74	BAAU01006425.1	-	310	139	HypD (pfam01924)→ **RNA**→
env-75	BABG01019422.1	+	349	526	←hypo **RNA**→ hypo→
Aca-1-1	NZ_ABAX03000010.1	-	68909	68756	hypo→ **RNA**→ ←hypo
env-76	BAAU01004259.1	-	2023	1862	**RNA**→ RplI (COG0359)→
env-77	BABG01022086.1	-	503	334	**RNA**→
env-78	BABG01022086.1	+	432	604	**RNA**→
env-79	BABG01022086.1	-	338	167	**RNA**→
env-80	BABG01022086.1	+	599	773	**RNA**→
env-81	hgutS8_s8_175420	+	669	843	FepC (COG1120)→ **RNA**→
env-82	BAAU01025016.1	-	257	80	hypo→ **RNA**→
env-83	BAAU01001327.1	+	473	650	←hypo **RNA**→ MelB (COG2211)→
env-84	BABB01016599.1	+	587	763	hypo→ **RNA**→ hypo→
env-85	BABD01030819.1	+	530	707	**RNA**→
env-86	BABA01021654.1	+	738	917	**RNA**→
env-87	BAAU01001711.1	-	3615	3436	**RNA**→ ←hypo
env-88	BAAU01018515.1	+	135	314	**RNA**→
env-89	BABA01021655.1	-	915	740	hypo→ **RNA**→ hypo→
env-90	BABG01001408.1	+	592	769	←hypo **RNA**→ ←hypo
env-91	hgutS8_s8_159931_1	-	2869	2692	←Hit (COG0537) **RNA**→ ←RecR (COG0353)
Csp-2-1	NZ_ABGC03000035.1	+	101575	101752	←Hit (COG0537) **RNA**→ ←RecR (COG0353)

env-92	BABG01003064.1	+	575	752	←HINT_subgroup (cd01277) **RNA**→ ←RecR (COG0353)
env-93	BAAV01013666.1	+	622	801	hypo→ **RNA**→ ←hypo
env-94	BAAX01013747.1	+	612	800	←hypo **RNA**→ hypo→
env-96	BABG01009698.1	+	320	498	←hypo **RNA**→ ←CorC (COG4535)
env-97	BAAU01018414.1	+	172	350	hypo→ **RNA**→ ←hypo
env-98	BABD01009573.1	-	298	120	COG2607 (COG2607)→ **RNA**→
env-99	BABG01004235.1	-	1830	1652	COG2607 (COG2607)→ **RNA**→ ModE (COG2005)→
env-100	hgutS8_s8_158962	+	686	864	←ABC-type sugar transport system, periplasmic comp onent **RNA**→ ←HTH_ARAC (smart00342)
env-101	BAAU01019504.1	+	336	499	hypo→ **RNA**→ hypo→
env-102	BABG01032637.1	-	818	657	**RNA**→ GD_AH_N (pfam04292)→
Ceu-1-2	NZ_ABEY02000008.1	-	42145	41983	OmpR (COG0745)→ **RNA**→ RluA (COG0564)→
env-103	BABB01014983.1	-	514	357	**RNA**→ hypo→
env-104	BAAX01013085.1	-	392	234	←hypo **RNA**→ hypo→
Ceu-1-3	NZ_ABEY02000023.1	-	60158	59997	←hypo **RNA**→ ←tRNA_bind_EcMetRS_like (cd02800)
env-105	hgutS8_s8_159061	+	771	932	←hypo **RNA**→ ←tRNA_bind_EcMetRS_like (cd02800)
env-106	BABG01015553.1	+	646	811	hypo→ **RNA**→ hypo→
env-107	BABA01025769.1	+	311	472	hypo→ **RNA**→ ←hypo
env-108	BAAZ01006092.1	+	658	819	GreA (COG0782)→ **RNA**→ hypo→
env-109	BABA01017275.1	+	327	488	←hypo **RNA**→
env-110	BABG01006513.1	-	1158	997	←YBL036c_PLPDEIII (cd00635) **RNA**→ ←hypo
env-111	BABG01006599.1	-	897	736	**RNA**→ ←hypo
env-112	BAAU01013082.1	-	887	727	GreA (COG0782)→ **RNA**→ ←hypo
env-113	BAAU01013081.1	+	474	635	COG1959 (COG1959)→ **RNA**→ ←hypo
env-114	BABG01034775.1	+	591	754	hypo→ **RNA**→
env-115	BAAU01019179.1	+	574	738	hypo→ **RNA**→ ←hypo
env-116	BAAU01026019.1	-	748	585	**RNA**→ ←HELICc2 (smart00491)
env-117	BABG01018910.1	+	823	983	SAICAR_synt_Sc (cd01414)→ **RNA**→
env-118	BAAU01018222.1	-	959	800	hypo→ **RNA**→ DnrI (COG3629)→
env-119	BAAU01026020.1	+	410	570	hypo→ **RNA**→
Dfo-1-3	NZ_AAXA02000006.1	+	32226	32385	hypo→ **RNA**→ hypo→
env-120	BABG01008432.1	+	584	749	Acm (COG3757)→ **RNA**→
env-121	BABG01026909.1	-	932	754	hypo→ **RNA**→ ←SASP (pfam00269)
Ceu-1-4	NZ_ABEY02000023.1	-	72091	71928	←LacZ (COG3250)Bgal_small_N (pfam02929)Bgal_small_C (pfam02930) **RNA**→ AraC_binding (pfam02311)HTH_ARAC (smart003
env-122	BAAZ01010534.1	+	457	622	COG1959 (COG1959)→ **RNA**→ hypo→
Csp-1-1	NZ_AAYW02000007.1	+	152558	152719	←tRNA-Pro **RNA**→ hypo→
env-123	BABG01021957.1	+	675	835	←hypo **RNA**→
env-124	BABG01021956.1	-	564	404	**RNA**→ hypo→
env-125	BABA01014690.1	+	426	585	←hypo **RNA**→ OmpR (COG0745)→
env-126	BABG01032678.1	-	440	280	hypo→ **RNA**→ hypo→
env-127	BABG01005904.1	+	388	548	hypo→ **RNA**→ hypo→
env-128	BAAZ01018676.1	-	282	125	hypo→ **RNA**→ hypo→

21.3 Conserved domains

Conserved domains found in protein-coding genes listed in Section 21.2 are listed, with the first sentence in their description from the Conserved Domain Database. Conserved domains associated with more than one Clostridiales-1 RNA are assigned a color, while others are shown in gray. The number in parentheses is the number of occurrences of the given gene.

u cd00037 (1) C-type lectin (CTL) and CTL-like domains; Many of these domains function as calcium-dependent carbohydrate binding modules and are known as carbohydrate-recognition domains (CRDs).
u cd00063 (1) Fibronectin type 3 domain; One of three types of internal repeats found in the plasma protein fibronectin.
uD cd00093 (4) Helix-turn-helix XRE-family like proteins.
u cd00211 (1) PTS_IIA, PTS system, fructose/mannitol specific IIA subunit.
u cd00260 (2) Sialidases or neuraminidases function to bind and hydrolyze terminal sialic acid residues from various glycoconjugates as well as playing roles in pathogenesis, bacterial nutrition and cellular interactions. [X]
u cd00315 (1) Cytosine-C5 specific DNA methylases; Methyl transfer reactions play an important role in many aspects of biology.
D cd00565 (1) ThiS (ThiaminS) is a sulfur carrier protein involved in thiamin biosynthesis in bacteria.
u cd00635 (1) PLP dependent enzymes class III (PLPDE_III).
D cd01017 (1) Metal binding protein AcdA.
D cd01158 (1) Short chain acyl-CoA dehydrogenase (SCAD).
u cd01173 (1) Pyridoxal kinase plays a key role in the synthesis of the active coenzyme pyridoxal-5'-phosphate (PLP), by catalyzing the phosphorylation of the precursor vitamin B6 in the presence of Zn2+ and ATP.
u cd01277 (1) HINT (histidine triad nucleotide-binding protein) subgroup: Members of this CD belong to the superfamily of histidine triad hydrolases that act on alpha-phosphate of ribonucleotides.
u cd01414 (1) Eukaryotic, prokaryotic and archaeal group of SAICAR synthetases represented by the Saccharomyces cerevisiae (Sc) SAICAR synthetase.
D cd01742 (1) Type 1 glutamine amidotransferase (GATase1) domain found in GMP synthetase.
d cd01948 (1) EAL domain.
u cd01949 (2) Diguanylate-cyclase (DGC) or GGDEF domain: Originally named after a conserved residue pattern, and initially described as domain of unknown function 1 (DUF1).
d cd02653 (1) NH_3: A subgroup of nucleoside hydrolases.
d cd02800 (2) tRNA-binding-domain-containing Escherichia coli methionyl-tRNA synthetase (EcMetRS)-like proteins.
D cd02803 (1) Old yellow enzyme (OYE)-like FMN binding domain.
D cd02869 (1) PseudoU_synth_RsuA/RluD: Pseudouridine synthase, RsuA/RluD family.
d cd03251 (1) MsbA is an essential ABC transporter, closely related to eukaryotic MDR proteins.
u cd03255 (1) This family is comprised of MJ0796 ATP-binding cassette, macrolide-specific ABC-type efflux carrier (MacAB), and proteins involved in cell division (FtsE) and release of liporoteins from the cytoplasmic membrane (LolCDE).
u cd03313 (1) Enolase: Enolases are homodimeric enzymes that catalyse the reversible dehydration of 2-phospho-D-glycerate to phosphoenolpyruvate as part of the glycolytic and gluconeogenesis pathways.
u cd03344 (1) GroEL_like type I chaperonin.
u cd03386 (1) PAP2_like proteins, Aur1_like subfamily.
D COG0002 (1) Acetylglutamate semialdehyde dehydrogenase [Amino acid transport and metabolism]
d COG0012 (1) Predicted GTPase, probable translation factor [Translation, ribosomal structure and biogenesis]
u COG0016 (1) Phenylalanyl-tRNA synthetase alpha subunit [Translation, ribosomal structure and biogenesis]
D COG0038 (1) Chloride channel protein EriC [Inorganic ion transport and metabolism]
u COG0047 (1) Phosphoribosylformylglycinamidine (FGAM) synthase, glutamine amidotransferase domain [Nucleotide transport and metabolism]
u COG0058 (1) Glucan phosphorylase [Carbohydrate transport and metabolism]
d COG0071 (1) Molecular chaperone (small heat shock protein) [Posttranslational modification, protein turnover, chaperones]
u COG0079 (1) Histidinol-phosphate/aromatic aminotransferase and cobyric acid decarboxylase [Amino acid transport and metabolism]
u COG0119 (1) Isopropylmalate/homocitrate/citramalate synthases [Amino acid transport and metabolism]
u COG0177 (1) Predicted EndoIII-related endonuclease [DNA replication, recombination, and repair]
u COG0178 (1) Excinuclease ATPase subunit [DNA replication, recombination, and repair]
D COG0204 (1) 1-acyl-sn-glycerol-3-phosphate acyltransferase [Lipid metabolism]
u COG0210 (1) Superfamily I DNA and RNA helicases [DNA replication, recombination, and repair]
u COG0296 (1) 1,4-alpha-glucan branching enzyme [Carbohydrate transport and metabolism]
d COG0297 (2) Glycogen synthase [Carbohydrate transport and metabolism]
d COG0353 (3) Recombinational DNA repair protein (RecF pathway) [DNA replication, recombination, and repair]
D COG0359 (1) Ribosomal protein L9 [Translation, ribosomal structure and biogenesis]
d COG0385 (1) Predicted Na+-dependent transporter [General function prediction only]
u COG0423 (1) Glycyl-tRNA synthetase (class II) [Translation, ribosomal structure and biogenesis]
D COG0436 (1) Aspartate/tyrosine/aromatic aminotransferase [Amino acid transport and metabolism]
u COG0444 (1) ABC-type dipeptide/oligopeptide/nickel transport system, ATPase component [Amino acid transport and metabolism / Inorganic ion transport and metabolism]
d COG0456 (1) Acetyltransferases [General function prediction only]
d COG0484 (1) DnaJ-class molecular chaperone with C-terminal Zn finger domain [Posttranslational modification, protein turnover, chaperones]
d COG0488 (1) ATPase components of ABC transporters with duplicated ATPase domains [General function prediction only]
u COG0495 (1) Leucyl-tRNA synthetase [Translation, ribosomal structure and biogenesis]
D COG0505 (1) Carbamoylphosphate synthase small subunit [Amino acid transport and metabolism / Nucleotide transport and metabolism]
u COG0516 (1) IMP dehydrogenase/GMP reductase [Nucleotide transport and metabolism]
D COG0519 (1) GMP synthase, PP-ATPase domain/subunit [Nucleotide transport and metabolism]
u COG0534 (1) Na+-driven multidrug efflux pump [Defense mechanisms]
u COG0537 (2) Diadenosine tetraphosphate (Ap4A) hydrolase and other HIT family hydrolases [Nucleotide transport and metabolism / Carbohydrate transport and metabolism / General function prediction only]
u COG0542 (1) ATPases with chaperone activity, ATP-binding subunit [Posttranslational modification, protein turnover, chaperones]
D COG0549 (1) Carbamate kinase [Amino acid transport and metabolism]
D COG0550 (1) Topoisomerase IA [DNA replication, recombination, and repair]

u COG0561 (1) Predicted hydrolases of the HAD superfamily [General function prediction only]
D COG0564 (1) Pseudouridylate synthases, 23S RNA-specific [Translation, ribosomal structure and biogenesis]
d COG0583 (1) Transcriptional regulator [Transcription]
D COG0624 (1) Acetylornithine deacetylase/Succinyl-diaminopimelate desuccinylase and related deacylases [Amino acid transport and metabolism]
u COG0656 (1) Aldo/keto reductases, related to diketogulonate reductase [General function prediction only]
u COG0662 (1) Mannose-6-phosphate isomerase [Carbohydrate transport and metabolism]
u COG0721 (2) Asp-tRNAAsn/Glu-tRNAGln amidotransferase C subunit [Translation, ribosomal structure and biogenesis]
uD COG0745 (2) Response regulators consisting of a CheY-like receiver domain and a winged-helix DNA-binding domain [Signal transduction mechanisms / Transcription]
u COG0782 (4) Transcription elongation factor [Transcription]
D COG0793 (1) Periplasmic protease [Cell envelope biogenesis, outer membrane]
d COG0796 (1) Glutamate racemase [Cell envelope biogenesis, outer membrane]
d COG0807 (1) GTP cyclohydrolase II [Coenzyme metabolism]
u COG1001 (1) Adenine deaminase [Nucleotide transport and metabolism]
u COG1032 (1) Fe-S oxidoreductase [Energy production and conversion]
d COG1040 (1) Predicted amidophosphoribosyltransferases [General function prediction only]
D COG1045 (1) Serine acetyltransferase [Amino acid transport and metabolism]
u COG1048 (1) Aconitase A [Energy production and conversion]
d COG1060 (1) Thiamine biosynthesis enzyme ThiH and related uncharacterized enzymes [Coenzyme metabolism / General function prediction only]
u COG1066 (1) Predicted ATP-dependent serine protease [Posttranslational modification, protein turnover, chaperones]
u d COG1120 (2) ABC-type cobalamin/Fe3+-siderophores transport systems, ATPase components [Inorganic ion transport and metabolism / Coenzyme metabolism]
u COG1171 (1) Threonine dehydratase [Amino acid transport and metabolism]
D COG1190 (1) Lysyl-tRNA synthetase (class II) [Translation, ribosomal structure and biogenesis]
u COG1283 (1) Na+/phosphate symporter [Inorganic ion transport and metabolism]
Dd COG1307 (2) Uncharacterized protein conserved in bacteria [Function unknown]
u COG1349 (1) Transcriptional regulators of sugar metabolism [Transcription / Carbohydrate transport and metabolism]
D COG1387 (1) Histidinol phosphatase and related hydrolases of the PHP family [Amino acid transport and metabolism / General function prediction only]
D COG1418 (1) Predicted HD superfamily hydrolase [General function prediction only]
u COG1420 (1) Transcriptional regulator of heat shock gene [Transcription]
u COG1434 (1) Uncharacterized conserved protein [Function unknown]
u COG1454 (1) Alcohol dehydrogenase, class IV [Energy production and conversion]
u COG1523 (1) Type II secretory pathway, pullulanase PulA and related glycosidases [Carbohydrate transport and metabolism]
D COG1561 (1) Uncharacterized stress-induced protein [Function unknown]
u COG1593 (1) TRAP-type C4-dicarboxylate transport system, large permease component [Carbohydrate transport and metabolism]
u COG1725 (1) Predicted transcriptional regulators [Transcription]
u COG1873 (1) Uncharacterized conserved protein [Function unknown]
u COG1917 (1) Uncharacterized conserved protein, contains double-stranded beta-helix domain [Function unknown]
u COG1959 (2) Predicted transcriptional regulator [Transcription]
d COG1966 (1) Carbon starvation protein, predicted membrane protein [Signal transduction mechanisms]
D COG2005 (1) N-terminal domain of molybdenum-binding protein [General function prediction only]
u COG2017 (1) Galactose mutarotase and related enzymes [Carbohydrate transport and metabolism]
d COG2070 (1) Dioxygenases related to 2-nitropropane dioxygenase [General function prediction only]
D COG2211 (1) Na+/melibiose symporter and related transporters [Carbohydrate transport and metabolism]
D COG2265 (1) SAM-dependent methyltransferases related to tRNA (uracil-5-)-methyltransferase [Translation, ribosomal structure and biogenesis]
u COG2267 (1) Lysophospholipase [Lipid metabolism]
u COG2607 (2) Predicted ATPase (AAA+ superfamily) [General function prediction only]
D COG2873 (1) O-acetylhomoserine sulfhydrylase [Amino acid transport and metabolism]
D COG2893 (1) Phosphotransferase system, mannose/fructose-specific component IIA [Carbohydrate transport and metabolism]
u COG3250 (1) Beta-galactosidase/beta-glucuronidase [Carbohydrate transport and metabolism]
u COG3335 (1) Transposase and inactivated derivatives [DNA replication, recombination, and repair]
u COG3415 (1) Transposase and inactivated derivatives [DNA replication, recombination, and repair]
u COG3481 (1) Predicted HD-superfamily hydrolase [General function prediction only]
D COG3629 (1) DNA-binding transcriptional activator of the SARP family [Signal transduction mechanisms]
u COG3711 (1) Transcriptional antiterminator [Transcription]
u COG3757 (1) Lyzozyme M1 (1,4-beta-N-acetylmuramidase) [Cell envelope biogenesis, outer membrane]
u COG3968 (1) Uncharacterized protein related to glutamine synthetase [General function prediction only]
d COG4535 (1) Putative Mg2+ and Co2+ transporter CorC [Inorganic ion transport and metabolism]
d COG4667 (1) Predicted esterase of the alpha-beta hydrolase superfamily [General function prediction only]
d COG5263 (1) FOG: Glucan-binding domain (YG repeat) [General function prediction only]
u COG5279 (1) Uncharacterized protein involved in cytokinesis, contains TGc (transglutaminase/protease-like) domain [Cell division and chromosome partitioning]
u pfam00144 (1) Beta-lactamase.
u pfam00180 (1) Isocitrate/isopropylmalate dehydrogenase.
d pfam00269 (1) Small, acid-soluble spore proteins, alpha/beta type.
u pfam00313 (1) 'Cold-shock' DNA-binding domain.
u pfam00725 (1) 3-hydroxyacyl-CoA dehydrogenase, C-terminal domain.
u pfam00885 (1) 6,7-dimethyl-8-ribityllumazine synthase.
D pfam00912 (1) Transglycosylase.
D pfam01047 (2) MarR family.
u pfam01205 (1) Uncharacterized protein family UPF0029.

D pfam01425 (1) Amidase.
u pfam01502 (1) Phosphoribosyl-AMP cyclohydrolase.
d pfam01741 (2) Large-conductance mechanosensitive channel, MscL.
u pfam01790 (1) Prolipoprotein diacylglyceryl transferase.
u pfam01924 (1) Hydrogenase formation hypA family.
uD pfam02311 (2) AraC-like ligand binding domain.
u pfam02503 (1) Polyphosphate kinase.
u pfam02620 (1) Uncharacterized ACR, COG1399.
u pfam02687 (1) Predicted permease.
u pfam02913 (1) FAD linked oxidases, C-terminal domain.
u pfam02929 (1) Beta galactosidase small chain, N terminal domain.
u pfam02930 (1) Beta galactosidase small chain, C terminal domain.
u pfam03313 (1) Serine dehydratase alpha chain.
D pfam03432 (1) Relaxase/Mobilisation nuclease domain.
u pfam03466 (2) LysR substrate binding domain.
u pfam03734 (1) ErfK/YbiS/YcfS/YnhG.
d pfam03880 (1) DbpA RNA binding domain.
u pfam04023 (1) FeoA domain.
D pfam04271 (1) DnaD-like domain.
D pfam04292 (1) D-galactarate dehydratase / Altronate hydrolase, N terminus.
u pfam06050 (1) 2-hydroxyglutaryl-CoA dehydratase, D-component. [4Fe-4S]
uDd smart00342 (3) helix_turn_helix, arabinose operon control protein;
d smart00491 (1) helicase superfamily c-terminal domain;

Duplicate sequences: the following putative homologs are not shown in the alignment because their sequences are identical to a homolog already shown: Rla-1-30

21.4 Multiple-sequence alignment

Each Clostridiales-1 RNA is denoted by an abbreviation (like "Eco-1-1") that refers to a taxonomy given in Section 21.1. The alignment includes sequences containing the RNA motif, as well as flanking sequence. The Clostridiales-1 RNA itself is denoted by the line underneath marked 5′ and 3′ on either end. Nucleotides in the flanking sequences (i.e., not part of the motif) are written in gray letters. Stems of predicted rho-independent transcription terminators are shaded yellow. (Note: terminator predictions have not been analyzed manually, and many are likely to be false positives.) Start codons are shaded green. (Note: start codons are frequently misannotated, especially in environmental samples.) Nucleotides proposed to basepair as part of the consensus structure are shaded in color when they comprise Watson-Crick or G-U pairs. Otherwise they are shaded gray. Conserved stems are also indicated at the bottom of the alignment by angle brackets, where matching < and > denote base-paired columns. Below these angle brackets, the symbol "2" denotes base pairs exhibiting covariation, "1" denotes base pairs exhibiting compatible mutations, "0" denotes base pairs that are not observed to mutate and "?" denotes base pairs that have a significant frequency of non-canonical nucleotides for Watson-Crick or G-U pairs (> 5%). Below these base pair annotation is the consensus sequence: "R" = "A" or "G", "Y" = "C" or "U", red nucleotides: nucleotide identity conserved more than 97% of the time, black nucleotides: 90%, gray nucleotides: 75%, red circle (•): nucleotide is present 97% of the time, black circle (•): 90%, gray circle (•): 75%, white circle (◦): 50%. All percentages of sequences just described (e.g. 97% conserved) assume that sequences have been weighted by the GSC algorithm implemented by the Infernal software.

alignment positions 1 · · · 157

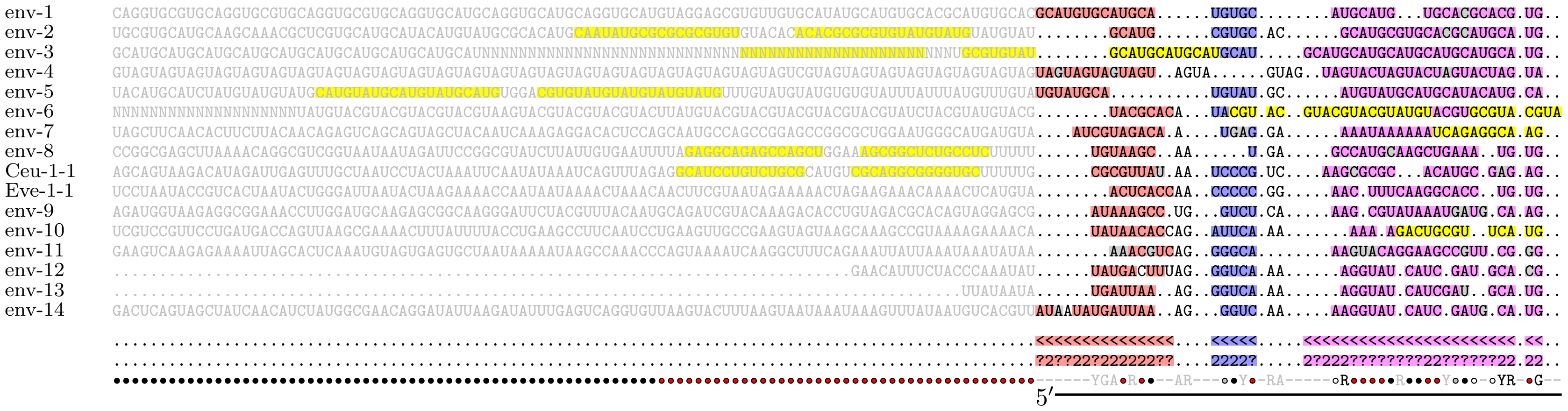

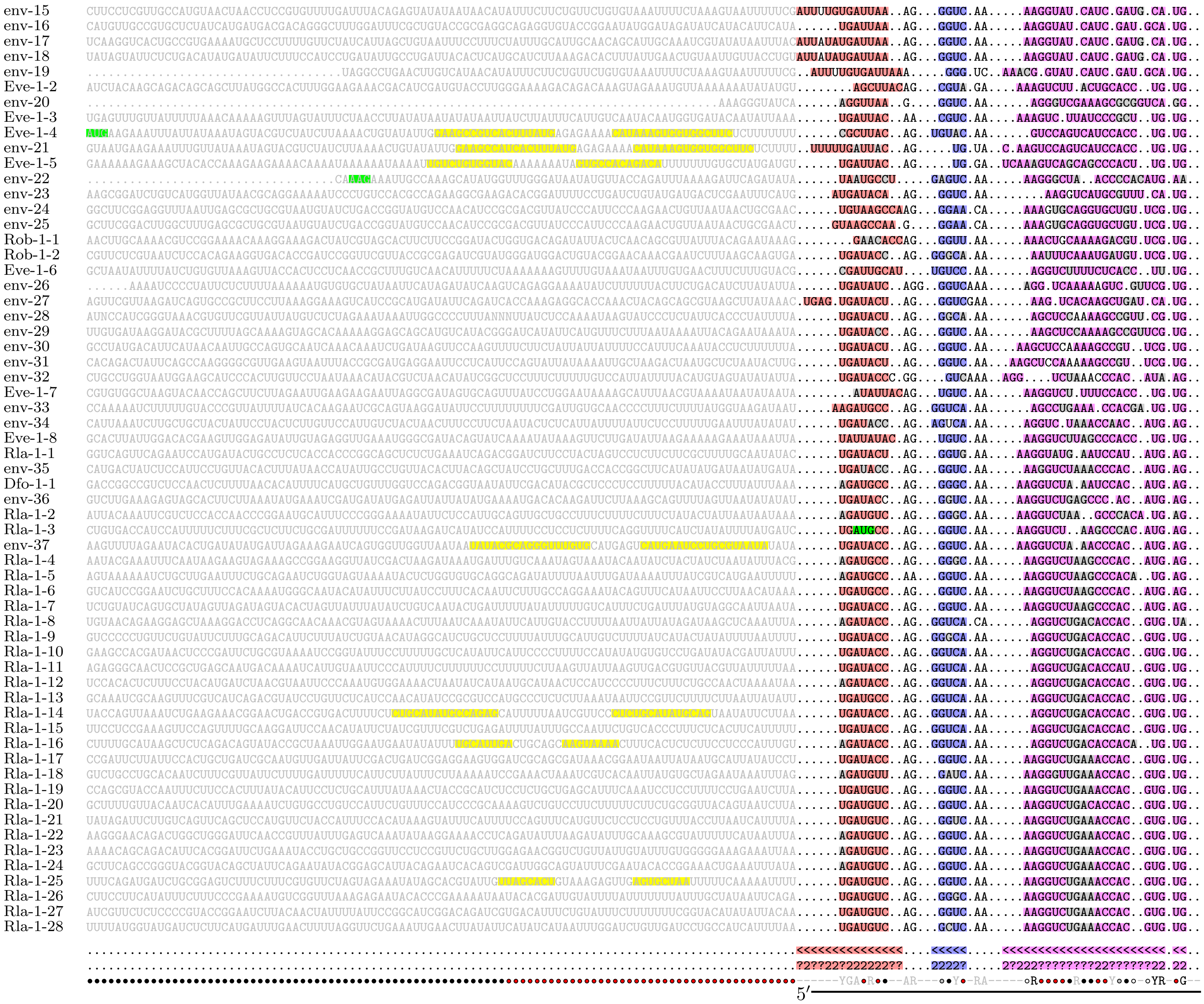
env-15
env-16
env-17
env-18
env-19
Eve-1-2
env-20
Eve-1-3
Eve-1-4
env-21
Eve-1-5
env-22
env-23
env-24
env-25
Rob-1-1
Rob-1-2
Eve-1-6
env-26
env-27
env-28
env-29
env-30
env-31
env-32
Eve-1-7
env-33
env-34
Eve-1-8
Rla-1-1
env-35
Dfo-1-1
env-36
Rla-1-2
Rla-1-3
env-37
Rla-1-4
Rla-1-5
Rla-1-6
Rla-1-7
Rla-1-8
Rla-1-9
Rla-1-10
Rla-1-11
Rla-1-12
Rla-1-13
Rla-1-14
Rla-1-15
Rla-1-16
Rla-1-17
Rla-1-18
Rla-1-19
Rla-1-20
Rla-1-21
Rla-1-22
Rla-1-23
Rla-1-24
Rla-1-25
Rla-1-26
Rla-1-27
Rla-1-28
5′

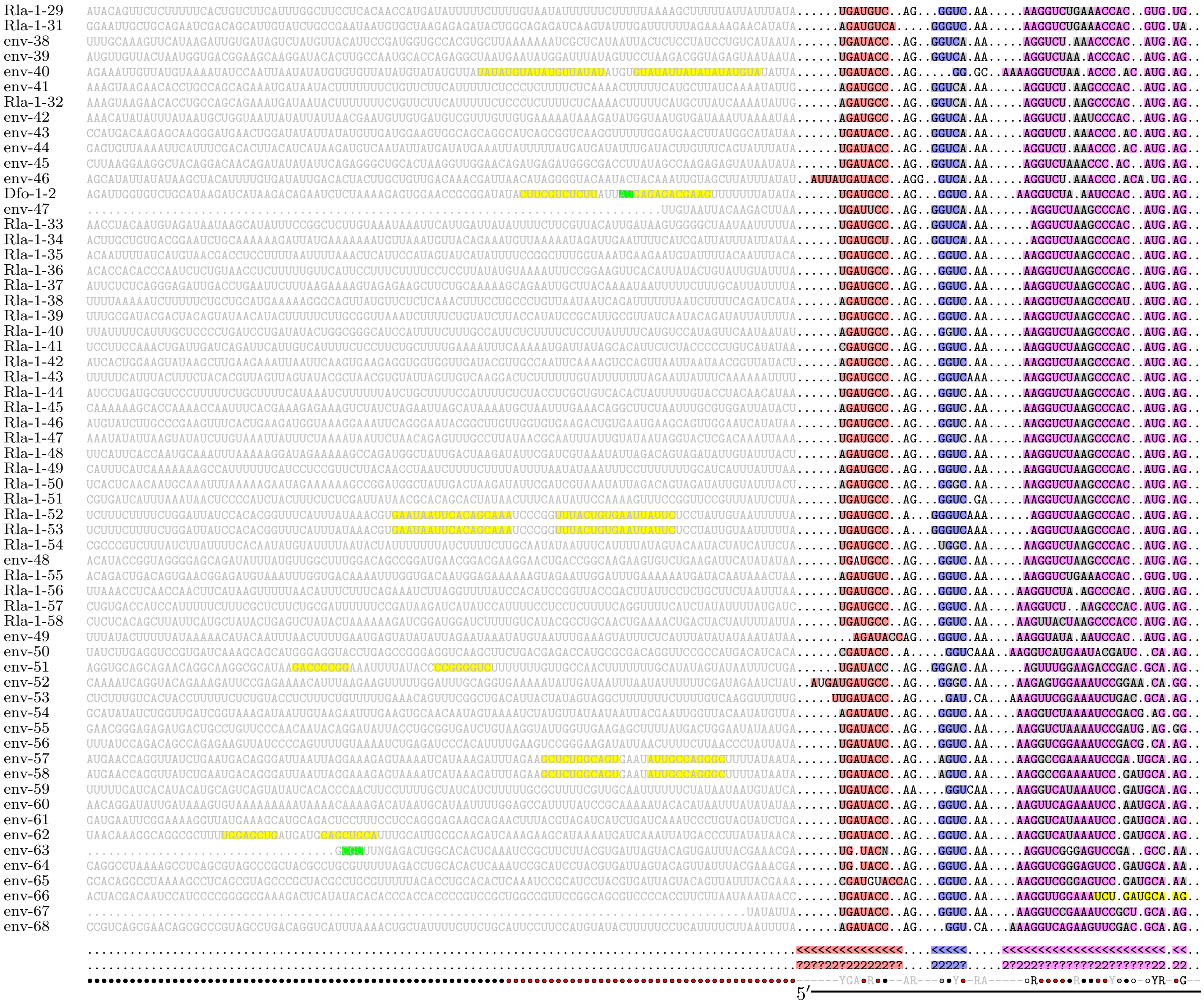

Rla-1-29
Rla-1-31
env-38
env-39
env-40
env-41
Rla-1-32
env-42
env-43
env-44
env-45
env-46
Dfo-1-2
env-47
Rla-1-33
Rla-1-34
Rla-1-35
Rla-1-36
Rla-1-37
Rla-1-38
Rla-1-39
Rla-1-40
Rla-1-41
Rla-1-42
Rla-1-43
Rla-1-44
Rla-1-45
Rla-1-46
Rla-1-47
Rla-1-48
Rla-1-49
Rla-1-50
Rla-1-51
Rla-1-52
Rla-1-53
Rla-1-54
env-48
Rla-1-55
Rla-1-56
Rla-1-57
Rla-1-58
env-49
env-50
env-51
env-52
env-53
env-54
env-55
env-56
env-57
env-58
env-59
env-60
env-61
env-62
env-63
env-64
env-65
env-66
env-67
env-68
5′

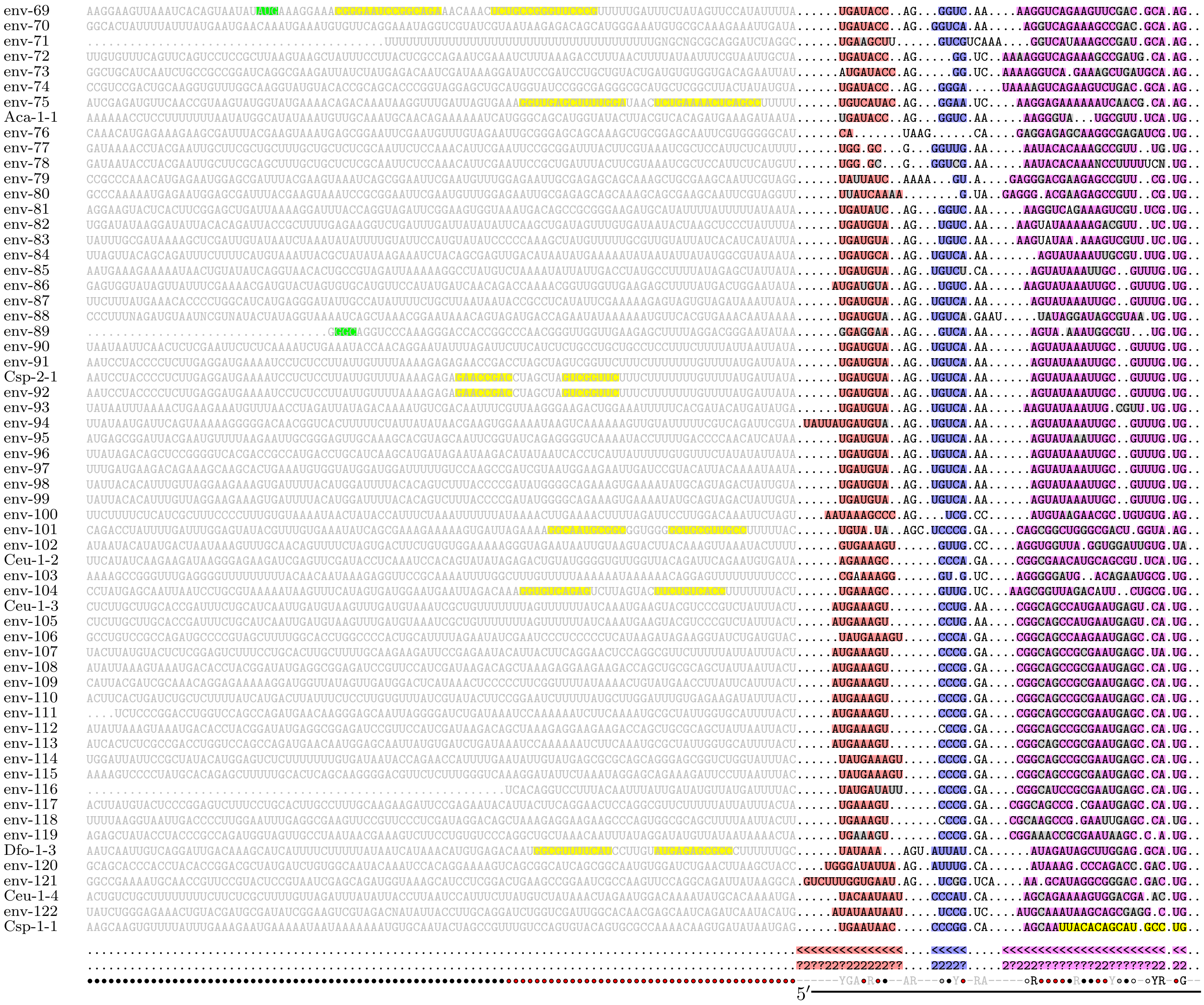
env-69
env-70
env-71
env-72
env-73
env-74
env-75
Aca-1-1
env-76
env-77
env-78
env-79
env-80
env-81
env-82
env-83
env-84
env-85
env-86
env-87
env-88
env-89
env-90
env-91
Csp-2-1
env-92
env-93
env-94
env-95
env-96
env-97
env-98
env-99
env-100
env-101
env-102
Ceu-1-2
env-103
env-104
Ceu-1-3
env-105
env-106
env-107
env-108
env-109
env-110
env-111
env-112
env-113
env-114
env-115
env-116
env-117
env-118
env-119
Dfo-1-3
env-120
env-121
Ceu-1-4
env-122
Csp-1-1
5′

```
env-123  AUAAAUAUGCCUCCUCUGUUGCCUACAGAUACUUCGGCUUUUCCUUAGGACUCUAAUAUCUCAAUUAUACAAAACUGUACCAAAAAAGGCAAUAAAUAUG......GUAUAAUA.AG.....CU.CACC...CAUA.AAAUCCUGCG.UGAC.UG..
env-124  AUAAAUAUGCCUCCUCUGUUGCCUACAGAUACUUCGGCUUUUCCUUAGGACUCUAAUAUCUCAAUUAUACAAAACUGUACCAAAAAAGGCAAUAAAUAUG......GUAUAAUA.AG.....CU.CACC...CAUA.AAAUCCUGCG.UGAC.UG..
env-125  CAAUCCUUUCCAAUAAUUCAUUUAAUAUAUGGUAUGAUCGGACGAAAAAAUAGUGCCAAAUAAAUAGAUGAUACAAAUUGGAUACAAAGAUGAGAUAUGC......UAUAAUA..AA...CUCA.CC.....CAUAGAAAGUCCAC.AUGAC.UG..
env-126  CAUCCGGGAAUUAGUUGCCAUGACACCGGAGGAGAAAGAGGCGAAGGGUGUCGUGAUUCAGGAAAUCUCCAUGAGCAAGCUGGCACUGGCCGCGUUUUGA......CAGAUGA..AG..CUCAC.CC......AUAGAAAGCAUGAGC.GAC.UG..
env-127  UUCCUGUCCAUCAACAUGAACAUGCAGUACGUGAUCAAGGGUCUCAUCAUUCUGGUUGCCUGUGCAAUCGAUAUGAGAAAGUACCUCGUAAGAAAGUAAU......GACAUAAG.A.....CUC.....AACCAUAGAAAACAUCCGUGA.C.UG..
env-128  CAACAGUGCGCACCGAAUUCCAGCAGACGGCGCAGGCCAUGGCACCGCUUCUUAAGACAGAAGGAAGUGUUCCGGUAGCACAAUAUGCCUUUGAUGUGAG......AACAU....AG....CUC.A...UCC.AUAGGAAACAGACGU.GAC.UG..
         ...................................................................................................<<<<<<<<<<<<<<<....<<<<<.....<<<<<<<<<<<<<<<<<<<<<<.<<..
         ...................................................................................................?2??22?2222222??....2222?.....2?222?????????22???????22.22..
         ••••••••••••••••••••••••••••••••••••••••••••••••••••••••••••••••••••••••••••••••••••••••••••••••••••••------YGA•R••--AR---○•Y•-RA-----○R•••••R••••Y○•○-○YR-•G--
                                                                                                            5′
```

alignment positions 158 · · · 312

```
env-1    C.AUG..CAUGUGCAUGCAGGUGCAUGCAU....GU.GCACG.CAGGUGCAU.GCAG.GUGCAU...GCAUGU.GC...........AU...............GC.ACGUGC...AUGCAG.....GUG.CAUGCA.....UGUGCA.UGC
env-2    C.AUG..CAUGCAUACAUG.CAUGCGUGC.....AU.GCACG.CAU..GCAU.GCAU...GCAU...GUAUGC.GUGUAUGCAUGCAUGCAGACAUGCAUGCAUGCAU.GC......AUGCAUGCAUAUAUG.CAUGC.....AUCC......
env-3    C.GUG..CGUGCAUGCAUG.CAUGCAUGUAUGUAUU.AUGUA.UGU..GCAU.GCAU.GCAUGCAU.G.CAUG.CA..........................UG.CAUGCA..UGCAUGA.....AUG.CAUGU.....A.UGCA.UAC
env-4    C.UAG..UACUAGUAUCAG.UACUAGUACUA...GCA......CGA......GUAC.GAGUAC....AAGU.AC...........GAGUACGA.........GU.AC.GA....GUACGAGUACGAGUG.C....G.....A.GUAC....
env-5    C.GUA..UGCAUGUAUGCA.UGUAUGCAU.....GU.AUGCA.UGU..AUGC.AUGU.AUGCAU...GUAUGC.ACGUA........UA.CA.........UGUGU.GCAUGU...AUGCAC.....GUA.UGCAUG....UAUGCA.UGC
env-6    C.UUG..CGUACGUACGUA.CGUACGUACGUAC.GU.AUGUA.UGU..ACGU.ACGU.ACGUAC...GCACGUACA.............UA............UGUAC......GUACGC.....ACG.UACGU.....ACGUAU....
env-7    C.UCG..CUUGCCUCUGAU.UUUUUAUUU.....GA.CGAA..UGU..CCGG.AUAU.GAGCAA...G.AAGC.AC...........CU.AA............GU.GC.GGA...UUGCGA.....AUA.UACGG.....A.GGCU.GGU
env-8    C.UUG..CACAGAUUUCAG.CUUAUAUGGC....GA.A.....UGUCAAUAA.U....GAACAAC..G.AAGG.UC...........GG..A............GA.CCGGGA..GUUGUGA.........AUUAU.....C.AAGG.CUC
Ceu-1-1  C.UUG..CUCGCGGCAUGU...AGCGCAUUU...GA.CGGGA.UGA..CGCG.ACAU.GAACAC...G.AAGG.CC...........GA.UA............GG.UC.GGA...GUGUGA.....AUG.CCGCGC.....A.GAAA.UGC
Eve-1-1  C.UUG..CACAAAGGUGCC.UUGAAAAGUU....UC.GGGGG.UGC..CUUA.UUAU.GAGCAUAAAA.GGGCGAC...........GA..A............GUCGCACGA..UAUGCGA.....AUA.AUAAGG....ACAGAU.UGC
env-9    C.UUG..CUUGAAAAAAUU.UGUACGCUUU....AG.AGAC.GCUA..AUGG.ACAU.GAGCAA...G.AAGC.AC...........GG.UA............GU.GC.GGA...UUGCGA.....AUG.UACAU.....A.GGAU.UGC
env-10   C.UUG..CAUGAAAACGCA.GUCUUUUU......UA.UGAAU.CGC..CAAA.AUAU.GAGAAA...A.UAGC.CC...........GA.UA............GG.GU.GGU...UUUCGA.....AUA.UUUUGU.....A.GAAU.UGC
env-11   C.UUG..CACGAGAAGGCU.UCCUGCCAUU.......UGCCC.CG...CCUAAUGCC.GAAAAC...G.UAAU.AAAAAGUU.....GUAAACAU....AACUUUUU.AUGGGA...CUUUGA.....GGU.AUGGG.........AU.UAC
env-12   C.UUG..CAUGCAAUUCGA.UGUAUACCU.....UAGUGACC.CGC..ACUA.CACC.GAGAAA...G.UAAC.AGUUUGCUGC....AGGGAC....GCAGAAAAUUAGC.GGA...CUUCGA.....GGU.GUAGU.....C.AGAU.UAU
env-13   C.UUG..CAUGCAAUUCGA.UGUAUACCU.....UAGUGACC.UGC..ACUA.CACC.GAGAAA...G.UAGC.AAUUUACUAUGC..GA..A...GC.UAGUAAAUUAGC.GGA...UUUCGA.....GGU.GGAGU.....C.AGAU.UAC
env-14   C.UUG..CAUGCAAUUCGA.UGUAUACCUU....AGUGACC..CGC..ACUA.CACU.GAGAAA...G.UAGC.AAUUUACUAUGC..GA..A...GC.UAGUAAAUUAGC.GGA...UUUCGA.....GGU.GGAGU.....C.AGAU.UAC
env-15   C.UUG..CAUGCAAUUCGA.UGUAUACCUU....AUUGACC..CGC..ACUA.CACC.GAGAAA...G.UAGC.AAUUUGCUGCG...GG.GA....CGUAGCAAAUUAGC.GGA...UUUCGA.....GGU.GGAGU.....C.AGAU.UAC
env-16   C.UUG..CAUGCAAUUCGA.UGUAUACCUU....AUUGACC..CGC..ACUA.CACC.GAGAAA...G.UAGC.AAUUUGCUGCG...GG.GA....CGCAGAAAAUUAGC.GGA...UUUCGA.....GGU.GUAGU.....C.AGAU.UAC
env-17   C.UUG..CAUGCAAUUCGA.UGUAUACCUU....AGUGACC..CGC..ACUA.CACC.GAGAAA...G.UAGC.AAUUUGCUGCG...GG.AA....CGCAGGAAAUUAGC.GGA...UUUCGA.....GGU.GUAGU.....C.AGAU.UAC
env-18   C.UUG..CAUGCAAUUCGA.UGUAUACCUU....AGUGACC..CGC..ACUA.CACC.GAGAAA...G.UAGC.AAUUUGCUGCG...GG.GA....CGCAGGAAAUUAGC.GGA...UUUCGA.....GGU.GUAGU.....C.AGAU.UAC
env-19   C.UUG..CAUGCAAUUCGA.UGUAUGCCUUAUU.GA.CCCG..CAC..UACA.CC...GAGAAA...G.UAGC.AAUUUGCUGCG...GG.GA....CGUAGCAAAUUAGC.GGA...UUUCGA.......G.GUGGAG....UCAGAU.UAC
Eve-1-2  C.UUG..CACAAAGGUGCA.GAAAAGACUUU...GA.GACG..CGCC.CCGA.ACAU.GAGCAAAAAAGCGAA.GC...........GA..A............GC.UG.AGUGAUAUGCGA.....AUG.UACGGG....AAGGAU.UGU
env-20   C.UUG..CAUGAUAAGGCU.UUUGAACUU.....GU.GACC..CGC...UAA.ACAU.GAGGAA...G.UAGC.GAUUUGC.......G..UA........GCAAAUCAGCGGGA...UUCCGA.....AAG.UUUAC.....A.GAAU.UGC
Eve-1-3  C.UUG..CACAAUAAAGGG.AUAAUGACUUU...GA.GACG..CGC..CC...ACAA.CA.......U.GC...............GAAUA.................................UUGAG..GGG....AAGGAU.UGU
Eve-1-4  C.UUG..CACAAAGGUGGA.UGACUGGAC.....UUAGGACA.CGCC.CCAA.GAAU.GAGCAU...AAAAGU.GAAGC.........GA.AA..........GCUUC.AC.GAU...AUGCGA.....AUG.CUUGGG....AAGGAU.UGC
env-21   C.UUG..CACAAAGGUGGA.UGACUGGACUUAG.GA.CA....CGCC.CCAA.GAAU.GAGCAU...A.AAGU.GAAGC.........GA.AA..........GCUUC.AC.GAU...AUGCGA.....AUG.CUUGGG....AAGGAU.UGC
Eve-1-5  C.UUG..CACAAGAGUGGG.GUGAUGACCUUGA.GA.CA....CGC..CCCCAACAU.GAGCAU...AAAAGU.GUAGC.........GC.AA..........GCUAC.AC.GAU...AUGCGA.....AUGCUGGGGG....A.GGAU.UGC
env-22   C.UUG..CUCAUAAGUGGU.AAAAGACCUU....AG.GACCCGCGC..CC...AUAU.GAGCAAAAAG.CGGG.AA...........GACUA............UC.CCACGA..UAUGCGA.....AUGUU..GGU....A.GAAUCAA.
env-23   C.UUG..CAUGAAAAAGCA.UGA.CUU.......AUUGACC..CGC..AAAA.CACC.GAANA....G.UAGC.CAUUUUUC.....G..UA.......GAAAAAUGAGC.GGA....UUUGA.....GGU.GUUUUU.....A.GGAU.UGC
env-24   C.UUG..CACGAUAAGGCA.UCUGAC.UUU....GG.UUCC..CGCC.AAAA.ACAU.GAAGAA...G.AAGC.GAUUUGC.......A..UC........GCAAAUCUGC.GGA...UUCUGA.....AUG.UUUUU.....A.GAAU.UGC
env-25   C.UUG..CACGAUAAGGCA.UCUGAC.UUU....GG.UUCC..CGCC.AAAA.ACAU.GAAGAA...G.AAGC.GAUUUGC.......AU..U........GCAAAUCUGC.GGA...UUCUGA.....AUG.UUUUU.....A.GAAU.UGC
Rob-1-1  C.UUG..CACGAUAAGGCU.UUUG.AGUUU....GG.AACC..CGC...CAA.AUAU.GAGAAA...G.UAGC.GAUUUAC.......GA.UA........GUAAAUCAGC.GGA...UUUCGA.....AUA.UUUGC.....A.GGAG.UGU
Rob-1-2  C.UUG..CACGAUAAAGCA.UUUGAAUUU.....GG.GGACC......UCCAAACAU.GAGGAA...G.UAGC.GAUUUAC........A.UA........GUAAAUCAGC.GGA...UUCCGA.....AUG.UUUGA.....A.GGAU.UGU
Eve-1-6  C.UUG..CACAAUGAUGGGAAAAUGACCU.....UG.GGACA.CGC..CCGG.AUAU.GAACAUAAAG.GUGAAGC...........GU.AA............GCUUCGCGA..UAUGCAA.....AUA.UCAGGG....A.GGAU.UGC
env-26   C.UUG..CACGAUAAGAC..UUUUGAACUG....GG.GACC..CGC..CAA..CCAU.GAGAAA...G.UAGC.GAUUUAC.......G..CA........GUAAAUCAGC.GGA...UUUCGA.....AUG..UUUG......A.GAAU.UGU
env-27   C.UUG..CAUGAAGAGGCU.UGUGACCUU.....GA.GACC..UGU..UAAA.ACAU.GAGGAU...G.CAGC.GAUUUUC.......AA..G........GAAAAUCAGC.GA....AUCCGA.....AUG.UUUUU.....A.GAAU.UGC
env-28   C..UG..CACGAUAAAGCU.U..UGAGCU.....NG.GGAC.CCGC..AAAA.ACAU.GAGGAU...G.CAGC.GAUUUUC.......AA..A........GAAAAUCAGC.GA....AUCCGA.....AUG.UUUUU.....A.GAAU.UGC
env-29   C.UUG..CACGAUUAGGUU.UUUGAGCUU.....GG.GACC..CGC..AAAA.ACAU.GAGGAU...G.CAGC.GAUUUUC.......AA..G........GAAAAUC.AG.CGA...AUCCGA.....AUG.UUUUU.....A.GAAU.UGC
env-30   C.UUG..CACGAUAAGGGU.UUU..GAGCUU...GG.GACC..CGC..AAAA.ACAU.GAGGAU...G.CAGC.GAUUUUC.......AA..G........GAAAAUC.AG.CGA...AUCCGA.....AUG.UUUUU.....A.GAAU.UGC
env-31   C.UUG..CACGAUAAGGGU.UU..UG.AGCUU..GG.GACC..CGCA.AAAA.ACAU.GAGGAU...G.CAGC.GAUUUUC.......AA..A........GAAAAUCAGC.GA....AUCCGA.....AUG.UUUUU.....A.GAAU.UGC
env-32   C.UCG..CUCAUAGGUGG....AUGAAAAACCU.UGGAACC..CGC..CCCA.AUAU.GAGCAUAAAGCAGG.AU...........GA.UA............AU.CC.CACGAUAUGCUA.....AUA.UUGGGU....A.GGUU.UGU
Eve-1-7  C.UUG..CACAAAGGUGGAAAAUGACCUU....GG.GACA..CGC..CCACAAUAU.GAGCAA...AAAAGU.GGAGC.........GU.AA..........GCUUC.AC.GAU...AUGCGA.....AUAUUGUGGU....A.GGAU.UGU
env-33   C.UUG..CACAGGAGUGGU.UUUAUGGCU.....UAAUGACC.CGC..CCCG.AUAU.GAGCAU...AAAAGU.GGAGC.........GU.AA..........GCUUC.AC.GAU...AUGCGA.....AUA.UUGGAU....A.GAAU.UGU
env-34   C.UUG..CUCAUAAGUGGG.UGAAUGACCU....UA.UG.CUGCGC..CCCA.AUAU.GAGCAUAAAGUGGG.AU...........GA.UA............AUCCCGUGA..UGUGCAA.....AUA.UUGGGG....A.GGAU.UGU
Eve-1-8  C.UUG..CACAAAGGUGGGAAAAUGACCUU....GA.GACA..CGC..UCCG.AUAU.GAGCAUAAA.GGGAAGC...........GU.AA............GCUUCACGA..UAUGCGA.....AUAAUCGGGG....A.GGAU.UGC
Rla-1-1  C.CUG..CUCAUAAGUGGA.UAAAAGACCUU...UG.AACC..CGU..CCCA.AUCU.GAGCAUAAA.UGGA.AU...........UG.UA............AU.CC.UGAGAUAUGCGA.....AUA.GUGGG.....AUGGAU.UAU
env-35   C.UUG..CUCAUAGGUGGGUGAAAGACAUU....GG.GACC..CGC..CCCG.AUAU.GAGCAUAAAGUGGG.AU...........GA.UA............AUCCCAUGA..UAUGCGA.....AUAUUGGG.....A.GAAU....

         ......>>>>>>>>>>>>.>>>>>>>>>>>>>....>>>>>.....<<<<.<<<<...<<<<<......<<.<<<<<<<<<<<<.........>>>>>>>>>>>>.>>......>>>>>........>>>.>>>>>.......<<<<.<<<
         ......2222????????22.?????????222?2....?2222.....2222.?222...22222......22.2222222?2?22.........22?2?2222222.22......22222........222.?2222.......2222.222
         C-UUG--C•YR•RRR•R••-•••••R••Y○----•R-•R•○--CG•--•••R-RYAU-GAG•A•---R-•AG•-R•○○○---------G•-○A----------○○○•Y-•C-RR•---•URCGA------AUR-•Y•••○------A-RRAU-UGY
```

```
Dfo-1-1    C.UUG..CUCAUAAGUGGA.UGAAAGACCUU...GG.GCCC..CGC..CCCA.AUAU.GAGCCUAAAAGUAGG.AU...........GA.CA............AUCCCACGA..UAUGCGA.....AUA.AUGGGG.....A.GGAU.UGU
env-36     C.UCG..CUCAUAGGUNGG.GGAAAGACCUU...GG.GAAC..CGN..CCCA.AUAU.GAGCAU.A.A.AAGU.GGGAU........GA.UG.........AUCCC.AC.GAU...AUGCGA.....AUA.UUGGGU.....C.AGAU.UGU
Rla-1-2    C.UUG..CACACUGGUGGU.AGAAUGACCUU...GA.GACC..CGC..CCCG.AUAU.GAGCAUAAAAGUGGG.GC...........GU.AA............GCACCAUGA..UAUGCGA.....AUA.GCGGGU.....A.GGAU.UGU
Rla-1-3    C.UUG..CUCAUAGGUGGA.UGAAAGACCUU...AG.GACC..CGC..CUCG.AUAU.GAGCAUAAAAGUGGG.GC...........GU.AA............GCACCAUGA..UAUGCGA.....AUA.GCGGGU.....A.GGAU.UGU
env-37     C.UUG..CUCAUAGGUGGG.UGAAAGACCUU...GG.GACC..CGC..CACG.AUAU.GC.......G.......................A...................................AUA.UUGGG......G.GAAU.UGU
Rla-1-4    C.UUG..UUCAUAGGUGGGUGAAAGACCUU....AG.GACC..CGC..CCCG.AUAU.UCGCAUAUCG.UGGG.AU...........GA.UA............AUCCCACGA..UAUGCGA.....AUA.UUGGGU.....A.GGAU.UGU
Rla-1-5    C.UUG..CUCACAGGUGGGUGAAAGACCUU....AG.GACC..CGC..CCCG.AUAU.GAGCAUAAAA.GUGGAAU...........GA.UA............AUCCCACGA..UAUGCGA.....AUA.UUGAGU.....A.GGAU.UGU
Rla-1-6    C.UUG..CUCAUAGGUGGGUGAAAGACCUU....AG.GACC..CGC..CCCG.AUAU.GAGCAUAAAA.AUGGGGC...........GU.AA............GCACCAUGA..UAUGCGA.....AUA.GCGGGU.....A.GGAU.UGU
Rla-1-7    C.UUG..CUCAUAGGUGGGUGAAAGACCUU....AG.GACC..CGC..CCCG.AUAU.GAGCAUAAAAGUGGGGGC...........GU.AA............GCACCACGA..UAUGCGA.....AUG.UUGGGU.....A.GGAU.UGU
Rla-1-8    C.UCG..CACACUAGUGGUAAAAUGACCU.....UG.UGACC.CGC..CCCA.AUAU.GAGCAUAAAAGCGGG.GC...........GU.AA............GCACUACGA..UAUGCGA.....AUG.UUGGGU.....A.GGAU.UGU
Rla-1-9    C.UCG..CACACCAGUGGUAGAAUGACCU.....UG.UGACC.UGC..CCCA.AUAU.GAGCAUAAAAGUAGG.GC...........GU.AA............GCACCACGA..UAUGCGA.....AUG.UUGGGU.....A.GGAU.UGU
Rla-1-10   C.UCG..CACACUAGUGGUAGAAUGACCU.....UG.UGACC.CGC..CCCA.AUAU.GAGCAUAAAA.GUGGCAC...........GU.AA............GCACCACGA..UAUGCGA.....AUG.UUGGGU.....A.GGAU.UGU
Rla-1-11   C.UCG..CACACUAGUGGUAGAAUGACCU.....UG.UGACC.CGC..CCCA.AUAU.GAGCAUAAAAGUGGG.GC...........GU.AA............GCACCAUGA..UAUGCGA.....AUG.UUGGGU.....A.GGAU.UGU
Rla-1-12   C.UCG..CACACGAGUGGUAGAAUGACCU.....UG.UGACC.CGC..CCCA.AUAU.GAGCAUAAAAGUGGG.GC...........GU.AA............GCACCACUA..UAUGCGA.....AUG.UUGGGU.....A.GAAU.UGU
Rla-1-13   C.UCG..CACACUAGUGGUAGAAUGACCU.....UG.UGACC.CGC..CCCA.AUAU.GAGCAUAAAAGUGGG.GC...........GU.AA............GCACCACGA..UAUGCGA.....AUG.UUGGGU.....A.GGAU.UGU
Rla-1-14   C.UCG..CACACUAGUGGUAGAAUGACCU.....UG.UGACC.CGC..CCCA.AUAU.GAGCAUAAAAGUGGG.GC...........GU.AA............GCACCACGA..UAUGCGA.....AUC.UUGGGU.....A.GGAU.UGU
Rla-1-15   C.UCG..CACACGAGUGGUAGAAUGACCU.....UG.UGACC.CGC..CCCA.AUAU.GAGCAUAAAAGUGGG.GC...........GU.AA............GCACCACGA..UAUGCGA.....AUG.UUGGGU.....A.GGAU.UGU
Rla-1-16   C.UCG..CACACUAGUGGUAGAAUGACUU.....UG.UGACC.CGC..CCCA.AUAU.GAGCAUAAAAGUGGG.GC...........GU.AA............GCACCACGA..UAUGCGA.....AUG.UUGGGU.....A.GGAU.UGU
Rla-1-17   C.UUG..CACACUGGUGGUAGAAUGACCUU....GA.GACC..CGC..CCCA.AUAU.GAGCAUAAAAGUGGG.GC...........GU.AA............GCACCACGA..UAUGCGA.....AUG.UUGGGU.....A.GGAU.UGU
Rla-1-18   C.UUG..CACACUGGUGGUAGAAUGACCUU....GA.GCCC..CGC..CCCG.AUAU.GAGCAUAAAAGUGGG.GC...........GU.AA............GCACCAUGA..UAUGCGA.....AUA.GCGGGU.....A.GGAU.UGU
Rla-1-19   C.UUG..CACACUGGUGGUAAAAUGACCUU....GA.GACC..CGC..CCCG.AUAU.GAGCAUAAAAGUGGG.GC...........GU.AA............ACGCCAUGA..UAUGCGA.....AUA.GCGGGU.....A.GGAU.UGU
Rla-1-20   C.CUG..CACACUGGUGGUAGAAUGACCUU....GA.GGCC..CGC..CCCG.AUAU.GAGCAUAAAAGUGGG.GC...........GU.AA............GCACCAUGA..UAUGCGA.....AUA.GCGGGU.....A.GGAU.UGU
Rla-1-21   C.UUG..CACACUGGUGGUAGAAUGACCUU....GA.GCCC..CGC..CCCG.AUAU.GAGCAUAAAAGUGGG.GC...........GU.AA............GCACCAUGA..UAUGCGA.....AUA.GCAGGU.....A.GGAU.UGU
Rla-1-22   C.UUG..CACACUGGUGGUAGAAUGACCUU....GA.GACC..CGC..CCCG.AUAU.GAGCAUAAAAGUGGG.GC...........GG.AA............GCACCAUGA..UAUGCGA.....AUA.GCGGGU.....A.GGAU.UCU
Rla-1-23   C.UUG..CACACUGGUGGUAGAAUGACCUU....GA.GACC..CGC..CCCG.AUAU.GAGCAUAAAAGUGGG.GC...........GU.AA............GCACCAUGA..UAUGCGA.....AUA.GCGGGU.....A.GGAU.UGU
Rla-1-24   C.UUG..CACACUGGUGGUAGAAUGACCUU....GA.GACC..CGC..CCAG.AUAU.GAGCAUAAAAGUGGG.GC...........GU.AA............GCACCAUGA..UAUGCGA.....AUA.GCGGGU.....A.GGAU.UGU
Rla-1-25   C.UUG..CACACUGGUGGUAGAAUGACCUU....GA.GACC..CGC..CCCG.AUAU.GAGCAUAAAAGUGGG.GC...........GU.AA............GCACCACGA..UAUGCGA.....AUA.GCGGGU.....A.GGAU.UGU
Rla-1-26   C.UUG..CACACUGGUGGUAGAAUGACCUU....GA.GACC..CGC..CCCG.AUAU.GAGCAUAAAAGUGGG.GC...........GU.AA............GCACCAUGA..UAUGCGA.....AUA.GCGGGU.....A.GGAU.UGU
Rla-1-27   C.UUG..CACACUGGUGGUAGAAUGACCUU....GA.GACC..CGC..CCCG.AUAU.GAGCAUAAAAGUGGG.GC...........GU.AA............GCACCAUGA..UAUGCGA.....AUA.GCGGGU.....A.GAAU.UGU
Rla-1-28   C.UUG..CACACUGGUGGUAGAAUGACCUU....GA.GACC..CGC..CCCG.AUAU.GAGCAUAAAAGUGGG.GC...........GU.AA............GCACCAUGA..UAUGCGA.....AUA.GCGGGU.....A.GGAU.UGU
Rla-1-29   C.UUG..CACACUGGUGGUAGAAUGACCUU....GA.GACC..CGC..CCCG.AUAU.GAGCAUAAAAGUGGG.GC...........GU.AA............GCACCACGA..UAUGCGA.....AUA.GCGGGU.....A.GGAU.UGU
Rla-1-31   C.UUG..CACACUGGUGGUAGAAUGACCUU....GA.GACCCGCCCCG.....AUAU.GAGCAUAAAAGUGGG.GC...........GU.AA............GCACCAUGA..UAUGCGA.....AUA.G..........C.GGGU....
env-38     C.UCG..CUCAUAUGUGGG.UGAAAGACCU....UG.GGACC.CGC..CCCA.AUAU.GAGCAU...A.AAAG.UGUGAU.......GA.UA........AUCCCA..U.GAU...AUGCGA.....AUA.UUGGGU.....A.GGAU.UGU
env-39     C.UUG..CUCAUAGGUGGG.UGAAAGACCU....UG.GGACC.CGC..CCCG.AUAU.GAGCAUAA.A.AGUG.GGAU.........GA.UA..........AUCC.CA.UGA..UAUGCGA.....AUA.UUGGGG.....A.GAAU.UGC
env-40     C.UCG..CUCAUAGGUGGG.UGAAAGACCUUGU.GA.CC....CACUCCCUG.AUAU.GAGCAU.A.A.AAGU.GGGAU........GA.UA.........AUCCC.AC.GAU...AUGCGA.....AUA.UUGGGG.....A.GAAU.UGU
env-41     C.UUG..CUCAUAGGUGGG.UGAAAGACCU....UA.GAACC.CGC..CCUG.CUAU.GAGCAU.A.A.AAGU.GGAU.........GA.UA.........AUCCC.AC.GAU...AUGCGA.....AUA.UUGGGU.....A.GGAU.UGU
Rla-1-32   C.UUG..CUCAUAGGUGGG.UGAAAGACCU....UA.GAACC.CGC..CCUG.CUAU.GAGCAU.A.A.AAGU.GGGAU........GA.UA.........AUCCC.AC.GAU...AUGCGA.....AUA.UUGGGU.....A.GGAU.UGU
env-42     C.UUG..CUCAUAAGUGGG.UGAAAGACCU....UG.GGACC.CGC..CCCA.AUAU.GAGCAU.A.A.AAGU.GGGAU........GA.CA.........AUCCC.GC.GAU...AUGUGA.....AUA.UUGGGU.....A.GAAU.UGU
env-43     C.UUG..CUCAUAGGUGGG.UGAAAGACCU....UG.GGACC.CGC..CCCG.AUAU.GAGCAU.A.A.AAGU.GGGAU........GA.UA.........AUCCC.AC.GAU...AUGCGA.....AUA.UUGGGG.....A.GAAU.UGU
env-44     C.UUG..CUCAUAGGUGGG.UGAAAGACCU....UG.GGACC.CGC..CCCG.AUAU.GAGCAU.A.A.AAGU.GGGAU........UA.UA.........AUCCC.AC.GAU...AUGCGA.....AUA.UUGGGG.....A.GAAU.UGU
env-45     C.UUG..CUCAUAGGUGGG.GGAAAGACCU....UG.GGACC.CGC..CCCG.AUAU.GAGCAU.A.A.AAGU.GGGAU........GA.UA.........AUCCC.AC.GAU...AUGCGA.....AUA.UUGGGG.....A.GAAU.UGU
env-46     C.UUG..CUCAUAGGUGGG.UGAAUGACCU....UG.GGAC.CCGC..CCCA.AUAU.GAGCAU...AAAAGU.GGGAU........GA.UA.........GUCCC.AC.GAU...AUGCGA.....AUA.UUGGGU.....A.GUAU.UGU
Dfo-1-2    C.UUG..CUCAUAAGUGGA.UGAAAGAUCUU...GG.GACC..CGC..CCCA.AUAU.GAGCCU...AGAAGU.GGGAU........GA.CA.........AUCCC.AC.GAU...AUGCGA.....AUAAUUGGG......A.GGAU.UGU
env-47     C.UUG..CUCAUAUGUGGG.GAAAGACU......UA.GGACC.CGC..CCCG.AUAU.GAGCAU.A.A.AAGU.GGGAU........GA.UA.........AUCCC.AC.GAU...AUGCGA.....AUA.UUGGGU.....A.GGAG.UGU
Rla-1-33   C.UUG..CUCAUAGGUGGGUGAAAGACCU.....UU.UGACC.CGC..CCCG.AUAU.GAGCAU...AAAAGU.GGGAU........GA.UA.........AUCCC.AC.GAU...AUGCGA.....AUA.UUGGGU.....A.GGAU.UGU
Rla-1-34   C.UUG..CUCAUAGGUGGGUGAAAGACCU.....UU.UGACC.CGC..CCCG.AUAU.GAGCAU...AAAAGU.GGGAU........GA.UA.........AUCCC.AC.GAU...AUGCGA.....AUA.UUGGGU.....A.GGAU.UGU
Rla-1-35   C.UUG..CUCAUAGGUGGGUGAAAGACCUU....AG.GAUC..UAC..CCCG.AUAU.GAGCAU...AAAAGU.GGGAU........GA.UA.........AUCCC.AC.GAU...AUGCGA.....AUA.UUGGGU.....A.GGAU.UGU
Rla-1-36   C.UUG..CUCAUAGGUGGGUGAAAGACCUU....AG.GACC..CGC..CCCG.AUAU.GAGCAU...AAAAGU.GGGAU........GA.UA.........AUCCC.AC.GAU...AUGCGA.....AGA.UUGGGU.....A.GGAC.UGU
Rla-1-37   C.UUG..CUCAUAGGUAGGUGAAAGACCUU....AG.GACC..CGC..CCCG.AUAU.GAGCAU...AAAAGU.GGGAU........GA.UA.........AUCCC.AC.GAU...AUGCGA.....AUA.UUGGGU.....A.GGAU.UGU
Rla-1-38   C.UUG..CUCAUAGGUGGGUGAAAGACCUU....AG.GGCC..CGC..CCCG.AUAU.GAGCAU...AAAAGU.GGGAU........GA.UA.........AUCCC.AC.GAU...AUGCGA.....AUA.UUGGGU.....A.GGAU.UGU
Rla-1-39   C.UUG..CUCAUAGGUGGGUGAAAGACCUU....AG.GACC..CGC..CCCG.AUAU.GAGCAU...AAAAGU.GGGAU........GU.UA.........AUCCC.AC.GAU...AUGCGA.....AUA.UUGGGU.....A.GGAU.UGU
Rla-1-40   C.UUG..CUCAUAGGUGGGUGAAAGACCUU....AG.GAAC..CGC..CCCG.AUAU.GAGCAU...AAAAGU.GGGAU........GA.UA.........AUCCC.AC.GAU...AUGCGA.....AUA.UUGGGU.....A.GGAU.UGU
Rla-1-41   C.UUG..CUCAUAGGUGGGUGAAAGACCUU....AG.GACC..CGC..CCCG.AUAU.GAGCAU...AAAAGU.GGGAU........GA.UA.........AUCCC.AC.GAU...AUGCGA.....AUA.UUGGGU.....A.GGAU.UGU
Rla-1-42   C.UUG..CUCAUAGGUGGGUGAAAGACCUU....AG.GACC..CGC..CCCG.AUAU.GAGCAU...AAAAGU.GGGAU........GA.UA.........AUCCC.AC.GAU...AUGCGA.....AUA.UUGGGU.....A.GGAU.UGU
Rla-1-43   C.UUG..CUCAUAGGUGGGUGAAAGACCUU....AG.GACC..CGC..CCCG.AUAU.GAGCAU...AAAAGU.GGGAU........GA.UA.........AUCCC.AC.GAU...AUGCGA.....AUA.UUGGGU.....A.GGAU.UGU
Rla-1-44   C.UUG..CUCAUAGGUGGGUGAAAGACCUU....AG.AACC..CGC..CCCG.AUAU.GAGCAU...AAAAGU.GGGAU........GA.UA.........AUCCC.AC.GAU...AUGCGA.....AUA.UUGGGC.....A.GGAU.UGU
Rla-1-45   C.UUG..CUCAUAGGUGGGUGAAAGACCUU....AG.AACC..CGC..CCCG.AUAU.GAGCAU...AAAAGU.GGGAU........GA.UA.........AUCCC.AC.GAU...AUGCGA.....AUA.UUGGGU.....A.GGAU.UGU
Rla-1-46   C.UUG..CUCAUAGGUGGGUGAAAGACCUU....AG.AACC..CGC..CCCG.AUAU.GAGCAU...AAAAGU.GGGAU........GA.UA.........AUCCC.AC.GAU...AUGCGA.....AUA.UUGGGU.....A.GGAU.UGU
Rla-1-47   C.UUG..CUCAUAGGUGGGUGAAAGACCUU....AG.AACC..CGC..CCCG.AUAU.GAGCAU...AAAAGU.GGGAU........GA.UA.........AUCCC.AC.GAU...AUGCGA.....AUA.UUGGGU.....A.GGAU.UGU
Rla-1-48   C.UUG..CUCAUAGGUGGGUGAAAGACCUU....AG.GACC..CGC..CCCG.AUAU.GAGCAU...AAAAGU.GGGAU........GA.UA.........AUCCC.AC.GAU...AUGCGA.....AUAUUCGGU......A.GGAU.UGU
Rla-1-49   C.UUG..CUCAUAGGUGGGUGAAAGACCUU....AG.GACC..CGC..CCCG.AUAU.GAGCAU...AAAAGU.GGGAU........GA.UA.........AUCCC.AC.GAU...AUGCGA.....AUAUUCGGU......A.GGAU.UGU

           .......>>>>>>>>>>>>.>>>>>>>>>>>>>...>>>>>......<<<<.<<<<...<<<<<......<<.<<<<<<<<<<<<.........>>>>>>>>>>>>.>>......>>>>>.......>>>.>>>>>........<<<<.<<<
           .......2222???????22.????????222?2...?2222......2222.?222...22222......22.2222222?2?22.........22?2?2222222.22......22222.......222.?2222........2222.222
           C-UUG--C•YR•RRR•R••-•••••R••Y○----•R-•R•○--CG•--•••R-RYAU-GAG•A•---R-•AG•-R•○○○---------G•-○A----------○○○•Y-•C-RR•---•URCGA------AUR-•Y•••○-----A-RRAU-UGY
```

```
Rla-1-50  C.UUG..CUCAUAGGUGGGUGAAAGACCUU....AG.GACC..CGC..CCCG.AUAU.GAGCAU...AAAAGU.GGGAU........GA.UA.........AUCCC.AC.GAU...AUGCGA.....AUAUUCGGU.....A.GGAU.UGU
Rla-1-51  C.UUG..CUCAUAGGUGGGUGAAAGACCUU....AG.GACC..CGC..CCCG.AUAU.GAGCAU...AAAAGU.GAGAU........GA.UA.........AUCCC.AC.GAU...AUGCGA.....AUA.UUGGGU.....A.GGAU.UGU
Rla-1-52  C.UUG..CUCAUAGGUGGGUGAAAGACCU.....UA.GGCCC.CGC..CCCG.AUAU.GAGCAU...AAAAGU.GGGAU........GA.UA.........AUCCC.AC.GAU...AUGCGA.....AUA.UUGGGU.....A.GGAU.UGU
Rla-1-53  C.UUG..CUCAUAGGUGGGUGAAAGACCU.....UA.GGCCC.CGC..CCCG.AUAU.GAGCAU...AAAAGU.GGGAU........GA.UA.........AUCCC.AC.GAU...AUGCGA.....AUA.UUGGGU.....A.GGAU.UGU
Rla-1-54  C.UUG..CUCAUAGGUGGGUGAAAAACCUU....AG.GACC..CGC..CCCG.AUAU.GAACAU...AAAAGU.GGGAU........GA.UA.........AUCCC.AC.GAU...AUGUGA.....AGA.UUGGGU.....A.GGAU.UGU
env-48    C.UUG..CUCAUAGGUGGGUGAAAGACCUU....AG.GACC..CGC..CCCG.AUAU.GAGCAU...AAAAGU.GGGAU........GA.UA.........AUCCC.AC.GAU...AUGCGA.....AUA.UUGGGU.....A.GGGU....
Rla-1-55  C.UUG..CACACUGGUGGUAGAAUGACCUU....GA.GACC..CGC..CCCG.AUAU.GAGCAU...AAAAGU.GGGAU........GA.UA.........AUCCC.AC.GAU...AUGCGA.....AUA.UUGGGU.....A.GGAU.UGU
Rla-1-56  C.UUG..CUCAUAGGUUGG.CGAAAGACCUU...AG.GACC..CGC..CCCG.AUAU.GAGCAU...AAAAGU.GGGAU........GA.UA.........AUCCC.AC.GAU...AUGCGA.....AUA.UUGGGU.....A.GGAU.UGU
Rla-1-57  C.UUG..CUCAUAGGUGGA.UGAAAGACCUU...AG.GACC..CGC..UCCG.AUAU.GAGCAU...AAAAGU.GGGAU........GA.UA.........AUCCC.AC.GAU...AUGCGA.....AUA.UUGGGU.....A.GGAU.UGU
Rla-1-58  C.UUG..CUCAUAGGUGGG.UGAAAGACCUU...AG.GACC..CGC..CCCG.ACUAUGAGCAUAAAAGUAGGGAU...........GA.UA............AUCCCACGA..UAUGCGA......AGAUUGGGU.....A.GGAU.UGU
env-49    C.UUG..CUCAUAGGUGGG.UGAAAGACCUU...GG.GACC..CGC..CCCG.AUAU.GAGCAU...AAAAGU.GGGAU........GA.UA.........AUCCC.AC.GAU...AUGCGA.....AUA.UUGGGG.....A.GA......
env-50    C.UCG..CUUGCAGGAGA..AUUCUAGAACUU.GGA.ACC...CGCCU.AAA.ACAU.GAGUAA...G.AAGC.CAU..GUU.....CGG.AA.......GAUGAAUGAGC.GGA...UUACGA.....AUG.UGUUC......A.GAAU.GGC
env-51    C.UUG..CUUGCAGGAGA.CUUGCAAAAU....UG.GGACC.CGC..CAAAACAU.GAGUAA...G.AAGC.CAUUUUU......GU.UA........AAAAAUGAGC.GGA...UUACGA.....AUG.UUUUCU.....A.GGAU.UGC
env-52    C.UUG..CCAGCAGGAGGA.UUUUUGGACUU...GG.GACC..UGCU.AAAA.ACAU.GAGUAA...G.AAGC.CAGUAUUU......AAAGAA.......AAAUA...AGC.GGA...UUAGGG.....AUG.UUUUU......A.GAAU.UGC
env-53    C.UUG..CUUGCACAAAGA.UUUAUGAACUU..GGA.AUC...CGCCA..AA.ACAU.GAGUAA...G.UAGC.CAUUUUU......CGA.AG........AAAAAUGAGC.GGA...UUACGA.....AUG.UUUUUG.....A.GGAU.UGC
env-54    C.UUG..CUUGAAGGAGGA.UUUUUGAACUU...GG.GACC..CGCC.AAAA.ACAU.GAGUAA...G.GAGC.CAUUUUUU.....GA..A.......GAAAAAUGAGC.GGA...UUACGA.....AUG.UUUUU......A.GAAU.UGC
env-55    C.UUG..CAUGAAGGAGGA.UUAUUGAACUU...GG.GACC..CGCC.UAAA.ACAU.GAGUAA...G.GAGC.CAUUUUUU.....GA..A.......GAUAAAUGAGC.GGA...UUACGA.....AUG.UUUUA......A.GAAU.UGC
env-56    C.UUG..CUUGCAGGAGGA.UUUUUGAAUUU...GG.GACC..CGCC.AAAA.ACAU.GAGUAA...G.GAGC.CAUUUUUU.....GA..A.......GAAAAAUGAGC.GGA...UUACGA.....AUG.UUUUU......G.GAAU.UGC
env-57    C.UUG..CUUGCAGUAGGA.UUUUUGAACUU...GG.GACC..CGAC.AAAA.ACAU.GAGUAA...G.AAGC.CAUUUUUU.....GA..A.......GAAAAAUGAGC.GGA...UUACGA.....AUG.UUUUU......A.GAAU.UGC
env-58    C.UUG..CUUGCAGGAGGA.UUUUUGAACUU...GG.GACC..CGAU.AAAA.ACAU.GAGUAA...G.GAGC.CAUUUUUU.....GA..A.......GAAAAAUGAGC.GGA...UUACGA.....AUG.UUUUU......A.GAAU.UGC
env-59    C.UUG..CUUGCAGGAGGA.UUUUUGAACUU...GGAAUC...CGCCAAAAA.GCAU.GAGUAA...G.AAGC.CAUUUUUU.....GA..A.......GAAAAAUGAGC.GGA...UUACGA.....AUG.UUUUU......A.GAAU.UGC
env-60    C.UUG..CUUGCAGGAGGA.UUUUUGAACUU...GG.GACC..CGCC.AAAA.ACAU.GAGUAA...G.AAGC.CAUUUUGG.....GA..A.......GAAAAAUGAGC.GGA...UUACGA.....AUG.UUUUU......A.GAAU.UGC
env-61    C.UUG..CUUGCAGGAGGA.UUUUUGAACUU...GG.GACC..CGCC.AAAA.ACAU.GAGUAA...G.AAGC.CAUUUUUU.....GA..A.......GAAAAAUGAGC.GGA...UUACGA.....AUG.UUUUU......A.GAAU.UGC
env-62    C.UUG..CUUGCAGGAGGA.UUUUUGAACUU...GG.GACC..CGCC.AAAA.ACAU.GAGUAA...G.AAGC.CAUUUUUU.....GA..A.......GAAAAAUGAGC.GGA...UUACGA.....AUG.UUUUU......A.GAAU.UGC
env-63    C.UGG..CUUGCAGGAGGA.UUG.GGACCU....UG.GACC..CGCC.AAAA.ACAU.GAGUAA...G.AANC.CAUUUUUN.....GA..A.......GAAAAAUGAGC.GGA...UUACGA.....AUG.UUUUU......A.GUAU.UAC
env-64    C.UUG..CUUGCAGGAGGA.UUUGUGACCUU...GG.GACC..CGCC.AAAA.ACAU.GAGUAA...G.AAGC.CAUUUUUU.....GA..A.......GAAAAAUGAGC.GGA...UUACGA.....AUG.UUUUU......A.GUAU.UAC
env-65    C.UUG..CUUGCAGGAGGA.UUUGUGACCUU...GG.GACC..CGCC.AAAA.ACAU.GAGUAA...G.AAGC.CAUUUUUU.....GA..A.......GAAAAAUGAGC.GGA...UUACGA.....AUG.UUUUU......A.GUAU.UAC
env-66    C.UUG..CUUGCAUGAAGA.UUUUUAAUCUU...GG.GACC..CGCC.AAAA.ACAU.GAGUAA...G.AAGC.CAUUUUUC.....GC..A.......GAAAAAUGAGC.GGA...UUGCGA.....AUG.UUUUUG.....A.GGAU.UGU
env-67    C.UUG..CUUGCAAGAGGA.UUUUUGACCUU...GG.GACC..CGCC.AAAA.ACAU.GAAUAA...G.CAGC.CAUUUUUC.......A.UA.......GAAAAAUGAGC.GGA...UUAUGA.....AUG.UUUUU......A.GGAU.UGU
env-68    C.UUG..CCUGCAGGAGAA.UUUUUGACCUUG..GG.ACC...CGCCA.AAA.ACAU.GAGUAA...G.UAGC.CAUUUUUC......AA..A.......GAAAAAUGAGC.GAA...UUACGA.....AUG.UUUUCG.....A.AGAU.UGC
env-69    C.UUG..CUUGCAGGAGAA.UUUUUGACCUU...GG.GACC..CGCC.AAAA.ACAU.GAGUAA...G.UAGC.CAUUUUUC......AA..A.......GAAAAAUGAGC.GAA...UUACGA.....AUG.UUUUUA.....A.GAAU.UGC
env-70    C.UUG..CUUGCAAGAGGU.UUUUUGAACU....CU.UGACC.CGCC.AAAA.ACAU.GAGUAA...G.UAGC.CAUUUUUC......AA..A.......GAAAAAUGAGC.GGA...UUACGA.....AUG.UUUUUG.....A.AGAU.UGC
env-71    C.UUG..CUUGCAAGAGGU.UUU.UGAAC.....UCUUGACC.CGCC.AAAA.AUAU.GAGUAA...G.UAGC.CAUUUUUC......AA..A.......GAAAAAUGAGC.GGA...UUACGA.....AUG.UUUUUG.....A.AGAU.UGC
env-72    C.UUG..CUUGCAAGAGGA.UUUUUGACCUCUU.GA.CC....CGCC.AAAA.ACAU.GAGUAA...G.UAGC.CAUUUUUC......AA..A.......GAAAAAUGAGC.GGA...UUACGA.....AUG.UUUUUG.....A.GGAU.UGC
env-73    C.UUG..CUUGCAAGAGGA.UUUUUGACCUUUU.GA.CC....CGACA.AAA.ACAU.GAGUAA...G.UAGC.CAUUUUUC......AA..A.......GAAAAAUGAGC.GGA...UUACGA.....AUG.UUUAUG.....A.GGAU.UGC
env-74    C.UUG..CUUGCAGGAAGA.CUUCUGACCUUUA....UCCC..CGC..CAAA.ACAU.GAGUAA...G.UAGC.CAUUUUAC.....GA..A.......GAAAAAUGAGC.GGA...UUACGA.....AUG.UUUUG......A.GGAU.UGU
env-75    C.UUG..CUUGCAGGGGAU.UUUUUCUCUUU...GA.UUCC..CGC..ACAA.ACAU.GAACAA...G.CAGC.CAUUUUUC.....GU.AA.......GAAAAAUGAGC.GGA...UUAUGA.....AUG.UUUGUG.....GAGAAU.CGC
Aca-1-1   C.UUG..UAUGAAAAAGCA....GGGCUUU....GG.GACC..CAU..AAAA..CAU.GAGAAA...G.CAGC.CC...........GAUUA............GG.GC.GGA...UUUCGA.....AUG..UUUUU.....A.GGAU.UGU
env-76    C.AUG..CACGAAAAAGCU.UUUAUAUUACC...GAC.....CCGC..CCAA.ACAU.GAGAAA...G.AAGC.GAUUUAC.......GA..A........GUAAAUCAGC.GGA...AUUCGA.....AUG.UUUGC......A.GAAU.UGC
env-77    N.UAG..CANGAAAAAGGN.UUUGUGUAUU....UU.CGACC.CGC..CCAA.ACAU.GAGAAU...G.GAGC.GAUUUAC.......GA..A........GUAAAUCAGC.GGA...AUUCGA.....AUG.UUUGG......A.GAAU.UGC
env-78    C.UAN..CAC..AAACGGC.UUUGUGUAUU....UU.CAACC.CGC..CCAA.AAAU.GAGAAU...G.GAGC.GAUUUAC.......GA..A........GUAAAUCCGC.GGA...AUUCGA.....AUG.UUUGG......A.GAAU.UGC
env-79    C.UAG..CACGAAAAAGGC.UUUAUGUAUUUU..CG.AC...CCG...CCAA.ACAU.GAGAAA...G.GAGC.GAUUUAC.......AA..A........GUAAAUCAGC.GGA...AUUCGA.....AUG.UUUGG......A.GAAU.UGA
env-80    C.UAC..CACGAAAAAGGC.UUUAUGUAUUCUC.GA.C.....CCG..UCCA.ACAU.GAGACA...G.GAGA.GAAUU.........ACAAAGUA.......AACUC.GC.GGA...AUUCGA.....AUGUUUGGAG.....A...AUUGGG
env-81    C.UUG..CACGAUAAGACU.UUG.GAGCUU....GG.GACU..CG...UCAA.ACAU.GAGAAA...G.UAGC.AAUUUAC.......GU..A........GUAAAUCAGA.GGA...UUUCGA.....AUG.UUUGAGUAUUGACAGGC.CAG
env-82    C.UUG..CAGAAUAAGACU.UUUUUA..CUU...GG.GACA..CGC..UAAA.CACC.GAAAAC...GCAAUA.AAAUGUCGU.....GAAU.......ACGGCAUUU.UA.UGAAA.GUUUGA.....GGU.GUUUA......A.GGAU.UGC
env-83    C.UUG..CAGAAAAAGACU.UUUUA..CUU....GG.GACA..UGC..UAAA.CACC.GAAAAC...GGAAUA.AAAUGUCGU.....GAAU.......ACGGCAUUU.UA.UGAAA.GUUUGA.....GGU.GUUUA......A.GGAU.UGC
env-84    C.UUG..CACAAUAAGNCA.UUUAUACU......UAUUGACA.CGCCGAACA.AC...GAGAAC...GGAAAU.AAAUGGUG.....UGAACA.......CACCAUUUUAU.GGGGA.GU.CGA........GGGUGUCG.....AGGGAUGUGA
env-85    C.UCG..CGCAAAUUAGCC.CUUUAUACU.....UUUUGACA.CGC..CGAA.CACC.GA........AAACGA.AAUAUAAUGGUGUGUA.CA...CGCCCUUUUAUU.UCGUGUUC............GGU.GUUCG......A.GGAU.AGC
env-86    C.UCG..CACAAAUAAGCA.AUUUGUACUU....AUUGACA..CGC..CGAA.CACC.GAAAAC...G.AAAUCACAUGGUGU.....GAAC.......AUGCCAUUUUAUGGGA...GUCUGA.....GGU.GUUCG......A.CGAU.UGC
env-87    C.UCG..CACAAAUAAGCA.AUUUAUACU.....UAUUGACA.CACC.GAAC.ACCU.CAAACU...CCCAUA.AAAUGGUGU.....GAAC.......ACGCCAUUU.UAUGGG...AGUUUG.....AGG.UGUUCG.....A.GGAU.UGC
env-88    C.UCG..CACAAAUAAGCA.AUAAUACU......UAUAGACA.CGC..CGAA.CACC.GAAAAC...G.AAAU.AAAAUGGAGU.....GA.AC......AUGCCAUUAU.AUGGGA...GUAAGA.....GGU.GUACG......A.GGAU.AGC
env-89    C.UCG..CACAAAUAAGCA.AUUGGUACU.....GAGUGAC.ACGC..CGAA.CUCC.GUAAAC...G.AAAU.AAAAUGGG......GUGAACAG....GCCAUUUU.AUGGGA...GUNUGA.....GGU.GUUCG......A.GGAU.UGC
env-90    C.UUG..CACAAAUAAGCA.AUUUAUACU.....UAUUGACA.CGCC.GAACAAC...GAGAAC...G.GAAU.AAAAUGGUGU....GAAC......GCACCAUUUU.AUGGGA...GUUCGA......GG.UGUUCG.....A.GGAU.UGU
env-91    C.UUG..CACAAAUAAGCA.AUUUAUACU.....UAUUGACA.CGCC.GAACAAC...GAGAAC...G.GAAU.AAAAUGGUGU....GAAC......ACACCAUUUU.AUGGGA...GUUCGA......GG.UGUUCG.....A.GGAU.UGC
Csp-2-1   C.UUG..CACAAAUAAGCA.AUUUAUACU.....UAUUGACA.CGC..CGAA.CAAC.GAGAAC...G.GAAU.AAAAUGGUGU....GA.AC.....ACACCAUUUU.AUGGGA...GUUCGA.....GGU.GUUCG......A.GGAU.UGC
env-92    C.UUG..CACAAAUAAGCA.AUUUAUACU.....UAUUGACA.CGC..CGAA.CAAC.GAGAAC...G.GAAU.AAAAUGGUGU....GA.AC.....ACACCAUUUU.AUGGGA...GUUCGA.....GGU.GUUCG......A.GGAU.UGC
env-93    C.UCG..CACAAAUAAGCA.AUUUAUACUU....UU.UGACA.CGC..CGAA.CACC.GAAAAC...G.GAAU.AAAAUGGUGU....GAAC......ACACCAUUUU.AUGGGA...GUUUGA.....GGU.GUUCG......A.GGAU.UGC
env-94    C.UUG..CACAAAUAAGCA.AUUUAUACU.....UAUUGACA.CGC..CGAA.CACC.GAAAAC...G.GAAU.AAAAUGGUGU....GAAU......ACGCCAUUUU.AUGGGA...GUUUGA.....GGU.GUUCG......A.GGAU.UGC
env-95    C.UCG..CACAAAUAAGCA.AGCUGUACU.....UAUUGACA.CGC..CGAA.CACC.GAAAAC...G.AAAU.AAAAUGGUGU....GAAC......AUGCCAUUUU.AUGGGA...GUUUGA.....GGU.GUUCG......A.GGAU.UGC
env-96    C.UCG..CACAAAUAAGCA.AUUUAUACU.....UAUUGACA.CGC..CGAA.CACC.GAAAAC...G.GAAU.AAAAUGGUGU....GAAU......ACGCCAUUUU.AUGGGA...GUUUGA.....GGU.GUUCG......A.GGAU.UGC
env-97    C.UCG..CACAAAUAAGCA.AUUUAUACU.....UAUUGACA.CGC..CGAA.CACU.GAAAAC...G.AAAU.AAAAUGGUGU....GAAC......ACGCCAUUUU.AUGGGA...GUUUGA.....GGU.GUUCG......A.GGAU.UGU

          .......>>>>>>>>>>>>.>>>>>>>>>>>>>....>>>>>......<<<<.<<<<...<<<<<......<<.<<<<<<<<<<<<..........>>>>>>>>>>>>.>>......>>>>>........>>>.>>>>>........<<<<.<<<
          .......2222????????22.??????????2222?2....?2222......2222.?222...22222......22.2222222?2?22..........22?2?2222222.22......22222........222.?2222........2222.222
          C-UUG--C•YR•RRR•R••-•••••R••Y○----•R-•R•○--CG•--•••R-RYAU-GAG•A•---R-•AG•-R•○○○---------G•-○A----------○○○•Y-•C-RR•---•URCGA------AUR-•Y•••○-----A-RRAU-UGY
```

```
env-98    C.UCG..CACAAAUAAGCA.AUUUAUACU.....UAUUGACA.CGC..CGAA.CACC.GAAAAC...G.AAAU.AAAAUGGUGU....GAAC......ACGCCAUUUU.AUGGGA...GUUUGA......GGU.GUUCG......A.GGAU.UGC
env-99    C.UCG..CACAAAUAAGCA.AUUUAUACU.....UAUUGACA.CGC..CGAA.CACC.GAAAAC...G.AAAU.AAAAUGGUGU....GAAC......ACGCCAUUUU.AUGGGA...GUUUGA......GGU.GUUCG......A.GGAU.UGC
env-100   C.UUG..CUCACAUAUGCG.UUCCUACAU.....GCGCGA...CGCUAACCC.GUAU.GAGCAA.A.ACAGGG.ACCGAGC......CAA.AA.......GCGAGGGA.CC.GAU...UUGCGA......AUG.CGGGU......A.GGAU.UGC
env-101   C.UUG..CUUGCCGAGACG.CACAGACAUUG...GA.CGGGG.CA...ACAG.GUAU.GAGCAC...G.CAGG.C............AUA.UGC.............G.CC.GGA...GUGCGA......AUG.CCUGUC.....G.GGAU.UGC
env-102   U.UUG..CAUGCCAGGAAA.CAUAAACAUUU...GG.CAGC..CAG..GCCG.GUAUUAAGUAA...G.CAGG.GC............AA.CA.............GC.CC.GGA...UCAUGA......AUA.GUGGAG.....G.CAGU.UGU
Ceu-1-2   C.UUG..CAUGAAACGCUG.CAUGUUCAUCG...GA.UGGG..CAG..AUAU.UCAU.GAGCAU...G.UAUG.CC............GA.UA.............GG.CA.GGA...AUGCGA......AUG.AAUGU......G.CGAU.UAC
env-103   C.UCG..CACGCAAGGAGU.UUCAAACAUUU...GA.CAAC..CAA..GCCGGGCAU.GAGUAA...G.CAGA.GC............GA.AA.............GC.CC.GGA...UUACGA......AUG.CCGGCG.....G.AGAU.UGC
env-104   C.CUG..CACGCAGUGAA..GAAUAGGCAUUU..GA.CGAC..CAA..GCCG.GUAU.G.GUAA...G.CAGG.GC............GA.UA.............GC.CC.GGA...UUACGA......AUG.CUGGCG.....G.UAGC.UGC
Ceu-1-3   C.UUG..CAUGAACGAAUU.CAUGGAUAUCG...GG.CAGG..CUA..AACU.GUAU.GAGUAA...G.CAGG.GC............AA.UC.............GC.CC.GGA...UUACGA......AUG.CAGUUG.....G.CAGU.UAC
env-105   C.UUG..CAUGAACGAAUU.CAUGGAUAUCG...GG.CAGG..CUA..AACU.GUAU.GAGUAA...G.CAGG.GC............AA.UC.............GC.CC.GGA...UUACGA......AUG.CAGUUG.....G.CAGU.UAC
env-106   CUUUG..CAUGAGCGAAUU.CUUGGAUAUCG...GA.UGGG..CUA..GACC.GUAU.GAGUAA...G.UAGG.GC............GA.UA.............GC.CC.GGA...UUACGA......AUG.CGGUCG.....G.CAGC.UGU
env-107   C.UUG..CAUGAGCGAAUU.CACGGGUAUCG...GA.CGGG..CAA..GCCG.GUAU.GAGCAA...G.GAGC.GC............GA.UA.............GC.GC.GGA...UUGCGA......AUG.CUGGCG.....A.CGAU.UGC
env-108   C.UUG..CAUGAGCGAAUU.CACGGGUAUCG...GA.CGGG..CAA..GCCG.GUAU.GAGCAA...G.GAGC.GC............GA.UA.............GC.GC.GGA...UUGCGA......AUG.CUGGCG.....A.CGAU.UGC
env-109   C.UUG..CAUGAGCGAAUU.CACGGGUAUCG...GA.CGGG..CAA..GCCG.GUAU.GAGCAA...G.GAGC.GC............GA.UA.............GC.GC.GGA...UUGCGA......AUG.CUGGCG.....A.CGAU.UGC
env-110   C.UUG..CAUGAGCGAAUU.CACGGGUAUCG...GA.CGGG..CAA..GCCG.GUAU.GAGCAA...G.GAGC.GC............GA.UA.............GC.GC.GGA...UUGCGA......AUG.CUGGCG.....A.CGAU.UGC
env-111   C.UUG..CAUGAGCGAAUU.CACGGGUAUCG...GA.CGGG..CAA..GCCG.GUAU.GAGCAA...G.GAGC.GC............GA.UA.............GC.GC.GGA...UUGCGA......AUG.CUGGCG.....A.CGAU.UGC
env-112   C.UUG..CAUGAGCGAAUU.CACGGGUAUCG...GA.CGG...CAA..GCCG.GUAU.GAGCAA...G.GAGC.GC............GA.UA.............GC.GC.GGA...UUGCGA......AUG.CUGGCG.....A.CGAU.UGC
env-113   C.UUG..CAUGAGCGAAUU.CACGGGGAUCG...GA.CGGG..CAA..GCCG.GUAU.GAGCAA...G.GAGC.GC............GA.UA.............GC.GC.GGA...UUGCGA......AUG.CUGGCG.....A.CGAU.UGC
env-114   C.UUG..CAUGAGCGAAUU.CACGGGUAUCG...GA.CGGG..CAA..GCAG.GUAU.GAGCAA...A.GAGC.GC............GA.UA.............GC.GC.GGA...UUGCGA......AUG.CUGGCG.....A.CGAU.UGC
env-115   C.UUG..CAUGAGCGAAUC.CACGGGUAUCG...GA.CGGG..CAA..GCCG.GUAU.GAGCAA...G.GAGC.GC............GA.UA.............GC.GC.GGA...UUGCGA......AUG.CUGGCG.....A.CAAU.AGC
env-116   C.AUG..CAUGAGCGAACU.CACGGGCAUCG...GU.CGGG..CAA..GCCG.GUAU.GAGCAA...G...GA.GC..........UCGA.UA.............GC.UC.GGA...UUGCGA......AUG.CUGGCG.....A.CGAU.UGC
env-117   C.UUG..CAUGAGCGAAUU.C.ACGGGUAUCG..GA.CGGG..CAA..GCCG.GUAU.GAGCAA...G.GAGC.GC............GA.UA.............AC.GCCGAA...UUGCG.......AUG.CUGGCG.....G.CCAA.UGC
env-118   C.UUG..CUUGAGCGA.UU.UCACGGGUAUCG...A.CGG...CAAA.GCCC.GUAU.GAGCAA...G.GAGC.GC............GA.UA.............GC.GC.GGA....UGCGA.....AAUG.CUGGCG.....A.CGAU.UGC
env-119   C.UUG..CAUAGAGCGAAU.UCACAGGCAUCG..AA.CGGG..CAA..ACCG.GUAU.GAGCAA...A.GAAC.GC............GA.UA.............GC.GC.GAA...UAGCGA......AUG.CAGGCA.....A.CGAU.AGC
Dfo-1-3   U.UUA..CAUGCAUUACAG.GCUAUCUAU.....UAGAUAAUCUGA..GCUG..CAU.GAACAA...G.AAGG.GC............GA.AA.............GC.CC.GGA...UUGUGA......AUG..UGGC......AGCAGA.UGC
env-120   C.UUG..CAGGCGAGGCUG.GGACUUUAU....UAG.CAAAU.CAA..ACAC.ACAU.GAGCAU...G.UAGG.AC............GA.UA.............GU.CC.GGA...AUGCGA......AUG.UGUGUC.....A.CGGA.UCC
env-121   C.UUG..CAGGCAGGAACG.CCUAUGCAUU...AGA.CGGA..CAA..ACAG.ACAU.GAGCAA...G.UAGG.GC............GA.CA.............GC.CC.GGA...AUGCGAAUGCGAAUG.GCUGUA.....G..CGG.UUC
Ceu-1-4   C.UUG..CAACAUCGGGCA.CUUUUCUAUU....UG.AUGGG.UAA..ACAG.UCAU.AAGCAC...G.CAGG.AC............GA.UA.............GU.CC.GGA...GUGCGC......AUG.GCUGGC.....A.CGAU.UUC
env-122   C.UUG..CAGACGGAGCUG.CUUAUUUAUAU...GA.CGGA..UAA..ACAG.ACAU.AAGCAU...G.AAGG.AC............GA.UA.............GU.CC.GGA...AUGCGC......AUG.GCUGUU.....A.CGAU.UUC
Csp-1-1   C.UAG..CAGGCAAAGCUG.UGUAAUUAUU....UG.CCGGG.CAA..ACAG.ACAU.GAGCAU...G.AAGG.AC............GA.AA.............GU.CC.GGA...AAGCGA......AUG.GCUGAG.....G.CGAU.UUC
env-123   C.UUG..CAGGCAAAGCAG.GAUUUCUAUG...AGG.GGAG..CAA..ACAG.ACAU.GAGCAA...G.AAGG.AC............GA.UA.............GC.CC.GGA...UUGCGA......AUG.UCUGUG.....A.CGAU.UUC
env-124   C.UUG..CAGGCAAAGCAG.GAUUUCUAUG...AGG.GGAG..CAA..ACAG.ACAU.GAGCAA...G.AAGG.AC............GA.UA.............GC.CC.GGA...UUGCGA......AUG.UCUGUG.....A.CGAU.UUC
env-125   C.UUG..CAGGCAAGGUGU.ACUUUCUAUG...AGG.GGAG..UAA..ACAG.ACAU.GAACAA...G.UAGG.AC............GG.UA.............GU.CC.GGA...UUGUGA......AUG.UCUGUU.....A.CGAU.UUC
env-126   C.UUG..CAGGCGGCGAAG.GCUUUCUAU.....GAGGGAG.CAA..ACGA.ACAU.GAGCAA...G.AAGG.AC............GA.UA.............GU.CC.GGA...UUGCGA......AUG.GUCGUU.....A.CGAU.UUC
env-127   C.UUG..CAGGUAAAGGAU.GUUUUCUAU.GUU.GG.GAG...UAA..AACA.AUAU.GAGUAU...G.AAGG.AC............GA.CA.............GU.CC.GGA...AUACGA......AUA.UUGGUU.....G.CAAU.UUC
env-128   C.UAG..CAGGCAGCGGAU.GUUUCCUAUGAGA.G..GAG...CGG..ACAA.ACAU.GAGUAU...G.CAGG.AC............GA.UA.............GU.CC.GGA...AUACGA......AUG.UUUGUU.....A.CGAU.UUC
          ......>>>>>>>>>>>>.>>>>>>>>>>>>>....>>>>>......<<<<.<<<<...<<<<<......<<.<<<<<<<<<<<<.........>>>>>>>>>>>>.>>......>>>>>........>>>.>>>>>.........<<<<.<<<
          ......2222????????22.????????22??2....?2222......2222.?222...22222......22.2222222?2?22.........22?2?2222222.22......22222........222.?2222.........2222.222
          C-UUG--C•YR•RRR•R••-•••••R••Y○----•R-•R•○--CG•--•••R-RYAU-GAG•A•---R-•AG•-R•○○○---------G•-○A---------○○○•Y-•C-RR•---•URCGA------AUR-•Y•••○-----A-RRAU-UGY
```

alignment positions 313 · · · 472

```
env-1     .......ACGUGCAU...GCAGGU.......GCAUG.CAGG....UGCAU.GCACGUGCAUGCAGGUCUCUUGGGCCUCUCUAGCGCCUUCGACUGGUGGCGGCGCAGCAGCUCGUCCAGCUCGCCAAACUCGAUCUUGCCCGUGCGCCGCGGGUCGAAU
env-2     GCGCAU.GC....UC.......GCAUG.UA.......UG......CAUGC.........GUGUAUGUAUGUUUGCAUGGGUGUGGGCAUGUAUGCGUGCAUGCAAGCAUGCCUGUAUAUAUGCAUGUAUGUGUAGAUGUGUGUGUAGGUGUGUAUGUAUG
env-3     ....AU.......GUGU.......AU.....GUAUG.UAUGUAUGUGUGU.........GUAUGUAUGUAUGUAUGUAUGUAUGUAUGUAUGUAUGUGUGUAUGUAUGCAUGCAUGCAUGCAUGUAUGUAUGUAUGUAUGUAUGUAUGUAUGUAUGUGUG
env-4     ..GAGU.AC....GA.......GUAC..AA....GU.ACUGGU..ACUAG.UACUAGUACUAGGACUAGGACUAGGACUAGGACUAGGACUAGGGCCAGGACUAGGACUAGGACUAGGACUGGGACUGGGACUAGGACUGGGACUGGGACUAGGACUAGG
env-5     ....AU.GC.......AU....GCAU.....GCAUG.CA............UGUAUGUAUGUAUGUACUCUACUGUACUGCACUGUGCUGUACUGUACUGUACUGUACUGUACUGCACUGUAUUGCAAUGUAAUGUGAUGUAAUGUGAUGUAAUGUAGUG
env-6     ....GU.AC....AUUAC....GUACU.UA...AGU.GC....GUGCGUG.........CGUGCGUAGGUAGGUAGGUAUGUAGGUAGGUAGGUAGGUAGGUAGGUAGGUAGGUAGGUAGGUAGGUAGGUAGGUUAGGUAGGGAGGUAGGUA
env-7     .............GUAA...............GCCAG.CCG....UGUCUA.CGAU....GUAGGUGGAAUAAUGCCACUGUCCGGAUAUGAGCAAGUAGCACCUACUGUGCGGACCACGAAUAUACGGAGGCUGGAGAAUCCAGCCGUGUCUACGAUGUA
env-8     GGGAGU.AGC...GA.A....GCUACG.GA.GAGAC.UUG.....GCUUA.CA......AAAAGCGCGAGUAACGAGGAGAGAAGACAUGGAAAUUUCACAGUCGUGCUUGCACGAACUGUGAAAUUUAUAUGGGUUCUCGAGGAGCACCGCGACAACGA
Ceu-1-1   GGGAGC.AC....GA.A.....GUGCG.AA.GCAAU.UCUGUG.UUAACG.CG......CAAUGCGUGUGGAGAAGUGCGAAGCAAUUCGGUAUUGGGACAUGGAUGGGGACAGGCACCUCCGUCCCCACUUGCCCCUUGGCGAGAAUUCUGUUAUAUUA
Eve-1-1   GGGAGC.GG....GA.A.....GCGCG.UA.GCAAU.CUGA..GGUGAGU.........UAUUUAAUCUCGAUUCCUUACUGACUUCCGACUUAAGUCAAUCAAUAAUUUUUUUCUUGACUUAAUGUACAAAAUAGUAUAUAUUACUGAAAUAAAUAGUU
env-9     GGGAGU.......GAAA.......GCG.UA.GCAAU.CCGUA..GGCUUU.AU......AUAACAUAUAUACGUUAUAAAAUAUAGGUCUUAUGUAGCGUAUAUAUAAACUAUAAAGUUAUAUAAGAAUUCAAUAACAAAACAAAAAUCAAGGAGAAUAA
env-10    GAGAGU.UC....GA.A.....GAACG.UA.GCAAU.UC.....GUGUUA.UA......UAUUUAUAUAAGAAUAUUCAUAGUCCGAUAGAACUGUUUUCUCAUAUAUUUUGUAGAAUUGCGAGAGUUCGAAGAACGUAGCAAUUCGUGUUAUACGUUGA
env-11    GGGAAU.UGC...GA.A....GCAAUG.GA.GUAAUCAG....GAGGUAG.........GUUAGGAACAAAAUACAUUUUCCCACAUGACCUAAUUACUCAGUUUCCUCGAAUUCAAGCCACUUAUCAGCUAAGAAUUUCUUUAUGUAAGAGUCAAAUGC
env-12    GGAAAU.GC....GU.A.....GCAUG.GA.GCAAU.CUGAUU.AA.UCA.UA......AAUGAGGGGCAAAAUACCUUUUGCCCCUCAUAUCAUUAUUAUCUUGUAUGAAUAAAAUAAACUAUGCAUUAUUACUUUCUAUGAAUGAAAUAAACUAUGCA
env-13    GGGAAU.GC....GG.A.....GCAUG.GA.GUAAU.CUGA....UUAAU.CA......UAAAUGAGGGCAAAAUACCUUUUGACCCUCAUAUCAUAAUAGAUGUAAGGUUCACAAGUUCAAAAGUCUUAUCGCACAGACACGCAUGACUUUGUAACCCU
env-14    GGGAAU.GC....GG.A.....GCAUG.GA.GUAAU.CUGA....UUAAU.CAUA.AAUGAGGGGCAAAAUACCUUUUGAACCUCAUAUCAUAAUAUUAAAUCGUAUGAUUAAUAUGAUAUUGAGGUUAAGCAGGUAAUUGCCAUAAUUGUACUUAGUAU
env-15    GGGAAU.GC....GU.A.....GCAUG.UA.GUAAU.CUGA....UUAAU.CAUA.AAUGAGGUGCAAAAUACCCCGCCAUGAAUGGCGGGGCAAAAUGUUAUGCAAAAAGCACAAUUUUACAAAAUAUUUUAUUCACCAAUAAGGUAAUUGUAUAAUCC

          ....<<.<<<<<<.....>>>>>>>>.....>>>>>.>>....>>>>>>>.>>>>>>>>...............................................................................................
          ....22.222212.....21222222.....22222.22....??22222.2?22??2?...............................................................................................
          GRGAGY-RY----R•-A-----GYRCG-•A-RCAAU-YY○○○---••Y•Y-YA------••R•••••RR••••••••••••••••••••••••••••••••••••••••••••••••••••••••••••••••••••••••••••••••••••••
                                                                  3′
```

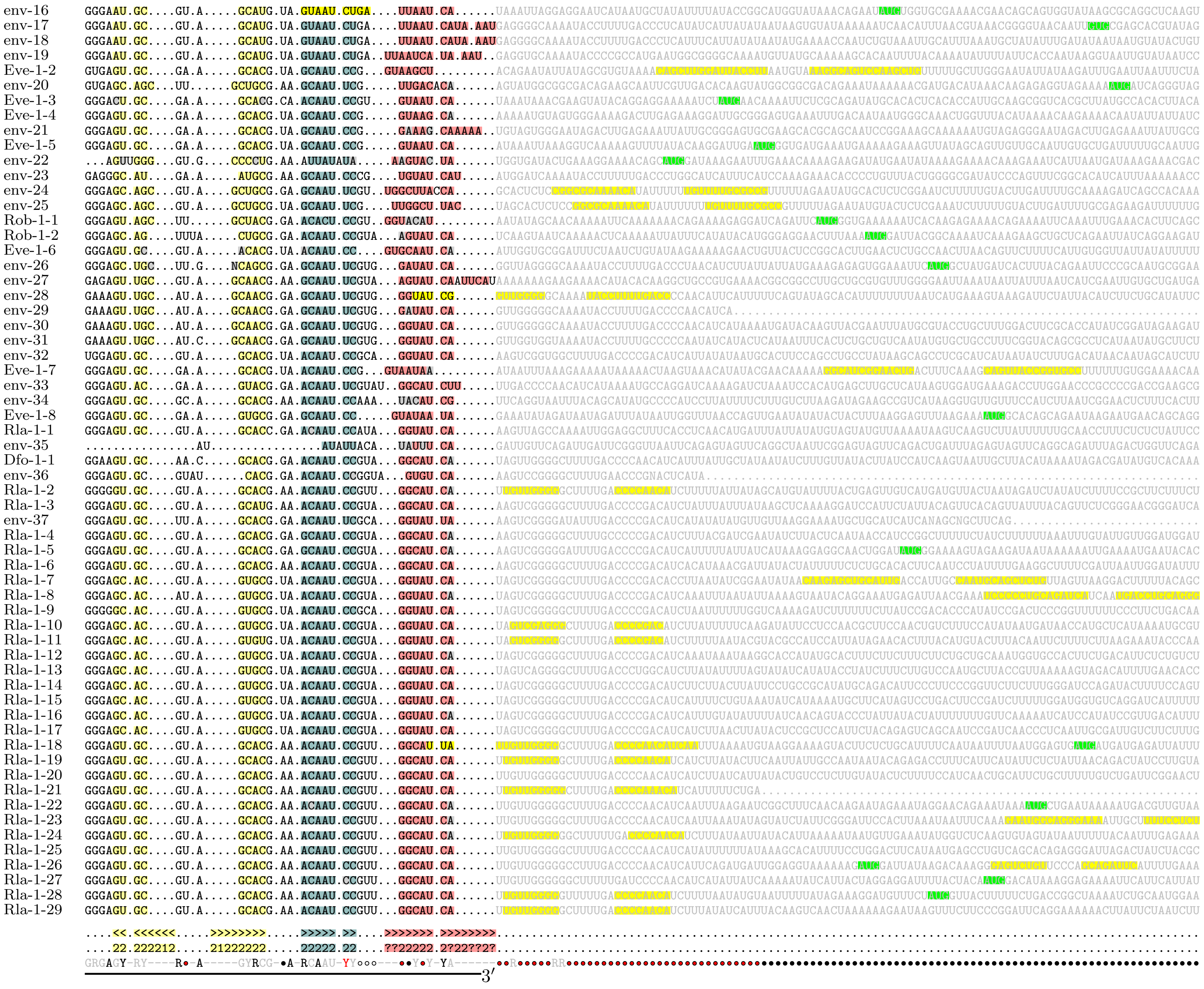

env-16
env-17
env-18
env-19
Eve-1-2
env-20
Eve-1-3
Eve-1-4
env-21
Eve-1-5
env-22
env-23
env-24
env-25
Rob-1-1
Rob-1-2
Eve-1-6
env-26
env-27
env-28
env-29
env-30
env-31
env-32
Eve-1-7
env-33
env-34
Eve-1-8
Rla-1-1
env-35
Dfo-1-1
env-36
Rla-1-2
Rla-1-3
env-37
Rla-1-4
Rla-1-5
Rla-1-6
Rla-1-7
Rla-1-8
Rla-1-9
Rla-1-10
Rla-1-11
Rla-1-12
Rla-1-13
Rla-1-14
Rla-1-15
Rla-1-16
Rla-1-17
Rla-1-18
Rla-1-19
Rla-1-20
Rla-1-21
Rla-1-22
Rla-1-23
Rla-1-24
Rla-1-25
Rla-1-26
Rla-1-27
Rla-1-28
Rla-1-29
3′

```
Rla-1-31  ...AGU.GC....GU.A.....GCACG.AA.ACAAU.CCGU...UGGCAU.CA......UUGUUGGGGGCUUUUGCCUCCAACAUCCAUAUAAGGAUAAGAUAAAAUGAGACAGACAGGGAAUACCCGAAAGGAGAAGGCAGUUCUCUGUCUGUCUUAUU
env-38    GGGAGU.GC....GU.A.....GCACG.GA.ACAAU.CCGUA...GGUAU.CA......AAGUCGGGGGCUUUUGACCCCGACAUCAUAAUAUAAUAUUUACUAUAUCCUUUAUACUACAGCUUCAAUACGAAAGUAUCUACUUAAAAUUAUAUUCAAUA
env-39    GGGAGU.GC....GU.A.....GCACG.GA.GUAAU.UCGUA...GGUAU.CA......AAGUCGGGGGAUUUUGACCCCGACAUCAUAAUAUUGUAUUUAAGUCAGGCAGAUGAUGGCUACAAUAUCGGGAUAUGUUAUGUACAAUAGCGAAAUGUAUU
env-40    GGGAGU.GC....GU.A.....GCACG.GA.ACAAU.UCGUA...GGUAU.CA......AAGUCGGGGGCUUUUGACCCCUACAUCAUGUUAUUUAUAUAUGCACAUUGUGUCAGGAUAUUUUUUAUGAUUGGCUGAUUAAAGUAUAGAAAUGCAUAUAA
env-41    GGGAGU.GC....GU.A.....GCACG.GA.GCAAU.CCGUA...GGCAU.CA......AAGUCGGGGGCUUUUGACCCCGACAUCAUUUUAUUUCAUUACAGGUCGUUCUUAUGGCUUCAUCCUCUCCCAUGCCAUAACUGCUGCCUCCUCCGAUAUCG
Rla-1-32  GGGAGU.GC....GU.A.....GCACG.GA.GCAAU.CCGUA...GGCAU.CA......AAGUCGGGGGCUUUUGACCCCGACAUCAUUUUAUUUCAUUACAGGUCGUUCUUACGGCUUCAUCCUCUCCCAUGCCAUAACUGCUGCCUCCUCCGAUAUCG
env-42    GGGAGU.GC....GU.A.....GCACG.AA.ACAAU.UCGUA...GGCAU.CA......AAGUCUGGGAUUUUGACAAUGACAUAAAAUAUUGUAACAGAAUGUAAGCCGGAUAAUCCGUGUAUGCAGGAGAUGAGUAGAUAAAAAUGAUUGGAUACAGU
env-43    GGGAGU.GC....GU.A.....GCACG.GA.ACAAU.UCGUA...GGUAU.CA......AAGUCGGGGGCUUUUGACCCCGACAUCAUAUAAUUAAUAAAUUAUAAAUAAUUAGUAAUCGUAUUGAAAUAUUUUAAGAUAUAAUACAUAAUUAAAAGCAG
env-44    GGGAGU.GC....GU.A.....GCACU.GA.GCAAU.UCGUA...GGUAU.CA......AAGUCGUGGGCUUUUGCCCCCGACAUCAUUUAUAUGAUAAUGGAGGUUGUUAUGGAUAAUAAGGAAGAUGAUAUAUCAAUCCAUACAAAAGAACAGAUAGC
env-45    GGGAGU.GC....GU.A.....GCACG.GA.GCAAU.UCGUA...GGUAU.CA......AAGUCGGGGGGCUUUUGACCACGACAUCAUAUAUAUUGAUAUACAUGUUAGGGAUAUGUUUAUAAAAGAAAUUGAUUUGCGAGGAGAAUGAUUAUGGUAAG
env-46    GGGAGU.GC....GU.A.....GCACG.GA.ACAAU.CCGUAG..GGUAU.CAUAGU..UGGGGGCUUUUGCCCCCAACAUCAUAUAAAAAUAUAGGAGUGUGAUAAUGUCAGAUAACAGACAGCGGCAUUCUGAUAAGAUAAUAUAUUACAGUUCCUAU
Dfo-1-2   GAGAGU.GC....AA.A.....GCACG.GA.ACAAU.CCGUA...GGCAU.CA......UAGUUGGGGCUUUUGCCCCAACAUCUAUAUAUUUGAUCAUUAUAUGUCAAGGGAGGAACUUAAAAUGGGAGUUGUAUCCUAUGUCAAGGAAGAAAUUCAGG
env-47    GGGAGU.GC....GU.A.....GCACG.AA.GCAAU.CCGUA...GGUAU.CA......AAGUCAGGGGCUUUUGCCCCGACAUCAUUAUAAUGAUGUCAUAUAAAAAGACAGGUUCGGCGGAACCUGUCUUUUUAUAUUUUAUAUCUCCGGAUACUGGA
Rla-1-33  GGGAGU.GC....GU.A.....GCACG.GA.GCAAU.CCGUA...GGCAU.CA......AAGUCGGGGGCUUUUGACCCCGACAUCAUUUUAUUCGACAAUUUUCUGCUUUUAAAGUAUUACAGGAAAGCAGACAGGAAUAAUUUUUAUCAUAUUUGUAU
Rla-1-34  GGGAGU.GC....GU.A.....GCACG.GA.GCAAU.CCGUA...GGCAU.CA......AAGUCGGGGACUUUUGACCCUGACAUCUUAUAAUGAUUGAUAUGAAUGGAACAAAAUGACCGGAUAUGUCUUUGACAGAAAAGUUUUUAAAAGAAGAUAGA
Rla-1-35  GGGAGU.GC....GU.A.....GCACA.GA.GCAAU.CCGUA...GGCAU.CA......AAGUCGGGGGGCUUUUGACCCCGACAUCUUUACAUUUUCUCUUUACAGAGGAGGUAUCCUUCCAAACGGCGUUGCAUUCUUUUCAUGUCGAUAAGCCAACG
Rla-1-36  GGAAGU.GC....GU.A.....GCACG.GA.GCAAU.CCGUA...GGCAU.CA......AAGUCGGGGGCUUUUGACCCCGACAUCUAUUUAUUCUUCUAAAAAACCAUUUUCCUGCAUCUGUCCGAUUAAACUCUGAACAUCUGCUGUGACCUUUUCCU
Rla-1-37  AGGAGU.GC....GU.A.....GCACG.GA.GCAAU.CCGUA...GGCAU.CA......AAGUCGGGGGCUUUUGACCCCGACAUCAUAAGAUAUAGAAAAAGACGUUCCGGUGACAGAGUCACCAAAACGCCUUUUCAAGGGGCUGUUCAUAAUUCCAG
Rla-1-38  GGGAGU.GC....GU.A.....GCACG.GA.GCAAU.CCGUA...GGCAU.CA......AAGUCGGGGGCUUUUGACCCCGACAUCAUCAUAAUUUUAUAUCUUGUUAUUAUGAAGUUUAUUUUACAAGUGCAAGUGUCUCACGUGCAAUAGCAAGCUCU
Rla-1-39  GGGAGU.GC....GU.A.....GCACG.GA.GCAAU.CCGUA...GGCAU.CA......AAGUCGGGGGCUUUUGACCCCGACAUAAUUUUAUAAUUUCAUAAUAUUUCCAGCACUUUUUUAUCACAUUUUCAUCCUAAAAUAACAUCUGACAUUAAGGG
Rla-1-40  GGGAGU.GC....GU.A.....GCACG.GA.GCAAU.CCGUA...GGCAU.CA......AAGUCGGGGGCUUUUAACCCUGGCAUCAUUAAAUUAUUUAUCCAUAUCAUUCAACAUCUUUUUCAGAUCAAUCAGCUUAUCACGCAGCUCUGCUGCCACCU
Rla-1-41  GGGAGU.GC....GU.A.....GCACG.GA.GCAAU.CCGUA...GGCAU.CA......AAGUCGGGGGCUUUUGACCCCGACAUCAUAUAACGGAUAAAAUACGCUUUGCGAUUUACCCCCGCUGCCCUCAUAAAAAACAGGACCAGGAACGCUCUGUU
Rla-1-42  GGGAGU.GC....GU.A.....GCACG.GA.GCAAU.CCGUA...GGCAU.CA......AAGUCGGGGACUUUUGACCCCGACAUCUUUACUAUUUGAUGGAGCAUUUAUCUUAGUCGAUAGCCAUCCGGCUCUUUAUUUCUUAUUGGAAUUUCGUGUUU
Rla-1-43  GGGAGU.GC....GU.A.....GCACG.GA.GCAAU.CCGUA...GGCAU.CA......AAGUCGGGGACUUUUGACCCCGACAUCAAUUUUUAUCAAUAACCUCUUUUUUCAUGAACUACAAAAAGAUGCUUUGCGUUAA..................
Rla-1-44  GGGAGU.GC....GU.A.....GCACG.GA.GCAAU.CCGUA...GGCAU.CA......AAGUCGGGGGCUUUUGACCCCGACAUCAUAUAAUUAUACAUAAUUUCUGCUGUUAUUGAAAUCUUCUCUUGCUGUCUCCUGCUUUCCGGGAGUAUAAUCUA
Rla-1-45  GGGAGU.GC....GU.A.....GCACG.GA.GCAAU.CCGUA...GGCAU.CA......AAGUCGGGGGCUUUUGACCCCGACAUCUUUACUAUUUGACUAAUCAUUUAUCUUAGUUGAUAGCCUUCCGGCUCUUUAUUUCUUAUUGAAAUUUCGUAUUU
Rla-1-46  GGGAGU.GC....GU.A.....GCACG.GA.GCAAU.CCGUA...GGCAU.CA......AAGUCGGGGGCUUUUGUCCCCGACAUCAUAUAAUGAUUAAAUGUGCAGAAAUUGUAAAAAAGACAGUUUCUGCAUUUUUUAUUUAUAUGAACAGUAUAUAA
Rla-1-47  GGGAGU.UC....GU.A.....GCACG.GA.GCAAU.CCGUA...GGCAU.CA......AAGUCGGGGGCUUUUGCCCCCGACAUCAUUAAAUGUCGUUAUAUAGAAAGGAUUCGAGAUUAUGGAAAUCUACAAAAAUCGCAGGAUGCUUACGGGAGCUG
Rla-1-48  GGGAGU.GC....GU.A.....GCACG.GA.GCAAU.CCGUA...GGCAU.CA......AAGUCGGGGGCUUUUGACCCCGACAUCUUUACUAUUGGACAGAUCAUUUAUCUUAGUUGAUAGCCAUCCGGCUCUUUAUUUCUUAUUGGAAUUUCGUCUUU
Rla-1-49  GGGAGU.GC....GU.A.....GCACG.GA.GCAAU.CCGUA...GGCAU.CA......AAGUCGGGGGCUUUUGACCCCGACAUCAUUUAAAUCGUAUCCGCAUCCACCACAAAAUCUGUCUUGACCAUCAAUUAAUUUCCACAGGAAGGACAAUAUC
Rla-1-50  GGGAGU.GC....CGUA.....GCACG.GA.GCAAU.CCGUA...GGCAU.CA......AAGUCGGGGGCUUUUGCCCCCGACAUCUUUACUAUUGGACAGAUCAUUUAUCUUAGUCGAUAGCCAUCCGGCUUUCUACUUCUUAUUUGAAAUUUCGUAUU
Rla-1-51  GGAAGU.GC....GU.A.....GCACG.GA.GCAAU.CCGCA...GGCAU.CA......CUUAAAAGGAAAAAAAACUCUUGAAUAUUAAAAUUCUUCAAUAAAAAAUCCUCUAAGACAAUAGGUAUUUCACUAUCUCUUAGAGGAUAUAUAUCAGAGGA
Rla-1-52  GGGAGU.GC....GU.A.....GCACG.GA.GCAAU.CCGUA...GGCAU.CA......AAGUCGGGGGCUUUUGCCCCCGACUUCAUUUUUAUUCUUUUAUAAAAGUUCUCUGAUUAUUUUAUCGUACCCUAUACUUCAGUACUUUAAUAAUCGUAAUU
Rla-1-53  GGGAGU.GC....GU.A.....GCACG.GA.GCAAU.CCGUA...GGCAU.CA......AAGUCGGGGACUUUUGACCCCGACAUCAAUUUUUAUCAAUAACCUCUUUUUUCAUGAACUACAAAAAGAUGCUUUGCGAAUUUCUCAGGUGUAAAAAAAAU
Rla-1-54  GGGAGU.GC....GU.A.....GCACG.GA.GCAAU.CCGUA...GGCAU.CA......AAGUCGGGGGCUUUUGACCCCGACAUCAUUCUAUUGUAUAAGCAGGCAUUAUGUGAGUCGAUCCUGUCGCUCAUCCAUGAACCACUCUGAAAGGAGCAGAU
env-48    .............GCUAU...............GUGCCAGU...GGCA..CA......UGUUUAGCACGGACCGAAGCGGAGCGGAGACGUGGGAGUGCGUAGCACGGAACAAUCCGUAGGCAUCAAAGUCGGGGGCUUUUGACCCCGACAUCAUAAUA
Rla-1-55  GGGAGU.GC....GU.A.....GCACG.GA.GCAAU.CCGUU...GGCAU.CA......UUGUU...................................................................................
Rla-1-56  GGGAGU.GU....GU.A.....GCACG.GA.GCAAU.CCGCA...GGCAU.CA......AAGUCGGGGGUUUUUGCCCCCGACAUCAUUUAAUUCAAAAAUAUACAUAUCAAAAGAUCUUUUUGCUGCCCUCAGAUCUUUCUCACCGUGCAGUGUCCACG
Rla-1-57  GGGAGU.GC....GU.A.....GCACG.GA.GCAAU.CCGUA...GGCAU.CA......AAGUCGGGGGCUUUUGCCCCCGACAUCAUGAUCUUAUUAUAUCGGAUUUUCCAGUUGCAGACUACCAAAUCAGGCUGAAAUUCUGUCAACCGGCAGUUGCA
Rla-1-58  UGGAGU.GC....GU.A.....GCACG.GA.GCAAU.CCGUA...GGCAU.CA......AAGUCGGGGGCUUUUGACCCUGACAUCUUUAUAUAUGAGGUGACAAAUUAUUAUGAAUAAAAAAAUCUGUAUUGGUAACUGGUUCUUCCCGCGGUAUCGGAA
env-49    ...AGU.......GUCA.......AUA.UA.......UAUAA.GUUGUUU.........AUUUGUUUUACUGCUGUUGCUUAUAAUACCAUGUUUAUAGGCAGCUGUCGAGUUUGAUAUAUAAGCAUACAACAGAUCCGGUUGUUUUUAUGAUGACUGAA
env-50    GAGAGA.GC....AA.A.....GCACG.UA.GCAAU.UC......GGUAU.CA......GAGGGGUCAAAAUACCCUUGGACCCCAACAUGAUCAUAUUCAGUCGGUCAUGUCCUGAAACAGCUCUGCGGCCAGGAAGACCAGACAGCCCAGAAACACGA
env-51    GGGAGC.AC....AC.A.....GUGCG.GA.GCAAU.CGA.....CGAAU.CA..............................................................................................
env-52    GGAAGU.GC....AA.A.....GCAUA.CA.GCAAU.UC......GGUAU.CAUC.U..GGAACGGUGAUCAAAAAAAUAAGAGCUUCUGAUGACUAAAAUCAUCAAAAACCCUUAAUAAAAAAAGAAAAUCGGAGUAACAGGAUUUGAACCUGCGACCU
env-53    GGGAGU.AC....AA.A.....GUACG.GA.GCAAU.CC......GGUAU.CAA.....AGGAGCAAAAUGCUUUUUGCCCCAACAUCUUUUACCGAAUAUCGUAAACGCUUCGCAGUUUCAGACCGUUCGCAAGCUGCUCUUUUAUCUGGUUCUCCCAG
env-54    GGGAGU.GC....AA.A.....GCACG.UA.GCAAU.UC......GGUAU.CA......GAGGGGUCAAAAUACCUUUUGACCCCAACAUCAUCAUAUGAAACUUUGAAAUAGAAAACAAAAAAAUACAUCAGGAAAGAAAGGAGUCGUUUUAUGGUUGA
env-55    GGGAGUUGC....AA.A.....GCACG.UA.GCAAU.UC......GGUAU.CA......GAGGGGUCAAAAUACCUUUUGACCCCAACAUCAUAAUGAUGUAAGUGUCAAAAGUAUAAAUUGCGUUUGUGCUCGCACAAAUAAGCAAGCUGUACUUAUUG
env-56    GGGUGU.GC....AA.A.....GCACG.CA.GCAAU.UC......GGUAU.CA......GAGGGGUCAAAAUAAUAACAACUGAAAGGUAAACCCAGCAAUGUUUCCAUAUUAUAUGAAGAUAACGAUAUUAUAGUUGUAAUAAAGCCAGCCGGCCUGG
env-57    GGGAGU.GC....AA.A.....GCACG.UA.GCAAU.UC......GGUAU.CA......GAGGGGUCAAAAUACCUUUUGAUCCCCACAUCAUAAUAUAAGGUUUUGUGCCUGAAAAGAAGUAUGCUGAAAAGAAGGCUGAGAAAAAGGGAUAUGCUAUA
env-58    GGGAGU.GC....AA.A.....GCACG.UA.GCAAU.UC......GGUAU.CA......GAGGGGUCAAAAUACCUUUUGACCCCAACAUCAUAUGAUGUAAGUGUCAAAAGUAUAAAUUGCGUUUGUGCUCGCACAAAUAAGCAAUUUAUACUUAUUGA
env-59    GAGAGU.GC....AA.A.....GCACG.UA.GCAAU.UC......GGUAU.CA......GAGGGGGGAUACCUUUUGACCCUAACAUCGUAUCAUGAUGUUAAAAAUAUUUCCUGAAAAGUGAGGUAAGCAUUAUGGCAAAAAAGAGGAUCGUCAUGGCC
env-60    GGAAGU.GC....AA.A.....GCACG.UA.GCAAU.UC......GGUGU.CA......GAGGGGUCAAAAAACCUUUUGACCCCAACAUCAU.......................................................
env-61    GAGAGU.GC....AA.A.....GCACG.UA.GCAAU.UC......GGUAU.CA......GAGGGGGCAAAAUACCUUUUGACCCCAACAUCAUCUGAUCCCCGCCACACUAUUAUAAAUAUAACUAUAACUAUAAAACUAUAAGGAGAAGCGUUAUGAGA
env-62    GAGAGU.GC....AA.A.....GCACG.UA.GCAAU.UC......GGUAU.CA......GAGGGGUCAAA...........................................................................
env-63    GGAAGU.GC....AA.A.....GCACG.UA.GCAAU.UC......GGUAU.CA......GAGGGGGUAAAAUACCUUUNGACUCCAACAUCUAAUAUGAUACCAGUUCCAAAAAAAGAUGACUCAGGACAGUUUUCCACUUCCCGGGUCAUCUUUUUUAU
env-64    GGAAGU.GC....AA.A.....GCACG.UA.GCAAU.UC......GGUAU.CA......GAGGGGGUAAAAUACCUUUUGACUCCNACAUCUAAUAUGAUACCAGUUCCAAAAAAAGAUGACUCAGGACAGUUUUCCACUUCCCGGGUCAUCUUUUUUAU
env-65    GGAAGU.GC....AA.A.....GCACG.UA.GCANU.UC....GGU..AU.CA......GAGGGGGUAAAAUACCUUUUGACUCCNACAUCUAAUAUGAUACCAGUUCCAAAAAAAGAUGACUCAGGACAGUUUUCCACUUCCCGGGUCAUCUUUUUUAU
env-66    GAGAGC.AC....GC.A.....GUGCG.GA.GCAAU.CC......GGUAU.CA......AAGGGGUCAAAAGUCCACUUGACCC.......................................................
env-67    GAGAAC.AC....GC.A.....GUGCG.AA.ACAAU.CC......GGUAU.CA......UAGUAUGUAAUUAUCAUUACUGGACCCAGACACCGUUUGCGUCUACAUUGUAACCAUCUGGAGUAGUGCAGUUUACAUACAGAGCACCGUCAGGAGCCAGG
env-68    GGGAGC.AU....AU.A.....GUGCG.GA.GCAAU.CU......GGUAU.CA......GAGGGGUCAAAACGUCUUUUGACCCCAACAUCAUUUUAAGCAACUUUAUAAUCUAACAUAUUCUCGCGGCAGAUACGACUGCCUGCGGCAUGAUUCUGGUU
env-69    GGGAGC.AC....GC.A.....GUGCA.GA.GCAAU.UC......GGUAU.CA......GAGGGGUCAAAAGCUUUUGACCCCAACAUCAUAUUUUACAGCAAUACUGCCCUUAUCUCCGGUUCGCCUUCUCCACAUGCACGCUCUUACCUUUGAUCUUC

          ....<<.<<<<<<.....>>>>>>>>.....>>>>>.>>....>>>>>>>.>>>>>>>>...................................................................................
          ....22.222212.....21222222.....22222.22....??22222.2?22??2?...................................................................................
          GRGAGY-RY----R•-A-----GYRCG-•A-RCAAU-YY○○○---••Y•Y-Y------••R•••••RR•••••••••••••••••••••••••••••••••••••••••••••••••••••••••••••••••••••••••••
          ———————————————————————————————————————————————————————————3′
```

```
env-70   GAGAGU.GC....AA.A.....GCACG.UA.GCAAU.CU......GGUAU.CA......AAGGGGUCAAAAUACCUUUUGACCCCAACAUCAUUGAUAUAAAUUAAGUGACAAAUUUGUCACGAAAGAGUAAGAGAGAUGUCACCUAGAUGCGGUAAAAU
env-71   GAGAGU.GC....AA.A.....GCACG.UA.GCAAU.CU......GGUAU.CA......AAGGGGUCAAAAUACCUUUUGACCCUGGUAUCAUUUAUAUUUCAGCGUAAUAAUAUCGCUGUCCUUUUUUUGUAUCCACAUGAAAAAAUGAUAUAUCAUAA
env-72   GAGAGU.GC....AA.A.....GCACG.UA.GCAAU.CU......GGUAU.CA......AAGGGGUCAAAAUACCUUUUGGCUCCAACAUCAUUGCUAUGUAAAAGAGGAAACAGAAAUUAUCACAUCUAUCCUGUUUCCUCUUUACAUGUUCUCCUACU
env-73   GAGAGU.GC....AA.A.....GCACG.UA.GCAAU.CU.....GGUAUC.A.......AAGGGGUCAAAAUACCUUUUGACCCCAACAUCAUUCUGUAUAAACAGUAUAACAAAAAACCAGUGGGGAUUGAUUUAUGGACAAUUACCACCUGCUCUUUA
env-74   GAGAGC.AC....GAAA.....GUGCG.GA.ACAAU.CC......GGUAU.CA......GGGAAACAAAGGAAUGGUACACAGACGCAAGCUUGCUUGCAGGAAGACUUCUGACCUUUAUACCCGCCAAUACAUGAGUAAGUAGCCAUUUUGCGAAUUAA
env-75   GGGAGC.AC....GA.A.....GUGCG.GA.GCGAU.UCUG...GUAUGA.CA......CUAUGAGACAUAUAUGGAGGUAAAAUCUUGAAAUAUUCCGGUAUCGGGGGACAGGCAGUAUUAGAGGGUAUCAUGAUGAAGAACAAGGACAAUUACGCCAC
Aca-1-1  GGGAGC.AC....AA.A.....GUGCG.GA.ACAAU.CCGCA...GGUAU.CU......GGGGGUCAAAAUACCUUUUGACCCUUACAUCACAAUAUGAAUAAGUUAAUAGAUGGAGGUAACAACAAUGAAAAAAUAUCUUUGCGAACCAUGCGGCUAUG
env-76   GGGAGC.AGC...GA.A....GCUGCG.GA.GCAAU.UCGCAG........UG......GCAUCAUAAGUAGCAGAGAGCAAACCCAAAAUAUAAAAUAAUACAGGAGGCAAAAAAAUGGAAGUUAUAUUAUUAGAGGAUGUAAAAACUCUUGGAAAGAA
env-77   GAGAGC.AGC...AA.A....GCUGCG.AA.GCAAU.UCGUA...GGUAU.UA......UCAAAAGUAGAGGGACGAAGAGCCGUUCGUGCUAGCACGAAAAAGGCUUUAUGUAUUUUCGACCCGCCAAACAUGAGAAAGGAGCGAUUUACAAAGUAAAU
env-78   GAGAGC.AGC...AA.A....GCAGCG.AA.GCAAU.UCGUAGGUUUUAU.CA......AAAGUAGAGGGACGAAGAGCCGUUCGUGCUACCACGAAAAAGGCUUUAUGUAUUCUCGACCCGUCCAACAUGAGACAGGAGAGAAUUACAAAGUAAACUCG
env-79   GGGAGC.AGC...AA.A....GCUGCG.GA.GCAAU.UCGUA...GGUAU.UA......UCAAAAGUGGAGGGACGAAGAGCCGUUCGUGCUAGCACGAAAAAGGCUUUGUGUAUUUUCGACCCGCCAAACAUGAGAAUGGAGCGAUUUACGAAGUAAAU
env-80   GGGAGC.AGC...AA.A....GCUGCG.GA.GCUAUUAGUAAGUAUUGAU.CA......AAGUGAAGGGACGAACAGCCGUUCGUUGAAAACUGAAAAGGCUUUGUUGCUAUUCGUGCUGCAAAUCAUAAAAAUGGAGGGGAUUUCCGAAGGGAUUACGG
env-81   ....AG.UCAGGAAA.AUUUUUGACU.....CUGGC.CU......GCUAUAUA......UGGAGGAAAAAGAUGAAUAAAAUAGGAAUUUUGACGUGUAUUCAUUCUAAUAAUGUAUGCGCCAGAGUCGGAUGCCUGGCAGCAUUUCAGAACAGAACUGA
env-82   GUGAAU.UGC...AU.A....GCAAUG.UA.GCAAU.CCUGA...UAUAU.CA......GUGAGUGGCAAAAUUUACUUUUGACACUCACAUCAUUUUAUUAUAUAUUAAUGGUUAUCCCGGAUGACAGGCAUUUACC......................
env-83   GUGAAU.UGC...AU.A....GCAAUG.UA.GCAAU.CCUGA...UACAU.CA......GUGAGUGGCAAAAUUUACUUUUGACACUCACAUCAUAUUAUAGCAUAAGUUGUAUAAAAAUAUUAUCUAUUAAUUAUUAUUUUAAAAUAAUUUUUAAAAU
env-84   ...AAUUGC....GUNA.....GCAUG.AA.GCAAU.CUGGA...UGCAU.CA......GAGAGUGGCAAAAUUUACUUUUGACCUCUCAUCAUAUAUUAGAGAUAUAUGAUGUAAG......................................
env-85   GGGAAUUGC....GU.AU....GCCUG.GA.GCAAU.CCGGU...UACAU.CA......GUGAGUGGCGGCCUUUUCUUUUGAGACUCCCCUCCUUUUUUUUUGAAAAGCCCGAA........................................
env-86   GCGGAU.UGC...AU.A....GCAUUG.GA.GCAUC.CGGU....UCCUU.CGU.....GAGUGCCAAAUUACUUUGACCCUCCCUCUUUUAUGUAUUAGCGCGACGAUUGCCCGCGCUGUAACUUACCUGUUUGCCCUUAUUUCCCUAUUUCGAGGGAA
env-87   GGGAAU.UGC...AU.A....GCAAUG.GA.GCAAU.CCGGU...UACAU.CA......GUGAGUGGCAAAAUUUACUUUUGACACUCACAUCAUUAUAUGCAUUUAAUUUCGCCAUAUGCAAUUCCAAACCCCAACUUUCUUCUCUCCCACUAUUUUCC
env-88   GGGAAU.UGC...AU.A....GCAAAG.GA.GCAAU.CCGGU...AACAU.CA......UAGAAUUAAAUACAAAAAGAUACCGGGAGUGUAUAACAGGAGAAAGGGGAUUACAGAUAGGAACUGAUUUAAAGGAAGCAAACAGAACAGAAAUUAGGAUA
env-89   GGGAAU.UGC...AU.A....GCAAUG.GA.GCAAU.CCGGU...UACAU.CA......GUGAGUGGCAAAAUUAACUUUUGACACUCACAUCAUAUAAGGUAAUAAGCGCAGCAGAGUGCAGCAGCGCAGGGAACCUGAGCCUGUAUGCACUAUAAUUU
env-90   G.GAAU.UGC...GU.A....GCAAUG.GA.ACAAU.CCGGG...UACAU.CA......GAGAGUGGCAAAAUUUACUUUUGACACUCUCAUCAUUAUAUAUUGAUAAACAUAUAUUUGCAGUUCAAUUUUUAAAUCUUUAAACCAAUACAUGUUAUAGC
env-91   GA.AAU.UGC...GU.A....GCAAUG.AA.GCAAU.CCGGG...UACGU.CA......GAGAGUGGCAAAAUUUACUUUUGACACUCUCAUCAUUAUAUAAUGUAAAAAGAAGCCUUGACUUAAGUUUUAUGUCGUUGAUCAACAUUUUUCUUUAGCCA
Csp-2-1  GA.AAU.UGC...GU.A....GCAAUG.AA.GCAAU.CCGGG...UACAU.CA......GAGAGUGGCAAAAUUUACUUUUGACACUCUCAUCAUUAUAUAAUGUAAAAAGAAGCCUUGACUUAAGUUUUAUGUCGUUGAUCAACAUUUUUCUUUAGCCA
env-92   GA.AAU.UGC...GU.A....GCAAUG.AA.GCAAU.CCGGG...UACAU.CA......GAGAGUGGCAAAAUUUACUUUUGACACUCUCAUCAUUAUAUAAUGUAAAAAGAAGCCUUGACUUAAGUUUUAUGUCGUUGAUCAACAUUUUUCUUUAGCCA
env-93   GGGAAU.UGC...AU.A....GCAAUG.GA.GCAAU.CCGGU...UACAU.CA......GUGAGUGGCAAAAUUUACUUUUGACACUCGCAUCAUAUCUUUUCUUUUCAGAAAGGCAAAAAAACUACGGUUUUACAUAUUGUUCUGUAAAAAUAUGGAAA
env-94   GGGAAU.UGC...AU.A....GCAAUG.GA.GCAAU.CCGUA...GGUAU.CAUAAUA.AAAAUAAGGUAAAAGUUGUCAGGGCAGAAAGGAAAGUUGAUAUGAAUUUUUCACAGCUUGGAAAGAUACAGGAAUUAAAAGGACAUAUGGAUACAUUUCGC
env-95   GGGAAU.UGC...AU.A....GCAAUG.GA.GCAAU.CCGGU...UACAU.CA......GUGAGUGGCAAAAUUUACUUUUGACACUCACAUCAUAUAAUGUAAUAAGCGCAGCAGAGUGCAGCAACGCAGGGAACCUGAGCCUGUAUGCACUAAUUUAC
env-96   GGGAAU.UGC...AU.A....GCAAUG.GA.GCAAU.CCGGU...UACAU.CA......GUGAGUGGCAAAAUUUACUUUUGACACUCACAUCAUUAUAUGAAAGCGUGCGUUGCCUGUGAAACGUCAAAAAAUGCCGGCUGCGAUAAUGCAGCCGACAU
env-97   GGGAAU.UGC...AU.A....GCAAUG.GA.GCAAU.CCGGU...UACAU.CA......GUGAGUGGCAAAAUUUACUUUUGACACUCACAUCAUAAUAUGAAAUGAGCCAGAUCACAGCCAUUUACCCAUGUUUUUGGCGGGUCUGAAGUAAAAAAGCC
env-98   GGGAAU.UGC...AU.A....GCAAUG.GA.GCAAU.CCGGU...UACAU.CA......GUGAGUGGCAAAAUUUACUUUUGACACUCACAUCAUUUAUAUGGCAAGUAAGUCUGACACUUUCUUGACCCGAUCAUGGCUUUCAGUCAUCUUUUUGUACU
env-99   GGGAAU.UGC...AU.A....GCAAUG.GA.GCAAU.CCGGU...UACAU.CA......GUGAGUGGCAAAAUUUACUUUUGACACUCACAUCAUUAAAAUAAGAAUUGCAUAAAUUAAAAAAACUGUUAUAAUGUAUUUAAGAAUGAACAAGCGCAGUU
env-100  GGGAAU.GC....GUCA.....GCAUG.GA.GCAAU.CCG...UGGGCUU.UAUU....CGUGGUGGACGUCAUGGCUGUAUGAGCAAAACAGGGUCCGGGCAGGCGGCGCAAAAAAGCUGCCCCCUUUUCAGGACAGCCCUUCUCUUAAUACUUUUUCA
env-101  GGGAGU.GC....GA.U.....GCACG.GG.GCAAU.CCGC...AGGUUA.UA......CUUUUUGGAAGUGCCGCCGACAGGCGGCAUAUGGGUUUGCGCGAUAAGCUGGAAGUGGUAUCGCGACAGAUAGGGAAGGAAGGCUUUGCGGAAGAUAAGCU
env-102  GGGAGU.GCC...AA.C....GGUAC..AU.ACAGC.UG.....ACUUUC.AU......UACUAUAAUAACAAGAUACCAGGGGCAAAAGGUCAUAAAUCCGAUGCAAGCUUGCUUGCAGGAGGAUUUAUGACAUUGGACCUGCCAAAAACAUGAGUAAG
Ceu-1-2  GAGAGC.CAC...GA.A....GUGGCG.AA.GUAAU.CGUAAG..GCUUU.CU......UAUCACAUUUAUAAUAAGCCCCAGACGGCGAAUGAGGAGUAAACAAAUGAAAUUAAACAUUUUACAUGAGGAUAGUGAUAUCAUUGUGGCUGUGAAACCGU
env-103  GGGAGC.AC....GC.A.....GUGCG.GA.GCAAU.CU.....CCUUU..CG......CGCACUUUGCCAACACCACCAUCAAUUCCUUAGUUUCUUAUCUGCGAGGGGCAGUUAAGAUAUAUACUUAUAAUCCAUCUAUUCUUAAAAGACUUUAGGAG
env-104  GGGAGU.GCC...GA.A....GCCACG.GA.GCAGC.UG......GCUUU.CA......GCAUUAUAUGAAACUACAGUUUCCCCACAUUAACAGUAAUAUAAAUUUGGAGGUAAGGUAUGGCAGUUAAAUACGUAUUCGUCACGGGAGGCGUUGUCUCC
Ceu-1-3  GUGAGU.GCC...AU.A....GGCACG.GA.GUAAC.UG......ACUUU.CAU.....AUAUUUACAUCUUAAUAGAGCUUAGCACCUGCCGGAAUGUGGUCAUCAACCAUCAGAAGAUGGAGCUUUUCUUCGCCUUCUUCUUCGUGGAUAGCACUGAG
env-105  GUGAGU.GCC...AU.A....GGCACG.GA.GUAAC.UG......ACUUU.CAU.....AUAUUUACAUCUUAAUAGAGCUUAGCACCUGCCGGAAUGUGGUCAUCAACCAUCAGAAGAUGGAGCUUUUCUUCGCCUUCUUCUUCGUGGAUAGCACUGAG
env-106  GGGAGC.GCC...UAUA....GGCGCG.GA.ACAGC.UG....ACUUUCA.UA......UAUGGUGAUGUCGCCGGCAGGCGACAUGUCCGAUUUGUGAGCAACGGGGGAUUUUUAUUGUAUGAUUAUGGUCUUAGCACCCUGACGCAAUACGGGAUCA
env-107  GUGAGC.ACC...AA.A....GGUGCG.GA.GCAAU.CG......GCUUU.CAU.....GAAUGGUGAUGCCUGCGUCAGCAGGCAUGUCCGUUUAAUUCCGGUCUCUGCCUGCGUCACCAGCCACACACUGACAUCCGCUUUUUAAUUCUGCAUAAAAG
env-108  GGGAGC.ACC...AA.A....GGUGCG.GA.GCAAU.CG......GCUUU.CAU.....GAAUGGUGAUGCCUGCGUCAGCAGGCAUGUCCGUUUAGAAACGGCGUCUUAUCACAGAAAGGCAGGAAAUGAUAUGGGCAAUGAUAUGGACAAAAAGGGAA
env-109  GGGAGC.ACC...AA.A....GGUGCG.GA.GCAAU.CG......GCUUU.CAU.....GAAUGGUGAUGCCUGCGUCAGCAGGCAUGUCCGUUUAGUUACAGUUCAGCCUUGCAUCUUCUUGCCCGCUCGCAGCGGACAAGAAGAUGCAAGGCUGAACA
env-110  GGGAGC.ACC...AA.A....GGUGCG.GA.GCAAU.CG......GCUUU.CAU.....GAAUGGUGAUGCCUGCGUCAGCAGGCAUGUCCGUUUAAUUCCGGUCUCUGCCUGCGUCACCAGCCACACACUGACAUCCGCUUUUUAAUUCAUGCAUAAAA
env-111  GGGAGC.ACC...AA.A....GGUGCG.GA.GCAAU.CG......GCUUU.CAU.....GAAUGGUGAUGCCUGCGUCAGCAGGCAUGUCCAUUUAGAUAUCCAUGUCAAAACAAGCGCCUGCUAUACUAUCAUGCAGACGCUUGUUUGUUUUAUUUCUA
env-112  GGGAGC.ACC...AA.A....GGUGCG.GA.GCAAU.CG......GCUUU.CAU.....GAAUGGUGAUGCCUGCGUCAGCAGGCAUGUCCGUUUAGAUAUCCAUGUCAAAACAAGCGCCUGCUAUACUAUCAUGCAGACGCUUGUUUAUUUUAUUUCUA
env-113  GGGAGC.ACC...AA.A....GGUGCG.GA.GCAAU.CG......GCUUU.CAU.....GAAUGGUGAUGCCUGCGUCAGCAGGCAUGUCCGUUUAAAUUGAAUGACCUGAAAACAGCCUUCUGAUAUGUCUGAUUAUCUCUUAAUUGGAGGAAAUAUCU
env-114  GGGAGC.ACC...AA.A....GGUGCG.GA.GCAAU.CG....GCUUUCA.UG......ACUGGUGAUGCCUGCGUCAGCAGGCCUGUCAGUUUAGAUCAGAAUCCUUUCGAAUGUACUCUCGAAGUCUUUCCUGCACUUGCCUUAGCGGCUGAUUUUCC
env-115  GGGAGC.ACC...AA.A....GGUGCG.GA.GCAAU.CG....GCUUUCAUUG......AAUGGUGAUAGCUGCGUCAGCAGGCAUGUCAGGUUACGCUAAGAUACAGUUGAUUCAGAACUUCCAUCUCCACCCAUUAACUGAGUAAUCUCAUCCAUGCU
env-116  GGGAUC.ACC...AA.A....GGUGCG.GA.GCAAU.CG....UCUUUCA.UG......UAUGGUUAUGCCUGCGUCAGCAGGCAUGUCAGUUUAGAGUAAAAGGUAUUCUAAAUACAUCCCUAAUCCGCUCACAGCCAGAUCACUAUACAAAUUGUUCC
env-117  GGGAGC.CAC...AA.A....GGGGCG.GA.GCAAU.CG......GCUUUCCA......GAAAUGGUGAUGCUGCGUCAGCCAGGAAGUCCCUUUUAAUUCCGGUCUCUGCCUGGGUCACCAGCCAA................................
env-118  GGGAGC.ACC...AA.A....GGUGCG.GA.GCAAU.CG......GCUUU.CAU.....GAAUGGUGAUGCCUGCGUCAGCAGGCAUGUCCGUUUAGAAACGGCGCCUUAUCACAGAAAGGCAGGAANUGAUAUGGGCAAUGAUAUGGACAAAAAGGGAA
env-119  GGGAGC.ACC...AA.A....GGUACA.GA.GCAAU.CG......GAUAA.CA......AGAAUAGAGAGCCUGCAAAAACAAGAUGUAAGUAAAAAGAAAAAGGUAUACAAAAAACAUACCUAAACCGAACACAAGCAAGAUAACUAUAACAAAAAGAA
Dfo-1-3  GGGAGU.GC....AA.A.....GCACG.GA.GCAUC.UGGA....UUUUA.UA......GAUUCAGAUAGUAAAAAUAAUACAAGCGGGAGGUUUCUUAUGAACAAUUACACACGCGAGGAUAUUUGAGGAUGGUUGAAGAGGAGGACGUUGGUUUCA
env-120  GAGAGC.AC....GA.A.....GUGCG.GA.GGAGC.CGUGU..UAAUA..CCCG....GCGCUAAGAAUGACGAUAGUCCGGAAUGCGAAUGUGUGUCACGGAUCCGAGAGCACGAAGUGCGGAGGAGCCGUGUUAAUACCCGGCGCUAAGAAUGCCGA
env-121  GAGAGU.GC....GA.A.....GCACG.GA.GAAACGCGUG...AUUCAC.CAAAGAU.ACUGACACAGUAAGAAGCUGUACAGAGAAUAUCUCUGUACAGCUUCUUUUGGGUUCUGUCGANUACGGCAGAGCUUAUUUCAUCUGCUCUUCCUGCUUCUU
Ceu-1-4  GAGAGU.UC....GA.A.....GAACG.AA.GAAAU.CGUG..AUUAUUG.UA......UCGAGUGAAAGGAGUGCUGCCCUAUGUACAUGAACACCGGUUAUUUGAACCAUUCACAUAUGGAUUUUAAGGAUAAAAGCCGACCACUGAUUGUCGGUAGC
env-122  GGGAGU.UC....GA.A.....GAACG.GA.GAAAU.CGUG..AUUAUUA.UAU.....GUAAUGUAUAAUGAUAAUCACGAGUUCGAAGAACGGAGAAAUCGUGAUUAUUAUAUGUAAUGUAUAAUGAUAAUCACGAGUUCGAAGAACGGAGAAAUCGU
Csp-1-1  GGGAGU.UC....GA.A.....GAACG.GA.GAAAU.CGUA...GUUAUU.CA......CGGAGAAGAAACAUGAGCAUGAAGGACGAAAGUCCGGAAAGCGAAUGACUGAAGCGAUUUCGGGAGUUCGAAGAACGGAGAAAUCGUAGUUAUUCACGGAG
env-123  GAGAGU.GC....GU.A.....GCACG.AA.AAAAU.CG.....UGUUAUUAU......GUAAACCAGCAUUGAGCAGUACCAAAAUCGAGAGAUGUCAAUUCGACUGCUUGCAGGCGGAAUUGACAGAUCCCAUUUUGAUAGGGCGAAAGCCCCGGCAA

         ....<<.<<<<<<.....>>>>>>>>.....>>>>>.>>....>>>>>>>.>>>>>>>>>...............................................................................................
         ....22.222212.....21222222.....22222.22....??22222.2?22??2?...............................................................................................
         GRGAGY-RY----R•-A-----GYRCG-•A-RCAAU-YYooo---••Y•Y-YA------••R•••••RR•••••••••••••••••••••••••••••••••••••••••••••••••••••••••••••••••••••••••••••••••••••••
         ______________________________________________________________3′
```

```
env-124  GAGAGU.GC....GU.A.....GCACG.AA.GAAAU.CG.....UGUUAUUAU......GUAAACCAGCAUUGAGCAGUACCAAAAUCGAGAGAUGUCAAUUCGACUGCUUGCAGGCGGAAUUGACAGAUCCGAUUUUGAUAGGGCGAAAGCCCGGCAGU
env-125  GAGAGU.GC....GAUA.....GCACG.GA.GAAAU.CG......UAUUA.UA......AAACAAGCGGCUGCGAAGCAGACAUUUGUUUUUCUUGCACUAUUAGCGAACGAAUAUCCUGCGAAGCAGGAUGCAGAGUGCGAUAGCACGAAUUCGUGAGC
env-126  GAGAGU.GC....GU.A.....GCACG.GA.GAAAU.CGA.....UUAAGAUA......GAAUAAAAGAGGACACAUCAGAAAUGAGAGUAGAAGAUUUAUCCACAUAUGAGAUUAUAGAGAAACGGCAGAUCCGGGACAUUAACAGCGUGACCUAUCUG
env-127  GAGAGU.GC....GC.A.....GCACA.UA.GAAAU.UG.....CUUAUGAUA......GAUUAGGAAAAGGAAAAAUUAUGAAUCAGGAACAAGUGACAACAGAGAAUACAGAAGAAAAGACAGCAGAAGUCACCACGCAGUCGGAAACGACGCAGCAG
env-128  GAGAGU.GC....GC.A.....GCACG.GA.GAAAA.CGUAU.....AUG.UU......AGAAGAGCAUAGAGGACAACAGAAUGGAUGACGAAAAAAAGAAAGUAAAACAGGCCGUAGCACUGGAAUACGAUCCCUCGGAUGAGGCACCAAGAGUCAUA
         ....<<.<<<<<<.....>>>>>>>>.....>>>>>.>>....>>>>>>>.>>>>>>>>.................................................................................................
         ....22.222212.....21222222.....22222.22....??22222.2?22??2?.................................................................................................
         GRGAGY-RY----R•-A-----GYRCG-•A-RCAAU-YYooo---••Y•Y-YA------••R•••••RR•••••••••••••••••••••••••••••••••••••••••••••••••••••••••••••••••••••••••••••••••••••
                                                                    3′
```

alignment positions 473 · · · 632

```
env-1    AUGUUCUCCCAGCAGGUGCGGAUGGCGAAGCUGGGCGCGCCCGUGCCGCCGGUUAGCACCUGCCAUGUUAGGAGUGCGGAUGGCGAAGCUGGGCGCGCACGUGCCGCCGGUUAGCACCUGCCAUAUUAGGAGUGCGGAUGGCGAAGCUGGGCGCGCCCGU
env-2    UAUGUGUGUAUCAUGUGUGUAUGCAUGUAUGCAUGCAUGCAUGCAUGUAUGUAUUUGCAUGUAUGAAUGCAUGCAUGUAUGUUUGUGUGCAUGCAUCUGUGUGUGCACGCGUGGGUGCAUGCAUGCAUGCAUCCAUGCAUGCAAUGUAUGUAUGCAUGUA
env-3    UAUGUAUGUGUGUAUGCAUGUAUGUAUGUGCGUACGCGCGUACGUGCGCACGUACGCACAUAUGUAUGAAUUAAUGUAUGUGUGUAUAUUUCUGUGUAGGUGUGUACGUGUAUGUAUGUGUGUGUAUGCAUGUGUAUGUGUAUGUAUUUGUGUAUCCAUG
env-4    ACUAGGACCAGGACUAGGACUAGGACUAGGACUAGAACUAGGACUAGGACUAGGCCUUGGACUAGGACUAGUACUAGUACUAGUACUAGUACUUUCAUUAGUACCUAGUUUUGGUGCUAGUACUAGUACUAGUCCUAGUACGCGGCGCCGCCCGCGGAGG
env-5    UAACGUAAUGCAAUACAAUGCAAUGCAAUGUAAUGUAAUGUACUGUAUUGUACUGUACUGUACUGUACUGUACUGUACUGUACUGNNNNNNNNNNNNNNNNNNNNNNNNNNNNNNNNNNNNNNNNNNNNNNNNNNNNNUAGGUUCCCGGAACUGUUCCCAGU
env-6    GGUAGGCAGGUAGGUAGGUAGGUAGGUAGGUUAGGUAGGUGUAGGUAGGUAGGUAGGUAGGUAGGUAGGUAGGUAGGUAGGUAGGUACGUAGGUACAUAGGUAUGUAUGUAGGUACGUAGGUAGGUAGGUAGGUAAGUAGGUAGGUAGGUAGGUAGGUAG
env-7    AGUAAAAUAACGCCACUGUCCAUCAAAAACUUCUUUUCAAACAAUUACACCUAUGGUAUAAUGAGCGGUGUUGGAAAAAAUACGACAAUGAAAAUUUAUAUCGGAGUGAGAAUUAAUGAGUAAGGUUAUAGUAGUUACCGAUUCAAACAGCGGUAUCACA
env-8    CAGGACGAAGUCCUGGAGUGUGAGUUGCUCGCGCAAAAAGGCGGAGAGAAGGAAAGGAGGCACAAUGGUACAGAUUAAUGGAGAACAACAGGAUGUUGCACUAGUCGCAGGAAAGACACUUCUUGCUUAUGUAACAGAAGCUGGGUAUGACCCGGUAGGU
Ceu-1-1  UAAAGAUGUGUUGUGAUGUAUUAUGGGCGGUUGCAUGAUGAUAGAAUAACGGGCAAUUGCCACCGGAACAGGAGUAGAAUUUGAAGAAUUUAUAUAUAGCAGAGAAACCCAGUGUUGCAAGACAGUUUGCCGAUGUGCUGGGGGUGAAGGAAAUGCCGG
Eve-1-1  CACAAUGUAAACUAUUCUGGGAUGCUAUCUUGUAUUGAACAGGAAACCUUUAAUGGCAUUUAUAAUUAGAAAAUAUAAAACAAAAAUAGGAAGUAGAAGAAAAAUGAAAGAUAAAAAAUGGAAAGACAUGAUAGAAAAACAUGGCGAGCUUAGAUUCUUU
env-9    AUCAUGGAUUUCACUUUAGAUCAGAAACAUGAGAUGGCUCGCGACCUUUUCAAACAGUUCGCAGAGACAGAAGUUAAACCUCUUGCACAGGAGACUGACGAGACAGAGGUAUUCCCAGCAGAGACAGUUGCUAAGAUGGGAAAAUAUGGAUUCAUGGGCA
env-10   ACUGUGCCAUAUACAAAUUAUAAUAUGCACCUUUCUUUUCCAAUAAUUGUCUAUGAUUUCCUUCUUCUAUUAUCUUUCCAUUAUCAACAACAAAUAUUCUAUCUGCUCUCUUUAUUGUAGAUAAUCUGUGUGCAAUUACAAAAGAAGUUCUUCCACUCAA
env-11   CGUAUCAAGUGUCUUAUCAAGGCUGAAUAUGUUGAGUGAAGUUCCUGUAAUGAUGUCGGCGUGUCCCUCCUGAUAUCGGAUAUUAAUGAAAAGACCAUUGAUGUUAUCAGUAGAUAAUCCGGCUUCAAUCUGGCUAAGAAGUGCUUCAGUAUCCUUAGUG
env-12   UUAUUACUUUCUAUGAAUGAAAUAAACUCAGCAGCAUUUCUCGGUUUUGAGAAAUAGUAGCCCUGAAUGUAAUCUAUUCCAAGCUCAUCCAGUUUUGCCAACUGCUCCUUCUCCUCGAUUCCCUCAGAAACAAUCUCAAGCUUUAGCUUUUGAAUCAUGC
env-13   UACAUUAAUACAUAAAUAUAACCAAUUCUUAUGUAUUACUUCAACAAAUUUUUGUUAUAAAAUUUCAGCAUAAAGGGAUUGCAAAAUCAACAUUAUACUUAUAAAAUAAGAAUAGACAUUUAUUGGACACGAUGUAUUAUUGAUAUAUAUUUGGCUCACA
env-14   GGUUAUGGUGUCCCUCAAUAUCGCAUAACAGGACAUCAUAAUAUAUUAAGAAACUAUUAACCAGAGAUUGCUAAUAUAGCCGAUUAGCGUGUAACUUAUUUAAUCAUCUGGAGUAGUUGUCUGUAACAGGCAGAAUAGCCGGAUGAUUAAUUGACGCAGA
env-15   CUCAUAUUUGAAUUUCGAAGCAUUCCAAGAGAAAUCAUUGGCCAUGCCCCUGUCCACAAUCUGGUUCCACUCACGCUUCUUAUCGAAGAAGAUGUGCUUAGAAUAAUUGAUGACUGCAAGCAUCUCAUCGGCAUUGUAGUUUGAGAAUGAGAAGCCAUCU
env-16   CAGAGUUUGAUGAGACUAUAAAGCAGAUUACACUCUUUCACAGAAAUGUGGACAGACCUUACAGGAUUCUCGUGCUCGGUUACUCGCCAAAUUUCAGGCAUUUCCUACACAGGCAGGGCAUGUACCGUUCGAAGUACUGGUCAUGCUUUGAUGCGAUAGC
env-17   GGCAGAUAGCUGCCGGCAGAUUUAAUGGCACGAAACCGAUUUUGGCGUUUUCAGAGGAGACAAUAGACCUUUCGGUCAUUGAGGGAAGAAGCGAAGCGGGAAGCUUUGUAAUAGAAAGUACGAAUCAGAUAAAAAUCUGUGGCAUAGUUUAUUCGACUAA
env-18   UUAUUUUCUAAUCACAGUAUAUACAAACAAGAUUACAAAUCUUUAUAAUGACAAGGAGGCCUAUAUAUGAAUUACGGCAAAAAAGCUACAAUGCAGCUGCUCCGGAAAUAUGACAAUAAGAGCUCUAAGGUCAAAAAUAAGGUCAAGCUCAUUGUUCUAA
env-19   CUCAUAUUUGAAUUUCGAAGCAUUCCAAGAGAAAUCAUUGGCCAUGCCCCUGUCCACAAUCUGGUUCCACUCACGCUUCUUAUCGAAGAAGAUGUGCUUAGAAUAAUUGAUGACUGCAAGCAUCUCAUCGGCAUUGUAGUUUGAGAACGAGAAUCCAUCU
Eve-1-2  CACCACACCAUUACACUUUCCUAAUCAGCUUCAACAGAUAUGGUAUUUCACUUUCGAAGUUCACAGCAUAGUUAACUGCCUCAGGGUCAUUGUAAGUGUUGUUUAUACAGGCUUUUGUAAAGUCAGCGGCAAUUUUUAAAGAAUCCUGAAUAUUGUAAUC
env-20   GAAUCAUCGGAGCAACCGGUUAUGCCGGAGGAGAACUGGUAAGGAUCCUGUUAAACCACAAGGAUGCCAAAAUCGUAUGGUACGGUUCCCGAAGCUAUAUAGAUAAGAAGUAUUAUCAGGUAUACCAGAACAUGUUCCAGCUGGUGGAGGAUAUCUGCAA
Eve-1-3  GAAAUGUGUAAAGAAGCAGCAAACAAAGGACUUGAAAUUUAUGGAAUCACUGAACACAGUUCAGGAAUCCCCGGAACUUGUGCUGAUAUUUAUUUUCAAAAUUUAAGAGUUAUUCCAAGAGACCUUUUUGGAUUAGAUUUAAGACUUGGUGCGGAAAUUA
Eve-1-4  GGAUAGCGUACCCCAAAUUUUUAAAAAUAGUGAAUUUUUGAGGUACGUUUAUUGCCAUUAAGCAAGUAUAAGUAACAUAGUGGUUUACAUAAUAAUUAAAAUGAAAGUCAAUAUGUGAUGAAUUGAAGACUGAAGCAAGAAUAUGUGAUGUGAUUAGUUU
env-21   GGAGUGAAAUUUGACAAUAAUGGGCAAACUGGUUUACAUAAAACAAGAAAACAAUAUUAUUAUCGGAUAGCGUACCCCAAAUUUUUAAAAAUAGUGAAUUUUUGUGGUACGCUUGCAAAAAUUACGAUAAGUUAUGUUGAGUUUUGCCAUCAACCCUUCU
Eve-1-5  CAGUGGUUAUAGGAAUAAAUUUUAACAAGAAGCAGUCUAAGAAUAACAAGCAGAGCAAUAGCGGUCAGGCAACGUCUACAUAUAAUGGUAAGUUGCCGGGGAUUGAUUUAUAUUAUGGAAGUCAGAAGGUUGGAAGUCUUAACGGCUACACAAUGGAUAU
env-22   AGAUGAAUCAUUAGACAAUACUAAUGAAACAAAAAAAGACUAUAUAAAAAAAGAAGACACAACUUCAACAGACAAAGAUGAUAAUGAAGAUGAAUAUGAAAAAAUUUGUUAUGUCUGCAGAAGACCUGAGAGCCGUGCAGGAAAGAUGGUAUCAAUGCCG
env-23   CAAUGGGGCUCGGUUUCUGGCCCUUGUUAGGGGGUUGCUUUACUGGGCCCAUGGGCCCGGUUGAAAUAAACGGGAAAAAAAAUUGGGAAAUCCCUCCGGGAACCUUUU.....................................................
env-24   GGCAAGUGCAGGANAGACUCCGAGAGUACAUGAAAUUUUCAAAGGAGGGCAUCCAUGAAAUUUAUCCAUACAGGCGACAUCCACUGGGGAAUGACUCCCGAUGCAGAUAAGCCCUGGGGAAGUGAACGGGCUCAGGCGAUCAAAGAUACCUUUAAGAAGA
env-25   UCAGUUGUGCCCAAAUUUCAGAGGCGUUGGCGUUGCGUCCGCCUCUGAAAUUCGGGUGCAGCUGGCGGAAAAUCAGCCGCAAAGGCAAGUGCAGGAAAGACUCCGAGAGUACAUGAAAUUCCAAAGGAGGUAUCCAUGAAAUUUAUCCAUACAGGCGACA
Rob-1-1  UUCACGUAGGACAGGAACAGCCGGAUCCGGUUACUGACGCAAGAGCCGUUCCAAUCUACCAGACUUCUUCUUAUGUAUUCCGUAACUGCGAUCAUGCAGCAGCUCGUUUCGGACUGGCAGAUGCAGGUAACAUCUAUGGUCGUCUGACAAACCCGACAGA
Rob-1-2  AUGGUCAAAUUCUUAAGAGCAAUCGUUAAAAAUCCUGGAGAAAGCUGCGACGAAAGAAAACAUAUCGAAACAAUCGAAGCUGAAAUGAAAAAGCUGAACUUCGAUGAAGUAACUAUUGAUCCACAGGGUAACGUCAUCGGUUACAUGGGAUCCGGUGAUA
Eve-1-6  AUUAUAGCUUUCAUCGUCAUUGCUAUUCCAACAUUUAUUAACUAAUUCUUUGCUUUUUCUACUGCUAUAAAUCCAAAUGUUUCAGGUCCAACAUGAGUUCCUAUUGUAGGUCCUAUCUGAUAACAUUCCUUAAACUUAAUUCCUUUUUCUUCAACUUUCU
env-26   GUAACUGUAUUAACCAACAAUUUCAUAGACAACUAUAUGCCUGGGGCCAAUGGAGAAUUUGUGAAAGUAUAUAUCUAUCUUCUGAGGCUUCUUUCUGACACCUCUGUACCCUUCAGUCUGGAGCAGAUGGCUGAUCAUUUCUUCUGUACAGAACGUGACA
env-27   UACGUAUAUUAUAUAUGGAAGGAAAACAAAAUGCGAAAAUUAACAGGUAAAUGCAAAUGGAUAUUGGCUGUUUUGAUUGUGGCAGUAUCUAUGGCUGCUGGUUGUAAUAAAACAAAAGCUACAGGUGUAAAUGCAGAUGAUUCAUCAGAAGCAUCACAUA
env-28   AAGAUGAUCACAUCUCCACGCAUCUGCUCCGCAGAAAACAAGACCAUUUUUUGCAUCGCCCGUGACUGAGGUAAUCAGAGCAUCUGUAAUACAAUAUGGAAUCUUAUCUGGACGGCAAUUUUUCAGACAGCCUCUGCAGCGCACUGGCGCUAUGCGUCCU
env-29   ................................................................................................................................................................
env-30   CAAGCUCAUCAGGAGUCUUAUUAAGCAGGCGCGAGAAAAAGAAGGUAAAGAAACGGACAGGAUCAGUCUGUGUCAACACCACGCUAUUAGAUUCCUUUUUAUAAAAAUUCAUAGAGUCAACAACUGUUUGACCGAUGCUUAUGCCUCCUGUUUCAACAGC
env-31   UUUUCGGGUGCACUGCUCUCAAAGCAGAUGUGCCCUUUUUCUUGAUCUGUACAGAGAUUUUUCGCAGCUAGCAGACAUUUUGCCUGUCUGGCAGUUCCCUCUCCUCCAUCAUAAAUGCGAACCGAUUCACCAACAACCUUUCCAAUCGCAGACUUUACAA
env-32   CUACAGGUCUUUCAUAAAAUCUUUUCCUUGUACCUAUAGGACUAUACCCAAGUUUUUCAUAAAGUUUUUUUGCUGCUUCAUUGCUUUGUCUGACUUCAAGAAAAAAAAAUUGUUACUGAUUUUUCUUUCGCCCUUCUUUCCAUUUCUUUAAGAAGUGCUUC
Eve-1-7  GAGAAUUGACACAUUUCCAUAAAUAUGCUAAGGUUUGACAUAGAUAUAAGAACAUAGAGAUGCAUAUGUUUGCAAUAAAUAAAAGGGAAUGAGGUUCUCCCCUCGAUUUAAUCGAAACCGCUUAUAACAAGCUGAUGACUUCUGUGCAAUACACAGGGGAA
env-33   GAGCGUAGAAAGAAUUGUGAAAGUACGAAGUACGAAACAAUUCAUAUGGCAUCUCACAACUUUCAUAACUUCACAAAAUUUGACAGGAGUAAUUAUGAUCUUAAAUGUAAAAAAUCUGACGCAUGGUUUGGUGACCGUGCGAUUUUCGACAAUGUUUCU
env-34   AUCUGCAUGCCAAAGUCUGAUAACUGAUAUACCUUAUCAGGAUUAUUGGUAAGAAGCCUUAAUGUCUUAGCGCCAAGGUCUUUUAGUAUCUGUGCGCCUAUGUAAUAUUCUCUCAUAUCACCCGGAAAGCCAAGUGCUAUAUUGGCAUCAAGUGUAUCCA
Eve-1-8  UUGACGUUAAUCAGCUUUUAAAGGUGCGUCGUGAGAAGCUUGCUGAUUUACAGGAAAGAGGAAAGGAUCCUUUCCAGAUCACAAAGUAUGAUGUAACAAACCAUACUACAGAGAUUAAGGAUAACUUUGAAAAGUUGAAUGUGAACAUGAUGAAGAAGG
Rla-1-1  UGAGCCUCUUGAUCAGACUUCCAUAACACGCCGCCAGUACCUGCUGAAAUAAUGUACCGAUCAUUACCGGAAACAUUACUUCUCCGGGAAAAUAAGCUGCUGCGAUCACCGCCCCCGCACUGAUAUUUCUCAUUCCUGAUCCAUAGAUCAUAGAAAUCCG
env-35   GUAGUUCAGGCUGGUUCGGAUUAAAUCAGAUUAAAUUAAGCGGAAAAUCCAUAGGUAAAUGUGGGUUUUCCGUUUUUUGAUUUUUACUUAAAAAAAUUUCUAUCAAAGUAUCGUGAAAAAUUCAUUUGUAUUGUAAUGCAAAUGGGGGUUAAGUAAAACA
Dfo-1-1  UUGUCAAAUUAUGCGAUUUUUUUGGAAUUAUAGCAUAUUUUGGGAAAACUCACAAUGAUUAUUCUUUUAUAAUCCACUCAUCUUUGAUGCACCACAGUCCUGCCUGCACACUGUGAACACCACUGUUAUCCCCCGCCUCGAUUGCUUGCUGAUGACCAAA
env-36   ................................................................................................................................................................

         ................................................................................................................................................................
         ................................................................................................................................................................
         ••••••••••••••••••••••••••••••••••••••••••••••••••••••••••••••••••••••••••••••••••••••••••••••••••••••••••••••••••••••••••oooooooooooooooooooooooooooooooooooooooo
```

```
Rla-1-2   CAUCAUGUAUUUUACUCCAUAUUAUAACUGUAUCGCAAUGCAAUUCAUUCUUUUUAAUGUGUAAUGCAAACUACUGUGGAGGUGAAUUACAAUGCAGGACAUAAAACAAAAUCUUGCCGAUGCAGUGCGAGAAGCUCGCACAAAACUCGGACUAUCACAG
Rla-1-3   CGUCACGGAUAUUCGACAUUCCUGUCAGAUACAUUACGCAACGCUCAAAUCCAAGUCCGAACCCUGCAUGUCUUGUAGAACCAUAUUUUCUGAGAUCAAGGUAGAAACCAUAAUCUUCCUCUUUCAGACCCAGCUCCUGCAUUCUUGCAAGAAGCUUUUC
env-37    ..........................................................................................
Rla-1-4   ACGCUGCGGAGCUAAAAAACUUCUUAUCGAGAUGGUCAGCGGAAAGCAGGGUGUUCCUGAGCAGGUGCAUUAGCAGGGAGCGUUCCGAAGACACUUUGCAGCGUUUGUUAGAGAAAGUAAAUCGAGUGCCAUUGCACGAAGAUUUCCACUUUCGAGUUAC
Rla-1-5   UACUUCAGACAGCAUUUGACCUUUUUACAGGAAAAGGCUUUGCAAAGACAACCGUAUCCGACAUUGUAAAUCAGGCCGGACUGGCAAAGGGAACUUUUUAUUUGUAUUUUAAAGAUAAAUAUGACUUGCGGGAUAAGCUGAUCGCAUUUAAAGCCAGCCA
Rla-1-6   AUGUCUGUUCUUCCAUUAAUUCCGUCCACGGAUCCCUGAUUCGUAUAUUGCCAUACUUCAAAAUGAUAUGGAGUCUGCGGCUUUAAUUCGUAAUCCGCAUACCAGACCGGAUAUGCGGAUAAUUUUUCCAGAUCCAGCUCAUAAGCUUCCCACAACAUAU
Rla-1-7   CUCAUUUUAUUUCUCAUAUUCUGUUUUUAUUAUUCUUCUGUAUGUUUUGCAGCGUAUUUUCUGCCAAGCAUAUAUACACCAACUGCAUAAACCGCUGUCAGAAUGGCUGCAAUGAUGUAUGCUACAUUCAUUGAAAGUCCGAAUCCGAUCUUUGCACUUA
Rla-1-8   GGAUUUGUAUUUCGUUUUUCAAUUACAUUUUAUGGUAUUUCUUCUGCUCCGGUAACCAGAUCAUGCUCCGCUUACAUUCGACACAUACGCCUGGUCUGCUUCCCAGACAUCAAAGCCACUUCCUCUUAAAAUAUCAACAACCCUUGCCGGAUCAUCGGUC
Rla-1-9   AUAGUUUUUCAAUGCUUUUUGCAGACUGGUGUUCUCAGGAUAUGCCUCCGGAUCUUCCAGUAUUCUUCCAAAAACCCUUUCCUCUGCCUGACGGUUUUCCAGAUUCACAGACUGAAUUUCAAAGCAAUUCUGAAACUGAAGGAUUCCGCUUUCUUUCUCC
Rla-1-10  AAAGAGCCUCUCGGUUCCGUUCCAGCCUUGAUACCGGCUUCACAUGGGGACGGAUCACAAAGAUCUCUCCCUUCCGCUCCAGCACAUCCAGAUAAUUCAUCGUCUUAUUAUACAUAAAACUUCUCCGUAAAAUCGUCCGCACCACAUUUGGAUAAGAACU
Rla-1-11  AAAAUUAUUUGUCCGGGUGAAAUUGCUGAAAAUGGAAGUUCCGAAUCAGACAAAAAUCUUUGCCAAAGAUGAAAAGAAAUACAAACUGAAAAAGGGGAAAAAGAUGAAGACUCAGUAUUAAAACUAUAUGAAAAGGUGGUGAGUUAGAUGGCAGUAUAUG
Rla-1-12  AAUUUUUAUACAUUAAAUCUGAACAGCAUGAUAUCGCCGUCCUGUACUACAUAGUCUUUUCCUUCCAGACGGAUCAGCCCUUUUUCUUUCGCUGCUGUAUGAGAACCGCAUGCAAUCAGAUCAUCAUAGCUUACAACUUCCGCACGGAUAAAUCCACGCU
Rla-1-13  UGCUCUUUUUAAUAUUCCUGCAGCAUGUUCCAUCGUACUUCCUGAUGUAUAAAUAUCGUCAAUCAGCAGGAUAUUUUCAGGAAAAUCGCUGCUGCUCCUCACUGCAAAUGCUCCCUGCAGUACUUCCAUCCGCUCUUUUCUGUCAAACAGCUUCAGUGGG
Rla-1-14  GCCUUUAAAUAGGUAGUUCCAAGUCUGCUUAUGCGGUUUUUCUGAUGCACGAUAUAUCCGAUCUUCAUUUCAUUCUCAUCUGCCAGCGGUACUGCAAUGAUAUCUUUCCCAUUUAAAUUUUCAUCAAUGACACCGCUGCAUACAGUAUAGCCAUUCAGCC
Rla-1-15  CCUGUCACGGUGAUUUCCGUUCCCUCCGUCAGUACUGCAUUUUUAUAGAUUGCAUUUUCACUUCCCGAUUCUACCAUGAUCGAAUAAUUCUGAUAAUUCAGGCUGUCAUAUUCCUCAUCAUACAGUUCUCCGAGAUAGAUCUCAUACUCCAUCACAGCCC
Rla-1-16  UUCUAAAUAUCCUGUAAAAUAUUGAUUCGUGCAGCCGGGGUGGUAGCGGUACCUUGAACCUGCAAUCCGCUAUAGCAGGGGUGAUAUUGUGCAGAGGGCGAAAGCUGGCAGAGCUGCCCCCGAUAAGUGACGUUGAUGUCCGGGUCUUACGCGACGGGAA
Rla-1-17  CCUCUACUGCUUUUGCAGGUUUCUUCGGAACAGACAAUUUCAGGAUACCGUUCUCAUAUUUUGCUUUGAUAUCUUCGUCUGUCAGAUUCUCACCGACAUAGAAGCUUCUCUGCAUUGCUCCGGCAUAACGCUCCUUACGGAUGUAUUUACCUUUCUUAUC
Rla-1-18  AAAAAAUAUAUUUCCGUUCAUAAGCCUGCAACGAUUAUUUAUGAUGAAACUUUCUAUCAGAUACUCAGAGAGACUGCCCGGCAUCCGAUUGUCCUGCGAAUGAAGCUUUAUCCCCACCACGGAACAACGAAUUGCUAUAUUCACUGCCUCCAUGUUGCUU
Rla-1-19  UAUUUUAUCAUUAUUUCAGGUUAAAUUUAAGGAUUUUUCUAAUUUUCAAACAUUUUCGUUAUUUUUCUACAAUUAACUCAUAUUUGCAUUUGUGCAAUUUUAGAAGAUUUUGGUCGUCCACUGGCUCAUAUCCCAGACUUCAUCCACCACAUCCUCAUAA
Rla-1-20  UUUUGUACUUUUGCACAAGUUAGUCAAUUAUAUUUUUCUUUUUCCUACAAAUUCACUUUGAAAUAGGGUAUUAUAAUAAGAUGUCGGGGUCAAAAGCCCCCGACUAUGAUACCUACGGAUUGUUACGCACUACGUGCUCCCACAAUCCUACCCAACAUUC
Rla-1-21  ..........................................................................................
Rla-1-22  CAGUAGAAAUAACGGAUAUUGGUGUGGGCGGUGAAGGAAUCGGCCGCGUGGAUGGAUAUACCCUGUUUAUUAAAGAUGCGAUCAUCGGGGAUGUAGUAGAAGCGAAAGUGAUGAAAGCAAAGAAAAAUUAUGGCUAUGCCCGGAUGAUGGAGAUUAUCCA
Rla-1-23  GCCAUUCUUUCUGCCUGAAACCUUAACUCACUAUUCAGGACUUUUUAUUCUUUAUCUUCAAAAACUUCUUUCACUUUUUCUUUGAAAGACUUCUUCUUUGACUUUUUGUCCUCUGCGGUUUCUGUAUUUUCACUGCGUUUCAGUGUAUUUCCUGUCAACU
Rla-1-24  GUGGGCGCUCUGGAACGAAAGACUCCAAGAGUGCCUUUACUAUGUCAAAGUAUGUAAGAGAAACUGCAGGUGGCAGAUUCUCCCUAUGCAAAGAUAAAUUUUACGAAGGGAAAGGGUGAACAGAUGAAAGCGUUUCUUAUUUUGGAAGACGGUACUGUGU
Rla-1-25  AUUCAGGGCAGCGACCACUGGGUUGUUACUAUGAAGGCAGAUGAUCCUCUUUCACAAAAAAGGAUCUGCUCGGUCUUCCGCUGAUUCUCCCGGAACGUACCACAAUCCAAAGUGAAUUAGCCAACUGGUUUGGCAAUGAUUUUGAUAAACUGGACAUUAU
Rla-1-26  UUGAAGAUAAUAAAAUCCACAACGUUUCUUUUGUUGGUGGAUGUAAUGGAAACCUUCAGGGAAUUUCCCAUCUGGUUGAAGGAAUGGAUGUAGAUGAAGCAAUUUCCAGACUGGAAGGUAUCCAAUGUGGAUUCAAGACUACUUCCUGUCCUGAUCAGCU
Rla-1-27  CAGAGGAUCUGCUCAAAAAUAUUGAACGUCUUGUUGUAAUCAAUUCGCAGCUCGGUACACCGGCUGAAGGAUGCCGUUCGGUCCAGGACCGGCAAAGGCCUUAGAUGAGGCUCUCAGCAUCGCCAGAGAGCUUGGUUUUAAAACGGUUAAUCUUGAUCA
Rla-1-28  CAAAAACAUCUCAAGCAGGUGGAUCUCAUCCGCAUUGCUUCACAGCAGGGCGUGAAGCUUGGGAAAAGUCAUAUCAGCCAGUAUGUAAGCGGGAAAACGAUUCCCCGGAAUGAUAUCCUUCAUUUUCUGGCAGAUACGCUGGAAGUAGAUGCCGGAUGGC
Rla-1-29  CACUCUGAUAUCUACAAUUUAUUUUUCUUCUGCAACAUUCUCUUCCAGAACAGAUGUACAAUGAGGGCAACGCGUUGCCUUGAUGCUGAUCUUGCUCUGACAGAACGGACAGACUUUUGUUGUCGGUGCUGCAGGCUUUUCUUCUGGCUUACUCAGAUUC
Rla-1-31  UUUAUGAGAAUCAAUCGUAUUACAGGUUAUCUUCAGUCUCAGAACCGGAAGUCUCUCCGAUUAUCCUGCUCAAUCAGUCUGAGGUUUUCAAGCACGAUCUUUCUUGCUUUUUCAUUCGGAACAUUCAGGACUUCCUUUUCAAUCAGCCGGUUUCCGAUGG
env-38    AUAAUCUUAUUACAUAUUUUUCACACUGCUUUUUCUGUGGUUACGACAUUUUACCAAUUACAUCUAAUAUAUAUUCUACCCUGUUUAUGUAGCUGUGCUUCUUACUUACAAGUUCAUGUCCUUUAGCAGCAAUCUGCCUUCGUUUAUCUUCGUGUAAAAG
env-39    AUGUACAAUAACAAAAUGUGUUAUGUGCAAUAACAAAAUAUAUGCAGGAAAGGAAAAAUGCUAUGAGUUUAUUAGAUUGUACAGCCGUUGAACUUGCUGCAAAGAUUAAGGCAGGUGAAGUGACAGCGGUGGAAGCUAUGCAGGCUGUUGUUGCUCAGAU
env-40    AUAUAUCUUCAACGCAAACUUAUGAUUAGGAAAGGACAAUAUGAAUCAGGAAAAGGUUAUAGUUAUUGACUUUGGCGGACAGUACAAUCAGUUAGUUGCACGUCGUGUCCGUGAAUGUAACGUAUAUUGUGAGAUAUAUUCUUACAAAACACCUCUUGA
env-41    UACAAUGCAUCUCAUAUAGCGGAAGUCUUUCUGCCAUCUGUUUAAUCAUCGAAAGUGUCUUUAACAUCUUGUCCUUUUCACCGGGACGGUAAGUCUGCUUAUACAGAUCCGGAUAUCCAUCUAUUGCAGAAAUUUUUCUGAUCAUAUUUUCUUCUCCUCU
Rla-1-32  UACAAUGCAUCUCAUAUAGCGGAAGUCUUUCUGCCAUCUGUUUAAUCAUCGAAAGUGUCUUUAACAUCUUGUCCUUUUCACCGGGACGGUAAGUCUGCUUAUACAGAUCCGGAUAUCCAUCUAUUGCAGAAAUUUUUCUGAUCAUAUUUUCUUCUCCUCU
env-42    ACCAAAAAUAAAAUUAUAUAUGUUAUACAAUUAGUAUUUAUCAUUGCUAUAUCAGUCAGCGGUGUUAUAUAUACACGAAAAUAUUGUGAUAUGCAGCAGGGAAUGUGUAUGUAUUCAUAUAUGGAUAUAUAUAUUAAUUCUGUCUGCACUGAUAUUAAUG
env-43    AUUGUUUUAUUGCAGGUAUGUAUGCAGUUACAUUCGAUNAUUAAUAUAGAGGAGUUCAUAUCUGAUGGAUAAGAAAGAAAAGAUAAGAAAUAUUAUAGUAUUUGGGGUGGUUAUGGCAGUCAGCCUUUCUAUUGCGCUUAACGUAGGAAAUAUAUUUGGU
env-44    AGGAAAUGAAGAUAACAUAUUUGAACAAGCAGCAAAGUAUGAGAAAAAAAGAGAAAAGUCUCAUUAUUUUAAGAAUGGAUUGCUGCUUGGUAUAGUAGCUGCAUUUCUUGUAAUGGUUAUAAUUGUUGUAAGUGUGGCUGGUGUGUGUAUACAGAAGGGA
env-45    UGAUAACGCUAGAAGUGGUAUGCAGCAGGCACCUGCUAGAAGUUUGUUUAAUGCACUUGGCUUCACAGCAGAAGAGAUGAAGAAGCCUAUGAUUGGUAUUGUUAGUUCAUAUAAUGAGAUUGUACCAGGUCAUAUGAAUAUAGAUAAGAUUGUUAAUGCA
env-46    UCACAGGAUGUCGUAAAGAGCAGUAAUCAGAAUUACAGGCUUAAAAAUGAUUAUAAGUGGAUUAAUAAUAACAUAUUUCAUAAGACAGGUGCAUAUAUCAUAUAUGGGAUUGCUAUAGUGGCAGGGUAUAUCGGCUGUAAGCUGUUCUUUGGAUUAAGCU
Dfo-1-2   UGAUUCGUGAGCGGGAUCCUGCAAUCAAAUCGAAUAUGGAAGUAUUUUUAUACCCUAGUUUUAAGGCAAUUCUUCAUUACAGAGUCGCACAUAAAUUAUACAAAAAGGGACAUUAUGUUAUGGCGCGCUGGAUUUCUCAAAGAGCAGUCAGAAAGACAGG
env-47    UCAUGUCCAGGAUCGGCGUCCGCAUUAUGUACUGAUAAGCUUCAAAAAUUUUCUCCGCAGGCACGUCUUUGGUAGAAGAGAGCCAAAGCUUCAGGAUCUCGAUCAGUAAUAGAGCAUUAUAAUGUGCCAGCGAUUGCGGAGUGAUCAGAUCGCAGGGCAU
Rla-1-33  AUAUUAUUUAUAGCCGUCUUUCUUAAUAGACAAUAAUAUUCUAAUAGUGUCUUGUGAAUAAGACACUAUUUAUAAUAAUUUGUCUUGUGGAGGAAAAUAAUUUAAUAUAUAAUGGUCUUAUAUGAUGGAGGUGACAAUAUUAUGAACAGAGAUACCCUGA
Rla-1-34  GCAGAAGAUGCAACCGCUUUUUUCAGGCAGUUUUUAUAGUUUUUAAGGAUGAUUCGUAUAAAUCUGUUGAAAAAAGCUGUCUGAUGUUGUAUCAUGAACGAUAUAUGUGGGGACAUAGAUAAUAUAAUAAGGAUUGGUGAAAAGAUGAAAUUAAAGAAGA
Rla-1-35  GAUAUUUCUUUGCAUACUGUAUAAAUCCUCUUCCAUAUUCAUCAUCUCUCAGCCUUGUAACAAUUUCCUGACGCUUCGCAUAAGAAAUUUCAUUCAGAUACAACAGAUAUUGGAUCUCUGCCCAUUUUGCCAUACCCUCAUAGAUCUCCAGCCUGUACUG
Rla-1-36  CACUGACUUCAUAAUCUUCAACCAUGCUGCGGAUAAUCUCUUCCUGAUCCAUGCCUUUCUGCAAUCUUAUCCAAAUUUCUUUGCCGGUAUCAUUCAGCAUAAUCAUGCCACGAAAUUUUUUGGCGGCUGCCCCUGUAGCAACUACAACGGAUCUGCCUGC
Rla-1-37  UGCUUCAUAUCUGCUCCACAGAUUGCAGCCGCUUUGAUCUCAAGGAUGAUAUCCUCCGGUCCGCAUGUUGGAUACGGUACUUCUACCAAUUUGUAGCUUCCAAAUUCUUUACCAAAUCUUGCGAUUGCCUUCAUUUUCAUAGUUCAUUUCUCCUCGUCUG
Rla-1-38  UCAUUUGUAGGAAUUACAAAGACUCUUACCUUGCAUCCAGGCUUUGUGAUCUCACCCUGCUCGCCUCUGAACUUCUCAUUCGCAUCAGGAUCAAGUUCAACACCAAGAUAUCCCAGAUAGCUGCAUACCUGCUCUCGUACAUAGCUUACAUUCUCACCGA
Rla-1-39  GAGUCUGUCAAUCGAAUUGAAGGGAGUAUAAAUUCUAUGACAAAUGAACAAUAUCAUACUUUGAUCCAUCCUUAUACAGAUGCCAUGAACCUGAUCCUUACCCGACUGGAGAUUUUAAAUCACUGUGCCGCUGACAAUGAGGAUGUCCGGCCAAUCCAUG
Rla-1-40  CGAAGUUCAGCUCUGCUGCCGCCUUCUUCAUCUGUUUUUCCAAAUCCCUGACCAGCUUCUCCAGUUCCUUGCGGCUCAUAGAUUCCGGAUCUUUCUCCAUCUUCAGUUCUUGUGCUGCCACCUUUUUGGAAAUGCUGAUCAGAUCACGUACUGAUUUCUG
Rla-1-41  CUGUCCCUGAUCCCACCGUAAUCUCAUAUAUGAUCUGAUAACCUGAUCUGAUCUUUAUUUUCUAAUGCUGCACCAUAAAAUACUCGAGGCUGAUCUCAUUCGUUGCCACAUCCGCAAGAUUUACGCAACUGUUGCCCAUACGGUCGAUCAGAUGAAGAAU
Rla-1-42  GUUGGGAGAAAAUGUUCCGGUGCGAGGAAAGUUAUCUGACUUGAAAAGCCUAAUAUCCACACUUGUUGUUGCGUGAGCAGUGCAUUGACAGGGAGCGUUCCGAAGACACUUUGCCCAACUUGUUAAGGAACGUAAACAGGAGUGCCUCUGCACGAGUGUU
Rla-1-43  ..........................................................................................
Rla-1-44  CUGUAAGCUCGUUGGAAUCUGUAUUGUUCCGGCAAUUGUAUUACAUGAUGUUUCAUACGAAAGCGUAACUGUAUCUGCUUUUUUCAUCUUCUUUUCAUAGAUAUUCCUUUCUUCUAAUCUUUUUGCUUCUUAAAUCUUACGCUUGUAAUAUUUAAAAGUC
Rla-1-45  GUUGAAAGAAAAUAUCCUGAUGCGAGGAAAGUUUUCUGACUUGAAAAGCCUAAUAUCCGCCCUUGCUGUUGCGUGAGCAGUGCAUUGACAGGGAGCGUUCCGAAGACACUUUGCCCAACUUGUUAAGGAACGUAAACAGGAGUGCCUCUGCACGAGUGUU
Rla-1-46  CAGUUGACAACAGUUUAACACUGUUAUAUACUGUUUGCAUAAAGGAGGCAGGUCAUGAAGAUUAUAAUUAACCAUUCAUCUAUGGUGCCGAUCUACGAGCAGAUCAUGGAGCAGAUCAAAGCACAGGUCAUUUCCGGUGAAUUAAAGGAAGGUGAUCUUC
Rla-1-47  CUCUCCUUUGCACCUUUGCAUUAACUCUUUCUGUUCUUCCGGUUCAUGCCACAGACAGUAUUGAGAGUCUGGAAAGCCAAACCUCCGCCCUUGAAAAUGAACUUGCAGGGAUCAACCAGGAUAUUCUGGAUUUGAGCAACCAGAUUUCUGAUACUGAGAU
Rla-1-48  GUUGGAAGAAAAUGUUCUGGCAUGAGGGAAAAUUUUCUGAUUUGAAAAGCCUAAUAUCCGCCCUUGCUGUUGCGUGAGCAGUGCAUUGACAAGGAGCGUUCCGAAGACACUUUGCCCAACUUGUUAAGGAACGUAAACAGGAGUGCCUCUGCACGAGUGU
Rla-1-49  UCUGUGCAGGAACGUCGUCCCUUUUCUGCUGACCCCUGCCUGCAGACAAGCCUUUUAUUUUUGUAAUUUUAUCAAAUAAAGGUAAUAACUGCGGUAAUGGAUCUUCAAAUGCUCCCUCAUAAUAGGCUUUCCCUAAUUUUCUGUAUAGUUCAUCUAAAGU
Rla-1-50  UGUUGAAAGAAAAUGUCCCGGUGCGAGAAAAGCAUUCUGAUUUGAAAAAUCCAAUAUCCGCCUUUGCUGUUGCGUGAGCAGUGCAUUGACAGGGAGCGUUCCGAAGACACUUUGCUCAACUUGUUAAGGAACGUAAACAGGAGUGCCUCUGCACGAGUGU
Rla-1-51  AACGAGAGAUGCAACGAAAUCAGCCUGCUCGGACUAAGAAAAAGUCACAUGAUAUGGAAUUGUAAAUUGGGAAGAGCCACUUUUGUAUUUUCUUUCAUAUCGCCAUCCUCAUUUGUGCCACAACUUGUGGCACAAUUCAGCUUUCACCAUCUGUUACUUU
Rla-1-52  AUUUCUUUGGUGUAAUUUCUUUAUUCCCACCCAUAUAUGGCUGAAGAACCUCAGGAAUCUUUACAGAUCCGUCUGCCUGAAGGUUAUUCUCAAGGAAUGCGAUCAACAUUCUCGGUGGAGCAACAACUGUAUUAUUCAAUGUAUGUGCAAAGUAGCUGCC

          ..........................................................................................
          ..........................................................................................
          ●●●●●●●●●●●●●●●●●●●●●●●●●●●●●●●●●●●●●●●●●●●●●●●●●●●●●●●●●●●●○○○○○○○○○○○○○○○○○○○○○○○○○○○○○○○○○○○○○○○○○○○○○○
```

Rla-1-53	GCAAAGACCUGUACUCUCAUACAAAUCUCUGCAUUUUUAUUCAUCCCGUUCCUGCAAAUUAUCCGGCCCGAAGCCAAACGGAAACAGUUCCCGUAAAGAACAGACUUUAUAAUUUUCCUCGUCCACCGCAAGAAUAAUCUGAAAAGUCUCCGGUUGACAA
Rla-1-54	UUAUUUGCAGAUCAAUAAUUAUCAGAAUUAUCACGAAACCAAGCAGCACACAACUGCAGAAUUUCCAUACAAUACUUAUCCUUGUUCCAUUCCACUGGAUUUUACCCGCGUCCCGCUUCACUGGCACGACGAACUGGAACUGAUUGUUAUAAAAAAAAGGG
env-48	UACGCCUAAUAAGAUAUAAUCACCAUCCAAACAUUCCUGUAUAGUUCUUUCAUCUGUAGGAUAAUAAUAUUCAUCAUGCAGCAAAGUCUUAUUAACUUCAUCCUGUAAUUUAAUAACAGGUUUCAAGUCUCUUUUUGGUUUCUUGAUCUAAAUCCUUCUU
Rla-1-55	....
Rla-1-56	CUGCCACCGGUGCAUGAAACAGCUUUUUAUUUACCAAAAAUCCCGGAUUUUUCCGGUCUGCCCAUUUAUAAGAAAUAAAAUCCGGUCUGCACAUACAGUUGCUCAGUAAAUAUUUUACGCAGAAUCUUAAUGCAUAAUGCGUUGUAUCCGGCUUCCUGGU
Rla-1-57	ACUGCAUAAAUAAUCCCUGAAGUGCUGCUAUCAGUGCAGCGAAAAGAAUGUUUCCAAUCGCAAUAUACCAACGGUUGACGGGUGCAACAAUAUUUUUAUACAAACUCUUGACUUUUUUUAUGAAAAGAAGGCAAACCAAAUGAACAAAAAAAGCGAAAAU
Rla-1-58	GAGCCUGUGCGAUUGCUUUUGCAAAAGCCGGAUAUCAUGUAUUUAUCAACUGCCGUCAAAAUAUCGCCCUUCUUCAUUCUUUACAGGAUGAGAUUCUUUCCUGUGGCGGUACCUGUACCCUUCUUCCCGGAGAUGUCGGUGAUCCGUCCCAGGUAAAAUC
env-49	AAAUGAUGGGGAAAUGUAAAAUACAUGAUAAGGUUACUUGUUUAGAAUAUGUAAAAUUGGUAUAAUAAGUUAUAAAACAUAAGUGAGGGAAUCAUAAUGAUAAAAAGCAUGACUGGUUUUGGCAGAAGUGAGAUUGCCAGUGGUAACCGUAAAAUAAUGG
env-50	AAAUAUCGCCAAGAUUGAAUAUCACAUUUUUAUAGUACGCUUAAACUCAAUAGGAAGAGUAGUNCACUACAGAACUCUUUACGCACGGCGGUGAUACAGAUUGGUGAUGGCGCCUCCUUAUACCAUGGGAUGGCCGAUCUUAUGGGCGCACACAGCCCUU
env-51	....
env-52	CUCGGCCCCCAGCCGAGCGCGCUACCAAGCUACGCCAUACUCCGUUACUAUUUGUGAACCUCUAGUAUUAUAGCAAAGAUUUUUCAUUUUGAAAAGCCCUAAUUUUAAUUUAUUCAGGAGAAUGUAAAAUGACACAGGAAAAUGCUGUUAAGAAACGGAU
env-53	UCUGCAGGAUCUUCCACUUUUCUUGAUGCUUUGCCAAGAAUCUUUACUCUCUUUUCUCCCGCACUCAUAUCAAAAUAGUUGUCCGACAGGAUCAGAUCUUCGGACAGAUUCAGGAUCUCCACGCCUUUCGCGUAGGCUUUCGCGCUGACUACAAUCUCGU
env-54	UAAACAGGAGCUUUCUUCUGCCGGCUUAAAAUUUAUGGAUUUUGCUAUCCGUUUUUUUAAUAUGGCUAAAUGCCAGUAUCAGCAGCAAAACCAGGUAAAAGCAAAUAAAGCAGGAUUUCAGGUUCUUUUUAUUUUAAGUAUGCCCGAAUAUACACAGCCU
env-55	ACACGCCGAACACCGAAAACGAAAUAAAAUGGUGUGAACAUGCCAUUUUAUGGGAGUUUGAGGUGUUCGAGGAUUGCGGGAAUUGCAUAGCAAUGGAGCAAUCCGGUUACAUCAGUGAGUGGCAAAAUUUACUUUUGACACUCACAUCAUAUAAUGUAAU
env-56	AAUCACAAAGCGGAAAAAGCUUUGCCCCGGACAUGGUCAGUGAGAUACGAAAACACCUGGCCAGUCAUCCACAAAAUAUCCACAAAUUAACAAAAGAAACAUCCACAGCUUCAAAAUAUCCACAGCCACCUUAUGUAGGAGUUAUCCACAGACUAGACCG
env-57	GGAAAUGGAAGUAGUAUAAUGAUGGUGUAUAAUCUUUGUGAACAAGAAGGAGAUCAUAAAAUGAGAGAGAUCCGUUGGGCAGUACUGGGAACAGGUGUGAUCGCCAAUGAGAUGGCAGCAGCACUGGAAAAAAAUGGGAAAAGCUUUAUGCAGUGGGUAC
env-58	CACGCCGAACACCGAAAACGAAAUAAAAUGGUGUGAAUACGCCAUUUUAUGGGAGUUUGAGGUGUUCGAGGAUUGCGGGAAUUGCAUAGCAAUGGAGCAAUCCGGUUACAUCAGUGAGUGGCAAAAUUUACUUUUGACACUCACAUCAUCAAUAUAAAGG
env-59	AUUGGCCAUAAAGAUAUGGGAACCAAUCUUCCAGAACAGAAGGAAGCCGUAAAGCGCACUGCAAAAGUGAUCGCAGACUUUAUUCAGGACGGCUGGCAGGUUGCCAUCGUUCACAGCAACGCACCACAGGUAGGCAUGAUACAUACAGCAAUGAAUGAAU
env-60	....
env-61	AGAUAUAUUAUCGCCUCCCAUCAUUUACUGGCCCACGGCCUGAAAGACACAUUAAAUUUUCUUACUUCCAUGGAUGGCAUCAUUGAUAUCAGUGCCUAUAUGGAUGACACCGAUCUUCCAACCCAGAUCAAAGAAUUAUUUGAUUCCUUUGACCCGGAGG
env-62	....
env-63	GAUCUGAUCUAUCAUCUGUUCUAUAACCUUUUAAAUCAGAUCUACCAUGAUCUUCUUCAUUUUCUUCCUACAAACUUUUUUAAAUAAAAUUUACAUAUUUGAUCAAUAGACAGGUACAUUUUAUAUUGAAAAUUUUAAAACCAGGCUAUAUAAUGUAUA
env-64	UAUCUGAUCUAUCAUCUUUUCUAUUACCUUUUAAUCAGAUCUACAUGAUCUUCUUCAUUUUCUUCCUACAAACUUUCUUAAAUACAAUUUACCUAUUUCUAUCACUAGACCGGUCCCUUUUAUCUUGAACAUUUUAAAACCCGGCUAUAUAUUGUCUCAC
env-65	UAUCUGAUCUAUCAUCUUUUCUAUAACCUUUUAAAUCAGAUCUACCAUGAUCUUCUUCAUUUUCUUCCUACAAACUUUUUUAAAUAAAAUUUACAUAUUUUUAUCAAUAGACAGGUACAUUUUAUAUUGAAAAUUUUAAAACCAGGCUAUAUAAUGUAUA
env-66	....
env-67	CAGUAGUCUAAGCCGCCUACUUUGAUCCAGCCGGUAGCCAUGUAGCCUUCUGCAUCGAAAUAGUACCACUUUCCGCUUACAUCUUUCCACUGAGAUGCCGGGUAGGAACCAUCCGCAUUCUGCCACCACCAGCGGAUACCAUCAGCCGCUUUCUGCCAGC
env-68	UCUACAAGCGUGUAUCUAUACAUUUGUGUUUCAUAAGCCGCACACUCCAAUGACGCAACAAGGAGACUGCGCCCGGACAGAUGUUUUUCCCAUUCUGCCCAACCACAGUCUCCUGUAUGUAUUUCAUUUUAAUUCUUCUCGACUGAGAUAAAUUGCCUUA
env-69	ACAUGGUUCAUUGCGUCCAUAACGAUAUCGGCCUGCUCCUUCGGAACUUCCACGAAGGUAUAUUUGUCAUACAUAUCGAUACUUCCAACGAGACGGC..................................
env-70	AAUGGAAACACUAAAAAGAAGGGAGAAGUUUUGGAGAAAGAAAAAAUGAAUGAAAAAAAACAAAAGCAAAGAGGCCUGAAACAGGGAUUUGAGCUGAUUUAUAAAUAUCGCUUUGUAUUAUCAUUUUUGCUGUUAAUCAUGUUGGUUUCAUUCAAAAUCA
env-71	AGUUUCUUUAAAUUUUCUUCACAUAGACAUUCAUUUGUAUUUCCUGCUGCAAUUACCUGCCCGUCUUUUAGCAUAAUCAGACGAUCACAAUAAAUUCCAGCCAGAUUGAUAUCAUGAAGCACCAUAAGCACAGAUACGCCUUCCUGUCUGUUUAAUUCCA
env-72	CAAUCAGUCCGAUUGCCUCGUUAAUGCUUUUCACUCCAAUGAUUUUGAUCCCACCAAUACUCUUCACGCUAUCCAGAGAUACUUCUGGCACAAUACACGUCUCAAAUCCAAGCUUCUUCGCCUCUGCCACCCGCUGUUCCGGCAUAUUUACCGCACGGAC
env-73	UUGCAUAUAUGUCACUAAUUAAUCCGGAGGACCAGUUAGAAGCCGGAUAGCACCAUGCAGAUGCUACACAGUAGUCAUACAGAUGUUCCAAGUGAAAAACAGCUCUUUCCUGAAUCACAGCAGGUACUGCACAGCGUAUACGGGGUU............
env-74	CUGAGCGCGGAUUCACCCGGAGAUCUGCUACUACGCG..
env-75	GGCUGUCAGAAAGCCGGACGGCGAGAUCGUUGUNCAGAAAGAUACCUAUGUGAGCAUGACAGAAAAAGUAAAGUUUUUCUCCCUGCCGUUUGUGAGAGGAAUCUUUAGUUUCGCGGACUCCAUGAUCCUGGGAAUGCGUUCCCUGACAUGGUCGGCGAGC
Aca-1-1	AAUACGAUCCGGCAGAAGGUGAUCCGAGUCAGGGAAUCGAACCGGGAACAGCUUUUGAGGAUCUUCCGGAAGAUUGGGUCUGCCCGAUCUGCGGUCUUGGAAAAGACGUAUUUGUAGAAGCAUAAAUUUUUCUUUAGCAUAGUAUUCAUUAUAACCGGAG
env-76	GGGGCAGAUUGUUAAGAUAAAUGAUGGAUAUGCAAGAAACUUUGUUUUACCUAAGAAGUUAGGUAUUGAGGCAACUCCUAAAAAUCUUAAUGACCUUAAGUUACAGAAGAAGAGAGAAGAAAAAGAAGCAGCAGAGGAGCUUGCAAGAGCUAAGGAACUU
env-77	CAGCGGAAUUCGAAUGUUUGGAGAAUUGAGGGAGCAGCAAAGCUGCGGAGCAAUUCGUAGGUAUUAUCAAAAGUGGAGGGACGAAGAGCCGUUCGUGCUAGCACGAAAAAGGCUUUGUGUAUUUCGACCCGCCAAACAUGAGAAUGGAGCGAUUUACGA
env-78	CGGAAUUCGAAUGUUUGGAGAAUUGGGGGGAGCAGCAAAGCUGCGGAGCUAUUAGUAAGUAUUGAUCAAAGUGAAGGGACGAACAGCCGUUCGUUGAAAACUGAAAAGGCUUUGUUGCUAUUCGUGCUGCAAAUCAUAAAAAUGGAGGGGAUUUCCGAAG
env-79	CAGCGGAAUUCGAAUGUUUGAAGAAUUGCGAGAGCAGCGAAGCAGCGGAGCAAUUCGUAGGUAUA..
env-80	GUAAUUCGAAAUGUAGGAAUAUUUCCAUCGGGAGCGAAGUAGCCGAAGUGAUUAGUUGUCCUUAACUGAUAUCCCCGAAAAGAACUUGUUUGUGGUACGCGUGGAAAUCAUCGCUACCC..........................
env-81	UUUCUUUCAGGAUUAUCCGGAAGAUACCUGUCUGGCAGCAAUGAUGACCUGCAAUGGAUGCAAAGACGCCAACCCGACAGAACCCAUAGAAGACAAAGGAAUCCUGGAAAAGAUAGACAGACUGGUCAGCGAAAAAAUCAGUACCAUCCACGUAGGUGUC
env-82	....
env-83	UAUGAUAUGAGCGUGAAACAUAUAUUUAAUGUAUGUAACAAUUAUAAAUCUAAAAAACUAACAUGUCACAAAUAAGAAUACUAACAUGUUAAUAGAGGUACAACUGACAUGUUAGUAUAAUUUAAAUGUAAAAGGGUAUAAUUUUUAGUAUAAAAAAUUGG
env-84	....
env-85	....
env-86	GGACUUGAAAGGAACCUCCUUGAUCCCUUGCCUCCGGCGGCUGGCGCGCAAACGGAACCCGACGUUCCAUUUUCGGUCCCUUUAUUUC..
env-87	UGACCUCUCGAANAAAUAUUUCUCCACUAGUGUACUGUAUCAUUCACUUUCAGCAAAAUGACGCUGAAUGAAUUUUCAUUCAGCUUCCUUGCACAUGAAAAUUUAUCCUGCGUGUAAGGGAUCGGUAAUUAUCGUGGAUUAUACCAGGUUCCCCUAAUUU
env-88	UGGAGAGUAUGACACCUUCAUAAAAACAGUAGACAUUACACCUAAUAUCAGCUAUAGAAAGCUGGAAGUAUGCUUCAAGAAAUCA..
env-89	ACCAAAAUUACGAGGAGAAGGAACAUGAAAAAGAAACAAUCAUUAGAUACCAUUUGCAUUCAGGGCGGCUGGCAGCCGAAGAACGGAGAACCGAGACAGCUUCCAAUCUAUCAGAGUACCACUUUUAAAUAUACCACCAGCGAACAGAUGGGACGUCUGU
env-90	CAACAACCUUCAGGAAUAAAUAGUUGAUCAGCCCAAACCGUAUGAGCUCGUCUGCACUUAGCGAGUGGAUGGUCGAAUGACCAUUCAGGAGCUUAGUACAGCUACGUGCGAAUCCGAGCGAGAGCGGGUUUGGGAUGAUCAACUAUUUAUUCACUUCUAU
env-91	AAGCUUCUUUUUUAUUUUUAUAUUUCGGUACGUCCUUCUAAUGCUCGAAGUAACGUUACUUCAUCUAUAUAUUCCAGAUCUCCUCCAACCGGAACUCCGCUUGCGAUCUUACUGACUUUGAUGCCUGUUGGUUUGACAAGUUUGCUGAUAUACAUCGCUG
Csp-2-1	AAGCUUCUUUUUUAUUUUUAUAUUUCGGUACGUCCUUCUAAUGCUCGAAGUAACGUUACUUCAUCUAUAUAUUCCAGAUCUCCUCCAACCGGAACUCCGCUUGCGAUCUUACUGACUUUGAUGCCUGUUGGUUUGACAAGUUUGCUGAUAUACAUCGCUG
env-92	AAACUUCUUUUUUAUUUUUAUAUUUCUGUACGUCCUUCUAAUGCUCGAAGUAACGUUACUUCAUCUAUAUAUUCCAGAUCUCCUCCAACCGGAACUCCGCUUGCGAUCUUACUGACUUUGAUACCUGUUGGUUUGACAAGUUUGCUGAUAUACAUCGCUG
env-93	AUUUCACUUCCAUGGUCAGUUGAAGUUCCAUAAUCAAAAAUGACCACUGCAAUCUCUUUCAUCUGGUCUUUCAUGCCUGUCAUGAUCUCUCCGGCAUAAGCCAGUUUUUUACCAUUUUUUUCCAAUUCUCCCGUAUCAGGAUAACAGUUCCACUCUUUAU
env-94	AGAAAUCAUCCGAAGUUUCCAAUGUUCUUAGGAGCAGUAGCUCAGAAUGCAGUUGAGGAAGGCACAAUCAUCGAGAUCAAUGUGACAGCACCGAACGGAGCUCAUUACGAGACAAAUCUGAAGCUUCGUGCAGAAGAUCUGGAAUUUAUCCGUGCACUGC
env-95	CAAAAUUACGAGGAGAAGGAACAUGAAAAAGAAACAAUCAUUAGAUACCAUUUGCAUUCAGGGCGGCUGGCAGCCGAAGAACGGAGAACCGAGACAGCUUCCAAUCUAUCAGAGUACCACUUUUAAAUAUACCACCAGCGAACAGAUGGGACGUCUGUUU
env-96	UUCAAAAAGUUCUAUUCAUGUGAGUGUUUGUCAGUUUCAGAAGGAUCUGAAUUCUCUUCAUCCGCUUCUUCCUCUGUAUCUGCUCUUUCAGGAAGAUAAACAUGUACCGUUUCUACGCGGUUCUUAUCCAACUUCUCGACCACCAGACGAAUUCCAUCUU
env-97	UCCUGCAAACUUGCUUGCAGGAGGUUUUUCUGACCUUUUGAACAUAGUAUUUUUGAACCUGGUAUCAUAAGAUCCAGGAUCAUCUUAAUUUCCAGAUAUCACGGUCAUACUCCCCGAUCGUUCUGUCAGAGGAGAAAUAUCCGGCCUGGCUGAUAUUGAC
env-98	AUGAAAGUGUUGCCAAUC..
env-99	UGGAGCUUUAUGCAAGCAGUGUAUCAGAUGUGGCUCUGUGGAUAGAGGAUCAUAUCUGUGAAAAAAGCCGGUCUGAUCAUGACCUGGGACAAAAGUGUCCUGGAAAUGAUUGGCCGGUUUUUUUGUUAAAGCGUUAUUCGCAAACGAAAAUAACGAAAG
env-100	UUUCCCGCAAAGACGCUGGCGCCACUCCGUAAUGCUGCUUGAACUUCCGGUAAAAGUAACUGGUAUCGGGAUAUCCAACUUCUUUCGCAAUGUCGUUGAUCUUCAUGUUCGUGUUCAGUAUUAAGUCUUUUGCAAUUUCUAUUUUCUUGCUGCUCAGAUA
env-101	UUAUUCACUCGAAUAUGACCUUUUCAGCGAAGUACGGGAACAGAUUGCAGCAGAGGUUGUAAGAAUACAGAAUACAGCAAAGUCUGUCGCUAUGGUGGAUGCGCUUGUUUCGCUUUCAGUUGUUGCGACACAGAAUAACUAUGUAAAACCAAAGAUUACU
	....
	....
	●●○○

```
env-102   AAGCCAUUUUUUGAAGAAAAAUGAGCGAAUUACGAAUGUUUUUAGAAUUGCGGGAGUGCAAAGCACGUAGCAAUUCGGUAUCAGAUGGGUCAAGAUACCUUUUGACCCCAACAUCAUAACACUAAACAAAGAAAGAUAGGAGAUUAAAGACCAUGCAGGA
Ceu-1-2   GUGGUGUGCCGACACAGCCGGACAAGACAAAUUCCGAGAGCAUGGUGAGCAUGCUCAAGCUUAGAAUAUAUGAGAGAGAAAACAGAAAAGAAGAGCCAUAUCUUGUUCCUAUACACAGGCUGGACAGACCUGUCGGAGGAGUUAUGGUAUUCGCAAAGAC
env-103   GUCAUUAUGAAAAAAAGUGCAAAAUUAUUAUCCGCCGUUAUGGCAGGCGCUAUGGUUCUUUCCNNNNNNNNNNNNNNNNNNNNNNNNNNNNNNNNNNNNNNNNNNNNNNNNNNNNNNNNNNNNNNNNNNNNNNNNNNNNNNNNNNNNNNNNNNNNNNNNNNNN
env-104   GGACUCGGCAAAGGUAUUACCGCCGCAUCCCUUGGACGUCUCUUAAAGGCAAGAGGUUAUCAGGUAACGAUGCAGAAGUUAGAUCCUUAUAUCAAUAUUGAUCCGGGUACCAGACUCUUAAAGGAGCAUGGA..........................
Ceu-1-3   GAGCAUACCGCAAGAGUCAAUGCCCAUCAUAGCUCUCGGUGGAAGAUUUGUGAUAGCGAUAAGAGUCUUUCCAACCAGUUCUUCCGGCUCAUAAAAGCUAUGAAUACCGCUUAAAAUAGUUCUGUCUGUGCCUGUUCCGUCAUCAAGAGUGAACUGUAAC
env-105   GAGCAUACCGCAAGAGUCAAUGCCCAUCAUAGCUCUCGGUGGAAGAUUUGUGAUAGCGAUAAGAGUCUUUCCAACCAGUUCUUCCGGCUCAUAAAAGCUAUGAAUACCGCUUAAAAUAGUUCUGUCUGUGCCUGUUCCGUCAUCAAGAGUGAACUGUAAC
env-106   CAGCCCAGUCUGCGUCUCGGACCCGCGGUGCACUUGUAUGCCGCACAGACCAGGGACUUCUGAUCCUGAAACAAUUUCACGGCUCGGAAAAAAAACUGCAGUUUCAGCAGGAGCUGCUUCAAAAGCUUGCCAAAGAGGGCUGCCAGGUGGAUACCUUCCU
env-107   GCCUUAAGCUGUUUUUCCAUAACCCACUUCAUAUACUCCCCUUCCAGAUACCUUCCCGGCCGGACAGACUGCAUAGCCUCAUUUCGCUUUAUAUCCUCUUUCAUCCAUUCCUCUAACUGCUCCAGACUCAUUUUUCCAGAUCGUUCCUUUAUCUUUUCCU
env-108   UACUGACUGUGCGGAUGAUGGGAACCAUAUCAGUGGAAUAUGAAGGGGAGUUUUUUCCUGUAGGGACUGCUCUUACAGGGAAGAUCCUGCAGCUUUUCCUGAUCCUUUUGUAUGCAGGAAAGAAAGGAGUGGGCAGGGAAGAACUGUUAGAUGUAUUUUA
env-109   CUUAUGUUUUGCAGCUGCUAUCCUGGAUCAUUAUUUGAUAUUUUAUAUUUGAUAUUUAAUAAUGUAUCCAUUUCGUGUUUUUAUAAUAAAACAGAACACCUUGAUUGUCAAUGGUCAUUUAACAAUUCGGCGUUCUGCAUACUCUUUUUGAUAUUUUAUU
env-110   GGCCUUAAGCUGUUUUUCCAUAACCCACUUCAUAUACUCCCCUUCCAGAUACCUUCCCGGCCAGACAGACUGCAUAGCCUCAUUUCGCUUUAUAUCCUCUUUCAGCCAUUCCUCUAACUGCUCCAGGCUCAUUUUUCCAGAUCGUGCCUUUAUCUUUUCC
env-111   UUUGUUUGAUCCCAUUUAUUUGAUCUUUAUUUGAUUUCUGGUUGUUUUAUUCUUACUUAUAAAGUGACAGUCCAUCAAAUAAAAUAACCGGAUCAUCCCAUUCAUUAUCCUUGAUCAGGGAUACUUCUACACGGAUCUGCUUCUGUCCGGAUACAUCCAC
env-112   UUUGUUUUAUUUCUAUUUAUAAAGUGACAGUCCAUCAAAUAAAAUAACCGGAUCAUCCCAUUCAUUAUCCUUGAUCAGGGAUACUUCUACACGGAUCUGCUUCUGUCCGGAUACAUCCACACCAAAAUAUACAUUCUUUUCAUUAUGAUGAAUAUCCUGU
env-113   UCAAUAACACCUUCUCCGAAUACCUCCUUCACAUCACUUACAAUAAGAAACGCCCGCUUGUCAUAUUCCCGGACAAUAUCCCUAAGCCAGACGAUCUCUUUUCUGGGAACAACACAGAACAGCAUGUCCCUUUCUGUUCCGGAAUACAUUCCUACUGACU
env-114   GACAGUCGCACCCGAAUUCUCGAGGCGGACGCAAAGCCUAAGCCUUCAGAAAUUGGGGCCACUUGACACAAAAUCUUCUCACCAAGUCAAGUACA.......................................................................
env-115   UUCCAAUGAGAAUAGCGGCUCGCAUAAAGGGCUUCAU....................................................................................................................................
env-116   CAGAAUCCAUCCACCGCCUGUUUUACAUUAAAGCGGUUCACUACUGUAUAACUUUCCCAUUCCCUUGGAAAUAAUGCCACAUAUUCAGGCAGCAGUUUACGGUUAUCUAAAAGGUCGAUCACCCCUUUAUCCUUUACGGUACGGAUGACCCGGCAUGCUG
env-117   .....................................................................................................................................................................
env-118   UACUGACUGUGCGGAUGAUGGGAACCAUAUCAGUGGAAUAUGAAGGGGAGUAUUUUCCUGUAGGAACUGCUCUUACAGGGAAGAUCCUGCAGCUUNUCCUGAUCCUUUUGUAUGCAGGAAAGAAAGGAGUGGGCAGGGAAGAACUGUUAGAUGUAUUUUA
env-119   CACAAGAUACCAUACAACCGACUAGAAAAAACAUAAAAAGCAGGAAACACAUACUAGUAAUAACCCUAUACCACAUAACCCCUUUGGGAAAAAUAACUAGCACAACAUAAUAAACAAGGACACGGACAACAAAACACGGUCAAAUCUAAAAAAAGGGGAC
Dfo-1-3   UUCGUCUUCAGUUUACAGAUAUGUAUGGAACUUUGAAAAAUGUAGCAAUCACCAGCAGCCAGCUUGAGAAGGCGCUGGAUAAUGAGUGCAUGUUCGAUGGUUCUGCCAUUGAAGGUUUUGCGAGAAUGGAAGAUUCGGAUAUGUAUCUUCGCCCGGAUCU
env-120   UAGUCCGGAAUGCGAAUGUGUGUCACGGAUCCGAGAGCACGAAGUGCGGAGGAGCCGUGUUAAUACCCGGCGCUAAGAAUGACGAUAGUCCGGAAUGCGAAUGGGUGUCACGGAUCCGAGAGCACGAAGUGCGGAGGAGCCGUGUUGAUAUCCUGCUUGU
env-121   GAUCAUACGACGGACCAUCUCACCGCCGAUGCUUCCUGCUUCCUUAGAAGUAAGGUCGCCGUUGUAACCCUCUUUCAGGUUAACACCCAGCUCAGAUGCUACCUCAGUCUUGAAACGGUCCAUUGCAGCCUUUGCUUCGGGAACUUCUACUCUAUUGGUU
Ceu-1-4   UGCGGAAUCUAUCGUUUGUCUGAGCACACAAAGAUGCCAACCUAUCGCCCAAGAGGACGUGUUGAUUUUCAGAUUAUAUAUAUUGCAGCCGGAUGCGGACAUUUUCAUUUUGAUACUGUUGAUAACGAAACAAUUGUUCCCGCUGGUAACAUUGUCAUAU
env-122   GAUUAUUAUAUGUAAUGUAUAAUGAUAAUCACGAGUUCGAAGAACGGAGAAAUCGUGAUUAUUAUAUGUAAUGUAUAAUGAUAAUCACGAGUUCGAAGAACGGAGAAAUCGUGAUUAUUAUGAAUAAGACGGAAUCGUGUUCAAUGAUGCAUCGGCAUAA
Csp-1-1   AAGAAAUAAGCGGAUGACAGCAUGCCUGCUAGCAGGCAAAGCUGUGUAAUUAUUUACACUAUAUAAAAAUUACGAAAGGAACAGCGGUUAUGAACGGAAAUCCAUCAAUAGAAAAAGGCGACUAUAUCAAACCGAAAAAAGCCUGGCGGUUGUUGAAGAC
env-123   UGAACUUGUACUGCGUAUUUUCAAUAAUAUUAAGGGCCCCCCAUGAACCCCAAAAUUACUCCCACUUUUAGAUAAAAUAAGAUCAUCCGACUUUCUCCCAAAAAAG.............................................................
env-124   GAGCUUGUACUGCGUAUUUUCAAUAAUAUUAAGGAGCCCCACAUGAACACAAAAGUACUCACCACUUUAGAAUAUAAUAAGAUCAUCGACCUUCUCACAGAGAAGGCCGACUCUGAGCCCGGUAAAAAACUAUGCAGAGAGCUGGUGCCUUCUACCGACC
env-125   UUAUGAAAUGUGGUCGAGGGACAGGGAAAUGCUUAGAAUAUUAAUUGCAGAAGAUGAGGAGCCCAUUGCCAAUCUGAUCCGGAUCAAUCUGACGAGAGCCGGGUACCAGUGCACCUGGGCACCGGACGGACAGACCGCAGCGGAUCUUAUGGAGCGGGAG
env-126   UGCCGUCACAAGAAGACCGGAGCAAGGGUGGCACUUGUAUCCAAUGAUGACGAGAACAAGGUAUUCUAUAUCGGUUUCCGCACCACACCCAAGGAUUCCACCGGUGUAGCCCAUAUUCUGGAACAUUCCGUAUUAUGCGGAUCUAAACGAUUCCGACAAG
env-127   CCGGAGGAAGCAAUCCGUCCGAAAGGAAAAUGGUUCGGCCGCGGCAUUUACGGAAGCAAGGAUGUUCCCAUCCGCAUUCUGGACGGCCUGAUCGGUGUCCUGAUCGUGGUGAUCGUGGGAAUGAUUAUUUUCUUCGCGGUACGCGGUGGAUUCAACAUUG
env-128   GCCAGCGGCAAGGGAAUUCGGCG..................................................................................................................................................
          .....................................................................................................................................................................
          .....................................................................................................................................................................
          ●●●●●●●●●●●●●●●●●●●●●●●●●●●●●●●●●●●●●●●●●●●●●●●●●●●●●●●●●●●●●●●●●●●●●●●●●●●●●●●●●●●●●●●●●●●●●●●●○○○○○○○○○○○○○○○○○○○○○○○○○○○○○○○○○○○○○○○○○○○○○○○○○○○○○○○○○○○○○
```

alignment positions 633 · · · 671

```
env-1     GCCGCCGGUGAGCACCUGCCAUAUUAGGGAGCAAGCCGA
env-2     UGUAUGCAUGCAUGCAUGCAUGUAUAUGUGUAUGUGUGU
env-3     CAUGCAUGCAUGCCUGCAUGCAUGCACGCACGCACGCAC
env-4     GCGAGGGCGCUGCGUGUGCCGCUCCCAAUCGCCGACGUC
env-5     UGGGUUCCCGGAACCUCAGAACCGUUCCUAGAACCUUGG
env-6     GUAGGUAGGUAGGUAGGUAGGUAGGUAAGUAGGUAGGUA
env-7     CCUGACGAGGCAAAGAAGCUUGGAGUUGAGGUGAUUCCA
env-8     AUUGUAAUUGAGAAGAAUAUGGAAAUAAUUCACAGAGAA
Ceu-1-1   AAAUGGUUAUUUGGAAUCAGAUAAGGCGAUCGUGACAUG
Eve-1-1   AGUAGCAUGGCAAUAAAGAAAUCACACAAAGGUCAGGGU
env-9     UUCCUGUACCAAAGGAGUACGGCGGACAGGGCUGUGAUC
env-10    CAAUUUAUCAAUCCCUUUUUGAACUAUUAAUUCUGUUUU
env-11    GACAUAAGCAUAACAAUCUUCAUUGAUGAAGGCACUUUG
env-12    CCAUUGCGGCAUCCAUAACAGGCUUGCCCUUUCCGUUGU
env-13    UAUCAUUUCAGGAAAUGGACUUCCUGUUAUUAUGCGCUG
env-14    CAUUUAAUCAGGAGUGAAGGCUAUGAGAGCAACAAAUAC
env-15    CCUGAAUUCUCAUACUCAUUGUAAGGCUUAACCGUAUCC
env-16    ACAGGUCAGGAGAAAGAAGGUCGCUGUACUGUCAUAUCA
env-17    UCCUAGGAUGGAGUGUCUGAAUCCGCACUUCGAGGGCGA
env-18    AGAUUCUUCUGGUUGUAGUAAUUGUAGCAKGCGCAGCCG
env-19    CCUGAAUUCUCAUACUCAUUGUAAGGCUUAACCGUAUCC
Eve-1-2   AUUCAUUAACGCUCCGGUAAAGGUGCUGGCAAAAACGUC
env-20    GGAUGACAAUCUGGAAGAACUGGCGAAGCAGGUAGAUGU
          .......................................
          .......................................
          ○○○○○○○○○○○○○○○○○○○○○○○○○○○○○○○○○○○○○○○
```

```
Eve-1-3    ACAUAAUUGACUACAAUGGUACACUUGACCUUGGUCAGG
Eve-1-4    ACAUAACUUGAAAACGUACCACAAAAUUUGGGAAAAUGC
env-21     ACUUAAGUGACACAUUUUUGUUUUUAUGCUAUAAUACUU
Eve-1-5    GGAAGAAGAACAUAUGCGUGAUGUUAUUGUCCCUGUUAA
env-22     GGAAACAUAUAUGUAUGUACAGAUUGUUUGCAGAGAAGU
env-23     .......................................
env-24     UAAUUGCCCAGGCUGGGAAAAUGCAGGCGGACUGUCUGU
env-25     UCCACUGGGGAAUGACUCCCGAUGCAGAUAAGCCCUGAC
Rob-1-1    AGAUGUUUUUGAAAAAAGAAUUGCAGCUCUUGAAGGUGG
Rob-1-2    AGAUUCUCGCUUUUGAUGCCCAUAUCGAUACGGUUGGUA
Eve-1-6    UUACAAAGUUAUCAAGAUUCUCUGUUCCGUCUGAAUAAA
env-26     UUAUCCGCGCUCUGAAAUACUGGGAGAAAGAGAAACUCC
env-27     AAAUUUCAGUUGUCGCAACGGUAUUUCCCUAUUAUGAUU
env-28     CCAGACAUAAUUGUAUCACUAAAUUUAUUUCUUACAGCU
env-29     .......................................
env-30     UGUGUAAGCAUCAAAACCACUGCAAAGUGAAUGAUCAGC
env-31     GCGGAAAAUGUGUACAGCCAAGAACUACUGCCUUAAUAC
env-32     AGCCACACCUUGUCUUCUAUAUGAUGGAUGCACAGCAAC
Eve-1-7    GUCAGCUUUUUACGCGUUAUGAGCUAAAUUUUAUGCGUU
env-33     UUUCGUCUCCUCAAAGGCGAACAUAUCGGCCUGAUCGGU
env-34     UUCCUUCAUCCUGAAGUGCAUAAGCCUUUAACUUAUUUA
Eve-1-8    AAAGCUUGUUAAAGAUCCUGCAAAGCUUGUUUCAAUUGC
Rla-1-1    GGUUUUCUCAUCCUGCUUUAACAGAACAGAGAUUCCCCA
env-35     UCCAGACAACCGAUAAACUUAUCAUAAAGAAAUAAAAAA
Dfo-1-1    CAGACGUUUCCAGCUUUCCUGAAUUCUCGCUUUCUGCUC
env-36     .......................................
Rla-1-2    GAACAAUGUCCCUUGUAAUCUGCUAGGUGGUCUAUAUUG
Rla-1-3    AUAGUCAUCCUCUCUCUGACUUCCUCCAAUGAUCUCACC
env-37     .......................................
Rla-1-4    AAACGGUCAACGGGCUGAGGUAAAGCGGGCCUGCACCGC
Rla-1-5    GCUUUUUGACGAUGCACAUAAGGCACUGGAUGCUGCCGU
Rla-1-6    UACUGUAGAUCAUAGCAUCAUAACCUGCUGCUUCAAUCG
Rla-1-7    AGAUAAAGGAAGAAAUUACGAACAUAUAAAAACUUCCCG
Rla-1-8    UUAAGAACAGCAGAAGCAUCUGCCCCGUUAGCAAAAGCA
Rla-1-9    AGCAGCUCUUUCAGUGCCGGAGAAAGCAGCCAGGAUUCG
Rla-1-10   GUAAGCCUUUCUGUAAAGCUUCUGCAUUGCAGGAGAAAG
Rla-1-11   GAAAUGUCAAUGUAAAAUAUGCUCCAUGCAAGUCUGCAG
Rla-1-12   CAAAGUCUGUAUGGAUCUUUCCUGCUGCCUGAGGUGCUU
Rla-1-13   CGGGUAUUCCUGAUCCUCAGCAACCGAUCCGUACACACC
Rla-1-14   CGAUCAAAAGAUUGAAAAGUGUUGCCCUGUCUCUGACUC
Rla-1-15   AGUCUUUUGUUCCCGAGACAUCAUACACCCCUGCCGGAA
Rla-1-16   UCUGUGAACCGUGUCAGGUCGGGAACGGAGCAGCACUAA
Rla-1-17   UUCCUCGUCUUUAUCAAGACCCUUAGCUGCAGAUACGGU
Rla-1-18   AUUUUAACUAUAAGUGGUGUAAGGCACUUGGACUUGAUU
Rla-1-19   AACUCCGGUUCAUGACAGAUCAGAAGGAUGCUUCCCCGG
Rla-1-20   GCAUAUCGUGGUGCUUACGCCCCACUUUUAUGCUCAUAU
Rla-1-21   .......................................
Rla-1-22   UCCGUCGGCAGAUCGUGUAGAACCGAAGUGUGCAUUUGC
Rla-1-23   CAUCAAAUCUUCUGAGAGCUUCUUUUGCUUCUGCACUCA
Rla-1-24   UUACAGGAACAAGCAUUGGUUCGACGAAGGAUAUGAUCA
Rla-1-25   AUUUACCAGCAAUCUCGGCACAAAUGCCGGAGUGAUGGC
Rla-1-26   UGCAAAAGCAUUGAAAGAAGCGGUUAAUAAGUAACGAAU
Rla-1-27   UUACUGCGGUUAUGCCGAAAUGGGGGAAGGAGAUGAUAU
Rla-1-28   UGAGCUGUAAAACGGCAGAAGAUCCUGCUCCGACCGGUU
Rla-1-29   ACAAGCUUAUUGAUCGCUUUCAUCAGACAGAACAAAAUA
Rla-1-31   CUGCCGUUUCAGGAGAGGCAUAGUCUGUCAGAUAUUCUU
env-38     AUAAUACUCGGCUUUUUUACAGCAGUCAUACAUAUCCUC
env-39     UGAAAAGUCUGAAGACAAGCUUAACUGCUAUGUGACAUU
env-40     GCAGAUUAAGGCGAUGAACCCUAAAGGAAUUAUCCUUAC
env-41     UCUCAAAAUACAGAUCCCCUGCACCGGCACUUCCACGUU
Rla-1-32   UCUCAAAAUACAGAUCCCCUGCACCGGCACUUCCACGUU
env-42     UUAUAGCCGCUUCGCAGAUAUUUUUAAUUAUUUAUCUUA

           .......................................
           .......................................
           ooooooooooooooooooooooooooooooooooooooo
```

env-43	GGUAUAGGAAGAUUUUUAGACAUAAUUAAUCCAAUAUUU
env-44	UAUAUUCAUAUAGGUGUUAAUGGUGAUGUAUAUAUCCAG
env-45	GUUAAGCUAGGAGUAGCUGAGGCUGGCGGUGUUCCAGUA
env-46	AUAAGAAUAAGAAAGUAUUAAAAGAGUGCAGGGAUAAAG
Dfo-1-2	AAUUGAAAUCCAUCCGGGUGCGACCAUUGGAAAAGGUCU
env-47	UUUCUUCAUGGGAUGUCUGGAUAGAAUACAGAAAAAAGU
Rla-1-33	AGCAGAUCAUGAUCGAUCAAAAAGAAAUUUAUCUCAACA
Rla-1-34	UUUGCAGUGUAGUUGUUGCAGCAGCGUUGACCUUUUCAC
Rla-1-35	CUUUCCAUAUUUACUGAGGAUCAUUUUUUCAUCCCAGUU
Rla-1-36	GACAUCCCGCAAUACAAAUCCCUUUUUUAUCUUCAUCUU
Rla-1-37	AAUUUUGUUCUUAAUGUUACAUUUACUUUUUUCUUUUUC
Rla-1-38	UUCCUGCUGUAAAUACAAUAUCAUCCACACCAUUCAUCG
Rla-1-39	GAAUCACCCACCGGAUCAAGGAAAAAAUAAGUAUGGAAA
Rla-1-40	AAUGGUCUUUGGCGUAAUUCCAUGCUCCUUAUUAUAUUG
Rla-1-41	AUUAGUAAAUGGUGCUGUCAUACUCGAUUUACAUUUUCC
Rla-1-42	UCCACAUUCCUGUUACAAGUUGUUAACGGGCUGAGGUAA
Rla-1-43	....
Rla-1-44	AAGUCUAUUUUCAGGAAUUGUAUACUCCAUAUUCACCAU
Rla-1-45	UCCACAUUCCUGUUACAAGUUGUCAACGGGCUGAGGUAA
Rla-1-46	UUCCAUCUGUAAGGGCGAUGGCAAAAGAGUUGAAGAUCA
Rla-1-47	GCAGAUUGAGAUCCUCAACAGCAGUAUAGACAAGACGCA
Rla-1-48	UUCCACGUUCCUGUUACAAGUUGUCAACGGGCUGAGGUA
Rla-1-49	AUCUCCUUCAUCCAAUCCUAAUUUGUCUGGAUUUACAAA
Rla-1-50	UUCCACGUUCCUGUUACAAGUUGUCAACGGGCUGAGGUA
Rla-1-51	UUUCCGUCAGUUUUCUGUUCCAGCAGCUUUAUCGAAUUC
Rla-1-52	UUUUUCACCUUUAAUACGGAUACCAAGUCUUCUUGCCUG
Rla-1-53	AAUUCCAUCAUCACCUGCCGGCACACACCACAGGGAGCU
Rla-1-54	CAUGGUCUGAUCUCAGUCGAUUUUCAGAAAAAGAUCGUC
env-48	AUUGAUAAAUCAAAAUAUUGACUCAUAUGAAACCUCCAU
Rla-1-55	....
Rla-1-56	AAGAUUUUCAGAAAGCUGUCCGCGGGGAAUCUCAUUUCU
Rla-1-57	UACUGGCUUUUAGACGCAGUCUGAUGAAUCCUCUAGUUC
Rla-1-58	UAUGUUCUGUCAGAUUGAAAAAGAAACCGGCGGUAUCGA
env-49	UUGAGAUGAAGUCUGUGAAUCACAGAUUUUUAGAAGCAA
env-50	UUUUGUACAAAGAGCCGCCAGUGCGCCUGCAGCUGCAGA
env-51	....
env-52	AUUCAGCUUUGAUUUGGAUAUGACGCUGCUGGACCAUGC
env-53	CUCCCUCGAUCCGAUAUGACAGCUGCGGAUCCAGAUAAU
env-54	ACUAUGAGUUUUCUGGCUGACGAGAUGGGGAUCACAAAA
env-55	AAGCGCAGCAGAGUGCAGCAACGCAGGGAACCUGAGCCU
env-56	UCCGGUAUCUGGUAUUAUGAUAUAUGCUAAGAAUCUCAA
env-57	AGGACCUAU..............................
env-58	AGAGAAAAAUAUUAUGACACACAAAUUUCCACAUCUUUG
env-59	UCGGUAAACAGCACCACGGAUACAGCUCCGCUCCUAUGU
env-60	....
env-61	AUGAAGUAGUCAUGAUGACUGAUAUGUUAGGUGGCAGUG
env-62	....
env-63	AAGAAGUUGUUAAGAAAUACUUCUGGUCAAUGAACCAUU
env-64	GAAGUUGCUUAGAAAUACUUCUGGUCCAUGAACCAUUNA
env-65	ACGAAGUUGUUAAGACAUACUUCUGGUCAAUGAACCAUU
env-66	....
env-67	CUUCGGAAACAGUAUUGCUGCCGUUGUCAGAAGUGGAGC
env-68	UGCUCUCGCUUCUACAGACAAUGCAUCCAGAAAACAUGC
env-69	....
env-70	GCGGAUCAUCCAUGGGAUGCUGGAAGUUGUUUUUGGGAG
env-71	AAAGCGUCUGCAUGAUUUCAAUUUGGUUUUUAAUGUCAA
env-72	UUCUCCACUUAAUCCAACUUCUCCAAACACAAUCGUCCG
env-73	....
env-74	....
env-75	UUCUUUGAGGACGAUUAGGAGGACGAGGAGCCGGGGAAG
Aca-1-1	UUGAAAACGUCCCGCAGAUUUUUGUGGGACGUUUUUCCG
env-76	GCCGCAAGCAUAGAGGAUAAGGCAGUUACUCUUUCUAUG
	....
	....
	°°°°°°°°°°°°°°°°°°°°°°°°°°°°°°°°°°°°°°°

env-77	AGUAAAUCAGCGGAAUUCGAAUGUUUGAAGAAUUGCGAG
env-78	GGAUUACGGGUAAUUCGAAAUGUAGGAAUAUUUCCAUCG
env-79	....
env-80	....
env-81	UGUCGUCUCCCGGAUGGAAAGCAUGAAUGUCCAAGGAUG
env-82	....
env-83	ACAGGUAAAAAUUGUACGAUAAAUACUGAAUAUAGUAAU
env-84	....
env-85	....
env-86	....
env-87	GUUCGCUCGCCGUCAGGCGUUUGCCGCCAUCAGGCGGCG
env-88	....
env-89	UUGAUCUGGAAGAGAAUGGUUAUUUCUAUACAAGACUGG
env-90	UUAUUCACUUCGAAAUGCUGAUAAUCCUUAGAGCUUCUC
env-91	UUGUCUCACCUUCCAGACUGGAGUUUGUUGCUAUGAUCA
Csp-2-1	UUGUCUCACCUUCCAGACUGGAGUUUGUUGCUAUGAUCA
env-92	UUGUCUCACCUUCCAGACUGGAGUUUGUUGCUAUGAUCA
env-93	CCCAUACUACCUGGUAUGUAUGGAUAUCUUCGCUGCUUU
env-94	AGGAGAUGGGAAAAUAAAAAAUAUAAGAAAAUUUAAAAA
env-95	GAUCUGGAAGAGAAUGGUUAUUUCUAUACAAGACUGGCA
env-96	CAGUGACAACUUCAUCACCCAUUUGAGGUAAACGGUCCA
env-97	UAACAUUUUUCUUGCCCAUGAGGUGCGGUCUUCGUAGUC
env-98	....
env-99	GAGAAUGGGAAUGGGGGAAAGCAGGCUUCACUACGGAAU
env-100	UUGGGUAAAGGAGCAGCCCACCUCCUUCUGAAAGAUCUG
env-101	GAUCAGGGCAUUAUAGACUUUAAGAACGGCAGACAUCCU
env-102	CAUUGUUAAGAUCAAUCAGAAUGAUAACGUUGCCGUUGC
Ceu-1-2	GCCUGAGGCGGCAGCAGCUCUCUCAAAGCAGAGUGAGGA
env-103	NNNNNNNNNNNNNNNNNNNNNNNNNNNNNNNNNNNNNNNN
env-104	....
Ceu-1-3	AGUUUCUUUGACUUCGGUACUGCAACACACUCUUUAACC
env-105	AGUUUCUUUGACUUCGGUACCGCAACACACU.........
env-106	GGAGAACCAGACCGGUUCUCUGAUCACCAAAGAUAAGGA
env-107	GAUACCGGGAAUAAAAGCUCAUAUNAUAUUCUUCCAGUU
env-108	CGGAAAUGGAGAGUAUGCUAAUCCAUCCGGCAGUUUAAG
env-109	GUAUGUUUUUUAUAAUACAACGUUUUACUUGUUAUUCUA
env-110	UGAUACCGGGAAUAAAAGCUCAUAUAAUAUUCUUCCAGU
env-111	ACCAAAAUAUACAUUCUUUUCAUUAUGAUGAAUAUCCUG
env-112	GACGUAUACAAUACCUGAUCUUCAUCACCAUAAACAGUU
env-113	UUACAGCAGUAACCCCUCUCUCCAUAUUUGUCAUGAUCG
env-114	....
env-115	....
env-116	CAUGCAUUACUUUAUUCAUUCCAGGAUAUUGGUAGGCAU
env-117	....
env-118	CGGAAAUGGAGAGUAUGCUAAUCCAUCCGGCAGUUUAAG
env-119	GAAAAAACCACCAAAUAACGACUAUAAAAAGGAAACCAG
Dfo-1-3	GAAUACAUUUGAAAUCUUUCCGUGGAGACCGCAGCAGGG
env-120	UUUAUGGUAAAAUAUCCCAAUCUAUAACAAAAAAUGUGC
env-121	UUACUUGCCAUUUCGUUCACCUCCGUUUGGAUUUUGGUU
Ceu-1-4	UCCGUCCAAAAGAGCUUCAGAAAUAUGAAUACUAUGGGG
env-122	AAAAAUUAUAUAUGGAAUAUUGAUCAUCUGUAUGGUGUU
Csp-1-1	GGCAGACGAUGAGAACGUAACCGCAUAUAUAUUUGGAGU
env-123	....
env-124	UUUCCGCCAUCAGAACAGCCCAGCGGGAGACCAAAGAUG
env-125	AAAUUCGAUCUGGCACUUUUGGAUAUCAUGCUGCCGGGA
env-126	CUUCAGCCUAGAUCCUGC......................
env-127	UGUACGAUACGGACGGCGGAAGUGAAGUGGCAGCCCGGA
env-128	....
	....
	....
	○○

22 COG2252

22.1 Taxa

The taxonomy of each organism containing a putative COG2252 RNA is listed, with abbreviations identifying each hit (e.g., "Eco-1-1" and "Eco-1-2" might hypothetically represent two distinct RNAs in *E. coli*). The abbreviations will be used to identify each individual COG2252 RNA in Sections 22.2 and 22.4.

abbrev. of hits	taxonomy of species
Pen-1-1	Bacteria Proteobacteria Gammaproteobacteria Pseudomonadales Pseudomonadaceae *Pseudomonas entomophila* L48
Pfl-1-1	Bacteria Proteobacteria Gammaproteobacteria Pseudomonadales Pseudomonadaceae *Pseudomonas fluorescens* Pf-5
Pfl-2-1	Bacteria Proteobacteria Gammaproteobacteria Pseudomonadales Pseudomonadaceae *Pseudomonas fluorescens* Pf0-1
Ppu-1-1	Bacteria Proteobacteria Gammaproteobacteria Pseudomonadales Pseudomonadaceae *Pseudomonas putida* F1
Ppu-2-1	Bacteria Proteobacteria Gammaproteobacteria Pseudomonadales Pseudomonadaceae *Pseudomonas putida* GB-1
Ppu-3-1	Bacteria Proteobacteria Gammaproteobacteria Pseudomonadales Pseudomonadaceae *Pseudomonas putida* KT2440
Ppu-4-1	Bacteria Proteobacteria Gammaproteobacteria Pseudomonadales Pseudomonadaceae *Pseudomonas putida* W619
Psy-1-1	Bacteria Proteobacteria Gammaproteobacteria Pseudomonadales Pseudomonadaceae *Pseudomonas syringae pv. phaseolicola* 1448A
Psy-2-1	Bacteria Proteobacteria Gammaproteobacteria Pseudomonadales Pseudomonadaceae *Pseudomonas syringae pv. syringae* B728a
Psy-3-1	Bacteria Proteobacteria Gammaproteobacteria Pseudomonadales Pseudomonadaceae *Pseudomonas syringae pv. tomato str.* DC3000
env-1	environmental sample

22.2 Gene contexts

Each COG2252 RNA (indicated by "RNA→") is listed with its surrounding genes. Environmental sequences and some RefSeq entries lack gene annotations, and no genes are listed for such sequences. The direction of each gene is indicated with an arrow (→), and each conserved domain in the gene is colored. Conserved domains associated with more than one COG2252 RNA are assigned a color; other domains are gray. Information about these conserved domains is given in Section 22.3. The accession number of the sequence containing each COG2252 RNA is given. Accessions beginning with "NC_" or "NZ_" are RefSeq, while other accession refer to environmental samples. Nucleotide coordinates are given for the 5′ and 3′ boundaries of each. If the 5′ coordinate is greater than the 3′ coordinate, the RNA is present on the reverse-complement strand of the containing genomic DNA sequence. Each hit is denoted by an abbreviation (like "Eco-1-1") that refers to a taxonomy given in Section 22.1.

abbrev.	Seq. accession		5′ at	3′ at	genes
env-1	JCVI_SCAF_1096627385357	+	1717	1779	**RNA**→ COG2252 (COG2252)tRNA_U5-meth_tr (pfam05958)? (GOS376)→
Pfl-2-1	NC_007492.1	+	5520810	5520872	**RNA**→ COG2252 (COG2252)→ tRNA_U5-meth_tr (pfam05958)→
Pen-1-1	NC_008027.1	+	5083735	5083796	**RNA**→ COG2252 (COG2252)→ tRNA_U5-meth_tr (pfam05958)→ LysR (COG0583)→
Ppu-4-1	NC_010501.1	-	877139	877077	**RNA**→ COG2252 (COG2252)→ tRNA_U5-meth_tr (pfam05958)→ LysR (COG0583)→
Ppu-3-1	NC_002947.3	+	5278425	5278487	**RNA**→ COG2252 (COG2252)→ tRNA_U5-meth_tr (pfam05958)→
Ppu-1-1	NC_009512.1	+	5040655	5040717	**RNA**→ COG2252 (COG2252)→ tRNA_U5-meth_tr (pfam05958)→
Ppu-2-1	NC_010322.1	+	5185631	5185693	**RNA**→ COG2252 (COG2252)→ tRNA_U5-meth_tr (pfam05958)→
Psy-2-1	NC_007005.1	+	5111437	5111499	**RNA**→ COG2252 (COG2252)→ tRNA_U5-meth_tr (pfam05958)→
Psy-3-1	NC_004578.1	+	5256253	5256315	**RNA**→ COG2252 (COG2252)→ tRNA_U5-meth_tr (pfam05958)→
Psy-1-1	NC_005773.3	+	4965899	4965961	**RNA**→ COG2252 (COG2252)→ tRNA_U5-meth_tr (pfam05958)→
Pfl-1-1	NC_004129.6	+	6139548	6139611	**RNA**→ COG2252 (COG2252)→ tRNA_U5-meth_tr (pfam05958)→

22.3 Conserved domains

Conserved domains found in protein-coding genes listed in Section 22.2 are listed, with the first sentence in their description from the Conserved Domain Database. Conserved domains associated with more than one COG2252 RNA are assigned a color, while others are shown in gray. The number in parentheses is the number of occurrences of the given gene.

COG0583 (2) Transcriptional regulator [Transcription]
COG2252 (11) Permeases [General function prediction only]
GOS376 (1) ?
pfam05958 (11) tRNA (Uracil-5-)-methyltransferase.

22.4 Multiple-sequence alignment

Each COG2252 RNA is denoted by an abbreviation (like "Eco-1-1") that refers to a taxonomy given in Section 22.1. The alignment includes sequences containing the RNA motif, as well as flanking sequence. The COG2252 RNA itself is denoted by the line underneath marked 5′ and 3′ on either end. Nucleotides in the flanking sequences (i.e., not part of the motif) are written in gray letters. Stems of predicted rho-independent transcription terminators are shaded yellow. (Note: terminator predictions have not been analyzed manually, and many are likely to be false positives.) Start codons are shaded green. (Note: start codons are frequently misannotated, especially in environmental samples.) Nucleotides proposed to basepair as part of the consensus structure are shaded in color when they comprise Watson-Crick or G-U pairs. Otherwise they are shaded gray. Conserved stems are also indicated at the bottom of the alignment by angle brackets, where matching < and > denote base-paired columns. Below these angle brackets, the symbol "2" denotes base pairs exhibiting covariation, "1" denotes base pairs exhibiting compatible mutations, "0" denotes base pairs that are not observed to mutate and "?" denotes base pairs that have a significant frequency of non-canonical nucleotides for Watson-Crick or G-U pairs (> 5%). Below these base pair annotation is the consensus sequence: "R" = "A" or "G", "Y" = "C" or "U", red nucleotides: nucleotide identity conserved more than 97% of the time, black nucleotides: 90%, gray nucleotides: 75%, red circle (•): nucleotide is present 97% of the time, black circle (•): 90%, gray circle (∘): 75%, white circle (∘): 50%. All percentages of sequences just described (e.g. 97% conserved) assume that sequences have been weighted by the GSC algorithm implemented by the Infernal software.

alignment positions 1 · · · 99

```
env-1     UCGGCGCAGUAUGCCUCAAGCCCAUGCGCCACACAGCCUCGGCUGCUGGUAUGAUGCGCCGCUUUUUCCGAUCCUCUCUGAAACCACAAGCGUUUGGCG
Pfl-2-1   CUUCGCCGACGAUCGACACCAACAUCCGCCAUCUUGCUCACGCUGCUGGUAUGAUGCGCGGCUUUUUCCGGCCCACCACAAUUUUCCAGGCGCUUGCGA
Pen-1-1   GUGCGAACCCCACCUCCCGAAUGGCCAUAGGCCCCCGCCAUCCAUGCUGGUAUGAUGCGCGGCUUUUUUCCGCCCCACACAAAACCACGGCAUCCGGUA
Ppu-4-1   AAUGGUUGACGGGGGCCUGAACGGCCAUAGGCCCCUGCCAGUCAUGCUGGUAUGAUGCGCGGCUUUUUUCCUCCCCACGCAAAACCACGGCAGCCGUUA
Ppu-3-1   CCAGAAAAACCCAUUCCCCAACGGCCAUAGGCCCCGCCAUGCAUGCUGGUAUGAUGCGCGGCUUUUUUCCUCCCCACACAAAACCACGGCAUCCGGUA
Ppu-1-1   CCAGAAAAACCCAUUCCCCAACGGCCAUAGGCCCCGCCAUGCAUGCUGGUAUCAUGCGCGGCUUUUUUCCUCCCUACACAAAACCACAGCAUCCGGUA
Ppu-2-1   AGAAUCGGCAGUAUAUCGCGACAUUGAUUACCGCUCACCGAGCAUGCUGGUAUGAUGCGCGGCUUUUUUCCUCCCCACACAAAACCACGGCAACCGGUA
Psy-2-1   UGUCAUUGAAAGGCCGUCAUCCACAGGCAGCCAUCCCGCCAAGCUGCUGGUAUGAUGCGCGGCUUUUUCCGACCCGCAAAAAUCAAACGGGCUGCCCUG
Psy-3-1   AUCAUCGAAAGGGCGUGAUCCACAGGCAGCCAUCCCGACACGCUGCUGGUAUGAUGCGCGGCUUUUUCCGACCCGCAAAAAUCAAACGGGCUGCCACUG
Psy-1-1   UGUCAUCGAAAGGCCCUUAUCCACAGGCAGCCAUCCUGACCCGCUGCUGGUAUGAUGCGCGGCUUUUUCCGACUCGCAGAAAUCAAACGGGCUGCCUCG
Pfl-1-1   GCGCGGCAAAACAUCGCCGACGAUCGCAGCCAUUGGCCCACGCUGCUGGUAUGAUGCGCGGCUUUUUCCGACCCAGAGCAAAUCCGCAGGCACCUUAUA
          ...................................................................................................
          ...................................................................................................
          •••••••RR•R•RY••Y•••••••••••••••Y•Y••••C••RC•••Y•G••••R••••••G•YUUUUYY••YCC••••••AA••••••GGC••YY••YR
```

alignment positions 100 · · · 259

```
env-1     CAGUUUGUGCUUUGACU.UAGAGGUCAAC.AAAUCACGACGCACAAU.AGCGUCACAGGGAGC.CGGCAUGCUGGAAAAGCUGUUUCAACUCAAAGCCCACAACACCAAUGUGCGCACCGAGAUUCUCGCGGGGAUCACCACCUUCCUGGCCAUGGCCUA
Pfl-2-1   CGGUCUGUGCUUUG.CUGUUGAGGUCGAUACAUUCACGGCGC.CACGC.GCGCCACGGGGAGC.AGACAUGCUGGAAAGGCUGUUUCAACUCAAGGCACACAACACCAACGUGCGGACCGAAAUUCUGGCGGGCGUCACGACCUUCCUGGCCAUGGCCUA
Pen-1-1   CGGUCUGUGCUUUG.CUGUUGGGGUCGAUACAUUCACGGCGC.GAGG..GCGCCCUGGGGAGC.AGGCAUGCUGGAAAGGCUGUUUCAACUAAGAGCACACAACACCAACGUGCGCACCGAGAUUCUCGCGGGCGUCACCACCUUCCUGGCCAUGGCCUA
Ppu-4-1   CGGUCUGUGCUUUG.CUGAUGGGGUCGAUACAUUCACGGCGCCGGG..GGCGCCUCGGGGAGC.GGGCAUGCUGGAAAGGCUGUUUCAACUAAAAGCACACAACACCAACGUGCGCACCGAGAUUCUUGCGGGCGUCACCACCUUCCUGGCGAUGGCCUA
Ppu-3-1   CGGUCUGUGCUUUG.CUGUUGGGGUCGAUACAUUCAUGGCGCCGGG..GGCGCCUUGGGGAGC.GGGCAUGCUGGAAAGGCUGUUUCAACUAAGAGCACACAACACCAACGUGCGCACCGAGAUUCUCGCGGGCGUCACCACCUUCCUGGCCAUGGCCUA

          ........<<<<<..<<<<<<<<.........>>>>.<<<<<<.....>>>>>>>>>>>>>>>.................................................................................................
          ........00001..01???01?.........?10?.02000?.....?00020??1010000.................................................................................................
          CRGUCUGUGCUUUG-CUGUUGRRGUCGAU∘CAUUCACGRCGC∘•R•--∘GCGYC•CGGRGAGC-RGRCAUGCUGGAAAGGCUGUUUCAACU•AARGCACACAACACCAACGUGCG•ACCGAGAUUCUYGCGGGCGUCAC•ACYUUCCUGGC•AUGGCCUA
           5′——————————————————————————————————————————————————————————————3′
```

```
Ppu-1-1  CGGUCUGUGCUUUG.CUGAUGGGGUCGAUACAUUCAUGGCGCCGGG..GGCGCCUUGGGGAGC.GGGCAUGCUGGAAAGGCUGUUUCAACUAAGAGCACACAACACCAACGUGCGCACCGAGAUUCUCGCGGGCGUCACCACCUUCCUGGCCAUGGCCUA
Ppu-2-1  CGGUCUGUGCUUUG.CUGAUGGGGUCGAUACAUUCAUGGCGCCGGG..GGCGCCUUGGGGAGC.GGGCAUGCUGGAAAGGCUGUUUCAACUAAAAGCACACAACACCAACGUGCGCACCGAGAUUCUUGCGGGCGUUACCACCUUCCUGGCCAUGGCCUA
Psy-2-1  CAGUCUGUGCAUUG.CUGUUGGAGUCGAU.CAUUCACGACGCGCGC..CGCGUCACGGAGAGCCAGACAUGCUGGAAAGGCUGUUUCAACUCAAGGCACACAACACCAACGUGCGAACCGAGAUUCUUGCGGGCGUCACGACUUUCCUGGCGAUGGCUUA
Psy-3-1  CAGUCUGUGCUUUG.CUGUUGGAGUCGAU.CAUUCACGACGCGCGC..CGCGUCACGGAGAGCCAGACAUGCUGGAAAGGCUGUUUCAACUCAAGGCACACAACACCAACGUGCGAACCGAGAUUCUUGCGGGCGUCACGACUUUCCUGGCGAUGGCCUA
Psy-1-1  CAGUCUGUGCUUUG.CUGUUGGAGUCGAU.CAUUCACGACGCGCGC..CGCGUCACGGAGAGCCAGACAUGCUGGAAAGGCUGUUUCAACUCAAGGCACACAACACCAACGUGCGGACCGAGAUUCUCGCGGGCGUCACGACUUUCCUGGCGAUGGCCUA
Pfl-1-1  AAGUCUGUGCUUUG.CUGUUGGAGUCGAUACAUUCACGGCGCCCGGC.GGCGCCACGGGGAGC.AGACAUGCUGGAAAGGCUGUUUCAACUCAAGGCACACAACACCAACGUGCGCACCGAGAUUCUUGCGGGCGUGACCACCUUCCUGGCCAUGGCCUA
         ........<<<<<..<<<<<<<<.........>>>>.<<<<<<.....>>>>>>>>>>>>>>>.................................................................................................
         ........00001..01???01?.........?10?.02000?.....?00020??1010000.................................................................................................
         CRGUCUGUGCUUUG-CUGUUGRRGUCGAU∘CAUUCACGRCGC∘•R•--∘GCGYC•CGGRGAGC-RGRCAUGCUGGAAAGGCUGUUUCAACU•AARGCACACAACACCAACGUGCG•ACCGAGAUUCUYGCGGGCGUCAC•ACYUUCCUGGC•AUGGCCUA
          5′———————————————————————————————————————————————————————3′
```

alignment positions 260 · · · 419

```
env-1    CAUCCUGUUCGUGAACCCGAGCAUCCUCGGCGAGACCGGCAUGGACAAGGGCGCGGUGUUUGUCGCCACCUGCCUGGCGGCCGCCAUCGGUUCGACCGUGAUGGGCCUGAUCGCCAACUACCCGAUUGCCCUGGCACCGGGCAUGGGUCUCAACGCCUUC
Pfl-2-1  CAUUCUGUUCGUCAACCCGAGCAUCCUCGGCGAAACCGGCAUGGACAAAGGCGCGGUGUUCGUCGCCACGUGCCUUGCGGCUGCCAUCGGCUCCACGGUGAUGGGCCUGAUCGCCAACUACCCGAUCGCCCUCGCGCCGGGCAUGGGCCUGAACGCCUUC
Pen-1-1  CAUCCUGUUCGUCAACCCGAGCAUCCUCGGCGAGACCGGCAUGGACAAGGGCGCGAUCUUCGUCGCCACGUGCCUUGCCGCGGCCAUCGGCUCGGUGACCAUGGGCAUCAUCGCCAACUACCCGAUCGCCCUGGCGCCGGGCAUGGGCCUGAACGCCUUC
Ppu-4-1  CAUCCUGUUCGUCAACCCGAGCAUUCUCGGCGAAACCGGCAUGGACAAGGGCGCGAUCUUCGUCGCCACCUGCCUGGCUGCCGCCAUCGGCUCGGCGACCAUGGGCCUGAUCGCCAACUACCCGAUCGCCUUGGCGCCGGGCAUGGGCUUGAACGCGUUC
Ppu-3-1  CAUCCUGUUCGUCAACCCGAGCAUCCUCGGCGAGACCGGCAUGGACAAGGGCGCGAUCUUCGUCGCCACCUGCCUGGCAGCGGCCAUCGGCUCCGCCACCAUGGGCCUGAUUGCCAACUACCCGAUCGCCCUGGCACCGGGCAUGGGCCUGAACGCGUUC
Ppu-1-1  CAUCCUGUUCGUCAACCCGAGCAUCCUCGGCGAGACCGGCAUGGACAAGGGCGCGAUCUUUGUCGCCACCUGCCUGGCAGCGGCCAUCGGCUCCGCCACCAUGGGCCUGAUUGCCAACUACCCGAUCGCCCUGGCACCGGGCAUGGGCCUGAACGCGUUC
Ppu-2-1  CAUCCUGUUCGUCAACCCGAGCAUCCUCGGCGAGACCGGCAUGGACAAAGGGGCGAUCUUCGUCGCCACCUGUCUGGCUGCAGCCAUCGGUUCCACCACCAUGGGCCUGAUCGCCAACUACCCGAUUGCCCUGGCACCGGGCAUGGGCCUGAACGCGUUC
Psy-2-1  CAUCCUGUUCGUCAACCCGAGCAUCCUCGGCGAAACCGGCAUGGACAAGGGCGCGGUAUUCGUCGCCACCUGCCUGGCGGCGGCCAUCGGCUCGACAGUGAUGGGCCUGAUCGCCAACUACCCGAUCGCCCUGGCACCGGGCAUGGGCCUGAACGCCUUC
Psy-3-1  CAUCCUGUUCGUCAACCCGAGCAUCCUCGGCGAAACCGGCAUGGACAAAGGCGCAGUGUUCGUUGCCACCUGCCUGGCAGCGGCCAUCGGCUCGACGGUGAUGGGCCUGAUCGCCAACUACCCGAUCGCCCUGGCACCGGGCAUGGGCCUGAACGCCUUC
Psy-1-1  CAUCCUGUUCGUCAACCCGAGCAUCCUCGGCGAAACCGGCAUGGACAAGGGCGCGGUAUUCGUCGCCACCUGCCUGGCAGCCGCCAUCGGCUCGACGGUGAUGGGGCUGAUCGCCAACUACCCGAUCGCCCUUGCACCGGGCAUGGGCCUGAACGCCUUC
Pfl-1-1  CAUCCUUUUCGUCAACCCGAGCAUUCUCGGCGAGACGGGCAUGGACAAGGGCGCGAUCUUCGUUGCCACCUGCCUGGCGGCGGCCAUCGGUUCCACCGUCAUGGGCCUGAUCGCCAACUACCCGAUCGCCCUGGCACCGGGCAUGGGCCUGAACGCCUUC
         ................................................................................................................................................................
         ................................................................................................................................................................
         CAUCCUGUUCGUCAACCCGAGCAUCCUCGGCGARACCGGCAUGGACAARGGCGCGRU•UUCGUCGCCACCUGCCUGGCRGC•GCCAUCGGYUC•AC•RY•AUGGGCCUGAUCGCCAACUACCCGAUCGCCCUGGCRCCGGGCAUGGGCCUGAACGC•UUC
```

alignment positions 420 · · · 467

```
env-1    UUCACCUACACCGUGGUCCUGCACAUGGGCCACACCUGGCAAGUGGCG
Pfl-2-1  UUCACCUACACCGUCGUGCUGCACAUGGGCCACACCUGGCAAGUGGCG
Pen-1-1  UUCACCUACACCGUGGUCCUGCACAUGGGCCACACCUGGCAGGUGGCA
Ppu-4-1  UUCACCUACACCGUGGUCCUGCACAUGGGCCACACCUGGCAGGUCGCG
Ppu-3-1  UUCACCUACACCGUGGUGCUGCACAUGGGCCACACCUGGCAGGUGGCA
Ppu-1-1  UUCACCUACACCGUGGUGCUGCACAUGGGCCACACCUGGCAGGUGGCA
Ppu-2-1  UUCACCUACACCGUGGUGCUGCACAUGGGCCACACCUGGCAGGUGGCG
Psy-2-1  UUUACCUACACCGUGGUCCUGCACAUGGGCCACAGCUGGCAGGUAGCG
Psy-3-1  UUUACCUACACCGUUGUGCUGCACAUGGGCCAUAGCUGGCAGGUGGCG
Psy-1-1  UUUACCUAUACCGUGGUCCUGCACAUGGGUCACAGCUGGCAGGUAGCG
Pfl-1-1  UUCACCUACACCGUGGUCCUGCACAUGGGCCACACCUGGCAGGUAGCC
         ................................................
         ................................................
         UUYACCUACACCGUGGU•CUGCACAUGGGCCACA•CUGGCARGURGCR
```

23 *Collinsella*-1

23.1 Taxa

The taxonomy of each organism containing a putative *Collinsella*-1 RNA is listed, with abbreviations identifying each hit (e.g., "Eco-1-1" and "Eco-1-2" might hypothetically represent two distinct RNAs in *E. coli*). The abbreviations will be used to identify each individual *Collinsella*-1 RNA in Sections 23.2 and 23.4.

abbrev. of hits	taxonomy of species
Cae-1-1	Bacteria Actinobacteria Coriobacteridae Coriobacteriales Coriobacterineae Coriobacteriaceae *Collinsella aerofaciens* ATCC 25986
uba-1-1	Bacteria environmental samples uncultured bacterium
env-1 to env-4	environmental samples

23.2 Gene contexts

Each *Collinsella*-1 RNA (indicated by "RNA→") is listed with its surrounding genes. Environmental sequences and some RefSeq entries lack gene annotations, and no genes are listed for such sequences. The direction of each gene is indicated with an arrow (→), and each conserved domain in the gene is colored. Conserved domains associated with more than one *Collinsella*-1 RNA are assigned a color; other domains are gray. Information about these conserved domains is given in Section 23.3. The accession number of the sequence containing each *Collinsella*-1 RNA is given. Accessions beginning with "NC_" or "NZ_" are RefSeq, while other accession refer to environmental samples. Nucleotide coordinates are given for the 5′ and 3′ boundaries of each. If the 5′ coordinate is greater than the 3′ coordinate, the RNA is present on the reverse-complement strand of the containing genomic DNA sequence. Each hit is denoted by an abbreviation (like "Eco-1-1") that refers to a taxonomy given in Section 23.1.

**abbrev.**	**Seq. accession**		**5′ at**	**3′ at**	**genes**
uba-1-1	NC_008154.1	+	4029	4216	←COG4186 (COG4186) **RNA**→ putative replication protein→
env-1	hgutS7_s7_166479	-	545	363	hypo→ **RNA**→ ←hypo
Cae-1-1	NZ_AAVN02000005.1	+	150266	150458	hypo→ **RNA**→ ←ADP_ribosyl_GH (pfam03747)
env-2	BAAV01020360.1	+	623	820	hypo→ **RNA**→
env-3	hgutS8_s8_179322_2	-	1637	1441	hypo→ **RNA**→ ←DUF1016 (pfam06250)
env-4	hgutS7_s7_179322_2	-	721	525	**RNA**→ ←Uncharacterized conserved protein

23.3 Conserved domains

Conserved domains found in protein-coding genes listed in Section 23.2 are listed, with the first sentence in their description from the Conserved Domain Database. Conserved domains associated with more than one *Collinsella*-1 RNA are assigned a color, while others are shown in gray. The number in parentheses is the number of occurrences of the given gene.

u COG4186 (1) Predicted phosphoesterase or phosphohydrolase [General function prediction only]

d pfam03747 (1) ADP-ribosylglycohydrolase.

d pfam06250 (1) Protein of unknown function (DUF1016).

23.4 Multiple-sequence alignment

Each *Collinsella*-1 RNA is denoted by an abbreviation (like "Eco-1-1") that refers to a taxonomy given in Section 23.1. The alignment includes sequences containing the RNA motif, as well as flanking sequence. The *Collinsella*-1 RNA itself is denoted by the line underneath marked 5′ and 3′ on either end. Nucleotides in the flanking sequences (i.e., not part of the motif) are written in gray letters. Stems of predicted rho-independent transcription terminators are shaded yellow. (Note: terminator predictions have not been analyzed manually, and many are likely to be false positives.) Start codons are shaded green. (Note: start codons are frequently misannotated, especially in environmental samples.) Nucleotides proposed to basepair as part of the consensus structure are shaded in color when they comprise Watson-Crick or G-U pairs. Otherwise they are shaded gray. Conserved stems are also indicated at the bottom of the alignment by angle brackets, where matching $<$ and $>$ denote base-paired columns. Below these angle brackets, the symbol "2" denotes base pairs exhibiting covariation, "1" denotes base pairs exhibiting compatible mutations, "0" denotes base pairs that are not observed to mutate and "?" denotes base pairs that have a significant frequency of non-canonical nucleotides for Watson-Crick or G-U pairs ($>$ 5%). Below these base pair annotation is the consensus sequence: "R" = "A" or "G", "Y" = "C" or "U", red nucleotides: nucleotide identity conserved more than 97% of the time, black nucleotides: 90%, gray nucleotides: 75%, red circle (•): nucleotide is present 97% of the time, black circle (•): 90%, gray circle (•): 75%, white circle (◦): 50%. All percentages of sequences just described (e.g. 97% conserved) assume that sequences have been weighted by the GSC algorithm implemented by the Infernal software.

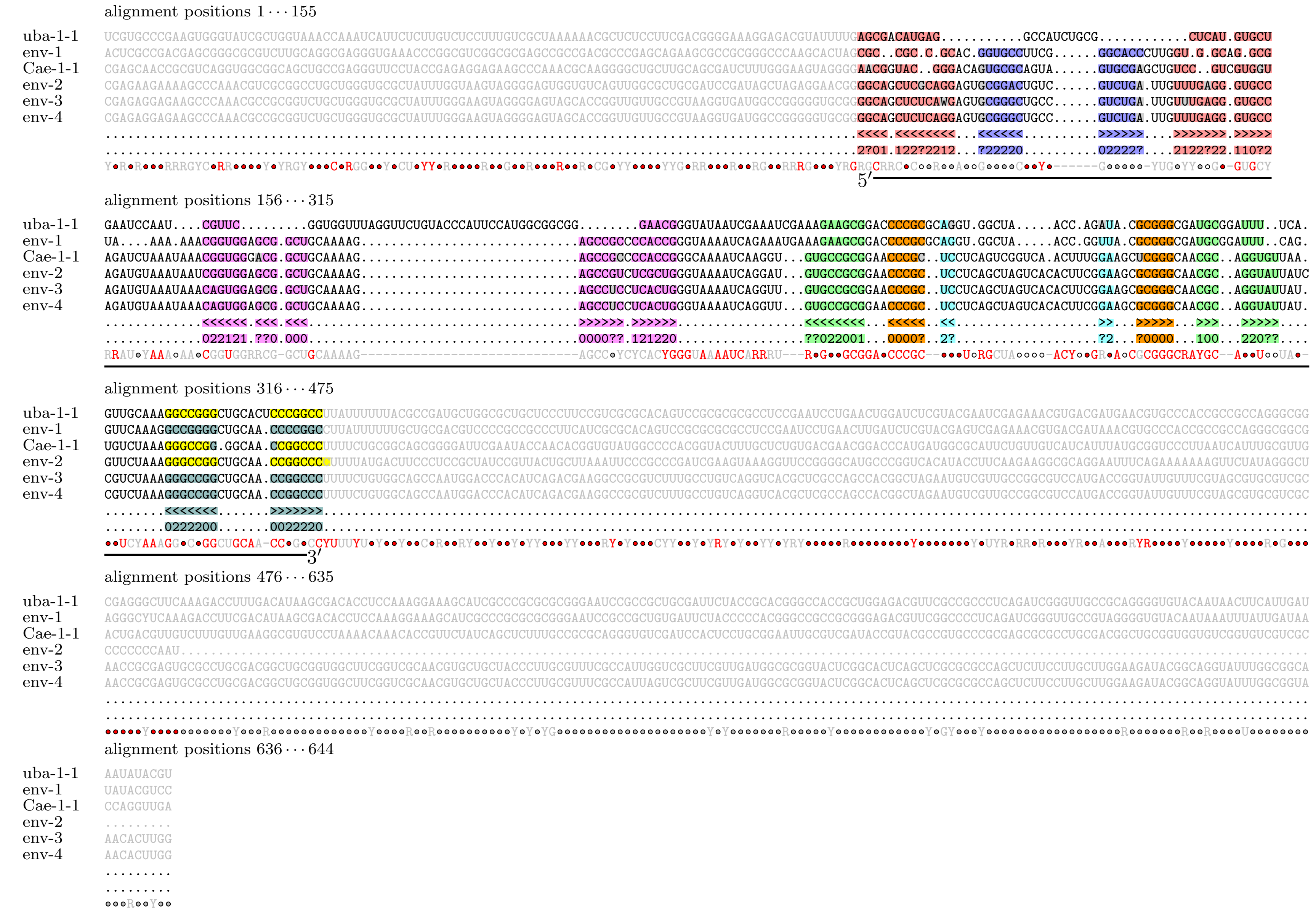

24 *crcB*

24.1 Taxa

The taxonomy of each organism containing a putative *crcB* RNA is listed, with abbreviations identifying each hit (e.g., "Eco-1-1" and "Eco-1-2" might hypothetically represent two distinct RNAs in *E. coli*). The abbreviations will be used to identify each individual *crcB* RNA in Sections 24.3 and 24.5.

**abbrev. of hits**	**taxonomy of species**
Mse-1-1	Archaea Crenarchaeota Thermoprotei Sulfolobales Sulfolobaceae *Metallosphaera sedula* DSM 5348
Sso-1-1	Archaea Crenarchaeota Thermoprotei Sulfolobales Sulfolobaceae *Sulfolobus solfataricus* P2
Abo-1-1 to Abo-1-4	Archaea Euryarchaeota *Aciduliprofundum boonei* T469
Afu-1-1	Archaea Euryarchaeota Archaeoglobi Archaeoglobales Archaeoglobaceae *Archaeoglobus fulgidus* DSM 4304
Msm-1-1	Archaea Euryarchaeota Methanobacteria Methanobacteriales Methanobacteriaceae *Methanobrevibacter smithii* ATCC 35061
Msm-2-1	Archaea Euryarchaeota Methanobacteria Methanobacteriales Methanobacteriaceae *Methanobrevibacter smithii* DSM 2374
Msm-3-1	Archaea Euryarchaeota Methanobacteria Methanobacteriales Methanobacteriaceae *Methanobrevibacter smithii* DSM 2375
Mst-1-1	Archaea Euryarchaeota Methanobacteria Methanobacteriales Methanobacteriaceae *Methanosphaera stadtmanae* DSM 3091
Mth-1-1	Archaea Euryarchaeota Methanobacteria Methanobacteriales Methanobacteriaceae *Methanothermobacter thermautotrophicus str.* Delta H
CMe-1-1 to CMe-1-2	Archaea Euryarchaeota Methanomicrobia Methanomicrobiales Generaincertae sedis *Candidatus* Methanoregula boonei 6A8
Mhu-1-1 to Mhu-1-3	Archaea Euryarchaeota Methanomicrobia Methanomicrobiales Methanospirillaceae *Methanospirillum hungatei* JF-1
Mac-1-1	Archaea Euryarchaeota Methanomicrobia Methanosarcinales Methanosarcinaceae *Methanosarcina acetivorans* C2A
Mba-1-1	Archaea Euryarchaeota Methanomicrobia Methanosarcinales Methanosarcinaceae *Methanosarcina barkeri str.* Fusaro
Pab-1-1	Archaea Euryarchaeota Thermococci Thermococcales Thermococcaceae *Pyrococcus abyssi* GE5
Pfu-1-1	Archaea Euryarchaeota Thermococci Thermococcales Thermococcaceae *Pyrococcus furiosus* DSM 3638
Pho-1-1	Archaea Euryarchaeota Thermococci Thermococcales Thermococcaceae *Pyrococcus horikoshii* OT3
Tba-1-1	Archaea Euryarchaeota Thermococci Thermococcales Thermococcaceae *Thermococcus barophilus* MP
Tko-1-1	Archaea Euryarchaeota Thermococci Thermococcales Thermococcaceae *Thermococcus kodakarensis* KOD1
Ton-1-1	Archaea Euryarchaeota Thermococci Thermococcales Thermococcaceae *Thermococcus onnurineus* NA1
Tsp-1-1	Archaea Euryarchaeota Thermococci Thermococcales Thermococcaceae *Thermococcus sp.* AM4
Pto-1-1	Archaea Euryarchaeota Thermoplasmata Thermoplasmatales Picrophilaceae *Picrophilus torridus* DSM 9790
Tvo-1-1 to Tvo-1-2	Archaea Euryarchaeota Thermoplasmata Thermoplasmatales Thermoplasmataceae *Thermoplasma volcanium* GSS1
Aba-1-1	Bacteria Acidobacteria Acidobacteriales Acidobacteriaceae *Acidobacteria bacterium* Ellin345
Mab-1-1	Bacteria Actinobacteria Actinobacteridae Actinomycetales Corynebacterineae Mycobacteriaceae *Mycobacterium abscessus*
Mma-1-1 to Mma-1-3	Bacteria Actinobacteria Actinobacteridae Actinomycetales Corynebacterineae Mycobacteriaceae *Mycobacterium marinum* M
Mul-1-1 to Mul-1-2	Bacteria Actinobacteria Actinobacteridae Actinomycetales Corynebacterineae Mycobacteriaceae *Mycobacterium ulcerans* Agy99
Nfa-1-1	Bacteria Actinobacteria Actinobacteridae Actinomycetales Corynebacterineae Nocardiaceae *Nocardia farcinica* IFM 10152
Ace-1-1 to Ace-1-2	Bacteria Actinobacteria Actinobacteridae Actinomycetales Frankineae Acidothermaceae *Acidothermus cellulolyticus* 11B
Lxy-1-1	Bacteria Actinobacteria Actinobacteridae Actinomycetales Micrococcineae Microbacteriaceae *Leifsonia xyli subsp. xyli str.* CTCB07
Bad-1-1	Bacteria Actinobacteria Actinobacteridae Bifidobacteriales Bifidobacteriaceae *Bifidobacterium adolescentis* ATCC 15703
Bad-2-1	Bacteria Actinobacteria Actinobacteridae Bifidobacteriales Bifidobacteriaceae *Bifidobacterium adolescentis* L2-32
Ban-1-1	Bacteria Actinobacteria Actinobacteridae Bifidobacteriales Bifidobacteriaceae *Bifidobacterium angulatum* DSM 20098
Blo-1-1 to Blo-1-2	Bacteria Actinobacteria Actinobacteridae Bifidobacteriales Bifidobacteriaceae *Bifidobacterium longum* DJO10A
Blo-2-1	Bacteria Actinobacteria Actinobacteridae Bifidobacteriales Bifidobacteriaceae *Bifidobacterium longum* NCC2705
Blo-3-1	Bacteria Actinobacteria Actinobacteridae Bifidobacteriales Bifidobacteriaceae *Bifidobacterium longum subsp. infantis* CCUG 52486
Rxy-1-1	Bacteria Actinobacteria Rubrobacteridae Rubrobacterales Rubrobacterineae Rubrobacteraceae *Rubrobacter xylanophilus* DSM 9941
Hsp-1-1 to Hsp-1-2	Bacteria Aquificae Aquificales Aquificaceae *Hydrogenobaculum sp.* Y04AAS1
Bca-1-1	Bacteria Bacteroidetes Bacteroidia Bacteroidales Bacteroidaceae *Bacteroides capillosus* ATCC 29799
Bco-1-1	Bacteria Bacteroidetes Bacteroidia Bacteroidales Bacteroidaceae *Bacteroides coprocola* DSM 17136
Bpl-1-1	Bacteria Bacteroidetes Bacteroidia Bacteroidales Bacteroidaceae *Bacteroides plebeius* DSM 17135
Bth-1-1	Bacteria Bacteroidetes Bacteroidia Bacteroidales Bacteroidaceae *Bacteroides thetaiotaomicron* VPI-5482
Lbl-1-1	Bacteria Bacteroidetes Flavobacteria Flavobacteriales Flavobacteriaceae *Leeuwenhoekiella blandensis* MED217
Psp-1-1 to Psp-1-2	Bacteria Bacteroidetes Sphingobacteria Sphingobacteriales Sphingobacteriaceae *Pedobacter sp.* BAL39
Cag-1-1	Bacteria Chloroflexi Chloroflexales Chloroflexaceae *Chloroflexus aggregans* DSM 9485
Cau-1-1	Bacteria Chloroflexi Chloroflexales Chloroflexaceae *Chloroflexus aurantiacus* J-10-fl
Csp-1-1	Bacteria Chloroflexi Chloroflexales Chloroflexaceae *Chloroflexus sp.* Y-400-fl
Rsp-1-1	Bacteria Chloroflexi Chloroflexales Chloroflexaceae *Roseiflexus sp.* RS-1
Sel-1-1	Bacteria Cyanobacteria Chroococcales *Synechococcus elongatus* PCC 6301
Sel-2-1	Bacteria Cyanobacteria Chroococcales *Synechococcus elongatus* PCC 7942
Ssp-1-1	Bacteria Cyanobacteria Chroococcales *Synechococcus sp.* JA-2-3B'a(2-13)
Ssp-2-1	Bacteria Cyanobacteria Chroococcales *Synechococcus sp.* JA-3-3Ab
Ssp-3-1	Bacteria Cyanobacteria Chroococcales *Synechococcus sp.* PCC 7002
Gvi-1-1	Bacteria Cyanobacteria Gloeobacteria Gloeobacterales *Gloeobacter violaceus* PCC 7421
Ava-1-1	Bacteria Cyanobacteria Nostocales Nostocaceae *Anabaena variabilis* ATCC 29413 (Anabaena flos-aquae UTEX 1444)
Nsp-1-1	Bacteria Cyanobacteria Nostocales Nostocaceae *Nostoc sp.* PCC 7120
Dge-1-1	Bacteria Deinococcus-Thermus Deinococci Deinococcales Deinococcaceae *Deinococcus geothermalis* DSM 11300
Taq-1-1	Bacteria Deinococcus-Thermus Deinococci Thermales Thermaceae *Thermus aquaticus* Y51MC23
Dth-1-1	Bacteria Dictyoglomi Dictyoglomales Dictyoglomaceae *Dictyoglomus thermophilum* H-6-12
Ban-3-1	Bacteria Firmicutes Bacillales Bacillaceae Bacillus Bacilluscereus group *Bacillus anthracis str.* 'Ames Ancestor'

Ban-4-1	Bacteria Firmicutes Bacillales Bacillaceae Bacillus Bacilluscereus group *Bacillus anthracis str.* A0174
Ban-5-1	Bacteria Firmicutes Bacillales Bacillaceae Bacillus Bacilluscereus group *Bacillus anthracis str.* A0193
Ban-6-1	Bacteria Firmicutes Bacillales Bacillaceae Bacillus Bacilluscereus group *Bacillus anthracis str.* A0389
Ban-7-1	Bacteria Firmicutes Bacillales Bacillaceae Bacillus Bacilluscereus group *Bacillus anthracis str.* A0442
Ban-8-1	Bacteria Firmicutes Bacillales Bacillaceae Bacillus Bacilluscereus group *Bacillus anthracis str.* A0465
Ban-9-1	Bacteria Firmicutes Bacillales Bacillaceae Bacillus Bacilluscereus group *Bacillus anthracis str.* A0488
Ban-10-1	Bacteria Firmicutes Bacillales Bacillaceae Bacillus Bacilluscereus group *Bacillus anthracis str.* A1055
Ban-11-1	Bacteria Firmicutes Bacillales Bacillaceae Bacillus Bacilluscereus group *Bacillus anthracis str.* A2012
Ban-12-1	Bacteria Firmicutes Bacillales Bacillaceae Bacillus Bacilluscereus group *Bacillus anthracis str.* Ames
Ban-13-1	Bacteria Firmicutes Bacillales Bacillaceae Bacillus Bacilluscereus group *Bacillus anthracis str.* Australia 94
Ban-14-1	Bacteria Firmicutes Bacillales Bacillaceae Bacillus Bacilluscereus group *Bacillus anthracis str.* CNEVA-9066
Ban-15-1	Bacteria Firmicutes Bacillales Bacillaceae Bacillus Bacilluscereus group *Bacillus anthracis str.* Kruger B
Ban-16-1	Bacteria Firmicutes Bacillales Bacillaceae Bacillus Bacilluscereus group *Bacillus anthracis str.* Sterne
Ban-17-1	Bacteria Firmicutes Bacillales Bacillaceae Bacillus Bacilluscereus group *Bacillus anthracis str.* Vollum
Ban-18-1	Bacteria Firmicutes Bacillales Bacillaceae Bacillus Bacilluscereus group *Bacillus anthracis str.* Western North America USA6153
Ban-2-1	Bacteria Firmicutes Bacillales Bacillaceae Bacillus Bacilluscereus group *Bacillus anthracis* Tsiankovskii-I
Bce-1-1	Bacteria Firmicutes Bacillales Bacillaceae Bacillus Bacilluscereus group *Bacillus cereus* 03BB108
Bce-2-1	Bacteria Firmicutes Bacillales Bacillaceae Bacillus Bacilluscereus group *Bacillus cereus* AH1134
Bce-3-1	Bacteria Firmicutes Bacillales Bacillaceae Bacillus Bacilluscereus group *Bacillus cereus* AH187
Bce-4-1	Bacteria Firmicutes Bacillales Bacillaceae Bacillus Bacilluscereus group *Bacillus cereus* AH820
Bce-5-1	Bacteria Firmicutes Bacillales Bacillaceae Bacillus Bacilluscereus group *Bacillus cereus* ATCC 10987
Bce-6-1	Bacteria Firmicutes Bacillales Bacillaceae Bacillus Bacilluscereus group *Bacillus cereus* ATCC 14579
Bce-7-1	Bacteria Firmicutes Bacillales Bacillaceae Bacillus Bacilluscereus group *Bacillus cereus* B4264
Bce-8-1	Bacteria Firmicutes Bacillales Bacillaceae Bacillus Bacilluscereus group *Bacillus cereus* E33L
Bce-9-1	Bacteria Firmicutes Bacillales Bacillaceae Bacillus Bacilluscereus group *Bacillus cereus* G9241
Bce-10-1	Bacteria Firmicutes Bacillales Bacillaceae Bacillus Bacilluscereus group *Bacillus cereus* G9842
Bce-11-1	Bacteria Firmicutes Bacillales Bacillaceae Bacillus Bacilluscereus group *Bacillus cereus* H3081.97
Bce-12-1	Bacteria Firmicutes Bacillales Bacillaceae Bacillus Bacilluscereus group *Bacillus cereus* NVH0597-99
Bce-14-1	Bacteria Firmicutes Bacillales Bacillaceae Bacillus Bacilluscereus group *Bacillus cereus subsp. cytotoxis* NVH 391-98
Bce-13-1	Bacteria Firmicutes Bacillales Bacillaceae Bacillus Bacilluscereus group *Bacillus cereus* W
Bth-2-1	Bacteria Firmicutes Bacillales Bacillaceae Bacillus Bacilluscereus group *Bacillus thuringiensis serovar israelensis* ATCC 35646
Bth-3-1	Bacteria Firmicutes Bacillales Bacillaceae Bacillus Bacilluscereus group *Bacillus thuringiensis serovar konkukian str.* 97-27
Bth-4-1	Bacteria Firmicutes Bacillales Bacillaceae Bacillus Bacilluscereus group *Bacillus thuringiensis str.* Al Hakam
Bwe-1-1	Bacteria Firmicutes Bacillales Bacillaceae Bacillus Bacilluscereus group *Bacillus weihenstephanensis* KBAB4
Gka-1-1	Bacteria Firmicutes Bacillales Bacillaceae *Geobacillus kaustophilus* HTA426
Gsp-1-1	Bacteria Firmicutes Bacillales Bacillaceae *Geobacillus sp.* G11MC16
Gsp-2-1	Bacteria Firmicutes Bacillales Bacillaceae *Geobacillus sp.* WCH70
Gsp-3-1	Bacteria Firmicutes Bacillales Bacillaceae *Geobacillus sp.* Y412MC10
Gth-1-1	Bacteria Firmicutes Bacillales Bacillaceae *Geobacillus thermodenitrificans* NG80-2
Psp-2-1	Bacteria Firmicutes Bacillales Paenibacillaceae *Paenibacillus sp.* JDR-2
Ame-1-1	Bacteria Firmicutes Clostridia Clostridiales Clostridiaceae *Alkaliphilus metalliredigens* QYMF
Aor-1-1	Bacteria Firmicutes Clostridia Clostridiales Clostridiaceae *Alkaliphilus oremlandii* OhILAs
Cac-1-1	Bacteria Firmicutes Clostridia Clostridiales Clostridiaceae *Clostridium acetobutylicum* ATCC 824
Cbo-1-1	Bacteria Firmicutes Clostridia Clostridiales Clostridiaceae *Clostridium bolteae* ATCC BAA-613
Cbo-2-1 to Cbo-2-2	Bacteria Firmicutes Clostridia Clostridiales Clostridiaceae *Clostridium botulinum* A str. ATCC 19397
Cbo-3-1 to Cbo-3-2	Bacteria Firmicutes Clostridia Clostridiales Clostridiaceae *Clostridium botulinum* A str. ATCC 3502
Cbo-4-1 to Cbo-4-2	Bacteria Firmicutes Clostridia Clostridiales Clostridiaceae *Clostridium botulinum* A str. Hall
Cbo-5-1 to Cbo-5-2	Bacteria Firmicutes Clostridia Clostridiales Clostridiaceae *Clostridium botulinum* A3 str. Loch Maree
Cbo-6-1	Bacteria Firmicutes Clostridia Clostridiales Clostridiaceae *Clostridium botulinum* B str. Eklund 17B
Cbo-7-1 to Cbo-7-2	Bacteria Firmicutes Clostridia Clostridiales Clostridiaceae *Clostridium botulinum* B1 str. Okra
Cbo-8-1	Bacteria Firmicutes Clostridia Clostridiales Clostridiaceae *Clostridium botulinum* Bf
Cbo-9-1	Bacteria Firmicutes Clostridia Clostridiales Clostridiaceae *Clostridium botulinum* E3 str. Alaska E43
Cbo-10-1 to Cbo-10-2	Bacteria Firmicutes Clostridia Clostridiales Clostridiaceae *Clostridium botulinum* F str. Langeland
Cbo-11-1 to Cbo-11-2	Bacteria Firmicutes Clostridia Clostridiales Clostridiaceae *Clostridium botulinum* NCTC 2916
Cbu-1-1	Bacteria Firmicutes Clostridia Clostridiales Clostridiaceae *Clostridium butyricum* 5521
Cdi-1-1	Bacteria Firmicutes Clostridia Clostridiales Clostridiaceae *Clostridium difficile* 630
Cdi-2-1	Bacteria Firmicutes Clostridia Clostridiales Clostridiaceae *Clostridium difficile* ATCC 43255
Cdi-3-1	Bacteria Firmicutes Clostridia Clostridiales Clostridiaceae *Clostridium difficile* CIP 107932
Cdi-4-1	Bacteria Firmicutes Clostridia Clostridiales Clostridiaceae *Clostridium difficile* QCD-23m63
Cdi-5-1	Bacteria Firmicutes Clostridia Clostridiales Clostridiaceae *Clostridium difficile* QCD-32g58
Cdi-6-1	Bacteria Firmicutes Clostridia Clostridiales Clostridiaceae *Clostridium difficile* QCD-37x79
Cdi-7-1	Bacteria Firmicutes Clostridia Clostridiales Clostridiaceae *Clostridium difficile* QCD-63q42
Cdi-8-1	Bacteria Firmicutes Clostridia Clostridiales Clostridiaceae *Clostridium difficile* QCD-66c26
Cdi-9-1	Bacteria Firmicutes Clostridia Clostridiales Clostridiaceae *Clostridium difficile* QCD-76w55
Cdi-10-1	Bacteria Firmicutes Clostridia Clostridiales Clostridiaceae *Clostridium difficile* QCD-97b34

Chi-1-1	Bacteria Firmicutes Clostridia Clostridiales Clostridiaceae *Clostridium hiranonis* DSM 13275
Ckl-1-1	Bacteria Firmicutes Clostridia Clostridiales Clostridiaceae *Clostridium kluyveri* DSM 555
Cpe-1-1	Bacteria Firmicutes Clostridia Clostridiales Clostridiaceae *Clostridium perfringens* ATCC 13124
Cpe-2-1	Bacteria Firmicutes Clostridia Clostridiales Clostridiaceae *Clostridium perfringens* B str. ATCC 3626
Cpe-3-1	Bacteria Firmicutes Clostridia Clostridiales Clostridiaceae *Clostridium perfringens* C str. JGS1495
Cpe-4-1	Bacteria Firmicutes Clostridia Clostridiales Clostridiaceae *Clostridium perfringens* CPE str. F4969
Cpe-5-1	Bacteria Firmicutes Clostridia Clostridiales Clostridiaceae *Clostridium perfringens* D str. JGS1721
Cpe-6-1	Bacteria Firmicutes Clostridia Clostridiales Clostridiaceae *Clostridium perfringens* E str. JGS1987
Cpe-7-1	Bacteria Firmicutes Clostridia Clostridiales Clostridiaceae *Clostridium perfringens* NCTC 8239
Cpe-8-1	Bacteria Firmicutes Clostridia Clostridiales Clostridiaceae *Clostridium perfringens* SM101
Cpe-9-1	Bacteria Firmicutes Clostridia Clostridiales Clostridiaceae *Clostridium perfringens str.* 13
Cph-1-1	Bacteria Firmicutes Clostridia Clostridiales Clostridiaceae *Clostridium phytofermentans* ISDg
Csp-2-1	Bacteria Firmicutes Clostridia Clostridiales Clostridiaceae *Clostridium sp.* SS2/1
Csp-3-1	Bacteria Firmicutes Clostridia Clostridiales Clostridiaceae *Clostridium sporogenes* ATCC 15579
Cth-1-1	Bacteria Firmicutes Clostridia Clostridiales Clostridiaceae *Clostridium thermocellum* ATCC 27405
Cth-2-1	Bacteria Firmicutes Clostridia Clostridiales Clostridiaceae *Clostridium thermocellum* DSM 4150
Cba-1-1	Bacteria Firmicutes Clostridia *Clostridiales bacterium* 1_7_47_FAA
Ahy-1-1	Bacteria Firmicutes Clostridia Clostridiales ClostridialesFamily XI. Incertae Sedis *Anaerococcus hydrogenalis* DSM 7454
Ala-1-1	Bacteria Firmicutes Clostridia Clostridiales ClostridialesFamily XI. Incertae Sedis *Anaerococcus lactolyticus* ATCC 51172
Fma-1-1	Bacteria Firmicutes Clostridia Clostridiales ClostridialesFamily XI. Incertae Sedis *Finegoldia magna* ATCC 29328
Eve-1-1	Bacteria Firmicutes Clostridia Clostridiales Eubacteriaceae *Eubacterium ventriosum* ATCC 27560
Hmo-1-1	Bacteria Firmicutes Clostridia Clostridiales Heliobacteriaceae *Heliobacterium modesticaldum* Ice1
Ceu-1-1	Bacteria Firmicutes Clostridia Clostridiales Lachnospiraceae *Coprococcus eutactus* ATCC 27759
Dlo-1-1	Bacteria Firmicutes Clostridia Clostridiales Lachnospiraceae *Dorea longicatena* DSM 13814
Rin-1-1	Bacteria Firmicutes Clostridia Clostridiales Lachnospiraceae *Roseburia intestinalis* L1-82
Dha-1-1	Bacteria Firmicutes Clostridia Clostridiales Peptococcaceae *Desulfitobacterium hafniense* DCB-2
Dha-2-1	Bacteria Firmicutes Clostridia Clostridiales Peptococcaceae *Desulfitobacterium hafniense* Y51
Pth-1-1	Bacteria Firmicutes Clostridia Clostridiales Peptococcaceae *Pelotomaculum thermopropionicum* SI
Rob-1-1	Bacteria Firmicutes Clostridia Clostridiales Ruminococcaceae *Ruminococcus obeum* ATCC 29174
Tca-1-1	Bacteria Firmicutes Clostridia Clostridiales Veillonellaceae *Thermosinus carboxydivorans* Nor1
Nth-1-1	Bacteria Firmicutes Clostridia Natranaerobiales Natranaerobiaceae *Natranaerobius thermophilus* JW/NM-WN-LF
Cpa-1-1 to Cpa-1-3	Bacteria Firmicutes Clostridia Thermoanaerobacterales Thermoanaerobacteraceae *Carboxydibrachium pacificum* DSM 12653
Chy-1-1	Bacteria Firmicutes Clostridia Thermoanaerobacterales Thermoanaerobacteraceae *Carboxydothermus hydrogenoformans* Z-2901
Mth-2-1	Bacteria Firmicutes Clostridia Thermoanaerobacterales Thermoanaerobacteraceae Moorella group *Moorella thermoacetica* ATCC 39073
Tps-1-1 to Tps-1-2	Bacteria Firmicutes Clostridia Thermoanaerobacterales Thermoanaerobacteraceae *Thermoanaerobacter pseudethanolicus* ATCC 33223
Tsp-2-1 to Tsp-2-2	Bacteria Firmicutes Clostridia Thermoanaerobacterales Thermoanaerobacteraceae *Thermoanaerobacter sp.* X514
Tte-1-1 to Tte-1-3	Bacteria Firmicutes Clostridia Thermoanaerobacterales Thermoanaerobacteraceae *Thermoanaerobacter tengcongensis* MB4
Csa-1-1 to Csa-1-2	Bacteria Firmicutes Clostridia Thermoanaerobacterales Thermoanaerobacterales Family III. Incertae Sedis *Caldicellulosiruptor saccharolyticus* DSM 8903
Ebi-1-1	Bacteria Firmicutes Erysipelotrichi Erysipelotrichales Erysipelotrichaceae *Eubacterium biforme* DSM 3989
Csp-4-1	Bacteria Firmicutes Lactobacillales Carnobacteriaceae *Carnobacterium sp.* AT7
Efa-1-1	Bacteria Firmicutes Lactobacillales Enterococcaceae *Enterococcus faecalis* OG1RF
Efa-2-1	Bacteria Firmicutes Lactobacillales Enterococcaceae *Enterococcus faecalis* V583
Efa-3-1	Bacteria Firmicutes Lactobacillales Enterococcaceae *Enterococcus faecium* DO
Lac-1-1	Bacteria Firmicutes Lactobacillales Lactobacillaceae *Lactobacillus acidophilus* NCFM
Lca-1-1 to Lca-1-2	Bacteria Firmicutes Lactobacillales Lactobacillaceae *Lactobacillus casei* ATCC 334
Lca-2-1 to Lca-2-2	Bacteria Firmicutes Lactobacillales Lactobacillaceae *Lactobacillus casei* BL23
Lga-1-1 to Lga-1-2	Bacteria Firmicutes Lactobacillales Lactobacillaceae *Lactobacillus gasseri* ATCC 33323
Lga-2-1 to Lga-2-2	Bacteria Firmicutes Lactobacillales Lactobacillaceae *Lactobacillus gasseri* MV-22
Lhe-1-1	Bacteria Firmicutes Lactobacillales Lactobacillaceae *Lactobacillus helveticus* DPC 4571
Ljo-1-1 to Ljo-1-2	Bacteria Firmicutes Lactobacillales Lactobacillaceae *Lactobacillus johnsonii* NCC 533
Lpa-1-1 to Lpa-1-2	Bacteria Firmicutes Lactobacillales Lactobacillaceae *Lactobacillus paracasei subsp. paracasei* 8700:2
Lpl-1-1	Bacteria Firmicutes Lactobacillales Lactobacillaceae *Lactobacillus plantarum* WCFS1
Lrh-1-1	Bacteria Firmicutes Lactobacillales Lactobacillaceae *Lactobacillus rhamnosus* HN001
Lla-1-1 to Lla-1-2	Bacteria Firmicutes Lactobacillales Streptococcaceae *Lactococcus lactis subsp. cremoris* MG1363
Lla-2-1 to Lla-2-2	Bacteria Firmicutes Lactobacillales Streptococcaceae *Lactococcus lactis subsp. cremoris* SK11
Lla-3-1 to Lla-3-2	Bacteria Firmicutes Lactobacillales Streptococcaceae *Lactococcus lactis subsp. lactis* Il1403
Tye-1-1	Bacteria Nitrospirae Nitrospirales Nitrospiraceae *Thermodesulfovibrio yellowstonii* DSM 11347
Bja-1-1	Bacteria Proteobacteria Alphaproteobacteria Rhizobiales Bradyrhizobiaceae *Bradyrhizobium japonicum* USDA 110
Nha-1-1 to Nha-1-2	Bacteria Proteobacteria Alphaproteobacteria Rhizobiales Bradyrhizobiaceae *Nitrobacter hamburgensis* X14
Nsp-2-1	Bacteria Proteobacteria Alphaproteobacteria Rhizobiales Bradyrhizobiaceae *Nitrobacter sp.* Nb-311A
Nwi-1-1	Bacteria Proteobacteria Alphaproteobacteria Rhizobiales Bradyrhizobiaceae *Nitrobacter winogradskyi* Nb-255
Oca-1-1	Bacteria Proteobacteria Alphaproteobacteria Rhizobiales Bradyrhizobiaceae *Oligotropha carboxidovorans* OM5
Rpa-1-1	Bacteria Proteobacteria Alphaproteobacteria Rhizobiales Bradyrhizobiaceae *Rhodopseudomonas palustris* HaA2
Oan-1-1	Bacteria Proteobacteria Alphaproteobacteria Rhizobiales Brucellaceae *Ochrobactrum anthropi* ATCC 49188
Mra-1-1	Bacteria Proteobacteria Alphaproteobacteria Rhizobiales Methylobacteriaceae *Methylobacterium radiotolerans* JCM 2831

Msp-1-1	Bacteria Proteobacteria Alphaproteobacteria Rhizobiales Methylobacteriaceae *Methylobacterium sp.* 4-46
Acr-1-1 to Acr-1-2	Bacteria Proteobacteria Alphaproteobacteria Rhodospirillales Acetobacteraceae *Acidiphilium cryptum* JF-5
Gdi-1-1 to Gdi-1-4	Bacteria Proteobacteria Alphaproteobacteria Rhodospirillales Acetobacteraceae *Gluconacetobacter diazotrophicus* PAl 5
Gox-1-1 to Gox-1-3	Bacteria Proteobacteria Alphaproteobacteria Rhodospirillales Acetobacteraceae *Gluconobacter oxydans* 621H
Rru-1-1	Bacteria Proteobacteria Alphaproteobacteria Rhodospirillales Rhodospirillaceae *Rhodospirillum rubrum* ATCC 11170
Zmo-1-1	Bacteria Proteobacteria Alphaproteobacteria Sphingomonadales Sphingomonadaceae *Zymomonas mobilis subsp. mobilis* ZM4
Bam-1-1	Bacteria Proteobacteria Betaproteobacteria Burkholderiales Burkholderiaceae Burkholderia Burkholderia cepacia complex *Burkholderia ambifaria* AMMD
Bam-2-1 to Bam-2-3	Bacteria Proteobacteria Betaproteobacteria Burkholderiales Burkholderiaceae Burkholderia Burkholderia cepacia complex *Burkholderia ambifaria* IOP40-10
Bam-3-1	Bacteria Proteobacteria Betaproteobacteria Burkholderiales Burkholderiaceae Burkholderia Burkholderia cepacia complex *Burkholderia ambifaria* MC40-6
Bam-4-1	Bacteria Proteobacteria Betaproteobacteria Burkholderiales Burkholderiaceae Burkholderia Burkholderia cepacia complex *Burkholderia ambifaria* MEX-5
Bce-15-1	Bacteria Proteobacteria Betaproteobacteria Burkholderiales Burkholderiaceae Burkholderia Burkholderia cepacia complex *Burkholderia cenocepacia* AU 1054
Bce-16-1	Bacteria Proteobacteria Betaproteobacteria Burkholderiales Burkholderiaceae Burkholderia Burkholderia cepacia complex *Burkholderia cenocepacia* HI2424
Bce-17-1	Bacteria Proteobacteria Betaproteobacteria Burkholderiales Burkholderiaceae Burkholderia Burkholderia cepacia complex *Burkholderia cenocepacia* J2315
Bce-18-1	Bacteria Proteobacteria Betaproteobacteria Burkholderiales Burkholderiaceae Burkholderia Burkholderia cepacia complex *Burkholderia cenocepacia* MC0-3
Bce-19-1	Bacteria Proteobacteria Betaproteobacteria Burkholderiales Burkholderiaceae Burkholderia Burkholderia cepacia complex *Burkholderia cenocepacia* PC184
Bmu-1-1 to Bmu-1-2	Bacteria Proteobacteria Betaproteobacteria Burkholderiales Burkholderiaceae Burkholderia Burkholderia cepacia complex *Burkholderia multivorans* ATCC 17616
Bsp-1-1	Bacteria Proteobacteria Betaproteobacteria Burkholderiales Burkholderiaceae Burkholderia Burkholderia cepacia complex *Burkholderia sp.* 383
Bvi-1-1	Bacteria Proteobacteria Betaproteobacteria Burkholderiales Burkholderiaceae Burkholderia Burkholderia cepacia complex *Burkholderia vietnamiensis* G4
Bgr-1-1	Bacteria Proteobacteria Betaproteobacteria Burkholderiales Burkholderiaceae *Burkholderia graminis* C4D1M
Bma-1-1	Bacteria Proteobacteria Betaproteobacteria Burkholderiales Burkholderiaceae *Burkholderia mallei* ATCC 10399
Bma-2-1	Bacteria Proteobacteria Betaproteobacteria Burkholderiales Burkholderiaceae *Burkholderia mallei* ATCC 23344
Bma-3-1	Bacteria Proteobacteria Betaproteobacteria Burkholderiales Burkholderiaceae *Burkholderia mallei* FMH
Bma-4-1	Bacteria Proteobacteria Betaproteobacteria Burkholderiales Burkholderiaceae *Burkholderia mallei* GB8 horse 4
Bma-5-1	Bacteria Proteobacteria Betaproteobacteria Burkholderiales Burkholderiaceae *Burkholderia mallei* JHU
Bma-6-1	Bacteria Proteobacteria Betaproteobacteria Burkholderiales Burkholderiaceae *Burkholderia mallei* NCTC 10229
Bma-7-1	Bacteria Proteobacteria Betaproteobacteria Burkholderiales Burkholderiaceae *Burkholderia mallei* NCTC 10247
Bma-8-1	Bacteria Proteobacteria Betaproteobacteria Burkholderiales Burkholderiaceae *Burkholderia mallei* PRL-20
Bma-9-1	Bacteria Proteobacteria Betaproteobacteria Burkholderiales Burkholderiaceae *Burkholderia mallei* SAVP1
Bph-1-1	Bacteria Proteobacteria Betaproteobacteria Burkholderiales Burkholderiaceae *Burkholderia phymatum* STM815
Bph-2-1 to Bph-2-2	Bacteria Proteobacteria Betaproteobacteria Burkholderiales Burkholderiaceae *Burkholderia phytofirmans* PsJN
Bsp-2-1	Bacteria Proteobacteria Betaproteobacteria Burkholderiales Burkholderiaceae *Burkholderia sp.* H160
Bub-1-1	Bacteria Proteobacteria Betaproteobacteria Burkholderiales Burkholderiaceae *Burkholderia ubonensis* Bu
Bxe-1-1	Bacteria Proteobacteria Betaproteobacteria Burkholderiales Burkholderiaceae *Burkholderia xenovorans* LB400
Bok-1-1	Bacteria Proteobacteria Betaproteobacteria Burkholderiales Burkholderiaceae Burkholderia pseudomallei group *Burkholderia oklahomensis* C6786
Bok-2-1	Bacteria Proteobacteria Betaproteobacteria Burkholderiales Burkholderiaceae Burkholderia pseudomallei group *Burkholderia oklahomensis* EO147
Bps-1-1	Bacteria Proteobacteria Betaproteobacteria Burkholderiales Burkholderiaceae Burkholderia pseudomallei group *Burkholderia pseudomallei* 1106a
Bps-2-1	Bacteria Proteobacteria Betaproteobacteria Burkholderiales Burkholderiaceae Burkholderia pseudomallei group *Burkholderia pseudomallei* 1106b
Bps-3-1	Bacteria Proteobacteria Betaproteobacteria Burkholderiales Burkholderiaceae Burkholderia pseudomallei group *Burkholderia pseudomallei* 112
Bps-4-1	Bacteria Proteobacteria Betaproteobacteria Burkholderiales Burkholderiaceae Burkholderia pseudomallei group *Burkholderia pseudomallei* 14
Bps-5-1	Bacteria Proteobacteria Betaproteobacteria Burkholderiales Burkholderiaceae Burkholderia pseudomallei group *Burkholderia pseudomallei* 1655
Bps-6-1	Bacteria Proteobacteria Betaproteobacteria Burkholderiales Burkholderiaceae Burkholderia pseudomallei group *Burkholderia pseudomallei* 1710a
Bps-7-1	Bacteria Proteobacteria Betaproteobacteria Burkholderiales Burkholderiaceae Burkholderia pseudomallei group *Burkholderia pseudomallei* 1710b
Bps-8-1	Bacteria Proteobacteria Betaproteobacteria Burkholderiales Burkholderiaceae Burkholderia pseudomallei group *Burkholderia pseudomallei* 305
Bps-9-1	Bacteria Proteobacteria Betaproteobacteria Burkholderiales Burkholderiaceae Burkholderia pseudomallei group *Burkholderia pseudomallei* 406e
Bps-10-1	Bacteria Proteobacteria Betaproteobacteria Burkholderiales Burkholderiaceae Burkholderia pseudomallei group *Burkholderia pseudomallei* 668
Bps-11-1	Bacteria Proteobacteria Betaproteobacteria Burkholderiales Burkholderiaceae Burkholderia pseudomallei group *Burkholderia pseudomallei* 7894
Bps-12-1	Bacteria Proteobacteria Betaproteobacteria Burkholderiales Burkholderiaceae Burkholderia pseudomallei group *Burkholderia pseudomallei* 9
Bps-13-1	Bacteria Proteobacteria Betaproteobacteria Burkholderiales Burkholderiaceae Burkholderia pseudomallei group *Burkholderia pseudomallei* 91
Bps-14-1	Bacteria Proteobacteria Betaproteobacteria Burkholderiales Burkholderiaceae Burkholderia pseudomallei group *Burkholderia pseudomallei* B7210
Bps-15-1	Bacteria Proteobacteria Betaproteobacteria Burkholderiales Burkholderiaceae Burkholderia pseudomallei group *Burkholderia pseudomallei* BCC215
Bps-16-1	Bacteria Proteobacteria Betaproteobacteria Burkholderiales Burkholderiaceae Burkholderia pseudomallei group *Burkholderia pseudomallei* DM98
Bps-17-1	Bacteria Proteobacteria Betaproteobacteria Burkholderiales Burkholderiaceae Burkholderia pseudomallei group *Burkholderia pseudomallei* K96243
Bps-18-1	Bacteria Proteobacteria Betaproteobacteria Burkholderiales Burkholderiaceae Burkholderia pseudomallei group *Burkholderia pseudomallei* NCTC 13177
Bps-19-1	Bacteria Proteobacteria Betaproteobacteria Burkholderiales Burkholderiaceae Burkholderia pseudomallei group *Burkholderia pseudomallei* Pasteur 52237
Bps-20-1	Bacteria Proteobacteria Betaproteobacteria Burkholderiales Burkholderiaceae Burkholderia pseudomallei group *Burkholderia pseudomallei* S13
Bth-5-1	Bacteria Proteobacteria Betaproteobacteria Burkholderiales Burkholderiaceae Burkholderia pseudomallei group *Burkholderia thailandensis* Bt4
Bth-6-1 to Bth-6-2	Bacteria Proteobacteria Betaproteobacteria Burkholderiales Burkholderiaceae Burkholderia pseudomallei group *Burkholderia thailandensis* E264
Bth-7-1	Bacteria Proteobacteria Betaproteobacteria Burkholderiales Burkholderiaceae Burkholderia pseudomallei group *Burkholderia thailandensis* MSMB43
Bth-8-1	Bacteria Proteobacteria Betaproteobacteria Burkholderiales Burkholderiaceae Burkholderia pseudomallei group *Burkholderia thailandensis* TXDOH
Rpi-1-1	Bacteria Proteobacteria Betaproteobacteria Burkholderiales Burkholderiaceae *Ralstonia pickettii* 12D
Rpi-2-1	Bacteria Proteobacteria Betaproteobacteria Burkholderiales Burkholderiaceae *Ralstonia pickettii* 12J
Rso-1-1	Bacteria Proteobacteria Betaproteobacteria Burkholderiales Burkholderiaceae *Ralstonia solanacearum* GMI1000
Rso-2-1	Bacteria Proteobacteria Betaproteobacteria Burkholderiales Burkholderiaceae *Ralstonia solanacearum* IPO1609
Rso-3-1	Bacteria Proteobacteria Betaproteobacteria Burkholderiales Burkholderiaceae *Ralstonia solanacearum* MolK2
Rso-4-1	Bacteria Proteobacteria Betaproteobacteria Burkholderiales Burkholderiaceae *Ralstonia solanacearum* UW551

Dac-1-1 to Dac-1-2	Bacteria Proteobacteria Betaproteobacteria Burkholderiales Comamonadaceae *Delftia acidovorans* SPH-1
Cvi-1-1	Bacteria Proteobacteria Betaproteobacteria Neisseriales Neisseriaceae *Chromobacterium violaceum* ATCC 12472
Neu-1-1 to Neu-1-3	Bacteria Proteobacteria Betaproteobacteria Nitrosomonadales Nitrosomonadaceae *Nitrosomonas europaea* ATCC 19718
Dol-1-1	Bacteria Proteobacteria Deltaproteobacteria Desulfobacterales Desulfobacteraceae *Desulfococcus oleovorans* Hxd3
Dpi-1-1	Bacteria Proteobacteria Deltaproteobacteria Desulfovibrionales Desulfovibrionaceae *Desulfovibrio piger* ATCC 29098
Dac-2-1	Bacteria Proteobacteria Deltaproteobacteria Desulfuromonadales Desulfuromonadaceae *Desulfuromonas acetoxidans* DSM 684
Sfu-1-1	Bacteria Proteobacteria Deltaproteobacteria Syntrophobacterales Syntrophobacteraceae *Syntrophobacter fumaroxidans* MPOB
Afe-1-1	Bacteria Proteobacteria Gammaproteobacteria Acidithiobacillales Acidithiobacillaceae *Acidithiobacillus ferrooxidans* ATCC 53993
Cko-1-1 to Cko-1-3	Bacteria Proteobacteria Gammaproteobacteria Enterobacteriales Enterobacteriaceae *Citrobacter koseri* ATCC BAA-895
Eta-1-1	Bacteria Proteobacteria Gammaproteobacteria Enterobacteriales Enterobacteriaceae *Erwinia tasmaniensis* Et1/99
Kpn-1-1	Bacteria Proteobacteria Gammaproteobacteria Enterobacteriales Enterobacteriaceae *Klebsiella pneumoniae subsp. pneumoniae* MGH 78578
Pat-1-1	Bacteria Proteobacteria Gammaproteobacteria Enterobacteriales Enterobacteriaceae *Pectobacterium atrosepticum* SCRI1043
Pca-1-1	Bacteria Proteobacteria Gammaproteobacteria Enterobacteriales Enterobacteriaceae *Pectobacterium carotovorum subsp. brasiliensis* PBR1692
Pca-2-1	Bacteria Proteobacteria Gammaproteobacteria Enterobacteriales Enterobacteriaceae *Pectobacterium carotovorum subsp. carotovorum* WPP14
Pal-1-1	Bacteria Proteobacteria Gammaproteobacteria Enterobacteriales Enterobacteriaceae *Providencia alcalifaciens* DSM 30120
Pru-1-1	Bacteria Proteobacteria Gammaproteobacteria Enterobacteriales Enterobacteriaceae *Providencia rustigianii* DSM 4541
Pst-1-1	Bacteria Proteobacteria Gammaproteobacteria Enterobacteriales Enterobacteriaceae *Providencia stuartii* ATCC 25827
Spr-1-1	Bacteria Proteobacteria Gammaproteobacteria Enterobacteriales Enterobacteriaceae *Serratia proteamaculans* 568
Sgl-1-1	Bacteria Proteobacteria Gammaproteobacteria Enterobacteriales Enterobacteriaceae *Sodalis glossinidius str. 'morsitans'*
Yin-1-1	Bacteria Proteobacteria Gammaproteobacteria Enterobacteriales Enterobacteriaceae *Yersinia intermedia* ATCC 29909
Ymo-1-1	Bacteria Proteobacteria Gammaproteobacteria Enterobacteriales Enterobacteriaceae *Yersinia mollaretii* ATCC 43969
Ype-1-1	Bacteria Proteobacteria Gammaproteobacteria Enterobacteriales Enterobacteriaceae *Yersinia pestis* Angola
Ype-2-1	Bacteria Proteobacteria Gammaproteobacteria Enterobacteriales Enterobacteriaceae *Yersinia pestis* Antiqua
Ype-9-1	Bacteria Proteobacteria Gammaproteobacteria Enterobacteriales Enterobacteriaceae *Yersinia pestis biovar* Antiqua str. B42003004
Ype-10-1	Bacteria Proteobacteria Gammaproteobacteria Enterobacteriales Enterobacteriaceae *Yersinia pestis biovar* Antiqua str. E1979001
Ype-11-1	Bacteria Proteobacteria Gammaproteobacteria Enterobacteriales Enterobacteriaceae *Yersinia pestis biovar* Antiqua str. UG05-0454
Ype-12-1	Bacteria Proteobacteria Gammaproteobacteria Enterobacteriales Enterobacteriaceae *Yersinia pestis biovar* Mediaevalis str. K1973002
Ype-13-1	Bacteria Proteobacteria Gammaproteobacteria Enterobacteriales Enterobacteriaceae *Yersinia pestis biovar* Microtus str. 91001
Ype-14-1	Bacteria Proteobacteria Gammaproteobacteria Enterobacteriales Enterobacteriaceae *Yersinia pestis biovar* Orientalis str. F1991016
Ype-15-1	Bacteria Proteobacteria Gammaproteobacteria Enterobacteriales Enterobacteriaceae *Yersinia pestis biovar* Orientalis str. IP275
Ype-16-1	Bacteria Proteobacteria Gammaproteobacteria Enterobacteriales Enterobacteriaceae *Yersinia pestis biovar* Orientalis str. MG05-1020
Ype-3-1	Bacteria Proteobacteria Gammaproteobacteria Enterobacteriales Enterobacteriaceae *Yersinia pestis* CA88-4125
Ype-4-1	Bacteria Proteobacteria Gammaproteobacteria Enterobacteriales Enterobacteriaceae *Yersinia pestis* CO92
Ype-5-1	Bacteria Proteobacteria Gammaproteobacteria Enterobacteriales Enterobacteriaceae *Yersinia pestis* FV-1
Ype-6-1	Bacteria Proteobacteria Gammaproteobacteria Enterobacteriales Enterobacteriaceae *Yersinia pestis* KIM
Ype-7-1	Bacteria Proteobacteria Gammaproteobacteria Enterobacteriales Enterobacteriaceae *Yersinia pestis* Nepal516
Ype-8-1	Bacteria Proteobacteria Gammaproteobacteria Enterobacteriales Enterobacteriaceae *Yersinia pestis* Pestoides F
Yps-1-1	Bacteria Proteobacteria Gammaproteobacteria Enterobacteriales Enterobacteriaceae *Yersinia pseudotuberculosis* IP 31758
Yps-2-1	Bacteria Proteobacteria Gammaproteobacteria Enterobacteriales Enterobacteriaceae *Yersinia pseudotuberculosis* IP 32953
Yps-3-1	Bacteria Proteobacteria Gammaproteobacteria Enterobacteriales Enterobacteriaceae *Yersinia pseudotuberculosis* PB1/+
Yps-4-1	Bacteria Proteobacteria Gammaproteobacteria Enterobacteriales Enterobacteriaceae *Yersinia pseudotuberculosis* YPIII
Csa-2-1	Bacteria Proteobacteria Gammaproteobacteria Oceanospirillales Halomonadaceae *Chromohalobacter salexigens* DSM 3043
Aba-2-1	Bacteria Proteobacteria Gammaproteobacteria Pseudomonadales Moraxellaceae *Acinetobacter baumannii* AB0057
Aba-3-1	Bacteria Proteobacteria Gammaproteobacteria Pseudomonadales Moraxellaceae *Acinetobacter baumannii* ACICU
Aba-4-1	Bacteria Proteobacteria Gammaproteobacteria Pseudomonadales Moraxellaceae *Acinetobacter baumannii* ATCC 17978
Aba-5-1	Bacteria Proteobacteria Gammaproteobacteria Pseudomonadales Moraxellaceae *Acinetobacter baumannii* AYE
Aba-6-1	Bacteria Proteobacteria Gammaproteobacteria Pseudomonadales Moraxellaceae *Acinetobacter baumannii* SDF
Asp-1-1	Bacteria Proteobacteria Gammaproteobacteria Pseudomonadales Moraxellaceae *Acinetobacter sp.* ADP1
Asp-2-1	Bacteria Proteobacteria Gammaproteobacteria Pseudomonadales Moraxellaceae *Acinetobacter sp.* ATCC 27244
Pae-1-1	Bacteria Proteobacteria Gammaproteobacteria Pseudomonadales Pseudomonadaceae *Pseudomonas aeruginosa* 2192
Pae-2-1	Bacteria Proteobacteria Gammaproteobacteria Pseudomonadales Pseudomonadaceae *Pseudomonas aeruginosa* C3719
Pae-3-1	Bacteria Proteobacteria Gammaproteobacteria Pseudomonadales Pseudomonadaceae *Pseudomonas aeruginosa* PA7
Pae-4-1	Bacteria Proteobacteria Gammaproteobacteria Pseudomonadales Pseudomonadaceae *Pseudomonas aeruginosa* PACS2
Pae-5-1	Bacteria Proteobacteria Gammaproteobacteria Pseudomonadales Pseudomonadaceae *Pseudomonas aeruginosa* PAO1
Pae-6-1	Bacteria Proteobacteria Gammaproteobacteria Pseudomonadales Pseudomonadaceae *Pseudomonas aeruginosa* UCBPP-PA14
Pen-1-1	Bacteria Proteobacteria Gammaproteobacteria Pseudomonadales Pseudomonadaceae *Pseudomonas entomophila* L48
Psy-1-1	Bacteria Proteobacteria Gammaproteobacteria Pseudomonadales Pseudomonadaceae *Pseudomonas syringae pv. maculicola*
Psy-2-1	Bacteria Proteobacteria Gammaproteobacteria Pseudomonadales Pseudomonadaceae *Pseudomonas syringae pv. syringae* B728a
Psy-3-1	Bacteria Proteobacteria Gammaproteobacteria Pseudomonadales Pseudomonadaceae *Pseudomonas syringae pv. tomato str.* DC3000
Vba-1-1	Bacteria Proteobacteria Gammaproteobacteria Vibrionales unclassified Vibrionales *Vibrionales bacterium* SWAT-3
Asa-1-1	Bacteria Proteobacteria Gammaproteobacteria Vibrionales Vibrionaceae *Aliivibrio salmonicida* LFI1238
Vfi-1-1 to Vfi-1-2	Bacteria Proteobacteria Gammaproteobacteria Vibrionales Vibrionaceae Aliivibrio *Vibrio fischeri* ES114
Vfi-2-1 to Vfi-2-2	Bacteria Proteobacteria Gammaproteobacteria Vibrionales Vibrionaceae Aliivibrio *Vibrio fischeri* MJ11
Ppr-1-1	Bacteria Proteobacteria Gammaproteobacteria Vibrionales Vibrionaceae *Photobacterium profundum* 3TCK
Ppr-2-1	Bacteria Proteobacteria Gammaproteobacteria Vibrionales Vibrionaceae *Photobacterium profundum* SS9

Psp-3-1	Bacteria Proteobacteria Gammaproteobacteria Vibrionales Vibrionaceae *Photobacterium sp.* SKA34
Val-1-1	Bacteria Proteobacteria Gammaproteobacteria Vibrionales Vibrionaceae *Vibrio alginolyticus* 12G01
Van-1-1	Bacteria Proteobacteria Gammaproteobacteria Vibrionales Vibrionaceae *Vibrio angustum* S14
Vca-1-1	Bacteria Proteobacteria Gammaproteobacteria Vibrionales Vibrionaceae *Vibrio campbellii* AND4
Vha-1-1	Bacteria Proteobacteria Gammaproteobacteria Vibrionales Vibrionaceae *Vibrio harveyi* ATCC BAA-1116
Vha-2-1	Bacteria Proteobacteria Gammaproteobacteria Vibrionales Vibrionaceae *Vibrio harveyi* HY01
Vsh-1-1	Bacteria Proteobacteria Gammaproteobacteria Vibrionales Vibrionaceae *Vibrio shilonii* AK1
Vsp-1-1	Bacteria Proteobacteria Gammaproteobacteria Vibrionales Vibrionaceae *Vibrio sp.* MED222
Vsp-2-1	Bacteria Proteobacteria Gammaproteobacteria Vibrionales Vibrionaceae *Vibrio splendidus* 12B01
Sma-1-1	Bacteria Proteobacteria Gammaproteobacteria Xanthomonadales Xanthomonadaceae *Stenotrophomonas maltophilia* K279a
Sma-2-1	Bacteria Proteobacteria Gammaproteobacteria Xanthomonadales Xanthomonadaceae *Stenotrophomonas maltophilia* R551-3
Xax-1-1	Bacteria Proteobacteria Gammaproteobacteria Xanthomonadales Xanthomonadaceae *Xanthomonas axonopodis pv. citri str.* 306
Xca-1-1	Bacteria Proteobacteria Gammaproteobacteria Xanthomonadales Xanthomonadaceae *Xanthomonas campestris pv. campestris str.* 8004
Xca-2-1	Bacteria Proteobacteria Gammaproteobacteria Xanthomonadales Xanthomonadaceae *Xanthomonas campestris pv. campestris str.* ATCC 33913
Xca-3-1	Bacteria Proteobacteria Gammaproteobacteria Xanthomonadales Xanthomonadaceae *Xanthomonas campestris pv. campestris str.* B100
Xca-4-1	Bacteria Proteobacteria Gammaproteobacteria Xanthomonadales Xanthomonadaceae *Xanthomonas campestris pv. vesicatoria str.* 85-10
Xor-1-1	Bacteria Proteobacteria Gammaproteobacteria Xanthomonadales Xanthomonadaceae *Xanthomonas oryzae pv. oryzae* KACC10331
Xor-2-1	Bacteria Proteobacteria Gammaproteobacteria Xanthomonadales Xanthomonadaceae *Xanthomonas oryzae pv. oryzae* MAFF 311018
Xor-3-1 to Xor-3-2	Bacteria Proteobacteria Gammaproteobacteria Xanthomonadales Xanthomonadaceae *Xanthomonas oryzae pv. oryzae* PXO99A
Xor-4-1	Bacteria Proteobacteria Gammaproteobacteria Xanthomonadales Xanthomonadaceae *Xanthomonas oryzae pv. oryzicola* BLS256
Mpi-1-1	Bacteria Thermotogae Thermotogales Thermotogaceae *Marinitoga piezophila* KA3
Tle-1-1	Bacteria Thermotogae Thermotogales Thermotogaceae *Thermotoga lettingae* TMO
Tpe-1-1	Bacteria Thermotogae Thermotogales Thermotogaceae *Thermotoga petrophila* RKU-1
Tsp-3-1	Bacteria Thermotogae Thermotogales Thermotogaceae *Thermotoga sp.* RQ2
Cfl-1-1	Bacteria Verrucomicrobia Spartobacteria *Chthoniobacter flavus* Ellin428
Min-1-1 to Min-1-2	Bacteria Verrucomicrobia unclassified Verrucomicrobia *Methylacidiphilum infernorum* V4
bEl-1-1	Bacteria Verrucomicrobia Verrucomicrobiae Verrucomicrobiales Verrucomicrobia subdivision 3 bacterium Ellin514
env-1 to env-45	environmental samples

24.2 Notes

Superscript numbers are used to annotate any *crcB* RNA that has special characteristics (described below). These numeric annotations will be used in Sections 24.3 and 24.5. The meaning of the numbers are as follows.

[1] This RNA is upstream of locus SSO1026.

24.3 Gene contexts

Each *crcB* RNA (indicated by "RNA→") is listed with its surrounding genes. Environmental sequences and some RefSeq entries lack gene annotations, and no genes are listed for such sequences. The direction of each gene is indicated with an arrow (→), and each conserved domain in the gene is colored. Conserved domains associated with more than one *crcB* RNA are assigned a color; other domains are gray. Information about these conserved domains is given in Section 24.4. The accession number of the sequence containing each *crcB* RNA is given. Accessions beginning with "NC_" or "NZ_" are RefSeq, while other accession refer to environmental samples. Nucleotide coordinates are given for the 5′ and 3′ boundaries of each. If the 5′ coordinate is greater than the 3′ coordinate, the RNA is present on the reverse-complement strand of the containing genomic DNA sequence. Each hit is denoted by an abbreviation (like "Eco-1-1") that refers to a taxonomy given in Section 24.1. Superscript numbers refer to annotations listed in Section 24.2

**abbrev.**	**Seq. accession**		**5′ at**	**3′ at**	**genes**
Archaea Crenarchaeota					
[1]Sso-1-1	NC_002754.1	-	885812	885728	**RNA**→ HyfC (COG0650)→ HyfE (COG4237)→ HyfB (COG0651)→ HycE (COG3261)→ COG3260 (COG3260)→ HyfB (COG0651)→ hypo→

Mse-1-1	NC_009440.1	+	1397941	1398019	**RNA**→ HyfB (COG0651)→ HycE (COG3261)→ COG3260 (COG3260)→ HyfB (COG0651)→ HyfC (COG0650)→ HyfE (COG4237)→
Archaea Euryarchaeota					
Pto-1-1	NC_005877.1	-	847940	847870	**RNA**→ HyfC (COG0650)→ HyfE (COG4237)→ HyfB (COG0651)→ HycE (COG3261)→ COG3260 (COG3260)→ HyfB (COG0651)→
Tvo-1-1	NC_002689.2	+	669722	669784	**RNA**→ MutS (COG0249)→ MutS (COG0249)→
Mhu-1-1	NC_007796.1	+	1236994	1237053	**RNA**→ COG1689 (COG1689)→ hypo→
Tvo-1-2	NC_002689.2	+	1528463	1528534	**RNA**→ HyfC (COG0650)→ HyfE (COG4237)→ HyfB (COG0651)→ HycE (COG3261)→ COG3260 (COG3260)→ HyfB (COG0651)→
CMe-1-1	NC_009712.1	+	914945	915010	**RNA**→ enolase→ COG0218 (COG0218)→
Mhu-1-2	NC_007796.1	-	1244505	1244443	**RNA**→ ←COG1993 (COG1993)
Abo-1-1	NZ_ABSD01000048.1	+	33401	33462	**RNA**→
Abo-1-2	NZ_ABSD01000051.1	-	73291	73230	**RNA**→
Abo-1-3	NZ_ABSD01000048.1	-	169379	169321	**RNA**→
Abo-1-4	NZ_ABSD01000050.1	+	82708	82766	**RNA**→
Mhu-1-3	NC_007796.1	+	1238338	1238398	**RNA**→ Era (COG1159)→
Msm-1-1	NC_009515.1	+	1167738	1167808	**RNA**→ KefB (COG0475)→
Msm-2-1	NZ_ABYV01000006.1	-	24382	24312	**RNA**→
Msm-3-1	NZ_ABYW01000007.1	+	311285	311355	**RNA**→
Mth-1-1	NC_000916.1	-	688929	688867	**RNA**→ NhaP (COG0025)→
CMe-1-2	NC_009712.1	-	914829	914764	**RNA**→ CrcB (COG0239)→ CrcB (COG0239)→ COG1993 (COG1993)→
Tko-1-1	NC_006624.1	+	440492	440567	**RNA**→ COG1689 (COG1689)→ CrcB (COG0239)→ COG1993 (COG1993)→ hypo→ Dfp (COG0452)→ COG4087 (COG4087)→ COG1739 (COG1739)→
Ton-1-1	NC_011529.1	+	737518	737592	**RNA**→ COG1689 (COG1689)→ hypo→ hypo→ hypo→ CrcB (COG0239)→ COG1993 (COG1993)→
Tba-1-1	NZ_ABSF01000003.1	+	198437	198512	**RNA**→
Tsp-1-1	NZ_ABXN01000016.1	-	250900	250825	**RNA**→
Pab-1-1	NC_000868.1	+	690134	690214	**RNA**→ COG1689 (COG1689)→ hypo→ hypo→ COG2520 (COG2520)→
Pfu-1-1	NC_003413.1	-	1333263	1333185	**RNA**→ COG1689 (COG1689)→ hypo→
Pho-1-1	NC_000961.1	-	1278365	1278285	**RNA**→ COG1689 (COG1689)→ hypo→ hypo→ COG2520 (COG2520)→
Afu-1-1	NC_000917.1	+	1391410	1391471	**RNA**→ COG1689 (COG1689)→ HTH_ARSR (smart00418)→ DUF432 (pfam04254)→ MS_channel (pfam00924)→ hypo→
Mst-1-1	NC_007681.1	+	1672674	1672743	**RNA**→ KefB (COG0475)→
Mac-1-1	NC_003552.1	+	4831358	4831427	**RNA**→ CrcB (COG0239)→ COG1993 (COG1993)→
Mba-1-1	NC_007355.1	-	4790369	4790299	**RNA**→ CrcB (COG0239)→ COG1993 (COG1993)→ pyrophosphatase (cd00412)→
Bacteria					
Gvi-1-1	NC_005125.1	-	2366225	2366144	**RNA**→ CrcB (COG0239)→
Ssp-2-1	NC_007775.1	-	520035	519935	**RNA**→ USP_Like (cd00293)NhaP (COG0025)→
Ssp-3-1	NC_010474.1	-	84698	84619	**RNA**→ USP_Like (cd00293)NhaP (COG0025)→
Sel-1-1	NC_006576.1	-	2053114	2053041	**RNA**→ USP_Like (cd00293)NhaP (COG0025)→
Sel-2-1	NC_007604.1	+	2263916	2263989	**RNA**→ USP_Like (cd00293)NhaP (COG0025)→
Ssp-1-1	NC_007776.1	-	171311	171206	**RNA**→ USP_Like (cd00293)NhaP (COG0025)→ CrcB (COG0239)→
Ava-1-1	NC_007413.1	+	118149	118230	**RNA**→ USP_Like (cd00293)NhaP (COG0025)→
Nsp-1-1	NC_003272.1	+	2723228	2723309	**RNA**→ USP_Like (cd00293)NhaP (COG0025)→
Dth-1-1	NC_011297.1	+	51886	51949	**RNA**→ CrcB (COG0239)→ CrcB (COG0239)→
Tye-1-1	NC_011296.1	+	1737215	1737284	**RNA**→ KefB (COG0475)→ USP_Like (cd00293)→
Taq-1-1	NZ_ABVK01000004.1	-	157546	157484	**RNA**→ DNA mismatch repair protein MutS domain protein→
Dge-1-1	NC_008010.2	+	504027	504091	**RNA**→ CrcB (COG0239)→ COG1993 (COG1993)→ COG1993 (COG1993)→ hypo→
Aba-1-1	NC_008009.1	-	3066978	3066906	**RNA**→ CrcB (COG0239)→ Acetyltransf_1 (pfam00583)→ GEWL (cd01021)→
Hsp-1-1	NC_011126.1	-	523442	523357	**RNA**→ CrcB (COG0239)→ COG1993 (COG1993)→ COG1993 (COG1993)→ EriC (COG0038)→ Pyruvate_Kinase (cd00288)→
Hsp-1-2	NC_011126.1	+	516815	516883	**RNA**→ hypo→ ISOPREN_C2_like (cd00688)→

Bacteria Actinobacteria					
Ban-1-1	NZ_ABYS01000027.1	-	262836	262758	**RNA**→
Lxy-1-1	NC_006087.1	-	68491	68420	**RNA**→ CrcB (COG0239)→ CrcB (COG0239)→
Mab-1-1	NC_010397.1	+	2498027	2498099	**RNA**→ CrcB (COG0239)→ CrcB (COG0239)→ Usp (pfam00582)→
Mma-1-1	NC_010612.1	-	5250157	5250088	**RNA**→ NarK (COG2223)→
Mul-1-1	NC_008611.1	-	976465	976395	**RNA**→ hypo→ NarK (COG2223)→
Mma-1-2	NC_010612.1	+	5239035	5239105	**RNA**→ hypo→
Ace-1-1	NC_008578.1	-	804142	804061	**RNA**→ HyfC (COG0650)→ hydrogenase subunit→ HyfB (COG0651)→ HycE (COG3261)Complex1_30kDa (pfam00329)→ COG3260 (COG3260)→ HyfB (COG0651)→
Mul-1-2	NC_008611.1	+	3045327	3045401	**RNA**→ COG1993 (COG1993)DUF190 (pfam02641)→ MalY (COG1168)→
Mma-1-3	NC_010612.1	+	4256142	4256216	**RNA**→ COG1993 (COG1993)DUF190 (pfam02641)→ MalY (COG1168)→
Ace-1-2	NC_008578.1	+	805841	805902	**RNA**→ MutS (COG0249)→ MutS (COG0249)→
Rxy-1-1	NC_008148.1	-	554775	554692	**RNA**→ CRCB (COG0239)→ CrcB (COG0239)→ COG1993 (COG1993)→ hypo→ hypo→ COG1993 (COG1993)→ major facilitator transporter→
Blo-3-1	NZ_ABQQ01000001.1	+	79958	80027	**RNA**→
Blo-2-1	NC_004307.2	+	925740	925809	**RNA**→ CrcB (COG0239)NOG22924 (NOG22924)→ CrcB (COG0239)→ hypo→
Blo-1-1	NC_010816.1	+	969434	969503	**RNA**→ CrcB (COG0239)→ CrcB (COG0239)→ GtrA (pfam04138)→
Blo-1-2	NZ_AABM02000006.1	+	7959	8028	**RNA**→ CrcB (COG0239)→ CrcB (COG0239)→ GtrA (pfam04138)→
Bad-1-1	NC_008618.1	+	922963	923040	**RNA**→ CrcB (COG0239)→ CrcB (COG0239)→
Bad-2-1	NZ_AAXD02000028.1	-	152193	152116	**RNA**→ CrcB (COG0239)→ CrcB (COG0239)→
Nfa-1-1	NC_006363.1	-	61342	61253	**RNA**→ ←putative transposase
Bacteria Bacteroidetes					
Bpl-1-1	NZ_ABQC02000019.1	+	404312	404380	**RNA**→ EriC (COG0038)→
Bco-1-1	NZ_ABIY02000058.1	+	8984	9052	**RNA**→ EriC (COG0038)→
Bth-1-1	NC_004663.1	-	5995735	5995673	**RNA**→ CrcB (COG0239)→ COG1357 (COG1357)→
Bca-1-1	NZ_AAXG02000029.1	+	31858	31932	**RNA**→ Na_H_Exchanger (pfam00999)→
Lbl-1-1	NZ_AANC01000009.1	-	2226	2162	**RNA**→ EriC (COG0038)→
Psp-1-1	NZ_ABCM01000001.1	-	151944	151879	**RNA**→ ←hypo
Psp-1-2	NZ_ABCM01000022.1	+	54798	54866	**RNA**→ PAP2_like_4 (cd03395)→ COG2510 (COG2510)→ enolase (cd03313)→ EriC (COG0038)→
Bacteria Chloroflexi					
Rsp-1-1	NC_009523.1	+	3080181	3080241	**RNA**→ CrcB (COG0239)→ COG1993 (COG1993)COG2524 (COG2524)CBS (pfam00571)→
Cau-1-1	NC_010175.1	+	429462	429523	**RNA**→ ABC_MutS_homologs (cd03243)→ ABC_MutS_homologs (cd03243)→
Csp-1-1	NZ_ABPG01000010.1	+	170766	170827	**RNA**→ ABC_MutS_homologs (cd03243)→ ABC_MutS_homologs (cd03243)→
Cag-1-1	NZ_AAUI01000037.1	+	30963	31024	**RNA**→ Acetyltransf_1 (pfam00583)→
Bacteria Firmicutes					
Ebi-1-1	NZ_ABYT01000096.1	-	44476	44400	**RNA**→
Bacteria Firmicutes Bacillales					
Gsp-2-1	NZ_ABLJ01000009.1	-	43939	43880	**RNA**→ CrcB (COG0239)→ CrcB (COG0239)→ COG2966 (COG2966)→ COG3610 (COG3610)→
Gth-1-1	NC_009328.1	-	2739339	2739277	**RNA**→ CrcB (COG0239)→ CrcB (COG0239)→
Gsp-1-1	NZ_ABVH01000009.1	-	150338	150276	**RNA**→ CrcB (COG0239)→ CrcB (COG0239)→
Gka-1-1	NC_006510.1	-	2726409	2726347	**RNA**→ CrcB (COG0239)→ CrcB (COG0239)→
Bth-2-1	NZ_AAJM01000092.1	-	4654	4595	**RNA**→ CrcB (COG0239)→ CrcB (COG0239)→
Bce-10-1	NZ_ABDJ01000008.1	-	214042	213983	**RNA**→ CrcB (COG0239)→ CrcB (COG0239)→
Bwe-1-1	NC_010184.1	+	4869306	4869365	**RNA**→ CrcB (COG0239)→ CrcB (COG0239)→
Bce-13-1	NZ_ABCZ02000002.1	-	119714	119655	**RNA**→ CrcB (COG0239)→ CrcB (COG0239)→
Bce-12-1	NZ_ABDK02000007.1	-	216428	216369	**RNA**→ CrcB (COG0239)→ CrcB (COG0239)→
Ban-16-1	NC_005945.1	+	4825068	4825127	**RNA**→ CrcB (COG0239)→ CrcB (COG0239)→
Ban-3-1	NC_007530.2	+	4823859	4823918	**RNA**→ CrcB (COG0239)→ CrcB (COG0239)→
Ban-12-1	NC_003997.3	+	4823733	4823792	**RNA**→ CrcB (COG0239)→ CrcB (COG0239)→

Ban-11-1	NZ_AAAC02000001.1	+	165548	165607	**RNA**→ CrcB (COG0239)→ CrcB (COG0239)→
Ban-14-1	NZ_AAEN01000018.1	-	235474	235415	**RNA**→
Ban-10-1	NZ_AAEO01000027.1	-	235458	235399	**RNA**→
Ban-17-1	NZ_AAEP01000006.1	-	11056	10997	**RNA**→
Ban-15-1	NZ_AAEQ01000042.1	+	19179	19238	**RNA**→
Ban-18-1	NZ_AAER01000034.1	-	116308	116249	**RNA**→
Ban-13-1	NZ_AAES01000020.1	+	17811	17870	**RNA**→
Ban-2-1	NZ_ABDN02000026.1	+	19393	19452	**RNA**→ CrcB (COG0239)→ CrcB (COG0239)→
Ban-9-1	NZ_ABJC01000004.1	+	17851	17910	**RNA**→ CrcB (COG0239)→ CrcB (COG0239)→
Ban-5-1	NZ_ABKF01000039.1	-	21440	21381	**RNA**→ CrcB (COG0239)→ CrcB (COG0239)→
Ban-7-1	NZ_ABKG01000033.1	+	18847	18906	**RNA**→ CrcB (COG0239)→ CrcB (COG0239)→
Ban-6-1	NZ_ABLB01000039.1	+	17548	17607	**RNA**→ CrcB (COG0239)→ CrcB (COG0239)→
Ban-8-1	NZ_ABLH01000033.1	+	18167	18226	**RNA**→ CrcB (COG0239)→ CrcB (COG0239)→
Ban-4-1	NZ_ABLT01000037.1	-	10125	10066	**RNA**→ CrcB (COG0239)→ CrcB (COG0239)→
Bth-3-1	NC_005957.1	+	4836662	4836721	**RNA**→ CrcB (COG0239)→ CrcB (COG0239)→
Bce-9-1	NZ_AAEK01000016.1	-	81808	81749	**RNA**→ CrcB (COG0239)→ CrcB (COG0239)→
Bce-8-1	NC_006274.1	+	4897977	4898036	**RNA**→ CrcB (COG0239)→ CrcB (COG0239)→
Bce-4-1	NZ_AAUE01000005.1	-	214692	214633	**RNA**→ CrcB (COG0239)→
Bce-7-1	NZ_ABDI01000008.1	-	206570	206511	**RNA**→ CrcB (COG0239)→ CrcB (COG0239)→
Bce-3-1	NZ_AAUF01000015.1	-	62117	62058	**RNA**→ CrcB (COG0239)→
Bce-11-1	NZ_ABDL02000014.1	-	72280	72221	**RNA**→ CrcB (COG0239)→ CrcB (COG0239)→
Bce-2-1	NZ_ABDA02000004.1	+	142970	143029	**RNA**→ CrcB (COG0239)→ CrcB (COG0239)→
Bth-4-1	NC_008600.1	+	4850198	4850257	**RNA**→ ←hypo
Bce-1-1	NZ_ABDM02000031.1	-	13426	13367	**RNA**→ CrcB (COG0239)→ CrcB (COG0239)→
Bce-5-1	NC_003909.8	+	4815585	4815644	**RNA**→ CrcB (COG0239)→ CrcB (COG0239)→
Bce-6-1	NC_004722.1	+	4975681	4975742	**RNA**→ CrcB (COG0239)→ CrcB (COG0239)→
Bce-14-1	NC_009674.1	+	3708919	3708978	**RNA**→ CrcB (COG0239)→ CrcB (COG0239)→
Gsp-3-1	NZ_ABRG01000006.1	-	47838	47778	**RNA**→ EriC (COG0038)→
Psp-2-1	NZ_ABKS01000027.1	+	41421	41481	**RNA**→ EriC (COG0038)→

Bacteria Firmicutes Clostridia

Mth-2-1	NC_007644.1	+	544530	544593	**RNA**→ CrcB (COG0239)→ camphor resistance protein CrcB→ COG1993 (COG1993)→ tRNA-Gly→ tRNA-Gly→
Tte-1-1	NC_003869.1	-	1840648	1840588	**RNA**→ KefB (COG0475)→ Usp (pfam00582)→ Era (COG1159)→
Cpa-1-1	NZ_ABXP01000064.1	-	2744	2684	**RNA**→
Cpa-1-2	NZ_ABXP01000064.1	-	2857	2798	**RNA**→
Tte-1-2	NC_003869.1	-	1840761	1840702	**RNA**→ KefB (COG0475)→ Usp (pfam00582)→ Era (COG1159)→
Cac-1-1	NC_003030.1	+	1730397	1730460	**RNA**→ CrcB (COG0239)→ CrcB (COG0239)→
Cdi-5-1	NZ_AAML04000007.1	+	96644	96720	**RNA**→ EriC (COG0038)→ NhaP (COG0025)→
Cdi-8-1	NZ_ABFD01000027.1	+	11837	11913	**RNA**→
Cdi-10-1	NZ_ABHF01000064.1	+	26742	26818	**RNA**→ EriC (COG0038)→ NhaP (COG0025)→
Cdi-7-1	NZ_ABHD01000009.1	-	19917	19841	**RNA**→ EriC (COG0038)→ NhaP (COG0025)→
Cdi-4-1	NZ_ABKL01000011.1	-	19174	19098	**RNA**→ EriC (COG0038)→
Cdi-9-1	NZ_ABHE01000037.1	+	26741	26817	**RNA**→ EriC (COG0038)→ NhaP (COG0025)→
Cdi-3-1	NZ_ABKK01000024.1	-	20543	20467	**RNA**→ EriC (COG0038)→ NhaP (COG0025)→
Cdi-2-1	NZ_ABKJ01000031.1	+	18054	18130	**RNA**→ EriC (COG0038)→ NhaP (COG0025)→
Cdi-6-1	NZ_ABHG01000008.1	-	19941	19865	**RNA**→ EriC (COG0038)→ NhaP (COG0025)→
Cdi-1-1	NC_009089.1	+	782179	782255	**RNA**→ EriC (COG0038)→ NhaP (COG0025)→
Cbu-1-1	NZ_ABDT01000004.2	-	28954	28883	**RNA**→ EriC (COG0038)→
Nth-1-1	NC_010718.1	-	285558	285483	**RNA**→ NhaP (COG0025)→
Aor-1-1	NC_009922.1	+	1242223	1242295	**RNA**→ Na_H_Exchanger (pfam00999)→
Ceu-1-1	NZ_ABEY02000020.1	+	92291	92375	**RNA**→ NhaP (COG0025)→ COG0637 (COG0637)→
Cba-1-1	NZ_ABQR01000024.1	-	52165	52090	**RNA**→

Rob-1-1	NZ_AAVO02000004.1	+	13914	13993	**RNA**→ NhaP (COG0025)→ COG3467 (COG3467)→
Dlo-1-1	NZ_AAXB02000001.1	+	235634	235709	**RNA**→ NhaP (COG0025)→
Ahy-1-1	NZ_ABXA01000043.1	+	134014	134086	**RNA**→
Cph-1-1	NC_010001.1	-	3806199	3806127	**RNA**→ NhaP (COG0025)→ MTHFR (cd00537)→ Acm (COG3757)→
Cbo-1-1	NZ_ABCC02000039.1	+	237860	237932	**RNA**→ NhaP (COG0025)→ CN_hydrolase (pfam00795)→ REC (cd00156)BaeS (COG0642)AtoS (COG2202)→
Fma-1-1	NC_010376.1	-	1641037	1640963	**RNA**→ Na_H_Exchanger (pfam00999)→
Ala-1-1	NZ_ABYO01000015.1	+	56802	56875	**RNA**→
Chi-1-1	NZ_ABWP01000024.1	-	12375	12304	**RNA**→ NhaP (COG0025)→
Rin-1-1	NZ_ABYJ01000311.1	-	4868	4795	**RNA**→
Eve-1-1	NZ_AAVL02000037.1	+	287218	287292	**RNA**→ EriC (COG0038)→
Csp-2-1	NZ_ABGC03000023.1	+	52156	52229	**RNA**→ EriC (COG0038)→
Csa-1-1	NC_009437.1	+	751276	751339	**RNA**→ CrcB (COG0239)→ CrcB (COG0239)→
Csa-1-2	NC_009437.1	+	782565	782643	**RNA**→ hypo→
Tps-1-1	NC_010321.1	-	2271016	2270957	**RNA**→ CrcB (COG0239)→ CrcB (COG0239)→ COG2206 (COG2206)→ hypo→ sigmaK-factor processing regulatory BofA→ hypo→
Tsp-2-1	NC_010320.1	+	40525	40583	**RNA**→ CrcB (COG0239)→ CrcB (COG0239)→
Tsp-2-2	NC_010320.1	+	1156972	1157031	**RNA**→ hypo→ hypo→
Tps-1-2	NC_010321.1	+	673881	673940	**RNA**→ hypo→
Cpa-1-3	NZ_ABXP01000064.1	-	9651	9591	**RNA**→
Tte-1-3	NC_003869.1	-	1847557	1847497	**RNA**→ hypo→ hypo→ hypo→ AraJ (COG2814)→
Tca-1-1	NZ_AAWL01000036.1	+	2586	2647	**RNA**→ Era (COG1159)→ KefB (COG0475)→ USP_Like (cd00293)→ COG1993 (COG1993)→
Ame-1-1	NC_009633.1	-	4518368	4518305	**RNA**→ NhaP (COG0025)→ MazG (COG1694)→ COG2846 (COG2846)→
Dha-2-1	NC_007907.1	+	2441450	2441510	**RNA**→ CrcB (COG0239)→ CrcB (COG0239)→
Dha-1-1	NZ_AAAW04000001.1	+	623601	623661	**RNA**→ CrcB (COG0239)→ CrcB (COG0239)→
Ckl-1-1	NC_009706.1	+	304406	304465	**RNA**→ CrcB (COG0239)→ CrcB (COG0239)→ COG1993 (COG1993)→
Cbo-5-1	NC_010520.1	-	3494982	3494923	**RNA**→ hypo→ COG3481 (COG3481)→
Cbo-11-1	NZ_ABDO02000002.1	+	363051	363110	**RNA**→ hypo→ COG3481 (COG3481)→
Cbo-3-1	NC_009495.1	-	3416950	3416891	**RNA**→ hypo→
Cbo-2-1	NC_009697.1	-	3392939	3392880	**RNA**→ hypo→
Cbo-4-1	NC_009698.1	-	3290046	3289987	**RNA**→ hypo→
Cbo-7-1	NC_010516.1	-	3485265	3485206	**RNA**→ hypo→ COG3481 (COG3481)→
Cbo-10-1	NC_009699.1	-	3509923	3509864	**RNA**→ hypo→ COG3481 (COG3481)→
Cpe-7-1	NZ_ABDY01000003.1	-	8838	8779	**RNA**→ EriC (COG0038)→
Cpe-1-1	NC_008261.1	+	678979	679038	**RNA**→ EriC (COG0038)→
Cpe-2-1	NZ_ABDV01000012.1	+	79943	80002	**RNA**→ EriC (COG0038)→
Cpe-6-1	NZ_ABDW01000033.1	-	8933	8874	**RNA**→ EriC (COG0038)→
Cpe-5-1	NZ_ABOO01000041.1	-	8519	8460	**RNA**→ EriC (COG0038)→
Cpe-8-1	NC_008262.1	+	656850	656909	**RNA**→ EriC (COG0038)→
Cpe-4-1	NZ_ABDX01000022.1	-	8803	8744	**RNA**→ EriC (COG0038)→
Cpe-3-1	NZ_ABDU01000025.2	+	239794	239853	**RNA**→ EriC (COG0038)→
Cpe-9-1	NC_003366.1	+	731613	731672	**RNA**→ EriC (COG0038)→
Cbo-9-1	NC_010723.1	-	1342444	1342385	**RNA**→ EriC (COG0038)→
Cbo-3-2	NC_009495.1	-	1975371	1975313	**RNA**→ NhaP (COG0025)→
Cbo-2-2	NC_009697.1	-	1904011	1903953	**RNA**→ NhaP (COG0025)→
Cbo-4-2	NC_009698.1	-	1904496	1904438	**RNA**→ NhaP (COG0025)→
Cbo-7-2	NC_010516.1	-	1975401	1975343	**RNA**→ NhaP (COG0025)→
Cbo-10-2	NC_009699.1	-	1991940	1991882	**RNA**→ NhaP (COG0025)→
Cbo-8-1	NZ_ABDP01000020.1	-	8552	8494	**RNA**→ transporter, monovalent cation:proton antiporter-2 (CPA2) family→
Cbo-5-2	NC_010520.1	-	2040077	2040019	**RNA**→ Na_H_Exchanger (pfam00999)→
Cbo-11-2	NZ_ABDO02000006.1	-	211703	211645	**RNA**→ NhaP (COG0025)→
Csp-3-1	NZ_ABKW02000004.1	+	1354900	1354958	**RNA**→ NhaP (COG0025)→
Cbo-6-1	NC_010674.1	+	2720217	2720276	**RNA**→ EriC (COG0038)→
Cth-1-1	NC_009012.1	+	234427	234485	**RNA**→ NhaP (COG0025)→ hypo→

Cth-2-1	NZ_ABVG01000004.1	+	46786	46844	**RNA**→ NhaP (COG0025)→
Hmo-1-1	NC_010337.1	-	1203106	1203045	**RNA**→ IscU (COG0822)→ hypo→ CrcB (COG0239)→ CrcB (COG0239)→ COG1993 (COG1993)→ hypo→
Pth-1-1	NC_009454.1	+	615295	615357	**RNA**→ Era (COG1159)→
Chy-1-1	NC_007503.1	-	1888854	1888795	**RNA**→ KefB (COG0475)→ hypo→ GTP_EFTU (pfam00009)→

Bacteria Firmicutes Lactobacillales

Efa-2-1	NC_004668.1	-	2384514	2384445	**RNA**→ EriC (COG0038)→ hypo→ IMPase_like (cd01637)→
Efa-1-1	NZ_ABPI01000001.1	-	1998814	1998745	**RNA**→
Lrh-1-1	NZ_ABWJ01000039.1	+	17614	17668	**RNA**→ CrcB (COG0239)→ Integral membrane protein for chromosome condensation→
Lpa-1-1	NZ_ABQV01000049.1	-	12339	12285	**RNA**→
Lca-1-1	NC_008526.1	-	2429871	2429817	**RNA**→ CrcB (COG0239)→ integral membrane protein for chromosome condensation→
Lca-2-1	NC_010999.1	-	2570801	2570747	**RNA**→ CrcB (COG0239)→ Protein crcB homolog→
Csp-4-1	NZ_ABHH01000009.1	-	50257	50182	**RNA**→ Na_H_Exchanger (pfam00999)→ EriC (COG0038)→
Lac-1-1	NC_006814.3	+	965933	965991	**RNA**→ CrcB (COG0239)→ CrcB (COG0239)→
Lhe-1-1	NC_010080.1	+	1061364	1061422	**RNA**→ CrcB (COG0239)→ CrcB (COG0239)→
Lga-1-1	NC_008530.1	+	283109	283169	**RNA**→ ←hypo
Lga-2-1	NZ_ABWH01000036.1	+	18440	18500	**RNA**→
Ljo-1-1	NC_005362.1	-	1172633	1172571	**RNA**→ enolase (cd03313)→
Lga-1-2	NC_008530.1	-	1279279	1279217	**RNA**→ Cof (COG0561)→ CrcB (COG0239)→ CrcB (COG0239)→
Lga-2-2	NZ_ABWH01000014.1	-	31911	31849	**RNA**→
Ljo-1-2	NC_005362.1	+	798633	798695	**RNA**→ Cof (COG0561)→ CrcB (COG0239)→ CrcB (COG0239)→
Lpl-1-1	NC_004567.1	-	1741235	1741170	**RNA**→ enolase (cd03313)→ MgtA (COG0474)→
Efa-3-1	NZ_AAAK03000106.1	+	3476	3542	**RNA**→ EriC (COG0038)→
Lca-2-2	NC_010999.1	+	2571522	2571586	**RNA**→ Enolase 2 (2-phosphoglycerate dehydratase 2) (2-phospho-D-glycerate hydro-lyase 2)→
Lpa-1-2	NZ_ABQV01000049.1	+	13062	13126	**RNA**→
Lca-1-2	NC_008526.1	+	2430592	2430656	**RNA**→ hypo→
Lla-3-1	NC_002662.1	+	1105212	1105286	**RNA**→ EriC (COG0038)→
Lla-1-1	NC_009004.1	-	1450312	1450238	**RNA**→ EriC (COG0038)→
Lla-2-1	NC_008527.1	+	1111074	1111148	**RNA**→ EriC (COG0038)→
Lla-3-2	NC_002662.1	+	274025	274097	**RNA**→ enolase (cd03313)→
Lla-2-2	NC_008527.1	+	262409	262481	**RNA**→ enolase (cd03313)→
Lla-1-2	NC_009004.1	+	272338	272410	**RNA**→ ←putative secreted protein

Bacteria Proteobacteria Alphaproteobacteria

Zmo-1-1	NC_006526.1	-	542156	542079	**RNA**→ EriC (COG0038)→
Gox-1-1	NC_006677.1	+	2355903	2355981	**RNA**→ EriC (COG0038)→
Gdi-1-1	NC_010125.1	-	3849897	3849819	**RNA**→ EriC (COG0038)→
Gdi-1-2	NC_011365.1	-	2909225	2909147	**RNA**→ EriC (COG0038)→
Gdi-1-3	NC_010125.1	-	3856660	3856597	**RNA**→ MutS (COG0249)→ ABC_MutS_homologs (cd03243)→
Gdi-1-4	NC_011365.1	-	2915994	2915931	**RNA**→ MutS (COG0249)→ ABC_MutS_homologs (cd03243)→
Nha-1-1	NC_007964.1	-	1049491	1049431	**RNA**→ ←hypo
Gox-1-2	NC_006672.1	-	152206	152146	**RNA**→ UspA (COG0589)→ hypo→
Oan-1-1	NC_009668.1	+	989075	989138	**RNA**→ CrcB (COG0239)→
Gox-1-3	NC_006672.1	+	152384	152449	**RNA**→ CrcB (COG0239)→ COG2510 (COG2510)→ hypo→
Mra-1-1	NC_010505.1	+	4759637	4759698	**RNA**→ CrcB (COG0239)→
Bja-1-1	NC_004463.1	-	2887392	2887315	**RNA**→ UhpC (COG2271)→ CrcB (COG0239)→ CrcB (COG0239)→ COG1993 (COG1993)→ ClpS (pfam02617)→
Oca-1-1	NC_011386.1	+	1408427	1408488	**RNA**→ CrcB (COG0239)→
Nwi-1-1	NC_007406.1	+	166253	166313	**RNA**→ CrcB (COG0239)→
Nha-1-2	NC_007964.1	-	237736	237676	**RNA**→ CrcB (COG0239)→
Nsp-2-1	NZ_AAMY01000002.1	+	145285	145345	**RNA**→ hypo→ CrcB (COG0239)→
Rpa-1-1	NC_007778.1	+	3360939	3360999	**RNA**→ hypo→
Rru-1-1	NC_007643.1	+	293075	293134	**RNA**→ CrcB (COG0239)→ COG1993 (COG1993)→ hypo→ hypo→ COG1051 (COG1051)→

Msp-1-1	NC_010511.1	+	4458865	4458926	**RNA**→ Na+/H+ antiporter→
Acr-1-1	NC_009484.1	-	351714	351653	**RNA**→ CrcB (COG0239)→ COG1993 (COG1993)→
Acr-1-2	NC_009484.1	+	677221	677283	**RNA**→ HyfC (COG0650)→

Bacteria Proteobacteria Betaproteobacteria

Neu-1-1	NC_004757.1	+	2158790	2158856	**RNA**→ AbrB (COG2002)→ hypo→
Dac-1-1	NC_010002.1	-	1926512	1926446	**RNA**→ EriC (COG0038)→ hypo→ hypo→
Cvi-1-1	NC_005085.1	-	2613318	2613254	**RNA**→ EriC (COG0038)→
Dac-1-2	NC_010002.1	+	3908908	3908971	**RNA**→ CrcB (COG0239)→
Neu-1-2	NC_004757.1	+	1756266	1756341	**RNA**→ VacB (COG0557)→ TonB (COG0810)→ AroE (COG0169)→ Transgly (pfam00912)→
Neu-1-3	NC_004757.1	-	1838968	1838902	**RNA**→ CrcB (COG0239)→ COG1993 (COG1993)NOG18330 (NOG18330)→
Bph-2-1	NC_010681.1	-	4147651	4147580	**RNA**→ EriC (COG0038)→ hypo→
Bxe-1-1	NC_007951.1	-	1757402	1757341	**RNA**→ CRCB (COG0239)→
Bph-2-2	NC_010681.1	-	1800329	1800268	**RNA**→ CrcB (COG0239)→
Bph-1-1	NC_010622.1	+	2012557	2012617	**RNA**→ CrcB (COG0239)→
Bgr-1-1	NZ_ABLD01000041.1	+	16968	17073	**RNA**→ CrcB (COG0239)→ PRC (pfam05239)→
Bsp-2-1	NZ_ABYL01000251.1	-	1837	1776	**RNA**→ CrcB (COG0239)→
Bub-1-1	NZ_ABBE01000034.1	+	547	651	**RNA**→ EriC (COG0038)→
Rpi-2-1	NC_010678.1	+	247334	247411	**RNA**→ EriC (COG0038)→
Rpi-1-1	NZ_ABDZ01000002.1	+	179749	179826	**RNA**→ EriC (COG0038)→
Bmu-1-1	NC_010086.1	-	2066773	2066667	**RNA**→ EriC (COG0038)→
Bmu-1-2	NC_010805.1	+	408531	408637	**RNA**→ EriC (COG0038)→
Rso-1-1	NC_003295.1	-	1941892	1941826	**RNA**→ EriC (COG0038)→
Rso-3-1	NW_002196564.1	+	565243	565309	**RNA**→ EriC (COG0038)→
Rso-2-1	NW_002196568.1	+	1142118	1142184	**RNA**→ EriC (COG0038)→
Rso-4-1	NZ_AAKL01000043.1	-	37416	37350	**RNA**→ EriC (COG0038)→
Bce-19-1	NZ_AAKX01000112.1	-	6305	6201	**RNA**→ EriC (COG0038)→ hypo→
Bce-18-1	NC_010515.1	-	639633	639529	**RNA**→ EriC (COG0038)→ hypo→
Bce-15-1	NC_008061.1	-	1826897	1826793	**RNA**→ EriC (COG0038)→ hypo→
Bce-16-1	NC_008543.1	+	654224	654328	**RNA**→ EriC (COG0038)→ hypo→
Bam-2-1	NZ_ABLC01000125.1	-	14642	14539	**RNA**→ EriC (COG0038)→
Bam-3-1	NC_010552.1	+	643438	643541	**RNA**→ EriC (COG0038)→
Bam-1-1	NC_008391.1	+	2589399	2589502	**RNA**→ EriC (COG0038)→ SK (cd00464)→ hypo→
Bam-4-1	NZ_ABLK01000079.1	+	2858	2961	**RNA**→ hypo→ EriC (COG0038)→
Bth-6-1	NC_007650.1	+	603169	603235	**RNA**→ EriC (COG0038)→
Bth-6-2	NZ_AACX01000033.1	+	18652	18718	**RNA**→
Bth-5-1	NZ_ABBH01000052.1	+	1772	1838	**RNA**→ EriC (COG0038)→
Bps-14-1	NZ_ABBN01001252.1	-	1464	1398	**RNA**→ EriC (COG0038)→
Bps-3-1	NZ_ABBP01001115.1	-	6777	6711	**RNA**→ EriC (COG0038)→
Bps-7-1	NC_007435.1	-	1222860	1222794	**RNA**→ EriC (COG0038)→
Bps-6-1	NZ_AAHS03000001.1	+	321518	321584	**RNA**→ EriC (COG0038)→
Bps-4-1	NZ_ABBJ01001663.1	-	1415	1349	**RNA**→ EriC (COG0038)→
Bps-12-1	NZ_ABBL01001544.1	-	1458	1392	**RNA**→ EriC (COG0038)→
Bps-5-1	NZ_AAHR02000024.1	+	6414	6480	**RNA**→ EriC (COG0038)→
Bok-2-1	NZ_ABBF01000636.1	-	6946	6880	**RNA**→ EriC (COG0038)→
Bps-17-1	NC_006351.1	-	2539005	2538939	**RNA**→ EriC (COG0038)→
Bps-19-1	NZ_AAHV02000023.1	+	4311	4377	**RNA**→ EriC (COG0038)→
Bps-13-1	NZ_ABBK01001514.1	-	879	813	**RNA**→ voltage-gated chloride channel→
Bth-7-1	NZ_ABBM01000878.1	-	872	806	**RNA**→ voltage-gated chloride channel→
Bok-1-1	NZ_ABBG01000437.1	-	4831	4765	**RNA**→ EriC (COG0038)→
Bps-16-1	NZ_ABBI01002081.1	-	1424	1358	**RNA**→ EriC (COG0038)→
Bps-10-1	NC_009075.1	-	2566222	2566156	**RNA**→ EriC (COG0038)→

Bps-9-1	NZ_AAMM02000004.1	+	6108	6174	**RNA**→ EriC (COG0038)→
Bps-11-1	NZ_ABBO01001356.1	-	911	845	**RNA**→ voltage-gated chloride channel→
Bps-15-1	NZ_ABBR01000883.1	-	6870	6804	**RNA**→ ←hypo
Bma-2-1	NC_006349.2	+	224010	224076	**RNA**→ ←hypo
Bma-9-1	NC_008784.1	+	1388196	1388262	**RNA**→ EriC (COG0038)→ hypo→
Bma-7-1	NC_009079.1	+	225172	225238	**RNA**→ EriC (COG0038)→
Bma-6-1	NC_008835.1	+	1653068	1653134	**RNA**→ EriC (COG0038)→ hypo→
Bma-1-1	NZ_AAHN02000008.1	+	103741	103807	**RNA**→ EriC (COG0038)→
Bma-4-1	NZ_AAHO01000134.1	+	6134	6200	**RNA**→ COG0038: Chloride channel protein EriC→
Bps-20-1	NZ_AAHW02000001.1	-	301150	301084	**RNA**→ EriC (COG0038)→
Bma-3-1	NZ_AAIQ02000003.1	+	20585	20651	**RNA**→ EriC (COG0038)→
Bma-5-1	NZ_AAIR02000026.1	+	4415	4481	**RNA**→ EriC (COG0038)→
Bma-8-1	NZ_AAZP01000053.1	-	29998	29932	**RNA**→ ←hypo
Bps-1-1	NC_009078.1	-	2493251	2493185	**RNA**→ EriC (COG0038)→
Bps-2-1	NZ_AAMB02000003.1	+	113680	113746	**RNA**→ ←hypo
Bps-8-1	NZ_AAYX01000002.1	-	364269	364203	**RNA**→ EriC (COG0038)→
Bps-18-1	NZ_ABBQ01000905.1	-	4347	4281	**RNA**→ ←hypo
Bth-8-1	NZ_ABBD01000040.1	+	1808	1874	**RNA**→ EriC (COG0038)→
Bsp-1-1	NC_007511.1	-	2629309	2629206	**RNA**→ EriC (COG0038)→ SK (cd00464)NOG33876 (NOG33876)→
Bce-17-1	NC_011001.1	+	913754	913857	**RNA**→ EriC (COG0038)→ hypo→
Bvi-1-1	NC_009255.1	+	985285	985390	**RNA**→ EriC (COG0038)→
Bam-2-2	NZ_ABLC01000046.1	-	24103	24037	**RNA**→ hypo→
Bam-2-3	NZ_ABLC01000083.1	+	20871	20937	**RNA**→ ←hypo

Bacteria Proteobacteria Deltaproteobacteria

Sfu-1-1	NC_008554.1	-	3591388	3591322	**RNA**→ TEF1 (COG5256)→
Dpi-1-1	NZ_ABXU01000089.1	+	23581	23642	**RNA**→
Dol-1-1	NC_009943.1	+	3573766	3573832	**RNA**→ CrcB (COG0239)→
Dac-2-1	NZ_AAEW02000001.1	-	156146	156076	**RNA**→ EriC (COG0038)→ NADPH_oxidoreductase_1 (cd02150)→

Bacteria Proteobacteria Gammaproteobacteria

Xca-1-1	NC_007086.1	+	2656872	2656947	**RNA**→ EriC (COG0038)→
Xca-3-1	NC_010688.1	-	2646740	2646665	**RNA**→ EriC (COG0038)→
Xca-2-1	NC_003902.1	-	2322664	2322589	**RNA**→ EriC (COG0038)→
Xor-1-1	NC_006834.1	+	2682944	2683020	**RNA**→ EriC (COG0038)→
Xor-2-1	NC_007705.1	+	2662419	2662495	**RNA**→ EriC (COG0038)→ hypo→ CrcB (COG0239)→ COG1993 (COG1993)→
Xor-3-1	NC_010717.1	+	2555457	2555533	**RNA**→ EriC (COG0038)→
Xor-3-2	NC_010717.1	+	2767544	2767620	**RNA**→ EriC (COG0038)→
Xor-4-1	NZ_AAQN01000001.1	-	2446869	2446793	**RNA**→ EriC (COG0038)→ hypo→
Ppr-1-1	NZ_AAPH01000002.1	+	303167	303249	**RNA**→ ←hypo
Ppr-2-1	NC_006370.1	-	1717779	1717696	**RNA**→ ←hypo
Psp-3-1	NZ_AAOU01000011.1	-	78540	78473	**RNA**→ IscU (COG0822)→ hypo→
Van-1-1	NZ_AAOJ01000002.1	+	263194	263261	**RNA**→ IscU (COG0822)→ hypo→
Eta-1-1	NC_010694.1	-	2891609	2891537	**RNA**→ CrcB (COG0239)→
Cko-1-1	NC_009792.1	-	1490183	1490109	**RNA**→ Usp (pfam00582)→ enolase (cd03313)→
Vfi-2-1	NC_011184.1	+	34842	34908	**RNA**→ EriC (COG0038)→ hypo→
Vfi-1-1	NC_006840.2	+	35443	35510	**RNA**→ EriC (COG0038)→
Sgl-1-1	NC_007712.1	-	1344619	1344556	**RNA**→ ←putative phage DNA adenine methylase
Csa-2-1	NC_007963.1	+	2902118	2902253	**RNA**→ CrcB (COG0239)→ COG1993 (COG1993)→
Vca-1-1	NZ_ABGR01000008.1	-	95568	95493	**RNA**→ IscU (COG0822)→ hypo→
Vha-2-1	NZ_AAWP01000027.1	+	44793	44868	**RNA**→ IscU (COG0822)→ hypo (cons)→
Vha-1-1	NC_009783.1	+	3337036	3337110	**RNA**→ IscU (COG0822)→ hypo→

Val-1-1	NZ_AAPS01000002.1	-	29498	29423	**RNA**→ IscU (COG0822)→ hypo→
Afe-1-1	NC_011206.1	+	2508708	2508771	**RNA**→ CrcB (COG0239)→ COG1993 (COG1993)→ COG1689 (COG1689)→
Sma-1-1	NC_010943.1	-	1735698	1735635	**RNA**→ CrcB (COG0239)→
Sma-2-1	NC_011071.1	-	1611287	1611224	**RNA**→ CrcB (COG0239)→
Vsp-1-1	NZ_AAND01000003.1	-	123453	123370	**RNA**→ IscU (COG0822)→ hypo→
Vba-1-1	NZ_AAZW01000073.1	-	10424	10341	**RNA**→ IscU (COG0822)→
Vsp-2-1	NZ_AAMR01000022.1	-	13784	13701	**RNA**→ IscU (COG0822)→ hypo→
Vfi-1-2	NC_006841.2	-	1258800	1258735	**RNA**→ IscU (COG0822)→ hypo→
Vfi-2-2	NC_011186.1	-	1365444	1365379	**RNA**→ IscU (COG0822)→ hypo→
Asa-1-1	NC_011313.1	-	1142244	1142180	**RNA**→ IscU (COG0822)→ hypo→
Vsh-1-1	NZ_ABCH01000002.1	+	2875	2939	**RNA**→ IscU (COG0822)→ hypo→
Pca-1-1	NZ_ABVX01000036.1	-	35509	35405	**RNA**→
Pca-2-1	NZ_ABVY01000154.1	-	6708	6604	**RNA**→
Psy-2-1	NC_007005.1	-	1675154	1675082	**RNA**→ EriC (COG0038)→ hypo→ COG1993 (COG1993)→ hypo→
Psy-3-1	NC_004578.1	-	5215709	5215637	**RNA**→ EriC (COG0038)→
Cko-1-2	NC_009792.1	+	1484941	1485003	**RNA**→ MutS (COG0249)→ MutS (COG0249)→
Psy-1-1	NC_002759.1	+	22906	22967	**RNA**→ CrcB (COG0239)→
Pae-3-1	NC_009656.1	-	5109630	5109568	**RNA**→ CrcB (COG0239)→
Pae-6-1	NC_008463.1	-	5078189	5078127	**RNA**→ CrcB (COG0239)→ COG1993 (COG1993)→
Pae-5-1	NC_002516.2	-	4914637	4914575	**RNA**→ CrcB (COG0239)→
Pae-1-1	NZ_AAKW01000074.1	+	187514	187576	**RNA**→ CrcB (COG0239)→ COG1993 (COG1993)→
Pae-2-1	NZ_AAKV01000114.1	+	24659	24721	**RNA**→ CrcB (COG0239)→ COG1993 (COG1993)→
Pae-4-1	NZ_AAQW01000001.1	+	1175247	1175309	**RNA**→ CrcB (COG0239)→ COG1993 (COG1993)→
Pen-1-1	NC_008027.1	-	3719897	3719836	**RNA**→ CrcB (COG0239)→
Xca-4-1	NC_007508.1	+	2350991	2351059	**RNA**→ CrcB (COG0239)→ hypo→
Xax-1-1	NC_003919.1	+	2352963	2353030	**RNA**→ CrcB (COG0239)→
Pst-1-1	NZ_ABJD02000101.1	+	724041	724112	**RNA**→ CrcB (COG0239)→
Asp-2-1	NZ_ABYN01000048.1	+	132548	132614	**RNA**→
Asp-1-1	NC_005966.1	+	406046	406112	**RNA**→ CrcB (COG0239)→
Aba-3-1	NC_010611.1	+	439864	439930	**RNA**→ CrcB (COG0239)→
Aba-4-1	NC_009085.1	+	421777	421843	**RNA**→ CrcB (COG0239)→
Aba-6-1	NC_010400.1	-	2879674	2879608	**RNA**→ CrcB (COG0239)→
Aba-5-1	NC_010410.1	-	3434387	3434321	**RNA**→ CrcB (COG0239)→
Aba-2-1	NZ_ABJM01000001.1	+	502200	502266	**RNA**→
Ymo-1-1	NZ_AALD01000017.1	-	44340	44289	**RNA**→ CrcB (COG0239)→
Yin-1-1	NZ_AALF01000008.1	-	82054	81992	**RNA**→ CrcB (COG0239)→
Yps-4-1	NC_010465.1	+	3331346	3331409	**RNA**→ CrcB (COG0239)→
Ype-13-1	NC_005810.1	+	1200714	1200777	**RNA**→ CrcB (COG0239)→
Ype-1-1	NC_010159.1	+	1924705	1924768	**RNA**→ CrcB (COG0239)→
Ype-2-1	NC_008150.1	+	2792665	2792728	**RNA**→ CrcB (COG0239)→
Ype-7-1	NC_008149.1	-	1244276	1244213	**RNA**→ CrcB (COG0239)→
Ype-8-1	NC_009381.1	+	3010272	3010335	**RNA**→ CrcB (COG0239)→
Ype-6-1	NC_004088.1	-	1314365	1314302	**RNA**→ CrcB (COG0239)→
Ype-4-1	NC_003143.1	-	2918233	2918170	**RNA**→ CrcB (COG0239)→
Ype-15-1	NZ_AAOS02000009.1	-	153684	153621	**RNA**→ CrcB (COG0239)→
Ype-5-1	NZ_AAUB01000309.1	+	2856	2919	**RNA**→ CrcB (COG0239)→
Ype-11-1	NZ_AAYR01000001.1	+	1051950	1052013	**RNA**→ CrcB (COG0239)→
Ype-16-1	NZ_AAYS01000013.1	+	76475	76538	**RNA**→ CrcB (COG0239)→
Ype-12-1	NZ_AAYT01000001.1	-	638230	638167	**RNA**→ CrcB (COG0239)→
Ype-9-1	NZ_AAYU01000004.1	+	393680	393743	**RNA**→ CrcB (COG0239)→
Ype-10-1	NZ_AAYV01000001.1	+	356990	357053	**RNA**→ CrcB (COG0239)→
Ype-14-1	NZ_ABAT01000003.1	-	35794	35731	**RNA**→ CrcB (COG0239)→

Ype-3-1	NZ_ABCD01000001.1	+	732214	732277	**RNA**→ CrcB (COG0239)→
Yps-2-1	NC_006155.1	-	1307162	1307099	**RNA**→ CrcB (COG0239)→
Yps-3-1	NC_010634.1	-	1289674	1289611	**RNA**→ CrcB (COG0239)→
Yps-1-1	NC_009708.1	+	3329098	3329161	**RNA**→ CrcB (COG0239)→
Spr-1-1	NC_009832.1	-	1310091	1310029	**RNA**→ CrcB (COG0239)→
Pru-1-1	NZ_ABXV01000084.1	+	33475	33542	**RNA**→
Pal-1-1	NZ_ABXW01000051.1	+	16406	16474	**RNA**→
Pat-1-1	NC_004547.2	-	1468958	1468853	**RNA**→ CrcB (COG0239)→
Cko-1-3	NC_009792.1	+	1409469	1409541	**RNA**→ PPX1 (COG1227)→ COG5383 (COG5383)→ PnbA (COG2272)→ hypo→
Kpn-1-1	NC_009651.1	+	81552	81622	**RNA**→ CrcB (COG0239)→
Bacteria Thermotogae					
Tpe-1-1	NC_009486.1	+	1794817	1794880	**RNA**→ ABC_MutS_homologs (cd03243)→ ABC_MutS_homologs (cd03243)→
Tsp-3-1	NC_010483.1	+	1853799	1853862	**RNA**→ ABC_MutS_homologs (cd03243)→ ABC_MutS_homologs (cd03243)→
Mpi-1-1	NZ_ABXR01000005.1	-	79537	79474	**RNA**→
Tle-1-1	NC_009828.1	-	982299	982238	**RNA**→ hypo→ COG0714 (COG0714)→ COG1721 (COG1721)→ hypo→
Bacteria Verrucomicrobia					
Cfl-1-1	NZ_ABVL01000003.1	+	298778	298860	**RNA**→ EriC (COG0038)→ hypo→
Min-1-1	NC_010794.1	+	1993796	1993891	**RNA**→ ←hypo
bEl-1-1	NZ_ABOX01000011.1	-	261745	261663	**RNA**→ hypo→ EriC (COG0038)→ UPF0132 (pfam03675)→
Min-1-2	NC_010794.1	+	1995740	1995815	**RNA**→ ←hypo
environmental					
env-1	JCVI_SCAF_1096627186085	-	22480	22408	**RNA**→ Phosphoesterase (pfam04185)? (GOS1882)→
env-2	amd_scaffold_498	-	3365	3303	**RNA**→ MutS (COG0249)→ Mismatch repair ATPase (MutS family)→
env-3	BABD01000872.1	-	1124	1056	**RNA**→ EriC (COG0038)→
env-4	soil_Contig38989	+	707	775	**RNA**→ CrcB (COG0239)→
env-5	soil_Contig18198	+	1168	1236	**RNA**→ hypo→
env-6	BAAV01024370.1	-	684	614	**RNA**→ hypo→
env-7	amd_scaffold_44	+	18357	18431	**RNA**→ ←hypo
env-8	amd_scaffold_352	+	2172	2233	**RNA**→ ←hypo
env-9	BABE01001448.1	-	1961	1891	**RNA**→ hypo→
env-10	BAAV01007000.1	+	1090	1152	**RNA**→
env-11	soil_Contig7507	-	664	577	**RNA**→ CrcB (COG0239)→ COG1993 (COG1993)→
env-12	BABC01000066.1	-	7792	7723	**RNA**→ CrcB (COG0239)→ CrcB (COG0239)→ GtrA (pfam04138)→
env-13	BABE01006493.1	+	378	447	**RNA**→ ←hypo
env-14	BABD01002470.1	-	1603	1534	**RNA**→ CrcB (COG0239)→ CrcB (COG0239)→ GtrA (pfam04138)→
env-15	BABB01014491.1	+	724	793	**RNA**→
env-16	BABE01000053.1	+	15068	15137	**RNA**→ CrcB (COG0239)→ CrcB (COG0239)→ COG2246 (COG2246)→
env-17	BABF01001201.1	-	3795	3726	**RNA**→ CrcB (COG0239)→ CrcB (COG0239)→ COG2246 (COG2246)→
env-18	BAAY01001852.1	+	271	348	**RNA**→ CrcB (COG0239)→ CrcB (COG0239)→
env-19	hgutS7_s7_167263	+	2364	2441	**RNA**→ ←hypo
env-20	BAAZ01017612.1	-	714	637	**RNA**→ CrcB (COG0239)→
env-21	amd_scaffold_1090	+	122	193	**RNA**→ HyfC (COG0650)→
env-22	amd_scaffold_653	+	2844	2904	**RNA**→ EmrA (COG1566)→
env-23	amd_scaffold_33	-	7896	7826	**RNA**→ AraJ (COG2814)→
env-24	amd_scaffold_470	+	2269	2327	**RNA**→ MutS (COG0249)→ MutS (COG0249)→
env-25	JCVI_SCAF_1101668140910	-	192	117	**RNA**→
env-26	BAAY01017851.1	-	254	182	**RNA**→ ←hypo
env-27	BABB01015950.1	-	548	449	**RNA**→ hypo→
env-28	BABF01003476.1	+	1904	1985	**RNA**→

env-29	BAAV01013884.1	-	268	196	**RNA**→ ←hypo
env-30	BABG01014606.1	-	762	690	**RNA**→ hypo→
env-31	BAAZ01021286.1	-	777	699	**RNA**→ Na_H_Exchanger (pfam00999)→
env-32	BAAV01021889.1	-	203	131	**RNA**→
env-33	BABG01014343.1	-	547	474	**RNA**→ hypo→
env-34	hgutS8_s8_174661	-	467	395	**RNA**→ Chloride channel protein EriC→
env-35	hgutS8_s8_165414	-	1469	1396	**RNA**→ EriC (COG0038)→
env-36	BABG01003409.1	-	431	358	**RNA**→ hypo→
env-37	soil_XZS55564_b1	-	357	289	**RNA**→ CRCB (COG0239)→
env-38	BABC01003766.1	-	468	402	**RNA**→ hypo→
env-39	BABF01015109.1	+	43	109	**RNA**→ EriC (COG0038)→
env-40	hgutS8_s8_178954_2	+	1879	1949	**RNA**→ KefB (COG0475)→
env-41	hgutS7_s7_178954_3	+	4537	4610	**RNA**→ KefB (COG0475)→
env-42	JCVI_SCAF_1096627184930	-	6761	6699	**RNA**→ CrcB (COG0239)? (GOS11882)→
env-43	JCVI_SCAF_1096627388964	+	899189	899292	**RNA**→ EriC (COG0038)? (GOS1383)→ ? (GOS1352)→ PAP2 (pfam01569)→
env-44	BAAW01000138.1	-	2195	2134	**RNA**→ EriC (COG0038)→
env-45	soil_XZS73310_b1	-	89	25	**RNA**→

24.4 Conserved domains

Conserved domains found in protein-coding genes listed in Section 24.3 are listed, with the first sentence in their description from the Conserved Domain Database. Conserved domains associated with more than one *crcB* RNA are assigned a color, while others are shown in gray. The number in parentheses is the number of occurrences of the given gene.

cd00156 (1) Signal receiver domain; originally thought to be unique to bacteria (CheY, OmpR, NtrC, and PhoB), now recently identified in eukaroytes ETR1 Arabidopsis thaliana; this domain receives the signal from the sensor partner in a two-component systems; contains a phosphoacceptor site that is phosphorylated by histidine kinase homologs; usually found N-terminal to a DNA binding effector domain; forms homodimers
cd00288 (1) Pyruvate kinase (PK): Large allosteric enzyme that regulates glycolysis through binding of the substrate, phosphoenolpyruvate, and one or more allosteric effectors.
cd00293 (9) Usp: Universal stress protein family.
cd00412 (1) Inorganic pyrophosphatase.
cd00464 (2) Shikimate kinase (SK) is the fifth enzyme in the shikimate pathway, a seven-step biosynthetic pathway which converts erythrose-4-phosphate to chorismic acid, found in bacteria, fungi and plants.
cd00537 (1) Methylenetetrahydrofolate reductase (MTHFR).
cd00688 (1) This group contains class II terpene cyclases, protein prenyltransferases beta subunit, two broadly specific proteinase inhibitors alpha2-macroglobulin (alpha (2)-M) and pregnancy zone protein (PZP) and, the C3 C4 and C5 components of vertebrate complement.
cd01021 (1) Goose Egg White Lysozyme domain.
cd01637 (1) Inositol-monophosphatase-like domains.
cd02150 (1) NAD(P)H:flavin oxidoreductase-like family 1.
cd03243 (10) The MutS protein initiates DNA mismatch repair by recognizing mispaired and unpaired bases embedded in duplex DNA and activating endo- and exonucleases to remove the mismatch.
cd03313 (6) Enolase: Enolases are homodimeric enzymes that catalyse the reversible dehydration of 2-phospho-D-glycerate to phosphoenolpyruvate as part of the glycolytic and gluconeogenesis pathways.
cd03395 (1) PAP2_like_4 proteins.
COG0025 (33) NhaP-type Na+/H+ and K+/H+ antiporters [Inorganic ion transport and metabolism]
COG0038 (109) Chloride channel protein EriC [Inorganic ion transport and metabolism]
COG0169 (1) Shikimate 5-dehydrogenase [Amino acid transport and metabolism]
COG0218 (1) Predicted GTPase [General function prediction only]
COG0239 (202) Integral membrane protein possibly involved in chromosome condensation [Cell division and chromosome partitioning]
COG0249 (11) Mismatch repair ATPase (MutS family) [DNA replication, recombination, and repair]
COG0452 (1) Phosphopantothenoylcysteine synthetase/decarboxylase [Coenzyme metabolism]
COG0474 (1) Cation transport ATPase [Inorganic ion transport and metabolism]
COG0475 (9) Kef-type K+ transport systems, membrane components [Inorganic ion transport and metabolism]
COG0557 (1) Exoribonuclease R [Transcription]
COG0561 (2) Predicted hydrolases of the HAD superfamily [General function prediction only]
COG0589 (1) Universal stress protein UspA and related nucleotide-binding proteins [Signal transduction mechanisms]
COG0637 (1) Predicted phosphatase/phosphohexomutase [General function prediction only]
COG0642 (1) Signal transduction histidine kinase [Signal transduction mechanisms]
COG0650 (7) Formate hydrogenlyase subunit 4 [Energy production and conversion]
COG0651 (10) Formate hydrogenlyase subunit 3/Multisubunit Na+/H+ antiporter, MnhD

subunit [Energy production and conversion / Inorganic ion transport and metabolism]
COG0714 (1) MoxR-like ATPases [General function prediction only]
COG0810 (1) Periplasmic protein TonB, links inner and outer membranes [Cell envelope biogenesis, outer membrane]
COG0822 (14) NifU homolog involved in Fe-S cluster formation [Energy production and conversion]
COG1051 (1) ADP-ribose pyrophosphatase [Nucleotide transport and metabolism]
COG1159 (5) GTPase [General function prediction only]
COG1168 (2) Bifunctional PLP-dependent enzyme with beta-cystathionase and maltose regulon repressor activities [Amino acid transport and metabolism]
COG1227 (1) Inorganic pyrophosphatase/exopolyphosphatase [Energy production and conversion]
COG1357 (1) Uncharacterized low-complexity proteins [Function unknown]
COG1566 (1) Multidrug resistance efflux pump [Defense mechanisms]
COG1689 (8) Uncharacterized protein conserved in archaea [Function unknown]
COG1694 (1) Predicted pyrophosphatase [General function prediction only]
COG1721 (1) Uncharacterized conserved protein (some members contain a von Willebrand factor type A (vWA) domain) [General function prediction only]
COG1739 (1) Uncharacterized conserved protein [Function unknown]
COG1993 (32) Uncharacterized conserved protein [Function unknown]
COG2002 (1) Regulators of stationary/sporulation gene expression [Transcription]
COG2202 (1) FOG: PAS/PAC domain [Signal transduction mechanisms]
COG2206 (1) HD-GYP domain [Signal transduction mechanisms]
COG2223 (2) Nitrate/nitrite transporter [Inorganic ion transport and metabolism]
COG2246 (2) Predicted membrane protein [Function unknown]
COG2271 (1) Sugar phosphate permease [Carbohydrate transport and metabolism]
COG2272 (1) Carboxylesterase type B [Lipid metabolism]
COG2510 (2) Predicted membrane protein [Function unknown]
COG2520 (2) Predicted methyltransferase [General function prediction only]
COG2524 (1) Predicted transcriptional regulator, contains C-terminal CBS domains [Transcription]
COG2814 (2) Arabinose efflux permease [Carbohydrate transport and metabolism]
COG2846 (1) Regulator of cell morphogenesis and NO signaling [Cell division and chromosome partitioning]
COG2966 (1) Uncharacterized conserved protein [Function unknown]
COG3260 (5) Ni,Fe-hydrogenase III small subunit [Energy production and conversion]
COG3261 (5) Ni,Fe-hydrogenase III large subunit [Energy production and conversion]
COG3467 (1) Predicted flavin-nucleotide-binding protein [General function prediction only]
COG3481 (4) Predicted HD-superfamily hydrolase [General function prediction only]
COG3610 (1) Uncharacterized conserved protein [Function unknown]
COG3757 (1) Lyzozyme M1 (1,4-beta-N-acetylmuramidase) [Cell envelope biogenesis, outer membrane]
COG4087 (1) Soluble P-type ATPase [General function prediction only]
COG4237 (4) Hydrogenase 4 membrane component (E) [Energy production and conversion]
COG5256 (1) Translation elongation factor EF-1alpha (GTPase) [Translation, ribosomal structure and biogenesis]
COG5383 (1) Uncharacterized protein conserved in bacteria [Function unknown]
GOS11882 (1) ?
GOS1352 (1) ?
GOS1383 (1) ?
GOS1882 (1) ?
NOG18330 (1)
NOG22924 (1)
NOG33876 (1)
pfam00009 (1) Elongation factor Tu GTP binding domain.
pfam00329 (1) Respiratory-chain NADH dehydrogenase, 30 Kd subunit.
pfam00571 (1) CBS domain.
pfam00582 (4) Universal stress protein family.
pfam00583 (2) Acetyltransferase (GNAT) family.
pfam00795 (1) Carbon-nitrogen hydrolase.
pfam00912 (1) Transglycosylase.
pfam00924 (1) Mechanosensitive ion channel.
pfam00999 (6) Sodium/hydrogen exchanger family.
pfam01569 (1) PAP2 superfamily.
pfam02617 (1) ATP-dependent Clp protease adaptor protein ClpS.
pfam02641 (2) Uncharacterized ACR, COG1993.
pfam03675 (1) Uncharacterized protein family (UPF0132).
pfam04138 (4) GtrA-like protein.
pfam04185 (1) Phosphoesterase family.
pfam04254 (1) Protein of unknown function (DUF432).
pfam05239 (1) PRC-barrel domain.
smart00418 (1) helix_turn_helix, Arsenical Resistance Operon Repressor;

Duplicate sequences: the following putative homologs are not shown in the alignment because their sequences are identical to a homolog already shown: Aba-2-1, Aba-6-1, Ban-10-1, Ban-11-1, Ban-12-1, Ban-13-1, Ban-14-1, Ban-15-1, Ban-17-1, Ban-18-1, Ban-2-1, Ban-3-1, Ban-4-1, Ban-5-1, Ban-6-1, Ban-7-1, Ban-8-1, Ban-9-1, Bce-1-1, Bce-11-1, Bce-16-1, Blo-1-2, Bma-1-1, Bma-3-1, Bma-4-1, Bma-5-1, Bma-6-1, Bma-7-1, Bma-8-1, Bma-9-1, Bmu-1-2, Bps-11-1, Bps-12-1, Bps-13-1, Bps-15-1, Bps-2-1, Bps-20-1, Bps-3-1, Bps-4-1, Bps-6-1, Bth-5-1, Bth-6-2, Cbo-2-1, Cbo-2-2, Cbo-4-1, Cbo-4-2, Cbo-7-2, Cdi-10-1, Cdi-3-1, Cdi-8-1, Cpe-2-1, Cpe-6-1, Csp-1-1, Cth-2-1, Dha-1-1, Gdi-1-4, Gsp-1-1, Lga-2-1, Lga-2-2, Mpi-1-1, Pae-1-1, Psy-3-1, Rpi-1-1, Rso-4-1, Sel-2-1, Tsp-3-1, Xca-2-1, Xca-3-1, Xor-2-1, Xor-3-1, Xor-3-2, Ype-1-1, Ype-10-1, Ype-11-1, Ype-12-1, Ype-14-1, Ype-15-1, Ype-16-1, Ype-2-1, Ype-3-1, Ype-4-1, Ype-5-1, Ype-6-1, Ype-7-1, Ype-8-1, Ype-9-1, Yps-3-1, env-14, env-18

24.5 Multiple-sequence alignment

Each *crcB* RNA is denoted by an abbreviation (like "Eco-1-1") that refers to a taxonomy given in Section 24.1. Superscript numbers refer to annotations listed in Section 24.2. The alignment includes sequences containing the RNA motif, as well as flanking sequence. The *crcB* RNA itself is denoted by the line underneath marked 5′ and 3′ on either end. Nucleotides in the flanking sequences (i.e., not part of the motif) are written in gray letters. Stems of predicted rho-independent transcription terminators are shaded yellow. (Note: terminator predictions have not been analyzed manually, and many are likely to be false positives.) Start codons are shaded green. (Note: start codons are frequently misannotated, especially in environmental samples.) Nucleotides proposed to basepair as part of the consensus structure are shaded in color when they comprise Watson-Crick or G-U pairs. Otherwise they are shaded gray. Conserved stems are also indicated at the bottom of the alignment by angle brackets, where matching < and > denote base-paired columns. Below these angle brackets, the symbol "2" denotes base pairs exhibiting covariation, "1" denotes base pairs exhibiting compatible mutations, "0" denotes base pairs that are not observed to mutate and "?" denotes base pairs that have a significant frequency of non-canonical nucleotides for Watson-Crick or G-U pairs (> 5%). Below these base pair annotation is the consensus sequence: "R" = "A" or "G", "Y" = "C" or "U", red nucleotides: nucleotide identity conserved more than 97% of the time, black nucleotides: 90%, gray nucleotides: 75%, red circle (•): nucleotide is present 97% of the time, black circle (•): 90%, gray circle (•): 75%, white circle (◦): 50%. All percentages of sequences just described (e.g. 97% conserved) assume that sequences have been weighted by the GSC algorithm implemented by the Infernal software.

alignment positions 1 · · · 159

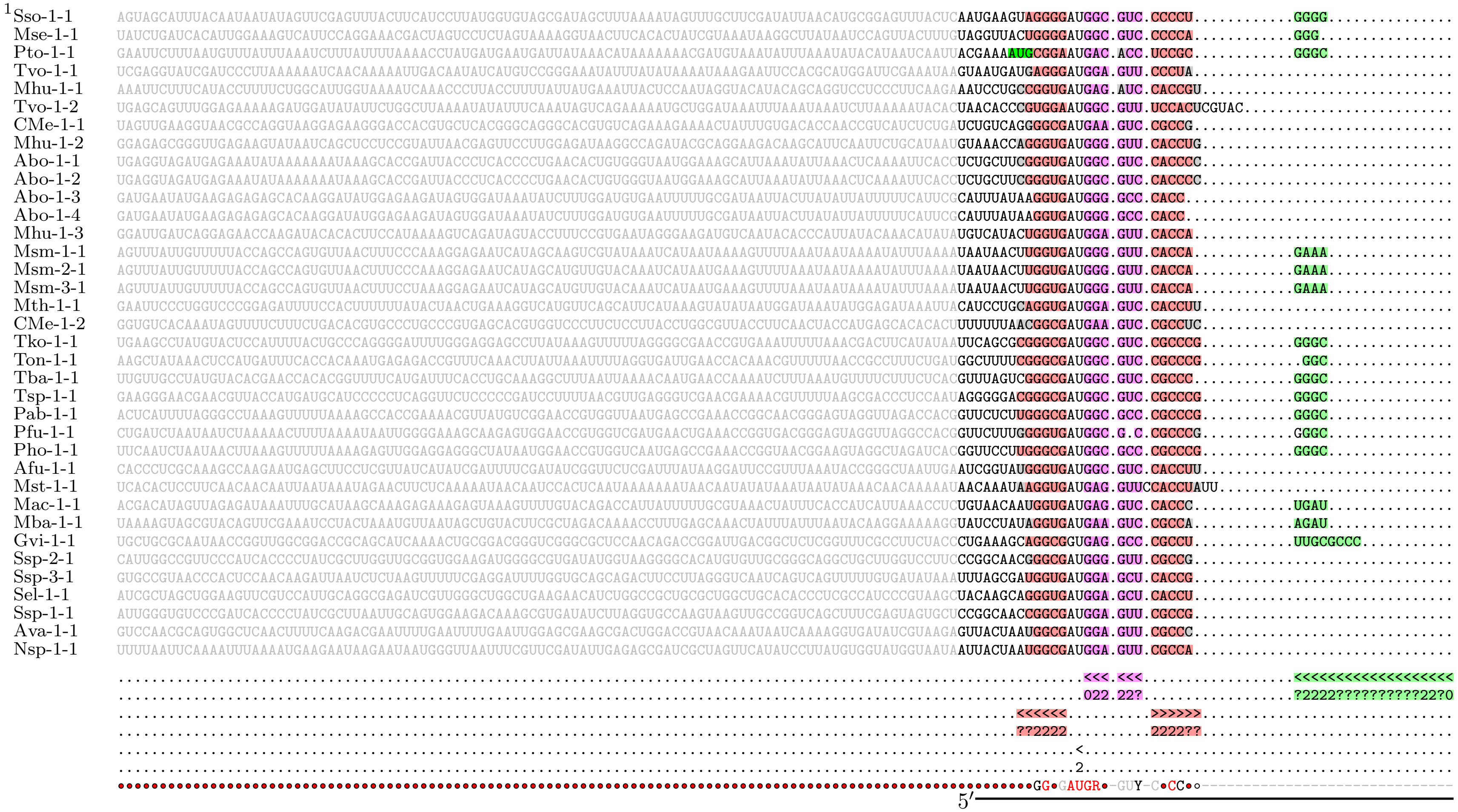

```
Dth-1-1   UUCAAAAGGACCUUCUGGCAUUUUAUAUUUCAUACAUUGUCCUCCUUUAAAAUUUUUUAAUAUAAAUAGAAUUGUUAUCUUCUUAUUUUGAUUAUAUAAUAUCACAAGUGGCGAUGGA.GUC.CGCCAUA.............................
Tye-1-1   UUCAAAAGAAGCAAUAGUUUUAGCCAGAGAAUUCUUAGAAAAAGCUAAGAAUAUCAUAAAAAUUUAAUUAUCAUUGACAGAUUAACUCAAAAUUUUUUAUUUUAAAAAAGGCGAUGGA.GUU.CGCCUG..............................
Taq-1-1   UCUCCUGGGGGGGCUUCUGGCCCUGGCCGUCUCGCGGGCCCUGGCUCGAGGAGAGGCCGGGUAGCGGGCCCAACCCCUUUCUCAUAUCGGUGGGCUAGAAUGCCCUUAGGGCGAUGGA.GUC.CGCCU...............................
Dge-1-1   CGUACGUCCUGGUCGUGAGUGCCGGGCUGCGGCCGAACGCGGAGAAAGGGUUUCCCUCCCAGGUCAGGCUUGCGCCAGCGCUCAGCCUUCCAGUACGGUGAGCAACACAGGCGAUGGC.AUG.CGCCUU..............................
Aba-1-1   CUGCGAUUGCGCACUUCGGUGAAGACGUGGAUUGGUCGGUGAUUGCGAAGGUCUCAGCGGAGCAUGCCGGCUUGAAGUAAAAUGCAGUACGGAAACACCUCAACUUCAUGGCGAUGGA.GUU.CGCCC...............................
Hsp-1-1   UUAUAGCACCAACACAAAAUAAACAAUUUUGUAUAAAAAAUUUUUUAUAUAUAAUGAUACCUAUCAUAGAAAGCAUUUAGAUGUUUUGUUAUAAUAACCUCAGGUUUAAGGAGAUGGC.GUU.CUCCU............UGAUGCU............
Hsp-1-2   AACAUUACCUCCUUUUAGGUUAGUAUUUAUUAUAUAAAAAAUUUUUUAUAUAUGCAAUGAGUUUUCUCAUAGUGAUUUUAUACCUUUUAAUGUUAAGAUAUAACCACAAGGAGAUGGC.GUU.CUCCUU..............................
Ban-1-1   UGGUGUGGACGCCUUCAUGGACCGAUAUCGGGCGUAAUCGGAAACGGUAUUGUUCGCAUGCCGGUCAAGGAGCAUGGAACAUGCCGCGGCGGUGAAUACACUUACCGGUGGCGAUGGG.ACU.CGCCUG...........GGG...CCU..........
Lxy-1-1   CGCGCACCUCGCGCACCGGCAGCACCGGCCCGUGGUCGUGGUGCCGCUGGCCCCGAUCUCCGGCGGUGAGAAGCUGCCGUGGGAGGGCCGACCGGUAUAGUGGCUCUGCGGCGAUGGA.UCU.CGCCG............GG.................
Mab-1-1   GCACGGAAAGCUUUCGGGGGAGCAAGCUCUCGAGUUCCGUAAGGAACUUGUGGCCGAAAAACGGUACGUCCGCGACGUGUACUGACGCACCUCGGCUAUAUUCGUCAGGCGCGAUGGA.UCU.CGCCA............GGG................
Mma-1-1   GAUCCAGUCUGCUGGAAAUCUCCUGUGCAUGCGCCGUAUCGAGUUGUUGAGCGCCCCGAAUUCGGUUUGACUAGUUGGACCAGCUAGCAAUCUUUUUUCGACCCAAGAUGGCGGUGGG.GCU.CGCCA...............................
Mul-1-1   CUGUGCAUCCCCCCGGUCGAGUUGUCGAAGGCCCAUAGCUCGGUUUAACUGUCAACCUAUAGCUUCAAGGAAGUUAUAAAGGUGCGCCGGGGCGAUUGCGGCGCAUAACGGCGGUGGG.GCU.CGCCA...............................
Mma-1-2   CUGUGCAUCCCCCCGGUCGAGUUGUCGAAGCACCAUAGCUCGGUUUAACUGUCAACCUAUAGCUUCAAGGAAGUUAUAAAGGUGCGCCGGGGCGAUUGCGGCGCAUAACGGCGGUGGG.GCU.CGCCA...............................
Ace-1-1   CGCCAUGCACGACCACACCGGCCGCGCCCUGCGCGGCGGCAUCGACCUGAUGCAGCGGUCCGAUGGACACCAUGGCUGAGCGUACGUCGGCAUCGGUUACCCUGAUGACGGCGAUGGA.GUC.CGCCU............GACCGGUGU..........
Mul-1-2   UGGUAGUCAACCUCGCCGCUCUGGGAUCGCACCGGCUGAGUAGUCGCGGGACCGACGGGCCUUCGAAUUCGGACUUGACCCACCGAGAGGCCGUCGGUUAACUAGCAGCGGCGAUGGG.GCU.CGCCA............GGAAG..............
Mma-1-3   UGGUAGUCAACCUCGCCGCUCUGGGAUCGCACCGGCUCAGUAGUCGCGGGACCGACGGGCCUUCGAAUUCGGACUUGACCCACCGAGAGGCCGUCGGUUAACUAGCAGCGGCGAUGGG.GCU.CGCCA............GGAAG..............
Ace-1-2   CCCGCGGUCGUUGCGCCGGGACCGGUCCCGGCGCCGUCUUCGCUCAGGACCGAUCCGUCCGUCGUCUCUGUGUCAGGGCCGAUCGAGCCGCCGCGGGUAGACUGAACGCGGCGAUGAA.GCC.CGCCGA..............................
Rxy-1-1   CCAGCGCCAAAGCUCCCUCCCGACGGGCAGGAUACACCGCCCGCCGGACGCCCGCACUUGCAGCGCCCCGCUCCCCGCUGUACAUUUCCGCACCACAGAAGACGAUACGGGCGAUGAG.GCC.CGCCU............UCCGGGGC...........
Blo-3-1   CCAAGUUCAUCGGCAUUGCAGAAGUCUCUUGCCGAAAUCCACCGCGAUAGGCCAAUAUCCGAGAUACUGCUGCCGUUUGUUGUCGCCGCCACGAGUAGGCUAAGGAACCGGCGAUGGA.ACC.CGCCU............GAA................
Blo-2-1   UUUCGCCAUACCCAUGAGGUAUGGCAACAACCGAGCAAUUCACUGCGAUAGGCCAAUAUCCGAGAUACGCUCACUGUUCGAUUCGUCGCCAUGAGUAAUCUGGAGAACCGGCGAUGGA.ACC.CGCCU............GAA................
Blo-1-1   UUUCGCCAUACCCAUGAGGUAUGGCAACAACCGAGCAAUUCACUGCGAUAGGCCAAUAUCCGAGAUACGCUCACUGUUCGAUUCGUCGCCAUGAGUAAUCUGGAGAACCGGCGAUGGG.ACC.CGCCU............GAA................
Bad-1-1   CCGCGCUAUUGGCAACGGUUAUGGAUCGUACGUUGCGGUAGUCCGGGGAAGUUGUCCGCAUCGUGAAUGAUGGUACGGAUGGCCUCGCGUGCCGGAUAUACUGGACUGCGGUGAUGGG.ACU.CGCCU............GAAGCC.............
Bad-2-1   CCGCGCUAUUGGCGACGGUUAUGGAUCGUACGUUGCGGUAGUCCGGGGAAGUUGUCCGCAUCGUGAAUGAUGGUACGGACGGCCUCGCGUGCCGGAUAUACUGGACUGCGGUGAUGGG.ACU.CGCCU............GAAGCC.............
Nfa-1-1   CAACCUCGCGCAGGACAGAUCUCCCACGAAAGGUGAUGUCACAGGCGCGCCGUCACGGACGCAGCGGGAAAGGGAGCGAACCGCACCGCAACGCUAUGAUGAGUCCGCAGGCGAUGGA.UCUCCGCCG............AGACGACC...........
Bpl-1-1   CGUGUUUAUUUUGUGUUUGUGUUGUGGCUGUUCUCCGACGAGAGUCGGGGAACAGUUUUUUAUGUUUACGGAUUUUAUUAUAUCUUUGCAGCCGAUUUUCGAAUCAAGGGAAUGGG.ACU.UCCCU...............................
Bco-1-1   UCGACGUGAGUCGGGAAACAGUUUUUUGUUUGCAGAAUUUUGUAUAACUUUGCAGCCGAUUUUGAAUUCUGUCCGAUUUUACCGAUUAUUCAGGCUGAUUCAAAGUCAGGGGAAUGGG.ACU.UCCCU...............................
Bth-1-1   CCAUUGUUUCCGAACGGAUAGAUAAGGUUUACACCGGAGGGUUCAAACAAUUUGAAAAAGAUUUUGAAGCCUUCUGCAAAUAGUUUUAUCUUUUUCUAUCUUUGCACCGGCUAUGGG.AUU.AGCCUU..............................
Bca-1-1   UCAGCGCAUUUUCUAACCCUAUGAUAUCCCUUUCCCGGGGAAUUUGCAAGGCUGGCCGGGGUUGACAGAGGGCGGAGAAUGUGGUAUCAUUCCUCCGCAAGUGAAUCAGGGGAAUGAA.GUG.CUCCCC...........GGGGC..............
Lbl-1-1   UGUUUGACCAGAAUAAAUAGAAUCGAAGCAUUAAAUCUUGAUUAUUCAGUAAAAGCUGUUUUCUUAAAGCACUUUAAAGAUUUAUUUUAUCUUUGUUGCUUCAAUCACAGGCAAUGAG.GUC.UGCCUC..............................
Psp-1-1   UGAUUCUCUUUCGCAUAAUCCGGUGGCGAAAAUAAUAAAAAAAUGGUAUUGGUCGAUUUAUGAGCCUCUUUAUUGGAAUAUUGGAACUGCAACCUUAUUUUUGCCUAAGGGAAAUGGU.GUCCUUCCAAUU............................
Psp-1-2   UAAGAAACUGGUUCUCGUAUUCAAAUAAGGAGGAAUAGUGAUAAAUUAAAUUCUCAACACCUACUUUAAGAUUUUGCACAUCAUUUUAUUAGUAUUAUAUUUGCAAUACGGAAAUGGU.GUCCUUCCGAUU............................
Rsp-1-1   UCGGAUCGUCGAGUCGCCGGUAACGUAUGAAGUAGAUCCGUCGGCUUCCAUUGCUGCACACUGAUGCUUGACGUUGCUGCAACCCGGCGGUAUACUGAGGCAGAGUUCACGCGAUGAG.GCU.CGCA................................
Cau-1-1   ACGCGUGGCUUUGAGAUACUAAAAGAAACGUAGAUCUGGUCUGUCAGGUUAAGAAAACAGAUUUGACACCGGCUGAGUAGCUGAUAAAAUGAGAUUAGACAAUCGAUUGGGUGAUGAG.GCU.CACCCU..............................
Cag-1-1   UGCGUGUGACGCAAGGCAACAGCCUGAUAGGCUAGGGCCUUGUGGGCAAAAUCACUCCCACAACUAUUGUCACCCCACUAUGUGCGUGGUAAACUAGGUGUAGCUCAUAGGUGAUAAG.GCU.CACCGUU.............................
Ebi-1-1   AAAUUUCUGAGAUUGUUGGACUAGAUUUAGAUGCAUUAAGAGAACUAAAAAAGAGUAUUUAAUUGAAAUACUAUUGAUGAAAACUUAGAUAUUGUAGUAUAAUCUUUUUGGGAAUGAA.GUU.CUCCCU...........UAGU...............
Gsp-2-1   GAAUAAGAAGAAAAAAACAGAUGGCUGGUAAAAACAUAGACAGCCAAGGGAGAAACUGCGCAAAUUGGCAAUGAGAUAUCUUUUUGUUAUAAUAACGGACAGAGUAAUAGGCGAUGGA.GUU.CGCU................................
Gth-1-1   UGAAGCAAUUUGAACAGUACGGGUUCCGCCGUGCCCACUAGCAUCCAAACGCACUUGUCUUCACACGAACAGGCCAUCAUUGUAAAAUAAAAGACAUAUCAACAUACUAGGCGAUGGG.GUU.CGCCA...............................
Gka-1-1   CGAACAAGCGCUCCAAAUAUUCGAACAACACGGGUUCCGACCAAGCCGUUGAAUCUGCUUCCCAUUGGGAGGGGAUAUAUGGUAAAAUAAAGGCGAUGCCAAUCGAAUAGGCGAUGGA.GUU.CGCCA...............................
Bth-2-1   CUCAUUUUAAUUACUAAAAAACCUUCAGACAUCAAAAAAAGAAAAGUUACACCCACCUCCUCACUAGAAAUUAGACGGUAUAUUUAGUACACUAUAUGUAAUAAUCAGAGGCGAUGGA.GUU.CGCCA...............................
Bce-10-1  CUCAUUUUAAUUACUAAAAAACCUUCAAACAUCAAAAAAAGAAAAGUUACACCCACCUCCUCACUAGAAAUUAAACGGUAUAUUUAGUACACUAUAUGUAAUAAUCAGAGGCGAUGGA.GUU.CGCCA...............................
Bwe-1-1   UUUAUGAGUAAGGAUUCCUUAUCUUAAACUAUUAUAUAAAUACAAAAUGCACCAACUUCUCCACUGGAAAUUAAACGCAAUGUUUAGUACACUAUAUGUAAUAAUCAUAGGUGAUGGA.GUU.CGCCA...............................
Bce-13-1  CAUUAUAUUUAGUUUAUAAAUAAUUGCUCUUGCCUUUAAAUGAUCAAAACUCCGACUCCUUCACUGGAAAUCAAGCGUAAUGUUUAGUACACUAUAUGUAAUAAUCAUAGGCGAUGGA.GUU.CGCCA...............................
Bce-12-1  CAUUAUAUUUAGUUUAUAAAUAACGAUUCUUGCCUUUAAAUGAUCAAAACUCCGACUCCUUCACUGGAAAUCAAGCGUAAUGUUUAGUACACUAUAUGUAAUAAUCAUAGGCGAUGGA.GUU.CGCCA...............................
Ban-16-1  CAUUAUAUUUAGUUUAUAAAUAAUUGUUCUUACCUUUAAAUGAUCAAAACUCCGACUCCUUCACUGGAAAUCAAGCGAAAUGUUUAGUACACUAUAUGUAAUAAUCAUAGGCGAUGGA.GUU.CGCCA...............................
Bth-3-1   CACUAUAUUUAGUUUAUAAAUAAUGAUUCUUACCUUUAAAUGAUCAAAACCCCAACUCCUUCACUGGAAAUCAAGCGUAAUGUUUAGUACACUAUAUGUAAUAAUCAUAGGCGAUGGA.GUU.CGCCA...............................
Bce-9-1   AGUUUAUAAGCGAUAUUUCUUACUCUUACAUUAUCAAAAAUACAAAACGCCCCAACUCCUUCACUGGAAAUCAAGCGCAAUGUUUAGUACACUAUAUGUAAUAAUCAUAGGCGAUGGA.GUU.CGCCA...............................
Bce-8-1   CAUUAUAUUUGGUUUAUAAACAAUGAUUCUUGCCUUUAAAUGAUCAAAACUCCGACUCCUUCACUGGAAAUCAAGCGUAAUGUUUAGUACACUAUAUGUAAUAAUCAUAGGCGAUGGA.GUU.CGCCA...............................
Bce-4-1   CAUUAUAUUUAGUUUAUAAAUAAUUUCUCUUGCCUUUAAAUGAUCAAAACUCCGACUCCUUCACUGGAAAUCAAGCGUAAUGUUUAGUACACUAUAUGUAAUAAUCAUAGGCGAUGGA.GUU.CGCCA...............................
Bce-7-1   CCAUUUUAAUUACUUGAAAAACCUUCAGACAUCAAAAAAAGAAAAGUUACACCCUCCUCCUCACUGGAAAUUAAACGGUAUAUUUAGUACACUAUAUGUAAUAAUCAUAGGCGAUGGA.GUU.CGCCA...............................
Bce-3-1   AGUUUAUAAACAAUAACUCUUGCUCUUACAUUAUCAAAAAUACAAAAUCCCCUAACUCCUUCACUGGAAAUCAAGCGCAAUGUUUAGUACACUAUAUGUAAUAAUCAUAGGCGAUGGA.GUU.CGCCA...............................
Bce-2-1   CCAUUUUAAUUACUUAAAAAGCCUUCAGACAUCAAAAAAAGAAAAGUUACACCCUCCUCCUCACUGGAAAUUAAACGGUAUAUUUAGUACACUAUAUGUAAUAAUCAUAGGCGAUGGA.GUU.CGCCA...............................
Bth-4-1   CAUUAUAUUUAGUUUAUAAAUAAUGAUUCUUACCCUUAAAUGAUCAAAACUCCGACUCCUUCACUGGAAAUCAAGCGUAAUGUUUAGUACACUAUAUGUAAUAAUCAUAGGCGAUGGA.GUU.CGCCA...............................
Bce-5-1   AGUUUAUAAACAAUAACUCUUGCUCUUACAUUAUCAAAAAUACAAAAUCCCCUAACUCCUUCACUGGAAAUCAAGCGCAAUGUUUAGUACACUAUAUGUAAUAAUUAUAGGCGAUGGA.GUU.CGCCA...............................
Bce-6-1   CCAUUUUAAUUACUUGAAAAACCUUCAGACAUCAAAAAAAGAAACGUUACACCCACCUCCUCACUGGAAUUUAAACGGUAUAUUUAGUACACUAUAUGUAAUAAUCCUAGGCGAUGGA.GUU.CGCCA...............................
Bce-14-1  UUAAAUAAAUAUUUCUGAUUGGUGCCUUUUCCACUCUUGACGAAAAAAUCUAUACGCACUUAUUGGAAAUCACCAAAAUAUUUAGUACACUAGUAAAUGGAAUGAAUACGGCGAUGGA.GUU.CGCCA...............................
Gsp-3-1   UUUUCCAGGUCAAUCGCAGCAAUCCGAAUUAAGUGUUCGUUUAAACCCUGAUUUGUUGUCUGUUGACAUAUAAGCCGCCGGUCCAUUACAAUAAAUGCUAAUUGAAUGAGGCGAUGGA.GUU.CGCCA...............................
Psp-2-1   AGUAAACCUCCUUUGUCAAGAGAAAUAACAGUCCCUCGAGUUUGGCCACCGCACGCAAAAAGAUUGACUAAGCAGCAAGAAAUAUUCUAAAAUGAAAAAAAGUGAAUAAGGCGAUGGA.GUU.CGCCAU..............................

          ...................................................................................................................<<<.<<<..................<<<<<<<<<<<<<<<<<<<
          ...................................................................................................................022.22?..................?2222??????????22?0
          ...........................................................................................................<<<<<<..........>>>>>>..............................
          ...........................................................................................................??2222..........2222??..............................
          ..................................................................................................................<............................................
          ..................................................................................................................2............................................
          •••••••••••••••••••••••••••••••••••••••••••••••••••••••••••••••••••••••••••••••••••••••••••••••••••••••••••••••GG•GAUGR•-GUY-C•CC•∘------------------------------
                                                                                                              5′
```

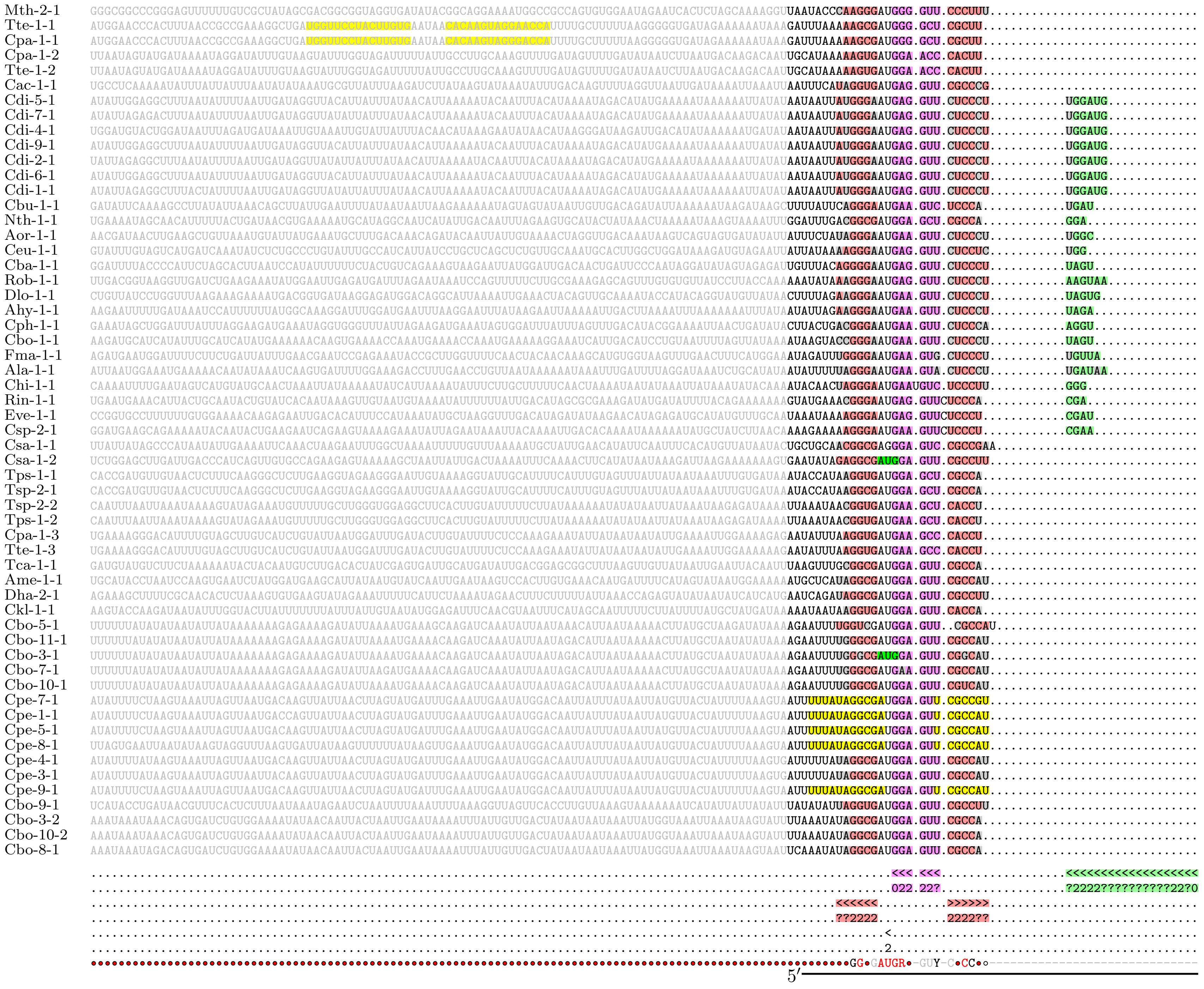
Mth-2-1
Tte-1-1
Cpa-1-1
Cpa-1-2
Tte-1-2
Cac-1-1
Cdi-5-1
Cdi-7-1
Cdi-4-1
Cdi-9-1
Cdi-2-1
Cdi-6-1
Cdi-1-1
Cbu-1-1
Nth-1-1
Aor-1-1
Ceu-1-1
Cba-1-1
Rob-1-1
Dlo-1-1
Ahy-1-1
Cph-1-1
Cbo-1-1
Fma-1-1
Ala-1-1
Chi-1-1
Rin-1-1
Eve-1-1
Csp-2-1
Csa-1-1
Csa-1-2
Tps-1-1
Tsp-2-1
Tsp-2-2
Tps-1-2
Cpa-1-3
Tte-1-3
Tca-1-1
Ame-1-1
Dha-2-1
Ckl-1-1
Cbo-5-1
Cbo-11-1
Cbo-3-1
Cbo-7-1
Cbo-10-1
Cpe-7-1
Cpe-1-1
Cpe-5-1
Cpe-8-1
Cpe-4-1
Cpe-3-1
Cpe-9-1
Cbo-9-1
Cbo-3-2
Cbo-10-2
Cbo-8-1
5′

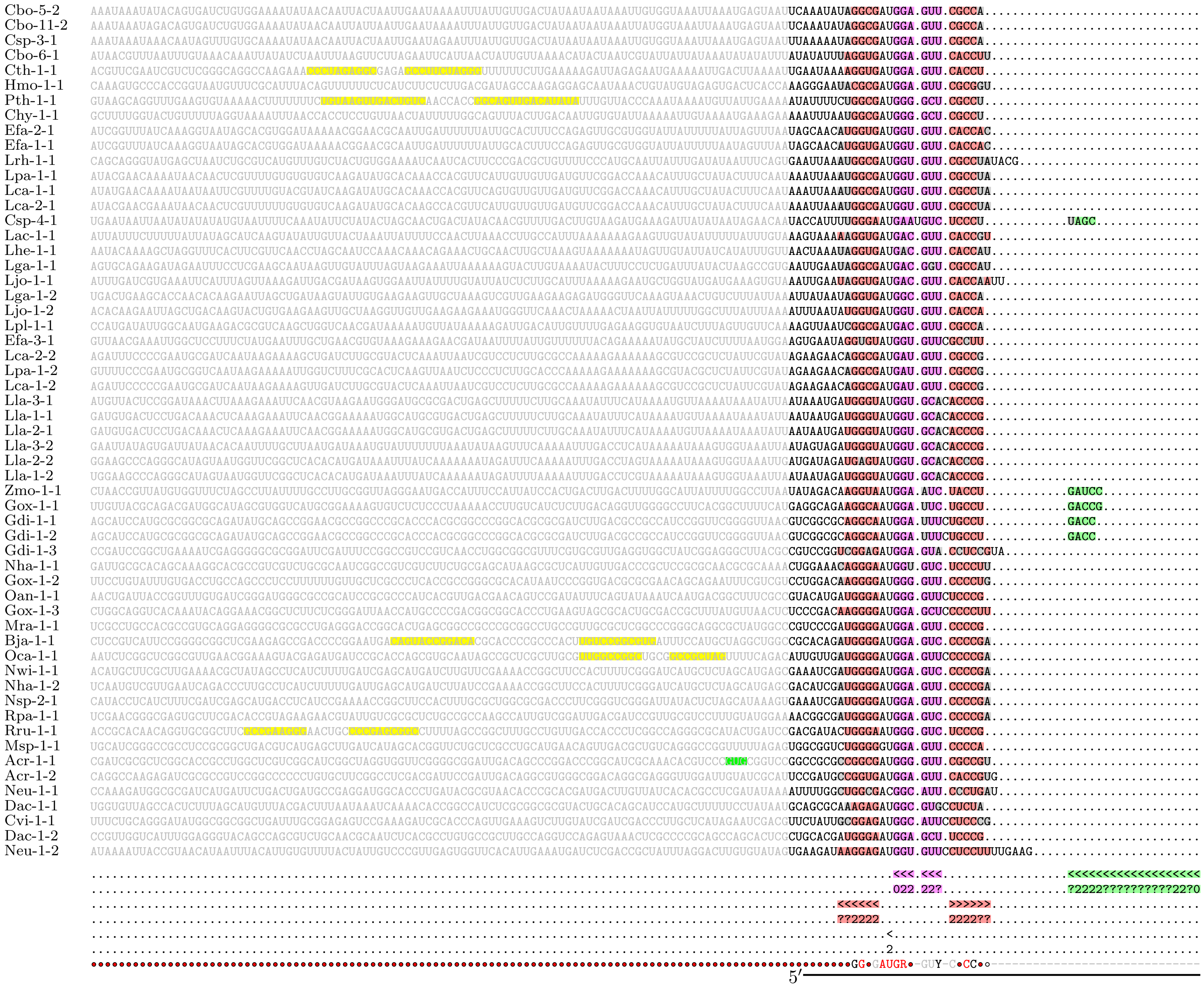
Cbo-5-2
Cbo-11-2
Csp-3-1
Cbo-6-1
Cth-1-1
Hmo-1-1
Pth-1-1
Chy-1-1
Efa-2-1
Efa-1-1
Lrh-1-1
Lpa-1-1
Lca-1-1
Lca-2-1
Csp-4-1
Lac-1-1
Lhe-1-1
Lga-1-1
Ljo-1-1
Lga-1-2
Ljo-1-2
Lpl-1-1
Efa-3-1
Lca-2-2
Lpa-1-2
Lca-1-2
Lla-3-1
Lla-1-1
Lla-2-1
Lla-3-2
Lla-2-2
Lla-1-2
Zmo-1-1
Gox-1-1
Gdi-1-1
Gdi-1-2
Gdi-1-3
Nha-1-1
Gox-1-2
Oan-1-1
Gox-1-3
Mra-1-1
Bja-1-1
Oca-1-1
Nwi-1-1
Nha-1-2
Nsp-2-1
Rpa-1-1
Rru-1-1
Msp-1-1
Acr-1-1
Acr-1-2
Neu-1-1
Dac-1-1
Cvi-1-1
Dac-1-2
Neu-1-2
5′

```
Neu-1-3   CUCGUUUGCUUACAUUUUUUCCUCACCCGCGCAGUGAGGCUUGUACUGUUUAUGCCAGGCUGAAACUGUCCGAUGACUCGUUCUUACUGCUUCCGAUAUAAUAAUAAUUGGAGAUGGC.AUUCCUCCAU..............................
Bph-2-1   GCUGCGAUUCUCUUUCUUACGCUGCUGUAGUCGUCGUAAUCUGCCCCAUAUUCUCGGCGACAAUCUUGUUGCAGUGCCAUAUCUUGUCGCAUAGAAUCACGAAGCUUUCGGAGAUGGC.AUUCCUCCCG..............................
Bxe-1-1   GCCUCGCUGCCGCACACGCCGGAUUCGGGCGUAAAGAGCGGGUGGGCAUGCUGCCCGCGUCCGCGCCGAACUGUCCCCACAUGCACUCGCGGUGUUAGACUGCUGCGCUGGAGAUGGC.AUU.CUCCUU..............................
Bph-2-2   GGCGCCGGAUGAAUAUGCGUCCCUCCAUCGGACGGCGCGUGCCAGCACCCCGCCGGUGAGCGCCCCGACCUGUCCCCACAUGCAUGCGCGGAUGUUAGACUUGCGCGCUGGAGAUGGC.AUU.CUCCCU..............................
Bph-1-1   CGGCCGCGACGCCCCGUGUAAGCUCGCGUUCCAGGGAUCGGCCCCCGCCACCGGCGAGCGGCCGGUUCCUUAUACGAUCGCACGCGCCGGUGUUAGACUUGCGCGCGCCGGAGAUGGC.AUU.CUCCU...............................
Bgr-1-1   GCCUCGGCUGAUGCAGCGGCUCGCGUGGGCACAAGCCGAUGCCAGUGGGCAAGCCGCGGCCUGGUCCGACCUGUCCCCAUAGAGCCGCCGAAUGUUAGACUUGCGUGCUGGAGAUGGC.AUU.CUCCA............AUGGGUGUCCGGCGUGCGC
Bsp-2-1   AGAUUUCGGCGACCGCGGAGUAAGAGGCGGCGACGCGAGAGGGAAACGCGACAGGUGCGGGCCACGCACCUGUCCCCGAAUCCGCUCGCCGAUGUUAGACUUCGCGGCUGGAGAUCGC.AUU.CUCCAU..............................
Bub-1-1   GAUCGCGCCGCAACUGAUCGCGCGCGUGAUGGGCCCAUCGCGCGGCUGAUUUCGCGGCCGGCCUCGCGCGUUGCGCGGGGCGUCGGCCGUCGCGUAAAAUGCGCGCUUCGGAGAUGGC.AUGCCUCCCC...........GGGGUCCGGGCUUGCUCG.
Rpi-2-1   AAGCCAAAAAGCCCGCCCGGUGUGAUGCGAGGCGGGCCUUUUUCUCGGGCGCGCGCGAUUGUGCCCGGUUGUGACCGCGGCCGCCUUCAGGCACAAUGCGCCCCGUUGUGGAGAUGGC.AUGCCUCCCU..............................
Bmu-1-1   CGCGUCGCGCGCCGCGCCGCGCGUCCGUGAGGCGGGCCGCGCGGUCCGGUUGCGUCCCGCGCAGCGCGUUGCGUGCGCGGACGGCGUCGCGUACAAUGGCCGCCUUGCGGAGAUGGC.AUGCCUCCG............UGCGGCGUUUUCCCGCGC.
Rso-1-1   CCGUGGGCGGCCCUGCGCAUCCUCCCGCUGGACGCGGCGCUCGCCGAGGGUUGAACGCGCGCACGGGUGUUGCGGCACCGCGGGCGUUCGGGCACAAUCGCCGGCCUGCGGAGAUGGC.AUGCCUCCCU..............................
Rso-3-1   GUCUCCCCGGAGAGAAUCCGCCGCCGGCUGUCUGCGCGCGAAAGCCACGCAGCCCCGCAUUCACCCGGGGUUGCCGCGCCGCCCGCCUUUGGGCACAAUGCCGGCUUGCGGAGAUGGC.AUACCUCCCU..............................
Rso-2-1   GUCUCCCCGGAGAGAAUCCGCUGCCGGCCGUCUGCGCGCGAAAGCCACGCAGCUCCGCAUUCACCCGGGGUUGCCGCCCCGCCCGCCUUUGGGCACAAUGCCGGCUUGCGGAGAUGGC.AUACCUCCCU..............................
Bce-19-1  GCAUUGACAUCGCGCGACUGACCGCGCAUCGGGCAGACGCCGUCGUUUCGGGGUGUGCCGGCGGCUGCAUUGCGUGCGUGGCGAGGCGUCGCGUACAAUUGCCGCCUGCGGAGAUGGC.AUGCCUCCC............UGCGGUCGUUCCG.GCGC.
Bce-18-1  GCAUUGACAUCGCGCGACUGACCGCGCAUCGGGCAGACGCCGUCGUUUCGGGGUGUGCCGGCGGCUGCAUUGCAUGCGUGGCGAGGCGUCGCGUACAAUUGCGGCCUGCGGAGAUGGC.AUGCCUCCC............UGCGGUCGUUCCG.GCGC.
Bce-15-1  GCACUGACAUCGCGCGACUGACCGUGCAUCGGGCAGACGCCGUCGUUUCGGGGUGUGCCGGCGGCUGCAUUGCGUGCGUGGCGAGGCGUCGCGUACAAUUGCGGCCUGCGGAGAUGGC.AUGCCUCCC............UGCGGUCGUUCCG.GCGC.
Bam-2-1   CGCAGCUGAUCUCGCGCGUGACGCGCGUGCCGCUCGACUGACGGUGCGCGGCGGCCCGGCCGGGCCGCGUUGCGUGCGAUGUGCGGCGUCGCGUACAAUUGCCUCCAGCGGAGAUGGC.AUGCCUCCCU...........GCGGUCGAUCCCG.GCGC.
Bam-3-1   CGCAGCUGAUCUCGCGCGUGGCGCGCGUGCCGCUCGACUGACGGCGUGCGGCGGCCCGGCGGGGCCGCAUUGCAUGCGAUGUGCGGCGUCGCGUACAAUCGCCGCCAGCGGAGAUGGC.AUGCCUCCCU...........GCGGUCGAUCCCG.GUGC.
Bam-1-1   CGCAGCUGAUCUCGCGCGUGACGCGCGUGCCGCUCGACUGACGGUGCGCGGCGGCCCGGCCGGGCCGCAUUGCGUGCGAUGUGCGGCAUCGCGUACAAUCGCCGCCAGCGGAGAUGGC.AUGCCUCCCU...........GCGGUCGAUCCCG.GCGC.
Bam-4-1   CGCAGCUGAUCUCGCGCGUGACGCGCGUGCCGCCCGACUGACGGUGCGCGGUGGCCCGGCCGGGCCGCAUUGCGUGCGAUAUGCGGCGUCGCGUACAAUUGCCGCCAGCGGAGAUGGC.AUGCCUCCCU...........GCGGUCGAUCCCG.GCGC.
Bth-6-1   CGCACCGGCCGGCCCGCCUCCUUCCUCGCGCCGACGCUCCGGCUCCGCGUGCGCCGUCGCCGCGACGGUUGCCGCGCGCGCCCGGCAUCGCGUACAAUCCGCGGCUACCGGAGAUGGC.AUGCCUCCGUA.............................
Bps-14-1  ACAUACCGGCCCACAUACCGGCCCGCGCGUCGGCCGGCCGGUGCGCGCGUGCGCCCUCAUCGCGCCGGUUGCCGCGUGCGCGCGGCAUCGCGUACAAUCCGCGGCGACCGGAGAUGGC.AUGCCUCCGUA.............................
Bps-7-1   ACAUACCGGCCCACAUACCGGCCCGCGCGUCGGCCGGCCGGUGCGCGCGUGUGCCCUCAUCGCGCCGGUUGCCGCGUGCGCGCGGCAUCGCGUACAAUCCGCGGCGACCGGAGAUGGC.AUGCCUCCGUA.............................
Bps-5-1   GUGCAUUGGCCCGCGCACCGGCCCGCGCGUCGGCCGGCCGGUGCGCGCGUGUGCCCUCAUCGCGCCGGUUGCCGCGUGAGCGCGGCAUCGCGUACAAUCCGCGGCGACCGGAGAUGGC.AUGCCUCCGUA.............................
Bok-2-1   CGAUGCGGCGCGCGGCCCGUCGAUCGGCCGUGCAUGCGGCGACGGCGCGCCCGCACCCGCCGCGACGGUUGCCGCUGCGCUCCGGCAUCGCGUACAAUCCGCGGCGACCGGAGAUGGC.AUGCCUCCGUA.............................
Bps-17-1  GUGCAUUGGCCCGCGCACCGGCCCGCGCGUCGGCCGGCCGGUGCGCGCGUGCGCCCUCAUCGCGCCGGUUGCCGCGUGCGCGCGGCAUCGCGUACAAUCCGCGGCGACCGGAGAUGGC.AUGCCUCCGUA.............................
Bps-19-1  ACAUACCGGCCCACAUACCGGCACGCGCGUCGGCCGGCCGGUGCGCGCGUGCGCCCUCAUCGCGCCGGUUGCCGCGUGCGCGCGGCAUCGCGUACAAUCCGCGGCGACCGGAGAUGGC.AUGCCUCCGUA.............................
Bth-7-1   GACGGCAUUCACGUCCACGUUUGCUUUCGCGCCCGUCUCCGUCCGCGCGCCCGCCGUCACCGCGCCGGUUGCCGCGUGCGCGCGGCAUCGCGUACAAUCCGCGGCGACCGGAGAUGGC.AUGCCUCCGUA.............................
Bok-1-1   CGAUGCGGCGCGCGGCCCGUCGAUCGGCCGUGCAUGCGGCGACGGCGCGCCCGCACCCGCCGCGACGGUUGCCGCUGCGCUCCGGCAUCGCGUACAAUCCGCGGCGACCGGAGAUGGC.AUGCCUCCGUA.............................
Bps-16-1  ...........................................CGCGCGUGCGCCCUCAUCGCGCCGGUUGCCGCGUGCGCGCGGCAUCGCGUACAAUCCGCGGCGACCGGAGAUGGC.AUGCCUCCGUA.............................
Bps-10-1  GCGCAUCGGCCCGCGCACCGGCCCGCGCGUCGGCCGGCCGGUGCGCGCGUGCGCCCUCAUCGCGCCGGUUGCCGCGUGCGCGCGGCAUCGCGUACAAUCCGCGGCGACCGGAGAUGGC.AUGCCUCCGUA.............................
Bps-9-1   GCAUACCGGCCCACAUACCGGCACGCGCGUCGGCCGGCCGGUGCGCGCGUGCGCCCUCAUCGCGCCGGUUGCCGCGUGAGCGCGGCAUCGCGUACAAUCCGCGGCGACCGGAGAUGGC.AUGCCUCCGUA.............................
Bma-2-1   ACAUACCGGCCCACAUACCGGCACGCGCGUCGGCCGGCCGGUGCGCGCGUGCGCCCUCAUCGCGCCGGUUGCCGCGUGAGCGCGGCAUCGCGUACAAUCCGCGGCGACCGGAGAUGGC.AUGCCUCCGUA.............................
Bps-1-1   GCAUACCGGCCCACAUACCGGCCCGCGCGUCGGCCGGCCGGUGCGCGCGUGCGCCCUCAUCGCGCCGGUUGCCGCGUGCGCGCGGCAUCGCGUACAAUCCGCGGCGACCGGAGAUGGC.AUGCCUCCGUA.............................
Bps-8-1   GCAUACCGGCCCACAUACUGGCCCGCGCGUCGGCCGGCCGGUGCGCGCGUGCGCCCUCAUCGCGCCGGUUGCCGCGUGAGCGCGGCAUCGCGUACAAUCCGCGGCGACCGGAGAUGGC.AUGCCUCCGUA.............................
Bps-18-1  ACAUACCGGCCCACAUACCGGCCCGCGCGUCGGCCGGCCGGUGCGCGCGUGCGCCCUCAUCGCGCCGGUUGCCGCGUGAGCGCGGCAUCGCGUACAAUCCGCGGCGACCGGAGAUGGC.AUGCCUCCGUA.............................
Bth-8-1   CGCACCGGCCGGCCCGCCUCCUUCCUCGCGCCGACGCUCCGGCUCCGCGUGCGCCGUCGCCGCGACGGUUGCCGCGUGCGCCCGGCAUCGCGUACAAUCCACGGCAACCGGAGAUGGC.AUGCCUCCGUA.............................
Bsp-1-1   UCGCGCAUCACGCGCCCGCUGCGCGACUGACGGCGUCCUGGGGGCGCGCGGCGCCAUCCCGUGGCCGCAUUGCGGGCGGCGCGGGGCGUCGCGUACAAUGGCGGCCUGCGGAGAUGGC.AUGCCUCCC............UGCGGUCGUAUGCG.GCGC
Bce-17-1  GCGCUCGCGCCGCGCGACUGACCGCGCGCCGGGCACGCGCUGUCGCUGCGUGGCGGCGCGGCGCCUGCAUUGCGUGCGUGGCGCGGCGUCGCGUACAAUGGCGGCCUGCGGAGAUGGC.AUGCCUCCC............UGCGGUCGUUGCCG.GCGC
Bvi-1-1   CUCGCGCGUGAUGCGUCGCUCGCCCGACUGAGAGAGCGCCCGGCGCGGCCGGCCGCGCUGGUCGUCAUGCUGGCGGCCGCGUCGCGCAUCGCGUACAAUCGCCGGCUGCGGAGAUGGC.AUGCCUCCCU...........GCGGUCGAGCACGGCGCG.
Bam-2-2   GUAAGGAUCGGGUCAUGUUUCUUGGCCCCGAAGCUGCCCAGGGCAGCAACGUGCCGCGCCGUGCGGUACCGAAUCCCUUGCAUUGGAAAGAUCCGGUAUCGUCACGCGUGGAGAUGGC.AUUCCUCCUU..............................
Bam-2-3   GCGGUCGUGCCGCUCCCAUAGCGGGCCGACACACGGCGGCAAAUAUGUUGCAUUUCGCCGGAUGUGAGACCUCCGUGUCGCUCGGCACUGGUCCGGCGCUGUUGGCCGUGGAGAUGGC.AUUCCUCCCU..............................
Sfu-1-1   GAGGAAUUCGAAUGAACCUCCGGGCAGCGCUUACCGCCGCCCGGGUCAAACCGCCGGUCGCCAGGCUUGACGUAUGGUGUGCCCGCUGGUAUUUAUAACCUUGAAUUGCGGCGAUGAA.GUU.CGCCCU..............................
Dpi-1-1   ACGGGAAAUGGCAGCCUGGACGGGGGAGGGCGGUGUGCUGUUCCUCCUGCCGGGGCUGGAUGCUGCCCGGAACUUGCGCUCCGGCGCGGGGACUGCUAAGGAAGGUCAAGGGGAUGGA.GUC.CCCCAU..............................
Dol-1-1   ACGCGGAAAGAACGGUUUUGACGUCUGUUUCCAUGCGGUUAUCAUACAACAGAUUGGGUAUUUCACGCUUGCCUUUUUCUGCUUCCUGCAUUAGGUUUUAAGAAACGAUGGGGAUGGG.GUC.CCCCGA..............................
Dac-2-1   GAAAACAGAUAAACGCAGCCUGCGCGAAAAACAUCUGUGAGGUAAACACCACCCCGAUAUGCUUGAAAAAAAGUGUCGGGAGGGCUAGAGUAUUCGACGACAUGUUAACGGGAAUGGA.GUUCUCCCGG..............................
Xca-1-1   CCACUGUCUCCGACCGAUCCAUUGCCGUCGGCAAGCGGACCGCUUGUCGUGCUAUGCCACAAGGCAGCGGCUGGACAGCCAAGCGACGCGGGAUUAUUCUGGCCAUCUCGGAGAUGGC.GUUCCUCCGCGCUGUACCAU....................
Xor-1-1   CCAUCGCUCGCCGGCCGUUUUCCAUGCGGAUGAAUGGCGUCGGACCAAGCGACUGUGGAUUCCCUGGCGGUCUGGACAGGACCGCGCACCAAGAUUACUCUCGGGAUUCGGAGAUGGC.GUUCCUCCGCGCAGUUCCAUC...................
Xor-4-1   CAAUCGCUCGCCGGCCGUUUUCCAUGCGGAUGAAUGGCGUCGGACCAAGCGACUGUGGAUUCCCUGGCGGUCUGGACAGGGCCGCGCACCAAGAUUACUCUCGGGAUUCGGAGAUGGC.GUUCCUCCGCGCAGUUCCAUC...................
Ppr-1-1   UUUGUUUGAAUAAUUAUCCCACUACAAAAUGAAAUAAAAGCGUAUAGUUACUUCAUCGGCAACGUGAACAACGCGUCGAAGUCACAAUAACCCACUUGUUAUUAUGACGGGAGAUGAU.GAUCCUCCUU..............................
Ppr-2-1   UUUGUUUGAUUAUUUAUCGCAACACACUCUCAGAUAACGGCGUAUAGUGACUUCAUCGGCAGGUUAUACACCGCGUCGAAGUCACAAUAACCCACUUGUUAUUAUGACGGGAGAUGAU.GUUCCUCCUU..............................
Psp-3-1   CUGUUUUUUAAAUCACACAGUAUUAAAAUAAAAGAGUAUAUUGAUACUAUAAGCGAAGGAGAUAUUUAUAUCUCUAUAUCGUUUGCCAUAAAUAAUAAAUAUUAUGGCGGGAGAUGAU.GUUCCUCCUU..............................
Van-1-1   UCUUUUAUUUAAAUAACGCAGUAUUAAAAUAAAAGAGUAUAUUUAUACUAUCAACGUUAUAGAUAAUUAUAUCUUUAUAUCGUUUGCCAUAAAUAAUAAAUUUAUGGCGGGAGAUGAU.GUUCCUCCUU..............................
Eta-1-1   UUGCGAACGCUCAUUUUACCUGCUCCUGCGGUAGCCACGUUUGAAUUAUCUUACCCCUUGAGUGACGGCUUGCAAAAUCAAUGUCGGCUGAUUAUCUUAAGUAUCACAAGGUGAUGGU.GCUCCACCU...............................
Cko-1-1   AUUUCCGCCGCAGAUUUCGUUGACUACCCUCUCCUUCUUCGCGAUCCAUGCGUAACAGAUUUACUUCUCAUUGAUUUCCGCCUGAACCUGCUAUACGCUUAGUUCACAAGGUGAUGGU.GUUCCACCU...............................
Vfi-2-1   UUUACUGAAUAGUUUGAAAGGGCAUACCUAAGGUGUGCCUUUUUUAUUUGCCAUUUGAAAAUGAUAGGAGUAGCAUUCCGCUUGUUGUUUGGUUUCUAUGCCAAAUACGGGUGAUGGA.UUUCCACCUU..............................
Vfi-1-1   UUUACUGAAUAGUUUGAAAGGGCAUACCUAAGGUGUGCCUUUUUUAUUUGCCAUUUGAAAAUGAUAGGAGUAGCAUUCCGCUUGUUGUUUGGUUCUUAUGCCAAAUACGGGUGAUGGA.UUUCCACCUU..............................

          ...................................................................................................................<<<.<<<..................<<<<<<<<<<<<<<<<<<<
          ...................................................................................................................022.22?..................?2222??????????22?0
          ............................................................................................................<<<<<<..........>>>>>>.............................
          ............................................................................................................??2222..........2222??.............................
          .................................................................................................................<.............................................
          .................................................................................................................2.............................................
          ••••••••••••••••••••••••••••••••••••••••••••••••••••••••••••••••••••••••••••••••••••••••••••••••••••••••••••••GG•GAUGR•-GUY-C•CC•∘-------------------------------
                                                                                                              5′─────────────────────────────────────────────────────────
```

```
Sgl-1-1   AAUGCGGUUGCAAUACAAGCGAUAUAUGUAACAGGUUGCCACCCGUUACGCCUGUUGCGUCGCGCCCUCUCAGCUGUCCCGCGCCAAAAAAUCUGAUAUACUUUCCCCAGGAGAUGAA.AUUACUCUUUA..........................
Csa-2-1   CCUCGGCACCACGCGAGUUGCGCCCCUACCGCAGCCUCUGGCUGGUACAGCGCCCCGGCGCGGAUUGAAGCACUUGCCGACGCGACAGGCGCUGGGUAGAGUGGAUGCCGGAGAUGGC.AUUCCUCCG...........GUCGCCGA.GCCCCGU...
Vca-1-1   UAUUUAUCGCUAUAUAUCUGUAGCAAUAUAAAGAACCAAUUUAAUAUCCCCAAUUACUCUAAGUUUUAUAUUGCAGGCUAAUGUGACUUAGAUCUAAUCUAUUGGGGUUGGUGAUGGG.GUGCCACCG...........GAAUC.............
Vha-2-1   AUUUAUCGCCAUAUAUCUCUAGCAAUAUAAAGAACCGAUUUAAUAUCUCAAAUUACUCUAAAUUUCAUAUUGCAGAUUAGUGUGACUUAGAUCUACUCUAAAUGCACACGGUGAUGGG.GUGCCACCA...........GAAUC.............
Vha-1-1   AUUUAUCGCCAUAUAUCUCUGAGAAUAUAAAGAACCGAUUUAAUAUCCCAAAUUGCUAUAAAUUUCAUGUUGCAGAUUAGUGUGACUUAGAUCUAAUCUAAAUGGGCACGGUGAUGGG.GUGCCACCG...........GAAUC.............
Val-1-1   GUUUAUCUCCAUAUAUCUCUGAGAAUAUAAAGAACCGAUUUAAUAUCCCAAAUUGCUCUAAAUUUCAUGUUGCAGAUUAGUGUGACUUAGAUCUAGUCUAAAUGGGUUCGGUGAUGGG.GUACCACCG...........GAAUC.............
Afe-1-1   CUGCGCUACGAGAUCUAUAACCACGAAAAUAUCGCCCUGUUGCGGAUCAGCCCGCGCACGAAAGGCCUUGACCAUGCCAUUGCGCCUUACUACCAUGAAUAAAACGUGUGGCGAUGGA.GUC.CGCCCAA..........................
Sma-1-1   CUGCUCGCGUUCGAACUGCUGAUGGAUGGCUUCAGCCGCAGCAAGGUGGCCCGCGUGCUGACCACCUCGAUGACGUGAAUGCACCGCCGCUGGUAGCAUGGACCGCGAUGGGGAUGGG.GCUCCCCCGA.........................
Sma-2-1   CUGCUCGCGUUCGAGCUGCUGAUGGAUGGCUUCAGCCGCAGCAAGGUCGCCCGCGUGCUGAUGACCUCGAUGACGUGAGCGCACCGCCGCUGGUAGCAUGGGCGACGAUGGGGAUGGG.GCUCCCCCGA.........................
Vsp-1-1   UGGCGUUUUUUUAUACUUAUCGAAAUAUAAUGAACUCUGUUUAUAGUCGAAAUCGCUCUAAACAUUCGCUUGCACAAAAGUGUGAUUAAGAUCUAAUCUAUAGCCAUCAGGUGAUGGG.GUUCCACCU...........AAGC..............
Vba-1-1   UUGGCGUUUUUUAUACUUAUCGAAAUAUAAUGAUGCUUGUUUAUAGUCGAAAUCGCUCUAAACAUUCGCUUGCACAAAAGUGUGAUAAAGAUCUAAUCUAUAGCCAUCAGGUGAUGGG.GUUCCACCU...........AAGC..............
Vsp-2-1   UUGGCGUUUUUUAUACUUGUUGAAAUAUAAUGAAGCCUGUUUAUAGUCGAAAUCGCUCUAAACAUUCGCUUGCAUGAAAGUGUGAUAAAGAUCUAAUCUAUAGCCAUCAGGUGAUGGG.GUUCCACCU...........AAGC..............
Vfi-1-2   CUCAAUCUUUAUGGUUUAUCUAAUAUAAAAGUUGGCUUUAAUAUAUAAAAUAAGUCUAAUAACCCUGUUGCGACUGCGUGUCUUUGGGUCUAAUAUUGAAUACGUAAAGGUGAUGGG.GUUCCACCUACU.........................
Vfi-2-2   CUCAAUCUUUAUGGUUUAUCUAAUAUAAAAAUUGGUAUUAAUAUAUAAAAUAAGUCUAAUAACCCUGUUGCGACUACGUGUUCUUUGGGUCUAAUAUUGAAUACGUAAAGGUGAUGGG.GUUCCACCUACU.........................
Asa-1-1   CUUUUUCUUUAUGGUUAUAUGAAUAUAAAACGCAACAUUUUUAUGUGAAUUUAAUCUAAUAACGUUGUUGCACAAUAGUGUGAUGUAGGUCUAAUAUAAAGGCGUACAAGGUGAUGGG.GUGCCACCUACU.........................
Vsh-1-1   AUCAUUAUGCUUAUUGCGUUAUGAAUAUAAAACUAAAUAUUUUUAUGAAUAAUGGUUCUAGGCAGAGGUCUUGCACAAAAAUGUGAUUUAGAUCUAAAAUUGCAACCAAGGUGAUGGG.GUUCCACCUACU.........................
Pca-1-1   GUAAGACGCGGCAAAAGCACUUUCGCCCGUAUUCAGAUGGCUUUACAACUUUCGCCUCGGCAGCACUACAAAAGUCCCUUCUGACGGCGUAGACUUUCCCUACUAAAGCGGAGAUGAC.AUUCCUCCC...........UACCCU............
Pca-2-1   GUAAGACGCGGCAAAAGUGCUUUCGCCCGCAUUCAGAUUGCUUUACAACUUUCGCCCCGACAUCACUACAAAAAUCCCCGCCUGACGGCGUACACUUUCCCUACUAAAGCGGAGAUGAC.AUUCCUCCC...........UACCCU............
Psy-2-1   CCUAGUUUGGCCCUCUUUCGUAAGCGGUGACUCGAAGACGCCUUCGCUUGCCUCGUACUCCGAAACACGAUUUUACAUUUUGGACAGACCUAGCUAAGAUCGGCGCAUUGGAGAUGGC.AUUCUCUCCAUUAAC........................
Cko-1-2   GAGCGUGACGAGAGCUGAUGAUCAUAUUUGCCAAUUUUCCGUUUGGUUAAUCCAAAAAAUCUUACACGAGAUCUGGCUAAUUACAGAGUUCAUUGCUACGUUUUUUACAGGAGAUGGC.AUU.CCUCCUCA.........................
Psy-1-1   UGGGUAGGCUCAGGUCACUAUGACAGUAGAGACAGCGUACCUAUAGGCCGCACUGAUUUCGGGGCGUGGAGGUUGAAGUUAUCGGUACUGUUGCUAUAGGAUCUGCACAGGAGAUGGC.AUU.CCUCCUUG.........................
Pae-3-1   UGGAGAGACUCCGGCGAGGGGGUUCUUGCUGUCAGACUGGCACGUUUUGCCCGUGCCUGCUGUACCCCGGGCUUUGAUAGGCGCUGCGCUUUGCCAAUCUAGCCGGCAGGAGAUGGC.AUUCCUCCUU.........................
Pae-6-1   GGGACUCCGGCGAAGGAAGGGGAAAGGUUCCCUAGACUGGCACGCUUCGGGCGAUCCGGCGUACUCCGGCCUUUGAUAGGUGCCUCGCUUUGUGCCAAUGUUGCCGACAGGAGAUGGC.AUUCCUCCUU.........................
Pae-5-1   GGGGCUCCGGCGAAGGAAGGGGGAAGGUUCCCUAGACUGGCACGCUUCGGGCGAUCCGGCGUACUCCGGCCUUUGAUAGGUGCCUCGCUUUGUGCCAAUGUUGCCGACAGGAGAUGGC.AUUCCUCCUU.........................
Pae-2-1   GGGGCUCCGGCGAAGGAAGGGGGAAGGUUCCCUAGACUGGCACGCUUCGGGCGAUCCGGCGUACUCCGGCCUUUGAUAGGUGCCUCGCUUUGUGCCAAUGUUGCCGACAGGAGAUGGC.AUUCCUCCUU.........................
Pae-4-1   GGGGCUCCGGCGAAGGAAGGGGAAAGGUUCCCUAGACUGGCACGCUUCGGGCGAUCCGGCGUACUCCGGCCUUUGAUAGGUGCCUCGCUUUGUGCCAAUGUUGCCGACAGGAGAUGGC.AUUCCUCCUU.........................
Pen-1-1   CAGGCGGCCAUUCUAACGAUGCGAACGAAGGGAUGUGCUGUAAUUUGCGUCCAAAUGUUGCCAAGCGCGCUGUCACCCUGGCAGGGGCCACGUAGCAUCGUGGCCCAGGAGAUGGC.AUUCCUCCUU.........................
Xca-4-1   CGCGCGGAUGGAGAUCAAGCUGAAGGAAAAGCUCGAUCGGCUGCGCGAGGCGCGCGCGCUGGCACGGGCUGACAAGGCCGGCAAGUCCACGGCAUAAUCGGCCGCACAGGAGAUGGC.AUUCCUCCUC.........................
Xax-1-1   CGCGCGGAAUGGAGAUCAAGCUGAAGGAAAGCUCGAUCGGCUGCGCGAGGCGCGCGCGCAGGCACGGGCUGACAAGGCCGGCAAGCCCACGGCAUAAUCGGCCGCACAGGAGAUGGC.AUUCCUCCUC.........................
Pst-1-1   UGUCAUUAAUGGUAGUGCAAAGAAAUUAACGUUACAUUGUGUUUAACAAGAGAAAUUCUUAAAGCUGGGGCUAUUUACCUUGUUGUUAAUUGCAGAUAUUCUAACAAAAGGAGAUGGC.AUUCCUCCUGUUUAA......................
Asp-2-1   UUUCGUCAGCGGACAAUCAUAACAUGAUGUAUCUGUGGAUAACUUUGCCUUUAUACACAGAUUAAAAGAUUCAUUGAGUUGUUUAAAAAUUAUGUUAUUUUAGGGCAAAGGAGAUGGC.AUUCCUCCUGUAAC.......................
Asp-1-1   AAACAAACGCUUAAAGAAGUUGAAAAGAUCACACGAUUUAAAUAAGCGUCCAAAUUGAUACGAUCACAGACUUCACAUUGUGAAAGUGCUAGGCUAAACUCGACAGAUAGGAGAUGAC.AUUCCUCCUCGCAA.......................
Aba-3-1   AGGCAGAGUGGGAAUGUAUCCAUGACGAUUUACCGCAAUACGAUGAACGUCCAUGAUUUAAAAAGUAUGUUUUAAUCAAAUCUUAGAUGAUUUAAUAUACCUAAAACAGGGAGAUGGC.AUUCCUCCCCUUGAA......................
Aba-4-1   AGGCAGAGUGGGAAUGCAUUCAUGACGAUUUACCGCAAUAUGAUGAACGUCCAUGAUUUAAAAAGUAUGUUUUAAUCAAAUUGUAGAUGAUUUAAUAUACUUAAAACAGGGAGAUGGC.AUUCCUCCCCUUGAA......................
Aba-5-1   AAGCAGAGUGGGAAAGCAUUUGUGACGAUUUACCGCAAUACGAUGAACGUCCAUGAUUUAAAAAGUAUGUUUUAAUCAAAUCUUAGAUGAUCUAAUAUACUUAAAACAGGGAGAUGGC.AUUCCUCCCCUUGAA......................
Ymo-1-1   GCAAAAGAUAAUGGCCGUAAUCGUAUUGAAUGGCGAGCUAAGUUGCGGGCUACAAAUUAGCAUUCACUCAGUGUAUUAUUAGCUUUCAAGCAUUGACCUUUAAAUGCUGGAGAUGAC.AUC.GCCCAU..........................
Yin-1-1   GUACCUAGCUAAAGAUAAUGGGCGUAAUCGUAUUGAGCUAGCCAGUUAAAAACUAUUCCCAAUAGAUUUCAAGAUGCAGGAAGUCAGUGUAUUAUAACCGCAAGAUCUGGAGAUGAC.AUUCCUCCA..........................
Yps-4-1   CCUUGCAUUAAUGGCCCUCUUGGGAAUAAUGCCAGCAAAAUGGGUCAUGAUAAAAACCGACAAGCUACAAAUUAAUAUUCAAGCAGUGUAAUAUCGCCCCUCAAGAUCUGGAGAUGAC.ACGCCUCCA..........................
Ype-13-1  CCUUGCAUUAAUGGCCCUAUUGGGAAUAAUGCCAGCAAAAUGGGUCAUGAUAAAAACCGACAAGCUACAAAUUAAUAUUCAAGCAGUGUAAUAUCGCCCCUCAAGAUCUGGAGAUGAC.ACGCCUCCA..........................
Yps-2-1   CCUUGCAUUAAUGGCCCUAUUGGGAAUAAUGCCAGCAAAAUGGGUCAUGAUAAAAACCGACAAGCUACAAAUUAAUAUUCAAGCAGUGUAAUAUCGCCCCUCAAGAUCUGGAGAUGAC.ACGCCUCCA..........................
Yps-1-1   CCUUGCAUUAAUGGCCCUAUUGGGAAUAAUGCCAGCAAAAUGGGUCAUGAUAAAAACCGACAGGCUACAAAUUAAUAUUCAAGCAGUGUAAUAUCGCCCCUCAAGAUCUGGAGAUGAC.ACGCCUCCA..........................
Spr-1-1   CACUGAGACCGGAGGAAAAAUUUCAGUUUCCAGUCACUUUUGAUACUACAAAAUAGGCAGCGCCGGGUGUAAGAUCCCGUCUGCGCGGCAAACAGCGGCGUCGAACGACGGAGAUGAC.AUUCCUCCU..........................
Pru-1-1   AUGCGCUAAUUUAGCCUAAAUUGACAUAAGGUUAUCAAUGGUAAUUUUAUAUAAAUUGAGUUUGCAUGGAAAAAAGGGUUGGAUUAUCAUUUCAAUAGAAAUUUAUUUGGAGAUGGC.AUUCCUCCUACCC.........................
Pal-1-1   UAUGCGCCAAUUUAGGCUAAAUAGACACCGCGUUAUGUGGAACAGAUUGGGUAAAUUAGAUUUGCAUGGCUAAAAUAGUUGCCUUAUUAUUUUCAGUAACGUCUUGUUAGGAGAUGGC.AUUCCUCCUA.........................
Pat-1-1   GUAACAAAGUAAGACACCGCUUUCGCAAGCAUUCAGAUUGCUUUACAACUUUCCCCUCUGCAUCACUACAAAAAAACGCUGACGGCGUAAACUUUUCACACUAAAGCGGAGAUGAC.AUUCCUCCC...........UACC..............
Cko-1-3   GAUUCACAGUGAUGUGAGAAUAAUGACAAUGCGUGGCGCAGAAAGCGACAGGUCAUAACGUGAGUUCAUACUUGAAAGAGACAUCAAGCGGGUUCACAAUCCUGUGCAGGUGAUGGC.GUUCCACCUU.........................
Kpn-1-1   UCAGGUCAAGCCUGAAAACGGGCUCCUCAUGCCCGCCGCAGGGGGAGUCGCACAAAAACAGUGGAUAGCCGCUUGAUUAACGCCCGUGACCGGGCCAGAAUUGCGGCGAGGUGAUGGC.GUUCCACCUUACC.........................
Tpe-1-1   GCUGGACUUUUCCAGAGACUACUUGAGGGAAAUAGUGAAUUUUGUUGCAGAAGAAAUUGAAAAGCUGGAGAUGGUAUAAUAACGUUUGGAAAAAAACAAUAACCCAACGGGCGAUGAG.GCC.CGCCC..........................
Tle-1-1   AUUCUUCAAAAAUAUUAAAACAGGAACUGAUAAAAACACGGUGAUGAAGGGCCCGCGGUUUGUGGUAGAAUAAUACUGUGUUUGAAGAGUUCAGGAUAUGAAUAAGCCUGGUGAUGGA.GUC.CACCGU.........................
Cfl-1-1   CUUCGGCGAAUUCCGCGUCCAUGGUGCGGGCAAAGUGCGCUACCGGCUCUGCAGCCCGGCGGGAUCCCUUGACAAAUCGCCGGUGAUCGCGUUCUCUUCCGCCCGUAACGGCAAUGGA.UUUCUGCCG...........CCCGA.............
Min-1-1   CAACCCGAACCCGAAGAGAACCGCUUCCUUCAGAGUGUAAAAUUUUUUUACAAAAAAAUUUGACAACUCUUUUUUAAAAGAAUAUUCGCUAGCAAGAAAACGAAAUCCCUGGCAAUGGA.GUU.UGCCG...........UCCCGCCGGU........
bEl-1-1   UUUCAAAAUCCGACCUAUGCCAGAAUUGUGCUUAAUGCGGUGAUCUACAAACCGUGAGAACAGGUUGUUCCUUGCCAAUUAACGGAUCUUUGUGUUCUCUAUAUGCAUUGGCAAUGGG.GUU.UGCCU...........UCUCG.............
Min-1-2   CUUCCAACAAAACCAGUCCAAAAACUGAUCAAAAAUGCAAAGAAAAGAGAACUUUUGAGUUGAAAAUAAAUCAAUCCAAGCUUAUAAAAAAAACAGCUUACUCUUAUUGGGCGAUGGG.GCU.CGCCGACCGU.........................
env-1     NNNNNNNNNNNNNNNNNNNNNNNNNNNNNNNNNNNNNNNNNNNNNNNNNNNNNNNNNNNNNNNNNNNNNNNNNNNAACUCGCGCGUCAUACACGAUCAGCGACAACUAUCACCAACCGGUGAUGGG.CGG.CACCGGUGC.........................
env-2     CCUACACACUGAGACUGUAGAUUUAAGGGAGAACCCAAAAAGAUCACUCACAAAAUUUAUAUAAAAUUCGCUGAUUUGGUGACAGGAAAGAUACACCGCAUGGUGCAGAGGGAUGGA.GUU.CCCAA..........................
env-3     UCGACGUGAGUCGGGAAACAGUUUUUUGUUUGCAGAAUUUUGUAUAACUUUGCAGCCGAUUUUGAAUUCUGUCCGAUUUUACUGACUGUUCAGGCUGAUUCAAAGUCAGGGGAAUGGG.ACU.UCCCU..........................
env-4     CGCGCGGAUGUUGCCGCCACGGCAGGUCACCGAGCGGUAGCGAUCGAUCCUGCACGUACCCGACGCGGUCAAUCACCAAGCCGGUUAUCAUCAAGCCAGCACGUCGCGUGGUGGUGGA.GUC.CACCU..........................

          ..............................................................................................................................<<<.<<<.................<<<<<<<<<<<<<<<<<<
          ..............................................................................................................................022.22?.................?2222??????????22?0
          ......................................................................................................................<<<<<<.........>>>>>>.............................
          ......................................................................................................................??2222.........2222??.............................
          ..........................................................................................................................<...................................................
          ..........................................................................................................................2...................................................
          ••••••••••••••••••••••••••••••••••••••••••••••••••••••••••••••••••••••••••••••••••••••••••••••••••••••••••GG•GAUGR•-GUY-C•CC•∘----------------------------
                                                                                                    5′
```

```
env-5   GCGCACGAGGCGCGAGGACGAGGCCAGUUCGGGGUCGGCCCCGAUCGGUUCUAGCGCCCCGGACAAUUGACAAUCGCGUCCUGCUCCGGUAAGUACCAGAAGUCCGAAGGCGGUGGA.GUC.CGCCA...............................
env-6   ...................CCAUUCUUUCUGGAAUACUUGACAGAUUCGCAAAUUCAUGNUAGCAUAGAAGCGCUGUCGGGUUUUGCUACAGUUUCACAUCAGNNAUCGGGAAUGAU.GUUCUCCCC............UGGGA..............
env-7   CUGAUGGGAGGAACCUUUUCCGUUGACAUAUGGAUAAAUUUCAAUCGAUGAUAGGAGGUGACGUUGGCGGGUAAGUUUUGACAGUGCCUGGUCGACAAGAUGACGAGGGGUGAUGGA.GUC.CACCU...............................
env-8   GAUUUUAACGAAAUAACAGGUUAUGCCACCAGAUACGUAAUAAAGAAAAGACACAGAGCAGAAAAGGAAGCCGAAUUCACAGGAGAAGUUGUAGAGUAAUUUCAAGAAAGGCUAUGAC.GUU.AGCCUU..............................
env-9   CCCCUUCUGGGGCGGGGGACGUCGCCCUUUUCAGGACGGGGAGAGCGACUUCGUCCGCACCUCGGAAGGAUUCGACGAGGUGGGGGACGGGGCUGGUAAAGUCCGUGACGGCGAUGGA.UCC.CGCCU............GGC................
env-10  CCAUUGUUUCCGAACGGAUAGAUAAGGUUUACACCGGAGGGUUCAAACAAUUUGAAAAAGAUUUUGAAGCCUUCUGCAAAUAGUUUUAUCUUUUUUCUAUCUUUGCACCGGCUAUGGG.AUU.AGCCUU..............................
env-11  UCUGUUGAUAGAUGUAUAGUAACAGAAUUUUUAUUUUGAGUGACUAAUUAAAAAUUCAUAAAGGUGUUUAAAAUUGUUUUCACUUAACCGGUUAUUAAUUUUGGGCGCGGUGAUGGA.GUU.CACCA............UCUACUA............
env-12  UAUGCCAUGCGGUCGUUGUGCUCAUAUCGACAACUCUAAAAACCUCAAGUACUUGAAGUAAAACGGCGACACACCCAUGGAUGUCUUCUGCACGAGUAGGCUGGGAUUCGGCGAUGGG.ACC.CGCCU............GAA................
env-13  CCAAGUUCAUCGGCGUUGCAGAAGUCUCUUGCCGAAAUCCACCGCGAUAGGCCAAUAUCCGAGAUACUGCUGCCGUUUGUUGUCGCCGCCACGAGUAGGCUAAGGAACCGGCGAUGGA.ACC.CGCCU............GAA................
env-15  UUUCGCCAUACCCAUGAGGUAUGGCAACAACCGAGCAAUUCACUGCGAUAGGCCAAUAUCCGAGAUACGCUCACUGUUCGAUUCGUCGCCAUGAGUAAUCUGGAGAACCGGCGAUGGG.ACC.CGCCU............GAA................
env-16  UUUCGCCAUACCCAUGAGGUAUGGCAACAACCGAGCAAUUCACUGCGAUAGGCCAAUAUCCGAGAUACGCUCACUGUUCGAUUCGUCGCCAUGAGUAAUCUGGAGAACCGGCGAUGGG.ACC.CGCCU............GAA................
env-17  UUUCGCCAUACCCAUGAGGUAUGGCAACAACCGAGCAAUUCACUGCGAUAGGCCAAUAUCCGAGAUACGCUCACUGUUCGAUUCGUCGCCAUGAGUAAUCUGGAGAACCGGCGAUGGG.ACC.CGCCU............GAA................
env-19  CCGCGCUAUUGGCGACGGUUAUGGAUCGUACGUUGCGGUAGUCCGGGGAAGUUGUCCGCAUCGUGAAUGAUGGUACGGACGGCCUCGCGUGCCGGAUAUACUGGACUGCGGUGAUGGG.ACU.CGCCU............GAAGCC.............
env-20  CCGCGCUAUUGGCGACGGUUAUGGAUCGUACGUUGCGGUAGUCCGGGGAAGUUGUCCGCAUCGUGAAUGAUGGUACGGACGGCCUCGCGUGCCGGAUAUACUGGACUGCGGUGAUGGG.ACU.CGCCU............GAAGCC.............
env-21  AUGAAUUCAGCCUUGUCUGAUAUGUGAUGAACGACGGGCAGACUGAGUGGUACCUUUUUAUGCGCUUGAAGAUGGAUUUAAAUAAAUCAUCAAAAUAUCCUGUAGUCCGUGGAAUGGC.GUU.UCCACUAAUUC.........................
env-22  CAGGGUUCCGGGAGCCUUCCAUUGAUAAAAUGAUCGGGGACAGUGGGAUCGCCCUGUGGACUCCCGGUCCCAUUCGGUGGUAGGAUGGGUUCGUCCGCUUCCGGCGGAUGGCGAUGGG.GUU.CGCCU...............................
env-23  UCUUUGGGAGGAGAGCACGCCUCCUUUCCCAAAUGCAUCGCGAUCCAUCAUCCCUUUCGAAAACAGGUUGUCUUAGGCGGCGCAUAAGAGCAUAAUCAUUGGGAAGACAGGCGAUGGG.GUU.CGCCA...............................
env-24  GUAUUAUAUGGCCAGGUAGAAACUUUUCACUCACUUUUAUCUCCCAGCUUACGAAAAUAGGAUUGCCACUCGCAAAUGUUAAAUCCGGAAUCAUAAUUAUGAUAAUAAUAUGGAUGGA.GUU.CCAU................................
env-25  GUUUAAGAGAGUACCAGACCCUAUUGGAGAGGGUCACGGAAGUAUUGAAUUAAGACGCGUAUAUUAAGAUGACAUUGACAAACCCAUCUAACAGUCUUAUAAUAGUGGCGGGAAUGAA.GUGCUCCCU............UCAU...............
env-26  GUAAACGACCCUUCAGCCAGAGUACUGGCAAACUACGGAGACAGCGGAAUGUGAUACGCCUAAAAAAUUUGUAUUGAAAAACAAAAAAAUCCAUGUUAAAAUAGCUGGCAGGGAAUGAA.GUUCUCCCC............AGGC...............
env-27  AAAUGGGCAGGCUAUAGAAUAAAUUUACUUUUCAAAGCCUAUAUAUUACUAUGAAUAUUGACAGUAUUCCAACAAUAUUGUAUACUUAACAAAUUGAUGAAAUAUUACUGGGAAUGAG.GUU.CUCCCA...........GGAACGAGA..........
env-28  UGAUACCGCCGGCGGGGCAGUGCUGCCGGAGUCAGAGAUUGCGAUGAUGAAGAGAUAGUGUUUGAUAAUCCAUACUUGCAUUUCCGGCCUCUGUCUGCUAUACUGUUACCCGGGAAUGAG.GUC.UCCCA............UGGUAGUGU..........
env-29  GAAAGAUGGCGAGAUUGUUAAUCUGUAUAAUGCAAUUAAAAAUGAAUAAUCUAACAUACUAUUCUUCUUACAUUUGACAAUCUGCCCCAAAGUAUUAUAAAUAUCAAAGGGAAUGAA.GUACUCCCU............UGG................
env-30  UAGAAUAACUACAGUGUGAUUUGCAGUUUUCGAUACAGUCAGUAUCCGUACAGCAAAUAUGGCGAUUGACAUAUCCAAAAACAGGUGGUAGUAUAAAGUUCGAAUGAGAGGGAAUGAA.GUUCUCCCU............AGG................
env-31  AGGGCUGGAAAAGAAGCAGAAAAUAUGCGGCAGAGUAAACCCCGGCGGCGUUGCGGAGCCCGGAUUUGCUUUGAGAGACAGGUAUGCUAUACUGCCAACAGAAUAAACGGGAAUGAA.GUU.CUCCCG...........AAGUAA.............
env-32  UACUGGUCACAGAGUGAGCAGUAACGUGCACAGCAGAAGUUGUUAUUUGAUUUAUAAUUUCUUCUUGCCACCACUUUUAUAAUGUGCUAUUAUACCACCAGAAACAAAUGGGAAUGAAUGUC.UCCCC............GGGA...............
env-33  CGGGAAAUUGAGAGGGGGUUCUGAUACAACAGAUAAGAGGGAGAAGUGGCUGUAACAGAUUUCGGCUUGACGAAUAUGCGGAAAAACUGUUAUACUUUCAUCCAGAACGGGAAUGAG.GUUCUCCCA............CGAU...............
env-34  AACCUGCCGAUUUGUUUAAUCAUAGCACACCUGUAACAAAUAAGAUACUCCGGAUUUCCGUCUGACUAUUGACGAUAUCAGCCUCGGAUGCUAUACUCUCAGGCAGAAAGGGAAUGAA.GUUCUCCCC............CGAU...............
env-35  GGAUGAAGCAGAAAAAUACAAUAUUGAAGAAUCAGAAGUAAAAGAAAUAUUAGAAUAAAUUACAAAAUUGACACAAAAUAAAAAAUAUGCUAUCUUAACAAAAGAGAAAGGGAAUGAA.GUUCUCCCU............CGAA...............
env-36  GGAUGAAGCAGAAAAAUACAAUAUUGAAGAAUCAGAAGUAAAAGAAAUAUUAGAAUAAAUUACAAAAUUGACACAAAAUAAAAAAUAUGCUAUCUUAACAAAAGAAAAAGGGAAUGAA.GUUCUCCCU............CGAA...............
env-37  GUGUAUCACGUGCCGGCGUCCGACGGCAAUGCGCCGCAUGCCGUUCCCAUGUGCGCUUUGCUGCUUGACUCGGCGCCGGCUUUGUACCCAUACUACAAAAAACGAUAACGGCGAUGGG.GUU.CGCCAGUC...........................
env-38  AGUUAACGAAAUUGGCUCCUUUCUAUGAAUUUGCUGAAUUGCGUGUAAAGAAAGAACGAUAAUUUUAUUGUUUUUUACAGAAAAAUAUGCUAUCUUUUAAUGGAAGUGAAUAGGUGAUGGU.GUU.CGCCUU..............................
env-39  ..........................................................CUUCCUUCCAAGAAGCGGCGAAAAAAUGCUAUCUUUCAAUGGAAGUGAAUAGGUGAUGGU.GUU.CGCCUU..............................
env-40  AGUUUAUUGUUUUUACCAACCAGUGUUAACUUUCCUAAAGGAGAAUCAUAGCAUGUUGUACAAAUCAUAAUGAAAGUUUAAAUAAUAAAAUAUUUAAAAUAAUAACUUGGUGAUGGG.GUU.CACCA............GAAA...............
env-41  AGUUUAUUGUUUUUACCAGCCAGUGUUAACUUUCCCAAAGGAGAAUCAUAGCAAGUCGUAUAAAUCAUAAUAAAAGUUUAAAUAAUAAAAUAUUUAAAAUAAUAACUUGGUGAUGGG.GUU.CACCA............GAAA...............
env-42  CGCUAGCCACGGUUGGCCGCAAAUCGGGCGCGGCGGCGCGCCGACUGCUGCUCGCCGCACGCCAUGCUCGUCUGUCCCCAAUGCCAGGCGGAUGCUAGACUUGCGCGCUGGAGAUGGC.AUU.CUCCAU..............................
env-43  CUCGCGCAUCACGCGUCCGCUGCGCGACUGACGGCGCCCCGGGGCGCGCGGCCGUGCCGUGGCCGCAUUGCGGCGGCGGGGCGUCGCGUACAAUGGCGGCCAGCGGAGAUGGC.AUGCCUCCC............UGCGGUCGUAGUCG.GCGC
env-44  UGGGCAACCAUCUCUCUUAAGCAUCGCCUGACUUGCCGUUUCCUGUCCGACUUUUGUGCAGAAAACGCUUCAGCUGACGCGUUUCACCUUGUAAAAUGAGCCUCACAGGGGAUGGC.AUUCCCCCUU..............................
env-45  UGUUGCAAGCGCUUGAUCACCGAUCUGAGGAACGGCGCCCCGCCCGCCCUGAAAAACAGCGAUUGACCCGAUCGGCCCGAUGUGCAAAAAUUUGACCGUCAACCCCAAGGGAAAUGGU.GUC.UUCCCUGCAC.........................
        ....................................................................................................................<<<.<<<...................<<<<<<<<<<<<<<<<<<<<
        ....................................................................................................................022.22?...................?2222???????????22?0
        ............................................................................................................<<<<<<..........>>>>>>..............................
        ............................................................................................................??2222..........2222??..............................
        ....................................................................................................................<......................................
        ....................................................................................................................2......................................
        ●●●●●●●●●●●●●●●●●●●●●●●●●●●●●●●●●●●●●●●●●●●●●●●●●●●●●●●●●●●●●●●●●●●●●●●●●●●●●●●●●●●●●●●●●●●●●●●●●●●●●●●●●GG●GAUGR●-GUY-C●CC●○-----------------------------
                                                                                                       5′
```

alignment positions 160 · · · 317

```
[1]Sso-1-1  AUAA.............................................CCCCGAA..CCGCCUC...................UUGAUUAAUGAUA.........................................GGGGCUGAUGACGCC
Mse-1-1     CUUGUU...........................................CCCGAA..CCGCCU....................GAUACAUGA............................................AGGCUGAUGACGCC
Pto-1-1     CAU..............................................GCCCAAA..CCGCC.....................GAAA..................................................GGCUGAUGGCGUC
Tvo-1-1     .................................................UGA..UUGUCU......................CAUUG.................................................AGACUGAUAACUCC
Mhu-1-1     .................................................AAA..CCGC........................CCGCU..................................................GCUAAUGACUUC
Tvo-1-2     .................................................AAA..CCGCCAGCU...................CC..................................................AGCUGACUGAUGACGCC
            ..................................>>>>>>>>>>>>>>>>>>>.......<<<<<<<<<<<<<<<<<<<<<<.................................>>>>>>>>>>>>>>>>>>>>>>...>>>>>>
            ..................................0?22???????????2222?.......22222??2?????????222122.................................221222??????????2??22222...?22220
            ...........................................................................................................................................................
            ...........................................................................................................................................................
            ....................................................................................................................................................>......
            ....................................................................................................................................................2......
            ----------------------------------------------------●AA--CCGCY○-------------------●●●●○------------------------------------------------○RGCUGAUGAC●YC
```

```
CMe-1-1  ........................................................UAA..CCGCCUUAC.................CCUAA....................................................AGGCUGAUGACUUC
Mhu-1-2  ........................................................UAA..CCGCUU....................UUUC.....................................................CAGCUGAUGACUCC
Abo-1-1  ........................................................GAA..ACGCC.....................CGCGU.....................................................GGCUGAUGACGCC
Abo-1-2  ........................................................GAA..ACGCC.....................CGCGU.....................................................GGCUGAUGACGCC
Abo-1-3  ........................................................AAA..CCGCC.....................GCAA......................................................GGCUGAUGGCUCC
Abo-1-4  ........................................................AAA..CCGCC.....................GCAA......................................................GGCUGAUGGCUCC
Mhu-1-3  ........................................................AAA..CCGCC.....................GUUCG.....................................................GGCUGAUAACUCC
Msm-1-1  CUUAU...............................................UUUCAAA..CCGC......................AAUU.......................................................GCUGAUAACUCC
Msm-2-1  CUUAU...............................................UUUCAAA..CCGC......................AAUU.......................................................GCUGAUAACUCC
Msm-3-1  CUUAU...............................................UUUCAAA..CCGC......................AAUU.......................................................GCUGAUAACUCC
Mth-1-1  ........................................................UAA..CCGCUU....................UUUC.....................................................UGGCUGAUGACUCC
CMe-1-2  ........................................................CAA..CCGCCAC...................GUAUC...................................................GUGACUGAUGACUUC
Tko-1-1  UUCGA...............................................GCCCGAA..CCGCUC....................GAAA.....................................................GGGCUGAUGACGCC
Ton-1-1  UUCGA...............................................GCC.GAA..CCGCCC....................GUUUU....................................................GGGCUGAUGACGCC
Tba-1-1  UUCGA...............................................GCCCGAA..CCGCCC....................GUUAA....................................................GGGCUGAUGACGCC
Tsp-1-1  UUCGA...............................................GCCCGAA..CCGCCC....................GCUC.....................................................GGGCUGAUGACGCC
Pab-1-1  UUCGA...............................................GCCCGAA..CCGCCUCCC.................AUG...................................................GGGAGGCUGAUGGCGCC
Pfu-1-1  UUCGA...............................................GCC.GAA..CCGCCUCCC.................GUU...................................................GGGAGGCUGAUGGCGCC
Pho-1-1  UUCGA...............................................GCCCGAA..CCGCCUCCC.................GUU...................................................GGGAGGCUGAUGGCGCC
Afu-1-1  ........................................................CAA..CCGCC.....................GCUAA.....................................................GGCUGAUGACGCC
Mst-1-1  ........................................................UAA..CUGCCAGUG.................AU....................................................UACUGGAUGAUGACUUC
Mac-1-1  AAUU................................................GUCAAAA..CCGC......................UUAU.......................................................GCUGAUGACUUC
Mba-1-1  UUACA...............................................GUCUAAA..CCGC......................UUAU.......................................................GCUGAUGACUUC
Gvi-1-1  CGAUCC..........................................GGGUGCAAGAA..CCGCC.....................CCCC......................................................GGCCAACGGUCUC
Ssp-2-1  ........................................................CAA..CCGCCUCAGGC...AGAUCCUAGGGAUAAAGUU..............................UCUCUAAGA.CUUAGAGCUGGGGCUGAUGACUCC
Ssp-3-1  ........................................................AAA..CCGCCCAGG.................AUUUUUCUUAAUUGCA......................................CCUCGGCUAAUGGCUCC
Sel-1-1  ........................................................CAA..CCGCGCACUCGG..............CAUGGC.............................................CCGAU..CGCUGAUGGCUCC
Ssp-1-1  ........................................................CAA..CCGCCUCA.CUU..............GGAAUUUUUCUAUAGAGGAAGAGGGUUCUAACCUCAA..............AAGCUGAGGCUGAUGACUCC
Ava-1-1  ........................................................AAA..CCGCUAUUCUAG..............UCAAAAAAUAAAU......................................CUAGAA.GGCUGAUGACUCC
Nsp-1-1  ........................................................AAA..CCGCUAUUCUAG..............UCAAAAAAUUAAU......................................CUAGAA.GGCUGAUGACUCC
Dth-1-1  ........................................................UAA..UUGCC.....................GAUAAA....................................................GGCUGAUGACUCC
Tye-1-1  ........................................................UAA..GUGCCUCUG.................AUUUU.................................................CAGGGGCUGAUAACUCC
Taq-1-1  ........................................................UAA..CCGCCC....................GCUUC....................................................GGGCUGAUGACUCC
Dge-1-1  ........................................................GAA..CCGCC.....................CCGCGUCC..................................................GGCUGAUUAUGCC
Aba-1-1  ........................................................UAA..CCGC.CGCAGC...............CACGUU..............................................GCUGCGUGCUGAUAACUCC
Hsp-1-1  CAUAAA...........................................AGCAUCAAAA..CCGCCU....................CACAUAUA.................................................AGGCUAAUAACGCC
Hsp-1-2  ........................................................UAA..GCGCCUCCU.................UCAA..................................................AAGAGGCUGAUAACGCC
Ban-1-1  GCA............................................AGGAUUCCCGAA..CCGC......................AAUUCC.....................................................GCUGAUGGUUCC
Lxy-1-1  GCGCUGA...............................................CCGAA..CCGUG.....................AUGGC.....................................................CGCUGAUAGUUCC
Mab-1-1  CUUGU................................................CCCGAA..CCGCC.....................ACUGAC....................................................GGCUGAUAGUUCC
Mma-1-1  ........................................................UAA..CCGCGCGUCG................AUUAU................................................CGACG.GCCAACGGUCCC
Mul-1-1  ........................................................UAA..CCGCCCGUCGC...............GAAA................................................GCGAU.GGCCAACGGUCCC
Mma-1-2  ........................................................UAA..CCGCCCGUCGC...............GAAA................................................GCGAU.GGCCAACGGUCCC
Ace-1-1  GAUCG..........................................ACACCGG.CGAA..GUGUC.....................ACCU......................................................GGCUGAUGGCUCC
Mul-1-2  CUCGA..............................................CUUCUGAA..CCGCC.....................AGCC......................................................GGCUGAUGGCACC
Mma-1-3  CUCGA..............................................CUUCUGAA..CCGCC.....................AGCC......................................................GGCUGAUGGCACC
Ace-1-2  ........................................................CAA.UGCGCCG....................CC.......................................................CGGCUGAUGGCUUC
Rxy-1-1  UCC............................................GCCUCCGGAGAA..CCGCGCC...................GGC.....................................................GGCGCUGAUGGCUUC
Blo-3-1  CUCGU................................................UUCGAA..CCGC......................ACACU......................................................GCUGAUAGUUCC
Blo-2-1  CUCGU................................................UUCGAA..CCGC......................ACACU......................................................GCUGAUAGUUCC
Blo-1-1  CUUGU................................................UUCGAA..CCGC......................ACACU......................................................GCUGAUAGUUCC
Bad-1-1  GUUACA............................................GGCUUCGAA..CCGC......................AAACCC.....................................................GCUGAUGGUUCC
Bad-2-1  GUUACA............................................GGCUUCGAA..CCGC......................AAACCC.....................................................GCUGAUGGUUCC
Nfa-1-1  GACCUAUG........................................GGCCGUCUGAA..CCGCCCC...................GGUCC...................................................GGGGCUGAUGGUUCC
Bpl-1-1  GUGAU...................................................AAA..CCGCUA....................AUGAAG...................................................UAGCUGAUAGUUCC
Bco-1-1  GUUAU...................................................AAA..CCGCUA....................AUAAAG...................................................UAGCUGAUAGUUCC
Bth-1-1  ........................................................UAA..CCGCCC....................UUUG.....................................................GAGCUGAUGAUCCC

         .....................................>>>>>>>>>>>>>>>>>>>.......<<<<<<<<<<<<<<<<<<<<<<<<.....................................>>>>>>>>>>>>>>>>>>>>>>>>....>>>>>>
         .....................................0?22??????????2222?.......22222??2??????????222122.....................................221222??????????2??22222....?22220
         ..............................................................................................................................................................
         ..............................................................................................................................................................
         .......................................................................................................................................................>......
         .......................................................................................................................................................2......
         --------------------------------------------------------•AA--CCGCY∘--------------------••••∘----------------------------------------------------∘RGCUGAUGAC•YC
```

```
Bca-1-1    GUG................................................GCCCCGAA..CCGCG.....................UUUGA.....................................................CGCUGAUGACUUC
Lbl-1-1    ........................................................AAA..CCGCUU....................CUUAGC...................................................AAGCUGAUGACUUC
Psp-1-1    ........................................................UAA..CCGCUU....................UUAG.....................................................AAGCUGAUGGCGCC
Psp-1-2    ........................................................CAA..CCGCUUU...................UUUCA...................................................AAAGCUGAUGGCGCC
Rsp-1-1    ........................................................GAA...UGCCG....................CGCGU....................................................CGGCUGAUAGCCUC
Cau-1-1    ........................................................CAA..CUGCC.....................AUUAC.....................................................GGCUGAUAGCCUC
Cag-1-1    ........................................................GAA..CUGCCCG...........................................................................CGGGCUGAUGGCCUC
Ebi-1-1    UUAAA...............................................ACUAAAA..CCGCUU....................UUUAU....................................................AGGCUGAUGACUUC
Gsp-2-1    ........................................................AAA..CUAUC.....................GUUCG.....................................................GGCUGAUGACUCC
Gth-1-1    ........................................................UAA..CCACCG....................GCUUC....................................................CUGCUGAUGACUCC
Gka-1-1    ........................................................UAA..CCGCCG....................GCUUC....................................................CGGCUGAUGACUCC
Bth-2-1    ........................................................UAA..CUGCU.....................GCUU......................................................AGCUAAUGACUCC
Bce-10-1   ........................................................UAA..CUGCU.....................GCUU......................................................AGCUAAUGACUCC
Bwe-1-1    ........................................................UAA..CCGCU.....................GCUU......................................................AGCUAAUGACUCC
Bce-13-1   ........................................................UAA..ACGCU.....................GCUU......................................................AGCUAAUGACUCC
Bce-12-1   ........................................................UAA..ACGCU.....................GCUU......................................................AGCUAAUGACUCC
Ban-16-1   ........................................................UAA..ACGCU.....................GCUU......................................................AGCUAAUGACUCC
Bth-3-1    ........................................................UAA..ACGCU.....................GCUU......................................................AGCUAAUGACUCC
Bce-9-1    ........................................................UAA..ACGCU.....................GCUU......................................................AGCUAAUGACUCC
Bce-8-1    ........................................................UAA..ACGCU.....................GCUU......................................................AGCUAAUGACUCC
Bce-4-1    ........................................................UAA..ACGCU.....................GCUU......................................................AGCUAAUGACUCC
Bce-7-1    ........................................................UAA..ACGCU.....................GCUU......................................................AGCUAAUGACUCC
Bce-3-1    ........................................................UAA..ACGCU.....................GCUU......................................................AGCUAAUGACUCC
Bce-2-1    ........................................................UAA..ACGCU.....................GCUU......................................................AGCUAAUGACUCC
Bth-4-1    ........................................................UAA..ACGCU.....................GCUU......................................................AGCUAAUGACUCC
Bce-5-1    ........................................................UAA..ACGCU.....................GCUU......................................................AGCUAAUGACUCC
Bce-6-1    ........................................................UAA..ACG.......................CGCUGCUUAU..................................................CUUAUGACUCC
Bce-14-1   ........................................................CAA..CCGCU.....................GCUU......................................................AGCUAAUGACUCC
Gsp-3-1    ........................................................UAA..CCGCU.....................UCCAU.....................................................AGCUAAUGACUCC
Psp-2-1    ........................................................UAA..CCGCC.....................UGCG......................................................GGCUAAUGACUCC
Mth-2-1    ........................................................AAA...UCUGCG...................CUGG....................................................UGCAGUGAUGACCCC
Tte-1-1    .........................................................AA...UGCA.....................GAAGAAA...................................................UGCUGAUGGCUCC
Cpa-1-1    .........................................................AA...UGCA.....................GAAGAAA...................................................UGCUGAUGGCUCC
Cpa-1-2    ........................................................UAA..CCGCC.....................GAAA......................................................GGCUGAUGGUUCC
Tte-1-2    ........................................................UAA..CCGCC.....................GAAA......................................................GGCUGAUGGUUCC
Cac-1-1    ........................................................UAA..AUAUCC....................UGUUU....................................................GGAUUGAUGACUUC
Cdi-5-1    AUAA..............................................UAUCCUAAA..CUGC......................CAAUGA.....................................................GCUGAUGACUUC
Cdi-7-1    AUAA..............................................UAUCCUAAA..CUGC......................CAAUGA.....................................................GCUGAUGACUUC
Cdi-4-1    AUAA..............................................UAUCCUAAA..CUGC......................CAAUGA.....................................................GCUGAUGACUUC
Cdi-9-1    AUAA..............................................UAUCCUAAA..CUGC......................CAAUGA.....................................................GCUGAUGACUUC
Cdi-2-1    AUAA..............................................UAUCCUAAA..CUGC......................CAAUGA.....................................................GCUGAUGACUUC
Cdi-6-1    AUAA..............................................UAUCCUAAA..CUGC......................CAAUGA.....................................................GCUGAUGACUUC
Cdi-1-1    AUAA..............................................UAUCCUAAA..CUGC......................CAAUGA.....................................................GCUGAUGACUUC
Cbu-1-1    AUA.................................................AUCUAAA..GUGCU.....................AUUAA.....................................................CGCUGAUGACUUC
Nth-1-1    UUAAAU...............................................UCCAAA..CUGCUUU...................CUUA....................................................GAAGCUAAUGGCUCC
Aor-1-1    AAUA................................................GCCUAAA..CCGCA.....................AAUA......................................................UGCUGAUGACUUC
Ceu-1-1    AAAACCUGAAUGGGCA..............................A......CCUAAA..CUGCU.....................AAUAU.....................................................AGCUGAUGACUUC
Cba-1-1    GUCAU...............................................ACUAAAA..CCGCUU....................AUUU.....................................................AAGCUGAUGACUUC
Rob-1-1    UAAAA.............................................UUACUUAAA..CCGCUU....................AUGA.....................................................AAGCUGAUGACUUC
Dlo-1-1    AUCA...............................................UACUAGAA..CCGCU.....................UAUAA.....................................................AGCUGAUGACUUC
Ahy-1-1    AAAA................................................UCUAAAA..CCGCA.....................UUUA......................................................UGCUGAUGACUUC
Cph-1-1    UUU.................................................ACCUAAA..CUGCU.....................UAUAA.....................................................AGCUGAUGACUUC
Cbo-1-1    CUG.................................................ACUAGAA..CCGCU.....................UAUAA.....................................................AGCUGAUGACUUC
Fma-1-1    U..................................................UAACUAAA..ACGCAA....................AAAUU....................................................UUGCUGAUGACUUC
Ala-1-1    UUA...............................................UAAUCUAAA..ACGC......................GUUG.......................................................GCUGAUGACUUC
Chi-1-1    AAA..................................................CCUAAA..CCGCU.....................UAUCG.....................................................AGCUGAUGACUUC
Rin-1-1    UUAAU................................................UCGAAA..CCGCUA....................AAUU.....................................................UAGCUGAUGACUUC

           .....................................>>>>>>>>>>>>>>>>>>>.......<<<<<<<<<<<<<<<<<<<<<<<<.....................................>>>>>>>>>>>>>>>>>>>>>>>>....>>>>>>
           .....................................0?22??????????2222?.......22222??2??????????222122.....................................221222??????????2??22222....?22220
           ..............................................................................................................................................................
           ..............................................................................................................................................................
           ......................................................................................................................................................>.......
           ......................................................................................................................................................2.......
           --------------------------------------------------------●AA--CCGCY○--------------------●●●●○----------------------------------------------------○RGCUGAUGAC●YC
```

```
Eve-1-1   UUA.................................................AUCGAAA..CCGCUU....................AUAAC....................................................AAGCUGAUGACUUC
Csp-2-1   GAGA................................................UUCGAAA..CCGCU.....................UAUUA.....................................................AGCUGAUGACUUC
Csa-1-1   ........................................................CAA..AUGCCA....................AUGA.....................................................UGGCUGAUGACUCC
Csa-1-2   ........................................................AAA..UUGCCACAAG................AAUGAAAAAUAC.........................................CUUGUGGCUGAUAACUCC
Tps-1-1   ........................................................UAA..AUGCG.....................GAAA......................................................CGCUGAUGGCUCC
Tsp-2-1   ........................................................UAA..AUGCG.....................GAA.......................................................CGCUGAUGGCUCC
Tsp-2-2   ........................................................UAU...UGCC.....................GUGAA.....................................................GGCUAAUGGCUUC
Tps-1-2   ........................................................UAU...UGCC.....................GUGAA.....................................................GGCUAAUGGCUUC
Cpa-1-3   ........................................................UAA..UUGCC.....................GUAAA.....................................................GGCUGAUGGCUUC
Tte-1-3   ........................................................UAA..UUGCC.....................GUAAA.....................................................GGCUGAUGGCUUC
Tca-1-1   ........................................................UAA..GUCCGC....................AUAA.....................................................GCGGUAAUGACUCC
Ame-1-1   ........................................................UAA..CCACUG....................GAUAA....................................................CAGUUAAUGACUCC
Dha-2-1   ........................................................UAA..CCGCC.....................GCUU......................................................GGCUAAUGACUCC
Ckl-1-1   ........................................................UAA..CCGCA.....................GAAA......................................................UGCUUAUGACUCC
Cbo-5-1   ........................................................UAA..AUGC......................GUAGA......................................................GUUAAUGACUCC
Cbo-11-1  ........................................................UAA..AUGC......................GUGAA......................................................GCUAAUGACUCC
Cbo-3-1   ........................................................UAA..AUGC......................GUGAA......................................................GCUAAUGACUCC
Cbo-7-1   ........................................................UAA..AUGC......................GUAAA......................................................GCUAAUGACUCC
Cbo-10-1  ........................................................UAA..AUGC......................GUAGA......................................................GUUAAUGACUCC
Cpe-7-1   ........................................................UAA..AUGC......................UUUAU......................................................GCUAAUGACUCC
Cpe-1-1   ........................................................UAA..AUGC......................UUUAU......................................................GCUAAUGACUCC
Cpe-5-1   ........................................................UAA..AUGC......................UUUAU......................................................GCUAAUGACUCC
Cpe-8-1   ........................................................UAA..AUGC......................UUUAU......................................................GCUAAUGACUCC
Cpe-4-1   ........................................................UAA..AUGC......................UUUAU......................................................GCUAAUGACUCC
Cpe-3-1   ........................................................UAA..AUGC......................UUUAU......................................................GCUAAUGACUCC
Cpe-9-1   ........................................................UAA..AUGC......................UUUAU......................................................GCUAAUGACUCC
Cbo-9-1   ........................................................UAA..ACGC......................GUAAU......................................................GCUAAUGACUCC
Cbo-3-2   ........................................................UAA..UUGC......................GUAAU......................................................GCUAAUGACUCC
Cbo-10-2  ........................................................UAA..UUGC......................GUAAU......................................................GCUAAUGACUCC
Cbo-8-1   ........................................................UAA..UUGC......................GUAAU......................................................GCUAAUGACUCC
Cbo-5-2   ........................................................UAA..UUGC......................GUAAU......................................................GCUAAUGACUCC
Cbo-11-2  ........................................................UAA..UUGC......................GUAAU......................................................GCUAAUGACUCC
Csp-3-1   ........................................................UAA..UCGC......................GUAAU......................................................GCUAAUGACUCC
Cbo-6-1   ........................................................UAA..CUGC......................GUAAU......................................................GCUAAUGACUCC
Cth-1-1   ........................................................GAA..AUGC......................GUAAU......................................................GCUGAUGACUCC
Hmo-1-1   ........................................................AAA....GCCG....................UUUUG....................................................CGGAUGAUGACUCC
Pth-1-1   ........................................................UAAUUUUGCU.....................GAAAA.....................................................AGCUGAUAGCUCC
Chy-1-1   .........................................................AA...CGCUC....................AUCU.....................................................GAGCUAAUAGCUCC
Efa-2-1   ........................................................GAA..CUAUUUAUU.................GGACG.................................................AAUAAAUUAAUGACGCC
Efa-1-1   ........................................................GAA..CUAUUUAUU.................GGACG.................................................AAUAAAUUAAUGACGCC
Lrh-1-1   ........................................................UAA..GU.....................................................................................UGAUGACACC
Lpa-1-1   ........................................................UA...CG........................CAAGU........................................................UGAUGACACC
Lca-1-1   ........................................................UA...CG........................CAAGU........................................................UGAUGACACC
Lca-2-1   ........................................................UA...CG........................CAAGU........................................................UGAUGACACC
Csp-4-1   AUACU...............................................GCUUAAA..CCGCUU....................AUUA.....................................................AAGCUGAUGACUUC
Lac-1-1   ........................................................UAA..UCGA......................GAAA.......................................................UCUAAUGACGUC
Lhe-1-1   ........................................................UAA..CCGA......................GAGA.......................................................UCUAAUGACGGC
Lga-1-1   ........................................................UAA..ACGA......................GCAAAA.....................................................UCUAAUGACGUC
Ljo-1-1   ........................................................AAA..CUGA......................GCAAAA.....................................................UCUAACGACGUC
Lga-1-2   AUU.....................................................UAA..CCGAU.....................UAAG......................................................AUCUGACGACGCC
Ljo-1-2   AUU.....................................................UAA..CCGAU.....................UAAU......................................................AUCUGACGACGCC
Lpl-1-1   CAUAA...................................................UAA..UUGAU.....................AAUCA.....................................................AUUUGAUGACGUC
Efa-3-1   ........................................................UAA..AUGCUA....................ACGAAAGA.................................................UAGCUAAUGACGCC
Lca-2-2   ........................................................CAA..AUGAUUG...................UGUAG...................................................CAAUCUGAUGACGUC
Lpa-1-2   ........................................................CAA..AUGAUUG...................UGUAG...................................................CAAUCUGAUGACGUC
Lca-1-2   ........................................................CAA..AUGAUUG...................UGUAG...................................................CAAUCUGAUGACGUC
Lla-3-1   ........................................................AAA..CCGCUUUAAGA...............AUAAAA..............................................UCUUAAAACUAAUGGCGCC

          .....................................>>>>>>>>>>>>>>>>>>>.......<<<<<<<<<<<<<<<<<<<<<<<<.....................................>>>>>>>>>>>>>>>>>>>>>>>>....>>>>>>
          .....................................0?22??????????2222?.......22222??2??????????222122.....................................221222???????????2?22222....?22220
          ..............................................................................................................................................................
          ..............................................................................................................................................................
          .......................................................................................................................................................>......
          .......................................................................................................................................................2......
          --------------------------------------------------------●AA--CCGCY○--------------------●●●●○--------------------------------------------------○RGCUGAUGAC●YC
```

```
Lla-1-1   ........................................................AAA..CCGCUUUAAGA...............AUAAAA..............................................UCUUAAAACUAAUGGCGCC
Lla-2-1   ........................................................AAA..CAGCUUUAAGA...............AUAAAA..............................................UCUUAAAACUAAUGGCGCC
Lla-3-2   ........................................................AAA..CCGUUUCAAGA...............AAUAG................................................CUUGAAUCUAAUGGCGCC
Lla-2-2   ........................................................AAA..CGGUUUCAAG................AAAUAA...............................................CUUGAAUCUAAUGGCGCC
Lla-1-2   ........................................................AAA..CCGUUUCAAG................AAAUAA...............................................CUUGAAUCUAAUGGCGCC
Zmo-1-1   UUUCUAU............................................GGAUCGAA..CCGCC.....................CUUUA.....................................................GGCUGAUGACUCC
Gox-1-1   UUCC...............................................CGGUCGAA..CCGCUUC...................AUCCG...................................................GAAGCUGAUGAUUCC
Gdi-1-1   GUCCG...............................................GGUCGAA..CCGCCUC...................UUUCG...................................................GGGGCUGAUGAUUCC
Gdi-1-2   GUCCG...............................................GGUCGAA..CCGCCUC...................UUUCG...................................................GGGGCUGAUGAUUCC
Gdi-1-3   ........................................................UAA..CCGCCC....................CCA......................................................GGGCUGAUGACUCC
Nha-1-1   ........................................................UCA..CCGCC.....................GACU......................................................GGCUGAUGACUCC
Gox-1-2   ........................................................AAA..CCGCC.....................GCAA......................................................GGCUGAUGACUCC
Oan-1-1   ........................................................AAA..CUGCCA....................GCAAU....................................................UGGCUGAUGACUCC
Gox-1-3   ........................................................CAA..CCGCCCU...................CGCA....................................................AGGGCUGAUGGCUCC
Mra-1-1   ........................................................AAA..CCGCCC....................GUCA.....................................................GGGCUGAUGACUCC
Bja-1-1   ........................................................CAA..CCGCCCG..GCGGU............AGGUG............................................ACCGUGAUGGGCUGAUGACUCC
Oca-1-1   ........................................................UAA..CCGCC.....................GCAA......................................................GGCUGAUGACUCC
Nwi-1-1   ........................................................UAA..CCGCC.....................GCAA......................................................GGCUGAUGACUCC
Nha-1-2   ........................................................UAC..CCGCC.....................GCAA......................................................GGCUGAUGACUCC
Nsp-2-1   ........................................................UAC..CCGCC.....................GCAA......................................................GGCUGAUGACUCC
Rpa-1-1   ........................................................CAA..CCGCC.....................GCGA......................................................GGCUGAUGACUCC
Rru-1-1   ........................................................AAA..CCGCC.....................GAAA......................................................GGCUGAUGACUCC
Msp-1-1   ........................................................UAA..CCGCCC....................ACCC.....................................................GGGCCGAUGACUCC
Acr-1-1   ........................................................GAA..CCGCCC....................GCG......................................................GGGCUGAUGACUCC
Acr-1-2   ........................................................UAA..CCGCCC....................GUG......................................................GGGCCGAUGACUCC
Neu-1-1   ........................................................UAA..CCGCCGC...................AGCCU...................................................GUGACUGGUGAUGCC
Dac-1-1   ........................................................AAA..CCGCCGCAC.................UA....................................................GUGCGGCUGAUGACGCC
Cvi-1-1   ........................................................UAA..CCACCC....................CUAGA....................................................GGGUUGAUGAUGCC
Dac-1-2   ........................................................AAA..CCUCCCU...................GCGA....................................................AGGGCUGAUGGCUCC
Neu-1-2   ........................................................AAA..CCGCAGCCG.................UUUAG.................................................CG.CUGCUGAUGACGCC
Neu-1-3   ........................................................UAA..CCGCCGC...................AAUCU...................................................GUGGCUGAUGAUGCC
Bph-2-1   ........................................................UAA..CCGCCCUGC.................AUUCCC................................................GCAGAGCUGAUGAUGCC
Bxe-1-1   ........................................................UAA..CCGCC.....................CUUAU.....................................................GGCUGAUGAUGCC
Bph-2-2   ........................................................UAA..CCGCC.....................CUCGU.....................................................GGCUGAUGAUGCC
Bph-1-1   ........................................................UAA..CCGCC.....................CUCGU.....................................................GGCUGAUGAUGCC
Bgr-1-1   UUCAACACAC...........................GCGCUCGCGCGA.ACCCAUUAA..CCGC......................CUCGU......................................................GCUGAUGAUGCC
Bsp-2-1   ........................................................UAA..CCGCCCC...................U.......................................................GGGGCUGAUGAUGCC
Bub-1-1   CCAC...................................GAGCACGCACAGGCCCUCAA..CCGCCGG...................UCGU....................................................CCGGCUGAUGAUGCC
Rpi-2-1   ........................................................UAA..CCGCCGGUUAGCG.............CGUAA.............................................CGAU.ACCGGCUGAUGAUGCC
Bmu-1-1   AUCGUGC...............................GCGCCGGUC..CGCUGCUCAA..CCGCCGG...................UUCGU...................................................CCGGCUGAUGAUGCC
Rso-1-1   ........................................................UAA..CCGCCGA...................UCCCA...................................................UCGGCUGAUGAUGCC
Rso-3-1   ........................................................UAA..CCGCCGA...................UCACC...................................................UCGGCUGAUGAUGCC
Rso-2-1   ........................................................UAA..CCGCCGA...................UCACC...................................................UCGGCUGAUGAUGCC
Bce-19-1  AUGGC.................................GCGCGCGGUGCGGCC.CUCAA..CCGCCGG...................UUUGC...................................................CCGGCUGAUGAUGCC
Bce-18-1  AUGGC.................................GCGCGCGGUGCGGCC.CUCAA..CCGCCGG...................UUCGC...................................................CCGGCUGAUGAUGCC
Bce-15-1  AUGGC.................................GCGCGCGGUGCGGCC.CUCAA..CCGCCGG...................UUUGC...................................................CCGGCUGAUGAUGCC
Bam-2-1   CUC...................................GCGUGCGGU.UCGGCCCUCAA..CCGCCGG...................UUUGC...................................................CCGGCUGAUGAUGCC
Bam-3-1   CUC...................................GCGUGCGGU.UCGGCCCUCAA..CCGCCGG...................UUUGC...................................................CCGGCUGAUGAUGCC
Bam-1-1   CUC...................................GCGUGCGGU.UCGGCCCUCAA..CCGCCGG...................UUCGC...................................................CCGGCUGAUGAUGCC
Bam-4-1   CUC...................................GCGUGCGGU.UCGGCCCUCAA..CCGCCGG...................UUUGC...................................................CCGGCUGAUGAUGCC
Bth-6-1   ........................................................CAA..CCGCCGG...................CGAG....................................................CCGGCUGAUGAUGCC
Bps-14-1  ........................................................CAA..CCGCCGG...................CGAG....................................................CCGGCUGAUGAUGCC
Bps-7-1   ........................................................CAA..CCGCCGG...................CGAG....................................................CCGGCUGAUGAUGCC
Bps-5-1   ........................................................CAA..CCGCCGG...................CGAG....................................................CCGGCUGAUGAUGCC
Bok-2-1   ........................................................CAA..CCGCCGG...................CGAG....................................................CCGGCUGAUGAUGCC
Bps-17-1  ........................................................CAA..CCGCCGG...................CGAG....................................................CCGGCUGAUGAUGCC
Bps-19-1  ........................................................CAA..CCGCCGG...................CGAG....................................................CCGGCUGAUGAUGCC

          .....................................>>>>>>>>>>>>>>>>>>>.......<<<<<<<<<<<<<<<<<<<<<<<<.....................................>>>>>>>>>>>>>>>>>>>>>>>>....>>>>>>
          .....................................0?22??????????2222?.......22222??2??????????222122.....................................221222??????????2??22222....?22220
          ..............................................................................................................................................................
          ..............................................................................................................................................................
          ......................................................................................................................................................>.......
          ......................................................................................................................................................2.......
          --------------------------------------------------------•AA--CCGCY○--------------------••••○----------------------------------------------------○RGCUGAUGAC•YC
```

```
Bth-7-1    .........................................................CAA..CCGCCGG..................CGAG..................................................CCGGCUGAUGAUGCC
Bok-1-1    .........................................................CAA..CCGCCGG..................CGAG..................................................CCGGCUGAUGAUGCC
Bps-16-1   .........................................................CAA..CCGCCGG..................CGAG..................................................CCGGCUGAUGAUGCC
Bps-10-1   .........................................................CAA..CCGCCGG..................CGAG..................................................CCGGCUGAUGAUGCC
Bps-9-1    .........................................................CAA..CCGCCGG..................CGAG..................................................CCGGCUGAUGAUGCC
Bma-2-1    .........................................................CAA..CCGCCGG..................CGAG..................................................CCGGCUGAUGAUGCC
Bps-1-1    .........................................................CAA..CCGCCGG..................CGAG..................................................CCGGCUGAUGAUGCC
Bps-8-1    .........................................................CAA..CCGCCGG..................CGAG..................................................CCGGCUGAUGAUGCC
Bps-18-1   .........................................................CAA..CCGCCGG..................CGAG..................................................CCGGCUGAUGAUGCC
Bth-8-1    .........................................................CAA..CCGCCGG..................CGAG..................................................CCGGCUGAUGAUGCC
Bsp-1-1    GUU..................................GCGCGCG.GUGCGGCC.CUCAA..CCGCCGG..................UUAGC.................................................CCGGCUGAUGAUGCC
Bce-17-1   GUA..................................GCGCGCGGU.GCGGCC.CUCAA..CCGCCGG..................UUUGC.................................................CCGGCUGAUGAUGCC
Bvi-1-1    UUGCG.................................CGCGCGAG..UCGGCCCUCAA..CCGCCGG..................UGAGC.................................................CCGGCUGAUGAUGCC
Bam-2-2    .........................................................UAA..CCGCCGA..................UUCGC.................................................UCGGCUGAUGAUGCC
Bam-2-3    .........................................................UAA..CUGCCGA..................UUCGU.................................................UCGGCUGAUGAUGCC
Sfu-1-1    .........................................................GAA..CCGCCCC..................GGGCCA................................................GGGGAUGAUGACUUC
Dpi-1-1    .........................................................GAA..CCGCA....................UGUUU...................................................UGCUGAUGACUCC
Dol-1-1    .........................................................CAA..CCGCC....................GCACUGGACA..............................................GGCUGAUGACUCC
Dac-2-1    .........................................................UAA..CCGCCUG..................UCGAUUUUU.............................................CAGGCUGAUGACUCC
Xca-1-1    .........................................................CAA..CCGUAGC..................GAUC..................................................GCUACUGAUGACGCC
Xor-1-1    .........................................................AAA..CCGUAGC..................GAUU..................................................GCUACUGAUGACGCC
Xor-4-1    .........................................................AAA..CCGUAGC..................GAUU..................................................GCUACUGAUGACGCC
Ppr-1-1    .........................................................UAA..CUGCCUUA.................CUGAAGUUUACCCUUCUUA..................................UAAGGAUGAUGACGUC
Ppr-2-1    .........................................................UAA..CUGCCUUA..CUGAAG.........UUUACC..........................................CUUCUUAAUAAGGAUGAUGACGUC
Psp-3-1    .........................................................AAA..CCGCCUU..................UCAAUC................................................AAGGAUAAUGACGUC
Van-1-1    .........................................................CAA..CCGCCUU..................UCAAUA................................................AAGGAUAAUGACGUC
Eta-1-1    UUCC.....................................................CAA..CCGCCCUGA................UUUUU.............................................UC.GGGCUGAUGACGCC
Cko-1-1    UUCC.....................................................CAA..CCGCCGGUUC...............GCUC...............................................GAAUCGGAUGAUGACGCC
Vfi-2-1    .........................................................UAA..CCGCUCU..................AUUUU.................................................AGAGAUAAUGAUUCC
Vfi-1-1    .........................................................UAA..CCGCUCUA.................UUUU.................................................UAGAGAUAAUGAUUCC
Sgl-1-1    .........................................................UAA..CCGCC....................GCUCU...................................................GGCUGAUGAUGUC
Csa-2-1    CAUGU...................................GCGGUGCCACCGCGACAAA..CCGC.CUGGCGUGAUC....CGAGACCAUGAC.....................GUCUCGACCACCGGCCGCCAGCGCUAAUGAUGCC
Vca-1-1    GAAU...........................................GAUUCAAA..CCGCU....................UGUAU...................................................AGCUGAUGACUCC
Vha-2-1    GAAA...........................................GAUUCAAA..CCGCU....................UUUAC...................................................AGCUGAUGACUCC
Vha-1-1    GAAA...........................................GAUUCGAA..CCGCU....................UUAA....................................................AGCUAAUGACUCC
Val-1-1    GAAA...........................................GAUUCGAA..CCGCU....................GUUAA...................................................AGCUAAUGACUCC
Afe-1-1    .........................................................UAA..CCGCCU...................UUAU...................................................AGGCUGAUGACUGC
Sma-1-1    .........................................................UAA..CCGCCU...................GAGA...................................................AGGCUGAUGGCUCC
Sma-2-1    .........................................................UAA..CCGCCU...................GAGA...................................................AGGCUGAUGGCUCC
Vsp-1-1    UUUU............................................GCUUCAA..CCGC..CCGUUC..............UUUU............................................GAACCGU.GCUAAUGACUCC
Vba-1-1    UUUU............................................GCUUCAA..CCGC..CCGUUC..............UUUU............................................GAACCGU.GCUAAUGGCUCC
Vsp-2-1    UUUU............................................GCUUCAA..CCGC.CCGUUC...............UUUC.............................................GAACAGUGCUAAUGACUCC
Vfi-1-2    .........................................................UAA..CCGCCA...................AUGU...................................................UGGCUGAUGACUCC
Vfi-2-2    .........................................................UAA..CCGCCA...................AUGU...................................................UGGCUGAUGACUCC
Asa-1-1    .........................................................UAA..CCGCC....................AAUUU...................................................GGCUGAUGACUCC
Vsh-1-1    .........................................................UAA..CCGCC....................AAAUC...................................................GGCUGAUGACUCC
Pca-1-1    CGCGCAGCCAAAACGCUGACAGCCUUAAAAAAC.................AGGGUACAA..CCGCC....................ACUC....................................................GGCUAAUGAUGUC
Pca-2-1    CGCGCAGCCAAAACGCUGACAGCCUUAAAAAAC.................AGGGUACAA..CCGCC....................ACUC....................................................GGCUAAUGAUGUC
Psy-2-1    .........................................................AAA..CCGCUGC..................GCCCGUA...............................................GCAGCUGAUGAUGCC
Cko-1-2    .........................................................UAA..CCGCC....................CUCU....................................................GGCUGAUGAUGCC
Psy-1-1    .........................................................AAA..CCGC.....................CUCGU....................................................GCUGAUGAUGCC
Pae-3-1    .........................................................CAA..CCGCCC...................CUG....................................................GGGCUGAUGAUGCC
Pae-6-1    .........................................................CAA..CCGCCC...................CUG....................................................GGGCUGAUGAUGCC
Pae-5-1    .........................................................CAA..CCGCCC...................CUG....................................................GGGCUGAUGAUGCC
Pae-2-1    .........................................................CAA..CCGCCC...................CUG....................................................GGGCUGAUGAUGCC
Pae-4-1    .........................................................CAA..CCGCCC...................CUG....................................................GGGCUGAUGAUGCC
Pen-1-1    .........................................................GAA..CCGC.....................CUCAGC...................................................GCUGAUGAUGCC

           .....................................>>>>>>>>>>>>>>>>>>>.......<<<<<<<<<<<<<<<<<<<<<<<<................................>>>>>>>>>>>>>>>>>>>>>>>>....>>>>>>
           .....................................0?22???????????2222?.......22222??2????????????222122................................221222????????????2??22222....?22220
           ......................................................................................................................................................................
           ......................................................................................................................................................................
           ...............................................................................................................................................>.......
           ...............................................................................................................................................2.......
           ---------------------------------------------------------•AA--CCGCY○--------------------•••••○--------------------------------------------○RGCUGAUGAC•YC
```

```
Xca-4-1    ...GAA..CCGCACGCA...CCC...UGCGCGCUGAUGAUGCC
Xax-1-1    ...GAA..CCGCACGC...ACCC...GCGCGCUGAUGAUGCC
Pst-1-1    ...AAA..CCGUCC...AAAUAAA...GGGCUGAUGAUGCC
Asp-2-1    ...AAA..CCGCC...AUUGU...GGCUAAUGAUGCC
Asp-1-1    ...AAA..CCGCC...GUUCU...GGCUAAUGAUGUC
Aba-3-1    ...AAA..CCGCC...GUAUU...GGCUAAUGAUGCC
Aba-4-1    ...AAA..CCGCC...GUAUU...GGCUAAUGAUGCC
Aba-5-1    ...AAA..CCGCC...GUAUU...GGCUAAUGAUGCC
Ymo-1-1    ...CAAG.GC...UGAUGAUGUC
Yin-1-1    ...UAA..CCGCC...CUUCAA...GGCUGAUGAUGUC
Yps-4-1    ...UAA..CCGCC...CUAACAA...GGCUAAUGAUGUC
Ype-13-1   ...UAA..CCGCC...CUAACAA...GGCUAAUGAUGUC
Yps-2-1    ...UAA..CCGCC...CUAACAA...GGCUAAUGAUGUC
Yps-1-1    ...UAA..CCGCC...CUAACAA...GGCUAAUGAUGUC
Spr-1-1    ...UAA..CCGCCU...UCAC...GGGCUGAUGAUGUC
Pru-1-1    ...AAA..CCGUCC...AUAUU...GGACUGAUGAUGCC
Pal-1-1    GUU...UAA..CCGUCC...UUUUGU...GGACUGAUGAUGCC
Pat-1-1    CUAACGCCGCCAAAAAUGCUGGCAGCCUGAAGUACAA...GGUACAA..CCGCC...ACCC...GGCUGAUGAUGUC
Cko-1-3    AUUC...CAA..CCGCCU...CGUUUCAGU...AGGCUGAUGACGCC
Kpn-1-1    ...CAA..CCGCUUC...CCCUGA...GAAGCUGAUGACGCC
Tpe-1-1    ...AAA..CUGCCCU...GAAG...AGGGCUGAUGGCCUC
Tle-1-1    ...UAU..UUGCC...UUAAA...GGCUGAUGACUCC
Cfl-1-1    GUGA...UCGGGGAA..CCGCUUGGU...AACG...GCCAGGCUGAUGAUUCC
Min-1-1    UCGAAGU...ACU..UGGGAAAA..CCUCCUUUC...UUGAAAA...GAAAGGCUGAUAACUCC
bEl-1-1    GUCAA...CGAGAGAA..CCGC.CUGA...UUAUU...UCGGUGCUGAUAACUCC
Min-1-2    ...UAA..CCGCCGUUCUU...UUCA...AAGAA.GGCUGAUAGCUCC
env-1      ...GAA..CAGC...AUCAUGAUCGGUACG...GCUGAUGCGCUC
env-2      ...UGA..UUGUCUC...CCU...GAGACUGAUAACUCC
env-3      GUUAU...AAA..CCGCUA...AUAAAG...UAGCUGAUAGUUCC
env-4      ...GAA..CCGCCGCUC...UGACU...GGACGGCCAAUGACUCC
env-5      ...CAA..CCGUCAUG...CGCCUGC...CGUGGCCAAUGACUCC
env-6      ...UUCCAAAA..CCGC...UGUUUC...GCUGAUGACGUC
env-7      ...GAA..CGGCCCUUUCUU...UCAUA...AGGAAAGGGAUGAUGACUCC
env-8      ...UAA..GCGCC...CUCCG...GGCUGAUAACGUC
env-9      CGAU...GCCGAA..CUGCC...GAUCU...GGCUGAUGGUUCC
env-10     ...UAA..CCGCCC...UUCG...GAGCUGAUGAUCCC
env-11     CAAA...UA.UGGAAAA..CCGUCCAACU...UCUUU...AGCCGGACUAAUAACUCC
env-12     CUUGU...UUCGAA..CCGC...ACACU...GCUGAUAGUUCC
env-13     CUCGU...UUCGAA..CCGC...ACACU...GCUGAUAGUUCC
env-15     CUUGU...UUCGAA..CCGC...ACACU...GCUGAUAGUUCC
env-16     CUUGU...UUCGAA..CCGC...ACACU...GCUGAUAGUUCC
env-17     CUUGU...UUCGAA..CCGC...ACACU...GCUGAUAGUUCC
env-19     GUUACA...GGCUUCGAA..CCGC...AAACCC...GCUGAUGGUUCC
env-20     GUUACA...GGCUUCGAA..CCGC...AAACCC...GCUGAUGGUUCC
env-21     ...AAA..CCGCCGGCU...GA...AGCUGGCUGAUGACGCC
env-22     ...AAU.CCCGC...AUUUC...GGGUGAUGACUCC
env-23     ...AAA..CCGCCCCGG...AGAACAG...CGGGAGCUGAUGACCCC
env-24     ...UAA..CUGC...CACGAU...GCUGAUGACUCC
env-25     AUGAU...AUGAAAA..CCGCA...AACACG...UGCUGAUGACUUC
env-26     AGU...GCCUAAA..CCGCU...AUUAC...AGCUGAUGACUUC
env-27     UAGAAAUAGCUAAAUC...UUUCGUUCAUAA..CCGCUU...GAUUAUA...AAGCUGAUGACUUC
env-28     AAU...AU.UUACCAGAA..CCGCU...UAUGAC...AGCUGAUGGCUUC
env-29     GAAA...CCUAAA..CUGCUU...AUUC...AAGCUGAUGACUUC
env-30     GAAA...CCAGAA..CUGCUG...AAUA...UAGCUGAUGACUUC
env-31     CCAGA...UUACUUGAA..CCGCU...UUAAA...AGCUGAUGACUUC
env-32     UCA...UCCCGAA..CCGCU...UAUUA...AGCUGAUGACUUC
env-33     UUUUG...AUCGAAA..CCGCC...AAAA...GGCUGAUGACUUC

           ...>>>>>>>>>>>>>>>>>>>......<<<<<<<<<<<<<<<<<<<<<<<<<<<...>>>>>>>>>>>>>>>>>>>>>>>>>....>>>>>>
           ...0?22???????????2222?......22222??2?????????????222122...221222???????????2??22222....?22220
           ...
           ...
           ...>.......
           ...2.......
           ---●AA--CCGCY○---●●●●○---○RGCUGAUGAC●YC
```

```
env-34   UUUU................................................AUCGAAA..CCGC......................CAAUGA.....................................................GCUGAUGACUUC
env-35   GAGA................................................UUCGAAA..CCGCU.....................UAUUA.....................................................AGCUGAUGACUUC
env-36   GAGA................................................UUCGAAA..CCGCU.....................UAUUA.....................................................AGCUGAUGACUUC
env-37   ........................................................AAA..CCACUCGC..................GGCG...................................................GCGAGUUGAUGACCCC
env-38   ........................................................UAA..AUGCUA....................ACGAAAGA.................................................UAGCUAAUGACGCC
env-39   ........................................................UAA..AUGCUA....................ACGAAAGA.................................................UAGCUAAUGACGCC
env-40   CUUAU...............................................UUUCAAA..CCGC......................AAUU.......................................................GCUGAUAACUCC
env-41   CUUAU...............................................UUUCAAA..CCGCA.....................AAAAU.....................................................UGCUGAUAACUCC
env-42   ........................................................UAA..CCGCC.....................CUUGAU....................................................GGCUGAUGAUGCC
env-43   GUU..................................GCGCACGG.UGCGGCC.CUCAA..CCGCCGG...................UCAGC...................................................CCGGCUGAUGAUGCC
env-44   ........................................................GAA..CCGCC.....................AUGU......................................................GGCUAAUGAUGCC
env-45   ........................................................AAA..CCGCU.....................UGAA......................................................AGCUGAUGACGCC
         .....................................>>>>>>>>>>>>>>>>>>>.......<<<<<<<<<<<<<<<<<<<<<<<<.....................................>>>>>>>>>>>>>>>>>>>>>>>>....>>>>>>
         .....................................0?22??????????2222?.......22222??2??????????222122.....................................221222??????????2??22222....?22220
         ..............................................................................................................................................................
         ..............................................................................................................................................................
         ......................................................................................................................................................>.......
         ......................................................................................................................................................2.......
         --------------------------------------------------------•AA--CCGCY◦--------------------••••◦----------------------------------------------------◦RGCUGAUGAC•YC
```

alignment positions 318 · · · 477

```
[1]Sso-1-1   UACUUCACCACAAAAGGGUGGGAAGUAUGGCGCCAAAUGAAGCUCAGAUAGUAGUUUUAACCAUAACUCAAGUUAUUGUAGUUAUUCUACUAUCCCCAUUAUAUCAAGGAAUUUACGAUAGGGGAAAAGCCAUAAUAGAAGGGCGUAAAGGCCCCAGUGU
Mse-1-1      UAUCUCUUGCCUAGGUGAGAUAGGAUGACUGGCCUAAACCUAGUGGGAUGGAUAAUACUAGUUCCCAUUUUGGGAAGUGCAGUAGUUUUUAAGGAAAAGUACUCCACGGUAGCCUCUGCUACGAUCACCCUAAUUCUCGUGCUAGCUCUCAAACCAUUUU
Pto-1-1      UACUCUUGCGGGUGGUGUCAUGCUGGUGCUUGAAACAUUAACACAGAUAGUUACGGUAAUUUUUAUAUCACCUUUAAUAGCCGGUAUCUACGAGAAUUUAAAGAGCAAGGUUGAAUUAAAAAAAGGGCCAUCGGUAUUCCAGCCAUAUUAUGACCUUUUU
Tvo-1-1      UGGACAGUAAUUGAUGCUUUAAGUAUGUUACGACAGGAGUGAUUGAAAUGGUCUUUAAUAGUAUUUUAUGGCCGGAUAAGAAGGCCGAAUCAUUGUUUGAUAGCGAUCCUGAUUUUUUUGCUGAUCUCAAUCUUGAUAUGAUUAUAGAUGGAAUAACUUC
Mhu-1-1      UACCCGGAAGACGUAUGUUAUCCGAAGGUGAAAAGGAAAAAACGAUUCGCUAUAUCUCUGAUCGUCGAUGUCAAAGUGGCGGAUAUUGUUUUUAUCAGCUCGAUGAGCCAAAUCUGUCUGAUACCUGGUAUGCCCUGGGCUGCUUAGAGGUUCUGCAAGC
Tvo-1-2      UGAUUGAAACUGGUGGCUUGAUAUGGGCGCCAUAACAUUAAUAGAGACCUUAAUGCAGUAUUUCUUCGUUGUUCUUACUGCUCCACUAUACACCGGCAUUUUAGAGAAGCUUAAGGCGAUGGUUGAAUCGCGUAAAGGGCCAAGCAUAUUUCAACCAUAC
CMe-1-1      UGUUUUAUGCGGGGAUUCACGUCUGAUCCCACAAACCCGGGCCCGGCUUGUGGUUUUCUUUCGUGAUGUGACCGACAGGCCGUUAAAUAGUGCAUCUGGAAAAAAAACACCGGAGGAAAACAGGAUGGACUCAUCGCUACAAUCGAUCCCUGCCCGGGAG
Mhu-1-2      UCUUCCUGUUUACCAGGAUUUACUUCUGUUUUCUUCCCAUCUUCACAUCAUGGGUGAUGACAAGCCCGUGUUCGACCAGUUCUUCCACUUCAUCAACUAUGCUCAUAACCCGUUCCUUUGUAUCAACGACAUCGAUGACCACCGGAAGGUCCAUUGAGAA
Abo-1-1      UUUUCGCAUGAGGUGACGCCAUAUGAUCCCUGAGAUAGUAGCAGGUUUAGUGCAGGUAUUGACCGUGUUAGCCCUUUCGCCGUUAAUGGUAGGUAUAUUGAAGAAAUGGGAAGCUAAAUUUGAAUCCAGAAAGGGUAUAUCCAUUUUCCAGCCGUACUAC
Abo-1-2      UUUUCGCAUGAGGUGACGCCAUAUGAUCCCUGAGAUAGUAGCAGGUUUAGUGCAGGUAUUGACCGUGUUAGUUCUUUCGCCCUUAAUGGUAGGCAUAUUGAAGAAAUGGGAAGCUAAAUUUGAAUCCAGAAAGGGUAUAUCAAUUUUCCAGCCGUACUAC
Abo-1-3      UUUUGAAAAAGGUGGUCCCUAUGCUCGAAAUAGAGCUCUCCAAGGAAUUGUUCGAAAAGAUUCGCAGCAUGAAAGAAGACGAUUUCAAUCGUUUUCUCUAUGAGAGAAUGGCUUCGAUGAACAGGUAUUUUGAGAUGAAUGAUAAAGAAGCCCUGCUCAA
Abo-1-4      UUUUGAAAAAGGUGGUCCCUAUGCUCGAAAUAGAGCUCUCCAAGGAAUUGUUCGAAAAGAUUCGCAGCAUGAAAGAAGACGAUUUCAAUCGUUUUCUCUAUGAGAGAAUGGCCUCUAUGAACAGGUAUUUUGAGAUGAAUGAUAAAGAAGCUCUGCUCAA
Mhu-1-3      UACAGGAUGUAUAUUAUUGGCUUCAUGGCCAUCAUUACAGAUGUGGAGUAUACUACAUGCACAAAGACGGACACGAUACCGGAUCCCUGCUAGCACAGGAUUAUGAACUCCUGCAAUCACUUGGUCCUGAUUAUACAUCAUACAAGGAACAAGUAUCCGG
Msm-1-1      UAUGUUAUAUAAAAAAUACUUAGGAGGAUAAUUUAAUGGCUGAUGUAUUUCCAUUAAUUUUAGGGAUAAUAAUACUUCUAGCUAGUGUAAUUUCUAUUAAAACUGGAAUUAGUGUAACAGUAGUUGAAAUUGCAAUAGGAGUAAUAAUAGGAAAUCUAGG
Msm-2-1      UAUGUUAUAUAAAAAAUAAUUAGGAGGAUAAUUUAAUGGCUGAUGUAUUUCCAUUAAUUAGGGAUAAUAAUACUUCUAGCUAGUGUAAUUUCUAUUAAAACUGGAAUUAGUGUAACAGUAGUUGAAAUUGCAAUAGGAGUAAUAAUAGGAAAUCUCGGAU
Msm-3-1      UAUGUUAUAUAAAAAAUACUUAGGAGGAUAAUUUAAUGGCUGAUGUAUUUCCAUUAAUUUUAGGGAUAAUAAUACUUCUAGCUAGUGUAAUUUCUAUUAAAACUGGAAUUAGUGUAACAGUAGUUGAAAUUGCAACAGGAGUAAUAAUAGGAAAUCUAGG
Mth-1-1      UGCAUAAUAAUGUGGGAGAAACUUUCCUCAAACUGGAGGUAUAAAUUUGGAGUACGUUGCAUUUAGCGUAGCUGUUAUCAUACUACUUGGUCUUCUAUUCAACAGGGUUUUCACCAGGGCAAGGCUCCCGGGAAUCCUGGGGAUGCUGAUACUGGGUAUC
CMe-1-2      UACCGGGGAUCAAUGUUUGGGGGAAGUGGCCGUUGUCGCUGUACAGCCUGAAGAAGGCGCUCUUAUCCUGCACCGUUGCCGGCACCGGUACGUACCGUUCUGGCGGCCGGACCGGUAAAUACCUCCCAGCGCUGAUCCGGGAGGGCCCGGCAUGGUAGAC
Tko-1-1      UGUUCUACUCCAUAGGAGGCGGUAGAAUGGGCUCGAAGCUUGAUGAGUUUGCACGCUUUGUUGAUGUGCACAAAGCACUUGAGUACGUCGAGAAGAGAAGGCACGAAGACGGCGGCUACUGCUUCGUCAGCGUCCUGAACGACACCAACGUGAACGACAC
Ton-1-1      UGUUCUCUGCCAGAUAUAGCAGGAGGCGGUAGAAUGGGCUCGAAGCUGAGUGAGAUUGGCAGGUUUAUUAACGCUGAUGCGCUCAUCAGCUACGUUGAGGAGAGGCGCCACGAGGACGGCGGCUACUGCUUCGUGAGCGUUUUGGACGAGACCAACAUUA
Tba-1-1      UGUUCUACCCAUUCAUGCUCAUAAAAAAUGGAAGGAGGCGGUAGAAUGGGCUCGAAGCUUAAUGAACUUGCGAGAUAUGUGAAUUUGAAUGCUGUUAUAAACUACAUCUCAAAGAGGCGUCAUGAAGAUGGCGGUUACUGCUUCGUCUCAAUGCUGAACGA
Tsp-1-1      UGUUCUACCACGGAAGGAGGCGGUAGAAUGGGCUCGAAGCUGUACGAACUCGGACGUUACGUUGACGUUGAUGCCGUCUUACGCUACGUUAAGGAGAGGCGCCACGAGGAUGGCGGCUACUGCUUCGUUAGCGUCCUCGACGACACCAACGUGAACGACA
Pab-1-1      UAUUCUUGAGAACUUUGGGAGGUGGUAGCAUGGGCUCGAAGCUCUCUAGGUAUGUGAAUAUAGAUAGGGUAAUCGAGUACAUUGAAAUGAGAAGGCACAAUGACGGAGGUUACUGCUUUGUCUCUCAGUUAGCCGAUACAAACAUUAACGACACGUAUUA
Pfu-1-1      UAUUCUGUAGAACUUGAGGGAGGUGGCAAUAUGGGCUCGAAGCUCUCUAGGUACAUAAAUUUGGAAAAGGUUUUGAACUAUAUUGAGAAAAGGCGUCAUGAGGAUGGAGGUUACUGUUUUGUCUCCCAACUUGAUGAUACAAACAUAAAUGAUACCUACU
Pho-1-1      UAUUCUGUAGGAACUUAGGGAGGUGGUAUUAUGGGCUCGAAGCUCUCUAGGUAUAUAAAUUUAGAAAAAGUAUUGGAGUACAUCGAGAAAAGACGACAUGAGGACGGUGGUUACUGCUUUGUAUCUCAGCUUGCUGAUACAAACAUAAAUGACACUUAUU
Afu-1-1      UGUUCUCAGAGAGGUGAGAGUGUGGACGCAAUCGACCUUAAGAGGCUCUCAGACUUCGUGAGGCAUAGGCAGAACGAGGAUGGUGGCUUUGCCUUCUGCAAACCCCUCCCAUCAACCCUUGCGGAGACCUUCUACGCUGUCAGCAUACUCACAUCCAUCG
Mst-1-1      UAUUUUUAAAGGUAAAAUUAAGUUUACCUAUAAAUCAUCAGAUAAUCACAAAAUAGGAUUAGGAGUUACCAUGCAAAUUUAUAGGAAAUUAACUUAUUUUAUCUUUAAUUUUAAAAAAAAAUUAAGGAGACUAUAAACUUGAUUAGUGAUAUUUACCCAG
Mac-1-1      UACACAACUAUCAUGGUGUUGACAUGUAUACAAUUCUAUUAAUAGGCAUAGGUGGUUUUAUAGGCGCCGUCCUGAGAUAUUCCUUAAGUGGGUGGGUGCAGAACAGUUUUGUUAAUUUUCCGUUAGGUACUUUGGUUGUAAACAUCGUGGGGAGUUUCUU
Mba-1-1      UACAUUAAUUAUUGAGGUUUAAUGUGUAUACGAUUUUGUUGGUAGGUAUAGGCGGCUUCAUAGGUGCUACCUUAAGAUACGUAUUCGGUGGAUGGAUUCAAAACAGUUUCGUUAAUUUUCCAGUAGGUACAUUAACCAUAAAUACAAUCGGAAGUUUCUU
Gvi-1-1      UAAGGCACGUUCGACGCGUAGGAGGCAUCCGGUGGUGCGGGAGUCGGUGUUGGUGAUGGUUGGUGGAGCGCUCGGCAGUCUGGCCCGCUACUGGGUCGGUCUUGGGAUAUCACAGUGGGCCGGUGCGCCGCCCUUCCUGUUCGGGACGCUGCUGGUCAAU
Ssp-2-1      UACUCUUCUGGGCAGAUCCAGGGGGUGGGAGUCUUUGAUGUUGAGUUCUCACCCCGCAGAAACGCGAACGGCUUUUUCUCCCUGGGCUGCCCUUCAACACGCGGACGGCGAGGAGAGAGGUUUUGUUGAUGCUGGGCAGUUGGCUGUGGAUCUUGGGGAU
Ssp-3-1      UACGCUUCCGACUUGUGGUGAAGCAGUAGGGCUAUUUUGAUCUAGGCUGCACUAAUCUUUUUCGCUACAAGUCACGUUGAUUAACCACCCUAACGACAACGUGAUCCCAUGUUAGAAAGUAUUAUUUGGAUUCUUUUGCUCGGUUUUUUUGGGGGGCAAA
Sel-1-1      UACAGUUCUGGCUAGGGCAAGAUCUGUAGCGAGUGCAUCGUUCAAGCUUUUCCCUAGCCACACACGCAAGCUGAAUUGAAGGAAUCAGCGUGAUGUUGGCGAAUUGGAUUGGAAUUUUAUUGGGCGGCUUUUUGGCCGGUCAAAUCGCGCGUCGGUUGG
             ................................................................................................................................................................
             ................................................................................................................................................................
             ................................................................................................................................................................
             ................................................................................................................................................................
             ................................................................................................................................................................
             ................................................................................................................................................................
             URC•••••••••••••••••••••••••••••••••••••••••••••••••••••••••••••••••••••••••••••••••••••••••••••••••••••••••••••••••••••••••••••••••••••••••••••••••••••••••••••
             3′
```

Ssp-1-1 UACACUUCUGGGCAAGUCCAGGGGGUAGGGGUCUUUGGCUUUGUUUGAGGCCCCACCCUGCGGACGCGAUGGUGCUCCCACUCCUGACGCGGAUGGCUUUUUCUCCCUGGGCUGCCCUUCAACACCCUGACGACAAGGAGAUGAGGAUCUGGUGAUGCUG
Ava-1-1 UACUACUCUUGCUUGCUGCGGGAGGGUAGGAGUUUGUUGUAUACAUUACCUAGCUUCCUUGUAGUAAGUUUUGAUCUACAAACCGCAACACUGGCAGUCAAGGUAUGUUAUUCAGCAUAUUGUGGAUUUUAAUCAUGGGCUUUUUUGCGGGACAACUUGC
Nsp-1-1 UACUUUUCUUGCUUGCUGCGGGAGGGUAGGAGUUUGUUGUAUACAUCACCUGGCUUCCUUGUAGUAAGUCUUGAUCUACAAAUCGCAACACUGGCAGUCAAGGUAUGUUAUUCAGCAUAUUGUGGAUUUUAAUCAUGGGCUUUUUUGCAGGUCAAAUUGC
Dth-1-1 UACUUAUGUUGUGUAAAGCAACAUAGGUAGGAGUUUUUUAUUUUUAUUGGAAAGGAGGCUAACUGGGUGAAAAAUAUUAUUGCAAUUUCUAUUGGAGCCUUCUUUGGAGCUGUUUGUAGAUUUGCUAUAUCUCGGUUAAAUUUUGGUUCUUUUCCUCUUA
Tye-1-1 UACCUUAAAGGUAGAGUUAUACCCUGAAAAGGAGGCGUAUUAUGUUGGAGAUCCUUCUUCAAGCAUCUUUUUGGUUUUUACUUGCUUUGAUUUCUACAAUCAUUGCAACAAAGCUCCGUUUAGCCACAGCUUUAACUGAAAUAGCAGUUGGAGCAAUUGC
Taq-1-1 UACCGGGUUUCGGGUAGGGGUUUUUGCUUGGGAGGGACCCGUGGAGAGAACGGCAGAAAGGCCAAGCCUCCUCGGCCCGGAGGGGCCCGUGGAGGAAGGGGAAAGGGGCGUCCCCGCCCACUUCCAGGACCUCCACCUGGACGAGCUGGAAGCCGCCCUC
Dge-1-1 UACCUUCCCAGGACGCCGGGAGGUCGUAUGCCGUUUUGGUUCGGAGUCGCGGUCGGUGGUGCCUUGGGUGCCCUCGCCCGGUAUGGCGUGAGUCUGCUGGUGGCGGGUCGGCUCGCCAGCACCGCCUGGGGGAACUUUCCCCUCGCCACGCUGCUCGUCA
Aba-1-1 UGCCGACACUACUGUGGGAGGACUCCAUCAUGGCCAUCCGUGAAUACCUCUGGGUAUCGCUGGGCGGAAUCGUAGGCGCGUGCGCGCGCUAUUUUCUGAGCCGUUUCACCGCGAAGAUCACGGGCACCAGCUUUCCGUGGGGCACGCUGCUGAUCAACAU
Hsp-1-1 UACCUGCUCUAUAUAUUUUUGAGGAGGCUAUGUGAACUACUUAGCGGUUCUUGUAGGUGGUGGUGUAGGUGCUUUGGUGAGGUAUCUAGUAUCUAUUUUUAUUCAGAAGUUUGUGCCAAACUUUCCUCUUGGUACAAUGGUGAUAAAUACAACGGGAGCG
Hsp-1-2 UACAAGCUCUAAGAUAUUUUAGGAGGUUUUAUGAACUAUCCUGCAUCGCUUGUAGGCGGCAUUGAAGCUUUGAUAAGACAUAUCUUCGUAGGUUUUGACCAAAAAGAUGUGUCCAAUUUUUUCUCUGGUACGUUUCCAAUAAAUAUUUCUGAAGCUUUCG
Ban-1-1 UAUUGACGCGUGGGUGUGUGGUAUCGCAUGCGUUCGGCGGAUGCGGUGCCGCCGUUGACGCCGGUCGACAUGUUUGCGGCACCCCGCCGAAAAGAGGGUAAAUAUGGCACGACAACCUAUUGUUCGUCUUGAUCUCAUAUGCGUGGCGUUCCUAGGGGGG
Lxy-1-1 UGCGCUUCUCUCCGAAGGGUGGAAUCUGUUUGUCCAGGAGGUCCUCGUUUCCCGUUCACUUGCACGGGCGUUCCAUGCUGCUCGUCUUCGUCGGCGGCGCUCUCGGCACGGCCGCCCGCGCUCUGCUCUCGGCGGCGGCGCCGACGGUCGCGGUCAUCUC
Mab-1-1 UGUGUUGAUGUGUCGUGACACGAUGGGACCGAAUGUGACAGCUGGAUACCGCCAAGAACCUCCGCGCCCUCGGCCAUAUUUACGUCCUUCCGCACUGUUGUGGGUGUUCCUCGGCGGUGCGUGUGGGACGGCGCUGCGCUACGGGUUCGAGCAGACGUGG
Mma-1-1 UGCGGAACAGAUCAAUAUUCGGCUUGCCAAACAUGCGGGCCACAUGUGACGCGGAGGAUGGCAAGUGUUCGUACAGUUUUCGUCAACAUGUGCUGACGGGUUGUUUAGGCCGGCCUCGGGCGCGGCCGGAUCAUCGGCGGGACUCCCCGCGGCGACGGCC
Mul-1-1 UGCGGAACAGUCAAUACGUGGCUGCCAAUUGUGCAGCCACCUGUGACGCGGGGAAGGUGGCCUUGUGACCGUUGGUUUUUCAUACUCGACUACGGUCGGCACCGCGUCGCACUUGCGCCGGCCCCCUCGCACAACUUCGGCUCGCACGACUUCGGCAGGC
Mma-1-2 UGCGGAACAGUCAAUACGUGGCUGCCAAUUGUGCAGCCACCUGUGACGCGGGGAAGGGGGCCUUGUGACCGUUGGUUUUUCAUACUCGACUACGGUCGGCACCGCGUCGCACUUGCGCCGGCCCCCUCGCACAACUUCGGCUCGCACGACUUCGGCAAGC
Ace-1-1 UACCUCCCAACCCCGCGGCGCGCGCAGCGCGCCACGUGUUGAGUCGAGAGGUAGGAGCGCAACAAUGCCCGGCAUCUCCUCAUCGCACCGCGCACUCCCCGCCCGCUGCUGCGCGUCAUCGACCCCGACGAGUGCACGCUGAUGAAUCCACGGCUCUC
Mul-1-2 UGCGAACAAAGGAGUCCCAGGUGGCAUAUCCAAACUCCCGCGCUUGUCCUGCGCCGCGCGUGGCCCGAAUCGAGCUGCGCCGCAACGAUGGUCAUCCGAUAGGCACACACCGACCCGAUGCCCACGAGGCGAACAUCGGUUGCAGCGGCGGCGCUGCGGC
Mma-1-3 UGCGAACAAAGGAGUCCCAGGUGGCAUAUCCAAACUCCCGCGCUUGUCCUGCGCCGCGCGUGGCCCGAAUCGAGCUGCGCCGCAACGGUGGUCAUCCCAUAGGCACACACCGACCCGAUGCCCACGAGGCGAACAUCGGUUGCAGCGGCGGCGCUGCGGC
Ace-1-2 UACCGAGUUCGGUGGAGGUCGUCAUGACGGUGCUCAAGGAGCGGGCCGCAAAACCGAGUUCUCUUCGUCGACCCGACGGCGUUGGCGGCGGUCGACGAGCAGCCCGAGUUCUUCAAGGACCUCCAUCUCGACGAGAUCCUGGACGUGAUUCUCGCUGG
Rxy-1-1 UGCCCCGCAGCCCUUGGUGGCCCGGGCAGGGGCUUUUUGUUGGCGAAAGGCGGUACGGAGGAGGUGGGACGGGUUGGAAACGGAGCGGACGGUGGCCCAGGCCACGGUGAGGCUGGCUCCGAGCUGCAGUGCGAGCGGGCGGUGCGCCUGGCGCAC
Blo-3-1 UAUACACGAUUUGAUAUUGCAUGGAAUCGUUGUGUCUUCAGCAGGAUUCAGCGGUUUCAGGAAAGGCUAUUAUGGAUUCGCAAUCCCCAUCCGCAGGUGCCCGGCCGAUUACGCACACGACCACUCCCCCGGCGCGCAAGCUCCCCGAUAUUCAUCUUGA
Blo-2-1 UACACACGAUUUGAUAUUGCAUGGAAUCGUUGUGUCUUCAGCAGGAUUCAGCGGUUUCAGGAAAGGCUAUUAUGGAUUCGCAAUCCCCAUCCGCAGGUGCCCGGCCGAUUACGCACACGACCACUCCCCCGGCGCGCAAGCUCCCCGAUAUUCAUCUUGA
Blo-1-1 UACACACGAUUUGACAUUGCAUGGGAUCGUUGUGUCUUCAGCAGGAUUCAGCGGUUUCAGGAAAGGCUAUUAUGGGCUCGCAAUCCCCAUCCGCAGAUGCCCGGCCGGGCACGCGCACGGCCACUCUCCGGCGCGCAAGCUCCCCGAUAUCCAUCUUGA
Bad-1-1 UACGACAUAGGGUGUGCUGUACCUUAUGGCUUUCAGCGGUUGUGGCAUCGCCAUUCUUCCAUGUCUGUCAUAUUGGUUCGGCACCUCGCUGGAAAGAAGAUACAUGGCACGACAACAUAUCAUUCGAGUCGAUCUCAUAUGCGUGGUGUUCCUUGGGGGA
Bad-2-1 UACGACAUAGGGUGUGCUGUACCUUAUGGCUUUCAGCGGUUGUGGCAUCGCCAUUCUUCCAUGUCUGUCAUAUUGGUUCGGCACCUCGCUGGAAAGAAGAUACAUGGCACGACAACAUAUCAUUCGAGUCGAUCUCAUAUGCGUGGUGUUCCUUGGGGGA
Nfa-1-1 UUCCCCACGAGGAAGGAGUCUGCUGUUGUCCGGUCACCGCGAACCUUCAGCUGGGCCCUACCCCCGGAUCUUGGACACGGUGGGUGAUUACGCAGCGGUUGGCAGCGUAGCGGCCCGGCGGGCCUCGUAGGUGGACGGGGACAGGUAGCCGCACCAGGA
Bpl-1-1 UACCGGAGCAGGUCUCUUUUUAGGCCAGCAGGGAGGGCUAUGUCUGUAUCCAUGCUAUGUGAAAGAGUUCUGGCUUUGGUUUUUGCGAAAACUUGAGAUAUGGAUACAAGAAAACUUAGAAAAAUAUGAAGACUUUAAGAAAUCAGAAUUACUGGUGGA
Bco-1-1 UGCCGGAGUUGGUCUUUGGAGUAAGACUGGCAGGAAAAUCUAUAUCUGCUUGUCUGAAAAAAGAAUCGGCUUCCGUUUUCGAAGAAGUUAUUUUUAUUGUUUGACAAAAUAUAGACAAGACAUGAAGAUUCAGAAAAAUCAAAACAUUGCGAUGAAACU
Bth-1-1 UACAAUUUGAAUGUAUAACUCUUAAAUUGUCAGCUUAUGCUCAAGACUUUACUCUUUAUCGGUAUGGGAAGUUUUACCGGAGGCGUUCUGCGUUAUCUCAUCUCGCGUUACGUUCAGAAUUUCUUGACACCUUCCUUCCCUUUGGGAACACUUCUGGUCA
Bca-1-1 UGCAUUUUGUGUGAUGCAGGGGUUAUCAGGUUUUUUGCUGUCUCCCCUGCGGAAGGGAUGGAAGUACCAUGUUGACAAGUCUGGCUAUAUCCUGCUCCUGGGAUUGCUCCUGGGGGCGCUGGCGGGGAAGCUGCGGCUGCCCCCCUCAUCGGCAUGAU
Lbl-1-1 UACUUAACCGGCCGCAAACGCGAGGUCAAAGUAGAAUCAUUUAUAUAUGGCAGUAUGAACUUAGACAAAUUCAAGCGCUCGCUCACUUCAAUCGAGCAAAUUCCUUCUCUGGUUUAUCUUUUUAAAUGGAUUAUCAUCUGCACCUUGAUCGGGGCGAUU
Psp-1-1 UGCAAUGAUACUAUUGGCAAUGAAGCCAGUAGAAAUAAACUGCUGCAGGUGAAAAAAAAUAAUAUUUUGAAAUAUCUCGUUCGCUGGACACUCAUGCUGUUCCCUGUUUCUGUUGCCAUCGGCACGGUUGUCGCACUGUUUAUGUGGCUGCUGAGCUGGG
Psp-1-2 UACAAUAGAAAUUCCAGCAGGUAAUAAAAUCUGCCGGUCAAAGUAUUGUAGGUGAAAAAAUUAAAAAUAAAUUCUCAACUCUUAUUGCUGGCAGCUAUCUUAAUUGCGGUUGUCUUACUUUGUCUGUCUGCCAAAGUGAGAUCAGUGUUAGCAAUGGUCC
Rsp-1-1 UACGGAAAAGGACCGUCACGGUCUGCCGUAGAGGUUUUUUGUUUGAAGAGGAGAAGCGCCUGUGAGGCUGGCGGGGAGACGUAUCGUGGUGAACAGCGCUUUCAUGAACAUCAUCGCCAUUGCUGUGGGAGCAGCCAUCGGCGCCAAUCUGCGCUACAGU
Cau-1-1 UACAGGGAAACGUUUUGUGUGUAGAGGGUUUUAUUUUGAAUCGAUAAGAGGCAUGAUCAUGGUUUCGCUCAGUAUUCUUCACUACCACGACGGUCUCCAAACAGAAGCAACCAGCCAGCUGAUUUCUUUCCCGAUCUAAACCUGGAUCAGGUGGUAGA
Cag-1-1 UACAGAAGUCACGUUCUGUAGAGGCUUUUGUUUUAGAGGCGAGGGAGAUGACGAUGGCAUUUCACAGCAUUCUGUUCAUCGAUACCCCCGAUGACACGCCGGCGCCGCAAUGCUUCGCCGAUUUGAACCUCGAUCAGGUGGUGGUUUUCAAGUUUGAGUUAA
Ebi-1-1 UGUGUUUUUAAAGGCGGAAACCAUCAGGUUUUCUGCGUUUUGAAAGGAGAGAUAAAAUGCUGAUCAGUUUAUCUUAUAUUUUUCUUGUAGGAAUGUUUCUAUCUUGGAUCUGUAAAAAGGUGGAAUUACCUGGACUAUUAGGAUGAUAUUUACAGGGAU
Gsp-2-1 UACCAGUAAAUCAAUUAUUUGGUAGGAGUUUGUUUUUUGGAUAAAUUGAAGGAAAAGGGGUAUUGUUCGUGAUUUAUGUCGUCGUUGGUCUUGCCGGCAUUGUCGGCGCGCUGCUUCGCUAUUAUUUAGGAUUGUUUGUGGGUGCUUAUGGAACGGUUG
Gth-1-1 UGCUGCGUUUGCGAGUGGCAGGAGUUUGCUUGUUUUGCAGAGGAGGGAUGUCUUAUGGUGUAUGUCGCGUUGUCGGCAUCGCUGGCAUGAUCGGCGCACUUGUCCGCUAUGCGCUCGGAUUGGCUGUUCCCGCUGCCGCUGUCGGCGGUUUUCCGCUCGGGA
Gka-1-1 UGCUGCAAAUUGAAGCGGCAGGAGUCUGUUGAUUUUUGGAAGGAGGGGUCCUUAUGGUCUAUCUCGCUGUCGGCAUCGCCGGCAUGAUCGGCGCGCUUGUCCGCUAUGGCCUCGGUUUGGUUGUUCCCGCCGCCGCCGUCGGCGGCUUUCCGCUCGGGA
Bth-2-1 UACCAGUAUAACUACUGGUAGGAGUCAUUUUUUUGAGCAAGCUAUUUAAAGAGGUGAAAAAAUUGAUUUAUAUUAUCGUUGGCAUAGCCGGUAUAUUAGGAGCCCUUUCUCGCUAUUAUUUAGGUCUUACUAUUCAUACUUCAUGGAAUCAUUCAUUUCC
Bce-10-1 UACCAGUAUAACUACUGGUAGGAGUCUAUUUUUUUGAGCAAGCUAUUUAAAGAGGUGAAGAAAUUGAUUUAUAUUAUCGUUGGCAUAGCCGGUAUAUUAGGAGCCCUUUCUCGCUAUUAUUUAGGUCUUACUAUUCAUACUUCAUGGAAUCAUUCAUUUC
Bwe-1-1 UACCAGUAUAACUACUGGUAGGAGUCUAUUUUUUUGAGCAAGCUAUUUAAAGAGGUGAGAAAAUUGAUUUAUAUUAUCGUUGGCAUCGCUGGCAUAUUAGGAGCCCUUUCCCGCUAUUUUUUAGGUCUUACUAUUCAUACAUUUUGGCAUCAUUCUUUUC
Bce-13-1 UACCAGUAUAACUACUGGUAGGAGUCUAUUUUUUUGAGCAAGCUAUUUAAAGAGGUGAGAAAAUUGAUUUAUAUUAUCGUCGGCAUUGCCGGUAUAUUAGGAGCCCUUUCUCGUUAUUAUUUAGGUCUUACUAUUCAUGAAUUUUGGCAUCAUACAUUUC
Bce-12-1 UACCAGUAUAACUACUGGUAGGAGUCUAUUUUUUUGAGCAAGCUAUUUAAAGAGGUGAAAAAAUUGAUUUAUAUUAUCGUCGGCAUUGCCGGUAUAUUAGGAGCCCUUUCUCGUUACUAUUUAGGUCUUACUAUUCAUGAAUUUUGGCAUCAUACAUUUC
Ban-16-1 UACCAGUAUAACUACUGGUAGGAGUCUAUUUUUUUGAGCAAGCUAUUUAAAGAGGUGAGAAAAUUGAUUUAUAUUAUCGUCGGCAUUGCCGGUAUAUUAGGAGCCCUUUCUCGUUAUUAUUUAGGUCUUACUAUUCAUGAAUUUUGGCAUCAUACAUUUC
Bth-3-1 UACCAGUAUAACUACUGGUAGGAGUCUAUUUUUUUGAGCAAGCUAUUUAAAGAGGUGAAAAAAUUGAUUUAUAUUAUCGUCGGCAUUGCCGGUAUAUUAGGAGCCCUUUCUCGUUACUAUUUAGGUCUUACUAUUCAUGAAUUUUGGCAUCAUACAUUUC
Bce-9-1 UACCAGUAUAACUACUGGUAGGAGUCUAUUUUUUUGAGCAAGCUAUUUAAAGAGGUGAGAAAAUUGAUUUAUAUUAUCGUUGGCAUUGCCGGUAUAUUAGGGGCCCUUUCUCGUUAUUAUUUAGGUCUUACUAUUCAUGAAUCUUGGCAUCAUACAUUUC
Bce-8-1 UACCAGUAUAACUACUGGUAGGAGUCUAUUUUUUUGAGCAAGCUAUUUAAAGAGGUGAGAAAAUUGAUUUAUAUUAUGGCUGCGGCAUUGCCGGUAUAUUAGGAGCCCUUUCUCGUUAUUAUUUAGGUCUUACUAUUCAUGAAUUUUGGCAUCAUACAUUUC
Bce-4-1 UACCAGUAUAACUACUGGUAGGAGUCUAUUUUUUUGAGCAAGCUAUUUAAAGAGGUGAGAAAAUUGAUUUAUAUUAUCGUCGGCAUUGCCGGUAUAUUAGGAGCCCUUUCUCGUUAUUAUUUAGGUCUUACUAUUCAUGAAUUUUGGCAUCAUACAUUUC
Bce-7-1 UACCAGUAUGGAUACUGGUAGGAGUCUAUUUUUUUGAGCAAGCUAUUUAAAGAGGUGAGAAAAUUGAUUUAUAUUAUCGUUGGCAUUGCUGGUAUAUUAGGAGCUCUUUCUCGUUAUUAUUUAGGUCUUAAAUCAACACAUUUUGGCAUCAUUCAUUUC
Bce-3-1 UACCAGUAUAACUACUGGUAGGAGUCUAUUUUUUUGAGCAAGCUAUUUAAAGAGGUGAGAAAAUUGAUUUAUAUUAUCGUCGGCAUUUCCGGUAUAUUAGGGGCCCUUUCUCGUUAUUAUUUAGGUCUUACGAUUCAUGAAUCUUGGCAUUAUACAUUUC
Bce-2-1 UACCAGUAUGGAUACUGGUAGGAGUCUAUUUUUUUGAGCAAGCUAUUUAAAGAGGUGAGAAAAUUGAUUUAUAUUAUCGUCGGCAUUGCUGGUAUAUUAGGAGCUCUUUCUCGUUAUUAUUUAGGUCUUAAAUCAACACAUUUUGGCAUCAUUCAUUUC
Bth-4-1 UACCAGUAUAACUACUGGUAGGAGUCUAUUUUUUUGAGCAAGCUAUUUAAAGAGGUGAGAAAAUUGAUUUAUAUUAUCGUCGGCAUUGCCGGUAUAUUAGGAGCCCUUUCUCGUUAUUAUUUAGGUCUUACUAUUCAUGAAUUUUGGCAUCAUACAUUUC
Bce-5-1 UACCAGUAUCACUACUGGUAGGAGUCUAUUUUUUUGAGCAAGCUAUUUAAAGAGGUGAGAAAAUUGAUUUAUAUUAUCGUUGGCAUUGCUGGCAUAUUAGGGGCCCUUUCUCGUUAUUAUUUAGGUCUUACGAUUCAUGAAUUUUGGCAUCAUACAUUUC
Bce-6-1 UACCAGUAUACAUACUGGUAGGAGUCUAUUUUUUUGAGCAAUCUAUUUAAAGAGGUGAGAAAAUUGAUUUAUAUUAUCGUCGGCAUUGCCGGUAUAUUAGGAGCUCUUUCUCGUUAUUAUUUAGGUCUUAAAUCACCACAUUUUGGCAUCAUUCAUUUC

URC

3′

```
Bce-14-1   UACCAGGAUACACACUGGUAGGAGUCUAUUUUUUUUGAGUAAACUAAGAGAGAGGUGAAGAAAAUGGUCUAUCUUAUUGUUGGAUUAGCUGGAAUAUUAGGUGCUCUUUCACGAUAUUAUUUAGGACUAAGUAUUGAUGUAUUGUGGCACCACCCUUUCC
Gsp-3-1    UACCAGAGGUCGCUUUCCUGGUAGGAGUCUGUUUGUGUAUGGGGGGUUAGCGGCGAAUCUAUAGAAACGGGUGAGCUUGCAUGAUGAAAAAAUGGAUUGAGAGCGCCGGGAAAUGGUCAUUGGCCAUGUUAUCCGGUUACUUCAUCAAAUGGGUCGUUCU
Psp-2-1    UACCAAUGUGUUUUUGCAUUGGUAGGAGUCUAUUUUUUGCCCAUAUUCACCUUUAACCAUUAACGAUUGGAGAGAAAAACGGAGAUGAACGUUCAAUCUAAAGCUCGGCAUUUUGCCGAAAAAGUCAUGCAUGUUGCCUUACUCGCGUCUUUAUUGAAAU
Mth-2-1    UACGCGGGGGGGUAAAGUUUCCCCGGGGUAGGGGUUUAUUUUUUGCCGCCAUGGGAGGAAUGAUAUUCACCAUGCUCUACCUUUACCUUGCCGUAGGAGGAUUUUGUGGCGCUGUCGGCCGUUAUUUCCUGGCUAGUUUUAUCAACCGUCUCUGGCCAGG
Tte-1-1    UACCUUUUUGUCCUACAAAAUGUUGUAAUAUUGAAAAUAAUCUACGUAGGAGGGUAUGUCUAUGUGGUUAACUUAUAGUUGGUGGUUAGGCUUGGCUUUAAUUGCAGGUAUAAUUGCCAAUUGGACAGGUAUAUCGAUCUCAUUAAUCGAGAUUUUGGUA
Cpa-1-1    UACCUUUUUGUCCUACAAAAUGUUGUAAUAUUGAAAAUAAUCUACGUAGGAGGGUAUGUCUAUGUGGUUAACUUAUAGUUGGUGGUUAGGCUUGGCUUUAAUUGCAGGUAUAAUUGCCAAUUGGACAGGUAUAUCGAUCUCAUUAAUCGAGAUUUUGGUA
Cpa-1-2    UACUUGUGAAUAACACAAGUAGGGACCAUUUUGCUUUUUAAGGGGGUGAUAGAAAAAAUAAAGAUUUAAAAAGCGAUGGGGCUCGCUUAAUGCAGAAGAAAUGCUGAUGGCUCCUACCUUUUUGUCCUACAAAAUGUUGUAAUAUUGAAAAUAAUCUACG
Tte-1-2    UACUUGUGAAUAACACAAGUAGGAACCAUUUUGCUUUUUAAGGGGGUGAUAGAAAAAAUAAAGAUUUAAAAAGCGAUGGGGCUCGCUUAAUGCAGAAGAAAUGCUGAUGGCUCCUACCUUUUUGUCCUACAAAAUGUUGUAAUAUUGAAAAUAAUCUACG
Cac-1-1    UGCAUAAUUAAUAUGUAGGGGUUUUUUGCUUUUUAUUAAUUGUUUUAGGAGGGAGUUAUGAAAAAAUAUAUUCUCAUUGGAUUAGGUGGAGCAAUUGGAGCUAUUUUAAGGUGUUUUAUAAGGAACACAAAAAUUCCUGUAUAUAAAGGUGAAUUUCCAA
Cdi-5-1    UAUGAUAUCAUAGAUGCCACCAGCUUUUUUUGUUGUUUAAAUCGAUAAAAUGGAGGUGAAUUACUUACUAAAUUUUUAUUUUUUAAAUUUGAAGAAAUAUUUAAUUAAUAGAAAAAACAGUAGAUUAUAUGUUAAUUAUUUUAUUAAGAAUGGGUUAAGA
Cdi-7-1    UAUGAUAUCAUAGAUGCCACCAGCUUUUUUUGUUGUUUAAAUCAAUAAAAUGGAGGUGAAGUACUUACUAAAUUUUUAUUUUUUAAAUUUGAAGAAAUAUUUAAUUAAUAGAAAAAAUAGUAGAUUAUAUGUUAAUUAUUUUAUUAAGAAUGGGUUAAGA
Cdi-4-1    UAUGAUAUCAUAGAUGCCACCAGCUUUUUUUGUUGUUUAAAUUGAUAAAAUGGAGGUGAAAUAGUUACUAAAUUCUUAUUGUUUGAAUUUGAAUAAAUAUUUAAAAGAUAGAAAAAAUGGCAUUUUAUAUGCUAAUUACUUUGUUAGUAUAGAGAUUAAG
Cdi-9-1    UAUGAUAUCAUAGAUGCCACCAGUCUUUUUUUGUUGUUUAAAUCGAUAAAAUGGAGGUGAAUUACUUACUAAAUUUUUAUUUUUUAAAUUUGAAGAAAUAUUUAAUUAAUAGAAAAAACAGUAGAUUAUAUGUUAAUUAUUUUAUUAAGAAUGGGUUAAG
Cdi-2-1    UAUGAUAUCAUAGAUGCCACCAGCUUUUUUUGUUGUUUAAAUCGAUAAAAUGGAGGUGAAUUACUUACUAAAUUUUUAUUUUUUAAAUUUGAAGAAAUAUUUAAUUAAUAGAAAAAAUAGUAGAUUAUAUGUUAAUUAUUUUAUUAAGAAUGGGUUAAGA
Cdi-6-1    UAUGAUAUCAUAGAUGCCACCAGUCUUUUUUUGUUGUUUAAAUCGAUAAAAUGGAGGUGAAUUACUUACUAAAUUUUUAUUUUUUAAAUUUGAAGAAAUAUUUAAUUAAUAGAAAAAACAGUAGAUUAUAUGUUAAUUAUUUUAUUAAGAAUGGGUUAA
Cdi-1-1    UAUGAUAUCAUAGAUGCCACCAGCUUUUUUUGUUGUUUAAAUCAAUAAAAUGGAGGUGAAGUACUUACUAAAUUUUUAUUUUUUAAAUUUGAAGAAAUAUUUAAUUAAUAGAAAAAAUAGUAGAUUAUAUGUUAAUUAUUUUAUUAAGAAUGGGUUAAGA
Cbu-1-1    UGUGGUUUAUUUUCCGCAGAAUUUAUCAGCUUUUUUUGUGUGCAUUUGUAAAUACUAAAUAAUAAUGACUCAAAAUAUAUAAAAUAACUUUAGAAAUACAAAUAGAGAAGAGGGAUAAUACGUAUGUUUAAAAAAAAUUUAUAAUCAGUAUAAGGAUAUUA
Nth-1-1    UACCCUAUCAGGGUAGGAGUCUAUUUUUUUAAGAAAGGGAUGAUUUACAAUGAUUGAAAGUAUUUUGAUUAUACUAAUUGCCGGCUUCAUUAUGGGAAAAGCUUUUGAAAAGUUAAACCUUCCAGGUUUGUUAGGAAUGCUUCUUGCGGGUAUUUUGUUA
Aor-1-1    UGCGAAAAUACGCAGGAAUUAUCAGUUUUUUUUAUUCCUGCCAAUAGGGCGGAGGAUUUUAUUAUGUUAACAAGUCUAGCGCUUAUUUUUUUAUUGGGGCUUACUCUAGGCAGUAUAUUGAACAAACUUAAACUGCCAAGCCUGCUAGGGAUGAUGAUUG
Ceu-1-1    UACGAUUAUUUUUGUCGCAGAAGUGUCAGCUUUUUUGUUUCCAUAAGAAGAAAAUGCUUUGAUACAUUUGCUAAAUUAAGGAGGUAUUGUCUAUCAUGUUGACAUCACUUGCACUCAUUUUCAUAGUCGGACUGGCGAUGGGCGCCAUCUGUCAGAGGCU
Cba-1-1    UGUGUGAUUUCACGCAGGAACCAUCGGGUUUUUUUGCGCUUUCAGGAGGAAUGGGAUAUGCUUUUAAGUAUUGCGUACAUGUUAAUGGUGGGGAUGGCUGCGGGCUGGGCGUGCAGGAGGCUGCGGCUGCCGGGCCUUGCAGGAAUGAUUGCCACAGGAA
Rob-1-1    UGCGAGUAAAUGAACGCAGAGAGUGUCAGCUUUUUCUGCGUAUAUGAGGAGGAAAAUUAUGUUAUUAAGUAUUUCGCUGAUUCUUAUUUUAGGGAUGUUUAUGGGAUGGAUAUGCCAGAAAAUCAAAUUGCCGAGUUUACUUGGCAUGCUGAUCACCGGU
Dlo-1-1    UGCGAAUAAUGACGCAGAAAGUCUCAGCUUUUUCUGCGUUUAUAGAUCGCUAUGCGGUUGAAAGAAACUGCCAGUGGCAGAUUCUGUAGAUAAGGAGGAAUAUAAAAUGUUACUAAGCGUUGCGCUGAUCCUGCUCUGUGGUAUGGGGAUGAGCUGGAUC
Ahy-1-1    UGUAACUAUUUAGGGACAGAUUUCAUCAGUUUUUUUGUCCCAAAAGGAGGAAUUUAUGUUAAUAUCACUUGGCCUAAUAAUAAUCAUUGGCCUUAUACUUGGAUUUAUUUGUAAAAAAUAAAAAUACCAGCCCUUAUAGGCAUGCUUUUUACAGGAAUUA
Cph-1-1    UGUGAAUUAUCACAGGGGGAAUCAGCUUUUUUGAUAUUUAAUACAAAUUUAGGAGGUUUUUAUGUUAAUAAGUUUAGCACUCAUUUUUUUGCUUUCUUUAUUAUUAGGAGGACUUGCACAAAAGUUAAAAUUACCAAGUUUAAUAGGAAUCCUAUUAGCU
Cbo-1-1    UGCAUUAUGUAUUAACGCAGAAAUGAUCGGCUUUUUCUGCGUUUAUAAGGAGGAAAAGGAUAUGCUGAUUAGUAUUGCUUUUAUGUUGAUGCUGGGAAUGUUUCUGGGGUGGGUGUGCAGGAAGCUGCGCCUGCCCGGCCUUAUGGGGAUGAUUUUUACC
Fma-1-1    UGUUUUUUACAGAUAUCAUCAGCUUUUUUAUUAUAUAAAGGAGAAAUUUUAUGUUAAAAAGUAUAGCUUUUAUAAUUAUUUUAUCGUUGAUUUUGGGAAAAUUUUUCAAAAAAAUAAAAUUGCCACCGAUAUUGGCAUUUUAAUAGUUGGAAUAACUCU
Ala-1-1    UACAUUUUUGUAGGGUUAUCAGCUUUUUUAUUGGAAAGGAGAUUUUAUGAUAUUAUCACUUGGACUUAUUGUUAUUGUGGGCAUGGUCUAUGCGAAAAUUUUUGAUAGGCUAAGACUACCUAGAAUAAUUGGUAUGCUUCUAACCGGAAUUAUCUUAGGA
Chi-1-1    UGCAACCACGCAGAAACCAUCAGCUUUUUUGCGUUUAAAAUAAAAUAAAAGGAGAAACAAAAUGUUAGUAUCACUUGCUUUAAUUUUUUUUAGUUGGAAUGAGCUUGGCAUCUAUAUGCGAAAAAAUUAAAAUUCCGAGAAUUAUAGGAAUGCUUGUAACU
Rin-1-1    UGUGUAGACUGCACGGGGGUAUCAGCUUUUUUUGUGAUUUCGAAAAAUAAUCAGAUAAAGAAAGGAUGUUUGUGCAAAAAAAUUGCACAGCGAAGGAUUAAACGGAUGGAAAACAAAUCACAAACGAUUGUUUUAUCUCUGAAAUAUGUAUUGCUUGCCA
Eve-1-1    UGUGCAAUACACAGGGGAAGUCAGCUUUUUACGCGUUAUGAGCUAAAUUUUAUGCGUUAUAUAUAUCGUUAGAGAGAAAAUGCCUUCAAACACGUUGAUUUUCUUAUUUUGCUCCGCCGUCUAUCGUACAGCGAAAACUUAAACUCGCCUUCGGCUCAAA
Csp-2-1    UGUGCGAUGAACACAGGGGGUUAUCAGCUUUUUUGAUGUUUGGAUCAUAUAAAAAAGAAAGGAUUUUAGGAAAAUGCAAAAGAAAGAUUAUAUGAUACAAUUGCUAAAAUAUUUUAUCAUGGCAGUCAUUGUAGGAAUCAUUGUAGGUGCGAUUGACGCC
Csa-1-1    UACAAAUAUUGCGUCUUUGUAAAAAGGCGUAAUAUUUGUAGGAGUUUUUUGUUUAUAUAGUCAAAAUUGUCCUUCUAAUUAUGAGCAACUUACCCAGAAGGAAGGAGAGACAAAAAAGAUGAAAAACAUAGCUGCAAUCUCAGUAGGUGCAUUUUUUGGU
Csa-1-2    UGUCCGUAUAAAGGUGCGGGCAGGAGUUUUUUGUUUUAUAUGCUCUUAUUUUUGAGUAUUUUGGGGGGAUGGACUUUGGAUAUUGUGUAUUUUGAAAUGAUAGAAAACUUCAAAGAAGAUAAGUGUAUAAUUUGCUAUUUAAAAAACAAGGCAAUGGAUA
Tps-1-1    UAUUAGGAGGAUAUAAGGCCUAAUAGGGGCCUUUUUAUUGCCCCAAAAUGUAAACGGAAAGGAGAUUUUCAUGGAAUACAUCUAUAUAGGGAUUGGAGGAUUUUUUGGAGCAGUACUGAGAUAUUUAAUAACAAUUUACUUUACAAAAAUGUAUCACACU
Tsp-2-1    UAUUAGGAGGAUAUAAGGCCUAAUAGGGGCCUUUUUAUUGCCCCAAAAUGUAAACGGAAAGGAGAUUUUCAUGGAAUACAUCUAUAUAGGGAUUGGAGGAUUUUUUGGAGCAGUACUGAGAUAUUUAAUAACAAUUUACUUUACAAAAAUGUAUCACACU
Tsp-2-2    UACGCGCUUUCGUAGAAGCCAUUUUUGUUUAUUUGACAAAAAAGAGAGGAUGAGGUUUUUGAAAAAUAUAAGGGAGGUUGUCCAAUAUGCAAGGUUAUAAAAGACGAAGAAAAAGCCUAUUGUGAGCAUUUGUUUGAAGAUGAGGUUUUAAAAGACCCUG
Tps-1-2    UACGCGCUUUCGUAGAAGCCAUUUUUGUUUAUUUGACAAAAAAGAGAGGAUGAGAAUCUGCAUGAAGUUAGAAUUUAUUCCAUUGUAUGAGGUUUUUGAAAAAUAUAAGGGAGGUUGUCCAAUAUGCAAGGUUAUAAAAGACGAAGAAAAAGCCUAUUGU
Cpa-1-3    UACGAAGAUUCGUAGAAGCCAUCUUUUAUUUUGAAAAAAUUAACUACAAAACGUCAAGGGGUGGCAUUUGGAUGAAAUUAGAAUUCAUUCCUUUAUAUGAAGCUUUUGAAAAAUACGAAGACAGAUGUCCUAUAUGUAAGAUUAUAGAACAGGAAGAAAA
Tte-1-3    UACGAAGAUUCGUAGAAGCCAUCUUUUAUUUUGGAAAAAUUAACUACAAAACGUCAAGGGGUGGCAUUUGGAUGAAAUUAGAAUUCAUUCCUUUAUAUGAAGCUUUUGAAAAAUACGAAGACAGAUGUCCUAUAUGUAAGAUUAUAGAACAGGAAGAAAA
Tca-1-1    UACCAGGACAGUGCUGGUAGGAGUUUUUGCAUUUUCGAAAGGAGUCAGGAAAUGCUGGAUAAUUACACUAAGUCCAAACAGGAAAUAGUAAGCGUUUUAGAUGAAAUCUUUCACAUGCUGUUAGGACUAAAAUUUAAUAAUGAUGCAUUGAAUGCUUUUA
Ame-1-1    UGCGAAACAAGGUGAACCCUUGUUUUGCAGGGGUUUUUUGUUUCCUAUCUGGGGGAUUUAAGCUGAAUGAGAAAAUAGAAUCAAAAGAAAUAGAGGGGCAAUAAUAUUAAAAGAGAAGGCGGUGUAGAUAUGGCUUUUAGUUUAGCAAUGAUUAUGGUAC
Dha-2-1    UGCCGGUUAUCUAUUGCCGGCAGGAGUCUUUUUAUCUUUAGGGAGUGAGCUGAACAUGAUUUAUUUAUGGGUAGGUUGUGCCGGAAUGUUCGGGGCAAUGCUCAGGUAUUUAAUCGGUAUUUCAUUCUUUGCUGAUAGUCGGUUUCCUUGGGCAACCUUA
Ckl-1-1    UACAAAUAACAUUCUGUUAUUUGUAGGAGUCAUUUUGUUUAAAGAGGUUAUGAAAGAAUACCUAUGUAGGACAAGGAGAAAAGAAAUGAGAGAGUAUAUAUUUAUUAGUAUUGGGGGUAUUCUAGGCGCUAUAUUAAGGUAUGUUAUCAGAAAUAUUCCU
Cbo-5-1    UACAAAUAGCAGUUUUAUAUUGCUAUUUGUAGGAGUAUUUUUUUUUGGCUUAACAAGUAGAUUAUUCAGUCAGUUAACUCAUAAAAAGCUAUAUAUUCUCUGGGCAUACUGGUAUCUAUACUUCUCAAAGGAUAGGUAUAAGUAAGAGUAAUUCAAUUAA
Cbo-11-1   UACAAAUAGCAGUUUUAUAUUGCUAUUUGGUGGAAAAAGAAGUUAUUGGUCUAGAAUUAGAAUUUGAUGGAUCUUUGCUUUUAAGUGGACAAGCAAUAGUAAAGAUAAACUAAAUAUAGUAAGAAAUAUAGAGAUAAAAACCGUACUAAAAAUGUACGGU
Cbo-3-1    UACAAAUAGCAGUUUUAUAUUGCUAUUUGCUGGAAAAAAAGUUAUUGGUCUAGAAUUAGAAUUUGAUGGAUCUUUGUUUUUAAGUGGACAAGCAAUAGUAAAGAUAAACUAAAUAUAGUAAGAAAUAUAGAGAUAAAAACCGUACUAAAAAUGUACGGUU
Cbo-7-1    UACAAAUAGCAGUUUUAUACUGCUAUUUGCUGGAAAAAGAAGUUAUUGGUCUAGAAUUAGAAUUUGAUGGCUCUUUGUUUUUAAGUGGACAAGCAAUAGUAAAGAUAAACUAAAUAUAGUAAGAAAUAUAGAGAUAAAAACCGUACUAAAAAUGUACGGU
Cbo-10-1   UACAAAUAGCAGUUUUAUAUUGCUAUUUGCUGGAAAAAGAAGUUAUUGGUCUAUAAUUAGAAUUUGAUGGCUCUUUGUUUUUAAGUGGACAAUCAAUAGUAAAGAUAAACUAAAUAUAGUAAGAAAUAUAGAGAUAAAAACCGUACUAAAAAUGUACGGU
Cpe-7-1    UACAAAAUACAAGUUAUAUGUUUAUAUAGCUUAUAUUUUGUAGGAGUUAUUUUUUUGCUCAAAAAUUAUUAAUAUCAAUGAAGGGAGAAUAUAAAUGAAGCAAAAAUCUAAGAAAUAUUUAAAGUAUAUAAGUAGAAAUAAUACCUUUCUUCUAGGAAAAA
Cpe-1-1    UACAAAAUACAAGUUAUAUGUUUAUAUAGCUUAUAUUUUGUAGGAGUUAUUUUUUUGCUCAAAAAUUAUUAAUAUCAAUGAAGGGAGAAUAUAAAUGAAGCAAAAAUCUAAGAAAUAUUUAAAGUAUAUAAGUAGAAAUAAUACCUUUCUUCUAGGAAAGA
Cpe-5-1    UACAAAAUACAAGUUAUAUGUUUAUAUAGCUUAUAUUUUGUAGGAGUUAUUUUUUUGCUCAAAAAUUAUUAAUAUCAAUGAAGGGAGAAUAUAAAUGAAGCAAAAAUCUAAGAAAUAUUUAAAGUAUAUUGGUAGAAAUAAUACCUUUCUUCUAGGAAAG
Cpe-8-1    UACAAAAUACAAGUUAUAUGUUUAUAUAGCUUGUAUUUUGUAGGAGUUAUUUUUUUGCUCAAAAAUUAUUAAUAUUAAUGAAGGGAGAAUAUAAAUGAAGCAAAAAUCUAAGAAAUAUUUAAAGUAUAUUAGUAGCAAUAAUACUUUUCUUCUAGGAAAGA
Cpe-4-1    UACAAAAUACAAGUUAUAUGUUUAUAUAGCUUAUAUUUUGUAGGAGUUAUUUUUUUGCUCAAAAAUUAUUAAUAUCAAUGAAGGGAGAAUAUAAGUGAAGCAAAAAUCUAAGAAAUAUUUAAAGUAUAUAAGUAGAAAUAAUACCUUUCUUCUAGGAAAG
Cpe-3-1    UACAAAAUACAAGUUAUAUGUUUAUAUAGCUUAUAUUUUGUAGGAGUUAUUUUUUUGCUCAAAAAUUAUUAAUAUCAAUGAAGGGAGAAUAUAAAUGAAGCAAAAAUCUAAGAAAUAUUUAAAGUAUAUUAGUAGCAAUAAUACUUUUCUUCUAGGAAAA
Cpe-9-1    UACAAAAUACAAGUUAUAUGUUUAUAUAGCUUAUAUUUUGUAGGAGUUAUUUUUUUGCUCAAAAAUUAUUAAUAUCAAUGAAGGGAGAAUAUAAAUGAAGCAAAAAUCUAAGAAAUAUUUAAAGUAUAUUAGUAGAAAUAAUACCUUUCUUCUAGGAAAGA
Cbo-9-1    UACAAAAAACGGGUAUACUGUUUUUUGUAGGAGUCAUUUUGUUUUUAAAUAUAUAAAUAAGGAGUAAAUUUAAUGAUUAAAGAGAAAUUUAUAAAAAACAAUAAGAGCUUAAGUACACUUAUUGGAAUAUCAUUUUUAAAAUGGAUUUUUCUAGGUACAG

           ..........................................................................................
           ..........................................................................................
           ..........................................................................................
           ..........................................................................................
           ..........................................................................................
           ..........................................................................................
           URC•••••••••••••••••••••••••••••••••••••••••••••••••••••••••••••••••••••••••••••••••••••••
           ———3′
```

```
Cbo-3-2    UACAAAUAAUAUAAUUAUUUGUAGGAUUUUUUGCGUUUUGAGUAAAAUUUAAAGGAGGAAAUUUUAUGGCAUCAAGUUUAGCUAUUAUUAUUCUAUUGGGAUUAAUCGCAAAUAAGCUCUUUGAAAAAUUAAAACUGCCAGGUCUUUUAGGAAUGUUAAU
Cbo-10-2   UACAAAUAAUAUAAUUAUUUGUAGGAUUUUUUGCGUUUUGAGUAAAAUUUAAAGGAGGAAAUUUUAUGGCAUCAAGUUUAGCUAUUAUUAUUCUAUUGGGAUUAAUCGCAAAUAAGCUCUUUGAAAAAUUAAAACUGCCAGGUCUUUUAGGAAUGUUAAU
Cbo-8-1    UACAAAUAAUAUAAUUAUUUGUAGGAUUUUUUGCGUUUUGAGUAAAAUUUAAGGGAGGAAGUUUUAUGGCAUCAAGUUUAGCUAUUAUUAUUCUAUUAGGGUUAAUUGCAAAUAAACUCUUUGAAAAAUUAAAACUGCCAGGUCUUUUAGGAAUGUUAAU
Cbo-5-2    UACAAAUAAUAUAAUUAUUAUUUGUAGGAUUUUUUGCGUUUUGAGUAAAAUUUAAGGGAGGAAAUUUUAUGGCAUCAAGUUUAGCUAUUAUUAUUCUAUUGGGAUUAAUCGCAAAUAAACUCUUUGAAAAAUUAAAACUGCCAGGUCUUUUAGGAAUGUU
Cbo-11-2   UACAAAUAAUAUAAUUAUUUGUAGGAUUUUUUGCGUUUUGAGUAAAAUUUAAGGGAGGAAAUUUUAUGGCAUCAAGUUUAGCUAUUAUUAUUCUAUUGGGAUUAAUUGCAAAUAAGCUCUUUGAAAAAUUAAAACUGCCAGGUCUUUUAGGAAUGUUAAU
Csp-3-1    UACAAAUAAUAUGAUUGUUAUUUGUAGGAUUUUUUGCGUUUGUAAUAAAAAUUUAAGGGAGGAGAUUUUAUGGCAGGAAGUUUAGCUAUUAUUAUUUUAUUAGGAUUAAUUGCAAAUAAACUCUUUGAAAAGUUAAAUCUACCAGGUCUUUUAGGAAUGU
Cbo-6-1    UACAAAAAACGGGUAUACUGUCUUUUGUAGGAGUCUUUUUAUUCUUUAGAAAUUAUAUUAUUAAACAAGUUAUAGCUAACUGCGUAAAAAAUCGAUGAUUCCACAAUUGCCUUAAUGAUUAAUUAUUAAAAAUAUAAAAAAGGAGAAAAGAAAUUAUAUG
Cth-1-1    UACAGGAAUAAUAAUACUGUUUCUGUAGGAGUUUUUUGUUACUUGGGAAGGGAGUAGUGUUUUAUGGCUGCGAGUUUAGCAAUUAUUUUAUUAUUGGGAUUACCGGCAAAACGAAUAUUUGAAAAAUUAAGAUUACCUGGUUUGUUGGGAAUGUUGAUAU
Hmo-1-1    UACUCGAUUUUUCGGGUGGGAGUCUGUCUUUUUGUGAAAGUGGUCAUUCUUUUGUAUGUAAGCAGCCAUUCUCUGUAGGAGGUUGUUCUUGUUUGACCAUCCUUUUAUUCGCUCUCGGUGGCUUUAUUGCCGCAGCCGUAUCGGGAGCAGUCGGAUUCGG
Pth-1-1    UACCCGUAAGUGUUAUGUUGCCGGUAGGAGCUUUUUUACUUGCUUUUCUGGAGGAGAUAAGAUUGGAUAUUGGCUUUGGUUACCUGAAAGAGAAGGUUCUCAAUGAACUGGAGGCGGUUGAUUUUUACGCGCAUCAAAGGGAAUCUUUGCAUCUUGCGGC
Chy-1-1    UACCGAAAGUAAAGUUUUCGGUAGGAGUUUUUUAUUUUCUUAGCUCCAUAAGGGGGGUAAAAUUAUGGAAAAUACCUGGCUGGUGGCCGCUCUUUGGAUAGGGCUUGCGUUAGUAAGCGGUUUAAUUGCUGGUUGGACCGGAAUCUCUAUUUCUUUAGUG
Efa-2-1    UACCAAACGGGAGAAGUGUACCCUUUGGUAGGCUUUUUCUGUACGCUAGGAUUGGCGAUAAGAAAAUUGUAAAGGAGCAUGUAAAGUGAAAAAAGAAACAAAGCAACUACUAUCAAUCGGCGGUUAUAUGGCAAAAUGGUUGCUCAUUACCGCAAUUAUU
Efa-1-1    UACCAAACGGGAGAAGUGUACCCUUUGGUAGGCUUUUUCUGUACGCUAGGAUUGGCGAUAAGAAAAUUGUAAAGGAGCAUGUAAAGUGAAAAAAGAAACAAAGCAACUACUAUCAAUCGGCGGUUAUAUGGCAAAAUGGUUGCUCAUUACCGCAAUUAUU
Lrh-1-1    UACCUUGUAAUUAACGGGGUAGGUGUCUUUUUUUGGACACAUGUACGGCAACGCGCGGAUGAAGAAAGGAGCAAUCUAUGCAACAACGGGAACAAGUACGGUUUAGCUGGUUACUGGCAGUAUUUGUCGGUGGUUUUAUCGGCGGGGUCAUGCGAUAUGG
Lpa-1-1    UACCUUGAGCAAAUCGGGGUAGGUGUCUUUUUUUGAGUAUGAGCUGGUGCGUUUAAAAAUUGCAGCAUAAAUGGCCUCAGGACAAAGUGACAGAUCUUAGCCUGAUAUGUUUAUGCAGAGGUUUCUGCGUCAGCGAGUGCGUUCCCACGAAAGGAGAUC
Lca-1-1    UACCUUGAGCAAAUCAGGGUAGGUGUCUUUUUUUGAGUAUGAGCUGGUGCGUUUAAAAAUUGCAGCAUAAAUGGUCUCAGGACAAAGUGACAGAUCUUAGCCUGAUAUGUUUAUGCAGAGGUUUCUGCGUCAGCGAGUGCGUUCCCACGAAAGGAGAUC
Lca-2-1    UACCUUGAGCAAAUCGGGGUAGGUGUCUUUUUUUGAGUAUGAGCUGGUGCGUUUAAAAAUUGCAGCAUAAAUGGCCUCAGGACAAAGUGACAGAUCUUAGCCUGAUAUGUUUAUGCAGAGGUUUCUGCGUCAGCGAGUGCGUUCCCACGAAAGGAGAUCA
Csp-4-1    UGCGCAUAGGCCGCAGUCUGUCUUCAGCUUUUUUUGUGGAGAGUGAGCAAAUUAUUUUUUUAUUUAGAAGGAGGUAGAAUAAAGAAUGCUUUUAAGUAUAGCUUUGAUAUUAAUUGUUGGUUUUAUAUUUAGUUCAAUUUUUAGUCGUUUCCGUUUACCA
Lac-1-1    UACUUUAUCGCAAGGGGUGGAGUGGACGUUUUUUGUUUGGAGGAAUUCGUUUUGUCUACAAAAACUGGUAAUUACAUUAGUAUUGCAAUUUUUGCUUUUUUUGGUGGAAUAGCACGUGCAUGGUUAAAUUCUAGUUUUGGCUUUUAUGGCACAUUUUGGG
Lhe-1-1    UACUUUAUCGCAGUGGGUAGAGUGGACGUUUUUGGUUUGGGGAUUUUUAAUGAAAAUUAGUAAUUAUAUUAGUAUUGCCUGUUUUGCCUUUUUAGGCGGAAUGGCUCGAUAUAGUUUAAAUGCUCAAUGGAGUUUCUAUGGGACGUUCUGGGGAAAUAU
Lga-1-1    UACUAUUUACCUACACAAGUUGUGUAAUUGAUAGUAGGCGUUUUUAAUUUUGCUCUUUUUUGUUUUGGGAGGUAAAAAUUAUGACUGUUUAUAUUGAAAAAGUUCGCGCAAUGGAAAUUUUUGAUUCUCGUGGCAAUCCAACUGUUGAAGUUCAUGUUGC
Ljo-1-1    UACUUCCUACAAACUAUUUUUGAGUAGCGGGUGGUAGGGGUCGUAUUUAUUUUGCUCUUUUUUGUAUAUCAGGAGGUAUUUGAAAUGACUGUUUAUGUAGAAAAAGUUAGAGCAUUAGAGAUUUUUGAUUCAAGAGGCAAUCCAACUGUAGAAGUUCAUG
Lga-1-2    UACUUUCUUAUGUGUAAUAAGGGAGUAGUGUCGUCUUUUUUUGUAUAAUAGAAAGGAAAGUGAACGAUUACAUAGGAGAAAAUUGAUGAAGACAAAAUUAAUUGCCGUUGACCUUGAUGGAACUUUAUUGAAUGAUAAACAAGAAAUAAGUGUUAAAACA
Ljo-1-2    UACUUUCUUGGAUCAACAGGGAGGUAGUGUCGUCUUUUUUUAUGCAUUUUUUGUAUAAUAGAAAGGAGAGUAACCGAUUACAUAAGGAGAAAAGCUAAUGAAUAUAAAAUUAAUUGCUGUAGAUCUUGAUGGAACAUUAUUAAAUGAUAAACAAGAAAUA
Lpl-1-1    UACUGUUUGUUUUUCAGACAGUAGGCGUCUUUUCUUUUUGGUUAAGAAGGAGAGAUUGAUAUGGAAAAACAAGUUAUUGAAACGGUAAAGGCUCGCGAAAUUUUUGAUUCACGGGGUAAUCCAACAGUUGAGGCUGAUGUUAUUUUGUCAGAUGGGACGC
Efa-3-1    UACUAAAGUCUGGGGACAGCCCCUUUCUUUAGUAGGCGUUUUUUUAUUGAGGAGGAAUUUUUUAUGGAAAAACAAUUACCGAGUCCAGUACGCAUUUUAGUGUAUAUGCUGAAGUGGCUGGUGAUCACAGCGAUUCUCGGUAUAUUCAUGGGGAGCUUGU
Lca-2-2    UACUGAAACGACUUUUAUGUCGCUUCGGUAGGCGUUUUUUUCGUUGUUACCGAAAGAGAAAAUAGACAUGGCAAUUUUUAUUGAUACGGUAAAGGCUCGCGAAAUUUUCGACUCACGCGGUAACCCAACAGUGGAAGCUGAUGUCGUCUUAUCUGAUGGC
Lpa-1-2    UACUGAAACGACUUUUAUGUCGCUUCGGUAGGCGUUUUUUCGUUGUUACCAAAAGGAGAGAUAUUAUGAUGGCACUUUUUAUUGAUACAGUGAAAGCUCGCGAAAUUUUCGACUCACGCGGCAACCCAACAGUGGAGGCUGAUGUCGUUUUAUCUGAUGG
Lca-1-2    UACUGAAACGACUUUUAUGUCGCUUCGGUAGGCGUUUUUUUCGUUGUUACCGAAAGGGGAAAUAGACAUGGCAAUUUUUAUUGAUACGGUAAAGGCUCGCGAAAUUUUCGACUCACGCGGCGCCUUCUCUAAUAUUGUUUUCCAAACAGAAUAAAAAAGU
Lla-3-1    UACAAACAAAAGGCUAUCUCCAAGUAGUCUUGUUUGUAGCGCCUUUUGUUUUGUAAAAUAUUAUAAAGUGAGUUAAACUCACAAACAAGGAGUUUCAAAAAUGAAUUUAUUUAAAGUAGGAAUUUCAAGAAGCGCAAUUUUAUAUGUCUUCUUAGCUGUA
Lla-1-1    UACAAACAAAAGGCUGUUUUUAAGCAGUCCUGUUUGUAGCGCCUUUUGUUUUGUAAAAUUCAGCUGCCCCUAAUCUGCUAUCAUUCUAAAUUUUGAAGCGAAAAAUCAAGUGAUAAGCAGAAAAGGGGCAGACGCGUCUCCCUAUGAGGAGAUGCUAGAG
Lla-2-1    UACAAACAAAAGGCUGUUUUUAAGCAGUCCUGUUUGUAGCGCCUUUUGUUUUGUAAAAUUCAGCUGCCCCUAAUCUGCUAUCAUUCUAAAUUUUGAAGCGACAAAAUCAAGUGAUAAGCAGAAAAGGGGCAGACGCGUCUCCCUAUGAGGAGAUGCUAGA
Lla-3-2    UACAAAGCAAAGUCUUUUUUUGCCUUGUAGUCUGCCAUUUUUUCUUUGACUUACAAGGCAUUAAAGGCAAAGAAAAGGAGAUUUAUUAUGACAGUCACAAUCGAAAACAUCCACGCAAGAGAAAUUUUUGAUUCUAGAGGAAAUCCAACCGUUGAGGUAG
Lla-2-2    UACAAAGCAAAGCCUUUUUUGCCUUGUAGUCUGCCAUUUUUUCUUUGGCUUACAAUGGCAUUAAAGGCAAAGAAAAGGAGAGUUAUUAUGACAGUCACAAUCGAAAACAUUCACGCAAGAGAAAUUUUUGAUUCCAGAGGAAAUCCAACCGUUGAGGUAG
Lla-1-2    UACAAAGCAAAGUCUUUUUUGCCUUGUAGUCUGCCAUUUUUUCUUUGGCUUACAACGGCAUUAAAGGCAAAGAAAAGGAGAGUUAUUAUGACAGUCACAAUCGAAAACAUUCACGCAAGAGAAAUUUUUGAUUCCAGAGGAAAUCCAACCGUUGAGGUAG
Zmo-1-1    UGCUCAAACAUAUUUUUAUGAUUGAGGAGUCAUGCCUGCUGAUGAUCGUUCUUGGCGGAACACGGUUUGGCUCCUUGAUUAAAGGAUGCCAGAUGUAUCAGUUAUUUUUAAAAUACAAACCUUAUCGGCUUAUUUUGCUGUUUAAAUGGCUCGCUAUCAU
Gox-1-1    UGUCCCUCGUCCCCUUUGGACAAGACAGGAGAACUGCGUCCGUUAUGAGUGAACUUCAGAAUCAGCCCCGUUUUCCCGGUAUUUUCAGCCUUAUGCGUCUGAGCGCGGUGCUUGUUGUGCUGUCGGUCUGUAUCGGGAGUGCCUGUGCCCUGUUCCUGUG
Gdi-1-1    UACCCACCGCCUUCGGGCAACAAGGGCAGGAAUCCGUUCGCAGCCAUGUACAUCCCUUUUUCGUUUCGUCCGCGCCGCGAUCGGUAUGUGGCCGUCAUGGCCCGGUUCGUGACGGGCUGCAUCCUCGCCGCCCGCGCCGCCGCCCGUUGGCUGUGCAUGC
Gdi-1-2    UACCCACCGCCUUCGGGCAACAAGGGCAGGAAUCCGUUCGCAGCCAUGUACAUCCCUUUUUCGUUUCGUCCGCGCCGCGAUCCGUAUGUGGCCGUCAUGGCCCGGUUCGUGACGGGCUGCAUCCUUGCCGCCCGCGCCGCCGCCCGUUGGCUGUGCAUGC
Gdi-1-3    UGCUCGCAUCCCGUGUGUUGCGGCAGGACGUCUUGCAUCCUUCCGUGACAGCAAUGCCGGAAGGAUACCGCACCAUGGCGGCGCACGAUGCCAUCGGUUUCGAAAGCAUUCUGAAUCCCGACCCCGCGAUGCAGGCCCGGACCGAGCCGGUGCCCGAUCG
Nha-1-1    UGCUGACUGAAAGCGCGGCAAGCGUCCGGUGCGUGAUGACCGGAUGACGACGUCAACAGCCGCGCAUUGUGGUGAGCGAGAGGUCGCGCGGCUCCAUUUCGUAAAGGUUUCCCAAGCAGACCAUUUGUCGUUGAGGUCAUCCGUUCGCGCAGGGGACCGC
Gox-1-2    UCUGUGCCGCAAUCGGUACGAGGAGACAUGUGCGCCUCUCUUCGUUCCGAAGGUCGUUUAUCUUUGGAGUGGAGAUCUAUCAUGCACAUACUGGCCAUUCUCGACCGGCCUGGGCCGGCGCUCCAGACCCUUCGCACCGUCGAUAGCCUCAUGGUGCGUU
Oan-1-1    UGCUUGAAUUGAAACAGGAGAAGAGUGCGUCGCAAGACCUCUCGAAGUGAGAUAUUCUAUCGAAUUUCCAGACGUCACCGACUAAUCUCUAUCCAUUAUUGAAAUGGACACAGAACAAUGCAAGCAACCCUCAUGGUAGCGCUCGGUGGAGCGAUCGGAA
Gox-1-3    UACCGCGACACAAUGGCGGUGGGAGUGCGUCUGCAUUUCGCCGCCCGAAACAGCGGGUUCCGUCCGAACUCGCGCAGAAAGGGAAUGGCAUGUCGUUUACAACCUGUCUUAUCGUCAUGGUCGGAGGCGCUCUCGGCACGUUGGCGCGUUACCUGGUUUC
Mra-1-1    UACCGCGAAACCGGCGGUGGGAGUGCGUCUUGCAAGCACCCGCCGCGACGGCGGGUUUCUCAUGCCCGCCCGACGAACUGGAGCGCGCAUGUCCUUCACCACCUGCAUCCUGGUCAUGAUCGGCGGGGCCCUCGGCACGCUGGCCCGCUACGUCGUCUCG
Bja-1-1    UGCUUGAGGUGGGGCAAUUUUUGAGCCUCUGCCUUUGGCGGGAGCGUGGACAAACCCGCCAAUGGCGGGUUUUUUGUUGCCUGAAGGCGAAGGCCGGGACGUGAAGGCCAAGGGGACGUUCGAGGGGAAGUCCAAGGCGUGACGACGACGACACCGAAUG
Oca-1-1    UACCGGACGCUUUCGCGUCGGGUAGGGCUUGUCCUCCCCCAGCGAUGCAUCCGGGAUGGGUGCAUGACAAAGGCUGGGUACCUCGCUCGAGGCUUCCUCAUUCGCUGCCAUCGCACUCCGGGCGUCGGCUUGGCCGGGCCUAACGAUCGUUGCGUAUCCG
Nwi-1-1    UACCGGGCGCUUUCGCGUCGGUAGGAGCUGUUUCCUCCCCGCUGCGCCCGAGAAAGGGCGCUGAUGAUCAAGUCUCUGAAUCCGCUGCAAGUCUGCCUGAGGGGAGACGAUGAUGGCUGGCGCACGGCCGGACAACCACGGGCGGAGCAAAAGCUUUGAA
Nha-1-2    UACCGGGCGCUUCGCGUCGGUAGGAGCUGUUUCCUCCCCCGUUGCGCCCGAGAAAGGGCGCUUAUGACCAAGCUUCCGAAUUCGCUGUUGGUCUCGUUAUGGGGCGUCGGAGUCUGCUGGGUUGCCGCAGCGAUCGCUUACCUGACCGAUUCCGUGACGC
Nsp-2-1    UACCGGGCGCUUCGCGUCGGUAGGAGCUGUAUCCUCCCUCGUUGCGCCCGAGAAAGGGCGCAUGAUGACCAGACUUCCGAGUUCUCUGUCGGUUUCGUUGUGGGGCGUCGGCGUCUGCUGGGUGGCUGUGGCGAUCGGCUACCUGAUGGAUUCCGCGACG
Rpa-1-1    UACCGGGCGCGUUCGCGUCGGUAGGAGUGUGUCCAACUCUCCUUCGACAGAACCCCCGACACACUUCAGUUUCGACGACCUGCAGAGGUCGCCGAGCGAUGUCGUUUGUCGCCGCGCUGCGUGCCUUGCUGGUCAUGCUCGCGGGACGUACUUGUGCGCU
Rru-1-1    UACCGCGUAGAAAUGCGUGGUGGGACCUUGUGACAACCCGCCAUGCCUGCCAAGCAGGAGGAGGCGGGUUUUUUUAUGCCCGCCCGGUCCCCAUCCAUCGGAGGUAAGUGCGCAGCGCCAUGACCUAUUUAUUCGUCGCCGCCGGGGGAGCCCUGGGCUC
Msp-1-1    UACCGCGUGACGACAUGCGGUGGGGGUGCGCCCGCAGACCCGCCGUCGGCGGGUUUUUCAUGCCGUCGCGCAUCCGCUUGAGCACCGGCCGAAGGCCGGAGGAGGCGUGUGAUGGCGGAUGUCUGGCUGGAGCCCGCGGUGUGGCUGGGGCUCGCGCUA
Acr-1-1    UGUUUCGAUCCGUCAACCGCGGAGGGACAGGCGCGCGUUUUCGACAUUUCCGAUCCGGCCAUGGCCGGCAGGCUUCCGCCCUGCCCCGCCGCCAUCCCCUCGACAUUCCCGACAUUUCCGCCACAACAAGAAGGCGUUUCAUGAUCAAGACACUUGCG
Acr-1-2    UGUUCAUAUCCGCUACAGCGGAAGAGGACGGGUGCCAUGGUGCGUAUUGGCCGGAACUGCCUUCAUUCAGCCCGACGGAACCAUCGGUUGCGCUCAUGACCGUGCGCGACGCCAUUCUGCAGGCCUGCCAGGUGGCGUUCACGAUCGCGGCGGCGCCGUU
Neu-1-1    UAUAUACGGGACUGGCCGGACAGGCAACGCGUGAGUCAUGGUUUGAGAAGUUCCUGGUUGGCGUAAGCAGAAUGGUAACAGUCGAAGUUAAUGCAUUUGCAUUGAAUAGACAAGGAAGUUGCAUUACCAUACAAGAACAUCCAUUCCUUUAGCAGGAGAA
Dac-1-1    UACAGAACCUGGCUUGUUCAGGGCUGUAGGCGCUCGCGCAUGCAACUCGUCGUUGGUCAGGCCGAUUAAUUCGAGCUACCUGACCAAUGCGCAAAUUCCCCCAUUUCGAACAGCUUGAACUGCUUCCCUAUCUGGGCAAGUGGUUCAUCUUCGCCACCCU
           ..................................................................................................................................................
           ..................................................................................................................................................
           ..................................................................................................................................................
           ..................................................................................................................................................
           ..................................................................................................................................................
           ..................................................................................................................................................
           URC●●●●●●●●●●●●●●●●●●●●●●●●●●●●●●●●●●●●●●●●●●●●●●●●●●●●●●●●●●●●●●●●●●●●●●●●●●●●●●●●●●●●●●●●●●●●●●●●●●●●●●●●●●●●●●●●●●●●●●●●●●●●●●●●●●●●●●●
           ———3′
```

Cvi-1-1 UACGGUAUUCCUGGCACGGAAGCUGUAGGCGCGCCCUAUCCCCUGUCCGUCCAGGCGUUUAGAUUCGACUGUAUUGGACAUUCCAUGAUGCAAAGAAAAUUGCCGGCUCUGUCAUUUGCGCUGUCUUUCAUCGGCAAAUGGUCGUUGCUUUCCGCGCU
Dac-1-2 UGCCUUGGUGGAUGGGUCCUGCCGGUGGCGGGGCCAUGUCUUGAUCCUGCCCGGCACGCCCGUCCCGGUUAUUCGAUGAAAGGACGGUCAUGGGUUCUACGGUUUCAUUCUGGUGUUCGUUGGCGGCGGCCUCGGCUCCGCGCUCAGGCAUGGCGCGA
Neu-1-2 UACAGGACCUGACCUUCGUUAGGGCUGUAGGCGUUCGUGUUUACAUCCUUUAGCAUUGUCAGCCGAUUGUUUCGAGCGAAUGACCAAAAUGUCCGAUGCAGAUAGACACCGACCAAGACGGAGACUCCAAUGACGACGUCUAGUACUCAAACCCUCCAU
Neu-1-3 UACGACUGGCCGGACAGGCCGCGCGUAGGUAUUGCUGCCUGUCCUCAAGAAAAUUUCAAGAGAGGACGAUACCGUGUGGAAACCUAUUCUCGCCAUUGCCCUGGGUUCAACGCUUGGCGGACUUCUGCGAUGGGGCUUGGGACUCAAGCUCAACAACCUG
Bph-2-1 UACGAGUUCCUGGGUGCAGGAGGUCGUAGGCCGUCCAUGCGACGCCCCCUGUCGCCCAGGUUUGACGCACACGCAAACCUGGACCGCAAUGAAACACCUGAAGUCUAUUGAGCAGUUCGCGAUGGUUCCGUACCUCGCGCGCUGGCUGCUGCUCUCGUGC
Bxe-1-1 UGCUCGCCCGGAUGAUUGCGGACGCAGCGUCGCGCUCCACGCUCAGCCCGGUUUGUCGACUCUCCGGUUUUGCCAGGCCUCCCAUGUAUUGGUCCAUUCUCGCCGUCGGAAUCGGCGGCGCGCUCGGUUCGUUAUUUCGCUGGUUUCUGGGCAUCCGCCU
Bph-2-2 UGCUUCGUCCGGCUGAACGCGGAGGUGGCAUCGCGCUCCUCGCUCUGCCCGGUUUGUCGACACUCCAGUUUUGCCAGGCCUCCUCCCAUGUAUUGGUCUAUUCUCGCUGUCGGCAUCGGCGGCGCGCUCGGUUCGUUAUUCCGCUGGUUUCUGGGCAUUC
Bph-1-1 UGCUUCGCCCGGAAGCUGCGGACGUUGCAGUGUCACGCUCCGCCGCAGCCCGGUUCGUCGACGUUUGGCUUUCCAGGCUUGCUCAUGUAUCUGUCCAUCCUUGCCGUCGGCAUCGGCGGCGCGCUCGGUUCGCUGUUCCGCUGGUUUCUCGGUCUGCGCC
Bgr-1-1 UGCUUCCCCGGACCCAUGCGGAGGCGGCAUCGCGUCUCCCCGCUCAGCCCGGCUUGUCGACUCCGGUUUCAGCAAAGUCCACUCAUGUACUGGUCCAUCUUGGCCGUCGCUAUCGGCGGUGCGCUCGGUUCGCUGUUUCGCUGGUUUCUCGGCCUGCGUC
Bsp-2-1 UGCUACGCCCGGCUAUGCGGACGCGGCAUCCACUCGCCCGCAUCAACCGGUUUGUCGACAUUCCGGUUUCGAUCCUCUUCCAUGUAUUGGUCUUUUCUCGCGGUCGCGAUUGGCGGCGCACUCGGCUCGCUGUUCCGCUGGUUCCUCGGCUUUCGCCUGA
Bub-1-1 UGCGCAUUCCUGGGUUUCGGGAGGUCGCAGGCCGUUCAUGCACCGUCCAUGCGGUAUCCCGCCGCCCAGGUUUGAUGUUCUGUCGAAUCUGGAAUCACCUGAUGCUCAACACUUCCGCCGACUUCCUCGCGACCGUGCGCUACGUGUGCCGCUGGCUCGC
Rpi-2-1 UACAAGUUCCUGGUCGAAGGAAGUUGUAGGCACACGCCCAAACGACGUUCUGUCGCUCAGGUUUGAGUUCUUCGAAUCUGGUCAUCAUGAAGAAAAUGGCUACACGCUCCGAGCCGCUGCUCAUGUUCGGGUAUCUGCUGCGCUGGCUUUCGCUCGGUUC
Bmu-1-1 UGCGUGUUCCUGGGUGUCAGGAGGUCGCAGGCCGUCCACGCGGUGUUCUGCCGCCCGGUUUGAUGUCCUGUCGAAUCUGGAAACCAUGUUUUUCUCGACUUCUGCCGAUCUUUUCGCGACGGUGCGCUACGUCUGCCGCUGGCUUGCGCUCUCGGCCCU
Rso-1-1 UACAGGUUCCUGGACAGGGAAGUUGUAGGCACGCGCCCAUACGGCGUUCCGCCGUCCAGGUUUGAGUGCUUCGAAUCUGGUUCUCAUGAAAAAGACAUACGCUCUUCCGGAGCAAGUGGCCAUGCUGCCGUAUCUGCUGCGCUGGCUGUUCCUGGCCUCG
Rso-3-1 UACAAGUUCCUGGACAGGGAAGUUGUAGGCGCACGUCCAUACGGCGUCCUGCCGUCCAGGUUUGAGUGCUUCGAAUCUGGUUAUCAUGAAGAAAAAGUUCGUUCCCCCGGAGCAAGUGGCCAUGCUGCCGUAUCUGGCGCGCUGGCUGUUCUUGGGCUCG
Rso-2-1 UACAAGUUCCUGGACAGGGAAGUUGUAGGCGCACGUCCAUACGGCGUCCUGCCGUCCAGGUUUGAGUGCUUCGAAUCUGGUUAUCAUGAAGAAAAAGUUCGUUCCCCCGGAGCAAGUGGCCAUGCUGCCGUAUCUGGCGCGCUGGCUGUUCUUGGGCUCG
Bce-19-1 UGCGUGUUCCUGGGUCGUCAGGAGGUCGCAGGCCGUCCACGCGGUAUUCUGCCGCCCAGGUUUUGAUAUCCCGUCGAAUCUGGAAAUCAUGUUUUUCACCACUUCUGCCGAUCUUUUCGCGACCGUGCGCUACGUGUGCCGCUGGCUCGCGCUUUCAGCC
Bce-18-1 UGCGUGUUCCUGGGUCGUCAGGAGGUCGCAGGCCGUCCACGCGGUAUUCUGCCGCCCAGGUUUUGAUAUCCCGUCGAAUCUGGAAAUCAUGUUUUUCACCACUUCUGCCGAUCUUUUCGCGACCGUGCGCUACGUGUGCCGCUGGCUCGCGCUUUCAGCC
Bce-15-1 UGCGUGUUCCUGGGUCGUCAGGAGGUCGCAGGCCGUCCACGCGGUAUUCUGCCGCCCAGGUUUUGAUAUCCCGUCGAAUCUGGAAAUCAUGUUUUUCACCACUUCUGCCGAUCUUUUCGCGACCGUGCGCUACGUGUGCCGCUGGCUCGCGCUUUCAGCC
Bam-2-1 UGCGUGUUCCUGGGUCGUCAGGAGGUCGCAGGCCAUCCAUGCGGUAUUCUGCCGCCCAGGUUUUGAUGUCCUGUCGAAUCUGGAAAUCAUGUUUUUCUCGACUUCUGCCGAUUUUUUCGCGACCGUGCGCUACGUGUGCCGCUGGCUCGCGCUCUCGGCC
Bam-3-1 UGCGUGUUCCUGGGUCGUCAGGAGGUCGCAGGCCGUCCAUGCGGUAUUCUGCCGCCCAGGUUUUGAUGUCCUGUCGAAUCUGGAAAUCAUGUUUUUCUCGACUUCUGCCGAUUUUUUCGCGACCGUGCGCUACGUGUGCCGCUGGCUCGCGCUCUCGGCC
Bam-1-1 UGCGUGUUCCUGGGUCGUCAGGAGGUCGCAGGCCGUCCAUGCGGUAUUCUGCCGCCCAGGUUUUGAUGUCCUGUCGAAUCUGGAAAUCAUGUUUUUCUCGACUUCUGCCGAUUUUUUCGCGACCGUGCGCUACGUGUGCCGCUGGCUCGCGCUCUCGGCC
Bam-4-1 UGCGUGUUCCUGGGUCGUCAGGAGGUCGCAGGCCAUCCAUGCGGUAUUCUGCCGCCCAGGUUUUGAUGUCCUGUCGAAUCUGGAAACCAUGUUUUUCUCGACUUCUGCCGAUUUUUUCGCGACCGUGCGCUACGUGUGCCGCUGGCUCGCGCUCUCGGCC
Bth-6-1 UACGCGUUCCUGGGUGCAGGAGGUCGUAGGCCAUCCGUGACAAGCGGCGUCCUGCCGCCCAGGUUUGAUGUCCUGUCGAAUCUGGAAAUUCAUGAAACGUCUGCACACUCUCGAGCCCGUCGCGGCGCUCGCGCAUCUGUGCCGCUGGCUCGCGCUGUCG
Bps-14-1 UACGCGUUCCUGGGUGCAGGAGGUCGUAGGCCAUCCGUGACAAGCGGCGUCCUGCCGCCCGGGUUUGAUGUCCUGUCGAAUCUGGAAAUUCAUGAAACGUCUGCACGCUUUCGAGCCCGUCGCGGCGCUCGCGCAUCUGUGCCGCUGGCUCGCGCUGUCA
Bps-7-1 UACGCGUUCCUGGGUGCAGGAGGUCGUAGGCCAUCCGUGACAAGCGGCGUCCUGCCGCCCGGGUUUGAUGUCCUGUCGAAUCUGGAAAUUCAUGAAACGUCUGCACGCUUUCGAGCCCGUCGCGGCGCUCGCGCAUCUGUGCCGCUGGCUCGCGCUGUCA
Bps-5-1 UACGCGUUCCUGGGUGCAGGAGGUCGUAGGCCAUCCGUGACAAGCGGCGUCCUGCCGCCCGGGUUUGAUGUCCUGUCGAAUCUGGAAAUUCAUGAAACGUCUGCACGCUUUCGAGCCCGUCGCGGCGCUCGCGCAUCUGUGCCGCUGGCUCGCGCUGUCA
Bok-2-1 UACGCGUUCCUGGGUGCAGGAGGUCGUAGGCCAUCCGUAAUACGCGGCGUCCUGCCGCCCGGGUUUGAUGUCCUGUCGAAUCUGGAAAUUCAUGAAACGUCUGCAAACCCUCGAGCCUGUCGUGAUGCUCGCGCAUCUGUGCCGCUGGCUCGCGCUGUCG
Bps-17-1 UACGCGUUCCUGGGUGCAGGAGGUCGUAGGCCAUCCGUGACAAGCGGCGUCCUGCCGCCCGGGUUUGAUGUCCUGUCGAAUCUGGAAAUUCAUGAAACGUCUGCACGCUUUCGAGCCCGUCGCGGCGCUCGCGCAUCUGUGCCGCUGGCUCGCGCUGUCA
Bps-19-1 UACGCGUUCCUGGGUGCAGGAGGUCGUAGGCCAUCCGUGACAAGCGGCGUCCUGCCGCCCGGGUUUGAUGUCCUGUCGAAUCUGGAAAUUCAUGAAACGUCUGCACGCUUUCGAGCCCGUCGCGGCGCUCGCGCAUCUGUGCCGCUGGCUCGCGCUGUCA
Bth-7-1 UACGCGUUCCUGGGUGCAGGAGGUCGUAGGCCAUCCGUGAGCAAGCAGCGUCCUGCCGCCCGGGUUUGAUGUCCUGUCGAAUCUGGAAAUUCAUGAAACGUCUGCACACCCUCGAGCCCGUCGCGGCGCUCGCGCAUCUGUGCCGCUGGCUCGCGCUGUCG
Bok-1-1 UACGCGUUCCUGGGUGCAGGAGGUCGUAGGCCAUCCGUAAUACGCGGCGUCCUGCCGCCCGGGUUUGAUGUCCUGUCGAAUCUGGAAAUUCAUGAAACGUCUGCAAACCCUCGAGCCUGUCGUGAUGCUCGCGCAUCUGUGCCGCUGGCUCGCGCUGUCG
Bps-16-1 UACGCGUUCCUGGGUGCAGGAGGUCGUAGGCCAUCCGUGACAAGCGGCGUCCUGCCGCCCGGGUUUGAUGUCCUGUCGAAUCUGGAAAUUCAUGAAACGUCUGCACGCUUUCGAGCCCGUCGCGGCGCUCGCGCAUCUGUGCCGCUGGCUCGCGCUGUCA
Bps-10-1 UACGCGUUCCUGGGUGCAGGAGGUCGUAGGCCAUCCGUGACAAGCGGCGUCCUGCCGCCCGGGUUUGAUGUCCUGUCGAAUCUGGAAAUUCAUGAAACGUCUGCACGCUUUCGAGCCCGUCGCGGCGCUCGCGCAUCUGUGCCGCUGGCUCGCGCUGUCA
Bps-9-1 UACGCGUUCCUGGGUGCAGGAGGUCGUAGGCCAUCCGUGACAAGCGGCGUCCUGCCGCCCGGGUUUGAUGUCCUGUCGAAUCUGGAAAUUCAUGAAACGUCUGCACGCUUUCGAGCCCGUCGCGGCGCUCGCGCAUCUGUGCCGCUGGCUCGCGCUGUCA
Bma-2-1 UACGCGUUCCUGGGUGCAGGAGGUCGUAGGCCAUCCGUGACAAGCGGCGUCCUGCCGCCCGGGUUUGAUGUCCUGUCGAAUCUGGAAAUUCAUGAAACGUCUGCACGCUUUCGAGCCCGUCGCGGCGCUCGCGCAUCUGUGCCGCUGGCUCGCGCUGUCA
Bps-1-1 UACGCGUUCCUGGGUGCAGGAGGUCGUAGGCCAUCCGUGACAAGCGGCGUCCUGCCGCCCGGGUUUGAUGUCCUGUCGAAUCUGGAAAUUCAUGAAACGUCUGCACGCUUUCGAGCCCGUCGCGGCGCUCGCGCAUCUGUGCCGCUGGCUCGCGCUGUCA
Bps-8-1 UACGCGUUCCUGGGUGCAGGAGGUCGUAGGCCAUCCGUGACAAGCGGCGUCCUGCCGCCCGGGUUUGAUGUCCUGUCGAAUCUGGAAAUUCAUGAAACGUCUGCACGCUUUCGAGCCCGUCGCGGCGCUCGCGCAUCUGUGCCGCUGGCUCGCGCUGUCA
Bps-18-1 UACGCGUUCCUGGGUGCAGGAGGUCGUAGGCCAUCCGUGACAAGCGGCGUCCUGCCGCCCGGGUUUGAUGUCCUGUCGAAUCUGGAAAUUCAUGAAACGUCUGCACGCUUUCGAGCCCGUCGCGGCGCUCGCGCAUCUGUGCCGCUGGCUCGCGCUGUCA
Bth-8-1 UACGCGUUCCUGGGUGCAGGAGGUCGUAGGCCAUCCGUGACAAGCGGCGUCCUGCCGCCCAGGUUUGAUGUCCUGUCGAAUCUGGAAAUUCAUGAAACGUCUGCACACUCUCGAGCCCGUCGCGGCGCUCGCGCAUCUGUGCCGCUGGCUCGCGCUGUCG
Bsp-1-1 UGCGUGUUCCUGGGUCGUCAGGAGGUCGCAGGCCGUCCAUGCGGGUUUCUGCCGCCCAGGUUUUGAUGUUCCGUCGAAUCUGGAAAUCAUGUUUUUCACGACUUCUGCCGAUCUUCUCGCGACCGUGCGCUAUGUGUGCCGCUGGCUCGCGCUCUCGGCC
Bce-17-1 UGCGUGUUCCUGGGUCGUCAGGAGGUCGCAGGCCGUCCACGCGGUAUUCUGCCGCCCAGGUUUUGAUGUCCCGUCGAAUCUGGAAAUCAUGUUUUUCACGACUUCUGCCGAUCUGUUCGCGACCGUGCGCUAUGUGUGCCGCUGGCUCGCGCUUUCAGCC
Bvi-1-1 UGCGUGUUCCUGGGUCGUCAGGAGGUCGCAGGCCGUGCAUUGGGGAUUCUGCGGGCCCAGGUUUUGAUGUUCCGUCGAAUCUGGAAAUCAUGUUUUUCUCGACCUCUGCCGAUUUCUUCGCGACCGUGCGCUACGUGUGCCGCUGGCUCGCCCUGUCGGC
Bam-2-2 UACGUGCCCUGGGGAAAGGGCGGUAGGACUAUUGGUCAUGCACCGCUCCCAAGGCAACCGAGAAAAGUGUAUUUUUCGUGUACUUUCGGAGAAAUCAUGCGGUGCGUUGGUCCUGCCUUUAAAAGGAAACUUCUCCGGCAACGGCGUCGAUCCGAACCUC
Bam-2-3 UACACACCCUGGGAACGGGUGGUAGGGCUAUUGGCUGUGCGCCGGUCCCCAAGAACGAGUAUUUGUUUUUCGGGGAAGUCAUGCGGUGCAUUGAUCUGCUUGUUAAAAGAAUGUCUUUCCGGCGUCGUGUGGCGCUGGCUGUAC
Sfu-1-1 UACUUCGAAUCAUGGAGUGGAAGUGUUUUUUUUAACUUGCCGCCGGUUCCCGAGAUUUCAGCUCAACACAGGGAGGCACACGAUGUUCGACUAUGCCCGGAUGCGCGAUGAUCUUUGUGAAGUGGCCGAACGCCUGGACAGCGCGGUGCGUGAAGAGA
Dpi-1-1 UGCCGGACCUGUGUGUCGGCAGGAGUCAUUUUUUUGUGCCGCGUAUAGGUCUUCGGCAGCUUCCUUGAAGGAGGGGACGAUGGGACGUGGCAUUUCACCGGCGUUCAUUUUUAUCUGCUCAUGGGCAUCGUCAGCCUGUUCGCGGACAUGGCCCA
Dol-1-1 UGCCGAUACCAUUAUAUAUAAUGUAUGGCAGGUGUUGUGAGGGCCUGCCGGAAGCGGUUUUUUUGUUUAUGGAAAGGCAUUGACAGGGGUGCAAAGGGUAUACACGGGCAAGCUGACGCUUGGCCCGUGCCACCCGGUAAAUAUGAUCCACACAAUAACG
Dac-2-1 UGUAUGAGGACUUUGUUCGCAAGGUUUUCGUACAGGAGUUUUUUGUGGAUAGCUUUUUUGUAAAAGGGUCUGUUUGAUCCAUUUUCCCGUGAUCAUUGUGGGAUCACAAUGGUGUUUAAGGACAAGGAGAAACUAUCAUGCCGAUACGCUGGGAUGUGCG
Xca-1-1 UACAAGAACUCUGGCCCGGCCAGGAUUGUAGGCGUGUCUCUGCUCACAUCCCCCUGCCGUGCCAGCCCUCACCUUCCAACCGACAGGCUGCCAUGUCCGGAUUCCGCCGCCCCGAGUAUUUCGAUCUUUUCCUACAGCUUGCCAAGUGGCUGGCGAUC
Xor-1-1 UACAAGAACUCUGGCUCGGUCCGGGGAUUGUAGGCGUGUUCGUGCUCACAUCUCCCUGCCGUGCCAGCCCUCAUGUCCACAGACGAUAGGCUGCCAUGUCACGAUUUCGUCGCCCCGAGCACCUCGACCUUUUCACUCAUCUCGCCAAGUGGCUGGUGAU
Xor-4-1 UACAAGAACUCUGGCUCGGUCCGGGGAUUGUAGGCGUGUUCGUGCUCAGAUCUCCCUGCCGUGCCAGCCCUGAUGUCCACAGACGAUAGGCUGCCAUGUCACGAUUUCGCCGCCCCGAGCACCUCGACCUUUUCACUCAUCUCGCCAAGUGGCUGGUGAU
Ppr-1-1 UAACAACAUUGGCAUAGAAUGGCCAGAGGAAGCUUUUCCGUGGAUCCCUACAUCCAUAGAAAACUGCAGUUGUUAGAUCGUAAUUAUCCUCUACCGUUCUCUGCCCUUUAUUUAAAGCACAAUCAUUCAGUUUGUACGGUCAAAUCGUUCGCCUUUAUU
Ppr-2-1 UAACAACAUUGGCAUAGAAUGGCCAGAGGAAGCUUUUCGUGGAUCCCUACAUCCAUAGAAAGCUGCAGUUGUUAGAUCGUAAUUAUCCUCUACCGUUCUCUGCCCUUUAUUUAAAGCACAAUCAUUCAGUUUGUACGGUCAAAUCGUUCGCCUUUAUU
Psp-3-1 UAACAACAUUCGACUUAAAAUAGUCGAGGACCGUGUAUUUGUUUAAAUUUAUAAUUUUAAUUAAUUAUAUUAAUUUUGCAUUCGUCGCGUUGUAGUUCAUUUUUGUCCUCUUCUAUUUUCUGCUCGAUAAAGAAUUAUCAAUUCAACACGGCUGAGCGU
Van-1-1 UAACAACAUUCGACUCUAAAUCGUCGAGGACCGUGUAUUUGUUAAAAUGGUUAAUUAUAAUUUUAAAUAGUUAUAAUAAUUUUUGCAUUCGGCGCGUUGUUAUUUCAUUUUUGUCCUCUUCUAUUUUCUGCUCGAUAAAGAAUUAUCAAUUCAACACGGC
Eta-1-1 UGAUAAAACCGACUCUUUUAACCGGAGGUCGGGGUAUCAGGCGAUUUUUUUGCCUGCUGACGGCCUCCUCAAUAACCACUGAGAAUUUUAUGAUAAAACCGUUAUUUAGCGGUGAUGAUCGGCGGCUGUGGCUGGCUGUGUUAUUCGCUGGCUGUUGGCCGU

....
....
....
....
....
....
URC•••

3′

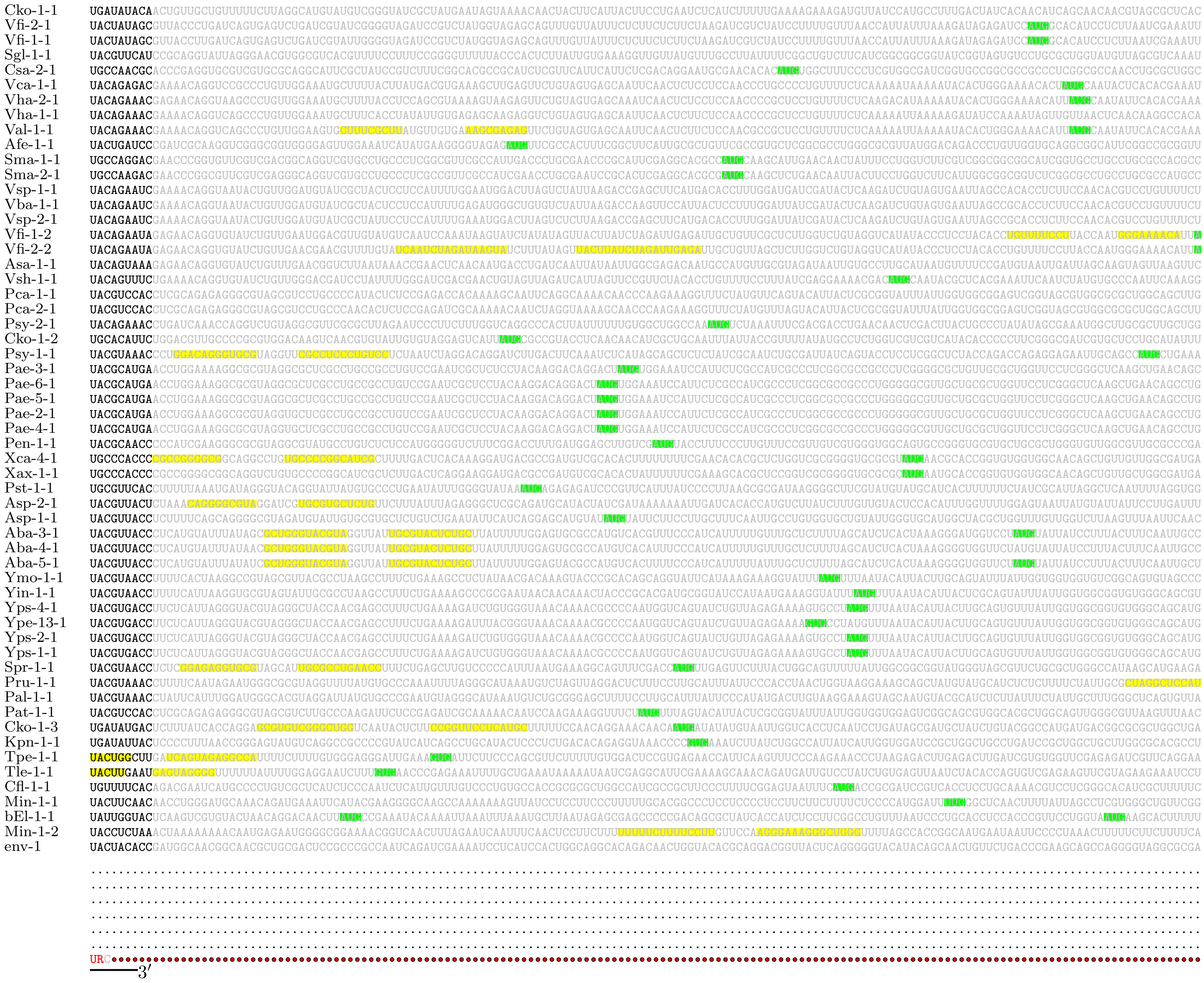

Cko-1-1 UGAUAUACA
Vfi-2-1 UACUAUAGC
Vfi-1-1 UACUAUAGC
Sgl-1-1 UACGUUCAU
Csa-2-1 UGCCAACGC
Vca-1-1 UACAGAGAC
Vha-2-1 UACAGAAAC
Vha-1-1 UACAGAAAC
Val-1-1 UACAGAAAC
Afe-1-1 UACUGAUCC
Sma-1-1 UGCCAGGAC
Sma-2-1 UGCCAAGAC
Vsp-1-1 UACAGAAUC
Vba-1-1 UACAGAAUC
Vsp-2-1 UACAGAAUC
Vfi-1-2 UACAGAAUA
Vfi-2-2 UACAGAAUA
Asa-1-1 UACAGUAAA
Vsh-1-1 UACAGUUUC
Pca-1-1 UACGUCCAC
Pca-2-1 UACGUCCAC
Psy-2-1 UACAGAAAC
Cko-1-2 UGCACAUUC
Psy-1-1 UACGUAAAC
Pae-3-1 UACGCAUGA
Pae-6-1 UACGCAUGA
Pae-5-1 UACGCAUGA
Pae-2-1 UACGCAUGA
Pae-4-1 UACGCAUGA
Pen-1-1 UACGCAACC
Xca-4-1 UGCCCACCC
Xax-1-1 UGCCCACCC
Pst-1-1 UGCGUUCAC
Asp-2-1 UACGUUACU
Asp-1-1 UACGUUACC
Aba-3-1 UACGUUACC
Aba-4-1 UACGUUACC
Aba-5-1 UACGUUACC
Ymo-1-1 UACGUAACC
Yin-1-1 UACGUAACC
Yps-4-1 UACGUGACC
Ype-13-1 UACGUGACC
Yps-2-1 UACGUGACC
Yps-1-1 UACGUGACC
Spr-1-1 UACGUAACC
Pru-1-1 UACGUAAAC
Pal-1-1 UACGUAAAC
Pat-1-1 UACGUCCAC
Cko-1-3 UGAUAUGAC
Kpn-1-1 UGAUAUUAC
Tpe-1-1 UACUGGCUU
Tle-1-1 UACUUGAAU
Cfl-1-1 UGUUUUCAC
Min-1-1 UACUUCAAC
bEl-1-1 UAUUGGUAC
Min-1-2 UACCUCUAA
env-1 UACUACACC
URC
3′

```
env-2   UUAGGGGCAACCGAUGGUUGUAGGGGGAGUAACGGCGAUGGGAUUCAACAGCAUUUUGUGGCCUGAUGGCCAGAAAGGAAAUCUUGCAGAGAAAGAUCCAGAGUUUUUUGUUGAUCUCAACAUUGAUCAGAUCAUAAACGGAGUAAUUGAGGGGUAUGGA
env-3   UGCCGGAGUUGGUCUUUGGAGUAAGACUGGCAGGAAAAUCUAUAUCUGCUUGUCUGAAAAAAAGAAUCCGGCUUCCGUUUUCGAAGAAGUUAUUUUUAUUGUUUGACAAAAUAUAGACAAGAGAUGAAGAUUCAGAAAAAUCAAAACAUUGCGAUGAAAC
env-4   UACGCAUAGCCGUAGGAGGGUAUGCGGUUGGCGCAAUUCCAGUCGGAGGCUGAAAUGACCUGGUUCUUGGUCGGCGUCGGUGGAGCCCUCGGAAGCAUCGCGCGGCACGGACUGAACCAUCUGGUUCAUCAGCGGAAUCUCAGCGCAACGUUUCCAGUAG
env-5   UGCGGGGUUGAACCGUGGGAGCCCGCCUGAAGGCCGCGCACCCCGAGCCCCGCACGAUACGCCGGCAACAGGGGCUCUCGAUGGAAUCCUGGUUUGGCAUCGCCACGCUGUGGUUUGGCCUCGCGCUGCUCGCGACCUUUCUCGCCACGCGGCUUCGCGU
env-6   UGCUUUUUUACGCAGAUGCUGUCGGCUUUUUUAUUUUUAUGGAGAUUCUGUUUAUGAAAUCAAUCAAACUGCAAUCCAUUCUCCCCACGCUCCUGNUGCCUCGCUCCGCCGGCGGUGUAAUGAAUGGGAUACUGCCGCUGUUCCGAGUGCUGUUCCUGUA
env-7   UGUGAGAGAUGGAUUUGUUUUUUCUUGCAGGAGACCGUUCGAGGUAGAUCUGUUGUCCCUUUUUUCUUGUGCCCGUUGGAGGGCUCCUGGGUCAGAUUCGAUUCCAGUCGGGCUGUCAUGCUGCUUGCUUUCGAUUGACAAUCGGGAAGCGAUGUCUUU
env-8   UGAUCUUUAAAUAGGUAGAGUUCAAAUGGACGUUCAGCUUGGAGAAACCAUAAUUCAGAUAUUUGGAGUUUUACUUUUAGCUCCGCUUGUGGCAGGUAUUUACGAAAACCUCAAGGCAAAAACCGAAUUCAGACGUGGGCCGAGUAUUUUUCAGCCAUAC
env-9   UGCCCCGUCAUGCGUGGGUGGGAGUCUGCCCUCGCACCAAUGAAUGAGCGAGAUGACGAUGGAUCCCGUGCAGUCGCGUGGCCUCCAAUGCGGAGGAUGGUCAUGAGUGGACACAUGGCGGCGAAAGAUGGCGCAGUGCCUCAGCCGCACCCCUCCCGAA
env-10  UACAAUUUGAAUGUAUAACUCUUAAUUGUCAGCUUAUGCUCAAGACUUGACAGCUUCAGCC.................................................................................................
env-11  UACCUAUAAAAUCUAUCAAUUGUACAGGAGAUUUUAAAUUGCAAAAUUAUUUAAUUGUGUUCUUAGGAGCCGGUUUAGGUGGUGUACUGCGUUAUUGGGGUUCAGAUUUUGUUUAUAAAUUUCUUCCUUUCACUUUUCCAUAUGGUACUUUAGUUGUCAA
env-12  UACACACGAUUUGACAUUGCAUGAAAUCGUUGUGUCUUCAGCAGGAUUCAGCGGUUUCAGGAAAGGCUACUAUGGGUUCGCAAUCCCCAUUCGCAGGCGCCCGGCCGAUUACGCACACGGCCACUCCCCCGGCGCGCAAGCUCCCCGAUAUUCAUCUUGA
env-13  UACACACGAUUUGAUAUUGCAUGGAAUCGUUGUGUCUUCAGCAGGAUUCAGCGGUUUCAGGAAAGGCUAUUAUGGAUUCGCAAUCCCCAUCCGCAGGUGCCCGGCCGAUUACGCACACGACCACUCUCCCGGCGCGCAAGCUCUCCGAUAUUCAUCUUGA
env-15  UACACACGAUUUGACAUUGCAUGGAAUCGUUGUGUCUUCCGCAGGAAUCAGCGGUUUCAGGAAAGGCUAUUAUGGGCUCGCAAUCCCCAUCCGCAGAUGCCCGGCCGGGCACGCGCACGGCCACUCCUCGGGGGGCAAGCUCCCCGAUAUAAUCUUGAAA
env-16  UACACACGAUUUGACAUUGCAUGGAAUCGUUGUGUCUUCAGCAGGAUUCAGCGGUUUCAGGAAAGGCUAUUAUGGGCUCGCAAUCCCCAUCCGCAGAUGCCCGGCCGGGCACGCGCACGGCCACUCCUCCGGCGCGCAAGCUCCCCGAUAUCCAUCUUGA
env-17  UACACACGAUUUGACAUUGCAUGGAAUCGUUGUGUCUUCAGCAGGAUUCAGCGGUUUCAGGAAAGGCUAUUAUGGGCUCGCAAUCCCCAUCCGCAGAUGCCCGGCCGGGCACGCGCACGGCCACUCCUCCGGCGCGCAAGCUCCCCGAUAUUCAUCUUGA
env-19  UACGACAUAAGGUGUGCUGUACCUUAUGGCUUUCAGCGGUUGUGGCAUCGCCAUUCUUCCAUGUCUGUCAUAUUGGUUCGGCACCUCGCUGGAAAGAAGAUACAUGGCACGACAACAUAUCAUUCGAGUCGAUCUCAUAUGCGUGGUGUUCCUUGGGGGA
env-20  UACGACAUAGGGUGUGCUGUACCUUAUGGCUUUCAGCGGUUGUGGCAUCGCCAUUCUUCCAUGUCUGUCAUAUUGGUUCGGCACCUCGCUGGAAAGAAGAUACAUGGCACGACAACAUAUCAUUCGAGUCGAUCUUAUAUGCGUGGUGUUCCUUGGGGGA
env-21  UGCUUGAAACUGGUGGUUAAACAUGGACAUGAUCACACUAGCAGAGACCUUAACUCAGUACUUCUUUGUUGUUCUCAGUGCCCCAUUAUACGCAGGAAUACUUGCCAAGCUCAAGGGCAUGGUUGAAUCAAGAAGAGGUCCCAGUAUAUUCCAGCCUUAC
env-22  UACCAUGGGUGAUCCUGGAUCAGUGUUCCGGCACAUGUGGUGGGAGAGGCACUUCCCUGUUGUCCCCCUUCCUGUUUCGCCGAUGCACUCCCCAGACUCUUCUCGCCCCAAGCUCCUGAAAAAAUAUUUGGAUGGUCUGCGCACUUUUUUGACCUGGACC
env-23  UACUCCAUUGAGGAGGAGGUGCGUCGUGCUCGAGGCCGUUCUGAUCGUUCUGUUUCCGUCCAGCUUUCGAUUUUGUCCUUUCGUCUUCUGACCCUUCUUCCCGCUCCAUUCAGGGAAACGCUCCUCAAGGCCGCAGGACUUCUGUUUCUGGGAACCCUC
env-24  UGCAGAAUAGAGUGGAAGUGAUACAUUGACAUUUGAAAGUAUAAUAUUCAGGAGUAAUAAAGCCAUUGAUGAAAUUGACCAUAAAAAUGAGUUAUUUGAAGAUUUGUCGCUUAAAAUAAUCUUUAAAGGCAUUCUGGGAAACACAAAUAACCAGGAUUUU
env-25  UACGAUUUUUUCGUAGAAGUCAUUAUUUUUUUGAAAAGAGGCAGUAUGUUAUUAUCGUUAUCACUCAUCAUUUUUACUGGACUGAGUCUAUCUAAACUUGCAGAUUUACUGAAACUCCCUAAACU.....................................
env-26  UGUUGUUUAUUACGACAGGGUUAUCAGCUUUUUUUAUUAUCAAAAUAACAGGAGGAAAUUAUGUUAUCAUCAUUGGCUUUUAUUUUCAUCUUCGGUCUUGCCUNUGCGGCGCUCUGCGAAAGGAUAUAGACUUCCAAGAUUCUCGUAUGCUGCAACGGCA
env-27  UGCAAAGCGCAGGAGCUGUCAGCUAUUUUUGCGCUAAUAUUUAUUCAGAAAAGAGGAUUGUUAUGUUAAAAAGUAUAGCGCUGAUAAUGUUAAUUGGAAUGUCUGCAGGCUGGGUAUGCCGCAAGGUAAAGCUGCCGGGACUGCUUGGAAUGUUAUUUAC
env-28  UGCAUUGUGCAGGAGUCACCAGCUUUUUUUGUGUCCGUCCUGCAGGUCAAACACGCCCUGAGGGGCAGCCAUUUUGCAGUGCUGCGGGCGGAAACAAAAUACAGGAAUUCGGAGGUUACACGUAUGCGAACAAGUUAUCACUGUUUUUCU.........
env-29  UACGAUUUUAUGUCGCAGGAAGCAUCAGCUUUUUUUGCGUUCAUAAAUGCAUUUACUAUAUUUUUAAGGAGGCUUAUUAUGCUGACAUCUUUAUCACUUAUCUUUCUGGUUGGGCUCGUCAUGGGCGCAAUCUGCCAGAAGUUGAAACUGCCAAGAAUCA
env-30  UACGAUUAUUUGAAAUUAUAGUCGCAGAAGGCAUCAGCUUUUUUGCGUCUAUAGCAAAAUAAGGAGGCUAAAUCCUCUGCUCCUGCAAGAGCAGUCGCAUUUUGCUUAGCAAAACAAGGAGGAUUUAUGUUAGCAUCAUUAUCACUUAUUUUUAUUGUAG
env-31  UGCGACGAAAACCACGCAGAAGGUGUCAGCUUUUUUGCGUGUUGUAAGUAAGGAGGAACUUUUAUGUUACUGAGUAUUUCGCUGAUUUUGAUCGUUGGGAUGUCUAUGGGGUGGCUUUGUCAGAAGUACAGGCUGCCGAGUCUGCUGGGAAUGCUGGCAA
env-32  UGUGAUAACAAUUGUCGCAGGAAGCAUCAGCUUUUCUGCGUAAAAAGGAGGAAUUUAUAACAAUGUUAACAUCUCUGGCUUUUAUUUUUCUUGUGGGACUUUCCCUGGCAGCGAUUUGCCAGAUACUGAAACUACCGCG....................
env-33  UGUGCAUGGCACGGGAGUAUCAGCCUUUUUUGCUACAGGAACUAUCAGUUAUUAGUUCGGCGGUUCAACUUUGAUUAUGCAGGGAAUGAGUCAGAUUCGCGAAGCGUAUUUUAUCUCAUUCAGGGGAGAAAUAAAUCAGAAGAACAUGGAGGAAAGAGAA
env-34  UGUGCAAUGCACGGGGUUACCAGCUUUUUUUGAUGGAUUUUUAAGAUCCGGGAGAUUGAAAGGAGUUACUAUGACAAAAGAAACUAUCCAAAAAGAACAUCAGAUCCUGCAGCUGAAAUAUGCUGUCCUUGCAGUGGUCGUGGAAUCUGCGUCGGGAUC
env-35  UGUGUAAUGAACACAGGGGUUAUCAGCUUUUUUGAUGUUUGGAUUAUAUAAAAAAGAAAGGAUUUUAGGAAAAUGCAAAAGAAAGACUAUAUUAUACAACUGCUAAAAUAUAUCAUCAUGGCAAUCGUUGUAGGAAUUAUUGUAGGUACGAUCGAUGCCU
env-36  UGUGUAAUGAACACAGGGGUUAUCAGCUUUUUUGAUGUUUGGAUUAUAUAAAAAAGAAAGGAUUUUAGGAAAAUGCAAAAGAAAGACUAUAUUAUACAACUGCUAAAAUAUAUCAUCAUGGCAAUCGUUGUAGGAAUUAUUGUAGGUACGAUCGAUGCCU
env-37  UACUUCGAGCGAGUUGAAGUAGGGGUUUUUUUUAUUUUCAGGAUGACAUCGGGAGGUCUAUGCUACCCAUGGUUCUAGUCGGCGUCGGCAGUUUGAUCGGCGGCGUGUUGCGUUACGGUCUAAGCAUGUGGGUUUAUCGCGUGCUCGAUAAUCCGUCGUU
env-38  UACUAAAGUUUGGGGACAGCCCCUUUCUUUAGUAGGCGUUUUUUUAUUGAGGAGGAAUUUUUAUGGAAAAACAAUUACCGAAUCCAGUACGCAUUUUAGUGUAUAUGCUGAAGUGGCUGGUGAUCACAGCAAUUCUCGGUAUAUUCAUGGGGAGCUUGU
env-39  UACUAAAGUCUGGGGACAGCCCCUUUCUUUAGUAGGCGUUUUUUUAUUGAGGAGGAAUUUUUAUGGAAAAACAAUUACCGAGUCCAGUGCGCAUUUUAGUGUACAUGCUGAAGUGGCUGGUGAUCACAGCGAUUCUCGGUAUAUUCAUGGGGAGCUUGU
env-40  UAGGUUAUAUAAAAAAUACUUAGGAGGAUAAUUUAAUGGCUGAUGUAUUUCCAUUAAUUUUAGGGACAAUAAUACUUCUAGCUAGUGUAAUUUCUAUUAAAACUGGAAUUAGUGUAACAGUAGUUGAAAUUGCAAUAGGAGUAAUAAUAGGAAAUCUAGG
env-41  UAUGUUAUAUAAAAAAUAAUUAGGAGGAUAAUUUAAUGGCUGAUGUAUUUCCAUUAAUUUUAGGGAUAAUAAUACUCCUAGCUAGUGUAAUUUCUAUUAAAACUGGAAUUAGUGUAACAGUAGUUGAAAUUGCAAUAGGAGUAAUAAUAGGAAAUCUAGG
env-42  UGCUUCGCCCGGACGACUGCGGACGCGGCAUCACGCCUGCCCCGCGAGCCCGGUUUGUCGAUCUUUCGGCUUCGCCAGAGUACUUUUCCAUGUAUCUGUCCAUUCUCGCGGUCGGCAUUGGCGGCGCGCUCGGCUCGCUAUUCCGCUGGUUUCUCGGCAU
env-43  UGCGUGUUCCUGGGUCGUCAGGAGGUCGCAGGCCGUCCAUGCGGGUUUCUGCCGCCCAGGUUUUGAUGUUCCGUCGAAUCUGGAAAUCAUGUUUUUCACGACUUCUGCCGAUCUUCUCGCGACCGUGCGCUAUGUAUGCCGCUGGCUCGCGCUCUCGGCC
env-44  UGCUUUUUAUUCCCGGACCGGGAAUGAGCAGGCAUAUCUAUCAUGCAGGCAACGCGUCCGGGAUGGUCAACCCUCUAAGGAUUCGUUCAUGCGAUCGCUUCAUACUUCACCUCUCACCACGCUCUUGUUCCUCGGACAGUGGCUUUUUAUCGCCUUUAUC
env-45  UUCAAAUCGUCCAGAUAACCCNNNNNAAACCCN..............................................................................................................................
        ...............................................................................................................................................................
        ...............................................................................................................................................................
        ...............................................................................................................................................................
        ...............................................................................................................................................................
        ...............................................................................................................................................................
        ...............................................................................................................................................................
        URC............................................................................................................................................................
        ———3′
```

alignment positions 478 · · · 626

```
[1]Sso-1-1  UCUGCAACCUUACUAUGAUAUAAUUAAACUACUAAGAAAAGAAACCGUUAUUUCAAAUAACUCAUUAUCUCUAUUUGUUUAUGCUCCUUACAUAGUUUUUGGAUUUAUCUCCUUAUUUCCUUCGUCAUCCCAGUAGUUUAUCCAGUGC
Mse-1-1     UACCCAUAGUCUCCCAAUUUUACGUUAGUAACUUAACUUGGUAUUUUUGGUCAUGGUGGCCGGUGUCUAUCUGCUCUCAUCCAUUUACUCCCUCUUCUACGUGGAGAAGAGGGGAGUUCAGGAAAGGAAUUACUAUUAUUUCCUCAAU
Pto-1-1     AAAUACAUUAAAAAGGAGACCAUAGUACCCUACAAUGCCGGAUUUUUAUUUAUAUACGGACCAUAUCUUGUCUUUGCAAUACUUGUAUUAAUAUCAUUUAUAAUACCUGUUGUCAUUCCUGAGCCGGUGUAUUUCACGGCAUCAGCAGA
Tvo-1-1     AGGCUAUACAGAUUAUAAUCUUAGGCCAUUUUUUAUCACAAUGCCUAGGAGCUUAGAUACAGUCAAAUACAGGCAAGAGAUCUUCAAGGAUCUCCUAAAUUCAGAAGUUUUUGCAUUGAUGAAGGAUUUUUCAGAAAAAAUGAAUACCA
            ......................................................................................................................................................
            ......................................................................................................................................................
            ......................................................................................................................................................
            ......................................................................................................................................................
            ......................................................................................................................................................
            ......................................................................................................................................................
            ......................................................................................................................................................
```

Mhu-1-1	GGCUUUUUCAGACAAUCUGACACAGGAGUAUCUAUCCCGCUUUGUUAAUCAAACCCGGGGAUUAUCAGGGUUAUACCGGCUGUGGUAUCUUUUCUGGUCAUUCCGGUAUCUAAAAAGAACAAUUCCAGAACAACUUAUUGCUCACCUGU
Tvo-1-2	UAUGAUCUCUGGAAGCUUUCCCGGAAAAUUACCGUAAUACCUGCCGGCUCUGGUAUAUUCUUCAGGUAUAUCCCCUAUGUGCUUUUUGGGAUUUAUUCCCUCAUCGCAUUAAUAAUACCAGUAGUGAUACCAGUUCCUAUAUACUUUAC
CMe-1-1	UUUCCCGACUCCCGAAGCAACCCUGCCAUUGAAGGAGAAAUUAUGAUCCGUGAUACUGUGCGGGCAGUGGAUCCGUCCGGGGCAUCGACCGGGAAAAACCAGGCAGUCGGGUUCCGGGACCGCUUAUCAUGACGCCCGGUGCCGCCCCG
Mhu-1-2	GUUCAGGACAUCAACGGUGUGAAUAACCUGUUCAUGUCCAUAUCCAACCAUACCCCGGAAUACCGUACACCCGGGAAAUCCUCUCUUCUGGAACAUCUCAACAAGAUGUCGAUACGCCGGCUUUUUUCCGAUUUUUGCUGAUUCCGCGA
Abo-1-1	GACCUUGCCAAGUCCUUCAAGAAGGAGAUAGUUGUGCCAGAAGAUGCGGGUUCUCUUUUAUCUUGGCGCCCAUGCUCUCAUUCACCGCAUACCUGAUUCUACCAUGGGUUGUGCCCAUAGUGUUCGGCUCUCCCUCGUGGUUCGCACC
Abo-1-2	GACCUUGCCAAGUUCUUCAAGAAGGAGAUAGUUGUGCCAGAAGAUGCGGGUUCUCUUUUAUCUUGGCGCCCAUGCUCUCCUUCACCGCGUACCUGAUUCUCCCCUGGGUUGUGCCGAUAGUGUUCGGCUCACCCUCGUGGUUCGCCCC
Abo-1-3	GAGAAAAAAGGAACUGGAAGAGAAGAUAAAGAGGAUGAAAGAAGAGCUUAAAGAACUUACGGAAUUCUGUGAUAAGGCCGAGAGGGACCAGAAAAUGAUGGAGCAGUGGAUUGACGAACUUGGUAUGGAAAAUAAAGCACUUUUGAUGG
Abo-1-4	GAGAAAAAAGGAACUGGAAGAGAAGAUAAAGAGGAUGAAAGAAGAGCUUAAAGAACUUACGGAAUUCUGUGAUAAGGCCGAGAGGGACCAGAAAAUGAUGGAGCAGUGGAUUGACGAACUUGGUAUGGAAAAUAAAGCACUUUUGAUGG
Mhu-1-3	UCUUAUCUCGCGACUGGAAAGCGGACGAUUUCAUCUUGCUAUCCUUGGUCAGUUCAAACGGGGGAAGAGCACAUUUUUAAACGCCCUUCUUGGUGAUGCAGUUCUCCCAAGUUCAGUCAUCCCACUGACAGCAGUUCCGACUCUGAUUA
Msm-1-1	AUUCUUCCACAGCGAAUCUUGGAUGAUUUAUAUAGCUAGUUUUGGAGGAAUAUUGCUUACAUUUCUAGCUGGAGUCGAAAUUGAUGUUGACUUAAUGAAAGAUAAUUUUAAAGAAAGUUUUACAAUAGGAUUUUUAUCAUUUCUAAUUC
Msm-2-1	UCUUCCACAGCGAAUCUUGGAUGAUUUAUAUAGCUAGUUUUGGAGGAAUAUUGCUUACAUUUCUAGCUGGAGUCGAAAUUGAUGUUGACUUAAUGAAAGAUAAUUUUAAAGAAAGUUUUACAAUAGGAUUUUUAUCAUUUCUAAUUCCU
Msm-3-1	AUUCUUCCACAGCGAAUCUUGGAUGAUUUAUAUAGCUAGUUUUGGAGGAAUAUUGCUUACAUUUCUAGCUGGAGUCGAAAUUGAUGUUGACUUAAUGAAAGAUAAUUUUAAAGAAAGUUUUACAAUAGGUUUUUUAUCAUUUCUAAUUC
Mth-1-1	CUUAUAGGCCCCCAUGGAUUGAACCUCAUAUCAAAGAGCAUAAUGGAUAUAUCCCCCGACCUCAGGGUUAUAGCCCUCAUAAUAAUCCUUCUGAGGGCAGGGUUUGGUAUAAACCUCGAGAGCCUCAGGAAGGUGGGAAUGACUGCAGU
CMe-1-2	ACCGCGGAAUGGAUGCCAUUUGCCGCAGUGGCUAUUGGCGGGUUUAUCGGCGCCGUGCUCCGGUACCUGACCGAUACCGUGCUCGUCUUCCCGCUUGCCGGCACGCUUGCGGUGAACACAAUCGGCUGCUUCUUUUUGGGCAUCUUCAU
Tko-1-1	CUACUAUGCAGUCAAGAUGUACGACCUGCUCGGCCUCGGAGUCCCUGAGAAGGAGAAGACGAUAGAGUUCCUUGAGAGGGCAAUUCAGCCCCAGACGGCCGUUGUAGCCAUAGCAAUGGCCCUCGAAGGCCUUGCGAUACUUGGAGCGA
Ton-1-1	ACGACACGUACUACGCGGUAAAGAUUUACAACCUGCUCGGGCUCGAAAUUCCCGAGAGGGAGAAGACCGUGGAGUUCCUUUACACUUCCGCCCAGAUGCAGACCGCGGUGGUGGCCGUUGCAAUGGCCAUCGAGGCACUGUCAUUACUG
Tba-1-1	GACAAAUAUAAAUGACACAUACUAUGCCGUUAAAAUCUAUGACCUGCUCAAUCUUGAAAUUCCAGAGAAAGAAAAAACAAUUGAAUUUUUAAAUAGCUCUGCUCAGCCGCACAGGGCUACAGUUGCUUUAGCAAUGGCCGGCGAAGCAC
Tsp-1-1	CCUACUACGCGGUCAAGAUACACAGGCUUCUGAAUGUGGAGUUCCCGGAACCCGAGAUGACCAUAGAGUUCCUGAGCAAUGCAAUCCAGCCCCAGACUGCGGUGGUGGCAAUAGCGAUGGCACUCGAGGCCUCGCCCUCCUUGGAGCC
Pab-1-1	UGCUAUUAAGAUAUACUCGCUUCUUGGGAUUGAAGUUCCAGAGAAAGAGAAGACCAUUGAGUUCCUAUACGAUUCAGCUCAGAUGCAGACAGCUACUGUGGGGGUGGCUAUGGCUAUCGAGGGGCUUGCAAUCCUAGGAGCUAAGGAUC
Pfu-1-1	AUGCCGUAAAGAUAUAUUCACUCCUGGGAAUUGAAGUUCCAGAAAAAGAAAAGACAAUUGAGUUCCUUUAUAGCUCAGCCAAACCACAGGCUGCUACUGUUGCUAUUGCCAUGGCAGUUGAGGCGCUUUCAAUUUUAGGAGCUAAGAAC
Pho-1-1	AUGCGGUUAAAAUAUACUCACUCCUGGGAAUGGAGGUUCCAGAGAAAGAGAAGACUAUAGAGUUCCUCUACAACUCGGCCCAAAUGCAGACCGCCGCUGUAGGAGUUGCCAUGGCAAUUGAGGCACUCUCAAUUUUAGGCGCUAAAGAU
Afu-1-1	GGGAGGAUGUUCCAAGAAGAGAGAAAGUAGUAGAAUUUCUGAAAUCAAGGAUUCAGACAGAGAUUAACUCCCUUUUUUACACGCUGCACUCGCUAAAUCUACUUGGAGAGGACCUUCCAGACUACUCUAGCUUUCUCCUGAAAAGGCUU
Mst-1-1	UUAUACUUGCAAUACUAAUAAUACUAGCAAGUAUGAUUUCCAUAAAAUGGAGCAUAUCCAUUUCAAUUGUUGAAAUAAUAAUGGGCGUAAUUGCAGGAAAUCUAGGAAUAUUACAUGUUGAACCAUGGAUGAUAUACAUAGCAAGUGUU
Mac-1-1	UUUAGGUUUGGUCAUGUAUCUUUCUGAAUACCAAGGACUGUUUAGCGAAGAAACAAGAAUACUCCUGACAAUCGGUUUGCUAGGUGCCUUUACAACAUUAUCAACAUUUAGUUACGAAUCAUUCAGAUUAUUAGAAAGUUCAAAAUUAA
Mba-1-1	UUUAGGUUUAAUAUGUACUUUUCUGAAUACCAGGGCUUGUUUAGUGAUCAAACCAGAAUAUUUCUGACAAUAGGUAUUUUGGGAGCUUUCACAACGUUGUCAACGUUUGGUUAUGAAUCAUUCCGAUUGCUUGAUGACUCAAAAUUAA
Gvi-1-1	CUGGUCGGCUCGUUUAUGCUCGGUGGAUUGUUUGCGUGGAGCGUUGCGCUGCGCAUCGACCCGGCAUUGCUGCUGUUGGCAGGCACCGGUUUUUGCGGCGGUUUUACUACCUUCAGUGCUUUGAGCAUUGAGUGCCUGGUGCUGUUGCA
Ssp-2-1	GGGCUUUUUUGUCGGCCAGAUCCUGGGCCGGCUGGGAAUGCCGCCGCUAGUGGGGAUGAUCCUGGUCGGGAUCCUGUUGCAAGAGGUCUUGUCCCCAGAGCUGUUGGCGGCAGCCGGGGAUCUGCGCUUGGUGGCCGUCACGAUUAUCC
Ssp-3-1	UUGCCCAACGGCUCAGGAUUCCGGCCCUGGUAGGAAUGGUGCUUGUUGGUAUUGUCCUCGGCCCCAGGUGGGGAAUGCGAUCGCCCCAGAAGUUUUAGCGAUCGCCGCUGAUCUGCGGACAAUGGCGGUGAUGGUGAUCUUGAUGAAA
Sel-1-1	UGCCCCAGCGCUAAUUGGCAUGAUUCUCUGGGGUAUCGUGUUGGGGCCGCAGGUUAGUCAGUGGUUGAGCCCUGCAGUGCUUGACCAGGCAGGCAUCUGGCGCACCUUGGCAGUGAUGGUCAUUCUGGUCAAGGCAGGUCUGGGACUAG
Ssp-1-1	GGCAGUUGGCUGUGGAUCUUGGUGAUGGGCUUUUUUGUCGGCCAAAUCGUGGGUCGGUGGGGAUGCCCCCCCUGCUGGGGAUGAUCCUCGUGGGGAUCUUGCUGGGAGAUGUCCUAUCCCCGGAGGUGCUGGCAGCGGCGGGGGAUCU
Ava-1-1	CCGCCGCUUAACAGCACCCCCUUUAAUUGGGAUGAUUUUAGUUGGGAUUAUUCUUGGCCCUCAAGUCCGUAACCUCAUCAGUCAAGAUGUGCUUGGUGCUGCGGAUGAAUUGAGAACGGUGGCGGUGACAAUUAUCCUGAUGAAAGCUG
Nsp-1-1	CCGUCGCUUAACAGCACCGCCGUUAAUUGGCAUGAUUCUAGUCGGGAUUUUUGUAGGCCCCCAAGUUGGUAAUGUCAUCAGUCAAGAUGUGCUUGGCGCUGCUGAUGAAUUAAGAACGGUGGCGGUGACAAUUAUCCUGAUGAAAGCUG
Dth-1-1	CUACACUUUUUGUAAAUGUUUUAGGUAGCUUUAUUUUAGGACUUAUUACAGAAAUAAUAUUAGAGCAUAUAAGGAUGACAGAGACAUUGAGACAUAUGAUAAUAACAGGUUUUAUAAGUAGUUUUACUACUUUUUCUAGUUUUAUCCUU
Tye-1-1	AAGUCUUGCUAUUACUGGAACAGGAAUAUCAAUUGGAGAAAAUGACCUCUGGAUUAAAUUUAUUGCCUCUGUUGGAGCUAUAAUGCUUACAUUUUUAGCUGGAGCAGAGCUUGAGCCCGAUGUAUUAAAGUCUCGCUGGAAAGAGACAA
Taq-1-1	UUCGCCCCUCACGGGGGGAUGGGCCUGGAGGCCAUCUUCCGCAGGCCCCUUCAGGAUGAAAAGGCCGUGGUCCACCGCCAGAGGGUGGCCCAGGACCUGGAGGCCGAGCCGCUGGCCCAGGCCUUCCGGACCUUCUUCCAGGGCAUGGA
Dge-1-1	AUGUCCUUGGGUCGUUCCUGCUGGCCUUCAUCACCACGCUGGCCCUGCGCGGCCUCGUGUCUCCAGCCUGGCGUCUCGCCGUUGGCACGGGCUUCAUCGGCGCCUUGACCACCUUUUCCACCUUCCGCCUGGGAAAGCGACCUGAUGGUG
Aba-1-1	CACCGGAAGCUUUGUGCUCGGUCUUUUCCUGGUCUACACGACCGAGCGCUCUUUGUUGAUCCGAAGUGGCGACUGCUGAUUGCGAUCGGUUUCUGCGGCGCAUACACGACGUUUUCGAGCUACGCGUACGAGAGCAUGGUCUAUUUCC
Hsp-1-1	UUUUUAAUAGGGUUUUUAAGCAUAUACCUAACUGAGGUGAUAGAUGCUCCUCCAAAUAUAAGACUUCUUCUUAUAACAGGCUUUCUUGGUGGAUAUACGACAUUUUCUACAUUUACAUUAGAAGGCAUAGGUCUUAUAAACAAUGGAGA
Hsp-1-2	CAAUUAAUAUAUGGAGGGGUAUAUAAAGAUGGAAAAAGCUAUUGAAAAAUCUGUAGAAUAUGUGAUGUCUAAGCUGUGUAGCGAUGGCGGGUAUUCGUUUUAUAGGCAUAUAUAUUUGGAAGAAUCAAACAUUUACGAUACUUACUAUG
Ban-1-1	UGCGUCGGUACGGCGCUGCGAUACGCGCUUUCCGCGAUUCCGGAGUCCGGAACGUUCCACAUCGGCACGUUUGCCGCCAAUAUGCUGGCAUGCCUCUGCUACGCGGCAUUAUCCGCGUGGCUGGGCGGCACGCGUAAGAUCGCCGGUCG
Lxy-1-1	GGUGAUCACGUUUGUGAUCAAUGUCAUCGGCGCGUUCGUCCUCGGCUGGUUGCUCGAGUCGCUGGCGCUGGCGGUCCCGAUGAGGGGCGACGUCGGGAUGUGCGCCUCUUCGCCGGCACCGGAGUUCUCGGCGGGUUCACGACCUACA
Mab-1-1	CCGGCCUCUGGUACGUCAUGGCCGUGGGGGACCUUCGCGGCCAAUGUGUCGGGGGCGUUCGUUCUCGGCGCCACCCUGGAGACUCUGACGCUGCUGGGGCCGGACGGCGGCUGGCGCUGGCACGCGCGGUUGUUUCUGGGAACGGUUU
Mma-1-1	CGGUAUGCUUGGACGCGCCCGAAGCGGAAUCCGGCGGUCCAAGAACAGCCCUCGUGUCGGUACAUCUCCACGCGCUGUGUGCGGGCAAGCCCGGUGUGGUAGCGAAAUGGCAACAACUCCAGCGGGUUUCGAUGAAGAGACCGACUCCC
Mul-1-1	AGCUCGACUGCGGCGAAUUCGGCCGCGGAUUAUACUGCUGUCAAGCAAUUCAUGCAACUCCCGUGCGGGACAACGCCGCCCCCCUGCGCAUUCCCCGGGUUCCCGUCGCUGAGACCGACGCCAACCCGCUGGACGCUCGAUUGCCUUUC
Mma-1-2	AGCUCGACUGCGGCGAAUUCGGCCGCGCACUGUACUGCUGUCAAGCAAUUCAUGCAACUCCCGUGCGGGACAACGCCUGCCCACGUGCGCAUUCCCCGGGUUCCCGUCGCUGAGACCGACGCCAACCCGCUGGACGCUCGAUUGCCUUU
Ace-1-1	CCCCGUUCUCCUCCAGGUUCUCCAGGUCGGGACGGUGGCCCUGGCCGCGCCCGGCCUCUCCGGGUUCAUCGCGCGUCUCGAGGCCCGGCUGCAAGGACGGCGCGGUCCACGGAUUCUCCAGCCCUACUACGACAUCGUGAAAUUCUUCC
Mul-1-2	AUGAAAACUCCUAGGCAGCACGGCUUCAGGGGCCGGCAUGGAUGACGAUUGCCUCAAGGUGAGCGCCUACUUGGGUGAGCGGCGCCGAACCGACGACGGCUUCCUGGCCGAUGUGCUGCUCAGCCUCUACCAAGAACACAACAUCGCGU
Mma-1-3	AUGAAAACUCCUAGGCAGCACGGCUUCAGGGGCCGGCAUGGAUGACGAUUGCCUCAAGGUGAGCGCCUACUUGGGUGAGCGGCGCCGAACCGACGACGGCUUCCUGGCCGAUGUGCUGCUCAGCCUCUACCAAGAACACAACAUCGCGU
Ace-1-2	AUACGAAGAAUAUGGGCUGCGGCCGUUGUUCUACCAACCGUUAGGUGACGUCGACGCCGUCCGGUAUCGGCACGAGGUCUUCCGGGAUCUUCAGGAUGCGGGCAUCCGCGAAGCGGUUGACGCGUUCACCGCUGCUCUGCGCGACAUGC
Rxy-1-1	CGCCGGGGCGUGGCCUCCGCGACGGCCUACUGGGGGCCCGAGACGGGGUCGGCGCGCCACGUCUUCCUGAUGCUCCUCGACGAGGAGGCAAGAUCGAGGGGUGCUCCCGGAGCUGCGCCUCUCCCGGACGCGGGCAUCUCGGU
Blo-3-1	UAUUGUGUUGGUCGUCUUCUGCGGCGGUGCGAUCGGCACCGCUAUCCGCUAUGCGUUCGCACAGAUUCCCGCGGCUGGCAGCUUCCAUACCGGCACGUUUGUGGCCAACAUGCUGGCUUGCUUCUGCUAUGCAGGUUGACCGCUUACU
Blo-2-1	UAUUGUGUUGGUCGUCUUCUGCGGCGGUGCGAUCGGCACCGCUAUCCGCUAUGCGUUCGCACAGAUUCCCGCGGCUGGCAGCUUCCAUACCGGCACGUUUGUGGCCAACAUGCUGGCUUGCUUCUGCUAUGCAGGUUGACCGCUUACU
Blo-1-1	UAUUGUGUUGGUCGUCUUCUGCGGCGGUGCCAUCGGCACCGCGAUUCGCUAUGCGUUCGCGCAGAUUCCCGCAGCCGGCAGCUUCCAUACCGGCACGUUUGUGGCCAACAUGCUGGCCUGCUUCUGCUAUGCGGGUCUGACCGCCUAUU
Bad-1-1	UGCAUCGGAACCGCAUUACGCUACGCUUGUUCGGUCAUUCCCGACUGCGGCGCAUUCCAUAUCGGCACAUUCGCGGCGAAUAUGGCCGCCUGCUUCGUAUACGCGGCGCUUUCGGCAUGGCUGGGAGGAACCCGCAGAAUCGUGGGACG
Bad-2-1	UGCAUCGGAACCGCAUUACGCUACGCUUGUUCGGUCAUUCCCGACUGCGGCGCAUUCCAUAUCGGCACAUUCGCGGCGAAUAUGGCCGCCUGCUUCGUAUACGCGGCGCUUUCGGCAUGGCUGGGAGGAACCCGCAGAACCGUGGGACG
Nfa-1-1	GUGACGGCGGCGGGUGUUGUAGCGGACGAGCCAGCGGAAGACCUCGCGGCGGCAGGUGGCCUCGUCGGGCCAGCAGGCGGCGUCUUGGAGGACCUCGCGCUUGAUGGUGGCGUUGAACGACUCGGCCAGAGCGUUGUCGGCCGAGGAGC
Bpl-1-1	AGUUUGAACAGCUUCCGUUACUCCUUUAUUUAUGUAAGUGGCUUUUCCUUUCAGUUGUGUCAGGGGCUUGUAUUGGGUCGGCUUCUGCCUUGCUGUUGGUAUCUCUGGAGUGGGCUACACAGUAUCGCGAACACCAUCUUUGGAUAAUU
Bco-1-1	GCAACAGUUUCCUUUACUUUUUUAUUUAUUCAAGUGGCUUUUUCUUUCUGCUAUAUCCGGAGCGUGUAUCGGUUCGGCUUCCGCCUUAUUGUUGGUUUCGCUGGAAUGGGCAACCCAAUAUCGUGAACACCAUCUGUGGAUUAUAGCCC

Bth-1-1	AUGUACUCGGUUGUUUCGCCAUCGGCCUGUUUUACGGACUCUUCGAACGGGGAAAUCUGAUGAACCCCAAUCUGAGAAUGUUUCUCACCGUAGGAUUCUGUGGAGGAUUCACCACCUUUUCAACCUUCAUGAAUGAGAACUUUCAGUUA
Bca-1-1	UGCCGCCGGCAUCCUGCUGGGGCCCUCCUGCCUGAACCUGCUCUCCGACAGCCUGCUGAACAUCUCCGCCGACCUGCGGCAGCUGGCCCUUAUCAUCAUCCUCACCCGUGCGGGCCUGACCCUAGACGUGGCCGCCCUGAAACGGUGG
Lbl-1-1	GCAGGCAGUAUUUCGGCAUUUUUUCUGCUCAGUUUAGAAUGGGCUACCAAUUAUAGGGAAGCUCAUUUAUGGAUUAUUGCGCUACUUCCUAUUGGCGGUUUUCUUGUUGGUCUUUCUUAUCACCUCUACGGAAACAGCGUCGUAAAAGG
Psp-1-1	CUACUCAUUUUCGCUUUCAGCAUACCUGGUUA**UUG**UAUCUGUUACCCCUUUCGGGCAUUCUCAUUCAUUUCAUCUAUCAAUCCGUGGGUAAGUCUUCUGAAAAAGGCAACAACCUGAUCAUUGAAGAAAUCCAUCAACCUGGGGGAGGU
Psp-1-2	UGCCGGUUGAAACUGAAUGGUUCUUGUGGAUAAAUCAGCACCAUAAUGCAUUCUGGGAUACGAUCAUGUACUGGGCCAGUGAUAAGCGUUUUUGGCUUCCUUUUUAUGCCUUCAUUAUUUAUUGCCUCUUCCAGAAUUUCUGCAAAAAA
Rsp-1-1	CUGUCGAUCUGGGCGGCGCAGCGUUGGGGGGCGUCGUUUCCGUAUGGUACACUGAUCGUCAACGUUAUUGGAAGUUUUGCCAUCGGGUUUGUGCUGGUUCUGGCAACAACACGCCUGAGCCUGAGCGAUACGGCGCGACUGCUGAUCGU
Cau-1-1	AGCCAUCACAAAACCCGGACAGGAGUACAACUUACAACCCUUUUUCUGGACUCCCUUACGCGAUGCUCAAGCCGUCCGCUACCGGCAAGAGGUGUUUCAGGACCUGGAAAAAGCUCCUAUCCUGGAAGCUAUCAAGCAGUUUGCGGAAG
Cag-1-1	UCUUAAAUAAGAUGGUCUGUUUUGUCAAUAUUAAAAAGCCACUAAUGCCCACAGUGGCCCAAUCCGUGGGCAUCCGUCUCAAUCCGCCUUCAUCCGUGUCCCAGUCAAAAUUUCGAGUAAAAUAAGCGGGACACAGCGUUGCUGUGUCC
Ebi-1-1	UCUUUUAGGACCUUAUUGUCUUGGCUUAAUUGAUUCAUCUCUAUUAAAUAUUUCUAGUGAAUUAAGAAAAAUUGCAUUAAUUAUUAUAUUGAUGAGAGCCGGAUUGAAUUUAGAUUUAAGUGAUUUAAAAAGAGUUGGAAGACCUGCUA
Gsp-2-1	UUUCCUUUGGCAACAUGGCUGGCCAAUAUAAUUGGCUGUUUUGCGUUAGGAUGGCUGACUGCCUAUUUGCCUCGGCUGCGCAAUCUUCCUCCUUAUGUUGUGACAGGACUAGGAACGGGAUUAGUAGGAUCGUUUACGACUUUUUCGAU
Gth-1-1	CACUGUUAAUGAACUGGACAGGCGCGUUUUUGCUUAGCUGGCUGACGGUUGUUUUCACCUGUCGCCCCACAUGGCCGACGUGGUGGAAAACGGCGAUGACGACCGGUUUUGUCGGCUCGUAUACAACGUUUUCUACGCUUAGCGUUGAG
Gka-1-1	CGCUUUUCAUCAACUGGACAGGCUCGUUUUUGCUCAGCUGGUUCACCGUGAUGUUCACCCGCCGCCCCGCGUGGCCGCCAUGGCUGAAAACAGCCGUAACGACCGGGUUCGUCGGUUCGUAUACGACGUUUUCGACGCUUAGCGUUGAA
Bth-2-1	GCUAGCUACAUUGCUCAUCAACUUACUCGGCUGCUUCCUAUUAGCGUGGCUAACUACCUAUAUCGCUCGGCGAAACAUCUUACCAUUAGAAAUUAUCACAGGCAUUGGGACUGGAUUCAUCGGUUCCUUUACGACGUUUUCAACAUUUA
Bce-10-1	CGCUAGCUACAUUGCUCAUCAACUUACUCGGCUGCUUCCUAUUAGCGUGGCUAACUACCUAUAUCGCUCGGCGAAACAUCUUACCAUUAGAAAUUAUCACAGGCAUUGGGACUGGAUUCAUUGGUUCCUUUACGACGUUUUCAACAUUU
Bwe-1-1	CAUUAGCUACAUUACUCAUUAACUUAGCCGGCUGCUUCUUAUUAGCAUGGCUAACUACGUAUAUCGCCCGGCUAAACAUCUUACCUUCAGAGGUUAUCACAGGCAUCGGGACUGGAUUCAUCGGUUCCUUUACGACGUUUUCAACAUUU
Bce-13-1	CACUAGCUACAUUACUUAUUAACUUAGCCGGAUGCUUCUUACUAGCUUGGCUGACUACGUAUAUCGCUAAGCUCAACAUCUUACCUUCAGAUGUAAUCACAAGUAUCGGGACUGGAUUCAUCGGUUCUUUUACGACGUUUUCAACAUUU
Bce-12-1	CACUAACUACAUUACUCAUUAACUUAUCCGGAUGCUUCUUAUUAGCAUGGCUGACUACGUAUAUCGCUAAGCGCAACCUCUUACCUUCAGAUGUAAUCACAGGUAUCGGGACUGGAUUCAUCGGUUCUUUUACGACGUUUUCAACAUUU
Ban-16-1	CACUAGCUACAUUACUUAUUAACUUAGCCGGAUGCUUCUUAUUAGCUUGGCUGACUACGUAUAUCGCUAAGCUCAACAUCUUACCUUCAGAUGUAAUCACAGGCAUUGGGACUGGAUUCAUCGGUUCUUUUACGACGUUUUCAACACUU
Bth-3-1	CACUAGCUACAUUACUCAUUAACUUAGCCGGAUGCUUCUUACUAGCUUGGCUGACUACGUAUAUCGCUAAGCGCAACCUCUUACCUUCAGAUGUAAUCACAGGUAUCGGGACUGGAUUCAUCGGUUCUUUUACGACGUUUUCAACAUUU
Bce-9-1	CACUAGCUACAUUACUGAUUAACUUAGUCGGCUGCUUCUUAUUAGCGUGUCUGACUACGUAUAUCGCUAAACUCAACAUCUUACCGUCAGAUGUAAUCACAGGCAUCGGGACUGGAUUCAUCGGUUCUUUUACGACGUUUUCAACAUUC
Bce-8-1	CACUAGCUACAUUACUUAUUAACUUAGCCGGAUGCUUCUUACUAGCUUGGCUGACUACGUAUAUCGCUAAGCUCAACAUCUUACCUUCAGAUGUAAUCACAGGUAUCGGGACUGGAUUCAUCGGUUCUUUUACGACGUUUUCAACAUUU
Bce-4-1	CACUAGCUACAUUACUUAUUAACUUAGCCGGAUGCUUCUUACUAGCUUGGCUGACUACGUAUAUCGCUAAGCUCAACAUCUUACCUUCAGAUGUAAUCACAAGUAUCGGGACUGGAUUCAUCGGUUCUUUUACGACGUUUUCAACAUUU
Bce-7-1	CAUUGGCAACAUUACUCAUUAACUUAAUCGGUUGCUUUUUCUUAGCGUGGCUAACUACUUAUAUCGCUCGGCUAAACAUCUUACCUUCAGAGGUUAUCACAGGCAUCGGAACUGGAUUCAUCGGUUCCUUUACGACUUUUUCAACAUUU
Bce-3-1	CACUAGCUACAUUACUGAUUAACUUAGUCGGCUGCUUCUUAUUAGCGUGGCUGACUACGUAUAUCGCUAAGCUCAACAUCUUAUCUUCAGAUGUAAUCACAGGCAUCGGGACAGGAUUCAUUGGUUCUUUUACGACGUUUUCAACAUUU
Bce-2-1	CAUUGGCAACAUUACUCAUUAACUUAAUCGGUUGCUUUUUCUUAGCGUGGCUAACUACUUAUAUCGCUCGGCUAAACAUCUUACCUUCAGAGGUUAUCACAGGCAUCGGAACUGGAUUCAUCGGUUCCUUUACGACUUUUUCAACAUUU
Bth-4-1	CACUAGCUACAUUACUCAUUAACUUAGCCGGAUGUUUCUUAUUAGCAUGGCUGACUACGUAUAUCGCUAAGCUCAACAUCUUACCUUCAGAUGUAAUCACAGGCAUCGGGACUGGAUUUAUCGGUUCUUUUACGACGUUUUCAACAUUU
Bce-5-1	CACUAGCUACAUUACUCAUUAACUUAGUUGGCUGCUUCUUAUUAGCAUGGCUGACUACGUAUAUCGCUCAGCGAAACAUCUUGCCUGCAGAAAUUAUCACAGGCAUCGGGACUGGAUUCAUCGGUUCCUUUACGACAUUUUCAACAUUU
Bce-6-1	CAUUAGCAACAUUACUCAUUAACUUAAUCGGUUGCUUCUUCUUAGCGUGGCUAACUACUUAUAUCGCCCGGCUAAACAUCUUACCUUCAGAGGUCAUCACAGGCAUCGGAACUGGAUUCAUCGGUUCCUUUACGACUUUUUCAACAUUU
Bce-14-1	CACUCGCCACAUUAUUUAUUAAUUUGAUUGGAUGCUUUUUAUUAAGCUGGAUUGCCACUUAUAUUUCUCGUCUAAAUAUUUUUCCUUCAGAGGUCGUUACGGGGAUUGGAACAGGAUUUGUAGGAUCUUUUACUACAUUUUCUACUUUU
Gsp-3-1	GGGAAGCCUCGUGGGAUUGAUGACGGGCAGUGCCUCCGCCUUCUUUCUCUACAGUCUGGACUACGUGACCUCCCUUCGCUUGGGAAAUCCAUGGCUGUUAUACCUUCUUCCCUUGGGAGGCGCCGCAGUCAGCUUCCUUUAUUACAAGU
Psp-2-1	GGAUUUUUUACGGAGGACUUGUCGGAAUACUCUCCGGCAGCGCGUCGGCAUUGUUUCUAGCCAGCCUCGACUACGCUACAGGGCAGCGGUUGAAUCAUCCGUGGCUGCUGUUCCUGCUUCCGUUCGGGGGAGCUCUUAUGAGCUUCUUU
Mth-2-1	UUCCUUUCCCCUGGCUACCUGGAUCAUUAACCUGGGCGGUUGCCUGGCCAUGGGCUUUAUCCUGACCUAUACCCUGGAACGACUGGUCAUGGGCCCGGAAUUGCGCCUGGGGCUUACUACAGGGAUGCUCGGUGCCUUCACCACCUUUU
Tte-1-1	GGAGUGAUUGCCGGUAAUUAUUUGGGAUUUCAGACGAAUGAUUGGAUAAAUUUUCUGGGUGGCGUAGGGAGUAUUUUGUUAACUUUUUUAGCAGGAGCAGAAGUAGACCCUAUAGUGUUGAGAACAAAAUUUAAAGAAAGUGUUACAAU
Cpa-1-1	GGAGUGAUUGCCGGUAAUUAUUUGGGAUUUCAGACGAAUGAUUGGAUAAAUUUUCUGGGUGGCGUAGGGAGUAUUUUGUUAACUUUUUUAGCAGGAGCAGAAGUAGACCCUAUAGUGUUGAGAACAAAAUUUAAAGAAAGUGUUACAAU
Cpa-1-2	UAGGAGGGUAUGUCUAUGUGGUUAACUUAUAGUUGGUGGUUAGGCUUGGCUUUAAUUGCAGGUAUAAUUGCCAAUUGGACAGGUAUAUCGAUCUCAUUAAUCGAGAUUUUGGUAGGAGUGAUUGCCGGUAAUUAUUUGGGAUUUCAGAC
Tte-1-2	UAGGAGGGUAUGUCU**AUG**UGGUUAACUUAUAGUUGGUGGUUAGGCUUGGCUUUAAUUGCAGGUAUAAUUGCCAAUUGGACAGGUAUAUCGAUCUCAUUAAUCGAGAUUUUGGUAGGAGUGAUUGCCGGUAAUUAUUUGGGAUUUCAGAC
Cac-1-1	UUAGUACACUUAUGAUAAAUUUAAGUGGAGCAUUUAUUUUAGCAGUAAUUUUGAUAACAGCAAAUGAAAUAUGGAGCUUUAAUGAAGAAAUAAGACUAGGUAUUGCAACUGGUUUUGUAGGAGCAUAUACUACUUUUUCGACUAUGUGC
Cdi-5-1	GUAUACCUAUAUCCUAUCAAUUUAAAAAACAAUUAUGAUAGUCAUUACAUUGACUACAGUUGAUGCAAUAAAACUUUCAAAAAAUCUUAUGAUGCAAAUAGAAAGGAAUUAAA**AUG**AUAGAAAAAUUUAAAACUUUAAUAUGGAUUAAA
Cdi-7-1	GUGUACCUAUAUCCUAUCAAUUUAAAAAACAAUUAUGAUAGUCAUUACAUUGACUACAGUUGAUGCAAUAAAACUUUCAAAAAAUCUUAUGAUGCAAAUAGAAAGGAAUUAAA**AUG**AUAGAAAAAUUUAAAACUUUAAUAUGGAUUAAA
Cdi-4-1	AGUGUGGCUAUAUGCUAUUAAUUAAAAAAAACAAUUAUUAUAGAAGCCACAUUGCCUAUAGUUGAUGCAAUAAUAUUUUCAAAAUAUCUUAUAAAGCAGGUAGAAAGGAAGUAAA**AUG**AUAACAAAAUUUAAAACUUUAAUAUGGAUUA
Cdi-9-1	AGUAUACCUAUAUCCUAUCAAUUUAAAAAACAAUUAUGAUAGUCAUUACAUUGACUACAGUUGAUGCAAUAAAACUUUCAAAAAAUCUUAUGAUGCAAAUAGAAAGGAAUUAAA**AUG**AUAGAAAAAUUUAAAACUUUAAUAUGGAUUAA
Cdi-2-1	GUGUACCUAUAUCCUAUCAAUUAAAAAAACAAUUACGAUAGUCAUUACAUUGACUACAGUUGAUGCAAUAAAACUUUCAAAAAAUCUUAUGAUGCAAAUAGAAAGGAAUUAAA**AUG**AUAGAAAAAUUUAAAACUUUAAUAUGGAUUAAA
Cdi-6-1	GAGUAUACCUAUAUCCUAUCAAUUUAAAAAACAAUUAUGAUAGUCAUUACAUUGACUACAGUUGAUGCAAUAAAACUUUCAAAAAAUCUUAUGAUGCAAAUAGAAAGGAAUUAAA**AUG**AUAGAAAAAUUUAAAACUUUAAUAUGGAUUA
Cdi-1-1	GUGUACCUAUAUCCUAUCAAUUUAAAAAACAAUUAUGAUAGUCAUUACAUUGACUACAGUUGAUGCAAUAAAACUUUCAAAAAAUCUUAUGAUGCAAAUAGAAAGGAAUUAAAAUGAUAGAAAAAUUUAAAACUUUAAUAUGGAUUAAA
Cbu-1-1	UCUUUAUGGGAAUAAUAUCAGUUUUCAUAGGAAUAAUUGUAGGUAUAAUAGAUACGUGUUUUGGAAAAGUAUUGAUUUUUAUAACAGAUUUUAGAAAUGAAAACCCAUUAAAAUUAAUUCCAUUUUUAUCAAUAGCAGGAGUAGCAAUU
Nth-1-1	GGACCAAAUUAUUUAGAUUUGAUUUCAAGUGAAAUUUUGGCUAUCUCUGAUGACAUUAGGUUGCUUGCAUUGAUUUUAAUCUUGCUUAGAGCGGGUCUUGGAUUAAGGCGUGAAACUUUAAAUAAGGUGGGAGUACUCUCUCUUAAGUU
Aor-1-1	CUGGGAUGAUUUUAAGUCCUUAUGGACUCAAUCUGUUGCACCCAUCUAUUUUAGAAAUAUCUUCUGAUUUAAGGCAACUAGCCCUAAUCAUUAUUCUAACGAGGGCAGGGUUAUCCUUUAAUAUUGGUGAUUUGAAAAAAGUUGGACGU
Ceu-1-1	UAAGCUGCCGCGCAUCAUAGGAAUGCUGGCGACGGGAAUCGUUCUUGGCCCGUAUGUGCUGGAUUUCCUUGAUCCGUCCAUAUUGUCCAUAUCUGCGGAUCUCAGAAAGAUAGCGCUUAUCAUCAUACUUAUCAAGGCAGGACUGUCCC
Cba-1-1	UCCUGCUGGGGCCUUAUGCGCUGGAUCUUAUUGACGGCUCCAUACUUGGGAUUUCAGCGGAGCUGCGCAGGAUUGCGCUUAUUAUCAUACUGAUGCGGGCAGGUCUGUCCCUGGAUGUGGAUGAUCUGAAGCGGGUGGCCGGCCGGCU
Rob-1-1	AUUGUUCUGGGACCAUAUGUGCUUAAUCUGUUGGAUGACAGUAUUCUUGGAAUAUCUGCAGAACUUCGUAAAAUAGCAUUGAUCAUUAUUCUCACAAGAGCAGGUCUUGGACUGGAUCUAUCUGGGCUUAAAAAGAUUGGAAGACCUGC
Dlo-1-1	UGUAAAAAAAUUAAAAACUGCCGGGGCUUCUGGGA**AUG**CUUCUCACCGGUAUCGUUCUGGGACCGUAUGUGCUGAAUCUUCUGGAUGAAAAUCUGUUAAAUAUUUCAUCGGAACUUAGGAAGAUGGCACUGAUCAUUAUUCUGAUGAGG
Ahy-1-1	UUUUGGACCCCAUGUCCUAAAUUUAAUUGAUCCUAAAACUUUAUCCAUAUCUGCUGAACUUAGGCAAAUUGCCCUUAUUAUUAUUUUAAUAAAGGCAGGACUUUCUUUAGAUAUUUCUGAUUUAAAAAAAGCUGGAAAAUCUGCAGUU
Cph-1-1	GGAAUGAUCUUAAGUCCUUAUGCUCUUAAUCUGCUGGAUGGAUCGAUUCUUACGAUUUCACCGGAUUUAAGAAAAUUAGCGUUGGUAAUCAUCUUGACGCGUGCUGGUUUGUCUCUUGAUUUAAAAUCCCUGAAAAAGGUUGGACGCCC
Cbo-1-1	GGGGUUCUUCUGGGUCCCUACGCCCUGAAUCUGAUUGAUGGGUCUAUAUUGAAUAUUUCUUCAGAGCUGAGACGGAUUGCCCUUAUUAUCAUAUUGAUGAGGGCAGGGCUUUCCCUGGAUUUAAAUGACCUUAAAAAGGUGGGGCGCCC
Fma-1-1	CGGACCGUUCGCGAUGAAUCUUAUUGACAAGAAAAUUCUUGACAUUUCAUCUGAACUGAGAACUAUUGCGCUGGUAAUAAUCUUGACUCGUGCAGGAUUGUCGCUGGACAUCGACGAUUUGAAAAAAAUCGGACGACCAGCGGUGUUAA
Ala-1-1	CCUCAAGUAUUAAACUUACUUGAUAAGAAUAUUUUAGAUAUUUCUGCCGACCUAAGAAGCAUAGCCUUGAUUGUUAUUUUAUUAAAAGCUGGACUCAGUCUUGAUAUAGGAGACCUCAAAAAAGUAGGACGCUCUGCUGUUUUACUAUC
Chi-1-1	GGGAUAAUUUUAGGUCCUUAUGUACUAGAUUUUUUAGAUAGCUCAAUUCUAAACAUAUCAUCAGAGCUUAGAAAAAUGGCUCUUAUCAUCAUCUUAAUCAAAGCUGGGUUAUCACUAGACUUAAAGGACUUAAAAAAAGUUGGAGACC
Rin-1-1	UUUUAACCGGUGUACUGGUUGGAAUCAUUGAUACAGUAUUUGGCAGAGGACUACUGACAAUUUCUGAUUUUCGGACAGGCCAUUAUUUGUAUCUGAUACCGUUUCUGCCGUUUGCUGGUCUUUUGAUCACAUGGCUGUAUUAUCAUUUC

....
....
....
....
....
....
....

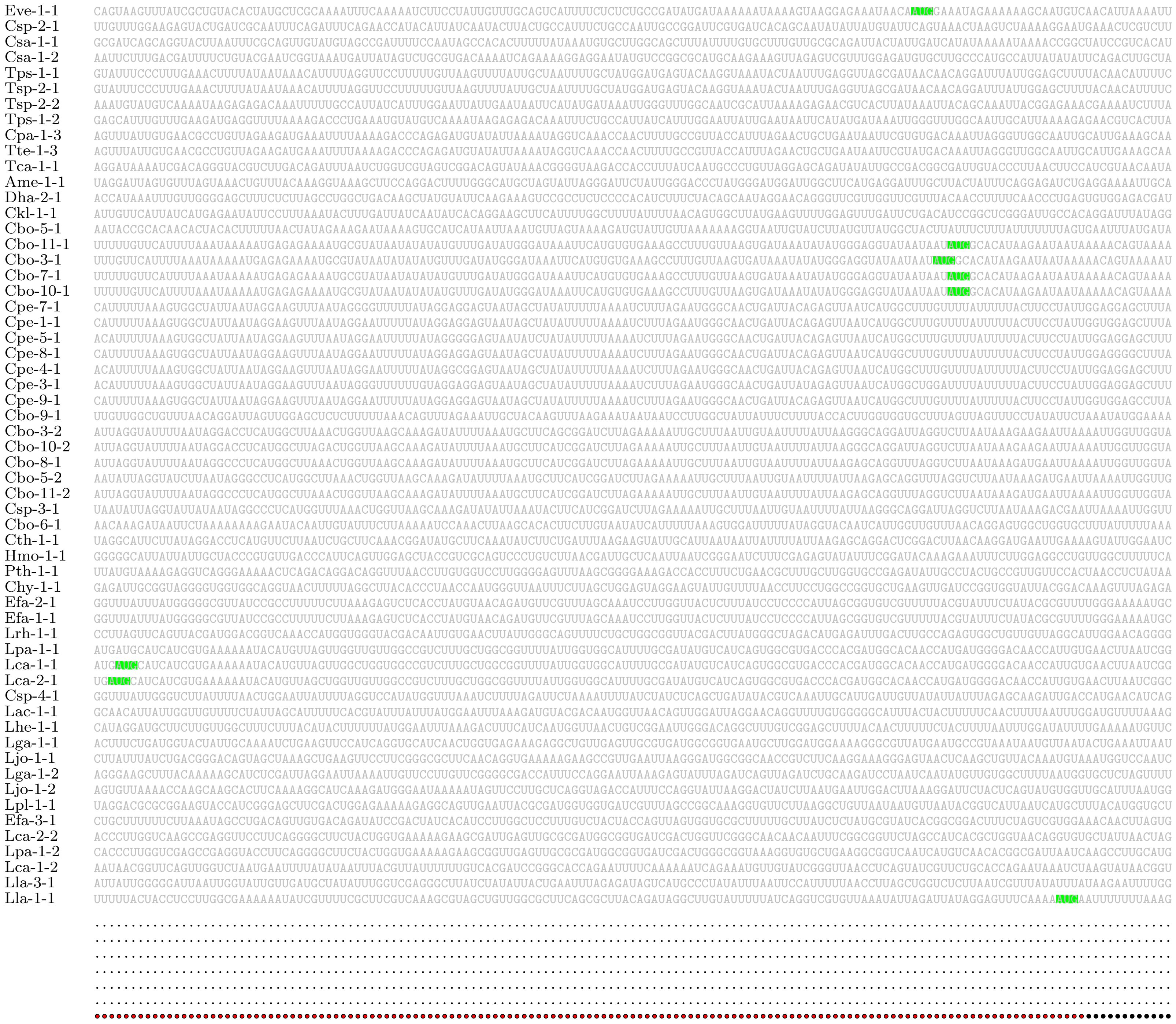
Eve-1-1
Csp-2-1
Csa-1-1
Csa-1-2
Tps-1-1
Tsp-2-1
Tsp-2-2
Tps-1-2
Cpa-1-3
Tte-1-3
Tca-1-1
Ame-1-1
Dha-2-1
Ckl-1-1
Cbo-5-1
Cbo-11-1
Cbo-3-1
Cbo-7-1
Cbo-10-1
Cpe-7-1
Cpe-1-1
Cpe-5-1
Cpe-8-1
Cpe-4-1
Cpe-3-1
Cpe-9-1
Cbo-9-1
Cbo-3-2
Cbo-10-2
Cbo-8-1
Cbo-5-2
Cbo-11-2
Csp-3-1
Cbo-6-1
Cth-1-1
Hmo-1-1
Pth-1-1
Chy-1-1
Efa-2-1
Efa-1-1
Lrh-1-1
Lpa-1-1
Lca-1-1
Lca-2-1
Csp-4-1
Lac-1-1
Lhe-1-1
Lga-1-1
Ljo-1-1
Lga-1-2
Ljo-1-2
Lpl-1-1
Efa-3-1
Lca-2-2
Lpa-1-2
Lca-1-2
Lla-3-1
Lla-1-1

Lla-2-1	GUUUUUACUACCUACUUGGCGAAAAAAUAUCGUUUUCGCUUCGUCAAAGCGUAGCUGUUGGCGCUUCAGCGCUUACAGAUAGGCUUGUAUUUUUAUCAGGUCGUGUUAAAUAUUAGAUUAUAGGAGUUUCAAAAAUGAAUUUUUUUAAA
Lla-3-2	AUGUUAGAUUAACAGAUGGAACAUUAGGACGCGCAGCAGUACCCUCAGGAGCAUCAACUGGAGACCGUGAAGCAGUUGAAUUAAGAGAUGGUGGAGACCGUCUUCAAGGGAAAGGCGUUUCUAAAGCCGUCGCAAAUGUCAAUGGCGAA
Lla-2-2	AUGUUAGAUUGACAGAUGGAACUUUGGGACGGGCGGCAGUUCCCUCAGGAGCAUCAACAGGAGAUCGCGAAGCGGUCGAACUAAGAGAUGGCGGAGCGCGACUUCAAGGAAAAGACGUUUCUAAAGCUGUUGCAAAUGUUAAUGGUGAA
Lla-1-2	AUGUUAGAUUGACAGAUGGAACUUUGGGACGGGCGGCAGUUCCCUCAGGAGCAUCAACAGGAGAUCGUGAAGCGGUCGAACUAAGAGAUGGCGGAGCGCGACUUCAAGGAAAAGGCGUUUCUAAAGCUGUUGCAAAUGUUAAUGGUGAA
Zmo-1-1	UUUCCCAUUGGCAGCUAUUAUCGGCAGUUGUUGUGCCCUAUUCCUCUGGUCAUUGGAAUGGGCGACAAAAACGCGUUUUCUCUAUCCAGCGCUUCUUUUCGGAUUGCCUAUUGCUGGUUUUAUCACUUCAUGGCUUUACAGCCGUUACG
Gox-1-1	GCUUCUGGAACGCGCCACUUCGCUGCGGAUCGAGCAUCCGGUGCUGCUGUUCGGGCUUCCGUUGGCGGGUGUGGCGGUCGGUCUGUGUUACUUCUGGUUCGGCCGCGACGUUGAGGCCGGGGCGAACCUGAUCGUGGAUGAAAUCCAUG
Gdi-1-1	UCCUGCCGAUGGCCGUCUGUGUCGGCAGCCUCUGCGCCCUGUUCCUCUGGCUGCUCGACCGGGUAACGGACGACCGUCUCCAUCAUCCGGACCUGCUCUACGCCCUGCCCCUGGCCGGGGUUGCCGUCGGACUGACGUAUCACGUCCUG
Gdi-1-2	UCCUGCCGAUGGCCGUCUGUGUCGGCAGCCUCUGCGCCCUGUUCCUCUGGCUGCUCGACCGGGUAACGGACGACCGUCUCCAUCAUCCGGACCUGCUCUACGCCCUGCCCCUGGCCGGGGUUGCCGUCGGACUGACGUAUCACGUCCUG
Gdi-1-3	UCCGGCACCCGAACCCCGCUGCUUCCACGACCUCUGCCUGGAUCAGAUCCUCGACCGGAUGACCGCCGGGCGGGAGAUGUUCGGGCUCGACCGGGUAUUCUGCACGCCGGCGCCGGAUCUCGGGACGAUCCGCUAUCGCCAGGCGUUCU
Nha-1-1	CGCCGGUCUUCGUCAUUUGUCCCGUCUGCCAUCACGACAGCGUCAGAGCGAGGACCAGGUACAUUGGUGCUCGUCCGACAUGGGAAAGUGAAUUUUCACGCCAAGGCCACGACAGCCUUCUUGUGGCUCGGCUUCGCGCGCCAACACCG
Gox-1-2	UGCAAGAUUGCAAGGUGACGAUCCUGCAUCCGCGGCAGGAGACCAAUCCGGACUUCCAGUCUGAGGACGAAGGCAUCGAGACGCAGAGCGAACACGCCGAGUUCGAAAAGCGCGAACAGAAUUUGAGUGGUACCUUGCGCGACAUCACC
Oan-1-1	GUGUCGCGCGGUAUUGGCUGGCCCUCUUGAUGCUGCCGAUAAGCCGCGAACUUCCCUGGGGCACCAUCGUCAUCAAUAUUGCCGGCUCUUUUGCAAUCUCGUUCUUCGGUGCACUGACACUGGAGCAAGGCCGUUUUCCCAUACCGGAA
Gox-1-3	CGUCGCGGCCAUGCCGAUCAGCCGCUUCAUCCCAUGGGGCACGAUCCUCCCCAUCAACGCGCUCGGCUCGUUCGUGAUCGGUUUCUUCGGCACGUUGACCCUUGCGGACGGGCGCUAUCCGGUGUCGGAAAACAUGCGUCUUUUCGUGA
Mra-1-1	GUGCUGUCGCUGCCCAUCAGCCGCGACCUGCCCUGGGGCACGAUCCUCAUCAACGUGACCGGCUCGUUCAUCAUCGGCCUGUUCGGCACGCUGACCCUGGCGCAGGGGCGAUUCCCGGUCUCCGAGAACGUGCGCCUGUUCGUGAUGAU
Bja-1-1	CUGCGCGCGCGGGGCUGCCCAGGGGGAUCUGGGUGCUCGGCUUCGUCUCGAUGCUGAUGGACGUCUCCUCCGAGAUGAUCCACGCGCUGCUGCCGGUCUAUCUCGUCACCGUGCUCGGCGCCUCGACGCUCACGGUCGGCUUCAUCGAG
Oca-1-1	CGUGCUGCGGAUACGGGUUCGGCGAUGAUGGAUGAUGUUCGGCGUGCUUCUGGCACGGCCGAAUGAGCGAUGAGGAAACGGACGUGAAUGUGAGUUUUAUUCUGGCGGUCGCGGUCGGCGGCGCACUCGGCUCGGUGGCGCGCUAUCUG
Nwi-1-1	GUGGACAUUCAUCCUGGCCGUCGCGGCCGGCGGGGCGCUGGGAUCGGUGGCCCGCUAUCUCGUCGGCAUCGGCUUCGGCAAAUGGCUGGGCCCGAAGUUUCCAUGGGGGACGCUGUUCAUCAAUGUCACGGGAUCGCUUCUGAUCGGAA
Nha-1-2	UCCCGGUGGUCCCUCUGCUGGUUUCGCUGGUGGUUCUUCUGCUGGUCCCUCUGCUGCUCAUCGGCAUCCUGGGCGGUAUGGCCGAAUGGCCGCAGGGGAGACAAAAACAUUGAAGUGGACGUUUAUUCUGGCCGUCGCGGCCGGCGGGU
Nsp-2-1	CUUCCGGUGACUUCCCUGUUGAUCUCUCUGGUCGUUCUUCUAGUGAUCCCUUUGGUGGUCAUCGGCGUCUUGGGCGGUAAUGACUGAAGGGCCGCAGAAGGAGCAAAAGCAUUGAAGUGGACGUUUAUUCUGGCCGUUGCGGCCGGCGG
Rpa-1-1	CUGCGCUUUGGCCGCCGUUGCGGCGAAAAUGAUGCCGGCGUUGGGCCCGGGUGCGAAGCAGAAGCUCGUGUUGUUGCCGCUUUGGGGAUCGAUGGCCAGGAUCGGGAACGCCCGCACGAUUGCAGACAUUGAAUUCGUCAUCGAUGCG
Rru-1-1	GACCCUGCGCUAUUGGCUUUCGGGGUUGAUCGCAGGCGCGAUCGGCCAAAGCUUUCCCUGGGGAACGCUGGUCAUCAAUAUCAGCGGAUCGAUCGUCAUCGGCGCCUUCGCCACCCUGACCGGACCCGAUGGCCGGGUGUUCAUCCCCG
Msp-1-1	CUCGCCGCGAUCAUUGCGCUGCGCGUGACGAUCUCGGUGGCCCUGGUCAAGAUGAUGGUAGGGGCCGUGGCCGGCAACACCAUCGGCCUGCACCUCACCGACUGGGUCAACUUCCUGGCCGGCUUCGGCGCAAUCCUGCUGACCUUCCU
Acr-1-1	GCCAUCGCCUUUUUCGGCUCGCUCGGCUGCUGGGCGCGCUACGGCCAGACCAUCUUCAUGCAGAACCUGUUCGGGCGCGGCUUUCCCGUCGCCGUGCUCAGCAUCAACGUGCUCGGCUCGUUCCUGAUCGGCUUUCUCUUCGUGCUCAC
Acr-1-2	CAUCCAGGGGUGCAUCGUCCAGUUCGAGGAGCGGAUCCAGCGCGGGCACGGCCCGGGGGUGCUGCAGCCCUACCGGGAUCUGUGGAAACUGUUCCACAAGGACAUCGUCGUGCCGGAGACCGCCUCGUGGCUGUUCUGGGUGACGCCCG
Neu-1-1	CAUUAUGCCAACCACCCUUGAAAUCGAAUCCACGUUGACCGACCGUUACCAGACCACAGUGCCUGAAUCCGUACGUCGCGCCCUUCAACUUGGUAAGCGCGACAAGAUUCACUACACCAUCCGCCCCGGCGGCGAAGUAGUACUGACCC
Dac-1-1	CGUGGCGGCCCUGGCCGGCACCGCUUCCGCGUUCUUCUUGCUGUCCCUGGACCUGGCGACGGCCUGGCGUGAGGCCCAUCCCUGGAUCAUCUGGCUGCUGCCCUUCGCAGGCAUGGCAGUGGGCCUCGUUUACUUCCUGUUGGGCAAGC
Cvi-1-1	GGUCGCCGCCUUGUCCGGCACGGCAUCCGCGUUUUUCCUGUUCGCGCUCGAGCAAGCUACGCGCACCCGACUCGCUCACCCAUGGCUGAUCUGGUUAUUGCCGCUUGGAGGCUUUGCCGUUGGAAUGGCCUAUCUGCGGCUGGGAAGG
Dac-1-2	ACCUGCUUGCACUGCGGCUGGUGGGCAGUGCCUAUCCGCUGGGCACCUUGGCCAUCAAUGUGCUGGGCGCUUUCGUGAUGGGGGUGGUGGUGGAGUGGUUUGCGCUCAAGACAUCGCUGCCGCAGAGUGCGGCGGCUGUUCAUCACCACG
Neu-1-2	ACGCGUGAAAUCCUAGACUCGCACGGCAAUCCCACUAUUGAGGUUGACGUUCAACUGGAAGGUGACGCAACAAGGCAUACCACGGUGCUAUACGGUCUGGAAAACAUUUUUUCACACGCUUUUCUGGGUUAAGCCGAGGGUUGAUUUGG
Neu-1-3	UUUCCUGACGUGCCGCCCGGUACAUUGGUGGCCAAUCUGAUAGCCGGCUAUGUCGUCGGCGUUGCAAUAGCCUUUUUUGCCCACAUGCCGAAUUUGUCACCAGAGUGGCGUUUGCUGGUCAUCACCGGCUUCUGCGGCGGGCUUUCUAC
Bph-2-1	AUUCUGGGUGCACUGGCGGGUAGCGCAUCCGCCUUGUUCCUCUAUGCGCUUGACAGCGCGACCGACGUUCGUGUUGGCCACGGCUGGCUGCUGUGGCUUUUGCCCAUUGCUGGCUUUGCGACCGGCUGGGUCUAUCACCGCGUCGGCAA
Bxe-1-1	GAACGGCCUGUUCAGCGGCCUGCCGCUCGGCACUUUCGCCGCCAACGUGAUUGCCGGCUACGUCAUCGGCGUCGCCGUCGCCGGGUUUGCGCGGGCGCCGCAGAUCGCACCGGAAUGGCGGCUCUUCGUCAUCACCGGCUUGAUGGGCG
Bph-2-2	GCCUGAACGGCGUCUUCAGCGGCUUGCCGCUCGGCACUUUCGCCGCCAAUGUCAUUGCCGGCUACGUCAUCGGCGUAGCGGUCGCAGGCUUCGCGCGGGCGCCGCAGAUCGCGCCCGAAUGGCGGCUCUUCGUCAUUACCGGUCUGAUG
Bph-1-1	UCAAUGCAUUGUUCCCGGCGCUGCCGCUCGGCACGCUCGCAUCCAAUGUGAUCGCCGGCUACAUCAUCGGCGCGGCGGUCGCGUACUUCGGGCGCAAUCCGCAGAUCGCGCCCGAAUGGCGCCUCUUCAUUAUCACGGGCCUGAUGGGC
Bgr-1-1	UGAAUGCGCUAUUUCCGUCGCUGCCGCUCGGCACCUUUGCCGCCAAUGUGAUCGCCGGCUACGUGAUCGGCGUGGCCGUCGCCGGCUUUGCGCGCGCGCCGCAGAUCGCGCCGGAAUGGCGGCUCUUCGUGAUCACCGGGAUGAUGGGC
Bsp-2-1	ACUCGAUCUUCAGUGACCUGCCUCUCGGCACGCUCGCCGCUAACGUGAUCGCAGGCUACAUCAUCGGCGUGGCGGUCGCGGGCUUCGCGCGCGUGCCUCAGCUCGCGCCGGAAUGGCGGCUGUUCGUCAUCACCGGUUUGAUGGGCGGU
Bub-1-1	GCUUUCGGCGCUGCUCGGCGCGCUCGCCGGGUCGGCGUCCGCGCUGUUGCUGAUCGCGCUCGACUGGGCGACCGGCGCGCGCGUCGCGCAUCCGUGGCUGCUGUGGGGGCUGCCCGCCGCCGGUUUCGCGACCUGCUGGAUCUACCACC
Rpi-2-1	GCUCGUCGGCAUGCUCGCCGGGCUUGGCUCUGCGGUGUUGCUGAUGGCGCUCGAUUGGGCAACCAACACGCGCACCGCACACCCUUGGCUGCUGUGGUUCCUGCCCGUGGCGGGGCUGGCCGUGGGCUUGCUCUACCACUACACGGGGC
Bmu-1-1	GCUCGGCGCGCUCGCCGGCUCGGCUUCCGCCCUGUUCCUGAUCGCGCUCGACUGGGCGACCGGUACGCGCGUCGCGCAUCCGUGGCUGCUCCGGGGAUUGCCCGCGGCCGGCUUCGCGACCGGCUGGGUCUACCAUCGCUUCGGCCAGC
Rso-1-1	CUGACCGGCGUGCUGGCCGGCCUCGGCUCGGCGGCGCUGCUGCUCGCGCUGGACUGGGCCACGCUCACGCGCACGGCCCACCCGUGGCUGCUCUGGCUCCUGCCGCUGGCCGGGCUGGCCGUUGGCCUGCUCUAUCACUACACGGGCCG
Rso-3-1	CUGAUCGGUAUCCUCGCCGGUGCCGGCUCCGCAGUGCUCCUGCUGGCGCUGGACUGGGCAACCGGCACGCGCACGGCGCAUCCGUGGCUGCUGUGGUGCCUGCCGCUUGCGGGCCUGGCCGUCGGCCUGCUCUAUCACUACGCCGGACG
Rso-2-1	CUGAUCGGCAUCCUGGCCGGCGCCGGCUCCGCAGUGCUCCUGCUGGCGCUGGACUGGGCAACCGGCACGCGCACGGCGCACCCGUGGCUGCUGUGGUGCCUGCCGCUGGCGGGCCUGGCCGUCGGCCUGCUCUAUCACUACGCCGGACG
Bce-19-1	CUGCUCGGCGCGCUCGCCGGCACGGCUUCCGCGCUGUUCCUGAUCGCGCUCGACUGGGCGACCGGCACGCGCGUCGCGCAUCCGUGGCUGCUGUGGGGGCUGCCGGCCGCCGGUUUCGCCACCGGCUGGGUCUACCAUCGGUUCGGCCA
Bce-18-1	CUGCUCGGCGCGCUCGCCGGCACGGCUUCCGCGCUGUUCCUGAUCGCGCUGGACUGGGCGACCGGCACGCGGGUCGCGCAUCCGUGGCUGCUGUGGGGGCUGCCGGCCGCCGGGUUCGCCACCGGCUGGGUCUACCAUCGGUUCGGCCA
Bce-15-1	UUGCUCGGCGCGCUCGCCGGCACGGCUUCCGCGCUGUUCCUGAUCGCGCUGGACUGGGCGACCGGCACGCGGGUCGCGCAUCCGUGGCUGCUGUGGGGGCUGCCGGCCGCCGGUUUCGCCACCGGCUGGGUCUACCAUCGGUUCGGCCA
Bam-2-1	UUGCUCGGCGCGCUGGCCGGCUCGGCUUCCGCGUUGUUCCUGCUCGCGCUCGACUGGGCGACCGGCACGCGCGUCGCGCAUCCGUGGCUGUUGUGGGGGCUGCCGGCCGCCGGCUUCGCGACCGGGUGGGUCUACCACCGGUUCGGCCA
Bam-3-1	UUGCUCGGCGCGCUGGCCGGCACGGCUUCCGCGUUGUUCCUGCUCGCCCUCGACUGGGCGACCGGCACGCGCGUCGCGCAUCCGUGGCUGUUGUGGGGGCUGCCGGCCGCCGGCUUCGCGACCGGCUGGGUCUACCACCGGUUCGGCCA
Bam-1-1	UUGCUCGGCGCGCUGGCCGGCACGGCUUCCGCGUUGUUCCUGCUCGCGCUCGACUGGGCGACCGGCACGCGCGUCGCGCAUCCGUGGCUGUUGUGGGGGCUGCCGGCCGCCGGCUUCGCGACCGGCUGGGUCUACCACCGGUUCGGCCA
Bam-4-1	UUGCUCGGUGCGCUGGCCGGCACGGCCUCCGCGUUGUUCCUGCUCGCGCUCGACUGGGCGACCGGCACGCGCGUCGCGCAUCCGUGGCUGUUGUGGGGGCUGCCGGCCGCCGGCUUCGCGACCGGCUGGGUCUACCACCGGUUCGGCCA
Bth-6-1	GCCGUCGUCGGCGUGCUCGCCGGCUCGGCAUCCGCGCUCUUUCUGCGGGCGCUCGAUCUCGCCACCGGCACGCGCGUCGCGCAUCCGUGGCUGCUGUGGCUGCUGCCCGCAGCGGGCUUCGCGACCGGCUGGGUCUAUCAUCGCGUCGG
Bps-14-1	GCCGUCGUCGGCGCGCUCGCCGGCUCGGCGUCCGCGCUCCUCCUGUGGGCGCUCGAUCUUGCCACCGGCACGCGCAUCGCGCAUCCGUGGCUGCUGUGGCUGCUGCCCGCGGCGGGUUUCGCGACGGGCUGGGUCUAUCAUCGCGUCGG
Bps-7-1	GCCGUCGUCGGCGCGCUCGCCGGCUCGGCGUCCGCGCUCUUCCUGUGGGCGCUCGAUCUUGCCACCGGCAAGCGCAUCGCGCAUCCGUGGCUGCUGUGGCUGCUGCCCGCGGCGGGUUUCGCGACGGGCUGGGUCUAUCAUCGCGUCGG
Bps-5-1	GCCGUCGUCGGCGCGCUCGCCGGCUCGGCGUCCGCGCUCCUCCUGUGGGCGCUCGAUCUUGCCACCGGCACGCGCAUCGCGCAUCCGUGGCUGCUGUGGCUGCUGCCCGCGGCGGGUUUCGCGACGGGCUGGGUCUAUCAUCGCGUCGG
Bok-2-1	GCGGUGGUGGGCACGCUCGCCGGCUCGGCGUCCGCGCUCUUUCUCCGGGCGCUCGAUCUCGCGACCGGCACGCGCGUCGCGCAUCCGUGGCUGCUGUGGCUGCUGCCCGUCGCGGGUUUUGCGACCGGCUGGAUCUAUCAUCGCGUCGG
Bps-17-1	GCCGUCGUCGGCGCGCUCGCCGGCUCGGCGUCCGCGCUCCUCCUGUGGGCGCUCGAUCUUGCCACCGGCACGCGCAUCGCGCAUCCGUGGCUGCUGUGGCUGCUGCCCGCGGCGGGUUUCGCGACGGGCUGGGUCUAUCAUCGCGUCGG
Bps-19-1	GCCGUCGUCGGCGCGCUCGCCGGCUCGGCGUCCGCGCUCUUCCUGUGGGCGCUCGAUCUUGCCACCGGCAAGCGCAUCGCGCAUCCGUGGCUGCUGUGGCUGCUGCCCGCGGCGGGUUUCGCGACGGGCUGGGUCUAUCAUCGCGUCGG
Bth-7-1	GCCGCCGUCGGCGCGCUCGCCGGAUCGGCGUCCGCGCUCUUCCUUUGGGCGCUCGAUCUCGCCACCGGCACGCGCGUCGCGCAUCCGUGGCUGCUGUGGCUGCUGCCCGCGGCGGGCUUCGCGACCGGCUGGGUCUAUCAGCGCGUCGG
Bok-1-1	GCGGUGGUGGGCACGCUCGCCGGCUCGGCGUCCGCGCUCUUUCUCCGGGCGCUCGAUCUCGCGACCGGCACGCGCGUCGCGCAUCCGCGGCUGCUGUGGCUGCUGCCCGUCGCGGGUUUUGCGACCGGCUGGAUCUAUCAUCGCGUCGG
	....
	....
	....
	....
	....
	....
	....

Bps-16-1	GCCGUCGUCGGCGCGCUCGCCGGCUCGGCGUCCGCGCUCCUCCUGUGGGCGCUCGAUCUUGCCACCGGCACGCGCAUCGCGCAUCCGUGGCUGCUGUGGCUGCUGCCCGCGGCGGUUUCGCGACGGGCUGGGUCUAUCAUCGCGUCGG
Bps-10-1	GCCGUCGUCGGCGCGCUCGCCGGCUCGGCGUCCGCGCUCUUCCUGUGGGCGCUCGAUCUUGCCGCCGGCACGCGCAUCGCGCAUCCGUGGCUGCUGUGGCUGCUGCCCGCGGCGGUUUCGCGACGGGCUGGGUCUAUCAUCGCGUCGG
Bps-9-1	GCCGUCGUCGGCGCGCUCGCCGGCUCGGCGUCCGCGCUCUUCCUGUGGGCGCUCGAUCUUGCCACCGGCACGCGCAUCGCGCAUCCGUGGCUGCUGUGGCUGCUGCCCGCGGCGGUUUCGCGACGGGCUGGGUCUAUCAUCGCGUCGG
Bma-2-1	GCCGUCGUCGGCGCGCUCGCCGGCUCGGCGUCCGCGCUCUUCCUGUGGGCGCUCGAUCUUGCCACCGGCACGCGCAUCGCGCAUCCGUGGCUGCUGUGGCUGCUGCCCGCGGCGGUUUCGCGACGGGCUGGGUCUAUCAUCGCGUCGG
Bps-1-1	GCCGUCGUCGGCGCGCUCGCCGGCUCGGCGUCCGCGCUCCUCCUGUGGGCGCUCGAUCUUGCCACCGGCACGCGCAUCGCGCAUCCGUGGCUGCUGUGGCUGCUGCCCGCGGCGGUUUCGCGACGGGCUGGGUCUAUCAUCGCGUCGG
Bps-8-1	GCCGUCGUCGGCGCGCUCGCCGGCUCGGCGUCCGCGCUCUUCCUGUGGGCGCUCGAUCUUGCCACCGGCACGCGCAUCGCGCAUCCGUGGCUGCUGUGGCUGCUGCCCGCGGCGGUUUCGCGACGGGCUGGGUCUAUCAUCGCGUCGG
Bps-18-1	GCCGUCGUCGGCGCGCUCGCCGGCUCGGCGUCCGCGCUCUUCCUGUGGGCGCUCGAUCUUGCCACCGGCACGCGCAUCGCGCAUCCGUGGCUGCUGUGGCUGCUGCCCGCGGCGGUUUCGCGACGGGCUGGGUCUAUCAUCGCGUCGG
Bth-8-1	GCCGUCGUCGGCGUGCUCGCCGGCUCGGCAUCCGCGCUCUUUCUGCGUGCGCUCGAUCUCGCCACCGGCACGCGCGUCGCGCAUCCGUGGCUGCUGUGGCUGCUGCCCGCAGCGGGCUUCGCGACCGGCUGGGUCUAUCAUCGCGUCGG
Bsp-1-1	CUGCUUGGCACGCUGGCCGGCACGGCUUCCGCCUUGUUCCUGAUCGCGCUCGACUGGGCGACCGGCACGCGCGUCGCGCAUCCGUGGCUGCUGUGGGGGCUGCCGGCCGCCGGUUUCGCAACCGGCUGGAUCUACCAUCGCUUCGGCCA
Bce-17-1	CUGCUUGGCGCGCUGGCCGGCACGGCUUCCGCGCUGUUCCUGAUCGCGCUCGACUGGGCGACCGGUACGCGCGUCGCGCAUCCCUGGCUGCUGUGGGGGCUGCCGGCCGCCGGUUUCGCCACCGGCUGGGUCUACCAUCGAUUCGGCCA
Bvi-1-1	CCUGCUCGGCAUCCUGGCCGGCACGGCUUCCGCAUUCUUCCUGAUCGCGCUCGACUGGGCGACCGGCACGCGCAUCGCGCAUCCGUGGCUGUUGUGGGGGCUGCCGGCCGCCGGCUUCGCGACCGGCUGGGUCUACCACCGUUUCGGCC
Bam-2-2	AUGCGAGCCUGUACGGCGUGCUGGGUGGCGGACUGGCUGCCGCGCUUGUCCAUGCGGCCGGGACACACCAUAUGCUCGGCCACCGGCGCGCUCAUACUGGCGGGAUUUAUAGCAGGCAUUGCUUUCGAUCGCGCGAAUCGAACGACAUCG
Bam-2-3	GGUGGGCUGGGCGGAGGACUGGCCGCAGUACUCGUGCACUGCGCCGCGACCCAGCAUCCUGGCAUCGCCGUAUCGAUGCUGGCGGCUUUUCUGGCAGGCGUGGCGUUCAGUUACACGGACGCGACGCCAUCGAAUGCCAACCCGCCGUC
Sfu-1-1	CGGUGCGGCACCUCGACGCCCUGAGAGCGAAGCUCCGGGAGGAGGCGUUCAACCUGGUAGUCCUCGGCCAGUUCAAGCGAGGGAAAUCCACGUUCAUCAACGCGCUGCUCGGCGCGAACAUCCUUCCCACCGCCAUUGUGCCCCUCACU
Dpi-1-1	CGAAGGGGCGCGCAGCGUGCUGGGGGAUUAUCUGGGACUGCUCGGGGCUUCCGCGGCCACCGUGGGCCUCGUGGCCGGUCUGGGCGAGUGCCUGGGCUAUGCCCUGCGCAUGUUCACGGGCAUGCUGGCCGACAGGACGCACUUAUACU
Dol-1-1	GGUGCCACGGGCGAACCGAAGGUUGCCCGUGAAAGGCCGAGGGUUGUAUCGUGCGUCAUUGCGAGCGAAGCGAAGCAAUCUUGUUCGUGUACUUACGUCAUUGCGAGCGAAGCGAAGCAAUCUUGUCCAGAAACGAAGGAGAUUGCCAC
Dac-2-1	UGAACAUUUGAGUCUCGGCUGGUUUGUGGUGCGCUGGUUGCUGAUGAUCGCACCGGUUGCCGUCCUGAUUGGUUCCAGCGUGGCUUUUUCCUCUGGUUGCUGGCUGAGGCCACUCAGACCCGUUGGGCGCACCCUGAGCUGGUGUUUG
Xca-1-1	GCCAGCGUGGUGGGCGUCCUGGCGGGUACGGCGUCGGCGCUGUUCUUGCUGGCGUUGGAGGCGGCGACCGCCUGGCGUGAAAGCCAUGCCUGGGUACUCUGGCUGUUGCCUGUGGCCGGAUUCGGUGUCGGGCUGGUCUACCACCUGCU
Xor-1-1	UGCCAGCAUUGUCGCGGCGCUGGCCGGCACCGCUUCGGCAUUUUUCUUGUGGGCACUGGAUUGGGCCACCAAUUGGCGCCCGGCACAUCCGCGCGCGAUUUGGCUGUUGCCGCUGGCCGGUUUUGCGGUCGGGCUGGUGUAUCUGCUGC
Xor-4-1	UGCCAGCAUUGUCGCGGCGCUGGCCGGCACCGCUUCGGCAUUUUUCUUGUGGGCACUGGAUUGGGCCACCAAUUGGCGCUUGGCACAUCCGCGCGCGAUUUGGCUGUUGCCGCUGGCCGGUUUUGCGGUCGGGCUGGUGUAUCUGCUGC
Ppr-1-1	AGACGCGAUUUCACAAUAACAACGAAUUGAACGGUACAACGCUAGAACAGCAAAUGAGGCAGAGUAAUGCAAUAUUCAGCAGAAGUUCAAAACAUGUGUCCUAUCUCUCGCGGACCACAACAUGCGUCUUCCCCCAUUCCUGUAGAAGG
Ppr-2-1	AGACGCGAUUUCACAAUAACAACGAAUUGAACGGUACAACGCUAGAACAGCAAAUGAGGCAGAGUAAUGCAAUAUUCAGCAGAAGUUCAAAACAUGUGUCCUAUCUCUCGCGGACCACAACAUGCGUCUUCCCCUAUUCCUGUAGAAGG
Psp-3-1	UAUACAACUCUUCCCUACUCUGAGUUUAUAAUUAGUAACGCGGUGUCUAGUGAGGCAGAGCAAUGCAAUACUCAAGUGAAGUACAAAAUAUGUGUCCUAUCUCUCGUGGUCCACAUCACGAGUCCGCACCCAUUCCUGUUGAAGGUCGC
Van-1-1	UGAGCGUUAUACAACUCUUCCCUACUCUGAGUUUUAUAAUUAGUAACGCGGUGUCUAGUGAGGCAGAGCAAUGCAAUACUCAACUGAAGUACAAAAUAUGUGUCCUAUCUCUCGUGGUCCACAUCACGAGUCUGCACCCAUUCCUGUUG
Eta-1-1	GCGCCUGAAUGCCUGGUUUCCAAAUUUACCGCCCGGCACCCUGCUGGUGAAUUUGGUGGGGGGUUGAUUAUUGGCGCGACCGUGGCGUGGUUUGCCCGUUAUCCGGGCAUCGAUCCAAACUGGAAGUUGCUCAUUACUACCGGGCUGU
Cko-1-1	CGGCUCUUGUUAUGUCCAUCGGCCCGUCUGCAACACAUUGUUCAUUUGCGAUGCUGAACGUUUCUCAUUACUUGAGAGGUGGCGUAUGCAUAUGUACAAACACGCAUUAUUACUGGUCCAGAAUGAAAUCGAUGGUGUGCUGUUGCUGC
Vfi-2-1	UUCAAAACUUAUUUCAAGUAGCUCGUUGGUCAUUACUAAUUCUACCUGUUACUUUAUUGGUAGGGUCAUUUAACGCAUUUUUCUUAUGGUUACUAAGUGAAGCGACGCAAGCUCGCAUUGCGAAUCCAUGGCUGAUUUAUUUACUACCA
Vfi-1-1	UUCAAAACUUAUUUCAAGUAGCUCGUUGGUCAUUACUAAUUCUACCUGUUGCUUUAUUGGUAGGGUCAUUUAAUGCAUUCUUCUUAUGGUUACUAAGUGAAGCAACGCAAGCUCGUAUUGCGAAUCCAUGGUUGAUUUAUUUAUUACCA
Sgl-1-1	UAAUCCAUUCGGUUCUGCCCUUCCCCGCCGGCACGCUGCUUGUCAAUUUGGUGGGGGCAUUUAUCAUCGGCUUAGCGCUUGUGCUCUUCCCGCGCAUGCCGCAGCUGGAUCCCGCCUGGAAGUUACUGCUCACAUCACCACCGGUUUUU
Csa-2-1	UACUCGGCAUGUGGCUCAAUGCCCUUUUCCCUGCCCUGCCCCCGGUACACUGGCGGCCAACUGGCUGGGCGCCUGGUUGAUCGGCAUCGCCAUUGCCCUGUUCGCCCAGCUGCCGCAGCUUUCACCGGAAUGGCGCCUGUUCGUGGUG
Vca-1-1	CCAAUCAAUGUGCCCGAUUCAACGUGGCGAUCUUCAUGCAUCUGCACCGAUCCCUGUCGAAGGCGCAAUGAUCAAUCCAAAAGACGUUAUCGCGAUUUCUGGUCUAAGCCACGGCGUUGGUGCUUGCGCUCCUCAACAGGGUGCAGCAA
Vha-2-1	UCCAAUCAAUGUGCCCGAUUCAACGCGGCGAUCUUCAUGCAUCUGCACCGAUCCCUGUUGAAGGCGCAAUGAUCAAUCCAAAAGACGUUAUCGCGAUUUCUGGUCUUAGCCACGGUGUUGGUGCUUGUGCUCCUCAACAAGGCGCAGCA
Vha-1-1	AACGCGUAAGCCUCCUGAACAUAGUUUUCCUAUUUGAUGGAGCGAAUAAGAGCAGUCAACGCCGUUGAGUCUGACCAAUAUGCUGGAUAUCGUACACUGGGAAAACAUUAUGCAAUACUCACACGAAAUCCAAUCAAUGUGCCCGAUUC
Val-1-1	UCCAAUCAAUGUGCCCGAUUCAACGCGGCGAUCUUCAUGCAUCUGCACCGAUCCCUGUUGAAGGCGCAAUGAUCAAUCCAAAAGACGUUAUCGCGAUUUCUGGUCUUAGCCACGGCGUUGGCGCUUGUGCUCCUCAACAAGGUGCAGCA
Afe-1-1	UUCCCUGGGCCACCCUGAGCAUCAAUGUCCUGGGCUGUUUUCUCAUGGGUUUUCUGUUUUUCGAGACCUUGGAACGUAUUUCCGUGAGCCCGGAAUUGCGCACGGGCAUGCUCACCGGCGGCCUGGGUGCCUACACCACCUUUUCGACA
Sma-1-1	UGCAACCUGAUCGGCGCCCGAGUGGCCGUCGGCAGCCCCUGGCCGUGGUCGACCUUCCUGAUCAACAUCAGCGGCGCCCUGCUGAUGGGCGUGGUGGUCGAGGUCUUCGCGAUGCGCAAUGGUGCCUCGCCGCAGCUUCGCCUGUUGCU
Sma-2-1	UGCAACCUGAUCGGCGCACGGGUGGCAGUCGGCAGCCCGUGGCCGUGGUCGACCUUCCUCAUCAACAUCAGCGGCGCACUGCUGAUGGGCGUGGUGGUCGAGGUCUUCGCGAUGCGCAAUGGCGCCUCACCGCAGCUUCGCCUGCUGCU
Vsp-1-1	CAACGCCCUAGGUCUUAAAUGGCUCUAUCAGUGAGAAAACAUUAUGCACUACUCUUCAGAAAUUCAAUCAAUGUGCCCUAUUCAAAGGGGUGAUCUUCACACUUCAGCUCCAAUUCCAGUUGAAGGCGCCAUGGUUAGCCCAAAAGAUG
Vba-1-1	CAACGCCCUAGGUCAAUACGACUUUAUUAGUGAGAAAACAUUAUGCAUUACUCUUCAGAAAUUCAAUCCAUGUGCCCUAUCCAAAGGGGUGAUCUUCACAACUCGGCUCCAAUCCCAGUUGAAGGCGCCAUGGUUAGUCCAAAAGAUGU
Vsp-2-1	CAACACCCUAGGUCUUCAAUGACUCUAACUGUGAGAAAACAUUAUGCAUUAUUCUUCAGAAAUUCAAUCAAUGUGCCCUAUUCAAAGGGGUGAUCUGCACACUUCAGCGCCAAUUCCAGUUGAAGGCGCUAUGGUUAGCCCAAAAGAUG
Vfi-1-2	UGCAAUACGCAUCUGAAAUUCAAACCAUGUGCCCUAUUCAAAGAGGCGAUCUUCACCCAUCAGCACCAAUUCCUGUUGAAGGAAGAAUGAUUAAUCCAAAAGAAGUAAUAGCGAUUUCUGGAUUAAGUCAUGGCGUAGGUGCUUGUGCU
Vfi-2-2	UGCAAUACGCAUCUGAAAUUCAAACCAUGUGCCCUAUUCAAAGAGGCGAUCUUCACCCAUCAGCACCAAUUCCUGUUGAAGGACGAAUGAUUAAUCCAAAAGAAGUGAUAGCGAUUUCUGGAUUAAGUCAUGGCGUAGGUGCUUGCGCU
Asa-1-1	GAGUUGAUCGCUCUUGGUCUGUAGGUUAUCAAUGCCCUCCUACACCUGUUUUCCUAAUCAUUGGGAAAAUAUUAUGCAAUACGCAUCAGAAAUUCAAACCAUGUGCCCUAUUCAAAGAGGCGAUCUCCACCCAUCAGCUCCUAUCCCUG
Vsh-1-1	GGCGACCUUCAUGCAUCCGCGCCAAUUCCUGUCGAAGGCCGCUGGAUCAAUCCCAAAGACGUUAUCGCAAUCUCUGGCCUGAGUCAUGGUGUUGGUGCUUGUGCUCCUCAACAAGGUGCGGCGAAGUUAACGCUUAACGUUAAAGAUGG
Pca-1-1	GCGUGAAGUUUAACAAUCUGUAUCCAACGCUGCCGCUGGGCACUCUGCUUGCCAACGUGAUCGGGGCUUUUGUUAUUGGCGGCGCACUCGCUUUCUUCCUGCGCCAUCCUCAUCUCGAUCAGGACUGGAAAAUAUUGAUUACUACCGGG
Pca-2-1	GGUGUGAAGUUUAACAAUCUGUAUCCAACGCUGCCGCUGGGCACGCUGCUUGCCAACCUGAUUGGUGCUUUCGUUAUUGGCGGCGCGUUAGCCUUCUUCCUGCGCCAUCCUCAUCUCGAUCAAGACUGGAAAAUAUUGAUUACCACCGG
Psy-2-1	UCUUGUAGCUCUUUUGGCAGGCUCUGCUUCUGCGUUAUUCCUGCUUUCUUUGGAUCAUGCCACCCAGUGGCGAGAAACCCAUCCCUGGGUAAUCUGGCUCCUGCCAGUGGCCGGCUUUGCUGUGGGACUUGCAUAUCACUUGAUAGGCA
Cko-1-2	UGUCGAUCUGAAUCUUGAUAAAAUCGUCGAUCGCAUUGUUCAGGGACGGGAAGAGUACGGCAUCAGCGCUUUCUUUUAUUCACCACUUGAAUGUCUGGAGGAUAUCCUCUAUCGACAGGAAAUUAUUCAGGAUGUGACAAUACAUGCGC
Psy-1-1	UCACUAUUAGUUAUCGCCAUCGGUGCAUCACUUGGUGCUUGGUUGCGCUGGCUCCUAGGCAUGAAGCUCAACGCACUCUUCCCGACAAUUCCCCCAGGCACUGUGGUGGCGAACAUGGUCGGGGGUUACAUCAUCGGCCUGGCCAUCGC
Pae-3-1	CUGCUGCCCAGCAUUCCCCCCGGCACGCUGCUGGCCAACCUGGUCGGCGGCUAUGCCAUUGGCGCGGCCAUCGCCUAUUUCGCCCAGGCGCCGGGCAUAGCGCCGGAGUGGCGGCUACUGAUCAUCACCGGGUUCUGCGGUGGCCUGAC
Pae-6-1	CUGUCCAGCAUUCCCCCCGGCACGCUGCUGGCCAACCUGGUCGGCGGCUAUGUCAUCGGUGCGGCGAUUGCCUAUUUCGCCCAGGCGCCGGGCAUCGCUCCGGAAUGGCGGCUACUGAUCAUCACCGGGUUCUGCGGCGGCCUGACCAC
Pae-5-1	CUGCCCAGCAUUCCCCCCGGCACGCUGCUGGCCAACCUGGUCGGCGGCUAUGUCAUCGGCGCGGCGAUUGCCUAUUUCGCCCAGGCGCCGGGCAUCGCUCCGGAAUGGCGGCUACUGAUCAUCACCGGGUUCUGCGGCGGCCUGACCAC
Pae-2-1	CUGCCCAGCAUUCCCCCCGGCACGCUGCUGGCCAACCUGAUCGGCGGCUAUGUCAUCGGCGCGGCGAUUGCCUAUUUCGCCCAGGCGCCGGGCAUCGCUCCGGAAUGGCGGCUACUGAUCAUCACCGGGUUCUGCGGCGGCCUGACCAC
Pae-4-1	CUGCCCAGCAUUCCCCCCGGCACGCUGCUGGCCAACCUGGUCGGCGGCUAUGUCAUCGGCGCGGCGAUUGCCUAUUUCGCCCAGGCGCCGGGCAUCGCUCCGGAAUGGCGGCUACUGAUCAUCACCGGGUUCUGCGGCGGCCUGACCAC
Pen-1-1	AUUCGCUGUUCGCCGGCAUGCCGUUCGGCACCUUGCUGGUCAACCUCAUCGGUGGCUUCGUGAUCGGUGGGGCGAUGGCCUUCUUCCUGCGUUUCCCCAACCUCGACCCGGCCUGGCGCCUGCUGAUCACCACCGGUUUUUGUGGCGGC
Xca-4-1	CCGGUGGCGCACUGGGAUCGGGCUUGCGUUUUGCGAUCGGUGCCUCGUUGAUCCAGCGCUUCGGUACCGGCUUCCCUUGGGGAACGCUGACGGUGAACCUGCUCGGGUCGUUCGUGGCCGGCGUGUUGCUGGUCUGGCUGGAUGCACGC
Xax-1-1	CCGGUGGUGCGCUGGGAUCCGGUUUGCGUUUUGCGAUCGGUGCCUCGUUGAUCCAGCGCUUCGGUAACGUCUUCCCGUGGGGAACGCUGACGGUGAACCUGCUCGGGUCGUUCGUUGCCGGCGUGUUGCUGGUCUGGCUGGAUGCACGC
Pst-1-1	CUGGCUAAGAUGGUUUGUGGGGCUGAAGCUGAAUAACUUAUACCCAAAUAUUCCGAUGGGAACUGUUUUUGUCAAUCUUGUCGGGGGAUUUGUUAUCGGUUUUGCCAUUUCUUAUUUUGCCAAUGCCAAUAUUAGCCCCAAUUAUAAAC

```
Asp-2-1   CAAUUGCAUUAGGUUCUGUGCUCGGCGCAUGGUUACGUUGGUUUAUUGGCUUAAAACUCAAUCCAAUUUUUCCGCAUAUUCCACUCGGGACCGUAAGUGUUAAUUUGGUUGGUGGUUUUAUGAUUGGUUUUGCGAUUUCAUAUUUUUCG
Asp-1-1   CUUUCAAAAUUUUCCGCUUGGUACCAUUUUAGUGAAUCUGGUGGGUGGUUUUAUCAUCGGCUUUGCAAUUGCCCUGUUUGCCAAUAUGCAGCUUAGUUCUAAUUAUAAAUUAUUUGUGAUUACUGGCUUUUGUGGUGCUCUAACCACAU
Aba-3-1   UUAGGUUCAGUGCUCGGGGCGUGGUUGCGUUGGCUUUUAGGUUUAAAACUCAACCCGAUUUAUCCGCAAAUUCCGUUGGGUACUGUUACUGUUAAUUUGGCCGGUGGCUUUAUUAUUGGUUUUGCGAUGGCUUAUUUUGCUCAUAGUGA
Aba-4-1   UUAGGUUCAGUUCUUGGGGCGUGGUUGCGUUGGUUUUUAGGUUUAAAACUGAACCCGAUUUAUCCGCAAAUUCCGUUGGGUACGGUUACUGUUAAUUUGGUCGGUGGCUUUAUUAUUGGUUUUGCGAUGGCUUAUUUUGCUCAUAGUGA
Aba-5-1   UUAGGCUCAGUUCUAGGGGCGUGGUUGCGUUGGUUUUUAGGUUUAAAACUCAACCCGAUUUAUCCGCAAAUUCCGUUGGGUACUGUUACUGUUAAUUUGGUCGGUGGCUUUAUUAUUGGUUUUGCGAUGGCUUAUUUUGCUCAUAGUGA
Ymo-1-1   GCUGGUUGGUCAGCAUGAAGCUAAACAGUCUAUCGCACAAUAUCCCGGUAGGGACACUGGUGGUAAACCUGAUUGGGGCCUUUAUUAUUGGCCUGACAUUGGCACUUUUUACCCGACUUACACAUAUUGAUCCCGUUUGGAAGUUACUG
Yin-1-1   CACCCGCUGGCUGAUCAGCCUGAAGCUAAACAGCCUAUCCAGCCAUGUCCCCGUAGGAACACUGUUGGUAAACCUGAUUGGUGCCUUUAUCAUCGGGCUGGCAUUAUCCCUGUUUACCCGCGUUACACAUAUUGACCCGGUAUGGAAGC
Yps-4-1   GCCCGUUGGUUGGUCAGUUUGAAGUUAAACAGUGCAUCGGCUCACUUACCGGUAGGUACGCUUAUCGUCAACCUGGUCGGUGCCUUUAUUAUUGGCCUGACACUGGCGCUUUUUAGCCGAAUGACUCAUAUUGAUCCCGUUUGGAAGCU
Ype-13-1  GCCCGUUGGUUGGUCAGUUUGAAGUUAAACAGUGCAUCGGCUCACUUACCGGUAGGUACGCUUAUUGUCAACCUGGUCGGUGCCUUUAUUAUUGGCCUGACACUGGCGUUUUUUAGCCGAAUGACUCAUAUUGAUCCCGUUUGGAAGCU
Yps-2-1   GCCCGUUGGUUGGUCAGUUUGAAGUUAAACAGUGCAUCGGCUCACUUACCGGUAGGUACGCUUAUCGUCAACCUGGUCGGUGCCUUUAUUAUUGGCCUGACACUGGCGCUUUUUAGCCGAAUGACUCAUAUUGAUCCCGUUUGGAAGCU
Yps-1-1   GCCCGUUGGUUGGUCAGUUUGAAGUUAAACAGUGCAUCGGCUCACUUACCGGUAGGUACGCUUAUCGUCAACCUGGUCGGUGCCUUUAUUAUUGGCCUGACACUGGCGCUUUUUAGCCGAAUGACUCAUAUUGAUCCCGUUUGGAAGCU
Spr-1-1   UGAAUCCGCUUAACGCGCACAUCCCGUUGGGGACGCUGGCAGUCAAUCUGCUGGGCGGCUUUAUUAUCGGUUUGGCUAUCGCCAUCUUCAGCCGUUUGACGCACCUGGAUCCCACCUGGAAGUUGCUGAUCACCACCGGUUUUUGCGGC
Pru-1-1   CUUGGGGGGCUGGUUAAGAUGGAUAAUUGGUUUAAAACUGAAUAACCUACAUCCGCAUAUUCCAUUUGGUACGGUGUUUGUUAAUCUCAUCGGUGGUUUUAUCAUCGGUUUUGCUGUGUCUUAUUUUGCGAGUGCCAAUAUCAAUCCCG
Pal-1-1   GGUGGCUGGUUAAGAUGGCUAAUCGGCUUGAAGCUCAAUAAUUUACAUCCUCAUAUCCCCUUUGGUACGGUAUUCGUCAAUUUAGUGGGCGGCUUUAUUAUUGGUUUUGCUGUCUCUUACUUUGCGAGUGCGAAUAUUAAUCCUGCAUA
Pat-1-1   AGUCUGUAUCCCACAUUGCCUUUGGGUACUCUACUUGCCAACUUGAUUGGUGCUUUCGUUAUUGGCGGCGCGCUAGCCUUCUUCUUGCGCCAUCCUCAUCUCGAUCAGGACUGGAAAAUACUGAUUACCACUGGGCUGUGCGGCGGCUU
Cko-1-3   CGUUACGCGGGCAGGAGGCAGUGGCGUUCCGCACGGGGAAGCCAACCGUGAGACGCAGUUUAUUUUUGCGCAGGCGGGGUUGCCCAUUCCUGAACGAUUAAGCGUUCCGCUCACCGAUCGGGACGUCUGGCUGGUUGAUUUUACCGAA
Kpn-1-1   GAACAGCCUGUUUCCUGACCUGCCACCGGGCACGCUGGUAGUGAACCUUGCGGGCGGAUUUAUCAUUGGUGCGUCGCUGGCCUGGUUCAUGCGCCAUCCUGACCUGGAUCCGGCCUGGCGAUUACUUGUUAUCACCGGGCUGUGCGGUG
Tpe-1-1   CGGAAUGAUAUUUUAGAGUAUUACCUCUCACCUGUUCAGGAUGAGAAAACCGUAAGGUACCGCCAGGAAGUGAUGAAAGAUUUGGAAAACAACGAAAAAUUGCUGGAAGCCAUCAGGAAGUUCGCAAAAGAUAUGGCAUCGUUUCAUCG
Tle-1-1   GUAUGAAUUCGUAAACGAUCCAAAUACAAGAUCCAGACUGAGGCGUGGUAUCUGCAGUAAACAUGUUCUGGCUAUUGAAAACACCCUUAAUAACCACCCAGAGCUUGGAUUACUUGGCUUAGCAAUAAUCUAUGAAGAUAUCAUGUCUG
Cfl-1-1   CCUCCGCUGGCUCAUCUAUAUCGUUCCCAUGGCGGCGGUUGUCGGUUCGGCGUGUGCUUUCUUUUUGUGGGGCCUUGAGCAAGUCACACACUGGCGUUUCGAGCAUCCAUGGCUGCUUUAUUUCCUGCCGCUGGCGGGCGUAGCGGUCG
Min-1-1   UCGGUCCCGAUUACAAGCCUCCCAAGCUCGAGGUUCCCCAAAACUGGAAAUGGGGAGAACAAAAACCCGAUCCCAAAGGCUCCCAUUCCAGGGAAAAGCCGCAAAUCGAAGAGAAAAAAACAGCCUGGGGAGGCAAUGACCUGCAAGGC
bEl-1-1   UAAUUUCUCGUUUCAUGCCGGAAGUUUGAAGCAACUGUUGGGGUGGUCGUUGCUCACAAUCCCUGUGGGAGUGCUGGCAGGUUCAGCGAGUGCUUUCUUCCUUUGGGCUCUGGAUCACGUUACUCACAUUCGCCAGCAACACGCAUGGU
Min-1-2   GCCUCUAUUUUAUCCUCCAGUGUUUCUGUUUUUUUCAAUCCGGCUCCUAUACCGACAUCAUUCCCUAUGCCACGGCUGAACUGGGACAAAGCCAAAGCCAUGGAGCAUGGACCAAUGGAUUUUUCUUCCUGGGGCAAGGUCUCGGACUG
env-1     UCCGCCAUUACCUGGGCACGCUGCCCUAUCACCCCGACGCGAACUGCGCGGCCAAUACCUACUAUCUGCUCAACAACUACAACCCAGGCUAUAACGGCGACGGCAGCGUCAACACGAGCAGUCCCUUCACGAUUCCGCCUUCGCCGGUC
env-2     GAUUACGACCUCAGGCCUGUAUUCGGUACCAUGCCUGAAAACAUACAUACCGUGAGAUACAGGCAGGAGGUCUUCAAGGACCUUCAGAACCCUGAUAAUAUUGCAUUUCUGAAGGGUUUCUCCUCAAAAAUGAAAGGUGUCAAUAUGAG
env-3     UGCAACAGUUUCCUUUACUUUUUUAUUUAUUCAAGUGGCUUUUUCUUUCUGCUAUAUCCGGAGCGUGUAUCGGUUCGGCUUCCGCCUUAUUGUUGGUUUCGCUGGAAUGGGCAACCCAAUAUCGUGAACACCAUCUGUGGAUUAUAGCC
env-4     GGAUCUUUCUCGUCAAUGUGGGCGGAUCGGUUCUGAUUGGUCUCGUCGCGGGACUCCUGGCAUCCGGACGAUGGCACUGGAGCUAUCAAGCUCGAACAUUUGUCAUCGUCGGUCUCCUGGGUGGGUUCACGACGUUCUCGUCGUUCAGC
env-5     CUCGAUCGCGCUCAUGGAAAUCGGCGUCGGGGUUGCGGCCGCGUUCGUCGUGGGCCGCAUCUGGGGUCCUGACGCCAUCGGAGCCGACGUGCCCUGGCUUCGGUUCGCCGCCGGGGCGGGUGCCGUCCUGCUCACGUUUCUCGCCGGGA
env-6     UAUUCAAAAGCUUUUGUCCGUUGCUCCGGAGCACAUGCUCCACCGCCCAGUGUGAUGCGAAGCUGCUCUUCCAUCUGUGUUCCCCCUGUUUUCACACUGCAAAGAGCCAGCAGUGCAGCAGCUUUCCCAGAAGCACACCGCCAAAACAG
env-7     UGGAAAGGACCAGGAAUGGGCGAGCCGUUCUUCGAAUCAAAGAUCCGGUCAGGUUGGAAGGAGUUCAUGAGCUGGCGCGUUCUCCUGCCACUGGCGGGAUUGCUCCUCACAUUGCUCUGGGGAGGCCAGCGCUGGCGUGAAGACCGACA
env-8     UAUGAUCUGGCAAAGUAUCUUAAAAAAGAGAAAACUUCAACAUAUGAUACAAAUCUUCUUUACCGCUGGGUACCAAUGCUGGUAUUUGGUGUUUUGUUCUCAAUUUCAUUUGUAAUUCCUGUUAUAAUACCAAUACCAACAUUAUUUGC
env-9     UGGUCGAGUUCCGCGGUCACCCCAUCGUCGUCGCGGUGGUUCCGGGGCAAUCGAGUCUGGUGGCCCUGACGGCUGCCUCACUGGCCCGUGCAACCAAGGCCUCCCAAAUUGUCUUCGCCCACGUUGAUCAGCGGCGCUGUGUCGUCGAG
env-10    ......................................................................................................................................................
env-11    CAUACUUGGCAGUUUUUUUCUUGGCAUAAUAAUGUUCUACCUGGAUGCAAAUCAAUUAAUUUCUCAGCAGCUUAGAAUUUUUUUAACUAUUGGGUUGUGCGGGGGACUUACUACUUUCUCAACAUUUUCCUUCGAAACAAUUAAUUUUU
env-12    UAUUGUGUUGGUCGUCUUCUGCGGCGGCACUAUUGGCACCGCGAUUCGCUAUGCGUUCGCGCAGAUUCCCGCGGCUGGCAGCUUCCACACCGGCACGUUUGUGGCCAACAUGCUGGCUUGCUUCUGCUAUGCAGGGCUGACCGCUUACU
env-13    UAUUGUGUUGGUCGUCUUCUGCNGCGGUGCGAUCGGCACCGCUAUCCGCUAUGCGUUCGCACAGAUUCCCGCGGCUGGCAGCCUUCAUACCGGCACCGUUUGCGGCCAACAUGCUGGCUUGCUUCUGCUAUGCAAGGGUGAACCGCCUA
env-15    UUGGGUUGGCGCCUUUCUGUGGG...............................................................................................................................
env-16    UAUUGUGUUGGUCGUCUUCUGCGGCGGUGCCAUCGGCACCGCGAUUCGCUAUGCGUUCGCGCAGAUUCCCGCAGCCGGCAGCUUCCAUACCGGCACGUUUGUGGCCAACAUGCUGGCCUGCUUCUGCUAUGCGGGUCUGACCGCCUAUU
env-17    UAUUGUGUUGGUCGUCUUCUGCGGCGGUGCGAUCGGCACCGCUAUCCGCUAUGCGUUCGCACAGAUUCCCGCGGCUGGCAGCUUCCAUACCGGCACGUUUGUGGCCAACAUGCUGGCUUGCUUCUGCUAUGCAGGGUUGACCGCUUACU
env-19    UGCAUCGGAACCGCAUUACGCUACGCUUGUUCGGUCAUUCCCGACUGCGGCGCAUUCCAUAUCGGCACAUUCGCGGCGAAUAUGGCCGCCUGCUUCGUAUACGCGGCGCUUUCGGCAUGGCUGGGAGGAACCCGCAGAAUCGUGGGACG
env-20    UGCAUCGGAACCGCAUUACGCUACGCUUGUUCGGUCAUUCCCGACUGCGGCGCAUUCCAUAUCGGCACAUUCGCGGCGAAUAUGGCCGCCUGCUUCGUAUACGCGGCGCUUUCGGCAUGGCUGGGAGGAACCCGCAGAACCGUGGGACG
env-21    UAUGAUCUCUGGAAACUUUUCCGGAAAGUUACCAUAAUUCCUGCGGGUUCAGGCGUAUUCUUCAGAUACAUUCCCUAUGUCCUCUUCGGCAUUUAUUCUCUCAUUGCGCUCAUAAUACCCGUACUUGUUCCCGUACCCAUAUAUUUCAC
env-22    UUGACCCCGGUCGGGGACGACCCGACUCCGAGGACUUCGAUGGCCGAUCCCGUUUCCCUCCCCUCCUGGCGAUCCCCCAGGAUCCUCAUUCCCCUCUUGGGGCUUGCGGCAACACUCGUAUGGGGACUCUUCCUUCUUGUGGACCGCGG
env-23    CUGACUUUUUUUCUCCUGUACGAAUCCUCUUUCCAGUGGCCGGAUUUUUCGUCCAGACUUCCCUCAUACAGUCUCUGGGCACCUUCGUGAAGCGUGGUCUUCUCCGCCGGGACAACCCCUGGUGGAUUCUUUUGACCGUCGUGAUUGGU
env-24    GAAGAAGUAUUAUAUAAACCAUUAGAUAGCAUAGAGGAUAUCCUAUACAGGCAGCAAAUUUUUAAUGAUUUACGAAGUCCACAAAUAUUUAAUGCUAUAAAACAAUUUUCCGAAACAUCAUCCACCAUUUUCUACAAUAUUGGGAAAUU
env-25    ......................................................................................................................................................
env-26    UUUACUGCCGACAGCUCAGCUAGAUCUGCG........................................................................................................................
env-27    AGGUAUUAUAUUAGGAGCGUUAAUUUUCAGAAUGGCAGGAGUCGCUUUGUGUAUGGCAGGAACUAAGCUGAAGCUGAAAGAAAGGGUAUUCUGUAUGCUUGCAUACACACCUAAGGCAACCGUUCAGGCGGCGAUAGGCGGAGUUCCAU
env-28    ......................................................................................................................................................
env-29    UCGGUAUGCUUGUUACCGGUAUUGUUCUGACAAGCUCAGGCCUA..........................................................................................................
env-30    GGCUUGCAAUGGGAGCCAUUUGCCAGAAACUGAAGCUGCCACGGAUCAUCGGAAUACUCGUCACCGGAAUUGUGUUGGGACCGUAUGUGCUCGAUGCGGUAUCUCCGGCGGUGGUCGUUCCAAGAAUGGUAACUUUUAUGGAGAAAAAA
env-31    CCGGCAUGGUUCUGGGACCCUACGUCCUGAACCUGCUGGACGGCAGCAUUCUGGGUAUCUCCCCGGAGCUGCGGAAGAUCGCCCUUAUCAUCAUUCUCACCCGUGCAGGGCUGGGGCUGGAUACUUCCGGGCUGAAAAAGCUGGGGCGA
env-32    ......................................................................................................................................................
env-33    AACGAAAUGAAACAGAAGAAAACAGGAAUAUCCGCAGAAAAACAGGAACAAUGGAUUCUGCAAGGAAAAUACGUGAUUCUUGCCGUAUUAGUAGGUAUCUGCGUGGGAAUCGUCGACACGAUUUUCGGAAGAGGGUUGCUGGCAAUUUC
env-34    GUAGAUACUAUCUUCGGCCGGGGCCUGCUGGCGAUCUCUGCUUUUCGCACUGCACACUGGCGCUGCCUGCUGCCCUUCCUGCCUCUGGCCGGACUUCUUAUUGUAUGGAUGUAUCAUCAUUUCAGCACCUUAAGCUUAAAGGGCAUGUC
env-35    UGUUUGGAAGAGUGCUGAUCGCAAUUUCAGAGUUCAGGACCAUACAUUAUCAAUAUUUACUGCCAUUCCUGCCAAUUGCUGGGCUGGUGAUCACAGCAAUGUAUUACGUAUUCAGUAAACUAAGUCUAAAAGGAAUGAAACUCAUUUUC
env-36    UGUUUGGAAGAGUGCUGAUCGCAAUUUCAGAGUUCAGGACCAUACAUUAUCAAUAUUUACUGCCAUUCCUGCCAAUUGCUGGGCUGGUGAUCACAGCAAUGUAUUACGUAUUCAGUAAACUAAGUCUAAAAGGAAUGAAACUCAUUUUC
env-37    UCCCUAUGGCACGCUCGCAGUCAAUGGCUUGGGUUGCCUGGUCAUCGGAUUUUUGGCGGGGCUGGCGGAGAGCCCGGUUGGCUUUCACGCCGGAAGCCCGUCUGUCAUUUGUCGACCAAGAUCAACCUUUUCCCNNN...........

          ......................................................................................................................................................
          ......................................................................................................................................................
          ......................................................................................................................................................
          ......................................................................................................................................................
          ......................................................................................................................................................
          ......................................................................................................................................................
          ......................................................................................................................................................
```

```
env-38   CUGCUUUUUUCUUAAAUAGCCUGACAGUUGUGACAGAUAUCCGACUAUCACAUCCUUGGCUCCUUUAUCUACUACCAGUUAGUGGUGCGCUUUUUGCUUAUCUCUAUGCGUAUCACGGCGGACUUUCUAGUCGUGGAAACAACUUAGUG
env-39   CUGCUUUUUUCUUAAAUAGCCUGACAGUUGUGACAGAUAUCCGACUAUCACAUCCUUGGCUCCUUUAUCUACUACCAGUUAGUGGUGCGCUUUUUGCUUAUCUCUAUGCGUAUCACGGCGGUCUUUCUAGUCGUGGAAACAACUUAGUG
env-40   AUUCUUCCACAGCGAAUCUUGGAUGAUUUAUAUAGCUAGUUUUGGAGGAAUAUUGCUUACAUUUCUAGCUGGAGUCGAAAUUGAUGUUGACUUAAUGAAAGAUAAUUUUAAAGAAAGUUUUACAAUAGGAUUUUUAUCAUUUCUAAUUC
env-41   AUUCUUCCACAGCGAAUCUUGGAUGAUUUAUAUAGCUAGUUUUGGAGGAAUAUUGCUUACAUUUCUAGCUGGAGUCGAAAUUGAUGUUGACUUAAUGAAAGAUAAUUUUAAAGAAAGUUUUACAAUAGGAUUUUUAUCAUUUCUAAUUC
env-42   CCGUCUGAACGGCCUGUUCACCGGUUUGCCGCUCGGCACUUUGGCCGCUAACGUGAUCGCCGGCUACGUCAUCGGCGUCGCGGUCGCGGGUUCGCGCGGGCGCCGCAGAUCGCCCCCGAAUGGCGGCUCUUCGUCAUCACGGGUUUGA
env-43   CUGCUCGGCACGCUGGCCGGCACGGCUUCCGCCCUGUUCCUGAUCGCGCUCGACUGGGCGACCGGCACGCGCGUCGCGCAUCCGUGGCUGCUGUGGGGGCUGCCGGCCGCCGGUUUCGCGACCGGCUGGAUCUACCAUCGCUUCGGCCA
env-44   GUUGCCGUUCUUGCGGGGAGCGCAUCCGCCGCCUUCCUCUUUUCCCUUGAGUGGGCAACGGCCAGCCGCGAAAGCCAUCGCUGGAUGAUUUGGCUGUUACCCGUUGCCGGCUUCAGCGUCGGAUGGGUUUAUCUCCGCUAUGGUAGCCG
env-45   ..........................................................................
         ..........................................................................
         ..........................................................................
         ..........................................................................
         ..........................................................................
         ..........................................................................
         ..........................................................................
         ..........................................................................
```

25 Cyano-1

25.1 Taxa

The taxonomy of each organism containing a putative Cyano-1 RNA is listed, with abbreviations identifying each hit (e.g., "Eco-1-1" and "Eco-1-2" might hypothetically represent two distinct RNAs in *E. coli*). The abbreviations will be used to identify each individual Cyano-1 RNA in Sections 25.2 and 25.4.

**abbrev. of hits**	**taxonomy of species**
Ama-1-1	Bacteria Cyanobacteria *Acaryochloris marina* MBIC11017
Cwa-1-1 to Cwa-1-2	Bacteria Cyanobacteria Chroococcales *Crocosphaera watsonii* WH 8501 (Synechocystis sp. WH 8501)
Csp-1-1	Bacteria Cyanobacteria Chroococcales *Cyanobium sp.* PCC 7001
Csp-2-1 to Csp-2-2	Bacteria Cyanobacteria Chroococcales *Cyanothece sp.* ATCC 51142
Csp-3-1 to Csp-3-2	Bacteria Cyanobacteria Chroococcales *Cyanothece sp.* CCY 0110
Csp-4-1	Bacteria Cyanobacteria Chroococcales *Cyanothece sp.* PCC 7424
Csp-5-1 to Csp-5-2	Bacteria Cyanobacteria Chroococcales *Cyanothece sp.* PCC 7822
Csp-6-1	Bacteria Cyanobacteria Chroococcales *Cyanothece sp.* PCC 8801
Csp-7-1	Bacteria Cyanobacteria Chroococcales *Cyanothece sp.* PCC 8802
Mae-1-1 to Mae-1-2	Bacteria Cyanobacteria Chroococcales *Microcystis aeruginosa* NIES-843
Sel-1-1	Bacteria Cyanobacteria Chroococcales *Synechococcus elongatus* PCC 6301
Sel-2-1	Bacteria Cyanobacteria Chroococcales *Synechococcus elongatus* PCC 7942
Ssp-1-1 to Ssp-1-2	Bacteria Cyanobacteria Chroococcales *Synechococcus sp.* BL107
Ssp-2-1 to Ssp-2-10	Bacteria Cyanobacteria Chroococcales *Synechococcus sp.* CC9311
Ssp-3-1 to Ssp-3-5	Bacteria Cyanobacteria Chroococcales *Synechococcus sp.* CC9605
Ssp-4-1 to Ssp-4-3	Bacteria Cyanobacteria Chroococcales *Synechococcus sp.* CC9902
Ssp-5-1	Bacteria Cyanobacteria Chroococcales *Synechococcus sp.* JA-2-3B'a(2-13)
Ssp-6-1	Bacteria Cyanobacteria Chroococcales *Synechococcus sp.* JA-3-3Ab
Ssp-7-1	Bacteria Cyanobacteria Chroococcales *Synechococcus sp.* RCC307
Ssp-8-1 to Ssp-8-2	Bacteria Cyanobacteria Chroococcales *Synechococcus sp.* RS9916
Ssp-9-1	Bacteria Cyanobacteria Chroococcales *Synechococcus sp.* RS9917
Ssp-10-1 to Ssp-10-2	Bacteria Cyanobacteria Chroococcales *Synechococcus sp.* WH 5701
Ssp-11-1 to Ssp-11-2	Bacteria Cyanobacteria Chroococcales *Synechococcus sp.* WH 7803
Ssp-12-1 to Ssp-12-2	Bacteria Cyanobacteria Chroococcales *Synechococcus sp.* WH 7805
Ssp-13-1 to Ssp-13-4	Bacteria Cyanobacteria Chroococcales *Synechococcus sp.* WH 8102
Ssp-14-1 to Ssp-14-3	Bacteria Cyanobacteria Chroococcales *Synechocystis sp.* PCC 6803
Tel-1-1 to Tel-1-2	Bacteria Cyanobacteria Chroococcales *Thermosynechococcus elongatus* BP-1
Ava-1-1 to Ava-1-3	Bacteria Cyanobacteria Nostocales Nostocaceae *Anabaena variabilis* ATCC 29413 (Anabaena flos-aquae UTEX 1444)
Npu-1-1	Bacteria Cyanobacteria Nostocales Nostocaceae *Nostoc punctiforme* PCC 73102 (Nostoc punctiforme ATCC 29133)
Nsp-1-1 to Nsp-1-5	Bacteria Cyanobacteria Nostocales Nostocaceae *Nostoc sp.* PCC 7120
Lsp-1-1	Bacteria Cyanobacteria Oscillatoriales *Lyngbya sp.* PCC 8106
Ter-1-1	Bacteria Cyanobacteria Oscillatoriales *Trichodesmium erythraeum* IMS101
Pma-1-1	Bacteria Cyanobacteria Prochlorales Prochlorococcaceae *Prochlorococcus marinus str.* AS9601
Pma-2-1	Bacteria Cyanobacteria Prochlorales Prochlorococcaceae *Prochlorococcus marinus str.* MIT 9211

Pma-3-1	Bacteria Cyanobacteria Prochlorales Prochlorococcaceae *Prochlorococcus marinus str.* MIT 9215
Pma-4-1 to Pma-4-2	Bacteria Cyanobacteria Prochlorales Prochlorococcaceae *Prochlorococcus marinus str.* MIT 9301
Pma-5-1 to Pma-5-2	Bacteria Cyanobacteria Prochlorales Prochlorococcaceae *Prochlorococcus marinus str.* MIT 9303
Pma-6-1 to Pma-6-2	Bacteria Cyanobacteria Prochlorales Prochlorococcaceae *Prochlorococcus marinus str.* MIT 9312
Pma-7-1 to Pma-7-2	Bacteria Cyanobacteria Prochlorales Prochlorococcaceae *Prochlorococcus marinus str.* MIT 9313
Pma-8-1 to Pma-8-2	Bacteria Cyanobacteria Prochlorales Prochlorococcaceae *Prochlorococcus marinus str.* MIT 9515
Pma-9-1 to Pma-9-4	Bacteria Cyanobacteria Prochlorales Prochlorococcaceae *Prochlorococcus marinus str.* NATL1A
Pma-10-1 to Pma-10-4	Bacteria Cyanobacteria Prochlorales Prochlorococcaceae *Prochlorococcus marinus str.* NATL2A
Pma-11-1 to Pma-11-3	Bacteria Cyanobacteria Prochlorales Prochlorococcaceae *Prochlorococcus marinus subsp. marinus str.* CCMP1375
Pma-12-1 to Pma-12-4	Bacteria Cyanobacteria Prochlorales Prochlorococcaceae *Prochlorococcus marinus subsp. pastoris str.* CCMP1986
env-1 to env-179	environmental samples

25.2 Gene contexts

Each Cyano-1 RNA (indicated by "RNA→") is listed with its surrounding genes. Environmental sequences and some RefSeq entries lack gene annotations, and no genes are listed for such sequences. The direction of each gene is indicated with an arrow (→), and each conserved domain in the gene is colored. Conserved domains associated with more than one Cyano-1 RNA are assigned a color; other domains are gray. Information about these conserved domains is given in Section 25.3. The accession number of the sequence containing each Cyano-1 RNA is given. Accessions beginning with "NC_" or "NZ_" are RefSeq, while other accession refer to environmental samples. Nucleotide coordinates are given for the 5′ and 3′ boundaries of each. If the 5′ coordinate is greater than the 3′ coordinate, the RNA is present on the reverse-complement strand of the containing genomic DNA sequence. Each hit is denoted by an abbreviation (like "Eco-1-1") that refers to a taxonomy given in Section 25.1.

abbrev.	Seq. accession		5′ at	3′ at	genes
Ssp-14-1	NC_000911.1	-	1558977	1558901	TxlA (cd02950)→ **RNA**→ hypo→
Sel-1-1	NC_006576.1	+	483196	483269	hypo→ **RNA**→ COG0398 (COG0398)→
Sel-2-1	NC_007604.1	-	1137608	1137535	hypo→ **RNA**→ COG0398 (COG0398)→
Ama-1-1	NC_009925.1	-	407448	407375	←tRNA-Pro **RNA**→ ←SRP RNA
Ter-1-1	NC_008312.1	-	4576095	4576023	MhpC (COG0596)→ **RNA**→ ←hypo
Tel-1-1	NC_004113.1	+	599896	599961	hypo→ **RNA**→ COG0606 (COG0606)→
Ava-1-1	NC_007413.1	+	2186368	2186439	tRNA-synt_1d_C (pfam05746)NOG25202 (NOG25202)→ **RNA**→ ←Cof (COG0561)
Nsp-1-1	NC_003272.1	-	4768549	4768478	tRNA-synt_1d_C (pfam05746)NOG25202 (NOG25202)→ **RNA**→ ←Cof (COG0561)
Pma-9-1	NC_008819.1	+	401851	401916	hypo→ **RNA**→ hypo→
Pma-2-1	NC_009976.1	+	1238813	1238882	←hypo **RNA**→ ←beta-lactamase
Pma-11-1	NC_005042.1	+	1298860	1298930	←hypo **RNA**→ ←hypo
Pma-11-2	NC_005042.1	-	636238	636172	←hypo **RNA**→ hypo→
Pma-11-3	NC_005042.1	-	556707	556642	hypo→ **RNA**→ Ribosomal_S21 (pfam01165)→
env-1	JCVI_SCAF_1096628057746	-	1221	1165	? (GOS11385446)→ **RNA**→ ? (GOS11326834)→
Ssp-13-1	NC_005070.1	-	1411144	1411088	high light inducible protein→ **RNA**→ hypo→
Ssp-14-2	NC_000911.1	-	3398354	3398286	hypo→ **RNA**→ COG4449 (COG4449)→
Ssp-8-1	NZ_AAUA01000001.1	+	379823	379889	hypo→ **RNA**→ ←FolC (COG0285)
env-2	JCVI_SCAF_1101668398476	-	679	615	**RNA**→ ←? (GOS12835)
env-3	JCVI_SCAF_1096627574863	+	1687	1755	? (GOS303)→ **RNA**→
Pma-6-1	NC_007577.1	-	1173349	1173281	tRNA-Ser→ **RNA**→ ←fer2 (cd00207)
Pma-8-1	NC_008817.1	+	45910	45978	←hypo **RNA**→ hypo→
env-4	JCVI_SCAF_1096626304682	-	260	197	? (GOS11457573)→ **RNA**→
env-5	JCVI_SCAF_1101668388273	-	763	700	**RNA**→ ←? (GOS12835)
env-6	JCVI_SCAF_1096627816644	-	901	832	**RNA**→ adh_short (pfam00106)? (GOS49)→
env-7	JCVI_SCAF_1096627600585	+	525	594	? (GOS11661723)→ **RNA**→ ←CobQ (COG1492)? (GOS3592)
Pma-12-1	NC_005072.1	-	972180	972111	←hypo **RNA**→ ←inward rectifier potassium channel

env-8	JCVI_SCAF_1096627537559	+	1508	1575	←? (GOS12183470) **RNA**→
env-9	JCVI_SCAF_1096626998670	-	696	627	Ser tRNA→ **RNA**→
env-10	JCVI_SCAF_1096627551526	-	1309	1240	**RNA**→ ←fer2 (cd00207)? (GOS9508)
env-11	JCVI_SCAF_1096627632931	-	234	167	? (GOS11053562)→ **RNA**→
env-12	JCVI_SCAF_1096627726923	+	1602	1671	? (GOS11661723)→ **RNA**→
Pma-12-2	NC_005072.1	+	383387	383453	←hypo **RNA**→ AceF (COG0508)→
env-13	JCVI_SCAF_1096627084234	+	850	917	←? (GOS11973280) **RNA**→ ←Asn tRNA
env-14	JCVI_SCAF_1101668306973	+	1040	1109	←? (GOS11385352) **RNA**→
env-15	JCVI_SCAF_1096627155230	+	115	182	**RNA**→ FpaA (COG0426)Flavin_Reduct (pfam01613)? (GOS395)→
env-16	JCVI_SCAF_1101668274677	-	485	417	? (GOS303)→ **RNA**→
env-17	JCVI_SCAF_1096628045981	+	1113	1182	←PiuC (COG3128)? (GOS1330) **RNA**→
Pma-12-3	NC_005072.1	+	346826	346893	HTH_MARR (smart00347)→ **RNA**→ malic enzyme→
env-18	JCVI_SCAF_1096628123936	+	329	396	**RNA**→ ←? (GOS13019159)
env-19	JCVI_SCAF_1101668304461	+	1115	1182	? (GOS303)→ **RNA**→
env-20	JCVI_SCAF_1096627549615	-	1297	1230	**RNA**→ ←CobQ (COG1492)? (GOS3592)
Pma-12-4	NC_005072.1	+	654509	654576	hypo→ **RNA**→ ←hypo
env-21	JCVI_SCAF_1096626931145	-	667	600	? (GOS303)→ **RNA**→ ? (GOS10697)→
env-22	JCVI_SCAF_1096627548057	+	1354	1423	? (GOS11661723)→ **RNA**→
env-23	JCVI_SCAF_1096628122610	+	1217	1286	Ser tRNA→ **RNA**→ ? (GOS10697)→
env-24	JCVI_SCAF_1096626989098	+	1363	1432	Rhomboid (pfam01694)? (GOS303)→ **RNA**→ ←fer2 (cd00207)? (GOS9508)
env-25	JCVI_SCAF_1096628273739	+	691	760	Rhomboid (pfam01694)? (GOS303)→ **RNA**→ ? (GOS13095788)→
env-26	JCVI_SCAF_1096627553294	-	1215	1145	**RNA**→ ←fer2 (cd00207)? (GOS9508)
env-27	JCVI_SCAF_1101668225625	+	844	913	**RNA**→ ? (GOS10697)→
env-28	JCVI_SCAF_1096626118376	+	371	440	**RNA**→
env-29	JCVI_SCAF_1101667201924	+	344	413	**RNA**→
env-30	JCVI_SCAF_1096626580640	-	77	10	? (GOS11661723)→ **RNA**→
env-31	JCVI_SCAF_1096627767908	-	556	489	Ser tRNA→ **RNA**→
env-32	JCVI_SCAF_1096627269409	-	1686	1617	? (GOS11661723)→ **RNA**→ ? (GOS10697)→
env-33	JCVI_SCAF_1096627581678	+	655	724	←? (GOS7134) **RNA**→ ←? (GOS1694)
env-34	JCVI_SCAF_1096627311661	-	1407	1338	Ser tRNA→ **RNA**→ ? (GOS10697)→
env-35	JCVI_SCAF_1096627591944	-	1486	1417	**RNA**→ SRA (smart00466)? (GOS2778)→
env-36	JCVI_SCAF_1101668221336	-	295	229	←? (GOS11385352) **RNA**→
env-37	JCVI_SCAF_1101667545738	+	700	769	Rhomboid (pfam01694)? (GOS303)→ **RNA**→
env-38	JCVI_SCAF_1096628120170	+	629	696	**RNA**→ ←? (GOS13534)
Pma-4-1	NC_009091.1	-	133115	133048	←hypo **RNA**→ ←tRNA-Asn
Pma-8-2	NC_008817.1	-	977450	977385	←hypo **RNA**→ ←inward rectifier potassium channel
env-39	JCVI_SCAF_1096627722494	+	1168	1235	←? (GOS11953) **RNA**→ DUF1499 (pfam07386)? (GOS7536)→
env-40	JCVI_SCAF_1096627764602	+	511	579	18S_rRNA→ **RNA**→ ? (GOS15049)→
env-41	JCVI_SCAF_1096626925644	+	987	1054	18S_rRNA→ **RNA**→ DUF1499 (pfam07386)? (GOS7536)→
env-42	JCVI_SCAF_1096627569082	-	1103	1036	? (GOS11907928)→ **RNA**→ ? (GOS15049)→
env-43	JCVI_SCAF_1096628094227	-	1965	1896	**RNA**→ DUF1499 (pfam07386)? (GOS7536)→
Pma-4-2	NC_009091.1	-	949300	949233	←hypo **RNA**→ DUF1499 (pfam07386)→
env-44	JCVI_SCAF_1096627732672	+	850	917	18S_rRNA→ **RNA**→ DUF1499 (pfam07386)? (GOS7536)→
env-45	JCVI_SCAF_1096626922070	+	106	173	**RNA**→ DUF1499 (pfam07386)? (GOS7536)→
env-46	JCVI_SCAF_1101667367890	+	504	571	**RNA**→ DUF1499 (pfam07386)? (GOS7536)→
env-47	JCVI_SCAF_1096626975232	-	2089	2022	**RNA**→ DUF1499 (pfam07386)? (GOS7536)→
env-48	JCVI_SCAF_1101668301977	+	598	665	←18S_rRNA **RNA**→ DUF1499 (pfam07386)? (GOS7536)→
env-49	JCVI_SCAF_1096626117883	+	357	425	18S_rRNA→ **RNA**→ DUF1499 (pfam07386)? (GOS7536)→
env-50	JCVI_SCAF_1096627546133	+	1102	1169	←18S_rRNA **RNA**→ ? (GOS7536)→
env-51	JCVI_SCAF_1096626991635	-	2734	2667	? (GOS11599787)→ **RNA**→ DUF1499 (pfam07386)? (GOS7536)→
env-52	JCVI_SCAF_1096627563490	-	954	887	←? (GOS19324) **RNA**→ ? (GOS15049)→
env-53	JCVI_SCAF_1096627520985	-	414	347	**RNA**→ ? (GOS7536)→

env-54	JCVI_SCAF_1096627549356	+	1513	1580	18S_rRNA→ **RNA**→
env-55	JCVI_SCAF_1096627766018	+	1345	1412	18S_rRNA→ **RNA**→ ? (GOS7536)→
env-56	JCVI_SCAF_1101667117829	+	720	787	18S_rRNA→ **RNA**→
env-57	JCVI_SCAF_1101667100941	+	211	278	**RNA**→ DUF1499 (pfam07386)? (GOS7536)→
env-58	JCVI_SCAF_1101668200313	+	1340	1407	18S_rRNA→ **RNA**→
env-59	JCVI_SCAF_1096627545220	-	403	336	←18S_rRNA **RNA**→
Pma-3-1	NC_009840.1	-	982248	982181	←hypo **RNA**→ DUF1499 (pfam07386)→
env-60	JCVI_SCAF_1096627622873	-	1456	1389	**RNA**→ COG4446 (COG4446)? (GOS7536)→
env-61	JCVI_SCAF_1096627530590	-	349	282	←? (GOS19324) **RNA**→
env-62	JCVI_SCAF_1101667146941	+	61	128	**RNA**→ DUF1499 (pfam07386)? (GOS7536)→
env-63	JCVI_SCAF_1096627062687	+	785	852	18S_rRNA→ **RNA**→ DUF1499 (pfam07386)? (GOS7536)→
env-64	JCVI_SCAF_1101668311145	-	1502	1435	**RNA**→ DUF1499 (pfam07386)? (GOS7536)→
env-65	JCVI_SCAF_1096627001989	-	749	682	←? (GOS19324) **RNA**→ DUF1499 (pfam07386)? (GOS7536)→
env-66	JCVI_SCAF_1096627705637	-	781	714	←? (GOS11953) **RNA**→ DUF1499 (pfam07386)? (GOS7536)→
env-67	JCVI_SCAF_1096626999505	+	210	277	**RNA**→ DUF1499 (pfam07386)? (GOS7536)→
env-68	JCVI_SCAF_1101667152469	+	849	916	18S_rRNA→ **RNA**→
env-69	JCVI_SCAF_1096628295458	-	641	574	ABC_Metallic_Cations (cd03235)? (GOS159)→ **RNA**→ DUF1499 (pfam07386)? (GOS7536)→
env-70	JCVI_SCAF_1101668293418	-	927	860	←? (GOS19324) **RNA**→ DUF1499 (pfam07386)? (GOS7536)→
env-71	JCVI_SCAF_1096628073177	+	535	602	18S_rRNA→ **RNA**→ ? (GOS7536)→
env-72	JCVI_SCAF_1101668290942	-	1389	1322	←18S_rRNA **RNA**→ DUF1499 (pfam07386)? (GOS7536)→
env-73	JCVI_SCAF_1096627562179	+	256	323	**RNA**→ ←OsmC (pfam02566)? (GOS10947)
env-74	JCVI_SCAF_1101668205915	-	398	331	←? (GOS19324) **RNA**→ ? (GOS7536)→
env-75	JCVI_SCAF_1096628227480	+	1746	1813	18S_rRNA→ **RNA**→
env-76	JCVI_SCAF_1101667224325	-	444	377	←18S_rRNA **RNA**→ ? (GOS7536)→
Pma-6-2	NC_007577.1	-	952144	952077	←hypo **RNA**→ DUF1499 (pfam07386)→
env-77	JCVI_SCAF_1101668197125	-	796	729	←? (GOS19324) **RNA**→ DUF1499 (pfam07386)? (GOS7536)→
env-78	JCVI_SCAF_1096627061123	-	706	639	←18S_rRNA **RNA**→ DUF1499 (pfam07386)? (GOS7536)→
env-79	JCVI_SCAF_1096627755302	-	374	307	←? (GOS19324) **RNA**→
env-80	JCVI_SCAF_1096627541626	+	556	623	? (GOS11907928)→ **RNA**→ ? (GOS7536)→
env-81	JCVI_SCAF_1096626485580	+	500	567	18S_rRNA→ **RNA**→
env-82	JCVI_SCAF_1096628286184	+	1714	1781	18S_rRNA→ **RNA**→
env-83	JCVI_SCAF_1096627758008	+	452	519	18S_rRNA→ **RNA**→ ? (GOS15049)→
env-84	JCVI_SCAF_1101668001054	+	132	199	**RNA**→ DUF1499 (pfam07386)? (GOS7536)→
env-85	JCVI_SCAF_1096627742934	-	1053	986	←? (GOS19324) **RNA**→
env-86	JCVI_SCAF_1096627739148	+	442	509	18S_rRNA→ **RNA**→ ? (GOS15049)→
env-87	JCVI_SCAF_1096627740395	-	1423	1356	←18S_rRNA **RNA**→ COG4446 (COG4446)? (GOS7536)→
env-88	JCVI_SCAF_1096627055327	-	1621	1554	**RNA**→ DUF1499 (pfam07386)? (GOS7536)→
env-89	JCVI_SCAF_1096627713867	+	632	699	18S_rRNA→ **RNA**→ DUF1499 (pfam07386)? (GOS7536)→
env-90	JCVI_SCAF_1096627048952	+	1057	1124	18S_rRNA→ **RNA**→ DUF1499 (pfam07386)? (GOS7536)→
env-91	JCVI_SCAF_1101668280001	-	1609	1542	**RNA**→ DUF1499 (pfam07386)? (GOS7536)→
env-92	JCVI_SCAF_1096626959190	+	716	783	18S_rRNA→ **RNA**→ COG4446 (COG4446)? (GOS7536)→
env-93	JCVI_SCAF_1096626968620	-	87	20	? (GOS11599787)→ **RNA**→
env-94	JCVI_SCAF_1101667134076	+	589	656	18S_rRNA→ **RNA**→
env-95	JCVI_SCAF_1101667543876	-	270	203	←? (GOS19324) **RNA**→
env-96	JCVI_SCAF_1096628374971	+	1470	1537	←18S_rRNA **RNA**→
env-97	JCVI_SCAF_1101668226838	-	832	765	←? (GOS19324) **RNA**→ DUF1499 (pfam07386)? (GOS7536)→
env-98	JCVI_SCAF_1096627207376	-	245	178	←? (GOS11953) **RNA**→
env-99	JCVI_SCAF_1096628297173	-	142	75	←18S_rRNA **RNA**→
env-100	JCVI_SCAF_1101668178152	+	203	270	**RNA**→ DUF1499 (pfam07386)? (GOS7536)→
env-101	JCVI_SCAF_1101668316227	+	308	375	18S_rRNA→ **RNA**→ DUF1499 (pfam07386)? (GOS7536)→
env-102	JCVI_SCAF_1096627226399	-	387	320	←? (GOS19324) **RNA**→
env-103	JCVI_SCAF_1096627629356	-	1256	1189	**RNA**→ DUF1499 (pfam07386)? (GOS7536)→

Pma-1-1	NC_008816.1	-	950815	950748	←hypo **RNA**→ DUF1499 (pfam07386)→
env-104	JCVI_SCAF_1096627724050	+	1423	1490	←18S_rRNA **RNA**→
env-105	JCVI_SCAF_1096628046754	-	1116	1049	←? (GOS19324) **RNA**→ ←? (GOS11036104)
env-106	JCVI_SCAF_1096628112325	-	1399	1332	←? (GOS19324) **RNA**→ DUF1499 (pfam07386)? (GOS7536)→
env-107	JCVI_SCAF_1101668193073	+	1424	1491	18S_rRNA→ **RNA**→
env-108	JCVI_SCAF_1096626961825	-	1162	1095	←18S_rRNA **RNA**→ DUF1499 (pfam07386)? (GOS7536)→
env-109	JCVI_SCAF_1096627566666	+	1137	1204	←18S_rRNA **RNA**→
env-110	JCVI_SCAF_1096627779711	+	83	150	**RNA**→ DUF1499 (pfam07386)? (GOS7536)→
env-111	JCVI_SCAF_1101667078247	-	316	249	18S_rRNA→ **RNA**→
env-112	JCVI_SCAF_1101668187194	+	1267	1334	18S_rRNA→ **RNA**→ DUF1499 (pfam07386)? (GOS7536)→
env-113	JCVI_SCAF_1101668318877	+	1250	1317	18S_rRNA→ **RNA**→ ? (GOS7536)→
env-114	JCVI_SCAF_1096626137142	-	346	279	? (GOS11599787)→ **RNA**→
env-115	JCVI_SCAF_1096628075878	+	1232	1299	←? (GOS11953) **RNA**→ DUF1499 (pfam07386)? (GOS7536)→
env-116	JCVI_SCAF_1101667155913	-	190	123	←? (GOS19324) **RNA**→
env-117	JCVI_SCAF_1096627543325	+	1488	1554	18S_rRNA→ **RNA**→
env-118	JCVI_SCAF_1096627526801	-	1092	1025	←? (GOS19324) **RNA**→ DUF1499 (pfam07386)? (GOS7536)→
env-119	JCVI_SCAF_1096627308669	+	557	624	18S_rRNA→ **RNA**→ DUF1499 (pfam07386)? (GOS7536)→
env-120	JCVI_SCAF_1096628291445	-	132	65	←? (GOS19324) **RNA**→
env-121	JCVI_SCAF_1096626973562	+	2739	2807	18S_rRNA→ **RNA**→
env-122	JCVI_SCAF_1096627800663	-	934	866	←? (GOS787) **RNA**→ ? (GOS10697)→
Pma-10-1	NC_007335.2	-	44898	44834	hypo→ **RNA**→ hypo→
Pma-9-2	NC_008819.1	-	53519	53454	hypo→ **RNA**→ hypo→
Pma-9-3	NC_008819.1	+	1037361	1037424	hypo→ **RNA**→ hypo→
Pma-10-2	NC_007335.2	+	386958	387022	high light inducible protein→ **RNA**→ hypo→
env-123	JCVI_SCAF_1096628087621	+	1053	1117	**RNA**→
env-124	JCVI_SCAF_1096628087621	+	450	514	**RNA**→
Pma-9-4	NC_008819.1	+	397455	397519	COG0398 (COG0398)→ **RNA**→ ←hypo
Pma-10-3	NC_007335.2	+	382482	382546	COG0398 (COG0398)→ **RNA**→ ←hypo
Pma-10-4	NC_007335.2	+	983401	983465	elongation factor Tu domain-containing protein→ **RNA**→ hypo→
env-125	JCVI_SCAF_1096628101613	-	1601	1537	**RNA**→ CobF (COG2243)? (GOS152)→
env-126	JCVI_SCAF_1096628040712	-	336	272	? (GOS13019008)→ **RNA**→
env-127	JCVI_SCAF_1101668539429	-	355	289	COG0398 (COG0398)? (GOS1258)→ **RNA**→
Ssp-3-1	NC_007516.1	-	1424714	1424654	hypo→ **RNA**→ ←hypo
Ssp-2-1	NC_008319.1	-	1487818	1487754	hypo→ **RNA**→ ←hypo
Ssp-2-2	NC_008319.1	+	1138022	1138086	SNase (pfam00565)→ **RNA**→ hypo→
Ssp-2-3	NC_008319.1	+	997952	998016	hypo→ **RNA**→ SNase (pfam00565)→
env-128	JCVI_SCAF_1096626611670	+	1400	1464	**RNA**→
Ssp-2-4	NC_008319.1	-	772842	772778	←hypo **RNA**→ ←hypo
Pma-5-1	NC_008820.1	+	356821	356885	hypo→ **RNA**→ hypo→
Pma-7-1	NC_005071.1	-	1667396	1667332	carbonic anhydrase→ **RNA**→ ←COG3152 (COG3152)
Ssp-2-5	NC_008319.1	+	1026369	1026433	←hypo **RNA**→ ←tRNA-Pro
Ssp-2-6	NC_008319.1	+	785499	785563	COG0412 (COG0412)→ **RNA**→ ←COG3386 (COG3386)
Ssp-2-7	NC_008319.1	+	979499	979563	hypo→ **RNA**→ putative lipoprotein→
Ssp-2-8	NC_008319.1	-	1490305	1490241	hypo→ **RNA**→ ←hypo
Ssp-10-1	NZ_AANO01000001.1	-	236655	236586	hypo→ **RNA**→ ←RplL (COG0222)
Ssp-2-9	NC_008319.1	+	2394765	2394834	hypo→ **RNA**→ ←RplL (COG0222)
Pma-7-2	NC_005071.1	-	2205028	2204960	hypo→ **RNA**→ ←RplL (COG0222)
Pma-5-2	NC_008820.1	-	2447837	2447769	hypo→ **RNA**→ ←hypo
env-129	JCVI_SCAF_1101668324115	+	456	524	? (GOS8033)→ **RNA**→ ? (GOS8033)→
Ssp-11-1	NC_009481.1	+	2191549	2191619	hypo→ **RNA**→ ←RplL (COG0222)
env-130	JCVI_SCAF_1096627998360	+	1284	1354	←RnhA (COG0328)? (GOS3931) **RNA**→ ? (GOS10654)→
Ssp-12-1	NZ_AAOK01000007.1	-	27940	27870	hypo→ **RNA**→ ←RplL (COG0222)

env-131	JCVI_SCAF_1101668230400	+	37	106	**RNA**→ ? (GOS10654)→
env-132	JCVI_SCAF_1096628046072	+	1220	1286	? (GOS10654)→ **RNA**→ ? (GOS8033)→
Ssp-7-1	NC_009482.1	+	2048577	2048642	←RnhA (COG0328) **RNA**→ ←RplL (COG0222)
Ssp-4-1	NC_007513.1	+	2054658	2054726	hypo→ **RNA**→ ←RplL (COG0222)
env-133	JCVI_SCAF_1096628043949	-	364	296	? (GOS13202071)→ **RNA**→ ? (GOS10654)→
env-134	JCVI_SCAF_1101667377116	-	115	47	? (GOS13096573)→ **RNA**→
env-135	JCVI_SCAF_1096627216867	+	473	541	? (GOS13202071)→ **RNA**→ ? (GOS10654)→
env-136	JCVI_SCAF_1096627197861	-	470	402	←? (GOS3931) **RNA**→ ? (GOS10654)→
Ssp-13-2	NC_005070.1	+	2242696	2242764	hypo→ **RNA**→ ←RplL (COG0222)
env-137	JCVI_SCAF_1101667184502	+	226	294	**RNA**→ ? (GOS8033)→
Ssp-3-2	NC_007516.1	+	2274186	2274254	hypo→ **RNA**→ ←Ribosomal_L12 (pfam00542)
env-138	JCVI_SCAF_1096627153447	+	3625	3693	? (GOS13096573)→ **RNA**→
env-139	JCVI_SCAF_1096628072039	+	978	1046	? (GOS2932)→ **RNA**→ ←RplL (COG0222)? (GOS996)
Ssp-10-2	NZ_AANO01000044.1	-	1123	1054	hypo→ **RNA**→ ←RplL (COG0222)
Ssp-1-1	NZ_AATZ01000002.1	-	532918	532850	hypo→ **RNA**→ ←RplL (COG0222)
env-140	JCVI_SCAF_1101668676589	-	444	376	? (GOS13096573)→ **RNA**→ ? (GOS8033)→
env-141	JCVI_SCAF_1101667122093	+	605	673	? (GOS13202071)→ **RNA**→
env-142	JCVI_SCAF_1096627383380	+	89	157	? (GOS8033)→ **RNA**→ ? (GOS8033)→
env-143	JCVI_SCAF_1096627969158	+	114	182	**RNA**→ ←RplJ (COG0244)? (GOS407)
env-144	JCVI_SCAF_1101667335056	+	528	596	? (GOS13202071)→ **RNA**→
env-145	JCVI_SCAF_1096627214382	+	918	986	←? (GOS3931) **RNA**→ ? (GOS10654)→
env-146	JCVI_SCAF_1096627824847	-	469	402	? (GOS13096573)→ **RNA**→ ? (GOS10654)→
Ssp-9-1	NZ_AANP01000001.1	-	504528	504459	hypo→ **RNA**→ ←RplL (COG0222)
env-147	JCVI_SCAF_1096627937755	+	23	92	? (GOS10654)→ **RNA**→ ? (GOS10654)→
env-148	JCVI_SCAF_1101668618928	-	1142	1073	? (GOS10654)→ **RNA**→ ? (GOS10654)→
env-149	JCVI_SCAF_1096627362115	-	1614	1545	? (GOS8033)→ **RNA**→ ? (GOS10654)→
env-150	JCVI_SCAF_1096627084801	+	994	1064	? (GOS13096573)→ **RNA**→ ? (GOS10654)→
env-151	JCVI_SCAF_1096627194150	-	880	813	←RnhA (COG0328)? (GOS3931) **RNA**→ ←RplL (COG0222)? (GOS996)
Ssp-8-2	NZ_AAUA01000003.1	-	154002	153938	←hypo **RNA**→ ←hypo
env-152	JCVI_SCAF_1096627074932	+	890	953	? (GOS11211626)→ **RNA**→ ←? (GOS15927)
env-153	JCVI_SCAF_1096627970216	+	3	65	**RNA**→ ? (GOS14642395)→
env-154	JCVI_SCAF_1096626970485	-	1112	1050	**RNA**→
env-155	JCVI_SCAF_1096628060967	+	1600	1660	ZnMc (cd00203)? (GOS4228)→ **RNA**→
env-156	JCVI_SCAF_1096627192266	+	5787	5848	? (GOS11820130)→ **RNA**→ ? (GOS11820130)→
env-157	JCVI_SCAF_1096628080391	-	572	511	←Ser tRNA **RNA**→ ? (GOS11820130)→
env-158	JCVI_SCAF_1096627183482	-	1345	1283	Soj (COG1192)? (GOS1716)→ **RNA**→
Ssp-13-3	NC_005070.1	+	1131767	1131827	hypo→ **RNA**→ ←hypo
env-159	JCVI_SCAF_1096627190267	-	899	838	? (GOS15836)→ **RNA**→
Ssp-3-3	NC_007516.1	+	534384	534444	hypo→ **RNA**→ GST_C_Zeta (cd03191)GST_N_Zeta (cd03042)→
env-160	JCVI_SCAF_1096627082690	-	719	656	? (GOS13547206)→ **RNA**→ ? (GOS11359600)→
env-161	JCVI_SCAF_1096627963120	-	495	432	? (GOS13547206)→ **RNA**→ ? (GOS11359600)→
env-162	JCVI_SCAF_1096627838359	+	250	313	**RNA**→
Ssp-4-2	NC_007513.1	-	1540528	1540466	←hypo **RNA**→ ←carbamoyl-phosphate synthase L chain
env-163	JCVI_SCAF_1096627282915	+	1522	1585	Pro tRNA→ **RNA**→
env-164	JCVI_SCAF_1096627828942	+	61	124	**RNA**→ AslB (COG0641)? (GOS255)→
env-165	JCVI_SCAF_1096628082578	+	1267	1332	←? (GOS11713070) **RNA**→
env-166	JCVI_SCAF_1096627303375	-	1736	1672	←? (GOS11177499) **RNA**→ ? (GOS13261968)→
env-167	JCVI_SCAF_1101668294323	+	1286	1350	? (GOS11249006)→ **RNA**→ ? (GOS11249006)→
env-168	JCVI_SCAF_1096628074362	+	174	238	? (GOS11249006)→ **RNA**→ ? (GOS11249006)→
env-169	JCVI_SCAF_1096627371490	+	2060	2124	? (GOS11249006)→ **RNA**→ ? (GOS11249006)→
env-170	JCVI_SCAF_1096628059788	+	1566	1630	←? (GOS11760973) **RNA**→
env-171	JCVI_SCAF_1096627516204	-	194	130	? (GOS11249006)→ **RNA**→ ? (GOS11249006)→

env-172	JCVI_SCAF_1101668668167	+	1286	1350	? (GOS11249006)→ **RNA**→ ? (GOS11249006)→
env-173	JCVI_SCAF_1096627369637	+	1187	1251	? (GOS14642395)→ **RNA**→ ? (GOS14642395)→
env-174	JCVI_SCAF_1096627961667	+	502	566	? (GOS14642395)→ **RNA**→ ? (GOS14642395)→
Ssp-3-4	NC_007516.1	+	1389620	1389683	hypo→ **RNA**→ hypo→
Ssp-4-3	NC_007513.1	+	1422400	1422465	←tRNA-Cys **RNA**→ membrane protein-like→
Ssp-1-2	NZ_AATZ01000003.1	-	191809	191743	hypo→ **RNA**→ ←hypo
env-175	JCVI_SCAF_1101668028160	-	650	584	**RNA**→
env-176	JCVI_SCAF_1096627794613	+	1686	1753	? (GOS11634178)→ **RNA**→
Ssp-3-5	NC_007516.1	-	524530	524469	hypo→ **RNA**→ ←hypo
env-177	JCVI_SCAF_1101668743973	-	770	706	? (GOS12184068)→ **RNA**→
env-178	JCVI_SCAF_1096627116428	-	1163	1099	? (GOS11249006)→ **RNA**→ ? (GOS11249006)→
Ssp-13-4	NC_005070.1	+	1127966	1128025	hypo→ **RNA**→ hypo→
env-179	JCVI_SCAF_1096627066253	-	975	909	? (GOS11335739)→ **RNA**→ ←? (GOS11395197)
Csp-1-1	NZ_ABSE01000014.1	+	50433	50504	**RNA**→
Tel-1-2	NC_004113.1	+	1092076	1092149	←tRNA-Pro **RNA**→ COG0742 (COG0742)→
Ssp-14-3	NC_000911.1	+	2730521	2730588	←COG3330 (COG3330) **RNA**→ YafJ (cd01908)→
Nsp-1-2	NC_003272.1	-	3740057	3739982	←REC (cd00156)BaeS (COG0642)AtoS (COG2202)PAC (pfam00785)PAS (smart00091) **RNA**→ **RNA**→ **RNA**→ ←hypo
Nsp-1-3	NC_003272.1	-	3740217	3740143	(shown above)
Nsp-1-4	NC_003272.1	-	3740423	3740349	(shown above)
Ava-1-2	NC_007413.1	+	4756984	4757059	←PAS (cd00130)REC (cd00156)BaeS (COG0642)AtoS (COG2202)PAS (smart00091) **RNA**→ **RNA**→ ←hypo
Ava-1-3	NC_007413.1	+	4756822	4756897	(shown above)
Npu-1-1	NC_010628.1	-	7794370	7794295	AICARFT_IMPCHas (pfam01808)MGS (pfam02142)→ **RNA**→ COG1896 (COG1896)→
Nsp-1-5	NC_003272.1	-	3744859	3744786	AICARFT_IMPCHas (pfam01808)MGS (pfam02142)→ **RNA**→ ←REC (cd00156)BaeS (COG0642)AtoS (COG2202)PAC (pfam[illegible]
Mae-1-1	NC_010296.1	-	3189581	3189508	hypo→ **RNA**→ PlsC (COG0204)→
Csp-2-1	NC_010546.1	-	4333822	4333760	←HCP (cd01914) **RNA**→ ←hypo
Cwa-1-1	NZ_AADV02000005.1	+	52634	52696	←DYNc (smart00053) **RNA**→ hypo→
Csp-2-2	NC_010546.1	+	3842870	3842932	Mfd (COG1197)→ **RNA**→ hypo→
Csp-3-1	NZ_AAXW01000004.1	+	185218	185280	←COG3335 (COG3335) **RNA**→ SgcQ (COG0434)→
Lsp-1-1	NZ_AAVU01000009.1	-	97263	97206	SecA (COG0653)→ **RNA**→ ←hypo
Ssp-6-1	NC_007775.1	+	383657	383722	hypo→ **RNA**→ ←Glyoxalase (pfam00903)
Ssp-5-1	NC_007776.1	-	729361	729297	hypo→ **RNA**→ BaeS (COG0642)→
Mae-1-2	NC_010296.1	-	3427434	3427352	←Transposase_2 (pfam01385)Transposase_35 (pfam07282) **RNA**→ GvpP (COG4980)→
Csp-5-1	NZ_ABVE01000004.1	+	406621	406694	CysE (COG1045)→ **RNA**→ COG2334 (COG2334)→
Csp-4-1	NZ_ABOY01000062.1	-	6593	6521	HTH_XRE (cd00093)→ **RNA**→ COG2334 (COG2334)→
Csp-5-2	NZ_ABVE01000004.1	-	807677	807604	PP2Ac (cd00144)→ **RNA**→ DUF159 (pfam02586)→
Cwa-1-2	NZ_AADV02000078.1	-	6931	6871	←hypo **RNA**→ ←Chorismate_synt (pfam01264)
Csp-7-1	NZ_ABVI01000008.1	+	27442	27506	←protein of unknown function DUF1824 **RNA**→ GlyRS_alpha_core (cd00733)→
Csp-6-1	NZ_ABLR01000007.1	+	51654	51718	←Domain of unknown function DUF1824 **RNA**→ GlyRS_alpha_core (cd00733)→
Csp-3-2	NZ_AAXW01000069.1	-	22055	21991	IbpA (COG0071)→ **RNA**→ ←hypo
Ssp-12-2	NZ_AAOK01000003.1	-	24359	24290	←hypo **RNA**→ ←COG4338 (COG4338)
Ssp-11-2	NC_009481.1	-	901908	901843	←hypo **RNA**→ ←COG4338 (COG4338)
Ssp-2-10	NC_008319.1	+	972621	972684	←hypo **RNA**→ ←hypo

25.3 Conserved domains

Conserved domains found in protein-coding genes listed in Section 25.2 are listed, with the first sentence in their description from the Conserved Domain Database. Conserved domains associated with more than one Cyano-1 RNA are assigned a color, while others are shown in gray. The number in parentheses is the number of occurrences of the given gene.

u cd00093 (1) Helix-turn-helix XRE-family like proteins.
u cd00130 (1) PAS domain; PAS motifs appear in archaea, eubacteria and eukarya.
u cd00144 (1) Protein phosphatase 2A homologues, catalytic domain.
u d cd00156 (3) Signal receiver domain; originally thought to be unique to bacteria (CheY, OmpR, NtrC, and PhoB), now recently identified in eukaroytes ETR1 Arabidopsis thaliana; this domain receives the signal from the sensor partner in a two-component systems; contains a phosphoacceptor site that is phosphorylated by histidine kinase homologs; usually found N-terminal to a DNA binding effector domain; forms homodimers
u cd00203 (1) Zinc-dependent metalloprotease; Neutral zinc metallopeptidases.
d cd00207 (4) 2Fe-2S iron-sulfur cluster binding domain.
D cd00733 (2) Class II Glycyl-tRNA synthetase (GlyRS) alpha subunit core catalytic domain.
D cd01908 (1) Glutamine amidotransferases class-II (Gn-AT)_YafJ-type.
u cd01914 (1) Hybrid cluster protein (HCP), formerly known as prismane, is thought to play a role in nitrogen metabolism but its specific function is unknown. [Fe4-S4] cubane cluster similar to that of carbon monoxide dehydrogenase (CODH). The second cluster, referred to as the hybrid cluster, is a hybrid [Fe4-S2-O2]
u cd02950 (1) TRX-like protein A (TxlA) family; TxlA was originally isolated from the cyanobacterium Synechococcus.
D cd03042 (1) GST_N family, Class Zeta subfamily; GSTs are cytosolic dimeric proteins involved in cellular detoxification by catalyzing the conjugation of glutathione (GSH) with a wide range of endogenous and xenobiotic alkylating agents, including carcinogens, therapeutic drugs, environmental toxins and products of oxidative stress.
D cd03191 (1) GST_C family, Class Zeta subfamily; GSTs are cytosolic dimeric proteins involved in cellular detoxification by catalyzing the conjugation of glutathione (GSH) with a wide range of endogenous and xenobiotic alkylating agents, including carcinogens, therapeutic drugs, environmental toxins, and products of oxidative stress.
u cd03235 (1) ABC-type Mn/Zn transport systems, ATPase component; This family is comprised of systems involved in the uptake of various metallic cations such as iron, manganese and zinc.
u COG0071 (1) Molecular chaperone (small heat shock protein) [Posttranslational modification, protein turnover, chaperones]
D COG0204 (1) 1-acyl-sn-glycerol-3-phosphate acyltransferase [Lipid metabolism]
d COG0222 (13) Ribosomal protein L7/L12 [Translation, ribosomal structure and biogenesis]
d COG0244 (1) Ribosomal protein L10 [Translation, ribosomal structure and biogenesis]
d COG0285 (1) Folylpolyglutamate synthase [Coenzyme metabolism]
u COG0328 (3) Ribonuclease HI [DNA replication, recombination, and repair]
uD COG0398 (5) Uncharacterized conserved protein [Function unknown]
u COG0412 (1) Dienelactone hydrolase and related enzymes [Secondary metabolites biosynthesis, transport, and catabolism]
D COG0426 (1) Uncharacterized flavoproteins [Energy production and conversion]
D COG0434 (1) Predicted TIM-barrel enzyme [General function prediction only]
D COG0508 (1) Pyruvate/2-oxoglutarate dehydrogenase complex, dihydrolipoamide acyltransferase (E2) component, and related enzymes [Energy production and conversion]
d COG0561 (2) Predicted hydrolases of the HAD superfamily [General function prediction only]
u COG0596 (1) Predicted hydrolases or acyltransferases (alpha/beta hydrolase superfamily) [General function prediction only]
D COG0606 (1) Predicted ATPase with chaperone activity [Posttranslational modification, protein turnover, chaperones]
D COG0641 (1) Arylsulfatase regulator (Fe-S oxidoreductase) [General function prediction only]
uDd COG0642 (4) Signal transduction histidine kinase [Signal transduction mechanisms]
u COG0653 (1) Preprotein translocase subunit SecA (ATPase, RNA helicase) [Intracellular trafficking and secretion]
D COG0742 (1) N6-adenine-specific methylase [DNA replication, recombination, and repair]
u COG1045 (1) Serine acetyltransferase [Amino acid transport and metabolism]
u COG1192 (1) ATPases involved in chromosome partitioning [Cell division and chromosome partitioning]
u COG1197 (1) Transcription-repair coupling factor (superfamily II helicase) [DNA replication, recombination, and repair / Transcription]
d COG1492 (2) Cobyric acid synthase [Coenzyme metabolism]
D COG1896 (1) Predicted hydrolases of HD superfamily [General function prediction only]
u d COG2202 (3) FOG: PAS/PAC domain [Signal transduction mechanisms]
D COG2243 (1) Precorrin-2 methylase [Coenzyme metabolism]
D COG2334 (2) Putative homoserine kinase type II (protein kinase fold) [General function prediction only]
u COG3128 (1) Uncharacterized iron-regulated protein [Function unknown]
d COG3152 (1) Predicted membrane protein [Function unknown]
u COG3330 (1) Uncharacterized protein conserved in bacteria [Function unknown]
u COG3335 (1) Transposase and inactivated derivatives [DNA replication, recombination, and repair]
d COG3386 (1) Gluconolactonase [Carbohydrate transport and metabolism]
d COG4338 (2) Uncharacterized protein conserved in bacteria [Function unknown]
D COG4446 (3) Uncharacterized protein conserved in bacteria [Function unknown]
D COG4449 (1) Predicted protease of the Abi (CAAX) family [General function prediction only]
D COG4980 (1) Gas vesicle protein [General function prediction only]
uD GOS10654 (14) ?
D GOS10697 (6) ?
d GOS10947 (1) ?
d GOS11036104 (1) ?
u GOS11053562 (1) ?
u GOS11177499 (1) ?
u GOS11211626 (1) ?
uD GOS11249006 (12) ?
D GOS11326834 (1) ?
u GOS11335739 (1) ?
D GOS11359600 (2) ?
u GOS11385352 (2) ?
u GOS11385446 (1) ?
d GOS11395197 (1) ?
u GOS11457573 (1) ?
u GOS11599787 (3) ?
u GOS11634178 (1) ?
u GOS11661723 (5) ?
u GOS11713070 (1) ?
u GOS11760973 (1) ?
uD GOS11820130 (3) ?
u GOS11907928 (2) ?
u GOS11953 (4) ?
u GOS11973280 (1) ?

u GOS12183470 (1) ?
u GOS12184068 (1) ?
u GOS1258 (1) ?
d GOS12835 (2) ?
u GOS13019008 (1) ?
d GOS13019159 (1) ?
D GOS13095788 (1) ?
u GOS13096573 (5) ?
u GOS13202071 (4) ?
D GOS13261968 (1) ?
u GOS1330 (1) ?
d GOS13534 (1) ?
u GOS13547206 (2) ?
uD GOS14642395 (5) ?
D GOS15049 (5) ?
D GOS152 (1) ?
u GOS15836 (1) ?
u GOS159 (1) ?
d GOS15927 (1) ?
d GOS1694 (1) ?
u GOS1716 (1) ?
u GOS19324 (16) ?
D GOS255 (1) ?
D GOS2778 (1) ?
u GOS2932 (1) ?
u GOS303 (7) ?
d GOS3592 (2) ?
u GOS3931 (4) ?
D GOS395 (1) ?
d GOS407 (1) ?
u GOS4228 (1) ?
D GOS49 (1) ?
u GOS7134 (1) ?
D GOS7536 (49) ?
u GOS787 (1) ?
uD GOS8033 (8) ?
d GOS9508 (3) ?
d GOS996 (2) ?
u NOG25202 (2)
D pfam00106 (1) short chain dehydrogenase.
d pfam00542 (1) Ribosomal protein L7/L12 C-terminal domain.
uD pfam00565 (2) Staphylococcal nuclease homologue. [SNase numbering in parentheses]
u d pfam00785 (2) PAC motif.
d pfam00903 (1) Glyoxalase/Bleomycin resistance protein/Dioxygenase superfamily.
D pfam01165 (1) Ribosomal protein S21.
d pfam01264 (1) Chorismate synthase.
u pfam01385 (1) Probable transposase.
D pfam01613 (1) Flavin reductase like domain.
u pfam01694 (3) Rhomboid family.
u pfam01808 (2) AICARFT/IMPCHase bienzyme.
u pfam02142 (2) MGS-like domain.
d pfam02566 (1) OsmC-like protein.
D pfam02586 (1) Uncharacterised ACR, COG2135.
u pfam05746 (2) DALR anticodon binding domain.
u pfam07282 (1) Putative transposase DNA-binding domain.
D pfam07386 (42) Protein of unknown function (DUF1499).
u smart00053 (1) Dynamin, GTPase; Large GTPases that mediate vesicle trafficking.
u d smart00091 (3) PAS domain; PAS motifs appear in archaea, eubacteria and eukarya.
u smart00347 (1) helix_turn_helix multiple antibiotic resistance protein;
D smart00466 (1) SET and RING finger associated domain.

Duplicate sequences: the following putative homologs are not shown in the alignment because their sequences are identical to a homolog already shown: Sel-2-1

25.4 Multiple-sequence alignment

Each Cyano-1 RNA is denoted by an abbreviation (like "Eco-1-1") that refers to a taxonomy given in Section 25.1. The alignment includes sequences containing the RNA motif, as well as flanking sequence. The Cyano-1 RNA itself is denoted by the line underneath marked 5′ and 3′ on either end. Nucleotides in the flanking sequences (i.e., not part of the motif) are written in gray letters. Stems of predicted rho-independent transcription terminators are shaded yellow. (Note: terminator predictions have not been analyzed manually, and many are likely to be false positives.) Start codons are shaded green. (Note: start codons are frequently misannotated, especially in environmental samples.) Nucleotides proposed to basepair as part of the consensus structure are shaded in color when they comprise Watson-Crick or G-U pairs. Otherwise they are shaded gray. Conserved stems are also indicated at the bottom of the alignment by angle brackets, where matching $<$ and $>$ denote base-paired columns. Below these angle brackets, the symbol "2" denotes base pairs exhibiting covariation, "1" denotes base pairs exhibiting compatible mutations, "0" denotes base pairs that are not observed to mutate and "?" denotes base pairs that have a significant frequency of non-canonical nucleotides for Watson-Crick or G-U pairs ($>$ 5%). Below these base pair annotation is the consensus sequence: "R" = "A" or "G", "Y" = "C" or "U", red nucleotides: nucleotide identity conserved more than 97% of the time, black nucleotides: 90%, gray nucleotides: 75%, red circle (●): nucleotide is present 97% of the time, black circle (●): 90%, gray circle (●): 75%, white circle (○): 50%. All percentages of sequences just described (e.g. 97% conserved) assume that sequences have been weighted by the GSC algorithm implemented by the Infernal software.

alignment positions $1 \cdots 99$

Ssp-14-1	CCCAGCUAGGAGCUUGAGCAACCAUAGCCAGAAACUAUGCCAAUCAUUAAACUGCUACAGGUUCGGCUAUCCCGCCCUUAGGCGAUCGGCUAACAUUUA
Sel-1-1	UUGAUAAGAGUUCUGCAAUCACGUUUGAGCAAAAAUCUGUGCCAAGUUGCCAAAGUGGCAUAGACUAAACCGAUGCUCUUGACCUAGCCGUUAGGCUAA
Ama-1-1	AUACCCUCGCACCUUAGCCUAGCAUCAUGAUGGCUUACCCUUCAGACUGGUAAACAGUUUGUUUAUAGAUGACAACUAGCAACAGAUUAUAUAUAAUCA
Ter-1-1	GAUUGGUUAGCUAAAAUGAUUAAUAUGUAAAACUUAUAAAAUUAAUAAAAAAAAAAUAUAUUAAUAGUCGUGACAAAAUUAACUUAGUAAGUUAUAAUAU
Tel-1-1	CUCCGCUAAAACUGGAGGCAGAAAUUGGGCUUUUGCAAAUCAACUUCAUGAAGUUCCUAUGCAGGGGUGACAAAUCGAGGAUUUUUCUGCUAAGGUCAU
Ava-1-1	UGUUUGGGCGUAAUUUUUGUCUAAAUCCUGUUAUUCAAAGAGACAAUAAAAACUUUUAUAACUAGGUAUUGACGCAUUACCUAAUGUCGGAUAUAUUAG
Nsp-1-1	GGUUUGGGCGUAAUUUUUGUCUAAAUCUUGUUAUUCAAAGAGACAAUAAAAACUUUUAUAACUAGGUAUUGACGCAUUACCUAAUGUCGGAUAUAUUAG
Pma-9-1	CUGUGCCGAAAAAGCAUCCAAUUGGGUGCUUUUUUAGUGCCUGUGCCAAUCACUAAACCCUCCUAUUAAGGGUUUAUUCCUAAGCCUGUUCAGAUAAAU
Pma-2-1	UCAUUUUUAACUUUGGUCAGUCACCUCCCCCAACUGUCACUGUGACAGUUGCCCUAGUUGCAGUAAGGAUGGUUGCAAAUAUAGAAACAUUGGCAAUAA
Pma-11-1	GUAAAAACUAGCUUUGAGCUAACGCCUUAUCUCAACUGUCACUGUGACAGUUGAGAUAGCUACCUUGAUCCUGCCCGUAAAAAUAAACUUUAGUAAUAA
Pma-11-2	CAAUUAUAGAGACUACUUCACGCAAACUAUUUCAUAAAAUGUCAUUUAAUGAGAACAAGGGGUCAUAUUCUUGCAAUAAAUCACUCAAUGCGUGAAAAU
Pma-11-3	UUCUUCAAAUCGAUAUAGAUAAGUAAAAAAGGCUUAUACAGAGCAAAUCCAAAAAAUCAUUGACGAGGAACUUGCAAAAUUAAAAUUAGUCAUUAUCAU
env-1	CUGGGCAUCGGCUGAAGCAUCCCCUGCAUCCAGCUGUCACUGUGACAGUUGACGGGCUUCCACUCUCGUUCUUUCAGACCCCUCGUCGGUUCGCGAGCG
Ssp-13-1	CUGGGCAUCGGCUGAAGCAUCCCCUGCAUCCAGCUGUCACUGUGACAGUUGACGGGCUUCCACUCUCGUUCUUUCAGAGCUCUCGUCGGUUCGCGAGCG
Ssp-14-2	AAAAGCUUCGGAGUUCUGCUUUAGGUCAAAGUUUCUUGAAAAUUAAGUUUUUUUCCAGGUCUGAAGCCUUGACAAUCCCCGUUGGUGAUUUAUACUUAG
Ssp-8-1	AGACUCGACUGCCCUUACUUUGGCGGGCCCUGGGAUUGGAUGGCGCGGAUUAGGAACUGGCCCAAGCGAUGAAGGGGCAAUUCCCCGGCCUGGCUAAUU
env-2	AUCCGAUAUUAAUAAAAUUCAUUUCUCAAUUGUCACAUGUGACAGUUGAAUUAGAUAAACACUACAUAAACAUCCAUCAACUAUCGCGGUUAUAUUAUG
env-3	UUUUUUCUUUAUGUCUAGAAAUGAAAAACUGAUAUAUGUGAAAGUUAAGCAACAUUCUAUUUAGCCCUUCCACGAAAAAGAAUUUUAUAAGAUAUCGUG
Pma-6-1	UAUUGCCAUAAAUCCUUUAAUACAUAACUGCUACAUGUGACAGUUACGUAACAUUCCAUGCGCCCCUUCCACGGAAAUGAAAUAUAUGGAAUAUUUUAA
Pma-8-1	UUUUUUAAUAGUAUUCACAAUGGGCAAUUAAGAUAUGUGACAAGUAAGUAACAUUACUCAUCCUCUUCCACGAAAAAGAAAUAUAUGUAAUAAAUUAUG
env-4	UGCUAUGGACGACUUUAAAUUGAAUAACUGUCACAUGUGACAGUUAAACUAGAUAUACAUUAAUCAUUCGCACUAUCAAAUUAAUCAAGCUAAUAUAAA
env-5	UGCGUUAGCCUACUUGAGAUACAACAACUGUCACAUAUGACAGUUGAACUAGAUAAACAUUCAUCACUCGCGCUAGUAAAUUAAUUAAGUUAAUAUUAG
env-6	UUUUAUUACGAUGAAACUCAAGACUUACAACUGGCACAUGUGACAAUUAAAUUACAUGCGGCAUCCCCCUUUCACCAAAGUAAAAUUUAUGACAUAUUU
env-7	UUAAUAGCAAAUUCAAUGCAAAAGACAAUUGGCACAUGUGACAGUUAAGUAACAUACCAUGGCCCCCUUCCACGGAAAAGAAAUAAUUGACAUAACAUA
Pma-12-1	ACUCGAAUAAUAAUUUGAGUACUUUUGGUUUUUAGCUUUGUGACAAUUAAUAUACUUGCCCAAUAAGUAUUUACUCUAUAAUAAAAUCAUGUGAAAUUU
env-8	UUUUGUCAGAAUUACUUAAAUCAAUAAACUGGCAUAUGUGACAUUUAGGUAAUAUUUUAUUCUCACUUUCCACGUGAAAGAAAUAAUUUGCCUACCUUA
env-9	UUUAUAGUCAAUAUUAUUUAAUUUCUAAACUGUCACAUGUGACAGUUUUAUAACAAUCCAUUCUCCCUUCCACGGAAAUUAAAUAUUUGUAAUAAUUUA
env-10	UUUUUUAUUUAUGUCAAGAAAUGAAAAACUGGCACAUGUGACAGUUAAGUAACAUUCCAUUCGACGCUCCCACGAAAAAGAAUUUAAUGGCAUAUUUUA
env-11	AAUGAUAAAUAUACUGAAAAAGAUAACUGUCACAUGUGACAGUUAGGUAACAUUUCUUUCGGCUCUUCCACGUAAGGGAAAUGCAUGAGAUAAAUUAAA
env-12	UUUUUUCGUUAGGUCCAAUAAUGGGAAACUGUCACAUAUAACAGUUAAGUAACAUUCCAUUUACCCCUUCCACGAAAAAGAAUUAAAUGACGUACCUUA
Pma-12-2	AAUGCAAUUAAGUCUGAAGAGGUAUAACUGUCAUAUGUGACAAUUAAGUAAUAUUACUCACCCCCUUCCACGAAAAAGAAAUAUAUGUAAUAGUUUAUU
env-13	GUGUUUUUUAAUGACUUUUCUAAAAAUUGGCACAUGUGACAGUUAGGUAACAUUCCAUUGCCCCCUUCCACAGAAAAAAAAUAUAUGGCAUAAAGUGUA
env-14	UUUUUUCAUUAUGUCCAGAAAUGGGAAACUGGUACAUGUGACAGUUAAGUAACAUUCCAUUUAGCCCUUCCACGAAAAAGAAUUUAAUGCAAUAGUUUG
env-15	UUUGCCAUAAAUCCUUUAAUGCACAACUGGCACAUGUGACAGUUUAGUAACAUACCAAUUACCCCUUCCACGAAAAAGAAAAAUAUGGCAUAAUUUAUA
env-16	UUUAUGAUAAACAUACUGCAAUAGACAACUGACACAUGUGACAGUUAAGUAACAUUCCAUUCGCCCCUUCCUCGGAAAAUGAAUAUAUAGAUUAAAUUA
env-17	UUAAUGACAAAUAAACUGUAAUAGACAACUGCCACAUGUGACAGUUAAGUAAAAUUCCAUUCGCCCCUUCCACGUAAAAGAAAUAAAUGUCAUAGUUUA
Pma-12-3	UUUAUUGACAUAAAAACAUAAAGUCAUAACUGGCACAUGUGACAAUUAAAUUACUUUUCUCAUCCCCUUCCGGUUUAGGAGAAUGUAUGGCAUAUUAAA
env-18	UUUAUUGACAUAAAAACAUAAAGUCAUAACUGGCACAUGUGACAAUUAAAUUACUUUUCUCAUCCCCUUCCGGUUUAGGAGAAUGUAUGGCAUAUUAAA
env-19	AAUGCAUUAUAUCCAGAAAUGGGAAAUUGGCACAUGUGACAGUUAAGUAACAUUCCAUCUAAUGCUUCCACGAAAAACAAUUUAAUGGCAUAAUCUAAG
env-20	AAUUAUAAAUUCACCGCAAUAAAUAAUUGGCACAUGUGACAGUUGAGUAACAUUGCAUUCUCCUCUUCCGCGGAUAAGAAAUAUUUGAAAUAAUAUGGG
Pma-12-4	AAAUAGUAAAUACUUGGUGAAUUACUUUUUUCAGCAUAUAACCAAUAAGUAACAAUACUCAUCAAUAUCCACGAAAAAAAAUAUCUUGUAUAUGUUAUA
env-21	UUUUUUCUUAAUGUCUAGGAAUAGAAAACUGUCAUAUGUGACAGUUAAGCAACAUUCCAUCAAGCGCUUCCACGAAAAAGAAUUUAAUGGCAUAAUUUA
env-22	UUUAUGACAAAUUUACUGCACCAAUUAACUGGUACAUGUGACAGUUAAGUAACAUUCCAUUCGCCUCCUAAACGGAAAAGAAAUAGAUGUCAUAGUCUA
env-23	UAUUUUAUUUAUGCCCAGAAAUGGGUAACUGUCACAUGUGACAGUUAAGUAACAUUCCAUCAAGCGCUUCCACGAAAAAGUAUUUAAUGGCAUAAUUUA
env-24	CUAUUCCAAUCUUUAUGAGCAAAAAAAGUUGGUACAUGUGACAGUUAAGUAACAUUCCAAUCGCUUAUUCCACGGAAAAAAAAUAGAUGUCAUAUCCUG
env-25	UGUUUUUUAUUCUUAAUUUAAUAGAUAACUAGCACAUAUGACAGUUAAGUAACAUUCUAUUCGCCCCUUCCACGGAUAAGAAAUACAUAGGAUACUUUA
env-26	UGCUUUUUCUUUAUGUCUAGAAAUGGAAAACUGUCACAUGUGACAGUUAAGUAACAUUCCAUUCAGCGCUUCCACGAAAAAGAAUUUAAUAGCAUAUUU
env-27	UUAUUGCUUUAUAUCCAAAGAUGGACAACUGGCCCAUGUGACAGUUAAGUAACAUUUCAUUCUCCCAUUCCACAAAAUAGAAUUUAAUGGAAUAUUGUA
env-28	UUUUUUUCUUAUAUGUAGGAAUGGAAAACUGUCACAUGUGACAGUUAAAUAACAUUCCAUUCAGCGCUUCCACGAAAAAGAAUUUAAUGGGAUAUCUUA
env-29	CUUUUUUGUUAUGUCCAAAAAUGAGAAACUGUCACAUGUGACAGUUAAGUAAAAUUAGAUUAAGCACUUCCACGAAAAAAAAUUUAUUGGCAUAAUCUG
env-30	AUUUUUCUUUAUGUCCACAAAUGGGAAACUGUCACAUGUGACAGUUAAGUAACAUUACAUUCAGCGCUUCCACGAAAUAGAAUUUAAUUGCAUAGUUUA
env-31	GAUGACAAAUAUCUCGUGCGAGGCAAUUGGCACAUGUGACAGUUUAGUAACAUUCCAUUUGGCACUUCCACGGAAAAGAAAACUAUGGCAUAGUUUAUA
env-32	AUAUUUCUUUAUGUCCAGAAAUGAAAAACUGGCACAUGUGACAGUUAAGCAACAUUCCAUUUAGCCCUUCCACAAAAAAGAAUUUAAUGAGAUAGUUUA
env-33	UUAUUGCCAUAAAUCCCUUAAUGCACAACUGGCACAUGUGACAGUUAAGUAACAUUCCAUUUUCCUCUUCCACGGAAAAGAAAAAUAUGGCAUAGUUUA
env-34	AAAUUCAAGAUAUUCAAAAAAAAAGAAACUGGCACAUGUGACAGUUAAGUAACAAUUCAAUCUUCUGUUCCACAAAAUAGAAUUUUAUGGAAUAUUUUA
env-35	UUAUUGCCAUAUAUUCCGAAAUGAAGAACUGGCACAUGUGACAGUUAAGUAACAUUCCAUUCGCCUCUUCCACGGAAAUGAAAAAUCUGGCAUAGUUUA
env-36	UUUCCUUUAUGUCUAGAAAUGAAAAACUGACACAUGAUCCAGUUAAGCAACAUUCUAUUUAGCUCAUCCACGAAAAAGAAUUUAAUGAGAUAGUGUAUG
env-37	CUUUUUAUUUAUUUCUAGAAAUGAGCAACUGUCACAUGUGACAGUUAAGUAACAUUCCAUUCAGCGCUUCCACGAAAAAGAAUUUAAUGGCAUAGUUUA
env-38	AAUGAAACUUAGUCCUAAAAGAGUUAAUUGGUAUAUGUGUCGAGUAAGUAACAUUACUUAUCCCCUUCCACCAAAAAAAAUUAUUUGUAAUACCUUAAA
Pma-4-1	AAUGCAACUAAGUCGUAAAAGGUAUAACUGGUAUAUGUGUCGAAUAAGUAACAUUACCAAACCCCUUUAACAAAAAAGAAAUAUGCGUAAUAAGUUAUA

```
Pma-8-2   ACUCAAGCGAUCGUUUGAGUACUUUUUAUUUAACCUUGUGACAGUUGUUAUACAUGCCCAUUAGGUAUUUCGUCUAUUGAGAAUUCAUGUGAUAUUUAA
env-39    AACUAGAUGUUUGAGUACUUUUUGUAAUUUUUGCCAUGUGACAGUUAAGAUAGAGGUCUAUUAAACAUUACAUUUUUUGAAUUUAGGUGUGAUAUUAGU
env-40    ACCGCAUUGUUAGAGUACUUUUUUAUAUUUUUGCGAUGUGACAGUUAAGAUAGAGGUCUAUUAGUCAUUACAUAUUUUGAAUUCAAGUGUGAUAGUAAG
env-41    ACCUUAAUGUUUGAGUACUUUUUGCAUUUUUAUAGAUGUGACAGUUAAAAUAGAGUCCUACGAUGUAUUACAUUUUUUGAAUUUAGAUGUGAUAUUAAG
env-42    UCUGCGGCAACGUUAGGGUACUUUUAUAUUUUAGGGAUGUGACAGUUAAUAUAGAGGUCCAGCAGGGAUGCCAAUUUUUUAAUUUGGUGUAAUAGUAAG
env-43    .............UUGAGUCUUUUUGUAUUUUUUGCGAUGUGACAAUUAAGAUAGAGGUCUAUUAAGCACUACAUAUUUUAAAAUUAAGUGUGAUAUUA
Pma-4-2   ACUGAAAUGUUAGGGUACUUUUUGUUUUUUUUGCUAUGUGACAGUUAAGAUAGAGGUCUAUUAGGCAUUACAUAUUUCAAAUUUAAGUGUGAUAUUAAG
env-44    UCCUCAUCGACGGCUAAUUUUNNNNNNNNNNNNNNNNNNNNNNNNNNNNNNNNNNNNNNNNNNNNNNNNNNNAUUUUCUAUUUAAGUGUGAUAUUAGG
env-45    ACCGUAAUGAUCGAGUACUUUUUGUAUUUUUAGAGAUGUGACAGUUAAGAUAGAGGUCUAAUAGGGAUUACAUUUUUGAUAUUUCGGUGUGAUAUUAAA
env-46    GACCGCCAAGUUUGAGUACUUUUUGUUUUUUAUCGAUGUGACAGUUCAAAUAGAGGACUAAUAGCUAUUACAUAUUUGAAAUUUAGAUGUGAUAUUGUA
env-47    ...........................................................................UUUUGAAUUUAGGUGUGAUAUUAAG
env-48    GACCGUAAUGUUUGAGUACUUUUUGCUUUUUAUGGAUGUGACAGUUAAAAUACAGGUCUAUGAUGGAUUACAUUUUUUGAAUUUAGAUGUGAUAUUAAG
env-49    CGACCUCGAUGUUUGAGUACUUUUUGUAUUUUUUGCCAUGUGACAGUUAAGAUAGAGGUCUAUUAAGCAUUACAUAUUUUGAAUUUAGGUGUGAUAUUA
env-50    GAACGCAAAGUUUGAGUACUUUUUGUUUUUUUGCGAUGUGACAGUUGAGAUAGAGGUCUAUUUGGCAUUACAUUUUUUGAAUUUUGGUGUGAUAUUAAG
env-51    AACGCAAAGUUUUAGUACUUUUUGUUUUUUUUGCGAUGUGACAGUUGAGAUAGAGGUCUAUUUGGCAUUACAUAUUUUGAAUUUUGGUGCGAUAUUAAG
env-52    ACCUCGGUGUUUGAGUACUUUUUGUAUUUUUUGCCAUGUGACAGUUAAGAUAGAGGUCUAUUAAGCAUUACAUAUUCUGAAUUUAGGUGUGAUAUUAGA
env-53    NNNNNNNNNNNNNNNNNNNNNNNNNNNNNNNNNNNNNNNNGACAGUUAAAAUAGAGGUCUAUUAAGCAUUACAUAUUUGAAUUUAAGUGUGAUAGUAAA
env-54    CCGCAAUGUUUGAGUACUUUUUUAUAUUUUUUGCGAUGUGACAGUUAAAAUAGAGGUCUAUUAAGCAUUACAUAUAUUCAAUUUAAGUGUGAUAUUAAG
env-55    ACCUCGAUGUUUGAGUACUUUUUGUAUUUUUUGCCAUGUGACAGUUAAGAUAGAGGUCUAUUAAGCAUUACAUAUUUUGAAUUUUGGUGUGAUAUUAGG
env-56    ACCUCGAUGUUUGAGUACUUUUUGUAUUUUUUGUCAUGUGACAGUUAAGAUAGAGGUCUAUUAGGUAUUACAUAUUUUGAAUUUAAGUGUGAUAUUAGG
env-57    CGACCUUGAUGUUUGAGUACUUUUUGUUUUUUGUCGUGUGACAGUUAAGAUAGAGGCCUAUUAAGCAUUACAUAUUUUGAAUUUAGGUGUGAUAUUAGU
env-58    CGCAAAGUUUGAGUACUUUUUGUAUUUUUUUUGAGAUGUGACAGUUAAAAUAGAGGUCUAUUAAGCAUUACAUAUUUUGAAUUUAAGUGUGAUAUUAGG
env-59    CUUCGAUGUUUGAGUACUUUUUUGGUAUUUUUGCCAUGUGACAGUUAAAAUAGAGGUCUAUUGAGCAUUACAUAUUUUAGAUUUUGGUGUGAUAUUUAU
Pma-3-1   ACUCGACCGUAAUGUUUGAGUACUUUUUGUUUUUUGGAUGUGACAAUUAAGAUAGAGGUCUAUGAGGCAUUACAUUUUCAUCAUUUAAGUUAUAUUAAA
env-60    AUACGCAAUGUUUGAGUACUUUUUGUUUUUUUCGGAUGUGACAGUUAAGAUAGAGGCCCAAUAGUCAUUACUUUUUUUAAAUUUAGGUGUGAUAGUGUA
env-61    ACCGCAAUGUUUGAGUACUUUUUGUAUUUUUUGCGAUGUGACAGUUAAGAUAGAGGCCUAUUAGGCAUUACAUAUUUUGGAAUUAGGUGUGAUAUUAGG
env-62    .........................................ACAGUUAAGAUAGAGGUCUAUUAGACAUUACAUAUUUGAAUUUAGAUGUGAUAUUAAG
env-63    CCUCGAUGUUUGAGUACUUUUUUGCAUUUUGUGCCAUGUGACAGUUAAGAUAGAGGUCUAUUAAGCAUUACAUAUUUUGAAUUUAGAUGUGAUAUUAGA
env-64    .......................................................................CAUAUUUGAAUUCAGGUGUCAUAUUAAA
env-65    ACCUCAAUGUUUGAGUACUUUUUGUAUUUUUUGCCAUGUGACAGUUAAGAUAGAGGUCUAUUGAGCAUUACAUAAUUUGAAUUUAGGUGUGAUAUUAGG
env-66    NNNNNNNNNNNNNNNNNNNNNNNNNNNNNNNNNNNNNNNNNACAGUUAAAAUAGAGGUCUAUUAAGCAUUACAUAUUUUAAUUUAAAUGUGAUAUUAAA
env-67    CCGCAAUGUUUGAGUACUUUUUGUAUUUUUUUGCGAUGUGACAGUUAAGAUAGAGGUCUAUUAGGCAUUACAUAUUUUGAAAUUAAGUGUGAUAUUAGA
env-68    CCGCAAUGUUUGAGUACUUUUUGUAUUUUUUUGCGAUGUGACAGUUAAGAUAGAGGUCUAUUAGGCAUUACAUAUUUUGAAAUUAAGUGUGAUAUUAGA
env-69    ACCGCGAUGUUCGAGUACUUUUUGUAUUUUUUGCGUUGUGACAGUUAAGAUAGAGGUCUAUUAGGCAUUACAUAUUUUGGAUUUAGGUGUGAUAGUAGG
env-70    UCGACCUUGAUGUUUGAGUACUUUUUGUUUUUUGAGUGUGACAGUUAAGAUAGAGGUCUAUUAAGCAUUACAUAUUUUGAAUUUAGGUGUGAUAUUAGG
env-71    ACCUCGAUGUUUGAGUACUUUUUGUAUUUUUUGCCAUGUGACAGUUAAGAUAGAGGUCUAAUAAGCAUUACAUAUUUUGAAUUUAGGUGUGAUAUUAGA
env-72    GCCGCAAAGUUUGAGUACUUUUUGUAUUUUUUGAGAUGUGACAGUUAAAAUAGAGGUCUAUUAAGCAUUACAUAUUUUGAAUUUAAGUGUGAUAUUAAG
env-73    ACCUUGAUGUUUGAGUACUUUUUGUAUUUUUUGCCAUGUGACAGUUAAGAUAGAGGUCUAUUAGACAUUACAUAUUUUGAAUUUAGGUGUGAUAUUAAG
env-74    ACCUCGAUGUUUGAGUACUUUUUGUAUUUUUUGCCAUGUGACAGUUAAGAUAGAGGUAUACUGAGCAUUACAUAUUUGGAAUUUAGGUGUGAUAGUAGG
env-75    ACCGCAAAGUUUGAGUACUUUUUGUAUUUUUUGAGAUGUGACAGUUAAAAUAGAGGUCUAUUAGGCAUUACAUAUUUUGAAAUUAAGUGUGAUAUUUAA
env-76    AAUCGCAUUGUUUGAGUACUUUUUGUAUUUUUGGAUGUGACAGUUAAGAUAGAGGUCUAAUAGACAUUACAUAUUUUAGAUUUAGGUGUGAUAUUAAA
Pma-6-2   ACCUCGAUGUUUGAGUACUUUUUGUUUUUUUUGCCAUGUGACAGUUAAGAUAGAGGUCUAAAAGGUAUUACGUAUCUUAUAAAUUGAUGUGAUAUUAAA
env-77    ACCGCAAUGUUUGAGUACUUUUUGUAUUUUUUGCGAUGUGACAGUUAAGAUAGAGGUCUAUUAGGCAUUACAUAUUUUGAAAUUAAGUGUGAUAUUAAG
env-78    ACCGCAAUGUUUGAGUACUUUUUGUAUUUUUUGCGAUGUGCCAGUUAAGAUAGAGGUCUAUUAAGCAUUACAUAUUUUGAAAUUAAGUGUGAUAUUAAA
env-79    ACCUCGAUGUUUGAGUACUUUUUGUAUUUUUUGCUAUGUGACAGUUAAGAUAGAGGUCUAAUAAGCAUUACAUAUUUUGAAUUUAGGUGUGAUAUUAAG
env-80    GGCCGCAAUGUUUGAGUACUUUUUGUAUUUUUUGCAAUGUGACAGUUAAGAUAGAGGUCCAGCAGGGAUUACUUUUUUUUGAAUUAAUGUAAUAUUUAA
env-81    CCGCAAAGUUUGAGUACUUUUUGUAUUUUUUUGAGAUGUGACAGUUAAAAUAGAGGUCUAUUAAGCAUUACAUAUUUUGAAUUUAGGUGUGAUAGUAAG
env-82    ACCUCGAUGGUUGAGUACUUUUUGUAUUUUUUACCAUGUGACAGUUAAGAUAGAGGUCUACUAAGCAUUACAUAUUUUGAAUUUAGGUGUGAUAGUAGG
env-83    ACUGCAAUGUUUGAGUACUUUUUGUGUUUUUUACGAUGUGACAGUUAAGAUAGAGGUCUAUUAGGCAUUACAUAUUUUGAAUUUAAGUGUGAUAUUAAG
env-84    CGCAAAGUUUGAGUACUUUUUGUAUUUUUUUUGAGAUGUGACAGUUAAAAUAGAGGUCUAUUAAGCAUUACAUAUUUUGAAUUCAGGUGUGAUAUUAAA
env-85    CCGCAAAGUUUGAGUACUUUUUAAAUUUUUUGAGAUGUGACAGUUAAAAUAGAGGUCUAUUAAGCAUUACAUAUUUUGAAUUUAGAUGUGAUAUUAGGG
env-86    CCGUAAUGUUUGAGUACUUUUUGUAUUUUUUUGAGAUGUGACAGUUAAAAUAGAGGUCUAUUAAGCGUUACAUAUUUUGAAUUUAAGUGUGAUAUUGGA
env-87    CCGCAAAGUUUGAGUACUUUUUGUAUUUUUUUGAGAUGUGACAGUUAAAAUAGAGGUCUAUUAAGCAUUACAUAUUUUGAAUUUAAGUGUGAUAUUAAA
env-88    GACCGCAAUGUUUGAGUACUUUUGUAUUUUUUGCGAUGUGACAGUUAAGAUAGAGGUCUAUUAAGCAUUACAUAUUUUAAAAUUAAGUGUGAUAUUAAG
env-89    ACCGUAAUGUUUGAGUACUUUUUGUCUUUUUUGCGAUGUGACAGUUAAGAUAGAGGUCUAUUGAGCAUUACACAUUUUGAAAUUAAGUGUGAUAUUAAA
env-90    AAUGCAAAGUUUGAGUACUUUUUGUAUUUUUUGCGAUGUGACAGUUGAGAUAGAGGUCUAUUUGGUAUUACAUAUUUUGAAUUUUGAUGUGAUAUUAAA
env-91    ..................................................................................UUUGGUGUGAUAUUUAU
env-92    ACCGCAAUGUUUGAGUACUUUUUGUAUUUUUUGCGAUGUGACAGUUAAGAUAGAGGUCUAUUAAGCAUUACAUAUUUUGAAAUUAAGUGUGAUAUUAGG
env-93    ACCGCUAUGUUUGAGUACUUUUUGUAUUUUUUGCCAUGUGACAGUUAAGAUAGAGGUCUAUAAGACAUUACAUAUUUUGAAUUUAAAUGUGAUAUUAGG
env-94    CCGUAAAGAUUGAGUACUUUUUGUAUUUUUUUGAGAUGUGACAGUUAAAAUAGAGGUCUAUUAAGCAUUACAUAUUUUGAAUUUAGGUGUGAUAUUGGG
env-95    CGCAAAGUUUGAGUACUUUUUGUAUUUUUUUUGAGAUGUGACAGUUAAAAUAGAGGUCUAUUAAGCAUUACAUGUUUUGAAUUUUAGUGUGAUAUUAAA
env-96    ACCUGGAUGUUUGAGUACUUUUUGUAUUUUUUGCCAUGUGACAGUUAAGAUAGAGGCCUAUUAAGCAUUACAUAUUUUGAAAUUAGGUGUGAUAUUAGA

          ...................................................................................................
          ...................................................................................................
          ●●●●●●●●●●●●●●●●●●●●●●●●●●●●●●●●●●●●●●●●●●●●●●●●●●●●●●●●●●●●●●●●●●●●●●●●●●●●●●●●●●●●●●●●●●●●●●●
```

env-97	GACCGCAAUGUUUGAGUACUUUUUGUAUUUUUGCGAUGUGACAGUUAAGAUAGAGGUCUAUUAGGCAUUACAUAUUUUGAAAUUAAGUGUGAUAUUAGG
env-98	ACCUCGAUGUUUGAGUACUUUUUGUAUUUUUUGCCAUGUGACAGUUAAGAUAGAGGUCUAUUAAGCAUUACAUAUUUUGAAUUUAGGUGUGAUAUUAGG
env-99	CCGCAAAGUUUGAGUACUUUUUGUAUUUUUUUGAGAUGUGACAGUUAAAAUAGAGGUAUAUUAAGCAUUACAUAUUUUGAAUUUAGGUGUGAUAUUAAA
env-100	CCGCAAUGUUUGAGUACUUUUUUAUUUUUUUUGCGAUGUGACAGUUAAGAUAGAGGUCUAUUAGACAUGACAUAUUUUGAAAUUAAGUGUGAUAUUAAG
env-101	GCCGCAAAGUUUGAGUACUUUUUGUAUUUUUUGAGAUGUGACAGUUAAAAUAGAGGUCUAUUAAGCAUUACAUAUUUUGAAUUUAAGUGUGAUAUUAGG
env-102	CCUUGAUGUUUGAGUACUUUUUGUAUUUUUUUGAGAUGUGACAGUUAAAAUAGAGGUCUAUUAAGCAUUACAUAUUUUGAAUUUAGGUGUGAUAUUAAA
env-103	.........................UUUUUUGAGAUGUGACAGUUAAAAUAGAGGUCUAUUAAGCAUUACAUAUUUUGAAUUUAAGUGUGAUAUUAGG
Pma-1-1	ACCGCAAUGUUUGAGUACUUUUUGUAUUUUUUGUGAUGUGACAGUUAAGAUAGAGGUCUAUUAGGUAUUACAUAUUUUGGAAAUAAGUGUGAUAUUAAG
env-104	CAGCAAUGUCAGAGUACUUUUUUGUAUUUCUUGCGAUGUGACAGUUAAUAUAGAGGUCUAAUAGGUAUUACUUUUUUUUAAUAAAGAUGACAUAUUAGG
env-105	CCGCAAUGUUUGAGUACUUUUUUAUAUUCUUUGCGAUGUGACAGUUAAGAUAGAGGUCUAUUAGGCAUUACAUAUUUUGAAAUUAAGUGUGAUAUUAGG
env-106	ACCGCAAUGUUUGAGUACUUUUUGUAUUUUUUGCGAUGUGACAGUUAAGAUAGAGGUCUAUUAGGCAUUACAUAUUUUGAAAUUAAGUGUGAUAUUUAA
env-107	ACCGCAAUGUUUGAGUACUUUUUGUAUUUUUUGCGAUGUGACAGUUAAGAUAGAGGUCUAUUAGGCAUUACAUAUUUUGAAAUUAAGUGUGAUAUUAAG
env-108	ACCUCGAUGUUUGAGUACUUUUUGUAUUUUUUGCCAUGUGACAGUUAAGAUAGAGGUCUAUUAAGCAUUACAUAUUUUGAAUUUAGGUGUGAUAUUGGA
env-109	CCUCGAUGAUUGAGUACUUUUUUGUAUUUUUUGCCAUGUGACAGUUGAGAUAGAGGUCUAUUAAGCAUUACAUAUUUUGAAUUUAGGUGUGAUAUUAGA
env-110	.................UUUUUGUAUUUUUUGGCAUGUGACAGUUAAAAUAGAGGUCUAUUAAGCAUUACAUAUUUUGAAUUAUGGUGUGAUAUUAGG
env-111	ACGCAAAGUUUGAGUACUUUUUGUUUUUUUUUGAGAUGUGACAGUUAAAAUAGAGGUCUAUUAACCAUUACAUAUUUUGAAUUUUGGUGUGAUAUUGGG
env-112	ACCUCGAUGUUUGAGUACUUUUUGUAUUUUUUGCCAUGUGACAGUUAAGAUAGAGGUCUAUUAAGCAUUACAUAUUUUGAAUUUAGGUGUGAUAUUAGG
env-113	CCGCAAUGUUUGAGUACUUUUUUAUAUUUUUUGCGAUGUGACAGUUAAGAUAGAGGUCUAUUAGGCAUUACAUAUUUUGAAAUUAAAUGUGAUAUUAAG
env-114	CGACCGAAAUGUUUGAGUACUUUUUGUUUUUUGCGAUGUGACAGUUUAGAUAGAGGUCUAUUAGGCAUUACAUAUUUUGAAUUUAAGUGUGAUAUUAAU
env-115	CCGCAAUGUUUGAGUACUUUUUUAUAUUCUUUGCGAUGUGACAGUUAAGAUAGAGGUCUAUUAGGCAUUACAUAUUUUGAAAUUAAGUGUGAUAUUAAG
env-116	ACCUCUAUGUUUGAGUACUUUUUAUAUUUUUUGCCAUGUGACAGUUAAGAUAGAGGUCUAUUAAGCAUUACAUAUUUUGAAUUUAGGUGUGAUAUUAGG
env-117	CUAUCGAAAUUUGAGUACUUUUUUUAUUUUUUGCGAUGUGACAGUUAGGAUAGAGGUCUAUUAGGUAUUACAUAUUUUGAAUUUAGGUGUGAUAUUGUA
env-118	ACCUCGAUGUUUGAGUACUUUUUGUAUUUUUUGCUAUGUGACAGUUAAUAUAGAGGUCUAUUAAGCAUUACAUUUAUUGAAUUAAGGUGUGAUAUUAAA
env-119	ACCUCAAUGUUUGAGUACUUUUUGUAUUUUUUACCAUGUGACAGUUAAGAUAGAGGCCUAUUAAGCAUUACAUAAUUUGAAUUUAGGUGUGAUAUUAGG
env-120	UGAAAUUGUUCGGGUACUUUUUGUAUUUUAUAGCAAUGUGACAGUUAAGAUAGAGGUCUAAUAGGCAUUACAUUUUUCAAGCUUAGAUGUGAUAUUAGA
env-121	CCGCAAUGUUCGAGUACUUUUUUAAUUUAAUUACGAUGUGACAGUUAAAAUAGAGGCCUAGUAGAUAUUACAAAUUUAAUUUAUUGGUGUGAUAUUAGA
env-122	UUUUUGCCAUAAAAACUAAAUUGGAAAACUGGCACAUGUGAUAGUUUAGUAACAUUCUAUUUACCUCUUCCACGAAAAAAAAAUAUAUGGGAUAGUUUA
Pma-10-1	AGCAGGGUGUUUUUUAAUGACUACUUUUGCAAAUUGUGACAUGUGCCACAAGAUAGACCAGCCUUCUAAUAUUUGAGUUUUUCAUUAGCUUAGUAAAUA
Pma-9-2	AGCAGGGUGCUUUUUAAUGACUACUUUUGCAAAUUGUGACCUUUGCCAAUAAAUAGACCAGCCAUCUCAUAUUUGAGUUUUUCAUUAGCUUCGUAAAUA
Pma-9-3	GUAGGGUGCUUUUUUAAUGUCUUUUUUUUAAAGUAGUCACAUGUGAAAGUUAAUAGACUCAUCUACUAAUAUUUGAAACAUCUAGAUCUUUAAAUAGUU
Pma-10-2	CUGUGCCGAAAGAGCAUCCAAUUGGGUGCUUUUUUAGUGCCUGUGCCAAUCAAUAAACCCUCCUAUUAGGGGUUUAUUCCUAAGCCUGUUCAGAUAAAU
env-123	UAGGGUGCUUUUUUAGUGCCUUUUUUCAAGUAUUGGCACAUGUGACAGUUGAGAAACUCAUCUGAUAAACCUUGAGUGCUCUAAUUGCUUCUAAUAAGU
env-124	UUAUUGUCCUUGCUGAAUAGAACUUUCUAAAUUCUUAUGUCUGUGACAGUUGAAAAACUUUACUGCUUGUUGCUUUCGUUAGUUUUAAUUACCUUAAUG
Pma-9-4	UAUUGUCCUUGCUGACGUGAAACUUUCGAAAUUUUAUGAUCUGUGACAGUUGAAAAACUUUACUGCUUCUUGUUUUAAUUAGUAUUGAACACCUUAAAG
Pma-10-3	UAUUGUCCUUGCUGAAGUGAACUUUCUAAAUUCUUAUGAUCUGUGACAGUUGAAAAACUUUACUGCUUGUUGCUUUCAUUGGUAUUAAUCAACUUAAUA
Pma-10-4	GCAGGGUGCUUUUUUAAUGUCUUAUUUAAAAGUAUGCACAUGUGACAGUUAAGAGACUCAUCUACUGAUACUUGAGACAUCUAGAUUUUUCAAAUAGUU
env-125	UAUUGUCCUUGCUGAAGGGAACUUUCUAAAUUCUUAUAAUCUGUGACAGUUGAAAAACUUUACUCCUUGUUGCUUUCAUUAGUAUUAAUCACCUUAAAA
env-126	UUGCAAUAAGUUGGGUGCUUUUUAAUGACUACUUUUGCCAAGUGUGACAAUCAAUAAACUAACCUUCUCAAAUUUGAGUUUUUCAUCAGAUGCGAAAUA
env-127	AAUAAUAUAAAUAAGAGAUAAUUUUAUUAAUGCUUAUAACUUGUGACAGUUGACAAACUUUACUGUCAUAAAUUGUUUAUAUCUCUAAUCCUUUAAAAU
Ssp-3-1	CGUCUUCUGACUCACGGCCUUGUGAUGGGUGCCCAGGCAGAGGGACAAGCCAAAGGCUGUCCAUGACGGGCUUCCCGAUUCCCUCUCUUGCAGACACGA
Ssp-2-1	UGUGUUGUCGGGAGUUGGCUGGACCAGUUCACUUGGAAACGGUGUGACAAUCGCUUUAGUGUUGGUGAUUACUGGCACAGUGCCAGUCCAGACGCAGAA
Ssp-2-2	CUUCACUUGAAGGCUGCUGCCACUAGCAAAUUCCAUCAAUCUUGUGCCAGUCACCCAAGUGUUGACGAACACUGGCACAGUGCCAGUUGCGAAGCAUAA
Ssp-2-3	UGGCGGUGCCAUAGGGCUGGCAGUUGGUUACGGAAUAGGUCGCAUUAGAAAAUGAACCCAGAGCUAAGGGUCUUGCUCAGCGAGUUUUCUGAGGCACGA
env-128	CCUUGUGAGGAGUUGGCCGAGCCCAUCCUGAGAAGAUUGCGUAGAUCAGUCAACCAACUGAUACGUAGGGUCUUGCUCUGUGGGAUUUCUGGGGCAUAG
Ssp-2-4	CCUUUUAGGGAAUUGACCAAGCACAUGCUGAGGAAAUACCGCAGAACAGUCAACCAACUGAUACGUAGGGGCUUGCUCAGCAUGAUUUCUGAGCCAUUA
Pma-5-1	GCUGAAUAGUCUUCAAUACAGCAACGCCAAGCGAACAGCUAGUGACGGAUGUGGAACUGACACGGUCAACUGGCACAGUGACAGUUGCGAUCGUUAUGG
Pma-7-1	GCUGAAUAGUCGUCAAUAGAGCAACCCCAAGCGAACAGCUAGUGACGGCUGUGGAACUGACACGGUCAACUGGCACAGUGACAGUUGCGAUCGUUAUGG
Ssp-2-5	CCUUGUAGGGAGUUGACCGAGCACAUGCUGAGGAAAUUCCGCAGAACAAUCAAUCAACUGAUACGAAGGGACUUGCUCAGCCGGAUUUCUCAGGCAUUA
Ssp-2-6	CUUGUGAGGAGUUGGCCACCACCAUGUUGAUGACGGCACUGCCGAUCAGUCAACCAACUGAUACGUAGGGGAUUGUUCUGCGAGGUUUCUGAGGCAUGA
Ssp-2-7	CCCUCUUAGGGGUUGGUCAACCACAUGGUGAGGACAUUCAGCAGAUCAGUCAAUCACCUGAUAUGAAGGGACUUGCUUAGAUGGUGUUCUGAGGCAAAA
Ssp-2-8	UCAGCAGUUGACGAUUUCUUUAUCAGUGAAUGGUCUGGUUUUAUGGCAAUCCAAUAAGUAACAAGACGUGUAUUGCAGGAAGUUUCCCAACAACUAAGA
Ssp-10-1	AGACCGGUCCGAUCGCUCUGCAGGUGAUCCCCUUCCGGGAGUGACAGUGAACACAGCGGCCCUUGGCGCUGGCACCUAACGGAUCCAGCCCAUACACUU
Ssp-2-9	CCGCCUCGAAGUGAUUCCAUUCCGCCCAUGAACGAAGCUUCAAGGACAGUUGGAUGAGCGACAACUGCUAUCACCCCAACCAAGCAAUUCUGCCUAUGU
Pma-7-2	UUGUGAUUCCUUAUCGUCCCUGACGUAGGCCCACUGCAGAGAGGACAGUUGAAGUAGCGCCCCAUAAUACUAGUAACUCUAAAAAUUGCCUCAUAUCAU
Pma-5-2	CUUGUGAUUCCUUAUCGUCCGUGACGCAGGCCACUGCAGAGAGGACAGUUGAAUUAGCGCCCCUUAAUACUAGUAACUCUAUAGAUUGCCUUAUAUCAU
env-129	CCGUGAUUCCCUUCCGCCCCUGAGCGAGGUCUUCACGGAAGGACAGUUGGCAGAGCGCCCCAGACCAGCGCGCAAUUCAAAGAGCAAUUUUUUAAUUUG
Ssp-11-1	CGAUCCGAUUGCAGGUGAUUCCUUUCCGCCCCUGAGGCGAGGACAGUUGAAGGAGCGCCCUGCGCAGGCGGGACAGACCCAGGAUCACUUCUUAAGUUG
env-130	CCGAUCAGGCUGACCGUCAUUCCUUUCAAACCCUGAAUUAGGGACAGUUGGAAGAGCGCACCAGUGAGACUCGCAAUCCAGGCAACAGUUUUUAACUUG
Ssp-12-1	CGAUCCGGCUGGAGGUGAUUCCCUUCCGUCCGUGACGCGAGGACAGUUGAGAGAGCGCCCUCCCCAAGCAAGACAGUGCCAGGAUCACUUCUUAUCUUG
env-131	....UUCGUCGUCGUGAAUGACUCCGAACUUCUAGUUUU
env-132	AGCUUUGGUAGGCAUCAAUGGAACCUAACCACCAGGCCGGACCACUCAGAAAACCGUCUUGCCAGGGGGUGGCACAUUUUCCUCUGAGGAAAUAGUUUU
Ssp-7-1	AGCUUUGGAAGGCAUCACUGGAACCUAAUCACCGGGGCAGACCACUCAGAGAACCGUCUCUCCAGGGGGUGGCACAUUUUCCUCUGAGGAAAUAGUUUU
	....
	....

```
Ssp-4-1    AAGUGAUCCCGUACAGCAGCCCUCGCCAAACACUGGCACAGUGACAGUUUUCAGAGAGGCCAGCGAGAUAACCAAUCACAUUUGAGGACUUUUAGGUUU
env-133    GGUGAUUCCUUACGGGCGGUAAAGCCGCAAAGUGUCACAGUGACACUUGAGAGAGCGACCUGAAAGGCGCACCAGUGCAGAACUGCCCUUCUAGUUUUU
env-134    AGGUGAUUCCGUACGGGCGGUGAACCCGCCAAGUGUCACAGUGACACUUGAGAGAGCGACCUGAACGGCGCGUCACUGCCGUACCGCGCUUCUAAGUUC
env-135    ACGUGAUUCCCUAUCGACCCUGAUCCCAGCGACUGUCACAGUGCCAGUUGGCAGGCCGACCUGUUGAAGUUGGUACUUCGUUUCUUCGCCCUUAAUUUG
env-136    AGGUCAUUCCCUAUCGACCCUGAGGCCAGCGACUGUCACAGUGCCAGUUGACAGGCCGACCUGUUGAAGUUGAAUAUUCGUUUCCUCGCCAUUAGUUUG
Ssp-13-2   AGGUCCUUCCCUAUCGACCCUGAGGCCAGCGACUGUCACAGUGCCAGUUGACAGGCCGACCUGUUGAAGUUGGAUAUUCGUUUCCUCGCCAUUAGUUUG
env-137    GCAGGUGAUUCCCUAUCGGCGCUGAGGGCCAACCGUCACAGUGCCAGUUAAGAGGGCGACCAGGCAGGGGCUGACAACAAAAUCCCCGCCCUUAAUUUG
Ssp-3-2    GCAGGUGAUUCCUUACCGCCGCUGAGGACAAACCGUCACAGUGCCAGUUGGGAGAGCGCACAGACAGGGGCUGAUAAUGGAAUCUCCGCCCUUAAUUUG
env-138    CAGGUGAUUCCCUAUCGCCGCUGAGGAUCAACCGUCACAGUGACAGUUGAGAGGGCGACCAGACAGGGGCUGACCAAUAGAUUUUCCACCCUUAGGUUA
env-139    NNNNNNNNNNNNNNNNNNNNNNNNNNNNNNNNNNNNNNNNNNAGUUGAGAGAGCGACCAGAAGGGGGCUGACAACAGAAGCAUCGCCCUUAGUUUUU
Ssp-10-2   AAGUGAUCCCUUACAAGAGCCAACGCUAGACAGUGGCACAGUGCCAGUUGCUAGAGAGUCCGGCAGAACAACCAAUCACAAUUUGAGGGCCCUAAUUUU
Ssp-1-1    AAGUGAUCCCUUACAAGAGCCAACGCUAGACAGUGGCACAGUGCCAGUUGCUAGAGAGUCCGGCAGAACAACCAAUCACAAUUUGAGGGCCCUAAUUUU
env-140    GCAGGUGAUUCCCUAUCGCCGCUGAGGACCAACCGUCACAGUGCCAGUUGAGAGGGCGACCAGACAGGGGCUGACAAUAGAUUCCCCACCCUUAGUUUG
env-141    GCAGGUGAUUCCUUACCGCCGCUGAGGACAAACCGUCACAGUGCCAGUUGGGAGAGCGAACAGACAGGUGCUGACAAUGGAAUUUCCGGUCUUAAUUUG
env-142    ............CCUACCGCCGUUAAGACCAACCGUCACAGUGCCAGUUGAAAGGGCGACCAGACAGGGGCUGACAACGUAACCCUGACCCUUAUUUUG
env-143    GCAGGUGAUUCCCUAUCGCCGCUGAGGACCAACCGUCACAGUGCCAGUUGAGAGGGCGACCAGACAGGGGCUGACAUUGGAUUCCCUGCCCUUAGUUUA
env-144    GCAGGUGAUUCCCUAUCGCCGCUGAGGACCAACCGUCACAGUGCCAGUUGAAAGGGCGACCAGACAGGGGCUGACAACGGAUUGCCCACCCUUAACUUG
env-145    AGGUCAUUCCGUACCGCCCGUGACGUGACCAACUGUCACAGUGACAGUCGGCAGGCUGACCUGCCAGGAUGUAAGAAGCCUUGACCUGCACUUAGGUUU
env-146    GGUCAUUUCCUUUCGACCCAGCACCCGCUUACUGGCACAGUGCCAGUUAAAAGAGCGUACUCACGCCAUAACGAAACAACUAUCAGGCCUUCUAAUUUC
Ssp-9-1    CCGAUCCGGCUGGAGGUGAUCCCCUAUCGCCCCUGAGGGCAGGGACAGUUCCAGGAGCGCCCCCGAAGGAGCACAUCCACCUGUUCACGCCCUUAAUUU
env-147    ...............................................................................CUUGUUCACGCCCUUAAUUUC
env-148    CGAUCCGGCUGGAGGUGAUCCCUUAUCGCCCCUGAGGGCAGGGACAGUUCCAGGAGCGCCCUCGAAGGAGCCCAUUCACUUGUUCACGCCCUUAAUUUC
env-149    CGAUCCGGCUGGAGGUGAUCCCUUAUCGCCCCUGAGGGCAGGGACAGUUCCAGGAGCGCCCUCGAAGGAGCCCAUUCACUUGUUCACGCCCUUAAUUUC
env-150    CAGUGAUUCCCUUUCGACCCUGAGCCGCGGCCUCACGGAAGGACAGUUGACAGAGCGCCCCAGCACAACGCGCAAUGUCCAAAGCAAUUUUUUACUUUG
env-151    GGCGUUGUGGGCAUGCCUUGAACCUAACCAUGUGCGGCGGACCACUCAGCAAACCGUCUGGGGUGGGGGUAGCUGAUUUUCCUCUGACGGAAUAGUUUU
Ssp-8-2    GGGAGUUGCACGGCCUGCAACAAAGCCCCACCAGCAGCUGUGCCAACCCAGCUUCUGGUCCAGACCCAACUGUCACAGUGCCAGUUCGUCUGGCUAGUA
env-152    UGUGAGGGAGAGUCAACGGCCCAAGGCUUAGUCAUGGUUUAUGCCGUUCUACAGACUGACCGCAAAACAACUGGCACGUUGCCUACUGGGUCGUUAUGU
env-153    ...................................................................................................C
env-154    CGGGGGGAGUUAUGGAGAUGGCUGAGCUCAAGAAACUGACUAAGGACAAGCGACAAGCCGUCCACCAACAACUGUCACAGUGCCAGUUGGGUAGAGAUA
env-155    CAAUUAGACCAACAAGAAAGAAGCCGAGGCUUGAUGGCUUAGGACAAGCCACAAGCUGUCCCCGCCCAACUGGCACAGUGACAGUUGGGACGCUUACAG
env-156    CAAGCAGACCGACAAGAAAGAAGUCGAGGCUUGAUCGCUUAGGACAAGCCACAAGGCGUCCACCCCCAGCUGGCACAGUGACAGUUGGGACGCUUACUG
env-157    ACAAGUAAGAAGUCGAGGCUUGAUCGCUUAGGACAAGCAAAAAGGCGUCACUACCAACUGGCACAGUGACAGUUGGUAUGGCCAUUCAUGUGGCUAGGU
env-158    GCCGUCGCCCCCUCAGCAGCGGUCUGCGUUUUGAUCGUUUAAGACAAACCCAAAGCCAUCCCCGCCUAACUGGCACAGUGACAGUUGGAACGCUUAUGG
Ssp-13-3   GGCAAGAAAAUUGCUUGCCUAUGAAGCAAGCGCGCUCUUCGGGACAGGCGGCAAAGCGUCUACUCGAAACUGGCACAGUGCCAGUUGGUAGAAGUAGGU
env-159    GGCAAGAAAAUUGCUUGCCUAUGAAGCAAGCGCGCUCUUCGGGACAGGCUGCAAAGCGUCUACUCGAAACUGGCACAGUGCCAGUUGGUAGAACUAGGU
Ssp-3-3    ACUGGAUCAACGCAGACCUCGUGACUCACAUCGUCCCCAGGGUUUGAAGCAGGCGCGCUCUUCUUUUUGCUUGCCUUUGAAUCAAGCGCGUGGCUAGUU
env-160    UGUGAGGAGAACCAACUGACCGAGUUCUAGCAGCUCCUUUGUGCCAUUCAAGAGACCGGCCUGCCAACAAGUGGCACUGUGCCAGUCAGGUGGCUAAAU
env-161    UGUGAGGAGAACCAACUAAAGGAGUUCUAGCAGCUCCUCUGUGCCAUUCAAGAGACCGGCCUCAUAACAAGUGGCACUGUGACAGUCGAUUGGCUAAAU
env-162    UGUGAGGAGAACCAACUGACCGAGUUCUAGCAGCUCCUAUGUGCCAUUCAAGAGACCGGCCUGCCAACAAGUGGCACUGUGCCAGUCAGGUGGCUAAAU
Ssp-4-2    UUGGAGUGCAGGCAUCGACAUGCCGAGCCCAAGGUCUGGGUUGUCAACUCCGAUGCCGUCCACUCCUAACUGGCACUGUGCCAAUUCCCUAGGCUAGGU
env-163    AAUAACCUGAAAAGGCGACCGCGGGUGACAAAUCACCAAAGAGACAAGUCGCGAUCUUUCCAUCCCUAACUGGCACUGUGCCAAUUUCCUGCGUUAAGC
env-164    ..........................................CCUAUGCCGUUUACCUCACUGGUCACAACUGGUUGCAUGCCUAUACCCAAGGAUACGA
env-165    ACGUAGCAAAGGGUGAUGAGCUGGGCUCAAGAAGCUGGCUGAGGACAAGCGACAAGCCGUCCACCAACAACUGUCACAGUGCCAGUUGGGUUGAGAUAG
env-166    UGGAGGAGUCAUGGAGAUGCCUGGGCUCAAGAAGCUGGCUGAGGACAAGGGACAAGCCGUCCACCAGCGACUGUCACAGUGCCAGUUGGGUAGAGAUAG
env-167    GGAGGAGUCAUGGAGAUGGGCUGAGCUCAAGAAACUGGCUAAGGACAAGCGACAAGCCGUCCACCCACGACUGUCACAGUGUCAGUUGAGUAGGGAUGC
env-168    AAGGAAGACGACACUUAAAGAUUGAGGGGAGCAGCUGGCUGAGGACAAGCGACAAGCCGUCCACCCACAACUGUCACAGUGCCAGUCGGGUAGAGAUAC
env-169    GGGAGGAGUUAUGGAGAUGGCUGAGCUAAAGAAACUGACUGAGGACAAGUGACAAGCCGUCCACCAACAACUGUCACAGUGCCAGUUGGGCAGAGAUAG
env-170    CGUAGCCAGAGGGUGAUGGGCUGGGCUCAAAAAGCUGGCUGAGGACAAGCGACAAGCCGUCCACCCAUGACUGUCACAGUGCCAGUUAGGUAGUGAUAG
env-171    GGGAGGAGUUAUGGAGAUGGCUGAGCUCAAGAAGCUGGCUAAGGACAAGCGACAAGCCGUCCACCCACAACUGUCACAGUGCCAGUUGAGUAGAGAAAA
env-172    GACGUAGCCACAGGGUGAUGGACUGGGCUCAAGAAGCGGGCUGAGGACAAGCGACAAGCCCCACCAACAACUGUCACAGUGCCAGUUGGGUAGAGAUAG
env-173    CCGGCCUGGGGCGACGCGGUUGAACUUUGAGACAGGUGGCUGGGGCCAACUGCAAGGCUGUCCAUCAGCAAUCCCAGCGCACGCGUCGGAUUGGCAUGU
env-174    CCGGCCUGGGGCGACGCGGUUGAACUUUGAGACAGGUCGCGGAGGCCAACCGCGAGGCUGUCCAUCAGCAAUCCCAGCAAACGCAUCGGAUUGGCAUGU
Ssp-3-4    CCGACCUGGGGGGACGCAGUUGAACUUUGAAAGAGGUCGCUGGGGCCAACUGCGAGGCUGUCCAUCAGCAACCCCAGCCCACGCAUUGGAUUGGCAAGU
Ssp-4-3    AAACAGAUCACGCACACAGCUGACAGAGGAUGCUGGGAAAAUCGCCAAUCAUCAAGCUGGUCCACGCUGGAAUGCCAUCGCAUUCCUUGAGAGGCAUAA
Ssp-1-2    AAACAGAUCCUGCACCGAGCUGUUAGAAGAUGCUAAGAAAAUUGCCAAUCAUCAAGCUGGUCCACGCUGGGACGCCAGUACAUGUUUGGAGAGACAUAA
env-175    AAACAGAUCCUGCACCGGGCUGUUAGAUGAUGCCAGGGAACUUGCCAAUCAUCAAGCUGGCCCACGCUGGGAUGCCAGUGCAUGCCUGGAGAGGGCAAA
env-176    GGAAUUCCCCCUGCCUGUAGAGCCAGGUCAGAAAUGCUGCAGGGCCAAUCACCAAGCUGAACCCUGAGCCUUGAUGACUCUGGUGGUGGAGAGCCAUAU
Ssp-3-5    CCGUUGUCGUCUCUCUGCUGCCGACGAUCGAAGGCGUCCUCCUGGACGAGUCAGCAGCUGUCCACUUUUGUAGUCGCAAUUCCAGUGGCGUUGCCAACU
env-177    AGGCUGAGCAGGAAAGCUGAGGCUUGCGUUCUGCCCGUGUCGGGCCAAUCACGAAUCCGAACCCAGGCCCUUGCCGAGGGCGCAGAUGGCGGGGCAUGU
env-178    ..................UUGCUACUGAAGCGGGAUAGCUAAGGCCAAGUGACAAGCCGUCCACCAACAACUGUCACAGUGCCAGUUGGGUAGAGAUAG
Ssp-13-4   GAAAAAGAGCGAGGAGCCUGGGGUAGAGGCUUGAUCGCUUAGGACAAGCCACAAGCUGUCCUCGCCUAACUGGCACAGUGACAGUUGGAACGCUUAUGA
env-179    AUUUGAUCCUGACCACGACACUGAUGAGCGGCAGCACGCUCAGGGUCAGUCGAGAAUCUGAACCCGUCACCUUGAUGCUUCCAGUGUUGCGGGCCAUAU

           ....................................................................................................
           ....................................................................................................
           ●●●●●●●●●●●●●●●●●●●●●●●●●●●●●●●●●●●●●●●●●●●●●●●●●●●●●●●●●●●●●●●●●●●●●●●●●●●●●●●●●●●●●●●●●●●●●●●●
```

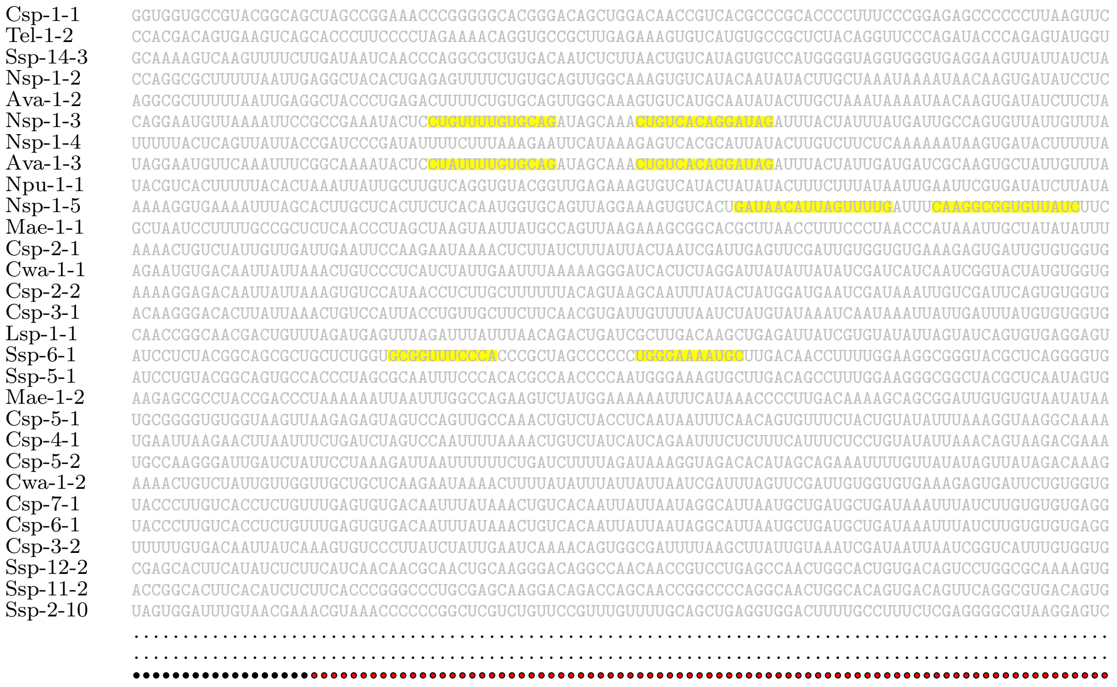

alignment positions 100 · · · 259

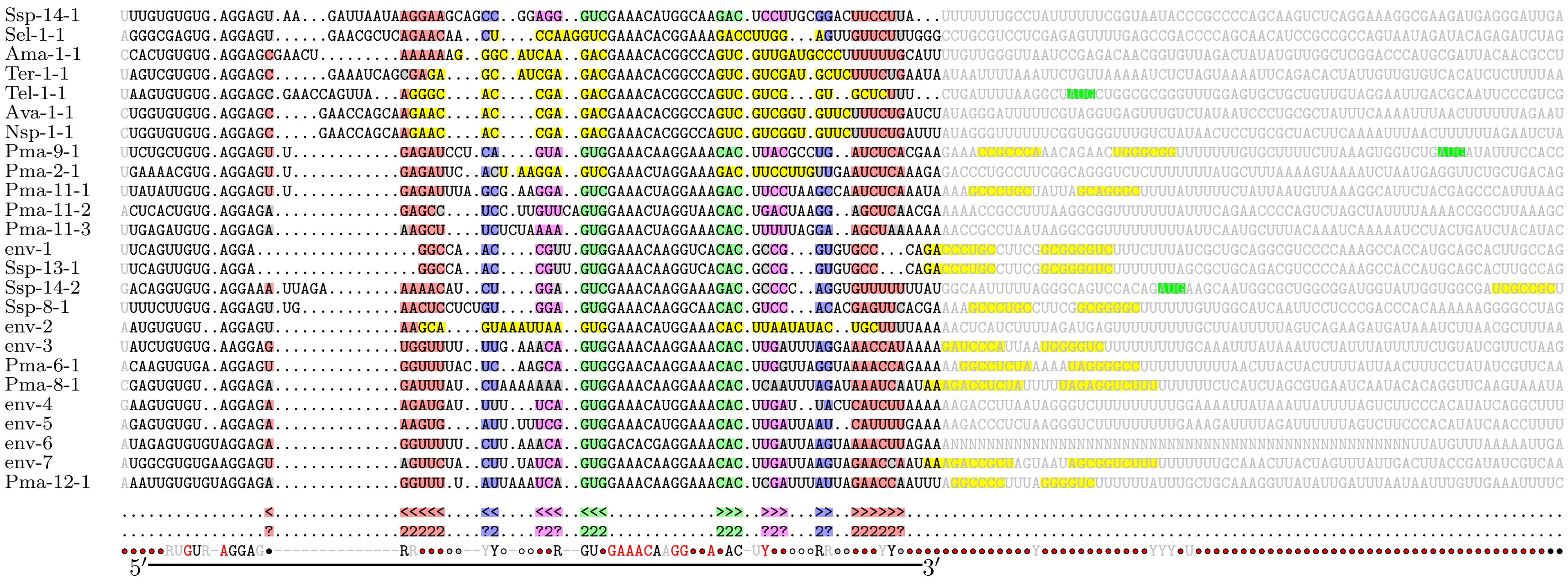

```
env-8      UAAAAGU.AG.AGGAGU..............UGUUUUA..UUG.AAUUA..GUGGAAACAAGGAAACAC.UUUAUUCAAUAAAACUAAAAAAGACCCCUGUAAUUAAGGGGUCUUUUUUUUUGCCUAUUUACUACUGUUGUUGGAAUUUUCAUAUCAUUA
env-9      UAUAUGUGUGAAGGAGU..............GGUUUUA..UUA.AAUCA..GUGGGAACAAGGAAACAC.AUGAUUUAAUAAAAUCAUCAAAGACCUCUAAAUAAUUGAGGUCUUUUUUUAUGCAUAUUAAUUAAAUUAGUGGACUUUCAUGUAUCUUCG
env-10     UAAGUGUGUGAAGGAGC..............GGUUUUA..CGG.AAUCA..GUGGAAACAAGGAAACAC.UUUAUUUCGUGAAUCCACGAAAAGACCCCUUAUAGGGGUCUUUUUUUAUGGAAAUCCAUUCCUUCUUUUACUUUGCUUUAUCUAAUCUAA
env-11     GUAGUGUGU..AGGAGA..............GGUUUUA..AUU.AAGCA..GUGGAAACAAGGAAACAC.UUGUUUAAUUAAAACCAAUAUAGACCUCUAAUAUAGAGGUCUUUUUUUAUGUUUUCUAAUUUUAUUUUCCUGUAUCAUUCCAAAUGAUCC
env-12     UGAAGGUGUGAAGGAGU..............GGUUCUA..CUG.AAAGA..GUGGAAACGAGGAAACAC.UUGAUUUGGUAAAACCAUGAAAGGCCCCAUUAUUAGGGGUUUUUUUUUUGCAAAUUCUUUGCUUCUAUUCACGUUCCUUCGUCUUCAAAAG
Pma-12-2   GUUGUGUGU..AGGAGA..............GUUUUUA..CUG.AAACA..GUGGAAACAAGGAAACAC.UUGAUUCGGU.AAAACCAGAAAAAGACCUCUAGAAAUAGGGGUCUUUUUUUGUGCAAAUGAUCUCUAAUCCACCUGUAACUCGAUAUCCU
env-13     UAUGUGUGU..AGGAGU..............GGUUUUA..CUG.AAUCA..GUGGAAACAAGGAUACAC.UUGAUUCAAUAAGACCUUAAAAAGACCUCUAGUAAUAGGGGUCUUUUUUUAUUAUCUUAGAAGUGAUAAAAAUAUGGACGAAGACUUUUA
env-14     AACGUGUGUGAAGGAGU..............GGUUUAU..CUG...AAACAGUGGAAACAAGGAAACAC.UUUAUUUAGUUAAACCAUAAUGGCCCCGUUAGAGGGGUCUUUUUUUACCAAAUUUACUACUUCUGUUUAUUUUCCUGUAUCGUUUCAAGG
env-15     CACGUGUGA..AGGAGU..............GGUUUUA..UUA.AAAAA..GUGGAAACAAGGAAACAC.UUUAUUUAGUAAAACCUUAAAAGACCUCUAGUAAUAGGGGUCUUUUUUUAAUGCGUAAUCACUACAUGCGAAUAAUGAACAAUAGGGAAU
env-16     AUCGAGUGUGAAGGAGU..............GGUUUAU..CU..AAACA..GUGGAAACAGGGAAACAC.UUGAUUUAGUUAAACCAGAAUGGCCUCUAAAGUAAUAGAGGCCUUUUUUAAUGCAUAAUCAUUACUUUUAUUCAAUGCUUAUUAUGUUCC
env-17     UAGAUGUGUGAAGGAGU..............GGCUUUA..CUA.AAACA..GUGGAAACAAGGAAACAC.UUGAUUUAGUAAUACCAAGAAAGACUUCUAGAAAUAGGGGUCUUUUUUUUAUGCAAACUCACUACUUUUAUUCACUAUUCCUAUUUCUUCC
Pma-12-3   AAGGUGUGU..AGGAGA..............GGUUUUA..CUG.AAACA..GUGGAAACAAGGAAACAC.UUGAUUUAGUUAAACCAAGGAAAGACCUCUAUUAGGGGUCUUUUUUUUUUAUGCUUCAAAAUAGAUUGAAUAUUAAAUUAUUUUAUUGGUG
env-18     AAGGUGUGU..AGGAGA..............GGUUUUA..CUG.AAACA..GUGGAAACAAGGAAACAC.UUGAUUUAGUUAAACCAAGGAAAGACCUCUAUUAGGGGUCUUUUUUUUUUAUGCUUCAAAAUAGAUUGAAUAUUAAAUUAUUUUAUUGGUG
env-19     CAAGUGUGA..AGGAGU..............GGUUUUA..CUU.AAGCA..GUGGAAACAAGGAAACAC.UUGUUUUAGUAAAAUCAUUAAAGACCCCAUUAUAAGGGGUCUUUUUUUAUGCAAAUUCUUUGCUUCUAUUUACUUUCCUGUAUCGUUCUA
env-20     UAAGUGUGA..AGGAGU..............GGUUUUA..CUU.AAUCA..GUGGAAACAAGGAAACAC.UUAAUUUAGUAAAACCAAUAAAGACCUCUGGUAAUAGGGGUCUUUUUUUAUGCAAAUUUACUAUUUUUAUUGACUUGCCGAUAUCCUCAA
Pma-12-4   UAUGUGUGU..AGGAGA..............AGUUUUA..CUG.AAACA..GUGGAAACAAGGAAACAC.UUGGUUUAGUAAAACUAUAUAAAGAUCUCUAGAAAUAGAGAUCUUUUUUAUUAUCUAGUUUUAAUUUGCAUAAAAAUGUACUUUUGACAG
env-21     GAAGUGUGC..AGGAGU..............GGUUUUA..CUG.AAGCA..GUGGAAACAAGGAAACAC.UUGAUUUGGUAGAACCGUAAAAGCCCCCAUUAUCGGGGGUCUUUUUUUUGCAAAUUCUUUGCUUCUAUUUACUUUGCUGGAUAGUUCCAA
env-22     AAUAUGUGUGAAGGAGU..............GGUUUUA..UUA.AAAUA..GUGGAAACAAGGAAACAC.UUGAUUUAGUAAAGCCAAAAGAGACCCACUAGAAAUAGGGGUCUUUUUUUUUAACUUAUUAAUUUUAUUGAGAUAAAUUUAGAAUAAAAU
env-23     GAUGUGUGUGAAGGAGU..............GGUUUUA..CUA.AUAUA..GUGGAAACAAGGAAACAC.UAGAUUUAGUAAAACCCAAAGAGACCUCUAGAAAUAGAGGUCUUUUUUUUUGCAUAUUCAUUCCUUUUAUUUAUUUCCCUAUAUUAUUCC
env-24     AAAAAGUGUGAAGGAGU..............GGUUUUA..CUA.AAACA..AUGGAAACAAGGAAACAU.UUGAUUUAGUAAAACUUAGAAAGACCUCUAAAAAUAGGGGUCUUUUUUUUUGUAAAAUCGCUACUUUUAUUAAUUCUUUCAUAUCAUAAA
env-25     UAUAAGUGUGAAGGAGU..............GGUUUUA..CUA.AAACA..GUGGAAACAAGGAAACAC.UAGAUUUAGUAAAACCUAGAGAGACCUCUAAAAUAGAGGUCUUUUUUUUAUGCAAAUUCAUUUCUUCUAUUCAAUGUCCUNNNNNNNNNN
env-26     UAAGUGUGUGAAGGAGU..............GGUUUUA..UCUGAAGCA..GUGGAAACAAGGAAACAC.UUGAUUUGGUAAAACUAUAAAAGACCCCAUUAUAAGGGGUCUUUUUUUUGUAAAUUAUUUGCUUCUAUUCACUUUCCUGCUUCUUCACAA
env-27     UAAAUGUGUGAAGAAGU..............AGAUUUA..CUG.AAACA..GUGGAAACAAGGAAACAC.UUGAUUUGGUAAAUCCAUAAAAGACCCCAUUAAAGGGUCUUUUUUUAUGCAAAUUAAUUACUUCUGUUUAUUUCCUUUAUCUUUCCAAG
env-28     AAAAUGUGUGAAGGAGU..............AGUUUUA..CUG.AAUCA..GUGGAAACAAGGAAACAC.UUGAUUUGGUAAAACUAUAAAAGACCCCAUUAUAAGGGGUCUUUUUUUUGCAAAUUAUUUGCUUCAAUUCACUUUCAUGCGUCUUCAGAA
env-29     UAUAUGUGUGAAGGAGU..............GGUUUUA..CUG.AAGCA..GUGGAAACAAGGGAACAC.UUGAUUUGGUAAGACCAUAAAGACCCCAUUAUAAGGGUCUUUUUUUAUGCAAAUUCUUUGCUUCUAUUCAUUUUCAUGUAUCUUUCUAAU
env-30     UAUAUGUGU..AGGAGU..............GGAUUUA..CUG.AAGCA..GUGGAAACAAGGAAACAC.UUGUUUUGGUAGAACCAUAAAAGACCCAAU..........................................................
env-31     GAUGUGUGA..AGGAGA..............GGUUUUU..CUG.AAACA..GUGGAAACAAGGAAACAC.UUGAUUUAGUAAAACCAAUAAGGCCUCUAAUUUUAGAGGUCCUUUUUUUUGUCAUCCCAUGUGUUCCCAUAUAUGCUGCAAAUGAAAUGG
env-32     AAUACGUGUGAAGGAGU..............GGUUUUA..CUG.AAACA..GUGGAAACAAGGAAACAC.UUGAUUUAGUAAAACCUUUAAAGACCCUAAUAAAGGGUCUUUUUUUUAUGCAAAUUCAUUUCCUCUAUUAACUUUCAUGUGUUUCCUCAA
env-33     UUUACGUGUGAAGGAGU..............GGUUUUA..CUG.AAACA..GUGGAAACAAGGAAACAC.UUGAUUUAGUAAAACCUCUAAAGACCUCUAGUAAUAGGGGUCUUUUUUAAUGGAAAUUAAAUUUAAAUAUUCAAAUUCUUAAUCCUUGUU
env-34     GGUAUGUGUGAAGGAGU..............GGUUUUA..CUG.AAACA..GUGGAAACAAGGAAACAC.UUGAUUUAGUAAAACCUUAAUAGACCCUAUUAUAAGGGUCUUUUUUUUAUGGAAAUUCAUCUCCUCUAUUAAUUUUCCUGUGUCUCCCCA
env-35     UUAACGUGUGAAGGAGU..............GGUUUUA..CUG.AAACA..GUGGAAACAAGGAAACAC.UUGAUUUAGUAAAACUAGAAAAAGACCUCUAGAAAUAGAGGUCUUUUUUUUUAUGUUUUCUAUUUGACAUGUAUUUAAAAUUAAAUUAUC
env-36     UCUGUGUGU..AGGAGU..............GGUUUUA..CUG.AAACA..GUGGAAACAAGGGAACAC.UUGAUUUAGU.AAACCAUAAAGGGCCCCGUAAAAAGGGUCUUUUUUUUUGCAAAUUUAUAACUUCUAUUUAUUUUUCUGUAUCGUUCCAA
env-37     AUCGUGUGUGAAGGAGU..............GGUUUUA..CUG.AAACA..GUGGAAACAAGGAAACAC.UUGAUUUAGUAAAACCAUAAAAGACCCUAUUAUUUGGGGUCUUUUUUUAUGCAAAUUCUAUGCUUCUAUUCACUUU..............
env-38     GAUGUGUGU..AGGAGA..............GGUUUUA..CUG.AAACA..GUGGAAACAAGGAAACAC.UUCAUUUAGUGAAACCAGAAAAAGACCUCUAGAAAUAGGGGUCUUUUUUUUCUAAAUUUAAUCUUAUGGUUUAUGUACCUCUUAAAAUUU
Pma-4-1    GAUGUGUGU..AGGAGA..............GGUUUUA..CUG.AAACA..GUGGAAACAAGGAAACAC.UUCAUUUAGUAAAACCAGAAAAAGAUCUCUAGAAAUAGGGGUCUUUUUUUAUGUCUUAUUAGUGAUAAAAAUAUGGACGAAUAUUUUCAC
Pma-8-2    UUUGUGUGU..AGGAG...............AGUUUUA..UUA.AAUCA..GUGGAAACAAGGAAACAC.UUGAUUUAAUAAAAUU.AUUCAAGAUCCCUUUUUAUAGGGGUCUUUUUUUUUUAUUUAUUGCCAAGAUUAUAUUUAACUAUUAGUUUGUUG
env-39     CUUGUGUGU..AGGAGG..............AAUUG.A..UUAAUCUCA..GUGGAAACAAGGAAACAC.UUGAUUUAAAUCAAUUCUCGAAAGAUCUCUCUUUGAGAGAUCUUUUUUUUUGGAUUAUUUGAAAUAAAUAUUAAAUUCUUAAUAUAAAA
env-40     UUUGUGUGU..AGGAGG.............AAUUGAUU..UUA.UUUCA..GUGGAAACAAGGAAACAC.UUGAAAUAAAUUCAAUUUUAAAAAGAUCUCUUUCUAAGAGAUCUUUUUUUUUUGAUUAUUAGCAAUUCAUACUAAAUCCUGAAUAUUAAAA
env-41     CAUGUGUGU..AGGAGG..............AACUGAU..UAA.UUUCA..GUGGAAACAAGGAAACAC.UUGAUUCUAAUCAGUUUUAAAAGAUCUCUCUCUGAGAGAUCUUUUUUUUUGGAUUAUUAGAAAAAGGUAUUAAAUCCUGAAUAUAAAAAC
env-42     AGAGUGUGU..AGGAGG..............AAUCGAU..UUA.UUUCA..GUGGAAACAAGGAAACAC.UUGAUUUAAUUCGAUUUUAAAAAGAUCUCUCAUUGAGAGAUCUUUUUUUUUUGGAUUAUUAGAAAGAUACAAAAGUAAUAUUUAAUCCAA
env-43     AGAUUGUGUGAAGGAGG..............AAUUGAU..UUA.UUUCA..GUGGAAACAAGGAAACAC.UUGAUGUAAAUCAAUUUUUAAAGAUCUCUCUCUGAGGGGUCUUUUUUUUUUAGCAAUACAUAUUAAAUUCUGAAUAUAAAAACAUAUAUGC
Pma-4-2    UUUGUGUGU..AGGAGG..............AAUUGAU..UUA.UUUCA..GUGGAAACAAGGUAACAC.UUGAUGUAAAUUAAUUUUAAAAAGAUCUCUCUUUGAGAGAUCUUUUUUUUUGGAUAUUAUUGGAAAUACAUAUUAAAUCCUGAAUAUAAA
env-44     UUUGUGUGU..AGGAGG..............AAUUAGU..UUA.UUUCA..GUGGAAACAAGGAAACAC.UUGAAAUAAAUUAAUUUUAAAAGAUCUCUCUUUGAGGGAUCUUUUUUUUUUGAUUAGAAAUAUACAAAAUGCAUACUUAAUUAUGUUUAUA
env-45     AAGGUGUGU..AGGAGG..............AAUUGGU..UUA.AUUCA..GUGGAAACAAGGAAACAC.UUGAUUUAAAUCAAUUUUAAAAGAUCUCUCUUAGAGAGAUCUUUUUUUUUGGGAUUAGAAAGACAUAAAAUACAUUUUUAAUACUUGAAA
env-46     UAUGUGUGU..AGGAGG..............AAUUGAU..UUU.UUUCA..GUGGAAACAAGGAAACAC.UUGAAUUAAAUUAAUUUUUAAAGAUCCCUUAGAUAGGGAUCUUUUUUUUUGGGUUCUUAUAAAUACAUAAAACAUAUUUAUGCCUGAAUU
env-47     UAUGUGUGU..AGGAGG..............AAUUGAU..UUA.UUUCA..GUGGAAACAAGGAAACAC.UUGAUAUAGAUCAAUUUAUUAAAAAGUCUCUCUCUGAGAGACUUUUUUUUUUGGGGAUUAUUAGAAAUACAUAUUAAAUUCUGAAUAUAA
env-48     CAUGUGUGU..AGGAGG..............AACUGAU..CUA.CUUCA..GUGGAAACAAGGAAACAC.UUGAUUUAAAUCAGUUUUAAAAGAUCUCUCUCUGAGAGAUCUUUUUUUUUGGAUUAUUAGAAAAAGGCAUUAAAUCCUGAAUAUAAAUAC
env-49     GGUAUGUGUGUAGGAG...............GAUUGAU..UUA.UUUCA..GUGGAAACAAGGAAACAC.UUGAUAUAAAUCAAUUUUAAAAAGAUCUCUCUCUGAGAGGUCUUUUUUUUUGGGAUUAUUAGAAAUACAUAUUAAAUUCUGACUAUAAAA
env-50     AUUGUGUGU..AGGAGA..............AAUUGAU..UUA.UUUCA..GUGGAAACAAGGAAACAC.UUGAUAUAGAUCAAUUUAAAAGAUCUCUCUUUGAGGGAUCUUUUUUUUUGCAGAUUAUUAGAAGUACAUAUUAAAUUCUGACUAUAAAAA
env-51     UUUGUGUGU..AGGAGA..............AAUUGAU..UUA.UUUCA..GUGGAAACAAGGAAACAC.UUGAUAUAGAUCAAUUUAAAAGAUCUCUCUUUGAGGGAUCUUUUUUUUUGCGGAUUUUUAGAAGUACAUAUUAAAUUCUGAAUAUAAAAAC
env-52     AUUGUGUGU..AGGAGG..............AAUUGAU..UUU.UUUCA..GUGGAAACAAGGAAACAC.UUGAGAUAAAUCAAUUUUAAAGAGAUCUCUCAGAGAGAGAUCUUUUUUUUUUGAAUUUNNNNNNNNNNNNNNNNNNNNNNNNNNNNNN
env-53     UCUGUGUGU..AGGAGG..............AAUUGAU..UUG.UUUCA..GUGGAAACAAGGUAACAC.UUGAUAUAAAUCAAUUUUAAAAAGAUCUCUCUUUGAGAGGUCUUUUUUUUUGGUGAUUAUUAGAAAUAAAUAUUAAAUUCUGAACAUAAAA
env-54     UUUGUGUGU..AGGAGG..............AAUUGAU..UUA.UUUCA..GUGGAAACAAGGAAACAC.UUGGCAUAAAUCAAUUUUAAAAGAUCUCUCUUUGAGGGAUCUUUUUUUUUGGUGAUUAUUAGAAAUAAAUAUUAAAUACUGAAUAUAGAA
env-55     UUUGUGUGU..AGGAGA..............AAUUGAU..UUA.UUUCA..GUGGAAACAAGGAAACAC.UUGUUGUAAAUCAAUUUAAAAAAGAUCUCUCUAUGAGAGAUCUUUUUUUUUGUAAUUAUUAGAAUACGUAUUAAAUCCUGAAUAUAAACAC
env-56     UUUGUGUGU..AGGAGG..............AAUUGAU..UUA.UUUCA..GUGGAAACAAGGAAACAC.UUGAUGUGAAUCAAUUUUAAAAGAUCUCUCUUUGAGGGGUCUUUUUUUU......................................
env-57     AAUGUGUGU..AGGAGC..............AAUUGAU..UUA.UUUCA..GUGGAAACAAGGAAACAC.UUGAUAUAAAUUAAUUUUUAAAAGAUCUCUCUUAGAGGGGUCUUUUUUUUUUGGGCAUUAUUAGAAAAAAAUAUUAAAUUCUGAAUAUAAA
env-58     UAUGUGUGU..AGGAGG..............AAUAGAU..UUA.UUUCA..GUGGAAACAAGGAAACAC.UUGAUAUAAAUCAAUUCUAAAAGAUCUCUCUUUGAGGGAUCUUUUUUUUUGGGGAUUAGAAAUAAAUAUUAAAUUCUGAAUAUAAAAA
env-59     AUUGUGUGU..AGGAGG..............AAUUGAU..UUA.UUUCG..GUGGAAACAAGGAAACAC.UUGAUUUAAAUUAAUUUUAAAAAGAUCUCUCUCUGAGGGAUCUUUUUUUUUGCCUUUAUUAGAAAUACAAAUUAAAUUCUAAAUAUAA
Pma-3-1    ACUGUGUGU..AGGAGG..............AAUUGGU..UUA.UUUCA..GUGGAAACAAGGAAACAC.UUGUUAUAAAUCAAUUUUAAAAAAGAUCUCUCGUCGAGAGAUCUUUUUUUUUGAUUACUAAGUUUAAAUAAAUAAAUAUUGAAUCCUGA
env-60     UAUGUGUGU..AGGAGG..............AAUUGAU..UUA.CUCCA..GUGGAAACAAGGAAACAC.UUGAUUUAAAUCAAUUUUAAAAGAUCUCUCUAGAGAGAUCUUUUUUUUUGGUAAUUAUUAGAAACAAAUAUAUUCUUCAUUUUUGAUACC
env-61     AAUGUGUGU..AGGAGG..............AAUUGAU..UUA.UUUCA..GUGGAAACAAGGAAACAC.UUGAUAUAAAUCAAUUUUUAAAGAUCUCUCUCUGAGGGAUCUUUUUUUUUUUUUUUUUUUGGAAUUUUUAGAAAUACAUAUAUGCUCUCU

           ................<..............<<<<<....<<....<<<..<<<............>>>..>>>...>>..>>>>>>.........................................................................
           ................?..............22222....?2....?2?..222............222..?2?...2?..22222?.........................................................................
           ●●●●●RUGUR-AGGAG●-------------RR●●●●○○--YY○-○○●●R--GU●GAAACAAGG●●A●AC-UY●●○○○RR○○●●●YY○●●●●●●●●●●●●●●Y●●●●●●●●●●●YYY●U●●●●●●●●●●●●●●●●●●●●●●●●●●●●●●●●●●●●●●●●●
           5′——————————————————————————————————————————————————————————————————————————3′
```

```
env-62    AAUGUGUGU..AGGAGG..............AAUUGAU..UUA.UUUCA..GUGGAAACAAGGAAACAC.UUGAUAUAAAUCAAUUUUUAAAAGAUCCCUUAGUGAGGGAUCUUUUUUUAUGGAGAUUAUUAGAAAUACAUAUUAAAUCCUUAAUACCAA
env-63    CUUGUGUGU..AGGAGG..............AAUUGAU..UUA.UUUCA..GUGGAAACAAGGAAACAC.UUGAUAUAAAUUAAUUUUUAAAAGAUCUCUCUCUGAGAGGUCUUUUUUUUUGAUUAUUAGAAAUACAUAUUAAAUUCUGAAUAUAAAAAC
env-64    UUUGUGUGU..AGGAGG..............AAUUGAU..UUA.UUUCA..GUGGAAACAAGGAAACAC.UUGAUUUAAAUUAAUUUUUAAAAGAUCUCUCUCAGAGGAUCUUUUUUUUUGGAUUAUUAGAAAUACAUAUCAAAUCCUGAAUAUAAAAC
env-65    UUUGUGUGU..AGGAGG..............AAUUGAU..UUA.UUUCA..GUGGAAACAAGGAAACAC.UCGAUAUAAAUCAAUUUUUAAAAGAUCUCUCUUUGAGAGAUCUUUUUUUUUAGGGAUUAUUAGAAUUACAUAUAAAAUUCUGAAUAUAAA
env-66    UUUGUGUGU..AGGAGG..............AAUUGAU..UUA.UUUCA..GUGGAAACAAGGAAACAC.UUGAUAUAAAUCAAUUUUUAAAGACUUCUCUCUUUGAGGGGUCUUUUUUUUUGGGAUUAUUAGAAACAAAUAUAAAAUUCUGAAUAUAAA
env-67    UUUGUGUGU..AGGAGG..............AAUUGAU..UUA.UUUCA..GUGGAAACAAGGAAACAC.UUGAUAUAAAUCAAUUUUUAAAAGAUCCCUCUCUGAGGGGUCUUUUUUUUUGGGUUAUGAGAAAUAAAUAUUAAAUUCUGAAUAUAAAAAA
env-68    UUUGUGUGU..AGGAGG..............AAUUGAU..UUA.UUUCA..GUGGAAACAAGGAAACAC.UUGAUAUAAAUCAAUUUUUAAAAGAUCCCUCUCUG....................................................
env-69    GAUGUGUGU..AGGAGG..............AAUUGAU..UUA.UUUCA..GUGGAAACAAGGAAACAC.UUGAUAUAAAUCAAUUUUAAAAAGAUCUCUCCUUGAGGGGUCUUUUUUUAAUGGAAUUUUGGAAAUACAUGUUAAAUCUAGAAUAUUAAA
env-70    AAUGUGUGU..AGGAGG..............AAUUGAU..UUA.UUUCA..GUGGAAACAAGGAAACAC.UUGAUAUAAAUCAAUUUUAAAAAGAUCUCUCUCUGAGAGGUCUUUUUUUUUGGAUUAUUAGAAAUACAUAUUAAAUUCUGACUAUAAAAA
env-71    UAUGUGUGU..AGGAGG..............AAUUGAU..UUA.UUUCA..GUGGAAACAAGGAAACAC.UUGAUAUAAAUCAAUUUUAAAAAGAUCUCUCUCUGAGAGGUCUUUUUUUUUUUGGAUUUUUAGAAAUACAUAUUUAAUUCUGACUAUA
env-72    UCUGUGUGU..AGGAGA..............AAUUGAU..UUA.UUUCA..GUGGAAACAAGGAAACAC.UUGAUAUAAAUCAAUUUUAAAAGAUCUCUCUUUGAGAGGUCUUUUUUUUUGGGAUUAUUAGAAAUAAAUAGUUAAAUUCUGAAUAUAAA
env-73    AUUGUGUGU..AGGAGU..............AAUUGAU..UUA.UUUCA..GUGGAAACAAGGAAACAC.UUGAUAUAAAUCAAUUUUAAAAGAUCUCUCUUAGAGGGAUCUUUUUUUUUGGGAUUAUUAGAAGUACAUAUUAAAUUCUGAAUACAAAAA
env-74    UUUGUGUGU..AGGAGA..............AAUUGAU..UUA.UUUCA..GUGGAAACAAGGAAACAC.UUGAUAUAAAUCAAUUUUAAAGAUCUCUCUUUGAGGGAUCUUUUUUUUUUGGUGAUUAUUAGAAAUACAUAUUAAAUUCUGAAUAUAAAA
env-75    UAUGUGUGU..AGGAGG..............AAUUGAU..CUA.UUUCA..GUGGAAACAAGGAAACAC.UUGAUUUAAAUCAAUUUUAAAAGAUCUCUCUUUGAGGGGUCUUUUUUUUUGAGAUUAUUAGAAA.......................
env-76    UGUGUGUGU..AGGAGG..............AAUUGAU..UUA.UUUCA..GUGGAAACAAGGAAACAC.UUGAUUUAAAUCAAUUUUAAAAGAUCUCUCUCUGAGAGGUCUUUUUUUUUGGGAUUUUUAGAAAAUUCAUAUUAAUUCCUGAAUAUAAAA
Pma-6-2   UAUGUGUGU..AGGAGG..............AAUUGAU..UUA.UUUCA..GUGGAAACAAGGAAACAC.UUGAUUUAAAUCAAUUUUAAAAAGAUCUCUUUAUUAGGAUCUUUUUUUUUGAGGAUUUUUAGAAAUAUAUAAAUACAUAUUAAAUCCUG
env-77    UCUGUGUGU..AGGAGG..............AAUUGAU..UUA.UUUCA..GUGGAAACAAGGAAACAC.UUGAUAUAAAUCAAUUUUAAAAAGAUCUCUCUAUGAGGGGUCUUUUUUUUGGGAUUUUUAGCAAUAUAUAUUAAAUUCUGAAUAUAAAA
env-78    UCUGUGUGU..AGGAGG..............AAUUGAU..UUA.UUUCA..GUGGAAACAAGGAAACAC.UUGAUAUAAAUCAAUUUUAAAAAGAUCUCUCUCUGAGGGGUCUUUUUUUUUUUUGGGAUUUUUAGCAAUACAUAUUAAAUUCUGAAUAUA
env-79    UCUGUGUGU..AGGAGG..............AAUUGAU..UUA.UUUCA..GUGGAAACAAGGAAACAC.UUGAUAUAAAUCAAUUUUAAAAAGAUCUCUCUCUGAGAGGUCUUUUUUUUUUGGAUUUUUAGAAAUACAUAUUUAAUUCUGACUAUAAAA
env-80    UGUGUGUGU..AGGAGG..............AAUUGAU..UUA.UUUCA..GUGGAAACAAGGAAACAC.UUGAUAUAAAUCAAUUUUAAAAAGAUCUCUCUCUGAGGGAUCUUUUUUUUUGGGGAUUUUUAGAAAUACAUAUUAAAUCCUGAAUAUAAA
env-81    UGUGUGUGU..AGGAGG..............AAUUGAU..UUA.CUUCA..GUGGAAACAAGGAAACAC.UUGAUAUAAAUCAAUUUUAAAAGAUCUCUCAAAGAGAGAUCUUUUUUUUUGGGGAUUAUUAGAAAUAAAUAUUAAAUUCUGAAUAUAAAA
env-82    UUUGUGUGU..AGGAGG..............AAUUGAU..UUA.UUUCA..GUGGAAACAAGGAAACAC.UUGUUGUAAAUCAAUUUUAAAAGAUCUCUCUUUGAGAGAUCUUUUUUUUUGAUGAUUAUUAGAAAUACAGAUUAAAU.............
env-83    UUUGUGUGU..AGGAGG..............AAUUGAU..UUA.UUUCA..GUGGAAACAAGGAAACAC.UUGUUAUAAAUCAAUUUUAAAAAGAUCUCUCUCUGAGGGGUCUUUUUUUUUUGGGGAUUUUUAGAAAUAAAUAUUAAAUCCUGAAUAUAA
env-84    UUUGUGUGU..AGGAGG..............AAUUGAU..UUA.UUUCA..GUGGAAACAAGGAAACAC.UUGAUAUAAAUCAAUUCUAAAAGAUCUCUCUUUGAGGGAUCUUUUUUUUUCGGAUUAUUAGAAAUAAAUAUUAGAUUCUGAAUAUAAAAA
env-85    UUUGUGUGU..AGGAGG..............AAUUGAU..UUA.UUUCA..GUGGAAACAAGGAAACAC.UUGAUAUAAAUCAAUUUUAANNNNNNNNNNNNNNNNNNNNNNNNNNNNNNNNNNNNNNNNNNNNNNNNNUGAAGUACUCAUAGACUGG
env-86    CUUGUGUGU..AGGAGG..............AAUUGAU..UUA.UUUCA..GUGGAAACAAGGAAACAC.UUGAUAUAAAUCAAUUUAAAAAGAUCUCUCUCUGAGAGAUCUUUUUUUUUGUAAUAUUAGAAUCGCAUAUUAAAUUCUGAAUAUAAAAAC
env-87    UUUGUGUGU..AGGAGG..............AAUUGAU..UUA.UUUCA..GUGGAAACAAGGAAACAC.UUGAUAUAAAUCAGUUUUAAAGGAUCUCUCUUUGAGGGAUCUUUUUUUUUGGGGAUUAUUAGAAAUAAAUAUUAAAUUCUGAAUAUAAAA
env-88    UUUGUGUGU..AGGAGG..............AAUUGAU..UUA.UUUCA..GUGGAAACAAGGAAACAC.UUGAUAUAGAUCAAUUUUAAAAAAGAUCUCUCUCUGAGGGGUCUUUUUUUUUGGGAUUUUUAGCAAUACAUACUAAAUUCUGAAUAUAAA
env-89    UUUGUGUGU..AGGAGG..............AAUUGAU..UUA.CUUCA..GUGGAAACAAGGAAACAC.UUGAUAUAAAUCAAUUUUAAAAGAUCUCUCUCUGAGGGGUCUUUUUUUUUUGGAUUUUUAGCNNNNNNNNNNNNNNNNNNNNNNNNNN
env-90    CUUGUGUGU..AGGAGG..............AAUUGAU..UUA.UUUCA..GUGGAAACAAGGAAACAC.UUGAUUUAAAUCGAUUUUAAAAGAUCUCUCUUAGAGAGAUCUUUUUUUUUGGGAAUAUUAGAAAUACAUAUUAAAUCCUGAAUAUAAAAA
env-91    AUUGUGUGU..AGGAGG..............AAUUGAU..UUA.UUUCA..GUGGAAACAAGGAAACAC.UUGACUUAAAUCAAUUUUAAAAGAUCUCUCUGAGGGGUCUUUUUUUUUGCUUAAUUAGAAAUACAUAUUUAAACCUGAAUAUAAAAAC
env-92    UUUGUGUGU..AGGAGG..............AAUUGAU..UUA.UUUCA..GUGGAAACAAGGAAACAC.UUGAUUUAAAUCAAUUUUAAAAGAUCUCUCUCUGAGGGGUCUUUUUUUUUUGGGGAUUAUUAGAAAUACAUAUUAAAUUCUAAAUAUAAAA
env-93    UUUGUGUGU..AGGAGG..............AAUUGAU..UUA.UUUCA..GUGGAAACAAGGAAACAC.UUGAUUUAAAUCAAUUUUAAAAGAUCUCUCUUUGAGGGAU...............................................
env-94    UUUGUGUGU..AGGAGG..............AAUUGAU..UUA.UUUCA..GUGGAAACAAGGAAACAC.UUGAUUUAAAUCAAUUUUAAAAGAUCUCUCUUUGAGGGAUCUUUUUUUUUGGGCUUAUUAGAAAUAAAUAUUAGAUUCUGAAUAUAAAAA
env-95    UUUGUGUGU..AGGAGG..............AAUUGAU..UUA.UUUCA..GUGGAAACAAGGAAACAC.UUGAUAUAAAUCAAUUUUAAAAGAUCUCUCUUUGAGGGGUCUUUUUUUUUGGGAUUAUUAGAAAUAAAUAUUAAAUUCUGAAUAUAAAA
env-96    UUUGUGUGU..AGGAGG..............AAUUGAU..UUA.UUUCA..GUGGAAACAAGGAAACAC.UUGAUAUAAAUCAAUUUUAAAAAGAUCUCUCUCUGAGAGAUCUUUUUUUUUGGGGAUUAUUAGAAAUACAUAUUAAAAUCUGAAUAUAAA
env-97    UUUGUGUGU..AGGAGG..............AAUUGAU..UUA.UUUCA..GUGGAAACAAGGAAACAC.UUGAUAUAAAUCAAUUUUAAAAAGAUCUCUCUAUGAGGGGUCUUUUUUUUGUGAUUUUUAGCAACAUAUAUUAAAUUCUGAAUAAAAAAA
env-98    AUUGUGUGU..AGGAGG..............AAUUGAU..UUA.UUUCA..GUGGAAACAAGGAAACAC.UUGAUAUAAAUCAAUUUUAAAAGAUCUCUCAAAGAGAGAUCUUUUUUUUUGGGGAUUAUUAGAAAUACAGAUUAAAUUCUGAAUAUAAAA
env-99    UUUGUGUGU..AGGAGG..............AAUUGAU..UUA.UUUCA..GUGGAAACAAGGAAACAC.UUGAUAUAAAUCAAUUUUAAAAGAUCUCUCUUUGAGGGAUCUUUUUUUUUGGGAUUAUUAGAAAUAAAUAUUAGAUUCUGAAUAUAAAAA
env-100   UUUGUGUGU..AGGAGG..............AAUUGAU..UUA.UUUCA..GUGGAAACAAGGAAACAC.UUGAUAUAAAUCAAUUUUAAAAGAUCUCUCAAAGAGAGAUCUUUUUUUUUGUGGAUUUUUAGAAAUACAUAUUAAAUUCUGAAUAUAAAA
env-101   CUUGUGUGU..AGGAGG..............AAUUGAU..UUA.UUUCA..GUGGAAACAAGGAAACAC.UUGAUAUAAAUCAAUUUUAAAAGAUCUCUCUUUGAGAGGUCUUUUUUUUUUUGGAUUAUUAGAAAUAAAUAGUGAAAUUCUGAAUAUAAA
env-102   UUUGUGUGU..AGGAGG..............AAUUGAU..UUA.UUUCA..GUGGAAACAAGGAAACAC.UUGAUAUAAAUCAAUUUUAAAAGAUCUUUCUUAGAGGGAUCUUUUUUUUUUGGGAUUAUUAGAAAUAAAUAUUAGAUUCUGAAUAUAAAA
env-103   UUUGUGUGU..AGGAGG..............AAUUGAU..UUA.UUUCA..GUGGAAACAAGGAAACAC.UUGAUAUAAAUCAAUUUUAAAAGAUCUCUCUUUGAGAGGUCUUUUUUUUUGGGAUAAUUAGAAAUAAAUAUUAAAUUCUGAAUAUAAAAA
Pma-1-1   UUUGUGUGU..AGGAGG..............AAUUGAU..UUA.UUUCA..GUGGAAACAAGGAAACAC.UUGAUAUAAAUCAAUUUUAAAAGAUCUCUCUCUGAGGGGUCUUUUUUUUUGGGAUUUUUAGCAAUACAUAUUAAAUUCUGAAUAUAAAAA
env-104   CUUGUGUGU..AGGAGG..............AAUUGAU..UUA.UUUCA..GUGGAAACAAGGAAACAC.UUGAUAUAAAUCAAUUUUAAAAAGAUCUCUCUUUGAGAGAUCUUUUUUUAUGAGGAUUUUUAGAAAUACAUAUUAAAAUCUGAAUAAAAA
env-105   AUUGUGUGU..AGGAGG..............AAUUGAU..UUA.UUUCA..GUGGAAACAAGGAAACAC.UUGAUAUAAAUCAAUUUUAAAAGAUCUCUCUUUGAGAGAUCUUUUUUUUUUGUGGAUUUUUAGAAAUACAUAUUAAAUUCUGAAUAUAAA
env-106   UUUGUGUGU..AGGAGG..............AAUUGAU..UUA.UUUCA..GUGGAAACAAGGAAACAC.UUGAUAUAAAUCAAUUUUAAAAAGAUCUCUCCCUGAGGGGUCUUUUUUUUUUUUGUAUUUUUUGCAAUACAUAUUAAAUUCUGAAUAUAAA
env-107   UUUGUGUGU..AGGAGG..............AAUUGAU..UUA.UUUCA..GUGGAAACAAGGAAACAC.UUGAUAUAAAUCAAUUUUAAAAAGAUCUCUCUUUGAGAGGUCUUUUUUUUUUUUGGGAUUUUUAGCAAUACAUAUUAAAUUCUGAAUAUAA
env-108   CUUGUGUGU..AGGAGG..............AAUUGAU..UUA.UUUCA..GUGGAAACAAGGAAACAC.UUGAUAUAAAUCAAUUUUAAAAAGAUCUCUCUCUGAGAGGUCUUUUUUUUUGGGGAUUAUUAGAAAUACAUAUUAAAUUCUGAAUAUAAA
env-109   UUUGUGUGU..AGGAGG..............AAUUGAU..UUA.UUUCA..GUGGAAACAAGGAAACAC.UUGAUAUAAAUCAAUUUUAAAAAGAUCUCUCUUUGAGGGAUCUUUUUUUUUGGGGAUUAUUAAAAAUACAUAUUAAAUUCUAAAUAUAAA
env-110   UUUGUGUGU..AGGAGG..............AAUUGAU..UUA.UUUCA..GUGGAAACAAGGAAACAC.UUGAUAUAAAUCAAUUUUAAAAAGAUCUCUCUCUGAGAGGUCUUUUUUUUUUUGGAUUAAUAGAAAUACAUAUUAAAUUCUGACUAUAAAA
env-111   AUUGUGUGU..AGGAGG..............AAUUGAU..UUA.UUUCA..GUGGAAACAAGGAAACAC.UUGAUAUAAAUCAAUUUUAAAAAGAUCUCUCUUCGAGGGGUCUUUUUUUUUGGAUUAUUAGAAAUAAAUAUUAAAUUCUGAAUAUAAAAC
env-112   UUUGUGUGU..AGGAGG..............AAUUGAU..UUA.UUUCA..GUGGAAACAAGGAAACAC.UUGAUAUAAAUCAAUUUUAAAAAGAUCUCUCUUUGAGAGGUCUUUUUUUUUGGGAAUUAUUAGAAAUACAUAUUAAAUACUGAAUAUAAA
env-113   UUUGUGUGU..AGGAGG..............AAUUGAU..UUA.UUUCA..GUGGAAACAAGGAAACAC.UUGAUAUAAAUCAAUUUUAAAAGAUCUCUCUUUGAGAGAUCUUUUUUUUUGUGGAUUUUUAGAAAUACAAAUUAAAUUCUGAAUAUAAAA
env-114   CUUGUGUGU..AGGAGG..............AAUUGAU..UUA.UUUCA..GUGGAAACAAGGAAACAC.UUGAUAUAAAUCAAUUUUAAAAGGUCUCUCUUUGAGAGACCUUUUUUUUUGGGAUUUUUGGAAAUACAUAUUAAAUUCUUAAUAAAAACA
env-115   UUUGUGUGU..AGGAGG..............AAUUGUU..UUA.UUUCA..GUGGAAACAAGGAAACAC.UUGAUAUAAAUCAAUUUUAAAAGAUCUCUCUUUGAGAGAUCUUUUUUUUUGUGGAUUUUUAGAAAUACAUAUUAAAUUCUGAAUAUAAAA
env-116   UUUGUGUGU..AGGAGG..............AAUUGGU..UUA.UUUCA..GUGGAAACAAGGAAACAC.UUGAUAUAAAUCAAUUUUAAAAAGAUCUCUCUUUGAGAGGUCUUUUUUUUUGUGCAUUAUUAGAAAUACAUAUUAAAUUCUGAAAAUAAAA
env-117   GUUGUGUGU..AGGAGG..............AAUUGAU..UU..AUUCA..GUGGAAACAAGGAAACAC.UUGAUUUAAAUCAAUUUUUUAAAGAUCUCUCUCUGAGGGGUCUUUUUUUUUGUUUAUUAAAAGUACAUAUUUAAUCCUGAAUAUAAAAA
env-118   UAUGUGUGU..AGGAGG..............AAUUG.A..UUCAUUUCA..GUGGAAACAAGGAAACAC.UUGAUGUAAAUCAAUUUUAUAAAGAUCUCUCUUUGAGAGAUCUUUUUUUUUGGGUUAUUAGAAAUUCAUAUUAAAUUCUGAAUAUACAAC
env-119   UUUGUGUGU..AGGAGG..............AAUUG.A..UUCAUUUCA..GUGGAAACAAGGAAACAC.UUGAUUUAAAUCAAUCUCAAAAGAUCUCUCUUUGAGAGAUCUUUUUUUUUGGGAUAAUAAGAAAUACAUAUUAAUUUCUGAGAAUAAAAG
env-120   UUUGUGUGU..AGGAGG..............AAUUGAU..UU..AUUUUA.GUGGAAACAAGGAAACAC.UAAAAUUAAAUCAGUUUUUAAAAGAUCUCUCUUUAAGGGAUCUUUUUUUUAUGAUUUAUUAGAAAUUUAUAAAAUACUUUUUAAUA.....

          ................<..............<<<<<....<<....<<<..<<<............>>>..>>>...>>..>>>>>>..................................................................
          ................?..............22222....?2....?2?..222............222..?2?...2?..22222?..................................................................
          ●●●●●RUGUR-AGGAG●--------------RR●●●●○○--YY○-○○●●R--GU●GAAACAAGG●●A●AC-UY●●○○○RR○○●●●YY○●●●●●●●●●●●●●●Y●●●●●●●●●●●●YYY●U●●●●●●●●●●●●●●●●●●●●●●●●●●●●●●●●●●●●●●●●
          5′————————————————————————————————————————————————————————————————————————————————3′
```

```
env-121    AGUGUGUGU..AGGAGG.............AAUUAAU..UUU.AAUCAA.GUGGAAACUAGGAAACAC.UUGAUCUAGAUUAAUUUAAAAGAUCUCUCUUUGAGAGAUCUUUUUUUUUUGGGU..................................
env-122    UUUAUGUGUGAAGGAGU.G...........GUUUU....CUA.AAAUG..GUGGAAACAAGGAAACAC.UUAUAUUAGCAAAAACCAUUAGAAAAGUCCUAGUAANNNNNNNNNNNNNNNNNNNNNNNNNNNNNNNNNNNNNNNNNNNNNNNNNAAU
Pma-10-1   UAUAACUGUG.AAGAGU.UG..........AGAUCCU..GC....GCA..GUGGAAACAAGGAAACAC.UUGU...GCCUGAUCUUAAAAAACCGUCUUGUUCAGAAUGGGGCGGUUUUUUUGUGCUUAUUUUGAUUUAGAAAAGCAAAAUCCAAAAC
Pma-9-2    UAUACCUGUG.AGAAGU.UG..........AGAUCCU..GC....GCA..UUGGAAACAAGGAAACAACUUGU...GCCUGAUCUUCAAAAAACCGCCUUGUUCAGAAUGGGGCGGUUUUUUGUGUUUAUUUUGAUUUAGAAAAGCUAAAUCCAAAAC
Pma-9-3    UUAAUUUGUG.AGGAGU.AG..........AGAUCCUCCGCA.......GUGGAAACAAGGAAACAC.UCA....GCCUGAUCUCACGAAGAAACGCGCUUGUUCAGAAACGGGCGGUUUUUUGUUUUUAUUUAGAAAACUAAUGAUGUUUCUAUAG
Pma-10-2   UCAUUUUGUG.AGGAGU.UG..........AGAUCCU..CC....GUA..GUGGAAACAAGGAAACAC.UUAC...GCCUGAUCUCACGAAGAACCGCGCCUAGCAGAACUGGGCGGUUUUUUGUGCCUUUCUUAAAGUGGUCUAAUGAUAUUUCCAC
env-123    UCGGUAUGUG.AGGAGU.UG..........AGAUCCU..CC....GUA..GUGGAAACAAGGAAACAC.UUAC...GCCUGAUCUCACGAAGAAACGCGUCCAAACAGAACUGGGCGGUUUUUUGUGCCUUUCUUAAAGUGGUCAAAUGAUAUUUCCAC
env-124    UUAACUCGUG.AGGAGU.UG..........AGAUCCU..CC....GCA..GUGGAAACAAGGAAACAC.UUGC...GACUGAUCUCACAAAGAAACGCGCUUGUUCAGAACGGGCGGUUUUUUGUACCUAUUAAUGCUCAUAUCCAAUAUUAUUUCAG
Pma-9-4    UUUAUUUGUG.AGGAGU.UG..........AGAUCCU..CC....GCA..GUGGAAACAAGGAAACAC.UUGC...GCCUGAUCUCACAAAUUAACGCGCUUGUUCAGAACGGGCGGUUUUUUGUACCUAUUAUCCUCGUAUCCAAUAUUAUUUCUCA
Pma-10-3   UUUAUAUGUG.AGGAGU.UG..........AGAUCCU..CC....GCA..GUGGAAACAAGGAAACAC.UUGC...GACUGAUCUCACAAAAAACGCGCUUGUUCAGAACGGGCGGUUUUUUGUGCCUAUUAAUGCUCGUAUCCAAUAUUGUUUUCAG
Pma-10-4   UUAAUUUGUG.AGGAGU.AG..........AGAUCCU..CC....GCA..GUGGAAACAAGGAAACAC.UUGC...GCCUGAUCUCACGAAUAAACGCGCUUGUUCAGAACGGGCGGUUUUUUGUUUUUAAUUAGAAAACUAAUGAUGUUUCUAUAG
env-125    UAUGACCGUG.AGGAGU.UG..........AGAUACU..CC....GCA..GUGGAAACAAGGAAACAC.UUGC...GACUGAUCUCACAAAGAAACGCGCUUGUUCAGAACGGGCGGUUUUUUGUACCUAUUAAUGCUCGUAUCCAAUAUUAUUUUCA
env-126    UCCAGUGUGUG.AGGAGU.UG.........AGAUCCU..CC....GCA..GUGUAAACAAGGAAACAC.UUGC...GCCCGAUCUUCAAAAACAGCCUGUUCAGGACGCGUCGGUUUUUGUGUUAAUUUAGAAAAAGCAAAAUCCAAGUAAUGA
env-127    UAUAGAUGUG.AGGAGU.UG..........GGAUC....CUC.CAGCA..GUGGAAACAAGGAAACAC.UCGCUUAAA..GAUCCACUAAAAAGCCGCCUAGAAUUUAGGCGGCUUUUUAGUGGAUCUUUAAGCUCAAUUUUAUAAGAUUAAGAAUCUU
Ssp-3-1    UUCAUAUGUG.AGGACA.............ACACC....UC..AUGGG..AUGGAAACAAGGUUGCAU.CCCU...GA..GAUUAUUUCAAGCCCUCUGCCUUUGGCGGAGGGCUUUUUGGGCUUCCCAAACAUGAAGAGAGUUUGUUGUUUCUAUCGG
Ssp-2-1    UGGUCGUGUG.AGGAGG.............GCUUC....UUC.ACGGA..GUGGAAACAAGGCAACAC.GCCCUACGA..GAAGCCGAGAAAACCCUGGCUUUGGCGGGGUUUUCUUUUGUCCGACGAUUCUGAGAUGCCCGGAAGGUGAAGAUCACUUC
Ssp-2-2    UGAAAUCGUG.AGGAAG.............ACUUC....UUC.GCGGA..GUGGAAACUAGGCAACAC.GCCCAAAGA..GAAGUCGAGAAAACCCUGGCUUUGGCGGGGUUUUCUUUUGUUUGAUCACCUUUUCAUGGCCGUGGAUCUCGCGCAUCCAU
Ssp-2-3    UUCACUUGUG.AGGAGG.............GCUUC.U..UC..GCGGA..GUGGAAACUAGGCAACAC.GCCCAACGA..GAAGCCGAGAAACCCUGCCUUUGGUUGGAGUUUUCUUUACUUGUGGCGUUGCUUGCAGCCGUCCCUGUGGCGGCCAUGC
env-128    UUCAUUUGUG.AGGAGG.............GCUUC.U..UC..ACGGA..GUGGAAACAAGGCAACAC.GCCCAAAGA..GAAGCCGAGAAAACCCUGGCUUUGGCGGGGUUUUCUUUUGGCCUUUUGGAGCUGAUGCGAACGUUGAUUCACUCGUAAGA
Ssp-2-4    UUCAUUUGUG.AGGAAG.............GCUUC.U..UC..ACGGA..GUGGAAACUAGGCAACAC.GCCCAACGA..GAAGCCGAGAGAACCCUGGCUUUGGCGGGGUUUUCUUUUGUCUAUUGCAGUGGGUAGUUUCAGCUUUGCUGGUGAUCCUG
Pma-5-1    UUCAUUUGUG.AGGAGG.............ACUUC....UCC.UCGGA..GUGGAAACAAGGAAACAC.GCCCAAAGA..GAAGUCUUGAAAACCCUGGCUUUGGCGGGGUUUCUUUUUGCCCUUACAUCUAGAGUGCUGAGCCCUACGAACAAUGGCGC
Pma-7-1    UUCGUUUGUG.AGGAGG.............ACUUC....UCC.UCGGA..GUGGAAACAAGGAAACAC.GCCCAAAGA..GAAGUCUUGAAAACCCUGGCUUUGGCGGGGUUUCUUUUUGCCCUUGCUUACAGCGCUGAGCCCGACGAACAAUGGCGC
Ssp-2-5    UUCAAAAGUG.AGGAGG.............GCUUC....UUC.ACGGA..GUGGAAACUAGGUAACAC.GCCCAAAGA..GAAGCCGAGAGAACCCUGGCUUUCGCGGGGUUUUCUUUUGUCUAAUGCAGUGGGUAGUUUCAGCUUUGUUGCUGCGCU
Ssp-2-6    UUGAUUUGUG.AGGAGG.............GCUUC....UUC.ACGGA..GUGGAAACUAGGAAACAC.GUCCAACUA..GAAGCCGAGAAAACCCUGGCUUUGGCGGGGUUUUCUUUUGCCUAAUCCAUUGGGCAUGGGCAGCUUUAGCUUUGCUGUUU
Ssp-2-7    UUCAUCCGUG.AGGAGG.............GCUUC....UUC.ACGGA..GUGGAAACAAGGAAACAC.GUCCAACGA..GAAGCCGAGAGAACCCUGGCUUAGCGGGGUUUUCUUUUGUCUGUGCAUAAAACGAUUUGUUUUUCACUCUGGCCUUCUG
Ssp-2-8    AAACUUUGUG.AGGAGA.............GCUUC....UUC.UUGAA..AUGGAAACGAGGCGACAC.GUCCUAAAA..GAAGCCGAGAAACUCUGCUUUUGGAGUGGUUUCUUUUGGUAUUUGCCUUCUCCAAGGCCUAGUCCACUCUGCUUCACA
Ssp-10-1   UCUUGUGUG.AGGAGUGCA...........GAACGCUCUCCU...GGG..GUCGAAACGAGGACAGAC.UCCCG..GCAUCGUUCCACCUCAAAAGCCUGCUUCGGCGGGGUUUUUUUUGGCCGUUUCAAGCCAGCAAAAAGCCGGCCCCGAGAGGC
Ssp-2-9    UCAUGACGUG.AGGAGU.GCUUUAC.....GCCUCUU..UC....AGG..GUCGAAACAAGGAAAGAC.UCCU...GAAUGAGUUCUUCUAAAAGACCUUGCCUUUGGCAGGGUUUUUUAUUGUCGAAAAGCGGGGGCCAUUAAGCCAUAAAAAAGCC
Pma-7-2    UAACAUCGUG.AGGGGU.GCGAAUC.....AACAG.U..UC....AGG..GUCGAAACAAGGCAAGAC.UCCU...GAUUCUGUCCCACCUAAAAAUCCCUGCCUUUGCGGGGGAUUUUUUUGCAUAUUUGCCACGCAAAAAAACACCGGCCCCGAAG
Pma-5-2    UAACAUUGUG.AGGGGU.GCGAAUC.....AACAG.U..UC....AGG..GUCGAAACAAGGAAAGAC.UCCU...GAAUCUGUCCCACCUAAAAAUCCCUGCCUUUGCGGGGGAUUUUUUUGCAUAUUGGCUACGCAAAAAAACACCGGCCCCGAAG
env-129    ACUUCGUGUG.AGGAGUGCA..........AAGCGCUC..UCU.UCUGG..GUCGAAACAAGGACAGAC.UCCA...GCAACGCGUCUCACUUCCGAGCUCUGCCUUUGGCAGAGUUUUUUUUUGCAACCUGACCAGGAUGUCGGGGUUCAUCAUCAGA
Ssp-11-1   CAUUCGUGUG.AGGAGU.GCU.........AAGCGCC..UCU.UCAGG..GUCGAAACAAGGACAGAC.UCCUGC.AACGCGCUUCACUUCUGAGCUCUGCCUUCGGCAGAGCUCUUUUUUUGCUCACAAAAAACCGACCCUGGGGUCGGUUGGUGG
env-130    AACUUGUGUG.AGGAGUGCU..........AAGCGCCUCUUC...UGG..GUCGAAACAAGGACAGAC.UCCAGC.AACGCGUUUCACUUCUGAGCUCUGCCUUCGGCAGAGGUUUUUUUUGUCAACACAGCAGGCUUUGCAGGAAUCAUGCAGGCAUA
Ssp-12-1   CGUGAGUGUG.AGGAGUGCU..........AAGCGCCUCUUC...AGG..GUCGAAACAAGGACAGAC.UCCUGC.AACGCGUUUCACUUCAGAGCUCUGCCUUCGGCAGGGCUCUUUUUUGACAACAAAAAAACCGACCCAAGGGUCGGUUGAGUG
env-131    AUGUUGUGUG.AGGAGUGAA..........GAGCAUCU..UCU.UCAGG..GUCGAAACAAGGACAGAC.UCCUG.GAUGUGUUCCGCUUGAUUCUUCCCCCUGCCUUCGGCGGGGGAUUUUUUUGCGUGAUUUCCAAAAAAAGACCGGCUCCUCAAG
env-132    AUCUCGUGUG.AGGAGUGCG..........GACGCUUC.UC....AGG..GUCGAAACAAGGACAGAC.UCCU...GAGUUCGUCCGAUUCCUGCCCCGGUUCGGCGGGGGUUUUUCUGGCAAAAAAAACCCUCCGCCGCAGCGGAGGCAUGAAUG
Ssp-7-1    AUUUCGUGUG.AGGAGU.GCG.........GACGCUUC.UC....AGG..GUCGAAACAAGGACAGAC.UCCU...GAGCUCGUC.CGAUUCCUGCCCCGGUUCGGCGGGGGUUUUUUUGGCAAAAAAAACCUCCGCCGGAGCGGAGGGCAUGAAG
Ssp-4-1    CUAGGGUGUG.AGGAGU.GCAA........AACACCUCUUC....AGG..GUCGAAACAAGGACAGAC.UCCU...GAACGUGUUCCACAAAAAACCCUGCCUUCGGCGGGGUUUUUUUAUUGCCGAGCCCAAUACACAACAAAAAAGCGACCCCCCGA
env-133    UAAAUGUGUG.AGGAGU.GCUG........UACGCCUCUUC....AGG..GUCGAAACAAGGACAGAC.UCCU...GAAUGCUUACCACCCAAAAAAACCCUGCCUUUGGCGGGGUUUUUUUAUUGCUGAACGAAAAUCAGACAAAAAAAUCGGUCCCU
env-134    UAGAUGUGUG.AGGAGU.GCUG........UACGCCUCUUC....AGG..GUCGAAACAAGGACAGAC.UCCU...GAAUGCAUACCACCCAAAAAAGCCUGCCUUUGGCGGGGUUUUUUAUUGCCAUACAAAAC.......................
env-135    UCAUGGUGUG.AGGAGU.GCUG........AAUGCCUCUUC....AGG..GUCGAAACAAGGACAGAC.UCCU...GAACGCGUUCCACUUAAAAAAAGCCCCUGCCUUCGGCGGGGUUUUUUAUUGAGCAGGUGUCCAACAAAAAAACCGGCCCCU
env-136    UGAAUGUGUG.AGGAGU.GCUG........AACGCCUCUUC....AGG..GUCGAAACAAGGACAGAC.UCCU...GAACGUGUUCCACUUAAAAAAAGCCCCUGCCUUCGGCGGGGUUUUUUAUUGAGCAGGUGUCCAACAAAAAACCGGCCCCUGG
Ssp-13-2   UGAAUGUGUG.AGGAGU.GCUG........AACGCCUCUUC....AGG..GUCGAAACAAGGACAGAC.UCCU...GAACGUGUUCCACUUAAAAAAAAGCCCUGCCUUCGGCGGGGGUUUUUUAUUGAGCAAGUGUCCAACAAAAAACCGGCCCCUGG
env-137    ACAAAGUGUG.AGGAGU.GCCG........AACGCCUCUUC....AGG..GUCGAAACAAGGACAGAC.UCCU...GAACGUGUUCCUCUCAAAAAAAGCCCCGGCUUUGGCGGGGGUUUUUAUUGGCCAGAGAAAACCACAAAAAAACCGGCCUUCG
Ssp-3-2    AUCUUGUGUG.AGGAGU.GCUG........AACGCUACUUC....AGG..GUCGAAACAAGGACAGAC.UCCU...GAACGUGUUCCUCUCGACAAAAGCUCCGCCUUUGGCGGGGCUUUUUUGUUGCUCAGACAAAAACACAAAAAAAAACCAGCUCU
env-138    GUCCUGUGUG.AGGAGU.GCUG........AACGCCUCUUC....AGG..GUCGAAACAAGGACAGAC.UCCU...GAACGUGUUCCUCUCAUGAAAAGCUCCGCCUUCGGCGGGGCUUU...............................
env-139    CAUUUGUGUG.AGGAGU.GCUG........AACGCCUCUAC....AGG..GUCGAAACAAGGACAGAC.UCCU...GUAUGUGUUCCUCUCGACAAAAGCUCCGCCUUUGGCGGGGCUUUUUUGUUGGCAAGAGAAAAACAUAAAAAAACCGGCCCUGG
Ssp-10-2   UAUCUGUGUG.AGGAGU.GCUG........AACGCCUCUUC....AGG..GUCGAAACAAGGACAGACUCCCU...GAACGUGUUCCUCAAAAAAACCCUGCCUUUGGCGGGGUUUUUUAUUGGCAGUCCCAGGCCACACCAAAAAAGCAACCCUCGAA
Ssp-1-1    UAUCUGUGUG.AGGAGU.GCUG........AACGCCUCUUC....AGG..GUCGAAACAAGGACAGAC.UCCU...GAACGUGUUCCUCAAAAAACCCUGCCUUUGGCGGGGUUUUUUAUUGGCAAGUCCAAGGCACCACAAAAAAGCAACCCUCGAA
env-140    AAUUUGUGUG.AGGAGU.GCUG........AACGCCUCUUC....AGG..GUCGAAACAAGGACAGAC.UCCU...GAACGUGUUCCUCUCAAUAAAGGCUCCGCCUUUGGCGGGGCUUUUUUGUUGACCAAAGAAACCACAAAAAAACCGGCCCUCG
env-141    AUUUUGUGUG.AGGAGU.GCCG........AACGCCUCUUC....AGG..GUCGAAACAAGGACAGAC.UCCU...GAACGUGUUCCUCUCGAAAAAAGCUCGGCCUUUGGCGGAGGUUUUUUGUUGCCAGAGAAAGCCAAAAAAAACCGGUCCUCGA
env-142    AAUUUGUGUG.AGGAGU.GCCG........AACGCCUCUUC....AGG..GUCGAAACAAGGACAGAC.UCCU...GAAUGUGUUCCUCUCAACAAAAGCUCCGCCUUUGGCGGGGCUUUUUUGUUGGCUAAACAAAAACCACAAAAAAACCGGUCCUC
env-143    AAUUUGUGUG.AGGAGU.GCUG........AGCGCCUCUUC....AGG..GUCGAAACAAGGACAGAC.UCCU...GAAUGUGCUCCUCUCUAAAAAGCUCCGGCUUUGGCGGGGGUUUUUAUUGGCCAGAGAAAACCACAAAAAAACCGGCCCUCGA
env-144    AACUUGUGUG.AGGAGU.GCUG........AGCGCCUCUUC....AGG..GUCGAAACAAGGACAGAC.UCCU...GAAUGUGUUCCUCUCUUAAAAAGCCCCGCCUUUGGCGGGGCUUUUUCAUUGGCCAAAACUACAAAAAACCGGCCCUCGAAAGG
env-145    UGUAUGUGCG.AGGAGU.GCAG........AGCAUCUCUUC....AGG..GUCGAAACAAGGACAGAC.UCCU...GAACGUGUUCCACUUAAAAAAGCCCCUGCCUUUGGCGGGGGUUUUUUAUUGCCAUGUGAGGCAGCAAAAAAGCCCGCCUUUGA
env-146    UGGCUGUGUG.AGGAGUGCA..........AGCGCCUCUUC....AGG..GUCGAAACAAGGACAGAC.UCCU...GAAUGUGCCCACUUUCCAAAGCAGUGGCUUCGGCAGUGGUUUUUUAUUGCCCAUUGAUCGUCGCUGCACAAAAAAGCCGACCC
Ssp-9-1    AAAUCGUGUG.AGGAGU.GCCG........AGCGCCU..CU..UCAGG..GUCGAAACAAGGACAGAC.UCCUGCAAC..GCGUUCCACUCAAGACCUCGGCUUCGGCGGGGUGUUUUUGUUGGACGGGUCACCGCUGCGACAGCGAAGACGUCGGCCC
env-147    UUAUUGUGUG.AGGAGU.GCCG........AGCGCCU..CU..UCAGG..GUCGAAACAAGGACAGAC.UCCUGCAAC..GCGUUCCACUCAAGACCUCGGCUUCGGCGGGGUGUUUUUGUUGGCCAGGUCACCGCUGCGACAGCGACAACGUCGCGGC
env-148    UUAUUGUGUG.AGGAGU.GCCG........AGCGCCU..CU..UCAGG..GUCGAAACAAGGACAGAC.UCCUGCAAC..GCGUUCCACUCAAGACCUCGGCUUCGGCGGGGUGUUUUUGUUGGCCAGGUCACCGCUGCGACAGCGACAACGUCGCGGC
env-149    CAAUUGUGUG.AGGAGU.GCCG........AGCGCCU..CU..UCAGG..GUCGAAACAAGGACAGAC.UCCUGCAAC..GCGUUCCACUCAAGACCUCGGCUUCGGCGGGGUGUUUUUGUUGGCCAGGUCACCGCUGCGACAGCGACAACGUCGCGGC
env-150    UCGAAGUGUG.AGGAGUGGA..........AAACGCU..UCU.UCCGG..GUCGAAACAAGGACAGAC.UCCGGUCGUG.CGUUUCACUUCUGAGCUCUGCCUUAAGGCAGAGGUUUUUUUUGCACAACCAAACCCAGUUGGCUGCAAUCGUCCAGACA

           ................<.............<<<<<....<<....<<<..<<<...........>>>..>>>...>>..>>>>>>......................................................................
           ................?.............22222....?2....?2?..222...........222..?2?...2?..22222?......................................................................
           •••••RUGUR-AGGAG•-------------RR••••∘∘--YY∘-∘∘∘•R--GU•GAAACAAGG••A•AC-UY•∘∘∘∘R∘∘∘∘•YY•••••••••••••••Y•••••••••••••YYY•U•••••••••••••••••••••••••••••••••••••••
           5′———————————————————————————————————————————————————————————————————————————3′
```

```
env-151   AUCUCGUGUG.AGGAGU.GC...........AGACGCU..AC..UCAGG..GUCGAAACAAGGACAGAC.UCCUGAGGU..UGUCUGAAUUCCUGCCCCGCUUUGCGGGGCUUUUUCUUGGUAAUUUUCAGACCAAAAAAAACCCCUGCCGCAGCAGG
Ssp-8-2   UUCGUUCGUG.AGGAGA.UA...........AAGCUUC..UC....GGG..GUGGAAACAAGGUCACAC.UCCU...GAGCAGCUUUCGGAACCCCUGCCUUCGGCGGGGGUUUCUUUUUGCCCUGGUGCCAGCGCUGCUGACGAGGCAUCAACACCAGG
env-152   UGCCUAAGUG.AGGAGA..............AAAGC....UC..ACGGG..GUGGAAACAAGGAAACAC.GCCGAGUGA..GUUUUUUAAACCCCUGCCUUUGGCGGGGGUUUAUUUUUGAAAUAGCGUCUGCUGGUCCGGCACCAUCAAAGGUGGGAA
env-153   UGGGUUUGUG.AGGAAA..............ACGCCUC..AC....AGG..GUGGAAACAAGGACACAC.UCCU...GCGAGCCGUUUUGAAACCCCUGCCUUUGGCGGGGGUUUCUUUUUGUCUGCCAGGGAGCCCAUGGAAUCGGUCAUCAACGAAAU
env-154   GCAAUGUGUG.AGGAAA..............ACGCC....UC..ACGGG..GUGGAAACAAGGUCACAC.UCCC...GAGGGACGUUUUGGAGCCCCUGCCUUCGGCGGGGGUUUCUUUUUGCCUCGCGUUAUCCACAGACCAAGUGGCUCUAACUACCG
env-155   UUCAUGUGUG.AGGGA...............AAACUUC..AC....GGG..UUGGAAACAAGGACACAC.CCCC...GUGAGGUUU.CGGAGCCCCUGCC............................................................
env-156   UUCAUGUGUG.AGGA.A..............AAACUUC..AC....GGG..UUGGAAACAAGGACACAA.CCCC...GUGAGAUUUUCGGAGCCCCUGCCUUUGGCGGGGGUUUCUUUUUGACCUCAGUUAUCCACAGGGUCAGUGGAUCGAAACUAUGU
env-157   UUUGGAAGUG.AGGA.A..............AAACCUC..GC....GGG..UUGGAAACAAGGACACAA.CCCC...GUGCGGUUUAGGAACCCCUGCCUUUGGCGGGGGUUUCUUUUUGACUCAGUUAUCCACAAGGUCAGUGGAUCCAAGCCUUGCCU
env-158   UUCGGGAGUG.AGGAAA..............AAAGCUA..AC....GGG..UUGGAAACAAGGACACAA.CCCC...GUGAGUUUUCGAAGCCCCUGCCUUUGGCGGGGGCUUCUUUUUGACCUCAGUUAUCCACAGGGUCAGUGGAUCAAAACUUUGCC
Ssp-13-3  UAUGGAAGUG.AGGA.A..............AAAUCUA..AC....GGG..UUGGAAACAAGGACACAA.CCCC...GUGAGGUUU.CGGAGCCCCUGCCUUUGGCGGGGGUUUCUUUUUGAACUCAGUUAUCCACAAGGUCAGUGGAUCCAAACCUUGC
env-159   UAUGUAAGUG.AGGA.A..............AAACCUA..AC....GGG..UUGGAAACAAGGACACAA.CCCC...GUGAGGUUUCGGAGCCCCUGCCUUUGGCGGGGGUUUCUUUUUGAACUCAGUUAUCCACAAGGUCAGUGGAUCCAAACCUUGCC
Ssp-3-3   UGUGGCAGUG.AGGA................AAAACCUU.GC....GGG..UUGGAAACAAGGACACAA.CCCC...GCGAGUUUU.CGAAACCUCUGCCUUUGGCGGGGGUUUCUUUUUGACCUCAGUUAUCCACAGGGUCGUGGAUCCAUACCGUGUC
env-160   UAAUUUGGUG.AGGAGA..............AAAGC....UC..ACGUG..GUGGAAACAAGGACACAC.UCACGUUGA..ACUUUUUGAACCCCUGCCUUUGGCGGGGGUUUCUUUUUUGCAGAUCAAAAACCAGCUUCAGCAAAAUCUUCAACGCUCA
env-161   UAAUCUCGUG.AGGAGA..............AAAGC....UC..ACGUG..GUGGAAACAAGGACACAC.UCACGUUGA..ACUUUCAGAACCCCUGCCUUCGGCGGGGGUUUCUUUUUGUUCAGUUCUCACUUGCUGCCUCAGUUAGAUCUCAGCGAUU
env-162   UAAAUUUGUG.AGGAGA..............AAAGC....UC..ACGUG..GUGGAAACAAGGACACAC.UCACGUUGA..ACUUUCAGAACCCCUGCCUUUGGCGGGGGUUUCUUUUUUGCAGAUCAAACCCAGCUUCAGCGAAUUCUUCAACGCUCAA
Ssp-4-2   UCAUGAGGUG.AGGAUA..............AAAAC....UU..ACGGG..GUGGAAACAAGGCCACAC.UCCCACUGA..GUUUU.CGGAACCCCUGCCUUUGGCGGGGGUUUGUUUUGGCUAUUCCUGCAGAAAAAUUAUCUGCUAGCUGGUGCAGU
env-163   UCAAUACGUG.AGGAAA..............AAAAC....UC..ACGGG..GUGGAAACAAGGUCACAC.UCCCGCUGA..GUUUUCGGAACCCCUGCCU............................................................
env-164   UUUACUUGUG.AGGAGA..............AACGC....UC..ACGGG..GUGGAAACAAGGACACAC.CCCCGCAGA..GUUUUCGAAGCCCUGCCUUUGGCGGGGGUUUCUUUUUUAGCGAUUUAUUUCUUGCUAUUGGCGAUGCCAAUUAACACCG
env-165   CGAGGUUGUG.AGGAA...............AAACGCG..CUC.AGGGG..GUGGAAACAAGGUCACAC.UCCCAAAAGA.CGUUU.UGGAGCCCCUGCCUUCGGCAAGGGUUUCUUUUUUGACUCGCGUUAUCCACAGACCAAGUGGCGCUACCUGCAG
env-166   UGAAAGUGUG.AGGAA...............AAACGCCAAAC....GGG..GUGGAAACAAGGUCACAC.UCCCGG.GAGACGUUU.UGGAGCCCCUGCCUUCGGCGGGGGUUUCUUUUUGUCUCGCGUUAUCCACAGACGAAAUGGCCCCCUGCCUGCA
env-167   UUGUCUUGUG.AGGAA...............AAACGCC..UC..ACGGG..GUGGAAACAAGGUCACAC.UCCCGA.GAGACGUUU.UGGAGCCCCUGCCUUCGGCGGGGGUUUCUUUUUGCCUCGCGUUAUCCACAGACCAGGUGGACCUACCUGCAGC
env-168   UGGGAUUGUG.AGGGA...............AAACGCC..UC..ACGGG..GUGGAAACAAGGUCACAC.ACCCGA.GAGACGUUU.UGGAGCCCCUGCCUUCGGCGGGGGUUUCUUUUUGGUUGAACUUGCUUCGAUACUCCUGAUAGCGGCUCGUGCC
env-169   UGAAGUCGUG.AGGAA...............AAACGCC..UC..ACGGG..GUGGAAACAAGGUCACAC.UCCCGA.GAGACGUUU.UGGAGCCCCUGCCUUCGGCGGGGGUUUCUUUUUGCCUCGCGUUAUCCACAGACCAAGUGGCCCUGCUUACAGC
env-170   UGAAGUCGUG.AGGAA...............AAACGCC..UC..ACGGG..GUGGAAACAAGGUCACAC.UCCCGA.GAGACGUUU.AGGAGCCCCUGCCUUCGGCGGGGGUUUCUUCUUGCCUGCAAUUAAGCGCCGAGAUAGCAGAAGAAGAUGGGCU
env-171   UGAAUCUGUG.AGGAA...............AAACGCC..UC..ACGGG..GUGGAAACAAGGUCACAC.UCCCGA.GAGACGUUU.UGGAGCCCCUGCCUUCGGCGGGGGUUUCUUUUUGCCUCGCGUUAUCCACAGACCAGGUGGACCUACCUGCAGC
env-172   UGAAUAUGUG.AGGGA...............AAACGCC..UC..ACGGG..GUGGAAACAAGGUCACAC.UCCCGA.GAGACGUUU.UGGAGCCCCUGCCUUCGGCGGGGGUUUCUUUUUGGCUCGCGUUAUCCACAGACCAAGUGGCGCUGCCUGCAGC
env-173   UGGGUUUGUG.AGGAA...............AAACGCC..UC..ACAGG..GUGGAAACAAGGACACAC.UCCUGC.GAGCCGUUU.UGGAACCCCUGCCUUUGGCGGGGGUUUCUUUUUGUCUGCCAGGGAGCCCAUGGAAUCGGUCAUCAACGAAAUC
env-174   UGGAUCUGUG.AGGAA...............AAACGCC..UC..ACAGG..GUGGAAACAAGGACACAC.UCCUGC.GAGCCGUUU.UGGAACCCCUGCCUUUGGCGGGGGUUUCUUUUUGUCUGCCAGGGAGCCCAUGGAAUCGGUCAUCAACGAAAUC
Ssp-3-4   UGGAUAUGUG.AGGA................AAACGCU..UC..ACAGG..GUGGAAACAAGGACACAC.UCCUAA.GAGUCGUUU.UGAAACCCCUGCCUUCGGCGGGGGUUUCUUUUUGUCUGCAGGAAAGCCCAUGGAAUCGGUCAUCAACGAAAUC
Ssp-4-3   UUAGCUCGUG.AGGAAA..............AAAUCCU..UC..AAAGG..GUGGAAACAAGGACACAC.UCCUGA.GAUUGAUUU.CGAAACCCCUGCCUUUGGCGGGGGUUUCUUUUUGGCGACCUGAUUGCCCUUUUGUUGAUCUCUCGGACGAGAA
Ssp-1-2   UUACCUUGUG.AGGAAA..............AAAUCCU..UC..ACAGG..GUGGAAACAAGGACACAC.UCCUGA.GAUUGAUUUCGUAACCCCUGCCUUCGGCGGGGGUUUCUUUUUGGGUCUGUGUUGCCCUUUUGUUGAUCUAUCAAAAAAGGAGC
env-175   AUAAAUUGUG.AGGAAA..............AAAUCCU..UC..ACAGG..GUGGAAACAAGGACACAC.UCCUGA.GAUUGAUUUCGAAACCCCUGCCUUUGGCGGGGGUUUCUUUUUGGAGACCUGAUUGUCCUUUUGUUGAUCUCUCGUACGAGAAG
env-176   UGUUUAUGUG.AGGA.A..............AAAUCGC..UUC.ACGGG..GUGGAAACAAGGAAACAU.UCCCGAGAAGCGUUUUUGGAACCCCUGCCUUUGGCGGGGGUUUCUUUUUGCCAUGUCAUCCAUAAAGAGAGGCCGCACUUGCACCUCU..
Ssp-3-5   UGAUCUUGUG.AGGAGG................CGC....CUC.ACGGGA.GUGGAAACAAGGUGACAC.UCCUGAAAC..GCG..UUCUGUGGCCCCUGCCUUUGGCGGGGCUUCUCAUUGGGAUGUGGCUCUCAAUGGCACAUCUAGCUAUCUGUUG
env-177   UUGUCGUGUG.AGGAAA..............AACGC....CUC.ACGGG..GUGGAAACAAGGUCACAC.UCCCGAGAA..GCGUUUUGAAGCCCCUGCCUUCGGCGGGGGUUUCUUUUUGCUCCUGCGAGAUCGGAGUGGCCUGCUCACCGCGUCCUGC
env-178   UGAAUGUGUA.AGGGGG..............AACGC....CUC.ACGGG..GUGGAAACAAGGUCACAC.UCCCGAGAG..ACGUUUUGGAGCCCCUGCCUUCGGCGGAGGUUUCUUUUUGCCUCGUGUUAUCCACAGACCAAGUGGCCCUAACUACCGC
Ssp-13-4  UCCGAAAGUG.AGGAAA.AA...........AUCUC....AC....GGG..UUGGAAACAAGGACACAA.CCCC...GU..GCGGU.UUCGGGACCCCUGCCUUUGGCGGGGGUUUCUUUUUGUGUAGGGACGCCGUCGCCUGCCAUCGCACACCUAUUG
env-179   UGUCCGCGUG.AGGAAA..............AAACG....CCACACGGA..GUGGAAACAAGGACACAC..UCCCUAGAAGCGUUUUAGAGCCCCUGCCUUCGGCGGGGGUUUCUUUUUGGCCGAAUGCCCCGACAUGAUUGGCUUGCUCCAAAGAGGA
Csp-1-1   AUGGGGUGUG.AGGAGU.GC...........GGAACGUC.UCU.UCAGG..GUCGAAACGAGGACAGAC.UCCCGAAGAUCGUUCCAACGUCCUGCCCCGCCUUCGGCGGGGCUUUUUUCUUGGCUGCCUACCGGCACUGGCGUCACUGACGAUCAGCCG
Tel-1-2   UGACUUAGUG.AGGAGU.GAUUCGCAAAC..AAAAGUC..CU....AGG..GUCGAAACACGGCAAGAC.UCCU...AGGGCUUUUUAUUUUGGGCUCAAGCCGUUACCUUAAAAAUAUGCCUUUGCGAAUUUCGGGCCAGCGAUCGCUCAAAACCCUUC
Ssp-14-3  GAGGUGUGUG.AGGAGU.AAGUU........GAAGCCC..CC....GGA..GUCGAAACAAGGAAAGAC.GCCCAG.GG..GCUUUUUCUUGCCUUUUUUUUUGCCCUGAAAGAUUUUUCAAGCUGGUCUAUGCUCCCCGUAAAAGUUCGGGAUGGGCAGC
Nsp-1-2   UAGUUGUGUG.AGGAGC.AAGUUGAAACUAAAAAGCAU..CU....GGG..GUGGAAACACGGCAACAC.UCCC...AGAUGCUUUUUAAUUAUUGGCAAAUUGUUACUUUUUAACGAAAGUCCACUCUAGCACCUUGAUUGGGGCGGUUUGUAGUGCUU
Ava-1-2   GUUGUGUGUG.AGGAGC.AAGUUAAAAAUAAAAAGCAU..CU....GGG..GUGGAAACACGGCAACAC.UCCC...AGGUGCUUUUUAGUUAUUGGCAAAUUGUUAAUUUUUAACGAAAGUCCACUCUAGCACCUUGAUGGGAGCGGUUUGUAGUGCUU
Nsp-1-3   UUUGUGUGUG.AGGAGC.AAGUUAAAAAUAAAAAGCGU..CU....GGG..GUGGAAACACGGCAACAC.UCCC...AGGCGCUUU.UUAAUUGAGGCUACACUGAGAGUUUUCUGUGCAGUUGGCAAAGUGUCAUACAAUAUACUUGCUAAAUAAAAUA
Nsp-1-4   UUUGUGUGUG.AGGAGC.AAGUUAAAAAUAAAAAGCGU..CU....GGG..GUGGAAACACGGCAACAC.UCCC...AGGCGCUUU.UUUAUUGCGCUGUGCGUGUCAGUUGUCCCAGUGGACAGGAAUGUUAAAAUUCCGCCGAAAUACUCCUGUUUUG
Ava-1-3   UUUGUGUGUG.AGGAGC.AAGUUAAAAAUAAAAAGCGC..CU....GGG..GUGGAAACACGGCAACAC.UCCC...AGGCGCUUUUUAAUUGAGGCUACCCUGAGACUUUUCUGUGCAGUUGGCAAAGUGUCAUGCAAUAUACUUGCUAAAUAAAAUAA
Npu-1-1   UUUGUGUGUG.AGGAGC.AAGUUAAAAGUAAAAAGCGU..UU....GGG..GUGGAAACACGGCAACAC.UCCC...AAGCGCUUUUUAAUUGGUCAGAAAUUGGAUUGCUUGUGCAGUUUUGAAAAUGUCACUCAAUAUACUUGCCAUUAGAUAAAAGU
Nsp-1-5   UAAUAGUGUG.AGGAGC.AAGUUGAAAUUA.AGAGCGC..UU....AGA..GUGGAAACACGGUAACAC.UUCU...GAGCGCUUU.UUAAUUUAUGGCUGUUGACAGUUGUCUGUUGUCUGUUGACAGACUUGAAAGCCUUUUAUUGUCCGGGUUUUAU
Mae-1-1   UAAUUGUGUG.AGGAGUGAGAAG........AAAGCAAAGUCC.UUGGG..GUCGAAACACGGCAAGAC.UCCC...GAGGGCUUUUCCAUGUCUAGGGAACAAUUGUCAGUCAUGAACCAGCCUCAAGAGCGAGAACCUUUUGCCAGUUUAGUUUUAUA
Csp-2-1   UGAGAGCUUA.AAGAGU..............AAAGCAC..UU....GGU..GUCGAAACAAGGCAAGAC.UUCC...AGGUGCUUUUCUCAUGCUAUUGGGUAACCUAUUUUUACCUGAGUUCGGGGUUAGGAGUUUGGAGUGCGACUACGUCGUGCUAC
Cwa-1-1   UGAGUGAGUU.AGUAGA..............AAAACAC..UU....GGG..GUCGAAACACGGCAAGAC.UCCC...GGGUGUUUUUCUCAUUUUGGCCAAACCACCCUAAAAAAAUAAUGUAUCUUAAUAAUUUUACACUUCGUAUUGUUGAAGGCAAA
Csp-2-2   UGAGAGAGUU.AGUAGG..............AAAGCAC..UU....GGG..GUCGAAACACGGCAAGAC.UCCC...AGGUGCUUUUCUCAAUAUUUGAGAUAAUAAAACAAGGUAAUCUUUUUCUUUUGGUACGAAUAUGAGUCAAGUUUUUCCAAUGG
Csp-3-1   UGAGAGAGCU.AGUAGA..............AAAGCAC..UU....GGG..GUCGAAACACGGCAAGAC.UCCC...AGGUGCUUUUCUCAAUAUUUGAGGUAAUAAAGUAGGAUAGUUUUUUUCCUUUGGUAGGAACAGGAGUCAAGUUUUUCCGAUUU
Lsp-1-1   GAGGUCUGU..AGAAGA..............AGUGC....UU....GGG..GUCGAAACACGGCCAGAC.UCCC...AG..GUACUUUUUCCAUUUUUAUUUAGGUCACGUUUAAUCGAGUCCUAAAGUGAGUUGGAGCGGACGUUGAUCUUUAGCCGUU
Ssp-6-1   GUGAGGAGUG.AAGA................GAAGAAG..CCU.CUGGG..GUCGAAACACGGACAGAC.GCCCAGAGGUUUUUUC.AUGACCUGCUCAGCCUCAACGGGGACACCGCCAUCCCUACCUAGCCUCCGAAGUUUUAACUAUUCUCAACCAC
Ssp-5-1   GUGAGGAGUG.AAGAGA..............AGAAG....CCU.CUGGG..GUCGAAACACGGACAGAC.GCCCAGGGG..UUUUUUCAUGUCCUAGGAGCUUAAGUGCUCCGCAUUAUAGUCAUCUUGAAUAGGAAUAAAACAUUUUCGUUCAUUGCUC
Mae-1-2   AUAUCGUGUG.AGGAGUAUAAGUUCAGCAUAAGAGGAA..CCU.UUGGG..GUCGAAACACGGCAAGAC.UCCCAGAGGUUCUUCUUUUUCACUUCCUUAUUGGCUCAAAGUCAGCCAUAAAAUCAGGUCUUACCCGAUCGCUAGUUUUUUCUCAAGUUA
Csp-5-1   GUGUGGUGUG.AGGAAAAAAGU.........AGAGAAG..UGC.UUGGG..GUCGAAACACGGAAAGAC.UCCCAGGCACUUUUCUCAUUUAAAGUCAUCGAUUUUGGUCGAGGCAACAGGCAACAAGCUCAAAACACAAAAACGCAAAUUUUACGCAAA
Csp-4-1   GUGUGGUGUG.AGGAAA.AAGU.........AGAGAAA..CGU.UUGGG..GUCGAAACACGGACAGAC.UCCCGAACGUUUUUCUCAUUUAAGUUCCUUGGCUAAAAUGUAGCCCAGUAUACGGUAAAAAAUAAGCGUUAAAGAGAUGAUUUAGGCCAG
Csp-5-2   AUUCGGUGUG.AGGAGA.AAUGU........AGAAAAG..CGU.UUGGG..GUCGAAACACGGCAAGAC.UCCCAGGCGCUUUUCUCAUUAAAAGAUUGAACUUAAAUCAGAUUUUUAAGCUGUUGUCCAUUUAAACUGGAUAUUAUGAUCUUAGGAAAC
Cwa-1-2   UGAGAAUGUG.AAGAGA..............AAAGC....AC....UGA.GGUCGAAACAAGGCAAGAC.UUCAA..GU..GCUUUUCUUGUUAUUAGUCGUUUCCAGUCAGUUUCAGCAAUUAACAAUUAUUCGGUGAGCCUCAUGCUCACUAAAUUAA

          ................<..............<<<<<....<<....<<<..<<<............>>>..>>>...>>..>>>>>>.........................................................................
          ................?..............22222....?2....?2?..222............222..?2?...2?..22222?.........................................................................
          •••••RUGUR-AGGAG•-------------RR•••••○○--YY○-○○••R--GU•GAAACAAGG••A•AC-UY••○○○RR○○•••YY○••••••••••••••Y••••••••••••YYY•U••••••••••••••••••••••••••••••••••••••••••
          5′——————————————————————————————————————————————————————————————————————————————————3′
```

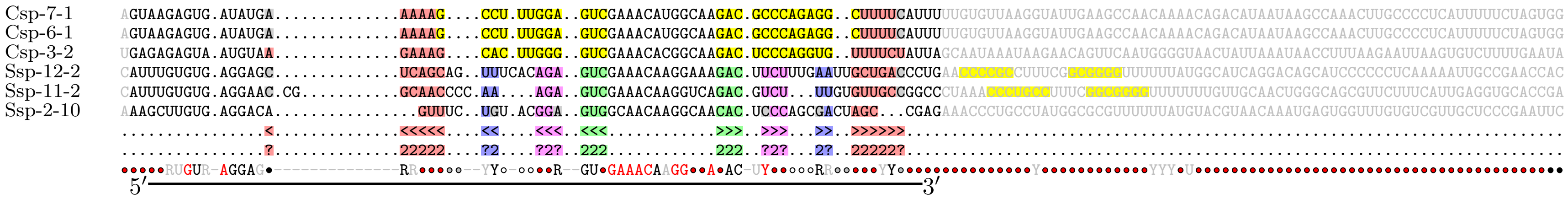

alignment positions 260 · · · 419

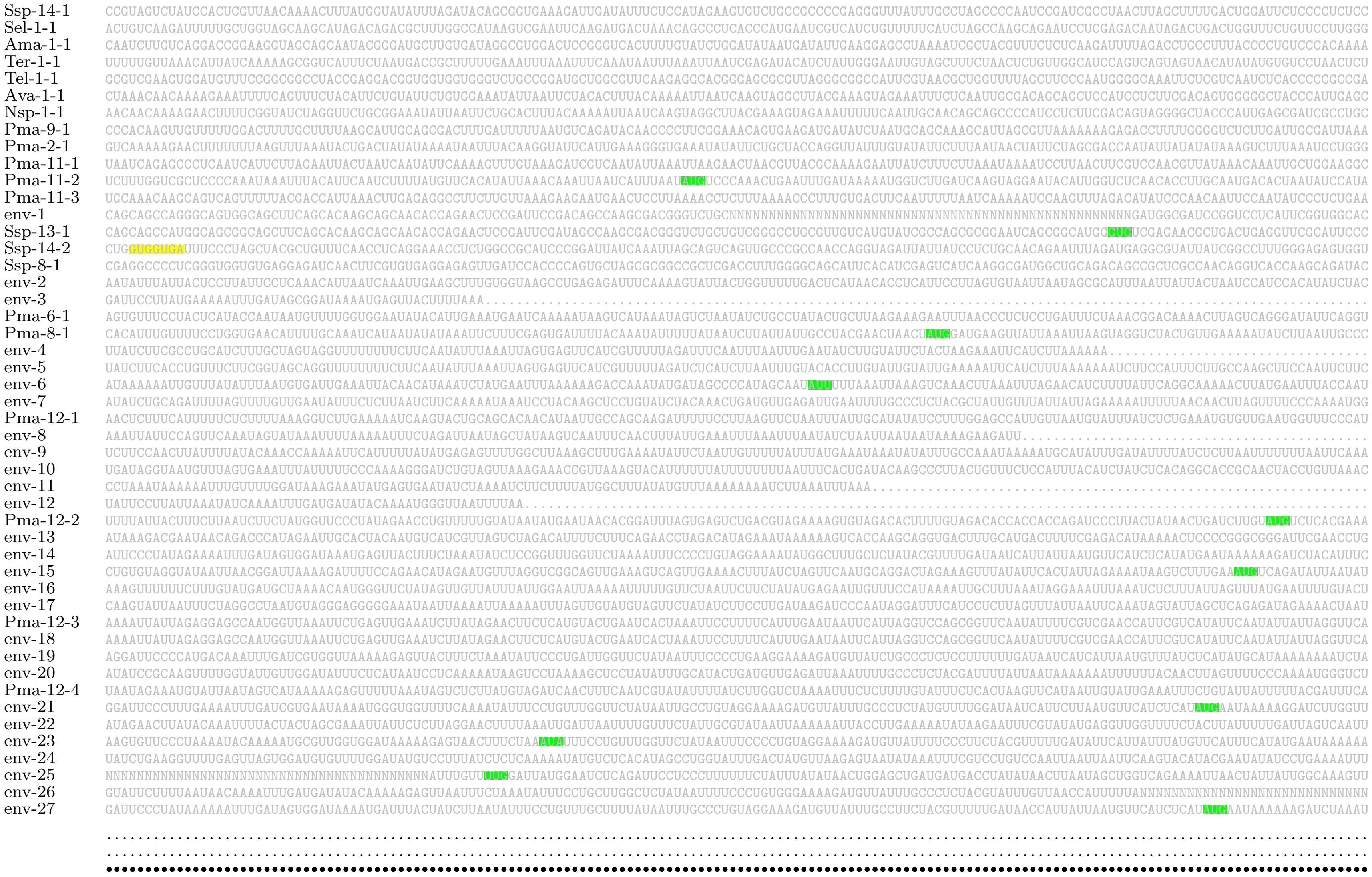

env-28 GUAUUCUUUUAACUACGAAAUUUGAUGAUAUACAAAAAGAGUAAAUUUCUAAAUAUUUCCUGCUUGGCUCUAUAAUUGUCCCUGUAGGAAAAAAUCUUAUUUGCCCUCUACGUUUUUGAUAACCAUAUGUAAUCUUCAUCUUAAAUGAAUAAAAAAGAUC
env-29 GAUUCCCUAUGAAAAGUUUGAUAGUCGAUAAAAUAAGUUGCUUUCUAAAUAAUUUCCUGUUUGGUUCUAUAAUUUCCUCUGUAUAAAAGAUGUUAUUUGCCUUCUACAUUUUUGAUAAUCAUUCUUAAUGUUCAUCUCAUAUGAAUAAAAAAGAUCUAAA
env-30 ....
env-31 GAUGCGUUAUGUAAGUUAAUUAUGAAUUGUUCUCAAAAUACUUACUUAUUUUUCUUUUCAGAAUUGCAGUAGUAUAAAUUUGUAAUUAAACCUUCUUAGGUUAUUAAUAAAAAUUAAGACAAGUUAAAUAAAGGAAACUAAUUCAAGAUUUGUUUAUAAA
env-32 GUAUUCCCUAUAAAUUAAAUUUGAUAGUGGAUAAAGUGAGUUACUUGCUAAAUAUUUCCUGUUAGGUUCUAUAAUUCCCCUAUAAGAAAAGAUGUUAUUUGCUCUCUACGUUUUUGAUAAUCAUCAUUAAUGUUCAUCUCAUAUGAAUCAAAAAGAUCUA
env-33 CGAUUAAUGUCUUAGACCACCAUUCCAUGAAAGUUCUUCUUUUAUCAAUCAGAGUUGAACGAUCAUAAUGACCCCGAGUGCCAUUUAAAUGACCCAUUCUUCCUAAUUGGCGAUCAAUAACCUCAUAAUCAAAAUUUGAUUUUUCUAAUGCUGAAGUUGU
env-34 AGUAUUCCCUAUAAAUUAAAUUUGAUAGUGGAUAAAAUGAGUUACUUGCUAAAUAUUUCCUGUUUGGUUCUAUAAUUCCCCAGUAAGAAAAGAUGUUAUUUGCCCUCUACUUUUUUGAUCAUCAUCAUAAUGUUCAUCUCAUAUGAAUAAAAAAGAUCUA
env-35 AAUUUAAAGUUUCUUUGGAAUGAAAGUUCAAUUUGGUCAUAUUCCUGGAACUAAAAUUGGACAACUUUUUAAGGACAGGACUGCUUUAAAAAAAUGCAGGAAUACAUGCUAAUACUGUCAAAGGCAUAUGGGGCGCAGGUACAGGUGCAUGUUCAAUAGUU
env-36 GGAUUCCCAAUGAAAAUUUUGAUAGUGGAAAAGAUCAGCAACUUUCUUAAUAUUUCCUGUUUGGUUCUAUAAUUUCCCGUAUAGAAAAGAUGUUAUCUGCUAUCUACGUAUUUGAUAAUCAUAAUUAAUGUCAUCUCAUAUGAAUAAAAAAGAUCUAA.
env-37 ....
env-38 UUUAGCUCUUUAUUCUUAUUUAGUUAGUNNNNNNNNNNNNNNNNNNNNNNNNNNNNNNNNNNNNNNNNNNNNNNNNNNNNNNNNNNNNNCUUUAGGACUAUUAACAAUAUAGAUAAUGGAGCAAAUAUUAAAGUAAUUUAUUGGAAACUGAAGUGUGAAGAGAACCCUUC
Pma-4-1 CAAAAGACGAAUAAAAAGACGAAUACUGGACUAAUAGAAUUGCACUACAAUGUCACCAUUCGUCUAGACAUUUUUCUCUUAUAACUGAUUGACUGACAUAAAAAAAGUCACCUAAGGGUGAGUUUGUAUGACUUUCGAGACUUUAAAACUCCCCGGGCG
Pma-8-2 AGUUUAUCAACUCUUUCAUUUUUCUCUUUUAAAGGUCUUGAUAAAUCAAGAACAGCAGCACAACAUAAAUGCCAACUUGAUUUUUCUUGAAGUUCUAAUUUCUUGCAUAUAUCCCUUGGAGCCAAAAUUAACGAAUUUAUCUCUGAUAUAUGUUGAAUUG
env-39 ACAUAUAUGCUUUCUUCCAAAAUAAUUAGGUCGUCAUUACAAAUUAGUAAUUUAAUUCGUUAGAUUAUGAAUCAUUAUAUUCUUCAGUUAAUGCAAAUACUUUUUUUAGCAAUUUUAAUUUGUUCAAACUUUUUAUUCCCUUCUUCAUCAUUUGCCUCAC
env-40 CAAAUAUGCUUUCUGACAAAAUAAAUGAGAUACUUAAUUGCAAACUAGUUAUUUAAUUCGUUAGAUUAUGAAUUAUUAAAAAACUAUAUUAAUGAAAAUAUUUUUUUAACAAUUUUAAUUUGUUCUAGCUUUUUAUUCCCUUCAUCAUCAUUUGCCUCA
env-41 CAUAUAUGCUUUCGAUAAAAAUAAUUAGGGAUGUCAUUACAAAUUAGUAAUUUGAUUCGUUAGAUUAUUAAUUGUUUAAUUUCUCUGUUAAUGAAAAUACUUUUUUUAGCAAUUUUAAUUUGUUCAAGCUUUCUAUUUCCUUCUUCAUCAUUUGCCUCAC
env-42 GAAUAUAGAAACAUAGAUGCUUUCUUCAAAAAUGAGAAACGUCAUUACAAAUAACUUAUUUAAUUCGUUAGAUUAUAUUAUGUUAAUUUCUUCUGUUAAUGAAAAUACUUUUUUUGACAAUUUNNNNNNNNNNNNNNNNNNNNNNNNNNNNNNNNNNNNNN
env-43 UUUCUUUCAAAAUAAUUAGAUAUGUCAUUACAAAUUAGUAAUUUAAUUCGUUAGAUUAUUGUUUGUUAAAUACUUCUGUUAAUGAAAAUACUUUUUUUAACAAUUUUAAUUUGUUCAAGCUUUUUAUUCCCUUCUUCAUCAUUUGCCUCACAUGUAGAAC
Pma-4-2 AAAAUAUAUGCUUUCUUAAAAAAAUAAUUAGAUACUUCAUUACAAAUUAGUAAUUUAUUUAGUUAGAUUAUGAUUUGUUUAAUUCUUCUGUUAAUGAAAAUACUUUUUUUAGCAAUUUUAAUUUGUUCAAGCUUUUUAUUCCCUUCUUCAUCAUUUGCCU
env-44 AAAAAUAUAUUUAAUAUUAAAUGUAAUGAUAAGCAUCAUUCAAAUUCGUAAUUUAAUUCGUUAGAUUAUGUUCUAAUUAUUUUCUUCUGUUAAUGAAAAUACUUUUGUUAGCCAUUGUAAUUUGUUCAAGUUUUUUAUUUCCCUCUUCAUCAUUUGCCUC
env-45 UAAAGAAUUAAAUGUUUGUUGAAUAAUAAAAAGAACUAUCAUUACAAAUUAUCUAUUUCAUUCGUUAGAUUAUUCCAUAUCAAUCUUUCUAUUUAAUGAAAAUAUUUUUUCUGGCAAUUUUAAUUUGUUCAGGCUUUUUAUUCCCUUCUUCAUCAUUUGC
env-46 AAAAAUAGGUUUUCUCAUCAAAGAACAAGAGACAUCUUUCAAAUUAGUAAUUUCAUUAGUUAGAUUAUCCCUAAUUAAUUUCUUCUUCUGAUGAGAGUACUUUUUUUUGCAAUUUUAAUUUGUUCAAGCUUUUUAUUUCCUUCUUCAUCAUUUGCCUCAC
env-47 AAACAUAUAUGCUUUGUUCCAAAAUAAUUAGGUCGUCAUUACAAAUUAGUAAUUUAAUUCGUUAGAUUAUGAGUCGUUAAAUUCUUCUGUUAAUGCAAAUACUUUUUUUAGCAAUUUUAAUUUGUUCAAACUUUUUAUUCCCUUCUUCAUCAUUUGCCUC
env-48 AUAUAUGCUUUUUAUAAAAAUAAUUUGUGAUGUCAAUACAAAUUAGUAAUUUGAUUCGUUAGAUUAUAAGUCGCUUAAUUUCUCUGUUAAUGAAAAUACUUUUUUUAGCAAUUUUAAUUUGUUCAAGCUUUUUAUUUCCUUCUUCAUCAUUUGCCUCACA
env-49 ACAUAUAUGCUUUCUUCCAAAAUAAUUAGGUCGUCAUUACAAAUUAGUAAUUUAAUUCGUUAGAUUAUGAGUCGUUAAAUUCUUCUAUUAAUGCAAAUACUUUUUUUAGCAAUUUUAAUUUGUUCAAACUUUUUAUUCCCUUCUUCAUCAUUUGCCUCAC
env-50 CAUAUAUGCUUUCUUUCAAAAUAAUUAGGUCGUCAUUACAAAUUAGUAAUUUAAUUCGUUAGAUUAUGAUUUGUUAAAUUCUUCUGUUAAUGCAAAUACUUUUUUUAGCAAUUUUAAUUUGUUCAAGCUUUUUAUUCCCUUCUUCAUCAUUUGCCUCACA
env-51 AUAUAUGCUUUCUUCAAAAUAAUUAGGUCGUCAUUACAAAUUAGUAAUUUAAUUCGUUAGAUUAUGAUUCGUUAAAUUCUUCUGUUAAUGCAAAUACUUUUUUUAGCAAUUUUAAUUUGUUCAAGCUUUAUAUUCCCUUCUUCAUCAUUUGCCUCACAU
env-52 NNNNNNNNNNNNNNNNNNNNNNAUCAAGAGUUGGAGAAAGUGAUUUGGGCGUGAAUCAAAAAAGAGUUGAUUUACUAAAAUCGAAAAUGUUUUAAGAUAAAAAGAAAAAAAAAUAAUUUGUUUUUAUUGUUUUUUGUAUAACUUUUCCAUUUUAAAAAAA
env-53 ACAAAUAUGCUUUCUUCCAAAAUAAUUAGGUCGUCAUUACAAAUUAGUAACUUAAUUCGUUAGAUUAUGAGUCGUUAAAUUCUUCUGUUAAUGCAAAUACUUUUUUUAGUAAUUUUAAUUUGUUCAAGCUUUUUAUUCCCUUCUUCAUCAUUUGCCUCAC
env-54 ACAAAAAUGCUUUCUUCCAAAAUAAUUAGAUACGUCAUUACAAAUUAGUAAUUUAAUUAGUUAGAUUAUGAUUCGUUAAAUUCUUCUGUUAAUGCAAAUACUUUUUUUAACAAUUUUAAUUUGUACAAGCUUUUUAUUCCCUUCUUCAUCAUUUGCCUCA
env-55 AUAUAUGCUUUCUUCAAAAAUAAUUAUGUCGUCAUUACAAAUUAUGAAUUCAAUUCGUUAGAUUAUGAGUUGUUAAAUUCUUCUUUUAAUGCAAAUACUUUUUUUAGCAAUUUUAAUUUGUUCAAGCUUUUUAUUCCCUUCUUCAUCAUUCGCCUCACAU
env-56 ....
env-57 UACAAAUAUGCUUUCUUCCAAAAUAAUUAUUUCGUCAUUACAAAUUUGUAAUUUAAUUCGUUAGAUUAUGAGUCGUUUAAUUCUUCUUUUAAUGCAAAUACUUUUUUUAGCAAUUUUAAUUUGUUCAAGCUUUUUAUUUCCUUCUUCAUCAUUUGCCUCA
env-58 CAAAUAUGCUUUCUUCUAAAAUAAUUGGGUCUUCAUUACAAAUUAGUAAUUUAAUUCGUUAGAUUAUGAGUUGUUAAAUUUUUCUGUUAAUGCAAAUAAUUUUUUUAGCAAUUUUAAUUUGUUCAAGCUUUUUAUUCCCUUCUUCAUCAUUUGCCUCACA
env-59 AAACAUAUAUUCUGUCUUCCAAAAUAAUUAGGACGACAUUACAAAUUAAUAAAUAAAUUCGUUAGAUUAUGAGUGAUUGAAAUCUUCUGUUAAUGCAAAUACUUUUUUUAGCAAUUUUAAUUUGUUCAAGCUUUUUAUUCCCUUCAUCAUCAUUUGCCUC
Pma-3-1 AUUUAAAUAAAUAAAUGUUUUCUUGAAAAUAUUGAGAUAGGUCUUUACAAACUACUAAUUAGAUUCUUAGAUUAUUAUAGUGUGUAAAUUUUUUUGAUAAUGAAAAUACUUUUUUUUGCAAUUUUGAUUUGUUCAAGCUUCUUAUUCCCUUCUUCAUCAUUU
env-60 UUGAUAUAAAAGGUUCAGAUGUUUUUUAUUUAAUAAUGAAAAUAACCUUCAUUACAAAUUGGUAGCUGAAUUAGUUAGAUUAGAUCUUAUUAAAUUUUUAUGUUAAUGAAAAUACUUUUUCUAUCAAUUUUAAUUUGUUCAAGCUUUUUAUUCCCUUCAU
env-61 UCUAAAAUAAUUAGACACUUCAUUACAAAUUAGUAAUUUAAUUCGUUAGAUUAUGGUUCGUUAAAUUCUUCUGUUAAUGAAAAUAAUUUUUUUAGCAAUUUUAGUUUGUUCAAGCUUUUUAUUCCCUUCUUCAUCAUUUGCCUCACAUGUAGAACUAAAA
env-62 AACAUAUAUACUUUCUUCCAAAAUAAUUAGAUACGUCAUUACAAAUUAGUAAUUUAAUUAGUUAGAUUAUGAUUCGUUAAAUACUUCUGUUAAUGAAAAUACUUUUUUUAACAAUUUUAAUUUGUACAAGCUUUUUAUUCCCUUCUUCAUCAUUUGCCUC
env-63 AUAUAUGCUUUCUUCCAAAAUAAUUAAGUUAAGUCUUCAUUACAAAUUAGUAAUUUAAUUAGUUAGAUUAUGAGUUGUUAAAUUCUUCUGUUAAUGCAAAUACUUUUUUUAGCAAUUUUAAUUUGUUCAAGCUUUUUAUUCCCUUCUUCAUCAUUUGCCU
env-64 CUAUAUGCUUUCUUCCAAAAUAAUUAGGUCUUAUUACAAAUUAGUAAUUUAAUUCGUUAGAUUAUAAGUCGUUAAAUUCUUCUGUUAAUGCUAAUACGUUUUUUAACAAUUCUAAUAUGUUCAAGCUUUCUAUUCCCUUCUUCAUCAUUUGCCUCACAU
env-65 AACAUAAAUGCUUUCUUCCGAAAUUAUUAGCUCUUCAUUACAAAUUAGUAAUUUAAUUCGUUAGAUUAUGAGUCGUUGAAUUCAUCUGUUAAUGCAAAUACUUUUUUUAGCAAUUUUAAUUUGUUCAAGCUUUUUAUUCCCUUAUUCAUCAUUUGCAUCA
env-66 AACAAAUAUACUUUCUUCAAAAAUAAUUAGGUCUUCAUUACAAAUUAGUAAUUUAAUUCGUUAGAUUAUGAGUCGUUAAAUUCUUCUGUUAAUGCAAAUACUUUUUUUAACAAUUUUAAUUUGUUCAAGCUUUUUAUUCCCUUCUUCAUCAUUUGCCUCA
env-67 CAUAUAUGCUUUCUUCCAAAAUAAUUAGAUACGUCAUUACAAAUUAGUAAUUUAAUUCGUUAGAUUAUGGUCUUUAAAUAUUUCUGUUAAUGAAAAUACUUUUUUUAGCAAUUUUAAUUUGUUCAAGCUUUUUAUUCCCUUCUUCAUCAUUUGCCUCACA
env-68 ....
env-69 CACAUAUGCUUUCUUCAAAAAUAAUGAGAGAGAUCAUUUCAAAUUUGUUAUUUAAUUCGUUAGAUUAUAAGCCGUUAAAUUCUUCUGUUAAUGAAAAUACUUUUUUUAGCAAUUUUUAUUUGUUCAAGCUUCUUAUUCCCUUCUUCAUCUUUUGCCUCAC
env-70 AAUAUAUGCUUUCUUCCAAAAUAAUUAGGUCGUCAUUACAAAUUAGUAAUUUAAUUCGUUAUAUUAUGAGUCGUUAAAUUCUUCUGUUAAUGCAAAUACUUUUUUUAGCAAUUUUAAUUUGUUCAAGCUUUUUAUUCCCUUCUUCAUCAUUUGCCUCACA
env-71 AAAACAUAUAUGCUUUCUUCCAAAAUAAUUUAGGUCUUCAUUACAAAUUAGUAAAUUAAUUCGUUAGAUUAUGAGUCGAUAAAUUCUUCUGUCAAUGCAAAUACUUUUUUUAGCAAUUUUAAUUUGUUCAAGCUUUUUAUUCCCUUCUUCAUCAUAUGCC
env-72 AACAAAUAUGCUUUCUUCCAAAAUAAUUAGGUCGUCAUUACAAAUUAGUAAUUAAAUUAGUUAGAUUAUGGAUUGUUAAAUUCUUCUGUUAAUGCAAAUACUUUUAUUAACAAUUUUAAUUUGUUCAAGCUUUUUAUUCCCUUCUUCAUCAUUUGCCUCA
env-73 CAAAUAUGCUUUCUUACAAAAUAAUUAGGUCAUCAUUACAAAUUAGUAAUUUAAUUCGUUAGAUUAUUAGUUCUUAAAUUCUUUUGUUAAUGCAAAUACUUUUUUUAGCAAUUUUAAUUUGUUCAAGCUUUAUAUUCCCCUCUUCAUCAUUUNNNNNNN
env-74 ACAUAUAUGCUUUCUUCAAAAAUAAUUAGUUCGACAUUACAAAUUAGUAAUUUAAUUCGUUAGAUUAUGAAUCGUUAAAUUCUUCUGUUAAUGCAAAUACUUUUUUUAGCAAUUUUAAUUUGUUCAAGCUUUUUAUUCCCUUCUUCGUCAUUUGCCUCAC
env-75 ....
env-76 ACAUAUACGCUUUCUUCCAAAUUAAUUAGAUAGGUCAUUACAAAUUAGUAAUUUAAUUCGUUAGAUUAUGAUUCGUUAAAUUCUAUUGCUAAUGAAAAUACUUUUUUUAUCAAUUUUAAUUUGUUCAAGCUUUUUAUUCCCUUCUUCAUCAUUUGCCUCA
Pma-6-2 AAUAUAAUAAACAUAUAUGUUUUCUUCAAAAAUAAUGAGAAACGUCAUUACAAAUUAGUAAUUUGAUUAGUUAGAUUAUGGAUUGUUAAAUUCUUCUGUUAAUGAAAAUACUUUUUCUAGCAAUUUUAAUUUGUUCAAGCUUUUUUAUUCCCUUCUUCAUC
env-77 ACAUAUAUGCUUUCUUCCAAAAUAAUUAGAUACGUCAUUACAAAUUAGUAAUUUAAUUCGUUAGAUUAUGGUCGUUAAAUACAUCUGUUAAUGAAAAUACUUUUUUUAGCAAUUUUAAUUUGUUCAAGCUUUUUAUUCCCUUCUUCAUCAUAUGCCUCA
env-78 AAAACAUAUAUGCUUUCUUCCAAAAUAAUUAGAUAUGUCAUUACAAAUUAGUAAUUUAAUUCGUUAGAUUAUGGUUCGUUAAAUACUUCUGUUAAUGAAAAUACUUUUUUUAGCAAUUUUAAUUUGUUCAAGCUUUUUAUUCCCUUCUUCAUCAUUUGCC
env-79 ACAUAUAUGAUUUCUUCCAAAAUAAUUAGAUACGUCAUUACAAAUUAGUAAUUUAAUUCGUUAGAUUAUGGUUGGUUAAAUACUUCUGUUAAUGAAAAUACUUUUUUUAGCAAUUUUAAUUUGUUCAAGCUUUUUAUUCCCUUCUUCAUCAUUUGCCUCA
env-80 AACUUAUAUGCUUUCUUCCAAAAUAAUUAGAUACGUCAUUACAAAUUAGUAAUUUAAUUCGUUAGAUUAUGUUUCGUUAAAUUCUUCUGUUAAUGAAAAUACUUUUUUUAGCAAUUUUAAUUUGUUCAAGUUUUUUAUUCCCUUCUUCAUCAUUUGCCUC
env-81 ACAAAAAUGCUUUCUUCCAAAAUAAUUAGGUCGUCAUUACAAAUUAGUAAUUUAAUUCGUUAGAUUAUAAGCCGUUAAAUUCUUCUG...
env-82 ....
env-83 AAACAUAUAUGCUUUCUUCCAAUAUAAUUACAUACAGCAUUACAAAUUAGUAAUUUAAUUCGUUAGAUUAUGACUCGUUAAAAUCUUCUUUUAAUGAAAAUACUUUUUUUAGCAAUUUUAAUUUGUUCAAGCUUUUUAUUCCNNNNNNNNNNNNNNNNN
env-84 CAAAUAUGCUUUCUUCCAAAAUAAUUAGGUCGUCAUUACAAUUUAGUAAUUUAAUUCGUUAGAUUAUGAUUUGUUAAAUUCUUCUGUUAAUGCAAAUACUUUUUUUAACAAUUUUAAUUUGUUCAAGCUUUUUAUUCCCUUCUUCAUCAUUUGCCUCACA

....
....
••

env-85	UUCAUCUGUUGUAACGGCUGUAAGCUGUUGCAUGAUCGGUCCAUGGUUGUAUGGGCGGAUGUUGAAUGAUUACUACUCGAUGAGGUUUUCCUUCUGGUCCAUCGAAGGUUGUGUGUUGAUCUAACUCUCCAUUCUCCAGAUGUGUUACCCAUCCACCAUC
env-86	AAAUAUGAUUUCUUCCAAAAUGAUAAGUUAUGUCAUUCCAAAUAAGUAAUUUAUUUCGUUAGAUUAUAGGUUGUUAAAUUUUUCGGUUAAUGAAAAUACUUUUUUUAACAAUUUUAAUUUGUUCAAGCUUUUUAUUCCCUUCUACAUCAUUUGCCUCACA
env-87	ACAAAAAUGCUUUCUUCCAAAAUAAUUAGGUCGUCAUUACAAAUAAGUAAUUUAAUUAGUUAGAUUAUAAGUCGUUAAAUUCUUCUGUUAAUGCAAAUACUUUUUUUAACAAUUUUAAUUUGUACAAGCUUUUUAUUCCCUUCUUCAUCAUUUGCCUCAC
env-88	AACAUAUAUGCUUUCUUCAAAAAUAAUUAGAUAUGUCAUUACAAAUUAGUAAUUUAAUUCGUUAGAUUAUUAUUCGUUAAAUACUUCUGUUAAUGAAAAUACUUUUUUUAGCAAUUUUAAUUUGUACAAGCUUUUUAUUCCCUUCUUCAUCAUUUGCCUC
env-89	NNNNNNNNNNNNNNNNNNNNNNNNACAUUUAUGGAAAACACUCCAAGAACUGAGAUUGUAGAAAUUGAUGGCGAUUAUCUUCAUGCUGAGGCAACCAGUAAAUGGAUGAAGUAUGUAGACGACUUAGAAGUAUCCUUUCUACCGGAAUCAAACAUCUUAU
env-90	CAUAAAAUGCUUCCUUCAAAAAUUAUAGAGACUUCAUUUCAAAUUAGUAAUAUGAUUCGUUAGAUUAUGAGUUGUUAAUUUCUUAUGUUAAUGAAAAUACUUUUUUUAGCAAUUUUAAUUUGUUCAAGCUUUUUAUUCCCUUCUUCAUCAUUUGCCUUCAC
env-91	AUAUAUGCUUUCUACAAAAAUAAUGAGAGACAUCAUUACAAAAUAGUAAUUUGAUUCGUUAGAUUAUUGCUUGUGAAUUCCUCUGUUAAUGAAAAUCCUUUUUUUAGUAAUUUUAAUUUGUUCGAGCUUCUUAUUCCCUUCUUCAUCAUUUGCCUCACA
env-92	ACAUAUAUGCUUUCUUCCAAAAUAAUUAAGUCGUCAUUACAAAUUAGUAAUUUAAUUCGUUAGAUUAUGUGUGGUUAAAUUCUUCUGUUAAUGCAAAUACUUUUUUUAGCUAUUUUAAUUUGUUCAAGCUUUUUAUUACCUUCUUCAUCAUUUGCCUCAC
env-93	....
env-94	CAAAUAUGCUUUCUUCCAAAAUAAUUAGGUCGUCAUUACAAUUUAGUAAUUUAAUUCGUUAGAUUAUUAUUUGUUAAAUUCUUCUGUUAAUGCAAAUACUUUUUUUAACAAUUUUAAUUUGUUCAAACUUUUUAUUCCCUUCUUCAUCAUUUGCCUCACA
env-95	ACAAAUAUGCUUUCUUCCAAAAUAAUUAGGUCGUCAUUACAAAUUAGUAAUUUAAUUCGUUAGAUUAUGAAUCGUUAAAUCCUUCUGUUAAUGCAAAUACUUUUUUUAACAAUUUUAAUUUGUUCAAGCUUUU..........................
env-96	AACAUAUAUGCUUUCUUCCAAAAUAAUUAAGUCUUCAUUACAAAUUAGUAAUUUAAUUCGUUAGAUUAUGGAUUGUUAAAUUCGUCUGUUAAUGCGAGUACUUUUUUUAGCAAUUUUAAUUUGUUCAGGCUUUUUAUUCCCUUCUUCAUCAUU.......
env-97	AUAUAUGCUUUCUUCCAAAAUAAUUAGAUACGUCAUUAUAAAUUAGUAAUUUAAUUCGUUAGAUUAUGGUUCGUUAAAUACUUCUGUUAAUGAAAAUACUUUUUUUAGCAAUUUUAAUUUGUUCAAGCUUUUUAUUCCCUUCUUCAUCAUUUGCCUCACA
env-98	ACAUAUAUGCUAUCUUUCAAAAUAAUUAGUCGUCGUCAUUACAAGUUAGUAAUUUAAUUCGUUAGAUUAUGAGUCGUUAAAUUCUUCUGCUAAUGCAAAUACUUUUUUUA...
env-99	CAAAU...
env-100	ACAUAUAUGCUUUCUUCCAAAAUAAUUAGAUACGUCAUUACAAAUUUGUAAUUUAAUUAGUUAGAUUAUGAUUCGUUAAAUACUUCUGUUAAUGAAAAUACUUUUUUUUACAAUUUUAAUUUGUACAAGCUUUUUAUUCCCUUCUUCAUCAUUUGCCUCA
env-101	AAAAAAAUAUGCUUUCUUCCAAAAUAAUUAGGUCGUCAUUACAAAUUAGUAAUUUAAUUAGUUAGAUUAUGAGCUGUAAAUUCUUCUGUUAAUGCAAAUACUUUUAUUAACAAUUUUAAUUUGUUCAAGCUUUUUAUUCCCUUCUUCAUCAUUUGCCUCA
env-102	ACAAAUAUGCUUUCUUCCAAAAUAAUUAGGUUGUCAUUACAAUUUAGUAAUUUAAUUCGUUAGAUUAUGAUUUGUUAAAUUCUUCUGUUAAUGCAAAUACUUUUUUUAACAAUUUUAAUUUGUUCAAGCUUUUUAUUCCCUUCUUCAUCAUUUGCCUCAC
env-103	CAAAUAUGCUUUCUUCAAAAAUAAUUAGGUCGUCAUUACAAAUUAGUAAUUAAAUUCGUUAGAUUAUGAGUCGUUAAAUUCUUCUGUAAAUGCAAAUACUUUUUUUAACAAUUUUAAUUUGUUCAAGCUUUUUAUUCCCUUCUUCAUCAUUUGCCUCACA
Pma-1-1	CAUAUAUGCUUUCUUCCAAAAUAAUUAGAUAUGUCAUUACAAAUUAGUAAUUUAAUUCGUUAGAUUAUGGUUCGUUAAAUACUUCUGUUAAUGAAAAUACUUUUUUUAGCAAUUUUAAUUUGUUCAAGCUUUUUAUUCCCUUCUUCAUCAUUUGCCUCAC
env-104	AACAUAUGUGCUUUCUUUCAAAAUAAUUAGGUCUUCAUUACAAAUUAGUAAUUUAAUUCGUUAGAUUAUGAGUCGUUAAAUUCUUUUGUCAAUGAAAAUAUUUUUUGCAAUUUUAAUUUGUUCAAGCUUUUUAUUCCCUUCUUCUUCAUUUGCCUCACAU
env-105	AACAUAUAUGCUUUCUUCCAAAAUAAUUAGAUACGUCAUUACAAAUUAGUAAUUUAAUUAGUUAGAUUAUCAUUCGUUAAAUACUUCUGUUAAUGAAAAUACUUUUUUUAACAAUUUUAAUUUGUACAAGCUUUUUAUUCCCUUCUUCAUCAUUUGCCUC
env-106	AACAUAUAUACUUUCUUCCAAAAUAAUUAGAUACGUCAUUACAAAUUAGUAAUUUAAUUCGUUAGAUUAUGGUUCGUUAAAUACUUCUGUUAAUGAAAAUACUUUUUUUAGCAAUUUUAAUUUGUUCAAGCUUUUUAUUCCCUUCUUCAUCAUUUGCCUC
env-107	AAACAUA...
env-108	AACAUAUAUACUUUCUUCCAAAAUAAUUAUGUCGUCAUUACAAAUUAGCAAUUUAAUUCGUUAGAUUAUGAGUCGUUAAAUUCUUCUGUUAAUGCAAAUACUUUUUUUAGCAAUUUUAAUUUGUUCAAGCUUUUUAUUCCCUUCUUCAUCAUUUGCCUCA
env-109	ACAUAUAUGCUUUCUUCCAAAAUAAUUAGGUCGUCAUUACAAAUUAGUAAUUUAAUUCGUUAGAUUAUGAGCUGUUAAAUUCUUUGUUAAUGCAAAUACUUUUUUUAGCAGUUUUAAUUUGUUCAAGCUUUUUAUUCCCUUCUUCAUCAUUUGCCUCUCA
env-110	ACAUAUAUGCUUUCUUCCAAAAUAAUUAGGUCGUCAUUACAAAUUAGUAAUUUAAUUCGUUAGAUUAUGAUUCGUUAAAUUCUUCUGUUAAUGCAAAUACUUUUUUUAGCAAUUUUAAUUUGUUCAAGCUUUUUAUUCCCUCCUCUCAUCAUUUGCCUCAC
env-111	ACAUAUGCUUUCUUCCAAAAUAAUUAGGACGUCAUUACAAAUUAGUAAUUUAAUUCGUUAGAUUAUUAGUCGUUAAAUUCUUCUGUUAAUGCAAAUACUUUUUUUAACAAUUUUAAUUUGUUCAAGCUUUUUAUUCCCUUCUUCAUCAUUUGCCUCACAU
env-112	AACAUAUAUGCUUUCUUCCAAAAUAAUUAGAUAUGUCAUUACAAAUUAGUAAUUUAAUUCGUUAGAUUAUGGUUCGUUAAAUACUUCUGUUAAUGAAAAUACUUUUUUUAGCAAUUUUAAUUUUUUCAAGCUUUUUAUUCCCUUCUUCAUCAUUUGCCUC
env-113	CAUAUAUGCUUUCUUCCUAAAUAAUUAGAUACGUCAUUACAAAUUAGUAAUUUAAUUAGUUAGAUUAUGAUUCGUUAAAUACUUCUGUUAAUGAAAAUACUUUUUUUAACAAUUUUAAUUUGUACUAGCUUUUUAUUCCCUUCUUCAUCAUUUGCCUCAC
env-114	UAUAUGCUUUCUUCCAAAAUAAUUAGCUCGACAUUACAAUUUAGUAAUUUAAUUCGUUAAAUUAUAAGUCGUUAAAUUCUUCUGUUAAUGCAAAUACUUUUUUUAGCAAUUUUAAUUUGUUCAAGCUUUUUAUUCCCUUCUGCAUCAUUUGCCUCACAUA
env-115	ACAUAUAUGCUUUCUUCCAAAAUAAUUAGAUACGUCAUUACAAAUUAGUAAUUUAAUUAGUUAGAUUAUGAUUCGUUAAAUACUUCUGUUAAUGAAAAUACUUUUUUUAACAAUUUUAAUUUGUACAAGCUUUUUAUUCCCUUCUUCAUCAUUUGCCUCA
env-116	ACAUAUAUGCUUUCUUCCAAAAUAAUUAGGUCGUCAUUACAAAUUAGUAAUUA..
env-117	CUUCUAUGCUUUCUUCCAAGAUAGUUGGAUAAGCGUAGUACCAAAUUAGUAAUUUAAUUCGUUAGAUU..
env-118	AUAUAUGCUUUAUUCCAAAAUAAUAAGNNNNNNNNNNNNNNNNNNNNNNNNNNNNNNNNNNNNNNNNNNNNNNNNNNNNNCUUCAUCAUUUGCCUCACAUAUAGAACUAAAACCUUGUGUAGAGAUUGCUCAUUGUGUACGAGAAGAAUGGGAGGUAAACAA
env-119	UACAUAUGCUUUCUACCAAAAUAAUUAGUUCGUCAUUACAAAUUAGUAAUUUAGUUCGUUAUAUUAUGAGUCAUUAAAUUCUUCUGUUAAUGAAAAUACUUUUAUUGGCAAUUUUAAUUUGUUCAAGCUUUUUAUUCCCUUCUUCAUCAUUUGCCUCACA
env-120	....
env-121	....
env-122	AUUUGAUUGCUUUAAAUAUGAUUUCUAUAUUUUUGGGUCUUCCUUUUUCAAUUAUUUUUGACGUUGUAUUAAUAAAGCUUUUUGGCUUGUACUAUAUUUUAUUUUUUUCUCCUGCUUUAACAAUAAUAAGUGUAUUUCAAGUUCUUAUUUUGAGAAAAAU
Pma-10-1	AAGUAAUGAGAACGGUAUUCACGACAAUUAUUGAAAUAAAUUCACCAACAAAUCAAUUAAUUUAAGGAAUUAACUAAUUUACAAAUGACCGAAGAGAAUAAAACAGACAAAAAAAAGUGCAGUAGAAAAGGCAAAGCUAUGUUUGCUUAUGGCAUUAUUC
Pma-9-2	AAGUAAUGAGAACGGUAUUCACGACAAUAAUUGAAAUAAAUCAACCAAAAAAUCAAUUAGUUUAUGAGAUUAACUAAUUUACAAAUGUCCGAAGAGAAUAAAACAGCCAAAAAAAAGUGCGGUGGAAAAGGCAAAGCGAUGUUUGCUUAUGGCAUUAUUC
Pma-9-3	GCACGUUUGAUUCAUUCAAUAUAGUUUUAUCUUUUCAAUGAGAUAGGUAUCAUGCAUGAGUUCUAUGAAAGAACCAAAAAGAGAAGCAUUGCAAAGUGCUCAAUCAAGACGCAACCAAAAACAAUUAGCAACUGAGAAGUGGAACCCUACUGAUGAUCAC
Pma-10-2	CCCCACAAGUUGUUUUUGGCCUUUUGCUUUUAAGCAUUGCAGCGACUUUGAUUUUUAAUGUCAGAUACAACCCCUUCGUAAACAGUGAAGAUGAUAUCUAAUACAGCAAUACAUUCGCCUUAAAAAAAGAUACCUUUUGGGUCCUAUUGAUUGCGAUUAA
env-123	CCCCACAAGUUGUUUUUGGACUUUUGCUUUUAAGCAAUGCAGUGACUUUGAUUUUUAAUCUCAGAUACAACCCCUUCGGAAACAGUGAAGAUGAUAUCUAAUACAGCAAAGCAUUAGCCUUAAAAAAAAGAGACCUUUUGGGGUCUCUUGAUUGCGAUUA
env-124	GAUUAGUAAUAAAGAGAAUCAUUUGAAAGACUGGGUUUGAAGGUAAUGAAAUUUUUUUAAGAAGCAAAAGAGUCAUGCCCUGCGUCACAUUGAACUUCUCCUUCUUCUUACUAAUGUCUUUUCUCCCAAAAUUUAUACAUAUUGGAGAAUUGUUUGCUCC
Pma-9-4	GGAUUAGUAAUAAAGAGAAUUAUUUGAAAAACUAGGUUUGAAGGUAGUGAAAUUUUUUUAAGAAGCAAAAGAGUCAUACCCUGCGUCACAUUGAACUUCUCUUUCUUCUUACUCAUGUCUUUUCUCCUGAAAUUUAUACAUGUUGGAGAAUUGUUUGCUC
Pma-10-3	GAUUAGUAAUAAAGAGAAUCAUUUGAAAGACUAGGUUUGAAGGUAAUGAAAUUUUUUUAAGAAGCAAAAGAGUUAUGCCCUGCGUCACAUUGAACUUCUCCUUCUUCUUAAUCAUGUCUUUUCUCCCAAAAUUUAUACAUGUUGGAGAAUUGUUUGCUCC
Pma-10-4	GCACGUUUGAUUCAUUCAAUAUAGUUUUAUCUUUUCAAUGAGAUAGGUAUCAUGCAUAAGUUCUAUGAAAGAACCAAAAAGAGAAGCAUUGCCAAUAGUUACAGGUUGGUUAGUUGCUCCAACAAGAGAUUUCUGUUUGUUCUUUAUUCGUGAUCCCAAA
env-125	GGGUUAGUAAUAAAGAGAAUCAAUUGAAAGACUAAGUUUGAAGGUAAUGAAAUUUUUUUAAGAAGCAAAAGAGUUAUGCCCUGCGUCACAUUAAACUUCUCCUUCUUCUUACUCAUGUCUUUUCUCCCAAAAUUUAUACAUGUUGGAGAAUUGUUUGCUC
env-126	GAACGGUAUUCACGACAGCUUUAAAAAUAUUUCAACAAAAAAUCAAUUAAAGUAGAUAAUUAAUUUAUUUACAAAUGACCGAAGACAAUAAAAUAAAGAAAAAAAAGUGCGGCGGAAAAGAGCAAAGCGAUGUUUGCUUAUGGCAUCAUUCAACUAGGCUC
env-127	GGUUUUUUAUUAAUAUUUUUAAUUGGAUGUAAUUAGGAAUUAUAAGAUGUUAGCGAGAGCAUAGAUUAAGUGAGGGAUAUGUAUAUAGAACUACUCCUCUUUUGCUUCUUGGCUAAAAAAAAUAGUUAAUAUUUCUUAUUAAGUAUCUUAAUUAGUGAGAA
Ssp-3-1	GUCUCAGGUAUCAGCAACUACUGCAGCCUGAGUUAGUCCUGCGAAGAUCAAUUAACCGCUCGAAGGUUGUGACGCUUGAAGAUCAAUACAUCACUCUGAUGGACGCAGCUGGAUGGGUGUCUGACGAAUGGUGUCGGAAAAGUUUAUUCAGGAAGCAGA
Ssp-2-1	AUUGGCUGGAUUCAGUCAACCAGUCGUAGUUGUUGAUCACGGACCAAAAAAACGAGCGGUUUCUCAGGCCGCUCGCGUUCUCCCUUGUGUCAGCAGCUCGACGCCGCUGGGCAAAGCAUCUUGGAUCUCCCUGCUUUUGUUUGUAGCGAUACACACCCAU
Ssp-2-2	UGCAGAUCUUCCCCUCUGAUUCAUUGGUUGGAACGAUGUUCGUCAAAGACCGUAUGUGGGUUUGACAUCAACCAACCAUCUCGUGAGAAUGGUCGUAAUCGAUUCUUCCAUCCAUGUCCCAAGCACGCGAGAUGAUUAAUGCUCAUCUGUUUCCUGUUUU
Ssp-2-3	CGUUGGUCACGAUCAGCAGCUGCUACGACGGCGAUACAUGUCGAACGAGCACAGGCGAAAAGAUCAGGCUGGCUUGUAUCGACACCGCAGAACUAAGGGGUAAACGAGCCGACCCGAUUCCAGCUCGUGCGGCUAGGGAUCAUCUGCGUGGAAUGGUGGU
env-128	AUGUCAGCCUGUGGCGGCAGCAGCUUGAACUCCAGGCCACAUGGCUG...
Ssp-2-4	AUGCAUUGAAGUGAAGUGAAAUCCUUGGUCGGAAGUGAUCGCCAGCUCUGAAUAACCUUGAGAAGACUCACCAACAAACAUGCUCCACGCUGAACGCGUCGCCUUGCAAGGCAUCUUUGAGCUGGAUUGUGCAGAAGAGGUUUAUCUAGCUGUGAUGGAC
Pma-5-1	CAGGCAUUUUUGCAGAGGGAGUAAGAGAACUGUCUGGUUAGCACCAAUGACUCCCCAAGCAUCCAGAUCUAGACCUAAAGAAAGCCACUACAACCCCCUUGGGCUCGAGGGGACCUGGGCGGGAUUCCCGGAUGAGGUGGCUUAGGGGCCCUACUUUCUC
Pma-7-1	CAGGCCUUUUUGCAUAGGGAGUAAGAGAACUGUCUGGUUAGCACCAAUGACUCCCCAAGCAUCCGGAUCUAGACCAAUAAAGCCACUACAACCCCCUUGGGCUCGAGGGGACCUGGGCGGGAUUCCCGGAUGAGGUGGCUUAGGGGCCCUAUUUCUCG
Ssp-2-5	CACAAGCGGUUGAAAGGCUCACACCAGCGCCAUCAGCAACCUGAGGUUCAGUUGAAAACAGUAUGGCUGUGAUGACAAGGGGUAGGUCCUGGUGCUCGGAAAGUUGUGCCAAAAACAGUCCACGACUAGAGGUCUAUUGGCACACAGUUGGCACAUCACU
Ssp-2-6	GUCGGUAAACAAUGAAAAACCCCGCUCGUGGCGGGGCUUGAUUGUUUUUUGGGUAGUCUGAUGAUAUGGGACAUAGGCUGAAAGCUACACAACAGCCAAAAAGAGCAUCGGUAACUGCCUGCACUGAAUAUCACCGCUAAGAAAUUUAUUUACAGGGAAA
Ssp-2-7	UCAUGUGUUUUGCCUGAGCAGCAGAUCACUGCUAUUUCAAGAAUGAUGUAUCCCUUGGAUCGAUCAUGUGUGUCGCGACACAGGCAAAGGCUGGAAGAAGCUCGACGGUAGGGCAGGCGAUCAUUCUUUAAGGGAGAAGGAAUCACCUAUUAGUGGACG

....

....

••

```
Ssp-2-8    UCCGUCAUGCAUCUUGUUCCCAGGGAUUGUCUUGAUUCACGCUUGGUCGAAGAGCCAGGCUGAUUGUUGACUAACAACCAGGGACUUGCGCAGCUUCAUCAAGUUGUUUCGAUGGAAAGGACAGUGCUGCUGACUCCAGCGGGACAACACACGUUUCUUG
Ssp-10-1   CGGCUUUGGUCGAUGAAGUUGGCGGAGGACAAACCUCCGCCGGGGCUGAUCACUUGAUGGUGACCUUGCCGCCCACUUCUUCAAUCGACUUCUUGAGAGCCUCGGCAUCGCCUUUGGCGAUGCCUUCCUUGACGGCCUUGGGGGCUGCCUCGACCAGGGC
Ssp-2-9    GGUUCAGAGAACCGGCAGAGAGGAUUGAAUGGGUGAGAGACUGUUUUUUAGAAGCAGGGGCUCCAACAGAUCACUUGAUUGUGACCUUGCCACCCACUUCUUCGAUGGCCUUUUUCAAGGCUUCAGCGUCGUCUUUGGAGACGCCUUCCUUGAUGGCCUU
Pma-7-2    GACCGGUGAAGAGUUAAAAAAACGGAUGCAUAGCAACCAAGCUGAAGGCCGCUAUCCGCUGGCAAAUCACUUGAGGCUGACCUUACCGCCAACCUCUUCGAUGGCCUUCUUGAGAGCCUCAGCAUCAUUCUUAGCAAUGCCUUCCUUGAUGGUCUUAGGA
Pma-5-2    GACCGGUUAAGAGUUAAAAAAACGGAUGCAUAGCAACCAAACUGAAGGCUGCUAUCCGCUGGCAAUCACUUGAGGCUGACCUUACCGCCAACCUCUUCGAUGGCCUUUUUGAGAGCCUCAGCAUCAUUCUUAGCAAUGCCUUCCUUGAUGGUCUUAGGAG
env-129    CAAAAAACCGACCCGAAGGUCGGUCGAGAAGCAGGAAAAGGAUCGGCAGCUUGCGCUUGCCGAUCCAGCGAAAUCACUUGAGGGUGACCUUGCCGCCCACCUCUUCAAUGGCCUUUUUGAGGGCUUCGGCGUCGUCCUUGGAGACACCUUCCUUGAUGGU
Ssp-11-1   AAUUGAAAUGGGGGGAAGGGUCGCGAUCCCGCAGGACACCGACCCAACACGAUCACUUGAGGGUGACCUUGCCGCCCACUUCUUCGAUGGCCUUCUUGAGGGCUUCGGCUUCGUCCUUGGACACGCCUUCCUUGAUGGCCUUGGGUGCAGCCUCGACCAU
env-130    AAAAAACCGACCCGCAGGUCGGUGAUGAAGCAGAGGAGGAACCGACAGCCGGAGGCUGACGGUUCUGCCGGAUCACUUGAGAGUGACCUUGCCGCCUGCCUCUUCGAUGGCCUUCUUGAGGGCCUCGGCAUCGUCCUUGGAGACGCCUUCCUUGAUGGCU
Ssp-12-1   UGAUGGAUGGUUGGCAUCCCGAAAGACGCCAACCCGAAGAGAUCACUUGAGAGUGACCUUUCCGCCCACCUCUUCGAUGGCCUUUUUGAGGGCCUCGGCUUCGUCCUUGGACACGCCUUCUUUGAUGGCCUUGGGUGCUGCCUCGACCAUGGCCUUGGCA
env-131    GCCGGCCUUGUCUGGUUUGAAGGGGAAUCGAGCUGGCAAACCAGCCAGCAGAAAUUACUUGAGGGUGACUUUGCCGCCUGCUUCUUCGAUCUCCUUCUUGAGGGCUUCGGCAUCGUCCUUGGAGAUGCCUUCCUUGACGGGCUUGGGAGCAGCCUCCAC
env-132    GGGUCCGAAGGGAGCGGGGCGAUCACUUGAGGGUGACUUUGCCGCCGGCUUCUUCGAUGGCCUUCUUCAUCUCUUCGGCAGCGGCCUUGGCCACGCCUUCCUUGACAGGGGUCGGAGCCUUUUCGACGAGGCCUUUGGCAUCGGCCAGGCCGAGACCAGU
Ssp-7-1    GUGGUCCGAGAGGACCGGGGCGAUCACUUGAGGGUGACCUUGCCGCCGGCUUCUUCGAUGGCCUUCUUCAUAUCUUCGGCAGCGGCCUUGGCCACGCCUUCCUUGACAGGGGUCGGAGCCUUUUCGACGAGGCCUUGGCAUCGGCCAGGCCGAGACCAG
Ssp-4-1    GGAGAGUCGCUUUUGUUGAAAAAAAUGAGAUGCGGAUCGGCGAAAAGCCGAUCCCACAGGAUCACUUGAUUGUGACCUUGCCACCCACUUCUUCGAUGGCCUUCUUGAGGGCUUCGGCAUCGUCCUUGGACGCACCUUCUUUGACCAGCUUUGGAGCGGC
env-133    GAAAGCAGGGACCGAUACCGGGCAUCGAGCCGGCUGAAAGAAUCACUUGAGCGUGACCUUUCCACCAACUUCUUCGAUUUCCUUUUUGAGAGCUUCGGCUUCGUCCUUGGAGAUGCCUUCUUUGAUGGGCUUGGGAGCAGCCUCCACAAGCGCUUUGGCA
env-134    ................................................................................................................................................................
env-135    GGGGACCGGCUUGAUUUUGUAGAAGACGUGAAUCGGGCCGGUGAGACCAGCCCGGACAGGAUCAUUUGAGGGUGACCUUGCCGCCCACUUCUUCGAUCUCCUUCUUGAGAGCUUCGGCUUCGUCCUUGGAGAUGCCUUCCUUGAUGGGCUUGGGAGCAGC
env-136    GACCGGCUUGGUUUUGUGGAAGAGGUGAAUCGGGCCGGUGAGACCAGCCCAAAAAGGAUCACUUGAGGGUGACCUUGCCGCCCACUUCUUCGAUCUCUUUCUUGAGAGCUUCGGCUUCGUCCUUGGAGAUGCCUUCCUUGAUGGGCUUGGGAGCAGCUUC
Ssp-13-2   GGACCGGCUUGGUUUUGUGGAAGAGGUGAAUCGGGCCGGUGAGACCAGCCCAAAAAGGAUCACUUGAGGGUGACCUUGCCGCCCACUUCUUCGAUCUCUUUCUUGAGAGCUUCGGCUUCGUCCUUGGAGAUGCCUUCCUUGAUGGGCUUGGGAGCAGCUU
env-137    AAAGGACCGGUGACUGAACGCAAACAGGGGGGAGAGGGGCAAGCCCUCUCCCAAGGCGAUCACUUGAGGGUGACCUUGCCGCCUGCUUCUUCGAUGGCCUUCUUGGCAGCUUCGGCAUCUUCCUUGGAGACACCUUCCUUGACGGCCUUGGGAGCAGCCU
Ssp-3-2    CAACAGAACCGGCAUCUGAAAGAGAACAGGGGGAGAGGGACAAGCCCUCUCCCAAGGCGAUCACUUGAGGGUGACCUUGCCGCCUGCUUCUUCGAUGGCCUUCUUGGCAGCCUCGGCGUCUUCCUUGGAGACACCUUCCUUGAAGGCCUUGGGAGCAGCC
env-138    ................................................................................................................................................................
env-139    AAGGACCGGUGACUGAACGAAACAGGGACGGGAGAGGCAUGCCCUCUCCCACCGCGAUCACUUGAGGGUGACUUUGCCGCCUGCUUCUUCGAUCUCUUUCUUGAGAGCUUCGGCGUCGGCUUUGAGAUGCCUUCCUUGACGGGCUUGGGGGCAGCUUCC
Ssp-10-2   GGAGGGUUGCUUUUGUUGAAAAGAAUGAGAUGCGGAUCGGCGGAAAGCCGAUCCCACAGGAUCACUUGAUCACUUGACCGUGACCUUGCCACCCACUUCUUCGAUGGCCUUCUUGAGGGCCUCGGCAUCGUCCUUGGACACACCUUCUUUGACCACCUUGGGAGCGGC
Ssp-1-1    GGAGGGUUGCUUUUGUUGAAAAGAAUGAGAUGCGGAUCGGCGGAAAGCCGAUCCCACAGGAUCACUUGACCGUGACCUUGCCACCCACUUCUUCGAUGGCCUUCUUGAGGGCCUCGGCAUCGUCCUUGGACACACCUUCUUUGACCACCUUGGGAGCGGC
env-140    AAAGGACCGGUUACUGAACAACACAGGGGGAGAGGGGCAAGCCCUCUCCCAAGGCGAUCACUUGAGGGUGACCUUGCCGCCUGCUUCUUCGAUGGCCUUCUUGGCAGCCUCGGCGUCUUCCUUGGAGACACCUUCCUUGACGGCCUUGGGAGCAGCUUCG
env-141    AAGGACCGGCGACUUAACGCAACAGGG.....................................................................................................................................
env-142    GAAAGGACCGGUUGCUGAACAAAACAGGGGGAGAGGGCAAGCCCUCUCCCAAGGCGAUCACUUGAGGGUGACUUUGCCGCCUGCUUCUUCGAUGGCCUUCUUGGCAGCCUCGGCGUCUUCCUUGGAGACACCUUCCUUGACGGCCUUGGGAGCAGCCUC
env-143    AANNNNNNNNNNNNNNNNNNNNNNNNNNNNNNNNNNNNNNNNNNNNNNNNNNCCAUGAGCUGCUCCUUGGAGGGGAGAUCGGCAAUGGCUUUAAUCUCGUCCUGAGACAGGAGCUUGCCUUCGAAAAGGCCGCCCUUGGUCUCGGACUUCUUGAGUUCC
env-144    ACCGGUAACUGAA...................................................................................................................................................
env-145    AGACCGGCUUUGUUGGUCUUGAAAAAGUUGGUCGGCCGGCAAAACCAGCCCGAUGGGAUCACUUGAGAGUGACCUUGCCGCCCACUUCUUCGAUCUCUUUCUUGAGAGCUUCGGCUUCGUCUUUGGAAAUGCCUUCCUUGACGGGCUUGGGAGCAGCCU
env-146    CCGAAGAGAUCGGCUGAAGGUUGAUGGAGGAAGGAUCGGCAACGGCCGAUCCAUGCCGAUCACUUGAGGGUGACUUUGCCGCCGGCCUCUUCGAUCUCUUUCUUGAGAGCUUCGGCAUCGUCCUUGGAGAUGCCUUCCUUGACGGGCUUGGGGGCAGCU
Ssp-9-1    ACCCAAAAAGGCCGGCCCGUGUGGGCCGGCCCUGUGCGCAUAACAGCAGGGGCCUCAGCCCCAGGCGAUCACUUGAGGGUGACCUUGCCGCCGACCUCUUCAAUGGCCUUCUUGAGGGCUUCAGCCUCAUCCUUCGACACACCUUCCUUGAUGGCCUUGG
env-147    AUCAAAAAAGCCGACCCUGAGGGCCGGCCCUGUGAACAAAACAGAAGGGGCUUUAGCCCCAGACGAUCACUUGAGGGUGACCUUGCCGCCGGCCUCUUCGAUGGCCUUCUUGAGGGCUUCAGCCUCAUCCUUCGACACACCUUCCUUGAUGGCCUUGGG
env-148    AUCAAAAAAGCCGACCCUGAGGGCCGGCCCUGUGAACAAAACAGAAGGGGCUUUAGCCCCAGACGAUCACUUGAGGGUGACCUUGCCGCCGGCCUCUUCGAUGGCCUUCUUGAGGGCUUCAGCCUCAUCCUUCGACACACCUUCCUUGAUGGCCUUGGG
env-149    AUCAAAAAAGCCGACCCUGAGGGCCGGCCCUUUGCGUACAACAGAAGGGGCCUCAGCCCCAGACGAUCACUUGAGGGUGACCUUGCCGCCGGCCUCUUCGAUGGCCUUCUUGAGGGCUUCAGCCUCAUCCUUCGACACACCUUCCUUGAUGGCCUUGGGA
env-150    AGAAAAAACCGACCCGAAGGUCGGUAUCGAUUGAAGGGAAAGGAUCGGCAACCGGAAGCCGCCGAUCCAACAAAAUCACUUGAGCGUGACCUUGCCGCCCACCUCUUCAAUGGCCUUCUUGAGGGCUUCGGCGUCGUCCUUGGAGACACCUUCUUUGAUG
env-151    GGGUUGACGGUCAUCGGCAGAAAGCCGCGGUGAUCACUUGAUGGUGACCUUGCCGCCCACUUCUUCGAUGGCCUUUUUGAGCUCUUCGGCAGCCUCUUUGGCAAUGCCUUCCUUGAUGGGGGUGGGAGCUUUUUCCACCAGACCCUUGGCGUCGGCCAGG
Ssp-8-2    GCAAAAAAAAGAGGCCCCUUCCGGCAGCCUCUUGGGGGUGUGAGGAGAAAAGCCCUGCUCGGGCCCUUCCGCCCACCUUCAUAGGGCCCCACCCGGGAUCAAGGGAAGCACUGAAAUACGACUCAAUACGACUGGGGGCUGACAGAGCUCAGCGGCCGA
env-152    GCAAUGCAUCUCUCCAGCUGGGCUCGAUGAACGCUGGGUGAGGAUCAGCUUUCGUCUACGCCAAGAAGUCAUUCCUUUUUCAGGAAGUCUUGAUAGGUUCAACGCCUUCCAACUCUGUCGCGAGUUGUUCCAGCAGCGCACGACGCGCAAACAUCAUCAC
env-153    CGAGUUCAACUUCGAGCAGACCGUCAGCAUUCGAACUGACGUGAAGCUGAGCAGGGACGACGUGAAGCAGAUCGUCUCAGAAGCUCCCGCAGAAACCCUGGAGGAGAUUGUCUACCAAAGCCUGGUGACCGAUGAGCUGUAUCUCAGGGCGAGGGCCAAG
env-154    CUUCGAGCCUCAAAACGCCUCGCAAGCACUACUACUAGUGCUUCAAGCCAUAAAAAAACCCGCUGUGACGCGUGCACAUCGGGCCAGGUGAUGGGGGAACUGACAGUAUGGCCCAGCUCUUAGCCACUGCCUAGCGCCACAUCUGGUGUCUUAAGAGUCGGU
env-155    ................................................................................................................................................................
env-156    CUUUGGGUCGCGACAGCCGCUCAGAGCACCAUCCCUAGCGCUUCAGGGCACAAAAAAACCCGGUGUGACUCGCACAACGGGCUGGGUGAUGGGGGAAACCUAAAUAUGGCUCAGCUACUUCGUGCUGACUAGCCCCACAUAUGGUGUUAAGAGGCUUGAG
env-157    CUGGGUCGCCACAGCCGCUCAGAGCACUAUCCCUAGUACUUCAGGGCACAAAAAAACCCGGUGUGACUCGCACAACGGGCUGGGUGAUGGGGGAAUCCUAAAUAUGGCUUAGCCGCUGAGUACUGACCAGCCCCACAUAUGGUGUCACCUACUACUGGUC
env-158    UGGGGCUCGCAAAAGCCGCUCAUGGCACCAUCCCUAGUGCUUCAAGCCACAAAAAAACCCGGUGUGACUCGCACAACGGGCUGGGUGAUGGGGGAAACCUAAAUAUGGCUUAGCCGCCAAGUACUGACCAGCCCCACAUAUGGUGUCACCCAUUACUGGU
Ssp-13-3   CUGGGGCUCGCAAAAGCCGCUCAUGGCACCAUCCCUAGUGCUUUAGGGCACAAAAAAACCCGGUGUGACUCGCACAACGGGCUGGGUGAUGGGGGAAACCUAAAUAUGGCUUAGCCGCCAAGUACUGACCAGCCCCACAUAUGGUGCCACCUAUUACUGG
env-159    UGGGGCUCGCAAAAGCCGCUCAUGGCACCAUCCCUAGUGCUUCAAGCUAUAAAAAAACCCGGUGUGACUCGCACAACGGGCUGGGUGAUGGGGGAAACCUAAAUAUGGCUUAGCCGCCAAGUACUGACCAGCCCCACAUAUGGUGCCACCUAUUACUGGU
Ssp-3-3    UUUGGAUCGCAACAGCUGCUCGGAGCACUACCCCUAGCGCUUCAGAGCACAAAAAAACCCGUGUGACUCGCACAACGGGCCGGGUGAUGGGGGAAACCUAAAUAUGGCCCAGCUACUACGUGCUGACCAGCCCCACAUAUGGUGUCACCCAAGAGUCUCA
env-160    AUGGAAGGCUCAAUGACCGAACAGGAGACAUUGGUCGAACAGGUUUUCGCAAAAUAUCGUUCCUACUGCAAAGACAUCGGCAUUACACCAGCCGAGAUGAUGCAACAGGCCUAUCUCAGCCACCUAAAAAGCCUCACAACAGAACAGCUCAAAGCAAAGC
env-161    GAUGGAACGCUCAAUGUCCGACAAGGAAACUCUGGUUGAGCAAGUCUUCGCCAAAUACCGCUCAUACUGCAAAAACAUCGGCAUCACACCAGCAGAGAUGAUGCAACAGGCUUACCUCAGCCACCUCCAGAGCCUGAGCAUUGAACAACUCAAAGCCAAG
env-162    UGGAAGGAUCAAUGACCGAAAAGGAGACAUUGGUCGAACAGGUUUUCGCAAAAUAUCGUUCCUACUGCAAAGACAUCGGCAUUACACCAGCCGAGAUGAUGCAACAGGCCUAUCUCAGCCACCUUAAAAAGCCUCACGACAGAACAGCUCAAAGCAAAGCU
Ssp-4-2    CUGACCGUGUAGCCCUGGCGCAACUAACGCCCACUUGGUUCUUUCUUUUGGAGUGGUCAGCCCGUGGCCUUUUCCUUCUGAAAUCAAACGAGCGUGUUUUAUAUAUCUUUCGAAUGGAUUUAAAUUAGCUAUUACAGAUCUUCAUAAACGUGAAUGUCGG
env-163    ................................................................................................................................................................
env-164    CUAACCACGUAACAGCGAUUAAGAGAUAAAGCACGGCCCUUAGUGAUGGAAUUAGAAAAAAGGCAGGGUCAUCAUUUCCGCCUCGAGCUGUCGCAUCCUUCAUCAAUACGACUUAUAAACCGUAGGAACAGAGAUUUGGGCGGCAUGAGUUCAUGGCUG
env-165    CCUCGAGCCUCGAAAUGCCUCAAAAGCGCUACUGCUAGUGCUUCAAGGCAUAAAAAAACCCUCUGUGACGUGCACAUCGGGCCAGGUGAUGGGGAACUGACAAUAUGGCUCAGCUCUUAACCACUGCCUAGCCCCACAUUUGGUGUCUUAAGAGUCGGUA
env-166    GCCUCGAGCCUCGAAAUGCCUCAAAAGCGCUACUGCUAGUGCUUCAAGGCAUAAAAAAACCCGCUGUGACGCGCACAUCGGGCCAGGUGAUGGGGGAACUGACAAUAUGGCCCAGCUCUUAGCCACUGCCUAGCCCCACAUUUGGUGUCUUAAGAGUCGG
env-167    UUCGAGCUUCAAAACGCCUCAAAAGCACUACUGCUAGUGCUUUGAGGCAUAAAAAAACCCGCUGUGACGCGCACAUCGGGCCAGGUGAUGGGGAACUGACAAUAUGGCCCAGCUCUUAGCCACUGCCUAGCCCCACAUUUGGUGUCUUUAGAGUCGGUA
env-168    AGCAGUUUUCAGCGGUGGCUUGAGUCGGCGGUGCCGAAAGCUUGAGCGGAAUGGUCUCCGAACAAUGCAUCUUGGUACUAGCGCACUAGUACAGAGUCAUGGGUUAAAGGGCUUCAUCCAAGAUCCGGCGACCAAGGAAACCAAGCGAUUCCACCGUGA
env-169    CUCGAGCCUCGAAAUGCCUCAAAAGCGCUACUGAUAGUGCUUCAAGGCAUAAAAAAACCCGCUGUGACGCGCACAUCGGGCCGGGUGAUGGGGGAACUGACAAUAUGGCCCAGCUCUUAGCCACUGCCUAGCCCCACAUGUGUGUGUCUUAAGAGUCGGUA
env-170    UAGCUACUGAGGCGAGCUAGCCCCAGUGGGGAAACCAGGCUGGAAGAAGCCCUGCACGAGCAAGGCCAAGUACGACUGCCAGUGUUGCCAGCUUCACUCCCGUCAGGAUUCCUGCCGUCUUCCAUGUGAACAGCGAUUCGCCAGGGGAU..........
env-171    UUCGAGCUUCAAAACGCCUCAAAAGCACUACUGCUAGUGCUUCAAGGCAUAAAAAAACCC....................................................................................................
env-172    UUCAAGCUUCAAAAUGCCUCAAAAGCACUACCACUAGUGCUUCAAGGCAUAAAAAACCCGGUGUGACGCGCACAUCGGGCCAGGUGAUGGGGGAACUGACAAUAUGGCCCAGCCCUUAGCCACUGCCUAGCCCCACAUUUGG.................

           ................................................................................................................................................................
           ................................................................................................................................................................
           ••••••••••••••••••••••••••••••••••••••••••••••••••••••••••••••••••••••••••••••••••••••••••••••••••••••••••••••••••••••••••••••••••••••••••••••••••••••••••••••••
```

```
env-173   GAGUUCAACUUCGAGCAGACCGUCAGCAUUCGAACUGACGUGAAGCUGAGCAAGGACGACGUGAAGCAGAUCGUCUCAGAAGCUCCCGCAGAAACCCUGGAGGAGAUCGUUUACCAAAGCCUGGUGACCGAUGAGCUGUAUCUGAGGGCGAGGGCCAAAG
env-174   GAGUUCAACUUCGAGCAGACCGUCAGCAUUCGAACUGACGUGAAGCUGAGCAAGGACGACGUNNNNNNNNNNNNNNNNNNNNNNNNNNNNNNNNNNNNNNNNNNNNNNNNNGAAACUCCCGCAGAAACCCUUGAGGAGAUCGUUUACCAAAGCCUGGU
Ssp-3-4   GAGUUCAACUUCGAGCAGACCGUCAGCAUCCGAACUGACGUGAAGCUGAGCAAGGACGACGUGAAGCAGAUCGUCUCGGAAGCCCCAGCAGAAACGCUGGAGGAGAUCGUCUACCAAAGCCUGGUCACCGAUGAGUUGUACCUGAGGGCGAGGGCAAAGG
Ssp-4-3   ACACGGUGACGGUGAGCGCUCAACAAUCCUGCUCAAUGGUGCUGAUCUGUUGAUGCAGGCAUCCAUCAGCUCCUCCCUACAAAUUGCACUAGCGGCCGUUCUUGCUUUGGCGACGGCCCAGGCUCUUCAGCUACCGGAUGCGUACUGGGCGCCAAUAUCC
Ssp-1-2   UCGGUCAUGAUGAGCAGGGCUUCGAUCAGCUCCUCCCUACAAAUUGCGAUCGCAGCCGUUCUUGCUUUAGCGACGGCCCAAGCGCUGAAGCUCCCAGAUGCGUACUGGGCGCCAAUAUCAGCCAUUGUUUGUUCUCUAGAGGCCUUUGAUCGUGCCUUUG
env-175   CACGGUGAUGGUGAGCGCUCAACAAUCUUGCUCAAUCGUGCUGAUCCGUUGAUGCAGGCAUCAAUCAGCUCCUCCCUACAAAUUGCACUAUCAGCCGUUCUUGCUUUGGCGACGGCCCAGGCUCUUCAGCUGCCAGAUGCGUACUGGGCACCAAUAUCCG
env-176   ......................................................................................................................................................
Ssp-3-5   AGUUCGACCAAUCGAUCUCCAUGAGCGCCGGUUUUCCAGAAGUGACCGAACAAGAUCCUUCGCUGCCUGAGCAGCUUGGUGAUCGAAAGGAGCGGCUCGCGAAGUUCGUGGUGAAGUUGUGGGCACAACAUCGCCACGUUGUAGAGACUCGGCGCCGGAU
env-177   CGGAGCUCCCAAAGAGGAAGGCCGCCCUUCCCGGUGGAAAGACGGCCUAGCUCGCUCGUUUGUGCAUUGCAAUCGACCUGUUCAGUGAAGCUGUAUCCGCGAAGCGUGAAGUGCCCUUCGAACGACAGUGAACUGAGAGAAGAGGUCGAAGCGUCUGGCU
env-178   UUCGAGCCUCAAAAAGCCUAGCAAGCACUACUGCUAGUGCUUCAAGCCAUAAAAAAACCCGCUGUGACGUGCACAUCGGGCCAGGUGAUGGGGGAAUUGACAAUAUGGCCCAGCUCCUAGCCACUGCCUAGCCCCACAUUUGGUGUCUUAAGAGUCGGUA
Ssp-13-4  UCAGCUUGUAGCAACAGCUACAAAACGUUCACCCCUCUUUCGGGGGGCGCAGACACUCCAAGCCACGGAACGGGGACUUGGAAUUAUCUGAGGAACUAUCCGAUGACUGCUCUUCUUUACAGAGGCCGUCCUUACACGACUCCACAAGCCUCACCCAAAU
env-179   AGGCCGCCCUUCCCGAUGGAAAGACGGCCUAGCUCGCUCGUUGGUUCAUAUUCCAACCCUGGAUAGGGAAACAGGAUUUUGUUGAGAGAGAGGCCAGCCCAUCAGUGCAGGAAUGGGAGUGACCUCUCAAUACUGUUCGCUUCCGACGCCCCCACUGAUC
Csp-1-1   ACACAACAAAGCCGGCCCCAAGGGGCCGGCUUUGUGCUCGGAAGCGGGGGGCCGGACAUGCCGGCCCCGGCUGCGAUCACUUGAUCGAAACCUUGCCGCCGGCUUCCUCGAUGGCCUUCUUGAGGGCCUCGGCAUCGGCCUUGGCGAUACCCUCCUUCAC
Tel-1-2   CCGGUCACCGUACCCGUCCCACAAGUGCCAAAGUUCGCCAAGCGAUUUUCAAUAUCUGGCAGGGUCGCCUACAGGGAUGUUGCUGGCUAGAUUUAUGUGCUGGUACCGGUGCAAUGGGAGCGGAAGCCCUACUGCGCGGUGCCCAGUGGGUAGUGGGUAU
Ssp-14-3  CACUGUCCUUUGGUUUCCCCGUCGGUAGGGAUGAUUUCAGCGGGCCGCCAAGAUUAUCAAAAGUUUUUUAUGUGUCGUCUUUUAGGGUAUCUGGGUCAACCACUGCGACCAGAGCAACUAAUUUAUCGCCCGGAACAUUCCCUGAUUGUCCAGAGCUAUC
Nsp-1-2   GAGUUAGUUUGCUGUUACUCGCAAAUUUGGCCUGUUGUAAGUCGCAAGCUUCGACAUUAACCGUAAACUUAUCGUCUUCAAAAGGCCAAGGUUUGACUGUGACUAAAUUAUCGCUACGUUGCAUGAUGUCAUAGCGUUGACCGUCGGGGCCUUUACUAAU
Ava-1-2   GAGUUAGUUUGGCGUUACUGGCAAAUUGAACCUUUUGUAAGUCGCAAGCUUCGACAUUGACCGUAAACUUAUCGUCUUCAAAGGGCCAGGGUUUAACUGUUACCAAAUUAUCGCUGCGUUGCAUGAUGUCAUAGCGUUGACCAUCAGGCCCUUGACUAAU
Nsp-1-3   ACAAGUGAUAUCCUCUAGUUGUGUGAGGAGCAAGUUGAAACUAAAAAGCAUCUGGGGUGGAAACACGGCAACACUCCCAGAUGCUUUUUAAUUAUUGGCAAAUUGUUACUUUUUAACGAAAGUCCACUCUAGCACCUUGAUUGGGGCGGUUUGUAGUGCU
Nsp-1-4   UGCAGAUAGCAAACUGUCACAGGAUAGAUUUACUAUUUAUGAUUGCCAGUGUUAUUGUUUAUUUGUGUGUGAGGAGCAAGUUAAAAAUAAAAAGCGUCUGGGGUGGAAACACGGCAACACUCCCAGGCGCUUUUUAAUUGAGGCUACACUGAGAGUUUUC
Ava-1-3   CAAGUGAUAUCUUCUAGUUGUGUGUGAGGAGCAAGUUAAAAAUAAAAAGCAUCUGGGGUGGAAACACGGCAACACUCCCAGGUGCUUUUUAGUUAUUGGCAAAUUGUUAAUUUUUAACGAAAGUCCACUCUAGCACCUUGAUGGGAGCGGUUUGUAGUGC
Npu-1-1   AGUGAUAUGUUUUAGUUGUGUGUGAGGAGCAAGUUAAGAAUAAAAAAUGCCUGGGGUGGAAACACGGCAACACUCCCGGGCAUUUUUAAUUUGCAUAUAAAAAUUGGAUUGUUGGUACACUUUUAAAAGUGUCACUCACUUUAAUCGUCUUUUCAUUAA
Nsp-1-5   CUUGGCGAAAUUACGAAUUACUUUGAAUUAGGUUGGCUAUGGCGGUUGCCAAUUCUGAUGGCUCUACGGGUUUGGUUAAAUGCCUUUGGAAACCAGCAGACAUAAUCUUUUGAAAGUUGGUUUCGCCAGCAAAUGCGGUGAGGGCGAUCGCUGGUAGUUU
Mae-1-1   UCAUCUCUUUAAAUGGUCGAUCGUUAGCCCAACCCUACACGGCUAUUUUCGCGGUCGCGUUUACGGAGUGGAAAAUGUCCCCCGGCGUCAUCCUCUGAUUAUCGUCUGCAAUCACGCCAGUUACUUCGAUCCACCCCUAUUAUCCUGUGCCGUGCGUCGU
Csp-2-1   GCAAAGGAGUUAGAGCAUAUGCAAAGAAAUAAUGGGUGUUUUAGGCAACGAUUUUAUUAACCUUUGAGCUUAUCCCGAAUUGACGUUAUUUUUCUUCAUUAAUCAUCAAGAGUCACAAUUAAAUCCAUUGCCUGAUCAAUGAUUACUGGUUUAUCUACUA
Cwa-1-1   GAAUUAGAAAACGGUUACGUUGAACUUAUUCACAAUACUCAAUAUAGAGUUAUUUUAGGUAAUCAAAAACCCGUUCGUUGUGAUGCUUACCUAGAAAUUGACGGUAAACACCUUGGGACUUGGCGACUUCACCCUUAUUAUUCUAUCACCUUAGAAAGAC
Csp-2-2   UUCCCGCCUCAUCGAAAACCCUUUGGUCAAUUGGAAUUUUGGCUGUUUUAAUGUUAGGGGUAUUAUGUCUUUGUUUAUUGAUCGCUUAUUCAUCCCAACAAGUUAAAUUUGAAAUUGAUCAGCAACAACUGAUGAUUAAAGGAGAUUUAUACGGUCGCAA
Csp-3-1   UACUGAACUUAGGAUGUAAAAUCAACUAGCAAAAUGCUAAUUUAAAAGGCUUAUAAUGCGAGAAUUAUCAAAAUUUAUAAAGCCAUAAGCUUUUCUUUUUUUUGAAUAUUUUCUAAAUAAAAAAGAUUCCUAUCACUUGAGUGAAGUGAUAAUUUUUAAC
Lsp-1-1   UGGGGUAGAGGUUGAGGAGAUUCUGUAAUUGCCGGACAGUAACCCGUAUAACUGCAUCGGUCACAGUGAUGUCCAGGAAUCGGUUCCCACUCACAAUCUGUUCUUAAUUGUGUUGCCAGUUCUCCAAUCGUUAAUUGGGCUUGUUGUUUGUGUUGGGGAG
Ssp-6-1   AGGGGAUGGGGGACUUUUCCCAACACCCUGGCCUCUUGCCCCAAUUGGGAAAAAGCCUGUCCCCCCAAACUGGCUACGACCCUUUGAAUUCAGGCUGGGACAUAGCACCUGCCACAACUUUUUUUGCUCGAAAAGGUUCCAAAGAGUCUCCCCUGAGCCU
Ssp-5-1   UCUAACUCCCCUCUCCCAAAAAGAGAGGGGCUUGGGGUGGUCGCGGAAGCGGCGCAGAGCGCUUGGGGAGUGUCUCAAUCUAGUUUGAAAUGGCUAUAAGAUGGCCAUAAUUGCUCGACGUUGGUCAUGCCCGGGGAUCAAGCUGCCUUUGCCGUGAUGU
Mae-1-2   UCCCUUCUAGGUCUGUUUUUGCUUACCAAGAGCAACAGGUCAAAAUUUUUGCCGAAGAAACUGCACAAGAGAAGUGUAAUUGCUCGUAAAAUUGGGAAUUAAGUACAAGGGCAAUCCUAUGGCAGAUAACAAUAACGGUAACGGUGGCUUAUUCGUCGUC
Csp-5-1   ACUUUAGCAAAAAUUUAGAAUUAAUGGCCCUUGGUUGUCGUUAAAAAUUCGGAUAAGAAUGUAGCAGACAAUACAGUAAAAACCCAGAGUUGAGGAGAAGAUAAAGUGGGGAAACUUAGCUAACCUGGAAGUGCAGACAGGAGAAACUGGCAAGCUCAUU
Csp-4-1   GGAAAGUAUACCUCCAAAUUUUGACUCCUAUCAUCACAAGGAAGGCAAGCUCAACUAUGAAAUUGAUGUUACAACCAAUUUUCCAAAAAGCCAAAGAUCAAGCCUGCCUCACUACACAACAGACAGAGGUUUUUCCAGUCCUGUCUUCCUUGUUAGCCCC
Csp-5-2   AAACCCUAAAACUCUUCCCCCUACACCCCGCUACAAAGAACAAAUUAAAUGCGUGACAGCUUAUGUGUGGACGAUUUACUUUAACUCACUCAGCCGCUACUAUUGGUGAUAAUUUUAAUUUACCUUUAGCGCUUGAACUAAAUCCUCGCUAUAAUCUCGC
Cwa-1-2   GACAAUUAAGUUAACACUUUACACUGGCCUUGCCAACGUAAUAAAUGAUCGCAUAAUACCAAGGCAACCAUCGCUUCUACCAUAGGAACAGCCCUAGAUAAAACACAAGGAUCGUGUCUUCCUUUGGCGGCUAAAGUUGUCUCUUCGCUCUCAGAAGUAA
Csp-7-1   UAUAUUUAGAUAAUCAGGUUUGAAGGAAUUUUGGAGGCCAUAGGGUCAGGUGUCGAUUAAUUUUCAGGAAAUUAUUGCGAAAUUACAUCAGUUUUGGAGCGAUCGCGGUUGCUUAAUUGCUCAACCUUACGAUACGGAAAAAGGGGCAGGAACCAUGAGU
Csp-6-1   UAUAUUUAGAUAAUCAGGUUUGAAGGAAUUUUGGAGGCCAUAGGGUCAGGUGUCGAUUAAUUUUCAGGAAAUUAUUGCGAAAUUACAUCAGUUUUGGAGCGAUCGCGGUUGCUUAAUUGCUCAACCUUACGAUACGGAAAAAGGGGCAGGAACCAUGAGC
Csp-3-2   GCUUGUAUCAUCAAAACCUGAUUCUCUUUUUCACGAGUUAGAACAUCAAUUAUUAAAUUAGUUUUCCGACGCUUUCUAGACUGUGAAUAGUCUCUGCUUGUAAAAACCGUCUUCCCCUUCAAUGAAUUAAACUGUUUCAUUAUUUGUUGAAGCUGUGUGA
Ssp-12-2  ACCACAUGCGGUGCACCGAACAAGACAAGCCACCGAAAGUGCUUUUCAUCCAUUCAGCCAGCCUCUGUCAACACUAGAUCUGGGGGUUGCGCAAACACUCACACCAGGCCUAUUCAUGAGCGCAAGGGUGGCGCUGAAGAAACUUCUCUUUAGCGUUGAU
Ssp-11-2  ACACCACAUGUGGGGCAUUCAAGAAACCAAGCGCCAGAGCAACUGCAAAUUUAUUCAUUGCCCCCUUGUCCACACCAGAUCUGGGGCUUGCGCAAAUCCUCACGCCAGGCCUAUUGAUAACUGCAAGAUUGGCACUGGAGACACUUCUCUCUAGCGUUCA
Ssp-2-10  UCUAUCUAUUCAGCUCGAGGGCGCUGCCGCAAAAAUGAUGUAUCAAUGCAAAUAUCUUGCGAUGGUACAUGCUCAUUUGGAGCCUUUGUCUGGGCCAUAGGCGAGCAAAACCAAAACCAUCACAACGUGCACGCCCAAGAUCCGCACUACCCCGUUUUCA
          ......................................................................................................................................................
          ......................................................................................................................................................
          ●●●●●●●●●●●●●●●●●●●●●●●●●●●●●●●●●●●●●●●●●●●●●●●●●●●●●●●●●●●●●●●●●●●●●●●●●●●●●●●●●●●●●●●●●●●●●●●●●●●●●●●●●●●●●●●●●●●●●●●●●●●●●●●●●●●●●●●●●●●●●●●
```

alignment positions 420 · · · 490

```
Ssp-14-1  AUCCUCGAACUUACUGCUUUAUGUUGGUACUUUCCGUCCUAUCUUUGGCCUUAGCCUCGUUUUCUGCCAUC
Sel-1-1   UAGCCUCACGAGUGCCUAUGCUCCUUUCAGCGAUCGCGCCAGCUUCAAUCAGUGUCUGGAAUCUGCAAGCA
Ama-1-1   CGAUUGGAUUCCUAAUAGGCAAUGAACGACCUUCCUGAUUUUGUCAAAAAGGCCUUUUACCUUGGAGUUGG
Ter-1-1   UUACCCUGAUAACUUCUUUUAGCAAACCGCCACCCAGGUUCAAGAAUAAUCUUAGUAAAUCCAUUUGCCAA
Tel-1-1   UUUACGCAAAGAAGGCCCCAGCUUUGAUCUCCCCAUUAGUAUUGGCAUCUUGGCCGCUUCAGGACAGGUGG
Ava-1-1   GAUCGCCUGUACUCCUGUGGGGGCGUUCCCCAUAGCGAUCCCAAUACCGGCGUAUUCCAGCAUUUCCAGAU
Nsp-1-1   ACUCCUGCGGGAGCGUUUCCCAUAGCGAUACCAAUACCAGCGUAUUCCAGCAUUUCCAAAUCAUUGAAGUU
Pma-9-1   UCUCUUUGAUGUCCCUCCGACGAUGCCCUCGUCGGGACUUGAACCCGAGACCUCUCCCUUACCAAGGGAGU
Pma-2-1   ACAUAGAAAUAAAGGCCAACUGCAACAAGCAGCAGCAUAUUUAAUGCUAGUACAAGAGCUCCUAGGAUAAU
Pma-11-1  UGGAAUAUAAAAAUACGCUGCUACUGCCAAAACAAGCAGUAGAUUCAACCCUAAAACAAUUGAACCUAUAA
Pma-11-2  CAAAUAUUUAUACUUGCUUUAGACAAAGCAAGUCCUGAUUUCCAUAAUUUUAUUUUACAUUGUUUGAUAAG
Pma-11-3  UUUUGCUGUCACCCUGUUAAGAUGGACAAUUAAUUAAUUUUGUUUUGACACAAGUCAUAGUUGGGGAAAAC
env-1     AAGACGUGGAUCAACGGCCUGAUGCGGCGCACCGGGAUGGGCCUGUACGGGCUGAUGUGGCUGUCCUUCGC
Ssp-13-1  UCAUUCGUGGAGCCAGUCACGGCAGAAUCAGAGGGUUGCCGUUGUCGCCCGAUGAAAGAGGUCAGCCUUCU

          .......................................................................
          .......................................................................
          ●●●●●●●●●●●●●●●●●●●●●●●●●●●●●●●●●●●●●●●●●●●●●●●●●○○○○○○○○○○○○○○○○○○○○○○
```

Ssp-14-2	UGGGUCGUUUAAUCCUGCCGACGGUGGAAGAAACUAAAGCCAGGACAGGAGAUUGGUUUGGUUGGAACUG
Ssp-8-1	AACGAUCCGGCAAUCACUGGCGUCGCAGCGGGCCAGCCUUGACGCUCCAGCUGCUCCAACACCCGAAUGAC
env-2	UUUUUCAUUUUUAAUUUCAUCAGUAUCCUUUUCUUGGCUUUCUGAUGAAUUUGUUUUAUAUUCAAUUUGCC
env-3	....
Pma-6-1	UGUCCAAAAUCACUUUUUUUACAGUGAAAAUCAGUGGUUUCUCUAAGUCAGUACACCCAAAACUUAUGACA
Pma-8-1	UAGAUGUUGCGUUCUUGAUAAAAAGCAUAAAAUAAAACCUCCAUCUUUACCCUUUACUCUAAAUAUUGCGG
env-4	....
env-5	CUCCUUACCAAUAGUAUUUUUAAUUUGAAGUGAAAUAUUUUCAAAAGCAAAACAUUUUAAAUCAUCAAUAU
env-6	AGAAAAACAAACCAUUUUAAUUACAGGCGCAGGUCGAGGAUUGGGUUCAGAAAUAGCAAAAUCAUUUCAUA
env-7	GUCUGUUGUAAGUGAAACAUUUAACGUUUGUUCUUUUUUACUUUUUAUAUUUGUUAUGCAAGCUCUUACAG
Pma-12-1	AAUUCUGCUUCUAGAAAGUCAAGUUCAGUACUAGACAAUAAUUCUGUGGCUAUAUAAUCUUUUAUUAAUUC
env-8	....
env-9	ACAUCUUUUCUUCGAUUAAUAACUUUUAAAAUUAAAUAUCUUAUUGAGCAUGAAUUUUAAAAGCAAUAUAA
env-10	AGCCAGUUGGAAUUUCUAACCCUGCUUUUUCUGCAGUUGAGAACCAAUCAUCUCCUUCAGAAACAAAUGUC
env-11	....
env-12	....
Pma-12-2	UAUUUAUGCCAGCUCUUAGUUCUACUAUGACUGAAGGCAAAAUUGUUGAAUGGUUAAAGAAUCCUGGAGAU
env-13	CGACCAAUCGGUUAACAGCCGAUCGCUCUACCGCUGAGCUACCGAGGAAUAUUAAAAACUUAACUCUUAUA
env-14	AAAUCAGUAAUUUUUAAAAUAUUUUUUACCUAUUUAGGGGUUUAUUUUUUUGCUCUAAUA...........
env-15	AGGCUUAGUUGCAGAAAAUCAAAAUUUAUCAGAAUUUGAAAUUACUGAAAAUUUUUCUUGUAUAAGAUUCU
env-16	AGUUCAGAGAUAGAAAACUACUAAACAUAUAGUAUUAAAAAAAAGAUUGUUAUUUUAUUACUUUAUAUUUU
env-17	UUAAGAUUUACUAAACAUAUUUUAUUAAAGAAAAACAAAAAGAUGCUAUCUUAAACUUUAUAUUUUAAUGA
Pma-12-3	GCAAAAUAAGUCAUAUUUAAUUAUUAGCUAUCAAAAAAUAUGUAAACGGUACAUGCUAUACAAAAUUAAAG
env-18	GCAAAAUAAGUCAUAUUUAAUUAUUAGCUAUCAAAAAAUAUGUAAACGGUACAUGCUAUACAAAUUUAAAG
env-19	AAUUUUAAAUCAGUAU...
env-20	GUGGUAAGUGAAACAUUUAAUAUUUGUUCUUUUUUUCUUUUAAUGUUUGUUACGCAAGCUCUUACAGUUUC
Pma-12-4	UCGAUAGCUUUUUCUGUUUCUUCUUUAGGUUUCAUGAUUUAAUAAACGGUGAAAGUAGUAAGAGUUUUAUU
env-21	UUAAAUCAGUAAUUUUAAAAAUAUUUUUUACCUAUUUAGUUGUUUAUUUUUUUGCUUUAAUAUUUAAGUAU
env-22	AUUAAACUUUAUUGGGGUAUUUGCAGAAAAAGUAAGAGAAGGUUCAUCGGGAUA.................
env-23	GGUCUAAAUUUUAAAUCAUUAAUUUUUAAAAUAUUUUUUUUCUAUUUAGUGGUUUAUUUUUUUGCUUUAAU
env-24	AAAGUAUGCUCUUCAUGAAUUAGUGCUCAUUAAUCUUCAAUAAUACUUUUGAUCUUGAUUUUUAAAUCUGA
env-25	ACGCACUUUCAUUUAAGAAAAAGUUAAUAGAUGAGAAAGAUUUUGAUUUUGGAUUGUCAUGGGUAUCCACU
env-26	NNNNNNNNNNNNNNNNNNNNNCUUCGUCAAAAAUCAAAAAAAAGAUUAUUCUAAUAAACACAUUUUUUGG
env-27	UUUAAAUCAGUAAUCUUUAAAAUAUUGUUUACCUAUUUAGGGGUUUAUUUUUUUGCUCUAAUAUUUAAGUA
env-28	UUAAUUUUAAAUCAGUAUUUUUUUAAAUAUUUUUUACCUAUUUAGGGGUUUAUUUUUUGCUCUAAUUUUC
env-29	UUUUAAAUCAGUAAUUUUAAAAAUAUUUUUUACCUAUUUAGGGGUUUAUUUUUUUGCUCUAAUAUUUAAGU
env-30	....
env-31	AAUUAUUAAGAAAAAGAUAUUAUUUUAAUAAUUUACAUUUCUACAAGUGUUACAUUAAAAGUAAUUUUUAA
env-32	AAUUUUAAAUCAGUAAUUUUUAAAAUAUUUUUUACCUAUUUAGGGGUUUAUUUUCUUGCUCUAAUAUUUAA
env-33	AAUUACACUUCGNNNNNNNNNNNNNNNNNNNNNNNNNNNNNNNNNNNNNNNNNNNNNNNNNNGCAUGAAA
env-34	AAUUUUAAAUCAGUAAUUUUUAAAAUAUUUUUUACCUAUUUAGGGGUUUAUUUUCUUGCUCUAAUAUUUAA
env-35	CUUUCCGGAGGUUAUCCAGAUGAUAAAGACUUUUUAGAGUACAUCAUUUAUACAGGAUUUGGUGGGCAAGA
env-36	....
env-37	....
env-38	AUAUCGCAGCUGCGAAAUUUAAUUGCUGAAACUUUAGAUCGACUGACAAUAAAUAUCUUAACGGUUCAAGU
Pma-4-1	GGAUUCGAACCUGCGACCAAUCGAUUAACAGUCGAUCGCUCUACCGCUGAGCUACCGAGGAAUAUAGAAUG
Pma-8-2	UUUCCCAUAGUUCUCCCUCUAGAAAGUCUAGUUCAAUAUUAGAGAGUAGUUCUGUGGCUACAUAAUCUCUU
env-39	AUAUAGAACUAAAACCUUGUAUAGAGAUUGCUCAUUGUGUACGAGAGGAAUGGGUGGUAAACAAUAUUGAG
env-40	CACGUAGAACUAAAACCCUGCGUACAGAUUGCUCAUUGUGUUCGAGAAGAAUGGGAGGUAAUGAUAUUGAG
env-41	AUAUAGAACUAAAACCUUGUGUAGAGAUUGCUCAUUGUGUAAGAGAGGAAUGGGAGGUUAAGAAUAUUGAG
env-42	NNNNNNNNNNNNNNAAUUAUCAUCAUGUCUUGAUAAUGGCAAAUUUAUUAGAUUUUCUAUAAAAAGAUGAU
env-43	UAAAACCUUGUGUAGAGAUCGCUCAUUGUGUACGAGAAGAAUGGGAGGUUAACAAUAUUGAGAAACCUUUU
Pma-4-2	CACAUGUAGAACUAAAACCUUGUGUAGAGAUUGCUCAUUGUGUACGAGAAGAAUGGAAGGUUAACAAUAUU
env-44	ACAUGUAGAACUAAAACCAUGUGUAGAGAUUGCUCAUUGUGUCCGGGAGGAAUGGGAUGUUAACAAUAUUG
env-45	CUCACAUGUAGAAUUAAAACCUUGUGUAGAAAUUGCUCAUUGCGUUCGUCAGGAAUGGGAGGUUAUGAAUA
env-46	AUGUAGAACUAAAACCUUGUGUAGAGAUUGCUCAUUGUGUACGAGAGGAAUGGGAGGUUAAGAAUAUUGAG
env-47	ACAUAUAGAACUAAAACCUUGUGUUGAGAUUGCUCAUUGUGUACGAGAAGAAUGGGUGGUAAACAAUAUUG
env-48	UGUAGAACUAAAACCUUGUGUAGAGAUUGCUCAUUGUGUACGAGAAGAAUGGGAGGUUAAGAAUAUUGAGG
env-49	AUAUAGAACUAAAACCUUGUGUAGAGAUUGCGCAUUGUGUACGAGAAGAAUGGCAGGUAAACAAAAUUGAG
env-50	UGUAGAACUAAAACCUUGUGUAGAGAUUGCUCAUUGUGUACGAGAAGAAUGGGAGGUUAACAAUAUUGAAA
env-51	GUAGAACUAAAACCUUGUGUAGAGAUUGCUCAUUGUGUACGAGAAGAAUGGGAGGUUAACAAUAUUGAAAA
env-52	AUUAGCAGAUAAAAAAGUGAUAAUUGUCAUCAUGCCUUGAUAUUGGCAAAUUUAUUAGAUUUUCUCAAAAA
	....
	....
	●●●○○○○○○○○○○○○○○○○○○○○○○

env-53	AUAUAGAACUAGAACCUUGUGUAGAAAUUGCUCAUUGUGUAAGAGAAGAAUGGGAGGUAAAAAAUAUUGAG
env-54	CAUAUAGAACUAAAACCUUGUGUAGAGAUUGCUCAUUGUGUACGAGAAGAAUGGGAGGUUAAUAAUA....
env-55	AUAGAACUAAAACCUUGUGUAGAGAUUGCUCAUUGUGUACGAGAAGAAUGGGAGGUAAACAAUAUUGAGAA
env-56	....
env-57	CAUGUAGAACUAAAACCUUGUGUAGAGAUUGCUCAUUGUGUACGAGAAGAAUGGAGGGUAAAUAAUAUUGA
env-58	UAUAGAACUAAAAC..
env-59	ACAUAUAGAACUAAAACCUUGUGUAGAGAUUGCUCAUUGUGUACGAGAAGAAUGGGAGGUAAACAAUAUUG
Pma-3-1	GCCUCACAUGUAGAACUAAAACCUUGUGUAGAGAUUGCACAUUGUGUAAGAGAGGAAUGGGAGGUUAAUAA
env-60	CAUCAUUUGCCUCUCAUGUAGAACUAAAACCUUGUAUUGAGAUUGCUCAUUGUGUACUAGAAGAAUGGGAG
env-61	CCAUGUGUAGAGAUUGCUCAUUGUGUACGAGAAGAAUGGGAGGUUAACAAUA..................
env-62	ACAUAUAGAACUAAAACCUUGUGUAGAGAUUGCUCAUUGUGUACGAGAAGAAUGGGAGGUUAAUAAUAUUG
env-63	CACAUAUAGAACUAAAACCUUGUGUAGAGAUUGCUCAUUGUGUACGAGAAGAAUGGGAGGUAAACAAUAUU
env-64	AUAGAACUAAAACCUUGUGUAGAGAUUGCUCAUUGUGUACGGGAAGAUUGGGAGGUAAAGGAUAUUGAGAA
env-65	CAUAUAGAACUAAAACCUUGUGUAGAGAUUUCUCAUUGUGUACGAGAAGAAUGGGAAGUGAAUGAUAUUGA
env-66	CAUAUAGAACUAAAACCUUGUGUAGAGAUUGCUCAUUGUGUACGAGAAGAAUGGGAAGUUAAUAAUAUUGA
env-67	UGUAGAACUAAAACCUUGUGUAGAGAUCGCUCAUUGUGUACGAGAAGAAUGGGAGGUUAACAAUAUGGAGA
env-68	....
env-69	AUGUAGAACUAGAACCUUGUGUAGAGAUUGCUCAUUGUGUACGAGAAGAAUGGGAUGUUAAGAAUAUUGAG
env-70	UAAAGAACUAAAACCUUGUGUAGAGAUUGCUCAUUGUGUACGAGAAGAAUGGGAGGUAAACAAUAUUGAUA
env-71	UCACAUAUAGAACUAAAACCUUGUGUAGAGAUUGCUCAUUGUGUACGAGAGGAAUGGGAGGUAAACGAUAU
env-72	CAUAUAGAACUAAAACCUUGUGUAGAGAUUGCUCAUUGUGUACGAGAAGAAUGGGAGGUCAAUAAUAUUGA
env-73	NNNNNNNNNNNNNNNNNNNNNNNNNNNNNNNNNNNNNNNNUGGUGCACUGUUGGAAUAAAUGUAAAUU
env-74	AUAUAGAACUAAAACCUUGCGUAGAGAUUGCUCAUUGUGUACUAGAAGAAUGGGAGGUAAACAAUAUUGAG
env-75	....
env-76	CAUGUAGAGCUAAAACCUUGUGUAGAGAUUGCUCAUUGUGUACGAGAAGAAUGGGAGGUUAACAAUAUUGA
Pma-6-2	AUUUGCCUCACAUAUAGAAUUAAUACCUUGUAUUGAAGUUGCUCAUUGUGUACGAGAAGAAUGGGAGGUUA
env-77	CAUGUAGAACUAAAACCUUGUGUAGAGAUCGCUCAUUGUGUACGAGAAGAAUGGGAGGUUAACAAUAUUGA
env-78	UCACAUGUAGAACUAAAACCUUGUGUAGAGAUCGCUCAUUGUGUACGAGAAGAAUGGGAGGUUAACAAUAU
env-79	CAUGUAAAACUAAAACCUUGUGUAGAGAUCGCUCAUUGUGUACGAGAAGAAUGGGAGGUUAACAAUAUUGA
env-80	ACAUGUAGAACUAAAACCUUGUGUAGAGAUUGCUCAUUGUGUACGAGAAGAAUGGGAGGUUAGCAAUAUUG
env-81	....
env-82	....
env-83	NNNNNNNNNNNNNNNNNNNNNNNNNNNNNNNNNNNAAUUGUUUUAAGAAAAAAUUAAAAAAAAUACUUUUUU
env-84	UAUUGAACUAAAACCUUGUGUAGAGAUUGCUCAUUGUGUACGAGAAGAAUGGGAGGUUAAUAAUAUUGAGA
env-85	UUCUUUCCAUUGUACUUUGAUCGUCAUCUUAUAUGUUCUAGCCCAUUGUGGUGGCGUAUUGACAUCUGUUU
env-86	UAUAGAACUAAAACCUUGUGUAGAGAUUGCUCAUUGUGUACGAGAAGAUUGNNNNNNNNNNNNNNNNNNN
env-87	AUAUUGAACUAAAACCUUGUGUAGAGAUUGCUCAUUGUGUACGGGAAGAAUGGCAGGUUAAUAAUAUUGAG
env-88	ACAUGUAGAACUAAAACCUUGUGUAGAGAUCGCUCAUUGUGUACGAGAAGAAUGGGAGGUUAACAAUAUUG
env-89	CAAUAAGAUCAGAAUCAAGAGUUGGAGAAAGUGAUUUGGGAGUAAAUCAAAAAAGAGUUGAUCUACUAAAA
env-90	AUAUAGAACUAAAACCUUGUGUAGAGAUUGCGCAUUGUGUACGAGAAGAAUGGGAGGUUAACAAUAUUGAG
env-91	UGUAGAACUUAAACCUUGUAUAGAGAUUGCUCAUUGUGUACGAGAGGAAUGGGAUGUUAGCAAUAUUGAAA
env-92	AUAUAGAACUAAAACCUUGUGUAGAGAUUGCUCAUUGUGUACGAGAAGAAUGGGAUGUAAACAAUAUUGAG
env-93	....
env-94	UAUAGAACUAAAACCUU...
env-95	....
env-96	....
env-97	UGUAGAACUAAAACCUUGUGUAGAGAUCGCUCAUUGUGUACGAGAAGAAUGGGAGGUUAACAAUAUUGAGA
env-98	....
env-99	....
env-100	CAUAUAGAACUAAAACCUUGUGUAGAGAUUGCUCAUUGUGUACGAGAAGAAUGGGAGGUUAAUAAUAUUGA
env-101	CAUAUAGAACUAAAACCUUGUGUAGAGAUUGCUCAUUGUGUACGAGAAGAAUGGGAGGUUAAUAAUAUUGA
env-102	AUAUAGAACUAAAACCUUGUGUAGAGAUUGCUCAUUGUGUACGAGAAGAAUGGGAGGUUAAUAAUAUUGAG
env-103	UAUAGAACUAAAACCUUGUGUAGAGAUUGCUCAUUGUGUAAGAGAAGAAUGGGAGGUUAAUAAUAUUGAGA
Pma-1-1	AUGUAGAACUAAAACCUUGUGUAGAGAUCGCUCAUUGUGUACGAGAAGAAUGGGAGGUUAACAAUAUUGAG
env-104	GUAGAACUAAAAC...
env-105	ACAUAUAGAACUAAANNNNNNNNNNNNNNNNNNNNNNNNNNNNNNNNNNNNNNNNNNNNNNNNNNNUAUCU
env-106	ACAUGUAGAACUAAAACCUUGUGUAGAGAUAGCUCAUUGUGUACGAGAAGAAUGGGAGGUUAACAAUAUUG
env-107	....
env-108	CAUAUAGAACUAAAACCUUGUGUAGAGAUUGCUCAUUGUGUACGAGAAGAAUGGGAGGUAAACAAUAUUGA
env-109	UAUAGAACUAAAACCUUGUGUAGAGAUUGCUCAUUGUGUACGGGAAGAAUGGGAGGUAAAUAAUAUUGAGA
env-110	AUAUAGAACUAAAACCUUGUGUAGAGAUUGCUCAUUGUGUACGAGAAGAAUGGGAGGUAAACAAUAUUGAG
env-111	AUAGAACUAAAACCUUGUG...
	....
	....
	●●●○○○○○○○○○○○○○○○○○○○○○

```
env-112    ACAUGUAGAACUAAAACCUUGUGUAGAGAUCGCUCAUUGUGUACGAGAAGAAUGGGAGGUUAACAAUAUUG
env-113    AUAUAGAACUAAAACCUUGUGUAGAGAUUGCUCAUUGUGUACGAGAAGAAUGGGAAGUUAAUAAUAUUGAG
env-114    UAGAACUAAAACCUUGUGUAGAGAUUGCUCAUUGUGUACGAGAAGAAUG.......................
env-115    CAUAUAGAACUAAAACCUUGUGUAGAGAUUGCUCAUUGUGUACGAGAAGAAUGGGAGGUUAAUAAUAUUGA
env-116    .......................................................................
env-117    .......................................................................
env-118    UAUUGAAAAACCUUUUGAAGAGAUUAAAACAUUUAUCGAAAACACUCCAAGAACUGAGAUUGUAGAAAUUG
env-119    UAUAGAACUAAAACCUUGUGUAGAGAUUGCUCAUUGUGUAAGAGAAGAAUGGGAGGUAAAUAAUAUUGAGA
env-120    .......................................................................
env-121    .......................................................................
env-122    GGAAUUGAAAUUUUCAAUAAAUAUCUUCUUUUUGGAAAGAAAAAAAAUAAAUAAUCUGUAUAAAUUUUUUG
Pma-10-1   AGCUAGGCUCUAGUGUAGUUUCUGCUCUCGCUCUUACUGCAAUCGCUCUAAGUUUUUGCUCACUUAAACAA
Pma-9-2    AGCUAGGCUCUAGUGUCGUUUCUGCUCUUGCUCUUACUGCAAUCGCUCUUAGUUUUUGCUCCCUCAAACAA
Pma-9-3    GCGAUUAACAUUAUUACUGAUUAUUUAAUUUGUGUUAGAGAAGAUAUGAACUCAAUGAUUGAAGAGCUGGG
Pma-10-2   AUCUCUUUGAUGUCCCUCCGACGAUGCCCUCGUCGGGACCACCACUCACAACACCUGAUAGUGCCUGACAA
env-123    AAUCUCUUUGAUGUCCCUCCGACGAUGCCCUCGUCGGGAUUUAGGUCGAUAGGCAACGCAUUCUUAUGACU
env-124    UUUCAACAAUAAAGGCAUAAGAAGAAUUUGNNNNNNNNNNNNNNNNNNNNNNNNNNNNNNNNNNNNNNNNN
Pma-9-4    CUAUCAACAAUAAAGGCAUAAAAUUUACCUCCUUUUGGUCAUUAGGAAUUUCUUCUGAUGCAGACCCUUUG
Pma-10-3   UUUCAACAAUAAAGGCAUAAGAAGAAUUUGCCUCCUUUUGGUCAUUAGGAAUUUCUUCUGAUGCAGACCCU
Pma-10-4   UCAUCCGUAGGUUAUCCAAGUGUGCUGAGUCAACUUUGGUAUUGCACUAUCGAAGGAAGUCCAACAAAGUU
env-125    CUUUCAACAAUAAAGGCAUAAGAAGAAUUUGCCUCCUUUUGGUCAUUAGGCAUUUCUUCUGAUGCAGAUCC
env-126    UAGUGUCGUUUCUGCUGUUGCUCUUGCUGCAAUCGCUCUUAG.............................
env-127    AAAAGACCAACUAAUGCUUGGUUUGAAAAAUUUUAGUUUAGAUAAUAUAGAUCCUUUAA............
Ssp-3-1    CAAUGUUUUGAUGCACAUCAAUGCUCAAGUUUUGAAGAAUCGGCAAGAGUUCAAUUCCAUGGCUGCAUGAG
Ssp-2-1    UGCUGAGCCGUUCAAACUUCAGUGAUGGGAAGUUUUCGAAGAGAUUCUGCUUGUCAUCGUUGAACUGAAGG
Ssp-2-2    GGCUGUUGUUGCAACCGUGAGUUCAGUUUCAGUUGCGAUUUCACUUCGACCUAUCGCUCAGCAUUCGGAAC
Ssp-2-3    GGGCCGUCAGGUACGUCUGCGCAGGAUCACAACUGAUCGCUAUGGGAGAACCGUUGGCGAGCUGUUCCUAA
env-128    .......................................................................
Ssp-2-4    AGCAAAAACCCCGAAGCCGUAGCUGCGGGGAUGCGAUUGCUGCUUACUCACAUGCACUCUUGGAGCCAUCA
Pma-5-1    GGGCCCCGCAAACAAUUGGCAACCCAUCAAGCGAUAGCCCACCUCCUAGCCUGAUUGGCUGAACAGCGAUA
Pma-7-1    GGCCCCGCAAACAGUUGGCAAUCCAUCAAGCGAUAGCCCACCUCCUCGCCUGAUUGGCUGAACAGCGGUAG
Ssp-2-5    CUCCAGGCAAUAAAAAAGCCACCCUUUCGAGUGGCGAAGAAUCCAGCUGGGGACUGGCUAAUUAGGCAUCG
Ssp-2-6    GGCGAGUACAUUUGCUUUCCAUGCACUUUUCCCAGUUGGCUUGUAAAGAGGAAGAUAUGUGAGUACUACUU
Ssp-2-7    UGCAAUGCGUCUCCUCGAUCAGUCACAUUGCACGAACGAACCAGUUUCGACAAUUAGUGAACAUCAAUGAC
Ssp-2-8    AGCCUGUCCAAAGCCAGUUGACUAAAGGCACGCGCUCGAGCUAGUACAGGUGUAUUGCUUGUGAGGGACUG
Ssp-10-1   CUUGGCUUCGCCCAGACCGAGGCCGGUGGCCUCGCGGACGGCCUUGAGCACCUUGAUCUUGGCGGCGGCGU
Ssp-2-9    UGGAGCAGCCUCGACCAUCGCCUUGGCAUCACCCAGGCCGAGACCUGUGGCUUCGCGAACAGCCUUGAGCA
Pma-7-2    GCUGCUUCGACCAUGGCUUUAGCGUCGCCCAGGCCGAGGCCGGUGGCUUCACGAACGGCCUUAAGGACCUU
Pma-5-2    CUGCUUCGACCAUGGCUUUAGCGUCGCCCAGGCCGAGGCCGGUGGCUUCACGAACAGCCUUAAGGACCUUG
env-129    CUUCGGAGCAGCCUCGACCAUGGCCUUGGCAUCGCCCAGGCCAAGGCCUGUGGCUUCGCGCACAGCCUUGA
Ssp-11-1   GGCCUUGGCAUCACCAAGACCCAGGCCGGUGGCCUCACGCACAGCCUUGAGGACCUUGAUCUUGGCCGAAG
env-130    UUCGGUGCGGCCUCGACCAUGGCCUUGGCAUCGCCAAGACCAAGGCCGGUGGCCUCACGCACAGCCUUGAG
Ssp-12-1   UCACCAAGACCCAGGCCGGUGGCCUCGCGCACAGCCUUGAGGACCUUGAUCUUGGAUGAAGCUUCAAAGCU
env-131    CAGAGCCUUGGCAUCGCCAAGACCCAGGCCGGUGGCAUUGCGCACAGCCUUGAGCACCUUGAUUUUGGCUG
env-132    GGCUUCGCGAACGGCCUUGAGCACCUUGAUCUUGGCGGCGGCAUCGAAGCUUUCGAGGAUGACGUCGAAUU
Ssp-7-1    UGGCUUCGCGAACAGCCUUCAGCACCUUGAUCUUGGCGGCGGCAUCGAAGCUUUCGAGGAUGACGUCGAAU
Ssp-4-1    CUCGACCAGAGCCUUGGCAUCGCCCAAGCCCAAACCGGUCGCUUCGCGAACGGCCUUGAGAACUUUGAUCU
env-133    UCGCCGAGGCCAAAACCAGUGGCAUUGCGCACGGCCUUGAGAACCUUGAUUUUGGCGGCAGCAUCA.....
env-134    .......................................................................
env-135    UUCCACCAGAGCCUUGGCAUCGCCAAGGCCCAGGCCGGUGGCGUUGCGCACAGCCUUGAGCACCUUGAUUU
env-136    CACCAGAGCCUUGGCGUCGCCAAGACCCAGGCCGGUGGCGUUGCGCACAGCCUUGAGCACCUUGAUCUUGG
Ssp-13-2   CCACCAGAGCCUUGGCGUCGCCAAGACCCAGGCCGGUGGCGUUGCGCACAGCCUUGAGCACCUUGAUCUUG
env-137    CGACCAGAGCCUUGGCGUCGCC.................................................
Ssp-3-2    UCGACCAGAGCCUUGGCGUCGCCCAGACCCAGGCCAGUGGCCUCGCGGACAGCUUUGAGGACUUUGAUCUU
env-138    .......................................................................
env-139    ACCAGAGCCUUGGCAUCGCCGAGGCCGAGGCCGGUGGCGUUGCGGACGGCCUUGAGGACCUUGAUCUUGGC
Ssp-10-2   CUCGACCAUGGCCUUGGCAUCGCCCAAGCCCAAGCCGGUCGCUUCGCGAACGGCCUUGAGAACUUUGAUCU
Ssp-1-1    CUCGACCAUGGCCUUGGCAUCGCCCAAGCCCAAGCCGGUCGCUUCGCGAACGGCCUUGAGAACUUUGAUCU
env-140    ACCAGAGCCUUGGCGUCGCCCAGACCCAGACCGGUGGCCUCACGAACGGCCUUGAGGACCUUGAUCUUGGC
env-141    .......................................................................
env-142    GACCAGAGCCUUGGCGUCGCCCAGACCCAGACCGGUGGCCUCACGGACGGCCUUGAGGACCUUGAUCUUGG
env-143    UUCUGGAAGGUCUGAACGGCCUUCACACCGGCGCCAACAUCGCCCUUCACCAGGACGAAGGCGUUGGUUCC

           .......................................................................
           .......................................................................
           ●●●●●●●●●●●●●●●●●●●●●●●●●●●●●●●●●●●●●●●●●●●●●●●●●●○○○○○○○○○○○○○○○○○○○○○
```

```
env-144    .......................................................................
env-145    CCACCAGAGCCUUGGCGUCGCCAAGACCCAGGCCGGUGGCGUUGCGCACAGCCUUGAGCACCUUGAUUUUG
env-146    UCCACCAAAGCUUUGGCAUCGCCGAGGCCGAGGCCAGUGGCGUUGCGGACAGCUUUAAGGACCUUGAUCUU
Ssp-9-1    GAGCGGCUUCCACCAUGGCUUUCGCAUCGCCGAGGCCGAGGCCGGUGGCUUCGCGAACAGCCUUGAGGACC
env-147    AGCGGCCUCCACCAUGGCUUUCGCAUCGCCGAGGCCGAGGCCGGUGGCUUCGCGAACAGCCUUGAGGACCU
env-148    AGCGGCCUCCACCAUGGCUUUCGCAUCGCCGAGGCCGAGGCCGGUGGCUUCGCGAACAGCCUUGAGGACCU
env-149    GCGGCUUCCACCAUGGCUUUCGCAUCGCCGAGGCCGAGGCCGGUGGCUUCGCGAACAGCCUUGAGGACCUU
env-150    GUCUUCGGAGCAGCCUCGACCAUGGCCUUGGCAUCGCCCAGGCCAAGGCCUGUUGCUUCGCGCACAGCCUU
env-151    CCGAGACCGGUGGCCUCGCGCACAGCCUUGAGCACCUUGAUCUUGGCGGCGGCAUCGAAGCCGUCGAGGAU
Ssp-8-2    UCAUGCGUUGACGCACCAGCGCAUCCCAGAGGCGCGUGGGCAUCAGCCAGCUGCUGGCGGUCGACGCCAUC
env-152    AUUGAGCUGGUCAUGGGUCAUCUGUAGUUGGGCAUCAGCCACUUGCAGACCUUUAAUCAGUUCUUUCGCUU
env-153    GAGAUCGCCGCCAAACUCAAACAAGACGACCUUGAAGUGAACAGAAUCGAGUACUGGGACGACGAAGGAAA
env-154    ACAGAACUUCACCGCACUAUGUCUGGAAUGUGGAUUUGUUGGCACUUCUCAGUCCAACAAGGCUAGUUUUG
env-155    .......................................................................
env-156    UCAGAUCUUCGCCACACAAUUGAAGAACCGUCCUGUUGUGGCACAAAUGAGUCCAACGAGGUUAGUUUUGU
env-157    AGAACUUCAAUACACAAUUAAGGAGUUCCACGGGGCACAAAUGAUGUGGCACAAAUGAGACCAAUGCGGCU
env-158    CAGAACUUCAAUACACAAUUAAGGAGUUCCACAGGGCACAAAUGAUGUGGCACAAAUGAGUCCAAUGCGGC
Ssp-13-3   UCAGAGCUUCAAUACACAAUUAAGGAGUUCCACAGGGCACAAAUGAUGUGGCACAAAUGAGUCCAAUGCGG
env-159    CAGGGCUUCAAUGCACAAUUAAGGAGUUCCACGGGGCACAAAUGAUGUGGCACAAAUGAGUCCAAUGCGGC
Ssp-3-3    CUACACAAUUCAAUAAAACGUUCCUUGUGGCACAAAUAAGUCCAAUGAGGCUAGAUUUGUCUUGUCCGGUU
env-160    UUUGACUGAUAGCCGUAUCGACAUGAACAACAGCCUGAGCUUCAUAGCUGAGUAGAAAUGCUCAAAACCAA
env-161    CUGUGACCCAGAGCAGCCGGGCAAUCUCAUCGGAUCGGAUCUGAGCUUACAAGCCUGGGUAAAAAUGCUCA
env-162    UUGACCGAUAGCGGUGUCGACAUGAGCAUCAGCCUGCGCUUCAUAGCUGAGUAGAAAUGCUCAAAACCNNN
Ssp-4-2    CUGUUGAACUUUUGUUCUUUAUAACGGCUUUUUUUGUGAAUGUCAUAAAAUCAUUACAUCAUCCCAGAAGC
env-163    .......................................................................
env-164    ACGGUUUACCCAACCUUCGCCAGAGACUCAUCAAAGUCAGGAAAUCACAUGGGUGACCAGGUCGGCAUACA
env-165    CAGAACUUCACCGCACUAUGUCCGGAAUAUGGAUUUGUUGGCACUUCUGAGUCCAACGAGGCUAGUUUUAU
env-166    UACAGAACUUCACCGCACUAUGUCCGGAAUAUGGAUUUGUUGGCACUUCUGAGUCCAACGAGGCUAGUUUU
env-167    CAGAACUUCACCGCACUAUGUCCGGAAUAUGGAUUUGUUGGCAUUUCUGAGUCCAACGAGGCUAGUUUUUG
env-168    CGAGAAGAGCUGGACACGAGACCCGAAGGUCGUUGUCGAUACGGGUGUGCCGGUACCUGACGAGCCACCAC
env-169    UAGAACUUCACCGCACUAUGUGCGGAAUAUACAUUUAAUGGCACUUCUGAGUCCAACGAGGCUAGUUUUGU
env-170    .......................................................................
env-171    .......................................................................
env-172    .......................................................................
env-173    AGAUCGCCGCCAAACUCAAACAAGACGACCUUGAAGUGAACAGAAUCGAGUACUGGGACGACGAAGGAAAC
env-174    GACCGAUGAGCUGUAUCUGAGGGCGAGGGCCAAGGAGAUCGCCGCCAAACUCAAACAAGACGAUCUUGAAG
Ssp-3-4    AGAUCGCCGCCAAACUCAAACAGGACACCCUUGAAGUGAACAGGAUCGAGUACUGGGACGACCAAGGGAAC
Ssp-4-3    GCCAUAGUUUGUUCCCUGGAGGCCUUUGAUCGUGCCUUUGAAACCGCUCGACGUCGCUUAAUUGGGACCCU
Ssp-1-2    AAACCGCCCGACGUCGCUUAAUUGGAACACUUCUCGGCGUUGUGUUGGCGGCUAUUCAAGUGAGUUUUGUU
env-175    CCAUUGUUUGUUCCCUGGAGGCCUUUGAUCGUGCCUUUGAAACCGCUCGACGUCGCUUAAUUGGGACCCUU
env-176    .......................................................................
Ssp-3-5    AGAUAAAAAGUUCGCGGACGAGAGCGAUUGGCUUGUCCGUAAGGACACGGAACUAGUCGACUAGAAAGUCG
env-177    UGUGGCAACCCUGGGGCCAUUGGAACCAGGUGUGAUGCAGGUCUUCUGCGGGGCACCUGAGACGGUGCAGA
env-178    CAGAACUUCACCGCACUAUGUCCGGAAUGUGGAUUUGUUGGCACUUCUCAGUCCAACGAAGCUAGUUUUGC
Ssp-13-4   CUUGCGUUGAGCUGACCUAUCGCCGCGGUCACUACAACACCUGCCGCGAAGAAGUGGCUCGCAACGUUCAU
env-179    UGUCCCGCUUUCGGGGGCCAUGCCACUUGCCAUUUCUGGCGUAGGGCGAAGUGUCGGCUUUCAGGUUCCUG
Csp-1-1    CGCCUUGGGAGCGGCUUCCACCAGUGACUUCGCGUCACCCAGGCCCAGGCCGGUGGCUUCGCGCACGGCCU
Tel-1-2    UGAGCAGUCUCCCCAAGCCUGCCAAAUCAUUCGUGAAAACUGGUCAGCCCUUGCCCAACCCCAUCAGCGCA
Ssp-14-3   AACCCCAGGAAAUGACAGCGGGGCUUUUGAAUGCUGACGGUUUUGGCUUGGGUUGGUUUGACCAGGAAAGU
Nsp-1-2    UUCUAAAAACCGUUCAUCCGCAGGUAGUUCUUUUUGGCAUAAGAUGAGAGACAGGCGAUCGCACCACUGCA
Ava-1-2    UUCUAAAAACCGUUCAUCGGCAGGUAAUUCUUUUUGACAUAAGAUGAGAGAUAGGCGAUCGCACCACUGCA
Nsp-1-3    UGAGUUAGUUUGCUGUUACUCGCAAAUUUGGCCUGUUGUAAGUCGCAAGCUUCGACAUUAACCGUAAACUU
Nsp-1-4    UGUGCAGUUGGCAAAGUGUCAUACAAUAUACUUGCUAAAUAAAAUAACAAGUGAUAUCCUCUAGUUGUGUG
Ava-1-3    UUGAGUUAGUUUGGCGUUACUGGCAAAUUGAACCUUUUGUAAGUCGCAAGCUUCGACAUUGACCGUAAACU
Npu-1-1    AGCAAUGAUAUUAUUUAAUCGUGUGUGAGGAGCAAGUUAAAAAUAAAAAGCGUUUGGGGUGGAAACACGGC
Nsp-1-5    UGUGCCGGAUGCUACUUCCAUACUUCUGAUUUGCUUAAUCAGCAUAUAGCCGUCAACUUCUGGCAUUCCAA
Mae-1-1    CCCGUAGCUUAUAUGGCUAAAGAAGAAUUAUUUACCAUCCCGAUUUUAAAACAGGCGAUCGCUCUUUAUGG
Csp-2-1    AUUUGAGUAAUUCUUGACGACUAUAACGCCCCUCUUCUACUUCUAAGGAUGCUUUAUUAAUAGCUUGUUUA
Cwa-1-1    CGGCACAUGAUGAUGGACGUUUUACAUUUUAUCAAUUAGGAACCACAGAAGCUUAUAGUGCUGGCUUAGUG
Csp-2-2    UAUUCCUUUAAAGACUUUAGUAAUUGAGGAAGCUAAAGUGAUUCAGUUAGAUCAAUCUUCUUCUUAUCGAC
Csp-3-1    UUUUAUUCUUUCUAGUUUGAGUAACUCGGUCAUUAACGCGAGUUCGAGAUUUGAUACCAUUUGUAUAAGUA
Lsp-1-1    UUGCCUCAAAAAUGAUGGAUUCUCGCCCGUUGCAGAUAAAUCAGACUCAUUUGCUGCAAACUCUUUUUAUA

           .......................................................................
           .......................................................................
           ●●●●●●●●●●●●●●●●●●●●●●●●●●●●●●●●●●●●●●●●●●●●●●●●●○○○○○○○○○○○○○○○○○○○○○○
```

```
Ssp-6-1    CAGGGGUCUUGGUUCAGUUGCAAGUCCCCUCCCAGGUCAUCGCCUGCCAGAAGGCCGCAGAAGGUUGGUGC
Ssp-5-1    CCACAGCUUUGCCAACCGGAAUACGGCGCGGCUUCUCUAAAGAAUGGGGUAGCUGGGUAUCCUAAAAGCUG
Mae-1-2    GGGGUUUUAGUGGGCAGUGCGAUCGGGGCAGUGGCGGGUUUAUUAGUAGCGCCCAGAUCUGGAAAAGAAAC
Csp-5-1    AAUGCUGAUUUAAUUGCUGCUGACACAGAUUAAAAAAAGUAGAGACACAGCUAGAAUUAACCUGAGUUGAA
Csp-4-1    UGAAGCGUUAAGUAAUCUCGUUCUUUGUCAUUAUGAUAUUGACAUCCCUACAAGUUGUCAUUUUUGGCAUC
Csp-5-2    CCCAACUCAACCAGUAUUAACGAUGGUUCAAGAGCUUAAUUCACCUCGAGAGUGGAAAAAAAUGCGCUGGG
Cwa-1-2    CUGUUUUUUGUUCUUUACCAAUGGUAGCAGUAGGUUUAAAAGCAACACGAAUAAUGAUAUUUUCUCCGUUA
Csp-7-1    CCUCAUACCUUCCUAAGAGCGAUCGGACCCGAACCCUGGUCUGUGGCCUAUGUCGAACCUUGUCGGCGGCC
Csp-6-1    CCCCAUACCUUCCUAAGAGCGAUCGGACCCGAACCCUGGUCUGUGGCCUAUGUCGAACCUUGUCGGCGGCC
Csp-3-2    CUGUUAAUUUUCGCACCUUGGAGAUGGCUUUCUUGACCUCCUCAUCGUGAGAUAAGCUAGAAUCAAACUCC
Ssp-12-2   CCCAAGGCCGGGUGAUGGGGUCUUACGACGCCGGCGAAGAGUCCUCCGACUUCAUUGCGGGGGAUUUUUUA
Ssp-11-2   UUCGACGGCCGGGUGAUGGGUUUCACGAAGCCGGCGAAGCAUCCUCCGACGUCAUGGCGGAGGAUUUUUUA
Ssp-2-10   UCACUCAGUCAAUCCAUCCAGUGAUCUGAGGAUCAGGGGCAUUGAGGGUUCGAGGUUUUGUACCCAACUGA
           .......................................................................
           .......................................................................
           ●●●●●●●●●●●●●●●●●●●●●●●●●●●●●●●●●●●●●●●●●●●●●●●●●○○○○○○○○○○○○○○○○○○○○○○
```

26 Cyano-2

26.1 Taxa

The taxonomy of each organism containing a putative Cyano-2 RNA is listed, with abbreviations identifying each hit (e.g., "Eco-1-1" and "Eco-1-2" might hypothetically represent two distinct RNAs in *E. coli*). The abbreviations will be used to identify each individual Cyano-2 RNA in Sections 26.2 and 26.4.

**abbrev. of hits**	**taxonomy of species**
Ssp-1-1 to Ssp-1-5	Bacteria Cyanobacteria Chroococcales *Synechococcus sp.* CC9605
Ssp-2-1 to Ssp-2-5	Bacteria Cyanobacteria Chroococcales *Synechococcus sp.* WH 8102
env-1 to env-47	environmental samples

26.2 Gene contexts

Each Cyano-2 RNA (indicated by "RNA→") is listed with its surrounding genes. Environmental sequences and some RefSeq entries lack gene annotations, and no genes are listed for such sequences. The direction of each gene is indicated with an arrow (→), and each conserved domain in the gene is colored. Conserved domains associated with more than one Cyano-2 RNA are assigned a color; other domains are gray. Information about these conserved domains is given in Section 26.3. The accession number of the sequence containing each Cyano-2 RNA is given. Accessions beginning with "NC_" or "NZ_" are RefSeq, while other accession refer to environmental samples. Nucleotide coordinates are given for the 5′ and 3′ boundaries of each. If the 5′ coordinate is greater than the 3′ coordinate, the RNA is present on the reverse-complement strand of the containing genomic DNA sequence. Each hit is denoted by an abbreviation (like "Eco-1-1") that refers to a taxonomy given in Section 26.1.

**abbrev.**	**Seq. accession**		**5′ at**	**3′ at**	**genes**
env-1	JCVI_SCAF_1096626604590	+	264	485	**RNA**→ ←? (GOS13939)
env-2	JCVI_SCAF_1096627196445	+	280	502	**RNA**→ ←? (GOS13939)
env-3	JCVI_SCAF_1101667386531	+	595	818	**RNA**→
Ssp-2-1	NC_005070.1	-	1051140	1050918	hypo→ **RNA**→ ←hypo
env-4	JCVI_SCAF_1096628040145	+	1264	1487	? (GOS11467169)→ **RNA**→ ←? (GOS13939)
env-5	JCVI_SCAF_1101668670117	-	764	566	? (GOS11039316)→ **RNA**→ ? (GOS11026)→
Ssp-1-1	NC_007516.1	+	1429118	1429312	hypo→ **RNA**→ COG1359 (COG1359)NOG29985 (NOG29985)→

env-6	JCVI_SCAF_1096626931592	-	1393	1187	**RNA**→ ? (GOS13122847)→
env-7	JCVI_SCAF_1096627964155	-	1030	832	? (GOS11250293)→ **RNA**→ ? (GOS13122847)→
env-8	JCVI_SCAF_1101668187095	+	1010	1208	? (GOS11250293)→ **RNA**→ ? (GOS12129995)→
env-9	JCVI_SCAF_1101668674017	-	1417	1219	**RNA**→ ? (GOS12129995)→
env-10	JCVI_SCAF_1096626917678	-	525	327	? (GOS11250293)→ **RNA**→ ? (GOS12129995)→
Ssp-1-2	NC_007516.1	+	1587566	1587776	←hypo **RNA**→ ←hypo
env-11	JCVI_SCAF_1101668673415	-	421	205	? (GOS11776927)→ **RNA**→
env-12	JCVI_SCAF_1096627155971	+	2428	2644	? (GOS11776927)→ **RNA**→ ? (GOS13966)→
env-13	JCVI_SCAF_1096627749470	-	426	212	←? (GOS11342131) **RNA**→
env-14	JCVI_SCAF_1101668675632	-	1073	860	**RNA**→
env-15	JCVI_SCAF_1096627965582	+	1119	1333	**RNA**→
env-16	JCVI_SCAF_1101667184623	-	634	419	**RNA**→
env-17	JCVI_SCAF_1096628048551	+	504	716	**RNA**→
Ssp-2-2	NC_005070.1	-	1129611	1129409	←hypo **RNA**→ ←hypo
env-18	JCVI_SCAF_1101667386135	-	214	12	←? (GOS11671314) **RNA**→
env-19	JCVI_SCAF_1096627526224	-	240	37	←? (GOS11944983) **RNA**→
env-20	JCVI_SCAF_1101667223140	+	321	525	**RNA**→
env-21	JCVI_SCAF_1096627961690	-	424	221	←? (GOS11944983) **RNA**→
env-22	JCVI_SCAF_1096626924384	-	2335	2132	**RNA**→ ? (GOS14498412)→
env-23	JCVI_SCAF_1096627515178	+	1041	1244	←DAP_epimerase (pfam01678)? (GOS5775) **RNA**→
env-24	JCVI_SCAF_1101668666528	-	448	245	? (GOS11760973)→ **RNA**→
env-25	JCVI_SCAF_1101668668167	-	629	425	? (GOS11116329)→ **RNA**→ ? (GOS11116329)→
env-26	JCVI_SCAF_1096627302430	+	48	252	? (GOS11116329)→ **RNA**→ ? (GOS11116329)→
env-27	JCVI_SCAF_1096627328368	-	1754	1551	←? (GOS14395810) **RNA**→ ←? (GOS13482625)
env-28	JCVI_SCAF_1096627158393	-	1145	942	**RNA**→
env-29	JCVI_SCAF_1101668672249	+	528	698	**RNA**→
env-30	JCVI_SCAF_1096627369623	-	628	424	? (GOS11116329)→ **RNA**→ ? (GOS11116329)→
Ssp-1-3	NC_007516.1	-	1291073	1290872	hypo→ **RNA**→ TFIIE (smart00531)→
env-31	JCVI_SCAF_1096628082578	-	727	523	**RNA**→ ? (GOS11713070)→
env-32	JCVI_SCAF_1096628257323	-	1683	1475	? (GOS11116329)→ **RNA**→ ? (GOS11116329)→
env-33	JCVI_SCAF_1096627794547	+	1128	1328	**RNA**→
env-34	JCVI_SCAF_1096628362723	+	603	802	**RNA**→
Ssp-1-4	NC_007516.1	-	535123	534921	←GST_C_Zeta (cd03191)GST_N_Zeta (cd03042) **RNA**→ ←hypo
Ssp-1-5	NC_007516.1	+	913565	913767	←hypo **RNA**→ hypo→
env-35	JCVI_SCAF_1096627540547	-	385	184	←DUF427 (pfam04248) **RNA**→
env-36	JCVI_SCAF_1096628046417	+	1622	1825	? (GOS13136162)→ **RNA**→
env-37	JCVI_SCAF_1101667383508	-	525	322	**RNA**→
env-38	JCVI_SCAF_1096627192266	-	6496	6293	**RNA**→ ←? (GOS11820130)
Ssp-2-3	NC_005070.1	-	1848644	1848441	←hypo **RNA**→ hypo→
env-39	JCVI_SCAF_1101667585229	+	234	437	**RNA**→
env-40	JCVI_SCAF_1096626459164	+	6	207	**RNA**→ ←? (GOS12231)
env-41	JCVI_SCAF_1101667380836	-	694	493	**RNA**→ ←? (GOS11820130)
env-42	JCVI_SCAF_1096628075702	+	466	667	**RNA**→ ←? (GOS11047726)
env-43	JCVI_SCAF_1096627183482	+	289	488	**RNA**→ ←Soj (COG1192)? (GOS1716)
Ssp-2-4	NC_005070.1	-	1132490	1132288	hypo→ **RNA**→ ←hypo
Ssp-2-5	NC_005070.1	-	372286	372086	←GAF (pfam01590) **RNA**→ ←COG3019 (COG3019)
env-44	JCVI_SCAF_1096627192266	-	3116	2919	? (GOS11402347)→ **RNA**→ **RNA**→ ←? (GOS13534)
env-45	JCVI_SCAF_1096627192266	-	3745	3548	(shown above)
env-46	JCVI_SCAF_1096628390651	-	1845	1648	**RNA**→
env-47	JCVI_SCAF_1096627798650	-	1673	1473	**RNA**→ ? (GOS13050596)→

26.3 Conserved domains

Conserved domains found in protein-coding genes listed in Section 26.2 are listed, with the first sentence in their description from the Conserved Domain Database. Conserved domains associated with more than one Cyano-2 RNA are assigned a color, while others are shown in gray. The number in parentheses is the number of occurrences of the given gene.

u cd03042 (1) GST_N family, Class Zeta subfamily; GSTs are cytosolic dimeric proteins involved in cellular detoxification by catalyzing the conjugation of glutathione (GSH) with a wide range of endogenous and xenobiotic alkylating agents, including carcinogens, therapeutic drugs, environmental toxins and products of oxidative stress.
u cd03191 (1) GST_C family, Class Zeta subfamily; GSTs are cytosolic dimeric proteins involved in cellular detoxification by catalyzing the conjugation of glutathione (GSH) with a wide range of endogenous and xenobiotic alkylating agents, including carcinogens, therapeutic drugs, environmental toxins, and products of oxidative stress.
d COG1192 (1) ATPases involved in chromosome partitioning [Cell division and chromosome partitioning]
D COG1359 (1) Uncharacterized conserved protein [Function unknown]
d COG3019 (1) Predicted metal-binding protein [General function prediction only]
D GOS11026 (1) ?
u GOS11039316 (1) ?
d GOS11047726 (1) ?
uD GOS11116329 (8) ?
u GOS11250293 (3) ?
u GOS11342131 (1) ?
u GOS11402347 (1) ?
u GOS11467169 (1) ?
u GOS11671314 (1) ?
D GOS11713070 (1) ?
u GOS11760973 (1) ?
u GOS11776927 (2) ?
d GOS11820130 (2) ?
u GOS11944983 (2) ?
D GOS12129995 (3) ?
d GOS12231 (1) ?
D GOS13050596 (1) ?
D GOS13122847 (2) ?
u GOS13136162 (1) ?
d GOS13482625 (1) ?
d GOS13534 (1) ?
d GOS13939 (3) ?
D GOS13966 (1) ?
u GOS14395810 (1) ?
D GOS14498412 (1) ?
d GOS1716 (1) ?
u GOS5775 (1) ?
D NOG29985 (1)
u pfam01590 (1) GAF domain.
u pfam01678 (1) Diaminopimelate epimerase.
u pfam04248 (1) Domain of unknown function (DUF427).
D smart00531 (1) Transcription initiation factor IIE;

26.4 Multiple-sequence alignment

Each Cyano-2 RNA is denoted by an abbreviation (like "Eco-1-1") that refers to a taxonomy given in Section 26.1. The alignment includes sequences containing the RNA motif, as well as flanking sequence. The Cyano-2 RNA itself is denoted by the line underneath marked 5′ and 3′ on either end. Nucleotides in the flanking sequences (i.e., not part of the motif) are written in gray letters. Stems of predicted rho-independent transcription terminators are shaded yellow. (Note: terminator predictions have not been analyzed manually, and many are likely to be false positives.) Start codons are shaded green. (Note: start codons are frequently misannotated, especially in environmental samples.) Nucleotides proposed to basepair as part of the consensus structure are shaded in color when they comprise Watson-Crick or G-U pairs. Otherwise they are shaded gray. Conserved stems are also indicated at the bottom of the alignment by angle brackets, where matching < and > denote base-paired columns. Below these angle brackets, the symbol "2" denotes base pairs exhibiting covariation, "1" denotes base pairs exhibiting compatible mutations, "0" denotes base pairs that are not observed to mutate and "?" denotes base pairs that have a significant frequency of non-canonical nucleotides for Watson-Crick or G-U pairs (> 5%). Below these base pair annotation is the consensus sequence: "R" = "A" or "G", "Y" = "C" or "U", red nucleotides: nucleotide identity conserved more than 97% of the time, black nucleotides: 90%, gray nucleotides: 75%, red circle (•): nucleotide is present 97% of the time, black circle (•): 90%, gray circle (•): 75%, white circle (◦): 50%. All percentages of sequences just described (e.g. 97% conserved) assume that sequences have been weighted by the GSC algorithm implemented by the Infernal software.

alignment positions 1 · · · 150

```
env-1    CUUGAUCCCAAAAAACCCUGGAACAUAGCGUGCGCGACCUGGGACAUCCACCUAAAAAGAGGACCUGCAACGCACCGUCCUGCAACCGGGUGGUCGUCGCAGCGGCACCGAGAGA.GCAA.......CUCGGGAGCUGGUAGAAACC..
env-2    CUUGAUCCCAAAAAACCCUGGAACAUCUCGUGCGCGGCCUGGGGCAUCCACCUAAAAAGGGGACCUGCAACACACCGUCCUGAAACCGGGUGGUCGUCGCAGCAGCACCGAGAGA.GCGA.......CUCGGGAGCUGGCAGAAACC..
env-3    UGAUCCCAAAAAACCCUGGAACAUCUCGUGCGCGGCCUGGGGCAUCCACCUAAAAAAAGAGGACCUGCAACACACCGUCCUGAAACCGGGUGGUCGUCGCAGCAGCACCGAAAGAGGCGA.......CUGGGGAGCUGGCAGAAACC..
Ssp-2-1  CUUGAUCCCAAAAAACCCUGGAACAUCUCGUGCGCGGCCUGGGGCAUCCACCUAAAAAGGGGACCUGCAACACACCGUCCUGAAACCGGGUGGUCGUCGCAGCAGCACCGAGAGA.GCGA.......CUCGGGAGCUGGCAGAAACCA.
env-4    CUUGAUCCCAAAAAACCCUGGAACAUCUCGUGCGCGGCCUGGGGCAUCCACCUAAAAAGAGAACCUGCAACACACCGUCCUGAAACCGGGUGGUCGUCGCAGCAGCACCGAGAGAGGCGA.......CUCGGGAGCUGGCAGAAACCAG
env-5    GUUUUUGGAGAUCCAGACAACCUCUCAAAAUGCCCACACAAACACCAUGGGGGUCCCCAUAGUUGGGUCCUUCGGAUCGCUGCGCUGGAUGUAGUGCCGAGGCAGUGCCGGGAGA.GCGA.......CCCGGUAUCUGGCGGAAGA...
Ssp-1-1  AUUUUUGGCGGAUCAGCACAUCUUCAGAAAUUUCCAGACAAGCCUGCUGAAUGGCCUCAUCAUUGAGCCCUAAGUUGUCCGCGCACCAGAUGUGGUGCUGAGCAGCACGGGGAGA.GCGA.......CCCUGGGGCUGGCAGACGAGU.
env-6    .........................................................................UGGCCUCAUUAUUGAACCCUCAGUUUCCUGCACACCAGAUGUGGUGCUGAGCAGUACGGGGAGA.GCGA.......CCCUGAUGCUGGCAGAAGAGU.
env-7    AUUUUUGGCGGAUCAGCACAUCUUCAAUAAUUUCCAGACAAGCCUGCUGAAUGGCCUCAUCAUUGAGCCCUCAGUCGCCCGCAUACCAGAUGUGGUGCUGAGCAGCACGGGGAGA.GCGA.......CCCUGGGGUUGGCAGACGAGU.
env-8    AUUUUUGGCGGAUCAGCACAUCUUCAGAAAUUUCCAGACAAGCCUGCUGCAUGGCCUCAUCAUUGAACCCUCAGUUACCUGCACACCAGAUGUGGUGCUGAGCAGCACGGGGAGA.GCGA.......CCCUGGGGCUGGCAGACGAGU.
env-9    ............................................................................................................CGCCCGCACACCAGAUGUGGUGCUGAGCAGCACGGGGAGA.GCGA.......CCCUGGGGCUGGCAGACGAGU.
env-10   AUUUUUGGCGGAUCAGCACAUCUUCAGAAAUUUCCAGACAAGCCUGCUGAAUGGCCUCAUCAUUGAGCCCUCAGUCGCCCGCAUACCAGAUGUGGUGCUGAGCAGCACGGGGAGA.GCGA.......CCCUGGGGCUGGCAGACGAGU.
Ssp-1-2  UUGAUCCCAAAGGUCUCCGAAAAAUGGUGUGCGGUGCCACUGGUGAGUCUCUAUUAAUGGUUCCUGCGGCACUUCCUCAUACGGCUGAGUGAGGCAUCGCAGCAGC.ACGGG..A.GCGA.......CCCGUUGGCUGGCAGACAAUC.
env-11   UUAAUCCCAAAGGUCUCCGAAAAAAGCUGUGCACGACCAAGGAACAUUCCCUAUCAAUGGUGUCUGCUUCAUCUCCUCUUACGGCUGAGUGAGCCAUCGCAGCAGC.ACGGGAGA.GCGA.......CCCGUUAGCUGGCAGAUAAUCC
env-12   UUGAUCCCAAAGGUCUCCGAAAAAAGCUGUGCACGACCAAGGAACAUUCCCUAUCUAUGGUCUCUGCGGCAUCUCCUCUUACGGCUGAGUGAGUCAUCGCAGCAGC.ACGGGAGA.GCGA.......CCCGUUAGCUGGCAGACAAUCC
env-13   UUGAUCCCAAAGGUCUCCGAAAAAAGAUGUGCAGCACCACUGGAGAUUACCUAUCAAUGGUUUCUGCGACAUCUCCUCUUACGGCUGAGUGAGCCAUCGCAGCAGC.ACGGGAGA.GCGA.......CCCGUUUGCUGGCAGACAAUCC
env-14   UUGAUCCCAAAGCUCUCCGAAAAAUGGUGUGCAACACCACUGGAGACUCCCUAUCAAUGGUUCCUGCGACAUCUCCUCUUACGGCUGAGUGAGCCAUCGCAGCAGC.ACGGGAGA.GCGA.......CCCGUG.GCUGGCAGACAAUCC
env-15   UUGAUCCCAAAGCUCUCCGAAAAAUGGUGUGCAACACCACUGAAGACUCCCUAUCAAUGGUUCCUGCGACAUCUCCUCUUACGGCUGAGUGAGCCAUCGCAGCAGC.ACGGGAGA.GCGA.......CCCGUG.GCUGGCAGACAAUCC
env-16   CUUGAUCCCAAAGCUCUCCGAAAAAUGGUGUGCAGCACCACUGGAGAUUCCCUAUCAAUGGUUCCUGCGACAUCUCCUCUACGGCUGAGUGAGGCAUCGUGGCAGC.ACGGGAGA.GCGA.......CCCGUUAGCUGGCAGACAAUCC
env-17   AUCCUUGAUCCCGAAGGUCUUCAGAAAUUGGUGUGCAAUGCCACCACCGAGCACCUAUCAAGGAGUCCCGCGACUCUCCUCUCACGGCUGAGUGAGUCAUCGCAGCAGCACGAGA.GCGA.......CCCAUUUGCUGGCAGACAAUCC
Ssp-2-2  UUCCGGAAACUAAAACCUGUAGAAAAAGACACUGCCAUCUCCCUCGCUUGCCCCCAUAUUUGGGGUCCAUGCAGACAACGCUUGAACGCGUAACUGUCCAGGCAGCACCGGGAGA.GCGA.......CCCGGCAGCUGGCAGAUAAAGC
env-18   CUGCCAUCUCCCUCGCUUGCCUCCAUAUUUGGAGUCCAGGCAGACAACGCUUGAACGCGUAACUGUCCAGGCAGACAACGCUUGAACGCGUAACUGUCCAGGCAGCACCGGGAGA.GCGA.......CCCGGCAGCUGGCAGAUAAAGC
env-19   UCCGGAAACUAAAACCUGUCCAAAAAGUCGCUGCAAACUUCAGCGUUAGCCCCCAUAUUUGGUGUCCAGGCAGCAGACACGUAUCACGUGUCACUGUCUAGGCAGCACCGGGAGA.GCGA.......CCUGGUGGCUGGCAGAAAUAGC
env-20   UGGAAACUAAAACCUGUCGAAAAAGUCGCUGCAAACUCCGGCGCUACCCCCCAUAGUUAGUGCCCAAGGCGUCCACUGCGUGACACCAGAGAAUUGUCUUGGCAGCACUGGGAGA.GCGA.......CCUGGUAGCUGGCAGAUAUAGC
env-21   UCUGGAAACUAAAACCUGUCCAAAAAGUCGAUGCAAACCCCGGCGUUAGCCCCCAUAUUUGGUGCCCAGGCAGCAGGCGCGUAUCACGUGUCACUGUCCAGGCAGUACCGGGAGA.GCGA.......CCUGGUAACUGGCAGAAAUAGC
env-22   UCUGGAAAGUAAAACCUGUCCAAAAAGUCGCUGCAAACCCGGGCGUUAGCCCCCAUAUUUGGUGCCCAGGCAGCAGGCGCGUAUCACGUGUCACUGUCCAGGCAGUACCGGGAGG.GCGA.......CCUGGUAACUGGCAGAAAUAGC
env-23   UCCGGAAACUAAAACCUGUCCAAAAAGUCGCUGCAAACCGUGUCACUUGCCUCCAUAUUUGGUGCCCAGGCAGCAGACACGUAUCACGUGGCACUGUCUAGGCAGCACCGGGAGA.GCGA.......CCUGGUGGCUGGCAGAAAUAGC
env-24   UGGAAACUAAAACCUGUCCGAAAAGUCGCUGCAAAGUCCGGCGAUAGCUCCCAUAUUUGGUGUCCAAGACCACCACUGCGUGACACCAGAGAUUCGUCUUGGCAGCACCGGGAGA.GCGA.......CCUGGUAGCUGGCAGAAAUAGC
env-25   UCUGGAAACUAAAACCUGUCCAAAAAGUCGCUGCAAACCCCGCCGUUAGCACCCAUAUUUGGAGCCCAGGCAGCAGAUGCGUAUCACGUGUCACUGUCCAGGCAGCACCGGGAGA.GCGA.......CCUGGUAGCUGGCAGACAAAGC
env-26   ............................................................................CAUAUUUGGUGUCCAGGCAGCAGACGCGUAUCACGUGUCACUGUCCAGGCAGUACCGGGAGA.GCGA.......CCUGGUAACUGGCAGAAAUAGC
env-27   GAACGUCGCUCAAUGGUUUUUGAGGGAAUUGAAAAAUCGCUGCAAGGCUCCCGAACUUCCCCCAUAUUUGGUGUCCGGACGGCGUACCUAUCACCGUCUCGGCAGC.ACGGGAGA.GCGA.......CCUGUUGGCUGGCAGUCAAAGC
env-28   AACGUCGCUCAGUCGUUUUUCAGGGAAUUGAAAAAUCGCUGCAAGGCUCCCGAACUUCCCCCAUAUUUGGUGUCCAGAUGGUGUCACCUAUCACCGUCUUGGCAGC.ACGGGAGA.GCGA.......CCCGUUGGCUGGCAGUCAAAGC
env-29   UCCAGCACUGGCAUCUGAGCGGUCCACCCGGCCCGCUUUUUUAGUGCAAAACAGCUAAACAUCGUUUUUUGUGCGCCAAAGUGUUCGCCAACUGCCCCAA........................................................AUAGUCAUGUC
env-30   AACGUCGCUCAAUGGUUUUUCAGGGAAUUGAAAAAUCGCUGCAAGGCUCCCGAACUUCCCCCAUAUUUGGUGUCCAGAUGGUGUCACCUAUCACCGUCUUGGCAGC.ACGGGAGA.GCGA.......CCCGUCGGCUGGCAGUCAAAGC
Ssp-1-3  GGCGUUUUUCAGGGAACUGAUAUUUGCCGGAGAAAUCGCUGCAGAUCCUCCCAACUGCCCCCAUAUUUGGUGUCCAGGCGGUGCCACUUGUCACUGUCUUGGCAGC.ACGGGAGA.GCGA.......CCCGUCAGCUGGCAGUCAAGGC
env-31   UUUAUGCAAACUAAAACCUGACCAAAAAGUCGCUGCAAACUCGGUGCUAGCCCUCAUAAUUAGCUCCCAGCAGCAGACGCGUAUCAUGUGUUACUGUCCAGGCAGUACCGGGAGA.CUGA.......CCCG.UAGCUAGCAGACAAUUC
env-32   UUGAUCCCAAAGGUCUCCGAAAAAUGGUGUGCGAAAGCACAGGAGAUUCCCUAUUAAAGAGACCUGCGACACCUCUCUUACGGCUGAGUGAGGCAUAGCAGCAGC.ACGGGAGA.GCGA.......CCCGUUAGCUGGCAGACAAUC.
env-33   AGAUUUUUUGGAAUCAAAAAAUGACCAAAAGACGUGUUGCGGAUCCUGCUGCUUGGCCCCAUAUUUGCCGAACGCGGAUAUGGGGAGUUCAUCCACACGCGACGGCCUUGGAAGA.GCGA.......UCCGAGAGCCGGCAGAACCAA.
env-34   AGAUUUUCUGGAAUCGAAAAAUGACCAAAUGAUGCGUUGCCAUCGACGUUGCCCCACCCCAUAGUUGUGCAGUGCGGACAAGGGGAGACCCUCCACACGCGACAGCCUUGGGAGA.GCGA.......CCCAUGAGCUGGCAGAACCCU.
Ssp-1-4  GAUUUUUUUGGAAUCAAAAAAUGACCAAAAGAGGUGUUGCAGAACACGCUGGUUACCCCCAUAUUUGCUGUGCGCGGAAAUGGGGAGUUCUUCCACACGCGACGACCUCGGAAGA.GCGA.......UCCGAGUGUUGGCAGAACCCC.
Ssp-1-5  GCAUUUUCUGGAAUCAAAAAAUGACCAAAAGACGCGUUGCGGAACACGCUGGAUACCCCCAUAUUUAGUGUGCGCGGAAAUGGGGAGUUCAUCCACACGCGUCGGCUUCGGAAGA.GCGA.......UCCGAUAGCUGGCAGAACCCC.
env-35   AGUUUUCCUGGAAUCAAAAAAUGACCAAAAGACGUGUUGCCGAACACGCUGCUUGGCUCCAUAUUUGCUGUGCGCGGACAUGGGGAGUUCAUCCACACGCGACGGCCUCGGAAGA.GCGA.......UCCGAGAGCUGGCAGAACCCC.
env-36   AGAUUUUCUGGAAUCAAAAAAUGACCAAAAGACGUGUUGCCGAACACGCUGCUUGGCCCCAUAUUUGCUGUGCGCGGACAUGGGGAGUUCAUCCACACGCGACGGCCUCGGAAGA.GCGA.......UCCGAGAGCUGGCAGAACCCC.
env-37   AAAUUUUCUGGAAUCCAAAAAUGACCAAAAGACGUGUUGCCGAACACGCUGCUUGACCCCAUAUUUGUUGGGCGCGGACAUGGGGAGUUCUUCCACACGCGACGGCCUCAGUAGA.GCGA.......UCUGAGAGCUGGCAGAACCCC.
env-38   AAAUUUUCUGGAAUCCAAAAAUGACCAAAAGACGUGUUGCCGAACACGCUGCUUGACCCCAUAUUUGUUGGGCGCGGACAUGGGGAGUUCUUCCACACGCGACGGCCUCAGUAGA.GCGA.......UCUGAGAGCUGGCAGAACCCC.
Ssp-2-3  AGAUUUUCUGGAAUCCAAAAAUGACCAAAAGACGUGUUGCCGAACACGCUGCUUGGCCCCAUAUUUGCUGUGCGCGGACAUGGGGAGUUCAUCCACACGCGACGGCCUCAGUAGA.GCGA.......UCUGAGAGCUGGCAGAACCCC.
env-39   AGAUUUUCUGGAAUCCAAAAAUGACCAAAAGACGUGUUGCCGAACACGCUGCUUGGCCCCAUAUUUGCUGUGCGCGGACAUGGGGAGUUCAUCCACACGCGACGGCCUCAGUAGA.GCGA.......UCUGAGAGCUGGCAGAACCCG.
env-40   .........................................................................................................................................CUCGCGGCAGCCUCGGGAGA.GCGA.......CCCAAGAGCUGACAGAAC...C
env-41   .................AUCAAAAAAUGACCAAAAGACGUGUUGCCGAACACGCUGCUUGACCCCAUAUUUGUUGGGCGCGGACAUGGGGAGUUCUUCCACACGCGACGGCCUCAGUAGA.GCGA.......UCUGAGAGCUGGCAGAACCCC.
env-42   AAAUUUUCUGGAAUCAAAAAAUGACCAAAAGACCUGUUGCCGAACACGCUGCUUGACCCCAUAUUUGUUGGGCGCGGACAUGGGGAGUUCUUCCACACGCGACGGCCUCAGUAGA.GCGA.......UCUGAGAGCUGGCAGAACCCC.
env-43   AAAUUUUCAGGAAUCCAAAAAUGACCAAAAGACGUGUUGCCGAACACGCUGCUUGAUCCCAUAGUUGUUGGGCGCGGACAUGUGGAGUUCAUCCACACGCGACGGCCUCAGUAGA.GCGA.......UCUGAGAGCUGGCAGAACAC..
Ssp-2-4  AAAUUUUCUGGAAUCAAAAAAUGACCAAAAGACGUGUUGCCGAACACGCUGCUUGACCCCAUAUUUGUUGGGCGCGGACAUGGGGAGUUCUUCCACACGCGACGGCCUCAGUAGA.GCGA.......UCUGAGAGCUGGCAGAACCCCC
Ssp-2-5  AGAUUCUCUGGAAUCAAAAAAUGACCAAAAGACGUGUUGCCGAAUAAGCUCCUUGGUUCCAUAUUUGCUUUGCGCGGACAUGUGGAGUUCAUCCACACGCGACGGCCUCGGAAGA.GCGA.......UCCGAGAGCUGGCAGAACCCC.
env-44   AAUUUUCUGGAAUCAAAAAAUGACCAAAAGACGCGUUGCCGAACACGCUGCUCGACCCCAUAUUUGUGCAACGCGGUGAUGGGGUCCUUCUUCCACUCGCGGCAGCCUCGGUAGA.GCGA.......UCCGAGAGCUGACAGAACAC..
env-45   GAUUUUGUUGAUCCCAAAAAACCUCGGGAGAAGUGUUGCCAGCUGAUCCGCUCGACCCCGUAUUUGUGCAACGCGGUGAUGGGGUCCUUCUUCCACUCGCGGCAGCCUCGGUAGA.GCGA.......UCCGAGAGCUGACAGAACAC..
env-46   ..............................................................................ACCCCAUAUUUGUGCAACGCGGUGAUGGGGUCCUUCUUCCACUCGCGGCAGCCUCGGUAGA.GCGA.......UCCGAGAGCUGACAGAGC...C
env-47   GAUUUUUUGGGUUCCAGGAAUUGUUCAAAAGAUCGGUUGCAAUUCCUUCGAAUGACCUUCAUAUUUGUGCGACGCGGACAUGGGGAGUUCAUCCACACGCGACGGUCACGGGAGA.GCGA.......UCCGAGGGCCGGCAGAACCCC.
         ...................................................................................................................................................<<<<<<<<<<................>>>>>>.>>>><<<<<<<....
         ...................................................................................................................................................0222?2222?................?2222?.22202?21??2....
         ∘∘∘∘∘∘∘∘∘∘R∘∘∘∘∘∘∘∘∘∘∘∘∘∘∘∘∘∘∘∘∘∘∘∘∘∘∘∘∘∘∘∘∘∘∘∘∘∘∘∘∘∘∘∘∘Y∙∙∙∙∙Y∙∙∙∙∙∙∙∙∙∙∙∙∙∙∙∙∙∙∙∙∙∙∙∙∙∙∙∙∙R∙∙∙∙∙∙∙∙∙∙∙∙∙∙∙C∙∙RGCAGC∘∙CGGRAGA-GCGA-------YCYG∙∙RGCUGGCAGA∙∙∙∙∘∘
                                                                                                                                                   5′
```

alignment positions 151 · · · 307

```
env-1    AGUCCCU.ACGG....CGUCCUAAGGGU...C....CGUAGGGACU.GU.UUUGCGCCUUC....AUGUCG....UCUUCCGAUAACAAGAAAGAUUCCGU..AGUUUGCAGCUUGUAUCGAGAUCGUUCGUCAGGCU.UUAAGAGCGGA..GUCGA
env-2    AGUCCCU.AUGGCCUCAG....AAGACU...C....CUUAGGGACU.G..UUU....UUGCGCCUUCAUAU....CGUCUUCCACUAACAAGAAAGAUUCC.GUAGUCUGCAGCUUGAUUCGAGAUCAUUCGUCAGGCUUGAAAUGCGGA..GUCGA
env-3    AGUCCCU.AAGGCCUCAG....AAGACU...C....CUUAGGGACUGUU.UUUGCGCCUUC.......AUAUCGUCUUCCACUAACAAGAAAGAUUCCGUAGUCUGCAGCUUAAAUCGAGAUCAUUAGUCAGGCUUG..GAAU.GCGGA..GUCGA
Ssp-2-1  .GUCCCU.AUGGCCUCAG....AAGACU...C....CUUAGGGACA.G..UUU....UUGC.GCCUUCAUAUCGUCUUCCACUAACAAGAAAGAUUCCGUA.GUCUGCAGCUUGAAUCGAGAUCAUUCGUCAGGCUUG..GAAU.GCGGA..GUCGA
env-4    UCCCUAA.GGCC....UC....AGAAGA...C....UCCUUAGGGA.GUGUUU....UUGC.GCCUUCAUAUCGUCUUCCACUAACAAGAAAGAUUCCGUA.GUCUGCAGCUUGAAUCGAGAUCAUUCGUCAGGCUUG..GAAU.GCGGA..GUCGA
env-5    GUCCUCU.GUUG....AG....AGCGAU...C....AACAGAGGAC....UUU....UCUU.............AAUACUGAAAUCUGAAUUUAAGUAGU.GCAUAUGGACGGUUUGCCUGUGCACUCAGGAACGUA.UUCAG.GUGGAU.ACCGC
Ssp-1-1  .UUCCCC.GUUG....AG....AGGGAU...C....AAUGGGGAAC.CC.UCU....CUCU.....UAAA....GCUGUAGAAAUUCAGUGCAUCAGGCG.CAAAUCAGUGUGAC.........GUGGCGAACGUG.UUUAU.GCGGAU.AUCGC
env-6    .UUCCCC.GUUG....AG....A.AGAU...C....AGCGGGGAAA.UC.UCU....CUGUG...CCUGAA....AGGUCUGAAAGCUUUAGACAUUCAGU.GCAUCAGGCACAAAUCAAUGUGCCGUCGCGAGCAUA.UUUAC.GCGGAA.ACCGC
env-7    .UUCCCC.GUUG....AG....AGCGAU...C....AAUGGGGGAA.CC.UCU....CUGU...............GUCUAAAAGCUGUAGACAUUCAGU.GCAUCGGGCACAAAUCAGUGUGACGAAGCGAACGUG.UUUAU.GCGGAU.ACCGC
env-8    .UUCCCC.GUUG....AG....AGCGCU...C....AAUGGGGGAA.CC.UCU....CUGU...............GUCUUAAAGCUGUAGACAUUCAGU.GCAUCAGGCGCAAAUCAGUGUGACGUGACGAACGUG.UUUAU.ACGGAU.ACCGC
env-9    .UUCCCC.GUUG....AG....AGCGAU...C....AAUGGGGGAA.CC.UCU....CUGU...............GUCUAAAAGCUGUAGAAAUUCAGU.GCAUCAGGCGCAAAUCAGUGUGACGUGACGAACGUG.UUUAU.GCGGAU.ACCGC
env-10   .UUCCCC.GUUG....AG....AGCGAU...C....AAUGGGGGAA.CC.UCU....CUGU...............GUCUAAAAGCUGUAGAAAUUCAGU.GCAUCAGGCGCAAAUCAGUGUGACGUGAUGAACGUG.UUUAU.GCGGAU.ACCGC
Ssp-1-2  CAGUCCC.AGCAC...AC....GGCAAG.CGU....GAUGGGGUUG.GC.UUU....GUGCU...UAUCCCCG..UCUUCCAAUAACGGGAAAGACUUAAU.GCAUCGCACCGAAAUACAUAAUGCCUCGUCAACGUU.AUGGA.ACGGCA.ACUGC
env-11   AGUCCUU.AUCC....CAC...GGCAUCUG.G....GACGAGGGCUGGC.UUU....CUGCG...CUUAAUCCCGUCUUCCAAUAAUGGGAAAGUUUCAGU.GCAUCGCACCGAAGUGCAUAACGCUUCACCAAGAUU.GUGGA.ACGGCA.ACCGC
env-12   AGUCCUC.AUCC....CAC...GGCAUCUG.G....GACGAGGGCUGGC.UUU....CUGCG...CUUAAUCCCGUCUUCCAAUAACGGGAAAGUUUCAGU.GCAUCGCACCGAAGUGCAUAACGCUUCACCAAGCUU.UUGGA.ACGGCA.ACCGC
env-13   AGUCCCC.AUCG....CAC...GGCAA....CUGUGGAUGGGGCUG.GC.UUU....CUGCG...CUUAACCCCGUCUUCCAAUAACGGUAAAGAUUCAGU.GCAUUGCACUUGAUUGCAGAACUCGUCGGCAGCGUU.AUGGA.ACGG...ACCGC
env-14   AGCCCCC.AUCC....CAC...GGCAACUGUG....GAUGGGGCUG.GC.UUU....CUGCG...CUUAACCCCGUCUUCCAAUAUCGGGAAAGAUUCAGU.GCAUUGCACUUGAUUGCACAACUGCUCGCCAACGUU.CGGGA.ACGGA...CCGC
env-15   AGCCCCC.AUCC....CAC...GGCAACUGUG....GAUAGGGGCUGGC.UUU....CUGCG...CUUAACCCCGUCUUCAAAUAAAGGGAAAGAUUCAGU.GCAUUGCACUUGAUUGCACAACUUCUCGCCAGCGUU.AGGGA.ACGG...ACCGC
env-16   AGGCCCC.AUCC....CAC...GGCAACUGUG....GAUGGGGCCUGGC.UUU....CUGCG...CUUAACCCCGUCUUCCAAUAACGGGAAAGAUUCAGU.GCAUUGCACUUGAUUGCAGAACGCCUCGGCAGCGUU.AUGGA.GCGGA...CCGC
env-17   AGUCUCA.UUAC....AA....GGCAAA..CG....UGAUGGGGCU.GU.UUU....CUGCG...CUUAACCCCGUCUUCCAAUAAUUGGAAAAAUGCAGU.GCAUUGCACUUGAUUGCACAACUCCUCGUCAGCGUU.AGGGA.ACGG...ACUGC
Ssp-2-2  .CUCCUU.GGCA....CG....AGCGAU...U....ACCAGGGAGA.GC.UUU....CUGC.......GA....UCUUUAGAUGUCUUAAAACACAUGGA.GUAUUUGACUCAAAGGUAUGUGCCGUUGCGAGCUUU.GUUAU.ACGUA..AUCGC
env-18   .CUCCUU.GGCA....CG....AGCGAU...U....GCCAGGGAGA.GC.UUU....CUGC.......GC....UCUUUAGAUGUCUUAAAAUAUAUGGU.GUAUUUGAUUCAAAAGUAUGUGCCGUUGCGAGCUUU.AUUUU.ACGUA..AUCGC
env-19   .UCCCUU.GGCA....UC....AGCGAU...U....GCCAGGGGGA.GC.UUU....CUGC.......GC....UCUUCAGAUCUCUGAAAAGAGUUGGU.GCAUUUGCAGCAAGGCCUUGUAUCGCGGCGAACUUG.UUCAA.GUGCGA.ACCGC
env-20   CCCCCUU.GGCA....UG....AGCGAU...U....GCCAUGGGGA.GC.UUU....CUGC.......GC....UCUUCAAAUCUCUGAAAAGAGUUAGU.GCAUUUGCUACAAGCCCGUGUAUCGCCGCGAGCUUG.UUCAU.GCGGAU.ACCGC
env-21   .UCCCUU.GGCA....CG....AGCGAU...U....GCCAGGGGGA.GC.UUU....CUGC.......GC....UCUUCAGAUCUCUGAAAAGAGUUAGU.GCAUUUGCAACAAGCACGUGUAUCGCGGCGAGCUUG.AUCAG.GCGGAU.ACCGC
env-22   .UCCCUU.GGCA....CG....AGCGAU...U....GCCAGGGGGA.GC.UUU....CUGC.......GC....UCUUCAAAUCUCUGAACAGAGUUAGU.GCAUUUGCUACAAGCCCGUGUAUCGCGGCGAGCUUG.UUCAU.GCGGAU.ACCGC
env-23   .UCCCUU.GGCA....CG....AGCGAU...U....GCCAGGGGGA.GC.UUU....CUGC.......AC....UCUUCAGAUCUCUGAAAAGAGUUGGU.GCAUUUGCGACAAGCACUUGUAUCGCAGCGAGCUUG.UUCAU.GCGGAU.ACCGC
env-24   .UCCCUU.GGCA....CG....AGCGAU...U....GCCAUGGGGA.GU.UUU....CUGC.......GC....UCUUCAAAUCUCUGAAAAGAGUUAGU.GCAUUUGCGACAAGCGCCUGUAUAGCGGCGAACUUG.UUUAA.GCGGAU.ACCGC
env-25   .CCCCAU.GGCA....CG....AGCGAU...U....GUCAUGGGGA.GC.UUU....CUGC.......GU....UCUUCAGAUCUCCGAAAAGAGUUGGU.GCAUUCGCACUAAAGCUGUGUAUUGCGACGAACUUG.UUUGCAGCGGAU.ACCGC
env-26   .UCCCUU.GGCA....CG....AGCGAU...U....GCCAGGGGGA.GC.UUU....CUGC.......GC....UCUUCAGAUCUCUGAAAAGAGUUGGU.GCAUUCGCGCUAAAGCCUUGUAUAGCGACGAACUUG.UUUUCAGCGGAU.ACCGC
env-27   AACCCUU.GGCA....CG....AGCGAU...U....GUCAGGGGUA.GC.UUU....CUGC.......GC....UUUUCAAAAAUCGGAAGAAUUUUGUA.CCUUUUGAGACGAAGGUUUGUAUACCGAGGAACGUG.UUUAA.ACGGAU.ACCGC
env-28   GACCCUU.GGCA....CG....AGCGAU...U....GUCAGGGGUG.GC.UUU....CUGC.......GC....UUUUCAAAAAUCGGAAGAAAUUUGUA.CCUUUUGAGACAAAGGUUUGUAUGCCGAGGAACUUG.UUUAA.GCGGAU.ACCGC
env-29   .ACCCUU.GGCA....CG....AGCGAU...U....GUCAGGGGUA.GC.UUU....CUGC.......GC....UUUUCAAAAAUCGGAAGAAAUUUGUA.CCUUUUGAGACGAAGGUUUGUAUACCGAGGAACGUG.UUUAA.GCGGAU.ACCGC
env-30   AACCCUU.GGCA....CG....AGCGAU...U....GUCAGGGGCG.GC.UUU....CUGC.......GC....UUUUCAGAAAUCGGAAGAAAUUUGUA.CCUUUUGAGACGAAGGUGUGUAUACCGACGAACUUGCUUUCA.GCGGAU.ACCGC
Ssp-1-3  .ACCCUU.GGCA....UG....AGCGAU........GUCAGGGGUU.GC.UUU....CUGC.......GC....UUAUCAAAAAUCUGAAGACAUUUGGU.GCAUUUGCGUAAGAGGUUUGUAACGCGCCGAACGUG.UUUAA.GCGGAU.ACUGC
env-31   .AGUCCC.AGUAC...UC....GGCAAG.CGU....GAUGGGGCUU.GC.UUU....CAGC.......GC....UCUUCAGAUCUCUGAAAAGAGUCGGU.GCAUU..CAUUAAAGCUACGUAAUGCGACGAUCUUG.UUUGCAGCUGAU.ACCGC
env-32   CAGUCCCCAUCC....CAC...GGCAACUGUG....GAUGGGACUG.GC.UUU....CUGC.......GC....UCUUCAGAUCUCUGAAAAGAGUUGGU.GCAUUCGCACUAAAUCUUUGUAUUGCGACGAACUUG.UUUGUAGCGGAU.ACCGC
env-33   UGUCUCC.CACG....CG....AGCGAU...C....GUGGGAGACA.AA.GUU....CUGU.............GUAAAUGAAUUCUGAAGAGACUUGGU.GCAAUUGCCAGCAAGUACUGGUUCGGACCGAGCUUG.UUGAA.GCAGAC.ACUGC
env-34   .GUCUCC.UGCG....UG....AGCGAU...C....GCGGGAGACA.UC.GUU....CUGU.............GGAACAGAA.CCCGAAGAGACUUGGU.GGGUUUGCAGCGAAGCUCUGUGUCAGGCCGAACUUG.UACAU.ACAGUC.ACCGC
Ssp-1-4  UGUCUCC.UGCG....UG....AGCGAU...C....GCGGGAGACA.CC.GUU....CUGU.............GCAACGGAAUUCUGAAGACACUUGGU.GUGUUCGUAGGGAAACUACGUAACACGGCGAACGUA.UGUUC.ACCGAU.GCCGC
Ssp-1-5  UGUCUCC.UGCG....UG....AGCGAU...C....GCGGGAGGCA.CC.GUU....CUGU.............GCAACGGAAUUCCGAAGAAAGUUGGU.GCGUUCGUGGAGAAACAAAGUAGCGCAGCGAACUUG.UAAUC.ACCGAU.GCCGC
env-35   UGUCUCC.UGCG....UG....AGCGAU...C....GUGGGAGACA.CA.GUU....CUGU.............GCAACGGA.UUCCGAAAAGACUUGGU.GGGUUCGCCAAGAAGCUUU.UAGCGCGGCGAAUUUG.GUUGU.CCCGAUGGUGGC
env-36   UGUCUCC.UGCG....UG....AGCGAU...C....GUGGGAGACA.CC.GUU....CUGU.............GCAACGGAAUCCUGAAAAGACUUGGC.GCGUUCGCCGAGAAGUUCAGUAGCGCGGCGAACUUG.GUCGU.ACCGACGGUCGC
env-37   UGUCUCC.UGCG....UG....UGCGAU...C....GCGGGGGACA.CC.GUU....UUGU.............GCAACGGAAUCCUGAAAAGACUUGGU.GCGUUCGCCGAGAAGUUCAGUAGCGCAGCGAAUUUG.GUUGU.ACCUACGGUCGC
env-38   UGUCUCC.UGCG....UG....AGCGAU...C....GCGGGGGACA.CC.GUU....UUGU.............GCAACGGAAUCCUGAAAAGACUUGGU.GCGUUCGCCGAGAAGUUCAGUAGCGCAGCGAAUUUG.GUUGU.ACCGACGGUCGC
Ssp-2-3  UGUCUCC.UGCG....UG....AGCGAU...C....GUAGGAGACA.CC.GUU....CUGU.............GCUUCGGAAUCCUGAAAAGACUUGGU.GCGUUCGCCAAAAAGUUCAGUGGUGCGGCGAACUUG.GUUGU.ACCGAUGGUCGC
env-39   UGUCUCC.UGCG....UG....AGCGAU...C....GUAGGAGACA.CC.GUU....CUGU.............GCUAUAGAAUCCUGAAAAGACUUGGU.GCGUUCGCCAAAAAGUUCAGUGGUGCGGCGAACUUG.GUUGU.ACCGAUGGUCGC
env-40   CGUCUCC.UGCG....UG....GGCGAU...C....GUGGGAGACG.GC.GUU....UUGU.............GAAAUGGAAUCCUGAAAAGACUUGGU.GCGUUCGCCGAGAAGCUCAGUAGCGCGGCAAACUUG.GUUGU.ACCGAUGGUCGC
env-41   UGUCUCC.CGCG....UG....AGCGAU...C....GCGGGGGACA.CC.GUU....CUGU.............GCAACGGAAACCUGAAAAGAGUCAGU.GCAUAUGACGCACAGCCCUGUAUCGGAACGAGCUUG.UGAAG.GCAGAC.ACCGC
env-42   UGUCUCC.CGCG....UG....AGCAAU...C....GCGGGGGACA.CC.GUU....CUGU.............GCAACGGAAACCUGAAAAGAGUCAGU.GCAUAUGACGCACAGCCCUGUAUCGGAACGAGCUUG.UGAAG.GCAGAC.ACCGC
env-43   .GUCUCC.UGCG....UG....AGCGAU...C....GCGGGGGACA.AC.GUU....CUGU.............GCAACGGAAUCCUGAAAAGAGUCAGU.GCAAACGACGCAUAGCUCUGUAUCACUACGAGCUUG.UGCAU.GCAGAC.ACCGC
Ssp-2-4  UGUCUCC.CGCG....UG....AGCGAU...C....GCGGGGGACA.CC.GUU....CUGU.............GCAACGGAAUGCUGAAAAGAGUCAGU.GCAUACGACGCAUGGCUCCGUAUCAGGAGGAGCUUG.UGUGU.GCAGAC.ACCGC
Ssp-2-5  UGUCUCC.UGCG....UG....AACGAU...C....GCGGGAGACA.AC.CUU....CUGU.............GCAACGGAAUGCUGAAAAGAGUCAGU.GCAUACGACGCAUGGCUCCGUAUCAGGAGGAGCUUG.UGUGU.GCAGAC.ACCGC
env-44   .GUCUCC.UGCG....UG....AGCGAU...C....GCGGGAGACA.CC.GUU....CUGU...............GCCAUAAACCUGAAAAGAGUUAGU.GCAUUUGAUGCACAGCUCUAUAUCAGUACGAGCUUG.UGAAU.GCAGAC.ACCGC
env-45   .GUCUCC.UGCG....UG....AGCGAU...C....GCGGGAGACA.CC.GUU....CUGU...............ACCAUAAACCUGAAAAGAGUUAGU.GCAUUUGAUGCACAGCUCUAUAUCAGUACGAGCUUG.UGAAU.GCAGAU.ACCGC
env-46   UGUCUCC.UGCG....UG....AGCGAU...C....GCGGGAGACA.CU.GUU....CUGU...............GUCACAAACCUGAAAAGAGUUAGU.GCUUUUGUUGCGCAGCUCCGUAUCGGGACGAGCUUG.CAUUU.GCAGAC.ACCGC
env-47   .GUCUCC.CGUG....CG....AGCGAU...C....GCGGGAGACA.AC.GUU....CUGC.............GCUGUUUAAAUCUGAAGAAAACUAGU.GCGUAUGGCCCAUCUGGGAACGGGUGAACGAGCUUU.ACCUU.UCAGUU.UCCGC
         <<<<<<<.<<<<...................>....>>>>>>>>>>....>>>....>>>>...........................................................................................<<<<<..<<<<<
         ?222222.2222...................2....222222222?....2??....12?2...........................................................................................21?0?..?2202
         ○●YCYYY-●●YR----YG----AGCGAU---Y----R●●RGRGR●●-●C-●UU----CUGY--------○○----○○●●C●RA●●●C●GRARARA●UYRGU-GCRUYYG●●●YR●R●●Y●YRUR●Y●●●RYGARC●UR-●●●R●-RCRGA○-ACCGC
```

alignment positions 308 · · · 467

env-1	AUCGCUCGGGAGACAUUGGCGAGA......CCGAUGUCACUCUCGGC.UCCGCAGUCAGCGGCAGCAGCCCCAACGACAUUGAGAGGCUUUUCUGCUUCAGCCUGCCUCAGGGGCCCAACCUGUCUGCUUCAGGUCCUGAAAGCGCACAGACCAAAGCUC
env-2	AUCGCUCGGGAAACGUCGGCGCGA......CUGAUGUCUCUCUCGGC.UCCGCGGUCAGUGGCAGCAGCUCCAACGGUAUUUAGGGGCUUUCCUGUUUCAGCCUGCCUCAGGUGCCCAACCUGUCUGCUUCAGGUCCUGAAAGCGAACAGACCAAAGUUC
env-3	AUCGCUCGGGAAACGUCGGCGCGA......CCGUUGUCUCUCUCGGC.UCCGCGGUCAGUGGCAGCAGCUCCAACGCUAUUUAGGGGCUUCCCUGUUUCAG...
Ssp-2-1	AUCGCUCGGGAAACGUCGGCGCGA......CCGAUGUCUCUCUCGGC.UCCGCGGUCAGUGGCAGCAGCUCCAACGGUAUUUAGGGGCUUUCCUGUUUCAGCUUGCCUCAGGUGCCCAACCUGUCUGCUUCAGGUCCUGAAAGCGAACAGACCAAAGUUC
env-4	AUCGCUCGGGAAACGUCGGCGCGA......CCGAUGUUUCUCUCGGC.UCCGCGGUCAGUGGCAGCAGCUCCAACGGUAUUUAGGGGCUUUCCUGUUUCAGCCUGCCUCAGGUGCCCAACCUGUCUGCUUCAGGUCCUGAAAGCGAACAGACCAAAGCUC
env-5	AUCGCUCGGGGAGGA....CAAGU..........UCCUCCUCGCGGUUCCCGUCUUUAAAUUUCGCCUAAAAGUCUUCGGCUCAGGAAGACGAGACGGAACGAAGCCAUUGGUCCUGUCGGCUACGCAGUGCAGACAGGUCAAGGCCAGUGGAAUGAGCU
Ssp-1-1	AUCGCUCGGGGAGGA....CUCGC..........UUCUCCUCGCGGU.UCCGUGGCUCCACAACGUUAAGUCGCAGCUUGGUUGAAGGUCAGCCAUCGCGGCAGAAGGAGCUGCUCAAAAACGCGGAUCACACCGCUCACAUCAUUCGUUUUCAUGAGAC
env-6	AUCGCUCGGGCAGGA....CCUGU..........UUCUCCUCGCGGU.UCCGUGGCUCCACAUCGUUAAGUCGCAGCUUGGUUGAAGGUCAGCCAUCGCGGCAGAGGGAGCUGCACGAAAAUGCCGGCCGCGCCUCUUCUCACCAAUCAAUUCAAUAAGG
env-7	AUCGCUCGGGGAGGA....CUCGC..........UUCUCCUUGCGGC.UCCGUGGCUCCACGUCGUUAAGUCGCAGCUUGGUUGAAGGUCAGCCAUCGCGGCAGAGGGAGCUGCUCAAAAACCUCGGCCACGCCCCUCCACACCNNNNNNNNNNNNNNNN
env-8	AUCGCUCGGGGAGGA....CUCGC..........UUCUCCUCGCGGU.UCCGUGGCUCCACAUCGUUGUGUCGCAGCUUGGUUGAAGGUCAGCCAUCGCGGCAGAGGGAGCUGCUCGAAAACGCCGAUCACACCGCUCGCAUCAUUCGAUUUCAUGAGAC
env-9	AUCGCUCGGGGAGGA....CUCGC..........UUCUCCUCGCGGU.UCCGUGGCUCCACAUCGUUAAGUCGCAGCUUGGUUGAAGGUCAGCCAUCGCGGCAGAGGGAGCUGCUCAAAAACCUCGGCCACGCCCCUCCACACCAAUCGAUUCAAUGUGU
env-10	AUCGCUCGGGGAGGA....CUCGC..........UUCUCCUCGCGGU.UCCGUGGCUCCACAUCGUUAAGUCGCAGCUUGGUUGAAGGUCAGCCAUCGCGGCAGAGGGAGCUGCUCAAAAACCUCGGCCACGCCCCUCCACACCAAUCGAUUCAAUGUGC
Ssp-1-2	AUCGCUCGGGGAGAA....UGCUG..........UUCUCCUCGCGGU.UCCGUCGCCCCACAUCAUUGCUUCGGUUAAGGCUGUUGGAAGUCAGCCCAACCAAUAGAAGGGGCUGCGCCGCAUCAAGAAGACGAGCAUCUCGGAAAUCACCGCACGUAGC
env-11	AUCGCUCGGGGAGAA....CGCAG..........UUCUCCUCGCGGU.UCCGUCGUUCCACAUCAUUGCUUUGGUUAAGGCUGUUGAAAGUCAGCCCAACCGAUAGAAGGGGCUGUUUUUGUAAUCACUGACUAGAUGUCGUUUAUGCGGCAGGCAUGAC
env-12	AUCGCUCGGGGAGAA....CGCUG..........UUCUCCUCGCGGU.UCCGUCGCCCCACAUCAUUGCUUCGGUUAAGGCUGUUGAAAGUCAGCCCAACCGAUAGAAGGGGCUGUUUUUGUAAUCACUGACUAGUUGUCGUUUAUGCGGCAGGCAUGAC
env-13	AUCGCUCAGGGCGAA....CAAGU..........UUCUCCUCGCGGU.UCCGUAGUCCCGCAUCUUUGAUUCGGUAACGGCUGUUGGAAGUCAGCCUCGCCGAUAGAGCGGGCUGCCAUCACGCAAUUAGCCAACUUCAGGAGCCCAGCCCAUCUCUCCU
env-14	AUCGCUCGGGGCGAA....CAUCU..........UUCUCCUCGCGGC.UCCGUAGUCCCGCAUCUUUAAUUCGGCAACGGCUGUUGGAAGUCAGCCACGCCGGUAGAGGGGACUGACAUCACGCAAUUAAUCAGACAUCACUAGGGUCUUGUCCCGCAGA
env-15	AUCGCUCGGGGCGAA....CAUCU..........UUCUCCUCGCGGU.UCCGUAGUCCCGCAUCUUUAAUUCGGCAACGGCUGUUGGAAGUCAGCCCCGCCGAUAGAGGGGACUGACAUCACGCAAUUAAUCAGACAU......................
env-16	AUCGCUCGGGGCGAA....CAAGC..........UUCUCCUCGCGGC.UCCGUAGUCCCGCAUCUUUGAUUCGGUAACGGCUGUUGGAAGUCAGCCCCGCCGAUAGAGGGGGCUGCCGUCACGCAAUUAGCCAGCUUCGUGAGCCCAGCCCAUCUCCUUC
env-17	AUUGCUCGGGGCGAA....CAUCG..........UUCUCCUCGCGGU.UCCGUAGUCCCGCAUCUUUAAUUCGGCAACGGAUGUUGGAAGUCAGCCCGGCCGAUAAAGGGGGGCUGACGCGAUGGUGCNNNNNNNNNNNNNNNNNNNNNNNNNNNNNNN
Ssp-2-2	AUCGCUCGGGGCAAA....CAUCG..........UUUUCCUCGCGGU.UCCGUGGUCCCACAUCAUUGAUUUGGUCAACGGCUGUGAGUCAGCCCAACAAAUAGAAGGGGCUGCCUUGGGAUACCCACUGGAGUUGGGCAGAGCGAUCGACAUGUAGUGC
env-18	AUCGCUCGGGGCGAA....CAUCG..........UUUUCCUCGCGGU.UCCGUGGUCCCACAUCAUUGAUU...
env-19	AUCGCUCGGGGAGAA....CAGCG..........UUCUCCUCGCGGU.UCCGCCGCCCCACAUCAUUGCUUCGGUGACGCUGUUGGAAGUCAGCCU..
env-20	AUCGCUCGAGGAGAA....CAGCG..........UUCUCCUCGCGGU.UCCGUGGCCCCACAUCUUUGAUUCGGUAAUGGCUGUUGGAAGUCAGCCCUGCCGAUGGAAGGGGCUGCAGAGCCGAGUGCUGAUUGCUAUCUGGUUUGGUUGCGAUUUAUUG
env-21	AUCGCUCGGGGAGAA....CAGCG..........UUCUCCUCGCGGU.UCCGUCGCCCCACAUCAUUCCGGUUAAGGCUGUUGGAAGUCAGCCCCGCCGAUAGAAGGGGCUGCUAAGCCGCAACUGGUUCGUGGAUUCUGGGCACGAUAUAAGUCACCAG
env-22	AUCGCUCGGGGAGAA....CAGCG..........UUCUCCUCGCGGU.UCCGUCGCCUAACAACUUUGAUUCGGUAAUGGCUGUUGGAAGUCAGCCCCGCCGAUGGAAGGGGCUGCAGAGCCGAGUGCUGAUUGCUAUCUGGUUUGGUUGCGAUUUAUUG
env-23	AUCGCUCGGGGAGAA....CAGCG..........UUCUCCUCGCGGU.UCCGUCGCCCCACAUCUUUGAUUCGGUAGUGGCUGUUGGAAGUCAGCCCCGCCGAUAGAAGGGGCUGCUAGGCCGCCACUGGUUCGUGGAUUCUUGGCACGAUAUGAGUCAC
env-24	AUCGCUCGGGGCGAA....CAUGU..........UUCUCCUCGCGGU.UCCGUAGUCCCGCAUCUUUGAUUCGGUAACGGCUGUUGGAAGUCAGCCCCACCGAUAGAGGGGGCUGACGUCACGCAAUUAGCCUGCUUCGGGAGCCCAGCCCAUUACCUUG
env-25	AUCGCUCGGGGAGAA....CGGCG..........UUCUCCUCGCGGU.UCCGUGGUCCCGCAUCUUUGAUUCGGUUAAGGCUGUUGGAAGUCAGCCCCGCCGAUAGAGGGGGCUGACAUCACGCAUAACCUUGAUCGCAUGACGCAUGAAAUCCCGAUCC
env-26	AUCGCUCGGGGAGAA....CGUCG..........UUCUCCUCGCGGU.UCUGUGGCCUCACAUCAUUGAUUCGGUAAGGCUGUUGGAAGUCAGCCCGACCGAUGGAAGGGGCUGCAGAGCCGAGUGCUGAUUGCUAUCUGGUUUGGUUGCGAUUUAUUGG
env-27	AUCGCUCGAGAAGAA....CAUCG..........UUCUUCUCGCGGU.UCCGUGGUCCCGCAUCUUUGAUUCGGUAACGGCUGUUAGAAGUCAGCCUCGCCGAUAGAGGGGGCUGCAAUCAGGACCUACAGCUAGGUGCAGACCCACCCACCAGAGCCGA
env-28	AUCGCUCGGGGAGAA....CGUCG..........UUCUCCUCGCGGU.UCCGUGGCCUCACAUCAUUGAUUCGGUAAGGCUGUUGGAAGUCAGCCCGACCGAUGGAAGGGGCUGCAGAGCCGAUAGCUGAUUUCUAUCUGGUUUGGUUGCGAUUUAUUGG
env-29	AUCGCUCGGGGA.......CGUCG..........UUCUCCUCGCGGU.UCCGUGGCCUCACAUCAUUGAUUCGGUAAGGCUGUUGGAAGUCAGCCAUCCGCCGAUGGAAUGGGCUGCAGAGACCGAUAACUACACGUGAACAACAAGCAAUCCCCUUGAU
env-30	AUCGCUCGGGGAGAA....CGCUG..........UUCUCCUCGCGGU.UCCGUCGCCCCACAUCAUUGCUUCGGUUAAGGCUGUUGAAAGUCAGCCCAACCGAUAGAAGGGGCUGUUUUUGUAAUCACUGACUAGUUGUCGUUUAUGCGGCAGGCAUGAC
Ssp-1-3	AUCGCUUGGGGAGGA....CUAGU..........UCCUCCUCGCAGU.UCCGUGGCCCCAAGUCCUUGCUUCGGUUGUUGACCUACCGACGGAGGGGUCUGUCAGUAAGUAAGGCGAUCUGGAACCGCUGAUCAGUGGUUAGGGAGAAGGAGGCAGCCGU
env-31	AUCGCUCGGGGAGAA....CGUCG..........UUCUCCUCGAGGG.UCCGUGGCCCCACUCCCUUGAUUCGGUUAGCGGCUGUUAGUCAGCCCAACCGAUAGAAGGGGCUCUCGUCACAGAUGUCGUCGAGAAUAUUGGCCAUCUUCAGGUUGGGCGU
env-32	AUCGCUCGGGGGGAA....CGCUG..........UUCUCCUCGCGGU.UCCGUCGCCCCACAUCAUUACUUCGGUUAAGGCUGUUGGAAGUCAGCCUGACCGAUUGAAGGGGCUGCGCGACAUCAAGAAGACGAGGAUCUCGGGAAUCACCACACGUAG
env-33	AUCGCUCGGGAAGAG....CA.AG..........CUCUUCUCGCAGU.UCUGUAGUCCUUUCCCUAUUUAGGUGGCAGGCAUGUUCGCCCCACCUAAUAGAGGGGGCUGUCUAAAGGUCCUUGUUUAAGCGGCCUCUUCAUCUCCUGCUUCAUGCAGAGA
env-34	AUCGCUCGGGAAGAG....CA.AG..........CUCUUCCCGCGGUUUCUGUGGUCCUUUUCUGCUCAGGUGCUCGGUCAUGCCUCACCUGUGGAGGGGGCUGCCGUGCGGCCUGAGCUGGUGGUCAAGCCCCACGUUGACCUUGCGUCGACGAAAGCC
Ssp-1-4	AUCGCUCGGGAAGGA....CAGAG..........UUCUUCUCGCGGCCUCAGUAGCCUCCCUCCCUCAGGAGCCGUUAGGCCCUGGAGGGUGCUGCCUGUUUUAGAGCAGAGUCAGAUAUCAAAGCGUUGUUCGCCUAGAACCCCCAACGUGUCCGACAU
Ssp-1-5	AUCGCUCGGGAAGGA....CAGAG..........UUCUUCUCGCGGCCUCAGUAGCCUCCCUUCCUUAGGAGCCGUUAAGCCCUUUGGAGGGUGCUGCCUUGUUGCUCUGGCCCAGCUGGAGCAAGGACAACCGAUGAUGUUUGAACCUCAACUGCUGAC
env-35	AUCGCUUGGGAGGGA....CAGCG..........UCCUUCUCGCGACCUCAGCAGCCCCCCUUCAUCAGGAGCCAUGGCGCCCUGCGGAGGGUGCUGCAUUGCUGUAUUGGCAGCCAAUACGAAUUGGCUAAAAAUGUUUAAUGGCAGAGGCCUUAAAUG
env-36	AUCGCUCGGGAAGAA....CAGCG..........UUCUUCUCGCGGCCUCAGUAGCCCCCCUCCCUGGAG..
env-37	AUCGCUCGGGAAGGA....CAGCG..........UUCUUCUCGCGGCCUCAGUAGCCCCCCUCCGUUAGGAGCCGUAAGGCCCUGGAGGAUGCUGCCUGGUGCCUGGAUCAACAUCGGGGACUCAGGCGGUCAAAAGAAACCCCGGAGAGAUCUCCGAG
env-38	AUCGCUCGGGAAGGA....CAGCG..........UUCUUCUCGCGGCCUCAGUAGCCCCCCUCCGUUAGGAGCCGUAAGGCCCUGGAGGAUGCUGCCUGGUGCCUGGAUCAACAUCGGCGACUCAGGCGGUCAAAAGAAACCCCGGAGAGAUCUCCGAG
Ssp-2-3	AUCGCUCGGGAAGGA....CAGCG..........UUCUUCUCGCGGCCUCAGUAGCCCCCCUCCCUUGGAGCCGUUAGGCCCUGGAGGGUGCUGCGCCUUAACUGGUUAGCCAGUUGGCCAGCUAUCCGAGUAGGCGGCCAUUUGGAUAGUCCGCUAGGG
env-39	AUCGCUCGGGAAGGA....CAGCG..........UUCUUCUCGCGGCCUCAGUAGCCCCCCUCCCUUGGAGCCGUUAGGCCCUGGAGGGUGCUGCGCCUUAACUGGUUAGCCAGUUGGCCAGCUAUCCGAGUAGGCGGCCAUUUGGAUAGUCCGCUAGGG
env-40	AUCGCUCGGGAAGAA....CCGCG..........UUCUUCUCGCGGCCUCAGUAGCCCCCCUCCCUUGGAGCCGUUAGGCCCUGGAGGGUGCUGCCUGGAUCGAAUCGGCAACUGAGGCGGCAAACCUUCCAAAUUUAGCUUGACCAGAAGCCAGACAAU
env-41	AUCGCUCGGGAAGAG....CA.AG..........CUCUUCUCGCGGUCUCUGCAGUCCCUUCCCUGAUCAGGUAGCUGGCUUGUAAGCACUGCCUGUAGAAGGGGCUGCCUGGAACGACAUCGGCGACUGAGGCGGCCAAAAAGAAACCCCGGAGUGAUC
env-42	AUCGCUCGGGAAGAG....CA.AG..........CUCUUCUCGCGGUCUCUGCAGUCCCUUCCCUGAUCAGGUAGCUGGCUUGUAAGCACUGCCUGUAGAAGGGGCUGCCUGGAACGACAUCGGCGACUGAGGCGGCCAAAAAGAAACCCCGGAGUGNNN
env-43	AUCGCUCGGGAGGAG....CA.AG..........CUCUUCUCGCGGUCUCUGCAGUCCUUUCCCUGAUCAGGUAGCUGGCUUGUGAGCCCUACCUGUAGAAGGGGCUGCCUGGAUCGACUCAGCAACUGAGGCGGCCAAAAAAAAACCCCGCCAAAGGCA
Ssp-2-4	AUCGCUCGGGAAGAG....CA.AG..........CUCUUCUCGCGGUCUCUGCAGUCCUUUCCUUGAUCAGGUAGCUGGCUUGUAAGCACUGCCUGUAGAAGGGGCUGCCUGGAACGACAUCGGCGACUGAGGCGGCCAAAAAGAAACCCCGGAGUGAUC
Ssp-2-5	AUCGCUCGGGAAGAG....CA.AG..........CUCUUCUCGCGGU.UCUGUAGUCCUUUCCCCGAUCAGGUAGCUGGCUUGUAAGCCCUACCUGUAGAAGGGGCUGCCUGGAUCGACAUCGGCAACUGAGGCGGUCAAAAAGAAACCCCGGAGAGAUC
env-44	AUCGCUCGGGGAGAA....CAGCG..........UUCUCCUCGCGGU.UCCGCCGCCCCACAUCGUUGCUUCGGUAAGGCUGUAGUGAGUCAGCCCAGCCGAUAGAAGGGGCUGUCAGACCCUCGGACAAAUGUGAGUCACGGGGUCAGCUGCUCAUGCU
env-45	AUCGCUCGGGGAGAA....CAGCG..........UUCUCCUCGCGGU.UCCGCCGCCCACAUCAUUGCUUCGGUAAGGCUGCAGAGAGUCAGCCCAGCCGAUAGAAGGGGCUGUAAGACCCUCGGACAAAUGUGAGUCACGAGGUCAGCUGCUCAUGCUC
env-46	AUCGCUCGGGAAGAG....CA.AG..........CUCUUCUCGCGGUCUCUGUAGCCCUUUCAACGAUUAGGCAGCUGGAUUGCCUGUAGAAGGGGCUUUGGGCGCCUCCCCCACGUGAUCGACGUCUGAAGCCACAUCAGAAUGGUCAUACCGAUCAA
env-47	AUCGCUCGGGAAGGG....CA.AC..........CUCUUCUCGCGGUCUCCGCAGUCCUUUCCCUGAUCAGACGGCCGGCAAUUGAGCCCCGUUUGUAGAGGGGGCUGCCCUACUGCGACUGAGGUGUUGUUAUUUCAGCUAUCAAGCUGGGGGUAGCUG
	.......<<<<<<<<<<<<...........>>>>>>>>>>>>>>>>>.>>>>>...
	.......1202?222?110...........011?222?20212022?.?0?12...
	AUCGCUCGGGRAGRR----CR•••----------UUCUYCUCGCGGY-UCYGURGYCCY•Y•YC•Y•••••••••••••••Y•••RR•••••••••••••••••R••R••

3′

alignment positions 468 · · · 627

```
env-1    UUCAUCGGUGACAUUGCCGUUGUCAUCCAACAAUUCAGGAUGGACCGUGCGUAGGCCUGUUCGGUCACUCUUGAACGCAGUCGAAGAGUUAGACGACAUGCUCAUCAUUCGAAAAGCCUGAAGCAGCAAACCAAUGAACGAUGCCGCGUAAAUCAAGGGA
env-2    UUCAUCGGUGACAUUGCCGUUGUCGUCCAACAAUUCAGGAUGGACAGUGCGUAAGCCUGUUCGGUCACUCUUGAACGCUGUCGAAGCGUUGGACGACAUGCUCAUCAUUCGAAAAGCCUGAAGCAGCAAACCAGUGAACAAUGCCGCGUAAAUCAAAGGG
env-3    ................................................................................................................................................................
Ssp-2-1  UUCAUCGGUGACAUUGCCGUUGUCGUCCAACAAUUCAGGAUGGACAGUGCGUAGGCCUGUUCGGUCACUCUUGAACGCUGUCGAAGCGUUGGACGACAUGCUCAUCAUUCGAAAAGCCUGAAGCAGCAAACCAGUGAACAAUGCCGCGUAAAUCAAAGGG
env-4    UUCAUCGGUGACAUUGCCGUUGUCGUCCAACAAUUCAGGAUGGACAGUGCGUAGGCCUGUUCGGUCACUCUUGAACGCUGUCGAAGCAUUGGACGACAUUCUCAUCAUUCGAAAAGCCUGAAGCAGCAAACCAGUGAACAAUGCCGCGUAAAUCAAAGGG
env-5    CAAUAUGCGAGAUAGCUUGUAAGCAAAGGAACGACCUUGAUGACCUCGCUUAAAUCUGUUCUUGCGUCGCUACUGAUUGGUAUAGCGGCAGCGCCAUUCGUUGGCCUUGCAGAUGCUGAAGCUCAAACUGCUUCGCAGCCUGGUUAUUCACGCACUACGC
Ssp-1-1  CUUGAUGGACGAAUCGUGAAAGAAAAAUAAUUACCUAUUUGCGAUAUUUAAAGCUUUAAAAUUAAACAAACAAUUAAGUGCUUAAGUCGUUCCGUUAAUUAAAGGAGGACAGUAUGCACAGUCAGAAGAACUCCCCGGCCUGCUUUUUGCGCUGGUCUGU
env-6    CCCUGACAAGCAAAUCAUGAAAGAAAUAUAAUUACCUAUUUGCGAUAUUUAAAGCUUUGAGAUGCAACAAACAAGCAAGUGCUUAAGUCGUCCCGUUAUUUCAUGGAGGACAGUAUGCAAAGUCAGAAGAACUCCCCGGCCUGCUUUUUGCGCUGGUCUG
env-7    NNNNNNNNNNNNNNNNNNNNNNNNNNNNNNNNNNAUCAUGAAAGAAAUAUAAUUACCUAUUUGCGAUAUUUAAAGCUUUGAAAUCAAACAAGUAAUCAAGUGCUUAAGUCGUCCCGUUAUUUCAUGGAGGACAGUAUGCACAGUCAGAAGAACUCCUCG
env-8    CGUGAGGAGGAAAUCAUGAAAGAAAAAUAAUUACCUAUUUGCGAUAUUUAAAGCUUUGAGAUCCAUCAAACAAGCAAGUGCUUAAGUCGUCCCGUUAUUUCAUGGAGGACAGUAUGCACAGUCAGAAGAACUCCCCGGC.....................
env-9    ACUUGACGAGCAAAUCGUGAAAGAAAAAUAAUUACCUAUUUACGAUAUUAAAAGCUUUAAAAUUAAAUGAUCAGGCAAGUGCUUAAGUCGUCCCGUUAUUUAAAGGAGGACAGUAUGCAAUGUCAGAAGAACUCCCCGGCCUGCUUUUUGCGCUGGUCUG
env-10   ACUUAAUGAGCAAAUCAUGAAAGAAAUAUAAUUACCUAUUUGCGAUAUUUAAAGCUUUGAAAUCAAACAAGUAAUCGAGUGCUUAAGUCGUCUCGUUAUUUCAUGGAGGACAGUAUGCACAGUCAGAAGAACUCCCCGGCCUGCUUUUUGCGCUGGUCUG
Ssp-1-2  GUGCGUAGCUCGGCAGUCGUUUCAAUCGAAUUUGAGUCGAGUAACCUGUCGGCGUGAUGAUAUUCACCACUUCAGGAGCAGGGAGACUCAGCGUGUAACCACGAUCGGUCAGCUUCUCCCAGGAUUGUUUGAGCAAUUCAGUAUCCAUAAGCUCUGAGUA
env-11   AACUGUUUGGUUAGACCAUCUACAUGCUCAAUAAGGAACGCAGCUUUCACGAGUCGCCACAGAAGGCUGGUGGUGAGGGGUGGCUGUAUCCGAGCAGCAACAGA........................................................
env-12   AACUGUUUGGUUAGACCAUCUACAUGCCCAAUAAGGACCGCAGCUUUCACGAAGCGCCAGAAAAGGCUGGUGGUGAAGGGUGGCUGUAUUCCGAGCAGCAGCAGAAGCUCUGUCACUUCAAGCCCGACACGGCAACGGUUCACGCUCAGUGGGUCGCGAU
env-13   GUGAUGUCUCGCUUGUGGCGUUCGCUGGGCUCGUAGACGUUUUUGCGGGGAACACUCAAGGCAGUCACGCCUGACCCAUGACCCAAGACACCCGCAUUGCACUGUCCCUGC.................................................
env-14   GCUGCGGAUUCUCUGGACUGCUCAUCUGGAGAAAAAGACAGAUGAGGCAGGGGGUGAGAUCCCUAACUUUUUAUUGCGAGGGCUCAUCCCACCUCAGGAACCCAGCCCAUCUGCUUCUGCUACGACAGAGCCCUGAGCUUGUACUUCUCCUUCGUGGGC
env-15   ................................................................................................................................................................
env-16   UGCAAGUCGGCGCCCAGAGCUUGCGUUUCUUUUUCUUCAGGUGCUCGCCAUUGCUGGAACUGCUGCUGCUGCCUGAGGUUGCAGCCCAACAGGGUGUGGCAGCGCUGAUCCACCCCGUAGGUGAAGUGCUGGCAGGUGAUGCAGACGCAGCCGCCCUUUC
env-17   NNNNNNNNNNNNNNNNNNACUAAGAGCCGAUGGGCACUAAAAAGAUACCUAAAGCCUCAAUGUCUUUAUGGCCAGGGUCAAGUGGGAAACAAUGACCGACGAAAGUCAGGCCAUGGUCAAGAAGGUCGGACUGAUCAGCCUUGGUGGAGUCAUCCUGCU
Ssp-2-2  CCGAUGGCGUUGCAGAUAUGAGUGAUGAGGAUUCAAAUUUCUGAAAAGCCCUAACGCCGAUACCGGUCAGGACUGAAAAACCAGACAGUCAUUCUGGAAGUGAACCAGGCGAGCAAGACGCCCAGGGCUAUUGAGACAAUCGCCCCAACAAAAGCCACCA
env-18   ................................................................................................................................................................
env-19   ................................................................................................................................................................
env-20   GGUCAGCUGCUUUGUGGUGAUGACUCGAGUUCUUCGUGACCAGUGGGCACCGAUGGCGAUUGCAGGAGCAUCACUAGUGGCGUUGGUGUGGCUUGAAGCCUUUGUUUGGUGAGUCGGCGGGGCAGUCUGAAGAAAACAGAGGG.................
env-21   AUCGGCGUUGAUCCAACGGAUCAAACCGGUGUCCGCGUUUGCCACUUGGAAGAGGGUCGGGACCUUGGUGUUCCUUGCUCCACCUUCGACAAAAACGACAUCGCCCAUCCACCAGUCACC........................................
env-22   GGUCAGCUGCUUUGUGGUGAUGACUCGAGUUCUUCGUGACCAGUGGGCACCGAUGGCGAUUGCAGGAGCAUCACUAGUGGCGUUGGUGUGGCUUGAAGCCUUUGUUUGGUGAGUCGGCGGGGCAGCCUGAAGAAAACAGAGGGCUUAAGUAGACCGCUCA
env-23   CAGAUCGGCGUUGAUCCAACGGAUCACAC...................................................................................................................................
env-24   UGCCACGUCGUCUCGACUGUGGCGGUGGGUCAGGGGGUAGCCCCCUUGAGCAGCUCUCUGAUUCAACCACUGACCACUUGGAGCUCCCUCGGCAGGCAUGGCUAUAAAAAGGUUGCUCAGAUGAUCUGGAUAUGAAAGAAGCUC................
env-25   UGAUGUGAUGCCAUAAAUUCCACCUUCAUUAGCCAUUCAAAGAUGGCAUCGACUGGACGAGAGCGUAAGAGUUUUCUCAAAAGCCAUCUUCAUCCAGAAGAAAUAGAUCACCGAGGAAUGGAUCAUUCGUACCAGAGUCAUUAUUACCCAGAACAACAUC
env-26   GUCAGCUGCUUUGUGGCGAUGACUCGCGUUCUUCGUGACCAGUGGGCACCGAUGGCGAUUGCAGGAGCAUCACUAGUGGCGUUGGUGUGGCUUGAAGCCUUUGUUUGGUGAGUCGGCGGGGCAGCCUGAAGAAAACAGAGGGCUUAAGUAGACCGCUCAA
env-27   UGACUCGGCGGCGGAUGCUGCGGCACAACGCGAUUGAGGCUUGGCAGACGAUGCAGAAGACAGGCUGGAGGCGUUGUCCGCCGCCUGUCAGGUGAAGUUCAACUGGAUGCACUGGUUGUUUGUUCGACCUAAUAAGAGGGAAGUUGAUCGACGAAUCCAU
env-28   GUCAGCUGCUUUGUGGUGAUGACUCGAGUUCUUCGUGACCAGUGGGCAUCGAUGGCGAUUGCAGGAGCAUCGCUAGUGGCGUUGGUGUGGCUUGAAGCCUUUGUUUGGUGAGUCGGCGGGGCAGCCUGAAGAAAACAGGGUGCUUAAGCAGACCGCUCAA
env-29   GUGGGAGGGUUUCAGGUAUGAGUGAAACCACGAGAGACAUUCCGAUUUGGGUCGGCGGAGAGUCAGGGCAGCGAAAGCGGUAUUUGAGAGCUUUAGAGAAGGACCUGGCAUCUGAGCUGGGGCCUGAUUGGCGGAACGUGAUUGAGCUGGCGAAGGGCAG
env-30   AACUGUUUGGUUAGACCAUCUACAUGCCCAAUAAGGACCGCAGCUUUCACGAGUCGCCAGAAAAGGCUGGUGGUGAGUGGUGGCUGUACUCCGAGCAGCAGCAGAAGCUCUGUCACUUCAAGCCCGACACGGCGACGGUCCACGCGCAGUGGGUAGCCAU
Ssp-1-3  AAGCCUCCCCUCCGCUACGGCAAGAAAGACCCCUGGUGGUUUCAGUCCCAGGGGUGCAGCUCUUCUCGCCGACCUCAAGUUGAUGCACCCAUGUGACCAGGUUGUGAUCGGCAGGCGCGUGGUUCAUGACCAGGUUUAACCCGCUGGCUGAUCCCGCCUC
env-31   GCGAAGAGCAUUGCUGGCGUUGUUGGUUCUGAUAGCACUGCCUGCACGGGGAGCGGAGCCCAUUCGCCUGCAGGUGAUUCCAUAUCAAGUGAGGAGGCAAGAGAUACCUGUCCUGCCAUUCUUGCUGCCGCCACCUCCCUUGCUCUUAGAACCAGCCAAA
env-32   CGGGCGUAGCUGGGCAAUCGCUUCAAUCGAAUCUGAGUCGAGUAACCCGUCGGCAUAAUGAUGUGCAUCACUUCAGGAGCAGGGCUAUUCAGCGUGUAACCACGUUGGGGCAACUUCUCCCAGGACUCUUUGAGUGAUUCAGUGUCCAUAAAUUUUCGAU
env-33   GUACAGAGCCUUGAGCUCAUCCAGCUCAGCCAUGAGGUAUUUGAUCUCAAGCCAGUCAGAUGAGAGGUCUAUAGAGGUUUCAAGCUGCUCGAUUUGGCGCUUGAGAAGCUCGGACAUGCCUAGGCAUUACUCAAAACCAUCAAACCCUGUAUCUGAUGAA
env-34   AACCGCCUCUGUCAGACCAGGUUCGGCUCGAUACGAAGCAAUCGUUCUGGUUGCGUCUGCGUCGGAUGGUGUCUUGAGAGAGUGGAGGGGGCUGAAUGCUGGGUUCCUUCUCGUUCCUGCCUAGAACUUCUGGUUGAACGUCUGGUCGUCAAUCCGUCGA
Ssp-1-4  CAGCAACUGAGUUGCCAAAAAGAAACCCCGGAGACAUCUCCGAGGUCAAAAUCCUUAUGGCUGAUCCCCAUCACCCAACCGGACAAGACAAAUCUAGCCUCAUUGGACUUAUUUGUGCCACAAGGAACGUUUUAUUGAAUUGUGUAGUGAGACUCUUGGG
Ssp-1-5  CGAGGAGGAAGCGCAAUAGCGUGCCUCCUGCGGUUGGCAUGUCGAUGAAUCAGCCGUCGUCCUUGCCCUUGUCUUUCGGCGGGUCUGUGGAUGCAAGCGUCUUCGCCUUACUGCUGAAGAUCACCUUUGCGAUGACUAAGGCAAUCGCAAUGGCGCCCAG
env-35   CCUGCUUAGCCAGUUAGCUGAUUAGGCAUUGACGCGACAGACAGAUGCCUUGUUCUCACCAAACUGAAGGCACAGAAGAGGGC.............................................................................
env-36   ................................................................................................................................................................
env-37   GUCCUUGAUCCAUUCGGAUGAUCCCCAUCACCCCGCCGGACCCGACAAAACUAACCUCGUUGGACUCAUUUGUGCCACAACAGGACGGUUCUUCAAUUGUGUGGCGAAGAUCUGACUCAAGCCUCUUAACACCAUAUGUGGGGCUAGUCAGCACGAAGUA
env-38   UUCCUUGAUCCAUUCGGAUGAUCCCCAUCACCCCGCCGGACUCGACAAAACUAACCUCGUUGGACUCAUUUGUGCCACAACAGGACGGUUCUUCAAUUGUGUGGCGAAGAUCUGACUCAAGCCUCUUAACACCAUAUGUGGGGCUAGUCAGCACGAAGUA
Ssp-2-3  AGCCCAAGAUGGGAUGCUCAAAGAGGAUCGUCGCCAGCUCUGAUUCGCUGAGCUUCAGGGGCUCAUAUUUUGCUUCUGCUCCUGUAAUGAGGCAUCCGGCAGUCAGAAGGCUUUCUCCAAGAAGCCAAGUGCAGCGCGUCAUGCGUUAAGUUAUGUAAAU
env-39   AGCCCAAGAUGGGAUGCUCAAAGAGGAUCGUCGCCAGCUCUGAUUCGCUGAGCUUCAGGGGCUCAUAUUUUGCUUCUGCUCCUGUAAUGAGGCAUCCGGCAGUCAGAAGGCUUUCUCCAAGAAGCCAAGUGCAGCGCGUCAUGCGUUAAGUUAUGUAAUU
env-40   GUGUCCUUCAGCAGAUACGAAUUUGCUAAUUCAGCAGUUACAGCAUUAGAUCAACUCUCCAUGAGUAAAAGCAAUUCUUUGAGAUGAAAGCUUUGCAGACAGGGUCUUGCCGACCUCGAUAGCAUCGCUUUCAUCCCCAGAUUCUAUAUCAUUUAUUUCU
env-41   CCCGAGGUCAUCAAUCCUUUCGGCUGAUCCCCAUCACCCGGCCGGAUACGGCAAAAUUAGCGGCAUUAGACUCAUUUGUGCCACAACUGGACGGUUCCACAAUUGUGUGGUGAAGAUCUGACCCACGCCUCUUGACACCAUAUGUGGGGCUAGUCAGCAC
env-42   NNNNNNNNNNNNNNNNNNNNNNNNNNNNNNNNNNNNNNNNNNNNNNNNNCAACGGAUGUCAAAAAGAAACCCCCGCCGGUAUCAGGGGCCAUGUCCUUUCGGUGCCUUGAUCACUUGUCGUAGGUGGCGCCUCUAUAAGUAAAGCUCUGAUGAACAUUGCG
env-43   GGGGUUCAGGAUCUUCUGAUUAAGCCUGAAUCACUUGGCGUAUGAAACGCCGCGAUAGGUCAGAGCAGGCUUGUUAGAGGAGCCAGCCACUUUCUGCUGGUCGUACUUCUGGCCGCGAUAGGUCAGGGUCAUUUGAGGUUCCUCAGAAAUCCAGGUCCCC
Ssp-2-4  CCCGAGGUCAUCAAUCCUUUCGGCUGAUCCCCAUCACCCGGCCGGACCCGACAAAACUAGCCGCAUUGGACUCAUUUGUGCCACAUCAUUUGUGCCCUGUGGAACUCCUUAAUUGUGUAUUGAAGCUCUGACCAGUAAUAGGUGGCACCAUAUGUGGGGC
Ssp-2-5  CCCGAGGUCUUCAAUCCUUUCGGCUGAUCCCCAUCACCCGGCCAGACCCGAUAAAGCUAGCCGCAUUGGACUCAUUUGUGCCACCCACAUCAUUUGUGCCAUGUGGAACGCCUCGAUUGUGUAUUGAAGUUCUGACCACUAAUAGGUGGCACCAUAUGUG
env-44   CUCUUCAAGCUCGACAUACUCCUCAUCGCUGAGACCAGGAGUAAUCGUCGCGGUAACGCCGAGACCCUUGGUCCAGGGGUAAGUGUGUUUCUGGACUGUUACGAGGUUGUCGGCCUCUACCAAAAGACAGCCACCACCGAGGUGAGGGUGGAUCUGACGU
env-45   UCUUCAAGCUCGACAUACUCCUCAUCGCUGAGACNNNNNNNNNNNNNNNNNNNNNNNNNNNNNNNNNNNNNNNNNNNNNNNNNNNUGAACGACCGACGUGAUCGACGUCUAACGGUUGGCCAUAGUUGAUGACCCACCGACCUUAUGAGAAGUCAGUCGAG
env-46   ACCGGUAUGGCGUUCCAAACGGGUCAUCGCAGGGGGGCAGGCCUGGGAUGACGUUCAGACUCUGGACAAGAUCAUUCACAACACCCGCUUGAACCUGUCCGGAAGCUGGGAGCCUUAGCGGUUGGUGUCCACACAACAACGACUAGUCAGUCGCAACAGG
env-47   GCCACUCCGCAGAGCUCUCCCCAUUCACCUGUUGGUCACGCAGAUGUAAGAAGCGUUUCAGACAAAAGGCCCCAGCGGGCAGGGACAGGCAAAUACGCCUAUUUACAUCUCGUAACAGAGCGCUUAACAUUUAUUCAUCUGCUUAAGCGUAAUGUCUUCG
         ................................................................................................................................................................
         ................................................................................................................................................................
         ●●●●●●●●●●●●●●●●●●●●●●●●●●●●●●●●●●●●●●●●●●●●●●●●●●●●●●●●●●●●●●●●●●●●●●●●●●●●●●●●●●●●●●●●●●●●●●●●●●●●○○○○○○○○○○○○○○○○○○○○○○○○○○○○○○○○○○○○○○○○○○○○○○○○○○○○○○○○
```

alignment positions 628 · · · 667

```
env-1     AAAAGGGCGCCAAGCACGGCCUUCGGUUACAAAUACUUAC
env-2     AAAAGGGCGCCAAGCACGGCAAUCAAUUUUCAAUGUUUAC
env-3     ........................................
Ssp-2-1   AAAAGGGCGCCAAGCACGGCAAUCAAUUUUCAAUGUUUAC
env-4     AAAAGGGCGCCAAGCACGGCAAUCAAUUUUCAACGUUUAC
env-5     AGUGCUUUAGAGACGAAUACAGGGAGGAAUACGUACCUGG
Ssp-1-1   GAGCAAGGUGACGGACAGGCUCUAUGCGGUUGCGGCAACG
env-6     UGAGCAAGGUUACUGACAAGCUCUAUGCGGUUGCGGCAAC
env-7     GCCUGCUUUUUGCGCUGGUCUGUGAGCAAGGUGACUGACA
env-8     ........................................
env-9     UGAGCAAGGUGACUGACAAGCUCUAUGCGGUUGCGGCGAC
env-10    UGAGCAAAGUUGCUGACAGGCUCUAUGCGGUUGCGGCAAC
Ssp-1-2   ACACAUCUCAUAGGGAUUCCGCCAAUCGAGGUCGUUAUGU
env-11    ........................................
env-12    CAGGACCUACAGCUACGUUCCGCCCCGCCCGCCUGAGCCG
env-13    ........................................
env-14    UCGCCUCACGGGCGUAGCCCACUACGCGAGUGAAUUCGUG
env-15    ........................................
env-16    AUCCCUGCAACUCCCCCUCGUCGAUAAAACUCCACUACUC
env-17    GGCCUUCAGCAAGAUAAUUCUUCCCUUAGUCCUGCUUGGU
Ssp-2-2   GAGACAAUCUCGAGGAGCCGGGUUGUCUCAAAAUGACGUG
env-18    ........................................
env-19    ........................................
env-20    ........................................
env-21    ........................................
env-22    AGUGGCAGGCACAUCUUGAACCUGCAGCCCGAGGGGGUCU
env-23    ........................................
env-24    ........................................
env-25    GCGUAUCGCUUGAUUUGCCUCGAAGUAAAGGAGAUCAUCU
env-26    GUGGCAGGCGCAUCUUGAACCUGCAGCCCGAGGGGGUCUG
env-27    GCCAAUCGCAAACCAGCAAUGCGCUUGCGAGCCUUGCGGC
env-28    GCAGUGAGCGCAUCUUGAGCCUGCAGCCCGAGGGGGUCUG
env-29    CUAAAGAUUGCGUUCCGCCGCACCCGCCUGAGCCGAUGAC
env-30    CAGGACGUACAGCUACGUUCCGCCCCGCCCUCCUGAGCCG
Ssp-1-3   UGGAGCCCCGCAGCGAUAGUUUGCAUUUUUUCUGAGAAUG
env-31    CCCAAGAAGCGCAGGCGAAAUAGCGGUAGGCACCAGGUCU
env-32    AACACUUCUCAUCAGACUUCCUAUAGUUGUGAUCAAUAUG
env-33    GCGCAACUAUUCACCGAUACCGAAUUGGUAAUCCAUUAGU
env-34    AGCGACACGAUUUCCAACCUCUUUCUGUUCGCCCCCAUUU
Ssp-1-4   UGACACCAUAUGUGGGGCUGGUCAGCACGUAGUAGCUGGG
Ssp-1-5   CAGAAAGCCAAGAAGCUCCAUUGCUGAUCAGGCUUCGACU
env-35    ........................................
env-36    ........................................
env-37    GCUGAGCCAUAUUUAGGUUUCCCCCAUCACCCAGCCCGUU
env-38    GCUGAGCCAUAUUUAGGUUUCCCCCAUCACCCAGCCCGUU
Ssp-2-3   AAAAUUUUUUAAUCCAAAUGAAUAGCUGGUUUGCAGCGAU
env-39    AAAUUUUUUUAAAUCCAAAUGAAUAGCUGGUUUGCAGCGA
env-40    UUCAAUGUUUUCAGGUAAGUAAUGCAGCUUUGCCGAAUCU
env-41    GAAGUAGCUGAGCCAUAUUUAGGUUUCCCCCAUCACCCAG
env-42    AGCCACUUCUUCGCGGCAGGUGUUGUAACGCCCGCGGCGA
env-43    GUUCCUUGGCCUGGUGAUCUGCGCCUUGUAGAAGCAAGGU
Ssp-2-4   UGGUCAGUACUUGGCGGCUAAGCCAUAUUUAGGUUUCCCC
Ssp-2-5   GGGCUGGUCAGUACUAGGCGGCUAAGCCAUAUUUAGGUUU
env-44    GCCAACACCUUGAAGCCCGCGUACUCAUCCAUAGGCUUUC
env-45    AAGUGUGAUUUGGAGGAUGGGAGUCCUGUUGUCGGUGUCG
env-46    CGAUCUCAGCUGCAGUUGGUAUCAACGCAGCAAAUGGUUG
env-47    GGCUCUGGUGAUAGCUGGUACCAGGAUGCAGCUGCCGCGC
          ........................................
          ........................................
          ∘∘∘∘∘∘∘∘∘∘∘∘∘∘∘∘∘∘∘∘∘∘∘∘∘∘∘∘∘∘∘∘∘∘∘∘∘∘∘∘
```

27 Desulfotalea-1

27.1 Taxa

The taxonomy of each organism containing a putative Desulfotalea-1 RNA is listed, with abbreviations identifying each hit (e.g., "Eco-1-1" and "Eco-1-2" might hypothetically represent two distinct RNAs in *E. coli*). The abbreviations will be used to identify each individual Desulfotalea-1 RNA in Sections 27.2 and 27.4.

abbrev. of hits	taxonomy of species
Dha-1-1	Bacteria Firmicutes Clostridia Clostridiales Peptococcaceae *Desulfitobacterium hafniense* Y51
Dps-1-1 to Dps-1-20	Bacteria Proteobacteria Deltaproteobacteria Desulfobacterales Desulfobulbaceae *Desulfotalea psychrophila* LSv54
Sba-1-1 to Sba-1-8	Bacteria Proteobacteria Gammaproteobacteria Alteromonadales Shewanellaceae *Shewanella baltica* OS155
Sba-2-1 to Sba-2-10	Bacteria Proteobacteria Gammaproteobacteria Alteromonadales Shewanellaceae *Shewanella baltica* OS185
Sba-3-1 to Sba-3-10	Bacteria Proteobacteria Gammaproteobacteria Alteromonadales Shewanellaceae *Shewanella baltica* OS195
Sba-4-1 to Sba-4-6	Bacteria Proteobacteria Gammaproteobacteria Alteromonadales Shewanellaceae *Shewanella baltica* OS223
Sde-1-1 to Sde-1-10	Bacteria Proteobacteria Gammaproteobacteria Alteromonadales Shewanellaceae *Shewanella denitrificans* OS217
Sfr-1-1 to Sfr-1-2	Bacteria Proteobacteria Gammaproteobacteria Alteromonadales Shewanellaceae *Shewanella frigidimarina* NCIMB 400
Sha-1-1 to Sha-1-2	Bacteria Proteobacteria Gammaproteobacteria Alteromonadales Shewanellaceae *Shewanella halifaxensis* HAW-EB4
Slo-1-1 to Slo-1-9	Bacteria Proteobacteria Gammaproteobacteria Alteromonadales Shewanellaceae *Shewanella loihica* PV-4
Son-1-1 to Son-1-8	Bacteria Proteobacteria Gammaproteobacteria Alteromonadales Shewanellaceae *Shewanella oneidensis* MR-1
Spe-1-1 to Spe-1-7	Bacteria Proteobacteria Gammaproteobacteria Alteromonadales Shewanellaceae *Shewanella pealeana* ATCC 700345
Spu-1-1 to Spu-1-11	Bacteria Proteobacteria Gammaproteobacteria Alteromonadales Shewanellaceae *Shewanella putrefaciens* 200
Spu-2-1 to Spu-2-10	Bacteria Proteobacteria Gammaproteobacteria Alteromonadales Shewanellaceae *Shewanella putrefaciens* CN-32
Sse-1-1 to Sse-1-10	Bacteria Proteobacteria Gammaproteobacteria Alteromonadales Shewanellaceae *Shewanella sediminis* HAW-EB3
Ssp-1-1 to Ssp-1-9	Bacteria Proteobacteria Gammaproteobacteria Alteromonadales Shewanellaceae *Shewanella sp.* ANA-3
Ssp-2-1 to Ssp-2-13	Bacteria Proteobacteria Gammaproteobacteria Alteromonadales Shewanellaceae *Shewanella sp.* MR-4
Ssp-3-1 to Ssp-3-9	Bacteria Proteobacteria Gammaproteobacteria Alteromonadales Shewanellaceae *Shewanella sp.* MR-7
Ssp-4-1 to Ssp-4-8	Bacteria Proteobacteria Gammaproteobacteria Alteromonadales Shewanellaceae *Shewanella sp.* W3-18-1
Swo-1-1 to Swo-1-15	Bacteria Proteobacteria Gammaproteobacteria Alteromonadales Shewanellaceae *Shewanella woodyi* ATCC 51908
Spr-1-1 to Spr-1-3	Bacteria Proteobacteria Gammaproteobacteria Enterobacteriales Enterobacteriaceae *Serratia proteamaculans* 568
Osp-1-1 to Osp-1-5	Bacteria Proteobacteria Gammaproteobacteria Oceanospirillales *Oceanobacter sp.* RED65
Epe-1-1 to Epe-1-2	Bacteria Proteobacteria Gammaproteobacteria sulfur-oxidizingsymbionts *Endoriftia persephone* 'Hot96_1+Hot96_2'
env-1 to env-41	environmental samples

27.2 Gene contexts

Each Desulfotalea-1 RNA (indicated by "RNA→") is listed with its surrounding genes. Environmental sequences and some RefSeq entries lack gene annotations, and no genes are listed for such sequences. The direction of each gene is indicated with an arrow (→), and each conserved domain in the gene is colored. Conserved domains associated with more than one Desulfotalea-1 RNA are assigned a color; other domains are gray. Information about these conserved domains is given in Section 27.3. The accession number of the sequence containing each Desulfotalea-1 RNA is given. Accessions beginning with "NC_" or "NZ_" are RefSeq, while other accession refer to environmental samples. Nucleotide coordinates are given for the 5′ and 3′ boundaries of each. If the 5′ coordinate is greater than the 3′ coordinate, the RNA is present on the reverse-complement strand of the containing genomic DNA sequence. Each hit is denoted by an abbreviation (like "Eco-1-1") that refers to a taxonomy given in Section 27.1.

abbrev.	Seq. accession		5′ at	3′ at	genes
Dps-1-1	NC_006138.1	-	2160819	2160757	←MdaB (COG2249)Flavodoxin_2 (pfam02525) **RNA→** **RNA→** **RNA→** **RNA→** ←5S ribosomal RNA
Dps-1-2	NC_006138.1	-	2159792	2159730	(shown above)
Dps-1-12	NC_006138.1	-	2160915	2160863	(shown above)
Dps-1-14	NC_006138.1	-	2159888	2159836	(shown above)
Dps-1-4	NC_006138.1	+	1458168	1458230	hypo→ **RNA→** **RNA→** **RNA→** hypo→

Dps-1-5	NC_006138.1	+	1458521	1458582	(shown above)
Dps-1-15	NC_006138.1	+	1458072	1458124	(shown above)
Dha-1-1	NC_007907.1	-	3254431	3254370	MGS_CPS_II (cd01424)CarB (COG0458)CPSase_L_D3 (pfam02787)→ **RNA**→ UbiB (COG0543)→
Epe-1-1	NZ_AASF01001432.1	+	2173	2233	←hypo **RNA**→ ←hypo
env-1	JCVI_SCAF_1096626308176	-	830	769	**RNA**→ ←ribokinase_group_A (cd01942)? (GOS4908)
env-2	JCVI_SCAF_1096627348977	+	2300	2361	NTP_transf_2 (pfam01909)? (GOS11052049)→ **RNA**→ ←Ala tRNA
Epe-1-2	NZ_AASF01000563.1	-	448	387	TOPRIM domain protein→ **RNA**→ TOPRIM domain protein→
env-3	wf1_AGZO6492_g2	-	920	859	hypo→ **RNA**→ hypo→
Sba-1-1	NC_009052.1	-	120367	120306	←hypo **RNA**→ ←HTH_XRE (smart00530)
Ssp-3-1	NC_008322.1	-	489145	489084	COG5635 (COG5635)→ **RNA**→ COG3046 (COG3046)→
Ssp-1-1	NC_008577.1	+	3929063	3929124	←hypo **RNA**→ ←COG0412 (COG0412)
Son-1-1	NC_004347.1	-	1172282	1172221	rve (pfam00665)→ **RNA**→ hypo→
Son-1-2	NC_004347.1	+	4341375	4341436	hypo→ **RNA**→ COG3046 (COG3046)→
Sba-4-1	NZ_ABAU01000009.1	-	74558	74497	←hypo (cons) **RNA**→ ←OmpA_membrane (pfam01389)
Ssp-4-1	NC_008750.1	-	620879	620818	Transgly (pfam00912)→ **RNA**→ hypo→
Sba-1-2	NC_009052.1	-	517146	517085	hypo→ **RNA**→ ←COG1853 (COG1853)
Son-1-3	NC_004347.1	-	488661	488600	←hypo **RNA**→ ←NUDIX (pfam00293)
Sba-3-1	NC_009997.1	-	1643237	1643176	COG1556 (COG1556)→ **RNA**→ AAA (pfam00004)→
Sba-4-2	NZ_ABAU01000042.1	+	9975	10036	COG1556 (COG1556)→ **RNA**→ AAA (pfam00004)→
Sba-3-2	NC_009997.1	+	1893207	1893268	hypo→ **RNA**→ ←hypo
Sba-1-3	NC_009052.1	-	1546107	1546046	COG1556 (COG1556)→ **RNA**→ ATPase central domain-containing protein→
Sba-2-1	NC_009665.1	-	1604137	1604076	COG1556 (COG1556)→ **RNA**→ AAA (pfam00004)→
Sse-1-1	NC_009831.1	-	3283522	3283462	←hypo **RNA**→ hypo→
env-4	soil_XZS79699_x1	+	711	771	Imidazolone-5PH (cd01296)→ **RNA**→ ←hypo
env-5	wf1_AGZO16092_g2	+	815	876	**RNA**→
Osp-1-1	NZ_AAQH01000003.1	+	35782	35843	tRNA-Arg→ **RNA**→ hypo→
env-6	JCVI_SCAF_1096627348411	-	5981	5920	←NtrB (COG3852)? (GOS336) **RNA**→ ←Gln-synt_C (pfam00120)Gln-synt_N (pfam03951)? (GOS3709)
env-7	wf1_Contig1252	+	513	574	Exopolyphosphatase(EC:3.6.1.40)→ **RNA**→ ←Phosphoenolpyruvate carboxykinase (ATP)
env-8	wf1_Contig1312	-	813	752	**RNA**→ ←PEPCK_ATP (cd00484)
env-9	wf1_Contig2174	-	228	167	Valyl-tRNA synthetase→ **RNA**→
env-11	wf1_Contig2174	-	280	219	(shown above)
env-10	wf2_AHAA10370_x1	-	323	263	←hypo **RNA**→
env-12	JCVI_SCAF_1101668564577	-	673	611	**RNA**→
Osp-1-2	NZ_AAQH01000022.1	+	41614	41675	←OraA (COG2137) **RNA**→ ←RecA (pfam00154)
Dps-1-6	NC_006138.1	-	827237	827176	←Nuc_deoxyrib_tr (pfam05014)NOG22301 (NOG22301) **RNA**→ ←5S ribosomal RNA
Dps-1-7	NC_006138.1	-	1424102	1424040	←Tar (COG0840)Cache (pfam02743) **RNA**→ ←AlsT (COG1115)
Dps-1-8	NC_006138.1	+	2324776	2324838	←hypo **RNA**→ ←hypo
Dps-1-9	NC_006138.1	+	1445432	1445494	←hypo **RNA**→ **RNA**→ ←hypo
Dps-1-11	NC_006138.1	+	1445882	1445944	(shown above)
Dps-1-10	NC_006138.1	+	1446411	1446473	←hypo **RNA**→ ←5S ribosomal RNA
Dps-1-13	NC_006138.1	-	812320	812268	←hypo **RNA**→ **RNA**→ ←hypo
Dps-1-3	NC_006138.1	-	812225	812163	(shown above)
Dps-1-16	NC_006138.1	-	2049951	2049888	←COG2968 (COG2968) **RNA**→ ←AccD (COG0777)AccA (COG0825)
Dps-1-17	NC_006138.1	-	2735941	2735879	←hypo **RNA**→ ←hypo
Dps-1-18	NC_006139.1	-	37032	36970	hypo→ **RNA**→ hypo→
Dps-1-19	NC_006138.1	+	2392578	2392640	←Met_dep_hydrolase_A (cd01299) **RNA**→ hypo→
Dps-1-20	NC_006138.1	+	1448411	1448470	hypo→ **RNA**→ hypo→
Osp-1-3	NZ_AAQH01000028.1	-	29628	29567	23S ribosomal RNA→ **RNA**→ 5S ribosomal RNA→
Osp-1-4	NZ_AAQH01000004.1	+	4024	4085	hypo→ **RNA**→ 5S ribosomal RNA→
Osp-1-5	NZ_AAQH01000014.1	-	77617	77556	hypo→ **RNA**→ 5S ribosomal RNA→
env-13	JCVI_SCAF_1096627235929	-	157	96	? (GOS14479710)→ **RNA**→ ? (GOS14479710)→
Spr-1-1	NC_009832.1	-	1300562	1300501	CSD (pfam00313)→ **RNA**→ ←GGDEF (cd01949)

Spr-1-2	NC_009832.1	+	3202354	3202415	←TauA (COG0715) **RNA**→ ←AmiR (COG3707)
Spr-1-3	NC_009832.1	+	2919065	2919126	←hypo **RNA**→ ←acidPPc (smart00014)
Sse-1-2	NC_009831.1	+	711934	711994	hypo→ **RNA**→ AlsT (COG1115)→
Sse-1-3	NC_009831.1	+	3777195	3777255	←UbiG (COG2227) **RNA**→ ProS (COG0442)→
Sse-1-4	NC_009831.1	+	2552830	2552889	hypo→ **RNA**→ GntR (COG1802)→
Slo-1-1	NC_009092.1	+	1060677	1060738	COG3547 (COG3547)→ **RNA**→ ←Ig family protein
Slo-1-2	NC_009092.1	-	2651592	2651531	←hypo **RNA**→ ←Abhydrolase_1 (pfam00561)
Swo-1-1	NC_010506.1	+	774208	774320	←COG1359 (COG1359) **RNA**→ ←PrfB (COG1186)
Slo-1-3	NC_009092.1	+	4021098	4021159	←hypo **RNA**→ SpeB (COG0010)→
Sse-1-5	NC_009831.1	-	5412662	5412549	DUF890 (pfam05971)→ **RNA**→ ←COG1910 (COG1910)
Swo-1-2	NC_010506.1	-	4595466	4595405	hypo→ **RNA**→ ←FBPase (cd00354)
Swo-1-3	NC_010506.1	-	4380115	4380054	hypo→ **RNA**→ **RNA**→ hypo→
Swo-1-14	NC_010506.1	-	4380230	4380170	(shown above)
Swo-1-4	NC_010506.1	-	5910922	5910861	Nucleoside_tra2 (pfam01773)→ **RNA**→ ←DUF1006 (pfam06224)
Swo-1-5	NC_010506.1	+	5927100	5927161	hypo→ **RNA**→ hypo→
Swo-1-6	NC_010506.1	+	4600300	4600361	hypo→ **RNA**→ ←Transposase_17 (pfam01797)
Swo-1-7	NC_010506.1	+	3624166	3624227	←hypo **RNA**→ ←hypo
Slo-1-4	NC_009092.1	-	3688293	3688232	hypo→ **RNA**→ ←rDP_like (cd01301)
Slo-1-5	NC_009092.1	-	1186360	1186299	hypo→ **RNA**→ Pfs (COG0775)→
Slo-1-6	NC_009092.1	-	864553	864492	PAS (cd00130)GGDEF (cd01949)→ **RNA**→ ←hypo
Sse-1-6	NC_009831.1	-	2551176	2551116	←hypo **RNA**→ ←GpmB (COG0406)
Spe-1-1	NC_009901.1	-	3591093	3591033	COG3547 (COG3547)→ **RNA**→ **RNA**→ hypo→
Spe-1-5	NC_009901.1	-	3590797	3590736	(shown above)
Sha-1-1	NC_010334.1	-	2712982	2712922	hypo→ **RNA**→ ←PMSR (pfam01625)
Spe-1-2	NC_009901.1	-	4953283	4953223	AbgB (COG1473)→ **RNA**→ ←hypo
Slo-1-7	NC_009092.1	-	3066234	3066173	hypo→ **RNA**→ ←COG1733 (COG1733)
Spe-1-3	NC_009901.1	+	816712	816773	Bcp (COG1225)→ **RNA**→ ←RIO1 (COG1718)
Sse-1-7	NC_009831.1	+	5439863	5439924	←OmpA_membrane (pfam01389) **RNA**→ ←COG2819 (COG2819)
Sfr-1-1	NC_008345.1	+	2377787	2377900	GreA (COG0782)→ **RNA**→ hypo→
Sfr-1-2	NC_008345.1	+	2432452	2432565	hypo→ **RNA**→ COG4584 (COG4584)→
env-14	JCVI_SCAF_1096627390099	-	160749	160688	? (GOS11588659)→ **RNA**→ ←? (GOS11627891)
Ssp-1-2	NC_008577.1	-	2788875	2788814	hypo→ **RNA**→ ←hypo
env-15	JCVI_SCAF_1096627388991	-	4053	3992	COG4271 (COG4271)? (GOS11522405)→ **RNA**→ COG4711 (COG4711)? (GOS12025990)→
Spu-2-1	NC_009438.1	+	1566542	1566603	hypo→ **RNA**→ ←decaheme cytochrome c
Ssp-4-2	NC_008750.1	-	3101842	3101781	hypo→ **RNA**→ ←decaheme cytochrome c
Spu-1-1	NZ_AAWY01000022.1	+	34792	34853	Transposase_32 (pfam04986)→ **RNA**→ ←decaheme cytochrome c
Slo-1-8	NC_009092.1	-	3928538	3928477	hypo→ **RNA**→ ←DUF481 (pfam04338)
Ssp-2-1	NC_008321.1	+	3905760	3905821	DegQ (COG0265)→ **RNA**→ COG1315 (COG1315)→
Ssp-3-2	NC_008322.1	-	794304	794243	DegQ (COG0265)→ **RNA**→ COG1315 (COG1315)→
Sde-1-1	NC_007954.1	-	334646	334533	←SSL2 (COG1061)COG3886 (COG3886) **RNA**→ ←COG2253 (COG2253)NOG14035 (NOG14035)
Spe-1-4	NC_009901.1	+	3911360	3911421	←hypo **RNA**→ ←hypo
Swo-1-8	NC_010506.1	-	4687364	4687303	←hypo **RNA**→ ←COG4146 (COG4146)
Sha-1-2	NC_010334.1	-	968451	968390	←hypo **RNA**→ ←SdhA (COG1053)
Spe-1-6	NC_009901.1	-	1415607	1415546	COG3547 (COG3547)→ **RNA**→ RecN (COG0497)→
Spe-1-7	NC_009901.1	+	898673	898734	←hypo **RNA**→ Med (COG1744)→
Sde-1-2	NC_007954.1	-	1309545	1309484	←COG2963 (COG2963) **RNA**→ INT_IntI (cd01193)→
Sba-4-3	NZ_ABAU01000046.1	+	18198	18259	Ada (COG0350)HTH_ARAC (smart00342)→ **RNA**→ COG1741 (COG1741)→
Ssp-3-3	NC_008322.1	+	1764328	1764389	UbiE (COG2226)→ **RNA**→ ←XthA (COG0708)
Sde-1-3	NC_007954.1	-	733198	733137	←putative lipoprotein **RNA**→ ←GST_C_Ure2p_like (cd03178)GST_N_Ure2p_like (cd03048)
Sde-1-4	NC_007954.1	-	4054983	4054922	←Transposase_11 (pfam01609)NOG07925 (NOG07925) **RNA**→ ←DadA (COG0665)
Sde-1-5	NC_007954.1	-	2843798	2843687	hypo→ **RNA**→ ←ManC (COG0662)
env-16	JCVI_SCAF_1096627389125	-	209051	208990	? (GOS14347974)→ **RNA**→ ←? (GOS13516489)

Ssp-4-3	NC_008750.1	-	3512493	3512432	hypo→ **RNA**→ DPBB_1 (pfam03330)→
Spu-1-2	NZ_AAWY01000014.1	+	74425	74486	hypo (cons)→ **RNA**→ ←Transposase_20 (pfam02371)
Spu-2-2	NC_009438.1	+	1220337	1220398	hypo→ **RNA**→ RlpA (COG0797)→
Sba-4-4	NZ_ABAU01000018.1	-	43511	43450	protein of unknown function DUF1568→ **RNA**→ ←COG3314 (COG3314)
Sba-3-3	NC_009997.1	-	659413	659352	←HSDR_N (pfam04313) **RNA**→ ←COG3258 (COG3258)
Sba-1-4	NC_009052.1	+	4423420	4423481	←HSDR_N (pfam04313) **RNA**→ ←COG2863 (COG2863)
Sba-2-2	NC_009665.1	-	3818743	3818682	CgeB family protein→ **RNA**→ ←COG3314 (COG3314)
Sde-1-6	NC_007954.1	+	2349930	2349991	hypo→ **RNA**→ ←Pgm (COG0033)
Spu-1-3	NZ_AAWY01000008.1	-	61270	61209	←Transposase_9 (pfam01548)Transposase_20 (pfam02371) **RNA**→ COG3673 (COG3673)→
Spu-1-4	NZ_AAWY01000024.1	-	27004	26943	SEC-C (pfam02810)→ **RNA**→ ←COG3150 (COG3150)
Ssp-4-4	NC_008750.1	-	2122035	2121974	UPF0167 (pfam03691)→ **RNA**→ ←COG3150 (COG3150)
Spu-2-3	NC_009438.1	-	4061889	4061828	hypo→ **RNA**→ ←hypo
Ssp-4-5	NC_008750.1	+	487038	487099	hypo→ **RNA**→ ←hypo
Spu-1-5	NZ_AAWY01000028.1	+	10812	10873	hypo→ **RNA**→ ←hypo (cons)
Sba-3-4	NC_009997.1	-	3353343	3353282	hypo→ **RNA**→ ←XthA (COG0708)
Sba-1-5	NC_009052.1	+	1266290	1266351	hypo→ **RNA**→ DPBB_1 (pfam03330)→
Sba-3-5	NC_009997.1	+	1414133	1414194	Transposase_32 (pfam04986)→ **RNA**→ DPBB_1 (pfam03330)→
Spu-2-4	NC_009438.1	-	3422364	3422303	Acetyltransf_1 (pfam00583)→ **RNA**→ COG3673 (COG3673)→
Spu-2-5	NC_009438.1	+	2495246	2495307	hypo→ **RNA**→ ←COG3150 (COG3150)
Sba-1-6	NC_009052.1	-	4071734	4071673	Ada (COG0350)HTH_ARAC (smart00342)→ **RNA**→ COG1741 (COG1741)→
Sba-3-6	NC_009997.1	+	1062421	1062482	←COG3344 (COG3344) **RNA**→ COG1741 (COG1741)→
Ssp-3-4	NC_008322.1	-	2627196	2627136	COG5293 (COG5293)→ **RNA**→ ←hypo
Son-1-4	NC_004347.1	+	3939830	3939891	←hypo **RNA**→ CsdB (COG0520)→
env-17	JCVI_SCAF_1096627385280	-	42059	41998	RbsB (COG1879)? (GOS5488)→ **RNA**→ predicted protein→
env-18	JCVI_SCAF_1096627195755	+	1607	1668	**RNA**→ ←GlnQ (COG1126)? (GOS2568)
Ssp-1-3	NC_008577.1	-	1067869	1067808	putative lipoprotein→ **RNA**→ ←dipeptidyl-peptidase 7
Ssp-3-5	NC_008322.1	-	1090676	1090615	hypo→ **RNA**→ ←dipeptidyl-peptidase 7
Ssp-2-2	NC_008321.1	-	1066577	1066516	hypo→ **RNA**→ COG3547 (COG3547)→
Ssp-2-3	NC_008321.1	+	3245705	3245766	S_TKc (cd00180)→ **RNA**→ ←DAP2 (COG1506)
env-19	JCVI_SCAF_1096627387715	-	52913	52852	? (GOS418)→ **RNA**→ ←XthA (COG0708)? (GOS6435)
env-20	JCVI_SCAF_1096627208462	-	192427	192366	←INT_Lambda_C (cd00800)? (GOS1694) **RNA**→ ←? (GOS11627891)
Ssp-2-4	NC_008321.1	+	3614162	3614223	hypo→ **RNA**→ COG3547 (COG3547)→
env-21	JCVI_SCAF_1096627191725	-	132397	132336	? (GOS11166260)→ **RNA**→ ←TrkG (COG0168)? (GOS6072)
Sba-4-5	NZ_ABAU01000030.1	-	9236	9175	Transposase_11 (pfam01609)→ **RNA**→ ←protein of unknown function DUF1379
Sde-1-7	NC_007954.1	+	1402477	1402538	Transposase_11 (pfam01609)NOG07925 (NOG07925)→ **RNA**→ ←hypo
Sba-2-3	NC_009665.1	+	1024503	1024564	Ada (COG0350)HTH_ARAC (smart00342)→ **RNA**→ COG1741 (COG1741)→
env-22	JCVI_SCAF_1096627390099	+	205614	205675	GGDEF (cd01949)? (GOS13550016)→ **RNA**→ ←Pro tRNA
Sse-1-8	NC_009831.1	-	2080558	2080497	←hypo **RNA**→ hypo→
Swo-1-9	NC_010506.1	-	5699016	5698955	←OLE1 (COG1398) **RNA**→ ←HTH_XRE (cd00093)
Swo-1-10	NC_010506.1	+	1288851	1288911	hypo→ **RNA**→ ←YcaC_related (cd01012)
Swo-1-11	NC_010506.1	+	567231	567291	hypo→ **RNA**→ Peptidase_M48 (pfam01435)→
Swo-1-12	NC_010506.1	-	1261348	1261288	←ANK (cd00204) **RNA**→ ←hypo
Swo-1-13	NC_010506.1	+	4870073	4870133	hypo→ **RNA**→ ←MdlB (COG1132)
Sse-1-9	NC_009831.1	-	248456	248395	hypo→ **RNA**→ ←Cytochrom_NNT (pfam03264)
Swo-1-15	NC_010506.1	+	1630791	1630852	←hypo **RNA**→ ←Peptidase_M28 (pfam04389)
Sde-1-8	NC_007954.1	-	4160783	4160723	←RecG (COG1200) **RNA**→ ←CitB (COG2197)
Sde-1-9	NC_007954.1	+	4438735	4438848	←UPF0153 (pfam03692) **RNA**→ ←tRNA-Asp
Sde-1-10	NC_007954.1	+	2847542	2847603	←hypo **RNA**→ ←COG3791 (COG3791)
Slo-1-9	NC_009092.1	+	681172	681233	hypo→ **RNA**→ UbiE (COG2226)→
Ssp-2-5	NC_008321.1	-	2558580	2558467	hypo→ **RNA**→ ←hypo
Sba-2-4	NC_009665.1	-	1891830	1891717	←COG3593 (COG3593) **RNA**→ hypo→
Sba-2-5	NC_009665.1	+	4934353	4934413	←hypo **RNA**→ ←UbiG (COG2227)

Sba-1-7	NC_009052.1	-	1261597	1261536	←Transposase_11 (pfam01609) **RNA**→ ←Aldedh (pfam00171)
Ssp-1-4	NC_008577.1	+	1069306	1069367	←putative lipoprotein **RNA**→ OmpA (COG2885)→
Spu-2-6	NC_009438.1	-	1219059	1218998	←hypo **RNA**→ ←Aldedh (pfam00171)
Spu-1-6	NZ_AAWY01000014.1	-	73147	73086	←hypo (cons) **RNA**→ ←aldehyde dehydrogenase
Son-1-5	NC_004347.1	-	2320864	2320751	hypo→ **RNA**→ ←LMWPc (cd00115)
env-23	JCVI_SCAF_1096627388974	-	239576	239515	Amidase (pfam01425)? (GOS672)→ **RNA**→ **RNA**→ COG1741 (COG1741)? (GOS5791)→
env-28	JCVI_SCAF_1096627388974	-	239343	239283	(shown above)
env-24	JCVI_SCAF_1096627388987	-	40406	40345	? (GOS14131473)→ **RNA**→ ←COG3314 (COG3314)? (GOS4580)
env-25	JCVI_SCAF_1096627388963	-	175755	175695	? (GOS12136901)→ **RNA**→ COG1741 (COG1741)? (GOS5791)→
env-26	JCVI_SCAF_1101668744010	-	402	342	? (GOS418)→ **RNA**→
env-27	JCVI_SCAF_1096627388998	-	175188	175128	? (GOS418)→ **RNA**→ ←MTH1175 (cd00851)? (GOS11736581)
Ssp-2-6	NC_008321.1	+	865136	865248	chromosome segregation ATPase→ **RNA**→ COG1741 (COG1741)→
Son-1-6	NC_004347.1	+	923243	923303	hypo→ **RNA**→ COG1741 (COG1741)→
env-29	JCVI_SCAF_1096627388993	-	179856	179743	COG4928 (COG4928)? (GOS11137131)→ **RNA**→ ←ProA (COG0014)? (GOS530)
env-30	JCVI_SCAF_1096627195142	+	9098	9158	? (GOS11452331)→ **RNA**→ ? (GOS11512624)→
env-31	JCVI_SCAF_1096627388986	-	17055	16994	←? (GOS14027118) **RNA**→ ←? (GOS13449409)
env-32	JCVI_SCAF_1096627388993	+	181868	181929	←COG4928 (COG4928)? (GOS11137131) **RNA**→ ←COG2856 (COG2856)HTH_XRE (smart00530)? (GOS1328)
Son-1-7	NC_004347.1	+	2135235	2135348	INT_IntI (cd01193)→ **RNA**→ ←EAL (cd01948)
Spu-2-7	NC_009438.1	+	159326	159387	hypo→ **RNA**→ ←hypo
Spu-1-7	NZ_AAWY01000007.1	-	24167	24106	hypo (cons)→ **RNA**→ ←leucine-rich repeat
Sba-3-7	NC_009997.1	+	826487	826548	HipA (COG3550)→ **RNA**→ hypo→
Sba-3-8	NC_009997.1	+	823256	823317	←putative esterase **RNA**→ HTH_XRE (cd00093)→
Sba-2-6	NC_009665.1	+	790094	790155	HEAT repeat-containing PBS lyase→ **RNA**→ HTH_XRE (cd00093)→
Ssp-1-5	NC_008577.1	-	2692065	2691952	←hypo **RNA**→ ←EAL (cd01948)
Ssp-1-6	NC_008577.1	+	4061579	4061640	←hypo **RNA**→ ←COG3529 (COG3529)
env-33	JCVI_SCAF_1096627390099	-	64909	64848	? (GOS12125594)→ **RNA**→ ←EAL (cd01948)? (GOS2819)
Ssp-4-6	NC_008750.1	-	4046106	4046045	hypo→ **RNA**→ hypo→
Sba-4-6	NZ_ABAU01000021.1	+	30672	30733	←ThrS (COG0441) **RNA**→ ←GcvP (COG0403)GcvP (COG1003)
Sba-1-8	NC_009052.1	-	707263	707202	AcrR (COG1309)→ **RNA**→ ←GcvP (COG0403)GcvP (COG1003)
Son-1-8	NC_004347.1	-	3779844	3779783	←hypo **RNA**→ ←hypo
env-34	JCVI_SCAF_1096627388971	+	117203	117264	COG2968 (COG2968)? (GOS7701)→ **RNA**→ ←? (GOS11679563)
Sba-3-9	NC_009997.1	+	1878992	1879053	←hypo **RNA**→ ←DUF1415 (pfam07209)
Spu-2-8	NC_009438.1	+	4063708	4063821	hypo→ **RNA**→ ←CspR (COG0219)
Ssp-4-7	NC_008750.1	-	485219	485106	←hypo **RNA**→ ←CspR (COG0219)
Spu-1-8	NZ_AAWY01000028.1	-	8977	8917	←hypo **RNA**→ ←CspR (COG0219)
Sba-2-7	NC_009665.1	+	165983	166044	glycosyl transferase family protein→ **RNA**→ ←putative lipoprotein
env-35	JCVI_SCAF_1096627389620	-	111480	111367	←? (GOS12137839) **RNA**→ PerM (COG0628)? (GOS5032)→
Ssp-2-7	NC_008321.1	-	1689515	1689402	hypo→ **RNA**→ ←Tar (COG0840)
Ssp-1-7	NC_008577.1	-	4728916	4728803	hypo→ **RNA**→ ←CspR (COG0219)
env-36	JCVI_SCAF_1101667373750	-	882	769	**RNA**→ ←CspR (COG0219)? (GOS493)
env-37	JCVI_SCAF_1101667400326	-	266	205	**RNA**→
env-38	JCVI_SCAF_1096627390098	-	45674	45561	←? (GOS418) **RNA**→ ←EAL (cd01948)? (GOS2819)
Ssp-2-8	NC_008321.1	+	2617054	2617115	←hypo **RNA**→ ←LMWPc (cd00115)
Ssp-3-6	NC_008322.1	+	2844817	2844930	←hypo **RNA**→ ←hypo
Ssp-3-7	NC_008322.1	+	2630493	2630554	←hypo **RNA**→ ←hypo
Ssp-1-8	NC_008577.1	-	3255357	3255296	←RimI (COG0456) **RNA**→ ←DUF6 (pfam00892)
env-39	JCVI_SCAF_1096627390075	-	63159	63098	←? (GOS14094631) **RNA**→ OmpA (COG2885)? (GOS11593935)→
Ssp-1-9	NC_008577.1	-	734223	734162	←SIR2-like (cd01406) **RNA**→ ←hypo
Ssp-2-9	NC_008321.1	+	2562107	2562168	←hypo **RNA**→ ←hypo
Ssp-2-10	NC_008321.1	-	2742481	2742420	COG3791 (COG3791)→ **RNA**→ ←G_glu_transpept (pfam01019)
Ssp-3-8	NC_008322.1	-	2809537	2809424	hypo→ **RNA**→ ←G_glu_transpept (pfam01019)
Ssp-2-11	NC_008321.1	-	3612319	3612258	←hypo **RNA**→ ←TPR repeat-containing protein

Ssp-3-9	NC_008322.1	-	3683793	3683732	←hypo **RNA**→ ←TPR repeat-containing protein
Ssp-2-12	NC_008321.1	+	2746294	2746407	←hypo **RNA**→ ←ArgE (COG0624)Peptidase_M20 (pfam01546)
Ssp-2-13	NC_008321.1	-	792103	792042	DUF469 (pfam04320)→ **RNA**→ ←MurA (COG0766)
env-40	JCVI_SCAF_1101667406492	+	78	139	**RNA**→ ←CspR (COG0219)? (GOS493)
Sba-2-8	NC_009665.1	-	1186168	1186109	COG4538 (COG4538)→ **RNA**→ ←DUF805 (pfam05656)
Ssp-4-8	NC_008750.1	-	1081336	1081276	hypo→ **RNA**→ MurI (COG0796)→
Spu-1-9	NZ_AAWY01000008.1	+	117088	117148	hypo→ **RNA**→ MurI (COG0796)→
Spu-2-9	NC_009438.1	+	3477323	3477383	hypo→ **RNA**→ MurI (COG0796)→
Sba-2-9	NC_009665.1	-	4215414	4215354	←hypo **RNA**→ ←MetK (COG0192)
Spu-2-10	NC_009438.1	-	4204629	4204568	hypo→ **RNA**→ COG4395 (COG4395)→
Spu-1-10	NZ_AAWY01000009.1	-	89170	89109	hypo (cons)→ **RNA**→ COG4395 (COG4395)→
env-41	JCVI_SCAF_1096627205315	+	46954	47015	? (GOS13258397)→ **RNA**→ Tim44 (pfam04280)? (GOS1170)→
Sba-2-10	NC_009665.1	+	605388	605449	←hypo **RNA**→ ←SAICAR_synt_Sc (cd01414)
Sba-3-10	NC_009997.1	-	5240430	5240369	SH3b (smart00287)→ **RNA**→ ←hypo
Spu-1-11	NZ_AAWY01000001.1	-	239916	239855	hypo→ **RNA**→ UvrD (COG0210)→
Sse-1-10	NC_009831.1	-	4566273	4566212	hypo→ **RNA**→ hypo→

27.3 Conserved domains

Conserved domains found in protein-coding genes listed in Section 27.2 are listed, with the first sentence in their description from the Conserved Domain Database. Conserved domains associated with more than one Desulfotalea-1 RNA are assigned a color, while others are shown in gray. The number in parentheses is the number of occurrences of the given gene.

Dd cd00093 (3) Helix-turn-helix XRE-family like proteins.
d cd00115 (2) Substituted updates: Aug 22, 2001
u cd00130 (1) PAS domain; PAS motifs appear in archaea, eubacteria and eukarya.
u cd00180 (1) Serine/Threonine protein kinases, catalytic domain.
u cd00204 (1) ankyrin repeats; ankyrin repeats mediate protein-protein interactions in very diverse families of proteins.
d cd00354 (1) Fructose-1,6-bisphosphatase, an enzyme that catalyzes the hydrolysis of fructose-1,6-biphosphate into fructose-6-phosphate and is critical in gluconeogenesis pathway.
d cd00484 (1) Phosphoenolpyruvate carboxykinase (PEPCK), a critical gluconeogenic enzyme, catalyzes the first committed step in the diversion of tricarboxylic acid cycle intermediates toward gluconeogenesis.
u cd00800 (1) Lambda integrase, C-terminal catalytic domain.
d cd00851 (1) This uncharacterized conserved protein belongs to a family of iron-molybdenum cluster-binding proteins that includes NifX, NifB, and NifY, all of which are involved in the synthesis of an iron-molybdenum cofactor (FeMo-co) that binds the active site of the dinitrogenase enzyme.
d cd01012 (1) YcaC related amidohydrolases; E.
uD cd01193 (2) IntI (E2) integrases, site-specific tyrosine recombinases, DNA breaking-rejoining enzymes, N- and C-terminal domains.
u cd01296 (1) Imidazolonepropionase/imidazolone-5-propionate hydrolase (Imidazolone-5PH) catalyzes the third step in the histidine degradation pathway, the hydrolysis of (S)-3-(5-oxo-4,5-dihydro-3H-imidazol-4-yl)propanoate to N-formimidoyl-L-glutamate.
u cd01299 (1) Metallo-dependent hydrolases, subgroup A is part of the superfamily of metallo-dependent hydrolases, a large group of proteins that show conservation in their 3-dimensional fold (TIM barrel) and in details of their active site.
d cd01301 (1) renal dipeptidase (rDP), best studied in mammals and also called membrane or microsomal dipeptidase, is a membrane-bound glycoprotein hydrolyzing dipeptides and is involved in hydrolytic metabolism of penem and carbapenem beta-lactam antibiotics.
u cd01406 (1) Sir2-like: Prokaryotic group of uncharacterized Sir2-like proteins which lack certain key catalytic residues and conserved zinc binding cysteines; and are members of the SIR2 superfamily of proteins, silent information regulator 2 (Sir2) enzymes which catalyze NAD+-dependent protein/histone deacetylation.
d cd01414 (1) Eukaryotic, prokaryotic and archaeal group of SAICAR synthetases represented by the Saccharomyces cerevisiae (Sc) SAICAR synthetase.
u cd01424 (1) Methylglyoxal synthase-like domain from type II glutamine-dependent carbamoyl phosphate synthetase (CSP).
d cd01942 (1) Ribokinase-like subgroup A.
d cd01948 (4) EAL domain.
u d cd01949 (3) Diguanylate-cyclase (DGC) or GGDEF domain: Originally named after a conserved residue pattern, and initially described as domain of unknown function 1 (DUF1).
d cd03048 (1) GST_N family, Ure2p-like subfamily; composed of the Saccharomyces cerevisiae Ure2p and related GSTs. [Ure3], an inactive, self-propagating and infectious amyloid. Ure2p displays a GST fold containing an N-terminal TRX-fold domain and a C-terminal alpha helical domain, with an active site located in a cleft between the two domains. The N-terminal TRX-fold domain is sufficient to induce the [Ure3]
d cd03178 (1) GST_C family, Ure2p-like subfamily; composed of the Saccharomyces cerevisiae Ure2p and related GSTs. [Ure3], an inactive, self-propagating and infectious amyloid. Ure2p displays a GST fold containing an N-terminal thioredoxin-fold domain and a C-terminal alpha

helical domain. The N-terminal thioredoxin-fold domain is sufficient to induce the [Ure3]
D COG0010 (1) Arginase/agmatinase/formimionoglutamate hydrolase, arginase family [Amino acid transport and metabolism]
d COG0014 (1) Gamma-glutamyl phosphate reductase [Amino acid transport and metabolism]
d COG0033 (1) Phosphoglucomutase [Carbohydrate transport and metabolism]
d COG0168 (1) Trk-type K+ transport systems, membrane components [Inorganic ion transport and metabolism]
d COG0192 (1) S-adenosylmethionine synthetase [Coenzyme metabolism]
D COG0210 (1) Superfamily I DNA and RNA helicases [DNA replication, recombination, and repair]
d COG0219 (6) Predicted rRNA methylase (SpoU class) [Translation, ribosomal structure and biogenesis]
u COG0265 (2) Trypsin-like serine proteases, typically periplasmic, contain C-terminal PDZ domain [Posttranslational modification, protein turnover, chaperones]
u COG0350 (3) Methylated DNA-protein cysteine methyltransferase [DNA replication, recombination, and repair]
d COG0403 (2) Glycine cleavage system protein P (pyridoxal-binding), N-terminal domain [Amino acid transport and metabolism]
d COG0406 (1) Fructose-2,6-bisphosphatase [Carbohydrate transport and metabolism]
d COG0412 (1) Dienelactone hydrolase and related enzymes [Secondary metabolites biosynthesis, transport, and catabolism]
u COG0441 (1) Threonyl-tRNA synthetase [Translation, ribosomal structure and biogenesis]
D COG0442 (1) Prolyl-tRNA synthetase [Translation, ribosomal structure and biogenesis]
u COG0456 (1) Acetyltransferases [General function prediction only]
u COG0458 (1) Carbamoylphosphate synthase large subunit (split gene in MJ) [Amino acid transport and metabolism / Nucleotide transport and metabolism]
D COG0497 (1) ATPase involved in DNA repair [DNA replication, recombination, and repair]
D COG0520 (1) Selenocysteine lyase [Amino acid transport and metabolism]
D COG0543 (1) 2-polyprenylphenol hydroxylase and related flavodoxin oxidoreductases [Coenzyme metabolism / Energy production and conversion]
d COG0624 (1) Acetylornithine deacetylase/Succinyl-diaminopimelate desuccinylase and related deacylases [Amino acid transport and metabolism]
D COG0628 (1) Predicted permease [General function prediction only]
d COG0662 (1) Mannose-6-phosphate isomerase [Carbohydrate transport and metabolism]
d COG0665 (1) Glycine/D-amino acid oxidases (deaminating) [Amino acid transport and metabolism]
d COG0708 (3) Exonuclease III [DNA replication, recombination, and repair]
u COG0715 (1) ABC-type nitrate/sulfonate/bicarbonate transport systems, periplasmic components [Inorganic ion transport and metabolism]
d COG0766 (1) UDP-N-acetylglucosamine enolpyruvyl transferase [Cell envelope biogenesis, outer membrane]
D COG0775 (1) Nucleoside phosphorylase [Nucleotide transport and metabolism]
d COG0777 (1) Acetyl-CoA carboxylase beta subunit [Lipid metabolism]
u COG0782 (1) Transcription elongation factor [Transcription]
D COG0796 (3) Glutamate racemase [Cell envelope biogenesis, outer membrane]
D COG0797 (1) Lipoproteins [Cell envelope biogenesis, outer membrane]
d COG0825 (1) Acetyl-CoA carboxylase alpha subunit [Lipid metabolism]
u d COG0840 (2) Methyl-accepting chemotaxis protein [Cell motility and secretion / Signal transduction mechanisms]
d COG1003 (2) Glycine cleavage system protein P (pyridoxal-binding), C-terminal domain [Amino acid transport and metabolism]
d COG1053 (1) Succinate dehydrogenase/fumarate reductase, flavoprotein subunit [Energy production and conversion]
u COG1061 (1) DNA or RNA helicases of superfamily II [Transcription / DNA replication, recombination, and repair]
Dd COG1115 (2) Na+/alanine symporter [Amino acid transport and metabolism]
d COG1126 (1) ABC-type polar amino acid transport system, ATPase component [Amino acid transport and metabolism]
d COG1132 (1) ABC-type multidrug transport system, ATPase and permease components [Defense mechanisms]
d COG1186 (1) Protein chain release factor B [Translation, ribosomal structure and biogenesis]
u COG1200 (1) RecG-like helicase [DNA replication, recombination, and repair / Transcription]
u COG1225 (1) Peroxiredoxin [Posttranslational modification, protein turnover, chaperones]
u COG1309 (1) Transcriptional regulator [Transcription]
D COG1315 (2) Predicted polymerase, most proteins contain PALM domain, HD hydrolase domain and Zn-ribbon domain [DNA replication, recombination, and repair]
u COG1359 (1) Uncharacterized conserved protein [Function unknown]
u COG1398 (1) Fatty-acid desaturase [Lipid metabolism]
u COG1473 (1) Metal-dependent amidase/aminoacylase/carboxypeptidase [General function prediction only]
d COG1506 (1) Dipeptidyl aminopeptidases/acylaminoacyl-peptidases [Amino acid transport and metabolism]
u COG1556 (4) Uncharacterized conserved protein [Function unknown]
d COG1718 (1) Serine/threonine protein kinase involved in cell cycle control [Signal transduction mechanisms / Cell division and chromosome partitioning]
d COG1733 (1) Predicted transcriptional regulators [Transcription]
D COG1741 (8) Pirin-related protein [General function prediction only]
D COG1744 (1) Uncharacterized ABC-type transport system, periplasmic component/surface lipoprotein [General function prediction only]
D COG1802 (1) Transcriptional regulators [Transcription]
d COG1853 (1) Conserved protein/domain typically associated with flavoprotein oxygenases, DIM6/NTAB family [General function prediction only]
u COG1879 (1) ABC-type sugar transport system, periplasmic component [Carbohydrate transport and metabolism]
d COG1910 (1) Periplasmic molybdate-binding protein/domain [Inorganic ion transport and metabolism]
u COG2137 (1) Uncharacterized protein conserved in bacteria [General function prediction only]
d COG2197 (1) Response regulator containing a CheY-like receiver domain and an HTH DNA-binding domain [Signal transduction mechanisms / Transcription]
uD COG2226 (2) Methylase involved in ubiquinone/menaquinone biosynthesis [Coenzyme metabolism]
u d COG2227 (2) 2-polyprenyl-3-methyl-5-hydroxy-6-metoxy-1,4-benzoquinol methylase [Coenzyme metabolism]
u COG2249 (1) Putative NADPH-quinone reductase (modulator of drug activity B) [General function prediction only]
d COG2253 (1) Uncharacterized conserved protein [Function unknown]
d COG2819 (1) Predicted hydrolase of the alpha/beta superfamily [General function prediction

only]
d COG2856 (1) Predicted Zn peptidase [Amino acid transport and metabolism]
d COG2863 (1) Cytochrome c553 [Energy production and conversion]
D COG2885 (2) Outer membrane protein and related peptidoglycan-associated (lipo)proteins [Cell envelope biogenesis, outer membrane]
u COG2963 (1) Transposase and inactivated derivatives [DNA replication, recombination, and repair]
u COG2968 (2) Uncharacterized conserved protein [Function unknown]
D COG3046 (2) Uncharacterized protein related to deoxyribodipyrimidine photolyase [General function prediction only]
d COG3150 (3) Predicted esterase [General function prediction only]
d COG3258 (1) Cytochrome c [Energy production and conversion]
d COG3314 (3) Uncharacterized protein conserved in bacteria [Function unknown]
u COG3344 (1) Retron-type reverse transcriptase [DNA replication, recombination, and repair]
d COG3529 (1) Predicted nucleic-acid-binding protein containing a Zn-ribbon domain [General function prediction only]
uD COG3547 (5) Transposase and inactivated derivatives [DNA replication, recombination, and repair]
u COG3550 (1) Uncharacterized protein related to capsule biosynthesis enzymes [General function prediction only]
u COG3593 (1) Predicted ATP-dependent endonuclease of the OLD family [DNA replication, recombination, and repair]
D COG3673 (2) Uncharacterized conserved protein [Function unknown]
d COG3707 (1) Response regulator with putative antiterminator output domain [Signal transduction mechanisms]
u d COG3791 (2) Uncharacterized conserved protein [Function unknown]
u COG3852 (1) Signal transduction histidine kinase, nitrogen specific [Signal transduction mechanisms]
u COG3886 (1) Predicted HKD family nuclease [DNA replication, recombination, and repair]
d COG4146 (1) Predicted symporter [General function prediction only]
u COG4271 (1) Predicted nucleotide-binding protein containing TIR -like domain [Transcription]
D COG4395 (2) Uncharacterized protein conserved in bacteria [Function unknown]
u COG4538 (1) Uncharacterized conserved protein [Function unknown]
D COG4584 (1) Transposase and inactivated derivatives [DNA replication, recombination, and repair]
D COG4711 (1) Predicted membrane protein [Function unknown]
u COG4928 (2) Predicted P-loop ATPase [General function prediction only]
u COG5293 (1) Uncharacterized protein conserved in bacteria [Function unknown]
u COG5635 (1) Predicted NTPase (NACHT family) [Signal transduction mechanisms]
u GOS11052049 (1) ?
u GOS11137131 (2) ?
u GOS11166260 (1) ?
u GOS11452331 (1) ?
D GOS11512624 (1) ?
u GOS11522405 (1) ?
u GOS11588659 (1) ?
D GOS11593935 (1) ?
d GOS11627891 (2) ?
d GOS11679563 (1) ?
D GOS1170 (1) ?
d GOS11736581 (1) ?
D GOS12025990 (1) ?
u GOS12125594 (1) ?
u GOS12136901 (1) ?
u GOS12137839 (1) ?
u GOS13258397 (1) ?
d GOS1328 (1) ?
d GOS13449409 (1) ?
d GOS13516489 (1) ?
u GOS13550016 (1) ?
u GOS14027118 (1) ?
u GOS14094631 (1) ?
u GOS14131473 (1) ?
u GOS14347974 (1) ?
uD GOS14479710 (2) ?
u GOS1694 (1) ?
d GOS2568 (1) ?
d GOS2819 (2) ?
u GOS336 (1) ?
d GOS3709 (1) ?
u GOS418 (4) ?
d GOS4580 (1) ?
d GOS4908 (1) ?
d GOS493 (2) ?
D GOS5032 (1) ?
d GOS530 (1) ?
u GOS5488 (1) ?
D GOS5791 (2) ?
d GOS6072 (1) ?
d GOS6435 (1) ?
u GOS672 (1) ?
u GOS7701 (1) ?
u NOG07925 (2)
d NOG14035 (1)
u NOG22301 (1)
D pfam00004 (3) ATPase family associated with various cellular activities (AAA).
d pfam00120 (1) Glutamine synthetase, catalytic domain.
d pfam00154 (1) recA bacterial DNA recombination protein.
d pfam00171 (2) Aldehyde dehydrogenase family.
d pfam00293 (1) NUDIX domain.
u pfam00313 (1) 'Cold-shock' DNA-binding domain.
d pfam00561 (1) alpha/beta hydrolase fold.
u pfam00583 (1) Acetyltransferase (GNAT) family.
u pfam00665 (1) Integrase core domain.
d pfam00892 (1) Integral membrane protein DUF6.
u pfam00912 (1) Transglycosylase.
d pfam01019 (2) Gamma-glutamyltranspeptidase.
u d pfam01389 (2) OmpA-like transmembrane domain.
u pfam01425 (1) Amidase.
D pfam01435 (1) Peptidase family M48.
d pfam01546 (1) Peptidase family M20/M25/M40.

u pfam01548 (1) Transposase.
u pfam01609 (4) Transposase DDE domain.
d pfam01625 (1) Peptide methionine sulfoxide reductase.
u pfam01773 (1) Na+ dependent nucleoside transporter.
d pfam01797 (1) Transposase IS200 like.
u pfam01909 (1) Nucleotidyltransferase domain.
u d pfam02371 (2) Transposase IS116/IS110/IS902 family.
u pfam02525 (1) Flavodoxin-like fold.
u pfam02743 (1) Cache domain.
u pfam02787 (1) Carbamoyl-phosphate synthetase large chain, oligomerisation domain.
u pfam02810 (1) SEC-C motif. [HC]
d pfam03264 (1) NapC/NirT cytochrome c family, N-terminal region.
D pfam03330 (3) Rare lipoprotein A (RlpA)-like double-psi beta-barrel.
u pfam03691 (1) Uncharacterised protein family (UPF0167).
u pfam03692 (1) Uncharacterised protein family (UPF0153).
d pfam03951 (1) Glutamine synthetase, beta-Grasp domain.
D pfam04280 (1) Tim44-like domain.
u pfam04313 (2) Type I restriction enzyme R protein N terminus (HSDR_N).
u pfam04320 (1) Protein with unknown function (DUF469).
d pfam04338 (1) Protein of unknown function, DUF481.
d pfam04389 (1) Peptidase family M28.
u pfam04986 (2) Putative transposase.
u pfam05014 (1) Nucleoside 2-deoxyribosyltransferase.
d pfam05656 (1) Protein of unknown function (DUF805).
u pfam05971 (1) Protein of unknown function (DUF890).
d pfam06224 (1) Protein of unknown function (DUF1006).
d pfam07209 (1) Protein of unknown function (DUF1415).
d smart00014 (1) Acid phosphatase homologues;
u smart00287 (1) Bacterial SH3 domain homologues;
u smart00342 (3) helix_turn_helix, arabinose operon control protein;
d smart00530 (2) Helix-turn-helix XRE-family like proteins ;

Duplicate sequences: the following putative homologs are not shown in the alignment because their sequences are identical to a homolog already shown: Spu-1-10, Ssp-4-2, Ssp-4-5, Ssp-4-7

27.4 Multiple-sequence alignment

Each Desulfotalea-1 RNA is denoted by an abbreviation (like "Eco-1-1") that refers to a taxonomy given in Section 27.1. The alignment includes sequences containing the RNA motif, as well as flanking sequence. The Desulfotalea-1 RNA itself is denoted by the line underneath marked 5′ and 3′ on either end. Nucleotides in the flanking sequences (i.e., not part of the motif) are written in gray letters. Stems of predicted rho-independent transcription terminators are shaded yellow. (Note: terminator predictions have not been analyzed manually, and many are likely to be false positives.) Start codons are shaded green. (Note: start codons are frequently misannotated, especially in environmental samples.) Nucleotides proposed to basepair as part of the consensus structure are shaded in color when they comprise Watson-Crick or G-U pairs. Otherwise they are shaded gray. Conserved stems are also indicated at the bottom of the alignment by angle brackets, where matching < and > denote base-paired columns. Below these angle brackets, the symbol "2" denotes base pairs exhibiting covariation, "1" denotes base pairs exhibiting compatible mutations, "0" denotes base pairs that are not observed to mutate and "?" denotes base pairs that have a significant frequency of non-canonical nucleotides for Watson-Crick or G-U pairs (> 5%). Below these base pair annotation is the consensus sequence: "R" = "A" or "G", "Y" = "C" or "U", red nucleotides: nucleotide identity conserved more than 97% of the time, black nucleotides: 90%, gray nucleotides: 75%, red circle (•): nucleotide is present 97% of the time, black circle (•): 90%, gray circle (•): 75%, white circle (◦): 50%. All percentages of sequences just described (e.g. 97% conserved) assume that sequences have been weighted by the GSC algorithm implemented by the Infernal software.

alignment positions 1 · · · 99

```
Dps-1-1   AAAGGACAUCGCCGCUCUUUGGCAUCCCUACCACCGCGGCAUACAAUCUAUAAAAAGAGUAGCACUGUACAGAUAAUUAAAUUAGCGGGUAGGGAAAAU
Dps-1-2   AAAGGGUGUCGCCGCUCUUUGGCAUCCCUGCCACCGCGGCAUACAAUCUAUAAAAAUAGGAGUACUGUACAGAUAAUUAAAUUAGCGGGUAGGGAAAAU
Dps-1-3   GAAAGGACAUCGCCGCUCUUUGGCAUCCCUGCCACCGCGGCAUACAAUCUAUAAAAUAGGAGUACUGUACAGAUAAUCAAACUAGCGGGUAGGGAAAAU
Dps-1-4   AAGGGGUGUCGCCGCUCCUUGGCAUCCCUGCCACCGCGGCAUACAAACUAUAAAAAUAGGAGUACUGUACAGAUAAUUAAAUUAGCGGGUAGGGAAAAU
Dha-1-1   UUGUUUGGGUCGUGUAGUUUUACGCACAACGCGUACUCCAUAGCUCCAGGACUGGUCAGUUUGCUUUCUUAACAGCUCAGCCAAACCCCGCUGCUUUCA
Epe-1-1   UUUGCAUCAUCUUCAUUGGGUACUUCUCAUCGUGGGAGCCUCUGAAUGAAUCUGGGUGAGCAGAAUUGUGAGGCUAAAUUUUCCGCUGCAAGGCGGAAA
env-1     GAUGGUCAGUAUGUCGCGAGAGGCACGACUACAGGGUUGUAGGAGUUAGGGCGACGCAGGAUGCCAAAGCCGAGGACGCCGAAGCGACGAUGGUUUGAA
env-2     AUCGAUUCAGAAAUAUUAUUAAAGCGGCAUACAUUCGAAUUCAGUAAGAUCCAAACAAAAAAGGCCACCUUACGGUAGCCAUUUUUAUUUGGUGGAGCC
Epe-1-2   GGCGGUGAUGUGCUGGAGCUCUGGGCUCGAGUAACAGGCAUGGACAGCCAGCGGGACUUUCCACAGGUGAUGGAAUCACUUGCGCGUUGGUUGGGUGAA
env-3     ................GGAAAGGGACGGGCUUUUUGCUUUUAAAAAUUAAAAAAAAACAAAAAAAAAUUAAUGGCUGCACCUGAAUUCAUUGCGACCUC

          ...................................................................................................
          ...................................................................................................
          ●●●●●●●●●●●●●●●●●●●●●●●●●●●●●●●●●●●●●●●●●●●●●●●●●●●●●●●●●●●●●●●●●●●●●●●●●●●●●●●●●●●●R●RR●●●YY●●
```

```
Sba-1-1    AACAAAAAAUAAAACAGGUAGUUCUCAGCUGGUCCUAACCCCGGCUCAAUUCCGCAGAAUGUAACUCCCCAAAAAAUUACCAUAAAAGUAUAUAUAUCU
Ssp-3-1    AAUUAAUAUCAAAUAUGAGCUUUUAAAGUGUAUGACAGGUAAUGAAUCCCCCAUCAUAUGCAAAGUUACUGACUGGCAUUGAAGAAAAUCGCGUGAGCG
Ssp-1-1    AUUUUUAUACCUUAACAAUCGACCAAUAACACCUGAGCCUAAGUAAUACACCAUCCCCUCGGAGUUGCCGCAGGGCGAAUAGGCAAAUUGCGUGAGUCU
Son-1-1    AGCUUUUAUGCCUUAAAAAUUACCGAUAAAACCAAAUCCUAAGGAAUACACCAGCCCCUCGGAGUUGCCGCAGGGCGAAUAGACAAAUUGCAUGAGUCU
Son-1-2    ACUCGGUUUGCUUUGUAUGACUUGCAAACAACGCAAAAGCUGAAGGAGUUCACCUCCCCUCGGAGUUGCCGCAGGGCAUUUGCGGUCGUUGCGUGAGCG
Sba-4-1    ACAUGCAUAACUAUCUUGGGAUUACGGCCAACACCGCGGUGACAGAAGUUCACCUCCCCUCGGAGUUGCCGCAGGGCAUUAGCGUUCGUUGCGUGAUCG
Ssp-4-1    ACCACGCUUUAAUGCGUUAAGUGACCAAUAAACAACUCAACAAUCGACGGAAUAUCCCCUCGAAGUGACCGCAGGGCAUUAGCGUUCGUUGCGUGAUCG
Sba-1-2    GGGACAUAACUCUUUUGGGAUUUACUGCCAACCCGAGAUCACAGAAGUUCACCUUCCCUUCGGAGUUGACGCAGGGCAUUUGCGUUCGUUGCGUGAGCG
Son-1-3    UCUUUAAUGCUUUAAGUGACCAAUAGACAACUCAACAAUCAAAGAAAUACACCAUCCCCUCGGAGUUGCCGCAGGGCAUUUGCGUUCUUUGCGUGAGCG
Sba-3-1    UGACGAGCAGUUUACAGGCUUGCCAUGGCAUUCACUCGCCGACUCGGUGAAUAGAAACCUAGGGCCUCUAAGCUUAUGAACGACAAGCUUAUGAACGAG
Sba-4-2    GACAAGCAGUUUACAGGCUUGCCAUGGCAUUCACUCACCGACUCGGUGAAUAGAAACCUAGGGCCUCUAAGCUUAUUGAACGACAAGCUUAUGAACGAG
Sba-3-2    AGGCUUAGCGUUUGUUGGUAUCCACUCAUAAAGGCUUGCCAUGGCAUUUACUCACCGACGCGGUGAAUAGAAACCUAGGGUUUUGAGCUUAUGAACGAG
Sba-1-3    UGACAAGUAGUUUACAGGCUUGCCAUGGCAUUCACUCGCCGACUCGGUGAAUAGAAACCUAGGGCCUCUAAGCUUAUGAACGACAAGCUUAUGAACGAG
Sba-2-1    CCAAUUAACGUUUAUUGACUAACAGUUUAAAGGCUUGACAUGGCAUUCACUCGCCGACUCGGUGAAUAGAAACCUAGGGCCUCUAAGCUUAUGAACGAG
Sse-1-1    UGUUAGCUGGCAGGCUUUUGCAUUGACAUCUACACUUGAAAGAUUCGAUUUUGGAUGUUCAGGAGGCAAGGCGAAGGUCGAAUGAGCGUUUUGAGUGAC
env-4      UGCGCGUGGGUAUGCGCGCUGACCUGGCAGUCUGGAAGCUACGUCACCCAGAACAACUCUGCGCCGAAGUCGGCACGCACAGGCCUGUGGAAAUCGCUG
env-5      NNNNNNNNNNCUGAUGAAGUGAACUAUGAAGUGAAUUGUAGGUAAGUUUGACUAACACUCGCAAGAGUGACACGUACAUAAAGCAUUGAAUAAGAAUUC
Osp-1-1    UCGGCGGGCCAAUCGCAAUACCUCCACUCAUAACAACAACCUCCAAAACUCCAUCAGUUUUGAUUCAAGCAAAAGCAUAGCCCUAGAUGUGAUGAGAAC
env-6      CAUGCACAAUAGAAUGGCAUAUCUGAAGAUAUAAUGCACCAAAUUGGUGCGCGGGUAAUUGAGGCACAUCCGUAUGCUCGCAGGAUACCGUCCGUCCUG
env-7      CCUCUGACCCAGACUGACCUGGAAAGCGAAGCGGAUUAUCUGAAAAGUAUCGAUUACAAACUGAAGUGCAGCUAACCCCACAUUGUGGGUAGCUUGUUU
env-8      CCUUUUGACCCAGACUGACCUGAAAGGGAAGCGGAUUAUCUGAAAAGUAUCGAUUACAAACUGAAGUGCAGCUAACCCCACAUGUGGGGUAGCUUGUUU
env-9      GCGCCUAUGCAGAAAAUGCAGGAGCAAUUUUCUGCCCGUACAUCCUGUACAUGCGCCGCUCCUGCGCAUCCAUGCGCCCGCGGCAUACCGUACAUCCUG
env-10     GGGCGAAAUGCCCCGGCGUACAAUCUGGAAAUCAAAUGCCGGACCGGAUUUGUCAUGUGACGGGGCCGGUCAACCCUUUUUCUAAUGCCGUGCGUAACU
env-11     GCAGCUUGCCCGUAUAAACCCUUAGAGCCCGCGGCAUAUAGCGCCAAGGAUGGCGCCUAUGCAGAAAAUGCAGGAGCAAUUUUCUGCCCGUACAUCCUG
env-12     CAGGAGCAAAAGCCAGUAUGCGCCCACGGGAUACGACCGCCAAGGAUGGCGGAAGUGCCGAUCAUGCAGGAGCAAUUAUCAGCCCGCCCAUCCCUGGAG
Osp-1-2    AACAUAUGCGCUCUUUGCCAUCCAUGAUAACCGCUAUACGAUGCACAAGGAUGUGCAAGUGUCGAAAAGAGGCAGGAGCCGGUUUGACCCGUUCUUCCU
Dps-1-5    GGUAAAGUCUCUUUUUUUGCUGUUGGGAAAAUAAGAAGAAAAGGGAAUGUCGGAUAAAUUUGCGGAGGUGCUUUUGGGGAAGAUCUUAGGAAAUGGAAA
Dps-1-6    GUAGGUUUGUAGGAAUUGCUUUUGGGGAAGGAAAAAGAAAAGGGAAUAUCGAAUAAAUUUGCGGGAGGUGCUUUUGGGGAAGGUCUUAGUAGGUGGAAG
Dps-1-7    CAGCUUUUCGUCUCAUCAACGCACCUAUACUUCAGAUUUCAGCUCAAAAUCAUCCAAUUAGAGAAUGAGAUGAGAAUUGGGCUGGCGAGGGGAACGUCC
Dps-1-8    GCCUUUUCUCUUCAAUGCCCUAGCAUCUACCCUCACCUUCAUAAAUUGCUGUCUAAGCACAUCUCCUACUCUUUGUUAGAUACCACAUAGUGGUACUCU
Dps-1-9    UAUAGGGGCAGGCUUAGAUGGGGGUGUUUUUGUUUGUGAGAAGAGAAAUUCUAGUCAUAGCUAUUUGGGAUAGGCUGUUGUCGAAAUGGAGUAUAGUGG
Dps-1-10   GUGGUAAAUGCUUUUUUACUGUUGAGAACAAUAAGAAGAAAAGGGAAUGUCGGAUAAGUUUUCGGGAGUUACUGCUGGGGGAGUUGUAACAUGAGGAUA
Dps-1-11   GUGGUAAAUGCUUUUUUACUGUUGAGAACAAUAAGAAGAAAAGGGAAUGUCGGAUAAGUUUUCGGGAGUUACUGCUGGGGGAGUUGUAACAUGAGGAUA
Dps-1-12   GCAGGUGCUUAGAACGCCUCUCCACCUUGACGAGGGGGAGAAUGAGAGGGGGUGCGCUUAUUUAGGGGAGGUUGGGUGGAGGUGUGCUUGUUUGGGAAA
Dps-1-13   UUUAGGAAAUCGAGAAUGUGCUAGAGCUGACAGGUGCUUAGAACGCCUCUCCACCUUGACGAGGGGGAGGAAUGAGAGGGGGUGUGCUUGUUUGGGAAA
Dps-1-14   AAUGGUAAAUGCUUUUCUGCUGUUGGGAAAGGAAGAAGAAAAGGGAAAGUUUGGUAGGUUUGCGGGAGGUGCUUUUGGGGAAAGUCUUGGUGGGGAAAA
Dps-1-15   CUGUGGUAAAUGUUUUUGCUGUUGGGAAAGGAAGAAGAAAAGGGAAAGUUUAGUAGAUUUGCGGGAGGUGCUUUUAGGGAAGGUCUUGGUAGGUGGAAG
Dps-1-16   UGUGGUCUCUGUGGUAAAUGCUUUUGCUGUUCAGCCGUUUCUGACAGCUGUUAUUCUUUCACAGCUUUCUGGACUAACAUGGGUGUUUGCUAAAGGACA
Dps-1-17   CUGUGGAGUAGGUAGGCGGGAUUUGCCAGAAAACGGAUAUCGGAGUAAAGUCCUAUCUCUGCCAUUGUGGCUGAUCAUGCAUGGUAACAGCCGCUCCCU
Dps-1-18   AGGGAGAAAAAAAGAGUCUCAAGGAUUCAUUCGUGUCCAUUGACAGUUGCCACCCUCCCGGCCGCAACCAUUUCACCAUAUUGGGUUGCGCACUGUCAU
Dps-1-19   UAAAGGUUUCUCAGGUUGACCCCUUAAGCUCUCCACUCUCCUGCGAAAGCCGUUAAGUCUUCUUUUAUUCUUAGCUAGAUACUACAGGGUAGCACCUCU
Dps-1-20   CACUGCCGCCAUUUCGCUAUGCUUCAUUGCUGCUCUUUUUUUGUCCUCCCAGGGUAUUGCGUCUCCAUGCCAUCCCUGGCACCGACGCGGCUCAUCCAU
Osp-1-3    CGCCAAAACUGAUCAAGCUGUUUGAAUGGAUCGCAAAGCGAUUGCUAAAGAAUUUGAAAGAGAAAAUUGUCCAACCGAUUUUGUGUAGGUUGUAGUAAC
Osp-1-4    UGUUUGAAUGGAUCGCAAAGCGAUUGCUAAAGAAUUUGAAAACGCUGAAAAGCAAAGUAGAGAAAAUUGUCCAACCGAUUUUGUGUAGGUUGUAGUAAC
Osp-1-5    UGUUUGAAUGGAUCGCAAAGCGAUUGCUAAAGAAUUUGAAAACGCUGAAAAGCAAAGUAGAGAAAAUUGUCCAACCGAUUUUGUGUAGGUUGUAGUAAC
env-13     AGGCAAAUACGGUGAAAUGCGUUUACCCACCCGUUGGCGUUUGGUGUUUUUAUCCACUGGCGAAGUGACGCUCGAAAGCCACCUCGCCAGUAUCGGCAA
Spr-1-1    AUACCUUCUCAGAACAUGAGAUCGACAUGGAUGAAACCCUGAAAGUGCUGAAAAGCCUGUAAUCACAACCACCGCCAGCCGCAUAUACCCUAUGAAUUU
Spr-1-2    CCAUUGGCCGGGCUACGUUUUAGUAAUGCAGCUUGCGUGCCAGAUCUGUCAUUUUACAUACCCCAAUUUAUAGUUAUGUAAUUUAUACCCUAUGAAUUU
Spr-1-3    AAUCACGAAAAUCACCAGAACCAACAAAAAAAUCAGCAAAUAUUUCACAUUCUUUCCCGCAAUGCCGAGUUAAUCGGAUGAAUUCUACCCUAUAGCUUU
Sse-1-2    ACGAUAUCCAUAUCUAACAGCCAGCUCGGCAUCCAUGCCUCUUGUUCAUAUACUCAGAAACCUAAGGUUUCUAUUCACCGUGUCGCAGACGUGUUUGCA
Sse-1-3    CCGAACUGGCAGUUAAACAAGGAUGUUGAUUGAAAUCGCAGAGUGCCCAGGGAUGACUCUUUUUGAAAUCAAGAACCAACGCUCUGAGAGGUUACUUAC
Sse-1-4    AAACAUUCAAGUGUAAACGCGGCUGUGACCGUCCGUGACAGGGAUGUCACGGCCGAAGCUAUAGGGACCCUUUCCGUUAAUAGGGAGGUUUACAUUUGC
Slo-1-1    CGGAUAUACGUACGCAGUAUUACUUUCUGUUACUCACAAAAUGUUUGAUCUAUCCGAGGUGUCCAUAUAUGUGCUUGCGGCGAGCCAAAAUAGUGUUUG
Slo-1-2    ACCGUAGAGCGGCCAAGGCCAAAAAUGUUCCCUACAUUUUUUGGCAUUCCCUACAUCCAUGUAGGUCAGAAGGCGCACAGCGUGUCACAGGCGUAUCUG
Swo-1-1    GUGUGAAUCCAAGUGUGAACUCGGUUUAUACUGUUGAAAUAUAUAGGCUUUAAUUGGCGGGUUAGGAAGUGUUUUACAGUGUCCCUCAGAAGUAUCUGC
Slo-1-3    CUCGAACAACCUCCCUGUUUAAAGGCCAGUUCGGCAUCCAUGCCUCUCGUUCGUAAGCACGAAGCUACGCUUCACUCACCGUGCCAAAAUAGUGUUUGC
Sse-1-5    UAGACGCGGCUGCGAGCUUCCAAAACAUGGAUGUUUUGGCUGAGCCUACAAGGAAGACUCUUUUGAAAUCAAGAGCCGGCGUCUAGCAGAGGUAUCUGC
Swo-1-2    AAAUAAUCGAGUGUGAACGCGGCUGUGACCUUCGAUAUCAAGGAUGAUGUCGCAGAGCGUAUAGGGACAUAUUUACAGCGUGUCGCAGAAGUGUUUACA
Swo-1-3    UGACAUCCUGUCACCACGGCAUUUGUGAAUCCAUUCACAUCAGAUGCCACCGUCGAGCUUAUAGGGACAUAUUUACAGCGUGUCGCAGAAGUGUUUACA
Swo-1-4    CUCUGACCCAUAAGGAUAUGGGAAAUGUCUUUAAAGCGUCAGGAACGCUUAAGACCAUGUAAGUCAGAUAGCUUCACAGCGUCCCGAGAGCUUACCUAC
Swo-1-5    AGAGAUAGGGAUAUCGAACUGGCUGUUUAACAUGGACGUUAUUGGCUUUGGUAGAGCUUACAGGGAGCGUACUUGCAGCGUGUCAUAGAAGUAUCGACA
Swo-1-6    GUAAAGCCCACAGGGCAGAAAAUGUUCCAGACAUUUUUCUGCAUUUCCUACUAUCCGUGUAGGUCAGACGUGCUAGUGGCACCUCGUAGAAGUAUCUGC

           ...................................................................................................
           ...................................................................................................
           ●●●●●●●●●●●●●●●●●●●●●●●●●●●●●●●●●●●●●●●●●●●●●●●●●●●●●●●●●●●●●●●●●●●●●●●●●●●●●●●●●●●●●●●●R●RR●●●YY●●
```

Swo-1-7	GUAAAGCCCACAGGGCAGAAAAUGUUCCAGACAUUUGUCUGCAUUUCCUACUAUCCGUGUAGGUCAGACGUGCUAGUGGCGUCUCGUAGAAGUAUCUAU
Slo-1-4	UGGAUAACUGGGUGUAAACAGGCUUUUGGCCUUCGAAAUCAGGGAUGAUUUCGCAGAGCAUCCAAGGAUGGACUUGCUGCGUGCCAAAAGCGUGUUUGC
Slo-1-5	UGAAAAACCGGGUGUAAACACGCUUUUGGCCUUCGAAAUCAUGGAUGAUUUCGCAGAGCAUCCAAGGAUGGACUCGCUGCGUGCCAAAAGCGUGUUUGC
Slo-1-6	UGUUCCCUACAUUUUUUGGCAUUCCCUCCAUCCAUGGAAGUCAGAUGUUUCGGCAGAGCCUACAAGGAAGUAUUCACGGCGUCUCACAGGCGUAUCUGC
Sse-1-6	GAAAUUAACCCAGUGUAAUGCGGCUGCGGACGUUGAUGGCAUGGAUGCCAUCGUCGAGCCUCCAUGGAAGGAUUUACGGUGUUUCGCAGGAGUAUCUGC
Spe-1-1	GCCGAACUGGCAGUUUAACAGGGAAGUUAUUGGAAACCGCAGAGCGUAUAGGGAUACUUUCCGUUAUAAAGAAGUACAGCGUCUCGCAGCAGUGUCUGC
Sha-1-1	CCAAUUAUUUAGUGCAGAUACGGCCGUGACCGUCCAUGAUAUGGACGUCAUGGCCGAGCUUCCAAGGACGGACUUGCAGCGUGUCACGGCAGUGUCUGC
Spe-1-2	CAGGCCAUUGGAACCAUUGAUGUUGGAAGAUACAUCAAAUGCUUCCUAAGCAUUGAUGAAGAAAAGGUUCUCUGCUUUGGGGGAACUUCUAAGUUCUGA
Slo-1-7	UAAGGCUUUAACUCGAAAAUGUCCAAAAAUGAUUUGGUGUUUGAUCGUCGCGGAUACAGAAGUCGCUCAAUCGAAGAGAUGGAUAACUAGGUGUUUGCA
Spe-1-3	GUUCCAACUCGGUGUAGAUACGGCUGAGACCUUCGAUGGCAUGGAUGCCAUCGUAGAGCUCACAUGGACGUGCUUGCAGCGAGUCUCAGAAGUGUCUGC
Sse-1-7	AGGUUAAAUUAGCUCCCGUUGGAAGGUGAAUAUGGCGUAGCCAUAAGCUCAUGAACGAGAGCUAGGGAUAGGGAACUGGCAGUUAAACAGGGAUGUUGU
Sfr-1-1	UUGAUUAAAAAUGUUUGAAUUCAACUCUAAUCUGAGGCCGUUGAAGACUCUUUAUAAAAGAAUGCGUGAGCUUGAUAGGACGUCAGGCUAGCUUUGGAG
Sfr-1-2	AGGAUAUUGGAAAUGUCAUUAGGAUGUAGGGAACAUCAUUGACCAUGUGCUACAAGGCAUUCAGGCAUCCUGCCUAUCAGAUGUGAGCUUAGCUUUCGA
env-14	ACACUUUUGUCUCAAAGCAACACUCAUAUCUUCUUAAAAACCUAAAGAUUAUGCAGAUAUUAAAAAGGUUAUCCAUGGUGAUUCGCGAGACAACCGUCC
Ssp-1-2	CACUUUUACCCCAAAACGAGACACAUAUUUUCUUUAAAACAUACAGAUUACGCCGCUGCUAAACAGGUGAGCCAUGGUGAUUAGCGAGACGACCGUCCG
env-15	GCUUUUGUCCACAAACGAGUUACUAAUCUUCUUUCAAAACAAACAGGCGACAUCGAUGUUAAACAGGAGAACCAUGGUGAUUAGUGAGACGACCAUCCG
Spu-2-1	UGAACAAUUUAGCAAAAUACUCAUGACUUAAUUUUUGAACCUACUGAUUACGCUAAUUAAGUGAAAGGUAAGCCAGGGAGAUUUGCGAGACGACCGUCC
Spu-1-1	UGAACAAUUUAGCAAAAUACUCAUGACUUAAUUUUUGAACCUACUGAUUACGCUAAUUAAGUGAAAGGUAAGCCAGGGAGAUUUGCGAGAUGACCGUCC
Slo-1-8	GUCCAAUAAUAACUUAGCUUUUGAUUGUUAGGGUCAUGACAAUAACGUCAAAUCGAAGAGGCGUUGGAUAACUAGUGCAGAUACGCCGGUGAGCUUCCG
Ssp-2-1	UACUUUUGCCCUAAAGUGAGAUUCAUAUCUCCUUUAAAACUCACAGGGUAUGCCAAUGCUAGGAAGGUGAGCCAUGGUAAUUAGCGAGACGACCGUCCG
Ssp-3-2	UACUUUUGCCCUAAAGUGAGAUUCAUAUCUCCUUUAAAACUCACAGGGUAUGCCAAUGCUAGGAAGGUGAGCCAUGGUAAUUAGCGAGACGACCGUCCG
Sde-1-1	CGCCAGUGCAUUAUGACUAUUAAUGAUAUUUAUCAAUAGUUAAACAUGUGACAAGGUACUGAAUAGAUGCGCCAUGGCAAUUUGCCAGCAGACCGUCAG
Spe-1-4	GGAAACACCAGUUCCCCAUCGGAGUUGCCGAAGUGCGUUGGAAGCUCUAUAUAAACCAGAGUUGCGUGAUAUCCACACGGACGUGGGCGUAGCUUUCGC
Swo-1-8	CUUCCUUAAAGUAGAAGAUGGUUCUCAGAGAUAAUUACAAUAUCUUUAAGUCAAUGAGGCUUGGUAAAUAUCGAGCACUGUUGCAACUGUAAGCGUUGA
Sha-1-2	AUCAUCUUUUGCUCCCCAUCGAAGUUGCCGAAAUGCGUUGGAAACUCUUUAUAAACCAGAGUCGCGUGAUGCCGACAUGGAUGUCGGCGUAGCUUUCGC
Spe-1-5	GGAAACACCCAUUCCCUAUAGGAGUUGCCGAAGUACGUUGAAGACUCUUUAUAAACAAGAGUACCGUGAUACCCACACGGAUGUGGGUAUAGCUUUCGC
Spe-1-6	GAAACACCCAUUCCCCAUCGGAGUUGCCGAAGUACGCAGAUGACACAUUACCUAAAAGAAUGCUGUAGAGCCAUCAUGGAUGAUGGCUCAGGCUCGGAG
Spe-1-7	GCUUUACUAACAGAAAACACCCAUUCCCCAUCGAAGUUGCCGAAGUACGCAGAUAACAAAUGCCGUAGAACCAUCAUGGAUGAUGGUUCAGGCUCGGGG
Sde-1-2	GUUAAGGUUAGCAUAGCUAUGGUUCUAGAUUUUUAAGAGCGAGAAGAACACACCUAAAUAUAUAAGGUGAGCCAUGAGUAUUUGCGAGCCGACCGUCCG
Sba-4-3	UCGGAGAUGACCAAAAGUGUGUUGCGUAUGUCUUUUAAAUCUACAGGUUACGCUAAAGCCAAAACGGUGAGCUAGGGUGAUUAGCGAGACGACCGUCCG
Ssp-3-3	CUCGAAAUGUCCCAAAACAUUUUGUGUAAUUCUUUUUUAAACUACAGACUAAGUCAAUGCUAGAAAGGUGGCCAUGGUGAUUAGUGAGACGACCGUCCG
Sde-1-3	GCUUAUCGAUCGCGAAAAUCAGGUUAGUAAUCUUUUAAGGCUAACAGAUACGCCAAUGCUAAAAAGGUGAACCAUGGUGAUUUGCGAGCCGACCGUCCG
Sde-1-4	CUGAUCACAAAGAGGGCGAUUAGUUCAGUUUUUUAUCCCGAGUUGAGGUUAACUAAUAUUAAAAAGGUGAACCAUGAGUGUUUGCGAGACGACCGUCCG
Sde-1-5	GUUAAAUGUUGGGUGUAGUCUUUUGAGUUAAAAGGUUACGCUAAUACUAAAAAGGUGAACCAUGGCGCUUCACUAGGAGAAAGGCGAGAAGACCGUCCA
env-16	ACGCUUUUGUCCCAAAGCAUACUGCGUAAAGCUUUUAAAUCUAAAGAUUAAGCUAAUGCUAAAAAGGUGAGGCAUGGUGAUUCGCGACACGACCGUCCG
Ssp-4-3	AGGUUUUUGUCCCAAAGCGUGCUGCGUAACGCUUUUAAAUCUGCAGGUUACGCUAAUGCCAAAAAGGUGAGCCAUGGUGAUUAGCGAGACGACCGUCCG
Spu-1-2	AAAUUUCUGUCCCAAAGCGUGCCGGGUAUCUCCUUCAAAACCGCAGGUUACACUAAUGCCAAAAAGGUGAGCCAUGGUGAUUAGCGAGACGACCGUCCG
Spu-2-2	AAAUUUCUGUCCCAAAGCGUGCCGGGUAUCUCCUUCAAAACCGCAGGUUACACUAAUGCCAAAAAGGUGAGCCAUGGUGAUUAGCGAGACGACCGUCCG
Sba-4-4	GCUAGCUUCUGUCCCGAAGUGCGUUACGUACGCUUUUAAAUCUACAAGCUACGCAAAUGCCAAAAGGUGAGCCAUGGUGAUUAGCGAGACGACCGUCCG
Sba-3-3	UGACUAAACAUAUACAUAAUCAGGUAUGGGACAAAGCUCCAAGACUUUGAUUUACCUCGACCGUAAGGCCGUAUUGAUCUUAUACAAGACAACCGCCUG
Sba-1-4	UGACUAAACAUAUACAUAAUCAGGUAUGGGACAAAGCUCCAAGACUUUGAUUUACCUCGACCGUAAGGCCGUAUUGAUCUUAUACAAGACAACCGCCUG
Sba-2-2	GCUAGCUUCUGUCCCUAAGUGCGUUACGUACGCUUUUAAAUCUACAAGCUACGCAAAUGCCCAAAGGUGAGCCAUGGUGAUUAGCGAGACGACCGUCCG
Sde-1-6	AAACCUGUCGUUUAAUCUUUCUAAACGAUAUAGCUGUAGCUAACUGAUACCACUAAAACUAUCGAGCCGAACCAUGGCAAUUUGCGAGACGACCGUCCG
Spu-1-3	CGCUUUUGUCCCAAAACUGUGAUGCGUAUGUCUUUUAAAUCUACAGGUUACGCUAAAGCCAAAACGGUGAGCCAUGGUGAUUGGCGAGACGACCGUCCG
Spu-1-4	CUCGGAAUUGUCCAAAAGUGCGUUACGUUUGUUUUUAAAUCUACAGAUUACGCUAAUGCUAAAAAGGUGAGCCAUGGUGAUUGGCGAGACGACCGUCCG
Ssp-4-4	CUCGGAAUUGUCCAAAAGUGCGUUACGUAUGUUUUUAAAUCUACAGAUUACGCGAAUGCUCGAAAGGUGAGCCAUGGUGAUUGGCGAGACGACCGUCCG
Spu-2-3	CAUUUAUGUCCACAAACGAGUCACAUAUCUACUUUAAAGCAAACAGGUUACACCGAUAUUAAAUAGGUGAGCCAUGGUGAUUGGCGAGACGACCGUCCG
Spu-1-5	CAUUUAUGUCCACAAACGAGUCACAUAUCUACUUUAAAGCAAACAGGUUACACCGAUAUUAAAUAGGUGAGCCAUGGUGAUUGGCGAGACGACCGUCCG
Sba-3-4	CUCGGAAUUGUCCUAAAGCUUGCUACGUAUUUCUUUAAAAAAACAGGCUUCGCCGAUGCUAAACAGGUGAGUCUUGGUGAUUUGCGAGACGACCGUCCG
Sba-1-5	AAGGUUUUGUCCCAAAGCGUGCUGCGUAAAGCUUUUAAAUCUAUAGGUUACGCUAAAGCCAAAAAGGUGAGCCAUGGUGAUUAGCGAGACGACCGUCCG
Sba-3-5	AUGCAUUUGUCCAAAAGCGUGCUGCGUAAGACCUUUAAAUCUACAGGUUACGCUAAAGCCAAAACGGUGAGCCAUGGUGAUUGGCGAGACGACCGUCCG
Spu-2-4	CGCUUUUGUCCCAAAACUGUGUUGCGUAUGUCUUUUAAAUCUACAGGUUACGCCAAAGCCAAAACGGUGAGCCAUGGUGAUUGGCGAGACAACCGUCCG
Spu-2-5	CAAGGUUCUGUCCAAAAGUGCGUUACGUAUGUUUUUAAAUCUACAGAUUACGCUAAUGCUAAAAAGGUGAGCCAUGGUGAUUGGCGAGACGACCGUCCG
Sba-1-6	AAGAAGCUUUUGUCCAAAAGUGUGUCGCGUAUGUCUUUUAAAUCUACAGGUUACGCUAAAGCCAAAACGGUGAGCUAGGGUGAUUAGCGAUGCGACCG
Sba-3-6	ACGUCGCACGUCCAAAAGCGUGUUGUGCAUGUCUUUUAAAUCUACAGGUACGUUAAUGCCAAAACGGUGAGCGAGGGUGAUUAGCGAGACGACCGUCCG
Ssp-3-4	CUCAUUCUUGUCCAAAAAGAUGUUCUGUAUUUCUUUAAAACCAACAGAUUAGCCGCUGCUAAACAGGUGAGCCAUGGUGAUUAGCGAGACGACCGUCCG
Son-1-4	AAACUUUUGUCCCAAAGCGUGCUGAGUAAGGUUAUUAAGUCUACAGGCUACGACAAUGCUAAACAGGUGAGCCAUGGUGAUUAGCGAGACGACAGUCCG
env-17	CAUGCUUUUGUCCCAAAGCAUGCUGUGUGUUUCUUUAAAACCUGCAAGCUACACGGAUGCUAAAAGGGUGAGCCAUGGUGAUUAGCGAGACGACCGUCC
env-18	ACGCUUUUGCCCCAAAGCUUGCUGAGUGUUUCUUUAAAACCUGCAAGCAACACCGAUGCUAAAAGGGUGAGCCAUGGUGAUUAGUGAGACGACCGUCCG
Ssp-1-3	ACGCUUUCGUCUCAAAACGAGUCACUAUCUGCUUUAAAACAAACAGGCCACACCGAUGUUAAACAGGAGAACCAUGGUAAUUAGCGAGACGACCGUCCG
Ssp-3-5	ACUCUAAAAUGCCCCUAGCGGACCGCGUAUUUUUUAAAACUUACUGACAAUGUCAAUGCUAAACAGGUGAGCCAUGGUGAUUAGCGAGACGACCGUCCG
Ssp-2-2	CACUCUUUUGUCAAAAAGUAUGUUACGUAAUCUUUUAAAUCUACAGGUUCCGCUAAUGCUAAAAAGGCGAGCUAUGGUGAUUGGCGAGACGACUGUCCG
	....
	....
	●●R●RR●●●YY●●

```
Ssp-2-3    UCGAAAAUGUCCCUAAGCAUACUUCGUAUUUCUUUAAAACUUACAGACUACGCUAAUGCUAAACAGGUGAGCCAUGGUGAUUAGCGAGACGACCAUCCG
env-19     UCGAAAAUGUCCCAAAACAUUUUGUGUAAUUCUUUUUUAAACUACAGACUAAGUCAAUGCUAGAAAGGUGGCCAUGGCAAUUAGCGAGACGACCGUCUG
env-20     UUUAUUCUGUUUGUCUGGUCGGCGAUGAACCACAUAAGGGCAAAGGAACCCAUCGCGACUCUUAUCGAUAAUGCGGGAUUACCAUUAGCGAGACGGCCG
Ssp-2-4    AAAAUGUCCCAAAACAUGCGGUGUAUUUCUUUGUUAAUACUCACAUGCGACGCCAAUGCUAAGCAGGUGAGCUAUGGUGGUUUGCGAGACGACCGUCCG
env-21     CCUGGAAAUAUCUCAAACAUACUACGUAUUUAUUUGAAACUUACAGAUUACGCUAAUACUAAAAAGGCGAGUGAUGGCGAUUAGAUAGAUGACCGUCUG
Sba-4-5    CACUGCCAACCAUAGUUCAAUAAAAACUCCUCCCUCUUGAACCUCUAGACUACGCAAAUACUAAAAGGUGCGCCAUGGUGAUUGGCGAGACGACCGUCC
Sde-1-7    UUAGCAUAGCUAUGGUUCUAGAUUUUUAAGAGCGAGAAGAACACACCUAAAUAUAUAAGGUGAGCCAUGCCGAUUUACCACGAGAAAGGCGAGCCGACC
Sba-2-3    AAGAAGCUUUCGUCCAAAAAGUGUGUUGCGUAUGUCUUUUAAAUCUACAGGUUACGCUAAAGCCAAAACGGUGAGCCAUGGUGAUUAGCGAGGCGAGCG
env-22     ACACGCUUUUGUCCAAAAACUGCUUAGCAUUUCUUUAGACCUACAGGCUAUACCGAUGCUAAACAGGUAAGCUGUGGUGAUUAGCAAGACGACCGUCUG
Sse-1-8    CUGCAACUCAUUUCGGGGCAGAGAUGAGUUACUAAACCUAGCAAUAAAGCCUAAUCGAAGAGAAAAACAACGCAGUGUAGAUACAGCUGUGACCGUCCG
Swo-1-9    CAGUACUGCAUAGGGCAAAUUACUGUCACUAAUGCAUAAAGAGAUAAAGCCAAAUCGAAGAGACAAAUAAACAGGUGUAGAUGCGGCAGUGACCAUCCG
Swo-1-10   CAAUUGGGUCUAACUCACUUCUGUUCAAAAAUGAGUUACAGCCCAAAACCUCAAUCGAAGAGAUAAAUACUCAGGUGUAGGUACGUCAGUGAGCUUCGA
Swo-1-11   UCCAGCCCCAAAACAAAGGGGUCGUAGCCCAUUGACCUGAAUCGAAGAGAUACCUAUGUAGUUGUCAGAGUCAGGUGUAGAUGCGGCAGUGAGUUUCGG
Swo-1-12   ACCCUAAGCACGAACUCUUUUAUGUCCAAUUGUUAGUCACAACAAGAGCCUAAUUCGAAGAGAUAAACAAUCAGGUGUGAAUGCGGCUGAGGGCGUUGG
Swo-1-13   AACAUCAAACACUUCUUAUUAAAGAGCAACUAGGUCGUAACUAAUCGGCUGAAGUCGAAGAGAUAAACACUCAGGUGUGAAUGCAGCUGUGGGCGUUGG
Swo-1-14   AGCCAUUCGCUCUAUAGUUGAUACUCUAACGUGUUUUUGUCCAAAAAUAUCAAAUCGAAGAGAUAAACAAUCAGGUGUGAAUGCGGCUGUGGGCGUUGG
Sse-1-9    GCUGAUUUCAACCAAGUUAACUUUUUAUAAAGCUCUAAGAUUAGAAACGAUUAAAUCGAAGAGAUAGAUCAACAAUGUAGGCACGGCUGUGACCGUCCG
Swo-1-15   GUUGGGGCGUACCAUCUUAUUCGUGAGUUACGAACACUGCUAAAUAAAGUCAAAUCGAAGAGAAAAAUAACCAAGUGUAGGUGCGGCUGUGACCGUCCG
Sde-1-8    AUGCUGUUGUUUUCCCCUCUUGGCCAGUGCAGAGUGGAACCCUGCGACUUUGAUCCAAACGGUGAAUAAUCAUCUUGGGUGAUUGCGCUUAUGAGCAAG
Sde-1-9    CGAAUUGCUAAUUUAAAAUUCAAACAAGCCCCGCCUUAUGACCAGGCCUCGAUGUAAGAUGGUGGGCGAUUAUUGCACAGCAAUACGUUUAUGAGCGCG
Sde-1-10   UUGUGGGUGGCCACAUUUUUUAAUGGGGGAGUGGAACCCUGCCACGUUAAUCCAAACGGUGAUGAAUAAUCACCUUGGGUGAUUAUGCUUAUGAGCGAG
Slo-1-9    CUUUUGUCCAAAAAUAAGUCAGCUUGUACGAGUUACACAAUCAACCCUAUAUCGAAGAGACGAUAGAGCCCCAGUGUAGAUACGCUAGUGAACUGUCCG
Ssp-2-5    UUUGAGUUAAGGCUUUAACUCGAAAAUGUCCCUUAGCUUUUUACGUACAUAUCGCAAAUAGGAAGUUAAAACUGGCAUUGGGGAACCAGUGACCGUUGG
Sba-2-4    AUGUGCCGACUUGAAAAUGGAAAUUGAUAGUGUGGAAACUGGCUAAAAAGGUACACAGGAAUUAACGAAAUUAAGCACACUCAUGGAAGUGACCGUUGG
Sba-2-5    GAACCAGACUAGGCAAGUCUGAUUCACGGAUUUUAACGAGCCCGUAUUUAAUUCUCGCCCUAAAAGAGGAUUAAGCACUGCCACGAAAGUAGCAGUCCA
Sba-1-7    CUGACAACAAAGUUAUCUUGCUUUGAGGCAAUAGCUUUUUCGGAGAACGGCAGCGAGAGGGUUGGUUAGGAUUGGCAUUGUUGUAACAGUGACCGUUAG
Ssp-1-4    AGCUAACGAGCCGAAAUACGGAAUGGGGCAGAAAGCUUUUAGCCAGAUGAACGGUUCAAUGGUCGUUAAGAUUACCACUGUUACAACAGUGACCGUUGG
Spu-2-6    AUCUUCGAGCCGUGGCACACUUAGGGGCAAUCGCUUUUUAGCUAAACGAACAGCGAUAGGGUUAGUUAAGAUUGUCACUGUUGCAACAGUGACCGUUGG
Spu-1-6    AUCUUCGAGCCGUGGCACACUUAGGGGCAAUAGCUUUUUAGCUAAACGAACAGCGAUAGGGUUAGUUAAGAUUGUCACUGUUGCAACAGUGACCGUUGG
Son-1-5    CACAGACAACUCGUUAACUUGCCUUGGUCAAUAGCUUUUUGGCCAGAUGAACCGUUCAAUGGUCGUUAAGAUUGGCAUUGUUGCGACUGUGACUGUUGG
env-23     UAGAGUUGGGAAUCUAACAUGCUUUUGUCCCUUAGCUUUUUUAGACUCAUACAGCAAUUGGGUAGAUGAAAUUGGCAUUGGGGCUACAGUGACCGUCGG
env-24     AUUCCCUGCAUUGAAAUUUGUAACACAGAGUGCCUUUAGUUUUUUAGACUCAUACAGGAAUUGGGGAGUUAUAAUUGGCAUUGGGGCAACAGUGACCGG
env-25     AAAUUAGAGUUCAAAAUCUACCAUGCUUUGGUCCCAUCAUUUUGAUGACUAAACGGUUGUAUGGAUGUGAAUUGCCAUUGAAGCACCAGUGACCGUUGG
env-26     UAGGUAUAACAAUCACUUGCAGGCAUACAGCUGGUGCUUUUUACGUACAUAUAGCAAAUGGAUAGUUAAAAUUAGCAUUGGGUCUACAGUGACCGUCGG
env-27     UAGGUAUAACAAUCACUUGCAGGCAUACAGCUGGUGCUUUUUACGUACAUAUAGCAAAUGGAUAGUUAAAAUUAGCAUUGGGUCUACAGUGACCGUCGG
Ssp-2-6    AAGCGUUUAGAUUAAACGGGGGACAUUUGAAUAGCUUUGUCCAAUCCUUAGAUGACGAGAUAGUUUGUGAAUUGCCAUUUUAGCACCAGUGACCGUUGG
env-28     GCAGAUAAAAUUGCUAUUAAGCUUUUGUCCCAUAUCUUCGGAUGACGAAACGGUUGUAUGUGGUGUGUGAAUUGCCAUUGAAGCACCAGUAACCGUUGG
Son-1-6    ACCUUUAGUGCAUUAAAUCUAACGCGCUUUUGUCCCAUCUUUAGAUGACUAAACGGUUGUAUGGAUGUGAAUUGCCAUUGAAGCACCAGUGACCGUUGG
env-29     UCUGAGACUUGCACCCAUCUUUAAAAGUUUAAUCAGAUAUUUAUCGCUAGAUUGUACGCAGAGAUCGAUGAUUCAGCGCUGGCAUACAAGCACCGUUGG
env-30     CCACCUGUUGUUAAAUUUCUAGGGUGUAUGUUUGUAUGAAGGGUUCAGAUUACUGAAGGGAUUGGUGAGAUUAAGCACUGGCAUGUUAGUGAUCGUUGG
env-31     AGUCACACUUAUAAAUCACUUUGGGGCAAAUAUCUUUUUAGCUGACCGAGCAGCUAGAGGGAUAGUUAGGAUUGGCAUUGUUGCAACAGUGACCGUUGG
env-32     ACCUCAGAAUAAAGCCGUUAUGUGUGCACUAUCAACUCAAUGAGCAAUACAGACAUCUGAGUUCGCUGAGUUAAACAUUCUCGCGACAGUGACCAUUGG
Son-1-7    AUUGUCAGUAACCCCGAUAACGCGCAAUGGGGCAAUACUUUUAGAGAUGAACAAUUCAAUAGUCGUUAAGAUUGGCAUUGUUGCAACUGUGACCGUUGG
Spu-2-7    AUUAUAUGUGAAGUUUAUCAAGGCAUUCAUUGUUCAAUUUGGCCACAGGUAUACUCAAAGGUUAAUGAGAUUAAGCACUGUCAUGGAAGUGACCGUUGG
Spu-1-7    AUUAUAUGUGAAGUUUAUCAAGGCAUUCAUUGUUCAAUUUGGCCACAGGUAUACUCAAAGGUUAAUGAGAUUAAGCACUGUCAUGGAAGUGACCGUUGG
Sba-3-7    CUAUCCAUUGUUAAAUUUGCAGAGUGUAUGCUUGUAAAAAGGCAUACAGAUUACUGGAAAGUUGGUUGAAUUAAGCAUUGGCAUGUAUGUGACCGUUGA
Sba-3-8    CCAUCCAUUGUGAAAUUUGCAGAGUGUAUGCUUAUAAAAAGGCAUACAGAUUACUGGAGAGUUGGUUGAAUUAAGCAUUGGCAUUUAAGUGACCGUUGA
Sba-2-6    CCAUCCAUUGUGAAAUUUGCAGCGUGUAUGCUUAUAAAAAGGCAUACAGAUUACUGGAGAGUUGGUUGAAUUAAGCAUUGGCAUUUAAGUGACCGUUGA
Ssp-1-5    GAGCUAACGAGCCGAAAUACAGAAUGGGGCAAAAAGCUUUUAGCCAAUGAACAGUUCAAUGAUCGUAAAGAUUGCUAUUGUUGCAACUGUGACUGUUGG
Ssp-1-6    GAGCCGAAACACGGAAUGGGCAAAUAGCUUUUAGUAGAACGAGCUGCGAGAAGGCUAGUUAGCUUUGGCCAUUGGCAAUUUUACGGAAGUGACCGUUGG
env-33     AGCCUCAACGAUCGUAACACCGAAUGGGGCAAAAAGCUUUUAGCCAAUGAACAGUUCAAUGAUCGUUAAGAUUGGCAUUGUUGCAACAGUGACCGUUGG
Ssp-4-6    CAACCAAUUGAACUUUAUUGAAACAUCCAUUGUUAAAUUUAGUGACAGGUAUACUCGGGAAUUAACGAGAUUAAGUACUUGCAUGGAAGUGACCGUUGG
Sba-4-6    UAAACUGAGAGCUACCUUGCCAAGCACCAACAGGCCACUUUUAGUGCAGAUAUACUCAAGGGCUAGCUAAAUUAGCACUUGCAUGUAAGUGACCGUUGG
Sba-1-8    CUGUGACCUUCUUAACAGAGAGCUACCUUGCCAAGCACUAACAGGCCACUUUUAGUGCAGACAUACUCAAGGGCUAGCUAAAUUAAGCACGACCGUUGG
Son-1-8    AUGCCAUUUUGCUAACGAGGUAUGGGGCAUUAGCUUUUUAGCUGAACGAGCAGCGAGAGGGUUAGUUAAGAUUGGCAUUGUUGCAACUGUGACCGUUGG
env-34     AUUGUUCGCUCAGUUUUACAUCACACUUAGAUGCUUUUCAGACUUACAGAAUACGAUUUAGCUCGCGGAAUUAAGCACUGUCAUGGAAGUGACCGUUGG
Sba-3-9    UCAUACUGAAUUAUUUAGAGUUUGGUCUUUUUGAUUCAUAUGUAGCAAAAAUUAUAGAAGGCUUGCUGAAUUAAGCUCUGUUAUGUAAGUUACCUUUUG
Spu-2-8    AUGCCACUUUGCUAACAAGGAAUGGGCAAUACCUUUUUAGCUAAACGAACAGUAUAAGGGGGUAGUUAAGAUUGCUACUGUUGCAACAGUGACCGUUGG
Spu-1-8    GAUGCCACUUUGCUAACAAGGAAUGGGCAAUACCUUUUUAGCUAAACGAACAGUUAAGGGGGUAGUUAAGAUUGCUACUGUUGCAACAGUGACCGUUGG
Sba-2-7    ACUCACGAUUAAAGUUUUUGUAUUUAUGUAUGUAAAAUUCGGGCAUUAAGGUGUGAGUGGAUUAAAGGAAUUAAGCACUGUCAUGGAAGUGACCGUUGG
env-35     CCUGACGACAAACGUAUCUCGUUUUGGGGGAAAAAGCUUUUAGCCAAUGAACAGUUCAAUGGACGUUAAGAUUGGCAUUGUUGUAACAGUGACCGUUGG
Ssp-2-7    CCUGACGACGAACUUAUCUCGUUUUGGGGCAAAAGCUUUUAGCCAGAUGAACAGUUCAAUGGUCAUUAAAAUUGCCAUUGUUACAACAGUGAUCGUUGG

           ...................................................................................................
           ...................................................................................................
           ••••••••••••••••••••••••••••••••••••••••••••••••••••••••••••••••••••••••••••••••••••••••R•RR•••YY••
```

```
Ssp-1-7    CCUGACGACAAACUUAUCUCGCUUUGGGGCAAAAGCUUUUAGCCAGAUGAACAGUUCAAUGGUCAUUAAAAUUGCCAUUGUUGCACCAGUGACCGUUGG
env-36     ...ACGAUCAGUCGUUUUGCUUUGGAGCAAAAAAGCUUUUAGCCAGAUGAACAGUUCAAUGGUCGUUAAAAUUGCCAUUGUUGCAACAGUGAUCGUUGG
env-37     AGAUAACGAGCCGAAACACAGAAUGGGGCAUUAGCUUUUUUGCCAAAUGAACAGUGCAAUGGCCGUCAAAAUUGCCAUUGCUGCAACAGUGACCGUUGG
env-38     AAAAAUGGAAUGCUGAAUUGCUUUGGGGCAAAAAGCUUUUAGCCUGAUGAACAGUUCAAUGGUCGUUAAGAUUGCCACUGUUGCAACUGUGACCGUUGG
Ssp-2-8    AGGAAUCUGAGGCUAGCAAGCUUUGGGGCAUUAGCUUUUUUGCCAAAUGAACAGUGCAAUGGCCGUCAAAAUUGCCAUUGUUGCAACAGUGACCGUUGG
Ssp-3-6    GAGCUAACGAGCCGAAGCAAGGAAUGGGGCAAAAGCUUUUAGCCAGAUGAACAGUUCAAUGAUCGUUAAGAUUGCCACUGUUGCAACAGUGACCGUUGG
Ssp-3-7    AGCUAAAGAUUUGAGUUGUUUUGGGCAAAAAAAUUUUUUAGCCAGGUGAACAGUUCGGUGGUCGUUAAGAUUGUCAUUGUUGCAACUGUGACCGUUGA
Ssp-1-8    GAGAUAACGAGCCGAAACACACUUUGGGGCAAAAGCUUUUAGCCAGAUGAACAGUUCAAUGGUCGUUAAGAUUGUCAUUGUUGCAACUGUGACCGUUGG
env-39     AGAUAACGAGCCGAAACACAGAAUGGGGCAUUAGCUUUUUUGCCAAAUGAACAGUGCAAUGGCCGUCAAAAUUGCCAUUAUUGCAACAGUGACCGUUGG
Ssp-1-9    AGCUAGAAAGCCGAAACAAGGAAUGGGGCGUUAGCUUUUUUGCCAAAUGGACAGUGCAAUGGCCGUCAAAAUUGCCAUUGUUGCAACAGUGACCGUUGG
Ssp-2-9    GAUCUAACGAGCCGAAACAAGGAAUGGGGCAAAAGCUUUUAGCCAGAUGAACAGUUCAAUGGCCGUAAAGAUUGUCAUUGUUGCAAUAGUGACCGUUGG
Ssp-2-10   AUGCAGCCGAUGUCCUGACACGUUUUUGUCCCUUUUCUUUUUACGUAUAUAUCGCAAAUGGGUAGUUAAAAUUGGCAUUGGGGCACCAUUGACCGUUGG
Ssp-3-8    UUAGAGUUAAUAACCCAACUCAUUUUUGUCCCUUUUCUUUUUACGUAUAUAUCGCAAAUGGGUAGUUAAAAUUGGCAUUGGGGCACCAUUGACCGUUGG
Ssp-2-11   CUAACGAGCCGAAACACACUUUGGAGCAAAUAUCUUUUUAGCUGAACGAGCAGCUAGAGGGAUAGUUAGGAUUGCCAUUGUUGCAACAGUGACCGUUGG
Ssp-3-9    CUGACGACGAACUUAUCUUGUUUUGGGGCAAAAAGCUUUUAGCCAGAUGAACAGUUCAAUGGCCGUAAAGAUUAGCAUAGUUGCAACUGUGACCGUUGG
Ssp-2-12   AACUAACGAGCCAAAACACGGAAUGGGGCAAAAAGCUUUUAGCAAGAUGAACGGUUCAAUGGUCGUAAAGAUUGCCAUUGUUGCAACUGUUACCGUUGG
Ssp-2-13   GUUAGAGUUAAGGCUUAACUCGAAAAUGUCCCUUUGCUUUUUACGUACAUAUCGUAAAUGGGUAGUUAAAAUUGGCAUUGGGGCACCAGUGACCGUUGG
env-40     .......................GGGGCAAUACCUUUUUAGCUGAACGAACAGCGAAAGGGUUAGUGAGGAUUGCCAUUGUUGCCACUGUGACCGUUGG
Sba-2-8    AGCAAAAGAGCGGACAGCCAUAAAUUUUAAAUAAUAAUCUGCUAUCAAAAAUACACAGGGAGUAACCAAAUUAAGCACAAUCAUAGAAGUAAUCGUUGG
Ssp-4-8    ACUUUGACUUUGACUUUGACUUUAGAAAUACAUCAAAUCAACUAUCAGACAUACACAGGGAUUAACCAAAUUAAGCACUAUCAUGGAAGUGACCGUCCG
Spu-1-9    ACUUUGACUUUGACUUUGAUUUUAGAAAUACAUCAAAUCAACUAUCAGACAUACACAGGGAUUAACCAAAUUAAGCACUAUCAUGGAAGUGACCGUCCG
Spu-2-9    ACUUUGACUUUGAUUUUGAUUUUAGAAAUACAUCAAAUCAACUAUCAGACAUACACAGGGAUUAACCAAAUUAAGCACUAUCAUGGAAGUGACCGUCCG
Sba-2-9    AUCUUAUCUAGCUCACUUUGGGGCAAUAGCUUUUUAGCUAAGCUAUCAGCGAAAAGGUUGGCUGAGAUUGGUAUGUAUUGUUGUACCAGUGACCGUUGG
Spu-2-10   UGCCUAUGUCAGGGUAUUGGACUUCGGUUAGGGGAGUUCUGGCUAUCAGGAUACAGGAUAGUUAACGGGAUUAAGCACUGGCAUCUAAGUGACCGUUGG
env-41     GCCACCCAUUAUUAAAUUUAAAGAGUGUAUGUUACUAAGAGAUCUCAGAUUACUGUGGGGAUUGGCAAGGUUAAGCACUGGCAUGUAAGUGACCGUUGG
Sba-2-10   UUAUUAUUUGUUAAAUUUGCAGAGUGCAUGCUUGUAAAAAGGCAUACAGAUUACUGAAGAGUUGGUUGAAUUAAGCAUUGGCAUGGAAGUGACCGUCCG
Sba-3-10   UACCGAACUAUCUAGGUCUAUUUGUAAAACUUGAAAGAGCGUACUUAAGGUUUGUAUUGAGUUGUUGAGAUUAAGCACUCGCAUGUAAGUGACCGUUGG
Spu-1-11   AAGAUAAGAGGGGGCCACUUUUCGCCUUUGAUCUUUGCUGGCUGGCAGAGUUACGCAAAGGUUAAUAGGAUUAAGCACUGUCAUGUAAGUGACCGUUGA
Sse-1-10   UGAGUCGUUAGGCGAUUUUUAUUGAAGUAUGAGGCCCGUACUCAAUGCAUGUAACUUCGCCGGGGCGUUAGGGUACUUUGGUUUACAGAGAAUCAAGGA
           ...................................................................................................
           ...................................................................................................
           ••••••••••••••••••••••••••••••••••••••••••••••••••••••••••••••••••••••••••••••••••••••••R•RR•••YY••
```

alignment positions 100 · · · 259

```
Dps-1-1    UAAGAUCACAGCUCUCUGCCAUCCA....................................................UGGCA.UCGCUUGCGUCC.CCUCAUCCCUGAGGGCAAAAAAAAACCAAGCUGAAAUUAAUCAACUUGGGGCUUUGGAUAUAAU
Dps-1-2    UAAGAUCACAGCUCUCUGCCAUCCA....................................................UGGCA.UCGCUUGCGUAC.CCUCAUCCCUGAGGGCAAAAAAAAGCCCAAACUAAAUUAAUAGCUUGGGCUUUAUAUAUAAAAU
Dps-1-3    UAAGAUCACAGCUCUCUGCCAUCCA....................................................UGGCA.UCGCUUGCGUAC.CCUCAUCCCUGAGGGCAAAAAAAAGCCCCAAGUCGAAAUUAAUCAACUUGGGGCUUUGGAUAUA
Dps-1-4    UAAGAUCACAGCUCUCUGCCAUCCA....................................................UGGCA.UCGCUUGCGUAC.CCUCAUCCCUGAGGGCAAAAAAAAGCCCCAAACUAAAUUAAUAGUUUGGGGCUUUGGAUAAAAU
Dha-1-1    GCAUGUGGCCGCUCCUGCACGUCCA....................................................UGUGC.CCGCGGCAUCA..GGACCUCCAUGUCCAAGCAACCGGUGGCUGCGCUAUGUUAAGAAAGCCUCAUUGACCUGACCGC
Epe-1-1    UCGUAGGGUCGCUCAAUGACAUCC.....................................................UGUCU.CCGCGACACUU..GCACAUCCUUGUGCGGCGUAAUAGUAGGCCUAUUGCCAAGAUUUCCAACACAGCAGCGGGAAAC
env-1      AGGUCAGGCCGUUCUCGAACAUCCC...................................................UGUUCUUCACGACACUC..GUACAUCC.UGUACAUCGUAUGUCGUAAGAUGCACACCGAUAGGGGUAUUAGGAGUUAGAUCGA
env-2      UAGCUUGGCCGUUCUCGGACAUCC.....................................................UGUCCUUCACGGCAUUC..GUACAUCCUUGUACUUCAUCGAACCACUUUCAGCGGUUCGGCAAUUUAACCAAACAAAAAAGGC
Epe-1-2    ACGCCGGGCCGCUCCUGUCCAUCCA....................................................UGGAC.CCGCGGCAUUC..GGACAUCCAUGUUCAUCAGCCUGUUACCCGACCUUACCAUUAAGAAACCGCCGGUCGAUGAACU
env-3      AGAAGUGACCGCUCUAGUACAUCCA....................................................UGUAC.CCACGGCAUUA..GUACAUCCAUGUACGUCAAACGUGUUAGCGCCGGUGGUAAAUAGUGUGUGGAUGCUCUUCUCUU
Sba-1-1    CACUAUGGUCGCUCAUGUGCAUCCA....................................................UGCACUUCACGACAUUC..AGACAUCC.UGCCUAUCAAAUAGCGAGCUAGCUUUCGCAGGUGCAGGGGCGAAUGAAAAUCGGU
Ssp-3-1    AGGUAUGGUCGUUCUCUUGCAUCGA....................................................UGCACCUCACGACAUUC..AGGCAUCC.UGCCUAUCAGAUGCCGAGAUAGCGUCAAUCGAAUCAGGGACGAUCGUCUGACGCG
Ssp-1-1    CGCCAUGGUCGUUAUCGUGCAUCCA....................................................UACACGUCACGACAUUC..AGGCAUCC.UGCCUAUCAGAUGGCGAGCUAGCUUUCGCAGGUGCUGGGACACAUCCUUCGGAAG
Son-1-1    CGCCAUGGUCGUUCUCGUGCAUCCA....................................................UGCACUUCACGACAUUC..AGGCAUCC.UGCCUAUCAUCAGAUGCCGAGAUAGCGUCAGUCGAACAUAAAAAAACAGGACUGG
Son-1-2    AGGCAUGGUCGUUCUCGUGCAUCCA....................................................UGCACUUCACGACAUUC..AGGCAUCC.UGCCUAUCAGAUGCCGAGAUAUCGUCAGUCGAAUCAGGGACGAGCGUCUGACGCG
Sba-4-1    AGGCAUGGUCGUUCUCGUGCAUCCA....................................................UGCACUUCACGACAUUC..AGGCAUCC.UGCCUAUCAGAUGCCGAGAUAGCGUCAGUCGAAUCAGGGAGUGAGCCCUACAACU
Ssp-4-1    AGGCAUGGUCGUUCUCGUGCAUCCA....................................................UGCACUUCACGACAUUC..AGGCAUCC.UGCCUAUCAGAUGCCGAGAUAGCGUCAGUCGAAUCAGAGAUGAGCGUCUGACGCG
Sba-1-2    AGGCAUGGUCGCUCAUGUGCAUCCA....................................................UGCACUUCACCACAUUC..AGGCAUCC.UGCCUAUCAGAUGCCGAGAUAGCGUCAGUCGAAUCAGGGACGAGCGUCUGACGCG
Son-1-3    AGGCAUGGUCGCUCACGUGCAUCCA....................................................UACACUUCACGACAUUC..AGGCAUCC.UGCCUAUCAGAUGUCGAGAUAGCGUCAGUCGAAUCAAGGACGAGCGUCUGACGCG
Sba-3-1    AGCCAUGGUCGUUCUCAUGCAUCCC...................................................UGCAUUGCACGACAUUC..AGGCGUCC.UGCCUAUCAGAUGGCGAACGGGCAGUUAAACACGGAUGUUGUUCAAGUCAAUCCG
Sba-4-2    AGCCAUGGUCGUUCUCAUGCAUCCC...................................................UGCAUUGCACGACAUUC..AGGCGUCC.UGCCUAUCAGAUGGCGAACGGGCUGUUAAACACUGAUGUUGUGCAAGUCAAUCCG
Sba-3-2    AGCCAUGGUCGUUCUCAUGCAUCCC...................................................UGCAUUGCACGACAUUC..AGGCGUCC.UGCCUAUCAGAUGGCGAACUGGCCGUUAAACAGGGAUGUUGUUCAAGUCCAUCCU
Sba-1-3    AGCCAUGGUCGUUCUCAUGCAUCCC...................................................UGCAUUGCACGACAUUC..AGGCGUCC.UGCCUAUCAGAUAGCGAACUGGCAGUUAAACACGGAUGUUGUGCAAGUCAAUCCG
Sba-2-1    AGCCAUGGUCGUUCUCAUGCAUCCC...................................................UGCAUUGCACGACAUUC..AGGCGUCC.UGCCUAUCAGAUAGCGAACUGGUAGUUAAACACGGAUGUUGUUCAAGUCAAUCCG
Sse-1-1    AGGCUAGGUCUUUCUCAUGCGUCCU....................................................UGCAU.UCAAGGCAUUC..AGGUGUCC.UACCUAUCAGAUGCCGAACUGGCUUUUUCAGAUGGAUGUAAUUUGCCUAGACCUC

           .........<<<....<<<<<.....<<<<<<....>>>>>>....<<<..<<<<<<<<.....>>>>>>>>>>>..>>>>>...>>>........<<<<<....>>>>>..................................................
           .........222....22211.....212020....020212....122..?01?1012.....2101?10?221..11222...222........2222?....?2222..................................................
           •••YA•GGYCGYUCYYU•RCAUCCR----------------------------------------------------UGY•A-YCRCGRCAUUY--GYR•RUCC∘U•YRCA•CARAYR••R•••Y•R•••••••••R•••••••••••••••••••••••
            5′ ——————————————————————————————————————————————————————————————————————————————————————————————————————————————— 3′
```

```
env-4     UAGGUCGGCCGCUCUAGCGCAUCC....................................................UGCGC.CCGCGGCAUUC..ACGCAUCCAUGCGCAUCAGGCUUCAGCCCGACACCCUUANAACGACACGCUGAUGUUCAGCGUC
env-5     GAAAAUCGUCGUUCCUUGGCAUCCA...................................................UGCCA.UCACGACAUUA..GUGCAUCCGUGCACGUCAACGUAUUUAUCAUCAUUAAAACAGUCUUUAUGAACGGGCUUUCAGA
Osp-1-1   CUCUCAGGCCGCUCCUGCGCAUCCA...................................................UGCGC.UCGCGGCAUUU..GUACAUCCUUGUACUUCGUUCGACCUAGCGAGGAACGAGCGUAGGAGACGAACGAAGUGAGAGU
env-6     GACAUACCCUGCUCCAUUGCAUCCG...................................................UGCAA.UCGCAGGAUACC.GUCCGUCC.UGGACAUAAAAAACCCGGCAGAUGCCGGGUUUUUUGAAUCGAUAAGAGUCUUAAA
env-7     UAGAUGCGCCGCGCCUGCGCAGCCA...................................................UGCGC.UCGCGGCAUAAC.GUACGUCC.CGUACAUAAAAAAGGGGAUCAAUUUCCAUUGACCCCCUUUUUUAUGCAACACCUA
env-8     UAGAUGCGCCGCGCCUGCGCAGCCA...................................................UGCGC.UCGCGGCAUAAC.GUACGUCC.CGUACAUAAAAAAGGGGAUCAAUUUCCAUUGACCCCCUUUUUUAUGCAACACNUA
env-9     UACAUGCGCCGCUCCUGCGCAUCCA...................................................UGCGC.CCGCGGCAUACC.GUACAUCC.UGUACAUAAAAAAACCUGCUGGAUGAGGCAGCAGGUUUUUACAGGGCACAAUUUU
env-10    GACACACGCCGCUCCUGCGCGUCC....................................................UGCGC.CCGCGGCAUACC.GUUCAUCC.UGAACAUAAAAAACCCGCUCAGCUGGCGCUAAAGCGGGUUUUGAAAUUGCCGCCG
env-11    UACAUGCGCCGCUCCUGCGCAUCCA...................................................UGCGC.CCGCGGCAUACC.GUACAUCC.UGUACAUGCGCCGCUCCUGCGCAUCCAUGCGCCCGCGGCAUACCGUACAUCCUGU
env-12    GGCAUGUCCUGCUCCUGCGCAUCCA...................................................UGCGC.ACGCAGGAUACC.GUCCAUCCCUGGACAUAAAAAAACCCGGAAGGGGUUGCCUUCCGGGUUUUAGGGUUUUUCAACU
Osp-1-2   GAACAUAUGCGCUCUUUGCCAUCCA...................................................UGGCA.UCGCGCUAUACC.GUUCAUCC.UGAACAUAAAAAAAGCCAACUUAGAAUCUCCAAGUUGGCUUUUUUCUACUGAAAA
Dps-1-5   GGGUGUCGCGUCUCCAUGCCGUCC....................................................UGGCA.CCGACGCAUACC.GCCCAUCCAUGGGCAUAAAAAAACCCCAAACUAAAUUAAUAGUUUGGGGCUUAUAUAUAUAAAA
Dps-1-6   GGAUGUCGCGUCUCCAUGCCGUCC....................................................UGGCA.CCGACGCAUACC.GCCCAUCCAUGGGCAUAAAAAAAGCCCCAAGUCGAAAUUAAUCAACUUGGGGCUUUAUAUAAAA
```
[truncated: 755,962 more chars]
